# Unveiling the presence of biosynthetic pathways for bioactive compounds in the *Thalassiosira rotula* transcriptome

**DOI:** 10.1038/s41598-019-46276-8

**Published:** 2019-07-09

**Authors:** Valeria Di Dato, Federica Di Costanzo, Roberta Barbarinaldi, Anna Perna, Adrianna Ianora, Giovanna Romano

**Affiliations:** Stazione Zoologica Anton Dohrn Napoli, Department of Marine Biotechnology, Villa Comunale, 80121 Napoli, Italy

**Keywords:** Gene ontology, Marine biology, Marine chemistry

## Abstract

Diatoms are phytoplankton eukaryotic microalgae that are widely distributed in the world’s oceans and are responsible for 20–25% of total carbon fixation on the planet. Using transcriptome sequencing here we show for the first time that the ubiquitous diatom *Thalassiosira rotula* expresses biosynthetic pathways that potentially lead to the synthesis of interesting secondary metabolites with pharmaceutical applications such as polyketides, prostaglandins and secologanin. We also show that these pathways are differentially expressed in conditions of silica depletion in comparison with standard growth conditions.

## Introduction

Diatoms are a major group of eukaryotic microalgae in the phytoplankton widely distributed in the world’s oceans, and capable through photosynthesis of fixing about 20–25% of the total carbon on the planet^1^. Their worldwide distribution is supported by a molecular tool-kit (M-T) that allows them to adapt to different conditions through the perception of environmental cues and the control of competitors and grazers^2–5^. Some of the metabolites which are part of this M-T have already been identified as having potential applications as pharmaceuticals or cosmeceuticals^6^. Nevertheless, the full potential of marine diatoms still needs to be unlocked. One way to unlock this potential is to expose diatoms to stressful conditions to force changes in their metabolism and activate biosynthetic pathways inducing the production of secondary metabolites. One of the stressful conditions to promote such changes is nutrient depletion during microalgae growth. For example, phosphorus (P) deficiency in *Thalassiosira rotula* induces a remodelling of the transcriptome including changes in cellular P allocation patterns, enzyme activity and lipid composition, whereas nitrogen (N) deprivation reduces primary carbon metabolism leading to accumulation of lipids^7^. In addition to P and N, also carbon deprivation, independently from the light intensity (30, 300 or 1000 μmol photon m^−2^ s^−1^), induces a rearrangement of the Carbon Concentration Mechanisms (CCM) toward the synthesis of pyruvate^8,9^ reorienting the carbon metabolism toward lipid accumulation. A similar remodelling of metabolic pathways occurs in conditions of silica depletion^10^. Diatoms require silica (Si) to generate the intricate frustules that surround the cell and allow the entrance of light to the chloroplasts and the transport of gases and solutes. The silica cell wall is believed to provide an ecological advantage over other phytoplankton groups due to its mechanical strength that protects diatoms against grazers. Deprivation of Si arrests progression of cell cycle events, such as cell division and DNA replication, attesting the importance of silica in the diatom life cycle^11^.

Here we analyse the transcriptome of the diatom *Thalassiosira rotula*, isolated from the Gulf of Naples in the Mediterranean Sea, comparing cultures grown in normal condition (CTR) versus cultures grown in conditions of stress such as silica depletion (STRESS). Our analysis shows that this diatom species has the potential to produce new metabolites, i.e. secologanin, polyketides and prostaglandins, the related pathways of which are differentially expressed under this stress condition.

We experimentally confirmed the expression of these pathways identified in the newly sequenced transcriptome of our clone and also compared their expression with those from other *T. rotula* clones, grown under different stress conditions and sequenced in the Marine Microbial Eukaryotic Transcriptome Sequencing Project (MMETSP)^12^ supported by the Moore Foundation. Our study also confirms that the identified pathways are actually present in the *T. rotula* genome and do not derive from bacteria commonly associated with diatom cells.

## Results

### *De Novo* transcriptome assembly and functional annotation

Total RNA from *Thalassiosira rotula* cultured in f/2 complete medium (control condition, CTR), and in f/2 silica depleted medium, (stress condition, STRESS), was obtained from cells collected at the beginning of the stationary phase of growth, when cells are more prone to produce secondary metabolites^13^. Table 1 shows the statistics for the total transcriptome assembly after filtering of unspecific sequences. The total assembly was composed by 142 Mbp in 107,289 transcripts grouped into 66,496 genes, with a N50 of 2,034 bp.

**Table 1:** Transcriptome sequencing statistics.

Assembly (Mbp)	142
Transcripts (n=)	107,289
Genes (n=)	66,496
Mean GC%	47.53
Average contig lenght (bp)	1,332.76
Median contig lenght (bp)	970
N50 (bp)	2,034
Proteins (n=)	60,172

The final dataset translated into proteins (minimum length 50aa) includes 60,172 protein sequences, 28,128 (46.7%) of which were identified as complete, i.e. having a start methionine and a stop codon.

The sequences were also analysed for the presence of repetitive elements and more than 3 Mbp were identified as repetitive elements including, for example, DNA transposons, retro-elements, satellites, and rRNA.

Functional annotation of the combined transcriptomes was performed on proteome sequences, 89% of which showed homology with other diatoms, in particular *T. pseudonana, T. oceanica* and *Phaeodactylum tricornutum* (Supplementary Data 1 and 2).

**Figure 1:**
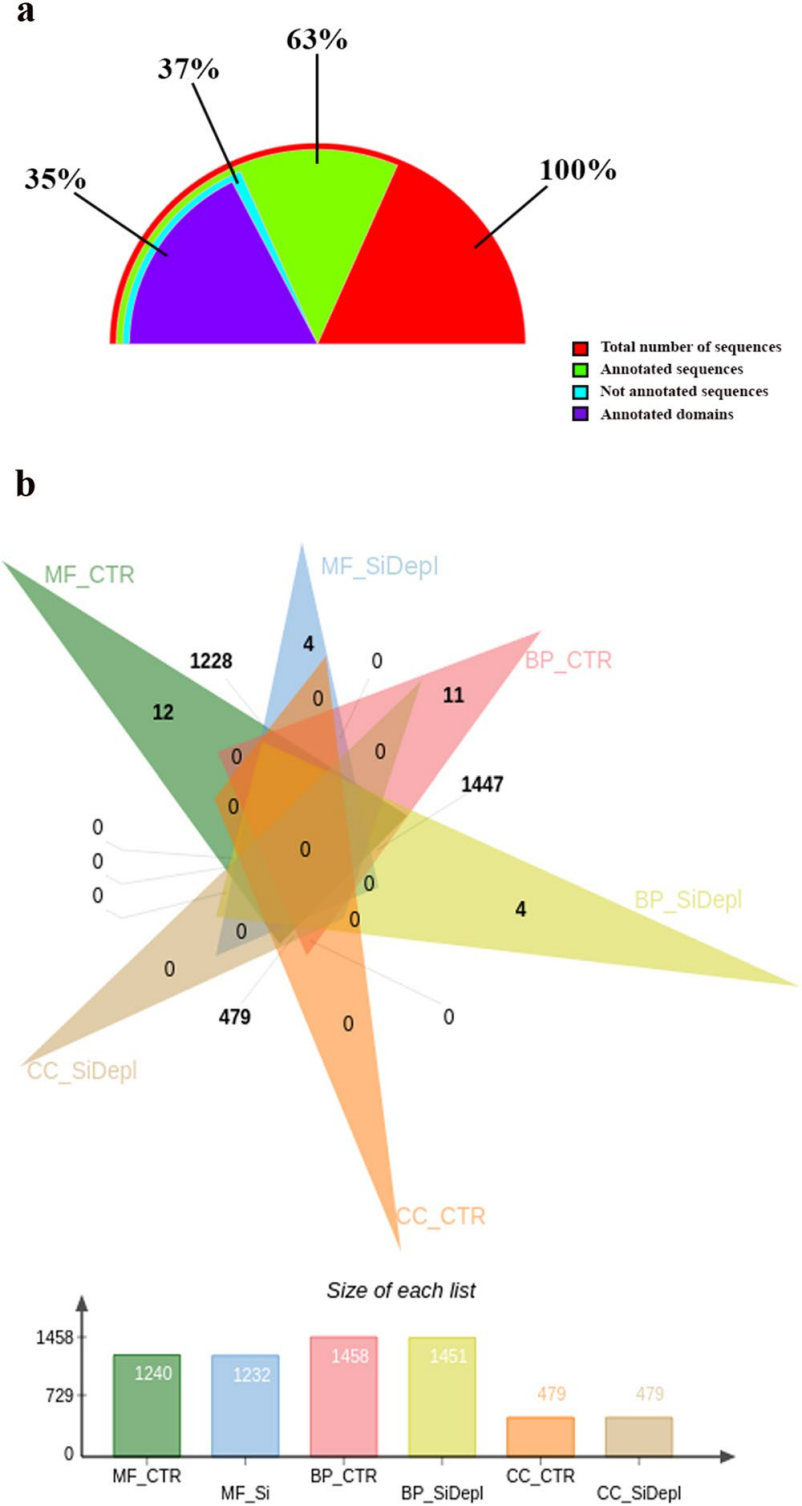
*Thalassiosira rotula* functional annotation. (**a**) Percentage of annotated sequences and domains of the total number of peptides in the diatom *Thalassiosira rotula*. (**b**) Venn diagram showing the number of common and unique GO terms between the two growth conditions for each category in the diatom *Thalassiosira rotula*. Abbreviations: MF: Molecular Functions; CTR: control condition; SiDepl: silica depleted medium corresponding to the STRESS condition; BP: Biological Processes; CC: CellularComponents.

Out of a total 60,172 peptide sequences, 63% had at least one blast hit, and almost half (35%) retrieved a blast hit against conserved domains (Fig. 1a).

The functional annotation of peptides assigned the majority of proteins and domains to the GO terms in the Molecular Functions (MF) category (Table 2). Except for the Cellular Components (CC) category, for both Biological Processes (BP) and MF categories, the number of associated domains was almost half the number of associated protein sequences.

**Table 2:** Number of sequences associated to each GO term category.

Number of sequences with at least one blast hit	37999
Number of peptides sequences associated to Molecular Functions GO terms	26255
Number of domains associated to Molecular Functions GO terms	11622
Number of peptides sequences associated to Biological Processes GO terms	15459
Number of domains associated to Biological Processes GO terms	7809
Number of peptides sequences associated to Cellular Components GO terms	17794
Number of domains associated to Cellular Components GO terms	2694

Subdivision of the sequencing retrieving at least one blast hit and an association with a Gene Ontology number in the three functional categories: Molecular Functions (MF), Biological Processes (BP) and Cellular components (CC).

The “ATP binding” GO term was the most represented in the MF category while “transmembrane transport” was the most represented GO term in the BP category, and “integral to membrane” was the most represented GO term in the CC category (Supplementary Data 3 to 8, SI1: Supplementary Fig. 2a–f).

### Functional annotation analysis: STRESS versus CTR

Principal Component Analysis showed that replicates 1 and 4, in the CTR and STRESS group, respectively, did not behave coherently within the group, so they were excluded from subsequent analysis (SI1: Supplementary Fig. 1).

Fisher test on the number of GO terms associated with each peptide sequence for each treatment did not reveal any significant difference. However, we were able to identify uniquely annotated GO terms. The Venn diagram in Fig. 1b shows the occurrence of 12 and 4 unique MF GO terms in CTR and STRESS, respectively. Similarly, 11 and 4 BP GO terms were uniquely annotated in CTR and STRESS, respectively. No unique GO term annotations were present in the CC category for both treatments (see Supplementary Data 9 for complete list of GO Terms). Table 3 lists the unique GO terms found.

**Table 3:** Unique GO terms.

Category	GO Number	GO Term	CTR	STRESS
MF	0004062	ATP-dependent helicase activity	+	−
	0008379	L-leucine transaminase activity		
	0008658	Metalloaminopeptidase activity		
	0015416	Oxidoreductase activity		
	0015430	Oxidoreductase activity, acting on a sulfur group of donors, disulfide as acceptor		
	0015604	Oxidoreductase activity, acting on iron-sulfur proteins as donors		
	0005391	Hydro-lyase activity		
	0008909	NAPDH binding		
	0034189	Pyrophosphatase activity		
	0043621	RNA strand-exchange activity		
	0015439	snRNA stem-loop binding		
	0015439	Oxidoreductase activity, acting on a sulfur group of donors, NAD or NADP as acceptor		
	0003983	Anion transmembrane-transporting ATPase activity	−	+
	0008320	Lipid transporter activity		
	0008761	Methyltransferase activity		
	0009002	Nicotinate phosphoribosyltransferase activity		
BP	0001407	Body fluid secretion	+	−
	0007281	Interleukin-8 biosynthetic process		
	0008354	Lactate biosynthetic process		
	001096	Negative regulation by host of viral transcription		
	0032802	Post-chaperonin tubulin folding pathway		
	0042744	Purine ribonucleoside salvage		
	004523	Regulation of exit from mitosis		
	0044458	Regulation of protein phosphatase type 2A activity		
	0050427	RNA modification		
	0051923	Sterol biosynthetic process		
	0006011	Cellular amino acid metabolic process	−	+
	001932	Peptidyl-tyrosine dephosphorylation		
	0043953	Regulation of mRNA stability		
	0000022	(1-3)-beta_D-glucan biosynthetic process		

Unique GO terms for each growth condition for each category: MF: Molecular Function; BP: Biological Processes; CC: Cellular Components.

Except for ‘Interleukin-8 biosynthetic process’, ‘lactate biosynthetic process’, CTR specific, and ‘1-3-beta-D-glucan biosynthetic process’, STRESS specific in the BP category, all other terms referred to more broad categories (Table 3).

### Differential expression analysis

*In silico* expression analysis on single transcripts identified 5,114 DET (Differential Expressed Transcripts) in STRESS vs CTR, with 2,949 upregulated and 2,165 downregulated transcripts (Supplementary Data 10). The GO terms associated to the DETs are reported in SI1: Supplementary Table 2 and Supplementary Fig. 3.

Enzymes in the chains for the sphingolipid, sterol, spermidine, secologanin, polyunsaturated fatty acids (PUFA), malonylCoA, lipotheicoic, L-ascorbate, L-cysteine biosynthesis and isoprenoid pathways were upregulated in STRESS condition.

Considering 2-fold change as the minimum value for significant results, the proportion of significant DET was 20% for downregulated and 34% for upregulated transcripts. Among these groups, 1,604 (54%) of the upregulated and 1,278 (60%) of the downregulated transcripts were not annotated but indicated as “protein”, or “predicted protein” or “hypothetical protein” or “NA” (not annotated at all). It is interesting to note that part of these “un-annotated” groups were considerably differentially expressed. Indeed, 21% of the “un-annotated”-upregulated transcripts had an expression fold change between +2.50 and +19.12, and 13% of the “un-annotated”-downregulated transcripts had an expression fold change between −2.50 and −20.03 indicating the presence in these groups of still unknown functions that are very important for the homeostasis of the cells (Supplementary Data 10).

Of the downregulated transcripts, it is interesting to note that the ‘ribosomal protein s6 kinase beta 2’ showed a fold change of −18, whereas the upregulated transcripts ‘cmp-sialic acid transporter’ and ‘peroxisomal trans-2-enoyl- reductase’ showed a fold change of +15 and +8, respectively.

### *In silico* pathway expression analysis

Total number of general pathways, for both growth conditions, was 68 with all essential and secondary metabolic pathways present. The most represented pathways, based on the number of associated sequences, are those involved in carbohydrate, lipid and protein metabolism (Table 4). Analysis of general pathways expressed in FPKM (Fragments Per Kilobase of transcript per Million mapped reads) indicated some differences between the two conditions, CTR and STRESS (Table 5).

**Table 4:** Top 10 most represented general pathways based on the number of associated sequences.

Pathway name	Number of associated sequences
Protein modification	456
Amino-acid biosynthesis	240
Cofactor biosynthesis	186
Lipid metabolism	175
Carbohydrate degradation	169
Amino-acid degradation	95
Porphyrin containing compounds metabolism	77
Purine metabolism	62
Sulfur metabolism	48
Carbohydrate biosynthesis	48
Carbohydrate metabolism	48

**Table 5:** Top 10 most expressed pathways in FPKM, Fragments Per Kilobase of transcript per Million mapped reads. Differentially expressed pathways are reported in bold.

Pathway Name	CTR_FPKM	STRESS_FPKM	Fold STRESS vs CTR
**Protein biosynthesis**	**88.73**	**245.92**	**+2.8**
**Carbohydrate degradation**	**42.97**	**26.17**	**−1.6**
**Thermoadapter biosynthesis**	**36,45**	**17.8**	**−1.9**
**Flavonoid metabolism**	**27.73**	**47**	**+1.7**
Organosulfur biosynthesis	24.26	19.77	
Carbohydrate biosynthesis	20.89	16.51	
Lipid metabolism	19.65	21.85	
Spore coat biogenesis	18.6	28.11	
Carbohydrate acid metabolism	18.07	17.59	
Nucleotide-sugar biosynthesis	15.07	21.1	

The ‘protein biosynthesis’ and ‘flavonoid metabolism’ pathways had almost three- and two-fold lower expression values in CTR with respect to STRESS, respectively. ‘Carbohydrate degradation’ and ‘thermoadapter’ biosynthesis on the contrary were almost two-fold higher in CTR with respect to STRESS.

Despite these differences, FPKM box plot expression analysis revealed no statistically significant differences between the two conditions (Supplementary Table 4 (in SI1), Supplementary Files 1 and 2).

The number of pathways identified in both conditions on the second level of annotation was 258 (Supplementary Data 11). The most represented pathways were ‘protein ubiquitination’ (293 sequences) followed by ‘protein glycosylation’ (127 sequences) and ‘glycolysis’ (105 sequences). Also within this level of pathway annotation there were no statistically significant differences in FPKM values between the two conditions (Supplementary Files 3 and 4).

The pipeline annotated also a third level with 327 pathways for CTR and 325 for STRESS (Supplementary Data 12). The best represented pathways were: ‘Pyruvate from D-glyceraldehyde 3-phosphate step 3/5’ with 26 associated sequences; ‘D-ribulose 5-phosphate from D-glucose 6-phosphate (oxidative stage) step 1/3’ with 25 associated sequences, ‘glutathione from L-cysteine and L-glutamate step 1/2’ with 21 associated sequences, ‘D-glyceraldehyde 3-phosphate and glycerone phosphate from D-glucose step 4/4’ with 20 associated sequences, ‘L-cysteine from L-serine’ step 2/2’ with 20 associated sequences. Again, no differences in FPKM values were appreciable (Supplementary File 5 and 6).

### *In silico* expression analysis of selected pathways

Despite the fact that no significant differences were evident in the FPKM expression of the annotated pathways, we focused our attention on single transcripts associated with pathways leading to secondary metabolites that have been poorly studied or that were previously unknown for diatoms: secologanin, prostaglandin and polyketides biosynthesis (Supplementary File 4, highlighted pathways).

### Secologanin biosynthesis

**Figure 2:**
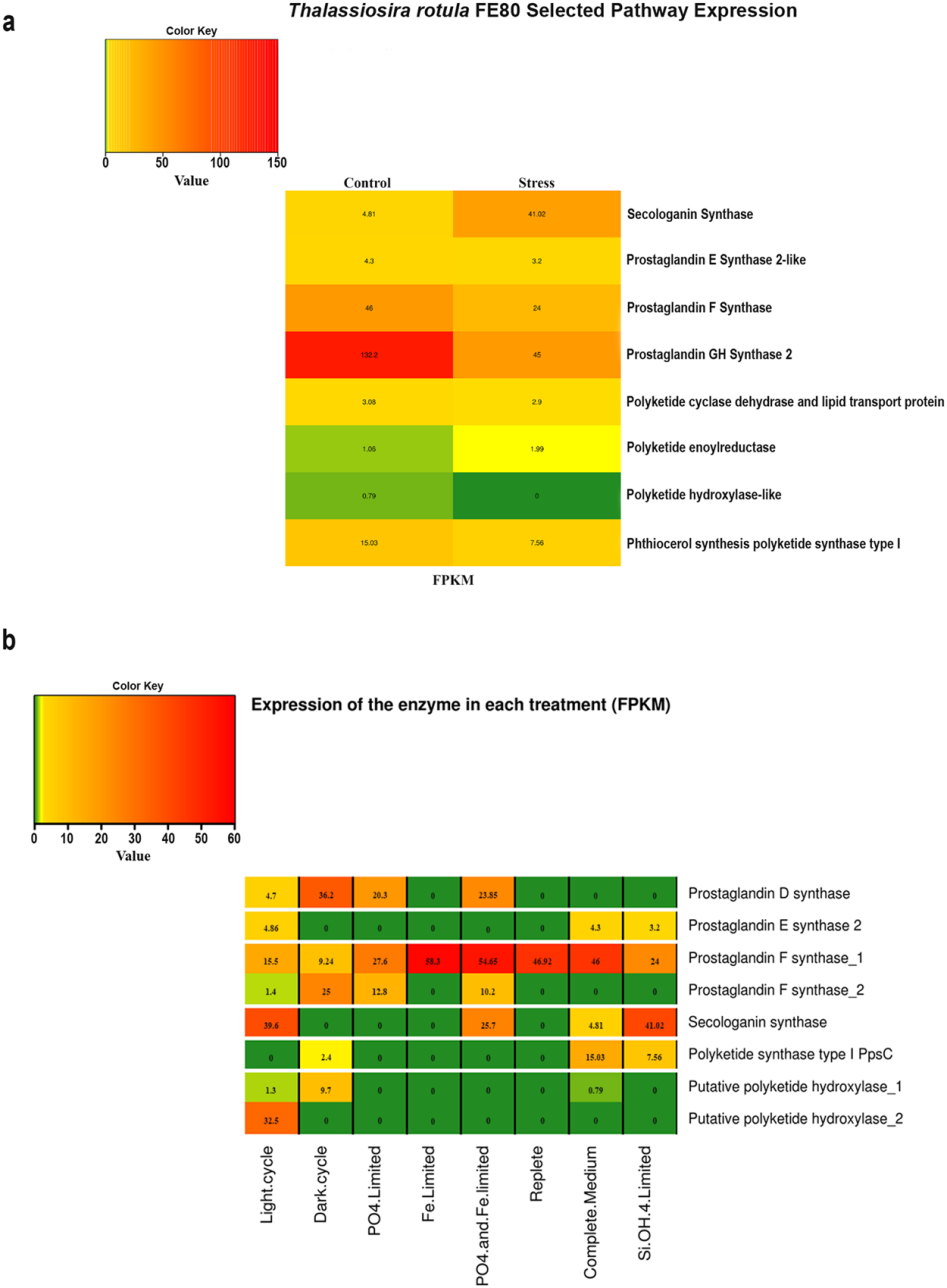
*In silico* expression analysis of selected pathways. (**a**) Heat map showing the presence and the expression levels, calculated in FPKM on the contig’s reads, of transcripts, belonging to our *Thalassiosira rotula* from the Mediterranean Sea, in the selected pathways for each growth condition considered. (**b**) Heat map showing the presence and the expression levels, calculated in FPKM on the contig’s reads of the transcripts in the selected pathways of the *Thalassiosira rotula* strains, grown in different conditions, from the MMETSP project in comparison with our clone from the Mediterranean Sea (Complete medium and SiOH4 limited).

The ‘Secologanin synthase (SLS)’ annotation in the transcriptome of *T. rotula* was associated to four peptides that when aligned matched one another, with the peptide TR26037|c1_g1_i1 being the longest (SI1: Supplementary Fig. 4). Heat map in Fig. 2a shows expression levels of the corresponding transcript, calculated in FPKM, which was ten times higher in STRESS with respect to CTR (Fig. 2). In addition, the related transcript was also present in the DET table with a logFC of +3.1 (Supplementary Data 10).

### Prostaglandin biosynthesis

Peptide TR754|c0_g1_i1 annotated as ‘Prostaglandin G/H synthase 2 (PGHS-2)’, TR25058|_c1_g1_i2 annotated as ‘Prostaglandin E synthase 2-like (mPGES-2)’ and TR11659|c0_g1_i1 annotated as ‘Prostaglandin F synthase (PGFS)’ were associated to the ‘Prostaglandin biosynthesis’ pathway (Supplementary Data 13).

In addition, three putative peptides and two transcripts were associated to the ‘Prostaglandin F synthase’ function. Table 6 shows the blast results of the five PGFS associated transcripts.

**Table 6:** Blast results of the prostaglandin F synthase related transcript.

Transcript	Annotation on:	First three BlastX results	Query cover (%)	Identity (%)
TR11659|c0_g1_i1	Peptide	Aldo-keto oxidoreductase *Thalassiosira pseudonana*;	76	63
		Hypothetical protein *Thalassiosira ocenanica*;	74	58
		Prostaglandin F synthase *Symbiodinium microadriaticum*.	70	45
TR3003|c0_g1_i1	Peptide	Hypothetical protein *Thalassiosira pseudonana*;	72	66
		Reductase with NAD or NADP as acceptor *Phaeodactylum tricornutum*;	76	48
		Aldo/keto reductase *Fragilariopsis cylindrus*.	69	48
TR17558|c0_g1_i1	Peptide	Hypothetical protein *Thalassiosira ocenanica*;	42	42
		Hypothetical protein *Fistulifera solaris*;	41	44
		Acetyl-CoA synthetase *Fistulifera solaris*.	41	43
TR22640|c0_g1_i1	Transcript	Predicted protein *Thalassiosira pseudonana*;	72	75
		Hypothetical protein *Thalassiosira oceanica*;	76	60
		Hypothetical protein *Fistulifera solaris*.	58	59
TR60026|c0_g1_i1	Transcript	Predicted protein *Thalassiosira pseudonana*;	67	82
		Hypothetical protein *Thalassiosira oceanica*;	71	74
		Hypothetical protein *Fistulifera solaris*.	55	54

TR11659|c0_g1_i1 was chosen for the following analysis since this transcript blasted to other *Thalassiosira* species and to a dinoflagellate PGFS function. Moreover, the match with *T. pseudonana* was a protein annotated with the more general function of aldo/keto oxidoreductase, which is a family of proteins that also include PGFS.

Furthermore, browsing the ‘Enzyme Description’ column of the annotation table (Supplementary Data 13) we found six peptides annotated as ‘Carbonyl reductase (NADPH)’ or ‘Prostaglandin E_2_ 9-reductase’ that reduces prostaglandin E_2_ (PGE_2_) to prostaglandin F_2__α_ (PGF_2α_) (SI1: Supplementary Table 4) differently from PGFS that produces PGF_2α_ starting from prostaglandin D_2_ (PGD_2_). However, we did not include this enzyme in our analysis as it was not annotated in the specific pathway.

Heat Map (Fig. 2a) shows no difference in mPGES-2 expression calculated in FPKM, between the two growth conditions, while PGFS and PGHS-2 expression were downregulated in STRESS by two- and three-fold, respectively. PGHS-2 was also present in the DET table with a LogFC of −1.57 (Supplementary Data 10).

### Polyketides biosynthesis

No specific pathway annotation exists for polyketides biosynthesis as they are included in the general lipid metabolism pathway. Searching in the complete annotation table (Supplementary Data 13), we found polyketide-related functional annotations of at least 4 putative enzymes, i.e. ‘Polyketide cyclase dehydrase and lipid transport protein’ (PK_Cyc, TR3754|c0_g1_i2), ‘Polyketide enoylreductase’ (PK_ER, TR30039|c0_g1_i1), ‘Polyketide hydroxylase-like’ (PK_Hxl, TR21208|c0_g1_i1), ‘Phthiocerol synthesis polyketide synthase type I’ (ppsC, TR40329|c0_g1_i6). Except for ppsC synthase, whose related transcript was downregulated in the STRESS samples by two-fold (in FPKM value) and had a LogFC value equal to −0.99 in DET table (Supplementary Data 10), the other three related transcripts were almost equal in expression with low FPKM values in both growth conditions (Fig. 2a).

Finally, we also found annotations for single modules associated to both polyketide synthesis and fatty acid elongation, like acyltransferase, acyl carrier protein, β-ketoacyl reductase and ketosynthase, methyl transferases, thioesterases and dehydrogenase domains (Supplementary Datas 13 to 19). These transcripts were not considered for the expression analysis due to their high number of redundant related transcripts.

### Comparative analysis of selected pathways with other strains of *Thalassiosira*

To understand if selected pathway expression in the transcriptome of *T. rotula* is a prerogative of the strain from the Mediterranean, we used the public available MMETSP transcriptomic dataset to verify their presence in other *T. rotula* strains, 3096 and GSO102, isolated from different geographical areas and grown in different conditions (Table 7 and Fig. 2b).

**Table 7:** Presence of the selected pathways in different *Thalassiosira rotula* strains. N= not present; Y= present.

*T. rotula* Strain	CCMP 3096	CCMP 3096	GSO 102	GSO 102	GSO 102	GSO 102	CCMP1647	CCMP1647
MMETSP code	0403	0404	910	911	912	913	—	—
Treatment	Light cycle	Dark Cycle	0.4 um PO4	4 nM Fe, 12.5 um EDTA	4 nM Fe, 12.5 um EDTA, 0.4 uM PO4	replete condition	Complete Medium	SiOH4 Limited
Site Name	Pacific Ocean	Pacific Ocean	Pacific Ocean	Pacific Ocean	Pacific Ocean	Pacific Ocean	Mediterranean sea	Mediterranean sea
Prostaglandin G/H synthase (PGHS-2)	N	N	N	N	N	N	Y	Y
Prostaglandin E synthase (mPGES-2)	Y	N	N	N	N	N	Y	Y
Prostaglandin F synthase (PGFS)	Y	Y	Y	Y	Y	Y	Y	Y
Prostaglandin D synthase (PTGDS)	Y	Y	Y	N	Y	N	N	N
Phthiocerol synthesis polyketide synthase type I (ppsC)	N	Y	N	N	N	N	Y	Y
Polyketide cyclase dehydrase and lipid transport protein (PYL5)	N	N	N	N	N	N	Y	Y
Polyketide enoylreductase (PK_ER)	N	N	N	N	N	N	Y	Y
Polyketide hydroxylase-like (PK_HxL)	Y	Y	N	N	N	N	Y	Y
Secologanin synthase (SLS)	Y	N	N	N	Y	N	Y	Y

The available conditions were: 3096LC = day portion of the day/night cycle; 3096DC = dark portion of the day/night cycle; GSO102Fe = iron depletion; GSO102P = phosphorus depletion; GSO102Fe2 = iron and phosphorus depletion; GSO102R = nutrient replete.

Secologanin synthase was present in the 3096LC and GSO102Fe2’s transcriptomes but not in those of 3096DC, GSO102Fe, GSO102P and GSO102R (Table 7, Fig. 2b). Of all the conditions considered in which we found SLS annotated, silica depletion and the light portion of the day/night cycle were similar and had the highest values (41.02 fpkm and 39.6 fpkm, respectively) compared to the other conditions.

Among the polyketide biosynthesis enzymes, PKS_ER annotation has not been reported for other strains. The synthase ppsC was present only in 3096DC and the best condition of expression was found in our clone in CTR. The hydrolase-lyase PK_HxL was present in both 3096LC and 3096DC. It was present with two isoforms, here named as PK_Hlx_1 and PK_Hlx_2, in 3096LC and with one isoform, PK_Hlx_1, in 3096DC and in our clone in CTR. The highest expression for PK_Hlx_1 was in the 3096DC condition. The PK-Hxl_2, present only in 3096LC, showed a higher FPKM value than PK_Hlx_1. The GSO102 strain, in all conditions tested, did not show any polyketide related enzyme annotation. Finally, all strains had annotations for the single modules associated to both polyketide synthesis and fatty acid elongation (Supplementary Data 13 to 19). Examining the prostaglandin pathway, we did not find any annotation for PGHS-2 in strains 3096 and GSO102. On the contrary, PGFS was present in all strains, together with a second isoform present in 3096LC, 3096DC, GSO102P and GSO102Fe2. The transcript for mPGES-2 was present only in the 3096LC transcriptome. Finally, annotation for prostaglandin D synthase (PTGDS) that was absent in our transcriptomes, was instead present in the 3096LC, 3096DC, GSO102P and GSOFe2 transcriptomes. The best condition of expression was 3096DC for PTGDS, 3096LC and CTR for mPGES-2, GSO102Fe and GSO102Fe2 for PGFS_1 and 3096DC for PGFS_2.

### Experimental confirmation of selected pathways

**Figure 3:**
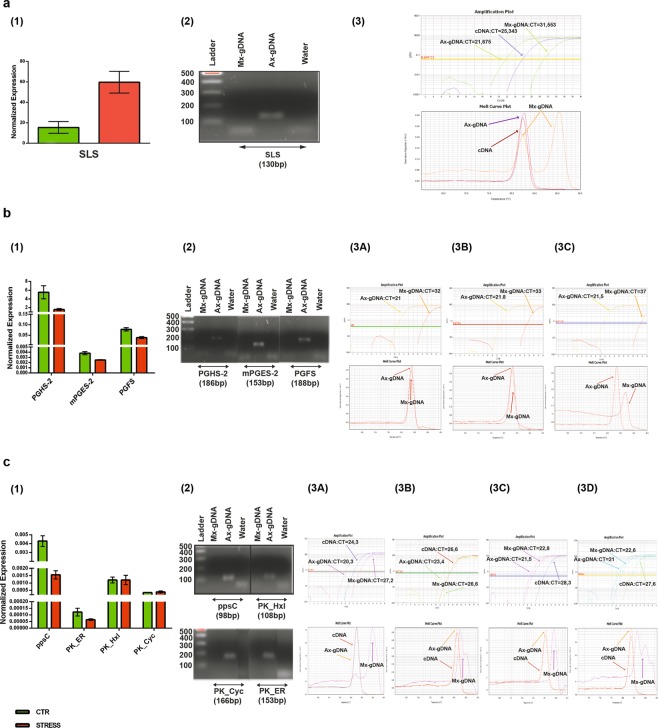
Experimental validation of the transcripts associated to the selected pathways in the *Thalassiosira rotula* from the Mediterranean Sea. (**a–c**) qPCR on cDNA (1), PCR on gDNA (2), qPCR on gDNA (3). (**a**) Secologanin synthase; (**b**) Prostaglandin biosynthesis; (**c**) Polyketides biosynthesis. Abbreviations: PGHS-2: Prostaglandin G/H synthase 2; mPGES-2: Prostaglandin E synthase 2-like; PGFS: Prostaglandin F synthase; ppsC: Phthiocerol synthesis polyketide synthase type I; PK_ER: Polyketide enoylreductase; PK_Cyc: Polyketide cyclase dehydrase and lipid transport protein; PK_Hxl: Polyketide hydroxylase-like; Ax: Axenic genomic DNA; Mx: mixed Bacterial-Algae genomic DNA; CT: cycle treshold. Original gel picture is shown in Supplementary Fig. 7.

In order to confirm *in silico* expression data, we performed quantitative Polymerase Chain Reaction (qPCR) amplification on coding DNA (cDNA) from the same sample used for RNA-seq and qPCR amplification on genomic DNA (gDNA) from an axenic *T. rotula* (Ax) and a non-axenic *T. rotula* (Mx) culture (Fig. 3) in which bacterial DNA was predominant (unpublished data).

### Secologanin synthase

qPCR on cDNA confirmed the significant overexpression of Secologanin synthase (SLS) in STRESS with respect to CTR with a p-value of 0.0062 (Fig. 3a(1)). PCR on Ax- and Mx-gDNA confirmed the presence of the corresponding gene exclusively in the algal genome excluding a co-partnership with the associated bacteria for the possible production of secologanin (Fig. 3a(2)). qPCR amplification on Ax-gDNA had a cycle threshold (CT) value of 21,6 compared to 31,5 of the amplification on the Mx-gDNA, confirming the algal specific expression of SLS (Fig. 3a(3)). Melt curve analysis showed one peak for the Ax-gDNA overlapping with the cDNA peak, while the Mx-gDNA showed two separated peaks. Sequencing of the corresponding amplicon definitively demonstrated that the additional amplicon from the Mx culture was non-specific.

### Prostaglandin biosynthesis

qPCR (Fig. 3b(1)) showed a significant downregulation of all three transcripts in STRESS with p-value = 0.2 for PGHS-2, 0.1 for mPGES-2 and 0.07 for PGFS. PGHS-2 had the highest level of expression in both conditions with respect to PGFS and mPGES-2 that had expression levels in the order of 10^−2^ and 10^−3^, respectively.

Amplifications on gDNAs (Fig. 3b(2)) and sequencing of the amplicons demonstrated the specific origin of the corresponding transcripts from the algae and not from the associated bacteria. All three transcript amplicons had a CT value equal to about 21 on the Ax-gDNA and equal to about 33 on the Mx-gDNA, confirming the specific derivation of the prostaglandin biosynthetic pathway transcripts from the alga and not from the associated bacteria (Fig. 3b(3A–C)). Differently from PGHS-2 and mPGES-2, PGFS melt curve on non-axenic Mx-gDNA showed one peak that did not overlap with that of the Ax-gDNA. Sequencing of the corresponding amplicon demonstrated its lack of specificity. The absence of a peak corresponding to the one present in the Ax-gDNA is probably due to its very low abundance also considering the low presence of eukaryotic DNA in the sample (unpublished data).

### Polyketides biosynthesis

qPCR (Fig. 3c(1)) confirmed the results of the FPKM calculation showing no regulation of PKS_ER, and PK_Hxl but a significant downregulation of the ppsC transcript with a p-value = 0.03.

Classical PCR amplification (Fig. 3c(2)), amplicon sequencing and qPCR analysis on gDNA (amplification and melt curve, Fig. 3c (3A–D) of ppsC, PK_Hxl, PKS_ER, of the Ax-gDNA, demonstrated the specificity of this transcript to the eukaryotic alga.

## Discussion

Expression analysis to identify transcripts and differentially expressed metabolic pathways between control and silica-limited growth conditions highlighted the presence of a large group of uncharacterized proteins in the diatom *Thalassiosira rotula*, which were differentially expressed between the two growth conditions, representing, as such, a large source of potentially still undiscovered new functions and correlated metabolites.

Among the differentially expressed annotated sequences, we found that Ribosomal protein s6 kinase beta 2 (S6K2) was the one with the highest downregulation (−18.03 fold change) in the silica-limited condition with respect to the control. S6K2 is an important cell growth regulator and metabolism and its downregulation is consistent with the status of starvation induced by silica limitation^14^. S6K2 is indeed activated in response to growth factors, cytokines and nutrients, together with other kinases of the same family. The activity of S6 kinases is linked to fundamental cellular processes, including protein synthesis, mRNA processing, glucose homeostasis, cell growth and survival. They also play an important role in several human pathologies, including obesity, diabetes, ageing and cancer^15^.

Upregulated functions in silica-limited conditions with respect to the control include upregulation of cmp-sialic acid transporter (+15.15) and peroxisomal trans-2-enoyl- reductase (+8), indicating that cells are coping with starvation by accumulating defensive molecules and lipids confirming again the results reported by Heydarizadeh in which light stress and carbon deprivation stress redirect the CCM toward lipids accumulation^8,9^. Cmp-sialic acid is one of a series of nucleotide sugar transporters, a class of transporters that have been associated with inflammation, tumours, virus infections, and other processes^16^. It transports CMP-sialic acid from the cytosol into Golgi vesicles where glycosyltransferases are activated and produce sialilated glycolipids that can act as defensive molecules. The presence of sialic acids has been ascertained only in animals, even though recently they have also been discovered in plants^17,18^ and in green microalgae^19^, but their role is still uncertain.

Peroxisomal trans-2-enoyl-reductase seems to be involved in the peroxisomal degradation of phytols, a product derived from chlorophyll degradation, and has also been proposed to participate in a peroxisomal fatty acid chain elongation system, indicating that cells are probably accumulating long fatty acids in response to starvation status^20^.

The CTR-specific uniquely annotated GO terms, ‘Interleukin-8 biosynthetic process’ and ‘lactate biosynthetic process’, and the STRESS specific ‘1-3-beta-D-glucan biosynthetic process’ are all involved in defensive systems and in pathogenic mechanisms in humans^21,22^. It would thus be very interesting to further investigate these processes to unveil their role in diatoms.

Worthy of mention is also the upregulation of the ‘flavonoid metabolism’ pathway in STRESS, which suggests that this condition could be useful to possibly increase the yield of this important class of molecules that are beneficial for human health^23^.

In addition to the above-mentioned differentially expressed transcripts, also enzymes in the chains for sphingolipid, sterol, spermidine, PUFA, malonylCoA, lipotheicoic, l-ascorbate, l-cysteine biosynthesis, and isoprenoid pathways were upregulated in STRESS confirming that the depletion of silica is a good condition to stimulate the synthesis of bioactive molecules.

Furthermore, with our study we show for the first time, that diatoms are capable of synthesising secondary metabolites such as polyketides and secologanin, the latter at increased levels under silica starvation. Moreover, we confirm the presence of genes that synthesize animal hormone-like molecules, such as prostaglandins also in this diatom species^24^, *Thalassiosira rotula*, other than *Skeletonema marinoi* already published in the 2017.

Secologanin is involved in a complex metabolic pathway leading to the synthesis of Monoterpenoid Indole Alkaloids (MIAs), a large class of chemically different bioactive molecules considered very attractive due to their activity against cancer and other severe diseases in humans^25^. Some of the most active anticancer drugs such as camptothecans and vinca alkaloids^26^ have this type skeleton. Secologanin is a precursor for many of these compounds that are present in low amounts in the Madagascar periwinkle *Catharanthus roseus* leaves^27,28^. The enzymes involved in its formation are target of increasing research efforts in the phytochemical area, but to date they have been identified only in a few plant species. Discovering secologanin synthase in the genome of a diatom species paves the way to a completely new research opportunity for the understanding of the synthetic pathway and the role of secologanin in eukaryotic unicellular photosynthetic organisms.

Surprisingly, we found the transcripts for enzymes involved in polyketide biosynthesis. Polyketides (PKs) are an important class of molecules with a broad range of biological and pharmacological activities^29^. They include toxins, siderophores, pigments, antibiotics, cytostatics, and immune-suppressants. PK Synthases (PKSs) are widely distributed in bacteria and sporadically in archaea and eukarya where they probably derive from bacteria via horizontal gene transfer^30–32^. There has been no report until now on the existence of polyketide enzymes in a diatom species even if this is not the first attempt to find them in diatoms. Kohli *et al*. (2016) made an evolutionary study on PKSs in marine microbial genera, including diatoms, utilizing the data in the MMETSP database. The species they considered also included the *T. rotula* strains we used in our *in silico* analysis. However, they were unable to find any PKS in their transcriptome, nor in all the Bacillariophytes they took into consideration^29^ whereas we found a low expression of PKS in the strain 3096 transcriptome, both in the dark (DC) and light portion (LC) of the day/night cycle. A possible reason for this discrepancy is the low sensitivity of the annotation pipeline used for data analysis in the MMETSP project, while for our analysis, we used Annocript^33^, an assembler pipeline specifically developed in our institute that differs from the ones used in the MMETSP project.

Prostaglandins are a very interesting class of PUFA-derived molecules acting as hormones that play a pivotal role in many physiological processes in animals^34^. They are present not only in mammals, but also in both marine and terrestrial invertebrates^35^ and only few studies report their occurrence in terrestrial plants^36^.

Some macroalgae also possess the prostaglandin pathway, but only a limited number of prostaglandin molecules have been described from this source^37,38^. Recently, we were able to demonstrate the presence of this pathway also in diatoms, showing that *Skeletonema marinoi* has the ability to synthesize the complete panel of three classes of prostaglandins^24^. With the present study, we demonstrate the expression of the prostaglandin pathway in another diatom species and the significant downregulation of the first enzyme, PGHS-2, induced by silica depletion, differently from *S. marinoi* in which we did not observe any regulation of the pathway by nutrient limitation^24^.

Overall, the results obtained from the transcriptomic analysis of *T. rotula*, provide evidence for the presence of biosynthetic pathways involved in the production of secondary metabolites with potentially interesting biotechnological applications and the possibility to modulate their expression by varying culturing conditions. Although the results do not definitely provide information on the conditions to stimulate secondary metabolite production, the use of different transcriptomes used together may facilitate the understanding of the most suitable conditions for the expression of the metabolites of interest. Another criticism to our work could be that our *T. rotula* strain from the Mediterranean Sea (Gulf of Naples, Italy) and *T. rotula* strains from other geographic regions do not represent the same species. However, a recent phylogenetic study by Whittaker *et al*.^39^ among different *Thalassiosira* species and strains, including a *T. rotula* strain from the Gulf of Naples, concluded that “*T. rotula* lineages should be considered as a single species”.

In conclusion, our results provide new stimuli to investigate the role and function of these newly discovered molecules in diatoms, especially as concerns their interactions with other phytoplankton species and with the surrounding environment.

## Methods

### Strain culturing and RNA extraction

*T. rotula*, strain CCMP1647, lab-name FE80, was isolated in 2011 in the Gulf of Naples (40°48.5′N, 14°15′E), Mediterranean Sea. Clonal cultures were established by isolating single cells from phytoplankton net samples collected from the surface layer of the water column. Cultures were grown in sterile filtered oligotrophic seawater amended with f/2^40^ nutrients at a temperature of 18–20 °C, at 12:12 h light:dark cycle, with a photon flux of 100 μ mol photons m^−2^ s^−1^, under agitation true filtered air influx.

2 L cultures, containing 2,5 × 10^3^ cells/mL, were grown in complete f/2 (CTR) and in silica depleted f/2 media with 36 μM Na_2_SiO_3_ (STRESS), in triplicates, until they reached the stationary growth phase (day 6 for CTR cultures and day 7 for the STRESS cultures). Cultures were harvested by filtration onto 1.2 μm pore size filters (RAWP04700 Millipore) and immediately frozen in liquid nitrogen.

10 Liter cultures of *T. rotula*, in triplicate, were used to follow their growth from day 3 to day 10. Every day, 250 mL of each culture was harvested by filtration onto 1.2 μm pore size filters (RAWP04700 Millipore) and immediately frozen in liquid nitrogen, and 100–200 mL of culture media recovered from the cell filtration was collected and stored at −80 °C until sample processing.

Total RNA was extracted with the RNeasy Micro Kit Quiagen according to the manufacturer’s instructions including a step with DNase digestion. RNA concentration was determined using a Qubit® 2.0 Fluorometer (Invitrogen) and a quality check was performed by gel electrophoresis (1% agarose w/v) and an Agilent2100 bioanalyzer.

### Antibiotic Treatment to produce axenic cultures

1 mL of exponentially growing culture was inoculated in F/2 medium containing final concentrations of 0.1 mg/mL Streptomycin (PanReac Applichem, A1852), 0.06 mg/mL Penicillin (PanReac Applichem, A1837), 1 mg/mL Ampicillin (PanReac Applichem, A0839), 0.1 mg/mL kanamicine (PanReac Applichem, A1493) and 0.02 mg/mL cefotaxime (Sigma-Aldrich, C7039) and allowed to grow for 5–6 days under standard growth conditions. Bacterial contamination was checked in two ways (SI1: Supplementary Fig. 6): (i) by staining DNA with DAPI and examining cultures under the microscope to check for the presence/absence of bacterial nucleoids; (ii) by performing peptone tests. For DAPI staining, 1 µL of DAPI stock solution (4′,6-diamidino-2-phenylindole, 1 mg/mL, Roche, Basel, Switzerland) was added to 1 mL of formalin preserved culture, incubated for 10 minutes and observed under the epifluorescence microscope. For peptone tests, 1 mL of diatom culture was added to a tube containing a peptone solution (1 mg/mL), incubated in the dark and checked after 2–3 days and 1–2 weeks; growth of bacteria in the tubes indicated contamination. If bacterial contamination persisted, the treatment was repeated. Large volume cultures used for DNA extraction were grown with antibiotics and the contamination tests were always performed on an aliquot of the culture.

### DNA extraction

Axenic *T. rotula* cells (strain FE80) were collected onto a 1.2 µm RAWP membrane filter (Millipore, Billerica, MA,US). The filter was rinsed with 1.5 mL seawater and cells were further collected into Eppendorf tubes and pelleted by centrifugation at 3,800 *g* at 4 °C for 5 minutes. The DNA was extracted following a Phenol-Chloroform extraction method^41^ with slight modifications that included cell disruption by adding 400 mg of 0.2–0.3 mm diameter silica beads and vortex mixing at 30 hertz for 85 seconds (3 times), and cooling the pellet on ice between the vortex mixings. The extracted DNA was ethanol precipitated, air dried, dissolved in 50 µL of sterile water and stored at −20 °C until sequencing.

### RNA sequencing

Next generation sequencing experiments, including sample quality control, were performed by Genomix4life S.R.L. (Baronissi, Salerno, Italy). Indexed libraries were prepared from 2 µg/each purified RNA with TruSeq Stranded mRNA Sample Prep Kit (Illumina) according to the manufacturer’s instructions. Libraries were quantified using the Agilent 2100 Bioanalyzer (Agilent Technologies) and pooled such that each index-tagged sample was present in equimolar amounts, with a final concentration of the pooled samples of 2 nM. The pooled samples were subject to cluster generation and sequencing using an Illumina HiSeq 2500 System (Illumina) in a 2 × 100 paired-end format at a final concentration of 8 pmol.

### Transcriptome assembly and annotation

Illumina paired-end 100 bp reads from 6 *T. rotula* samples were processed to produce the transcriptome assembly. Raw reads were trimmed and clipped with BBDuk (https://jgi.doe.gov/data-and-tools/bbtools/) setting a minimum Phred-like quality of 35 and a minimum length of 35 nucleotides. The quality of the reads before and after trimming was checked with the software FASTQC (http://www.bioi http://www.bioinformatics.babraham.ac.uk/projects/fastqc/nformatics.babraham.ac.uk/projects/fastqc/). Possible human contaminant reads were removed after mapping the high quality reads against the reference human genome (GRCh38) with STAR (v 2.4.0j)^42^. High quality reads were then normalized with Trinity^43^ using the options: –SS_lib_type RF–pairs_together–max_cov 50. *De novo* transcriptome assembly was then performed with Trinity using the options: –SS_lib_type RF–no_normalize_reads–min_kmer_cov 1–KMER_SIZE 32. Transcriptome redundancy was removed with CD-HIT-EST^44^ using the following options: -r 0 -g 1. A filter for contaminants was performed by BLASTing the transcripts against the NCBI nr database, discarding all the sequences having a significant hit (evalue < = 0.0001) against bacteria or metazoa.

The completeness of the assembly was checked against the Core Eukaryotic Genes database (http://korflab.ucdavis.edu/Datasets/genome_completeness/ and http://korflab.ucdavis.edu/datasets/cegma/).

*In silico* translation was performed with TransDecoder^45^ whereas Functional Annotation was performed with Blast2GO software^46^.

### Transcriptome expression quantification and differential expression analysis

Transcript expression quantification was performed using Express (v 1.5.1)^47^ after mapping the reads against the assembly with STAR^42^. Posterior counts were used as input to perform transcript differential expression analysis with EBSeq^48^ transcripts with a probability of being differentially expressed higher than 0.95 were considered significant.

The contigs and peptide sequences for *T. rotula* FE80 (CCMP1647) can be found in Supplementary Files 7 and 8. Peptide sequences were used for the subsequent analyses. To annotate the translated transcriptome we used the custom pipeline Annocript^33^. We used the Swiss-Prot (SP) and UniRef90^49^ (version: August 2013) for the blastp against proteins with the following parameters: word_size = 4; e-value = 10−5; num_descriptions = 5; num_alignments = 5; threshold = 18.

For each sequence the best hit, if any, was chosen. Rpsblast parameters, to identify domains composition of putative proteins in the Conserved Domains Database, were: e-value = 10−5; num_descriptions = 20; num_alignments = 20.

The software returned GO functional classification^50^, the Enzyme Commission IDs^51^ and Pathways^52^ descriptions associated to the resulting best matches.

R scripts were used to perform further analyses and graphs (http://www.r-project.org/).

Venn diagram was done using the freely available software interactivenn (http://www.interactivenn.net/).

We used the t-test to compare the FPKM for groups of peptides associated to the same GO/pathway and the Fisher exact test to compare the corresponding proportions of transcripts.

### Bioinformatic identification and selection of pathways of interest

Pathways annotated as ‘prostaglandin biosynthesis’ and ‘secologanin biosynthesis’ were found among the second level pathways list generated within the Annocript pipeline annotation of the proteome from the *T. rotula* CCMP 1647 RNA-seq. Transcripts associated to the above pathways were extrapolated from the total proteome annotation table (Supplementary Data 13). Transcript and enzymes involved in polyketide biosynthesis were found by searching in the transcriptome and proteome blast description table, using the term ‘polyketide’ as search term.

### Comparative analysis

Fasta files and contig reads of *T. rotula* strains CCMP3096 and GSO102’s RNA-seq were retrieved from the public database CAMERA (http://camera.crbs.ucsd.edu/mmetsp/list.php) and iMicrobes interactive query tool for microbial data (http://data.imicrobe.us/sample/view/1867). Corresponding proteome were annotated with Annocript and the output files for pathway and global annotation were used to search for the selected pathways. For comparisons among the proteomes, we used FPKM. Paired aligned reads were used to calculate the Fragments Per Kilobase of transcript per Million fragments mapped (FPKM) of each sequence for each transcriptome as a measure of expression levels.

The FPKM were calculated as follows: FPKM = [mapped reads pairs]/([length of transcript]/1000)/([total reads pairs]/10^6^).

### Reverse transcription

1 μg of total RNA used for RNA-seq was retro-transcribed in the T100 Thermal cycler (Bio-Rad Laboratories, Hercules, CA, USA) following the manufacturer’s instructions of the sensiFAST™ cDNA synthesis kit (Bioline, Cat. No. BIO-65054).

### Primer design and real time quantitative PCR

Candidate reference genes and genes of interest were selected considering the annotation of the peptides reported in the annotated transcriptome of *T. rotula* FE80 (CCMP1647) (Supplementary Data 13).

Primers for amplification of selected transcripts, designed using Primer3 program V. 0.4^53,54^, are listed in SI1-Supplementary Table 5, together with their relative sequences and characteristics.

Each sequence was initially tested by standard PCR. Reactions were carried out in 25 μL volume with 2,5 μL of 10× PCR reaction buffer (Roche, Basel, Switzerland), 2,5 μL of 10 × 2 mM dNTP, 0.3 μL of 5 U/μL Taq (Roche, Basel, Switzerland), 1 μL 10 μΜ of each oligo, 1 μL of cDNA templates and nuclease-free water up to 25 μL. The PCR program consisted of a denaturation step at 95 °C for 3 min, 40 cycles at 95 °C for 30 s, 53 °C 30 s, 72 °C for 30 s, and a final extension step at 72 °C for 7 min. Amplified PCR products were analysed by agarose gel electrophoresis. The resulting bands were excised from the gel and extracted according to the GenElute Gel Extraction Kit protocol (Sigma-Aldrich, St. Louis, MO, USA). Sequences were obtained by BigDye Terminator Cycle Sequencing Technology (Applied Biosystems, Foster City, CA, USA) and purified using the Agencourt CleanSEQ Dye terminator removal Kit (Agencourt Bioscience Corporation, Beverly, MA, USA) in automated robotic station Biomek FX (Beckman Coulter, Pasadena, CA, USA). Products were analysed on the Automated Capillary Electrophoresis Sequencer 3730 DNA Analyser (Applied Biosystems, Foster City, CA, USA). Alignments were performed with BioEdit software V. 7.0.5.3 (http://www.mbio.ncsu.edu/BioEdit/bioedit.html).

Reverse transcription-quantitative PCR (rt-qPCR) experiments were performed in MicroAmp Optical 384-Well reaction plate (Applied Biosystems, Foster City, CA, USA) with Optical Adhesive Covers (Applied Biosystems, Foster City, CA, USA) in a Viia7 Real Time PCR System (Applied Biosystem, Foster City, CA, USA). Five serial dilutions of mixed cDNAs were used to determine primer reaction efficiency using the formula: E = 10^−1/slope^ (SI1-Supplementary Fig. 5). The PCR volume for each sample was 10 μL, with 5 μl of SensiFAST^TM^ SYBR^®^ Lo-ROX Kit (BIO_94020, Bioline), 1 μL of cDNA template (1 to 5 dilution each template) and 4 μL of 0.7 μM oligo mix (forward and reverse). Program reaction used was: 95 °C for 20 s, 40 cycles of 95 °C for 1 s and 60 °C for 20 s. The program was set to reveal the melting curve of each amplicon from 60 °C to 95 °C, and read every 0.5 °C. Single peaks for all genes confirmed gene-specific amplification and the absence of primer-dimers. All RT-qPCR reactions were carried out in triplicate to capture intra-assay variability. Each assay included three no-template negative controls for each primer pair.

The normalized expression levels of each gene of interest relative to the most stable reference genes (see Supplementary Information 2 and 3 for reference genes analysis), actin and TBP, were calculated by using the Q-Gene tool^55^. Only TBP normalized values were reported in the main text and figure results.

Relative expression ratios above two fold were considered significant. Statistical analysis was performed using the unpaired t-test with Welch’s correction for comparison between conditions, using GraphPad Prim statistic software V. 6.01 (GraphPad Software Inc., San Diego, CA, USA).

Three different algorithms were utilized to identify the best reference genes in our experimental design: BestKeeper^56^; NormFinder^57^ and geNorm^58^.

### Statistics

Using GraphPadPrism6 software t-test was performed between CTR and STRESS sample for qPCR amplification data to determine significant differences between treatments.

R software was used for bioinformatic analysis of the transcriptome performing the following tests: Fisher test to identify unique GO terms; t-test between the CTR and STRESS condition considering the FPKM expression value of each pathway; heatmaps in different conditions for the selected pathways; boxplot analysis of the expression in FPKM of each pathway in each level of annotation for each replicate and group.

## Supplementary information

Supplementary Information

## Data Availability

The sequencing data described in this article, i.e. the transcript and peptide sequences of *T. rotula*, strain CCMP1647, lab-name FE80, are in supplementary files 7 and 8 respectively. The sequencing data relative to the *T. rotula* strains CCMP3096 and GSO102 used for the comparative analysis are available at https://www.imicrobe.us/#/projects/104.
