# Supplementary material for: Unveiling the presence of biosynthetic pathways for bioactive compounds in the Thalassiosira rotula transcriptome
Source: Sci Rep. 2019 Jul 9;9:9893. doi: 10.1038/s41598-019-46276-8 (PMC6616357; doi:10.1038/s41598-019-46276-8)
Supplement: Supplementary file 1 — Supplementary Information [file 41598_2019_46276_MOESM1_ESM.pdf]

Unveiling the presence of biosynthetic pathways for bioactive compounds in the *Thalassiosira rotula* transcriptome

Valeria Di Dato*, Federica Di Costanzo, Roberta Barbarinaldi, Anna Perna, Adrianna Ianora, Giovanna Romano

Supplementary Information 1 (SI1)

Supplementary Figures Page

Supplementary Figure 1: Principal component analysis. 4

Supplementary Figure 2: Representation of the GO term categories. 5

Supplementary Figure 3: Representation of the GO terms categories associated to the Differential Expressed Transcripts (DET). 6

Supplementary Figure 4: Representation of the Monoterpenoid biosynthetic pathway. 7

Supplementary Figure 5: Alignment of the five peptides annotated as Secologanin synthase. 8

Supplementary Figure 6: Representation of the Prostaglandin biosynthetic pathway. 9

Supplementary Figure 7: Representation of the Polyketides biosynthetic pathway. 10

Supplementary Figure 8: Oligo pairs standard curve of the transcripts in the selected pathways analysed by qPCR. 11

Supplementary Figure 9: Dapi staining and peptone assay for genomic DNA axenicity verification. 12

Supplementary Figure 10: Agarose gel of PCR amplification of *Thalassiosira rotula* FE80 clone transcripts in figure 6, in main text. 13

Supplementary Tables

Supplementary table 1: 15

1a. Transcriptome assembly statistics.
1b. General transcriptome statistics.
1c. General proteome statistics.

Supplementary table 2: Top Ten DET-associated GO terms. 16

Supplementary table 3: Level 1 Pathway Annotation. 17

Supplementary table 4: Prostaglandin annotation in *Thalassiosira rotula* FE80 (CCMP1647). 20

Supplementary table 5: List of primers used for amplification of selected transcripts. 21

Supplementary Figures

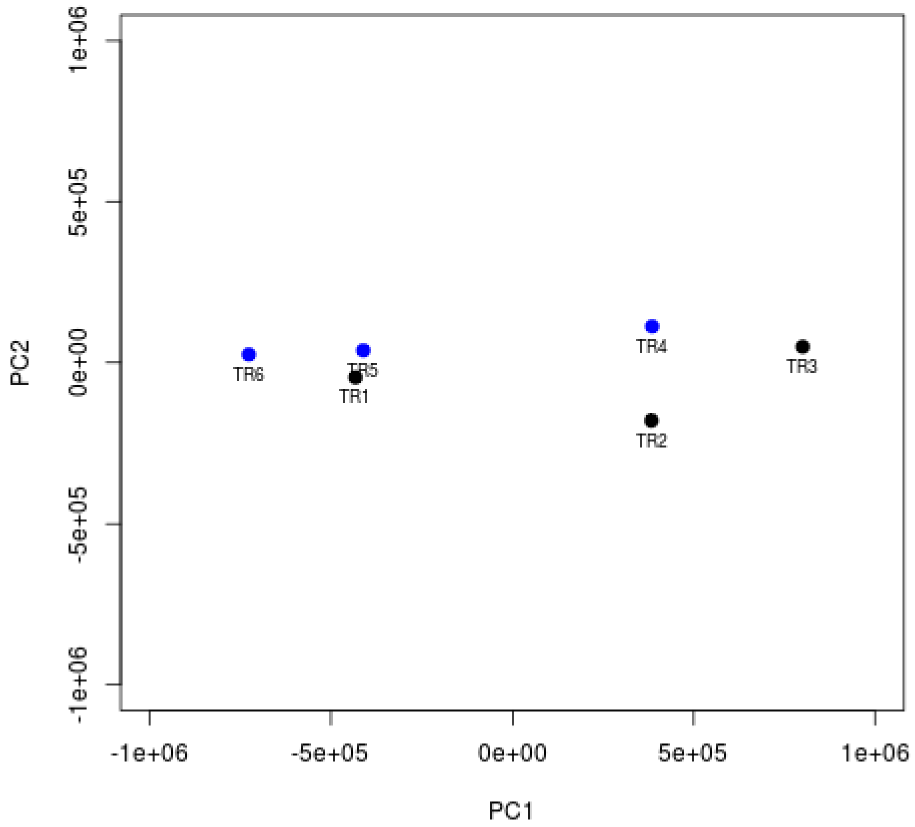

Supplementary Figure 1. Principal component analysis (PCA) of each Thalassiosira rotula replicate, with PC1 plotted against PC2. In total, 6 cultures were used: three cultivated in normal condition and three cultivated in a medium depleted of silica.
TR1, TR2 and TR3 correspond to the control replicate, alias cultures cultivated in complete medium. TR4, TR5 and TR6 correspond to stress replicate, alias cultures cultivated in silica depleted medium (36µM SiOH4).

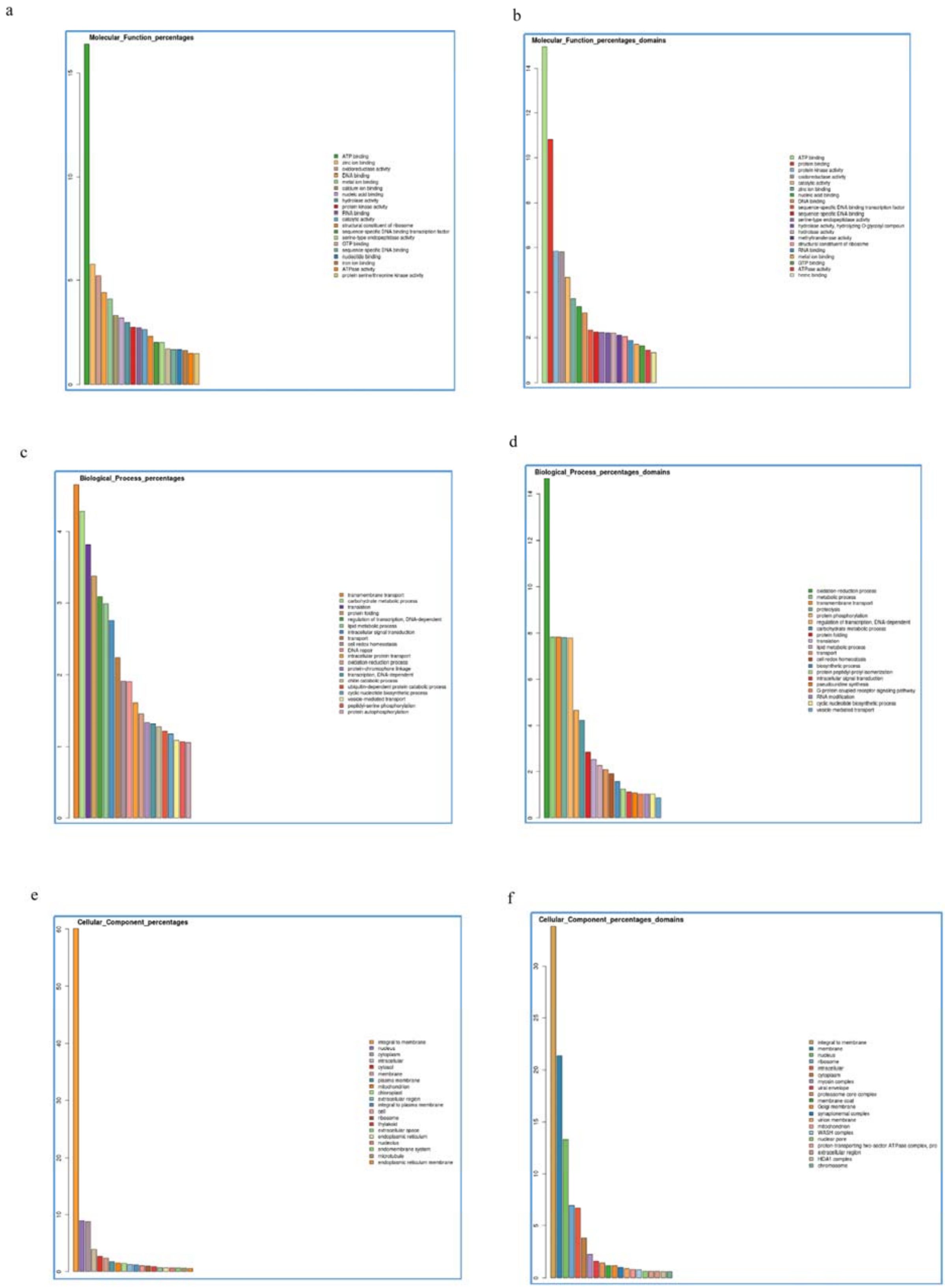

Supplementary figure 2. Histograms representing the top 20 peptide sequences and domains associated to the GO term categories: a and b, Molecular Function category; c and d, Biological Processes category; e and f, Cellular Components category.

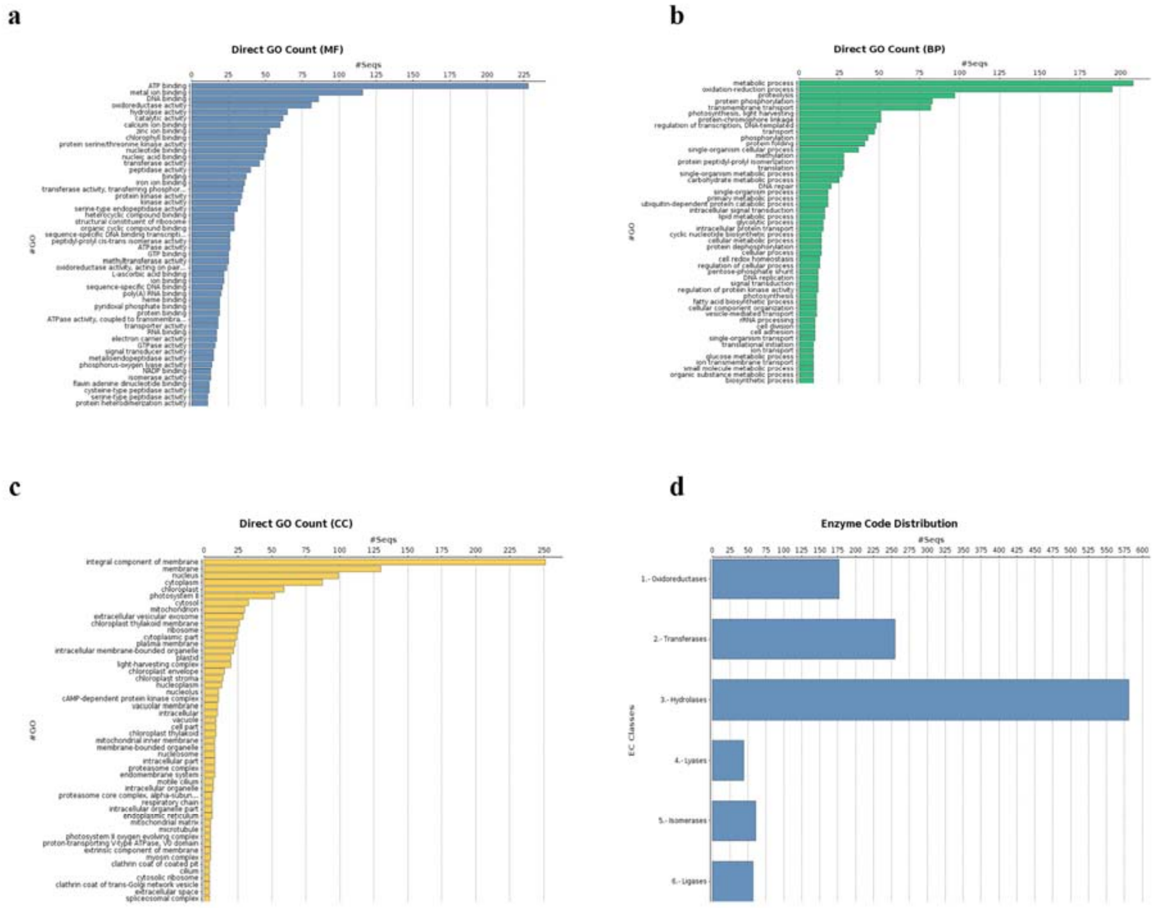

Supplementary figure 3. Histograms representing the distribution and number, in the different categories, of the GO terms associated to the DET (Differential Expressed Transcripts). a) Molecular Function category; b) Biological Processes category; c) Cellular Components category, d) Enzyme classes.

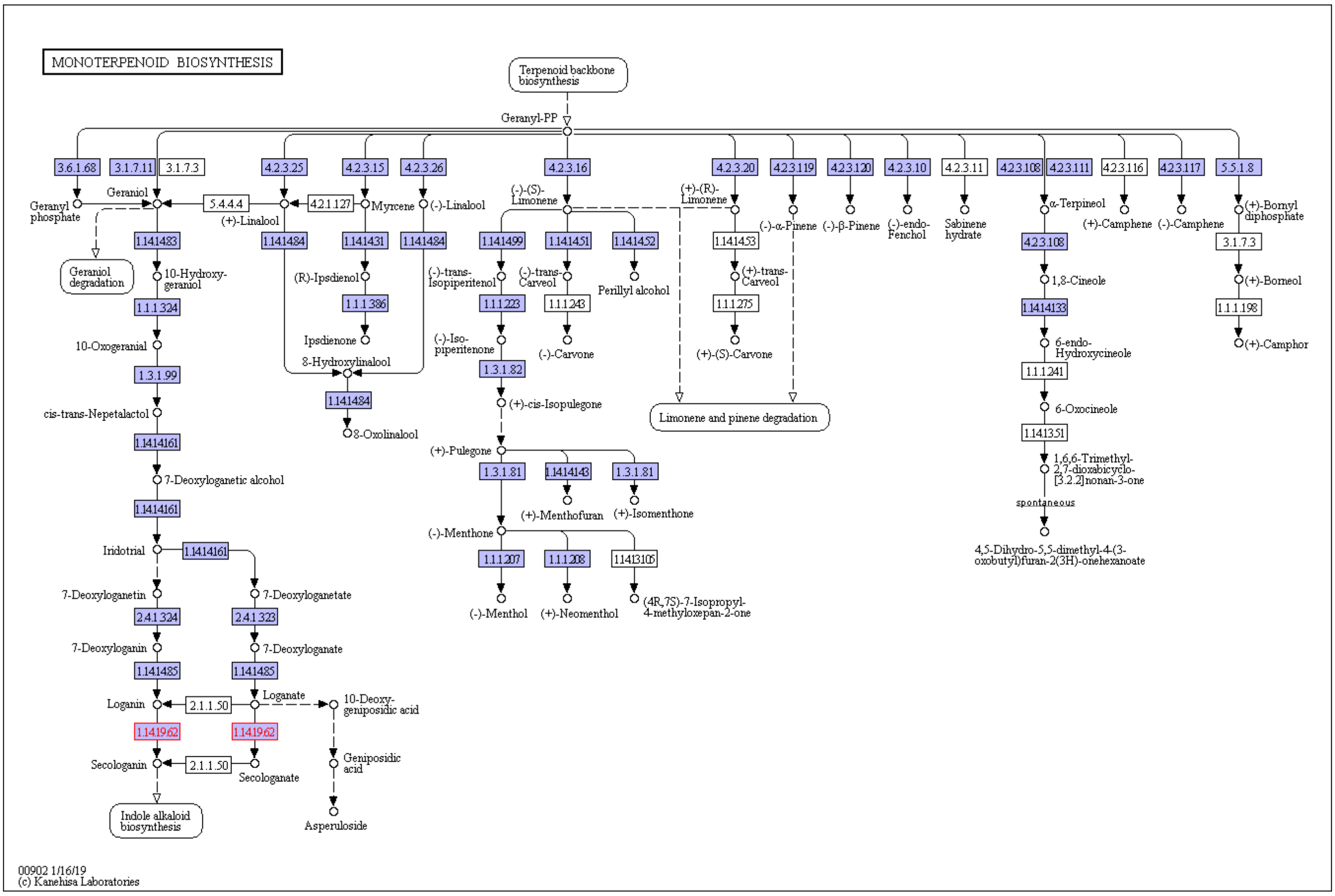

Supplementary figure 4: pattern of monoterpenoid biosynthesis of which secologanin synthase, indicated in red, is part. Font: KEGG: Kyoto Encyclopedia of Genes and Genomes.

Secologanin Synthase's alignments

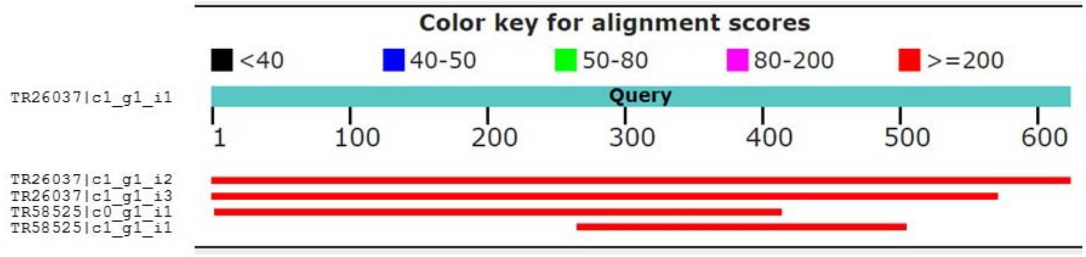

Description	Max score	Total score	Query cover	E value	Ident	Accession
TR26037|c1_g1_i2|m.127109	1288	1288	100%	0.0	99%	Query_24922
TR26037|c1_g1_i3|m.127112	1024	1024	91%	0.0	87%	Query_24923
TR58525|c0_g1_i1|m.284670	455	455	66%	2e-159	54%	Query_24924
TR58525|c1_g1_i1|m.284673	351	351	38%	6e-122	66%	Query_24925

Supplementary figure 5. Alignment of the five peptides annotated as Secologanin synthase.

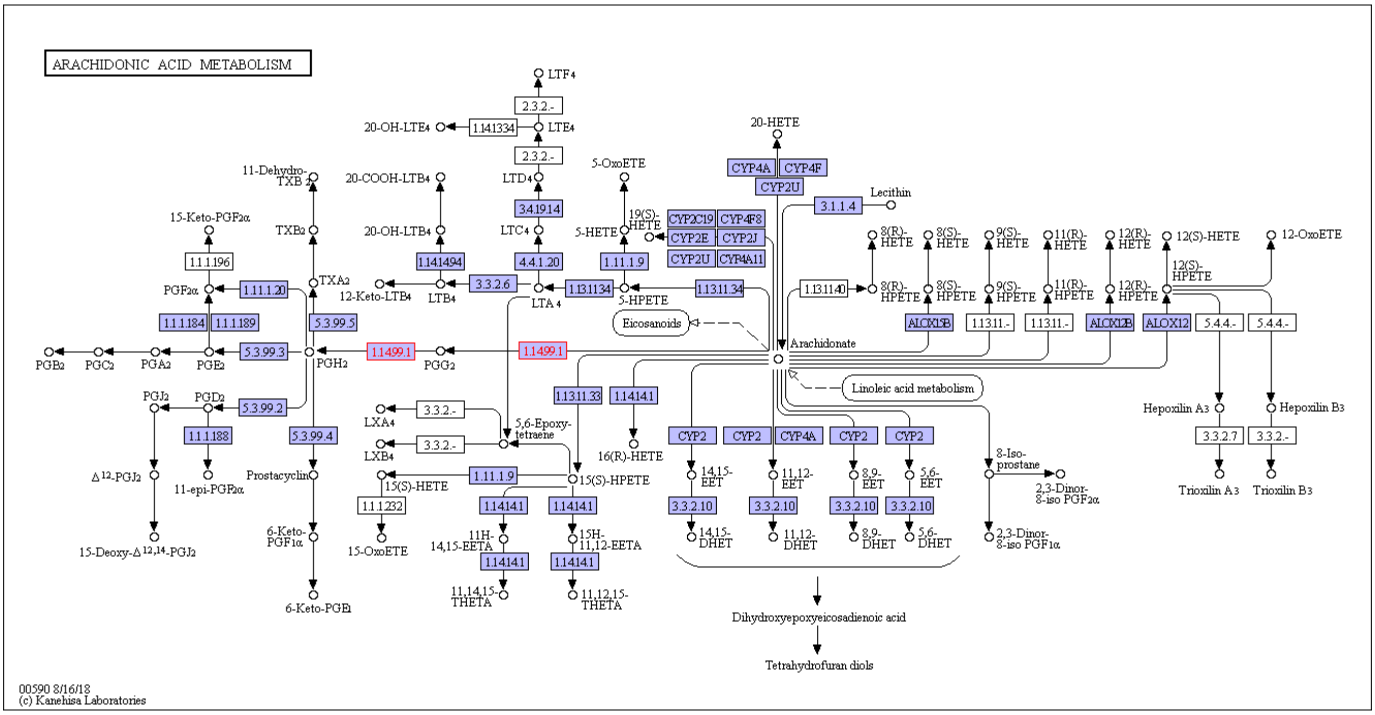

Supplementary figure 6: Pattern of arachidonic acid biosynthesis of which prostaglandin enzymes are part. In red the prostaglandin G/H synthase or cyclooxygenase, from which prostaglandins derive. 5.3.99.2: microsomal prostaglandin-E synthase 1; 1.11.1.20: prostaglandin F2alpha synthase. Font: KEGG: Kyoto Encyclopedia of Genes and Genomes.

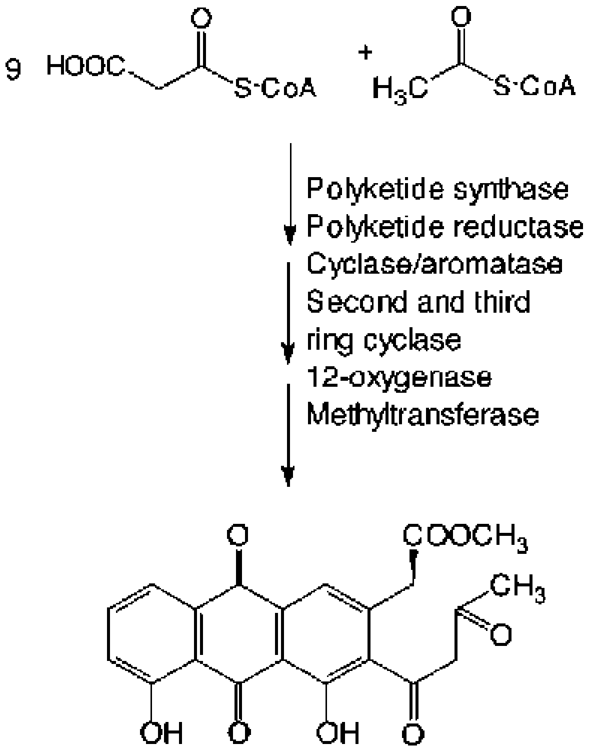

Supplementary figure 7: Example of iterative reactions for the synthesys of polyketides. Modified from: Sultana A. et al., 2004. Structure of the polyketide cyclase SnoaL reveals a novel mechanism for enzymatic aldol condensation. The EMBO journal,volume 23, 9, pages 1911-21.

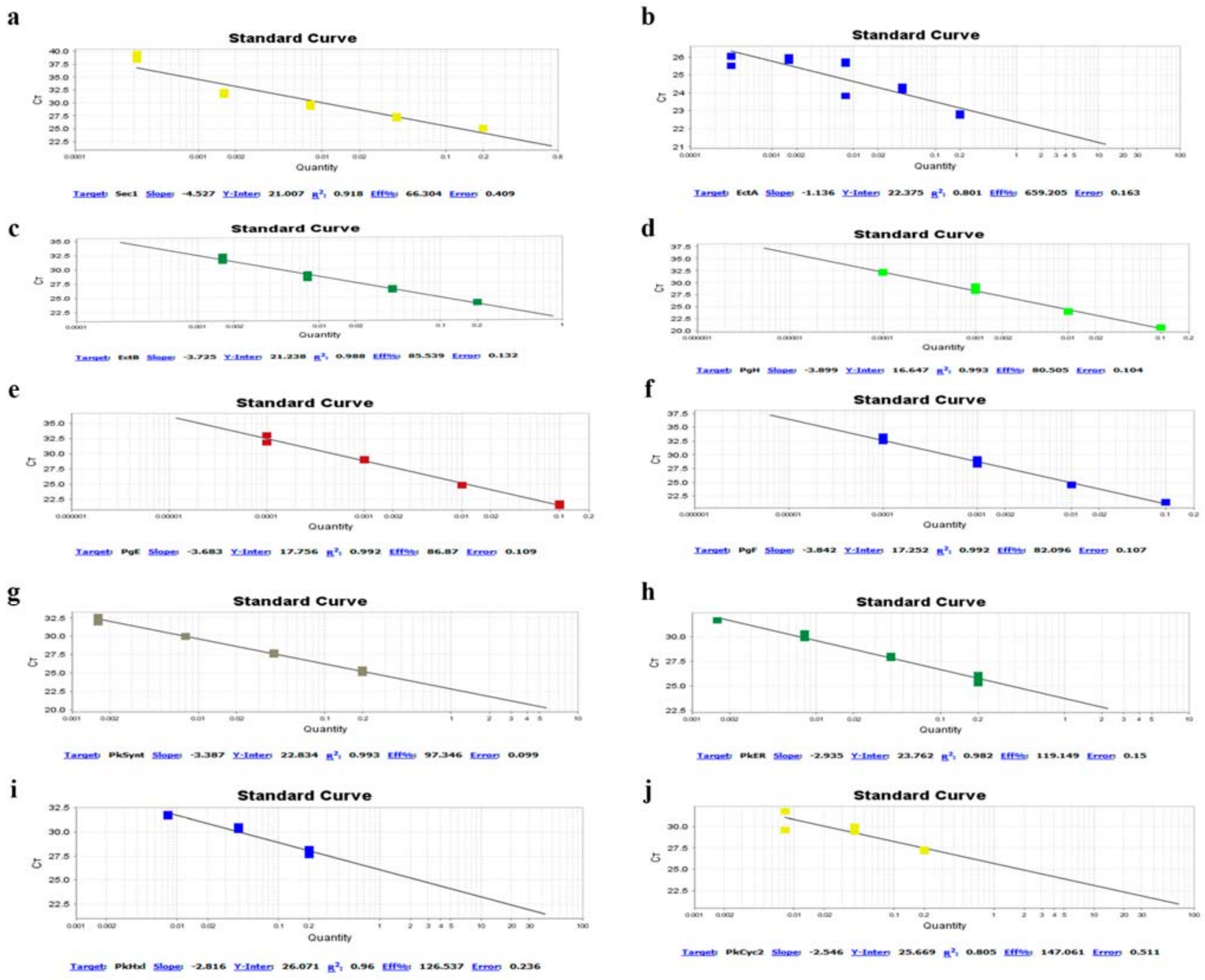

Supplementary figure 8. Oligo pairs standard curve of the transcripts in the selected pathways analysed by qPCR. a. Secologanin synthase; b. Ecotine A; c. Ectoine B; d. PgG/Hs; e. PgEs2; f. PgFs; g. PKS_Synt; h. PKS_ER; i. PKS_Hxl; j. PKS_Cyc.

a

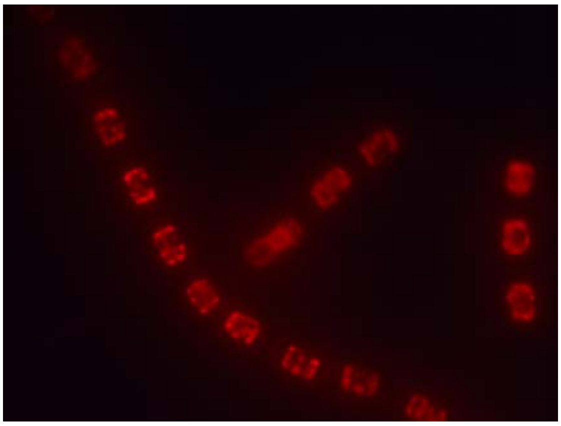

b

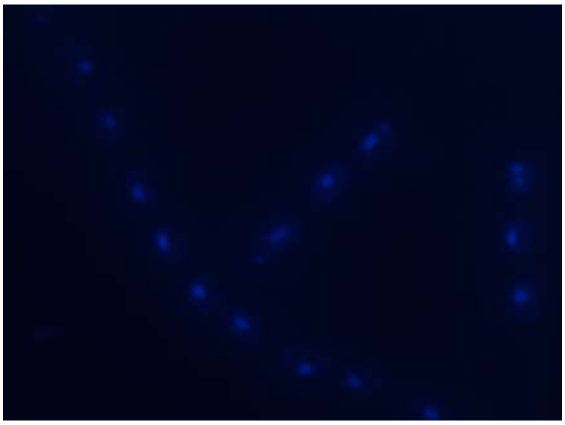

c

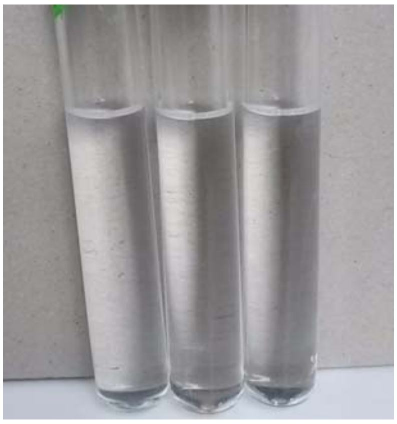

Supplementary Figure 9: Dapi staining and peptone assay to verify if genomic DNA was axenic.

a. **Dapi staining to mark the nucleus to see the presence of bacteria associated to the cell. Only the cell nuclei are visible;**

b. **Spontaneous Cholorophyll autofluorescence to assess normal cell shape;**

c. **Peptone assay on three replicates to verify bacterial growth. The solutions are transparent indicating no bacterial growth.**

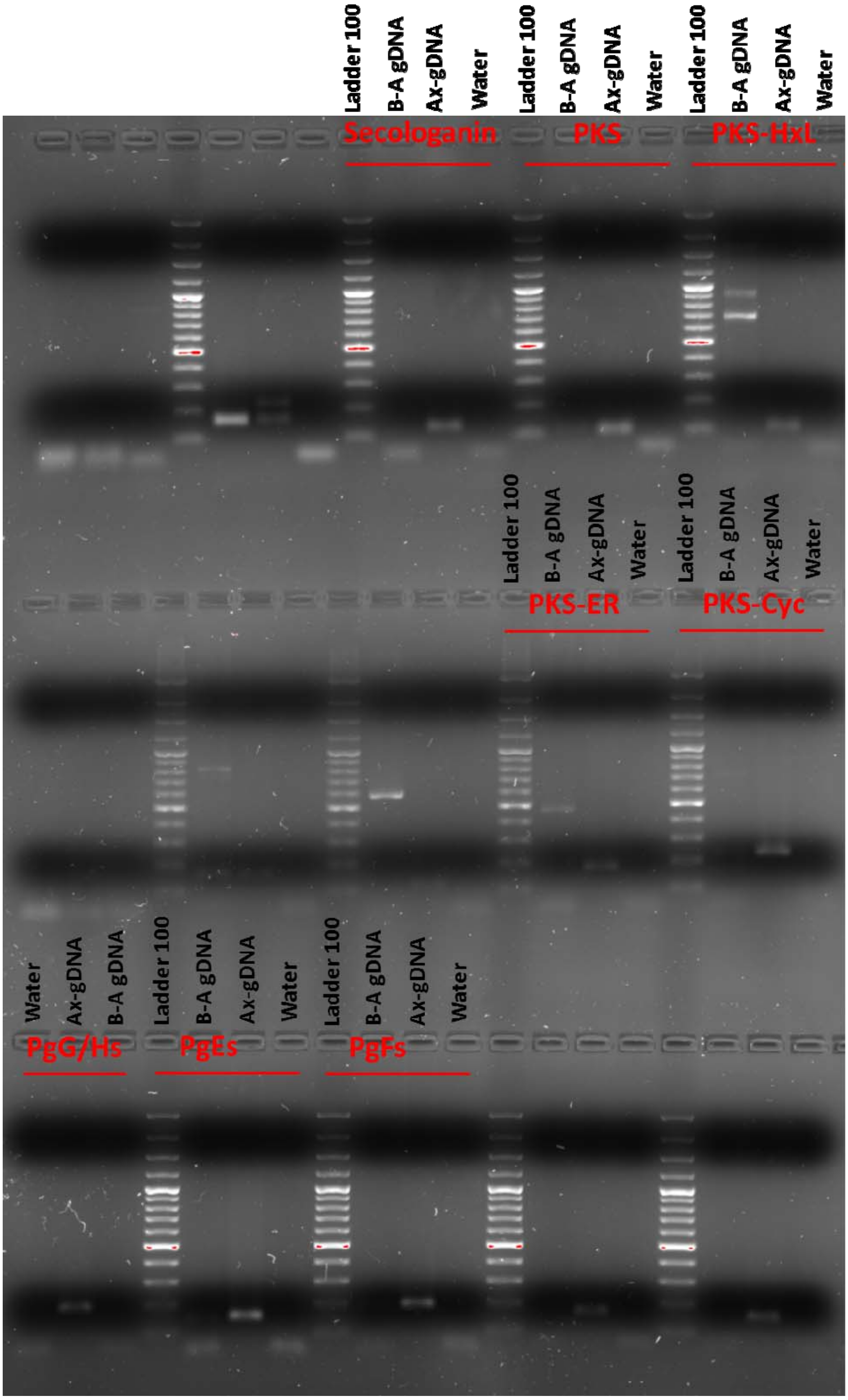

Supplementary Figure 10: Agarose Gel of PCR amplification of the *Thalassiosira rotula,* clone FE80, transcripts showed in the figure 6 of the main text.

- **B-A gDNA: genomic DNA of a mixed culture *Thalassiosira rotula*/associated bacteria.**
- **Ax-gDNA: genomic DNA of an axenic *Thalassiosira rotula* culture.**

Ladder100: from bottom to top: 100, 200, 300, 400, 500, 600, 700, 800, 900, 1000, 1200, 1500, 2000, 3000 bps.

Supplementary Tables

Supplementary table 1a. Transcriptome assembly statistics.

Sample Name	Treatment	Reads Before Trimming	Reads After Trimming
TR1	COMPLETE MEDIA	45735814	44763298
TR2	COMPLETE MEDIA	55689770	54219334
TR3	COMPLETE MEDIA	54231050	52909044
TR4	Silica DEPLETED MEDIA	50028032	48504904
TR5	Silica DEPLETED MEDIA	41982052	41063084
TR6	Silica DEPLETED MEDIA	35165342	34181574

Supplementary table 1b. General transcriptome statistics.

	BEFORE FILTRATION FOR CONTAMINATION	AFTER FILTRATION FOR CONTAMINATION
Mbp	152	142
TRANSCRIPT NUMBER	125135	107289
GENES	79330	66496
% GC	47.55	47.53
AVERAGE CONTIG LENGTH (BP)	1217	1332.76
MEDIAN CONTIG LENGTH (BP)	810	970
N50 (BP)	1940	2034

Supplementary table 1c. General proteome statistics.

		BEFORE FILTRATION FOR CONTAMINATION	AFTER FILTRATION FOR CONTAMINATION
PROTEIN SEQUENCES		67222	60172
COMPLETE (STARTING METHIONINE AND STOP CODON)		29304	28128
STARTING METHIONINE, NO STOP CODON		8669	7616
NO STARTING METHIONINE, YES STOP CODON		14628	13291
NO STARTING METHIONINE, NO STOP CODON		14621	11137
MEAN PEPTIDE LENGTH		-	281
MINIMUM PEPTIDE LENGTH		-	49
MAXIMUM PEPTIDE LENGTH		-	3952
Number of BLAST results	Swiss-Prot	-	15601
	UniRef	-	37717
	Domains	-	21011
	PEPTIDES WITH AT LEAST ONE BLAST RESULT	-	37999

Supplementary Table 2: Top Ten DET-associated GO terms.

Biological Processes	Cellular Components	Molecular Functions
Metabolic Process	Integral component of membrane	ATP binding
Oxidation-Reduction Process	Nucleus	Metal Ion Binding
Proteolysis	Cytoplasm	DNA Binding
Protein Phosphorylation	Chloroplast	Oxidoreductase activity
Transmembrane transport	Photosystem II	Hydrolase activity
Photosynthesis, light harvesting	Cytosol	Catalytic activity
Protein-chromophore linkage	Mitochondrion	Calcium Ion Binding
Regulation of transcription, DNA-templated	Extracellular vesicular exosome	Zinc Ion Binding
Transport	Chloroplast thylakoid membrane	Chlorophyll Binding
Phosphorylation	Ribosome	Protein Serine/Threonine Kinase Activity

Supplementary table 3. Level 1 Pathway Annotation. Mean expression value, in FPKM, and statistics data of the pathways on first level of annotation on the total proteome.

# : pathways numeration; # of ass. Seqs: number of peptide sequences associated to each pathway; Pathways on Level 1: pathway's name; CTR MEAN: mean FPKM value among all the corresponding transcript of the complete medium cultures associated to the pathway; CTR SD: standard deviation of the CTR mean; STRESS FPKM MEAN: mean FPKM value among all the corresponding transcript of the silica depleted medium cultures associated to the pathway; STRESS SD: standard deviation of the STRESS mean; p-value CTR vs STRESS : ttest on the FPKM mean of the CTR versus STRESS for each pathway; p-adj value CTR vs STRESS: pvalue adjusted on the total number of associated sequences.

#	# of ass. seqs	Pathways on Level 1	CTR FPKM MEAN	CTR SD	STRESS FPKM MEAN	STRESS SD	p-value CTR vs STRESS	p-adj value CTR vs STRESS
**1**	**7**	**Alkaloid biosynthesis**	1.93819087357143	1.93100775440389	12.3622564214286	14.9041172497516	0.114564625127527	1
**2**	**3**	**Alkaloid degradation**	1.9967997	1.48837117146259	2.60919641666667	2.14566014688563	0.707797304611788	1
**3**	**7**	**Alkene biosynthesis**	3.0170001	3.57221627966652	2.01524596428571	2.02850639235049	0.534072564311056	1
**4**	**46**	**Amine and polyamine biosynthesis**	10.7085171301143	15.0839619677234	9.77146942633582	15.6913691264389	0.759141265109627	1
**5**	**13**	**Amine and polyamine degradation**	1.57156235322269	1.64110401961755	1.61593673070014	1.68325362842774	0.946304022362477	1
**6**	**2**	**Amine and polyamine metabolism**	4.02284685	3.99030772306456	5.44424825	0.232285638279952	0.702901173816272	1
**7**	**240**	**Amino-acid biosynthesis**	9.6266325170581	32.4637595818077	11.2866950575017	52.8568136964496	0.656232622029268	1
**8**	**95**	**Amino-acid degradation**	5.6716736557636	11.218993623446	4.87511562220517	8.61322524297467	0.562695290408778	1
**9**	**4**	**Amino-acid metabolism**	13.243841875	19.6965739004505	8.3848496375	12.3864094255887	0.565898107703054	1
**10**	**4**	**Aminoacyl-tRNA biosynthesis**	5.444138975	6.31044116618844	6.26338385	7.83919532348209	0.876245938475542	1
**11**	**32**	**Antibiotic biosynthesis**	10.9575223058901	17.1491798977856	11.3091498875625	17.8616027224739	0.936233460295997	1
**12**	**4**	**Aromatic compound metabolism**	2.27103873763088	4.07718996467948	1.64405210003583	2.79758487981645	0.809329477753681	1
**13**	**12**	**Bacterial outer membrane biogenesis**	9.07707028302721	12.9355798436961	11.3307976828702	10.7835107069336	0.647645874467103	1
**14**	**4**	**Capsule biogenesis**	3.473348	4.33567149842042	6.3905280125	7.96247669281662	0.550361698290555	1
**15**	**6**	**Carbohydrate acid metabolism**	18.0743664166667	23.9999559141408	17.5919465	19.1559628760125	0.97009745943686	1
**16**	**48**	**Carbohydrate biosynthesis**	20.8858513833617	43.6280500889786	16.5137272622563	36.3145331426027	0.594898683302364	1
**17**	**169**	**Carbohydrate degradation**	42.9728390495802	226.42491928468	26.1735629970953	101.447625525475	0.378245239853317	1
**18**	**48**	**Carbohydrate metabolism**	11.5114837273302	16.3308996710298	11.3171064160177	18.1685114337505	0.956156619827766	1
**19**	**19**	**Carotenoid biosynthesis**	3.9948779495122	4.83838511854079	3.36496709079836	3.25374585209978	0.615374133775705	1
**20**	**2**	**Catecholamine biosynthesis**	2.01856175	0.533213313790199	1.808283	0.402181123935473	0.702558791913895	1
**21**	**8**	**Cell wall biogenesis**	5.26537841875	6.4327968739877	5.0421862625	5.98166237031681	0.943730291791584	1
**22**	**186**	**Cofactor biosynthesis**	8.4263563238636	25.8026840511522	7.61973078395479	19.4338939703801	0.727517781932475	1
**23**	**6**	**Cofactor degradation**	6.91392311666667	12.1431704362354	8.13644774166667	13.3013763416456	0.871287996676324	1
**24**	**3**	**Cofactor metabolism**	1.94363667125	1.71070792005389	1.4350754625	0.873441316787731	0.621685095970365	1
**25**	**26**	**Energy metabolism**	11.5748235052116	18.0531535068561	11.0145811675983	16.8634257577519	0.90840615899133	1
**26**	**16**	**Fermentation**	0.397935310579678	0.704412451264113	0.885105205625	1.91650883318659	0.351897372773782	1
**27**	**1**	**Flavonoid metabolism**	27.731895	NA	46.99605	NA	10	1

#	# of ass. seqs	Pathways on Level 1	CTR MEAN	CTR SD	STRESS MEAN	STRESS SD	p-value CTR vs STRESS	p-adj value CTR vs STRESS
28	10	Genetic information processing	8.309927715	7.5362047500817	5.48287793	3.48224541669386	0.301611232918807	1
29	1	Glycan biosynthesis	2.2356625	NA	3.128675	NA	10	1
30	20	Glycan degradation	7.2114768525	10.728644651084	4.8022975257675	8.80311121422541	0.442530192577737	1
31	18	Glycan metabolism	2.21121046901364	2.822066444023	2.16918962655682	2.63783765463647	0.959550834346818	1
32	19	Glycerolipid metabolism	5.58599072026316	8.20476744902123	6.04173952631579	8.62839832602791	0.868427745579636	1
33	28	Glycolipid biosynthesis	3.42656811932143	6.77484974711564	3.94197677283591	8.46621660637824	0.802412639261149	1
34	3	Hormone biosynthesis	1.58883798166667	1.87897594804455	1.53535429333333	1.24958862937039	0.969499137381665	1
35	21	Isoprenoid biosynthesis	8.08461845735294	13.0375577599331	9.09789294558824	12.37164096636	0.743402187863152	1
36	7	Ketone metabolism	2.75663957106605	2.90248527588007	2.27429664674354	2.29461346544345	0.736443822783611	1
37	175	Lipid metabolism	19.653383597443	77.0040678340764	21.8519716131275	105.980158295988	0.824443293554184	1
38	4	Membrane lipid metabolism	3.3040355	2.06912854027273	5.116475125	3.10595097012977	0.374198350328475	1
39	27	Metabolic intermediate biosynthesis	7.20515494715714	11.953221005346	6.9181116620466	10.3579764811837	0.914821263838417	1
40	10	Metabolic intermediate metabolism	6.75222531815	7.99860877282214	5.5872351644951	6.89036276021084	0.731257767515254	1
41	16	Nitrogen metabolism	2.93282775240625	4.29816271070079	3.7027467718125	6.97339241219183	0.710126837069234	1
42	2	Nucleoside biosynthesis	2.77259525	1.54572799905259	2.76126105	3.35109988059195	0.997067883355439	1
43	35	Nucleotide-sugar biosynthesis	15.0664536510462	20.2615260954377	21.0973595339467	28.0400371748146	0.299517010219956	1
44	34	One-carbon metabolism	4.3652897082669	8.97176633435821	4.22715016741417	8.49042459715027	0.948205158148916	1
45	5	Organic acid metabolism	2.939366604	2.57301072759137	2.7137586459581	3.12923916010463	0.904085053178901	1
46	7	Organosulfur biosynthesis	24.2572826571429	43.6444289237238	19.7665988571429	28.8121068192813	0.824683760954756	1
47	43	Phospholipid metabolism	4.72428288846705	5.69588511867248	5.53593137490795	7.60717240030549	0.572627363100781	1
48	10	Photosynthesis	1.979514526	1.77363261163519	1.4482921781964	1.15504667948054	0.439390337888341	1
49	2	Phytoalexin biosynthesis	12.59049875	11.3292241895309	8.8297445	6.7948796813086	0.73360541508224	1
50	7	Plant hormone biosynthesis	2.44234762142857	3.40077574119786	1.24640577142857	1.07377775759095	0.40366725583701	1
51	24	Polyol metabolism	7.6744324291875	8.95201620215457	9.00329173261382	8.31275298910688	0.596685711930388	1
52	77	Porphyrin-containing compound metabolism	5.51203371967617	10.138413377452	6.15737972043181	12.0970801479157	0.703416690572848	1
53	7	Protein biosynthesis	88.731714595	228.281078664317	245.919347917536	643.421291668945	0.560446907179927	1
54	3	Protein degradation	11.4585388333333	8.5407517994378	11.7043961666667	10.0797161999054	0.975870621104043	1
55	456	Protein modification	9.15065204228311	17.5006443204081	9.68016677262585	19.1038948893162	0.662621983834879	1
56	62	Purine metabolism	9.45315776836404	17.5755754183667	8.16373798801145	10.6739617156226	0.606128817994198	1
57	34	Pyrimidine metabolism	6.42380451179622	12.8555357231255	5.72509374014706	10.2303485577813	0.762010544473349	1
58	14	Quinol/quinone metabolism	6.13504992176471	4.77106920981642	7.08846534676471	6.07587124849835	0.614539376892254	1
59	1	Secondary metabolite biosynthesis	1.887811	NA	2.0184605	NA	10	1
60	8	Secondary metabolite metabolism	8.01349330484776	13.0120863166746	15.1421185014724	31.5947931505467	0.569180660718774	1
61	1	Signal transduction	10.61557	NA	8.9021045	NA	10	1
62	1	Spore coat biogenesis	18.60315	NA	28.106485	NA	10	1

#	# of ass. seqs	Pathways on Level 1	CTR MEAN	CTR SD	STRESS MEAN	STRESS SD	p-value CTR vs STRESS	p-adj value CTR vs STRESS
**63**	**18**	**Steroid biosynthesis**	5.57185156421053	7.5054417087592	6.80928485352205	10.9452695542686	0.687146803764157	1
**64**	**4**	**Steroid metabolism**	12.77444025	14.7488280129856	14.2216669125	18.5241955574504	0.906895024120955	1
**65**	**48**	**Sulfur metabolism**	8.05854475010417	38.5316113141322	7.9326679588125	31.4647990229458	0.986051748274181	1
**66**	**3**	**Terpene metabolism**	3.68994991666667	5.82481319632118	3.69068256666667	5.06165386582286	0.999876811908281	1
**67**	**1**	**Thermoadapter biosynthesis**	36.453995	NA	17.79681	NA	10	1
**68**	**22**	**tRNA modification**	1.73801389812273	2.17021583703122	2.01134279954547	1.72551912375392	0.646306823900338	1

Supplementary Table 4: Prostaglandin annotation in *Thalassiosira rotula* FE80 (CCMP1647)

Transcript Name	DescriptionSP	EnzymeDescs	PwLev1	PwLev2	PwLev3	DescriptionUf
TR11659|c0_g1_i1	Prostaglandin F synthase	Prostaglandin-F synthase.	Lipid metabolism	prostaglandin biosynthesis	-	Aldo-keto oxidoreductase
TR13003|c0_g1_i1	Prostaglandin F synthase	Prostaglandin-F synthase.	Lipid metabolism	prostaglandin biosynthesis	-	Putative uncharacterized protein
TR17558|c0_g1_i1	Prostaglandin F synthase	Prostaglandin-F synthase.	Lipid metabolism	prostaglandin biosynthesis	-	Uncharacterized protein
TR19490|c1_g4_i3	Carbonyl reductase NADPH 1	Carbonyl reductase (NADPH).]---[Prostaglandin-E(2) 9-reductase.]---[15-hydroxyprostaglandin dehydrogenase (NADP( )).	-	-	-	Uncharacterized protein
TR19490|c1_g4_i4	Carbonyl reductase NADPH 1	Carbonyl reductase (NADPH).]---[Prostaglandin-E(2) 9-reductase.]---[15-hydroxyprostaglandin dehydrogenase (NADP( )).	-	-	-	Uncharacterized protein
TR19490|c1_g4_i5	Carbonyl reductase NADPH 1	Carbonyl reductase (NADPH).]---[Prostaglandin-E(2) 9-reductase.]---[15-hydroxyprostaglandin dehydrogenase (NADP( )).	-	-	-	Uncharacterized protein
TR25058|c1_g1_i2	Prostaglandin E synthase 2	Prostaglandin-E synthase.	Lipid metabolism	prostaglandin biosynthesis	-	Predicted protein
TR28149|c0_g1_i1	Carbonyl reductase NADPH 1	Carbonyl reductase (NADPH).]---[Prostaglandin-E(2) 9-reductase.]---[15-hydroxyprostaglandin dehydrogenase (NADP( )).	-	-	-	Uncharacterized protein
TR31063|c0_g1_i1	Carbonyl reductase NADPH 1	Carbonyl reductase (NADPH).]---[Prostaglandin-E(2) 9-reductase.]---[15-hydroxyprostaglandin dehydrogenase (NADP( )).	-	-	-	Uncharacterized protein
TR31063|c0_g1_i2	Carbonyl reductase NADPH 1	Carbonyl reductase (NADPH).]---[Prostaglandin-E(2) 9-reductase.]---[15-hydroxyprostaglandin dehydrogenase (NADP( )).	-	-	-	Uncharacterized protein
TR754|c0_g1_i1	Prostaglandin G/H synthase 2	Prostaglandin-endoperoxide synthase.	Lipid metabolism	prostaglandin biosynthesis	-	Heme peroxidase

Supplementary Table 5: List of primers used for amplification of selected transcripts.

Gene	Primer Sequence Forward	Reverse	Length (bp)	Tm °C	GC content (%)	Amplicon Length (bp)
**COX-1 or PgG/Hs2**	TCATCAAGGGAGGAGAATGG	CTTCCACCAAGAGCGAAGAC	20	58.4/60.5	50/55	170
**PgEs2**	TTCCAAACAGGGCAAGTTAC	TTGCACGAGACAGATTGGAG	20	56.4/58.4	45/50	183
**PgFs**	TCTCCCCTATCGAGGGTTCT	AGCTCCACTCTGCTATCC	20/18	60.5/56.3	55/56	114
**RPS**	GCGCCTTGACAATACCGTCGATG	GACCTCATTGGACAGGTTCTTCC	23	66.6/64.6	57/52	148
**H4**	GACAACATCCAGGGCATCAC	CTCGGTGTAGGTAACGGAGTC	20/21	60.5/63.2	55/57	153
**TUBa**	GTATCGCCAGCTCTACCATCC	GTGGCGTGGAAGATGAGGAATC	21/22	63.2/64.2	57/55	176
**TUBb**	GAAACGGCCGATATGTTCCC	CAGTGTAATGTCCCTTGGCCC	20/21	60.5/63.2	55/57	159
**TBP**	CCTTCTTCAACCCCTCCACCAAC	GTTCGCTCATCCCACGTTTTCG	23/22	66.6/64.2	57/55	161
**GAPDH**	CTGCGAAGGTCCATCCACCGTC	CCACAAGGAGTACGAGAACAGC	22	67.9/64.2	64/55	172
**ACTIN**	TCGGCCCTTGAGAAGAGTTTCG	GATGGTCTGGAAAGTGGAGTCC	22	64.2/64.2	55/55	147
**COPa**	CCCATGCACCTGACTTGATGC	GTGAACTGACAGCACCCAAG	21/20	63.2/60.5	57/55	156
**UBI**	GGGAGCGATGCGATAATACG	GAGGGAACAACGGAGGAAGAAC	20/22	60.5/64.2	55/55	156
**EF1a**	GGGAGCGATGCGATAATACG	GTTGTGCATCGAGGGGAATC	21/20	60.5/60.5	55/55	151
**EctA**	GATCTACACAGCGATGCGAC	GCAACCTGATCCAATGCGAT	20/20	60.5/58.4	55/50	160
**EctB**	CCGAAGCGACCCTGAAATC	GTCTCTGATGCGCAATGGTC	19/20	59.5/55	58/60.5	162
**Secologanin**	ACAGGGGTCGTATTGTGCTG	CGACTCCTCTTGCACACTCA	20/20	55/55	60.5/60.5	130
**PKS_Synt**	AGATGGAGGGCTTTTGGTGG	CAGAGGATATGCGTCGCTGT	20/20	55/55	60.5/60.5	100
**PKS_ER**	TAGTATCCAACCCCATGCCG	CGGTTTCTTGTTCGCTTCCA	20/20	55/50	60.5/58.4	153
**PKS_HxL**	CACAATATCGCGTGGAGGC	GTCCAAGTACGACGCGGAA	19/19	58/58	59.5/59.5	108
**PKS_Cyc**	CATACTTTCGGGTTTGGGCC	ACCGTATCGCAATGCCATTC	20/20	55/50	60.5/58.4	166

Unveiling the presence of biosynthetic pathways for bioactive compounds in the *Thalassiosira rotula* transcriptome

Valeria Di Dato*, Federica Di Costanzo, Roberta Barbarinaldi, Anna Perna, Adrianna Ianora, Giovanna Romano

Supplementary Information 2:

- **Analysis of the best reference gene.**
- **qPCR efficiency and stability in condition of silica starvation.**

Analysis of the best reference gene.

Putative reference genes have never been identified before in *Thalassiosira rotula*.

To study the best genes to be used for data normalization in RT-qPCR experiments, a set of ten genes commonly used as internal controls for diatom gene expression analysis[1] were selected: Actin, Ubiquitin, TBP, COPα, TUBα, TUBβ, RPS, GAPDH, H4.

Three different softwares, BestKeeper[2]; NormFinder[3] and geNorm[4] were used to identify the best reference genes in condition of silica starvation, taken as reference condition to analyse all the genes set. Considering that each individual algorithm has some drawbacks, we decided to use all three algorithms and then compare the results.

In table 1 is reported the qPCR analysis of each reference gene in terms of standard curve efficiency and melting curve consistency.

Table1: qPCR analysis

Gene	Standard Curve			Tm Peak
	**Slope**	**Efficiency**	**R^2**	
Actin	-3.553	1.9118	0.999	Single
Ubiquitin	-3.256	2.0283	0.99	Multiple
TBP	-3.694	1.8651	0.984	Single
COPA	-3.125	2.0893	0.997	Single
TUBα	-3.33	1.9966	0.992	Single
TUBβ	-3.227	2.0412	0.992	Multiple
RPS	-3.09	2.1068	0.991	Single
GAPDH	-2.928	2.1955	0.996	Single
H4	-0.908	12.6276	0.798	Multiple
EF1α	-2.85	2.2432	0.964	Single

Couple of oligo for ubiquitin, tubulin beta and histon4 were not able to amplify well as indicated by multiple temperature of melting that are synonymous of multiple amplicons. These genes were excluded from the analysis.

Table 2 show that each algorithm utilized gave GAPDH as the third better gene putting it at the second position in the final ranking of better genes. However, even if several papers have reported GAPDH as an appropriate reference gene because of its constitutively expression and its involvement in basic functions required for cell maintenance, we decided to not use it in our analysis. In fact, GAPDH has been shown to be sometimes unstable in different experimental conditions and in diatoms also seems be regulated by light, chemical stresses and along the growth curve[1].

Table2: Ranking Order (Better--Good--Average)_7 genes

Method	1	2	3	4	5	6	7
Delta CT	ACTIN	EF1α	GAPDH	TUBα	TBP	RPS	COPα
BestKeeper	ACTIN	EF1α	GAPDH	TBP	RPS	COPα	TUBα
Normfinder	ACTIN	EF1α	GAPDH	TUBα	TBP	RPS	COPα
Genorm	GAPDH | RPS		ACTIN	TBP	EF1α	TUBα	COPα
Recommended comprehensive ranking	**ACTIN**	**GAPDH**	**EF1α**	**RPS**	**TBP**	**TUBα**	**COPα**

The genes finally analysed were six: actin, tubulinα, TBP (Tata Binding Protein), RPS (Ribosomal Protein Small subunit 30S), EF1α (Elongation Factor), COPα (Coatomer subunit alpha).

Table 3 show the best ranking with the different methods, and figures 1 to 5 shows the results for each methods. All algorithms indicated Actin as the most stable gene followed by EF1α, TBP, TUBα, COPα and finally RPS.

Table3: Ranking Order (Better--Good--Average)_6 genes

Method	1	2	3	4	5	6
Delta CT	ACTIN	EF1α	TBP	TUBα	COPα	RPS
BestKeeper	ACTIN	EF1α	TBP	RPS	COPα	TUBα
Normfinder	ACTIN	EF1α	TBP	TUBα	COPα	RPS
Genorm	EF1α | TBP		ACTIN	TUBα	COPα	RPS
Recommended comprehensive ranking	**ACTIN**	**EF1A**	**TBP**	**TUBα**	**COPα**	**RPS**

Figure 1: Comprehensive gene stability.

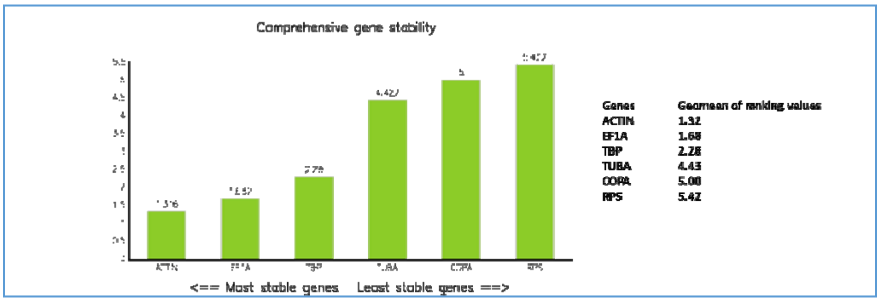

Figure 2: Delta CT method.

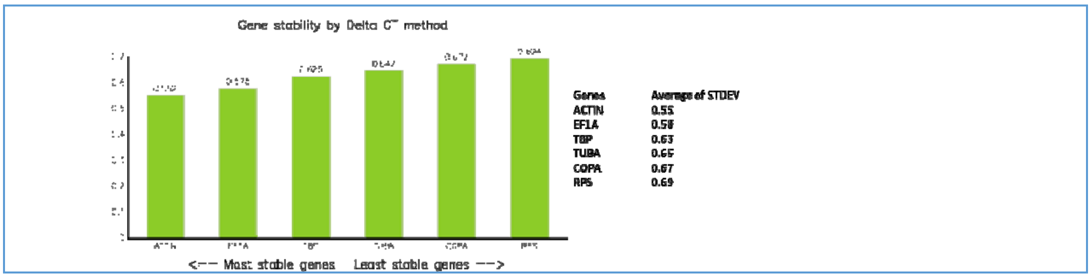

Figure 3: BestKeeper.

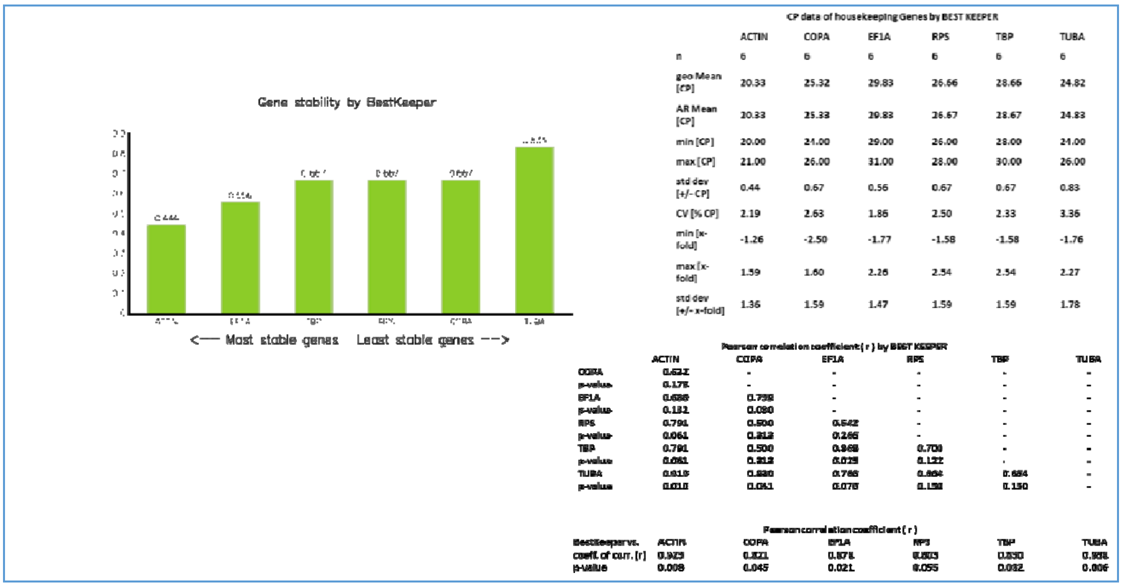

CP data of housekeeping Genes by BEST KEEPER

	ACTIN	COPA	EF1A	RPS	TBP	TUBA
n	6	6	6	6	6	6
geo Mean [CP]	20.33	25.32	29.83	26.66	28.66	24.82
AR Mean [CP]	30.33	35.33	39.83	36.67	38.67	34.83
min [CP]	20.00	24.00	29.00	26.00	28.00	24.00
max [CP]	21.00	26.00	31.00	28.00	30.00	26.00
std dev [+/- CP]	0.44	0.67	0.56	0.67	0.67	0.83
CV [% CP]	2.19	2.63	1.86	2.50	2.33	3.36
min [x-fold]	-1.26	-2.50	-1.77	-1.58	-1.58	-1.76
max [x-fold]	1.59	1.60	2.26	2.54	2.54	2.27
std dev [+/- x-fold]	1.36	1.59	1.47	1.59	1.59	1.78

Pearson correlation coefficient (r) by BEST KEEPER

	ACTIN	COPA	EF1A	RPS	TBP	TUBA
COPA	0.622	-	-	-	-	-
p-value	0.178	-	-	-	-	-
EF1A	0.686	0.798	-	-	-	-
p-value	0.132	0.080	-	-	-	-
RPS	0.791	0.500	0.642	-	-	-
p-value	0.061	0.212	0.266	-	-	-
TBP	0.791	0.500	0.868	0.700	-	-
p-value	0.061	0.212	0.025	0.122	-	-
TUBA	0.919	0.880	0.766	0.664	0.684	-
p-value	0.010	0.041	0.076	0.150	0.150	-

Pearson correlation coefficient (r)

BestKeeper vs.	ACTIN	COPA	EF1A	RPS	TBP	TUBA
coeff. of corr. [r]	0.929	0.821	0.878	0.803	0.850	0.938
p-value	0.008	0.045	0.021	0.055	0.032	0.006

Figure 4: Normfinder.

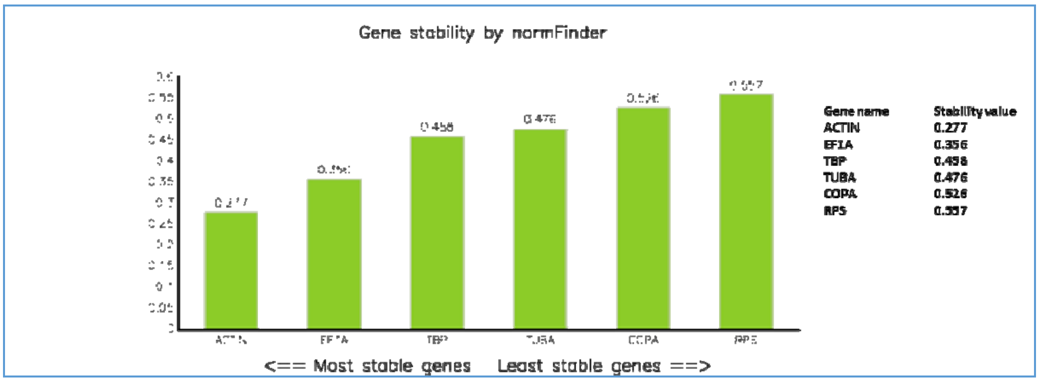

Figure 5: Genorm.

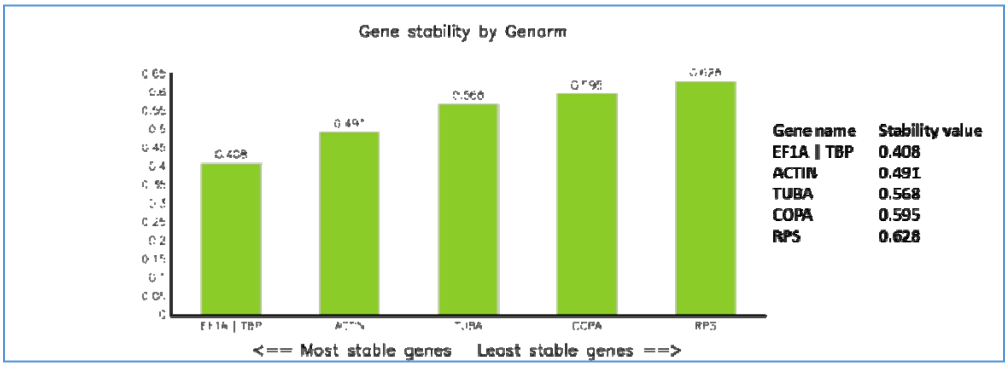

For our qPCR normalization, we decide to use Actin, EF1α and TBP. However, even designing different oligo's couple, EF1α in our subsequent experimental conditions was always instable, so we decide to use only Actin and TBP as reference genes.

qPCR efficiency and stability in condition of silica starvation.

qPCR standard curves:

Gene	Standard Curve			Ct range
	Slope	Efficiency	R^2	
Actin	-3.424	1.9591	0.999	19-21.5
TBP	-3.381	1.9759	0.976	26.8-29.1
PgG/Hs2	-3.271	2.0217	0.979	23.9-27.6
PgEs2	-2.578	2.4429	0.944	26.6-28.8
PgFs	-2.796	2.2785	0.974	25.2-28.5

Reference genes stability:

Ranking Order (Better--Good--Average)

Method	1	2
Delta CT	TBP	Actin
BestKeeper	Actin	TBP
Normfinder	TBP	Actin
Genorm	Actin | TBP	
**Recommended comprehensive ranking**	**TBP**	**Actin**

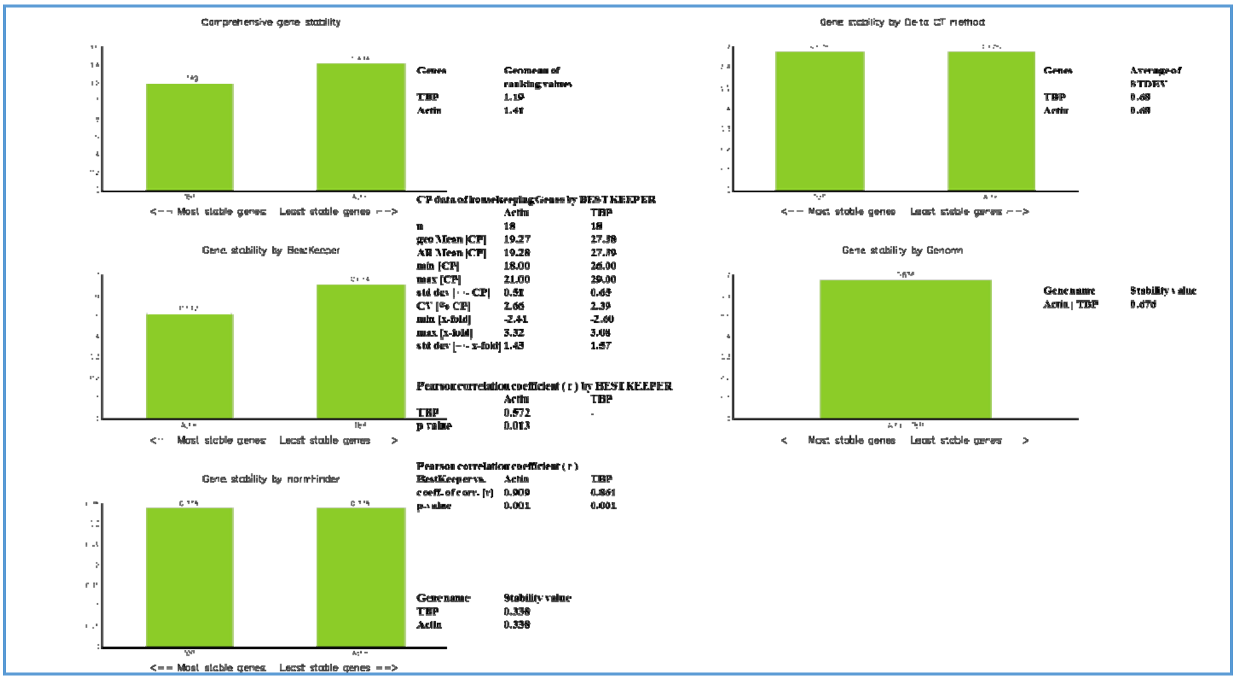

qPCR Relative Expression Analysis by REST tool on Complete Media:

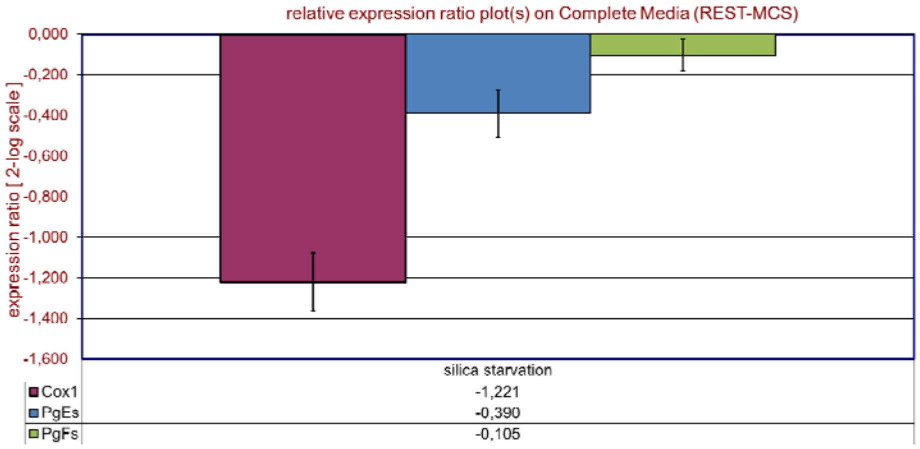

Unveiling the presence of biosynthetic pathways for bioactive compounds in the *Thalassiosira rotula* transcriptome

Valeria Di Dato*, Federica Di Costanzo, Roberta Barbarinaldi, Anna Perna, Adrianna Ianora, Giovanna Romano

Supplementary Information 3:

- **Sequences of the Reference genes and of the Transcripts in the Selected Pathways.**

S ribosomal protein-RPS
>TR58414|c0_g1_i2
TATCTAGAGCAGGGTCCTAAACTAGTTCGTAGTTCGAGCCTATGGTAATGATCGCATGGA
CGTGATGTTTTACGACACATTATTATTATTTTCAATTTCCGTCCCATTTACAATTCCAGTGC
TTCTTCAGCTCAGGAGTGAATCAAGATTATCACTGGCATCATCCGAACTCTCTTTATCGC
CACTATCCATGCCGACGCTCAAATCATCCAATGCATCTCCCAATCCCAAAACAATATCCTT
CGCGCTAGCCTCTCTCTTCTTGCGTTCCGCCTCTTGTTTCTCCAGGTACACGCGAGCCGT
CTCGTCAGCCATCTCATAAACGCGTTCCGGATCCTTGATCATGTCACCAGGCTCCGGTTC
CAACGTCTTGGTGCTGAGTGCTATGCGGCCATTCACCTTATCATGATCGATAATCATACAC
TTGATCTTCTGCCCCGGTTGGAGGACGCGCTCAAGATCGTTGATGCGATCCATGGAAATT
TGAGAAATGTGCAACAGACCCGACATGCCACCAACCTCGACGAAGGCTCCGTAAGGCT
TGAGCGCCTTGACAATACCGTCGATGACATCGCCCCGCGATAGGTCCTCCATCTGAGCCT
CAACAACAGCACGGCGGTTACTCACAACAAGCTTGTTATCCTCCTCATTGACCTCCAAA
AACTTCAGCGGAAGAACCTGTCCAATGAGGTCCTCGTCAGGCAGGCGTCCCGTTAGGT
GGGATCCGGGGAGGAATGCACGAAGCCCCTCCACGAGGAAGATGGCTCCTCCGCGGTT
GACGCTGACGCACTCGGCCTCAAAGGCCTCGTCCGTATCCTGCTTGGCCACAACCTTCT
CCCATGCCTCGCGGTACTGAATCCTTCGCACGGATACCATGAGCTGGCCGTTCTCATCCT
CCTCGGAGATGATCTGGAAGTTTCGCTCTTGGTCGATTTCGATGAGGGATTCGATATTCT
CACCCTGGACCTGGATGAGAGCAGCCTCCTGGAGTGGGAGGAAGGCGCTGGCCTTGGC
TCCAATGTCGACGATGCAGCCACCCTTGTCGTACTGGACGACGGTTCCCTTAACGACAT
CATTGCGGTTGAAGGAGTAGTCGGTGGCGGCGACGGCCTCGTCGAAGGAGGCGTAGGT
GAAGCCAACTTCGTCTTCAATTTCGGGGGAGATTTCGAGTTGGGGGCCCTTATATTTGGA
CTTGATGCGGTCAAATCGGGTCATTTGTTGTACCTTAGGCTTTTCCGCCACTTCCGAGAA
GAGGGCTGTGGAATGGCGAGTTTGTCCAATGGTAGAGAACGCCGACACGGCACTCCCT
GTGAGAGCTACTAATGCGAATCCAAGCTTCATCATTCAATAATAAAACCGTATGAACTGG
GGGATTTATCGTGCCTTAATTGCGATATCAGACTATTAATAGTGGGGATATGGCGCCGTGT
CGTTGTATTGATACGGTGTATGAAGGCGGACGGTGGGGGAGATGGTAAGTCGATTATGTT
GGGAGGGCGTTGGTTGATGCGTGAG

HISTONE H4
>TR7889|c0_g1_i1
TAACATACTGTCTCTAGTGTATAAATGACAAGTGGAATCTCAAATTAGGAGAACTGACAT
AAATTAGAAATCCCATTGACACTGATCAAATCGGCCTGGTTGGATCACCATACTTGTTAC
CCTATTTTTTTTTTCCACAAACATGAGGTAGTACATCTCTCGGGTGAAAAATCATGATCAA
CTGGCCGTTCAAGCAAGTTCTTACATGACACACAGTGTATGTAATTTGGTATCAGATCTA
ATCGAAGTACGAGCATCGGCTGTGAACAAGGATACAACAGTCCGTCACTGCTGAGATGC
ACATTTGCTTGGTGATATTGATTGTCGTCACCTTTATTGTAATCATGGAGAGTTACCCTGT
CTTCAGCACACACATTCTCACACAAAATCGCAATCGTCATAAACAAAAGAAGTTAATCA
TGTCGTGGGTTCGGATTGCGTTCGAGAGAGATAGTTCGCACCAACTAAGGCGCAAAAAT
ACATGCGTACAAATGTTTCAGGGGTTTCGATTTTTGGTCTTATTGGATTTTCAGTTTCTGT
CCTGGTTGCTAACCACAACTCTATTCATATTTCCATATCCCATAGTCTCCAACGAAACCCT
AGTCTCCCTGTCAATTCCAATCGTCTCCTGCTCCAAGGCACTGACCTCAGGAACAAAGT
CCAACCTGCGCTCACATTCCTCCTCCCGCAACTTCAACGAAATGTTACGCACGAGTCCC
TGAGTAATACGCTTCATCAAATGGGTAGTGAATCCGGCAATCTTATTACGCATGCGCTTCG
AGGGGGTGATGGCAATTTCATCGATGATACGCTTGTTGGTGTAGAAGTCGAGGGTGAGA
CGAGGGTAGTACTTCTCGACGATGGTACGAGCGGACTTCTTAACAGTCTTGTTCGACAT
GTCTGCTGGATAAAACATTAAAAAAGCGCGCGAGTGTTTCTTTGGCAGTTGGGTGGATT
TCAGCAAATCCGGTTGCAGGGGGGTAGAGGCATCATGTCGCCATAATATCTCAATTTTAT
CTCTCTGTCACGGCAGGTAAATTATCCACCGAAGCCATAAAGAGTCTTTCCCTGGCGCTT
GAGGGCATAAACAACGTCCATAGCGGTGACAGTCTTGCGCCTGGCGTGCTCCGTGTAGG
TGACGGAGTCACGGATGACATTCTCAAGGAAGACCTTGAGCACGCCACGCGTTTCCTCA
TAAATGAGTCCGGAGATGCGCTTGACACCGCCACGACGGGCGAGGCGACGGATGGCGG

GCTTGGTGATGCCTTGGATGTTATCGCGAAGGACCTTGCGATGACGCTTGGCGCCTCCCT
TTCCTAGACCTTTGCCTCCTTTTCCGCGACCAGACATGATGAAGTGATGTTGTTGTTTGG
GTTACTGTGACGATGACAATCGTTTTATGGCAAACGGAATGTAGGACCCATATATCGTAC
CTAAGGTAATCGTGAGA

Tubulin alpha chain
>TR11532|c0_g1_i10
CATATTCAATGATGTGAAGGGTGGCGCATAAAATAGATACGCATATTAATCAGCACCCGC
AACTCTTGTCACGAGAGCTTTAGCGTCTGCGAATCAAATACTCTTATCCCCTTCAAATCC
TCTACCTTTCATCATGCGCGAATGTATCTCCATCCATATGGGCCAAGCAGGTGTCCAAAC
CGGTAACGGCTGCTGGGAACTCTACTGCCTCGAACACGGCATCCAACCCGATGGTCAAA
TGCCTTCCGACAAGACCGTCGGAGGCGGAGACGACGCCTTCAACACCTTCTTCAGCGA
GACGGGCGGAGGGAAACACGTCCCTCGCGCCATCTACGTCGATCTCGAACCCACCGTCT
GCGATGAGGTTCGTTCCGGGACGTATCGCCAGCTCTACCATCCCGAGCAGATCATCAGC
GGGAAAGAGGATGCGGCCAACAACTACGCCAGGGGACACTACACCGTCGGCAAGGAG
ATCGTGGATCAAGTATCGGACAGGATCCGCAAGCTGGCCGACAATTGCACAGGCCTGCA
GGGATTCCTCATCTTCCACGCCACTGGCGGCGGAACCGGATCCGGCTTCGGCGCCCTCC
TCCTGGAGCGTCTGTCCGTGGACTTCGGTCGCAAGTCCAAGTTGTCGTTCGCTGTATCG
CCCTCTCCCCAGGTATCCACCGCCGTGGTCGAGCCATACAACTCCGTCCTTTCCACCCAC
GCTCTCCTGGAGCACACGGACGTCACCTTCTGCCTCGACAACGAGGCCCTGTACGACGT
CTGTAAGCATAGCCTCGACATAGAGCGTCCCACCTACACCAATCTCAATCGCCTCATCGC
CCAGGTCACCTCCTCCCTCACGGCCTCCCTTCGCTTCGACGGCGCGCTTAATGTCGACGT
GACCGAGTTCCAGACCAACTTGGTGCCCTTCCCCCGCATCCACTTCATGCTCACCTCGTA
CGCGCCCATCATCTCGTCTGAGAAGGCCCATCACGAGAACCTCTCTGTGGCCGAGATCA
CATCGTCGGTCTTCGAGCCTCAATCCGCCATGAGCAAGTGCGATGCTCGCACTGGCAAG
TACATGGCGTGCTGCCTCATGTACCGTGGGGATGTGGTGCCCACGGACGTCAGCGCGGC
TGTGACCACTGTGAAGACCAAGCGCACGATTCAGTTCGTGGATTGGTGTCCCACAGGAT
TCAAGTGCGGGATCAACTACCAGCCGCCGACGGTGGTGCCCGGGGGTGATCT

Tubulin beta chain
>TR22371|c1_g1_i1
CTTCCGATCTCAACGCCAAGCCGAAATCTTTTGACTGCTTTGCCTTTGCCAAGATCCCAA
CCATTACTCCCCCCACGCCGTATCGCCAGAGCAATCGATGCCTATAACCACACAAGAGA
GAAATAATTAATCAATTCAACCATGAGAGAAATCGTTCATCTTCAAGCGGGACAATGCGG
AAACCAAATCGGGTCCAAATTCTGGGAGGTTATGGCCCAGGAGCATGGGATCAACGACC
AAGGAGCCTACCATGGTGACAACGACCTCCAACTTCAGCGCATCAACGTCTACTTCCAT
GAGGGCGGAAACGGCCGATATGTTCCCCGTGCTGTTCTCACTGATCTTGAACCGGGCAC
CATGGACGCCATTCGCGCTGGACCATTCGGGGGAGTTTTCCGACCTGACAACTTTATATT
TGGGCAGTCTGGGGCAGGTAATAATTGGGCCAAGGGACATTACACTGAGGGTGCTGAGC
TGGTGGAGTCCATCATGGATGTGACGCGTAAAGAGGCAGAGGCATGTGATATGCTCCAG
GGATTTCAGATCACACATTCCATGGGAGGTGGAACCGGAAGTGGAATGGGAACGCTTCT
TGTGAGCAAGATCAGGGAGGAATTTCCGGATAGGATCATGACCACTTACAGTGTTGTGC
CCTCCCCGAAGGTGTCTGATACCGTGGTAGAGCCTTACAATGCGACACTATCGATCCACC
AGCTTGTGGAAAACGCAGATCAGTGCTACGCTCTCGACAACGAGGCCCTCTACGATATC
TGCTTCCGCACATTGAATCTTACCAACCCAAGCTACGGCGACCTCAACTGCCTCATCGCG
GCGGCCATCACTGGGACGACTTGCTCCCTCCGCTTCCCTGGTCAATTAAACTGCGACTT
GCGTAAGCTGTCGGTGAACATGGTCCCATTCCCCCGTCTTCACTTCTTCCTCGTGGATT
CGCTCCCTTGACCTCGCATCGCTCCCAGGCCTTCCGTGTTCTCACCGTCCCTGAACTGAC
CCAACAGGCCTTCGATGCCAAAAACATGATGTGCGCTGCTGACCCCCGCCATGGACGAT
ACCTCACGTGCGCGATGATGTTCCGTGGAGCAATGTCTAGCAAGGAGGTTGATGATGAG
ATGCTTAAGATGACGCTGAAGAATTCTGGATACTTTGTGGAGTGGATTCCCAATAACCTC
AAGGCAAGCATTTGTGATGTACCCCCAACGGGATTTAAAATGTCGAGTGTCTTCATTGGT

AACTCTACATGCATTCAGGAAGCCTGGAAGCGTGTGGCTGAGCAGTTCACTGTTATGTT
CCGTCGTAAGGCCTTCCTTCATTGGTACACTGGAGAGGGTATGGATGAGATGGAGTTCA
CTGAGGCAGAGAGCAACCTGAATGATCTTGTGTCAGAGTATCAGCAATATCAGGATGCT
ACGGTTGGGGATGACGATATTTCGGGGGAGTTTGAGTATGAGGGTGATGACCAAAACTT
TAATGATGACGTCGCAAACAGCCTTGAAAACAATACGGACAAAGTGGCCGATGAATTCA
TTGGCATTGTGGACCAACAAACTGACCAGCTGGAATTTGATATCCCTACCGAACAACCA
TTGGAATCAGAACCTTAAGGATGAGAAACATGTAGACTTTTCGAAAGAATTTTTGTGGT
TGAGGTAGACTTTGTTGGAATGTGAATGATATTATTTGGGGTGCTATGAATATTGCGCAAG
CATTCTCTAGTGATCGATTTTGTGGGTTGGCGTGTGACATGTGATTGTGAGTCTTTAGTAC
TTGACTGTGAATGTGTTGTTTGATGAGACAACTTGAGCGGGCCAAGATTGACACCCTTC
GTGTTGGGACACCTTATTGCACCAATAAGTGGCTAAATATGTAGATAGCTATCGGTTCGAT
GGGCATGTTTTGAAAGCATGGATACGATTTTTCGACAATTCTCCTGCAAAGTACATGCTG
AAGTGCGTTGTAAAGGATATCAATGAACACATAGATGTCAGGTCAAATCGAAATAGTACA
GTAAACACTATATTTGTTGAAAAACGGTGCTTCGGTGGGTTCATGGTGGTTCAAATTGTG
GTATTCAATAAAAAATTAAGTCCTACTACAGACAAAAAAAAA

TBP (TATA box binding protein)
>TR38216|c0_g1_i2
GCTCTTCCGATCTCCTACCTCTCCCAGACGATGATGCGGCCGCATTCTTCGCACTCGCAG
CCGCCGCACTCGCTGCCTCCTCCATCGTGGGCAAACTCGCCACATTGGCCTTGCGAAAT
TCCAAAAGAACCGGATAAACCTTCTTCAACCCCTCCACCAACTGCTCTTCCGTCTTGGC
ACCGGTAATAACCACCTTGCCGCTCACGAACACCAACAGCACTACCCTGGGCTGGACGA
GCCTGTATATCAACCCCGGGAACAACTCCGGCTCGTACGACGAAAACGTGGGATGAGCG
AACGCCACTCCCTCCAACCGAATGGGATACCCCACATCCACCGTTCCCACGATGTTCTGC
ACTTTGAAGTTAATGTAACTCGACGGTTGGAATCCCACCCTTTCGAGGATGTAGGCGATT
TTCTTCGTTGCCAAACTGGAATTGTGACTGCTTTTGGTTCCCGTGATGACTAATTTCCCC
GAGGCGAATACGAGTGCGGTGGCGCGGGGTTCGCGGAGGCGCATTATGACGGCGGCGA
AACGACGGGGGTTGAATTCGGTGTTTCTGCATCGGAGTGCTATTTGTTTTAAATCTAATC
GCACGCCCAGGTCGGCTATTTTTGACATATGTACGTGGAAGGGGAGAGATTCATGTGATG
ATGATGAGCGTTTTCGATAGAGTGTTAAGGATTGGAAAATCGTTTCATTATACAAAAATC
ATCCCATCATATATCTGCACAAACATACATGCAATCCTGATCTCTCTCAC

GAPDH
>TR4255|c0_g1_i1
TCTTCCGATCTTTTTTATTCAAAACTGGCTCCTATTTTATCTATCCTTCGTAGCTTCAATCA
CATCCATTACTTATTTCTGACTCTCCATGTGCGAAATCAAATCAAGGACACGGTTCGAGT
ATCCGGCCTCGTTATCATACCAGGAAACCATCTTGACAAAGTTATCATTCAATGCAATACC
TGCCTTCTTGTCAAAGATACTGGAATGGGTATCACCAATGAAGTCGGACGAGACAATGT
CGTCATCATGGAATCCCAACACTCCCTTCATCTCATTCTCGGACGCATCCTTGATCTTCTG
GCAAATGGTCTCGTAATCCGCACCCTTGTCCAACACCGCCGTCAAATCCACCACACTGA
CGTTGGCGGTAGGCACACGGAACGCCATGCCCGTCAACTTCCCATTCAAATCCGGAATG
ACCTTACCCACCGCCTTGGCAGCACCGGTACCACTGGGAATAATGTTGTAACACGCGGC
GCGTCCTCCCCTCCAATCCTTCTGCGAAGGTCCATCCACCGTCTGCTGCGTGGCGGTAAC
GGCGTGCACCGTCGTCATGAGCGCCTCCTTGATTCCGAACTCGTCATTGACCACCTTGG
CCATGGGAGCCAGACAGTTGGTCGTACAGGAGGCGTTGGAGACAACATCCATGCTGTTC
TCGTACTCCTTGTGGTTGACTCCCATGACGAACATGGGAGCGTCGCCGGAGGGAGCGGA
GATGACGACCTTCTTGGCCCCGCCGGCCATGTGCATGCTGGCCTTTTCGGTGGTGGTGA
AGACTCCCGTGGATTCGATGACGTAGTCGGCTCCAGCGTCGCCCCAGGCGATTTTGGAG
GGGTCCATCTCGCCGTAGATTTGGATGGGTTTGCCATCGATGGAGAGGGTCTTGGCGTCC
TTGTCGTGGGTGACGGTTTGACCGCATCGGCCGTGGACGGTGTCGTACTTGTACATGTAC
TCCATGTAATCGACCGAGATGAAGGGATCATTGACGGCGACAATGTTCACATTCGGCTTG
TTGGCAGCCGCGCGCATCACCAGGCGCCCGATGCGCCCAAATCCGTTGATTCCAATATTG

ATAGACATGATGCTGATTATAGAGTTCTATGAAAAAGTGAATATGAGCTAAATT

Actin
>TR18494|c0_g1_i4
CCCGACAGCCTGGAATCATGGTGCGTGACTGAGACTCGGACATTTCCATTGATGATGTTG
GCGCTCGCCCTTATCTGTCCAAGGATCTTTTGCTCACATCACACACCTCTTTTTTCACATG
CGTCTAACAGGTGGGAATGGAACAAAGAGACGCCTACGTCGGAGACGAAGCCCAGGCC
AAGCGTGGTGTCCTCACCCTGAAATACCCCATTGAGCACGGAATTGTCACCAACTGGGA
CGATATGGAGAAAATCTGGCATCACACCTTCTACAACGAGCTTCGCGTCGCTCCCGAGG
CCCACCCGGTCCTCCTCACAGAAGCTCCCCAAAATCCCAAGGCGAACAGGGAGCGCAT
GACCCAAATCATGTTCGAAACCTTCAACGTCCCCGCCATGTACGTGAACATCCAGGCCG
TCCTTTCCCTCTACGCCTCCGGACGTACCACGGGATGCGTCCTCGACTCCGGAGACGGT
GTCACCCACACCGTCCCCATTTACGAGGGGTACGCGCTCCCCCACGCCGTCGTCCGTCT
CGATCTCGCCGGACGCGATCTCACCGACTACCTCATGAAAATCCTCACGGAGCGTGGTT
ATTCCCTCACCACCACCGCCGAGCGCGAGATCGTCCGCGATATCAAGGAGTCTCTGTGC
TTTGTCGCTGTGGACTTCGAGGAGGAGATGAAGAAGGCTGCGGAGTCCTCGGCCCTTG
AGAAGAGTTTCGAGCTTCCCGATGGTAACATTATTGTTATTGGTAACGAGCGTTTCCGTT
GTCCTGAGGTCCTCTTCCAGCCTAACTTGACTGGACTTGAGATGGATGGAATTGCGGAC
TCCACTTTCCAGACCATCATGAAGTGCGATGTCGATATCCGTAAGGATTTGTATGCCAAC
ATTGTTCTGTCGGGAGGTACCACCATGTTCCCTGGAATCAGTGAGCGTATGAGCAAGGA
GATCACGGCGTTGGCCCCTGCATCTATTAAGGTGAAGATTGTTGCCCCACCAGAGCGTA
AGTACTCTGTGTGGATTGGAGGAAGTATTTTGGCAAGTCTTTCGACTTTCCAGAGTATGT
GGATTAGCAAGGAGGAGTATGATGAGAGTGGTCCCAGTATTGTGCACAGGAAGTGCTTC
TAAGTAGGCCTGAGCGCAGATGTTTCTTGGATATGTGACATCATGCGGCCCCAGAGGCG
GAATTTGTGGTATTTGTTGTGAAGCACAATAGATGAGCGTACTGGTGAATAGTGATAGTG
TTCCACAGAACAATCATCTAGGTAATTAAGTCCTACTGTTATCAAATCAAAAAAAAAAGA
TCGGAAGAGCGTCG

COPA
>TR6988|c0_g1_i2
CCATTAGACATGGTATCTAGTCATAAGACAATACGACAAACTATGTAGCTTAGGTGACCA
AAACTAATAGATATCATCAGGTGGGCGAGCTATTGGTTTGCCTCTGAAAAGATTTTTTGA
AGATGAAATGCTAAGGGGGGCTTAGACTTGACCCTGTGGCTCAGTGATGTGATATCTGCT
TGGCCTAAGCGAAATAGACGGATGACCAACAAGCAAAAATTAGCAAAATACTATCCAGT
TACAAGCAGTGTTTCAACGCCTTATCATAGTGCGTAAGACATCTCTCAATAAGAGTAAAT
AATAATTTCTTTCTGGATGCAAGTAATAGCAGAGGAATATCATATACTCTATCATCAATTTT
ATATGATCAATTTTTCGTTAATGATAGGGCATCAGTGGGACAGCCAACAACTTATTTTCCA
GTGACAAGCCCAATCGTTTCAACACCAATTGTGCAGAAGCCATCTGTCAGATCGACATG
TCCTTTCATTTCTGATCCCTTGTAAGAGGATCCAGAGTAGGAGCATTTGACACTCTCTTC
TCCTGTGTAAATGGGAACAAAATCTTTACAATCGATTACAAATGCAGCGTTCTCATTGTA
GTTGAGGGTGTGTTCATTGCGTGCCATCTGTTCACTCTTTTGAAGCACCTTTGTGGCCTT
CATCCTAAGATCAGCATTTTTCGCGCTTCTCATGTCAGGGAGCTCCAGTAGACGCCTGGC
AAAACTAGCTGCAACAATGTAGTTCTTGTGTTTGAAGGAAGTTCCCATGGCAGAGCGAA
GGGCAAGAAGTTTGTGTACAGGCTGCAAGTCGCAGTGAGTAAAAAGGGCAGCAAGTTC
TGTCGATCGGACAGGATCCGCACCAGCCTCGGCCATTGCTGCCTTGATGCGAATTGCAG
TGATATATTCGCGACAGATCCCTAGCATCTCCTTCATTTCACTGGCTTCGTTTTTGTTCTCT
GCAGCAGTGAGAGGGATCTGGGCCATGACTTCAGTAAATGCATTCTTCGCATCGTTGAAT
TTTCCAGTTTGGAAGAACCTGTATCCCTGGCGGATACCTGTGACAAGAGTCCTCATGTTA
AGTGACGTGCGAGGTAAGCTGTCATTTCCTGGATGGCCTGACGCGTCATTCCTCATGAG
AGGCACTACAATGCTTCCGCTGCCAGGGATACCTGGTAAGCTCACAGTGGAGCCAAGAT
AACAGCCAAGCATGGACCCTTTAAGGACAGAAAAGTCACTGGCGGCAATTTGTCGATG
AAGCAATTGCATAGCCGATGAAGCACCTCCAGCTGCCATGTGATCAGCGGCATGGGAAC

TGTTCGCTGCCCAGCATCCAGCGGGAGGGCGCCCAGCAGCAGGCATTTGAAACCCTGC
GTCATCTGAAATGTCATTGAGACCCGCCATTTCGTCAACTGGGGCAGCCTTGGGAGTATC
ATCTCCGAAATCGTCTCCAAGATCATCCAAGTCATCACCCCAGCCCATATCATCATCATCA
CCCATATCAAAATCATCAGCTGCTCCGCCCAGGCCTCCATCAGCGTCACCATCCCCCGCG
GCAAAATCGTCCTCCCAAACAGCTGCTCCAGCAGTGTCAGCAACTGGAGCAGCTTCTTC
TTCTTCATACTCATCACCAGCATCTGCAGCTGATAAGTCTTCAAGTGTTGTCTTCTTGATC
TCAAGAGTTGGCCAGTTACCACCTCTGATGATAGGTGTTGGTGGTTGGAGGAGGCGACC
ACCCTTCTTTGTGCCAACAGGGACCTTGTCCATCAGTCCTTCCACTGAACCTTCCTGGGT
TTCAATGGCAACCTTGATTCTTTCCGCAACATCTACCAACCCATGAAGATTGGCACTGAT
GTAGGCCAATGGCAAGTTTCCACTGGCCTCCAAGACAGCAACACGCTCAGCAGCATCA
CCAAGAAGGAGGGCATTGTGGTAACGGCCCATGATATCATTGCGCATGTTGGAGATCTTG
AGCATTTTTCTCAACTTCTCAGAGTCACCAGTGAGCATGTATAAGAAAGATAGTCGATCG
AAATCCTTCTTGCGTTGATAGCTCATCTCCACAACCTGATGGTTTCCTTGGCGGAGAGCT
TCGCTTCCAAGTTCCCCCCAGACATCGCGTGCTTTGCCATCCAATCCTTGCTGCTGCTCC
AAAGAAAAAGCACTCTCCATCGCAGCCTCAATATTTCCACAAGCAAGAGCAAGACGGA
ACCGAGTTTGCGGATCACGGACAAAGTGGAGTGCCACTTCAGGAAAGCCTTTAGATTG
AAGGTAGGCGACAATTGCCCGGCCACAGAGACGGCTGTGTTTAACCATGTGCATAACTT
GTCCATACTTTTTGTTTGCAAGAGCAAGCTTGAAACGGGCTTCAGTTGTGTCGATGCTGA
TGACTCTGGACCTGGCTTCCCTGTCAAGGCAGAAGAGTTGATCCTTAACCACTCGTGTA
GCATAAATGGGATTGTCCAATGTACGAATAGTACCAGTATCGCCAGTAGAAAGGCAATAT
TTCACGTGGTGAAGGGTGGTGTAAATGAAAAGATTGGAGCTGGTGCCGCCAGACGGAC
TGACATCCCATGCACCTGACTTGATGCGAACAGTGTCGCTGATTGAGCATAGCTGTTCAA
GCTGACGGTTGGCAATAACGACTCCATACTTGCAGAGGAGGGCAACCTTACTGCCGTCG
GGAGACCATACAACGTTCTTAATCTTGGGTGCTGTCAGTTCACCAATGACGCGACGACT
TTGGACTTCGAAGAGGACGGCGCGATCGTCCGCACGAAGAATGATTCGACCCGAGGCA
CCACCATCATAAAGGCAATCAACATTGGGCAAGGGCGCCTGAACACGCTTTGTGGTTTC
GTTGTTGAGGTTCTTGACAACAATTTCACGCTTGTGCCGATCAAGGATCGCAAAGCGGT
TACGTCCAAGGAAGACTCCAGGCCCAAGACAGGATCCTCGTTTGCCATCAGTGACAGAT
CCCCCAGCATTAGTGAGAGAGAAGGTGATGAGTTCATAGCATCCACCATCAACTTCAGA
GCAGACTAGAACATTTCCTTCGGTGGGGTTGTGCATATTGTAAGAGAGGAAGCGAGGAC
CAGACCCAATTCCATCCGTCTGCGCCTGCTGTCCCACCCTGCGGAGGGATGCAATGGGC
ACATCCACGCCAGAACTTCCGCCCGTGGCCCGACCATAATCGTGGCAGAAGAGCTCACG
TCCGCGAACATAATACAATTTGTCTCCTGGTCCGTTGGCGGATGCAGGACGCTCCCTCTC
AAGCTTGAACACGATCATACCGGAGTCATGACCAGCCGCAAGCAGATTCTGAGAGCGAT
GAGCGGCTAAGATCCAGAAGCGGTCCCCCTCCCGACGGAAGGTTTGCACGCCAACACG
TTTGGACACATCCCAGACGCGAATGCTGCGATCTTCTGAGTTGGAGACGACGAGTTCTT
GTTTCGGGTGGAAGAGACAGCACGAAACATTATTGGCATGGCCACGGAGGGTGTCAAC
CTCCCAAGCCTTGGTCTCACTCATTCTCCAGAGTTTGACCTGACGATCATCAGCAGCACT
TGCCAATAAAGGCAGGGTAGGATGGAAGGCTGCCCAATTCACGCCACGGTCGTGTCCCT
CCAAAACATATTTGACCACAACGTCATTGGTACCAAAGAGCTCTGCCTGGACATTCAAA
GCAGTTGCCGCCGCAGCCGATTGACCACGATTGCCATTGGAGTCCGTCATGCCCGGATAT
CCTTGGTCTGCTGCCGCGGGTCCCCCTAGCTGCTTTTTACGAAGACCAGTCGTGTCCCA
CACTCGAACCGTCTGATCCAAACTGGCACTAACAATAAGATCTTCCGATGGGTGGAAGG
CTGCACACATGACATAATGGTTGTGACCCGTCAAGACACTCAAACAGCTCCTCTTTTGA
AAATCCCACAACCTCAAAGTCTGATCGTCACTAGCACTCAAAATCCATGGAAATTGGGT
GGCATTGGGGTGGAACTGCACCGTACGGATGTAATCCAAGTGACCCAACAATGTGAAAA
GGCACCTCCTAAGTTTGTAATCCCAAACCTTGATTTTGTAATCATCTCCACCAGTGACGA
TGAGAGGTTCGTGGAGATGAAAGTCTACGCCACGGACGGGACCATCGTGTTCTTCAAA
GCGGTCTATGACGGTGCCTACGCGGTAGTCCCATAGTTGAATGACACCATTGTGTAATGA
GGCGCATATCCACGGCCGCACCGGGTGGAATGCGAGGCCCTTCACGCGGGCTGATTTAC
TCTCAAACTTCGTTAGC

Ubiquitin like protein
>TR31138|c0_g1_i1
TGAAGCTTGAAATACGCTGAATGCAAGCTAGTTTAATTCTGCTACTTTTCAGACGCGATT
TCAAACAGAATACCCCGGCCCATGATGATGAGGAGGTTTCTTCAATTTGTAAAAAGATCA
TACATTTTTCTACCACAGACCTACTTTCAAACCAAAACACGCCATCTCCTTTTGAATTTA
GGCGAGCTTCATTCTATTCAACGCCCGCAATAAAGTCCCTCGATTATCCCTCCTCTTGTAA
ATATTCGTCAACTTCGTCTCATCCTTCCCCAATAATCCAAATGCGCTCTTGTGCTCCACCT
CCACCAACCTCGACCCCCTGCCGCCCGGCAAATCAAACGCCCTATCACGAGGCTGGGCC
GTGCGCATGCCATACTCCAATTCCTCCAGGGGGGACGTGTCGCACGTACGGCGAGGTAG
GGGAGCGATGCGATAATACGCGCCCAGGACGGGCGCCACGCCGTCAACGACCGCCGCC
TCGACTTCTTCCGCTTCGTCCTCCTCGCTGTTGGTGCGGCCGAGGCTTTGGTCGATAAAC
ATGTTCATCAATTCGCGTTCTTCCTCCGTTGTTCCCTCCAAATTGGACTTGACGAAGGAT
TCGTCCACGCGAAAAACGCCCTTCCCGAGCGCCTTTTCGAATGCGCTCTTGACACCTCC
GCCGCCACGCAGCCTCAAAACCAAATGAAGCGTGGCGTCATCTCCGATATTGTAATCGT
CGACCGTCTTCCCGTCCTGGAGCTGCTGGCCACCGAATATGATCCGTTGTTGTTCAGCCG
GGATGCCCTCCTTCTCGGCAATCTTTGCCTTGACGTCCTCGATGGATTCGCCCTCCTCGA
TTTCGATGGAGACTGTCTTGCCGGTGAGAGTTTTGACGAAGAGTTGCATCCCGCCGCGA
AGGTCGAGGGCGGTGGTGAGTGCGGACGAGGGGCGGACGAGGGCGCGGCTGCTGCCG
ATGAGGGGGAGACGATTACCACTGCATGATTGAGTTACCAGGAGAGATGTTGCCAGGAA
GAGGCAGGATTTGGAGGAAACCATAGTGCTATTGAAATGGCCTGGACCTTGAGATTGAG
CAGAACGATGACGACTTAGTTCT

Translation elongation factor-1-alpha/EF1a
>TR44161|c0_g1_i2
CCATCATCCAGCCATCACCATGATCATCGTCACTGGCCTCCGCCGTAGAGCAATGCGGTA
GTATGATCATCGCCTGCGGCCCTGACGGAAGGTGACGATGAAACCAAAACACACGAGAT
AAGAGTGTCAATGCATATCACCTGCTGATGGGCGAATCAACGAAGAAGGGCTCAAGCTC
AACAATGCCACATCATCTCACCCTCATCAAGCTAGCCATCTTCTTCCAAACAGCCTCGCA
GGCAGGATGGAGTCGTTTCAGCAGCATCTACATGCTCAGCCAGGGATTCACTCCATCTC
AAATCGGAAGAATAAAATCAACCAGTCAAATCGCCAAGGCGCTGGCTCAGCCCATTTGG
GGTGTAATGAGCGACATAACCGATCCCCTTAGTACCATCATCGTGTCCACCGTTCTCGGT
GCAGTGACACTAGCGGGTGTTCGGGAAGCGATTGATGCGCAGAGTTTTTCCAAGCTCAT
GACCTGGCGAATTATACGCTCTGCGGCCACGGCAGCATCTCCGGCGCTCGATGCGCTTGT
ATTACGTCTAGTCGAGAAATCCTCCGAGGGATATGGACCGCAGCGACTTTGGGGGAGTG
TGGCATGGGGTTTATCATCGCTTTGTGCTGGTGCAATCCTGGATCGGTTTGGCGAAGGCG
CCATTTTTGGGTACACATACGTTATCAGCGGTGTCCTGCTGGCATTTTTCCTGGCGCTGA
GAAGGGTAATTCCCAGAAGTAGTACGAAACGGAGTGACGACGAGTCTCCCCAGAAGAA
AGAATCTCAATCTACTGGGTTGATGTTGCGGGTATGTGGATTTATTCGCACTGTAATCAGT
GATCTCTTGAGAGGGACGACTTTGACAGTTTGGGCGATTTTGATTCACAGTTTTGGATAC
GGCGTCATTATGGTCTTATTCAATACCATCCTTATGCTGCAACTCGAACGTGACTTTGGAA
TATCACGATCCGTACAGGGTATATTCACCTTGGTGTCAATACTCAGCACATTGCCAGTGTA
CCATTATTCGAGCAAGCTCAGAGAACGCATTGGACATTTAGGGATGATTCAATCTTCAAT
AATTGTCGCAACCTTCCGCCTCTGCCTGACAAGGTTTGCTTGCAGCGAAGCTGTCTCCG
ATGGCTCAAGAGCAAAATGGCTTTTGTTGCTGCAACTTCTTCATGGCTATCATTTTGCTG
CAGATTGGGTTGCATCGGTAGAGTTACTAGACACCCTGGCAAGCCAACGACTTCGTAGT
TCTATGCAATTTCTTTTAAATATGGCATATTTCACGGCAGGGTCTGGGGTTGGCAATTTAT
GGTTTGGTGTCTTATACGAGCAACAAGGGGGTCACACAACTTATACCAGGGGTCTCTGG
TTTTGCTTGATAGATTTCGCTTTCATTTCGGCATTAAAAGCGTACAATGCGCGAGTCATGT
TTACCAGGAATGATCCATTAGAAGTGGGGATGTCAGAACATGTAAGCAGCAATCCATGAT
TGGGGTGATTAATAAAAAAGAAGAAGTCTGACTGCGCAATACATGTTATCTTCTTCCATC
AAAAACTCAGCCCCTGATGAGACTGACATGCCAGTAATGATAGTTGAGGTTGAGCAGAG
CAAGCACTACAGAAGGAGTACCTAGGTGCCTCAATCCTCAATCATGAGACATAAAAAGT

CTAGCTGCTTGTTGTAAAAAAATCAACATGAGTTCTCAGGGTAGGGTCCGAACGGACAA
AGGCCAAACAGTGCCCTACTCATTCAGTCTTAGATTGATATAGGGCATCAACTCAATAAT
AGGCATGCCCCGCCGAAGATCCAACATAAGAACCTTTGTACTGTGTAATTTATTAAACTC
AACACACTATACCTTACTTCGGCATGGTGTTGTGGATTTGTAAAGCCATAACCTATCAGC
ACTCACCAGCACACAGTATTGGTCTTTGGAATGAATAATAGAGCTATGTGCTAGTTATAC
CAAAAATTGAAGACTCCGCTCAAAACAACAGGTTGTTCAAACATAACCTTCTTCGCACT
TCTGCGTTTCACCTTTTCCGGCAACTCGTTAGATTCATACGCCTCGTGATCTTTCGCGCAC
CATATGGTAACATGCGGGAACGTATTCAGCGGCGCAGGAAAGGAGGAAGAATGCGAGT
TGTTGTCGTTGTTCTTCTTCTTCGGAATTTCCAACTCGATTGCGGCCGATTCATCGCTGTA
AAGCAAGGACACCGCTTTCACCTCGTGCTTGGTACCAATGACATGTTGAAATGTCGTTAT
CATACTTTCCTGCGTTGCTTGAGAAGCATGTGCAAACGTACAATGCAACGAAGTTATGAA
TCTATTCTGGCTCTTGTCATTTTCGTCGTTGTTGTTTTTGTTATGCTCCTCCCGATCGGCGA
AGTATTGCTCTACTTCCGGGAAGCTCTTCCGAAGCTTAGCAATAATTTCGGATTGCACCG
CTTTGCGATCGAGGTCCAGCGATGCAATTTTGATTGTTCCGGGCTTATTATTCGCTTCGGC
AGCGGCCAGGATTGTTTTCGAATCTAATAATTTGTCTGGCAGGGATGCAATCACTCTGGA
CAGCTCTGAGACAAATACCGTTTTAGATTCCTCCAGCGATACTGTCAAATTCTCAATATAA
CTTTGATTACCGTGAATGGACGAACGAAGTCTTTTTCCATATCAACAACGGCATCCTTG
CCAACTTTAAAGTTGTACACATCCTCTCGCGTTTGCAATACGATGGCGTCCTCAAGAGCC
ATCTTTAGATCTTTCGGTAGATCCGGAGGCATTTCTTCTCTGAAGAATGGAGTAGTCACT
GCTACAATCTCCTTCCCTCCTCGATCATGACCAATACCCAGCAGCTTTTCCTTCAACGCC
GACGTAGACATGTTACGATAAAAACAATAAAACTTCACGACCACCATACAAGCATTCTC
GACTGCCGAATCGAGTTTGCCCTTGTGGGTATTCGGCGGACGACGTAACACACGAGCTA
TGCAGACTGCCAAGTGTTGCAGAGAAAATGGATACACGTGCGATGATTTCGATGCCCCA
TTAGTTCCGACTACCAATGTGTCTTTCATTCCCGAAGGAAGGACGGGCAGCGCGATGGA
CTGATTCCCCTCGATGCACAACGTGTGAACAGCCGGAAAGGAGGAAGGGGGGACGTTC
TTATCCAGAATCAAAATTGAATCGAGGTTTGTCAAAATTTCCTTCTGAGCAACGTTGAAA
AATTTCCCTTTCCCCCGTTTCTTTATTTCGTCTCCTTCCATCAGTATTAGCTTTCTATTGTC
GTTCATAATACCGATGGATGTAATGTTTTGACATAATGATGATTTGCCCGATCCTGGTAAT
GAAGGAAAATAT

Prostaglandin E synthase 2-like_mPGES-2
>TR25058|c1_g1_i2
AGGCGGAGAAAACGGAGAGAAACCCGCTATCACGCAACCATCATTGACCGAACGACGC
AAAAGACGAATAGAAGAACAATCATTGAGAACTTCCAACTTCCAGCCGCCTCTATCATG
ACCATCTCCAAAGTCACCCCAATGGCGGGCCGCATAATCGGTAGGCACTCTTTTCTCGCG
ACATCCAGGTCATCAACGGCGCTCATCGGATTGGAAACACCTTCGATATCAGCCACTCTC
CCATCTGCAGCAGCATCTCCATCATCGCGTCCAGTGCGTGCCCCATCGACTTTCAATTTT
TGTTCAACTTATTCTACTACACGATGTTTTACAGCTGCGTCATCAACAACAATCCAACCA
CCCGAGATAAAACTCTACCAATACCACATATGCCCATTCTGCAACATTACCAAAGCACTC
CTATCCTATTCCAAAGTCAACTATCAATCAGTAGAAGTCAATCCATTGACAAAGGCAGAG
TTAAAACCATGGTCGGGAGATTACAAAAAAGTTCCGATTGCTTTGATAGATGAAGGACA
AATCAATGGCTCGGATAAAATTATCGATTCTATACTAGAGTCGCCTCATGTCCAAACTATA
CTGGAGGCACGATGGGCAGAACAGTGCAGTGGCAGTAATGACGACAACGAAAGAATGA
CCATGCAACAATTCCACACGTCCGAAAATGTCCAAAAATGGCTTCAATTCGCTCGTGAC
GACCTAGCCGCGATACTGTATCCTAATATTTGCGGCACTCTAGGCGATTCTTACGATGCAT
TCGGATACGTCAATAATGTGGATTCATTTACGGCAATGCAAAAAACTTCCATTCGATGGA
TGGGTTCGCTGGCAATGTATTTTGCAGCGTCCAAGATTAAATCAAAGCGAAACATCACC
GATGAACGAGCAAGCCTCCATGAAGCTCTCGATAAATTCGAAAGCGAAGGACTTCAGGA
TGGAAAGATGGCGTATTTATCAGGAGTGGATTGTCCGGATATGGGGGATGTGGCTGTGTT
TGGCGTTCTCCACTCTGTGAAGGGATTGAATGCGCATGATGATGCAATTGGATCACGAG
GGGGTCACGTGAAGGATTGGTATGATAGGATGCATGTGCAGGTGGTGGGAGAAGAAGC
AGAATAATAAAATACCTGATCATTGATCAACATTAGAACGGATTTCATACTTAATCGGATG

TCATGAACTCTGGTGCAACGACTCTATGGGACTGTATTCTGAACAATCCAATTATCGTTCT
CTCAAACTGTATTGGTACGCCATCGTATCAAGAGGAAAGATGATCAAATTGGAAGTGGAT
AAAAGTATCACATACCTAATGGCATCACTTTAATCGCAATGATTCCAAACAGGGCAAGTT
ACGATTAAAATTAGACGCCGAGTTTTGCAGCAGAGAGTTCATCGCAAAGAATCCTTCCA
TTTTATCATCACACATCCCTGCCAAACTCTCCTAATATGTTACCAATTCCTTCACCTTTTCT
CTATCCTCACCAACGCTGTCATCCCTCCAATCTGTCTCGTGCAACTCTTCTTCCTTCACCT
ACAAAATAATCTACAGAGGCATCATCACTGCAACTGTTGTCATTTTACCAACCAAGTAAT
TATTTGGAAATGTGCGATTGAGTAGGTGGTAGTGATGTGTAATCCATGGTGAAAGT

Prostaglandin F synthase_PGFS
>TR60026|c0_g1_i1
GCTTTTAGTATCGTACCAACGATACCATTGCAATTACCATCATTGGCGTTGTGAACTAACA
CCCAACCATTACTCCTACGCAGCAACAAAATCTATTGCGCATCCCGCTATCCCTTCGAAA
TGAAGCTATTTATAAAGTTAGCGATAGCGCTGACGCCAGTCGTAACAGCTCTCCAATTCC
CATCGCCCACTTCTTCATCCAATGGTGACCAGATATGTCGGCGATCGGCTGTATCAAAGT
TGATCACATCCTCCACAGCGGCCGTGGCATCTGGCTCGGCTTCCTCTCTTTTGATGCCAA
CAGGTGTGGCAAATGCAGTTGATTCTGTTACTCAAGTAACTTTGAATAGCCAAAACAAA
AAGCCTTTTCCATTGGCTTCTTTCGGTTTACAAGTTTATAGCGATGATATAGCCTATAAGC
TGACTCTTACTGCGCTGGAAGTGGGATATCGAAACTTCTTTGCAAGTGTGTTGGCAAAC
AATCAGAAGGGATTTGCGAAGGCGGTGAAAGCGTCTGGAATCCCTCGCGATGAGCTGTA
CATATGTGGAACTGTCCTATCCAATCGTGTTAATGGAGAAAAAGCTGCATACCAAAAAA
ACGAAGCAGGGCTGTCTCGAAAACATGGCAGCAATGGCCGCTGGCAATATTGACAAGC
TCGACATGATCATGCTTGATTATCCTGGGCCAAATGATGAGAGCGTCCGCGGTCAATGGC
AGGCATTCGAGGAAATGCATTCTAAAGACGGAACTGTAGATGATTTGGCAGTGAGCAAC
TTCAGCCCAAATCAACTCGACGCAATATTGGTAAACAAGGATGCGATTAAGCCAACCGT
CAATCAGCTCCCGTTCTCCGTCGCATATCACCCCGAGGGAATTTTGGACTACAATGCCAA
ACGTGATATATTGGTGCAATCGTGGTCTCCCCTATCGAGGGTTCTCGGGCGCTATGGAAAT
ACCCTGGATGCAATTGGGAAAAATTATGGAAAGAGTGCAGCGCAGGTGGGACTTCGGT
GGATAGTGCAGAGTGGAGCTGCGTTTTCCACGCAATCGAAGAGTAAAAGCCATTTTGAA
GAGGACTTGAACGTATTTGATTTCGCTCTTTCTGATGACGAAATGGCGAAGATTGCTCAA
TTGGCAGCTGCTTGAGTTTTGTAAGAAAATGCAGGAGCAAGTTCAGAGCAGGGCGACT
GATCATTCTTGATCCGTAGGTACACTAAATCCGTACCGAAATTTAAGATAATGTTTGTACT
TTCAAGTTGAGTAAGAACCCCCTGATCAAGCTGACTGAGCGCTGCTATGTCTGTGCACA
CGTCAACCCTAAAGCGCCCATGAAAACCAATAGATTCGCAAATAAACGCAAAACTGAGA
ATCAAAAAAATAATCCGAAGGTACTGAGATCGGA

Prostaglandin G/H synthase 2_PGHS-2
>TR754|c0_g1_i1
TCCGATCTGTGATTTCAAAGTTATCTTCAACCATTCCAACATGAAGGCTGTGCTTATTAAC
GACAAGAGACTCCCCGAAGATTCAGTGGAGTACACCTACCCCCATTCCAAGTTGACCCA
TTTCAAGGAAAATATCCTCCAAAAGACCTTTTCGGCTTTGCTCAGGGCACCCATTGTCGG
CAAGCCACTTAACCGCAAATTGACCAACAAAGCCGCAAGCAGCGGAACCAACCGCCCC
TATGTACTCTCCTGCAAGACTGACTACGCATCGTATGATTCTCTCACCGATTACTCACACT
ATGGAAGGCACTTGAAGCCCGCCTCGAAGGATTGGTTGGAAAGCCTTCCCCCCATCGAT
GACGTTCTCAACATGTTCGAGCGCAGAAAAAAATAAGGAGGGTAAGGACGTCCAGGTCA
TGTGCCCCAAGAGTACCATGCTTTTCCCCACCTTCGCGCAGCACTTGATTGATTCCTTCA
TTGTTACCGCCATCAAGGAAAATACATCTGAAGGTGTAGAGTTCGACTGGAGGAAGACG
GGATCCCCCCACGACATCGGACTTCTTCCCCTATACGGAAAAACCATCGAACAAACTAC
CCAACTCCGTTTAAAGTCCGAGTTGAAGGGCTCAAAGGGAAAATTGAAGACTCAAATC
ATCAAGGGAGGAGAATGGTCTCCATACCTCTTCGACGCCAAGGGAAACAAGAAGCCTG
AATTCAGCTCCCTTCCCGACCCTGAAGGATTGGAATTTGCCTTGGGAATGGCTAAACAG
CATGGCGTAGACGTCAAGAAGAACAAGGCAACTGTCTTCGCTCTTGGTGGAAGCAGAG

CCAATCTTACTCCCAACATCGTGGCATGGAATACTTTGTTGCTTCGTGAGCACAACCGTA
TCGCATCGAAGATCGAGGAAGAAAACCCCAAGTGGGATGATGAGCGTGTGTTCCAGAC
CGCGCGTAATGTGAATCTTGCTGTGTACCTCAGACTTGTGATTGAGGAGTACATCAACCA
CATTACTGGATTTGGTGTTGACTTTACGGTGGAGCCAGGGAAGTGGATGTGGGATGCCC
CATGGTACAAGAGGAACTGGATCAGTGCCGAGTTCGCTGTCCTCTACCGCTGGCATGCT
GTTATCCCCAACTGCATGAAGTGGGGTGAGAAGACATTGCCCACTGCCTCATACCTTTAC
TCAAACCACCTTCTTCTTGATGAGGATGGTATGAAGGGAGATCTTCGCGAGGCATTCATC
AATATTAGTGACCACCGTGCGACTAGTATGCAGATCCATAACACAGAAAGTTGGATGAA
AGGTCGTGATGCCAGGGCACTTCAGATGAGTCGAGCATGTGAATTGAGATCCTTCACTG
AGTACTGTGCCTATTTAGGAGCAAAGGTTCCCAAGACATTCGCCGATATCACCTCTGATG
TGGAGGTGCAGAACGAGTTGAAGGCCCTGTACGGAAAGGTGGAGAATATTGAGTTCTG
GGTCGGTTTGATTGCGAAGGACCATCCCACTGAGTCTATCATGTCTGCGGAGTTGACATC
TTTTGTCGCGAATGATGCCTTCAATCAGGCATTGACCCATCCTCTTCTTTCAGAGCATGTA
TGGGCAGCTGGACCCGAGACTTTCTCGAAGGTTGGATGGGAGATGGTAATTAAGGTGCC
ATCAATCAAGGACATGCTTCAGAGGAACACTGGAGGAGCCCCAATTGAGGGTTTCGTTG
GTATGACAAATCCTCACTACAAGATTCCTCTTTCTGAGATTACCACAAAGATTCTCCTTAC
CGCTTTCGTTGTCCTGGCCGGTGTTGCTTACAAGGCTTACACTGAAATGTAAAAGCCTTG
TTACACCAAATGCATCCATGTCTCTTTCACAGTGTACTTTTGAACTTCTCATGATGCATGA
TTTCAGGGAGAGAGACAACGAATGGAACAATAGATGGGCACGTTTTTGTTTGAATGTTG
AAAAACCTCTAGGTAGTGAAGTAGGTATCGTAAGCAAGAGTATTAAGATACTATTCGATC
TAGGAAAAAAAAAAAGATCGGAAGAG

Secologanin synthase_SLS
>TR26037|c1_g1_i1
ATTTCTCTCGTGTTCTGATCCGTGTCAATAAGGCTTTGTTCTCTCAATTAAGAGTAATCCA
GTAAGCTATGGCGGTCATAATTCCCAGATCTGCCGATTACAACTTCACCCATAGTTAGAA
GAAAATAACAACGTCATGAACGTTCTGAACCGATCCGATGCACTTTGCAGTGCTCCACA
AGAAGTAGTTGCTTCAATCAATATTCCGCGACTGAGTGACACACCCATCCTGGTCAACAT
TCAATCACCGTCCGCGTCAAAGTCTAATTCGAATGGCTCAATCGCACTTCAGGCTCCAAC
ACTTCATTATTCCATGCTCTCTATTCCGCTACTACTTCTATTTACCATAGCACTAATCGCCG
CGACGCTTCGAAAACTCCTACGGCATTACATGCAGCAGCAACAGCCCCATGTCTTCCCA
GCCTCGACAGGATTACCCCCCTGCGACGTGCCGTCCCATCCAATTTACGGACATCTGTGG
CATGTCCTCAGCTCCCCCGACAGTGCCAAGTACAATTCCATATTTGGCGATCATGCGAAC
TCTTCTGGCTTGTCGACATTACGGTTCCTGAATACTCCTTGTATCGGCGTTTTGAAGGCC
GAGCATGCGAGGTTCGTTCTTAGGAATAGCATAGAACGGACTGGGACGAGCATGTTCTC
TCGACATTTTAAGAGGGCTCTGGGCAAAAATTCGATAGTGATGATTGACTGCGGAGAAA
CCAGCAAAGAGTTATGGAGGACTCACAGGAACTTAATCAAGGTGGGATTCACCAAAAA
TGCTGTGAATAATATGGCCAACAAGGTTTGGCAAGTAGCCAACGGGTTCACTTCTTCCTT
ATTGCGTGAATGCGCCAAGAATGATCAAGGTACAAAACAGGGGTCGTATTGTGCTGAGG
CAGCAGATATCTTCAAATGGGTGACCCTCGACATCTTTGGCAAGGTTGCCTTCAACTACA
ATTTTGGATGCACCGATTCGTTGTCAACGACTCCTCTTGCACACTCACTCAACTATACAC
TCGAGGATAGCAATACTCGATGCAAGGCGGCCAACTTACTCAATCCTGCGTATCAACTCT
ATTGGCTTCCTACCAAAAGAAATAGAGACTACAAGCACCACAGCCAGAATGTTCGAGGC
CTGATGAGAGAGCTCTGCCAGCAACGGGCGCAAGAGATAGACAACCAAAAGAATGTCC
CGGAAGGCCATACGAATCTACCGAAGAAAGATGATCTCTTGACCATTCTTCTCAAATCAA
AAATAGAATCCCAAGGCGCCCAGAGGGATGGCACTCACGATGAATTGGTCAAAATGCTC
CTGACAGTCTTCTTTGCAGGATTTGATACATCATCCGTCCTACTCTCCATGGCCATGTGGT
CCATAGCCACAAATCCCAGTATTCAACAAGAATGCGCCATGGAGGCGCAAGACGCAAGC
AGTACTTCCTCCGAGAACGAACCAAGTCTTCATGAGGATGCCTCACATTGGGAGTCGAG
ATTAGCCTACTGCCGCGCAGTAATCTTGGAGACGTTGCGGTTGCACCCTCCGGTGCATAT
GAACAGTCGCATTCTCAACAAGGATGTAAAGTTGGGCGGATGCACCATACCAAAGAGG
ACGCGCGTGTACCTCCCAATAACGCAGATCCATACAGATGAGAGGAACTTTGCAAGGCC

AACAGAGTTTTTGCCCGAACGTTGGGTTCGTAGAAATGCGACCAACAACAGATGGGTTG
CCCGAGACTACAAGACAGAACCATCATTTGTAGAGAATGACCTGTCGTATATCCCTCCAG
CCAACCCCCAAAATATATTTACCTTCAGTGATGGTGCCCGTAACTGTGTAGGACACCGCC
TGGCCCTTCAAGAGAGCACCATGATATTTGCATGTGTTGTACGGGATTTGACTGTGAATG
TACCGAAGGGCTTGGTCATGCAAAAACGAAAAAAGTTTGCGTTGGCGCCTCCAATGGA
GATGCCACTGATTTTAAAGAGAAAAGGAAGGCAAAGCTAAGAGACTGAGAGAGGAAAT
CACATTTTTTTGGTTGGCCTAACTGCGAGTTGATCAGGCATGATCTATGATCTGATGTGCT
CTCCAGTTGCTGCTGCCACGACCCTTCCCCAGTTTTTCCACAGGGCGTCCCAATTTTGCT
TTGTGTATATGTAAATAGATATTTTCTCCAGTTGTTGTAGCCAAGGCTCTCTCTATTCCTTG
CTATCCCTCATAGGGAGGTGTAAGTTGTCTTGATGTATAGGAGTAGTTACTTAGATAAGTG
TAACAATTAACTTGGCCTGAATAATTAAAACACACACACTACAAAAAAA

Phthiocerol synthesis polyketide synthase type I _ ppsC
>TR40329|c0_g1_i6
ACAGCAAAACCAGGATCAATCTATGCCAATGTACTTGGTACAAGTGTCATGTCTGATGGA
AAGTCAGCTTCAATCACAGCACCCAATGGCTCTGCCCAGGAAGAGTTGATCAAGAGGG
CTCTGGATGTGTCAGGCATCAAGCCCAATGATGTAGATTACATTGAATCTCATGGGACTG
GAACTCCACTTGGTGACCCCATTGAGGTTGAAGCTTTGGCAGAAGTTTTCGCAAATTCA
AAGACTGAATCACATCCATTGATGATGGGCTCAGTGAAGAGCAACATTGGGCACCTAGA
AGGTGCAGCTGGTATTGCAGGTCTGATCAAGGCTGTTTTGGTACTAGCTCATGAGCGTGT
TCCATCCAATGTTGGCCTGAAGACATTGAACCCTCTCATTGAGAAAACAATTGAATCTCA
TTCATTCCACATCAAGTTTCCTACAGAGTCTCAGCCAATTATGAAAGAAGAAGATGGAG
GGCTTTTGGTGGCAGGAGTGAGCTCATTTGGCTATTCAGGCACAATTGCCCATGCAATCA
TTCAACAAGCTCCAGAGGATATGCGTCGCTGTACTGCTAATGCGAACCATTTTGGTTGTG
AAGTTGATCATAATAGTTTAGTGTTTTTGTTTACAGGTCAAGGCTCTCAATATGCAGGGAT
GGGGAAAGAGTTGTACCACAAAAATGAATCATTTCGTGAAGCTATGGATAAATGTGAAA
GCATATACAAGTCCCTGGTGGATGGGGAATCTCTTCTGGATATAATTTTTAGTTCAGGAAA
TGAAGGACTTGTGACATCAAATGCTCAACCATCTCTGATTGCCCTTGAGTGGAGTCTGG
CCAGAATGTGGCAAACAAAAGGTGTTACTCCCAGCATTGTGCTTGGTCATAGTGTGGGT
GAGATAGCTGCTGCCTGTGTTGCTGGTGCAATGACAATTGAAACAGCATTGAAGCTGGC
TGTTGCTAGACAAAGACTAGTGAATCATCTTCCTCAAAATAATGGCGCAATGGTTGCAGT
GAAATGCAGCAAGGAAGAGGCTAACTCTACAATATCTTACTGTTTAAGTGAGGAAGAGC
AAGATCTTGTTGGTGTAGCTAGTGTGAATGGCCCCAACAGCATTGTCCTCTCAGGTGTAC
ATGATGTATTGGAAAAGCTGCTTCTTGAGCTAGAAAGGAAAGGTGTTTATTTACAGGTAT
CCCATGCCTTTCATTCGCCTTTGATGAGAGGAATGGAGGATGATTTCAGGCGTGTCATTG
GTTCACTTGACCTCCAACAGCCTCTATCTATTCCACTTGCAAGCACAGTGACCGGTCATG
TTATGACTCCTGGCAAAGTAATTGACTTAGAACATTGGGTGCAACAGCTTGCTTCTCCAG
TTCTGTTTGCAGATGCCTTCACAGAGGTCATCAATGGGAACAAGTGTGACATTTTAGTTG
AGGTTGGACCAAAGCCAATTCTATCCAAGATGGCTCAAAGCTGGTGGAAGCCAAAAAA
GAAAATTTCTCAGCCGTTGTGGGTTGCTTCTCTTGAGAAAGGGAAATTGTCGAGTCTGG
AAGAATCTTTTTTAGCTGTCGATGAGGCACTTGGCACACCGACTCATGCGGCGTCACCA
AAACTGTGTAGTGTCTTCCCCAATCGTACAAGGTTGCCTTGGCCTGAAAGCCCACCCCA
CCCACTTTTACAGCTTACTATTCCTATTGGATCACATGGCACAGAG

Polyketide Cyclase dehydrase and lipid transport protein_ PYL5
>TR3754|c0_g1_i2
CGCAGTCAATGAATCATGAACGGCACGAACACATGGCGCATGCATGGAACAACAGACGT
AATCAGACAAAGAAAATAACAAAGAACAATGGTGACAACTGATAATATGAGGCAGCAG
GGCAGGAATGACTCTGACCAACAACATGATGACACAGCAAGTCGAAATACGGGCGCAA
TAAGTTTTATCATTGCACCCCAATAGCTGAAACCCCCTTCTTCATAATAAGCTCCCAACCT
CATTAACACCAGTACAGCGGTCCCGTACTTACTTTTACATTAGAAAAACAATGAAATTCG
TGTGTCTGATCCTAGCTCTCTCAGTACTTAAAGCCGATGCTTTCACTACGCCATCGCACC

CTTTGATTCCATCCAAAAATGCAGCCCATAATAATATCGTCACTCAGAAAACACAGCAGT
CATCCACGTCCCTAAACGTATGGTGGTTCGGCGGACCCCTGGGGCAGGATCAGAAACG
TCCACATCTGGCGAAGAATGCGAACTCGTCGCCGTCCGAATTGACCGCACATCTGCTAA
CTCGAGACGCATCGCTGGAGAGATCGTTGTAAACGCCCCTCCGATCGATGTATGGGCCAT
TCTCACTGACTACGACAATTTAACGACTCATGTTCCAAATTTAGTGAAAAGTAAGATAGT
TAGTGATGGACCCGGGGAAGCAGGAGATGGATCACACAAGTGCAGATTGTATCAGAGA
GGAGCTCAGAAAATTATAGGATTCGAGTTTGGAGCAGATGTAACCATGGACATGACAGA
GAAGATTATCGTGGGTGGAAAGTCGGGTAAAATTACAACATCTCAAGATACAACCTTGG
GCAATGGTGTGTCCTCGACTGGACTATTTCCCGAGGAACGACGTATTGATTTCAAGTGCG
TGGATTCACAGTTCTTCTCAGAGTTTGACGGTACATGGCGCGTGGTACCGATGCCTGAC
AATCCCTTCACGGGGGAGGCTGAAACGACTGTGAGCTACACTGTGGAGGTAAGGCCGA
AAGGGCCTGTTCCAGTAGCGGCACTAGAATGGAGAATACGAGAGGATGTGCCAACAAA
TTTAAGGGCAGTGAAAAAGGCTGCGATGGAGGTAGGCGCAGAAGGGGTCTGGTCAAAG
AAAAATGGAGGCAAGATTGGAGATGCTTTGGCGAAGTTGACGGGCGGGACAGGGCACG
ACGAGAGCACGCCACAGGGGAACGAGTTTGCTGATTTCATTCTTGATTATGAAAATGAG
ACATTAGGGGCGTATTTGGACTGAAGCTCACATGCGGATAAGAATTTTGAGTTACTTAAC
TTTTACTGACAAACCTGAAGGTATTGGAAAAATGTTGTGATAGAATAGTGTAAGATATGT
GAAATTTTACGAGCGCATCAGCAACGATGCGAGAGAGAGTTAGTTGTGGGGGAGTTTGT
CCTATGATATCTTTCCTGACCACAAATTGAACCACAACTGAGGCGCTTGACGAAAATAAG
ATTTAAGAGTTGTATCGCTATGATGAACTACAGATACATTCCGACGAAATAGGTCACACT
GTTTATTTTAGATGATACTTGACGCCACACAAAATGTATTTGTGACCCCTGAGCGTGATC
GAAATCAAGTAGCTCGTTAGATCCAGCTTGTCAATTTCTCTCCGTTTGTACCTCTGCATCT
GTTTTCCATGAGTATTCTGTTTTCCGTGAGTGAATAGTACCCAAACCGGCACAGAGTTTA
AACACTGTATCCCCCTTATCTTTTTTTCGCTCCAGCTTTTACTTTGTTCATAAGTAAGTAA
GATCCTTCAGTCCACCAGCATTCATAACTTTTGTATATCCCTTCTGTTCAAGCATTTCCTTC
GCTTTTGCAGCACGGCGTCCAGAACGGCAGAATACGATCACAGGAGCTATGGTAGGATG
CAAGATGTATTGTTATGATGAAACATATGATACGAGGTTCACAAAATAAAATACATAGATC
TGATCTGACCACCATTTTTAATCAACGTACCATTTTTGTCGGGCATGAGTTCTTCAGCTTT
TGCCATCAACTCCGAACAATCGTCTAAATGGCAGCTCGCGTGGCGGAACTTCCTTGGAG
TGAGCTGTTCCTCTTCCAATTCCTCTTTGGTTCTCACATCGAGAATTATGGCGCCAGATAC
ATCGACGGCGGCTTTTACTTCGTCAGGCGTGCTGAAGGATGTCATACTTTCGGGTTTGGG
CCAGATCCAAAGTTGAGCAGGAAAAGGAGATGTGCAGGCTGGGTTGACGTCTAGGGCG
TCGAACGAGATAATTCGGTAATAGGAGTAGACGACAGACTGGCCCTCGGTTCATGAAAT
AATGTGACATCACCGTATCGCAATGCCATTCTCGATTCTCGGCGGCAATTTCGATTCTATT
TCGGGT

Polyketide Enoylreductase_PKS_ER
>TR30039|c0_g1_i1
TTTTTTTTTAAAAGTAAAGTAAATTTGAAACGCTTAAATCGGAGCAGTATCTACTTGTGG
GGTTCATTATTGTACACTTTTAGGATACCGAGACTCCTATCATTATCATCCATCTCCATTCT
ATTCGTGTTGCCACTTTCATTTGTTGCAGAAGGTTGGTACTATCACAGTGTTTTGTCACAT
CTCCTACAGATTACTCTTGACGTTACTTTCTGTGAGGCTATGGTCCAATACAACCTTTCCA
GAAGATTCTCCCTTCAAAAACTTCTTCAGATAGTCGTCCAGTTGATGTCGTCCGATACTG
ACAGCAGAAGCCTCTGTCTTGGGACAAGACCACTCGCCCGCCAGCTTCTCCCAAACAG
CTACTTTCTGTAGCAAGGGAATCTCGACCGAGTCCACTCCCAGCAAATTGACGCCTCGC
AGAATAAAGGGCAAAACAGTAGTATCCAACCCCATGCCGGCCACCAAACCGCAGCAGG
CCACCGAACCACCTGGCCGAATTTGTTTTAACAATTCTGCTAGGGGACCTCCACCCACG
GTATCTACGGCATGCGCAAACAGCGGCTTGACCAGCGGTTTCTTGTTCGCTTCCAAAGC
TTCTCTGCCCACCACTTCCGTTGCGCCCAATTCCAGAAGTCTGCCTTTTTCAGATACTTT
CCCCGAAATGGCAACAACTTTGAATCCTAGTTTGGACAGCAACTCCACGGCCACGCTTC
CCACGGCGCCGCTGGCCCCCGTCACGGCCACCCGGCCTCCATGTTGGGACAGATCG

Polyketide hydroxylase-like_ PK_Hxl
>TR21208|c0_g1_i1
TCGAGGAACGTCGCGTTGGTGGGGGATGCGGCGCATTCGTTCCCTCCCGCGGGAGGATT
CGGGATGAATACCGGTTTGCAGGACGCGCACAATATCGCGTGGAGGCTCGCTTTGTTGC
TGCTCGACGGAGGGAGAGGGAATTCGTCCGAGGCGTCCTTCTTCTCGGAGGACGTGCT
GTCCAAGTACGACGCGGAACGAAGACCCATCGCCACCCAAAACGCGGCGCTCAG

Unveiling the presence of biosynthetic pathways for bioactive compounds in the *Thalassiosira rotula* transcriptome

Valeria Di Dato*, Federica Di Costanzo, Roberta Barbarinaldi, Anna Perna, Adrianna Ianora, Giovanna Romano

Supplementary File 1:

Box Plot Analysis of the expression, in FPKM, of the pathways annotated on level 1 for each replicate in each condition

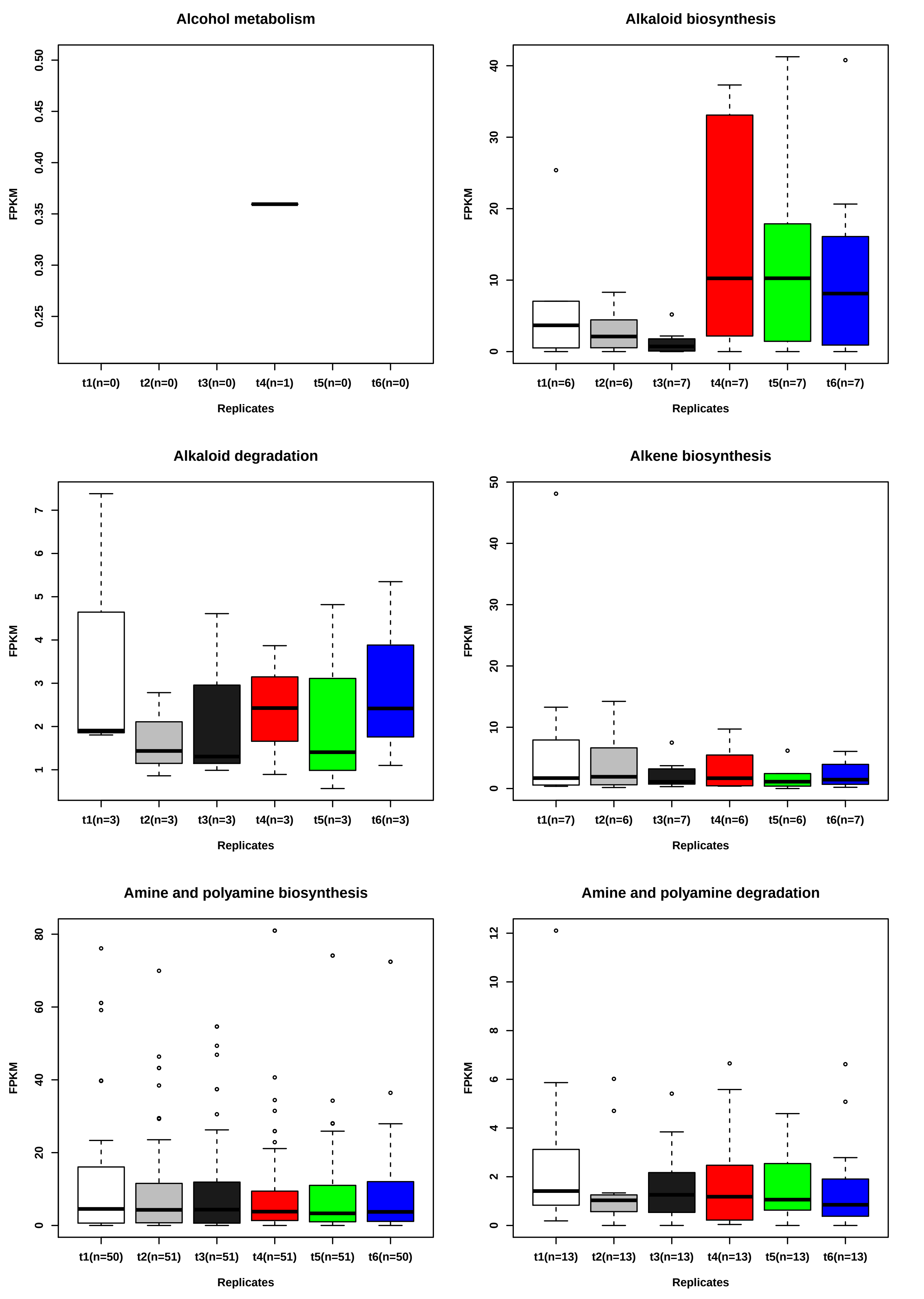

Alcohol metabolism
FPKM
0.25 0.30 0.35 0.40 0.45 0.50
t1(n=0) t2(n=0) t3(n=0) t4(n=1) t5(n=0) t6(n=0)
Replicates
Alkaloid biosynthesis
FPKM
0 10 20 30 40
t1(n=6) t2(n=6) t3(n=7) t4(n=7) t5(n=7) t6(n=7)
Replicates
Alkaloid degradation
FPKM
1 2 3 4 5 6 7
t1(n=3) t2(n=3) t3(n=3) t4(n=3) t5(n=3) t6(n=3)
Replicates
Alkene biosynthesis
FPKM
0 10 20 30 40 50
t1(n=7) t2(n=6) t3(n=7) t4(n=6) t5(n=6) t6(n=7)
Replicates
Amine and polyamine biosynthesis
FPKM
0 20 40 60 80
t1(n=50) t2(n=51) t3(n=51) t4(n=51) t5(n=51) t6(n=50)
Replicates
Amine and polyamine degradation
FPKM
0 2 4 6 8 10 12
t1(n=13) t2(n=13) t3(n=13) t4(n=13) t5(n=13) t6(n=13)
Replicates

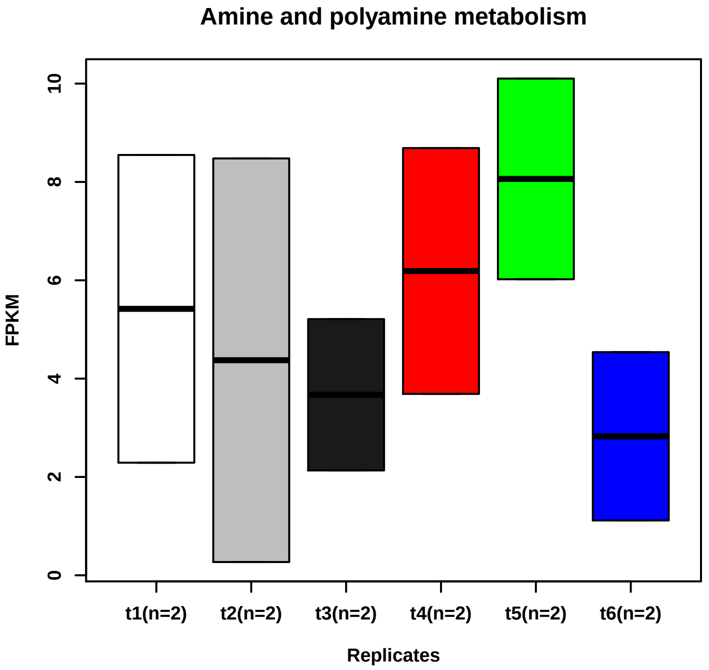
Amine and polyamine metabolism
FPKM
0
2
4
6
8
10
t1(n=2)
t2(n=2)
t3(n=2)
t4(n=2)
t5(n=2)
t6(n=2)
Replicates

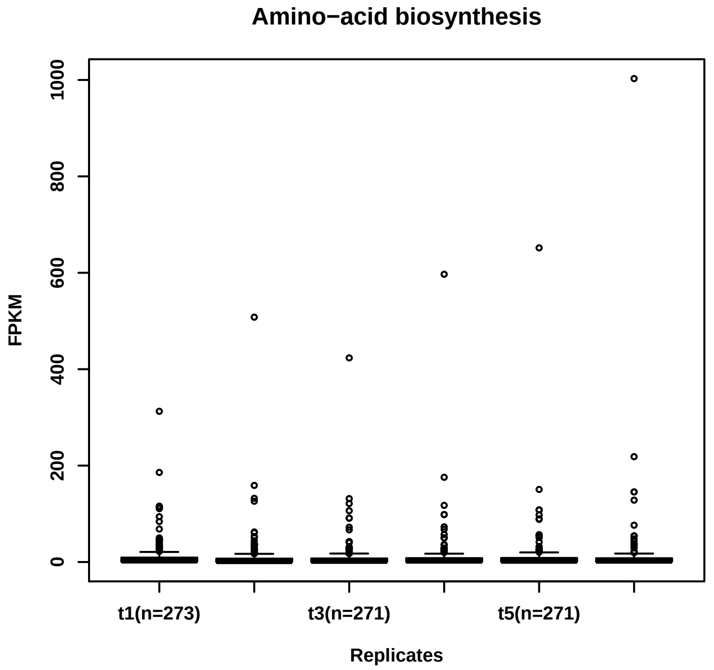
Amino−acid biosynthesis
FPKM
0
200
400
600
800
1000
t1(n=273)
t3(n=271)
t5(n=271)
Replicates

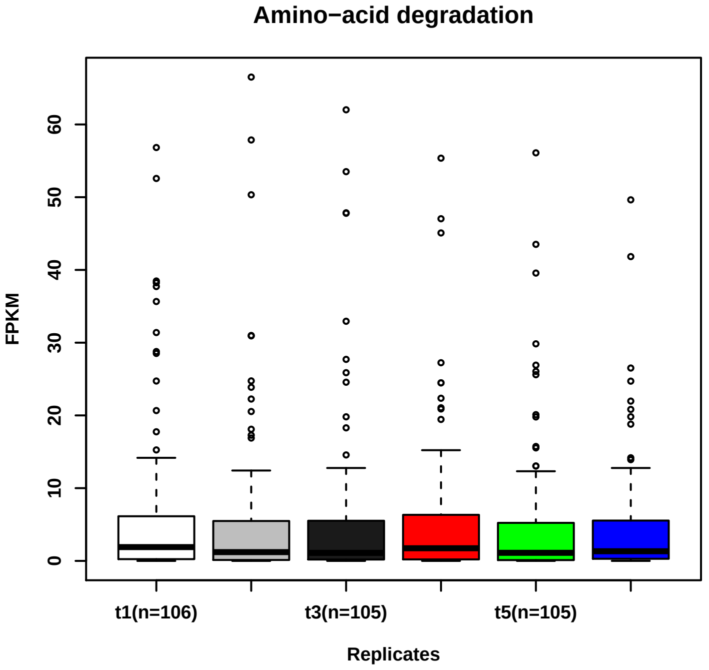
Amino−acid degradation
FPKM
0
10
20
30
40
50
60
t1(n=106)
t3(n=105)
t5(n=105)
Replicates

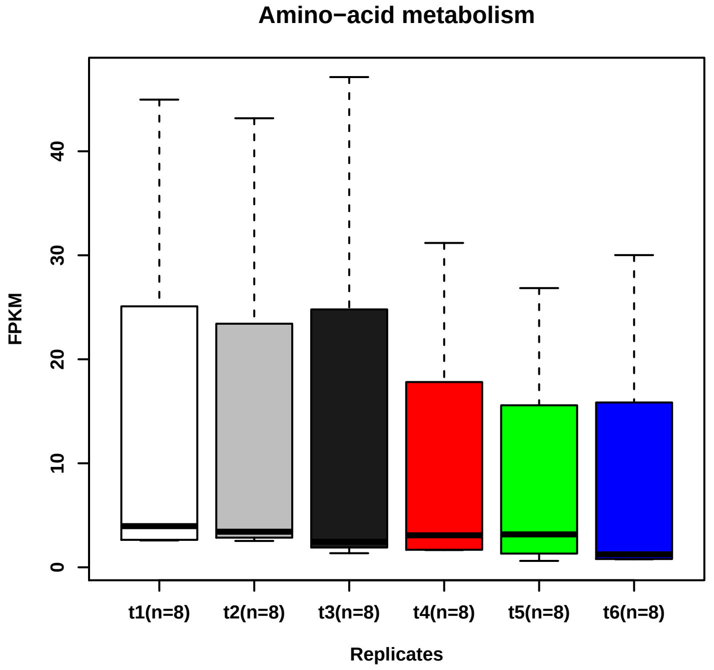
Amino−acid metabolism
FPKM
0
10
20
30
40
t1(n=8)
t2(n=8)
t3(n=8)
t4(n=8)
t5(n=8)
t6(n=8)
Replicates

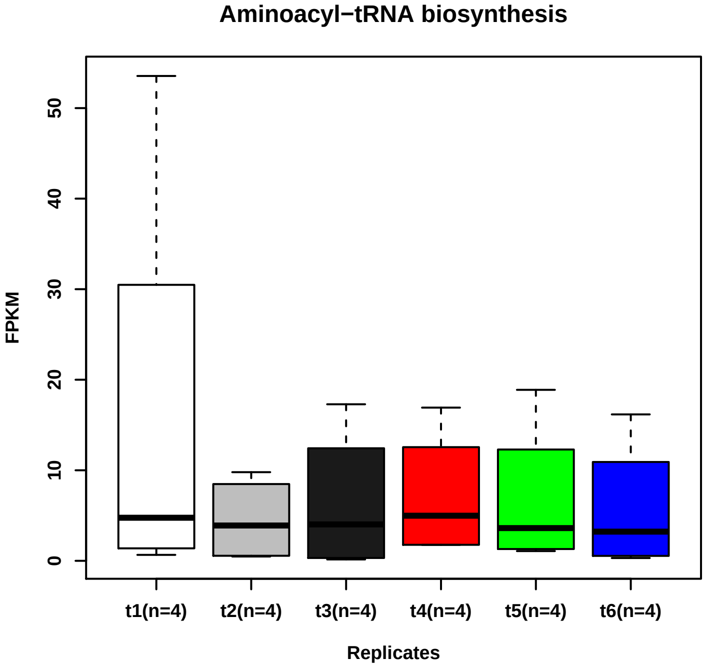
Aminoacyl−tRNA biosynthesis
FPKM
0
10
20
30
40
50
t1(n=4)
t2(n=4)
t3(n=4)
t4(n=4)
t5(n=4)
t6(n=4)
Replicates

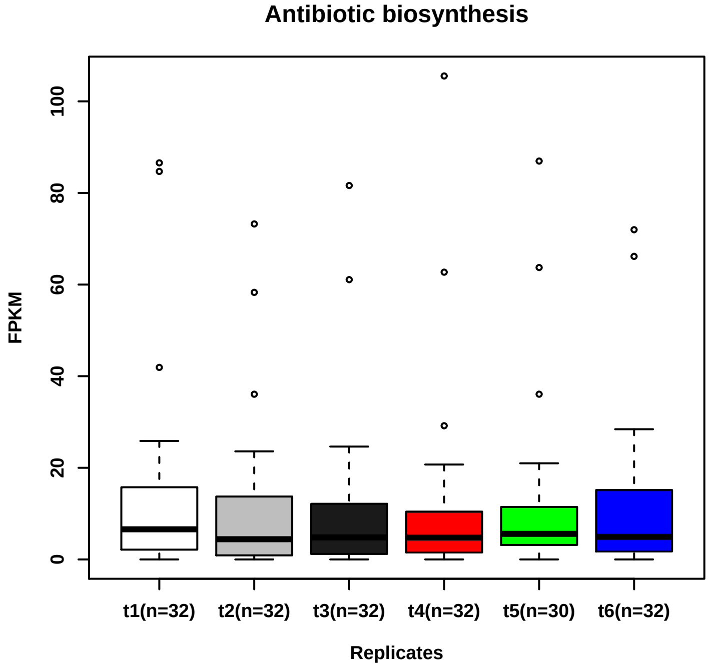
Antibiotic biosynthesis
FPKM
0
20
40
60
80
100
t1(n=32)
t2(n=32)
t3(n=32)
t4(n=32)
t5(n=30)
t6(n=32)
Replicates

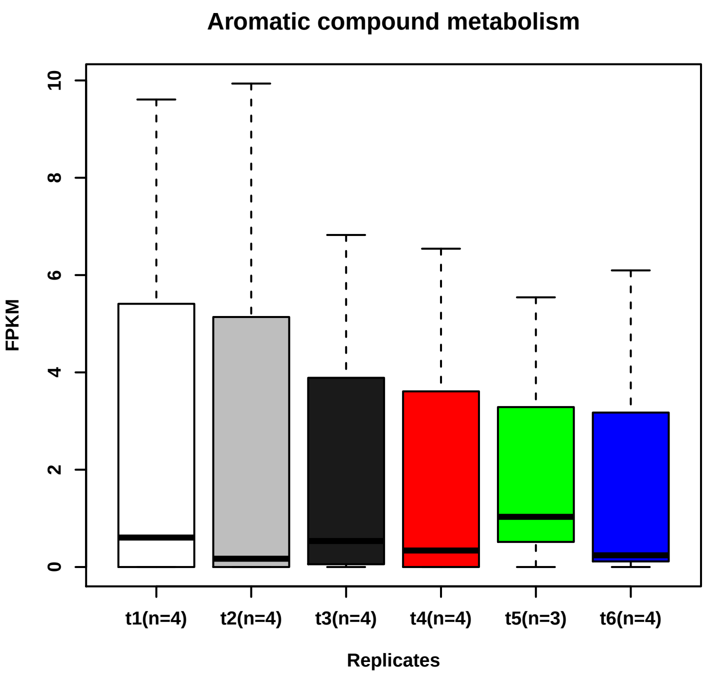
Aromatic compound metabolism
FPKM
0
2
4
6
8
10
t1(n=4)
t2(n=4)
t3(n=4)
t4(n=4)
t5(n=3)
t6(n=4)
Replicates

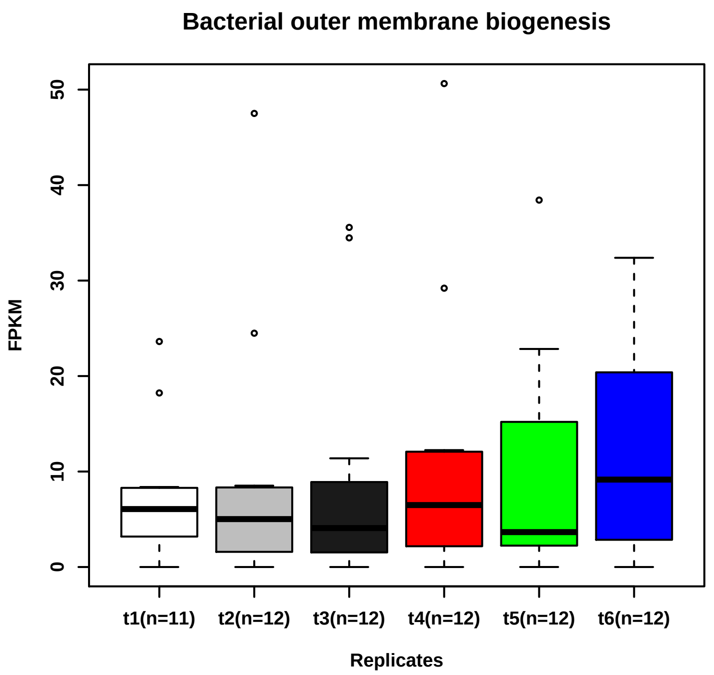
Bacterial outer membrane biogenesis
FPKM
0
10
20
30
40
50
t1(n=11)
t2(n=12)
t3(n=12)
t4(n=12)
t5(n=12)
t6(n=12)
Replicates

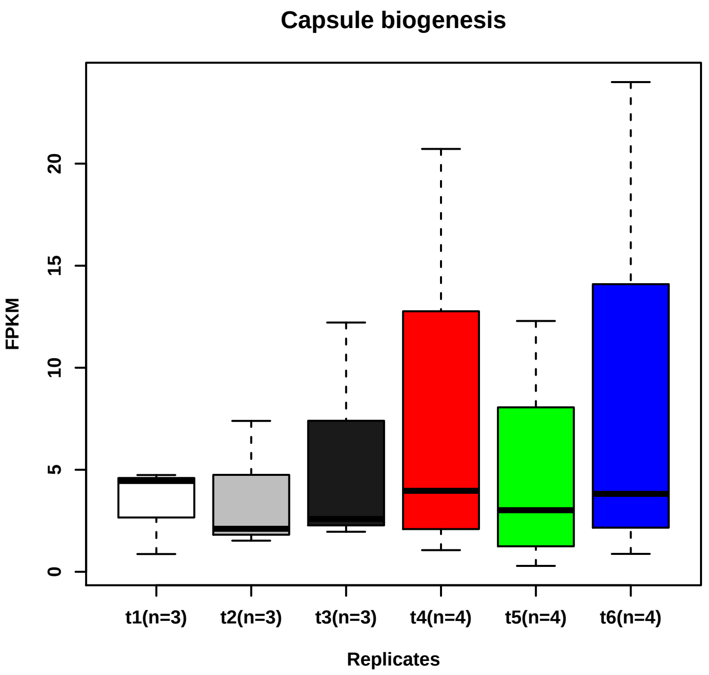
Capsule biogenesis
FPKM
0
5
10
15
20
t1(n=3)
t2(n=3)
t3(n=3)
t4(n=4)
t5(n=4)
t6(n=4)
Replicates

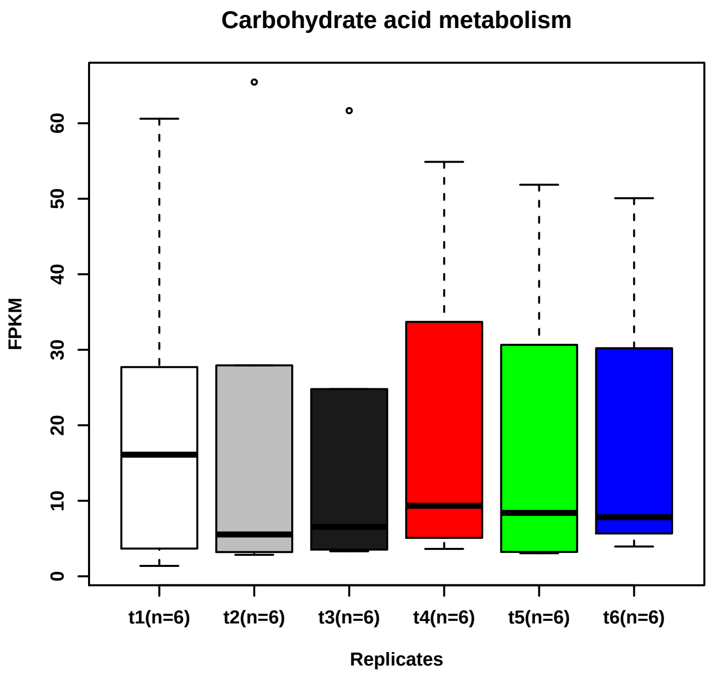
Carbohydrate acid metabolism
FPKM
0
10
20
30
40
50
60
t1(n=6)
t2(n=6)
t3(n=6)
t4(n=6)
t5(n=6)
t6(n=6)
Replicates

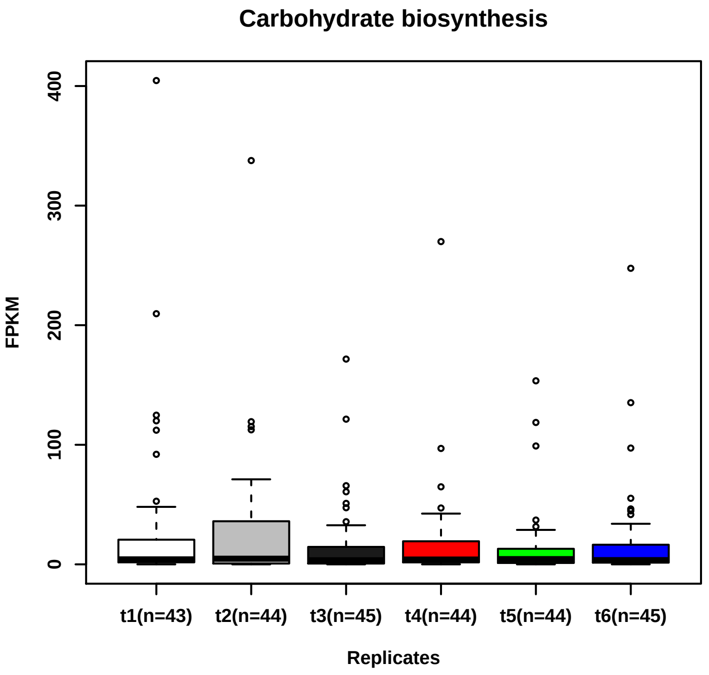
Carbohydrate biosynthesis
FPKM
0
100
200
300
400
t1(n=43)
t2(n=44)
t3(n=45)
t4(n=44)
t5(n=44)
t6(n=45)
Replicates

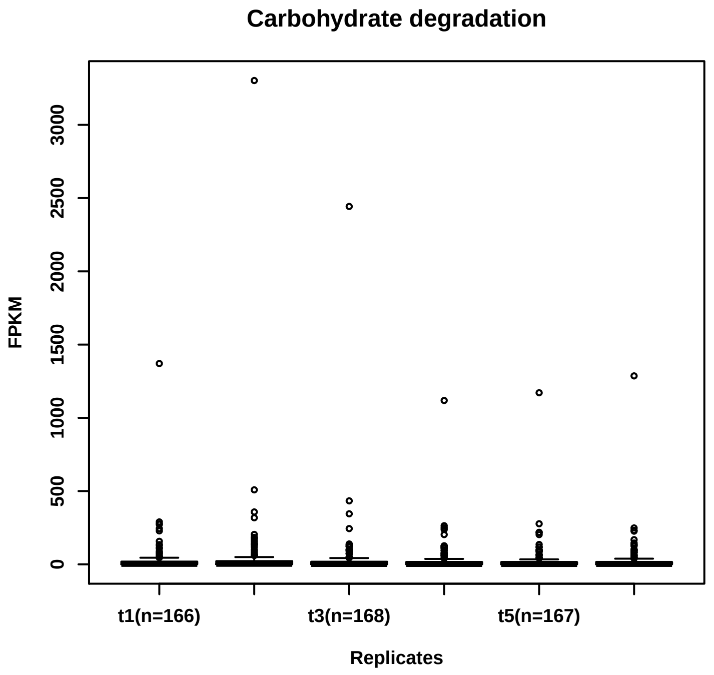
Carbohydrate degradation
FPKM
0
500
1000
1500
2000
2500
3000
t1(n=166)
t3(n=168)
t5(n=167)
Replicates

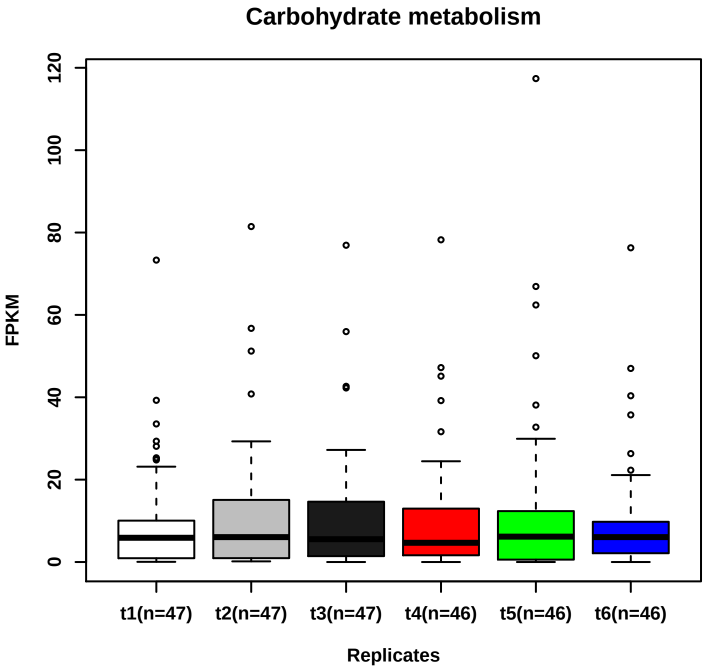
Carbohydrate metabolism
FPKM
0
20
40
60
80
100
120
t1(n=47)
t2(n=47)
t3(n=47)
t4(n=46)
t5(n=46)
t6(n=46)
Replicates

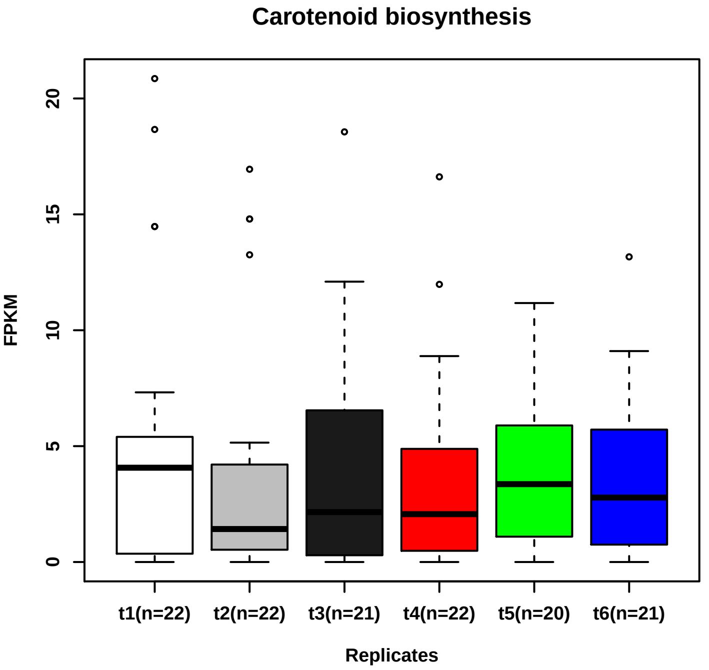
Carotenoid biosynthesis
FPKM
0
5
10
15
20
t1(n=22)
t2(n=22)
t3(n=21)
t4(n=22)
t5(n=20)
t6(n=21)
Replicates

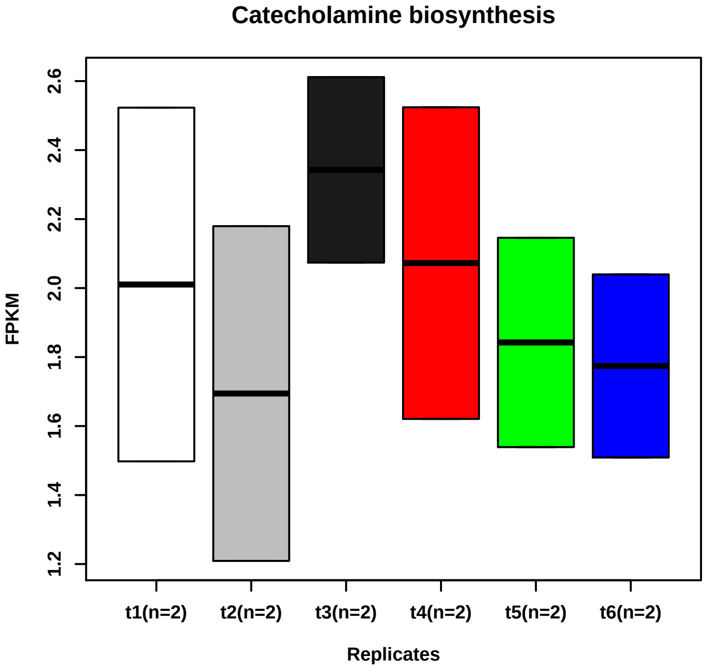
Catecholamine biosynthesis
FPKM
1.2
1.4
1.6
1.8
2.0
2.2
2.4
2.6
t1(n=2)
t2(n=2)
t3(n=2)
t4(n=2)
t5(n=2)
t6(n=2)
Replicates

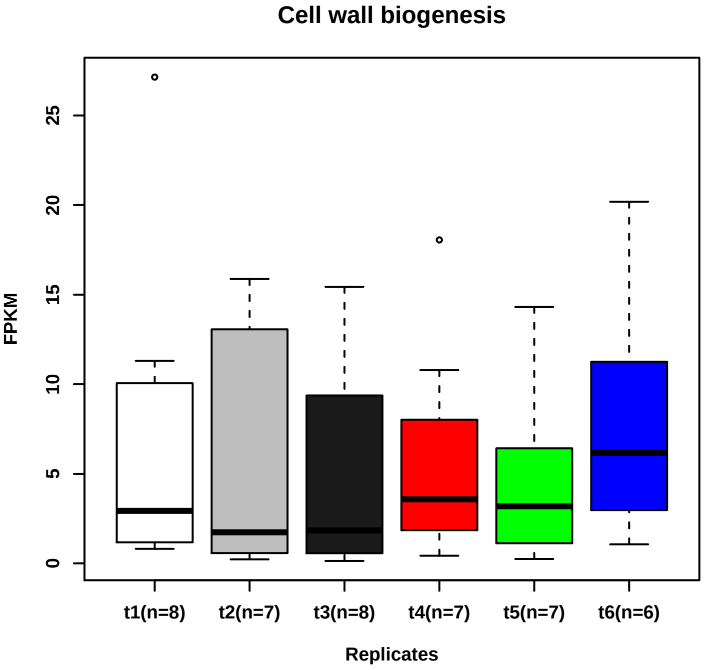
Cell wall biogenesis
FPKM
0
5
10
15
20
25
t1(n=8)
t2(n=7)
t3(n=8)
t4(n=7)
t5(n=7)
t6(n=6)
Replicates

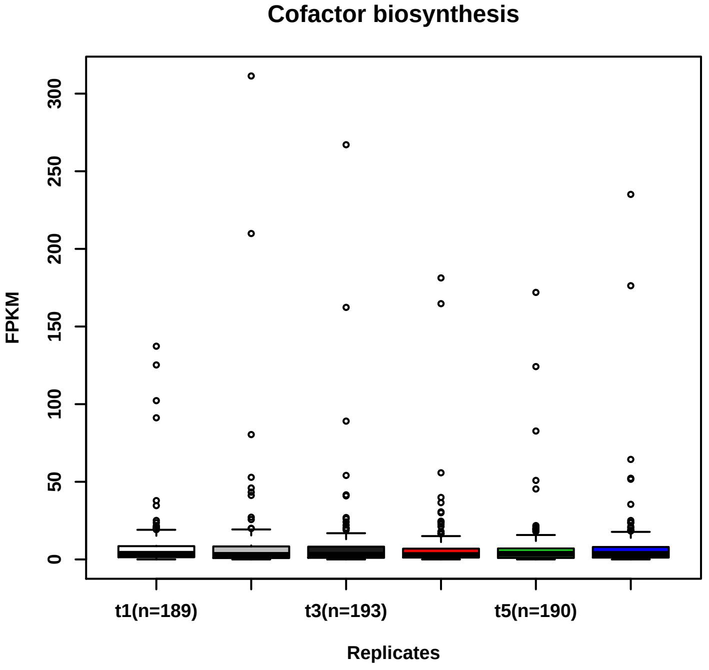
Cofactor biosynthesis
FPKM
0
50
100
150
200
250
300
t1(n=189)
t3(n=193)
t5(n=190)
Replicates

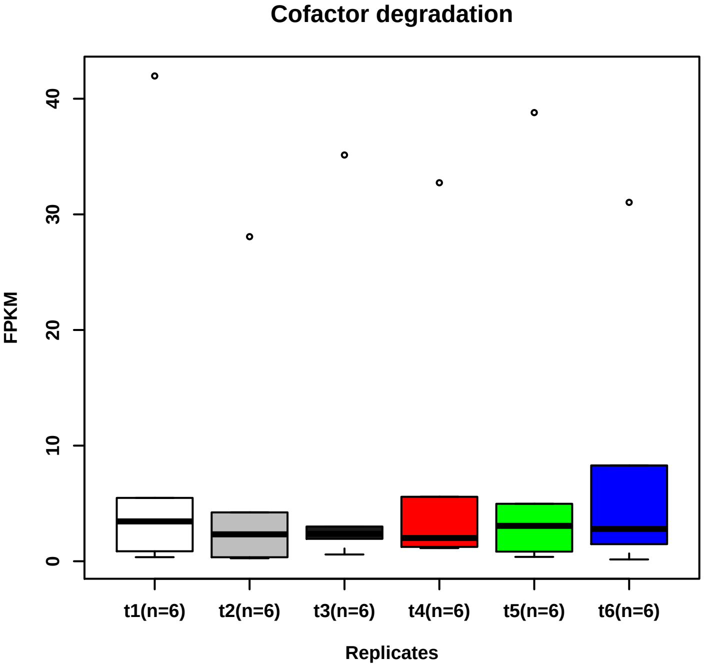
Cofactor degradation
FPKM
0
10
20
30
40
t1(n=6)
t2(n=6)
t3(n=6)
t4(n=6)
t5(n=6)
t6(n=6)
Replicates

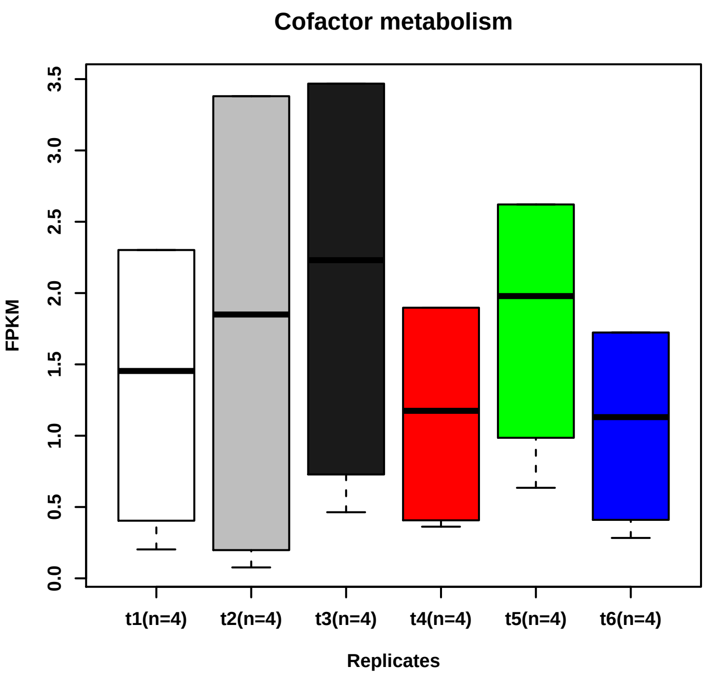
Cofactor metabolism
FPKM
0.0
0.5
1.0
1.5
2.0
2.5
3.0
3.5
t1(n=4)
t2(n=4)
t3(n=4)
t4(n=4)
t5(n=4)
t6(n=4)
Replicates

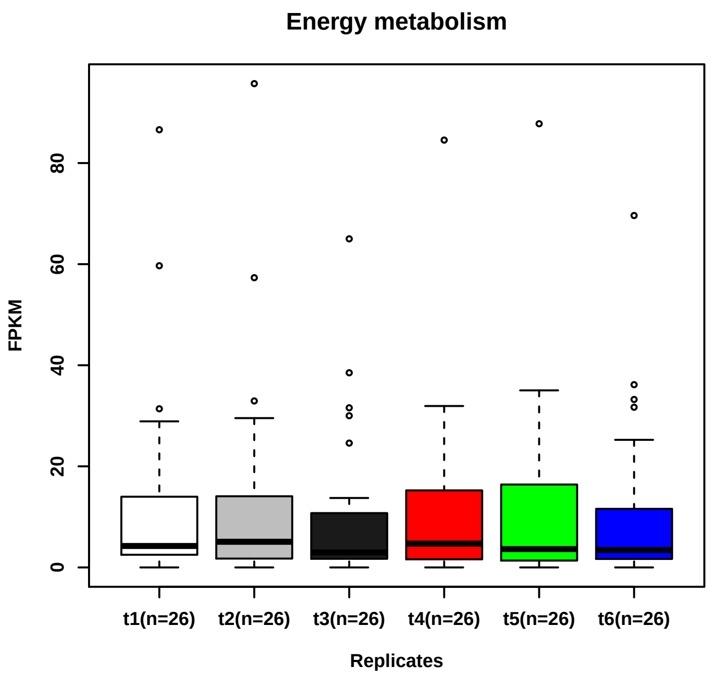
Energy metabolism
FPKM
0
20
40
60
80
t1(n=26)
t2(n=26)
t3(n=26)
t4(n=26)
t5(n=26)
t6(n=26)
Replicates

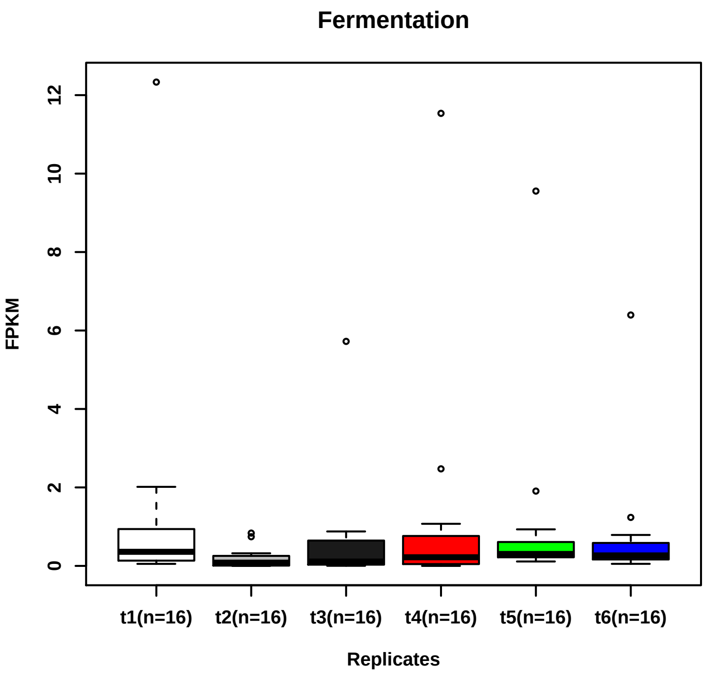
Fermentation
FPKM
0
2
4
6
8
10
12
t1(n=16)
t2(n=16)
t3(n=16)
t4(n=16)
t5(n=16)
t6(n=16)
Replicates

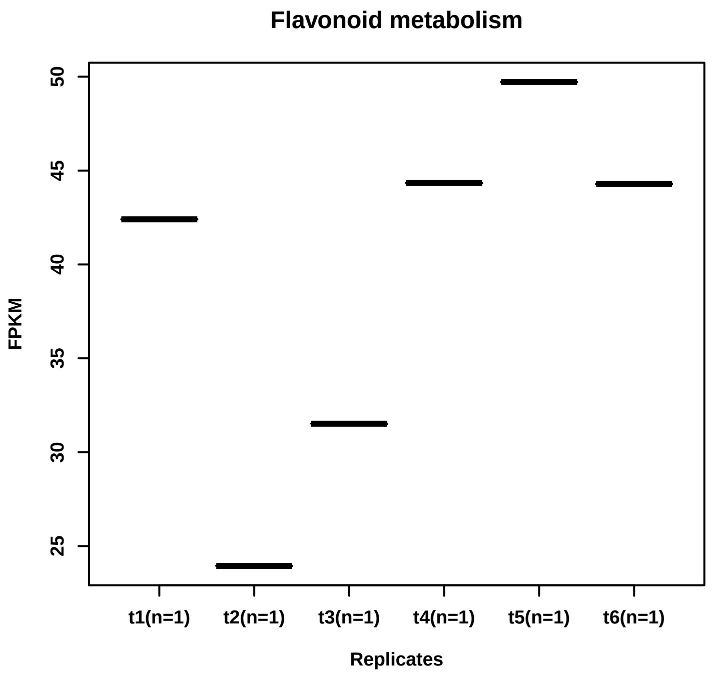
Flavonoid metabolism
FPKM
25
30
35
40
45
50
t1(n=1)
t2(n=1)
t3(n=1)
t4(n=1)
t5(n=1)
t6(n=1)
Replicates

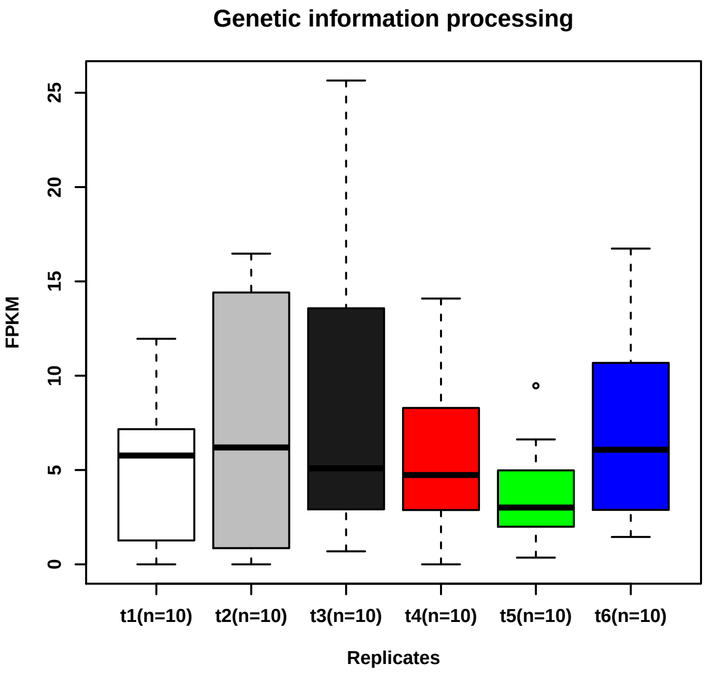
Genetic information processing
FPKM
0
5
10
15
20
25
t1(n=10)
t2(n=10)
t3(n=10)
t4(n=10)
t5(n=10)
t6(n=10)
Replicates

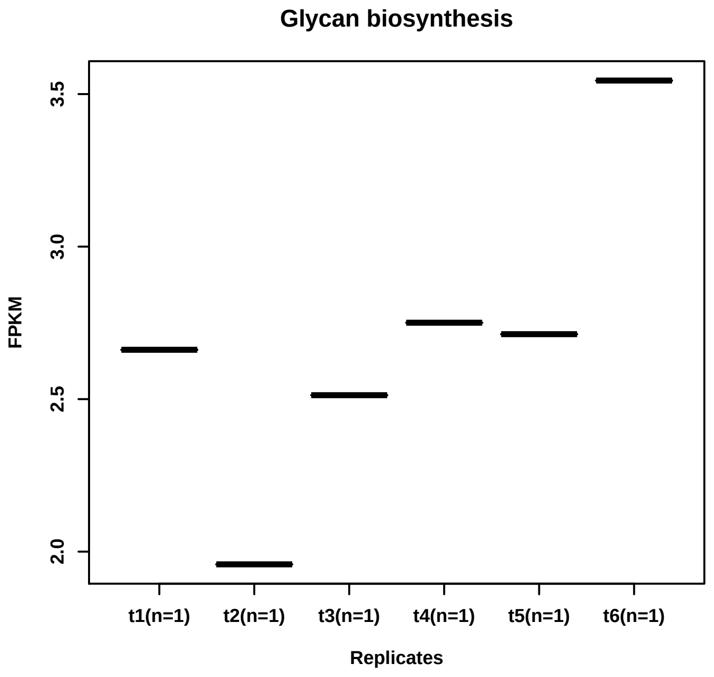
Glycan biosynthesis
FPKM
2.0
2.5
3.0
3.5
t1(n=1)
t2(n=1)
t3(n=1)
t4(n=1)
t5(n=1)
t6(n=1)
Replicates

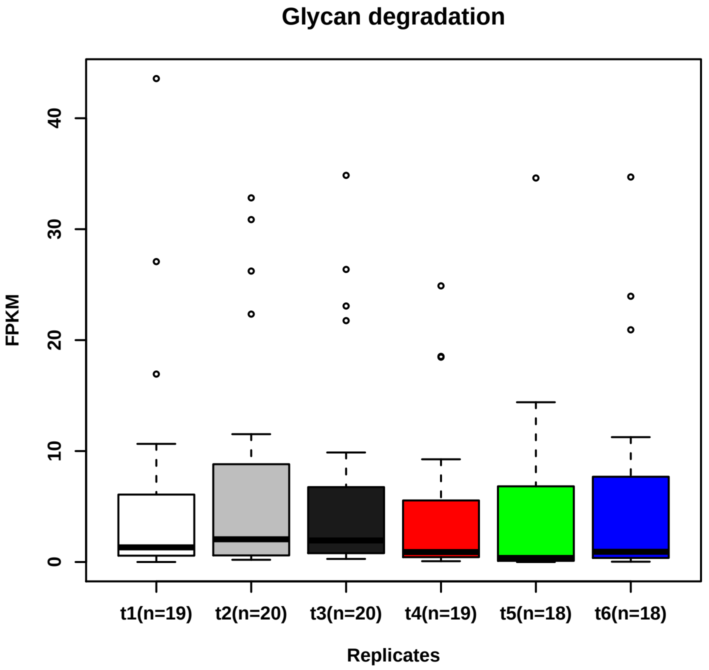
Glycan degradation
FPKM
0
10
20
30
40
t1(n=19)
t2(n=20)
t3(n=20)
t4(n=19)
t5(n=18)
t6(n=18)
Replicates

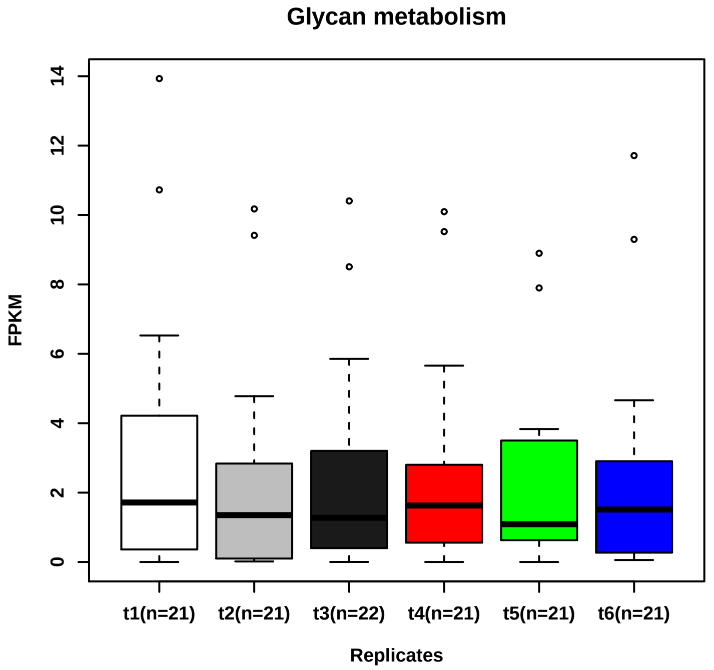
Glycan metabolism
FPKM
0
2
4
6
8
10
12
14
t1(n=21)
t2(n=21)
t3(n=22)
t4(n=21)
t5(n=21)
t6(n=21)
Replicates

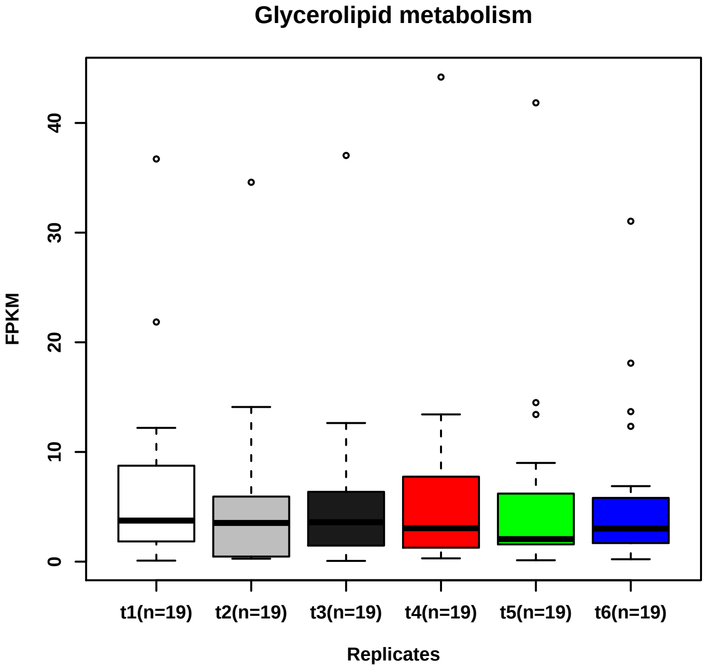
Glycerolipid metabolism
FPKM
0
10
20
30
40
t1(n=19)
t2(n=19)
t3(n=19)
t4(n=19)
t5(n=19)
t6(n=19)
Replicates

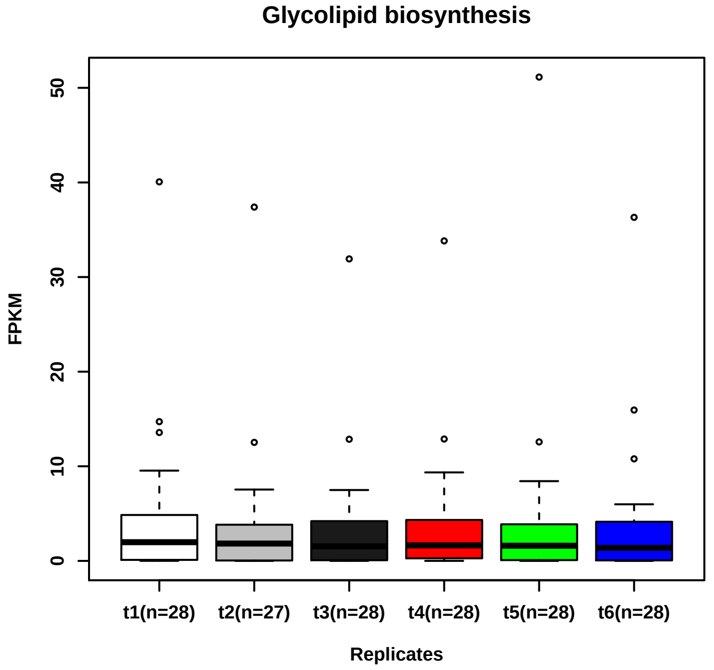
Glycolipid biosynthesis
FPKM
0
10
20
30
40
50
t1(n=28)
t2(n=27)
t3(n=28)
t4(n=28)
t5(n=28)
t6(n=28)
Replicates

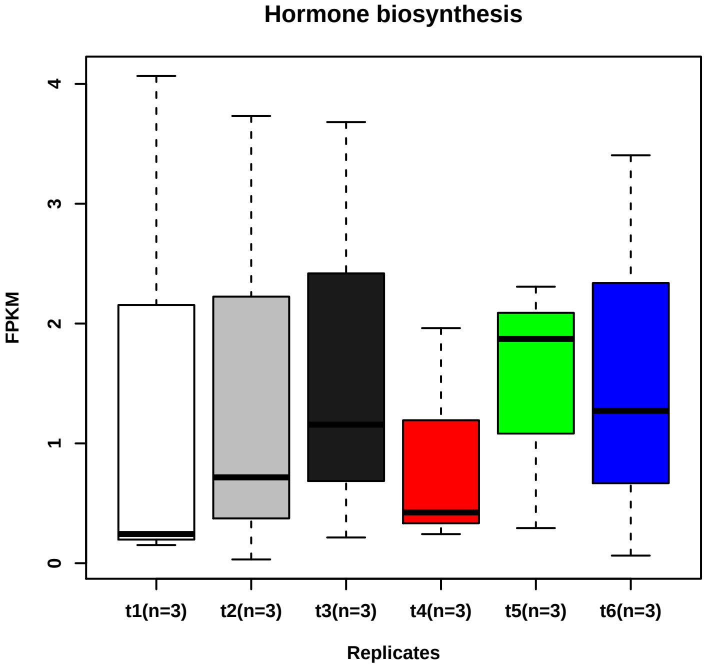
Hormone biosynthesis
FPKM
0
1
2
3
4
t1(n=3)
t2(n=3)
t3(n=3)
t4(n=3)
t5(n=3)
t6(n=3)
Replicates

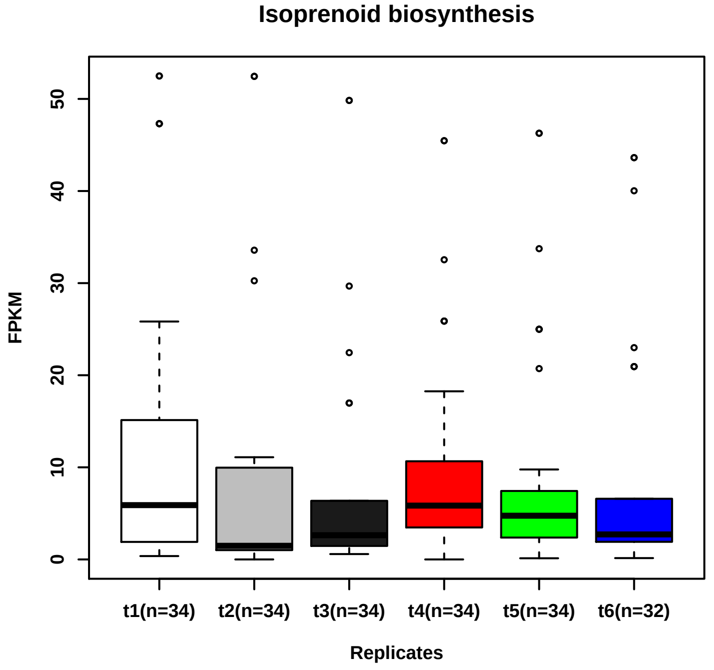
Isoprenoid biosynthesis
FPKM
0
10
20
30
40
50
t1(n=34)
t2(n=34)
t3(n=34)
t4(n=34)
t5(n=34)
t6(n=32)
Replicates

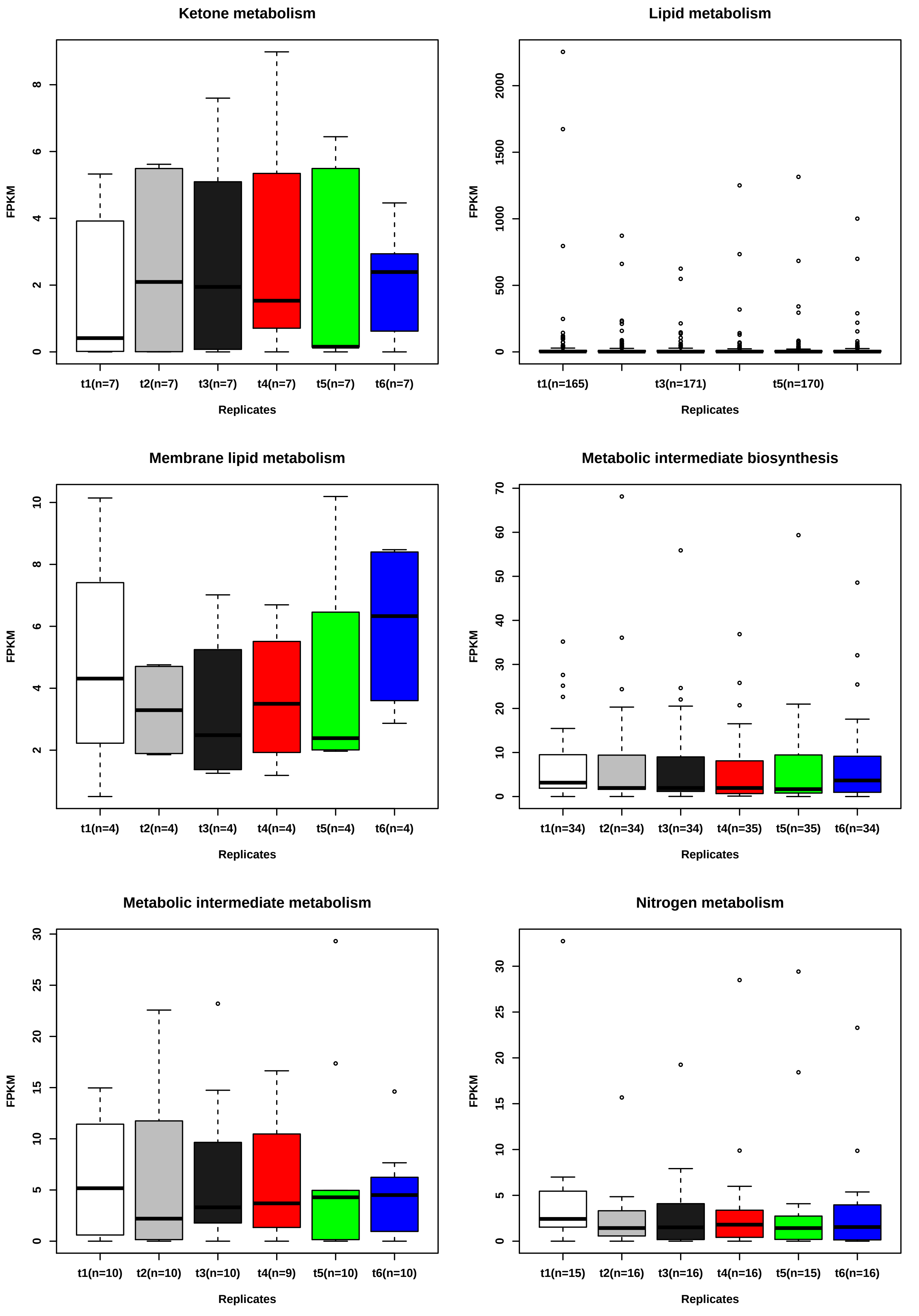
Ketone metabolism
FPKM
0 2 4 6 8
t1(n=7) t2(n=7) t3(n=7) t4(n=7) t5(n=7) t6(n=7)
Replicates
Lipid metabolism
FPKM
0 500 1000 1500 2000
t1(n=165) t3(n=171) t5(n=170)
Replicates
Membrane lipid metabolism
FPKM
2 4 6 8 10
t1(n=4) t2(n=4) t3(n=4) t4(n=4) t5(n=4) t6(n=4)
Replicates
Metabolic intermediate biosynthesis
FPKM
0 10 20 30 40 50 60 70
t1(n=34) t2(n=34) t3(n=34) t4(n=35) t5(n=35) t6(n=34)
Replicates
Metabolic intermediate metabolism
FPKM
0 5 10 15 20 25 30
t1(n=10) t2(n=10) t3(n=10) t4(n=9) t5(n=10) t6(n=10)
Replicates
Nitrogen metabolism
FPKM
0 5 10 15 20 25 30
t1(n=15) t2(n=16) t3(n=16) t4(n=16) t5(n=15) t6(n=16)
Replicates

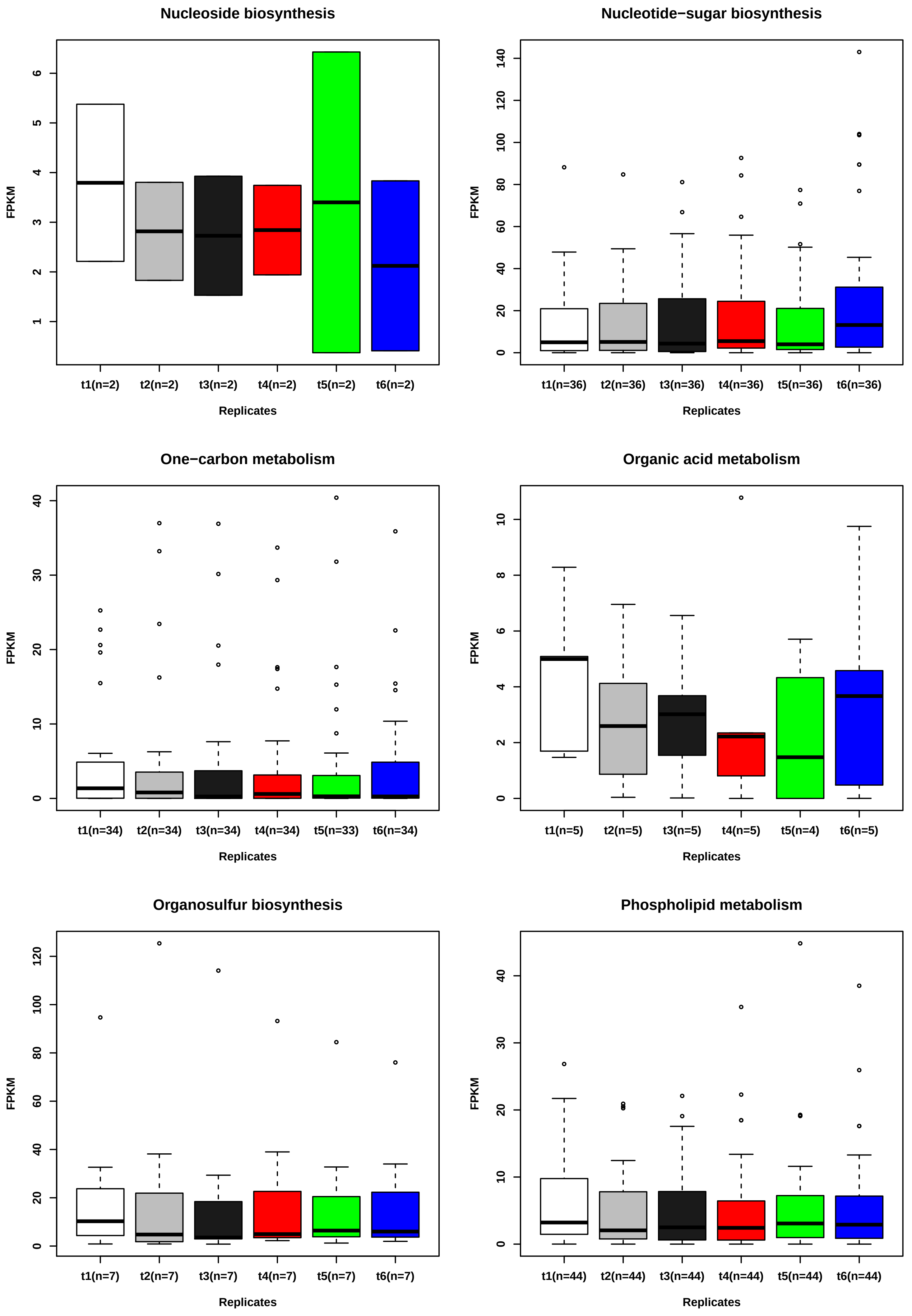

Nucleoside biosynthesis
FPKM
Replicates
t1(n=2) t2(n=2) t3(n=2) t4(n=2) t5(n=2) t6(n=2)
Nucleotide−sugar biosynthesis
FPKM
Replicates
t1(n=36) t2(n=36) t3(n=36) t4(n=36) t5(n=36) t6(n=36)
One−carbon metabolism
FPKM
Replicates
t1(n=34) t2(n=34) t3(n=34) t4(n=34) t5(n=33) t6(n=34)
Organic acid metabolism
FPKM
Replicates
t1(n=5) t2(n=5) t3(n=5) t4(n=5) t5(n=4) t6(n=5)
Organosulfur biosynthesis
FPKM
Replicates
t1(n=7) t2(n=7) t3(n=7) t4(n=7) t5(n=7) t6(n=7)
Phospholipid metabolism
FPKM
Replicates
t1(n=44) t2(n=44) t3(n=44) t4(n=44) t5(n=44) t6(n=44)

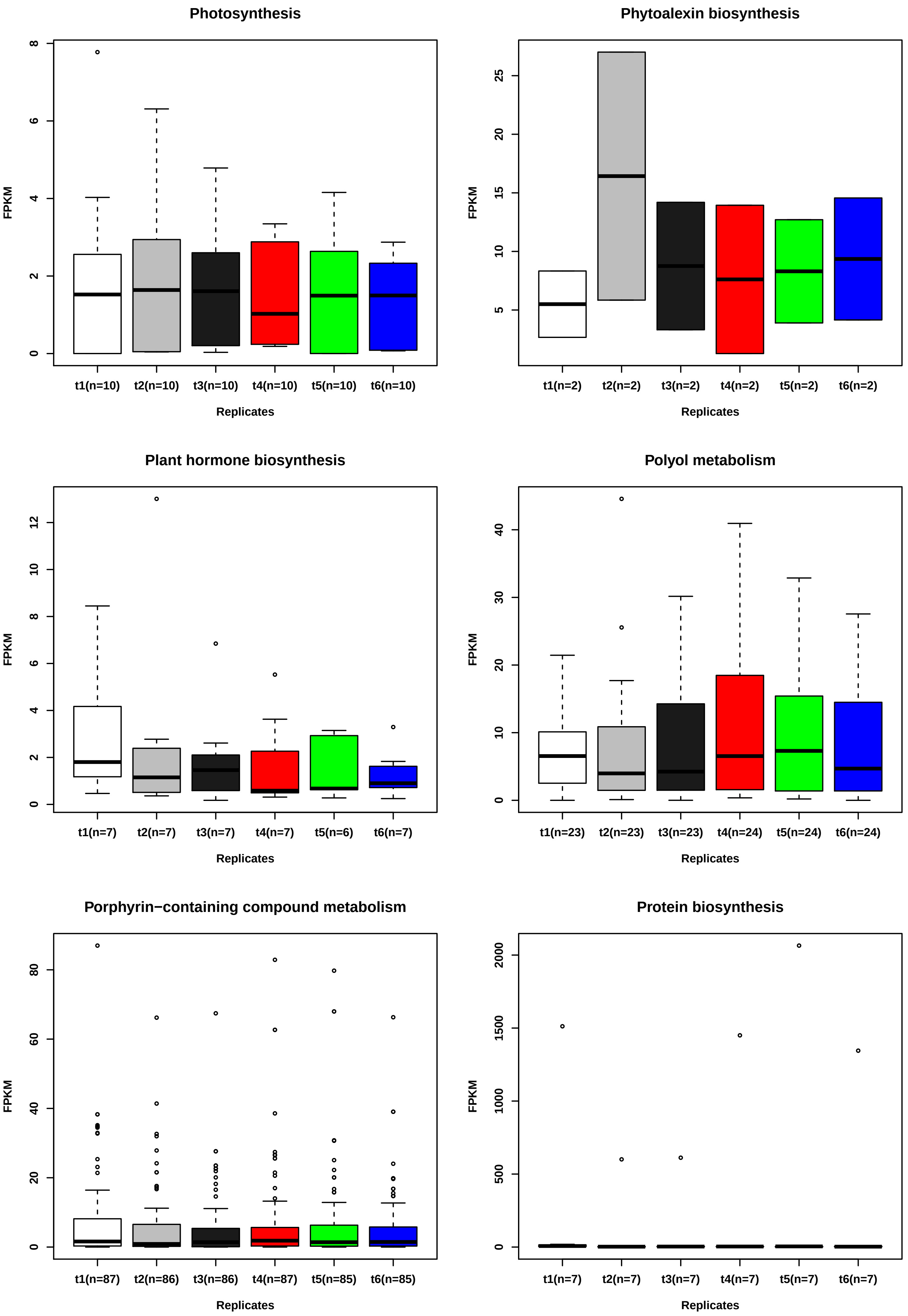
Photosynthesis
FPKM
0
2
4
6
8
t1(n=10)
t2(n=10)
t3(n=10)
t4(n=10)
t5(n=10)
t6(n=10)
Replicates
Phytoalexin biosynthesis
FPKM
5
10
15
20
25
t1(n=2)
t2(n=2)
t3(n=2)
t4(n=2)
t5(n=2)
t6(n=2)
Replicates
Plant hormone biosynthesis
FPKM
0
2
4
6
8
10
12
t1(n=7)
t2(n=7)
t3(n=7)
t4(n=7)
t5(n=6)
t6(n=7)
Replicates
Polyol metabolism
FPKM
0
10
20
30
40
t1(n=23)
t2(n=23)
t3(n=23)
t4(n=24)
t5(n=24)
t6(n=24)
Replicates
Porphyrin−containing compound metabolism
FPKM
0
20
40
60
80
t1(n=87)
t2(n=86)
t3(n=86)
t4(n=87)
t5(n=85)
t6(n=85)
Replicates
Protein biosynthesis
FPKM
0
500
1000
1500
2000
t1(n=7)
t2(n=7)
t3(n=7)
t4(n=7)
t5(n=7)
t6(n=7)
Replicates

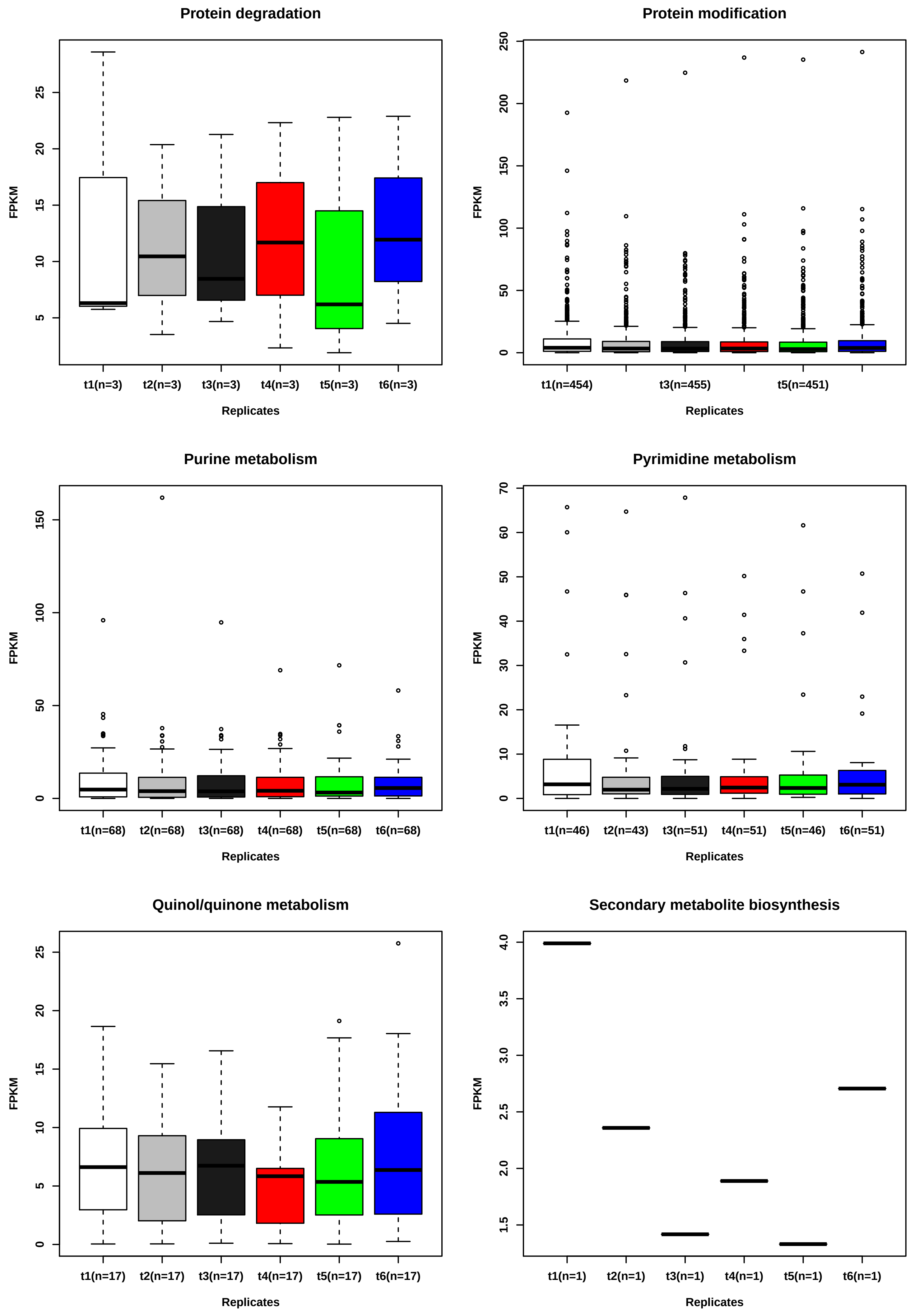

Protein degradation
FPKM
5
10
15
20
25
t1(n=3)
t2(n=3)
t3(n=3)
t4(n=3)
t5(n=3)
t6(n=3)
Replicates
Protein modification
FPKM
0
50
100
150
200
250
t1(n=454)
t3(n=455)
t5(n=451)
Replicates
Purine metabolism
FPKM
0
50
100
150
t1(n=68)
t2(n=68)
t3(n=68)
t4(n=68)
t5(n=68)
t6(n=68)
Replicates
Pyrimidine metabolism
FPKM
0
10
20
30
40
50
60
70
t1(n=46)
t2(n=43)
t3(n=51)
t4(n=51)
t5(n=46)
t6(n=51)
Replicates
Quinol/quinone metabolism
FPKM
0
5
10
15
20
25
t1(n=17)
t2(n=17)
t3(n=17)
t4(n=17)
t5(n=17)
t6(n=17)
Replicates
Secondary metabolite biosynthesis
FPKM
1.5
2.0
2.5
3.0
3.5
4.0
t1(n=1)
t2(n=1)
t3(n=1)
t4(n=1)
t5(n=1)
t6(n=1)
Replicates

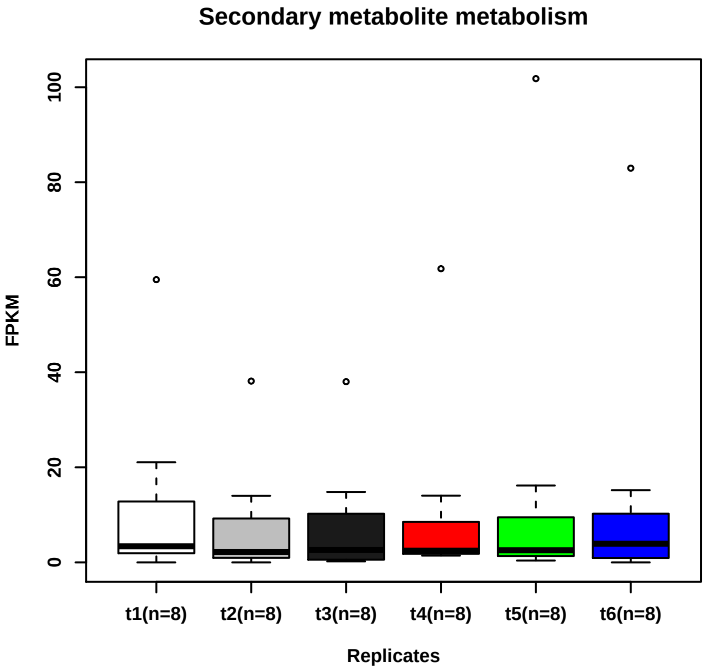
Secondary metabolite metabolism
FPKM
0
20
40
60
80
100
t1(n=8)
t2(n=8)
t3(n=8)
t4(n=8)
t5(n=8)
t6(n=8)
Replicates

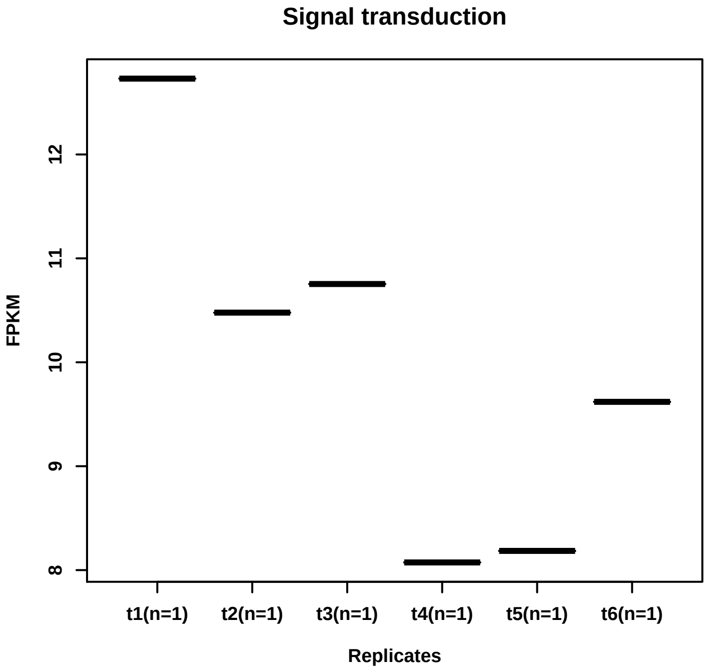
Signal transduction
FPKM
8
9
10
11
12
t1(n=1)
t2(n=1)
t3(n=1)
t4(n=1)
t5(n=1)
t6(n=1)
Replicates

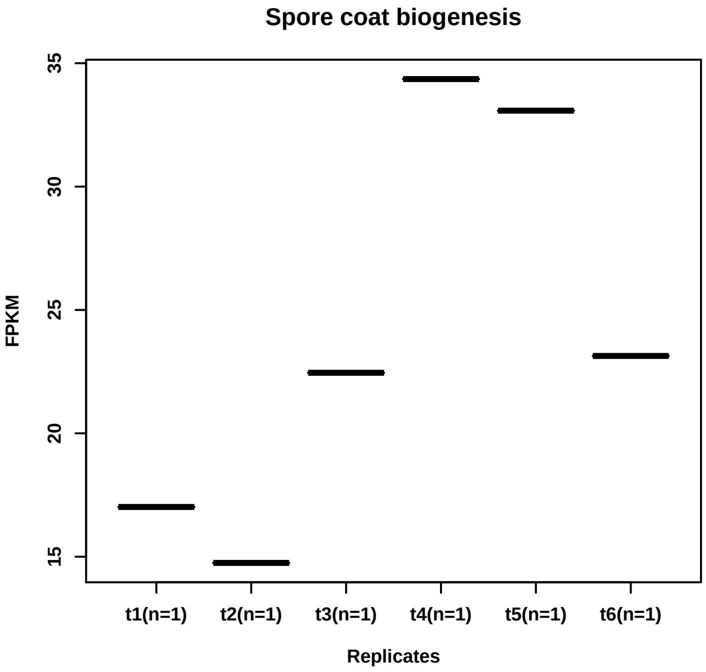
Spore coat biogenesis
FPKM
15
20
25
30
35
t1(n=1)
t2(n=1)
t3(n=1)
t4(n=1)
t5(n=1)
t6(n=1)
Replicates

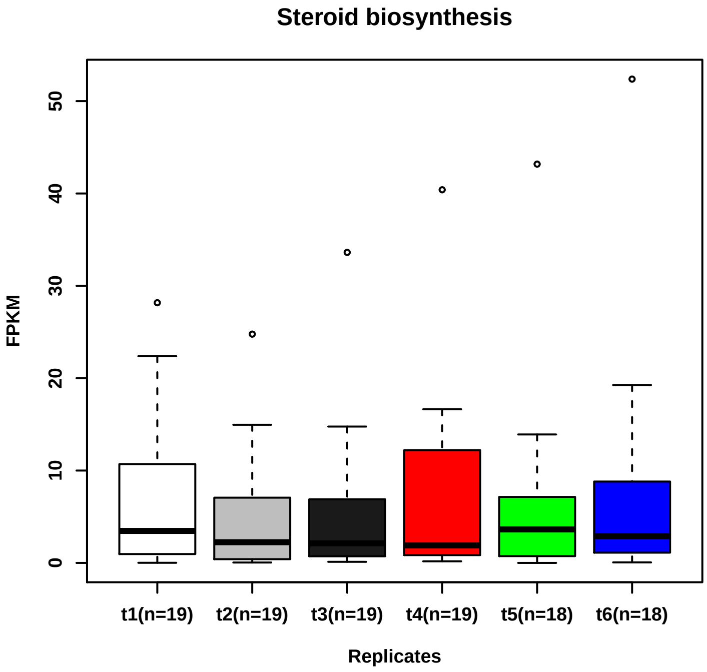
Steroid biosynthesis
FPKM
0
10
20
30
40
50
t1(n=19)
t2(n=19)
t3(n=19)
t4(n=19)
t5(n=18)
t6(n=18)
Replicates

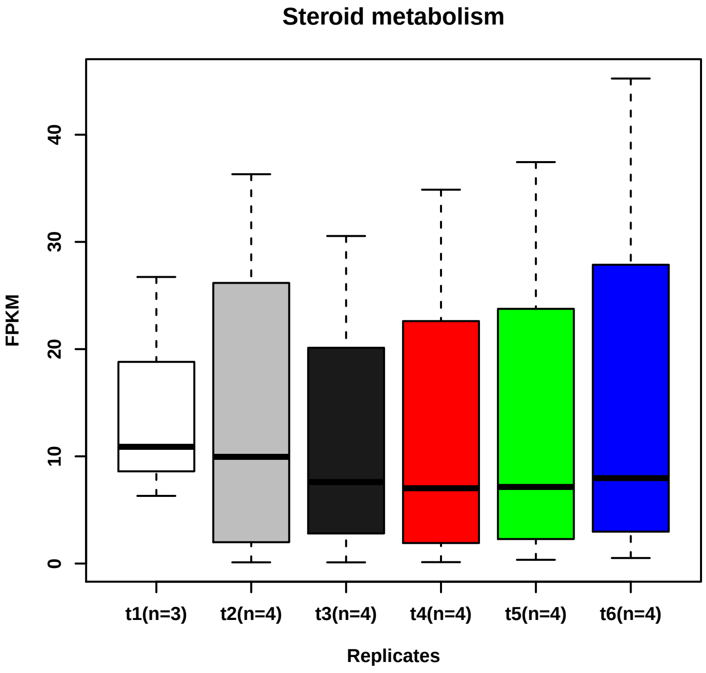
Steroid metabolism
FPKM
0
10
20
30
40
t1(n=3)
t2(n=4)
t3(n=4)
t4(n=4)
t5(n=4)
t6(n=4)
Replicates

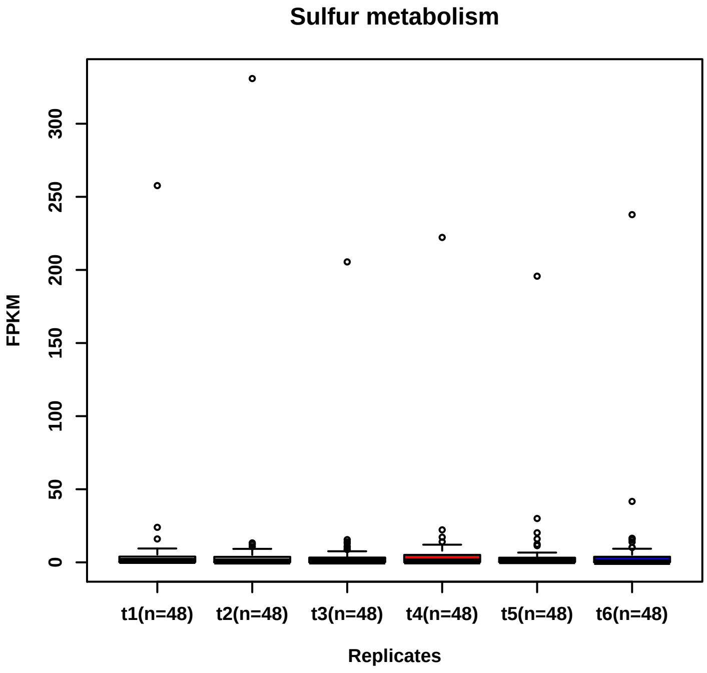
Sulfur metabolism
FPKM
0
50
100
150
200
250
300
t1(n=48)
t2(n=48)
t3(n=48)
t4(n=48)
t5(n=48)
t6(n=48)
Replicates

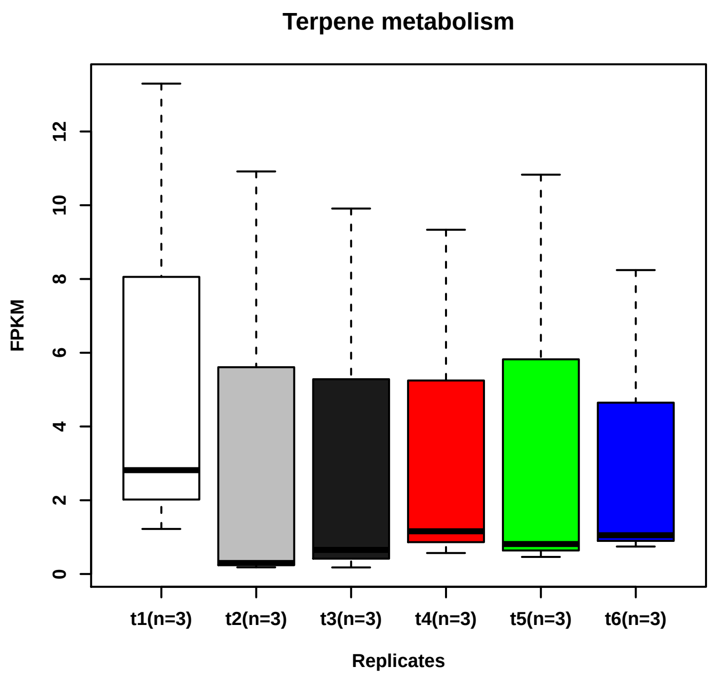

Terpene metabolism
FPKM
0
2
4
6
8
10
12
t1(n=3)
t2(n=3)
t3(n=3)
t4(n=3)
t5(n=3)
t6(n=3)
Replicates

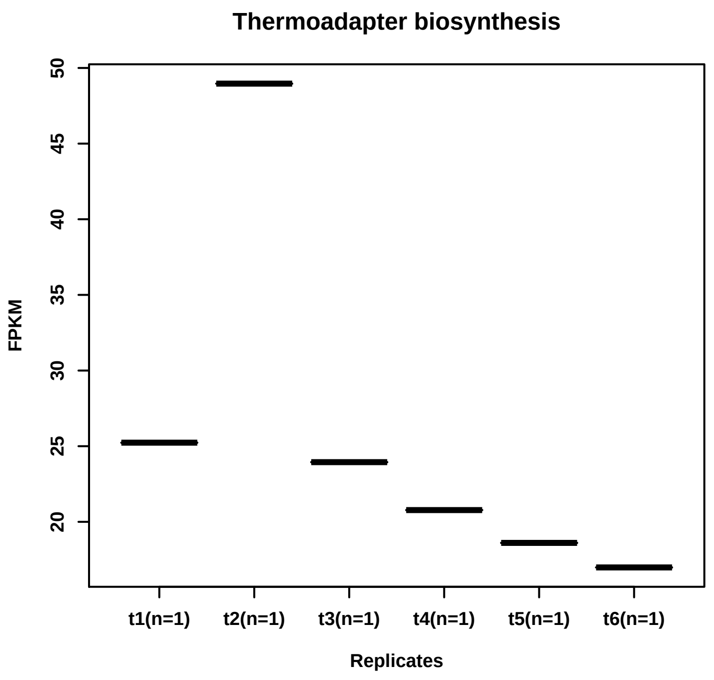

Thermoadapter biosynthesis
FPKM
20
25
30
35
40
45
50
t1(n=1)
t2(n=1)
t3(n=1)
t4(n=1)
t5(n=1)
t6(n=1)
Replicates

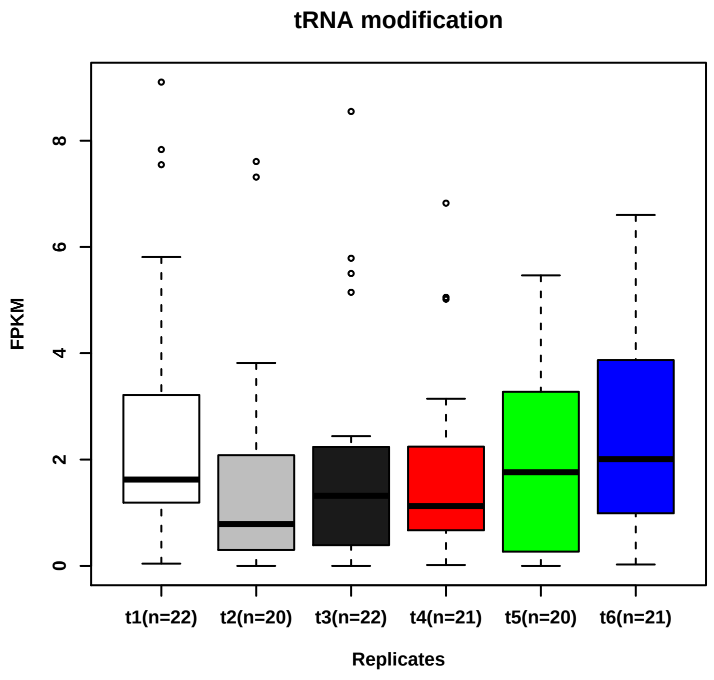

tRNA modification
FPKM
0
2
4
6
8
t1(n=22)
t2(n=20)
t3(n=22)
t4(n=21)
t5(n=20)
t6(n=21)
Replicates

Unveiling the presence of biosynthetic pathways for bioactive compounds in the *Thalassiosira rotula* transcriptome

Valeria Di Dato*, Federica Di Costanzo, Roberta Barbarinaldi, Anna Perna, Adrianna Ianora, Giovanna Romano

Supplementary File 2:

Box Plot Analysis of the expression, in FPKM, of the pathways annotated on level 1 for each condition (mean of two replicates).

Alcohol metabolism

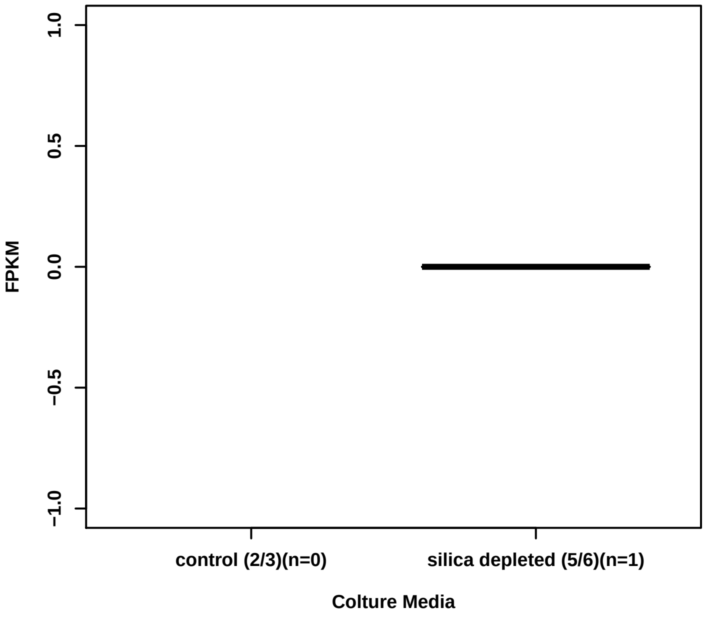

Alkaloid biosynthesis

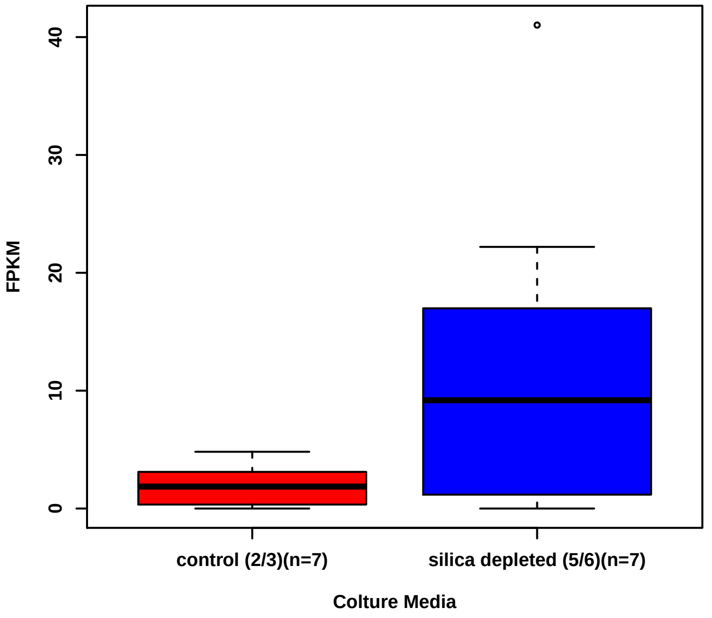

Alkaloid degradation

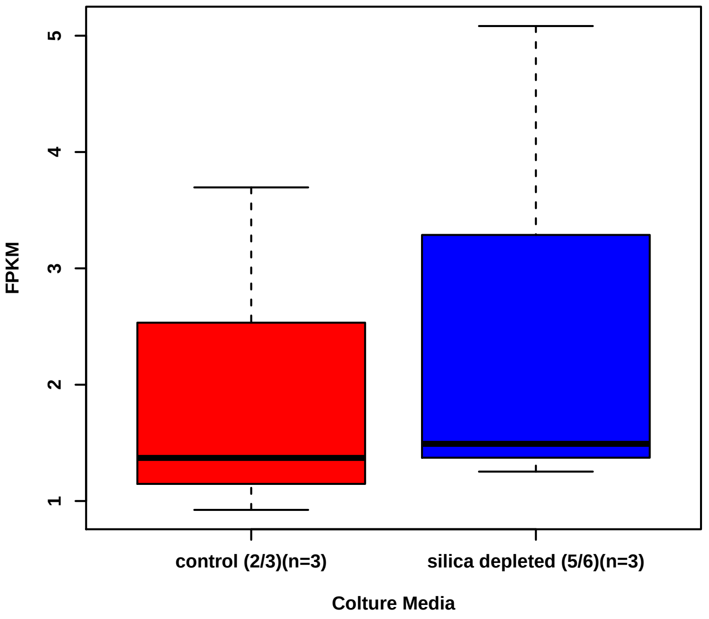

Alkene biosynthesis

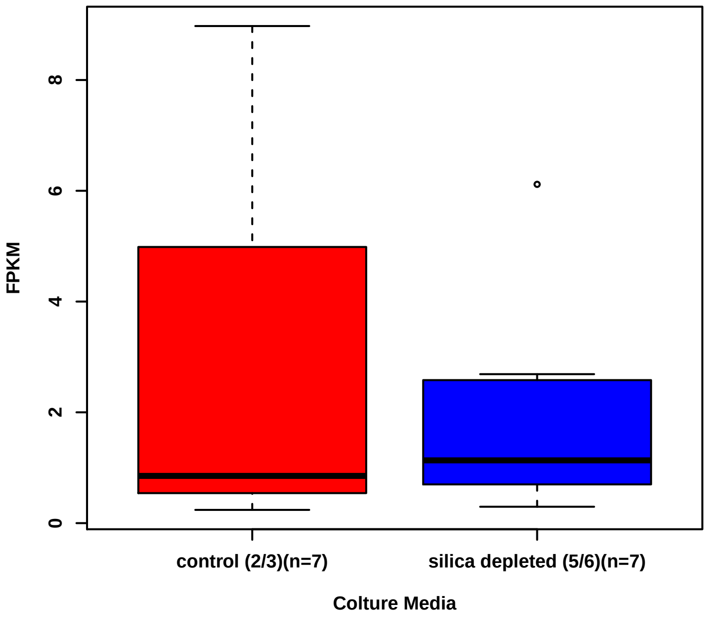

Amine and polyamine biosynthesis

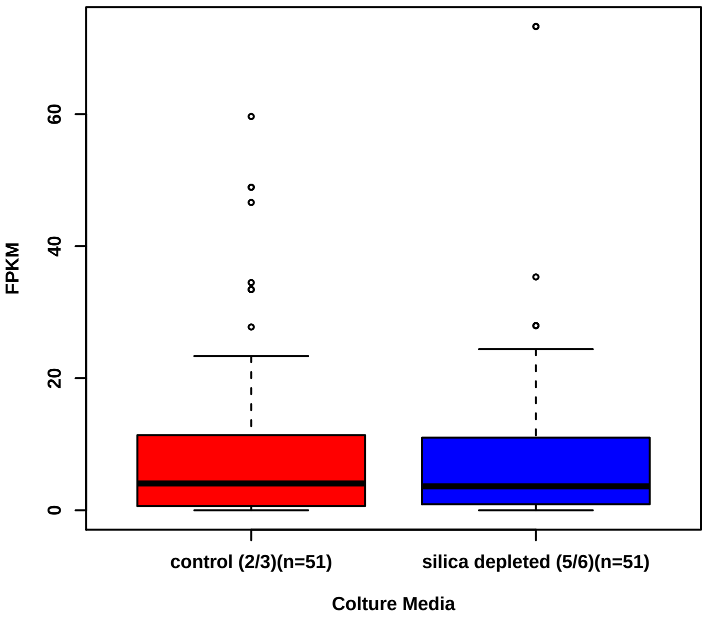

Amine and polyamine degradation

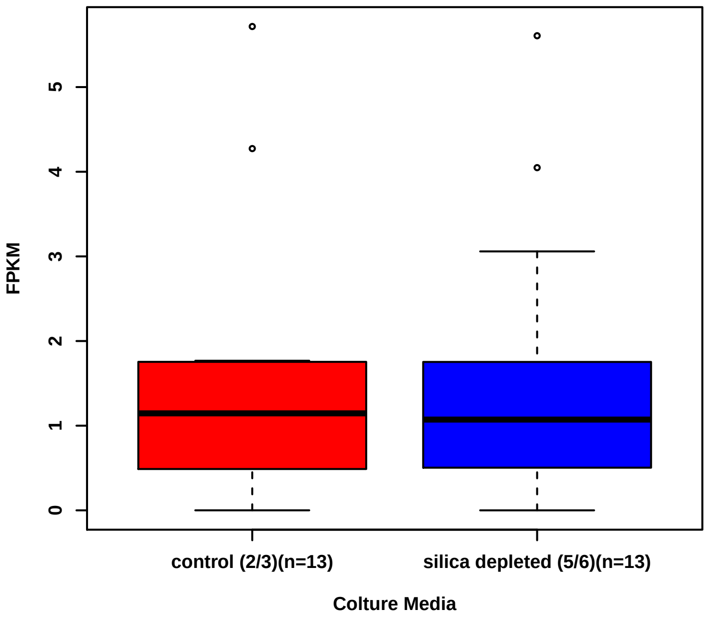

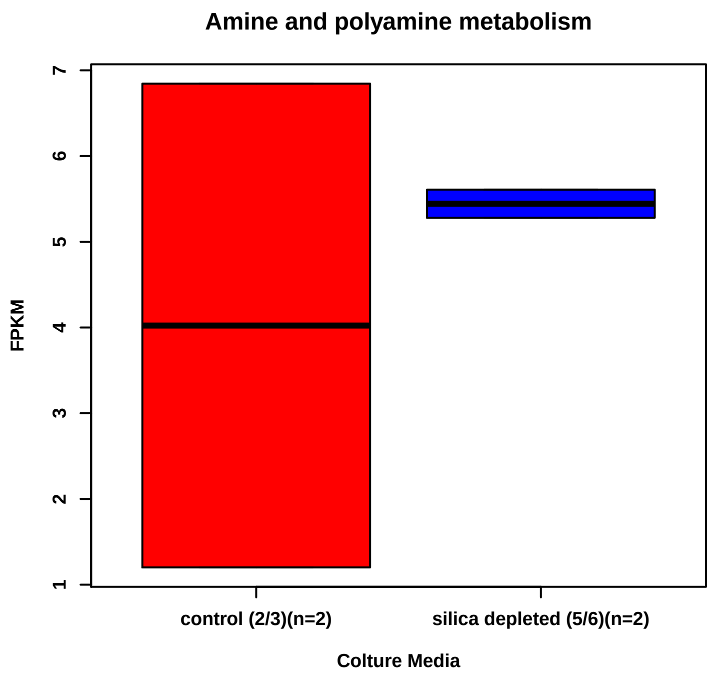
Amine and polyamine metabolism
FPKM
1
2
3
4
5
6
7
control (2/3)(n=2)
silica depleted (5/6)(n=2)
Colture Media

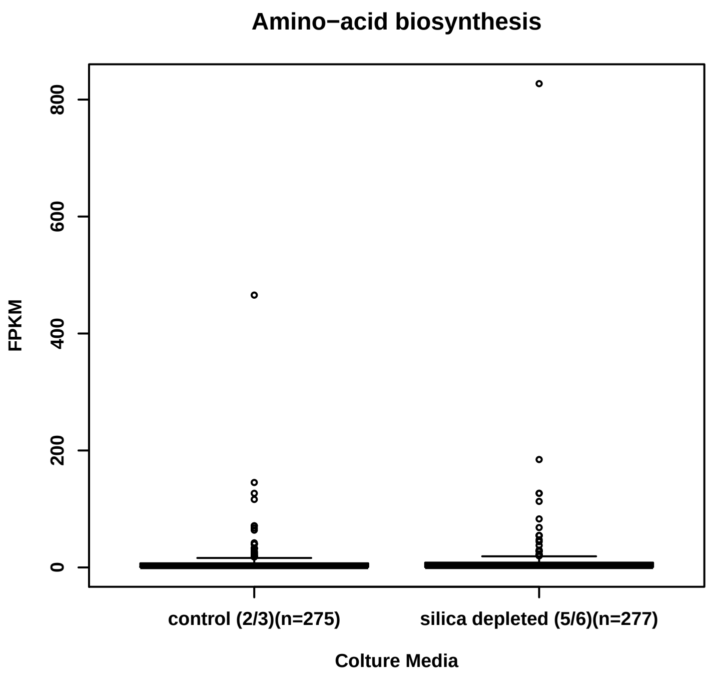
Amino−acid biosynthesis
FPKM
0
200
400
600
800
control (2/3)(n=275)
silica depleted (5/6)(n=277)
Colture Media

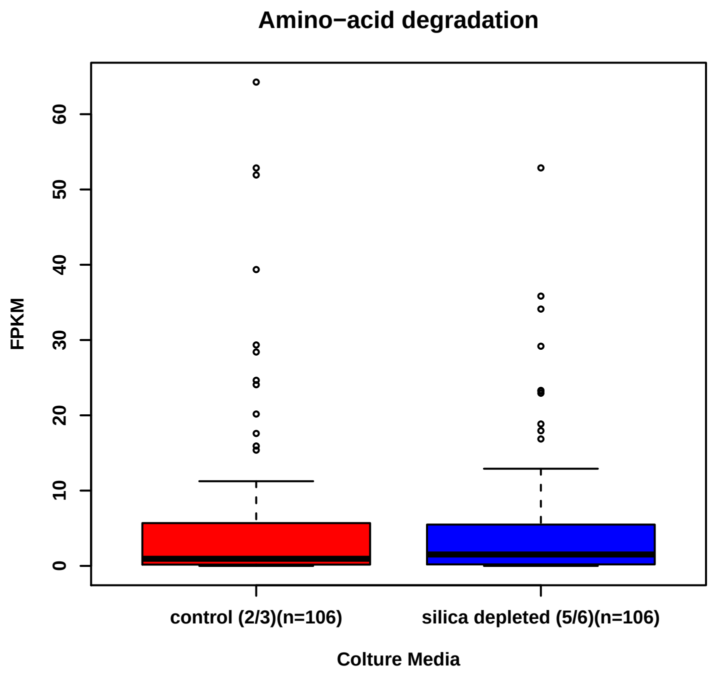
Amino−acid degradation
FPKM
0
10
20
30
40
50
60
control (2/3)(n=106)
silica depleted (5/6)(n=106)
Colture Media

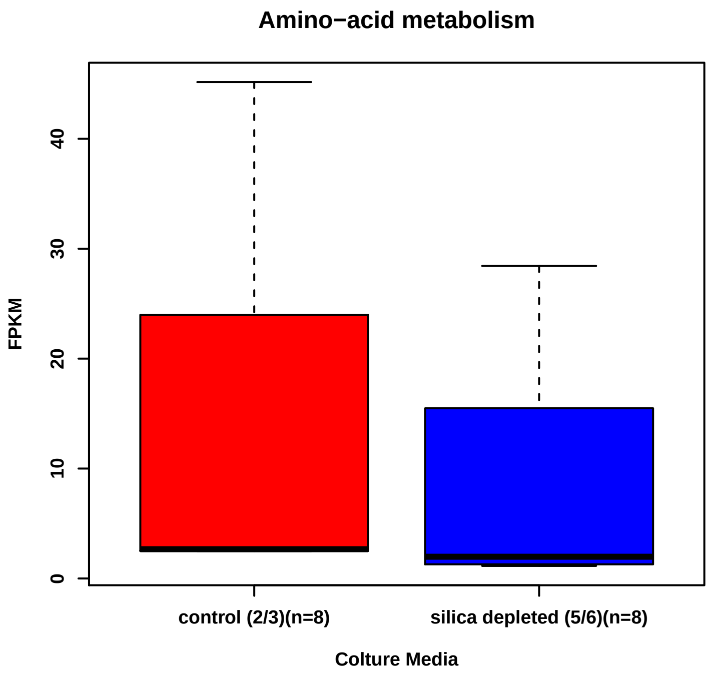
Amino−acid metabolism
FPKM
0
10
20
30
40
control (2/3)(n=8)
silica depleted (5/6)(n=8)
Colture Media

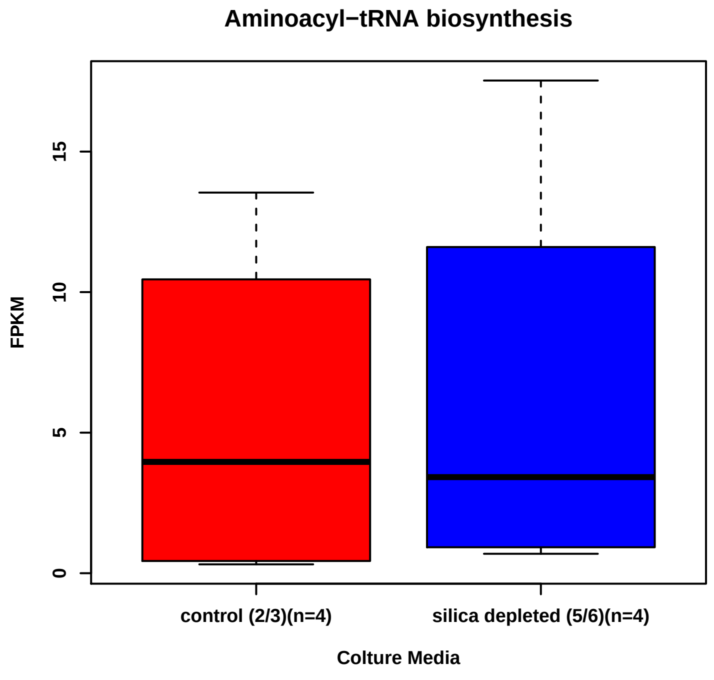
Aminoacyl−tRNA biosynthesis
FPKM
0
5
10
15
control (2/3)(n=4)
silica depleted (5/6)(n=4)
Colture Media

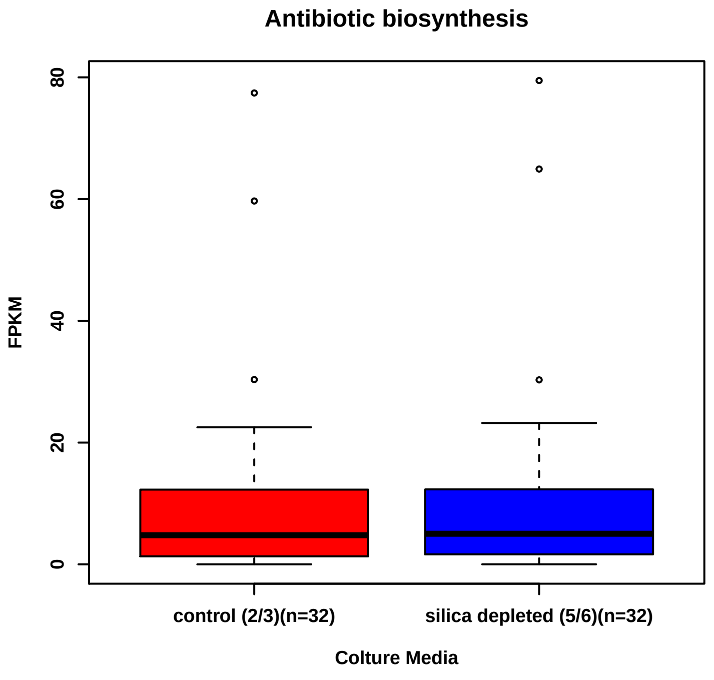
Antibiotic biosynthesis
FPKM
0
20
40
60
80
control (2/3)(n=32)
silica depleted (5/6)(n=32)
Colture Media

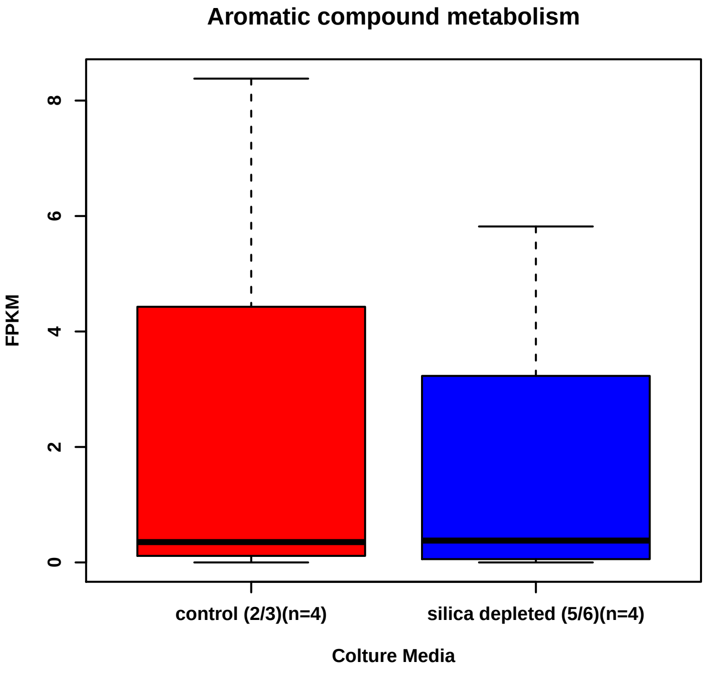
Aromatic compound metabolism
FPKM
0
2
4
6
8
control (2/3)(n=4)
silica depleted (5/6)(n=4)
Colture Media

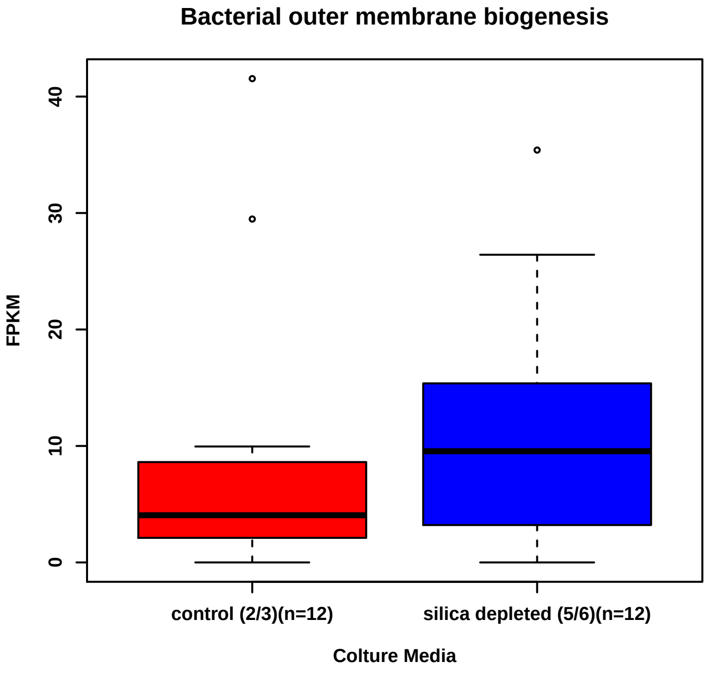
Bacterial outer membrane biogenesis
FPKM
0
10
20
30
40
control (2/3)(n=12)
silica depleted (5/6)(n=12)
Colture Media

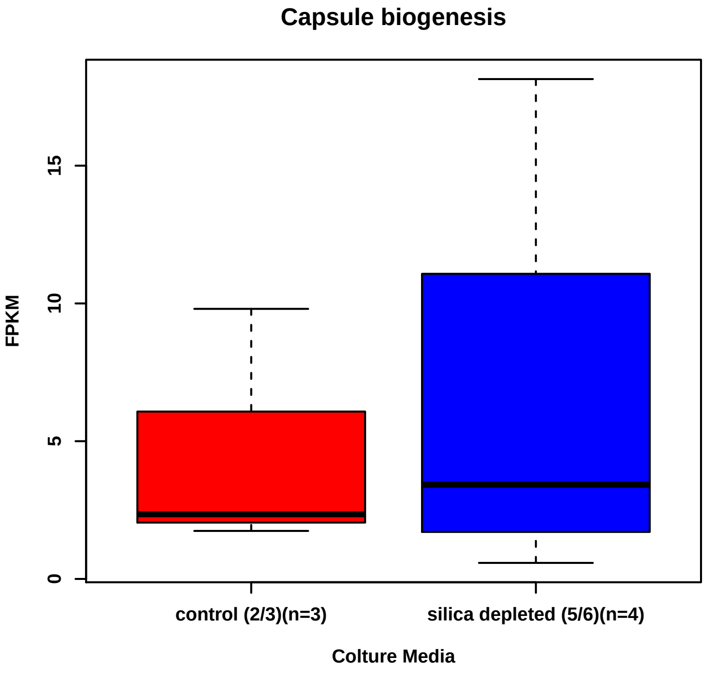
Capsule biogenesis
FPKM
0
5
10
15
control (2/3)(n=3)
silica depleted (5/6)(n=4)
Colture Media

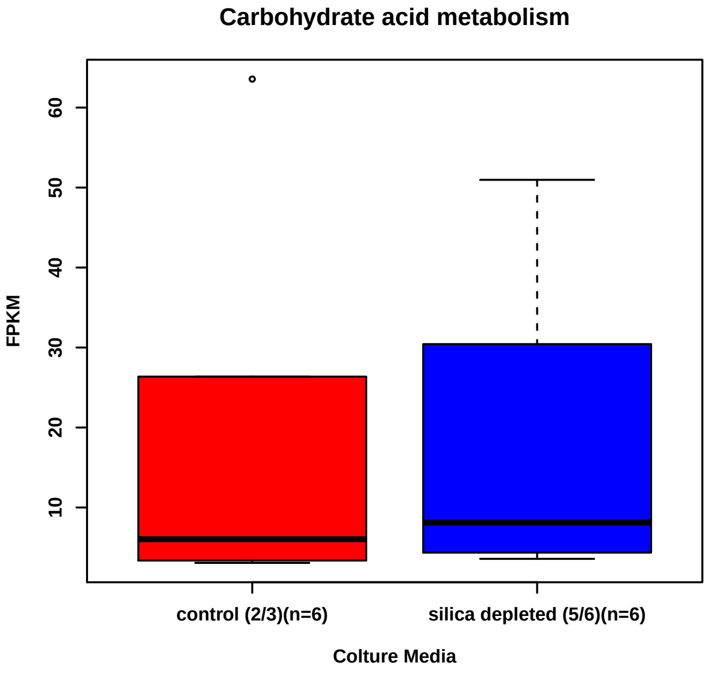
Carbohydrate acid metabolism
FPKM
10
20
30
40
50
60
control (2/3)(n=6)
silica depleted (5/6)(n=6)
Colture Media

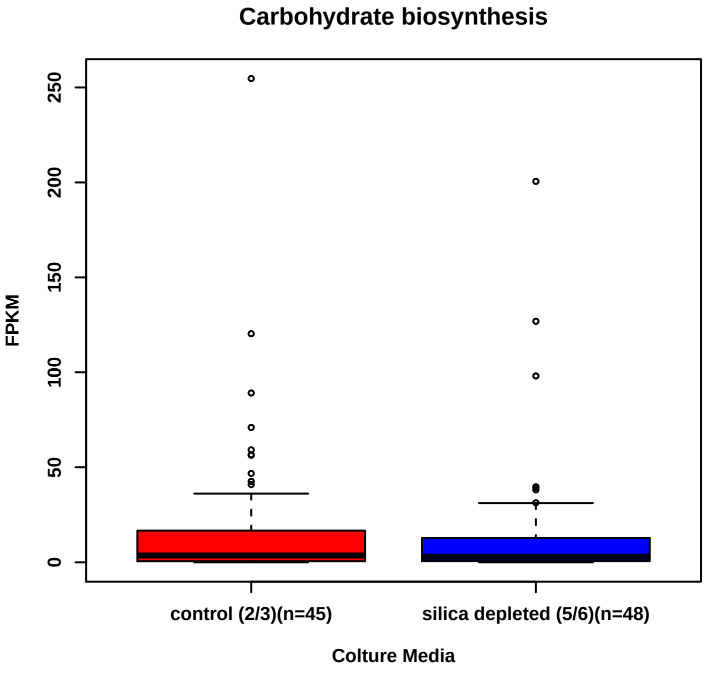
Carbohydrate biosynthesis
FPKM
0
50
100
150
200
250
control (2/3)(n=45)
silica depleted (5/6)(n=48)
Colture Media

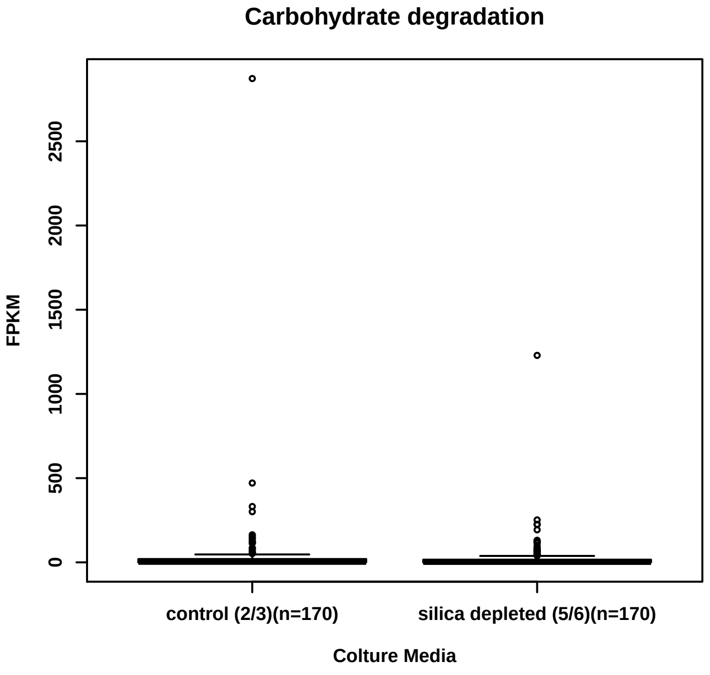
Carbohydrate degradation
FPKM
0
500
1000
1500
2000
2500
control (2/3)(n=170)
silica depleted (5/6)(n=170)
Colture Media

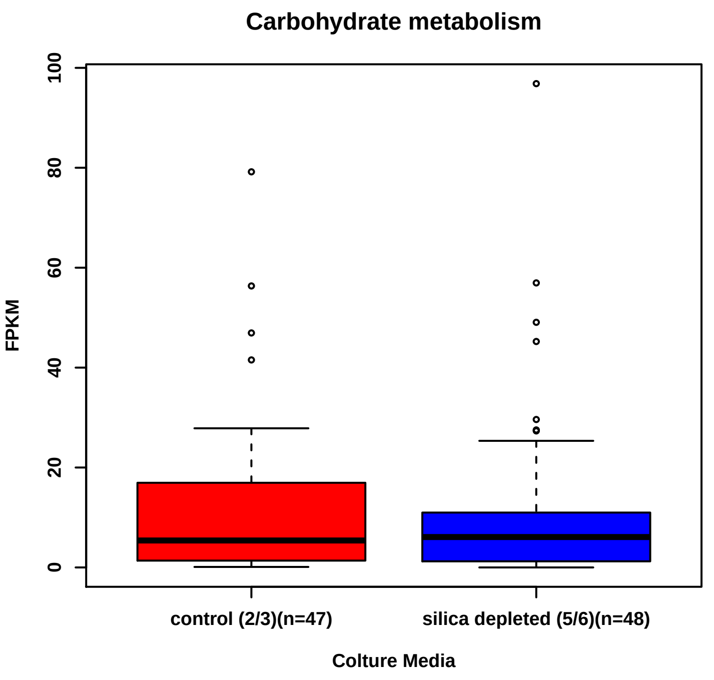
Carbohydrate metabolism
FPKM
0
20
40
60
80
100
control (2/3)(n=47)
silica depleted (5/6)(n=48)
Colture Media

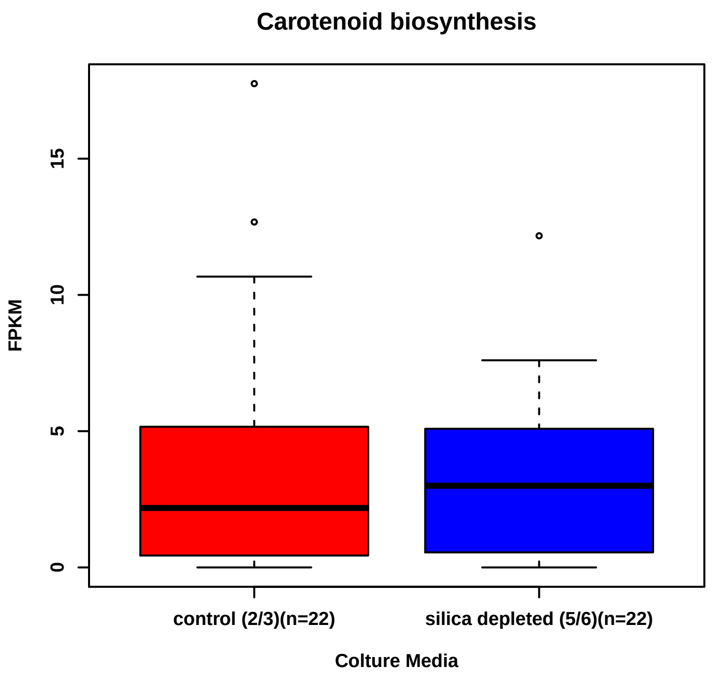
Carotenoid biosynthesis
FPKM
0
5
10
15
control (2/3)(n=22)
silica depleted (5/6)(n=22)
Colture Media

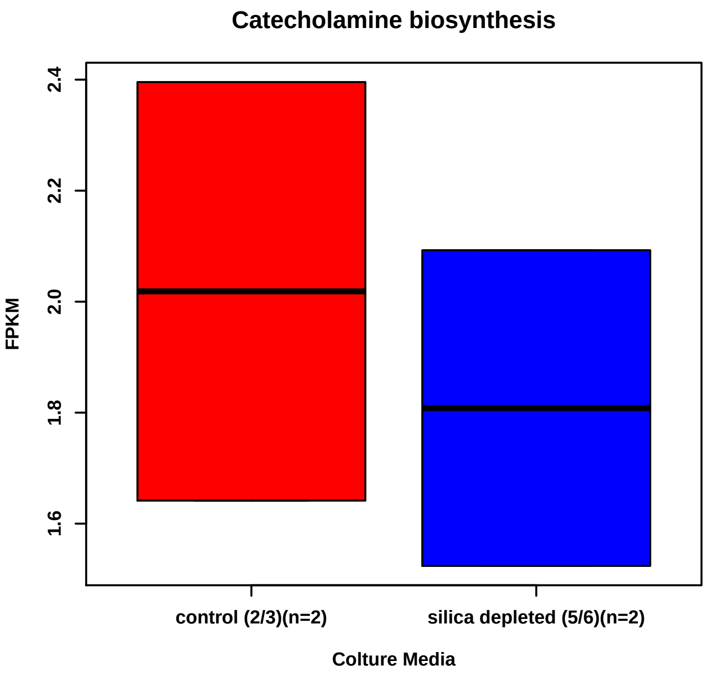
Catecholamine biosynthesis
FPKM
1.6
1.8
2.0
2.2
2.4
control (2/3)(n=2)
silica depleted (5/6)(n=2)
Colture Media

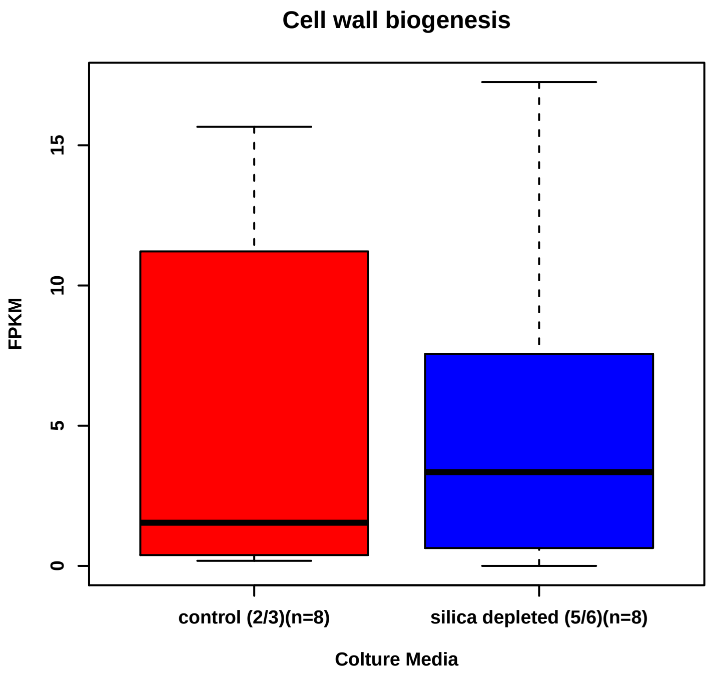
Cell wall biogenesis
FPKM
0
5
10
15
control (2/3)(n=8)
silica depleted (5/6)(n=8)
Colture Media

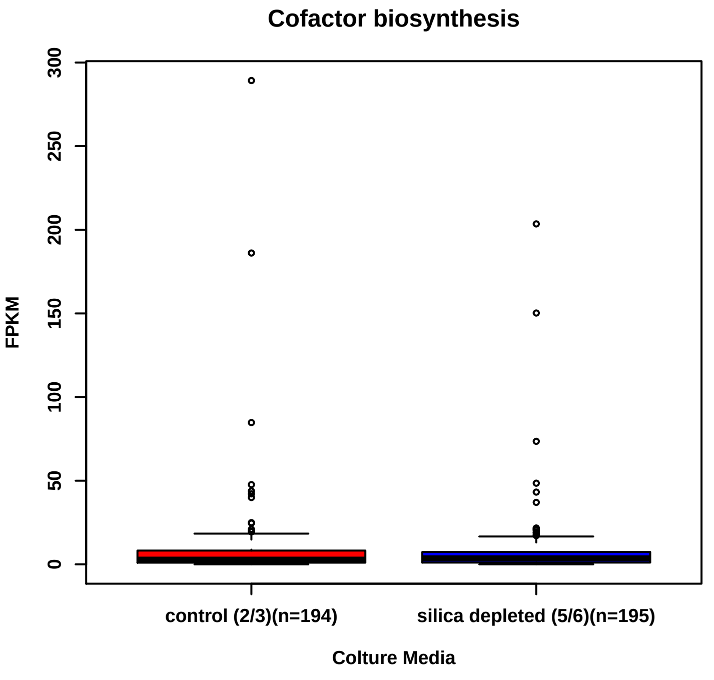
Cofactor biosynthesis
FPKM
0
50
100
150
200
250
300
control (2/3)(n=194)
silica depleted (5/6)(n=195)
Colture Media

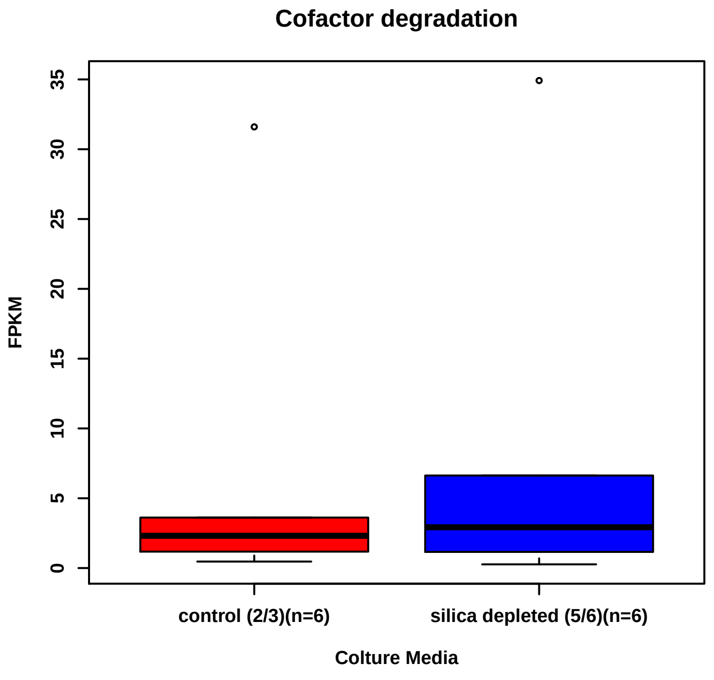
Cofactor degradation
FPKM
0
5
10
15
20
25
30
35
control (2/3)(n=6)
silica depleted (5/6)(n=6)
Colture Media

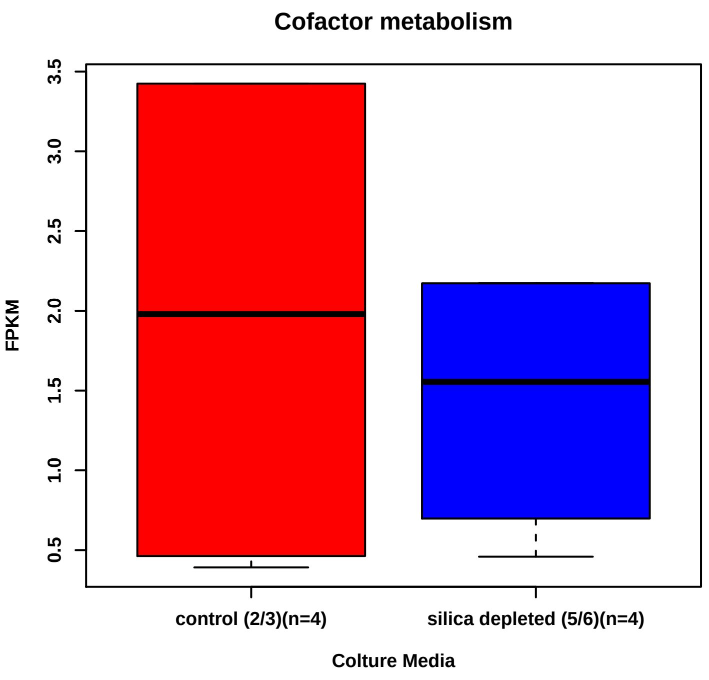

Cofactor metabolism
FPKM
0.5
1.0
1.5
2.0
2.5
3.0
3.5
control (2/3)(n=4)
silica depleted (5/6)(n=4)
Colture Media

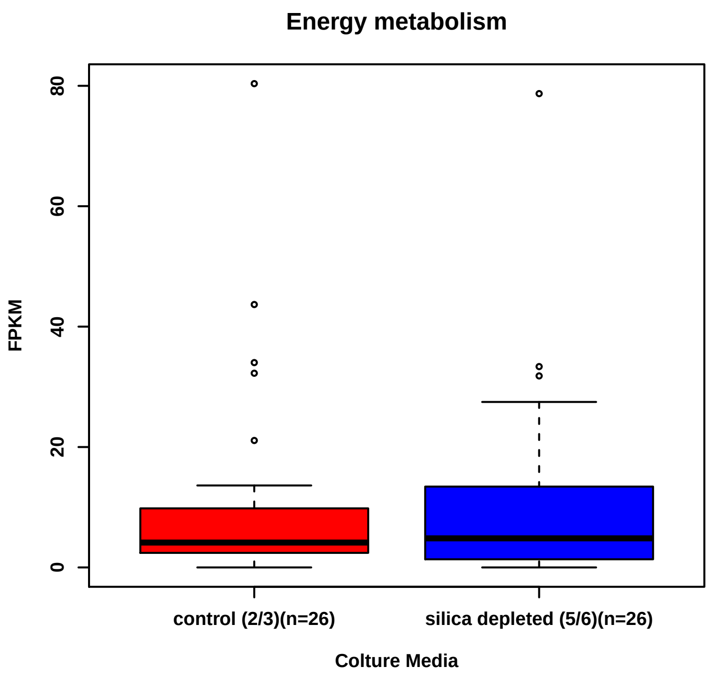

Energy metabolism
FPKM
0
20
40
60
80
control (2/3)(n=26)
silica depleted (5/6)(n=26)
Colture Media

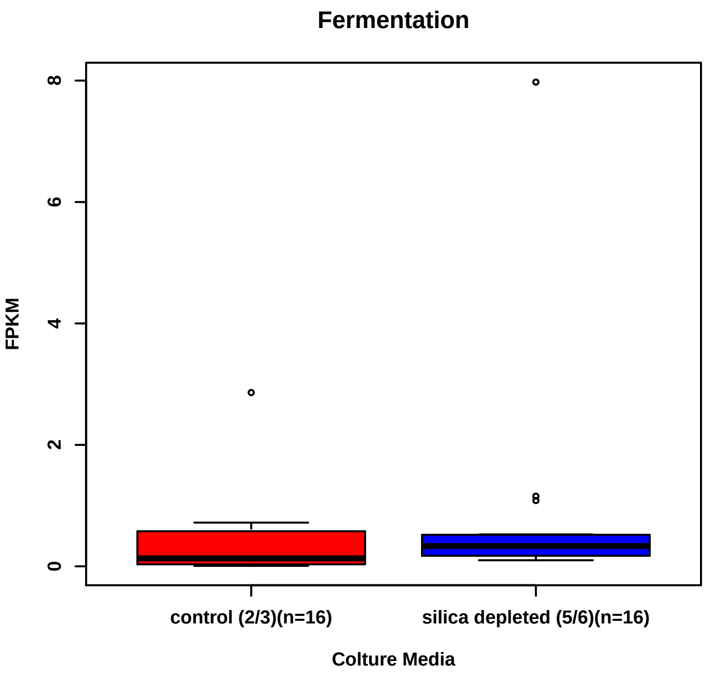

Fermentation
FPKM
0
2
4
6
8
control (2/3)(n=16)
silica depleted (5/6)(n=16)
Colture Media

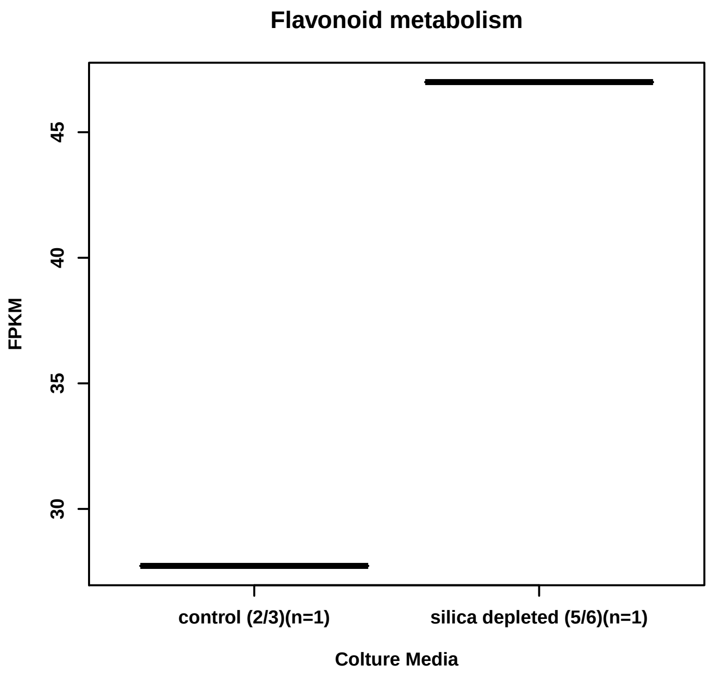

Flavonoid metabolism
FPKM
30
35
40
45
control (2/3)(n=1)
silica depleted (5/6)(n=1)
Colture Media

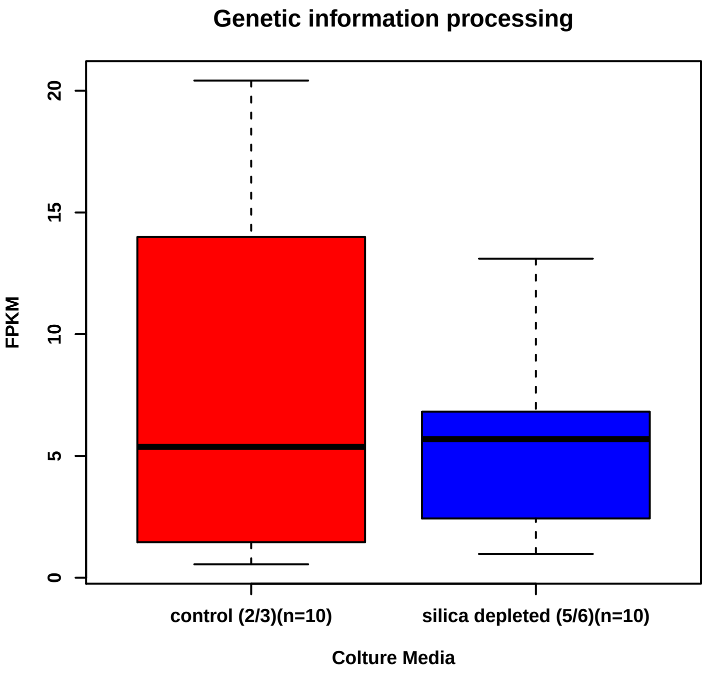

Genetic information processing
FPKM
0
5
10
15
20
control (2/3)(n=10)
silica depleted (5/6)(n=10)
Colture Media

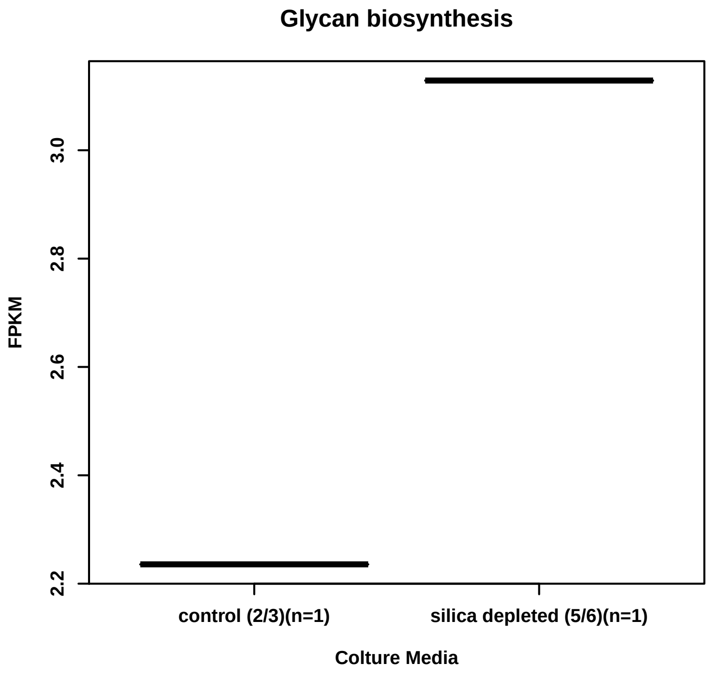

Glycan biosynthesis
FPKM
2.2
2.4
2.6
2.8
3.0
control (2/3)(n=1)
silica depleted (5/6)(n=1)
Colture Media

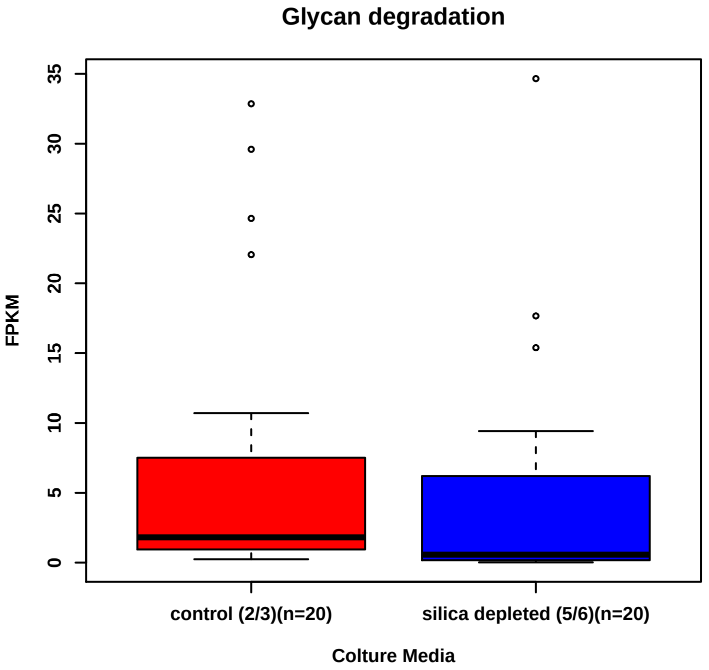
Glycan degradation
FPKM
0
5
10
15
20
25
30
35
control (2/3)(n=20)
silica depleted (5/6)(n=20)
Colture Media

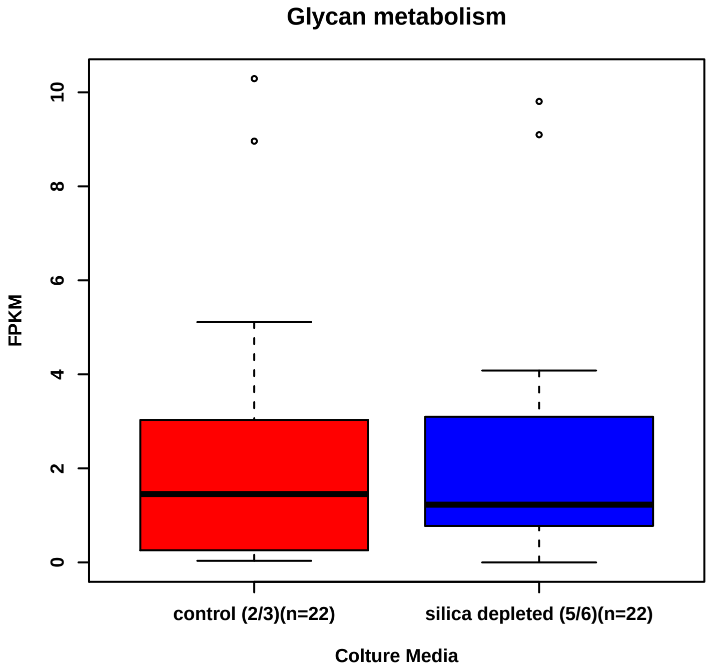
Glycan metabolism
FPKM
0
2
4
6
8
10
control (2/3)(n=22)
silica depleted (5/6)(n=22)
Colture Media

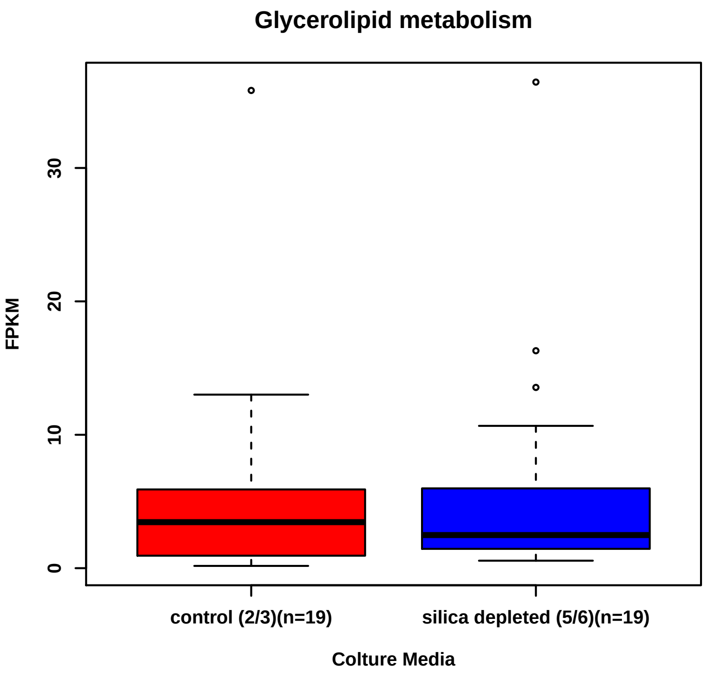
Glycerolipid metabolism
FPKM
0
10
20
30
control (2/3)(n=19)
silica depleted (5/6)(n=19)
Colture Media

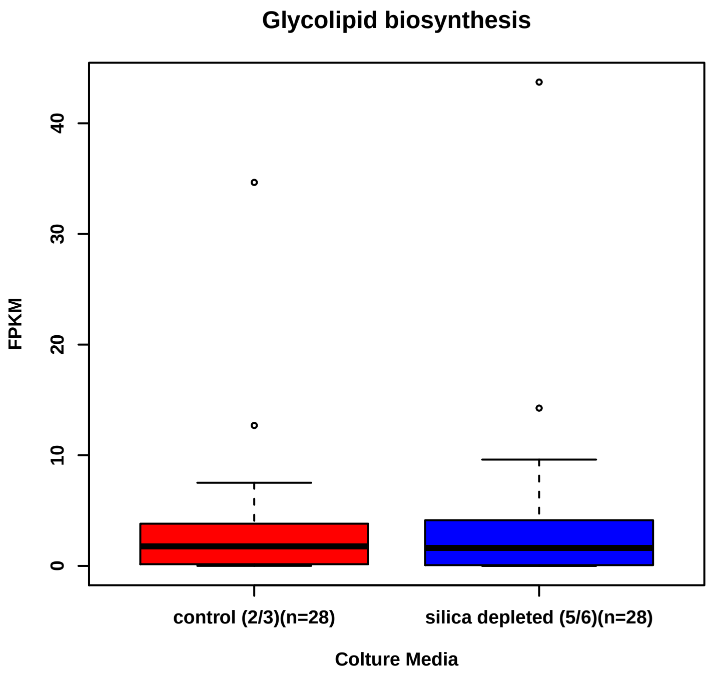
Glycolipid biosynthesis
FPKM
0
10
20
30
40
control (2/3)(n=28)
silica depleted (5/6)(n=28)
Colture Media

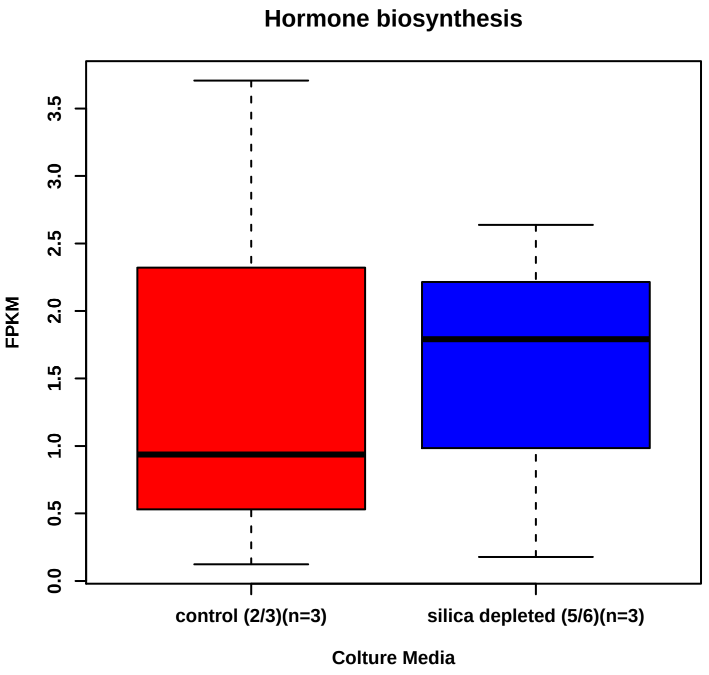
Hormone biosynthesis
FPKM
0.0
0.5
1.0
1.5
2.0
2.5
3.0
3.5
control (2/3)(n=3)
silica depleted (5/6)(n=3)
Colture Media

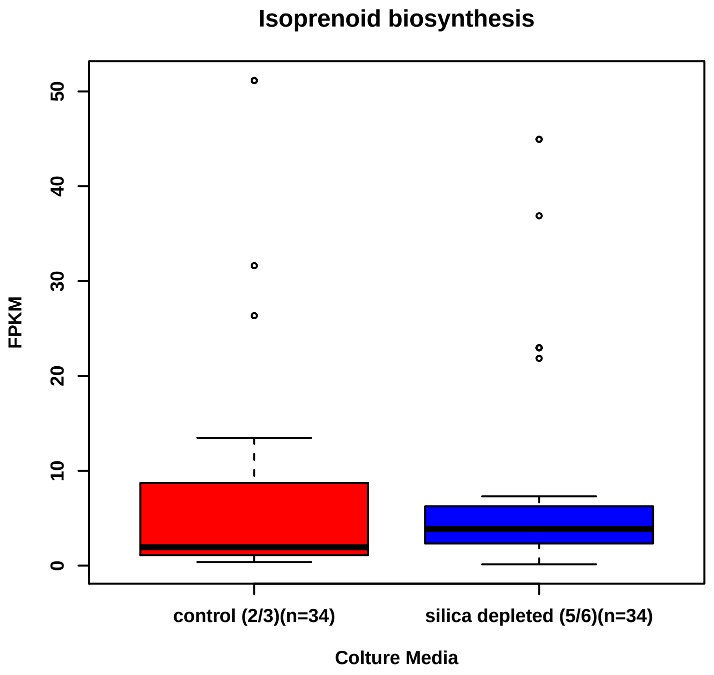
Isoprenoid biosynthesis
FPKM
0
10
20
30
40
50
control (2/3)(n=34)
silica depleted (5/6)(n=34)
Colture Media

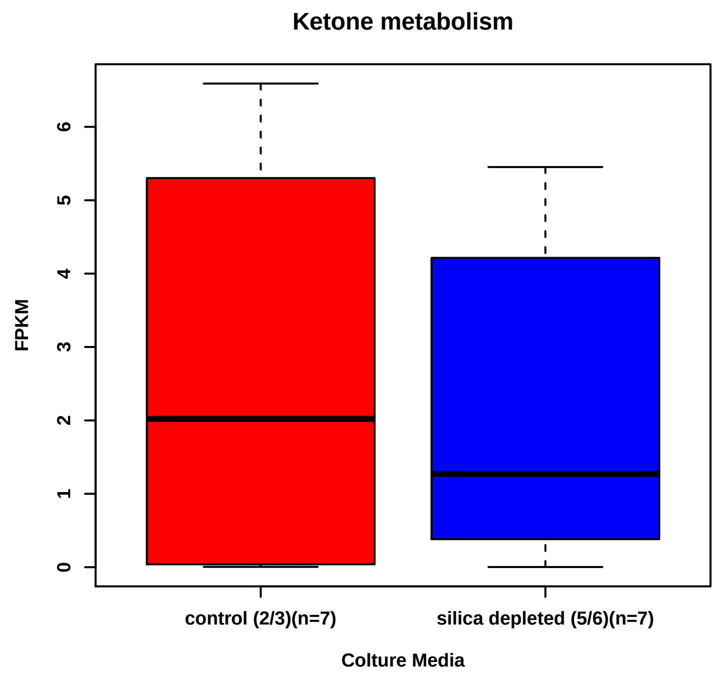
Ketone metabolism
FPKM
0
1
2
3
4
5
6
control (2/3)(n=7)
silica depleted (5/6)(n=7)
Colture Media

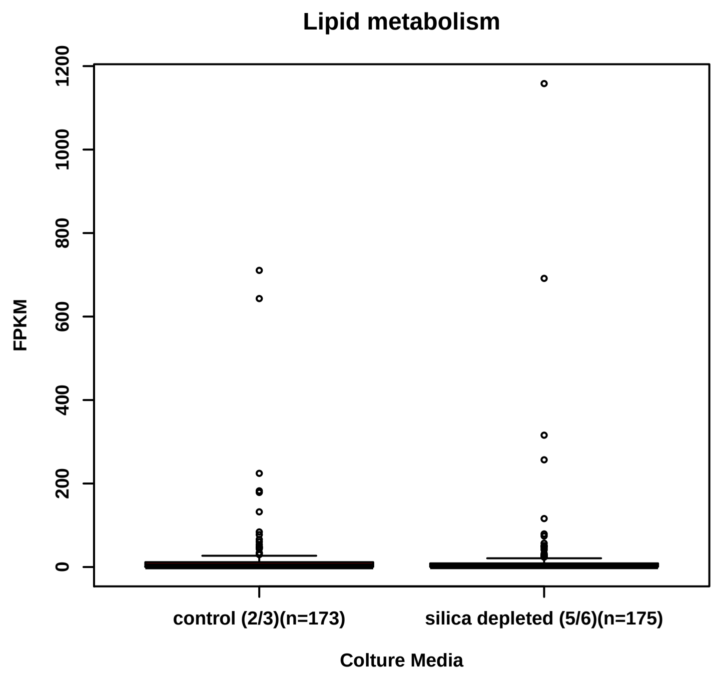
Lipid metabolism
FPKM
0
200
400
600
800
1000
1200
control (2/3)(n=173)
silica depleted (5/6)(n=175)
Colture Media

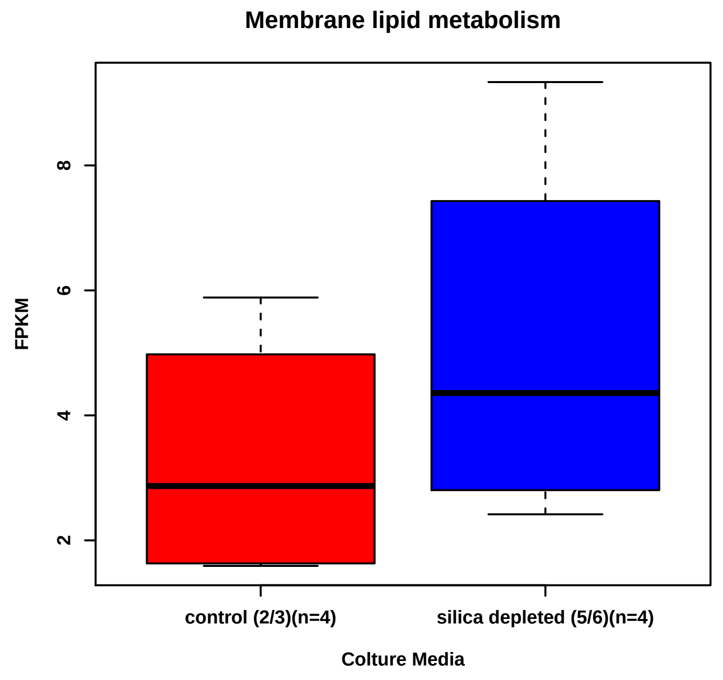
Membrane lipid metabolism
FPKM
2
4
6
8
control (2/3)(n=4)
silica depleted (5/6)(n=4)
Colture Media

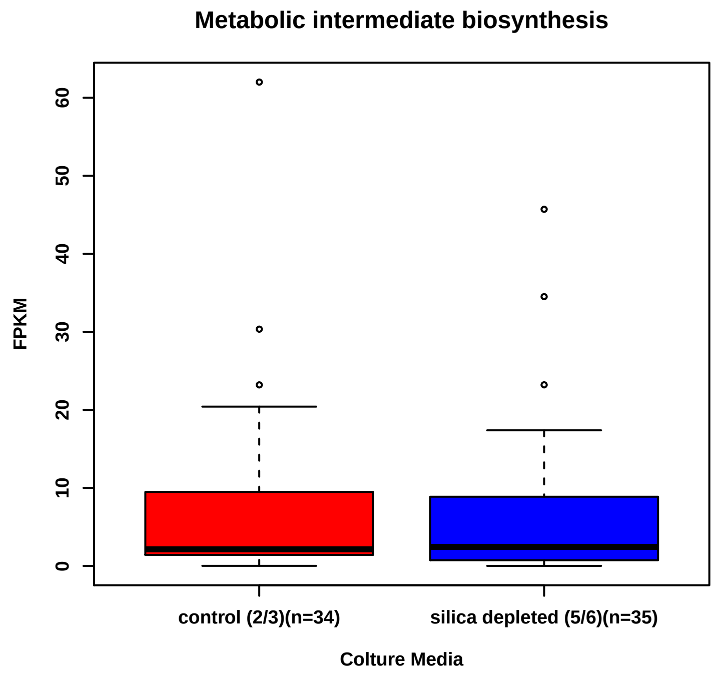
Metabolic intermediate biosynthesis
FPKM
0
10
20
30
40
50
60
control (2/3)(n=34)
silica depleted (5/6)(n=35)
Colture Media

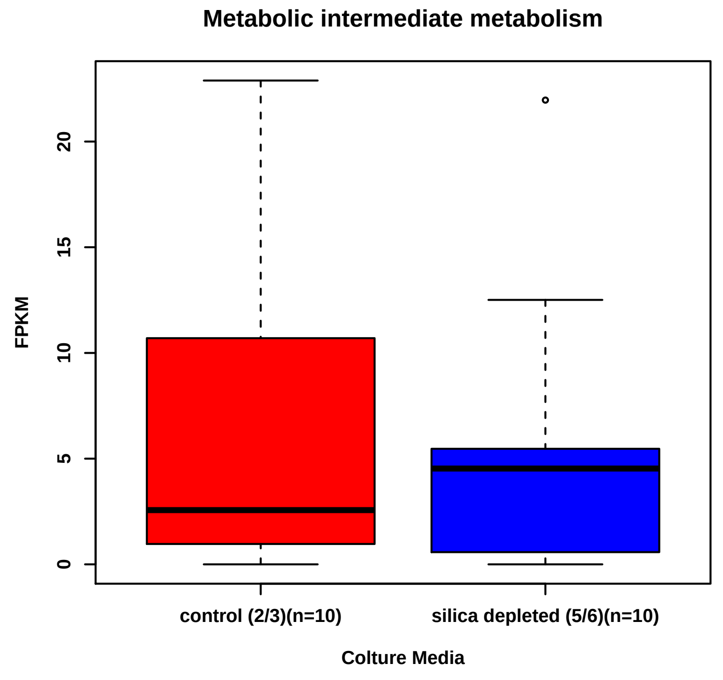
Metabolic intermediate metabolism
FPKM
0
5
10
15
20
control (2/3)(n=10)
silica depleted (5/6)(n=10)
Colture Media

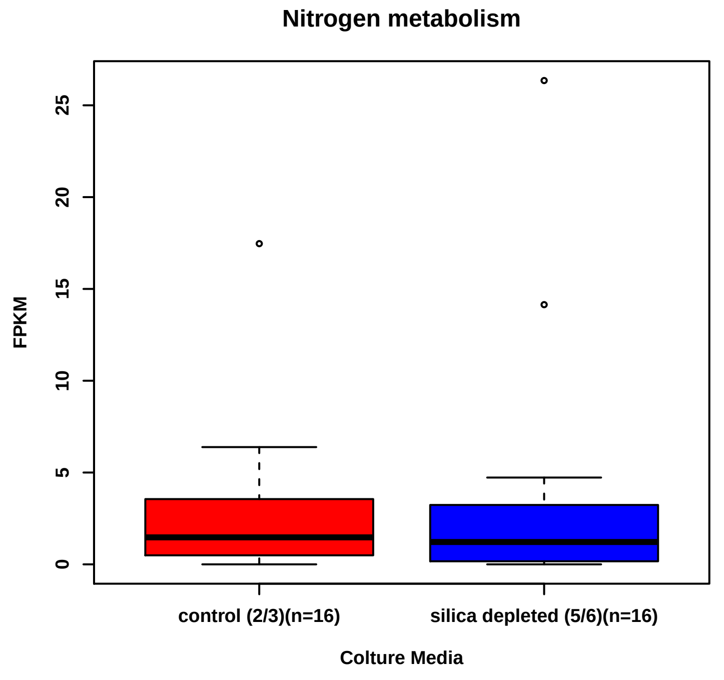
Nitrogen metabolism
FPKM
0
5
10
15
20
25
control (2/3)(n=16)
silica depleted (5/6)(n=16)
Colture Media

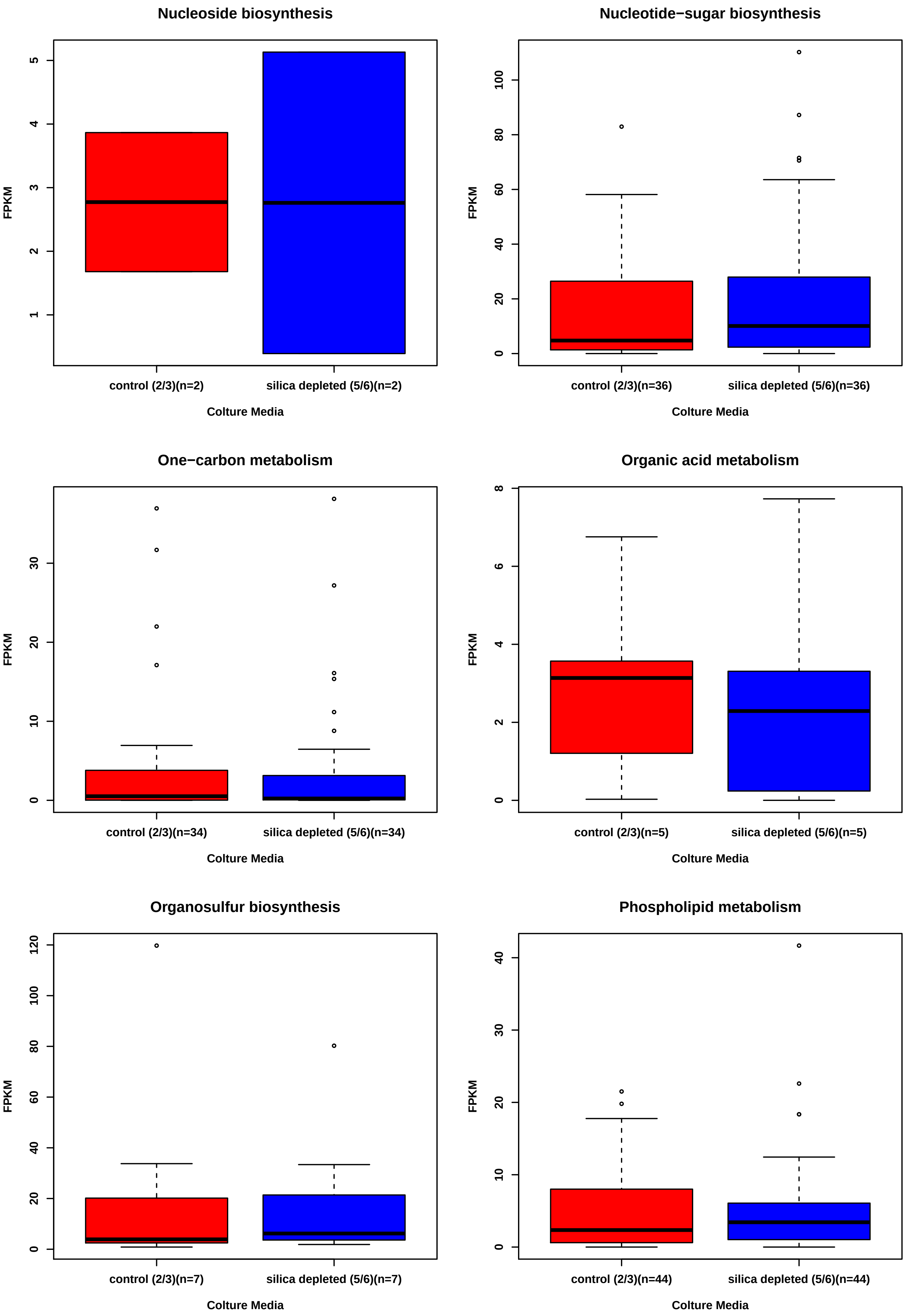
Nucleoside biosynthesis
FPKM
1
2
3
4
5
control (2/3)(n=2)
silica depleted (5/6)(n=2)
Colture Media
Nucleotide−sugar biosynthesis
FPKM
0
20
40
60
80
100
control (2/3)(n=36)
silica depleted (5/6)(n=36)
Colture Media
One−carbon metabolism
FPKM
0
10
20
30
control (2/3)(n=34)
silica depleted (5/6)(n=34)
Colture Media
Organic acid metabolism
FPKM
0
2
4
6
8
control (2/3)(n=5)
silica depleted (5/6)(n=5)
Colture Media
Organosulfur biosynthesis
FPKM
0
20
40
60
80
100
120
control (2/3)(n=7)
silica depleted (5/6)(n=7)
Colture Media
Phospholipid metabolism
FPKM
0
10
20
30
40
control (2/3)(n=44)
silica depleted (5/6)(n=44)
Colture Media

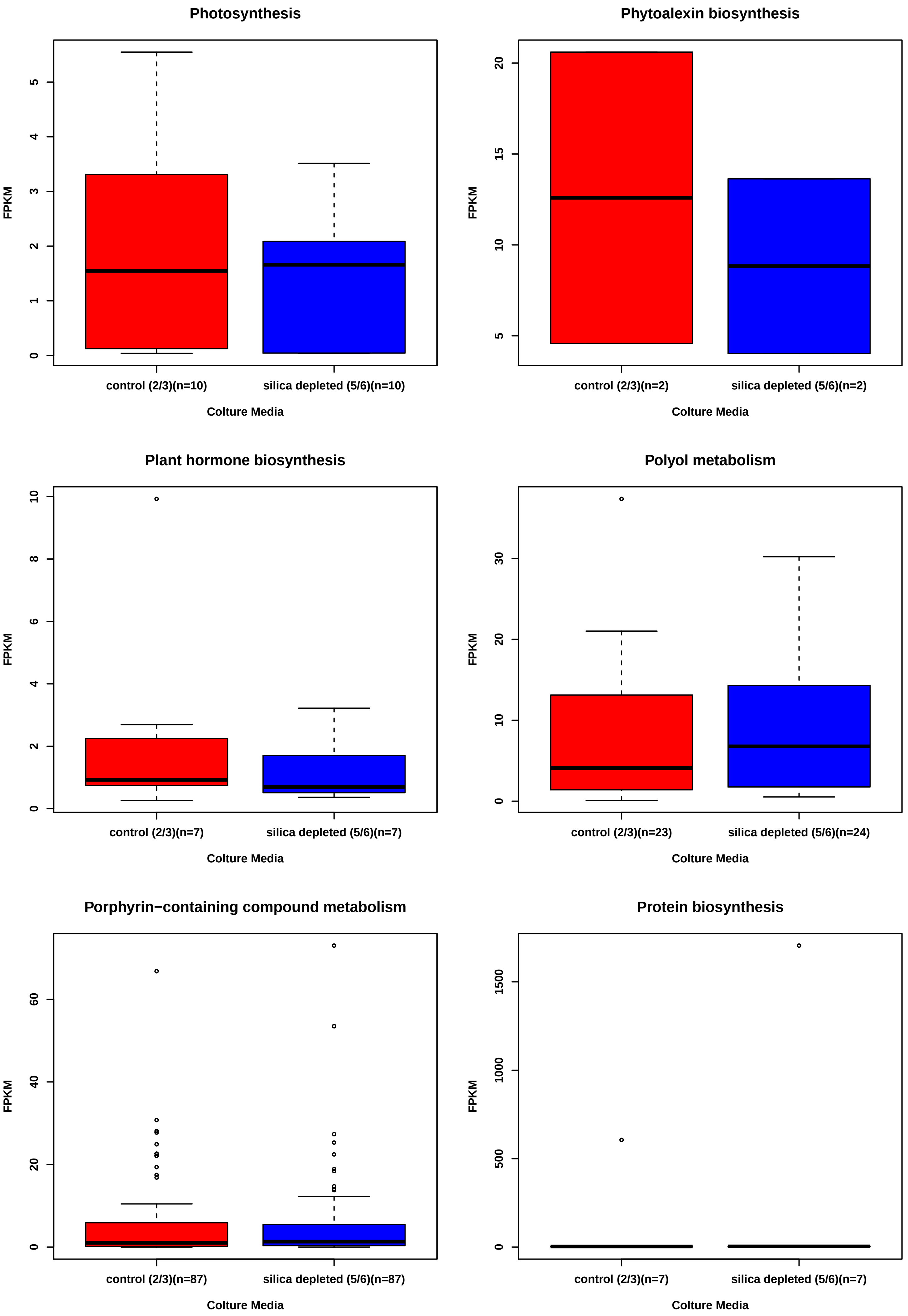

Photosynthesis
FPKM
0
1
2
3
4
5
control (2/3)(n=10)
silica depleted (5/6)(n=10)
Colture Media
Phytoalexin biosynthesis
FPKM
5
10
15
20
control (2/3)(n=2)
silica depleted (5/6)(n=2)
Colture Media
Plant hormone biosynthesis
FPKM
0
2
4
6
8
10
control (2/3)(n=7)
silica depleted (5/6)(n=7)
Colture Media
Polyol metabolism
FPKM
0
10
20
30
control (2/3)(n=23)
silica depleted (5/6)(n=24)
Colture Media
Porphyrin−containing compound metabolism
FPKM
0
20
40
60
control (2/3)(n=87)
silica depleted (5/6)(n=87)
Colture Media
Protein biosynthesis
FPKM
0
500
1000
1500
control (2/3)(n=7)
silica depleted (5/6)(n=7)
Colture Media

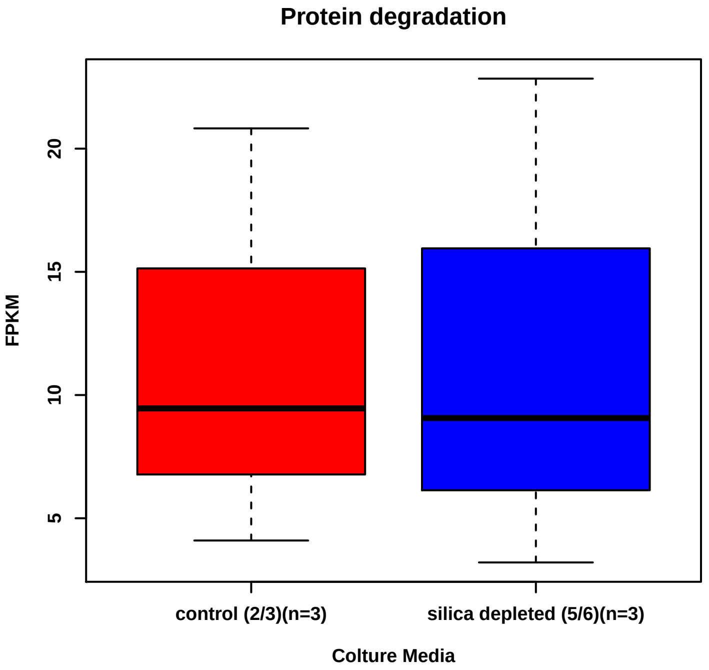
Protein degradation
FPKM
5
10
15
20
control (2/3)(n=3)
silica depleted (5/6)(n=3)
Colture Media

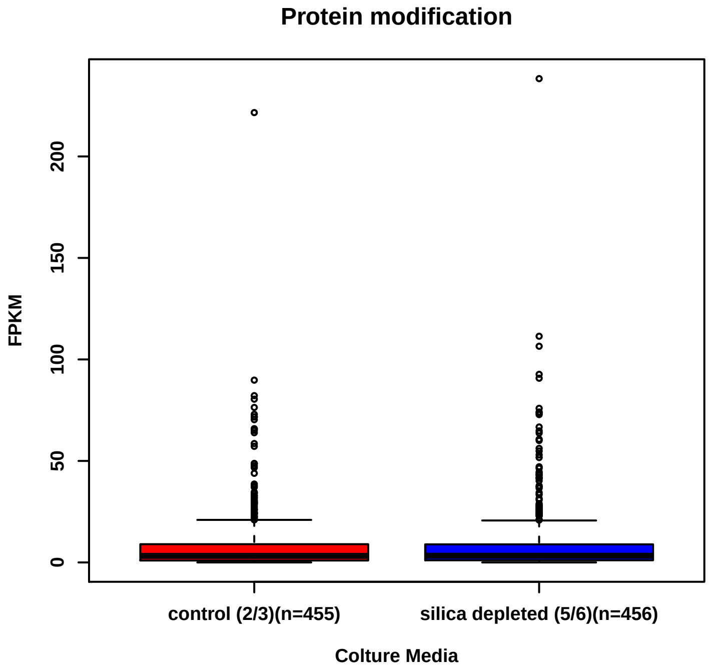
Protein modification
FPKM
0
50
100
150
200
control (2/3)(n=455)
silica depleted (5/6)(n=456)
Colture Media

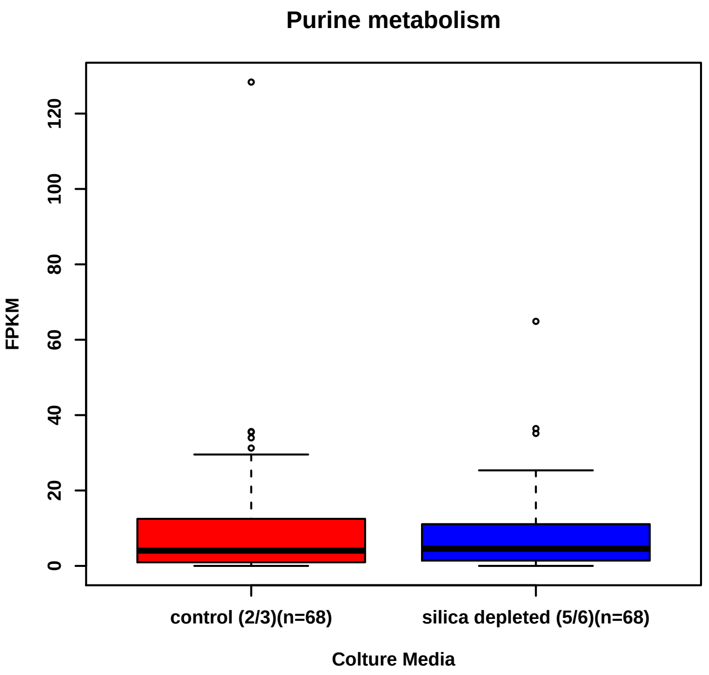
Purine metabolism
FPKM
0
20
40
60
80
100
120
control (2/3)(n=68)
silica depleted (5/6)(n=68)
Colture Media

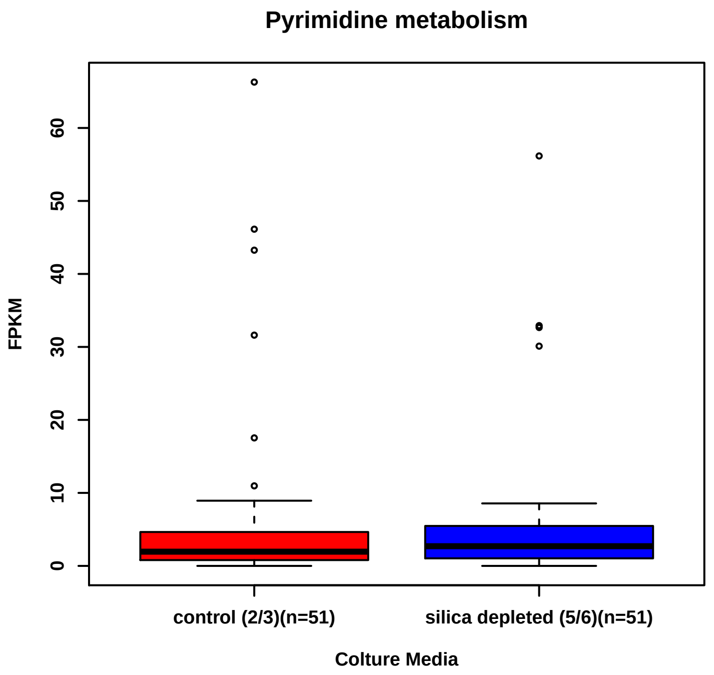
Pyrimidine metabolism
FPKM
0
10
20
30
40
50
60
control (2/3)(n=51)
silica depleted (5/6)(n=51)
Colture Media

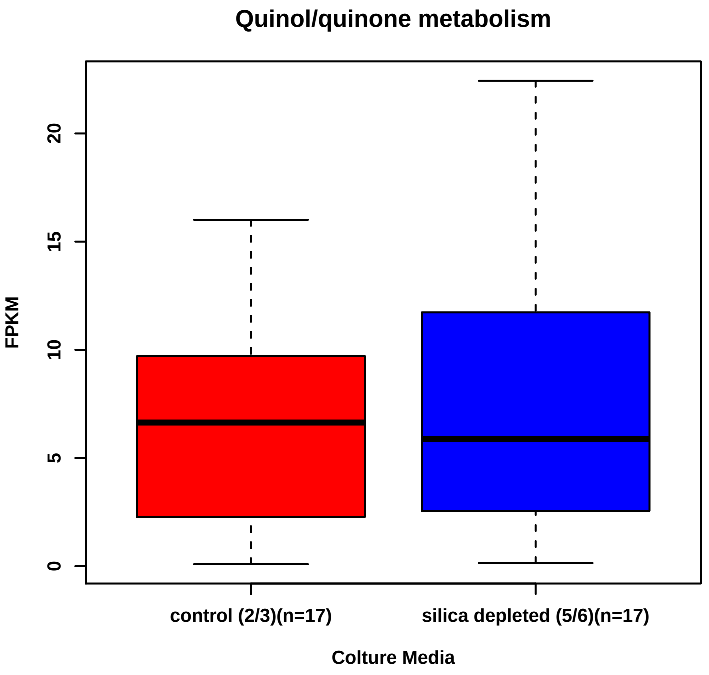
Quinol/quinone metabolism
FPKM
0
5
10
15
20
control (2/3)(n=17)
silica depleted (5/6)(n=17)
Colture Media

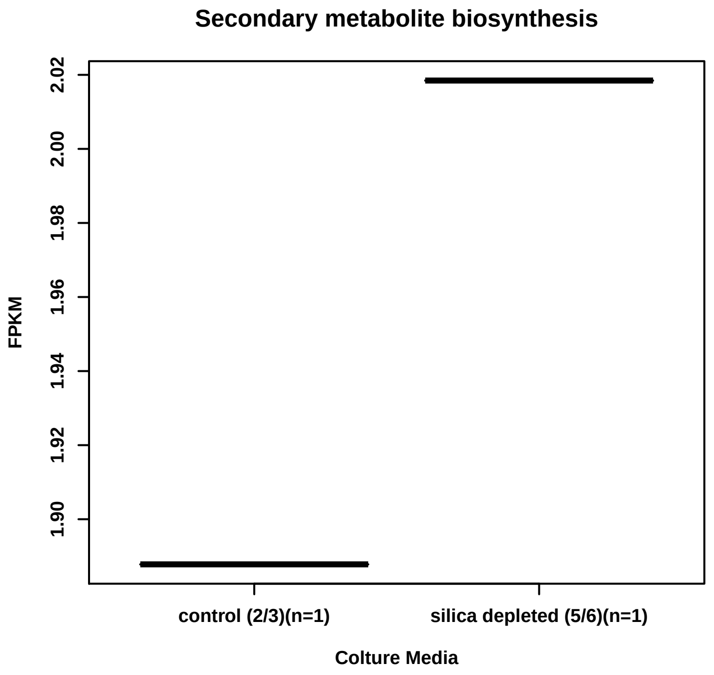
Secondary metabolite biosynthesis
FPKM
1.90
1.92
1.94
1.96
1.98
2.00
2.02
control (2/3)(n=1)
silica depleted (5/6)(n=1)
Colture Media

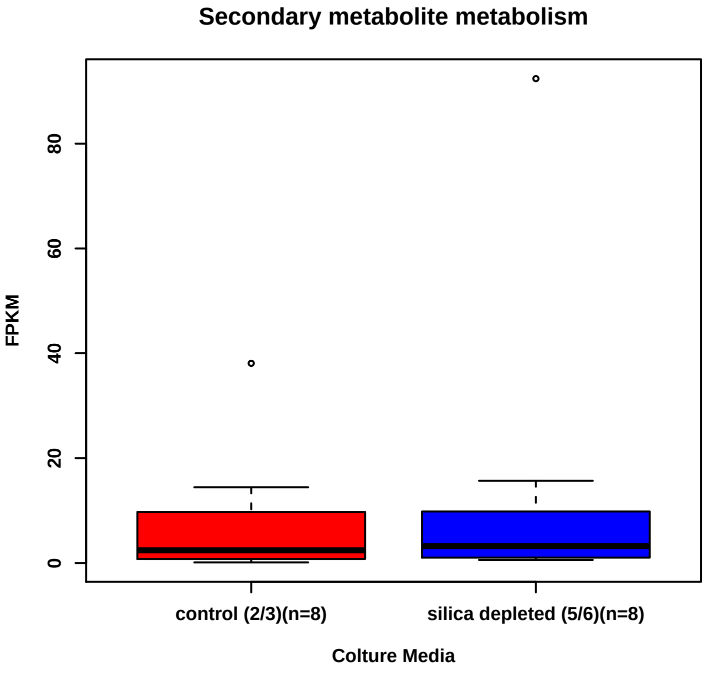

Secondary metabolite metabolism
FPKM
0
20
40
60
80
control (2/3)(n=8)
silica depleted (5/6)(n=8)
Colture Media

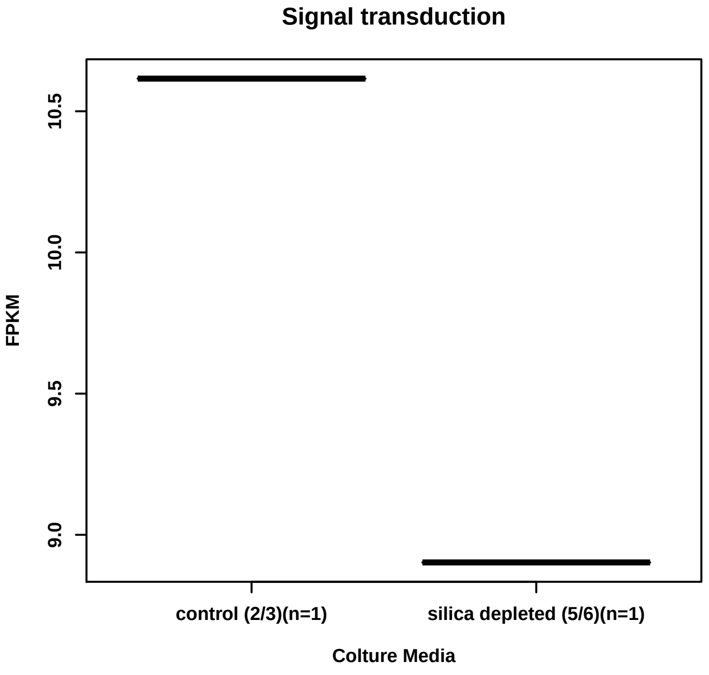

Signal transduction
FPKM
9.0
9.5
10.0
10.5
control (2/3)(n=1)
silica depleted (5/6)(n=1)
Colture Media

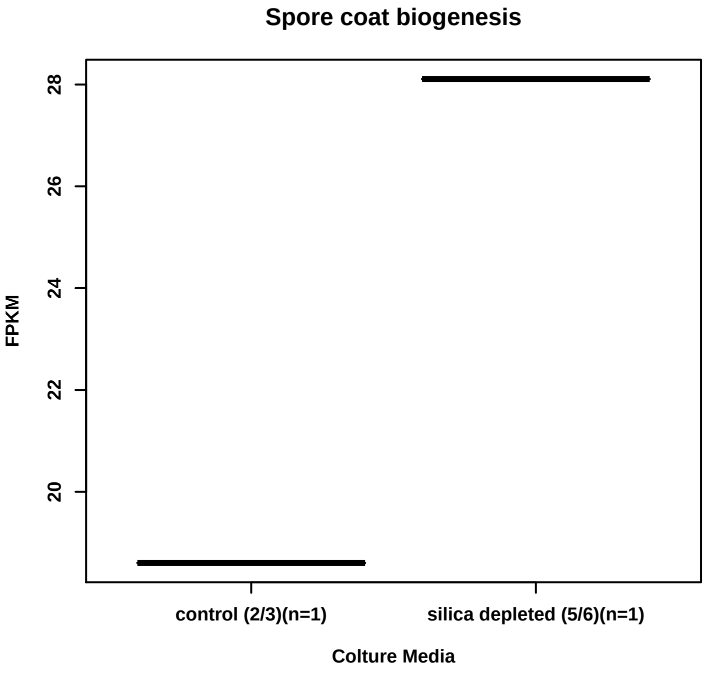

Spore coat biogenesis
FPKM
20
22
24
26
28
control (2/3)(n=1)
silica depleted (5/6)(n=1)
Colture Media

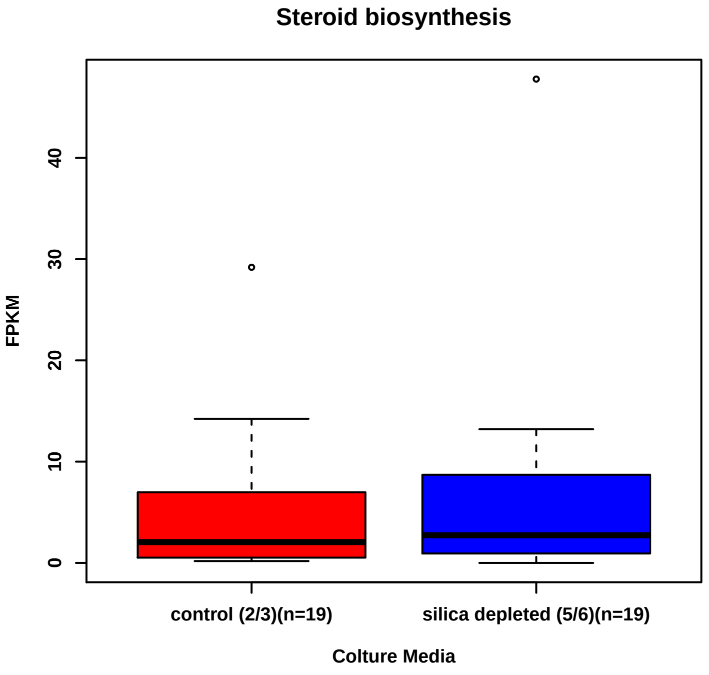

Steroid biosynthesis
FPKM
0
10
20
30
40
control (2/3)(n=19)
silica depleted (5/6)(n=19)
Colture Media

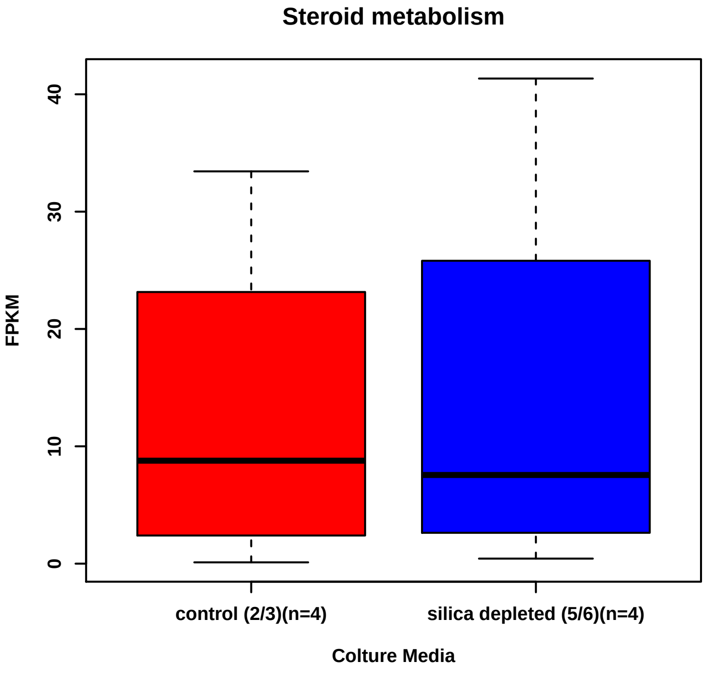

Steroid metabolism
FPKM
0
10
20
30
40
control (2/3)(n=4)
silica depleted (5/6)(n=4)
Colture Media

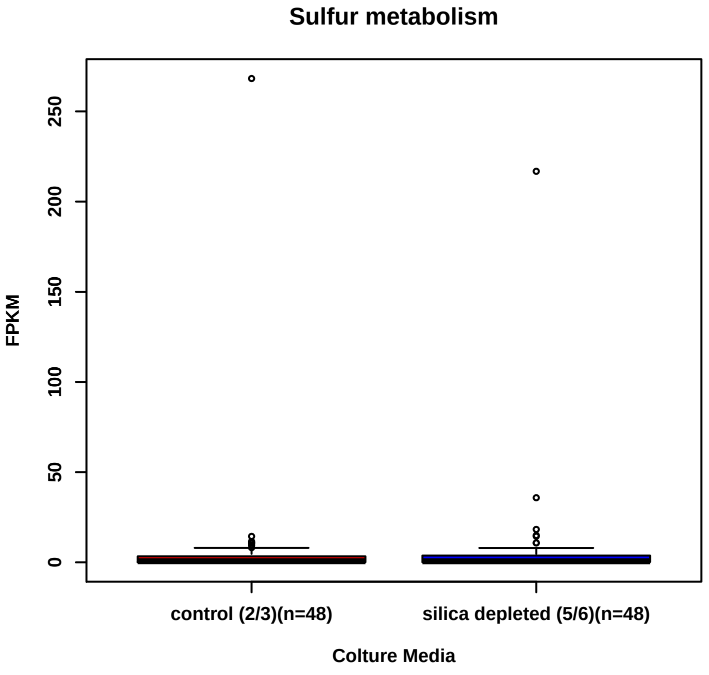

Sulfur metabolism
FPKM
0
50
100
150
200
250
control (2/3)(n=48)
silica depleted (5/6)(n=48)
Colture Media

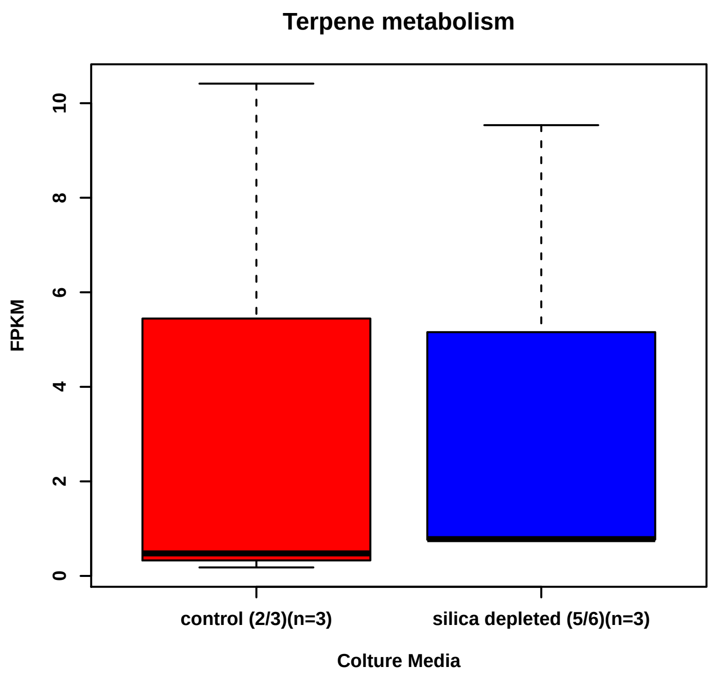
Terpene metabolism
FPKM
0
2
4
6
8
10
control (2/3)(n=3)
silica depleted (5/6)(n=3)
Colture Media

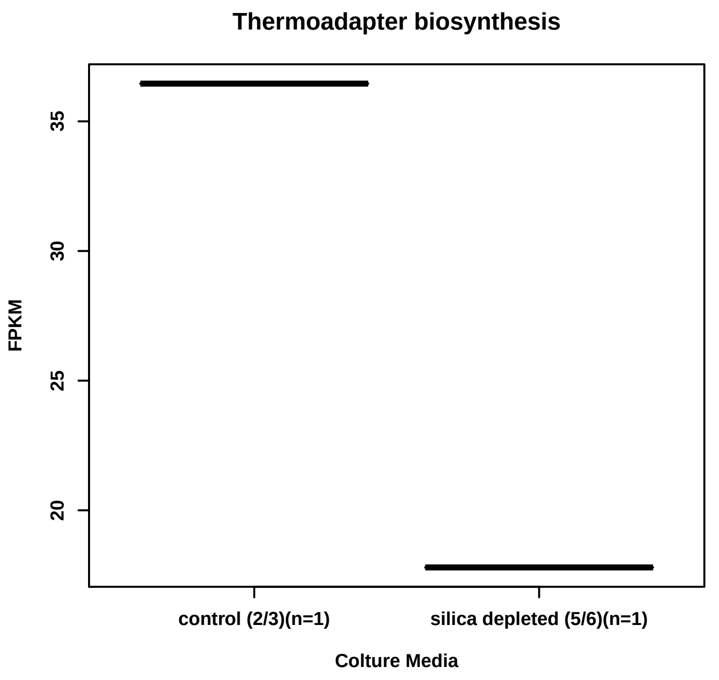
Thermoadapter biosynthesis
FPKM
20
25
30
35
control (2/3)(n=1)
silica depleted (5/6)(n=1)
Colture Media

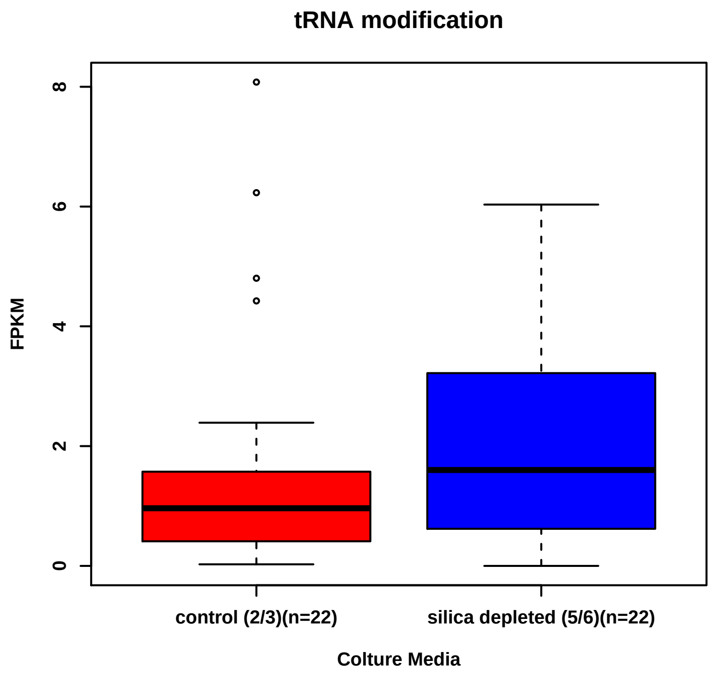
tRNA modification
FPKM
0
2
4
6
8
control (2/3)(n=22)
silica depleted (5/6)(n=22)
Colture Media

Unveiling the presence of biosynthetic pathways for bioactive compounds in the *Thalassiosira rotula* transcriptome

Valeria Di Dato*, Federica Di Costanzo, Roberta Barbarinaldi, Anna Perna, Adrianna Ianora, Giovanna Romano

Supplementary File 3:

Box Plot Analysis of the expression, in FPKM, of the pathways annotated on level 2 for each replicate in each condition

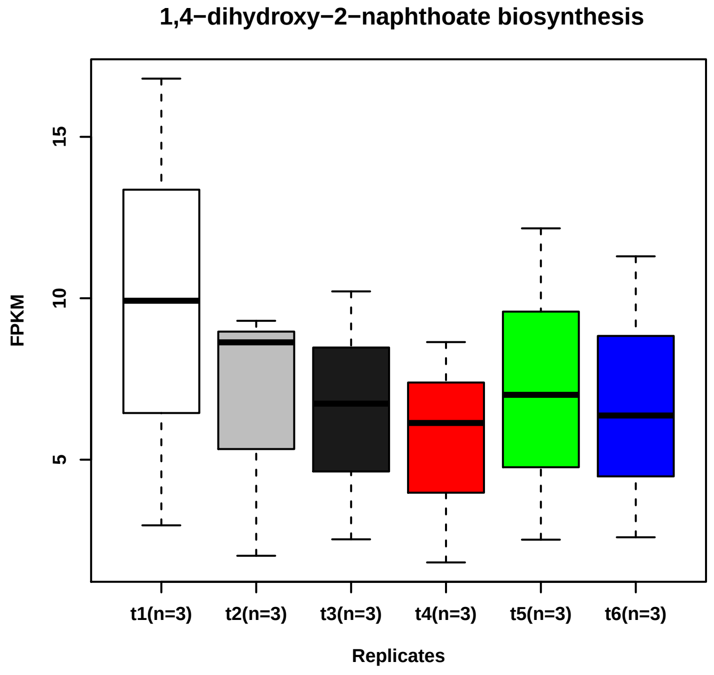
1,4−dihydroxy−2−naphthoate biosynthesis
FPKM
5
10
15
t1(n=3)
t2(n=3)
t3(n=3)
t4(n=3)
t5(n=3)
t6(n=3)
Replicates

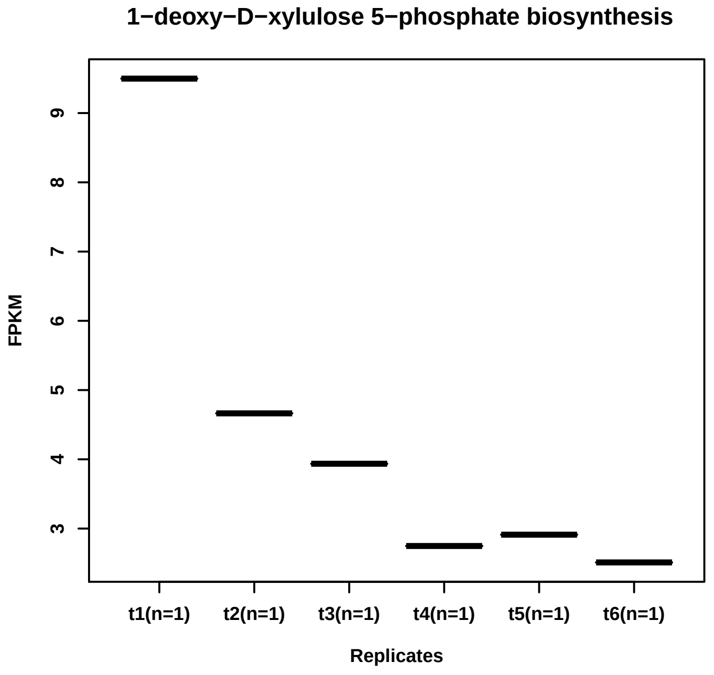
1−deoxy−D−xylulose 5−phosphate biosynthesis
FPKM
3
4
5
6
7
8
9
t1(n=1)
t2(n=1)
t3(n=1)
t4(n=1)
t5(n=1)
t6(n=1)
Replicates

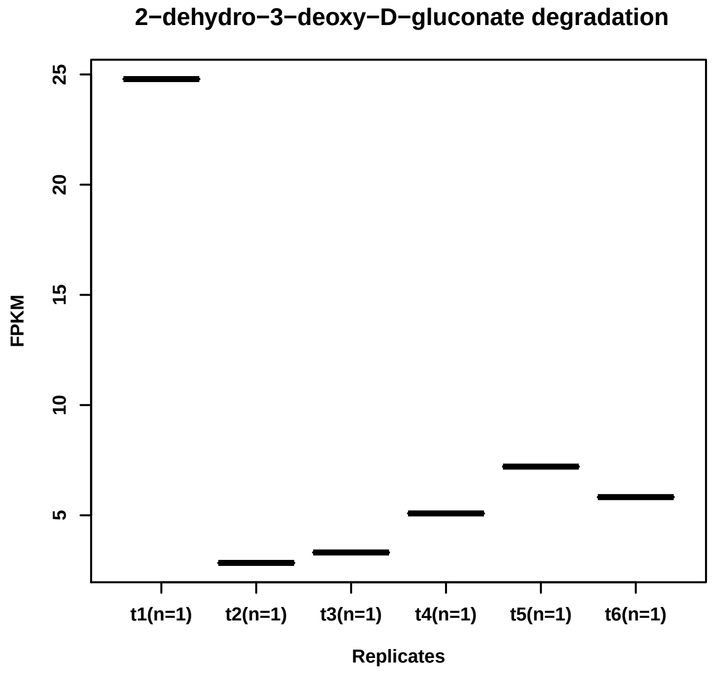
2−dehydro−3−deoxy−D−gluconate degradation
FPKM
5
10
15
20
25
t1(n=1)
t2(n=1)
t3(n=1)
t4(n=1)
t5(n=1)
t6(n=1)
Replicates

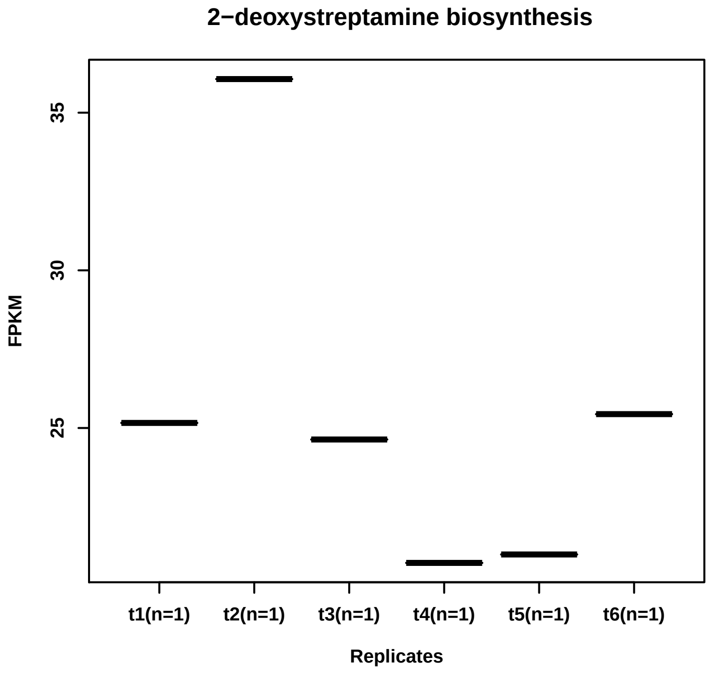
2−deoxystreptamine biosynthesis
FPKM
25
30
35
t1(n=1)
t2(n=1)
t3(n=1)
t4(n=1)
t5(n=1)
t6(n=1)
Replicates

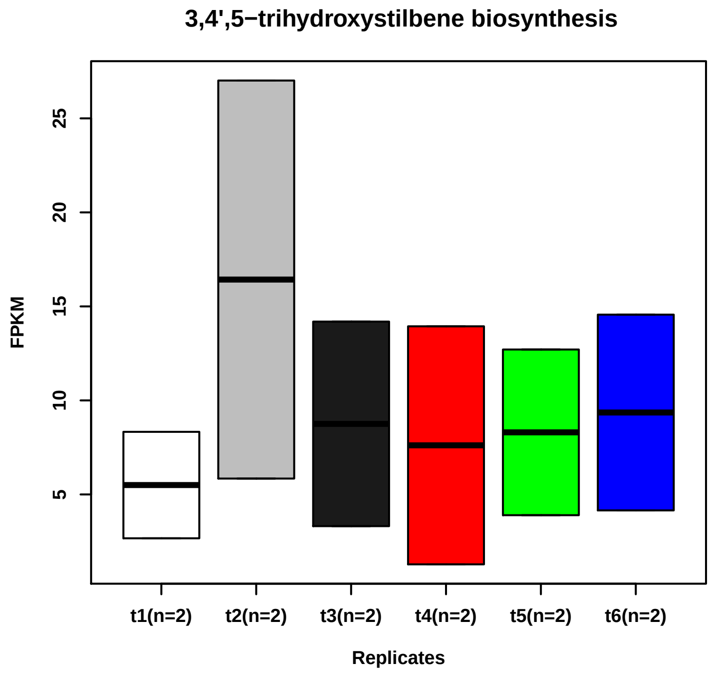
3,4',5−trihydroxystilbene biosynthesis
FPKM
5
10
15
20
25
t1(n=2)
t2(n=2)
t3(n=2)
t4(n=2)
t5(n=2)
t6(n=2)
Replicates

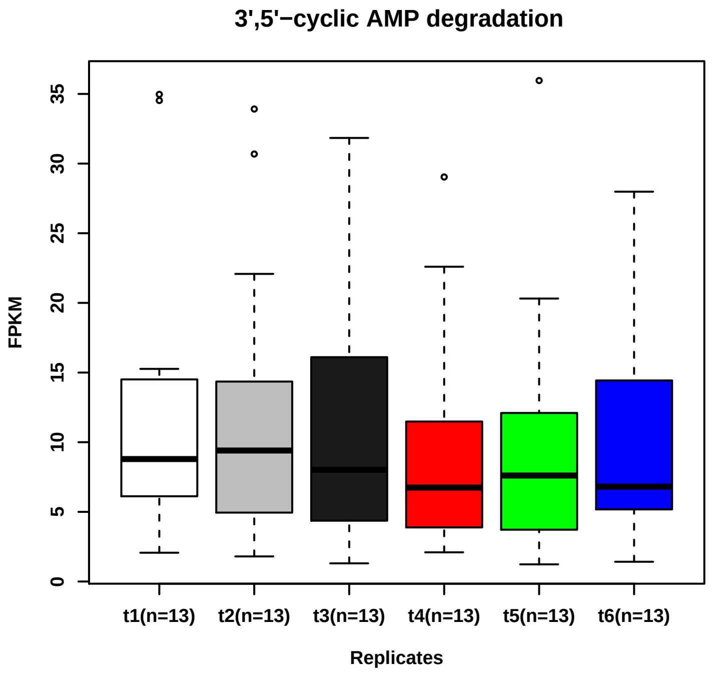
3',5'−cyclic AMP degradation
FPKM
0
5
10
15
20
25
30
35
t1(n=13)
t2(n=13)
t3(n=13)
t4(n=13)
t5(n=13)
t6(n=13)
Replicates

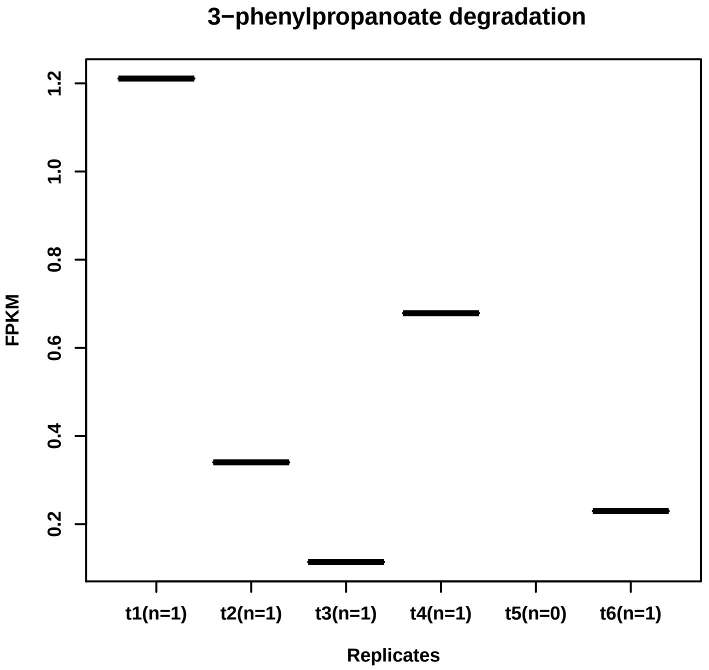
3−phenylpropanoate degradation
FPKM
0.2
0.4
0.6
0.8
1.0
1.2
t1(n=1)
t2(n=1)
t3(n=1)
t4(n=1)
t5(n=0)
t6(n=1)
Replicates

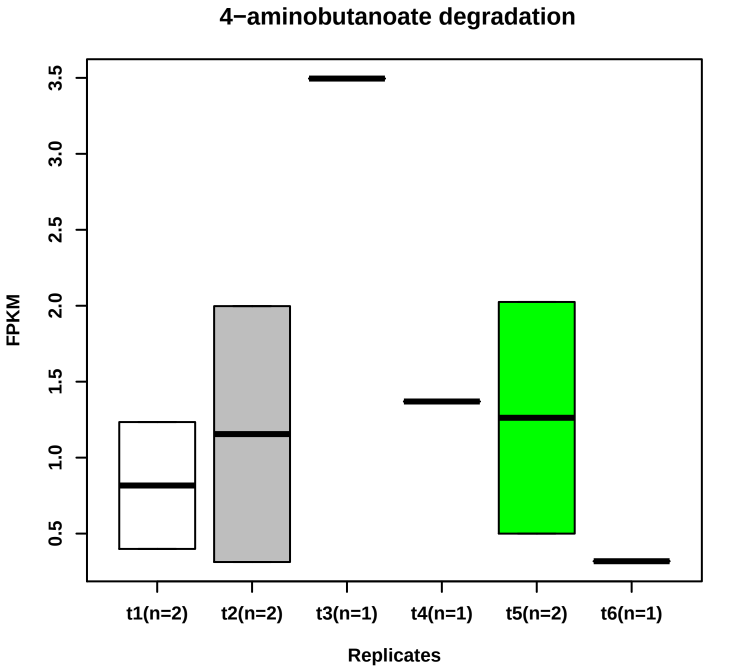
4−aminobutanoate degradation
FPKM
0.5
1.0
1.5
2.0
2.5
3.0
3.5
t1(n=2)
t2(n=2)
t3(n=1)
t4(n=1)
t5(n=2)
t6(n=1)
Replicates

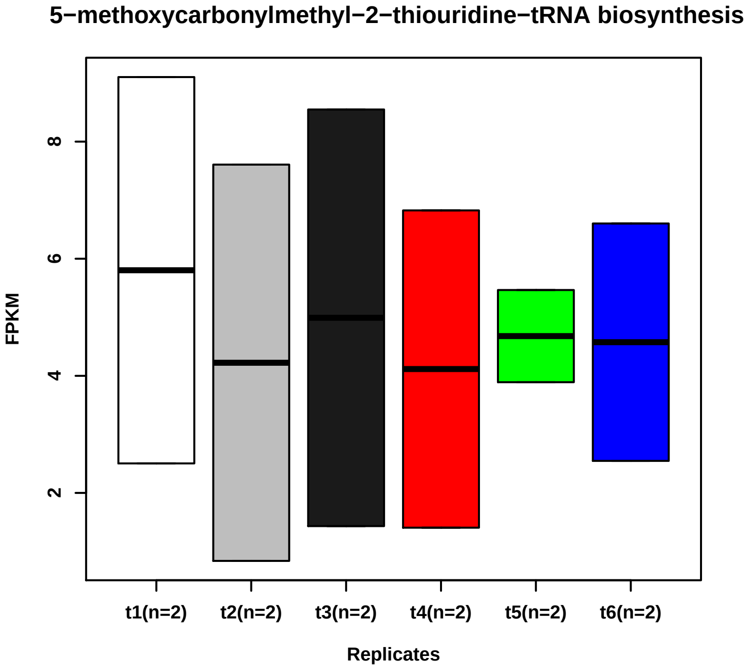
5−methoxycarbonylmethyl−2−thiouridine−tRNA biosynthesis
FPKM
2
4
6
8
t1(n=2)
t2(n=2)
t3(n=2)
t4(n=2)
t5(n=2)
t6(n=2)
Replicates

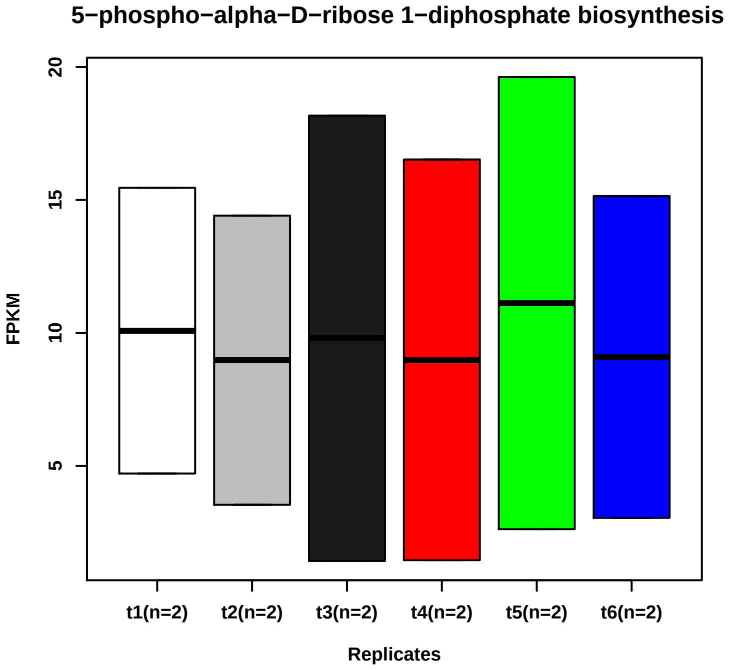
5−phospho−alpha−D−ribose 1−diphosphate biosynthesis
FPKM
5
10
15
20
t1(n=2)
t2(n=2)
t3(n=2)
t4(n=2)
t5(n=2)
t6(n=2)
Replicates

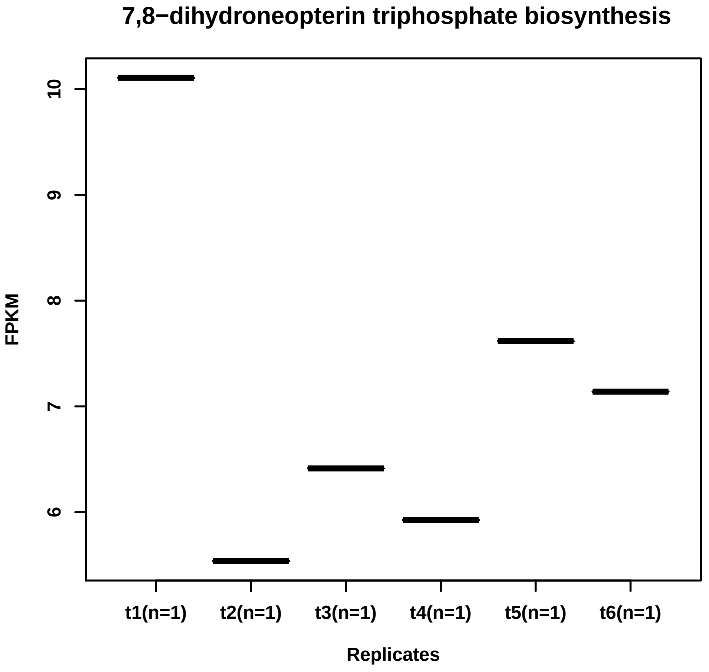
7,8−dihydroneopterin triphosphate biosynthesis
FPKM
6
7
8
9
10
t1(n=1)
t2(n=1)
t3(n=1)
t4(n=1)
t5(n=1)
t6(n=1)
Replicates

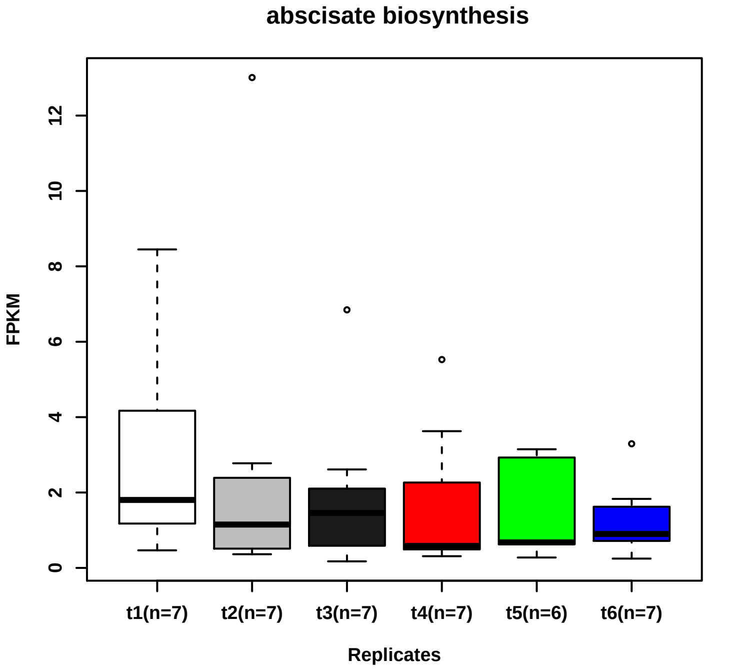
abscisate biosynthesis
FPKM
0
2
4
6
8
10
12
t1(n=7)
t2(n=7)
t3(n=7)
t4(n=7)
t5(n=6)
t6(n=7)
Replicates

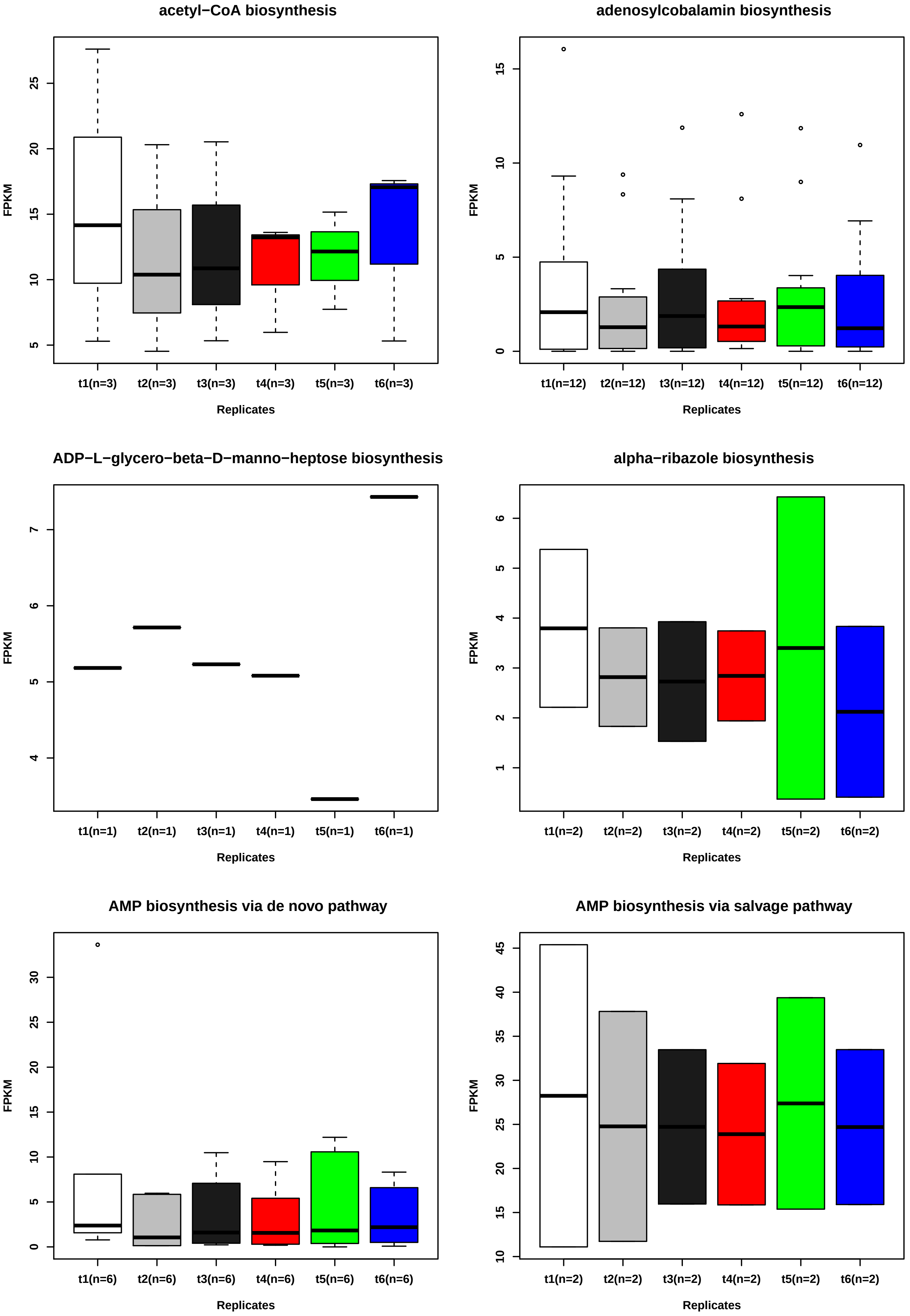
acetyl−CoA biosynthesis
FPKM
5
10
15
20
25
t1(n=3)
t2(n=3)
t3(n=3)
t4(n=3)
t5(n=3)
t6(n=3)
Replicates
adenosylcobalamin biosynthesis
FPKM
0
5
10
15
t1(n=12)
t2(n=12)
t3(n=12)
t4(n=12)
t5(n=12)
t6(n=12)
Replicates
ADP−L−glycero−beta−D−manno−heptose biosynthesis
FPKM
4
5
6
7
t1(n=1)
t2(n=1)
t3(n=1)
t4(n=1)
t5(n=1)
t6(n=1)
Replicates
alpha−ribazole biosynthesis
FPKM
1
2
3
4
5
6
t1(n=2)
t2(n=2)
t3(n=2)
t4(n=2)
t5(n=2)
t6(n=2)
Replicates
AMP biosynthesis via de novo pathway
FPKM
0
5
10
15
20
25
30
t1(n=6)
t2(n=6)
t3(n=6)
t4(n=6)
t5(n=6)
t6(n=6)
Replicates
AMP biosynthesis via salvage pathway
FPKM
10
15
20
25
30
35
40
45
t1(n=2)
t2(n=2)
t3(n=2)
t4(n=2)
t5(n=2)
t6(n=2)
Replicates

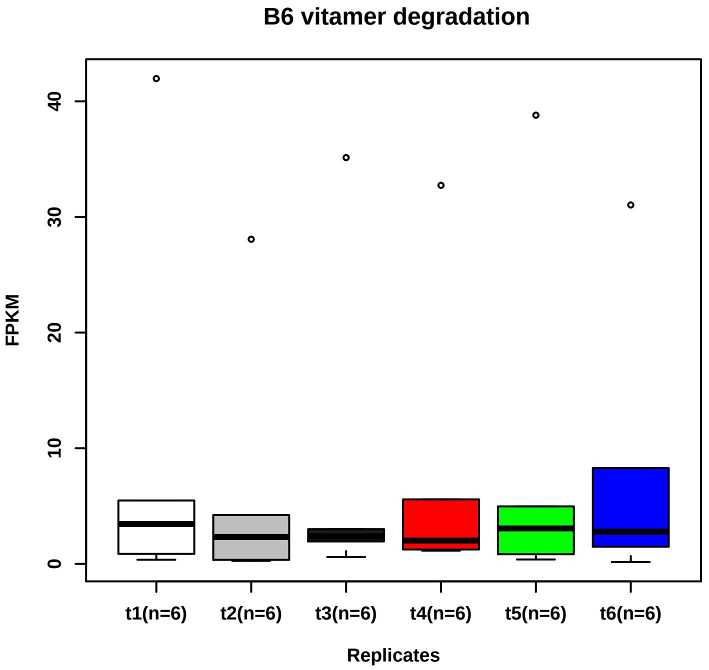

B6 vitamer degradation
FPKM
0
10
20
30
40
t1(n=6)
t2(n=6)
t3(n=6)
t4(n=6)
t5(n=6)
t6(n=6)
Replicates

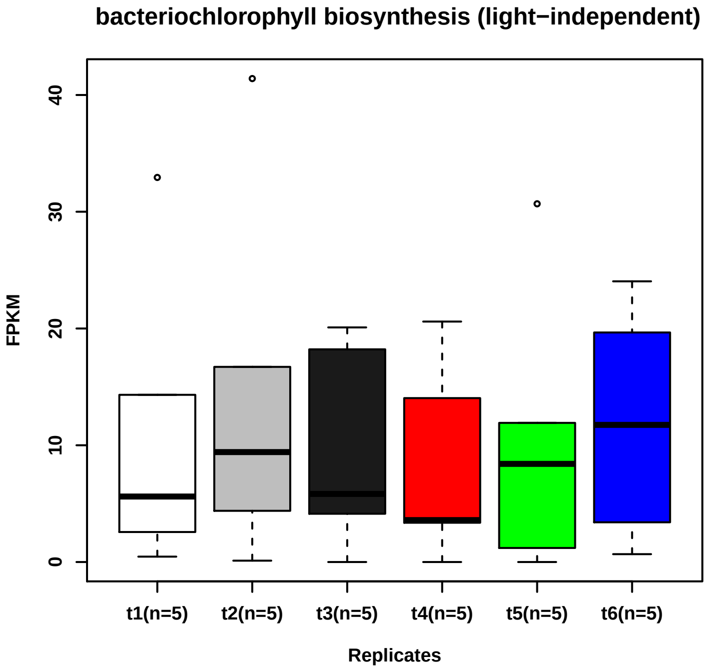

bacteriochlorophyll biosynthesis (light−independent)
FPKM
0
10
20
30
40
t1(n=5)
t2(n=5)
t3(n=5)
t4(n=5)
t5(n=5)
t6(n=5)
Replicates

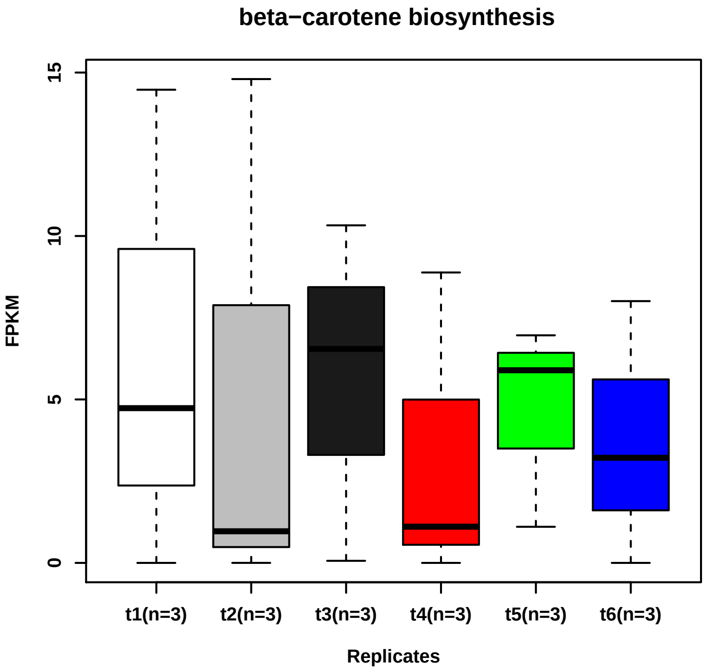

beta−carotene biosynthesis
FPKM
0
5
10
15
t1(n=3)
t2(n=3)
t3(n=3)
t4(n=3)
t5(n=3)
t6(n=3)
Replicates

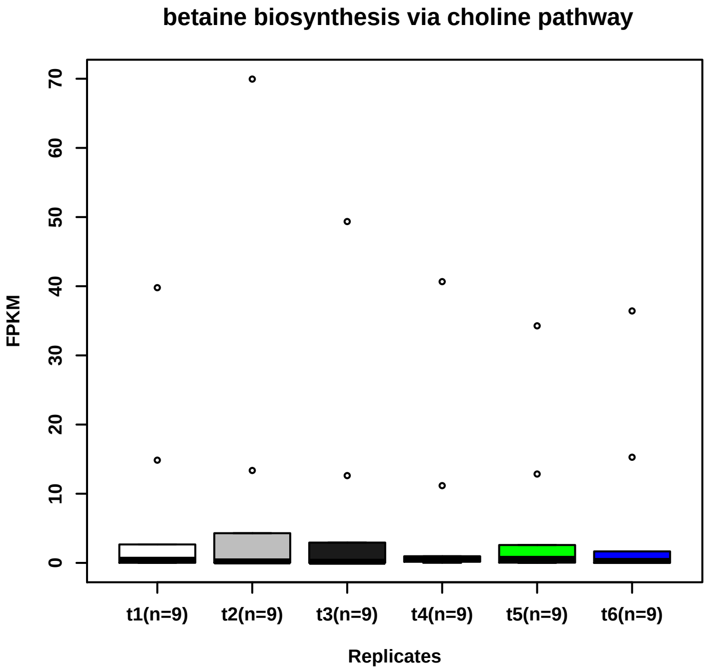

betaine biosynthesis via choline pathway
FPKM
0
10
20
30
40
50
60
70
t1(n=9)
t2(n=9)
t3(n=9)
t4(n=9)
t5(n=9)
t6(n=9)
Replicates

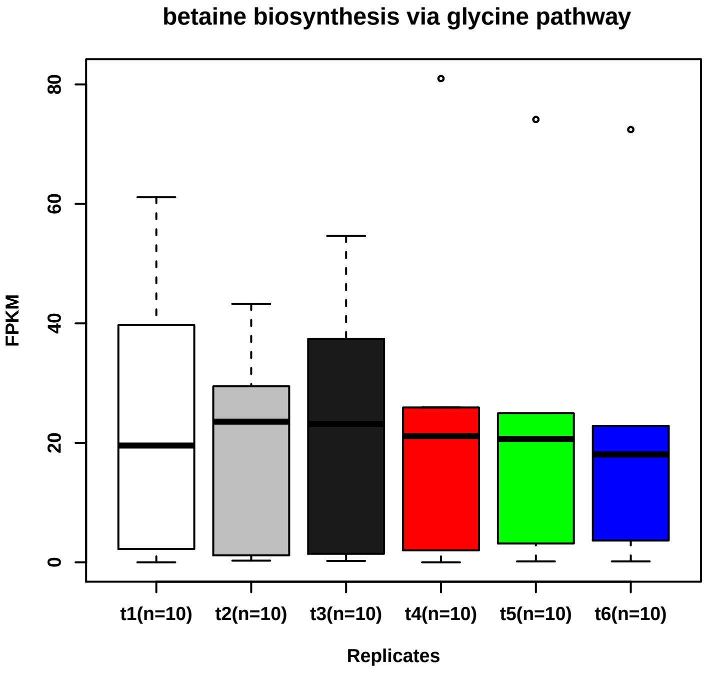

betaine biosynthesis via glycine pathway
FPKM
0
20
40
60
80
t1(n=10)
t2(n=10)
t3(n=10)
t4(n=10)
t5(n=10)
t6(n=10)
Replicates

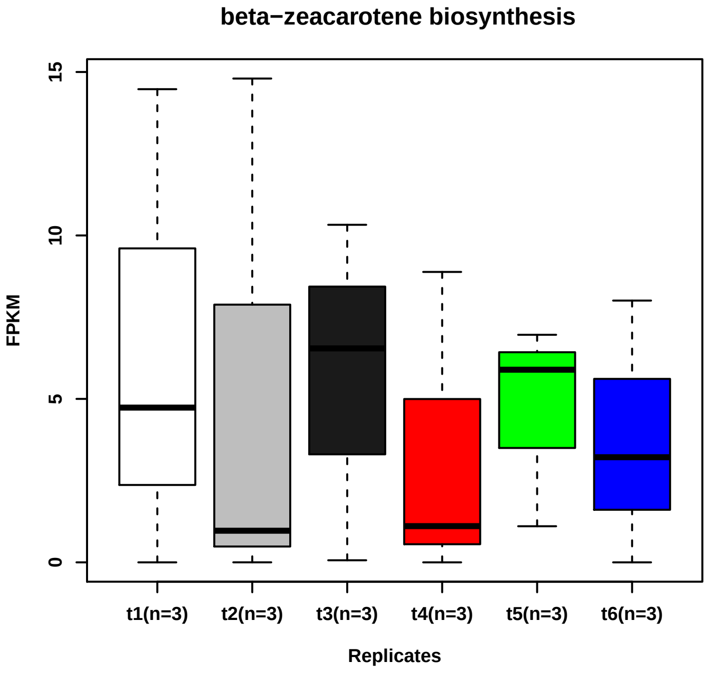

beta−zeacarotene biosynthesis
FPKM
0
5
10
15
t1(n=3)
t2(n=3)
t3(n=3)
t4(n=3)
t5(n=3)
t6(n=3)
Replicates

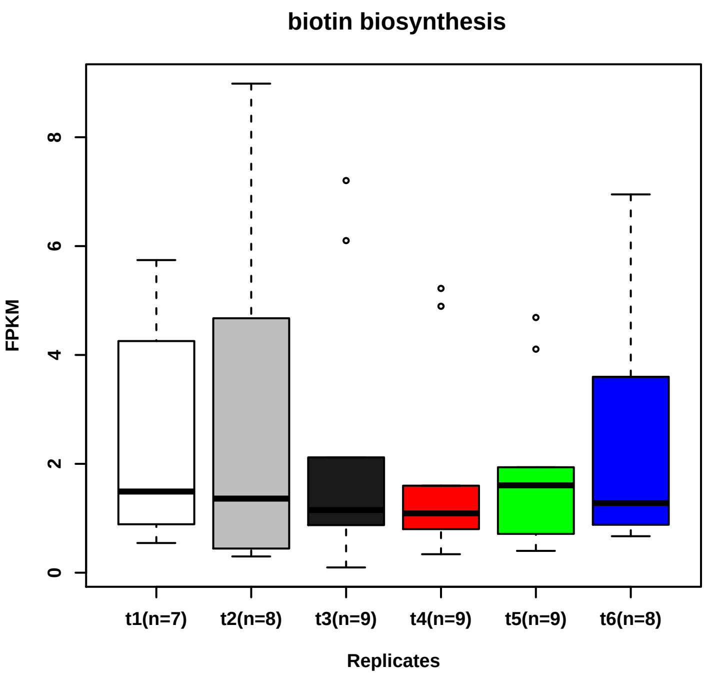
biotin biosynthesis
FPKM
0
2
4
6
8
t1(n=7)
t2(n=8)
t3(n=9)
t4(n=9)
t5(n=9)
t6(n=8)
Replicates

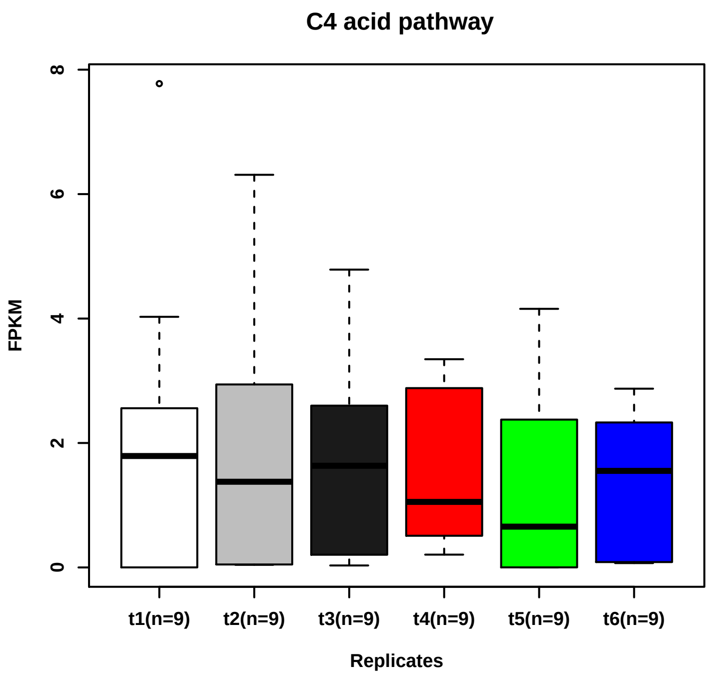
C4 acid pathway
FPKM
0
2
4
6
8
t1(n=9)
t2(n=9)
t3(n=9)
t4(n=9)
t5(n=9)
t6(n=9)
Replicates

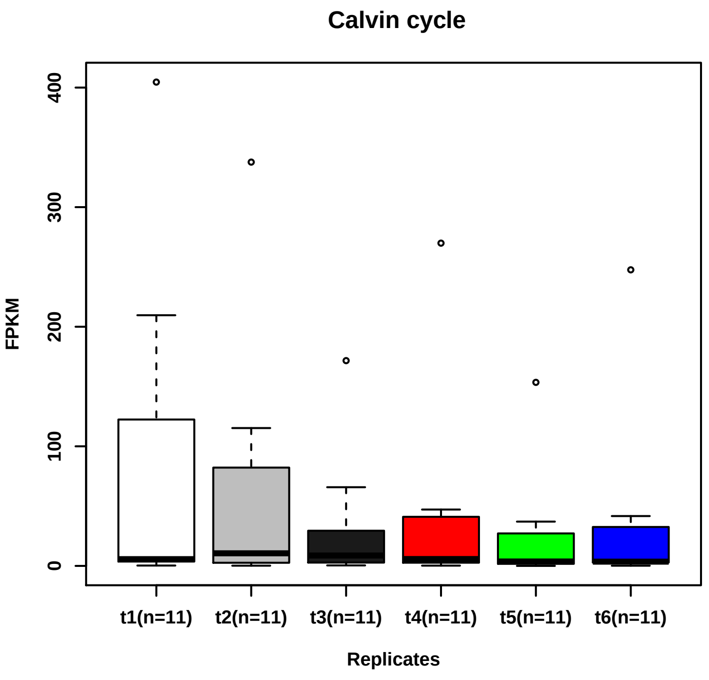
Calvin cycle
FPKM
0
100
200
300
400
t1(n=11)
t2(n=11)
t3(n=11)
t4(n=11)
t5(n=11)
t6(n=11)
Replicates

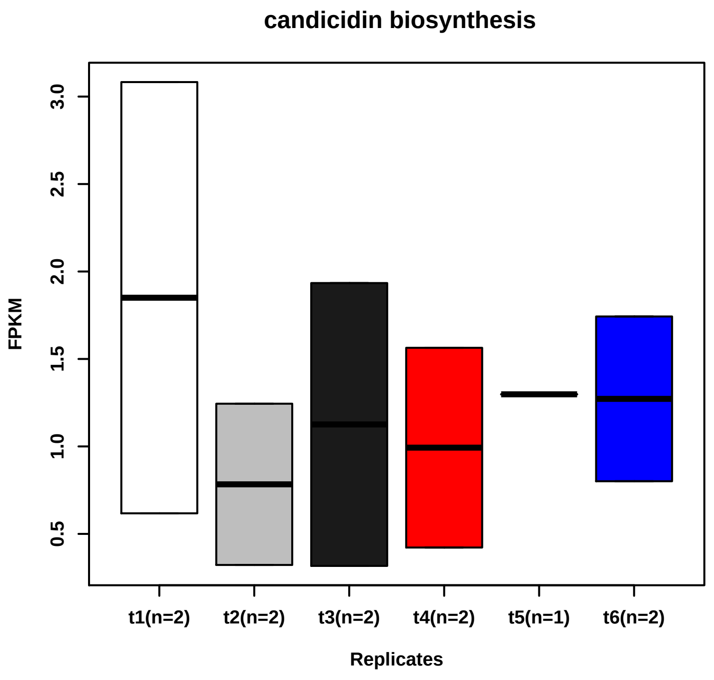
candicidin biosynthesis
FPKM
0.5
1.0
1.5
2.0
2.5
3.0
t1(n=2)
t2(n=2)
t3(n=2)
t4(n=2)
t5(n=1)
t6(n=2)
Replicates

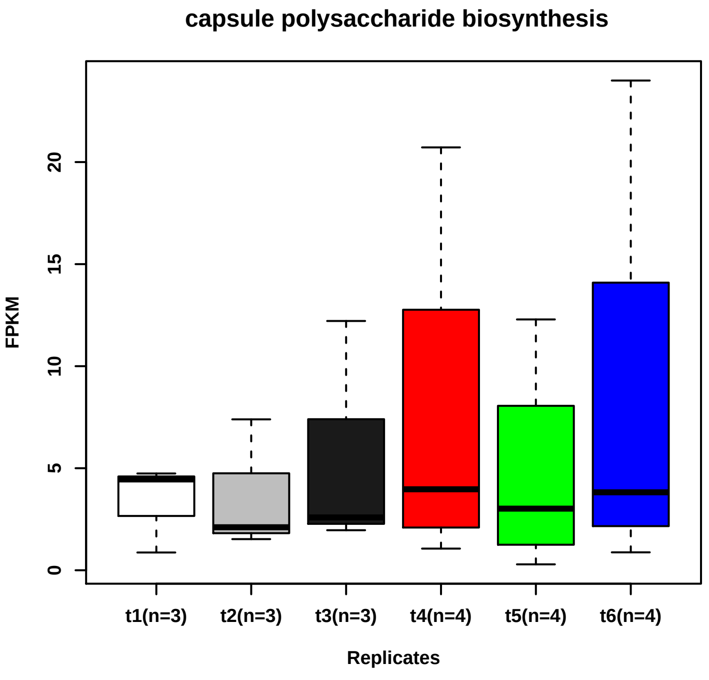
capsule polysaccharide biosynthesis
FPKM
0
5
10
15
20
t1(n=3)
t2(n=3)
t3(n=3)
t4(n=4)
t5(n=4)
t6(n=4)
Replicates

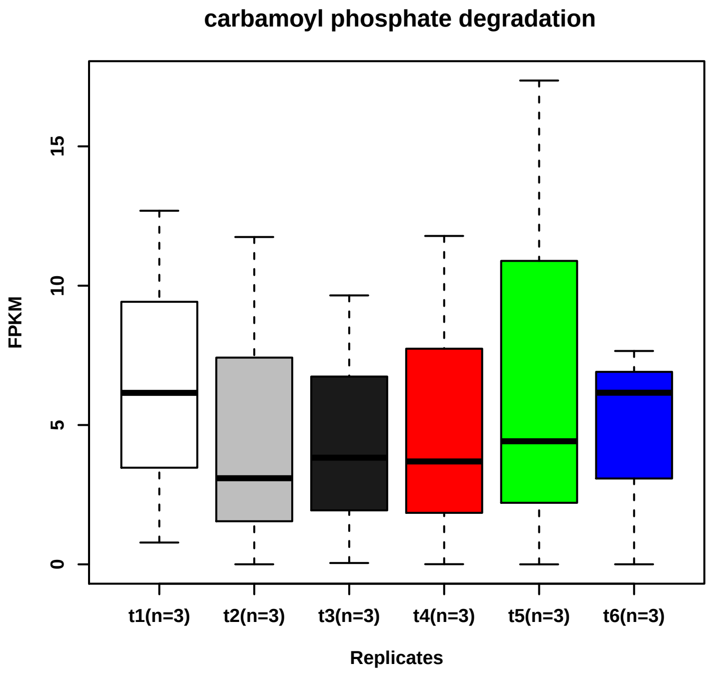
carbamoyl phosphate degradation
FPKM
0
5
10
15
t1(n=3)
t2(n=3)
t3(n=3)
t4(n=3)
t5(n=3)
t6(n=3)
Replicates

carnitine biosynthesis

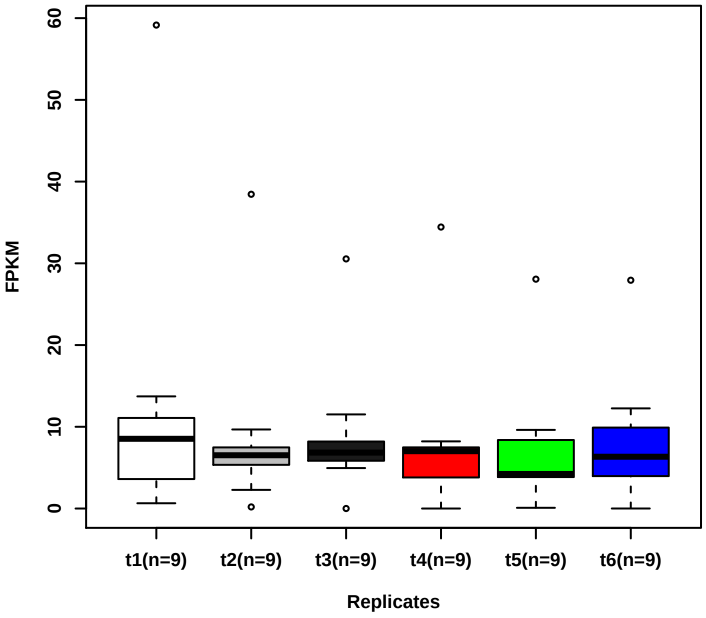

carnitine metabolism

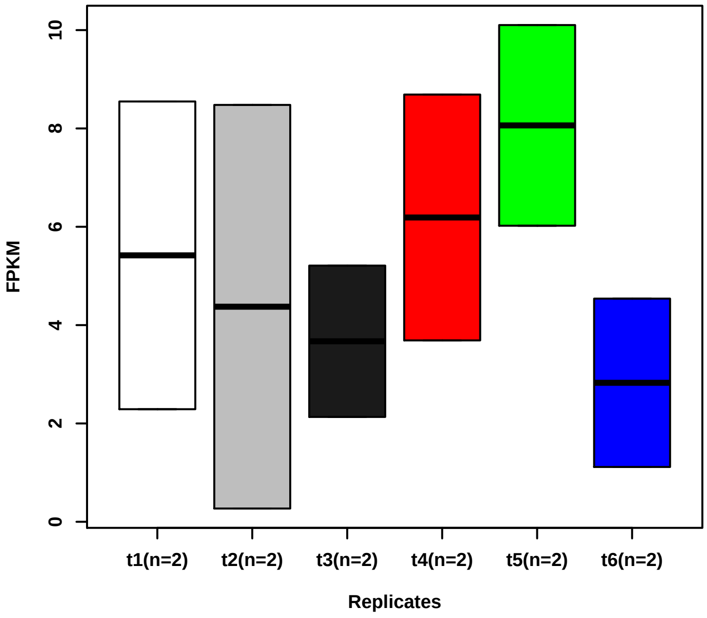

CDP−diacylglycerol biosynthesis

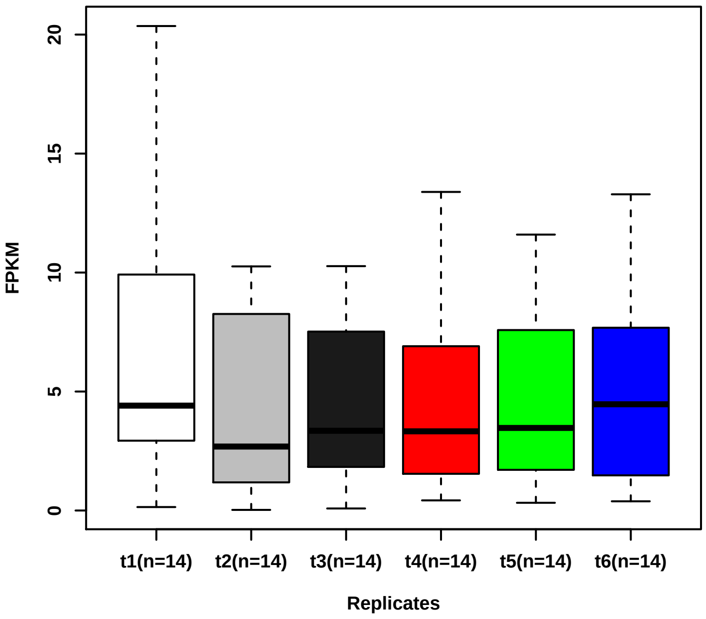

cellulose degradation

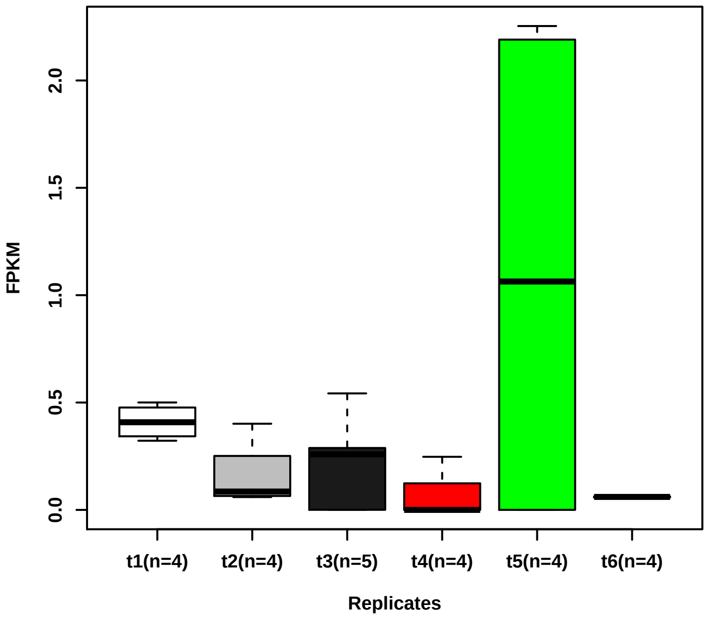

chitin degradation

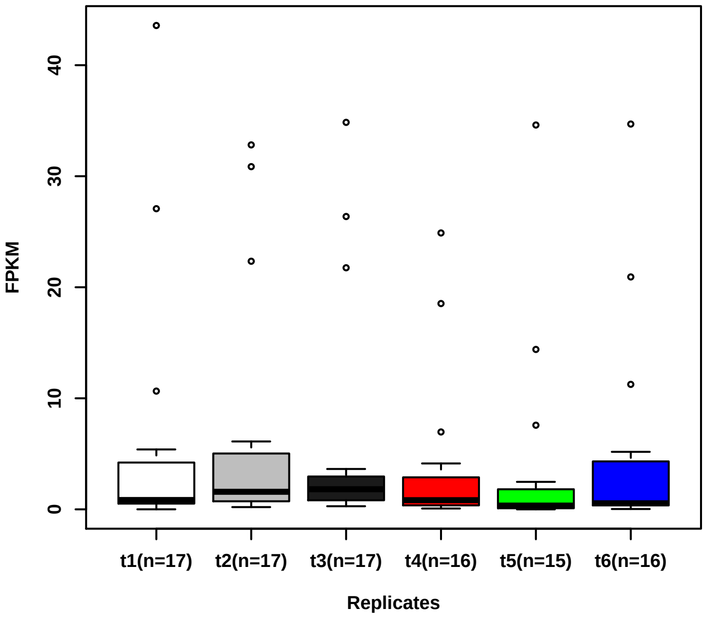

chlorophyll biosynthesis

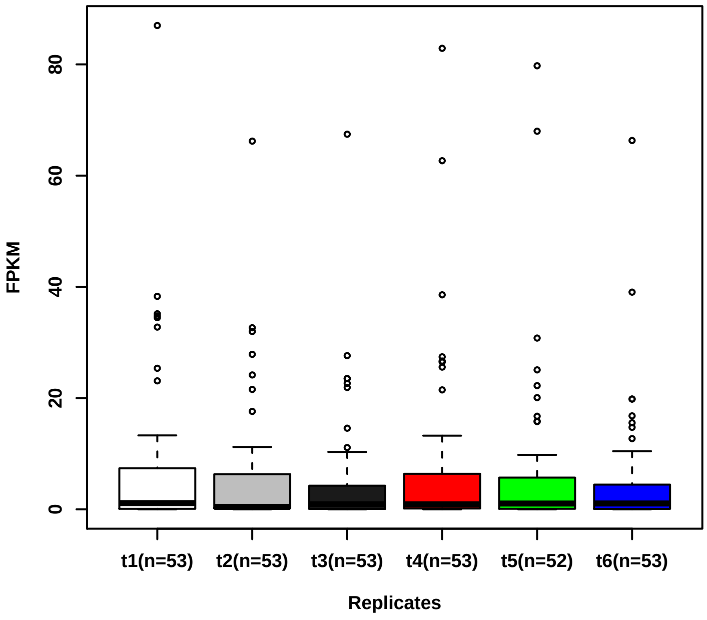

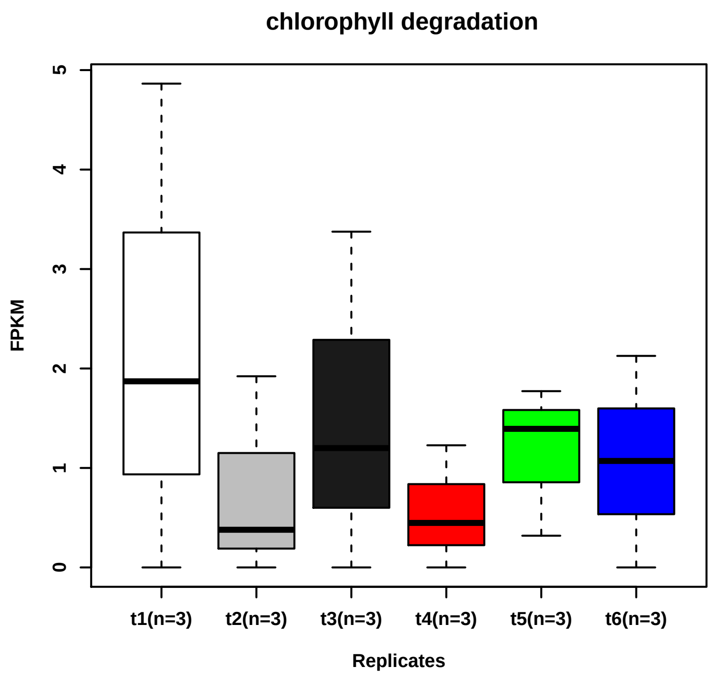
chlorophyll degradation
FPKM
0
1
2
3
4
5
t1(n=3)
t2(n=3)
t3(n=3)
t4(n=3)
t5(n=3)
t6(n=3)
Replicates

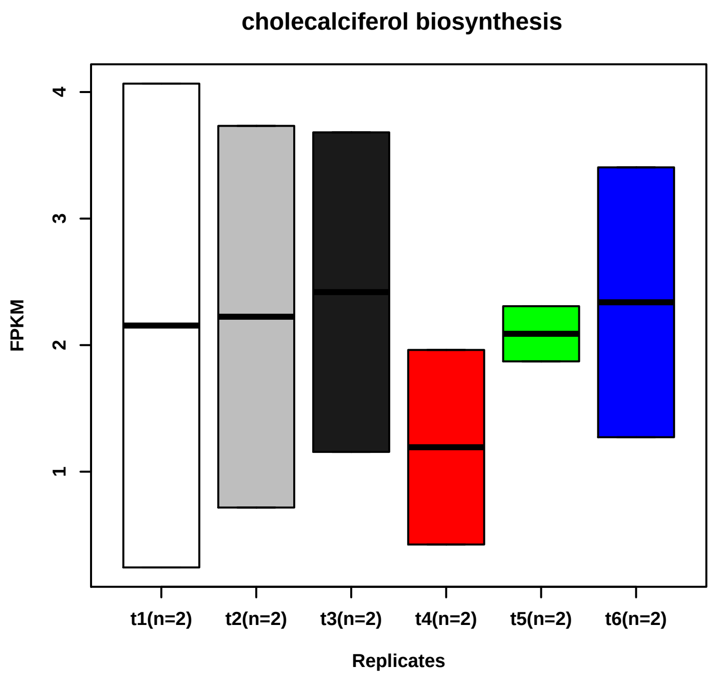
cholecalciferol biosynthesis
FPKM
1
2
3
4
t1(n=2)
t2(n=2)
t3(n=2)
t4(n=2)
t5(n=2)
t6(n=2)
Replicates

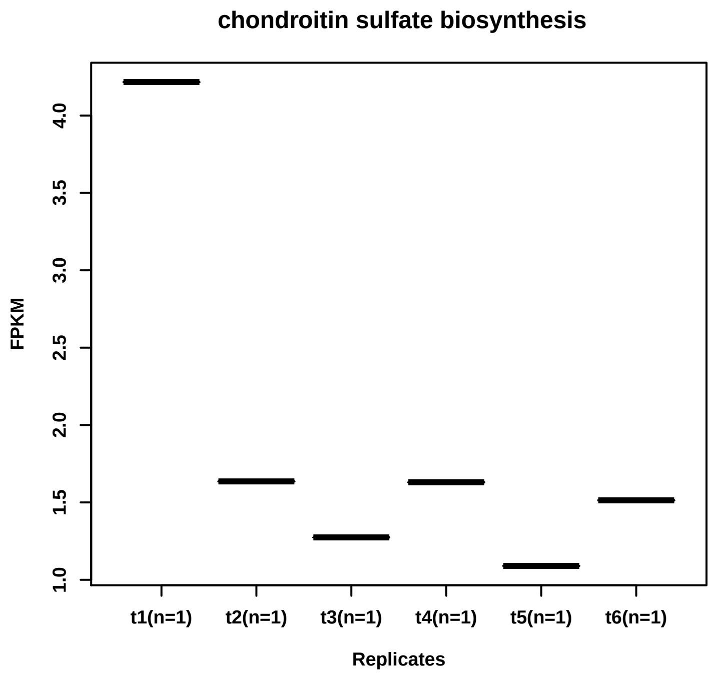
chondroitin sulfate biosynthesis
FPKM
1.0
1.5
2.0
2.5
3.0
3.5
4.0
t1(n=1)
t2(n=1)
t3(n=1)
t4(n=1)
t5(n=1)
t6(n=1)
Replicates

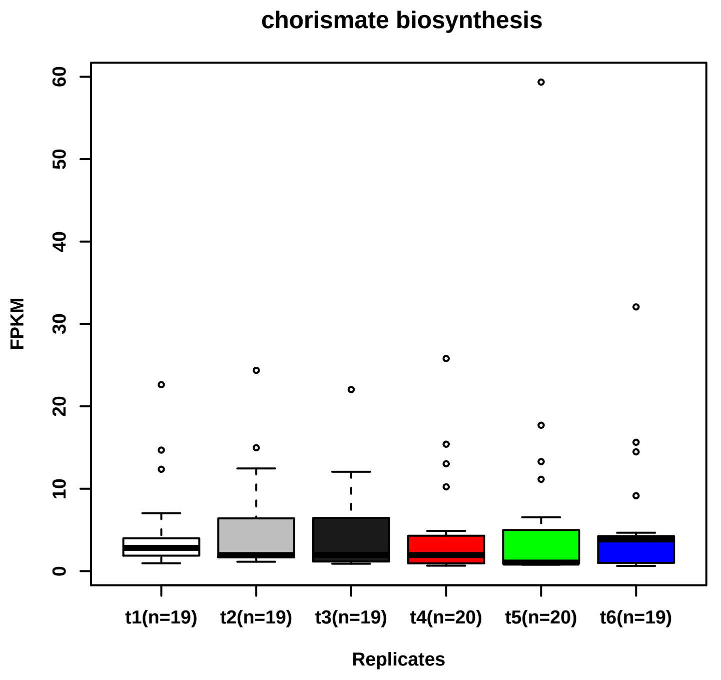
chorismate biosynthesis
FPKM
0
10
20
30
40
50
60
t1(n=19)
t2(n=19)
t3(n=19)
t4(n=20)
t5(n=20)
t6(n=19)
Replicates

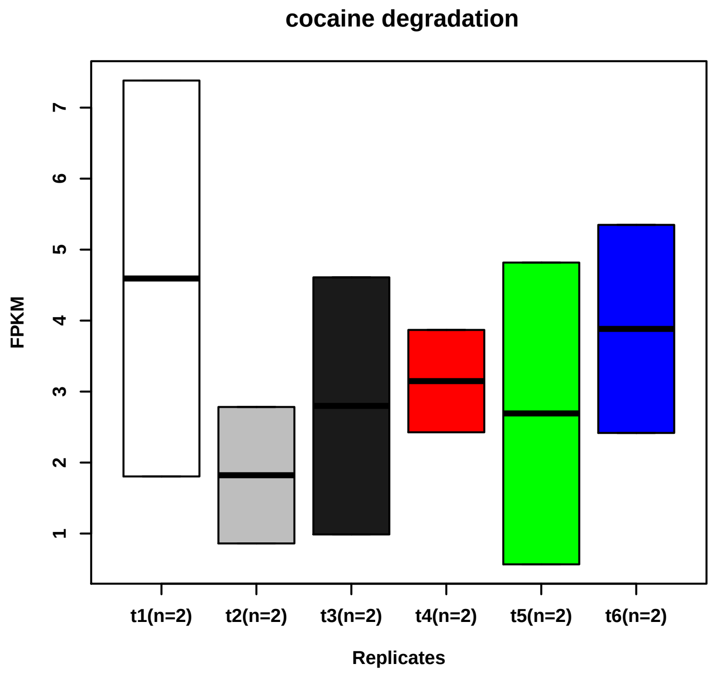
cocaine degradation
FPKM
1
2
3
4
5
6
7
t1(n=2)
t2(n=2)
t3(n=2)
t4(n=2)
t5(n=2)
t6(n=2)
Replicates

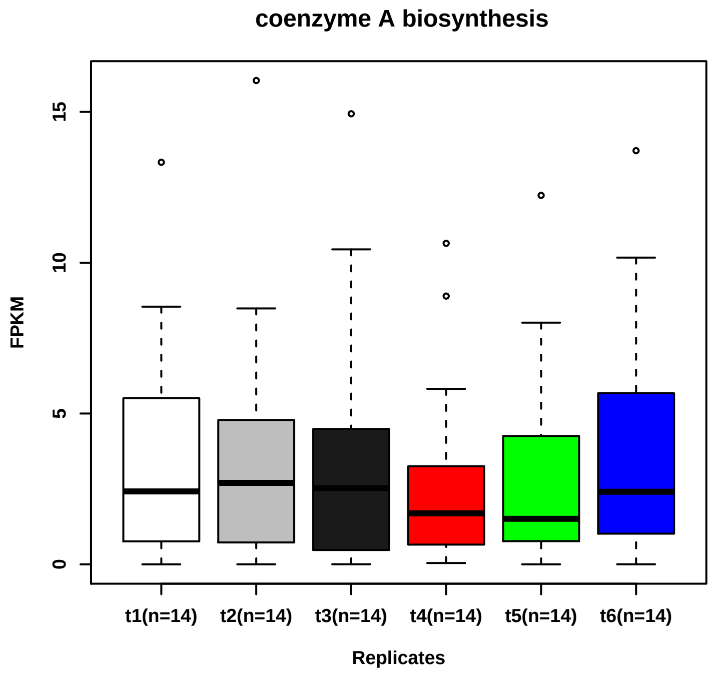
coenzyme A biosynthesis
FPKM
0
5
10
15
t1(n=14)
t2(n=14)
t3(n=14)
t4(n=14)
t5(n=14)
t6(n=14)
Replicates

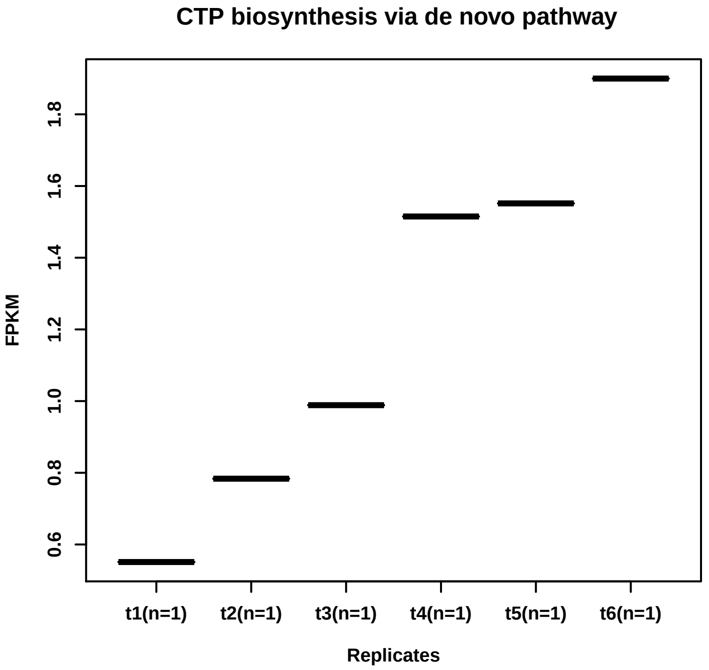
CTP biosynthesis via de novo pathway
FPKM
0.6
0.8
1.0
1.2
1.4
1.6
1.8
t1(n=1)
t2(n=1)
t3(n=1)
t4(n=1)
t5(n=1)
t6(n=1)
Replicates

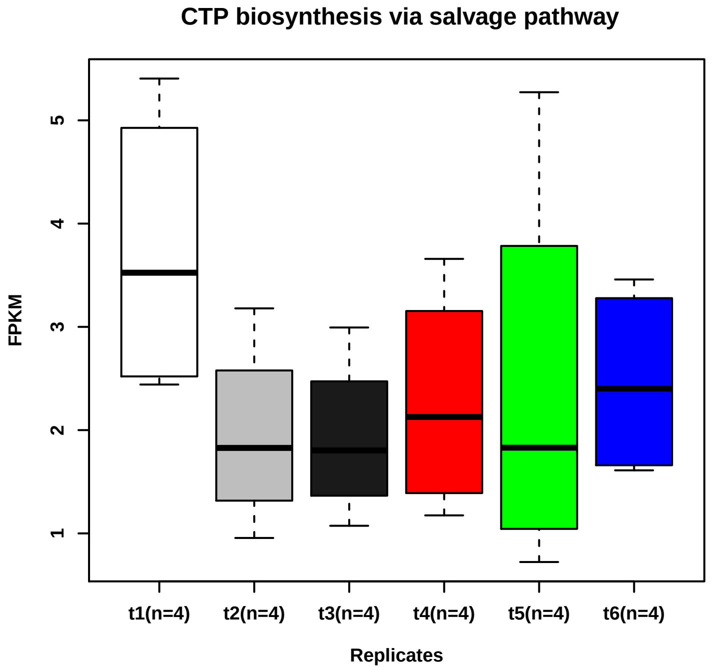
CTP biosynthesis via salvage pathway
FPKM
1
2
3
4
5
t1(n=4)
t2(n=4)
t3(n=4)
t4(n=4)
t5(n=4)
t6(n=4)
Replicates

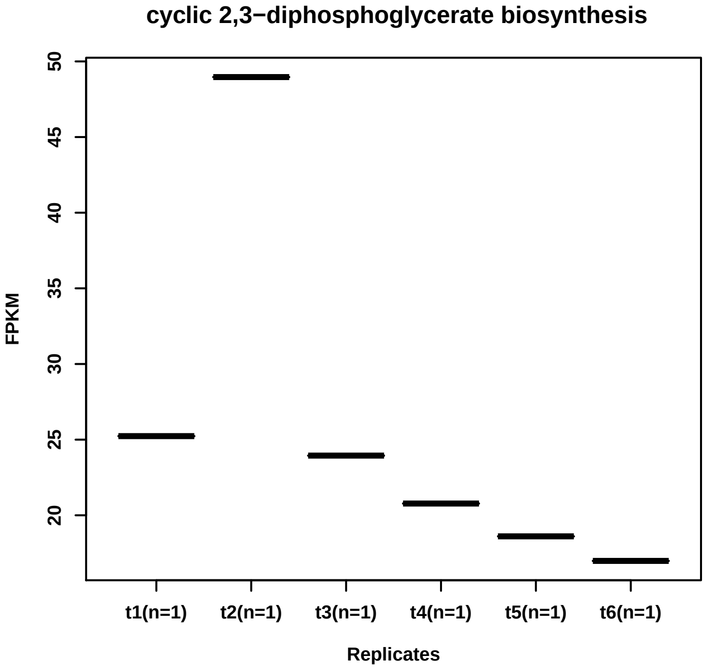
cyclic 2,3−diphosphoglycerate biosynthesis
FPKM
20
25
30
35
40
45
50
t1(n=1)
t2(n=1)
t3(n=1)
t4(n=1)
t5(n=1)
t6(n=1)
Replicates

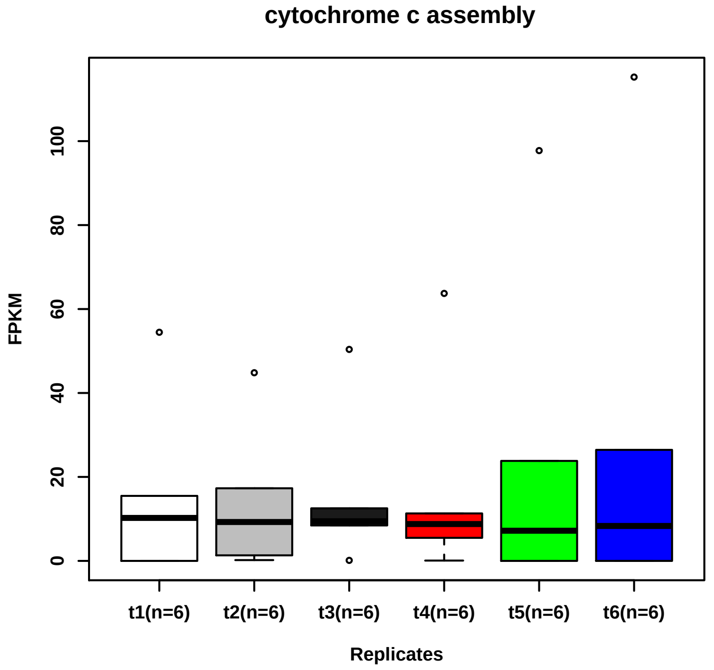
cytochrome c assembly
FPKM
0
20
40
60
80
100
t1(n=6)
t2(n=6)
t3(n=6)
t4(n=6)
t5(n=6)
t6(n=6)
Replicates

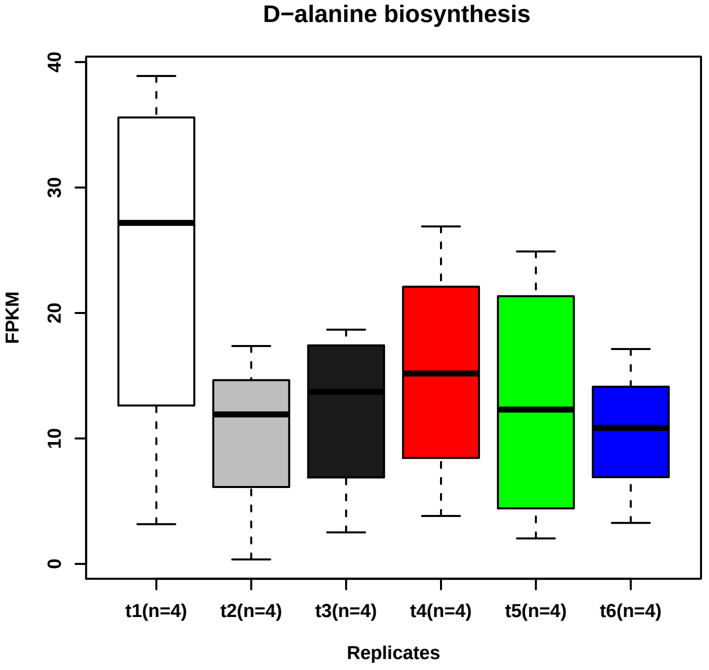
D−alanine biosynthesis
FPKM
0
10
20
30
40
t1(n=4)
t2(n=4)
t3(n=4)
t4(n=4)
t5(n=4)
t6(n=4)
Replicates

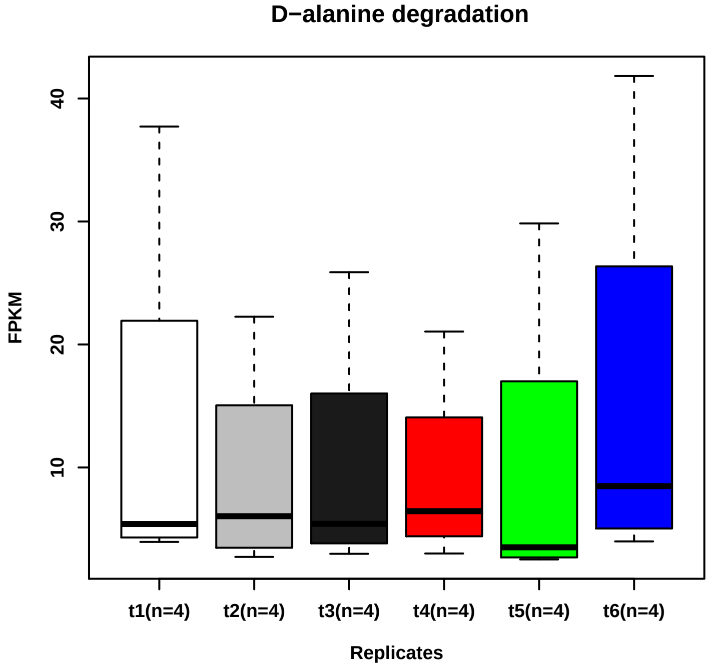
D−alanine degradation
FPKM
10
20
30
40
t1(n=4)
t2(n=4)
t3(n=4)
t4(n=4)
t5(n=4)
t6(n=4)
Replicates

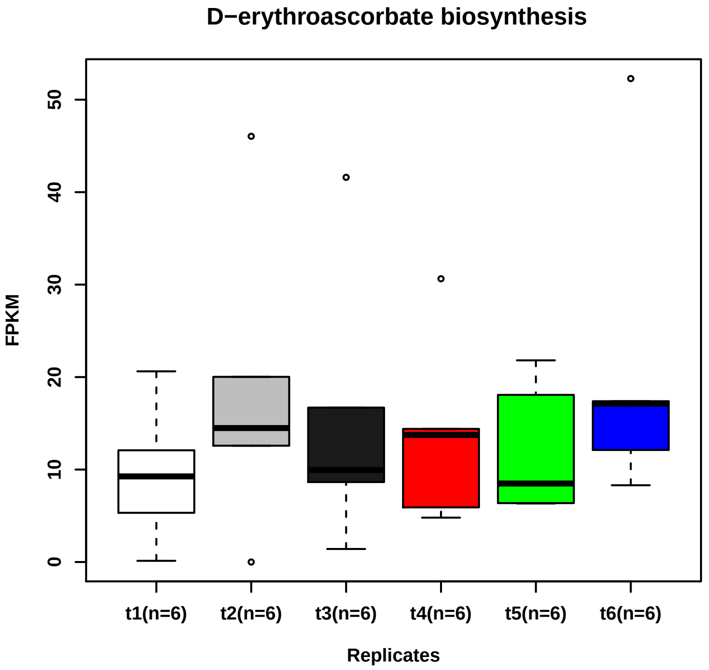
D−erythroascorbate biosynthesis
FPKM
0
10
20
30
40
50
t1(n=6)
t2(n=6)
t3(n=6)
t4(n=6)
t5(n=6)
t6(n=6)
Replicates

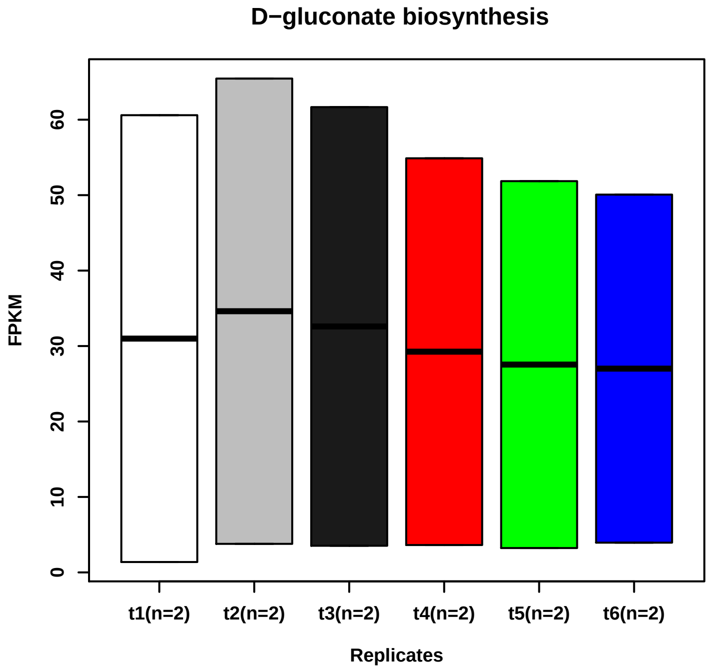
D−gluconate biosynthesis
FPKM
0
10
20
30
40
50
60
t1(n=2)
t2(n=2)
t3(n=2)
t4(n=2)
t5(n=2)
t6(n=2)
Replicates

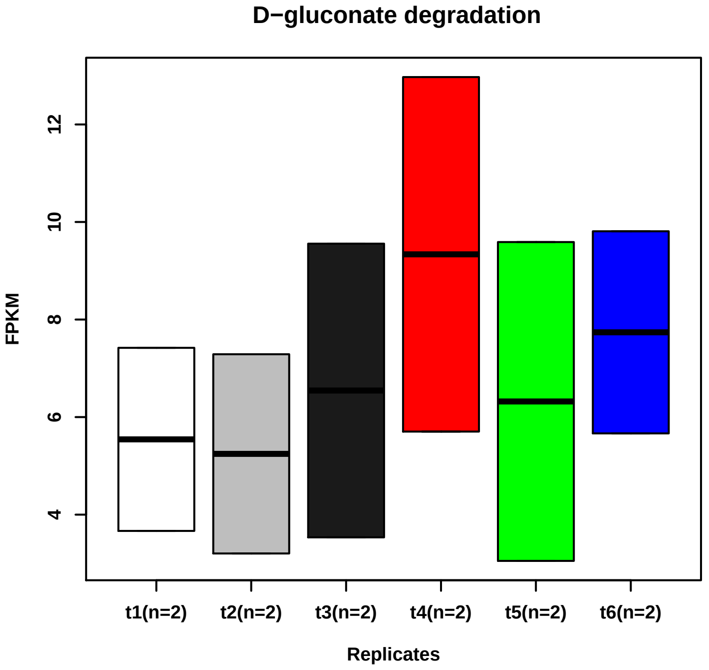
D−gluconate degradation
FPKM
4
6
8
10
12
t1(n=2)
t2(n=2)
t3(n=2)
t4(n=2)
t5(n=2)
t6(n=2)
Replicates

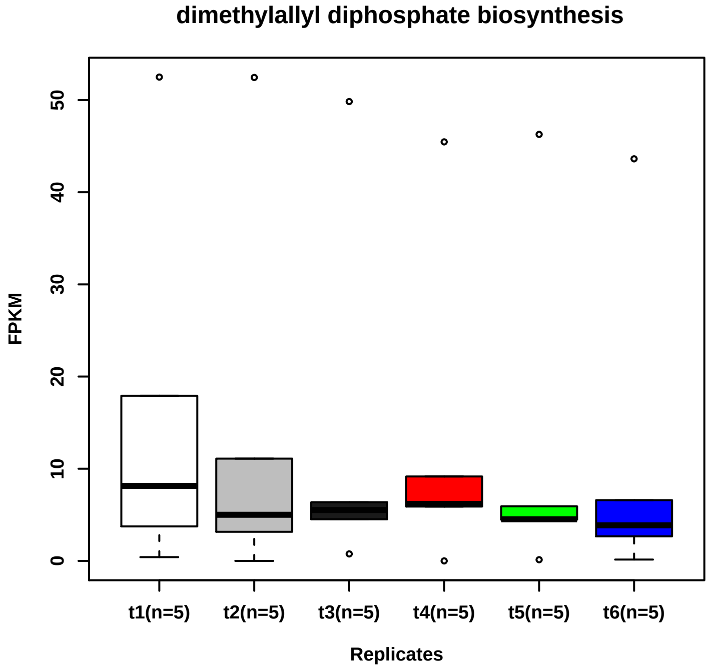
dimethylallyl diphosphate biosynthesis
FPKM
0
10
20
30
40
50
t1(n=5)
t2(n=5)
t3(n=5)
t4(n=5)
t5(n=5)
t6(n=5)
Replicates

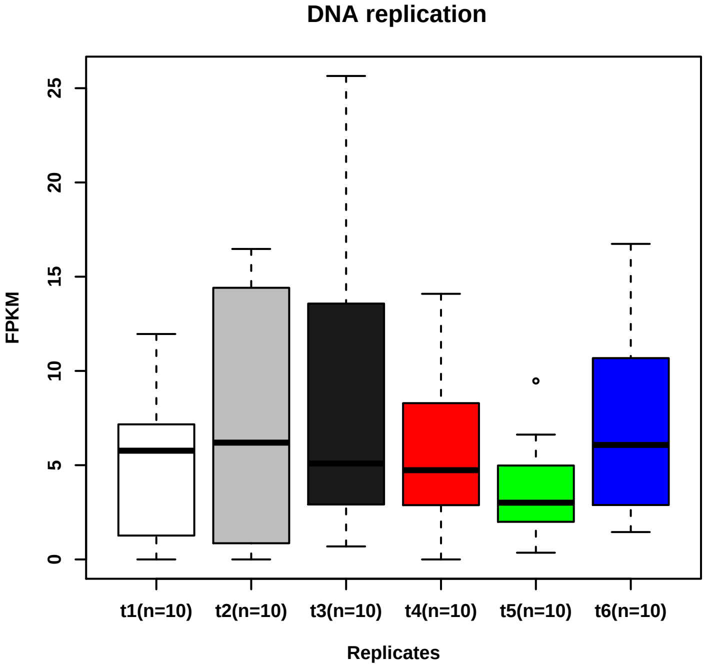
DNA replication
FPKM
0
5
10
15
20
25
t1(n=10)
t2(n=10)
t3(n=10)
t4(n=10)
t5(n=10)
t6(n=10)
Replicates

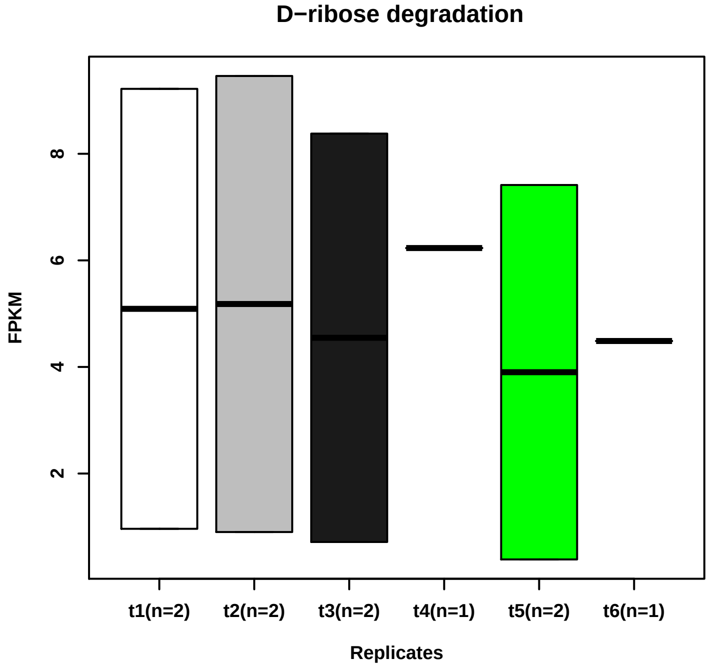
D−ribose degradation
FPKM
2
4
6
8
t1(n=2)
t2(n=2)
t3(n=2)
t4(n=1)
t5(n=2)
t6(n=1)
Replicates

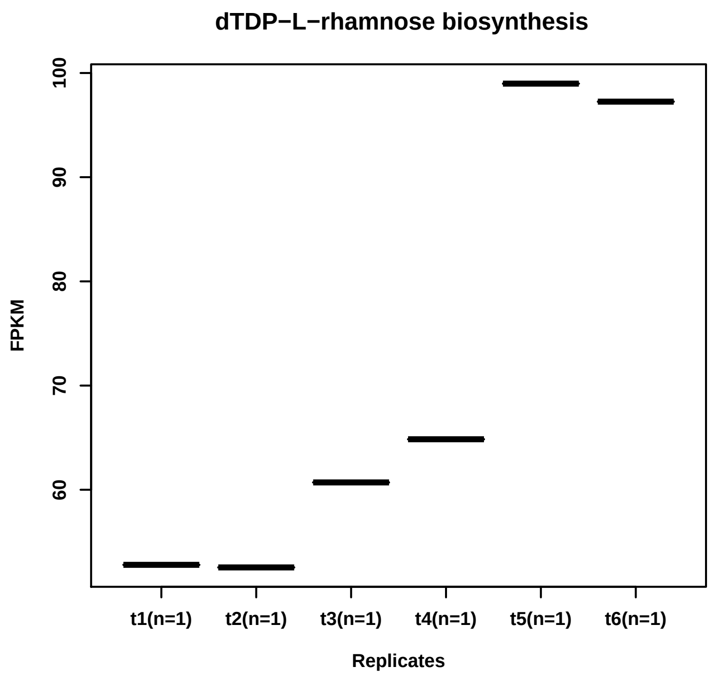

dTDP−L−rhamnose biosynthesis
FPKM
60
70
80
90
100
t1(n=1)
t2(n=1)
t3(n=1)
t4(n=1)
t5(n=1)
t6(n=1)
Replicates

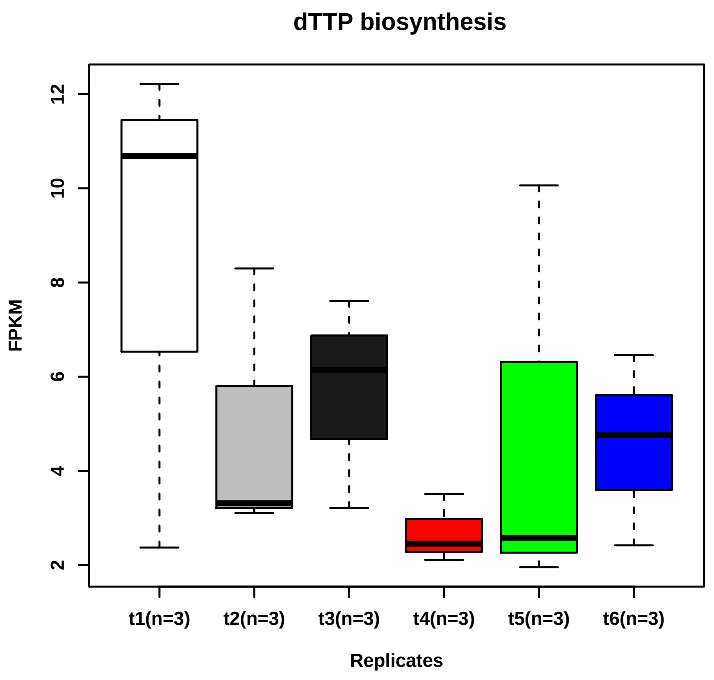

dTTP biosynthesis
FPKM
2
4
6
8
10
12
t1(n=3)
t2(n=3)
t3(n=3)
t4(n=3)
t5(n=3)
t6(n=3)
Replicates

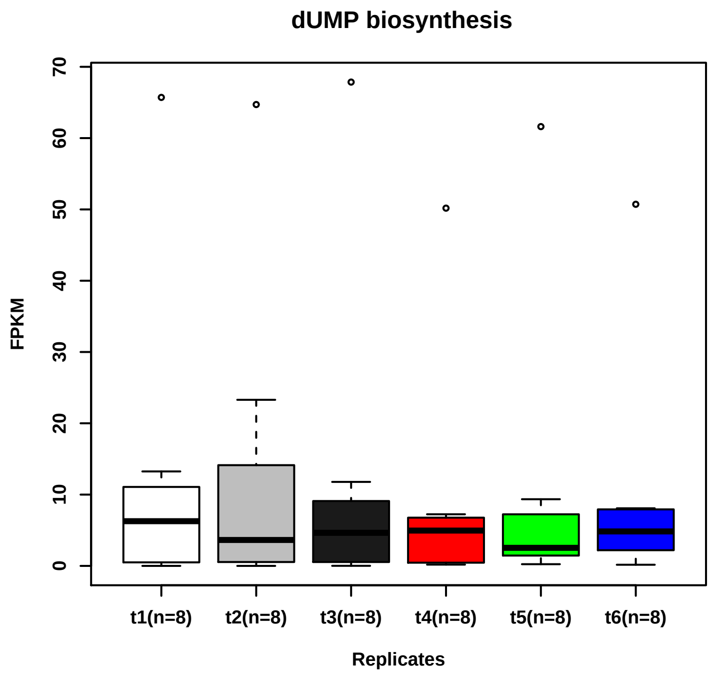

dUMP biosynthesis
FPKM
0
10
20
30
40
50
60
70
t1(n=8)
t2(n=8)
t3(n=8)
t4(n=8)
t5(n=8)
t6(n=8)
Replicates

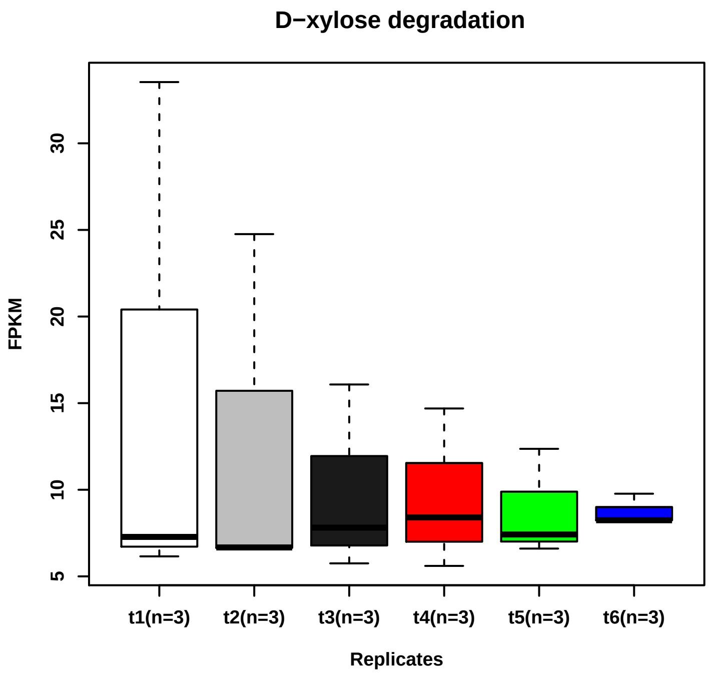

D−xylose degradation
FPKM
5
10
15
20
25
30
t1(n=3)
t2(n=3)
t3(n=3)
t4(n=3)
t5(n=3)
t6(n=3)
Replicates

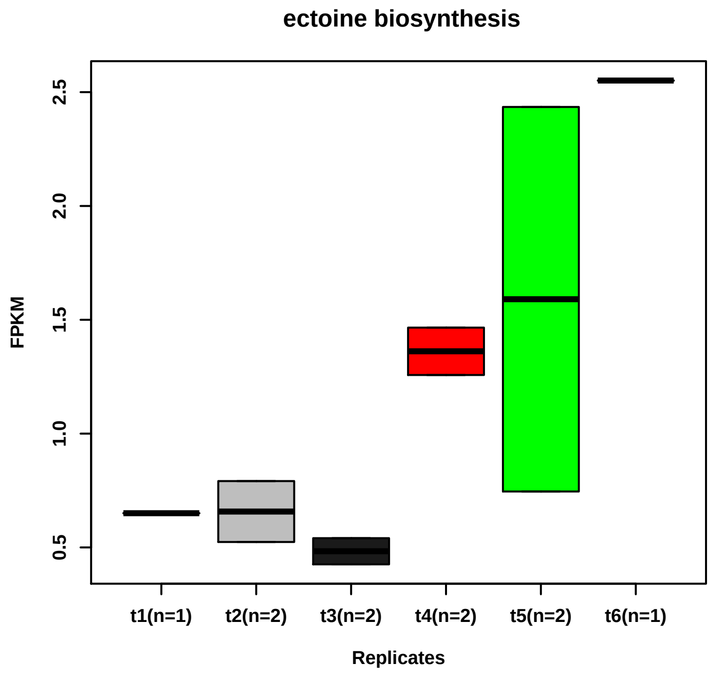

ectoine biosynthesis
FPKM
0.5
1.0
1.5
2.0
2.5
t1(n=1)
t2(n=2)
t3(n=2)
t4(n=2)
t5(n=2)
t6(n=1)
Replicates

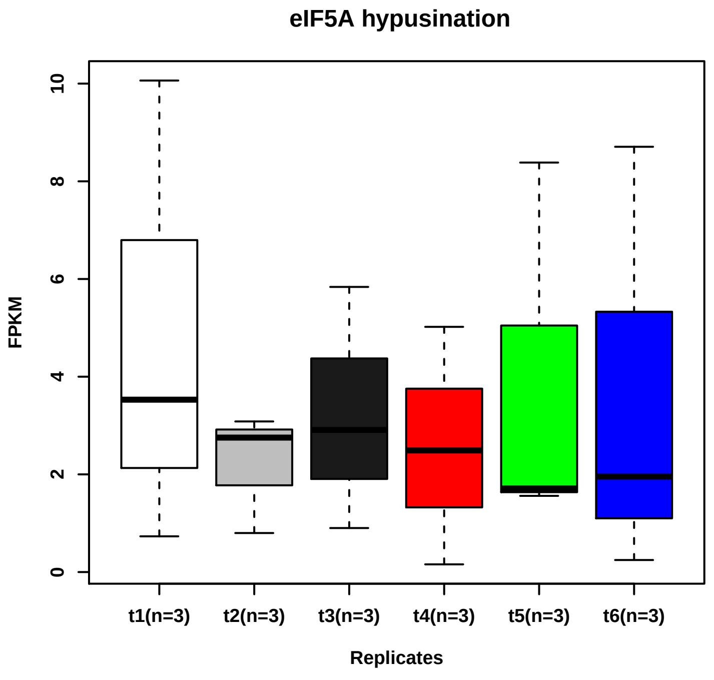

eIF5A hypusination
FPKM
0
2
4
6
8
10
t1(n=3)
t2(n=3)
t3(n=3)
t4(n=3)
t5(n=3)
t6(n=3)
Replicates

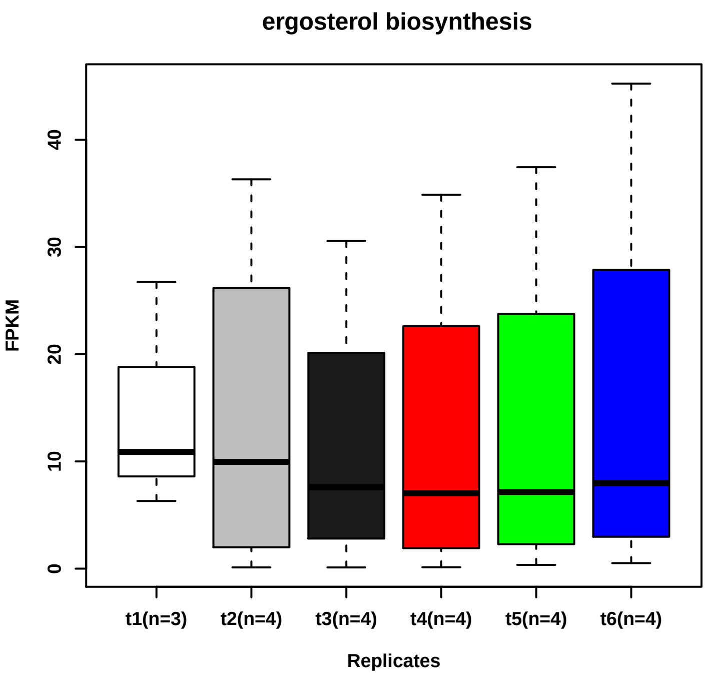

ergosterol biosynthesis
FPKM
0
10
20
30
40
t1(n=3)
t2(n=4)
t3(n=4)
t4(n=4)
t5(n=4)
t6(n=4)
Replicates

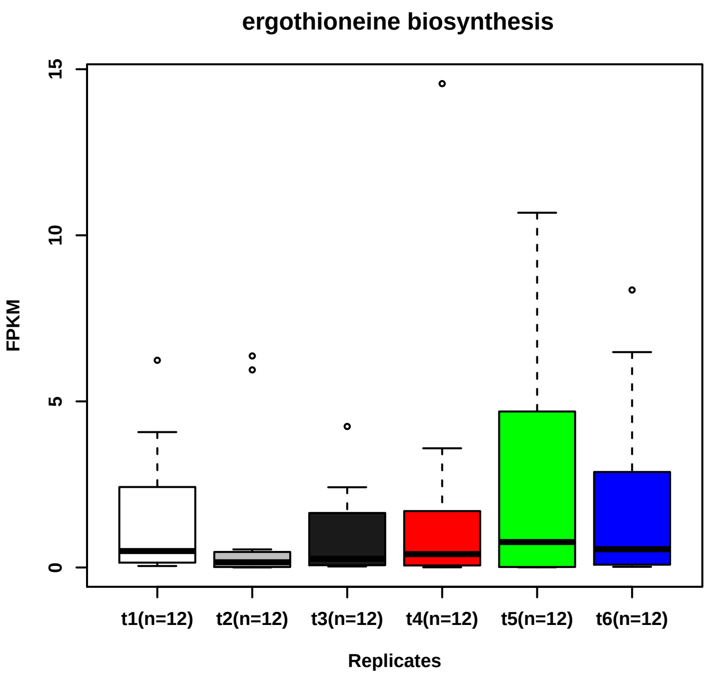

ergothioneine biosynthesis
FPKM
0
5
10
15
t1(n=12)
t2(n=12)
t3(n=12)
t4(n=12)
t5(n=12)
t6(n=12)
Replicates

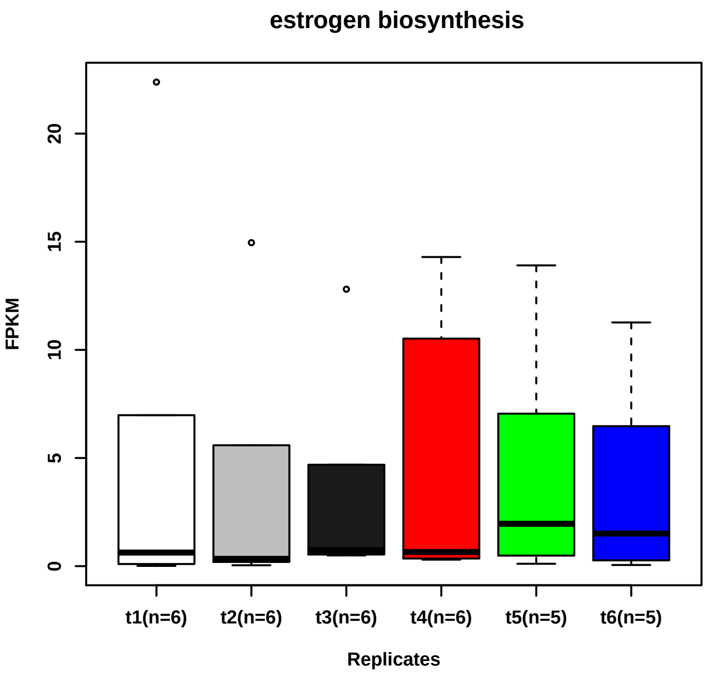

estrogen biosynthesis
FPKM
0
5
10
15
20
t1(n=6)
t2(n=6)
t3(n=6)
t4(n=6)
t5(n=5)
t6(n=5)
Replicates

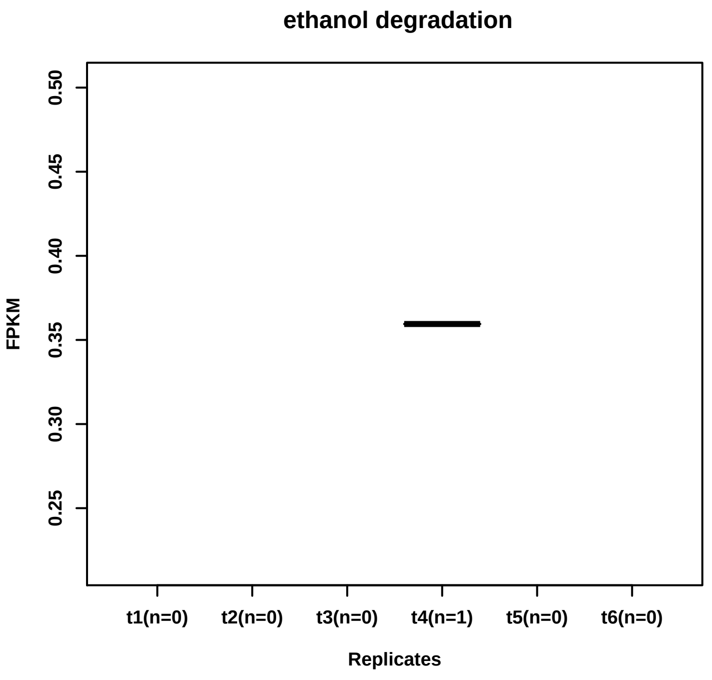

ethanol degradation
FPKM
0.25
0.30
0.35
0.40
0.45
0.50
t1(n=0)
t2(n=0)
t3(n=0)
t4(n=1)
t5(n=0)
t6(n=0)
Replicates

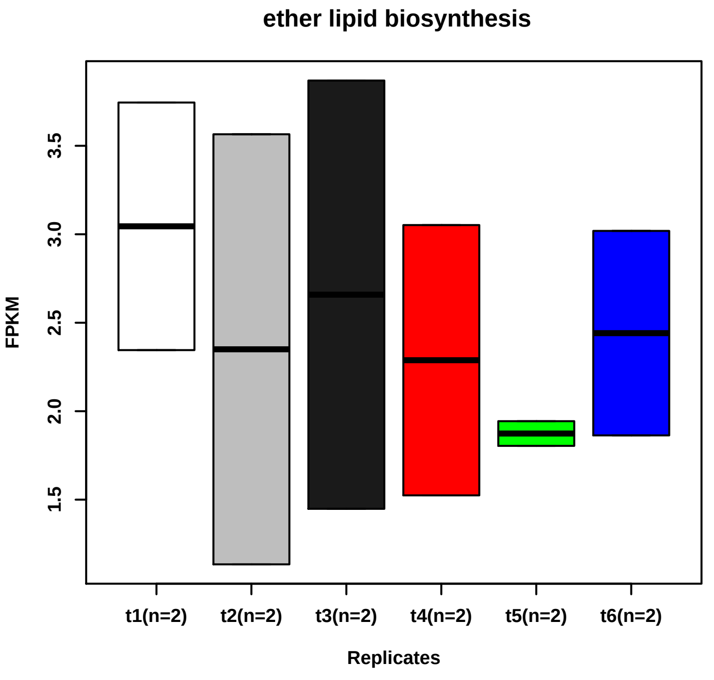

ether lipid biosynthesis
FPKM
1.5
2.0
2.5
3.0
3.5
t1(n=2)
t2(n=2)
t3(n=2)
t4(n=2)
t5(n=2)
t6(n=2)
Replicates

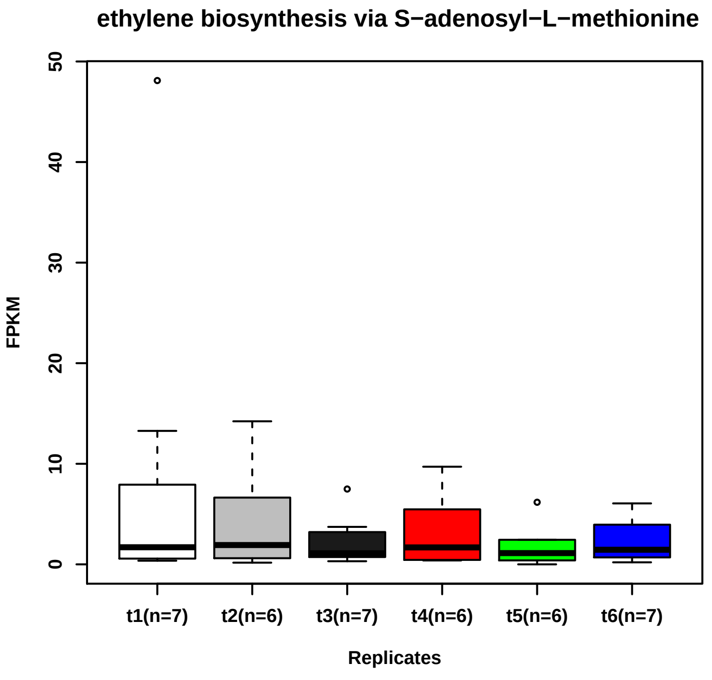

ethylene biosynthesis via S−adenosyl−L−methionine
FPKM
0
10
20
30
40
50
t1(n=7)
t2(n=6)
t3(n=7)
t4(n=6)
t5(n=6)
t6(n=7)
Replicates

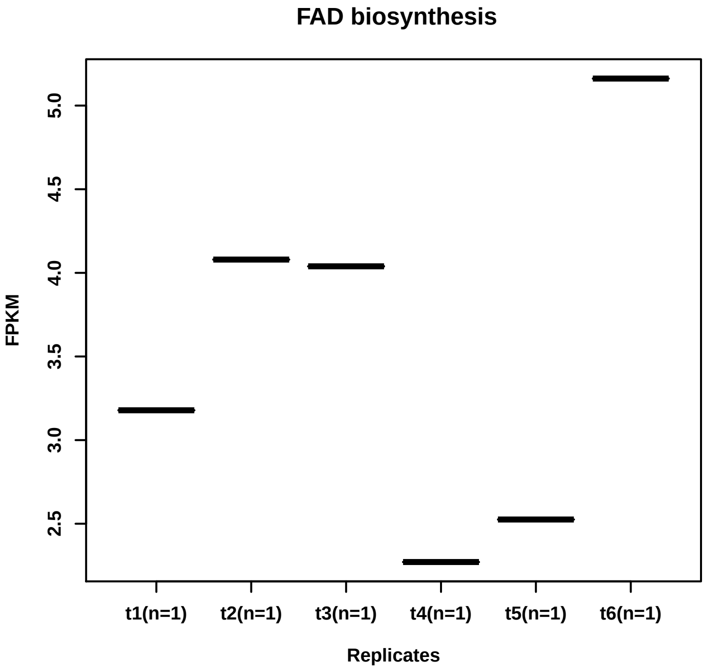
FAD biosynthesis
FPKM
Replicates
t1(n=1)
t2(n=1)
t3(n=1)
t4(n=1)
t5(n=1)
t6(n=1)

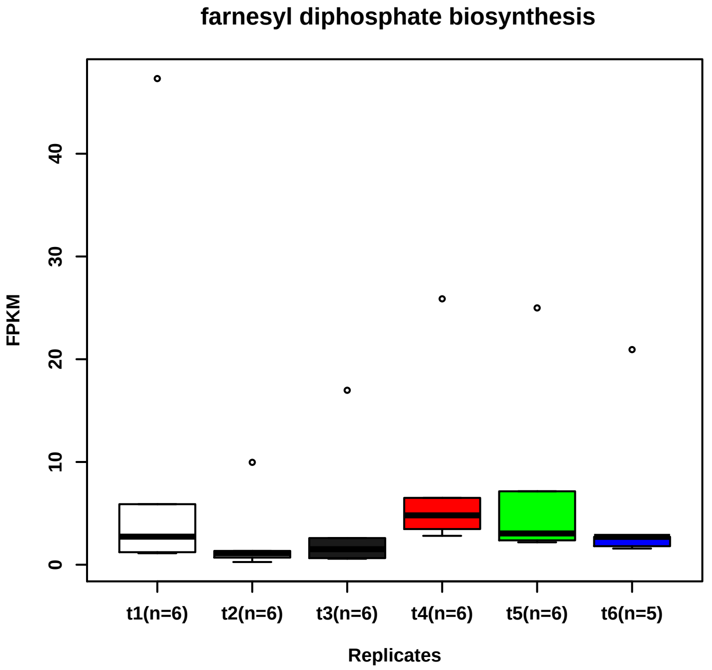
farnesyl diphosphate biosynthesis
FPKM
Replicates
t1(n=6)
t2(n=6)
t3(n=6)
t4(n=6)
t5(n=6)
t6(n=5)

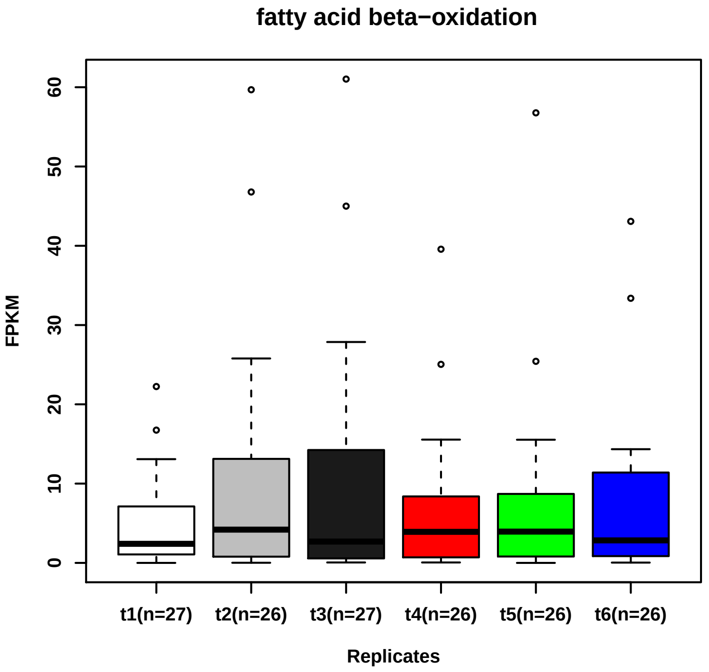
fatty acid beta−oxidation
FPKM
Replicates
t1(n=27)
t2(n=26)
t3(n=27)
t4(n=26)
t5(n=26)
t6(n=26)

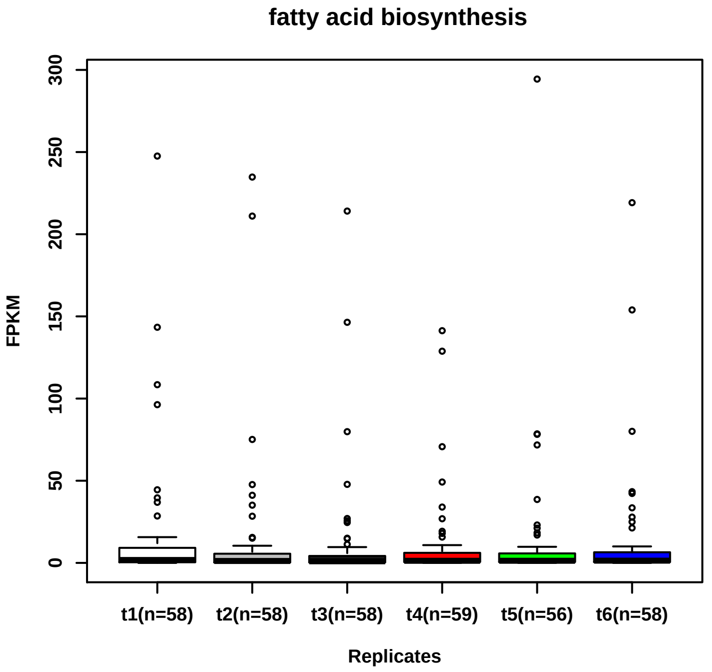
fatty acid biosynthesis
FPKM
Replicates
t1(n=58)
t2(n=58)
t3(n=58)
t4(n=59)
t5(n=56)
t6(n=58)

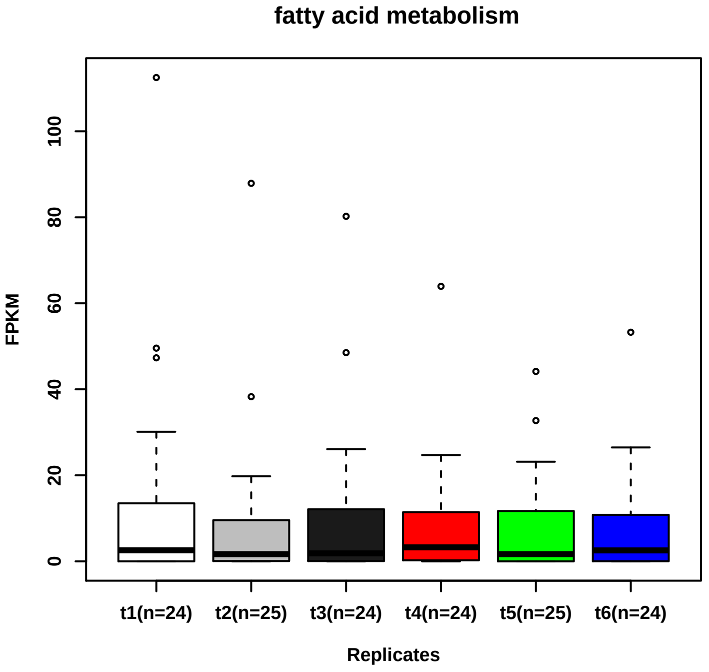
fatty acid metabolism
FPKM
Replicates
t1(n=24)
t2(n=25)
t3(n=24)
t4(n=24)
t5(n=25)
t6(n=24)

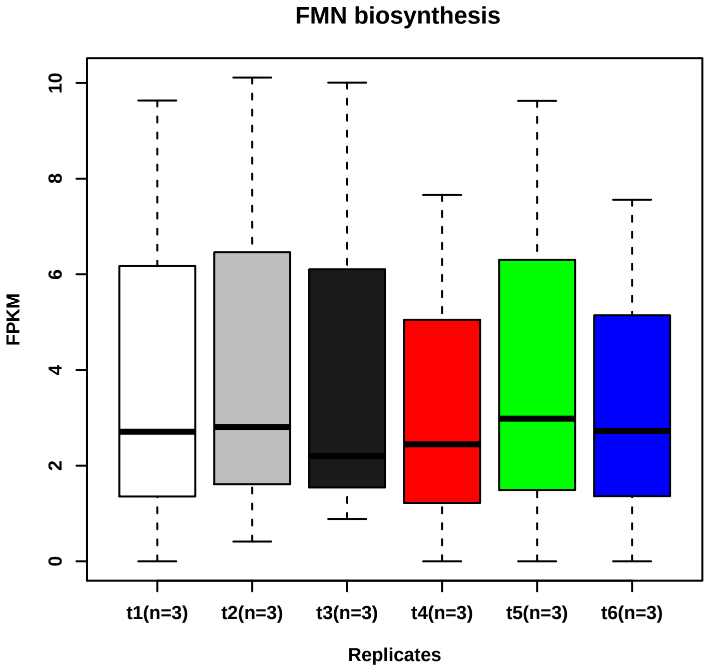
FMN biosynthesis
FPKM
Replicates
t1(n=3)
t2(n=3)
t3(n=3)
t4(n=3)
t5(n=3)
t6(n=3)

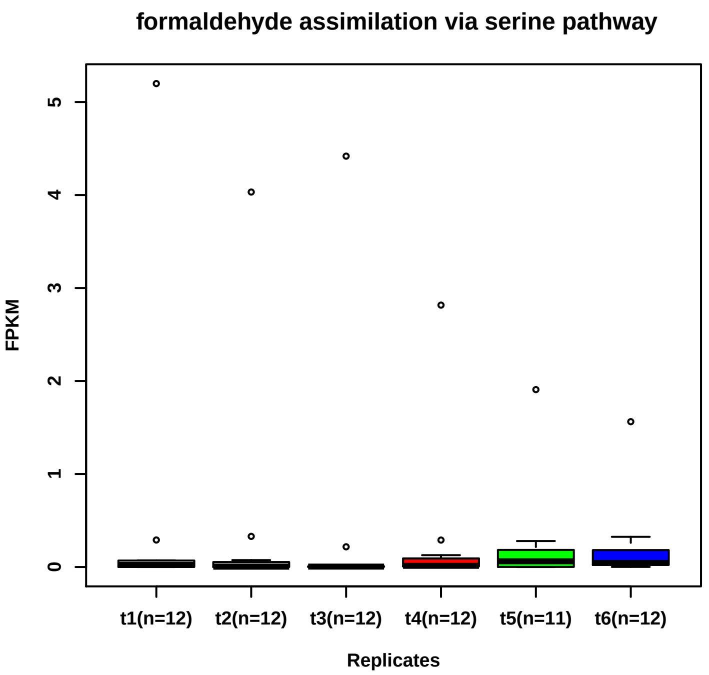
formaldehyde assimilation via serine pathway
FPKM
0
1
2
3
4
5
t1(n=12)
t2(n=12)
t3(n=12)
t4(n=12)
t5(n=11)
t6(n=12)
Replicates

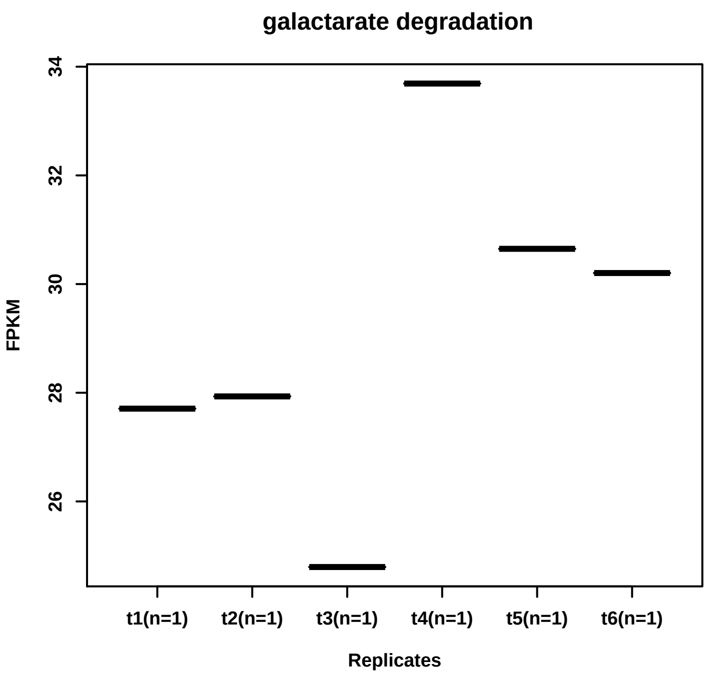
galactarate degradation
FPKM
26
28
30
32
34
t1(n=1)
t2(n=1)
t3(n=1)
t4(n=1)
t5(n=1)
t6(n=1)
Replicates

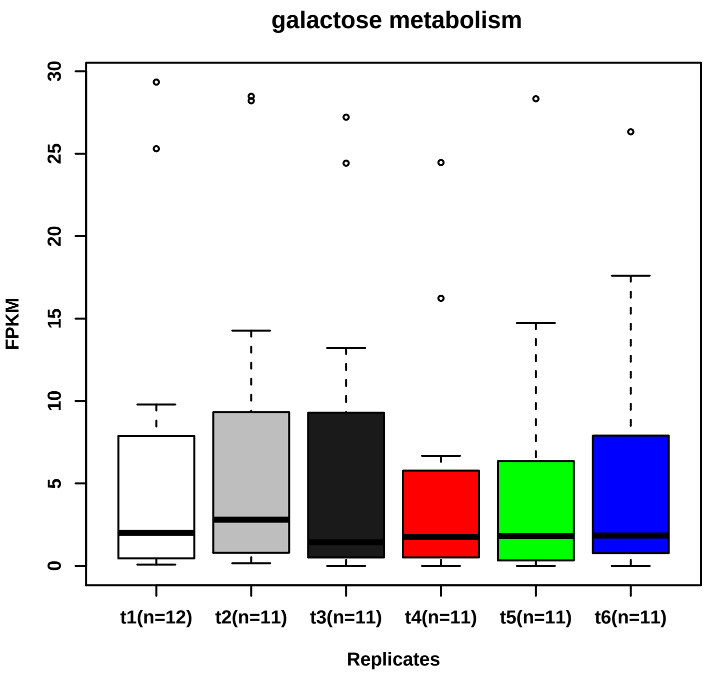
galactose metabolism
FPKM
0
5
10
15
20
25
30
t1(n=12)
t2(n=11)
t3(n=11)
t4(n=11)
t5(n=11)
t6(n=11)
Replicates

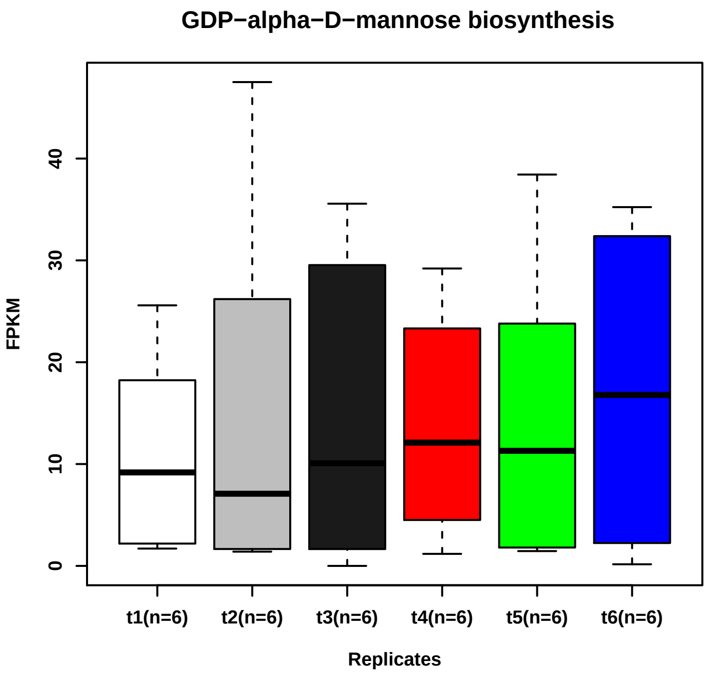
GDP−alpha−D−mannose biosynthesis
FPKM
0
10
20
30
40
t1(n=6)
t2(n=6)
t3(n=6)
t4(n=6)
t5(n=6)
t6(n=6)
Replicates

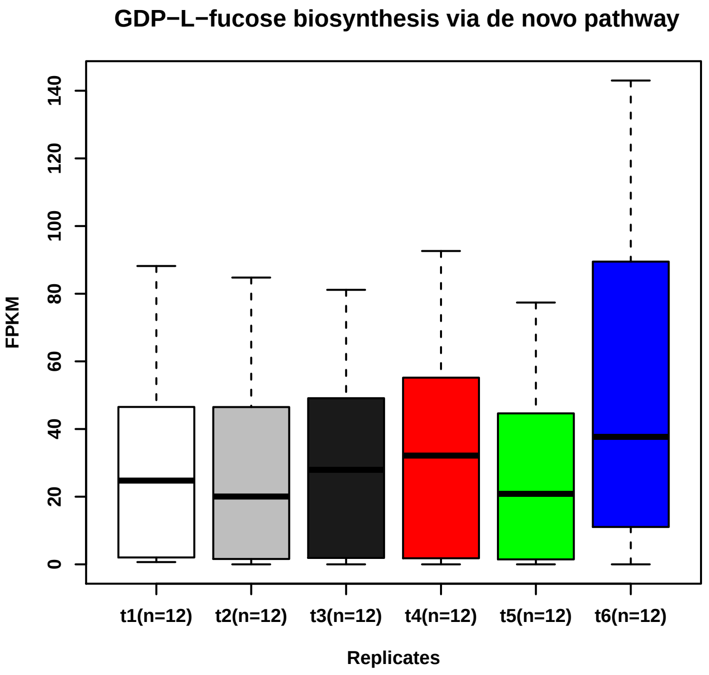
GDP−L−fucose biosynthesis via de novo pathway
FPKM
0
20
40
60
80
100
120
140
t1(n=12)
t2(n=12)
t3(n=12)
t4(n=12)
t5(n=12)
t6(n=12)
Replicates

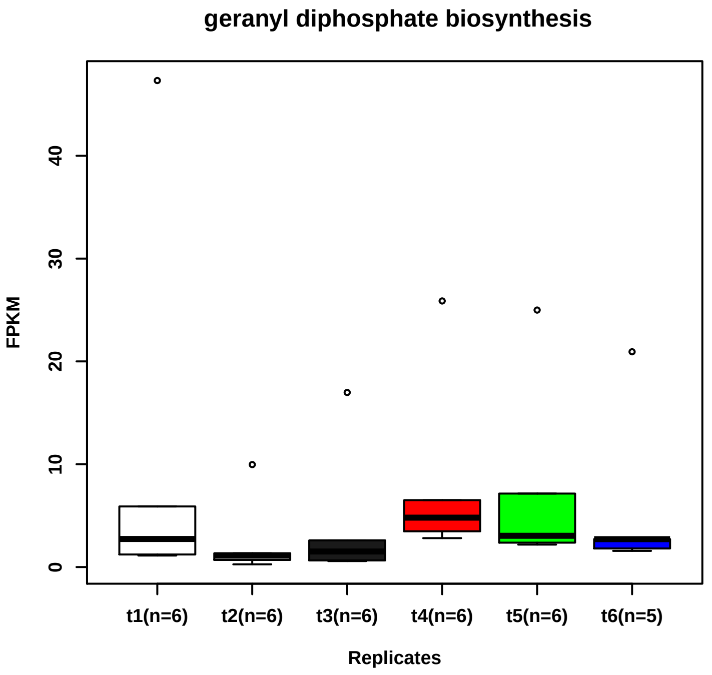
geranyl diphosphate biosynthesis
FPKM
0
10
20
30
40
t1(n=6)
t2(n=6)
t3(n=6)
t4(n=6)
t5(n=6)
t6(n=5)
Replicates

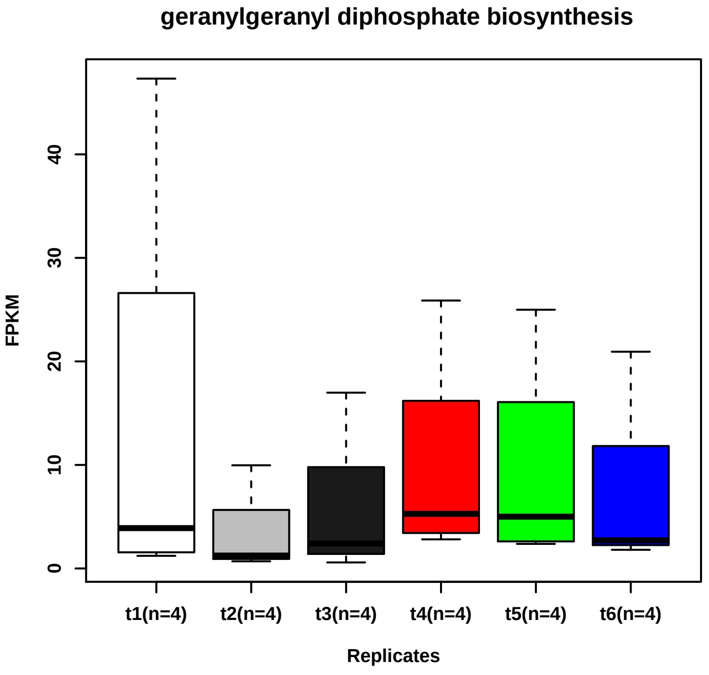
geranylgeranyl diphosphate biosynthesis
FPKM
0
10
20
30
40
t1(n=4)
t2(n=4)
t3(n=4)
t4(n=4)
t5(n=4)
t6(n=4)
Replicates

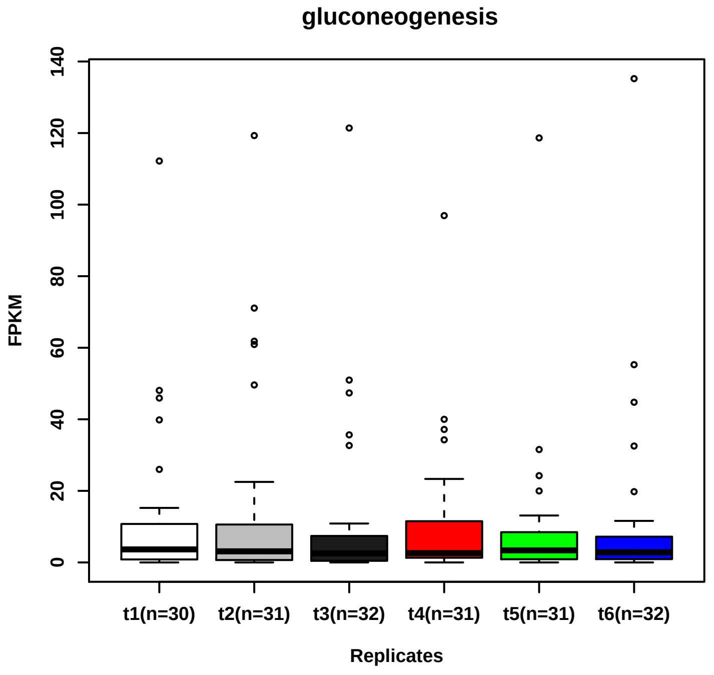
gluconeogenesis
FPKM
0
20
40
60
80
100
120
140
t1(n=30)
t2(n=31)
t3(n=32)
t4(n=31)
t5(n=31)
t6(n=32)
Replicates

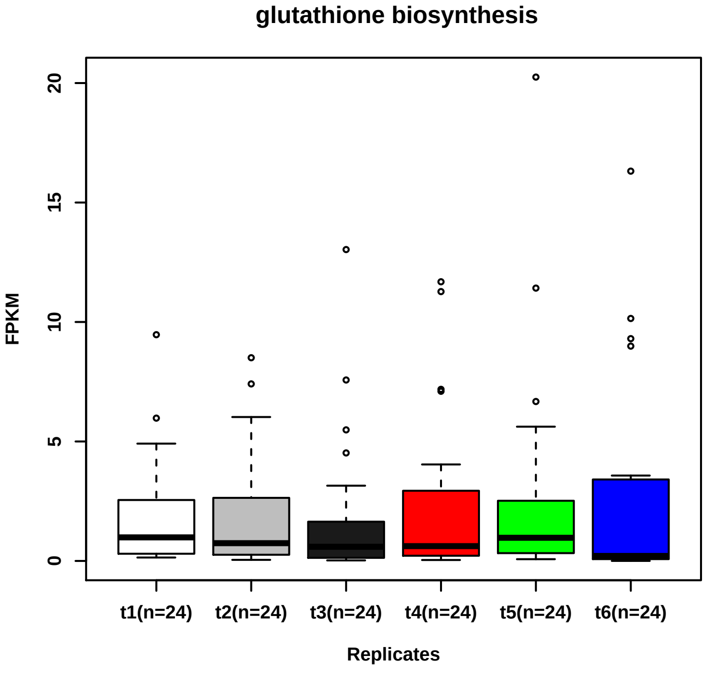
glutathione biosynthesis
FPKM
0
5
10
15
20
t1(n=24)
t2(n=24)
t3(n=24)
t4(n=24)
t5(n=24)
t6(n=24)
Replicates

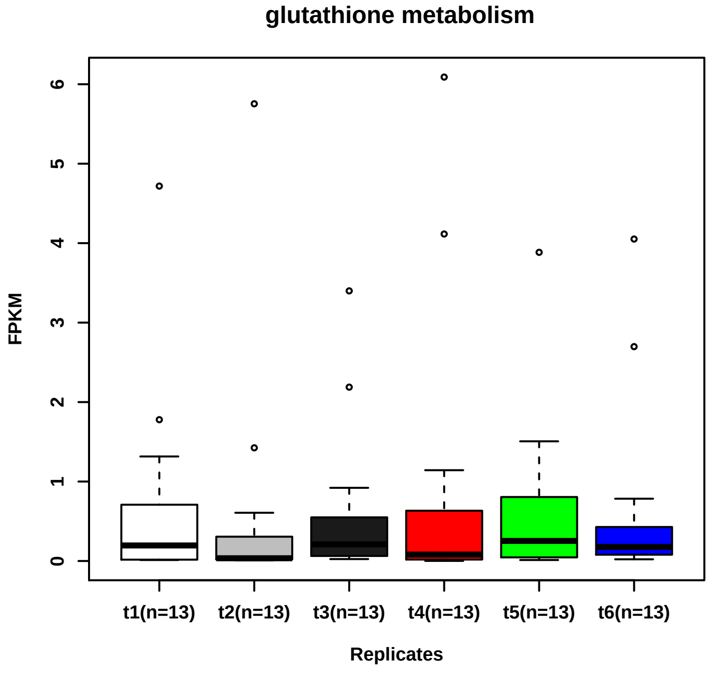
glutathione metabolism
FPKM
0
1
2
3
4
5
6
t1(n=13)
t2(n=13)
t3(n=13)
t4(n=13)
t5(n=13)
t6(n=13)
Replicates

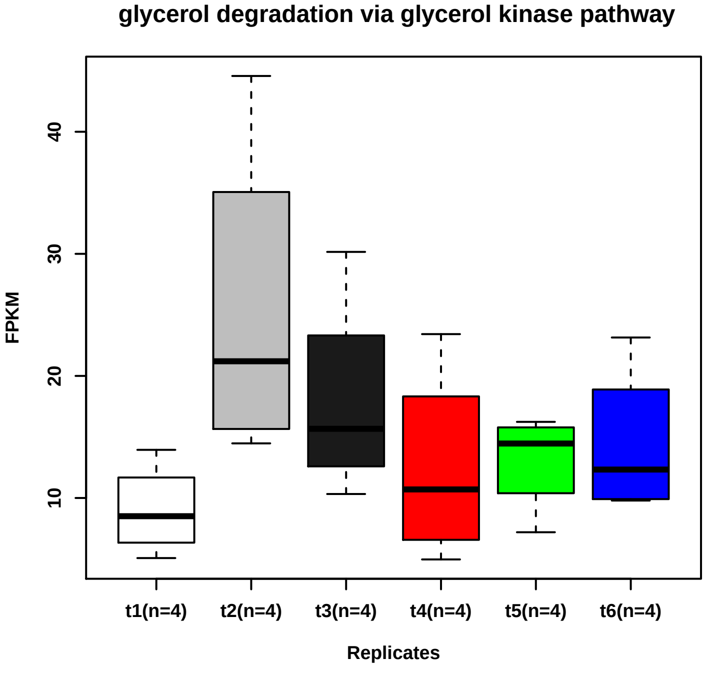
glycerol degradation via glycerol kinase pathway
FPKM
10
20
30
40
t1(n=4)
t2(n=4)
t3(n=4)
t4(n=4)
t5(n=4)
t6(n=4)
Replicates

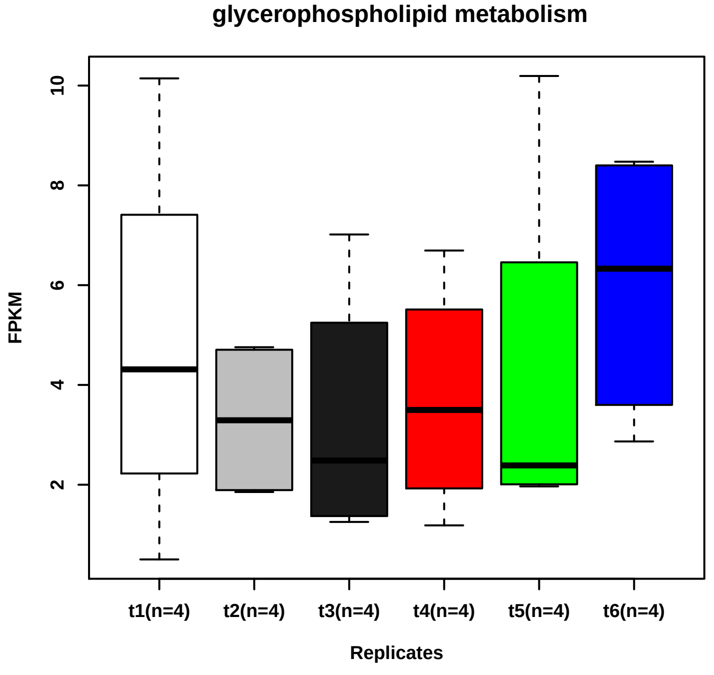
glycerophospholipid metabolism
FPKM
2
4
6
8
10
t1(n=4)
t2(n=4)
t3(n=4)
t4(n=4)
t5(n=4)
t6(n=4)
Replicates

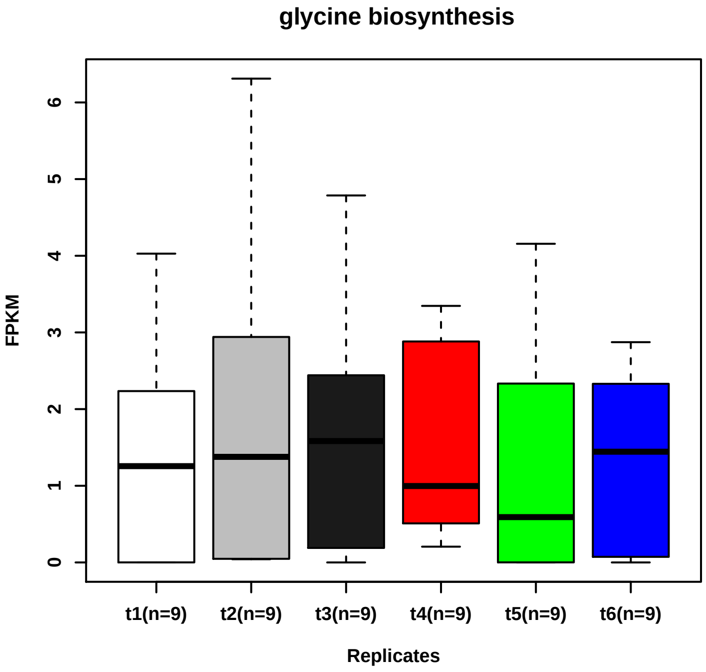
glycine biosynthesis
FPKM
0
1
2
3
4
5
6
t1(n=9)
t2(n=9)
t3(n=9)
t4(n=9)
t5(n=9)
t6(n=9)
Replicates

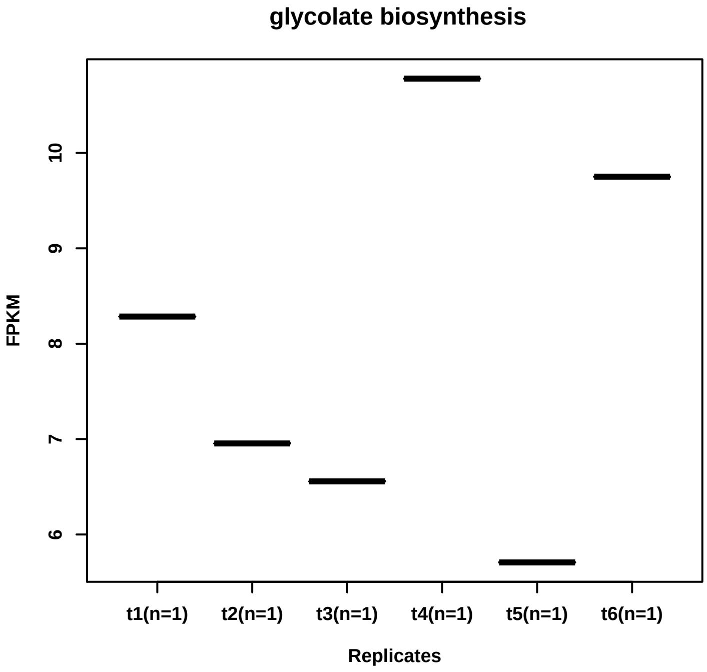
glycolate biosynthesis
FPKM
6
7
8
9
10
t1(n=1)
t2(n=1)
t3(n=1)
t4(n=1)
t5(n=1)
t6(n=1)
Replicates

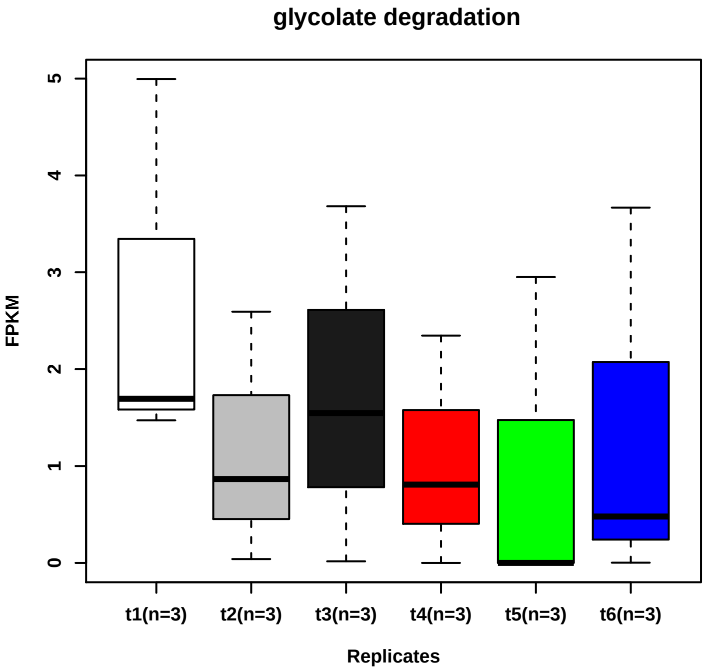
glycolate degradation
FPKM
0
1
2
3
4
5
t1(n=3)
t2(n=3)
t3(n=3)
t4(n=3)
t5(n=3)
t6(n=3)
Replicates

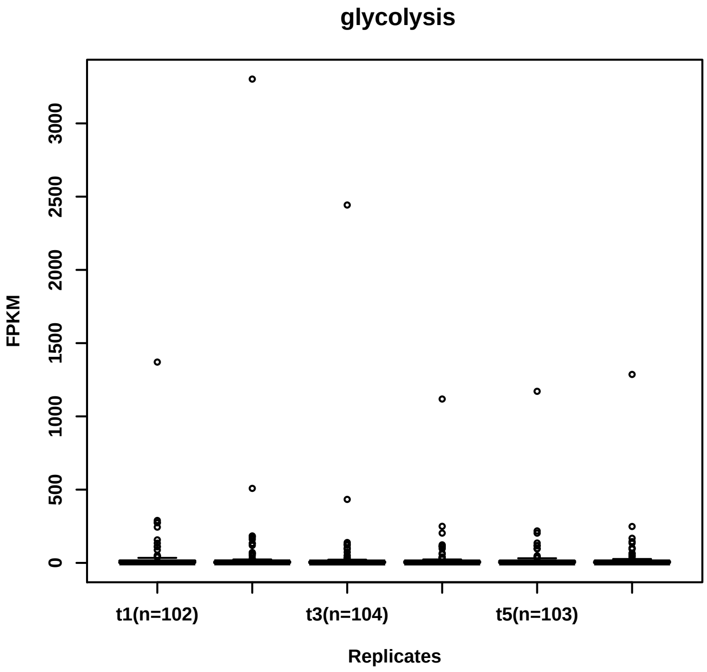
glycolysis
FPKM
0
500
1000
1500
2000
2500
3000
t1(n=102)
t3(n=104)
t5(n=103)
Replicates

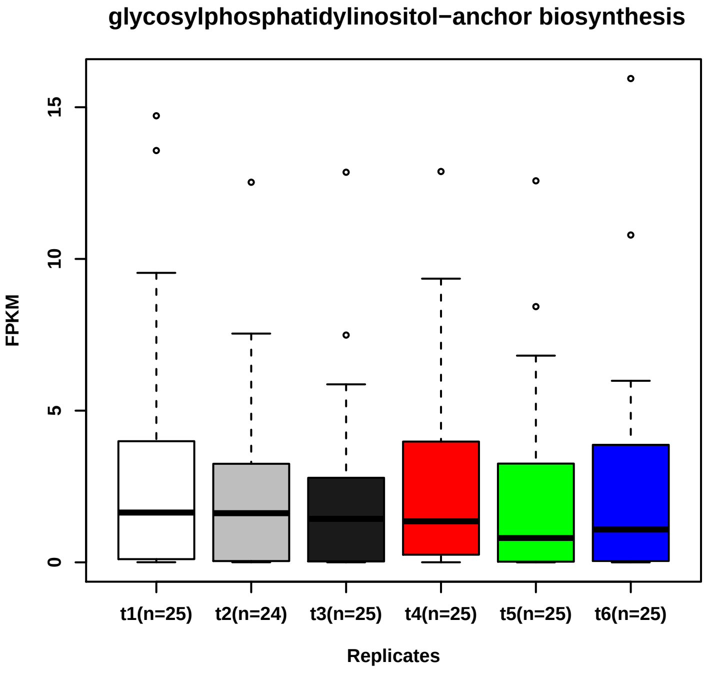
glycosylphosphatidylinositol−anchor biosynthesis
FPKM
0
5
10
15
t1(n=25)
t2(n=24)
t3(n=25)
t4(n=25)
t5(n=25)
t6(n=25)
Replicates

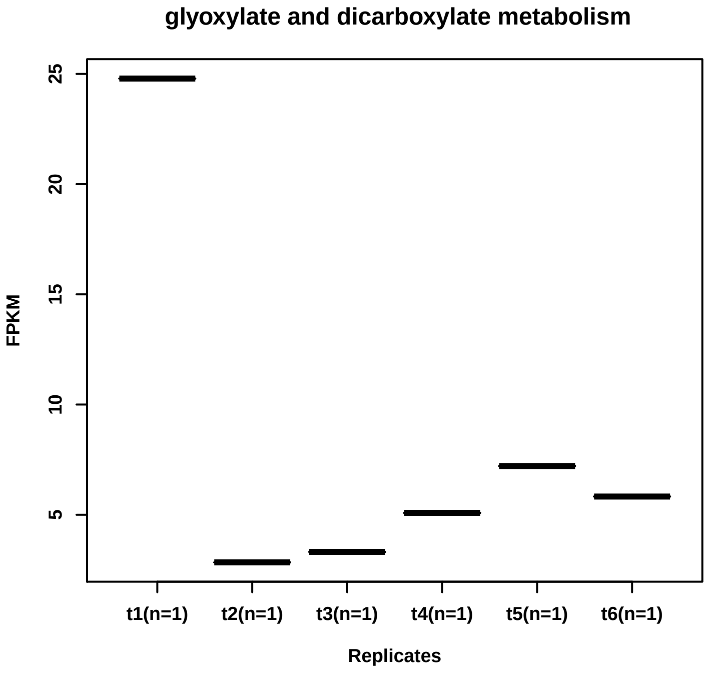
glyoxylate and dicarboxylate metabolism
FPKM
5
10
15
20
25
t1(n=1)
t2(n=1)
t3(n=1)
t4(n=1)
t5(n=1)
t6(n=1)
Replicates

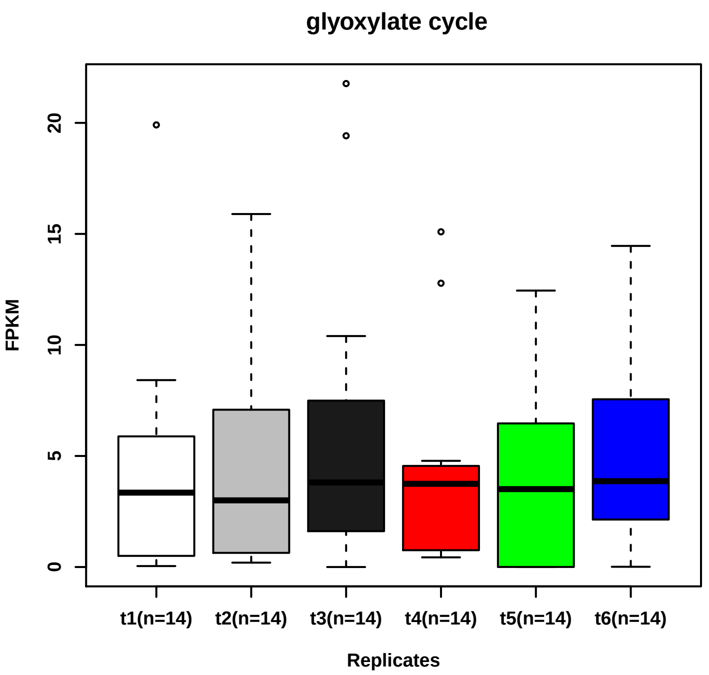
glyoxylate cycle
FPKM
0
5
10
15
20
t1(n=14)
t2(n=14)
t3(n=14)
t4(n=14)
t5(n=14)
t6(n=14)
Replicates

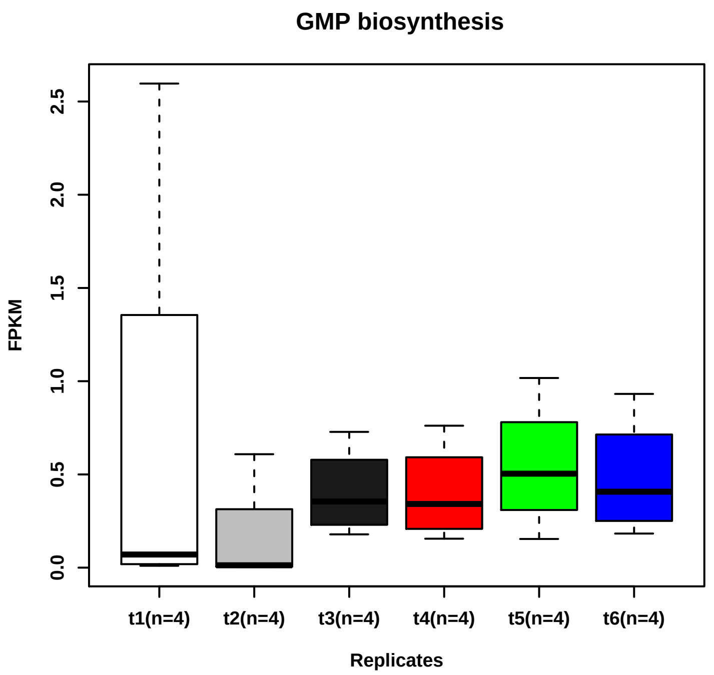
GMP biosynthesis
FPKM
0.0
0.5
1.0
1.5
2.0
2.5
t1(n=4)
t2(n=4)
t3(n=4)
t4(n=4)
t5(n=4)
t6(n=4)
Replicates

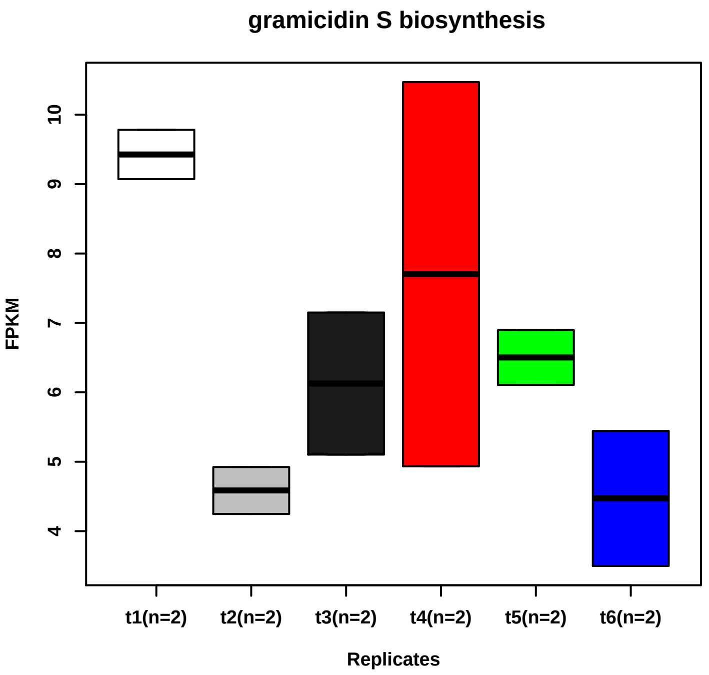
gramicidin S biosynthesis
FPKM
4
5
6
7
8
9
10
t1(n=2)
t2(n=2)
t3(n=2)
t4(n=2)
t5(n=2)
t6(n=2)
Replicates

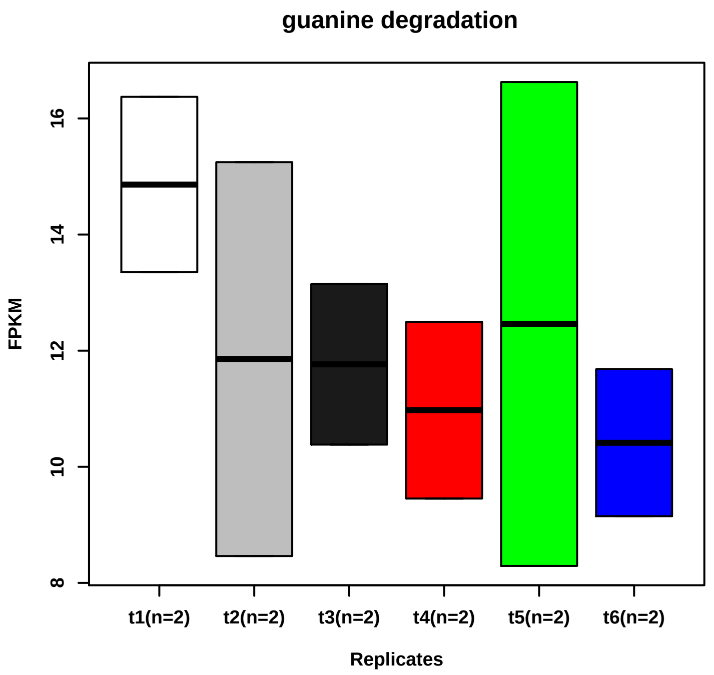
guanine degradation
FPKM
8
10
12
14
16
t1(n=2)
t2(n=2)
t3(n=2)
t4(n=2)
t5(n=2)
t6(n=2)
Replicates

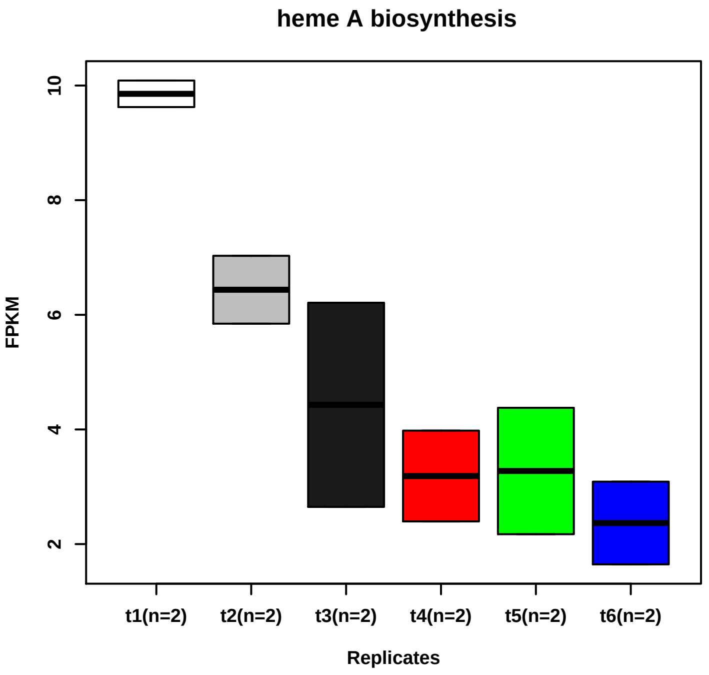
heme A biosynthesis
FPKM
2
4
6
8
10
t1(n=2)
t2(n=2)
t3(n=2)
t4(n=2)
t5(n=2)
t6(n=2)
Replicates

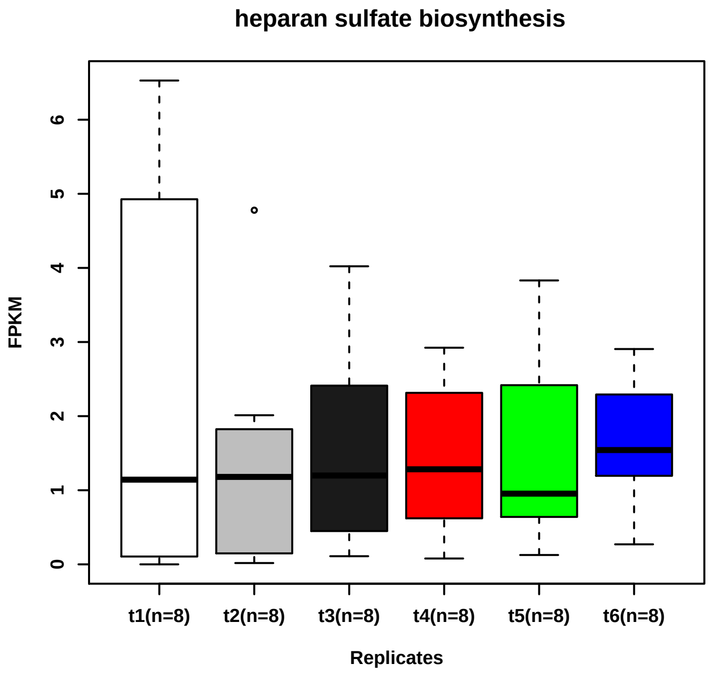
heparan sulfate biosynthesis
FPKM
0
1
2
3
4
5
6
t1(n=8)
t2(n=8)
t3(n=8)
t4(n=8)
t5(n=8)
t6(n=8)
Replicates

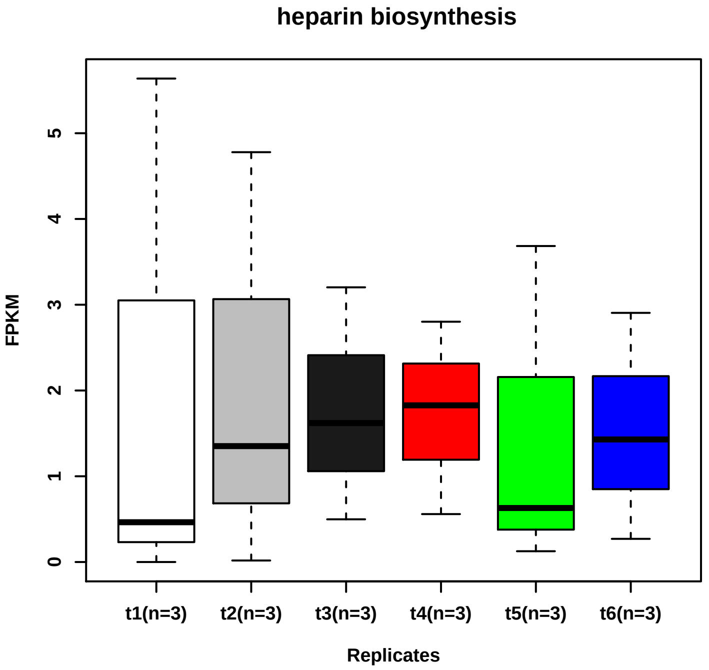
heparin biosynthesis
FPKM
0
1
2
3
4
5
t1(n=3)
t2(n=3)
t3(n=3)
t4(n=3)
t5(n=3)
t6(n=3)
Replicates

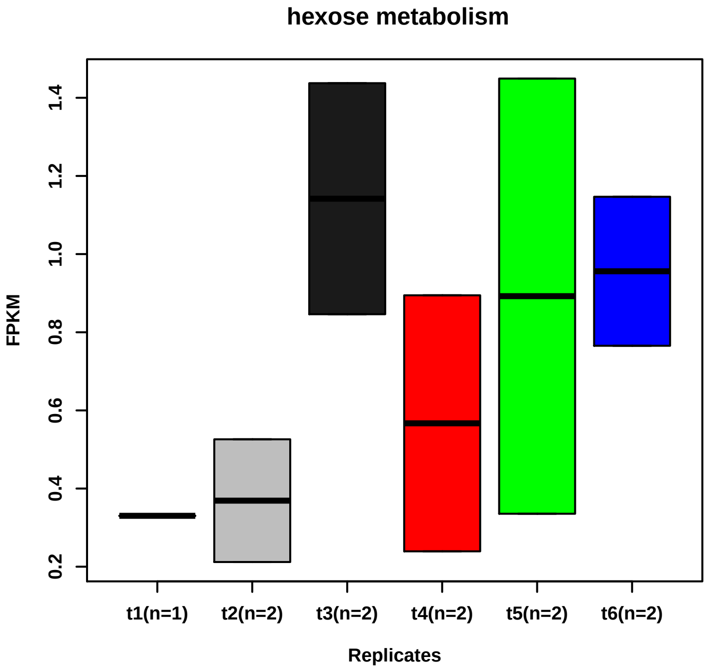
hexose metabolism
FPKM
0.2
0.4
0.6
0.8
1.0
1.2
1.4
t1(n=1)
t2(n=2)
t3(n=2)
t4(n=2)
t5(n=2)
t6(n=2)
Replicates

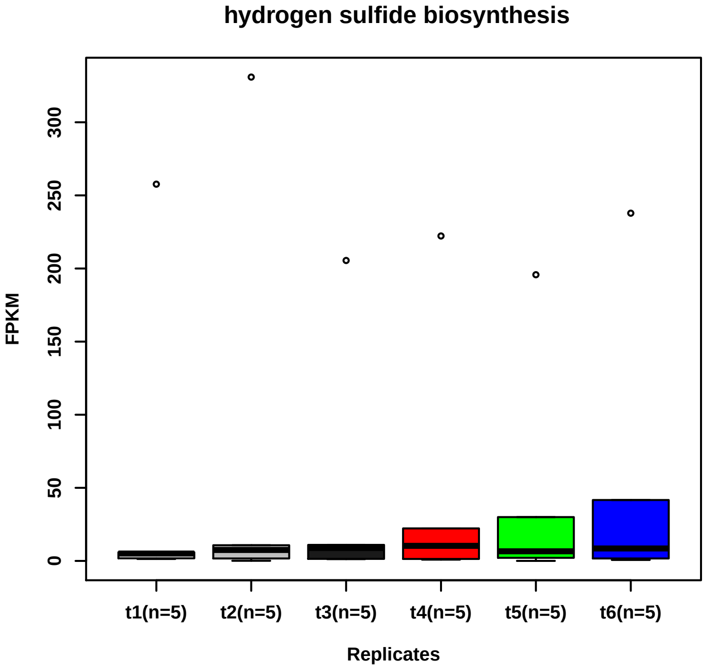
hydrogen sulfide biosynthesis
FPKM
0
50
100
150
200
250
300
t1(n=5)
t2(n=5)
t3(n=5)
t4(n=5)
t5(n=5)
t6(n=5)
Replicates

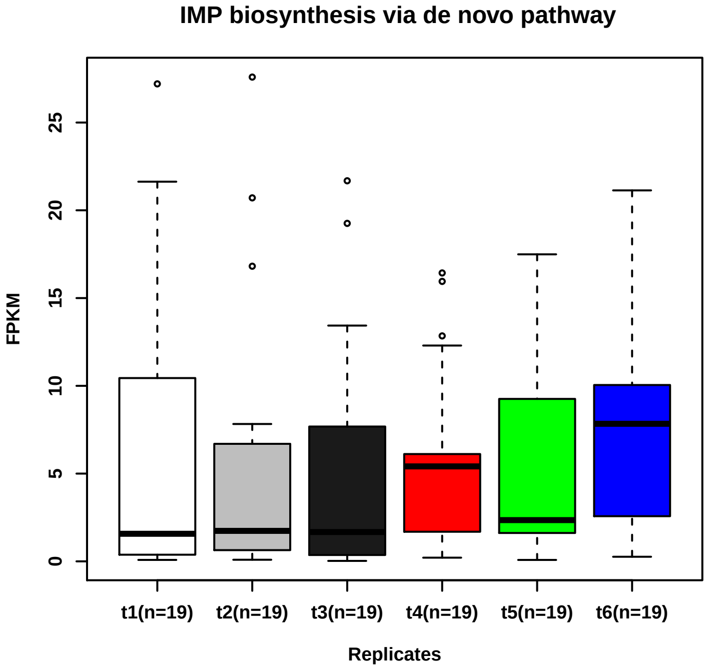
IMP biosynthesis via de novo pathway
FPKM
0
5
10
15
20
25
t1(n=19)
t2(n=19)
t3(n=19)
t4(n=19)
t5(n=19)
t6(n=19)
Replicates

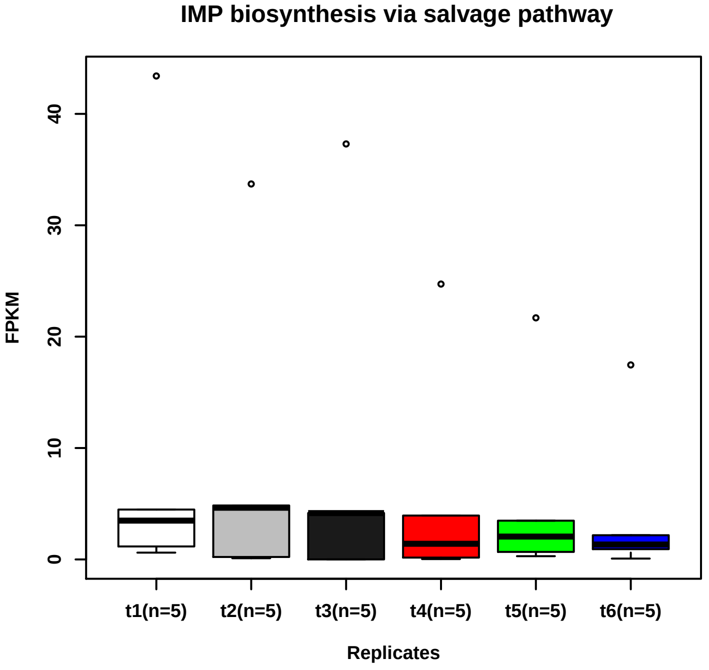
IMP biosynthesis via salvage pathway
FPKM
0
10
20
30
40
t1(n=5)
t2(n=5)
t3(n=5)
t4(n=5)
t5(n=5)
t6(n=5)
Replicates

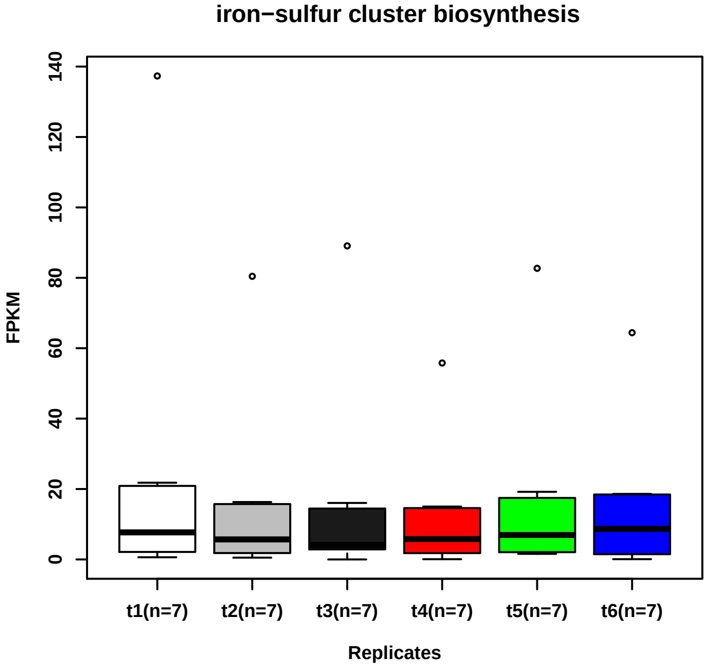
iron−sulfur cluster biosynthesis
FPKM
0
20
40
60
80
100
120
140
t1(n=7)
t2(n=7)
t3(n=7)
t4(n=7)
t5(n=7)
t6(n=7)
Replicates

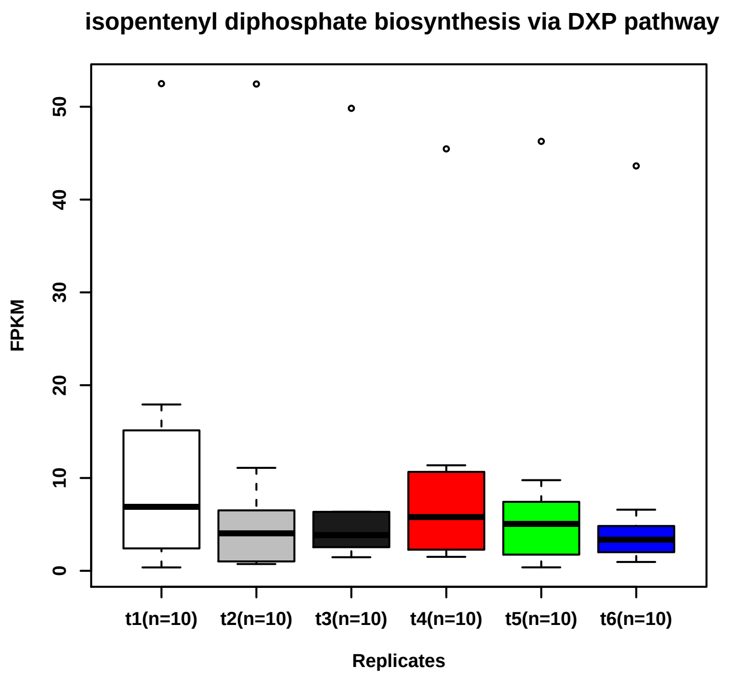
isopentenyl diphosphate biosynthesis via DXP pathway
FPKM
0
10
20
30
40
50
t1(n=10)
t2(n=10)
t3(n=10)
t4(n=10)
t5(n=10)
t6(n=10)
Replicates

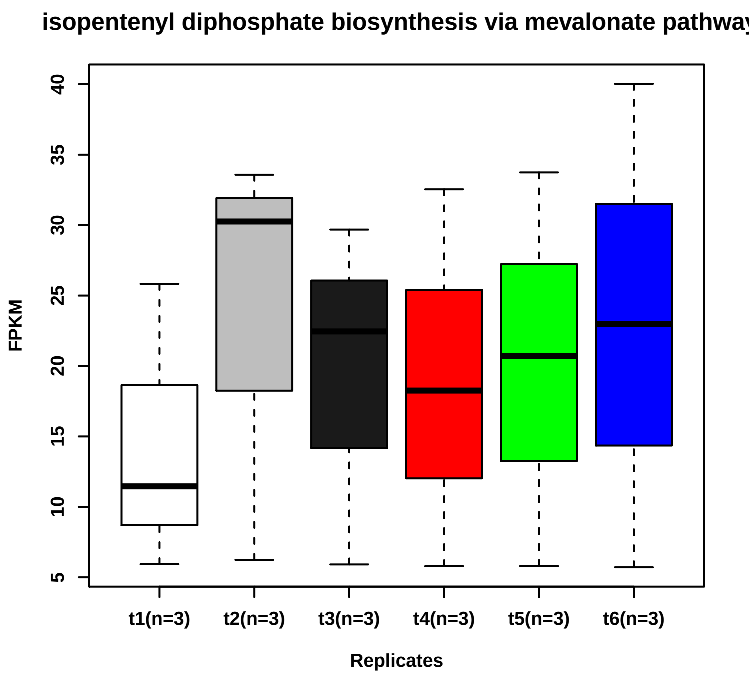
isopentenyl diphosphate biosynthesis via mevalonate pathway
FPKM
5
10
15
20
25
30
35
40
t1(n=3)
t2(n=3)
t3(n=3)
t4(n=3)
t5(n=3)
t6(n=3)
Replicates

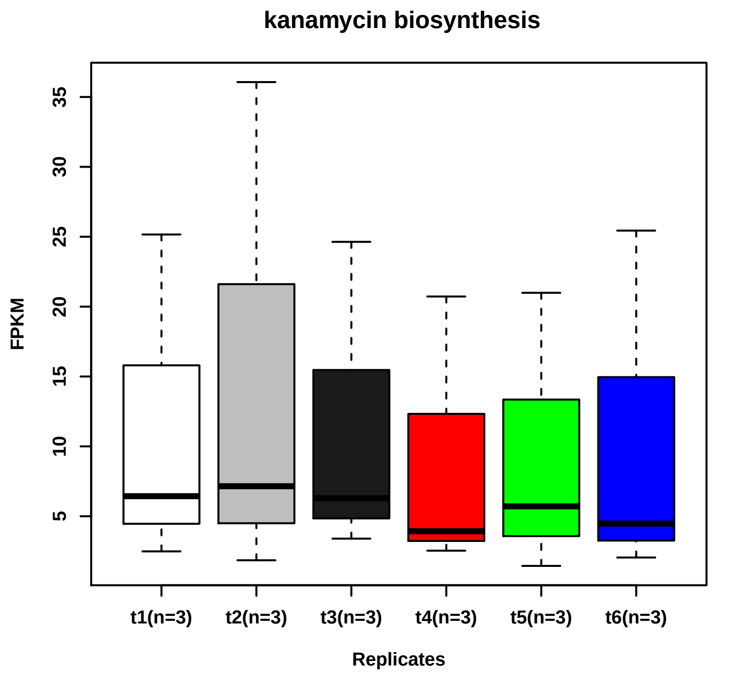
kanamycin biosynthesis
FPKM
5
10
15
20
25
30
35
t1(n=3)
t2(n=3)
t3(n=3)
t4(n=3)
t5(n=3)
t6(n=3)
Replicates

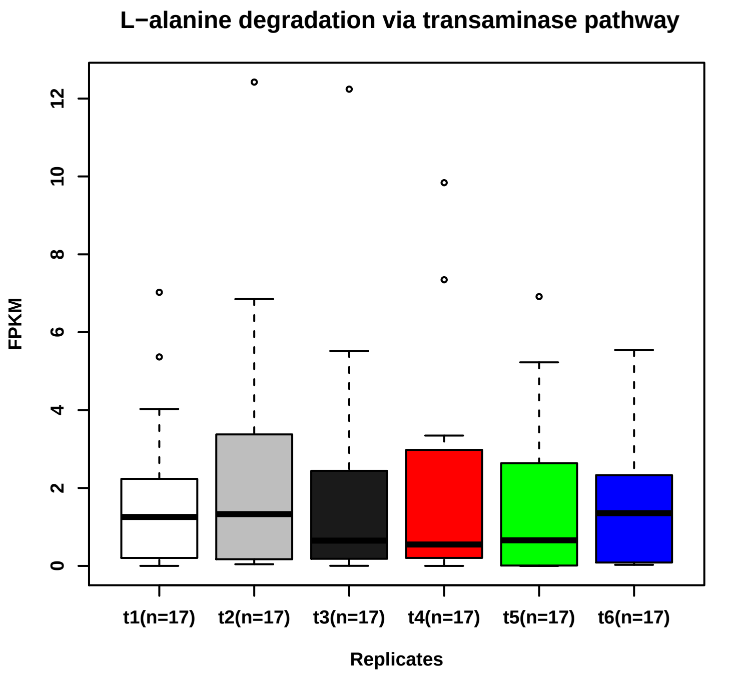
L−alanine degradation via transaminase pathway
FPKM
0
2
4
6
8
10
12
t1(n=17)
t2(n=17)
t3(n=17)
t4(n=17)
t5(n=17)
t6(n=17)
Replicates

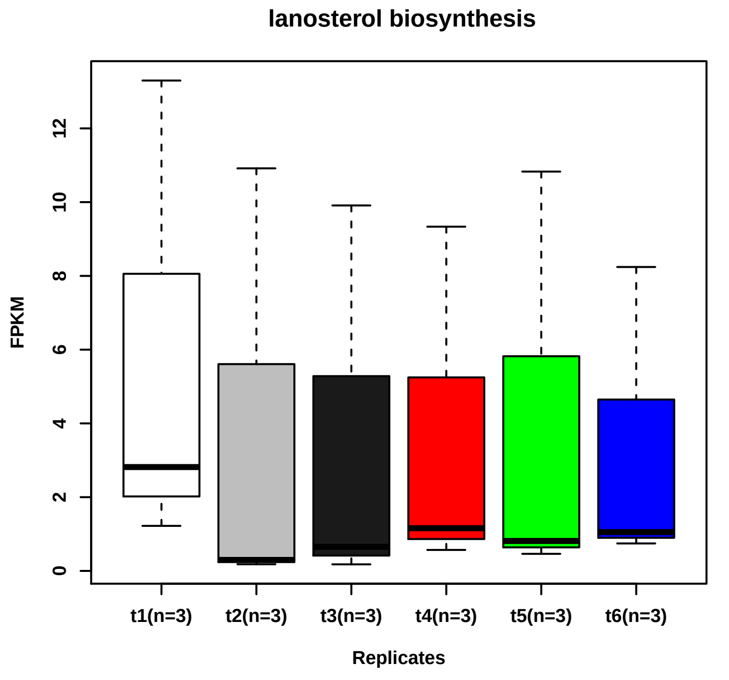
lanosterol biosynthesis
FPKM
0
2
4
6
8
10
12
t1(n=3)
t2(n=3)
t3(n=3)
t4(n=3)
t5(n=3)
t6(n=3)
Replicates

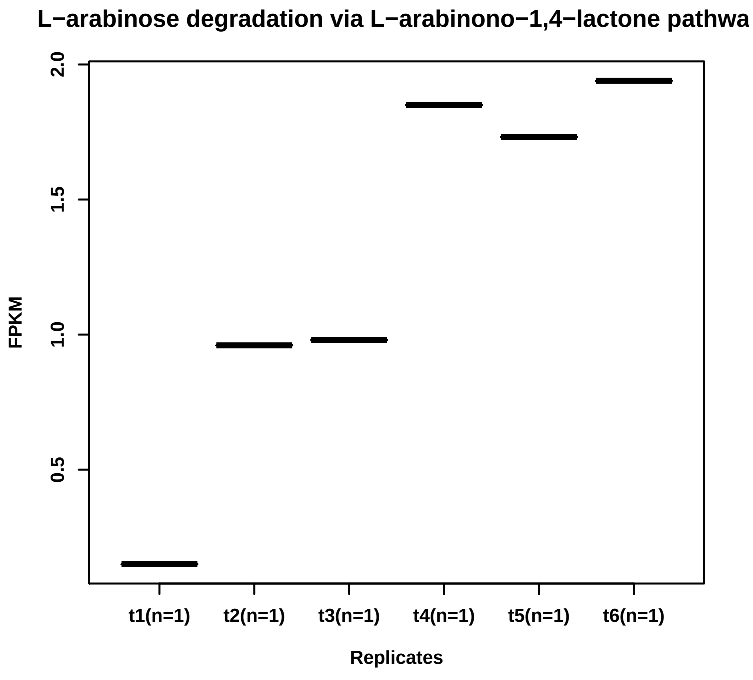
L−arabinose degradation via L−arabinono−1,4−lactone pathwa
FPKM
0.5
1.0
1.5
2.0
t1(n=1)
t2(n=1)
t3(n=1)
t4(n=1)
t5(n=1)
t6(n=1)
Replicates

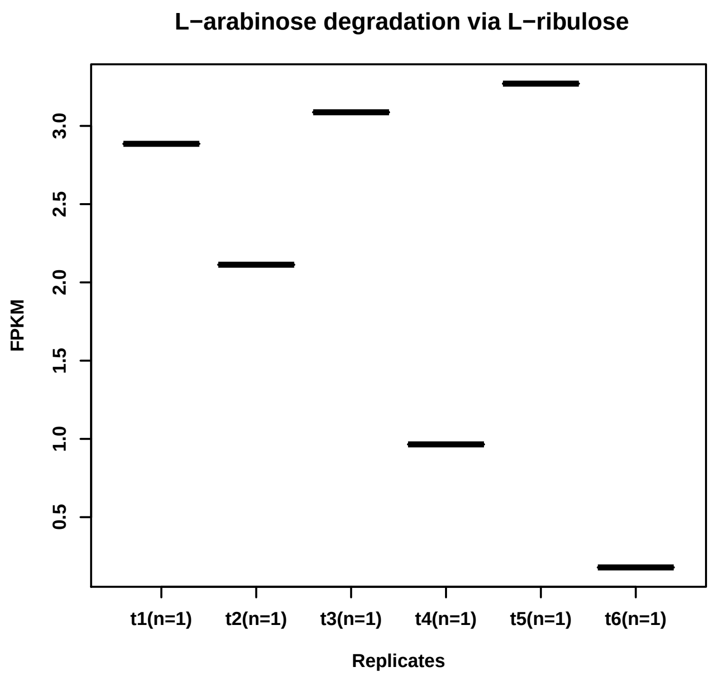
L−arabinose degradation via L−ribulose
FPKM
0.5
1.0
1.5
2.0
2.5
3.0
t1(n=1)
t2(n=1)
t3(n=1)
t4(n=1)
t5(n=1)
t6(n=1)
Replicates

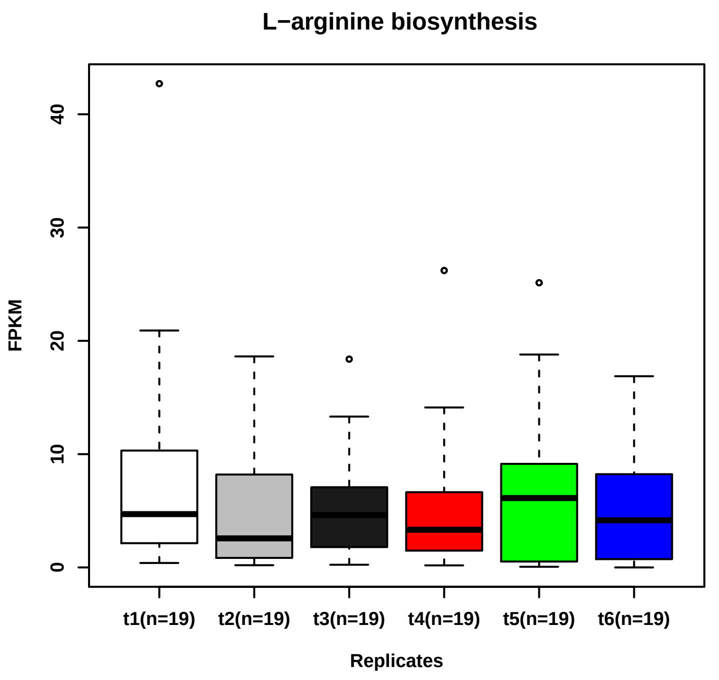
L−arginine biosynthesis
FPKM
0
10
20
30
40
t1(n=19)
t2(n=19)
t3(n=19)
t4(n=19)
t5(n=19)
t6(n=19)
Replicates

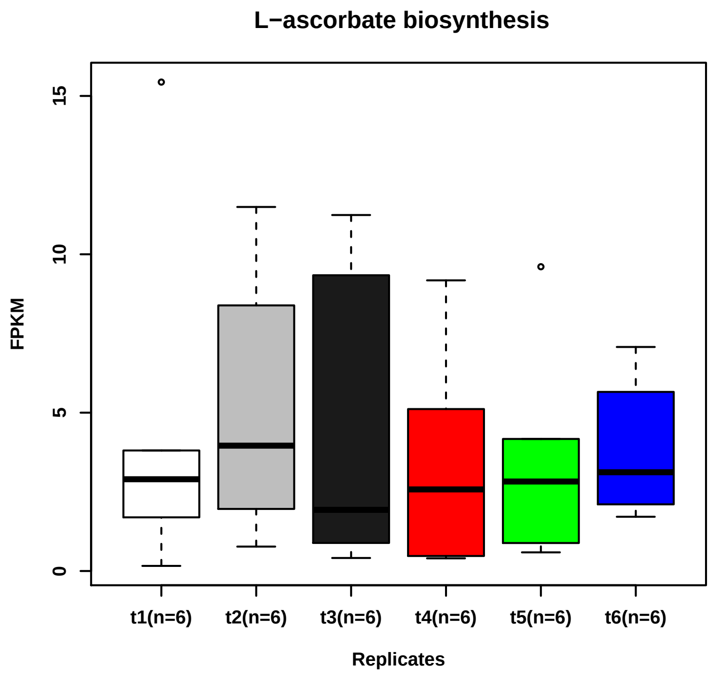
L−ascorbate biosynthesis
FPKM
0
5
10
15
t1(n=6)
t2(n=6)
t3(n=6)
t4(n=6)
t5(n=6)
t6(n=6)
Replicates

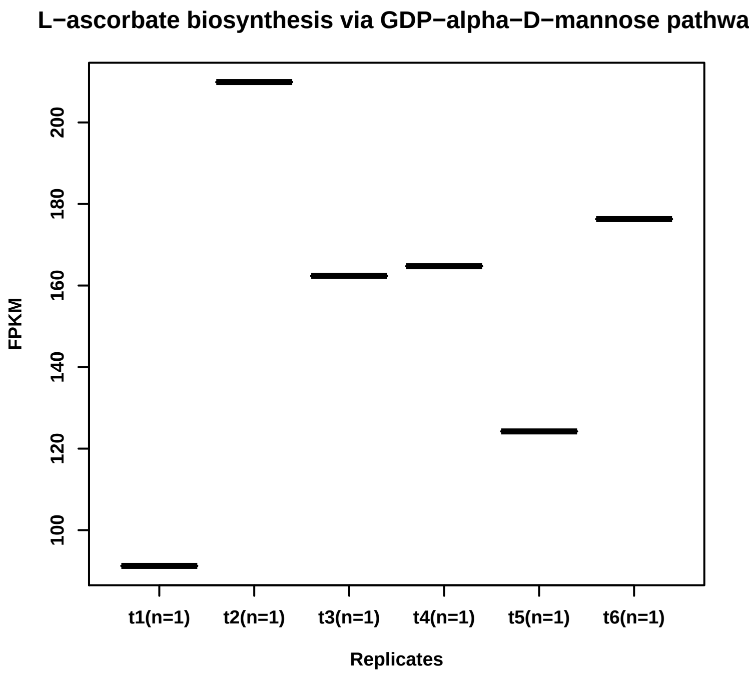
L−ascorbate biosynthesis via GDP−alpha−D−mannose pathwa
FPKM
100
120
140
160
180
200
t1(n=1)
t2(n=1)
t3(n=1)
t4(n=1)
t5(n=1)
t6(n=1)
Replicates

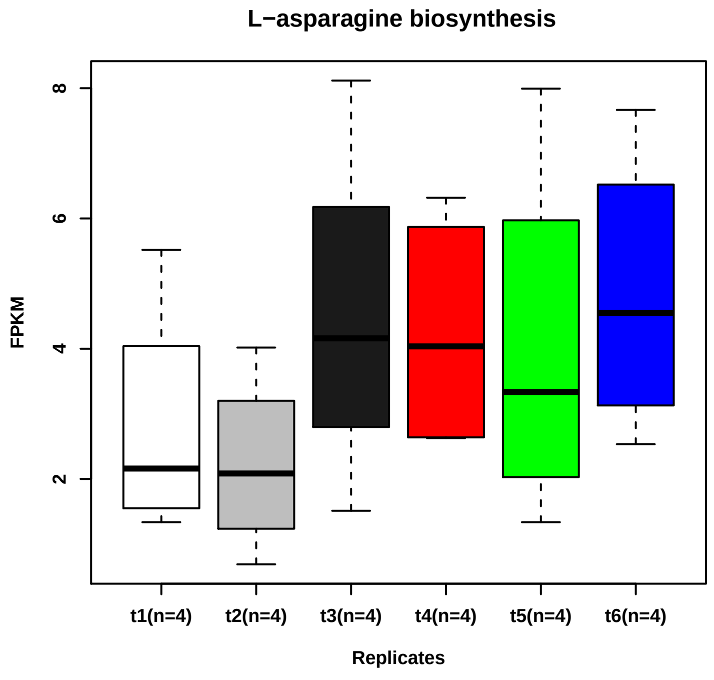
L−asparagine biosynthesis
FPKM
2
4
6
8
t1(n=4)
t2(n=4)
t3(n=4)
t4(n=4)
t5(n=4)
t6(n=4)
Replicates

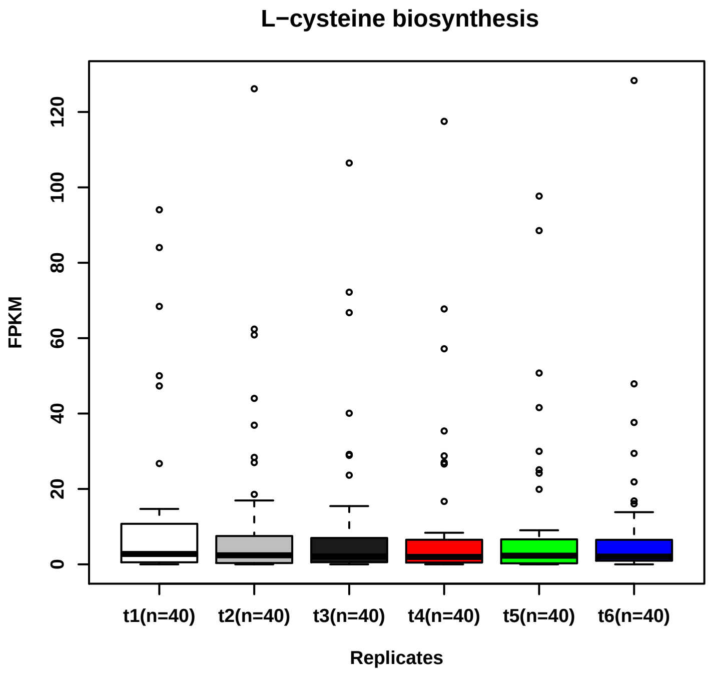
L−cysteine biosynthesis
FPKM
0
20
40
60
80
100
120
t1(n=40)
t2(n=40)
t3(n=40)
t4(n=40)
t5(n=40)
t6(n=40)
Replicates

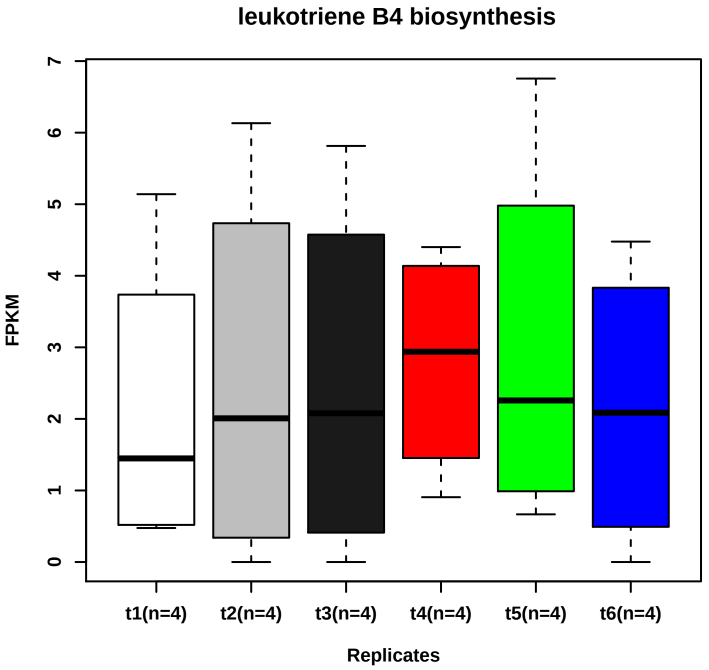
leukotriene B4 biosynthesis
FPKM
0
1
2
3
4
5
6
7
t1(n=4)
t2(n=4)
t3(n=4)
t4(n=4)
t5(n=4)
t6(n=4)
Replicates

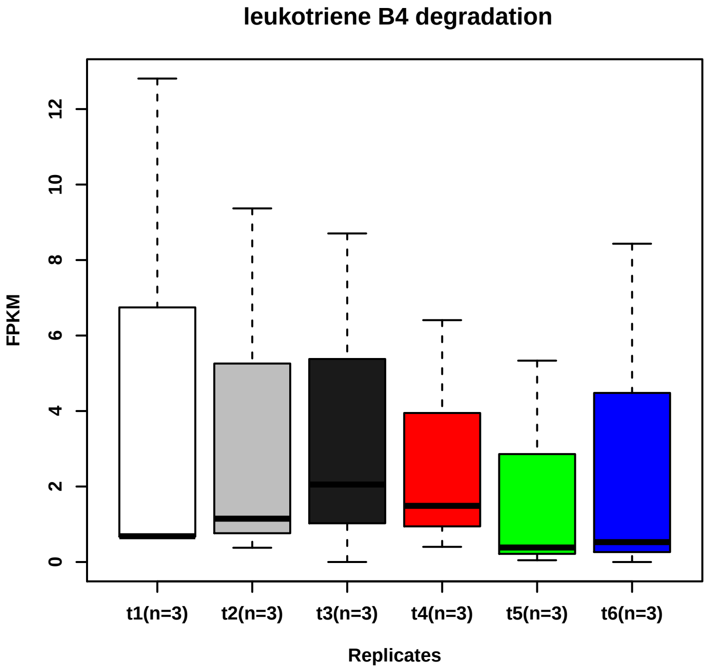
leukotriene B4 degradation
FPKM
0
2
4
6
8
10
12
t1(n=3)
t2(n=3)
t3(n=3)
t4(n=3)
t5(n=3)
t6(n=3)
Replicates

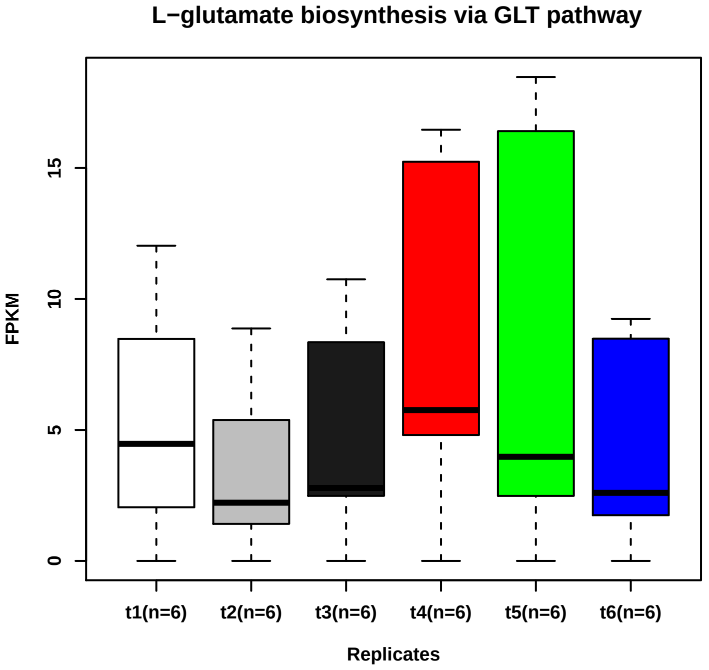
L−glutamate biosynthesis via GLT pathway
FPKM
0
5
10
15
t1(n=6)
t2(n=6)
t3(n=6)
t4(n=6)
t5(n=6)
t6(n=6)
Replicates

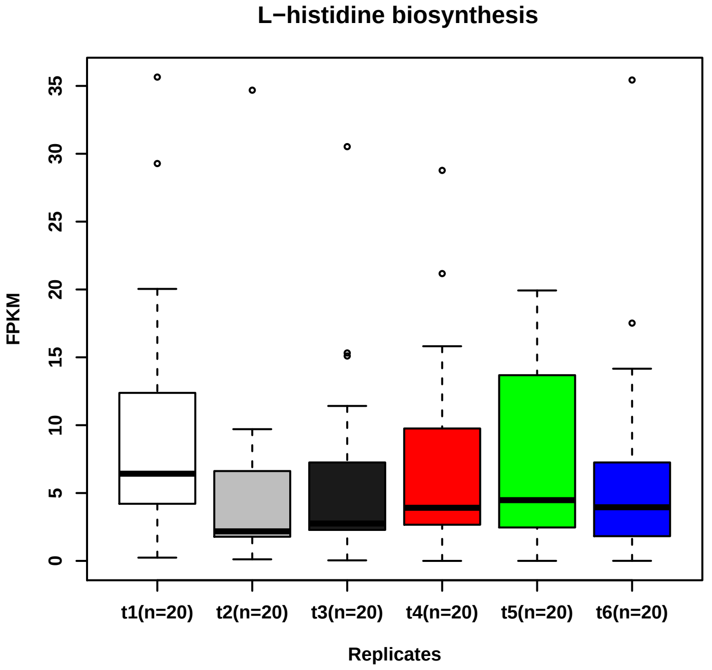
L−histidine biosynthesis
FPKM
0
5
10
15
20
25
30
35
t1(n=20)
t2(n=20)
t3(n=20)
t4(n=20)
t5(n=20)
t6(n=20)
Replicates

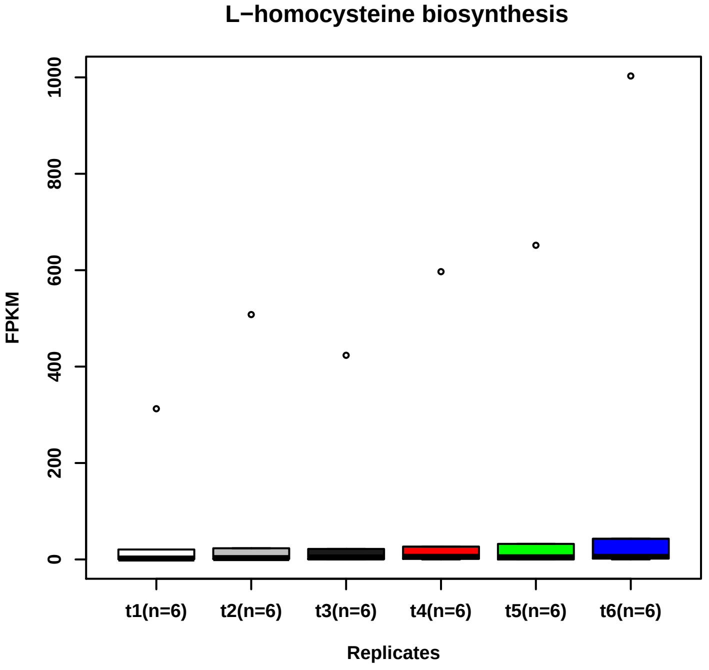
L−homocysteine biosynthesis
FPKM
0
200
400
600
800
1000
t1(n=6)
t2(n=6)
t3(n=6)
t4(n=6)
t5(n=6)
t6(n=6)
Replicates

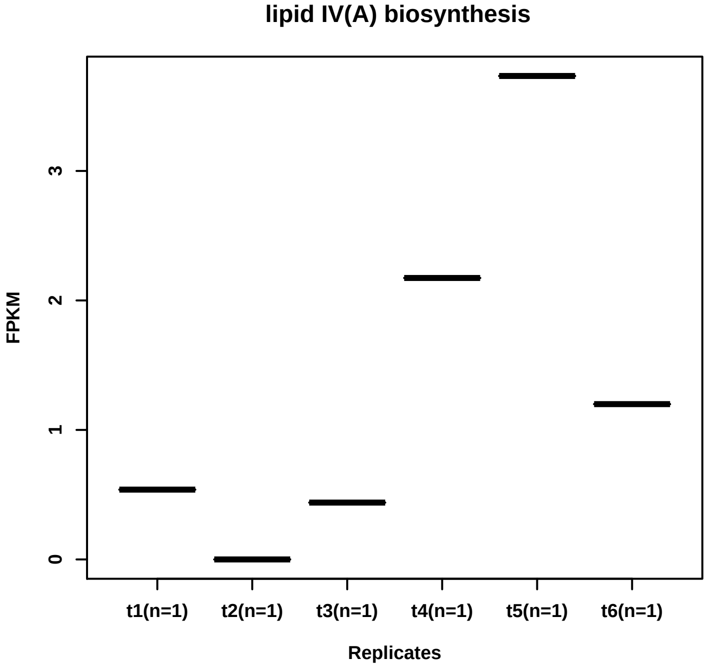
lipid IV(A) biosynthesis
FPKM
0
1
2
3
t1(n=1)
t2(n=1)
t3(n=1)
t4(n=1)
t5(n=1)
t6(n=1)
Replicates

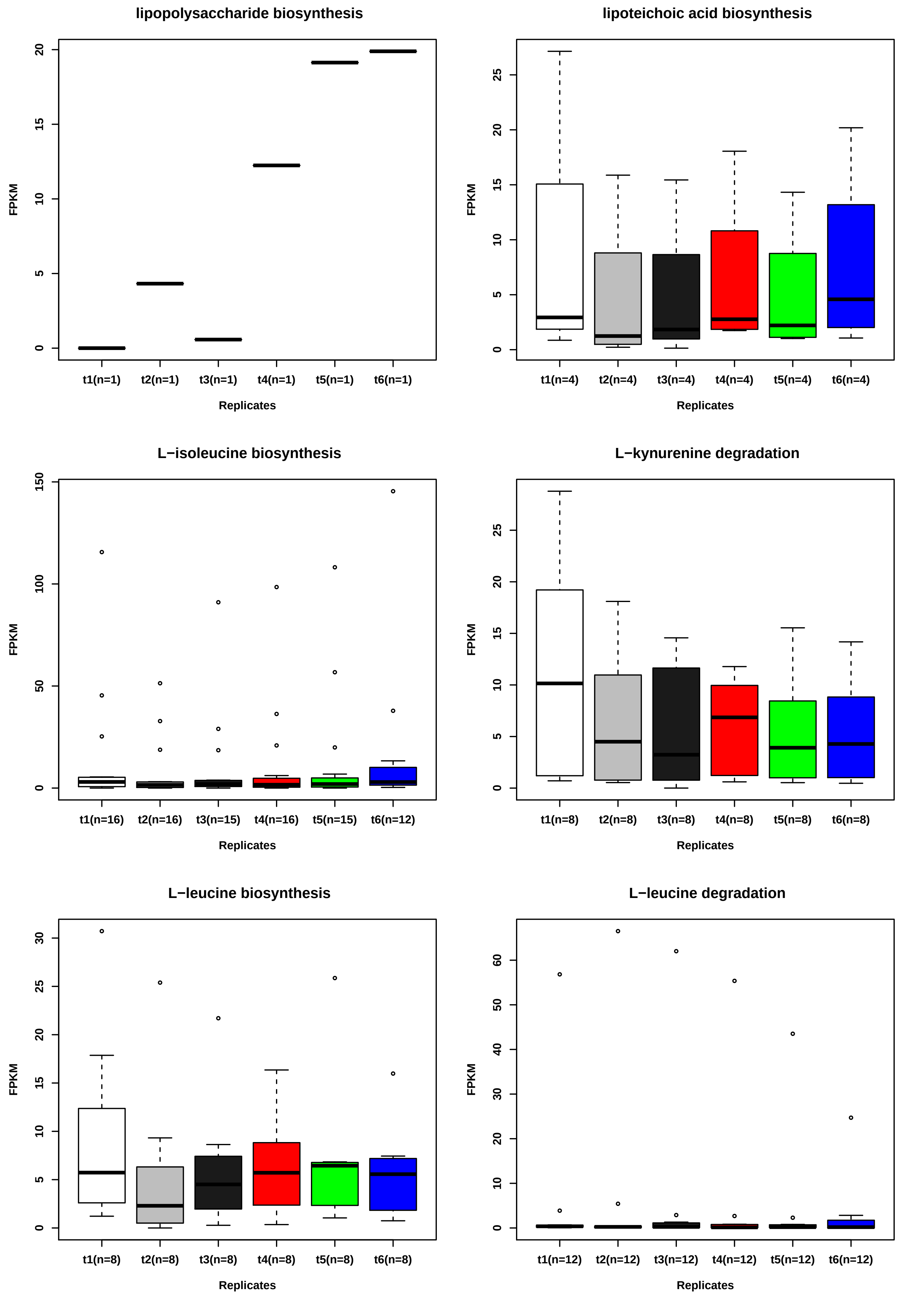

lipopolysaccharide biosynthesis
lipoteichoic acid biosynthesis
L−isoleucine biosynthesis
L−kynurenine degradation
L−leucine biosynthesis
L−leucine degradation
FPKM
Replicates
t1(n=1) t2(n=1) t3(n=1) t4(n=1) t5(n=1) t6(n=1)
t1(n=4) t2(n=4) t3(n=4) t4(n=4) t5(n=4) t6(n=4)
t1(n=16) t2(n=16) t3(n=15) t4(n=16) t5(n=15) t6(n=12)
t1(n=8) t2(n=8) t3(n=8) t4(n=8) t5(n=8) t6(n=8)
t1(n=12) t2(n=12) t3(n=12) t4(n=12) t5(n=12) t6(n=12)

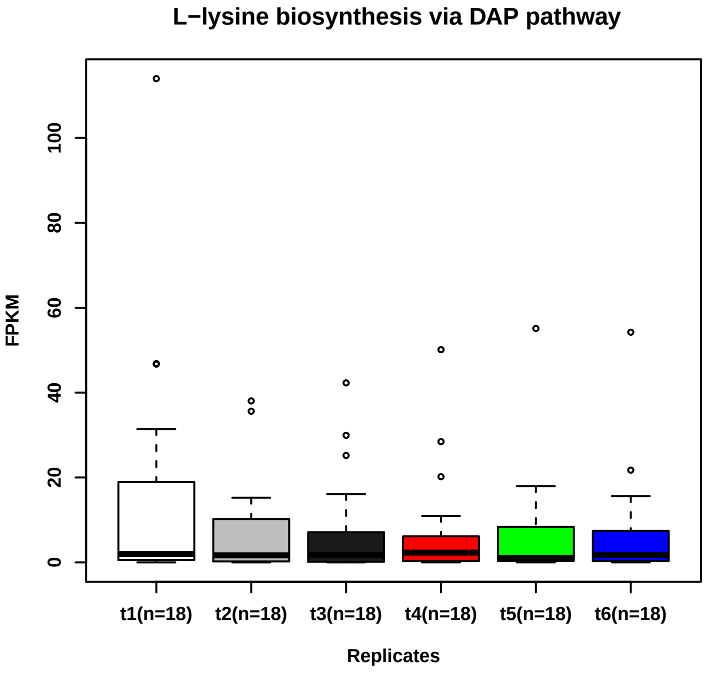
L−lysine biosynthesis via DAP pathway
FPKM
0
20
40
60
80
100
t1(n=18)
t2(n=18)
t3(n=18)
t4(n=18)
t5(n=18)
t6(n=18)
Replicates

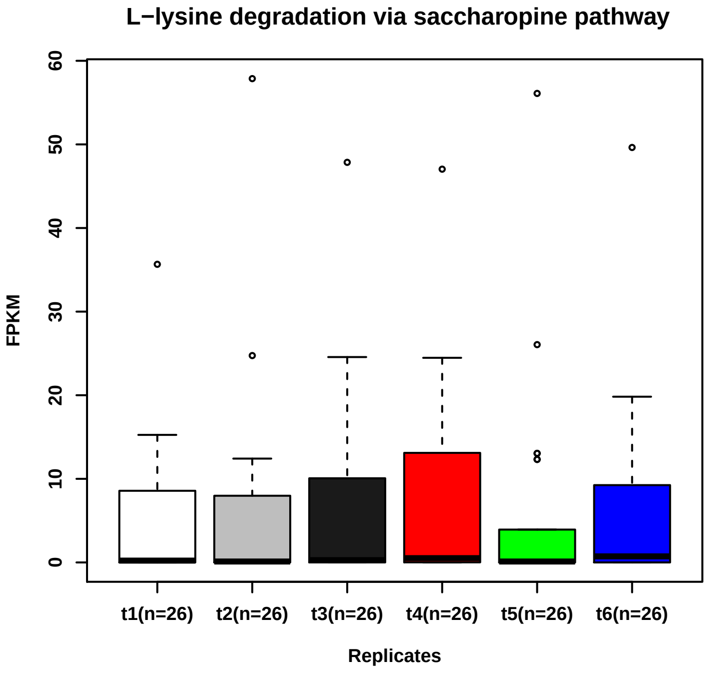
L−lysine degradation via saccharopine pathway
FPKM
0
10
20
30
40
50
60
t1(n=26)
t2(n=26)
t3(n=26)
t4(n=26)
t5(n=26)
t6(n=26)
Replicates

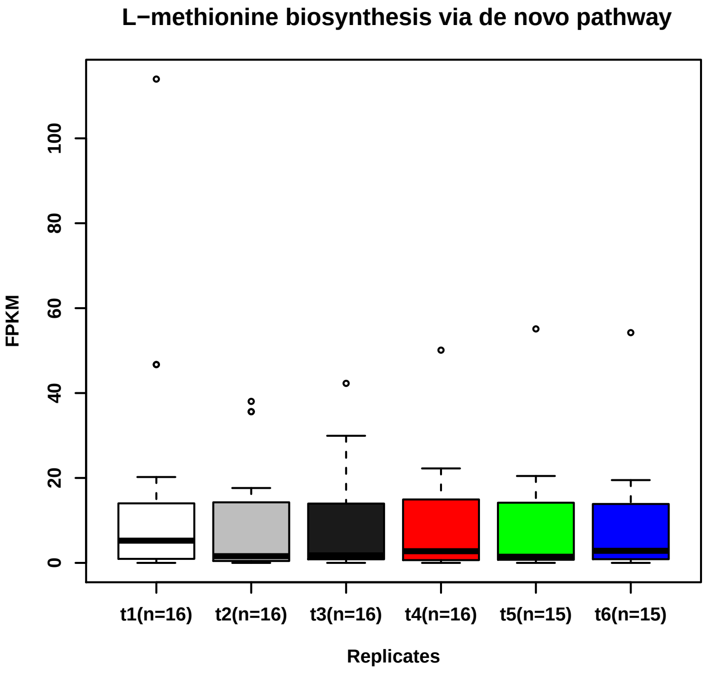
L−methionine biosynthesis via de novo pathway
FPKM
0
20
40
60
80
100
t1(n=16)
t2(n=16)
t3(n=16)
t4(n=16)
t5(n=15)
t6(n=15)
Replicates

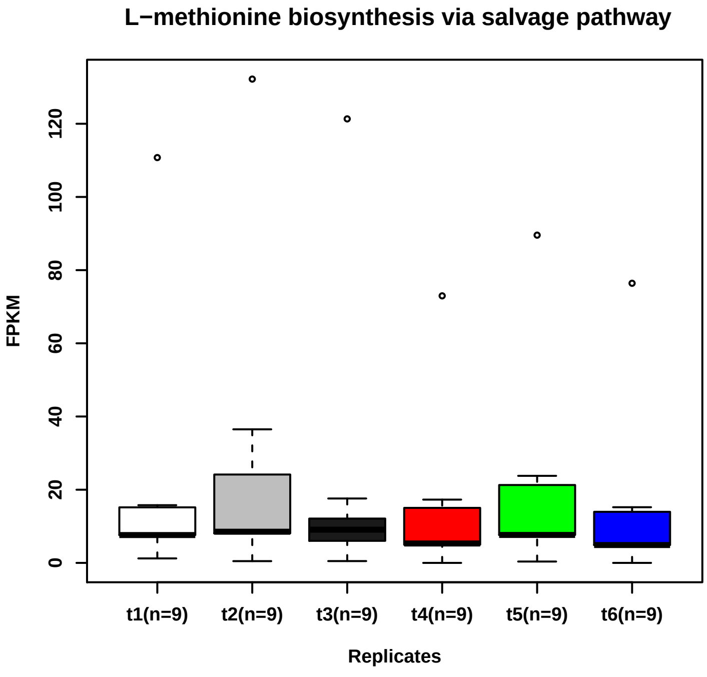
L−methionine biosynthesis via salvage pathway
FPKM
0
20
40
60
80
100
120
t1(n=9)
t2(n=9)
t3(n=9)
t4(n=9)
t5(n=9)
t6(n=9)
Replicates

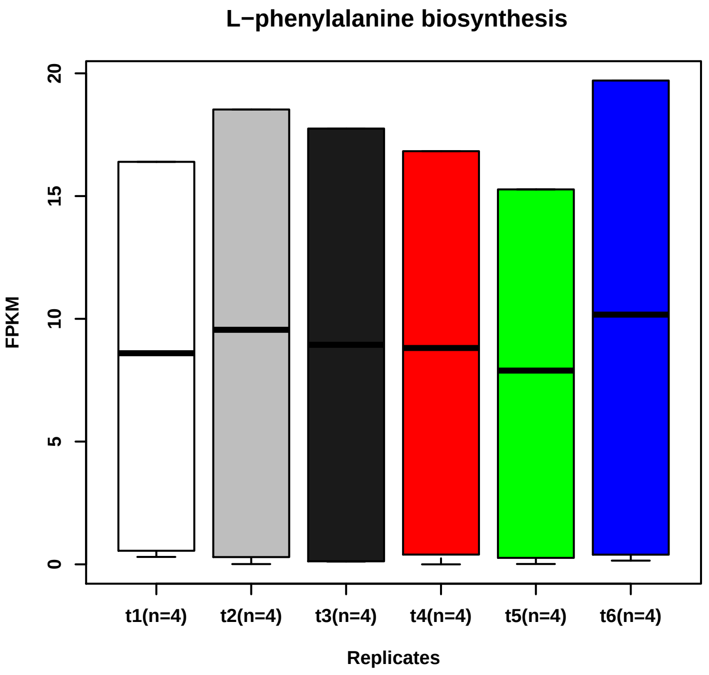
L−phenylalanine biosynthesis
FPKM
0
5
10
15
20
t1(n=4)
t2(n=4)
t3(n=4)
t4(n=4)
t5(n=4)
t6(n=4)
Replicates

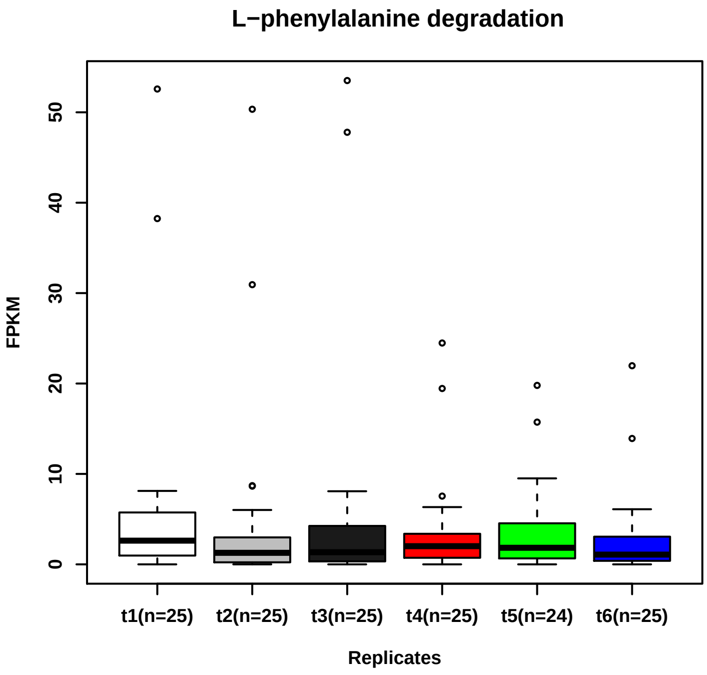
L−phenylalanine degradation
FPKM
0
10
20
30
40
50
t1(n=25)
t2(n=25)
t3(n=25)
t4(n=25)
t5(n=24)
t6(n=25)
Replicates

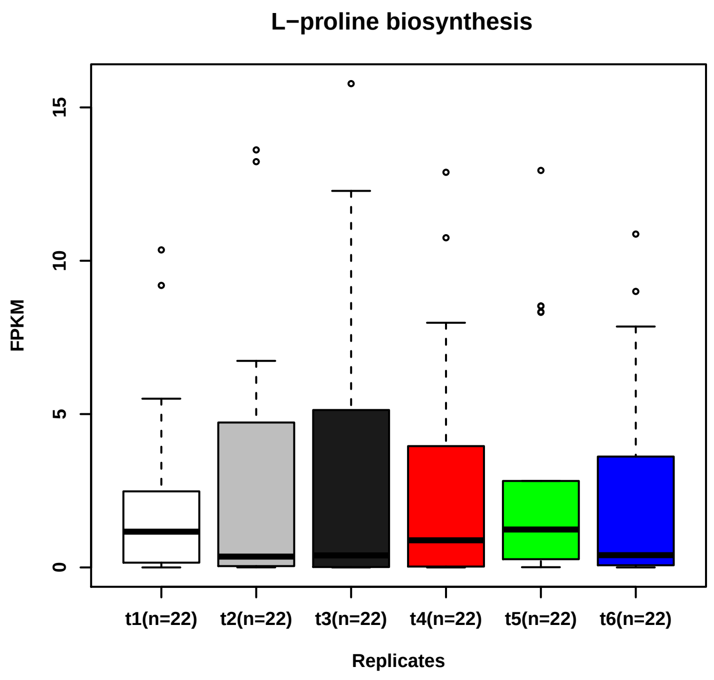
L−proline biosynthesis
FPKM
0
5
10
15
t1(n=22)
t2(n=22)
t3(n=22)
t4(n=22)
t5(n=22)
t6(n=22)
Replicates

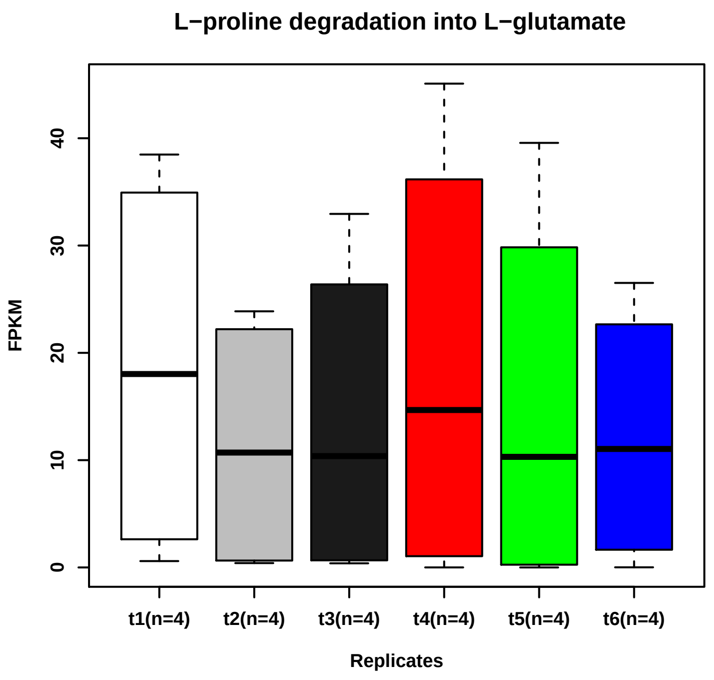
L−proline degradation into L−glutamate
FPKM
0
10
20
30
40
t1(n=4)
t2(n=4)
t3(n=4)
t4(n=4)
t5(n=4)
t6(n=4)
Replicates

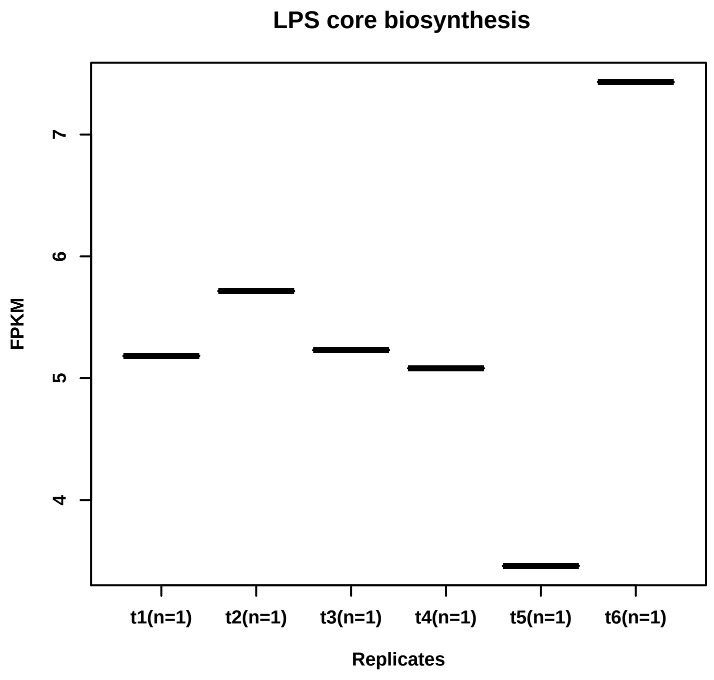
LPS core biosynthesis
FPKM
4
5
6
7
t1(n=1)
t2(n=1)
t3(n=1)
t4(n=1)
t5(n=1)
t6(n=1)
Replicates

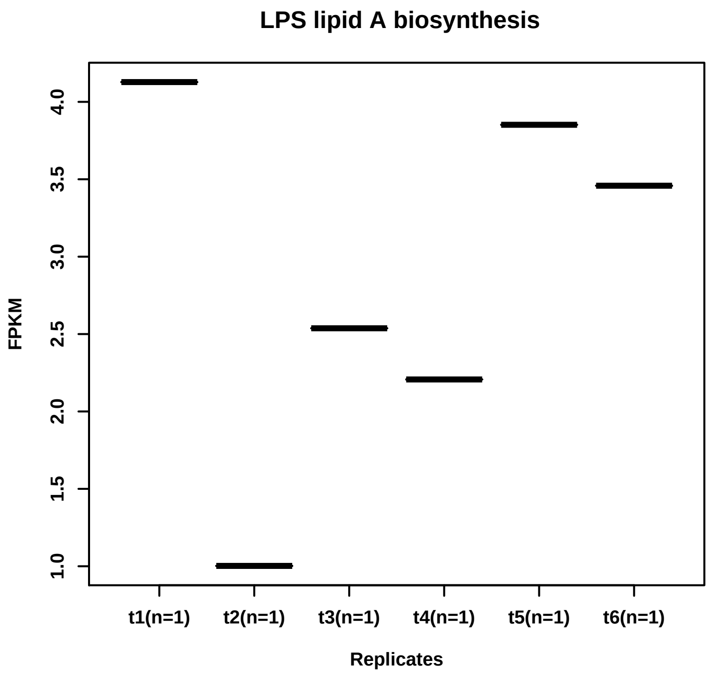
LPS lipid A biosynthesis
FPKM
1.0
1.5
2.0
2.5
3.0
3.5
4.0
t1(n=1)
t2(n=1)
t3(n=1)
t4(n=1)
t5(n=1)
t6(n=1)
Replicates

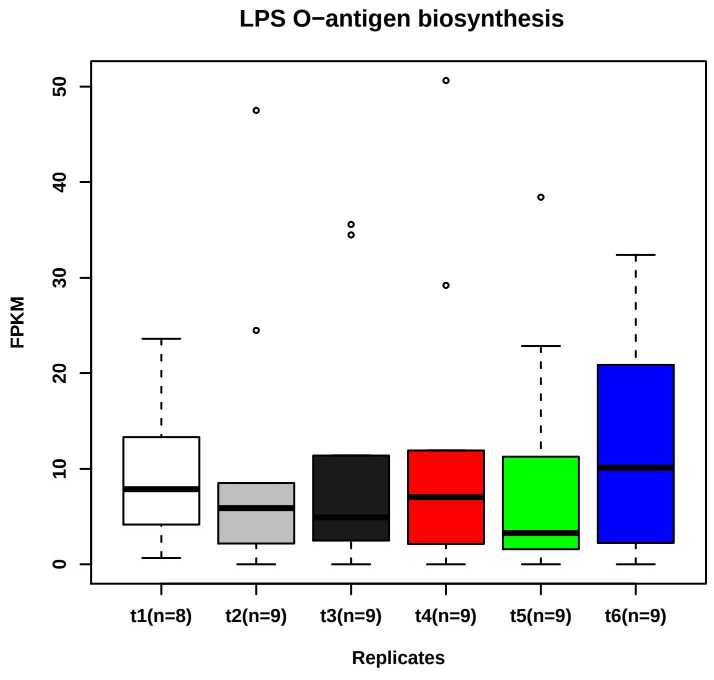
LPS O−antigen biosynthesis
FPKM
0
10
20
30
40
50
t1(n=8)
t2(n=9)
t3(n=9)
t4(n=9)
t5(n=9)
t6(n=9)
Replicates

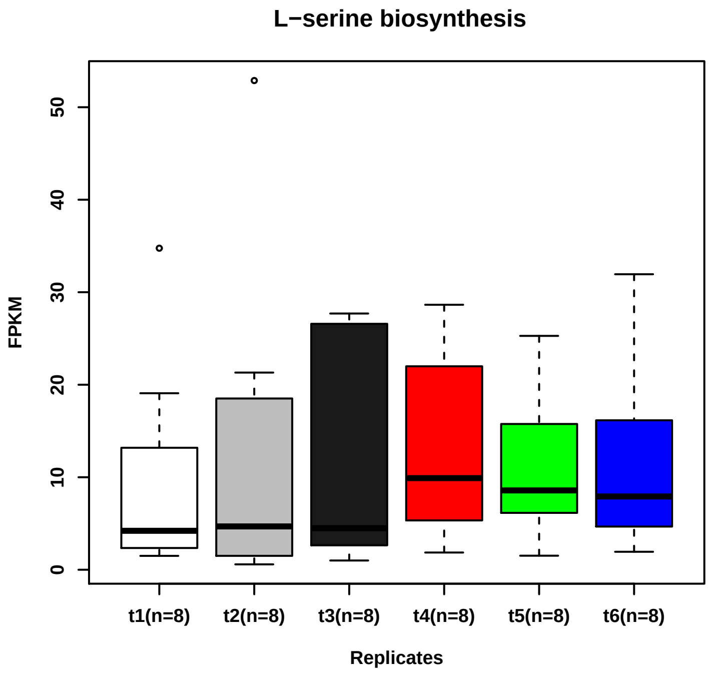
L−serine biosynthesis
FPKM
0
10
20
30
40
50
t1(n=8)
t2(n=8)
t3(n=8)
t4(n=8)
t5(n=8)
t6(n=8)
Replicates

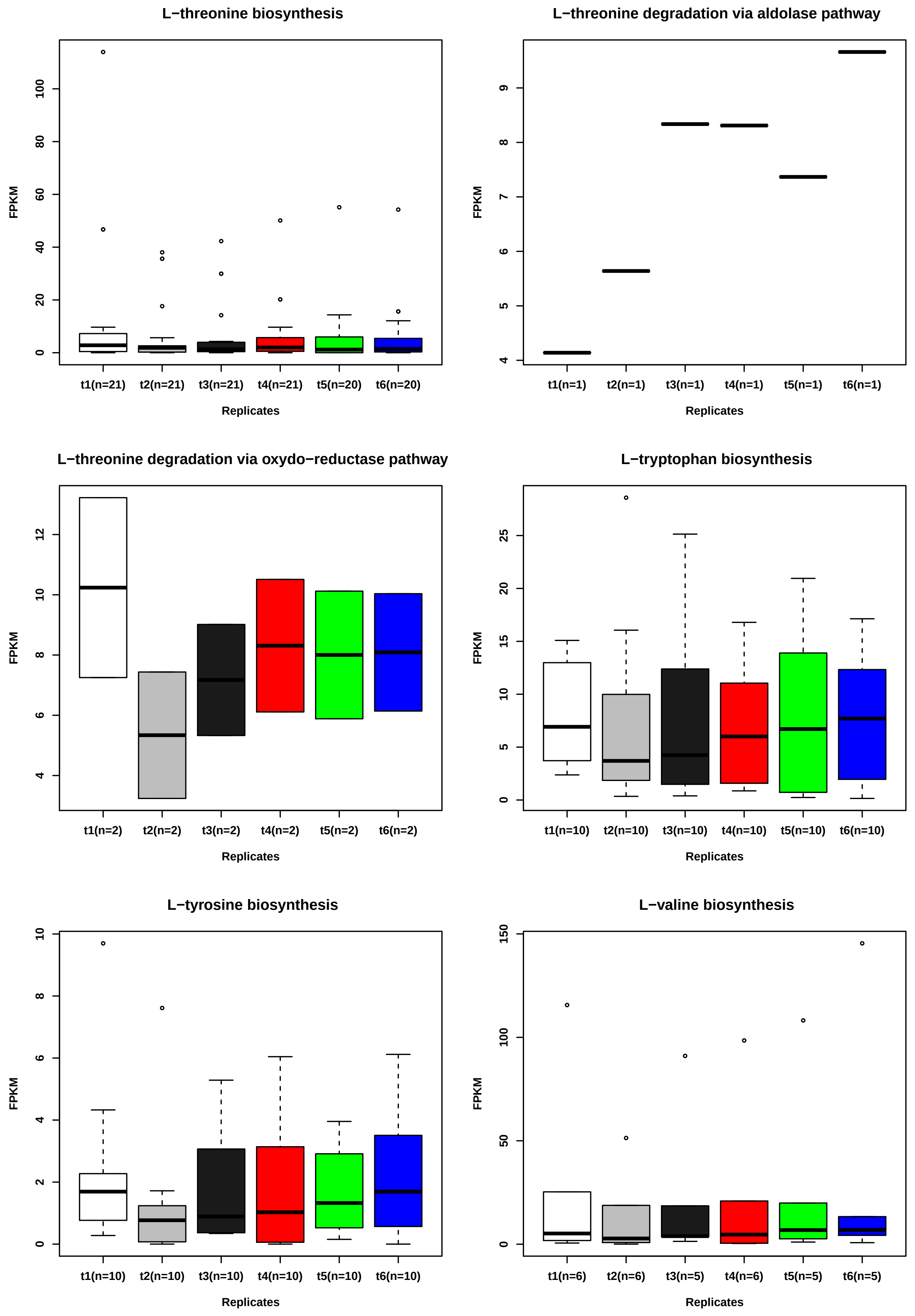

L−threonine biosynthesis
FPKM
0
20
40
60
80
100
t1(n=21)
t2(n=21)
t3(n=21)
t4(n=21)
t5(n=20)
t6(n=20)
Replicates
L−threonine degradation via aldolase pathway
FPKM
4
5
6
7
8
9
t1(n=1)
t2(n=1)
t3(n=1)
t4(n=1)
t5(n=1)
t6(n=1)
Replicates
L−threonine degradation via oxydo−reductase pathway
FPKM
4
6
8
10
12
t1(n=2)
t2(n=2)
t3(n=2)
t4(n=2)
t5(n=2)
t6(n=2)
Replicates
L−tryptophan biosynthesis
FPKM
0
5
10
15
20
25
t1(n=10)
t2(n=10)
t3(n=10)
t4(n=10)
t5(n=10)
t6(n=10)
Replicates
L−tyrosine biosynthesis
FPKM
0
2
4
6
8
10
t1(n=10)
t2(n=10)
t3(n=10)
t4(n=10)
t5(n=10)
t6(n=10)
Replicates
L−valine biosynthesis
FPKM
0
50
100
150
t1(n=6)
t2(n=6)
t3(n=5)
t4(n=6)
t5(n=5)
t6(n=5)
Replicates

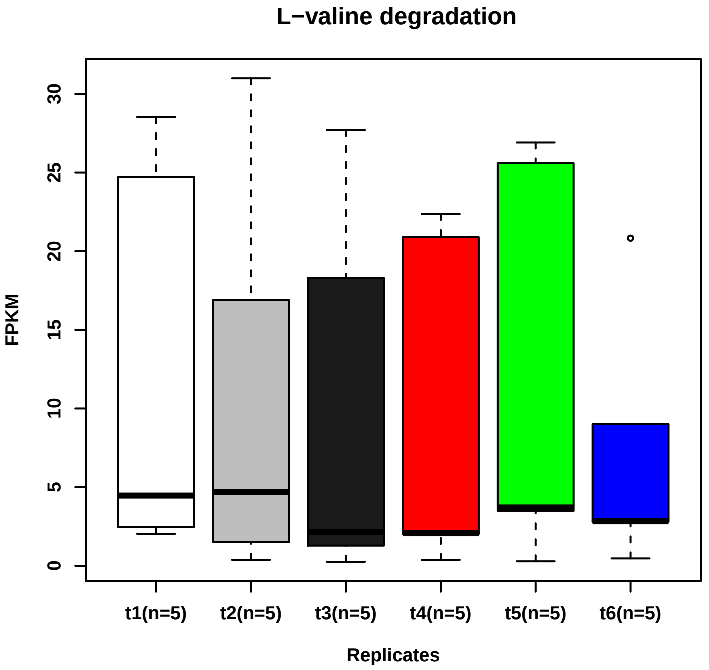
L−valine degradation
FPKM
0
5
10
15
20
25
30
t1(n=5)
t2(n=5)
t3(n=5)
t4(n=5)
t5(n=5)
t6(n=5)
Replicates

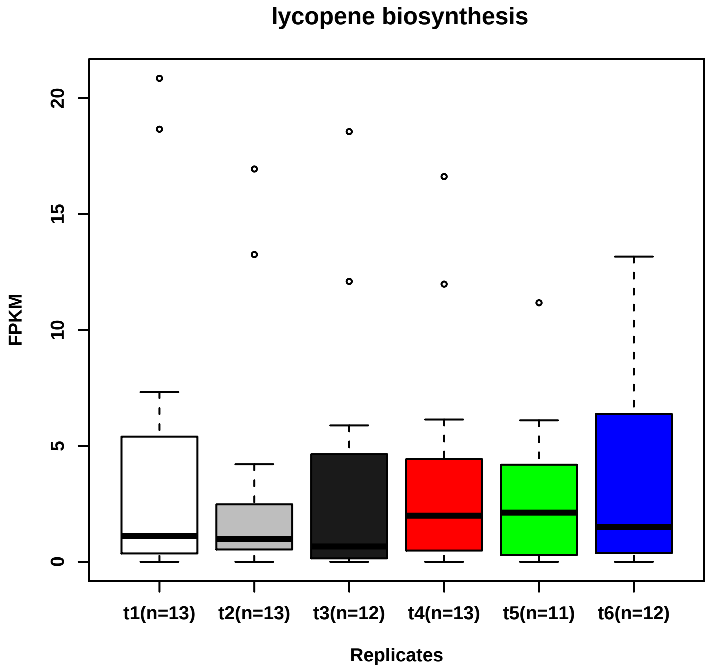
lycopene biosynthesis
FPKM
0
5
10
15
20
t1(n=13)
t2(n=13)
t3(n=12)
t4(n=13)
t5(n=11)
t6(n=12)
Replicates

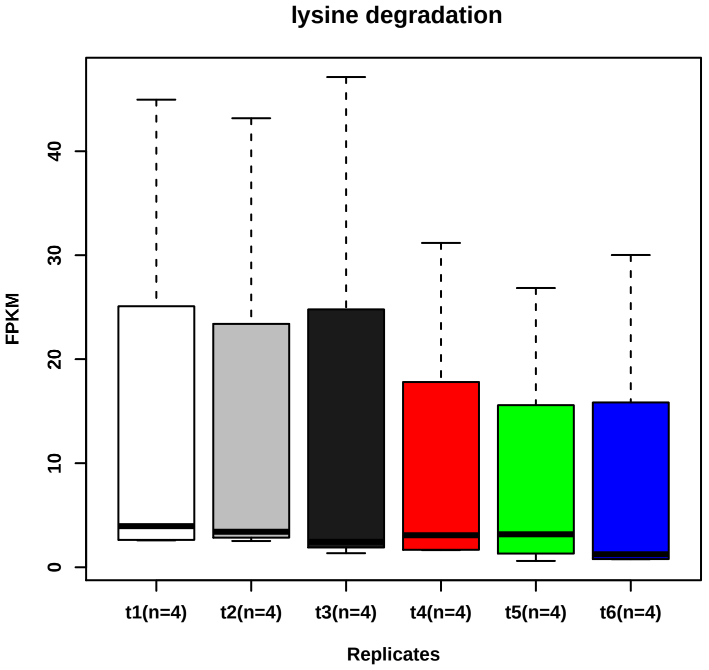
lysine degradation
FPKM
0
10
20
30
40
t1(n=4)
t2(n=4)
t3(n=4)
t4(n=4)
t5(n=4)
t6(n=4)
Replicates

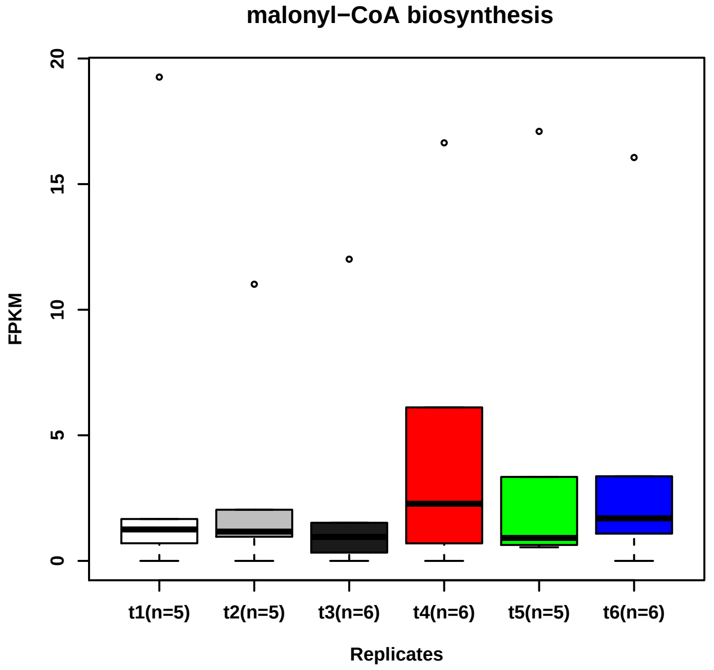
malonyl−CoA biosynthesis
FPKM
0
5
10
15
20
t1(n=5)
t2(n=5)
t3(n=6)
t4(n=6)
t5(n=5)
t6(n=6)
Replicates

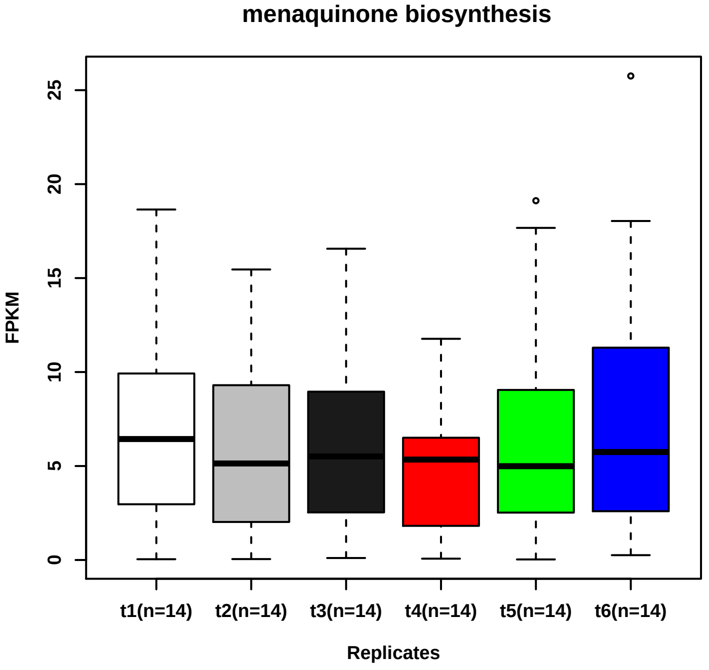
menaquinone biosynthesis
FPKM
0
5
10
15
20
25
t1(n=14)
t2(n=14)
t3(n=14)
t4(n=14)
t5(n=14)
t6(n=14)
Replicates

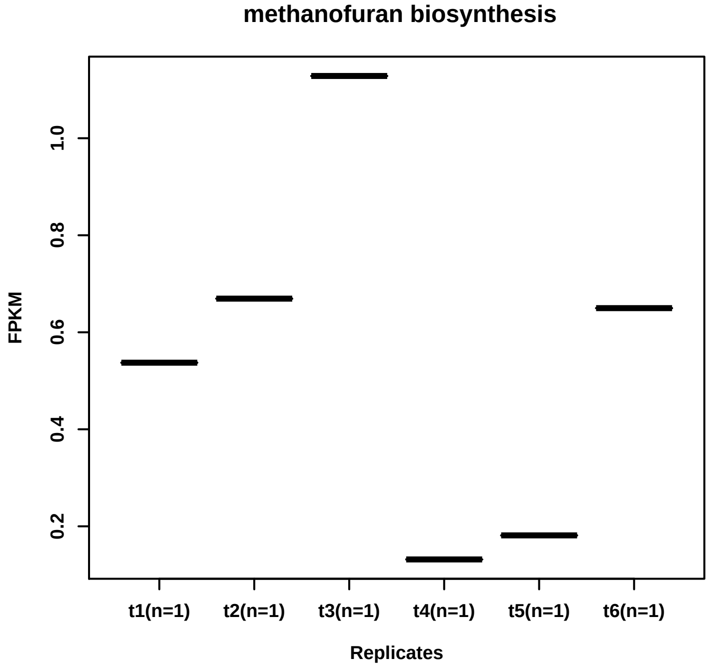
methanofuran biosynthesis
FPKM
0.2
0.4
0.6
0.8
1.0
t1(n=1)
t2(n=1)
t3(n=1)
t4(n=1)
t5(n=1)
t6(n=1)
Replicates

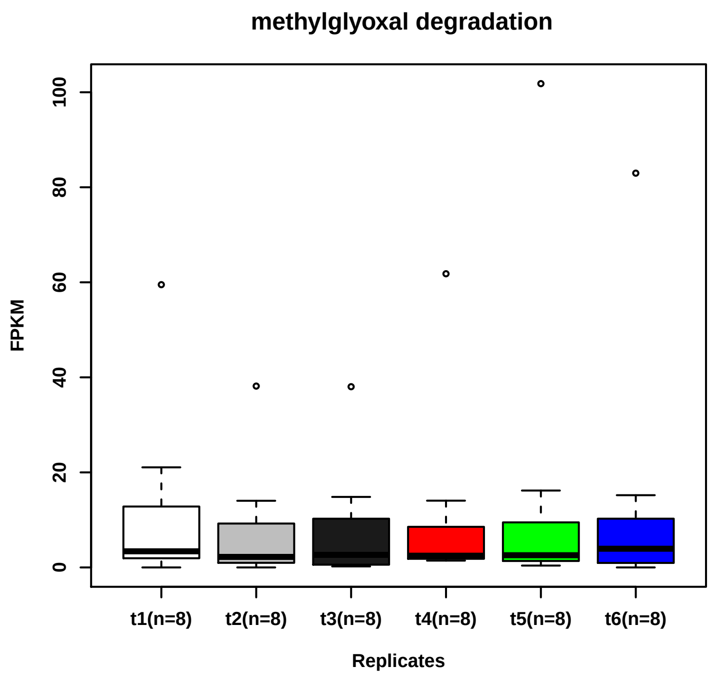
methylglyoxal degradation
FPKM
0
20
40
60
80
100
t1(n=8)
t2(n=8)
t3(n=8)
t4(n=8)
t5(n=8)
t6(n=8)
Replicates

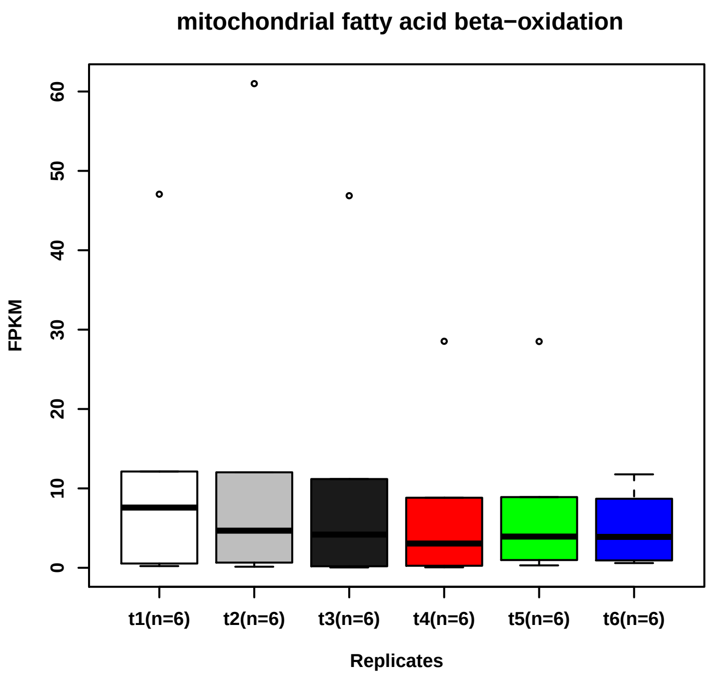
mitochondrial fatty acid beta−oxidation
FPKM
0
10
20
30
40
50
60
t1(n=6)
t2(n=6)
t3(n=6)
t4(n=6)
t5(n=6)
t6(n=6)
Replicates

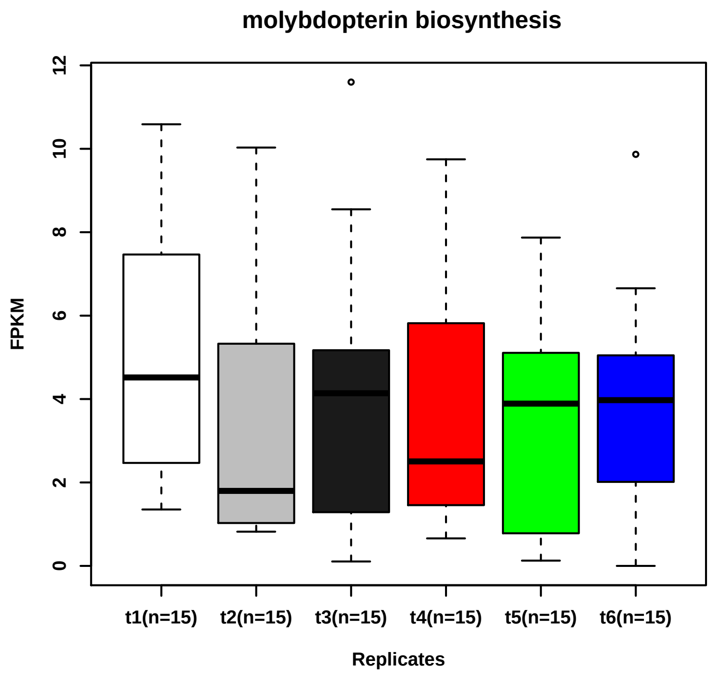
molybdopterin biosynthesis
FPKM
0
2
4
6
8
10
12
t1(n=15)
t2(n=15)
t3(n=15)
t4(n=15)
t5(n=15)
t6(n=15)
Replicates

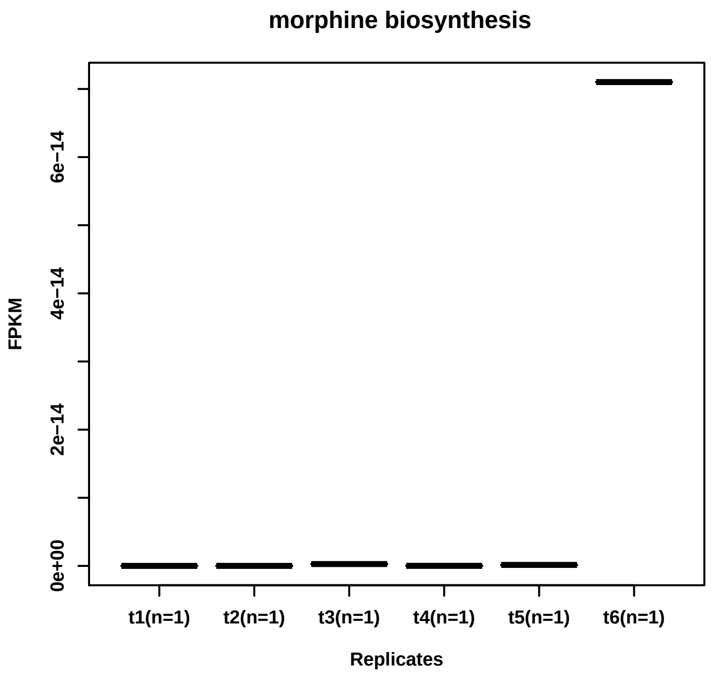
morphine biosynthesis
FPKM
0e+00
2e−14
4e−14
6e−14
t1(n=1)
t2(n=1)
t3(n=1)
t4(n=1)
t5(n=1)
t6(n=1)
Replicates

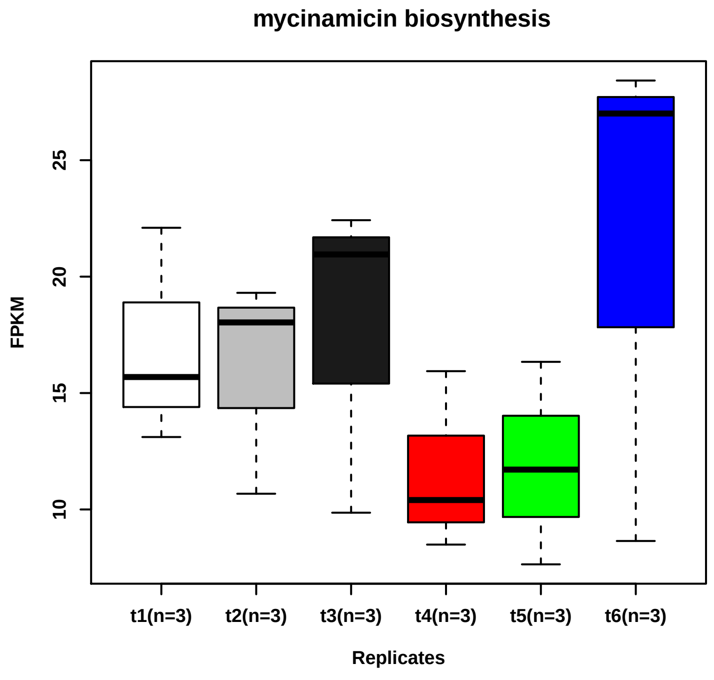
mycinamicin biosynthesis
FPKM
10
15
20
25
t1(n=3)
t2(n=3)
t3(n=3)
t4(n=3)
t5(n=3)
t6(n=3)
Replicates

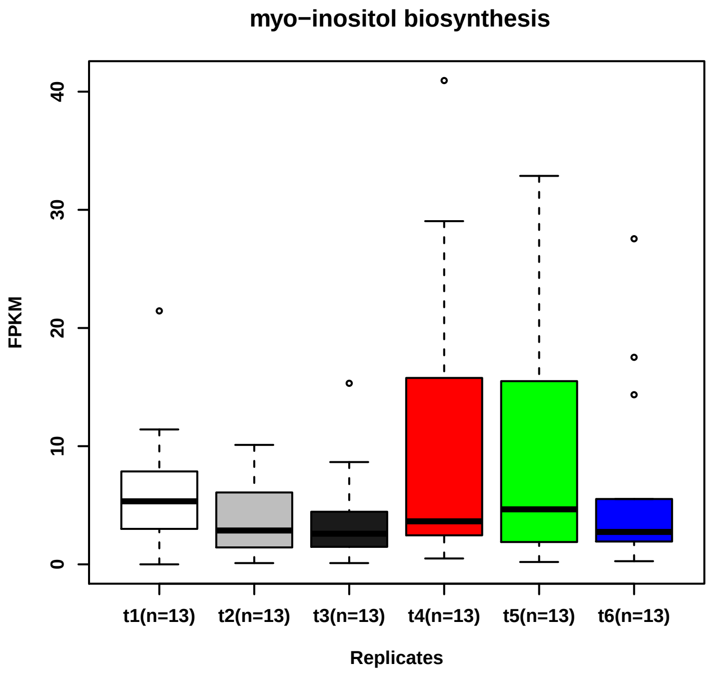
myo−inositol biosynthesis
FPKM
0
10
20
30
40
t1(n=13)
t2(n=13)
t3(n=13)
t4(n=13)
t5(n=13)
t6(n=13)
Replicates

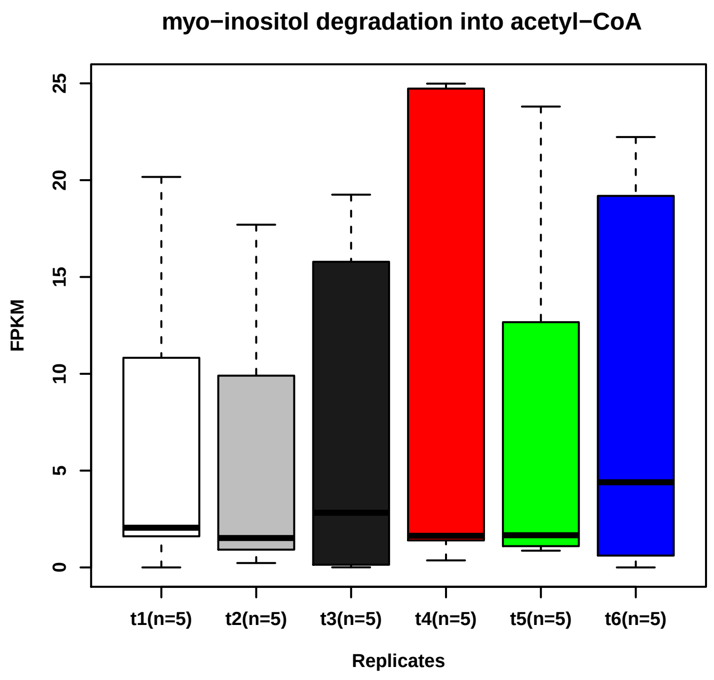
myo−inositol degradation into acetyl−CoA
FPKM
0
5
10
15
20
25
t1(n=5)
t2(n=5)
t3(n=5)
t4(n=5)
t5(n=5)
t6(n=5)
Replicates

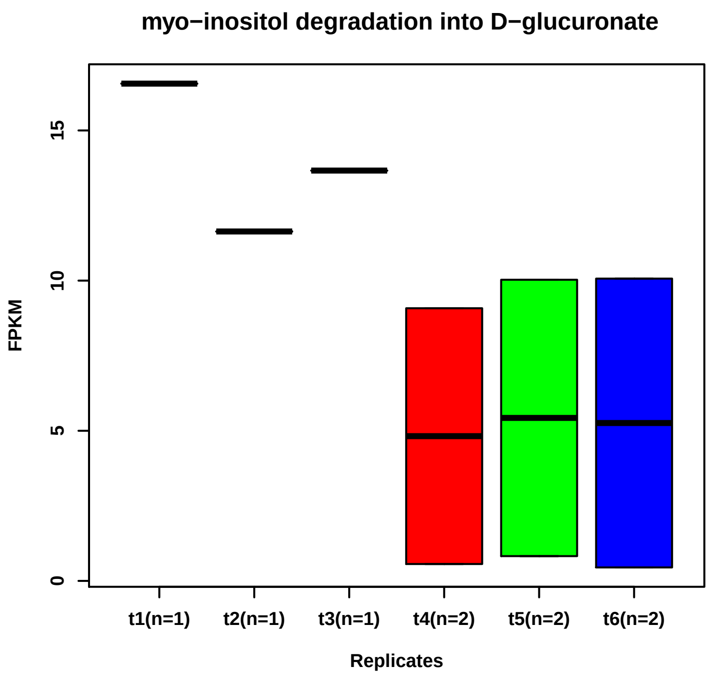
myo−inositol degradation into D−glucuronate
FPKM
0
5
10
15
t1(n=1)
t2(n=1)
t3(n=1)
t4(n=2)
t5(n=2)
t6(n=2)
Replicates

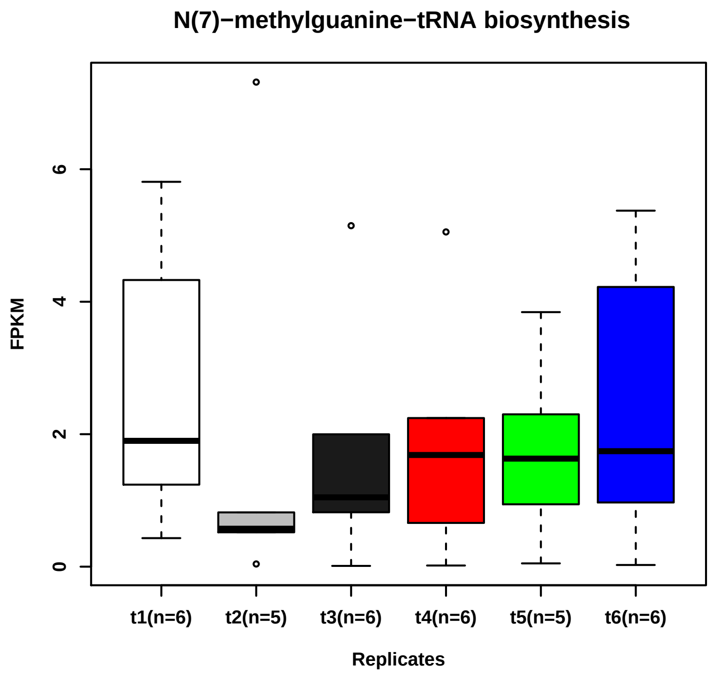
N(7)−methylguanine−tRNA biosynthesis
FPKM
0
2
4
6
t1(n=6)
t2(n=5)
t3(n=6)
t4(n=6)
t5(n=5)
t6(n=6)
Replicates

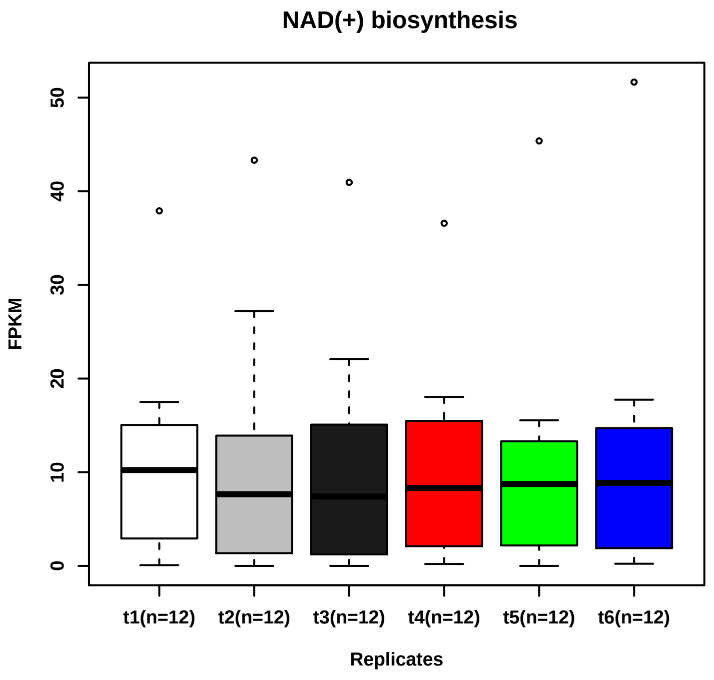
NAD(+) biosynthesis
FPKM
0
10
20
30
40
50
t1(n=12)
t2(n=12)
t3(n=12)
t4(n=12)
t5(n=12)
t6(n=12)
Replicates

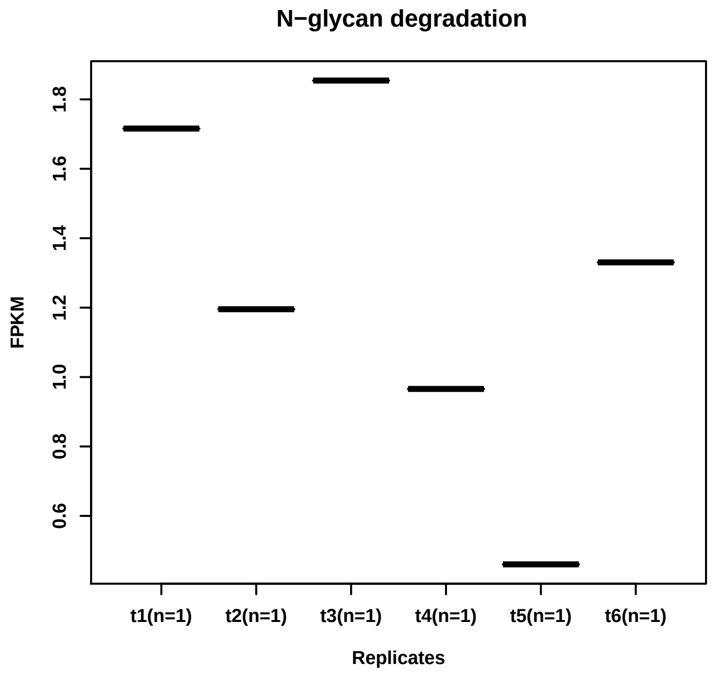
N−glycan degradation
FPKM
0.6
0.8
1.0
1.2
1.4
1.6
1.8
t1(n=1)
t2(n=1)
t3(n=1)
t4(n=1)
t5(n=1)
t6(n=1)
Replicates

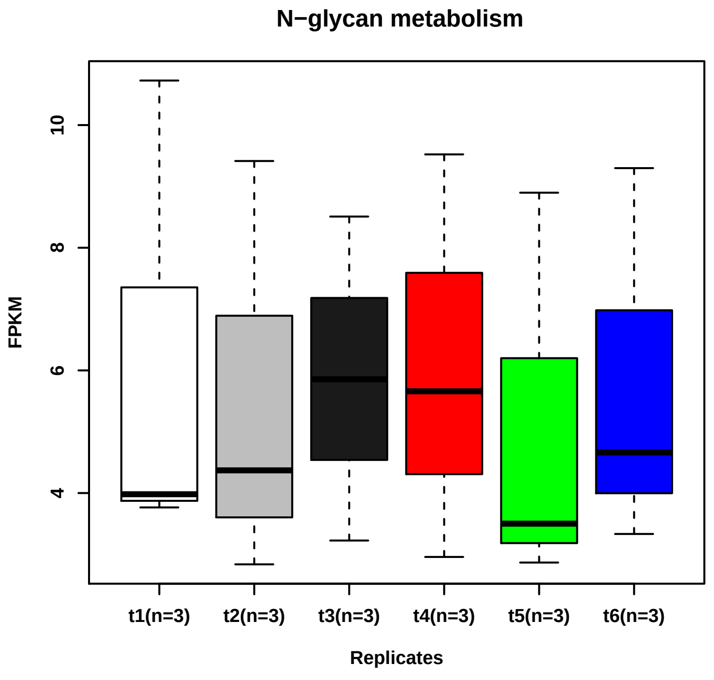
N−glycan metabolism
FPKM
4
6
8
10
t1(n=3)
t2(n=3)
t3(n=3)
t4(n=3)
t5(n=3)
t6(n=3)
Replicates

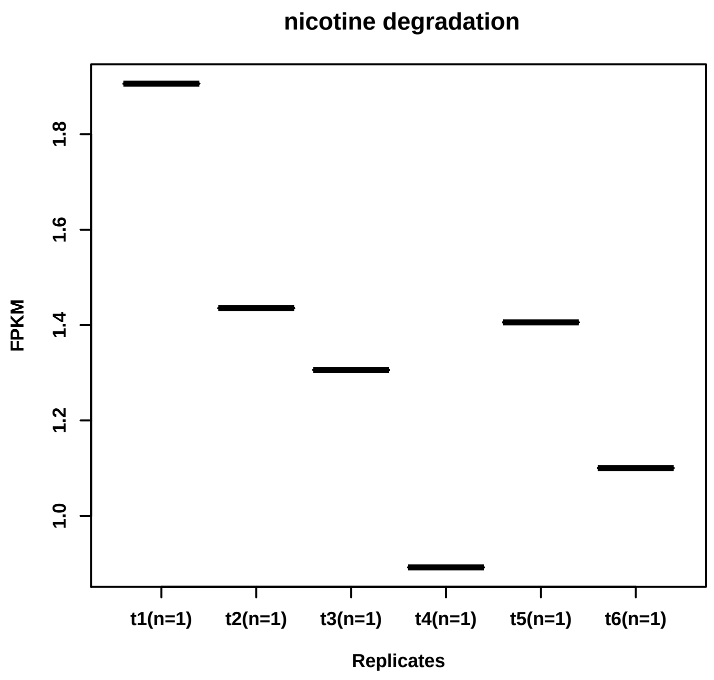
nicotine degradation
FPKM
1.0
1.2
1.4
1.6
1.8
t1(n=1) t2(n=1) t3(n=1) t4(n=1) t5(n=1) t6(n=1)
Replicates

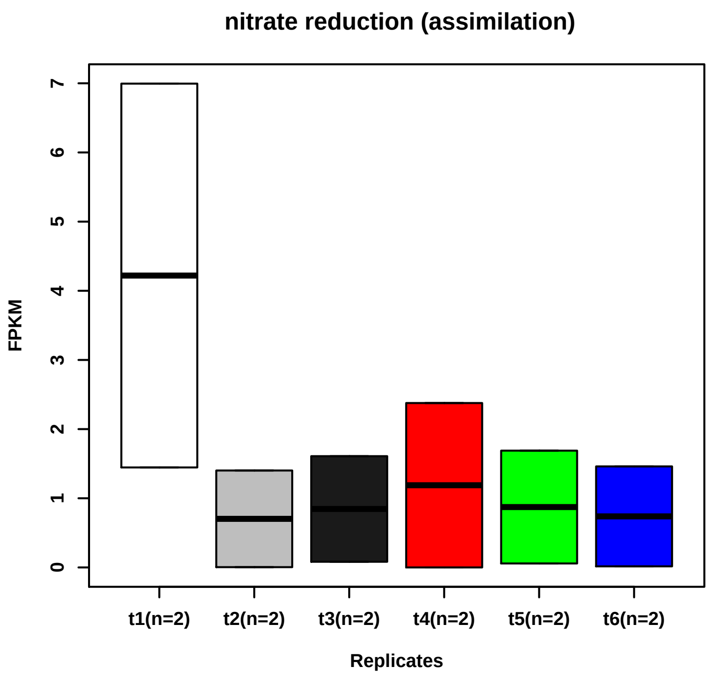
nitrate reduction (assimilation)
FPKM
0 1 2 3 4 5 6 7
t1(n=2) t2(n=2) t3(n=2) t4(n=2) t5(n=2) t6(n=2)
Replicates

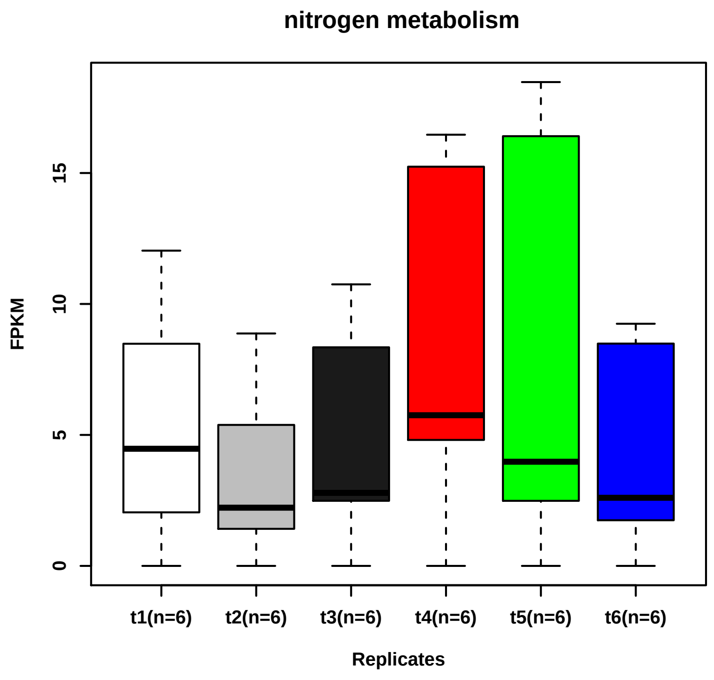
nitrogen metabolism
FPKM
0 5 10 15
t1(n=6) t2(n=6) t3(n=6) t4(n=6) t5(n=6) t6(n=6)
Replicates

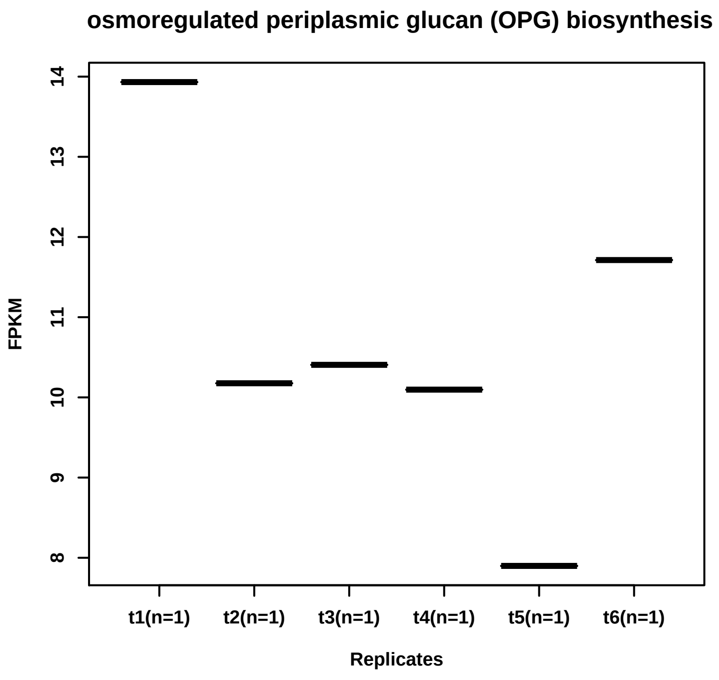
osmoregulated periplasmic glucan (OPG) biosynthesis
FPKM
8 9 10 11 12 13 14
t1(n=1) t2(n=1) t3(n=1) t4(n=1) t5(n=1) t6(n=1)
Replicates

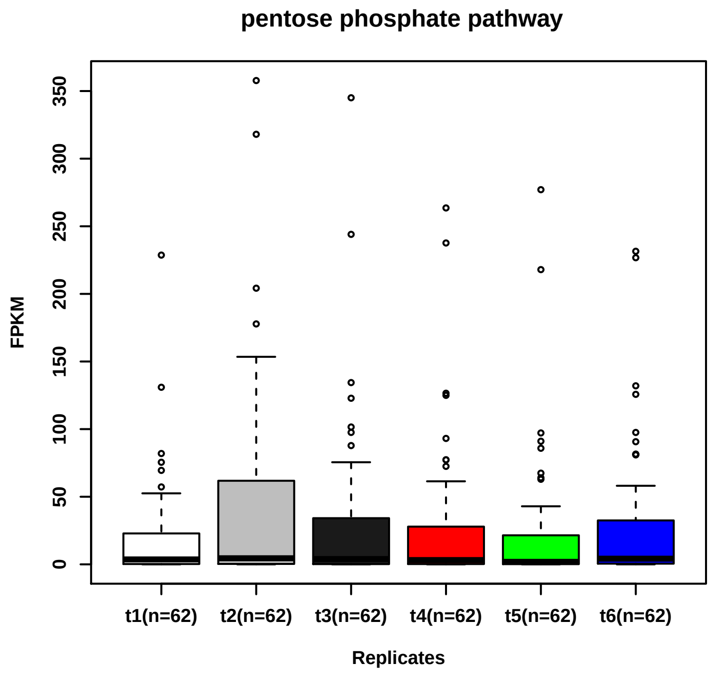
pentose phosphate pathway
FPKM
0 50 100 150 200 250 300 350
t1(n=62) t2(n=62) t3(n=62) t4(n=62) t5(n=62) t6(n=62)
Replicates

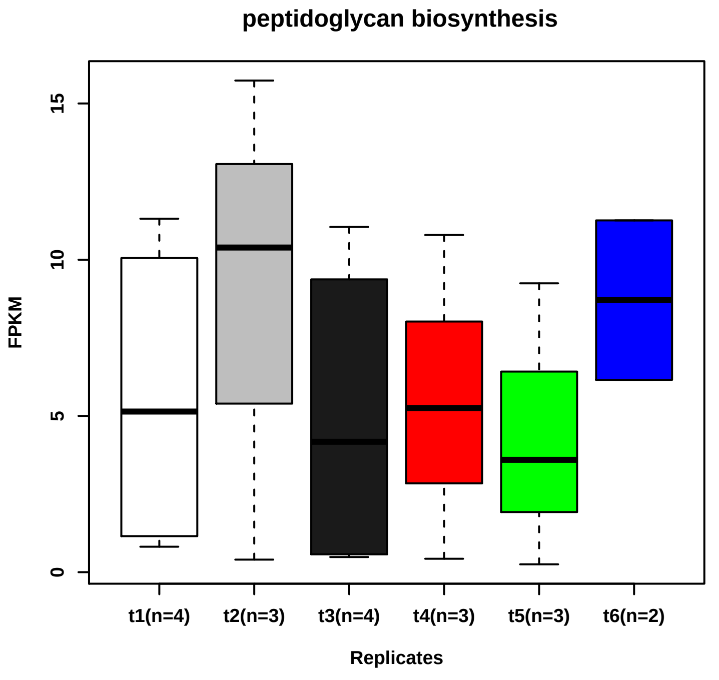
peptidoglycan biosynthesis
FPKM
0 5 10 15
t1(n=4) t2(n=3) t3(n=4) t4(n=3) t5(n=3) t6(n=2)
Replicates

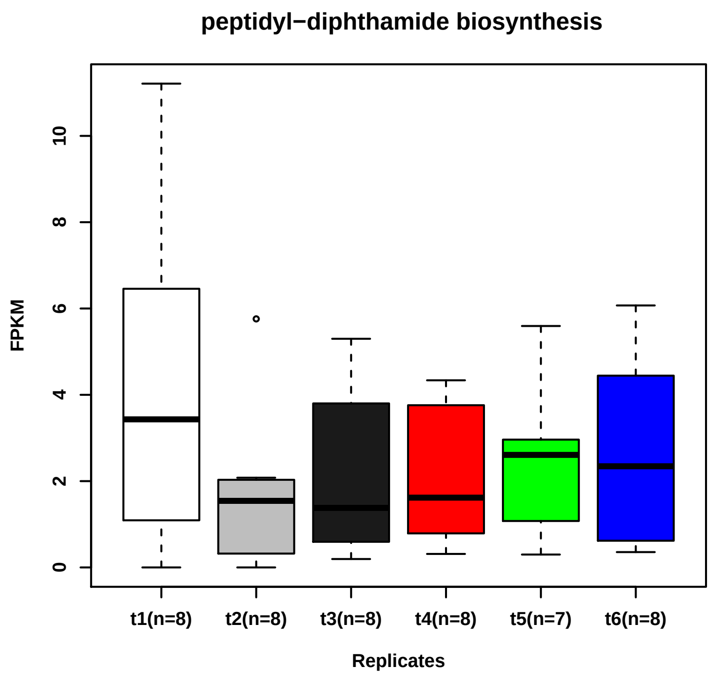
peptidyl−diphthamide biosynthesis
FPKM
0
2
4
6
8
10
t1(n=8)
t2(n=8)
t3(n=8)
t4(n=8)
t5(n=7)
t6(n=8)
Replicates

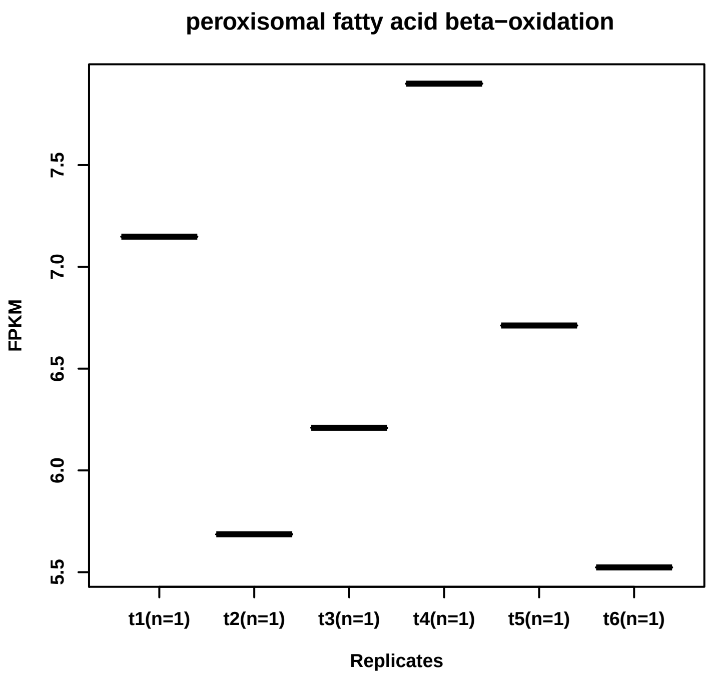
peroxisomal fatty acid beta−oxidation
FPKM
5.5
6.0
6.5
7.0
7.5
t1(n=1)
t2(n=1)
t3(n=1)
t4(n=1)
t5(n=1)
t6(n=1)
Replicates

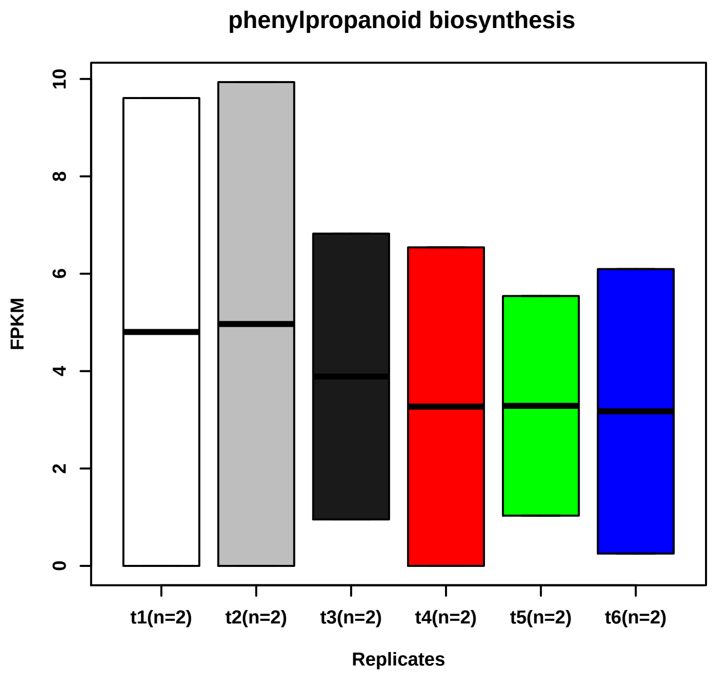
phenylpropanoid biosynthesis
FPKM
0
2
4
6
8
10
t1(n=2)
t2(n=2)
t3(n=2)
t4(n=2)
t5(n=2)
t6(n=2)
Replicates

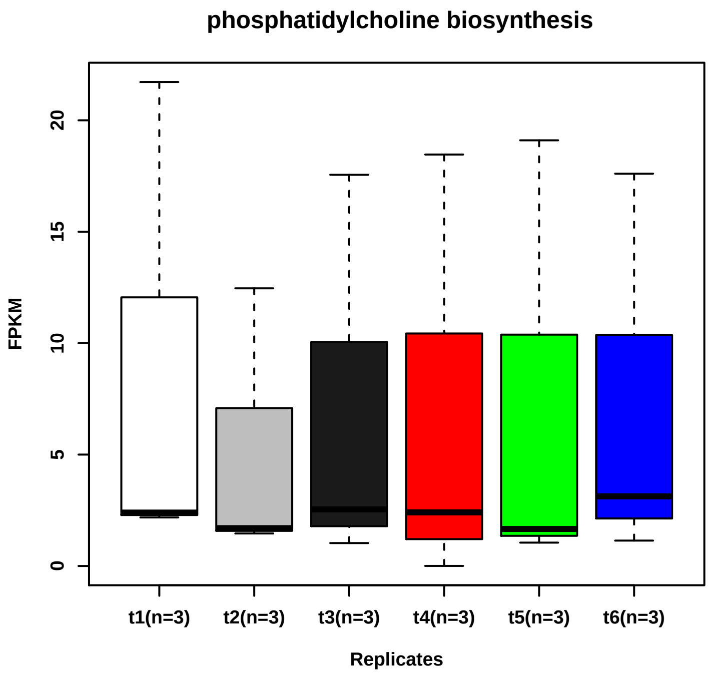
phosphatidylcholine biosynthesis
FPKM
0
5
10
15
20
t1(n=3)
t2(n=3)
t3(n=3)
t4(n=3)
t5(n=3)
t6(n=3)
Replicates

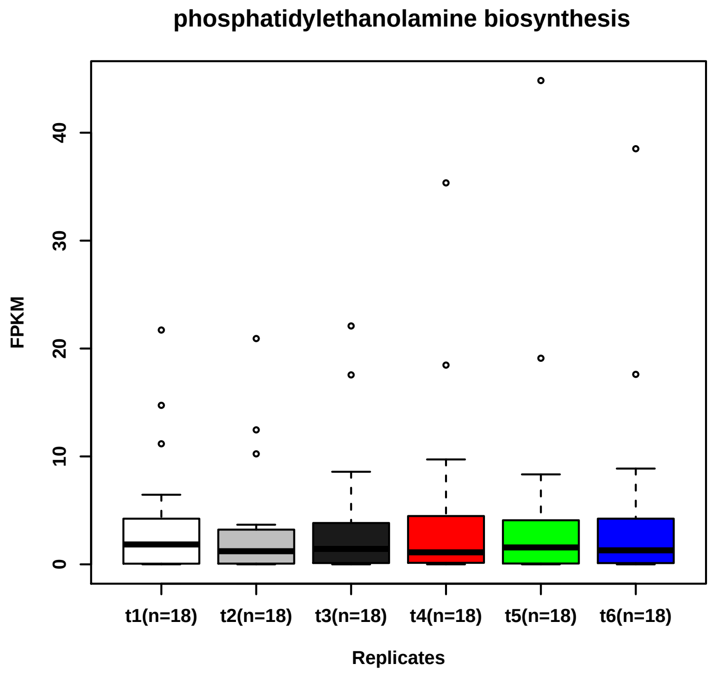
phosphatidylethanolamine biosynthesis
FPKM
0
10
20
30
40
t1(n=18)
t2(n=18)
t3(n=18)
t4(n=18)
t5(n=18)
t6(n=18)
Replicates

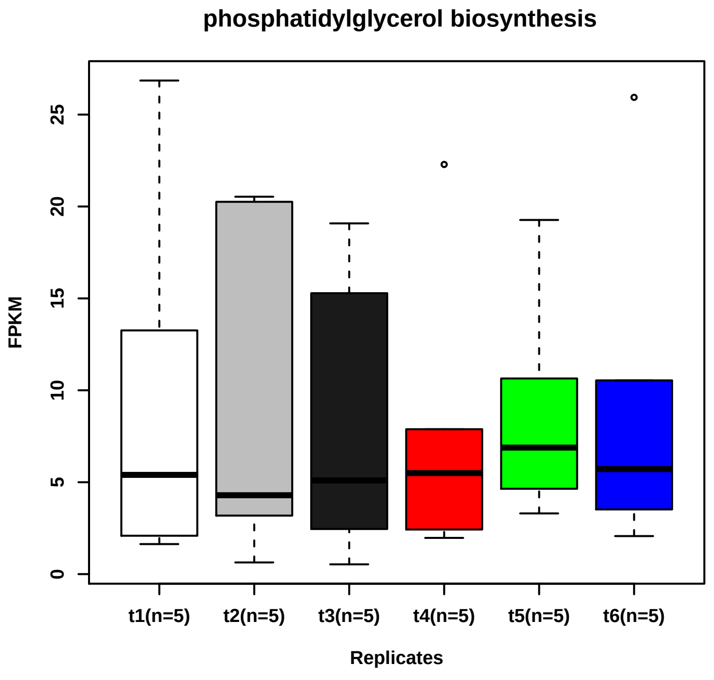
phosphatidylglycerol biosynthesis
FPKM
0
5
10
15
20
25
t1(n=5)
t2(n=5)
t3(n=5)
t4(n=5)
t5(n=5)
t6(n=5)
Replicates

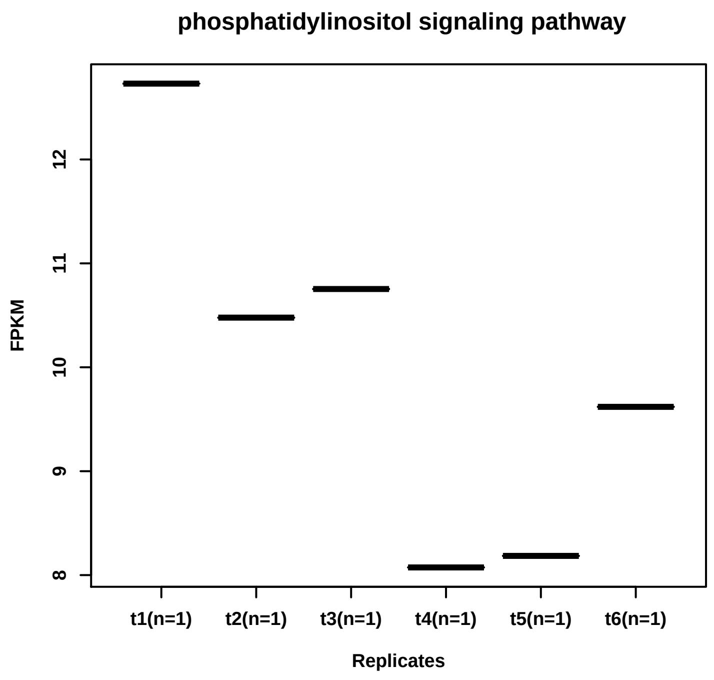
phosphatidylinositol signaling pathway
FPKM
8
9
10
11
12
t1(n=1) t2(n=1) t3(n=1) t4(n=1) t5(n=1) t6(n=1)
Replicates

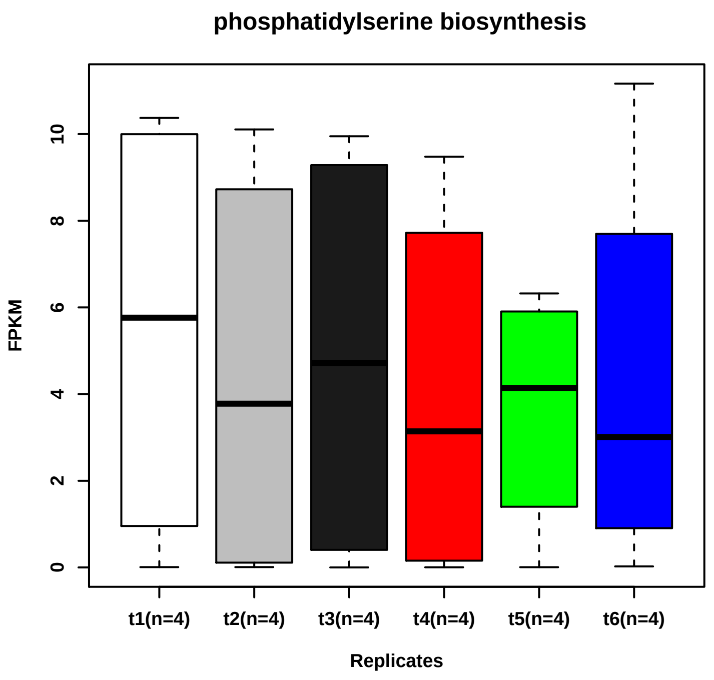
phosphatidylserine biosynthesis
FPKM
0
2
4
6
8
10
t1(n=4) t2(n=4) t3(n=4) t4(n=4) t5(n=4) t6(n=4)
Replicates

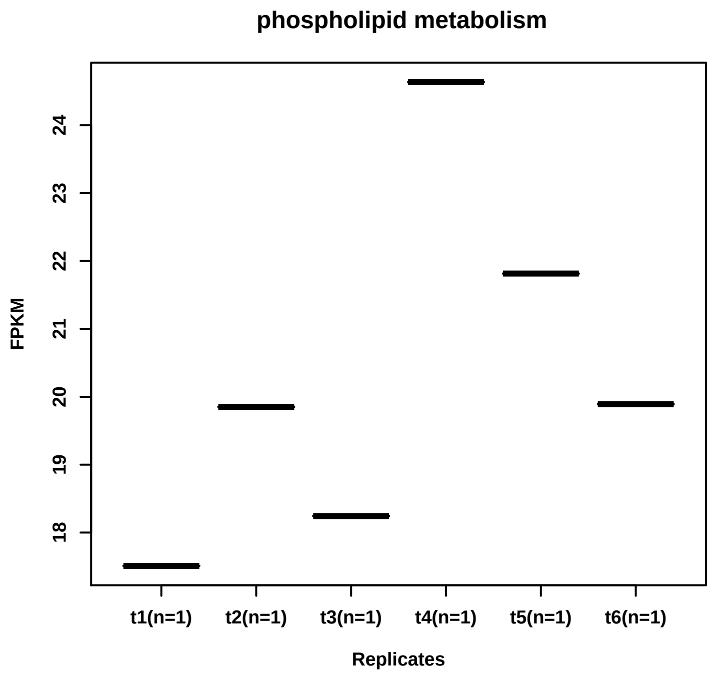
phospholipid metabolism
FPKM
18
19
20
21
22
23
24
t1(n=1) t2(n=1) t3(n=1) t4(n=1) t5(n=1) t6(n=1)
Replicates

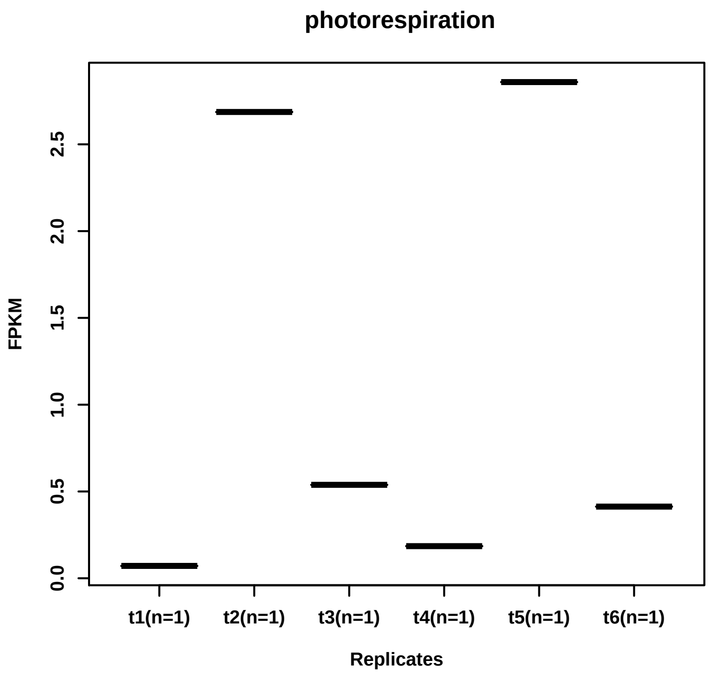
photorespiration
FPKM
0.0
0.5
1.0
1.5
2.0
2.5
t1(n=1) t2(n=1) t3(n=1) t4(n=1) t5(n=1) t6(n=1)
Replicates

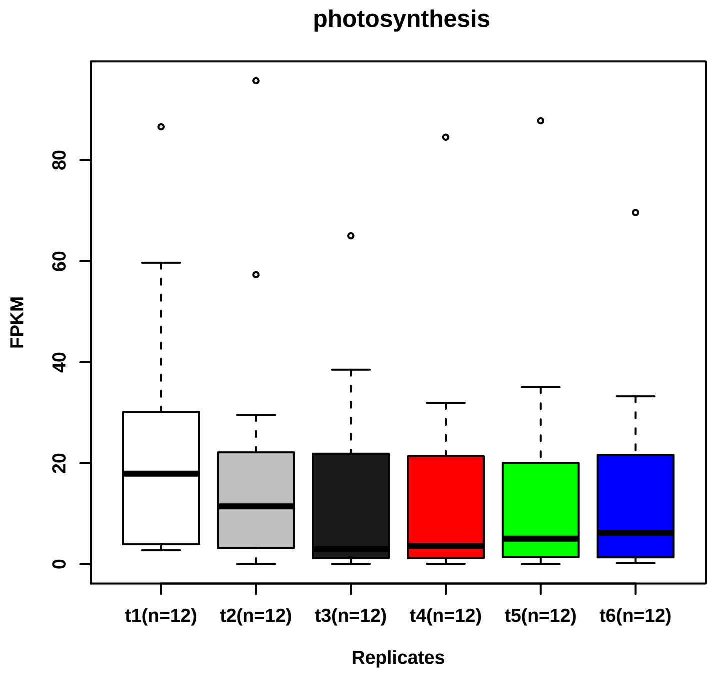
photosynthesis
FPKM
0
20
40
60
80
t1(n=12) t2(n=12) t3(n=12) t4(n=12) t5(n=12) t6(n=12)
Replicates

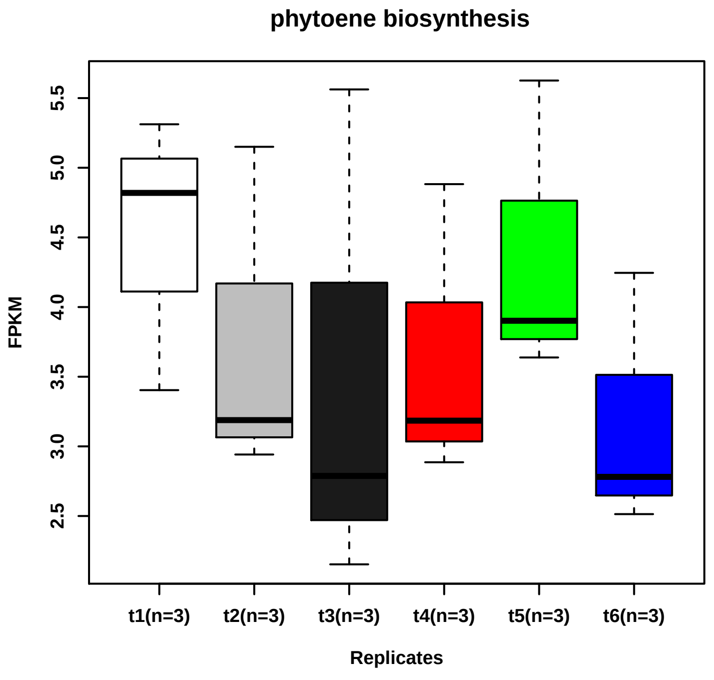
phytoene biosynthesis
FPKM
2.5
3.0
3.5
4.0
4.5
5.0
5.5
t1(n=3) t2(n=3) t3(n=3) t4(n=3) t5(n=3) t6(n=3)
Replicates

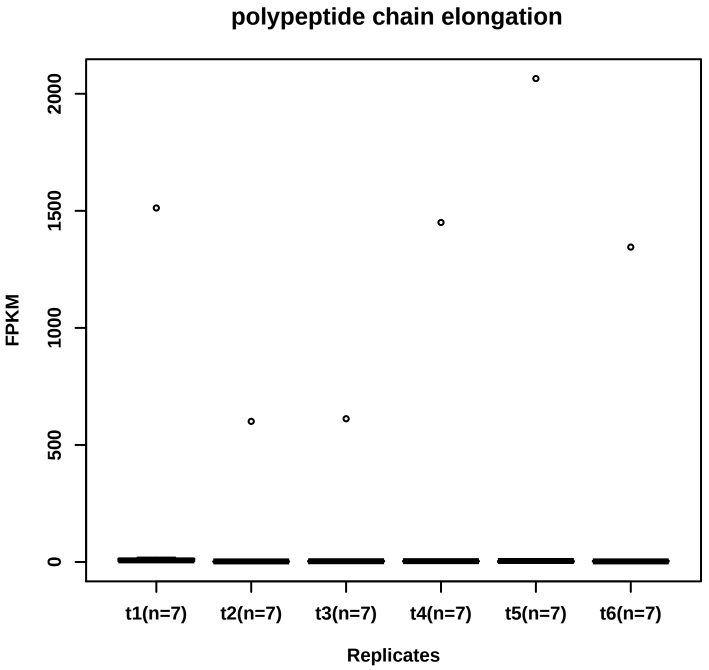
polypeptide chain elongation
FPKM
0
500
1000
1500
2000
t1(n=7)
t2(n=7)
t3(n=7)
t4(n=7)
t5(n=7)
t6(n=7)
Replicates

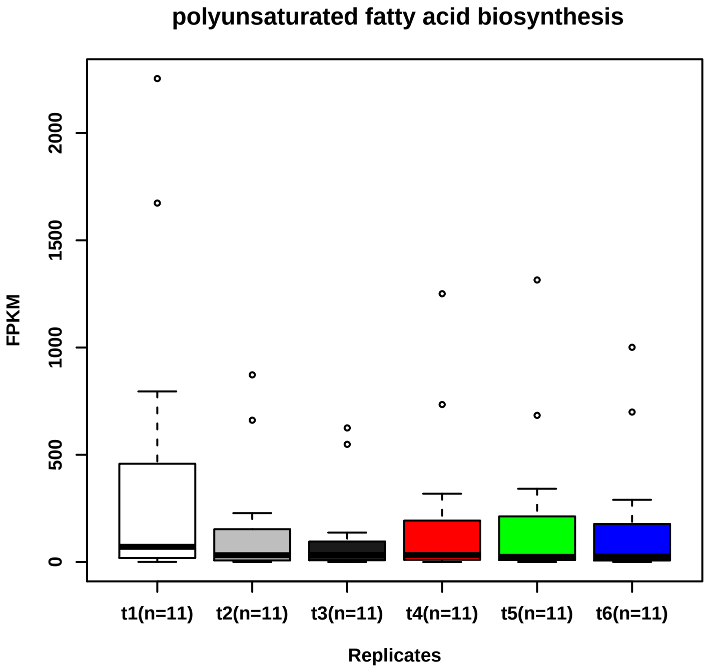
polyunsaturated fatty acid biosynthesis
FPKM
0
500
1000
1500
2000
t1(n=11)
t2(n=11)
t3(n=11)
t4(n=11)
t5(n=11)
t6(n=11)
Replicates

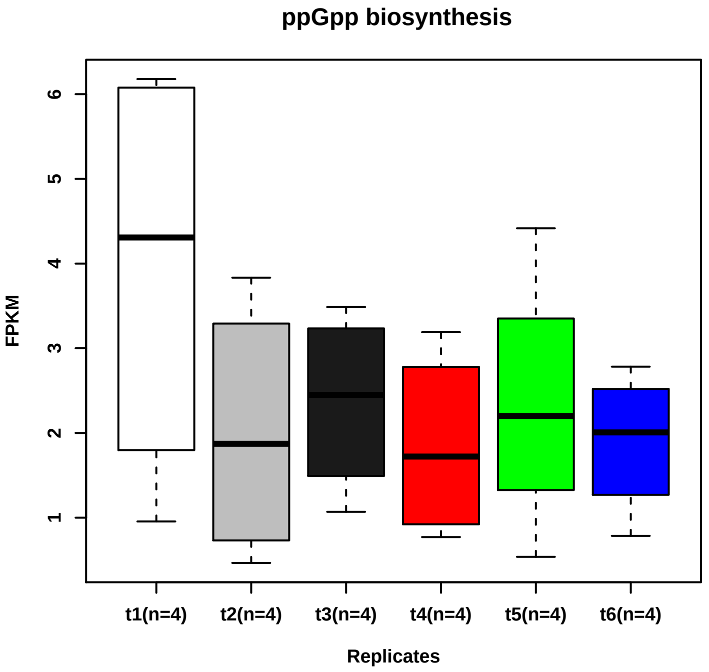
ppGpp biosynthesis
FPKM
1
2
3
4
5
6
t1(n=4)
t2(n=4)
t3(n=4)
t4(n=4)
t5(n=4)
t6(n=4)
Replicates

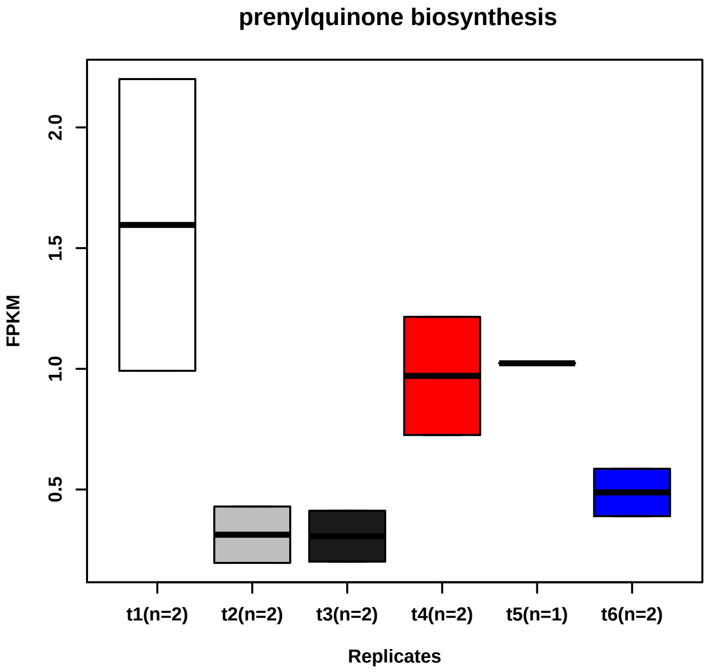
prenylquinone biosynthesis
FPKM
0.5
1.0
1.5
2.0
t1(n=2)
t2(n=2)
t3(n=2)
t4(n=2)
t5(n=1)
t6(n=2)
Replicates

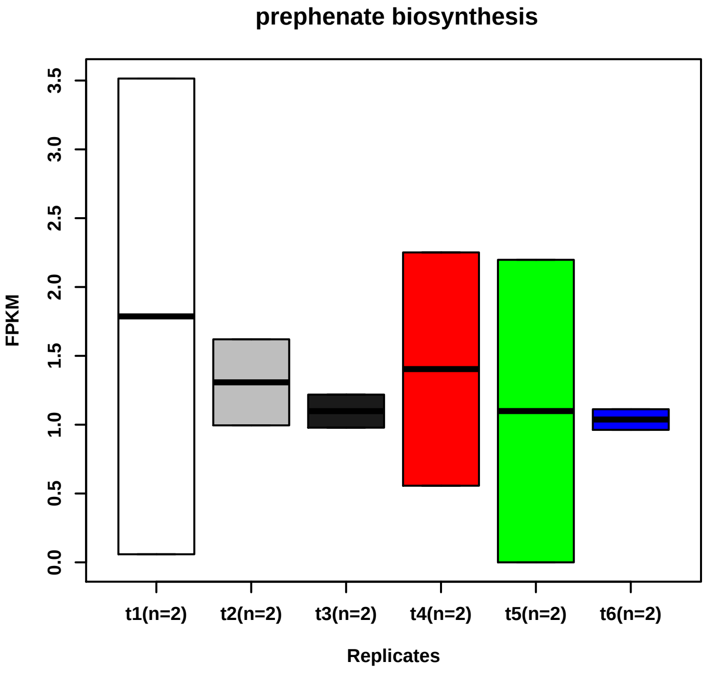
prephenate biosynthesis
FPKM
0.0
0.5
1.0
1.5
2.0
2.5
3.0
3.5
t1(n=2)
t2(n=2)
t3(n=2)
t4(n=2)
t5(n=2)
t6(n=2)
Replicates

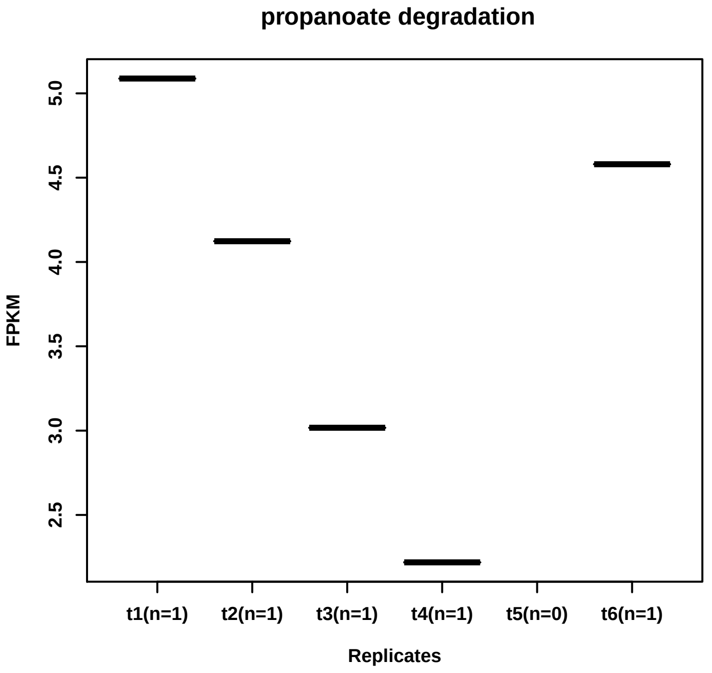
propanoate degradation
FPKM
2.5
3.0
3.5
4.0
4.5
5.0
t1(n=1)
t2(n=1)
t3(n=1)
t4(n=1)
t5(n=0)
t6(n=1)
Replicates

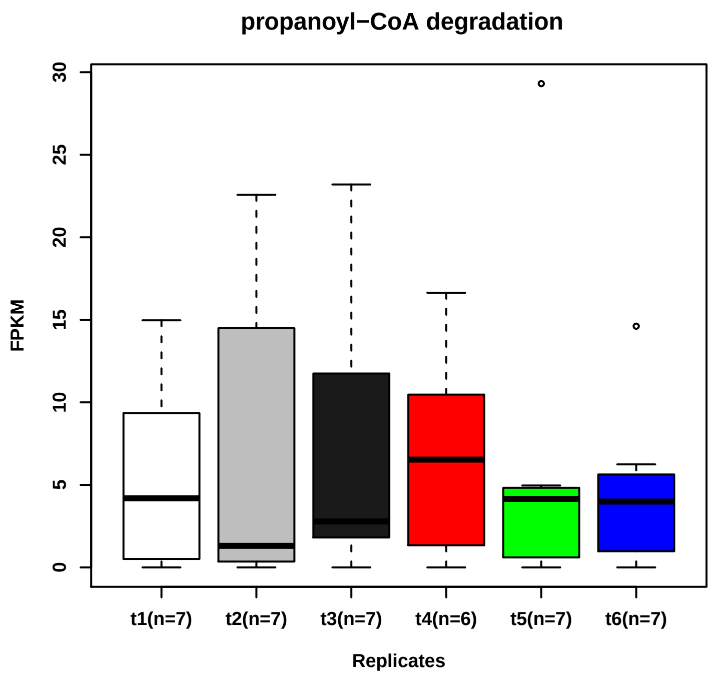
propanoyl−CoA degradation
FPKM
0
5
10
15
20
25
30
t1(n=7)
t2(n=7)
t3(n=7)
t4(n=6)
t5(n=7)
t6(n=7)
Replicates

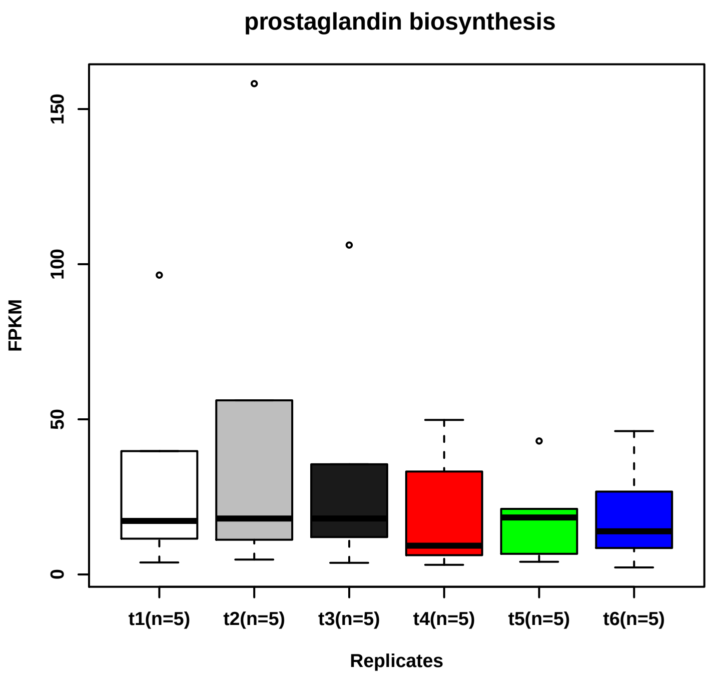
prostaglandin biosynthesis
FPKM
0
50
100
150
t1(n=5)
t2(n=5)
t3(n=5)
t4(n=5)
t5(n=5)
t6(n=5)
Replicates

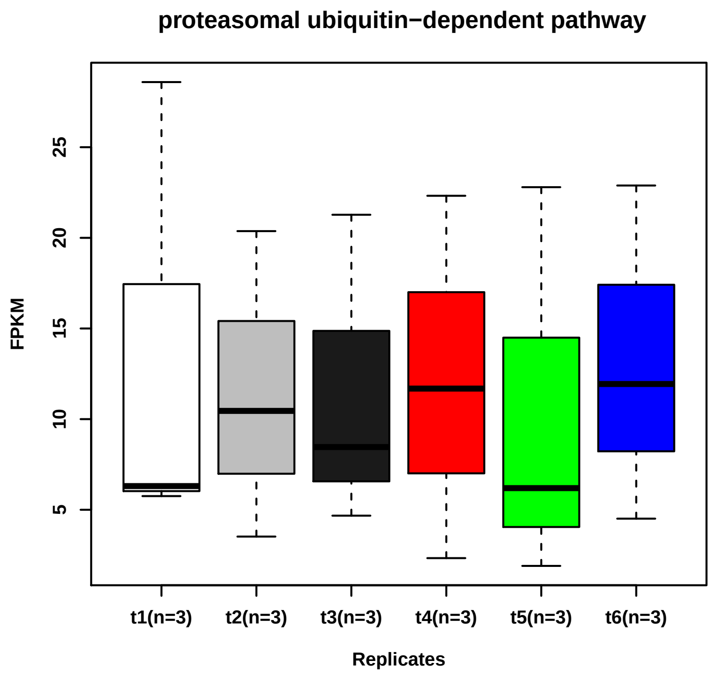
proteasomal ubiquitin−dependent pathway
FPKM
5
10
15
20
25
t1(n=3)
t2(n=3)
t3(n=3)
t4(n=3)
t5(n=3)
t6(n=3)
Replicates

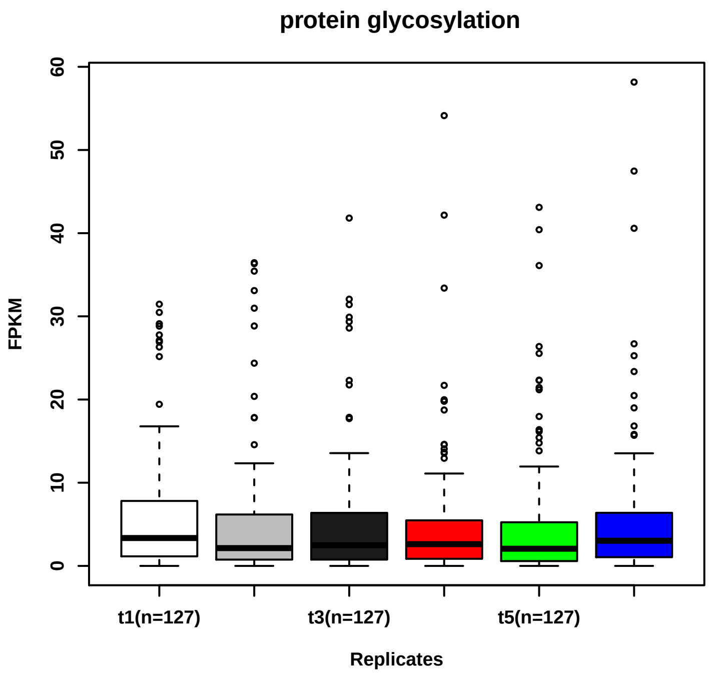
protein glycosylation
FPKM
0
10
20
30
40
50
60
t1(n=127)
t3(n=127)
t5(n=127)
Replicates

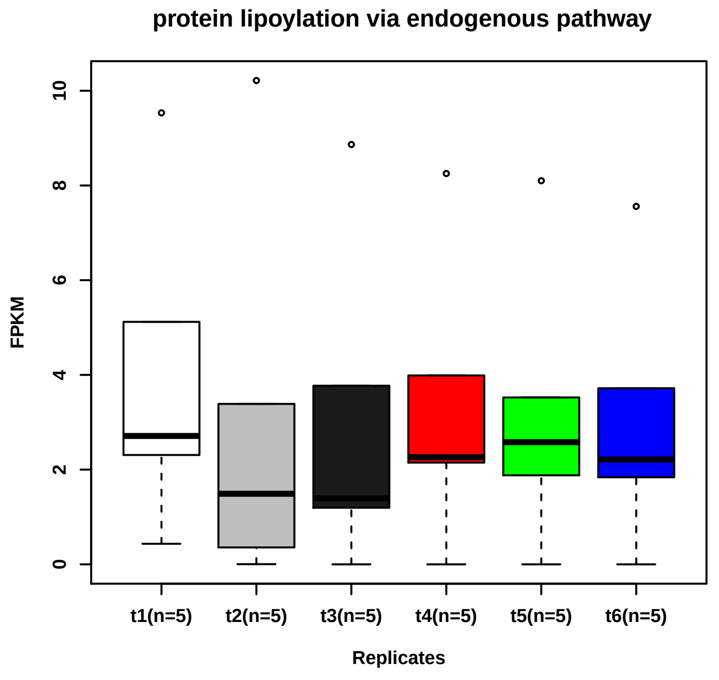
protein lipoylation via endogenous pathway
FPKM
0
2
4
6
8
10
t1(n=5)
t2(n=5)
t3(n=5)
t4(n=5)
t5(n=5)
t6(n=5)
Replicates

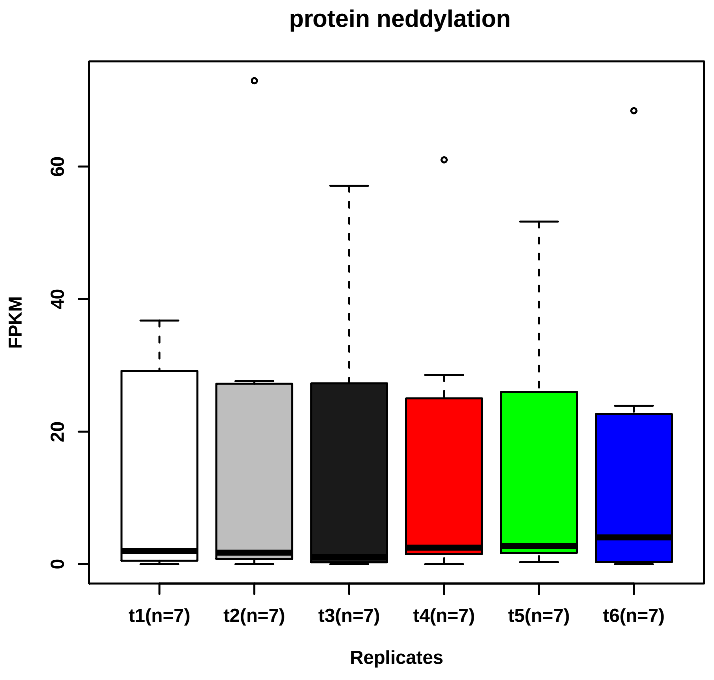
protein neddylation
FPKM
0
20
40
60
t1(n=7)
t2(n=7)
t3(n=7)
t4(n=7)
t5(n=7)
t6(n=7)
Replicates

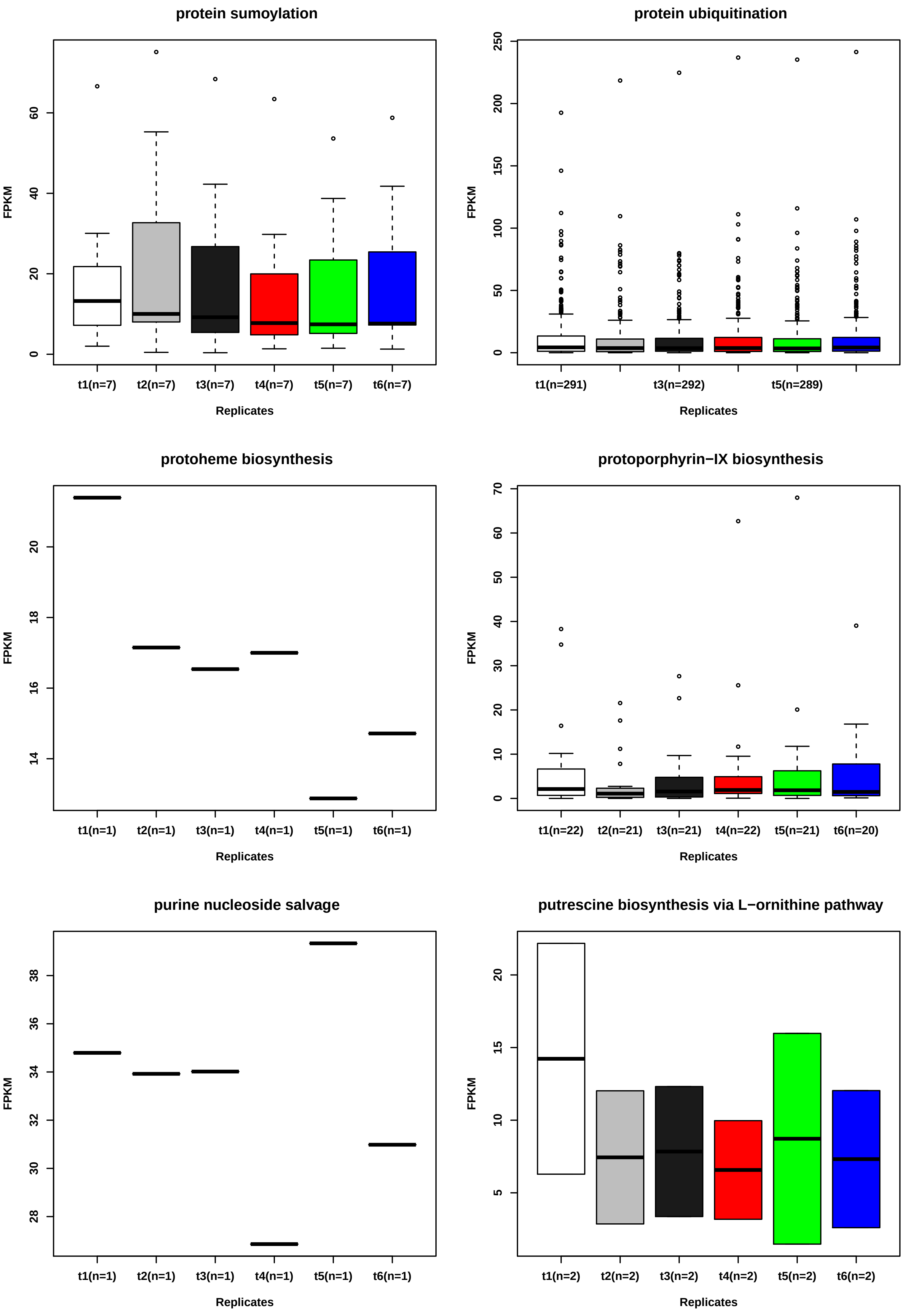
protein sumoylation
FPKM
0
20
40
60
t1(n=7)
t2(n=7)
t3(n=7)
t4(n=7)
t5(n=7)
t6(n=7)
Replicates
protein ubiquitination
FPKM
0
50
100
150
200
250
t1(n=291)
t3(n=292)
t5(n=289)
Replicates
protoheme biosynthesis
FPKM
14
16
18
20
t1(n=1)
t2(n=1)
t3(n=1)
t4(n=1)
t5(n=1)
t6(n=1)
Replicates
protoporphyrin−IX biosynthesis
FPKM
0
10
20
30
40
50
60
70
t1(n=22)
t2(n=21)
t3(n=21)
t4(n=22)
t5(n=21)
t6(n=20)
Replicates
purine nucleoside salvage
FPKM
28
30
32
34
36
38
t1(n=1)
t2(n=1)
t3(n=1)
t4(n=1)
t5(n=1)
t6(n=1)
Replicates
putrescine biosynthesis via L−ornithine pathway
FPKM
5
10
15
20
t1(n=2)
t2(n=2)
t3(n=2)
t4(n=2)
t5(n=2)
t6(n=2)
Replicates

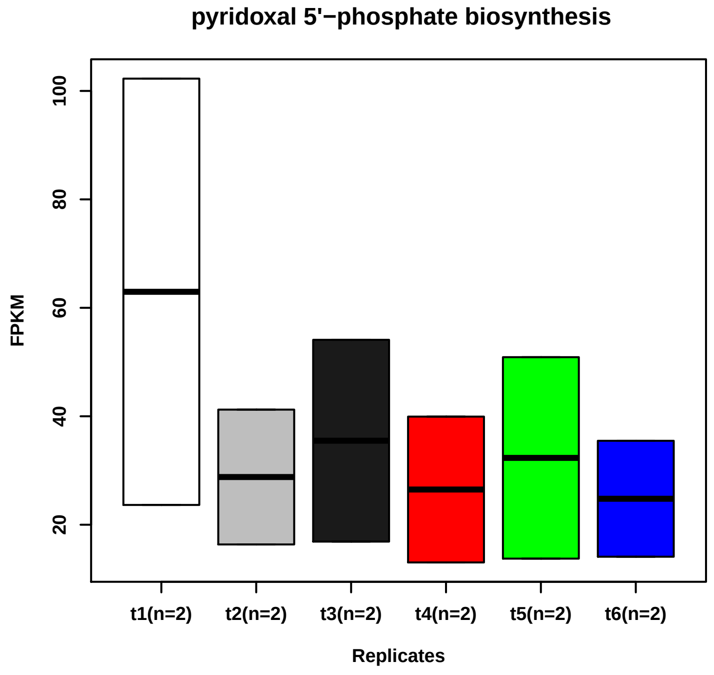

pyridoxal 5'−phosphate biosynthesis
FPKM
20
40
60
80
100
t1(n=2)
t2(n=2)
t3(n=2)
t4(n=2)
t5(n=2)
t6(n=2)
Replicates

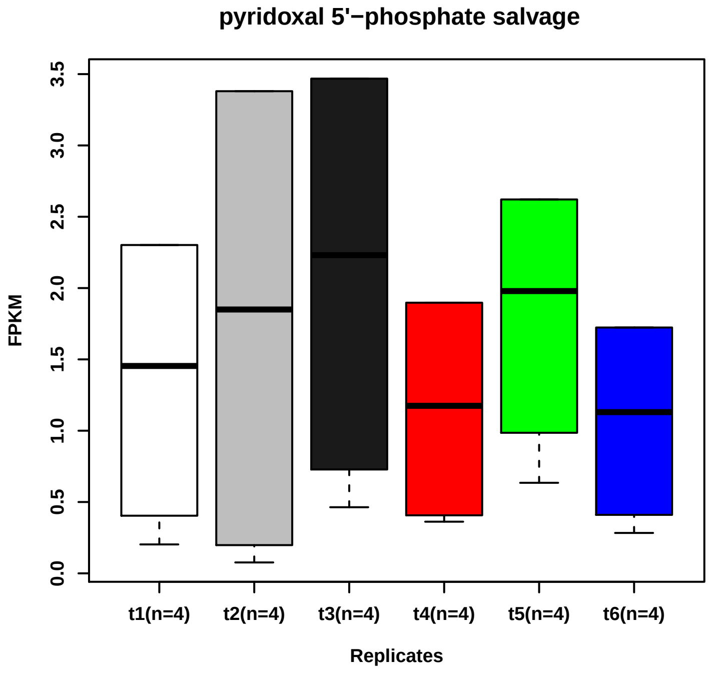

pyridoxal 5'−phosphate salvage
FPKM
0.0
0.5
1.0
1.5
2.0
2.5
3.0
3.5
t1(n=4)
t2(n=4)
t3(n=4)
t4(n=4)
t5(n=4)
t6(n=4)
Replicates

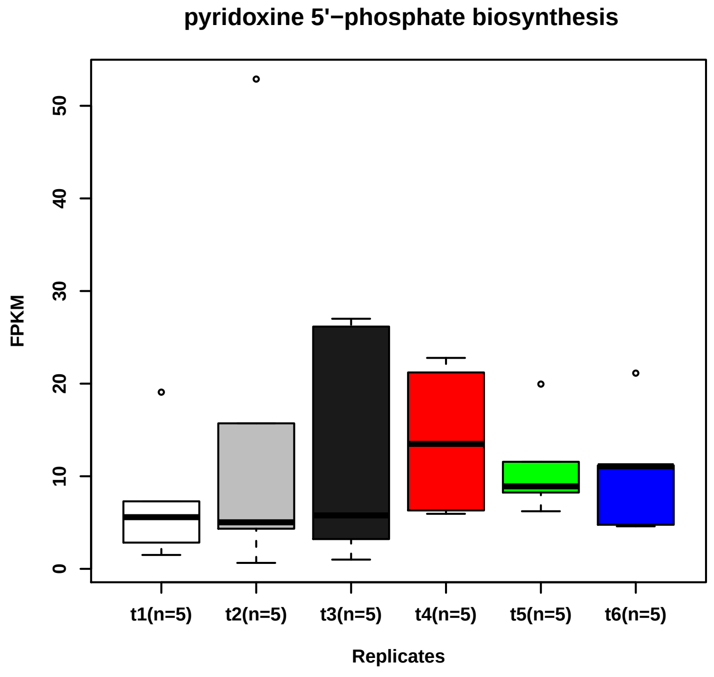

pyridoxine 5'−phosphate biosynthesis
FPKM
0
10
20
30
40
50
t1(n=5)
t2(n=5)
t3(n=5)
t4(n=5)
t5(n=5)
t6(n=5)
Replicates

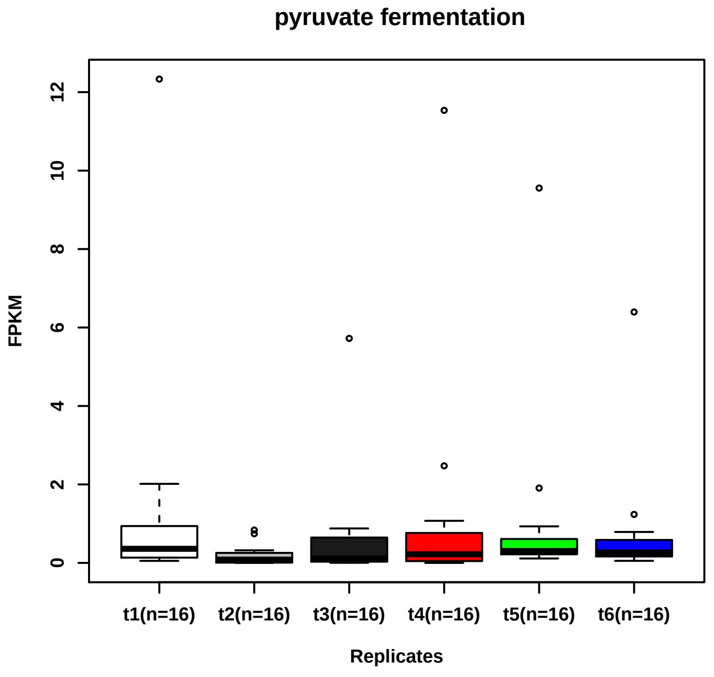

pyruvate fermentation
FPKM
0
2
4
6
8
10
12
t1(n=16)
t2(n=16)
t3(n=16)
t4(n=16)
t5(n=16)
t6(n=16)
Replicates

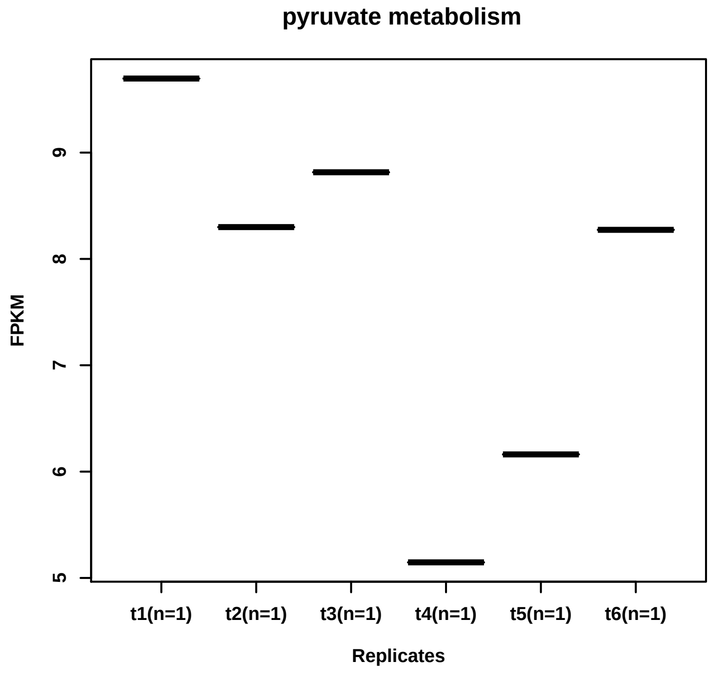

pyruvate metabolism
FPKM
5
6
7
8
9
t1(n=1)
t2(n=1)
t3(n=1)
t4(n=1)
t5(n=1)
t6(n=1)
Replicates

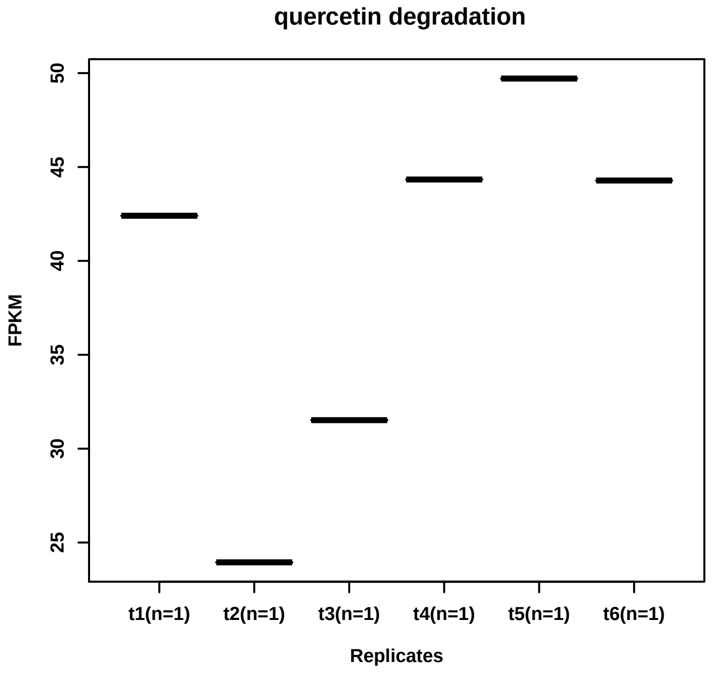

quercetin degradation
FPKM
25
30
35
40
45
50
t1(n=1)
t2(n=1)
t3(n=1)
t4(n=1)
t5(n=1)
t6(n=1)
Replicates

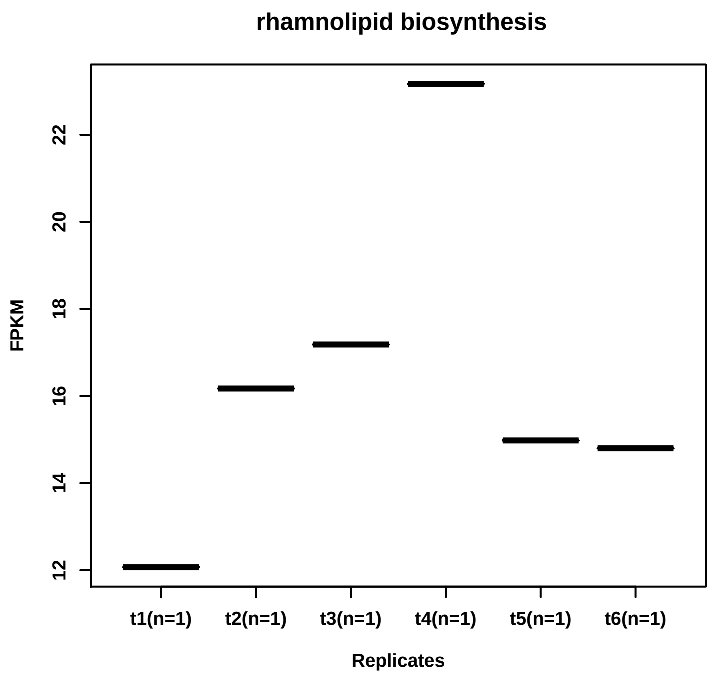

rhamnolipid biosynthesis
FPKM
12
14
16
18
20
22
t1(n=1)
t2(n=1)
t3(n=1)
t4(n=1)
t5(n=1)
t6(n=1)
Replicates

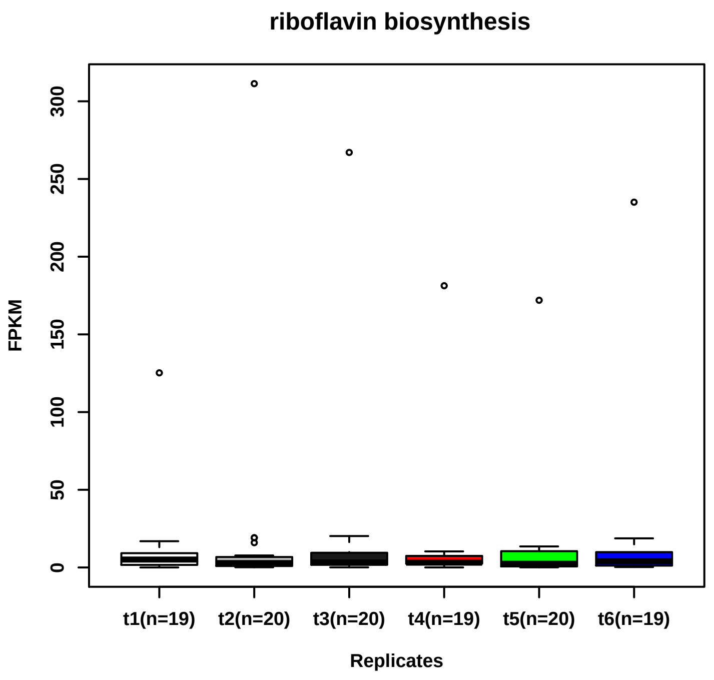

riboflavin biosynthesis
FPKM
0
50
100
150
200
250
300
t1(n=19)
t2(n=20)
t3(n=20)
t4(n=19)
t5(n=20)
t6(n=19)
Replicates

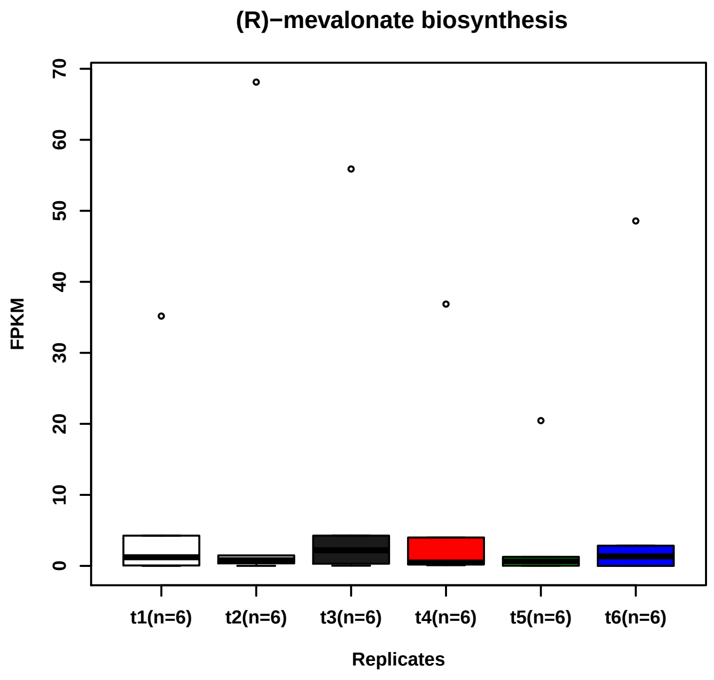

(R)−mevalonate biosynthesis
FPKM
0
10
20
30
40
50
60
70
t1(n=6)
t2(n=6)
t3(n=6)
t4(n=6)
t5(n=6)
t6(n=6)
Replicates

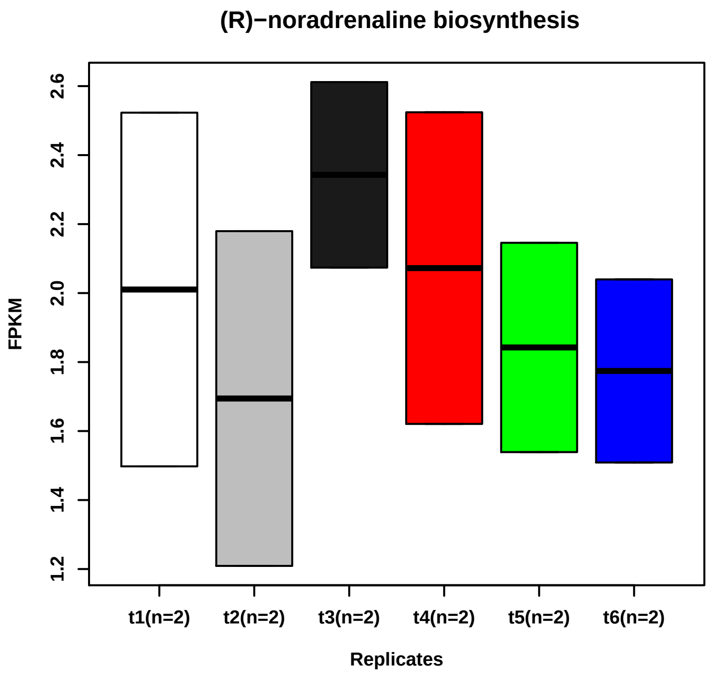

(R)−noradrenaline biosynthesis
FPKM
1.2
1.4
1.6
1.8
2.0
2.2
2.4
2.6
t1(n=2)
t2(n=2)
t3(n=2)
t4(n=2)
t5(n=2)
t6(n=2)
Replicates

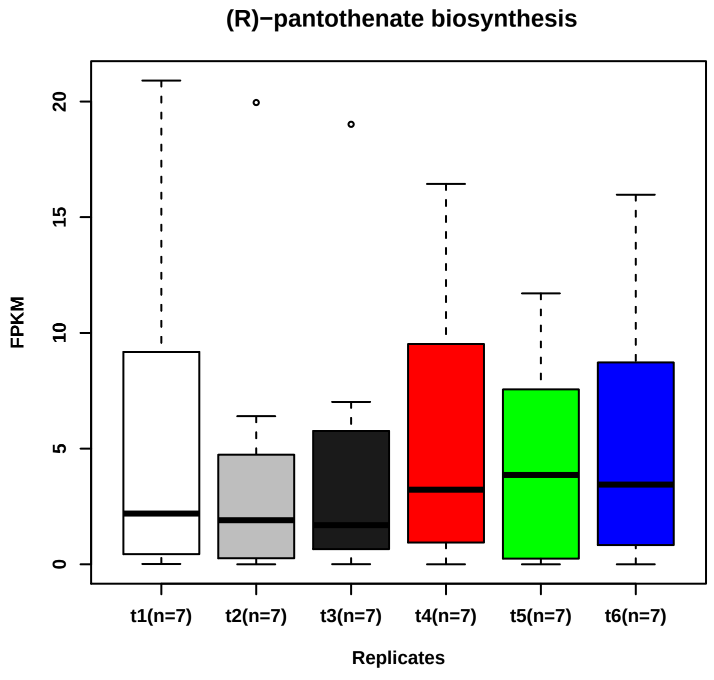

(R)−pantothenate biosynthesis
FPKM
0
5
10
15
20
t1(n=7)
t2(n=7)
t3(n=7)
t4(n=7)
t5(n=7)
t6(n=7)
Replicates

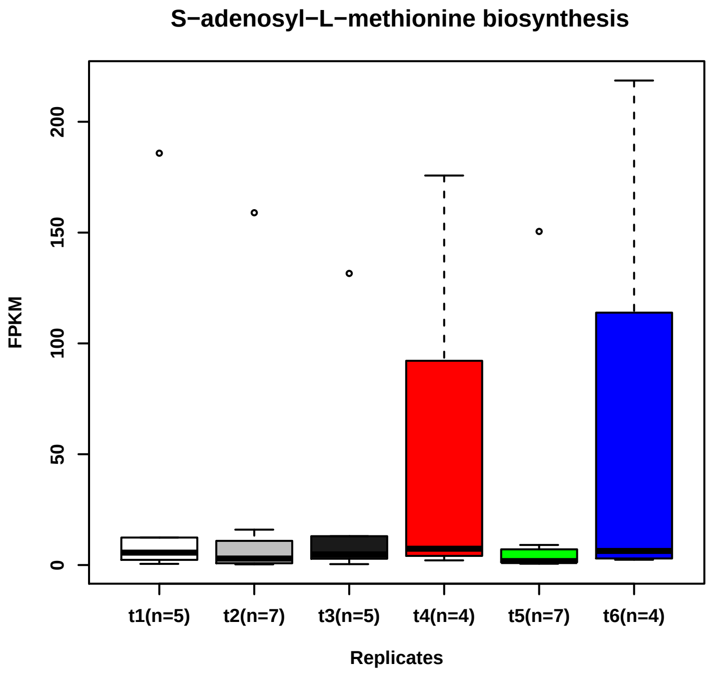

S−adenosyl−L−methionine biosynthesis
FPKM
0
50
100
150
200
t1(n=5)
t2(n=7)
t3(n=5)
t4(n=4)
t5(n=7)
t6(n=4)
Replicates

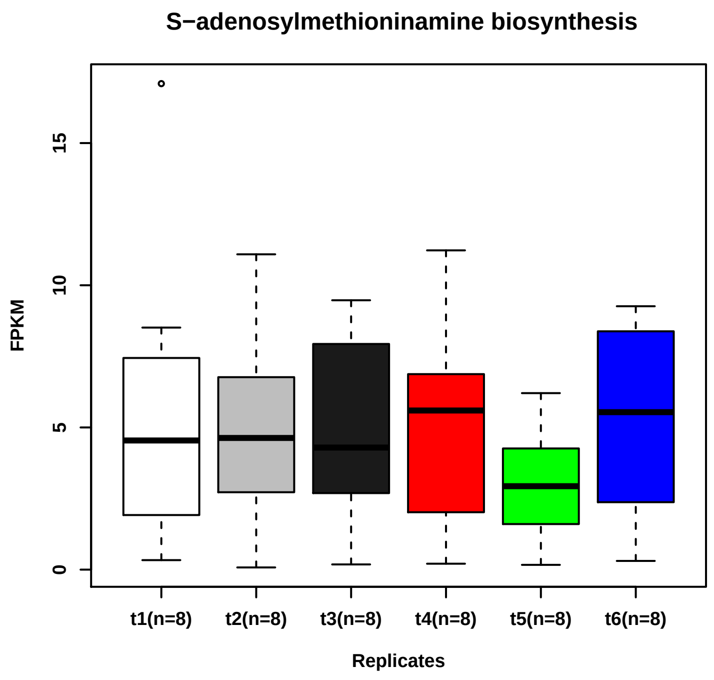
S−adenosylmethioninamine biosynthesis
FPKM
0
5
10
15
t1(n=8)
t2(n=8)
t3(n=8)
t4(n=8)
t5(n=8)
t6(n=8)
Replicates

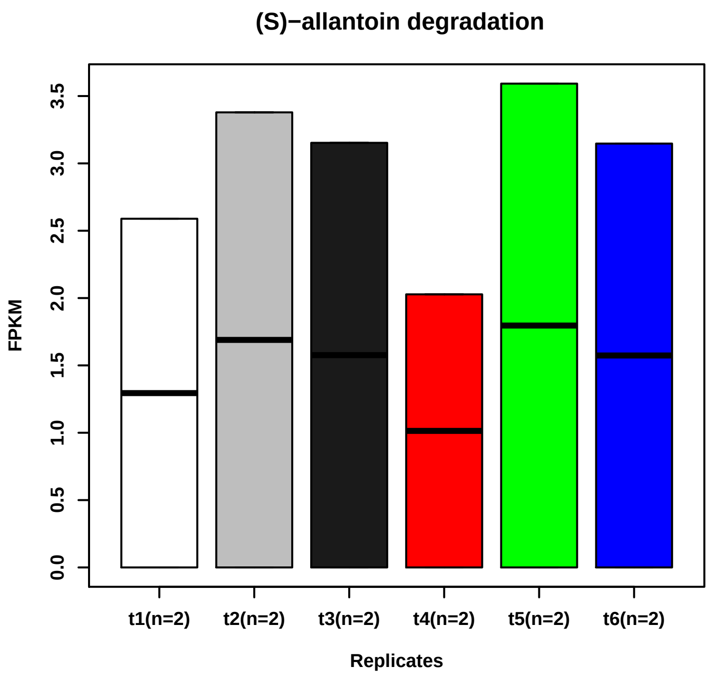
(S)−allantoin degradation
FPKM
0.0
0.5
1.0
1.5
2.0
2.5
3.0
3.5
t1(n=2)
t2(n=2)
t3(n=2)
t4(n=2)
t5(n=2)
t6(n=2)
Replicates

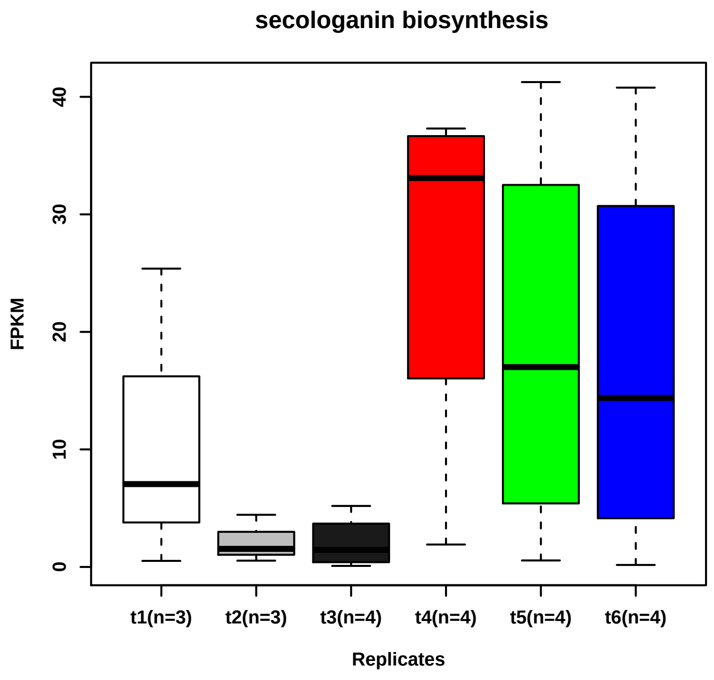
secologanin biosynthesis
FPKM
0
10
20
30
40
t1(n=3)
t2(n=3)
t3(n=4)
t4(n=4)
t5(n=4)
t6(n=4)
Replicates

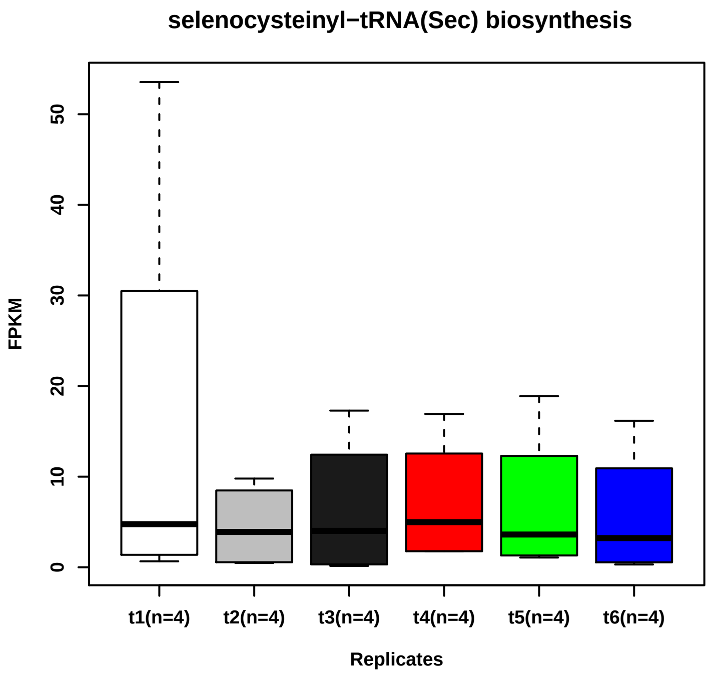
selenocysteinyl−tRNA(Sec) biosynthesis
FPKM
0
10
20
30
40
50
t1(n=4)
t2(n=4)
t3(n=4)
t4(n=4)
t5(n=4)
t6(n=4)
Replicates

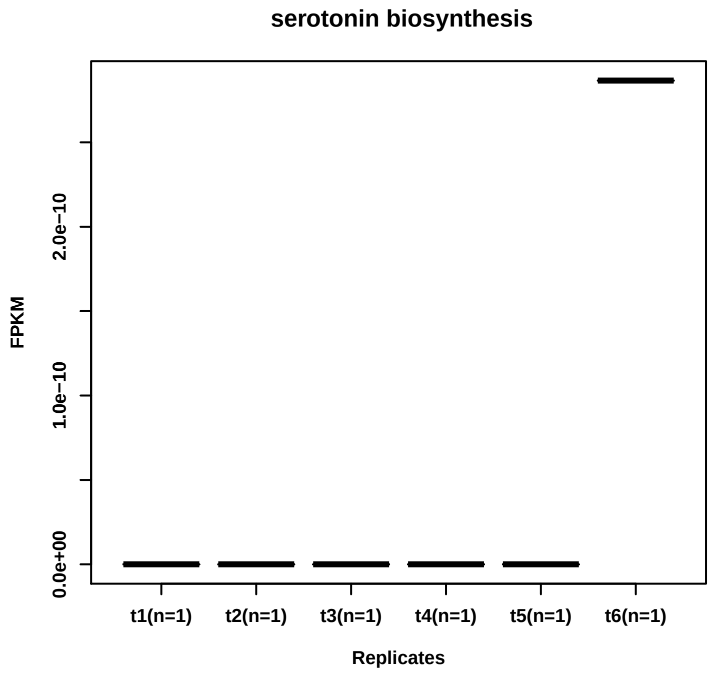
serotonin biosynthesis
FPKM
0.0e+00
1.0e−10
2.0e−10
t1(n=1)
t2(n=1)
t3(n=1)
t4(n=1)
t5(n=1)
t6(n=1)
Replicates

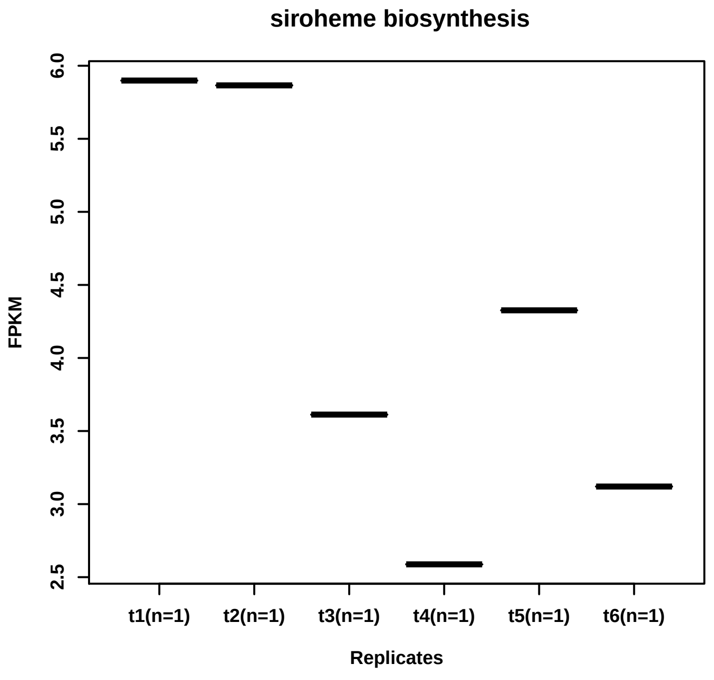
siroheme biosynthesis
FPKM
2.5
3.0
3.5
4.0
4.5
5.0
5.5
6.0
t1(n=1)
t2(n=1)
t3(n=1)
t4(n=1)
t5(n=1)
t6(n=1)
Replicates

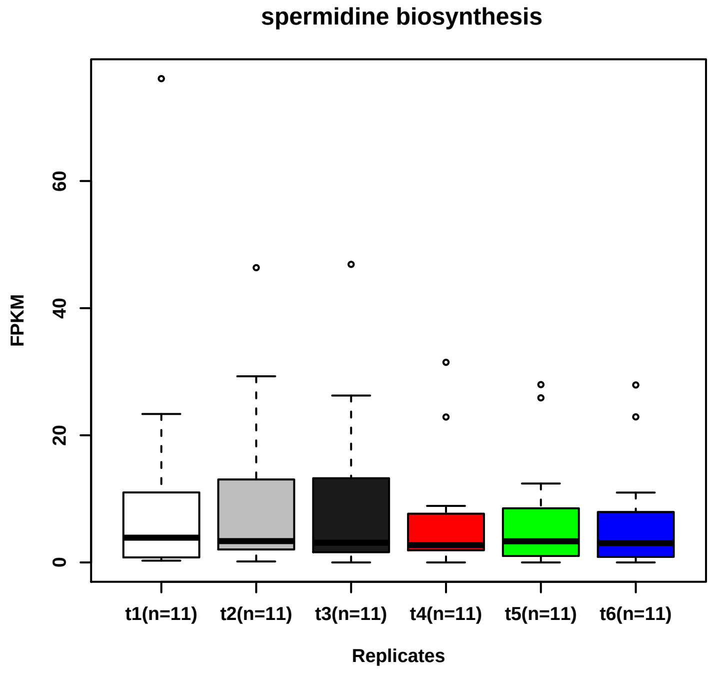

spermidine biosynthesis
FPKM
0
20
40
60
t1(n=11)
t2(n=11)
t3(n=11)
t4(n=11)
t5(n=11)
t6(n=11)
Replicates

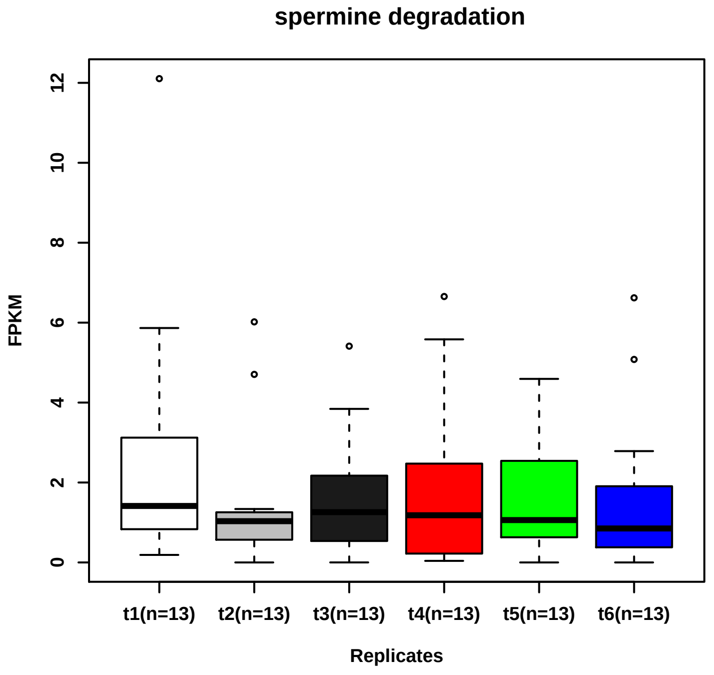

spermine degradation
FPKM
0
2
4
6
8
10
12
t1(n=13)
t2(n=13)
t3(n=13)
t4(n=13)
t5(n=13)
t6(n=13)
Replicates

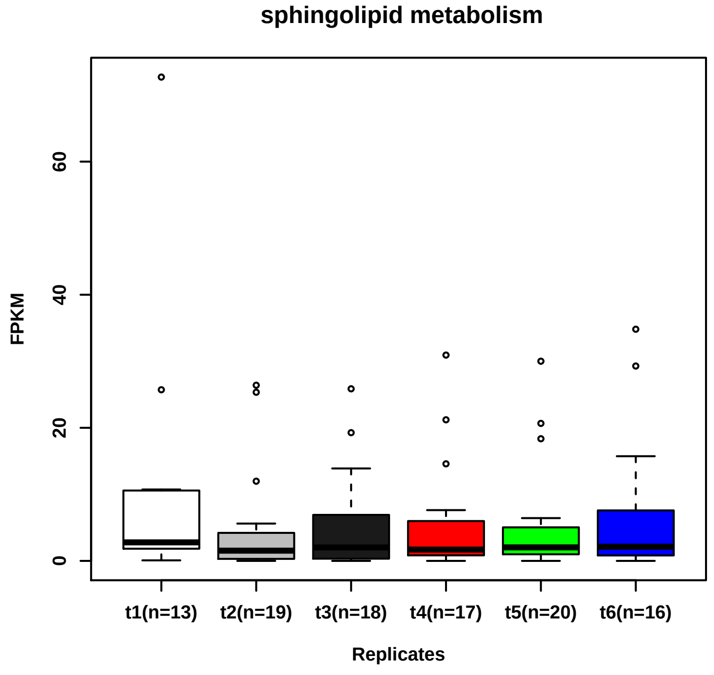

sphingolipid metabolism
FPKM
0
20
40
60
t1(n=13)
t2(n=19)
t3(n=18)
t4(n=17)
t5(n=20)
t6(n=16)
Replicates

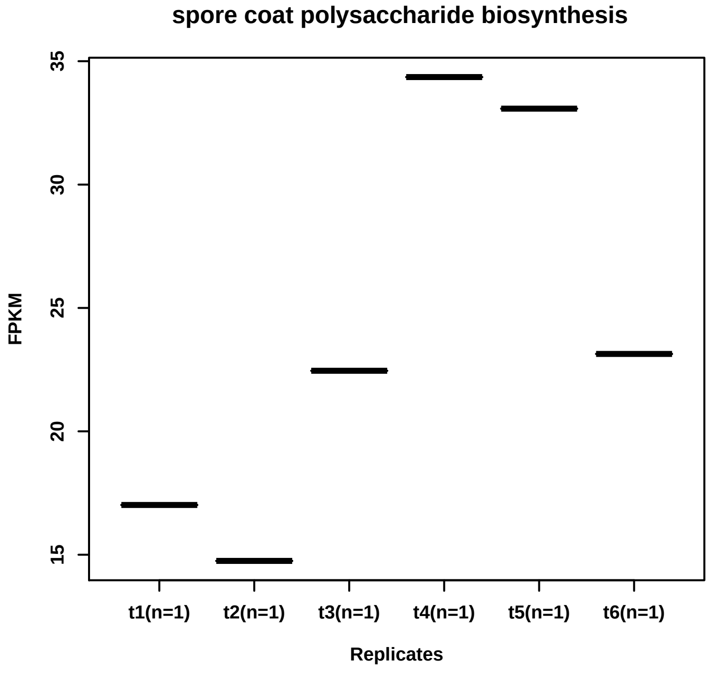

spore coat polysaccharide biosynthesis
FPKM
15
20
25
30
35
t1(n=1)
t2(n=1)
t3(n=1)
t4(n=1)
t5(n=1)
t6(n=1)
Replicates

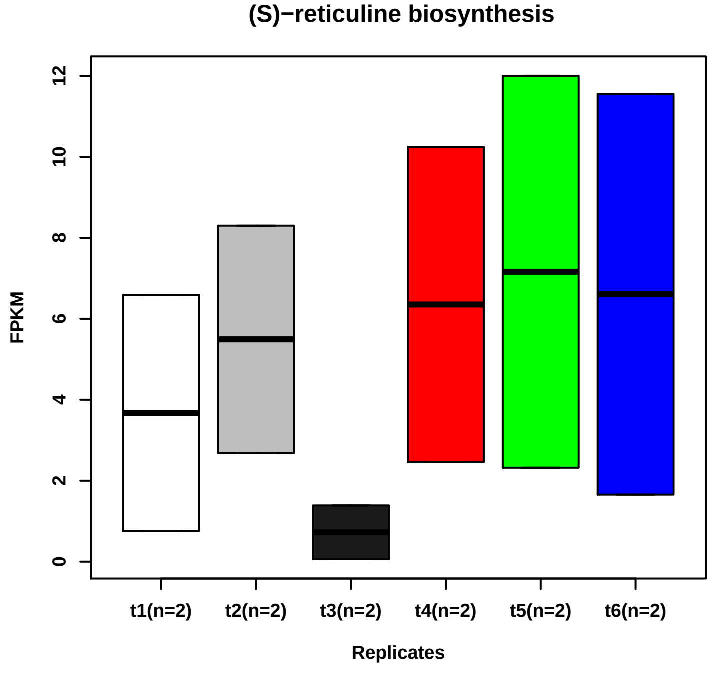

(S)−reticuline biosynthesis
FPKM
0
2
4
6
8
10
12
t1(n=2)
t2(n=2)
t3(n=2)
t4(n=2)
t5(n=2)
t6(n=2)
Replicates

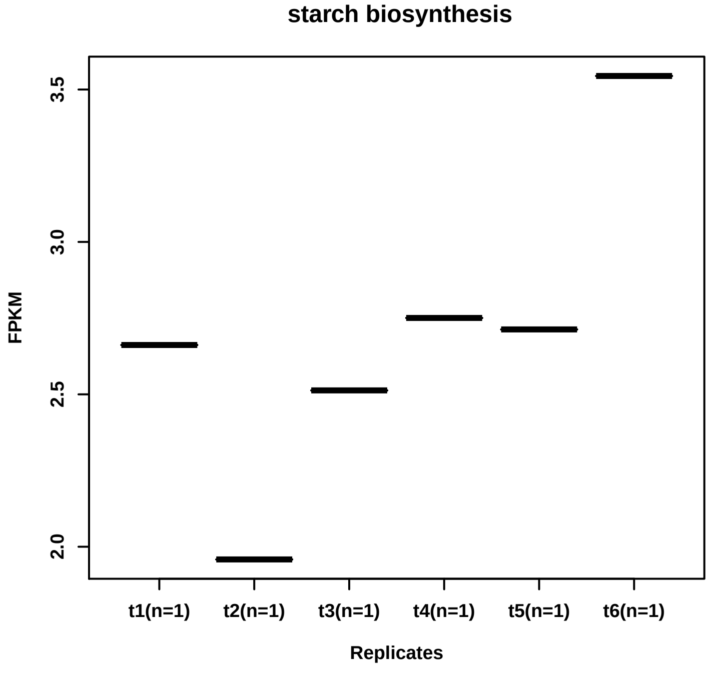

starch biosynthesis
FPKM
2.0
2.5
3.0
3.5
t1(n=1)
t2(n=1)
t3(n=1)
t4(n=1)
t5(n=1)
t6(n=1)
Replicates

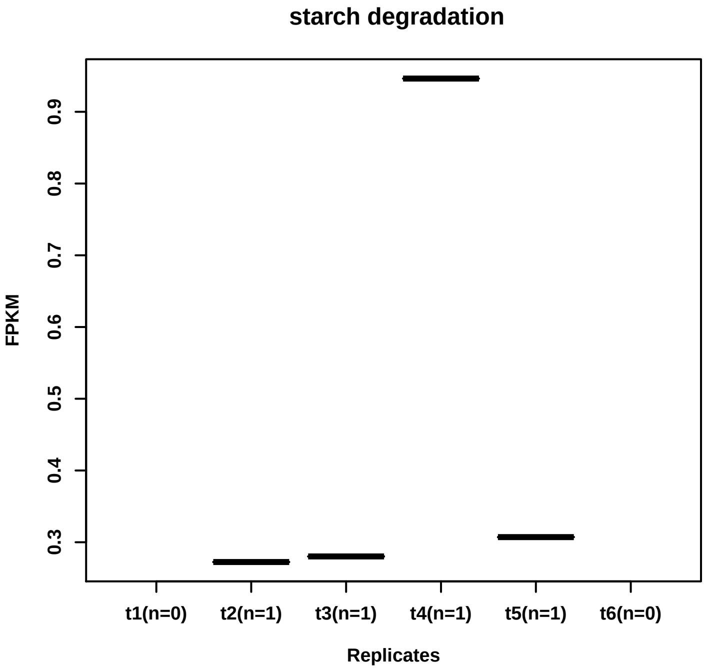
starch degradation
FPKM
0.3
0.4
0.5
0.6
0.7
0.8
0.9
t1(n=0)
t2(n=1)
t3(n=1)
t4(n=1)
t5(n=1)
t6(n=0)
Replicates

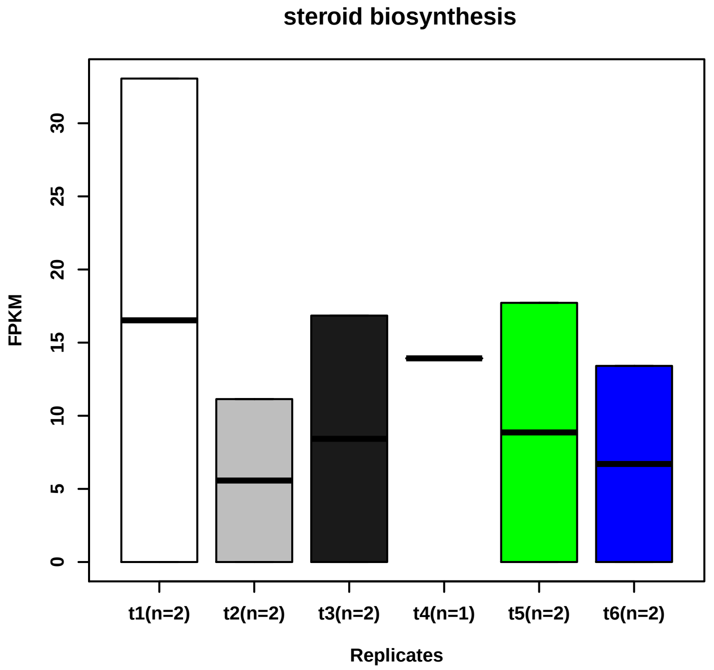
steroid biosynthesis
FPKM
0
5
10
15
20
25
30
t1(n=2)
t2(n=2)
t3(n=2)
t4(n=1)
t5(n=2)
t6(n=2)
Replicates

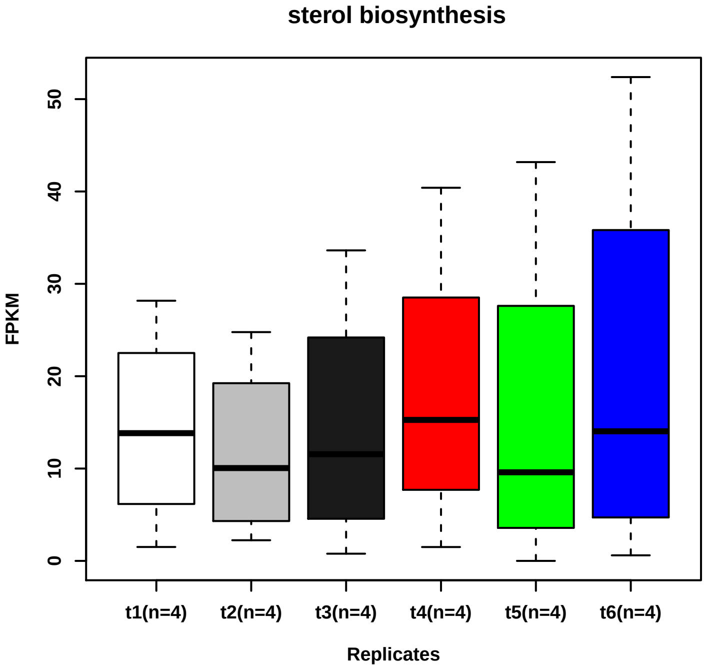
sterol biosynthesis
FPKM
0
10
20
30
40
50
t1(n=4)
t2(n=4)
t3(n=4)
t4(n=4)
t5(n=4)
t6(n=4)
Replicates

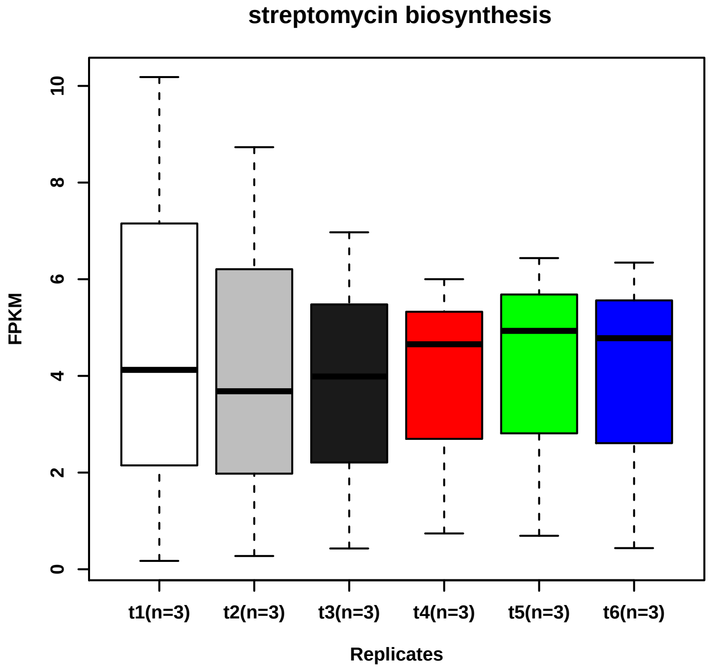
streptomycin biosynthesis
FPKM
0
2
4
6
8
10
t1(n=3)
t2(n=3)
t3(n=3)
t4(n=3)
t5(n=3)
t6(n=3)
Replicates

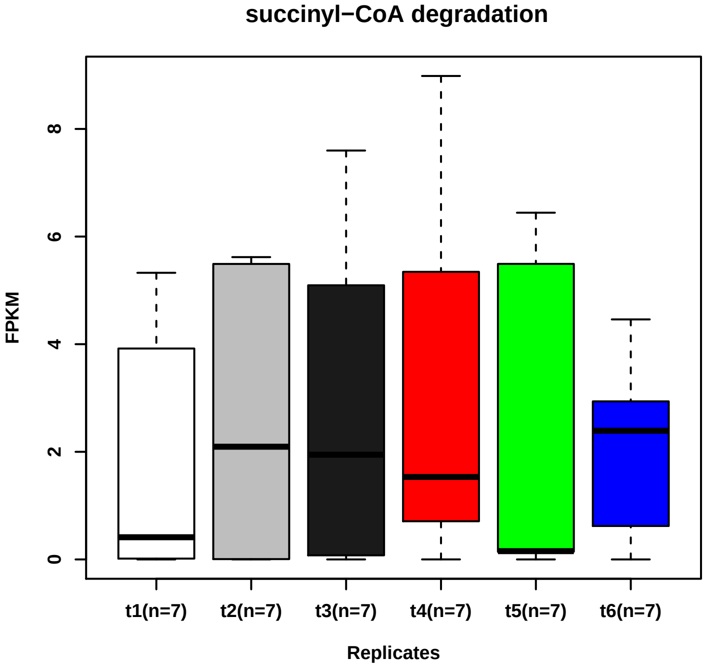
succinyl−CoA degradation
FPKM
0
2
4
6
8
t1(n=7)
t2(n=7)
t3(n=7)
t4(n=7)
t5(n=7)
t6(n=7)
Replicates

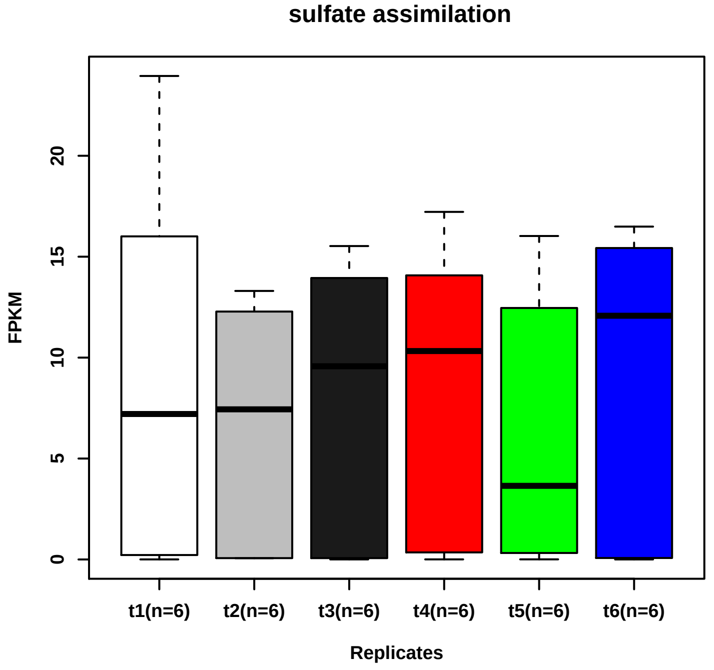
sulfate assimilation
FPKM
0
5
10
15
20
t1(n=6)
t2(n=6)
t3(n=6)
t4(n=6)
t5(n=6)
t6(n=6)
Replicates

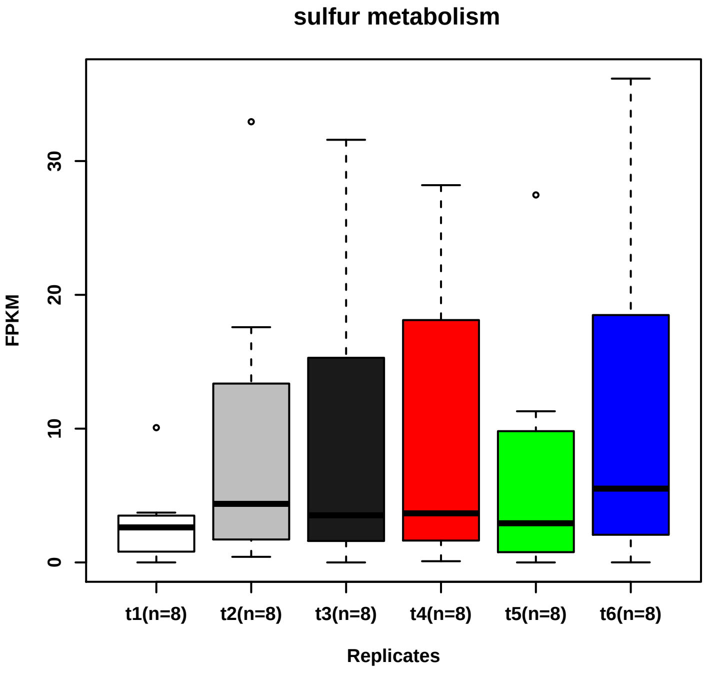
sulfur metabolism
FPKM
0
10
20
30
t1(n=8)
t2(n=8)
t3(n=8)
t4(n=8)
t5(n=8)
t6(n=8)
Replicates

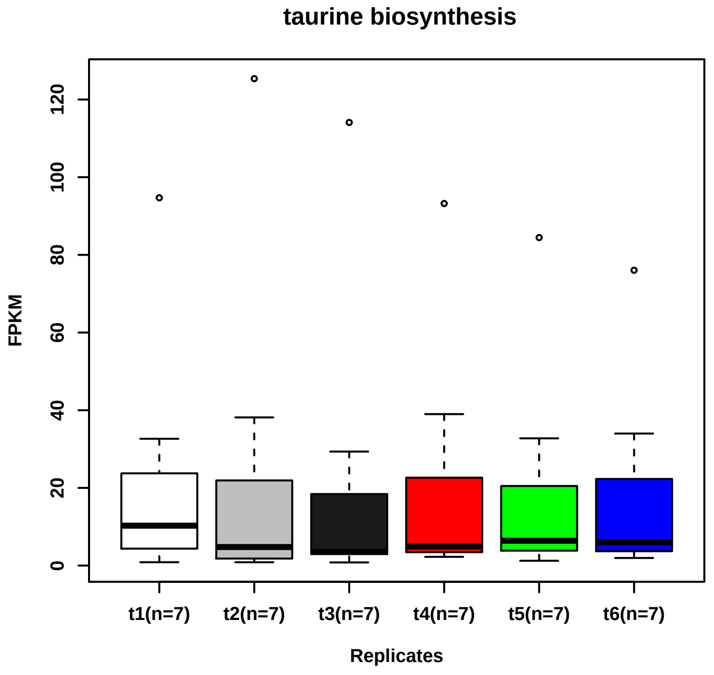
taurine biosynthesis
FPKM
0
20
40
60
80
100
120
t1(n=7)
t2(n=7)
t3(n=7)
t4(n=7)
t5(n=7)
t6(n=7)
Replicates

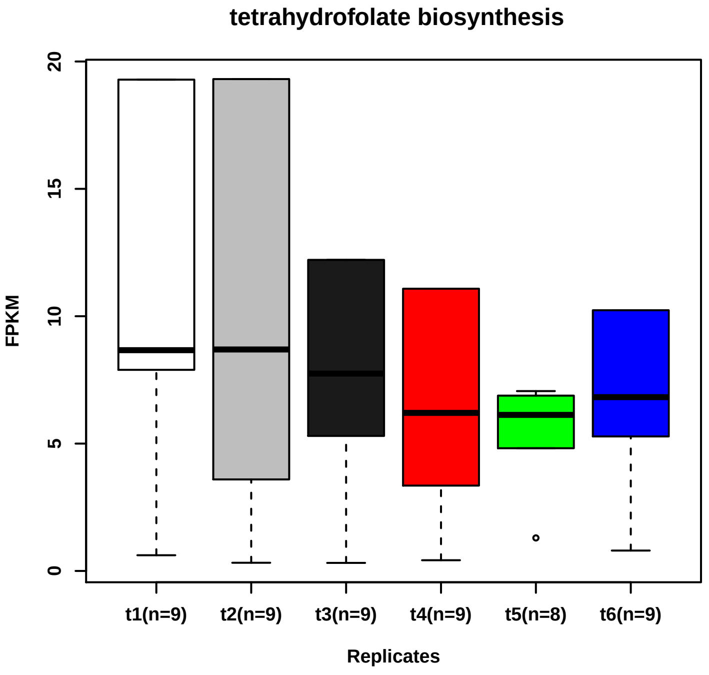
tetrahydrofolate biosynthesis
FPKM
0
5
10
15
20
t1(n=9)
t2(n=9)
t3(n=9)
t4(n=9)
t5(n=8)
t6(n=9)
Replicates

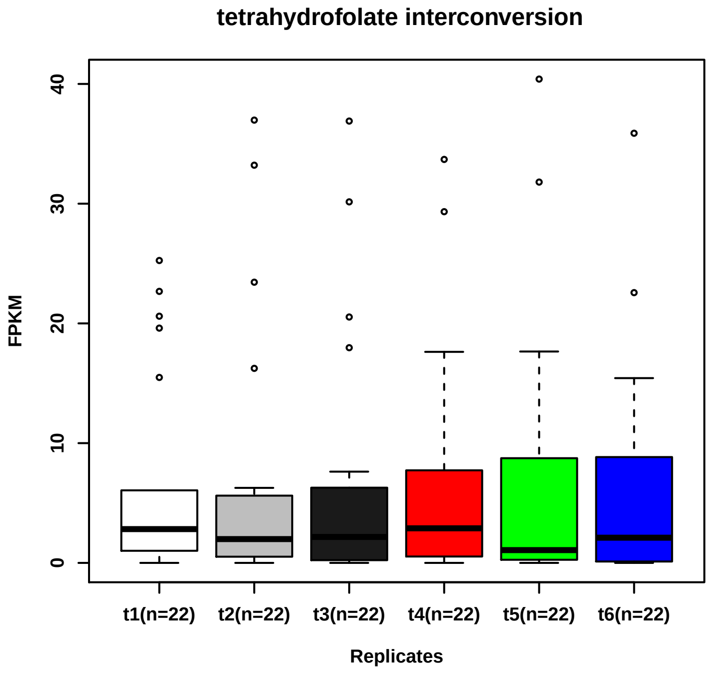
tetrahydrofolate interconversion
FPKM
0
10
20
30
40
t1(n=22)
t2(n=22)
t3(n=22)
t4(n=22)
t5(n=22)
t6(n=22)
Replicates

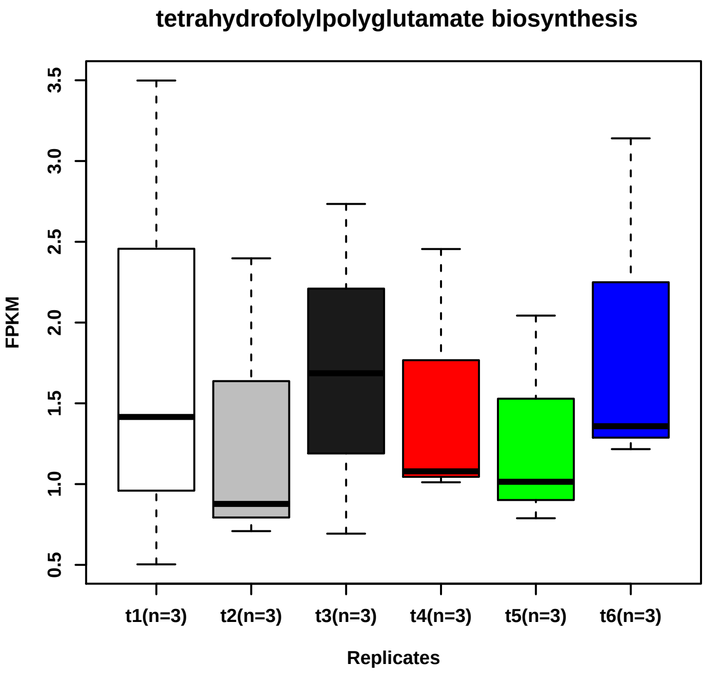
tetrahydrofolylpolyglutamate biosynthesis
FPKM
0.5
1.0
1.5
2.0
2.5
3.0
3.5
t1(n=3)
t2(n=3)
t3(n=3)
t4(n=3)
t5(n=3)
t6(n=3)
Replicates

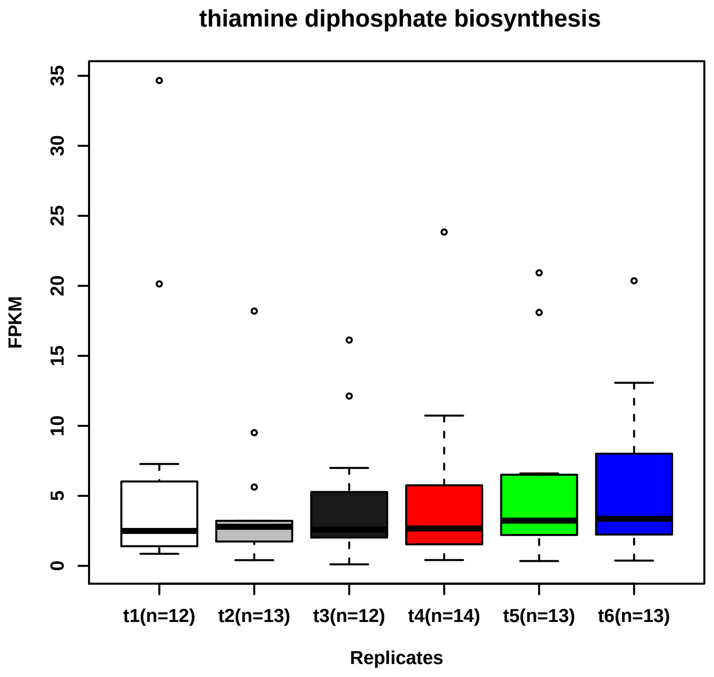
thiamine diphosphate biosynthesis
FPKM
0
5
10
15
20
25
30
35
t1(n=12)
t2(n=13)
t3(n=12)
t4(n=14)
t5(n=13)
t6(n=13)
Replicates

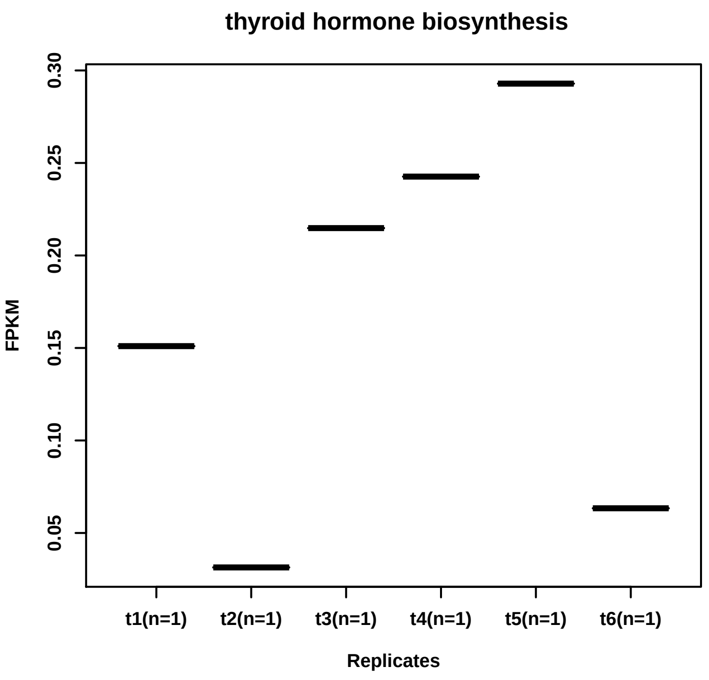
thyroid hormone biosynthesis
FPKM
0.05
0.10
0.15
0.20
0.25
0.30
t1(n=1)
t2(n=1)
t3(n=1)
t4(n=1)
t5(n=1)
t6(n=1)
Replicates

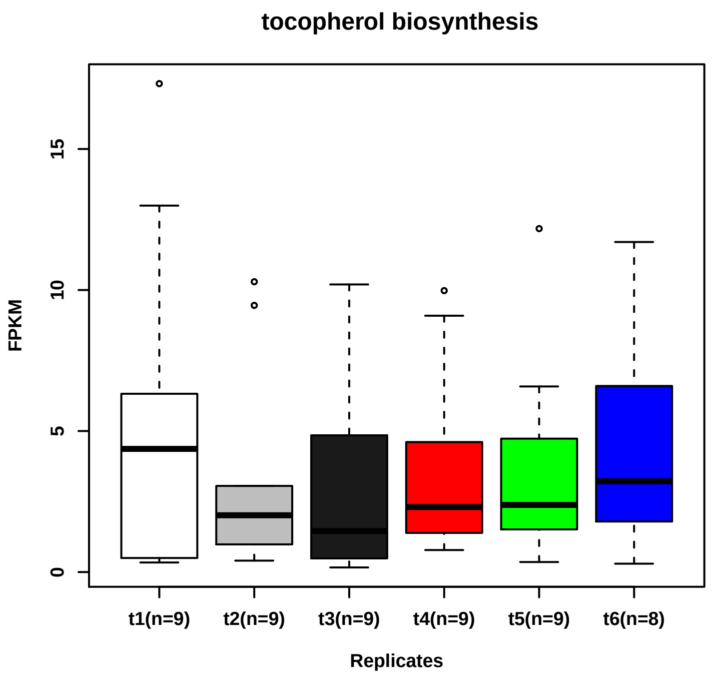
tocopherol biosynthesis
FPKM
0
5
10
15
t1(n=9)
t2(n=9)
t3(n=9)
t4(n=9)
t5(n=9)
t6(n=8)
Replicates

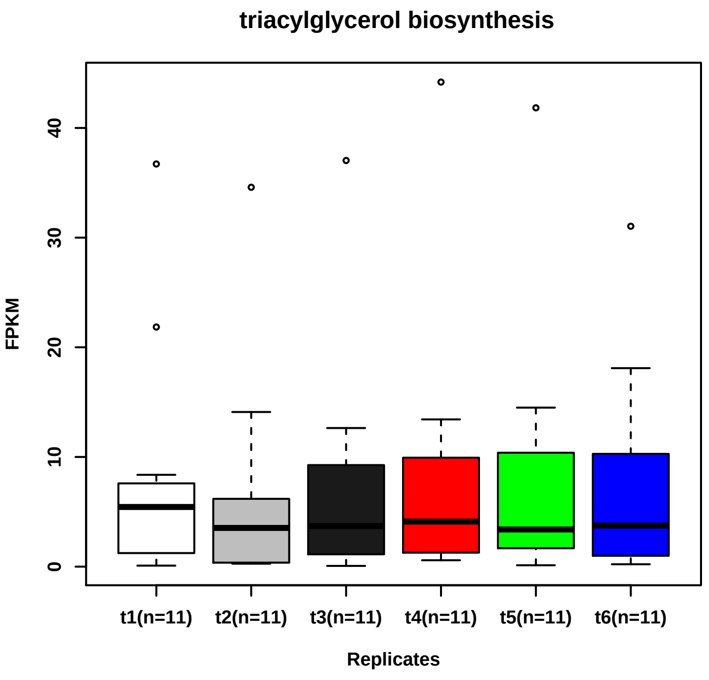
triacylglycerol biosynthesis
FPKM
0
10
20
30
40
t1(n=11)
t2(n=11)
t3(n=11)
t4(n=11)
t5(n=11)
t6(n=11)
Replicates

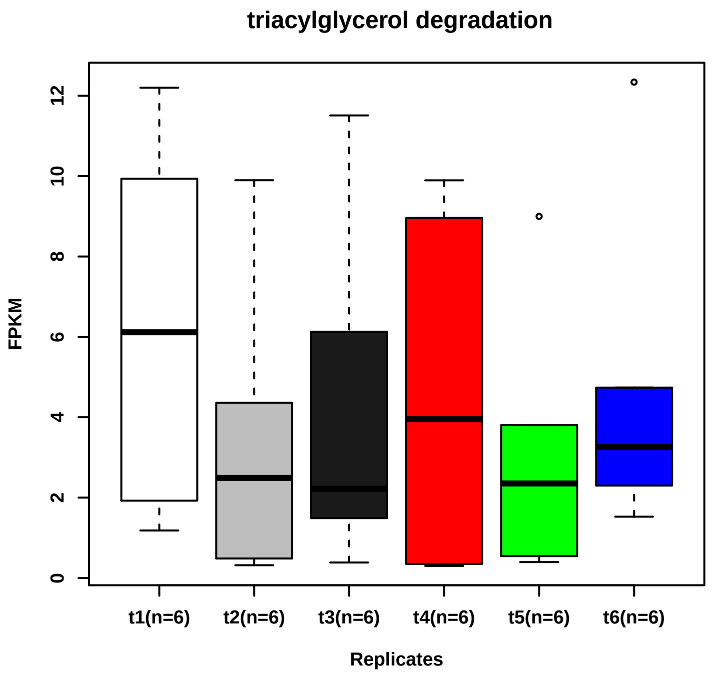
triacylglycerol degradation
FPKM
0
2
4
6
8
10
12
t1(n=6)
t2(n=6)
t3(n=6)
t4(n=6)
t5(n=6)
t6(n=6)
Replicates

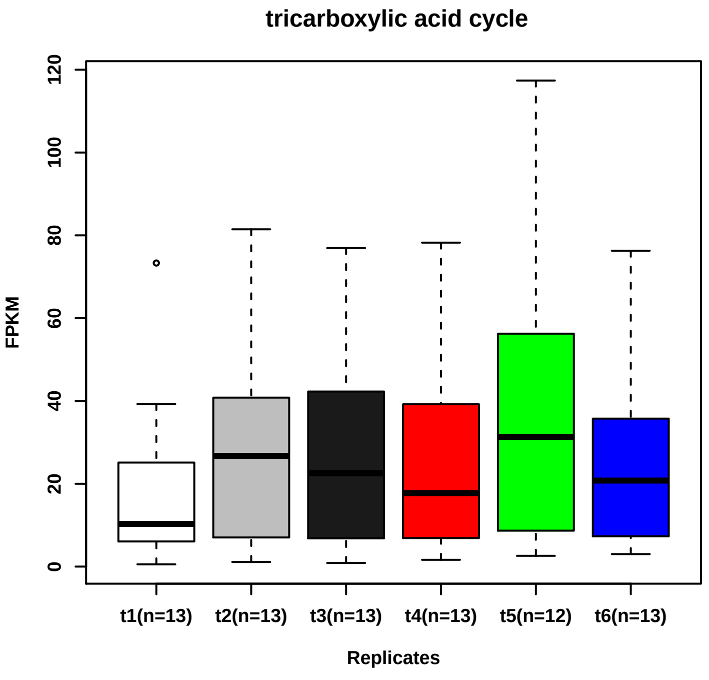
tricarboxylic acid cycle
FPKM
0
20
40
60
80
100
120
t1(n=13)
t2(n=13)
t3(n=13)
t4(n=13)
t5(n=12)
t6(n=13)
Replicates

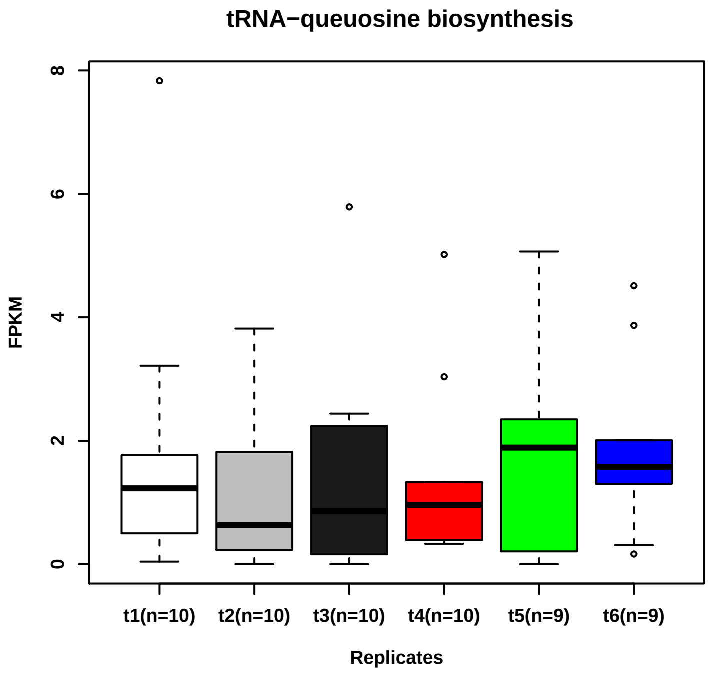
tRNA−queuosine biosynthesis
FPKM
0
2
4
6
8
t1(n=10)
t2(n=10)
t3(n=10)
t4(n=10)
t5(n=9)
t6(n=9)
Replicates

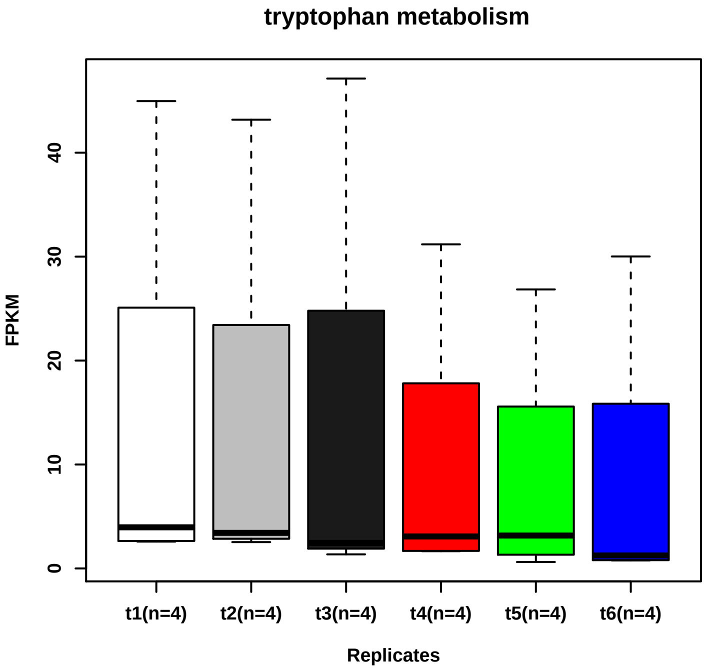
tryptophan metabolism
FPKM
0
10
20
30
40
t1(n=4)
t2(n=4)
t3(n=4)
t4(n=4)
t5(n=4)
t6(n=4)
Replicates

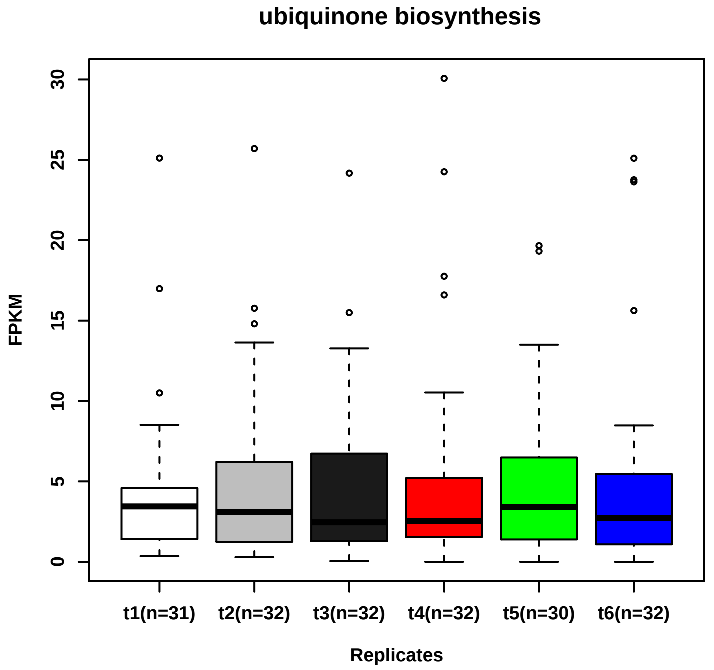
ubiquinone biosynthesis
FPKM
0
5
10
15
20
25
30
t1(n=31)
t2(n=32)
t3(n=32)
t4(n=32)
t5(n=30)
t6(n=32)
Replicates

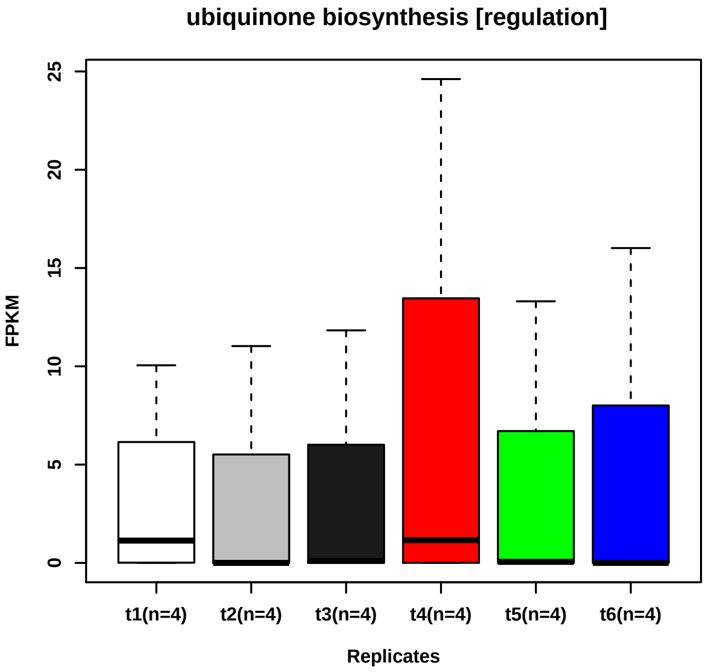
ubiquinone biosynthesis [regulation]
FPKM
0
5
10
15
20
25
t1(n=4)
t2(n=4)
t3(n=4)
t4(n=4)
t5(n=4)
t6(n=4)
Replicates

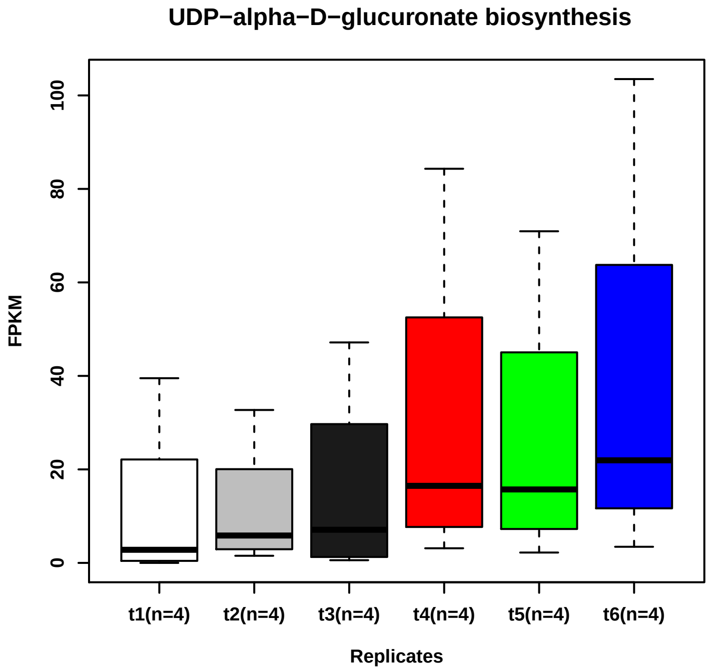
UDP−alpha−D−glucuronate biosynthesis
FPKM
0
20
40
60
80
100
t1(n=4)
t2(n=4)
t3(n=4)
t4(n=4)
t5(n=4)
t6(n=4)
Replicates

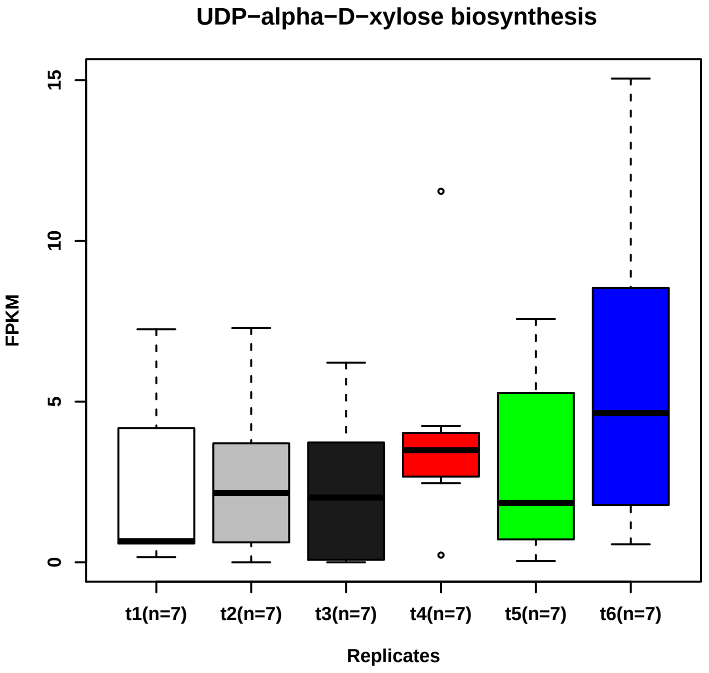
UDP−alpha−D−xylose biosynthesis
FPKM
0
5
10
15
t1(n=7)
t2(n=7)
t3(n=7)
t4(n=7)
t5(n=7)
t6(n=7)
Replicates

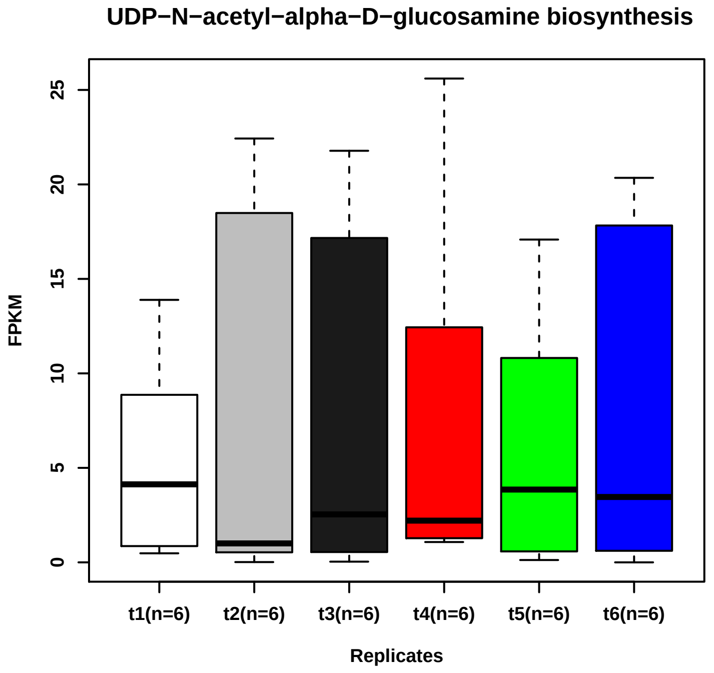
UDP−N−acetyl−alpha−D−glucosamine biosynthesis
FPKM
0
5
10
15
20
25
t1(n=6)
t2(n=6)
t3(n=6)
t4(n=6)
t5(n=6)
t6(n=6)
Replicates

UMP biosynthesis via de novo pathway

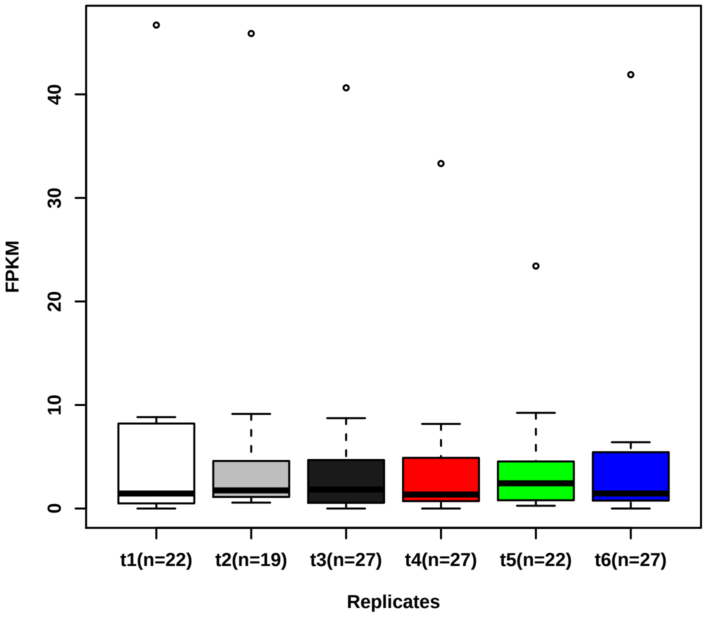

UMP biosynthesis via salvage pathway

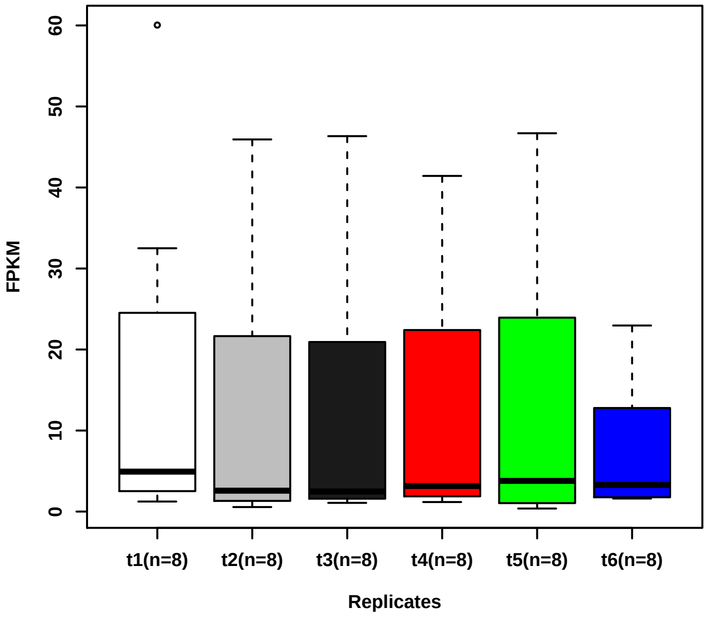

urate degradation

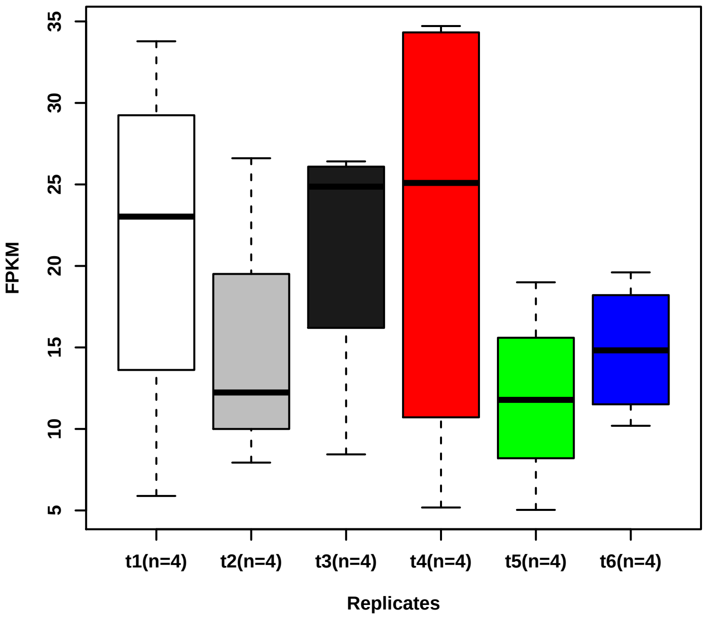

urea cycle

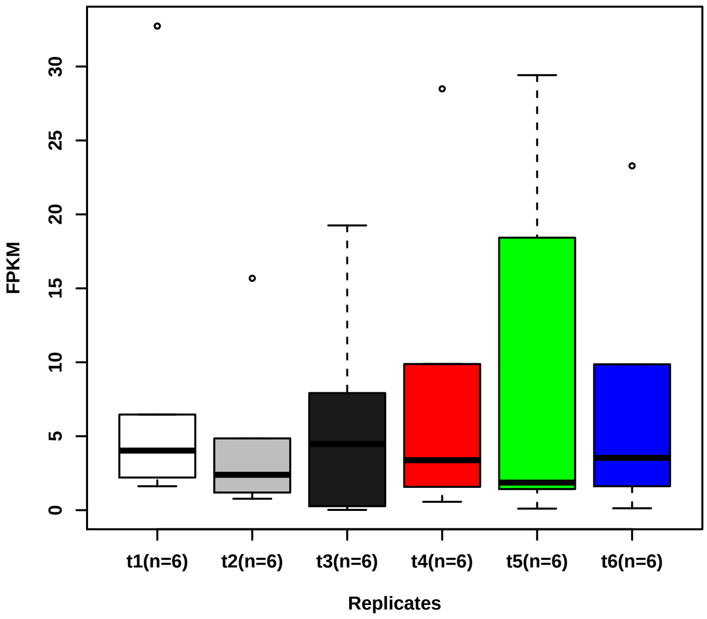

urea degradation

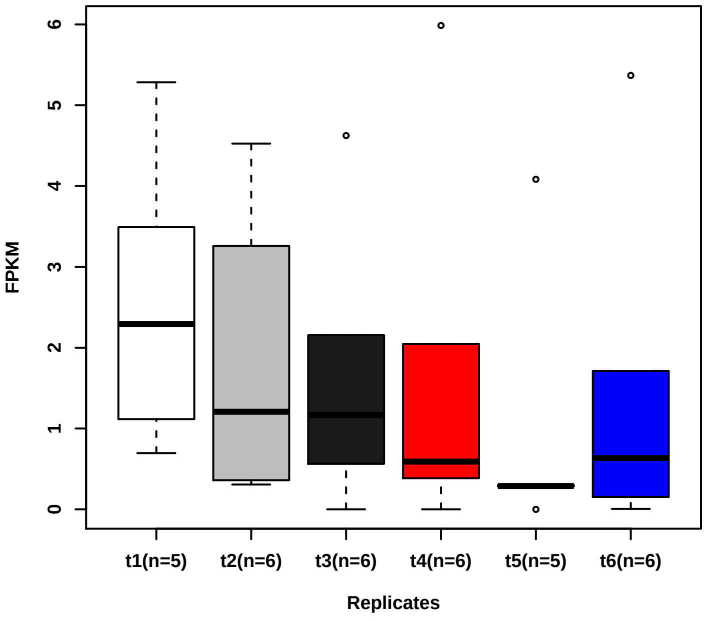

wybutosine−tRNA(Phe) biosynthesis

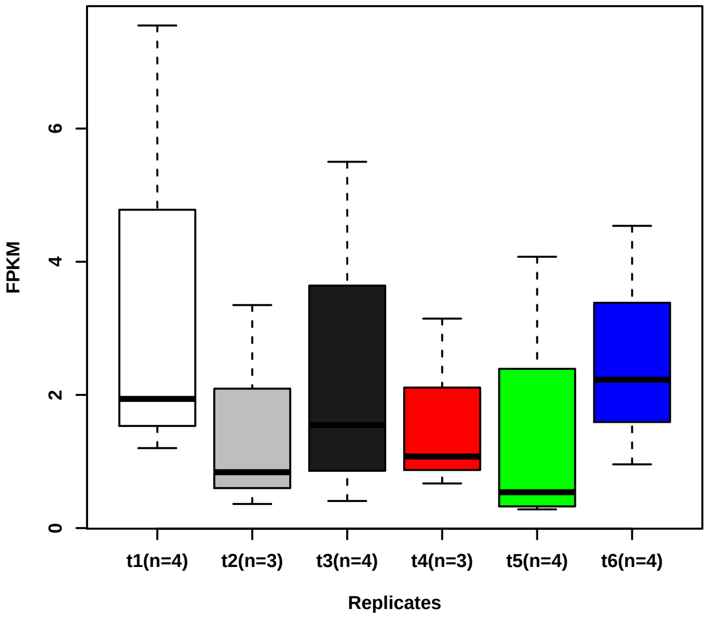

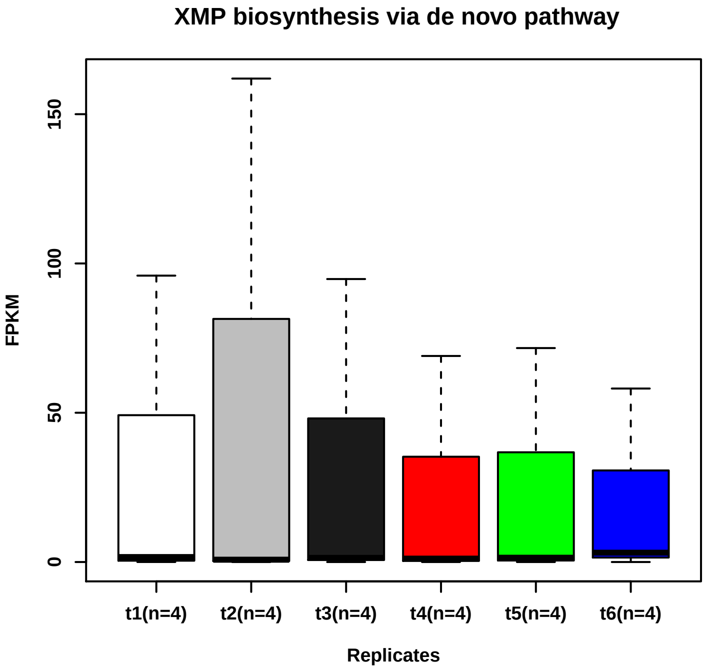
XMP biosynthesis via de novo pathway
FPKM
0
50
100
150
t1(n=4)
t2(n=4)
t3(n=4)
t4(n=4)
t5(n=4)
t6(n=4)
Replicates

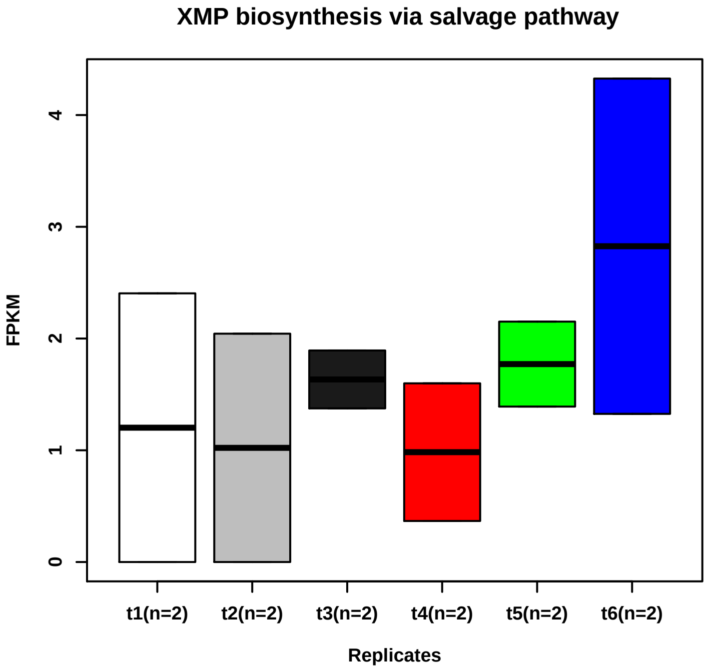
XMP biosynthesis via salvage pathway
FPKM
0
1
2
3
4
t1(n=2)
t2(n=2)
t3(n=2)
t4(n=2)
t5(n=2)
t6(n=2)
Replicates

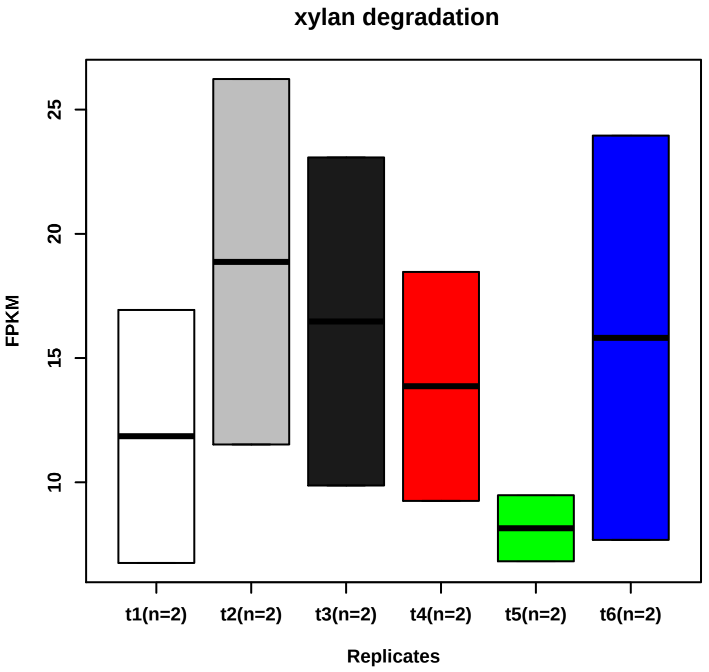
xylan degradation
FPKM
10
15
20
25
t1(n=2)
t2(n=2)
t3(n=2)
t4(n=2)
t5(n=2)
t6(n=2)
Replicates

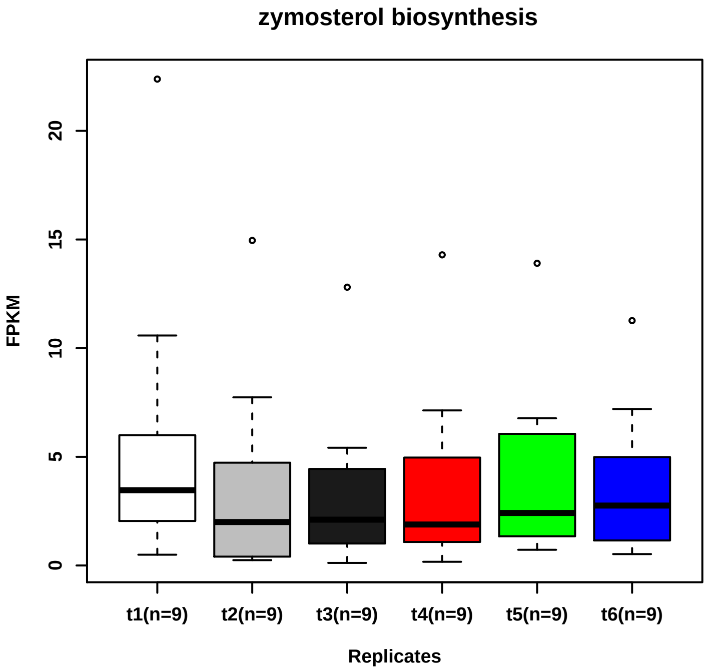
zymosterol biosynthesis
FPKM
0
5
10
15
20
t1(n=9)
t2(n=9)
t3(n=9)
t4(n=9)
t5(n=9)
t6(n=9)
Replicates

Unveiling the presence of biosynthetic pathways for bioactive compounds in the *Thalassiosira rotula* transcriptome

Valeria Di Dato*, Federica Di Costanzo, Roberta Barbarinaldi, Anna Perna, Adrianna Ianora, Giovanna Romano

Supplementary File 4:

Box Plot Analysis of the expression, in FPKM, of the pathways annotated on level 2 for each condition (mean of two replicates).

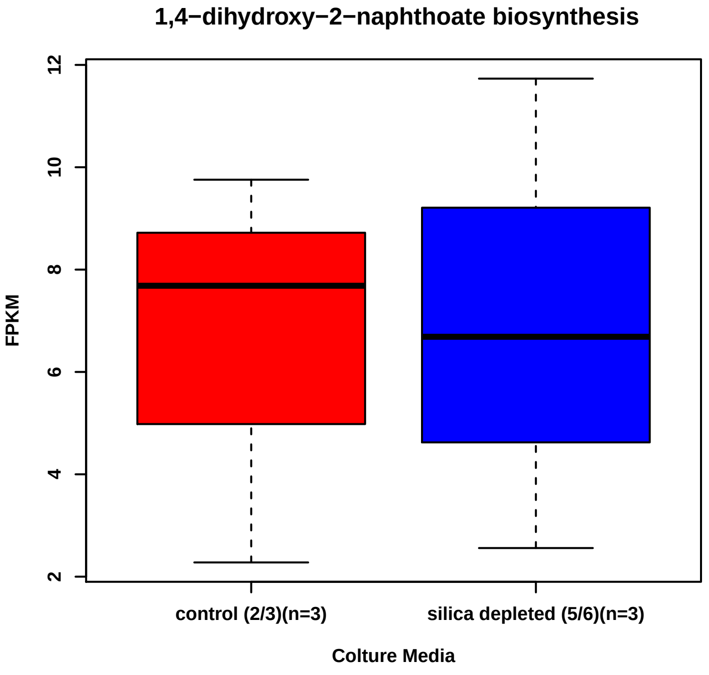
1,4−dihydroxy−2−naphthoate biosynthesis
FPKM
2
4
6
8
10
12
control (2/3)(n=3)
silica depleted (5/6)(n=3)
Colture Media

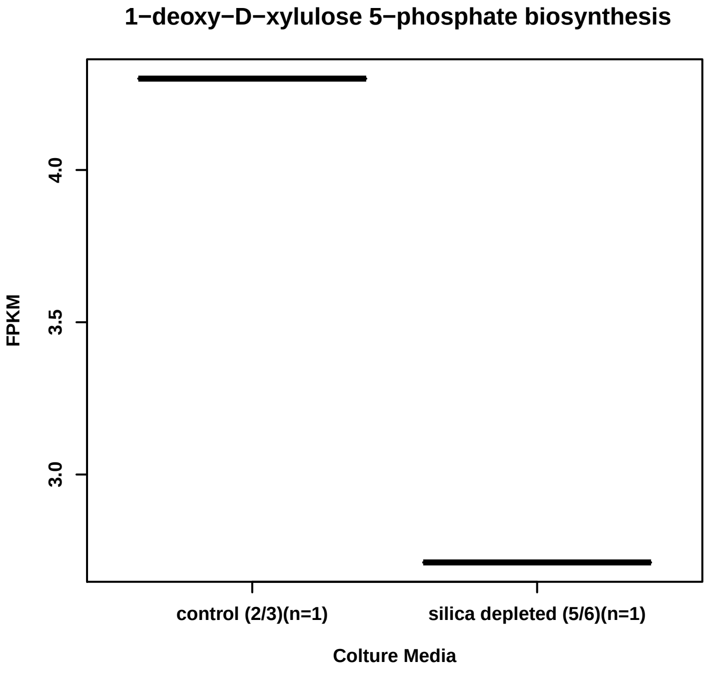
1−deoxy−D−xylulose 5−phosphate biosynthesis
FPKM
3.0
3.5
4.0
control (2/3)(n=1)
silica depleted (5/6)(n=1)
Colture Media

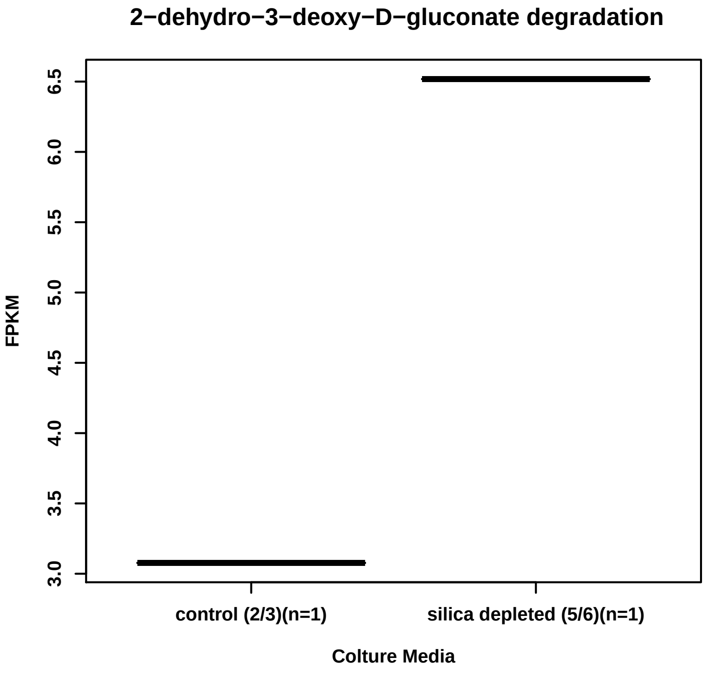
2−dehydro−3−deoxy−D−gluconate degradation
FPKM
3.0
3.5
4.0
4.5
5.0
5.5
6.0
6.5
control (2/3)(n=1)
silica depleted (5/6)(n=1)
Colture Media

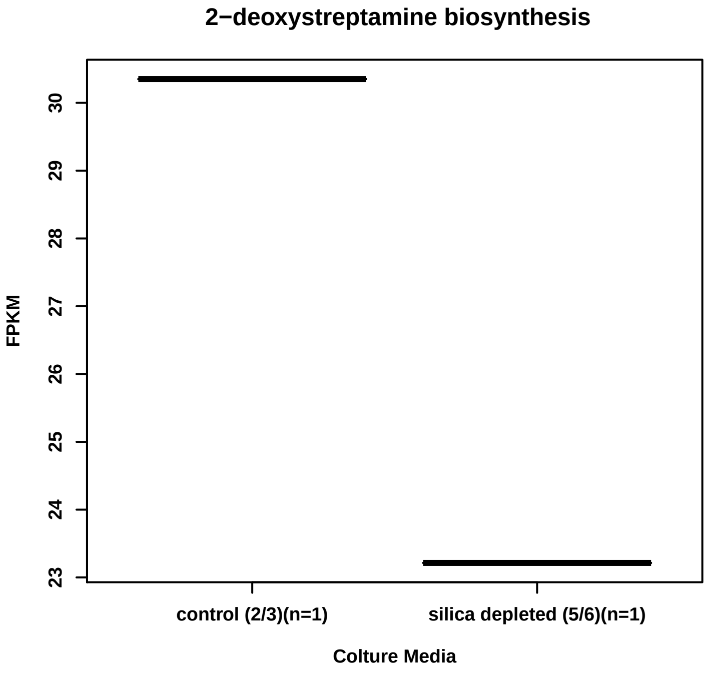
2−deoxystreptamine biosynthesis
FPKM
23
24
25
26
27
28
29
30
control (2/3)(n=1)
silica depleted (5/6)(n=1)
Colture Media

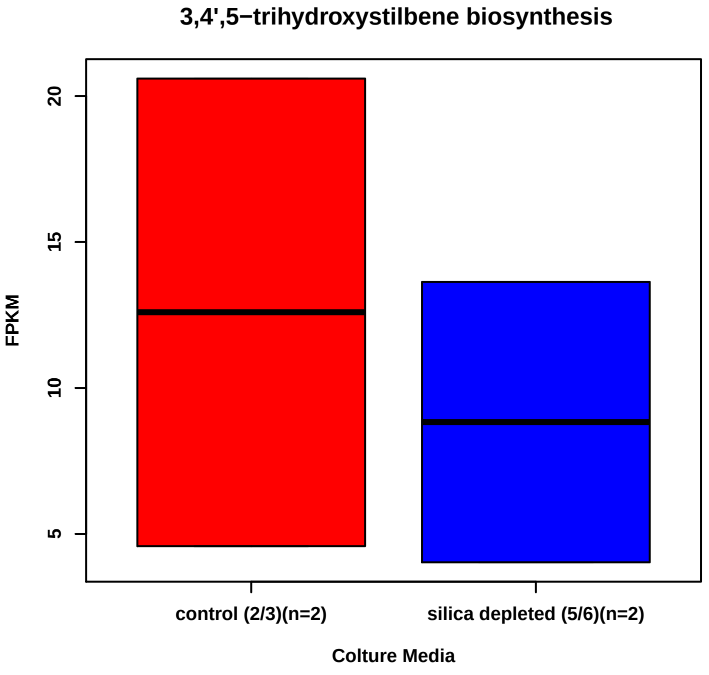
3,4',5−trihydroxystilbene biosynthesis
FPKM
5
10
15
20
control (2/3)(n=2)
silica depleted (5/6)(n=2)
Colture Media

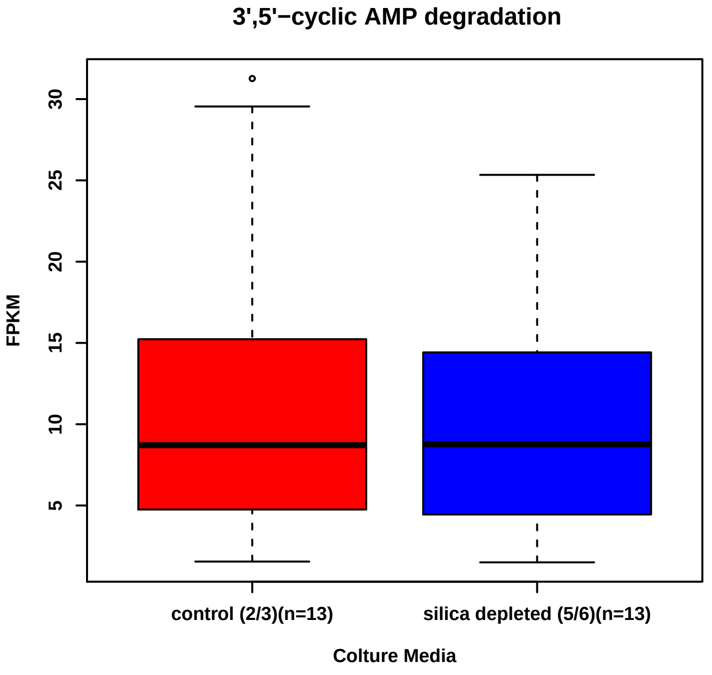
3',5'−cyclic AMP degradation
FPKM
5
10
15
20
25
30
control (2/3)(n=13)
silica depleted (5/6)(n=13)
Colture Media

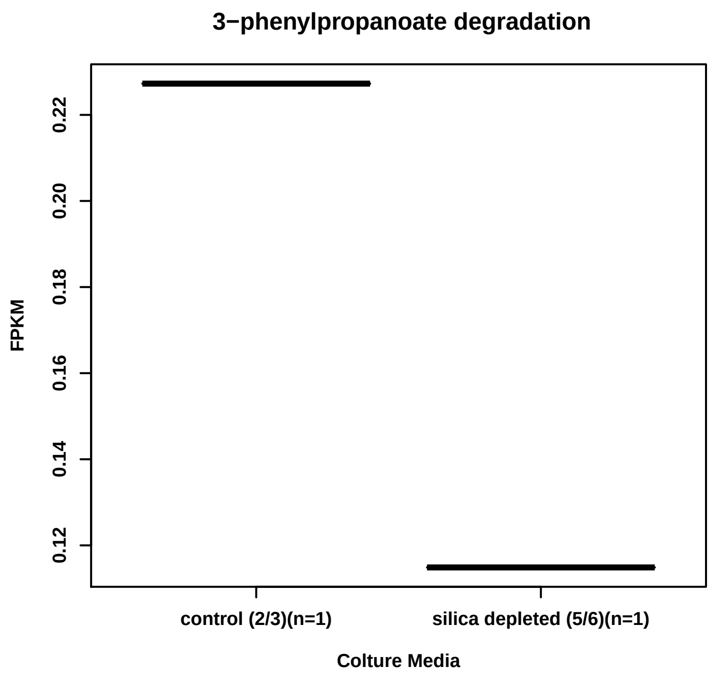
3−phenylpropanoate degradation
FPKM
0.12
0.14
0.16
0.18
0.20
0.22
control (2/3)(n=1)
silica depleted (5/6)(n=1)
Colture Media

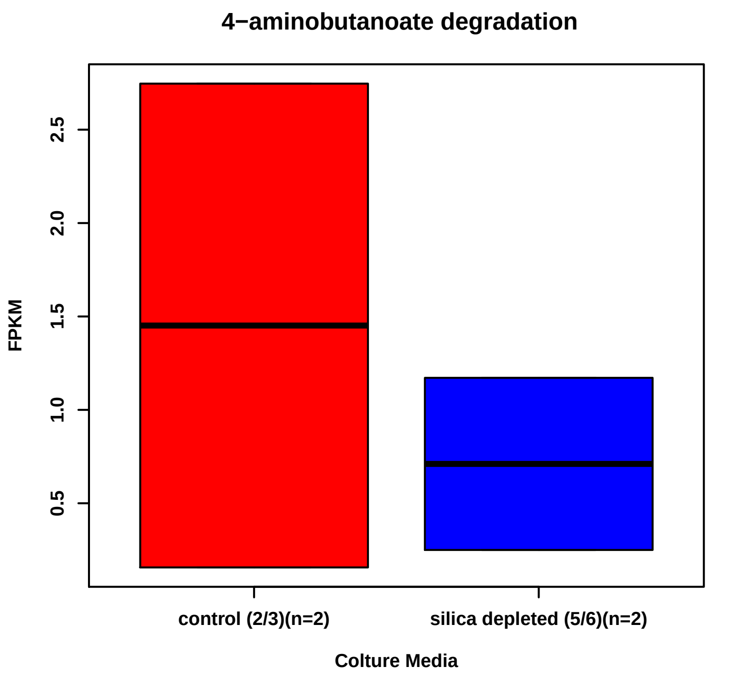
4−aminobutanoate degradation
FPKM
0.5
1.0
1.5
2.0
2.5
control (2/3)(n=2)
silica depleted (5/6)(n=2)
Colture Media

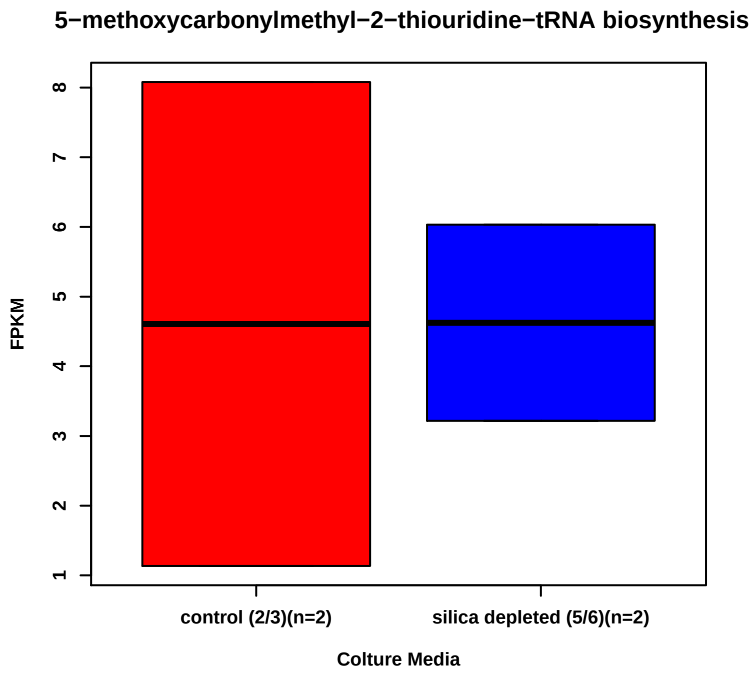
5−methoxycarbonylmethyl−2−thiouridine−tRNA biosynthesis
FPKM
1
2
3
4
5
6
7
8
control (2/3)(n=2)
silica depleted (5/6)(n=2)
Colture Media

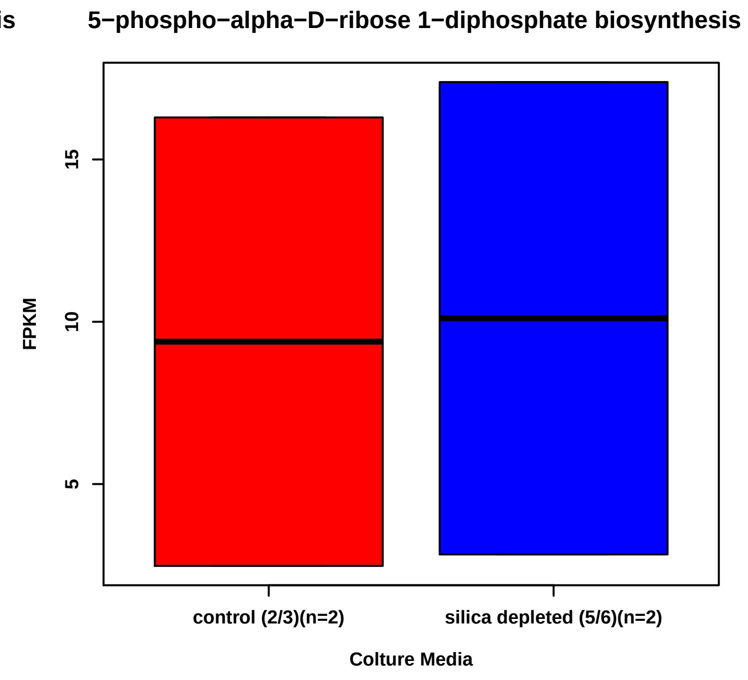
5−phospho−alpha−D−ribose 1−diphosphate biosynthesis
FPKM
5
10
15
control (2/3)(n=2)
silica depleted (5/6)(n=2)
Colture Media

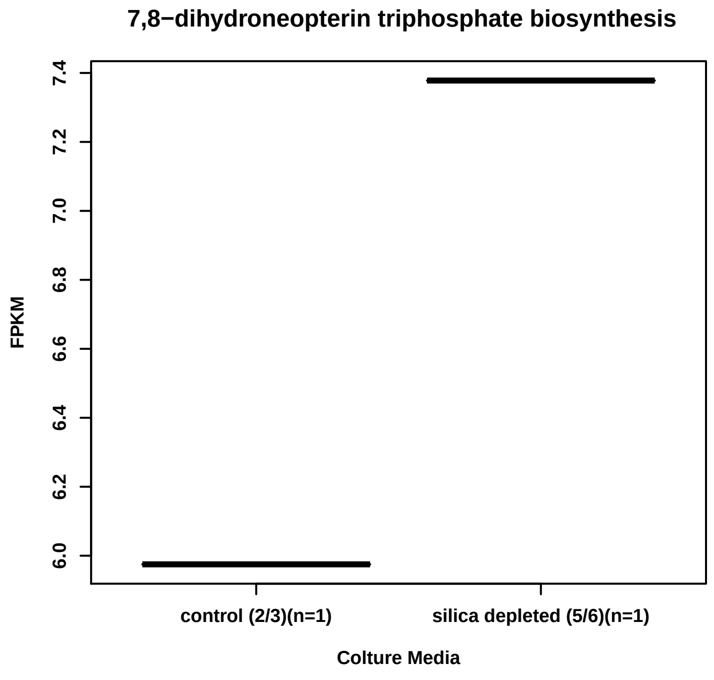
7,8−dihydroneopterin triphosphate biosynthesis
FPKM
6.0
6.2
6.4
6.6
6.8
7.0
7.2
7.4
control (2/3)(n=1)
silica depleted (5/6)(n=1)
Colture Media

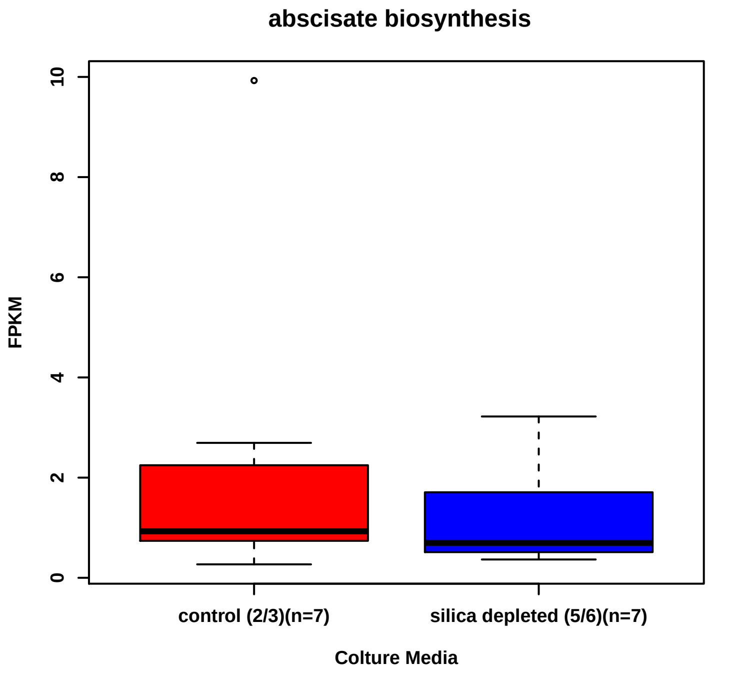
abscisate biosynthesis
FPKM
0
2
4
6
8
10
control (2/3)(n=7)
silica depleted (5/6)(n=7)
Colture Media

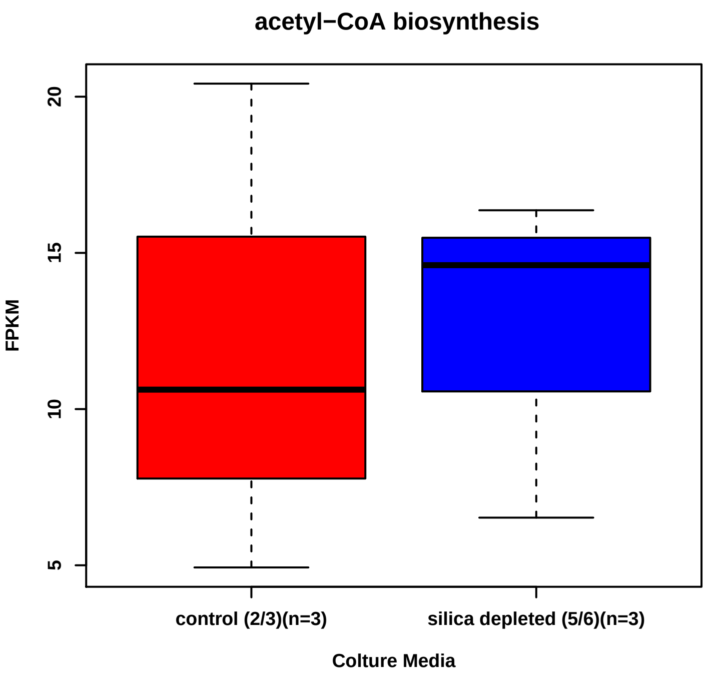
acetyl−CoA biosynthesis
FPKM
5
10
15
20
control (2/3)(n=3)
silica depleted (5/6)(n=3)
Colture Media

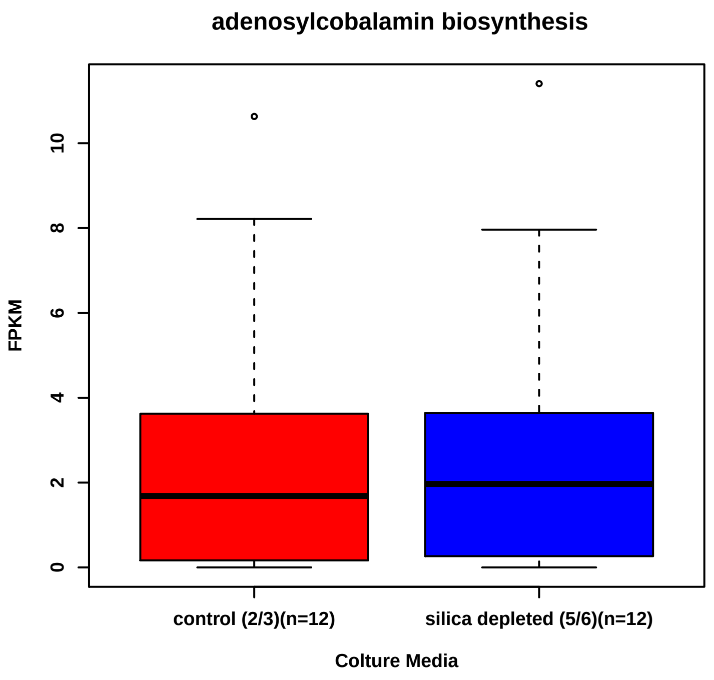
adenosylcobalamin biosynthesis
FPKM
0
2
4
6
8
10
control (2/3)(n=12)
silica depleted (5/6)(n=12)
Colture Media

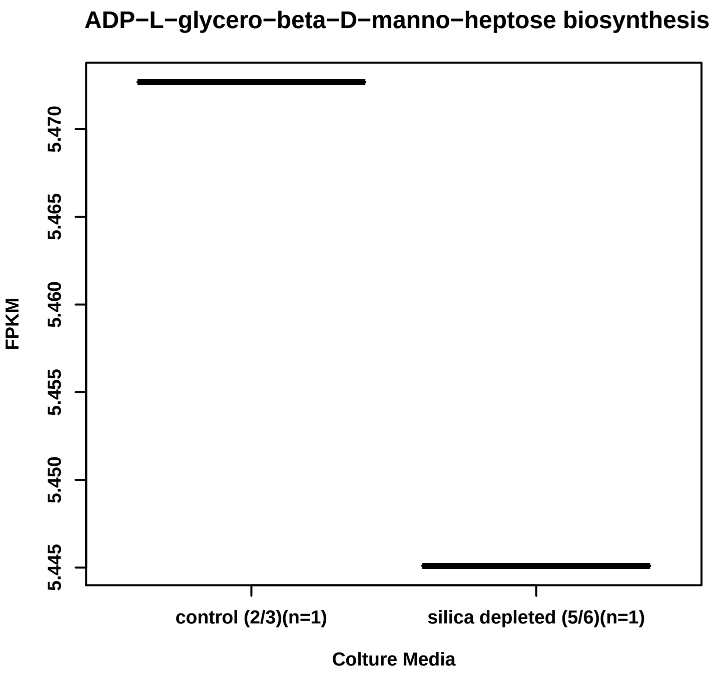
ADP−L−glycero−beta−D−manno−heptose biosynthesis
FPKM
5.445
5.450
5.455
5.460
5.465
5.470
control (2/3)(n=1)
silica depleted (5/6)(n=1)
Colture Media

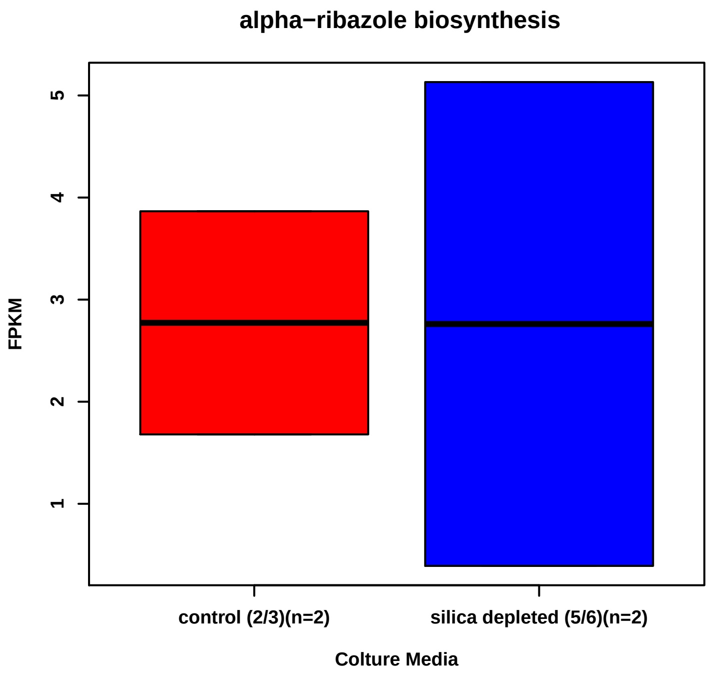
alpha−ribazole biosynthesis
FPKM
1
2
3
4
5
control (2/3)(n=2)
silica depleted (5/6)(n=2)
Colture Media

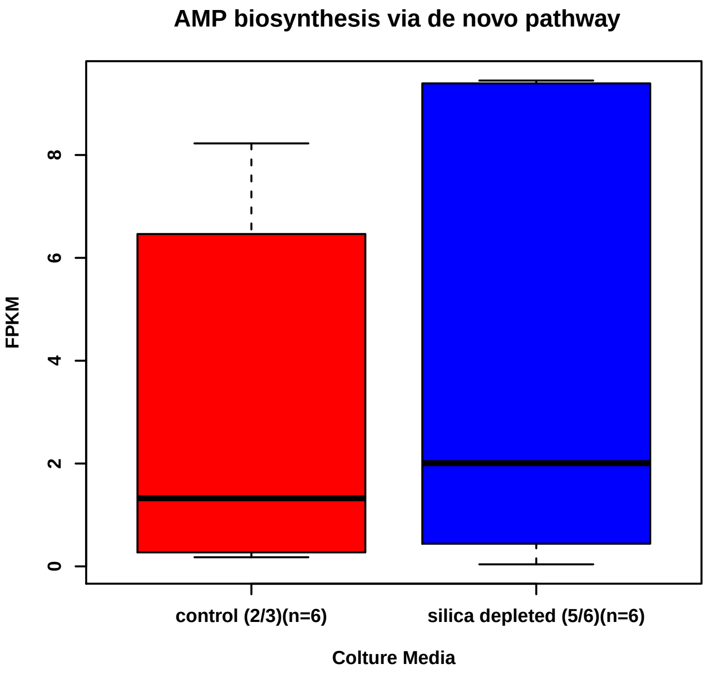
AMP biosynthesis via de novo pathway
FPKM
0
2
4
6
8
control (2/3)(n=6)
silica depleted (5/6)(n=6)
Colture Media

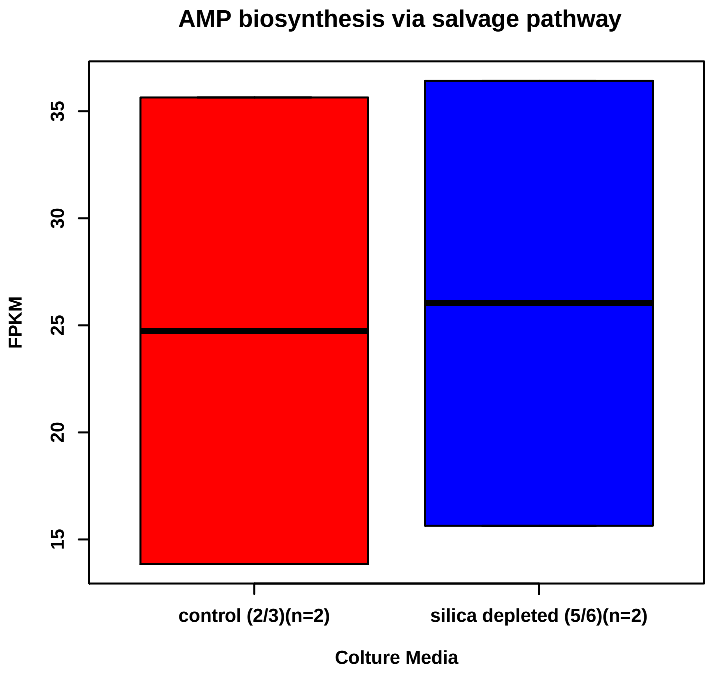
AMP biosynthesis via salvage pathway
FPKM
15
20
25
30
35
control (2/3)(n=2)
silica depleted (5/6)(n=2)
Colture Media

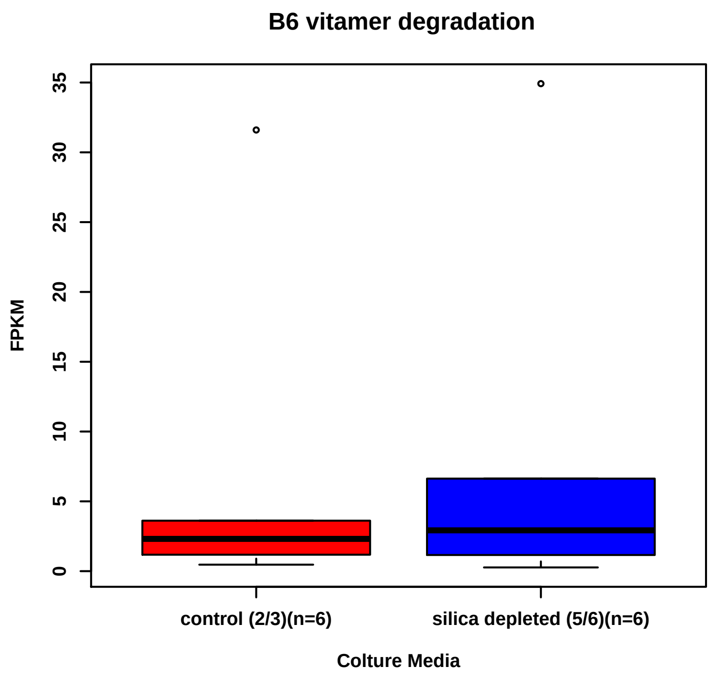
B6 vitamer degradation
FPKM
0
5
10
15
20
25
30
35
control (2/3)(n=6)
silica depleted (5/6)(n=6)
Colture Media

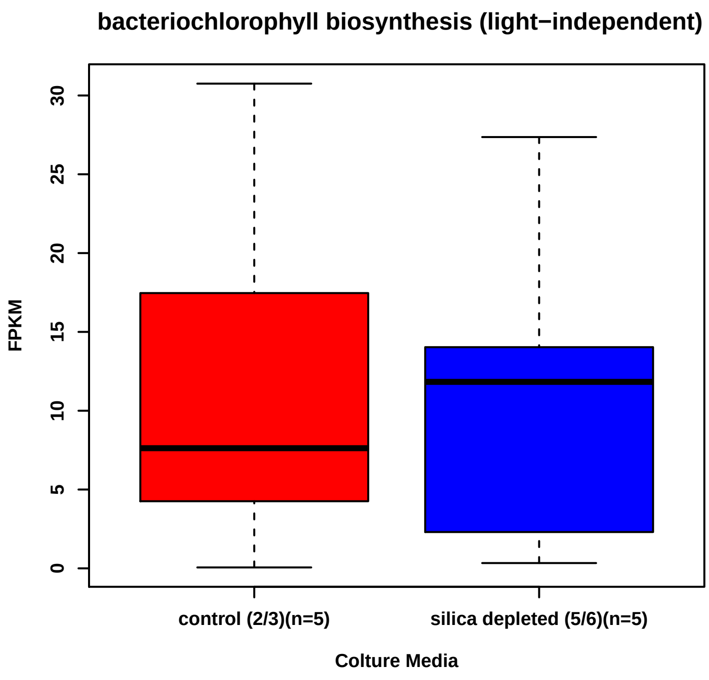
bacteriochlorophyll biosynthesis (light−independent)
FPKM
0
5
10
15
20
25
30
control (2/3)(n=5)
silica depleted (5/6)(n=5)
Colture Media

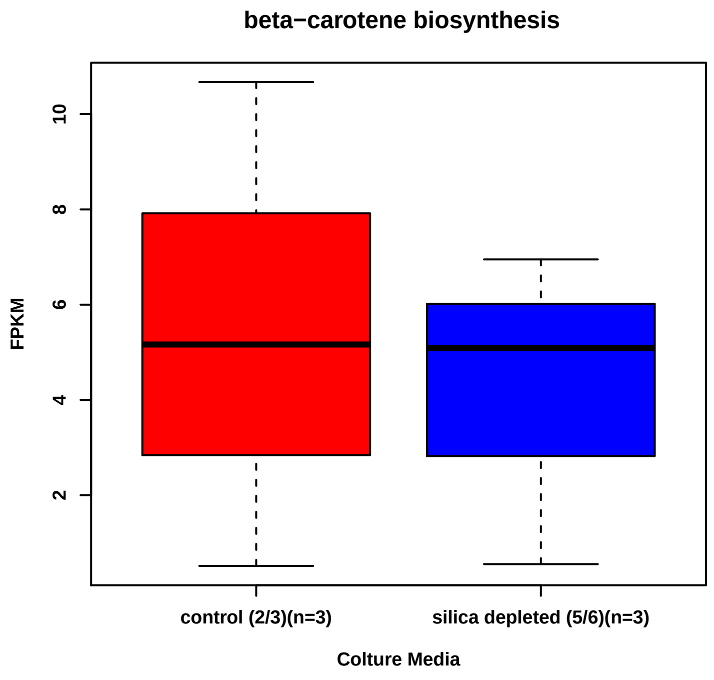
beta−carotene biosynthesis
FPKM
2
4
6
8
10
control (2/3)(n=3)
silica depleted (5/6)(n=3)
Colture Media

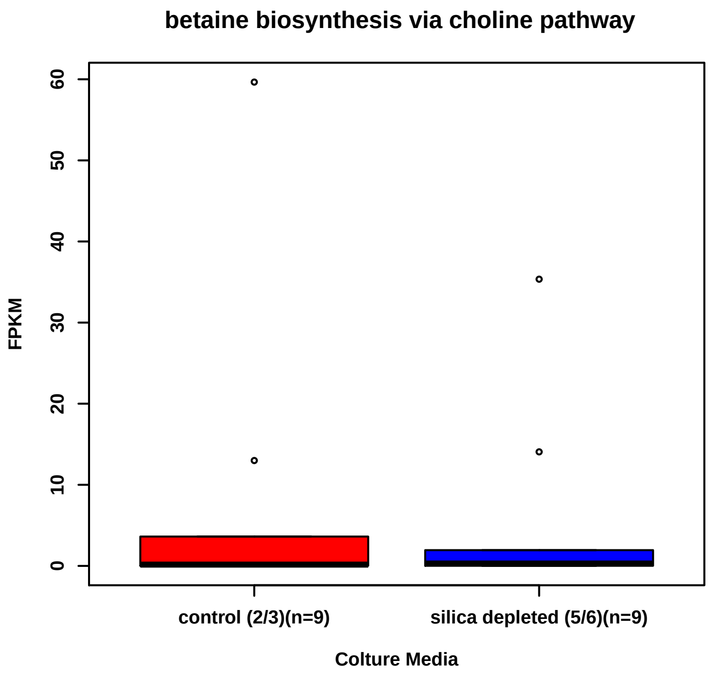
betaine biosynthesis via choline pathway
FPKM
0
10
20
30
40
50
60
control (2/3)(n=9)
silica depleted (5/6)(n=9)
Colture Media

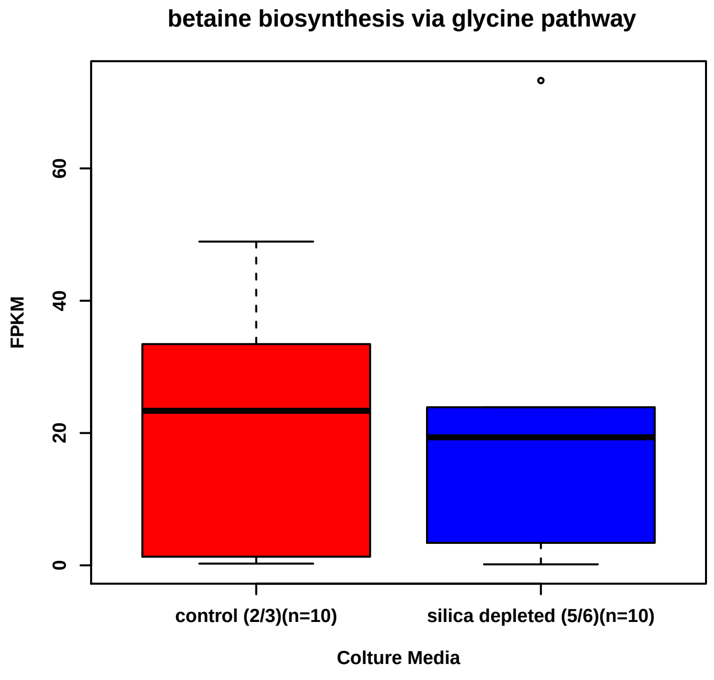
betaine biosynthesis via glycine pathway
FPKM
0
20
40
60
control (2/3)(n=10)
silica depleted (5/6)(n=10)
Colture Media

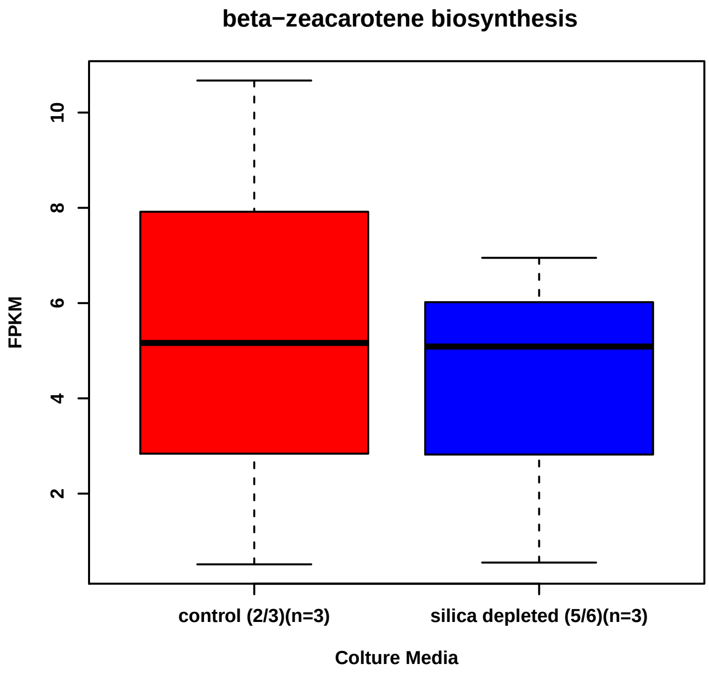
beta−zeacarotene biosynthesis
FPKM
2
4
6
8
10
control (2/3)(n=3)
silica depleted (5/6)(n=3)
Colture Media

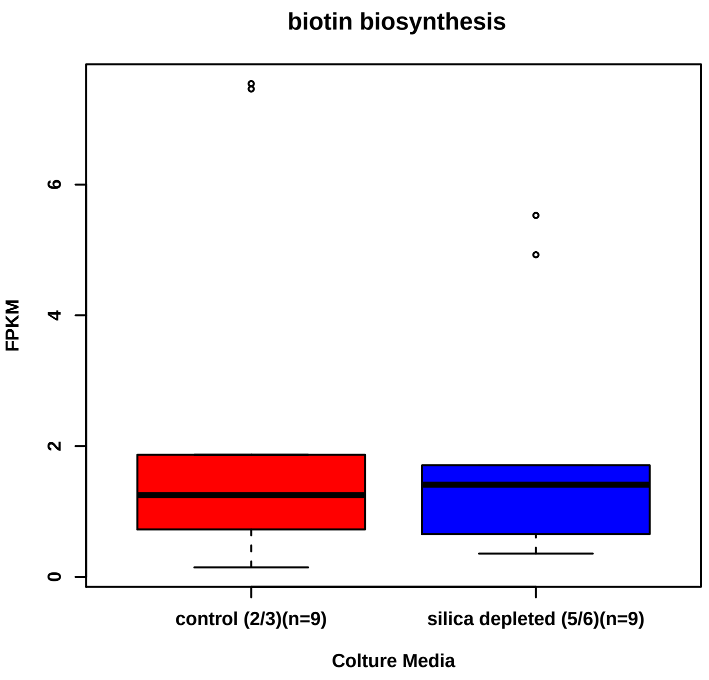

biotin biosynthesis
FPKM
0
2
4
6
8
control (2/3)(n=9)
silica depleted (5/6)(n=9)
Colture Media

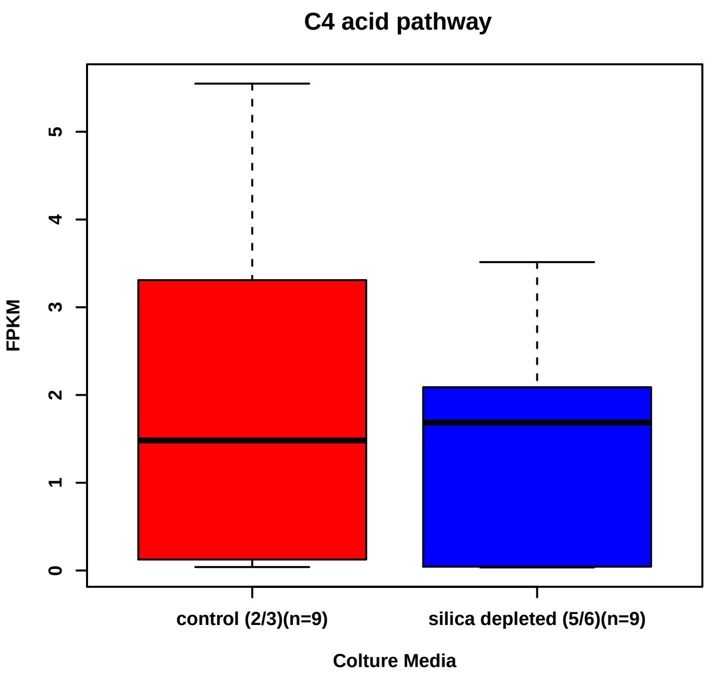

C4 acid pathway
FPKM
0
1
2
3
4
5
control (2/3)(n=9)
silica depleted (5/6)(n=9)
Colture Media

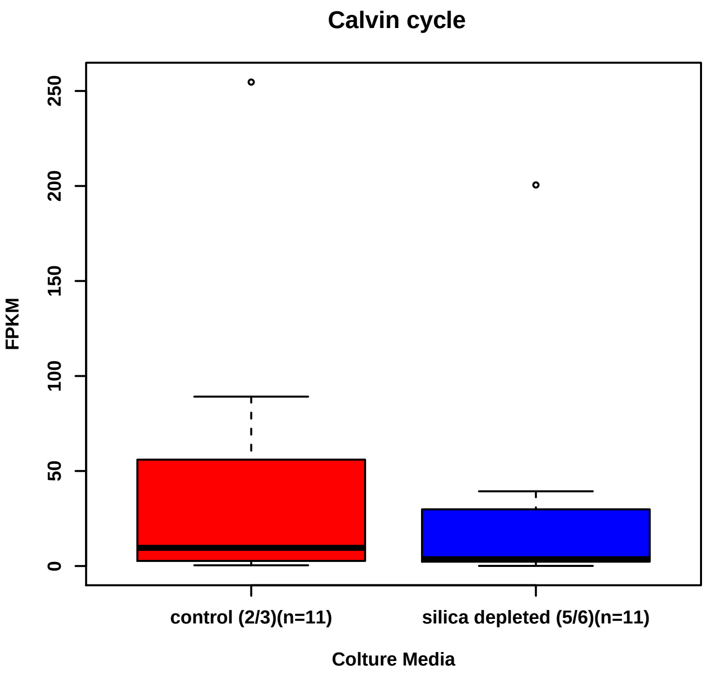

Calvin cycle
FPKM
0
50
100
150
200
250
control (2/3)(n=11)
silica depleted (5/6)(n=11)
Colture Media

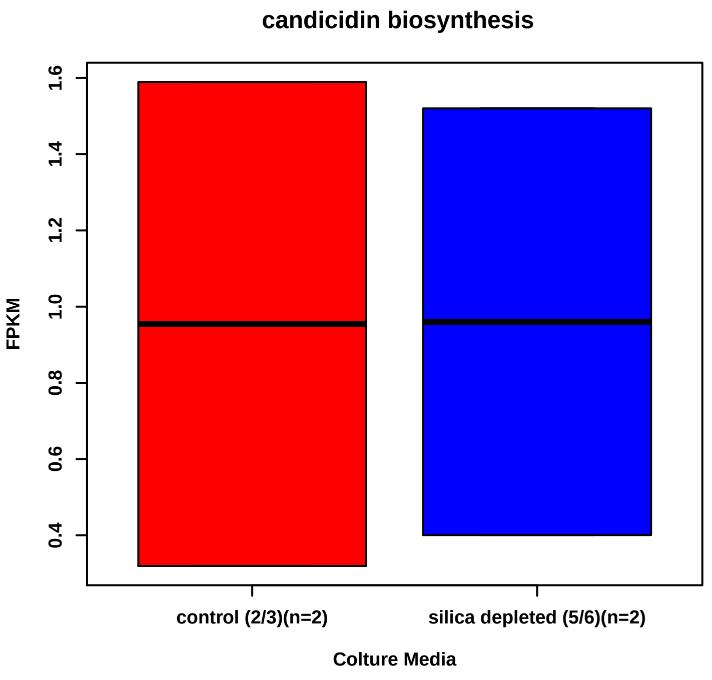

candicidin biosynthesis
FPKM
0.4
0.6
0.8
1.0
1.2
1.4
1.6
control (2/3)(n=2)
silica depleted (5/6)(n=2)
Colture Media

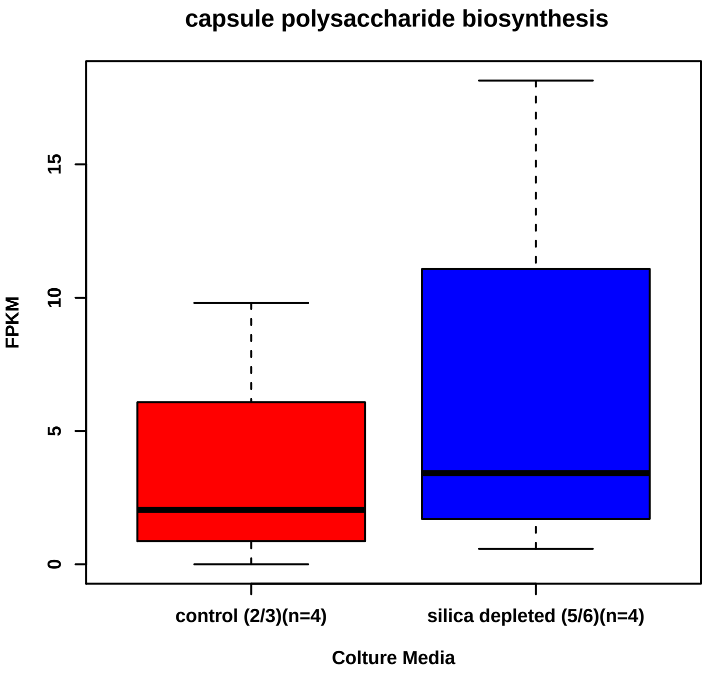

capsule polysaccharide biosynthesis
FPKM
0
5
10
15
control (2/3)(n=4)
silica depleted (5/6)(n=4)
Colture Media

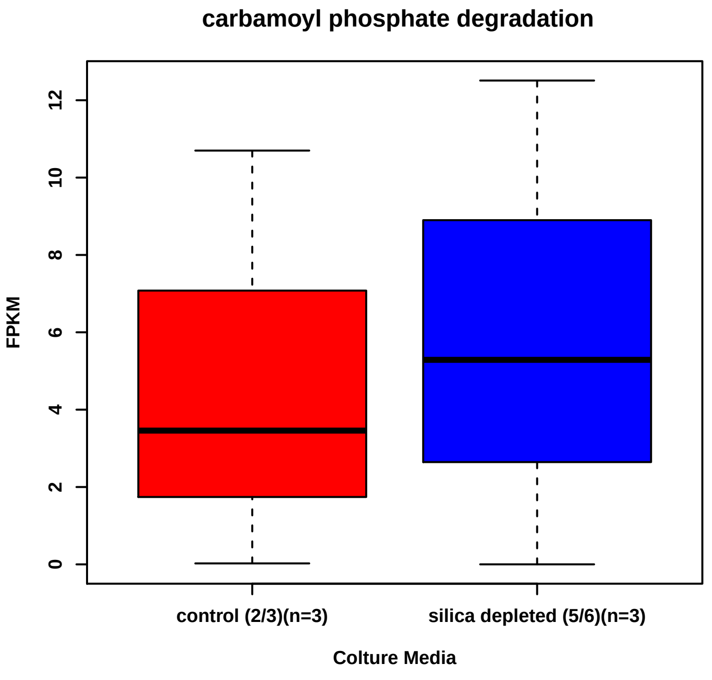

carbamoyl phosphate degradation
FPKM
0
2
4
6
8
10
12
control (2/3)(n=3)
silica depleted (5/6)(n=3)
Colture Media

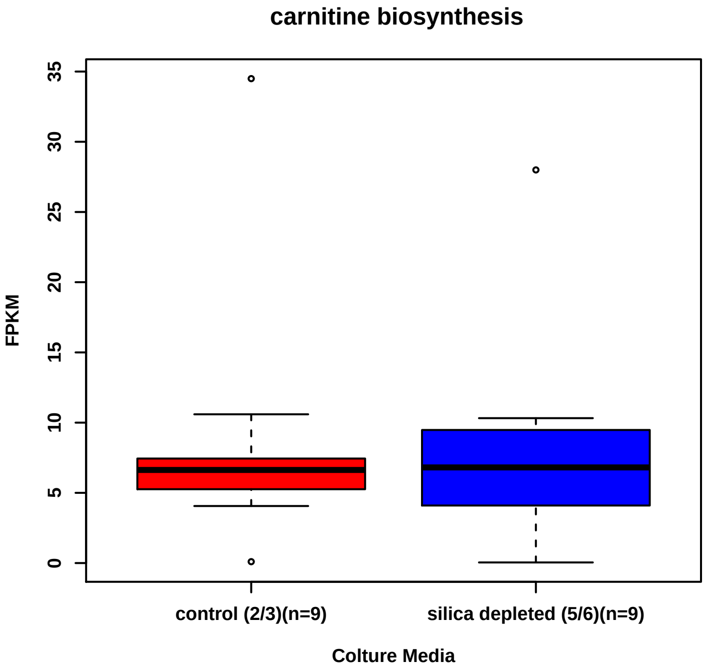
carnitine biosynthesis
FPKM
0
5
10
15
20
25
30
35
control (2/3)(n=9)
silica depleted (5/6)(n=9)
Colture Media

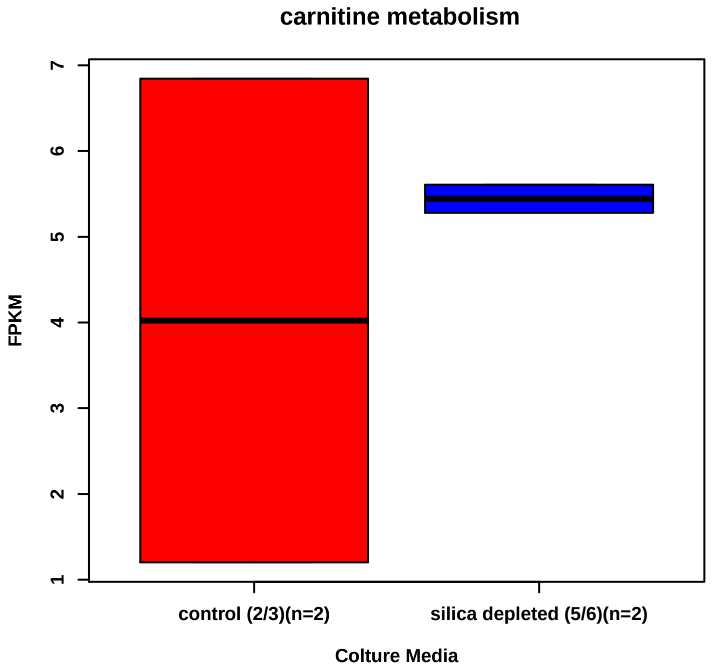
carnitine metabolism
FPKM
1
2
3
4
5
6
7
control (2/3)(n=2)
silica depleted (5/6)(n=2)
Colture Media

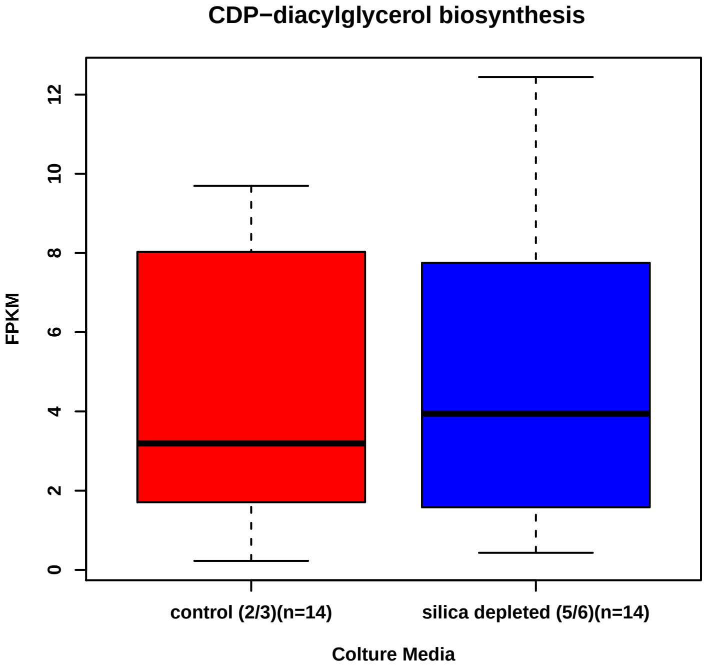
CDP−diacylglycerol biosynthesis
FPKM
0
2
4
6
8
10
12
control (2/3)(n=14)
silica depleted (5/6)(n=14)
Colture Media

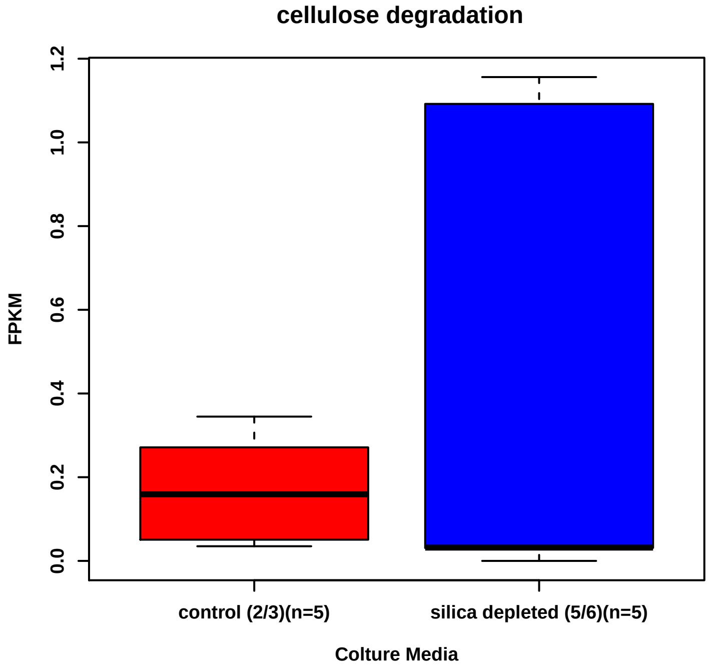
cellulose degradation
FPKM
0.0
0.2
0.4
0.6
0.8
1.0
1.2
control (2/3)(n=5)
silica depleted (5/6)(n=5)
Colture Media

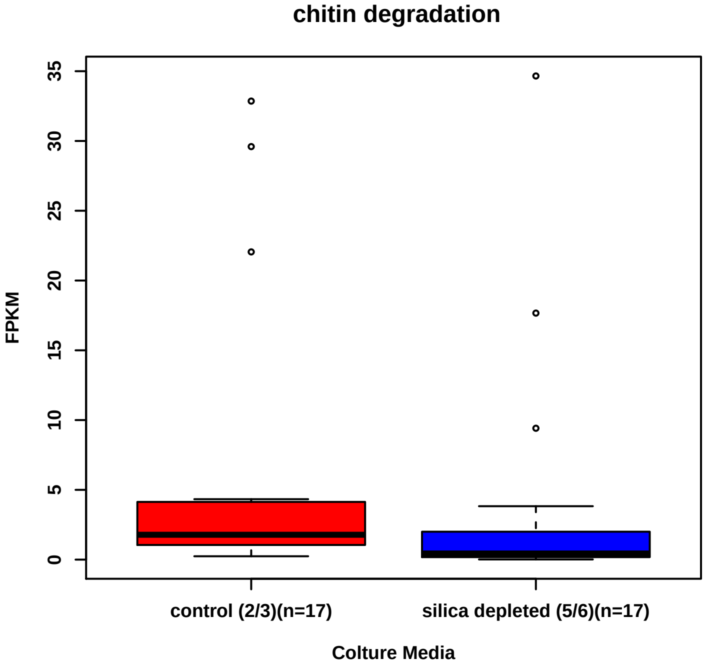
chitin degradation
FPKM
0
5
10
15
20
25
30
35
control (2/3)(n=17)
silica depleted (5/6)(n=17)
Colture Media

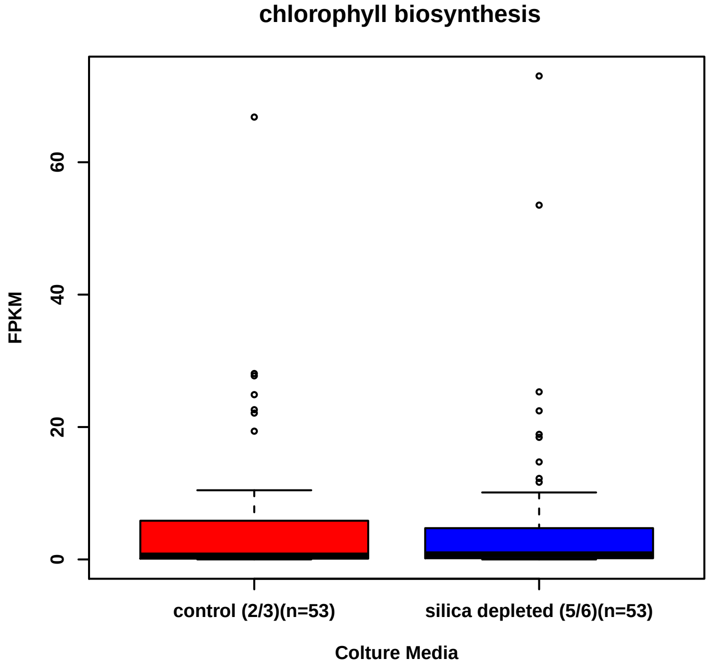
chlorophyll biosynthesis
FPKM
0
20
40
60
control (2/3)(n=53)
silica depleted (5/6)(n=53)
Colture Media

chlorophyll degradation

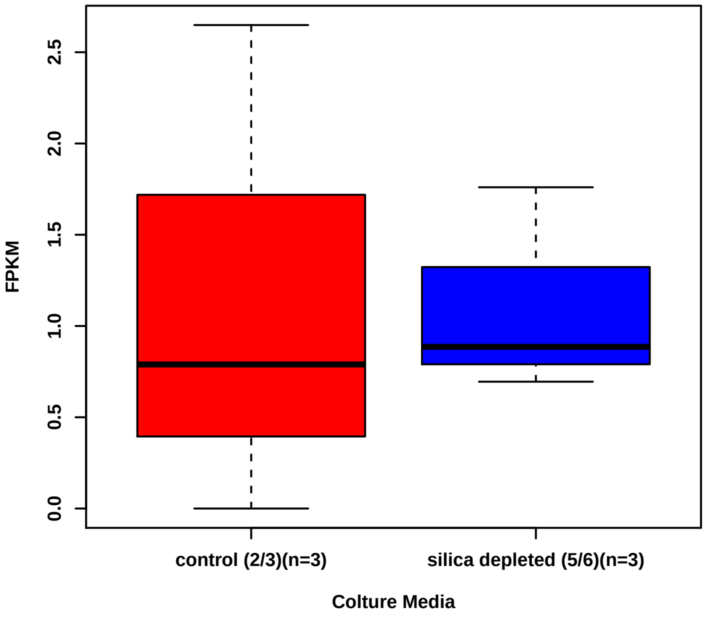

cholecalciferol biosynthesis

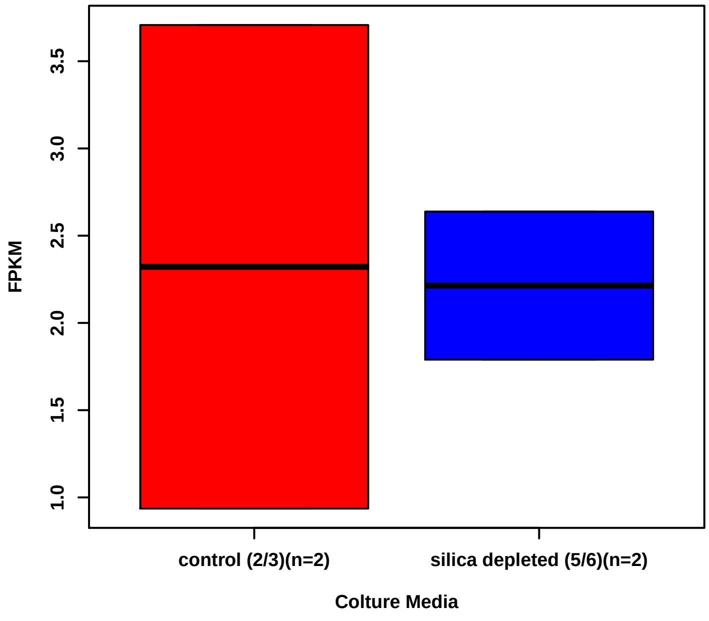

chondroitin sulfate biosynthesis

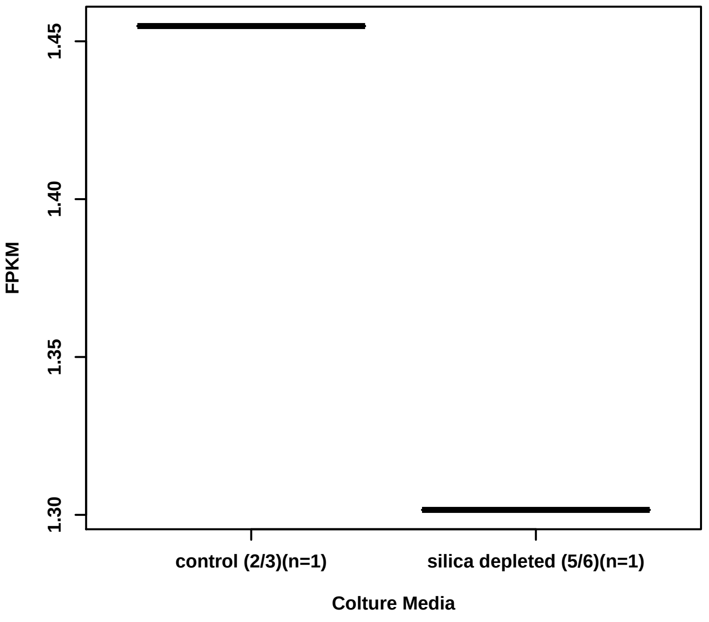

chorismate biosynthesis

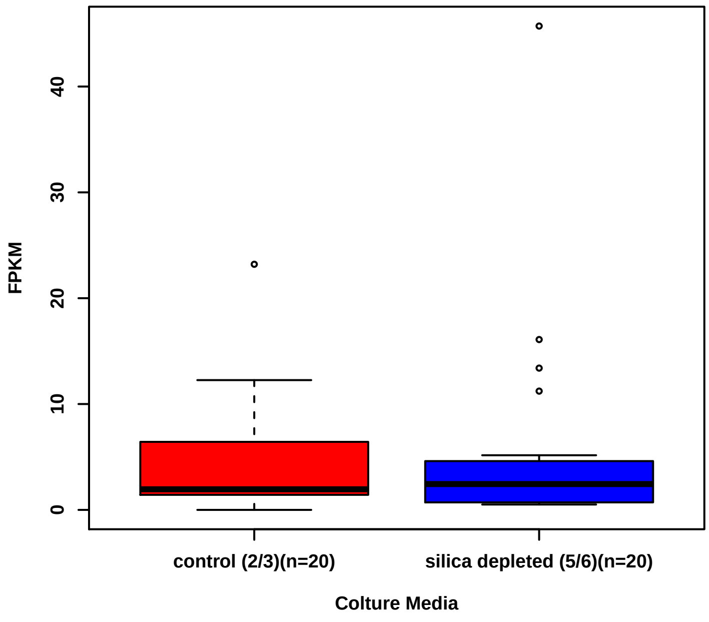

cocaine degradation

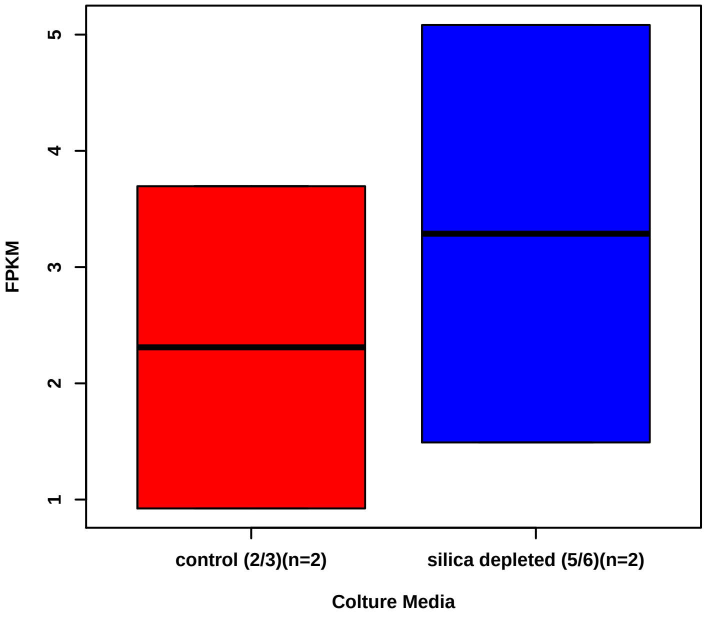

coenzyme A biosynthesis

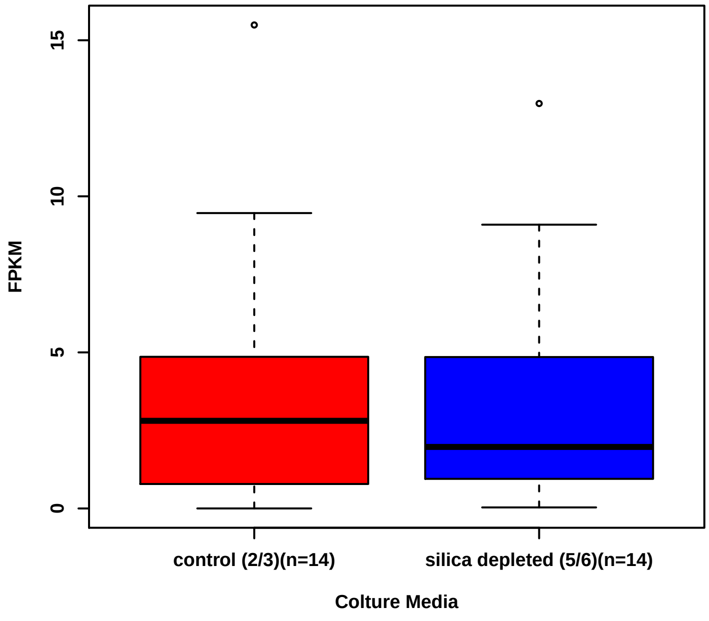

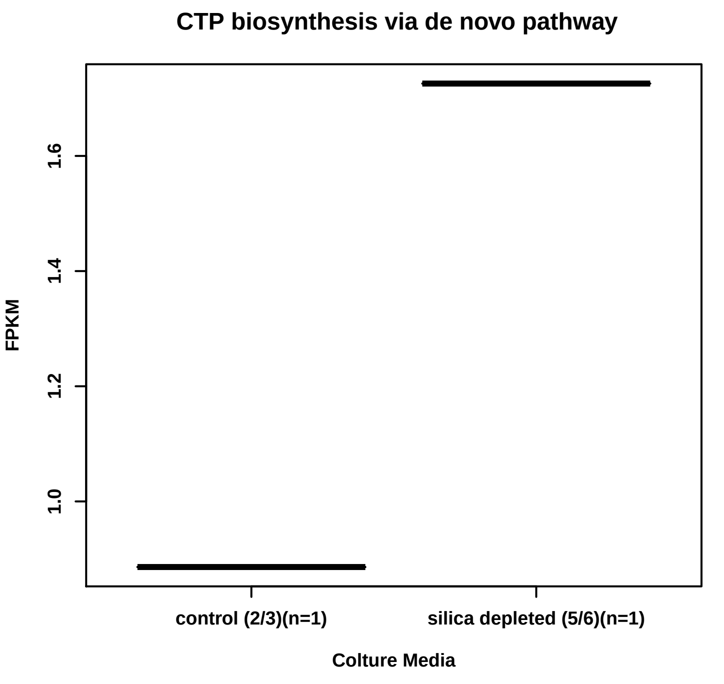
CTP biosynthesis via de novo pathway
FPKM
1.0
1.2
1.4
1.6
control (2/3)(n=1)
silica depleted (5/6)(n=1)
Colture Media

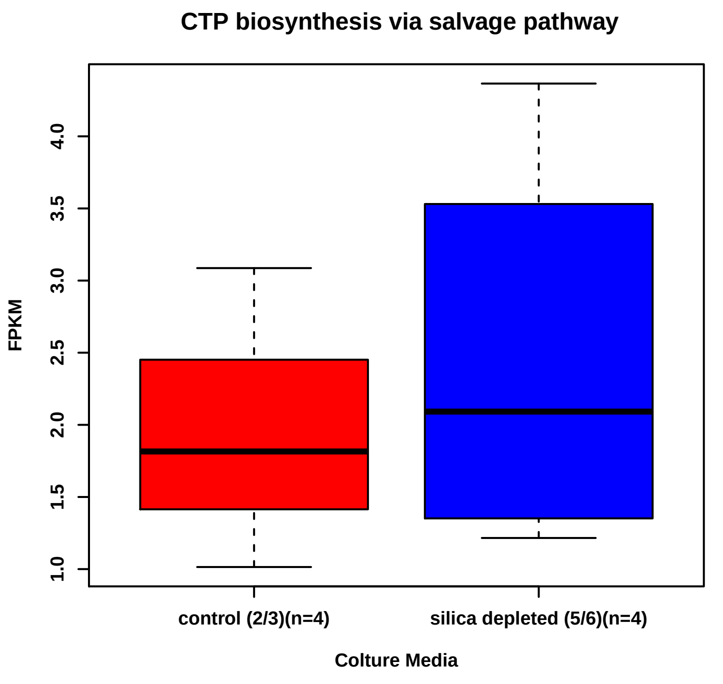
CTP biosynthesis via salvage pathway
FPKM
1.0
1.5
2.0
2.5
3.0
3.5
4.0
control (2/3)(n=4)
silica depleted (5/6)(n=4)
Colture Media

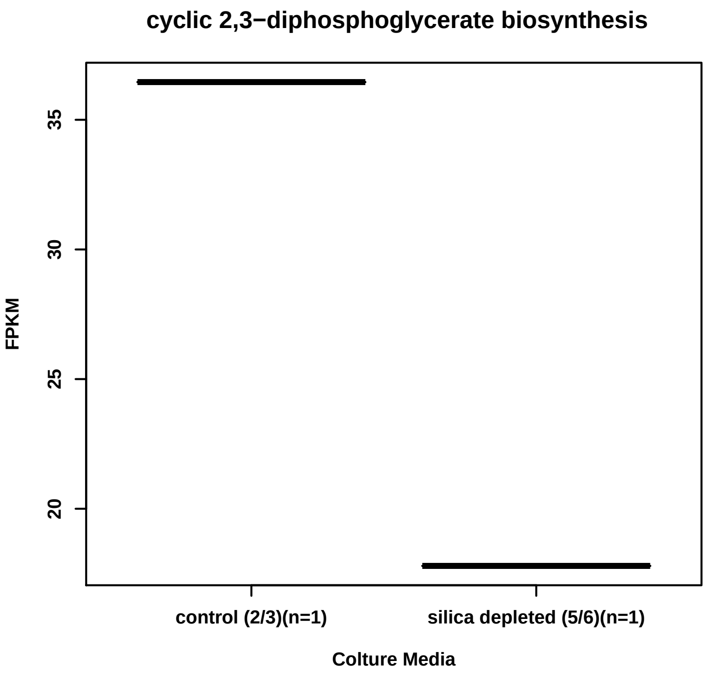
cyclic 2,3−diphosphoglycerate biosynthesis
FPKM
20
25
30
35
control (2/3)(n=1)
silica depleted (5/6)(n=1)
Colture Media

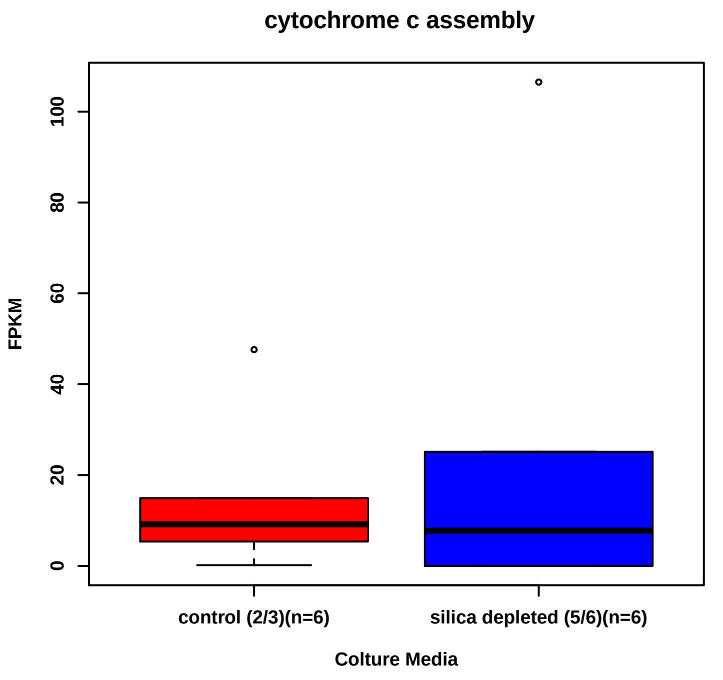
cytochrome c assembly
FPKM
0
20
40
60
80
100
control (2/3)(n=6)
silica depleted (5/6)(n=6)
Colture Media

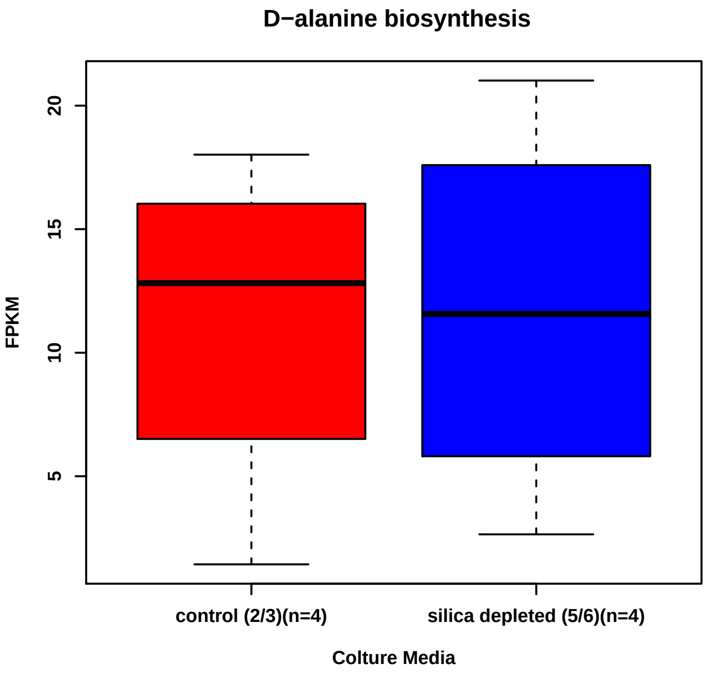
D−alanine biosynthesis
FPKM
5
10
15
20
control (2/3)(n=4)
silica depleted (5/6)(n=4)
Colture Media

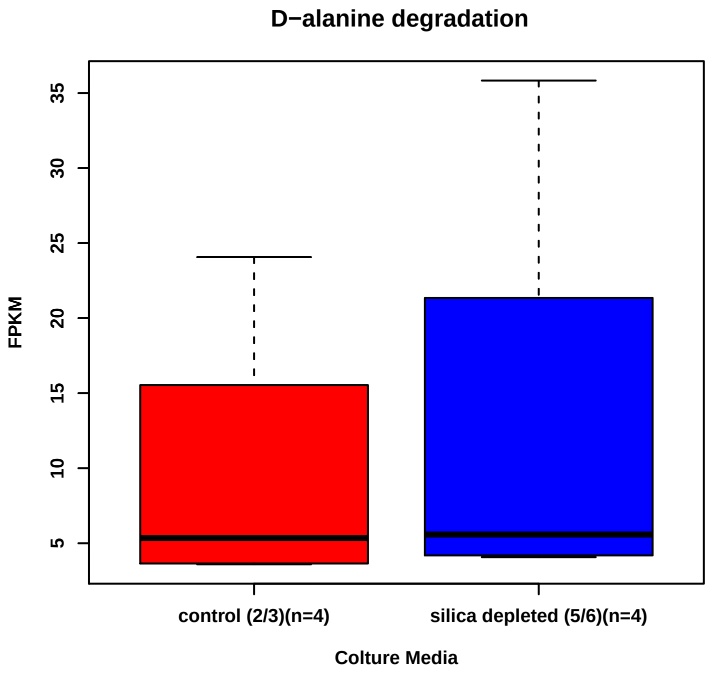
D−alanine degradation
FPKM
5
10
15
20
25
30
35
control (2/3)(n=4)
silica depleted (5/6)(n=4)
Colture Media

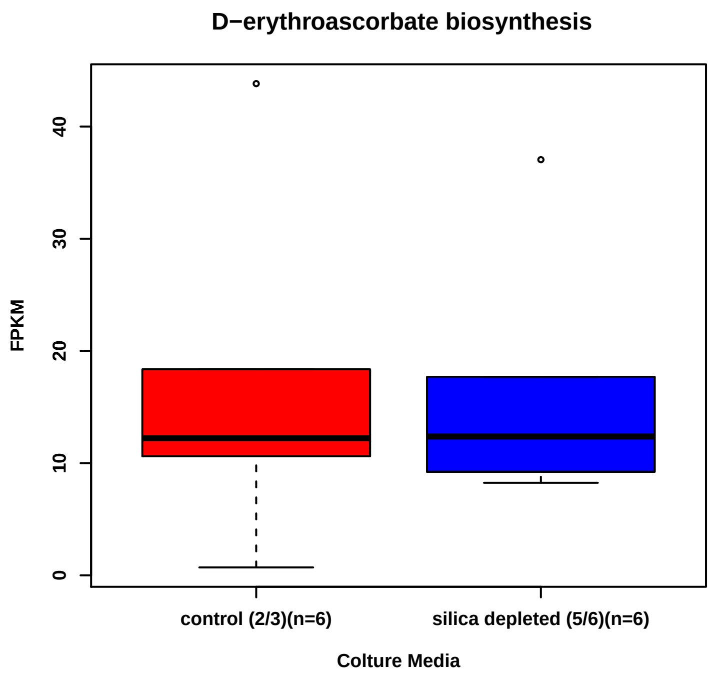

D−erythroascorbate biosynthesis
FPKM
0
10
20
30
40
control (2/3)(n=6)
silica depleted (5/6)(n=6)
Colture Media

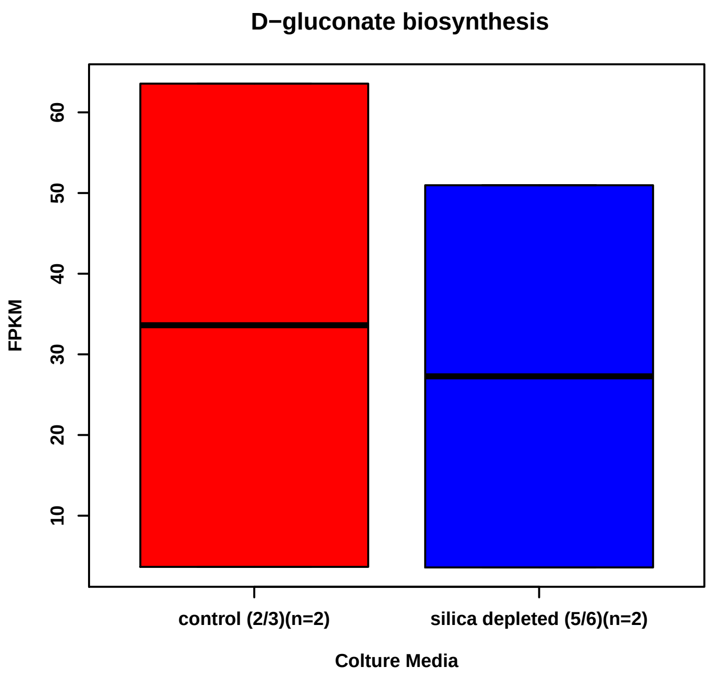

D−gluconate biosynthesis
FPKM
10
20
30
40
50
60
control (2/3)(n=2)
silica depleted (5/6)(n=2)
Colture Media

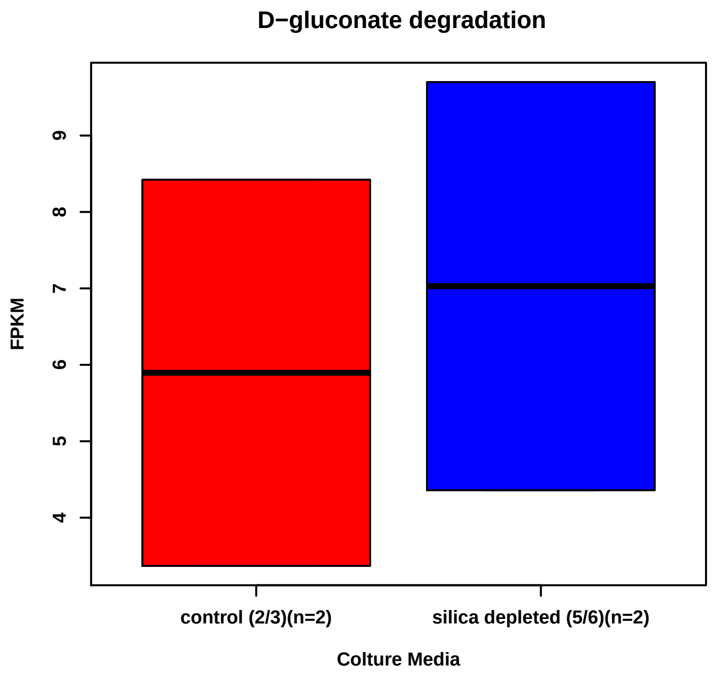

D−gluconate degradation
FPKM
4
5
6
7
8
9
control (2/3)(n=2)
silica depleted (5/6)(n=2)
Colture Media

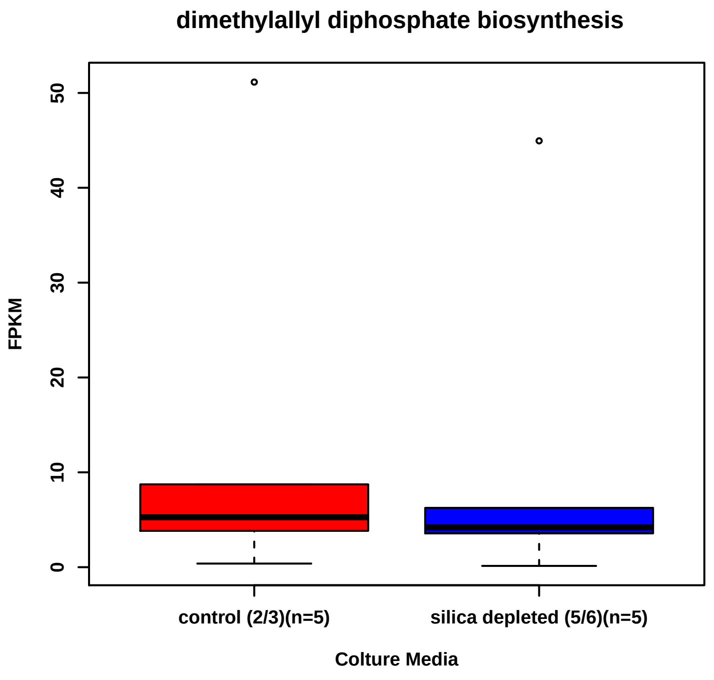

dimethylallyl diphosphate biosynthesis
FPKM
0
10
20
30
40
50
control (2/3)(n=5)
silica depleted (5/6)(n=5)
Colture Media

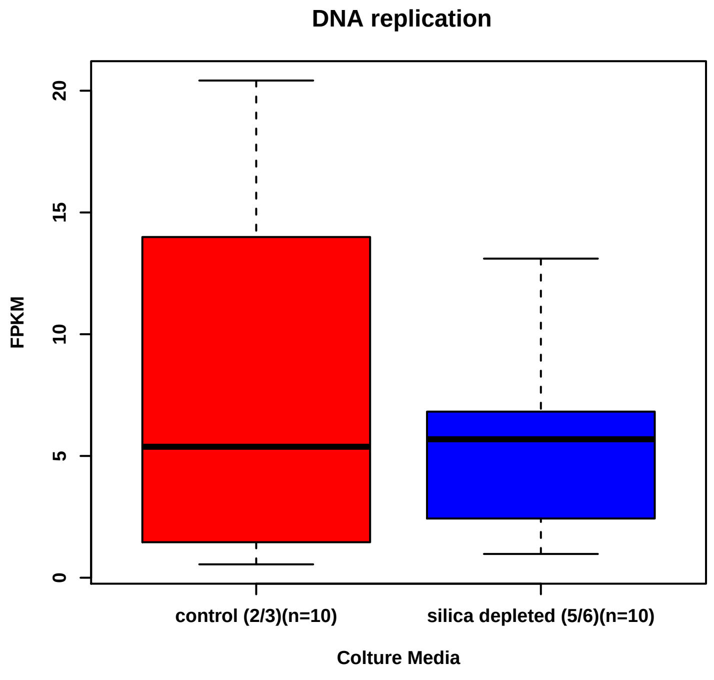

DNA replication
FPKM
0
5
10
15
20
control (2/3)(n=10)
silica depleted (5/6)(n=10)
Colture Media

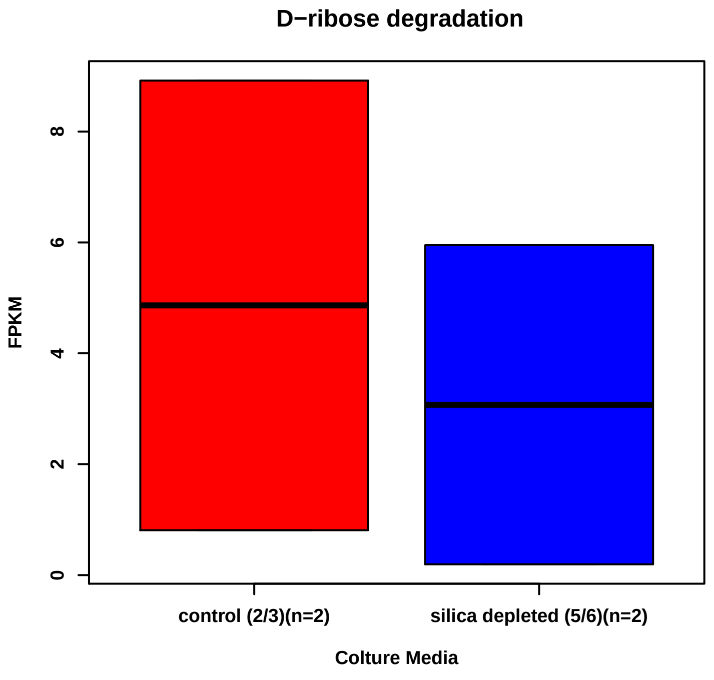

D−ribose degradation
FPKM
0
2
4
6
8
control (2/3)(n=2)
silica depleted (5/6)(n=2)
Colture Media

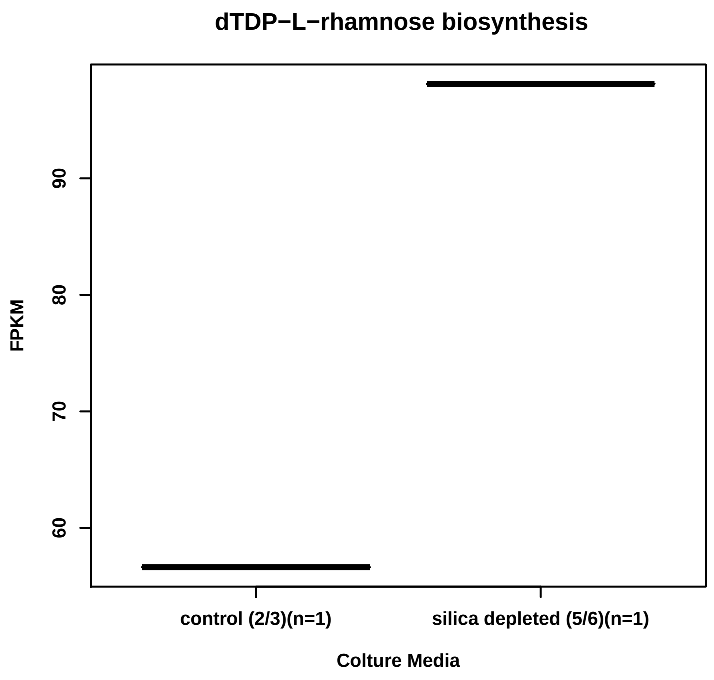
dTDP−L−rhamnose biosynthesis
FPKM
60
70
80
90
control (2/3)(n=1)
silica depleted (5/6)(n=1)
Colture Media

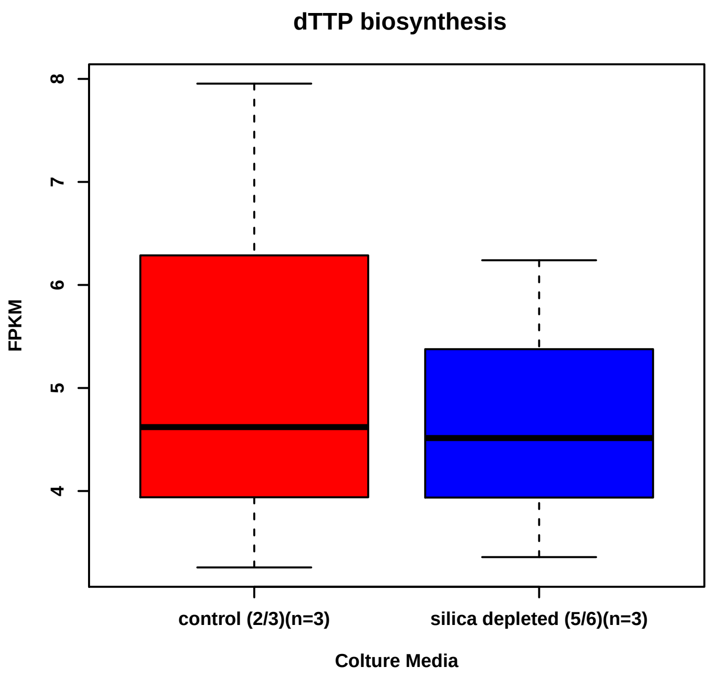
dTTP biosynthesis
FPKM
4
5
6
7
8
control (2/3)(n=3)
silica depleted (5/6)(n=3)
Colture Media

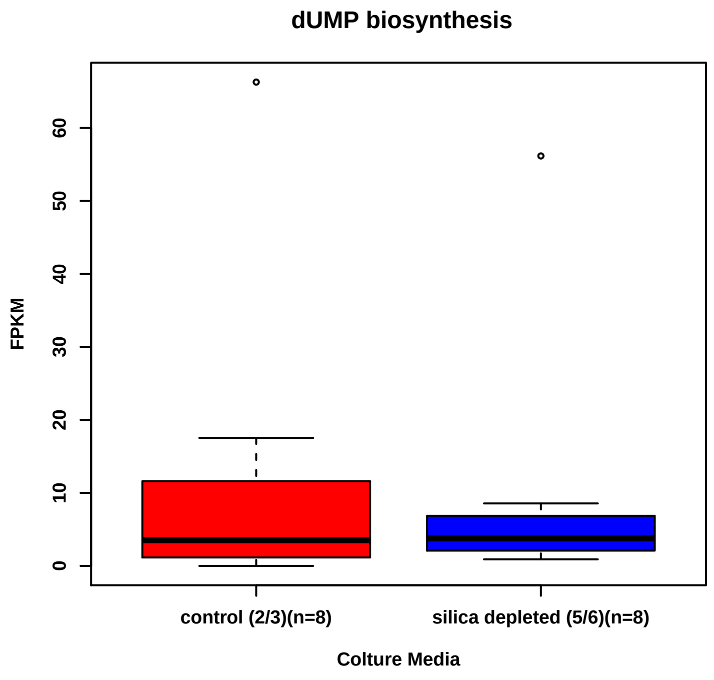
dUMP biosynthesis
FPKM
0
10
20
30
40
50
60
control (2/3)(n=8)
silica depleted (5/6)(n=8)
Colture Media

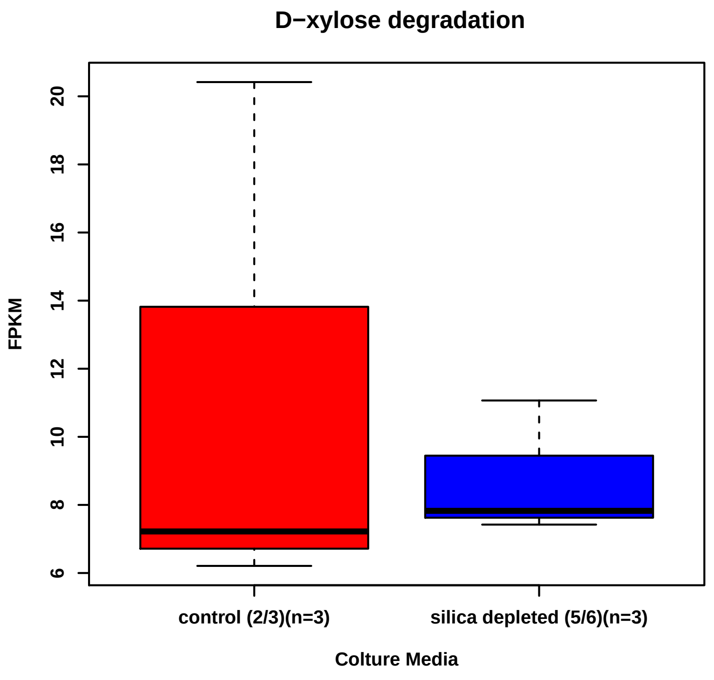
D−xylose degradation
FPKM
6
8
10
12
14
16
18
20
control (2/3)(n=3)
silica depleted (5/6)(n=3)
Colture Media

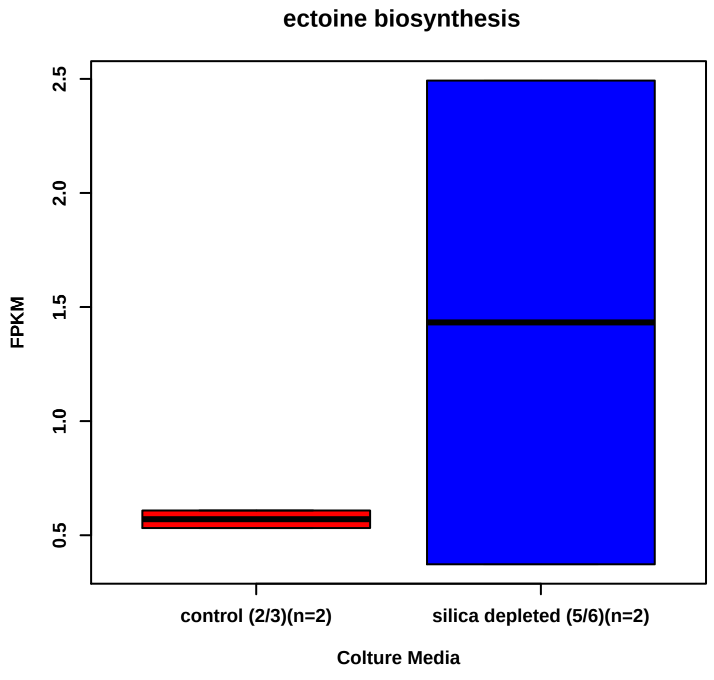
ectoine biosynthesis
FPKM
0.5
1.0
1.5
2.0
2.5
control (2/3)(n=2)
silica depleted (5/6)(n=2)
Colture Media

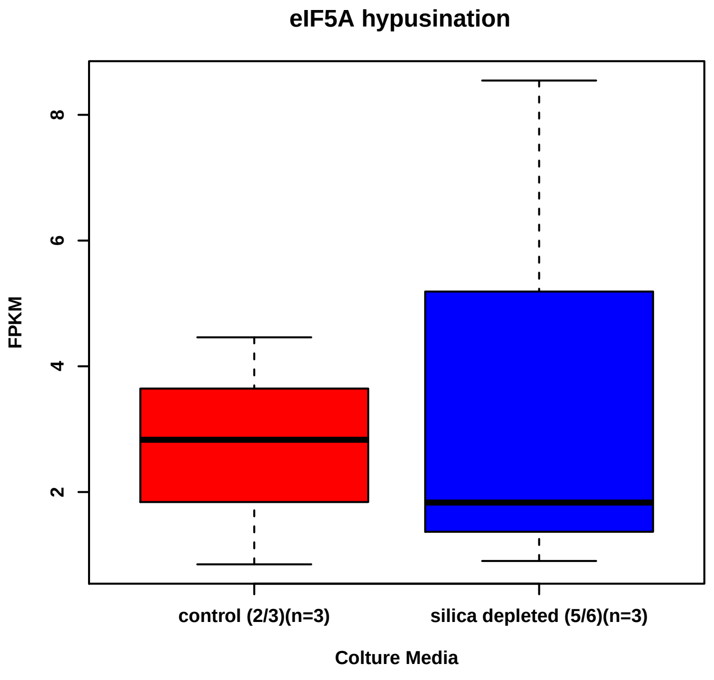
eIF5A hypusination
FPKM
2
4
6
8
control (2/3)(n=3)
silica depleted (5/6)(n=3)
Colture Media

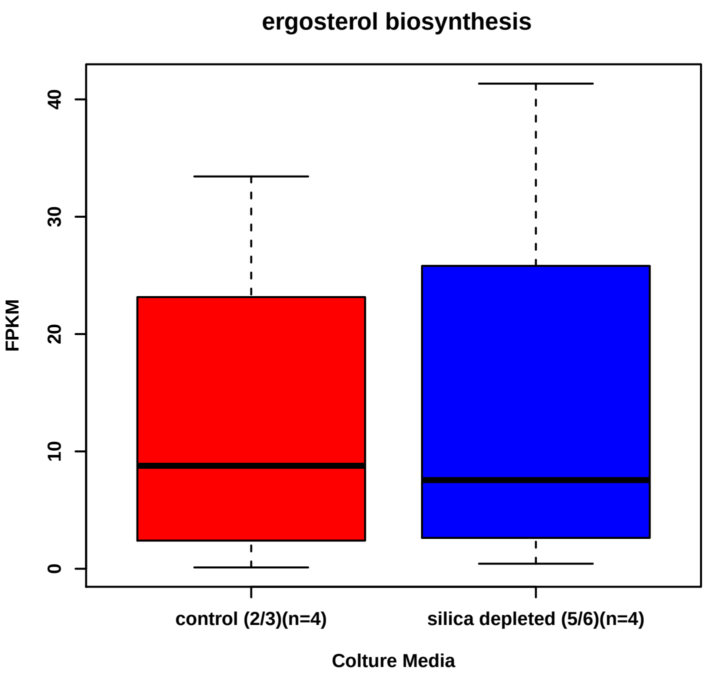
ergosterol biosynthesis
FPKM
0
10
20
30
40
control (2/3)(n=4)
silica depleted (5/6)(n=4)
Colture Media

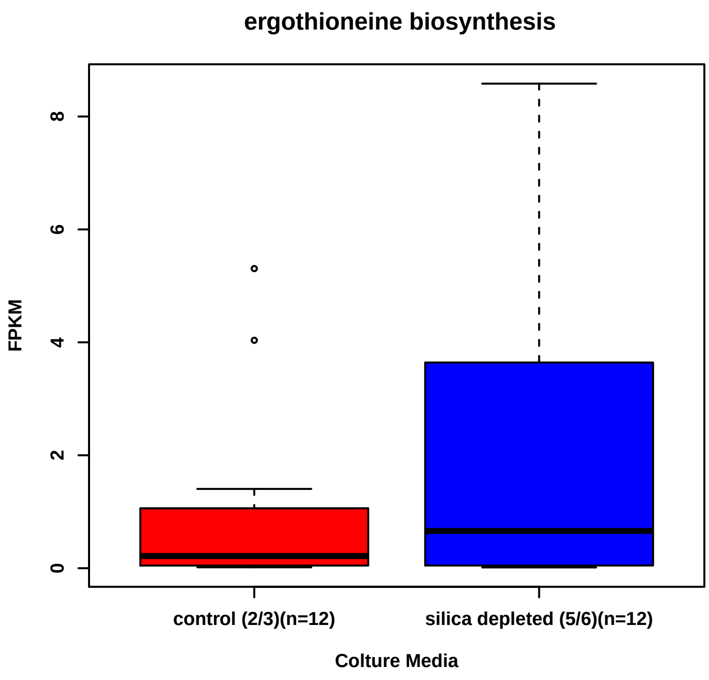
ergothioneine biosynthesis
FPKM
0
2
4
6
8
control (2/3)(n=12)
silica depleted (5/6)(n=12)
Colture Media

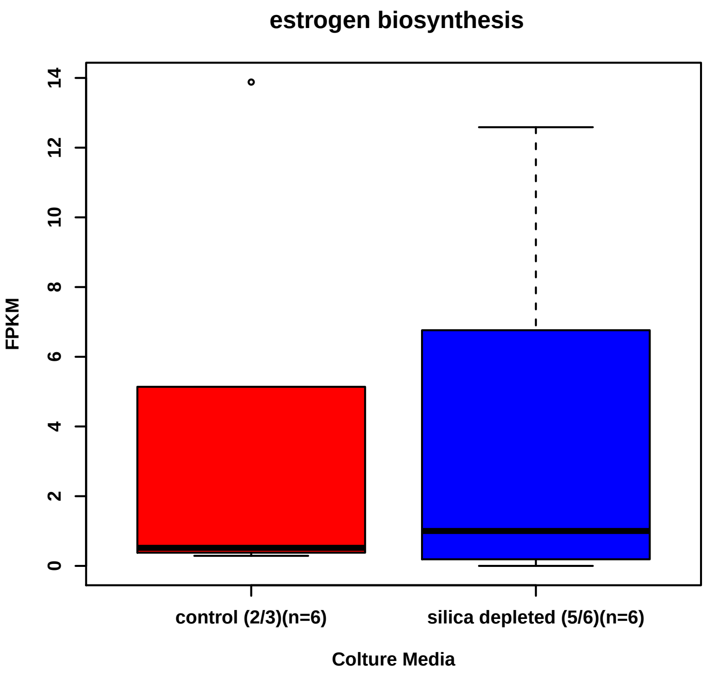
estrogen biosynthesis
FPKM
0
2
4
6
8
10
12
14
control (2/3)(n=6)
silica depleted (5/6)(n=6)
Colture Media

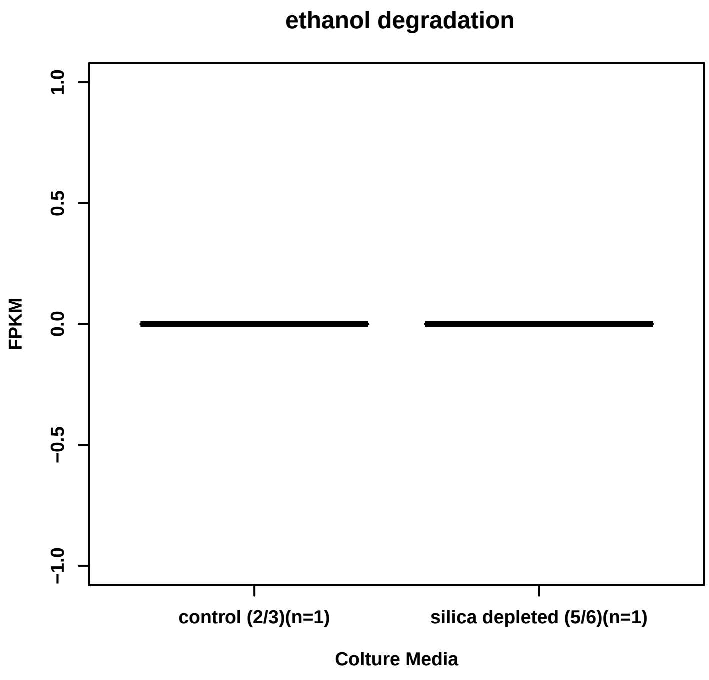
ethanol degradation
FPKM
−1.0
−0.5
0.0
0.5
1.0
control (2/3)(n=1)
silica depleted (5/6)(n=1)
Colture Media

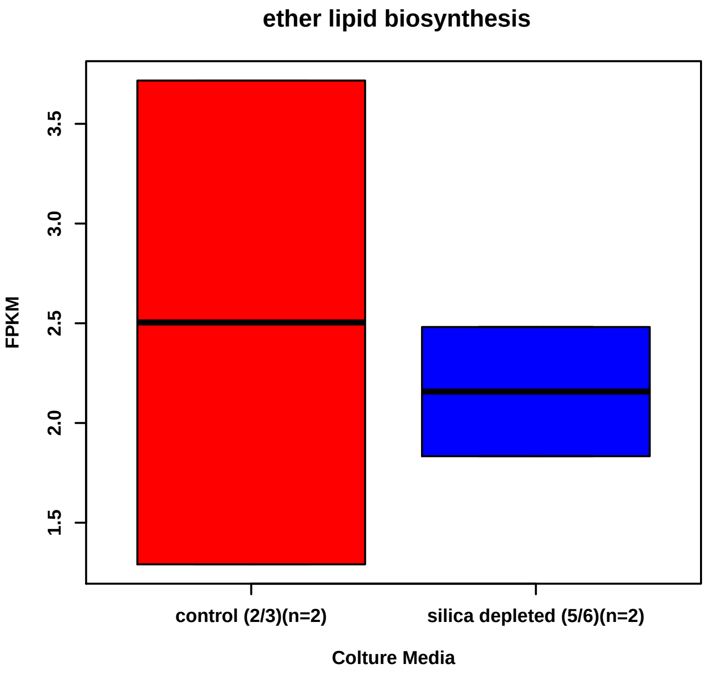
ether lipid biosynthesis
FPKM
1.5
2.0
2.5
3.0
3.5
control (2/3)(n=2)
silica depleted (5/6)(n=2)
Colture Media

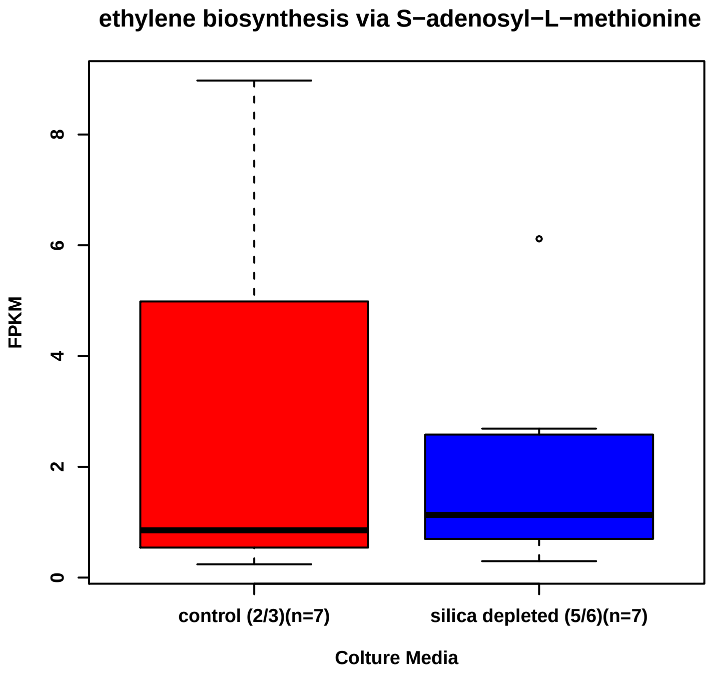
ethylene biosynthesis via S−adenosyl−L−methionine
FPKM
0
2
4
6
8
control (2/3)(n=7)
silica depleted (5/6)(n=7)
Colture Media

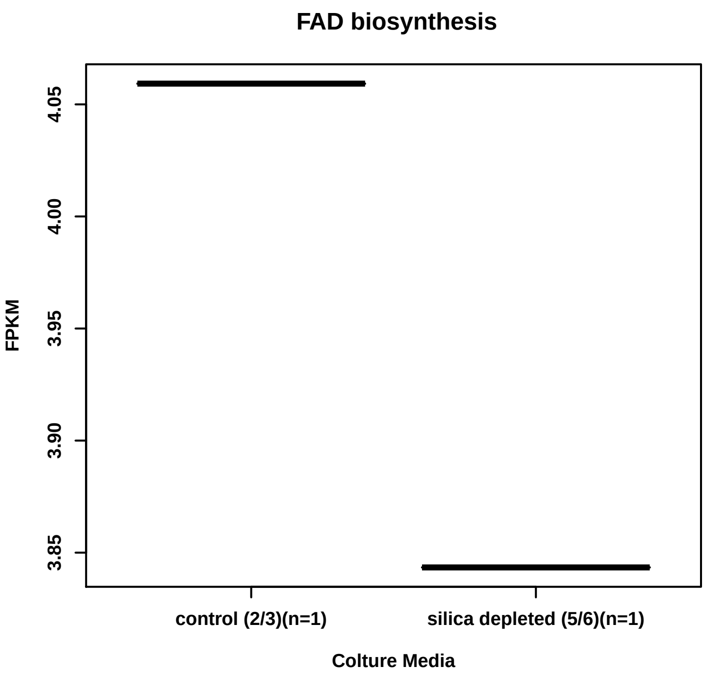
FAD biosynthesis
FPKM
4.05
4.00
3.95
3.90
3.85
control (2/3)(n=1)
silica depleted (5/6)(n=1)
Colture Media

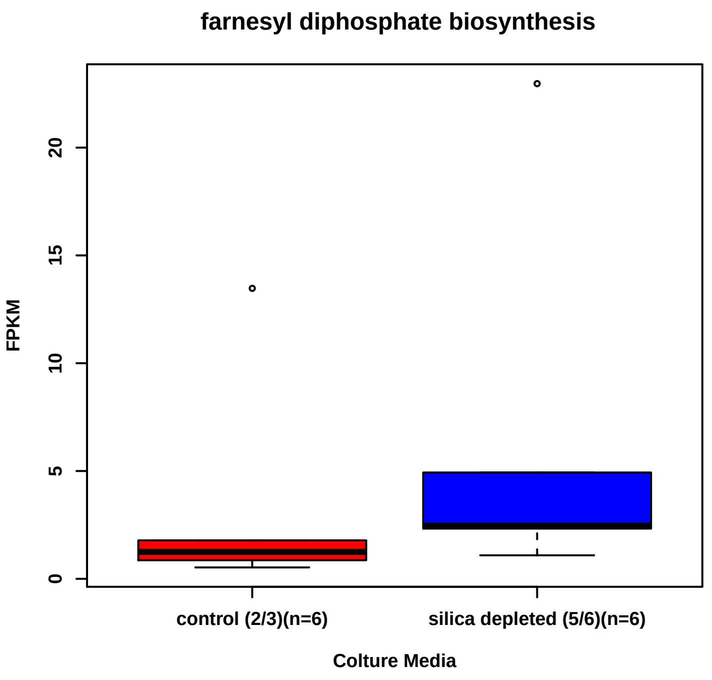
farnesyl diphosphate biosynthesis
FPKM
20
15
10
5
0
control (2/3)(n=6)
silica depleted (5/6)(n=6)
Colture Media

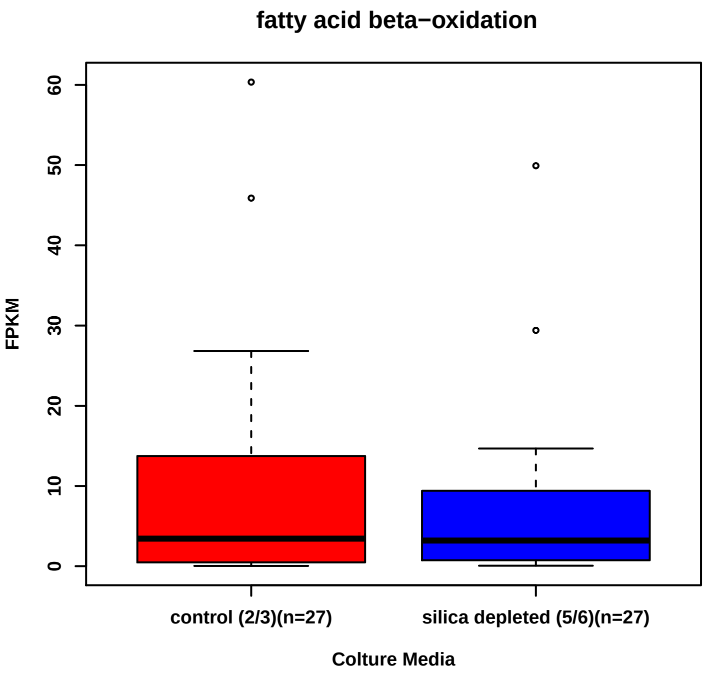
fatty acid beta−oxidation
FPKM
60
50
40
30
20
10
0
control (2/3)(n=27)
silica depleted (5/6)(n=27)
Colture Media

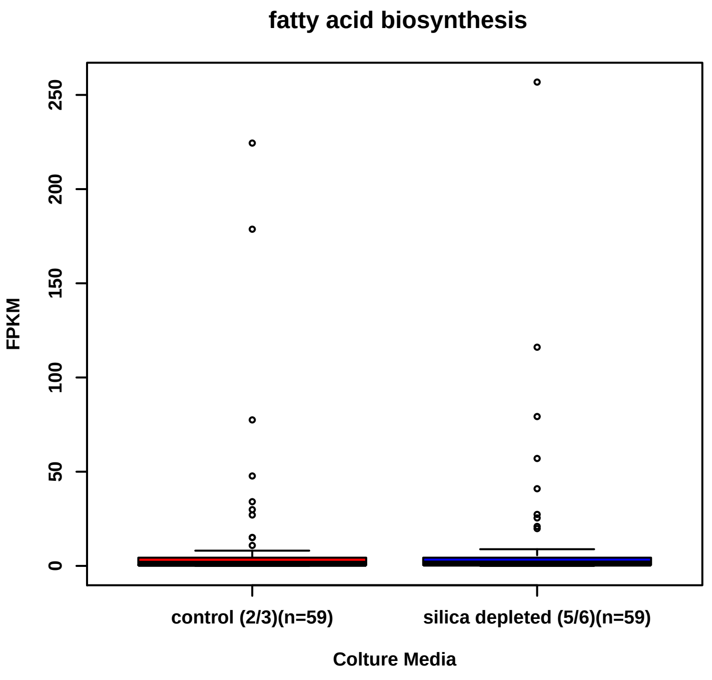
fatty acid biosynthesis
FPKM
250
200
150
100
50
0
control (2/3)(n=59)
silica depleted (5/6)(n=59)
Colture Media

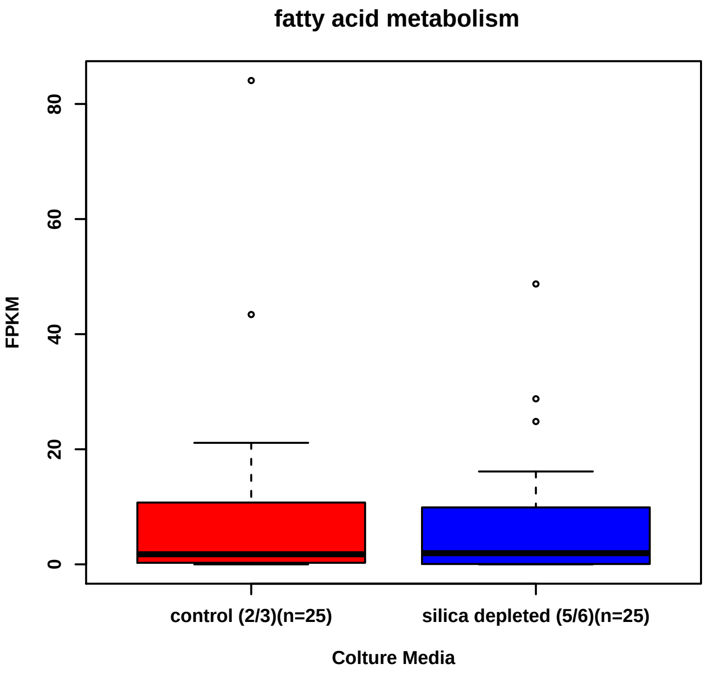
fatty acid metabolism
FPKM
80
60
40
20
0
control (2/3)(n=25)
silica depleted (5/6)(n=25)
Colture Media

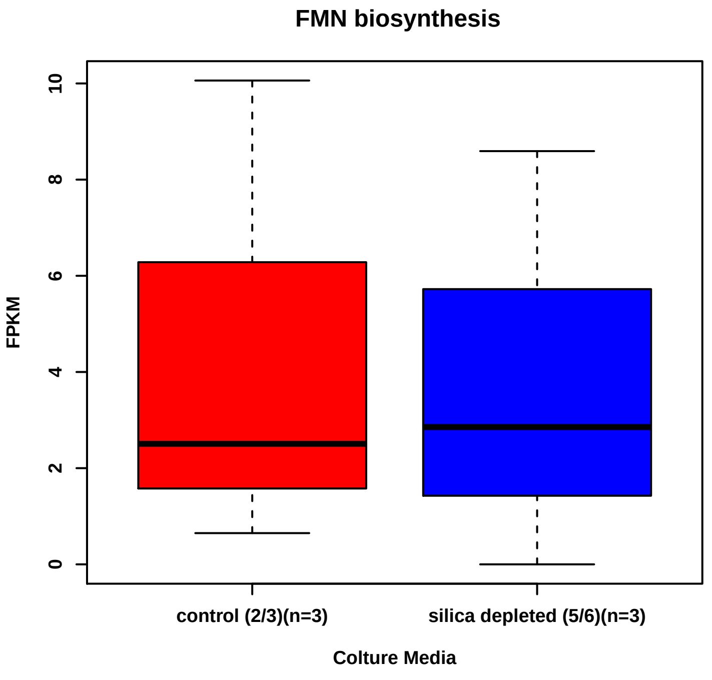
FMN biosynthesis
FPKM
10
8
6
4
2
0
control (2/3)(n=3)
silica depleted (5/6)(n=3)
Colture Media

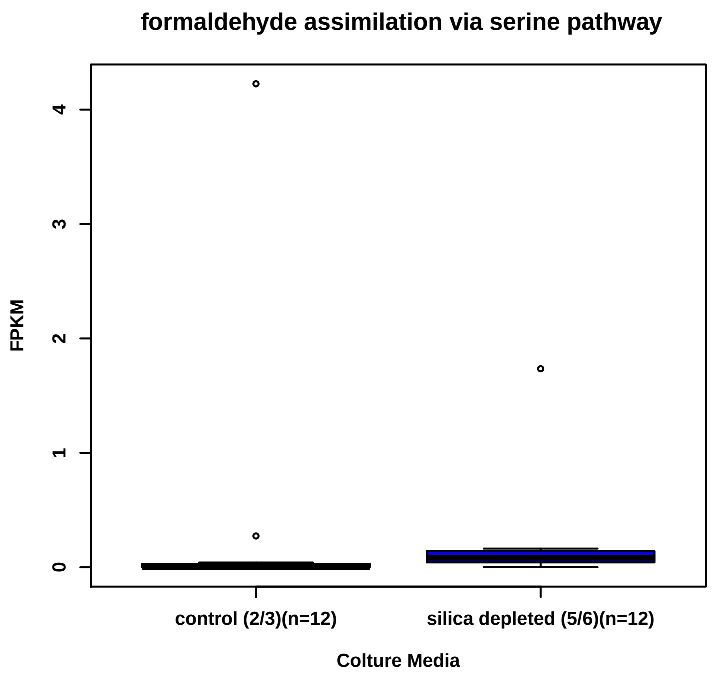
formaldehyde assimilation via serine pathway
FPKM
0
1
2
3
4
control (2/3)(n=12)
silica depleted (5/6)(n=12)
Colture Media

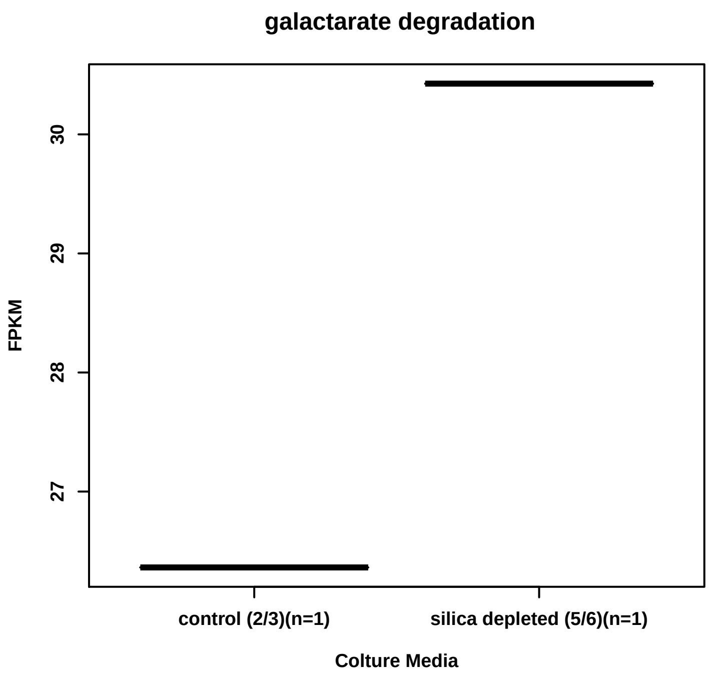
galactarate degradation
FPKM
27
28
29
30
control (2/3)(n=1)
silica depleted (5/6)(n=1)
Colture Media

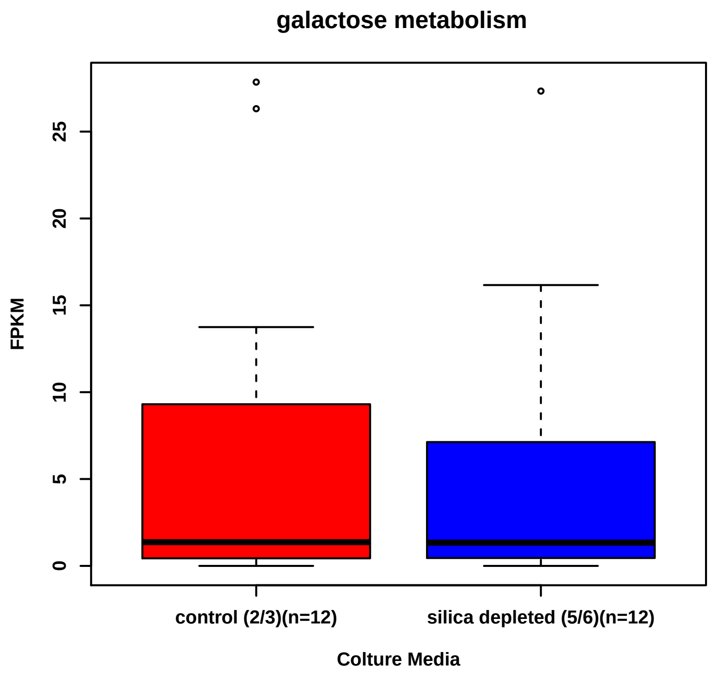
galactose metabolism
FPKM
0
5
10
15
20
25
control (2/3)(n=12)
silica depleted (5/6)(n=12)
Colture Media

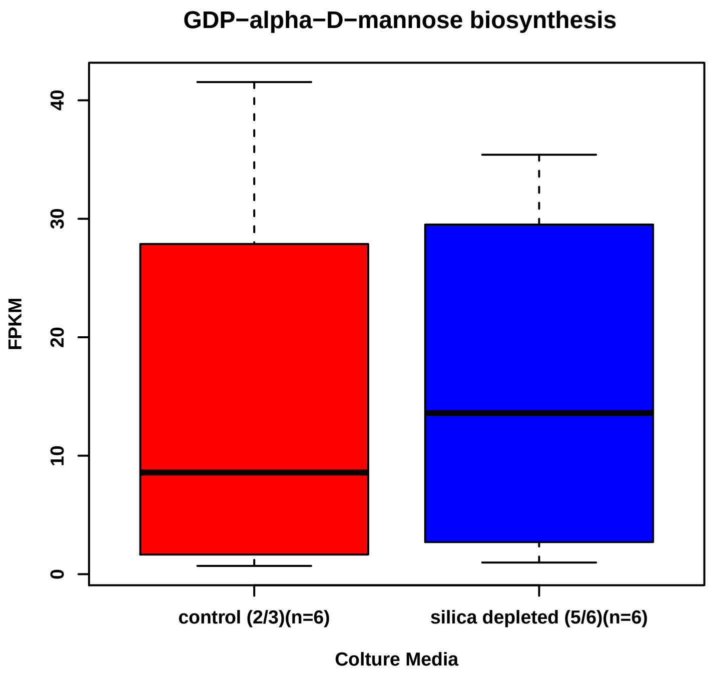
GDP−alpha−D−mannose biosynthesis
FPKM
0
10
20
30
40
control (2/3)(n=6)
silica depleted (5/6)(n=6)
Colture Media

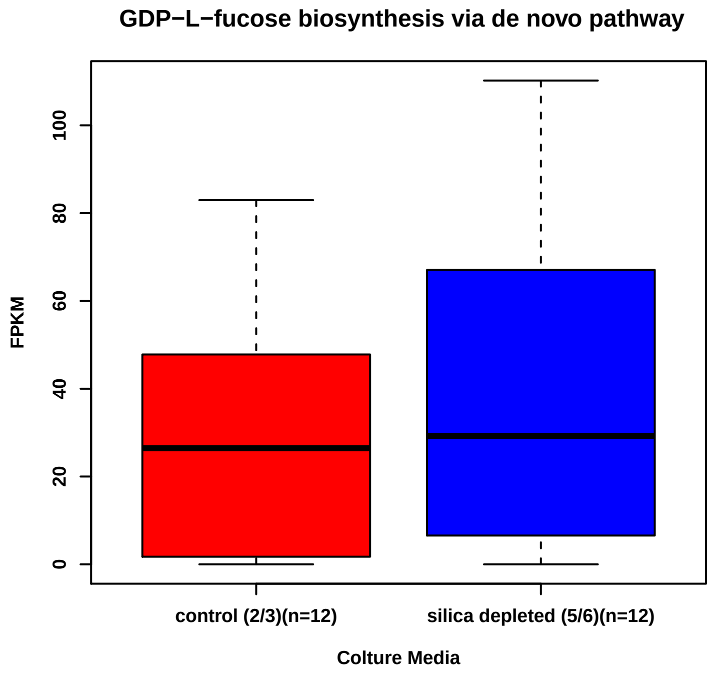
GDP−L−fucose biosynthesis via de novo pathway
FPKM
0
20
40
60
80
100
control (2/3)(n=12)
silica depleted (5/6)(n=12)
Colture Media

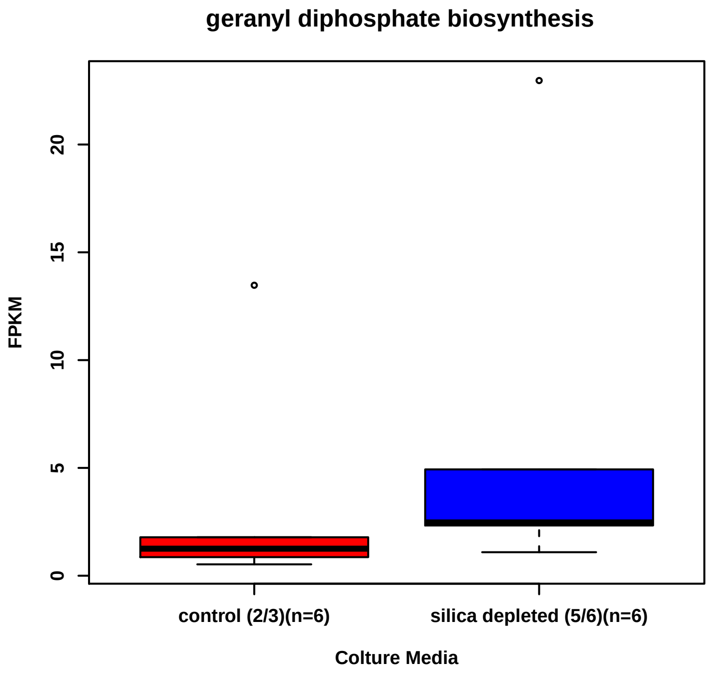
geranyl diphosphate biosynthesis
FPKM
0
5
10
15
20
control (2/3)(n=6)
silica depleted (5/6)(n=6)
Colture Media

geranylgeranyl diphosphate biosynthesis

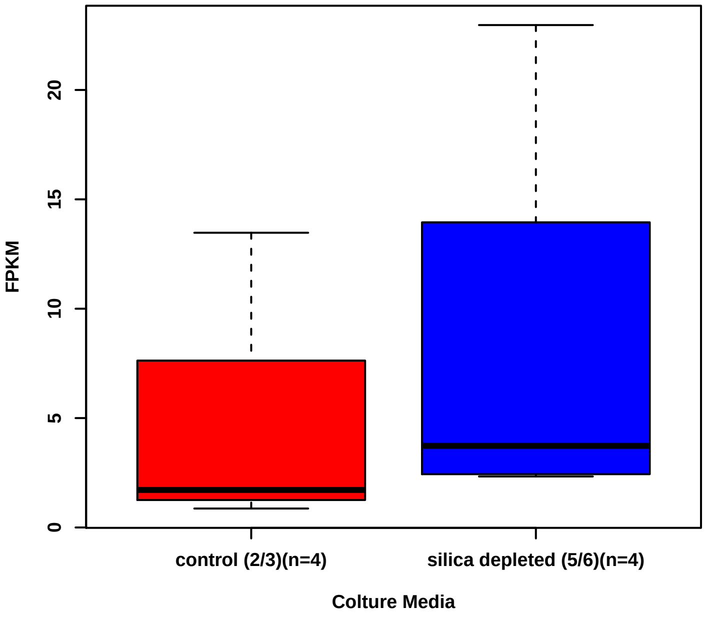

gluconeogenesis

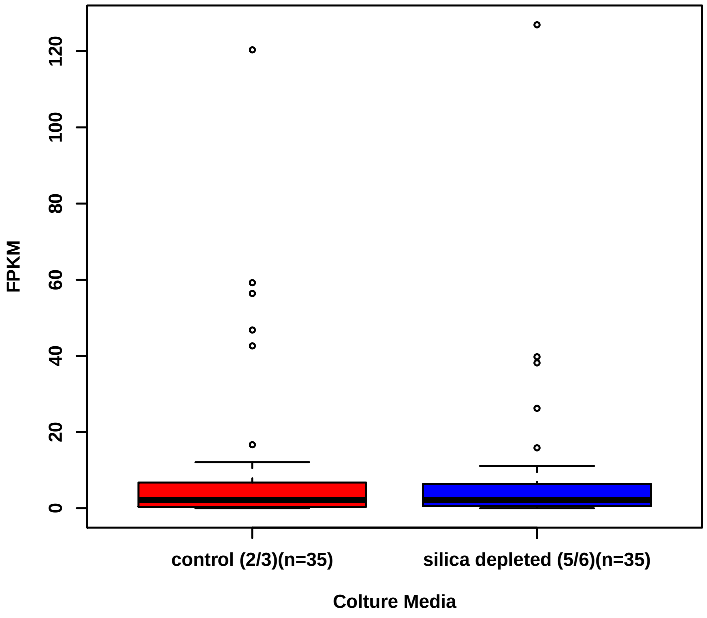

glutathione biosynthesis

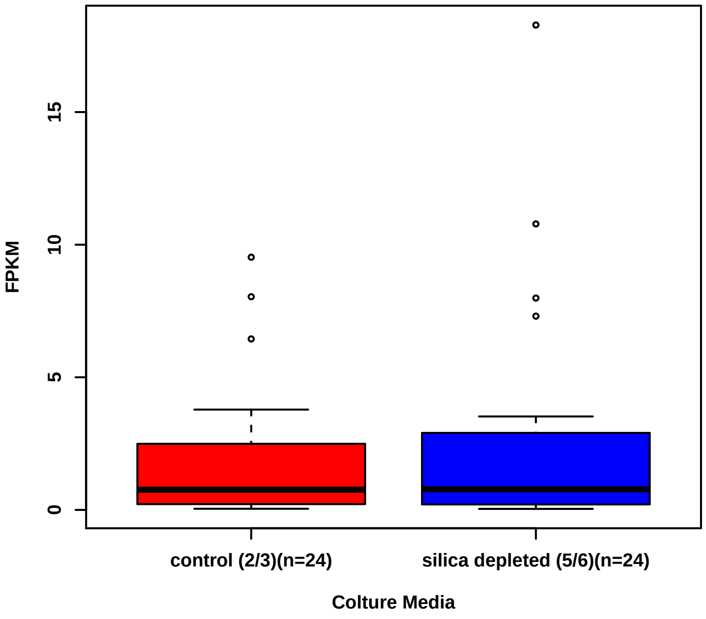

glutathione metabolism

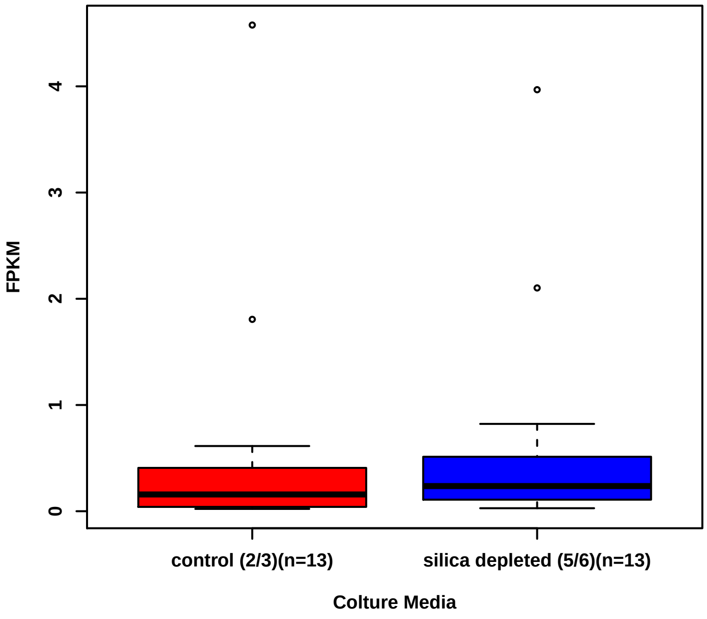

glycerol degradation via glycerol kinase pathway

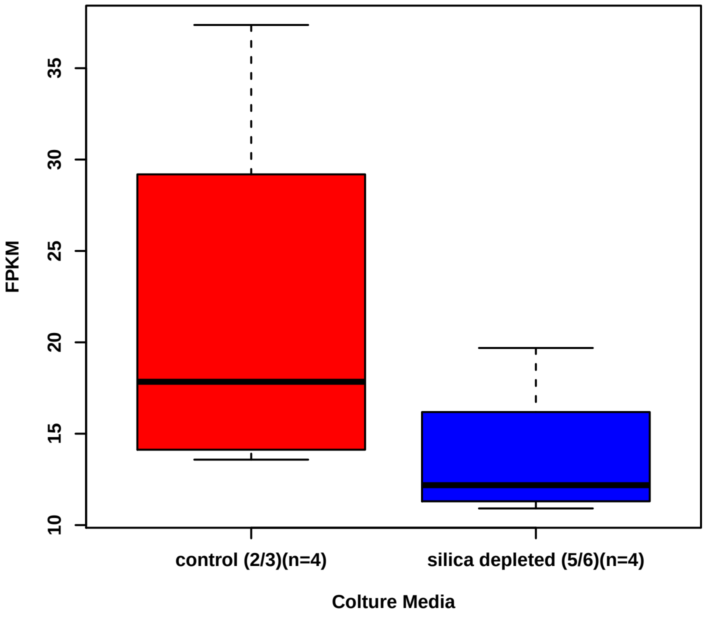

glycerophospholipid metabolism

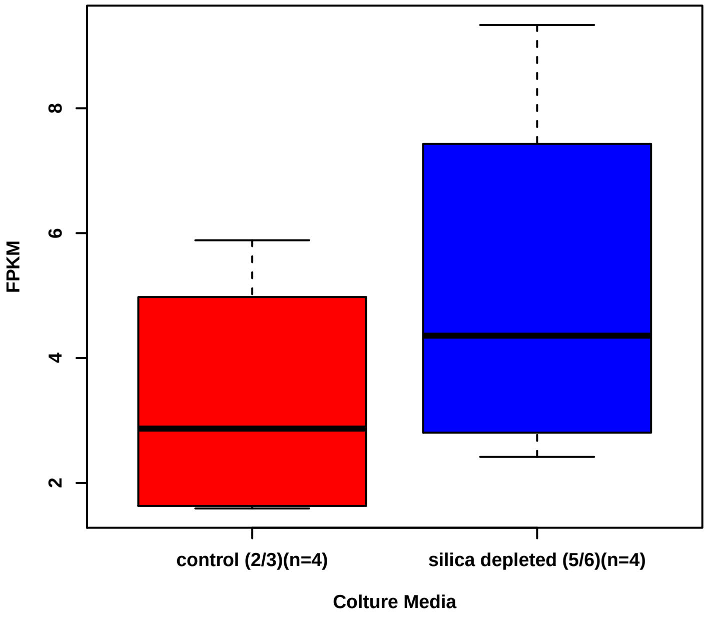

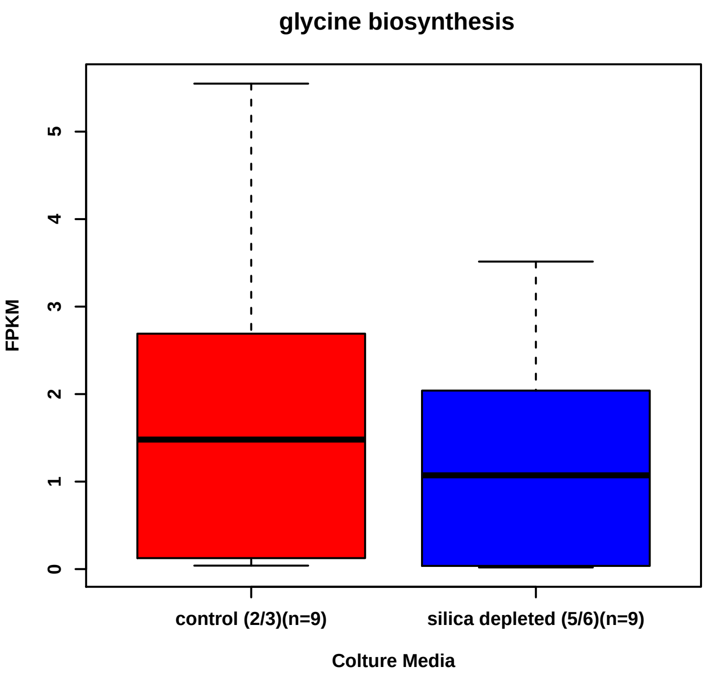
glycine biosynthesis
FPKM
0
1
2
3
4
5
control (2/3)(n=9)
silica depleted (5/6)(n=9)
Colture Media

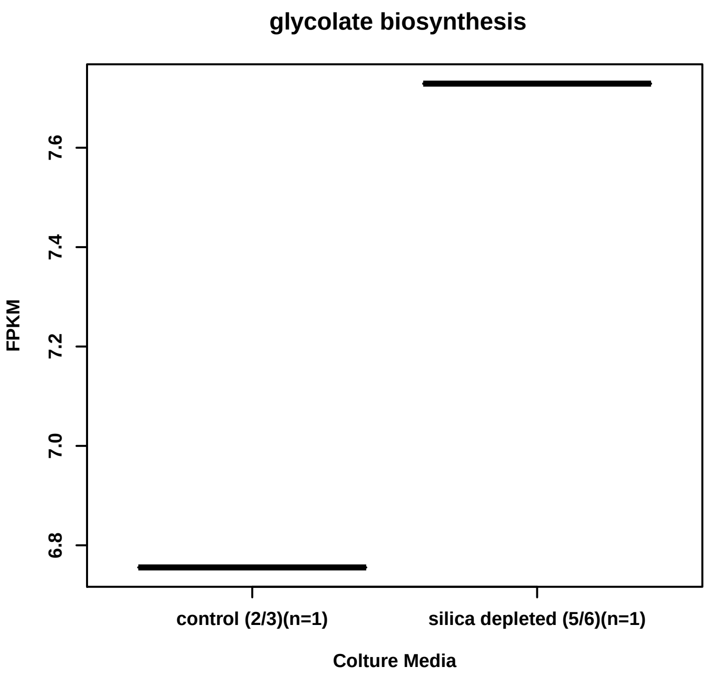
glycolate biosynthesis
FPKM
6.8
7.0
7.2
7.4
7.6
control (2/3)(n=1)
silica depleted (5/6)(n=1)
Colture Media

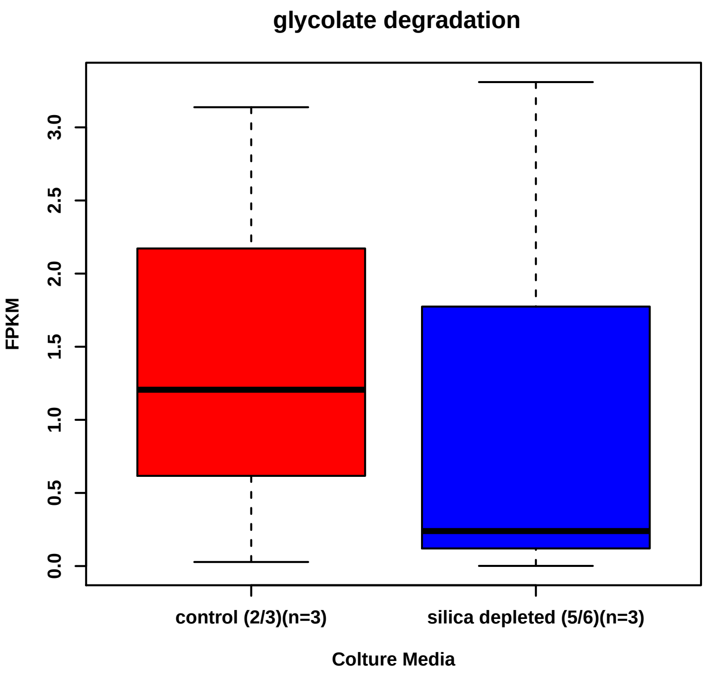
glycolate degradation
FPKM
0.0
0.5
1.0
1.5
2.0
2.5
3.0
control (2/3)(n=3)
silica depleted (5/6)(n=3)
Colture Media

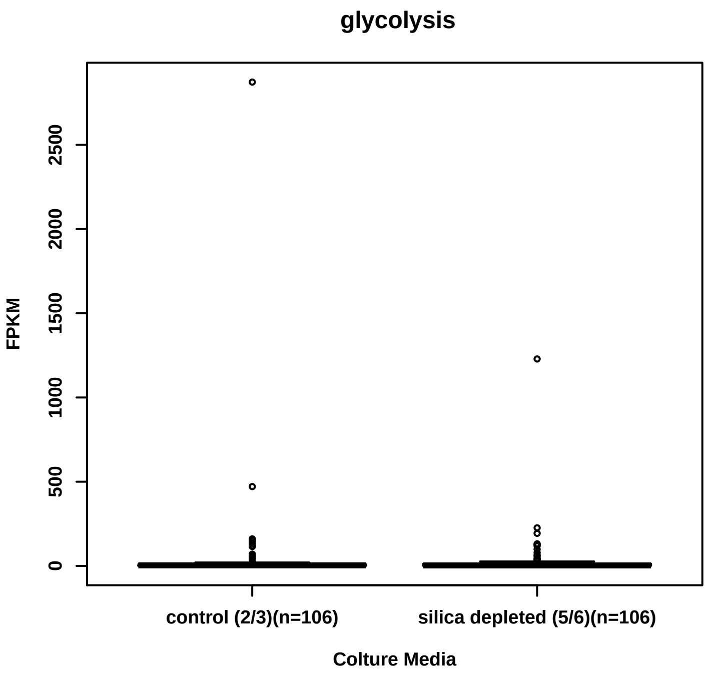
glycolysis
FPKM
0
500
1000
1500
2000
2500
control (2/3)(n=106)
silica depleted (5/6)(n=106)
Colture Media

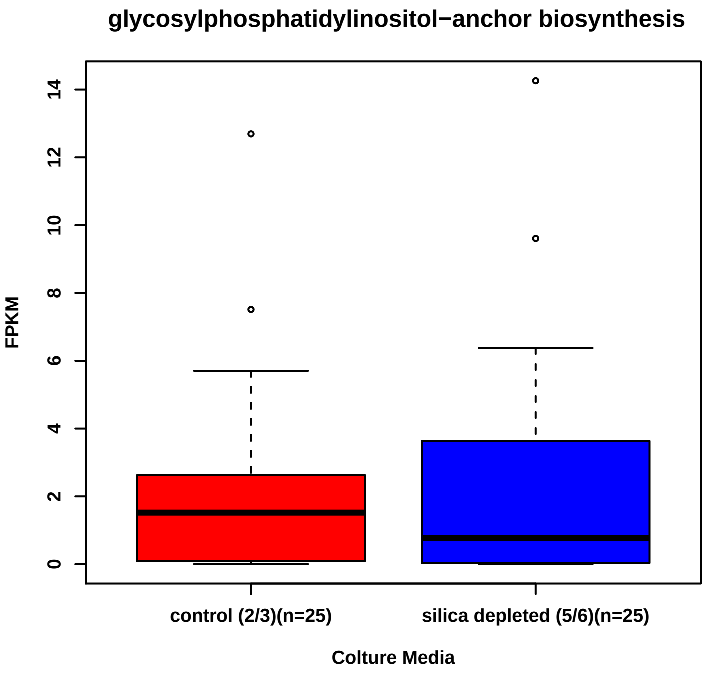
glycosylphosphatidylinositol−anchor biosynthesis
FPKM
0
2
4
6
8
10
12
14
control (2/3)(n=25)
silica depleted (5/6)(n=25)
Colture Media

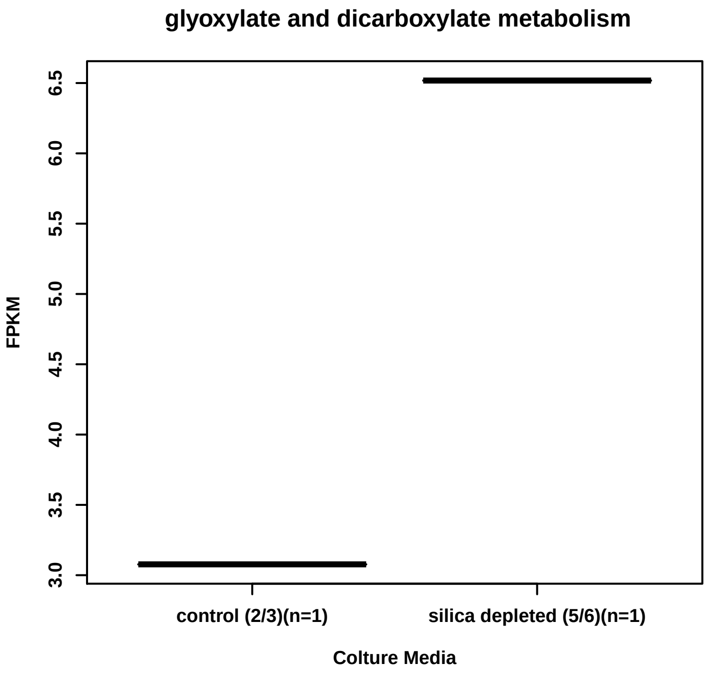
glyoxylate and dicarboxylate metabolism
FPKM
3.0
3.5
4.0
4.5
5.0
5.5
6.0
6.5
control (2/3)(n=1)
silica depleted (5/6)(n=1)
Colture Media

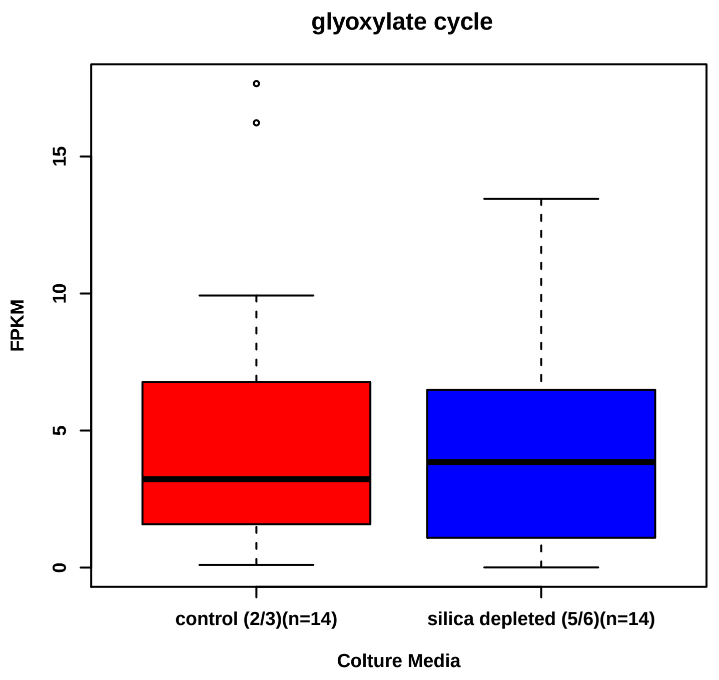

glyoxylate cycle
FPKM
0
5
10
15
control (2/3)(n=14)
silica depleted (5/6)(n=14)
Colture Media

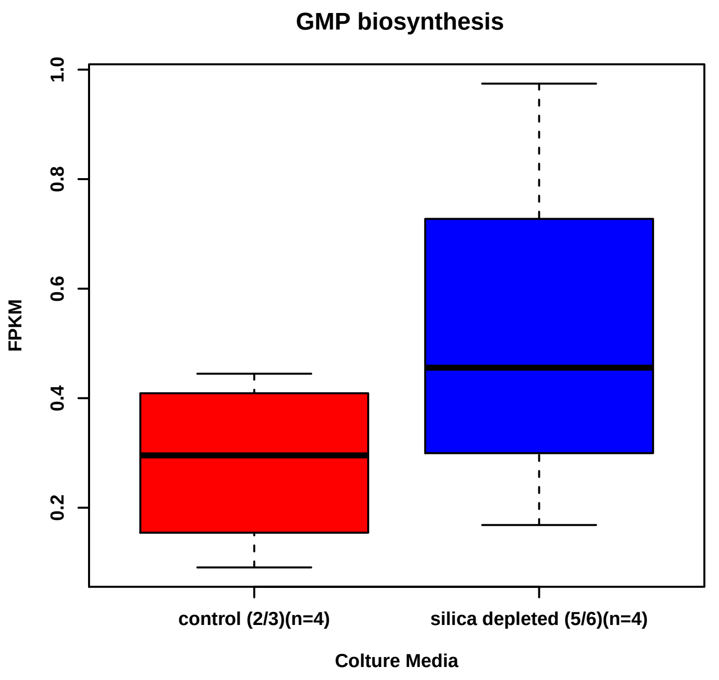

GMP biosynthesis
FPKM
0.2
0.4
0.6
0.8
1.0
control (2/3)(n=4)
silica depleted (5/6)(n=4)
Colture Media

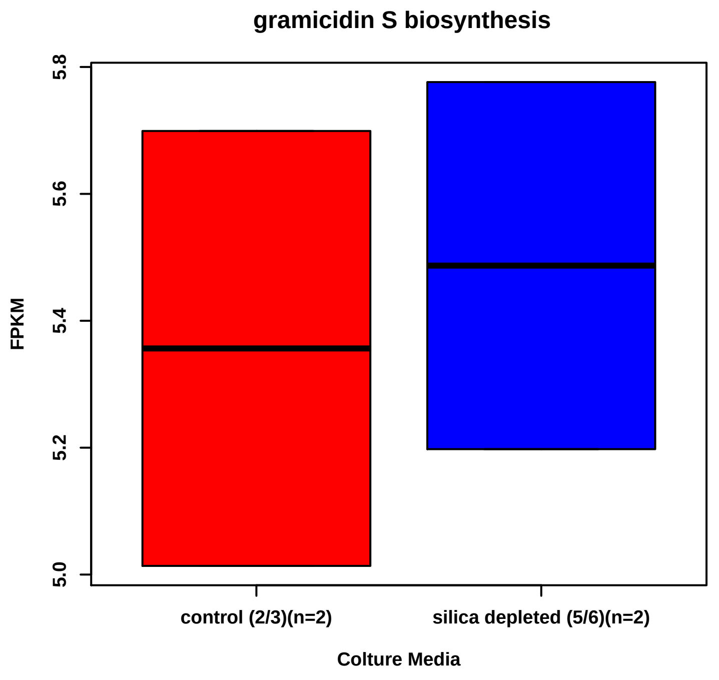

gramicidin S biosynthesis
FPKM
5.0
5.2
5.4
5.6
5.8
control (2/3)(n=2)
silica depleted (5/6)(n=2)
Colture Media

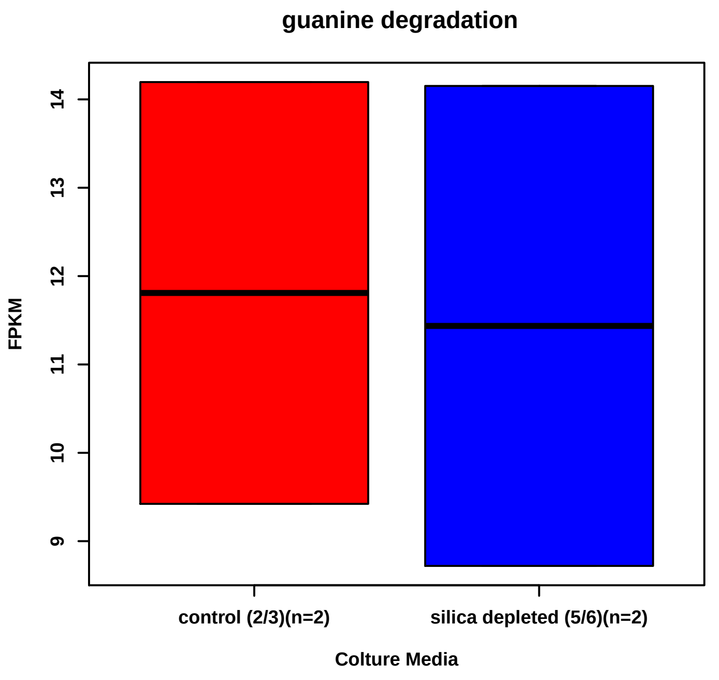

guanine degradation
FPKM
9
10
11
12
13
14
control (2/3)(n=2)
silica depleted (5/6)(n=2)
Colture Media

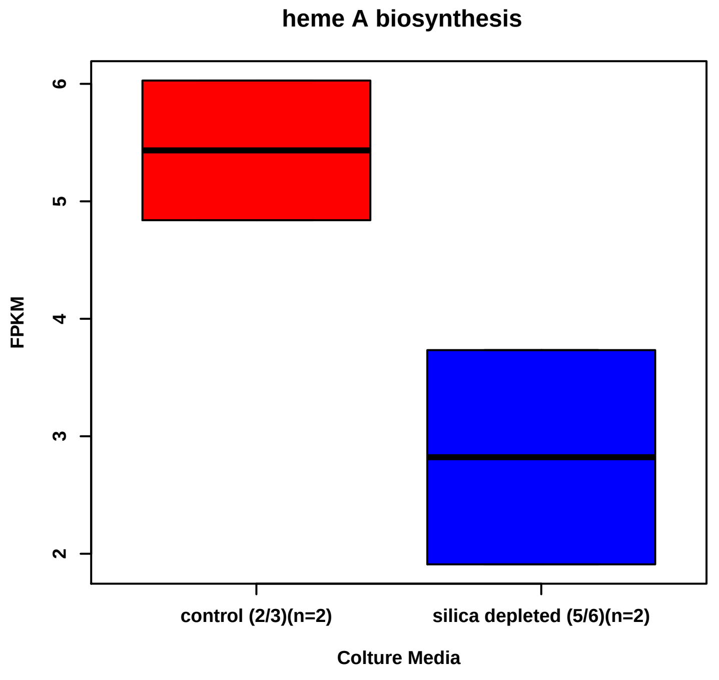

heme A biosynthesis
FPKM
2
3
4
5
6
control (2/3)(n=2)
silica depleted (5/6)(n=2)
Colture Media

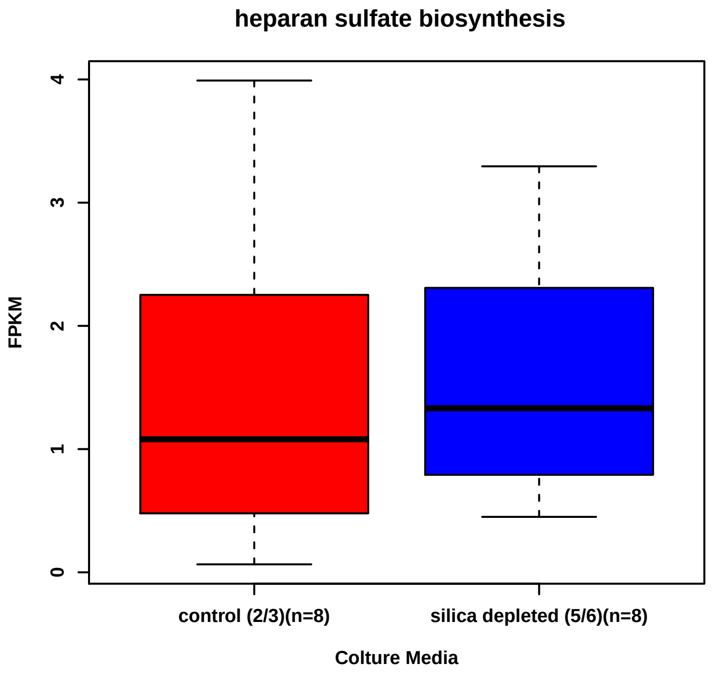

heparan sulfate biosynthesis
FPKM
0
1
2
3
4
control (2/3)(n=8)
silica depleted (5/6)(n=8)
Colture Media

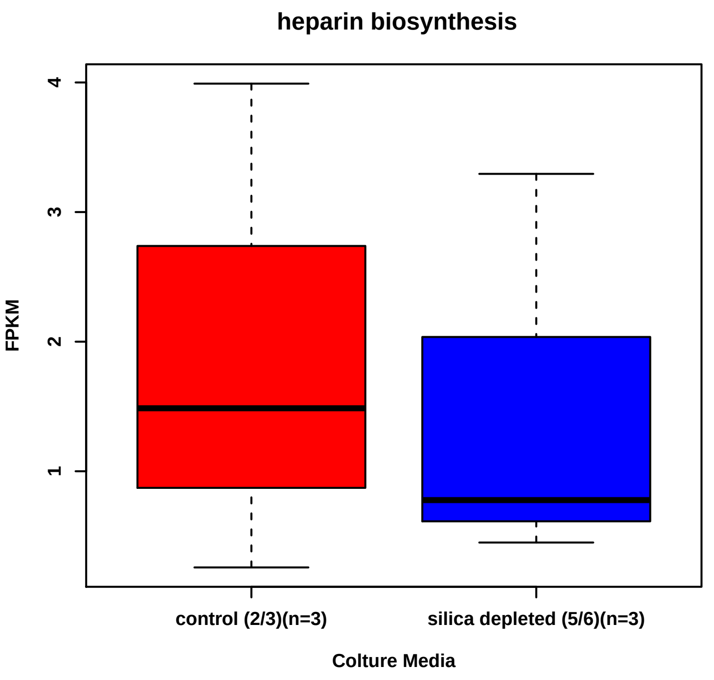
heparin biosynthesis
FPKM
1
2
3
4
control (2/3)(n=3)
silica depleted (5/6)(n=3)
Colture Media

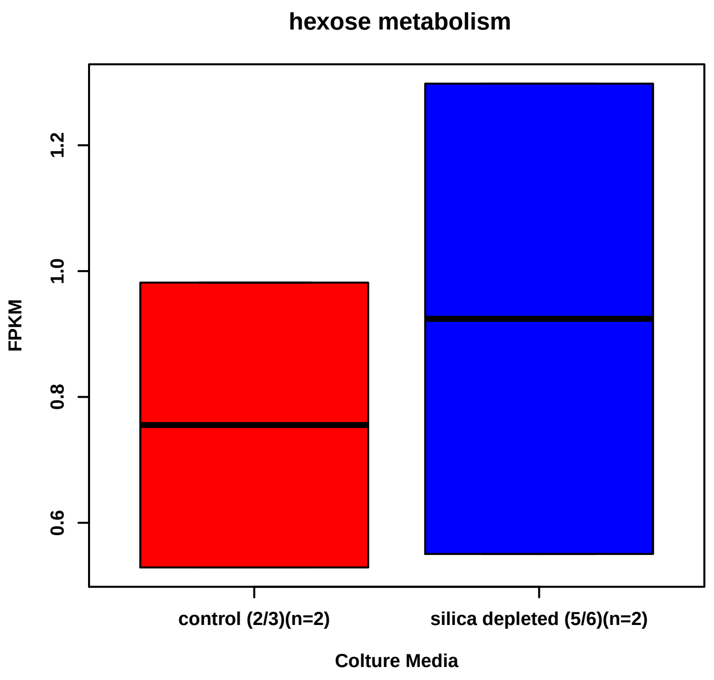
hexose metabolism
FPKM
0.6
0.8
1.0
1.2
control (2/3)(n=2)
silica depleted (5/6)(n=2)
Colture Media

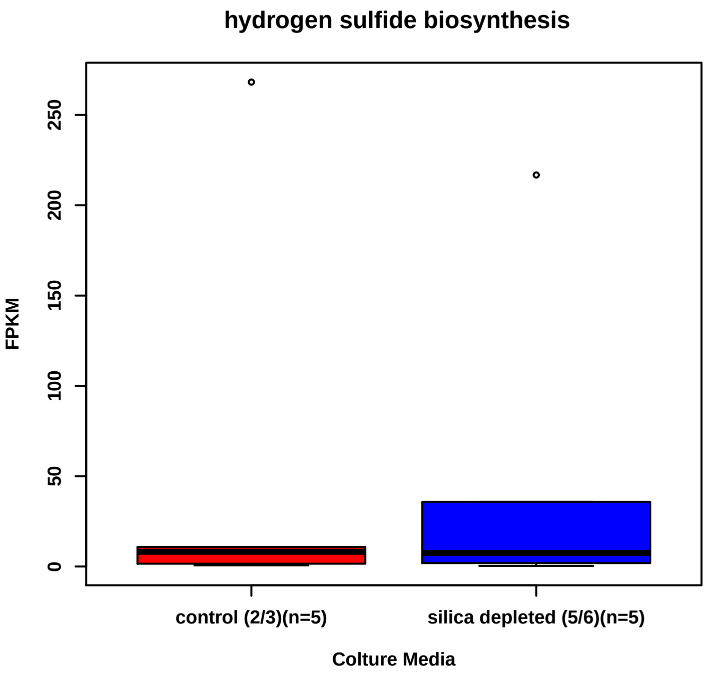
hydrogen sulfide biosynthesis
FPKM
0
50
100
150
200
250
control (2/3)(n=5)
silica depleted (5/6)(n=5)
Colture Media

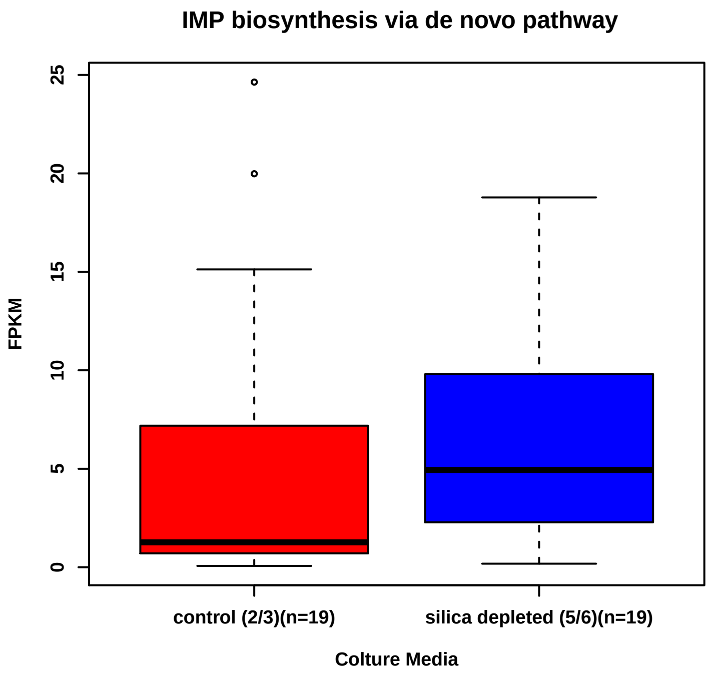
IMP biosynthesis via de novo pathway
FPKM
0
5
10
15
20
25
control (2/3)(n=19)
silica depleted (5/6)(n=19)
Colture Media

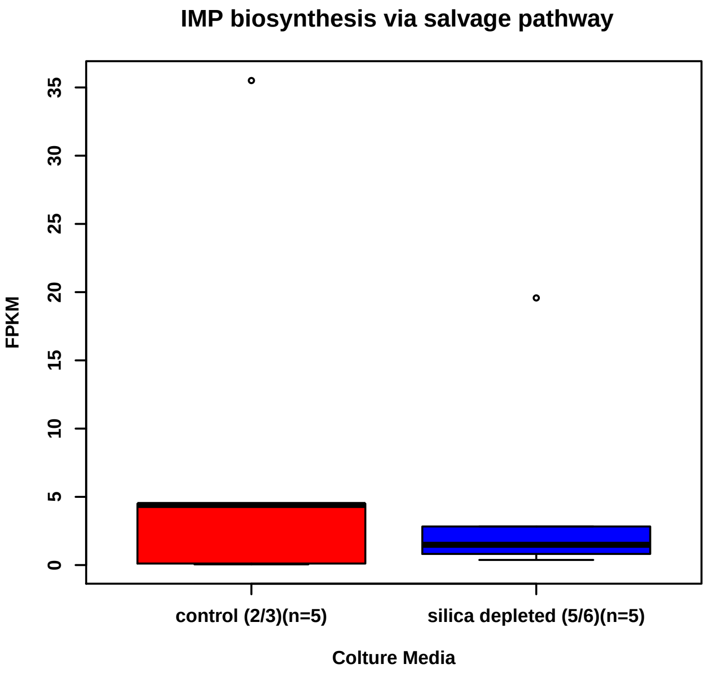
IMP biosynthesis via salvage pathway
FPKM
0
5
10
15
20
25
30
35
control (2/3)(n=5)
silica depleted (5/6)(n=5)
Colture Media

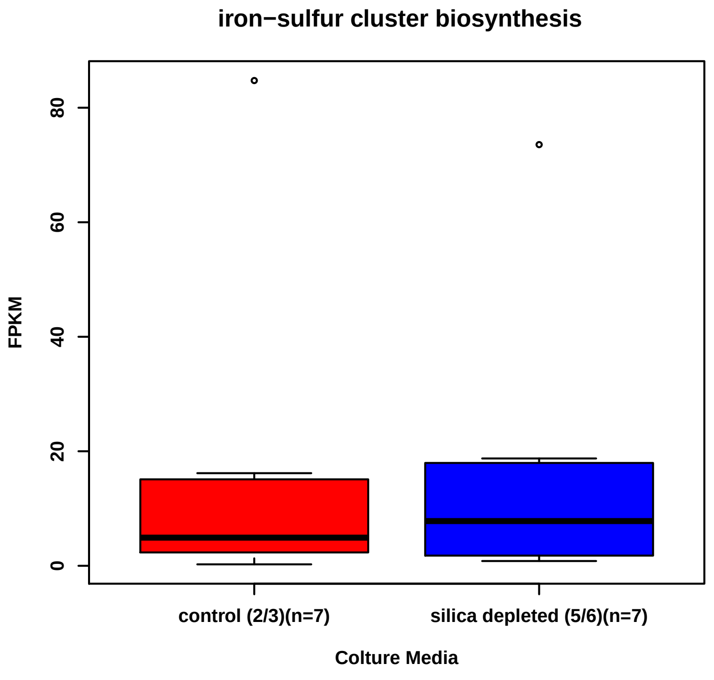
iron−sulfur cluster biosynthesis
FPKM
0
20
40
60
80
control (2/3)(n=7)
silica depleted (5/6)(n=7)
Colture Media

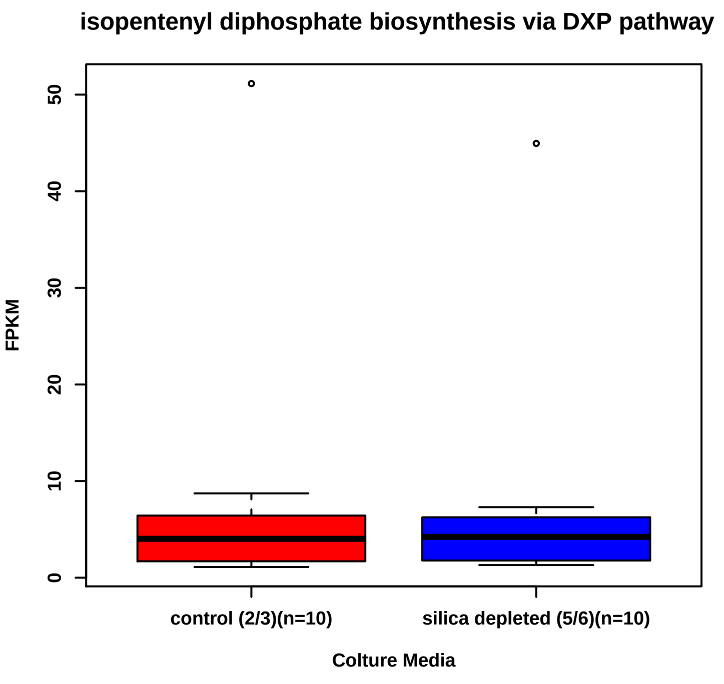
isopentenyl diphosphate biosynthesis via DXP pathway
FPKM
0
10
20
30
40
50
control (2/3)(n=10)
silica depleted (5/6)(n=10)
Colture Media

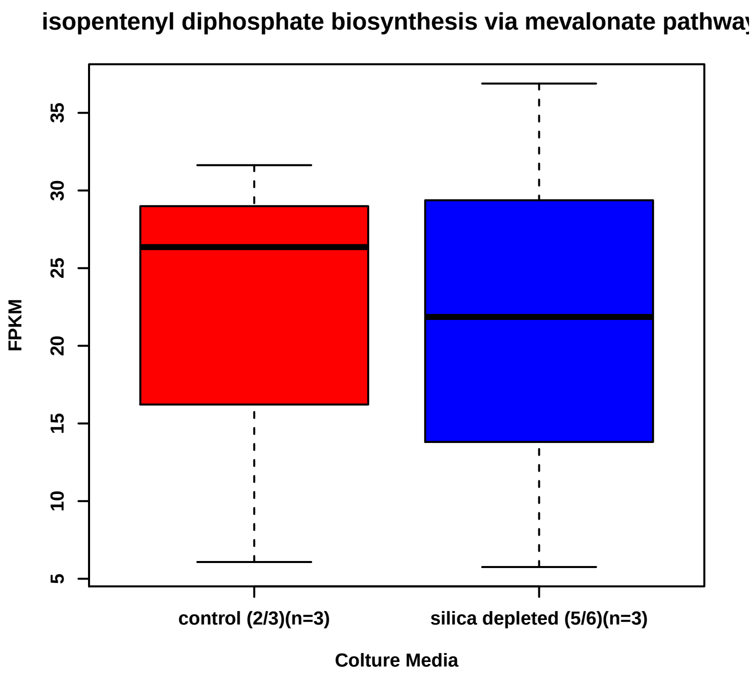
isopentenyl diphosphate biosynthesis via mevalonate pathway
FPKM
5
10
15
20
25
30
35
control (2/3)(n=3)
silica depleted (5/6)(n=3)
Colture Media

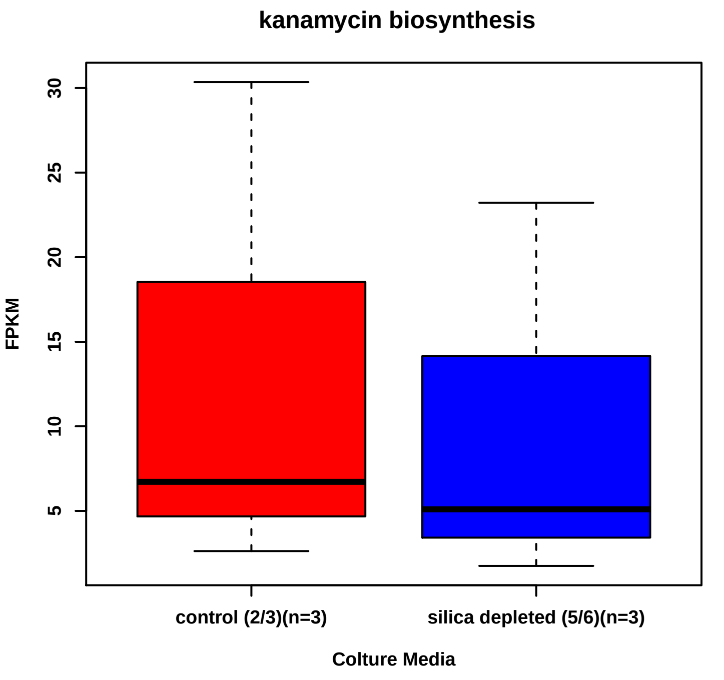
kanamycin biosynthesis
FPKM
5
10
15
20
25
30
control (2/3)(n=3)
silica depleted (5/6)(n=3)
Colture Media

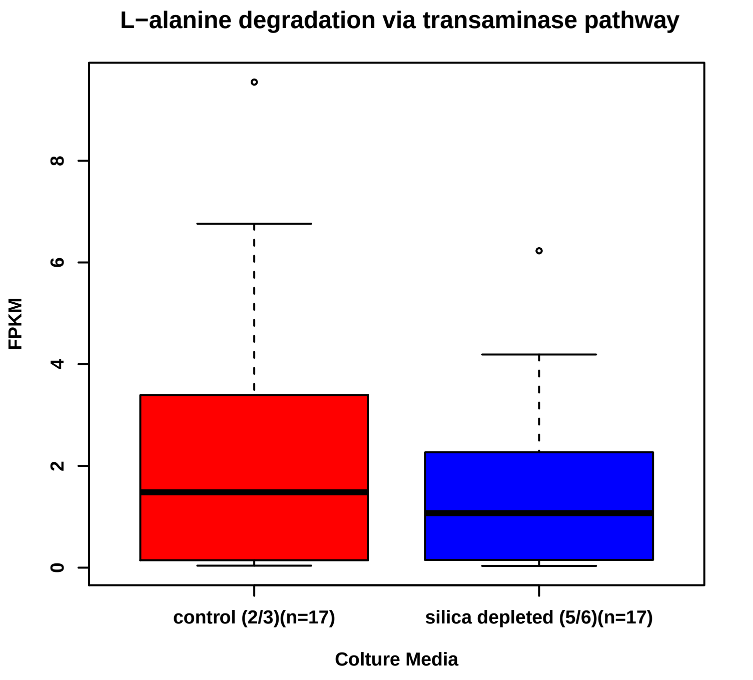
L−alanine degradation via transaminase pathway
FPKM
0
2
4
6
8
control (2/3)(n=17)
silica depleted (5/6)(n=17)
Colture Media

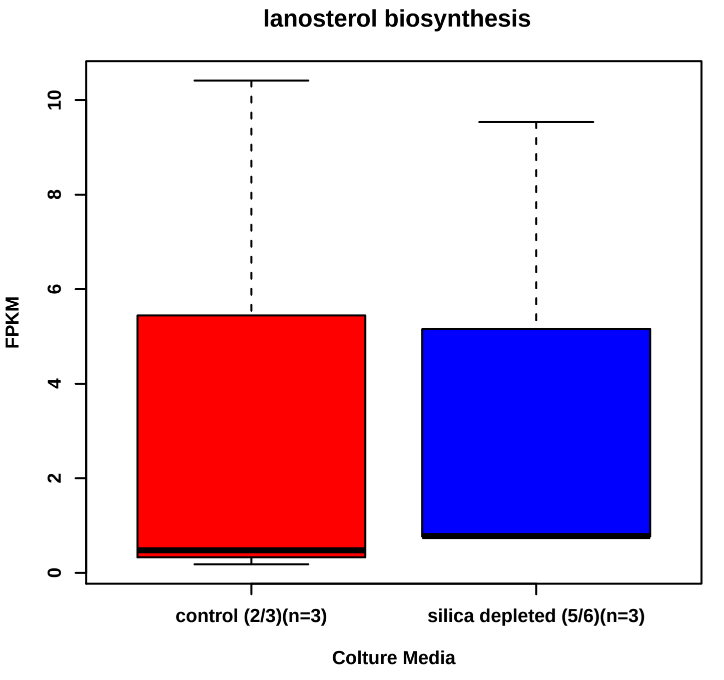
lanosterol biosynthesis
FPKM
0
2
4
6
8
10
control (2/3)(n=3)
silica depleted (5/6)(n=3)
Colture Media

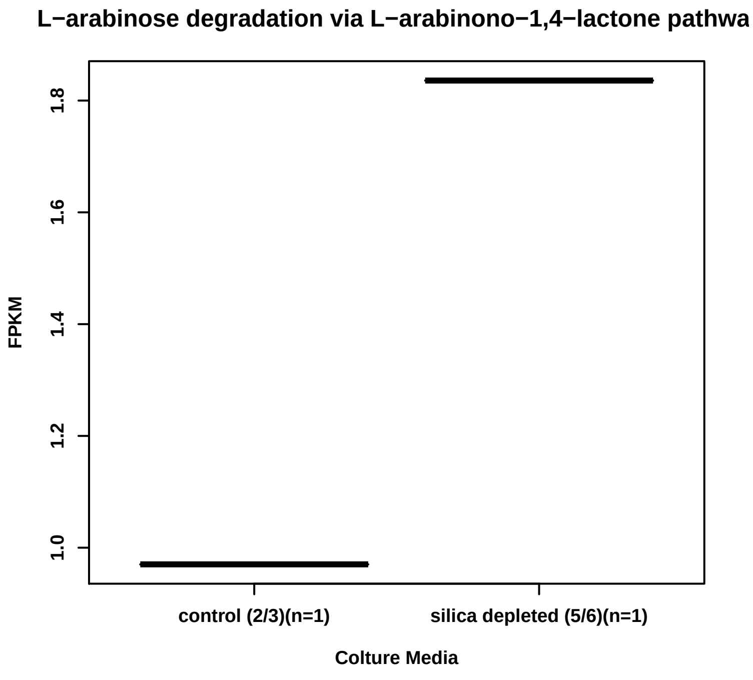
L−arabinose degradation via L−arabinono−1,4−lactone pathwa
FPKM
1.0
1.2
1.4
1.6
1.8
control (2/3)(n=1)
silica depleted (5/6)(n=1)
Colture Media

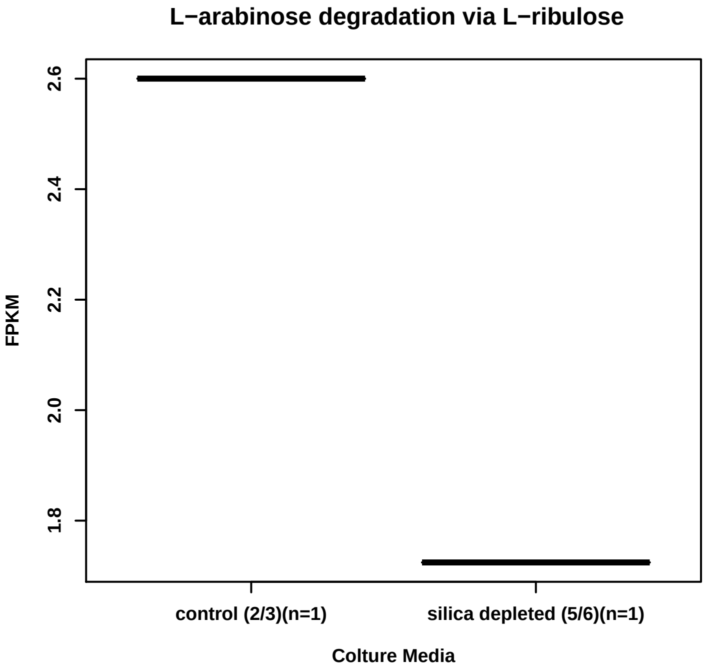
L−arabinose degradation via L−ribulose
FPKM
1.8
2.0
2.2
2.4
2.6
control (2/3)(n=1)
silica depleted (5/6)(n=1)
Colture Media

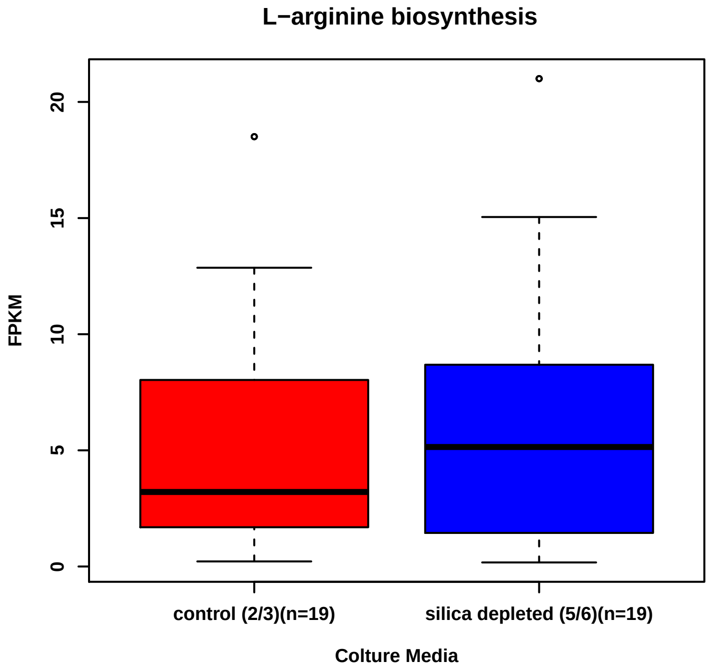
L−arginine biosynthesis
FPKM
0
5
10
15
20
control (2/3)(n=19)
silica depleted (5/6)(n=19)
Colture Media

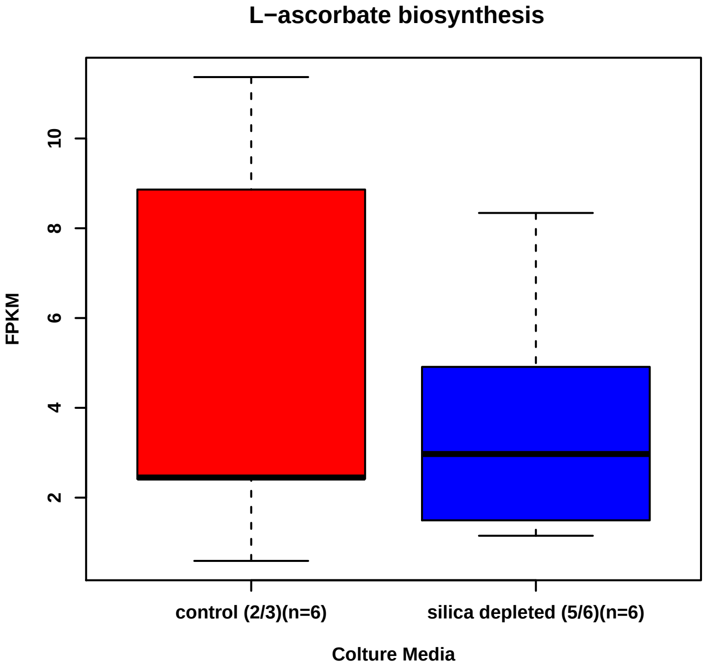
L−ascorbate biosynthesis
FPKM
2
4
6
8
10
control (2/3)(n=6)
silica depleted (5/6)(n=6)
Colture Media

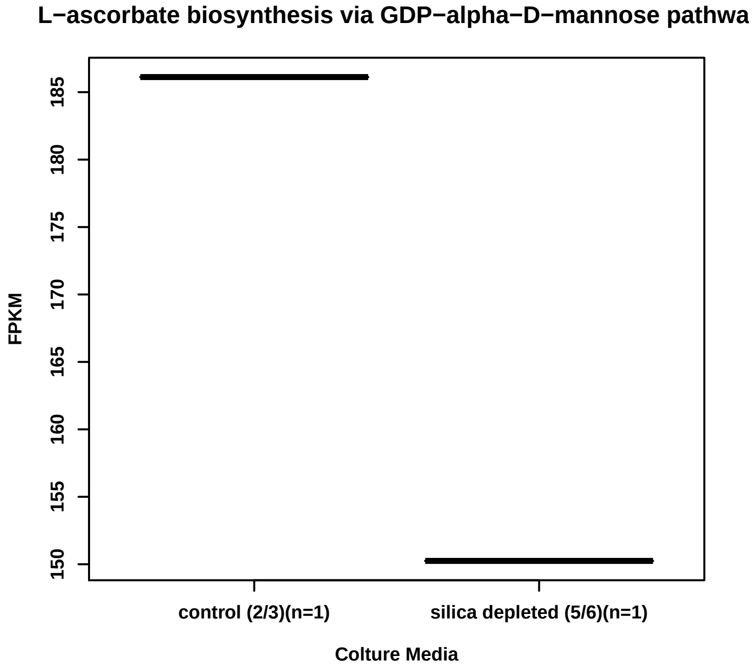
L−ascorbate biosynthesis via GDP−alpha−D−mannose pathwa
FPKM
150
155
160
165
170
175
180
185
control (2/3)(n=1)
silica depleted (5/6)(n=1)
Colture Media

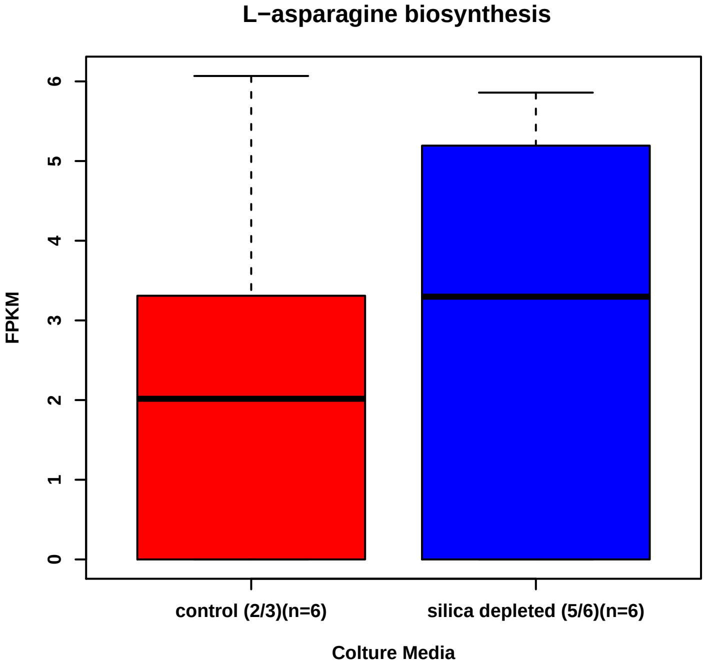
L−asparagine biosynthesis
FPKM
0
1
2
3
4
5
6
control (2/3)(n=6)
silica depleted (5/6)(n=6)
Colture Media

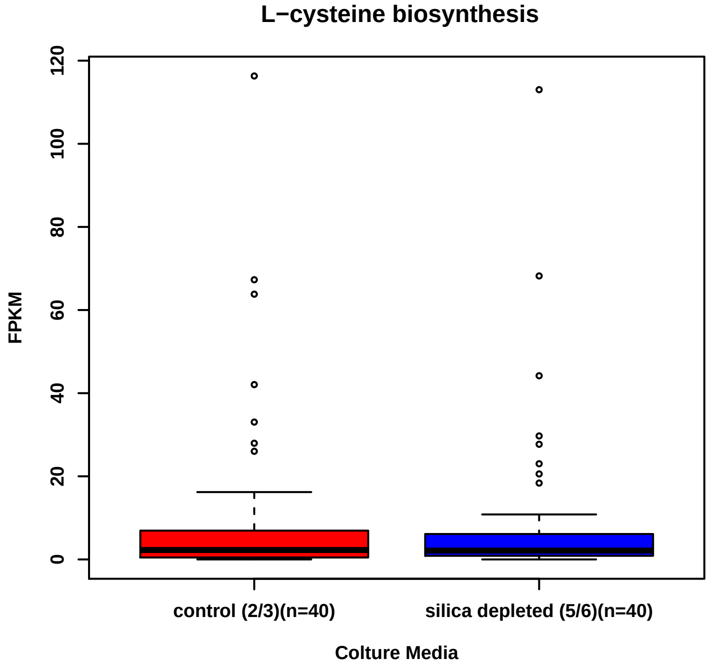
L−cysteine biosynthesis
FPKM
0
20
40
60
80
100
120
control (2/3)(n=40)
silica depleted (5/6)(n=40)
Colture Media

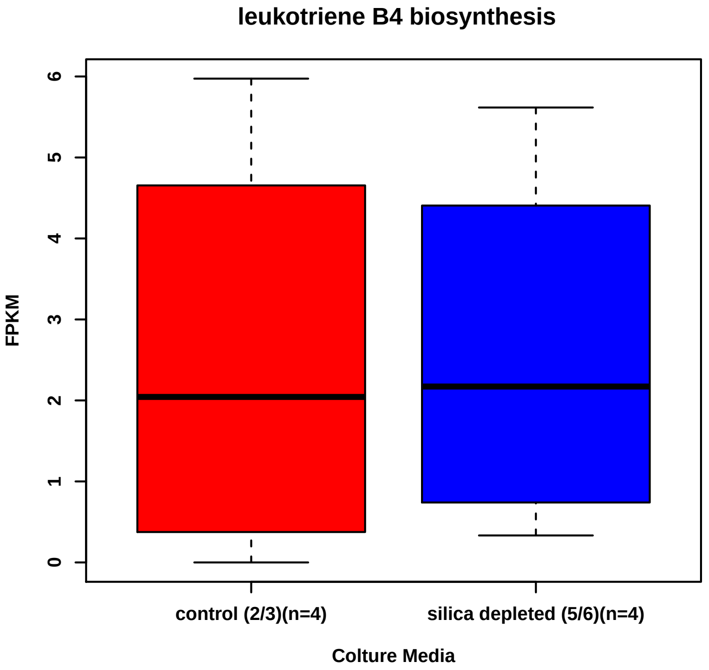
leukotriene B4 biosynthesis
FPKM
0
1
2
3
4
5
6
control (2/3)(n=4)
silica depleted (5/6)(n=4)
Colture Media

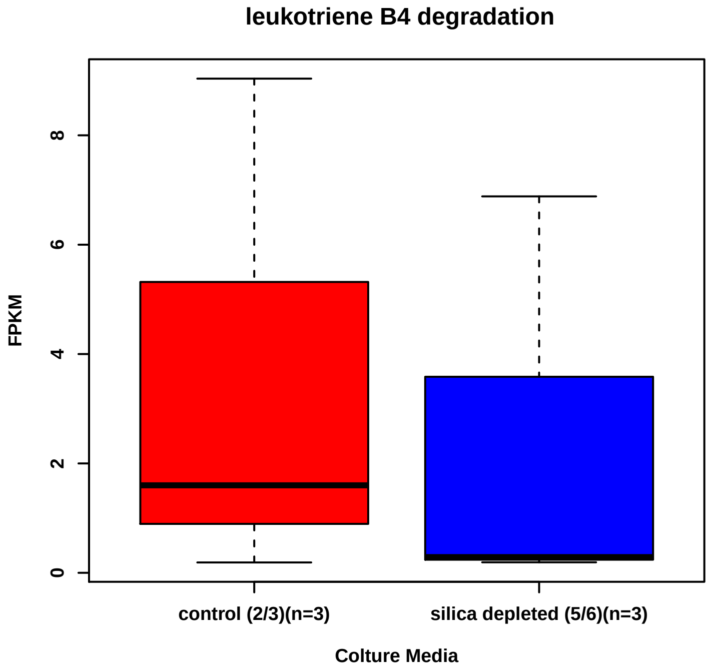
leukotriene B4 degradation
FPKM
0
2
4
6
8
control (2/3)(n=3)
silica depleted (5/6)(n=3)
Colture Media

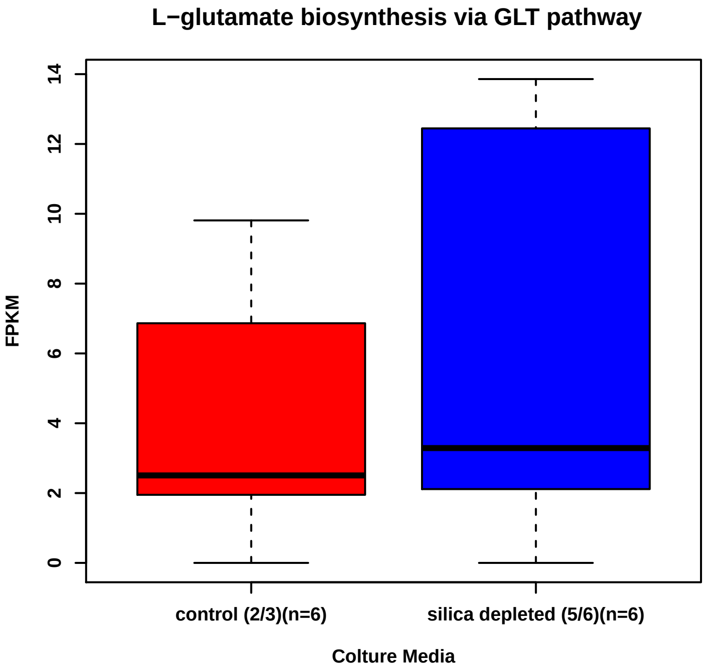
L−glutamate biosynthesis via GLT pathway
FPKM
0
2
4
6
8
10
12
14
control (2/3)(n=6)
silica depleted (5/6)(n=6)
Colture Media

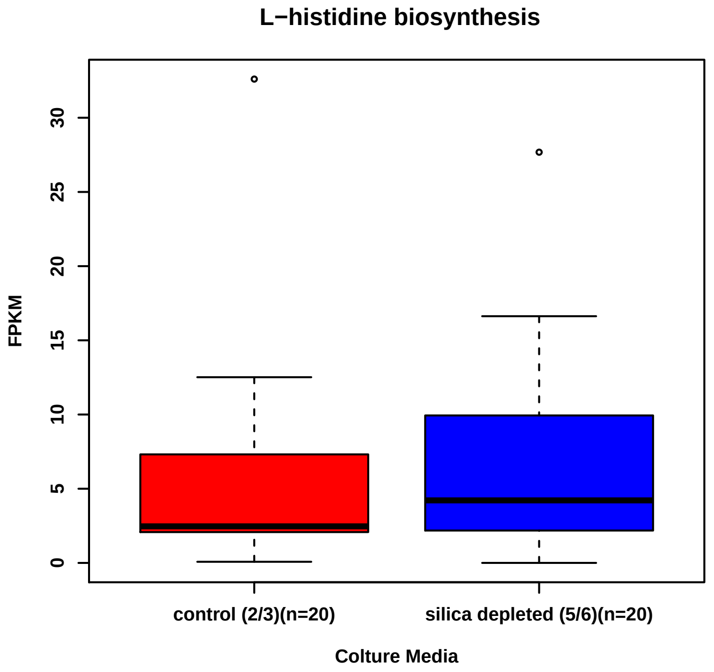
L−histidine biosynthesis
FPKM
0
5
10
15
20
25
30
control (2/3)(n=20)
silica depleted (5/6)(n=20)
Colture Media

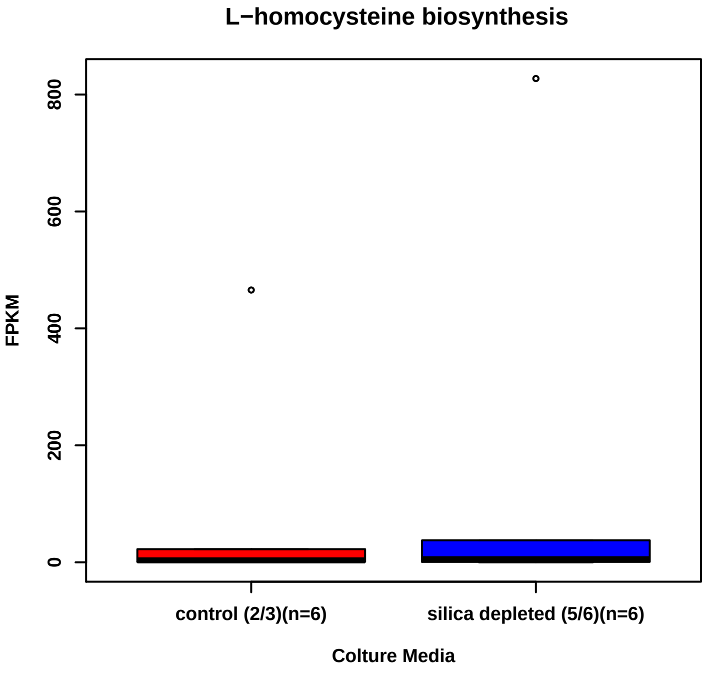
L−homocysteine biosynthesis
FPKM
0
200
400
600
800
control (2/3)(n=6)
silica depleted (5/6)(n=6)
Colture Media

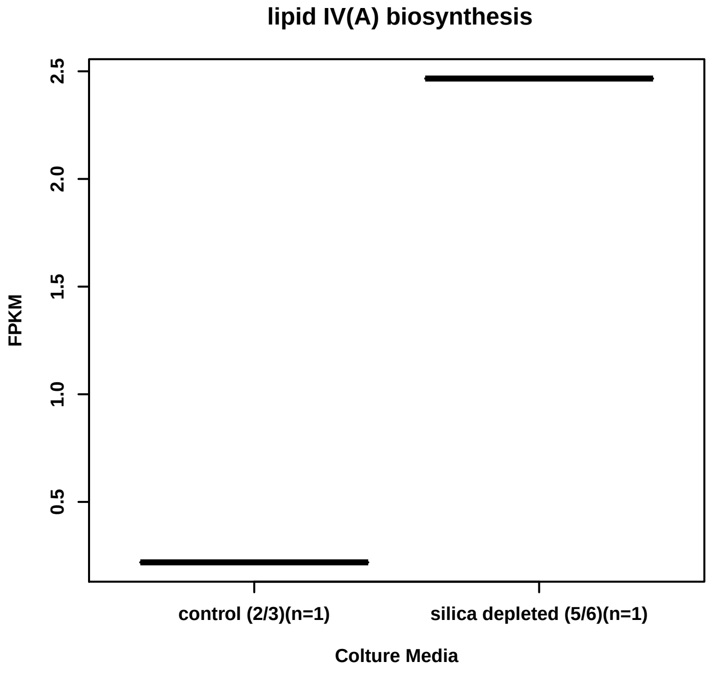
lipid IV(A) biosynthesis
FPKM
0.5
1.0
1.5
2.0
2.5
control (2/3)(n=1)
silica depleted (5/6)(n=1)
Colture Media

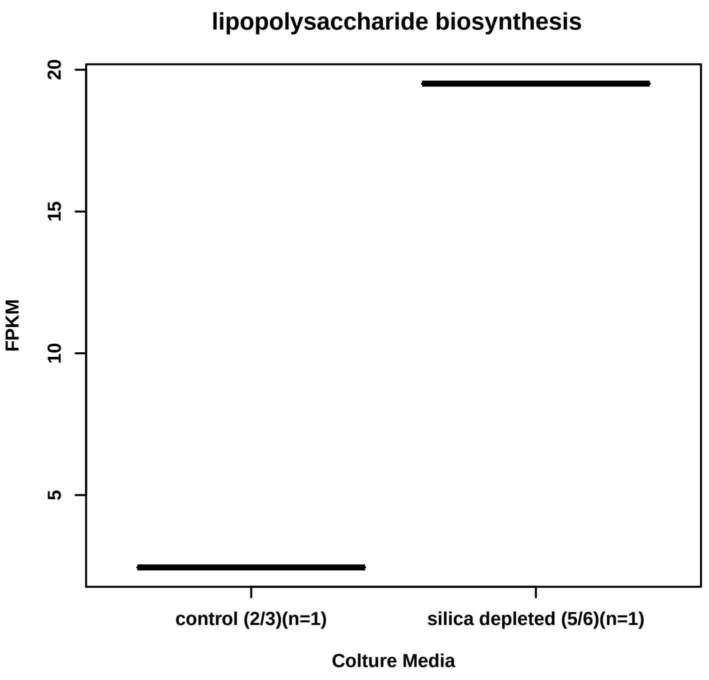
lipopolysaccharide biosynthesis
FPKM
5
10
15
20
control (2/3)(n=1)
silica depleted (5/6)(n=1)
Colture Media

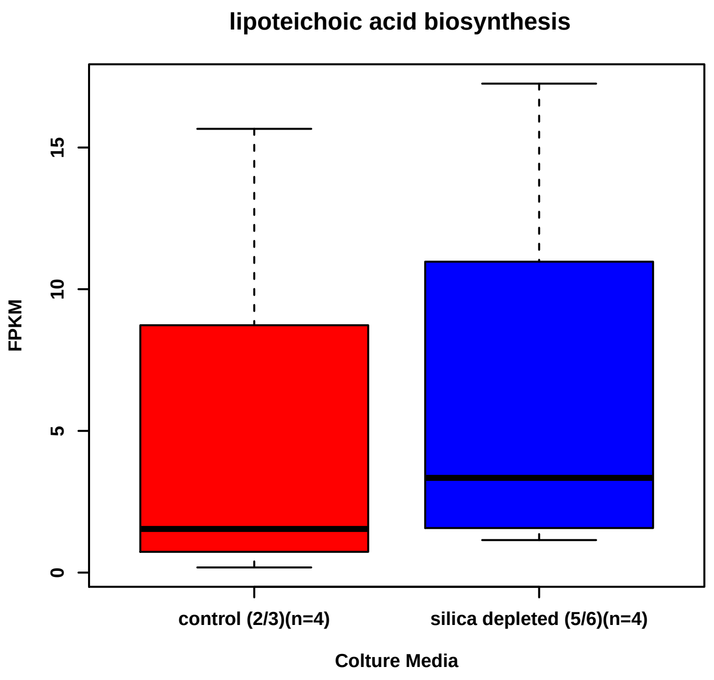
lipoteichoic acid biosynthesis
FPKM
0
5
10
15
control (2/3)(n=4)
silica depleted (5/6)(n=4)
Colture Media

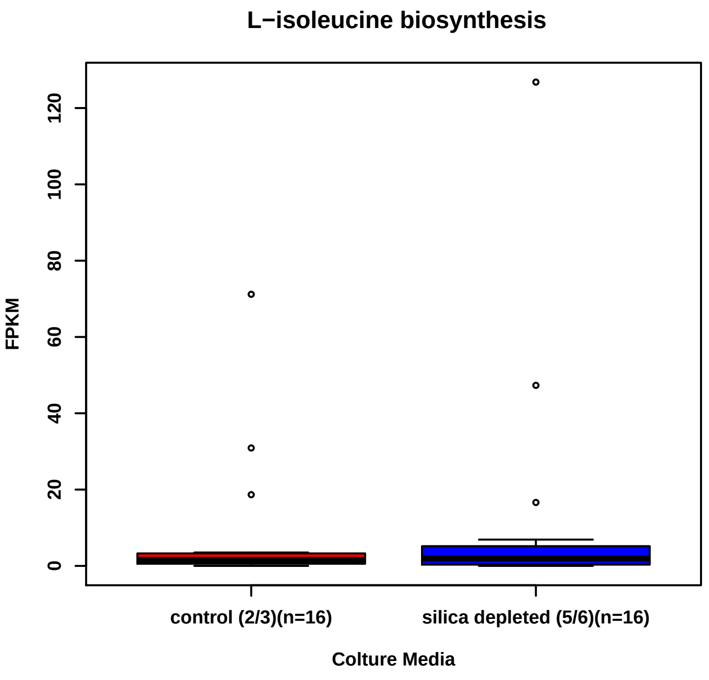
L−isoleucine biosynthesis
FPKM
0
20
40
60
80
100
120
control (2/3)(n=16)
silica depleted (5/6)(n=16)
Colture Media

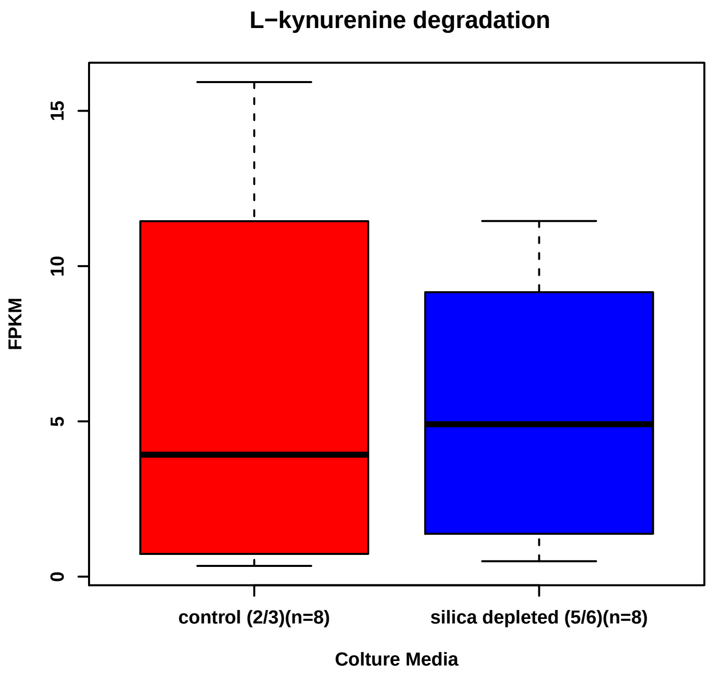
L−kynurenine degradation
FPKM
0
5
10
15
control (2/3)(n=8)
silica depleted (5/6)(n=8)
Colture Media

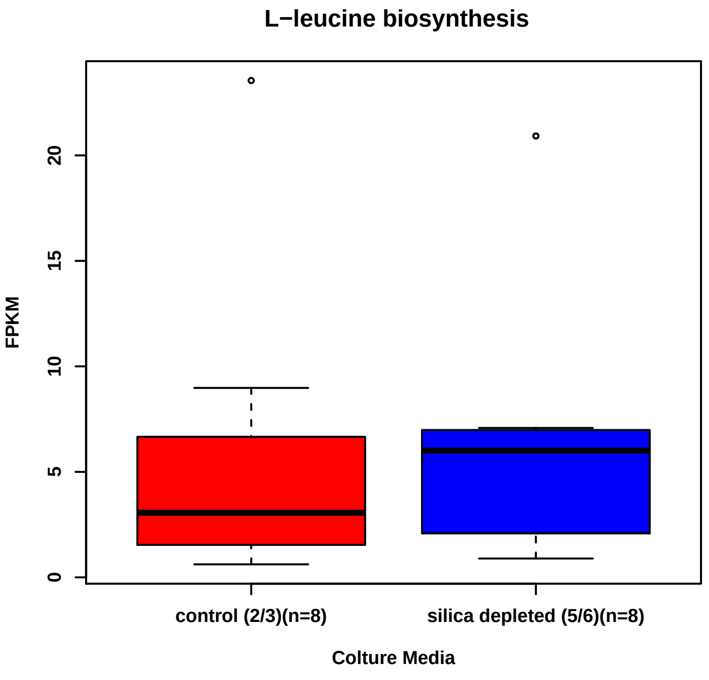
L−leucine biosynthesis
FPKM
0
5
10
15
20
control (2/3)(n=8)
silica depleted (5/6)(n=8)
Colture Media

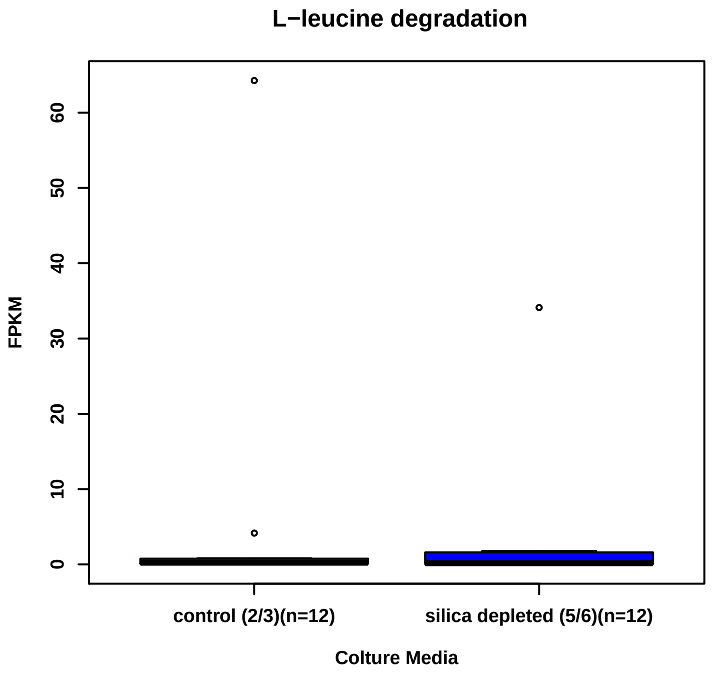
L−leucine degradation
FPKM
0
10
20
30
40
50
60
control (2/3)(n=12)
silica depleted (5/6)(n=12)
Colture Media

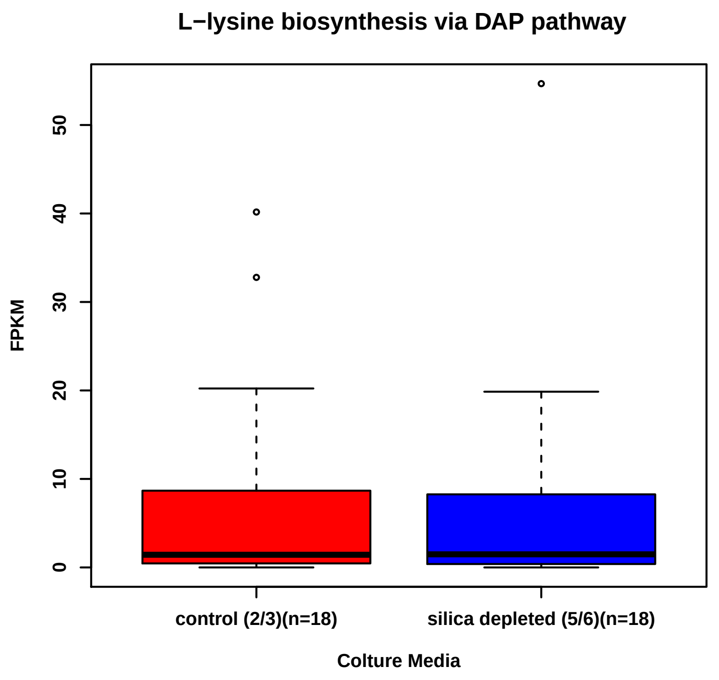

L−lysine biosynthesis via DAP pathway
FPKM
0
10
20
30
40
50
control (2/3)(n=18)
silica depleted (5/6)(n=18)
Colture Media

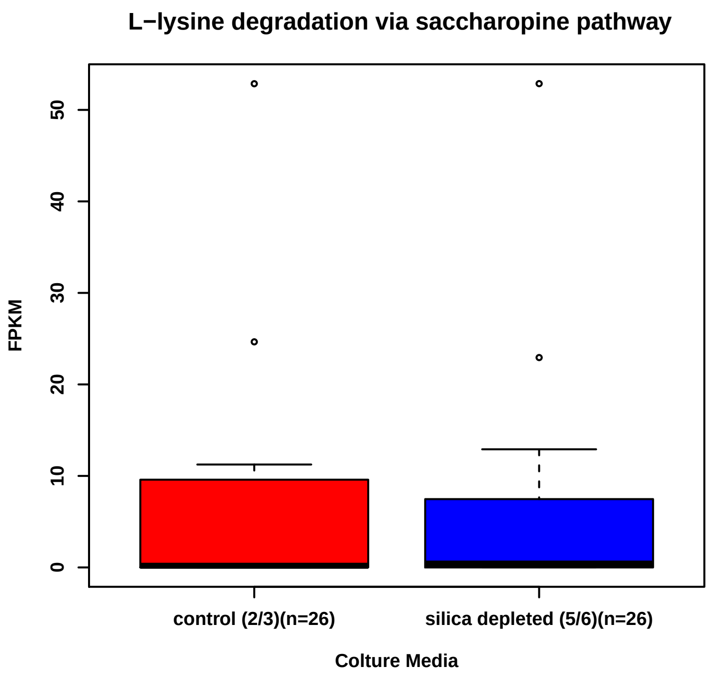

L−lysine degradation via saccharopine pathway
FPKM
0
10
20
30
40
50
control (2/3)(n=26)
silica depleted (5/6)(n=26)
Colture Media

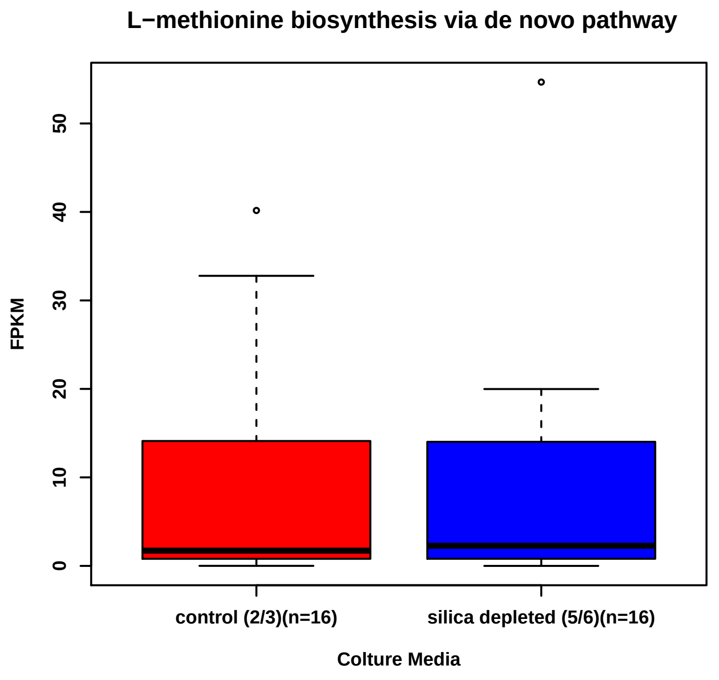

L−methionine biosynthesis via de novo pathway
FPKM
0
10
20
30
40
50
control (2/3)(n=16)
silica depleted (5/6)(n=16)
Colture Media

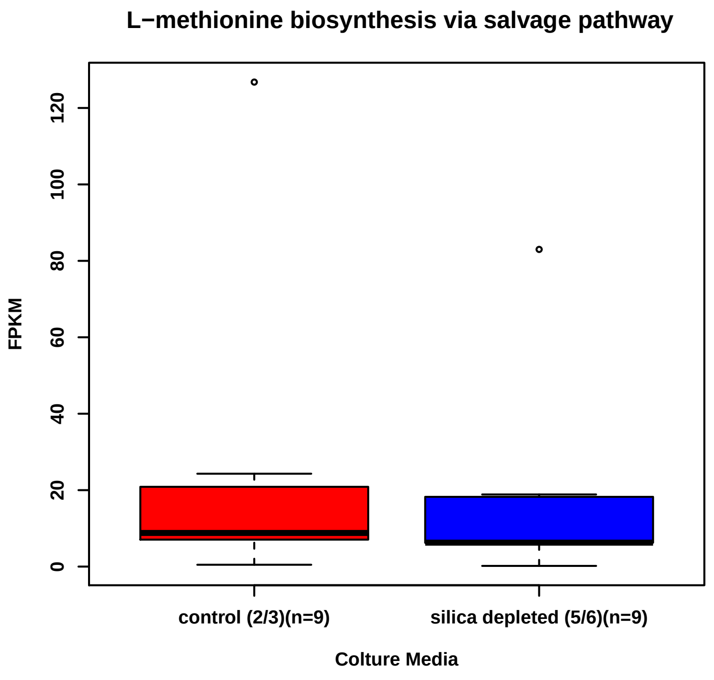

L−methionine biosynthesis via salvage pathway
FPKM
0
20
40
60
80
100
120
control (2/3)(n=9)
silica depleted (5/6)(n=9)
Colture Media

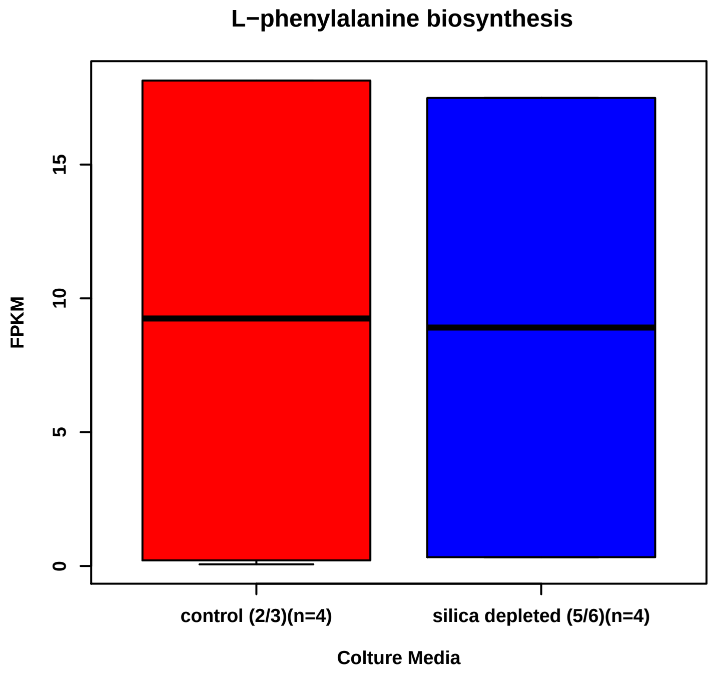

L−phenylalanine biosynthesis
FPKM
0
5
10
15
control (2/3)(n=4)
silica depleted (5/6)(n=4)
Colture Media

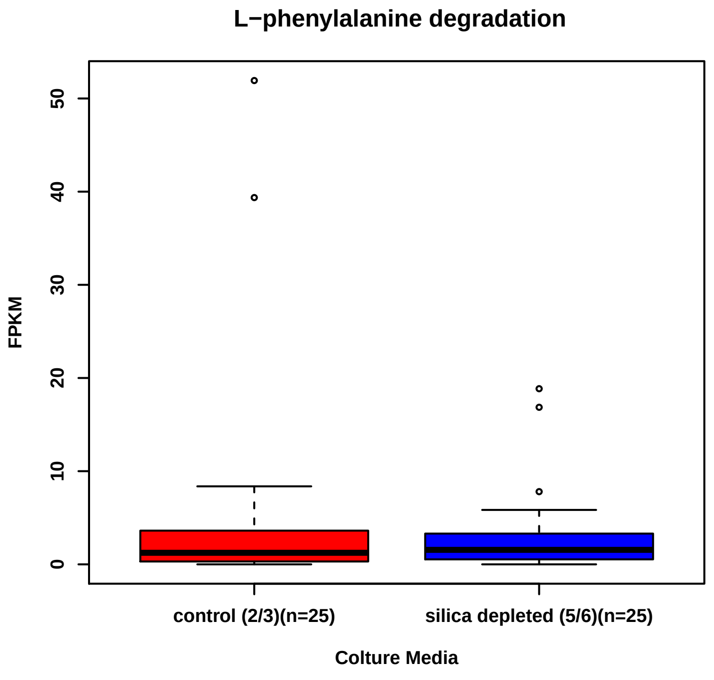

L−phenylalanine degradation
FPKM
0
10
20
30
40
50
control (2/3)(n=25)
silica depleted (5/6)(n=25)
Colture Media

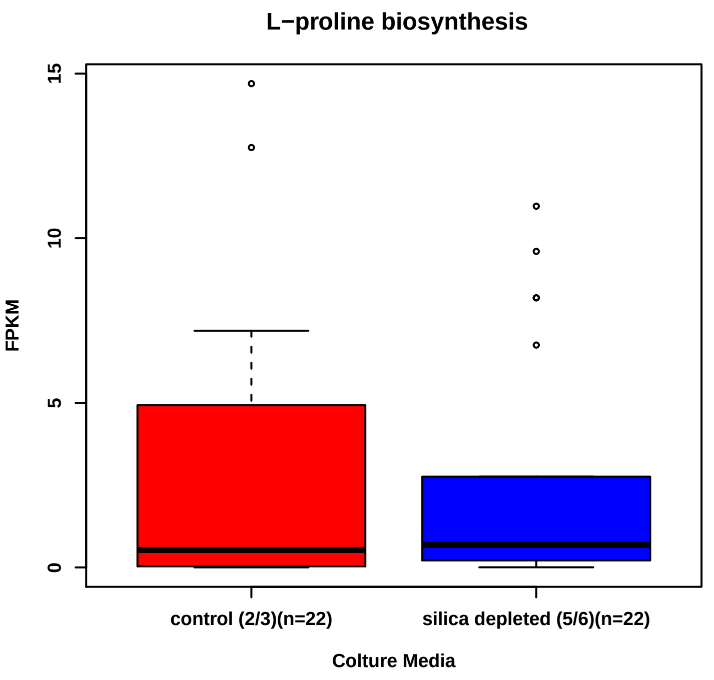
L−proline biosynthesis
FPKM
0
5
10
15
control (2/3)(n=22)
silica depleted (5/6)(n=22)
Colture Media

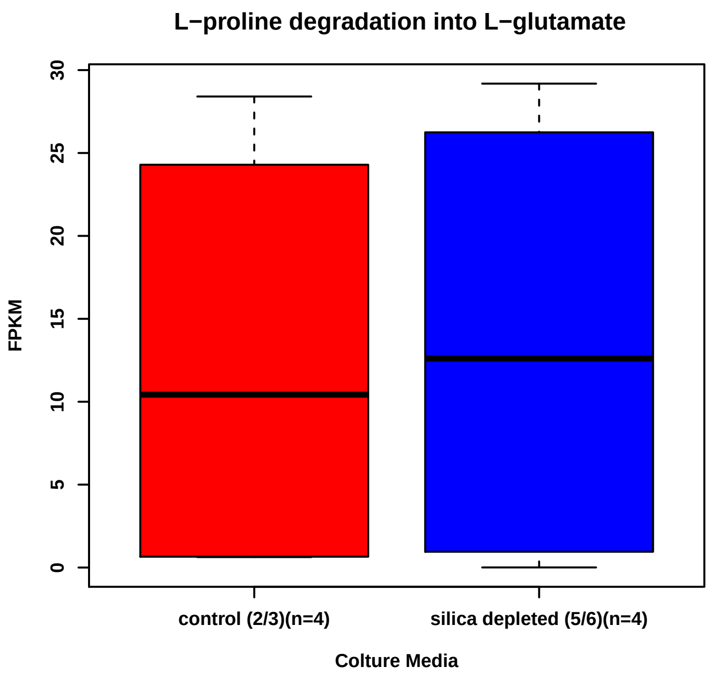
L−proline degradation into L−glutamate
FPKM
0
5
10
15
20
25
30
control (2/3)(n=4)
silica depleted (5/6)(n=4)
Colture Media

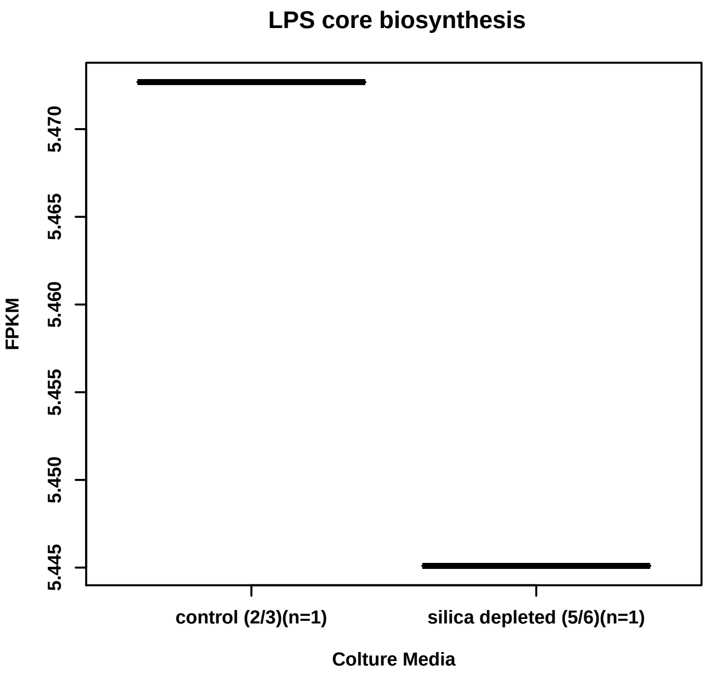
LPS core biosynthesis
FPKM
5.445
5.450
5.455
5.460
5.465
5.470
control (2/3)(n=1)
silica depleted (5/6)(n=1)
Colture Media

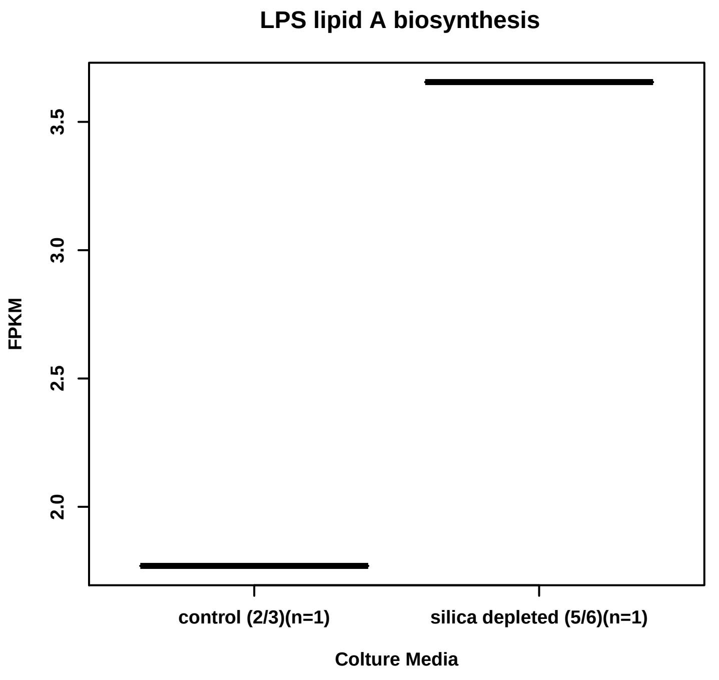
LPS lipid A biosynthesis
FPKM
2.0
2.5
3.0
3.5
control (2/3)(n=1)
silica depleted (5/6)(n=1)
Colture Media

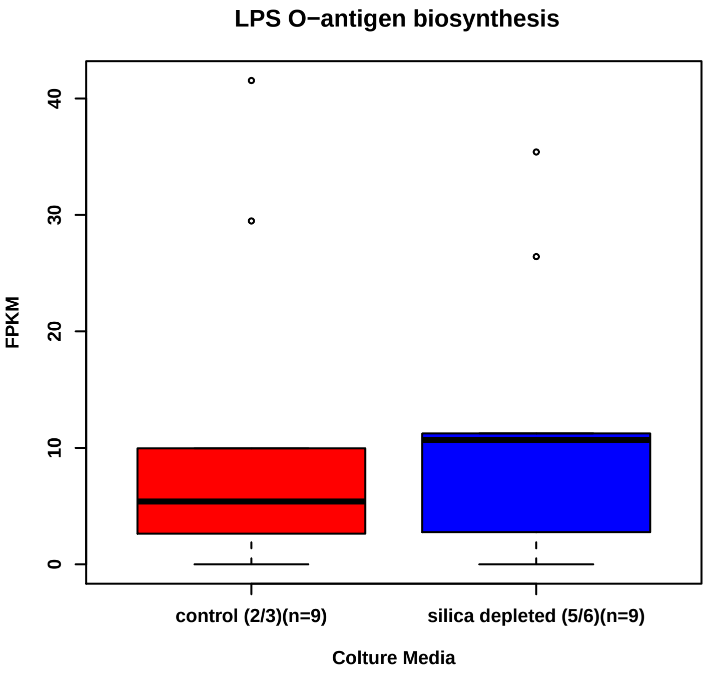
LPS O−antigen biosynthesis
FPKM
0
10
20
30
40
control (2/3)(n=9)
silica depleted (5/6)(n=9)
Colture Media

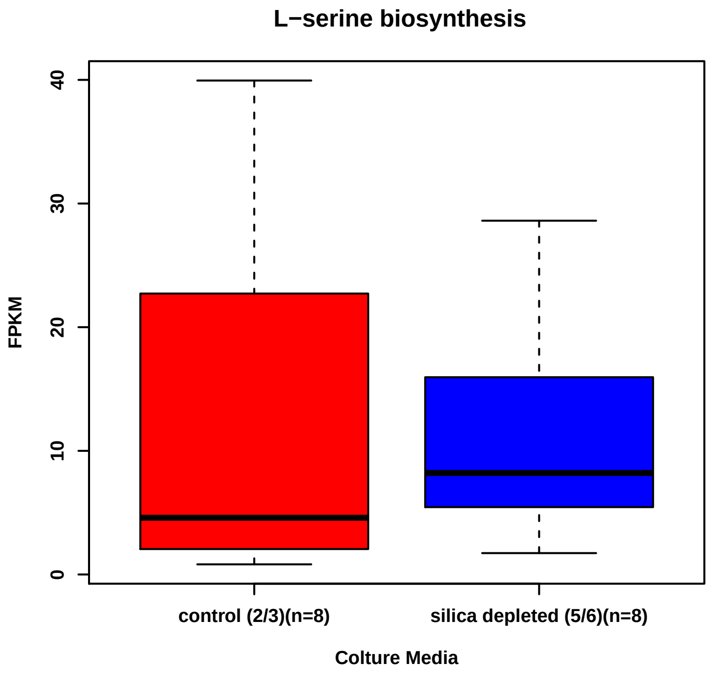
L−serine biosynthesis
FPKM
0
10
20
30
40
control (2/3)(n=8)
silica depleted (5/6)(n=8)
Colture Media

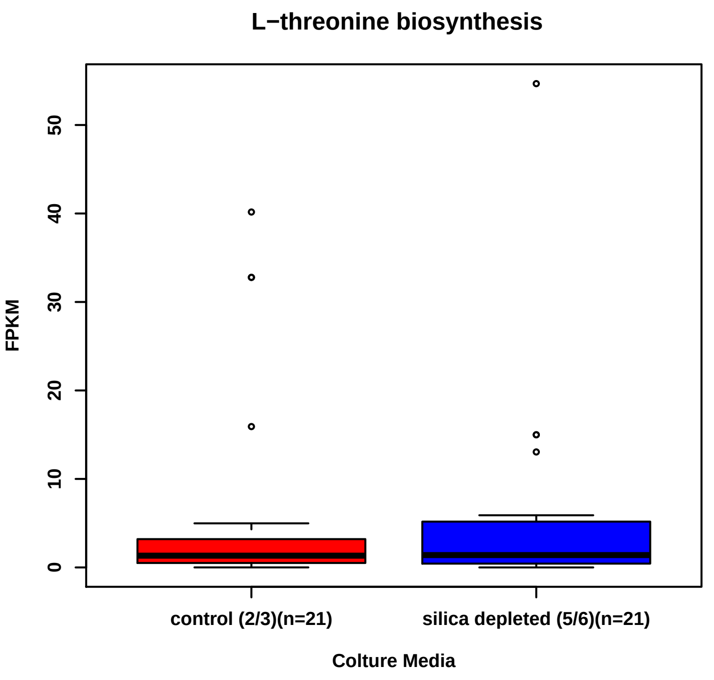

L−threonine biosynthesis
FPKM
0
10
20
30
40
50
control (2/3)(n=21)
silica depleted (5/6)(n=21)
Colture Media

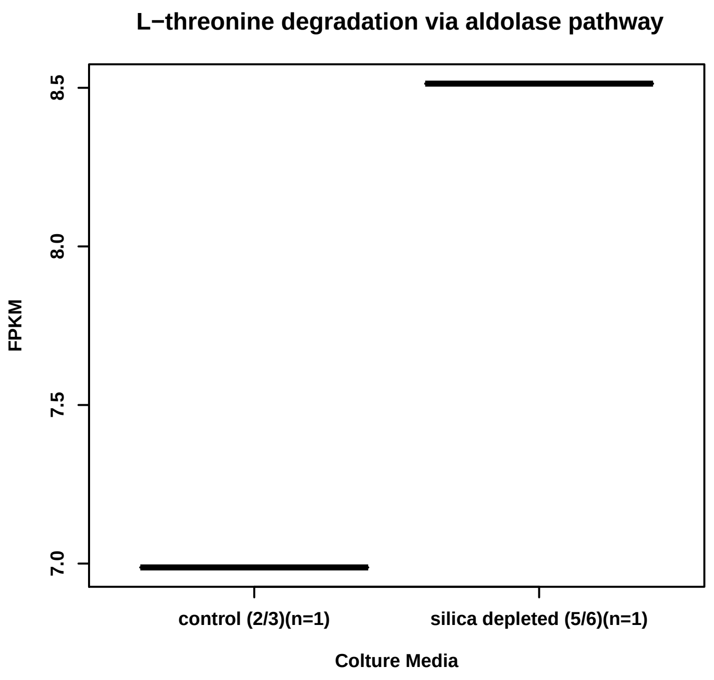

L−threonine degradation via aldolase pathway
FPKM
7.0
7.5
8.0
8.5
control (2/3)(n=1)
silica depleted (5/6)(n=1)
Colture Media

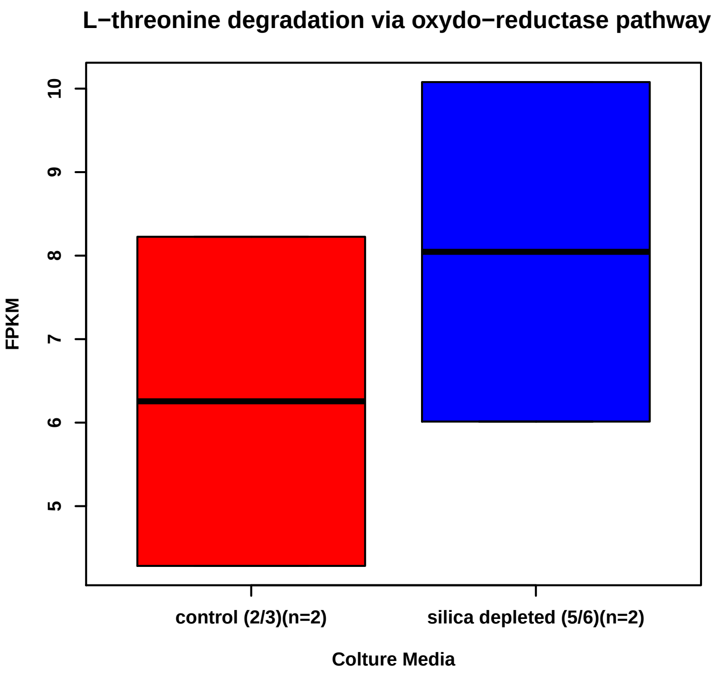

L−threonine degradation via oxydo−reductase pathway
FPKM
5
6
7
8
9
10
control (2/3)(n=2)
silica depleted (5/6)(n=2)
Colture Media

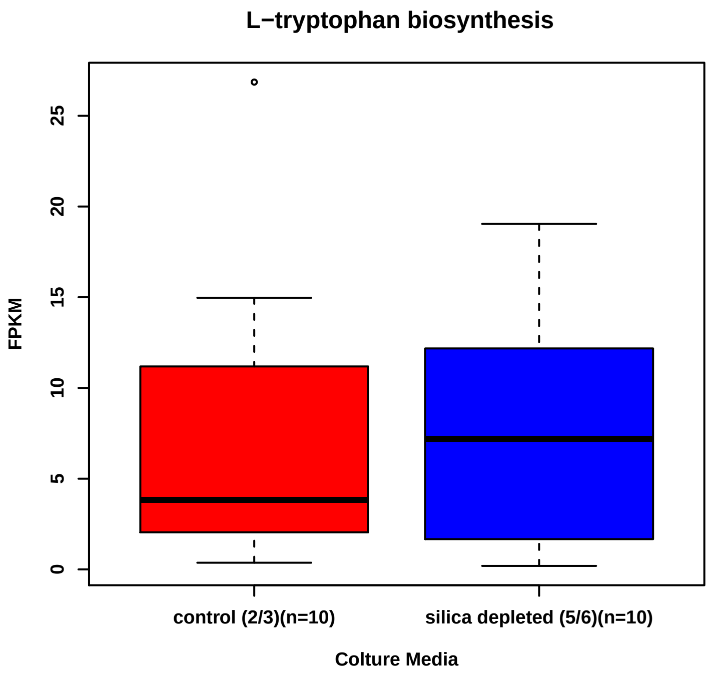

L−tryptophan biosynthesis
FPKM
0
5
10
15
20
25
control (2/3)(n=10)
silica depleted (5/6)(n=10)
Colture Media

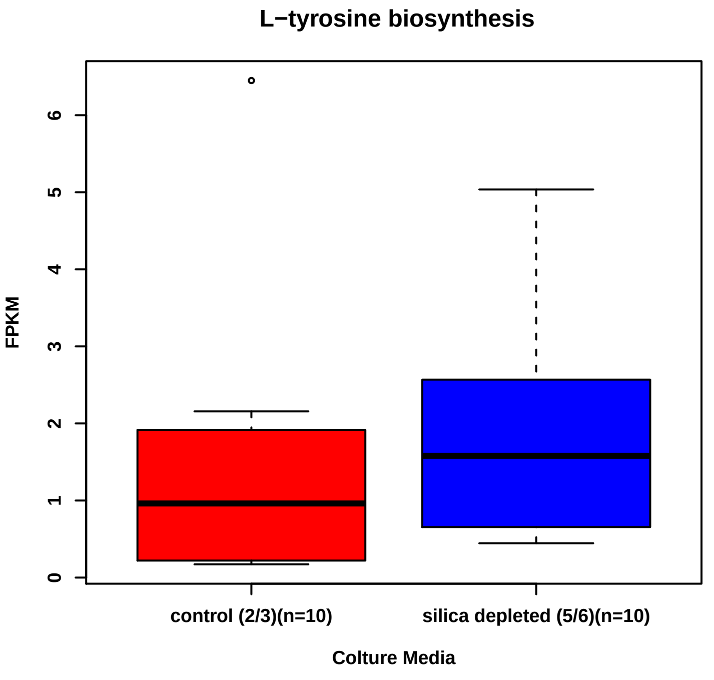

L−tyrosine biosynthesis
FPKM
0
1
2
3
4
5
6
control (2/3)(n=10)
silica depleted (5/6)(n=10)
Colture Media

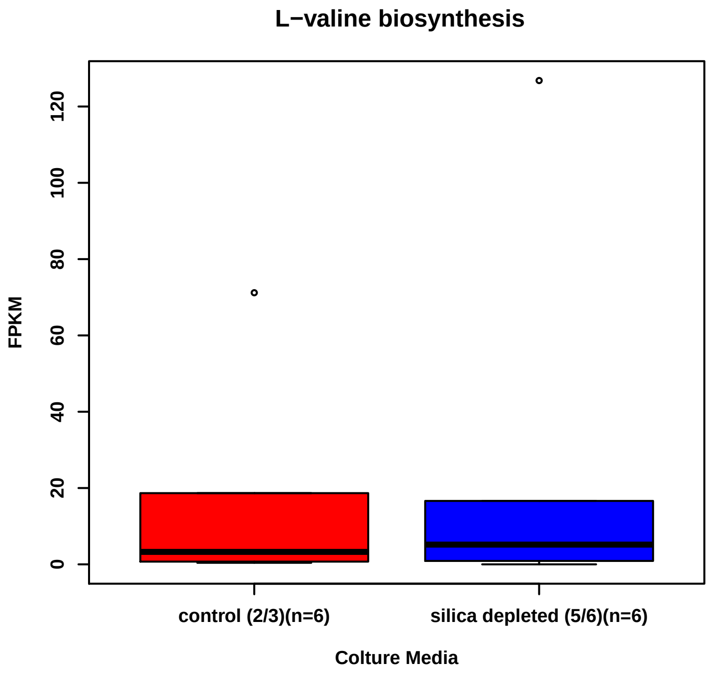

L−valine biosynthesis
FPKM
0
20
40
60
80
100
120
control (2/3)(n=6)
silica depleted (5/6)(n=6)
Colture Media

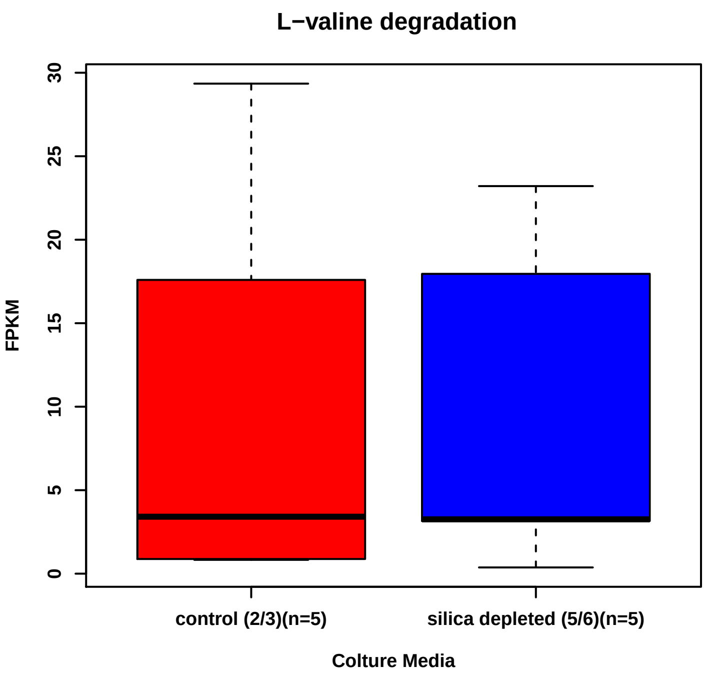
L−valine degradation
FPKM
0
5
10
15
20
25
30
control (2/3)(n=5)
silica depleted (5/6)(n=5)
Colture Media

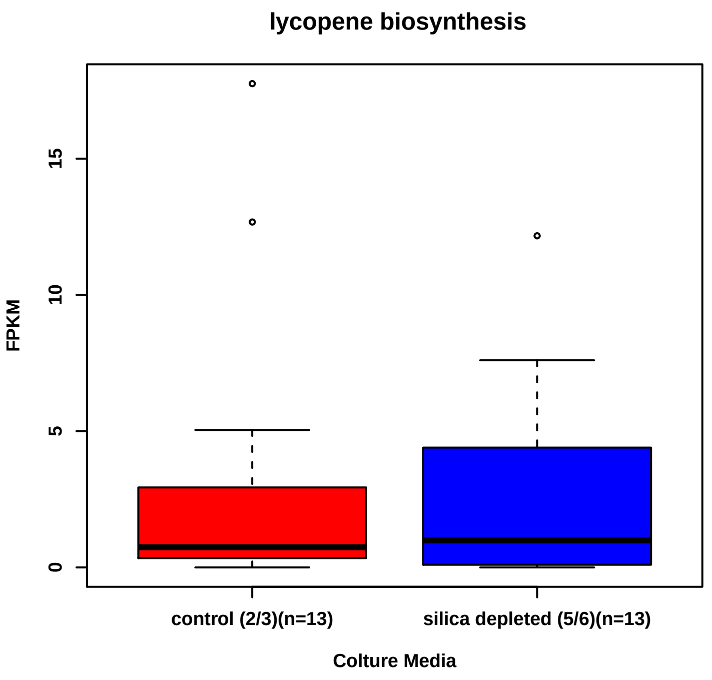
lycopene biosynthesis
FPKM
0
5
10
15
control (2/3)(n=13)
silica depleted (5/6)(n=13)
Colture Media

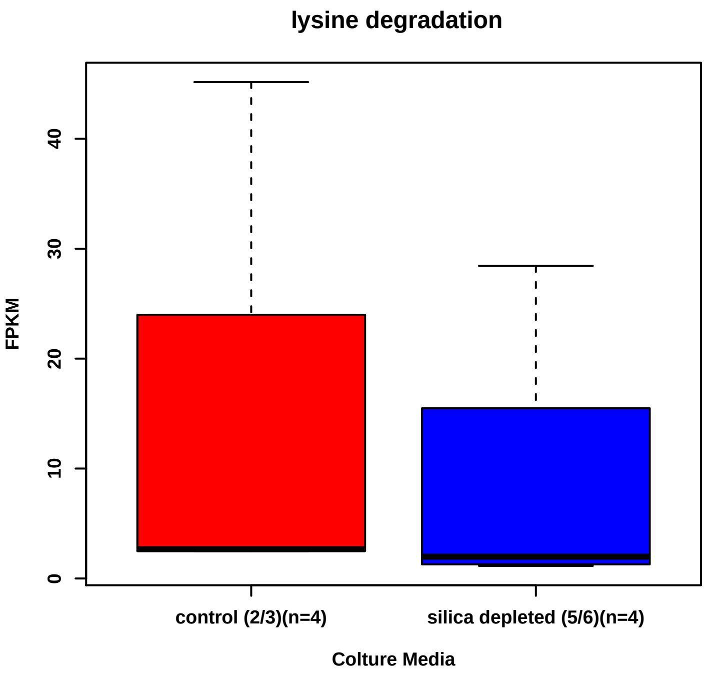
lysine degradation
FPKM
0
10
20
30
40
control (2/3)(n=4)
silica depleted (5/6)(n=4)
Colture Media

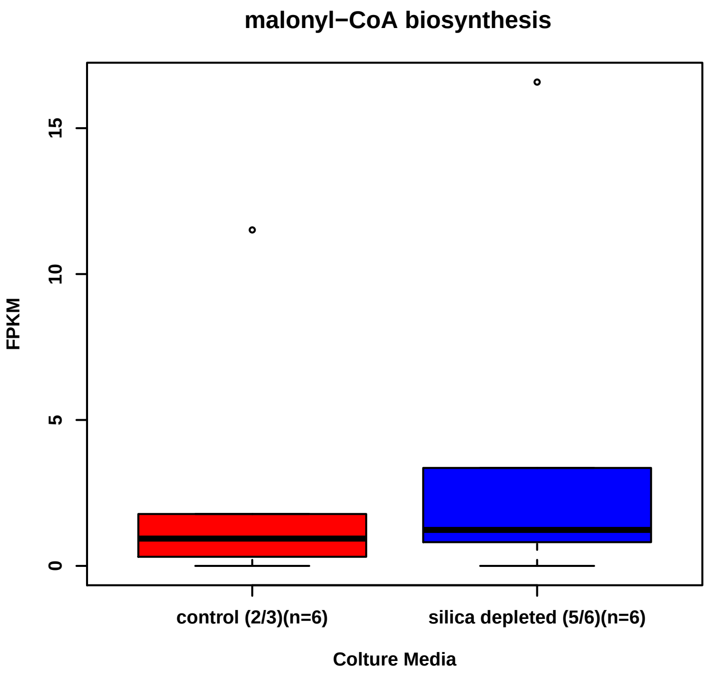
malonyl−CoA biosynthesis
FPKM
0
5
10
15
control (2/3)(n=6)
silica depleted (5/6)(n=6)
Colture Media

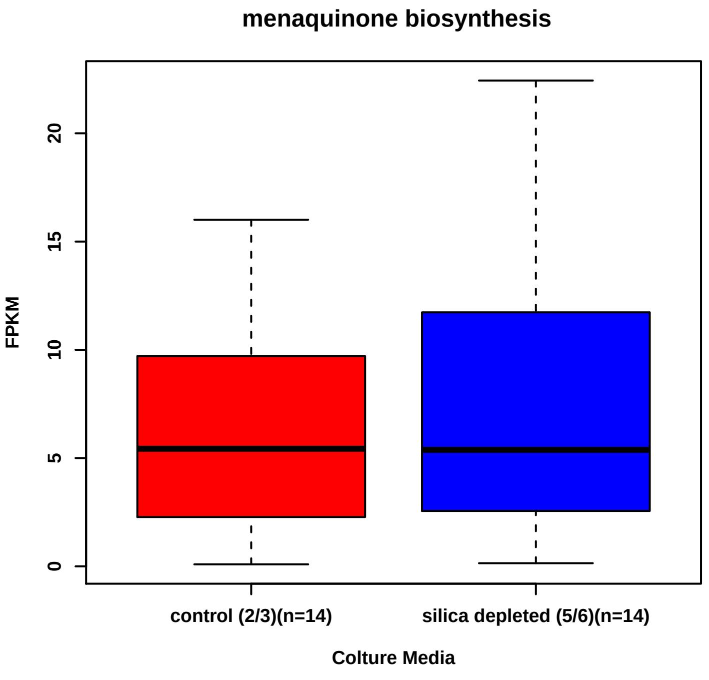
menaquinone biosynthesis
FPKM
0
5
10
15
20
control (2/3)(n=14)
silica depleted (5/6)(n=14)
Colture Media

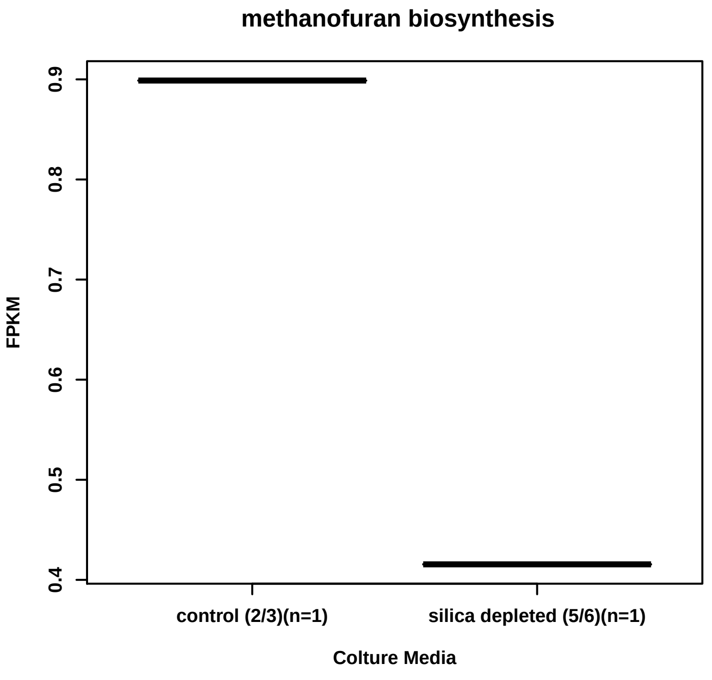
methanofuran biosynthesis
FPKM
0.4
0.5
0.6
0.7
0.8
0.9
control (2/3)(n=1)
silica depleted (5/6)(n=1)
Colture Media

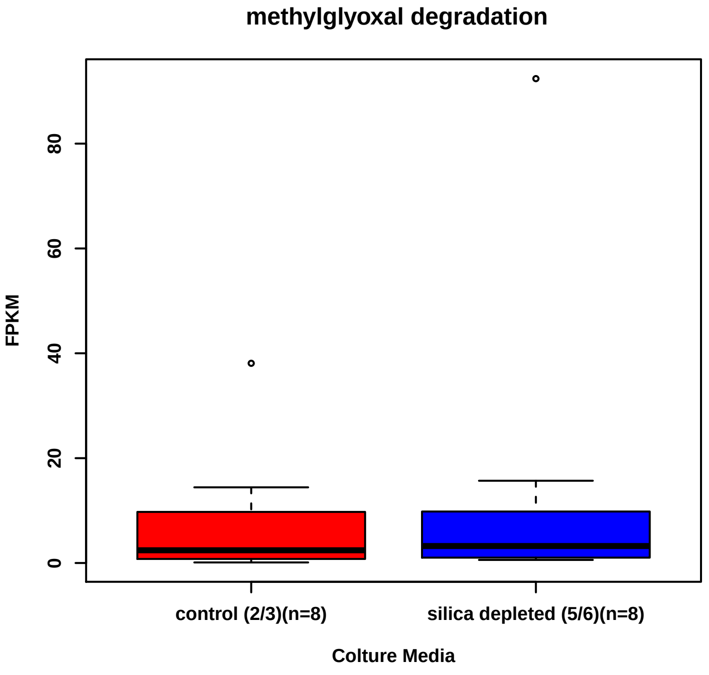

methylglyoxal degradation
FPKM
0
20
40
60
80
control (2/3)(n=8)
silica depleted (5/6)(n=8)
Colture Media

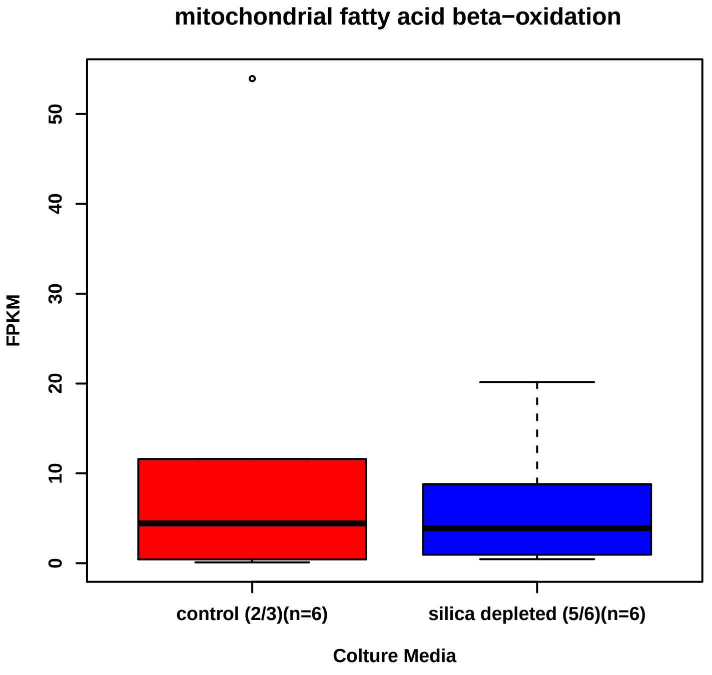

mitochondrial fatty acid beta−oxidation
FPKM
0
10
20
30
40
50
control (2/3)(n=6)
silica depleted (5/6)(n=6)
Colture Media

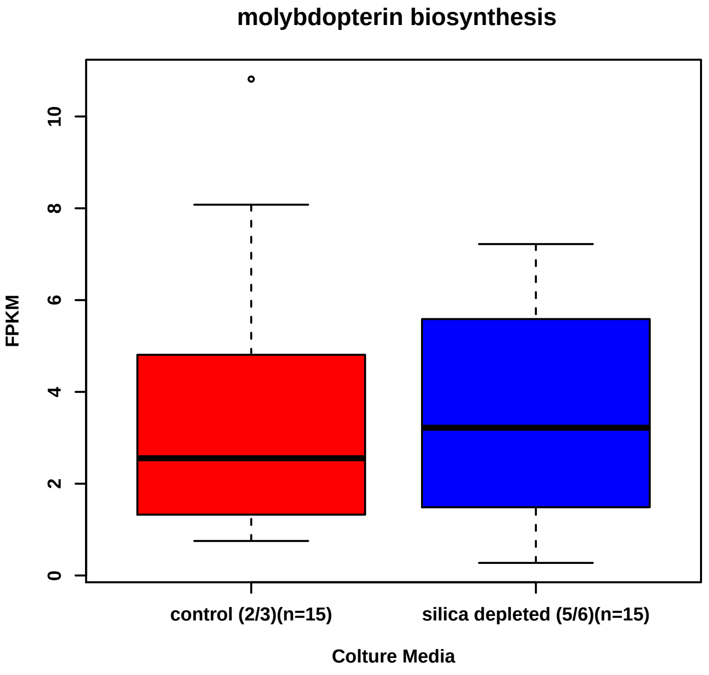

molybdopterin biosynthesis
FPKM
0
2
4
6
8
10
control (2/3)(n=15)
silica depleted (5/6)(n=15)
Colture Media

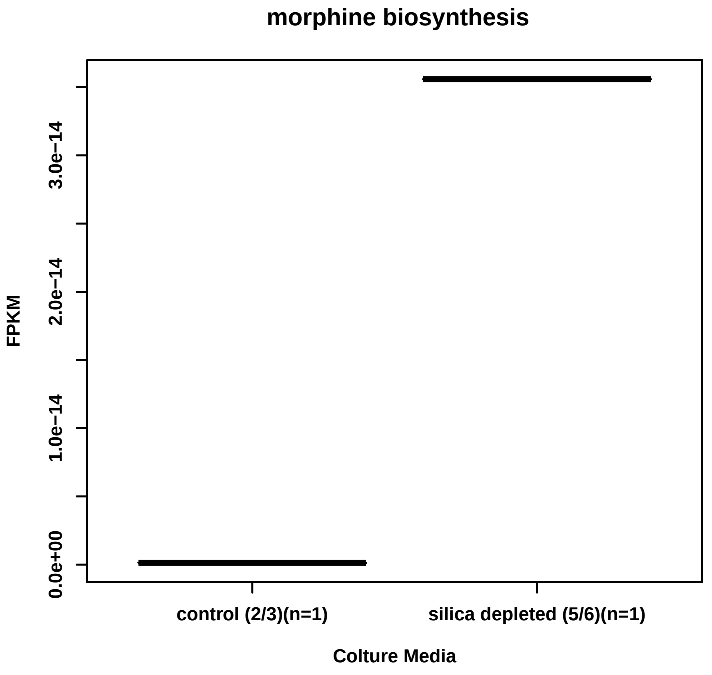

morphine biosynthesis
FPKM
0.0e+00
1.0e−14
2.0e−14
3.0e−14
control (2/3)(n=1)
silica depleted (5/6)(n=1)
Colture Media

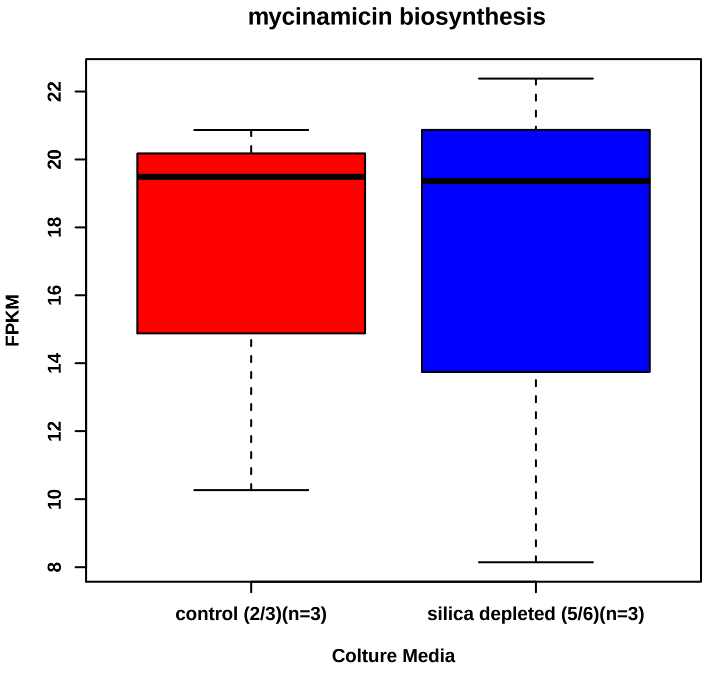

mycinamicin biosynthesis
FPKM
8
10
12
14
16
18
20
22
control (2/3)(n=3)
silica depleted (5/6)(n=3)
Colture Media

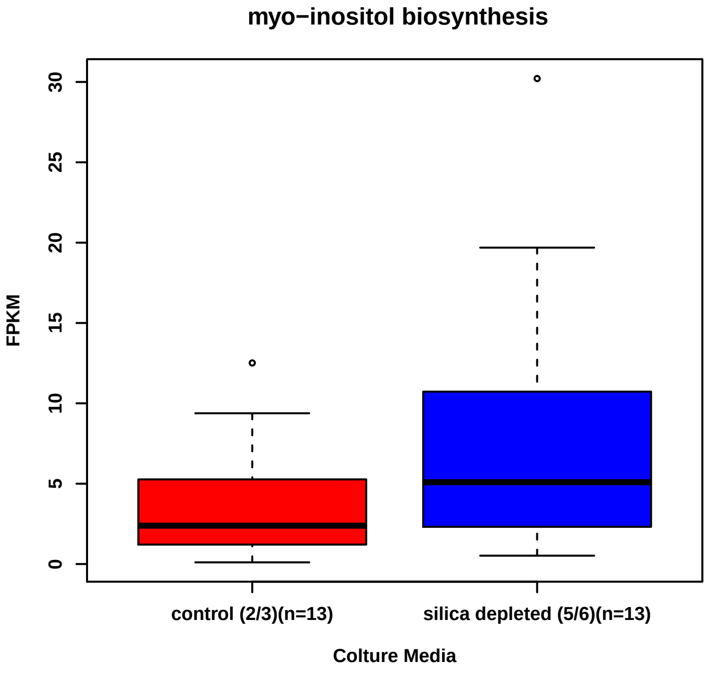

myo−inositol biosynthesis
FPKM
0
5
10
15
20
25
30
control (2/3)(n=13)
silica depleted (5/6)(n=13)
Colture Media

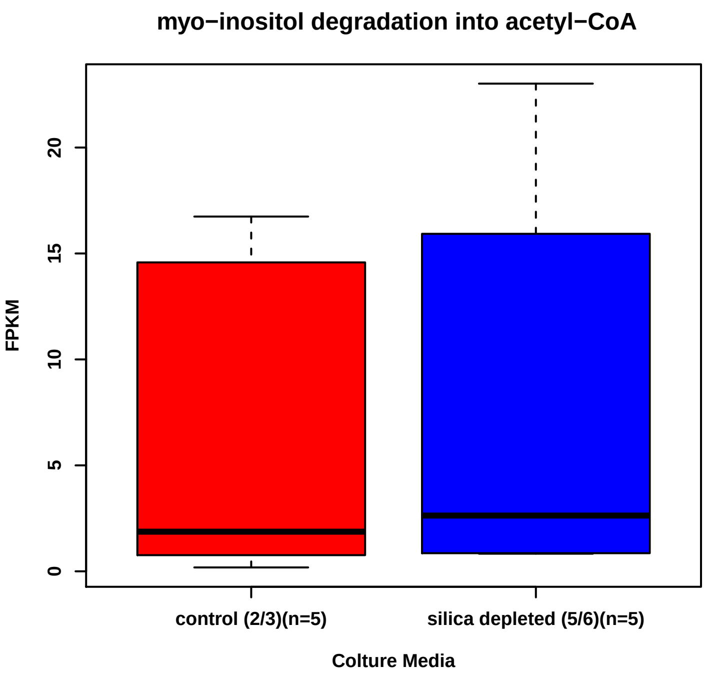
myo−inositol degradation into acetyl−CoA
FPKM
0
5
10
15
20
control (2/3)(n=5)
silica depleted (5/6)(n=5)
Colture Media

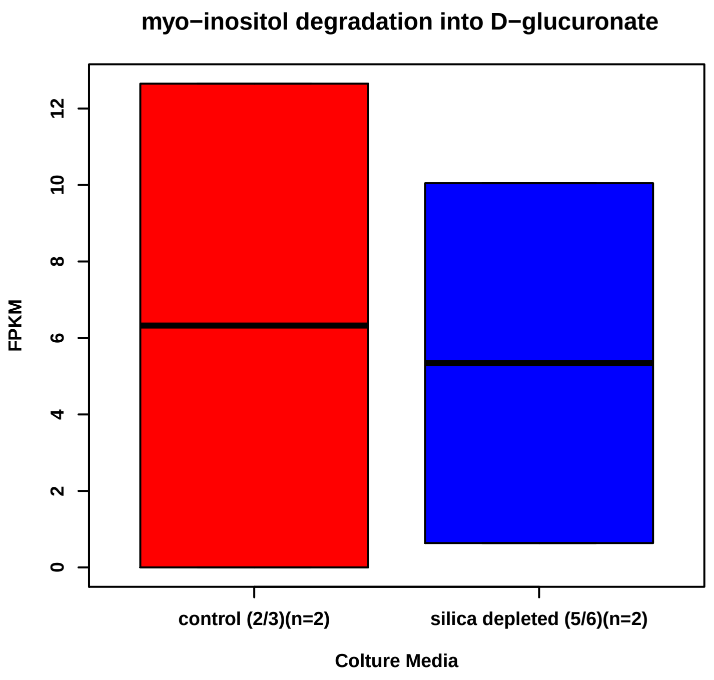
myo−inositol degradation into D−glucuronate
FPKM
0
2
4
6
8
10
12
control (2/3)(n=2)
silica depleted (5/6)(n=2)
Colture Media

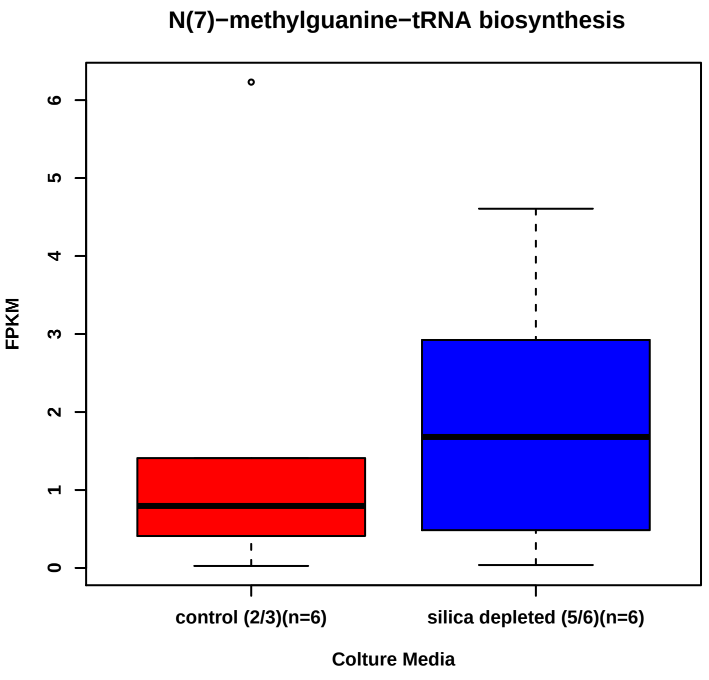
N(7)−methylguanine−tRNA biosynthesis
FPKM
0
1
2
3
4
5
6
control (2/3)(n=6)
silica depleted (5/6)(n=6)
Colture Media

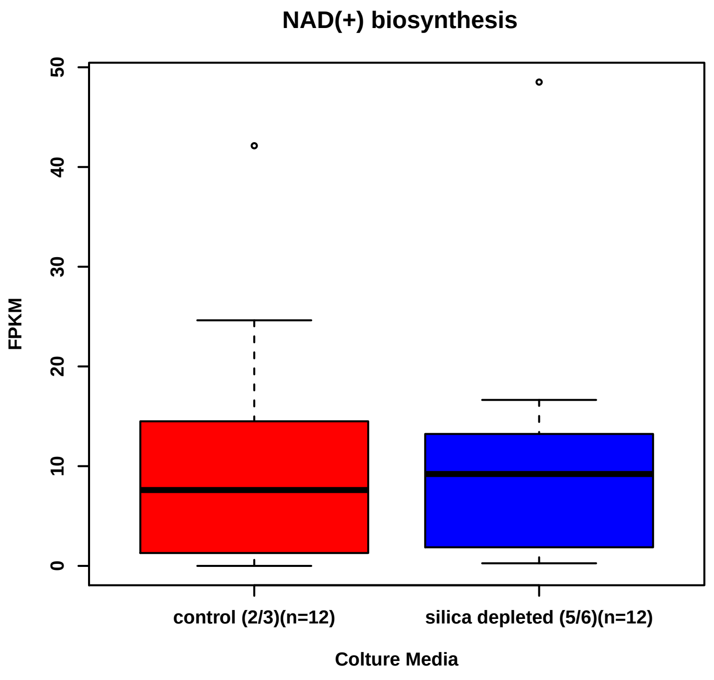
NAD(+) biosynthesis
FPKM
0
10
20
30
40
50
control (2/3)(n=12)
silica depleted (5/6)(n=12)
Colture Media

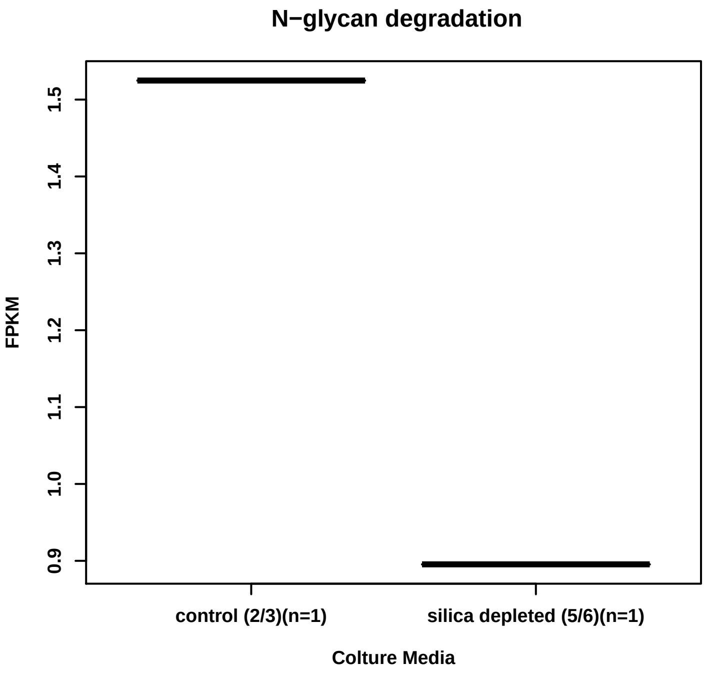
N−glycan degradation
FPKM
0.9
1.0
1.1
1.2
1.3
1.4
1.5
control (2/3)(n=1)
silica depleted (5/6)(n=1)
Colture Media

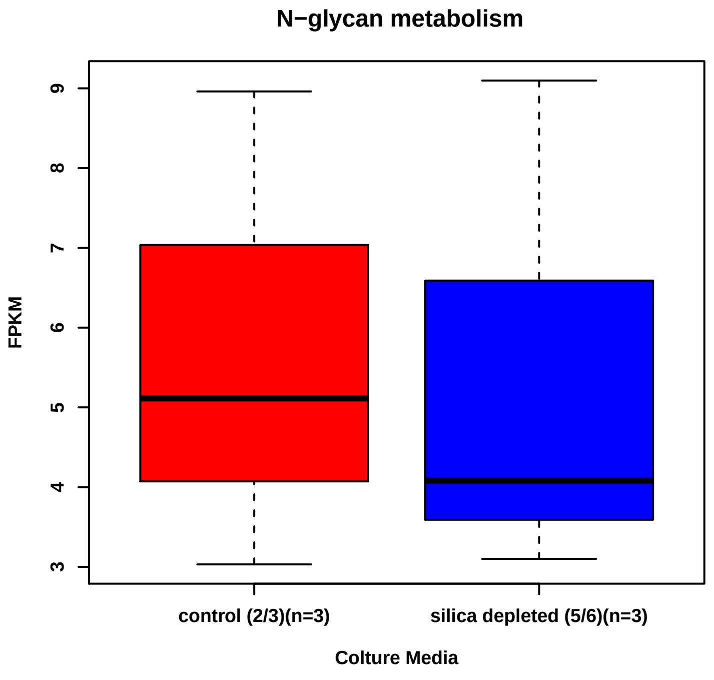
N−glycan metabolism
FPKM
3
4
5
6
7
8
9
control (2/3)(n=3)
silica depleted (5/6)(n=3)
Colture Media

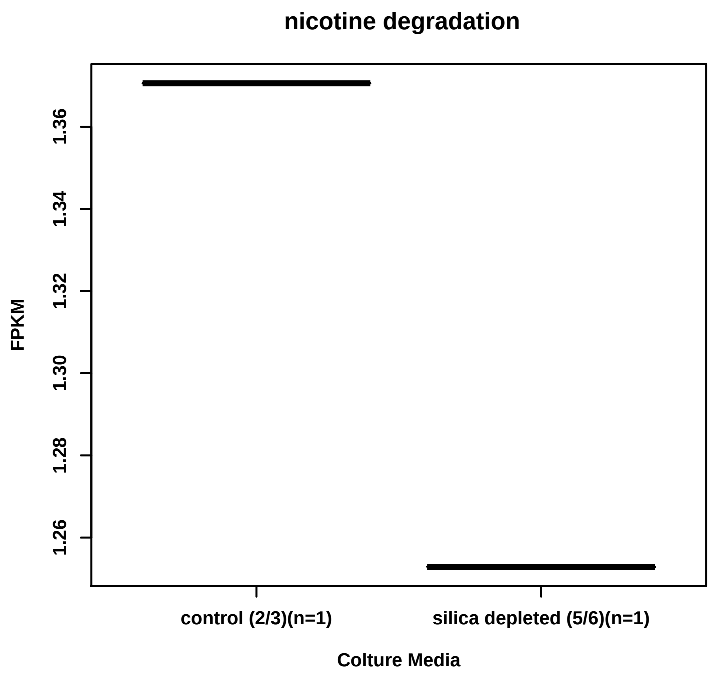
nicotine degradation
FPKM
1.26
1.28
1.30
1.32
1.34
1.36
control (2/3)(n=1)
silica depleted (5/6)(n=1)
Colture Media
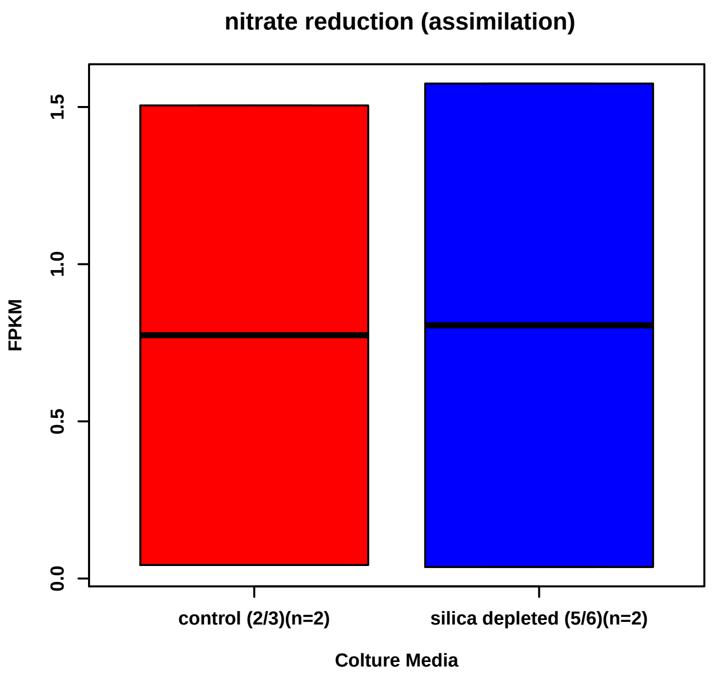
nitrate reduction (assimilation)
FPKM
0.0
0.5
1.0
1.5
control (2/3)(n=2)
silica depleted (5/6)(n=2)
Colture Media
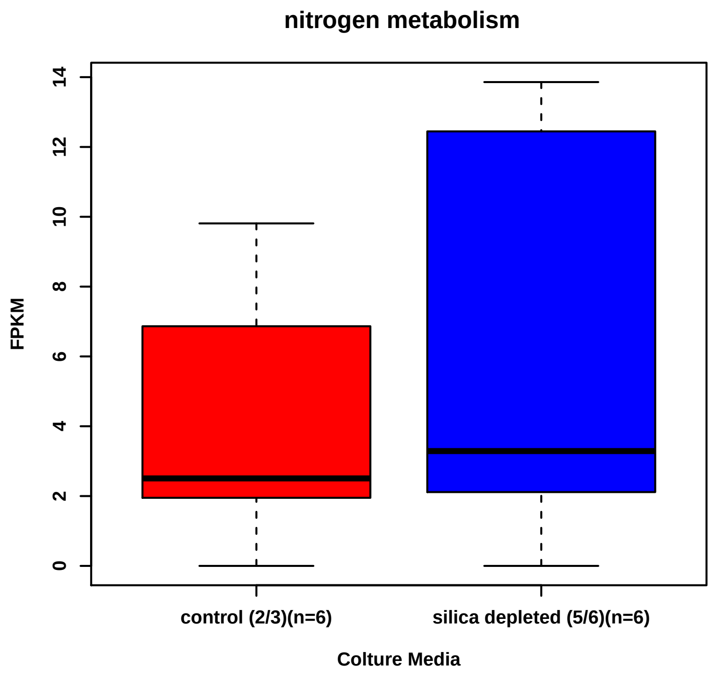
nitrogen metabolism
FPKM
0
2
4
6
8
10
12
14
control (2/3)(n=6)
silica depleted (5/6)(n=6)
Colture Media
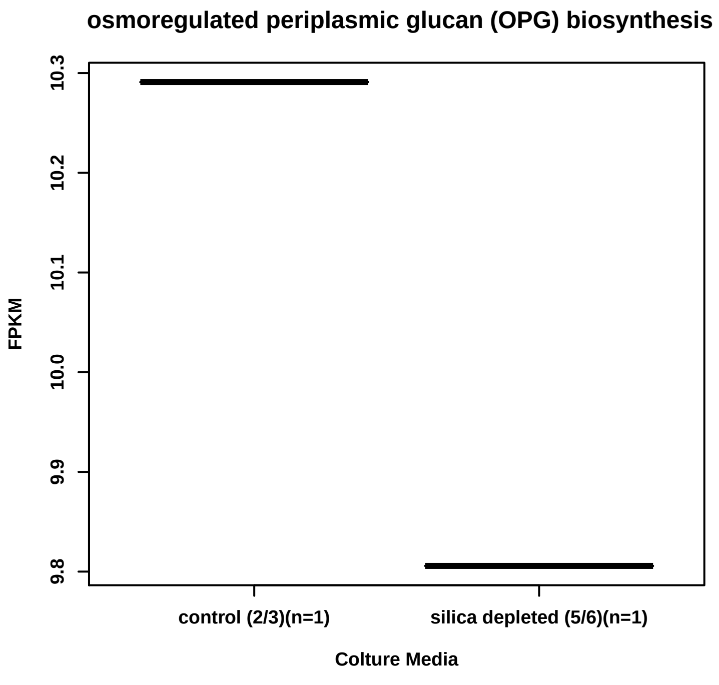
osmoregulated periplasmic glucan (OPG) biosynthesis
FPKM
9.8
9.9
10.0
10.1
10.2
10.3
control (2/3)(n=1)
silica depleted (5/6)(n=1)
Colture Media
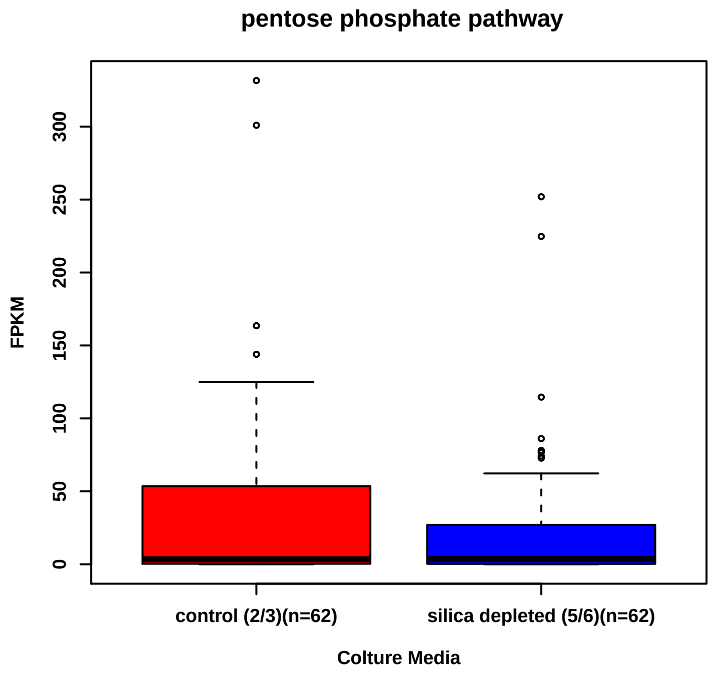
pentose phosphate pathway
FPKM
0
50
100
150
200
250
300
control (2/3)(n=62)
silica depleted (5/6)(n=62)
Colture Media
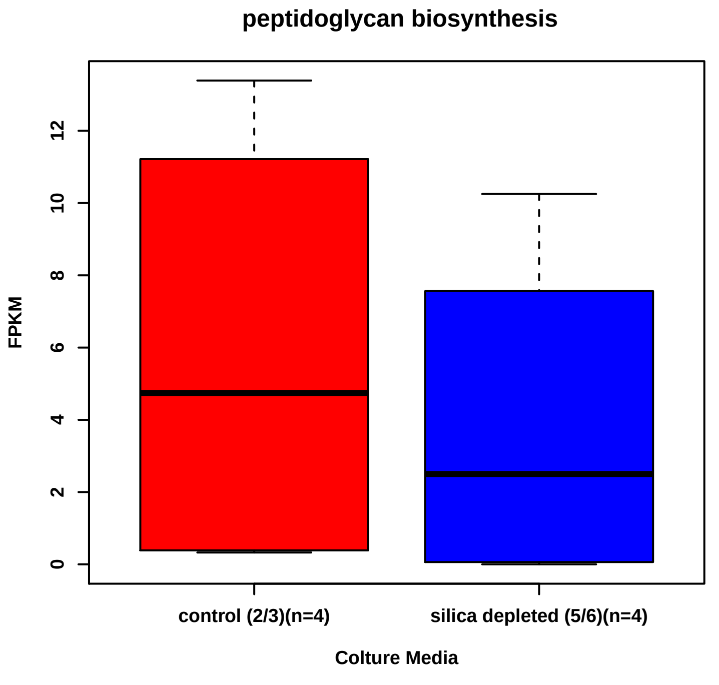
peptidoglycan biosynthesis
FPKM
0
2
4
6
8
10
12
control (2/3)(n=4)
silica depleted (5/6)(n=4)
Colture Media

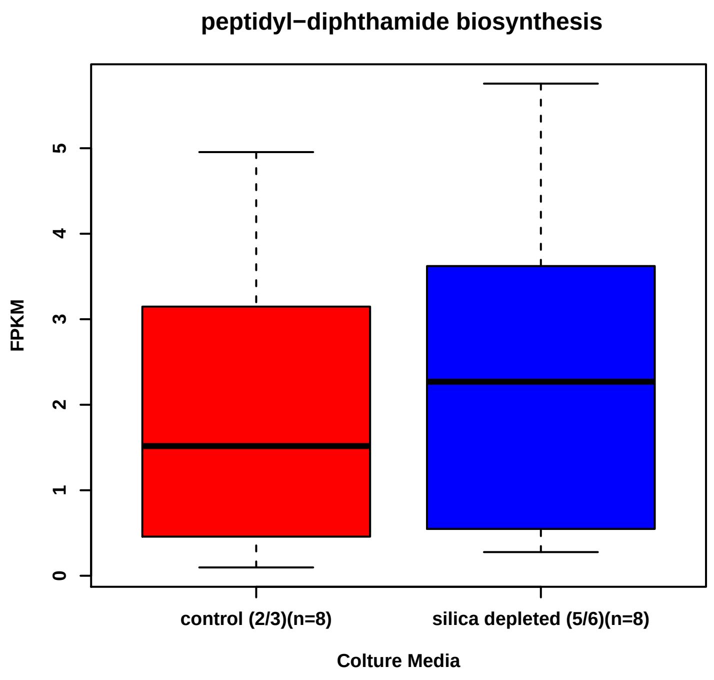
peptidyl−diphthamide biosynthesis
FPKM
0
1
2
3
4
5
control (2/3)(n=8)
silica depleted (5/6)(n=8)
Colture Media

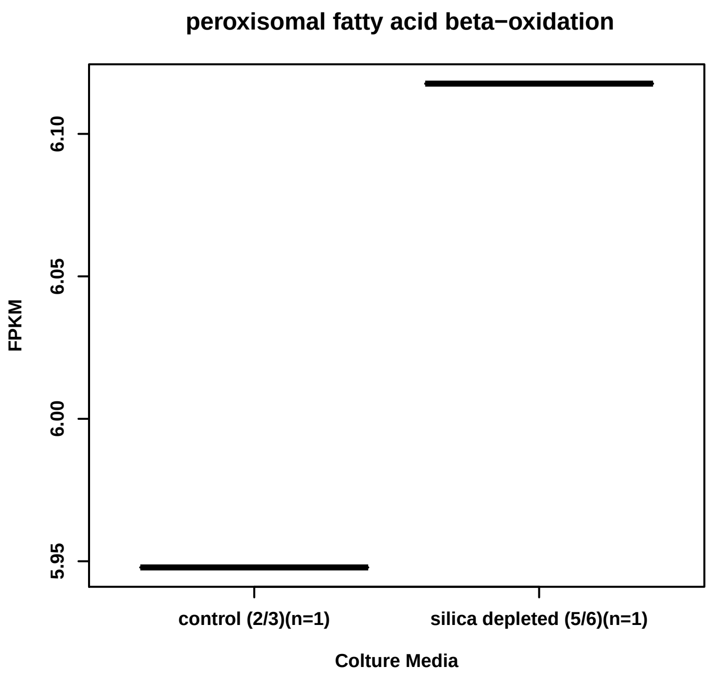
peroxisomal fatty acid beta−oxidation
FPKM
5.95
6.00
6.05
6.10
control (2/3)(n=1)
silica depleted (5/6)(n=1)
Colture Media

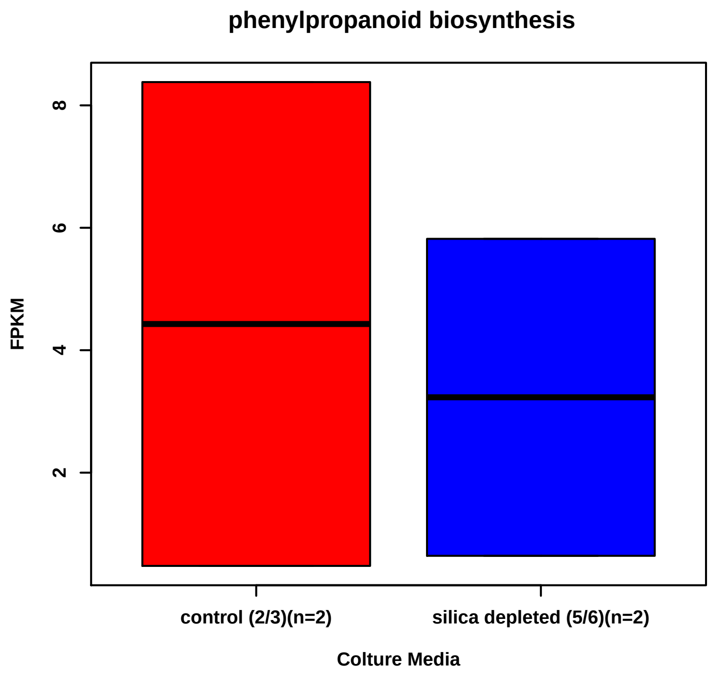
phenylpropanoid biosynthesis
FPKM
2
4
6
8
control (2/3)(n=2)
silica depleted (5/6)(n=2)
Colture Media

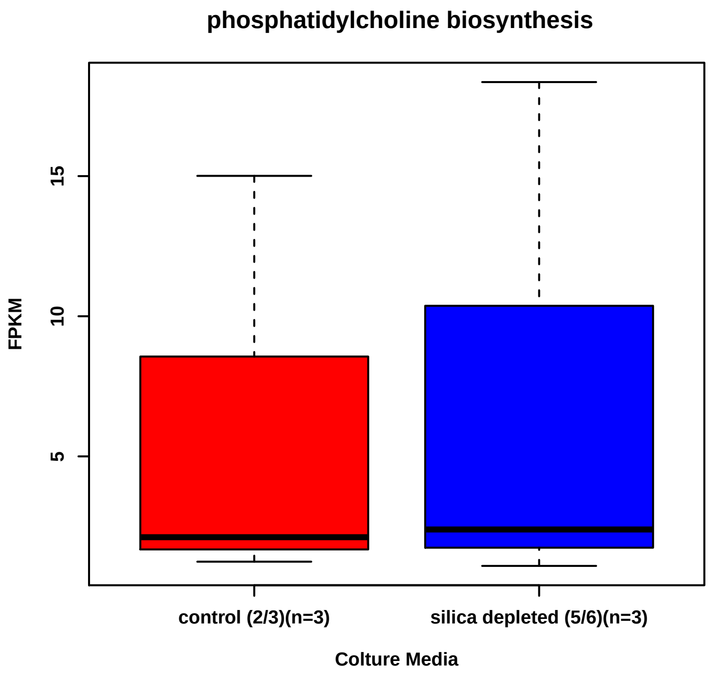
phosphatidylcholine biosynthesis
FPKM
5
10
15
control (2/3)(n=3)
silica depleted (5/6)(n=3)
Colture Media

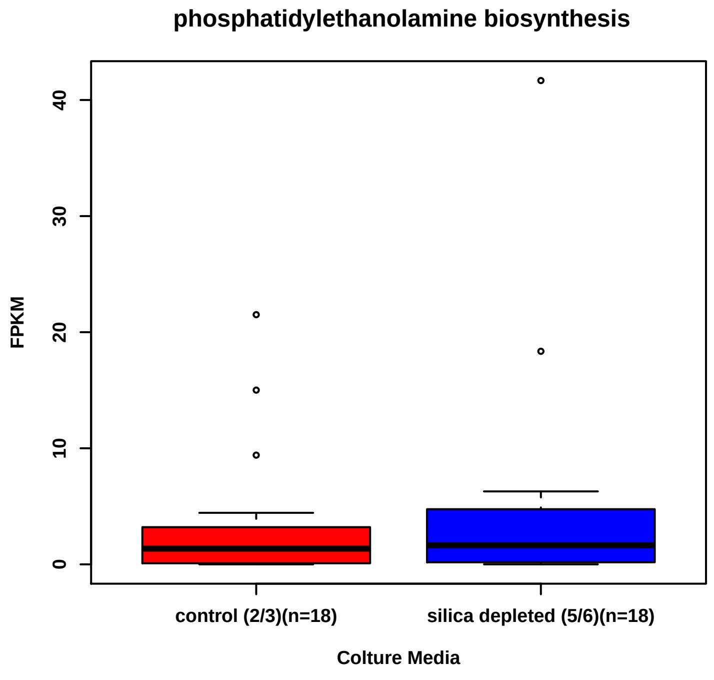
phosphatidylethanolamine biosynthesis
FPKM
0
10
20
30
40
control (2/3)(n=18)
silica depleted (5/6)(n=18)
Colture Media

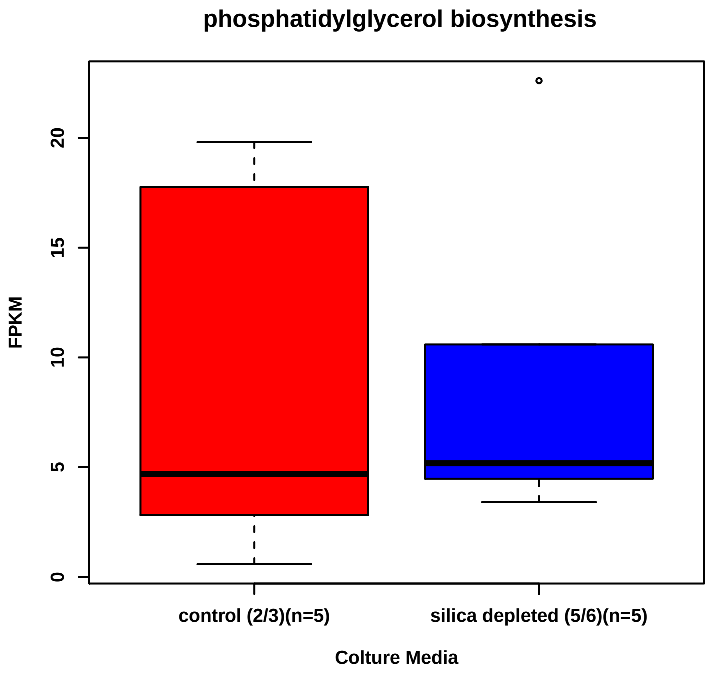
phosphatidylglycerol biosynthesis
FPKM
0
5
10
15
20
control (2/3)(n=5)
silica depleted (5/6)(n=5)
Colture Media

phosphatidylinositol signaling pathway

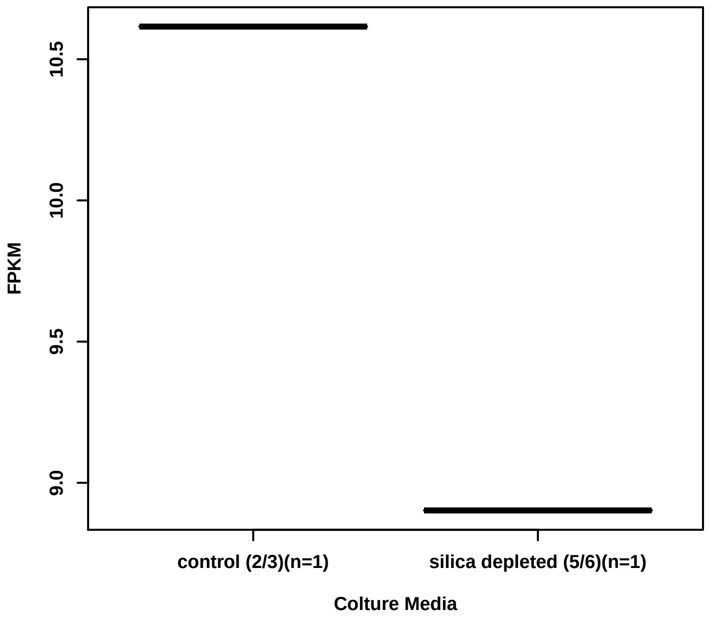

phosphatidylserine biosynthesis

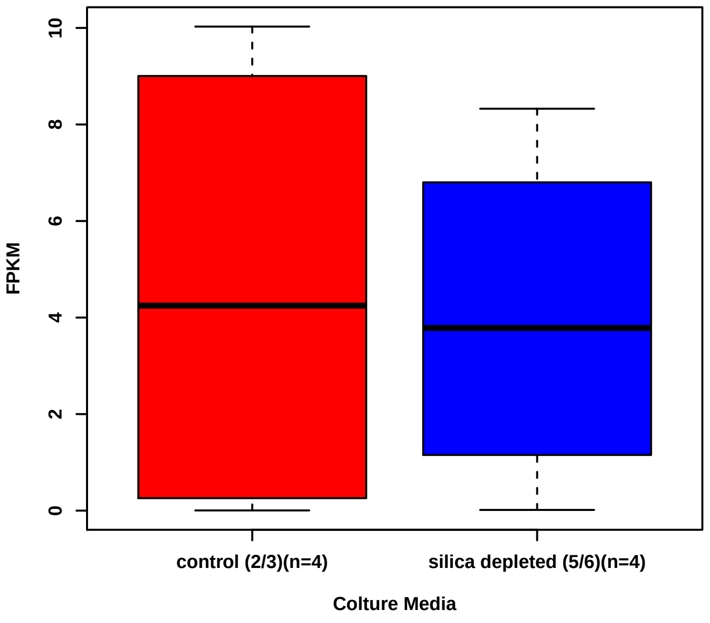

phospholipid metabolism

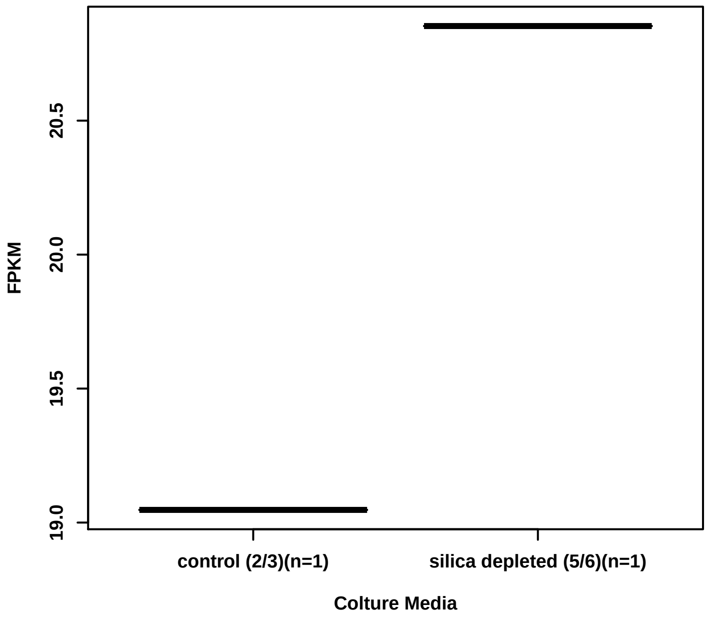

photorespiration

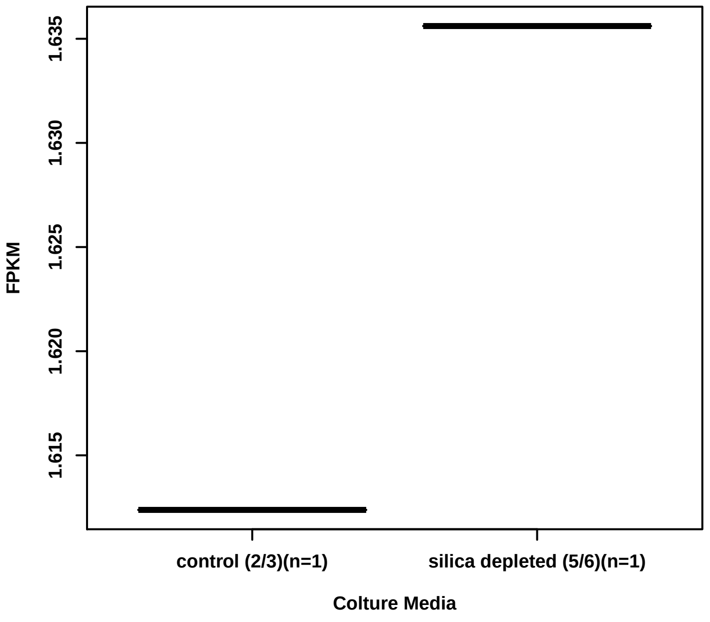

photosynthesis

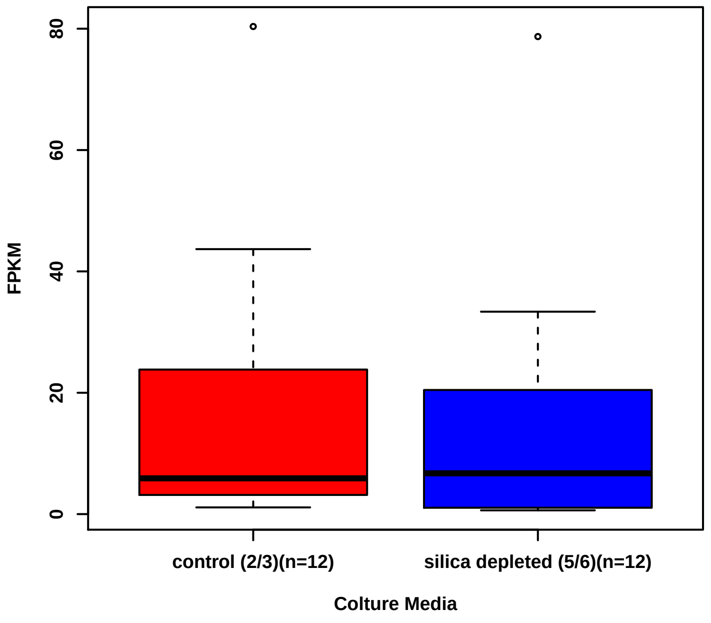

phytoene biosynthesis

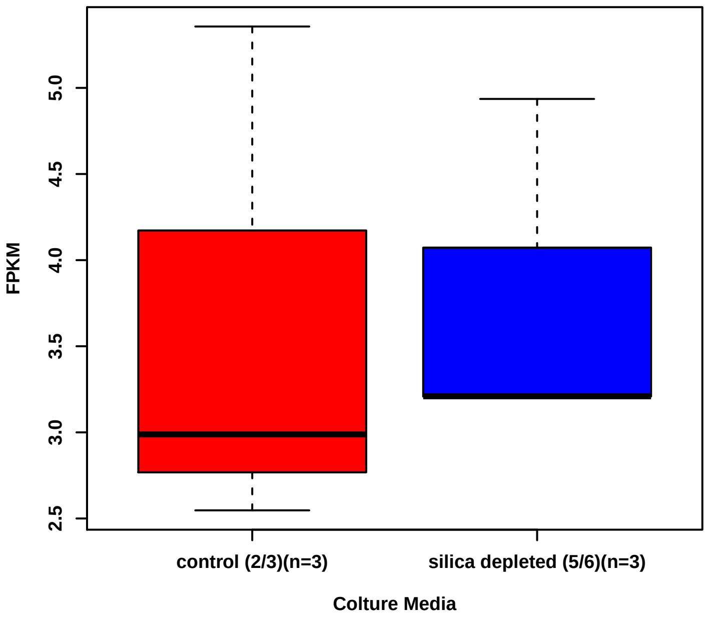

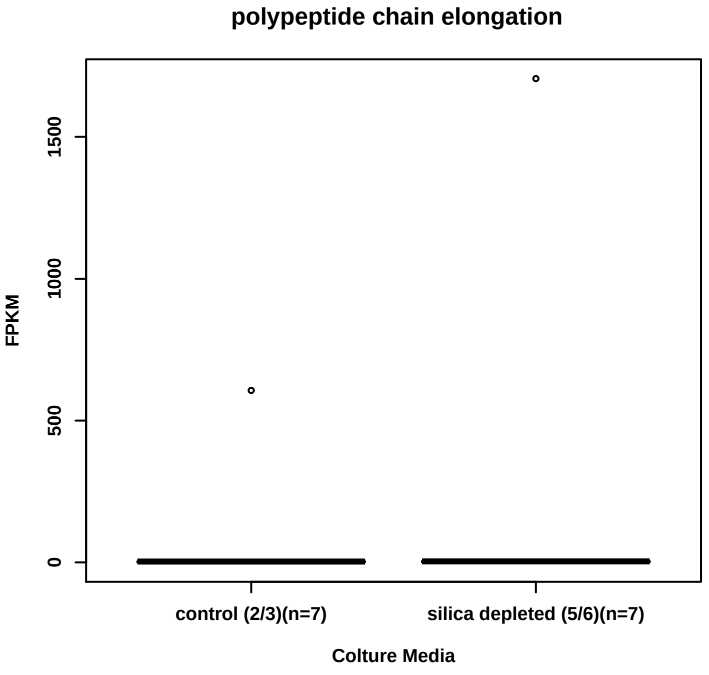
polypeptide chain elongation
FPKM
0
500
1000
1500
control (2/3)(n=7)
silica depleted (5/6)(n=7)
Colture Media

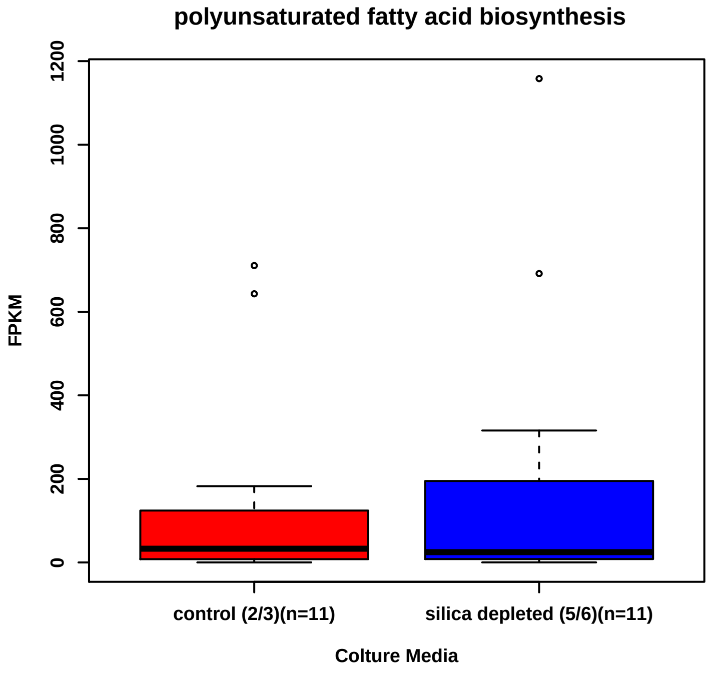
polyunsaturated fatty acid biosynthesis
FPKM
0
200
400
600
800
1000
1200
control (2/3)(n=11)
silica depleted (5/6)(n=11)
Colture Media

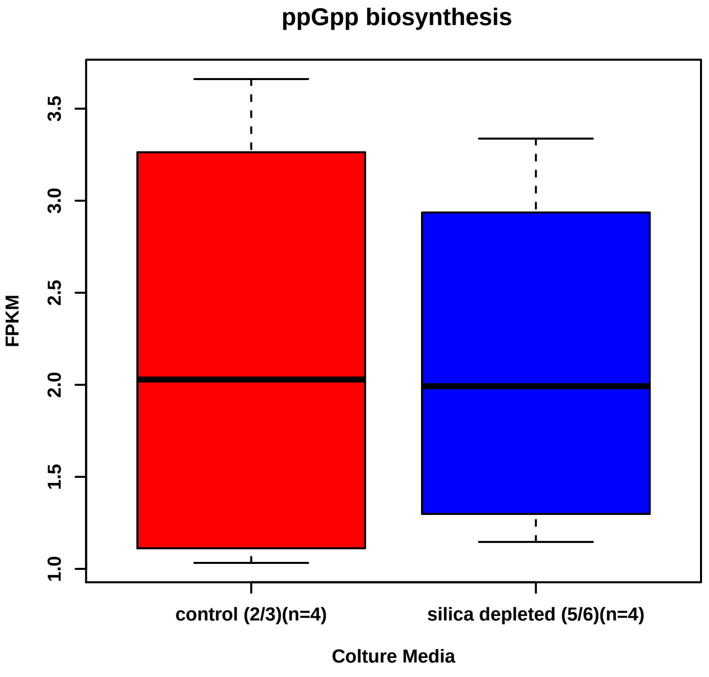
ppGpp biosynthesis
FPKM
1.0
1.5
2.0
2.5
3.0
3.5
control (2/3)(n=4)
silica depleted (5/6)(n=4)
Colture Media

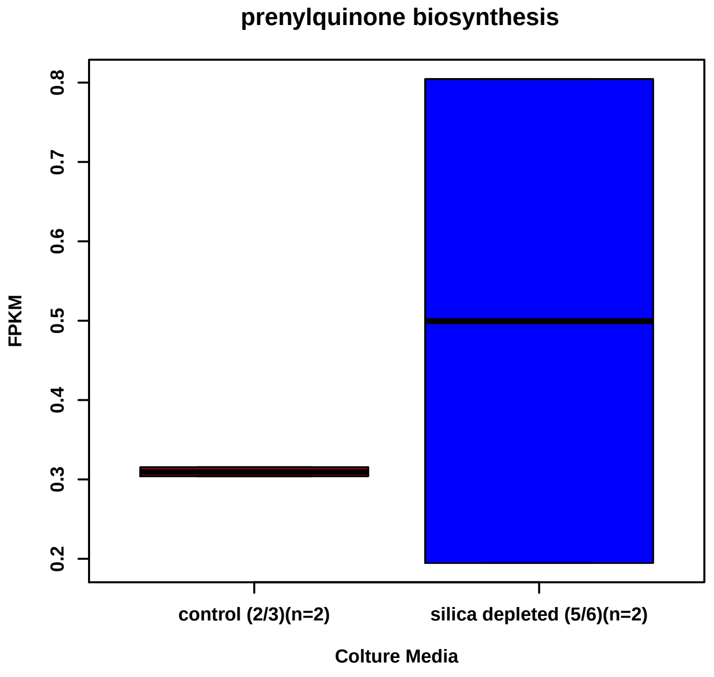
prenylquinone biosynthesis
FPKM
0.2
0.3
0.4
0.5
0.6
0.7
0.8
control (2/3)(n=2)
silica depleted (5/6)(n=2)
Colture Media

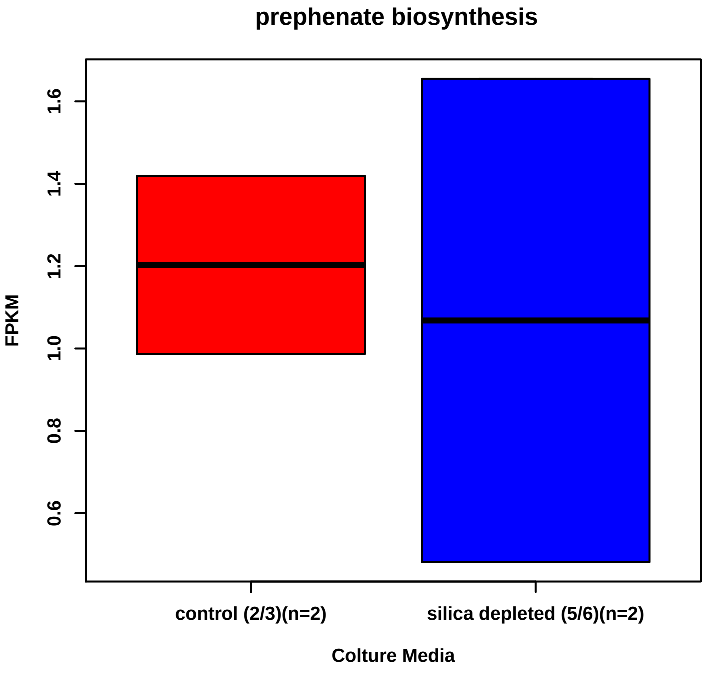
prephenate biosynthesis
FPKM
0.6
0.8
1.0
1.2
1.4
1.6
control (2/3)(n=2)
silica depleted (5/6)(n=2)
Colture Media

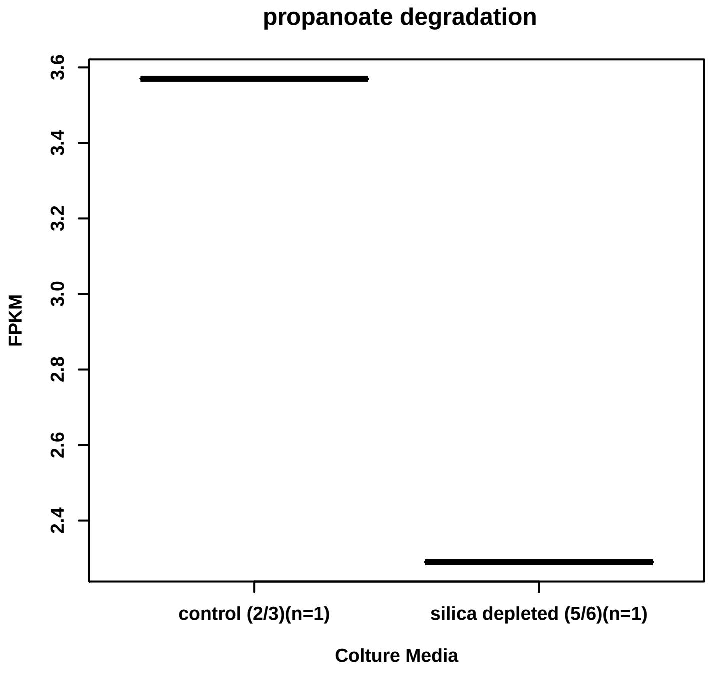
propanoate degradation
FPKM
2.4
2.6
2.8
3.0
3.2
3.4
3.6
control (2/3)(n=1)
silica depleted (5/6)(n=1)
Colture Media

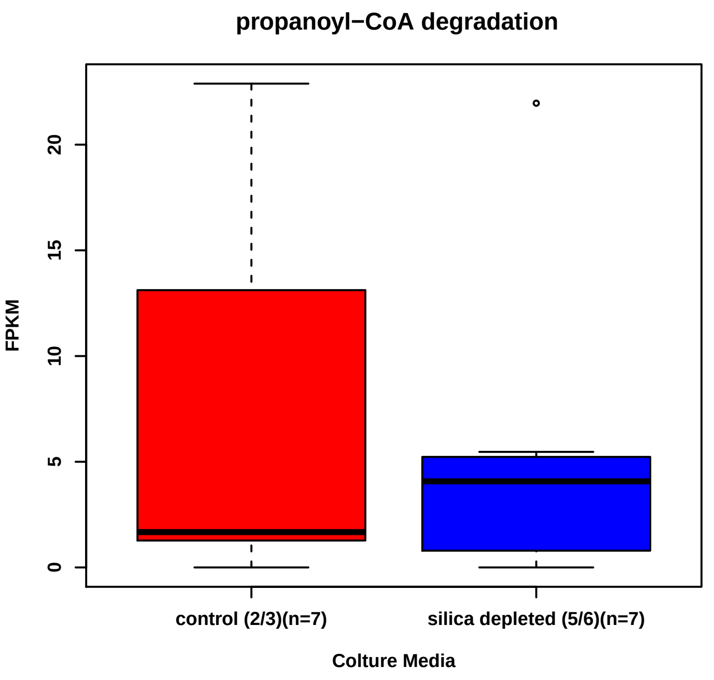
propanoyl−CoA degradation
FPKM
0
5
10
15
20
control (2/3)(n=7)
silica depleted (5/6)(n=7)
Colture Media

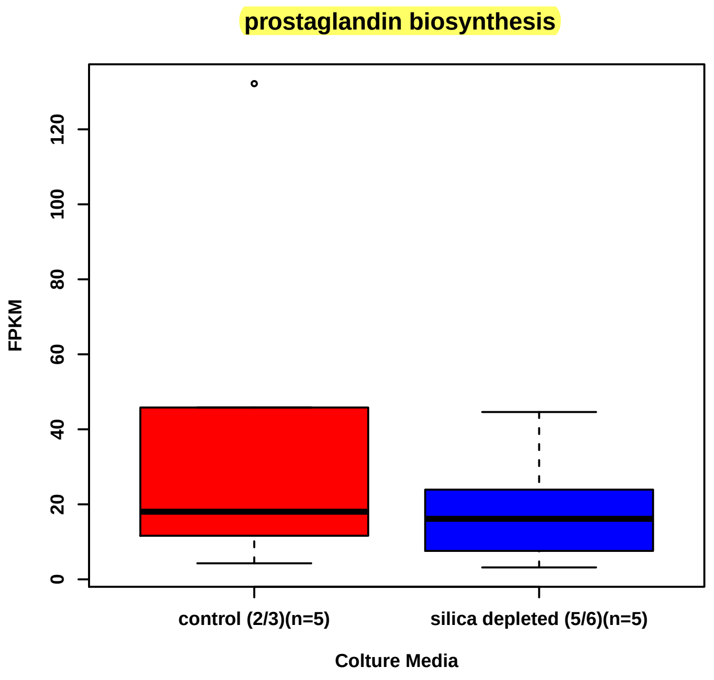
prostaglandin biosynthesis
FPKM
0
20
40
60
80
100
120
control (2/3)(n=5)
silica depleted (5/6)(n=5)
Colture Media

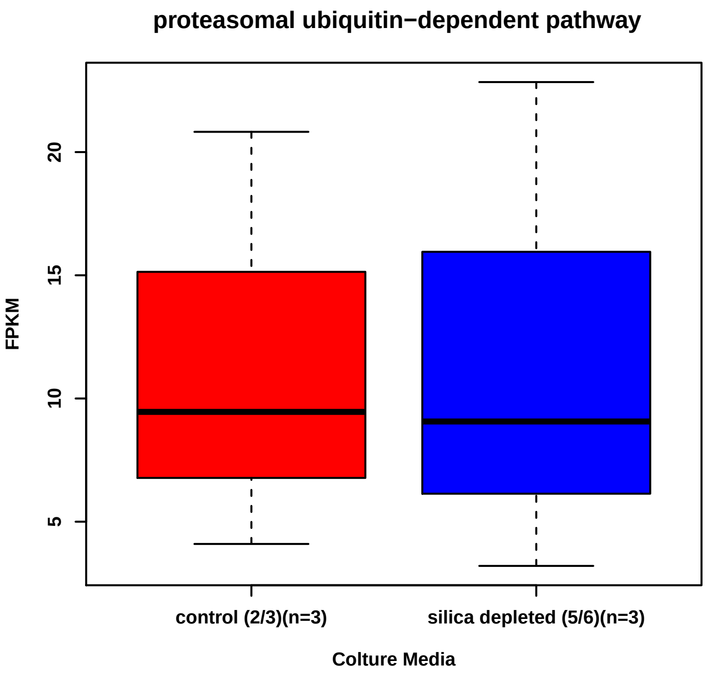
proteasomal ubiquitin−dependent pathway
FPKM
5
10
15
20
control (2/3)(n=3)
silica depleted (5/6)(n=3)
Colture Media

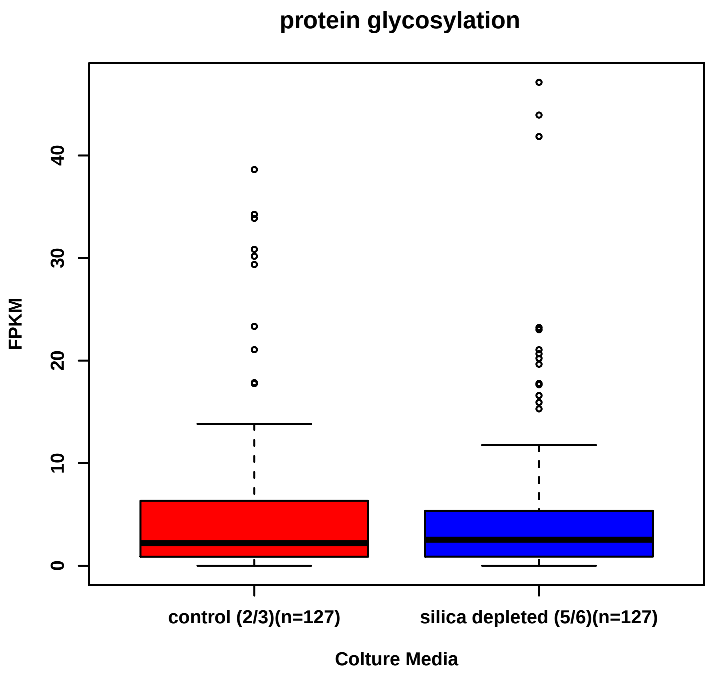
protein glycosylation
FPKM
0
10
20
30
40
control (2/3)(n=127)
silica depleted (5/6)(n=127)
Colture Media

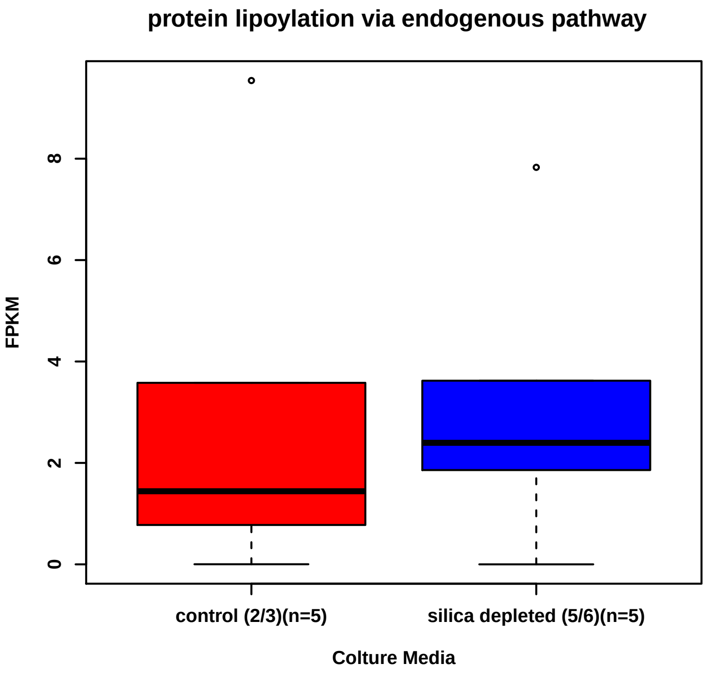
protein lipoylation via endogenous pathway
FPKM
0
2
4
6
8
control (2/3)(n=5)
silica depleted (5/6)(n=5)
Colture Media

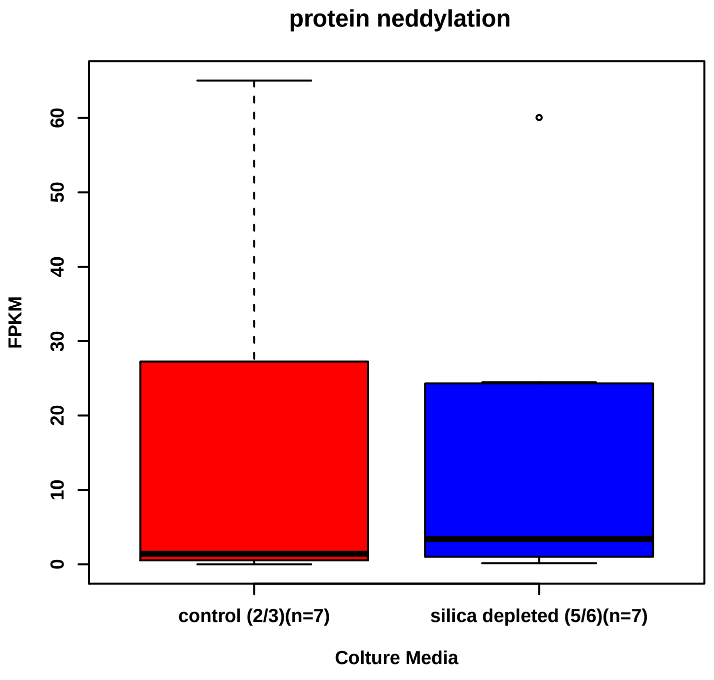
protein neddylation
FPKM
0
10
20
30
40
50
60
control (2/3)(n=7)
silica depleted (5/6)(n=7)
Colture Media

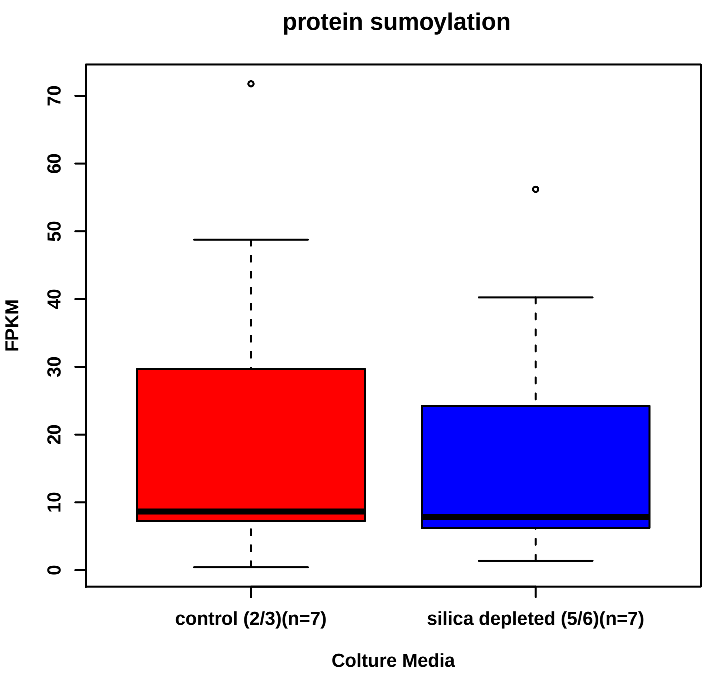
protein sumoylation
FPKM
0
10
20
30
40
50
60
70
control (2/3)(n=7)
silica depleted (5/6)(n=7)
Colture Media

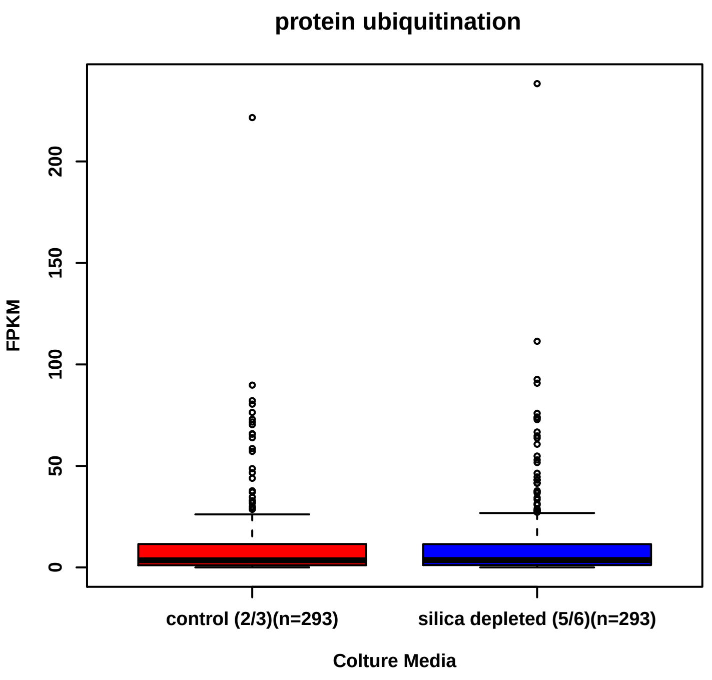
protein ubiquitination
FPKM
0
50
100
150
200
control (2/3)(n=293)
silica depleted (5/6)(n=293)
Colture Media

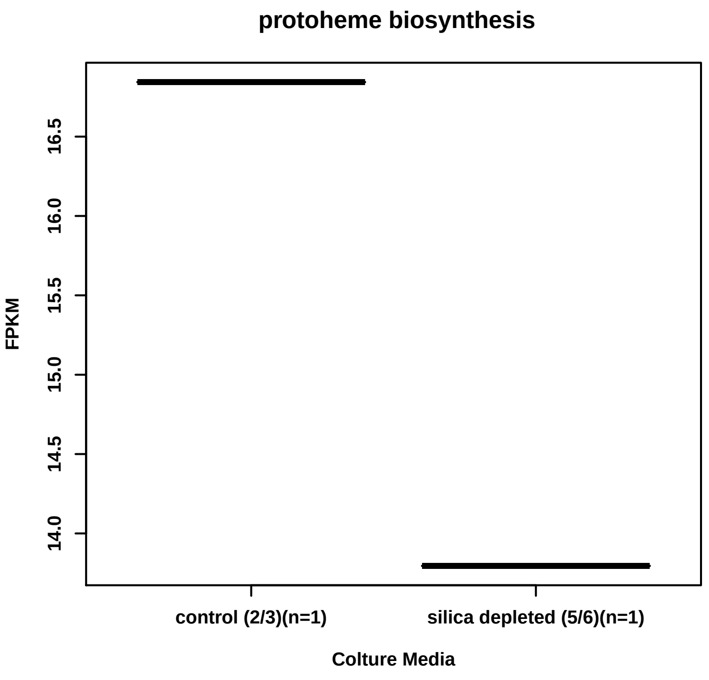
protoheme biosynthesis
FPKM
14.0
14.5
15.0
15.5
16.0
16.5
control (2/3)(n=1)
silica depleted (5/6)(n=1)
Colture Media

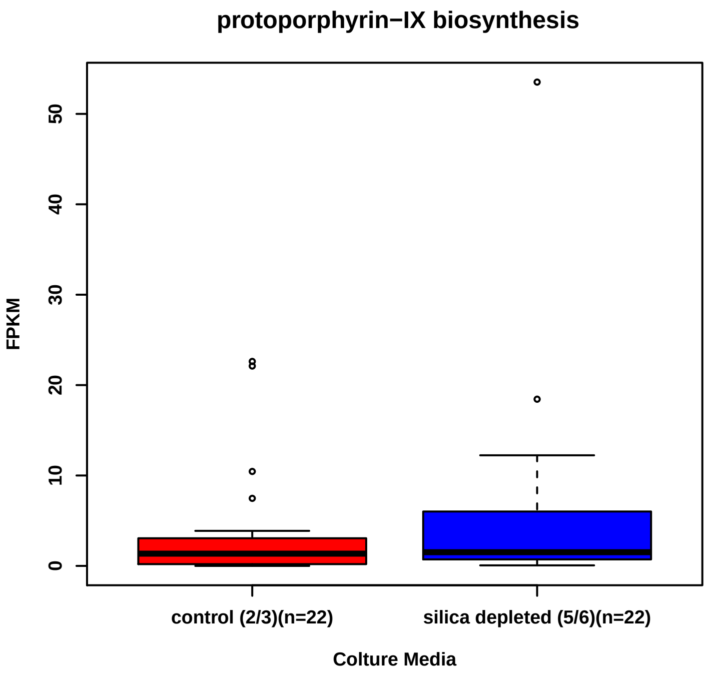
protoporphyrin−IX biosynthesis
FPKM
0
10
20
30
40
50
control (2/3)(n=22)
silica depleted (5/6)(n=22)
Colture Media

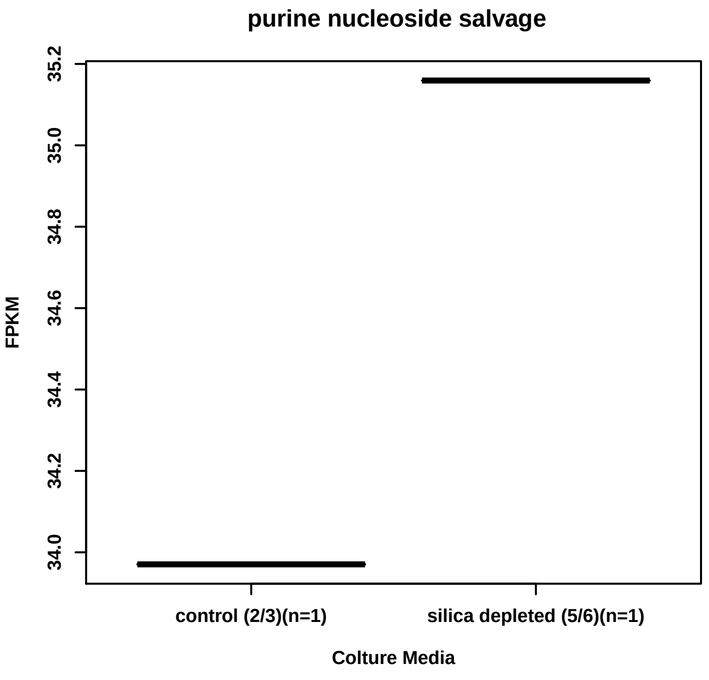
purine nucleoside salvage
FPKM
34.0
34.2
34.4
34.6
34.8
35.0
35.2
control (2/3)(n=1)
silica depleted (5/6)(n=1)
Colture Media

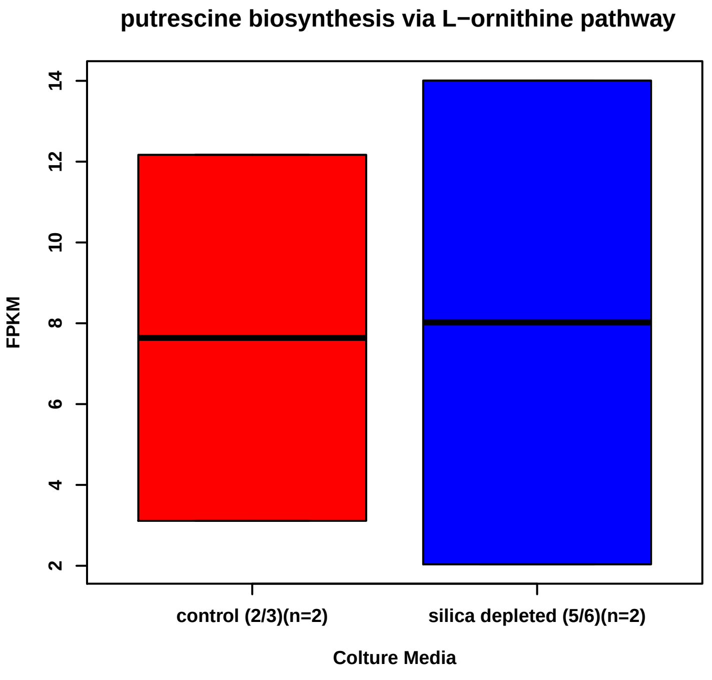
putrescine biosynthesis via L−ornithine pathway
FPKM
2
4
6
8
10
12
14
control (2/3)(n=2)
silica depleted (5/6)(n=2)
Colture Media

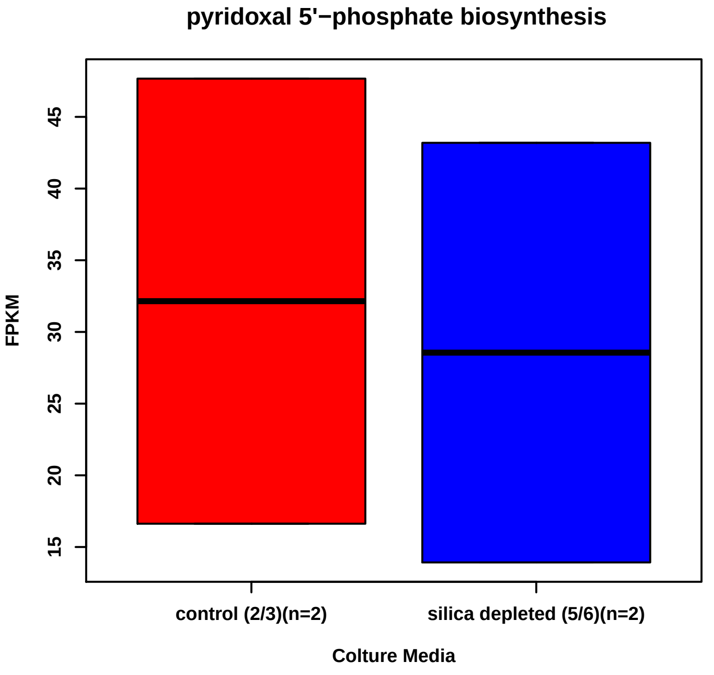
pyridoxal 5'−phosphate biosynthesis
FPKM
15
20
25
30
35
40
45
control (2/3)(n=2)
silica depleted (5/6)(n=2)
Colture Media

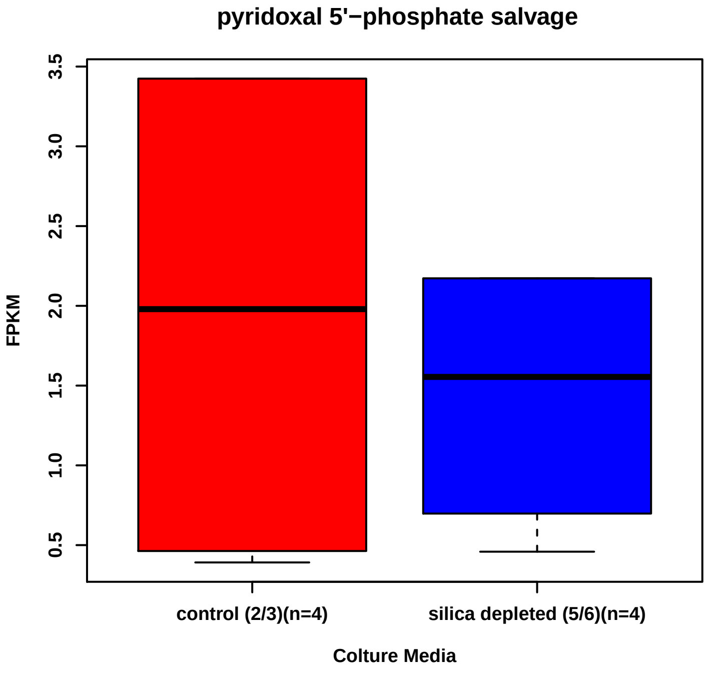
pyridoxal 5'−phosphate salvage
FPKM
0.5
1.0
1.5
2.0
2.5
3.0
3.5
control (2/3)(n=4)
silica depleted (5/6)(n=4)
Colture Media

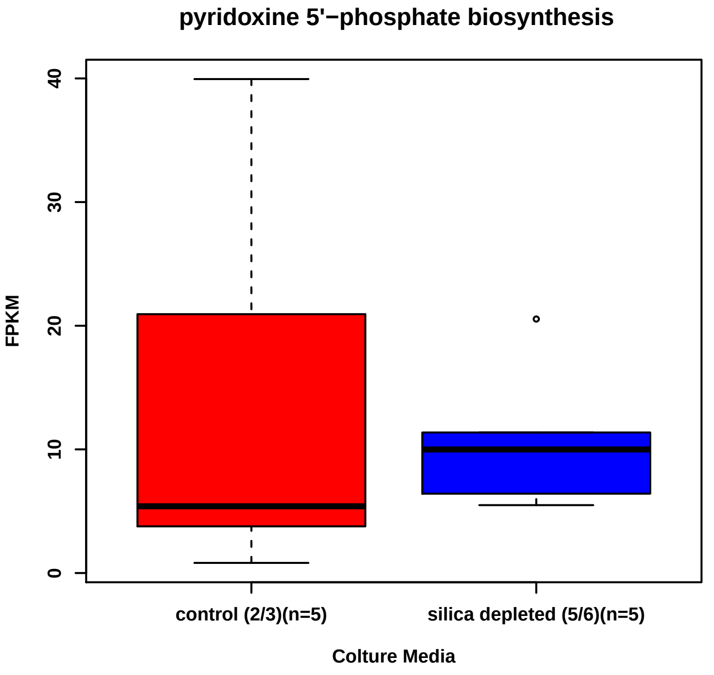
pyridoxine 5'−phosphate biosynthesis
FPKM
0
10
20
30
40
control (2/3)(n=5)
silica depleted (5/6)(n=5)
Colture Media

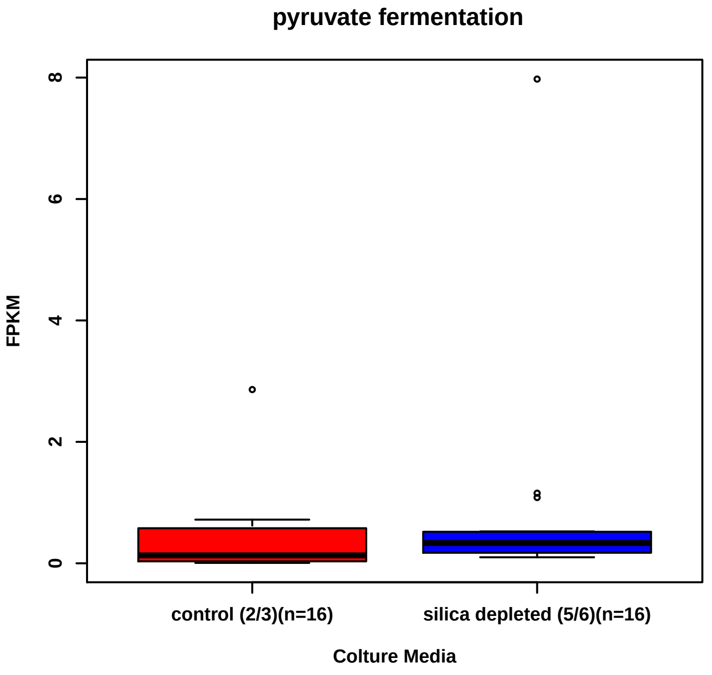
pyruvate fermentation
FPKM
0
2
4
6
8
control (2/3)(n=16)
silica depleted (5/6)(n=16)
Colture Media

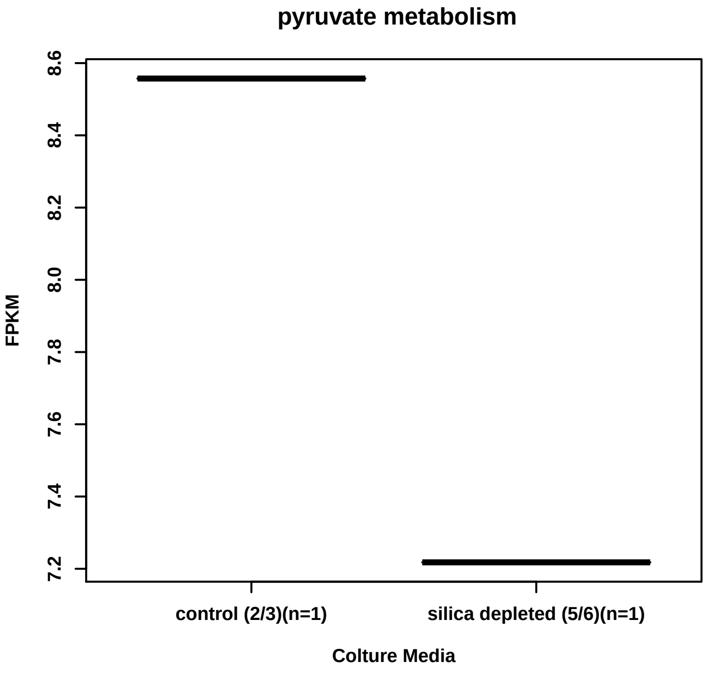
pyruvate metabolism
FPKM
7.2
7.4
7.6
7.8
8.0
8.2
8.4
8.6
control (2/3)(n=1)
silica depleted (5/6)(n=1)
Colture Media

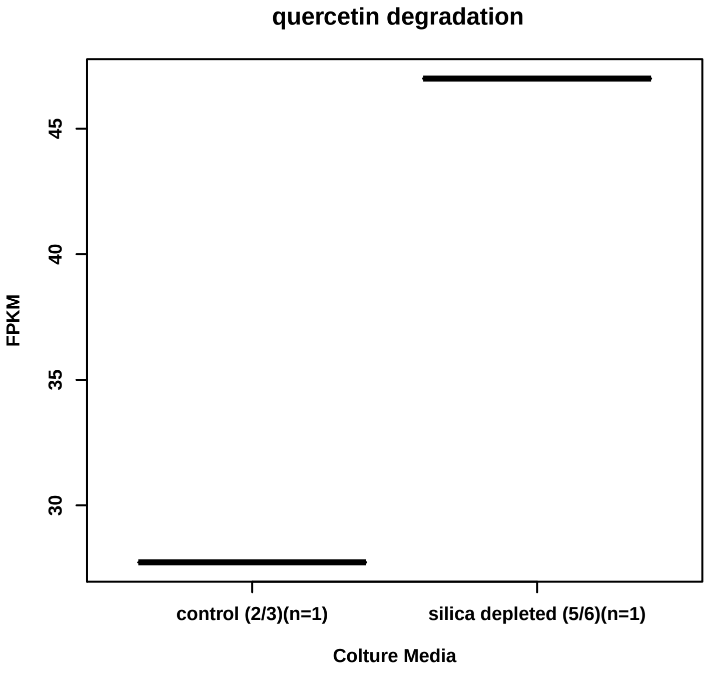
quercetin degradation
FPKM
30
35
40
45
control (2/3)(n=1)
silica depleted (5/6)(n=1)
Colture Media

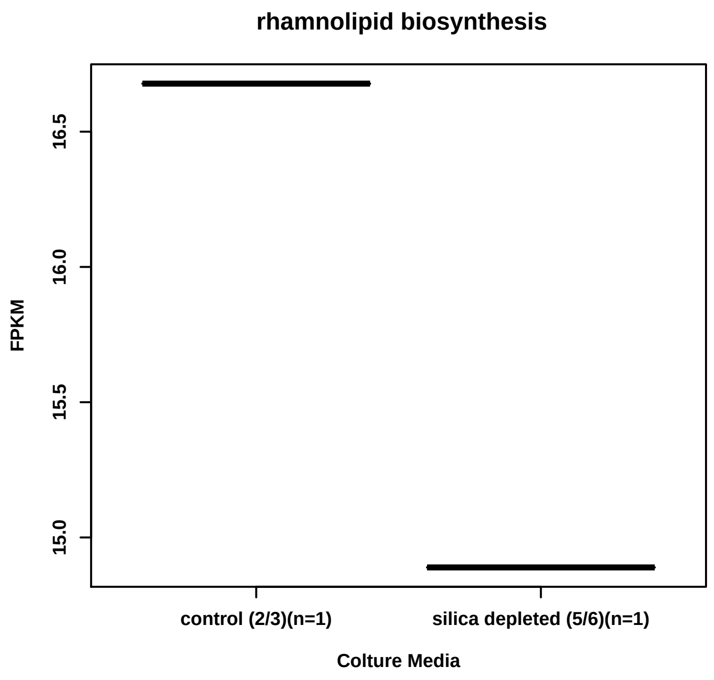
rhamnolipid biosynthesis
FPKM
16.5
16.0
15.5
15.0
control (2/3)(n=1)
silica depleted (5/6)(n=1)
Colture Media

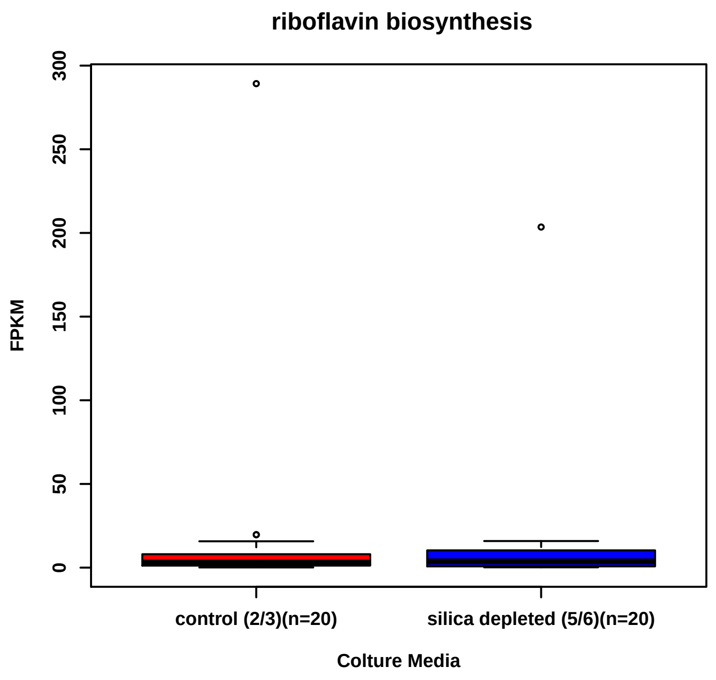
riboflavin biosynthesis
FPKM
300
250
200
150
100
50
0
control (2/3)(n=20)
silica depleted (5/6)(n=20)
Colture Media

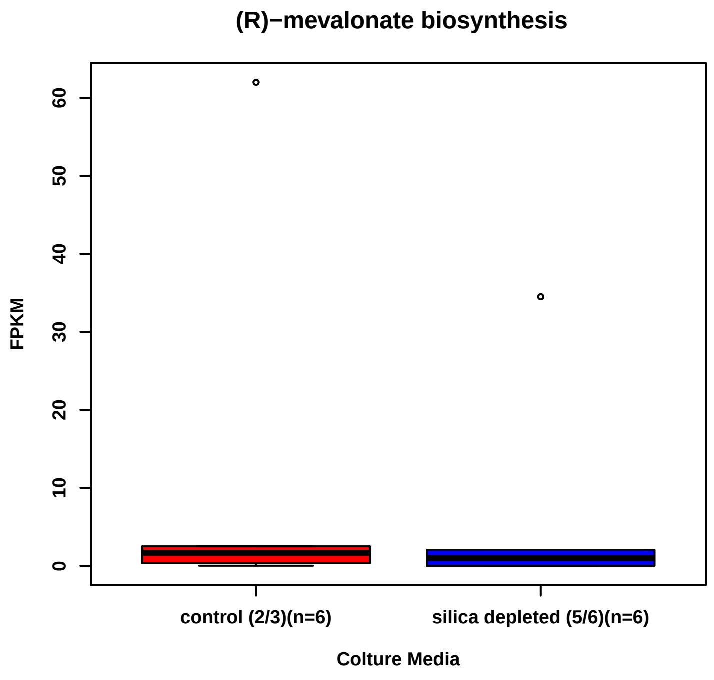
(R)−mevalonate biosynthesis
FPKM
60
50
40
30
20
10
0
control (2/3)(n=6)
silica depleted (5/6)(n=6)
Colture Media

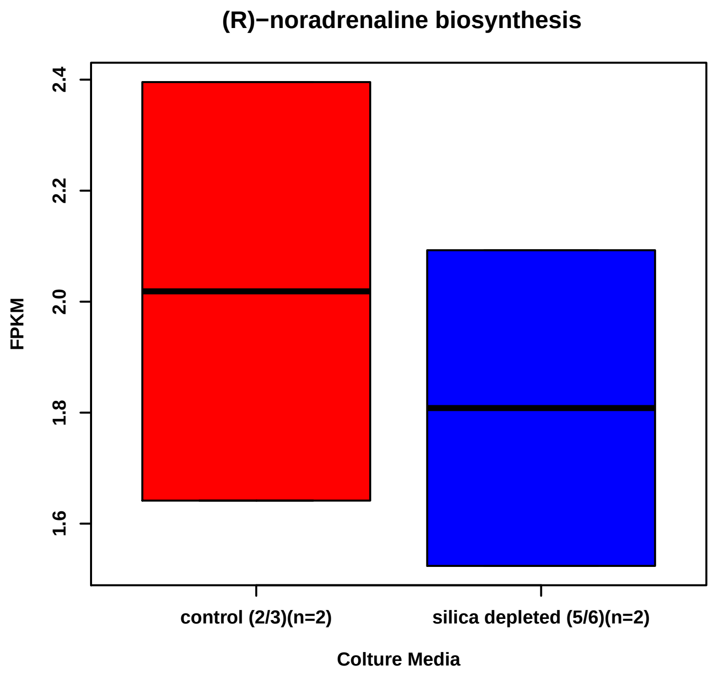
(R)−noradrenaline biosynthesis
FPKM
2.4
2.2
2.0
1.8
1.6
control (2/3)(n=2)
silica depleted (5/6)(n=2)
Colture Media

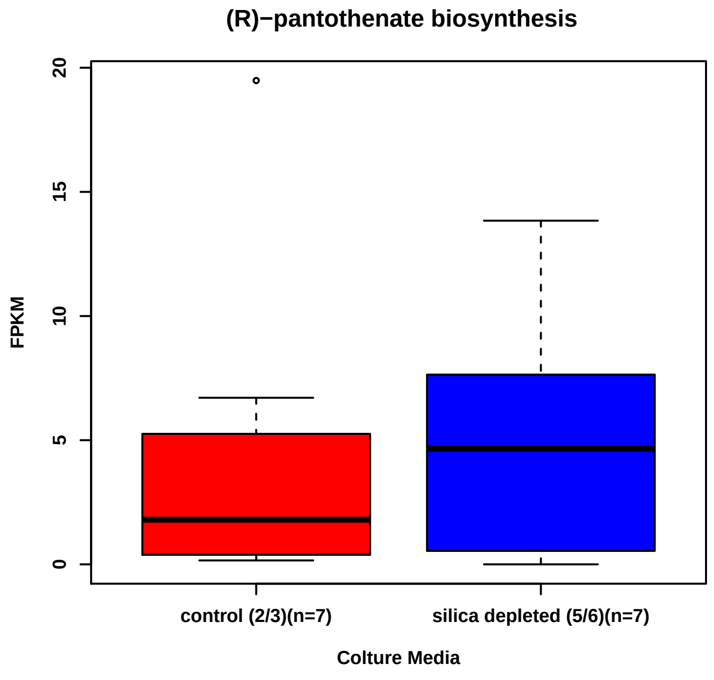
(R)−pantothenate biosynthesis
FPKM
20
15
10
5
0
control (2/3)(n=7)
silica depleted (5/6)(n=7)
Colture Media

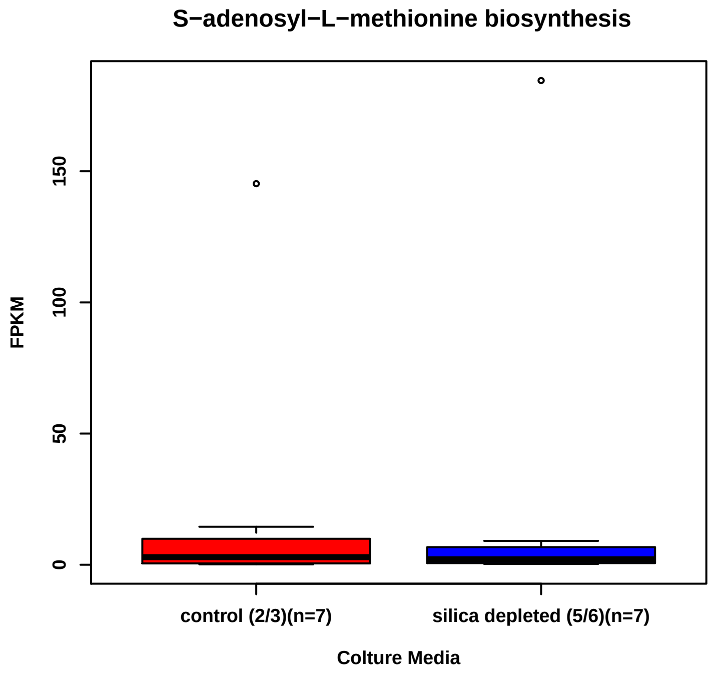
S−adenosyl−L−methionine biosynthesis
FPKM
150
100
50
0
control (2/3)(n=7)
silica depleted (5/6)(n=7)
Colture Media

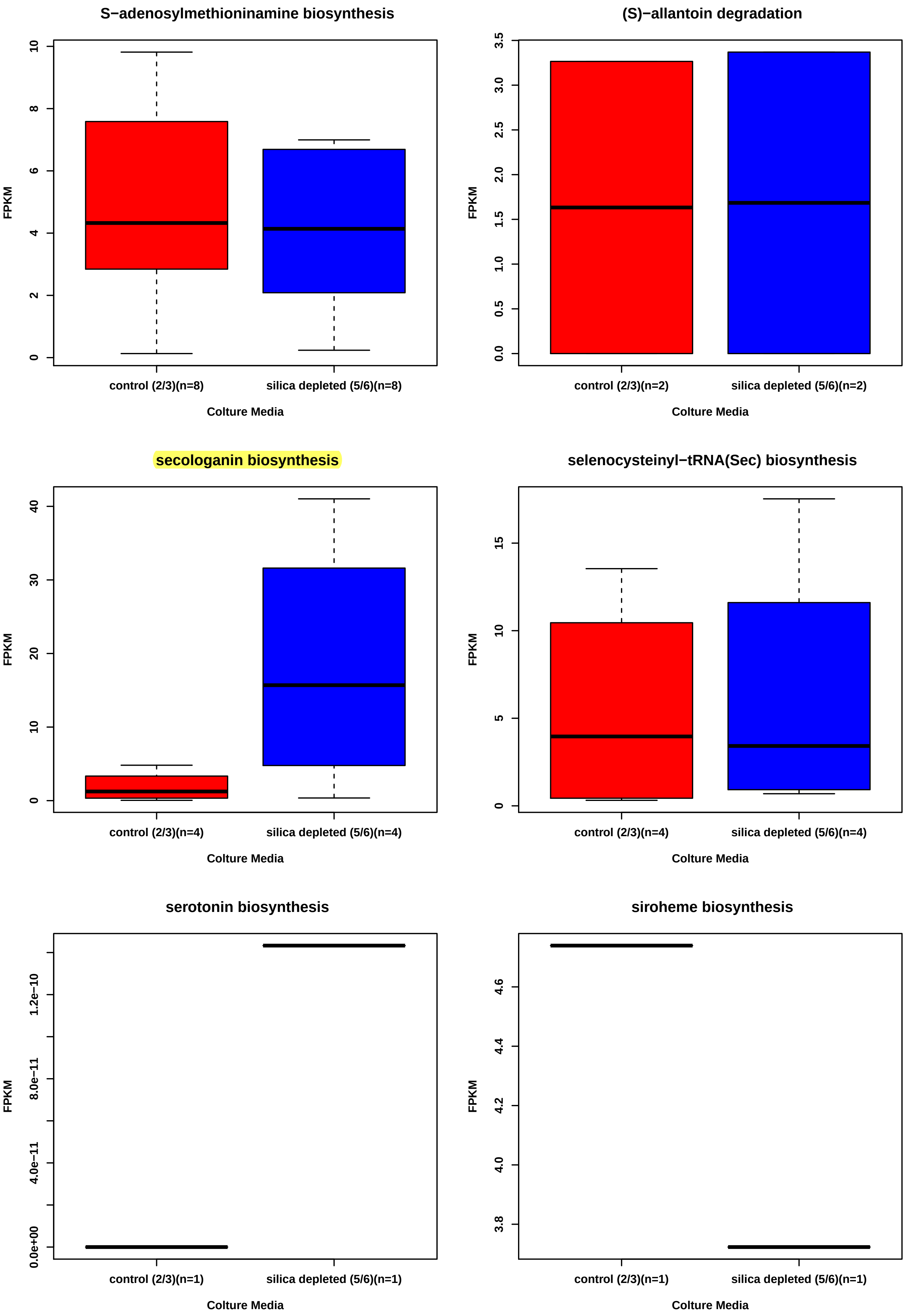
S−adenosylmethioninamine biosynthesis
FPKM
0
2
4
6
8
10
control (2/3)(n=8)
silica depleted (5/6)(n=8)
Colture Media
(S)−allantoin degradation
FPKM
0.0
0.5
1.0
1.5
2.0
2.5
3.0
3.5
control (2/3)(n=2)
silica depleted (5/6)(n=2)
Colture Media
secologanin biosynthesis
FPKM
0
10
20
30
40
control (2/3)(n=4)
silica depleted (5/6)(n=4)
Colture Media
selenocysteinyl−tRNA(Sec) biosynthesis
FPKM
0
5
10
15
control (2/3)(n=4)
silica depleted (5/6)(n=4)
Colture Media
serotonin biosynthesis
FPKM
0.0e+00
4.0e−11
8.0e−11
1.2e−10
control (2/3)(n=1)
silica depleted (5/6)(n=1)
Colture Media
siroheme biosynthesis
FPKM
3.8
4.0
4.2
4.4
4.6
control (2/3)(n=1)
silica depleted (5/6)(n=1)
Colture Media

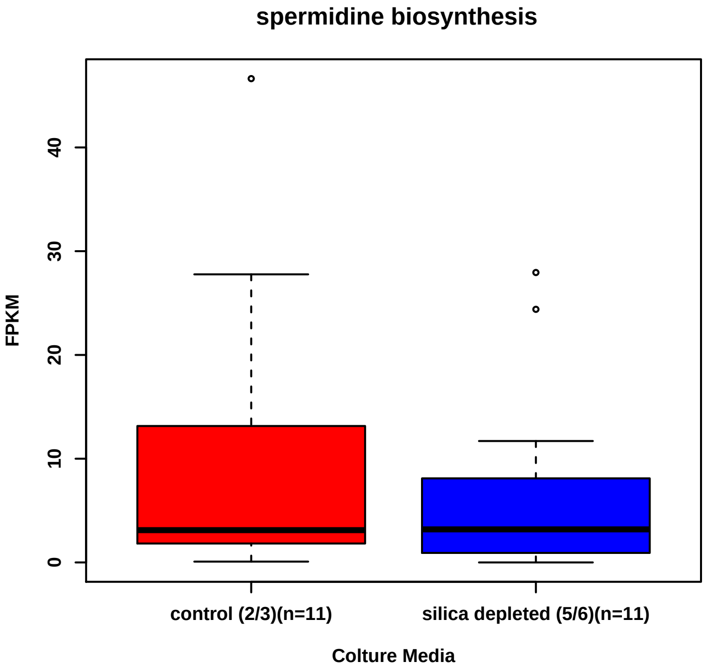
spermidine biosynthesis
FPKM
0
10
20
30
40
control (2/3)(n=11)
silica depleted (5/6)(n=11)
Colture Media

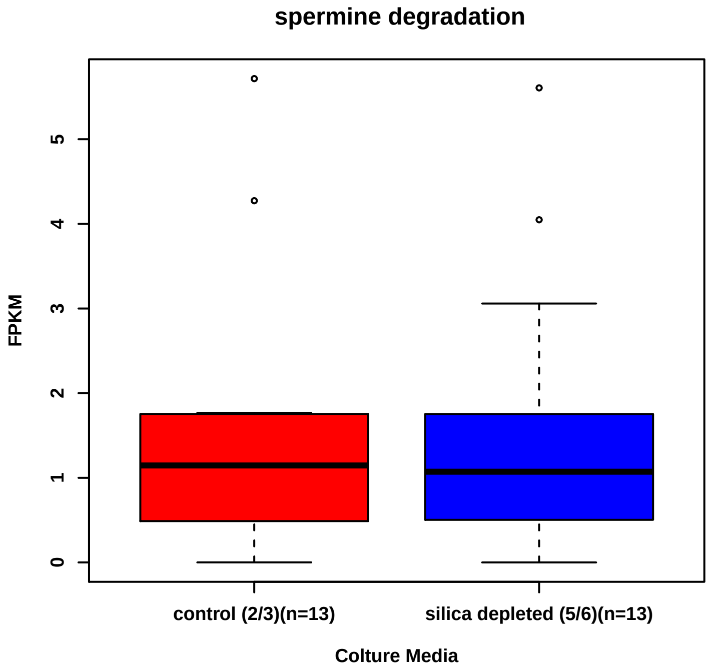
spermine degradation
FPKM
0
1
2
3
4
5
control (2/3)(n=13)
silica depleted (5/6)(n=13)
Colture Media

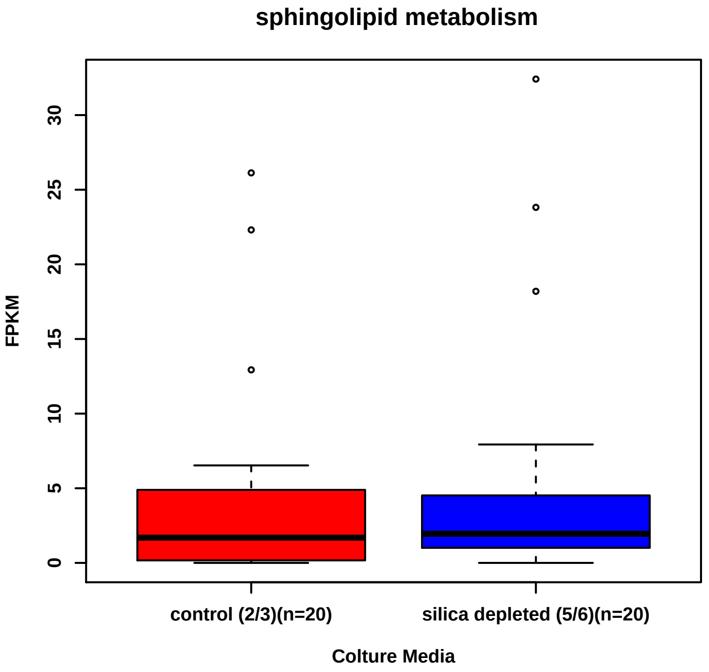
sphingolipid metabolism
FPKM
0
5
10
15
20
25
30
control (2/3)(n=20)
silica depleted (5/6)(n=20)
Colture Media

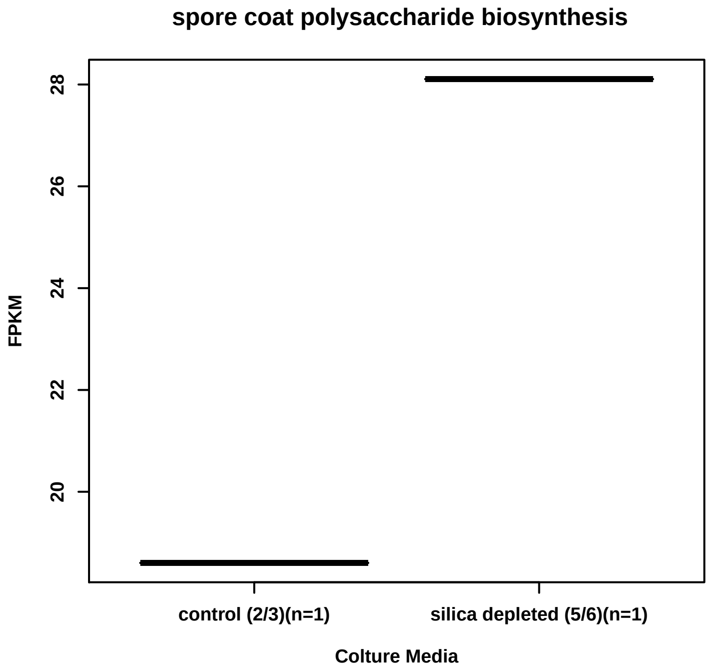
spore coat polysaccharide biosynthesis
FPKM
20
22
24
26
28
control (2/3)(n=1)
silica depleted (5/6)(n=1)
Colture Media

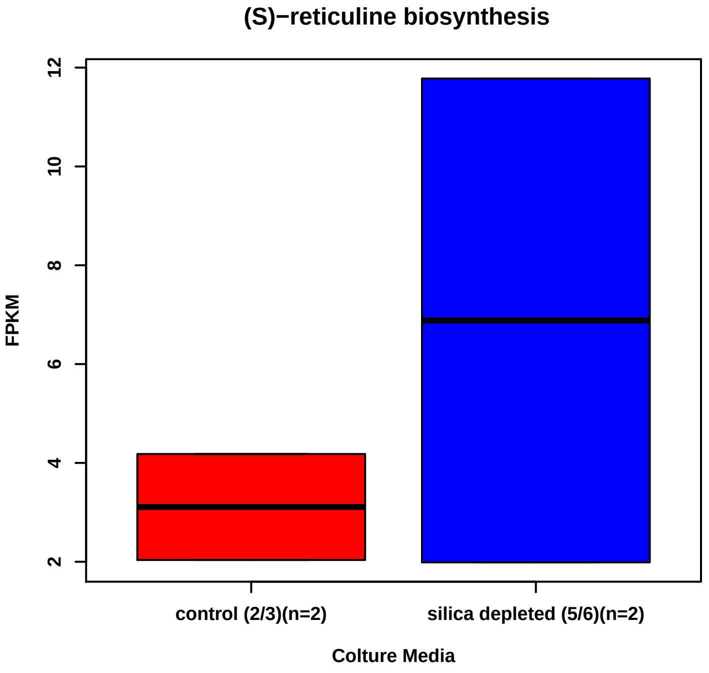
(S)−reticuline biosynthesis
FPKM
2
4
6
8
10
12
control (2/3)(n=2)
silica depleted (5/6)(n=2)
Colture Media

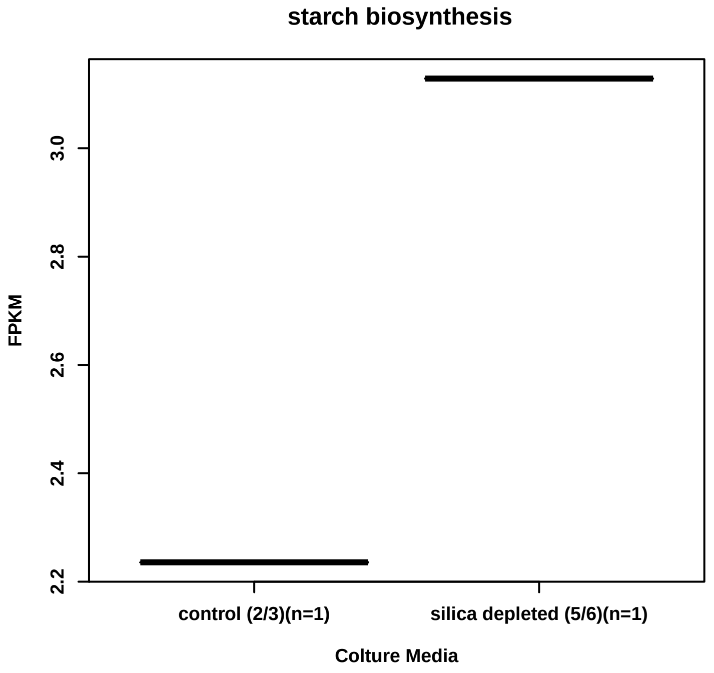
starch biosynthesis
FPKM
2.2
2.4
2.6
2.8
3.0
control (2/3)(n=1)
silica depleted (5/6)(n=1)
Colture Media

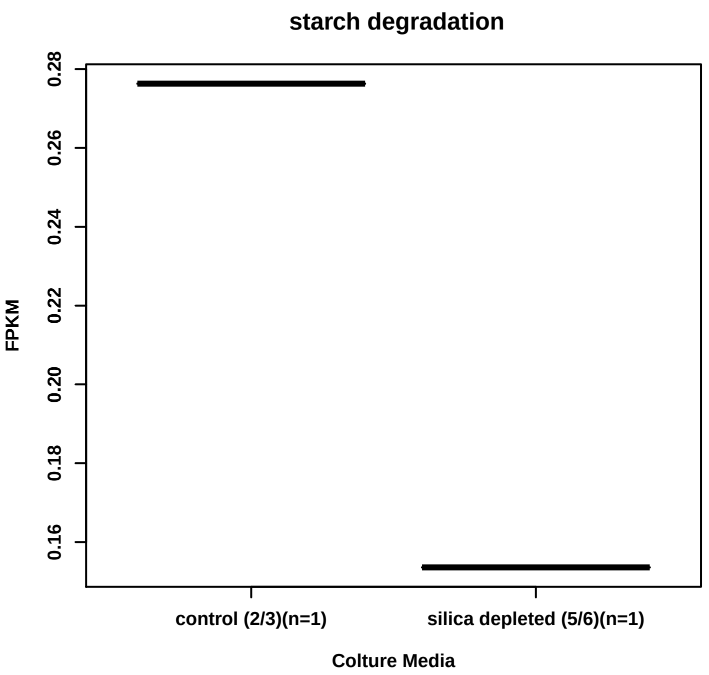
starch degradation
FPKM
0.16
0.18
0.20
0.22
0.24
0.26
0.28
control (2/3)(n=1)
silica depleted (5/6)(n=1)
Colture Media

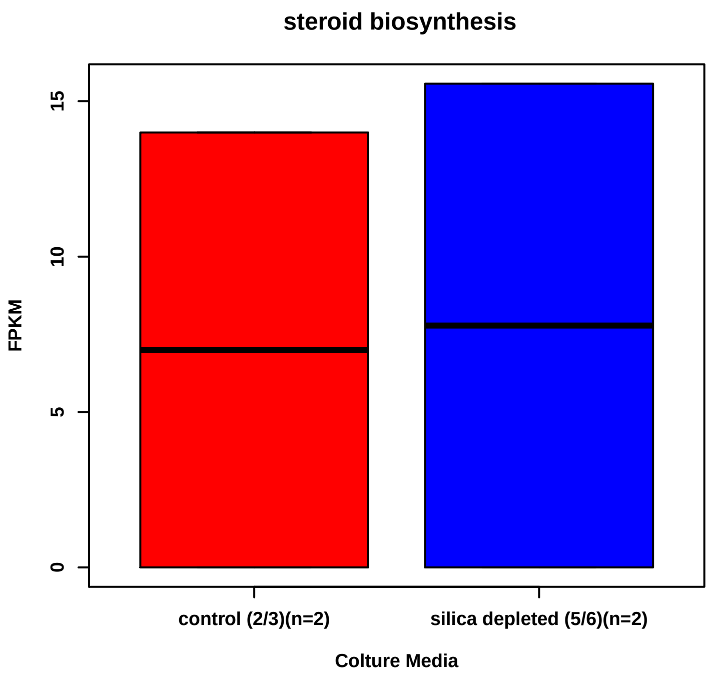
steroid biosynthesis
FPKM
0
5
10
15
control (2/3)(n=2)
silica depleted (5/6)(n=2)
Colture Media

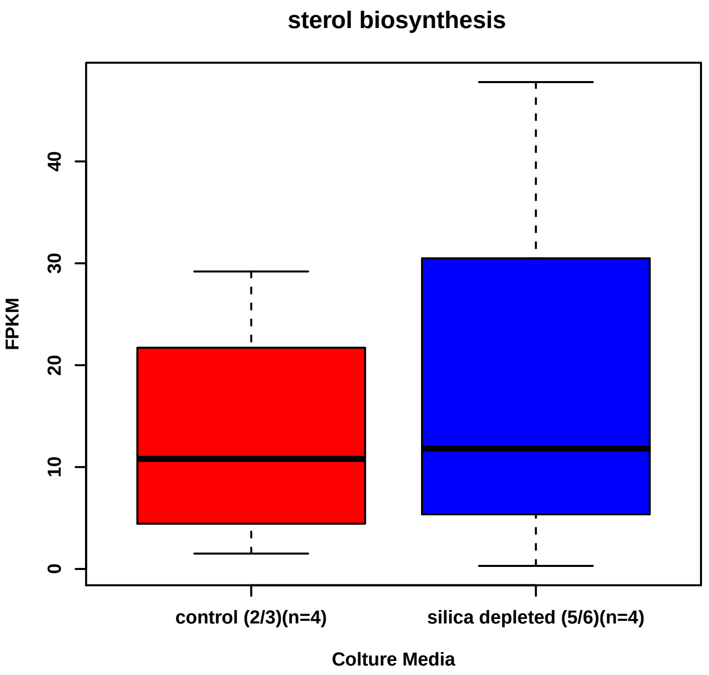
sterol biosynthesis
FPKM
0
10
20
30
40
control (2/3)(n=4)
silica depleted (5/6)(n=4)
Colture Media

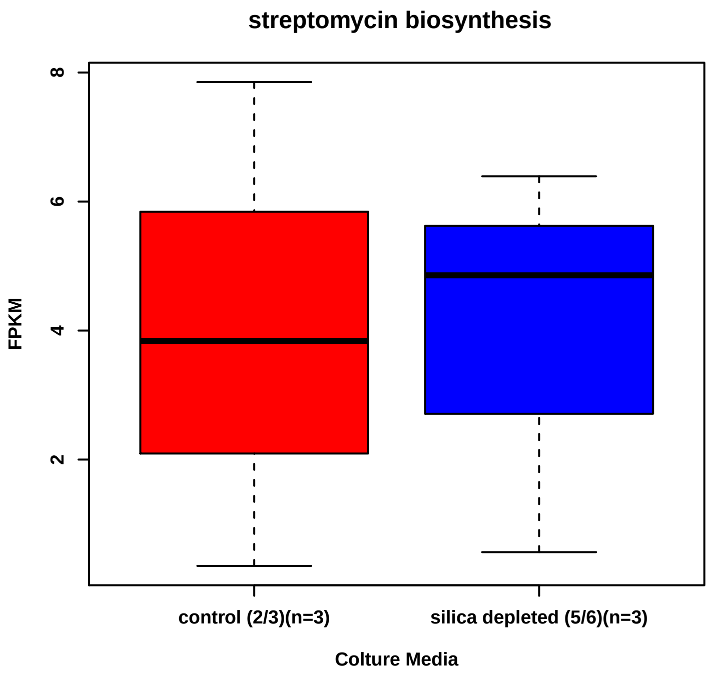
streptomycin biosynthesis
FPKM
2
4
6
8
control (2/3)(n=3)
silica depleted (5/6)(n=3)
Colture Media

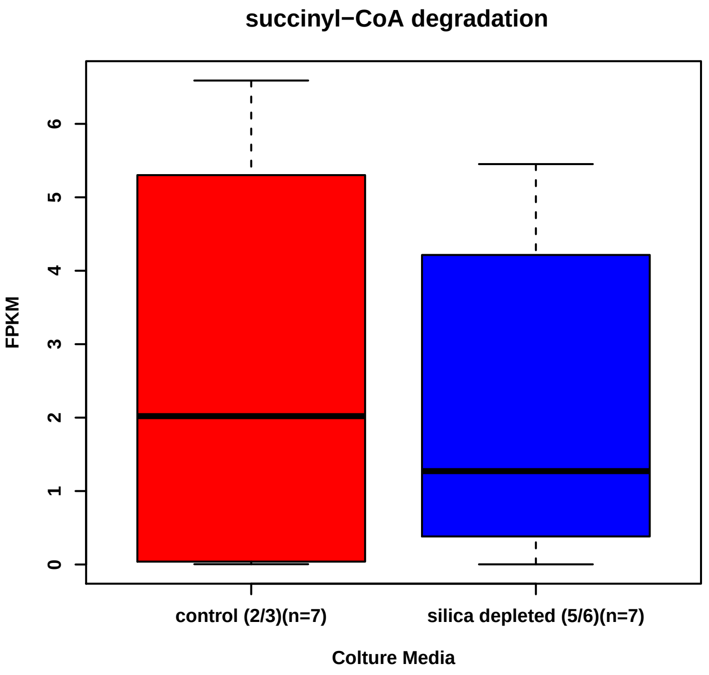
succinyl−CoA degradation
FPKM
0
1
2
3
4
5
6
control (2/3)(n=7)
silica depleted (5/6)(n=7)
Colture Media

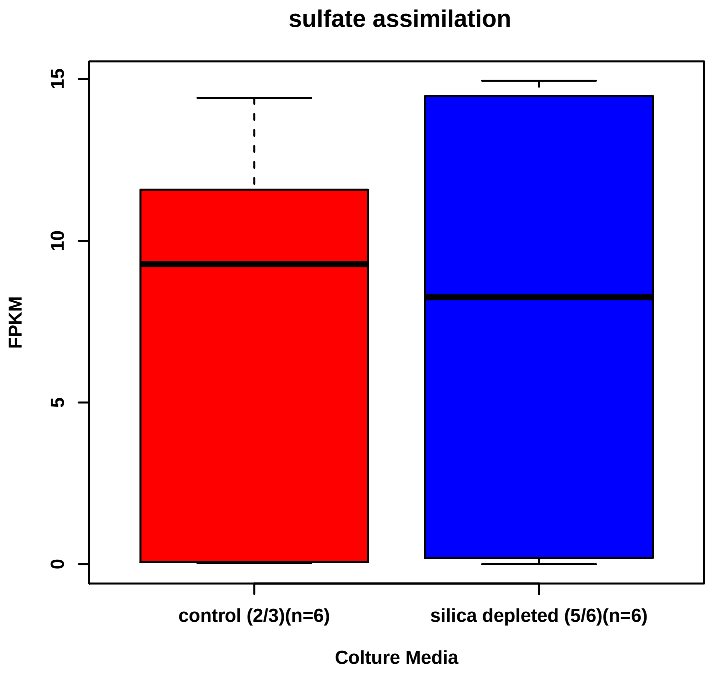
sulfate assimilation
FPKM
0
5
10
15
control (2/3)(n=6)
silica depleted (5/6)(n=6)
Colture Media

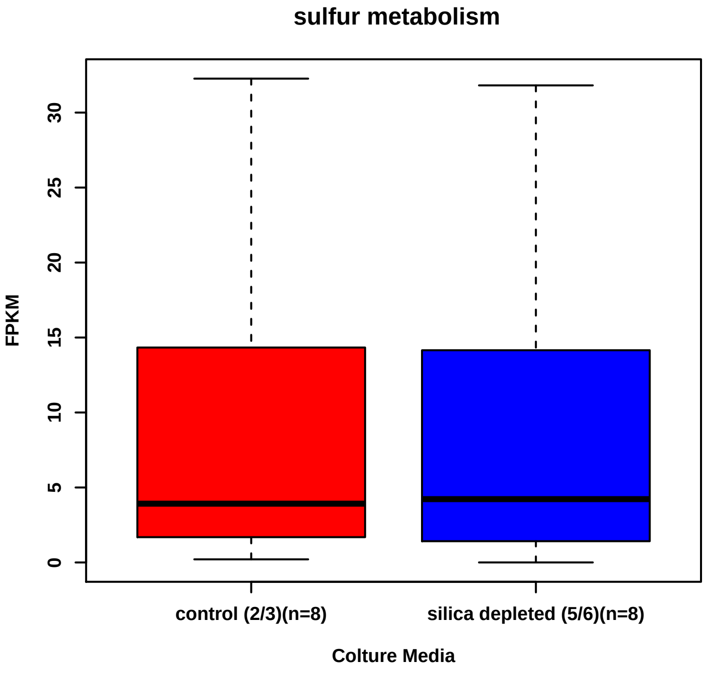
sulfur metabolism
FPKM
0
5
10
15
20
25
30
control (2/3)(n=8)
silica depleted (5/6)(n=8)
Colture Media

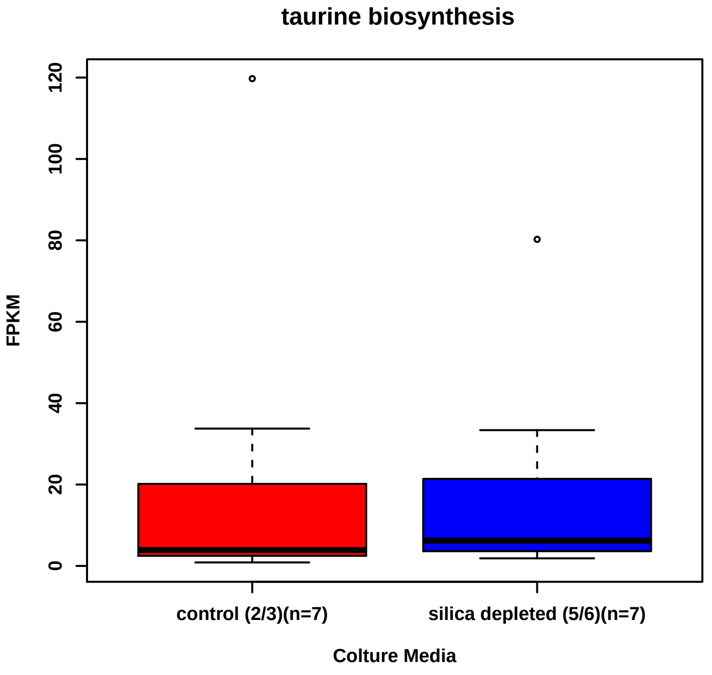
taurine biosynthesis
FPKM
0
20
40
60
80
100
120
control (2/3)(n=7)
silica depleted (5/6)(n=7)
Colture Media

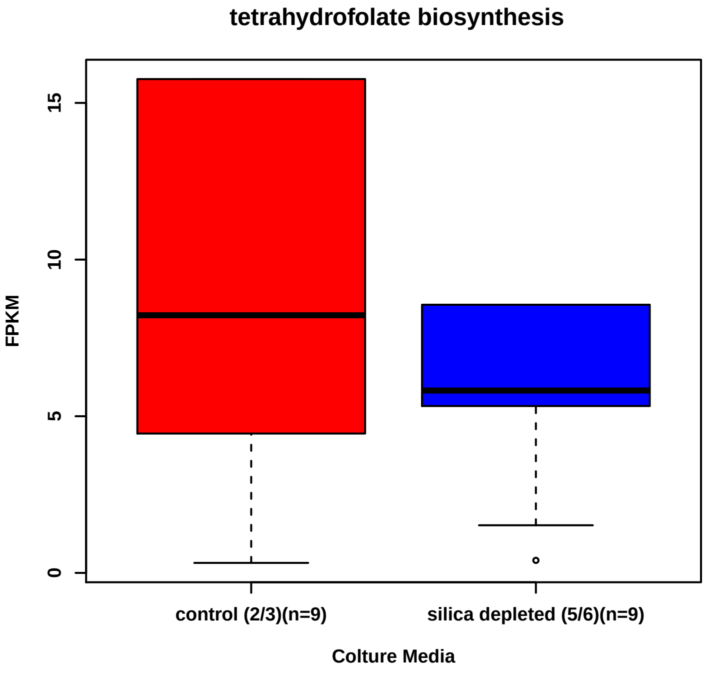
tetrahydrofolate biosynthesis
FPKM
0
5
10
15
control (2/3)(n=9)
silica depleted (5/6)(n=9)
Colture Media

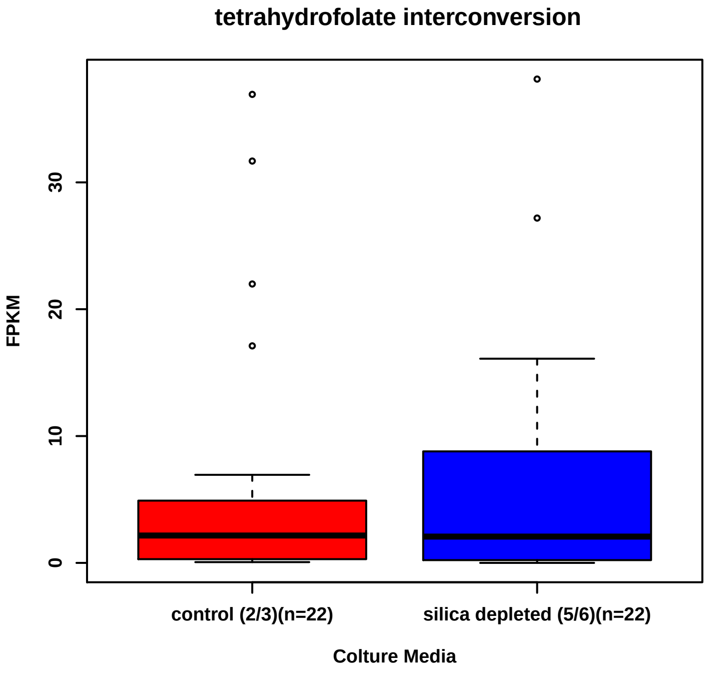
tetrahydrofolate interconversion
FPKM
0
10
20
30
control (2/3)(n=22)
silica depleted (5/6)(n=22)
Colture Media

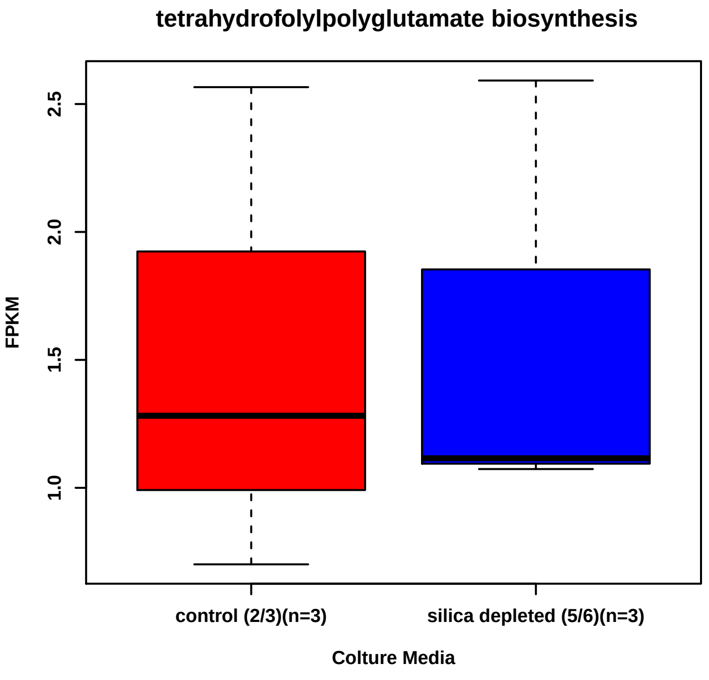
tetrahydrofolylpolyglutamate biosynthesis
FPKM
1.0
1.5
2.0
2.5
control (2/3)(n=3)
silica depleted (5/6)(n=3)
Colture Media

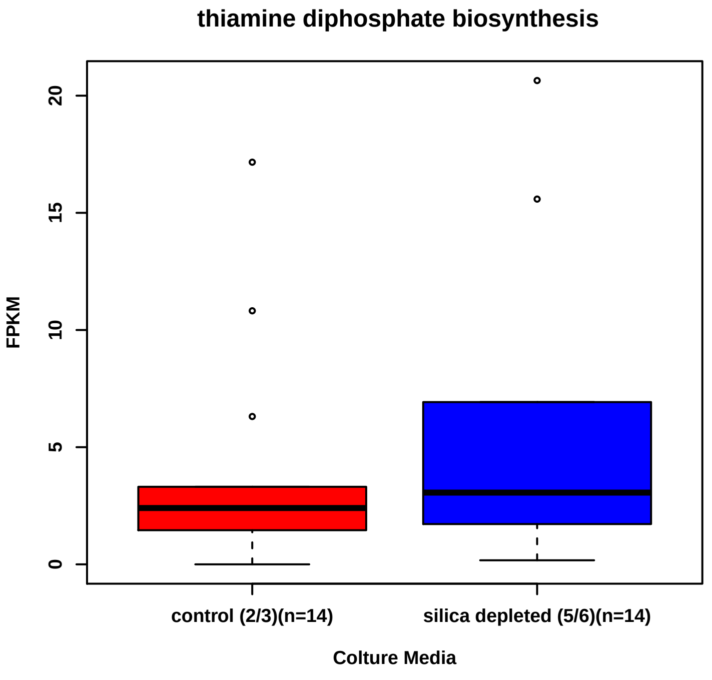
thiamine diphosphate biosynthesis
FPKM
0
5
10
15
20
control (2/3)(n=14)
silica depleted (5/6)(n=14)
Colture Media

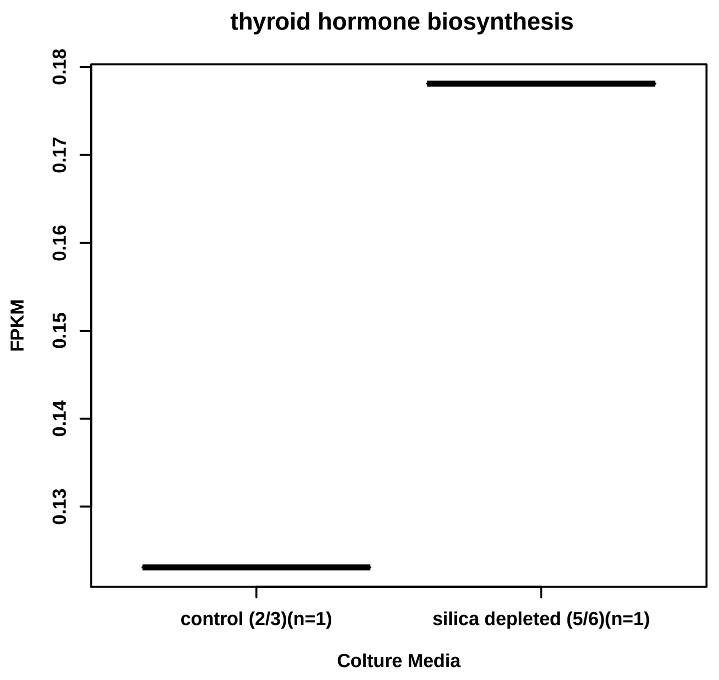
thyroid hormone biosynthesis
FPKM
0.13
0.14
0.15
0.16
0.17
0.18
control (2/3)(n=1)
silica depleted (5/6)(n=1)
Colture Media

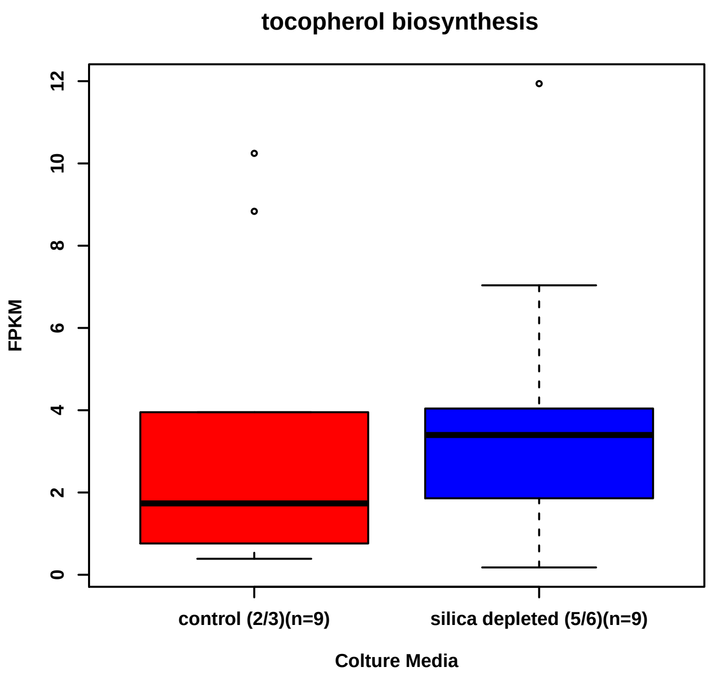
tocopherol biosynthesis
FPKM
0
2
4
6
8
10
12
control (2/3)(n=9)
silica depleted (5/6)(n=9)
Colture Media

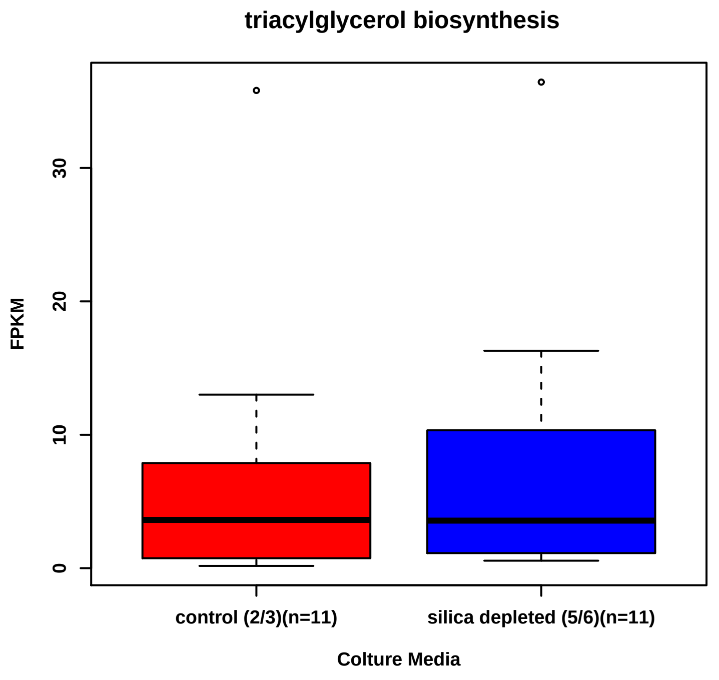
triacylglycerol biosynthesis
FPKM
0
10
20
30
control (2/3)(n=11)
silica depleted (5/6)(n=11)
Colture Media

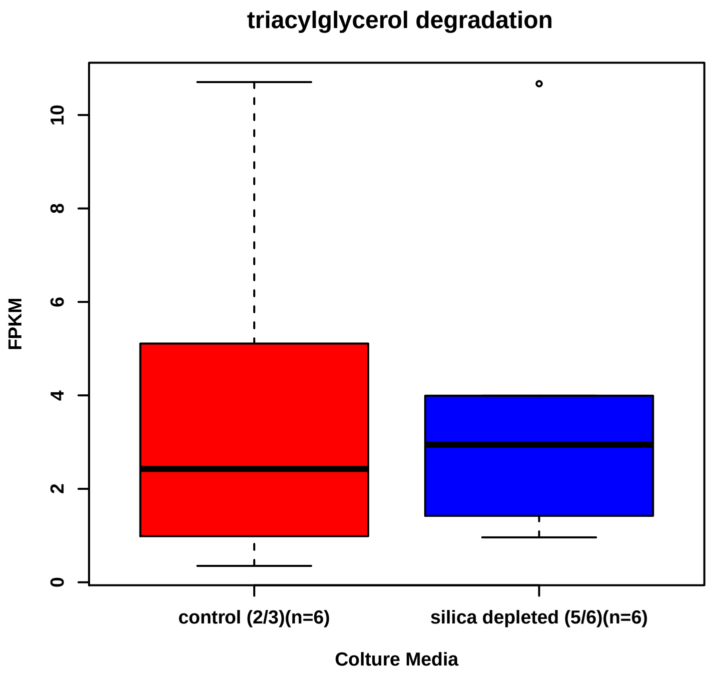
triacylglycerol degradation
FPKM
0
2
4
6
8
10
control (2/3)(n=6)
silica depleted (5/6)(n=6)
Colture Media

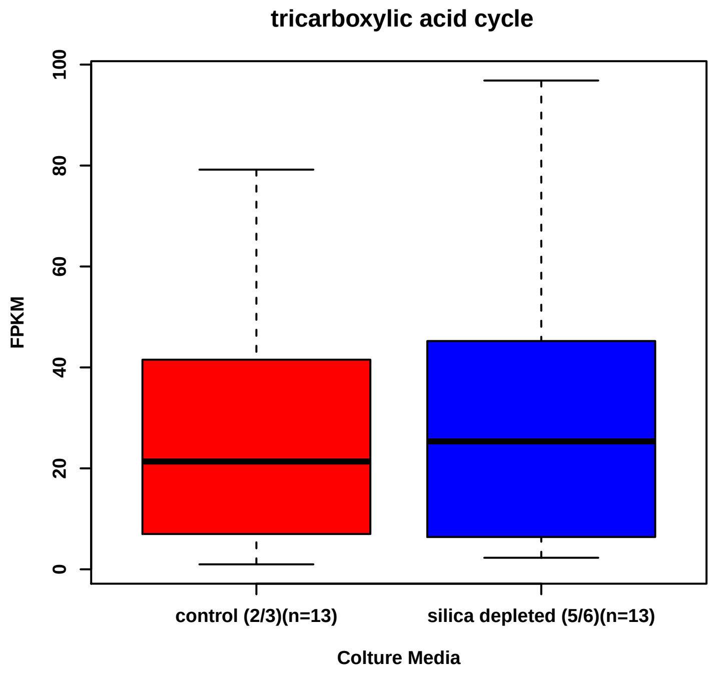
tricarboxylic acid cycle
FPKM
0
20
40
60
80
100
control (2/3)(n=13)
silica depleted (5/6)(n=13)
Colture Media

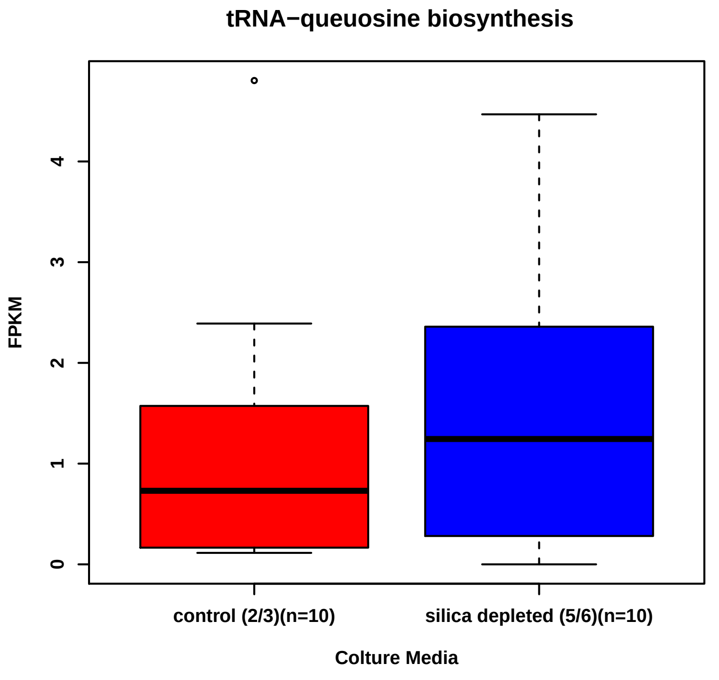
tRNA−queuosine biosynthesis
FPKM
0
1
2
3
4
control (2/3)(n=10)
silica depleted (5/6)(n=10)
Colture Media

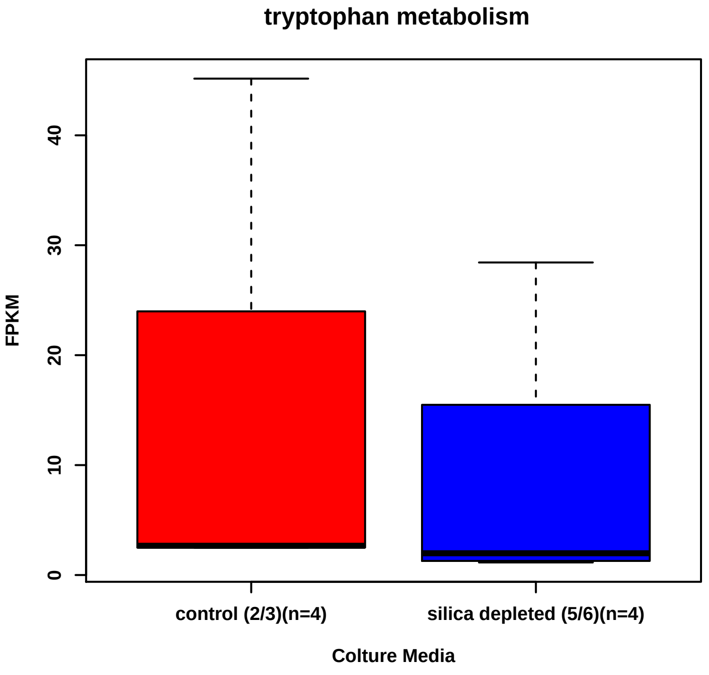
tryptophan metabolism
FPKM
0
10
20
30
40
control (2/3)(n=4)
silica depleted (5/6)(n=4)
Colture Media

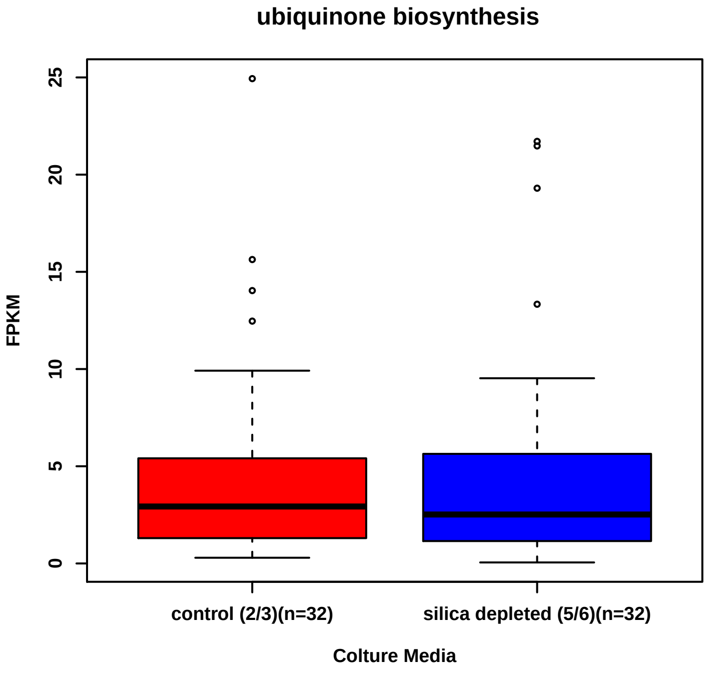
ubiquinone biosynthesis
FPKM
0
5
10
15
20
25
control (2/3)(n=32)
silica depleted (5/6)(n=32)
Colture Media

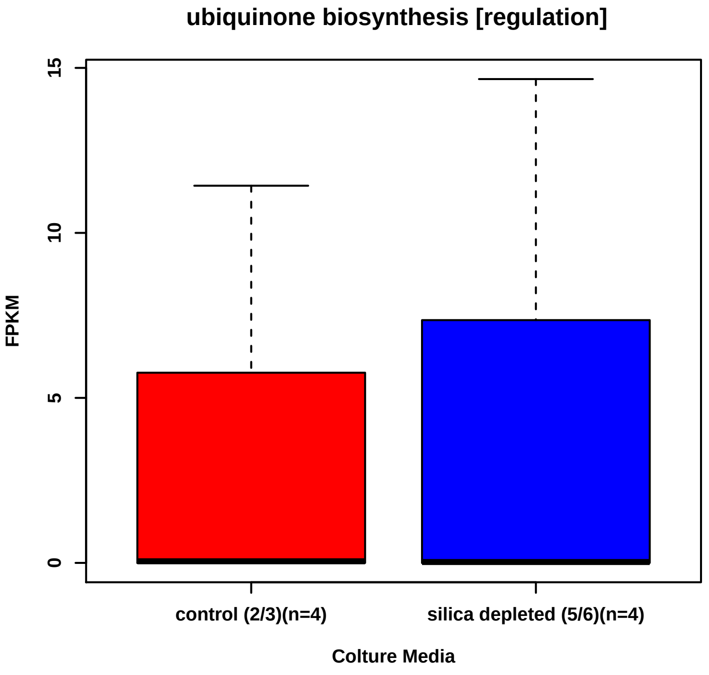
ubiquinone biosynthesis [regulation]
FPKM
0
5
10
15
control (2/3)(n=4)
silica depleted (5/6)(n=4)
Colture Media

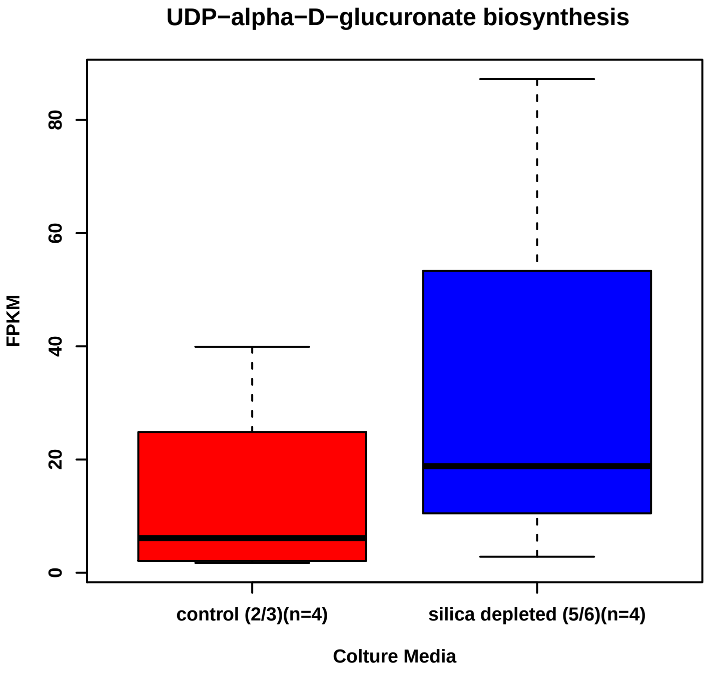
UDP−alpha−D−glucuronate biosynthesis
FPKM
0
20
40
60
80
control (2/3)(n=4)
silica depleted (5/6)(n=4)
Colture Media

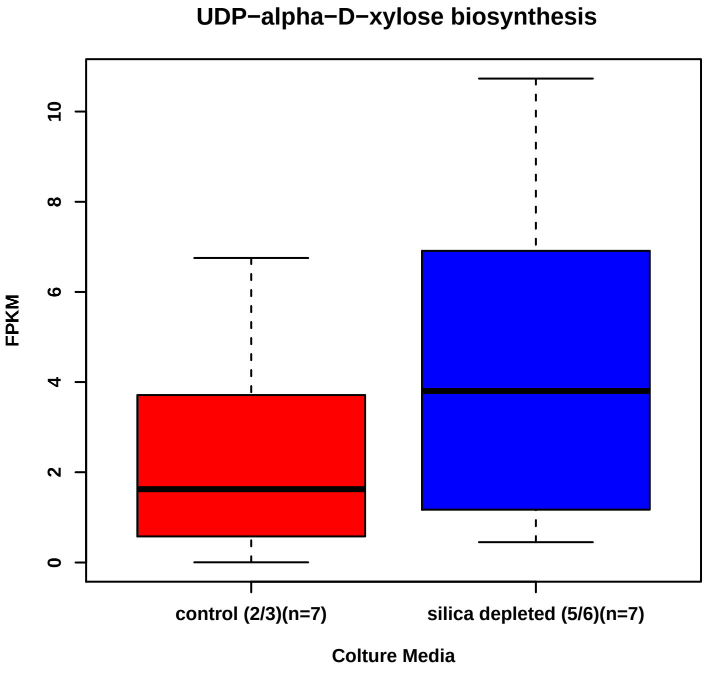
UDP−alpha−D−xylose biosynthesis
FPKM
0
2
4
6
8
10
control (2/3)(n=7)
silica depleted (5/6)(n=7)
Colture Media

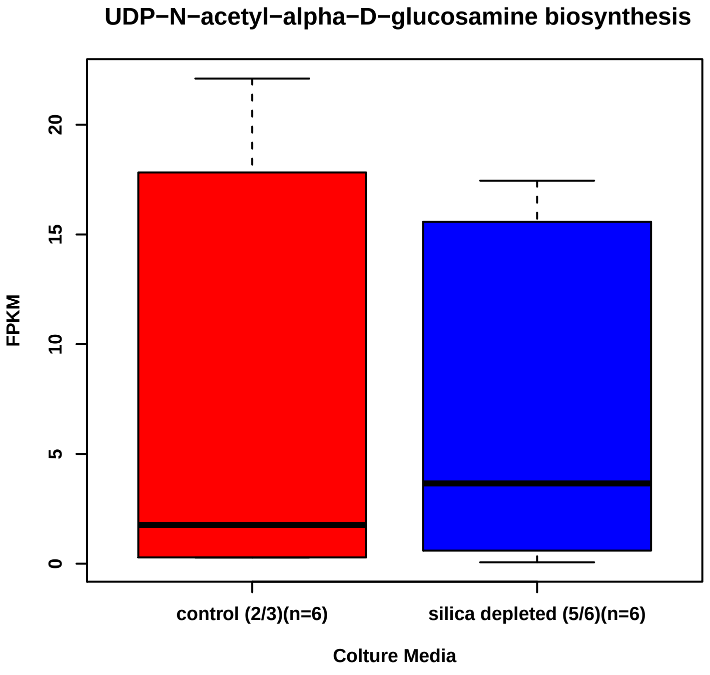
UDP−N−acetyl−alpha−D−glucosamine biosynthesis
FPKM
0
5
10
15
20
control (2/3)(n=6)
silica depleted (5/6)(n=6)
Colture Media

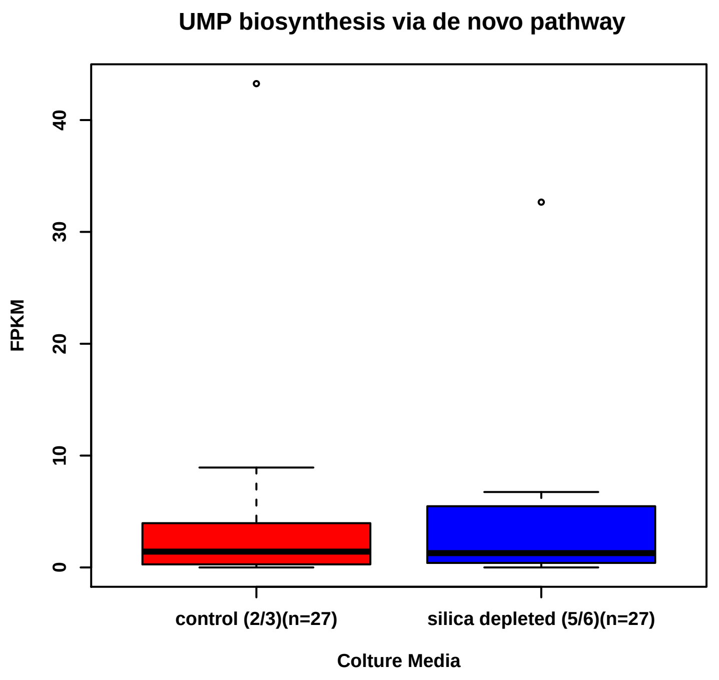
UMP biosynthesis via de novo pathway
FPKM
0
10
20
30
40
control (2/3)(n=27)
silica depleted (5/6)(n=27)
Colture Media

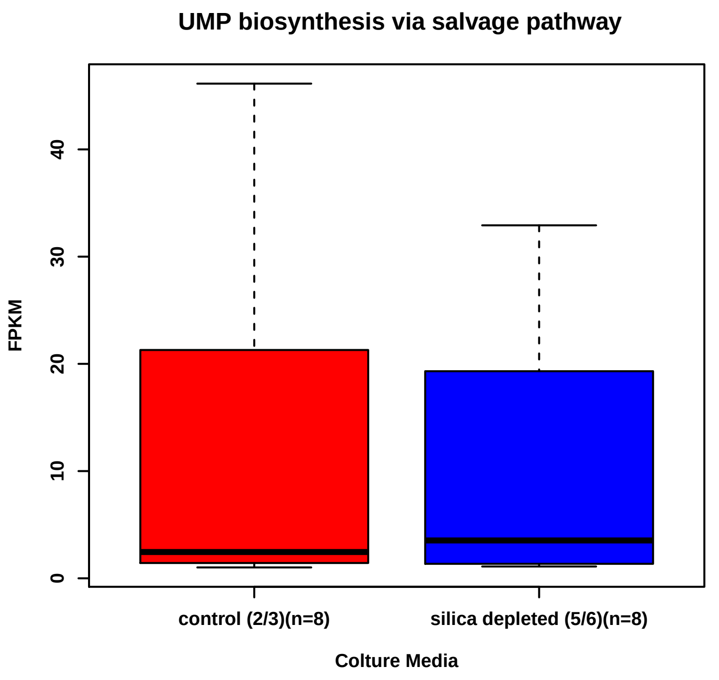
UMP biosynthesis via salvage pathway
FPKM
0
10
20
30
40
control (2/3)(n=8)
silica depleted (5/6)(n=8)
Colture Media

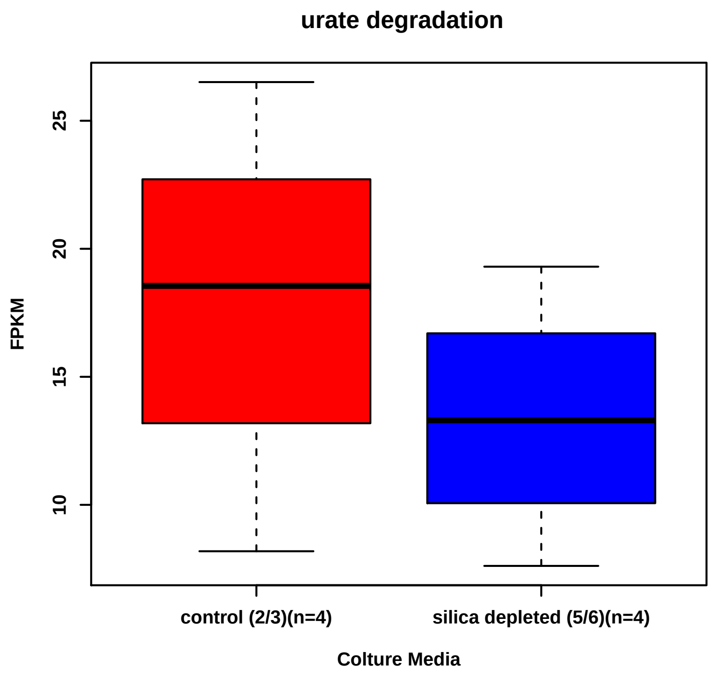
urate degradation
FPKM
10
15
20
25
control (2/3)(n=4)
silica depleted (5/6)(n=4)
Colture Media

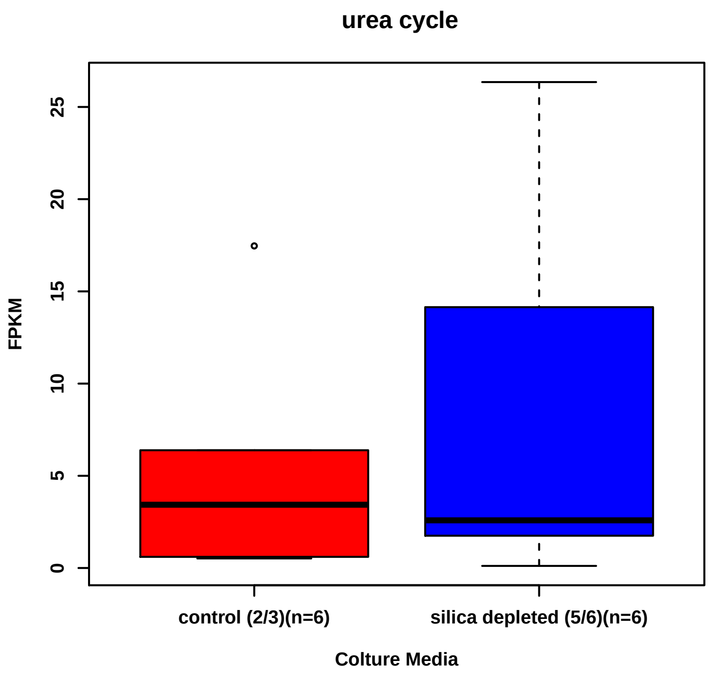
urea cycle
FPKM
0
5
10
15
20
25
control (2/3)(n=6)
silica depleted (5/6)(n=6)
Colture Media

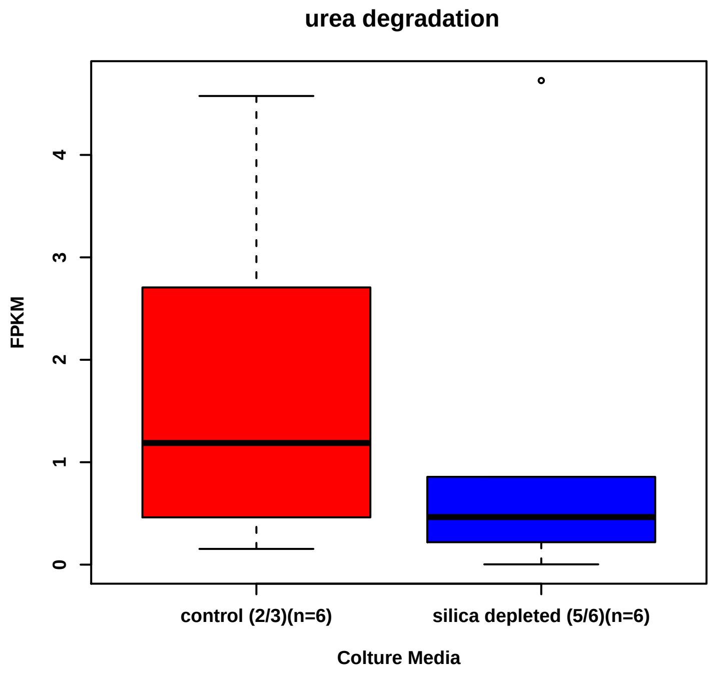
urea degradation
FPKM
0
1
2
3
4
control (2/3)(n=6)
silica depleted (5/6)(n=6)
Colture Media

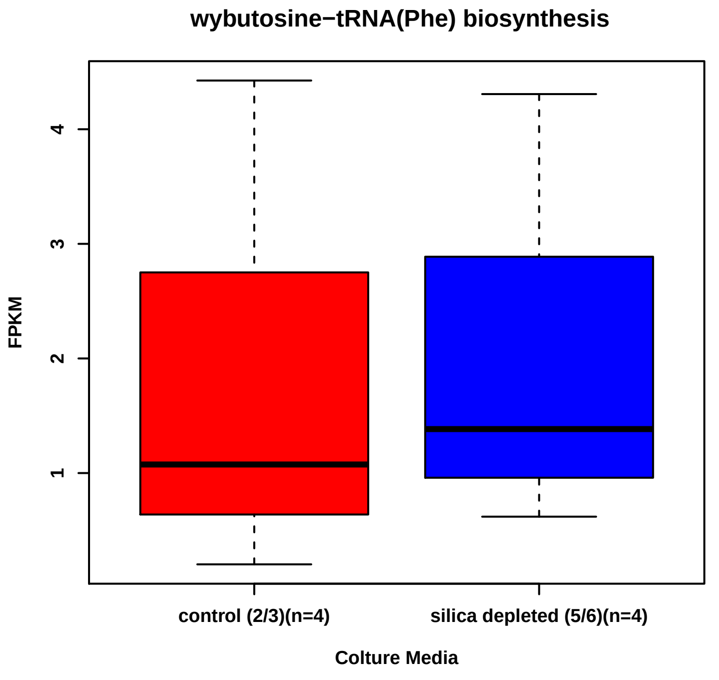
wybutosine−tRNA(Phe) biosynthesis
FPKM
1
2
3
4
control (2/3)(n=4)
silica depleted (5/6)(n=4)
Colture Media

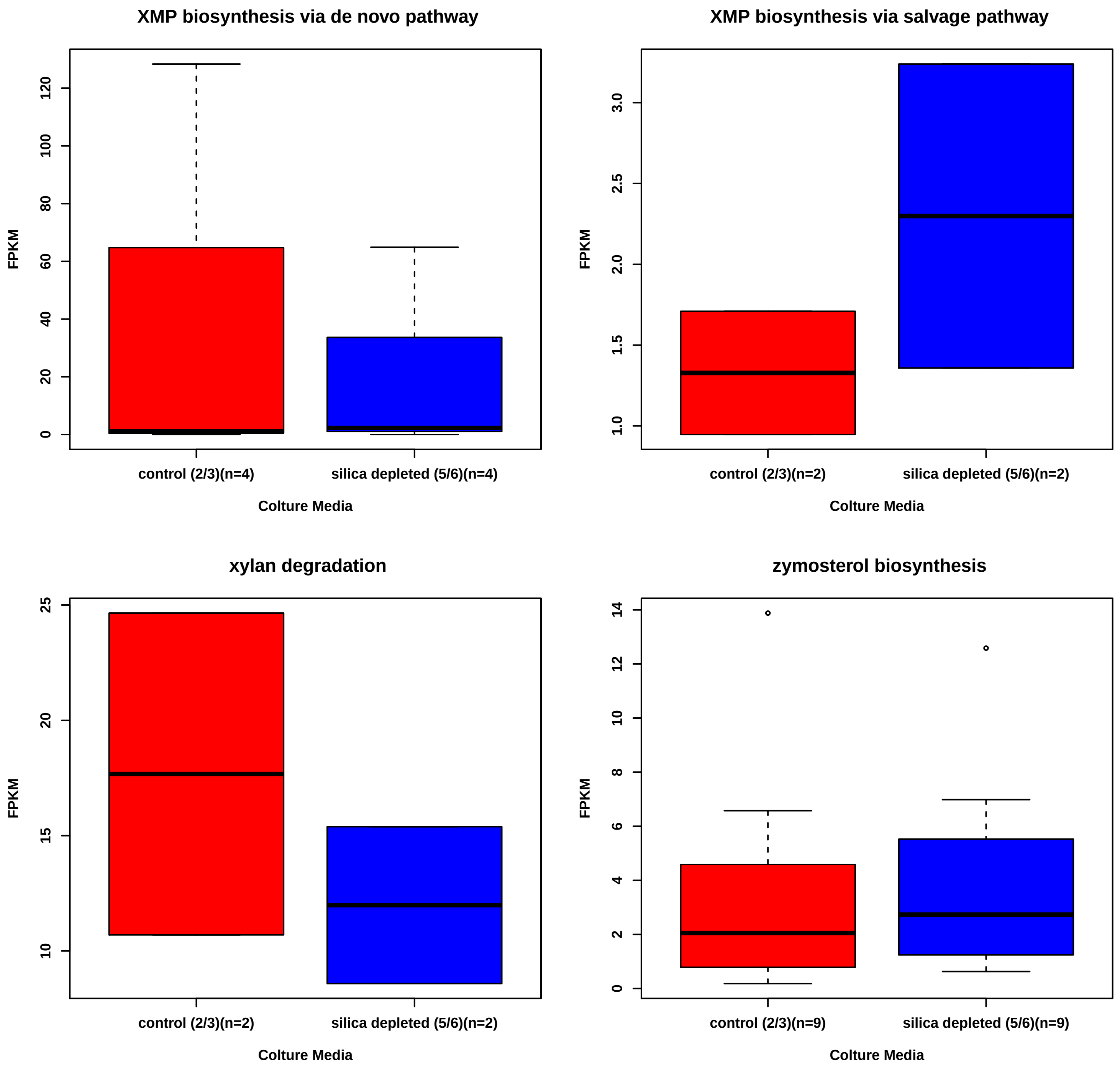

XMP biosynthesis via de novo pathway
FPKM
0
20
40
60
80
100
120
control (2/3)(n=4)
silica depleted (5/6)(n=4)
Colture Media
XMP biosynthesis via salvage pathway
FPKM
1.0
1.5
2.0
2.5
3.0
control (2/3)(n=2)
silica depleted (5/6)(n=2)
Colture Media
xylan degradation
FPKM
10
15
20
25
control (2/3)(n=2)
silica depleted (5/6)(n=2)
Colture Media
zymosterol biosynthesis
FPKM
0
2
4
6
8
10
12
14
control (2/3)(n=9)
silica depleted (5/6)(n=9)
Colture Media

Unveiling the presence of biosynthetic pathways for bioactive compounds in the *Thalassiosira rotula* transcriptome

Valeria Di Dato*, Federica Di Costanzo, Roberta Barbarinaldi, Anna Perna, Adrianna Ianora, Giovanna Romano

Supplementary File 5:

Box Plot Analysis of the expression, in FPKM, of the pathways annotated on level 3 for each replicate in each condition

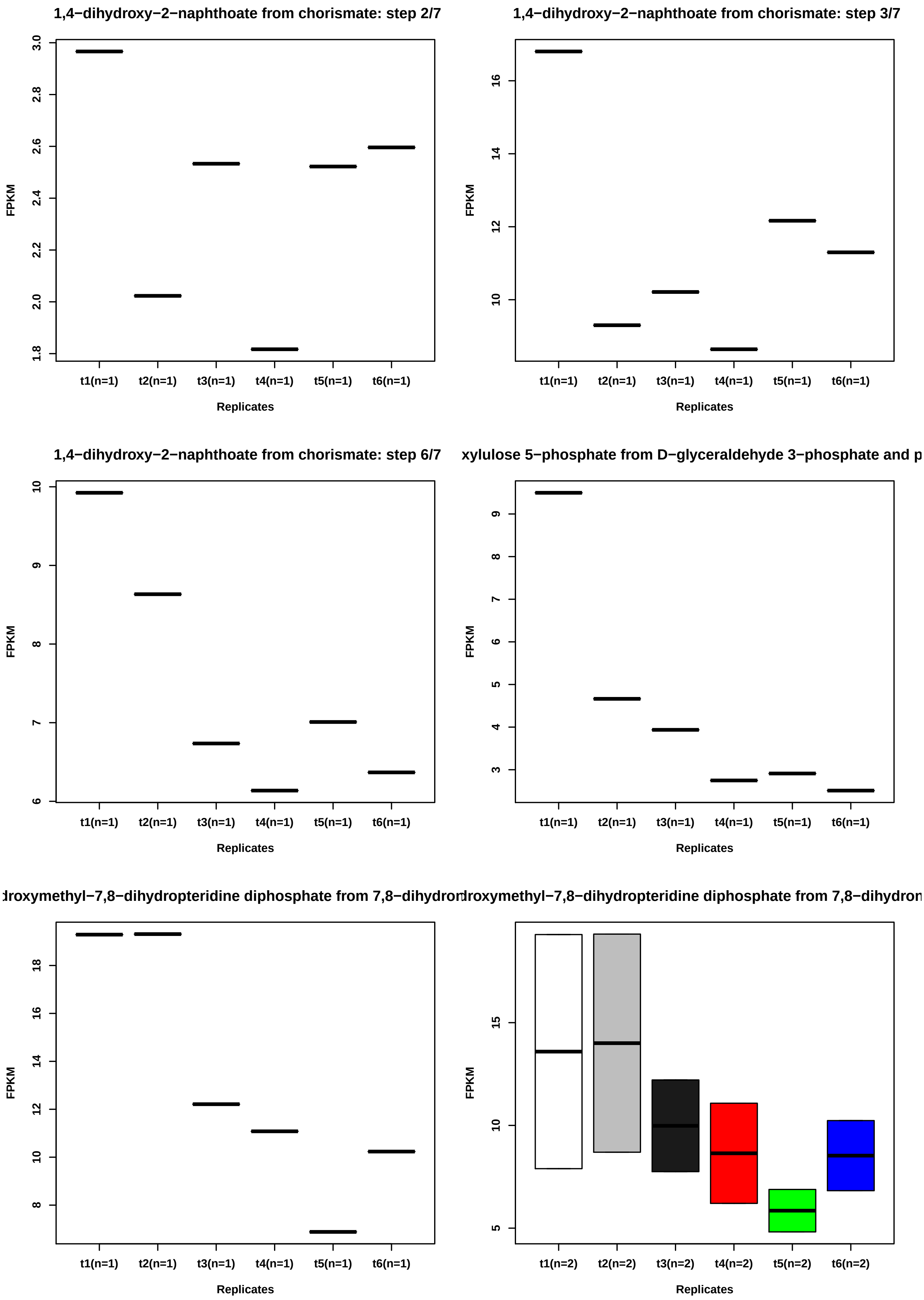
1,4−dihydroxy−2−naphthoate from chorismate: step 2/7
1,4−dihydroxy−2−naphthoate from chorismate: step 3/7
1,4−dihydroxy−2−naphthoate from chorismate: step 6/7
xylulose 5−phosphate from D−glyceraldehyde 3−phosphate and p
droxymethyl−7,8−dihydropteridine diphosphate from 7,8−dihydron
roxymethyl−7,8−dihydropteridine diphosphate from 7,8−dihydron
FPKM
Replicates
t1(n=1)
t2(n=1)
t3(n=1)
t4(n=1)
t5(n=1)
t6(n=1)
t1(n=2)
t2(n=2)
t3(n=2)
t4(n=2)
t5(n=2)
t6(n=2)

2−deoxystreptamine from D−glucose 6−phosphate: step 3/4

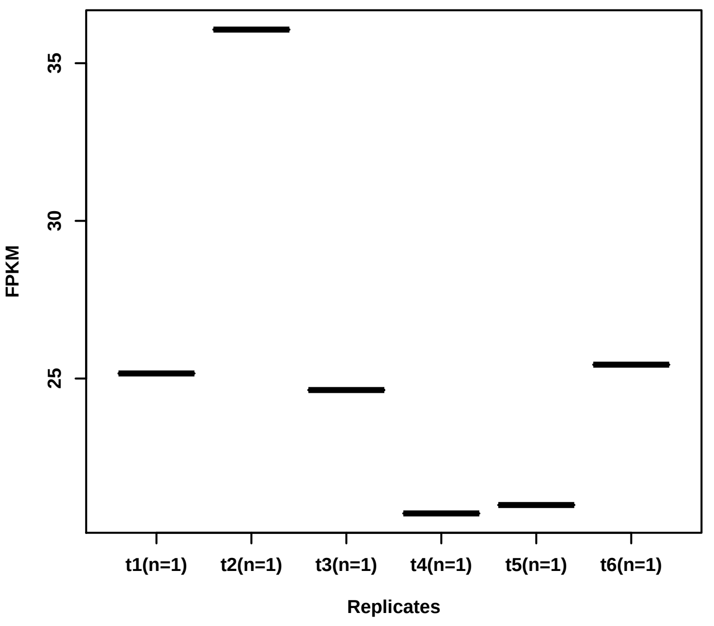

-hydroxy−3−oxobutyl phosphate from D−ribulose 5−phosphate: st

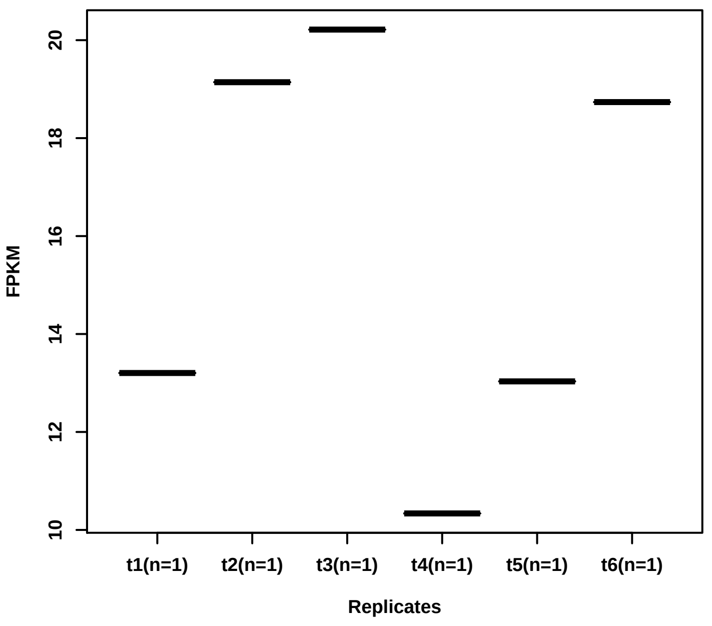

2−oxobutanoate from L−threonine: step 1/1

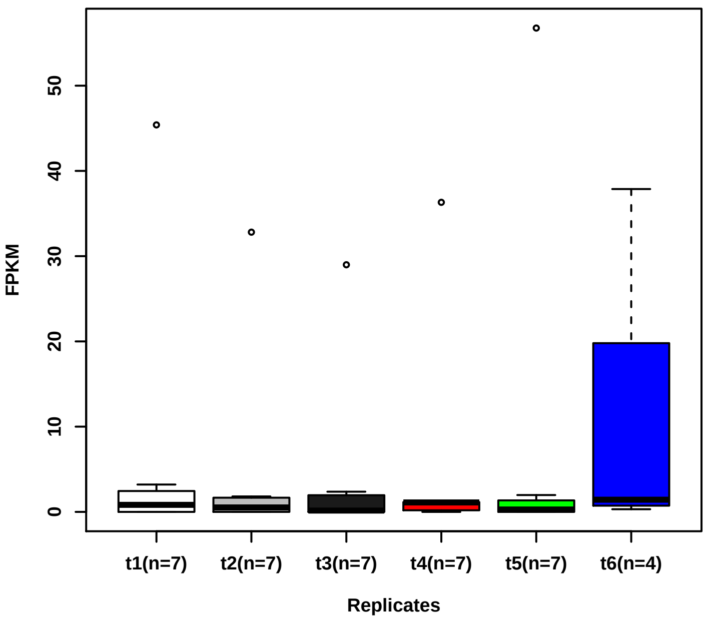

2−oxobutanoate from pyruvate: step 1/3

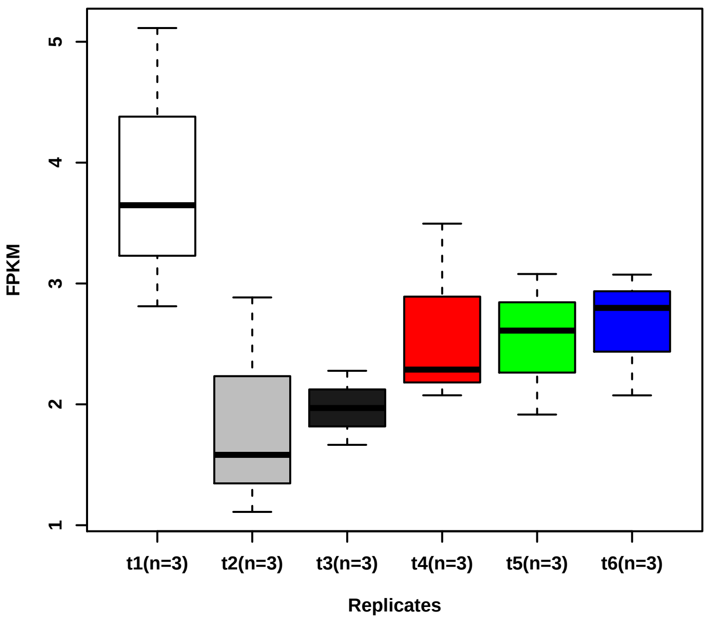

3,4',5−trihydroxystilbene from trans−4−coumarate: step 1/2

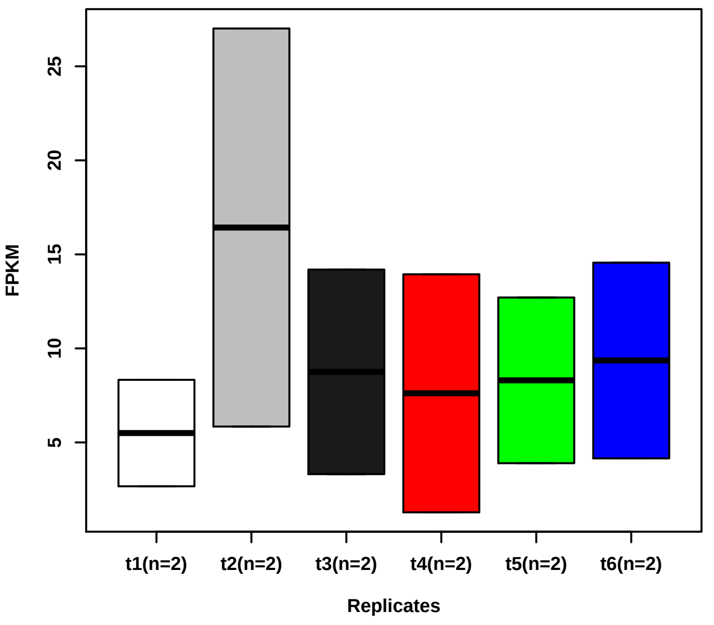

3−phospho−D−glycerate from glycolate: step 1/4

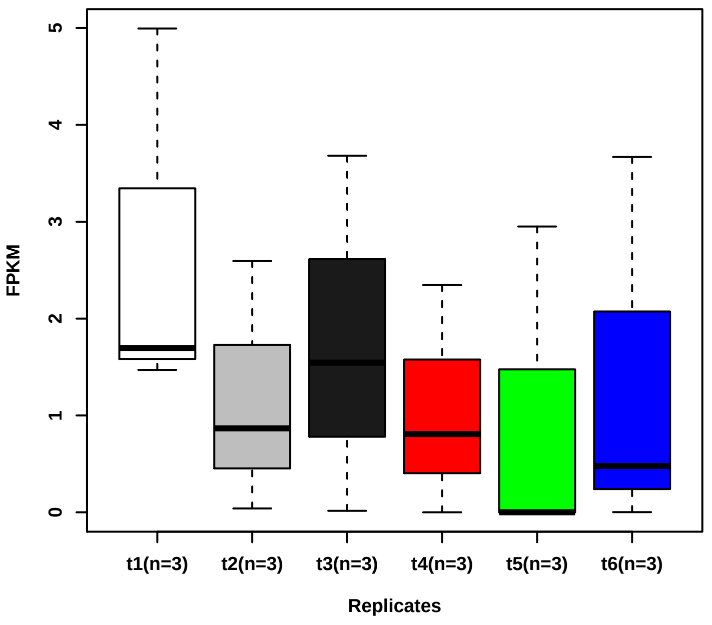

5−diphosphomethylpyrimidine from 5−amino−1−(5−phospho−D−ri

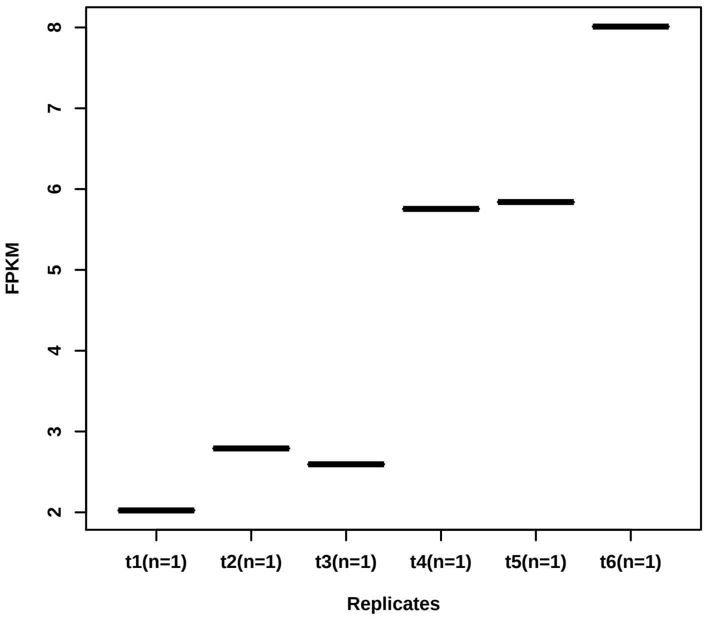

5−diphosphomethylpyrimidine from 5−amino−1−(5−phospho−D−ri

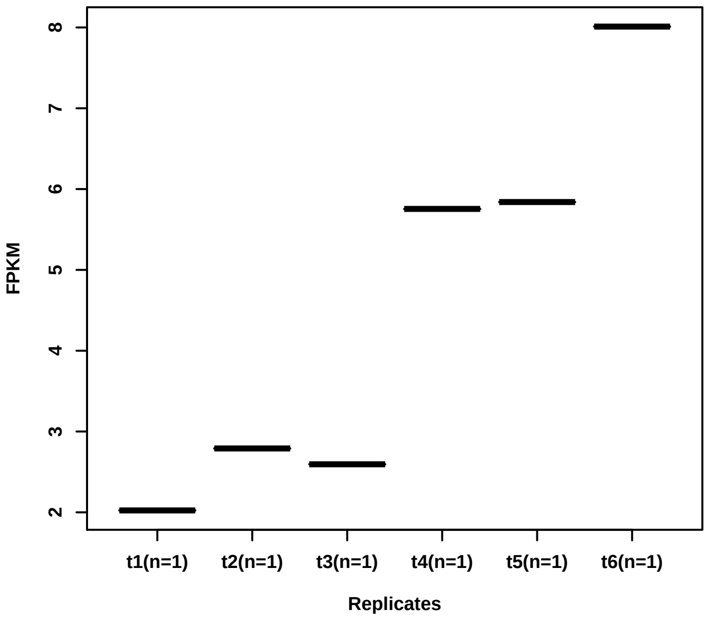

4−aminobenzoate from chorismate: step 1/2

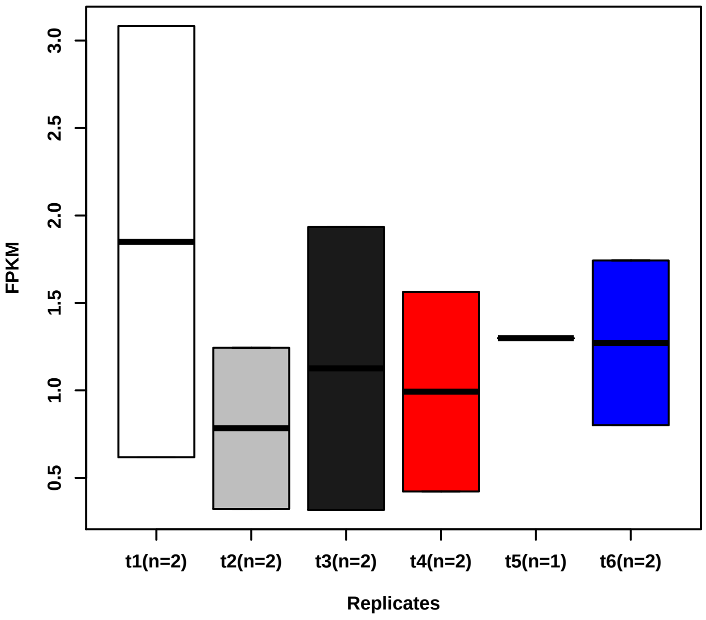

5,6,7,8−tetrahydrofolate from 7,8−dihydrofolate: step 1/1

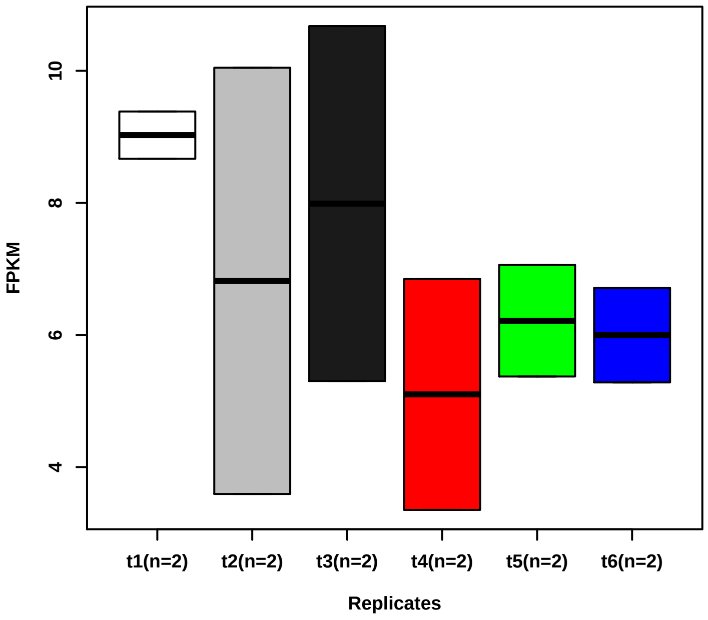

/l)imidazole−4−carboxamide from 5−amino−1−(5−phospho−D−ribo

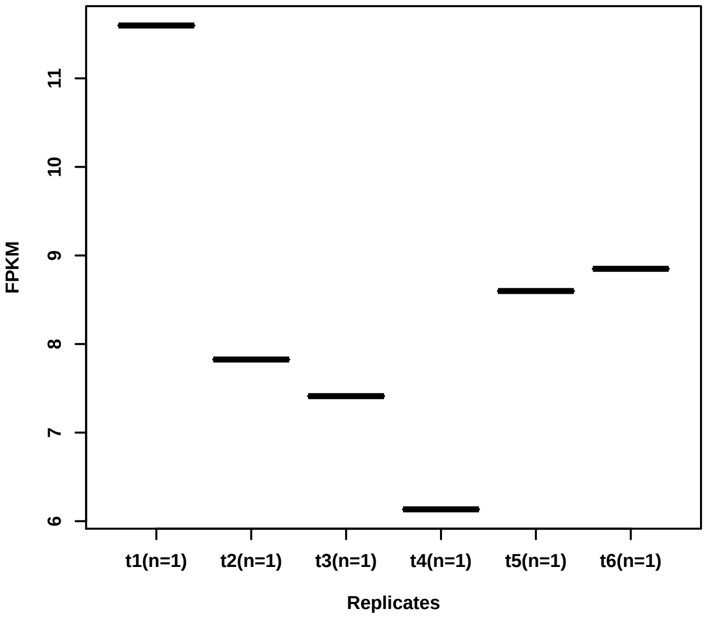

/l)imidazole−4−carboxamide from 5−amino−1−(5−phospho−D−ribo

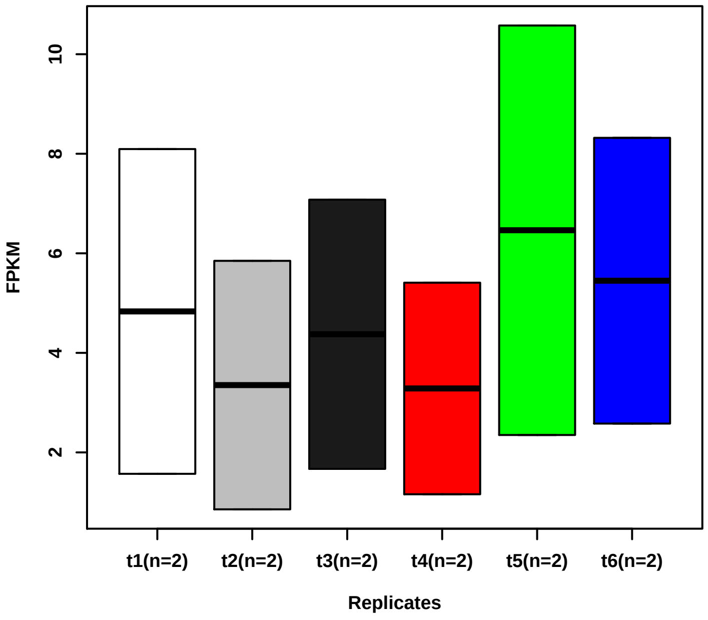

ı imidazole−4−carboxylate from 5−amino−1−(5−phospho−D−ribosyno−D−ribosyl)imidazole from N(2)−formyl−N(1)−(5−phospho−D−rib

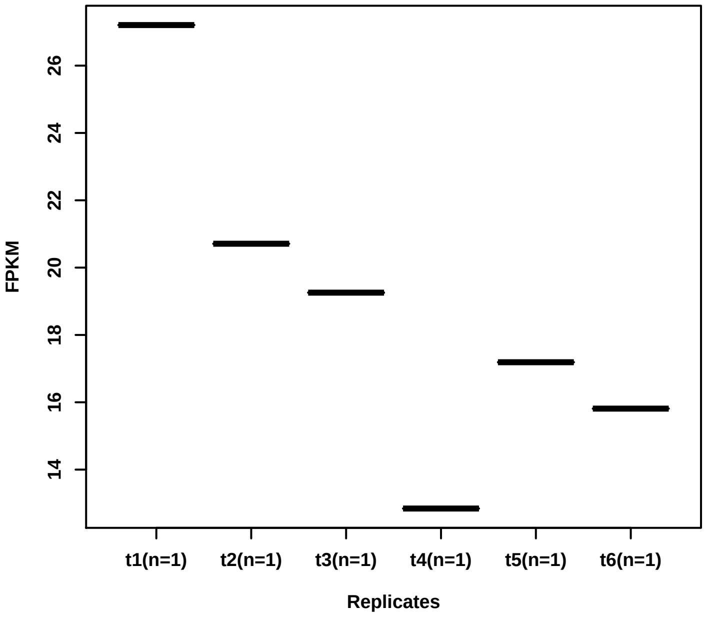

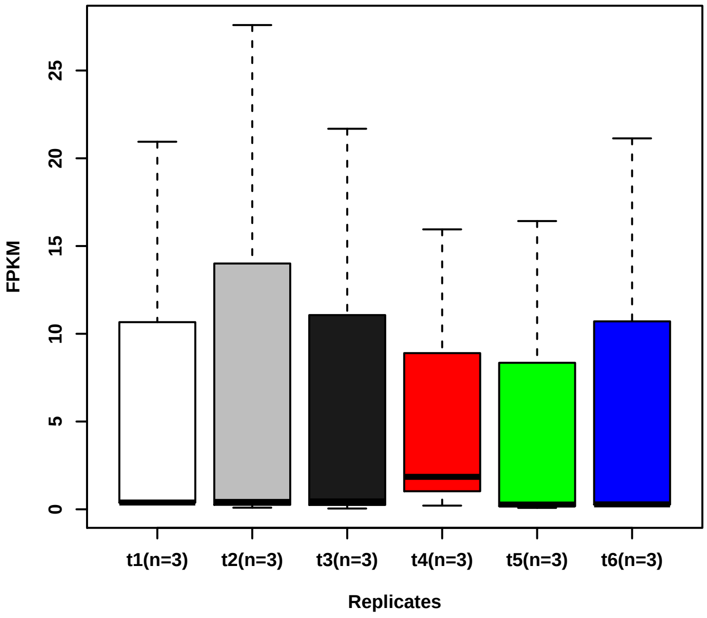

ıo−D−ribosyl)imidazole from N(2)−formyl−N(1)−(5−phospho−D−rib

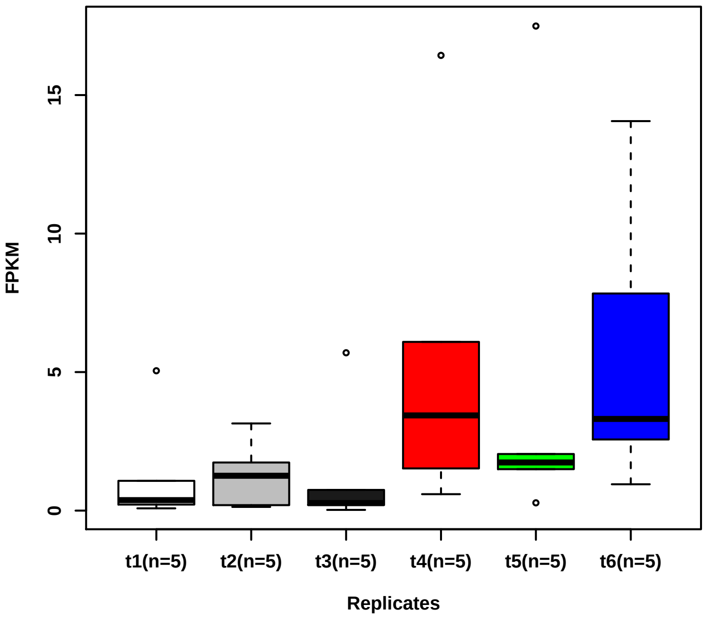

5−amino−6−(D−ribitylamino)uracil from GTP: step 1/4

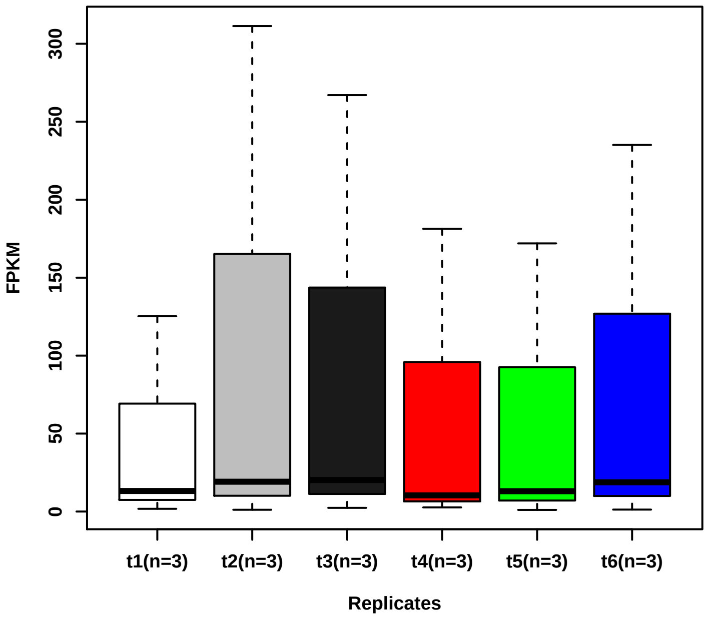

5−amino−6−(D−ribitylamino)uracil from GTP: step 2/4

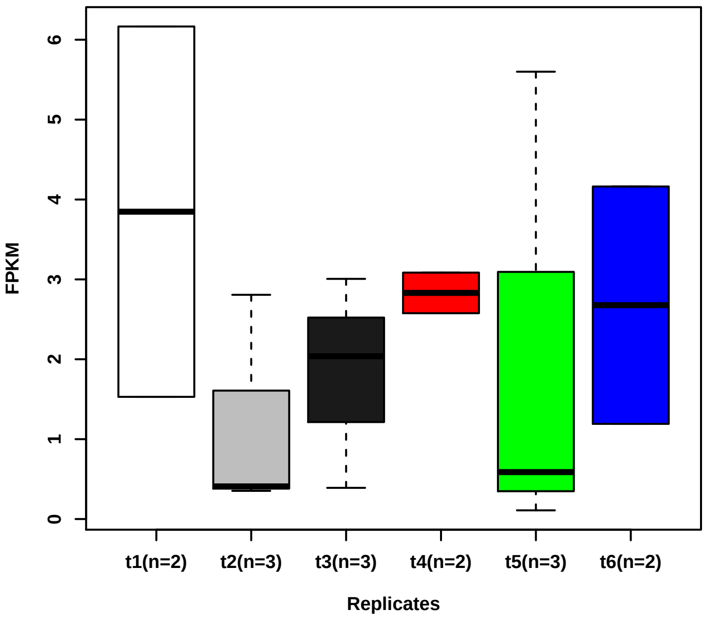

5−amino−6−(D−ribitylamino)uracil from GTP: step 3/4

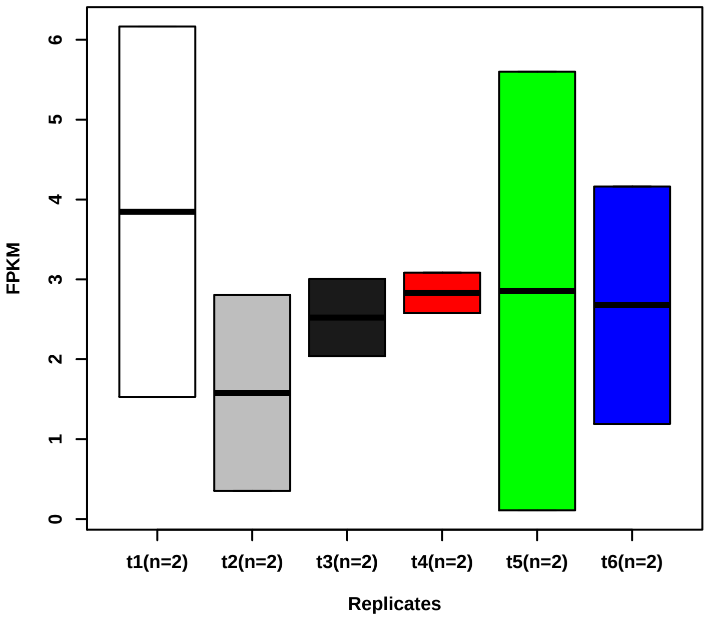

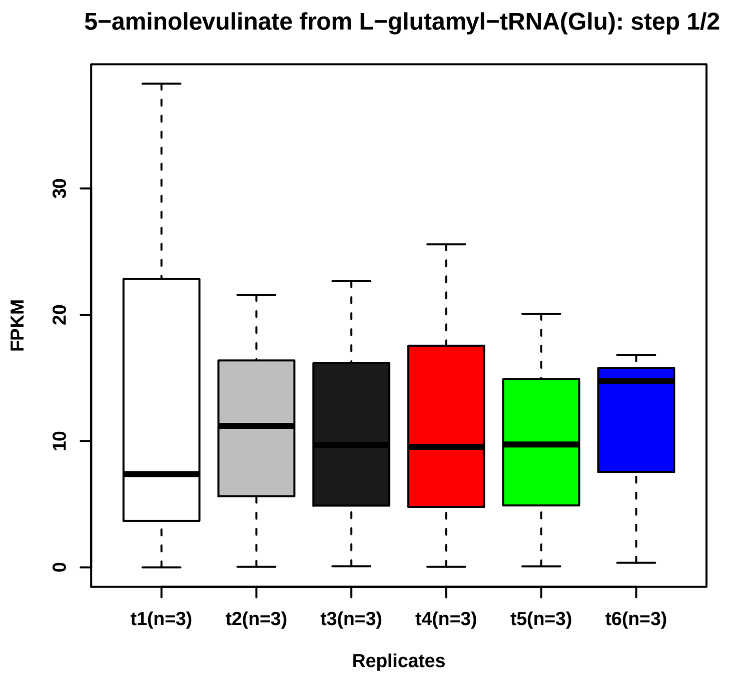
5−aminolevulinate from L−glutamyl−tRNA(Glu): step 1/2
FPKM
0
10
20
30
t1(n=3)
t2(n=3)
t3(n=3)
t4(n=3)
t5(n=3)
t6(n=3)
Replicates

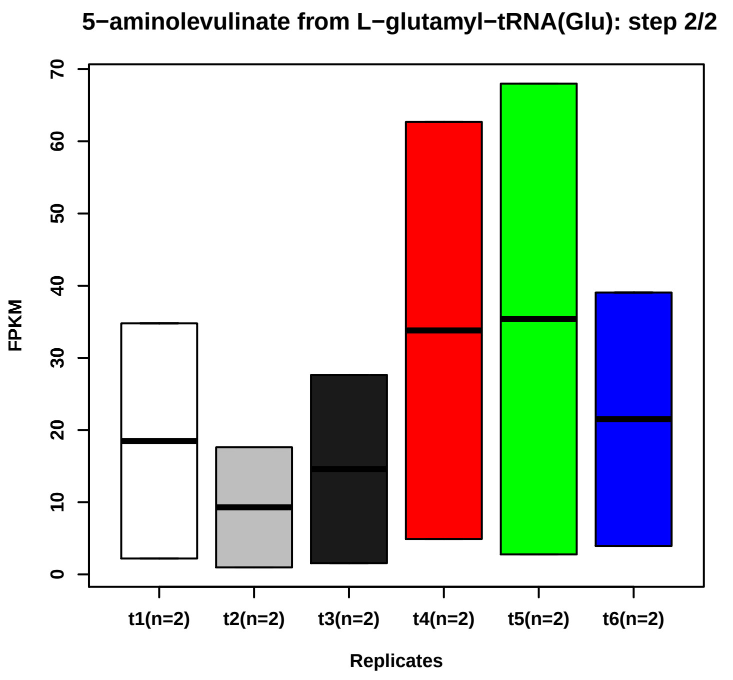
5−aminolevulinate from L−glutamyl−tRNA(Glu): step 2/2
FPKM
0
10
20
30
40
50
60
70
t1(n=2)
t2(n=2)
t3(n=2)
t4(n=2)
t5(n=2)
t6(n=2)
Replicates

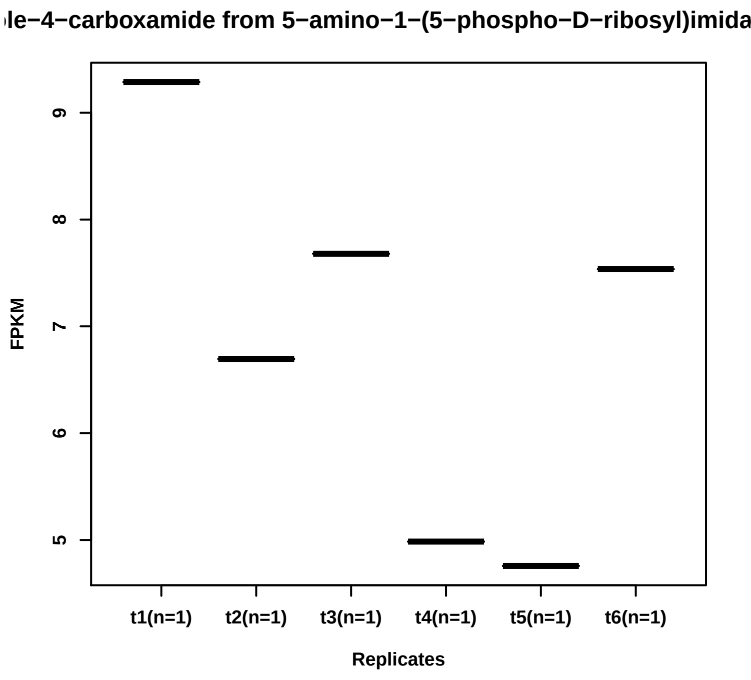
le−4−carboxamide from 5−amino−1−(5−phospho−D−ribosyl)imida
FPKM
5
6
7
8
9
t1(n=1)
t2(n=1)
t3(n=1)
t4(n=1)
t5(n=1)
t6(n=1)
Replicates

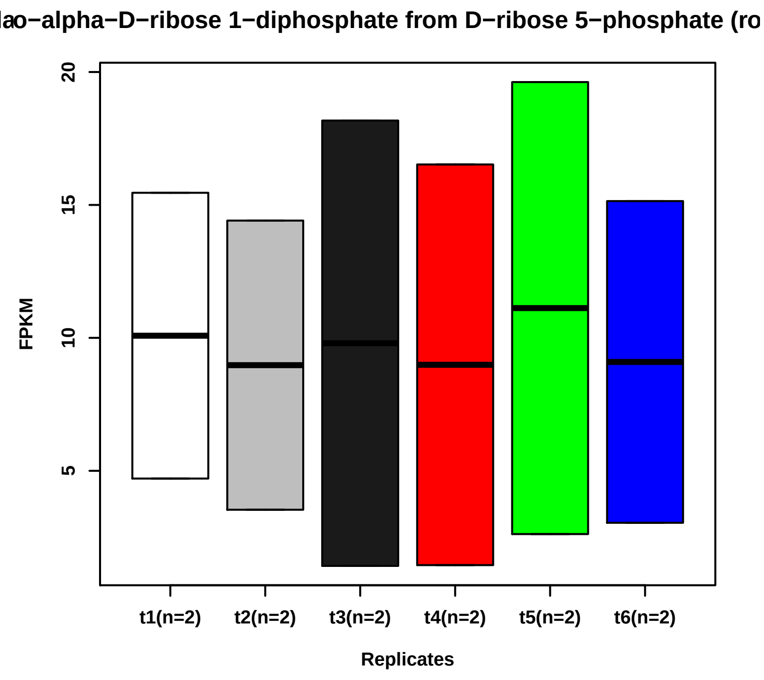
o−alpha−D−ribose 1−diphosphate from D−ribose 5−phosphate (ro
FPKM
5
10
15
20
t1(n=2)
t2(n=2)
t3(n=2)
t4(n=2)
t5(n=2)
t6(n=2)
Replicates

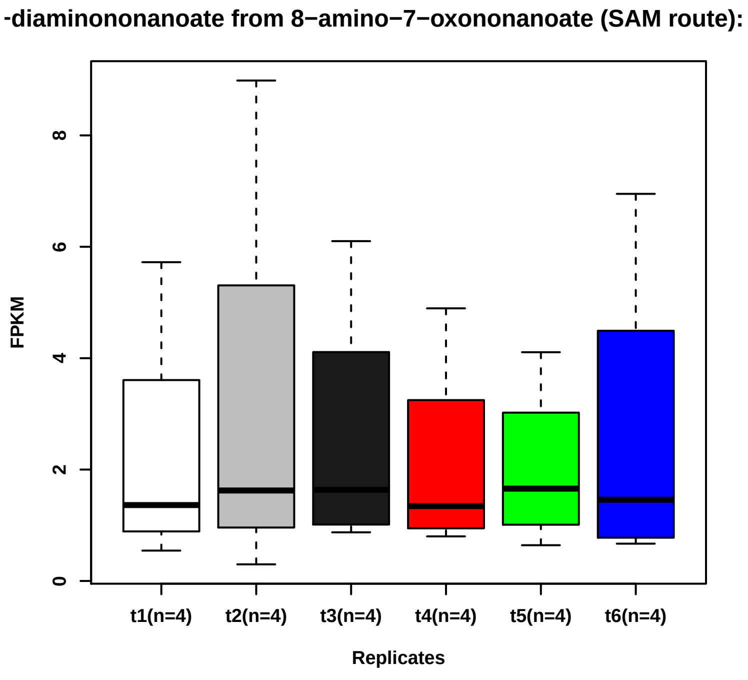
-diaminononanoate from 8−amino−7−oxononanoate (SAM route):
FPKM
0
2
4
6
8
t1(n=4)
t2(n=4)
t3(n=4)
t4(n=4)
t5(n=4)
t6(n=4)
Replicates

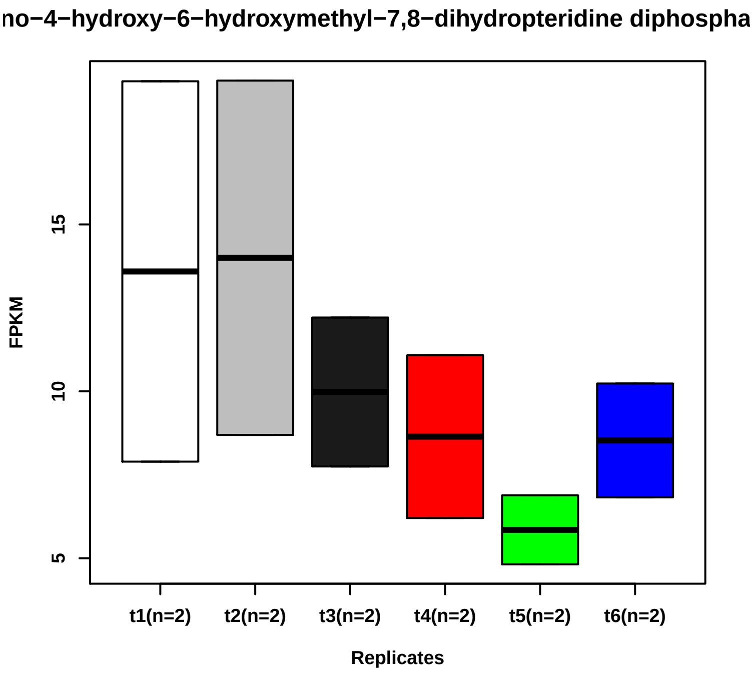
no−4−hydroxy−6−hydroxymethyl−7,8−dihydropteridine diphospha
FPKM
5
10
15
t1(n=2)
t2(n=2)
t3(n=2)
t4(n=2)
t5(n=2)
t6(n=2)
Replicates

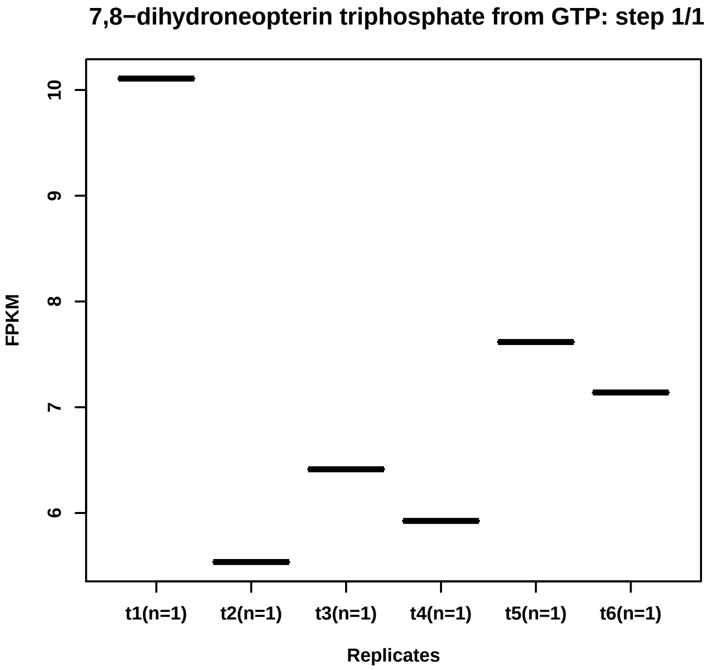
7,8−dihydroneopterin triphosphate from GTP: step 1/1
FPKM
6
7
8
9
10
t1(n=1)
t2(n=1)
t3(n=1)
t4(n=1)
t5(n=1)
t6(n=1)
Replicates

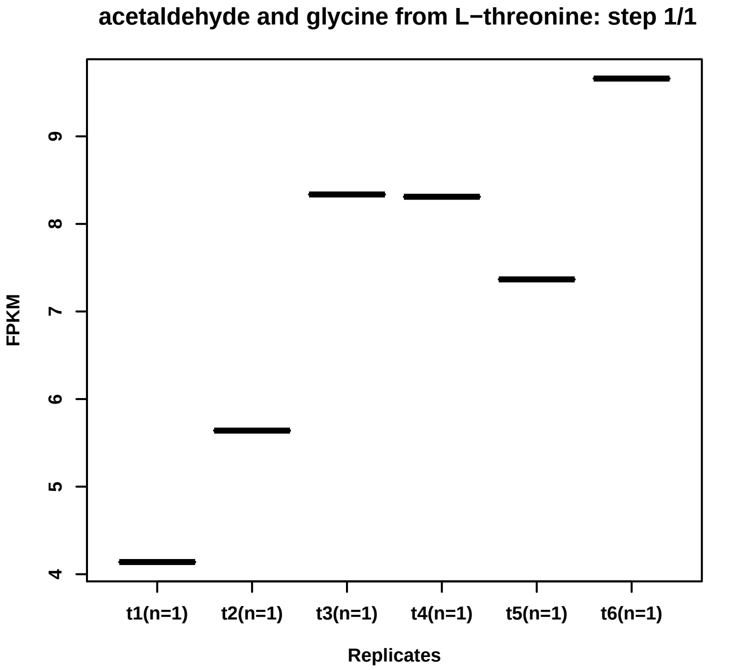
acetaldehyde and glycine from L−threonine: step 1/1
FPKM
4
5
6
7
8
9
t1(n=1)
t2(n=1)
t3(n=1)
t4(n=1)
t5(n=1)
t6(n=1)
Replicates

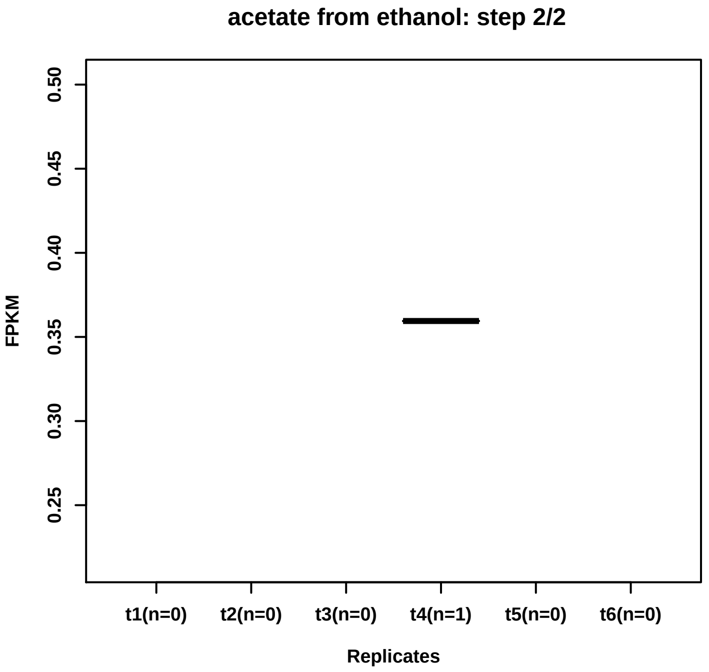
acetate from ethanol: step 2/2
FPKM
0.25
0.30
0.35
0.40
0.45
0.50
t1(n=0)
t2(n=0)
t3(n=0)
t4(n=1)
t5(n=0)
t6(n=0)
Replicates

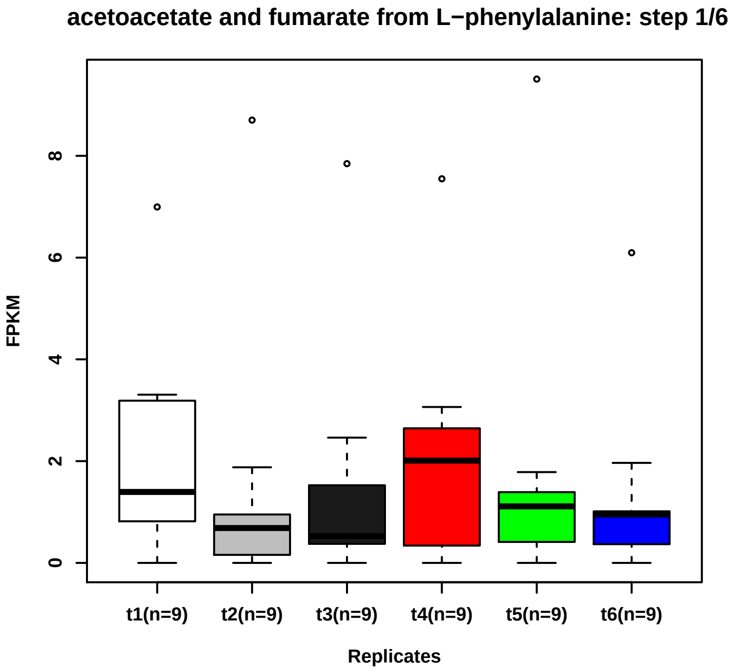
acetoacetate and fumarate from L−phenylalanine: step 1/6
FPKM
0
2
4
6
8
t1(n=9)
t2(n=9)
t3(n=9)
t4(n=9)
t5(n=9)
t6(n=9)
Replicates

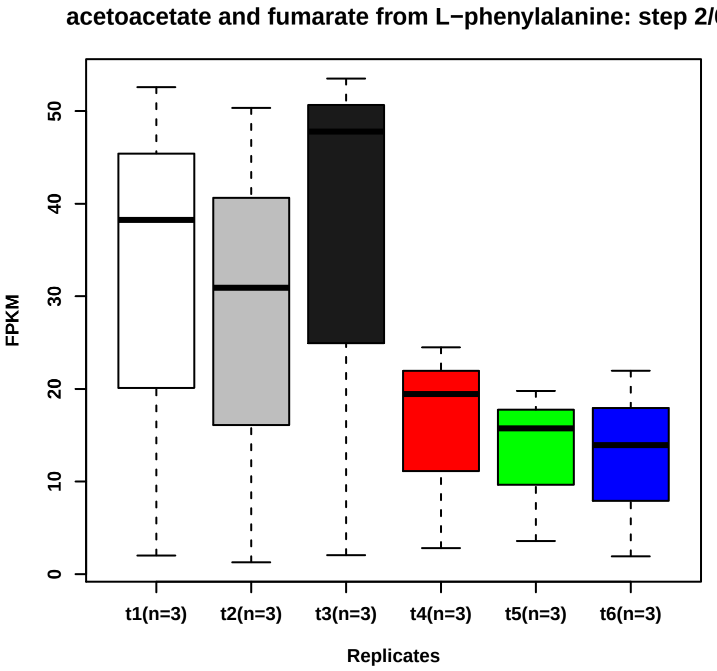
acetoacetate and fumarate from L−phenylalanine: step 2/6
FPKM
0
10
20
30
40
50
t1(n=3)
t2(n=3)
t3(n=3)
t4(n=3)
t5(n=3)
t6(n=3)
Replicates

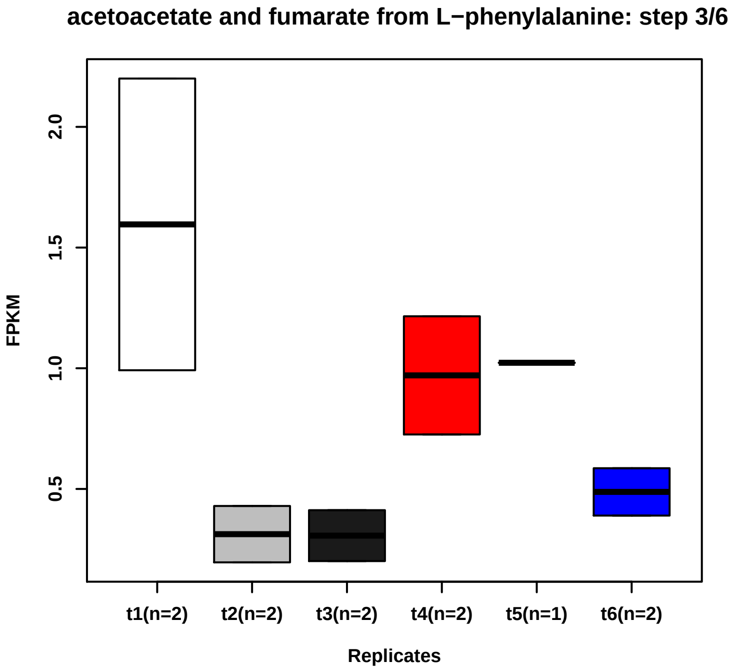
acetoacetate and fumarate from L−phenylalanine: step 3/6
FPKM
0.5
1.0
1.5
2.0
t1(n=2)
t2(n=2)
t3(n=2)
t4(n=2)
t5(n=1)
t6(n=2)
Replicates

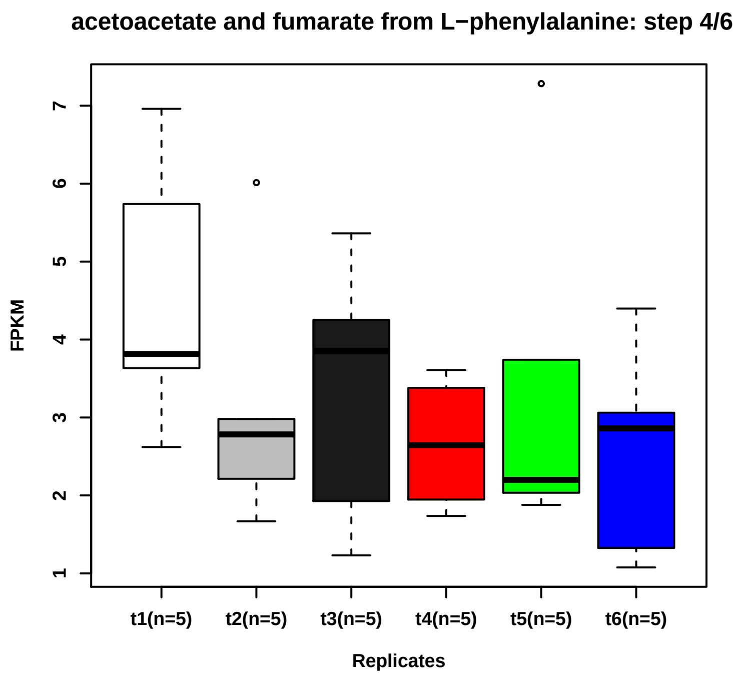
acetoacetate and fumarate from L−phenylalanine: step 4/6
FPKM
Replicates
t1(n=5)
t2(n=5)
t3(n=5)
t4(n=5)
t5(n=5)
t6(n=5)

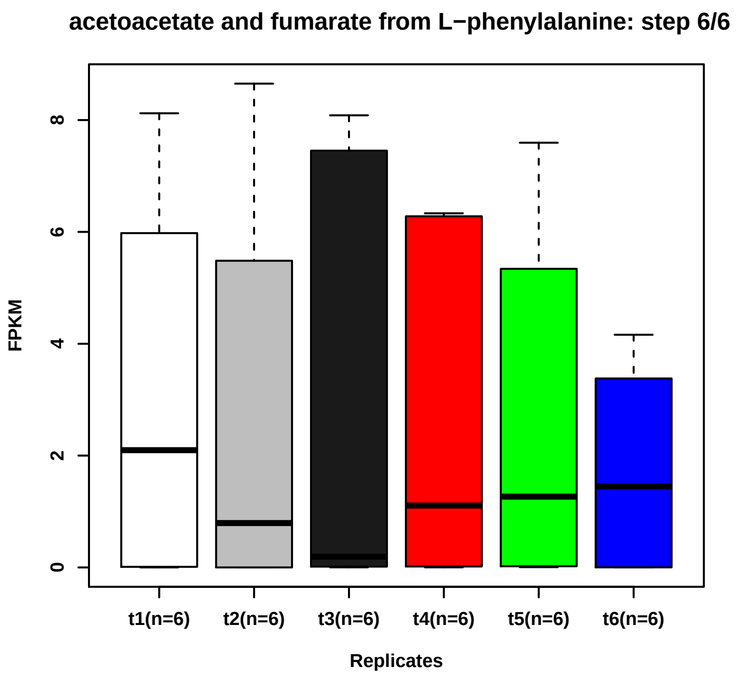
acetoacetate and fumarate from L−phenylalanine: step 6/6
FPKM
Replicates
t1(n=6)
t2(n=6)
t3(n=6)
t4(n=6)
t5(n=6)
t6(n=6)

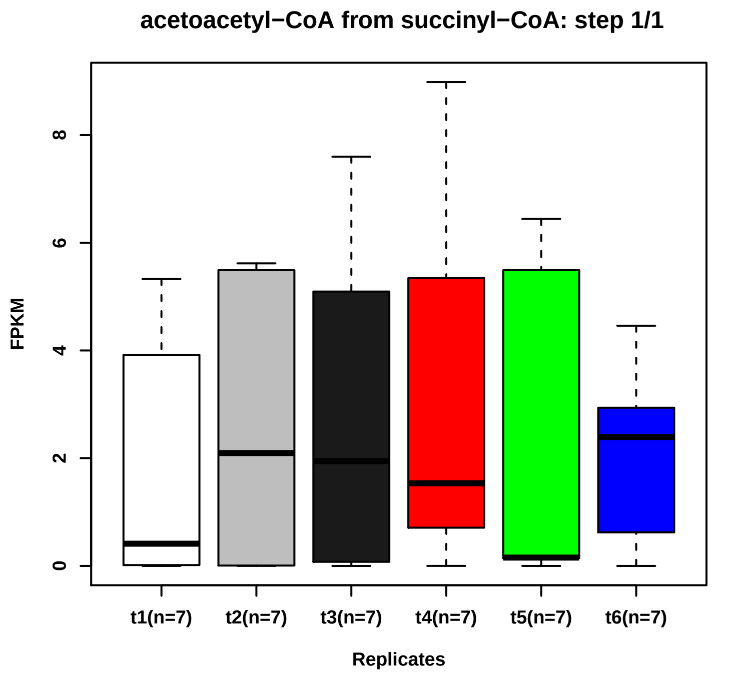
acetoacetyl−CoA from succinyl−CoA: step 1/1
FPKM
Replicates
t1(n=7)
t2(n=7)
t3(n=7)
t4(n=7)
t5(n=7)
t6(n=7)

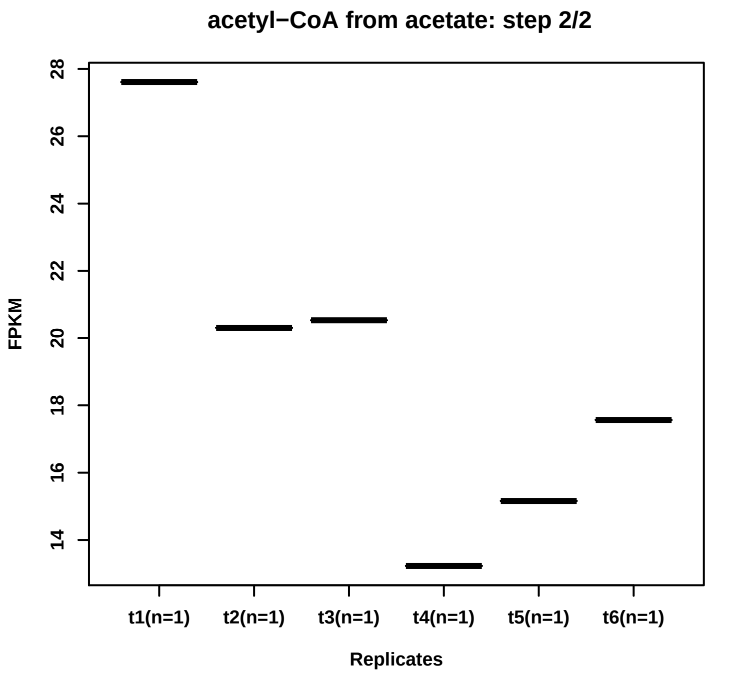
acetyl−CoA from acetate: step 2/2
FPKM
Replicates
t1(n=1)
t2(n=1)
t3(n=1)
t4(n=1)
t5(n=1)
t6(n=1)

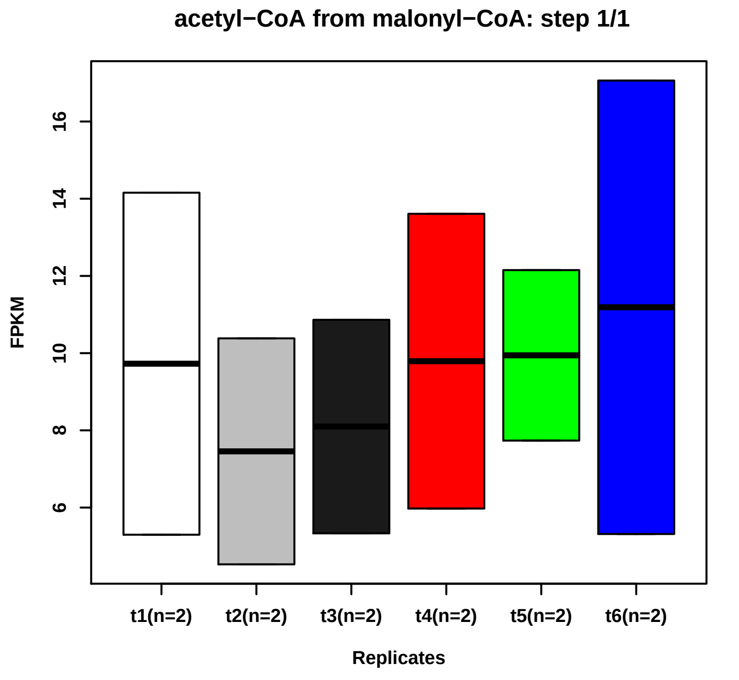
acetyl−CoA from malonyl−CoA: step 1/1
FPKM
Replicates
t1(n=2)
t2(n=2)
t3(n=2)
t4(n=2)
t5(n=2)
t6(n=2)

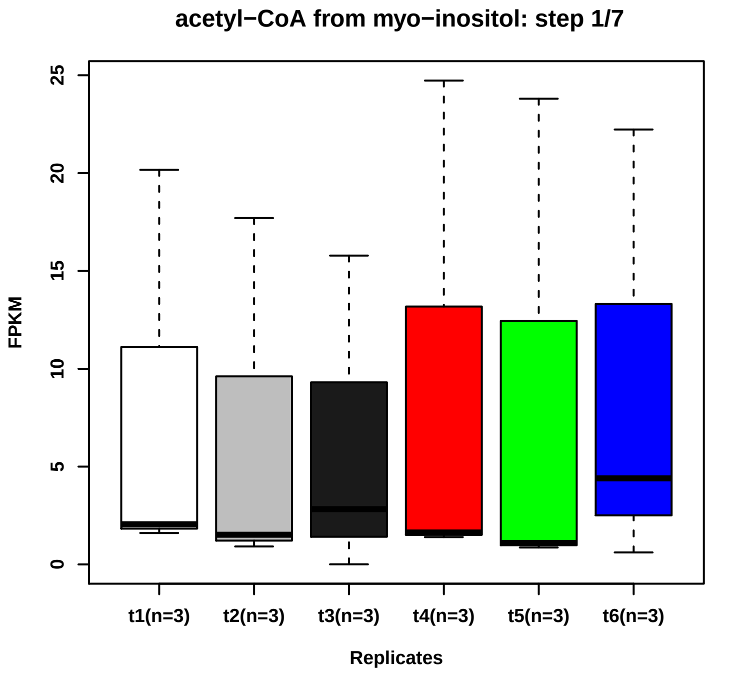
acetyl−CoA from myo−inositol: step 1/7
FPKM
Replicates
t1(n=3)
t2(n=3)
t3(n=3)
t4(n=3)
t5(n=3)
t6(n=3)

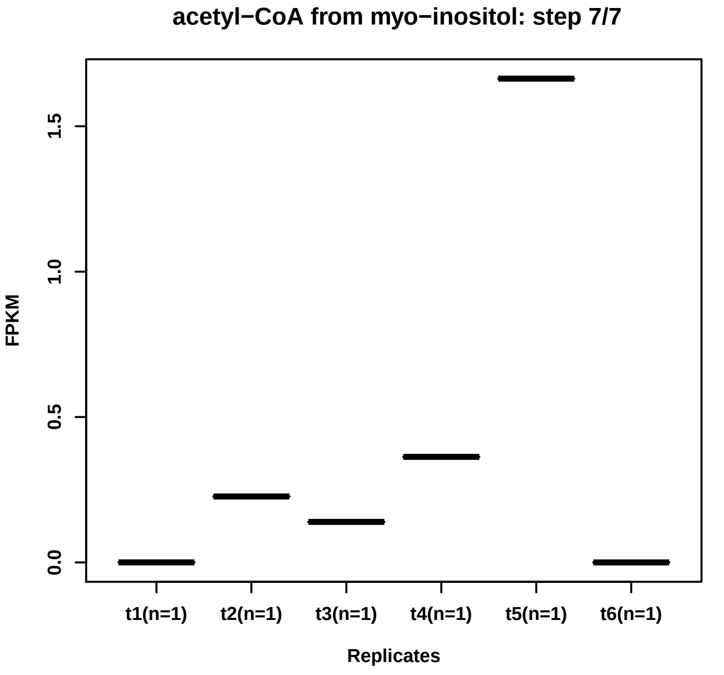
acetyl−CoA from myo−inositol: step 7/7
FPKM
0.0
0.5
1.0
1.5
t1(n=1)
t2(n=1)
t3(n=1)
t4(n=1)
t5(n=1)
t6(n=1)
Replicates

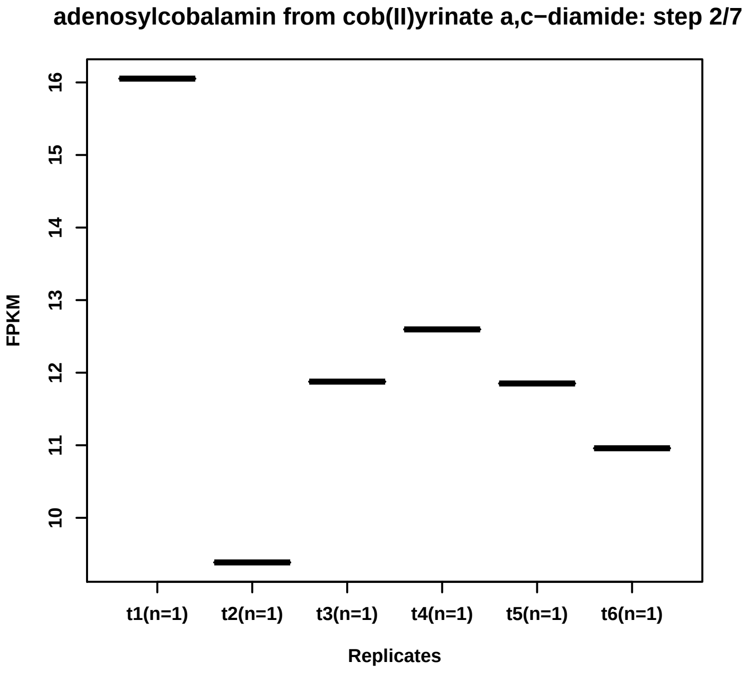
adenosylcobalamin from cob(II)yrinate a,c−diamide: step 2/7
FPKM
10
11
12
13
14
15
16
t1(n=1)
t2(n=1)
t3(n=1)
t4(n=1)
t5(n=1)
t6(n=1)
Replicates

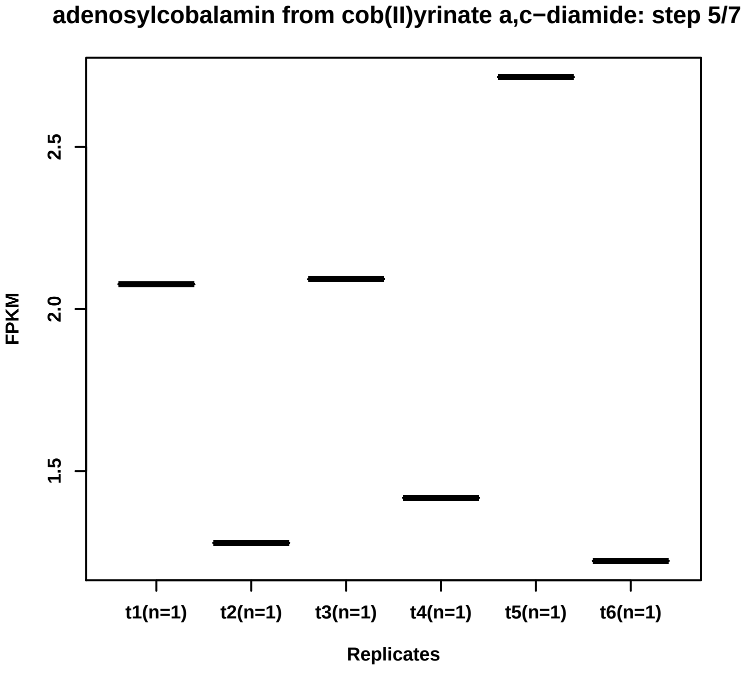
adenosylcobalamin from cob(II)yrinate a,c−diamide: step 5/7
FPKM
1.5
2.0
2.5
t1(n=1)
t2(n=1)
t3(n=1)
t4(n=1)
t5(n=1)
t6(n=1)
Replicates

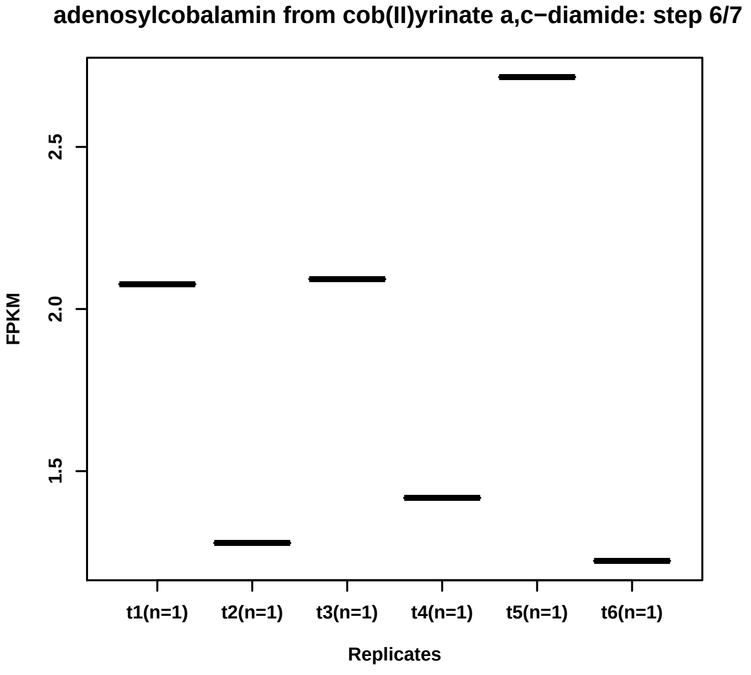
adenosylcobalamin from cob(II)yrinate a,c−diamide: step 6/7
FPKM
1.5
2.0
2.5
t1(n=1)
t2(n=1)
t3(n=1)
t4(n=1)
t5(n=1)
t6(n=1)
Replicates

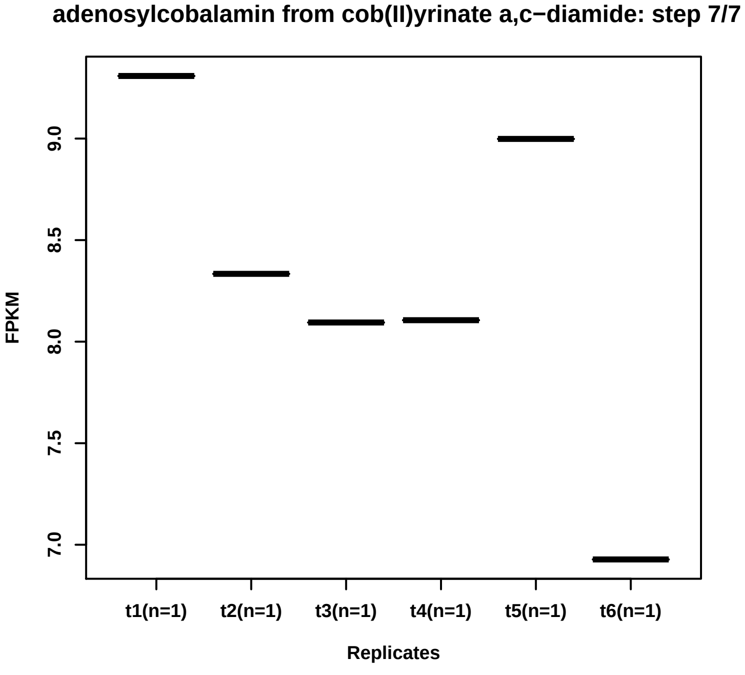
adenosylcobalamin from cob(II)yrinate a,c−diamide: step 7/7
FPKM
7.0
7.5
8.0
8.5
9.0
t1(n=1)
t2(n=1)
t3(n=1)
t4(n=1)
t5(n=1)
t6(n=1)
Replicates

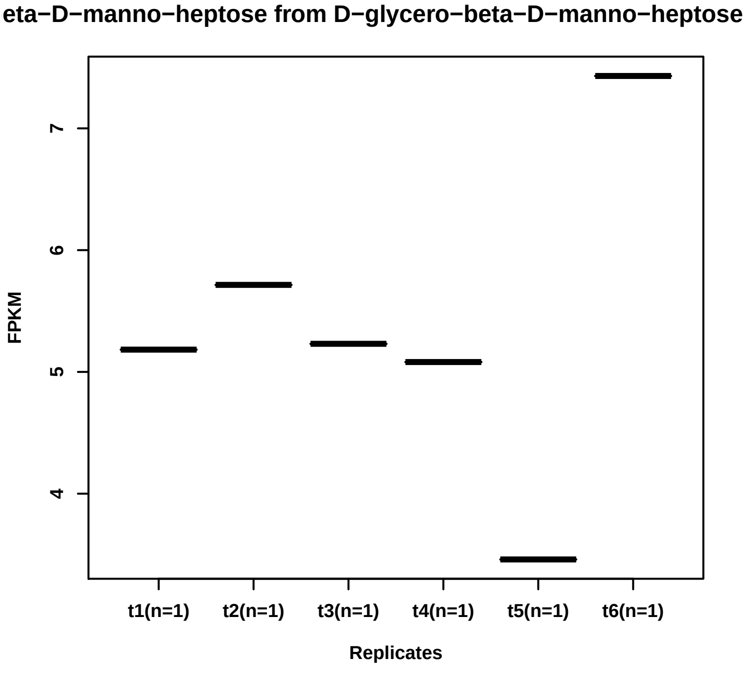
eta−D−manno−heptose from D−glycero−beta−D−manno−heptose
FPKM
4
5
6
7
t1(n=1)
t2(n=1)
t3(n=1)
t4(n=1)
t5(n=1)
t6(n=1)
Replicates

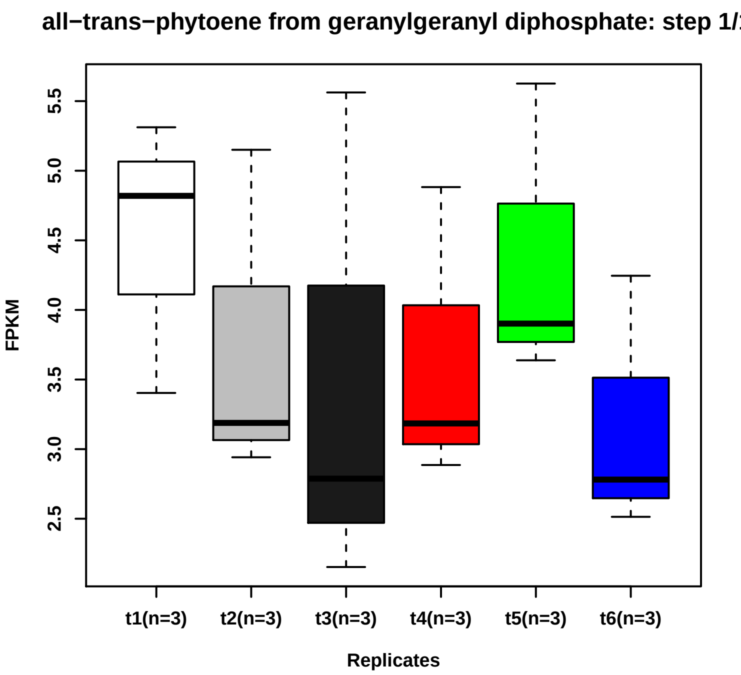
all−trans−phytoene from geranylgeranyl diphosphate: step 1/1
FPKM
2.5
3.0
3.5
4.0
4.5
5.0
5.5
t1(n=3)
t2(n=3)
t3(n=3)
t4(n=3)
t5(n=3)
t6(n=3)
Replicates

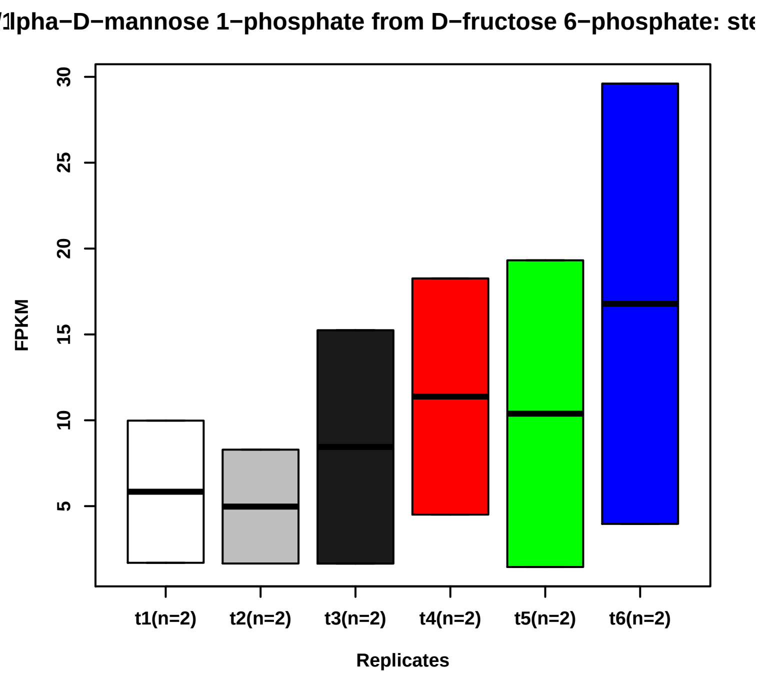
lpha−D−mannose 1−phosphate from D−fructose 6−phosphate: ste
FPKM
5
10
15
20
25
30
t1(n=2)
t2(n=2)
t3(n=2)
t4(n=2)
t5(n=2)
t6(n=2)
Replicates

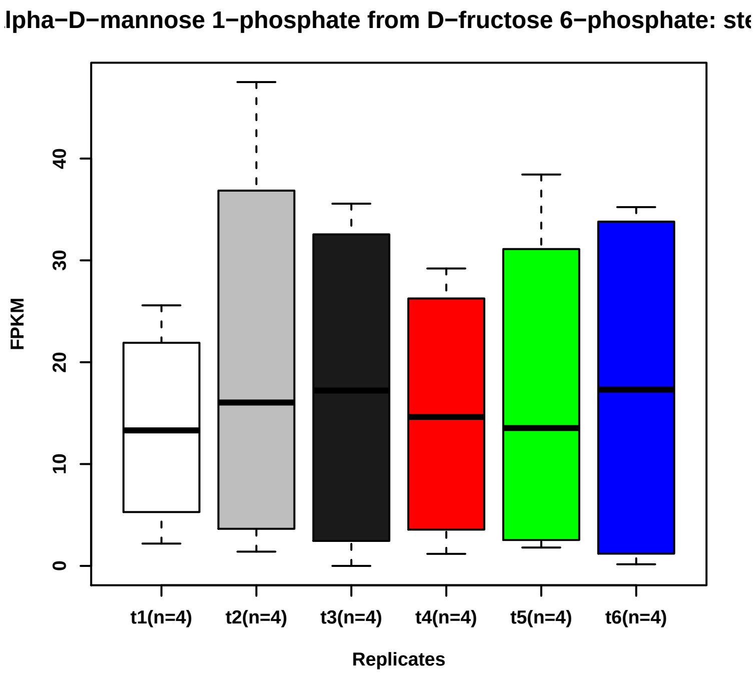
lpha−D−mannose 1−phosphate from D−fructose 6−phosphate: ste
FPKM
0
10
20
30
40
t1(n=4)
t2(n=4)
t3(n=4)
t4(n=4)
t5(n=4)
t6(n=4)
Replicates

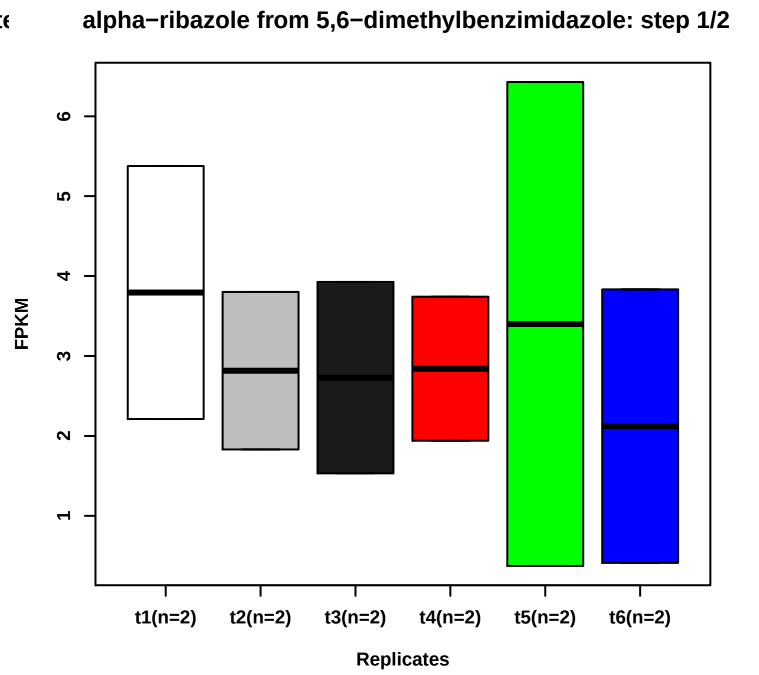
alpha−ribazole from 5,6−dimethylbenzimidazole: step 1/2
FPKM
1
2
3
4
5
6
t1(n=2)
t2(n=2)
t3(n=2)
t4(n=2)
t5(n=2)
t6(n=2)
Replicates

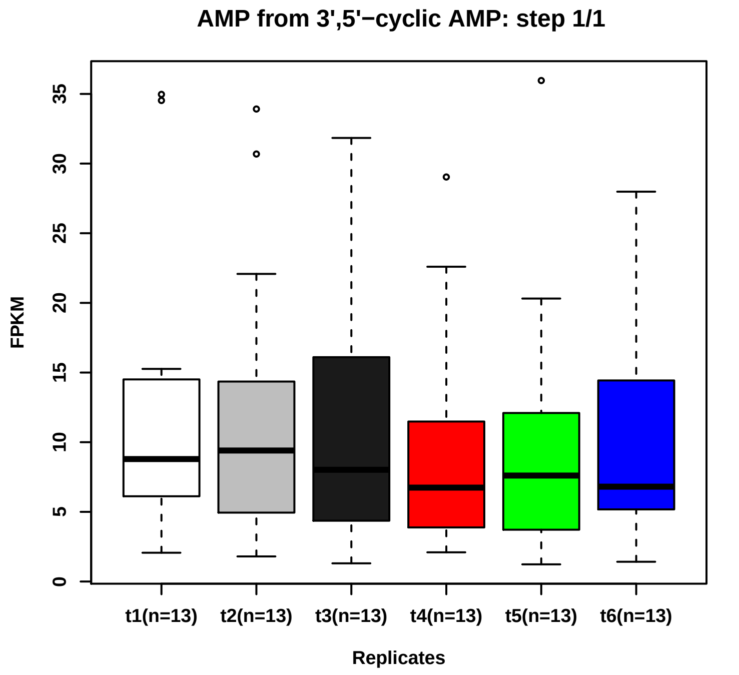
AMP from 3',5'−cyclic AMP: step 1/1
FPKM
0
5
10
15
20
25
30
35
t1(n=13)
t2(n=13)
t3(n=13)
t4(n=13)
t5(n=13)
t6(n=13)
Replicates

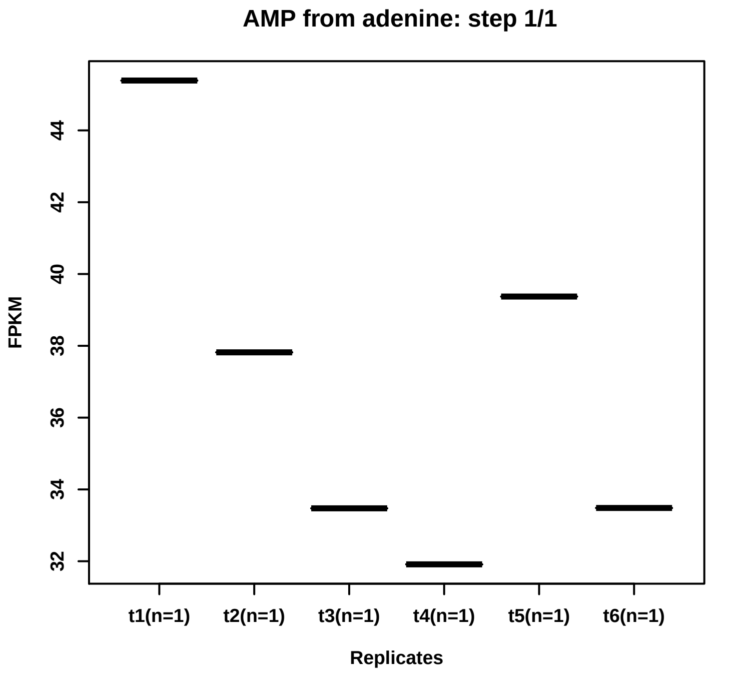
AMP from adenine: step 1/1
FPKM
32
34
36
38
40
42
44
t1(n=1)
t2(n=1)
t3(n=1)
t4(n=1)
t5(n=1)
t6(n=1)
Replicates

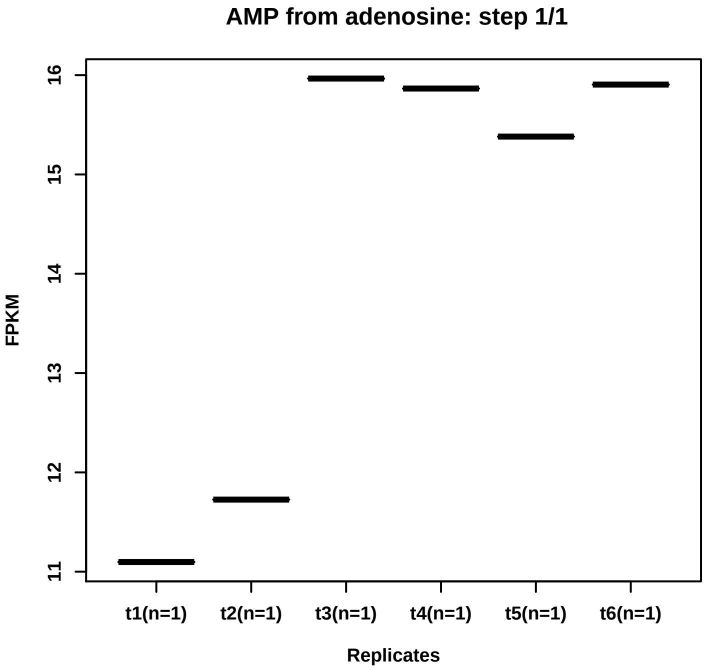
AMP from adenosine: step 1/1
FPKM
11
12
13
14
15
16
t1(n=1)
t2(n=1)
t3(n=1)
t4(n=1)
t5(n=1)
t6(n=1)
Replicates

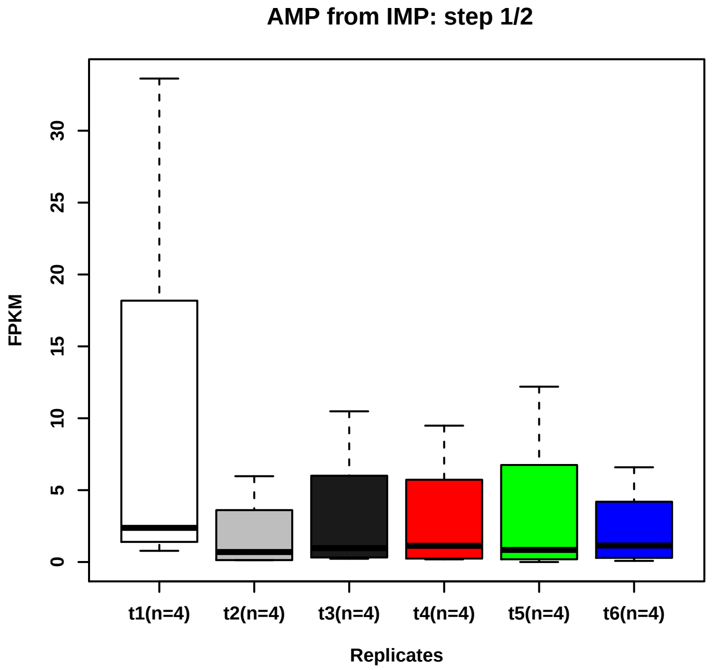
AMP from IMP: step 1/2
FPKM
0
5
10
15
20
25
30
t1(n=4)
t2(n=4)
t3(n=4)
t4(n=4)
t5(n=4)
t6(n=4)
Replicates

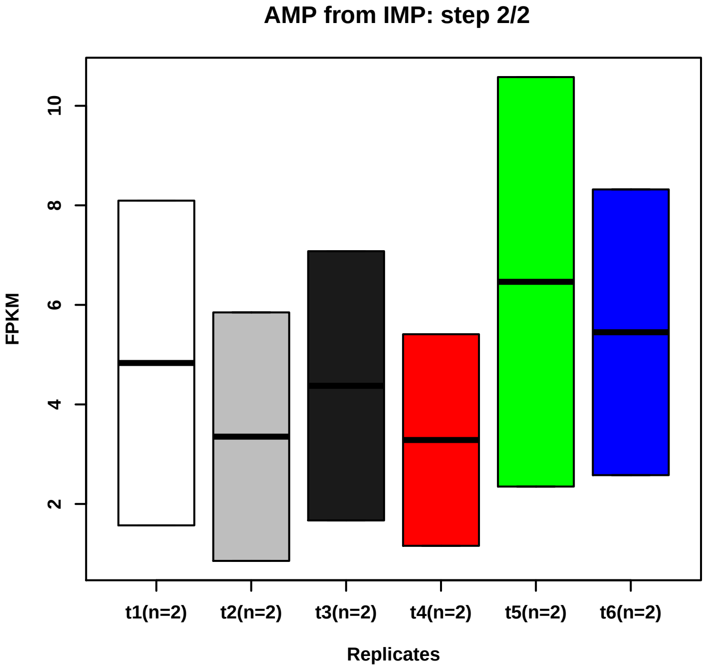
AMP from IMP: step 2/2
FPKM
2
4
6
8
10
t1(n=2)
t2(n=2)
t3(n=2)
t4(n=2)
t5(n=2)
t6(n=2)
Replicates

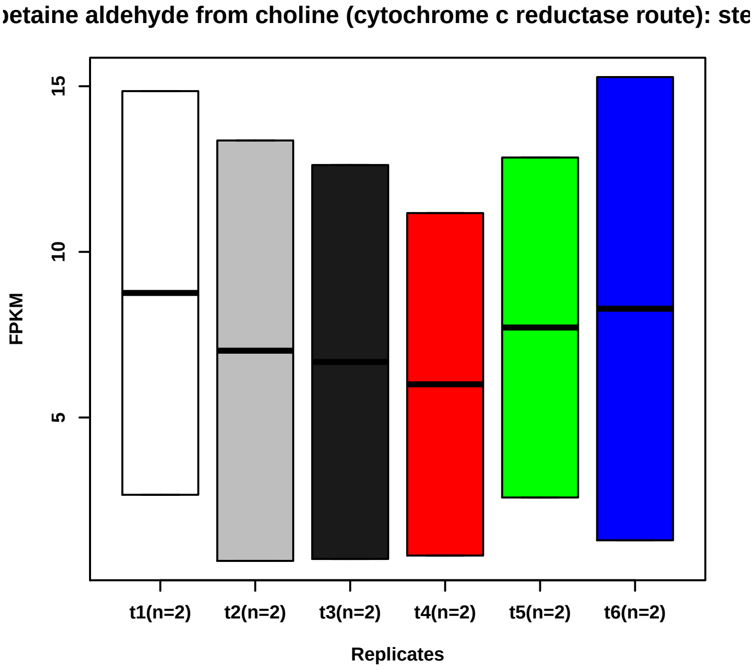
etaine aldehyde from choline (cytochrome c reductase route): ste
FPKM
5
10
15
t1(n=2)
t2(n=2)
t3(n=2)
t4(n=2)
t5(n=2)
t6(n=2)
Replicates

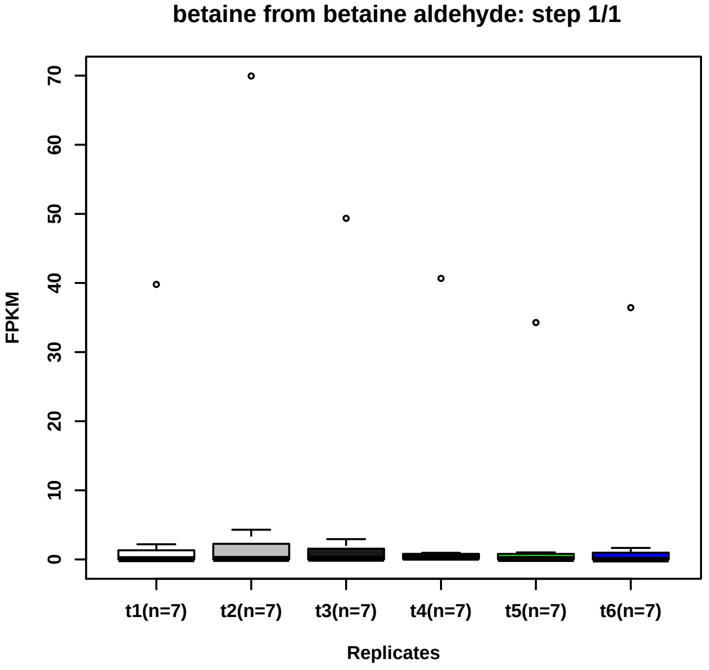
betaine from betaine aldehyde: step 1/1
FPKM
0
10
20
30
40
50
60
70
t1(n=7)
t2(n=7)
t3(n=7)
t4(n=7)
t5(n=7)
t6(n=7)
Replicates

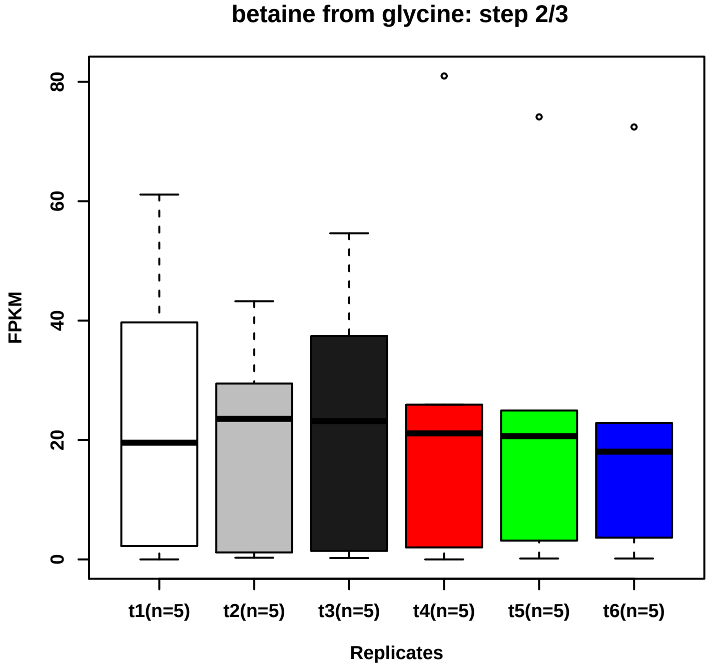
betaine from glycine: step 2/3
FPKM
0
20
40
60
80
t1(n=5)
t2(n=5)
t3(n=5)
t4(n=5)
t5(n=5)
t6(n=5)
Replicates

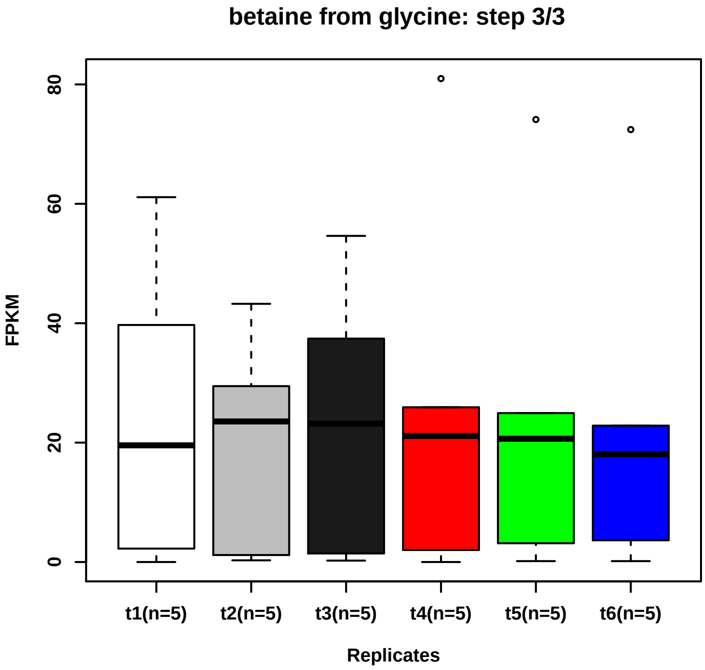
betaine from glycine: step 3/3
FPKM
0
20
40
60
80
t1(n=5)
t2(n=5)
t3(n=5)
t4(n=5)
t5(n=5)
t6(n=5)
Replicates

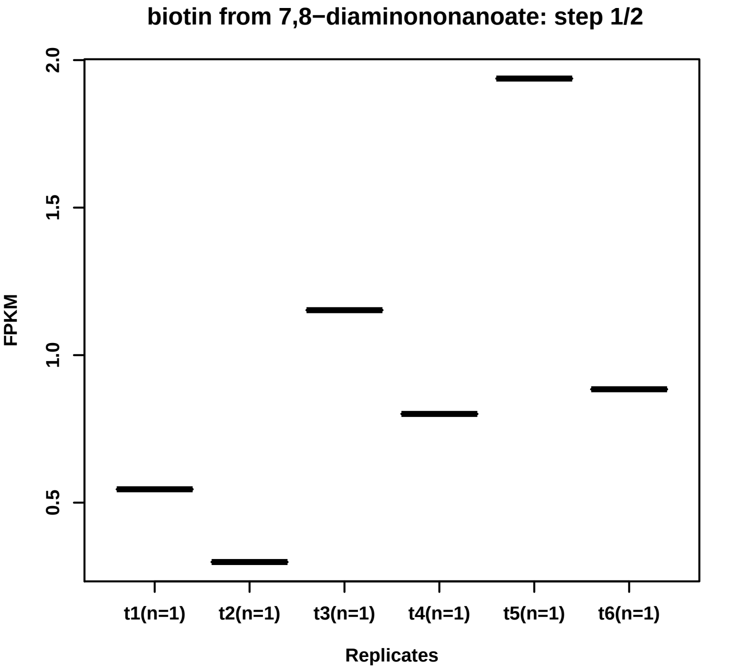
biotin from 7,8−diaminononanoate: step 1/2
FPKM
0.5
1.0
1.5
2.0
t1(n=1)
t2(n=1)
t3(n=1)
t4(n=1)
t5(n=1)
t6(n=1)
Replicates

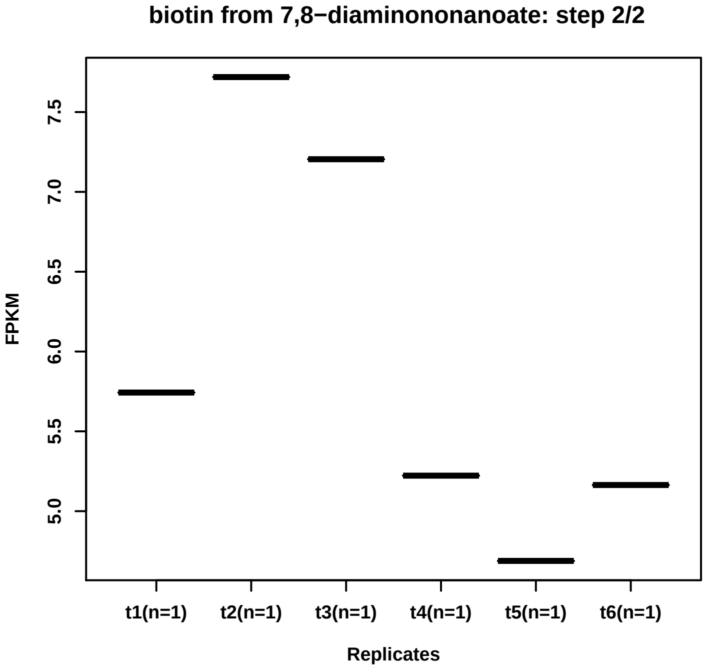
biotin from 7,8−diaminononanoate: step 2/2
FPKM
5.0
5.5
6.0
6.5
7.0
7.5
t1(n=1)
t2(n=1)
t3(n=1)
t4(n=1)
t5(n=1)
t6(n=1)
Replicates

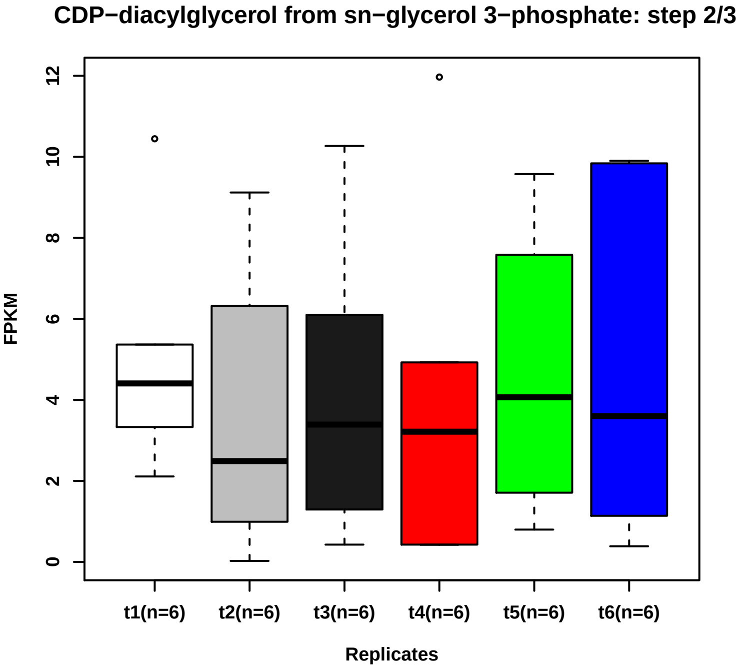
CDP−diacylglycerol from sn−glycerol 3−phosphate: step 2/3
FPKM
0
2
4
6
8
10
12
t1(n=6)
t2(n=6)
t3(n=6)
t4(n=6)
t5(n=6)
t6(n=6)
Replicates

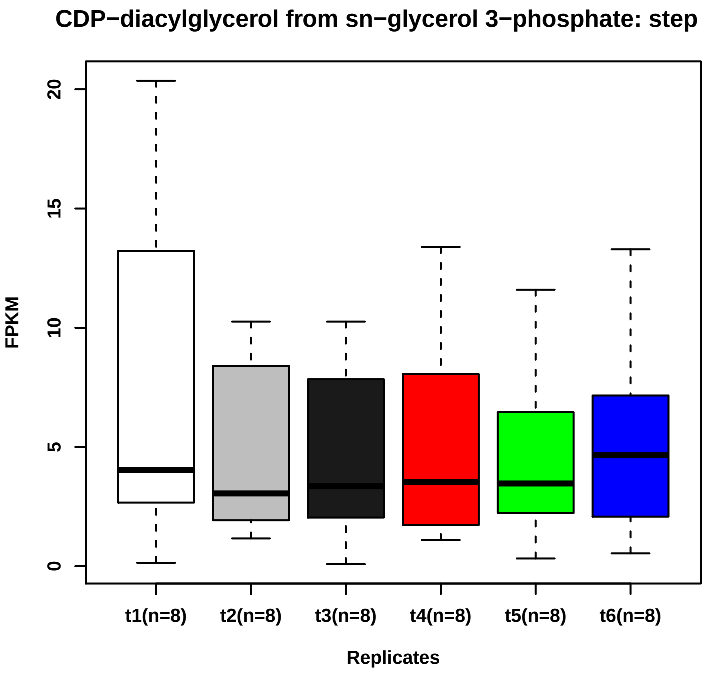
CDP−diacylglycerol from sn−glycerol 3−phosphate: step 3/3
FPKM
0
5
10
15
20
t1(n=8)
t2(n=8)
t3(n=8)
t4(n=8)
t5(n=8)
t6(n=8)
Replicates

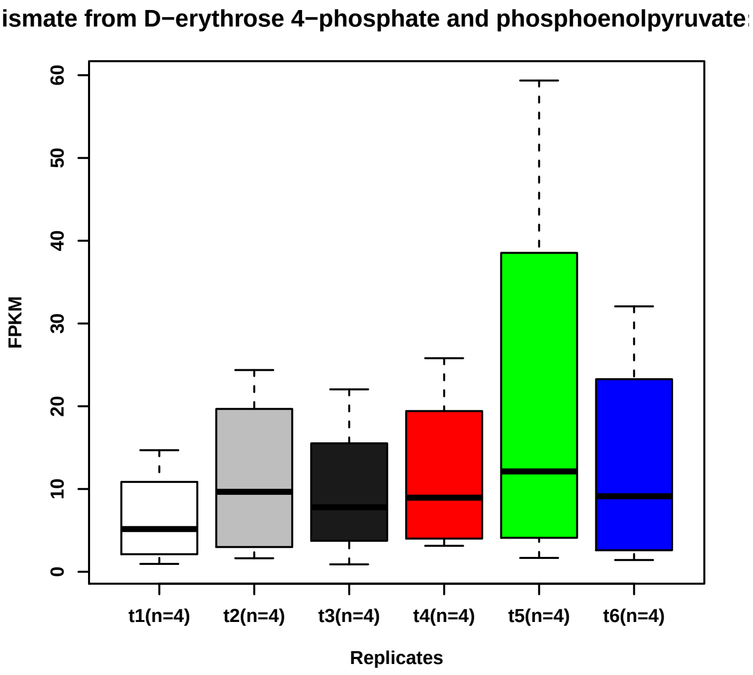
ismate from D−erythrose 4−phosphate and phosphoenolpyruvate
FPKM
0
10
20
30
40
50
60
t1(n=4)
t2(n=4)
t3(n=4)
t4(n=4)
t5(n=4)
t6(n=4)
Replicates

ismate from D−erythrose 4−phosphate and phosphoenolpyruvate

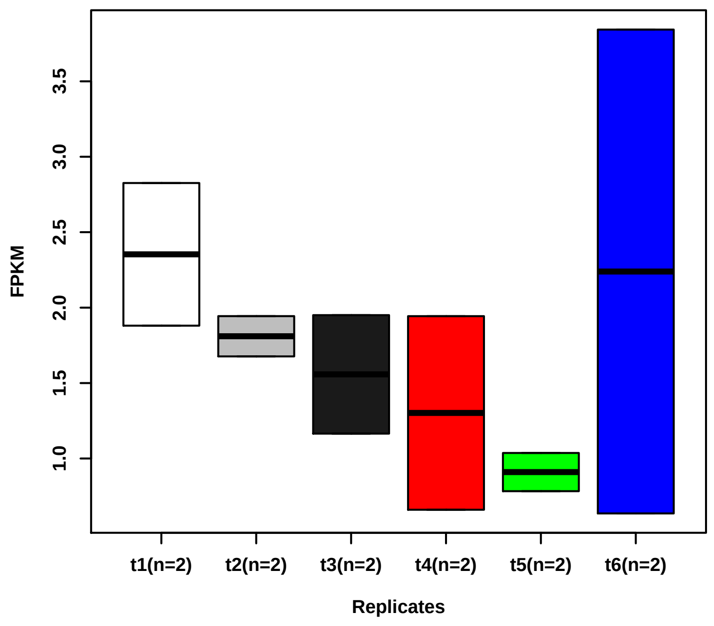

ismate from D−erythrose 4−phosphate and phosphoenolpyruvate:

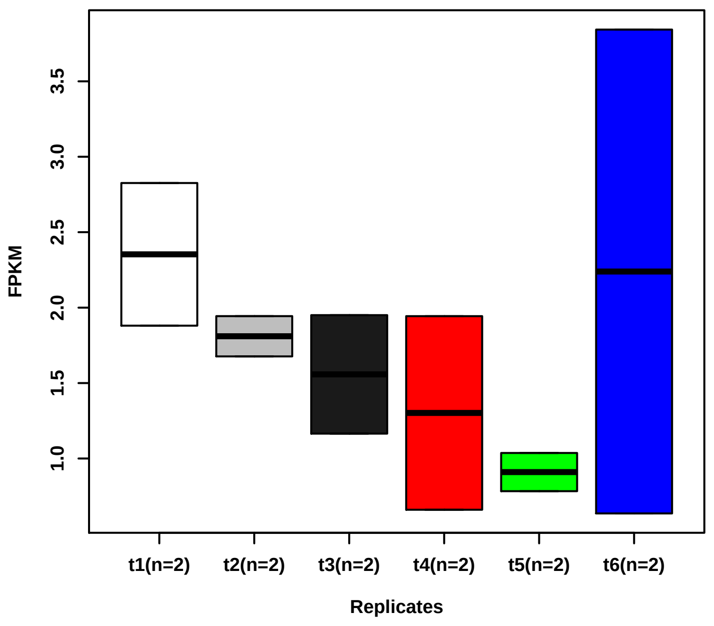

ismate from D−erythrose 4−phosphate and phosphoenolpyruvate

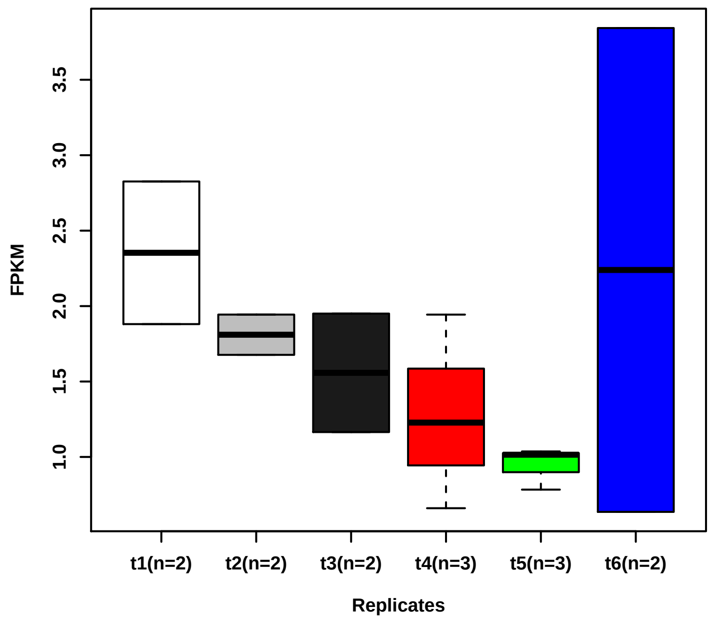

ismate from D−erythrose 4−phosphate and phosphoenolpyruvate:

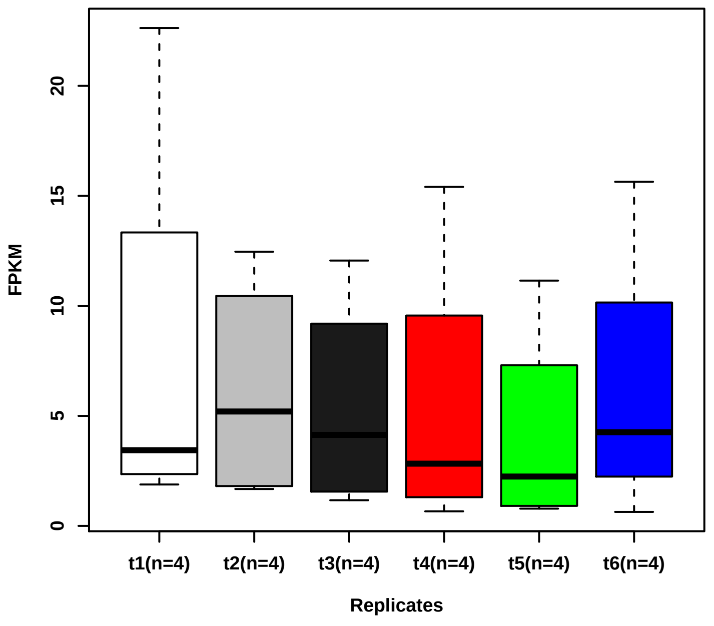

ismate from D−erythrose 4−phosphate and phosphoenolpyruvate

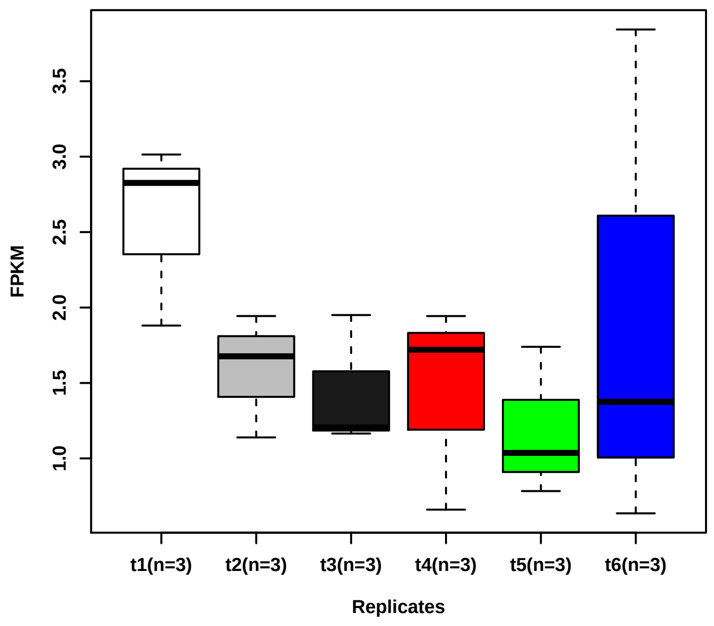

ismate from D−erythrose 4−phosphate and phosphoenolpyruvate:

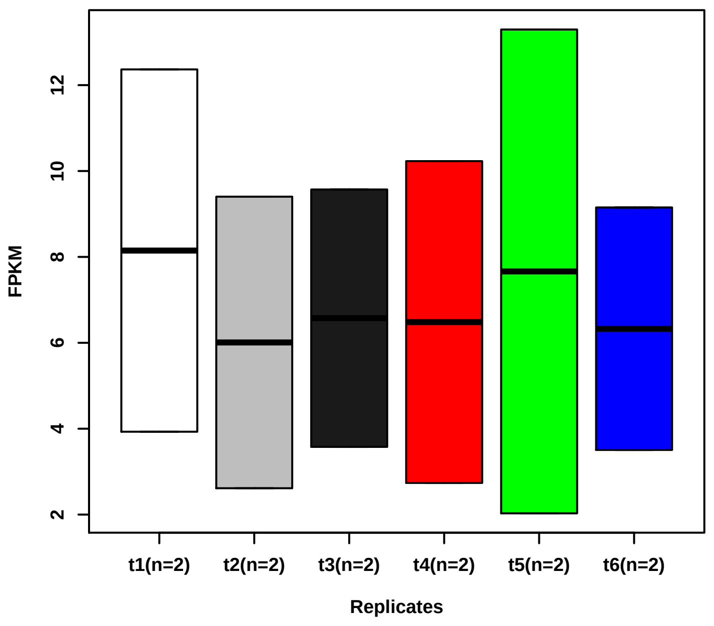

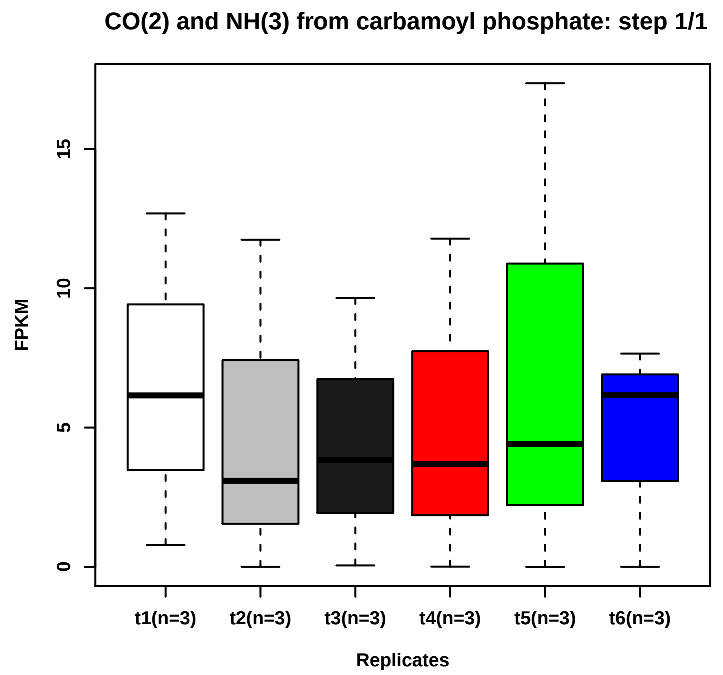
CO(2) and NH(3) from carbamoyl phosphate: step 1/1
FPKM
0
5
10
15
t1(n=3)
t2(n=3)
t3(n=3)
t4(n=3)
t5(n=3)
t6(n=3)
Replicates

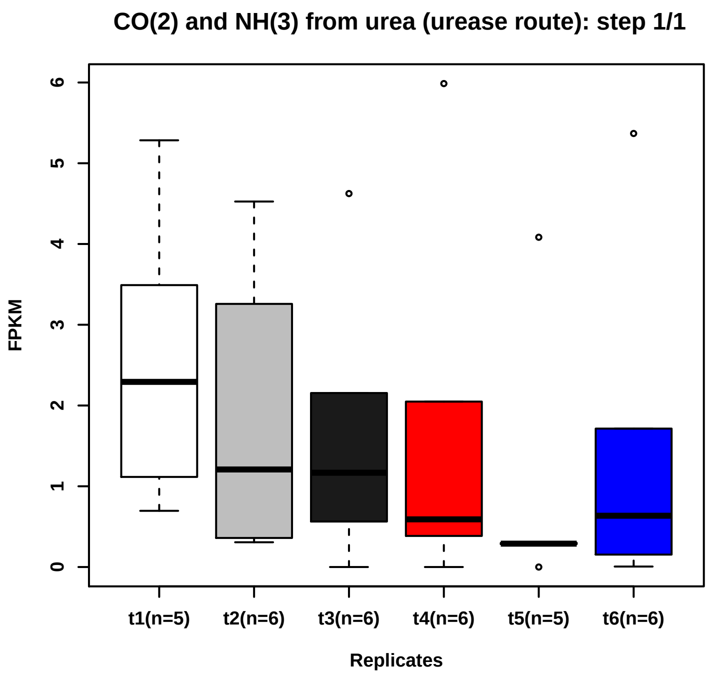
CO(2) and NH(3) from urea (urease route): step 1/1
FPKM
0
1
2
3
4
5
6
t1(n=5)
t2(n=6)
t3(n=6)
t4(n=6)
t5(n=5)
t6(n=6)
Replicates

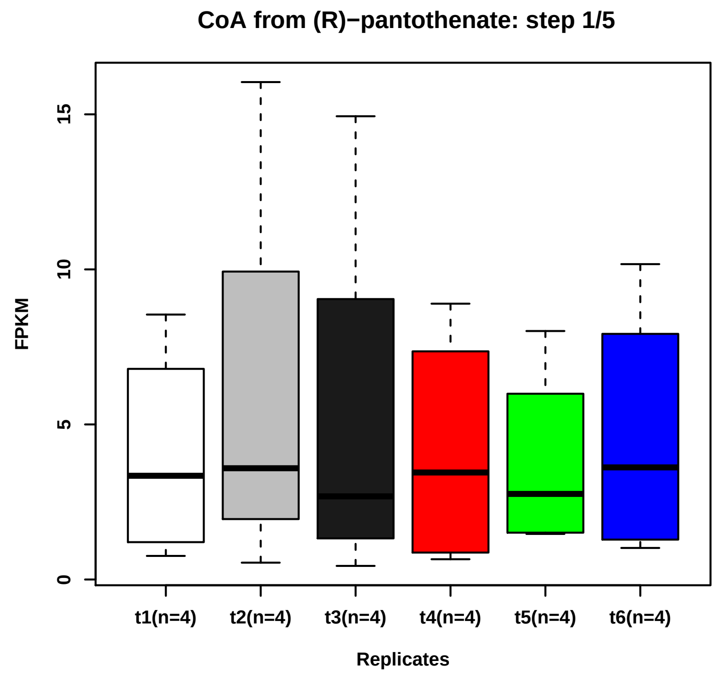
CoA from (R)−pantothenate: step 1/5
FPKM
0
5
10
15
t1(n=4)
t2(n=4)
t3(n=4)
t4(n=4)
t5(n=4)
t6(n=4)
Replicates

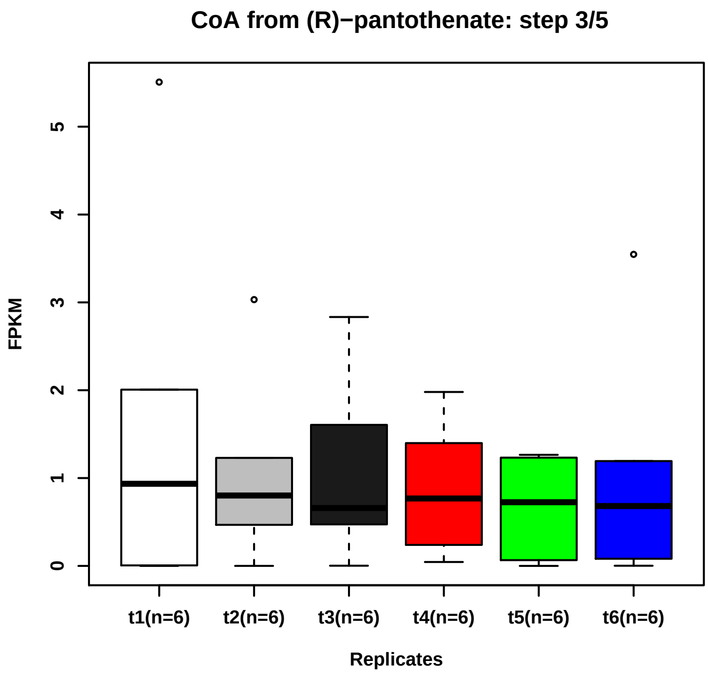
CoA from (R)−pantothenate: step 3/5
FPKM
0
1
2
3
4
5
t1(n=6)
t2(n=6)
t3(n=6)
t4(n=6)
t5(n=6)
t6(n=6)
Replicates

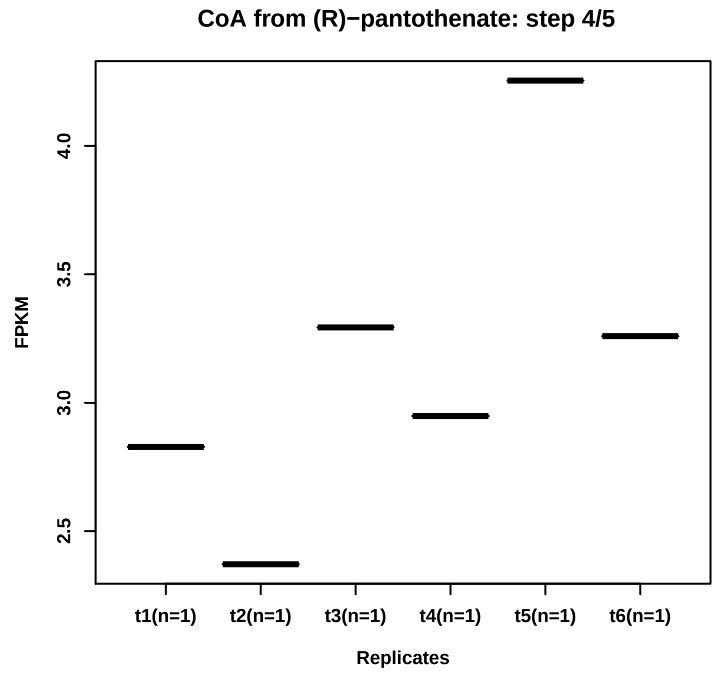
CoA from (R)−pantothenate: step 4/5
FPKM
2.5
3.0
3.5
4.0
t1(n=1)
t2(n=1)
t3(n=1)
t4(n=1)
t5(n=1)
t6(n=1)
Replicates

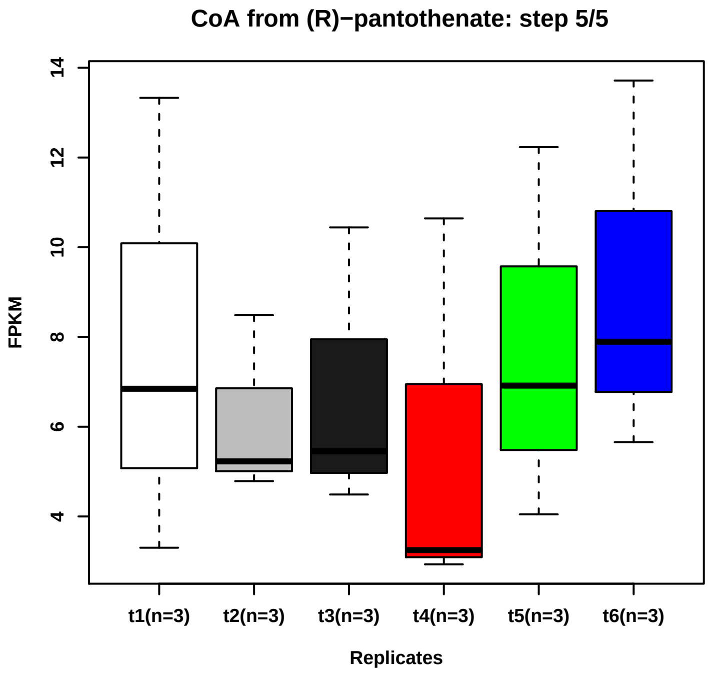
CoA from (R)−pantothenate: step 5/5
FPKM
4
6
8
10
12
14
t1(n=3)
t2(n=3)
t3(n=3)
t4(n=3)
t5(n=3)
t6(n=3)
Replicates

cob(II)yrinate a,c−diamide from precorrin−2 (aerobic route): step 1

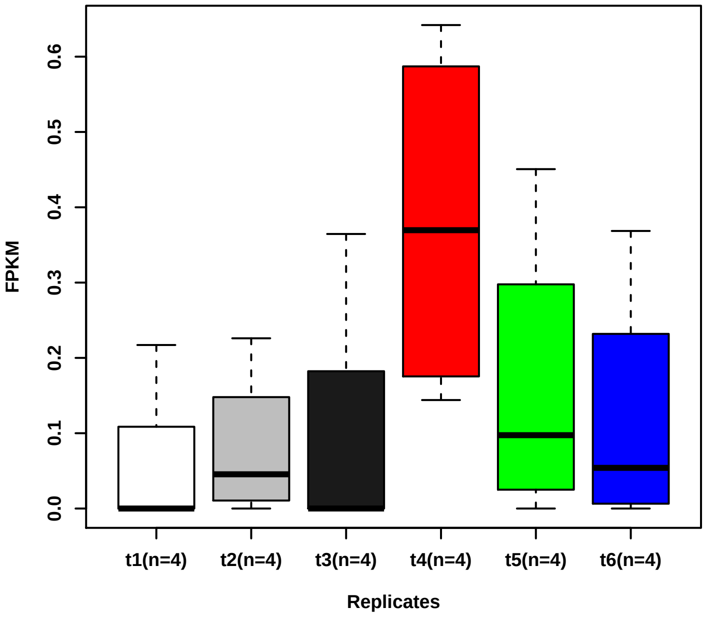

cob(II)yrinate a,c−diamide from precorrin−2 (aerobic route): step [illegible]

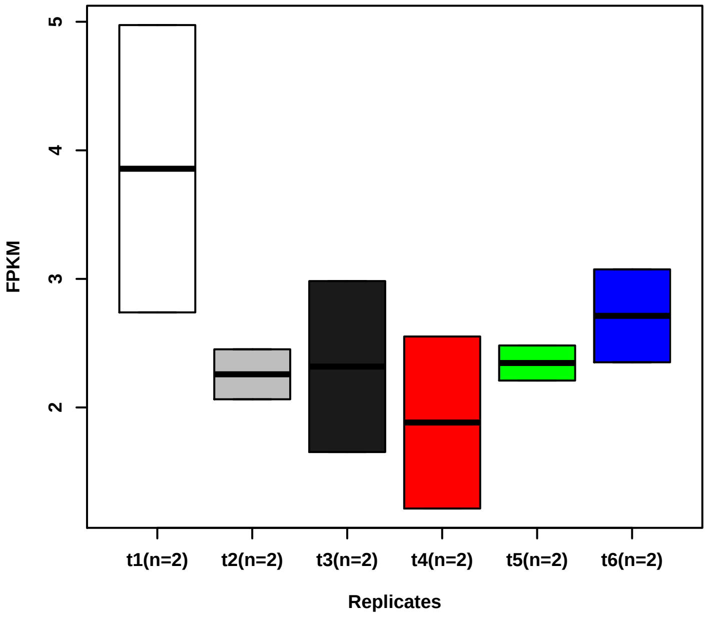

coproporphyrinogen−III from 5−aminolevulinate: step 2/4

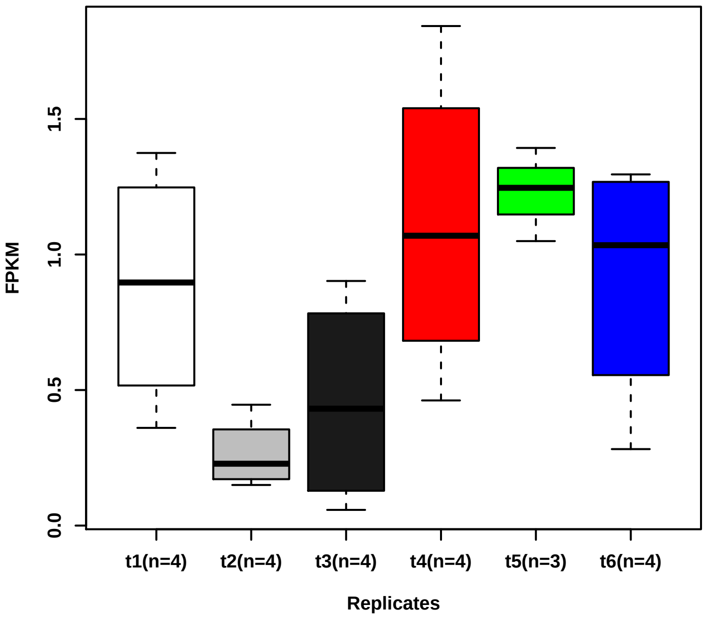

coproporphyrinogen−III from 5−aminolevulinate: step 3/4

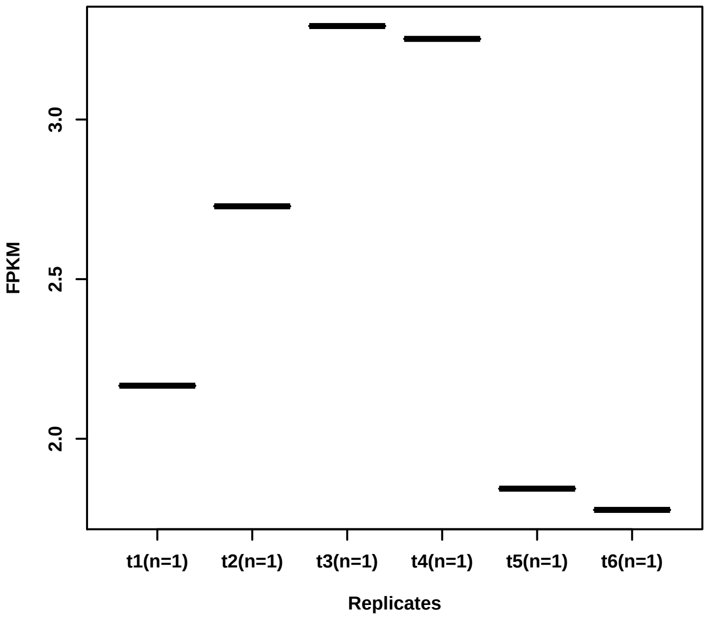

coproporphyrinogen−III from 5−aminolevulinate: step 4/4

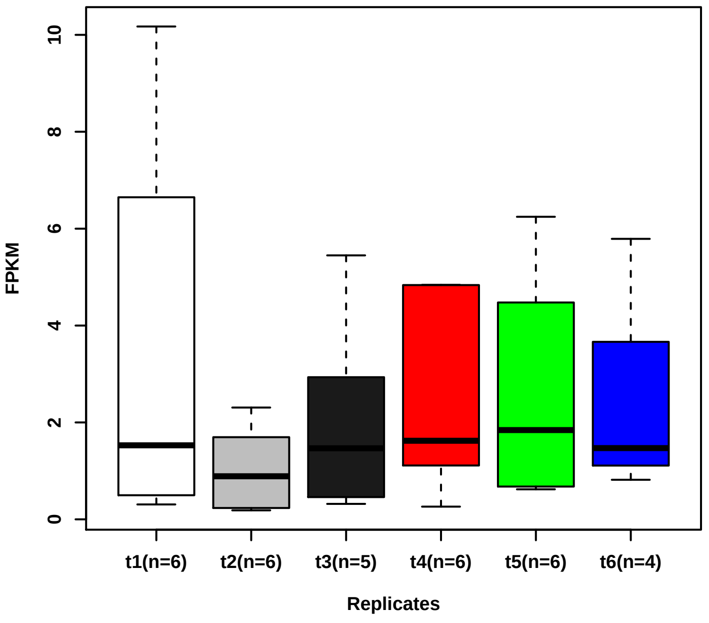

CTP from cytidine: step 1/3

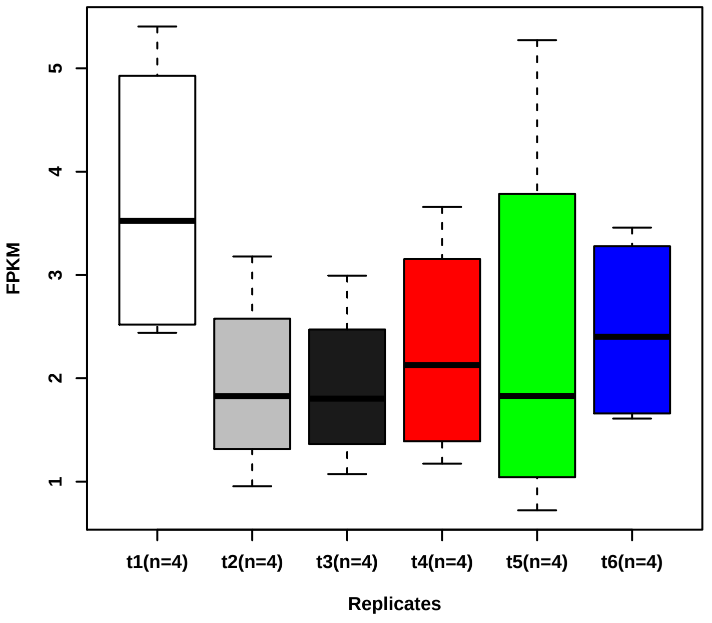

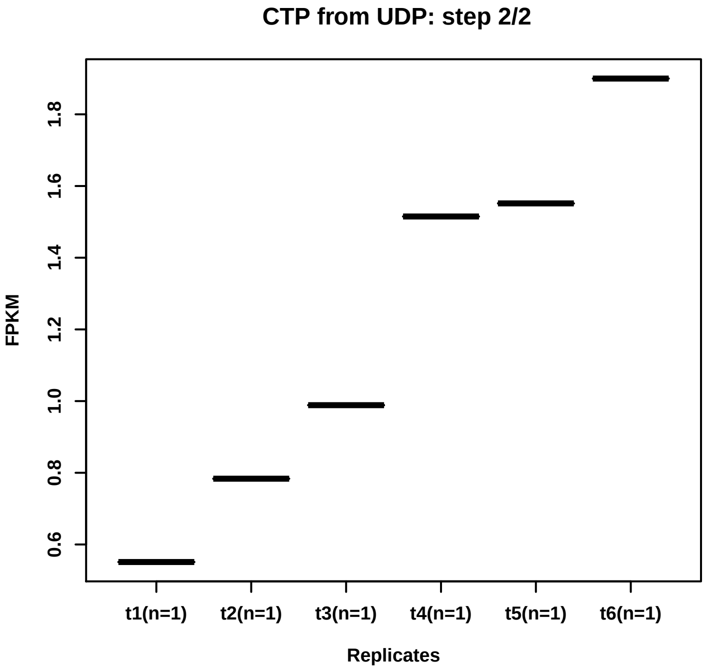
CTP from UDP: step 2/2
FPKM
0.6
0.8
1.0
1.2
1.4
1.6
1.8
t1(n=1)
t2(n=1)
t3(n=1)
t4(n=1)
t5(n=1)
t6(n=1)
Replicates

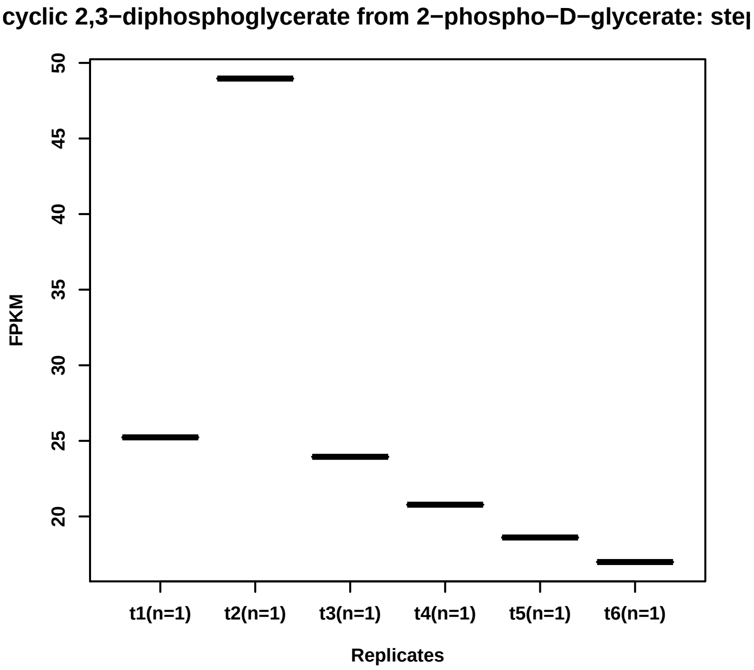
cyclic 2,3−diphosphoglycerate from 2−phospho−D−glycerate: ste
FPKM
20
25
30
35
40
45
50
t1(n=1)
t2(n=1)
t3(n=1)
t4(n=1)
t5(n=1)
t6(n=1)
Replicates

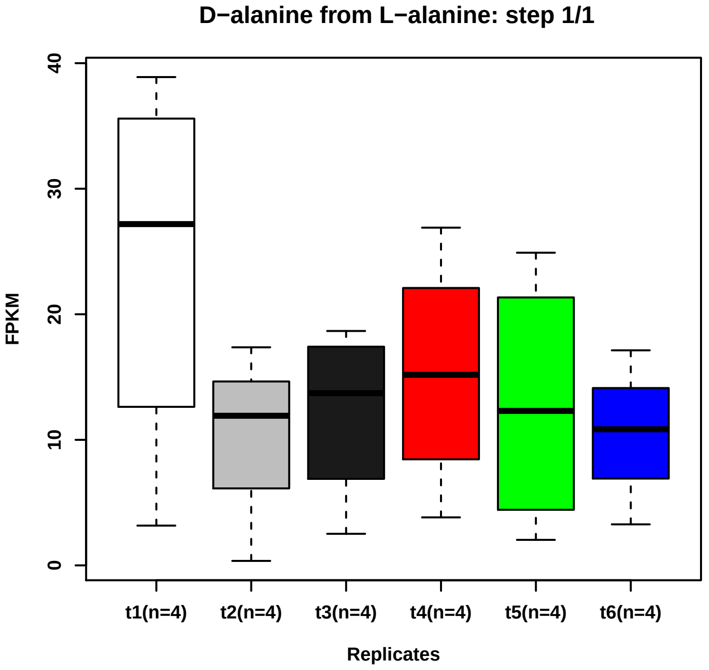
D−alanine from L−alanine: step 1/1
FPKM
0
10
20
30
40
t1(n=4)
t2(n=4)
t3(n=4)
t4(n=4)
t5(n=4)
t6(n=4)
Replicates

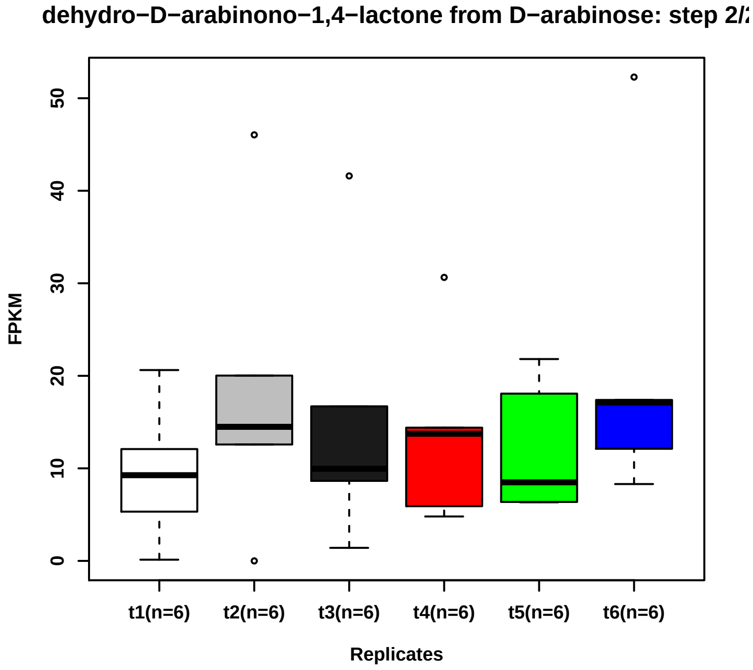
dehydro−D−arabinono−1,4−lactone from D−arabinose: step 2/
FPKM
0
10
20
30
40
50
t1(n=6)
t2(n=6)
t3(n=6)
t4(n=6)
t5(n=6)
t6(n=6)
Replicates

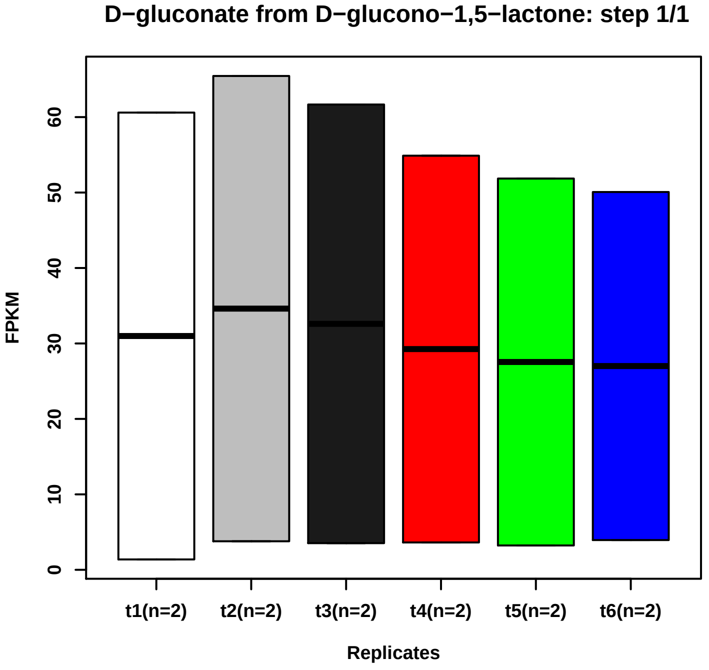
D−gluconate from D−glucono−1,5−lactone: step 1/1
FPKM
0
10
20
30
40
50
60
t1(n=2)
t2(n=2)
t3(n=2)
t4(n=2)
t5(n=2)
t6(n=2)
Replicates

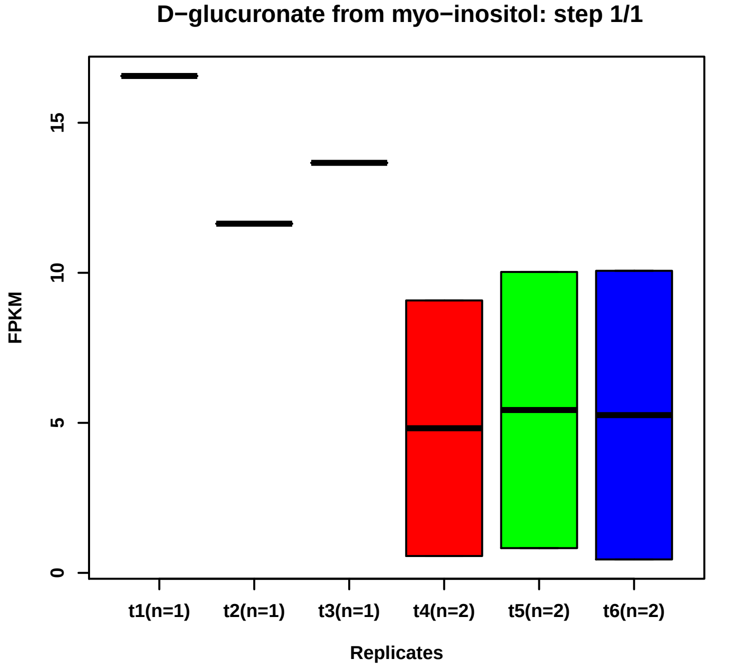
D−glucuronate from myo−inositol: step 1/1
FPKM
0
5
10
15
t1(n=1)
t2(n=1)
t3(n=1)
t4(n=2)
t5(n=2)
t6(n=2)
Replicates

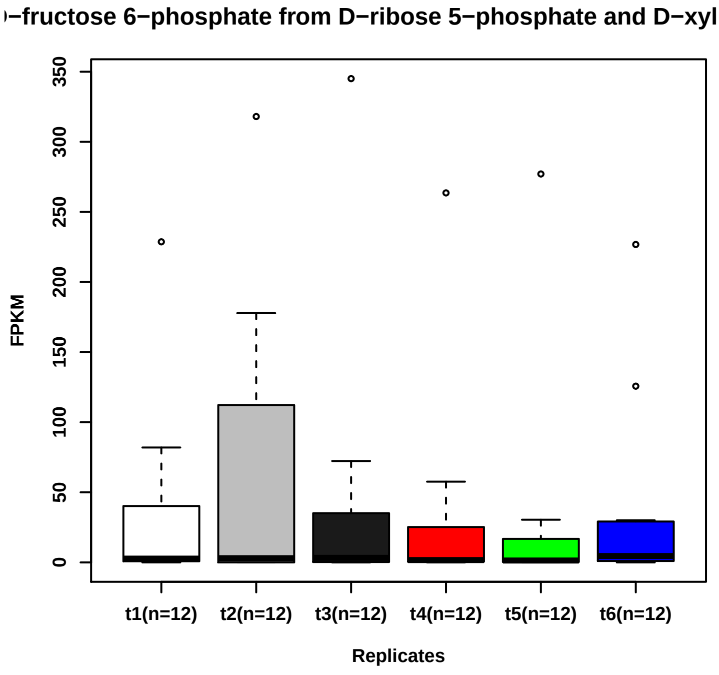
)−fructose 6−phosphate from D−ribose 5−phosphate and D−xylulo
FPKM
0
50
100
150
200
250
300
350
t1(n=12)
t2(n=12)
t3(n=12)
t4(n=12)
t5(n=12)
t6(n=12)
Replicates

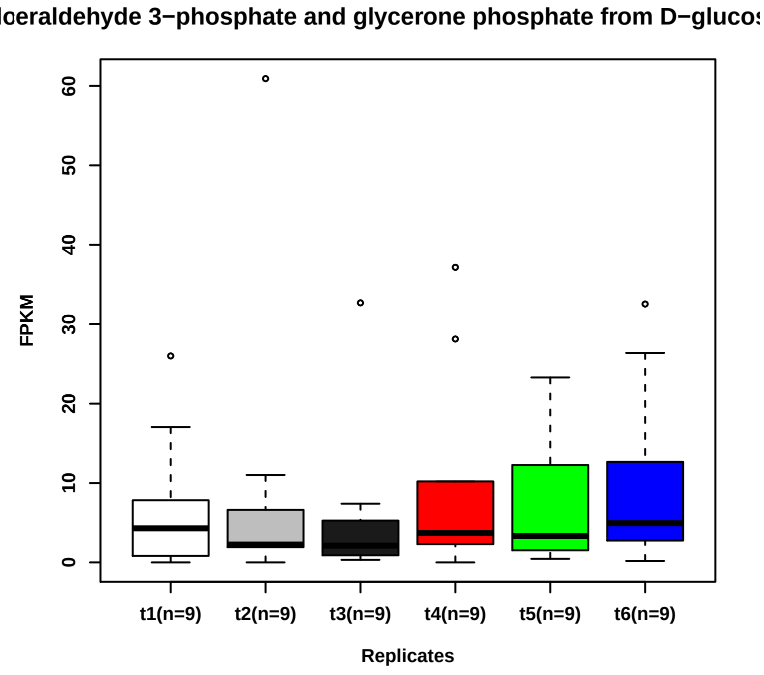
ceraldehyde 3−phosphate and glycerone phosphate from D−glucos
FPKM
0
10
20
30
40
50
60
t1(n=9)
t2(n=9)
t3(n=9)
t4(n=9)
t5(n=9)
t6(n=9)
Replicates

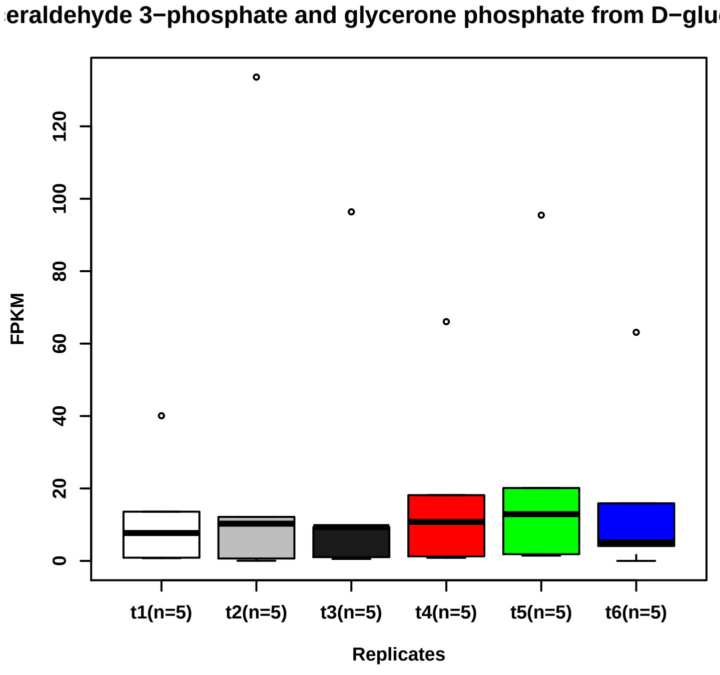
eraldehyde 3−phosphate and glycerone phosphate from D−gluco
FPKM
0
20
40
60
80
100
120
t1(n=5)
t2(n=5)
t3(n=5)
t4(n=5)
t5(n=5)
t6(n=5)
Replicates

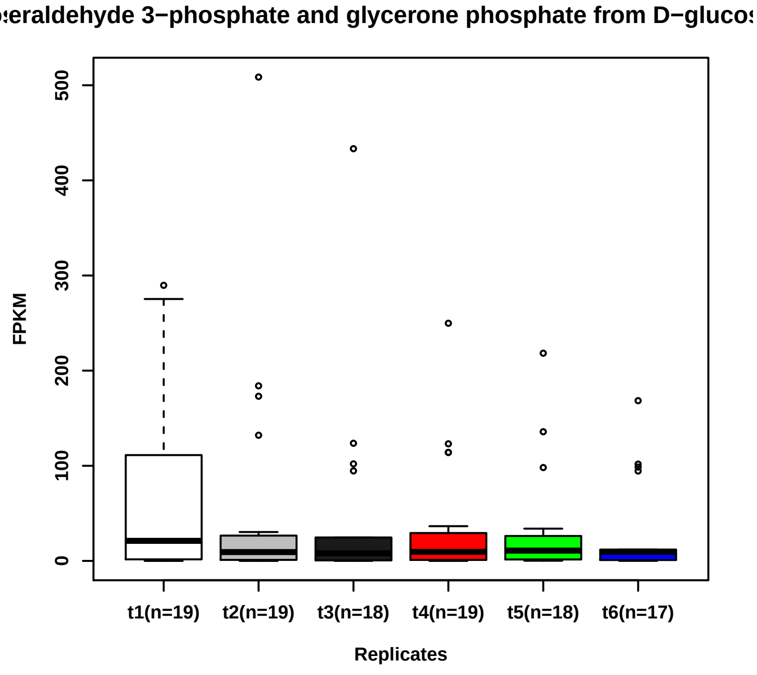
eraldehyde 3−phosphate and glycerone phosphate from D−glucos
FPKM
0
100
200
300
400
500
t1(n=19)
t2(n=19)
t3(n=18)
t4(n=19)
t5(n=18)
t6(n=17)
Replicates

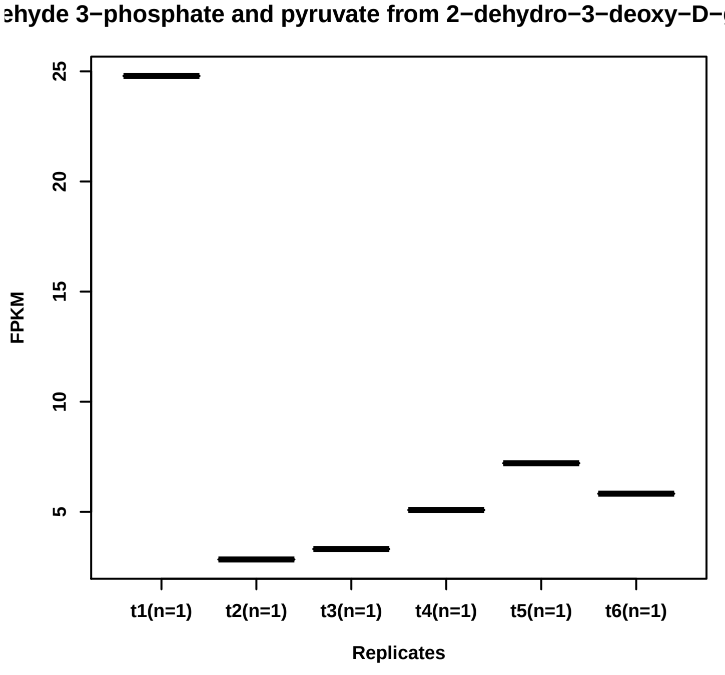
ehyde 3−phosphate and pyruvate from 2−dehydro−3−deoxy−D−glu
FPKM
5
10
15
20
25
t1(n=1)
t2(n=1)
t3(n=1)
t4(n=1)
t5(n=1)
t6(n=1)
Replicates

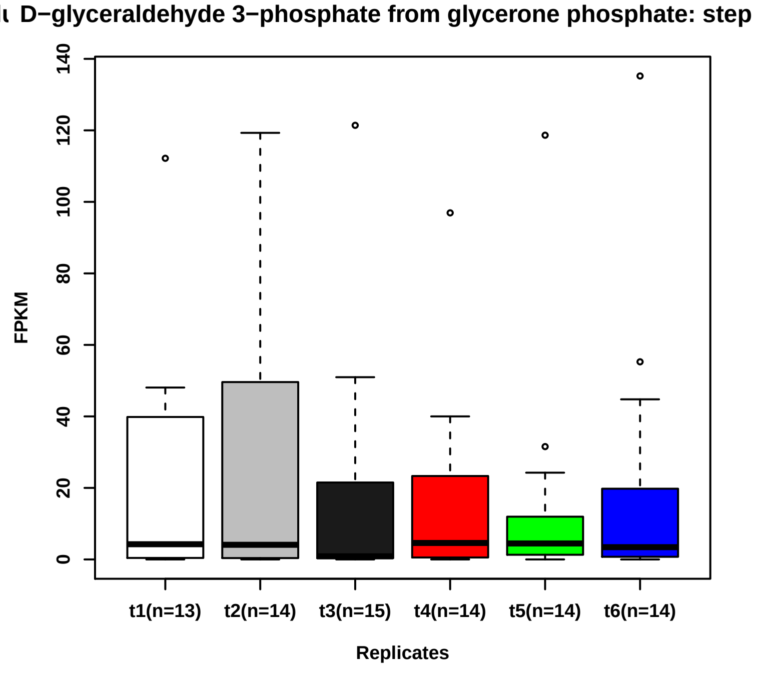
D−glyceraldehyde 3−phosphate from glycerone phosphate: step
FPKM
0
20
40
60
80
100
120
140
t1(n=13)
t2(n=14)
t3(n=15)
t4(n=14)
t5(n=14)
t6(n=14)
Replicates

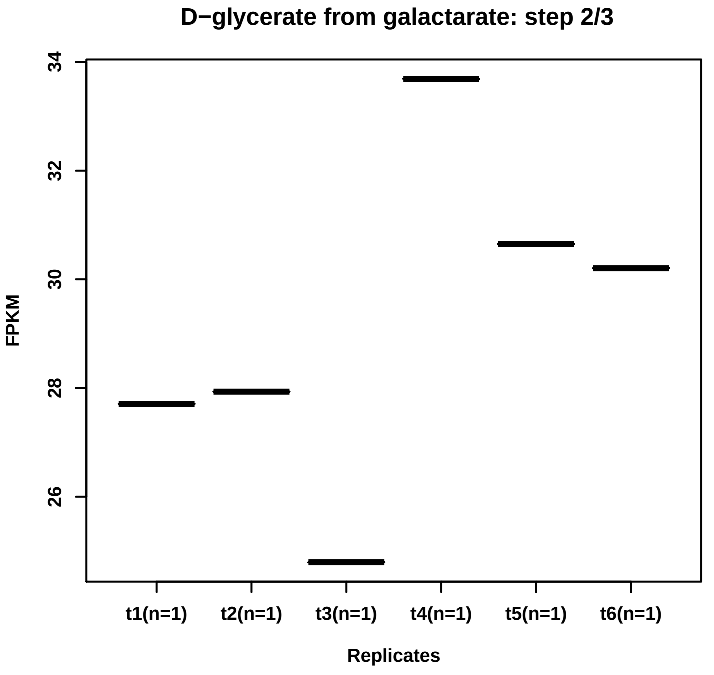
D−glycerate from galactarate: step 2/3
FPKM
26
28
30
32
34
t1(n=1)
t2(n=1)
t3(n=1)
t4(n=1)
t5(n=1)
t6(n=1)
Replicates

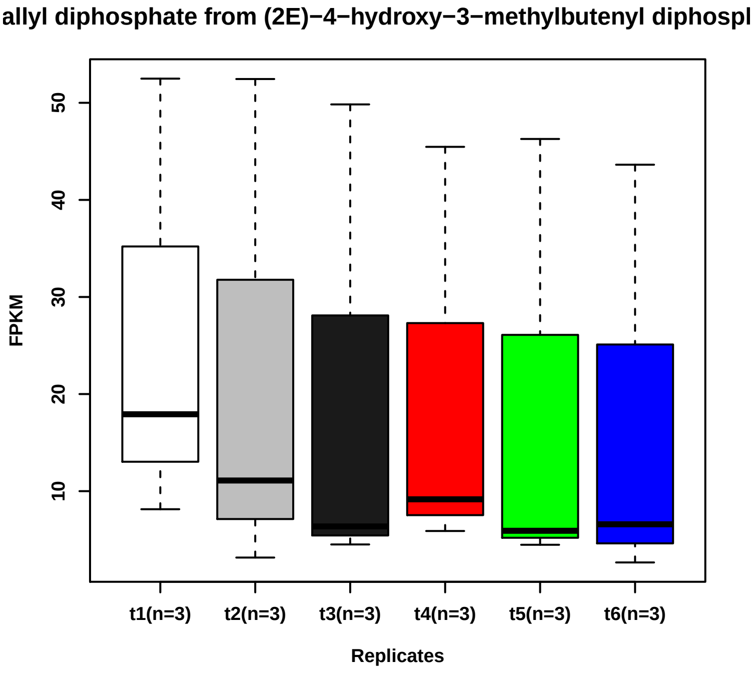
allyl diphosphate from (2E)−4−hydroxy−3−methylbutenyl diphospl
FPKM
10
20
30
40
50
t1(n=3)
t2(n=3)
t3(n=3)
t4(n=3)
t5(n=3)
t6(n=3)
Replicates

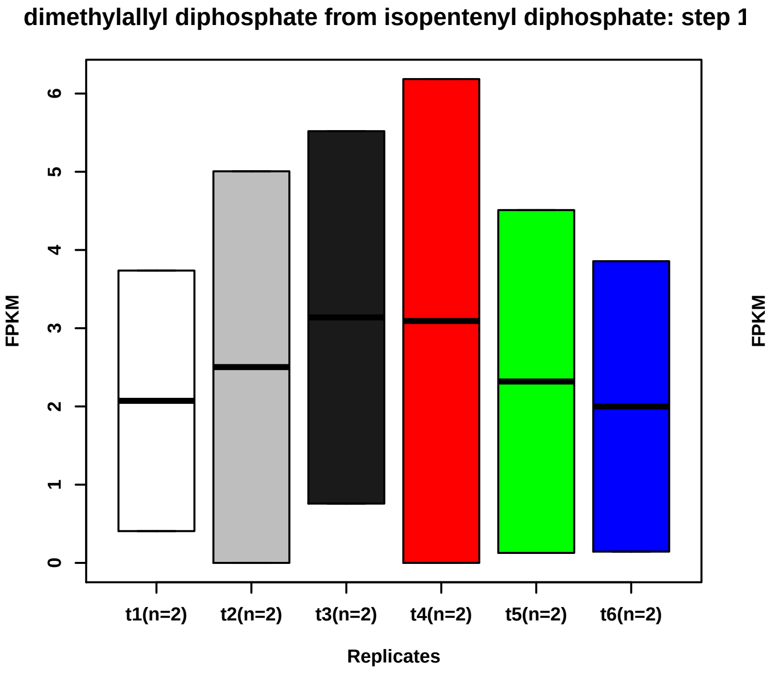
dimethylallyl diphosphate from isopentenyl diphosphate: step 1
FPKM
0
1
2
3
4
5
6
t1(n=2)
t2(n=2)
t3(n=2)
t4(n=2)
t5(n=2)
t6(n=2)
Replicates

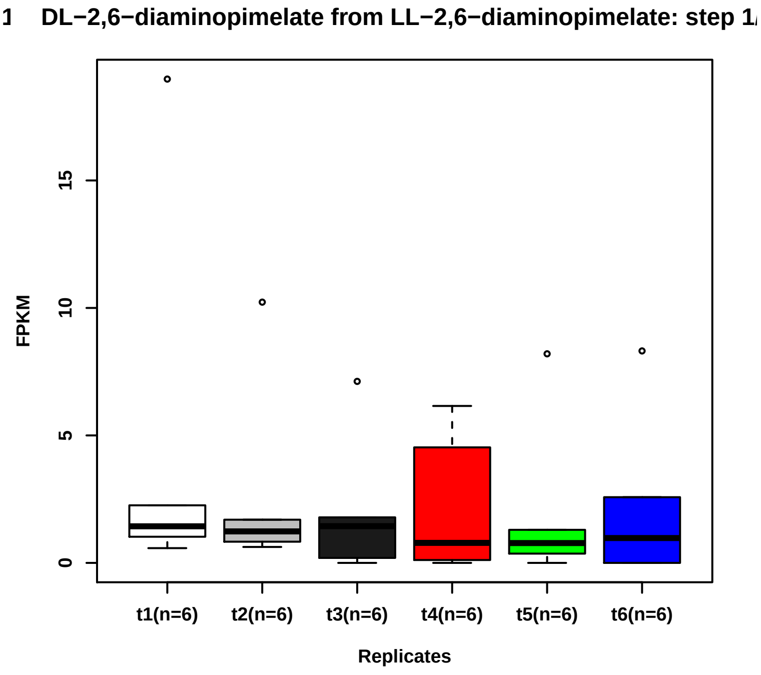
DL−2,6−diaminopimelate from LL−2,6−diaminopimelate: step 1/
FPKM
0
5
10
15
t1(n=6)
t2(n=6)
t3(n=6)
t4(n=6)
t5(n=6)
t6(n=6)
Replicates

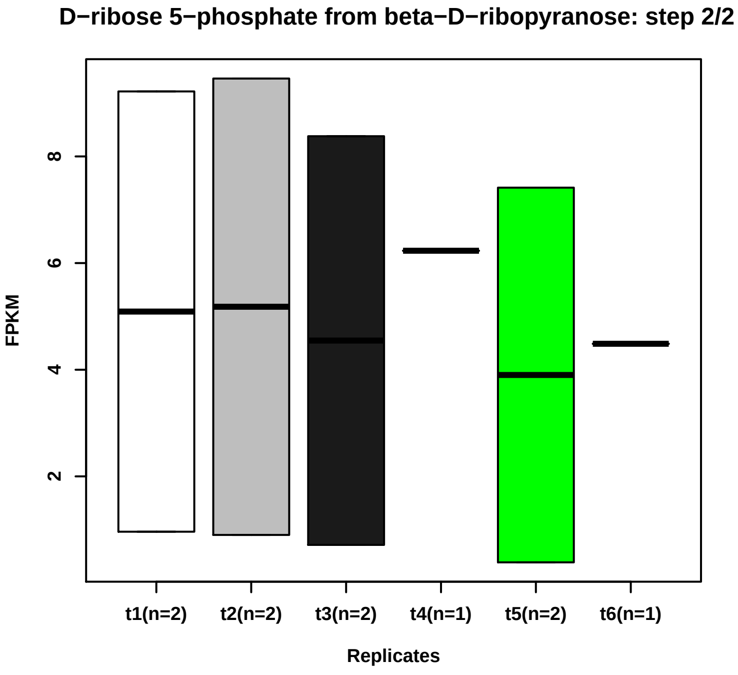
D−ribose 5−phosphate from beta−D−ribopyranose: step 2/2
FPKM
2
4
6
8
t1(n=2)
t2(n=2)
t3(n=2)
t4(n=1)
t5(n=2)
t6(n=1)
Replicates

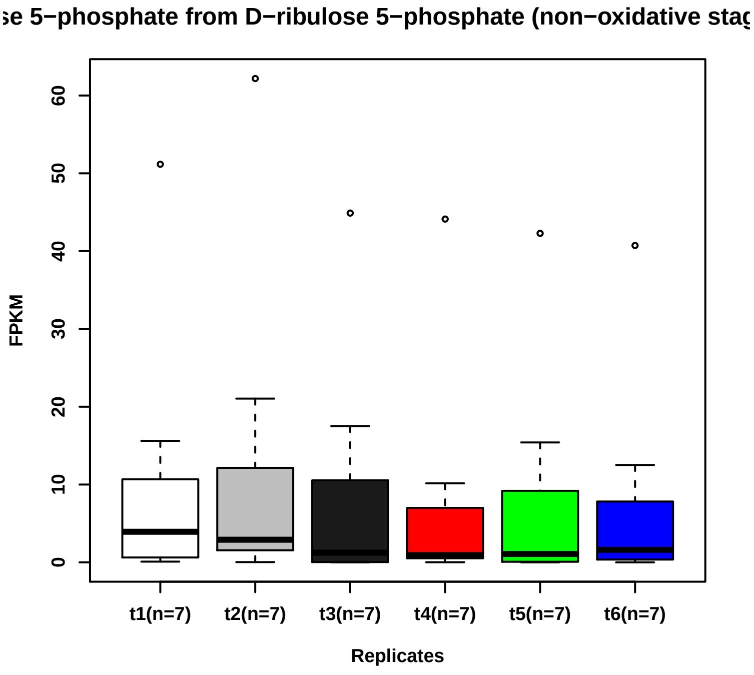
se 5−phosphate from D−ribulose 5−phosphate (non−oxidative stag
FPKM
0
10
20
30
40
50
60
t1(n=7)
t2(n=7)
t3(n=7)
t4(n=7)
t5(n=7)
t6(n=7)
Replicates

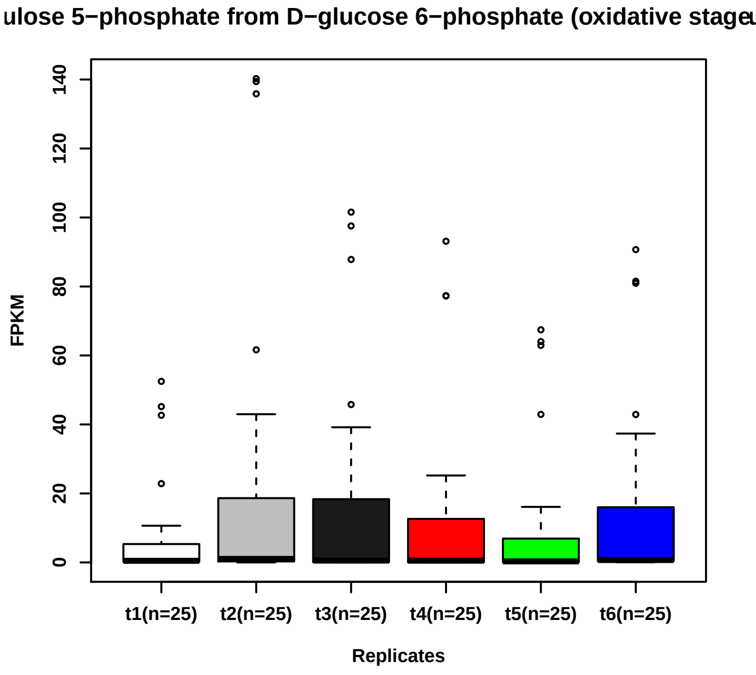
ulose 5−phosphate from D−glucose 6−phosphate (oxidative stage
FPKM
0
20
40
60
80
100
120
140
t1(n=25)
t2(n=25)
t3(n=25)
t4(n=25)
t5(n=25)
t6(n=25)
Replicates

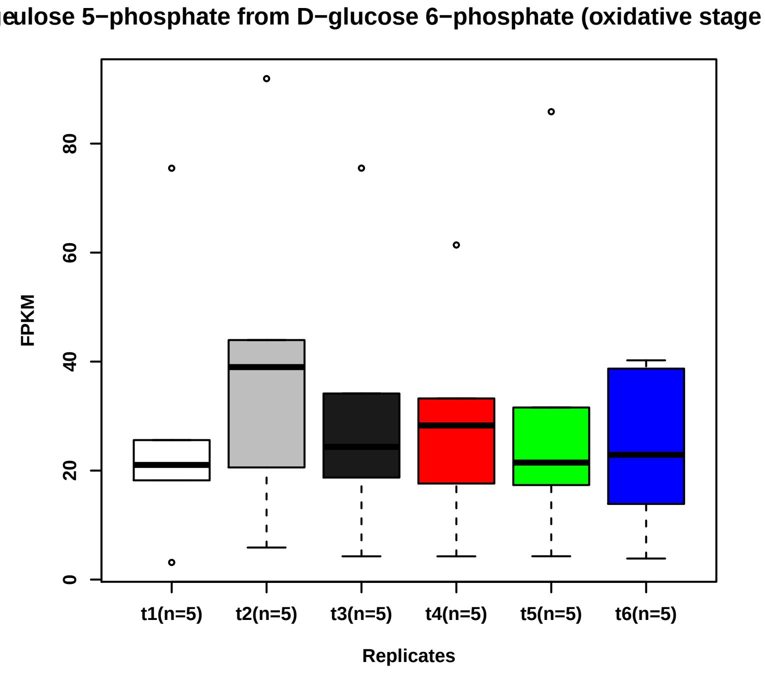
ulose 5−phosphate from D−glucose 6−phosphate (oxidative stage
FPKM
0
20
40
60
80
t1(n=5)
t2(n=5)
t3(n=5)
t4(n=5)
t5(n=5)
t6(n=5)
Replicates

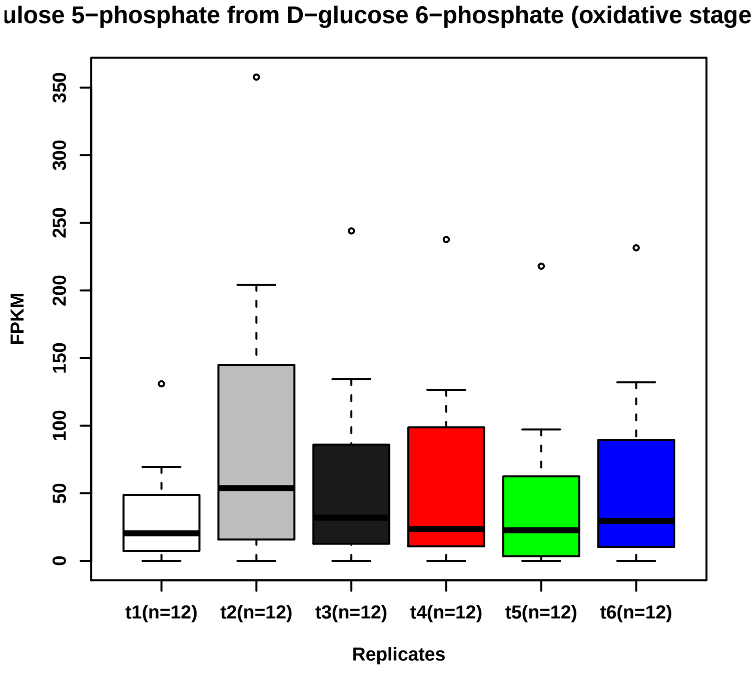
ulose 5−phosphate from D−glucose 6−phosphate (oxidative stage
FPKM
0
50
100
150
200
250
300
350
t1(n=12)
t2(n=12)
t3(n=12)
t4(n=12)
t5(n=12)
t6(n=12)
Replicates

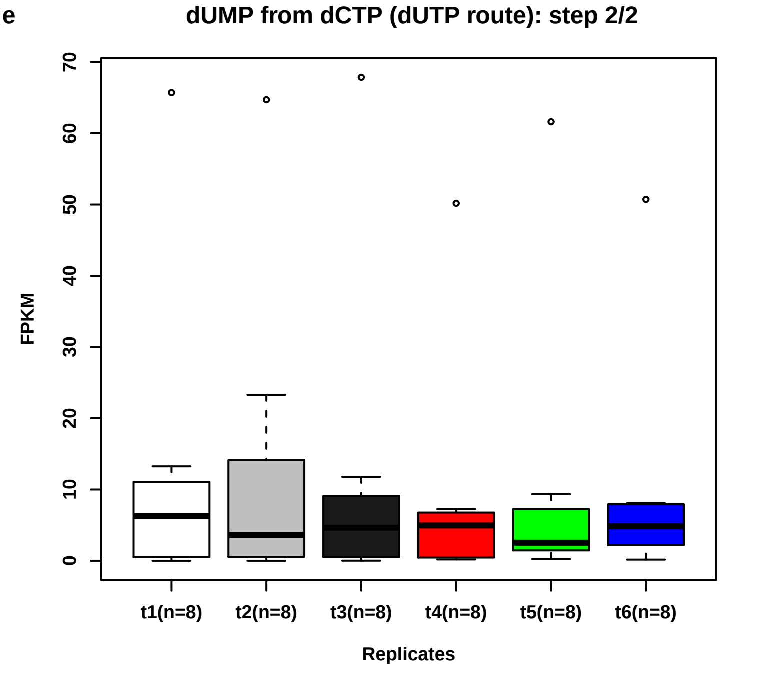
dUMP from dCTP (dUTP route): step 2/2
FPKM
0
10
20
30
40
50
60
70
t1(n=8)
t2(n=8)
t3(n=8)
t4(n=8)
t5(n=8)
t6(n=8)
Replicates

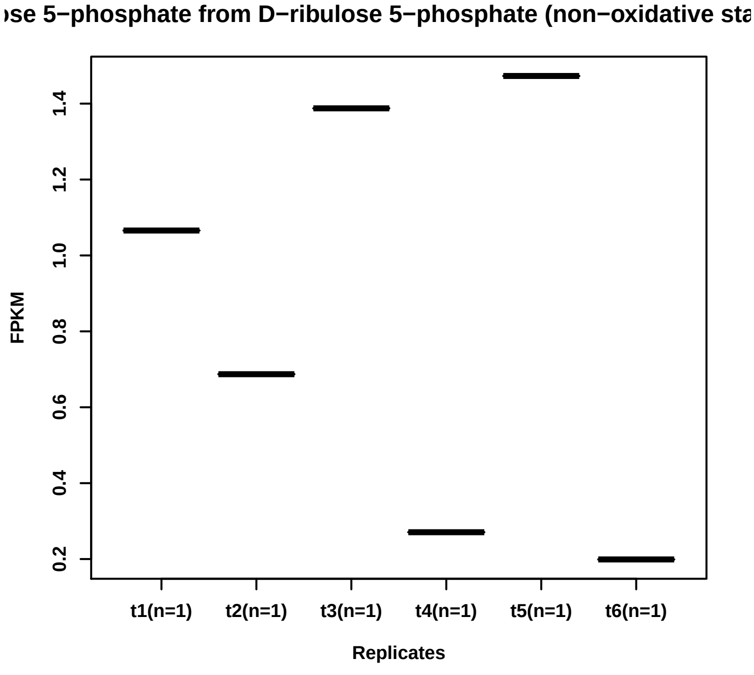
›se 5−phosphate from D−ribulose 5−phosphate (non−oxidative sta
FPKM
0.2
0.4
0.6
0.8
1.0
1.2
1.4
t1(n=1)
t2(n=1)
t3(n=1)
t4(n=1)
t5(n=1)
t6(n=1)
Replicates

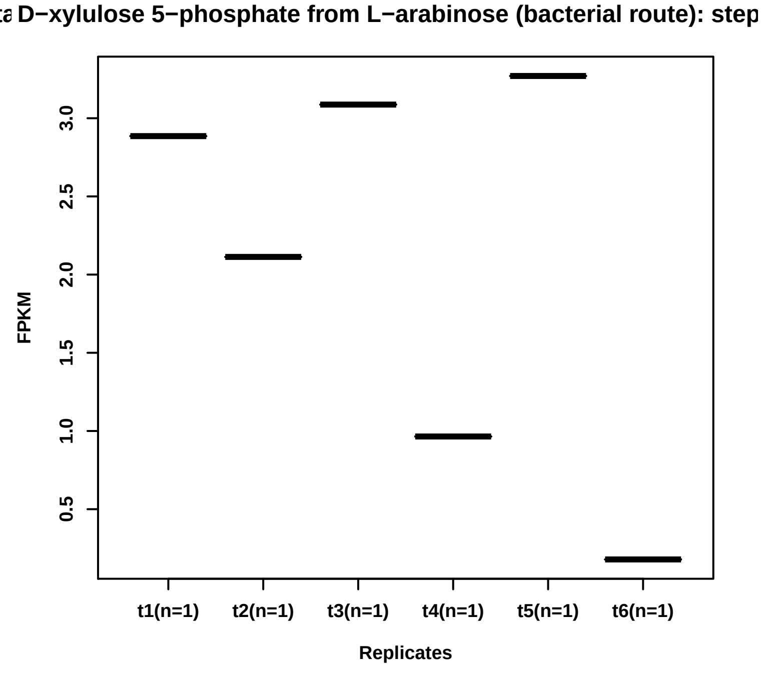
D−xylulose 5−phosphate from L−arabinose (bacterial route): step
FPKM
0.5
1.0
1.5
2.0
2.5
3.0
t1(n=1)
t2(n=1)
t3(n=1)
t4(n=1)
t5(n=1)
t6(n=1)
Replicates

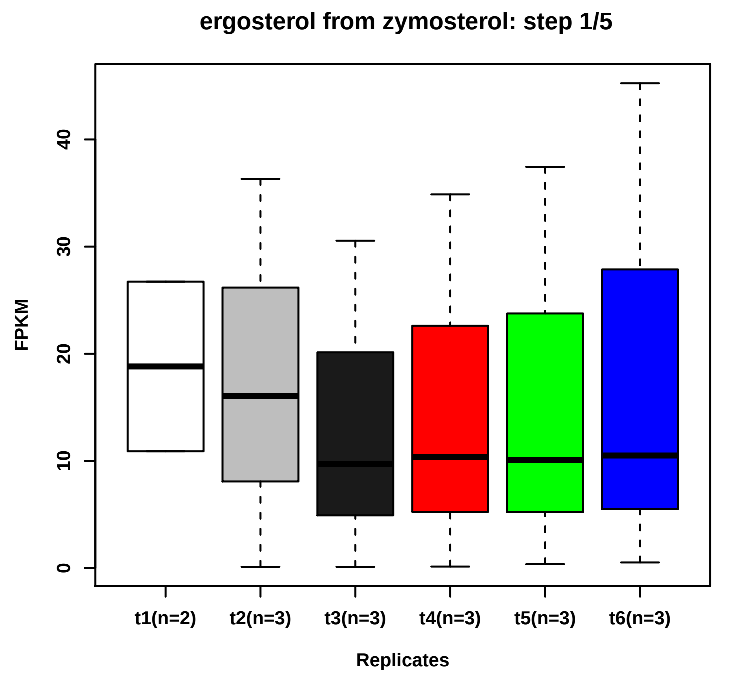
ergosterol from zymosterol: step 1/5
FPKM
0
10
20
30
40
t1(n=2)
t2(n=3)
t3(n=3)
t4(n=3)
t5(n=3)
t6(n=3)
Replicates

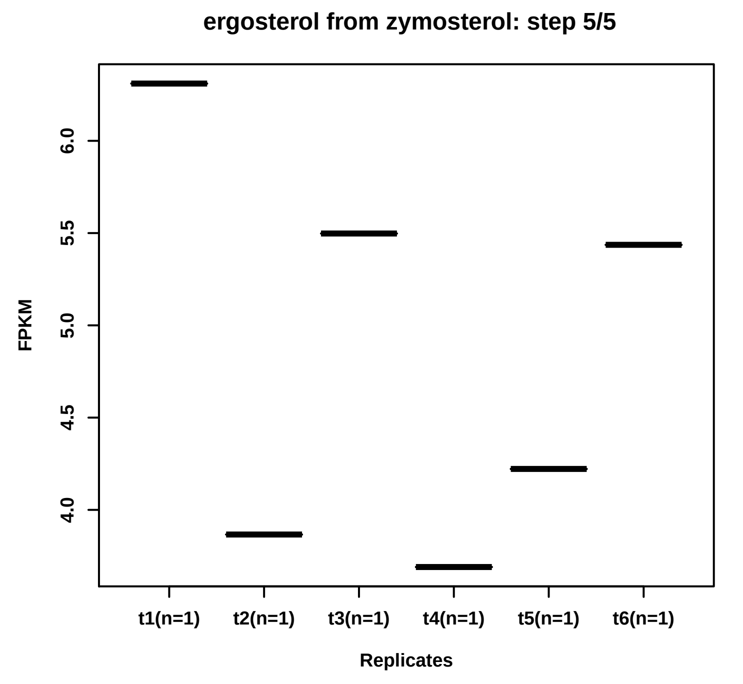
ergosterol from zymosterol: step 5/5
FPKM
4.0
4.5
5.0
5.5
6.0
t1(n=1)
t2(n=1)
t3(n=1)
t4(n=1)
t5(n=1)
t6(n=1)
Replicates

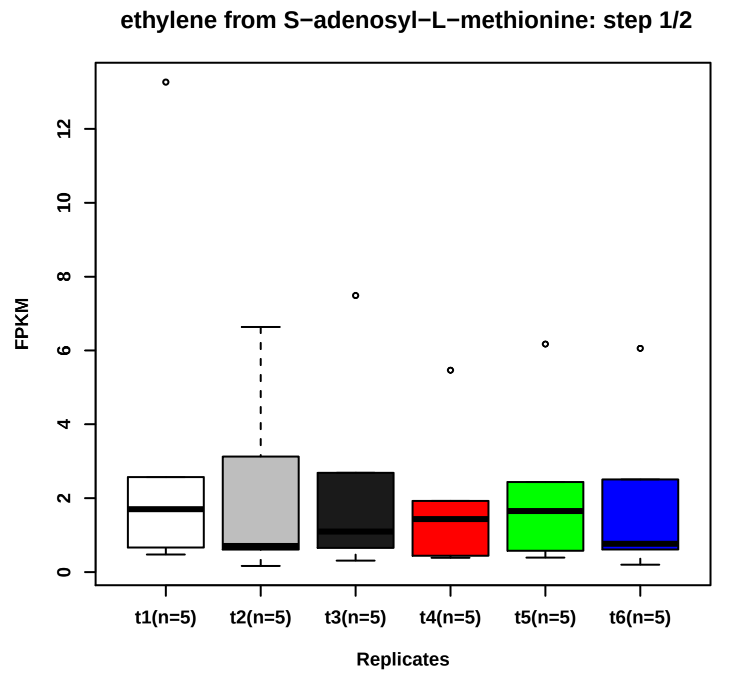
ethylene from S−adenosyl−L−methionine: step 1/2
FPKM
0
2
4
6
8
10
12
t1(n=5)
t2(n=5)
t3(n=5)
t4(n=5)
t5(n=5)
t6(n=5)
Replicates

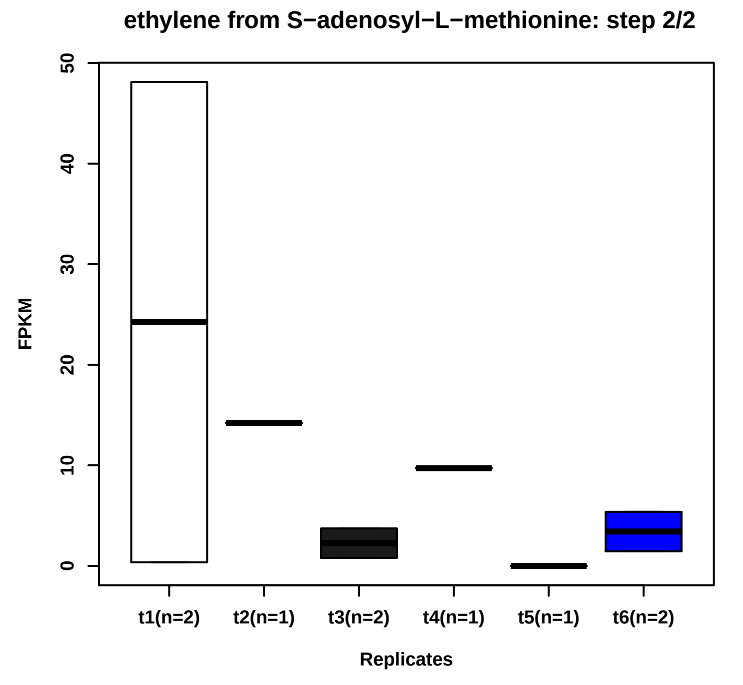
ethylene from S−adenosyl−L−methionine: step 2/2
FPKM
0
10
20
30
40
50
t1(n=2)
t2(n=1)
t3(n=2)
t4(n=1)
t5(n=1)
t6(n=2)
Replicates

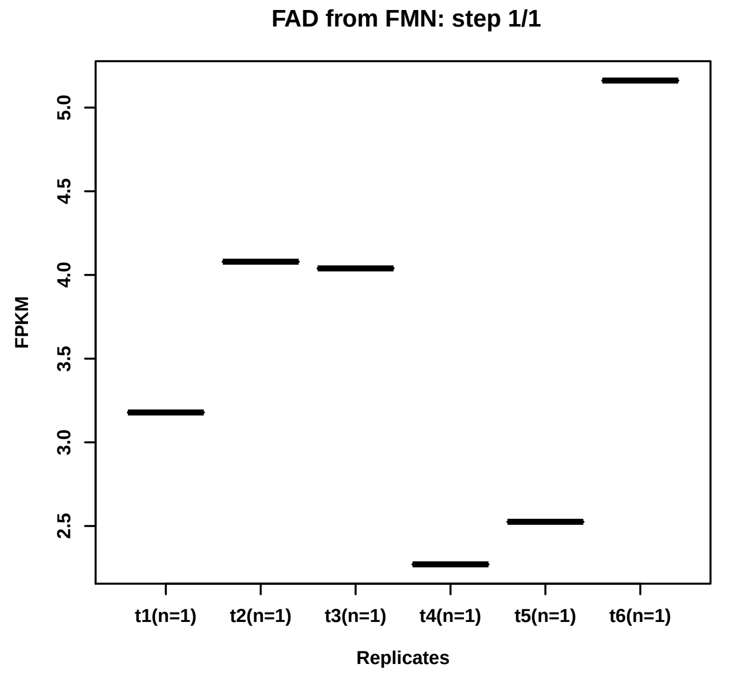
FAD from FMN: step 1/1
FPKM
2.5
3.0
3.5
4.0
4.5
5.0
t1(n=1)
t2(n=1)
t3(n=1)
t4(n=1)
t5(n=1)
t6(n=1)
Replicates

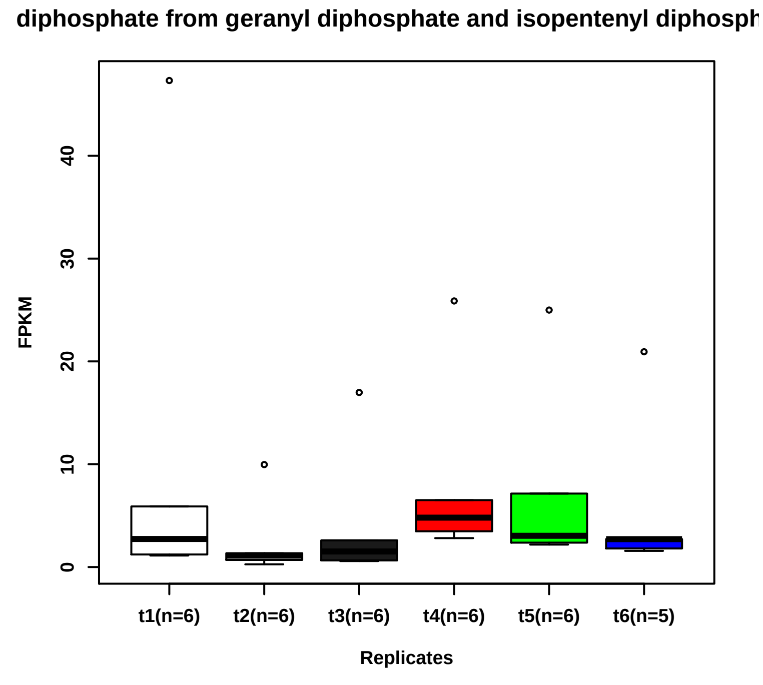
diphosphate from geranyl diphosphate and isopentenyl diphosph
FPKM
0
10
20
30
40
t1(n=6)
t2(n=6)
t3(n=6)
t4(n=6)
t5(n=6)
t6(n=5)
Replicates

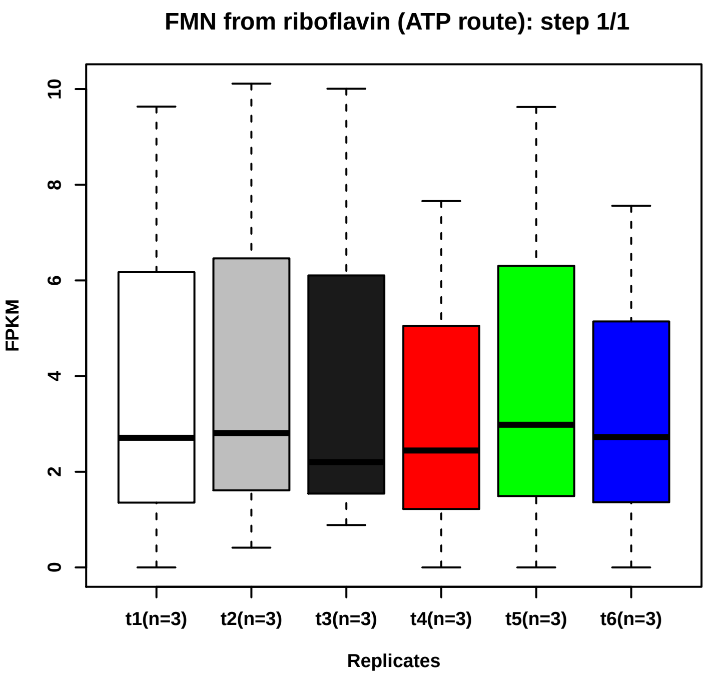

FMN from riboflavin (ATP route): step 1/1
FPKM
0
2
4
6
8
10
t1(n=3)
t2(n=3)
t3(n=3)
t4(n=3)
t5(n=3)
t6(n=3)
Replicates

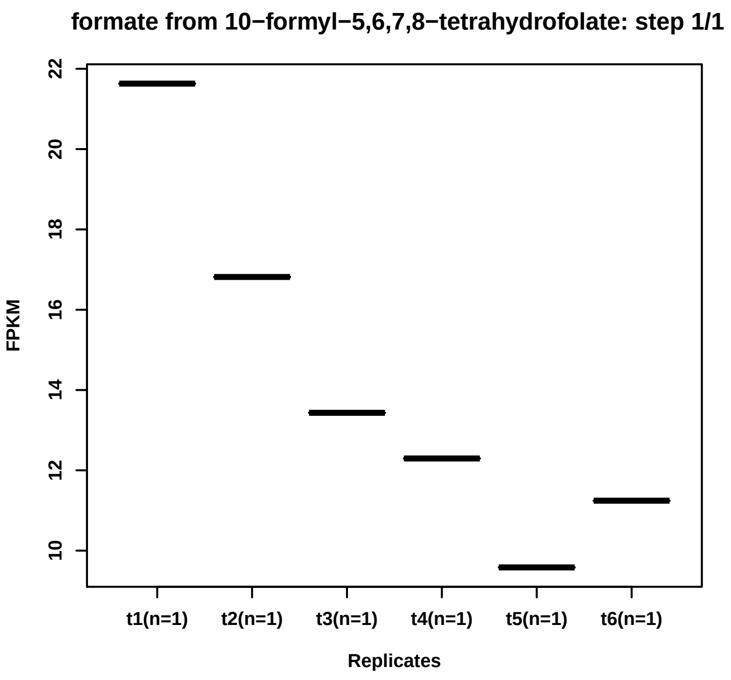

formate from 10−formyl−5,6,7,8−tetrahydrofolate: step 1/1
FPKM
10
12
14
16
18
20
22
t1(n=1)
t2(n=1)
t3(n=1)
t4(n=1)
t5(n=1)
t6(n=1)
Replicates

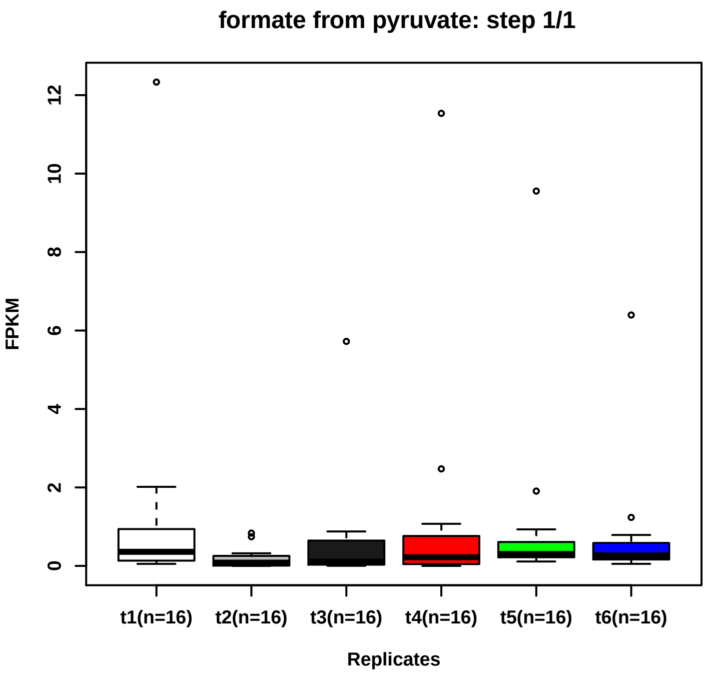

formate from pyruvate: step 1/1
FPKM
0
2
4
6
8
10
12
t1(n=16)
t2(n=16)
t3(n=16)
t4(n=16)
t5(n=16)
t6(n=16)
Replicates

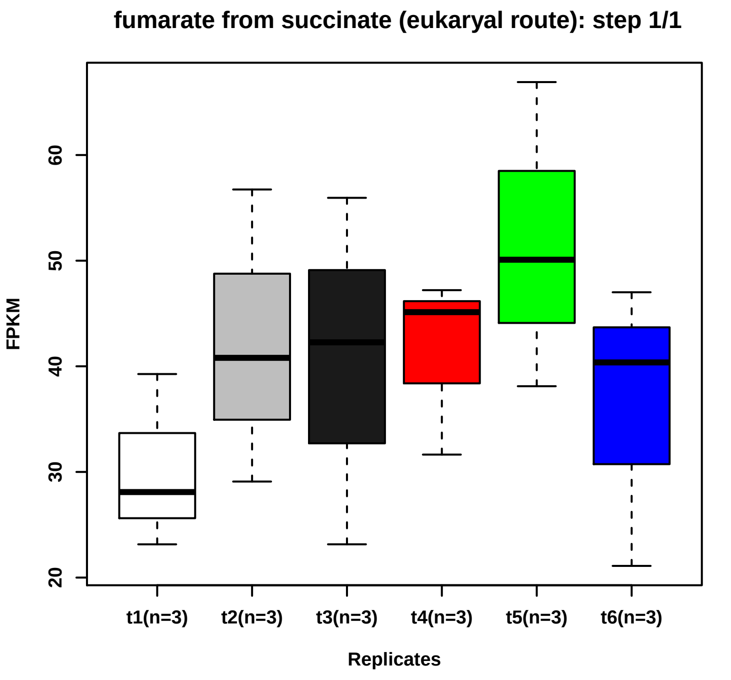

fumarate from succinate (eukaryal route): step 1/1
FPKM
20
30
40
50
60
t1(n=3)
t2(n=3)
t3(n=3)
t4(n=3)
t5(n=3)
t6(n=3)
Replicates

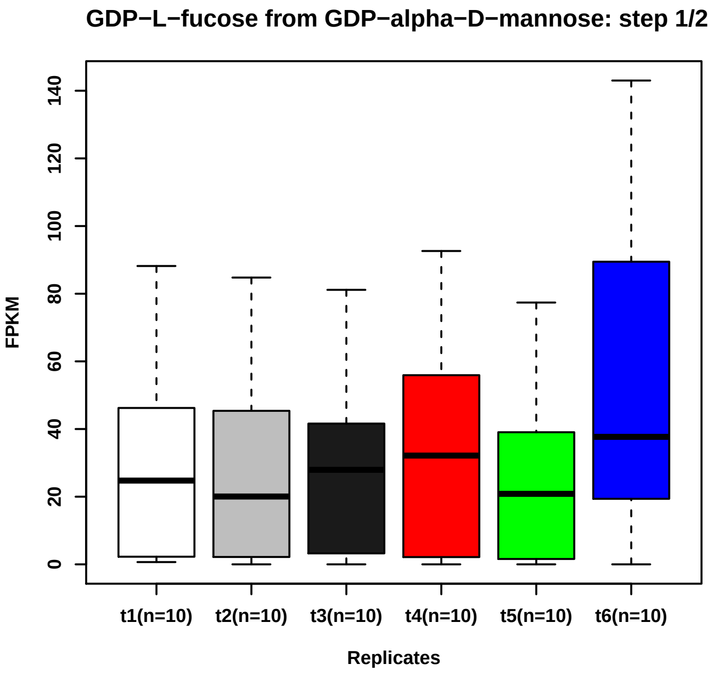

GDP−L−fucose from GDP−alpha−D−mannose: step 1/2
FPKM
0
20
40
60
80
100
120
140
t1(n=10)
t2(n=10)
t3(n=10)
t4(n=10)
t5(n=10)
t6(n=10)
Replicates

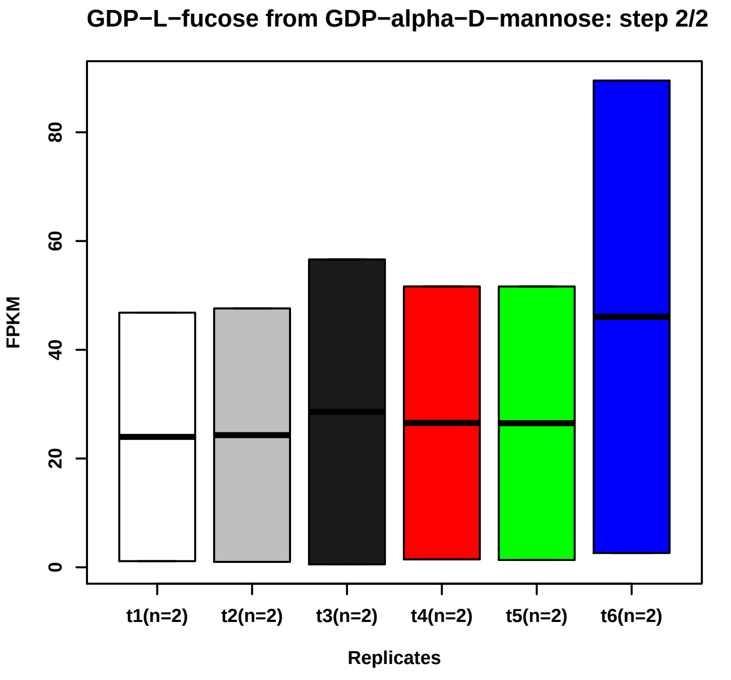

GDP−L−fucose from GDP−alpha−D−mannose: step 2/2
FPKM
0
20
40
60
80
t1(n=2)
t2(n=2)
t3(n=2)
t4(n=2)
t5(n=2)
t6(n=2)
Replicates

hosphate from dimethylallyl diphosphate and isopentenyl diphos

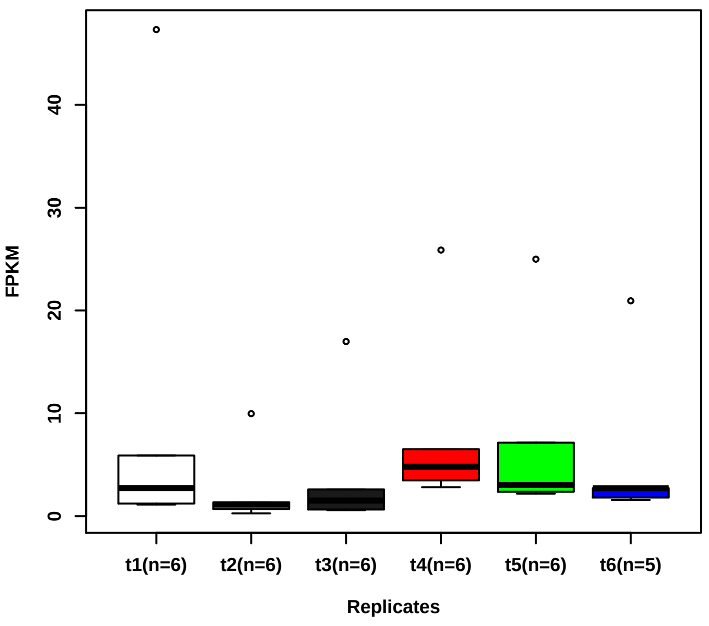

nyl diphosphate from farnesyl diphosphate and isopentenyl diphc

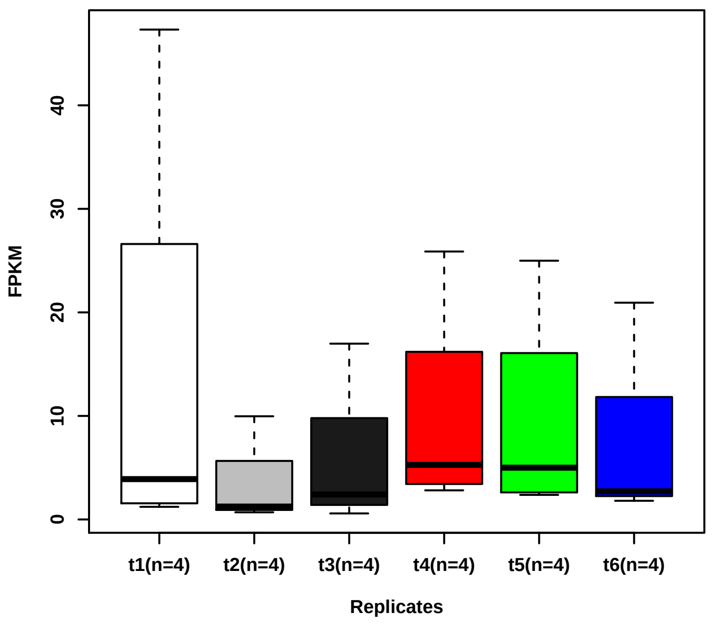

glutaryl−CoA from L−lysine: step 1/6

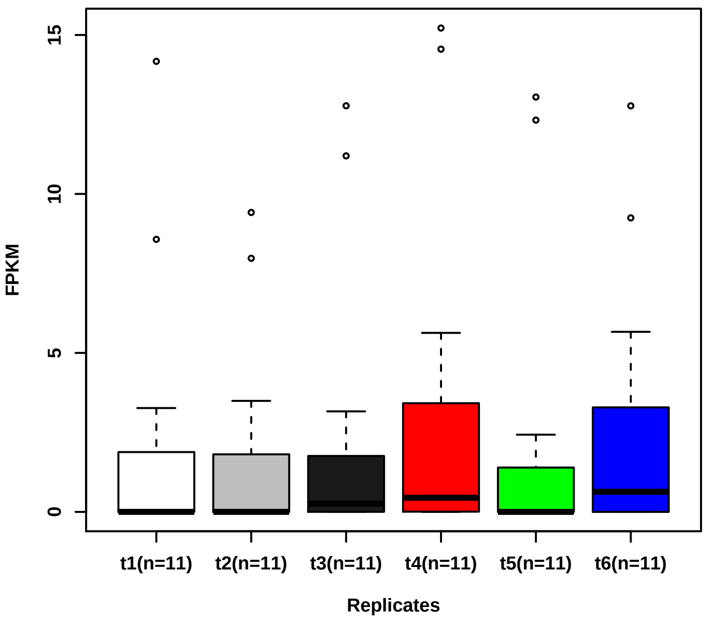

glutaryl−CoA from L−lysine: step 2/6

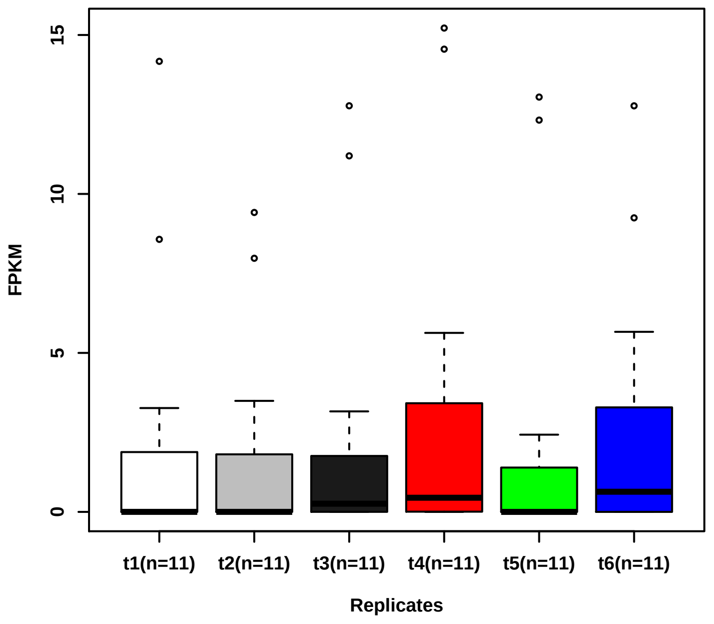

glutaryl−CoA from L−lysine: step 6/6

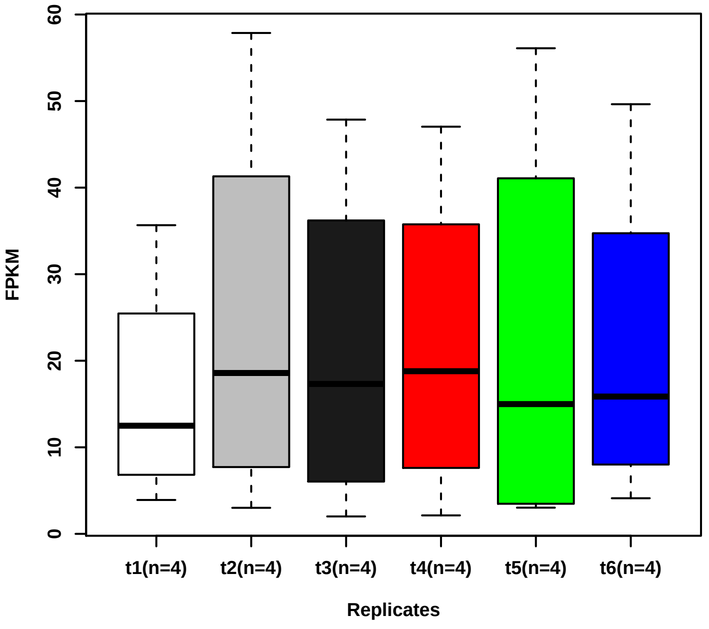

glutathione from L−cysteine and L−glutamate: step 1/2

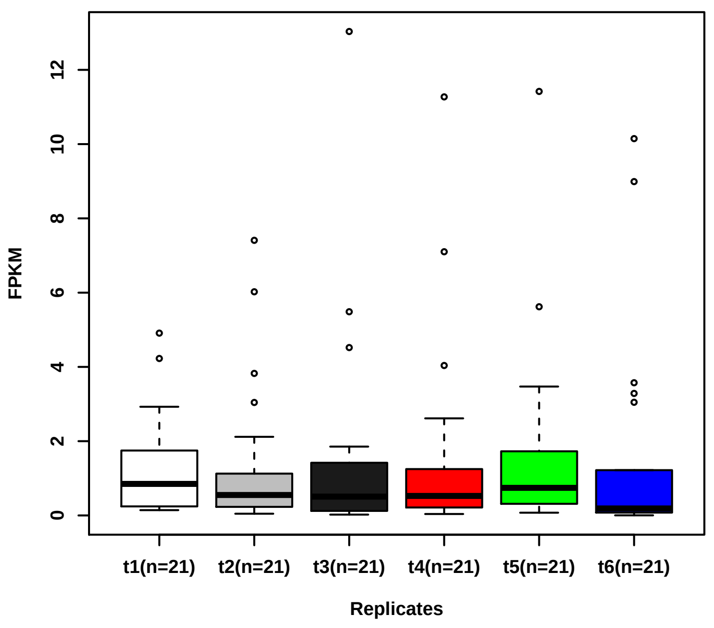

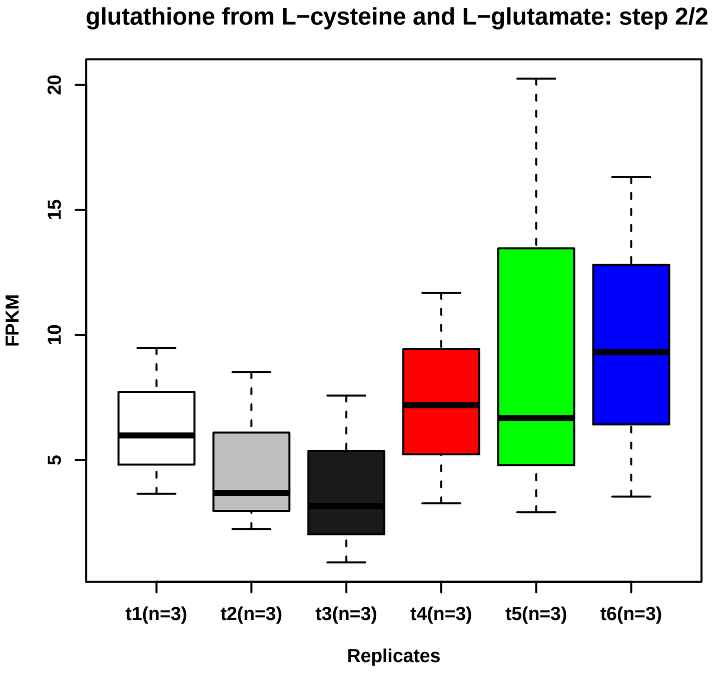
glutathione from L−cysteine and L−glutamate: step 2/2
FPKM
Replicates
t1(n=3) t2(n=3) t3(n=3) t4(n=3) t5(n=3) t6(n=3)

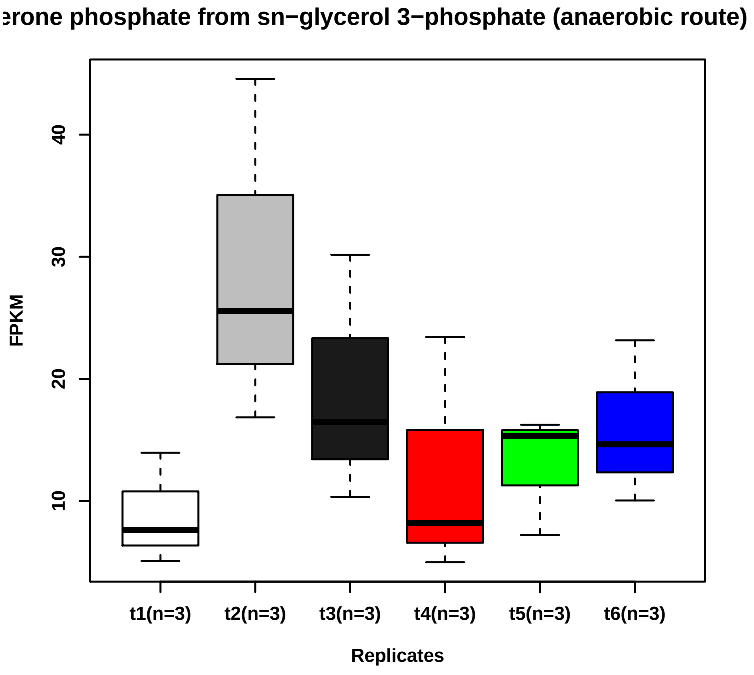
erone phosphate from sn−glycerol 3−phosphate (anaerobic route)
FPKM
Replicates
t1(n=3) t2(n=3) t3(n=3) t4(n=3) t5(n=3) t6(n=3)

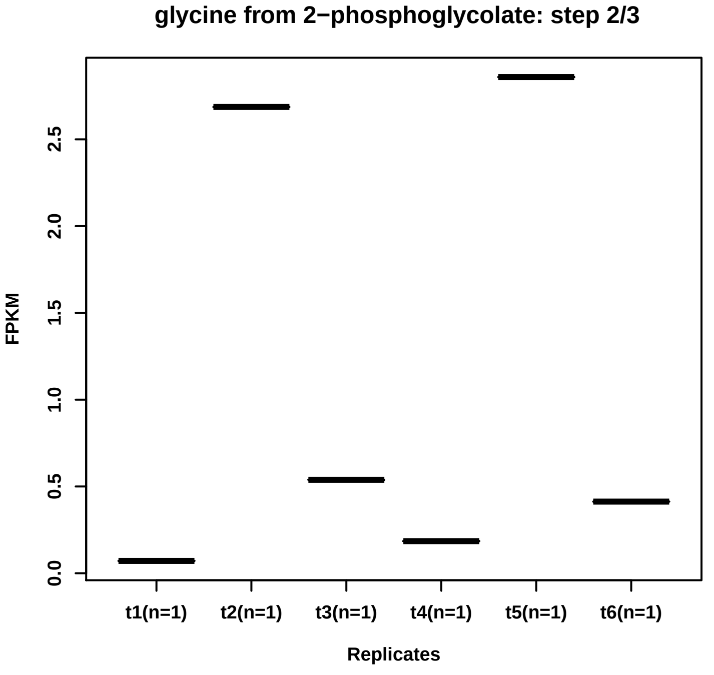
glycine from 2−phosphoglycolate: step 2/3
FPKM
Replicates
t1(n=1) t2(n=1) t3(n=1) t4(n=1) t5(n=1) t6(n=1)

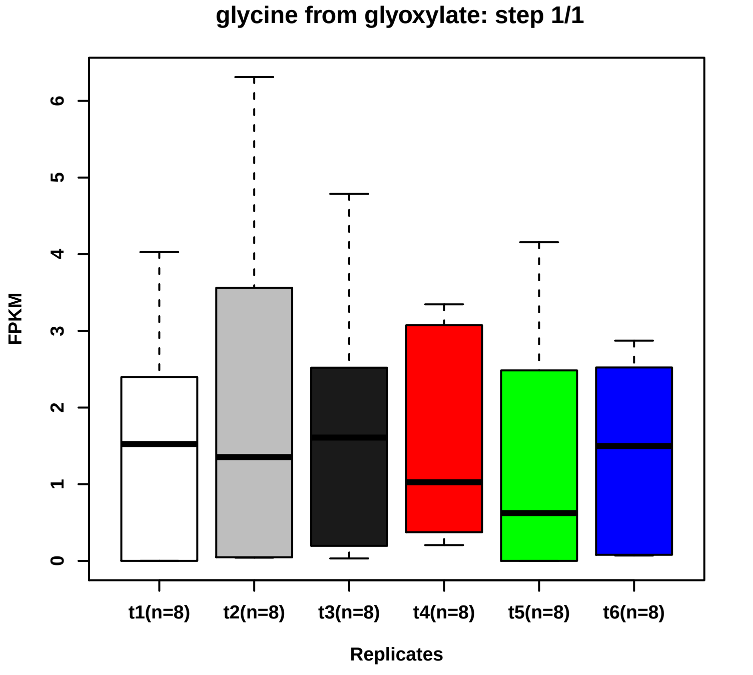
glycine from glyoxylate: step 1/1
FPKM
Replicates
t1(n=8) t2(n=8) t3(n=8) t4(n=8) t5(n=8) t6(n=8)

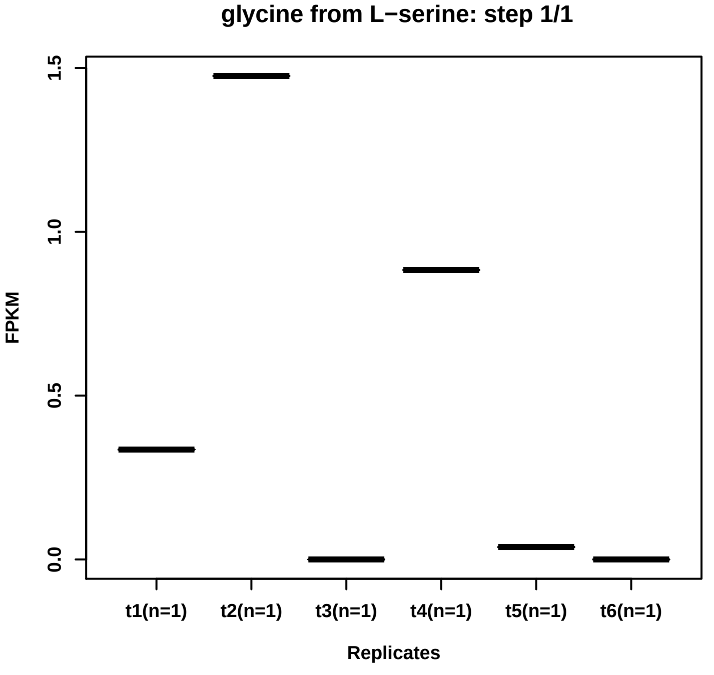
glycine from L−serine: step 1/1
FPKM
Replicates
t1(n=1) t2(n=1) t3(n=1) t4(n=1) t5(n=1) t6(n=1)

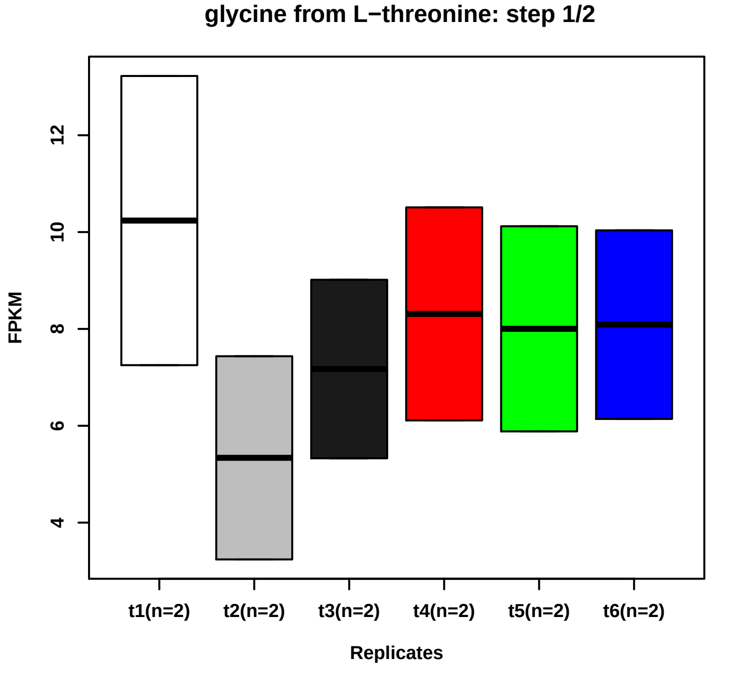
glycine from L−threonine: step 1/2
FPKM
Replicates
t1(n=2) t2(n=2) t3(n=2) t4(n=2) t5(n=2) t6(n=2)

glycolate from 2−phosphoglycolate: step 1/1

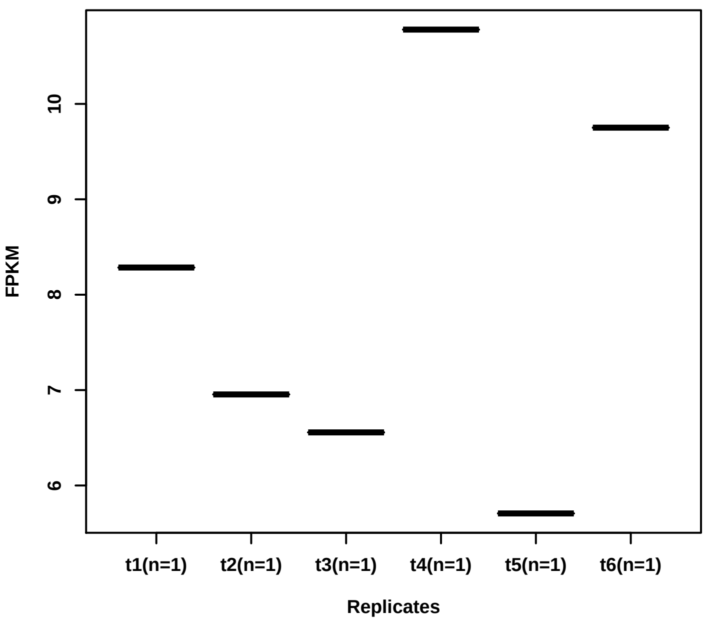

GMP from XMP (L−Gln route): step 1/1

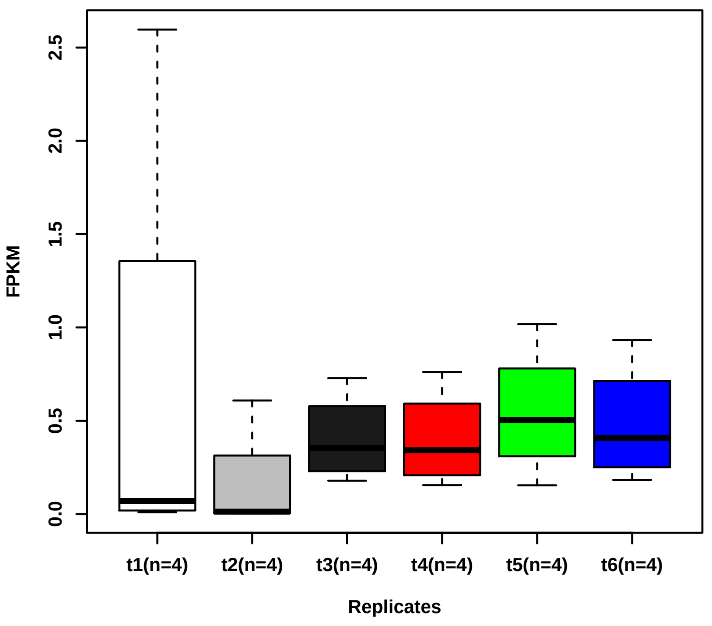

heme A from heme O: step 1/1

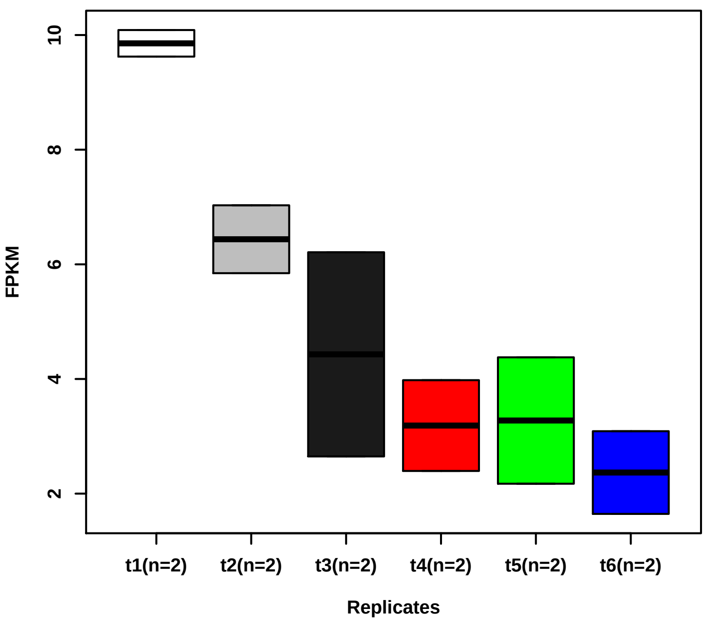

hydrogen sulfide from sulfite (NADPH route): step 1/1

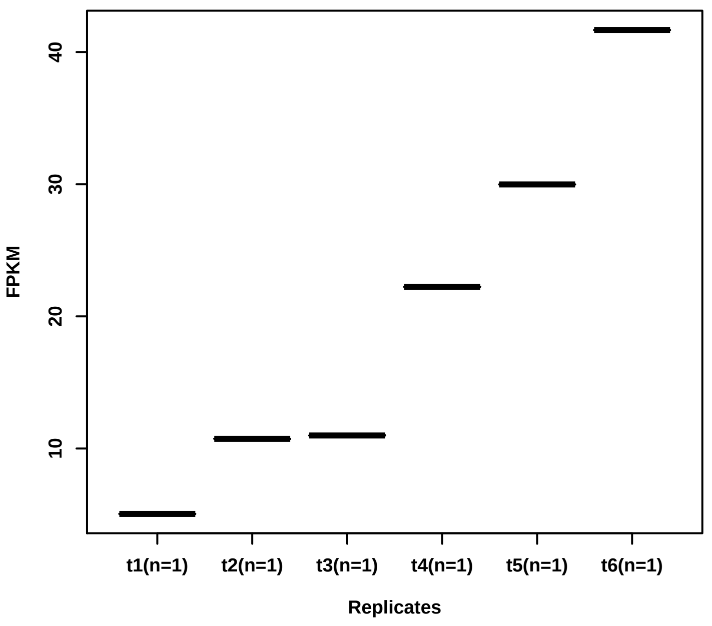

hypotaurine from L−cysteine: step 1/2

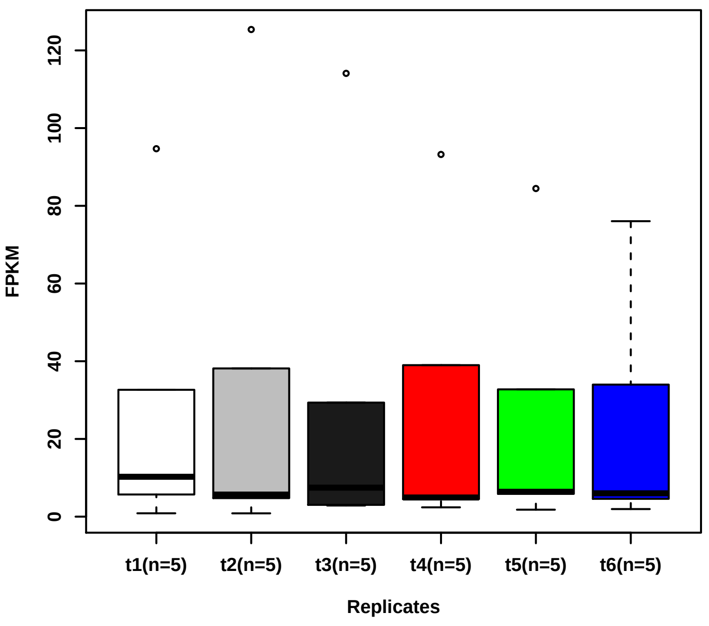

hypotaurine from L−cysteine: step 2/2

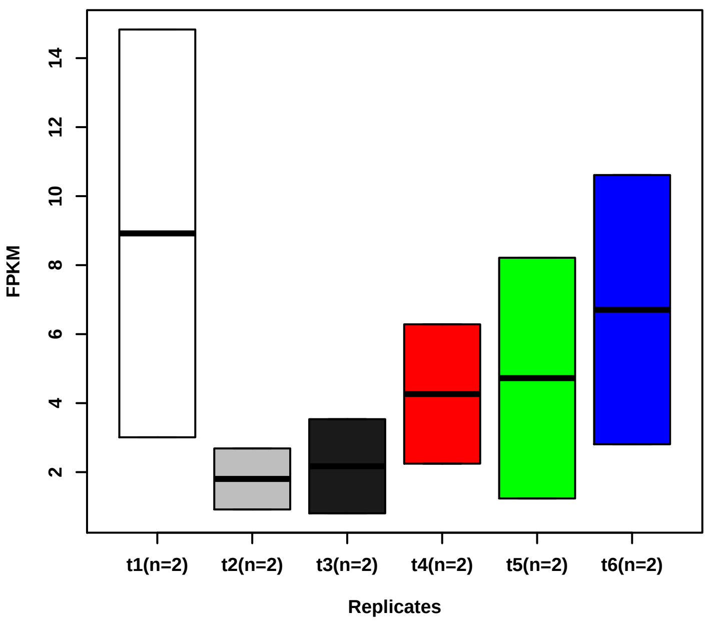

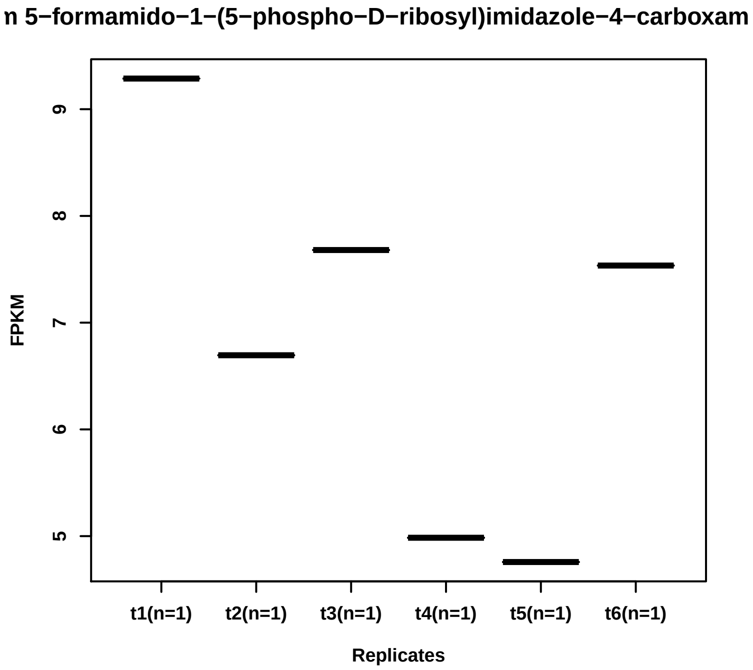
n 5−formamido−1−(5−phospho−D−ribosyl)imidazole−4−carboxam
FPKM
5
6
7
8
9
t1(n=1)
t2(n=1)
t3(n=1)
t4(n=1)
t5(n=1)
t6(n=1)
Replicates

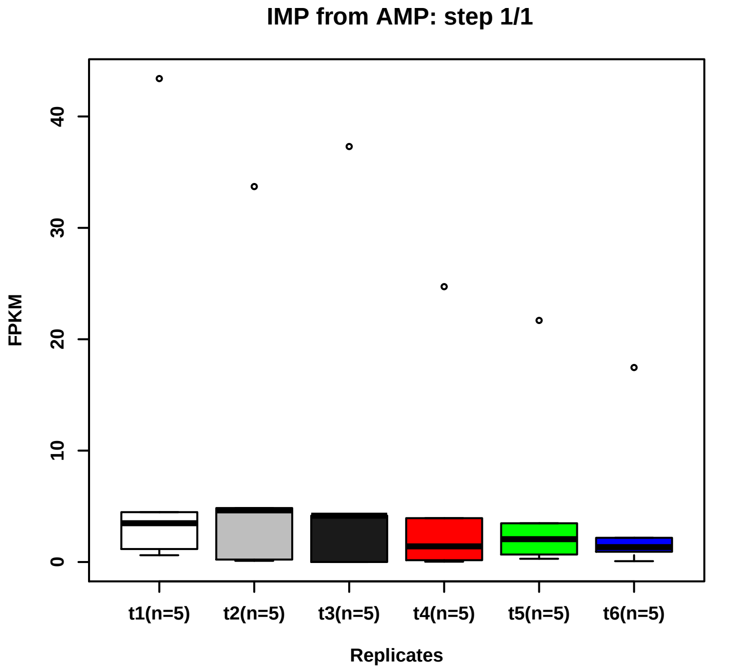
IMP from AMP: step 1/1
FPKM
0
10
20
30
40
t1(n=5)
t2(n=5)
t3(n=5)
t4(n=5)
t5(n=5)
t6(n=5)
Replicates

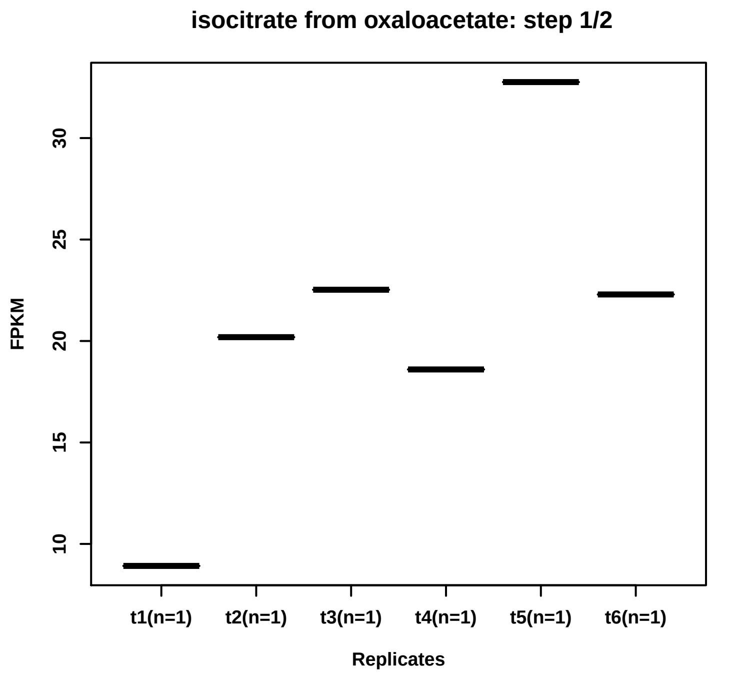
isocitrate from oxaloacetate: step 1/2
FPKM
10
15
20
25
30
t1(n=1)
t2(n=1)
t3(n=1)
t4(n=1)
t5(n=1)
t6(n=1)
Replicates

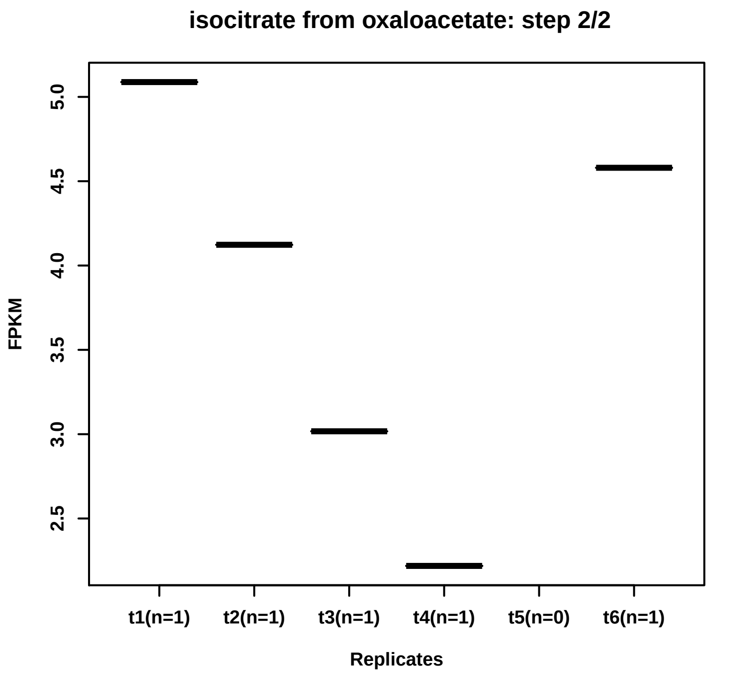
isocitrate from oxaloacetate: step 2/2
FPKM
2.5
3.0
3.5
4.0
4.5
5.0
t1(n=1)
t2(n=1)
t3(n=1)
t4(n=1)
t5(n=0)
t6(n=1)
Replicates

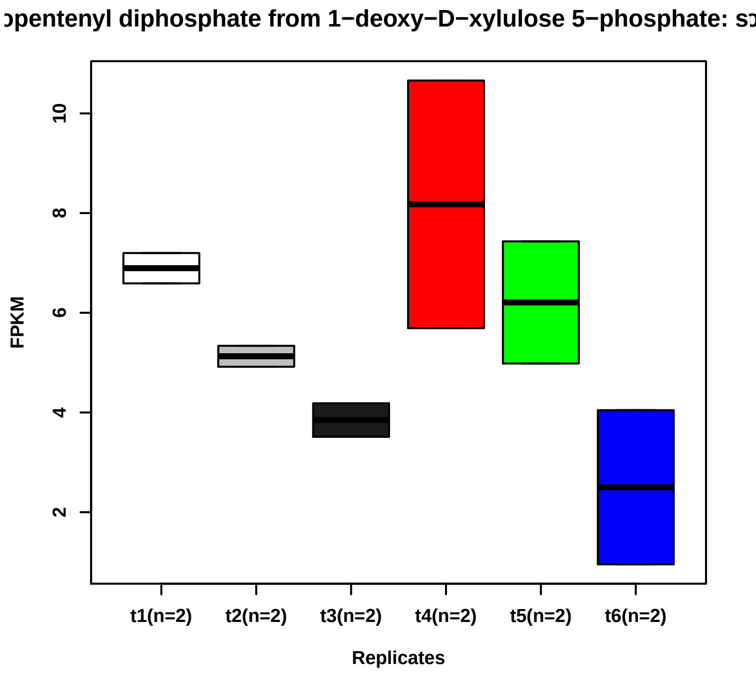
ɔpentenyl diphosphate from 1−deoxy−D−xylulose 5−phosphate: s
FPKM
2
4
6
8
10
t1(n=2)
t2(n=2)
t3(n=2)
t4(n=2)
t5(n=2)
t6(n=2)
Replicates

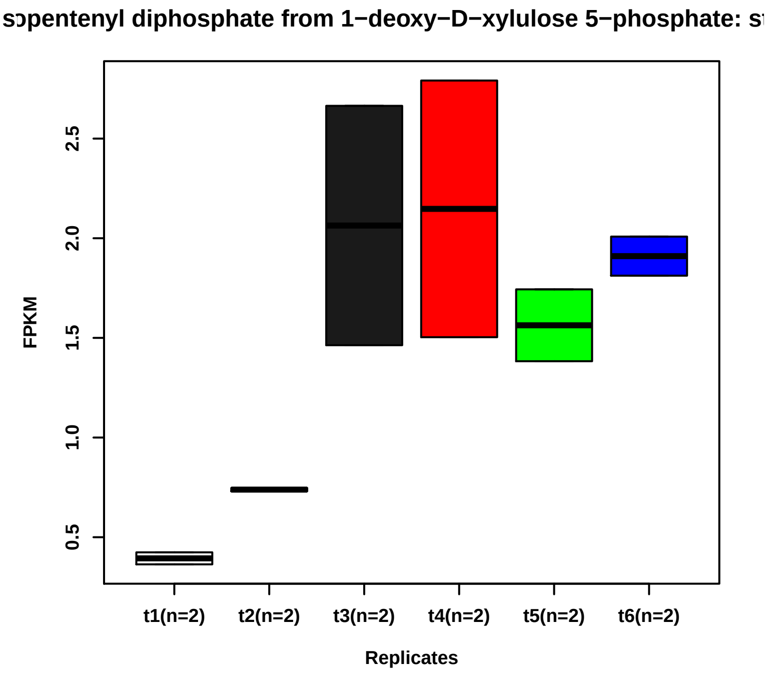
ɔpentenyl diphosphate from 1−deoxy−D−xylulose 5−phosphate: s
FPKM
0.5
1.0
1.5
2.0
2.5
t1(n=2)
t2(n=2)
t3(n=2)
t4(n=2)
t5(n=2)
t6(n=2)
Replicates

pentenyl diphosphate from 1−deoxy−D−xylulose 5−phosphate: s

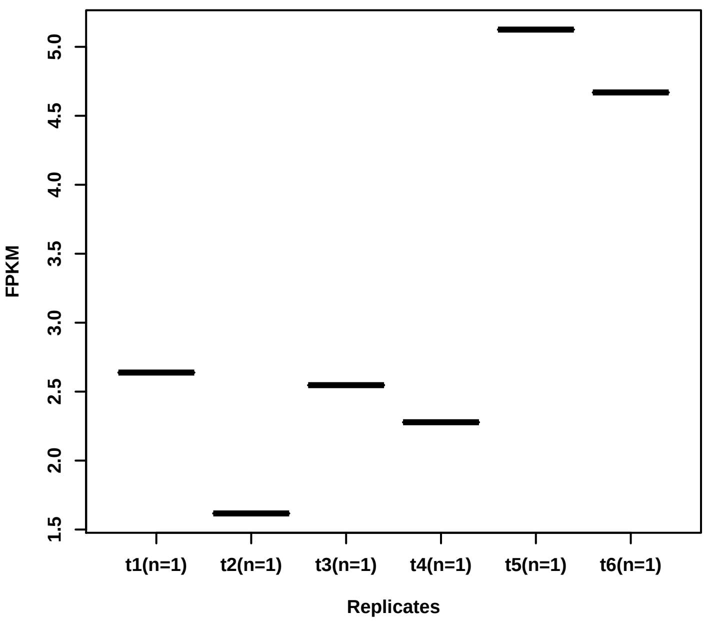

pentenyl diphosphate from 1−deoxy−D−xylulose 5−phosphate: s

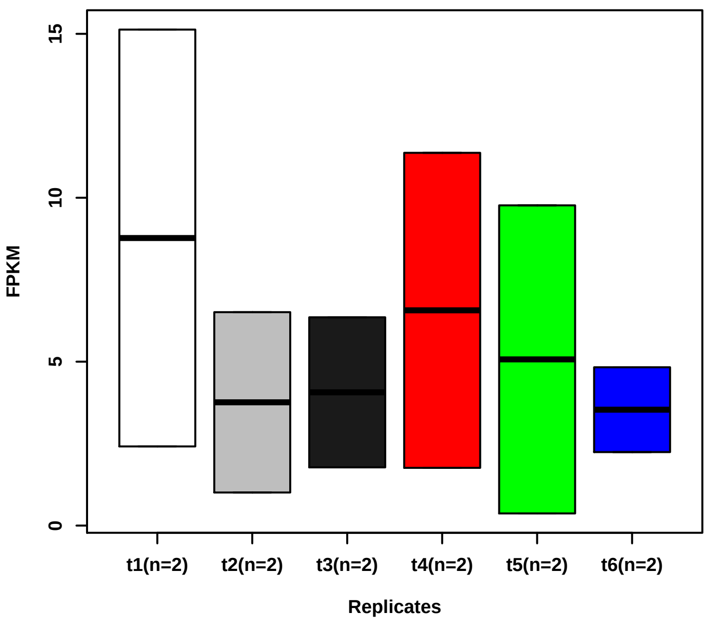

pentenyl diphosphate from 1−deoxy−D−xylulose 5−phosphate: s

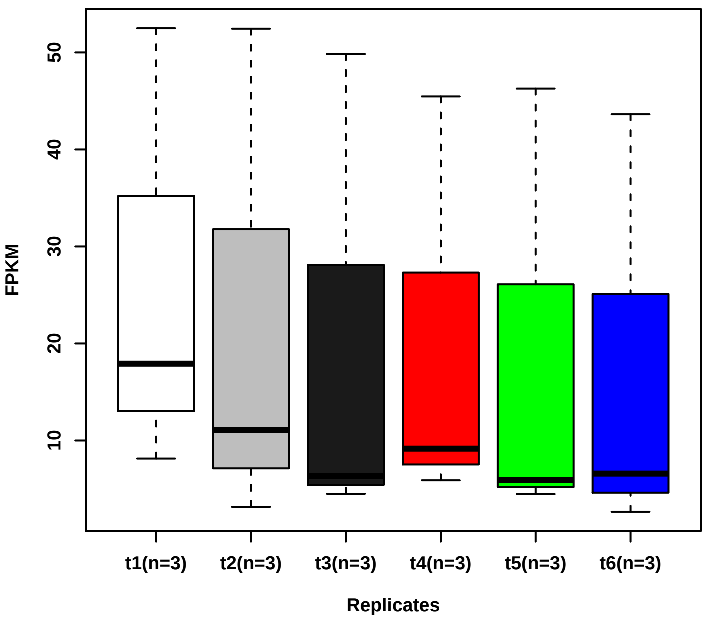

isopentenyl diphosphate from (R)−mevalonate: step 1/3

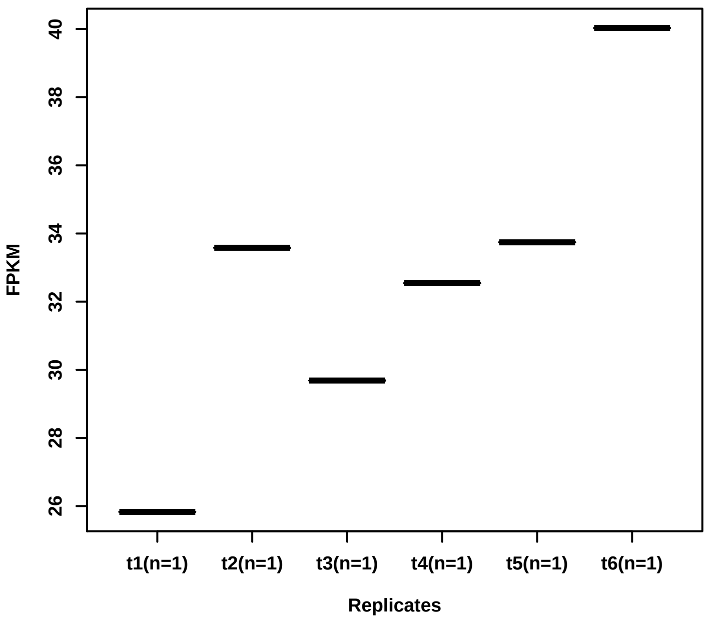

isopentenyl diphosphate from (R)−mevalonate: step 2/3

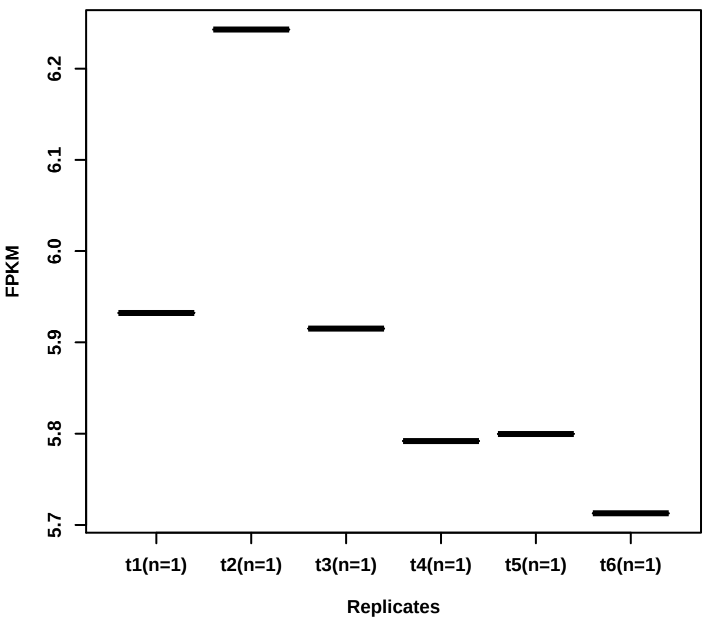

isopentenyl diphosphate from (R)−mevalonate: step 3/3

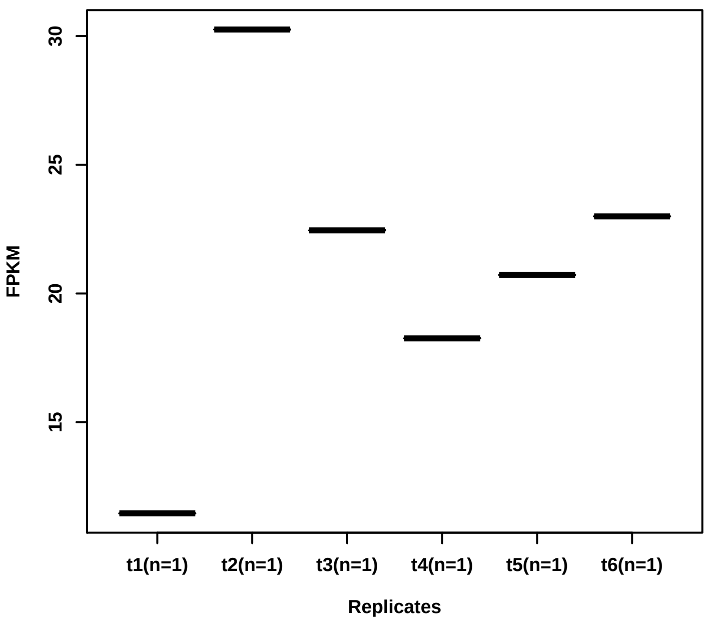

kynurenate from L−kynurenine: step 1/2

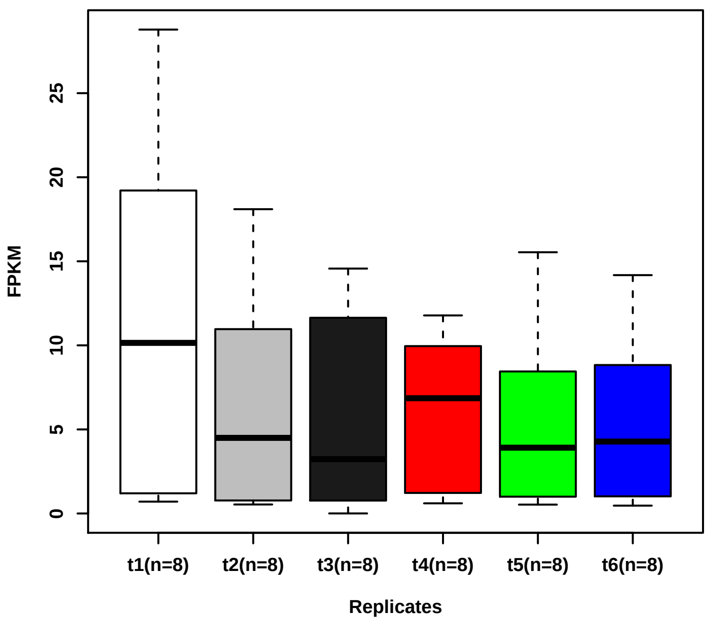

lanosterol from farnesyl diphosphate: step 1/3

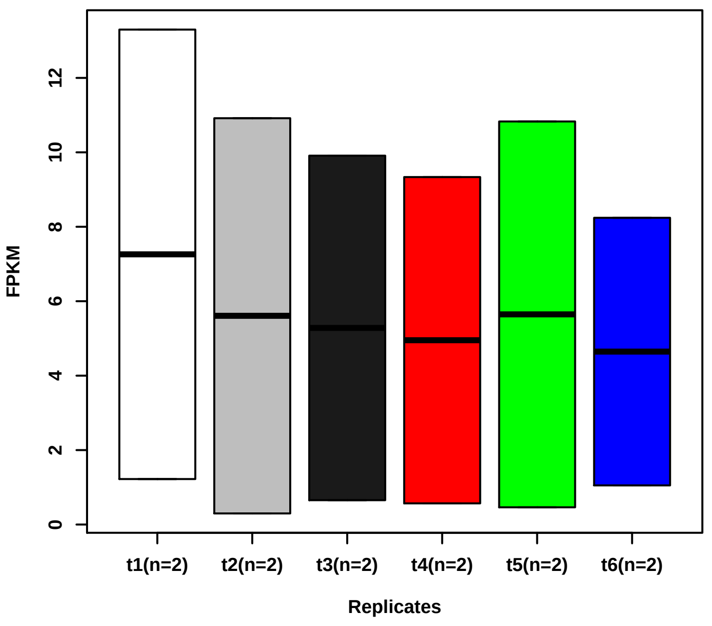

lanosterol from farnesyl diphosphate: step 3/3

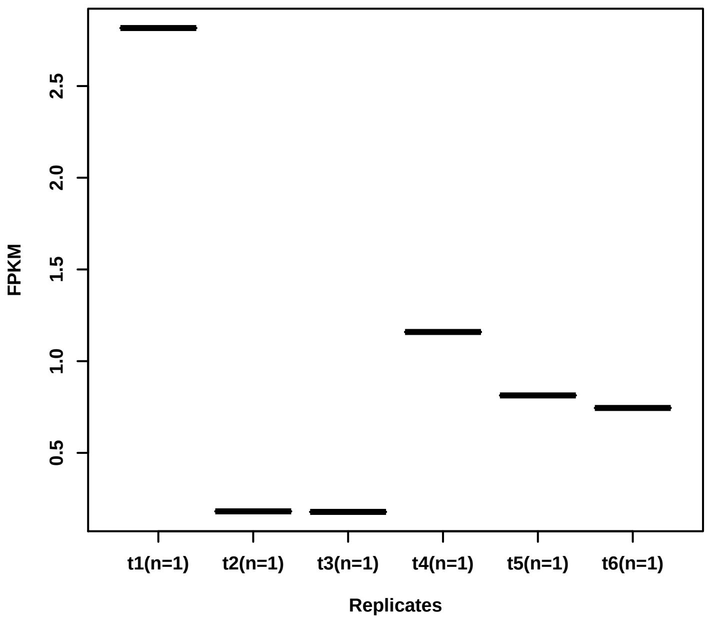

L−arginine from L−ornithine and carbamoyl phosphate: step 2/

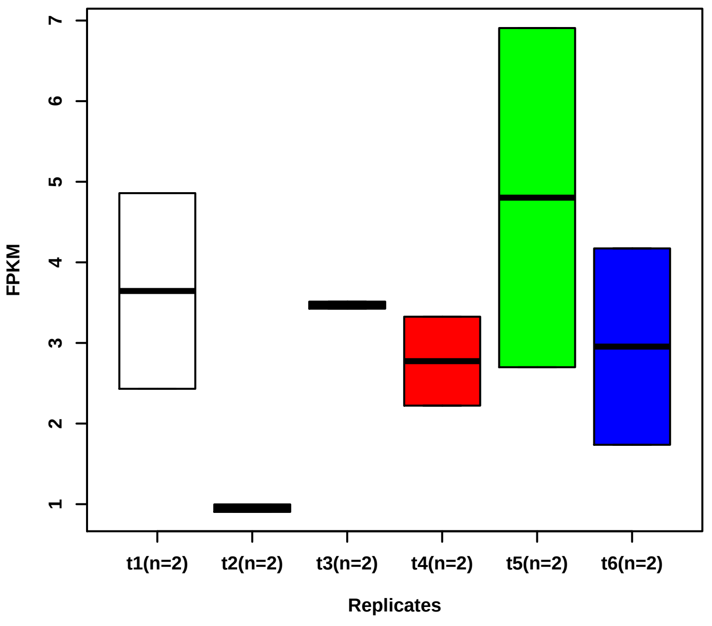

L−arginine from L−ornithine and carbamoyl phosphate: step 3/

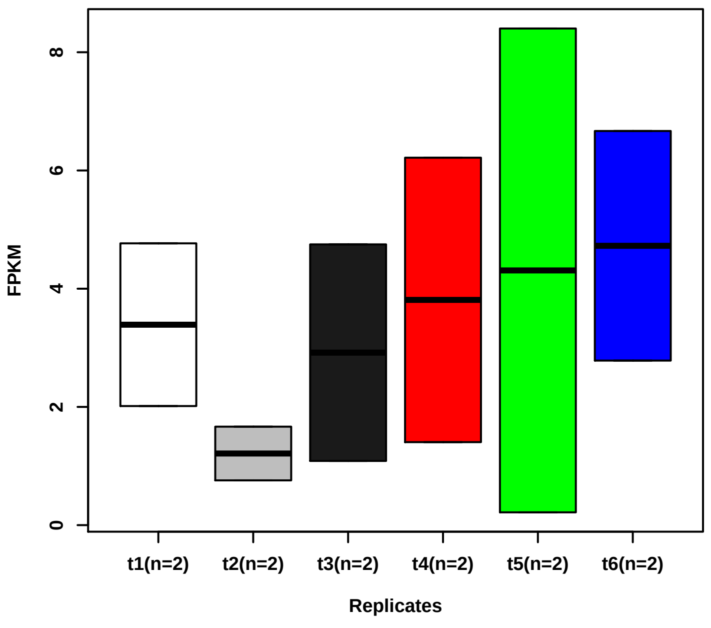

L−ascorbate from GDP−alpha−D−mannose: step 1/5

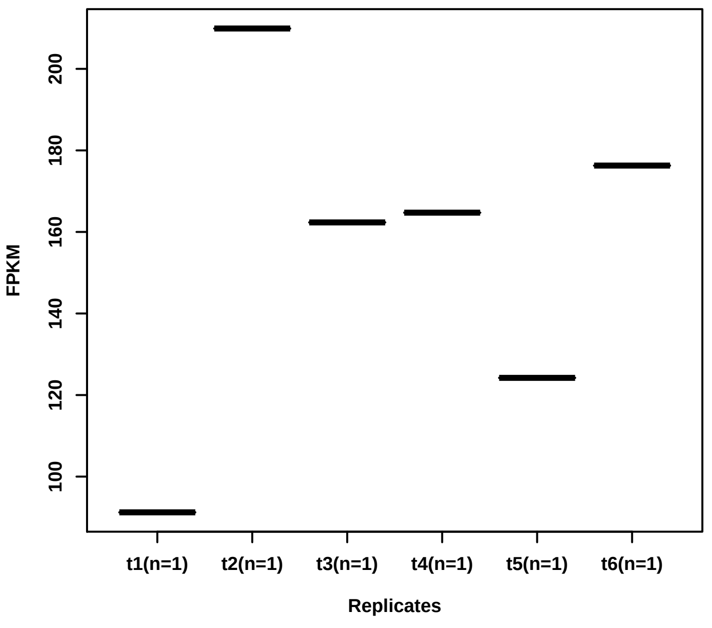

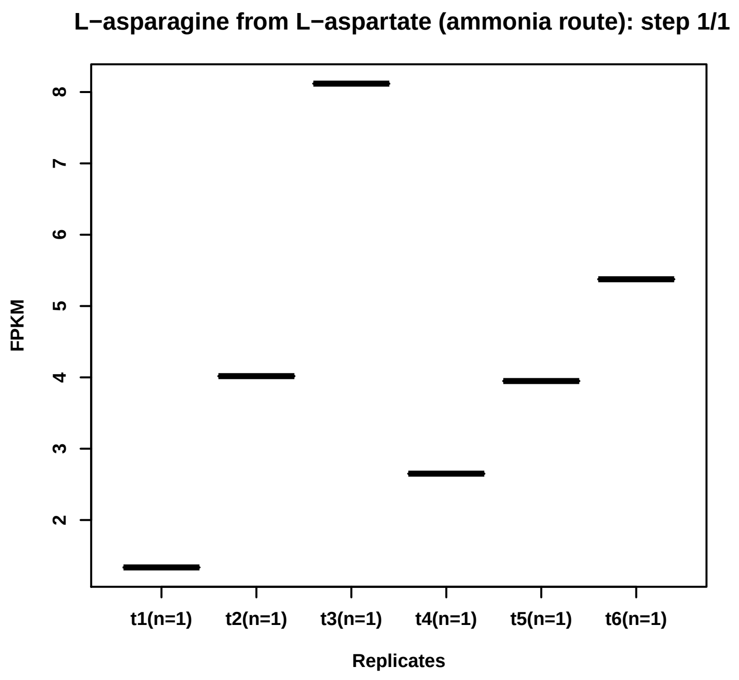
L−asparagine from L−aspartate (ammonia route): step 1/1
FPKM
2
3
4
5
6
7
8
t1(n=1)
t2(n=1)
t3(n=1)
t4(n=1)
t5(n=1)
t6(n=1)
Replicates

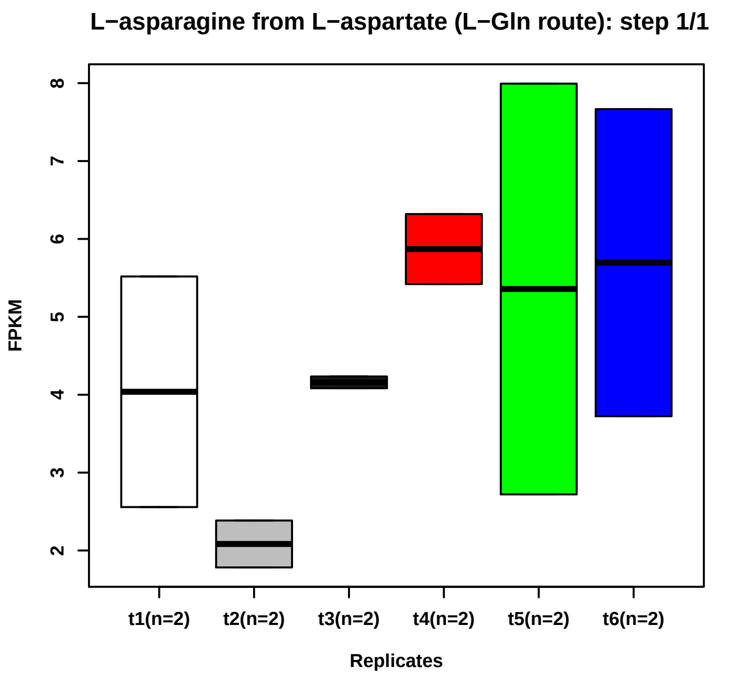
L−asparagine from L−aspartate (L−Gln route): step 1/1
FPKM
2
3
4
5
6
7
8
t1(n=2)
t2(n=2)
t3(n=2)
t4(n=2)
t5(n=2)
t6(n=2)
Replicates

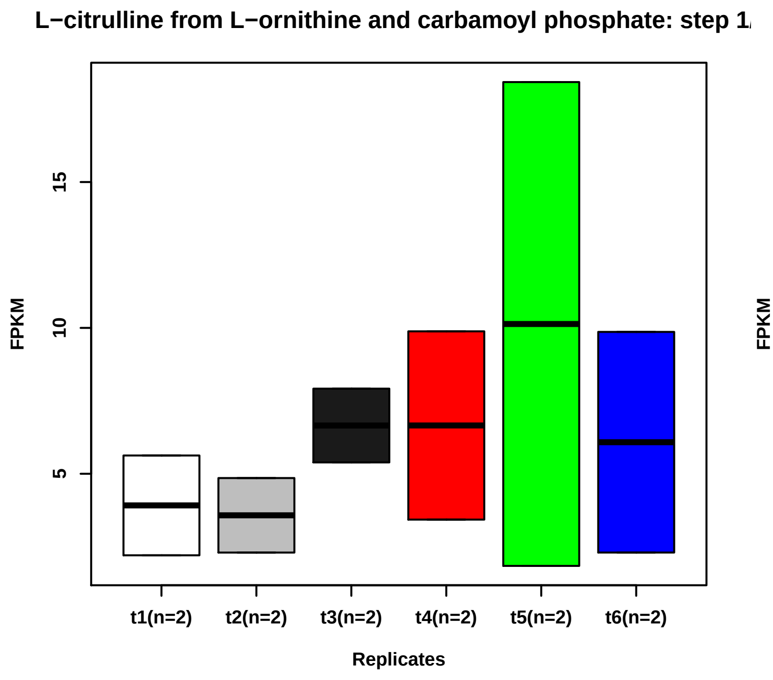
L−citrulline from L−ornithine and carbamoyl phosphate: step 1/
FPKM
5
10
15
t1(n=2)
t2(n=2)
t3(n=2)
t4(n=2)
t5(n=2)
t6(n=2)
Replicates

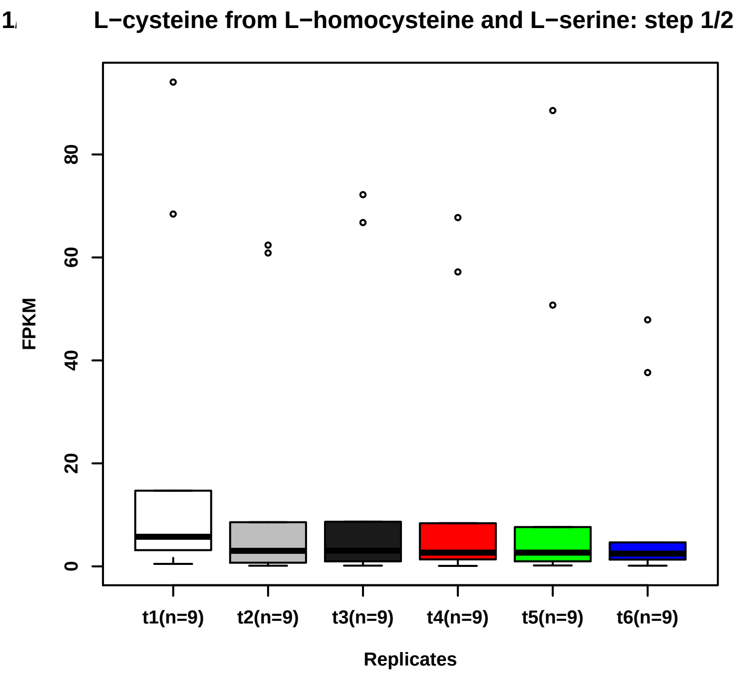
L−cysteine from L−homocysteine and L−serine: step 1/2
FPKM
0
20
40
60
80
t1(n=9)
t2(n=9)
t3(n=9)
t4(n=9)
t5(n=9)
t6(n=9)
Replicates

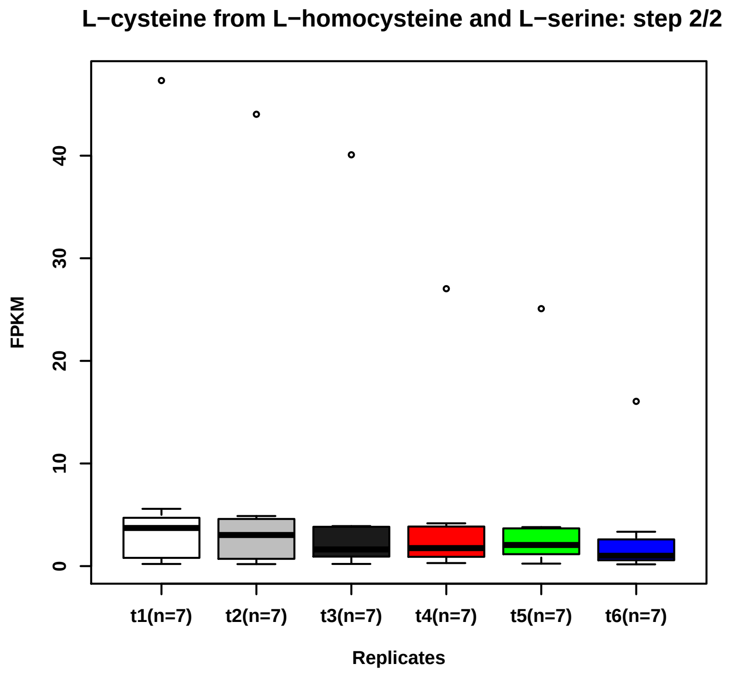
L−cysteine from L−homocysteine and L−serine: step 2/2
FPKM
0
10
20
30
40
t1(n=7)
t2(n=7)
t3(n=7)
t4(n=7)
t5(n=7)
t6(n=7)
Replicates

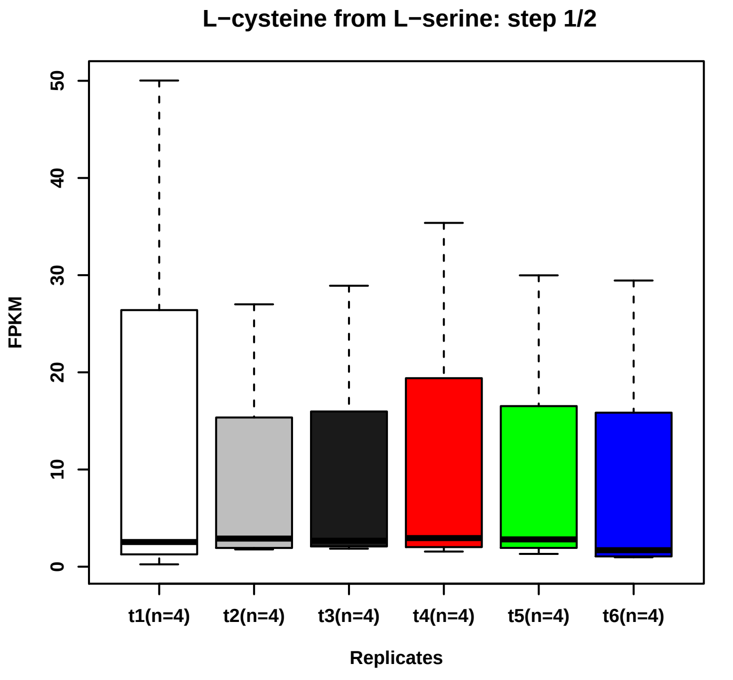
L−cysteine from L−serine: step 1/2
FPKM
0
10
20
30
40
50
t1(n=4)
t2(n=4)
t3(n=4)
t4(n=4)
t5(n=4)
t6(n=4)
Replicates

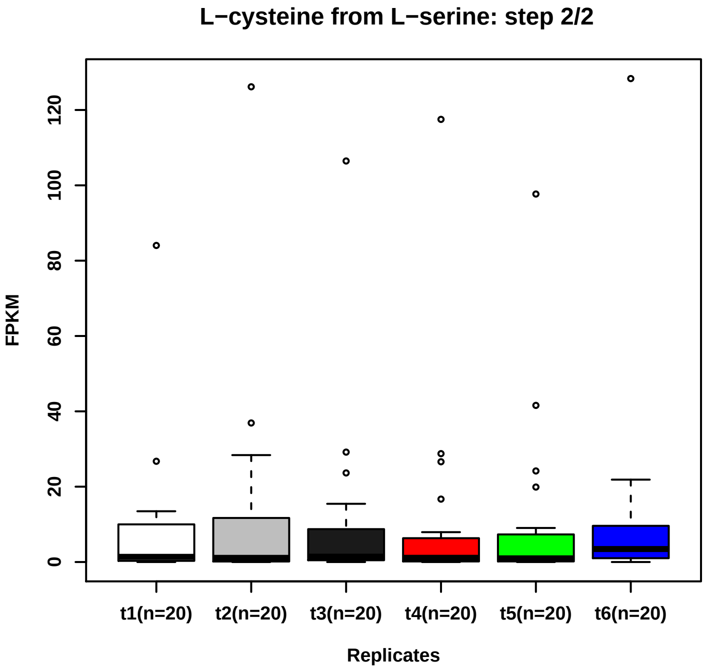
L−cysteine from L−serine: step 2/2
FPKM
0
20
40
60
80
100
120
t1(n=20)
t2(n=20)
t3(n=20)
t4(n=20)
t5(n=20)
t6(n=20)
Replicates

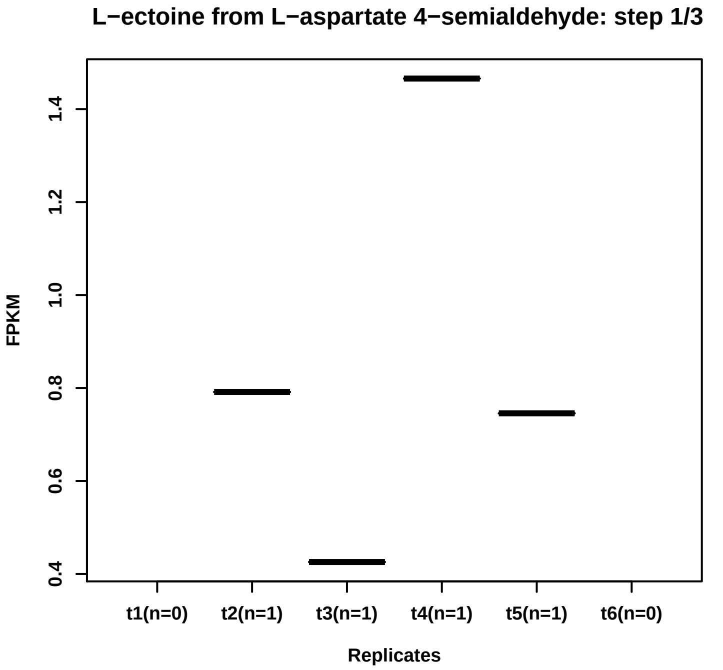
L−ectoine from L−aspartate 4−semialdehyde: step 1/3
FPKM
0.4
0.6
0.8
1.0
1.2
1.4
t1(n=0)
t2(n=1)
t3(n=1)
t4(n=1)
t5(n=1)
t6(n=0)
Replicates

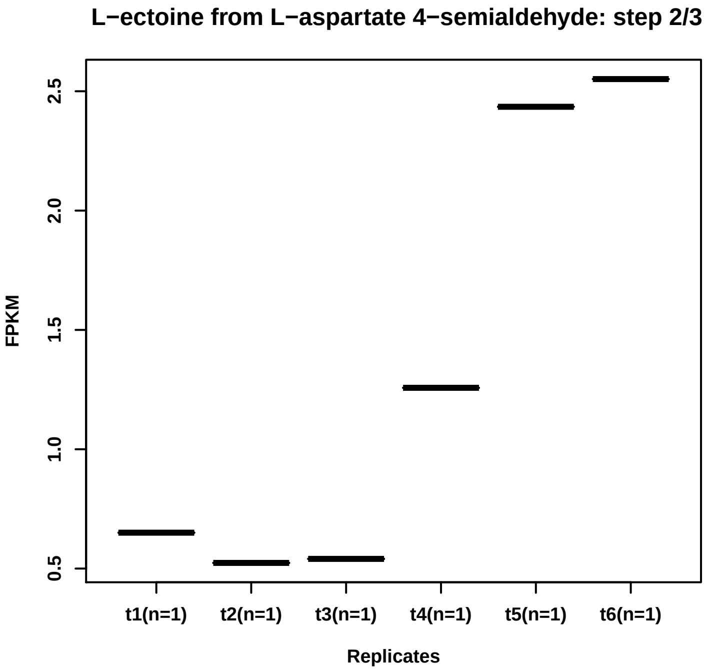
L−ectoine from L−aspartate 4−semialdehyde: step 2/3
FPKM
0.5
1.0
1.5
2.0
2.5
t1(n=1)
t2(n=1)
t3(n=1)
t4(n=1)
t5(n=1)
t6(n=1)
Replicates

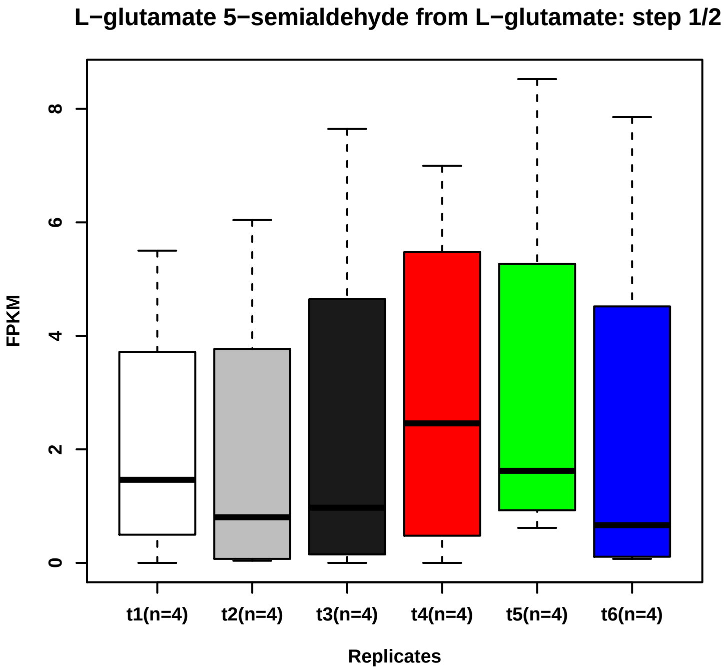
L−glutamate 5−semialdehyde from L−glutamate: step 1/2
FPKM
0
2
4
6
8
t1(n=4)
t2(n=4)
t3(n=4)
t4(n=4)
t5(n=4)
t6(n=4)
Replicates

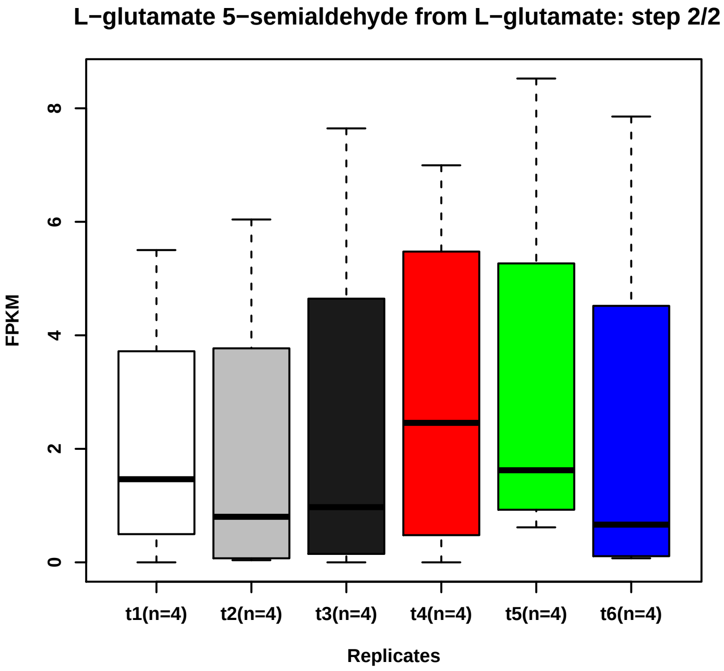
L−glutamate 5−semialdehyde from L−glutamate: step 2/2
FPKM
0
2
4
6
8
t1(n=4)
t2(n=4)
t3(n=4)
t4(n=4)
t5(n=4)
t6(n=4)
Replicates

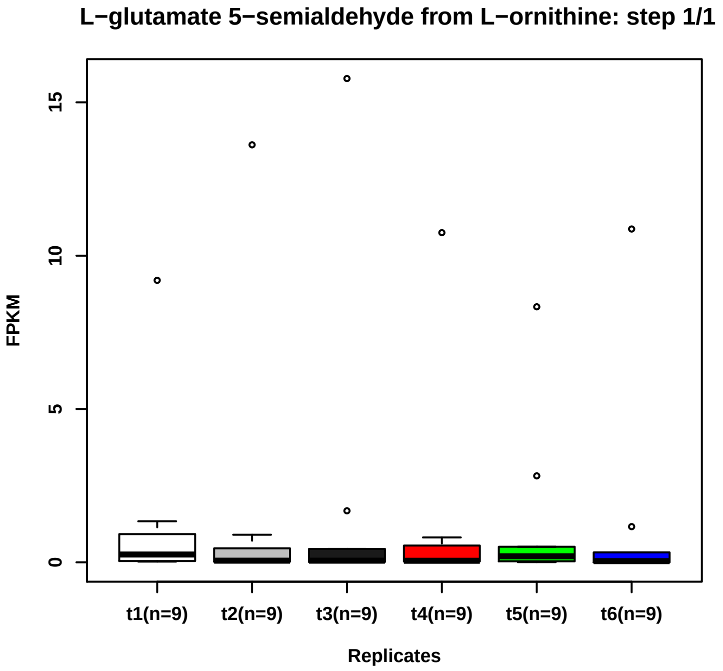
L−glutamate 5−semialdehyde from L−ornithine: step 1/1
FPKM
0
5
10
15
t1(n=9)
t2(n=9)
t3(n=9)
t4(n=9)
t5(n=9)
t6(n=9)
Replicates

lutamate from 2−oxoglutarate and L−glutamine (ferredoxin route):

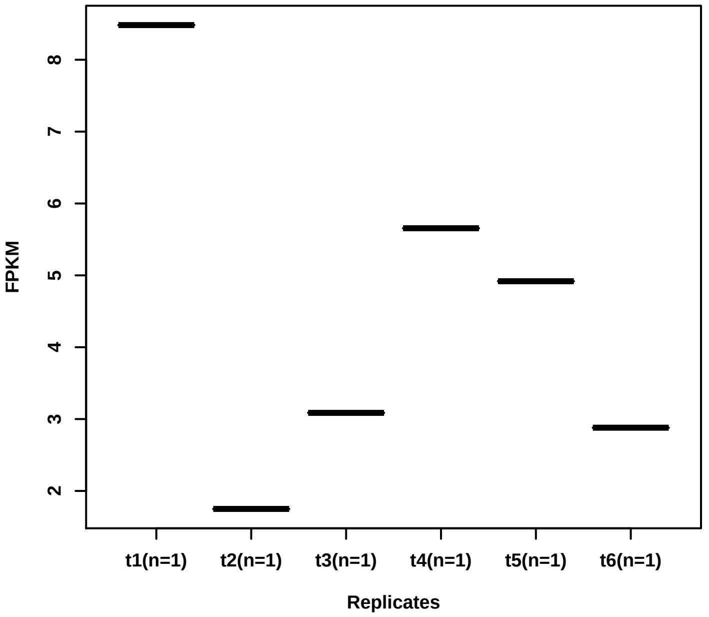

glutamate from 2−oxoglutarate and L−glutamine (NAD(+) route): s

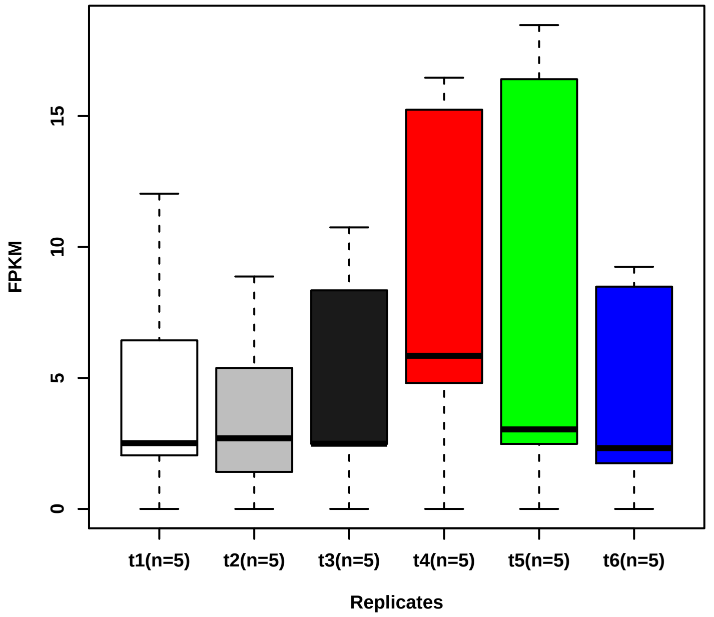

L−glutamate from L−proline: step 1/2

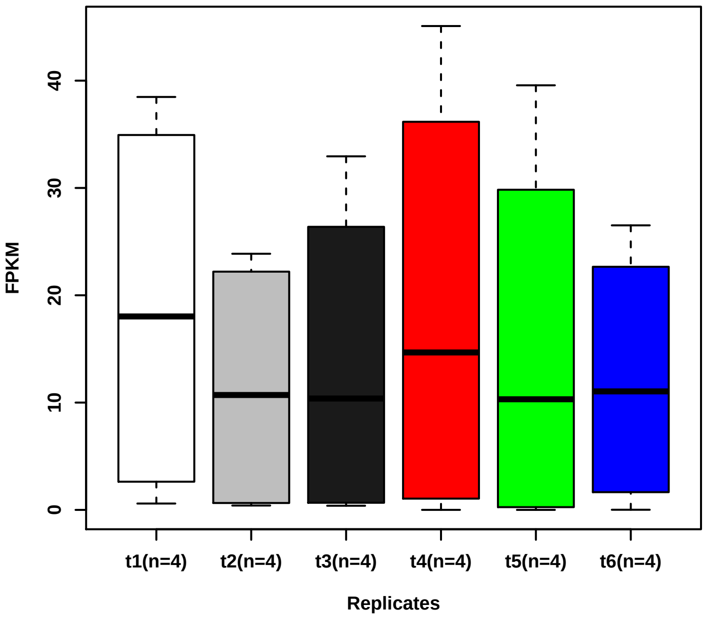

L−histidine from 5−phospho−alpha−D−ribose 1−diphosphate: step

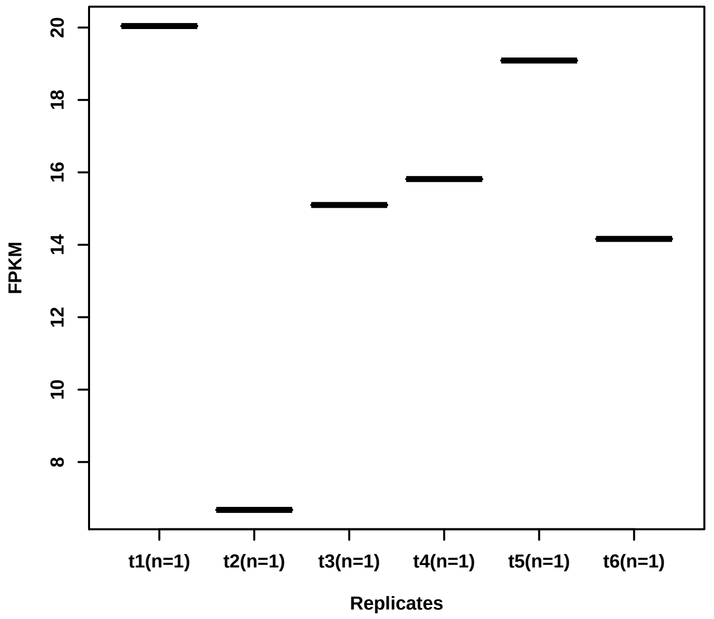

L−histidine from 5−phospho−alpha−D−ribose 1−diphosphate: step

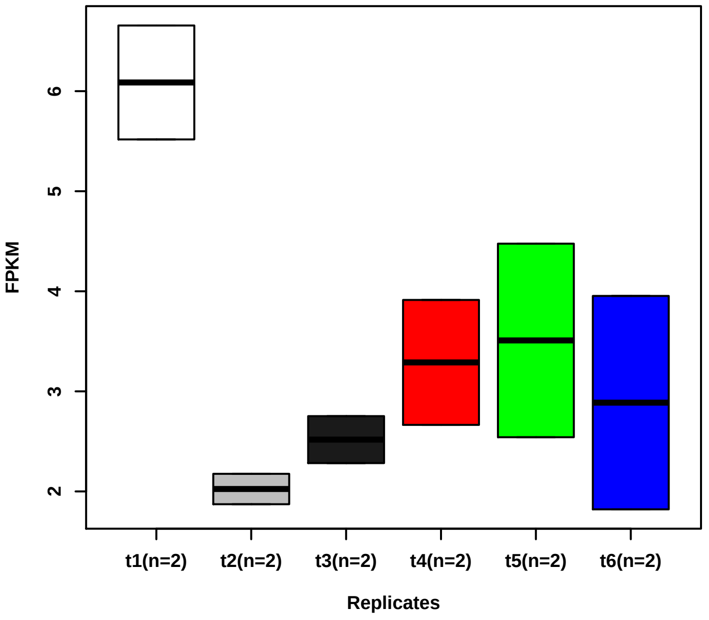

L−histidine from 5−phospho−alpha−D−ribose 1−diphosphate: step

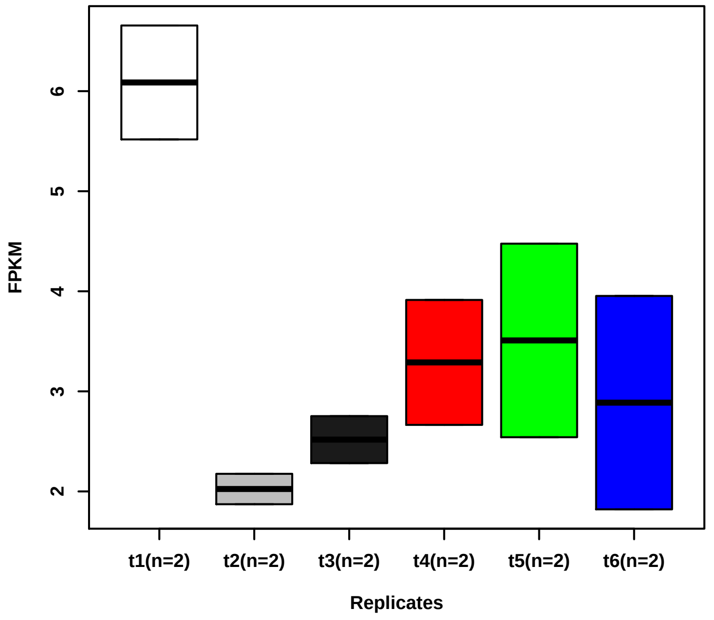

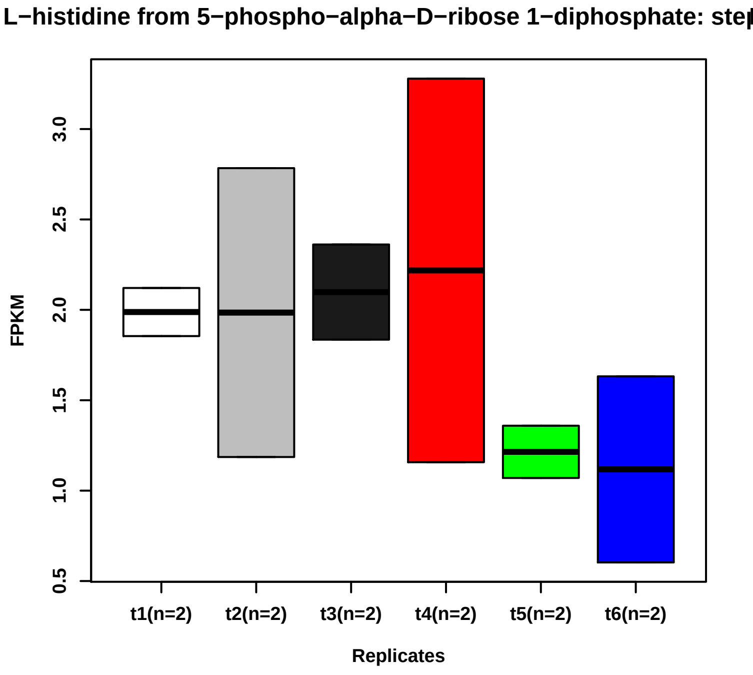
L−histidine from 5−phospho−alpha−D−ribose 1−diphosphate: step
FPKM
Replicates
t1(n=2) t2(n=2) t3(n=2) t4(n=2) t5(n=2) t6(n=2)

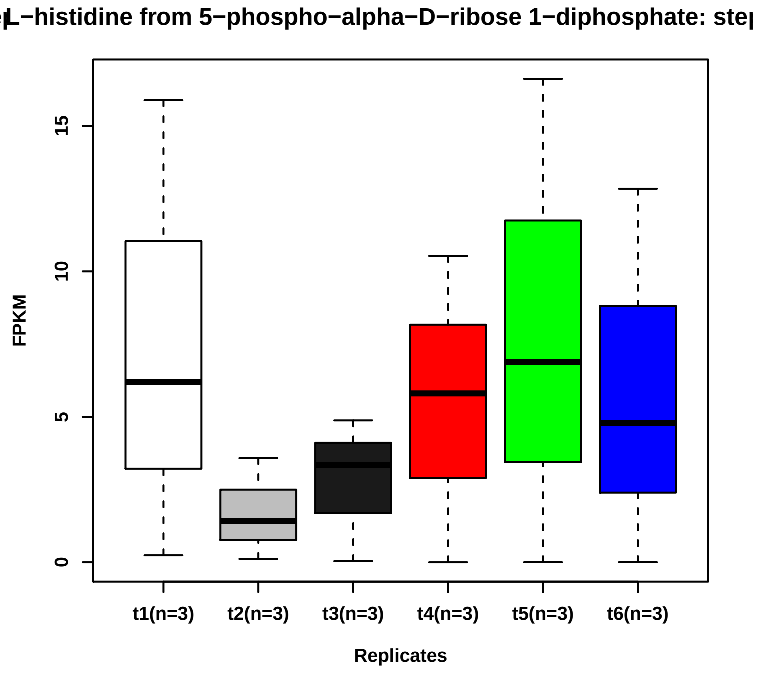
L−histidine from 5−phospho−alpha−D−ribose 1−diphosphate: step
FPKM
Replicates
t1(n=3) t2(n=3) t3(n=3) t4(n=3) t5(n=3) t6(n=3)

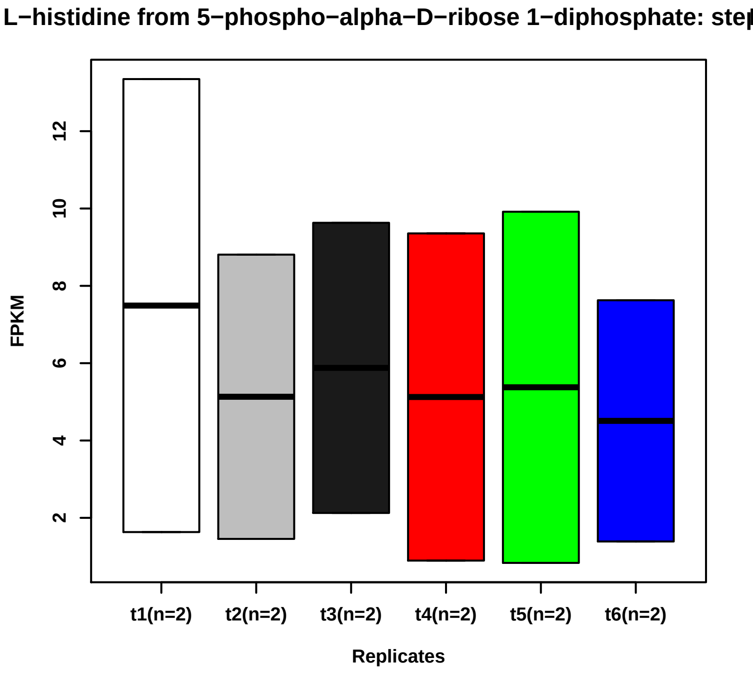
L−histidine from 5−phospho−alpha−D−ribose 1−diphosphate: step
FPKM
Replicates
t1(n=2) t2(n=2) t3(n=2) t4(n=2) t5(n=2) t6(n=2)

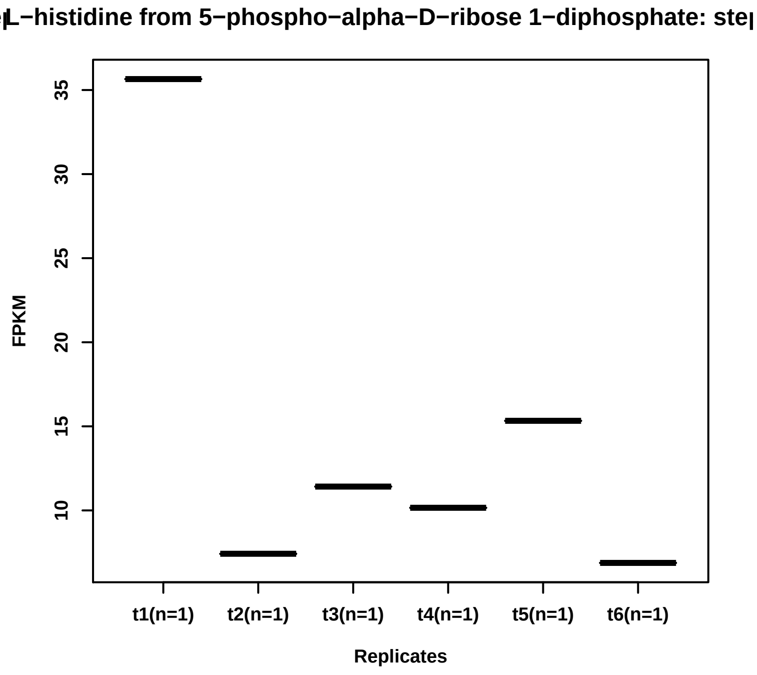
L−histidine from 5−phospho−alpha−D−ribose 1−diphosphate: step
FPKM
Replicates
t1(n=1) t2(n=1) t3(n=1) t4(n=1) t5(n=1) t6(n=1)

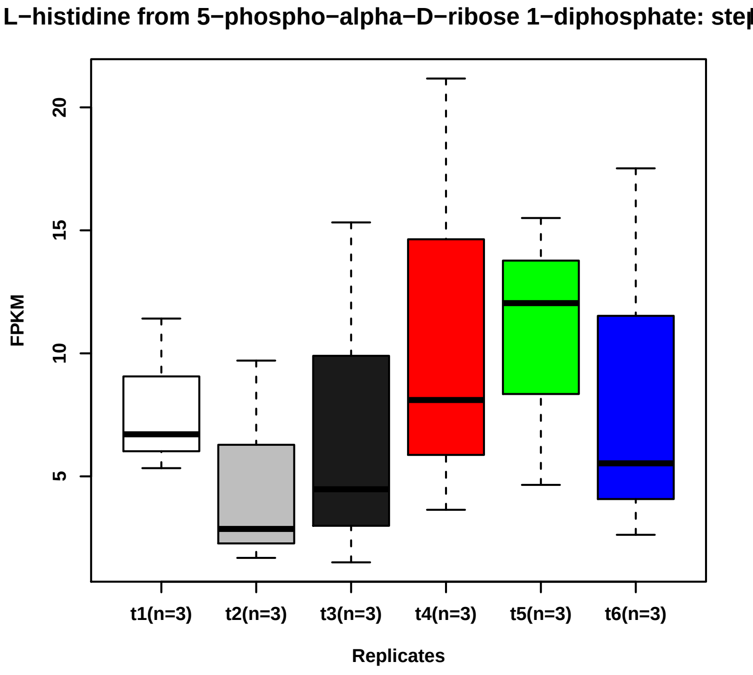
L−histidine from 5−phospho−alpha−D−ribose 1−diphosphate: step
FPKM
Replicates
t1(n=3) t2(n=3) t3(n=3) t4(n=3) t5(n=3) t6(n=3)

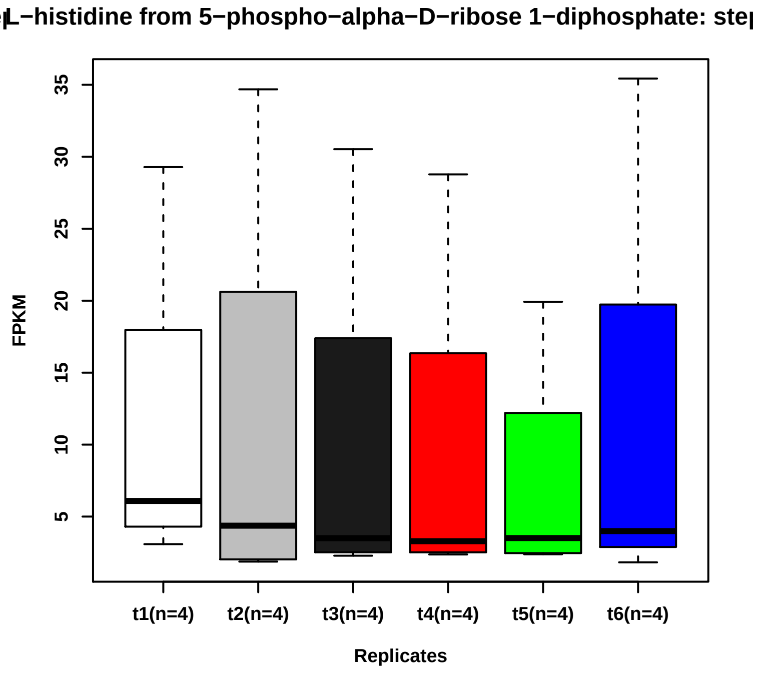
L−histidine from 5−phospho−alpha−D−ribose 1−diphosphate: step
FPKM
Replicates
t1(n=4) t2(n=4) t3(n=4) t4(n=4) t5(n=4) t6(n=4)

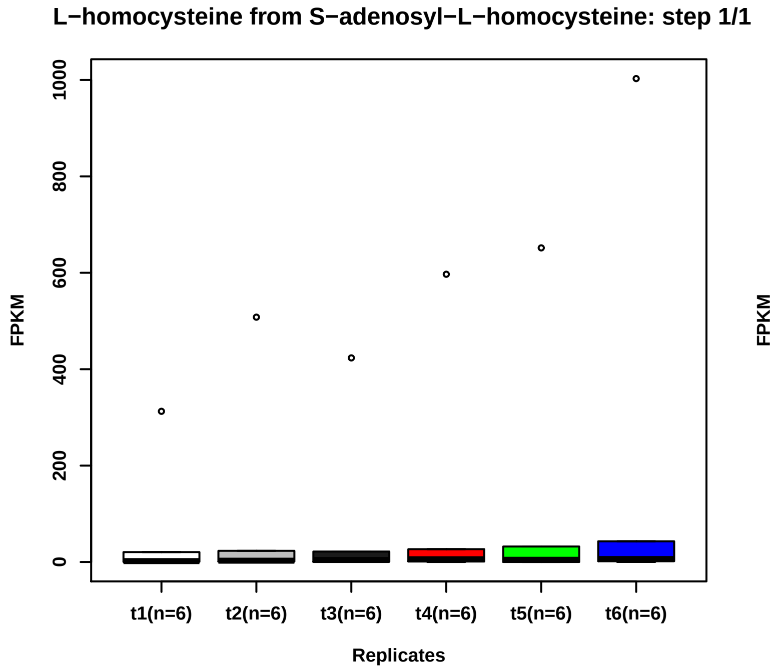
L−homocysteine from S−adenosyl−L−homocysteine: step 1/1
FPKM
0
200
400
600
800
1000
t1(n=6)
t2(n=6)
t3(n=6)
t4(n=6)
t5(n=6)
t6(n=6)
Replicates

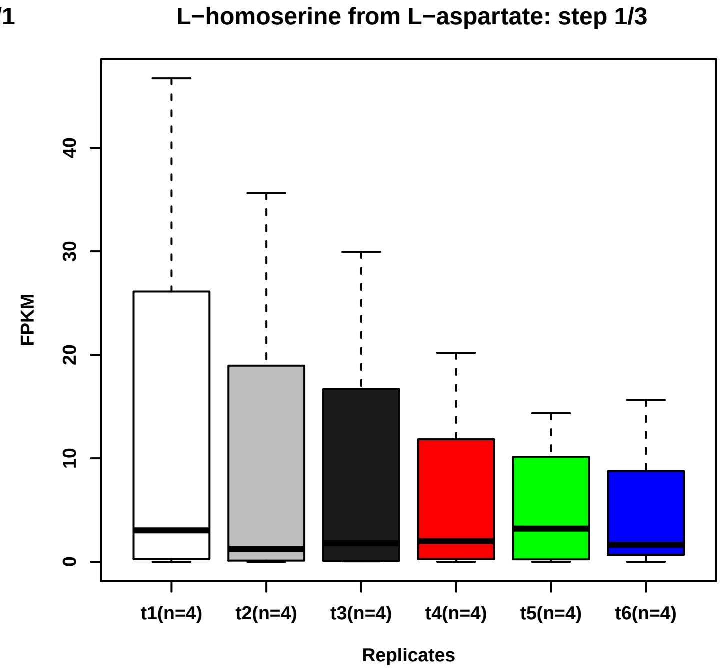
L−homoserine from L−aspartate: step 1/3
FPKM
0
10
20
30
40
t1(n=4)
t2(n=4)
t3(n=4)
t4(n=4)
t5(n=4)
t6(n=4)
Replicates

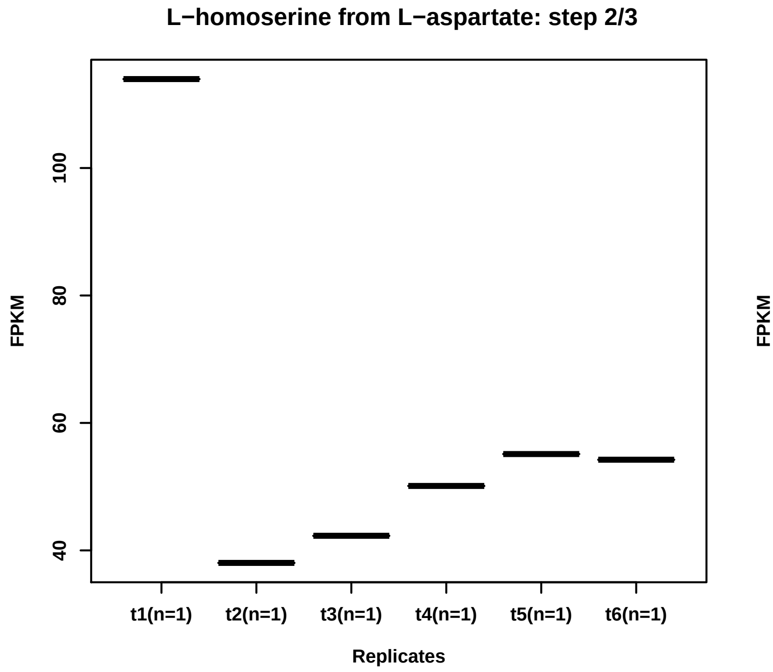
L−homoserine from L−aspartate: step 2/3
FPKM
40
60
80
100
t1(n=1)
t2(n=1)
t3(n=1)
t4(n=1)
t5(n=1)
t6(n=1)
Replicates

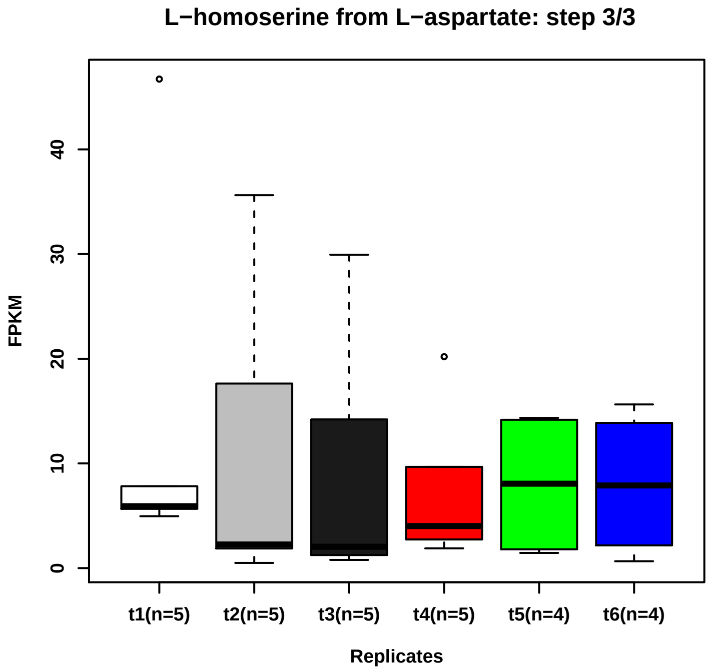
L−homoserine from L−aspartate: step 3/3
FPKM
0
10
20
30
40
t1(n=5)
t2(n=5)
t3(n=5)
t4(n=5)
t5(n=4)
t6(n=4)
Replicates

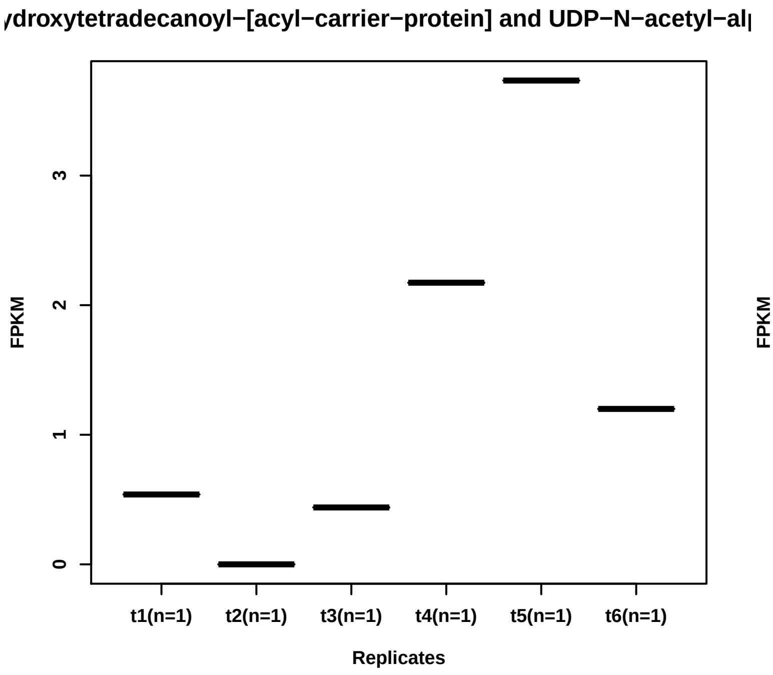
ydroxytetradecanoyl−[acyl−carrier−protein] and UDP−N−acetyl−al
FPKM
0
1
2
3
t1(n=1)
t2(n=1)
t3(n=1)
t4(n=1)
t5(n=1)
t6(n=1)
Replicates

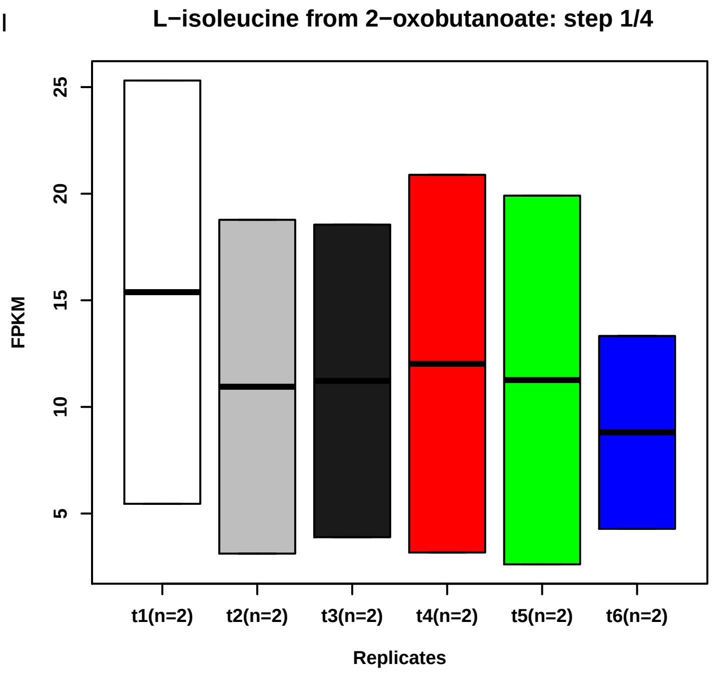
L−isoleucine from 2−oxobutanoate: step 1/4
FPKM
5
10
15
20
25
t1(n=2)
t2(n=2)
t3(n=2)
t4(n=2)
t5(n=2)
t6(n=2)
Replicates

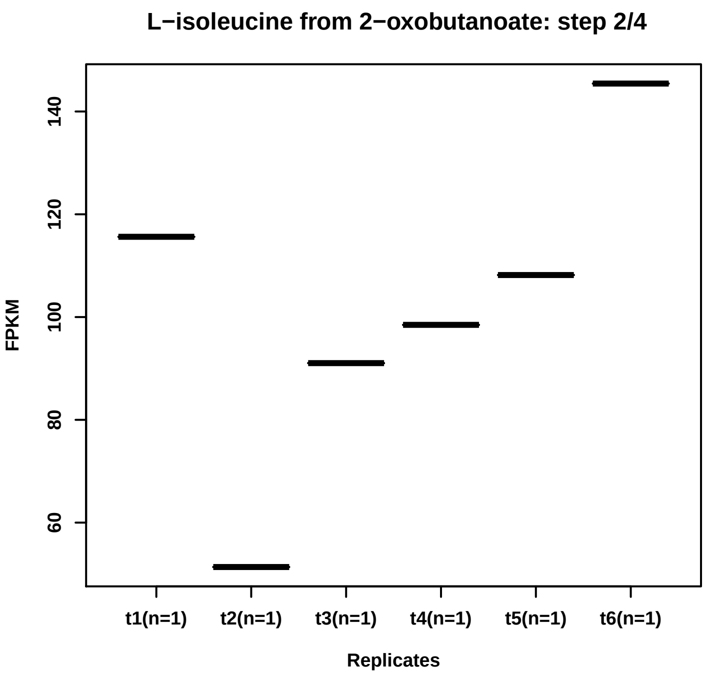
L−isoleucine from 2−oxobutanoate: step 2/4
FPKM
60
80
100
120
140
t1(n=1)
t2(n=1)
t3(n=1)
t4(n=1)
t5(n=1)
t6(n=1)
Replicates

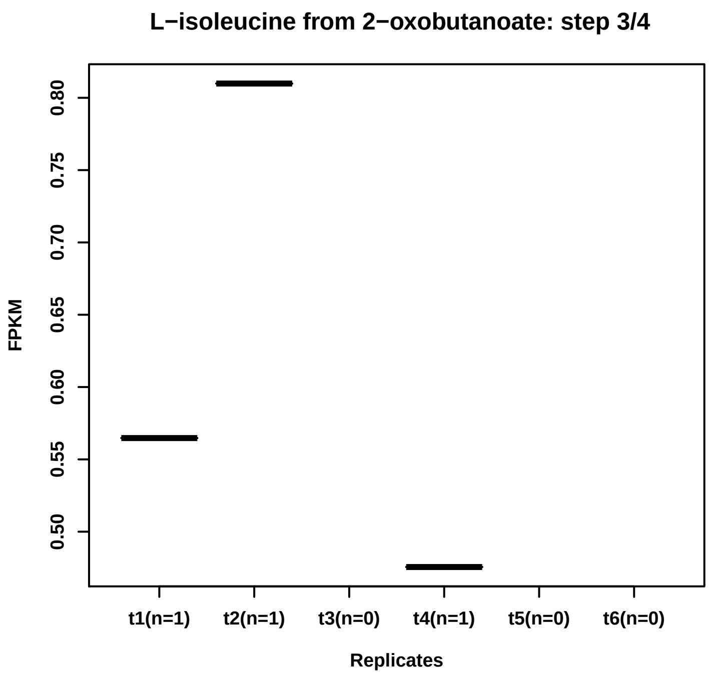
L−isoleucine from 2−oxobutanoate: step 3/4
FPKM
0.50
0.55
0.60
0.65
0.70
0.75
0.80
t1(n=1)
t2(n=1)
t3(n=0)
t4(n=1)
t5(n=0)
t6(n=0)
Replicates

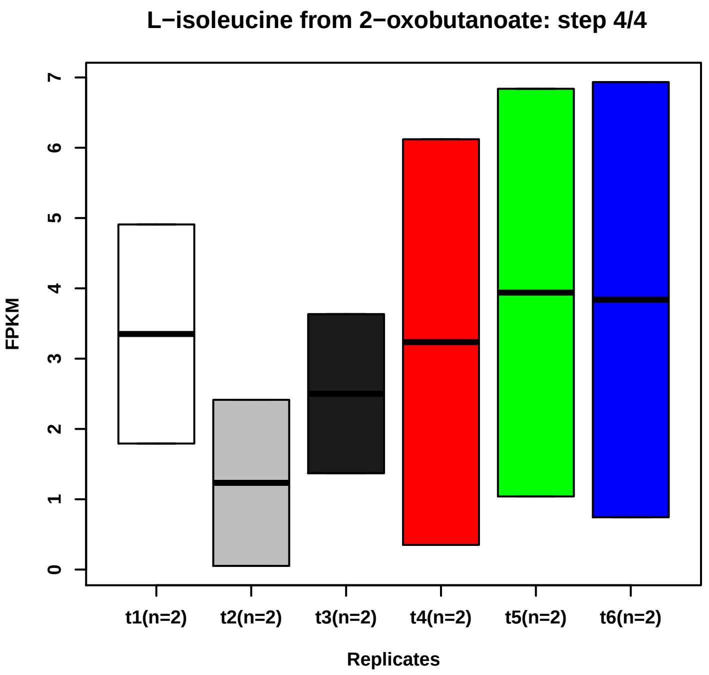
L−isoleucine from 2−oxobutanoate: step 4/4
FPKM
0
1
2
3
4
5
6
7
t1(n=2)
t2(n=2)
t3(n=2)
t4(n=2)
t5(n=2)
t6(n=2)
Replicates

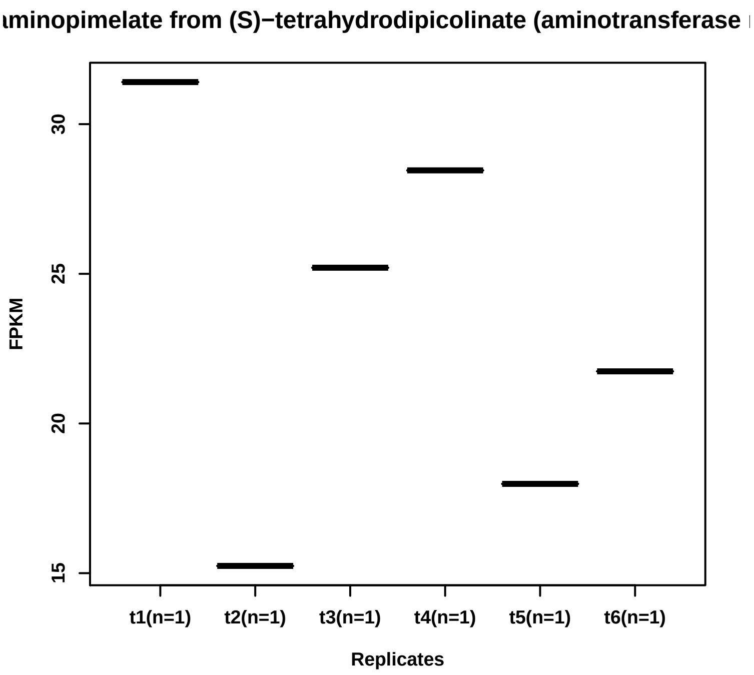
ıminopimelate from (S)−tetrahydrodipicolinate (aminotransferase
FPKM
15
20
25
30
t1(n=1)
t2(n=1)
t3(n=1)
t4(n=1)
t5(n=1)
t6(n=1)
Replicates

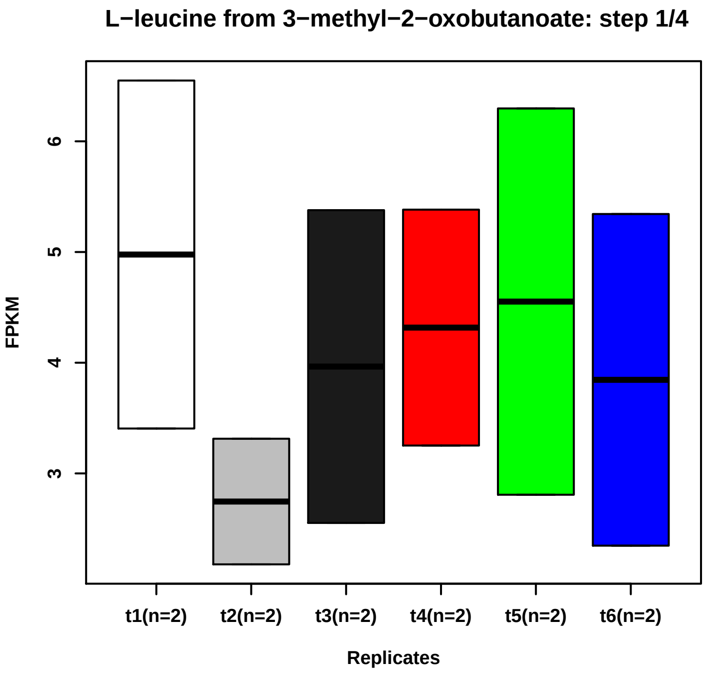
L−leucine from 3−methyl−2−oxobutanoate: step 1/4
FPKM
3
4
5
6
t1(n=2)
t2(n=2)
t3(n=2)
t4(n=2)
t5(n=2)
t6(n=2)
Replicates

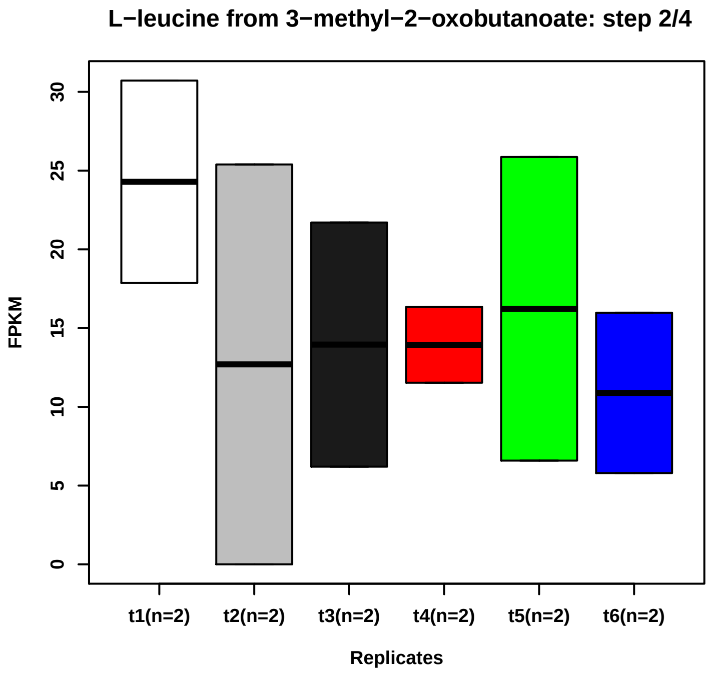
L−leucine from 3−methyl−2−oxobutanoate: step 2/4
FPKM
0
5
10
15
20
25
30
t1(n=2)
t2(n=2)
t3(n=2)
t4(n=2)
t5(n=2)
t6(n=2)
Replicates

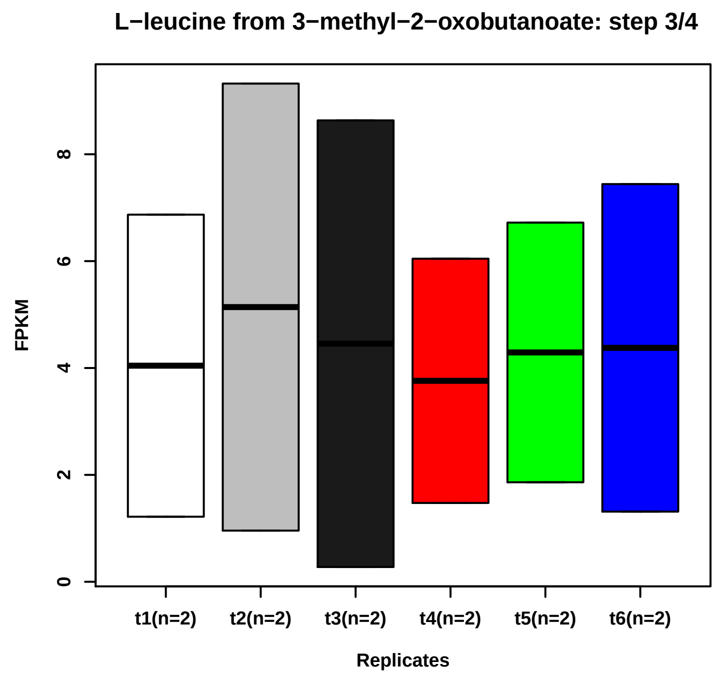
L−leucine from 3−methyl−2−oxobutanoate: step 3/4
FPKM
0
2
4
6
8
t1(n=2)
t2(n=2)
t3(n=2)
t4(n=2)
t5(n=2)
t6(n=2)
Replicates

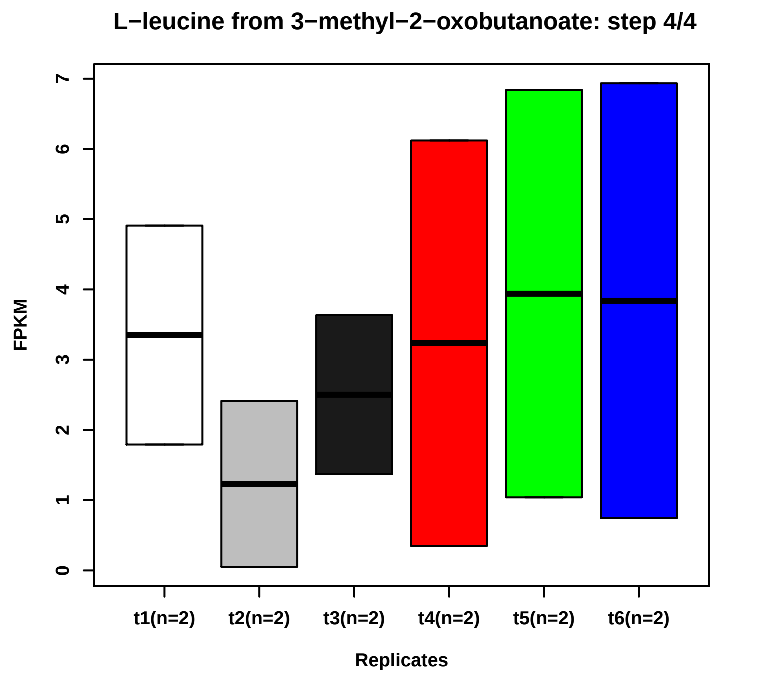
L−leucine from 3−methyl−2−oxobutanoate: step 4/4
FPKM
0
1
2
3
4
5
6
7
t1(n=2)
t2(n=2)
t3(n=2)
t4(n=2)
t5(n=2)
t6(n=2)
Replicates

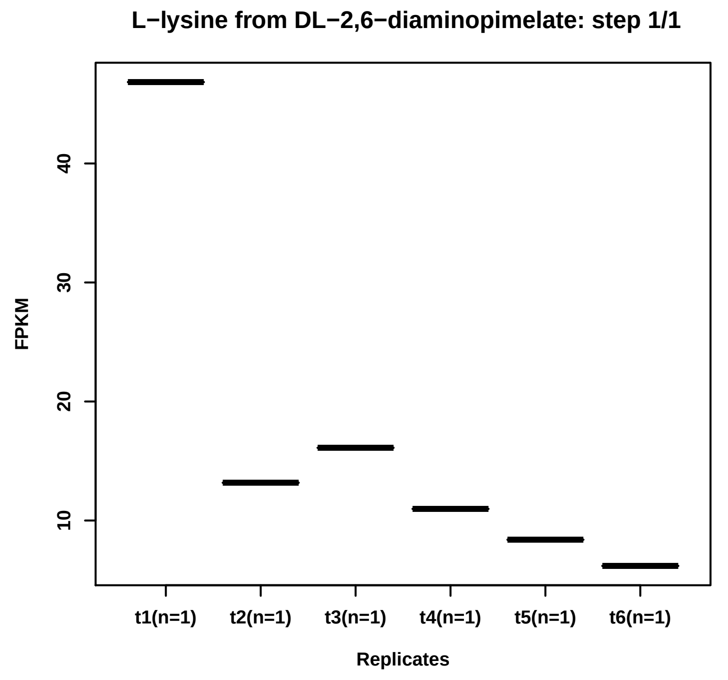
L−lysine from DL−2,6−diaminopimelate: step 1/1
FPKM
10
20
30
40
t1(n=1)
t2(n=1)
t3(n=1)
t4(n=1)
t5(n=1)
t6(n=1)
Replicates

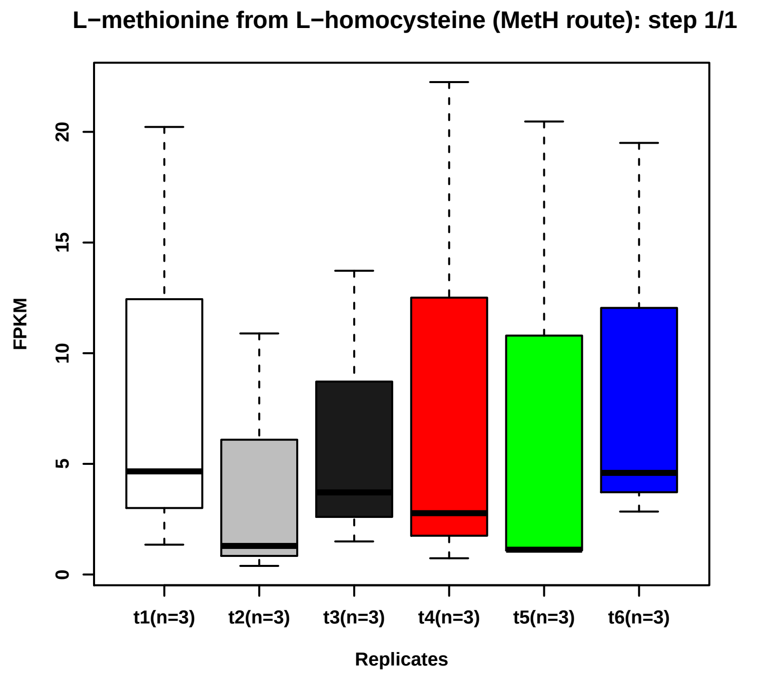
L−methionine from L−homocysteine (MetH route): step 1/1
FPKM
0
5
10
15
20
t1(n=3)
t2(n=3)
t3(n=3)
t4(n=3)
t5(n=3)
t6(n=3)
Replicates

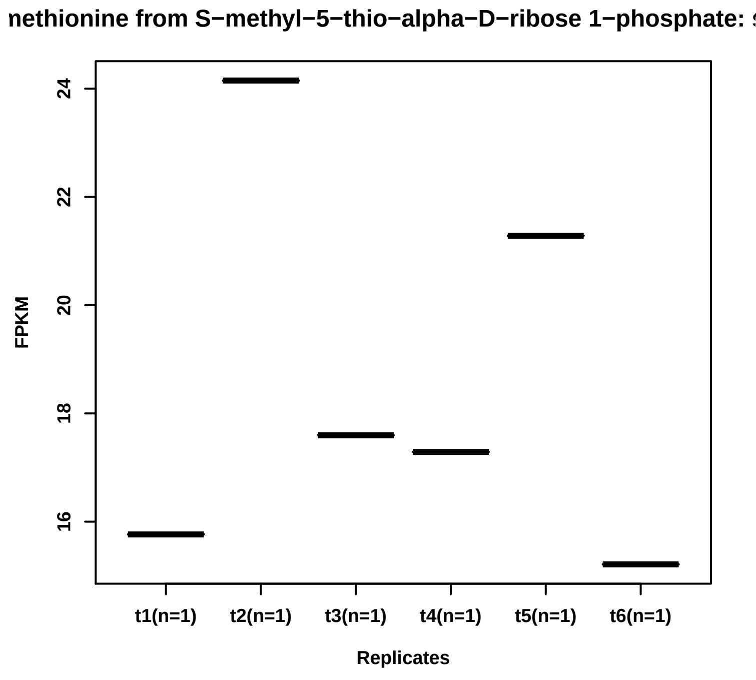
nethionine from S−methyl−5−thio−alpha−D−ribose 1−phosphate:
FPKM
16
18
20
22
24
t1(n=1)
t2(n=1)
t3(n=1)
t4(n=1)
t5(n=1)
t6(n=1)
Replicates

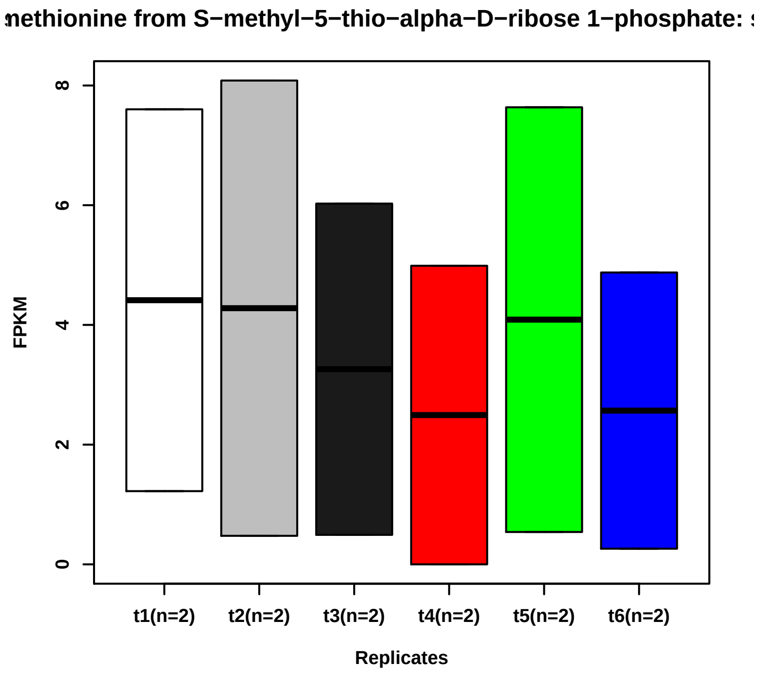
nethionine from S−methyl−5−thio−alpha−D−ribose 1−phosphate:
FPKM
0
2
4
6
8
t1(n=2)
t2(n=2)
t3(n=2)
t4(n=2)
t5(n=2)
t6(n=2)
Replicates

nethionine from S−methyl−5−thio−alpha−D−ribose 1−phosphate:

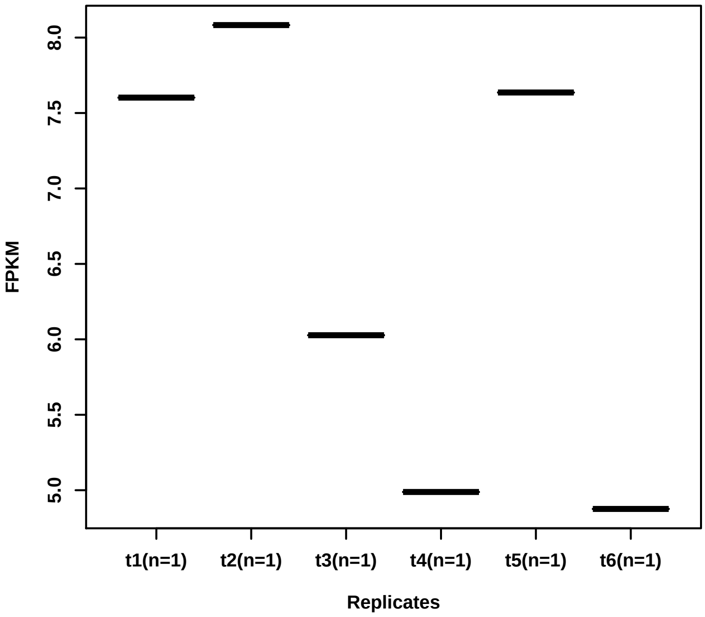

nethionine from S−methyl−5−thio−alpha−D−ribose 1−phosphate: s

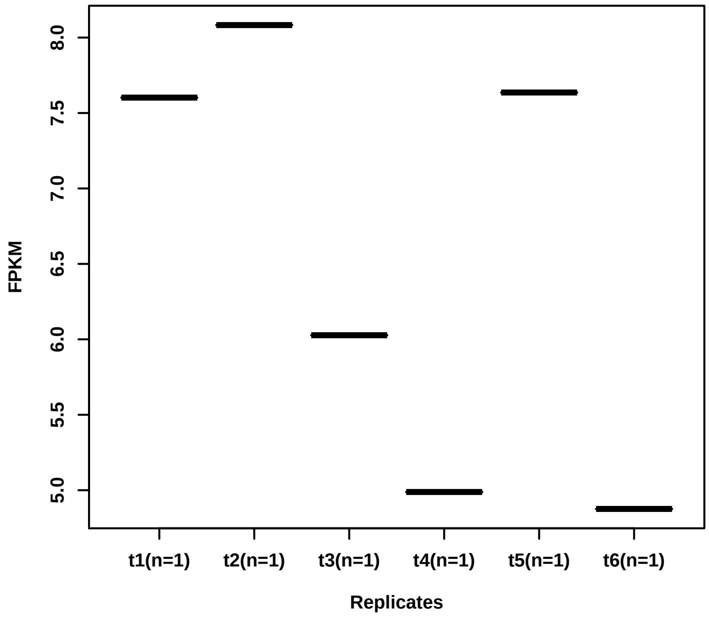

nethionine from S−methyl−5−thio−alpha−D−ribose 1−phosphate:

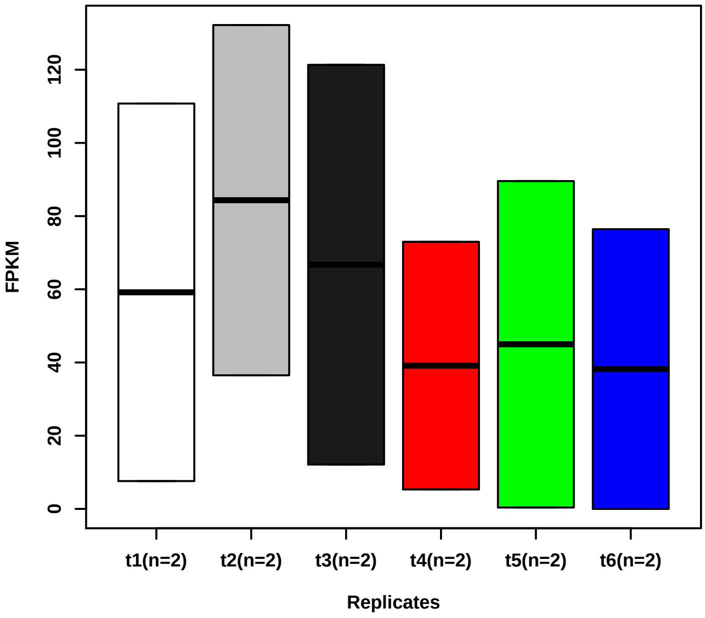

N−acetyl−L−glutamate from L−glutamate and N(2)−acetyl−L−ornitl

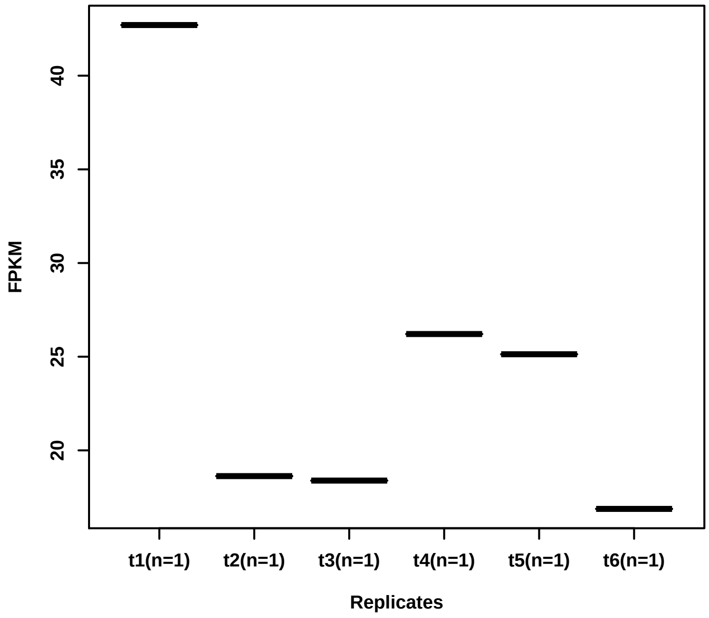

L−ornithine and urea from L−arginine: step 1/1

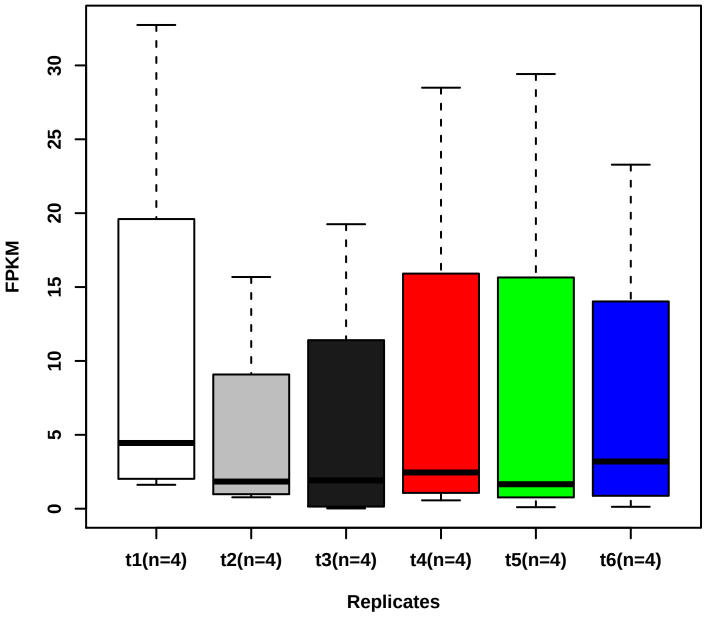

L−phenylalanine from L−arogenate: step 1/1

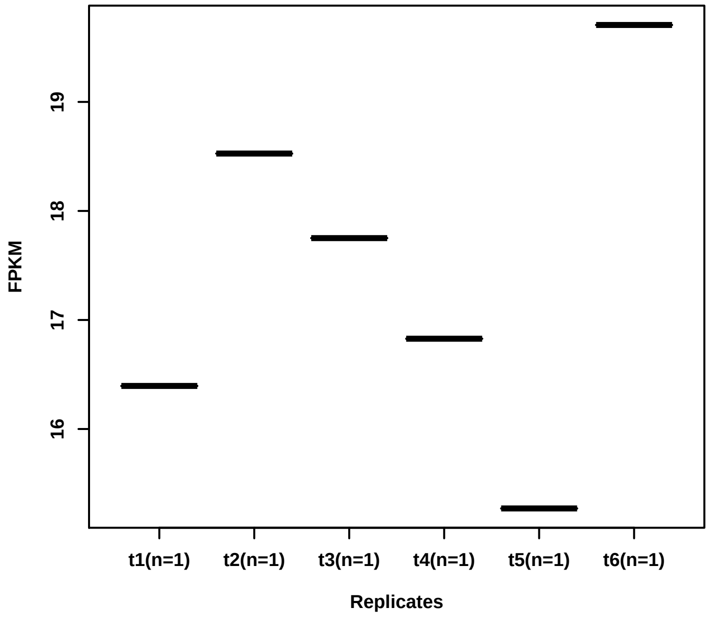

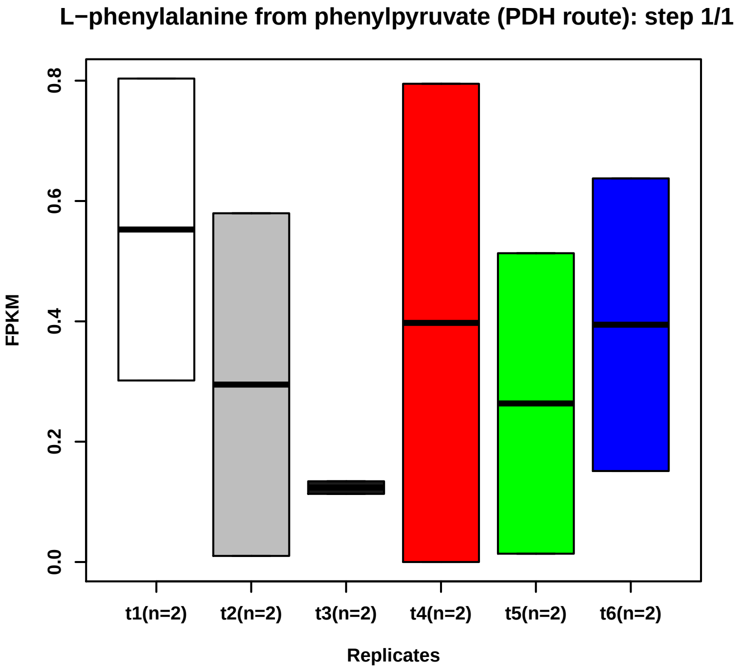
L−phenylalanine from phenylpyruvate (PDH route): step 1/1
FPKM
0.0
0.2
0.4
0.6
0.8
t1(n=2)
t2(n=2)
t3(n=2)
t4(n=2)
t5(n=2)
t6(n=2)
Replicates

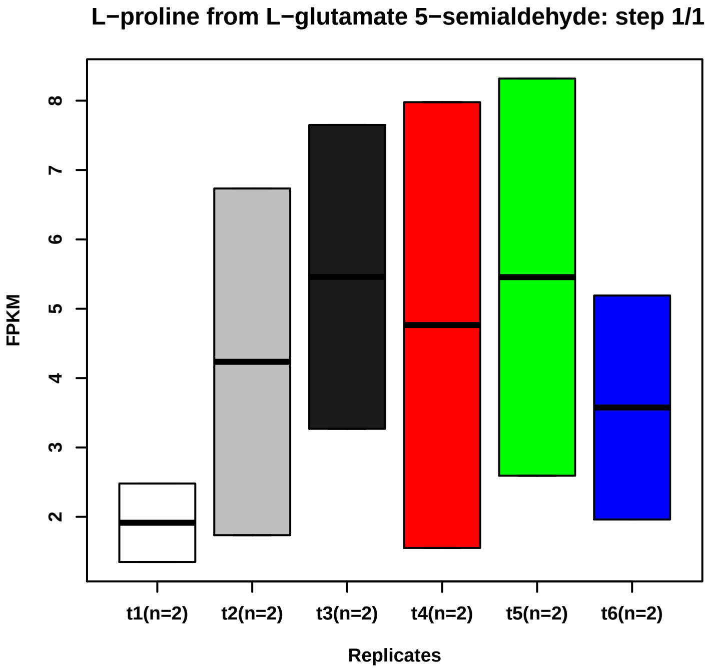
L−proline from L−glutamate 5−semialdehyde: step 1/1
FPKM
2
3
4
5
6
7
8
t1(n=2)
t2(n=2)
t3(n=2)
t4(n=2)
t5(n=2)
t6(n=2)
Replicates

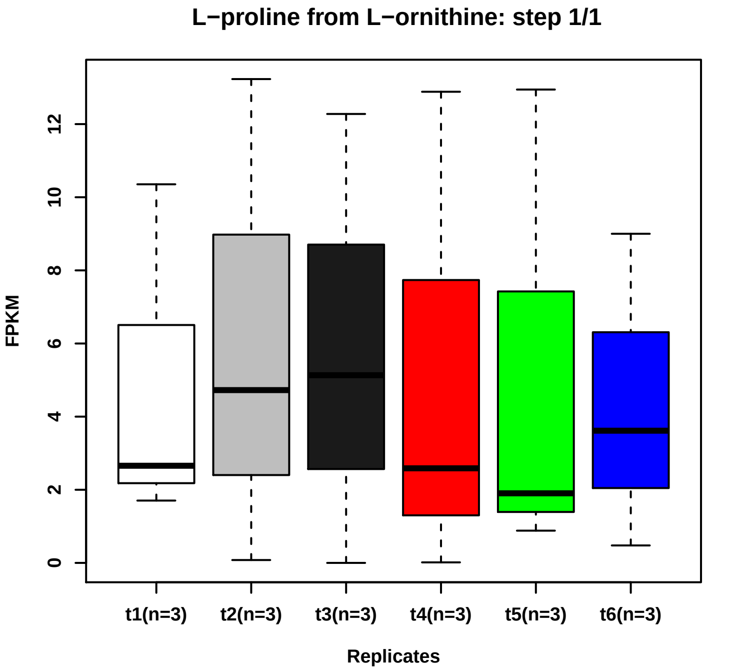
L−proline from L−ornithine: step 1/1
FPKM
0
2
4
6
8
10
12
t1(n=3)
t2(n=3)
t3(n=3)
t4(n=3)
t5(n=3)
t6(n=3)
Replicates

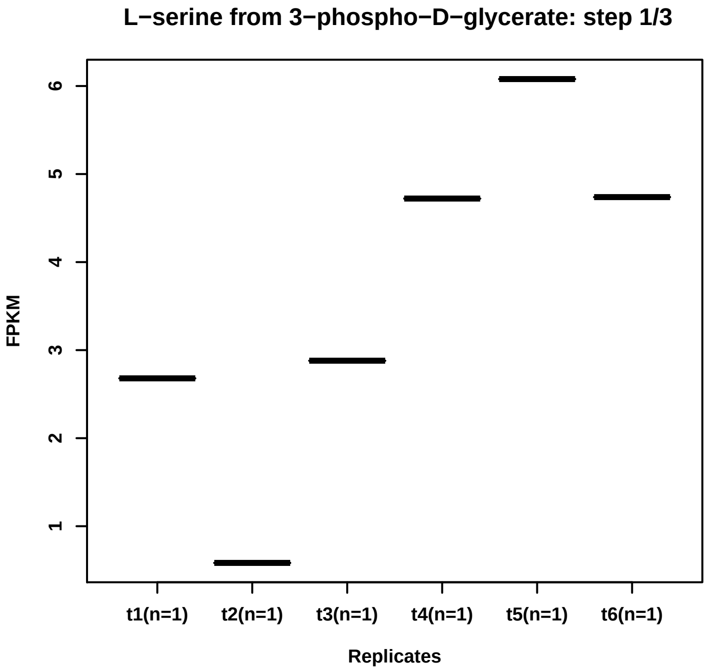
L−serine from 3−phospho−D−glycerate: step 1/3
FPKM
1
2
3
4
5
6
t1(n=1)
t2(n=1)
t3(n=1)
t4(n=1)
t5(n=1)
t6(n=1)
Replicates

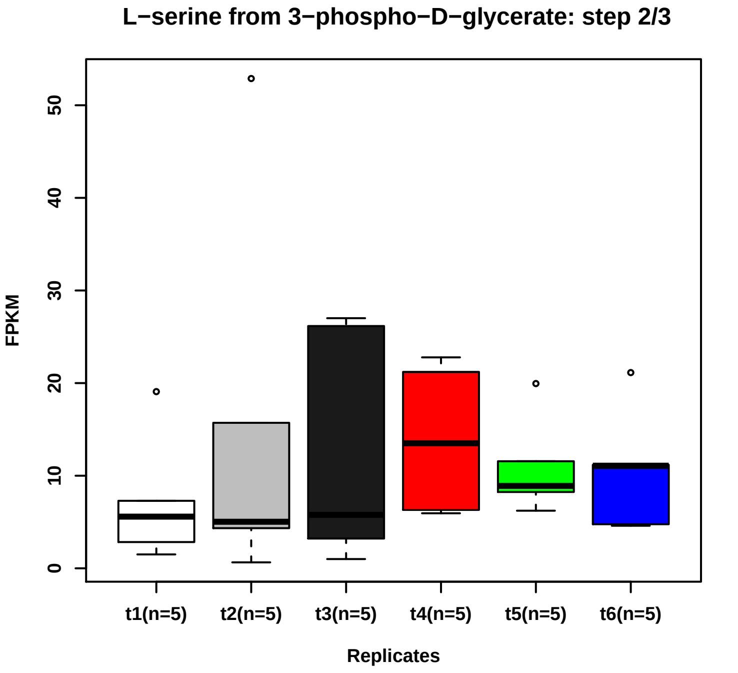
L−serine from 3−phospho−D−glycerate: step 2/3
FPKM
0
10
20
30
40
50
t1(n=5)
t2(n=5)
t3(n=5)
t4(n=5)
t5(n=5)
t6(n=5)
Replicates

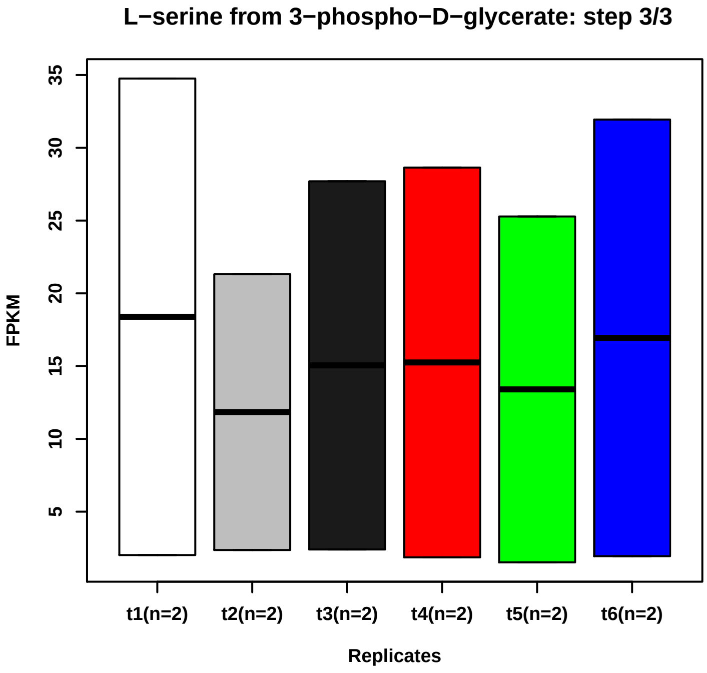
L−serine from 3−phospho−D−glycerate: step 3/3
FPKM
5
10
15
20
25
30
35
t1(n=2)
t2(n=2)
t3(n=2)
t4(n=2)
t5(n=2)
t6(n=2)
Replicates

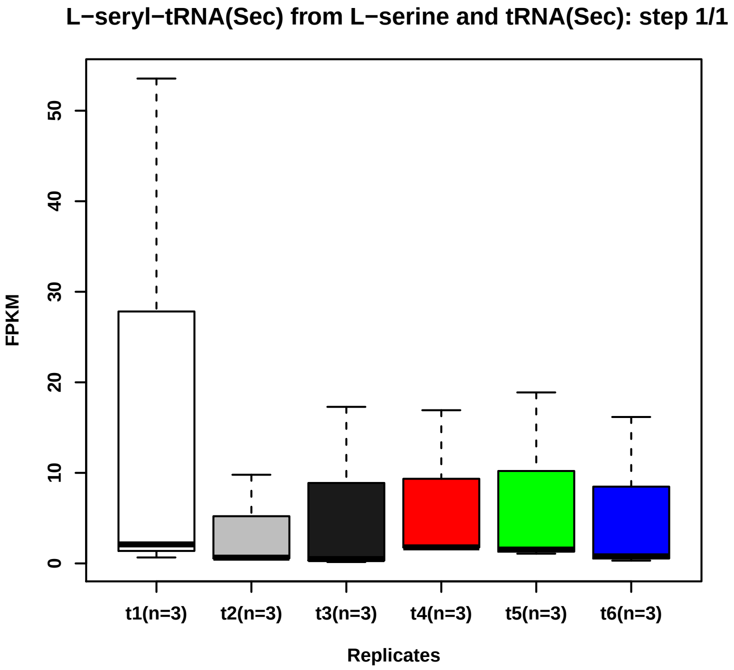
L−seryl−tRNA(Sec) from L−serine and tRNA(Sec): step 1/1
FPKM
0
10
20
30
40
50
t1(n=3)
t2(n=3)
t3(n=3)
t4(n=3)
t5(n=3)
t6(n=3)
Replicates

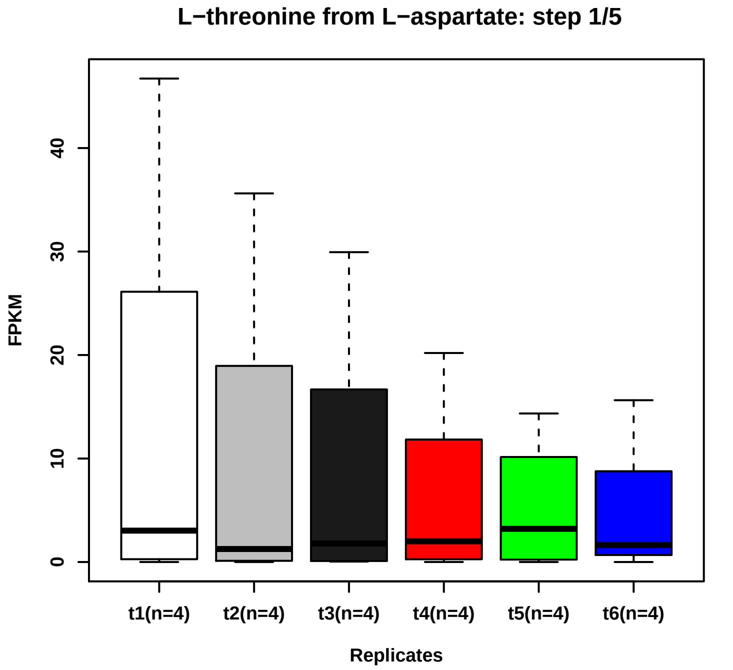
L−threonine from L−aspartate: step 1/5
FPKM
0
10
20
30
40
t1(n=4)
t2(n=4)
t3(n=4)
t4(n=4)
t5(n=4)
t6(n=4)
Replicates

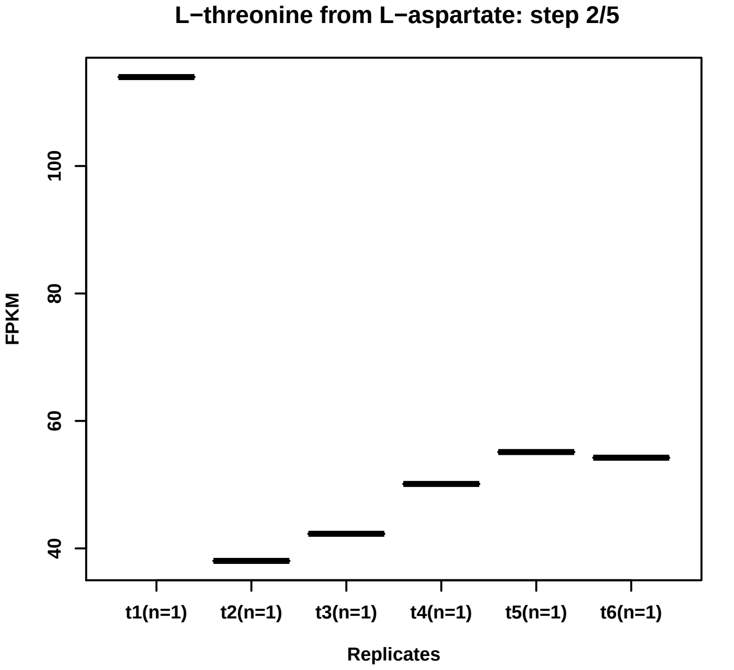
L−threonine from L−aspartate: step 2/5
FPKM
40
60
80
100
t1(n=1)
t2(n=1)
t3(n=1)
t4(n=1)
t5(n=1)
t6(n=1)
Replicates

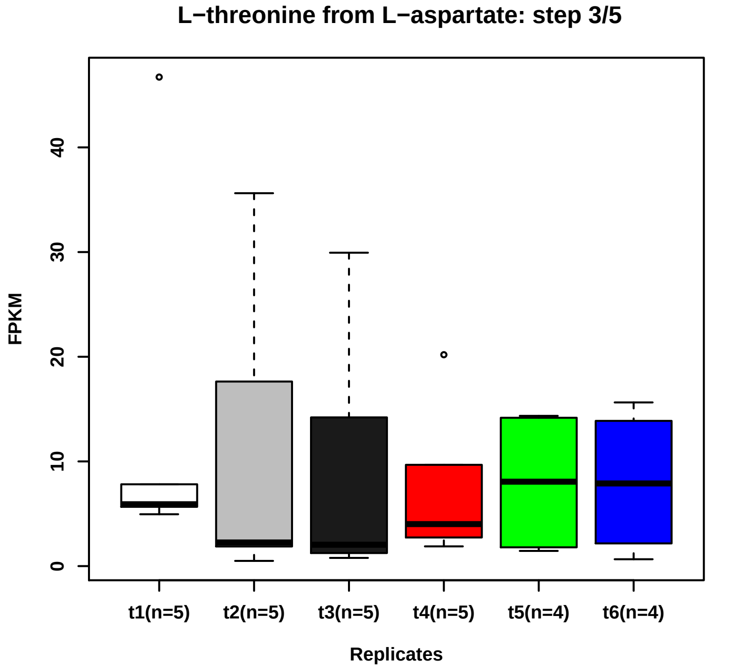
L−threonine from L−aspartate: step 3/5
FPKM
0
10
20
30
40
t1(n=5)
t2(n=5)
t3(n=5)
t4(n=5)
t5(n=4)
t6(n=4)
Replicates

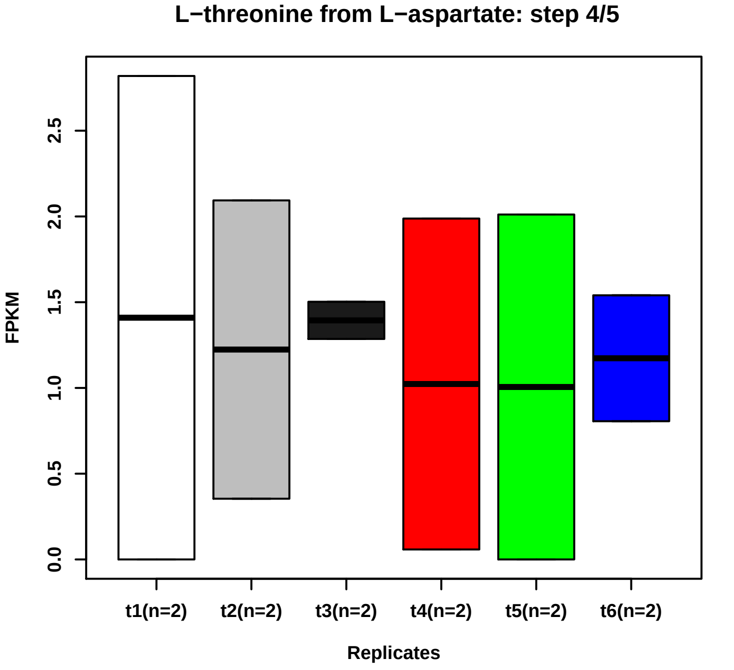
L−threonine from L−aspartate: step 4/5
FPKM
0.0
0.5
1.0
1.5
2.0
2.5
t1(n=2)
t2(n=2)
t3(n=2)
t4(n=2)
t5(n=2)
t6(n=2)
Replicates

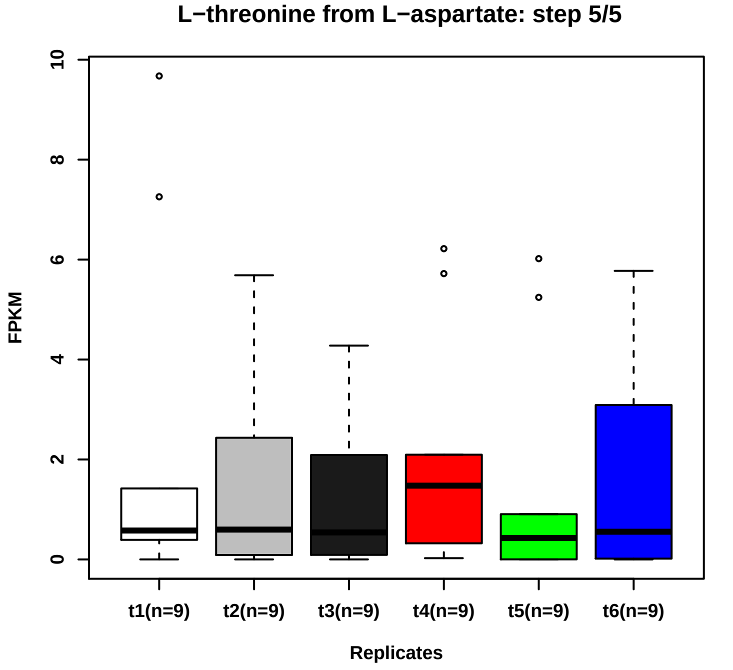
L−threonine from L−aspartate: step 5/5
FPKM
0
2
4
6
8
10
t1(n=9)
t2(n=9)
t3(n=9)
t4(n=9)
t5(n=9)
t6(n=9)
Replicates

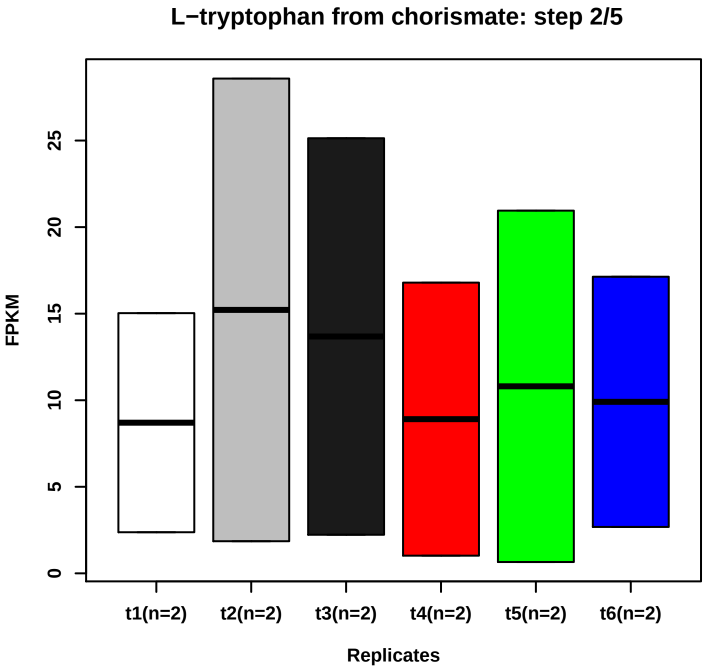
L−tryptophan from chorismate: step 2/5
FPKM
0
5
10
15
20
25
t1(n=2)
t2(n=2)
t3(n=2)
t4(n=2)
t5(n=2)
t6(n=2)
Replicates

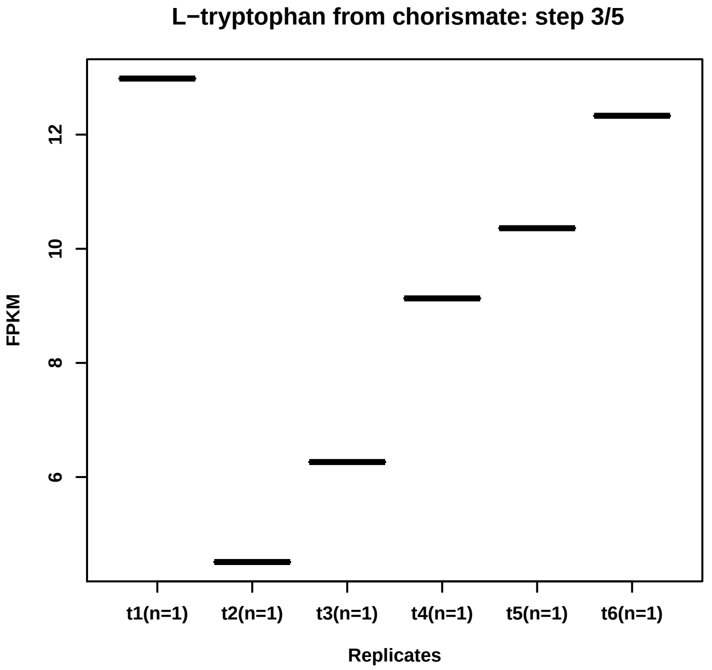
L−tryptophan from chorismate: step 3/5
FPKM
6
8
10
12
t1(n=1)
t2(n=1)
t3(n=1)
t4(n=1)
t5(n=1)
t6(n=1)
Replicates

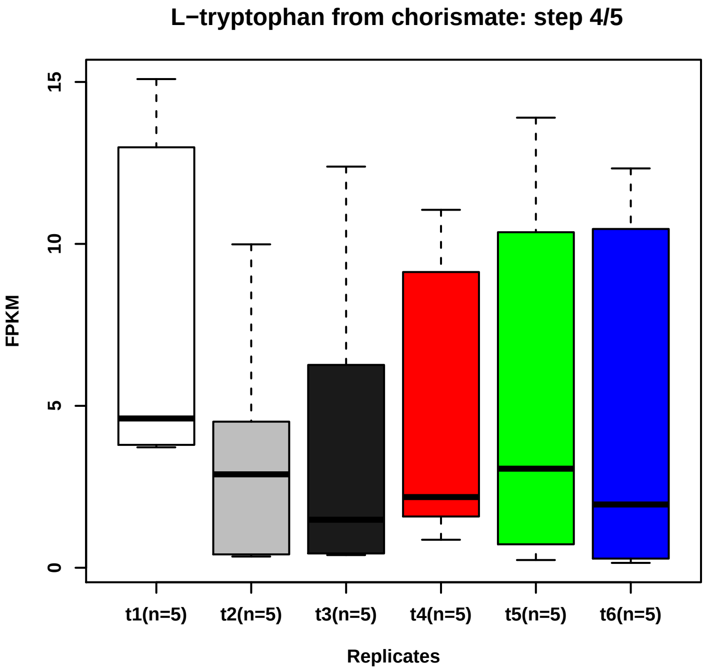
L−tryptophan from chorismate: step 4/5
FPKM
0
5
10
15
t1(n=5)
t2(n=5)
t3(n=5)
t4(n=5)
t5(n=5)
t6(n=5)
Replicates

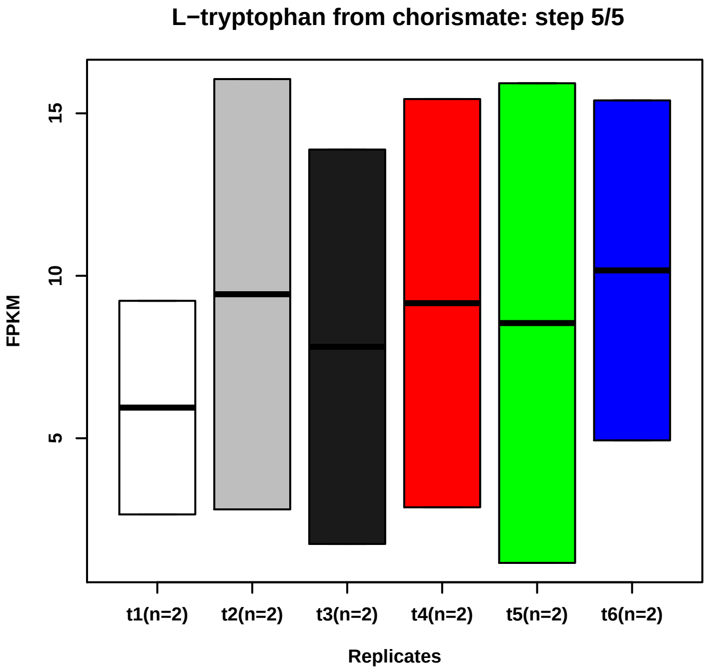
L−tryptophan from chorismate: step 5/5
FPKM
5
10
15
t1(n=2)
t2(n=2)
t3(n=2)
t4(n=2)
t5(n=2)
t6(n=2)
Replicates

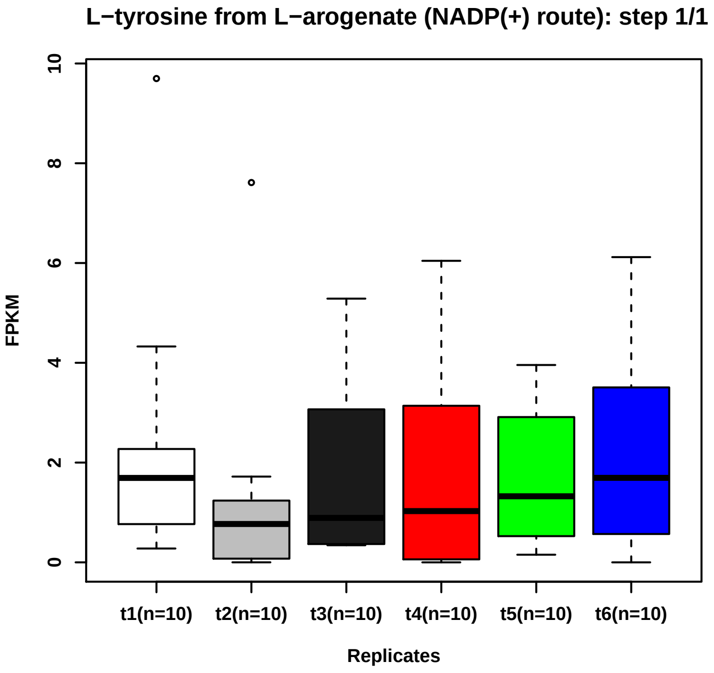
L−tyrosine from L−arogenate (NADP(+) route): step 1/1
FPKM
0
2
4
6
8
10
t1(n=10)
t2(n=10)
t3(n=10)
t4(n=10)
t5(n=10)
t6(n=10)
Replicates

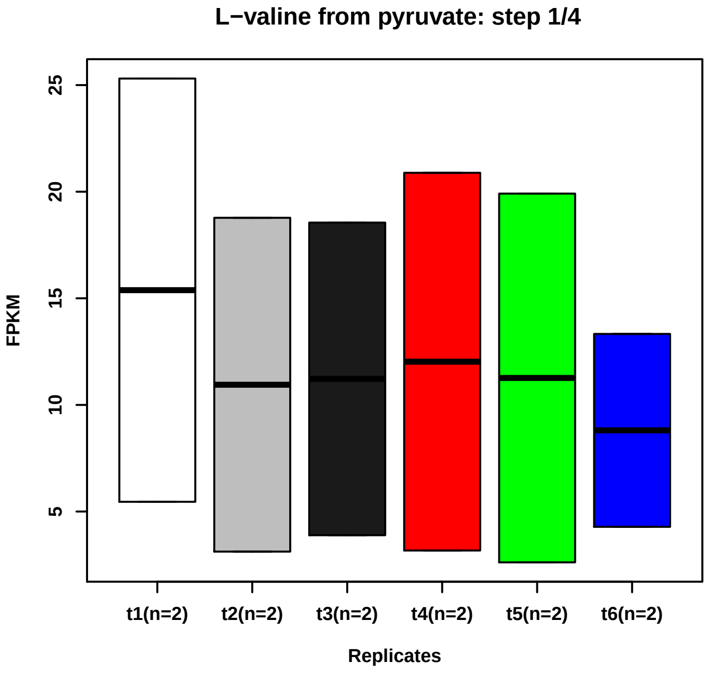
L−valine from pyruvate: step 1/4
FPKM
5
10
15
20
25
t1(n=2)
t2(n=2)
t3(n=2)
t4(n=2)
t5(n=2)
t6(n=2)
Replicates

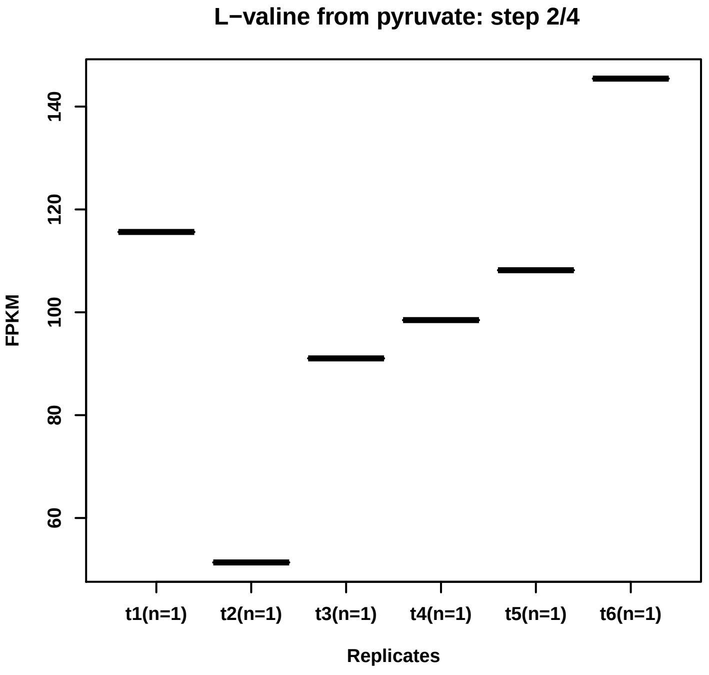
L−valine from pyruvate: step 2/4
FPKM
60
80
100
120
140
t1(n=1)
t2(n=1)
t3(n=1)
t4(n=1)
t5(n=1)
t6(n=1)
Replicates

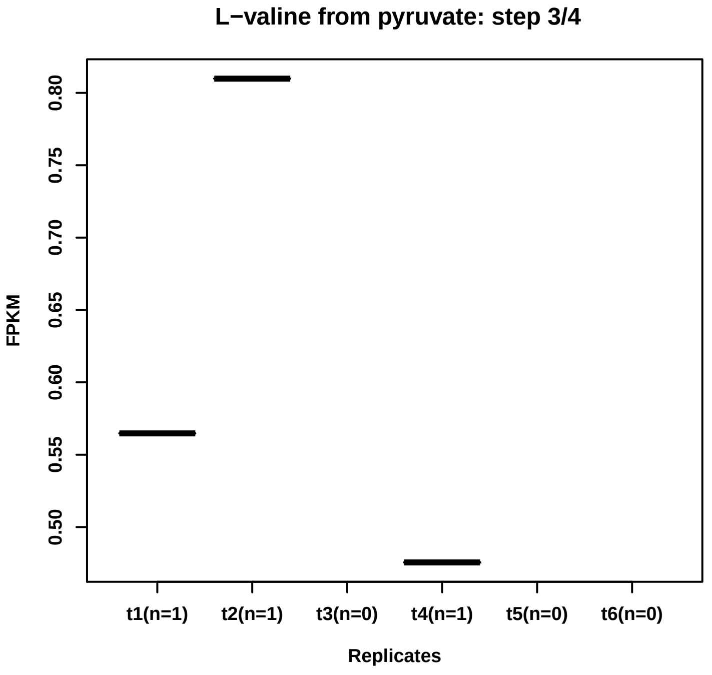
L−valine from pyruvate: step 3/4
FPKM
0.50
0.55
0.60
0.65
0.70
0.75
0.80
t1(n=1)
t2(n=1)
t3(n=0)
t4(n=1)
t5(n=0)
t6(n=0)
Replicates

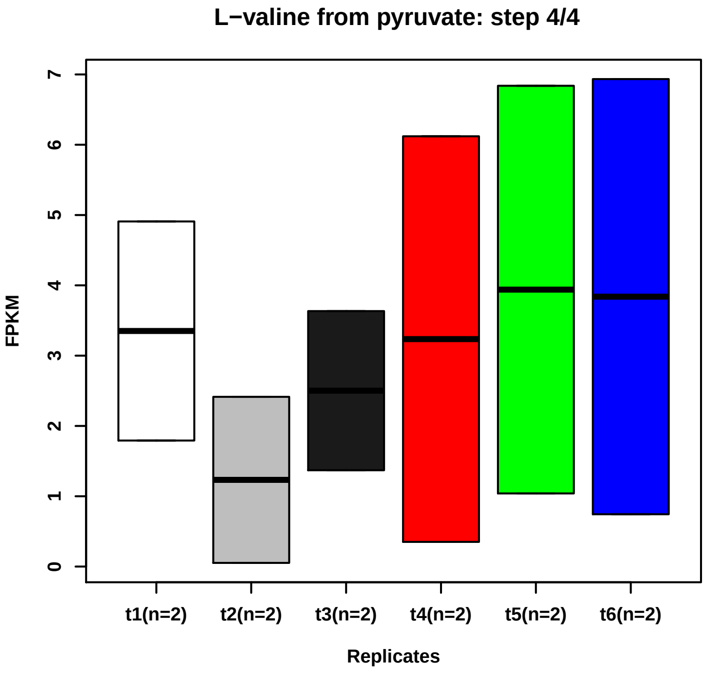
L−valine from pyruvate: step 4/4
FPKM
0
1
2
3
4
5
6
7
t1(n=2)
t2(n=2)
t3(n=2)
t4(n=2)
t5(n=2)
t6(n=2)
Replicates

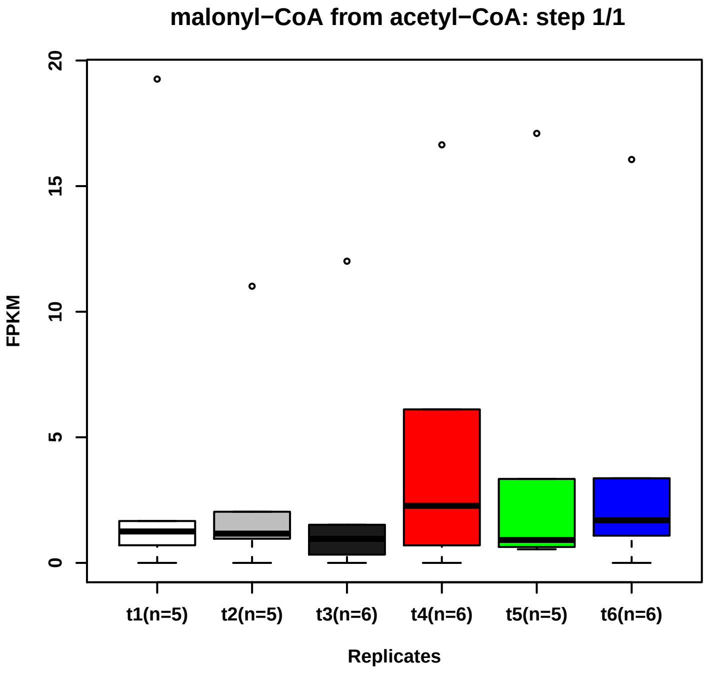
malonyl−CoA from acetyl−CoA: step 1/1
FPKM
0
5
10
15
20
t1(n=5)
t2(n=5)
t3(n=6)
t4(n=6)
t5(n=5)
t6(n=6)
Replicates

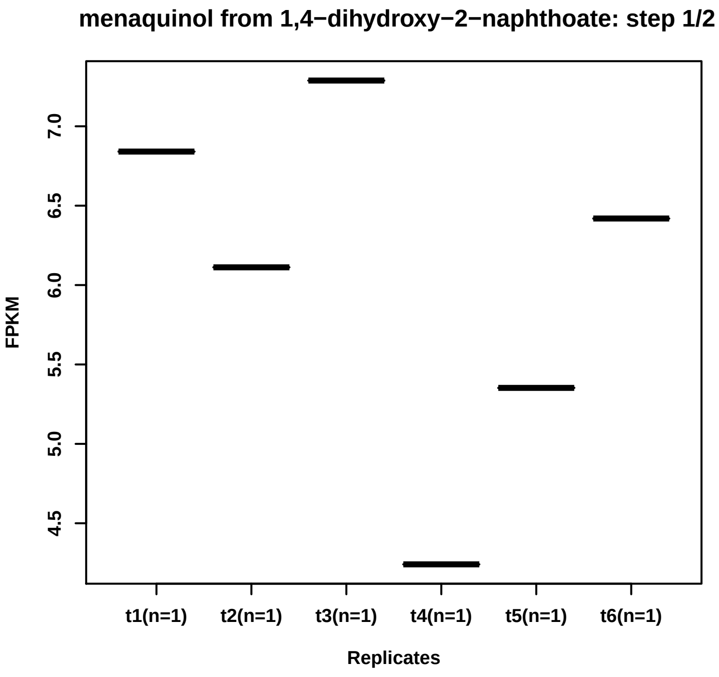
menaquinol from 1,4−dihydroxy−2−naphthoate: step 1/2
FPKM
4.5
5.0
5.5
6.0
6.5
7.0
t1(n=1)
t2(n=1)
t3(n=1)
t4(n=1)
t5(n=1)
t6(n=1)
Replicates

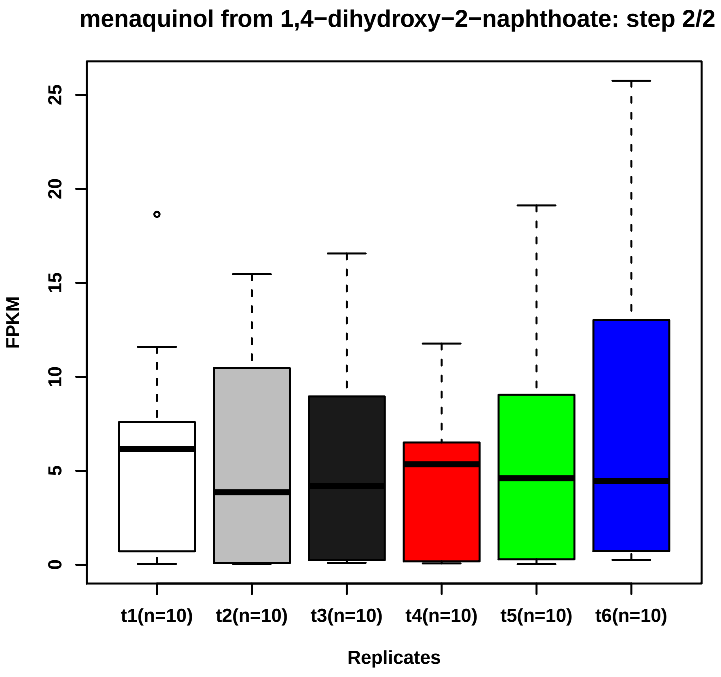
menaquinol from 1,4−dihydroxy−2−naphthoate: step 2/2
FPKM
0
5
10
15
20
25
t1(n=10)
t2(n=10)
t3(n=10)
t4(n=10)
t5(n=10)
t6(n=10)
Replicates

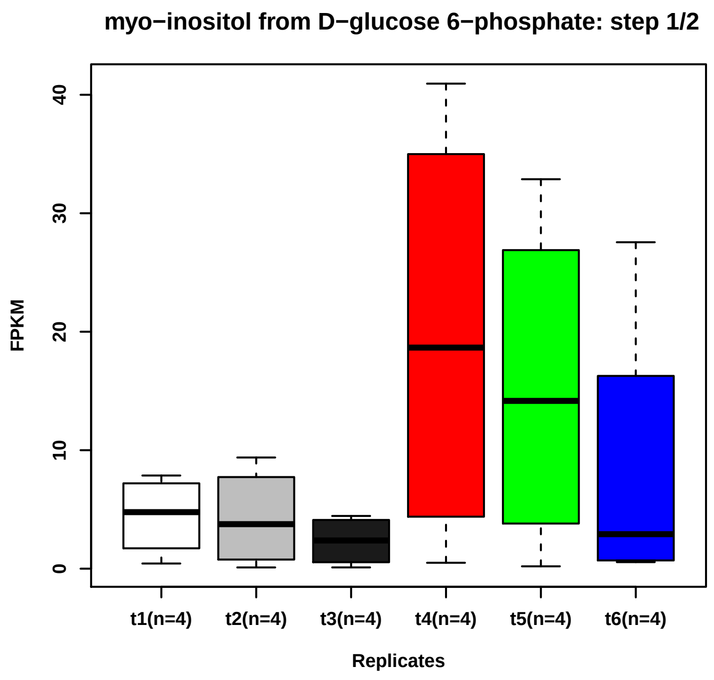
myo−inositol from D−glucose 6−phosphate: step 1/2
FPKM
0
10
20
30
40
t1(n=4)
t2(n=4)
t3(n=4)
t4(n=4)
t5(n=4)
t6(n=4)
Replicates

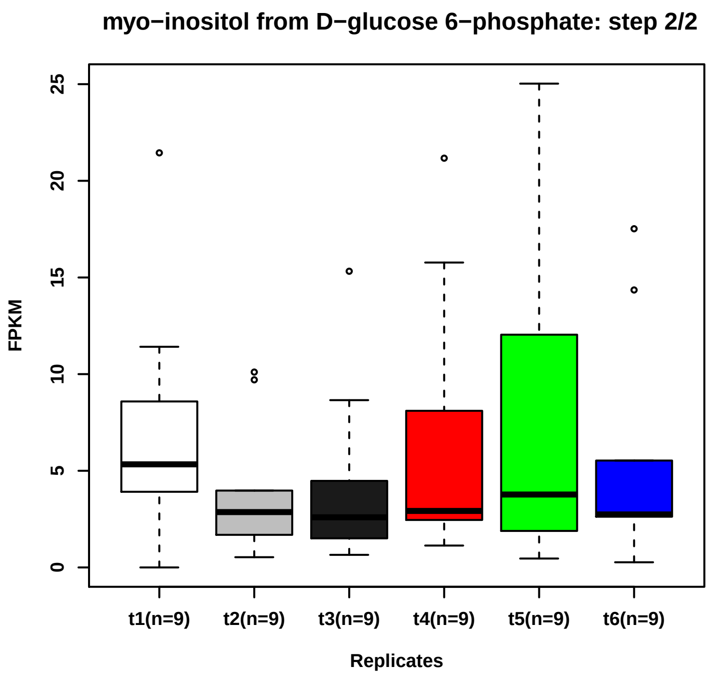
myo−inositol from D−glucose 6−phosphate: step 2/2
FPKM
0
5
10
15
20
25
t1(n=9)
t2(n=9)
t3(n=9)
t4(n=9)
t5(n=9)
t6(n=9)
Replicates

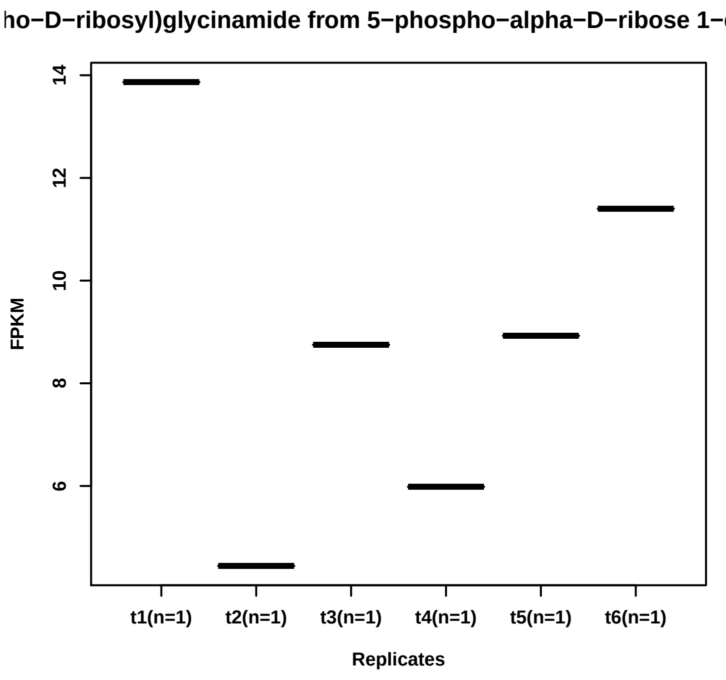
ho−D−ribosyl)glycinamide from 5−phospho−alpha−D−ribose 1−di
FPKM
6
8
10
12
14
t1(n=1)
t2(n=1)
t3(n=1)
t4(n=1)
t5(n=1)
t6(n=1)
Replicates

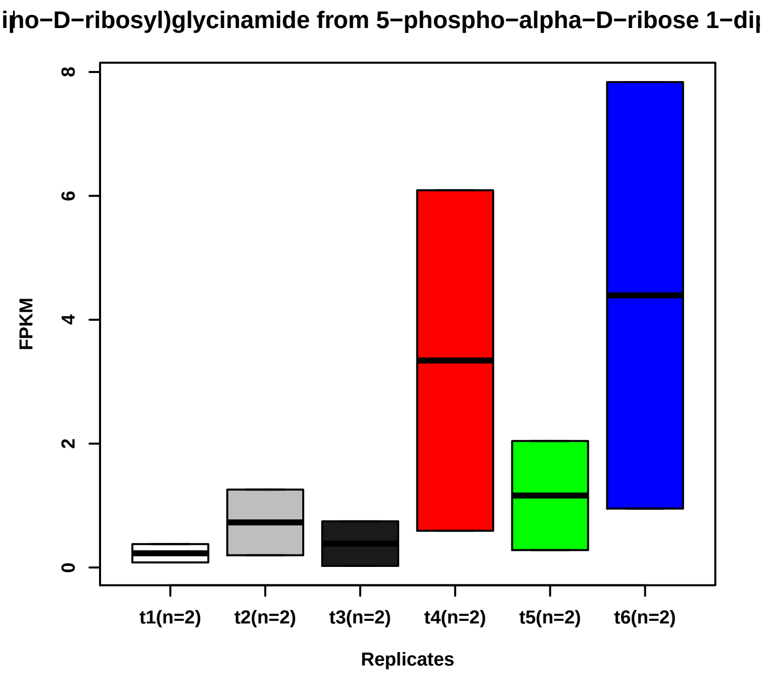
ho−D−ribosyl)glycinamide from 5−phospho−alpha−D−ribose 1−di
FPKM
0
2
4
6
8
t1(n=2)
t2(n=2)
t3(n=2)
t4(n=2)
t5(n=2)
t6(n=2)
Replicates

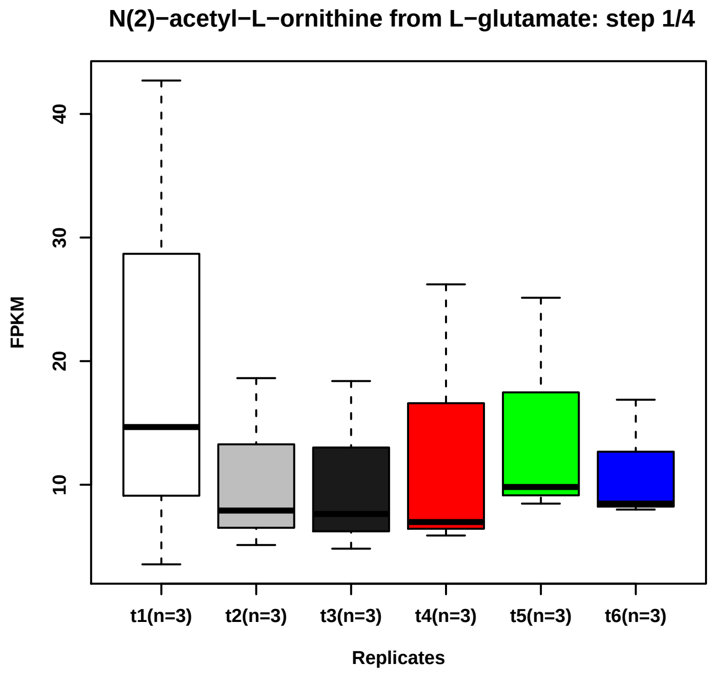
N(2)−acetyl−L−ornithine from L−glutamate: step 1/4
FPKM
10
20
30
40
t1(n=3)
t2(n=3)
t3(n=3)
t4(n=3)
t5(n=3)
t6(n=3)
Replicates

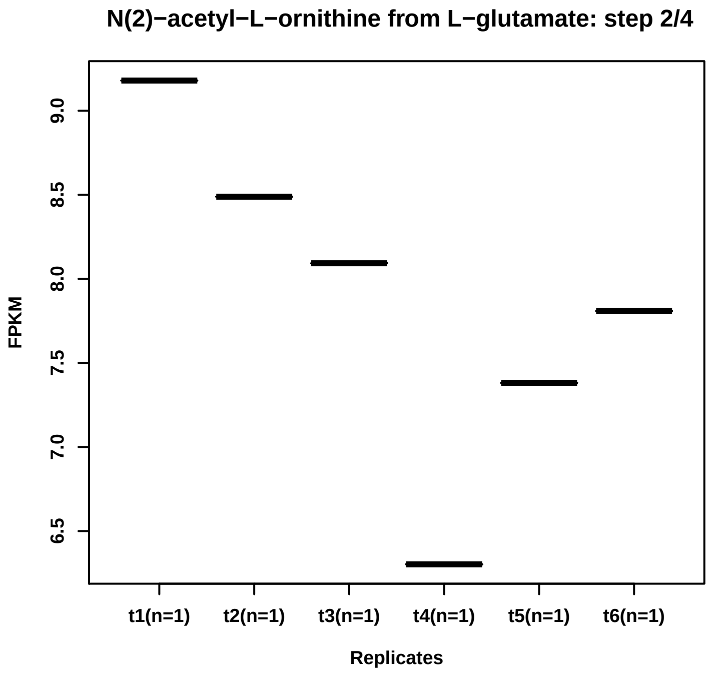
N(2)−acetyl−L−ornithine from L−glutamate: step 2/4
FPKM
6.5
7.0
7.5
8.0
8.5
9.0
t1(n=1)
t2(n=1)
t3(n=1)
t4(n=1)
t5(n=1)
t6(n=1)
Replicates

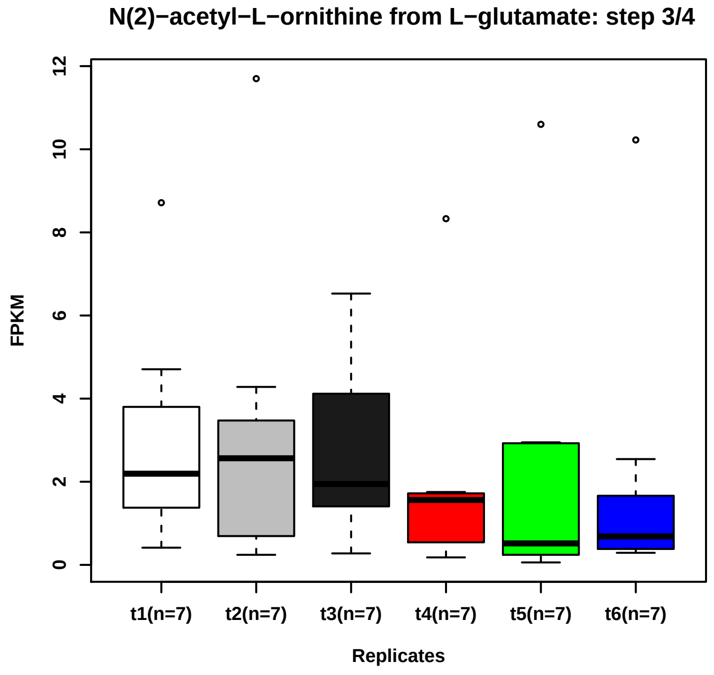
N(2)−acetyl−L−ornithine from L−glutamate: step 3/4
FPKM
Replicates
t1(n=7)
t2(n=7)
t3(n=7)
t4(n=7)
t5(n=7)
t6(n=7)

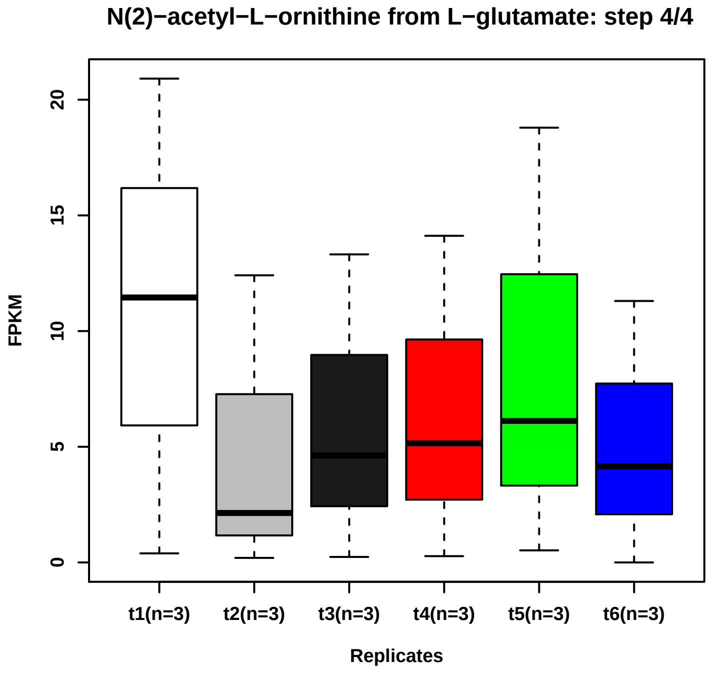
N(2)−acetyl−L−ornithine from L−glutamate: step 4/4
FPKM
Replicates
t1(n=3)
t2(n=3)
t3(n=3)
t4(n=3)
t5(n=3)
t6(n=3)

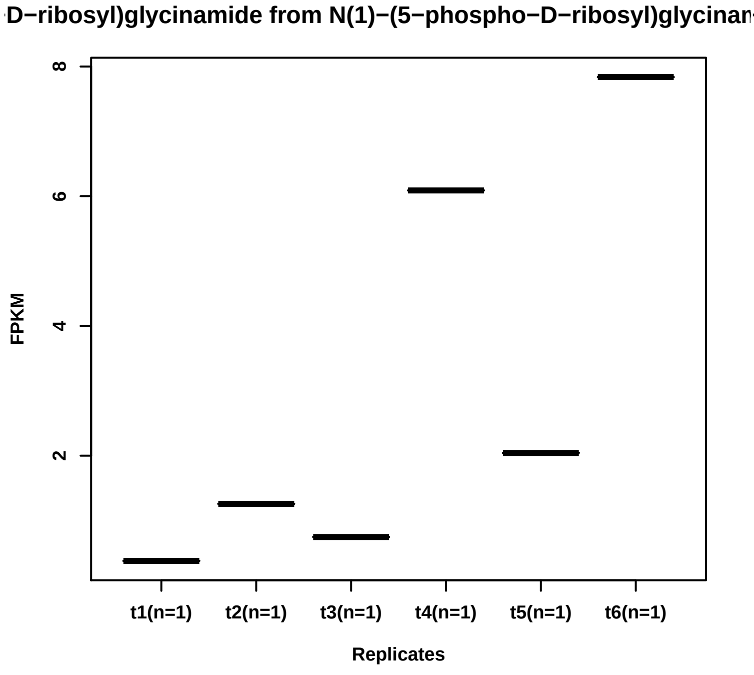
D−ribosyl)glycinamide from N(1)−(5−phospho−D−ribosyl)glycinan
FPKM
Replicates
t1(n=1)
t2(n=1)
t3(n=1)
t4(n=1)
t5(n=1)
t6(n=1)

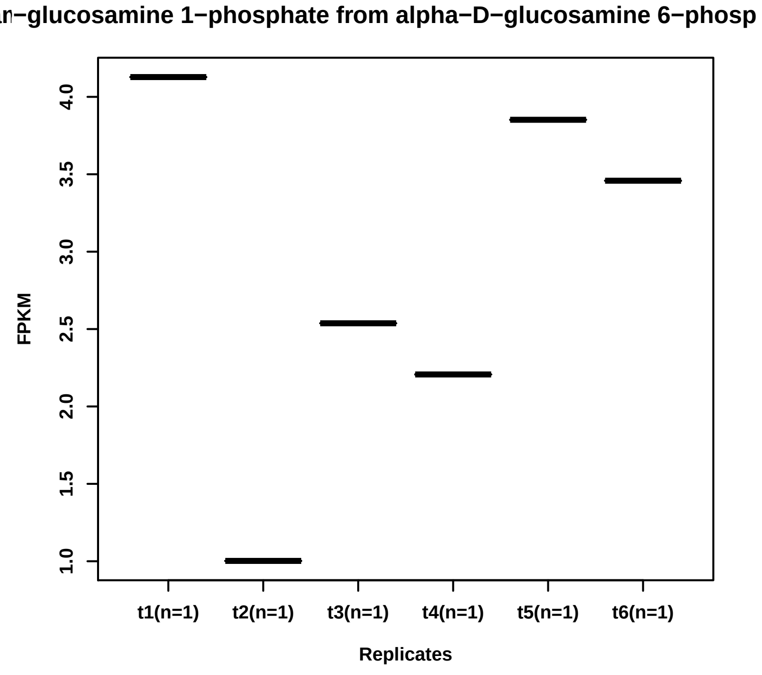
−glucosamine 1−phosphate from alpha−D−glucosamine 6−phosp
FPKM
Replicates
t1(n=1)
t2(n=1)
t3(n=1)
t4(n=1)
t5(n=1)
t6(n=1)

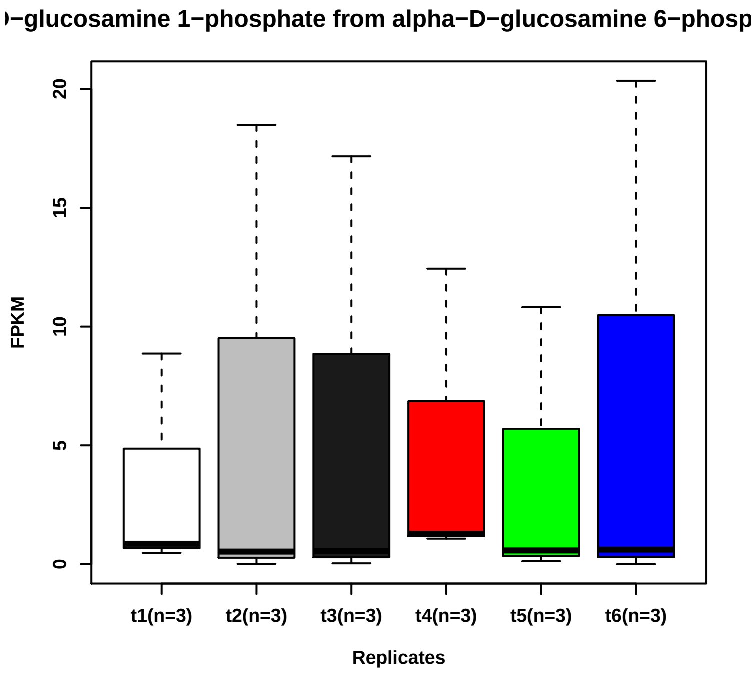
)−glucosamine 1−phosphate from alpha−D−glucosamine 6−phosp
FPKM
Replicates
t1(n=3)
t2(n=3)
t3(n=3)
t4(n=3)
t5(n=3)
t6(n=3)

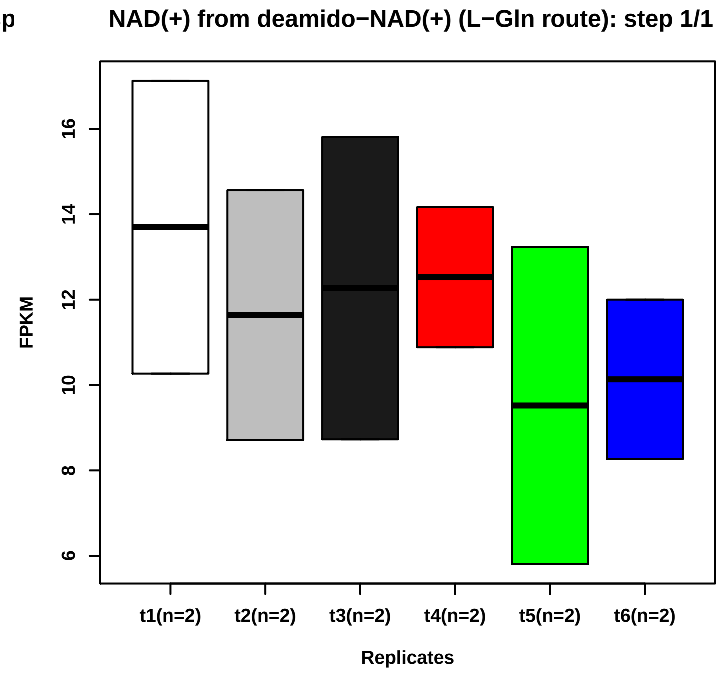
NAD(+) from deamido−NAD(+) (L−Gln route): step 1/1
FPKM
Replicates
t1(n=2)
t2(n=2)
t3(n=2)
t4(n=2)
t5(n=2)
t6(n=2)

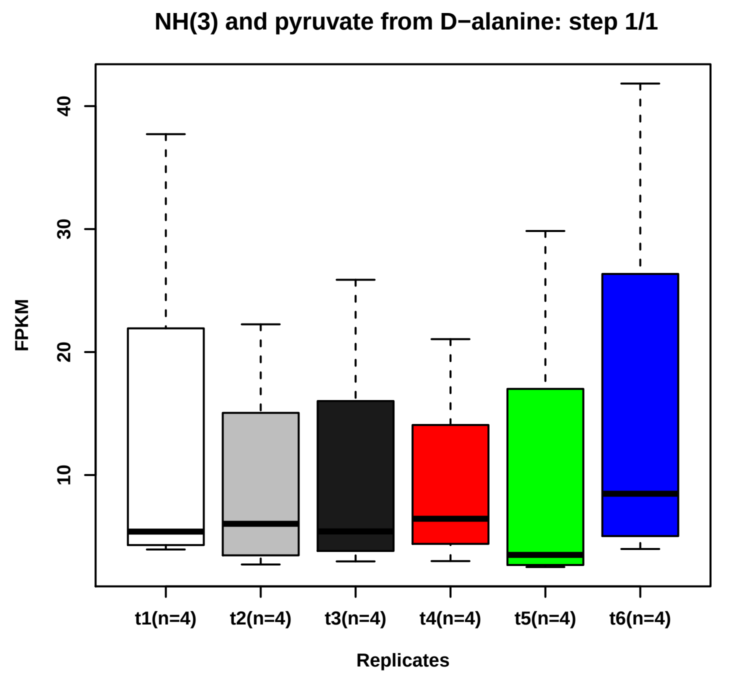

NH(3) and pyruvate from D−alanine: step 1/1
FPKM
t1(n=4) t2(n=4) t3(n=4) t4(n=4) t5(n=4) t6(n=4)
Replicates

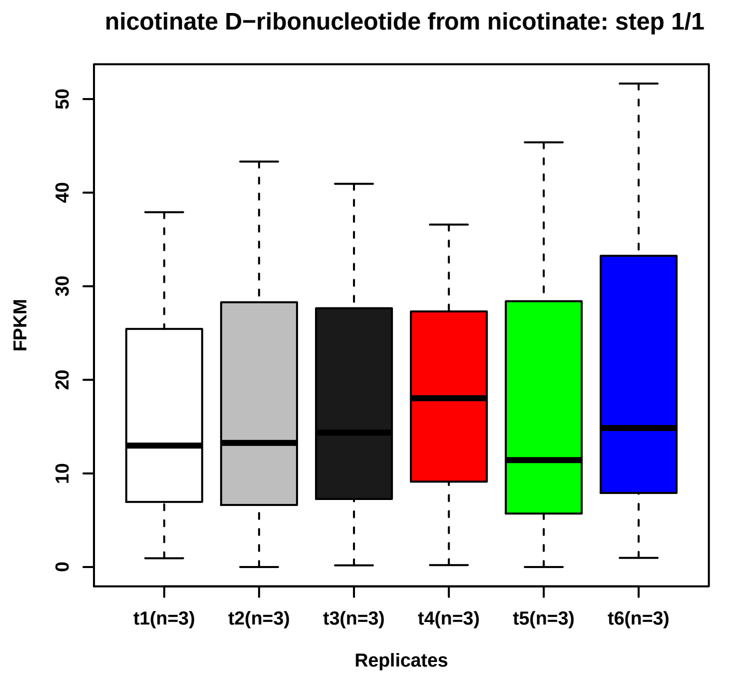

nicotinate D−ribonucleotide from nicotinate: step 1/1
FPKM
t1(n=3) t2(n=3) t3(n=3) t4(n=3) t5(n=3) t6(n=3)
Replicates

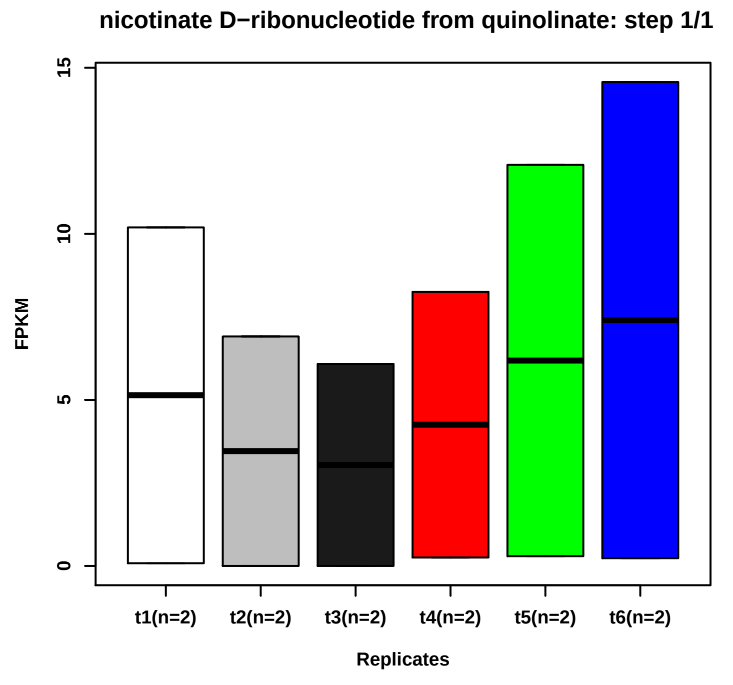

nicotinate D−ribonucleotide from quinolinate: step 1/1
FPKM
t1(n=2) t2(n=2) t3(n=2) t4(n=2) t5(n=2) t6(n=2)
Replicates

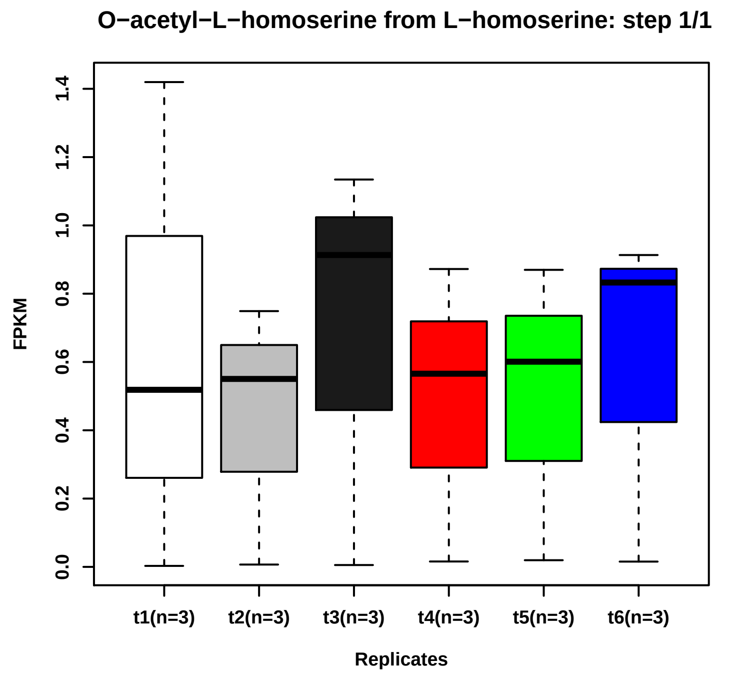

O−acetyl−L−homoserine from L−homoserine: step 1/1
FPKM
t1(n=3) t2(n=3) t3(n=3) t4(n=3) t5(n=3) t6(n=3)
Replicates

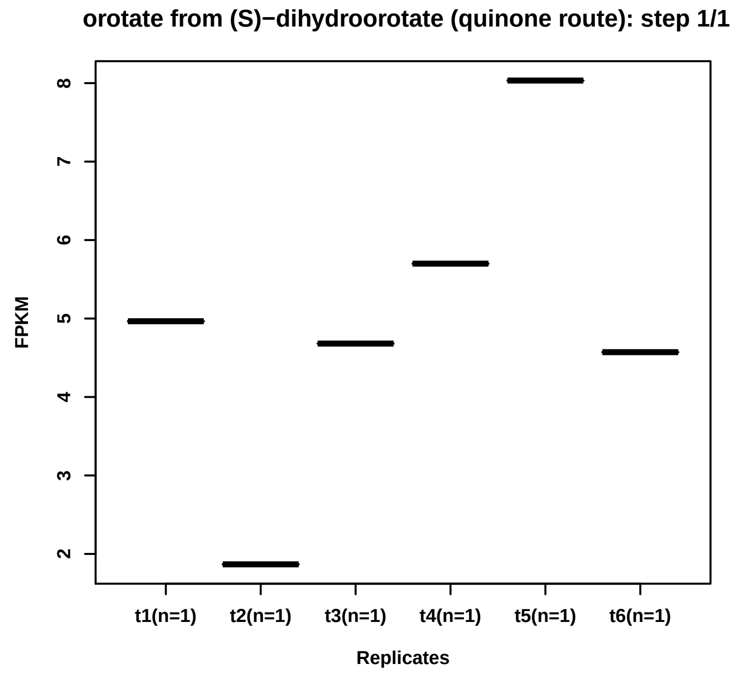

orotate from (S)−dihydroorotate (quinone route): step 1/1
FPKM
t1(n=1) t2(n=1) t3(n=1) t4(n=1) t5(n=1) t6(n=1)
Replicates

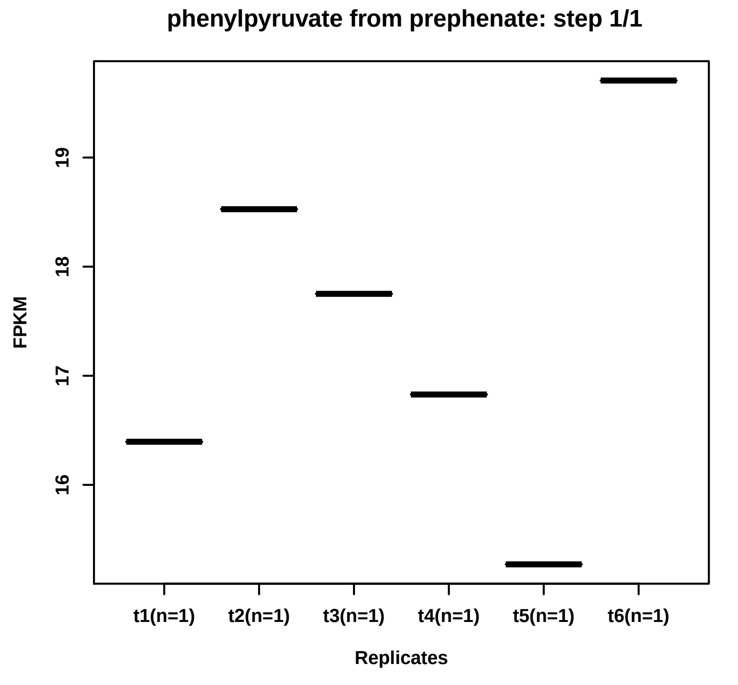

phenylpyruvate from prephenate: step 1/1
FPKM
t1(n=1) t2(n=1) t3(n=1) t4(n=1) t5(n=1) t6(n=1)
Replicates

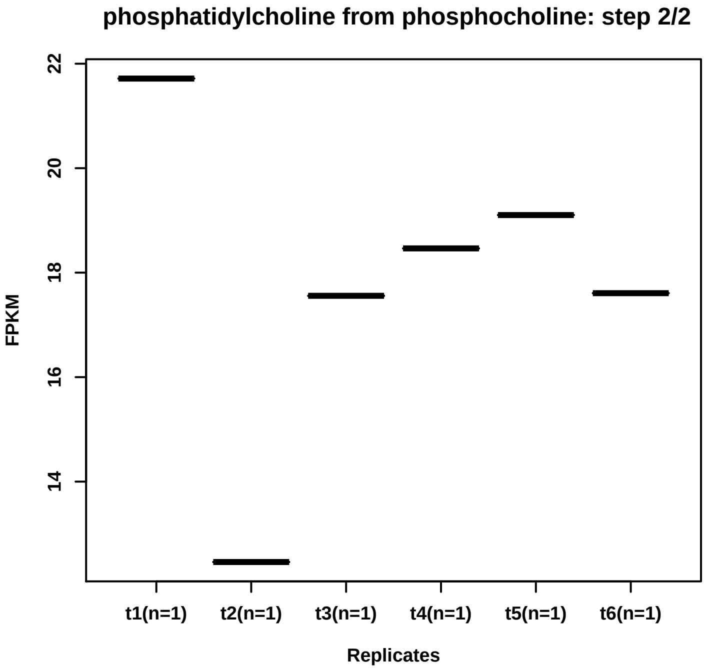
phosphatidylcholine from phosphocholine: step 2/2
FPKM
22
20
18
16
14
t1(n=1)
t2(n=1)
t3(n=1)
t4(n=1)
t5(n=1)
t6(n=1)
Replicates

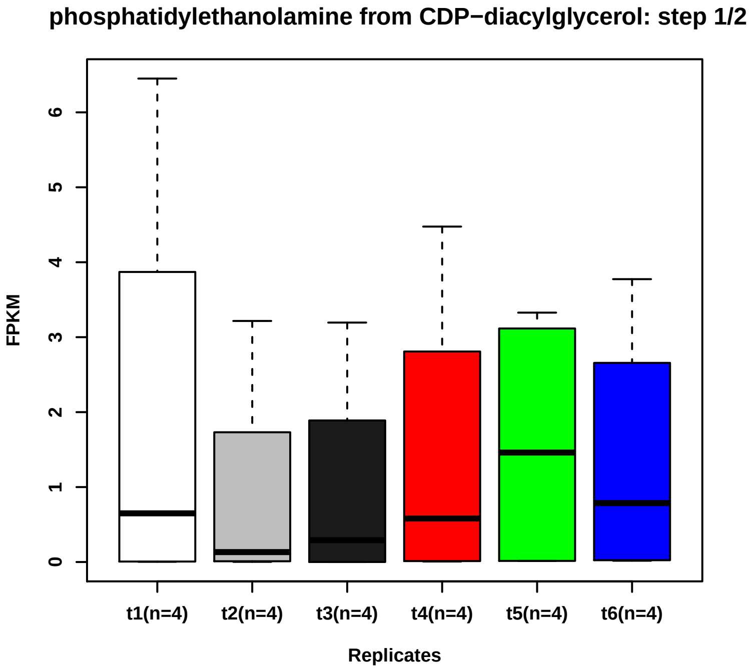
phosphatidylethanolamine from CDP−diacylglycerol: step 1/2
FPKM
6
5
4
3
2
1
0
t1(n=4)
t2(n=4)
t3(n=4)
t4(n=4)
t5(n=4)
t6(n=4)
Replicates

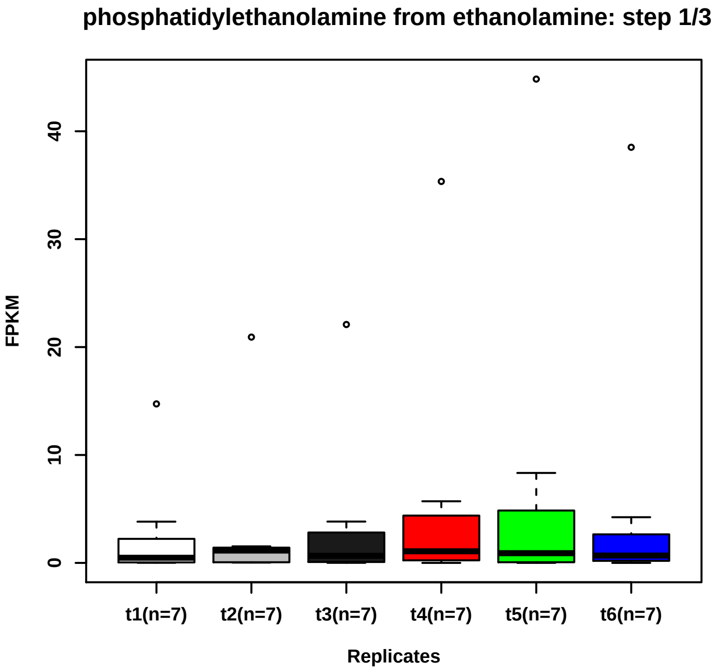
phosphatidylethanolamine from ethanolamine: step 1/3
FPKM
40
30
20
10
0
t1(n=7)
t2(n=7)
t3(n=7)
t4(n=7)
t5(n=7)
t6(n=7)
Replicates

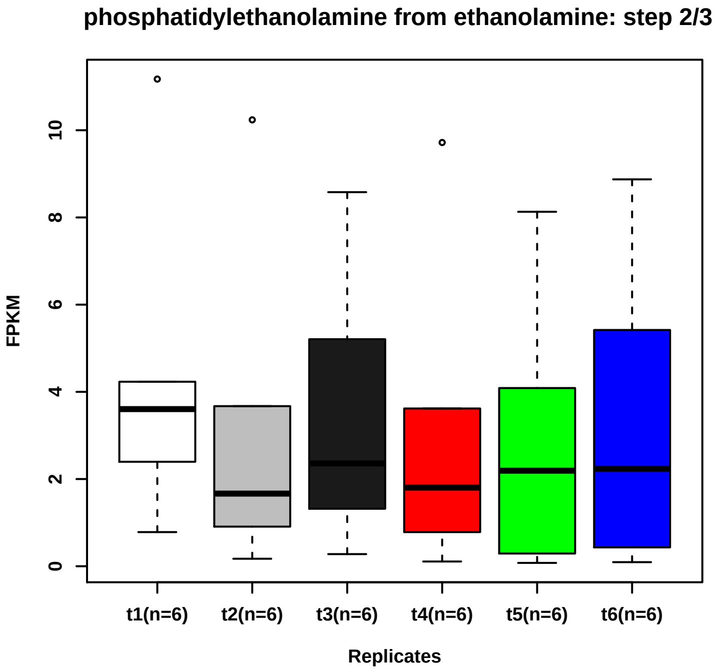
phosphatidylethanolamine from ethanolamine: step 2/3
FPKM
10
8
6
4
2
0
t1(n=6)
t2(n=6)
t3(n=6)
t4(n=6)
t5(n=6)
t6(n=6)
Replicates

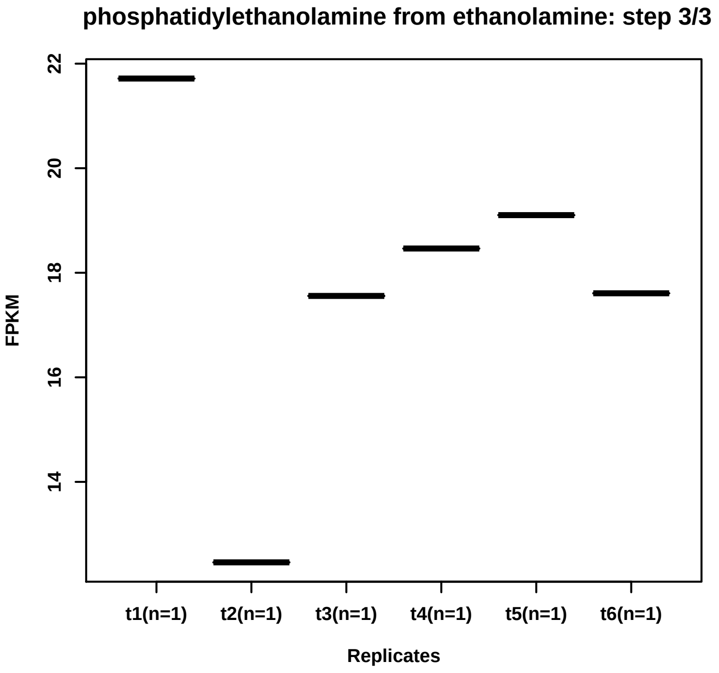
phosphatidylethanolamine from ethanolamine: step 3/3
FPKM
22
20
18
16
14
t1(n=1)
t2(n=1)
t3(n=1)
t4(n=1)
t5(n=1)
t6(n=1)
Replicates

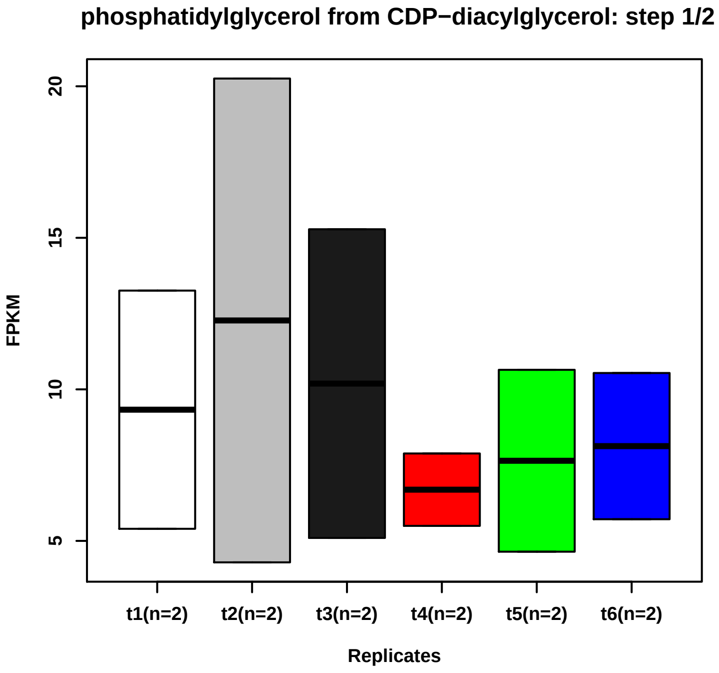
phosphatidylglycerol from CDP−diacylglycerol: step 1/2
FPKM
20
15
10
5
t1(n=2)
t2(n=2)
t3(n=2)
t4(n=2)
t5(n=2)
t6(n=2)
Replicates

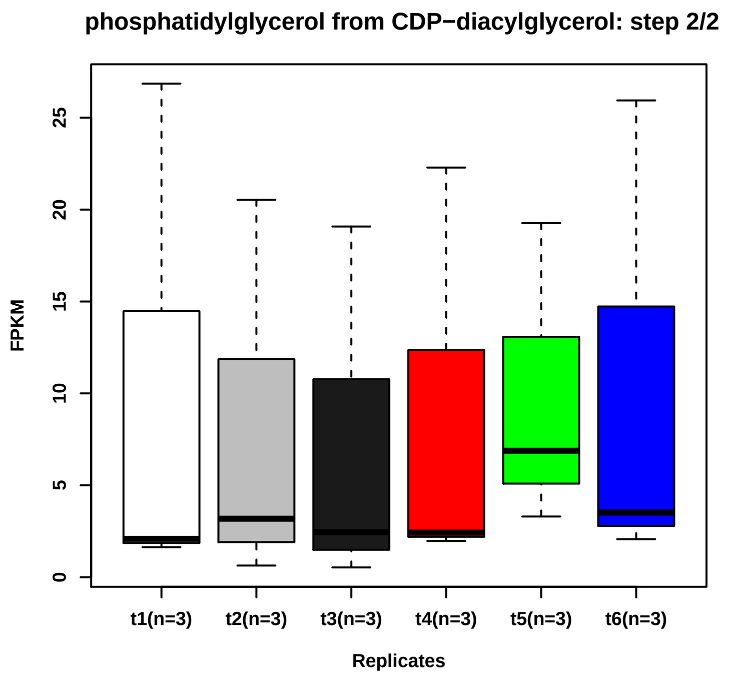
phosphatidylglycerol from CDP−diacylglycerol: step 2/2
FPKM
0
5
10
15
20
25
t1(n=3)
t2(n=3)
t3(n=3)
t4(n=3)
t5(n=3)
t6(n=3)
Replicates

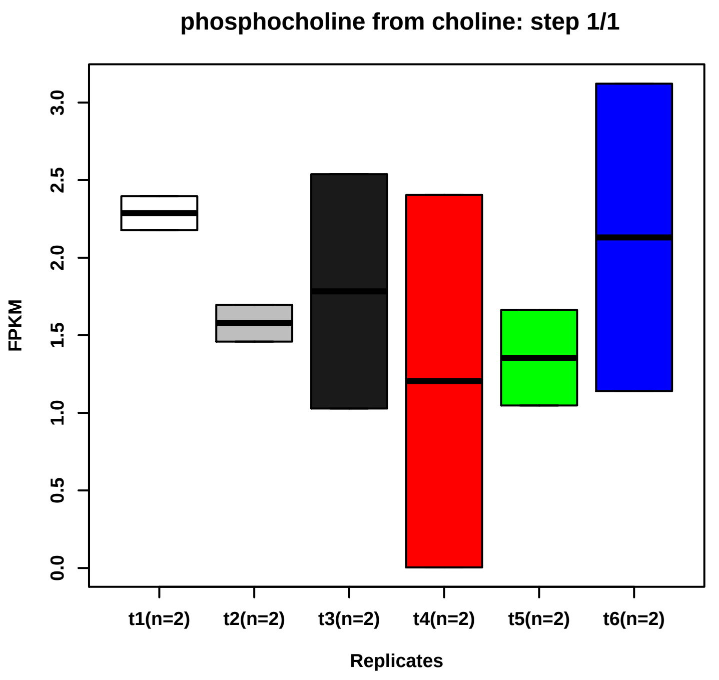
phosphocholine from choline: step 1/1
FPKM
0.0
0.5
1.0
1.5
2.0
2.5
3.0
t1(n=2)
t2(n=2)
t3(n=2)
t4(n=2)
t5(n=2)
t6(n=2)
Replicates

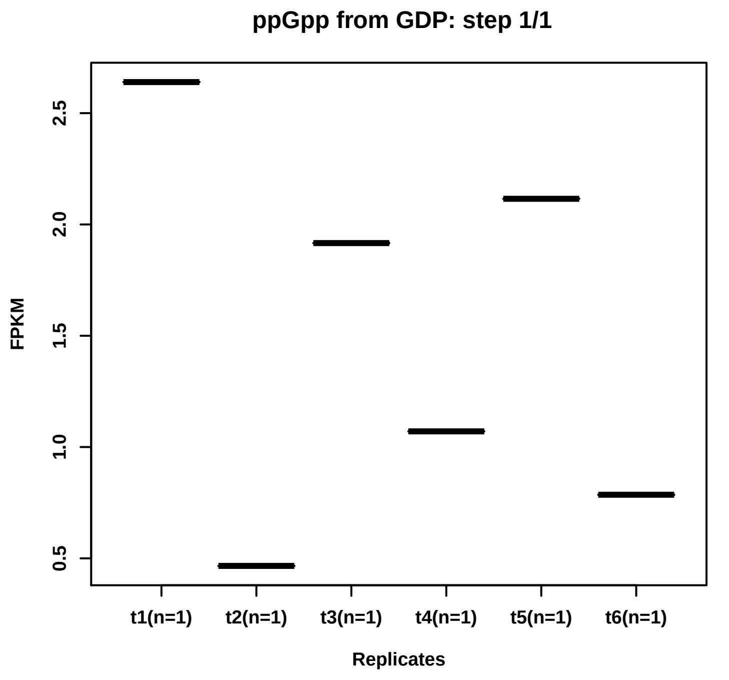
ppGpp from GDP: step 1/1
FPKM
0.5
1.0
1.5
2.0
2.5
t1(n=1)
t2(n=1)
t3(n=1)
t4(n=1)
t5(n=1)
t6(n=1)
Replicates

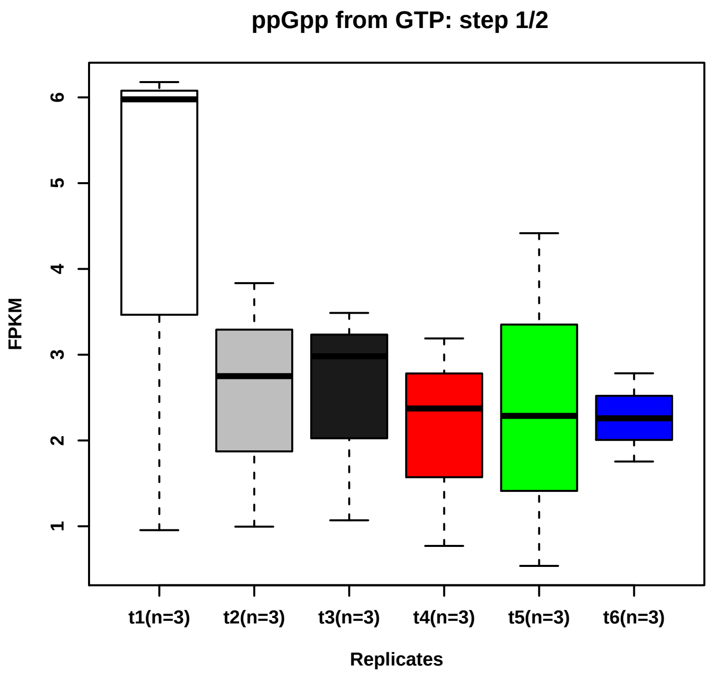
ppGpp from GTP: step 1/2
FPKM
1
2
3
4
5
6
t1(n=3)
t2(n=3)
t3(n=3)
t4(n=3)
t5(n=3)
t6(n=3)
Replicates

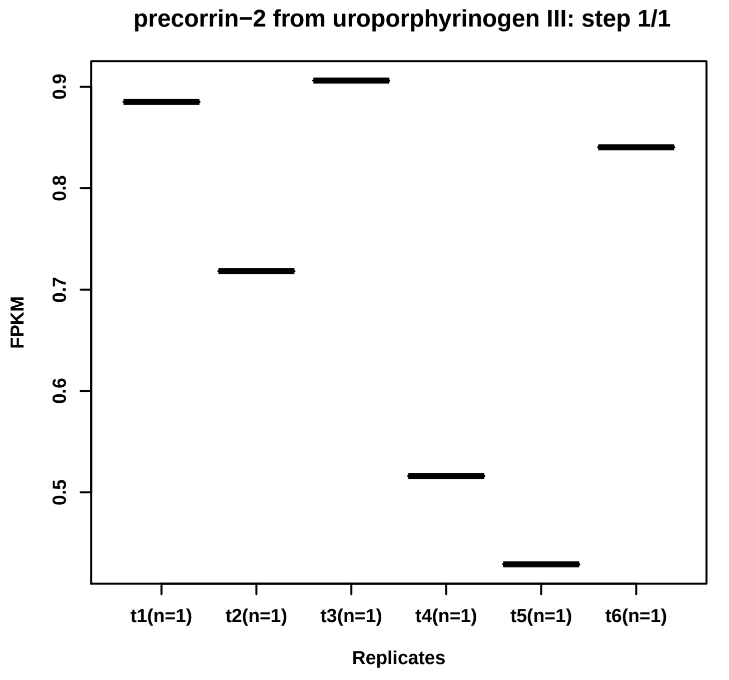
precorrin−2 from uroporphyrinogen III: step 1/1
FPKM
0.5
0.6
0.7
0.8
0.9
t1(n=1)
t2(n=1)
t3(n=1)
t4(n=1)
t5(n=1)
t6(n=1)
Replicates

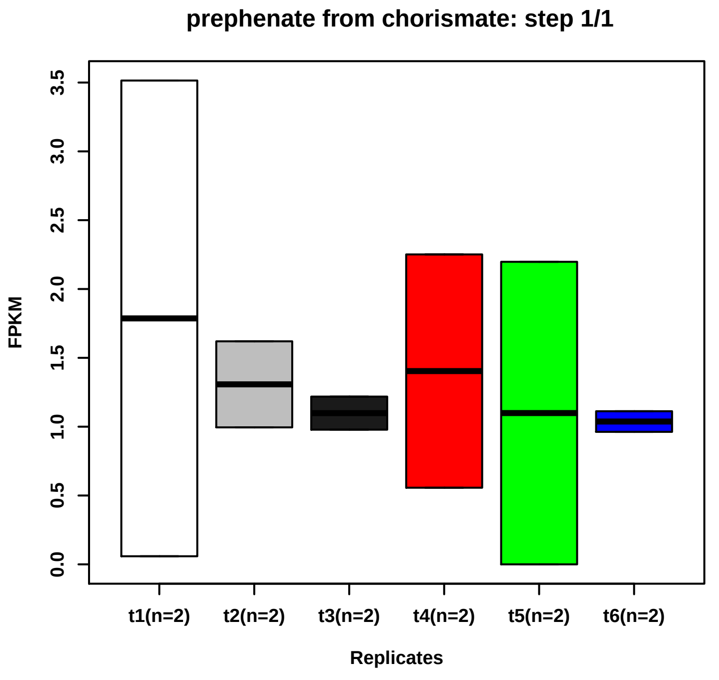
prephenate from chorismate: step 1/1
FPKM
0.0
0.5
1.0
1.5
2.0
2.5
3.0
3.5
t1(n=2)
t2(n=2)
t3(n=2)
t4(n=2)
t5(n=2)
t6(n=2)
Replicates

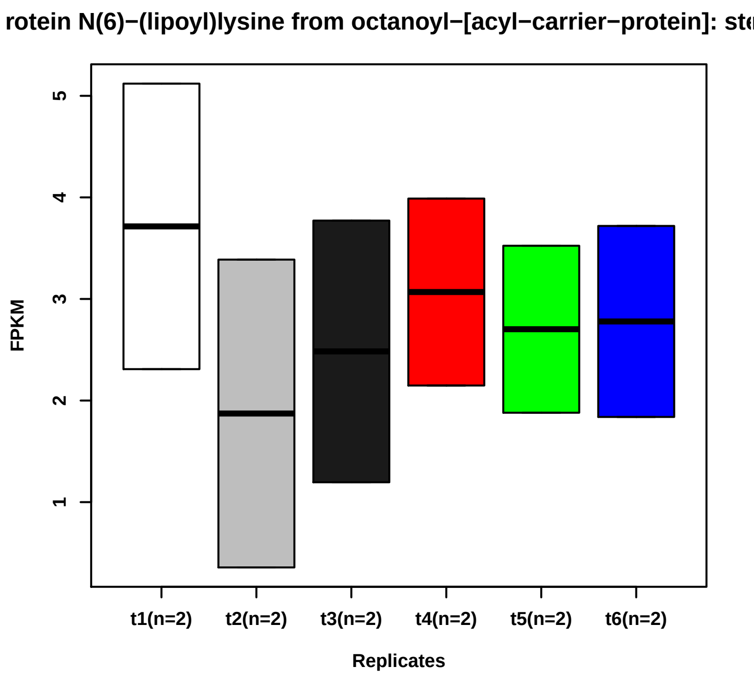
rotein N(6)−(lipoyl)lysine from octanoyl−[acyl−carrier−protein]: st
FPKM
Replicates
t1(n=2) t2(n=2) t3(n=2) t4(n=2) t5(n=2) t6(n=2)

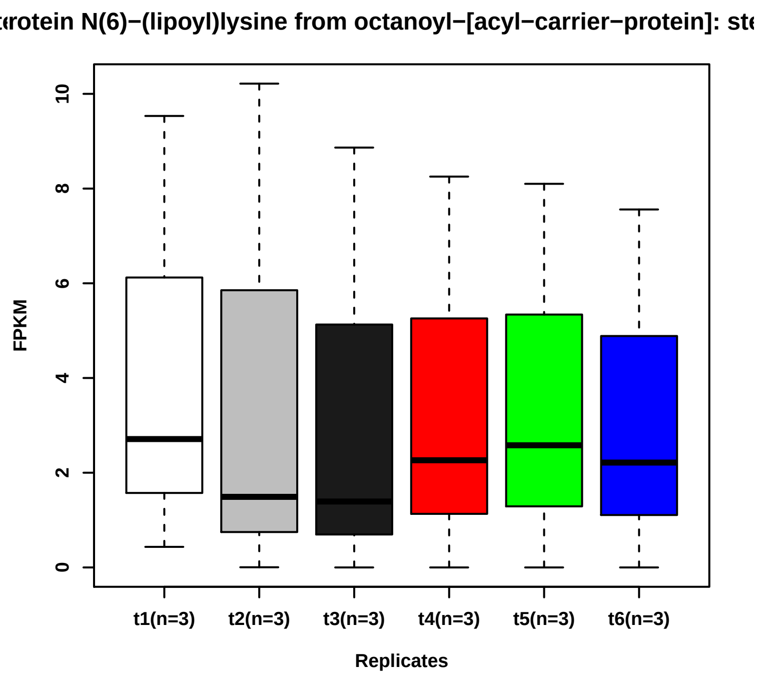
rotein N(6)−(lipoyl)lysine from octanoyl−[acyl−carrier−protein]: ste
FPKM
Replicates
t1(n=3) t2(n=3) t3(n=3) t4(n=3) t5(n=3) t6(n=3)

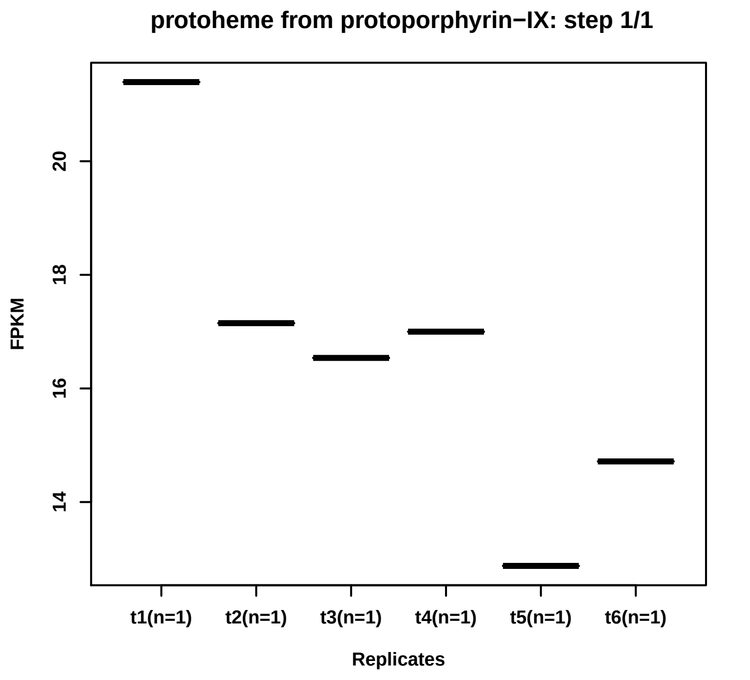
protoheme from protoporphyrin−IX: step 1/1
FPKM
Replicates
t1(n=1) t2(n=1) t3(n=1) t4(n=1) t5(n=1) t6(n=1)

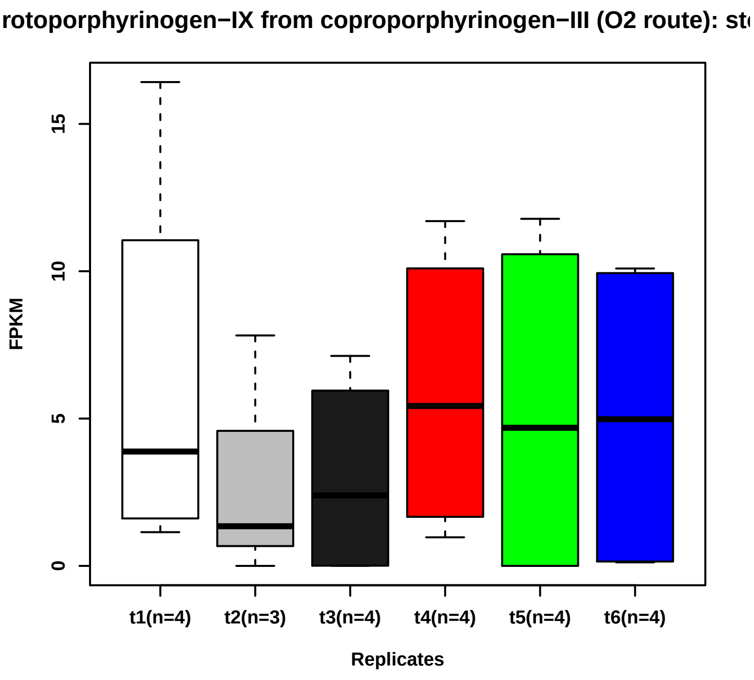
rotoporphyrinogen−IX from coproporphyrinogen−III (O2 route): st
FPKM
Replicates
t1(n=4) t2(n=3) t3(n=4) t4(n=4) t5(n=4) t6(n=4)

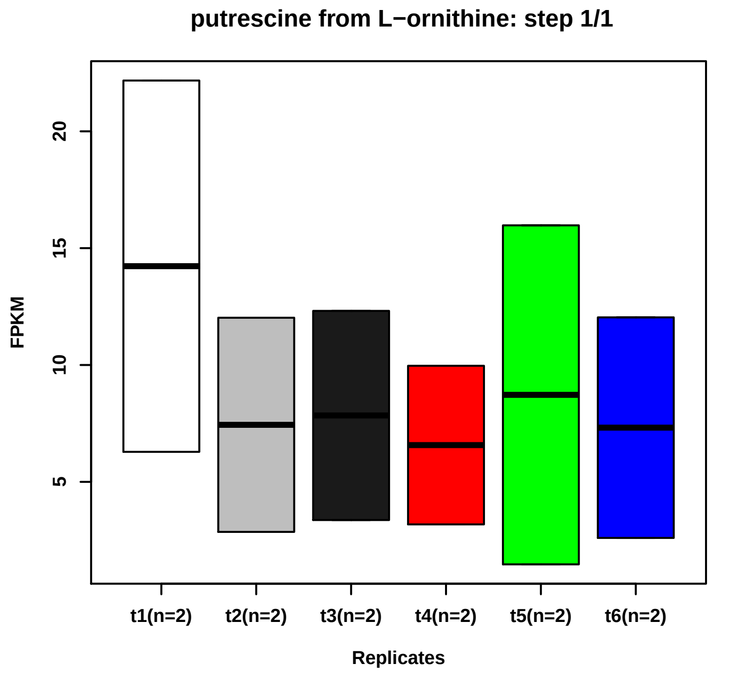
putrescine from L−ornithine: step 1/1
FPKM
Replicates
t1(n=2) t2(n=2) t3(n=2) t4(n=2) t5(n=2) t6(n=2)

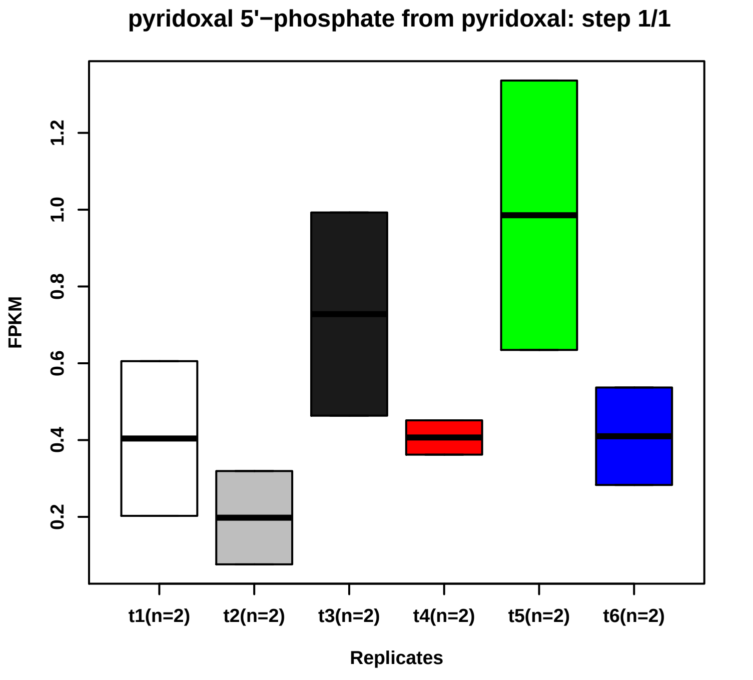
pyridoxal 5'−phosphate from pyridoxal: step 1/1
FPKM
Replicates
t1(n=2) t2(n=2) t3(n=2) t4(n=2) t5(n=2) t6(n=2)

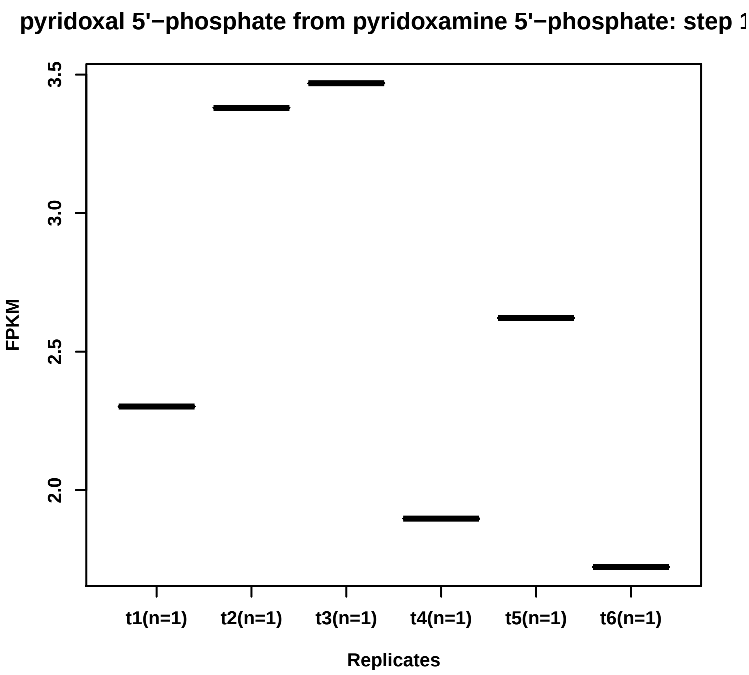
pyridoxal 5'−phosphate from pyridoxamine 5'−phosphate: step 1
FPKM
2.0
2.5
3.0
3.5
t1(n=1)
t2(n=1)
t3(n=1)
t4(n=1)
t5(n=1)
t6(n=1)
Replicates

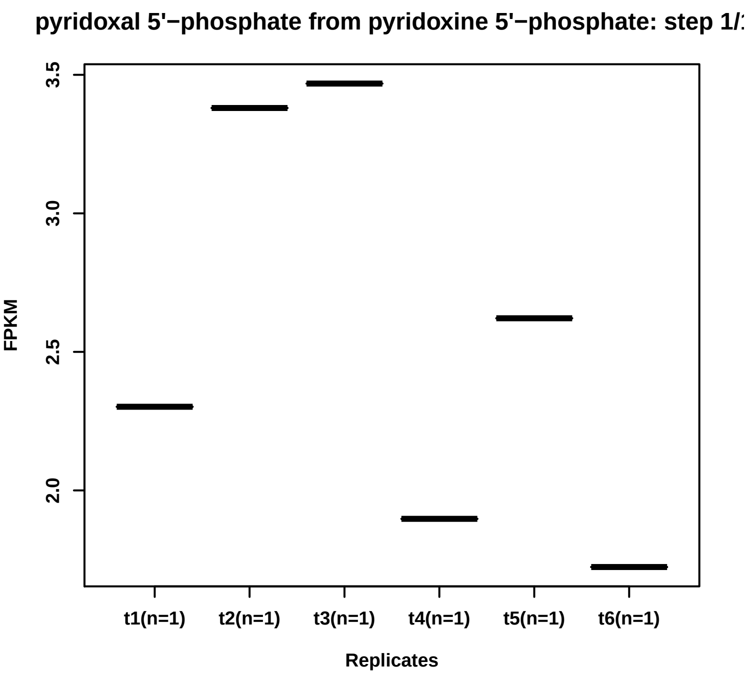
pyridoxal 5'−phosphate from pyridoxine 5'−phosphate: step 1/
FPKM
2.0
2.5
3.0
3.5
t1(n=1)
t2(n=1)
t3(n=1)
t4(n=1)
t5(n=1)
t6(n=1)
Replicates

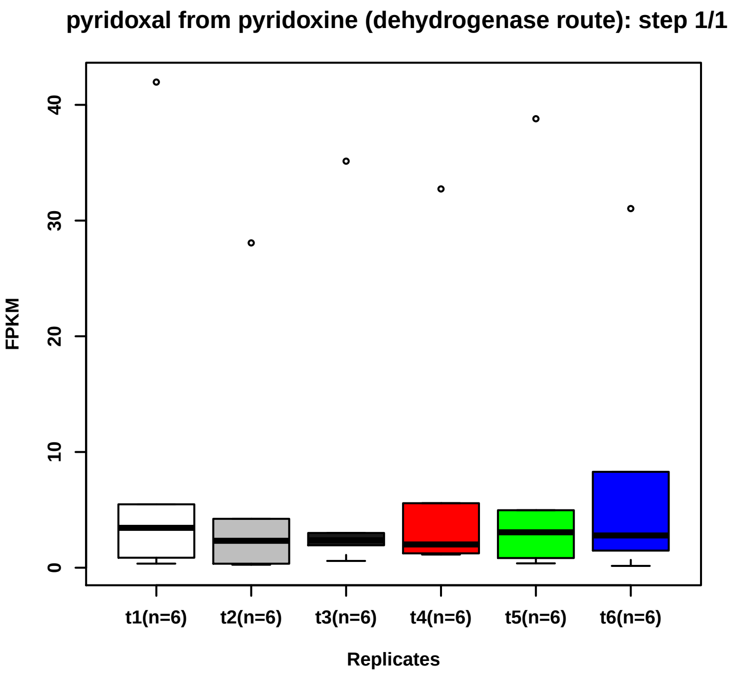
pyridoxal from pyridoxine (dehydrogenase route): step 1/1
FPKM
0
10
20
30
40
t1(n=6)
t2(n=6)
t3(n=6)
t4(n=6)
t5(n=6)
t6(n=6)
Replicates

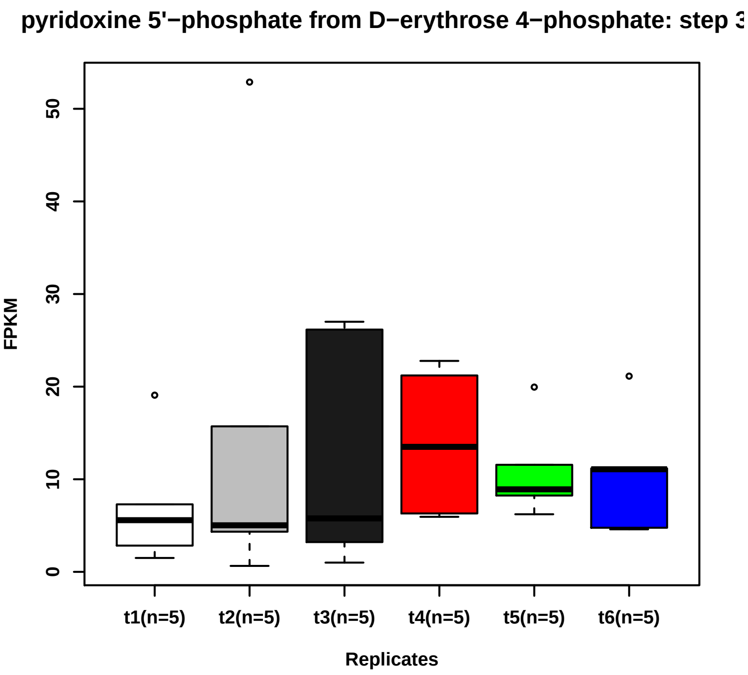
pyridoxine 5'−phosphate from D−erythrose 4−phosphate: step 3
FPKM
0
10
20
30
40
50
t1(n=5)
t2(n=5)
t3(n=5)
t4(n=5)
t5(n=5)
t6(n=5)
Replicates

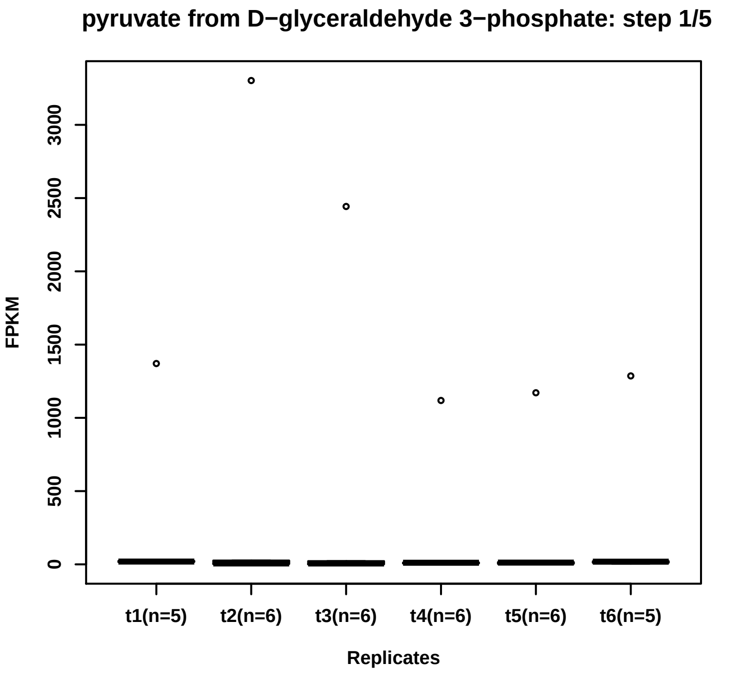
pyruvate from D−glyceraldehyde 3−phosphate: step 1/5
FPKM
0
500
1000
1500
2000
2500
3000
t1(n=5)
t2(n=6)
t3(n=6)
t4(n=6)
t5(n=6)
t6(n=5)
Replicates

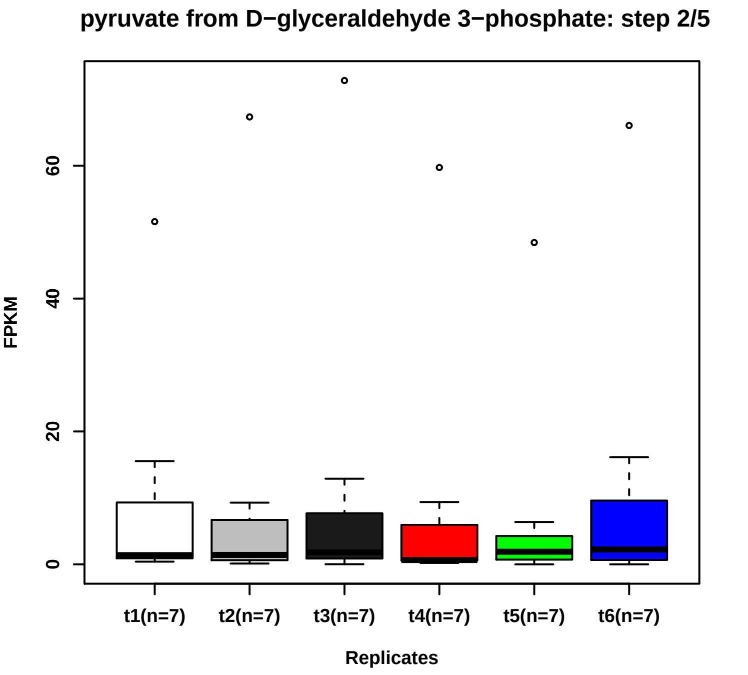
pyruvate from D−glyceraldehyde 3−phosphate: step 2/5
FPKM
0
20
40
60
t1(n=7)
t2(n=7)
t3(n=7)
t4(n=7)
t5(n=7)
t6(n=7)
Replicates

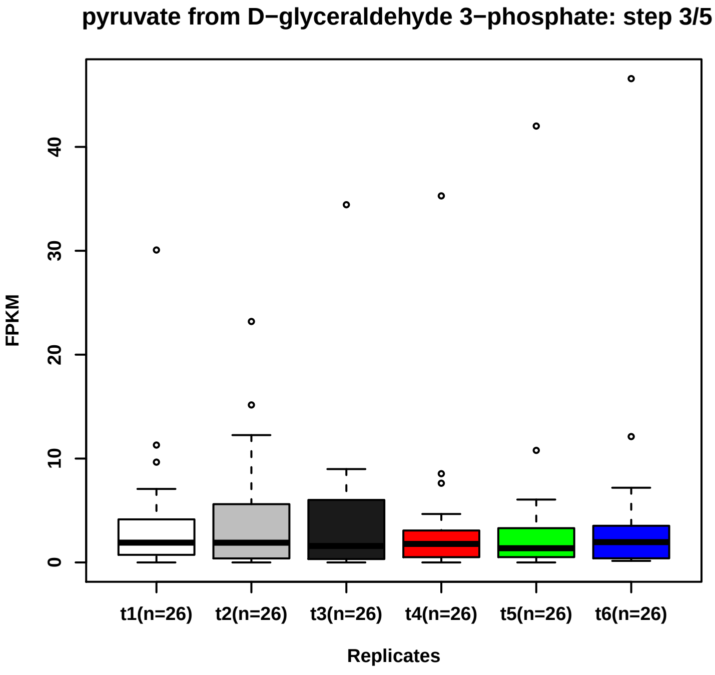
pyruvate from D−glyceraldehyde 3−phosphate: step 3/5
FPKM
0
10
20
30
40
t1(n=26)
t2(n=26)
t3(n=26)
t4(n=26)
t5(n=26)
t6(n=26)
Replicates

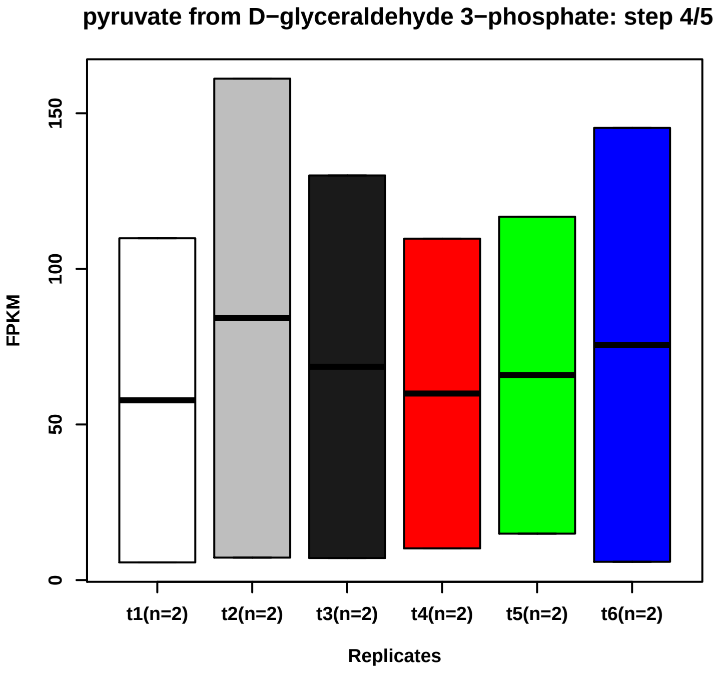
pyruvate from D−glyceraldehyde 3−phosphate: step 4/5
FPKM
0
50
100
150
t1(n=2)
t2(n=2)
t3(n=2)
t4(n=2)
t5(n=2)
t6(n=2)
Replicates

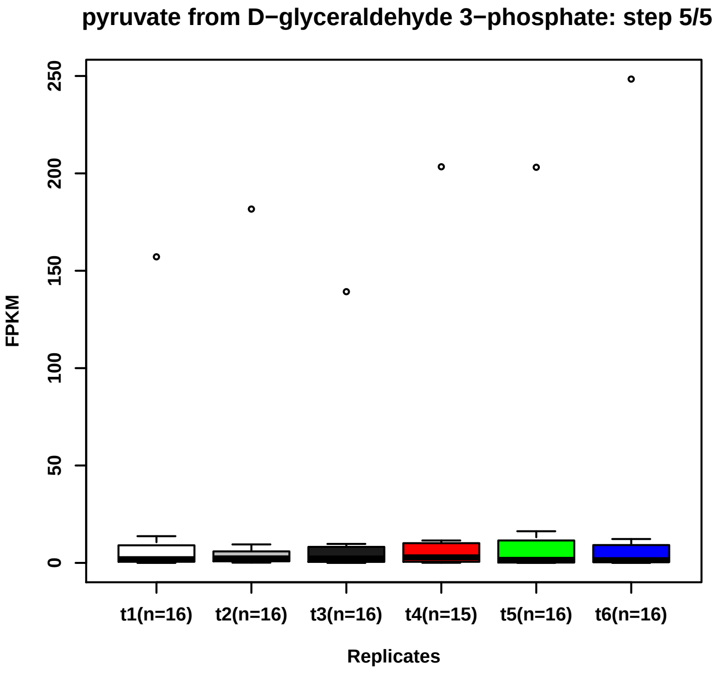
pyruvate from D−glyceraldehyde 3−phosphate: step 5/5
FPKM
0
50
100
150
200
250
t1(n=16)
t2(n=16)
t3(n=16)
t4(n=15)
t5(n=16)
t6(n=16)
Replicates

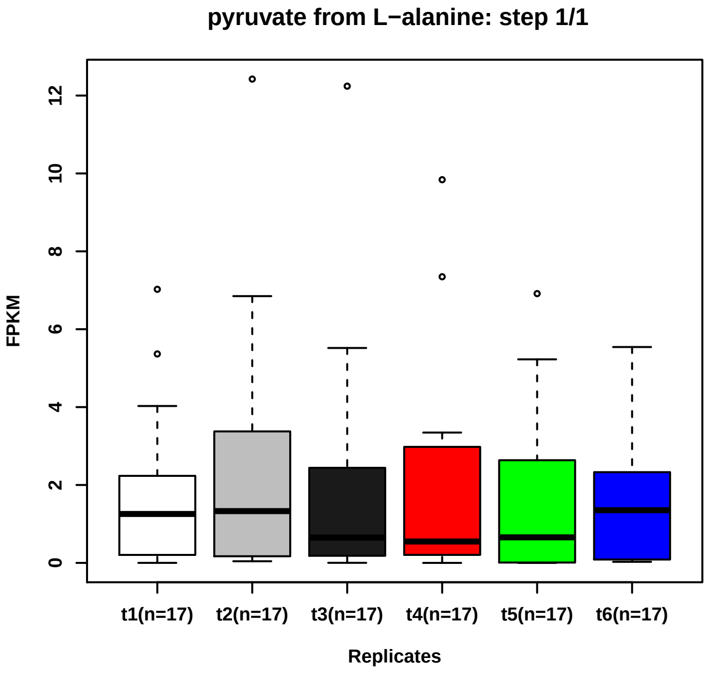
pyruvate from L−alanine: step 1/1
FPKM
0
2
4
6
8
10
12
t1(n=17)
t2(n=17)
t3(n=17)
t4(n=17)
t5(n=17)
t6(n=17)
Replicates

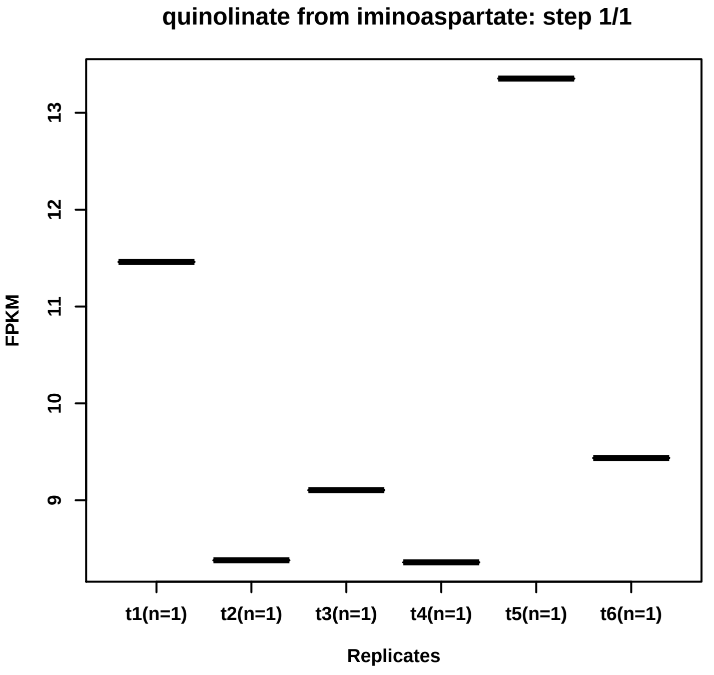
quinolinate from iminoaspartate: step 1/1
FPKM
9
10
11
12
13
t1(n=1)
t2(n=1)
t3(n=1)
t4(n=1)
t5(n=1)
t6(n=1)
Replicates

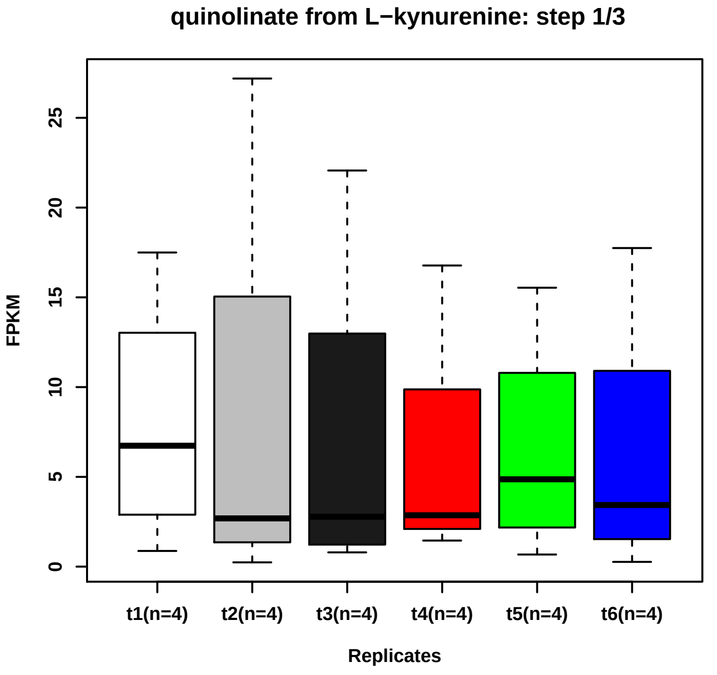
quinolinate from L−kynurenine: step 1/3
FPKM
0
5
10
15
20
25
t1(n=4)
t2(n=4)
t3(n=4)
t4(n=4)
t5(n=4)
t6(n=4)
Replicates

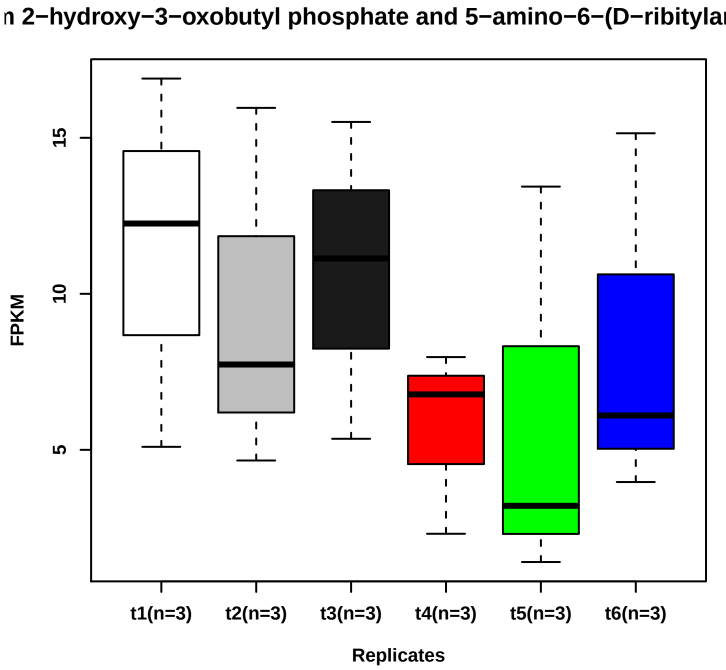
n 2−hydroxy−3−oxobutyl phosphate and 5−amino−6−(D−ribitylamin
FPKM
5
10
15
t1(n=3)
t2(n=3)
t3(n=3)
t4(n=3)
t5(n=3)
t6(n=3)
Replicates

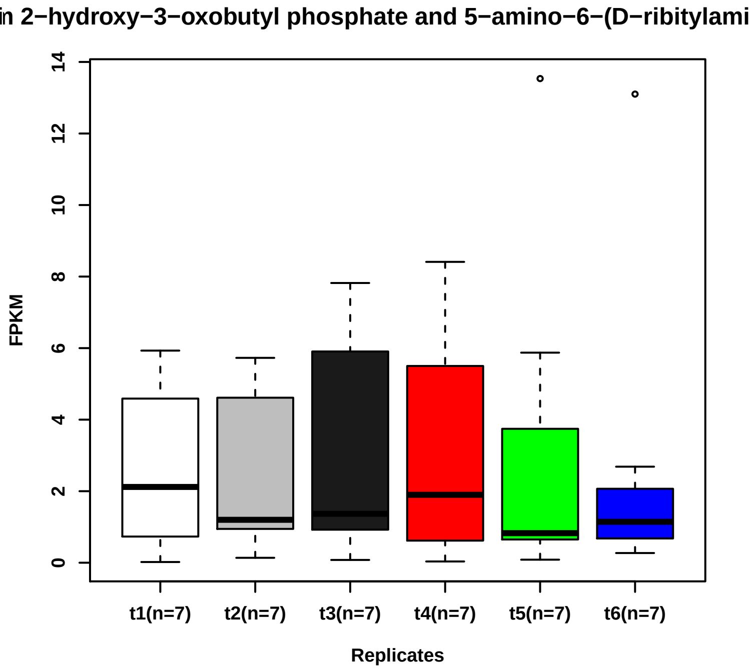
n 2−hydroxy−3−oxobutyl phosphate and 5−amino−6−(D−ribitylami
FPKM
0
2
4
6
8
10
12
14
t1(n=7)
t2(n=7)
t3(n=7)
t4(n=7)
t5(n=7)
t6(n=7)
Replicates

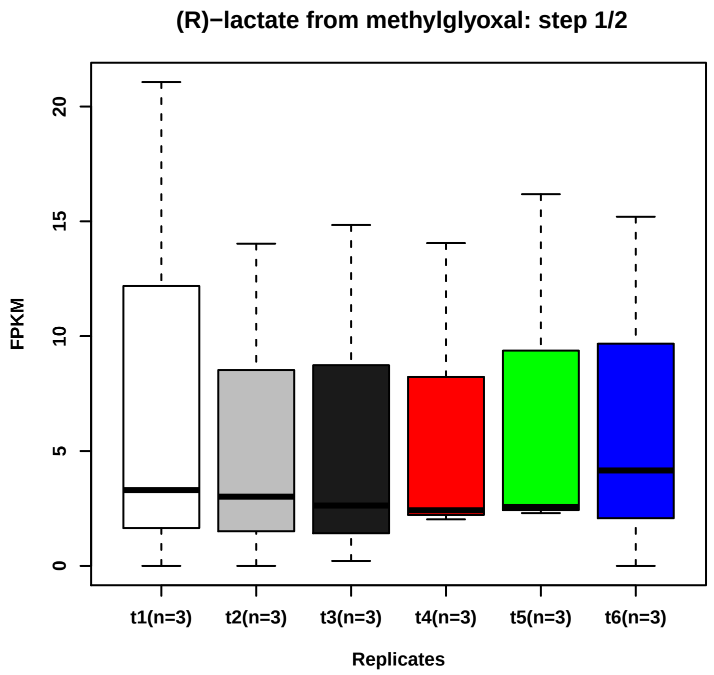
(R)−lactate from methylglyoxal: step 1/2
FPKM
0
5
10
15
20
t1(n=3)
t2(n=3)
t3(n=3)
t4(n=3)
t5(n=3)
t6(n=3)
Replicates

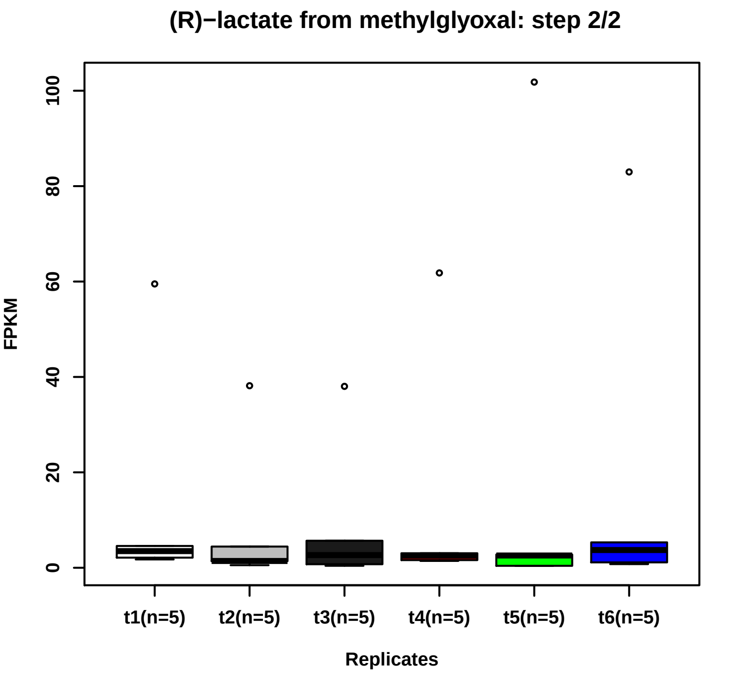
(R)−lactate from methylglyoxal: step 2/2
FPKM
0
20
40
60
80
100
t1(n=5)
t2(n=5)
t3(n=5)
t4(n=5)
t5(n=5)
t6(n=5)
Replicates

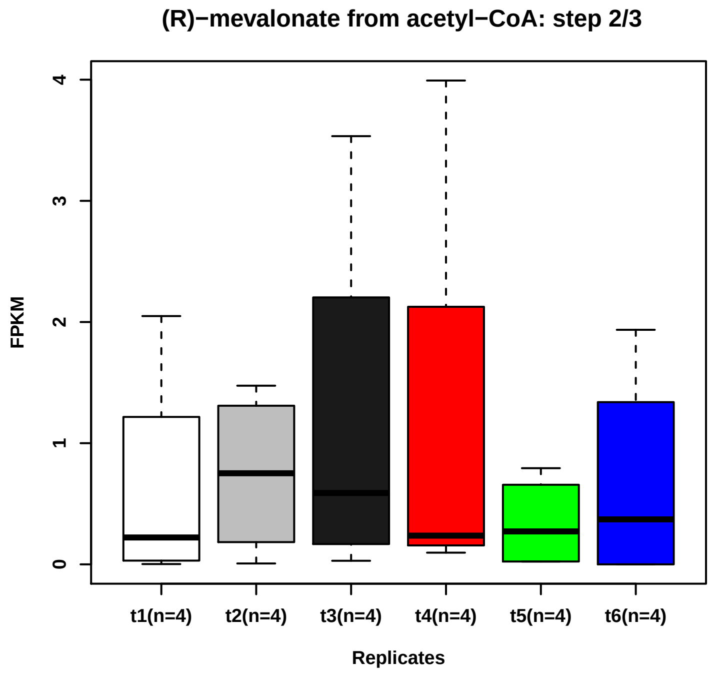
(R)−mevalonate from acetyl−CoA: step 2/3
FPKM
0
1
2
3
4
t1(n=4)
t2(n=4)
t3(n=4)
t4(n=4)
t5(n=4)
t6(n=4)
Replicates

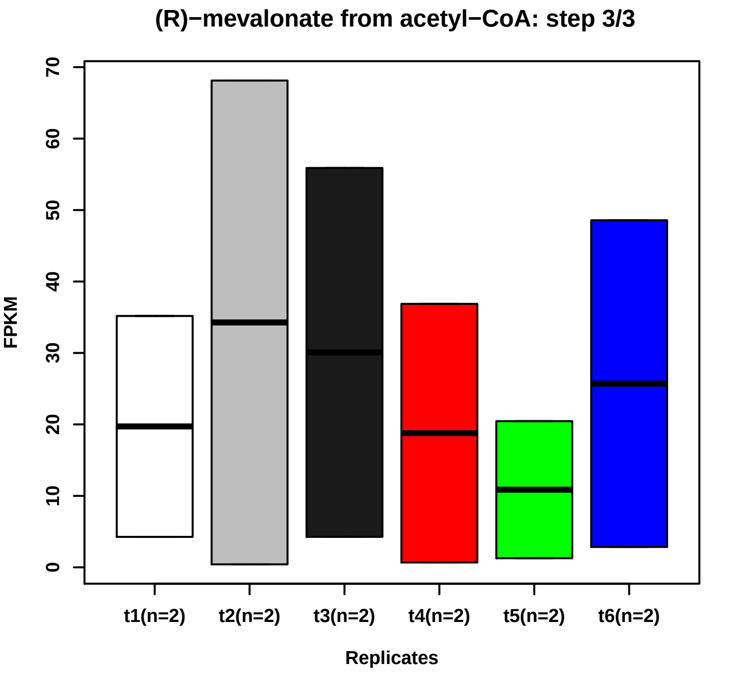
(R)−mevalonate from acetyl−CoA: step 3/3
FPKM
0
10
20
30
40
50
60
70
t1(n=2)
t2(n=2)
t3(n=2)
t4(n=2)
t5(n=2)
t6(n=2)
Replicates

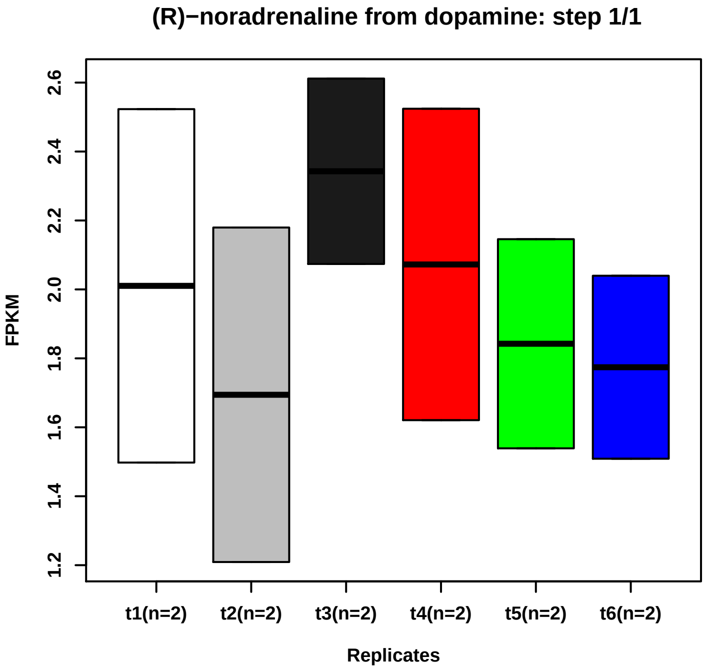
(R)−noradrenaline from dopamine: step 1/1
FPKM
1.2 1.4 1.6 1.8 2.0 2.2 2.4 2.6
t1(n=2) t2(n=2) t3(n=2) t4(n=2) t5(n=2) t6(n=2)
Replicates

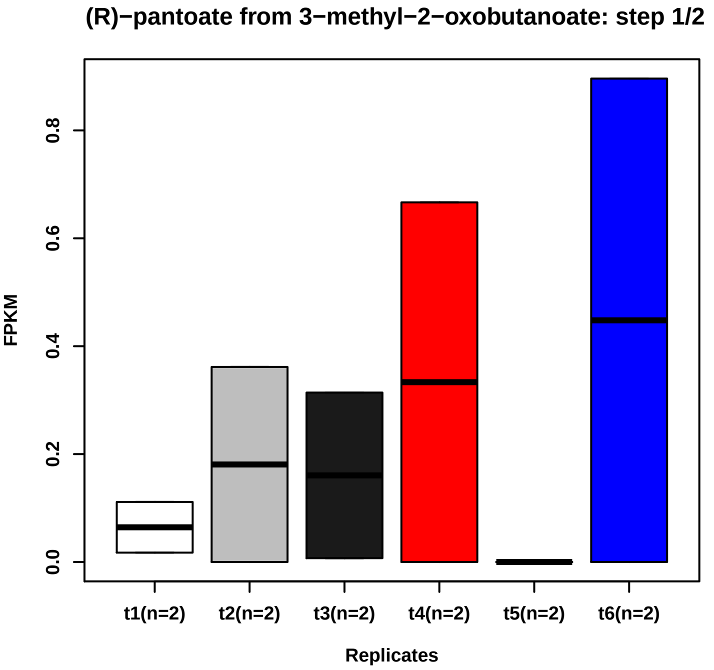
(R)−pantoate from 3−methyl−2−oxobutanoate: step 1/2
FPKM
0.0 0.2 0.4 0.6 0.8
t1(n=2) t2(n=2) t3(n=2) t4(n=2) t5(n=2) t6(n=2)
Replicates

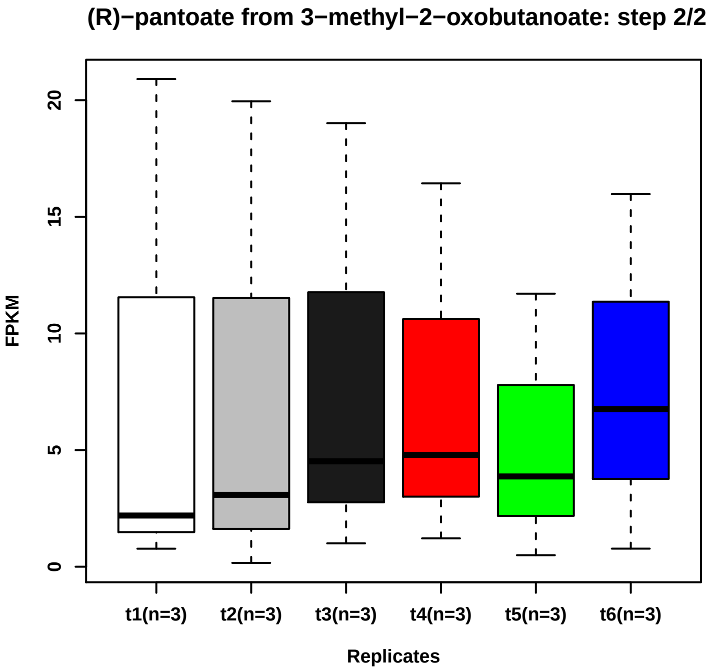
(R)−pantoate from 3−methyl−2−oxobutanoate: step 2/2
FPKM
0 5 10 15 20
t1(n=3) t2(n=3) t3(n=3) t4(n=3) t5(n=3) t6(n=3)
Replicates

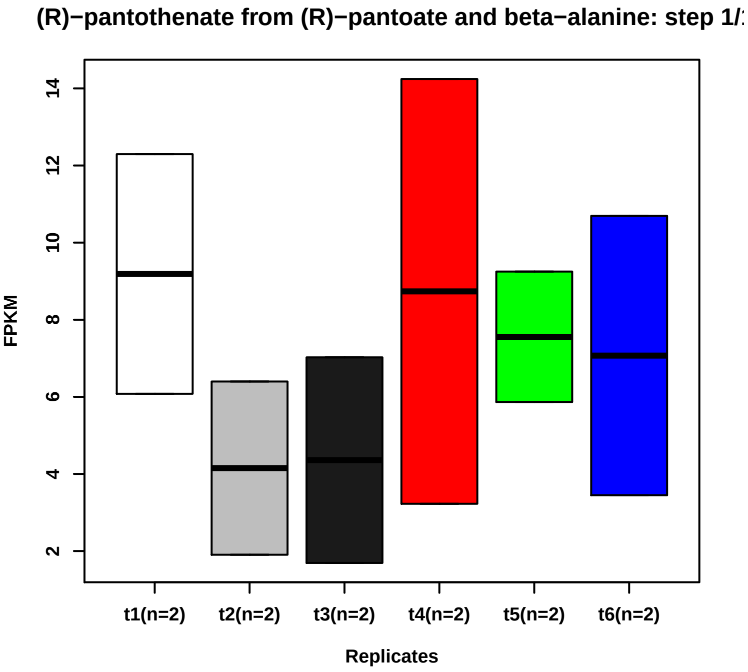
(R)−pantothenate from (R)−pantoate and beta−alanine: step 1/
FPKM
2 4 6 8 10 12 14
t1(n=2) t2(n=2) t3(n=2) t4(n=2) t5(n=2) t6(n=2)
Replicates

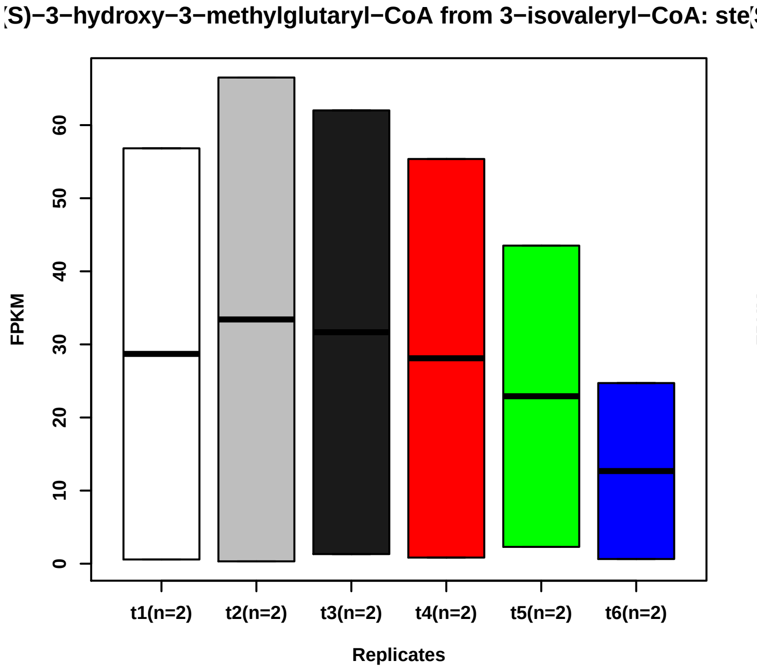
S)−3−hydroxy−3−methylglutaryl−CoA from 3−isovaleryl−CoA: ste
FPKM
0 10 20 30 40 50 60
t1(n=2) t2(n=2) t3(n=2) t4(n=2) t5(n=2) t6(n=2)
Replicates

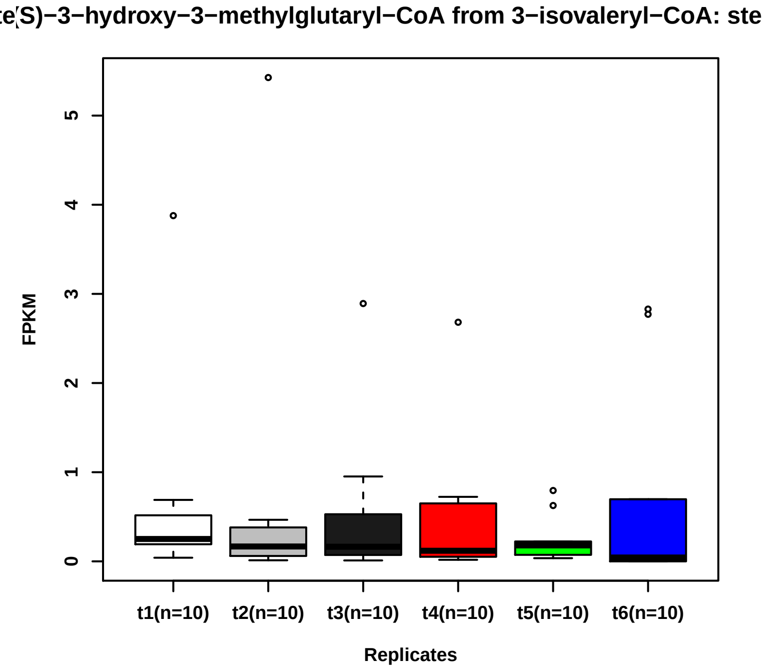
S)−3−hydroxy−3−methylglutaryl−CoA from 3−isovaleryl−CoA: ste
FPKM
0 1 2 3 4 5
t1(n=10) t2(n=10) t3(n=10) t4(n=10) t5(n=10) t6(n=10)
Replicates

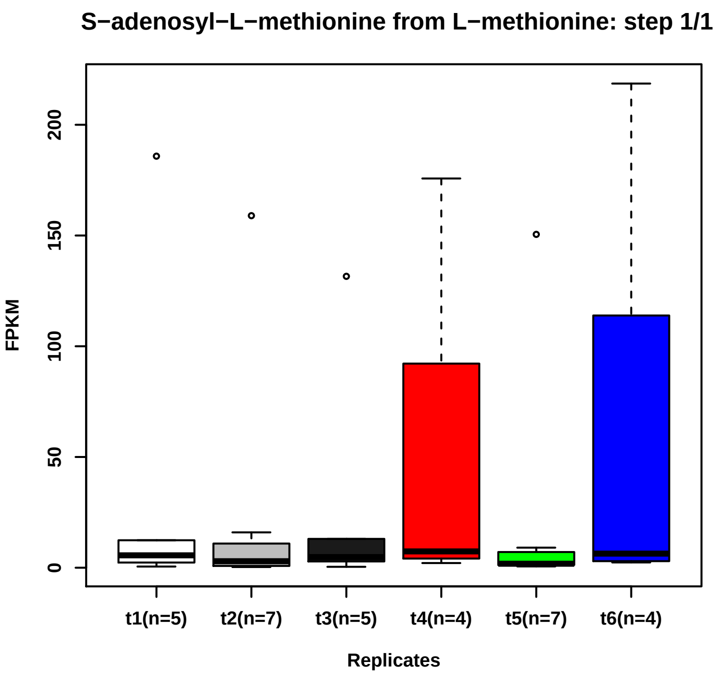
S−adenosyl−L−methionine from L−methionine: step 1/1
FPKM
0
50
100
150
200
t1(n=5)
t2(n=7)
t3(n=5)
t4(n=4)
t5(n=7)
t6(n=4)
Replicates

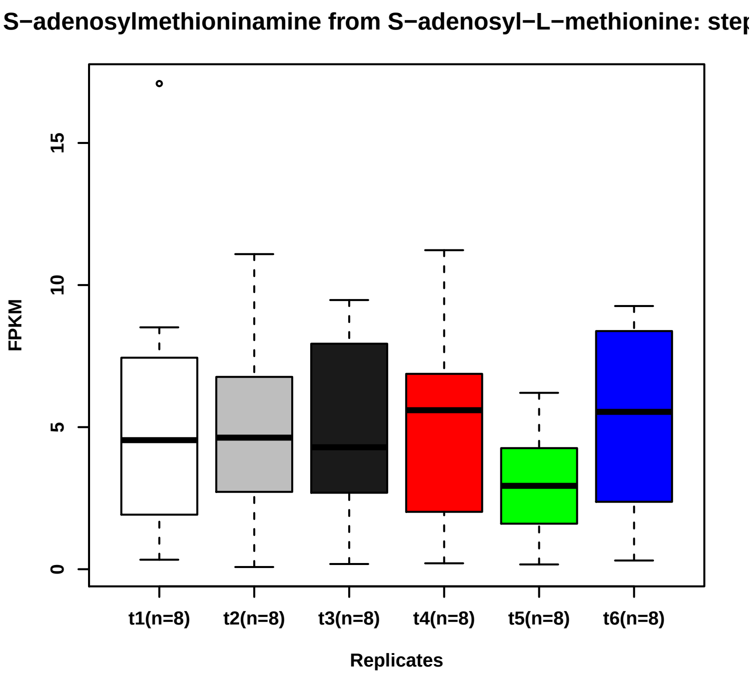
S−adenosylmethioninamine from S−adenosyl−L−methionine: step
FPKM
0
5
10
15
t1(n=8)
t2(n=8)
t3(n=8)
t4(n=8)
t5(n=8)
t6(n=8)
Replicates

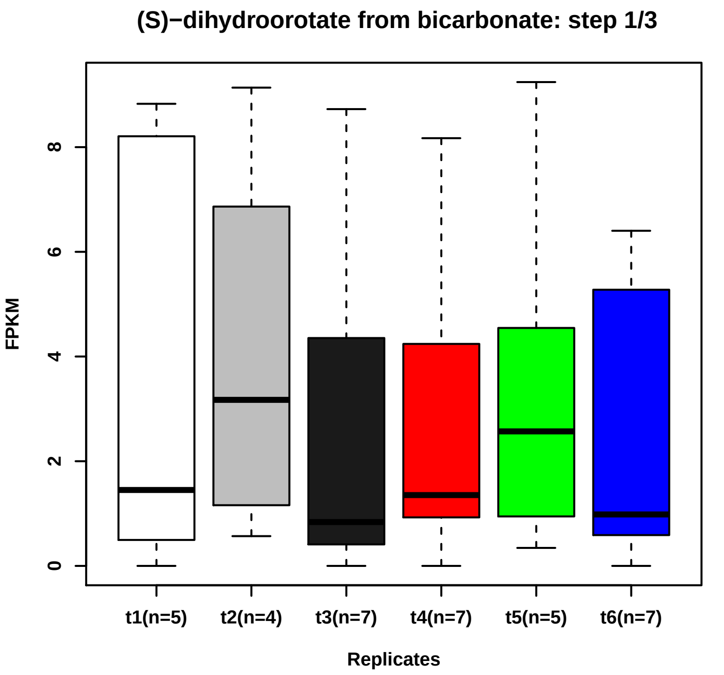
(S)−dihydroorotate from bicarbonate: step 1/3
FPKM
0
2
4
6
8
t1(n=5)
t2(n=4)
t3(n=7)
t4(n=7)
t5(n=5)
t6(n=7)
Replicates

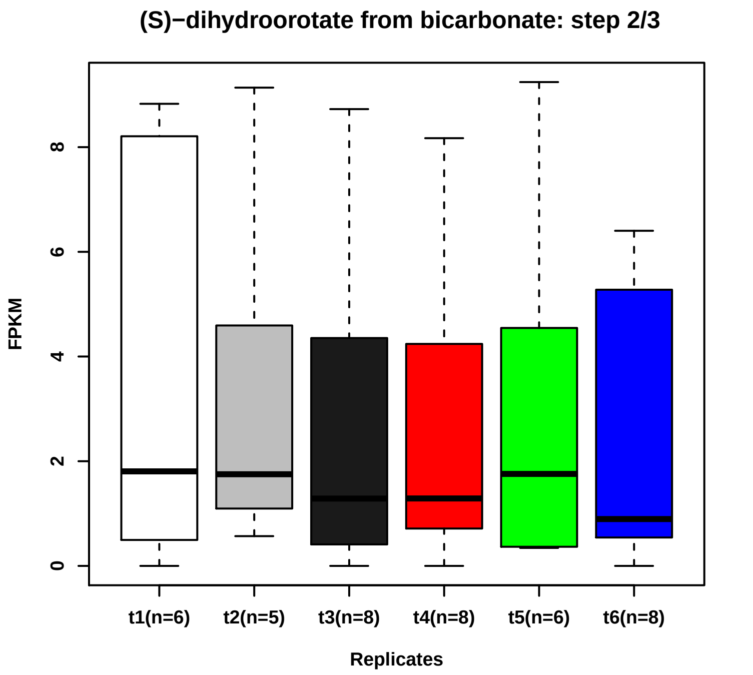
(S)−dihydroorotate from bicarbonate: step 2/3
FPKM
0
2
4
6
8
t1(n=6)
t2(n=5)
t3(n=8)
t4(n=8)
t5(n=6)
t6(n=8)
Replicates

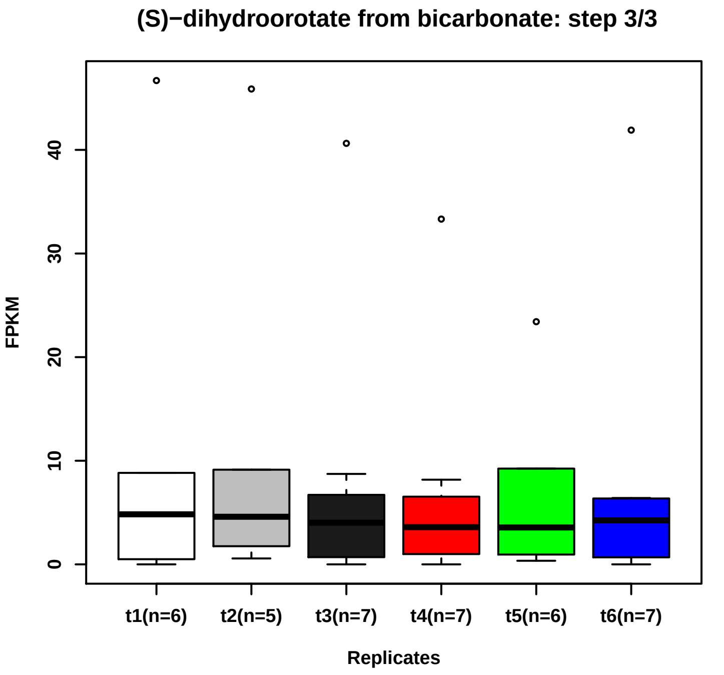
(S)−dihydroorotate from bicarbonate: step 3/3
FPKM
0
10
20
30
40
t1(n=6)
t2(n=5)
t3(n=7)
t4(n=7)
t5(n=6)
t6(n=7)
Replicates

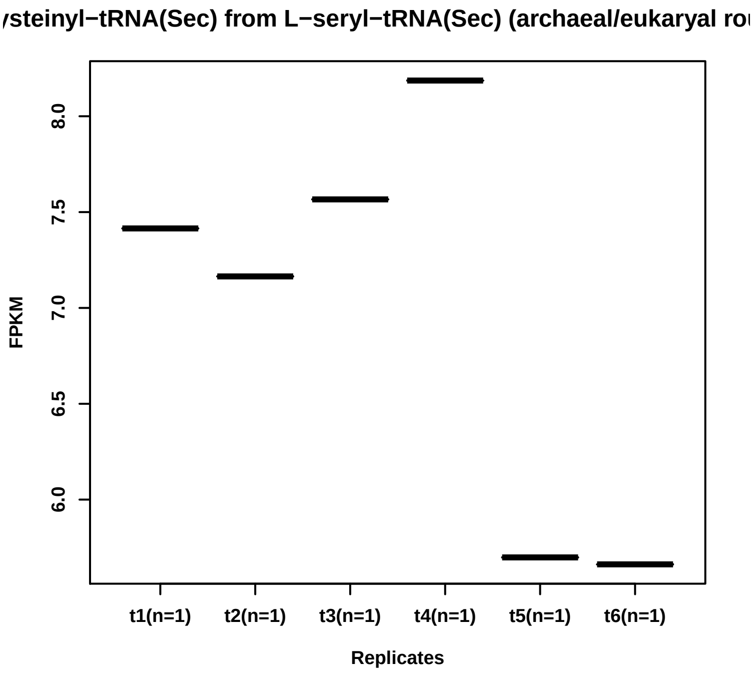
steinyl−tRNA(Sec) from L−seryl−tRNA(Sec) (archaeal/eukaryal ro
FPKM
6.0
6.5
7.0
7.5
8.0
t1(n=1)
t2(n=1)
t3(n=1)
t4(n=1)
t5(n=1)
t6(n=1)
Replicates

serotonin from L−tryptophan: step 1/2

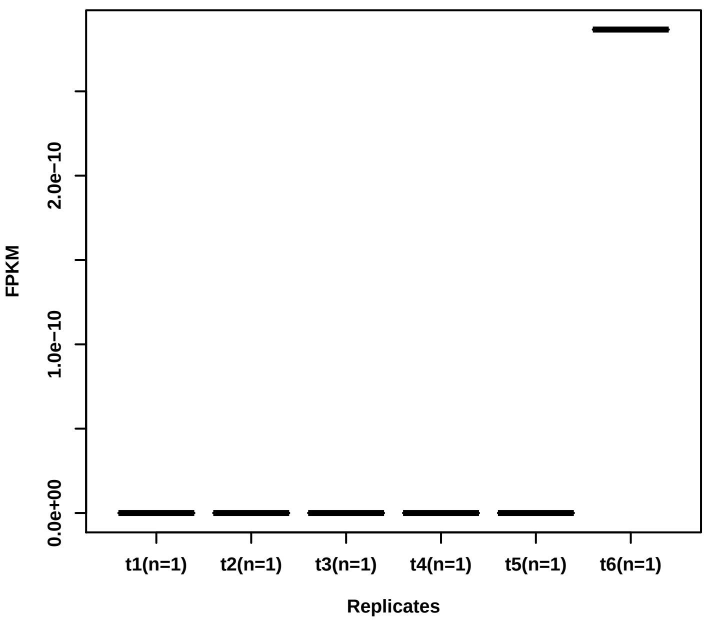

siroheme from sirohydrochlorin: step 1/1

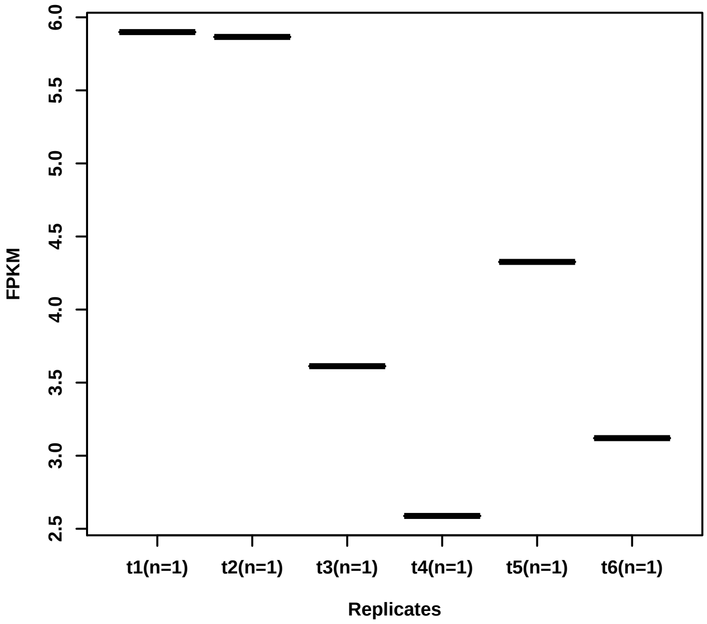

(S)−malate from fumarate: step 1/1

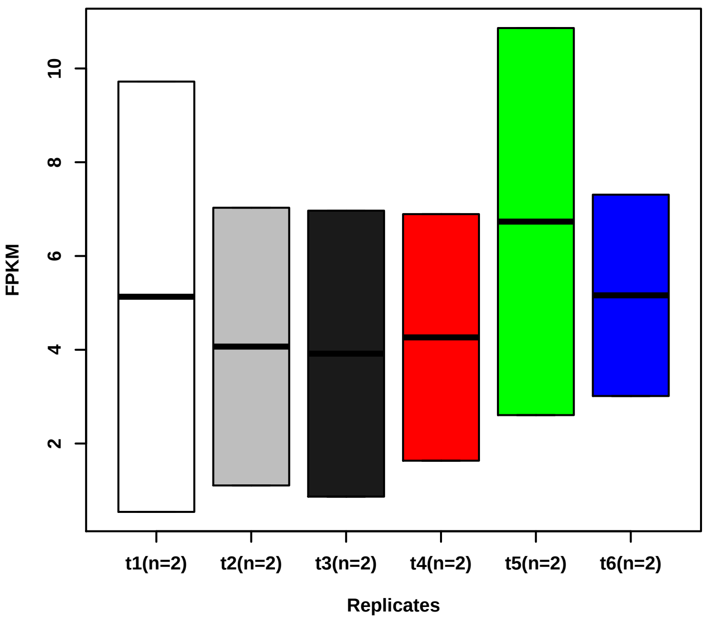

(S)−malate from isocitrate: step 1/2

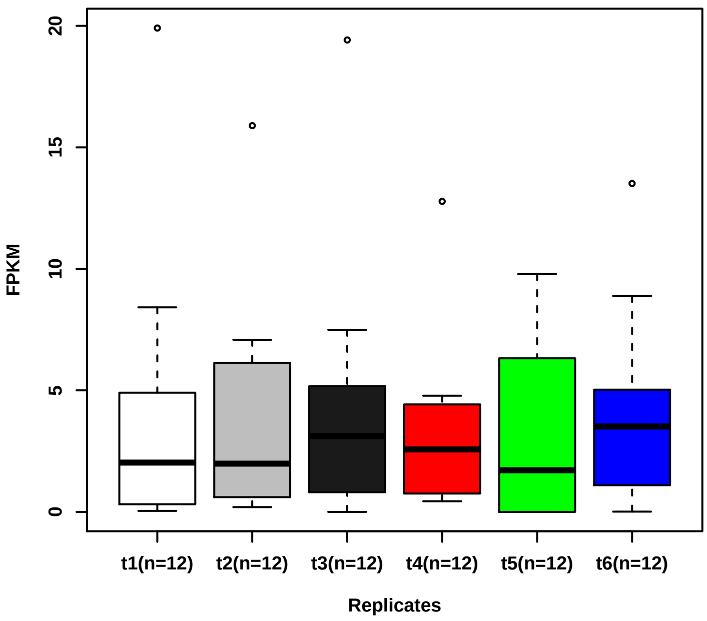

(S)−malate from isocitrate: step 2/2

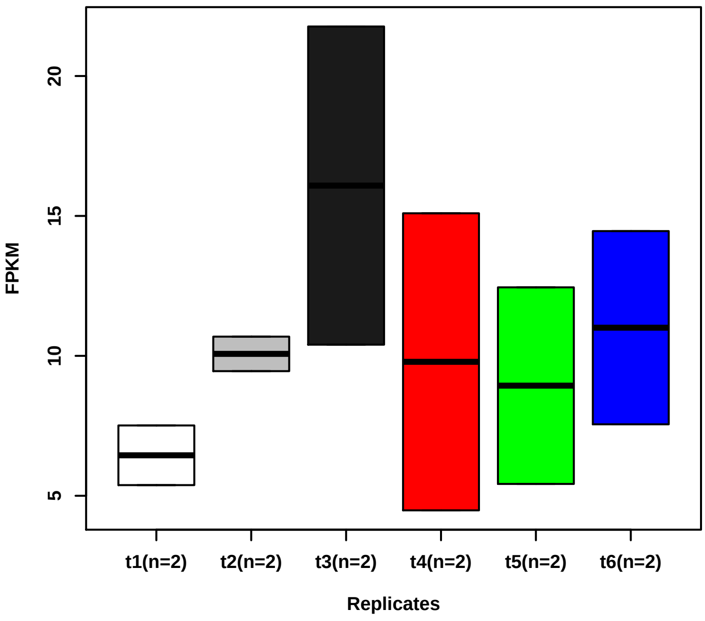

lpha−D−ribose 1−phosphate from S−methyl−5'−thioadenosine (hy

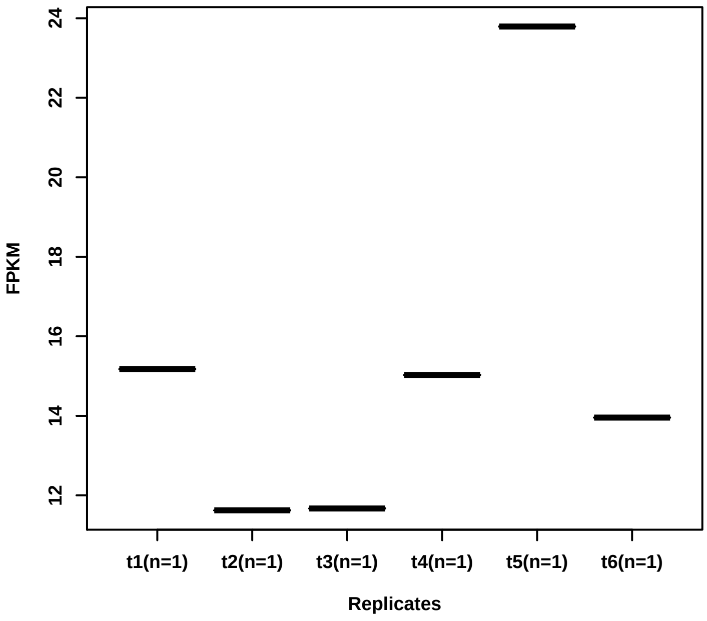

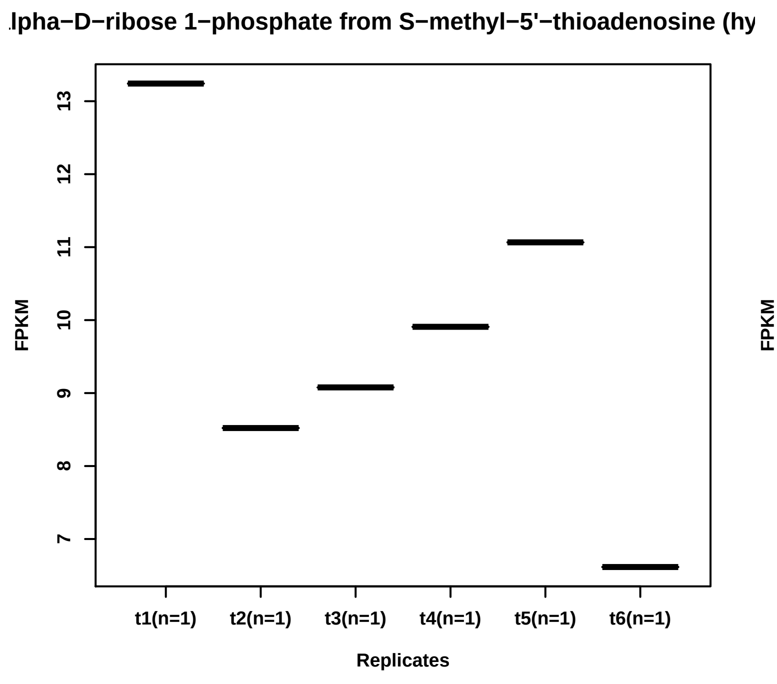

lpha−D−ribose 1−phosphate from S−methyl−5'−thioadenosine (hy
FPKM
Replicates
t1(n=1) t2(n=1) t3(n=1) t4(n=1) t5(n=1) t6(n=1)

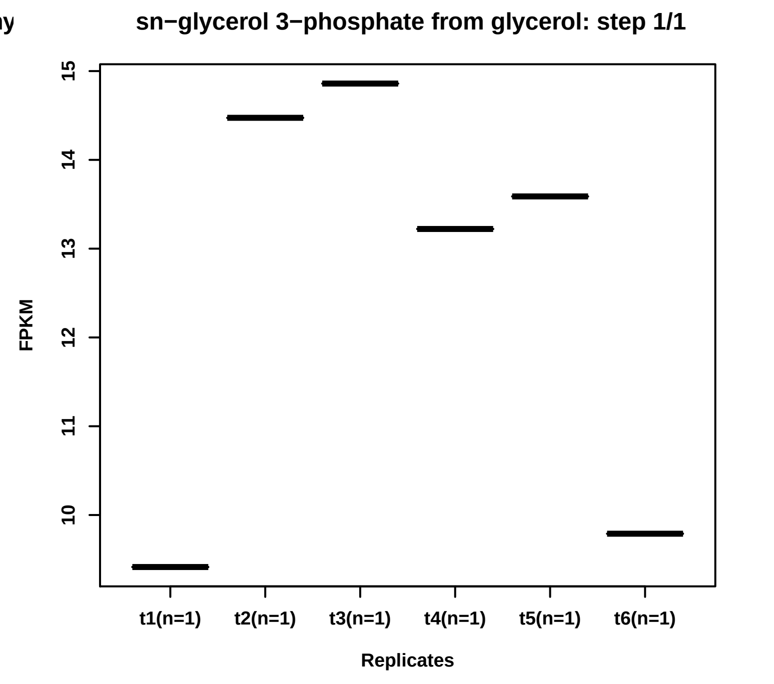

sn−glycerol 3−phosphate from glycerol: step 1/1
FPKM
Replicates
t1(n=1) t2(n=1) t3(n=1) t4(n=1) t5(n=1) t6(n=1)

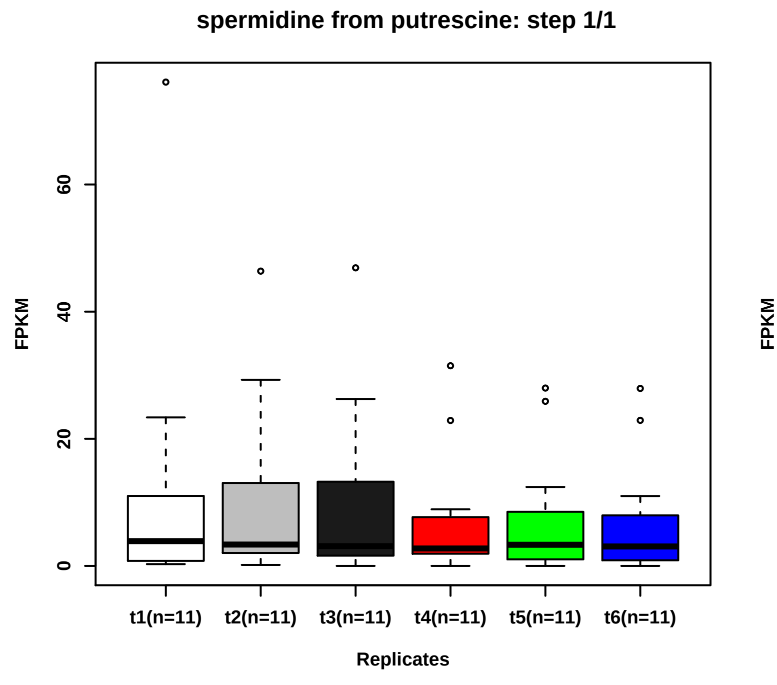

spermidine from putrescine: step 1/1
FPKM
Replicates
t1(n=11) t2(n=11) t3(n=11) t4(n=11) t5(n=11) t6(n=11)

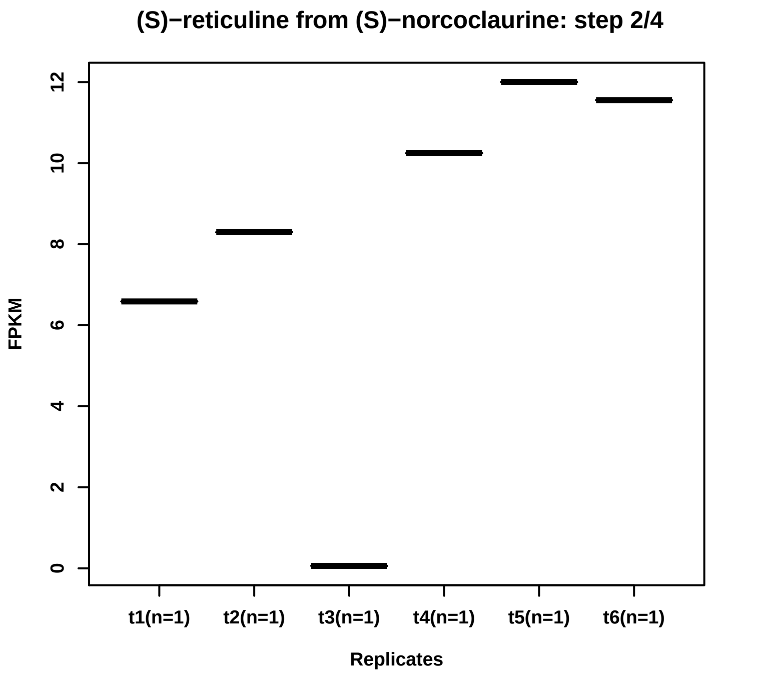

(S)−reticuline from (S)−norcoclaurine: step 2/4
FPKM
Replicates
t1(n=1) t2(n=1) t3(n=1) t4(n=1) t5(n=1) t6(n=1)

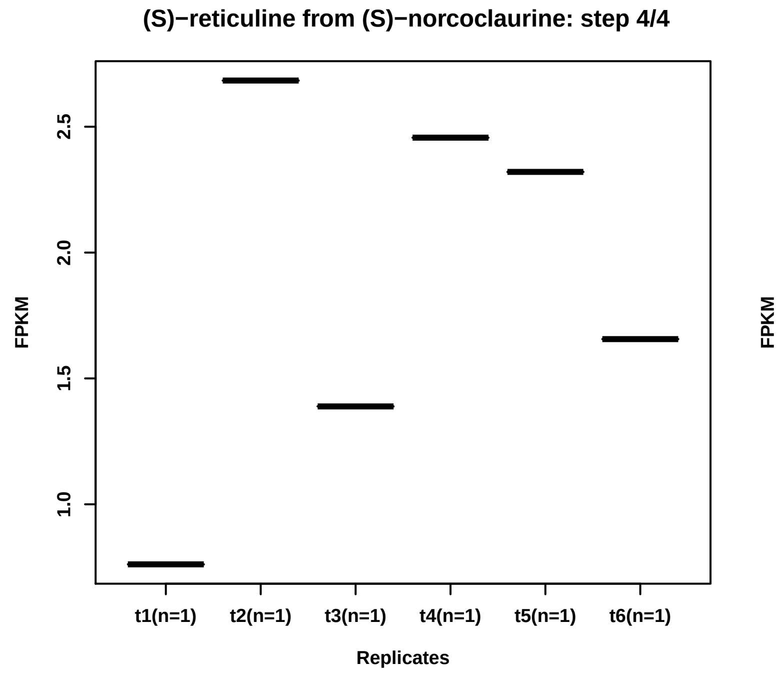

(S)−reticuline from (S)−norcoclaurine: step 4/4
FPKM
Replicates
t1(n=1) t2(n=1) t3(n=1) t4(n=1) t5(n=1) t6(n=1)

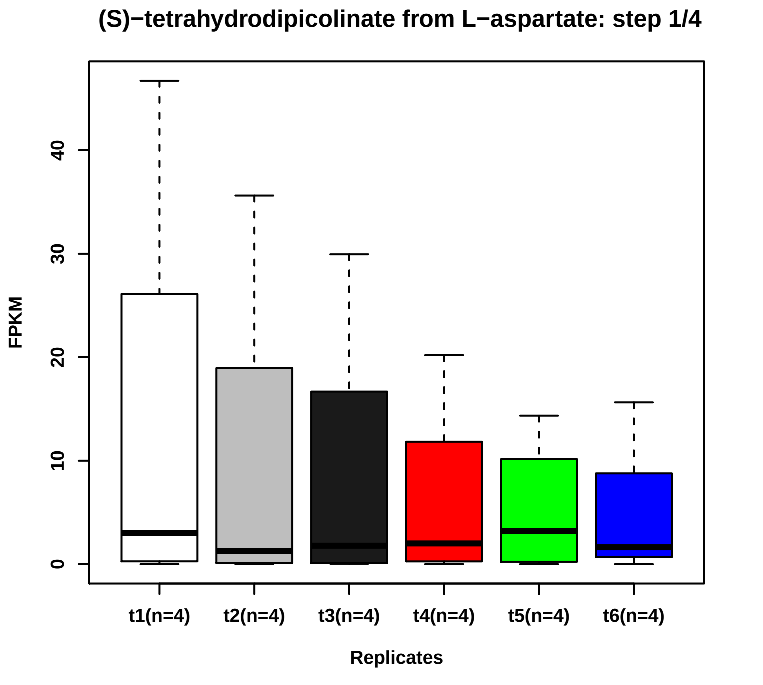

(S)−tetrahydrodipicolinate from L−aspartate: step 1/4
FPKM
Replicates
t1(n=4) t2(n=4) t3(n=4) t4(n=4) t5(n=4) t6(n=4)

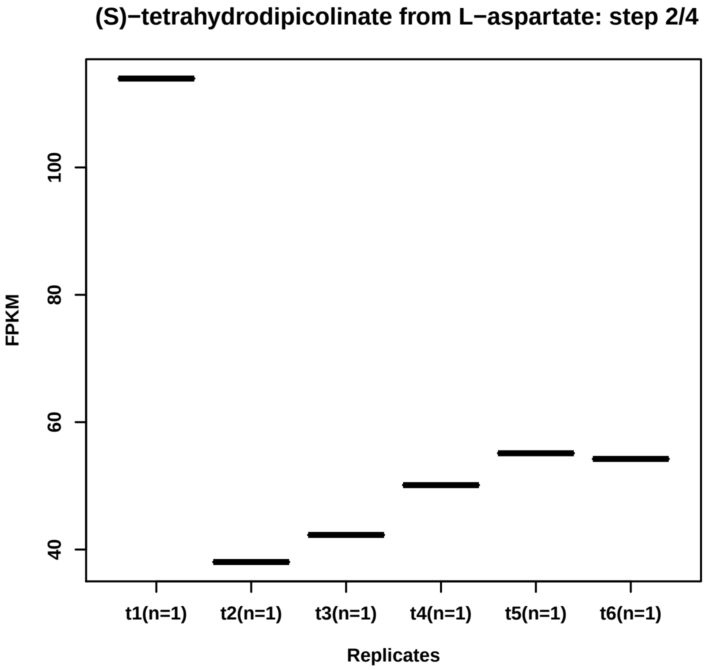
(S)−tetrahydrodipicolinate from L−aspartate: step 2/4
FPKM
40
60
80
100
t1(n=1)
t2(n=1)
t3(n=1)
t4(n=1)
t5(n=1)
t6(n=1)
Replicates

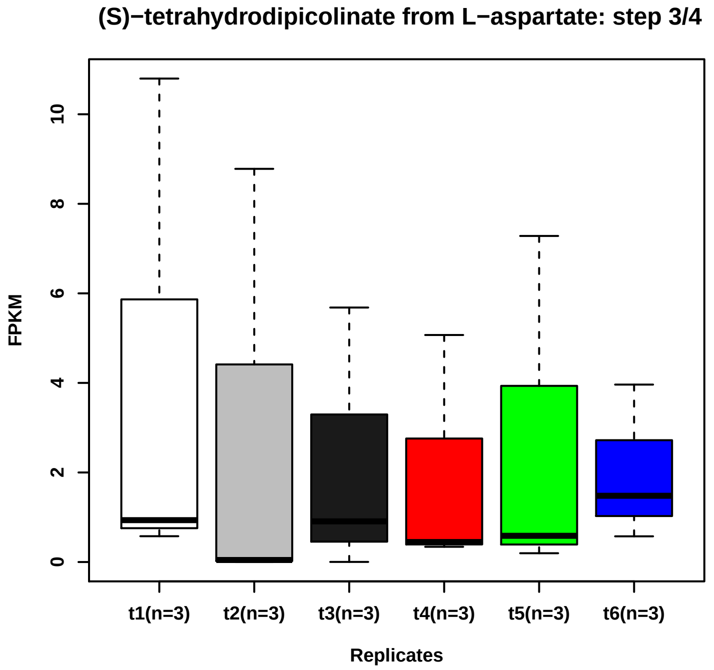
(S)−tetrahydrodipicolinate from L−aspartate: step 3/4
FPKM
0
2
4
6
8
10
t1(n=3)
t2(n=3)
t3(n=3)
t4(n=3)
t5(n=3)
t6(n=3)
Replicates

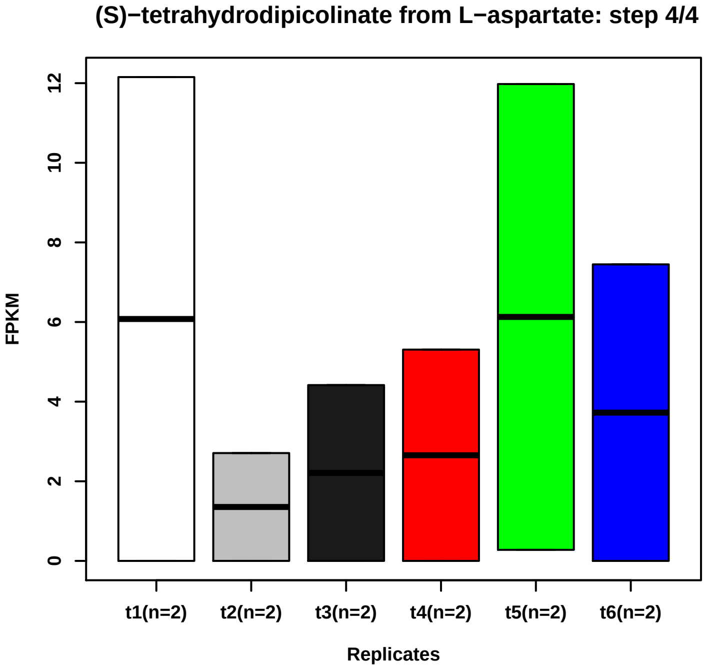
(S)−tetrahydrodipicolinate from L−aspartate: step 4/4
FPKM
0
2
4
6
8
10
12
t1(n=2)
t2(n=2)
t3(n=2)
t4(n=2)
t5(n=2)
t6(n=2)
Replicates

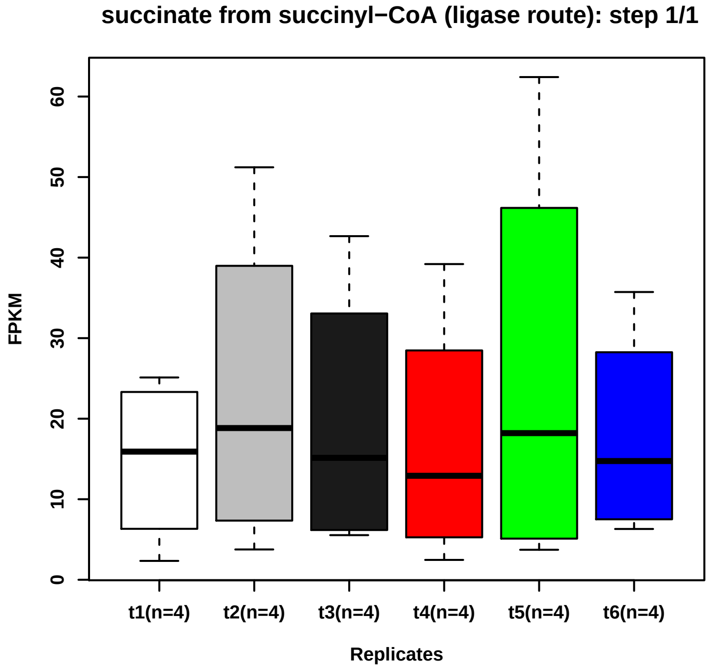
succinate from succinyl−CoA (ligase route): step 1/1
FPKM
0
10
20
30
40
50
60
t1(n=4)
t2(n=4)
t3(n=4)
t4(n=4)
t5(n=4)
t6(n=4)
Replicates

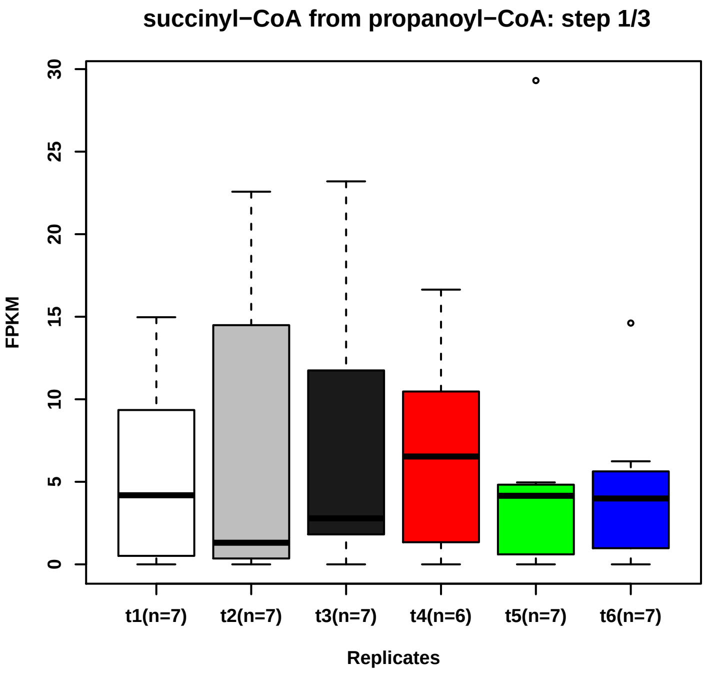
succinyl−CoA from propanoyl−CoA: step 1/3
FPKM
0
5
10
15
20
25
30
t1(n=7)
t2(n=7)
t3(n=7)
t4(n=6)
t5(n=7)
t6(n=7)
Replicates

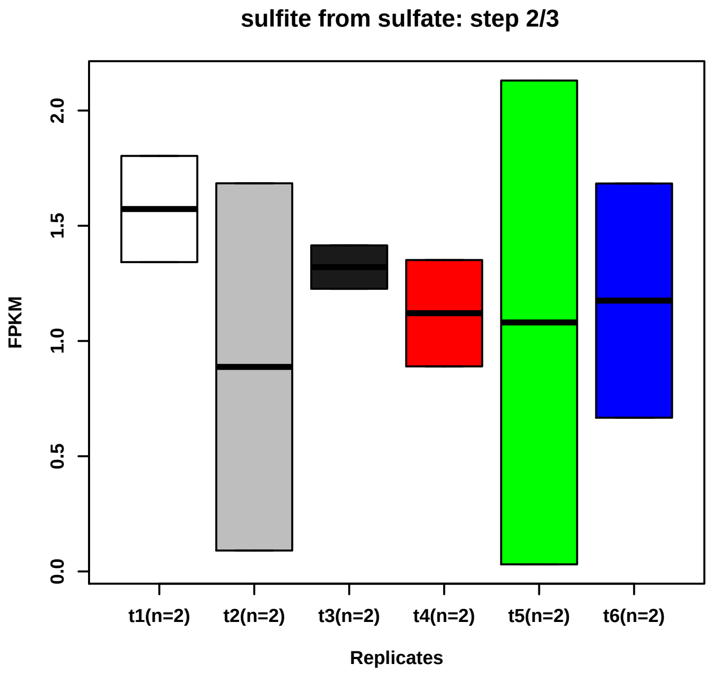
sulfite from sulfate: step 2/3
FPKM
0.0
0.5
1.0
1.5
2.0
t1(n=2)
t2(n=2)
t3(n=2)
t4(n=2)
t5(n=2)
t6(n=2)
Replicates

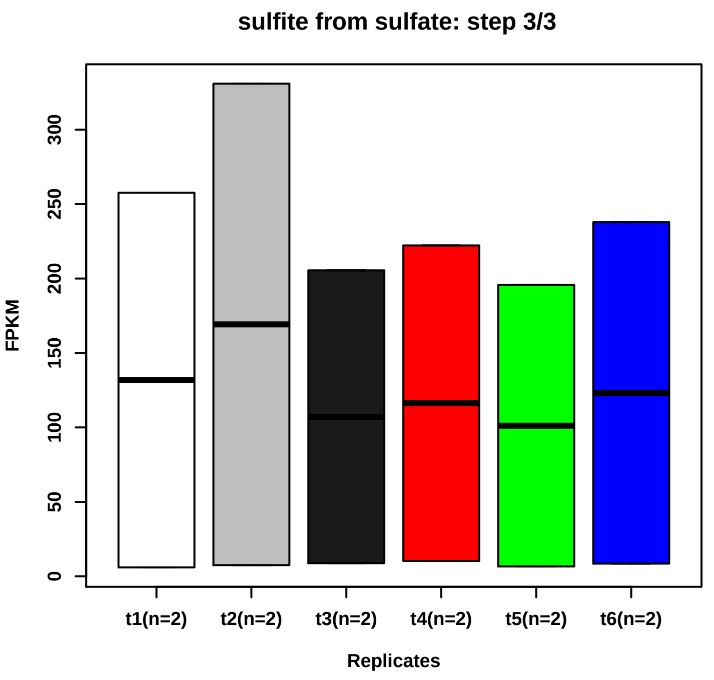
sulfite from sulfate: step 3/3
FPKM
0
50
100
150
200
250
300
t1(n=2)
t2(n=2)
t3(n=2)
t4(n=2)
t5(n=2)
t6(n=2)
Replicates

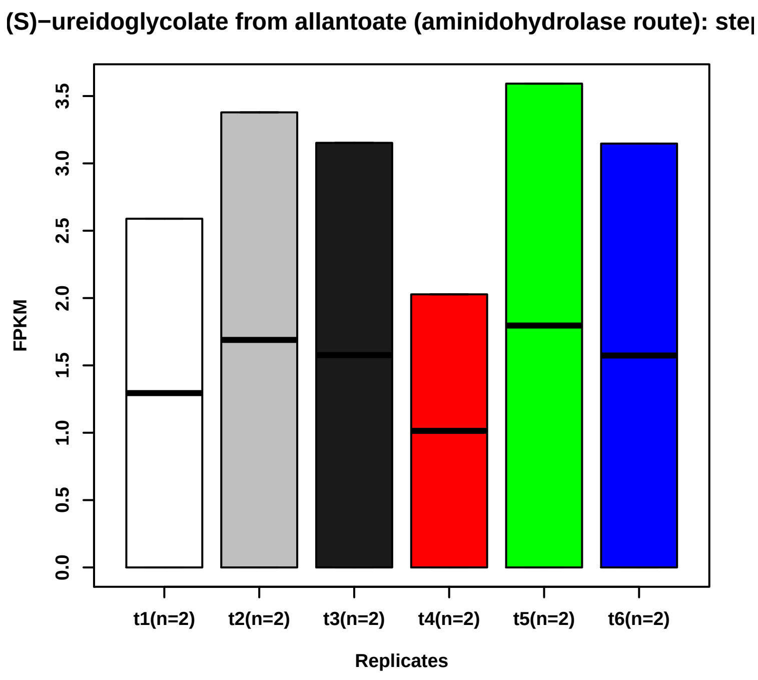
(S)−ureidoglycolate from allantoate (aminidohydrolase route): ste
FPKM
0.0
0.5
1.0
1.5
2.0
2.5
3.0
3.5
t1(n=2)
t2(n=2)
t3(n=2)
t4(n=2)
t5(n=2)
t6(n=2)
Replicates

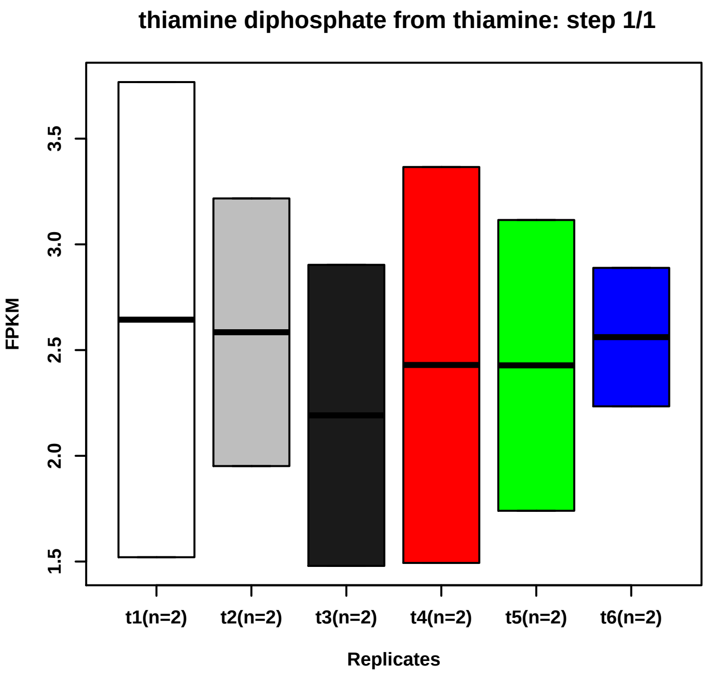
thiamine diphosphate from thiamine: step 1/1
FPKM
1.5
2.0
2.5
3.0
3.5
t1(n=2)
t2(n=2)
t3(n=2)
t4(n=2)
t5(n=2)
t6(n=2)
Replicates

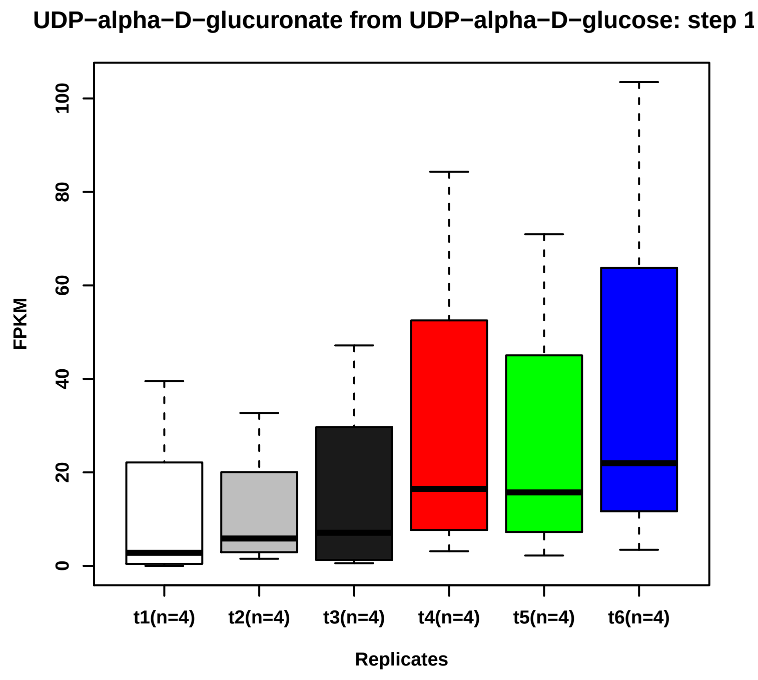
UDP−alpha−D−glucuronate from UDP−alpha−D−glucose: step 1
FPKM
0
20
40
60
80
100
t1(n=4)
t2(n=4)
t3(n=4)
t4(n=4)
t5(n=4)
t6(n=4)
Replicates

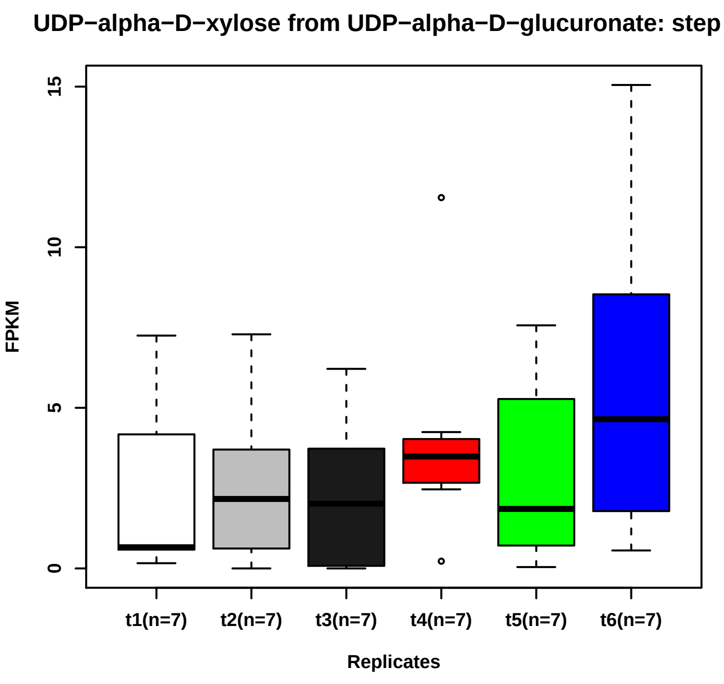
UDP−alpha−D−xylose from UDP−alpha−D−glucuronate: step 1/
FPKM
0
5
10
15
t1(n=7)
t2(n=7)
t3(n=7)
t4(n=7)
t5(n=7)
t6(n=7)
Replicates

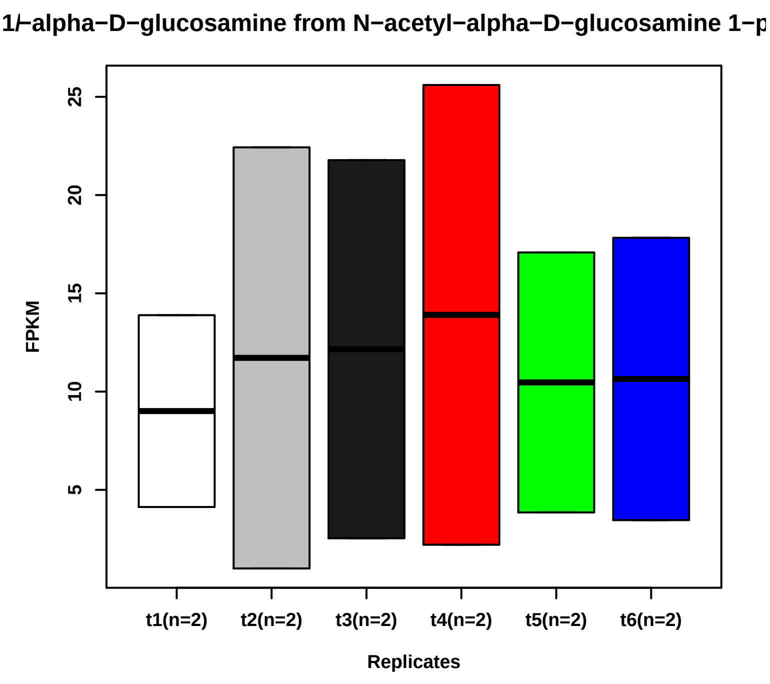
−alpha−D−glucosamine from N−acetyl−alpha−D−glucosamine 1−p
FPKM
5
10
15
20
25
t1(n=2)
t2(n=2)
t3(n=2)
t4(n=2)
t5(n=2)
t6(n=2)
Replicates

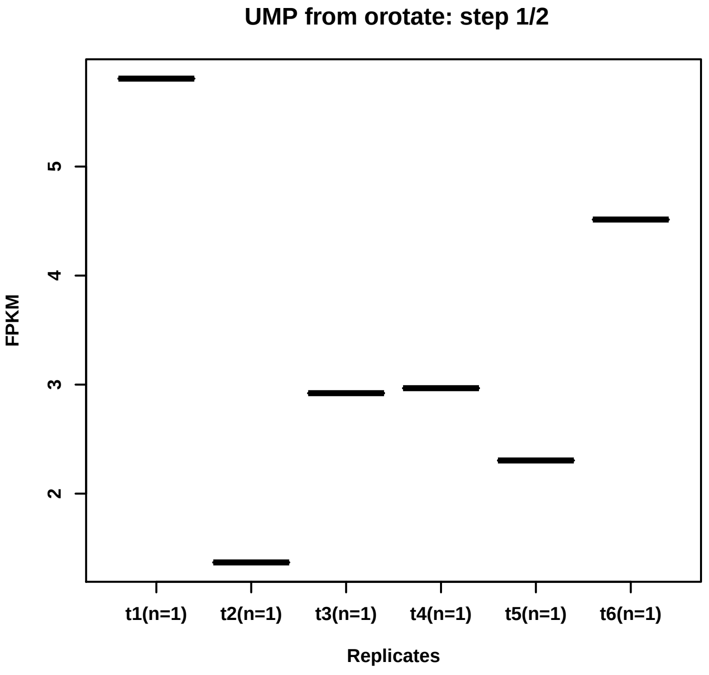
UMP from orotate: step 1/2
FPKM
2
3
4
5
t1(n=1)
t2(n=1)
t3(n=1)
t4(n=1)
t5(n=1)
t6(n=1)
Replicates

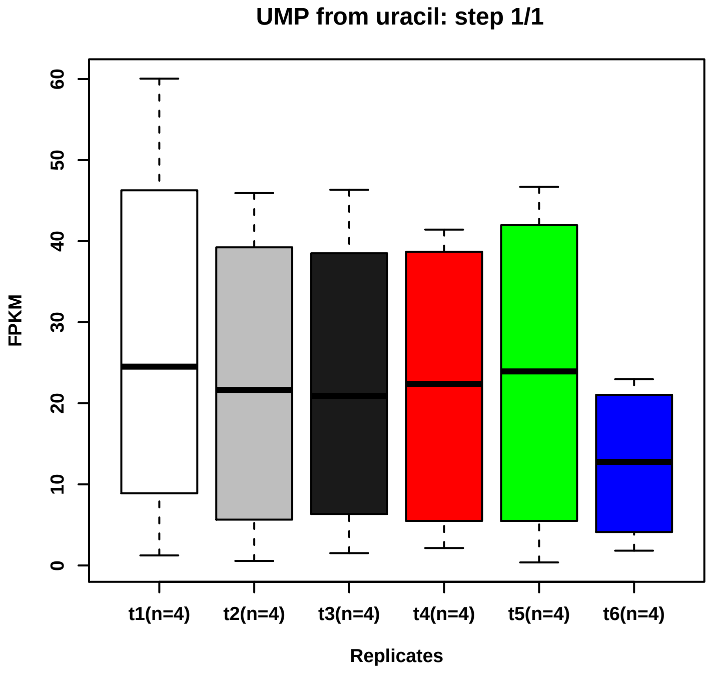
UMP from uracil: step 1/1
FPKM
0
10
20
30
40
50
60
t1(n=4)
t2(n=4)
t3(n=4)
t4(n=4)
t5(n=4)
t6(n=4)
Replicates

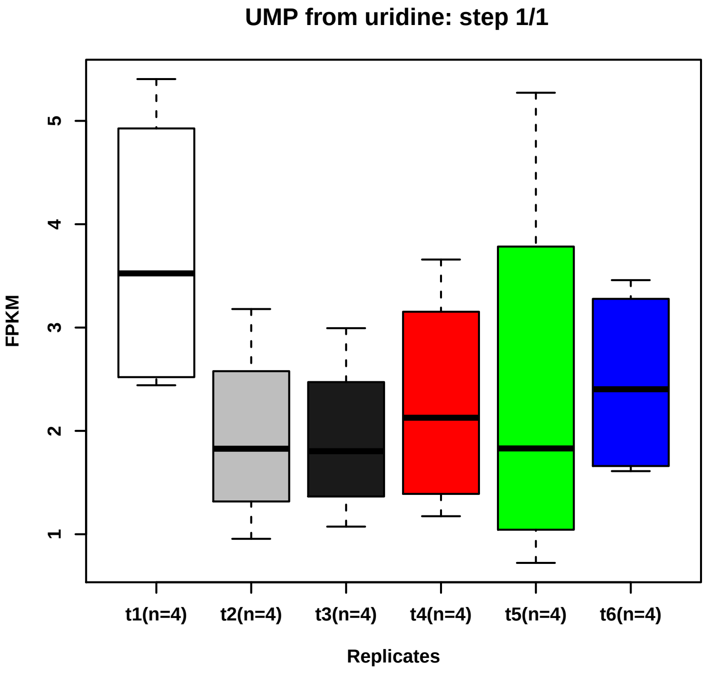
UMP from uridine: step 1/1
FPKM
1
2
3
4
5
t1(n=4)
t2(n=4)
t3(n=4)
t4(n=4)
t5(n=4)
t6(n=4)
Replicates

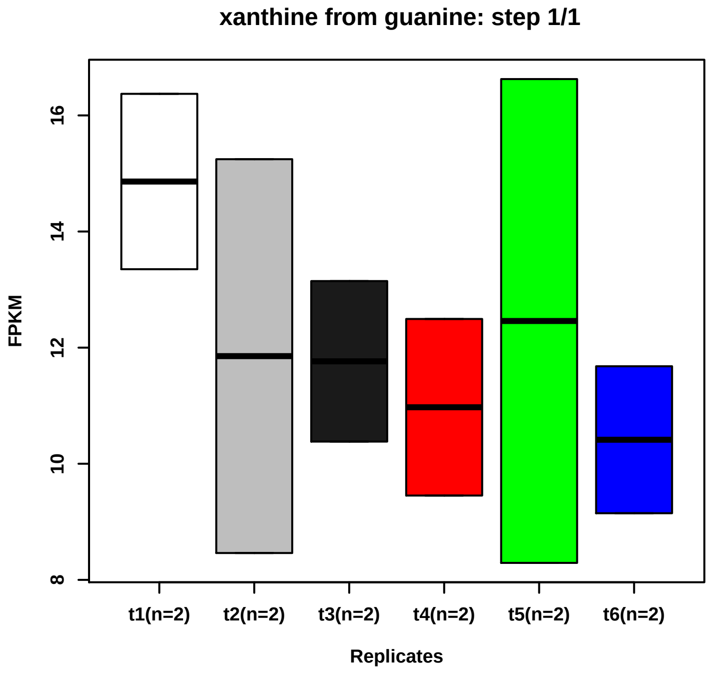
xanthine from guanine: step 1/1
FPKM
8
10
12
14
16
t1(n=2)
t2(n=2)
t3(n=2)
t4(n=2)
t5(n=2)
t6(n=2)
Replicates

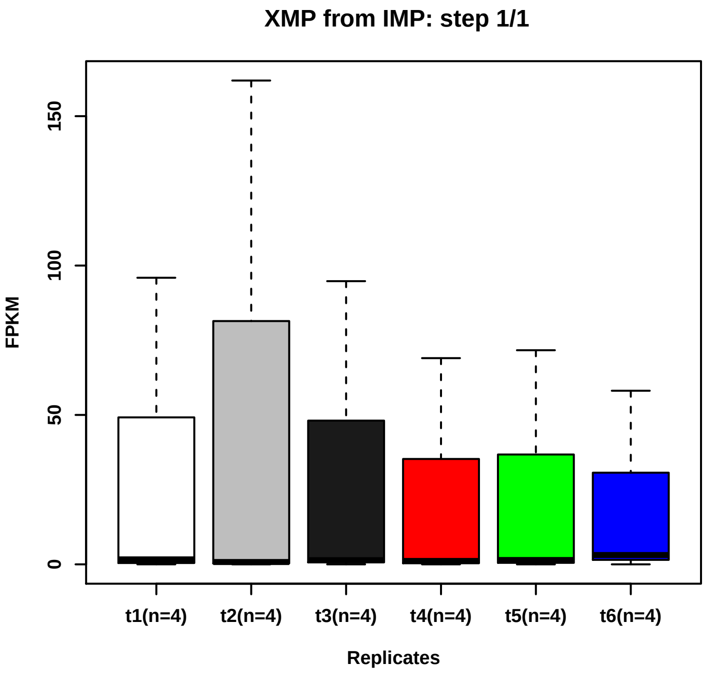
XMP from IMP: step 1/1
FPKM
0
50
100
150
t1(n=4)
t2(n=4)
t3(n=4)
t4(n=4)
t5(n=4)
t6(n=4)
Replicates

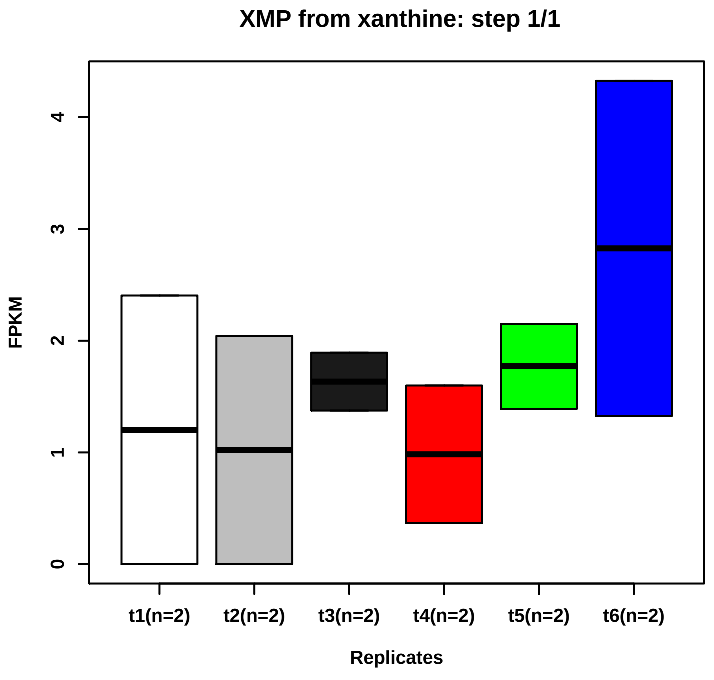
XMP from xanthine: step 1/1
FPKM
0
1
2
3
4
t1(n=2)
t2(n=2)
t3(n=2)
t4(n=2)
t5(n=2)
t6(n=2)
Replicates

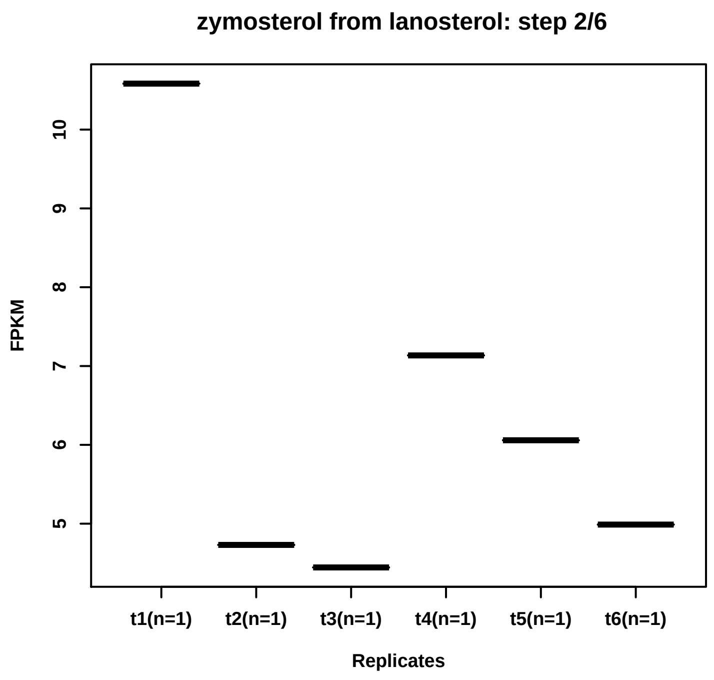
zymosterol from lanosterol: step 2/6
FPKM
5
6
7
8
9
10
t1(n=1)
t2(n=1)
t3(n=1)
t4(n=1)
t5(n=1)
t6(n=1)
Replicates

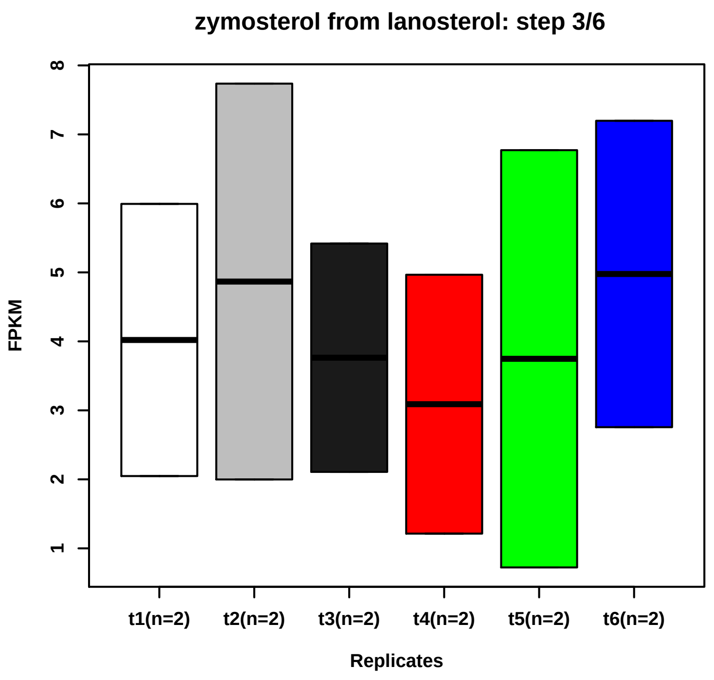
zymosterol from lanosterol: step 3/6
FPKM
1
2
3
4
5
6
7
8
t1(n=2)
t2(n=2)
t3(n=2)
t4(n=2)
t5(n=2)
t6(n=2)
Replicates

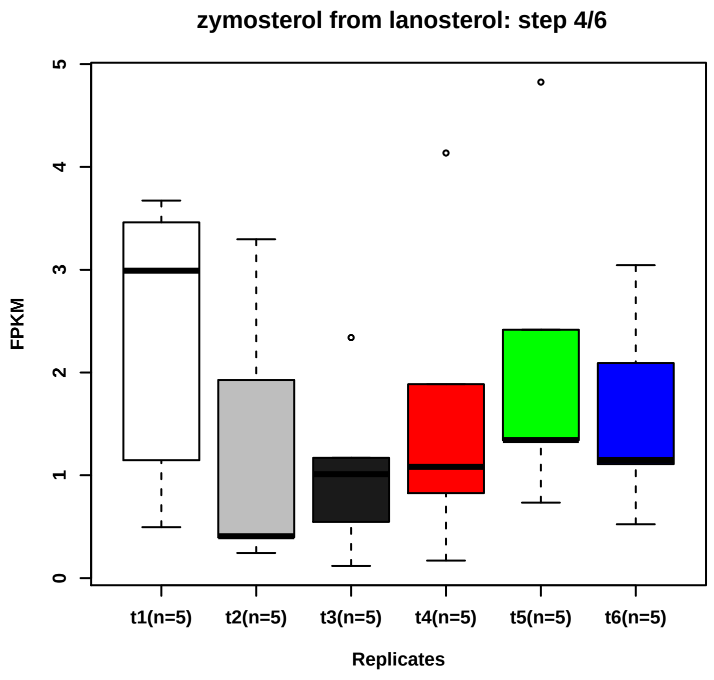
zymosterol from lanosterol: step 4/6
FPKM
0
1
2
3
4
5
t1(n=5)
t2(n=5)
t3(n=5)
t4(n=5)
t5(n=5)
t6(n=5)
Replicates

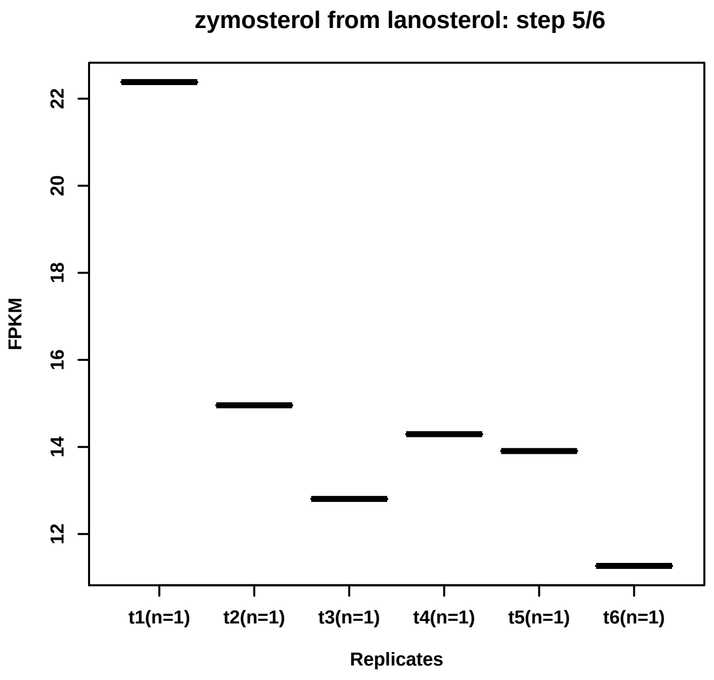
zymosterol from lanosterol: step 5/6
FPKM
12
14
16
18
20
22
t1(n=1)
t2(n=1)
t3(n=1)
t4(n=1)
t5(n=1)
t6(n=1)
Replicates

Unveiling the presence of biosynthetic pathways for bioactive compounds in the *Thalassiosira rotula* transcriptome

Valeria Di Dato*, Federica Di Costanzo, Roberta Barbarinaldi, Anna Perna, Adrianna Ianora, Giovanna Romano

Supplementary File 6:

Box Plot Analysis of the expression, in FPKM, of the pathways annotated on level 3 for each condition (mean of two replicates).

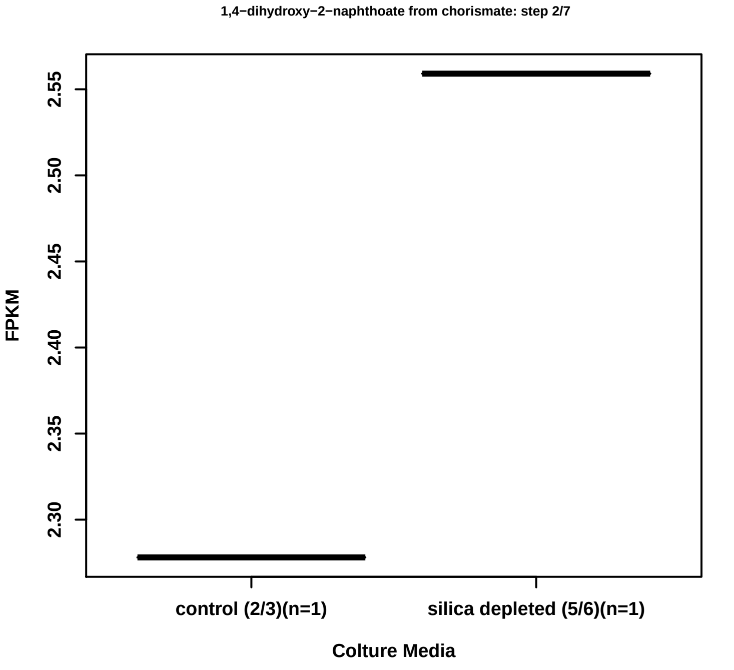
1,4−dihydroxy−2−naphthoate from chorismate: step 2/7
FPKM
2.30
2.35
2.40
2.45
2.50
2.55
control (2/3)(n=1)
silica depleted (5/6)(n=1)
Colture Media

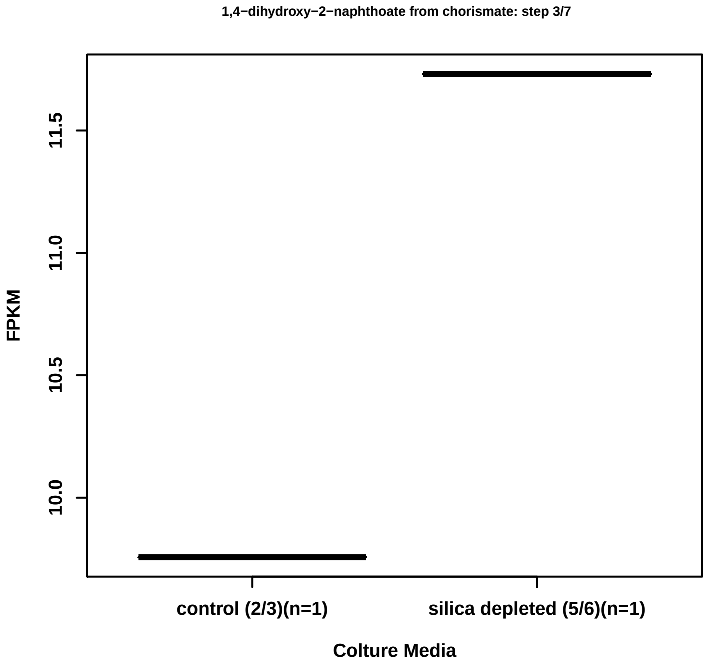
1,4−dihydroxy−2−naphthoate from chorismate: step 3/7
FPKM
10.0
10.5
11.0
11.5
control (2/3)(n=1)
silica depleted (5/6)(n=1)
Colture Media

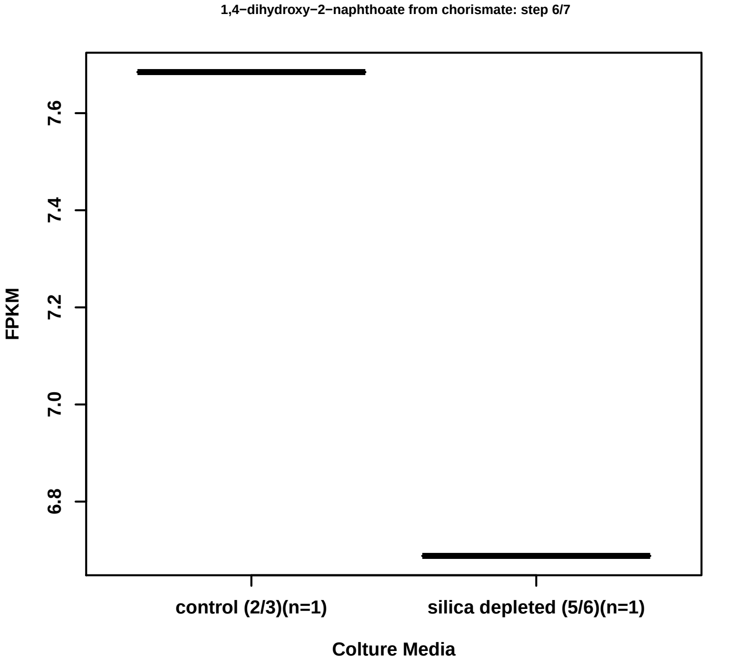
1,4−dihydroxy−2−naphthoate from chorismate: step 6/7
FPKM
6.8
7.0
7.2
7.4
7.6
control (2/3)(n=1)
silica depleted (5/6)(n=1)
Colture Media

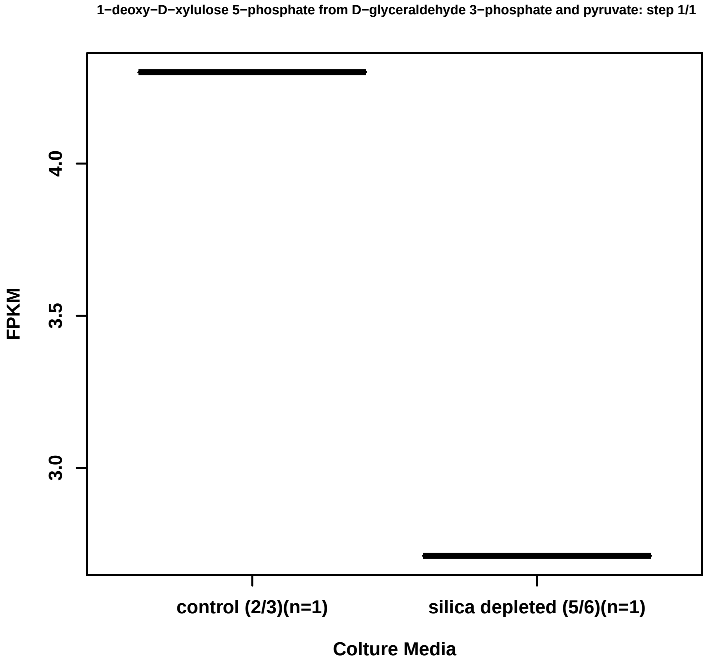
1−deoxy−D−xylulose 5−phosphate from D−glyceraldehyde 3−phosphate and pyruvate: step 1/1
FPKM
3.0
3.5
4.0
control (2/3)(n=1)
silica depleted (5/6)(n=1)
Colture Media

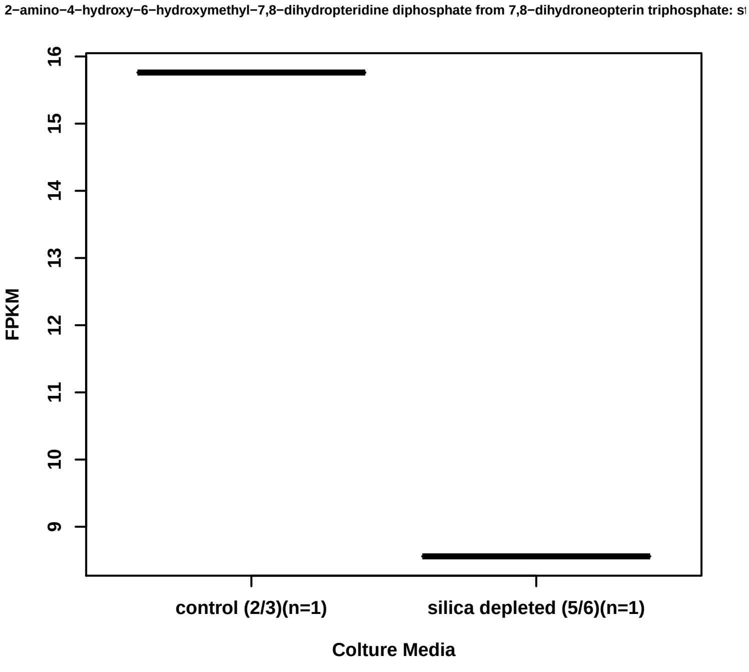
2−amino−4−hydroxy−6−hydroxymethyl−7,8−dihydropteridine diphosphate from 7,8−dihydroneopterin triphosphate: st
FPKM
9
10
11
12
13
14
15
16
control (2/3)(n=1)
silica depleted (5/6)(n=1)
Colture Media

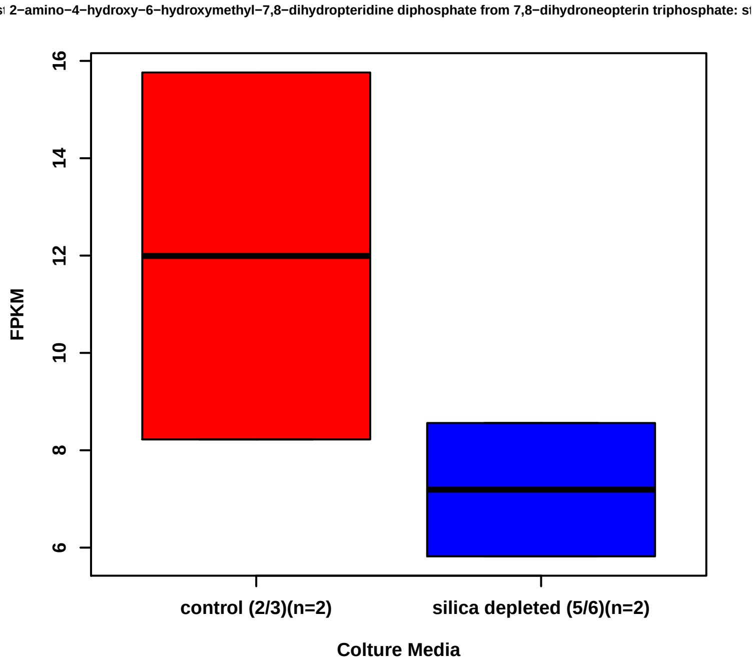
2−amino−4−hydroxy−6−hydroxymethyl−7,8−dihydropteridine diphosphate from 7,8−dihydroneopterin triphosphate: st
FPKM
6
8
10
12
14
16
control (2/3)(n=2)
silica depleted (5/6)(n=2)
Colture Media

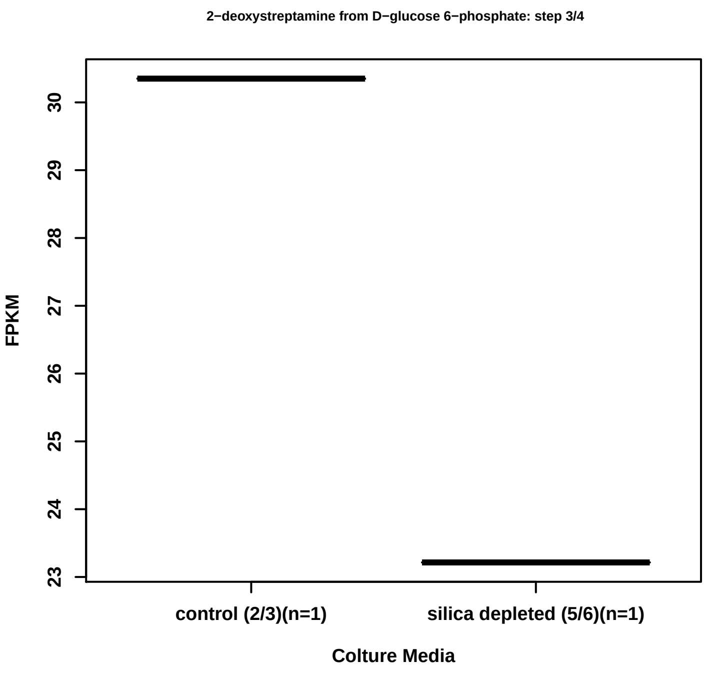
2−deoxystreptamine from D−glucose 6−phosphate: step 3/4
FPKM
23
24
25
26
27
28
29
30
control (2/3)(n=1)
silica depleted (5/6)(n=1)
Colture Media

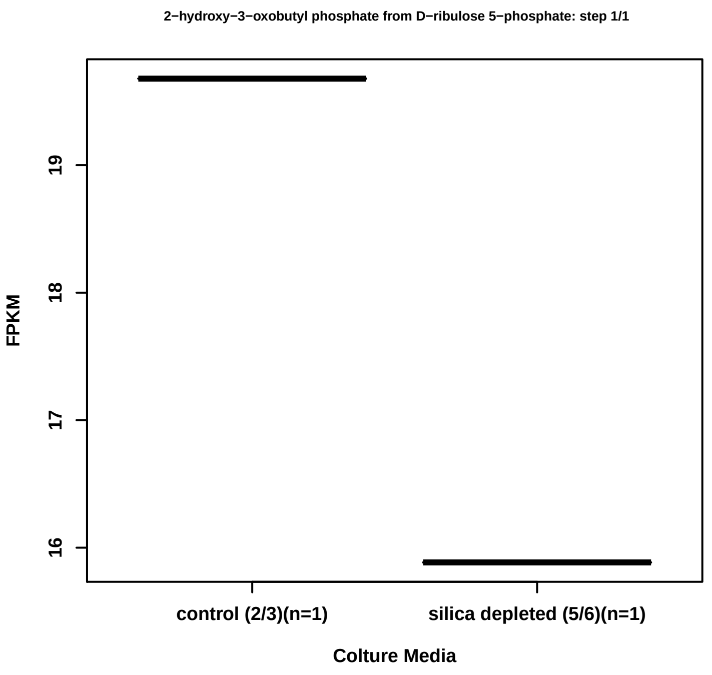
2−hydroxy−3−oxobutyl phosphate from D−ribulose 5−phosphate: step 1/1
FPKM
16
17
18
19
control (2/3)(n=1)
silica depleted (5/6)(n=1)
Colture Media

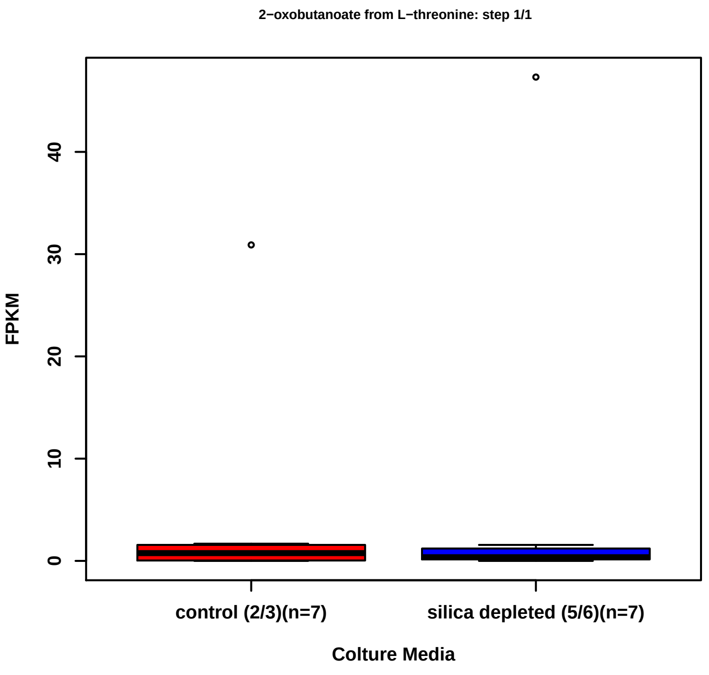
2−oxobutanoate from L−threonine: step 1/1
FPKM
0
10
20
30
40
control (2/3)(n=7)
silica depleted (5/6)(n=7)
Colture Media

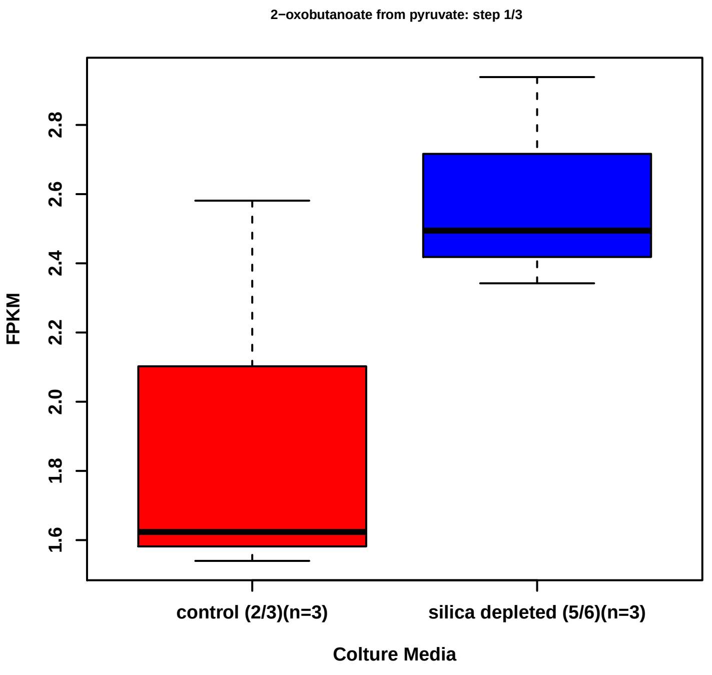
2−oxobutanoate from pyruvate: step 1/3
FPKM
1.6
1.8
2.0
2.2
2.4
2.6
2.8
control (2/3)(n=3)
silica depleted (5/6)(n=3)
Colture Media

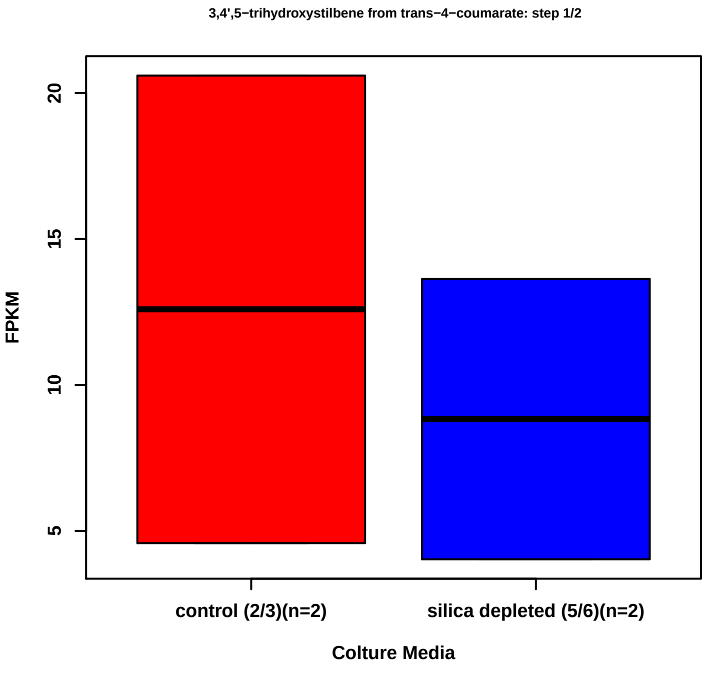
3,4',5−trihydroxystilbene from trans−4−coumarate: step 1/2
FPKM
5
10
15
20
control (2/3)(n=2)
silica depleted (5/6)(n=2)
Colture Media

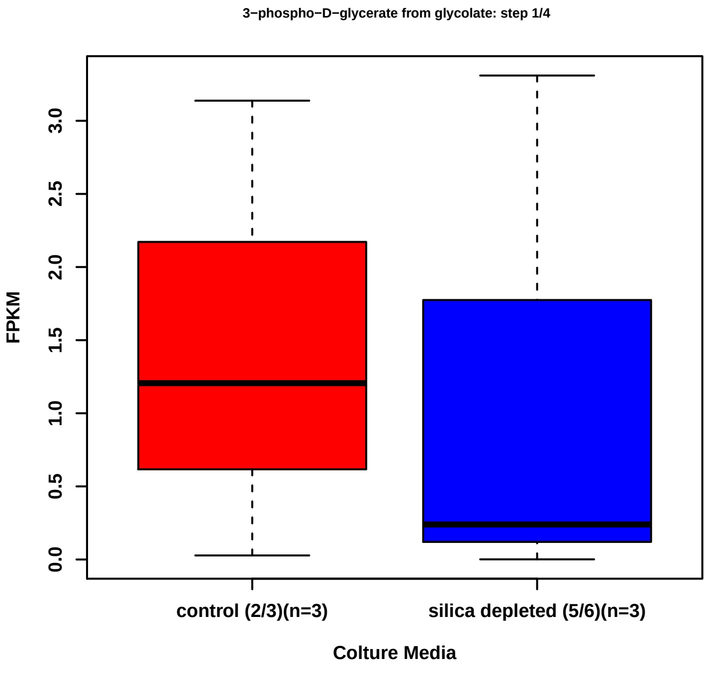
3−phospho−D−glycerate from glycolate: step 1/4
FPKM
0.0
0.5
1.0
1.5
2.0
2.5
3.0
control (2/3)(n=3)
silica depleted (5/6)(n=3)
Colture Media

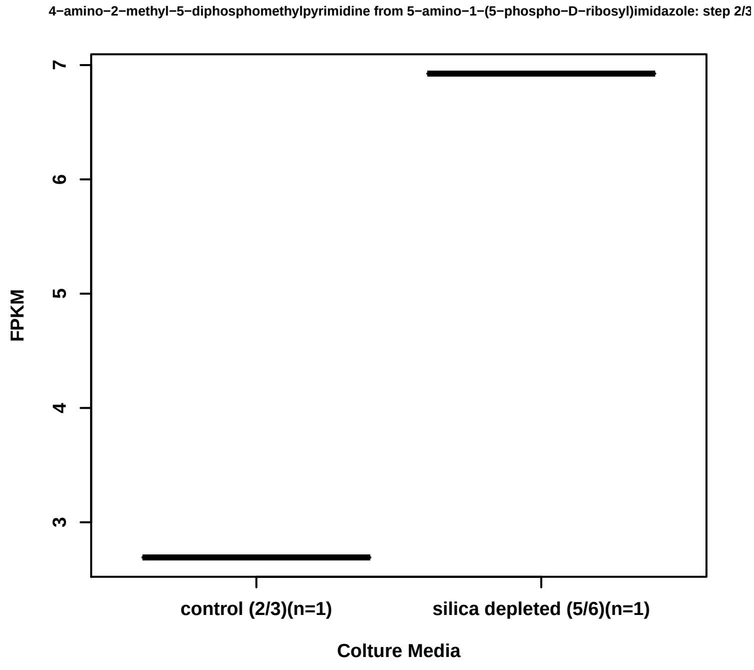
4−amino−2−methyl−5−diphosphomethylpyrimidine from 5−amino−1−(5−phospho−D−ribosyl)imidazole: step 2/3
FPKM
3
4
5
6
7
control (2/3)(n=1)
silica depleted (5/6)(n=1)
Colture Media

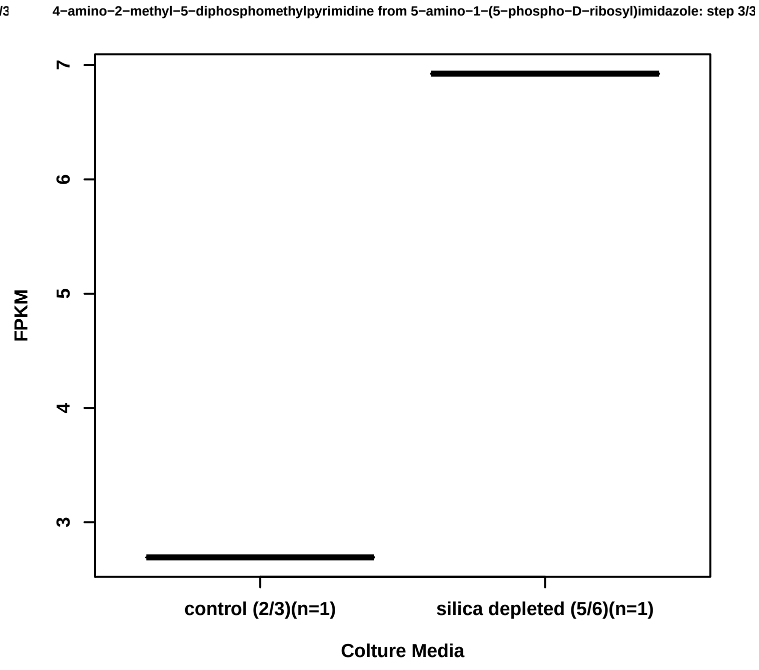
4−amino−2−methyl−5−diphosphomethylpyrimidine from 5−amino−1−(5−phospho−D−ribosyl)imidazole: step 3/3
FPKM
3
4
5
6
7
control (2/3)(n=1)
silica depleted (5/6)(n=1)
Colture Media

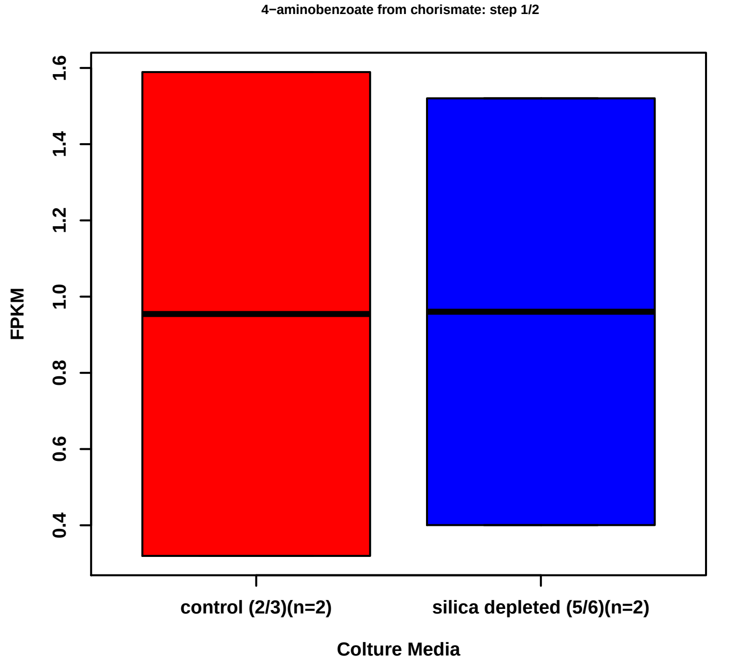
4−aminobenzoate from chorismate: step 1/2
FPKM
0.4
0.6
0.8
1.0
1.2
1.4
1.6
control (2/3)(n=2)
silica depleted (5/6)(n=2)
Colture Media

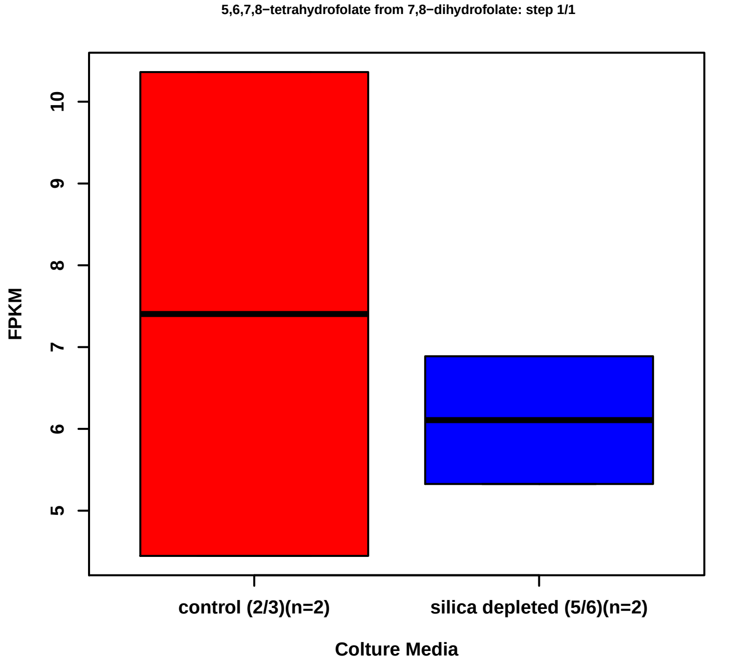
5,6,7,8−tetrahydrofolate from 7,8−dihydrofolate: step 1/1
FPKM
5
6
7
8
9
10
control (2/3)(n=2)
silica depleted (5/6)(n=2)
Colture Media

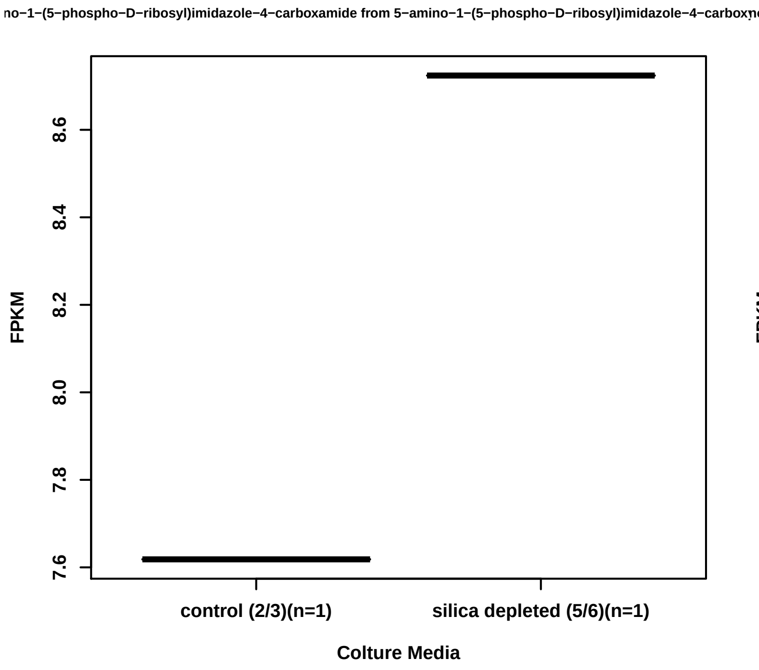
no−1−(5−phospho−D−ribosyl)imidazole−4−carboxamide from 5−amino−1−(5−phospho−D−ribosyl)imidazole−4−carbox
FPKM
7.6
7.8
8.0
8.2
8.4
8.6
control (2/3)(n=1)
silica depleted (5/6)(n=1)
Colture Media

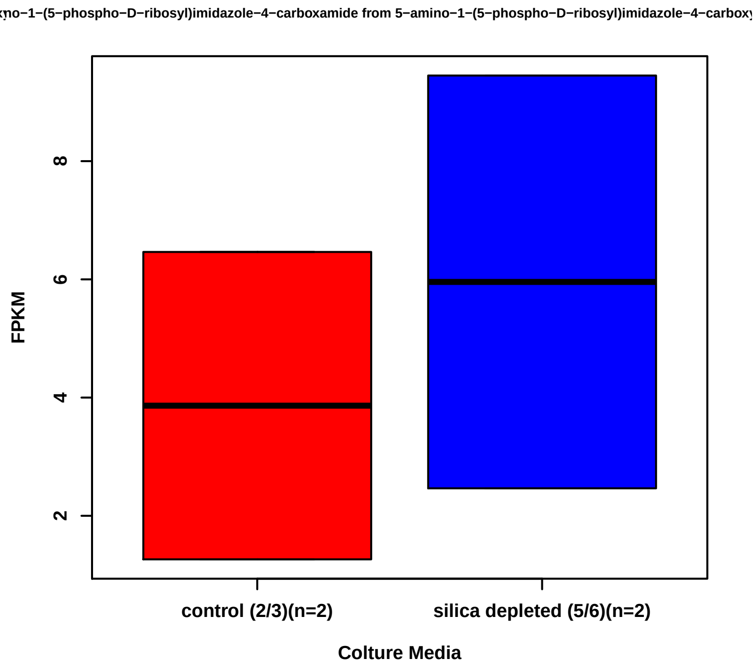
no−1−(5−phospho−D−ribosyl)imidazole−4−carboxamide from 5−amino−1−(5−phospho−D−ribosyl)imidazole−4−carbox
FPKM
2
4
6
8
control (2/3)(n=2)
silica depleted (5/6)(n=2)
Colture Media

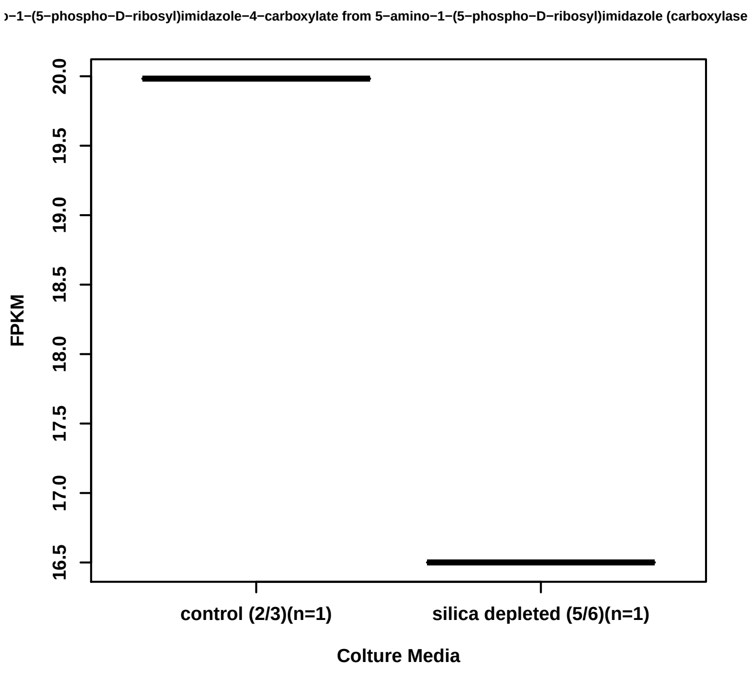
)−1−(5−phospho−D−ribosyl)imidazole−4−carboxylate from 5−amino−1−(5−phospho−D−ribosyl)imidazole (carboxylase
FPKM
16.5
17.0
17.5
18.0
18.5
19.0
19.5
20.0
control (2/3)(n=1)
silica depleted (5/6)(n=1)
Colture Media

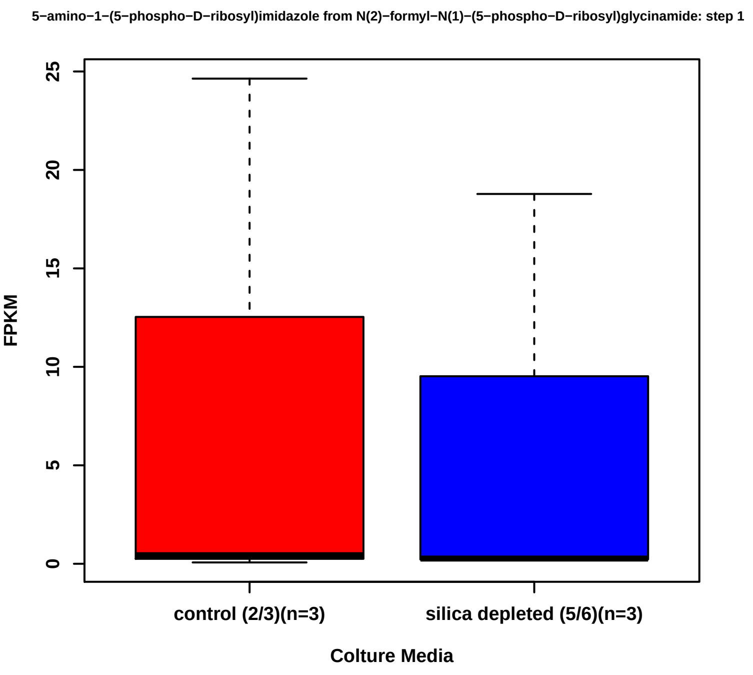
5−amino−1−(5−phospho−D−ribosyl)imidazole from N(2)−formyl−N(1)−(5−phospho−D−ribosyl)glycinamide: step 1
FPKM
0
5
10
15
20
25
control (2/3)(n=3)
silica depleted (5/6)(n=3)
Colture Media

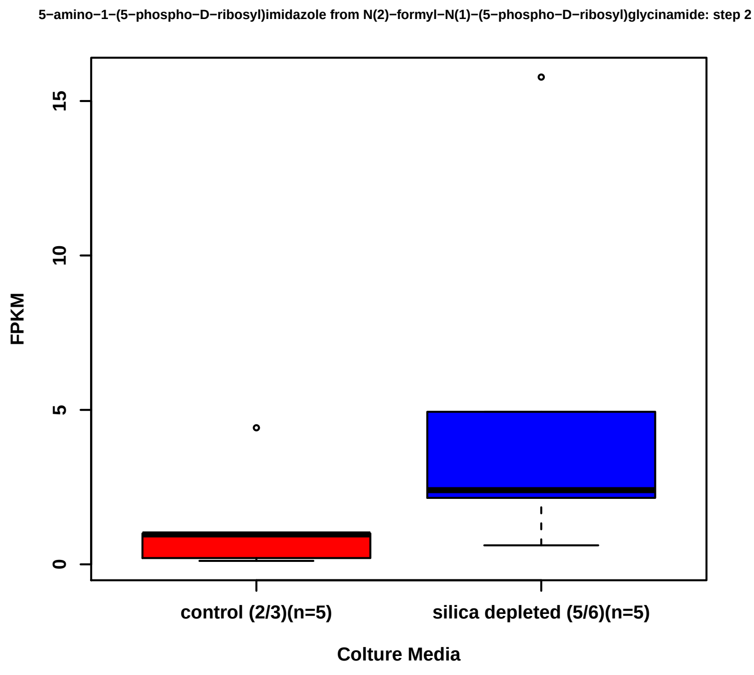
5−amino−1−(5−phospho−D−ribosyl)imidazole from N(2)−formyl−N(1)−(5−phospho−D−ribosyl)glycinamide: step 2
FPKM
0
5
10
15
control (2/3)(n=5)
silica depleted (5/6)(n=5)
Colture Media

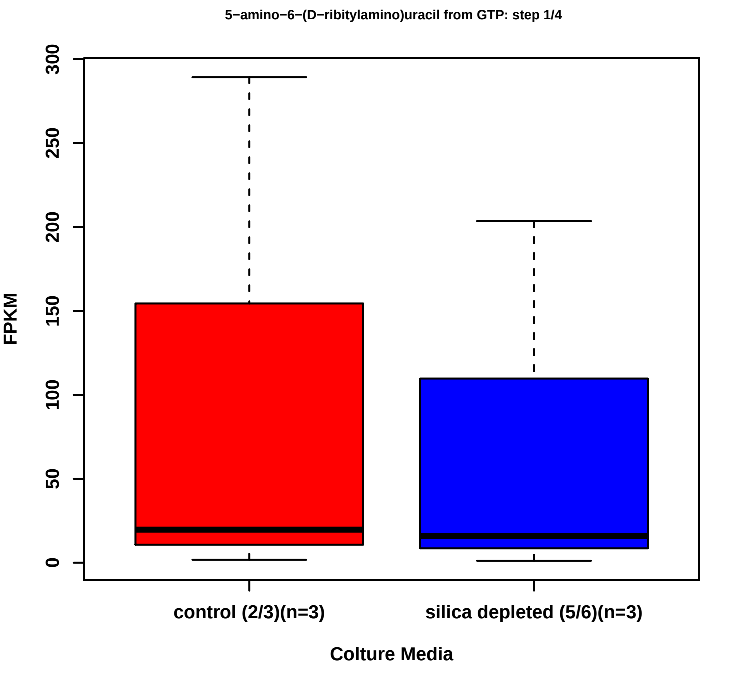
5−amino−6−(D−ribitylamino)uracil from GTP: step 1/4
FPKM
0
50
100
150
200
250
300
control (2/3)(n=3)
silica depleted (5/6)(n=3)
Colture Media

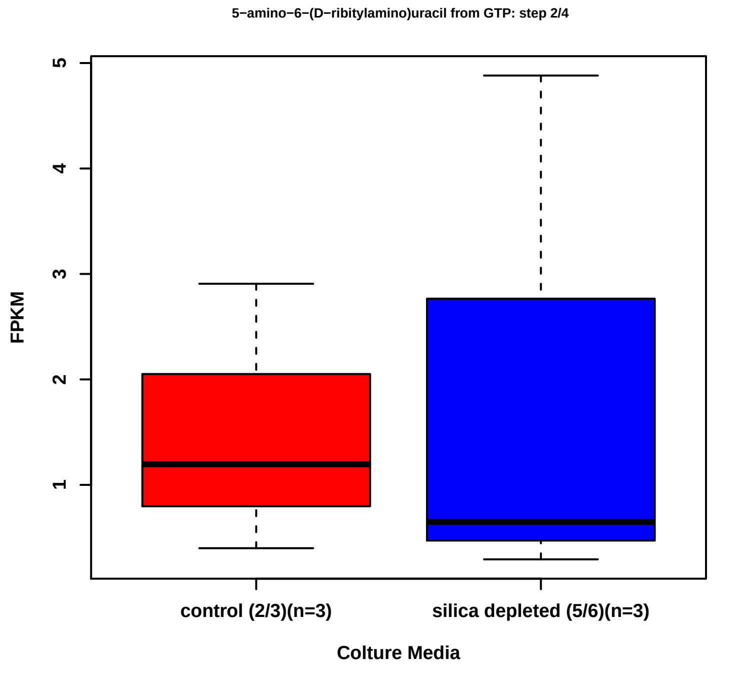
5−amino−6−(D−ribitylamino)uracil from GTP: step 2/4
FPKM
1
2
3
4
5
control (2/3)(n=3)
silica depleted (5/6)(n=3)
Colture Media

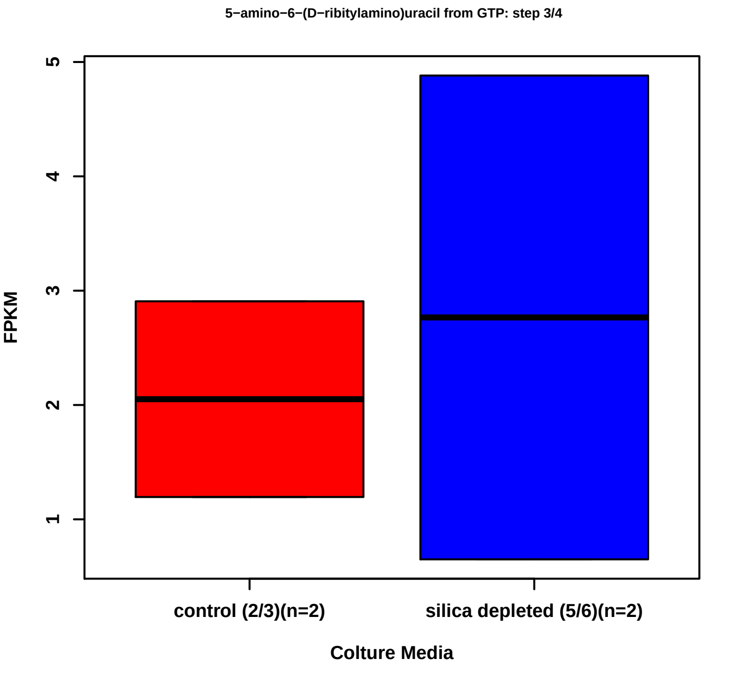
5−amino−6−(D−ribitylamino)uracil from GTP: step 3/4
FPKM
1
2
3
4
5
control (2/3)(n=2)
silica depleted (5/6)(n=2)
Colture Media

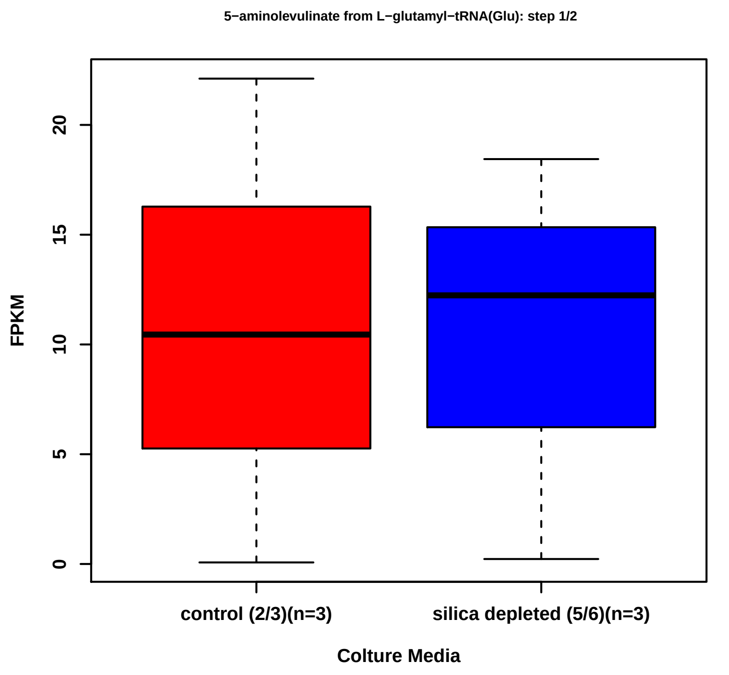
5−aminolevulinate from L−glutamyl−tRNA(Glu): step 1/2
FPKM
0
5
10
15
20
control (2/3)(n=3)
silica depleted (5/6)(n=3)
Colture Media

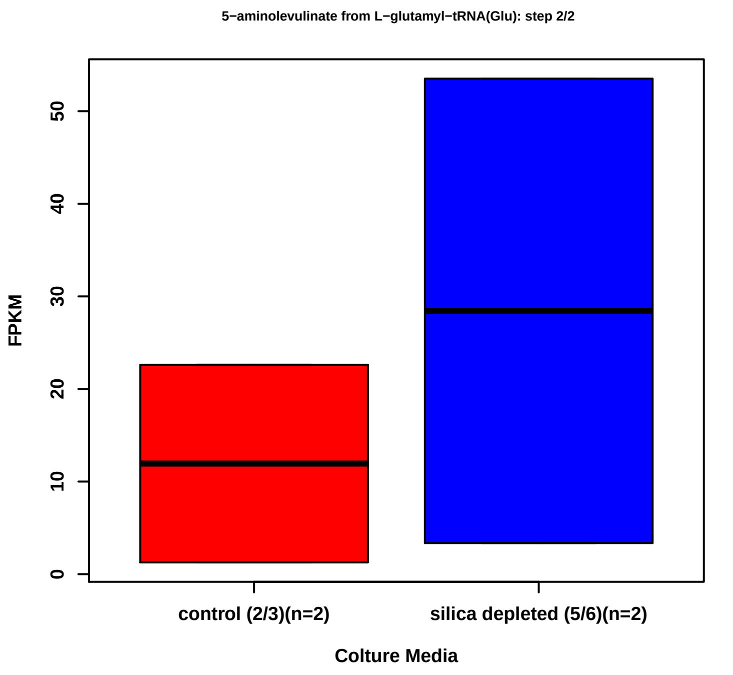
5−aminolevulinate from L−glutamyl−tRNA(Glu): step 2/2
FPKM
0
10
20
30
40
50
control (2/3)(n=2)
silica depleted (5/6)(n=2)
Colture Media

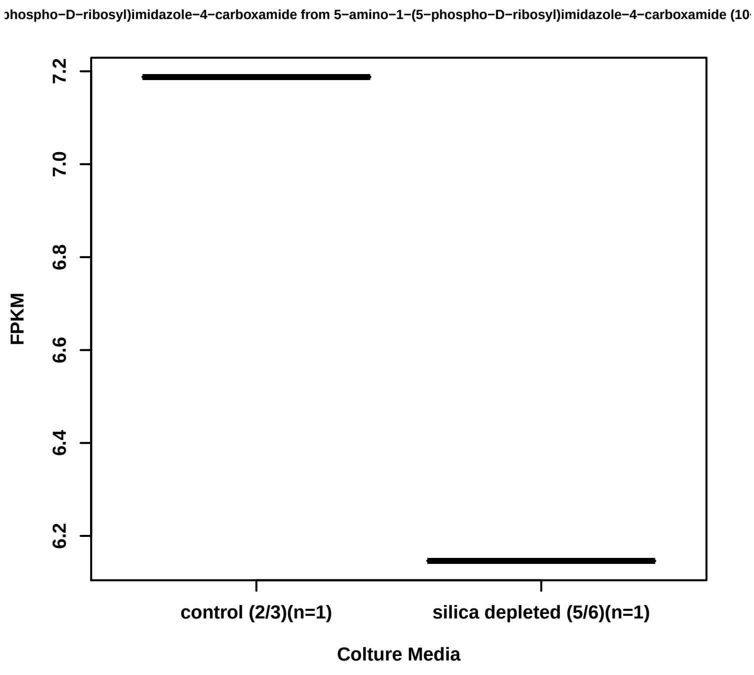
phospho−D−ribosyl)imidazole−4−carboxamide from 5−amino−1−(5−phospho−D−ribosyl)imidazole−4−carboxamide (10
FPKM
6.2
6.4
6.6
6.8
7.0
7.2
control (2/3)(n=1)
silica depleted (5/6)(n=1)
Colture Media

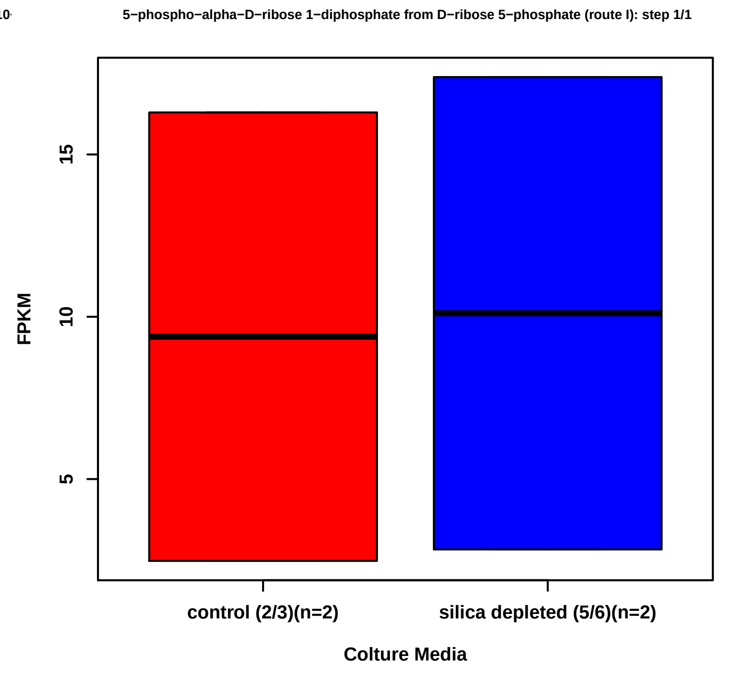
5−phospho−alpha−D−ribose 1−diphosphate from D−ribose 5−phosphate (route I): step 1/1
FPKM
5
10
15
control (2/3)(n=2)
silica depleted (5/6)(n=2)
Colture Media

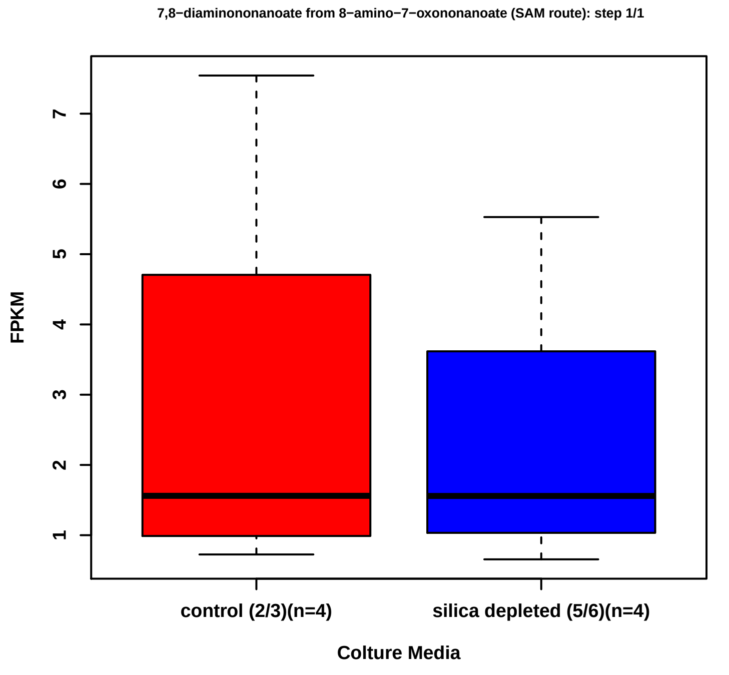
7,8−diaminononanoate from 8−amino−7−oxononanoate (SAM route): step 1/1
FPKM
1
2
3
4
5
6
7
control (2/3)(n=4)
silica depleted (5/6)(n=4)
Colture Media

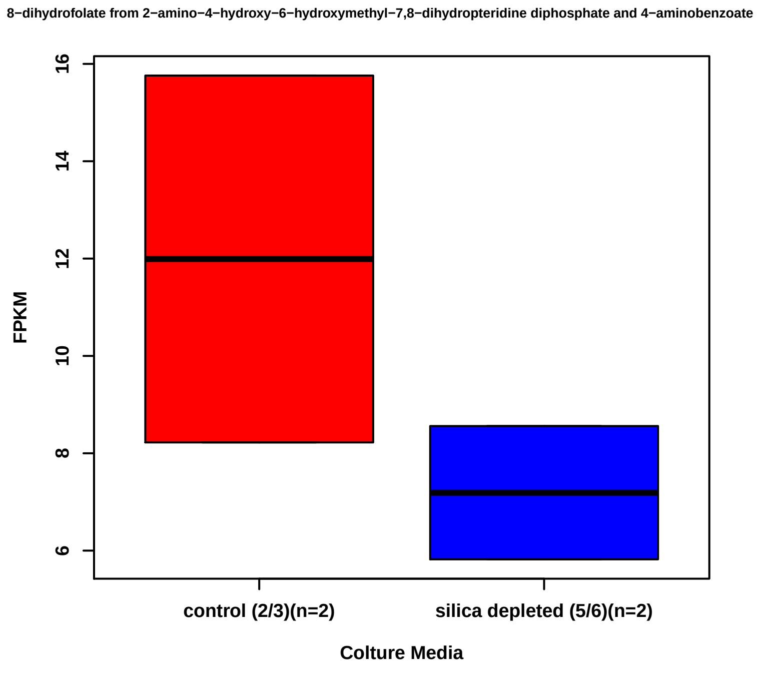
8−dihydrofolate from 2−amino−4−hydroxy−6−hydroxymethyl−7,8−dihydropteridine diphosphate and 4−aminobenzoate
FPKM
6
8
10
12
14
16
control (2/3)(n=2)
silica depleted (5/6)(n=2)
Colture Media

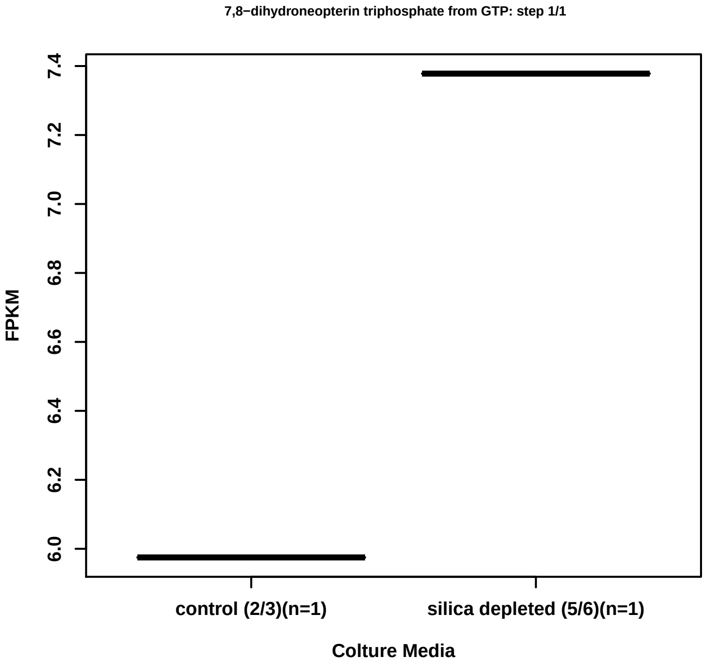
7,8−dihydroneopterin triphosphate from GTP: step 1/1
FPKM
6.0
6.2
6.4
6.6
6.8
7.0
7.2
7.4
control (2/3)(n=1)
silica depleted (5/6)(n=1)
Colture Media

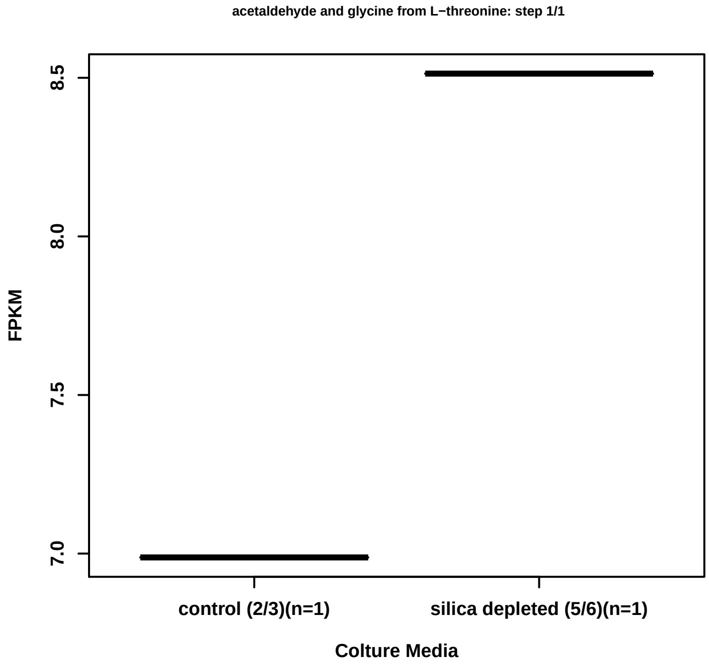
acetaldehyde and glycine from L−threonine: step 1/1
FPKM
7.0
7.5
8.0
8.5
control (2/3)(n=1)
silica depleted (5/6)(n=1)
Colture Media

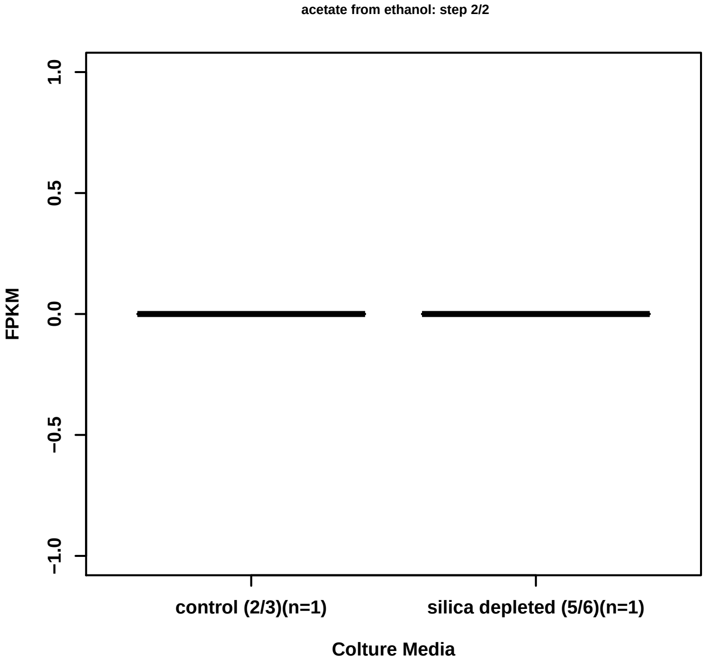
acetate from ethanol: step 2/2
FPKM
−1.0
−0.5
0.0
0.5
1.0
control (2/3)(n=1)
silica depleted (5/6)(n=1)
Colture Media

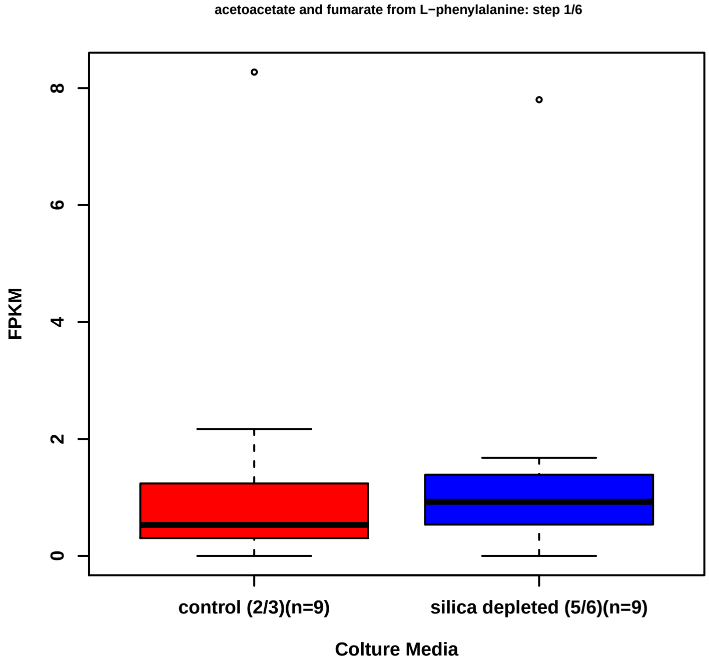
acetoacetate and fumarate from L−phenylalanine: step 1/6
FPKM
0
2
4
6
8
control (2/3)(n=9)
silica depleted (5/6)(n=9)
Colture Media

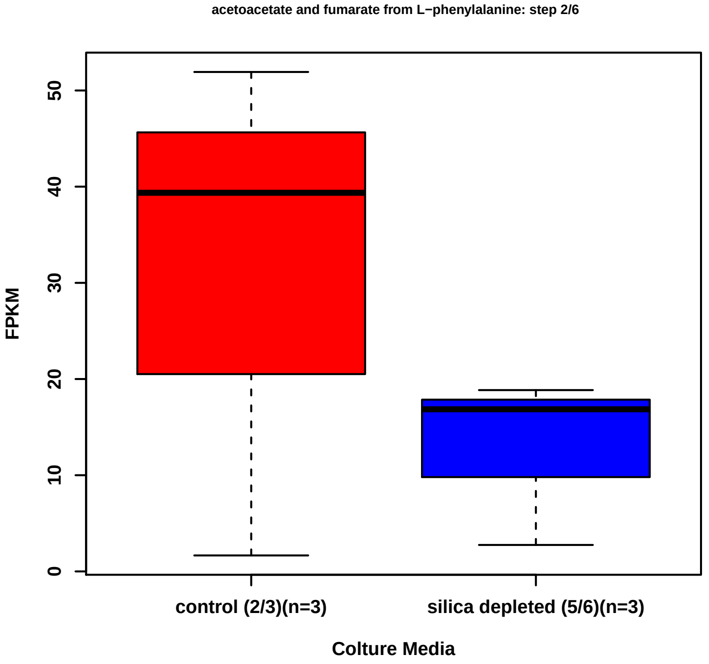
acetoacetate and fumarate from L−phenylalanine: step 2/6
FPKM
0
10
20
30
40
50
control (2/3)(n=3)
silica depleted (5/6)(n=3)
Colture Media

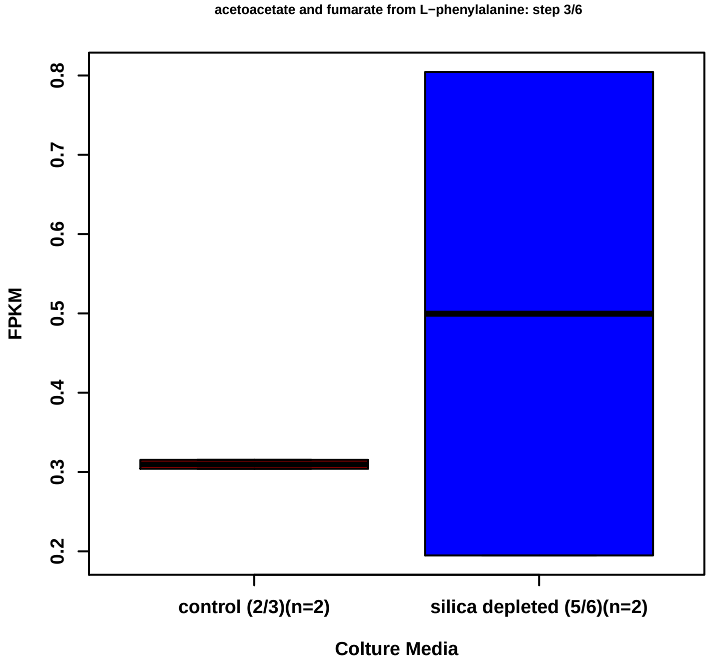
acetoacetate and fumarate from L−phenylalanine: step 3/6
FPKM
0.2
0.3
0.4
0.5
0.6
0.7
0.8
control (2/3)(n=2)
silica depleted (5/6)(n=2)
Colture Media

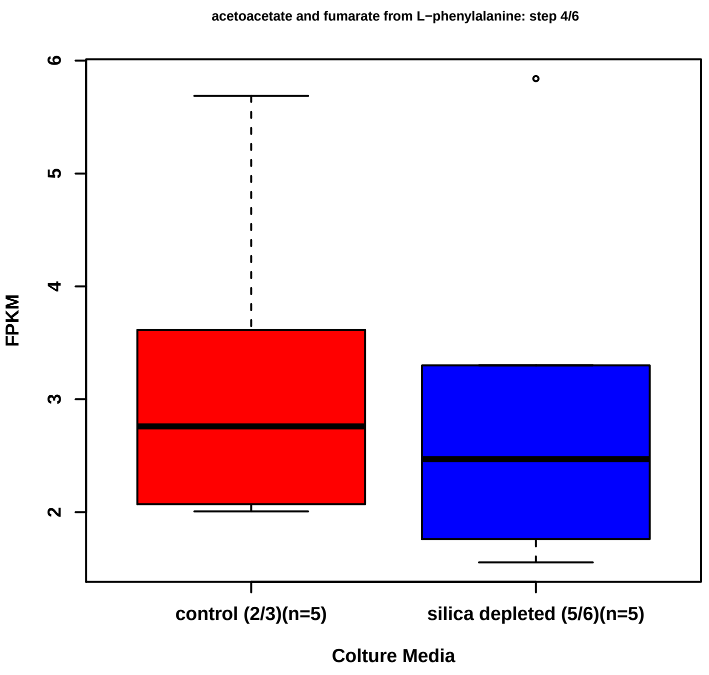
acetoacetate and fumarate from L−phenylalanine: step 4/6
FPKM
control (2/3)(n=5)
silica depleted (5/6)(n=5)
Colture Media

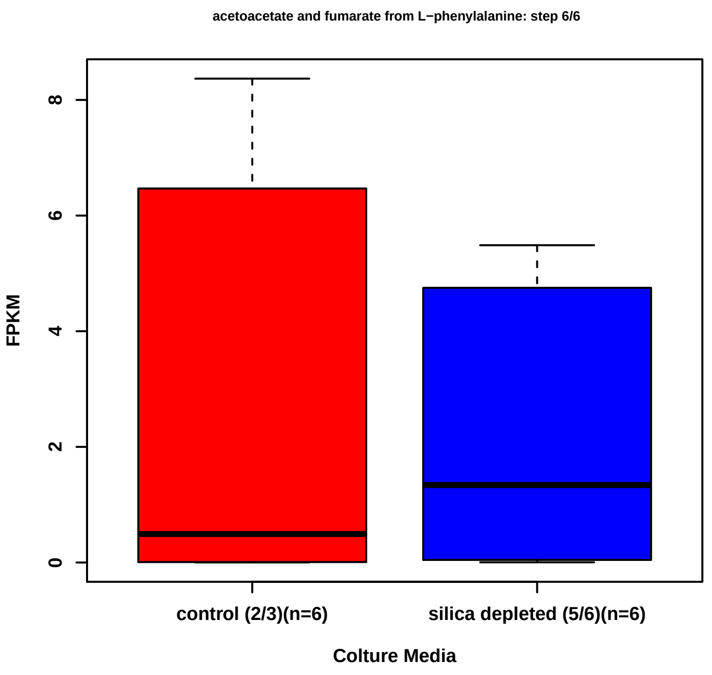
acetoacetate and fumarate from L−phenylalanine: step 6/6
FPKM
control (2/3)(n=6)
silica depleted (5/6)(n=6)
Colture Media

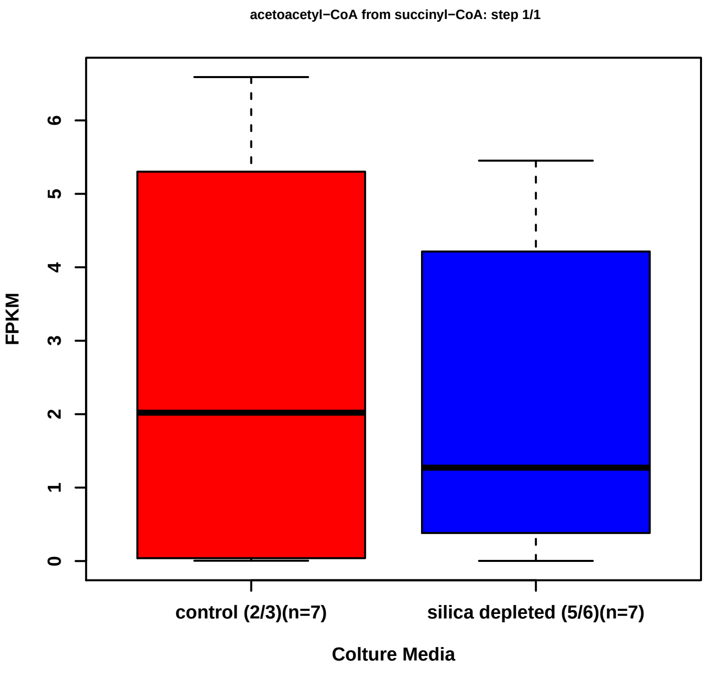
acetoacetyl−CoA from succinyl−CoA: step 1/1
FPKM
control (2/3)(n=7)
silica depleted (5/6)(n=7)
Colture Media

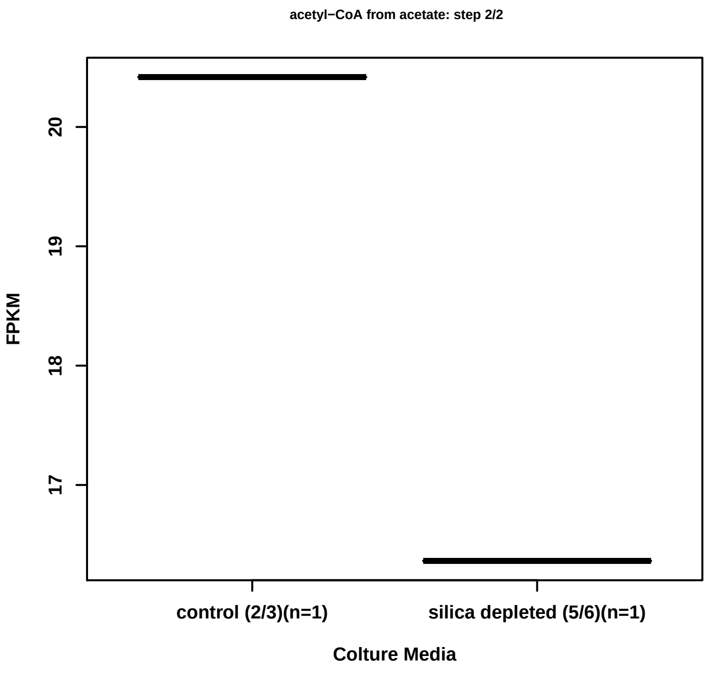
acetyl−CoA from acetate: step 2/2
FPKM
control (2/3)(n=1)
silica depleted (5/6)(n=1)
Colture Media

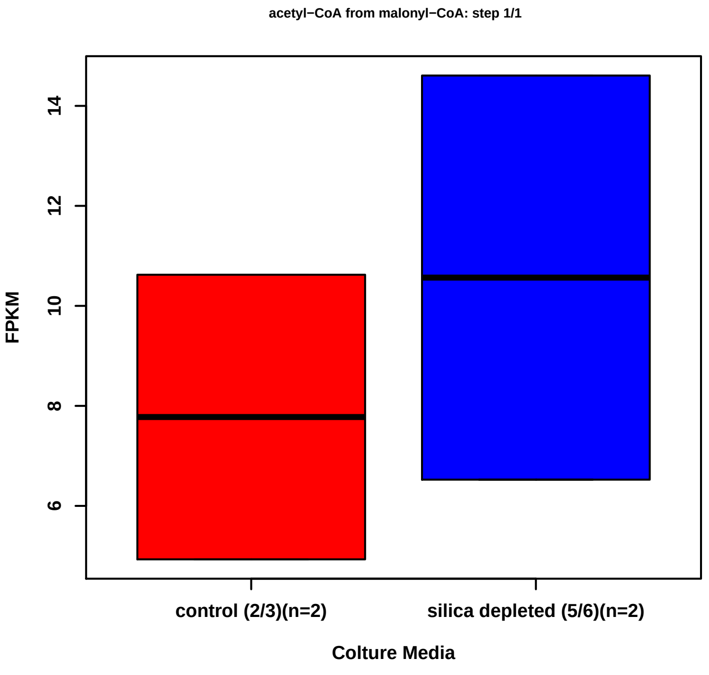
acetyl−CoA from malonyl−CoA: step 1/1
FPKM
control (2/3)(n=2)
silica depleted (5/6)(n=2)
Colture Media

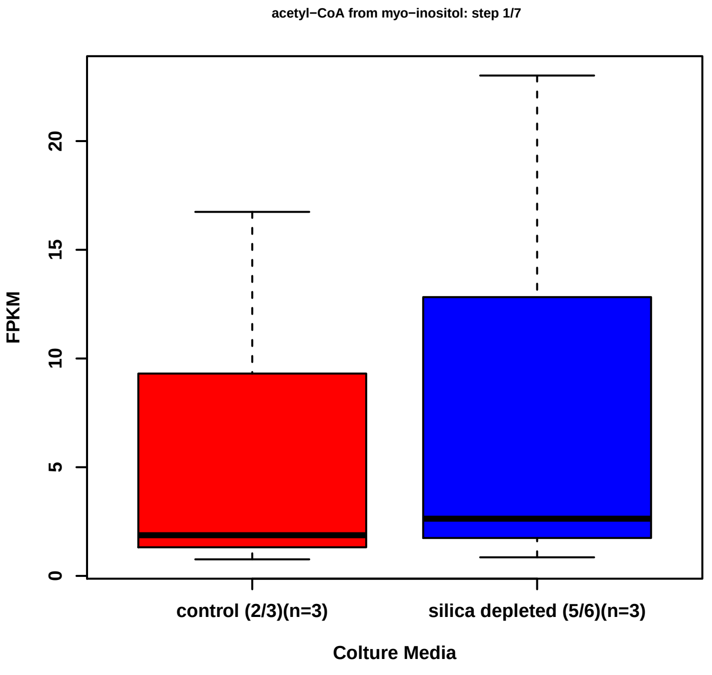
acetyl−CoA from myo−inositol: step 1/7
FPKM
control (2/3)(n=3)
silica depleted (5/6)(n=3)
Colture Media

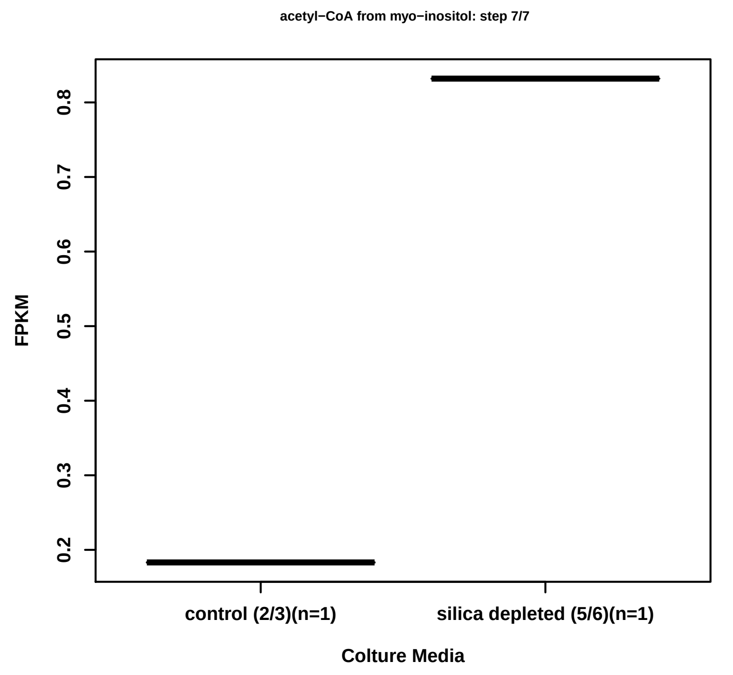
acetyl−CoA from myo−inositol: step 7/7
FPKM
0.2
0.3
0.4
0.5
0.6
0.7
0.8
control (2/3)(n=1)
silica depleted (5/6)(n=1)
Colture Media

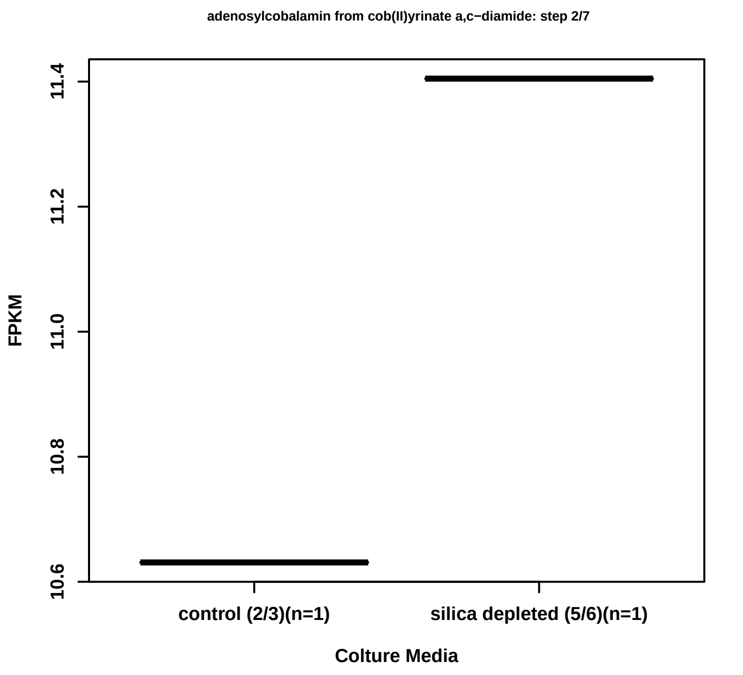
adenosylcobalamin from cob(II)yrinate a,c−diamide: step 2/7
FPKM
10.6
10.8
11.0
11.2
11.4
control (2/3)(n=1)
silica depleted (5/6)(n=1)
Colture Media

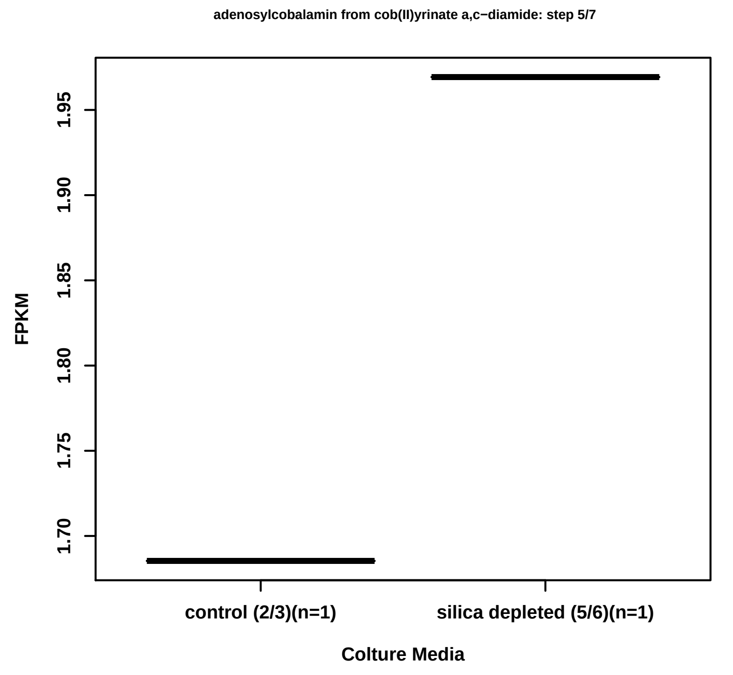
adenosylcobalamin from cob(II)yrinate a,c−diamide: step 5/7
FPKM
1.70
1.75
1.80
1.85
1.90
1.95
control (2/3)(n=1)
silica depleted (5/6)(n=1)
Colture Media

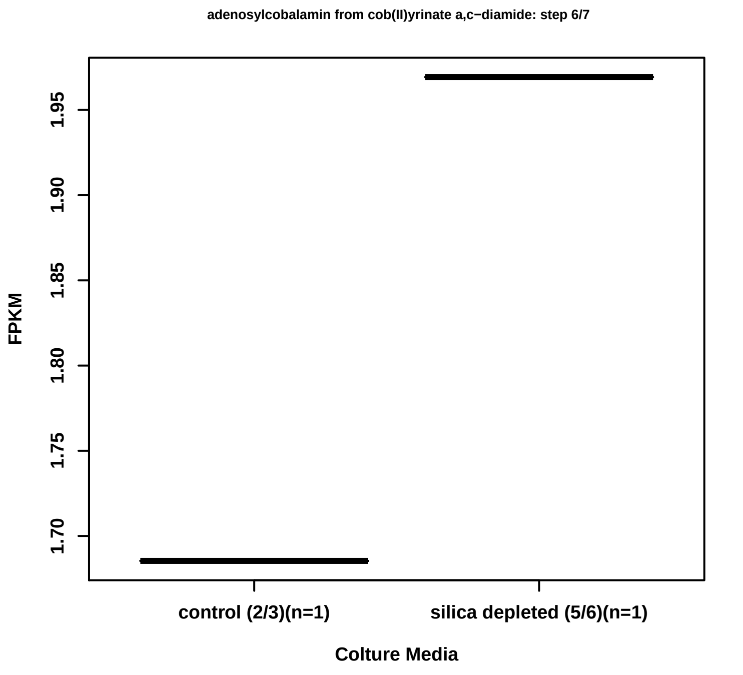
adenosylcobalamin from cob(II)yrinate a,c−diamide: step 6/7
FPKM
1.70
1.75
1.80
1.85
1.90
1.95
control (2/3)(n=1)
silica depleted (5/6)(n=1)
Colture Media

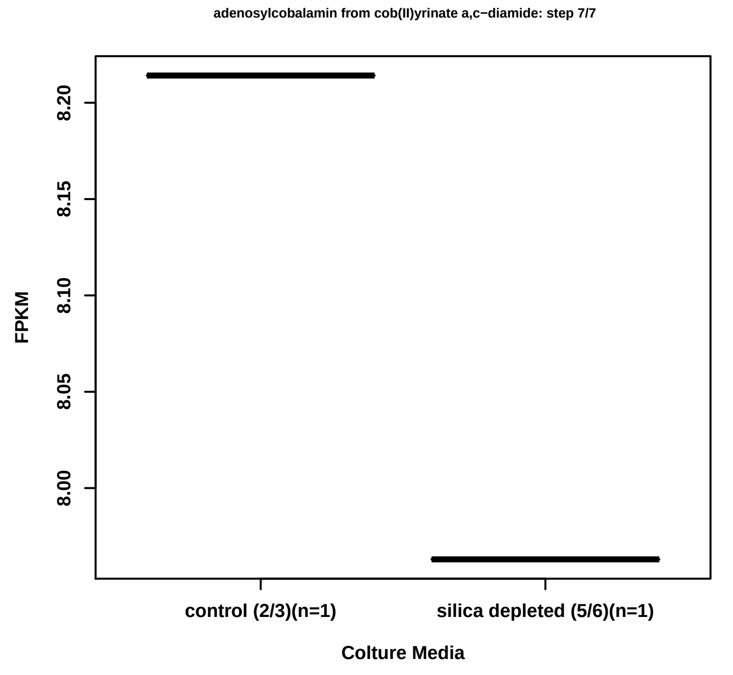
adenosylcobalamin from cob(II)yrinate a,c−diamide: step 7/7
FPKM
8.00
8.05
8.10
8.15
8.20
control (2/3)(n=1)
silica depleted (5/6)(n=1)
Colture Media

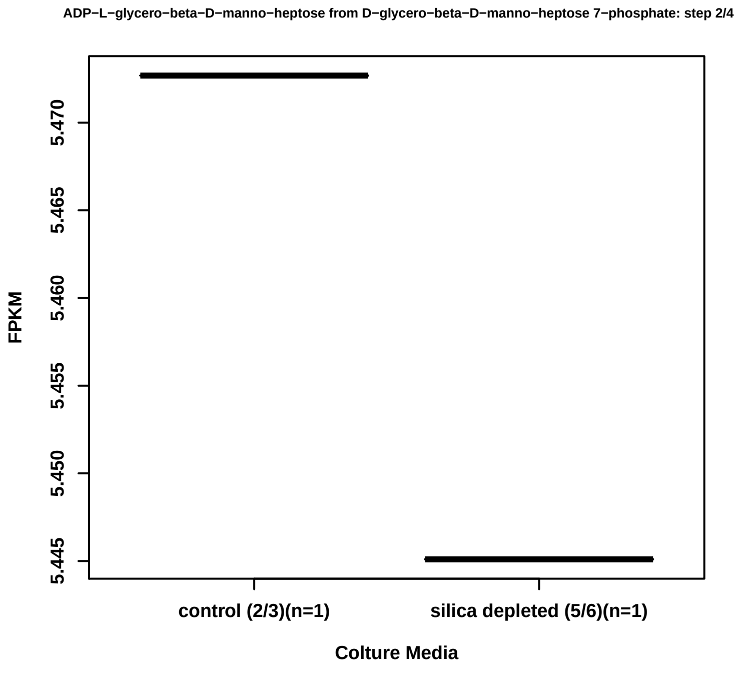
ADP−L−glycero−beta−D−manno−heptose from D−glycero−beta−D−manno−heptose 7−phosphate: step 2/4
FPKM
5.445
5.450
5.455
5.460
5.465
5.470
control (2/3)(n=1)
silica depleted (5/6)(n=1)
Colture Media

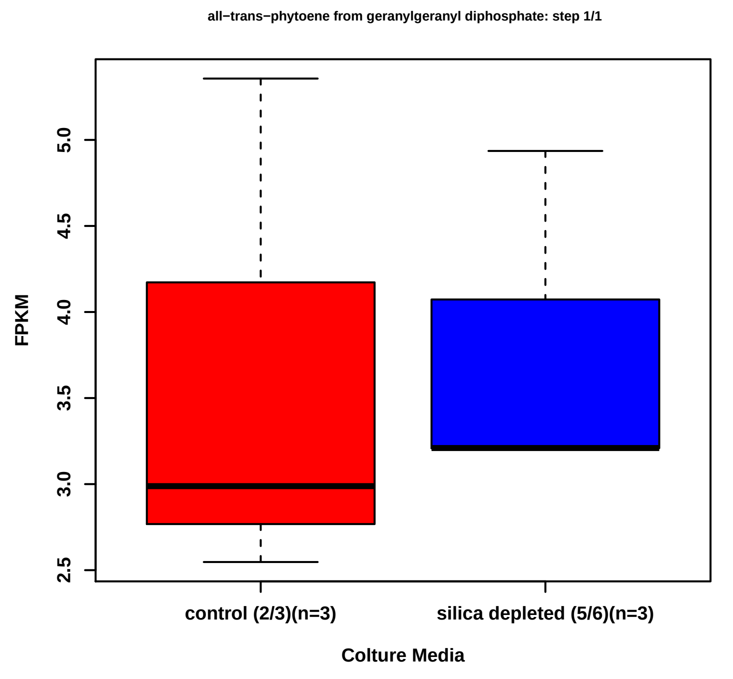
all−trans−phytoene from geranylgeranyl diphosphate: step 1/1
FPKM
2.5
3.0
3.5
4.0
4.5
5.0
control (2/3)(n=3)
silica depleted (5/6)(n=3)
Colture Media

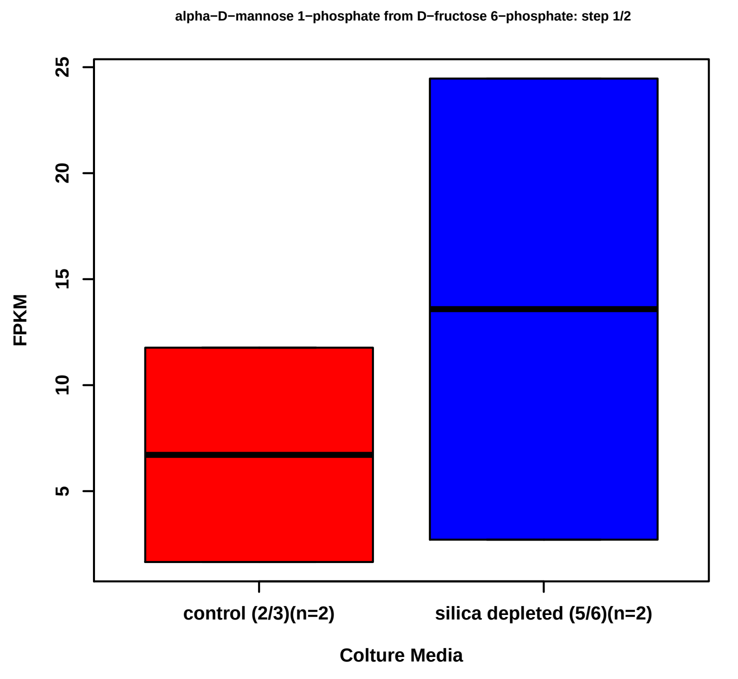
alpha−D−mannose 1−phosphate from D−fructose 6−phosphate: step 1/2
FPKM
5
10
15
20
25
control (2/3)(n=2)
silica depleted (5/6)(n=2)
Colture Media

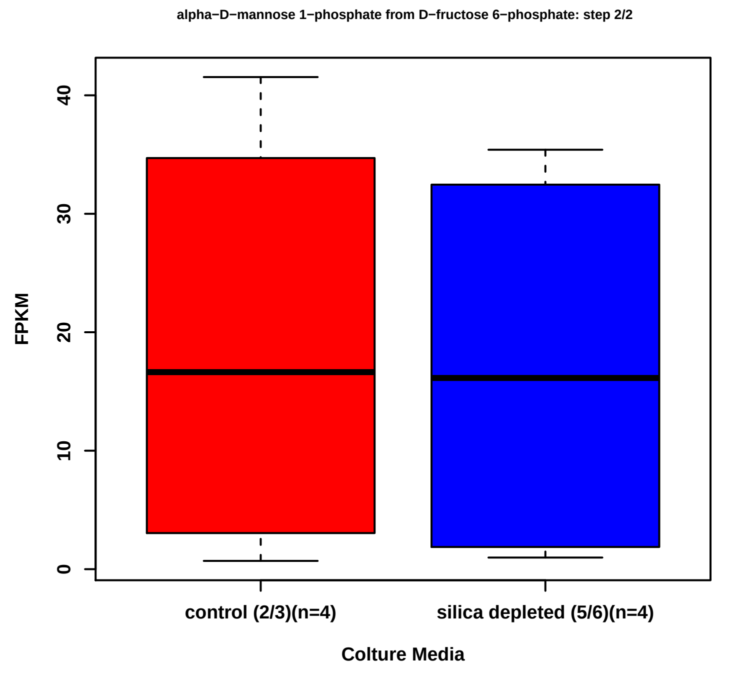
alpha−D−mannose 1−phosphate from D−fructose 6−phosphate: step 2/2
FPKM
0
10
20
30
40
control (2/3)(n=4)
silica depleted (5/6)(n=4)
Colture Media

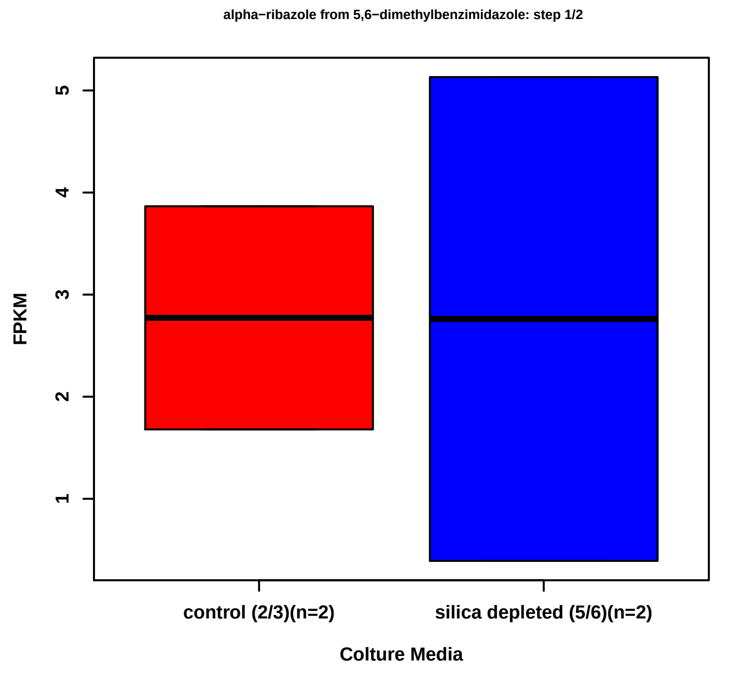
alpha−ribazole from 5,6−dimethylbenzimidazole: step 1/2
FPKM
1
2
3
4
5
control (2/3)(n=2)
silica depleted (5/6)(n=2)
Colture Media

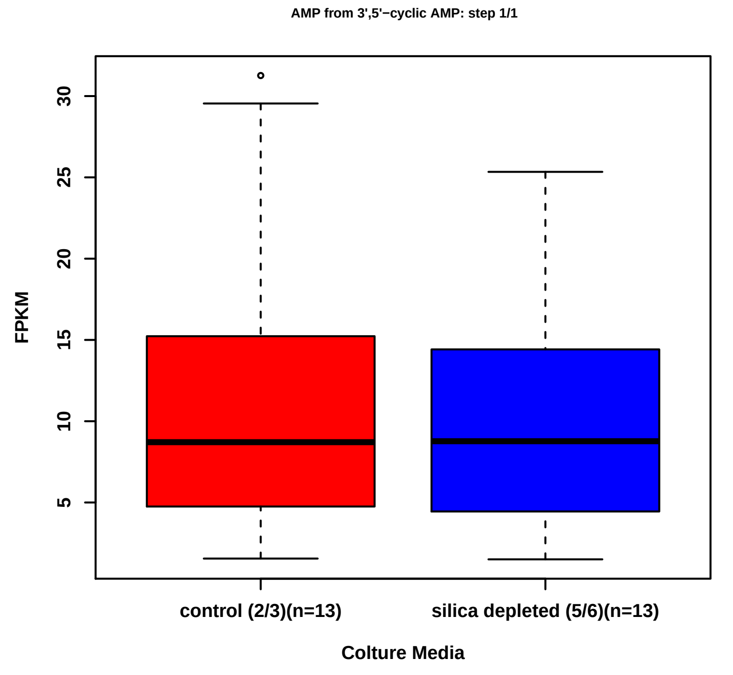
AMP from 3',5'−cyclic AMP: step 1/1
FPKM
5
10
15
20
25
30
control (2/3)(n=13)
silica depleted (5/6)(n=13)
Colture Media

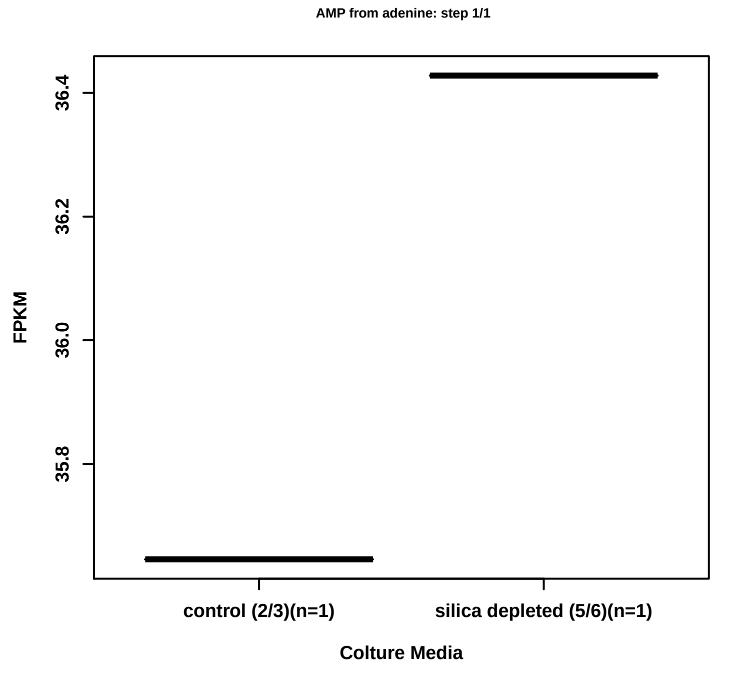
AMP from adenine: step 1/1
FPKM
35.8
36.0
36.2
36.4
control (2/3)(n=1)
silica depleted (5/6)(n=1)
Colture Media

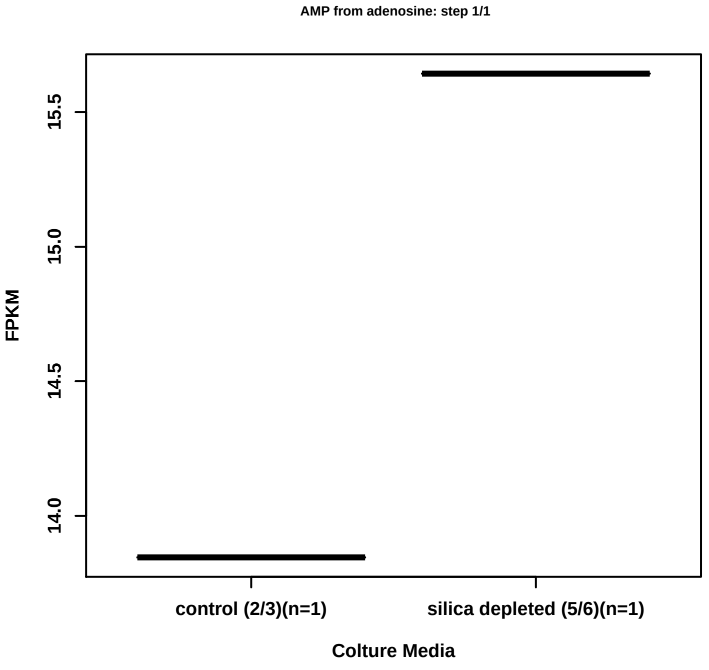
AMP from adenosine: step 1/1
FPKM
14.0
14.5
15.0
15.5
control (2/3)(n=1)
silica depleted (5/6)(n=1)
Colture Media

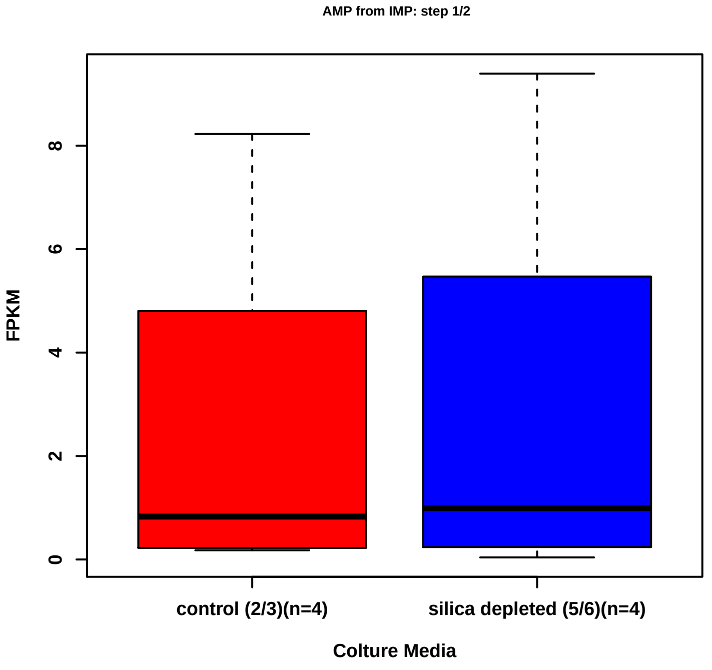
AMP from IMP: step 1/2
FPKM
0
2
4
6
8
control (2/3)(n=4)
silica depleted (5/6)(n=4)
Colture Media

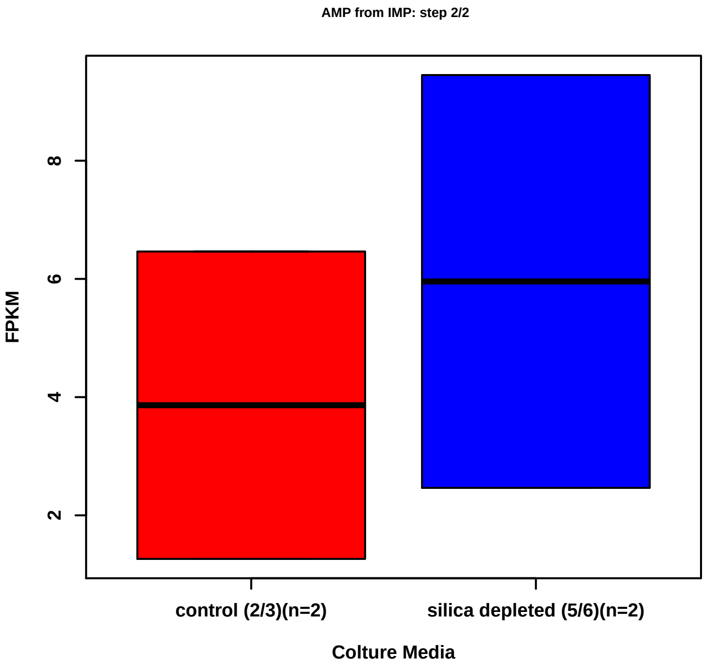
AMP from IMP: step 2/2
FPKM
2
4
6
8
control (2/3)(n=2)
silica depleted (5/6)(n=2)
Colture Media

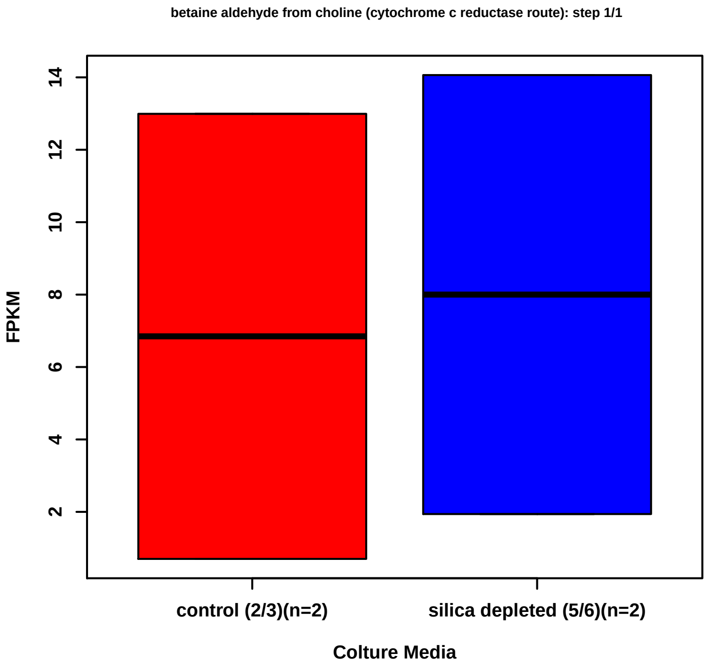
betaine aldehyde from choline (cytochrome c reductase route): step 1/1
FPKM
2
4
6
8
10
12
14
control (2/3)(n=2)
silica depleted (5/6)(n=2)
Colture Media

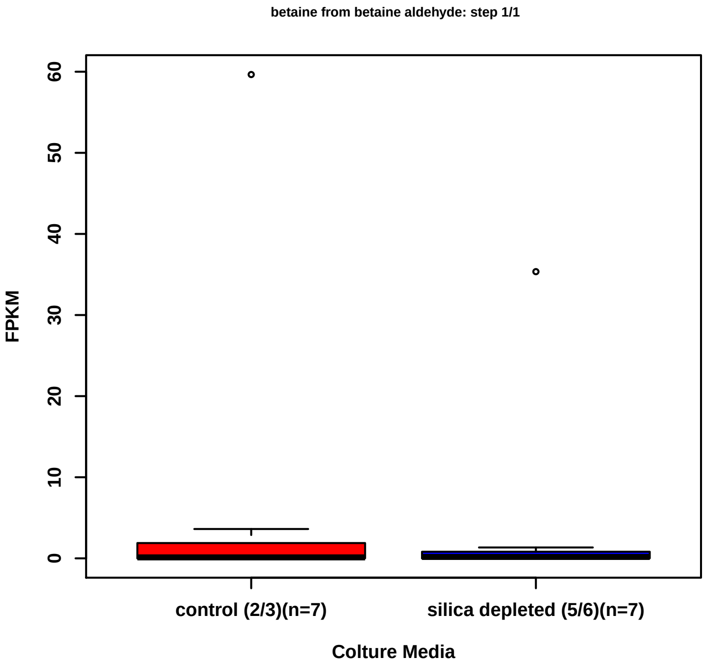
betaine from betaine aldehyde: step 1/1
FPKM
0
10
20
30
40
50
60
control (2/3)(n=7)
silica depleted (5/6)(n=7)
Colture Media

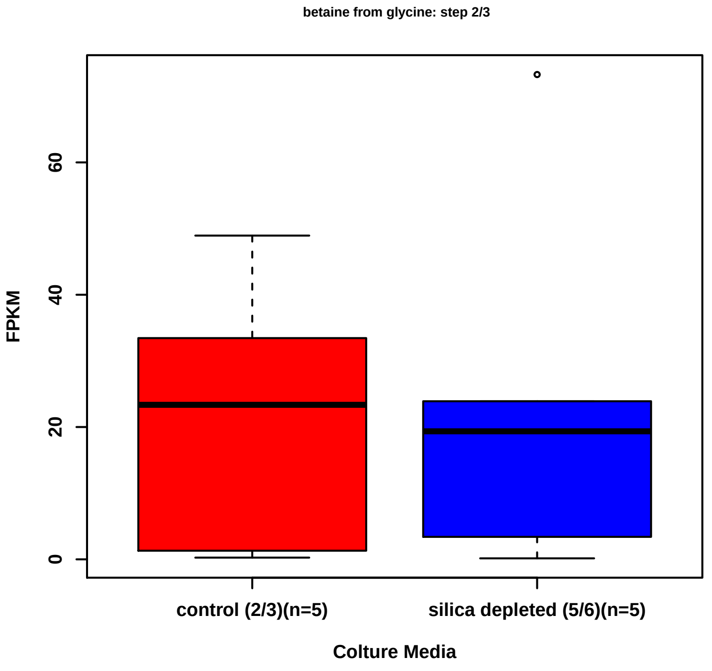
betaine from glycine: step 2/3
FPKM
0
20
40
60
control (2/3)(n=5)
silica depleted (5/6)(n=5)
Colture Media

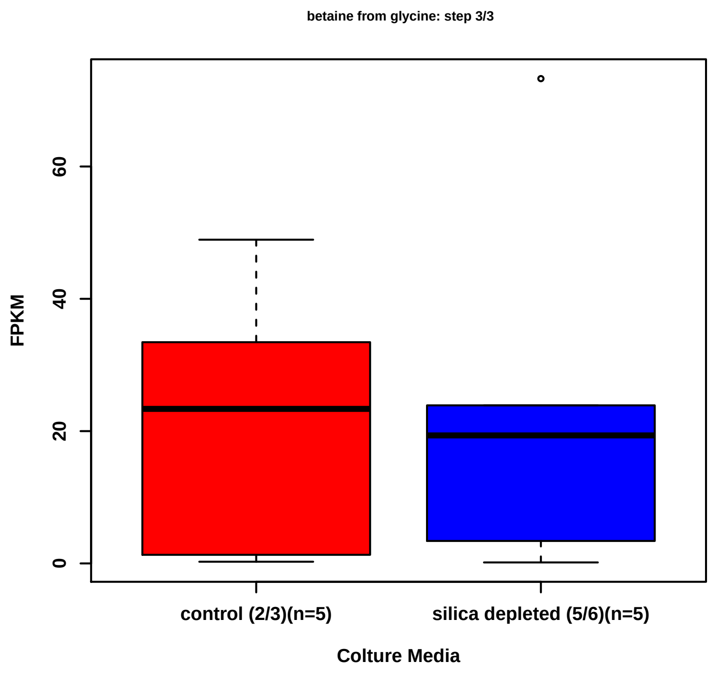
betaine from glycine: step 3/3
FPKM
0
20
40
60
control (2/3)(n=5)
silica depleted (5/6)(n=5)
Colture Media

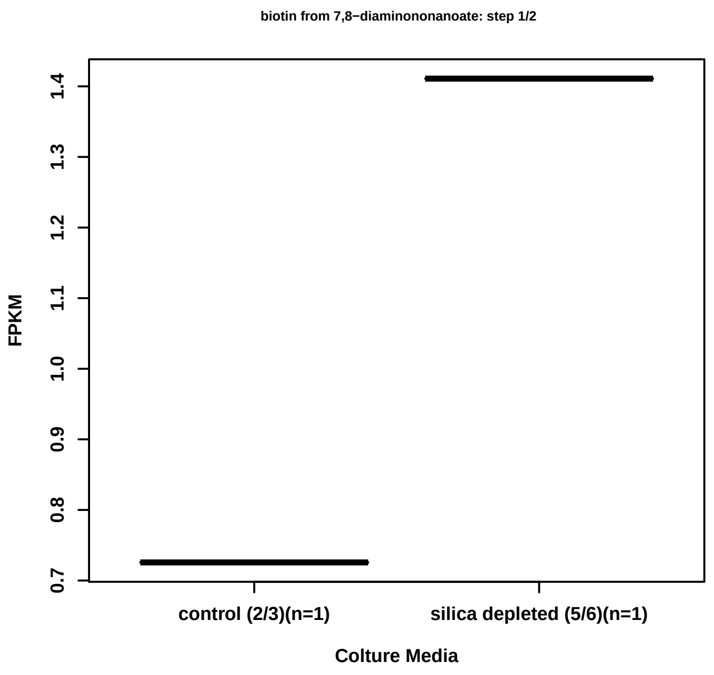
biotin from 7,8−diaminononanoate: step 1/2
FPKM
0.7
0.8
0.9
1.0
1.1
1.2
1.3
1.4
control (2/3)(n=1)
silica depleted (5/6)(n=1)
Colture Media

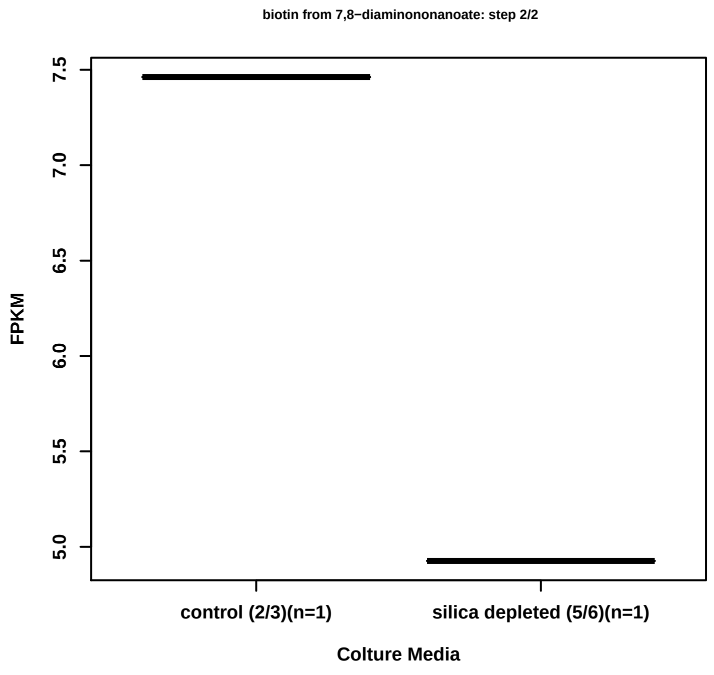
biotin from 7,8−diaminononanoate: step 2/2
FPKM
5.0
5.5
6.0
6.5
7.0
7.5
control (2/3)(n=1)
silica depleted (5/6)(n=1)
Colture Media

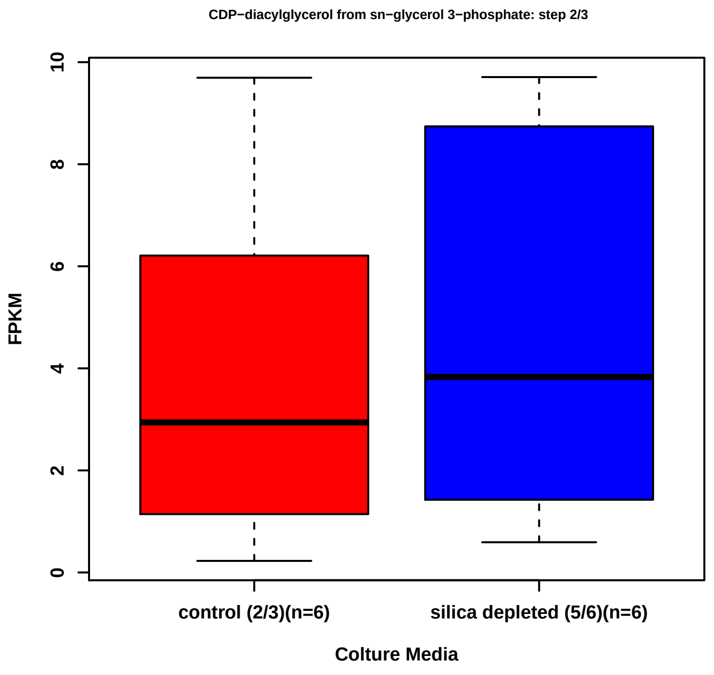
CDP−diacylglycerol from sn−glycerol 3−phosphate: step 2/3
FPKM
0
2
4
6
8
10
control (2/3)(n=6)
silica depleted (5/6)(n=6)
Colture Media

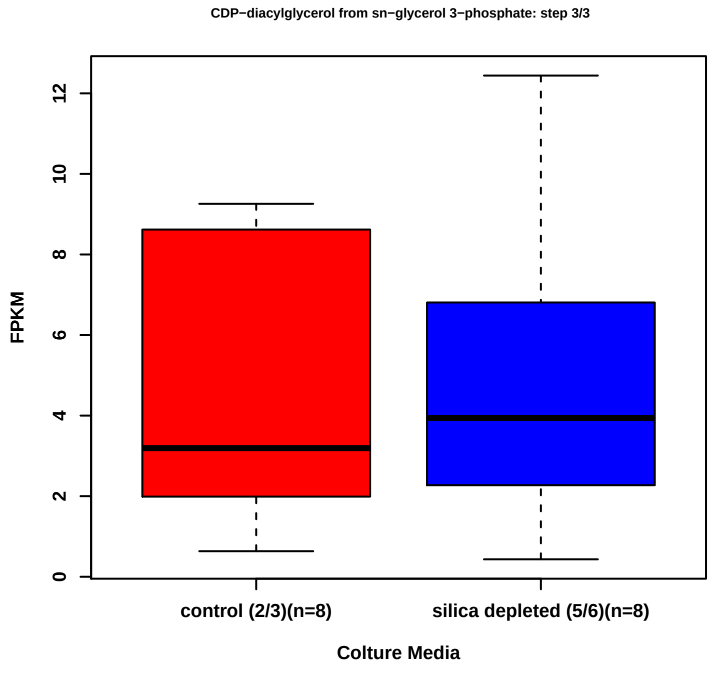
CDP−diacylglycerol from sn−glycerol 3−phosphate: step 3/3
FPKM
0
2
4
6
8
10
12
control (2/3)(n=8)
silica depleted (5/6)(n=8)
Colture Media

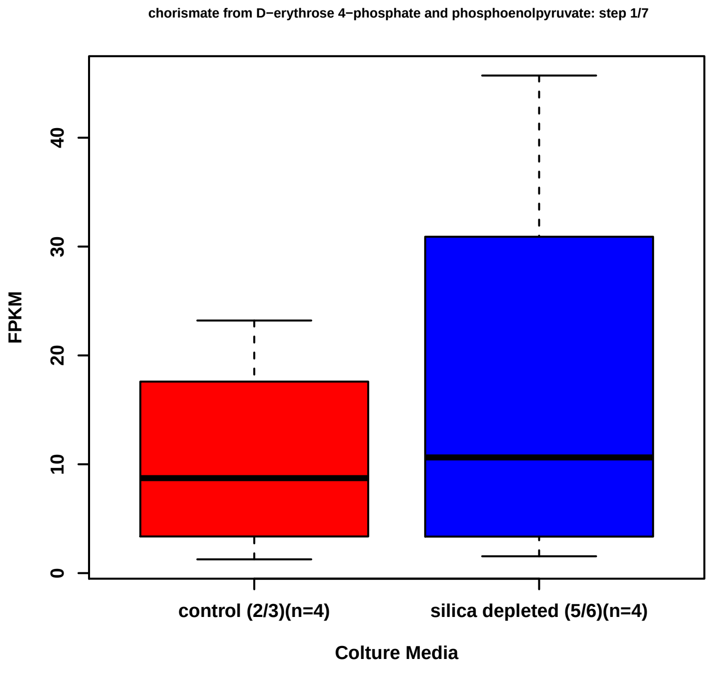
chorismate from D−erythrose 4−phosphate and phosphoenolpyruvate: step 1/7
FPKM
0
10
20
30
40
control (2/3)(n=4)
silica depleted (5/6)(n=4)
Colture Media

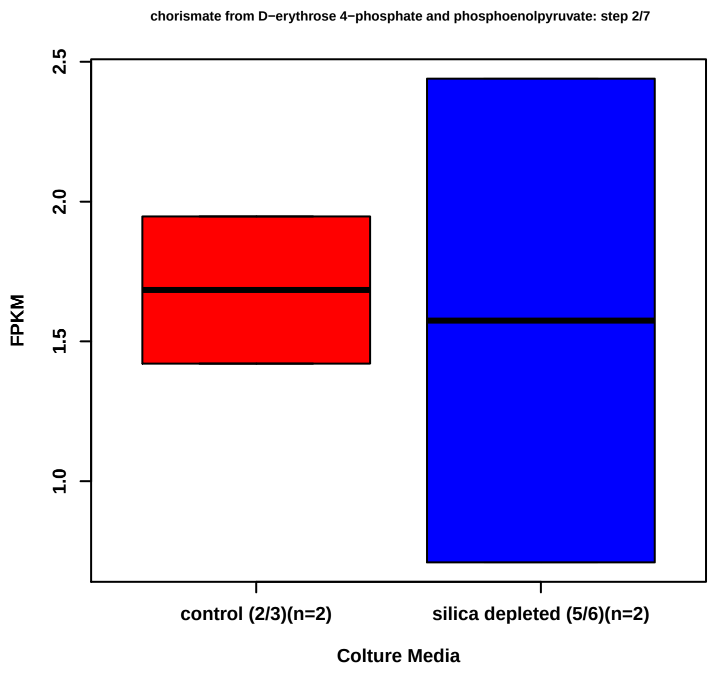
chorismate from D−erythrose 4−phosphate and phosphoenolpyruvate: step 2/7
FPKM
1.0
1.5
2.0
2.5
control (2/3)(n=2)
silica depleted (5/6)(n=2)
Colture Media

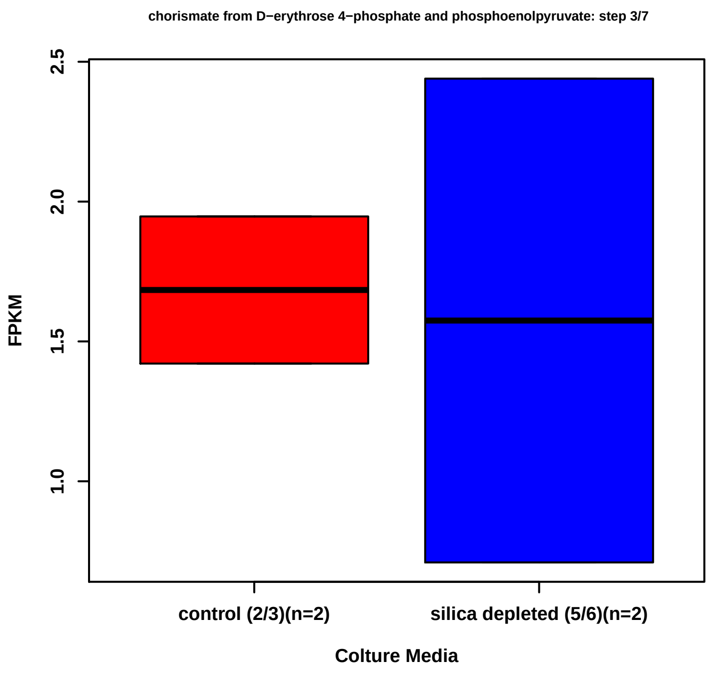
chorismate from D−erythrose 4−phosphate and phosphoenolpyruvate: step 3/7
FPKM
1.0
1.5
2.0
2.5
control (2/3)(n=2)
silica depleted (5/6)(n=2)
Colture Media

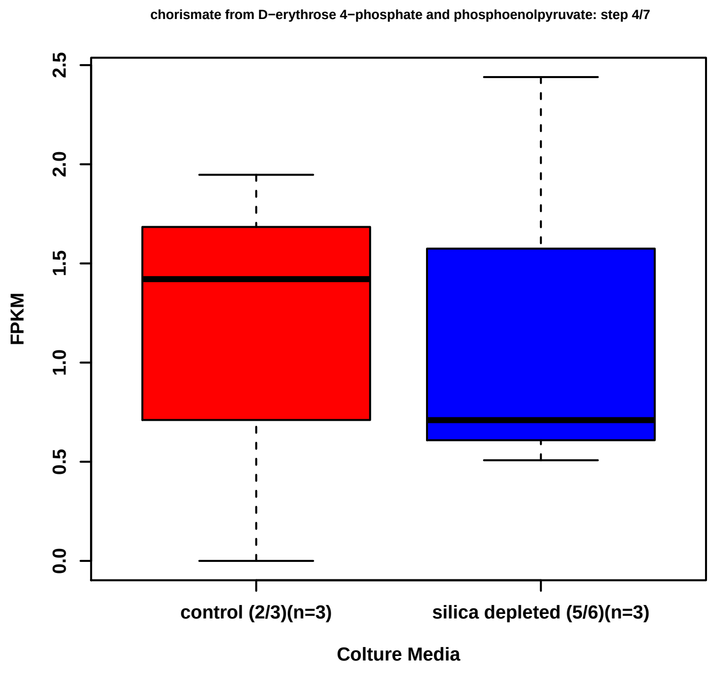
chorismate from D−erythrose 4−phosphate and phosphoenolpyruvate: step 4/7
FPKM
0.0
0.5
1.0
1.5
2.0
2.5
control (2/3)(n=3)
silica depleted (5/6)(n=3)
Colture Media

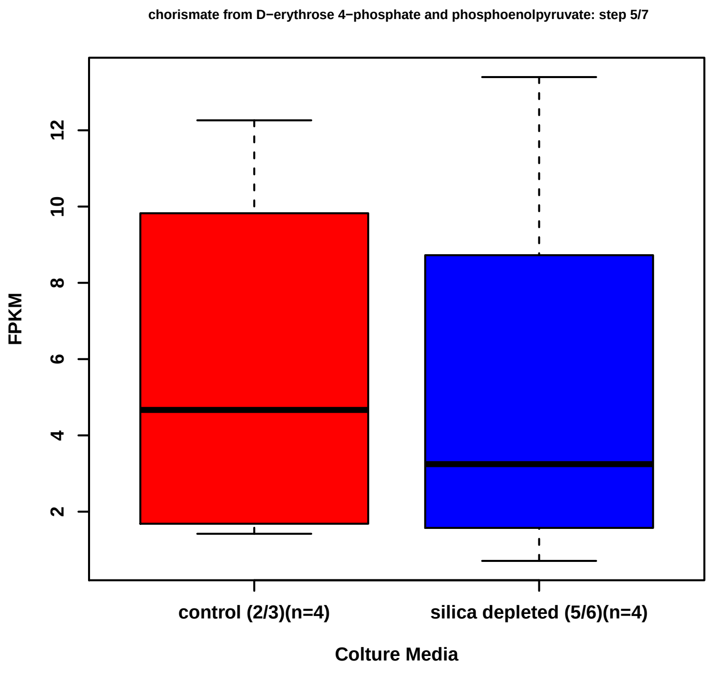
chorismate from D−erythrose 4−phosphate and phosphoenolpyruvate: step 5/7
FPKM
2
4
6
8
10
12
control (2/3)(n=4)
silica depleted (5/6)(n=4)
Colture Media

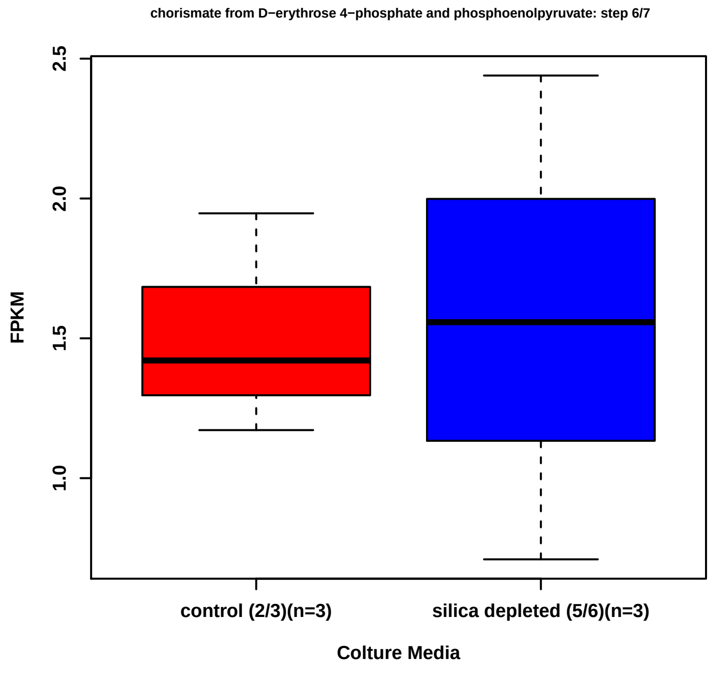
chorismate from D−erythrose 4−phosphate and phosphoenolpyruvate: step 6/7
FPKM
1.0
1.5
2.0
2.5
control (2/3)(n=3)
silica depleted (5/6)(n=3)
Colture Media

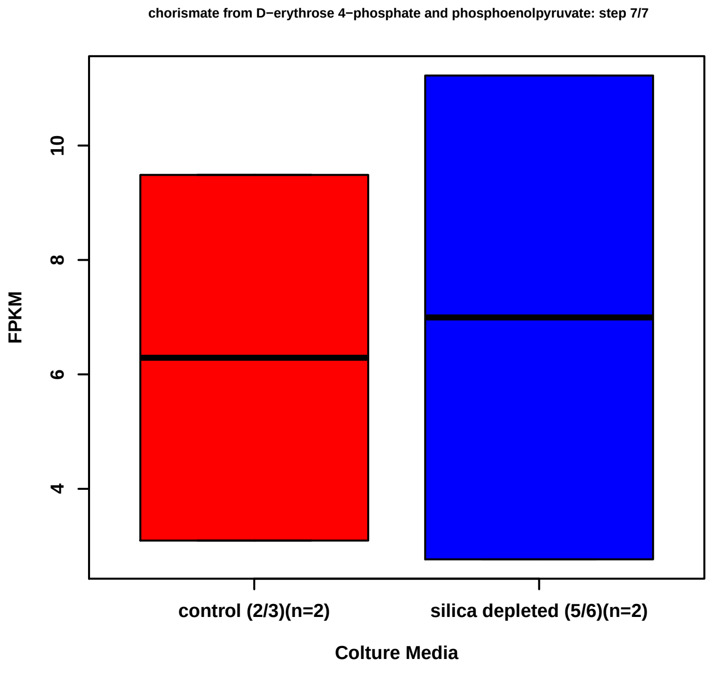
chorismate from D−erythrose 4−phosphate and phosphoenolpyruvate: step 7/7
FPKM
4
6
8
10
control (2/3)(n=2)
silica depleted (5/6)(n=2)
Colture Media

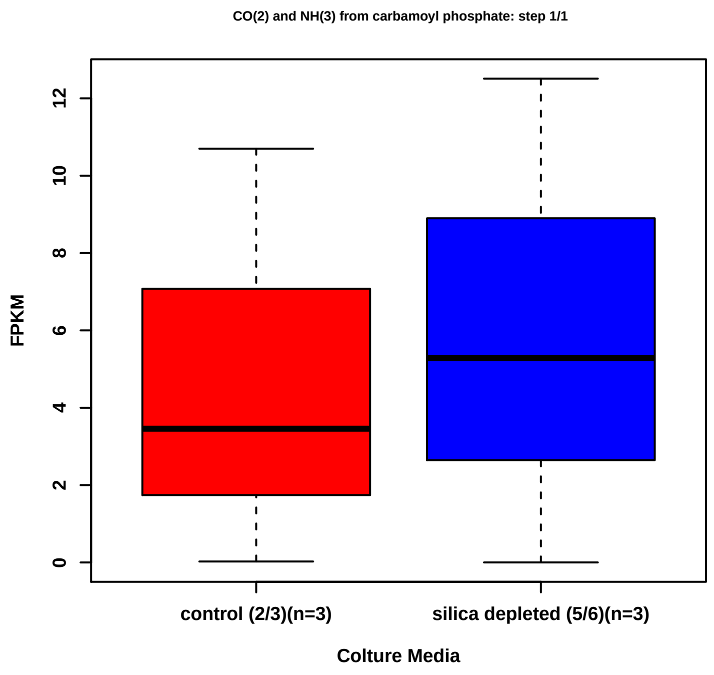
CO(2) and NH(3) from carbamoyl phosphate: step 1/1
FPKM
0
2
4
6
8
10
12
control (2/3)(n=3)
silica depleted (5/6)(n=3)
Colture Media

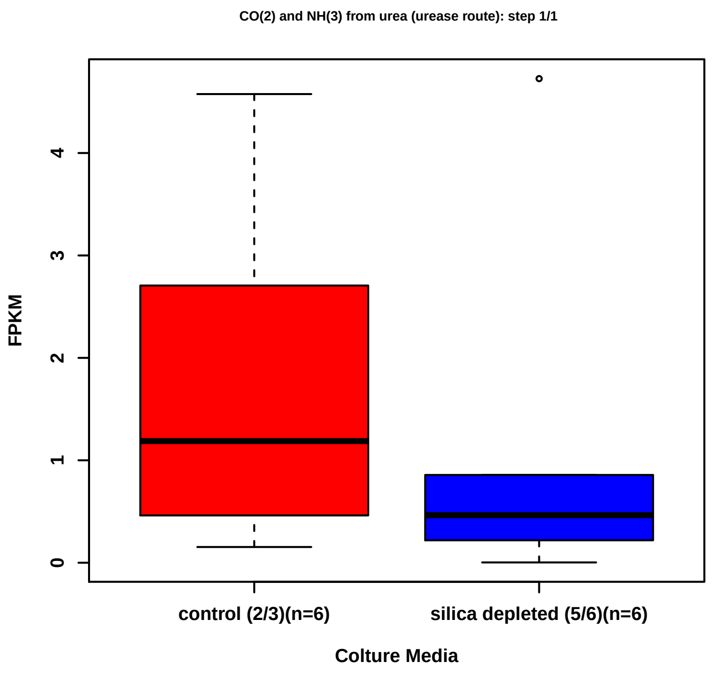
CO(2) and NH(3) from urea (urease route): step 1/1
FPKM
0
1
2
3
4
control (2/3)(n=6)
silica depleted (5/6)(n=6)
Colture Media

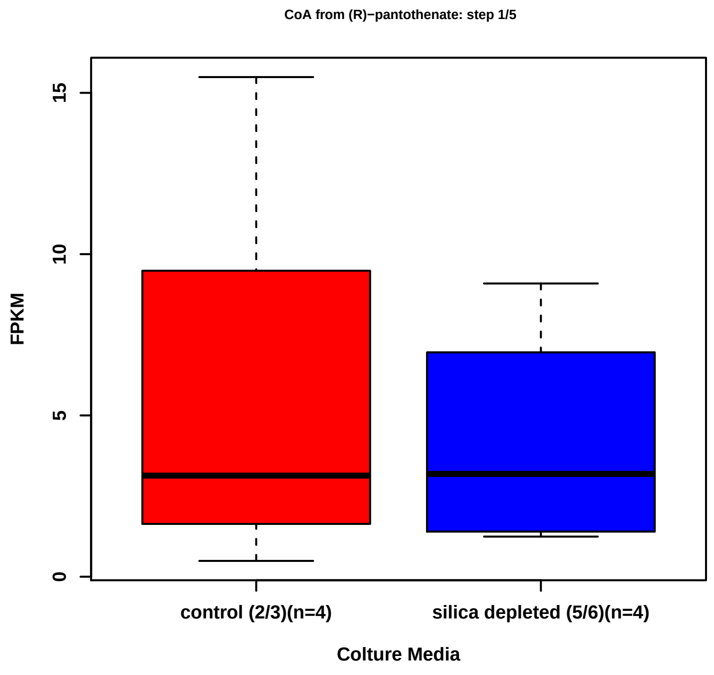
CoA from (R)−pantothenate: step 1/5
FPKM
0
5
10
15
control (2/3)(n=4)
silica depleted (5/6)(n=4)
Colture Media

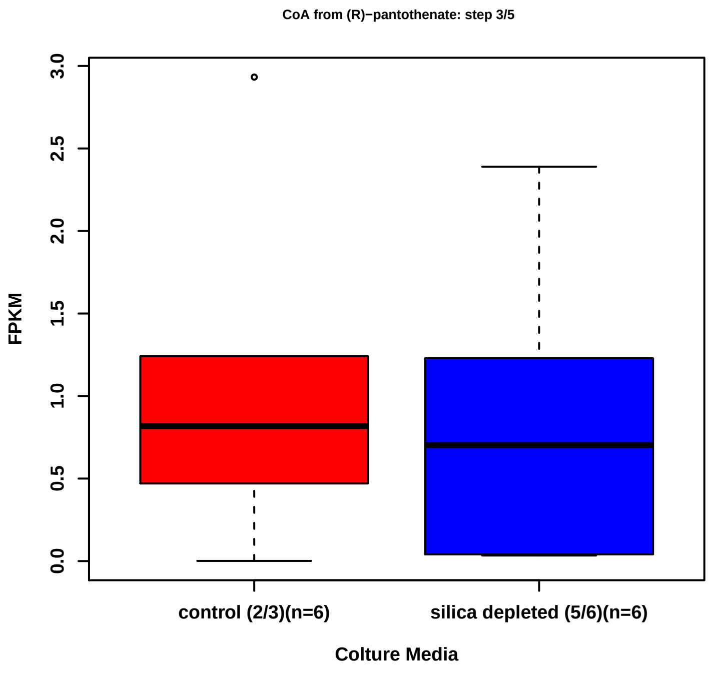
CoA from (R)−pantothenate: step 3/5
FPKM
0.0
0.5
1.0
1.5
2.0
2.5
3.0
control (2/3)(n=6)
silica depleted (5/6)(n=6)
Colture Media

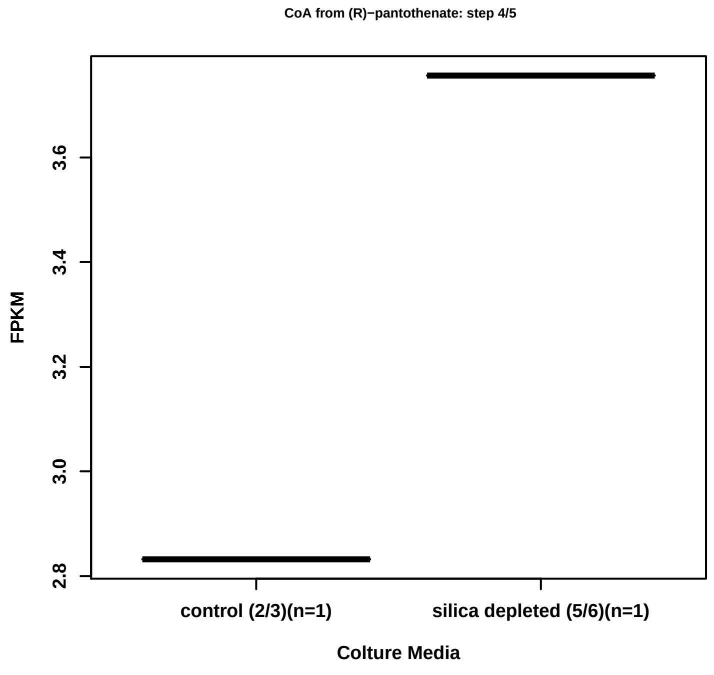
CoA from (R)−pantothenate: step 4/5
FPKM
2.8
3.0
3.2
3.4
3.6
control (2/3)(n=1)
silica depleted (5/6)(n=1)
Colture Media

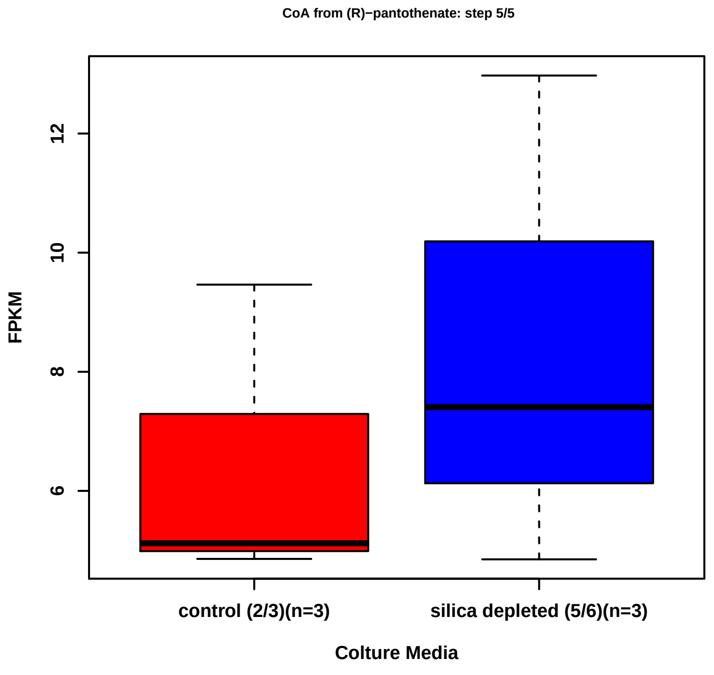
CoA from (R)−pantothenate: step 5/5
FPKM
6
8
10
12
control (2/3)(n=3)
silica depleted (5/6)(n=3)
Colture Media

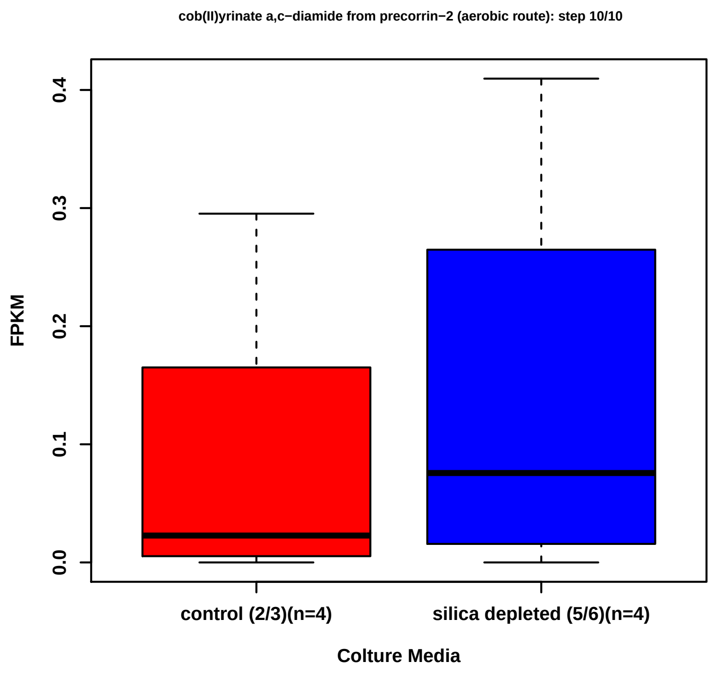

cob(II)yrinate a,c−diamide from precorrin−2 (aerobic route): step 10/10
FPKM
0.0
0.1
0.2
0.3
0.4
control (2/3)(n=4)
silica depleted (5/6)(n=4)
Colture Media

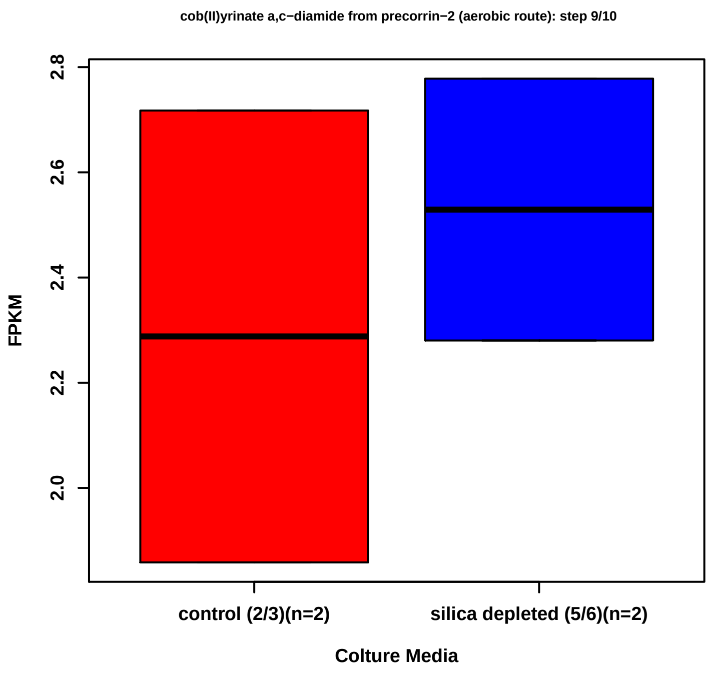

cob(II)yrinate a,c−diamide from precorrin−2 (aerobic route): step 9/10
FPKM
2.0
2.2
2.4
2.6
2.8
control (2/3)(n=2)
silica depleted (5/6)(n=2)
Colture Media

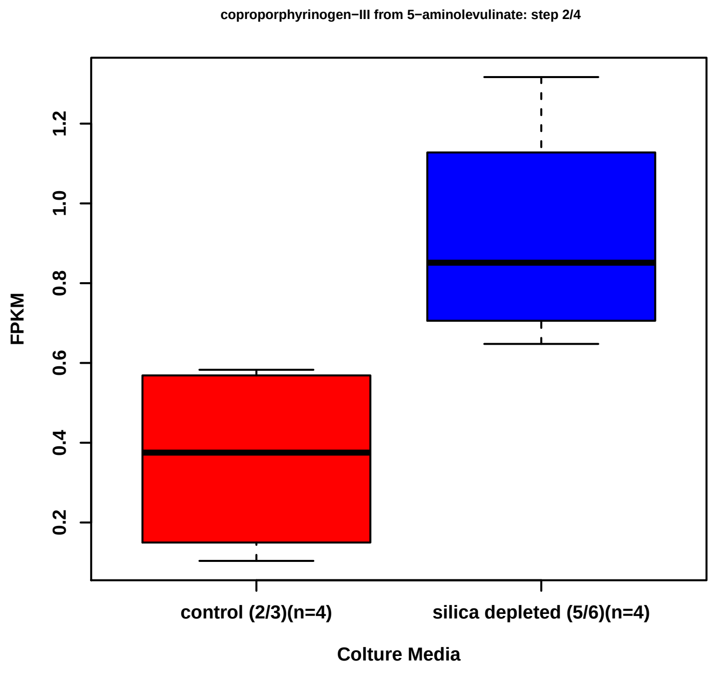

coproporphyrinogen−III from 5−aminolevulinate: step 2/4
FPKM
0.2
0.4
0.6
0.8
1.0
1.2
control (2/3)(n=4)
silica depleted (5/6)(n=4)
Colture Media

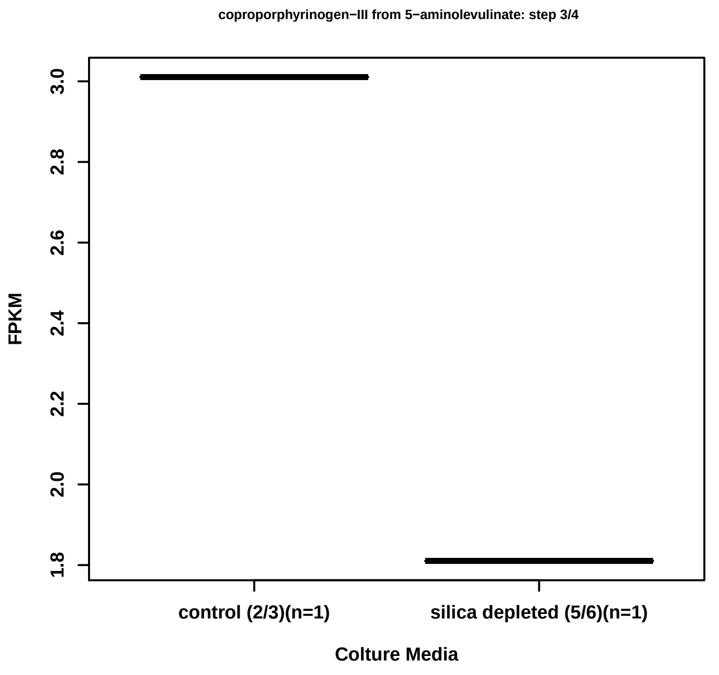

coproporphyrinogen−III from 5−aminolevulinate: step 3/4
FPKM
1.8
2.0
2.2
2.4
2.6
2.8
3.0
control (2/3)(n=1)
silica depleted (5/6)(n=1)
Colture Media

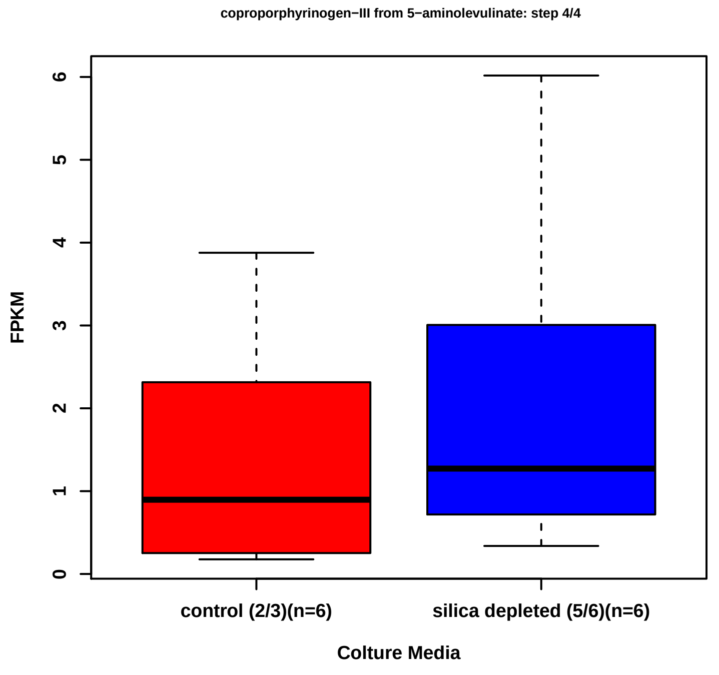

coproporphyrinogen−III from 5−aminolevulinate: step 4/4
FPKM
0
1
2
3
4
5
6
control (2/3)(n=6)
silica depleted (5/6)(n=6)
Colture Media

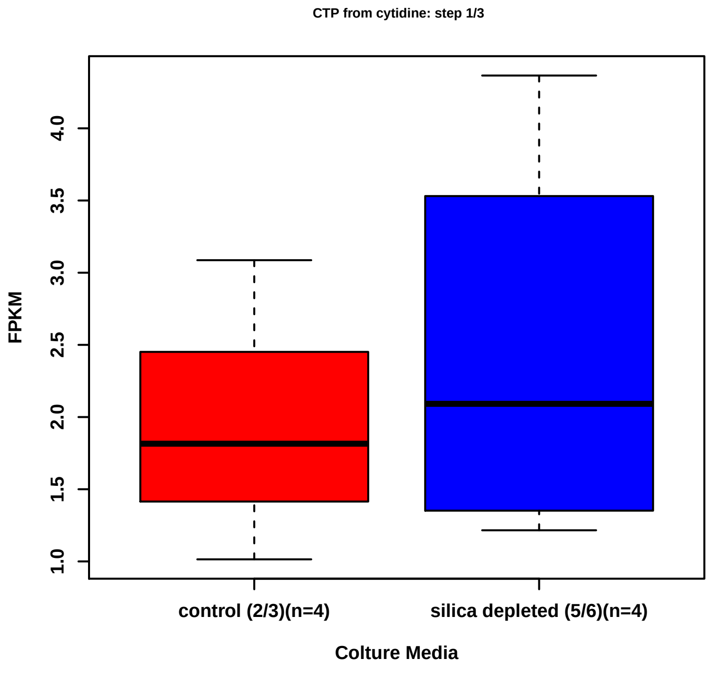

CTP from cytidine: step 1/3
FPKM
1.0
1.5
2.0
2.5
3.0
3.5
4.0
control (2/3)(n=4)
silica depleted (5/6)(n=4)
Colture Media

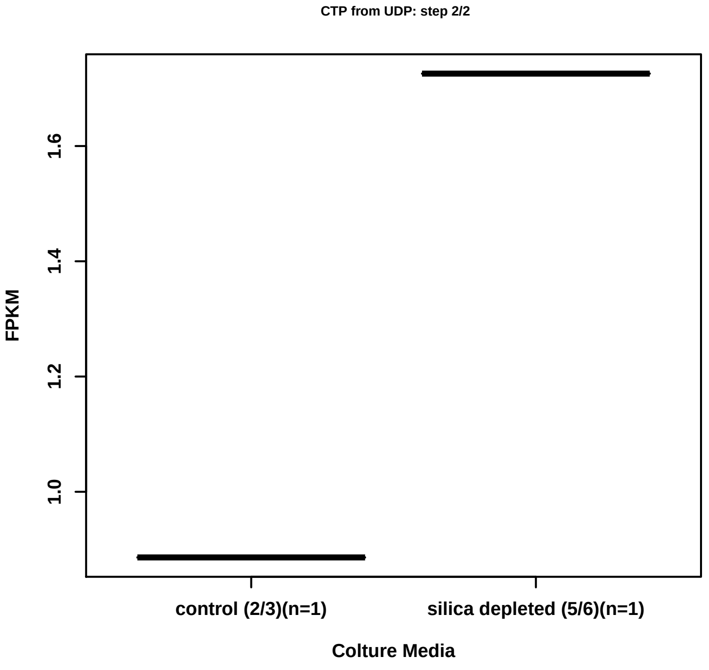
CTP from UDP: step 2/2
FPKM
1.0
1.2
1.4
1.6
control (2/3)(n=1)
silica depleted (5/6)(n=1)
Colture Media

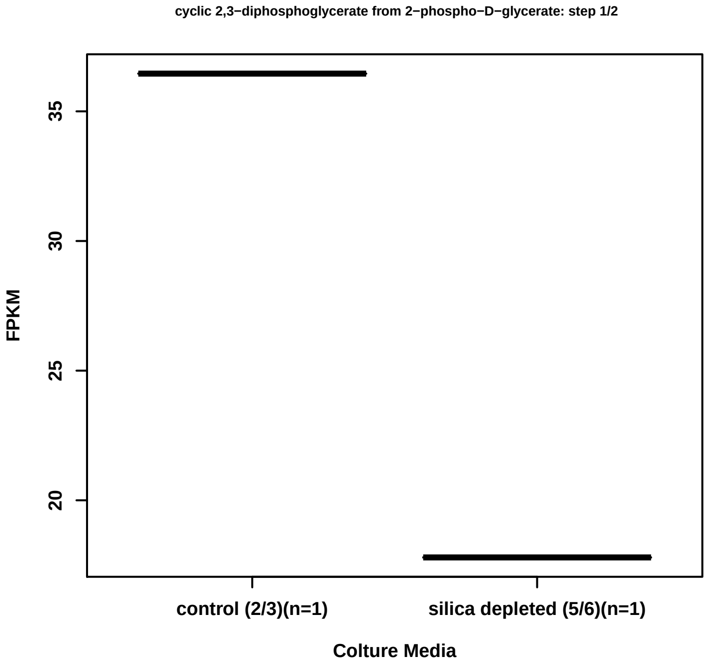
cyclic 2,3−diphosphoglycerate from 2−phospho−D−glycerate: step 1/2
FPKM
20
25
30
35
control (2/3)(n=1)
silica depleted (5/6)(n=1)
Colture Media

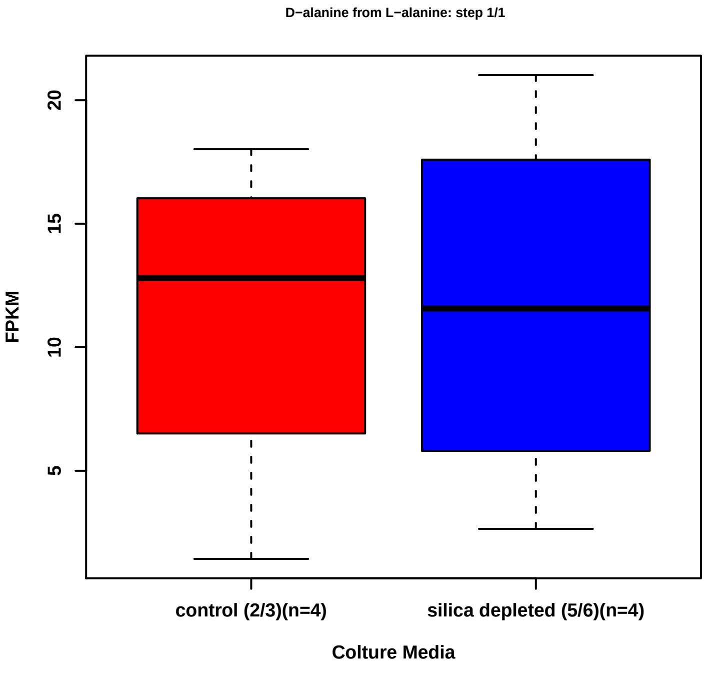
D−alanine from L−alanine: step 1/1
FPKM
5
10
15
20
control (2/3)(n=4)
silica depleted (5/6)(n=4)
Colture Media

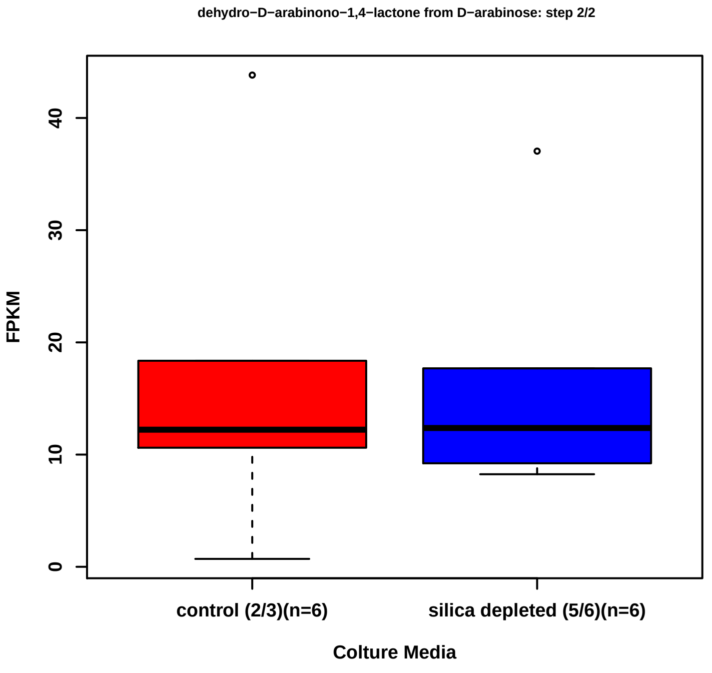
dehydro−D−arabinono−1,4−lactone from D−arabinose: step 2/2
FPKM
0
10
20
30
40
control (2/3)(n=6)
silica depleted (5/6)(n=6)
Colture Media

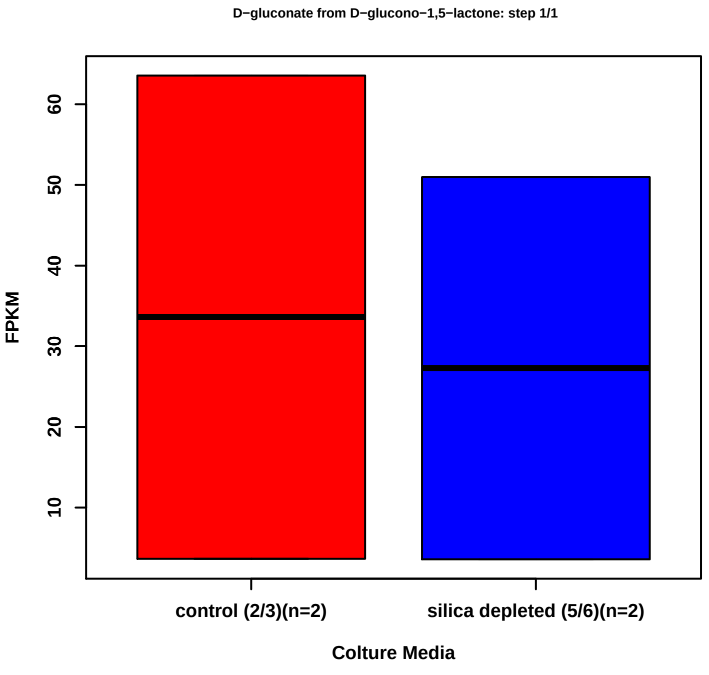
D−gluconate from D−glucono−1,5−lactone: step 1/1
FPKM
10
20
30
40
50
60
control (2/3)(n=2)
silica depleted (5/6)(n=2)
Colture Media

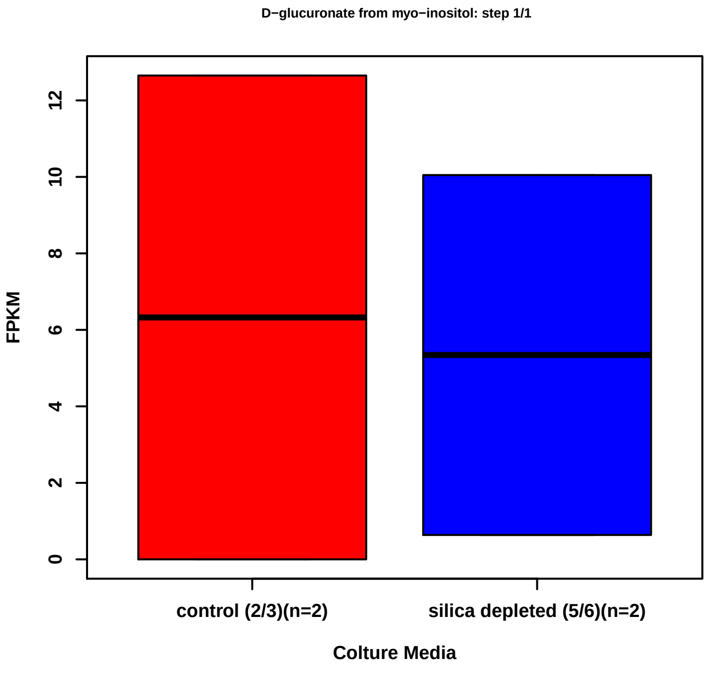
D−glucuronate from myo−inositol: step 1/1
FPKM
0
2
4
6
8
10
12
control (2/3)(n=2)
silica depleted (5/6)(n=2)
Colture Media

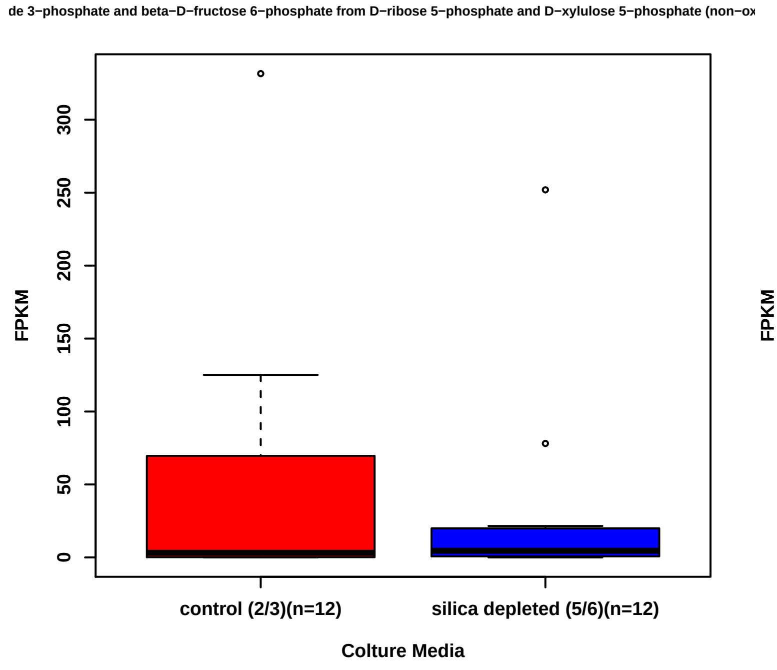
de 3−phosphate and beta−D−fructose 6−phosphate from D−ribose 5−phosphate and D−xylulose 5−phosphate (non−ox
FPKM
0
50
100
150
200
250
300
control (2/3)(n=12)
silica depleted (5/6)(n=12)
Colture Media

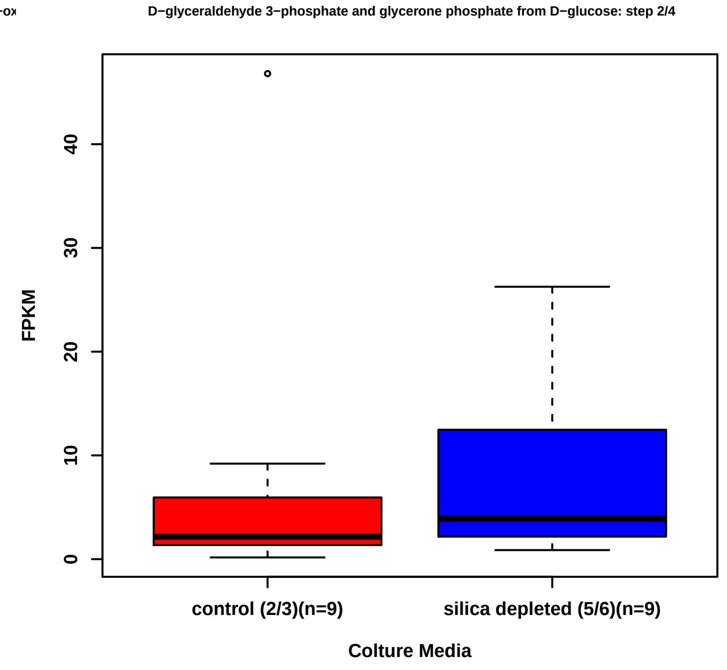
D−glyceraldehyde 3−phosphate and glycerone phosphate from D−glucose: step 2/4
FPKM
0
10
20
30
40
control (2/3)(n=9)
silica depleted (5/6)(n=9)
Colture Media

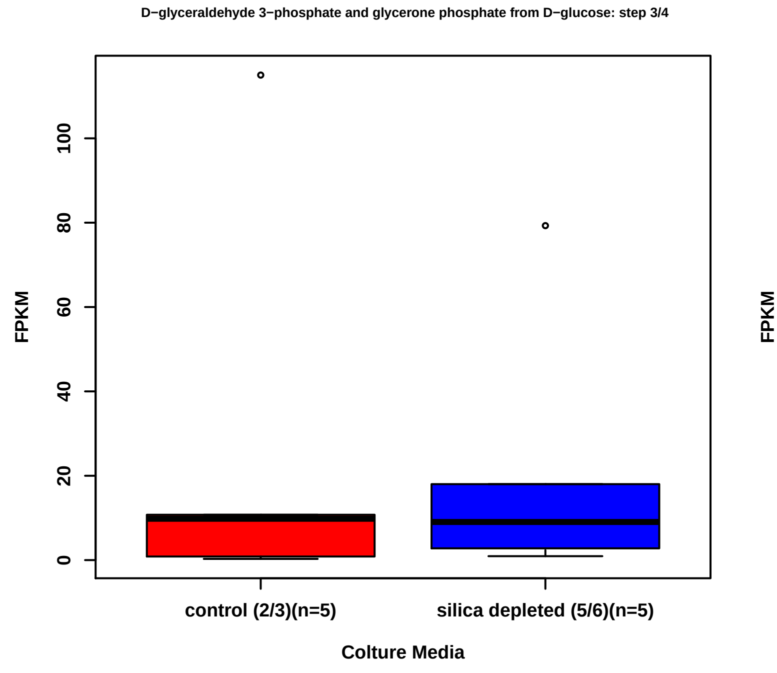
D−glyceraldehyde 3−phosphate and glycerone phosphate from D−glucose: step 3/4
FPKM
0
20
40
60
80
100
control (2/3)(n=5)
silica depleted (5/6)(n=5)
Colture Media

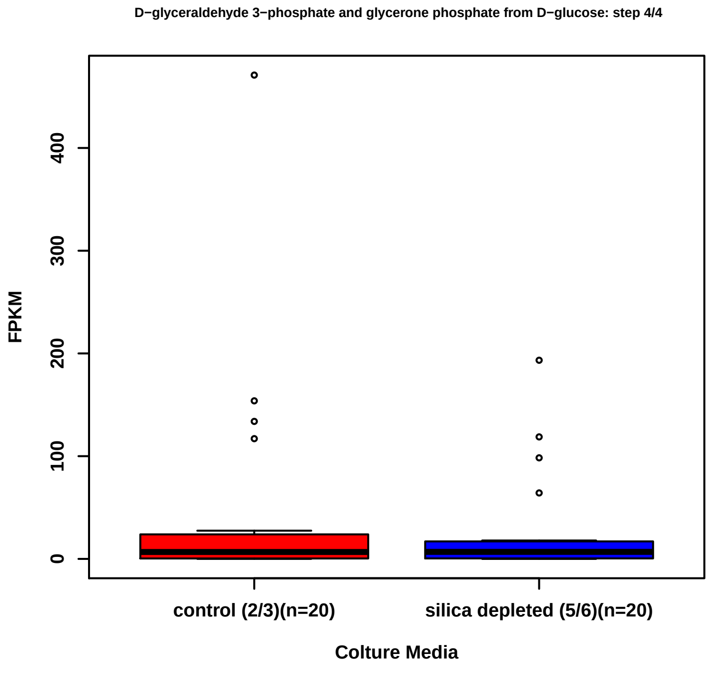
D−glyceraldehyde 3−phosphate and glycerone phosphate from D−glucose: step 4/4
FPKM
0
100
200
300
400
control (2/3)(n=20)
silica depleted (5/6)(n=20)
Colture Media

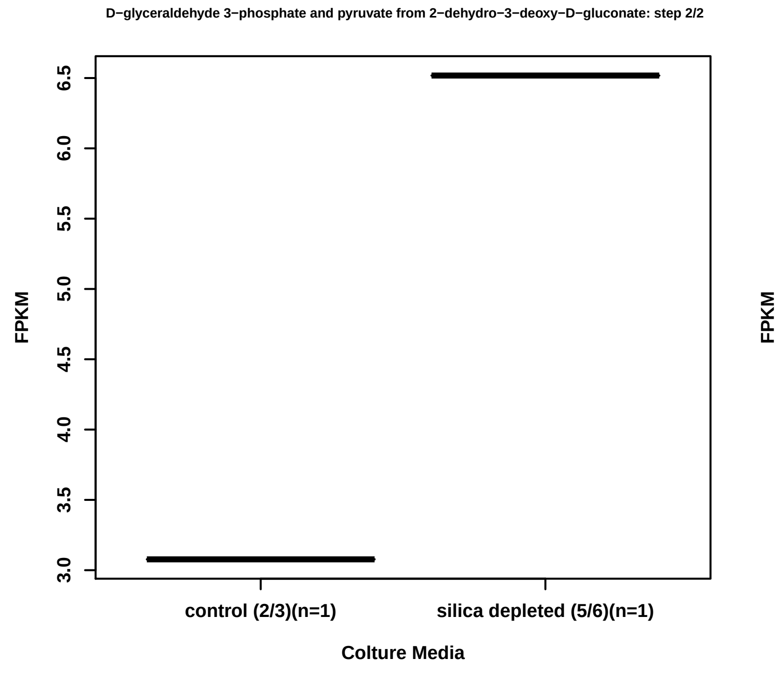
D−glyceraldehyde 3−phosphate and pyruvate from 2−dehydro−3−deoxy−D−gluconate: step 2/2
FPKM
3.0
3.5
4.0
4.5
5.0
5.5
6.0
6.5
control (2/3)(n=1)
silica depleted (5/6)(n=1)
Colture Media

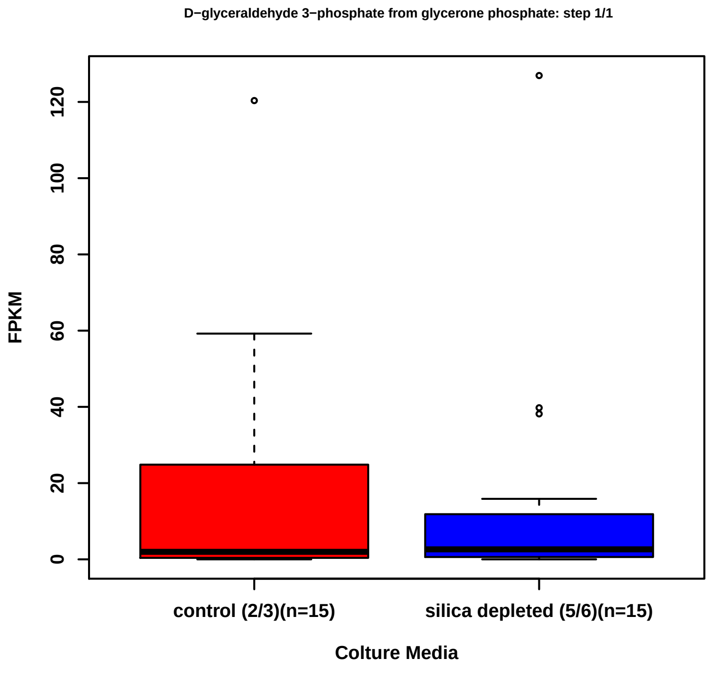
D−glyceraldehyde 3−phosphate from glycerone phosphate: step 1/1
FPKM
0
20
40
60
80
100
120
control (2/3)(n=15)
silica depleted (5/6)(n=15)
Colture Media

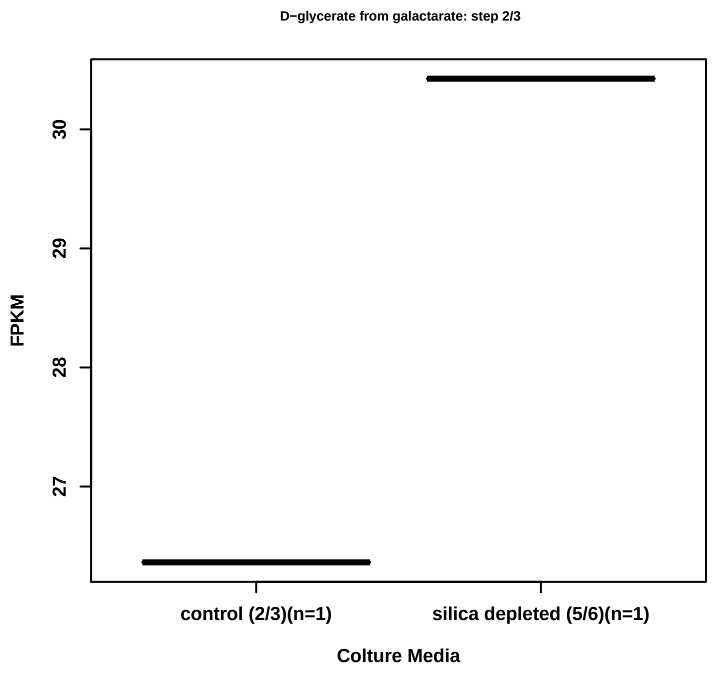
D−glycerate from galactarate: step 2/3
FPKM
27
28
29
30
control (2/3)(n=1)
silica depleted (5/6)(n=1)
Colture Media

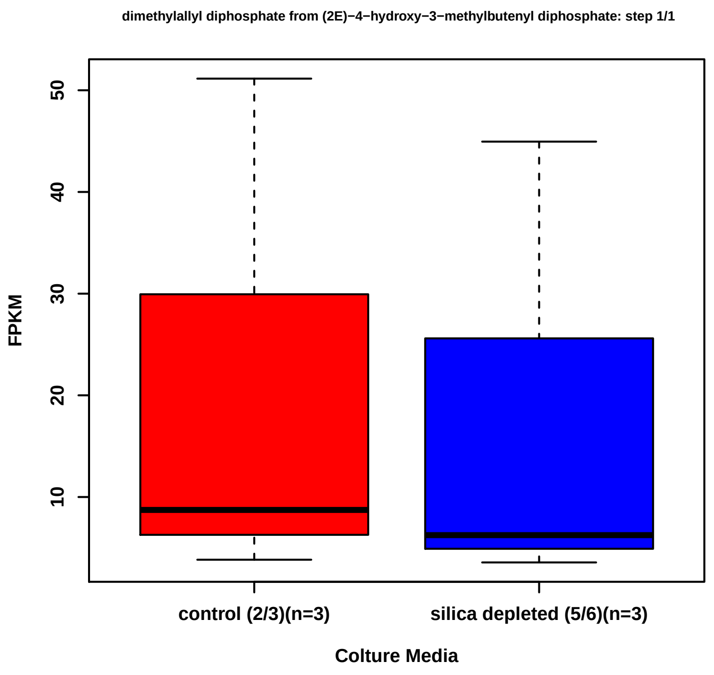
dimethylallyl diphosphate from (2E)−4−hydroxy−3−methylbutenyl diphosphate: step 1/1
FPKM
10
20
30
40
50
control (2/3)(n=3)
silica depleted (5/6)(n=3)
Colture Media

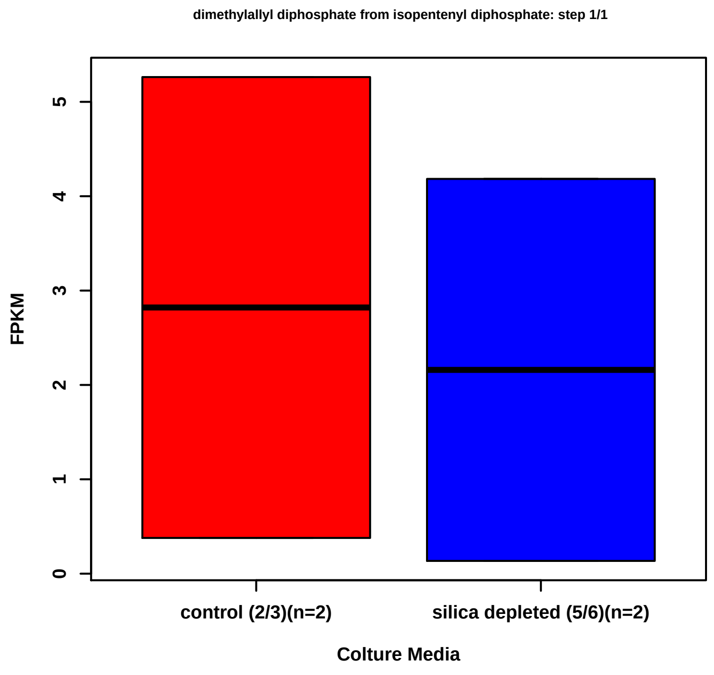
dimethylallyl diphosphate from isopentenyl diphosphate: step 1/1
FPKM
0
1
2
3
4
5
control (2/3)(n=2)
silica depleted (5/6)(n=2)
Colture Media

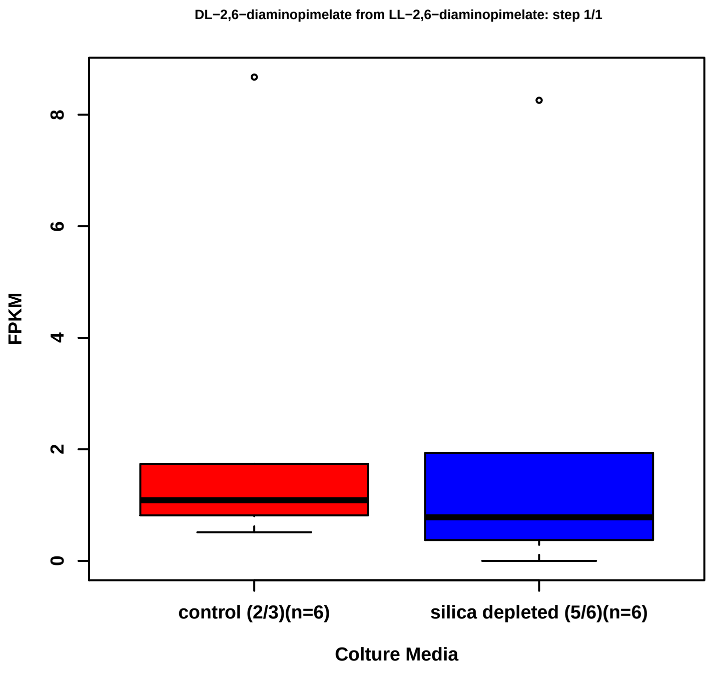
DL−2,6−diaminopimelate from LL−2,6−diaminopimelate: step 1/1
FPKM
0
2
4
6
8
control (2/3)(n=6)
silica depleted (5/6)(n=6)
Colture Media

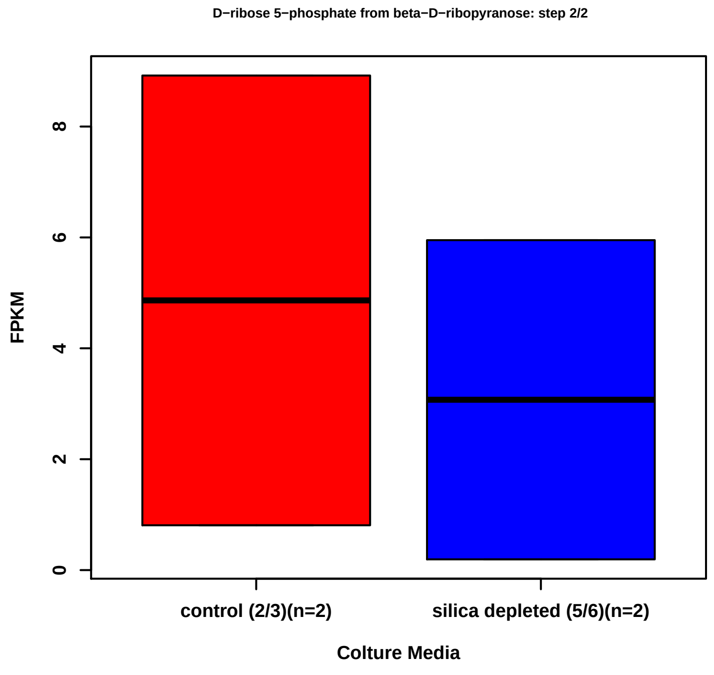
D−ribose 5−phosphate from beta−D−ribopyranose: step 2/2
FPKM
0
2
4
6
8
control (2/3)(n=2)
silica depleted (5/6)(n=2)
Colture Media

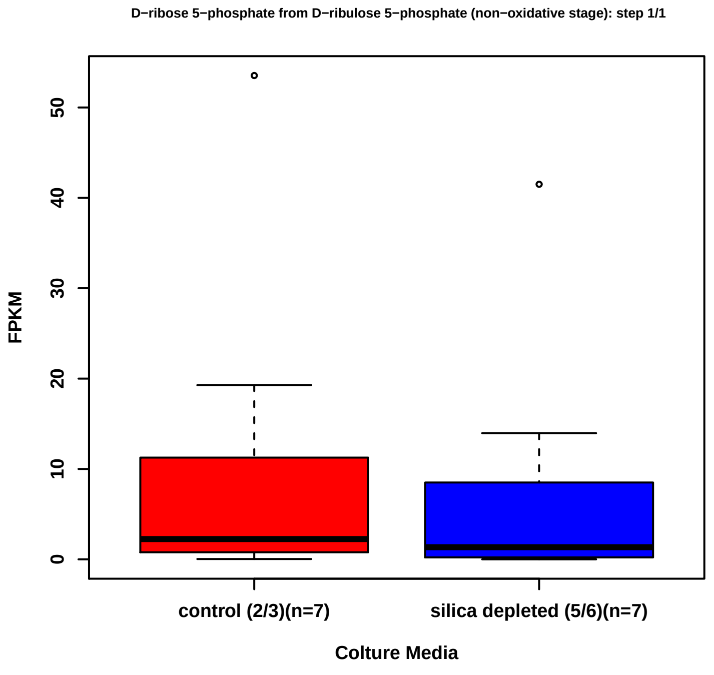
D−ribose 5−phosphate from D−ribulose 5−phosphate (non−oxidative stage): step 1/1
FPKM
0
10
20
30
40
50
control (2/3)(n=7)
silica depleted (5/6)(n=7)
Colture Media

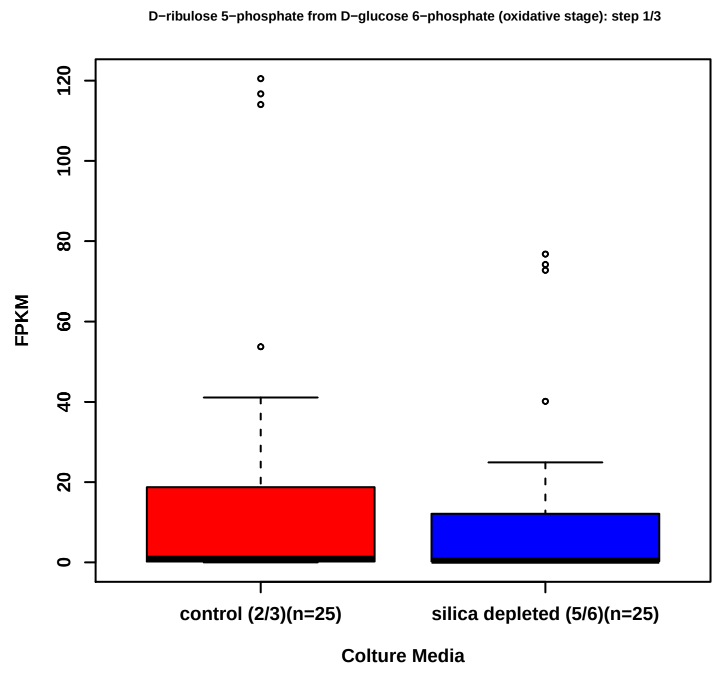
D−ribulose 5−phosphate from D−glucose 6−phosphate (oxidative stage): step 1/3
FPKM
0
20
40
60
80
100
120
control (2/3)(n=25)
silica depleted (5/6)(n=25)
Colture Media

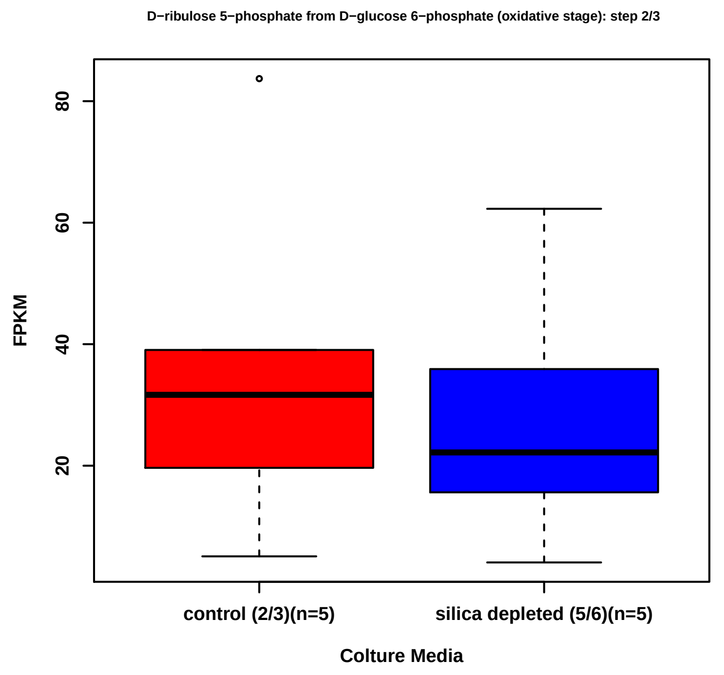
D−ribulose 5−phosphate from D−glucose 6−phosphate (oxidative stage): step 2/3
FPKM
20
40
60
80
control (2/3)(n=5)
silica depleted (5/6)(n=5)
Colture Media

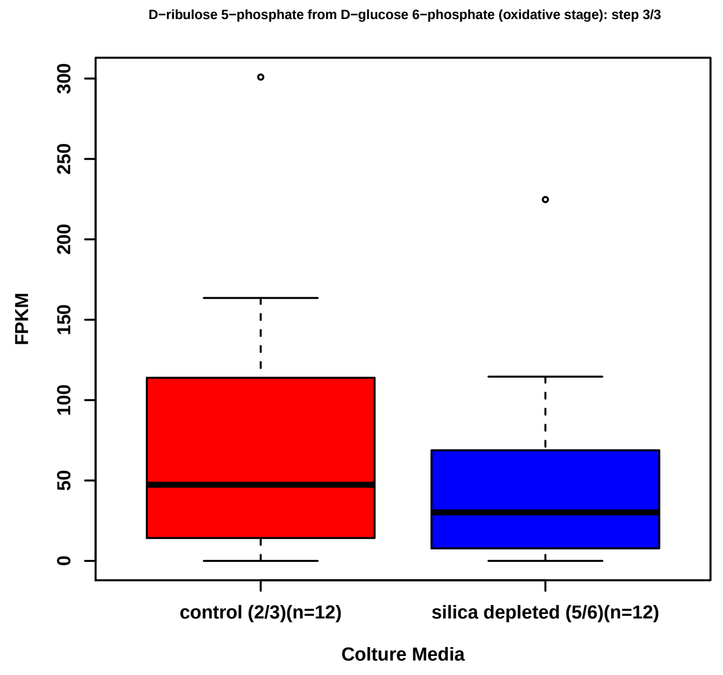
D−ribulose 5−phosphate from D−glucose 6−phosphate (oxidative stage): step 3/3
FPKM
0
50
100
150
200
250
300
control (2/3)(n=12)
silica depleted (5/6)(n=12)
Colture Media

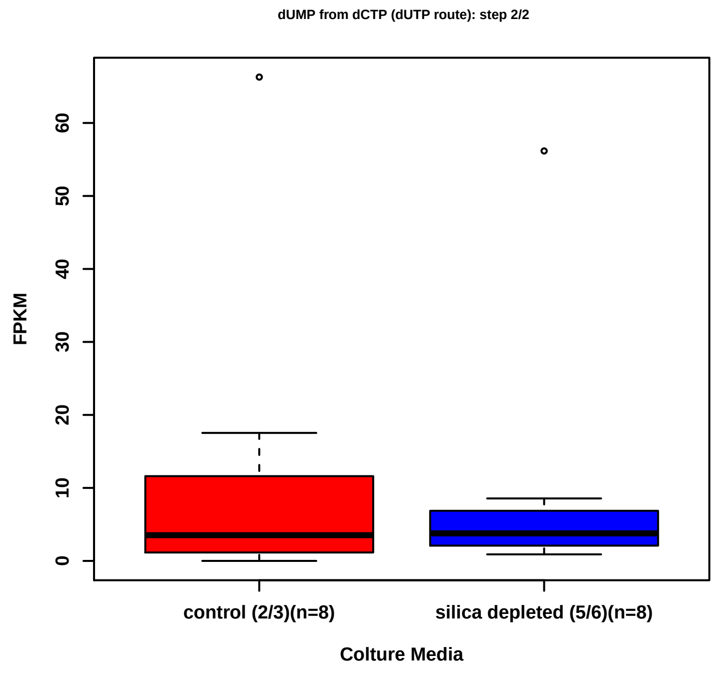
dUMP from dCTP (dUTP route): step 2/2
FPKM
0
10
20
30
40
50
60
control (2/3)(n=8)
silica depleted (5/6)(n=8)
Colture Media

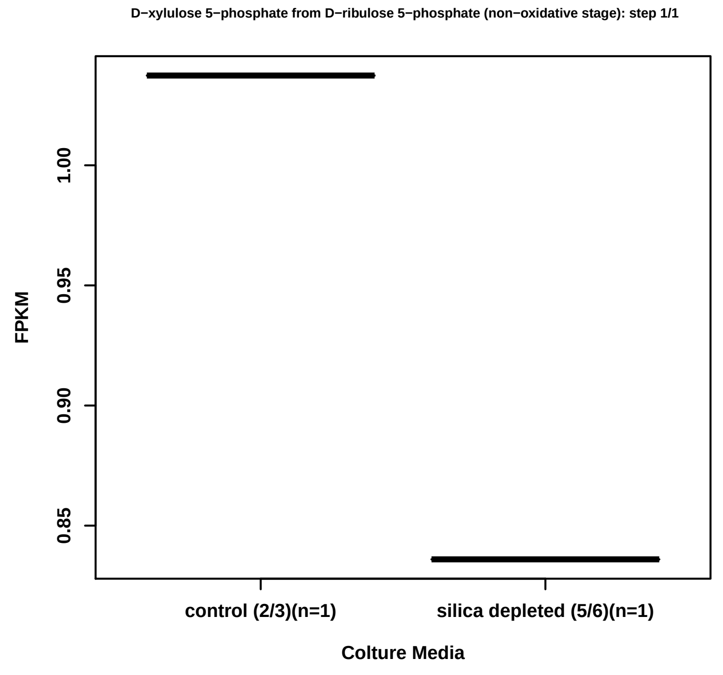
D−xylulose 5−phosphate from D−ribulose 5−phosphate (non−oxidative stage): step 1/1
FPKM
0.85
0.90
0.95
1.00
control (2/3)(n=1)
silica depleted (5/6)(n=1)
Colture Media

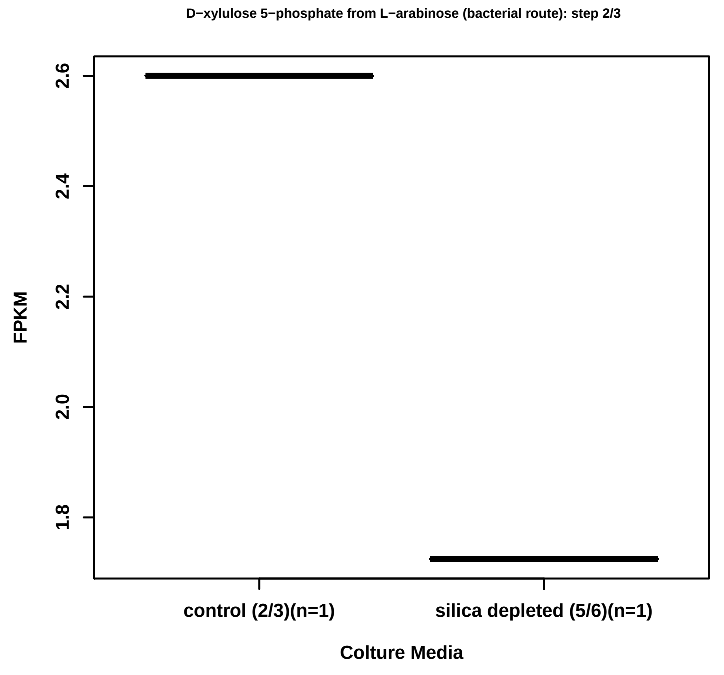
D−xylulose 5−phosphate from L−arabinose (bacterial route): step 2/3
FPKM
1.8
2.0
2.2
2.4
2.6
control (2/3)(n=1)
silica depleted (5/6)(n=1)
Colture Media

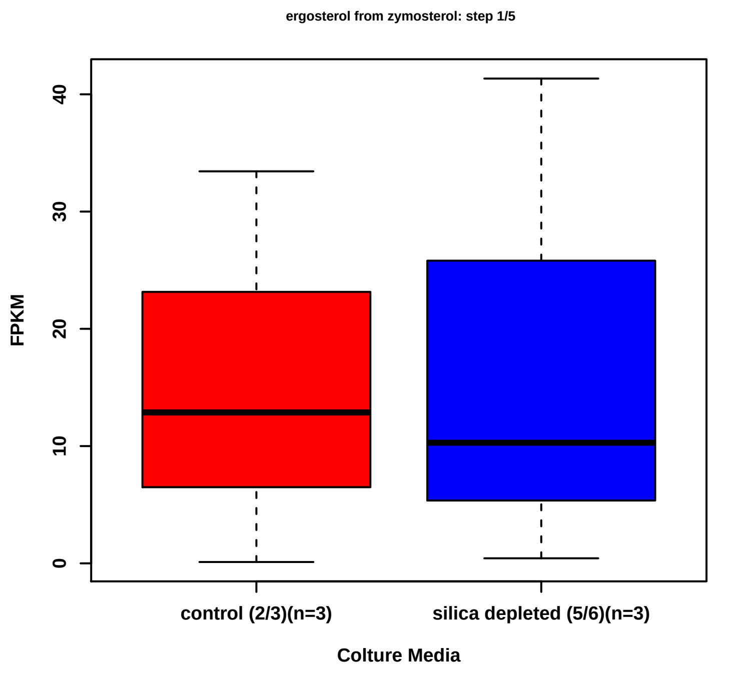
ergosterol from zymosterol: step 1/5
FPKM
0
10
20
30
40
control (2/3)(n=3)
silica depleted (5/6)(n=3)
Colture Media

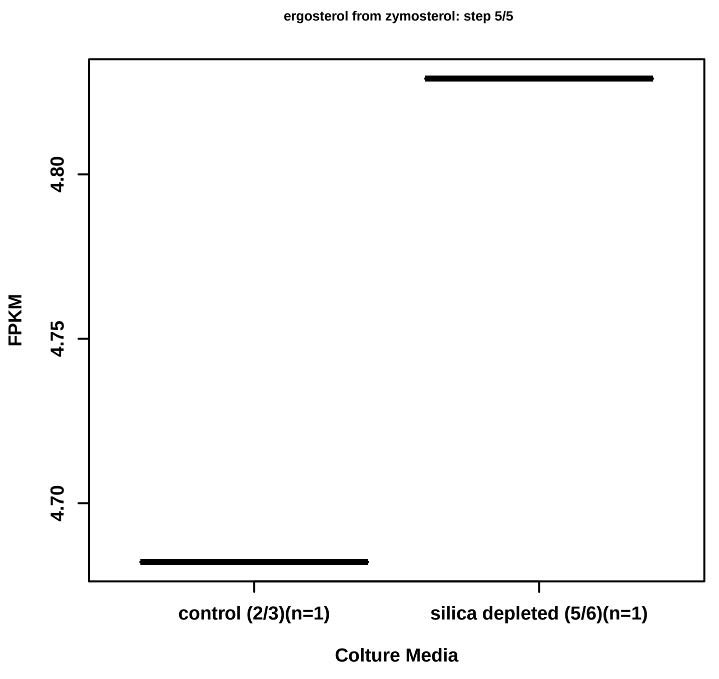
ergosterol from zymosterol: step 5/5
FPKM
4.70
4.75
4.80
control (2/3)(n=1)
silica depleted (5/6)(n=1)
Colture Media

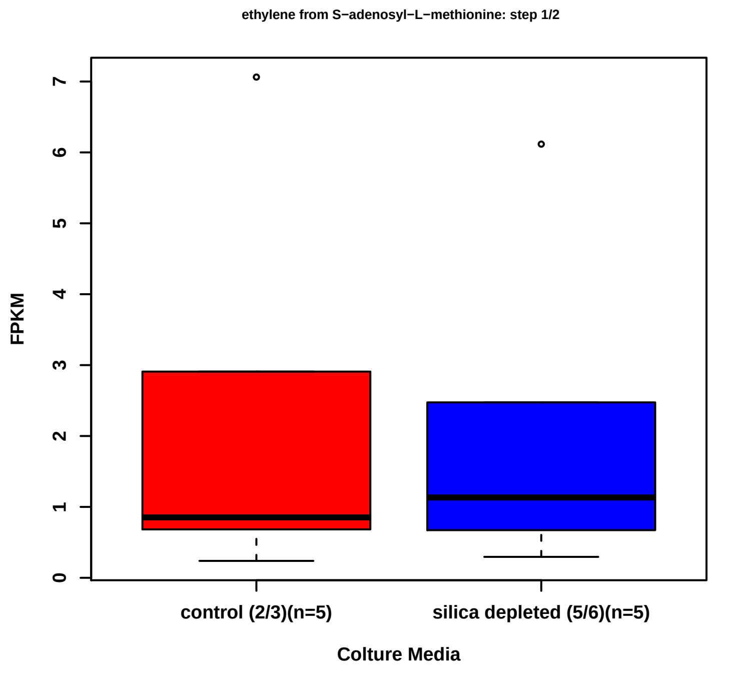
ethylene from S−adenosyl−L−methionine: step 1/2
FPKM
0
1
2
3
4
5
6
7
control (2/3)(n=5)
silica depleted (5/6)(n=5)
Colture Media

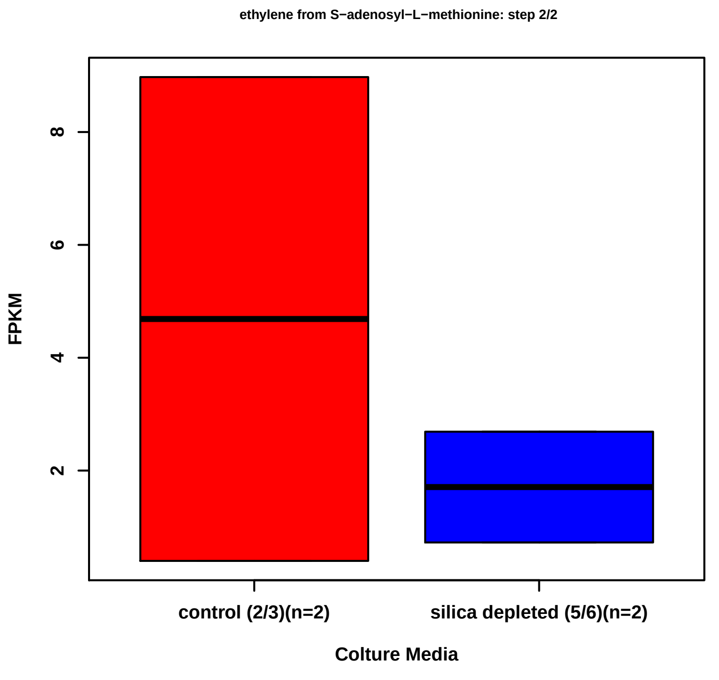
ethylene from S−adenosyl−L−methionine: step 2/2
FPKM
2
4
6
8
control (2/3)(n=2)
silica depleted (5/6)(n=2)
Colture Media

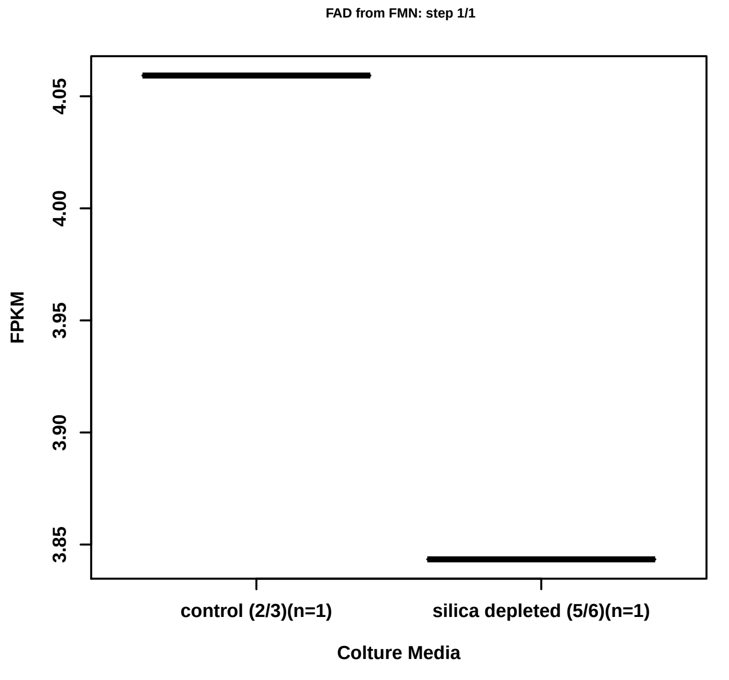
FAD from FMN: step 1/1
FPKM
3.85
3.90
3.95
4.00
4.05
control (2/3)(n=1)
silica depleted (5/6)(n=1)
Colture Media

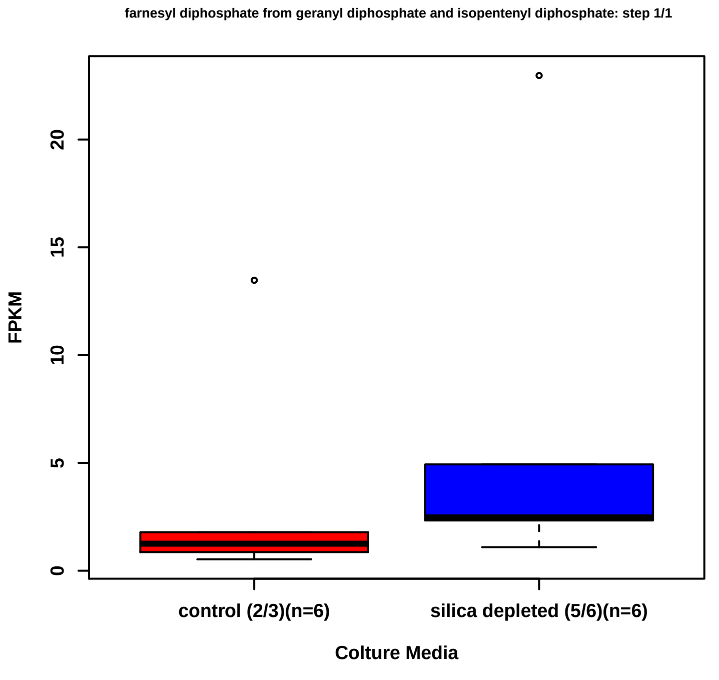
farnesyl diphosphate from geranyl diphosphate and isopentenyl diphosphate: step 1/1
FPKM
0
5
10
15
20
control (2/3)(n=6)
silica depleted (5/6)(n=6)
Colture Media

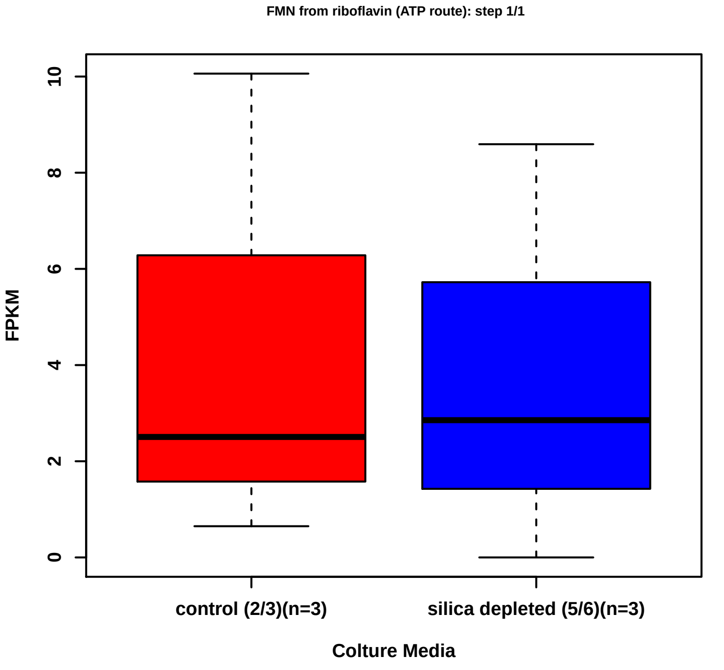
FMN from riboflavin (ATP route): step 1/1
FPKM
0
2
4
6
8
10
control (2/3)(n=3)
silica depleted (5/6)(n=3)
Colture Media

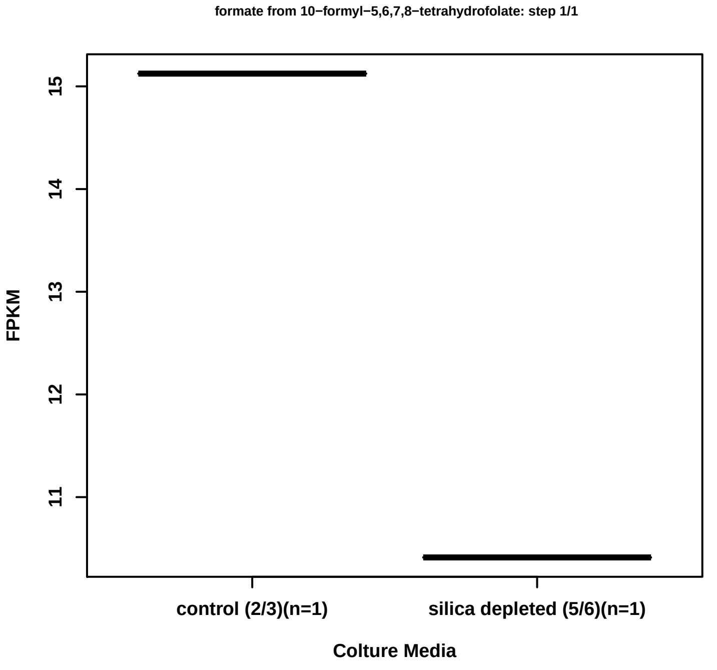
formate from 10−formyl−5,6,7,8−tetrahydrofolate: step 1/1
FPKM
11
12
13
14
15
control (2/3)(n=1)
silica depleted (5/6)(n=1)
Colture Media

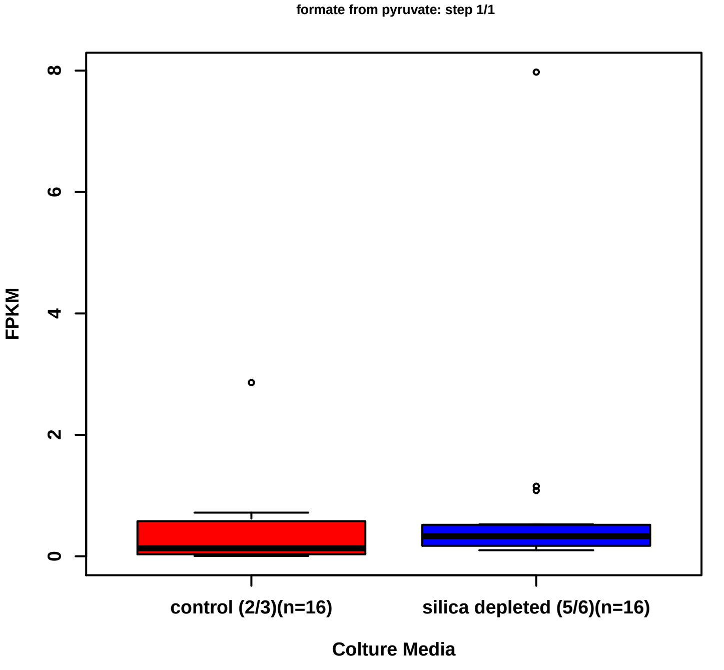
formate from pyruvate: step 1/1
FPKM
0
2
4
6
8
control (2/3)(n=16)
silica depleted (5/6)(n=16)
Colture Media

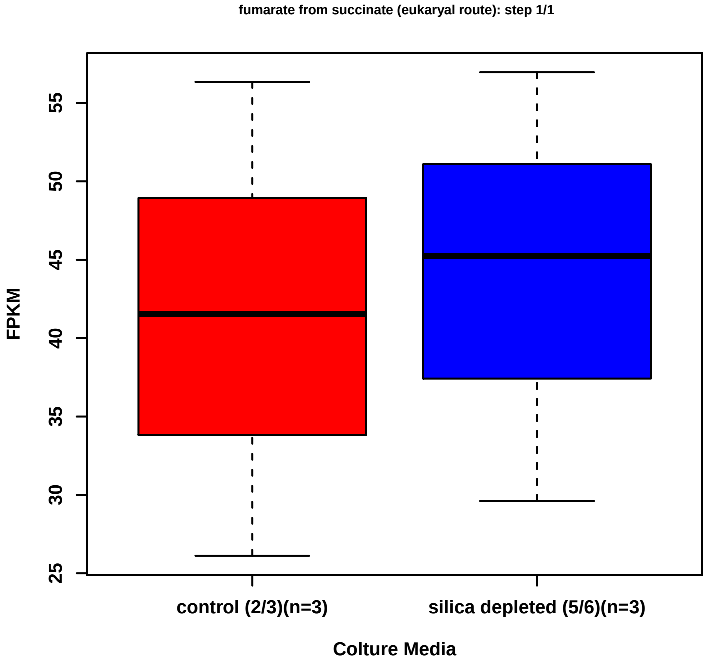
fumarate from succinate (eukaryal route): step 1/1
FPKM
25
30
35
40
45
50
55
control (2/3)(n=3)
silica depleted (5/6)(n=3)
Colture Media

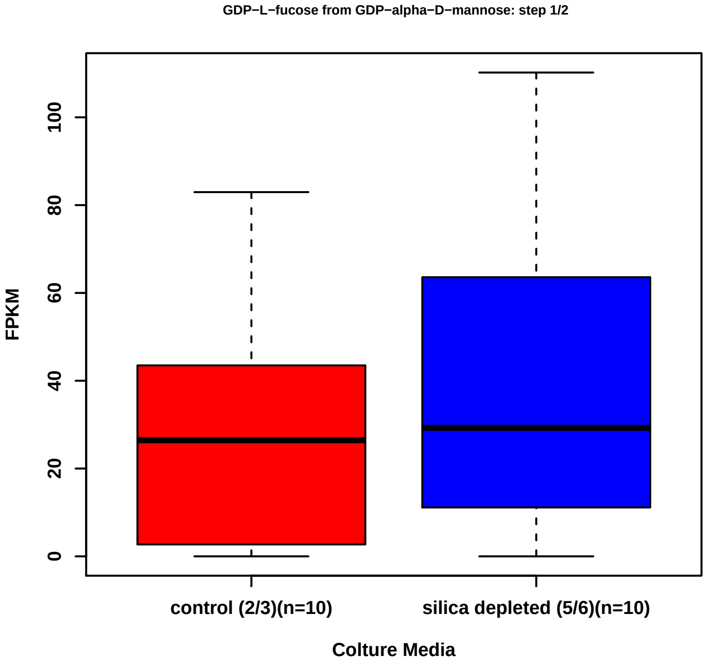
GDP−L−fucose from GDP−alpha−D−mannose: step 1/2
FPKM
0
20
40
60
80
100
control (2/3)(n=10)
silica depleted (5/6)(n=10)
Colture Media

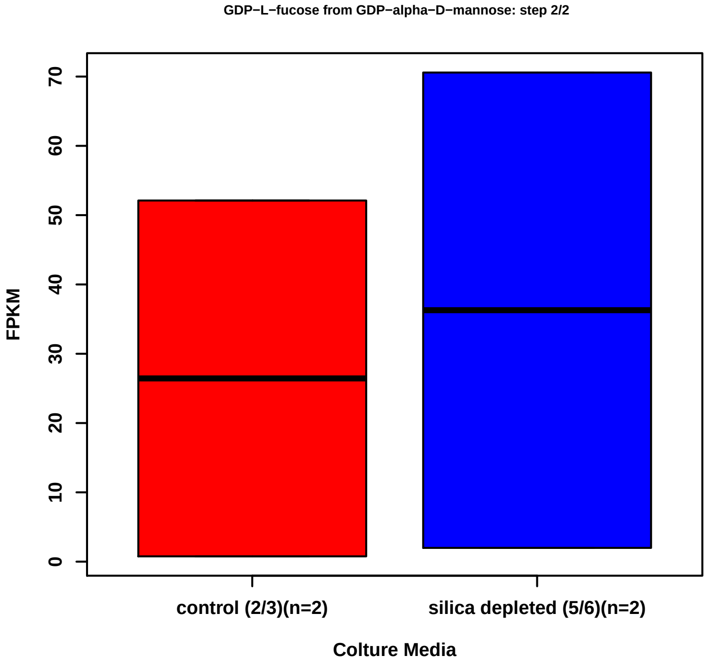
GDP−L−fucose from GDP−alpha−D−mannose: step 2/2
FPKM
0
10
20
30
40
50
60
70
control (2/3)(n=2)
silica depleted (5/6)(n=2)
Colture Media

geranyl diphosphate from dimethylallyl diphosphate and isopentenyl diphosphate: step 1/1

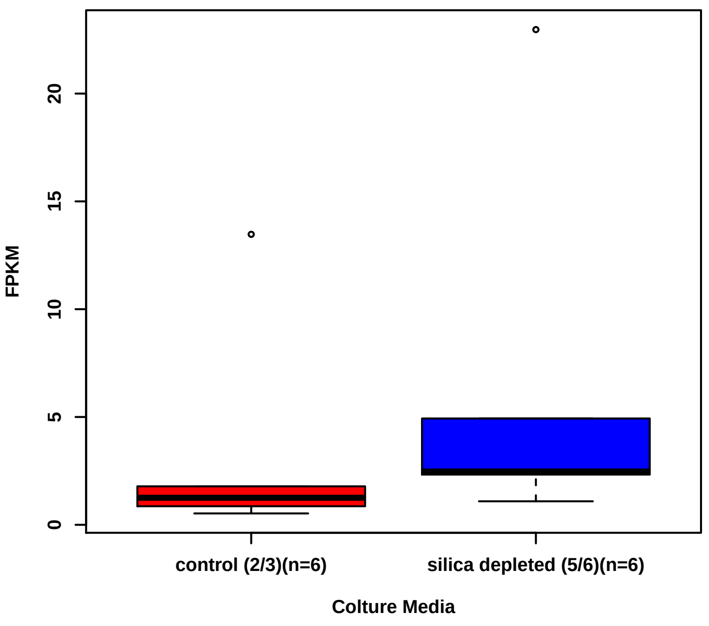

geranylgeranyl diphosphate from farnesyl diphosphate and isopentenyl diphosphate: step 1/1

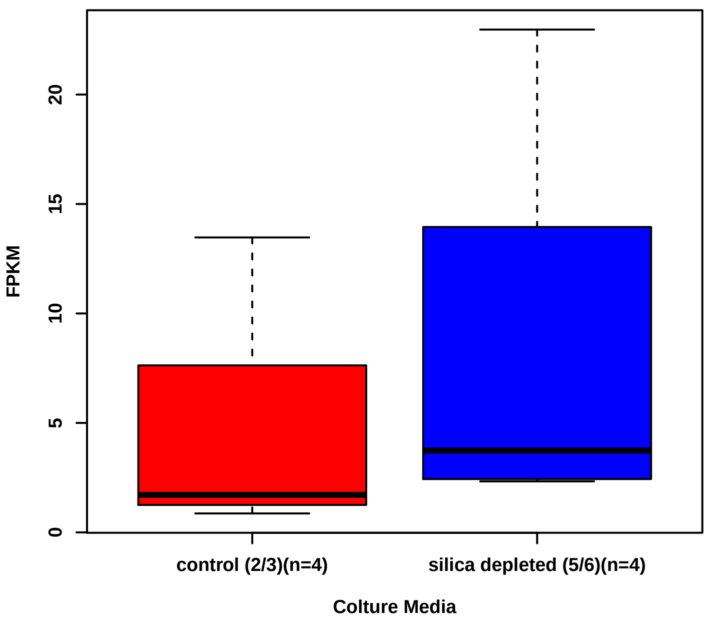

glutaryl−CoA from L−lysine: step 1/6

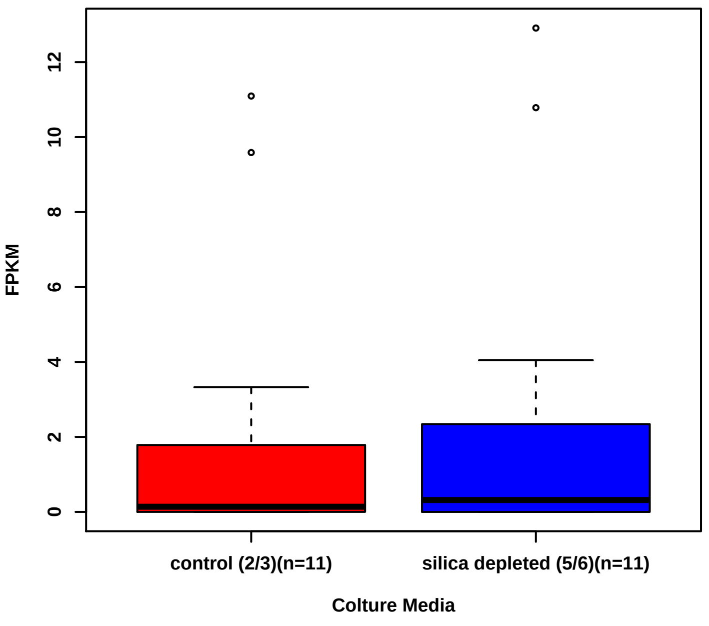

glutaryl−CoA from L−lysine: step 2/6

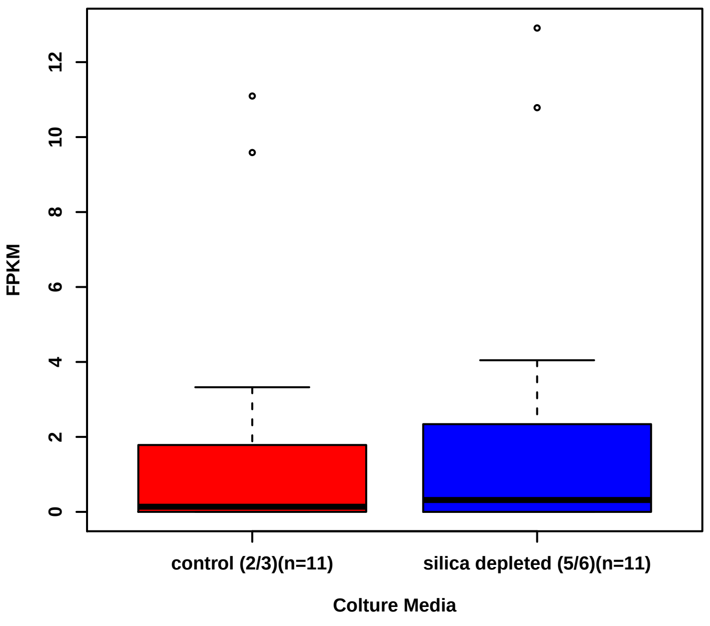

glutaryl−CoA from L−lysine: step 6/6

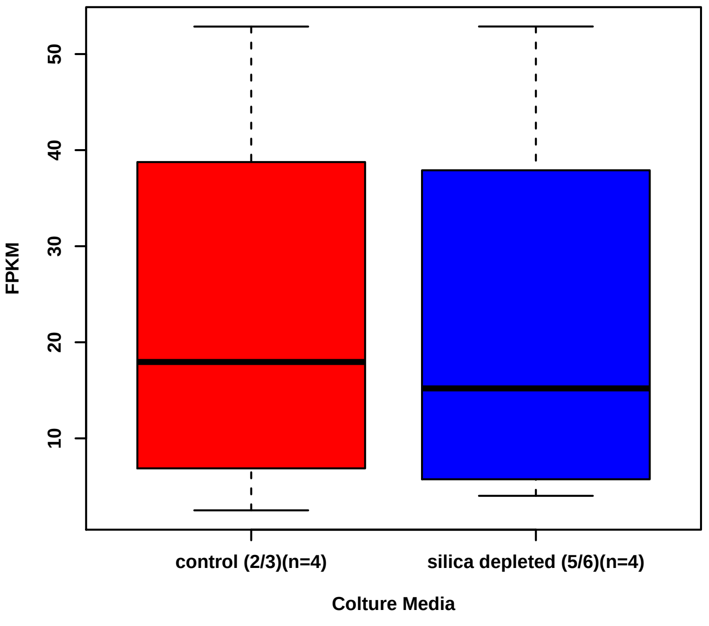

glutathione from L−cysteine and L−glutamate: step 1/2

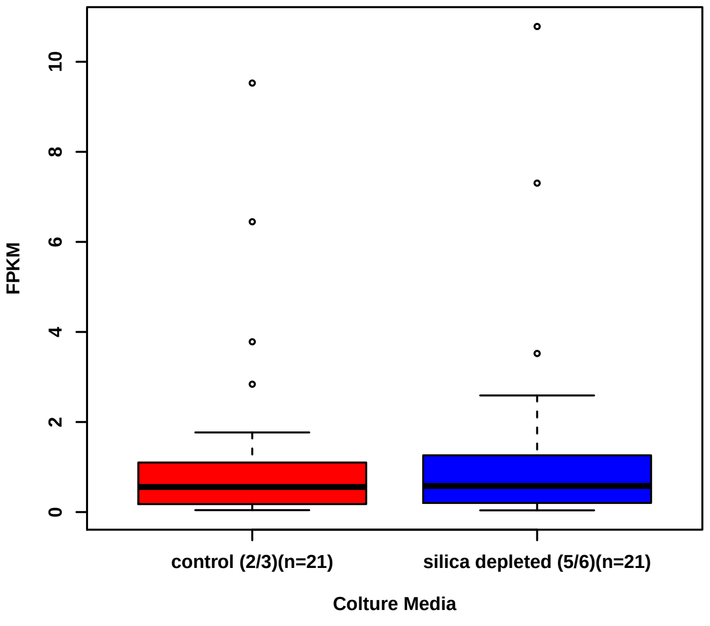

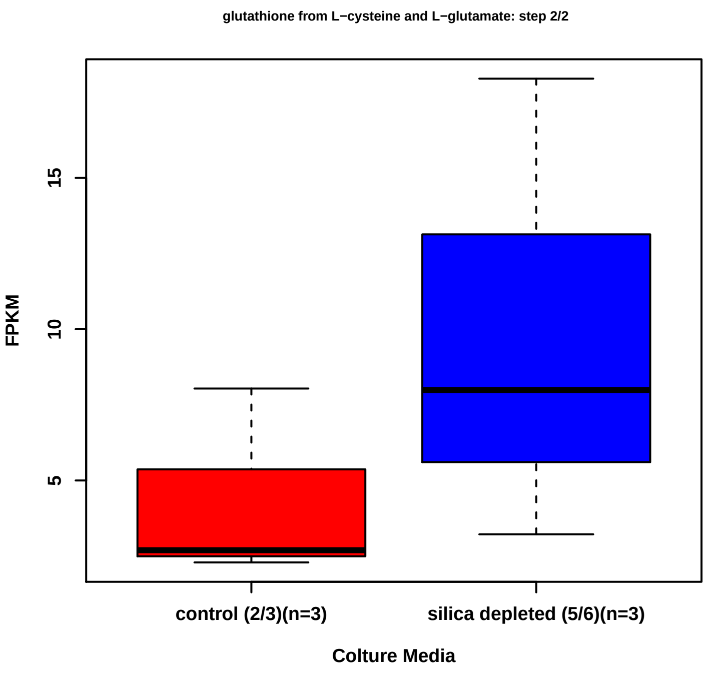
glutathione from L−cysteine and L−glutamate: step 2/2
FPKM
5
10
15
control (2/3)(n=3)
silica depleted (5/6)(n=3)
Colture Media

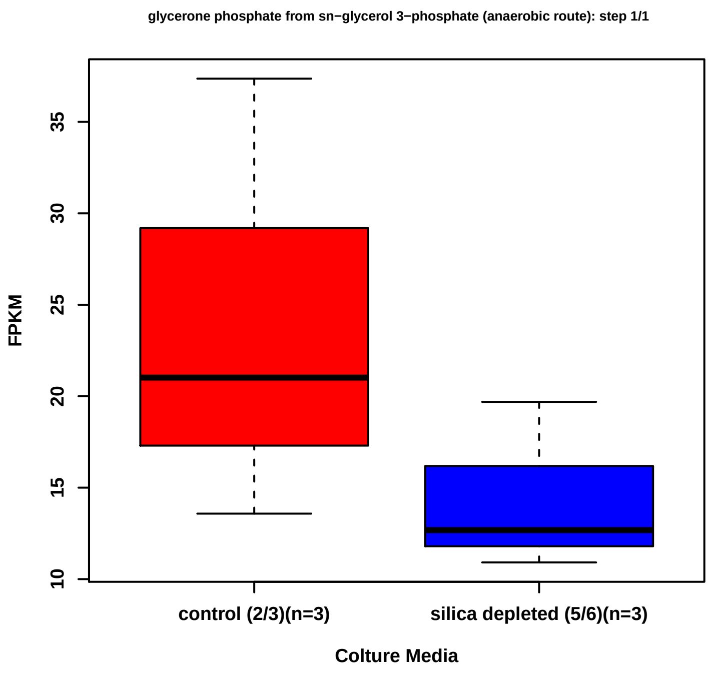
glycerone phosphate from sn−glycerol 3−phosphate (anaerobic route): step 1/1
FPKM
10
15
20
25
30
35
control (2/3)(n=3)
silica depleted (5/6)(n=3)
Colture Media

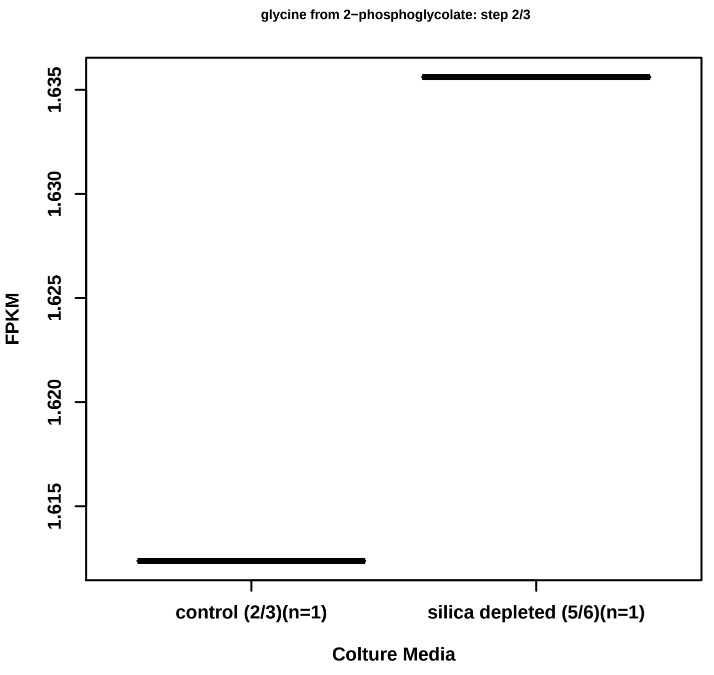
glycine from 2−phosphoglycolate: step 2/3
FPKM
1.615
1.620
1.625
1.630
1.635
control (2/3)(n=1)
silica depleted (5/6)(n=1)
Colture Media

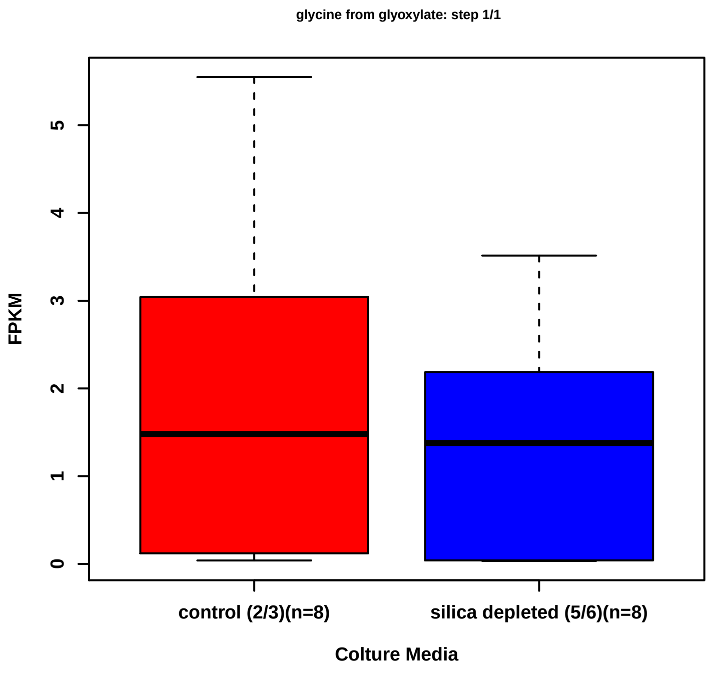
glycine from glyoxylate: step 1/1
FPKM
0
1
2
3
4
5
control (2/3)(n=8)
silica depleted (5/6)(n=8)
Colture Media

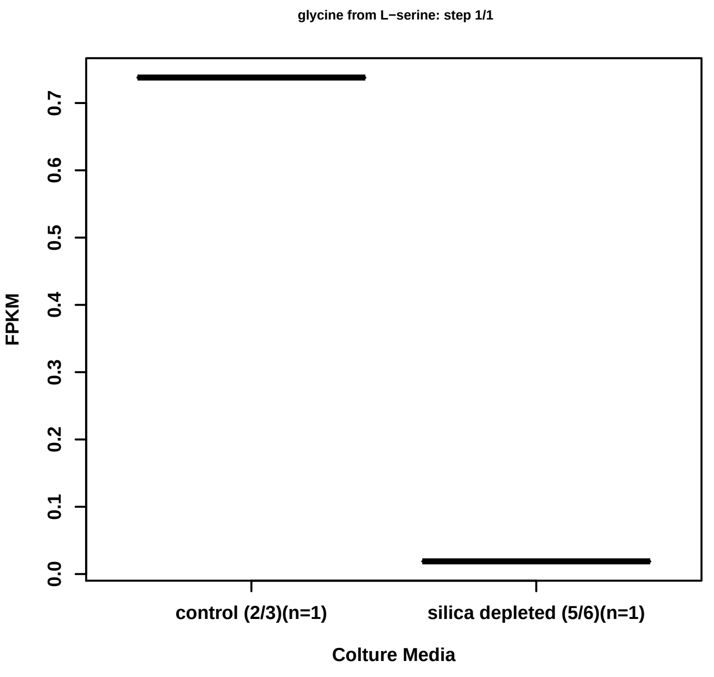
glycine from L−serine: step 1/1
FPKM
0.0
0.1
0.2
0.3
0.4
0.5
0.6
0.7
control (2/3)(n=1)
silica depleted (5/6)(n=1)
Colture Media

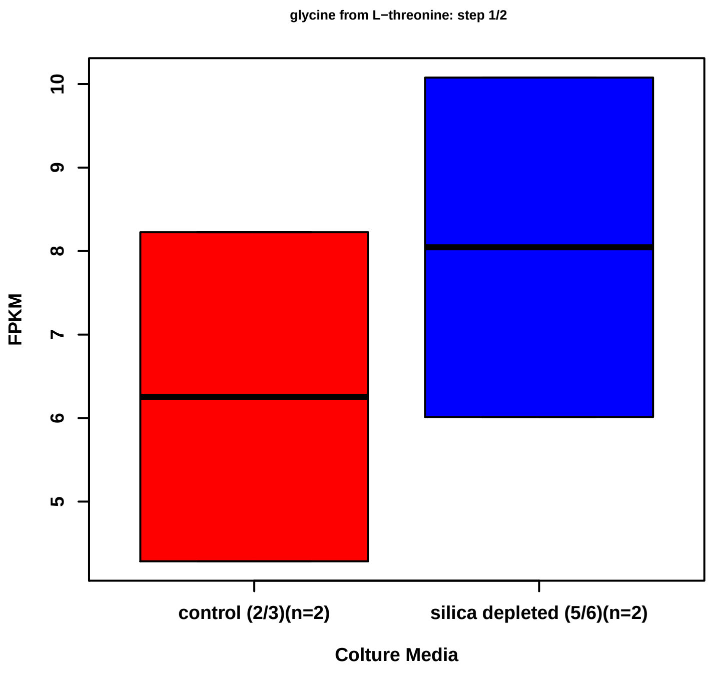
glycine from L−threonine: step 1/2
FPKM
5
6
7
8
9
10
control (2/3)(n=2)
silica depleted (5/6)(n=2)
Colture Media

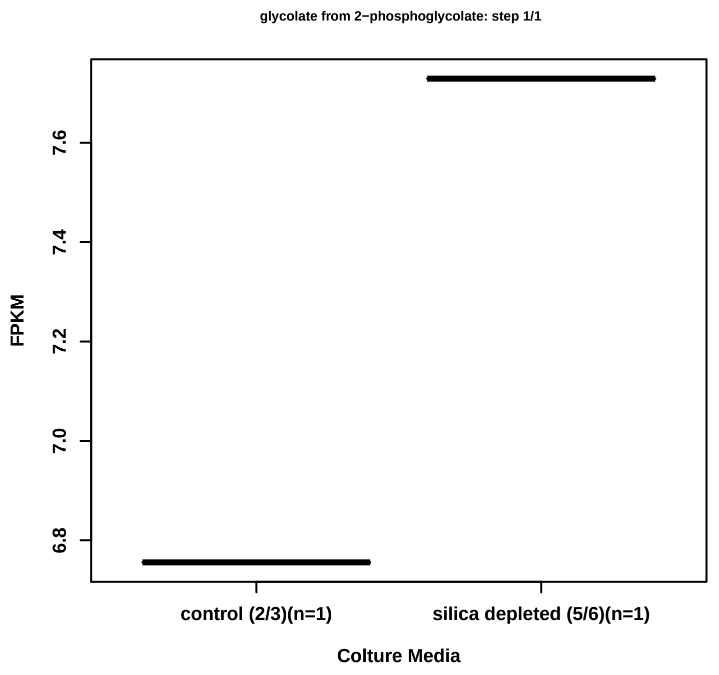
glycolate from 2−phosphoglycolate: step 1/1
FPKM
6.8
7.0
7.2
7.4
7.6
control (2/3)(n=1)
silica depleted (5/6)(n=1)
Colture Media

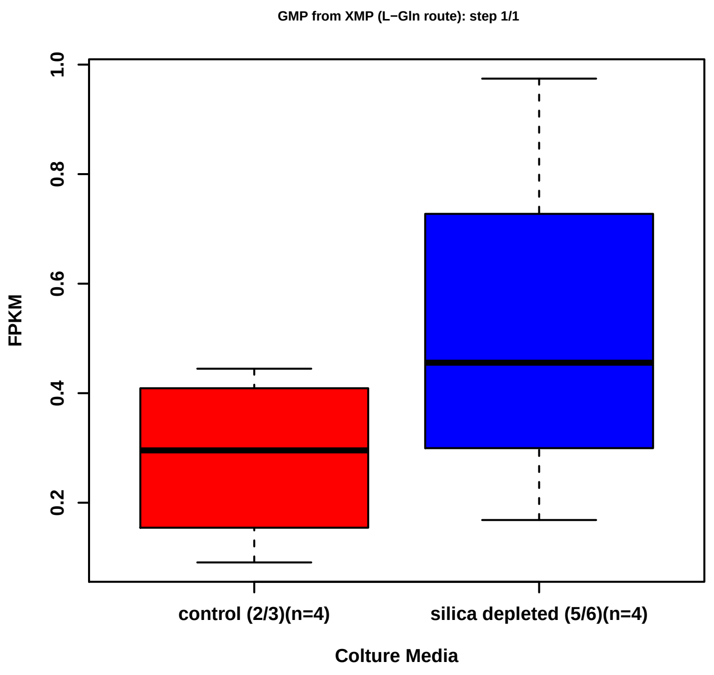
GMP from XMP (L−Gln route): step 1/1
FPKM
0.2
0.4
0.6
0.8
1.0
control (2/3)(n=4)
silica depleted (5/6)(n=4)
Colture Media

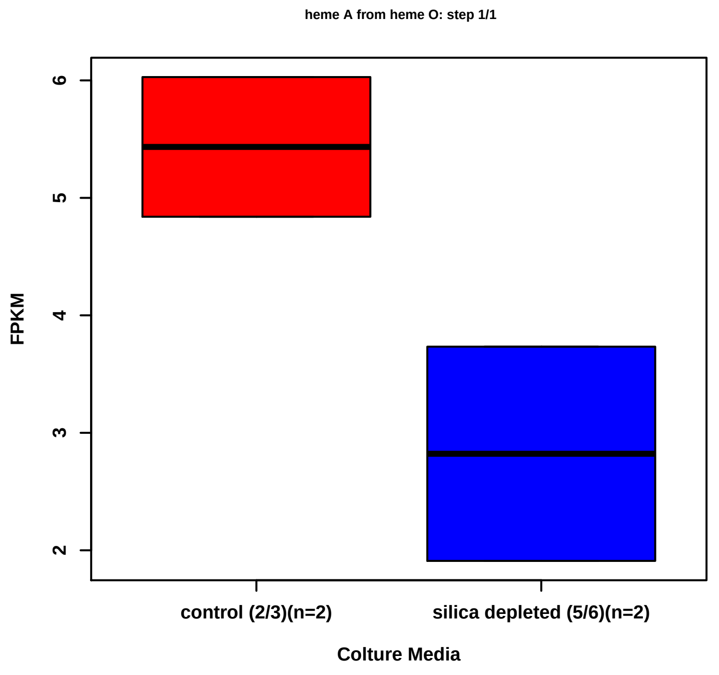
heme A from heme O: step 1/1
FPKM
2
3
4
5
6
control (2/3)(n=2)
silica depleted (5/6)(n=2)
Colture Media

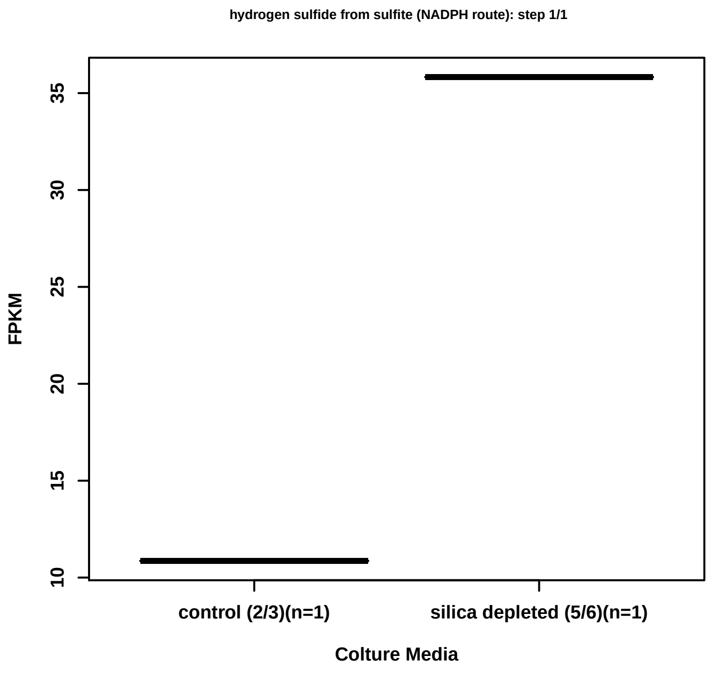
hydrogen sulfide from sulfite (NADPH route): step 1/1
FPKM
10
15
20
25
30
35
control (2/3)(n=1)
silica depleted (5/6)(n=1)
Colture Media

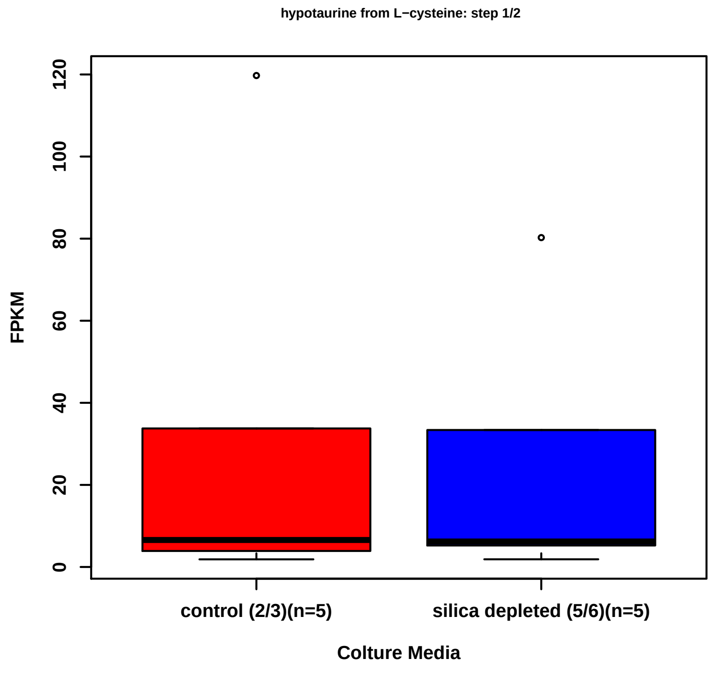
hypotaurine from L−cysteine: step 1/2
FPKM
0
20
40
60
80
100
120
control (2/3)(n=5)
silica depleted (5/6)(n=5)
Colture Media

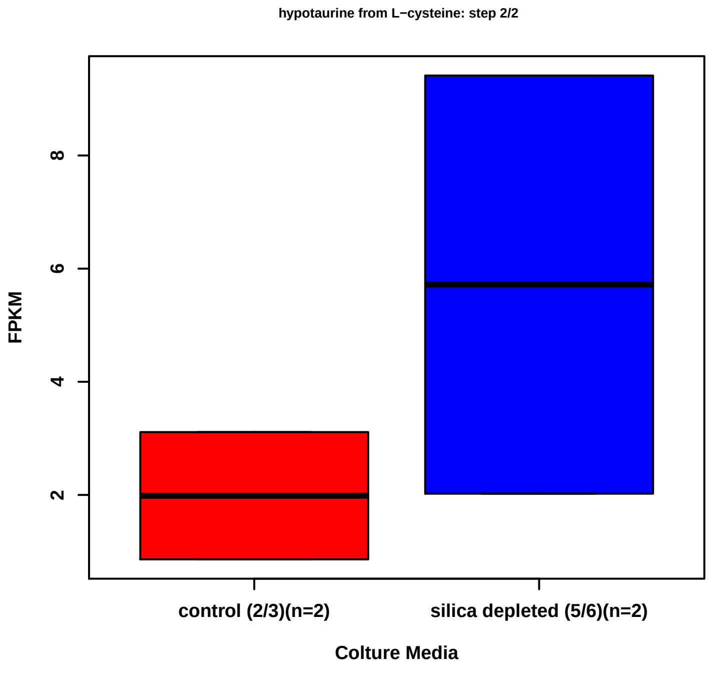
hypotaurine from L−cysteine: step 2/2
FPKM
2
4
6
8
control (2/3)(n=2)
silica depleted (5/6)(n=2)
Colture Media

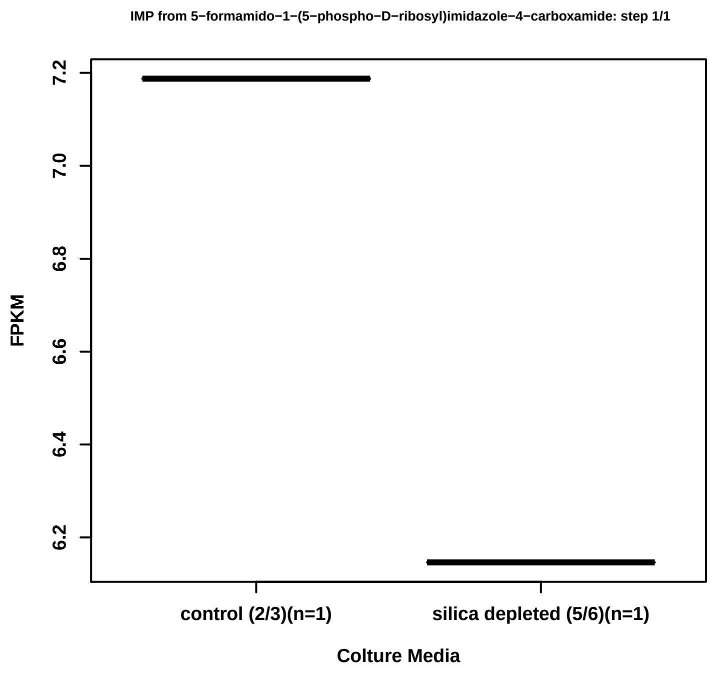
IMP from 5−formamido−1−(5−phospho−D−ribosyl)imidazole−4−carboxamide: step 1/1
FPKM
6.2
6.4
6.6
6.8
7.0
7.2
control (2/3)(n=1)
silica depleted (5/6)(n=1)
Colture Media

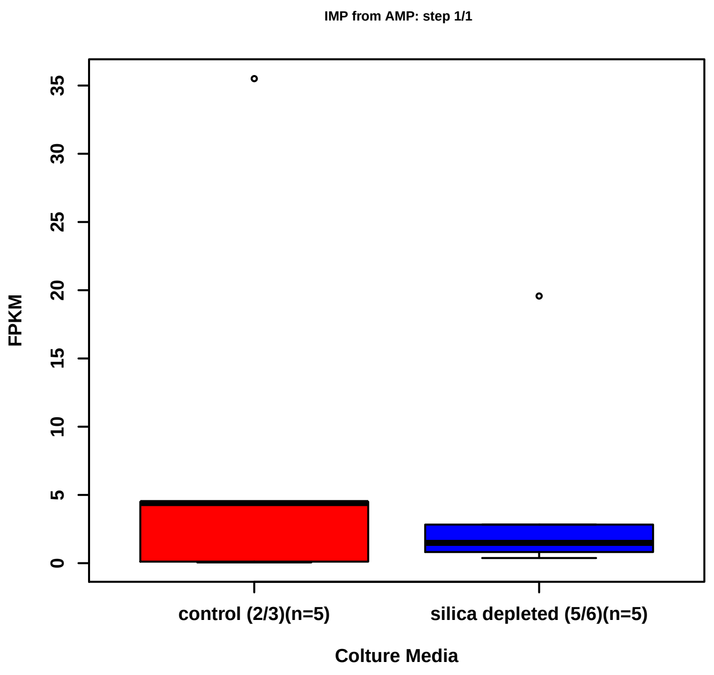
IMP from AMP: step 1/1
FPKM
0
5
10
15
20
25
30
35
control (2/3)(n=5)
silica depleted (5/6)(n=5)
Colture Media

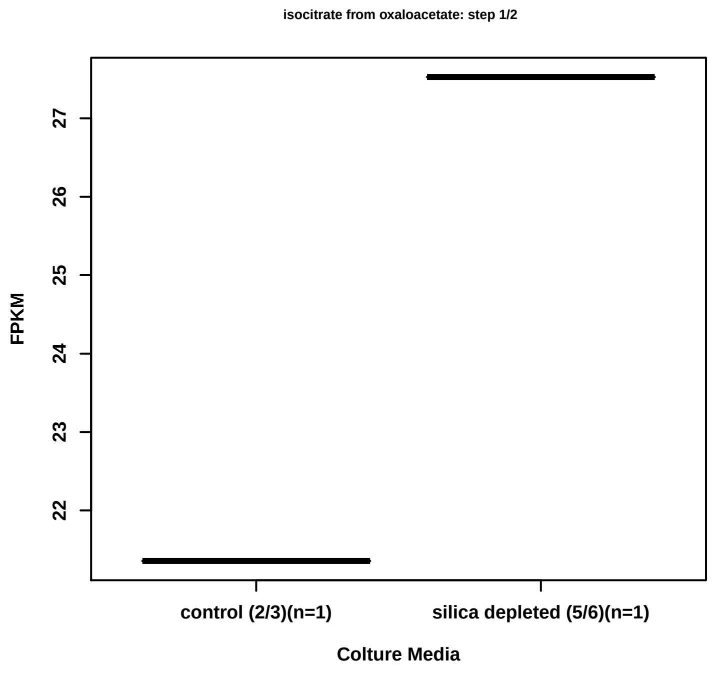
isocitrate from oxaloacetate: step 1/2
FPKM
22
23
24
25
26
27
control (2/3)(n=1)
silica depleted (5/6)(n=1)
Colture Media

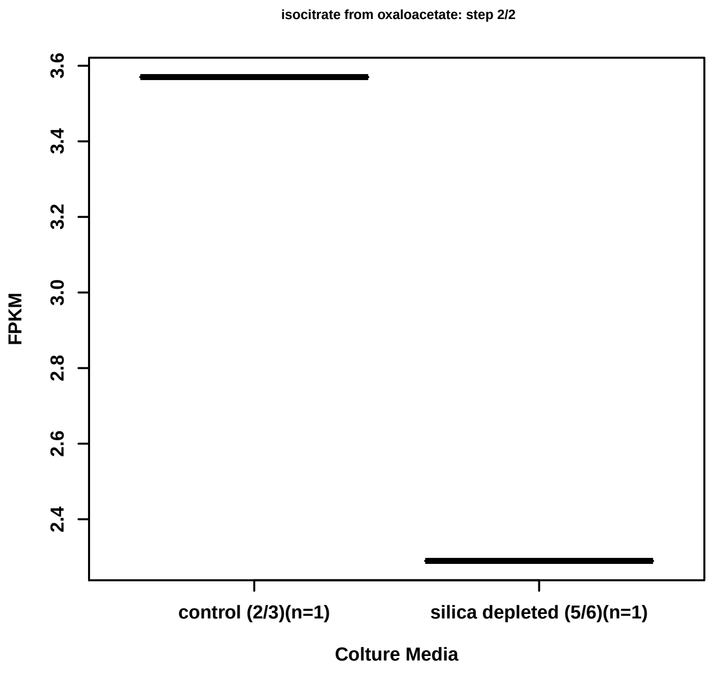
isocitrate from oxaloacetate: step 2/2
FPKM
2.4
2.6
2.8
3.0
3.2
3.4
3.6
control (2/3)(n=1)
silica depleted (5/6)(n=1)
Colture Media

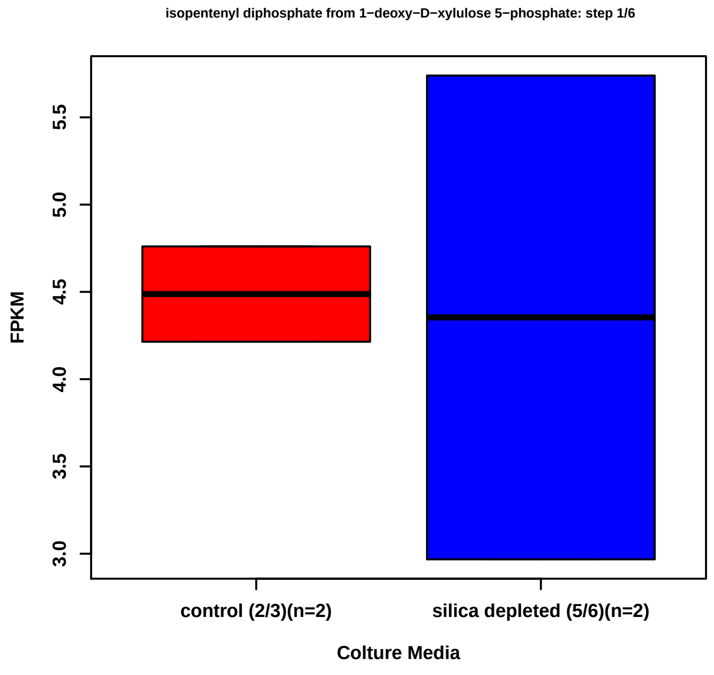
isopentenyl diphosphate from 1−deoxy−D−xylulose 5−phosphate: step 1/6
FPKM
3.0
3.5
4.0
4.5
5.0
5.5
control (2/3)(n=2)
silica depleted (5/6)(n=2)
Colture Media

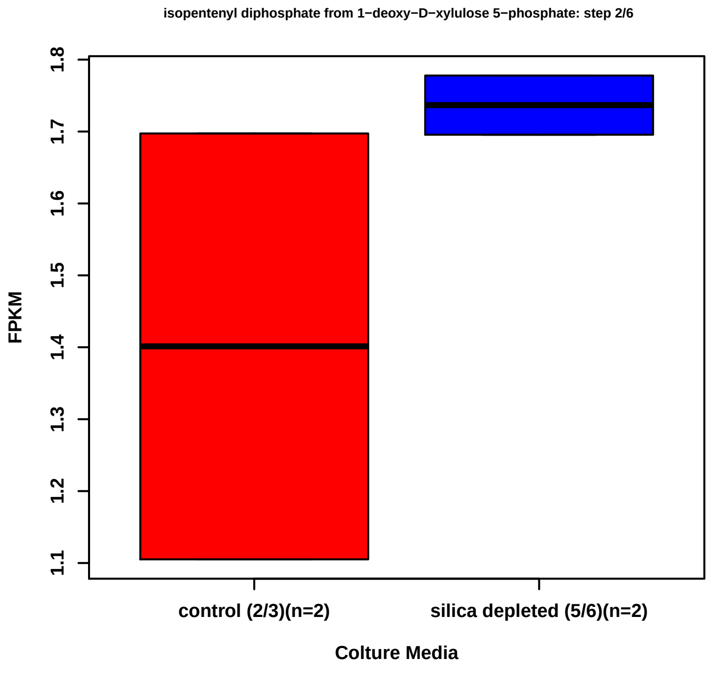
isopentenyl diphosphate from 1−deoxy−D−xylulose 5−phosphate: step 2/6
FPKM
1.1
1.2
1.3
1.4
1.5
1.6
1.7
1.8
control (2/3)(n=2)
silica depleted (5/6)(n=2)
Colture Media

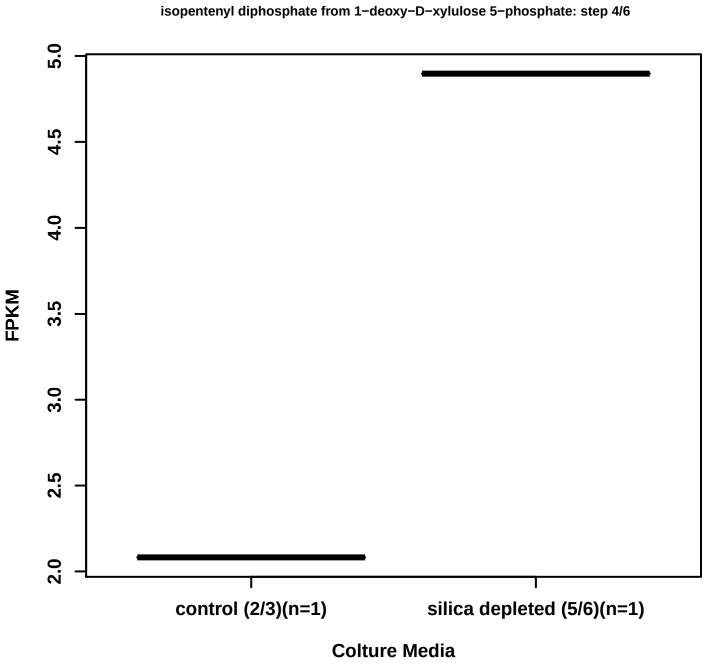
isopentenyl diphosphate from 1−deoxy−D−xylulose 5−phosphate: step 4/6
FPKM
2.0
2.5
3.0
3.5
4.0
4.5
5.0
control (2/3)(n=1)
silica depleted (5/6)(n=1)
Colture Media

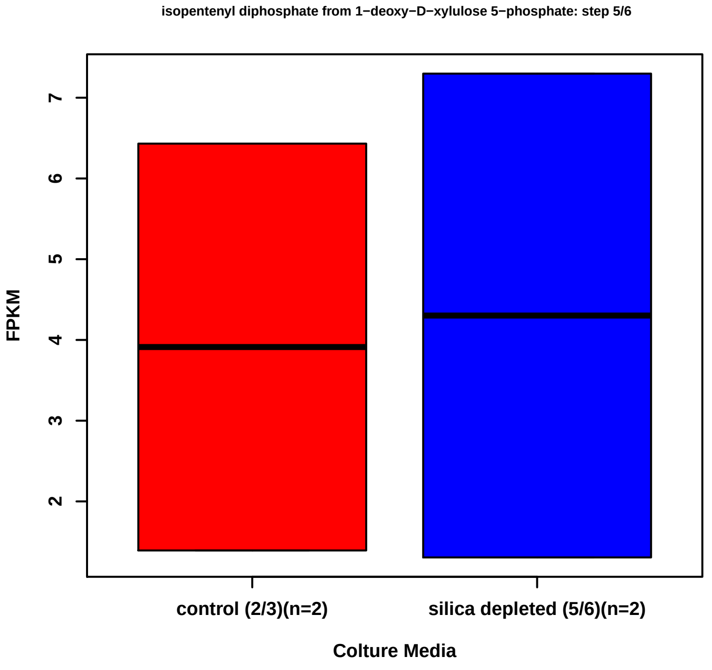
isopentenyl diphosphate from 1−deoxy−D−xylulose 5−phosphate: step 5/6
FPKM
2
3
4
5
6
7
control (2/3)(n=2)
silica depleted (5/6)(n=2)
Colture Media

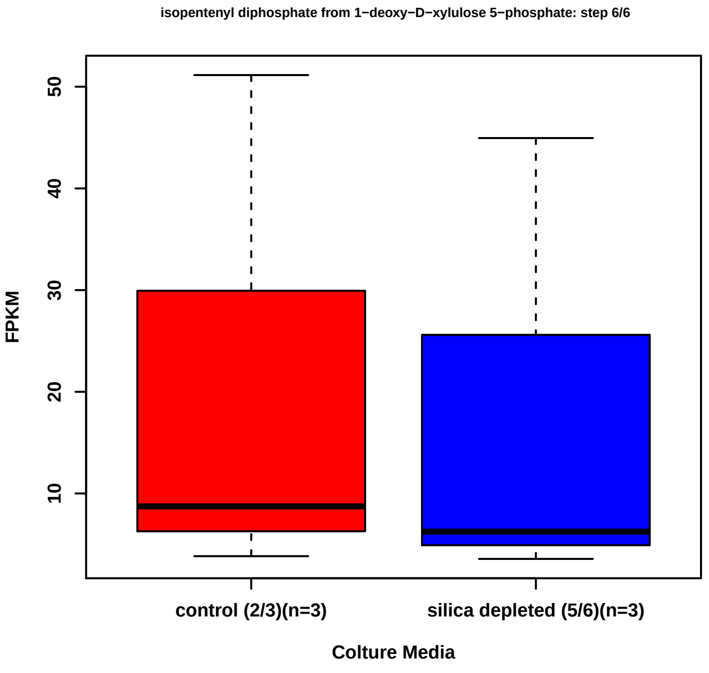
isopentenyl diphosphate from 1−deoxy−D−xylulose 5−phosphate: step 6/6
FPKM
10
20
30
40
50
control (2/3)(n=3)
silica depleted (5/6)(n=3)
Colture Media

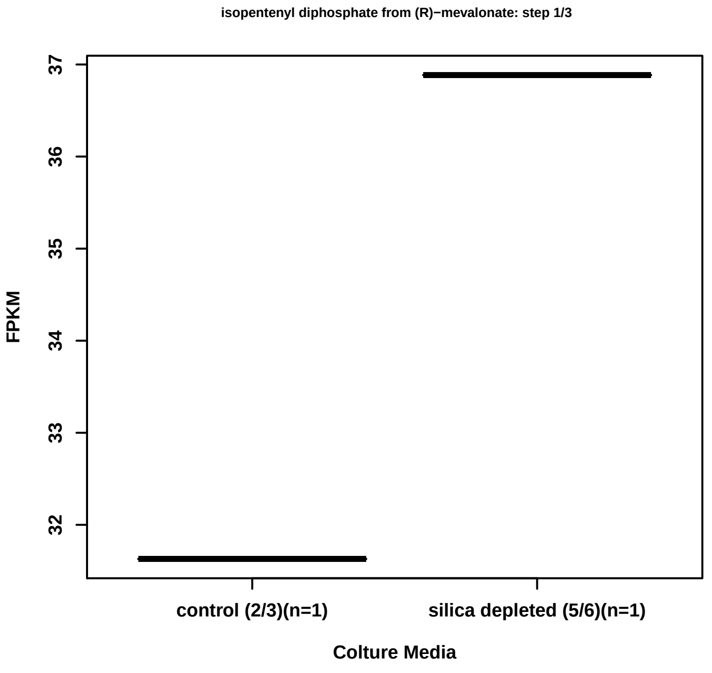
isopentenyl diphosphate from (R)−mevalonate: step 1/3
FPKM
32
33
34
35
36
37
control (2/3)(n=1)
silica depleted (5/6)(n=1)
Colture Media

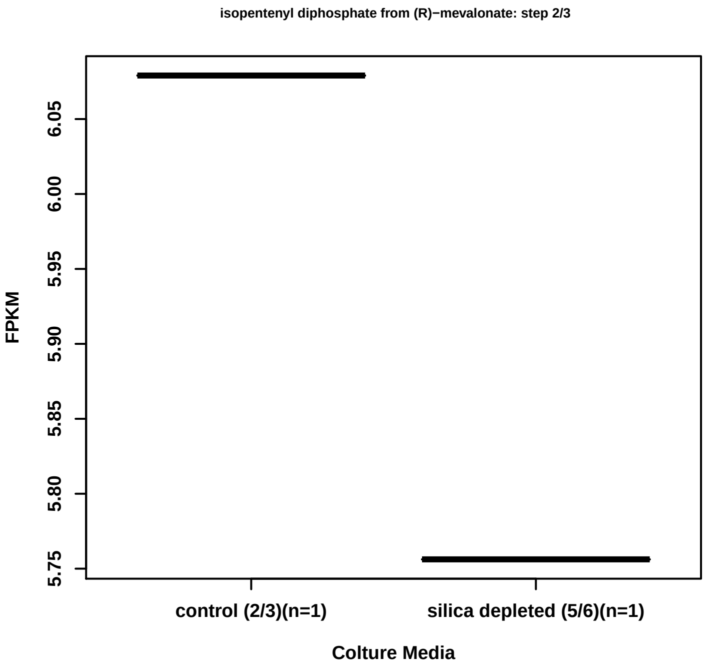
isopentenyl diphosphate from (R)−mevalonate: step 2/3
FPKM
5.75
5.80
5.85
5.90
5.95
6.00
6.05
control (2/3)(n=1)
silica depleted (5/6)(n=1)
Colture Media

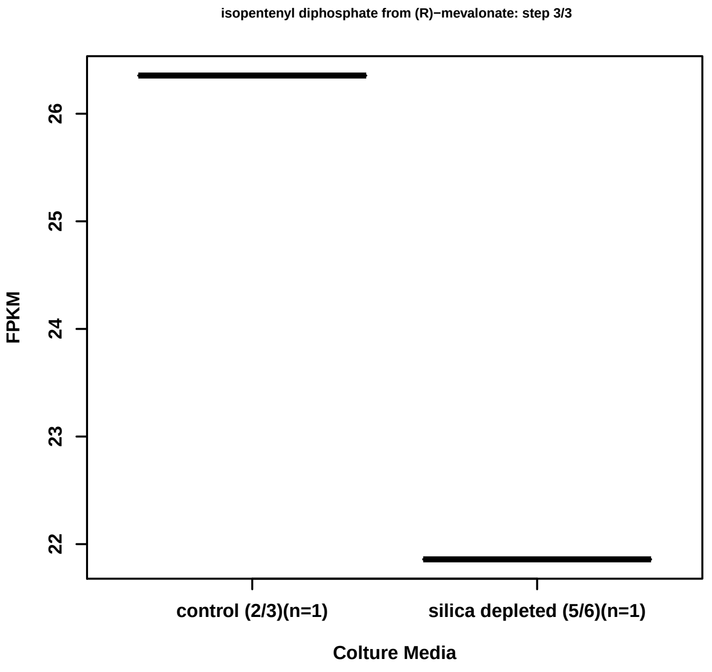
isopentenyl diphosphate from (R)−mevalonate: step 3/3
FPKM
22
23
24
25
26
control (2/3)(n=1)
silica depleted (5/6)(n=1)
Colture Media

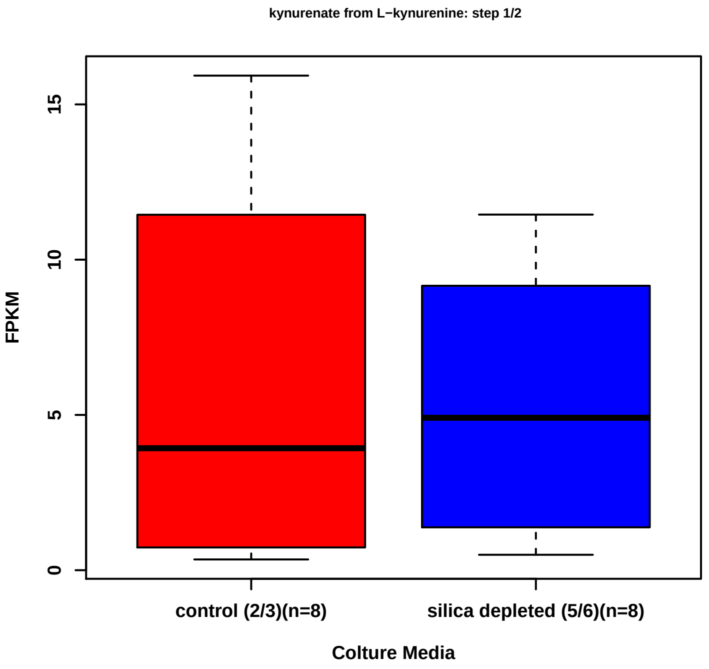
kynurenate from L−kynurenine: step 1/2
FPKM
0
5
10
15
control (2/3)(n=8)
silica depleted (5/6)(n=8)
Colture Media

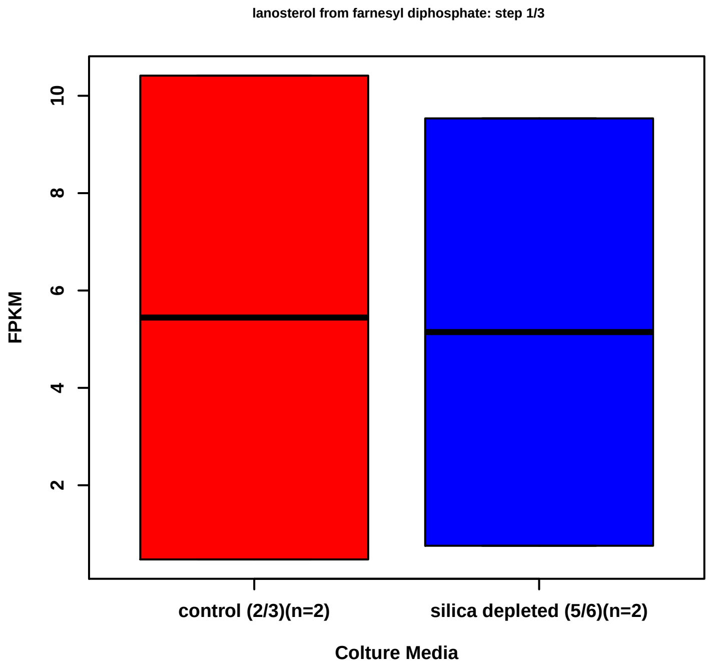
lanosterol from farnesyl diphosphate: step 1/3
FPKM
2
4
6
8
10
control (2/3)(n=2)
silica depleted (5/6)(n=2)
Colture Media

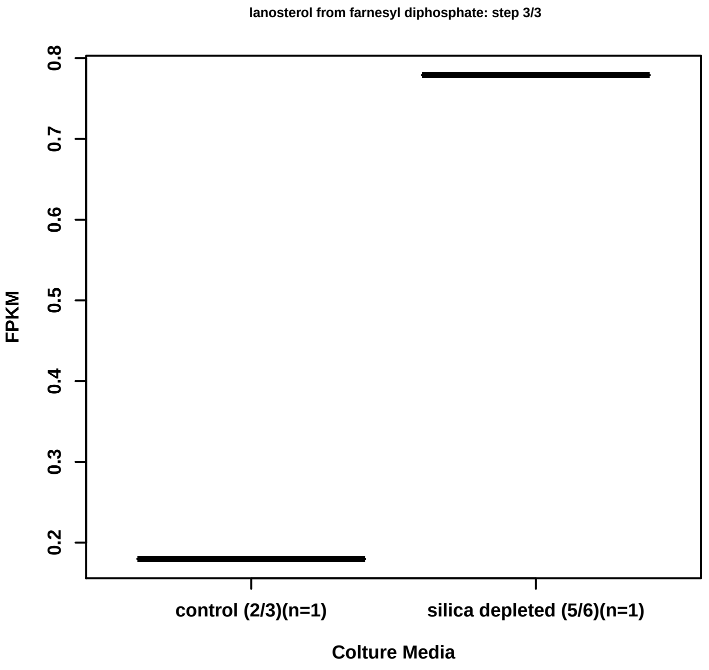
lanosterol from farnesyl diphosphate: step 3/3
FPKM
0.2
0.3
0.4
0.5
0.6
0.7
0.8
control (2/3)(n=1)
silica depleted (5/6)(n=1)
Colture Media

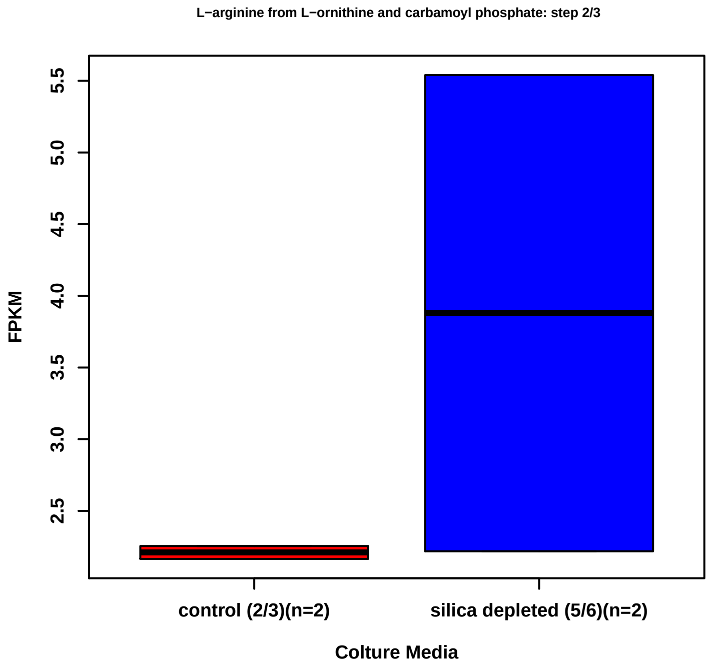
L−arginine from L−ornithine and carbamoyl phosphate: step 2/3
FPKM
2.5
3.0
3.5
4.0
4.5
5.0
5.5
control (2/3)(n=2)
silica depleted (5/6)(n=2)
Colture Media

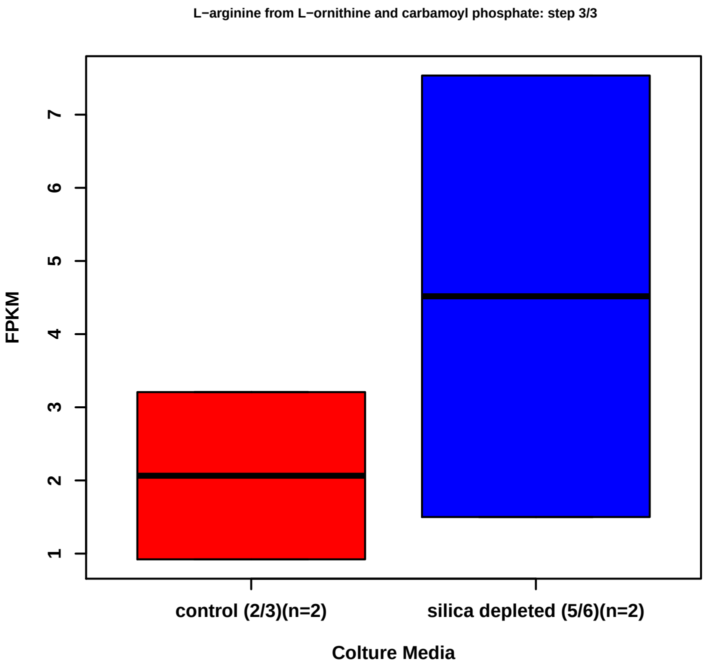
L−arginine from L−ornithine and carbamoyl phosphate: step 3/3
FPKM
1
2
3
4
5
6
7
control (2/3)(n=2)
silica depleted (5/6)(n=2)
Colture Media

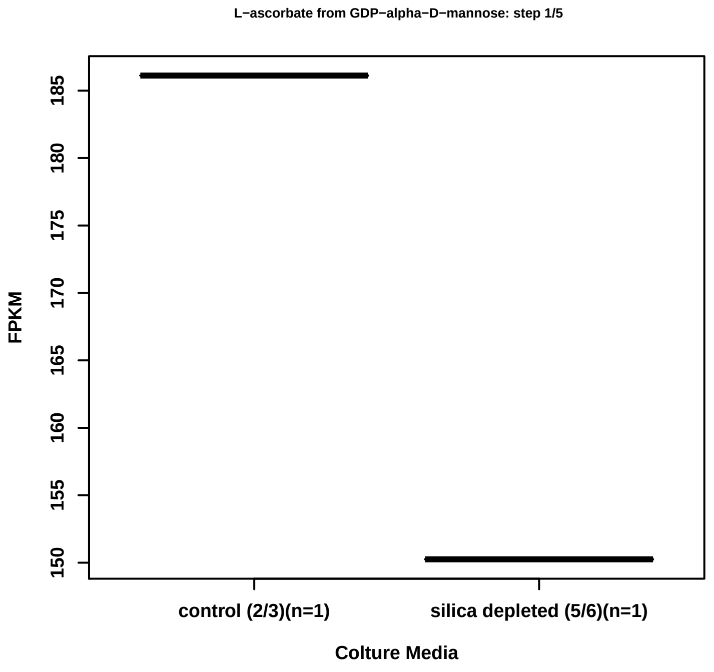
L−ascorbate from GDP−alpha−D−mannose: step 1/5
FPKM
150
155
160
165
170
175
180
185
control (2/3)(n=1)
silica depleted (5/6)(n=1)
Colture Media

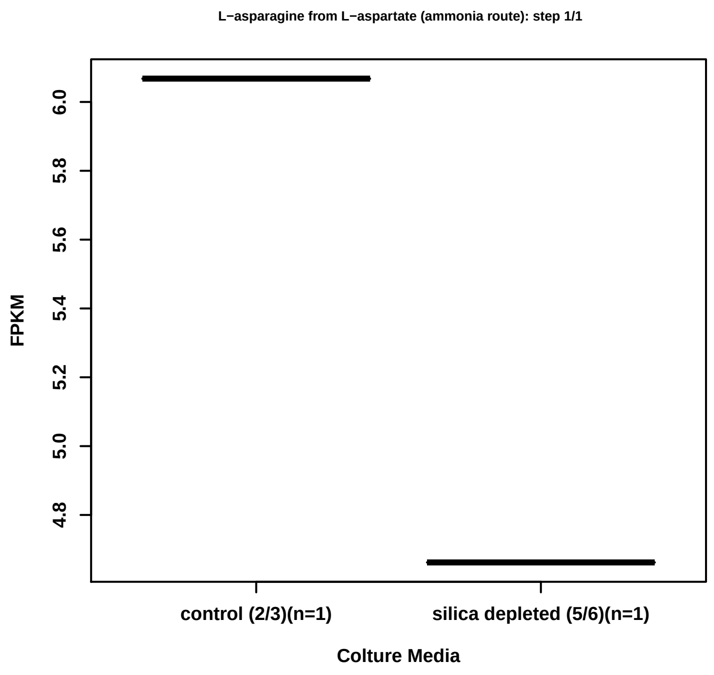

L−asparagine from L−aspartate (ammonia route): step 1/1
FPKM
6.0
5.8
5.6
5.4
5.2
5.0
4.8
control (2/3)(n=1)
silica depleted (5/6)(n=1)
Colture Media

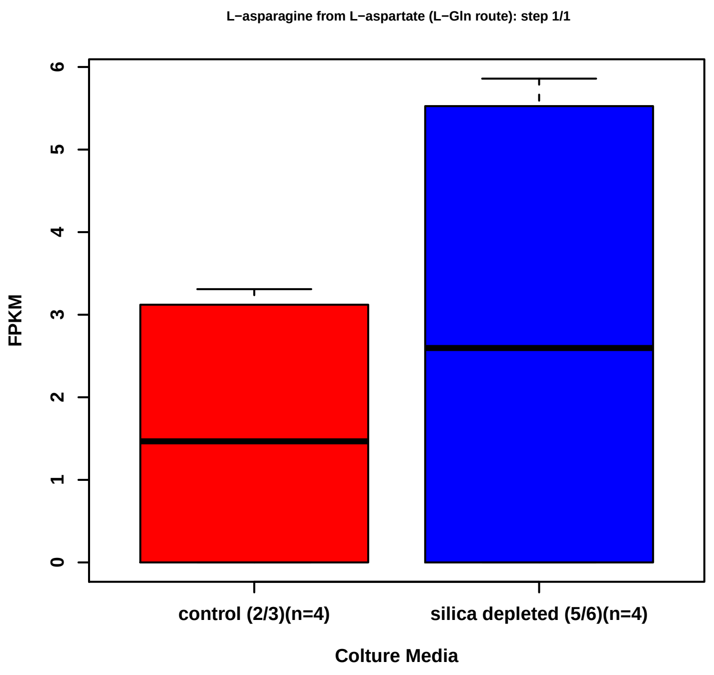

L−asparagine from L−aspartate (L−Gln route): step 1/1
FPKM
0
1
2
3
4
5
6
control (2/3)(n=4)
silica depleted (5/6)(n=4)
Colture Media

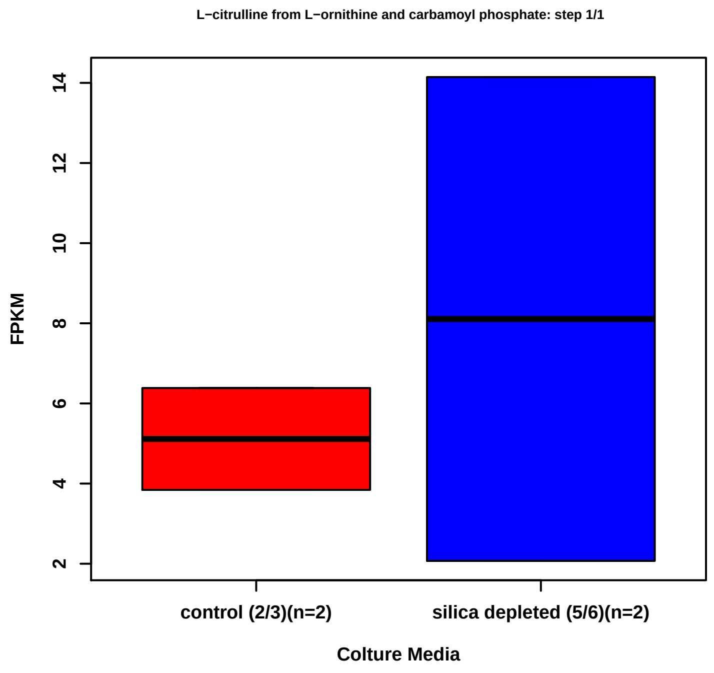

L−citrulline from L−ornithine and carbamoyl phosphate: step 1/1
FPKM
2
4
6
8
10
12
14
control (2/3)(n=2)
silica depleted (5/6)(n=2)
Colture Media

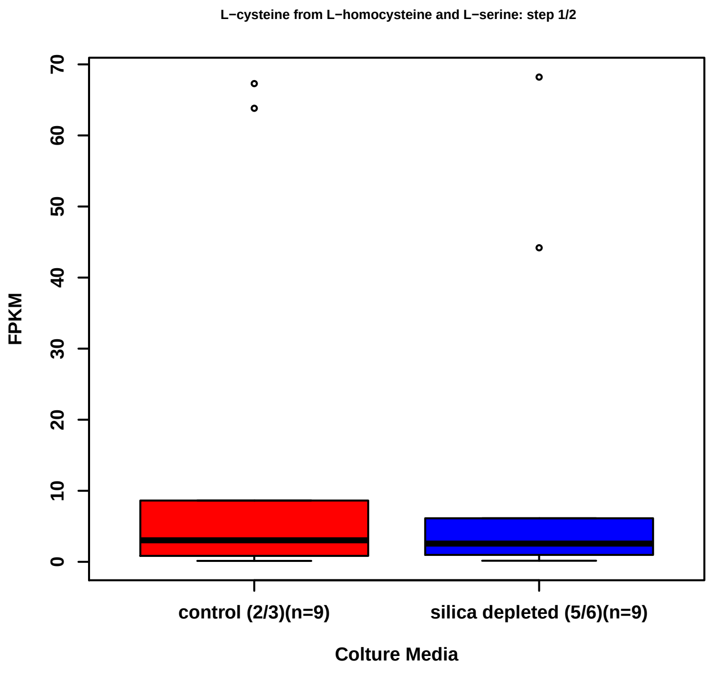

L−cysteine from L−homocysteine and L−serine: step 1/2
FPKM
0
10
20
30
40
50
60
70
control (2/3)(n=9)
silica depleted (5/6)(n=9)
Colture Media

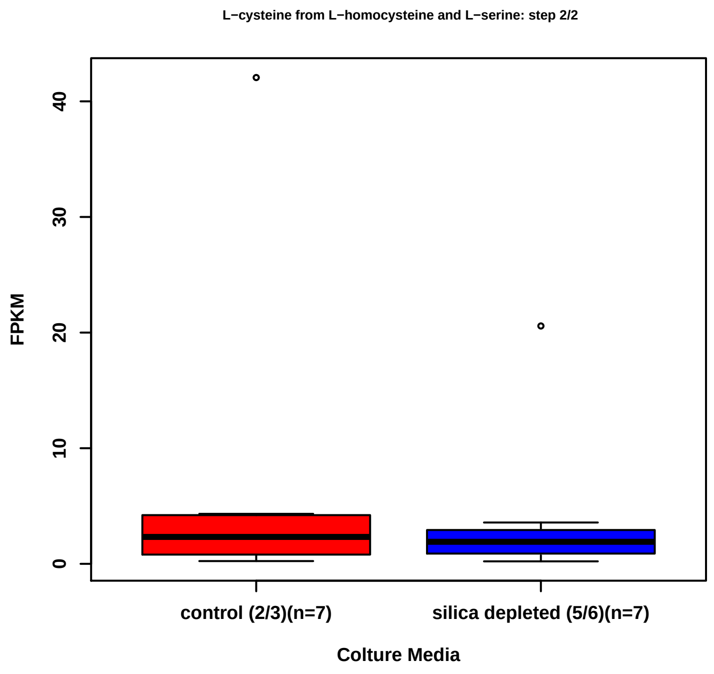

L−cysteine from L−homocysteine and L−serine: step 2/2
FPKM
0
10
20
30
40
control (2/3)(n=7)
silica depleted (5/6)(n=7)
Colture Media

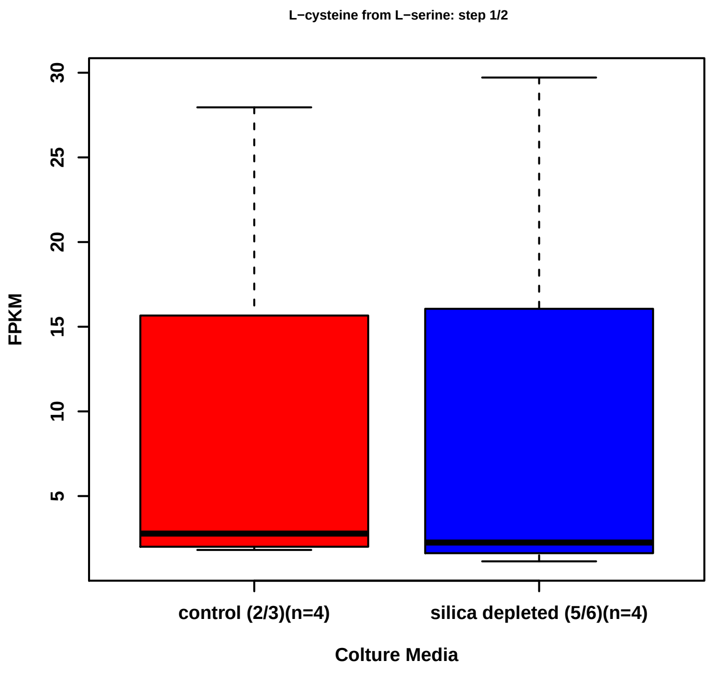

L−cysteine from L−serine: step 1/2
FPKM
5
10
15
20
25
30
control (2/3)(n=4)
silica depleted (5/6)(n=4)
Colture Media

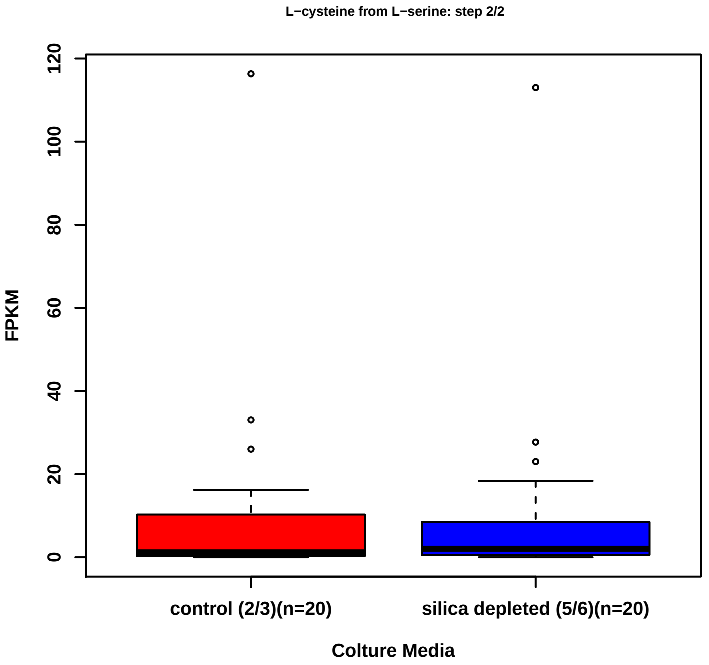
L−cysteine from L−serine: step 2/2
FPKM
0
20
40
60
80
100
120
control (2/3)(n=20)
silica depleted (5/6)(n=20)
Colture Media

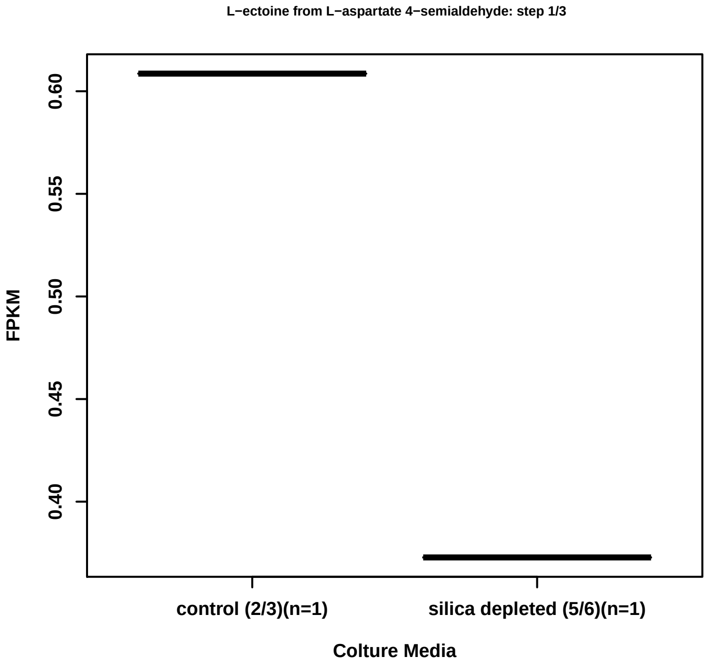
L−ectoine from L−aspartate 4−semialdehyde: step 1/3
FPKM
0.40
0.45
0.50
0.55
0.60
control (2/3)(n=1)
silica depleted (5/6)(n=1)
Colture Media

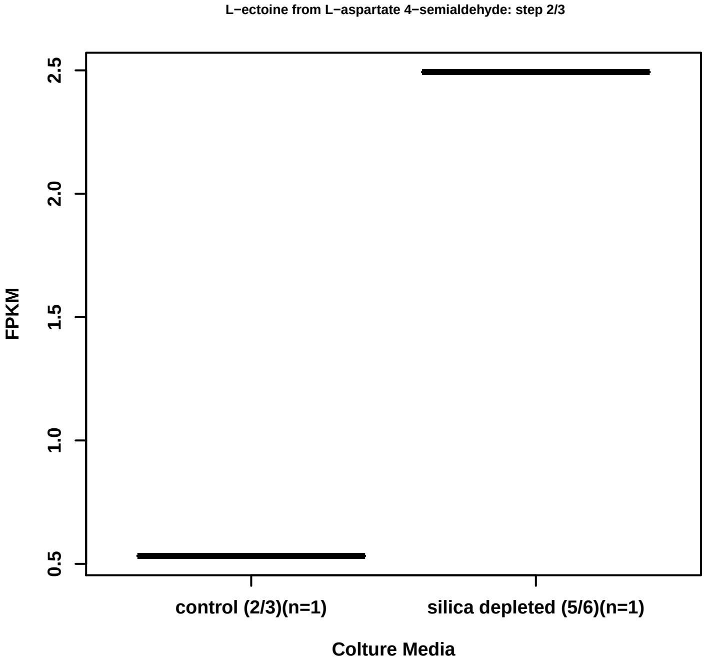
L−ectoine from L−aspartate 4−semialdehyde: step 2/3
FPKM
0.5
1.0
1.5
2.0
2.5
control (2/3)(n=1)
silica depleted (5/6)(n=1)
Colture Media

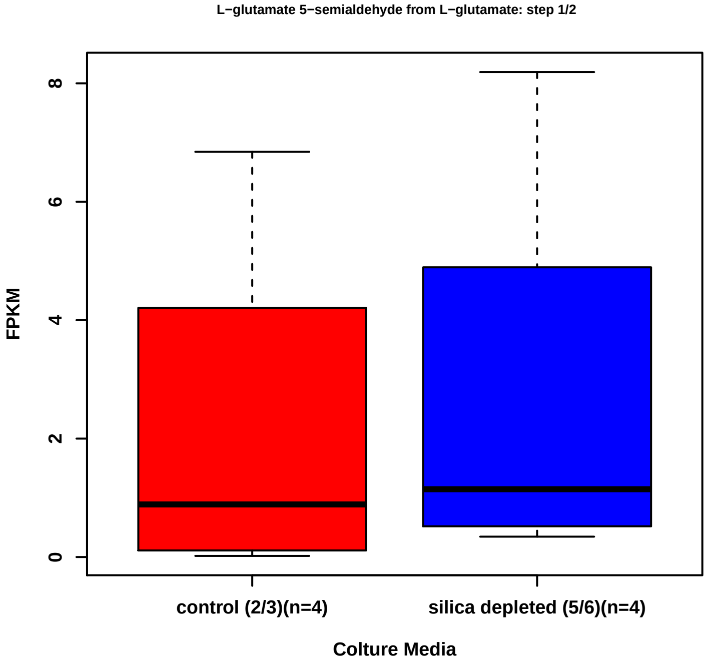
L−glutamate 5−semialdehyde from L−glutamate: step 1/2
FPKM
0
2
4
6
8
control (2/3)(n=4)
silica depleted (5/6)(n=4)
Colture Media

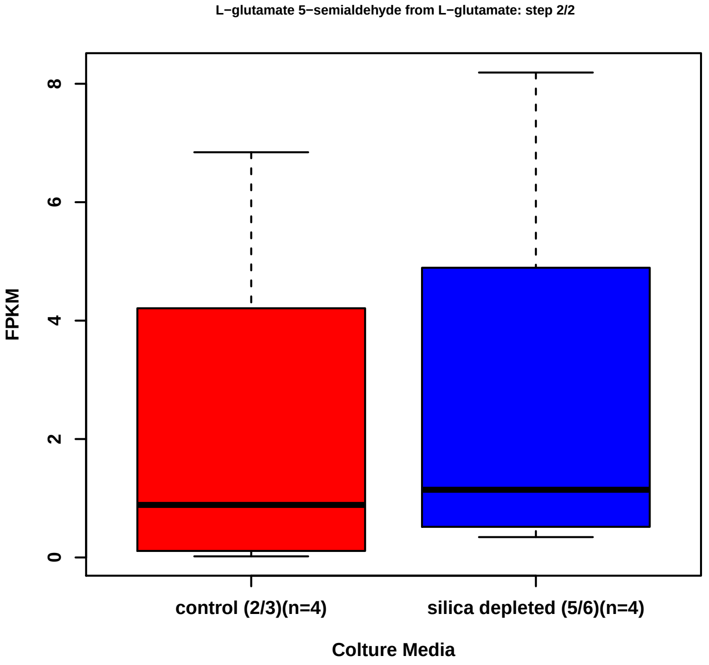
L−glutamate 5−semialdehyde from L−glutamate: step 2/2
FPKM
0
2
4
6
8
control (2/3)(n=4)
silica depleted (5/6)(n=4)
Colture Media

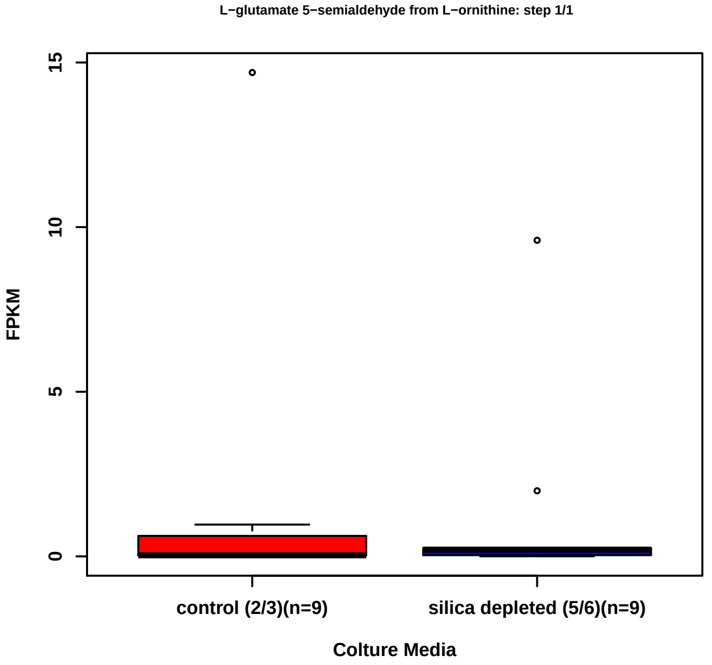
L−glutamate 5−semialdehyde from L−ornithine: step 1/1
FPKM
0
5
10
15
control (2/3)(n=9)
silica depleted (5/6)(n=9)
Colture Media

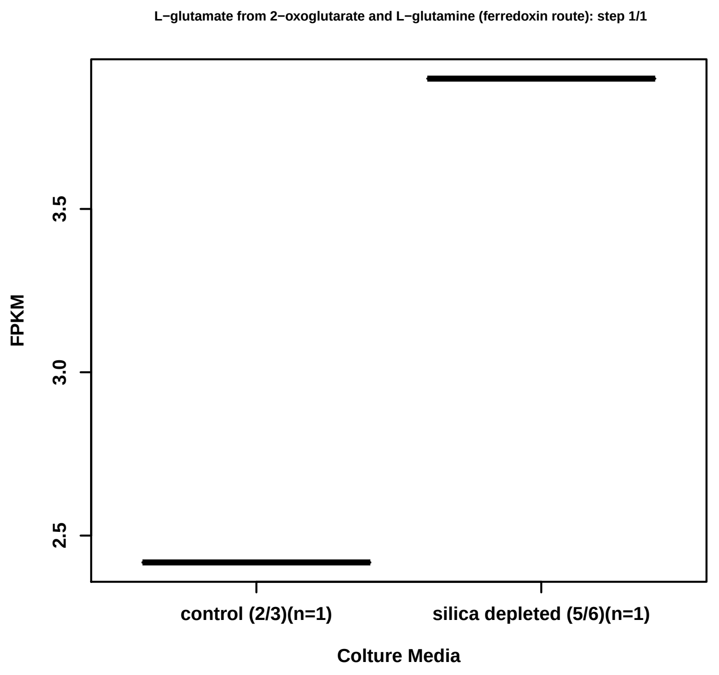
L−glutamate from 2−oxoglutarate and L−glutamine (ferredoxin route): step 1/1
FPKM
2.5
3.0
3.5
control (2/3)(n=1)
silica depleted (5/6)(n=1)
Colture Media

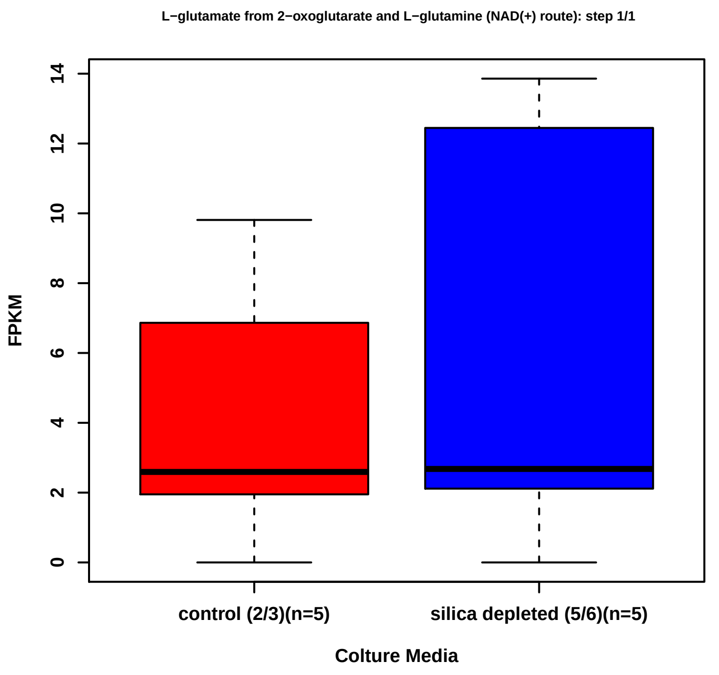
L−glutamate from 2−oxoglutarate and L−glutamine (NAD(+) route): step 1/1
FPKM
0
2
4
6
8
10
12
14
control (2/3)(n=5)
silica depleted (5/6)(n=5)
Colture Media

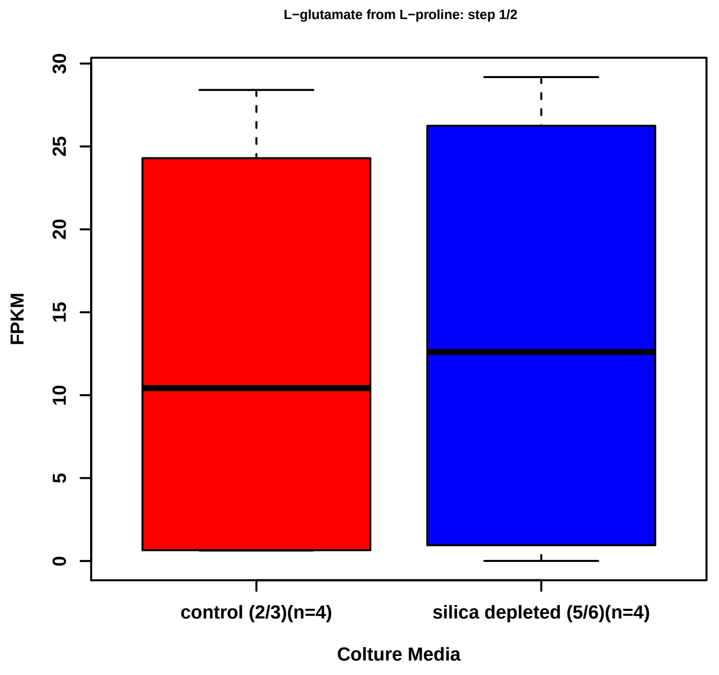
L−glutamate from L−proline: step 1/2
FPKM
0
5
10
15
20
25
30
control (2/3)(n=4)
silica depleted (5/6)(n=4)
Colture Media

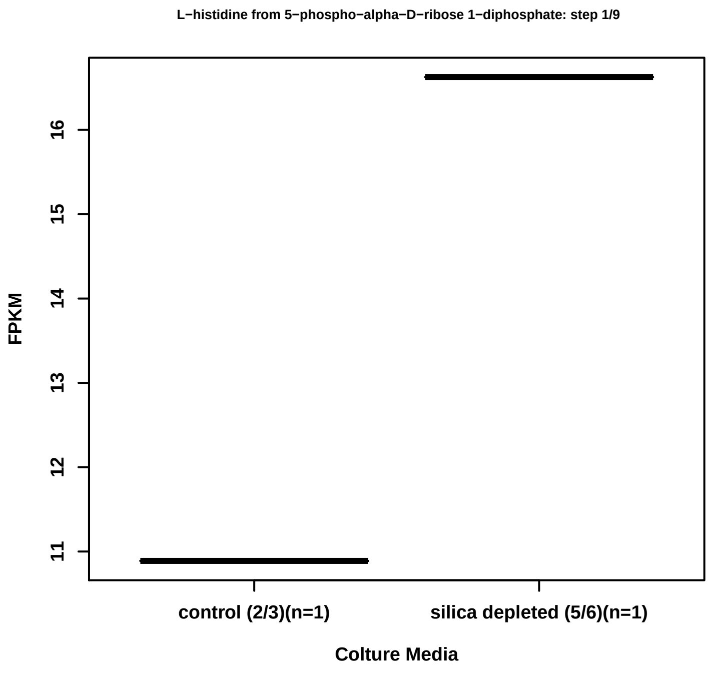
L−histidine from 5−phospho−alpha−D−ribose 1−diphosphate: step 1/9
FPKM
11
12
13
14
15
16
control (2/3)(n=1)
silica depleted (5/6)(n=1)
Colture Media

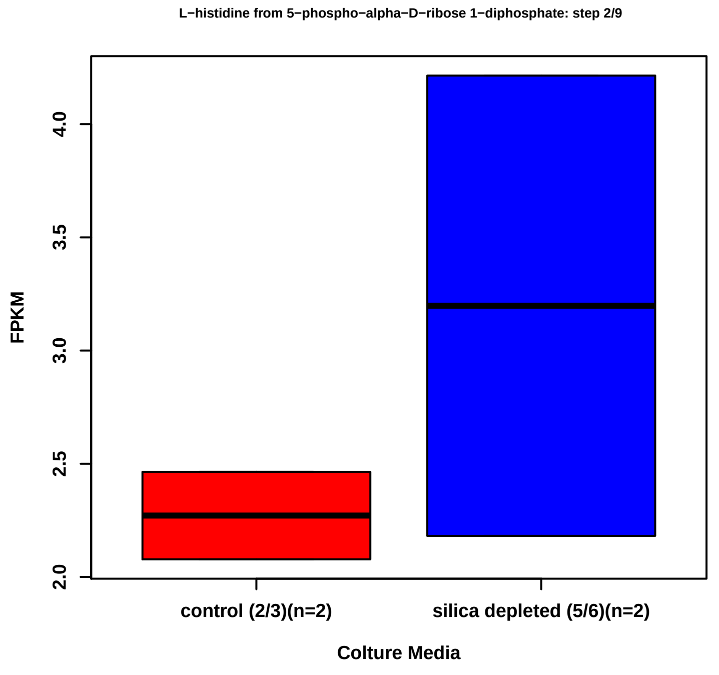
L−histidine from 5−phospho−alpha−D−ribose 1−diphosphate: step 2/9
FPKM
2.0
2.5
3.0
3.5
4.0
control (2/3)(n=2)
silica depleted (5/6)(n=2)
Colture Media

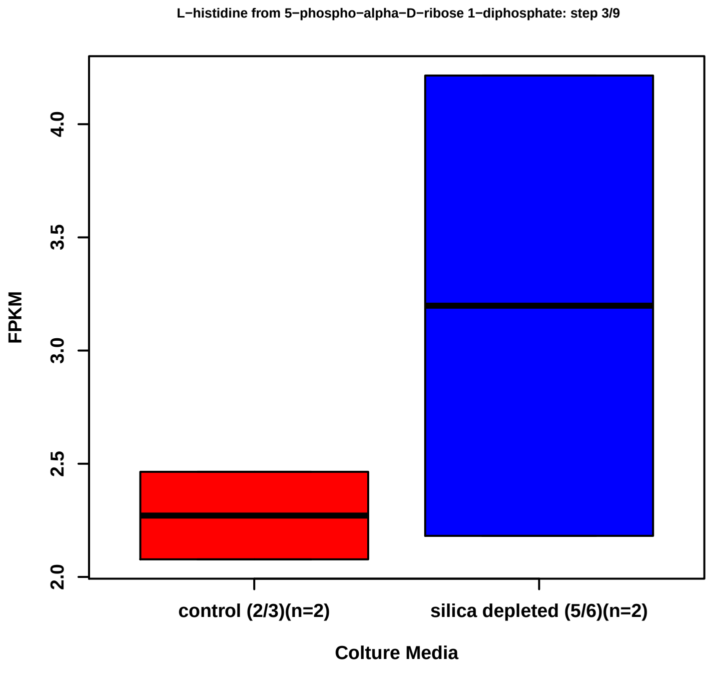
L−histidine from 5−phospho−alpha−D−ribose 1−diphosphate: step 3/9
FPKM
2.0
2.5
3.0
3.5
4.0
control (2/3)(n=2)
silica depleted (5/6)(n=2)
Colture Media

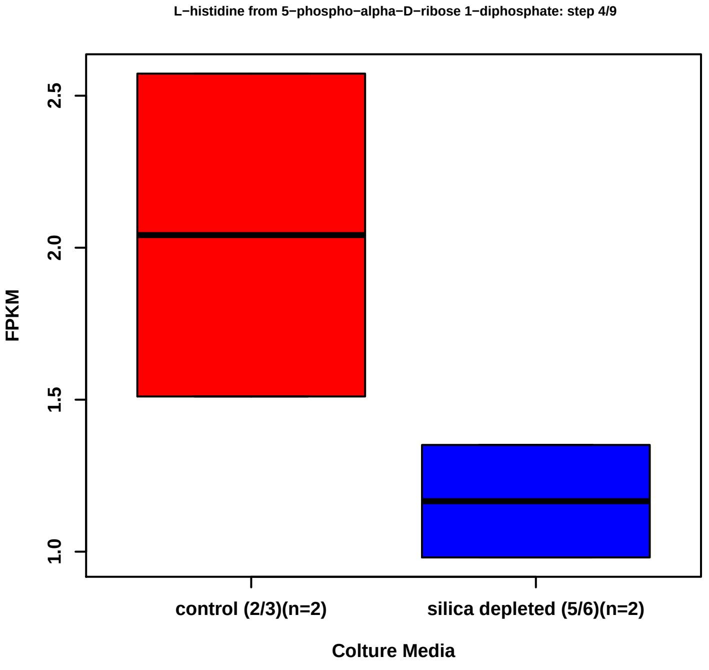
L−histidine from 5−phospho−alpha−D−ribose 1−diphosphate: step 4/9
FPKM
1.0
1.5
2.0
2.5
control (2/3)(n=2)
silica depleted (5/6)(n=2)
Colture Media

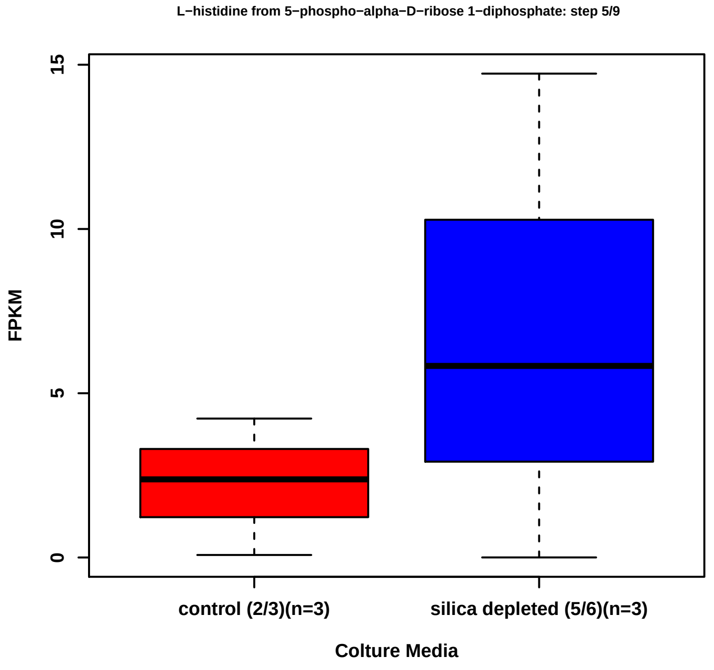
L−histidine from 5−phospho−alpha−D−ribose 1−diphosphate: step 5/9
FPKM
0
5
10
15
control (2/3)(n=3)
silica depleted (5/6)(n=3)
Colture Media

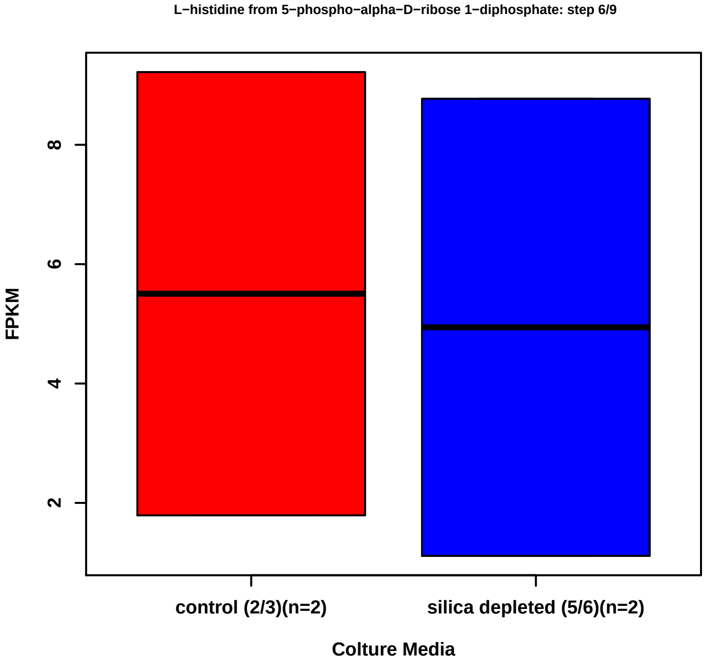
L−histidine from 5−phospho−alpha−D−ribose 1−diphosphate: step 6/9
FPKM
2
4
6
8
control (2/3)(n=2)
silica depleted (5/6)(n=2)
Colture Media

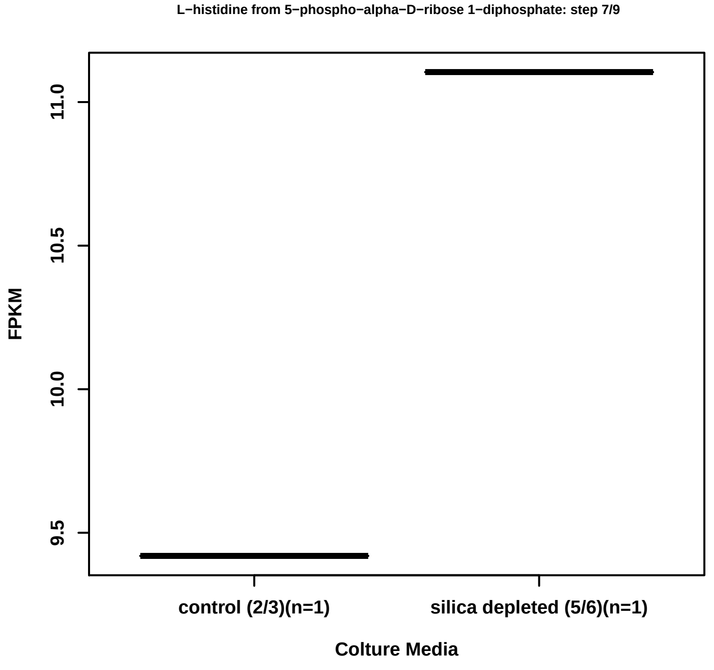
L−histidine from 5−phospho−alpha−D−ribose 1−diphosphate: step 7/9
FPKM
9.5
10.0
10.5
11.0
control (2/3)(n=1)
silica depleted (5/6)(n=1)
Colture Media

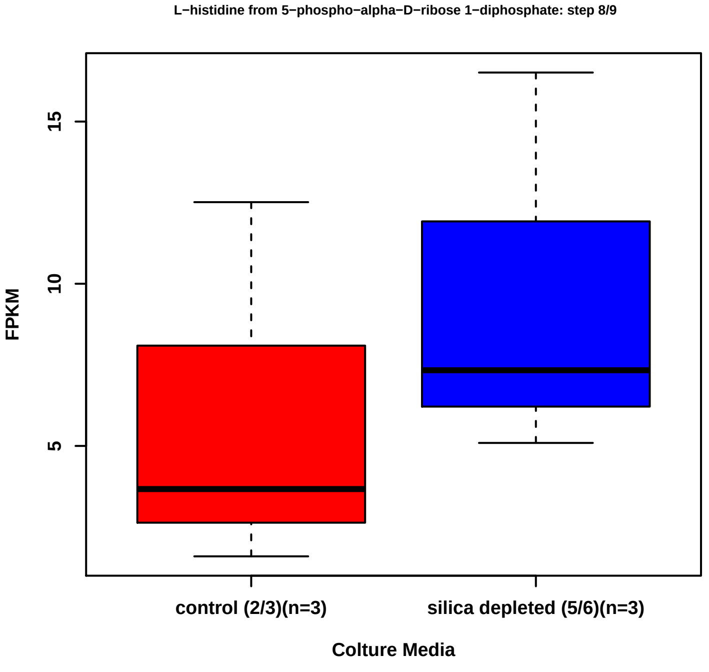
L−histidine from 5−phospho−alpha−D−ribose 1−diphosphate: step 8/9
FPKM
5
10
15
control (2/3)(n=3)
silica depleted (5/6)(n=3)
Colture Media

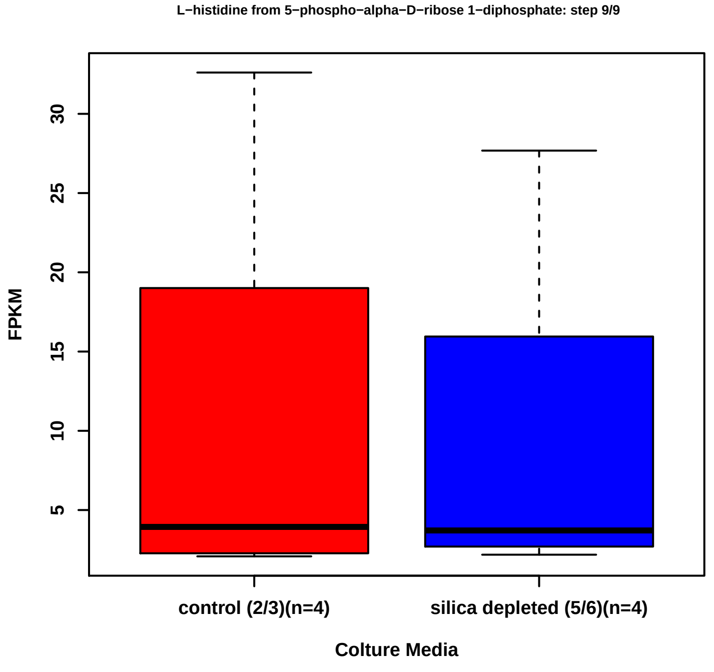
L−histidine from 5−phospho−alpha−D−ribose 1−diphosphate: step 9/9
FPKM
5
10
15
20
25
30
control (2/3)(n=4)
silica depleted (5/6)(n=4)
Colture Media

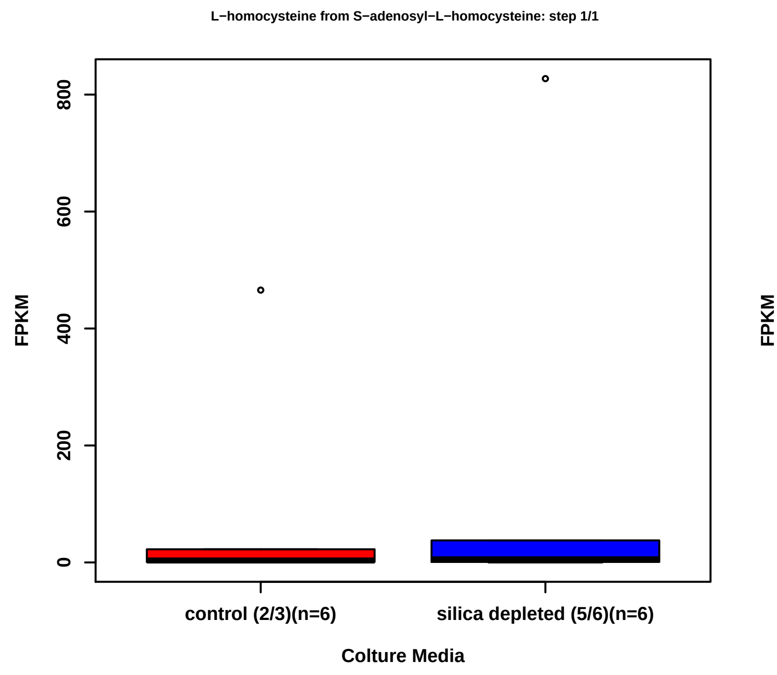

L−homocysteine from S−adenosyl−L−homocysteine: step 1/1
FPKM
0
200
400
600
800
control (2/3)(n=6)
silica depleted (5/6)(n=6)
Colture Media

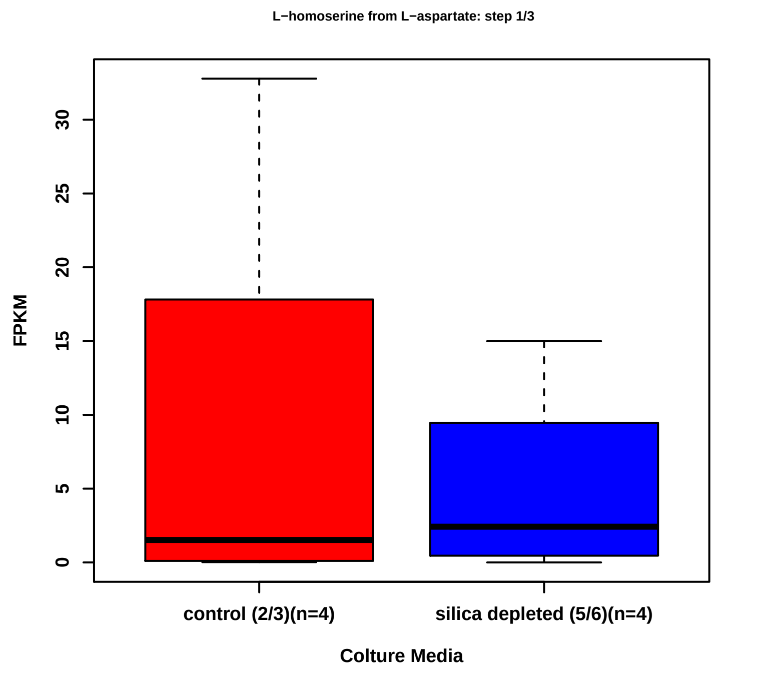

L−homoserine from L−aspartate: step 1/3
FPKM
0
5
10
15
20
25
30
control (2/3)(n=4)
silica depleted (5/6)(n=4)
Colture Media

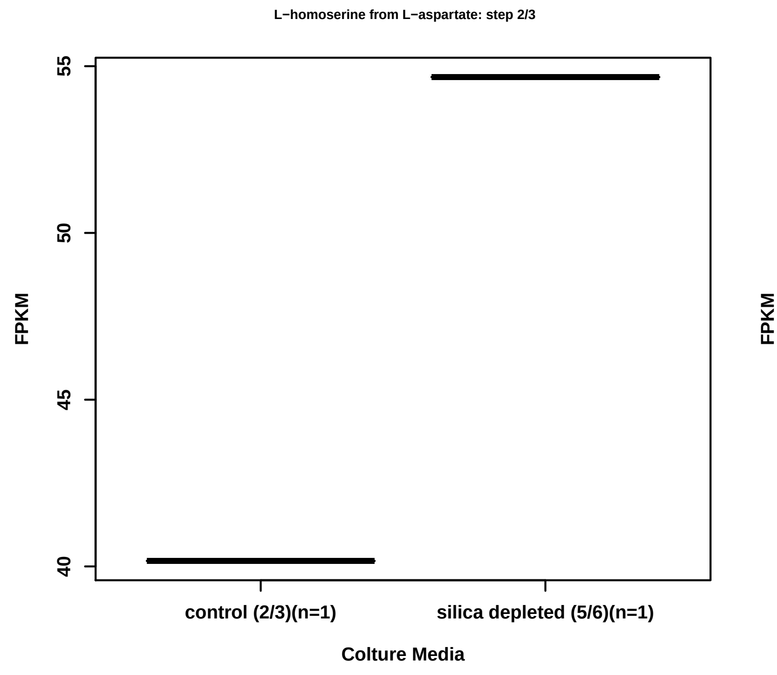

L−homoserine from L−aspartate: step 2/3
FPKM
40
45
50
55
control (2/3)(n=1)
silica depleted (5/6)(n=1)
Colture Media

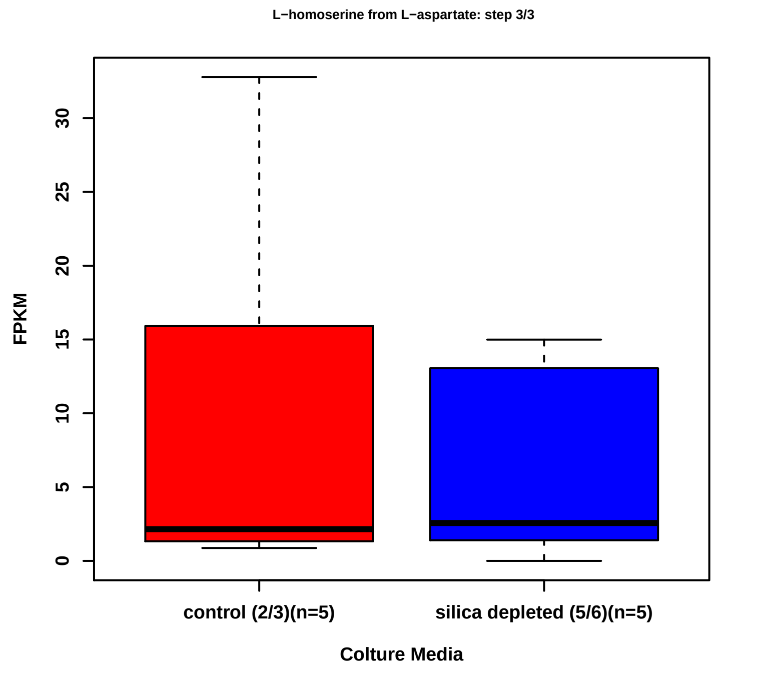

L−homoserine from L−aspartate: step 3/3
FPKM
0
5
10
15
20
25
30
control (2/3)(n=5)
silica depleted (5/6)(n=5)
Colture Media

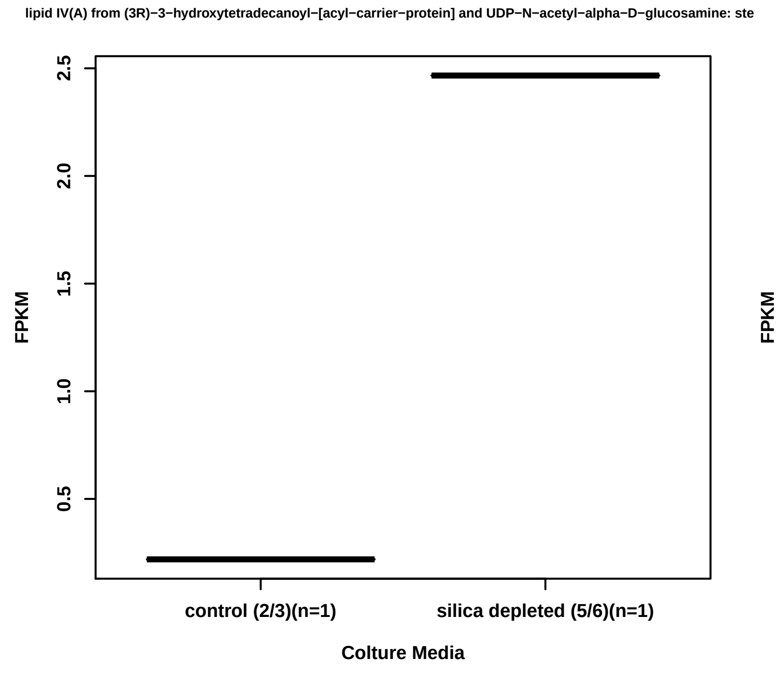

lipid IV(A) from (3R)−3−hydroxytetradecanoyl−[acyl−carrier−protein] and UDP−N−acetyl−alpha−D−glucosamine: ste
FPKM
0.5
1.0
1.5
2.0
2.5
control (2/3)(n=1)
silica depleted (5/6)(n=1)
Colture Media

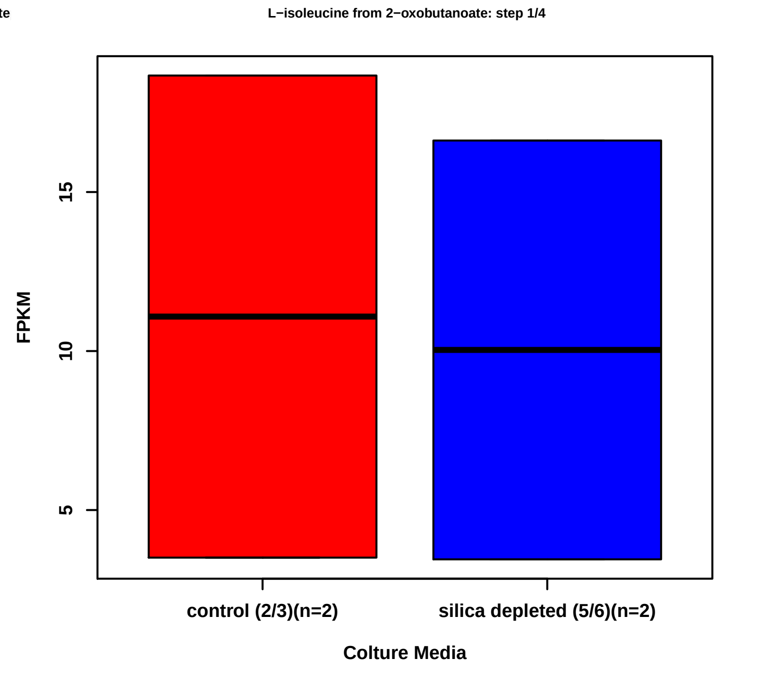

L−isoleucine from 2−oxobutanoate: step 1/4
FPKM
5
10
15
control (2/3)(n=2)
silica depleted (5/6)(n=2)
Colture Media

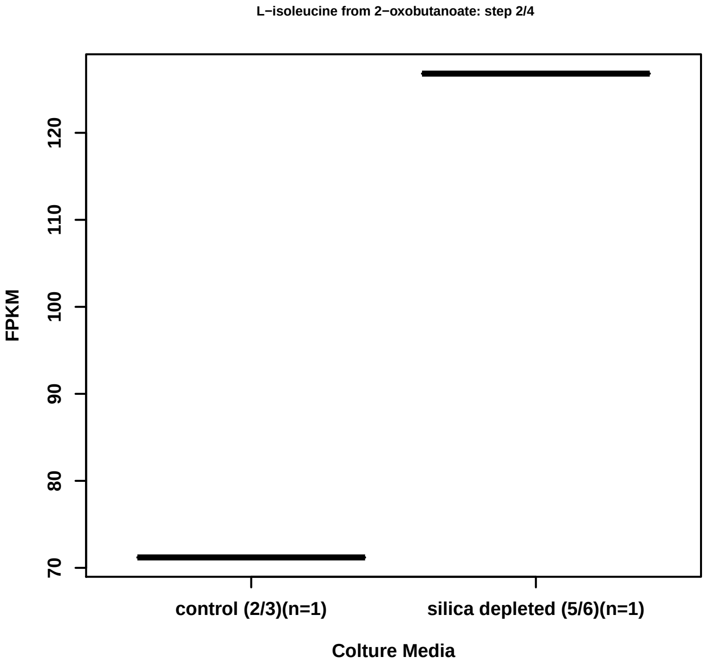

L−isoleucine from 2−oxobutanoate: step 2/4
FPKM
70
80
90
100
110
120
control (2/3)(n=1)
silica depleted (5/6)(n=1)
Colture Media

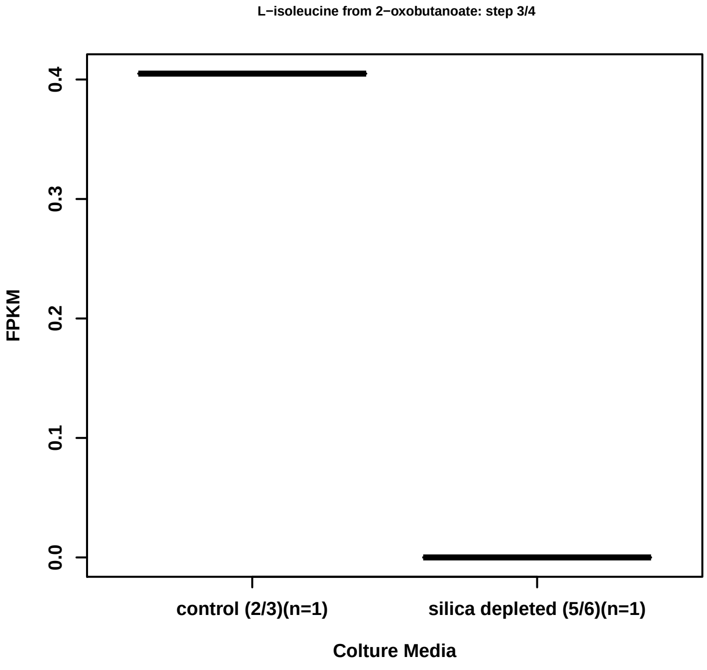

L−isoleucine from 2−oxobutanoate: step 3/4
FPKM
0.0
0.1
0.2
0.3
0.4
control (2/3)(n=1)
silica depleted (5/6)(n=1)
Colture Media

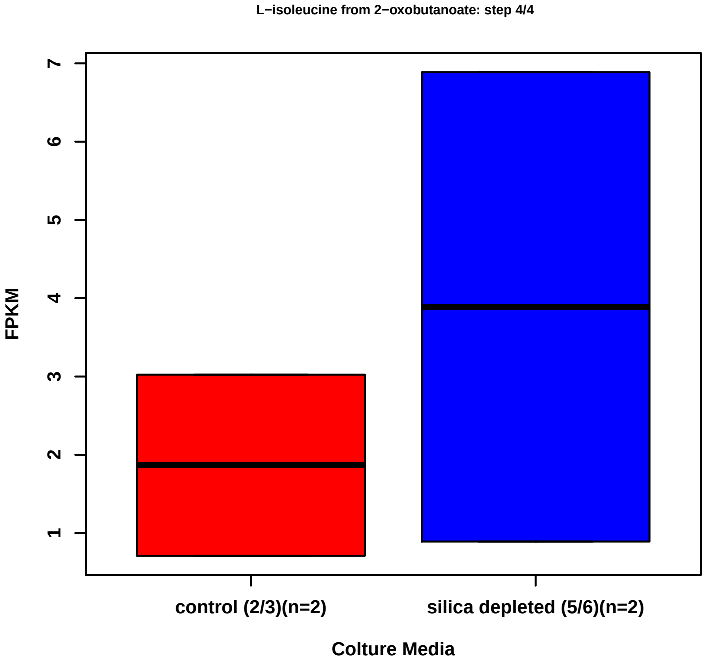

L−isoleucine from 2−oxobutanoate: step 4/4
FPKM
1
2
3
4
5
6
7
control (2/3)(n=2)
silica depleted (5/6)(n=2)
Colture Media

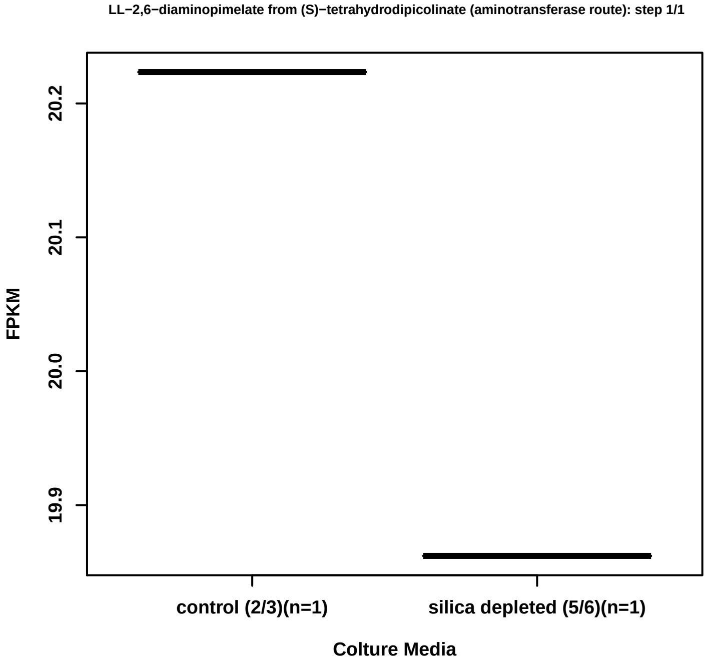

LL−2,6−diaminopimelate from (S)−tetrahydrodipicolinate (aminotransferase route): step 1/1
FPKM
19.9
20.0
20.1
20.2
control (2/3)(n=1)
silica depleted (5/6)(n=1)
Colture Media

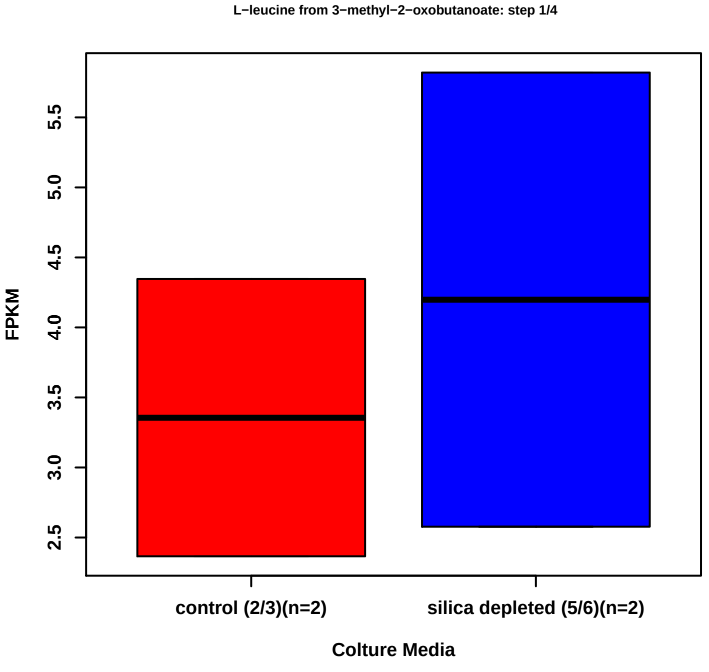

L−leucine from 3−methyl−2−oxobutanoate: step 1/4
FPKM
2.5
3.0
3.5
4.0
4.5
5.0
5.5
control (2/3)(n=2)
silica depleted (5/6)(n=2)
Colture Media

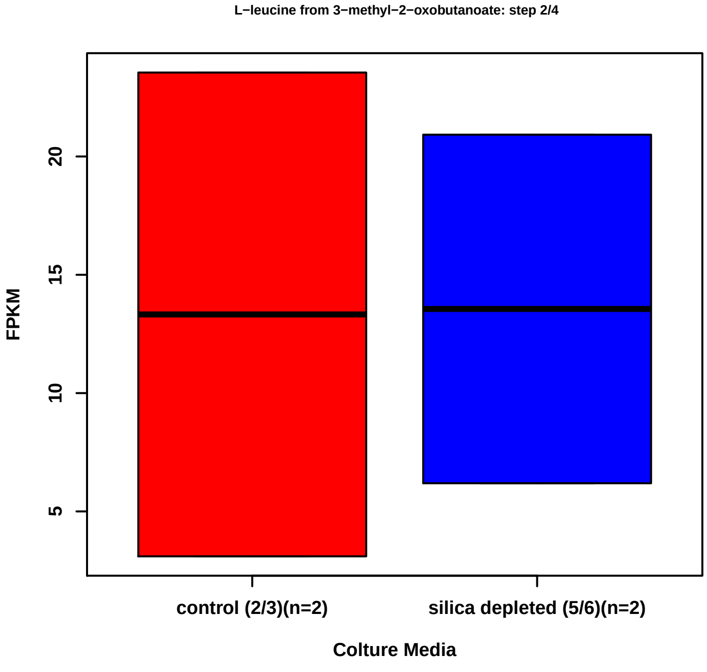

L−leucine from 3−methyl−2−oxobutanoate: step 2/4
FPKM
5
10
15
20
control (2/3)(n=2)
silica depleted (5/6)(n=2)
Colture Media

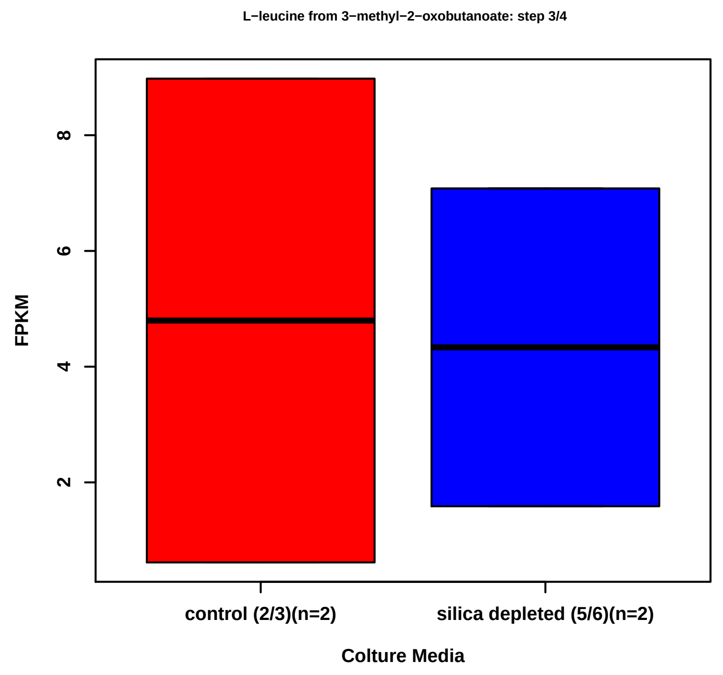
L−leucine from 3−methyl−2−oxobutanoate: step 3/4
FPKM
2
4
6
8
control (2/3)(n=2)
silica depleted (5/6)(n=2)
Colture Media

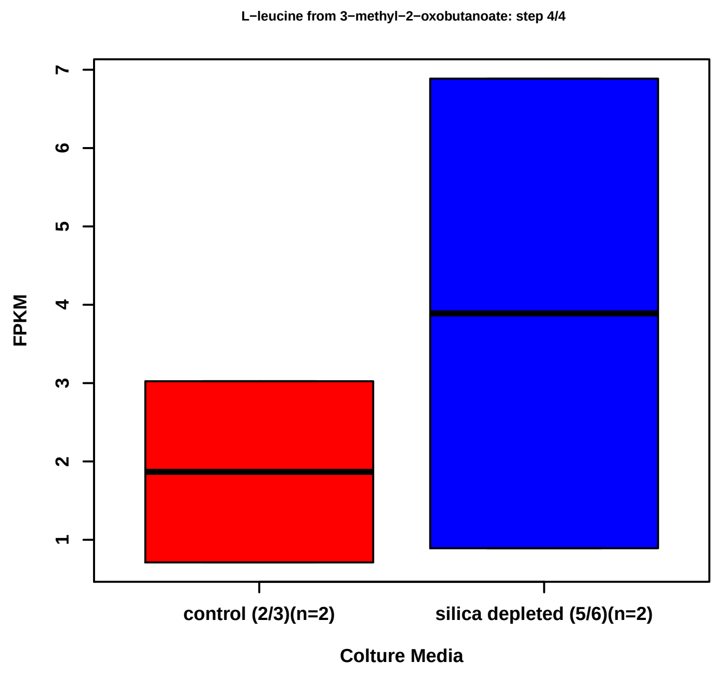
L−leucine from 3−methyl−2−oxobutanoate: step 4/4
FPKM
1
2
3
4
5
6
7
control (2/3)(n=2)
silica depleted (5/6)(n=2)
Colture Media

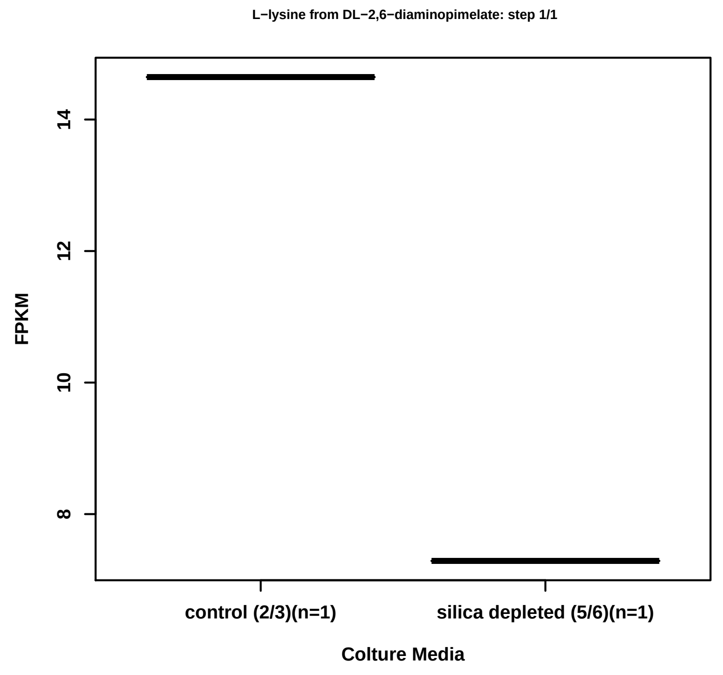
L−lysine from DL−2,6−diaminopimelate: step 1/1
FPKM
8
10
12
14
control (2/3)(n=1)
silica depleted (5/6)(n=1)
Colture Media

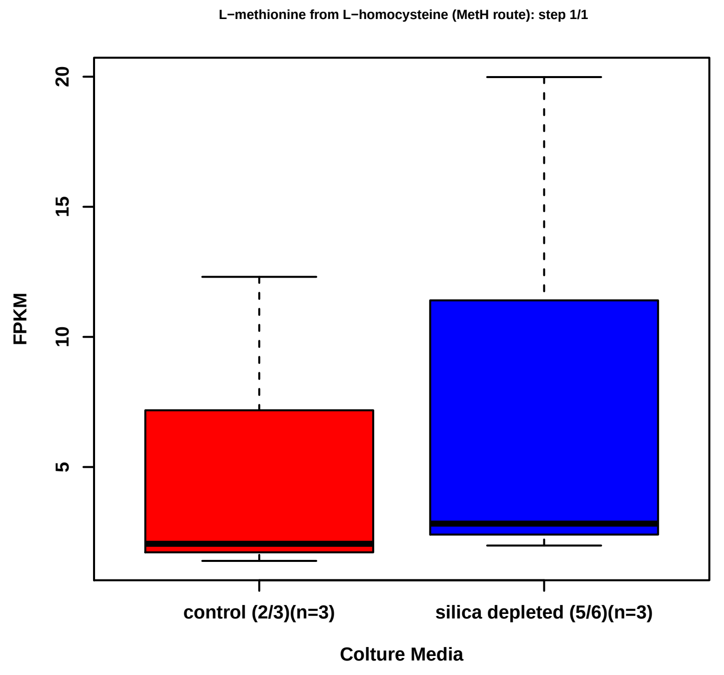
L−methionine from L−homocysteine (MetH route): step 1/1
FPKM
5
10
15
20
control (2/3)(n=3)
silica depleted (5/6)(n=3)
Colture Media

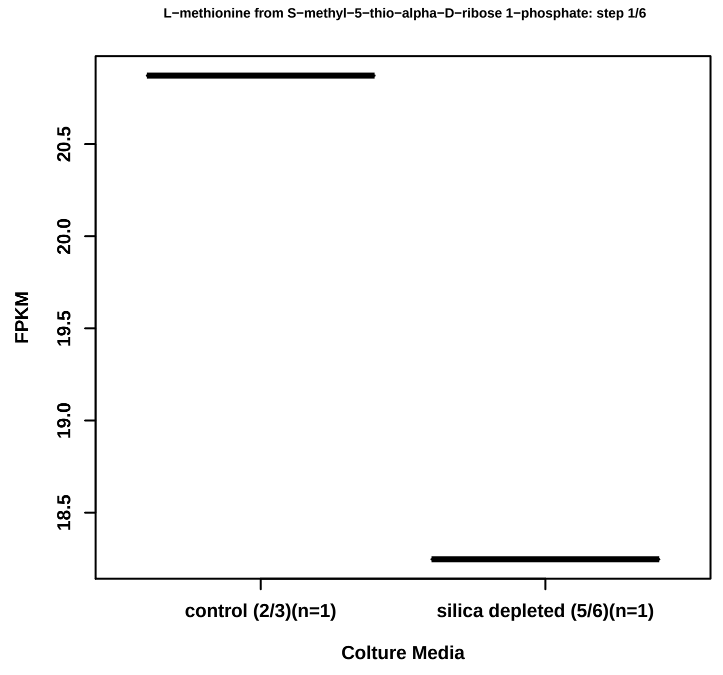
L−methionine from S−methyl−5−thio−alpha−D−ribose 1−phosphate: step 1/6
FPKM
18.5
19.0
19.5
20.0
20.5
control (2/3)(n=1)
silica depleted (5/6)(n=1)
Colture Media

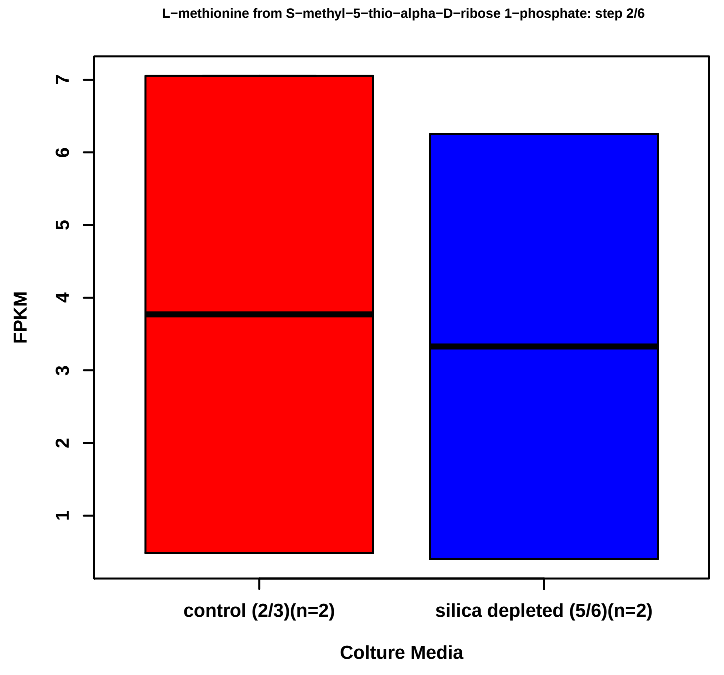
L−methionine from S−methyl−5−thio−alpha−D−ribose 1−phosphate: step 2/6
FPKM
1
2
3
4
5
6
7
control (2/3)(n=2)
silica depleted (5/6)(n=2)
Colture Media

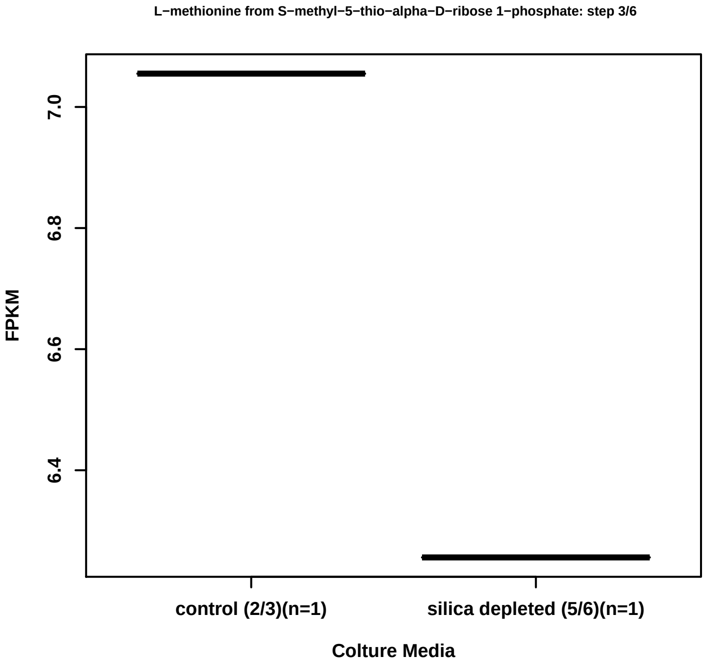
L−methionine from S−methyl−5−thio−alpha−D−ribose 1−phosphate: step 3/6
FPKM
6.4
6.6
6.8
7.0
control (2/3)(n=1)
silica depleted (5/6)(n=1)
Colture Media

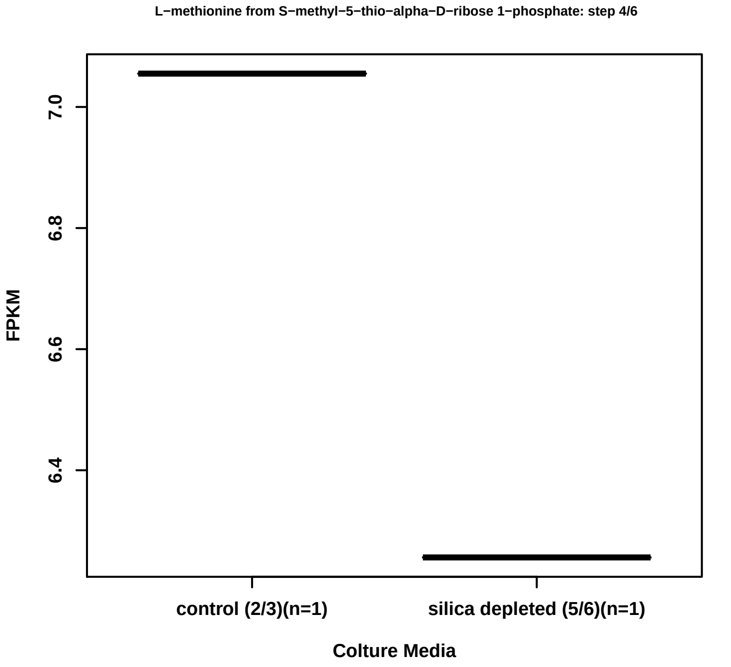
L−methionine from S−methyl−5−thio−alpha−D−ribose 1−phosphate: step 4/6
FPKM
6.4
6.6
6.8
7.0
control (2/3)(n=1)
silica depleted (5/6)(n=1)
Colture Media

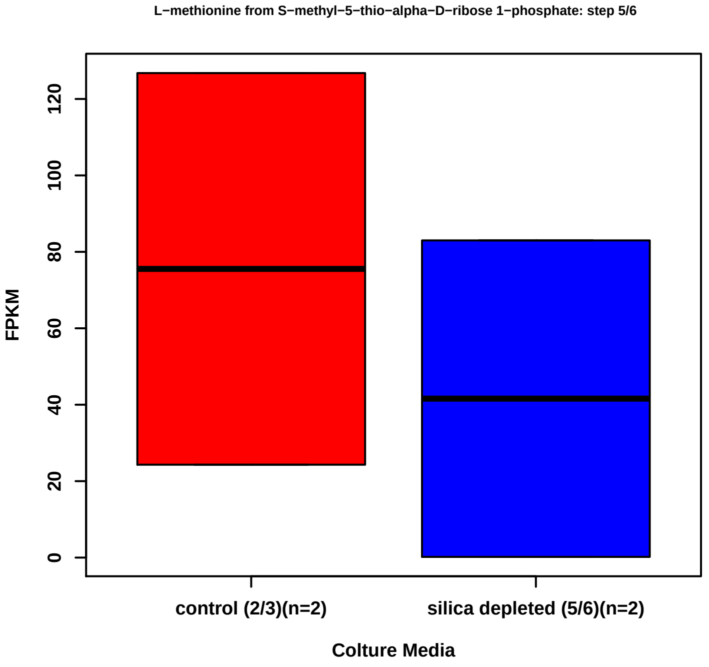
L−methionine from S−methyl−5−thio−alpha−D−ribose 1−phosphate: step 5/6
FPKM
0
20
40
60
80
100
120
control (2/3)(n=2)
silica depleted (5/6)(n=2)
Colture Media

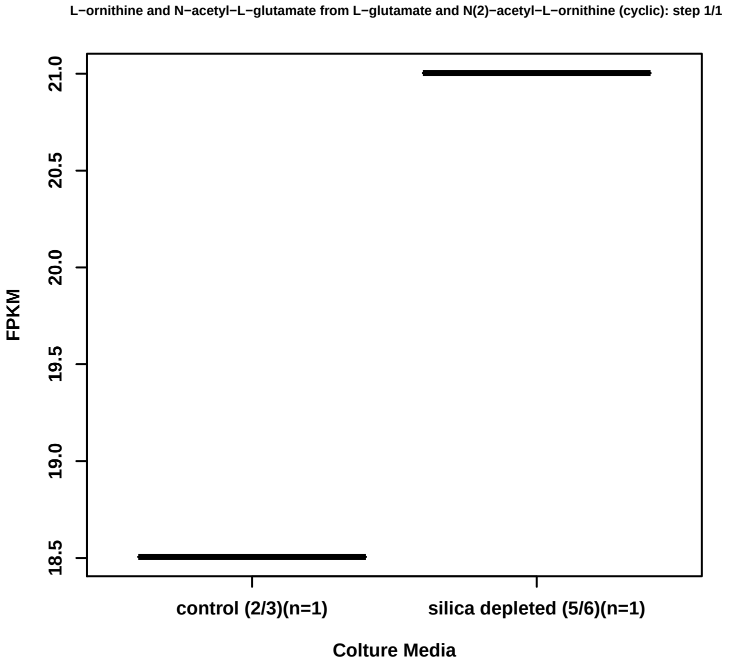
L−ornithine and N−acetyl−L−glutamate from L−glutamate and N(2)−acetyl−L−ornithine (cyclic): step 1/1
FPKM
18.5
19.0
19.5
20.0
20.5
21.0
control (2/3)(n=1)
silica depleted (5/6)(n=1)
Colture Media

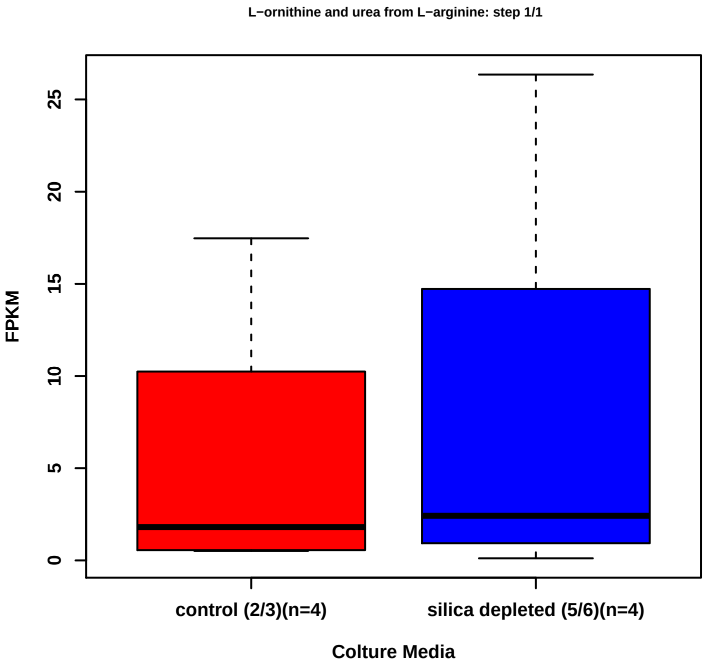
L−ornithine and urea from L−arginine: step 1/1
FPKM
0
5
10
15
20
25
control (2/3)(n=4)
silica depleted (5/6)(n=4)
Colture Media

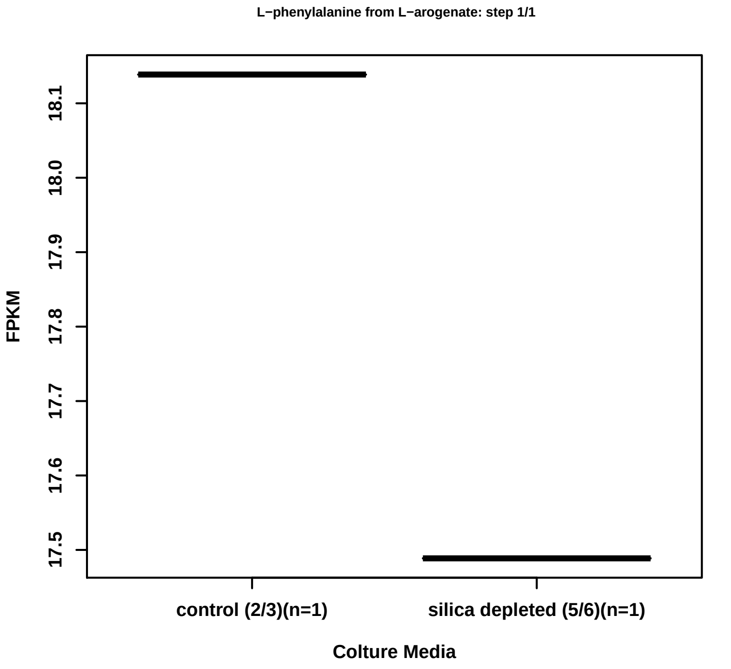
L−phenylalanine from L−arogenate: step 1/1
FPKM
17.5
17.6
17.7
17.8
17.9
18.0
18.1
control (2/3)(n=1)
silica depleted (5/6)(n=1)
Colture Media

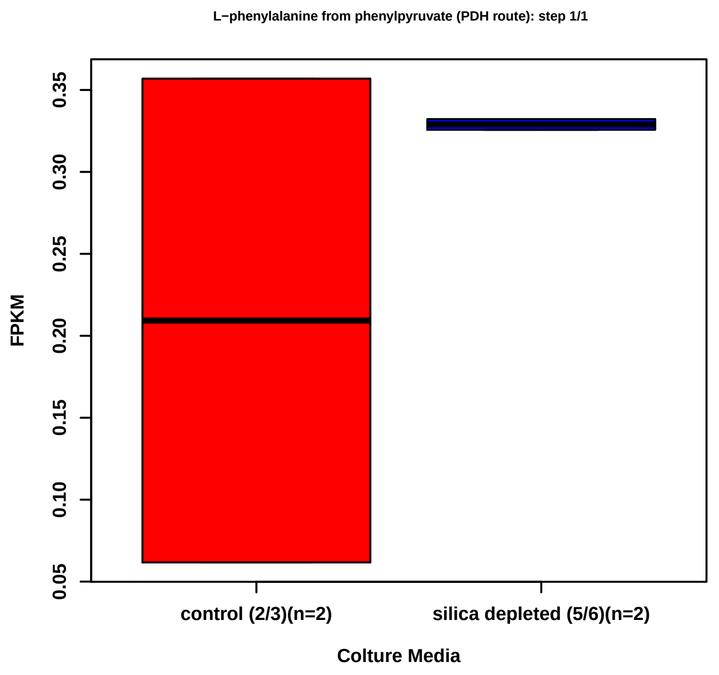

L−phenylalanine from phenylpyruvate (PDH route): step 1/1
FPKM
0.05
0.10
0.15
0.20
0.25
0.30
0.35
control (2/3)(n=2)
silica depleted (5/6)(n=2)
Colture Media

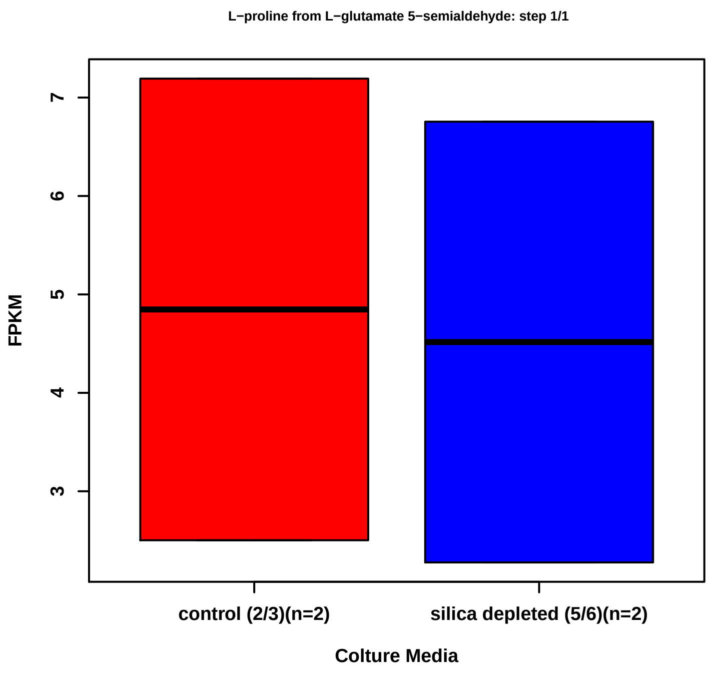

L−proline from L−glutamate 5−semialdehyde: step 1/1
FPKM
3
4
5
6
7
control (2/3)(n=2)
silica depleted (5/6)(n=2)
Colture Media

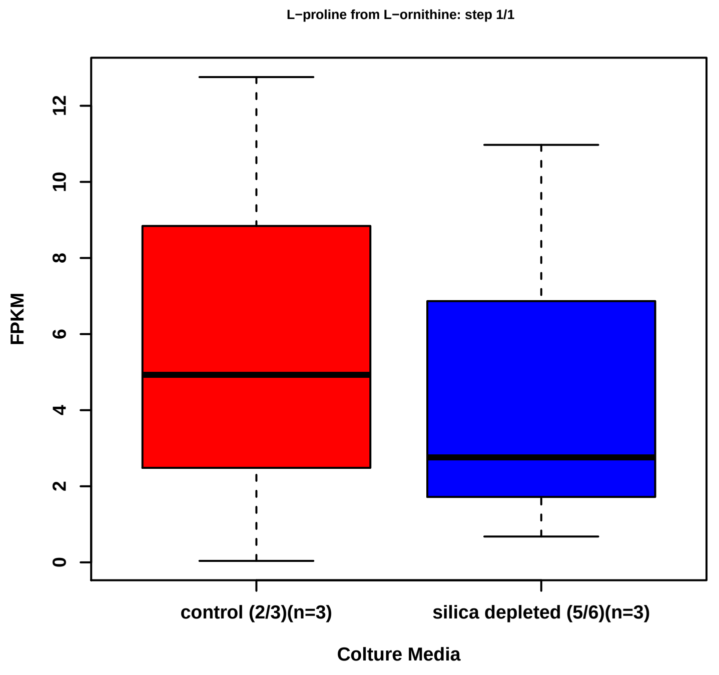

L−proline from L−ornithine: step 1/1
FPKM
0
2
4
6
8
10
12
control (2/3)(n=3)
silica depleted (5/6)(n=3)
Colture Media

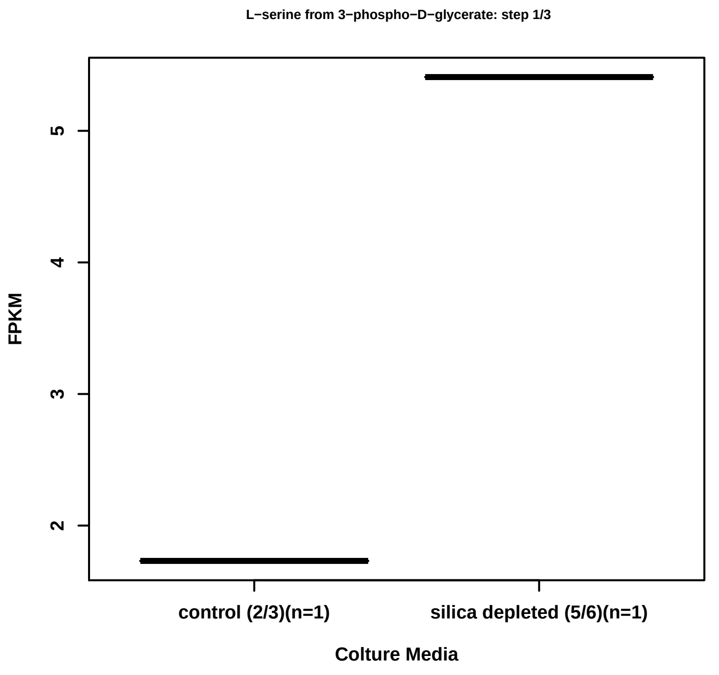

L−serine from 3−phospho−D−glycerate: step 1/3
FPKM
2
3
4
5
control (2/3)(n=1)
silica depleted (5/6)(n=1)
Colture Media

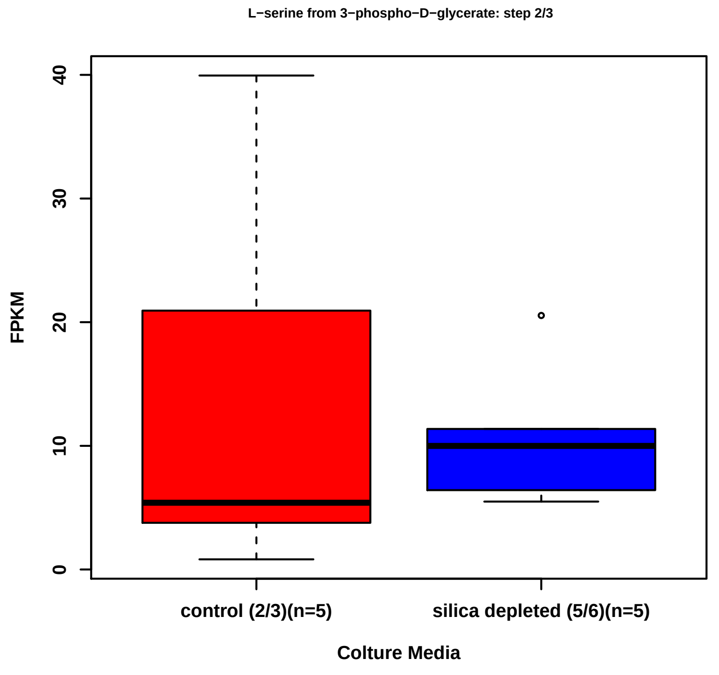

L−serine from 3−phospho−D−glycerate: step 2/3
FPKM
0
10
20
30
40
control (2/3)(n=5)
silica depleted (5/6)(n=5)
Colture Media

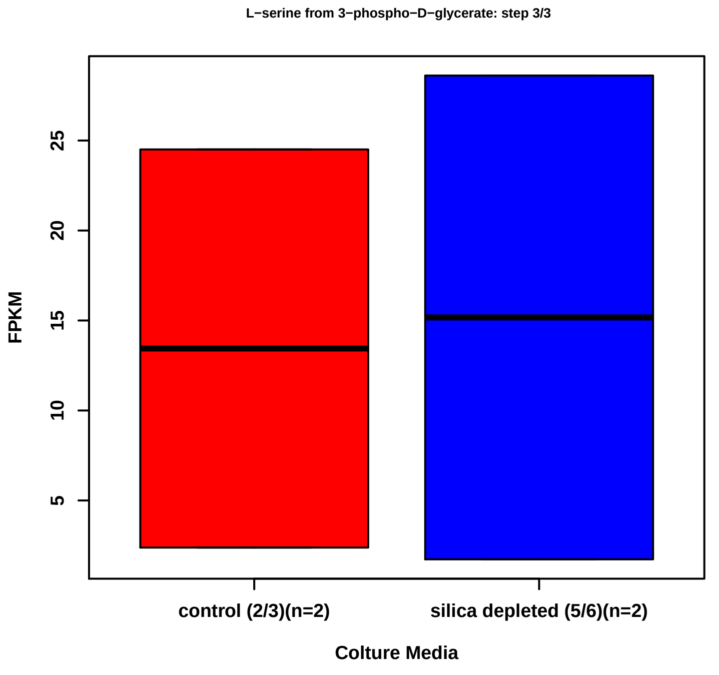

L−serine from 3−phospho−D−glycerate: step 3/3
FPKM
5
10
15
20
25
control (2/3)(n=2)
silica depleted (5/6)(n=2)
Colture Media

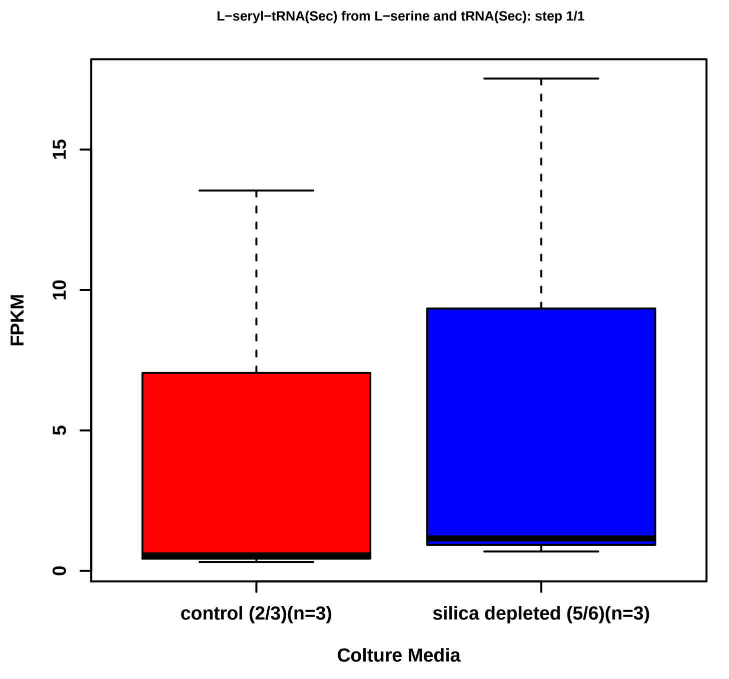
L−seryl−tRNA(Sec) from L−serine and tRNA(Sec): step 1/1
FPKM
0
5
10
15
control (2/3)(n=3)
silica depleted (5/6)(n=3)
Colture Media

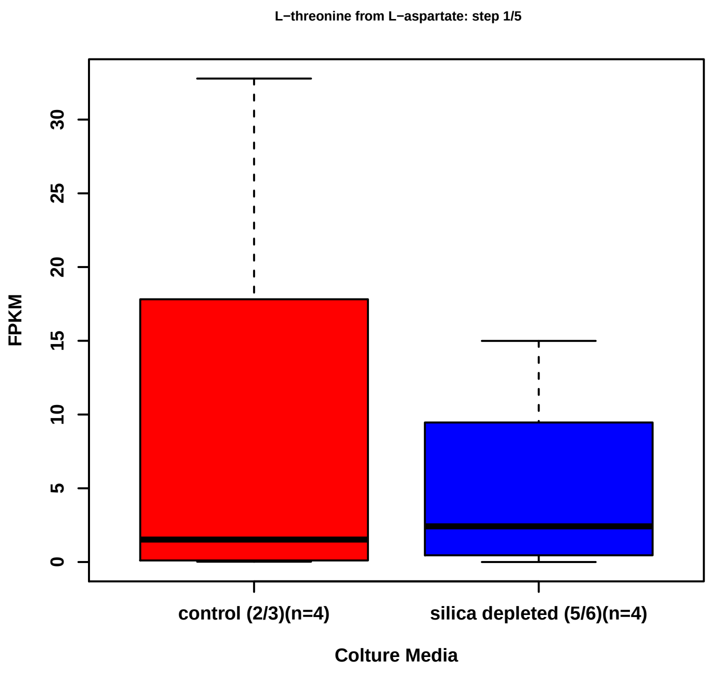
L−threonine from L−aspartate: step 1/5
FPKM
0
5
10
15
20
25
30
control (2/3)(n=4)
silica depleted (5/6)(n=4)
Colture Media

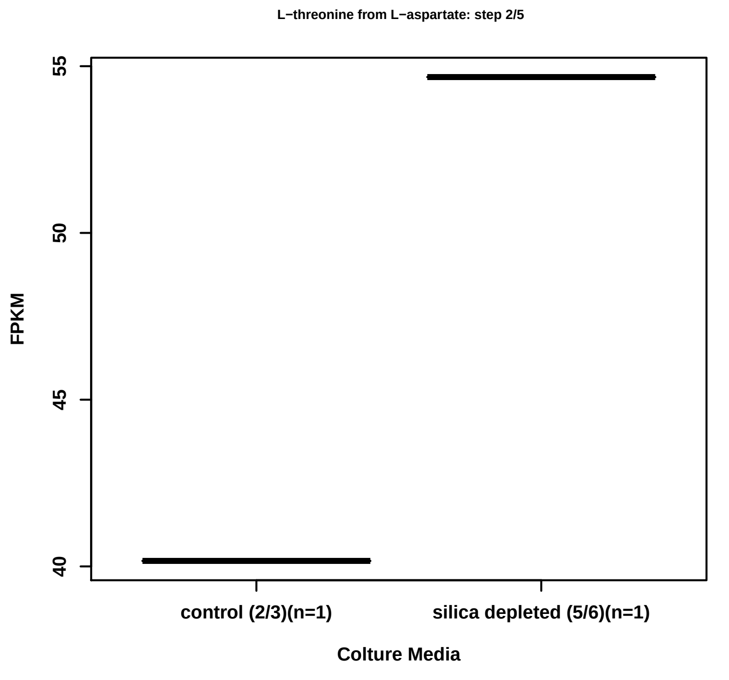
L−threonine from L−aspartate: step 2/5
FPKM
40
45
50
55
control (2/3)(n=1)
silica depleted (5/6)(n=1)
Colture Media

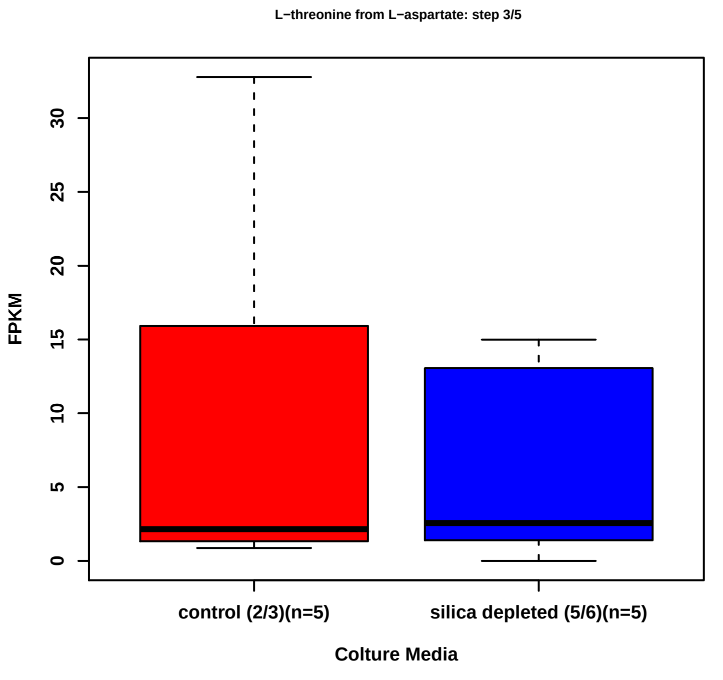
L−threonine from L−aspartate: step 3/5
FPKM
0
5
10
15
20
25
30
control (2/3)(n=5)
silica depleted (5/6)(n=5)
Colture Media

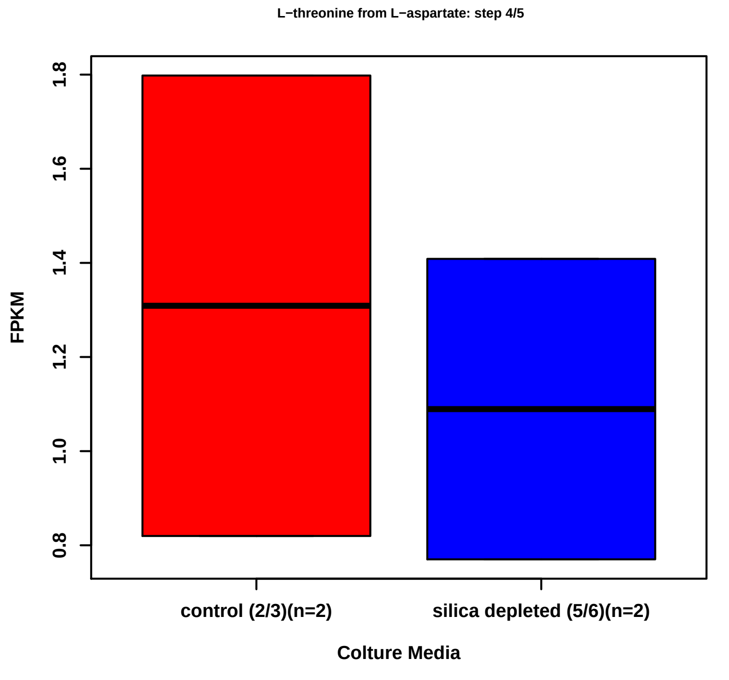
L−threonine from L−aspartate: step 4/5
FPKM
0.8
1.0
1.2
1.4
1.6
1.8
control (2/3)(n=2)
silica depleted (5/6)(n=2)
Colture Media

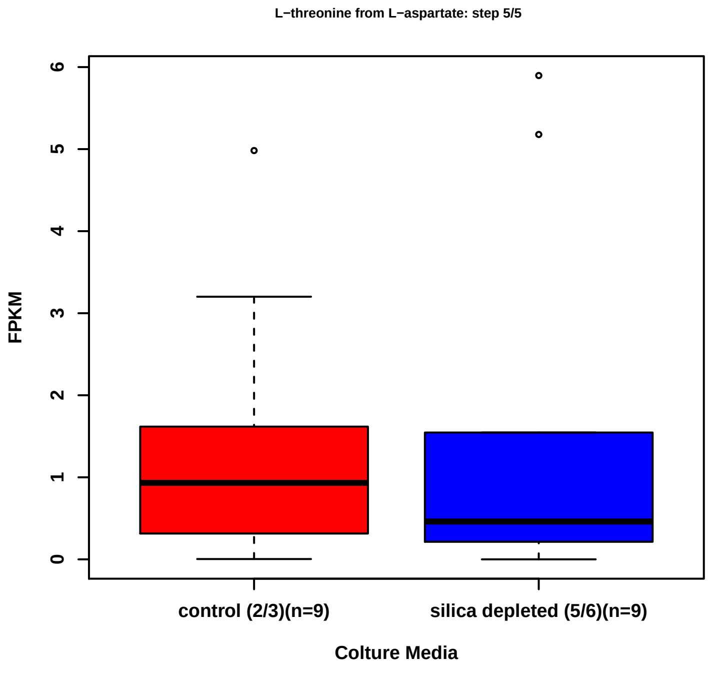
L−threonine from L−aspartate: step 5/5
FPKM
0
1
2
3
4
5
6
control (2/3)(n=9)
silica depleted (5/6)(n=9)
Colture Media

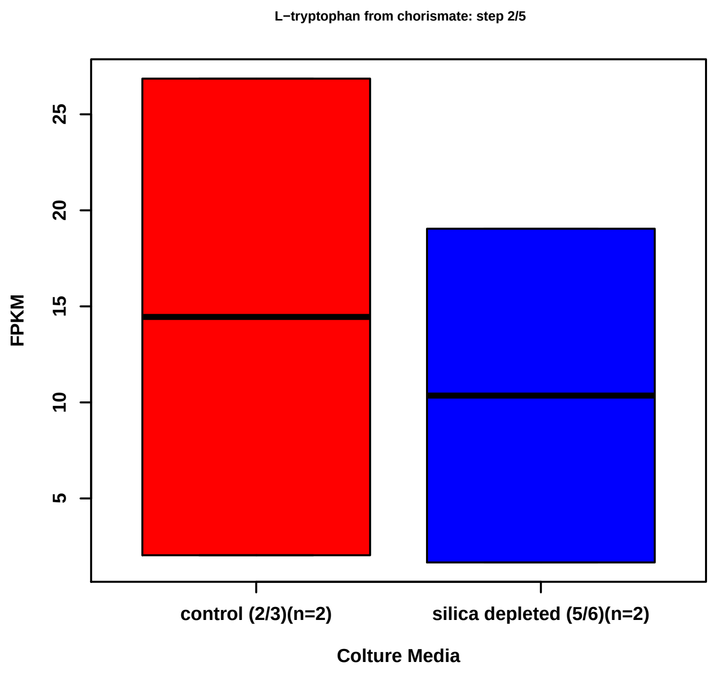
L−tryptophan from chorismate: step 2/5
FPKM
5
10
15
20
25
control (2/3)(n=2)
silica depleted (5/6)(n=2)
Colture Media

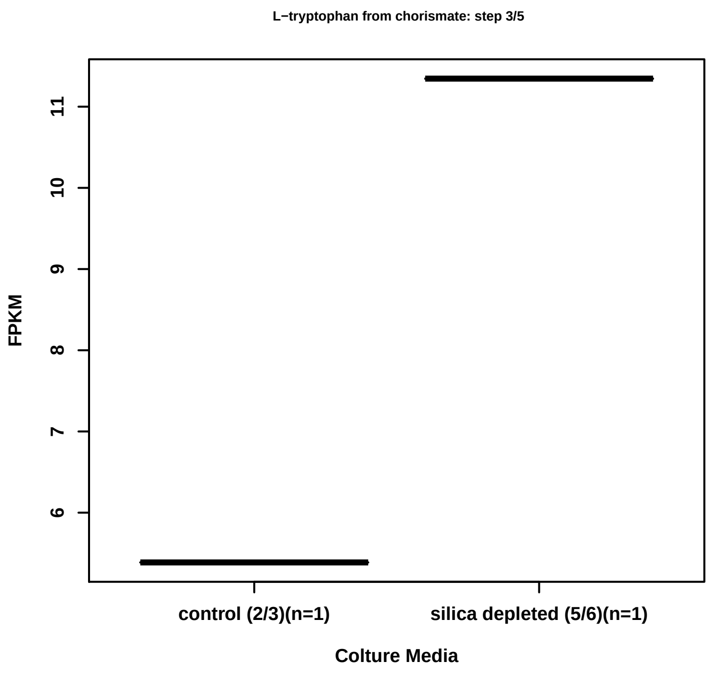
L−tryptophan from chorismate: step 3/5
FPKM
6
7
8
9
10
11
control (2/3)(n=1)
silica depleted (5/6)(n=1)
Colture Media

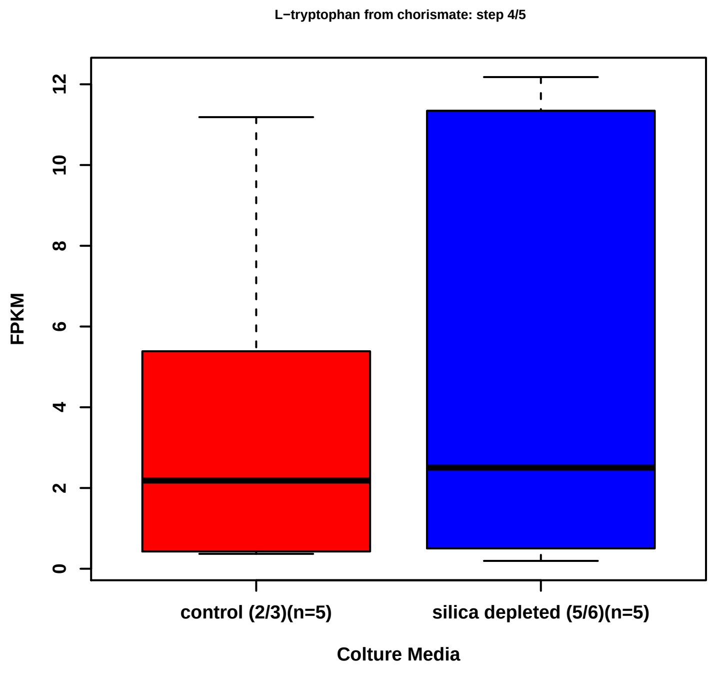
L−tryptophan from chorismate: step 4/5
FPKM
0
2
4
6
8
10
12
control (2/3)(n=5)
silica depleted (5/6)(n=5)
Colture Media

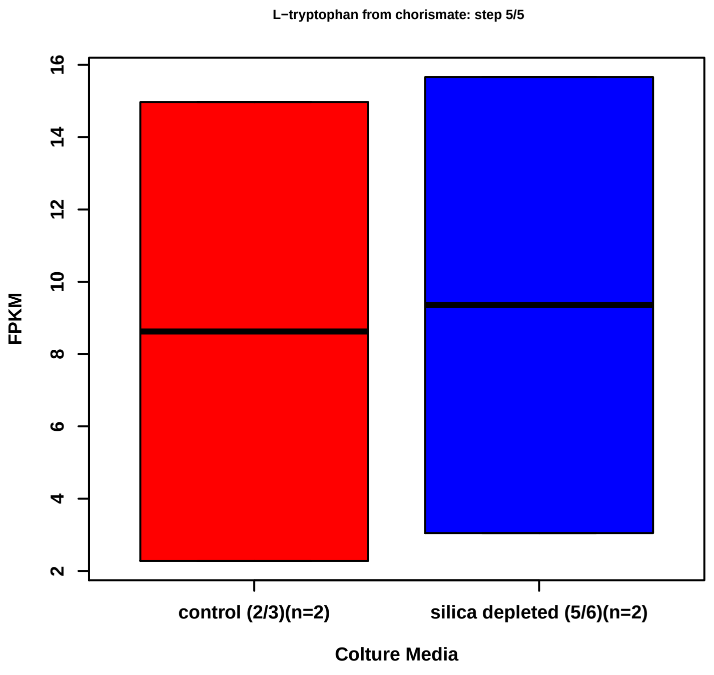
L−tryptophan from chorismate: step 5/5
FPKM
2
4
6
8
10
12
14
16
control (2/3)(n=2)
silica depleted (5/6)(n=2)
Colture Media

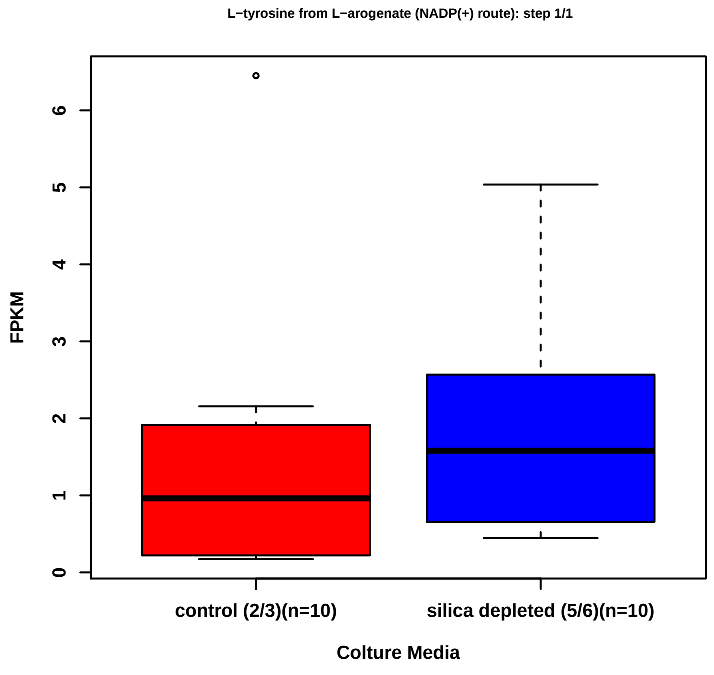
L−tyrosine from L−arogenate (NADP(+) route): step 1/1
FPKM
0
1
2
3
4
5
6
control (2/3)(n=10)
silica depleted (5/6)(n=10)
Colture Media

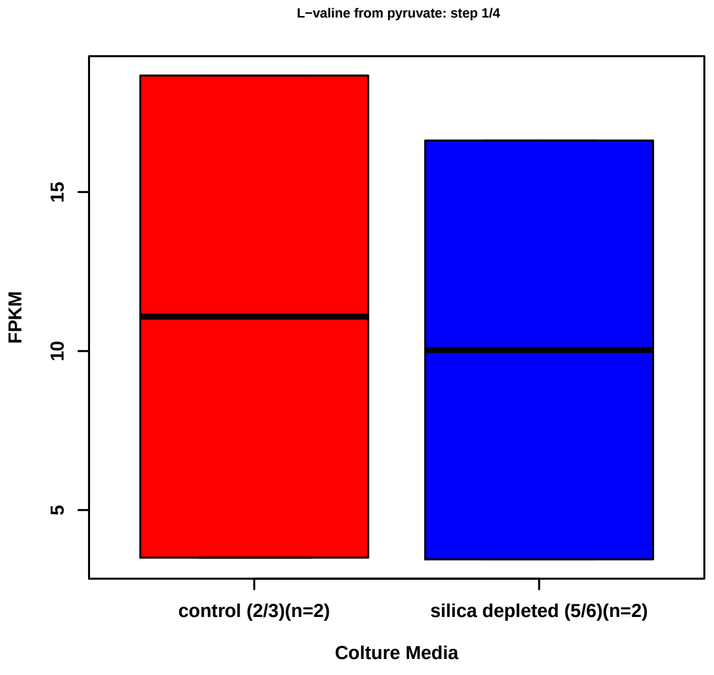
L−valine from pyruvate: step 1/4
FPKM
5
10
15
control (2/3)(n=2)
silica depleted (5/6)(n=2)
Colture Media

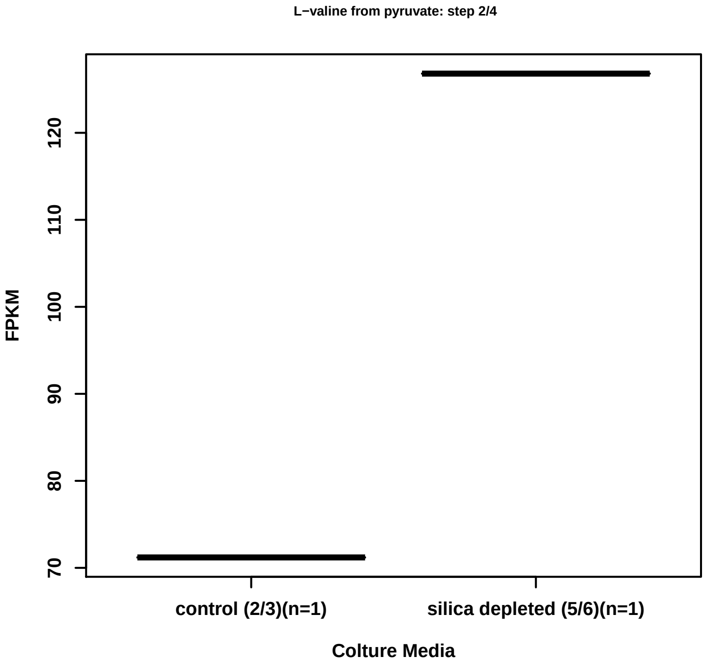
L−valine from pyruvate: step 2/4
FPKM
70
80
90
100
110
120
control (2/3)(n=1)
silica depleted (5/6)(n=1)
Colture Media

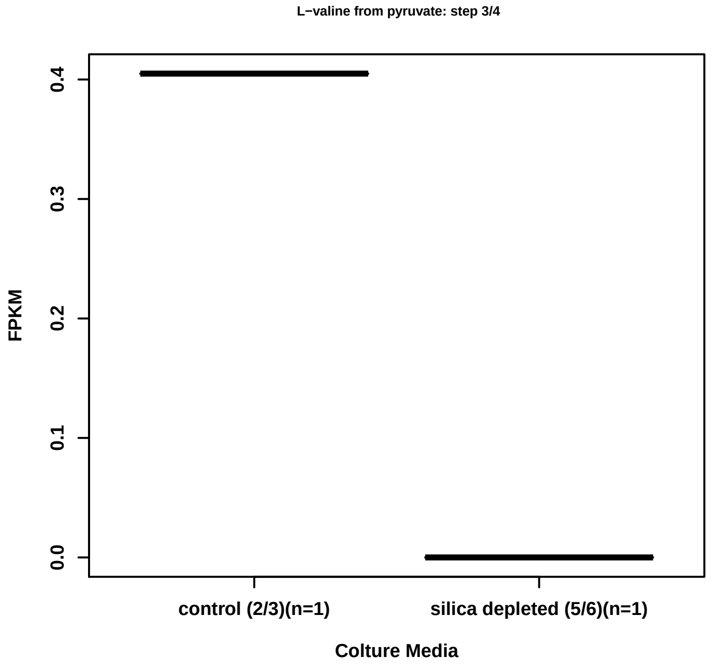
L−valine from pyruvate: step 3/4
FPKM
0.0
0.1
0.2
0.3
0.4
control (2/3)(n=1)
silica depleted (5/6)(n=1)
Colture Media

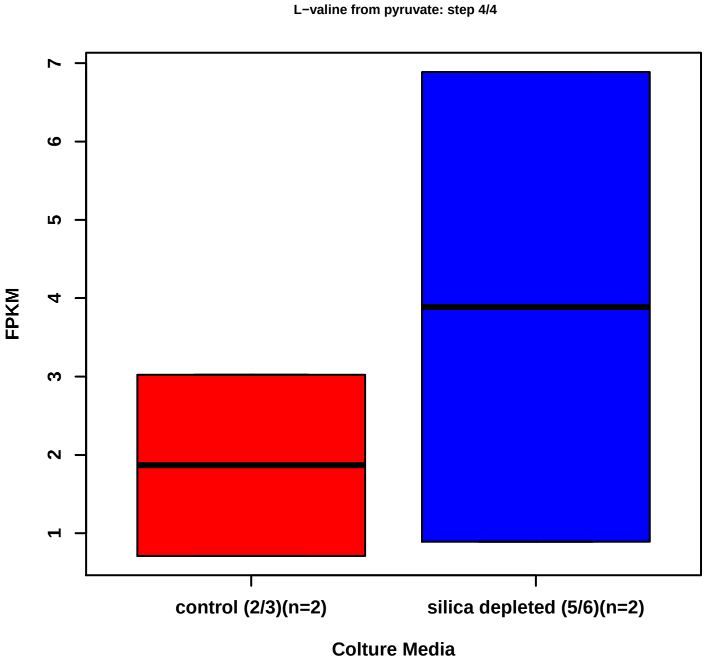
L−valine from pyruvate: step 4/4
FPKM
1
2
3
4
5
6
7
control (2/3)(n=2)
silica depleted (5/6)(n=2)
Colture Media

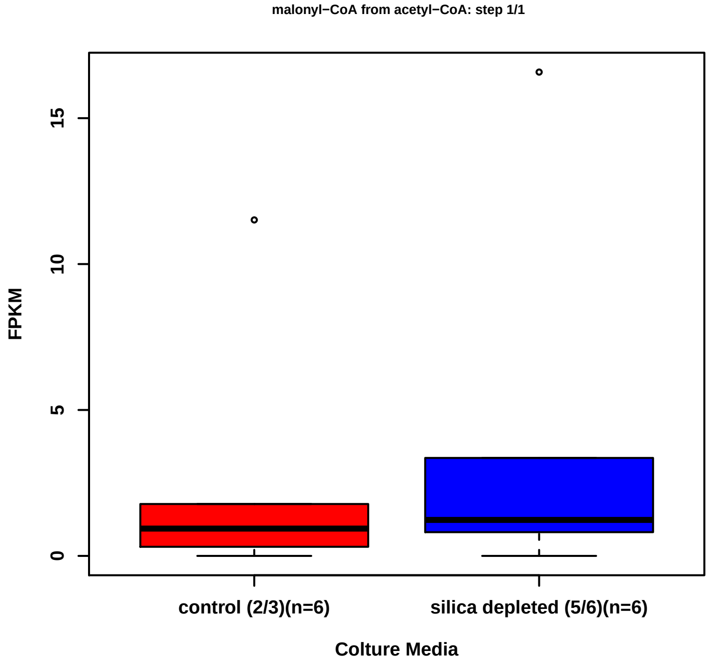
malonyl−CoA from acetyl−CoA: step 1/1
FPKM
0
5
10
15
control (2/3)(n=6)
silica depleted (5/6)(n=6)
Colture Media

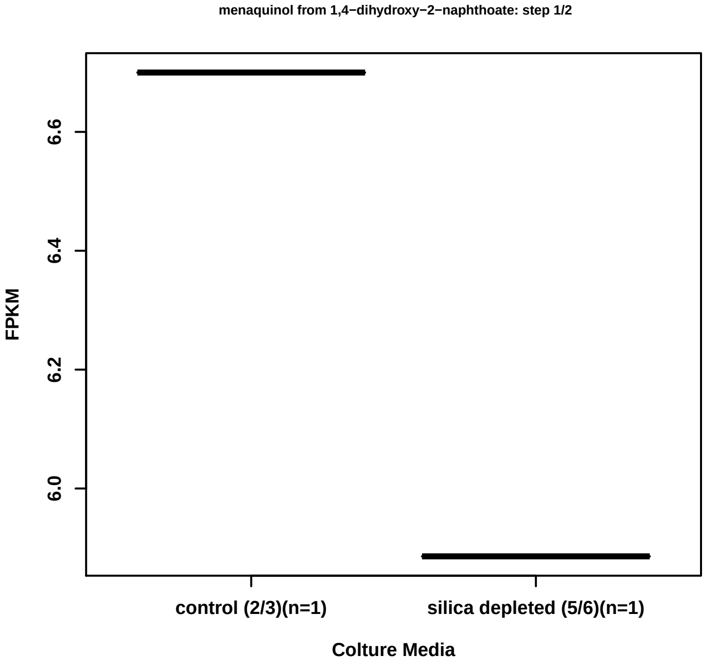
menaquinol from 1,4−dihydroxy−2−naphthoate: step 1/2
FPKM
6.0
6.2
6.4
6.6
control (2/3)(n=1)
silica depleted (5/6)(n=1)
Colture Media

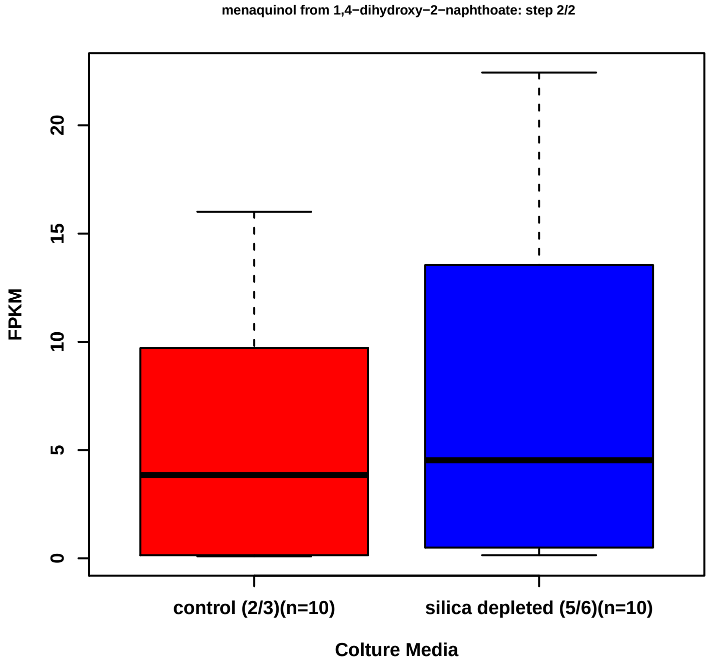
menaquinol from 1,4−dihydroxy−2−naphthoate: step 2/2
FPKM
0
5
10
15
20
control (2/3)(n=10)
silica depleted (5/6)(n=10)
Colture Media

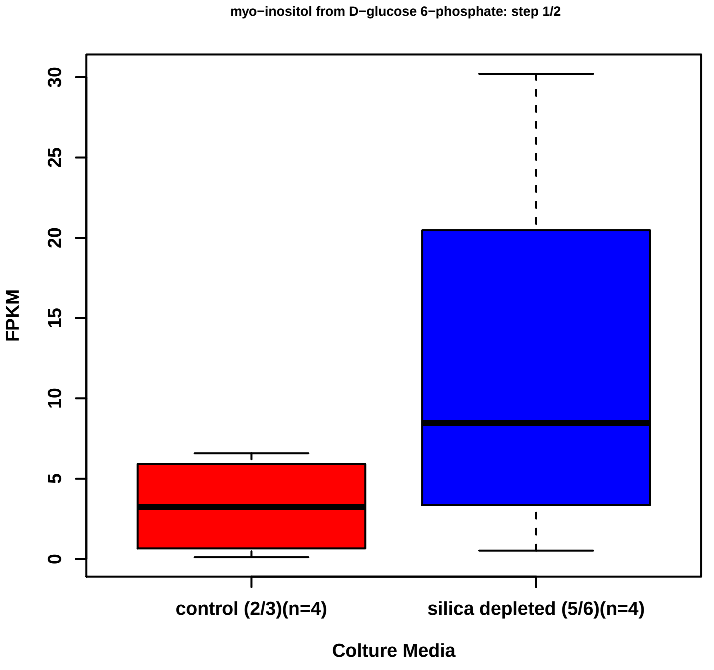
myo−inositol from D−glucose 6−phosphate: step 1/2
FPKM
0
5
10
15
20
25
30
control (2/3)(n=4)
silica depleted (5/6)(n=4)
Colture Media

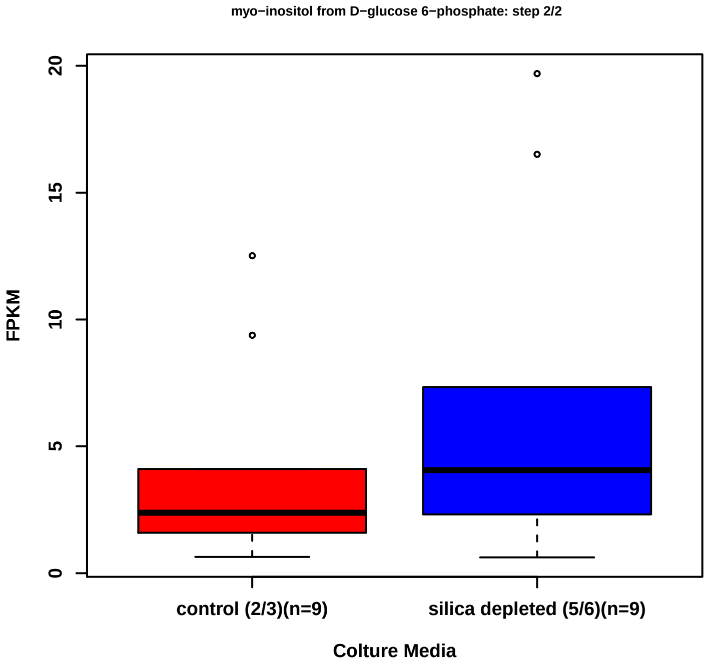
myo−inositol from D−glucose 6−phosphate: step 2/2
FPKM
0
5
10
15
20
control (2/3)(n=9)
silica depleted (5/6)(n=9)
Colture Media

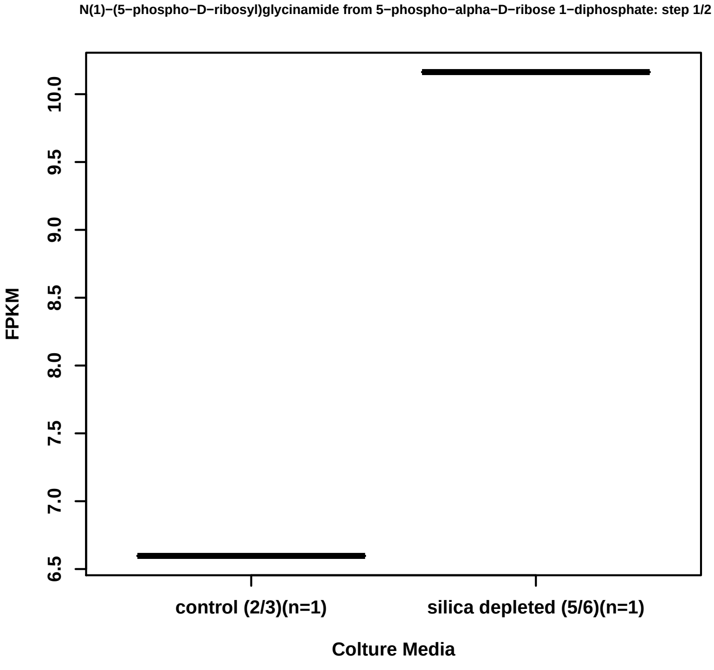
N(1)−(5−phospho−D−ribosyl)glycinamide from 5−phospho−alpha−D−ribose 1−diphosphate: step 1/2
FPKM
6.5
7.0
7.5
8.0
8.5
9.0
9.5
10.0
control (2/3)(n=1)
silica depleted (5/6)(n=1)
Colture Media

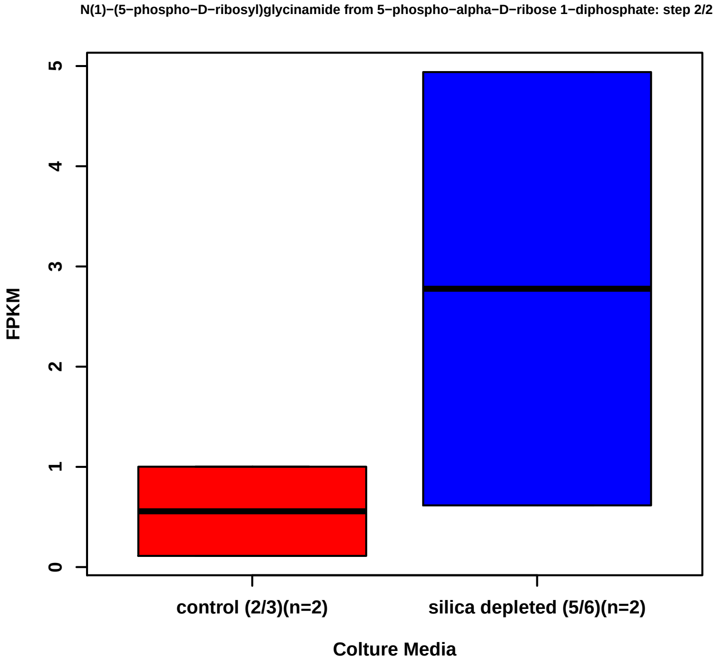
N(1)−(5−phospho−D−ribosyl)glycinamide from 5−phospho−alpha−D−ribose 1−diphosphate: step 2/2
FPKM
0
1
2
3
4
5
control (2/3)(n=2)
silica depleted (5/6)(n=2)
Colture Media

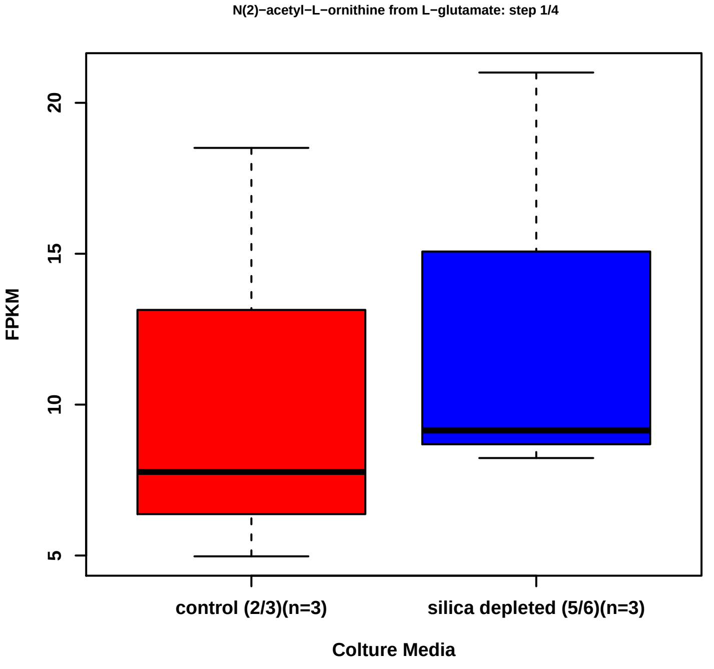
N(2)−acetyl−L−ornithine from L−glutamate: step 1/4
FPKM
5
10
15
20
control (2/3)(n=3)
silica depleted (5/6)(n=3)
Colture Media

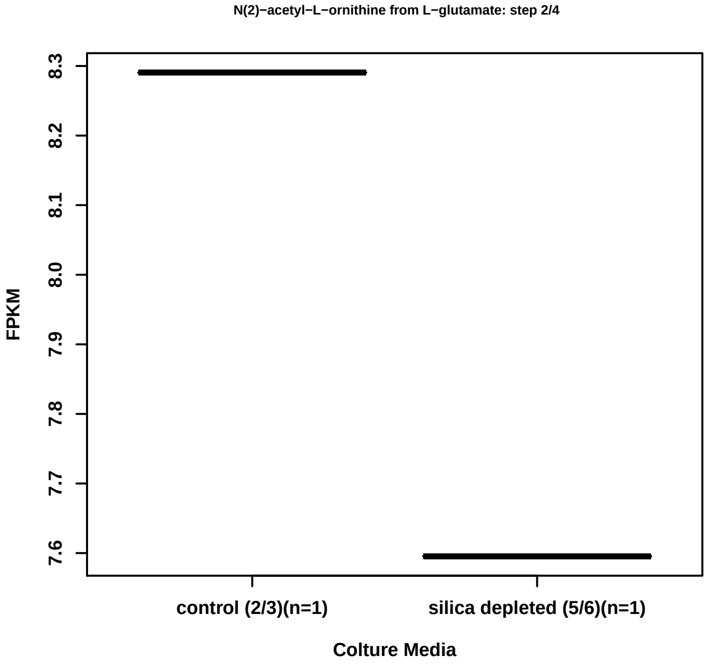
N(2)−acetyl−L−ornithine from L−glutamate: step 2/4
FPKM
7.6
7.7
7.8
7.9
8.0
8.1
8.2
8.3
control (2/3)(n=1)
silica depleted (5/6)(n=1)
Colture Media

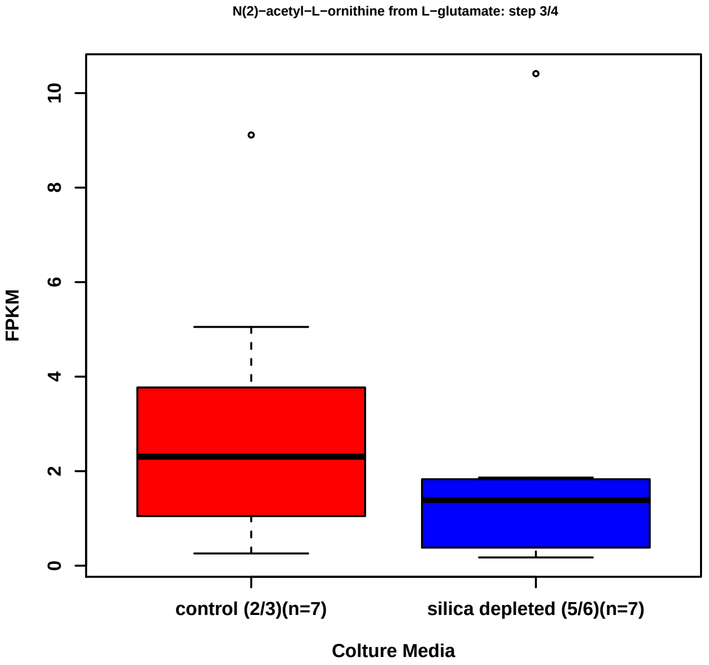

N(2)−acetyl−L−ornithine from L−glutamate: step 3/4
FPKM
0
2
4
6
8
10
control (2/3)(n=7)
silica depleted (5/6)(n=7)
Colture Media

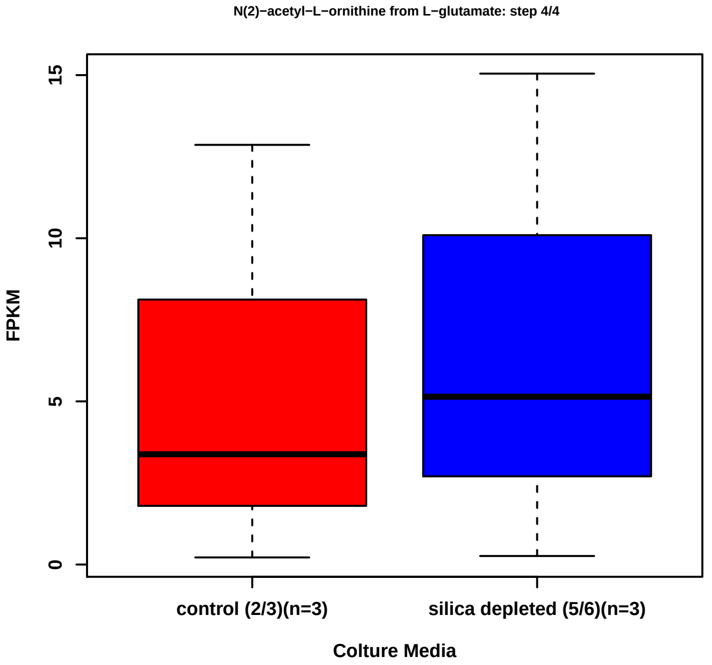

N(2)−acetyl−L−ornithine from L−glutamate: step 4/4
FPKM
0
5
10
15
control (2/3)(n=3)
silica depleted (5/6)(n=3)
Colture Media

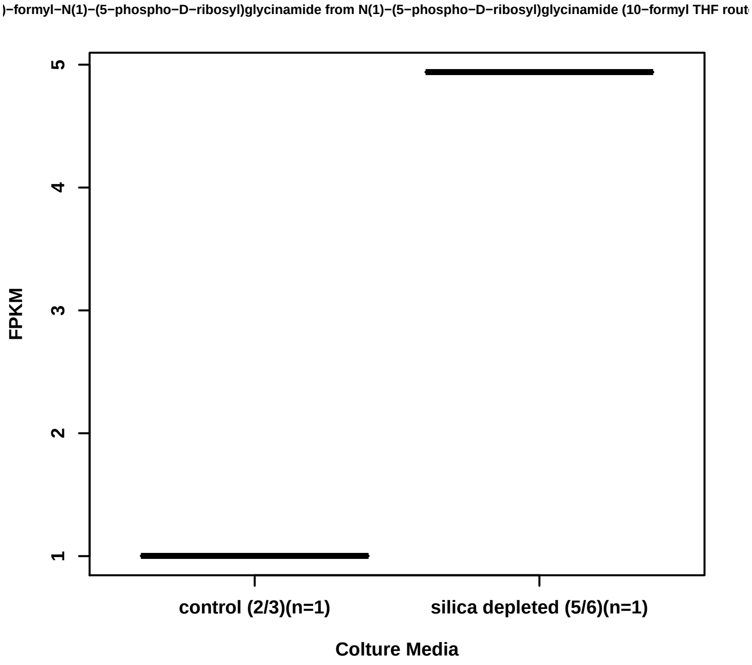

)−formyl−N(1)−(5−phospho−D−ribosyl)glycinamide from N(1)−(5−phospho−D−ribosyl)glycinamide (10−formyl THF rout
FPKM
1
2
3
4
5
control (2/3)(n=1)
silica depleted (5/6)(n=1)
Colture Media

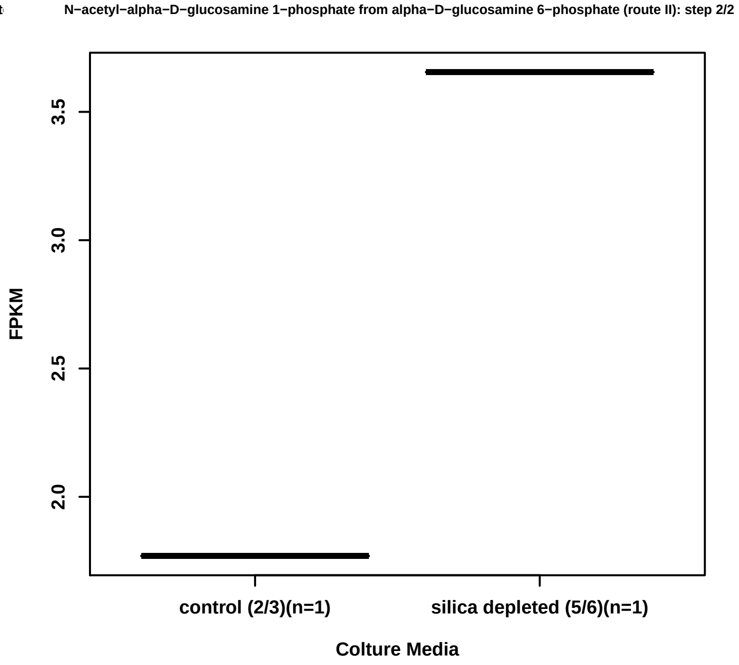

N−acetyl−alpha−D−glucosamine 1−phosphate from alpha−D−glucosamine 6−phosphate (route II): step 2/2
FPKM
2.0
2.5
3.0
3.5
control (2/3)(n=1)
silica depleted (5/6)(n=1)
Colture Media

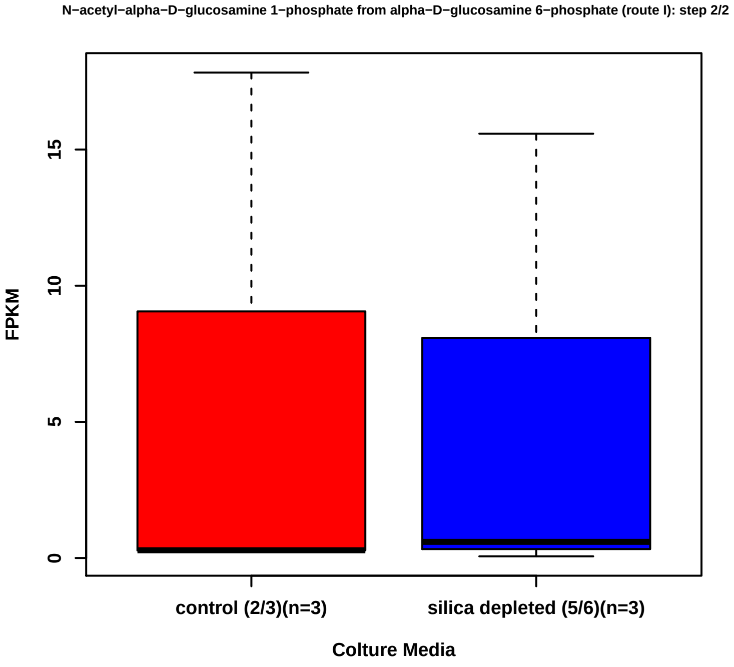

N−acetyl−alpha−D−glucosamine 1−phosphate from alpha−D−glucosamine 6−phosphate (route I): step 2/2
FPKM
0
5
10
15
control (2/3)(n=3)
silica depleted (5/6)(n=3)
Colture Media

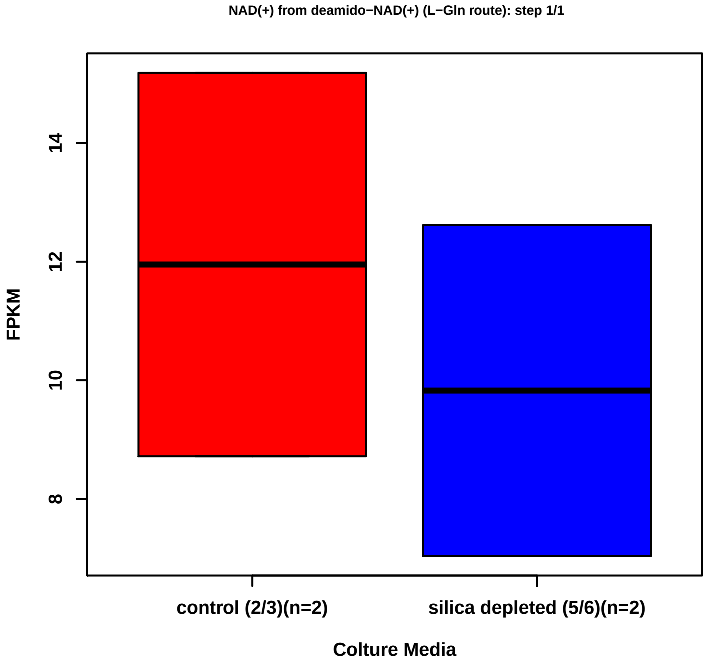

NAD(+) from deamido−NAD(+) (L−Gln route): step 1/1
FPKM
8
10
12
14
control (2/3)(n=2)
silica depleted (5/6)(n=2)
Colture Media

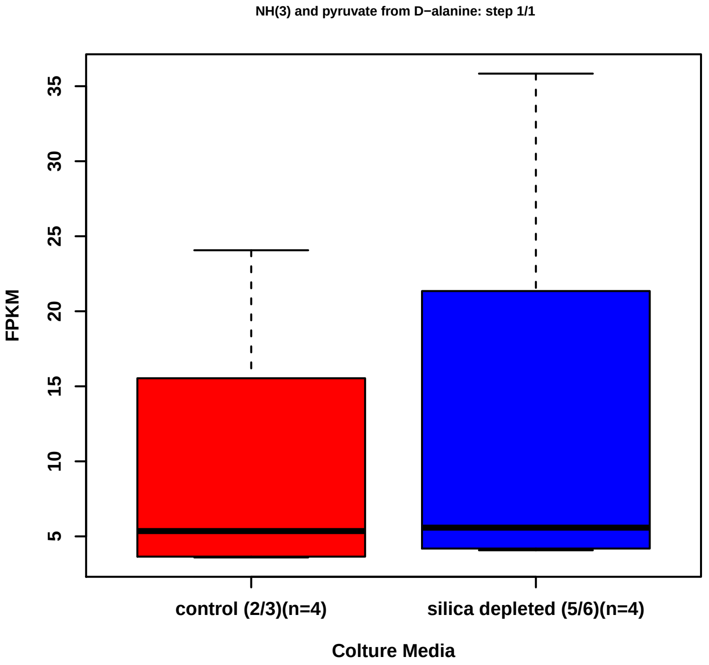
NH(3) and pyruvate from D−alanine: step 1/1
FPKM
5
10
15
20
25
30
35
control (2/3)(n=4)
silica depleted (5/6)(n=4)
Colture Media

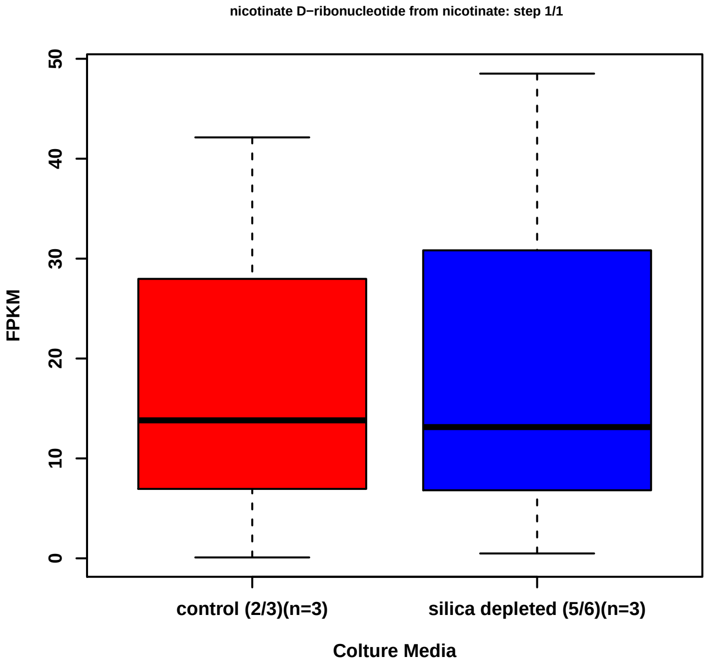
nicotinate D−ribonucleotide from nicotinate: step 1/1
FPKM
0
10
20
30
40
50
control (2/3)(n=3)
silica depleted (5/6)(n=3)
Colture Media

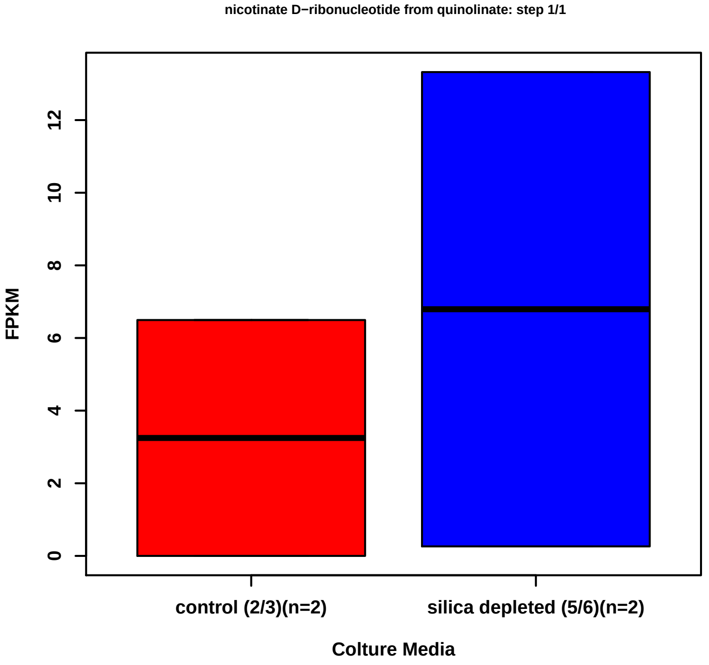
nicotinate D−ribonucleotide from quinolinate: step 1/1
FPKM
0
2
4
6
8
10
12
control (2/3)(n=2)
silica depleted (5/6)(n=2)
Colture Media

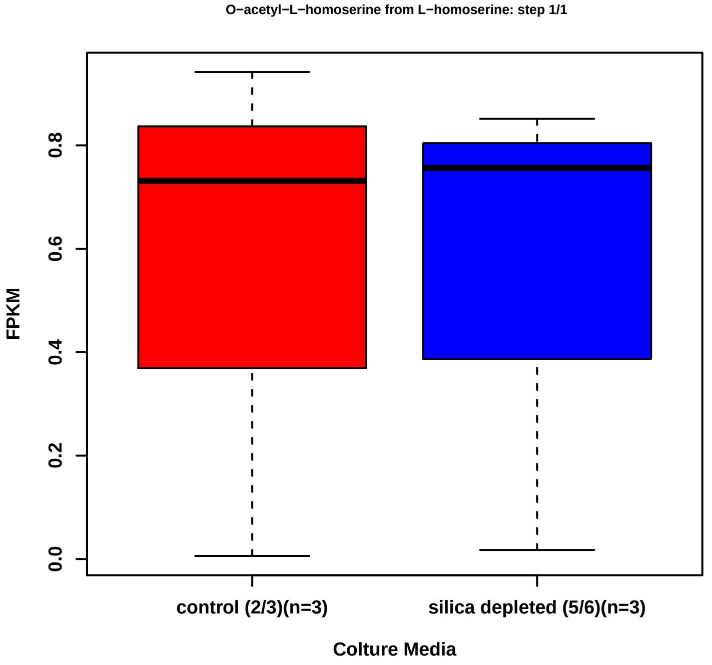
O−acetyl−L−homoserine from L−homoserine: step 1/1
FPKM
0.0
0.2
0.4
0.6
0.8
control (2/3)(n=3)
silica depleted (5/6)(n=3)
Colture Media

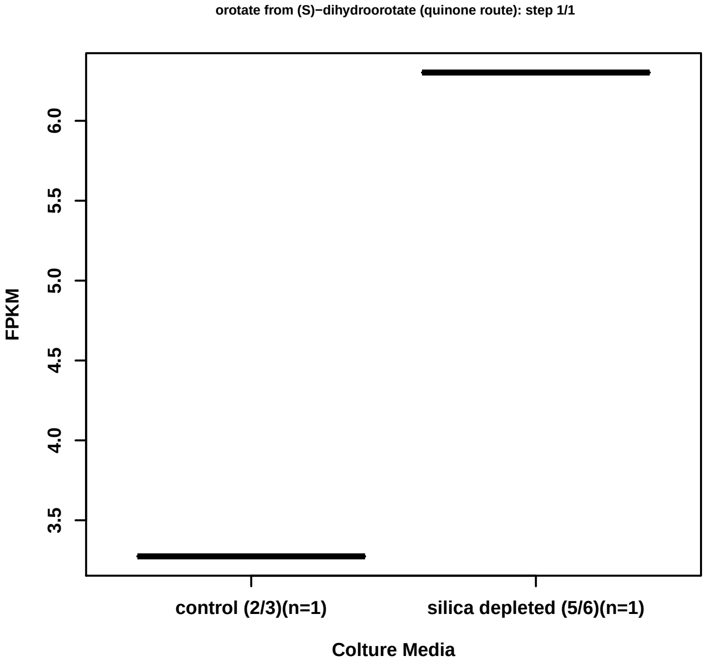
orotate from (S)−dihydroorotate (quinone route): step 1/1
FPKM
3.5
4.0
4.5
5.0
5.5
6.0
control (2/3)(n=1)
silica depleted (5/6)(n=1)
Colture Media

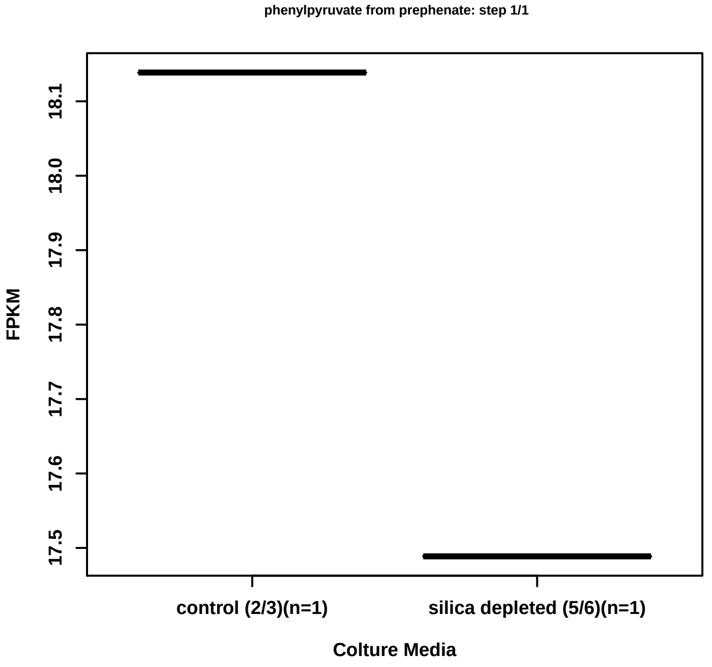
phenylpyruvate from prephenate: step 1/1
FPKM
17.5
17.6
17.7
17.8
17.9
18.0
18.1
control (2/3)(n=1)
silica depleted (5/6)(n=1)
Colture Media

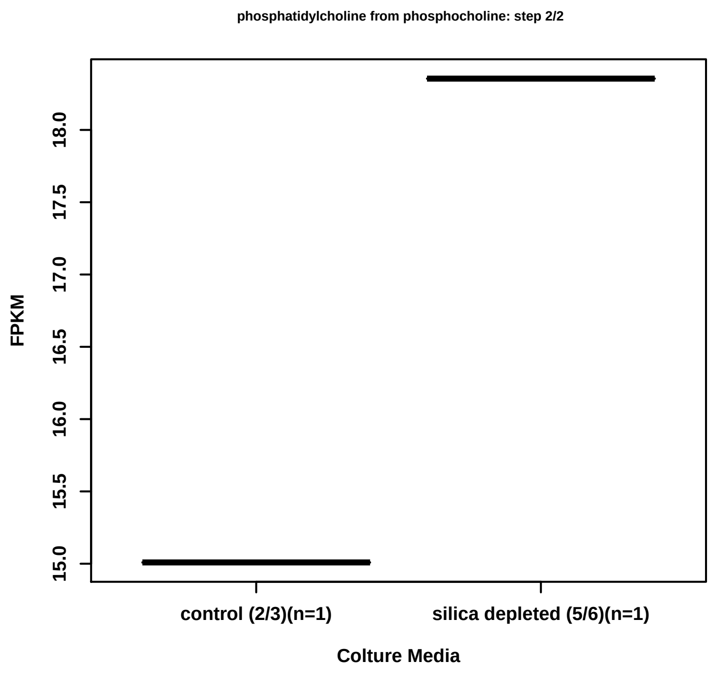
phosphatidylcholine from phosphocholine: step 2/2
FPKM
15.0
15.5
16.0
16.5
17.0
17.5
18.0
control (2/3)(n=1)
silica depleted (5/6)(n=1)
Colture Media

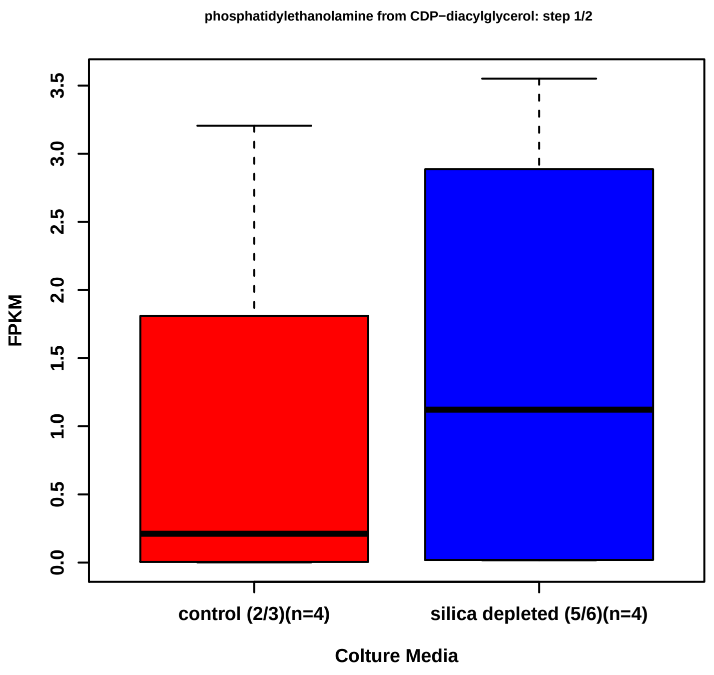
phosphatidylethanolamine from CDP−diacylglycerol: step 1/2
FPKM
0.0
0.5
1.0
1.5
2.0
2.5
3.0
3.5
control (2/3)(n=4)
silica depleted (5/6)(n=4)
Colture Media

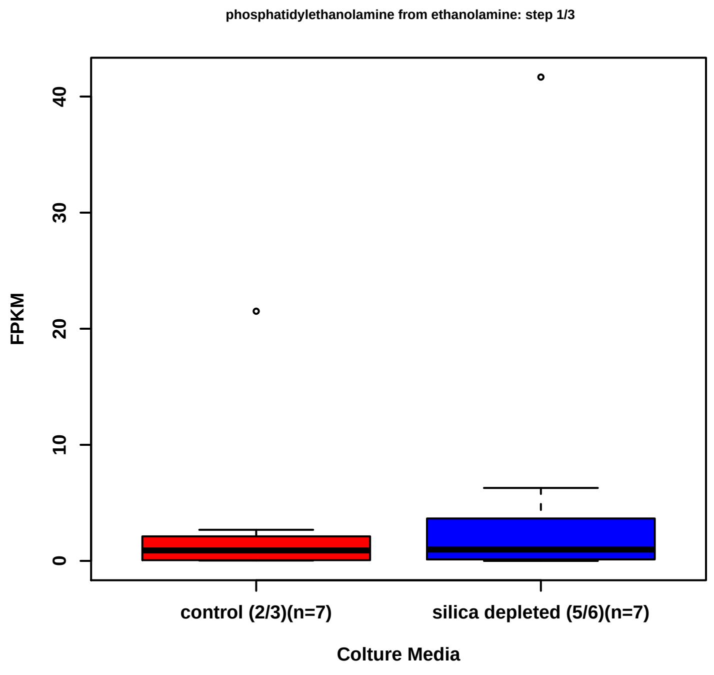
phosphatidylethanolamine from ethanolamine: step 1/3
FPKM
0
10
20
30
40
control (2/3)(n=7)
silica depleted (5/6)(n=7)
Colture Media

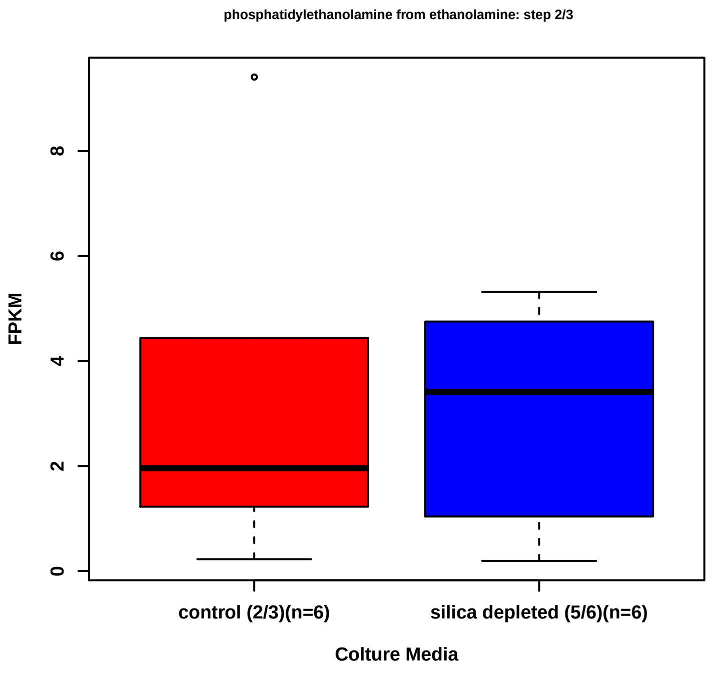
phosphatidylethanolamine from ethanolamine: step 2/3
FPKM
0
2
4
6
8
control (2/3)(n=6)
silica depleted (5/6)(n=6)
Colture Media

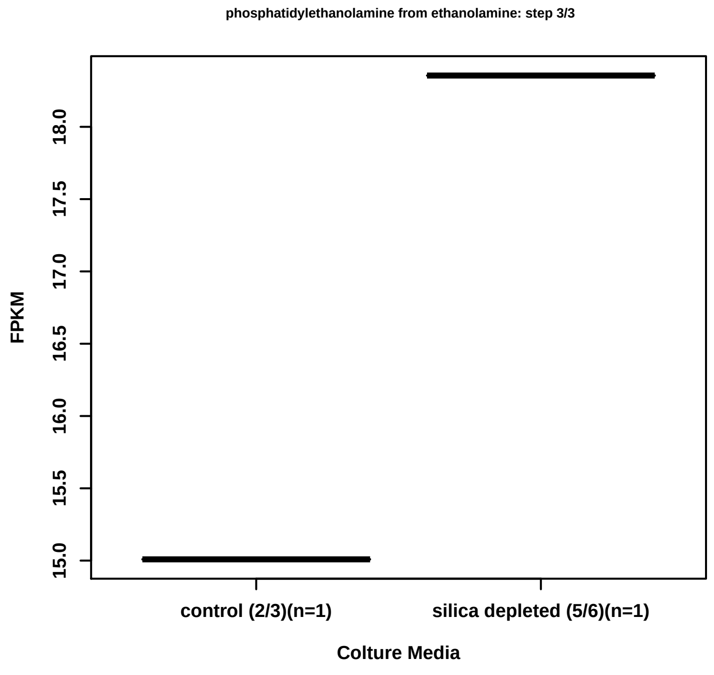
phosphatidylethanolamine from ethanolamine: step 3/3
FPKM
15.0
15.5
16.0
16.5
17.0
17.5
18.0
control (2/3)(n=1)
silica depleted (5/6)(n=1)
Colture Media

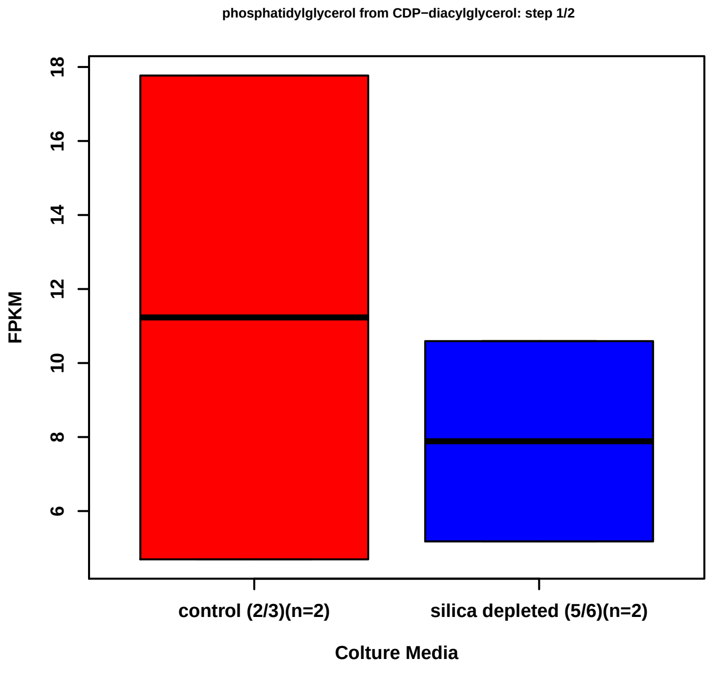
phosphatidylglycerol from CDP−diacylglycerol: step 1/2
FPKM
6
8
10
12
14
16
18
control (2/3)(n=2)
silica depleted (5/6)(n=2)
Colture Media

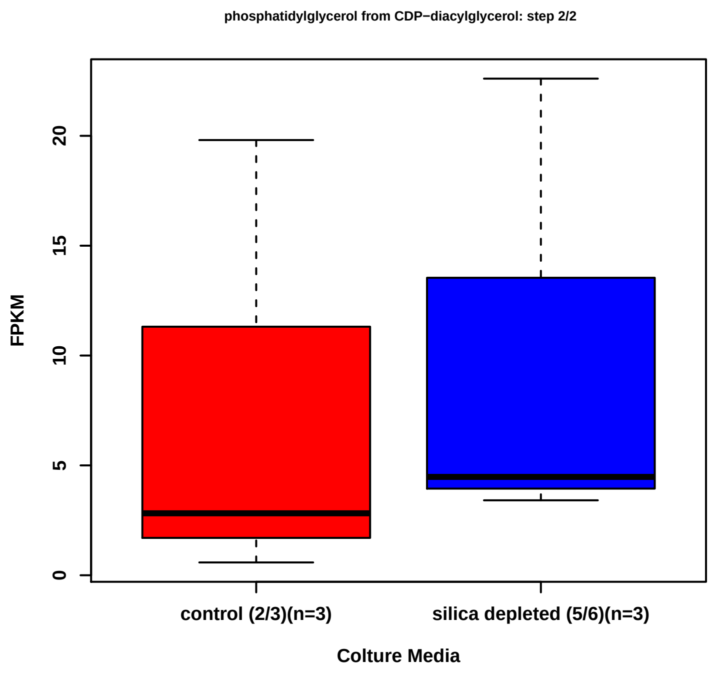
phosphatidylglycerol from CDP−diacylglycerol: step 2/2
FPKM
0
5
10
15
20
control (2/3)(n=3)
silica depleted (5/6)(n=3)
Colture Media

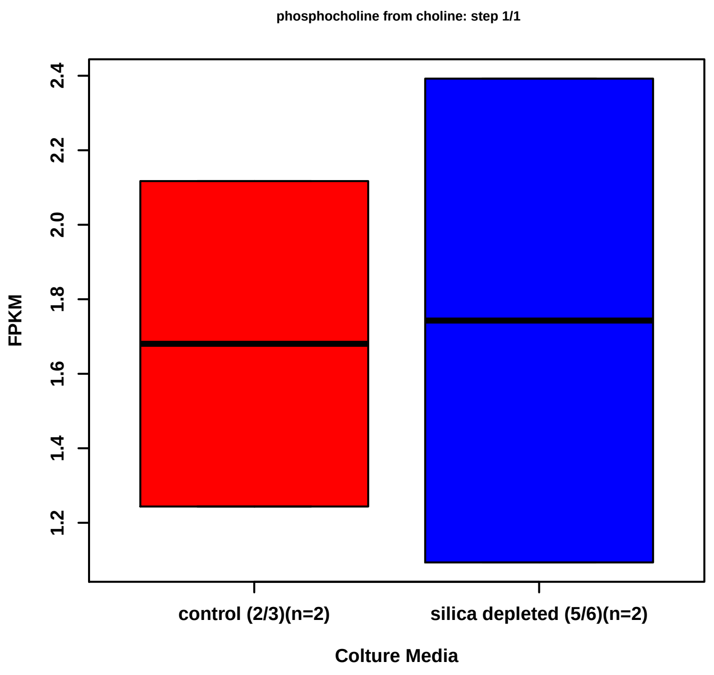
phosphocholine from choline: step 1/1
FPKM
1.2
1.4
1.6
1.8
2.0
2.2
2.4
control (2/3)(n=2)
silica depleted (5/6)(n=2)
Colture Media

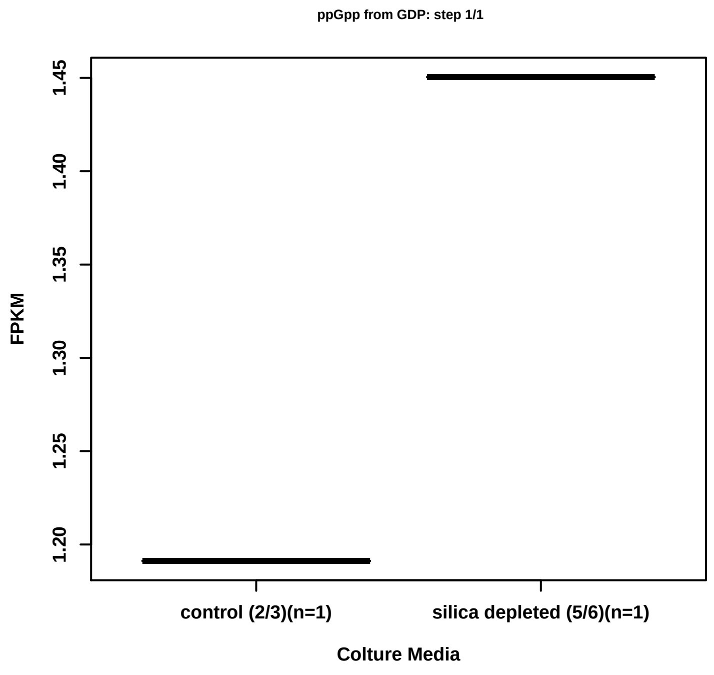
ppGpp from GDP: step 1/1
FPKM
1.20
1.25
1.30
1.35
1.40
1.45
control (2/3)(n=1)
silica depleted (5/6)(n=1)
Colture Media

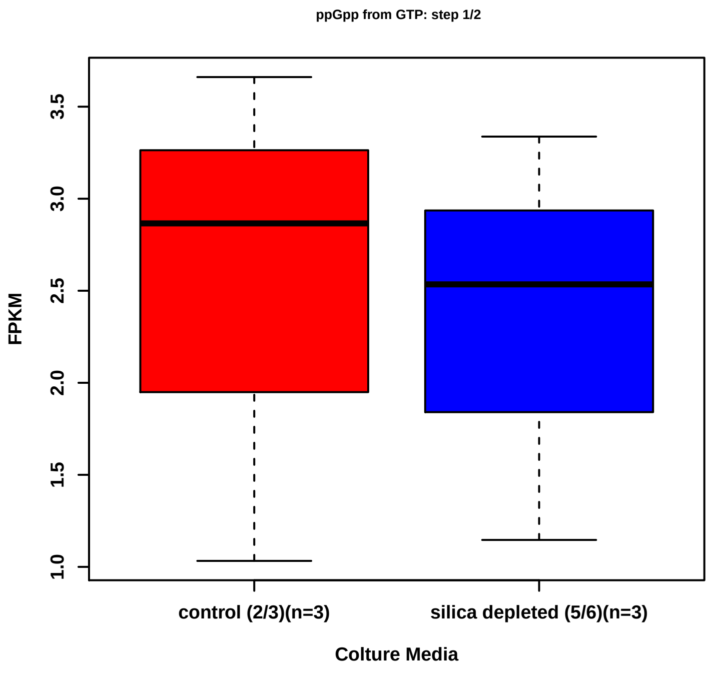
ppGpp from GTP: step 1/2
FPKM
1.0
1.5
2.0
2.5
3.0
3.5
control (2/3)(n=3)
silica depleted (5/6)(n=3)
Colture Media

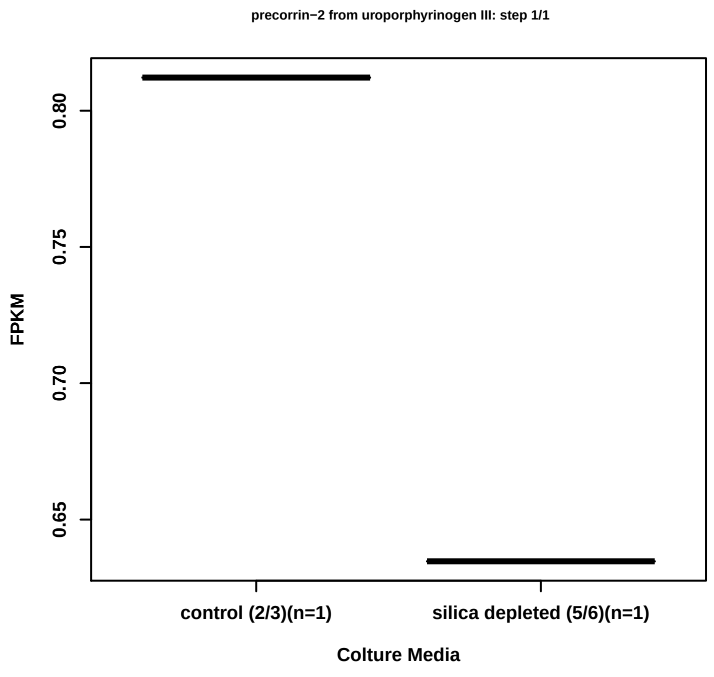
precorrin−2 from uroporphyrinogen III: step 1/1
FPKM
0.65
0.70
0.75
0.80
control (2/3)(n=1)
silica depleted (5/6)(n=1)
Colture Media

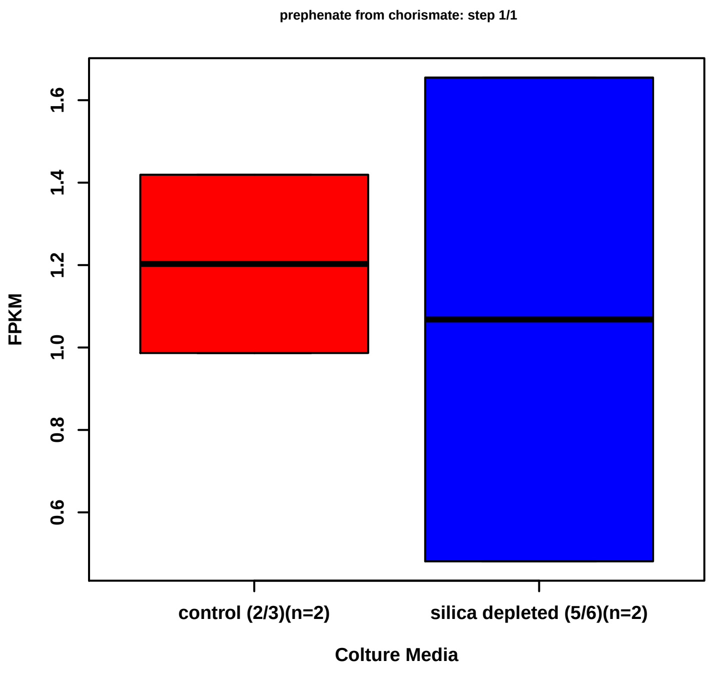
prephenate from chorismate: step 1/1
FPKM
0.6
0.8
1.0
1.2
1.4
1.6
control (2/3)(n=2)
silica depleted (5/6)(n=2)
Colture Media

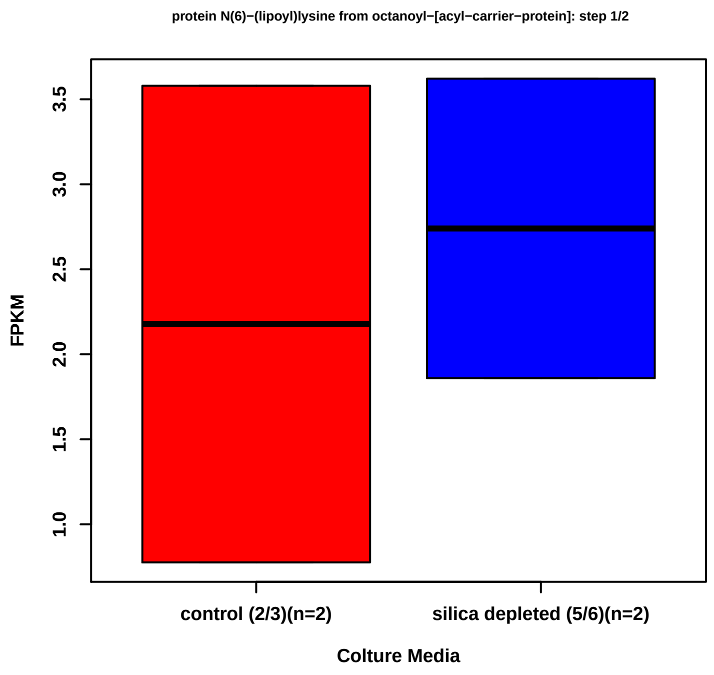
protein N(6)−(lipoyl)lysine from octanoyl−[acyl−carrier−protein]: step 1/2
FPKM
1.0
1.5
2.0
2.5
3.0
3.5
control (2/3)(n=2)
silica depleted (5/6)(n=2)
Colture Media

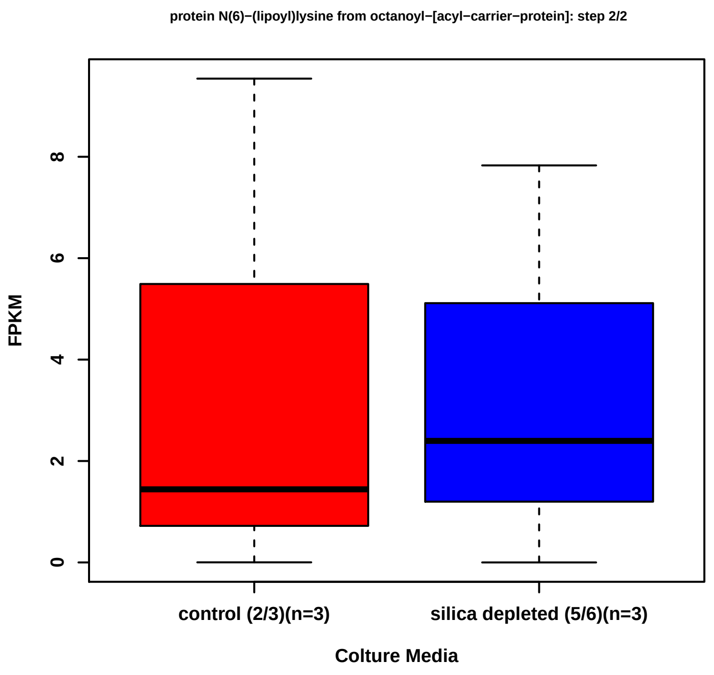
protein N(6)−(lipoyl)lysine from octanoyl−[acyl−carrier−protein]: step 2/2
FPKM
0
2
4
6
8
control (2/3)(n=3)
silica depleted (5/6)(n=3)
Colture Media

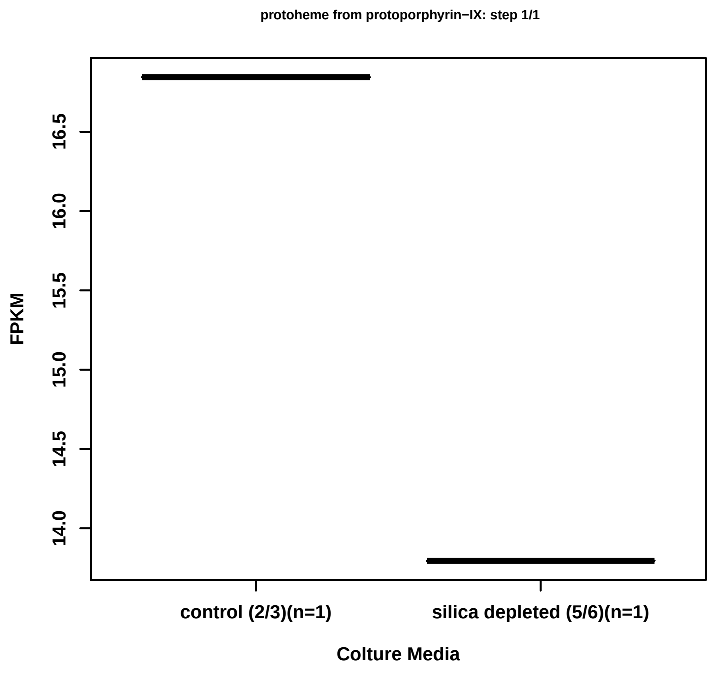
protoheme from protoporphyrin−IX: step 1/1
FPKM
14.0
14.5
15.0
15.5
16.0
16.5
control (2/3)(n=1)
silica depleted (5/6)(n=1)
Colture Media

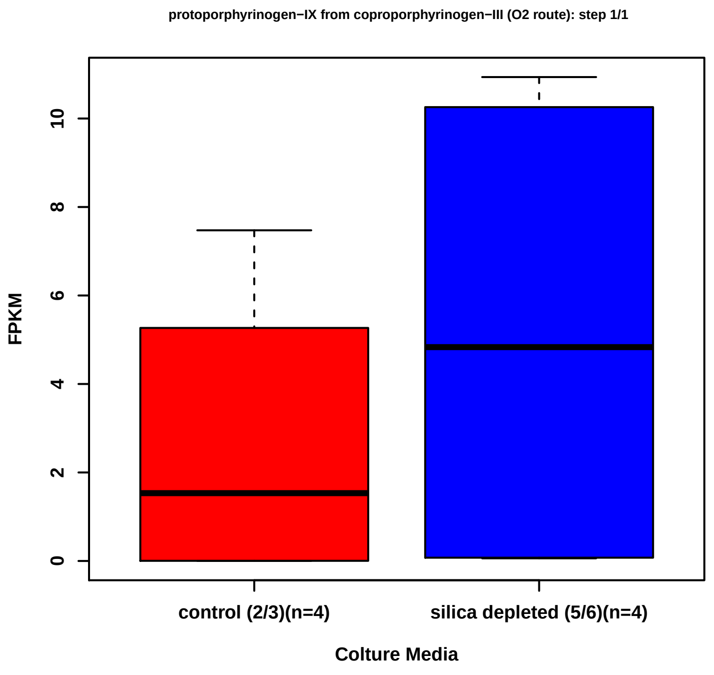
protoporphyrinogen−IX from coproporphyrinogen−III (O2 route): step 1/1
FPKM
0
2
4
6
8
10
control (2/3)(n=4)
silica depleted (5/6)(n=4)
Colture Media

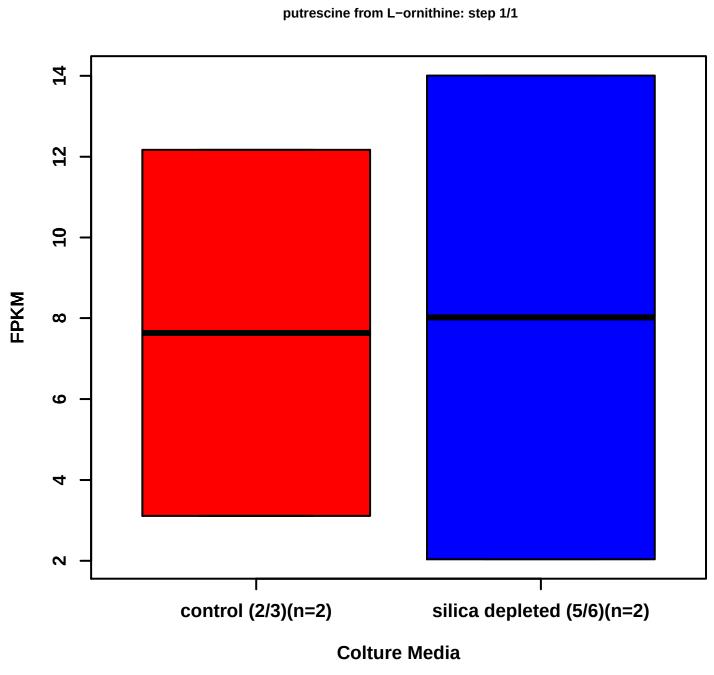
putrescine from L−ornithine: step 1/1
FPKM
2
4
6
8
10
12
14
control (2/3)(n=2)
silica depleted (5/6)(n=2)
Colture Media

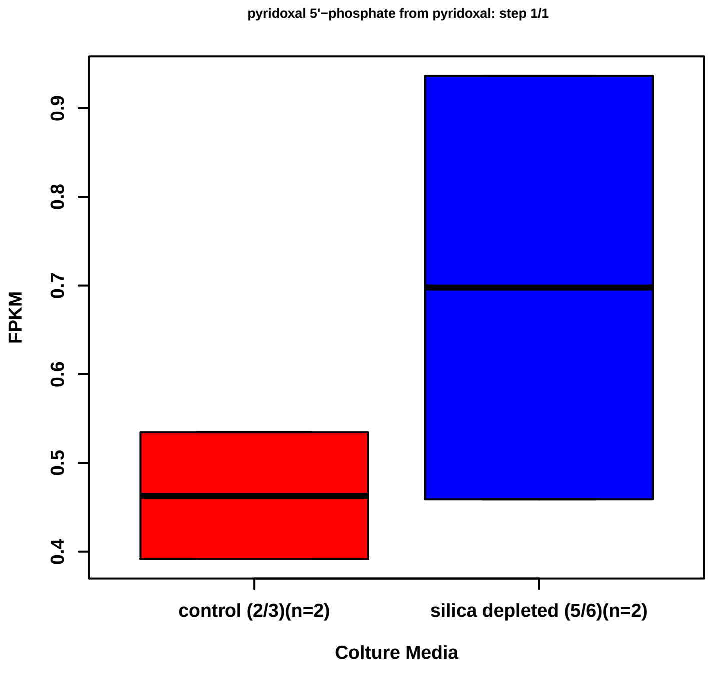
pyridoxal 5'−phosphate from pyridoxal: step 1/1
FPKM
0.4
0.5
0.6
0.7
0.8
0.9
control (2/3)(n=2)
silica depleted (5/6)(n=2)
Colture Media

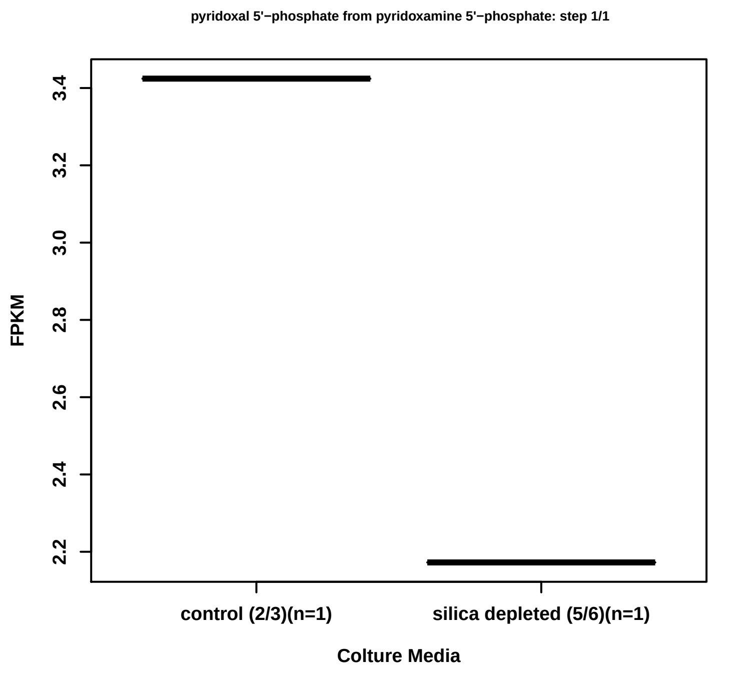
pyridoxal 5'−phosphate from pyridoxamine 5'−phosphate: step 1/1
FPKM
2.2
2.4
2.6
2.8
3.0
3.2
3.4
control (2/3)(n=1)
silica depleted (5/6)(n=1)
Colture Media

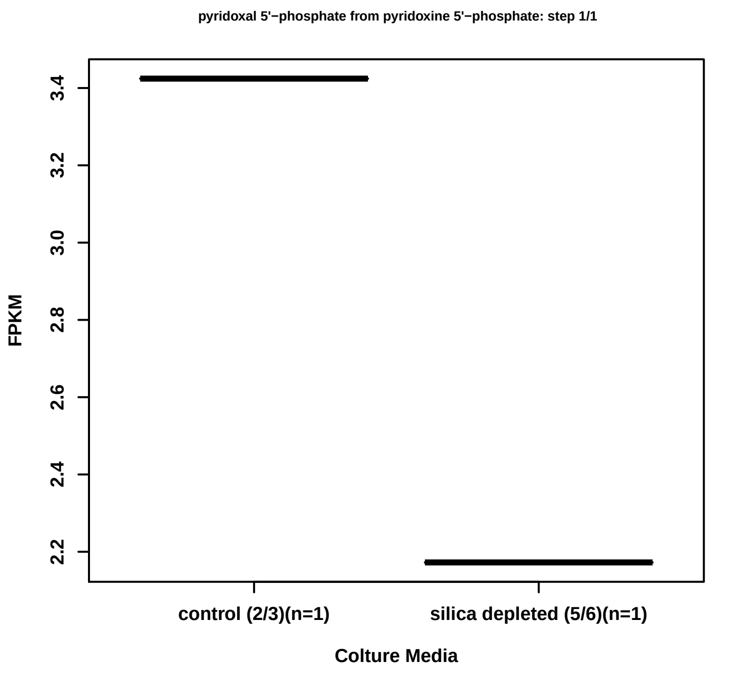
pyridoxal 5'−phosphate from pyridoxine 5'−phosphate: step 1/1
FPKM
2.2
2.4
2.6
2.8
3.0
3.2
3.4
control (2/3)(n=1)
silica depleted (5/6)(n=1)
Colture Media

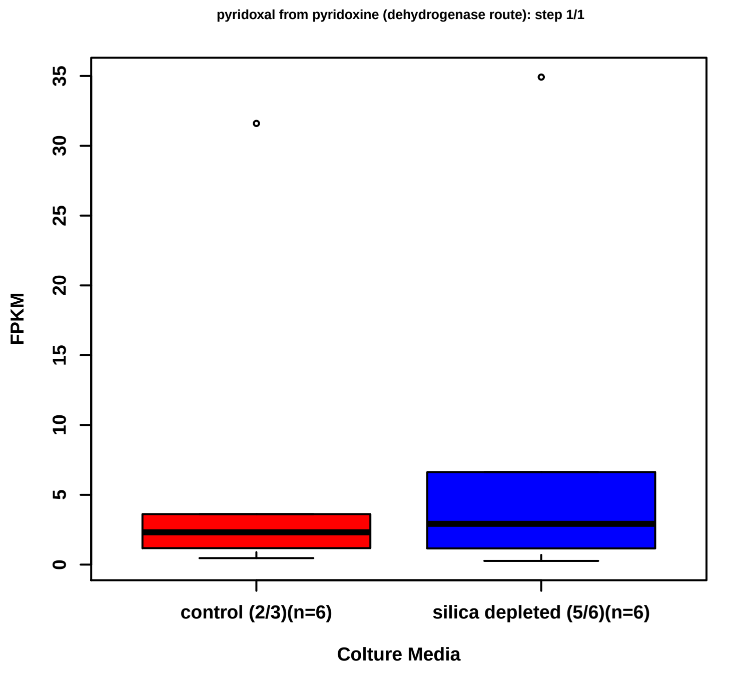
pyridoxal from pyridoxine (dehydrogenase route): step 1/1
FPKM
0
5
10
15
20
25
30
35
control (2/3)(n=6)
silica depleted (5/6)(n=6)
Colture Media

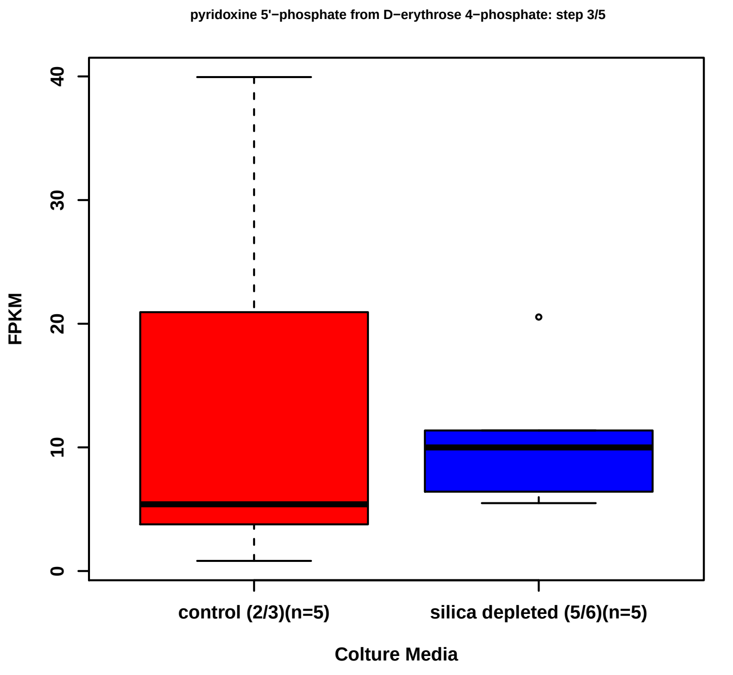
pyridoxine 5'−phosphate from D−erythrose 4−phosphate: step 3/5
FPKM
0
10
20
30
40
control (2/3)(n=5)
silica depleted (5/6)(n=5)
Colture Media

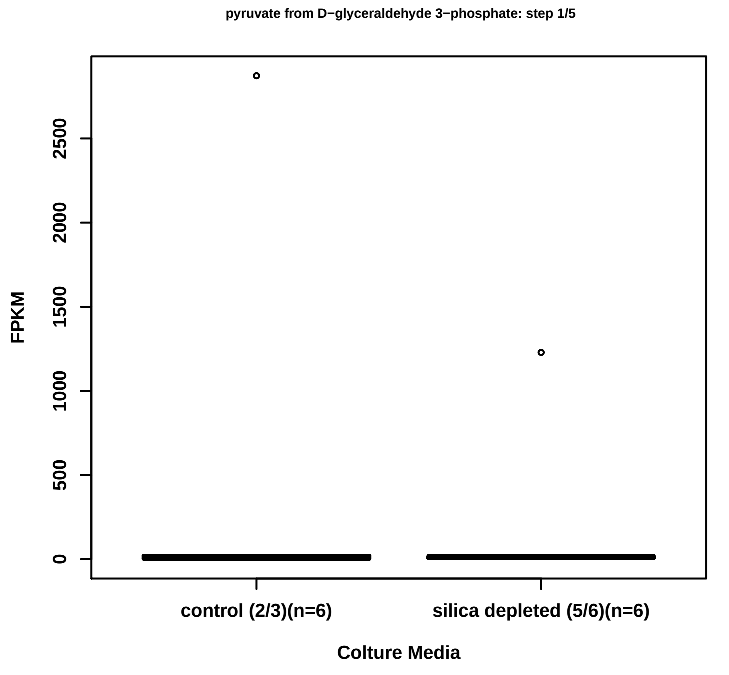
pyruvate from D−glyceraldehyde 3−phosphate: step 1/5
FPKM
0
500
1000
1500
2000
2500
control (2/3)(n=6)
silica depleted (5/6)(n=6)
Colture Media

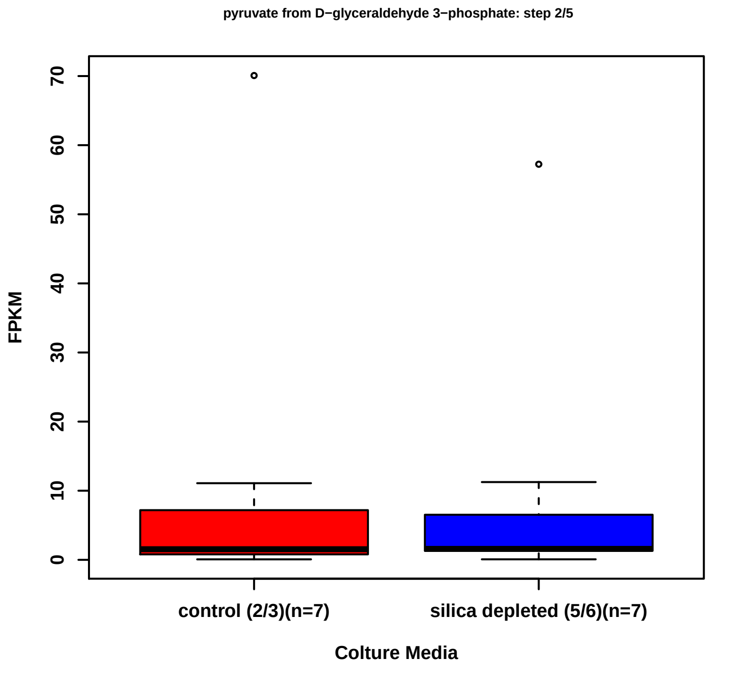
pyruvate from D−glyceraldehyde 3−phosphate: step 2/5
FPKM
0
10
20
30
40
50
60
70
control (2/3)(n=7)
silica depleted (5/6)(n=7)
Colture Media

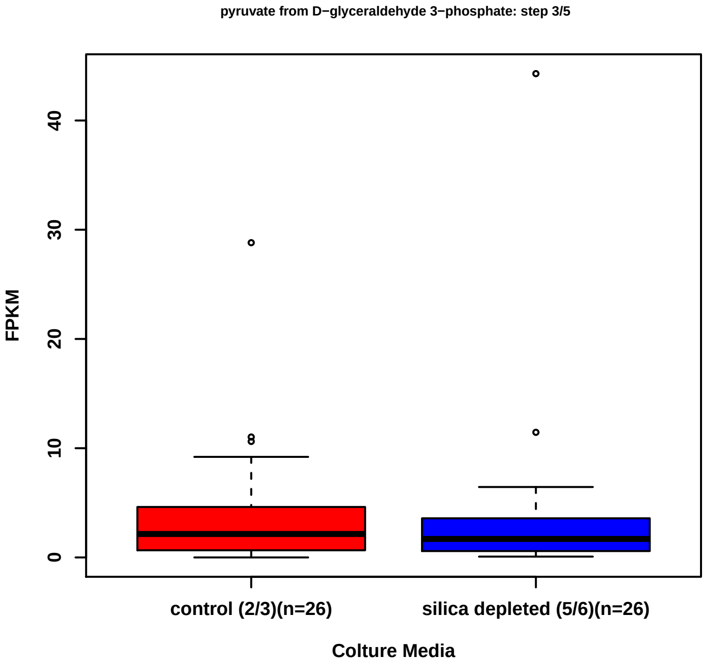
pyruvate from D−glyceraldehyde 3−phosphate: step 3/5
FPKM
0
10
20
30
40
control (2/3)(n=26)
silica depleted (5/6)(n=26)
Colture Media

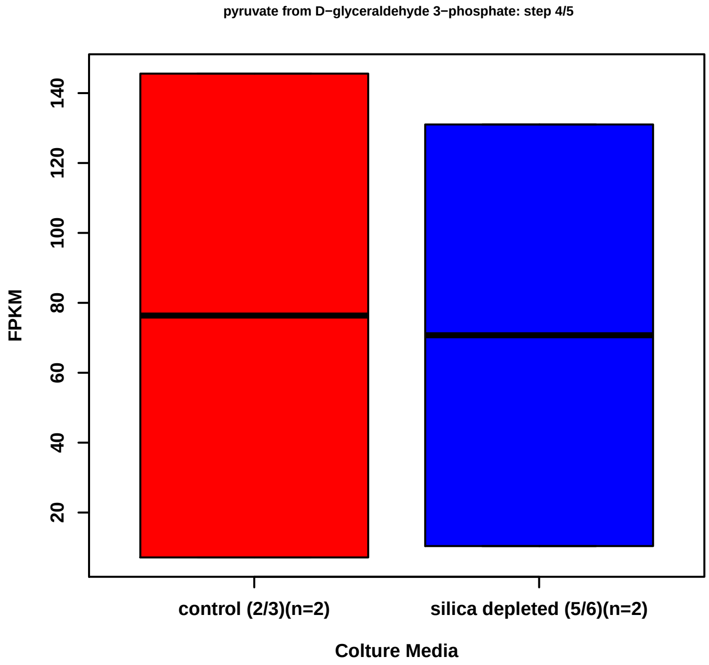
pyruvate from D−glyceraldehyde 3−phosphate: step 4/5
FPKM
20
40
60
80
100
120
140
control (2/3)(n=2)
silica depleted (5/6)(n=2)
Colture Media

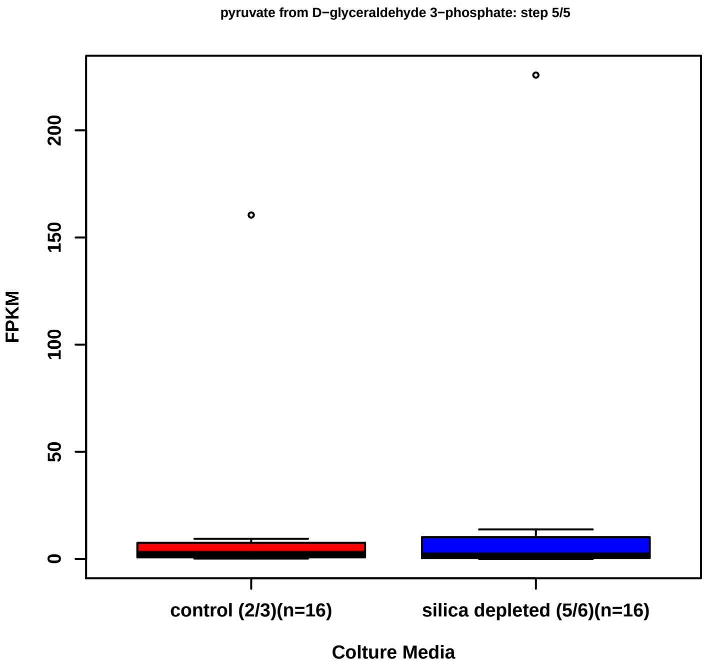
pyruvate from D−glyceraldehyde 3−phosphate: step 5/5
FPKM
0
50
100
150
200
control (2/3)(n=16)
silica depleted (5/6)(n=16)
Colture Media

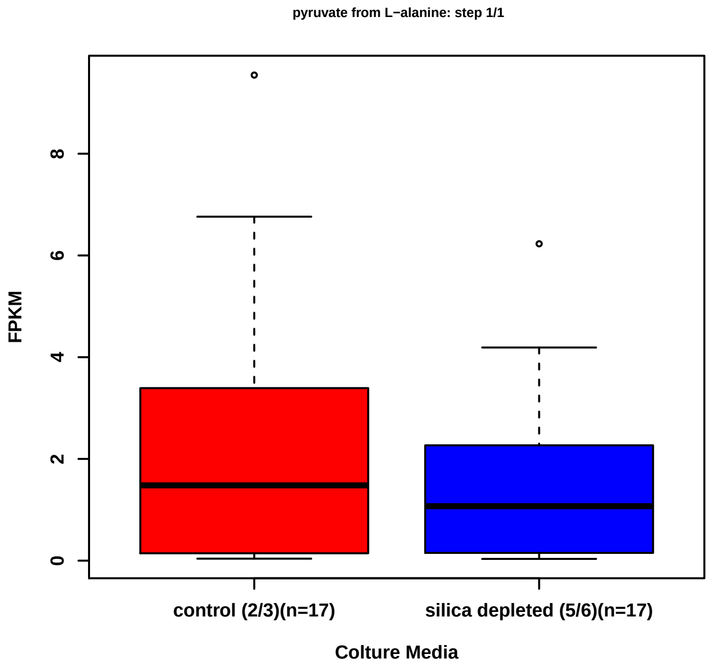
pyruvate from L−alanine: step 1/1
FPKM
0
2
4
6
8
control (2/3)(n=17)
silica depleted (5/6)(n=17)
Colture Media

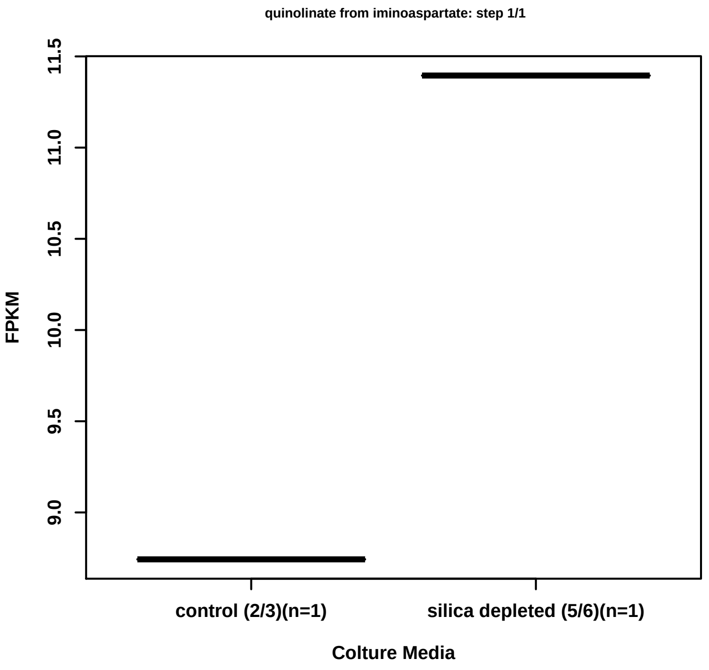
quinolinate from iminoaspartate: step 1/1
FPKM
9.0
9.5
10.0
10.5
11.0
11.5
control (2/3)(n=1)
silica depleted (5/6)(n=1)
Colture Media

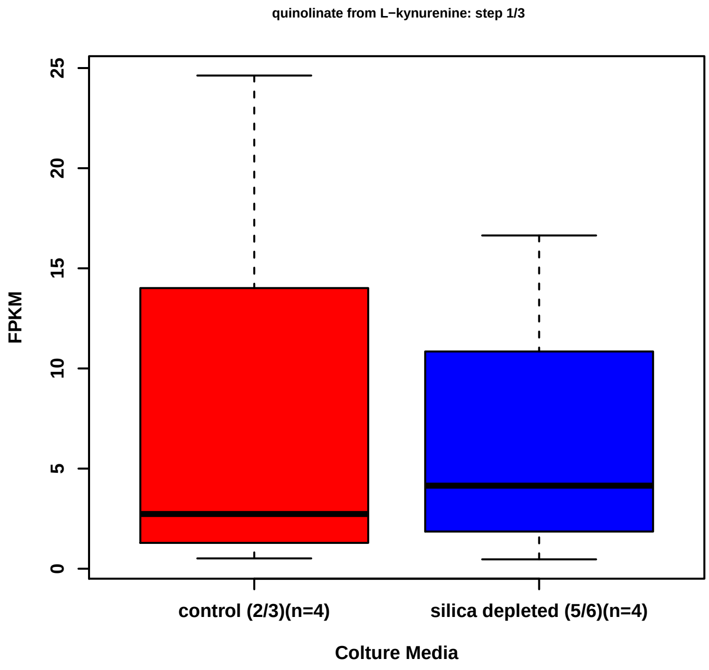
quinolinate from L−kynurenine: step 1/3
FPKM
0
5
10
15
20
25
control (2/3)(n=4)
silica depleted (5/6)(n=4)
Colture Media

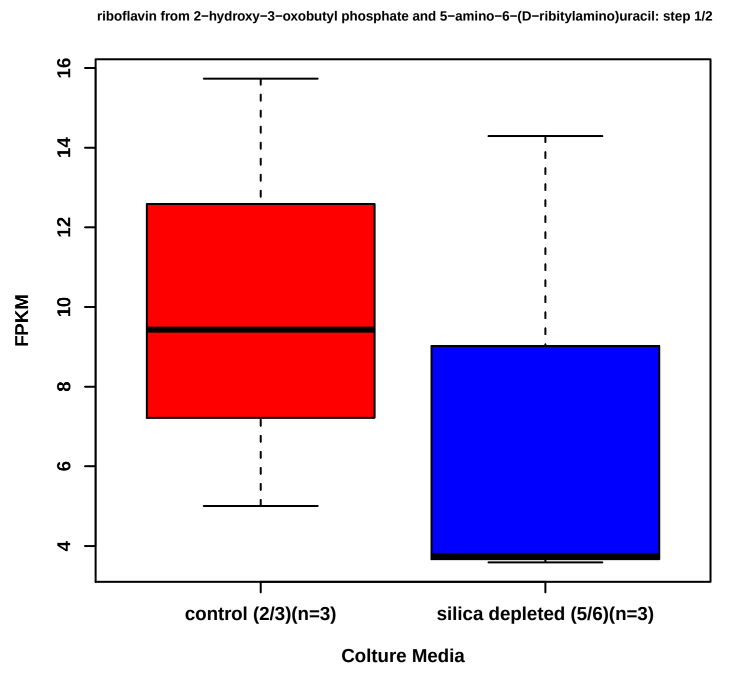
riboflavin from 2−hydroxy−3−oxobutyl phosphate and 5−amino−6−(D−ribitylamino)uracil: step 1/2
FPKM
4
6
8
10
12
14
16
control (2/3)(n=3)
silica depleted (5/6)(n=3)
Colture Media

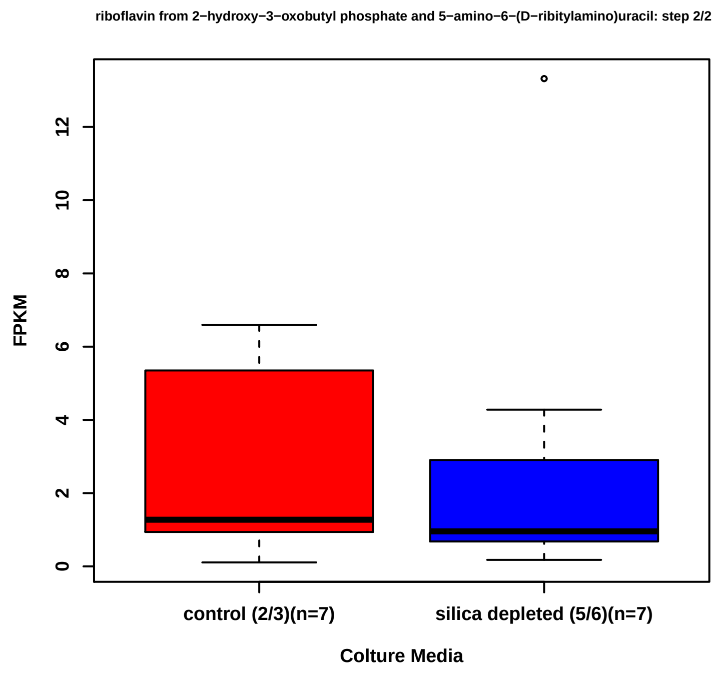
riboflavin from 2−hydroxy−3−oxobutyl phosphate and 5−amino−6−(D−ribitylamino)uracil: step 2/2
FPKM
0
2
4
6
8
10
12
control (2/3)(n=7)
silica depleted (5/6)(n=7)
Colture Media

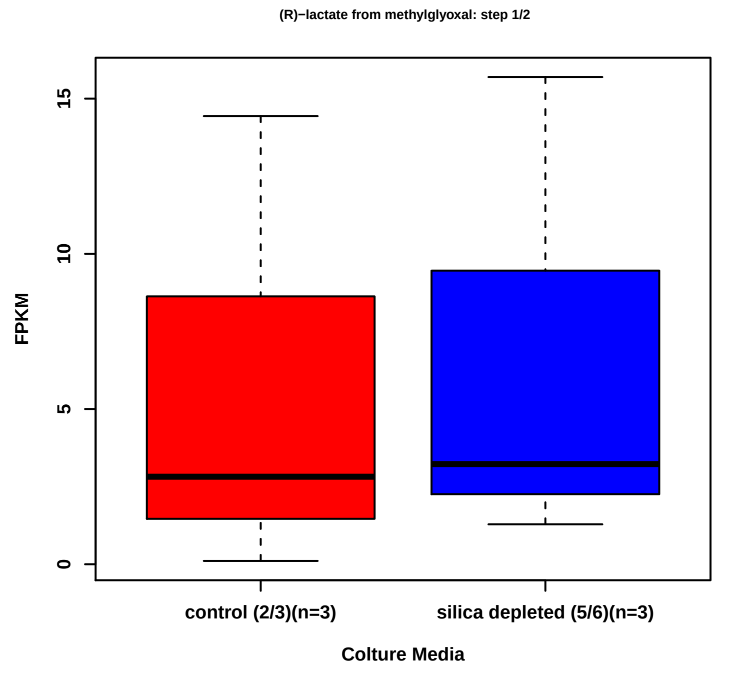
(R)−lactate from methylglyoxal: step 1/2
FPKM
0
5
10
15
control (2/3)(n=3)
silica depleted (5/6)(n=3)
Colture Media

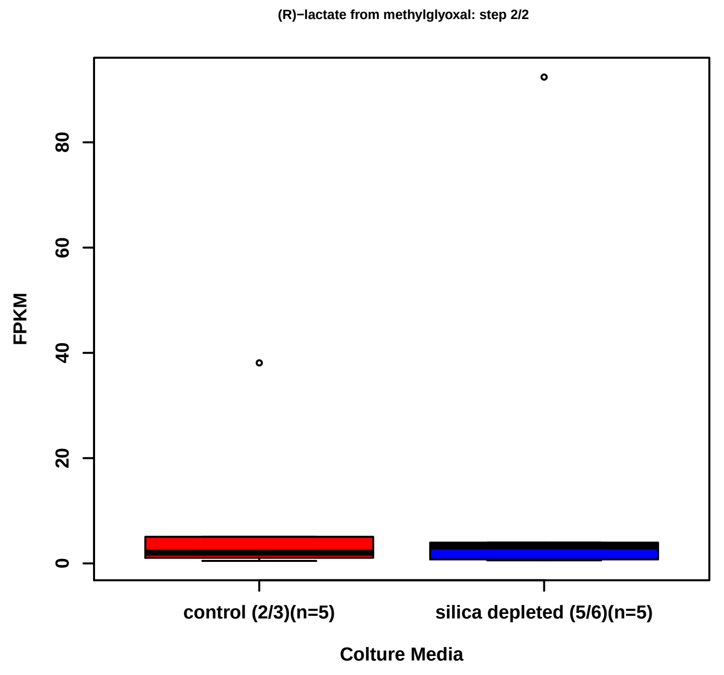
(R)−lactate from methylglyoxal: step 2/2
FPKM
0
20
40
60
80
control (2/3)(n=5)
silica depleted (5/6)(n=5)
Colture Media

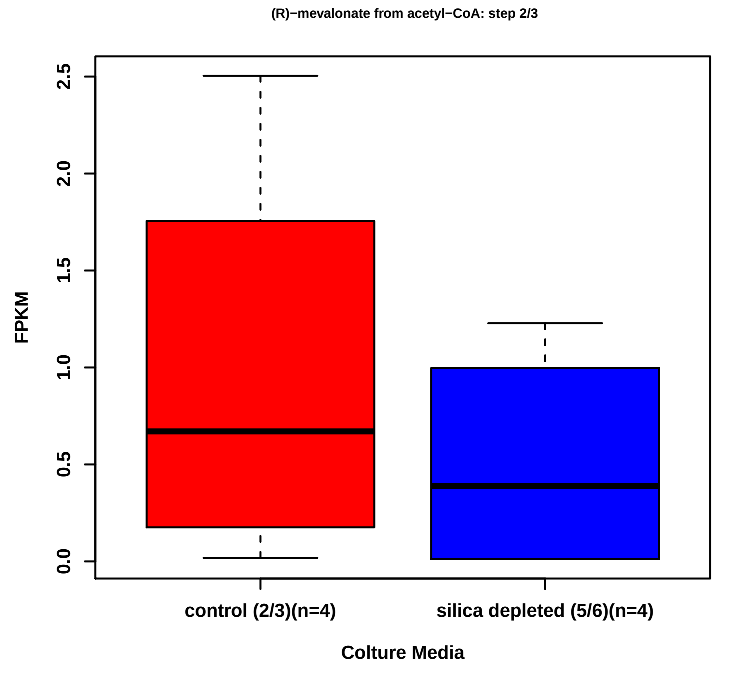
(R)−mevalonate from acetyl−CoA: step 2/3
FPKM
0.0
0.5
1.0
1.5
2.0
2.5
control (2/3)(n=4)
silica depleted (5/6)(n=4)
Colture Media

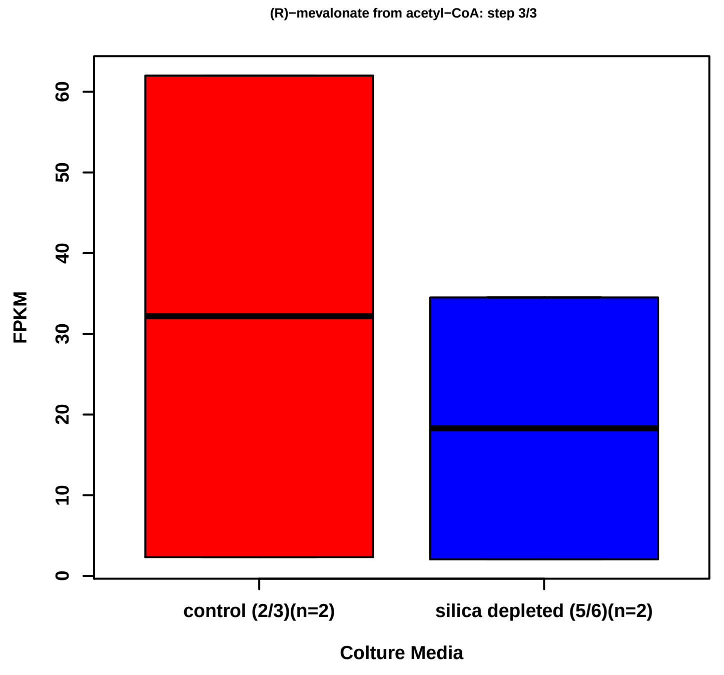
(R)−mevalonate from acetyl−CoA: step 3/3
FPKM
0
10
20
30
40
50
60
control (2/3)(n=2)
silica depleted (5/6)(n=2)
Colture Media

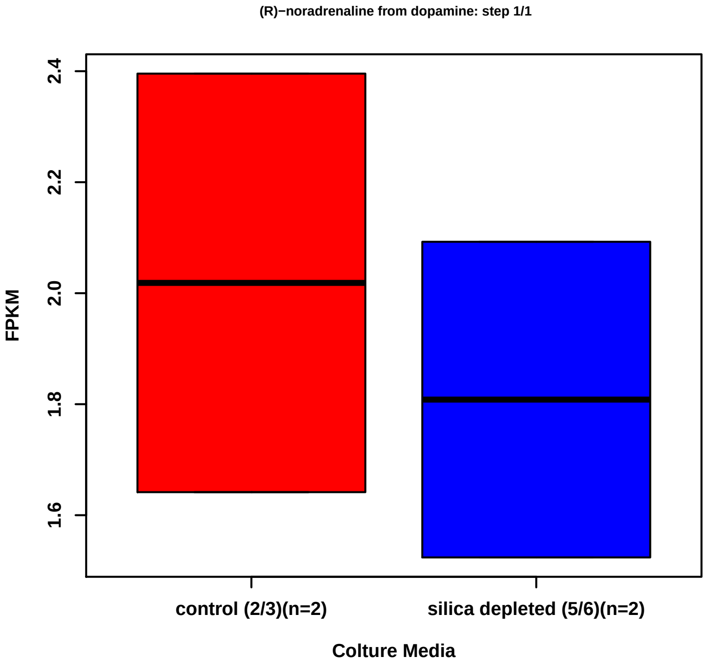
(R)−noradrenaline from dopamine: step 1/1
FPKM
1.6
1.8
2.0
2.2
2.4
control (2/3)(n=2)
silica depleted (5/6)(n=2)
Colture Media

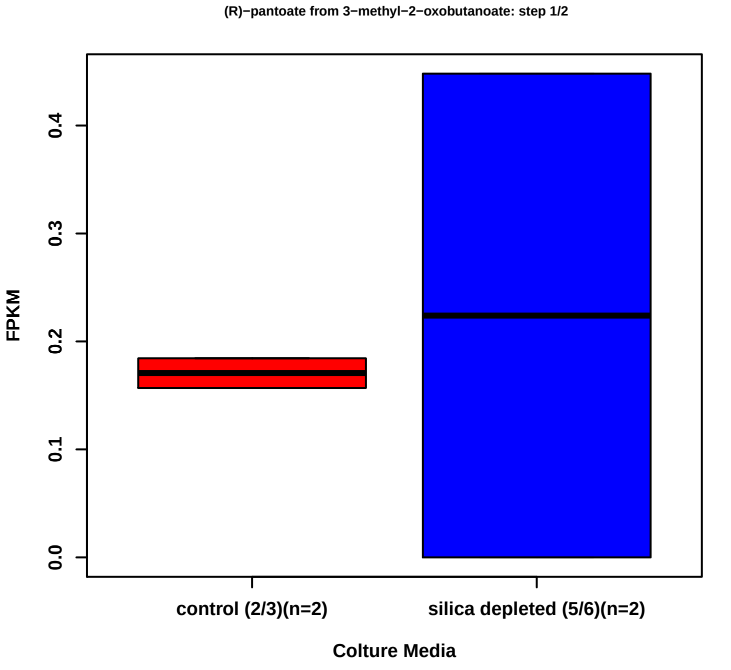
(R)−pantoate from 3−methyl−2−oxobutanoate: step 1/2
FPKM
0.0
0.1
0.2
0.3
0.4
control (2/3)(n=2)
silica depleted (5/6)(n=2)
Colture Media

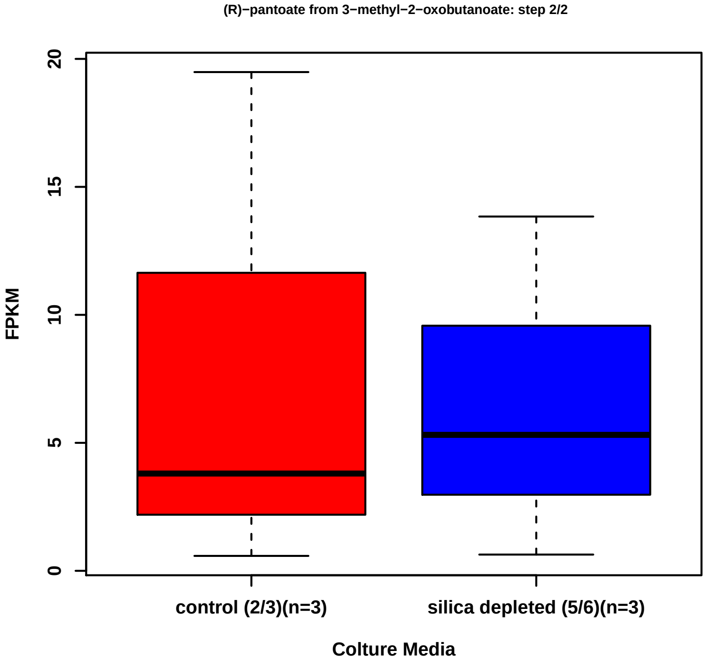
(R)−pantoate from 3−methyl−2−oxobutanoate: step 2/2
FPKM
0
5
10
15
20
control (2/3)(n=3)
silica depleted (5/6)(n=3)
Colture Media

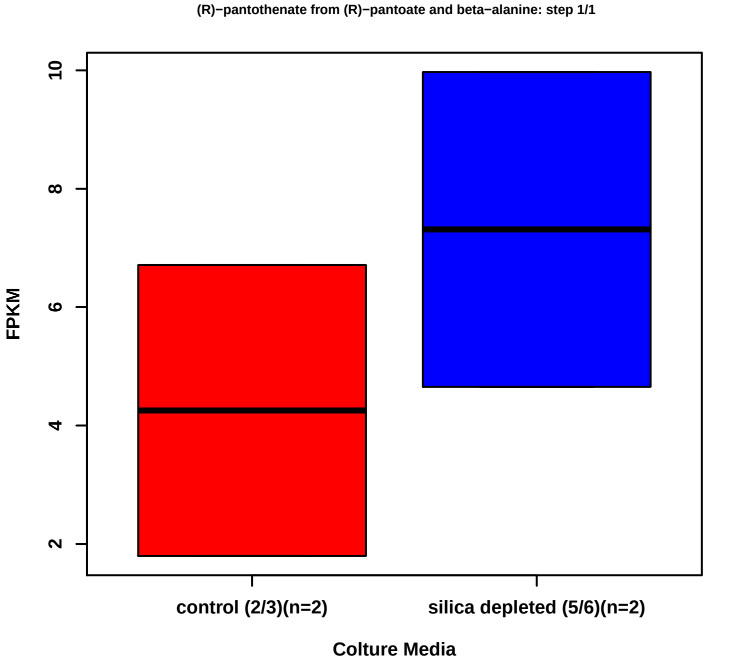
(R)−pantothenate from (R)−pantoate and beta−alanine: step 1/1
FPKM
2
4
6
8
10
control (2/3)(n=2)
silica depleted (5/6)(n=2)
Colture Media

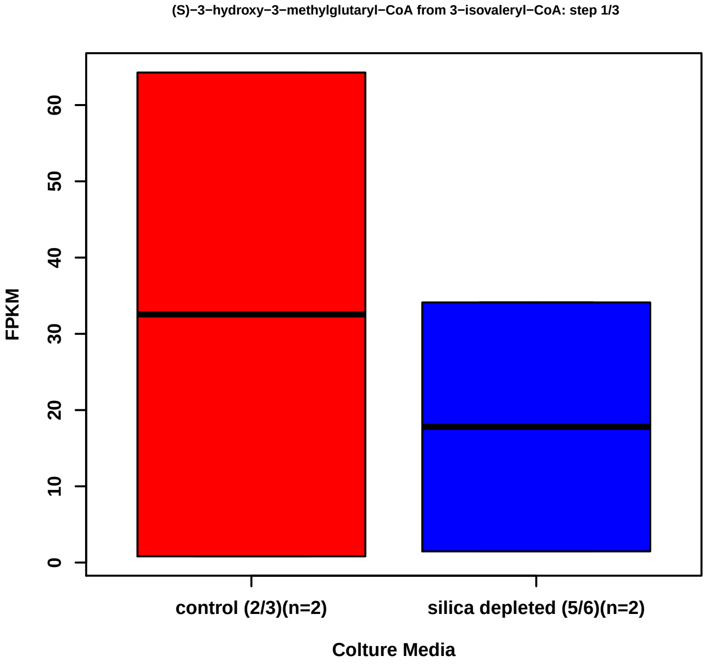
(S)−3−hydroxy−3−methylglutaryl−CoA from 3−isovaleryl−CoA: step 1/3
FPKM
0
10
20
30
40
50
60
control (2/3)(n=2)
silica depleted (5/6)(n=2)
Colture Media

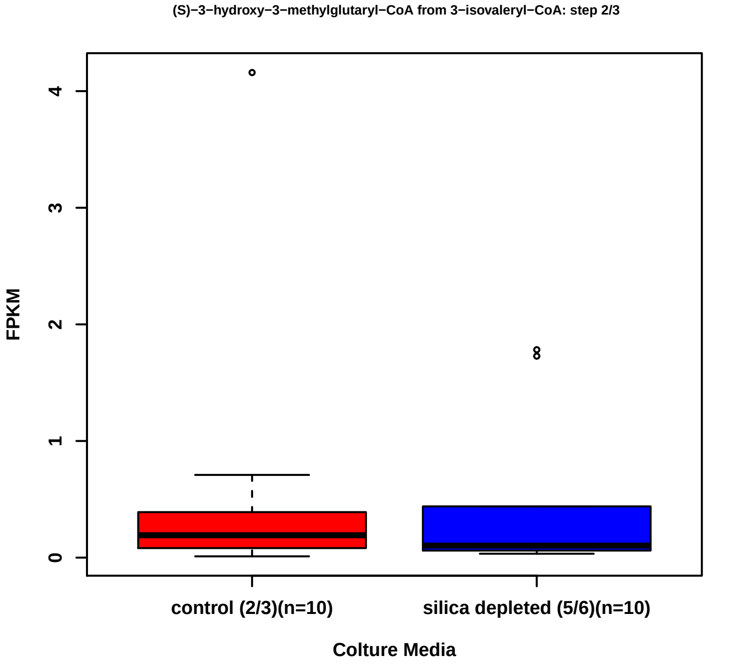
(S)−3−hydroxy−3−methylglutaryl−CoA from 3−isovaleryl−CoA: step 2/3
FPKM
0
1
2
3
4
control (2/3)(n=10)
silica depleted (5/6)(n=10)
Colture Media

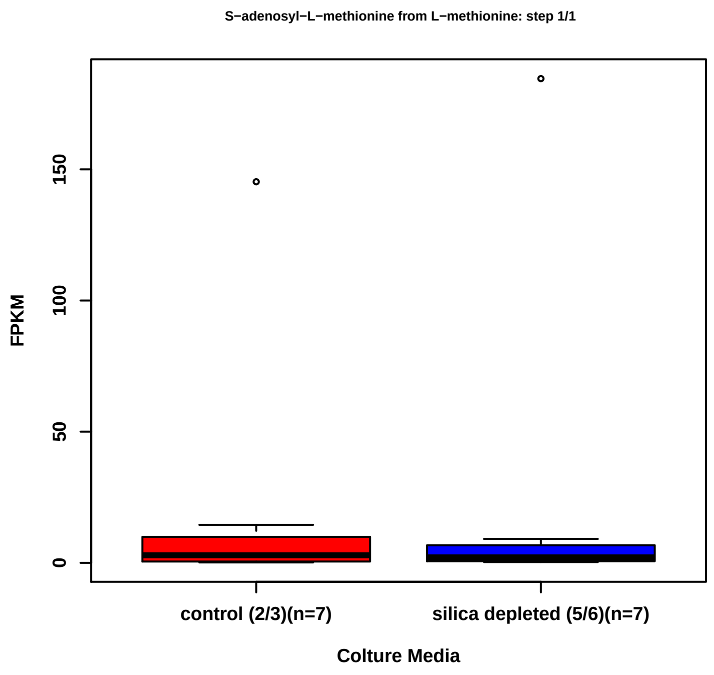
S−adenosyl−L−methionine from L−methionine: step 1/1
FPKM
0
50
100
150
control (2/3)(n=7)
silica depleted (5/6)(n=7)
Colture Media

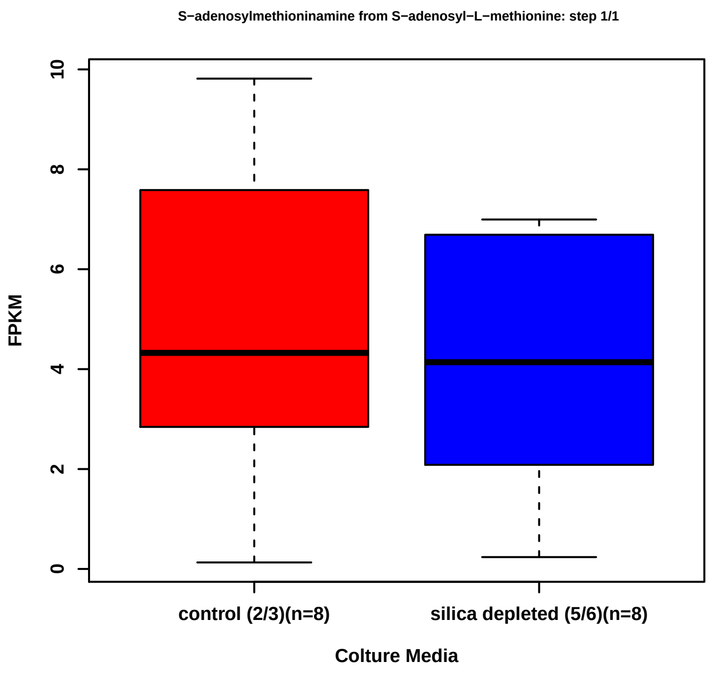
S−adenosylmethioninamine from S−adenosyl−L−methionine: step 1/1
FPKM
0
2
4
6
8
10
control (2/3)(n=8)
silica depleted (5/6)(n=8)
Colture Media

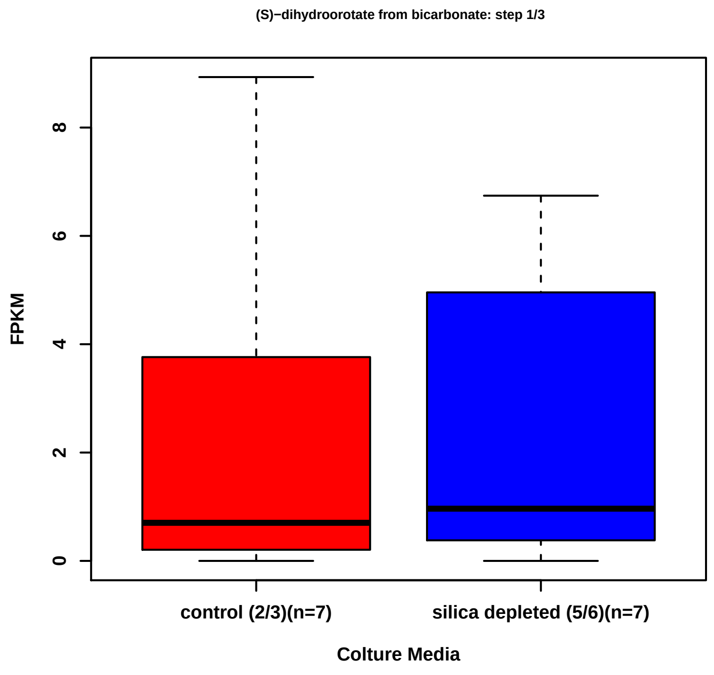
(S)−dihydroorotate from bicarbonate: step 1/3
FPKM
0
2
4
6
8
control (2/3)(n=7)
silica depleted (5/6)(n=7)
Colture Media

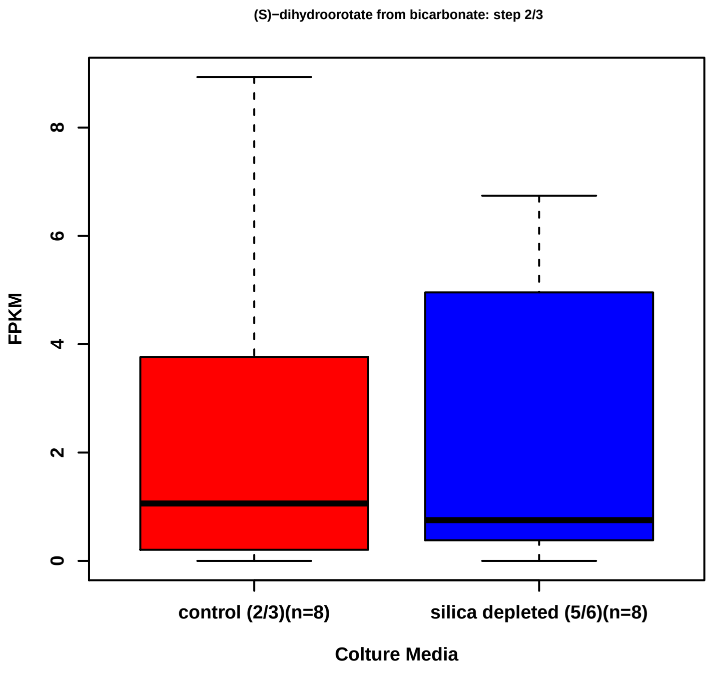
(S)−dihydroorotate from bicarbonate: step 2/3
FPKM
0
2
4
6
8
control (2/3)(n=8)
silica depleted (5/6)(n=8)
Colture Media

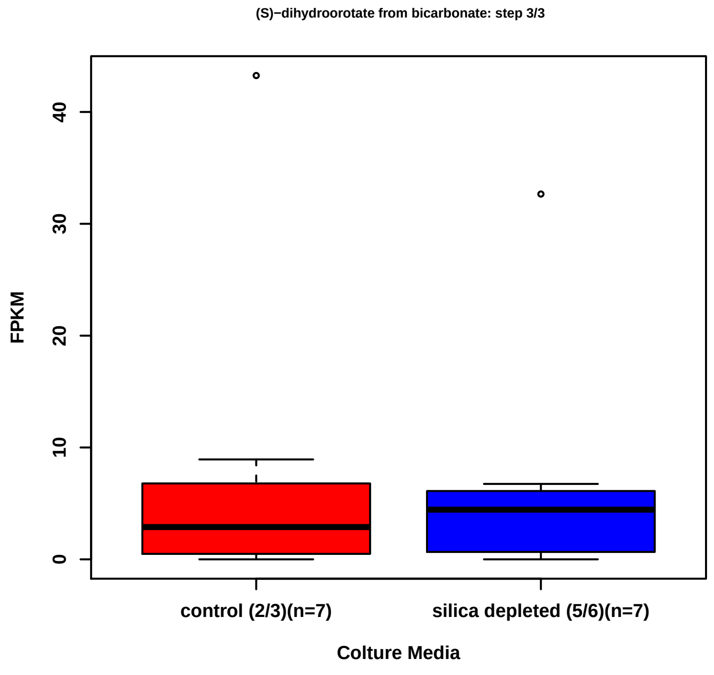
(S)−dihydroorotate from bicarbonate: step 3/3
FPKM
0
10
20
30
40
control (2/3)(n=7)
silica depleted (5/6)(n=7)
Colture Media

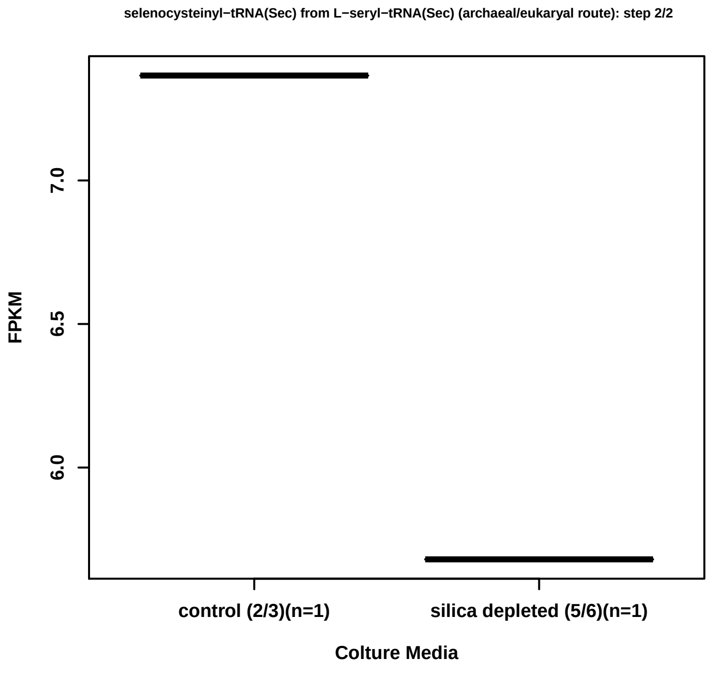
selenocysteinyl−tRNA(Sec) from L−seryl−tRNA(Sec) (archaeal/eukaryal route): step 2/2
FPKM
6.0
6.5
7.0
control (2/3)(n=1)
silica depleted (5/6)(n=1)
Colture Media

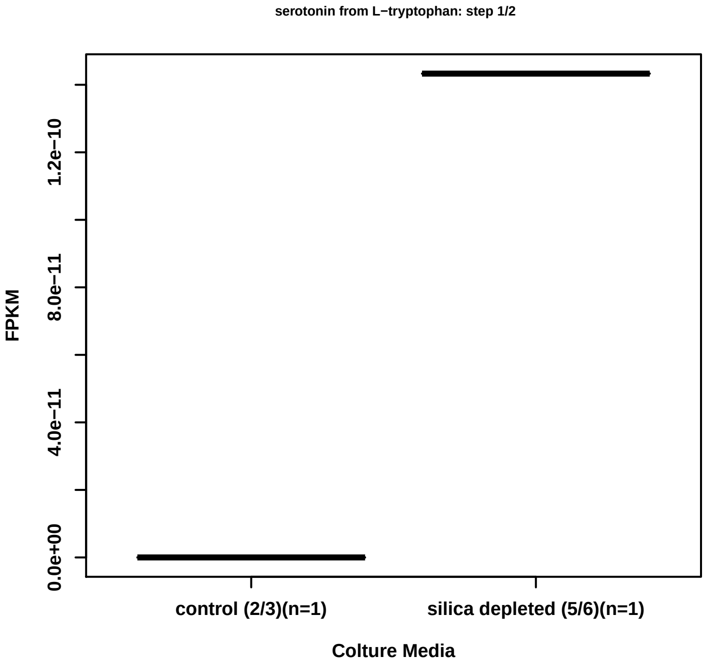
serotonin from L−tryptophan: step 1/2
FPKM
0.0e+00
4.0e−11
8.0e−11
1.2e−10
control (2/3)(n=1)
silica depleted (5/6)(n=1)
Colture Media

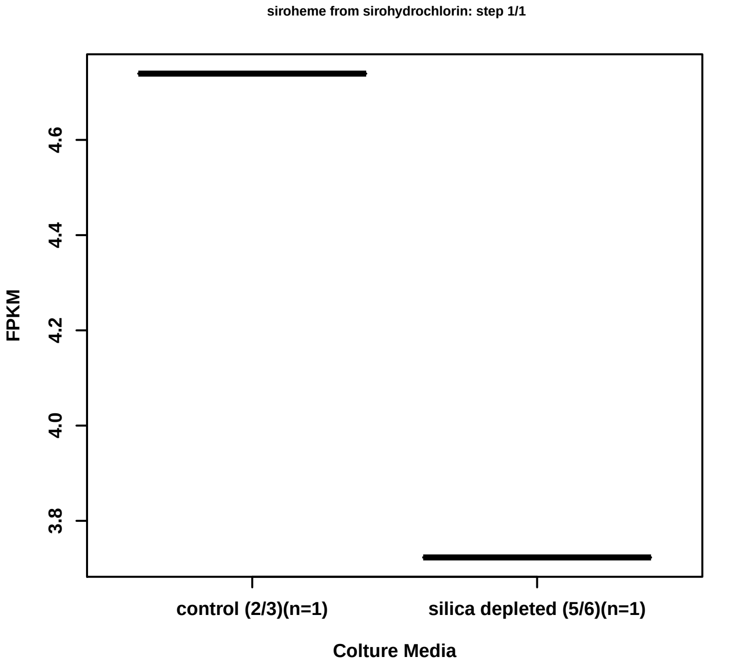
siroheme from sirohydrochlorin: step 1/1
FPKM
3.8
4.0
4.2
4.4
4.6
control (2/3)(n=1)
silica depleted (5/6)(n=1)
Colture Media

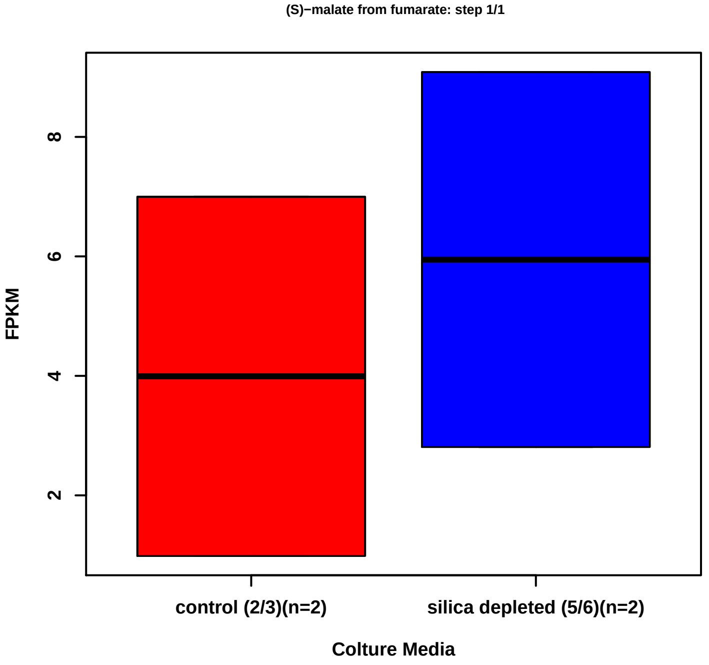
(S)−malate from fumarate: step 1/1
FPKM
2
4
6
8
control (2/3)(n=2)
silica depleted (5/6)(n=2)
Colture Media

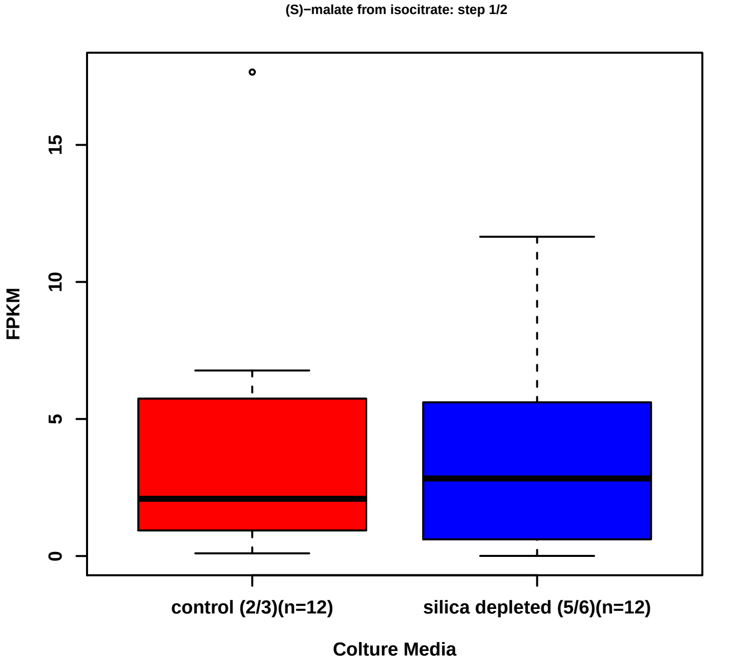
(S)−malate from isocitrate: step 1/2
FPKM
0
5
10
15
control (2/3)(n=12)
silica depleted (5/6)(n=12)
Colture Media

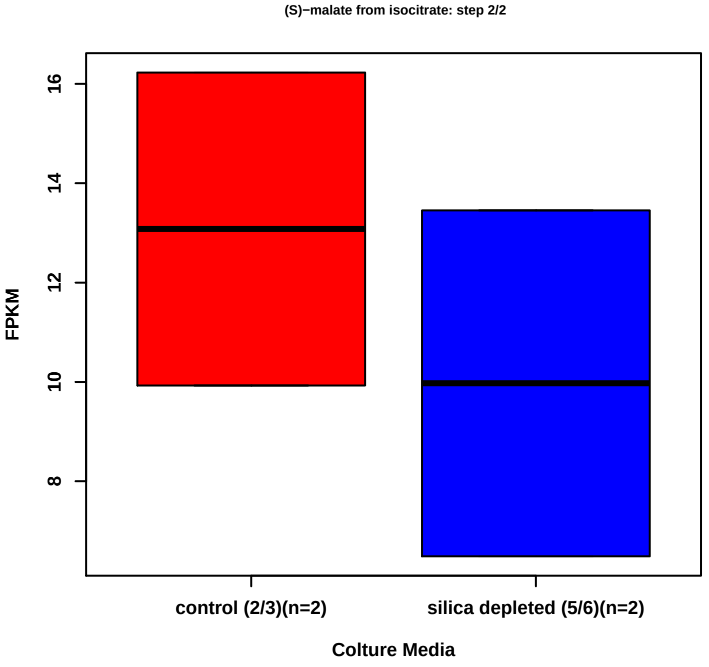
(S)−malate from isocitrate: step 2/2
FPKM
8
10
12
14
16
control (2/3)(n=2)
silica depleted (5/6)(n=2)
Colture Media

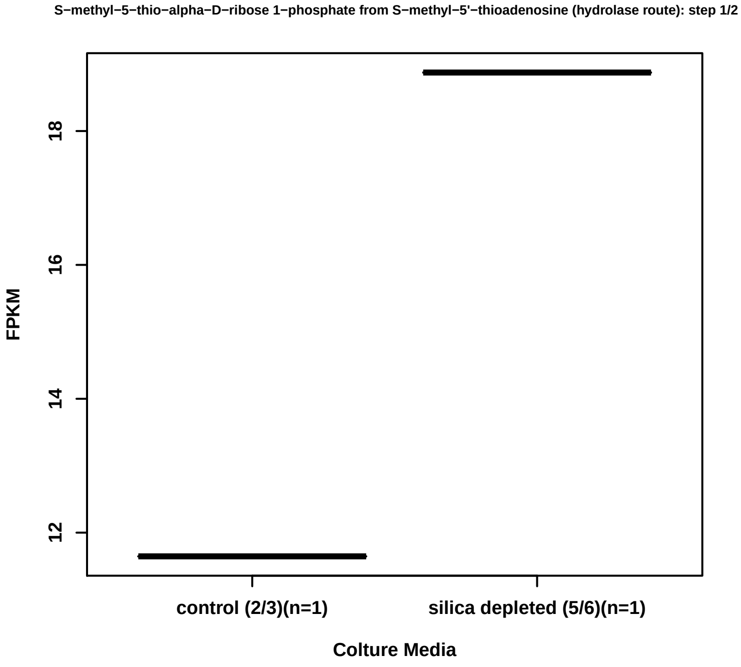
S−methyl−5−thio−alpha−D−ribose 1−phosphate from S−methyl−5'−thioadenosine (hydrolase route): step 1/2
FPKM
12
14
16
18
control (2/3)(n=1)
silica depleted (5/6)(n=1)
Colture Media

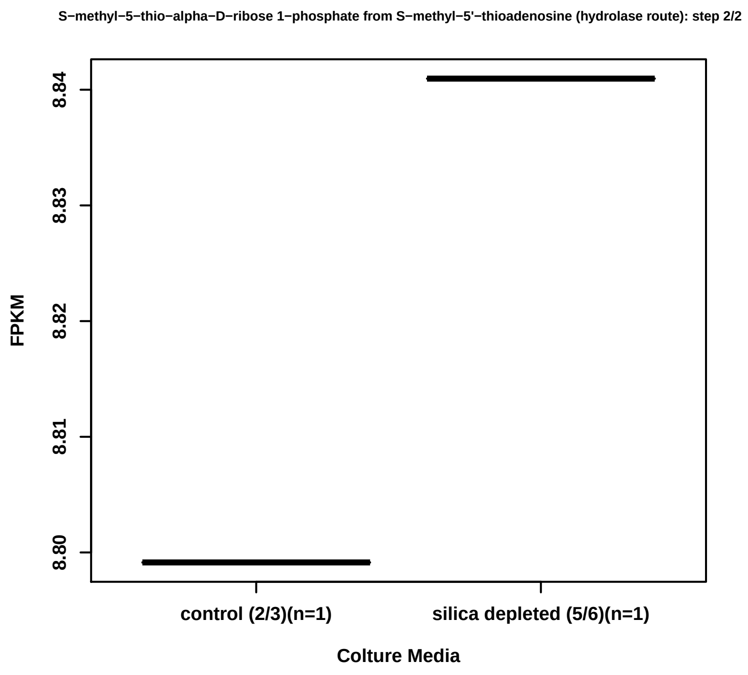
S−methyl−5−thio−alpha−D−ribose 1−phosphate from S−methyl−5'−thioadenosine (hydrolase route): step 2/2
FPKM
8.80
8.81
8.82
8.83
8.84
control (2/3)(n=1)
silica depleted (5/6)(n=1)
Colture Media

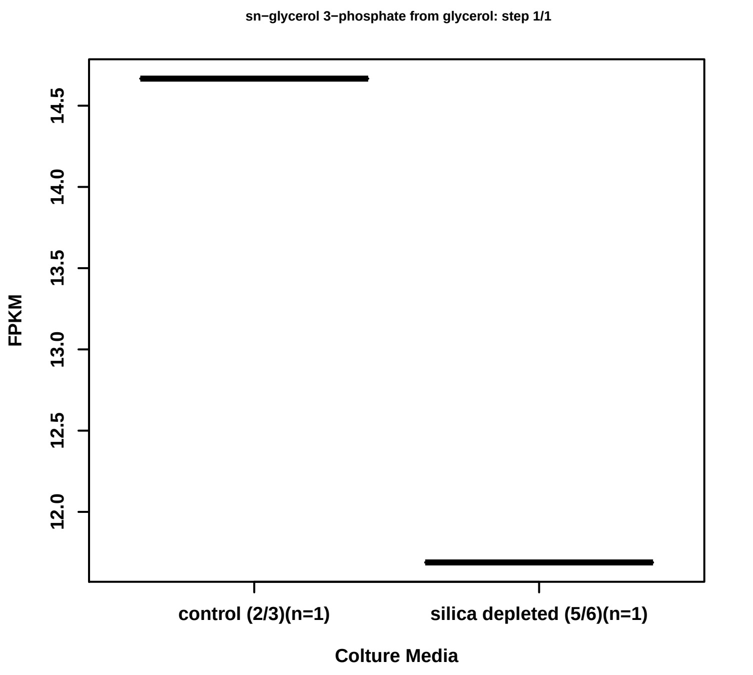
sn−glycerol 3−phosphate from glycerol: step 1/1
FPKM
12.0
12.5
13.0
13.5
14.0
14.5
control (2/3)(n=1)
silica depleted (5/6)(n=1)
Colture Media

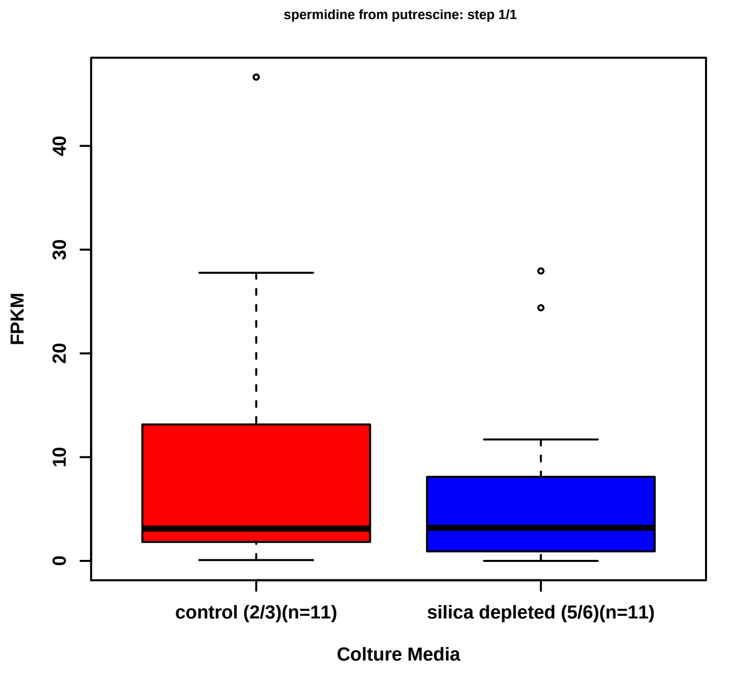
spermidine from putrescine: step 1/1
FPKM
0
10
20
30
40
control (2/3)(n=11)
silica depleted (5/6)(n=11)
Colture Media

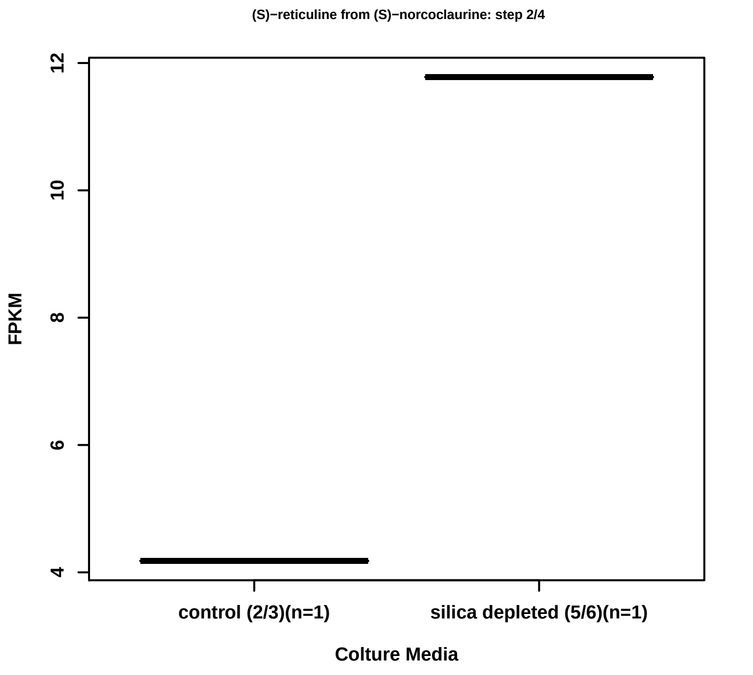
(S)−reticuline from (S)−norcoclaurine: step 2/4
FPKM
4
6
8
10
12
control (2/3)(n=1)
silica depleted (5/6)(n=1)
Colture Media

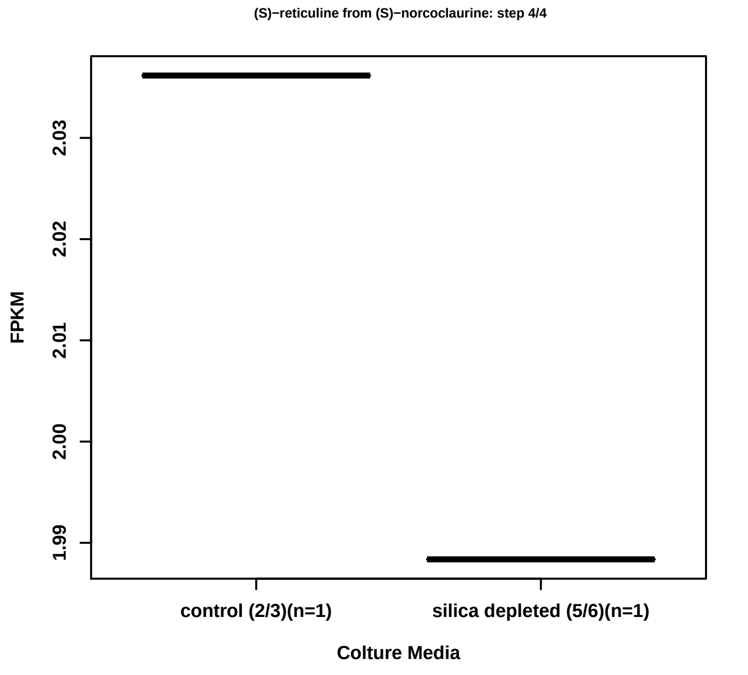
(S)−reticuline from (S)−norcoclaurine: step 4/4
FPKM
1.99
2.00
2.01
2.02
2.03
control (2/3)(n=1)
silica depleted (5/6)(n=1)
Colture Media

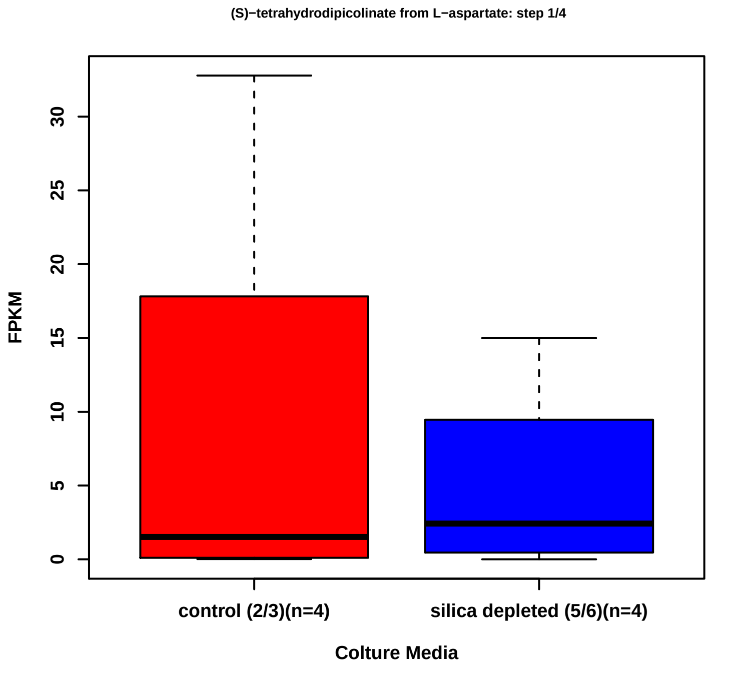
(S)−tetrahydrodipicolinate from L−aspartate: step 1/4
FPKM
0
5
10
15
20
25
30
control (2/3)(n=4)
silica depleted (5/6)(n=4)
Colture Media

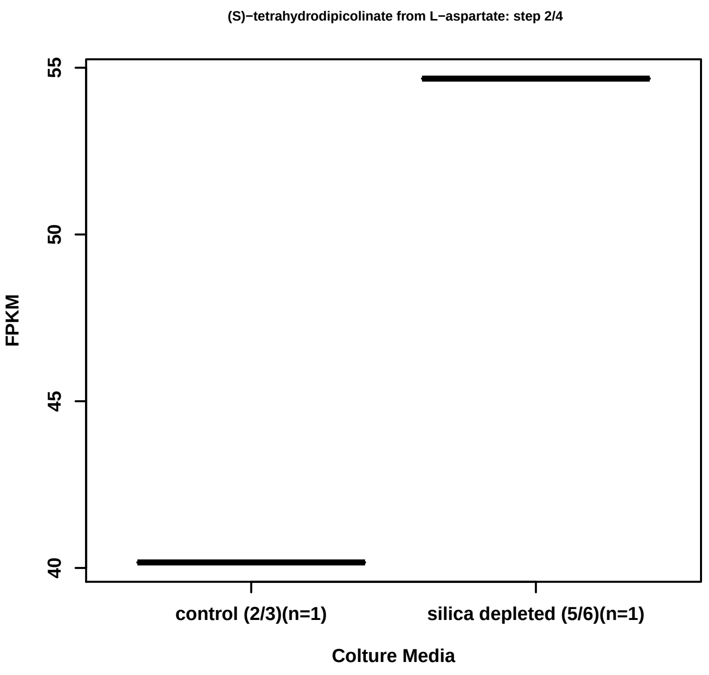

(S)−tetrahydrodipicolinate from L−aspartate: step 2/4
FPKM
40
45
50
55
control (2/3)(n=1)
silica depleted (5/6)(n=1)
Colture Media

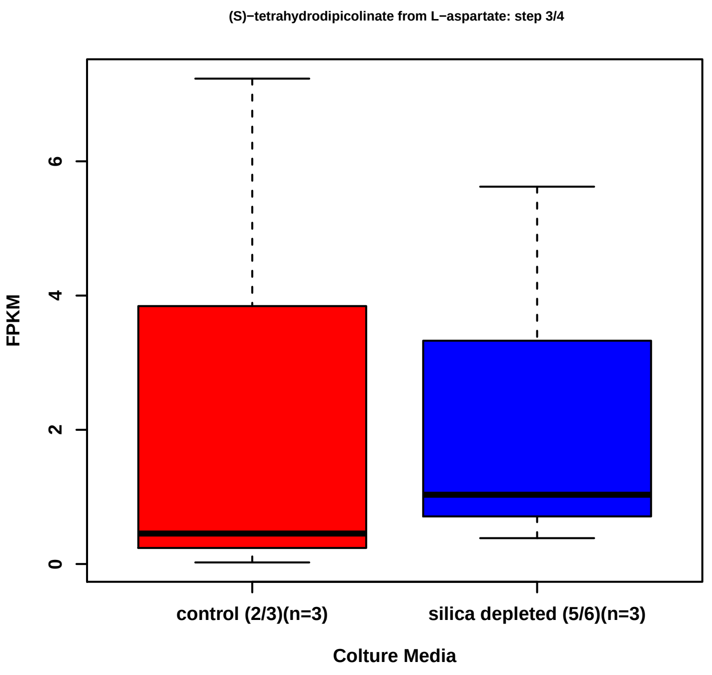

(S)−tetrahydrodipicolinate from L−aspartate: step 3/4
FPKM
0
2
4
6
control (2/3)(n=3)
silica depleted (5/6)(n=3)
Colture Media

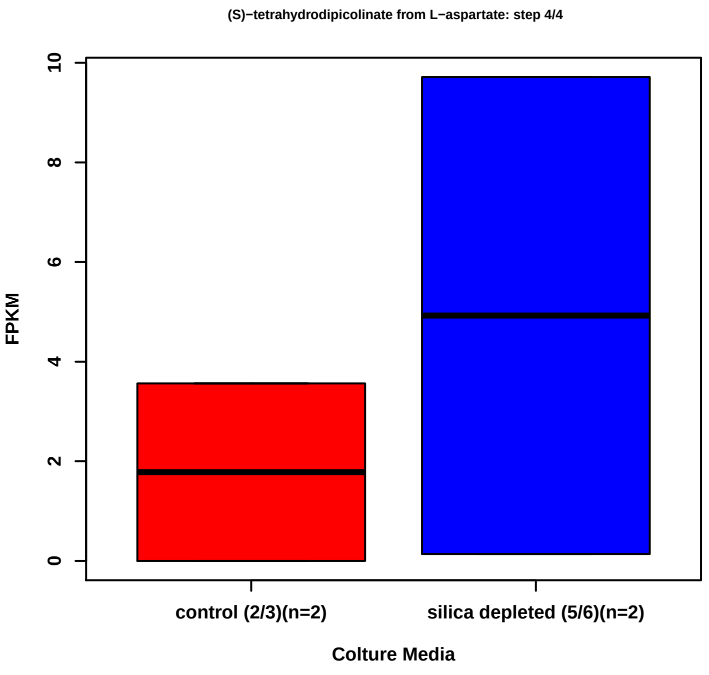

(S)−tetrahydrodipicolinate from L−aspartate: step 4/4
FPKM
0
2
4
6
8
10
control (2/3)(n=2)
silica depleted (5/6)(n=2)
Colture Media

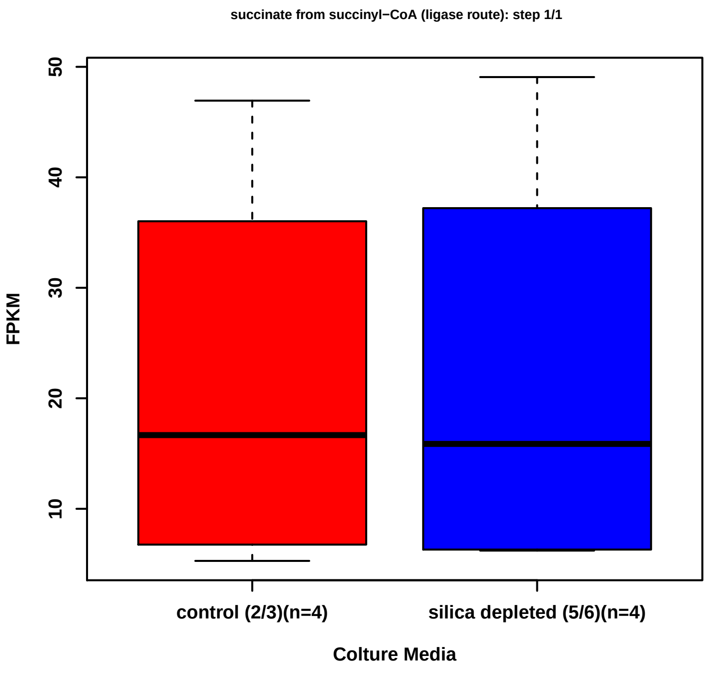

succinate from succinyl−CoA (ligase route): step 1/1
FPKM
10
20
30
40
50
control (2/3)(n=4)
silica depleted (5/6)(n=4)
Colture Media

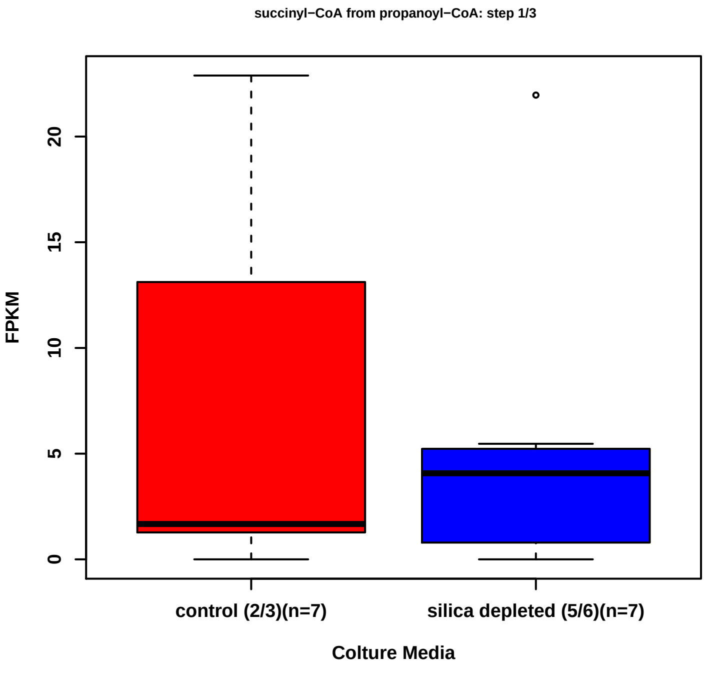

succinyl−CoA from propanoyl−CoA: step 1/3
FPKM
0
5
10
15
20
control (2/3)(n=7)
silica depleted (5/6)(n=7)
Colture Media

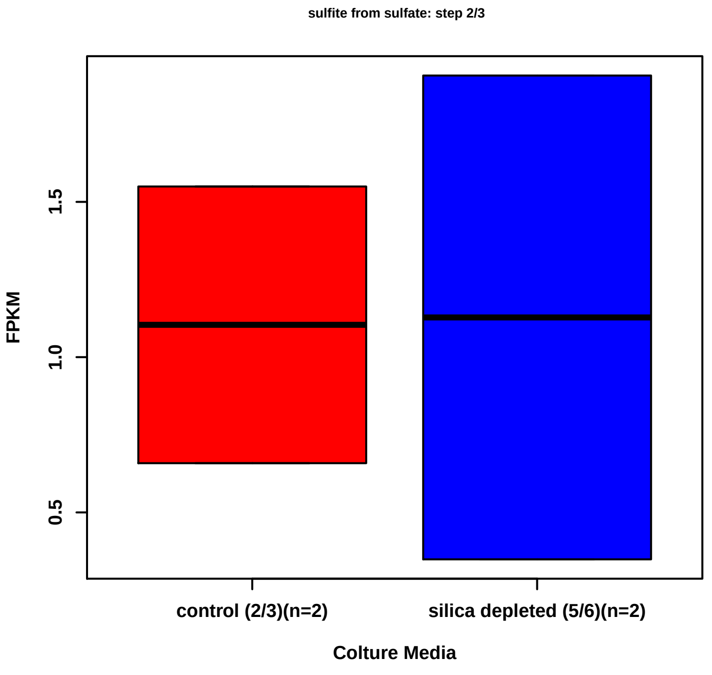

sulfite from sulfate: step 2/3
FPKM
0.5
1.0
1.5
control (2/3)(n=2)
silica depleted (5/6)(n=2)
Colture Media

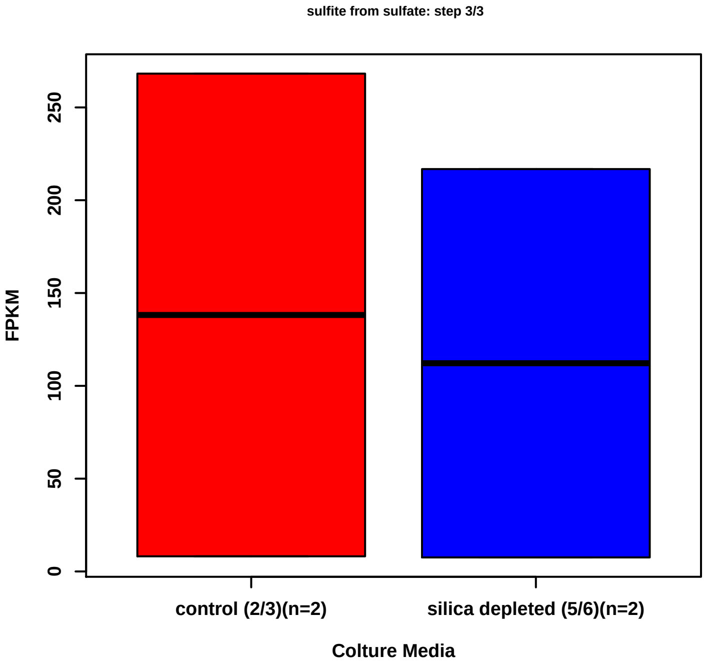

sulfite from sulfate: step 3/3
FPKM
0
50
100
150
200
250
control (2/3)(n=2)
silica depleted (5/6)(n=2)
Colture Media

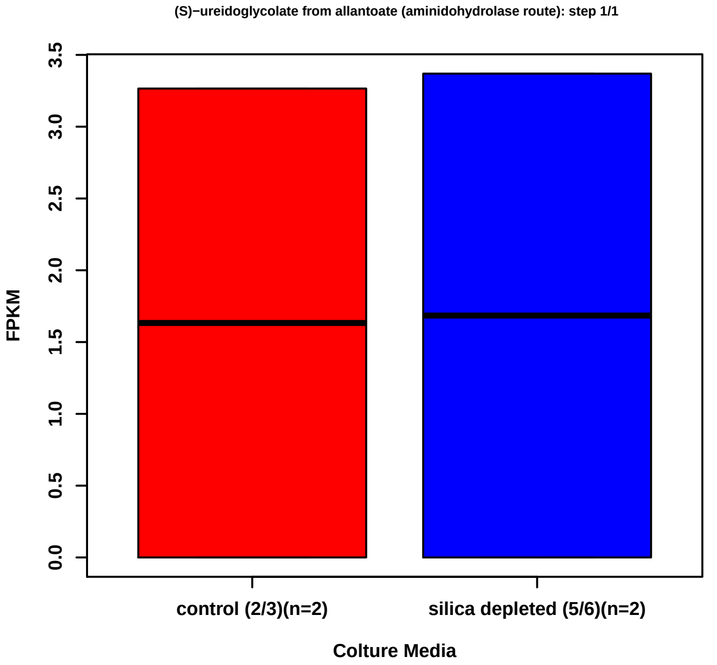

(S)−ureidoglycolate from allantoate (aminidohydrolase route): step 1/1
FPKM
0.0
0.5
1.0
1.5
2.0
2.5
3.0
3.5
control (2/3)(n=2)
silica depleted (5/6)(n=2)
Colture Media

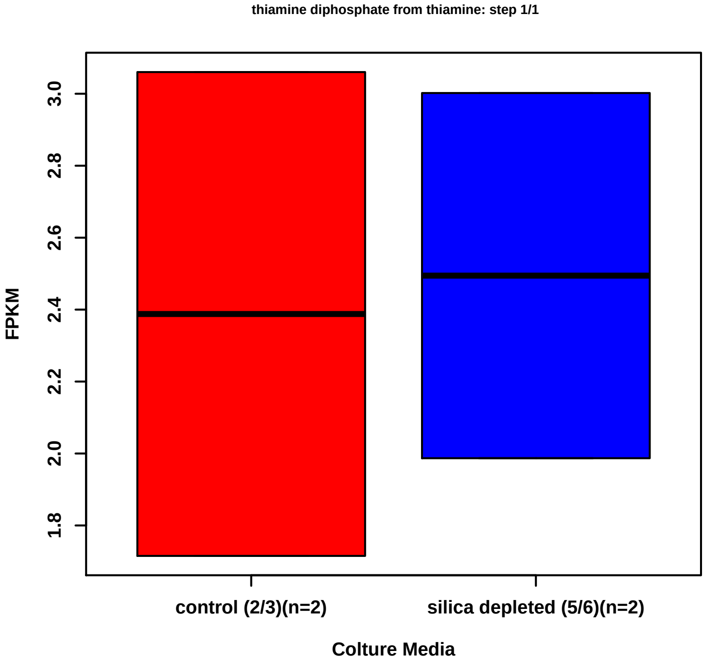

thiamine diphosphate from thiamine: step 1/1
FPKM
1.8
2.0
2.2
2.4
2.6
2.8
3.0
control (2/3)(n=2)
silica depleted (5/6)(n=2)
Colture Media

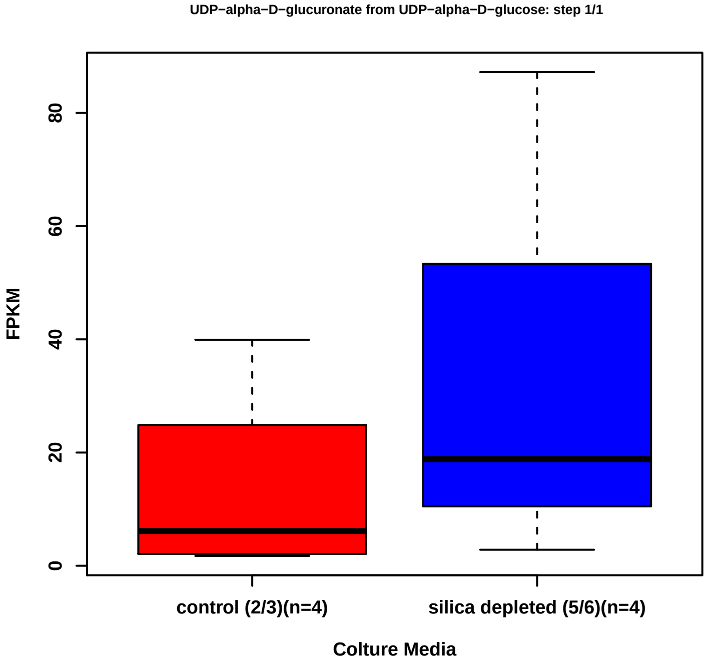

UDP−alpha−D−glucuronate from UDP−alpha−D−glucose: step 1/1
FPKM
0
20
40
60
80
control (2/3)(n=4)
silica depleted (5/6)(n=4)
Colture Media

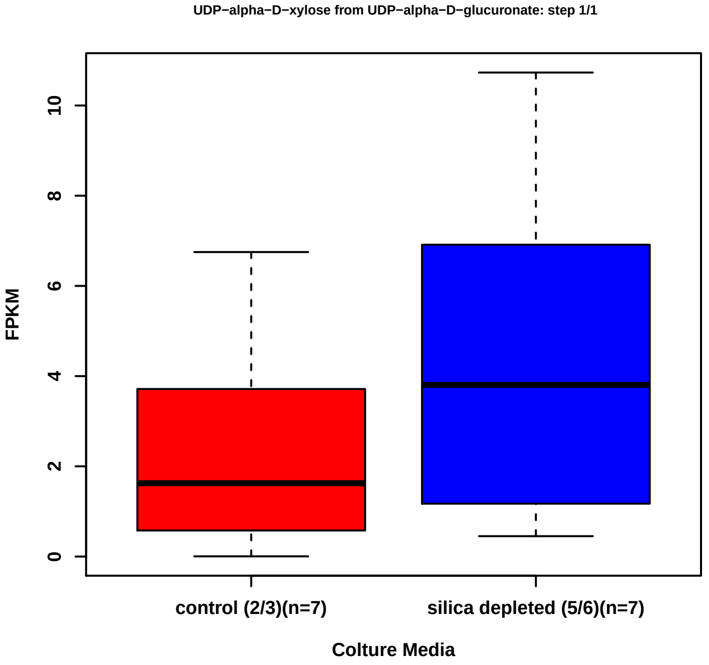

UDP−alpha−D−xylose from UDP−alpha−D−glucuronate: step 1/1
FPKM
0
2
4
6
8
10
control (2/3)(n=7)
silica depleted (5/6)(n=7)
Colture Media

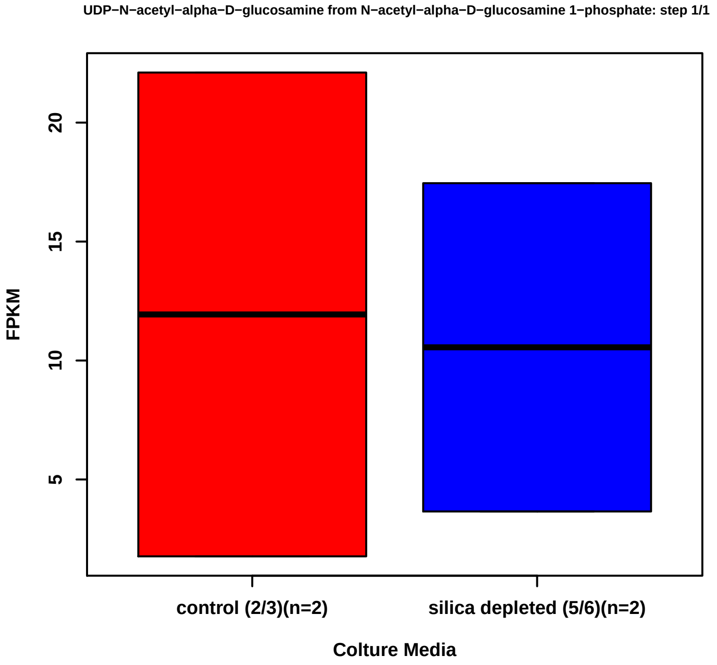

UDP−N−acetyl−alpha−D−glucosamine from N−acetyl−alpha−D−glucosamine 1−phosphate: step 1/1
FPKM
5
10
15
20
control (2/3)(n=2)
silica depleted (5/6)(n=2)
Colture Media

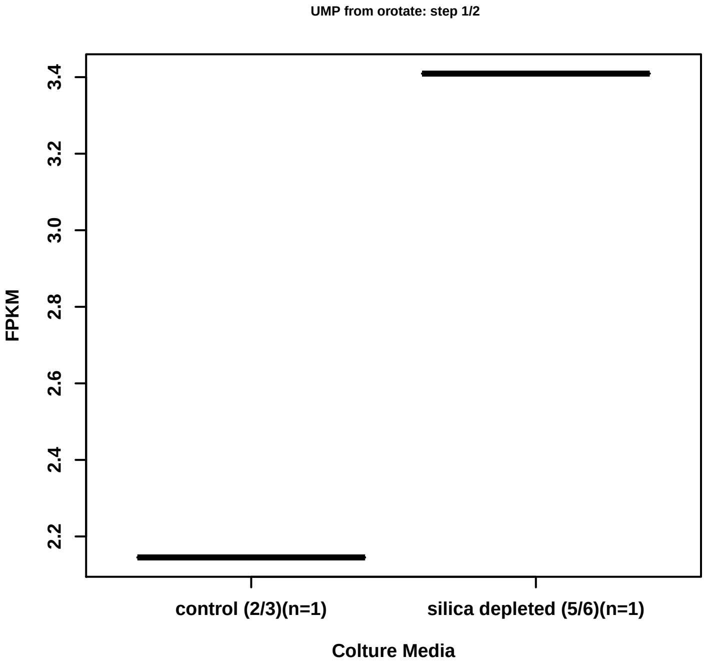
UMP from orotate: step 1/2
FPKM
2.2
2.4
2.6
2.8
3.0
3.2
3.4
control (2/3)(n=1)
silica depleted (5/6)(n=1)
Colture Media

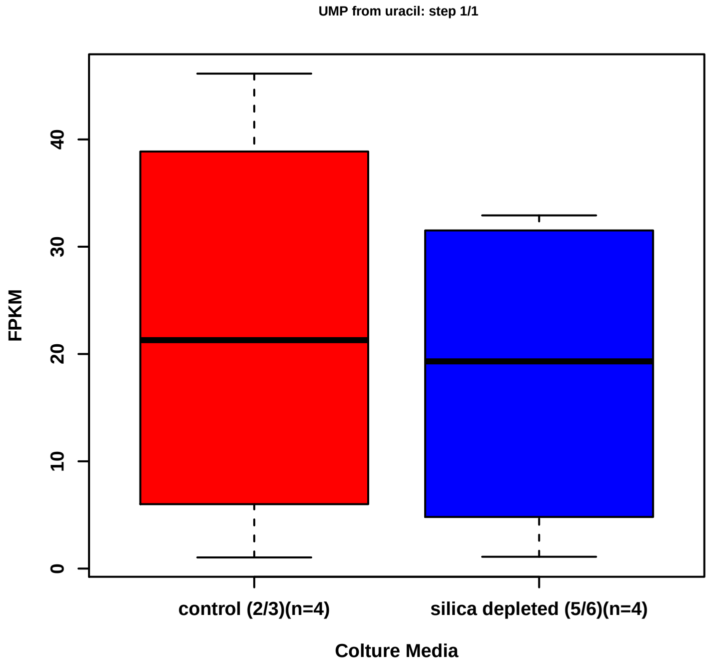
UMP from uracil: step 1/1
FPKM
0
10
20
30
40
control (2/3)(n=4)
silica depleted (5/6)(n=4)
Colture Media

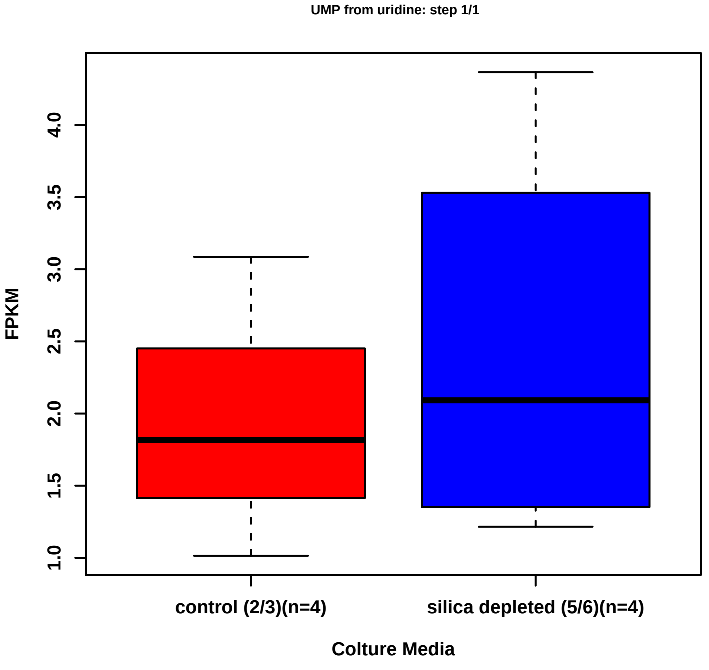
UMP from uridine: step 1/1
FPKM
1.0
1.5
2.0
2.5
3.0
3.5
4.0
control (2/3)(n=4)
silica depleted (5/6)(n=4)
Colture Media

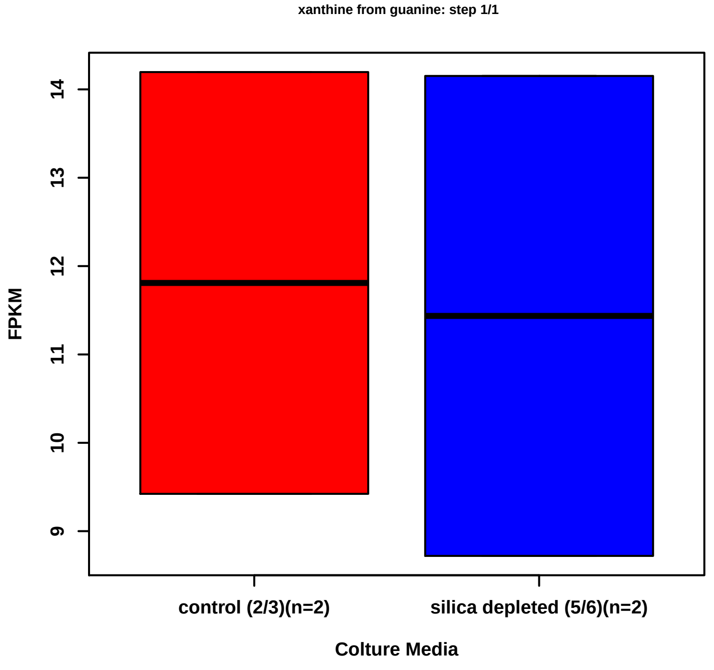
xanthine from guanine: step 1/1
FPKM
9
10
11
12
13
14
control (2/3)(n=2)
silica depleted (5/6)(n=2)
Colture Media

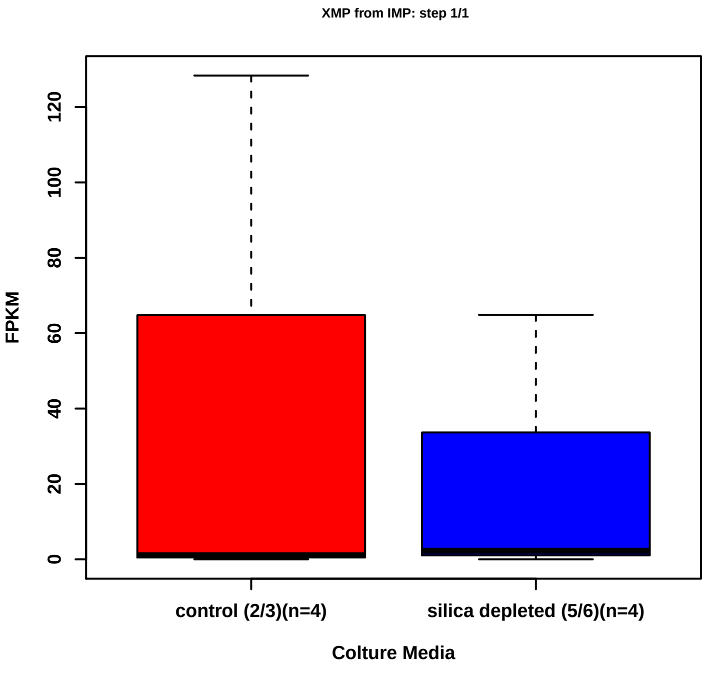
XMP from IMP: step 1/1
FPKM
0
20
40
60
80
100
120
control (2/3)(n=4)
silica depleted (5/6)(n=4)
Colture Media

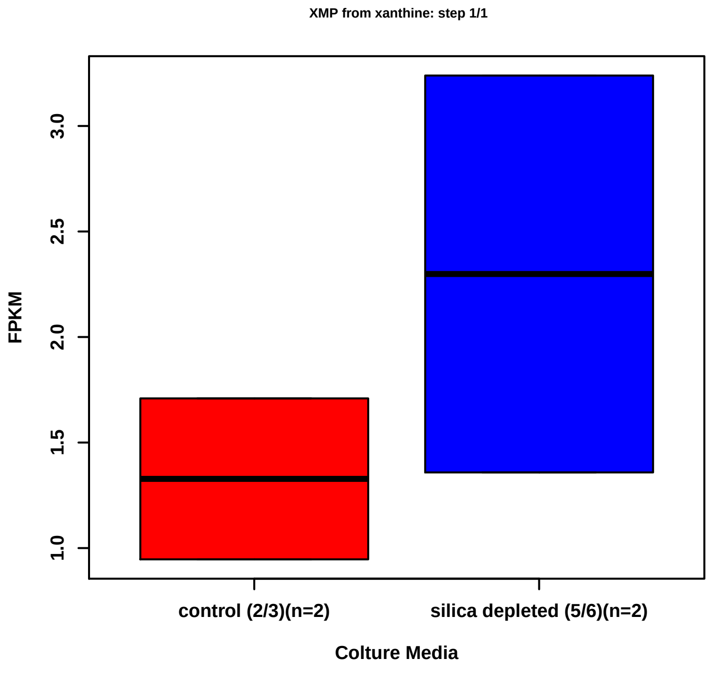
XMP from xanthine: step 1/1
FPKM
1.0
1.5
2.0
2.5
3.0
control (2/3)(n=2)
silica depleted (5/6)(n=2)
Colture Media

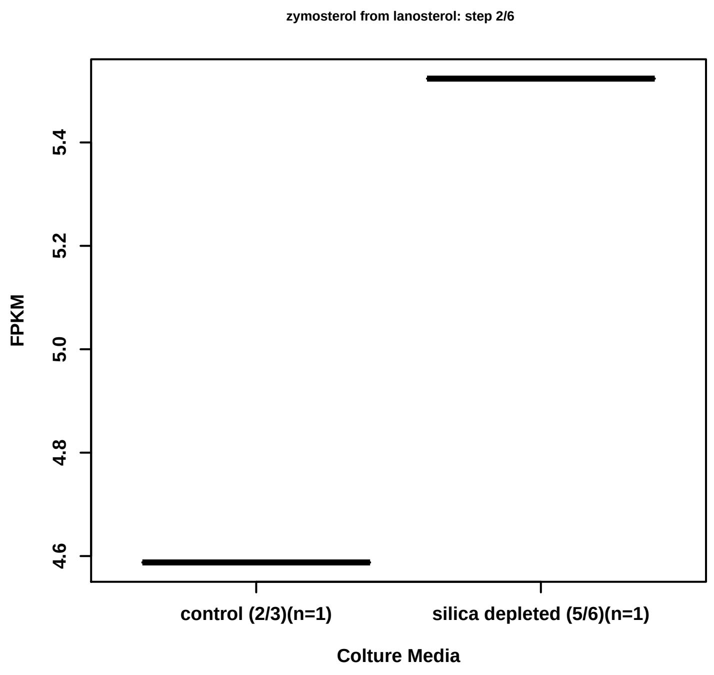

zymosterol from lanosterol: step 2/6
FPKM
4.6
4.8
5.0
5.2
5.4
control (2/3)(n=1)
silica depleted (5/6)(n=1)
Colture Media

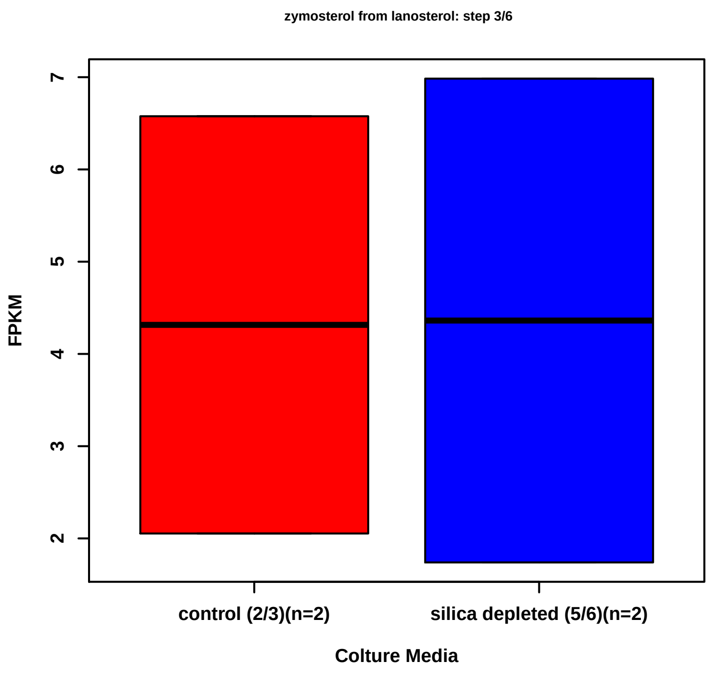

zymosterol from lanosterol: step 3/6
FPKM
2
3
4
5
6
7
control (2/3)(n=2)
silica depleted (5/6)(n=2)
Colture Media

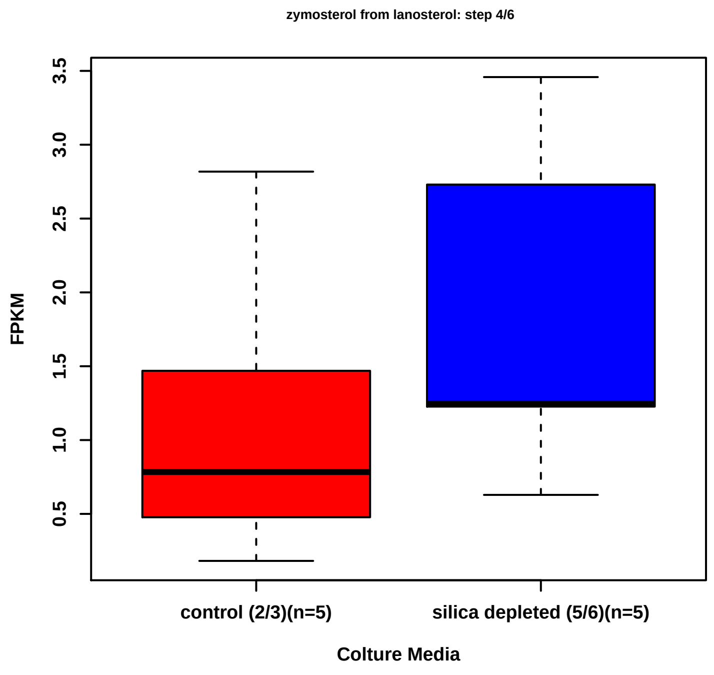

zymosterol from lanosterol: step 4/6
FPKM
0.5
1.0
1.5
2.0
2.5
3.0
3.5
control (2/3)(n=5)
silica depleted (5/6)(n=5)
Colture Media

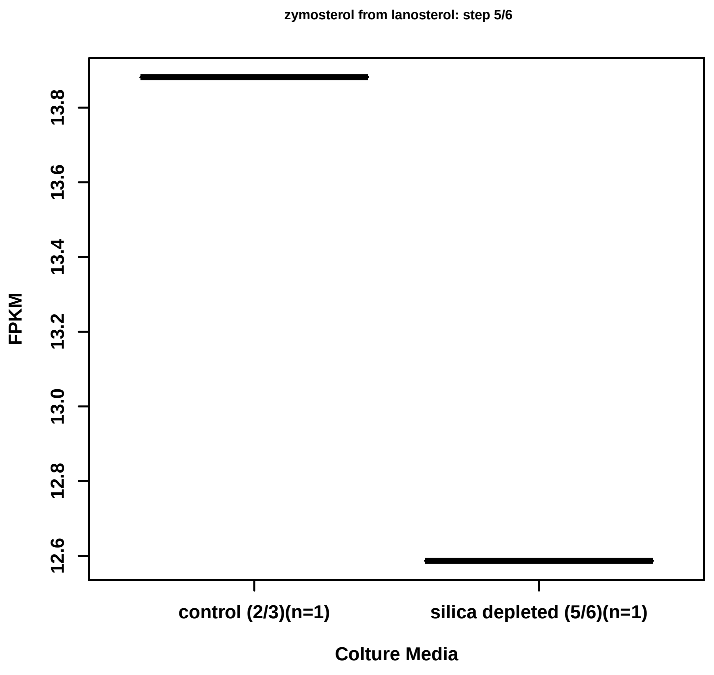

zymosterol from lanosterol: step 5/6
FPKM
12.6
12.8
13.0
13.2
13.4
13.6
13.8
control (2/3)(n=1)
silica depleted (5/6)(n=1)
Colture Media

Unveiling the presence of biosynthetic pathways for bioactive compounds in the *Thalassiosira rotula* transcriptome

Valeria Di Dato*, Federica Di Costanzo, Roberta Barbarinaldi, Anna Perna, Adrianna Ianora, Giovanna Romano

Supplementary File 7:

Nucleotide sequences of the transcripts in the *Thalassiosira rotula* clone FE80 transcriptome.

>TR1|c0_g1_i1 len=586 path=[589:0-294 590:295-318 591:319-585] [-1, 589,
590, 591, -2]
CGACACGGCGCGGAGGAGGTCCGACGCGTCGTGAACCTCGGCAATGCCGTCCGTCAATAATGCCACCTCGTCG
TCGGTGATGGGGGGCAACGGGACGGTGAGGAAGAGATGATGGTAGTACCGCAATACGCGATGGTACTGCGCTC
CTCCGTAGAGCTTGGCATTGGGCGTGAGTCCCCAGTAGGATAGGAGGGCGGGATCTTCGATGTGACGGAAGGA
TGGATCGGGGGAGAACGAATACGTATCCACGTCCATGCCATTCTCGTCCTCCTCCTCGCTTCCTCCTTCTCCT
CCTCCCGAAGAAGACGAAGCCGAGGCCATCGATGGAGATTCCAACAATTCGCCCCTGTGTCTCTCCTCCCTCC
TCCTCCTCTCGTCCTCCAACACGTTCCTCCACCTCTCGTCGCACGCCGCCCTCTCCATCGCTATCGATTGACC
GAACAACGCGGGGGGAACGTGCGCGGCATTTCCCCGAATCAACGACTGCACCGCCGACACGCGAGAGTCGTGC
AACGTCAATGCCAGGTCGGACAGCAATTGTTGACGTTCGTCTTCGATGGCGACGTGACGTTCGTGAGATCGGA
AG
>TR2|c0_g2_i1 len=248 path=[294:0-173 295:174-247] [-1, 294, 295, -2]
GCTTTTTCGGCGCTCCAGCGCGGATCTCGGATGAAATCAACCGTCTAACCGGCGGCAAGTTGTTTCTTGACGG
TCTTGTGTCGGTTTGGCAGGTTGGTGGAGAAGAGGCGGCGGAAAACTAGGGGAAAACAGATGATCAAGTCGGA
ACTGGTTCAGGCGGTTGCCGCGCGCAACAGAAAAGAAAAAAAGTAAAACAGAAGGCCAACCACAATCAAGCAC
CCAAAAAAAAACGAAGAAAAACACCGAAAG
>TR4|c0_g1_i1 len=923 path=[922:0-417 929:418-565 928:566-922] [-1, 922,
929, 928, -2]
TCTCGTGAATGTAATTCTATGATTATTACTGAAAAATTGTTTGTCCGAGTGAAAATCCACTTCCAAAGTAAAA
TCCACAAACGGATACAGCCATCTTAGTGGAGTACACTGACCCCCCCCCCCGCTCTGATGGCCCAAAACAAATA
ACATTTACAGCCGTTTGAGTATAAAGAGTCTATCATTGGCCTGGAGTATTATACGGTGTATATTTACTCTTCG
TCGTCATCTTCATCATCGTAAAGATCGGCATCGTACACTCCCGGCAAGAGTCCATTCACCTTCAGCGCAATGG
CCTTAACGATGGCGGAACGATGAGCCGAAATTCCCTTGTCTTGCAACCAATACCAAAATTTCTCGCAGTGTTC
CATGGATTCAACCTTATTATCGTCCGTGTCTGGTCCCCTGAGCATGAGAAACTGTTTTATTGCCCCGGCCGAG
TAAATAACGTCGCGCACACACCATAGTGTATCCAATCGTGAACAAAATTGACATTAGAGACACATCTTATTGT
AACAGGGACACCACTTAAACACAAAATCGTACCTTTCCAATCAACTGATACGTATTTGTGACCGCTTCGTCAT
TCTCACCCATCGCCATCTTCTTCGCCGCCGCTGGTCCGATGCCGGGCACCTCGGTAATGTCACCGGTTATGGA
TCCGCGAATAAAATCCATCATCGTGTCATCATTCACGCGGGAGCGTTTGGGGTCGTATCCTTGTTCCGCCATG
GTTGTGTTTTAGAGTTGCGATTGGTTGAGATTGTATGTGTCGTCGTCGCGTCGCGTCGTTGAGGAGGGGTTCT
GATTGTGGATTTTATGACCGTTGGCGGTTTGCGGGCGGACGACTACTACTTTCCAAATACGATTGGTCACGAT
AGTTCTCTCATCACGTAGGAATAATATATGTTTTCGATACACAACTT
>TR4|c0_g2_i1 len=796 path=[924:0-417 925:418-438 926:439-795] [-1, 924,
925, 926, -2]
TCTCGTGAATGTAATTCTATGATTATTACTGAAAAATTGTTTGTCCGAGTGAAAATCCACTTCCAAAGTAAAA
TCCACAAACGGATACAGCCATCTTAGTGGAGTACACTGACCCCCCCCCCCGCTCTGATGGCCCAAAACAAATA
ACATTTACAGCCGTTTGAGTATAAAGAGTCTATCATTGGCCTGGAGTATTATACGGTGTATATTTACTCTTCG
TCGTCATCTTCATCATCGTAAAGATCGGCATCGTACACTCCCGGCAAGAGTCCATTCACCTTCAGCGCAATGG
CCTTAACGATGGCGGAACGATGAGCCGAAATTCCCTTGTCTTGCAACCAATACCAAAATTTCTCGCAGTGTTC
CATGGATTCAACCTTATTATCGTCCGTGTCTGGTCCCCTGAGCATGAGAAACTTTCCAATCAACTGATACGTA
TTTGTGACCGCTTCGTCATTCTCACCCATCGCCATCTTCTTCGCCGCCGCTGGTCCGATGCCGGGCACCTCGG
TAATGTCACCGGTTATGGATCCGCGAATAAAATCCATCATCGTGTCATCATTCACGCGGGAGCGTTTGGGGTC
GTATCCTTGTTCCGCCATGGTTGTGTTTTAGAGTTGCGATTGGTTGAGATTGTATGTGTCGTCGTCGCGTCGC
GTCGTTGAGGAGGGGTTCTGATTGTGGATTTTATGACCGTTGGCGGTTTGCGGGCGGACGACTACTACTTTCC
AAATACGATTGGTCACGATAGTTCTCTCATCACGTAGGAATAATATATGTTTTCGATACACAACTT
>TR5|c0_g1_i1 len=259 path=[313:0-68 329:69-100 327:101-101 325:102-134
320:135-258] [-1, 313, 329, 327, 325, 320, -2]
CGATCTCTCCTCCATCGCCGAAGCACTACTCGTAAACGTGCTTACTTTCATCGACGCCAACTGCGCAAAATCA
TGTGAACTTTGCAGCACTTCGCGCTTCCATCAGTACGACGACACTGGCCATCCGGGACTTTGTTACTCTAAGC
CTTGGGTGAGTTCAAATTTCAATGTGTTCACTTTCGCAATATACACCTCAACTTGTATAAACTATAGGCAACT
ACGTTGTAATTTTTCCAGTGGTGTAGATCCTCCCCCGCGC
>TR6|c0_g1_i1 len=1298 path=[1361:0-688 1362:689-715 1363:716-1297] [-1,
1361, 1362, 1363, -2]
TCCGATCTGAGAATGATGCGAACAAACGACGTGTGCAACTTGAACCAGCTTGATCGCTGGTGAAGGCGTGATC
TGTTTACGCAATTTGAGGTATCCTGAACAGCACGTCAGTCCAAAGATCTGAAAACATCTCTGACGAGGGTCAT
AATATTCCTTCTCCAAACACTAAAAATGAAGTCTTTTGTCGTTGCCACCCTTGTTGCTTCTGCTGCCGCGTTT
GCCCCCGTCAGTCAACGGGCTTCCTCCCCCACCGCCATTTCAGCAGCAAACAAGTTCGCCAATGAACTCGGGG
CCCAGGCTCCTCTAGGATTCTTCGATCCCATGGGATTTTTGGGACCAGAAGATGACACCCAATTCCAGTGGCT
GAGGGAGGCAGAAATCAAGCATGGCCGTATTTCTATGCTCGCTGTTGTCGGCTACCTCACCACCTATGCTGGA
TTCCGCATCCCTGGCATGGAGGATGTTCCCTGTGGATTTGGAGTCTTTGACAAGACAGTCTACACCACGGACC
TCGCGCTGAATAATGTTAGGTGGACCATTGCCACCATGTTGATTCTAGAAACTGCAATTGTGAAGGATGGATA

TGATTTGGCGGAATTCCCAGGTGACTACCTTAATGGATTTGGAAAAATTCGATGGGATGGACGTTCTGCTGAT
TTCCAATACTCATCGCGTGCCAAAGAATTGAATAACGGCCGTGCTGCCATGATGGGAATTCTTGGACTCATGG
TACACGAAAGCATGGGAAATGTTGACCAAATTCTTCCGTTTTTGAACAAGTAGGCTATCATGGCTCCCATGCA
TCGTCGTGGTGCTGAGGGAAACACTGAATAAACCAGTGTGTAGCATATAACGAACTAATTGCATTCCGACTCG
CACCACAAAAGCAGATGTACTTCCACTTTTATCTTATGGTATGATATTCCAGAGAATCAGTCCAGGAGGCATT
GCGCGAATTAACAGCTTTCTTCGACCAAATCGTCATATTGTACGAGATAACGACGATGGCGATGCCACATGCA
TATGACTCGTTTGTTACTGCCACACTCATCTCGAGTACAGCACTAGTAGACAGGTAGAAGTCAAAATAGCATC
ATTATTTGGAAATCTGCTCTCTATTCCAACCAGCCAGAGTTACTCCGAAACACAGCGAAAACGACAGGGCAAA
ATGGAAGGGCCATCTTACAAACAATTAATCCTGTATTTTGAGATATCTTGTATGCGAAGGGCGATAAAAATAA
GCTCCCAACTTCTTCTGTTTGCATGCTAGCTAAATAGCGTTATAAATGTTACTAAGC
>TR7|c0_g1_i1 len=1421 path=[1535:0-43 1536:44-67 1537:68-82 1550:83-118 @1549@!:119-1248 1527:1249-1272 1528:1273-1420] [-1, 1535, 1536, 1537, 1550, 1549, 1527, 1528, -2]
TTGCTGTTGTTGTTGTTTTCAAGAGACCATCCCTCGTCATCGAGTTCCATGCCCTCGCCGTCCCTGTCGTACG
CCTTCTTCTTCGTTCGCAGCCCGGACACCCAGCTTCCCAACTGGATATCCTTCTGATACTTGACGGGCACGTT
GGGGTGGCCGTGCTCTTCCTTGTAGACGAGGAGCTTTTCGAACATAATGTCGAAGTCGAGGGATTCGCCGCTG
GGCTTCTTTTTGTTGGAGAATGGTTCGTACTTCATTTCGGACCTGGCCTTCTTCCGCGCTTCTTCGTCTTTGG
CTGCCAAGATGGTCATTTCTCTCACGAGTGATTCTTCCAGTATCGGTATCTTTGGAACACCATCCTCGTTGGC
GTTGCCATTGCCATTATCATCGTCCTCCTCCTCTTCCTCATCGTCTTGACCATTCTCAAGTTTCAAAGCTTTG
GCCATCTTCTTCTGCCCAATGACTTTGAATGTCGTCTCCAACTTGAGCAATAATCCCTTAAGGCCCGCCGCCG
GCAAATCGGCCTTCTTCACAGCCCGCCTCCCCGCCTCGCTCGTTTCCAACTCGGCCACCCTCTCGCTCAGCCA
CGAAAAGTAATTGTAAATTTCCGAATGCAATTGACGACGCTCCCCATTGCTCAACAACGTGTTGGCCAATCGA
TTGTTATTCGCGCCGCCCTTCTTCTTGCTATTCGTATTCGTGACTGCCGCCGTCCGTCCGCTCCCGACCTTCC
CCACCAATATCGAGCATCCCCCGCCCATGCTGGCGATGGCATCGGCGGTAGAAATTTGCATCGTCCCATGCAC
GATTTCGGGAGGTATGTCCAACGCCGACTGCGCCTGCGGACTCCAGTGGGGATAACCCGTCACCGCTCCCAGT
TCGTCGCAGTGCATGTACGGAAATGCGTGCGGGTACTTGGTAACCCATTCCGCCTTGGACGAGTACGGGAGGA
ATTGGATCTTGTCGCGGGGAAAGCCGTAGCATTCGAGATGGGAGCACGTTGCGTCGATGGAGGCGTCGGTGTG
ATTGGCGATCCAGGCGAACGCGTGTTCTTCGCGGTTGTTGTTGGGGGAGGAACGTAACGCGTGGAGGACGCCG
CAGGGATTCGAATCGAGTTGGGATTTGCGGAGGTTGGTCTTGAACGTGAAGGTGGTTCGTTGCACCTTTGGGG
GAGGCTCTCGTGTGTCGGTCGCCATTGTGGATCTCATCGGCTGGGGGGGGGGGGAATTATTAGTGTTGCTGAA
TAGTATGGCACGGTGATTTGCTCTAGCTTTCTCGATTGATCATAAAGTTGTGCCGCCGCGCCATGGTCAAAAG
GCAGAGCTTTGGTTGGGGTTGTTGCGCGTTGCGCGGTCGGTGGTTGATGTTAGTTTTGGAGAATATAATGCAG
CAGCGGGAATGCTGCATCCTAGAGAGATCGGAAG
>TR7|c0_g2_i1 len=227 path=[1539:0-64 1540:65-78 1541:79-226] [-1, 1539, 1540, 1541, -2]
TTGAATCTCGCCGACTTTCGTCGACCCATGTAAGTATCTCACCTGAATAGTATGGTACGGTGATTTGCTCTAG
CTTTCTCGATTGATCATAAAGTTGTGCCGCCGCGCCATGGTCAAAAGGCAGAGCTTTGGTTGGGGTTGTTGCG
CGTTGCGCGGTCGGTGGTTGATGTTAGTTTTGGAGAATATAATGCAGCAGCGGGAATGCTGCATCCTAGAGAG
ATCGGAAG
>TR8|c0_g1_i1 len=371 path=[582:0-324 583:325-370] [-1, 582, 583, -2]
GATGGTGATTGGCCTAGGCCATCATCGTTGTTGTTGTTGTTGCTGAGACTTGCGTAGGCTAGGGATAGACCCA
CTGAAATACCGGTAGCCGTTCCACCACTTCCCGTAGCAAAAACGACATGATCCAAAGCAAAATCCTTCTCCTC
CTCTTGGATAGTTTCAATCTGTTGCATCAGCTCGTCCACGGCGTTGATGTAACCCCACGTTCCTAGAGCATTC
GATCCTCCTACCGGTATTGGATAGGGATTGCTGTCGTTGTCGTTGTTGGATTCGATGTGGTTGCAGACGTCTT
CAACTAATTTTTGGGAACCCAGCCTTCCGTACTTTATATAAGTTTGTGTGAGAGATCGTCATTTTTGATGCAC
AAACCA
>TR8|c0_g2_i1 len=391 path=[579:0-324 580:325-376 581:377-390] [-1, 579, 580, 581, -2]
GATGGTGATTGGCCTAGGCCATCATCGTTGTTGTTGTTGTTGCTGAGACTTGCGTAGGCTAGGGATAGACCCA
CTGAAATACCGGTAGCCGTTCCACCACTTCCCGTAGCAAAAACGACATGATCCAAAGCAAAATCCTTCTCCTC
CTCTTGGATAGTTTCAATCTGTTGCATCAGCTCGTCCACGGCGTTGATGTAACCCCACGTTCCTAGAGCATTC
GATCCTCCTACCGGTATTGGATAGGGATTGCTGTCGTTGTCGTTGTTGGATTCGATGTGGTTGCAGACGTCTT
CAACTAATTTTTGGGAACCCAGCCTTCCGTACTCTCCAGGAGTGCACGTGTAAATGGTACTTCCCACCATTCG
ATCGAATAAGATAGATCGGAAGAGCA
>TR8|c0_g3_i1 len=517 path=[584:0-324 585:325-376 591:377-460 590:461-516] [-1, 584, 585, 591, 590, -2]
GATGGTGATTGGCCTAGGCCATCATCGTTGTTGTTGTTGTTGCTGAGACTTGCGTAGGCTAGGGATAGACCCA
CTGAAATACCGGTAGCCGTTCCACCACTTCCCGTAGCAAAAACGACATGATCCAAAGCAAAATCCTTCTCCTC
CTCTTGGATAGTTTCAATCTGTTGCATCAGCTCGTCCACGGCGTTGATGTAACCCCACGTTCCTAGAGCATTC
GATCCTCCTACCGGTATTGGATAGGGATTGCTGTCGTTGTCGTTGTTGGATTCGATGTGGTTGCAGACGTCTT

CAACTAATTTTTGGGAACCCAGCCTTCCGTACTCTCCAGGAGTGCACGTGTAAATGGTACTTCCCACCATTCG
ATCGAATAAGATGTTGCCTACCCATCCAATATCATCCTTCTTGTCGTGGATTGCATCTGCCCTGCGTGTGCGT
AGAATCAAGTGGGGAGACAGTCCTACCATTCGACTGGCAGCAGCAGTCGCACGACAGTGATTACTCTGCTCCC
CCCCGA
>TR9|c0_g1_i1 len=1183 path=[1697:0-264 1724:265-295 1722:296-328
1719:329-1033 1696:1034-1182] [-1, 1697, 1724, 1722, 1719, 1696, -2]
GAGGCGAGTAGTTATGAGGAGACGTGAGTTTGCTGAAGTTGTTGGTAGTCTTGTTGATGTGCCGACATTGTTG
ATATGCGTTTGATTGGGCTCCTATCGCGGTGCTAGCTCAATATTTTGCCGCTTTGTTGGCATTTTTATTTCTC
ATTTTTCCCTCGTCTCTTAAATTTCATTCCAATATCTTGACGGATCACTACACATGATATCATACCATCTCTA
CAACAAAACAACCATATCCACAGCTACGGCACCAAAATCCCCCCAACAACACTTGCCGCCCGTCTCTCCACCT
ACGTCCACTACTTCACCCTCCACGGAGCCCTCCGTCCCTTCGGCGCCTCCACCCTCGTCGCCGCGTACGATCC
CGCCACCAAAAACCACTCCCTCCACATGGTCGAACCCAGCGGAGTAAGCTACGAATACTTCGGAGCCGCGGCA
GGACGAGGACGACAGAGCGCACGCACGGAGATGGAAAAGTTACCCACCAATCCCAAGACCGCCGGCGGAGCCG
AAGGAGGAGAGGAGAATCTGATTACCGTGGCAGAGGGAGTAAAGCAGCTTGCCAAAATAATCCACACATTGCA
CGACGAAGCGAAGGATAAGCCGTTTGAGTTGGAAATGTCGTGGCTTTCGGAGGGGACTGGGTGGAGACATGCG
GGTGTGCCGCGTGCCACGATTGCCGAGGCGGTGGCGTGGGCGAAGAAGGAGATCGAGGAGGCAGAGGAGGAGA
GCGAGGACGATGAGATGGAGGAGTAAGCGAGAATGAATATAATATCACGGAAGGAAATGAGACAAGTTGCATA
AGATAGGGATTAGTTATTTTCCAGTGCGAATGAAAGAAATAGGGTATATCGGTTGGTTTTAGGCATGGACATA
CTATTTGTTGAAGTGCAATGTATGTCGCATTATTAACGTTGGGGTCCTGCCTTTATAACACTCACAATGGGAA
AGGATTTATTGACATTCAATGGCGTTTTTGATGGAGAGGACAGACTTGTAACCAATTTGGCCTTTCTTCTTTG
TCCCCTACCTTTTGATGGCAACGCCAACTGGTAGACACATGGCATATGCTTACCAGGGGAATTTCATCATGGT
CTTCTTACTGACGGATAGCCTGAATCTTGATTAAATATTCGACCAATTAAGCATCGTAGACATCCCTCTGTGC
GCCCGAAAAAAAAAAA
>TR9|c0_g1_i2 len=1391 path=[1690:0-440 1691:441-464 1720:465-472
1724:473-503 1722:504-536 1719:537-1241 1696:1242-1390] [-1, 1690, 1691,
1720, 1724, 1722, 1719, 1696, -2]
CTCTTCATCTCACATCTTCGACAGCGACAGCATCCATCGGCATCTTTCAATCCTACAGATTCTGGCCCGATAT
CTCCCATCCTTCATCAAAACATTAGCACCAGTACAAACAATCCGATATACAACAAACTAAATTTAAAAAATGG
CATCCTCCGGCTCAGGCTACGATCTCTCCTCCTCCACCTTCTCCCCCGACGGCCGCATCTTCCAAATCGAATA
CGCCGCCAAGGCCGTCGAAAATGCCGGCACGGCCCTCGGTTTGCGATGTCGAGATGGCGTAGTGTTGGCCGTG
GAAAAACCGTTGCAAAATAAGATGCTCGTGGCAGCGACGACGGGGAGAAGGATTCACACGGTCGATGAACACG
CGGGGGTGGCCATGACGGGATTTGTAAGCGATGGGAGGCAGATTGTGAATCGGGCGAGGGAGGAGGCGAGTAG
TTACGAGGAGACCTACGGCACCAAAATCCCCCCAACAACACTTGCCGCCCGTCTCTCCACCTACGTCCACTAC
TTCACCCTCCACGGAGCCCTCCGTCCCTTCGGCGCCTCCACCCTCGTCGCCGCGTACGATCCCGCCACCAAAA
ACCACTCCCTCCACATGGTCGAACCCAGCGGAGTAAGCTACGAATACTTCGGAGCCGCGGCAGGACGAGGACG
ACAGAGCGCACGCACGGAGATGGAAAAGTTACCCACCAATCCCAAGACCGCCGGCGGAGCCGAAGGAGGAGAG
GAGAATCTGATTACCGTGGCAGAGGGAGTAAAGCAGCTTGCCAAAATAATCCACACATTGCACGACGAAGCGA
AGGATAAGCCGTTTGAGTTGGAAATGTCGTGGCTTTCGGAGGGGACTGGGTGGAGACATGCGGGTGTGCCGCG
TGCCACGATTGCCGAGGCGGTGGCGTGGGCGAAGAAGGAGATCGAGGAGGCAGAGGAGGAGAGCGAGGACGAT
GAGATGGAGGAGTAAGCGAGAATGAATATAATATCACGGAAGGAAATGAGACAAGTTGCATAAGATAGGGATT
AGTTATTTTCCAGTGCGAATGAAAGAAATAGGGTATATCGGTTGGTTTTAGGCATGGACATACTATTTGTTGA
AGTGCAATGTATGTCGCATTATTAACGTTGGGGTCCTGCCTTTATAACACTCACAATGGGAAAGGATTTATTG
ACATTCAATGGCGTTTTTGATGGAGAGGACAGACTTGTAACCAATTTGGCCTTTCTTCTTTGTCCCCTACCTT
TTGATGGCAACGCCAACTGGTAGACACATGGCATATGCTTACCAGGGGAATTTCATCATGGTCTTCTTACTGA
CGGATAGCCTGAATCTTGATTAAATATTCGACCAATTAAGCATCGTAGACATCCCTCTGTGCGCCCGAAAAAA
AAAA
>TR10|c0_g1_i1 len=3778 path=[4228:0-340 4229:341-364 4268:365-377
4274:378-402 4282:403-419 4266:420-425 4281:426-443 4278:444-484
4264:485-651 4255:652-675 4271:676-771 4240:772-795 4263:796-1008
4205:1009-1032 4277:1033-1312 4226:1313-1334 4261:1335-2140 4198:2141-
2164 4280:2165-2184 4276:2185-2208 4269:2209-3320 4202:3321-3337
4203:3338-3777] [-1, 4228, 4229, 4268, 4274, 4282, 4266, 4281, 4278,
4264, 4255, 4271, 4240, 4263, 4205, 4277, 4226, 4261, 4198, 4280, 4276,
4269, 4202, 4203, -2]
TTCCGATCTACGCAATCAATCACAGAGGCCTTTTACGTAGAATGTCAAGAACCAAGCAGGACAGGATGGCGTC
ATTCAACAAAAAGCGACGATTCTTCTTTGGCTTTCCTCAACGTCAATGAAAGAACAAAAGCAAAAGAACGAAC
AATGAGGCGATCGCATCGATTCGCAATTGATCATTAATGTAAAGCAATCGTTTCGTCGAAAAAACCGTGGCGT
GGTCGACCGTCGGAAGTGCTCCTGCGATAAAATTAGACGAGTCCCAAAATCATTGATACATACACCCTTCGCT
CCCTTCGCTCTCTAGATACGCGGAGTCGTCAGTCGCGGAGTCCACGACCAACATTCACTTGAAAATCGAGCAA
CTTCCAGTTCAGTCGCATTAAATTCGCTTTGGACAAATTGGTTGAAAGTGACGGTGATCTATTGAACTGAGTA

CATTTTTATCTCTCTCATGAACCGAATGTCCAACAAAAGCAAATGGTAGAAATTTAATATCGATGGTATTGTA
CGTAATTGATTGCATCGAAACACCCAACTGAACGACTTCAACTTCACCGCATCACGACCGACCAATCAAAATG
GATCACCACATGAATATGATCGACCAATCATCCGCCCATCCAGGAAGTACCACTCTGAATAGCAACGTAAAAG
TTGCAGCAGGATACGTTCTGCAAGAGCGTTTGGGATCGGGTTCTTTTGCCACTGTCTACAAAGGAATACGATT
GGCAGACTATCAGCGGGCTATCAGTGATGCCTCGCATCAGACACCGCCGCCAGCAAATTCTATCACCGCCATC
AAGGCCATTTCTCGATCGTCCAAAAAGCTTACCAAGAAGGTTCTCGAAAATCTTGATATGGAAATTGCCATCT
TGCGAACTTATAGACATCCTAATATTGTCTGCCTCCACGAAGTCCAAAAGACTGATAGACACTTCTATCTAGT
CCTCGAATACTGCGGGGGAGGGGATCTTCAACGACTCATTCGCACGCGTCAAAAGGGTCGATTGTCCGAACGG
TTATGCCGACGATTAGTGAGAGATTTGGCGGCAGGATTGGGATTTTTGTGGGGGAAGGAGCTCGTGCATAGAG
ATATCAAGCCGCAGAATTTGTTGTTGACGGGACCGTTGCCCGCGGAGGACGAGGTGGACAATCCGCCGAGGGA
GGATGCGAATCAAGCGTTGCGGGGGATGACTGGGGAGAGGTTCATGTTGAAGATTGCTGACTTTGGGTTCGCC
AGGCATTTGTCAGGAGTTGACTTGGCGGAGACCATGTGTGGGAGTCCTCTTTACATGGCTCCTGAAATATTGT
CGGGAGAAAAGTACGATGCAAAAGCAGATCTATGGTCAGTTGGAGCAGTATTATTTGAAATGATTGCCGGGAA
AACACCGTTTCACGGAGAGAATCATCTGGACTTGTTGAGAAATATAAAAACAAAAGCTGTTCGTTTGCCATCT
AATGTTCGGATATCAAGGGAATTCGTCAAACTGTTGAAGTTACTCCTAGATCGCAAACCTCGCAGTCGGGCGG
GATTCAGAGCTTTCTTTGAAGCAAGTGAAGCTTGCGTGGCACTTGGATGCAATGGTACTCCTGCCGTTCCTCC
ATCCAGTCTGGTCGGTCCAAATGCTACAACTAATCCATTGGATCCATTGCAGGTACCCAGTTCTGGTCAGCAA
ATAGTGGACAGGGAAGTATGCGCCCAAATGAATTTGTGTGCCATTTCAGAAAACGAAGGTGCCGGAGAATCAT
TCCATCAGGGGTATGGCAATGCGAGCTTGGCCACTGTTGCGACATTAGGAACGGTCAATCATCAGCAATTGCA
CCAGGTCCGGCCTCAACCTACTCAGCTTCCCCATGGTGCGCAAGGTGCAAACTACGTTTCGAACATGGCCACA
TCACAGACAAACGCTGTGAATGCCGTGGTTCAGATCCGACCTGACCATATGAATCATGCAGAGGTTGTAACGC
CCCCTTTCAACCCAGTCTCGGCACCGCAGCCGCCTCCCGCTCTACCGCCCGGAATGCAAGGGGTTGGCAGGCA
GCATGGTAGGCCATCTGTTTTTACTCCTCTTCAAGGAAGCCCTGTCCTTCCCAGAACCCGAACTATTCCTAGT
GCTCCTCAGTATTCACTTGGAGGAACCCCTGTCGGTCTTAGTGCGCATCCATTCCCAGGCACACTTCCACCCC
CGCCGATGCAGTTCCGTAACCAGGGAGATGGTGGTTTCCCAATGACGCAGCAGCAACAAATGATTGGTCATCC
AACTCCAGTAAACAACCAAAACACTCTTGTACCAAACAGAAGGACTTCGATCCCCAAAACATCCAGTCATGAA
CCTTCTGATTCTGGTTTTGTTTTGGTTGAGCATAGTGGCTGCAGAAGCAGGCCAGGTAGTGGCCACAACTCTC
CTGCTGGTTCAATTGGGGGTATTGCACGCTCGCCTTCAACGTCTCCACACAACTCTTCCGGTAGGGTCACCAT
CCTGAATGCCAGACATAACATGGGAATCCTTGGTACTAGTCCAAATACAGGACGCGCTCTTGTTGGGAAAATG
ATGATAGGCTCTCCGTCGCCATCTGCCTTGACTCCTCCGTCCGCCTCTGGTGGAGGAAGATTCAACCTGTCTC
CACGGGCTGCTCTTCGCAGTGGAGGATGCTTGGCTCATATTGACGCTCTTGCTCGTATGTTGGCGACCGCAGA
AGATATCGGGCGTAGGTCCATAACTGTTGCTCATCTGGGAGATGTGCGAGCATACTTGGCAATGGAGCTTTTG
GTAGCTCAAAGGGAAGAAAGTCAGTTATCGTCAATGTCGACAGGCACGGGAACACCCATGGAGGGTGTGGATG
AGGAAGGTAATAGTGAAACACCCAATCCAAAGAGGCCGTCGTCTAGAAAAGCAGCTGCCGAAGAAGATGATGA
AGTAGAATTACCCTTTACCATGACGTCATCGTCCGATGGTATGTCTCATCCTTTGAACAACGATATTCAAAAC
CCCGCACCAATGGCAGGCAAGAGTTCTGTGAATGAGTCCCCTAAAGAGGAAATAACAACCTCGATGATTCAAG
TCCGTTTTCGGGAGGCTCTGACGTGCTACTTCAAAACTCTAACCATGATGAAAGGGTCCATTTGTGCAGCTCA
AAAGGTTATGAAGGAAGTTGAAGAGGTTCTTCCAGCTGGCCAACCCGCATCGAACAATAAAAACCCATACACA
CCCTTGAAGGAACGTTGCTCTGCAAGCGTAGTGTGGCTTCGGGGACAATTTTCTGCAGTTCTAGAAAGAGCAG
ATGCGGCAAGTGAGCAAATTTCTAAGCTGCAAAAAACTAATACTTCGCGAGAAGGGGGAGTAGAAGTATCACT
TTGCGTAGAGGAACTGATTTATAATCATTCTCTTAAATGTGGCCGTGAAGGAGCGGTCAAACAAATTCTTGGT
CACTACGATGTGGCAAGATCCTGCTATCGTTCAGCTGGATTGCTGGCGGAGACGTTGCTCATGGAATCGAAAG
TGGGGGATGACGACCGGGTTGTGCTCGAGGGCTATATTCACAGTTTTGCTGATCAAATCCTCGAACTCGATGG
CTTGATTCTTCTTGAAACGAGGAAATCCCGTGCATCTGTTGTGCCTGGTCATGAAGGAAATACTTCGTCAGGG
ACGAGACGTCCAAGTGCTGCCGCAAATCACTAGCTTGTCATGGACGTTTGGGGATTGTATCAATTATTGTTTT
GCATATTATGTAGGAAGATAAATATTAGAGTATGTCTGAATAGAAAAAAAAAGAT
>TR10|c0_g1_i2 len=3873 path=[4220:0-340 4221:341-364 4275:365-377 4267:378-402 4282:403-419 4273:420-443 4278:444-484 4264:485-651 4255:652-675 4271:676-771 4240:772-795 4263:796-1008 4205:1009-1032 4277:1033-1312 4262:1313-1371 4251:1372-1395 4252:1396-1429 4261:1430-2235 4198:2236-2259 4280:2260-2279 4276:2280-2303 4269:2304-3415 4202:3416-3432 4203:3433-3872] [-1, 4220, 4221, 4275, 4267, 4282, 4273, 4278, 4264, 4255, 4271, 4240, 4263, 4205, 4277, 4262, 4251, 4252, 4261, 4198, 4280, 4276, 4269, 4202, 4203, -2]
TTCCGATCTACGCAATCAATCACAGAGGCCTTTTACGTAGAATGTCAAGAACCAAGCAGGACAGGATGGCGTC
ATTCAACAAAAAGCGACGATTCTTCTTTGGCTTTCCTCAACGTCAATGAAAGAACAAAAGCAAAAGAACGAAC
AATGAGGCGATCGCATCGATTCGCAATTGATCATTAATGTAAAGCAATCGTTTCGTCGAAAAAAACCGTGGCGT
GGTCGACCGTCGGAAGTGCTCCTGCGATAAAATTAGACGAGTCCCAAAATCATTGATACATACACCCTTCGCT
CCCTTCGCTCTCTAGATACGCGGAGTCGTCAGTCGCGGAGTCCACGACCAACATTCACTTGAAAATCGAGCAA
CTTCCAGTTCAGTTTCATTAAATTCGCTTTGGACAAATTGGTTGAAAGTGACGGTTATCTATTGAACTGAGTA

CATTTTTATCTCTCTCATGAACCGAATGTCCAACAAAAGCAAATGGTAGAAATTTAATATCGATGGTATTGTA
CGTAATTGATTGCATCGAAACACCCAACTGAACGACTTCAACTTCACCGCATCACGACCGACCAATCAAAATG
GATCACCACATGAATATGATCGACCAATCATCCGCCCATCCAGGAAGTACCACTCTGAATAGCAACGTAAAAG
TTGCAGCAGGATACGTTCTGCAAGAGCGTTTGGGATCGGGTTCTTTTGCCACTGTCTACAAAGGAATACGATT
GGCAGACTATCAGCGGGCTATCAGTGATGCCTCGCATCAGACACCGCCGCCAGCAAATTCTATCACCGCCATC
AAGGCCATTTCTCGATCGTCCAAAAAGCTTACCAAGAAGGTTCTCGAAAATCTTGATATGGAAATTGCCATCT
TGCGAACTTATAGACATCCTAATATTGTCTGCCTCCACGAAGTCCAAAAGACTGATAGACACTTCTATCTAGT
CCTCGAATACTGCGGGGGAGGGGATCTTCAACGACTCATTCGCACGCGTCAAAAGGGTCGATTGTCCGAACGG
TTATGCCGACGATTAGTGAGAGATTTGGCGGCAGGATTGGGATTTTTGTGGGGGAAGGAGCTCGTGCATAGAG
ATATCAAGCCGCAGAATTTGTTGTTGACGGGACCGTTGCCCGCGGAGGACGAGGTGGACAATCCGCCGAGGGA
GGATGCGAATCAAGCGTTGCGGGGGATGACTGGGGAGAGGTTCATGTTGAAGATTGCTGACTTTGGGTTCGCC
AGGCATTTGTCAGGAGTTGACTTGGCGGAGACCATGTGTGGGAGTCCTCTTTACATGGCTCCTGAAATATTGG
TAAGTTTTCTTTCCATCGTGGCTCTTTGATTGACGTGTTTCATGCGTTTATTCTCATTGAGCTGCACACCAAA
TTCTGCTTCGCCTAATTGTAGTCGGGAGAAAAGTACGATGCAAAAGCAGATCTATGGTCAGTTGGAGCAGTAT
TATTTGAAATGATTGCCGGGAAAACACCGTTTCACGGAGAGAATCATCTGGACTTGTTGAGAAATATAAAAAC
AAAAGCTGTTCGTTTGCCATCTAATGTTCGGATATCAAGGGAATTCGTCAAACTGTTGAAGTTACTCCTAGAT
CGCAAACCTCGCAGTCGGGCGGGATTCAGAGCTTTCTTTGAAGCAAGTGAAGCTTGCGTGGCACTTGGATGCA
ATGGTACTCCTGCCGTTCCTCCATCCAGTCTGGTCGGTCCAAATGCTACAACTAATCCATTGGATCCATTGCA
GGTACCCAGTTCTGGTCAGCAAATAGTGGACAGGGAAGTATGCGCCCAAATGAATTTGTGTGCCATTTCAGAA
AACGAAGGTGCCGGAGAATCATTCCATCAGGGGTATGGCAATGCGAGCTTGGCCACTGTTGCGACATTAGGAA
CGGTCAATCATCAGCAATTGCACCAGGTCCGGCCTCAACCTACTCAGCTTCCCCATGGTGCGCAAGGTGCAAA
CTACGTTTCGAACATGGCCACATCACAGACAAACGCTGTGAATGCCGTGGTTCAGATCCGACCTGACCATATG
AATCATGCAGAGGTTGTAACGCCCCCTTTCAACCCAGTCTCGGCACCGCAGCCGCCTCCCGCTCTACCGCCCG
GAATGCAAGGGGTTGGCAGGCAGCATGGTAGGCCATCTGTTTTTACTCCTCTTCAAGGAAGCCCTGTCCTTCC
CAGAACCCGAACTATTCCTAGTGCTCCTCAGTATTCACTTGGAGGAACCCCTGTCGGTCTTAGTGCGCATCCA
TTCCCAGGCACACTTCCACCCCCGCCGATGCAGTTCCGTAACCAGGGAGATGGTGGTTTCCCAATGACGCAGC
AGCAACAAATGATTGGTCATCCAACTCCAGTAAACAACCAAAACACTCTTGTACCAAACAGAAGGACTTCGAT
CCCCAAAACATCCAGTCATGAACCTTCTGATTCTGGTTTTGTTTTGGTTGAGCATAGTGGCTGCAGAAGCAGG
CCAGGTAGTGGCCACAACTCTCCTGCTGGTTCAATTGGGGGTATTGCACGCTCGCCTTCAACGTCTCCACACA
ACTCTTCCGGTAGGGTCACCATCCTGAATGCCAGACATAACATGGGAATCCTTGGTACTAGTCCAAATACAGG
ACGCGCTCTTGTTGGGAAAATGATGATAGGCTCTCCGTCGCCATCTGCCTTGACTCCTCCGTCCGCCTCTGGT
GGAGGAAGATTCAACCTGTCTCCACGGGCTGCTCTTCGCAGTGGAGGATGCTTGGCTCATATTGACGCTCTTG
CTCGTATGTTGGCGACCGCAGAAGATATCGGGCGTAGGTCCATAACTGTTGCTCATCTGGGAGATGTGCGAGC
ATACTTGGCAATGGAGCTTTTGGTAGCTCAAAGGGAAGAAAGTCAGTTATCGTCAATGTCGACAGGCACGGGA
ACACCCATGGAGGGTGTGGATGAGGAAGGTAATAGTGAAACACCCAATCCAAAGAGGCCGTCGTCTAGAAAAG
CAGCTGCCGAAGAAGATGATGAAGTAGAATTACCCTTTACCATGACGTCATCGTCCGATGGTATGTCTCATCC
TTTGAACAACGATATTCAAAACCCCGCACCAATGGCAGGCAAGAGTTCTGTGAATGAGTCCCCTAAAGAGGAA
ATAACAACCTCGATGATTCAAGTCCGTTTTCGGGAGGCTCTGACGTGCTACTTCAAAACTCTAACCATGATGA
AAGGGTCCATTTGTGCAGCTCAAAAGGTTATGAAGGAAGTTGAAGAGGTTCTTCCAGCTGGCCAACCCGCATC
GAACAATAAAAACCCATACACACCCTTGAAGGAACGTTGCTCTGCAAGCGTAGTGTGGCTTCGGGGACAATTT
TCTGCAGTTCTAGAAAGAGCAGATGCGGCAAGTGAGCAAATTTCTAAGCTGCAAAAAACTAATACTTCGCGAG
AAGGGGGAGTAGAAGTATCACTTTGCGTAGAGGAACTGATTTATAATCATTCTCTTAAATGTGGCCGTGAAGG
AGCGGTCAAACAAATTCTTGGTCACTACGATGTGGCAAGATCCTGCTATCGTTCAGCTGGATTGCTGGCGGAG
ACGTTGCTCATGGAATCGAAAGTGGGGGATGACGACCGGGTTGTGCTCGAGGGCTATATTCACAGTTTTGCTG
ATCAAATCCTCGAACTCGATGGCTTGATTCTTCTTGAAACGAGGAAATCCCGTGCATCTGTTGTGCCTGGTCA
TGAAGGAAATACTTCGTCAGGGACGAGACGTCCAAGTGCTGCCGCAAATCACTAGCTTGTCATGGACGTTTGG
GGATTGTATCAATTATTGTTTTGCATATTATGTAGGAAGATAAATATTAGAGTATGTCTGAATAGAAAAAAAA
AGAT
>TR11|c0_g1_i1 len=609 path=[1875:0-61 1876:62-92 1914:93-171 1912:172-
219 1907:220-276 1899:277-300 1911:301-309 1905:310-333 1913:334-357
1908:358-387 1902:388-414 1894:415-446 1881:447-608] [-1, 1875, 1876,
1914, 1912, 1907, 1899, 1911, 1905, 1913, 1908, 1902, 1894, 1881, -2]
ACTGGTACGAAAGAGGAAAAATCAAAATTGATACGAGCGGACAGTGTGCCCGACGTGAGGAGCGTGATTGCGA
GTGATATGGGATTGAGGTATCTGTTGAGAAATGACGTGTTGACGGCATGCGATGATGCCAGGGCCGAGTTGAA
GTACCTCCTGAAACTGCAGGAGGGGGGAGGCTCAAAGGAAACGGATGAGGGGGCTTTGGATGGGAAGGAGTTG
TTGGAGTTGTTACTGGGAGCGCAAAAGGCTTTGGGACAGTGGTTTGATTTGATCGAGGGGAAGGATGTGTTGG
CTGCTGTGGATATTGTCAAGCGGGAAATGTGATTGTGCTCGATACTGCAATACATTGATACTTCGATGGCCAT
ATATGACAAAGATCGAGGTCAGAGAAACATCAGCATTGTTGCATGATGTGGGAGGTCGTTTATAGTTTCTGCT
CCAGAATATGTGTTTTCGTTACCCTATCTGGTGGATGAGTTACAACCGTAAGTGATTTCATACAAAAGCACCT

AATCAACTATTACTCTGTTTTTACCTTTCAATGCAGAATAGACATAGTGTGTAGAGTAATTCAAATCACATGA
TTACATAAAATAGATGATCAAGATC
>TR11|c0_g2_i1 len=994 path=[1866:0-161 1867:162-185 1900:186-224
1860:225-248 1906:249-383 1873:384-407 1898:408-770 1846:771-794
1910:795-815 1904:816-839 1896:840-920 1854:921-951 1855:952-993] [-1,
1866, 1867, 1900, 1860, 1906, 1873, 1898, 1846, 1910, 1904, 1896, 1854,
1855, -2]
TGCTGATGATGTCACTGGCGCCTGTGACTCACTGATAACTAAAGCCGCAAACACGAAGAACAGCGTAAATATG
TCGAAAGGATGAATGTTTGCCATGGGCAGCAGGGGTGATAATACTGGGCGTAAAGTTAACGATGGCCCCTCTT
CCATCGACACTCCCACACAATCGCTATGTTACTTTGAGTTAAAGCGCCCACCATTTTCTTTACGCCACGCTCA
TTGAATGTCGTACCTGAGTATGCATAGTTCATCGTTGCGATGGTCGTCCATCGGCATCTTCTCCATGGCGCTA
ATGTCTGCTCCCACGGATGTGTCAGCATTTGGCATAATGACAACCGATAGGAGATCCGCGATTCGTGAATTAT
TAACAACAACAACGTCCAGTACCGCTGCTGCACTGATAATCTGCACAACAAATAAACAACCTGCTTTGGCCGT
GGCTCCATTCGCTCCCATCGATGCTCTATTGCCCGCGACGAGAGTCAAGATAACAATCGATCGGGCTGTTGAT
ATTGCTTCGAATTTAGTCGCCGAGGAGAAGACCAACGAATCTACTCGAACAGAAATGCTACACAATCTCGAAC
GCCTGCTGTCCACTCCCCAAAACTACAATCGCGATACAACACCCCTCTCCATACCTCAAAAACCGGCCCAGTC
CTACCTAGACGCCTACGCCCAATATCGTAATCGAGTGACCATCCTCGAAAAGCCCGGGGCCATGTTAGTACAA
AACGGAGAAATCAACGCCTGGAGAGATCTCAAGAAGGACGAGAAGGCGAGGGAGGAAAAGGACGAAATTAGAG
CGGCGTTGAATTACTACACTAGTAATTTAAACTTTGATCCGGATCGATTCAGTTTGACTGGTACGAAAGAGGA
AAAATCGAAGTTGATACGAGCAGACAGTGTGCCCGACGTGAGGAGCGTGATTGCGAGTGATATGGGATTGAGG
TATTTGTTGAGAAATGATGTGTTGACGGCATGCGATGACGCCAGG
>TR12|c0_g1_i1 len=1864 path=[2245:0-220 2246:221-265 2265:266-452
2255:453-476 2273:477-603 2264:604-1338 2269:1339-1430 2221:1431-1454
2272:1455-1525 2278:1526-1550 2275:1551-1574 2271:1575-1612 2267:1613-
1636 2274:1637-1655 2270:1656-1679 2266:1680-1752 2202:1753-1760
2203:1761-1784 2204:1785-1863] [-1, 2245, 2246, 2265, 2255, 2273, 2264,
2269, 2221, 2272, 2278, 2275, 2271, 2267, 2274, 2270, 2266, 2202, 2203,
2204, -2]
AACATGAGAATGCAGTGCTTCTCCACAGCACAGACATCATGCAGTGCGCATAGTAGAGGCAAAGTTCGTAGCG
TAGCTGACAAGGACATCCACCAATCTTTCATCACTCGAATAAAACATAGATTCTCCAATTCAAGAACAGACCG
GCATCGGCAATAGAATGGCAGTGCGCTTCAGCTTCAAGTAGGAGAAGAATTCACGGTTCCAAAACAACCGAGC
CAATCGATACATTTGCAACATCAGGTTTCTCATCATAAAATGGCATCGCGCCCTAGCCCCGGCTTCCCTCGGG
CGCATGCATCGACTTCGCCAAAGGCGGCGTCGGTCGGCGCTACTCATGTCAATAATCTCCTCAAGAACTATTC
GCACGAGCAAGTAGTCCATGCGTTGATGTCCCAACTACGGGAAAAGGAAACAGAGCTGACGGTACTTTCTGAA
CAGCACCAGGTTATGCTGCGGCATCAGCAAATCACAAAAGAAAGCAACAAGCAGCTGAAGAGCAAGATCGACG
TCCACAATAAGTTAGCGGCCGAAAAAGACACAAGGATAGCAGTGCTGGAGAAGAAACTAGCTCAACAGGAGAA
GAACAGAGGTTCGGAAGCATTAGCAGAGGCCAAGATGAAGGCCATCACTGAAATAGCCGCTGAGTCGAAGCGT
ATGCTCAAAGAACGCGATGACGAGCTGTGTCGCCTCAAACCCGTATTGAAAGATTTAACAGCACTGGTTGATG
AATTAGAAAATAACGCGGACGTAAAGGCGTTCGCACAGCTTGAAGAGGAACTGGTCAAAATCGAGTTAGAACT
TGCAACAGCGAAGAAGGAAAATCTTAAATCGATCAGTGCAATCGAAGAATTGGAAAAGAAATCCAAAGGGAAA
GAGTGGGTGATTAAATCACTCAAAATGGATAGCAATGATCAACGCAGCAGAGAGAATCATTTGCTTGCACATA
TTAAGGCATTGAATGAGAAAATTGACACATATGAGACGAAGTTCAACGGGAAGGGAACTGATGTACCTATATT
ACTTGCAAAGCTGAAGGATTACGAAGTTCGGGTTAAGGATTTGCAAGGTCAAGTGAGAAGGCTGACCAATAAG
AAACTGAATGAAATGGTCCTGCGATCCTCCCCACTTCCCAGCGAGGCCCACCTAATTCCTGAGGAGCCGATCG
TGACCGCCTCCCTAACAACCTCACGCAGCAAACTGCTACAAGACGAGGAAGACAACGAGAGTAAGGCGTCCGA
GAACTCCTATTCTTTGGTCAATAGCGAACAGTTTGACGAGGAAGACGCCTCATTTGCCTCTAACGTGTCTAAC
GATATCGATCAATTTTTTCCCAAGTCTAAACAAAAAGAGGATGCATTCGGTGAATTTCTAACGGACGTTAAGG
TGGGGATTGAATCACTGCATACAGAAGGCCTCTGTTGTGCGCAGGCACCAATGCCGAACGAATGACGTCCCCT
CTCAACGAACTCTCCCCTACCAGCTATAGCACGTCGCAGGAACGGTCTCCTAGAAGCTACAATGGTAGTAACT
CCACTTCTCTTCACATCCACAAGTCGGAGTGATGTTTAGAAAACGAGAACATTCCGTTTACTCGGGGATATAT
CGAAATTTTTACCCTTCTCCCCTCTAGCTACGACCCCAATGCTCTCCTGCGAAGCTTAACAGCAGACTTTTGT
TTGGACGTCAGTAGCATTATAGGAAGGGTGCTGTGGCTCTTGATACTCAGGTAAAGAGAACCAGTCGTTTCTC
TTTGGAGTGTACGAAGTGTAACTGCCAACGAAATCCATGTTCGTGTGTAAAGTTTAGACCTTATTTGTTCATC
ATCTATAATACCATCAGTTTACCGAGTAAAAAAAGATCG
>TR13|c0_g1_i1 len=5478 path=[5888:0-1227 5889:1228-1251 5948:1252-1285
5943:1286-1309 5934:1310-2016 5846:2017-2040 5950:2041-2058 5942:2059-
2075 5947:2076-2436 5952:2437-2445 5954:2446-2486 5931:2487-2528
5946:2529-2568 5939:2569-2577 5930:2578-2596 5951:2597-2607 5949:2608-
2631 5945:2632-2862 5953:2863-2884 5901:2885-2886 5902:2887-2901
5928:2902-3044 5843:3045-3068 5937:3069-3604 5920:3605-3628 5944:3629-

3755 5863:3756-3773 5936:3774-4062 5926:4063-4235 5923:4236-4259 5935:4260-4515 5886:4516-4539 5925:4540-5231 5883:5232-5255 5884:5256-5477] [-1, 5888, 5889, 5948, 5943, 5934, 5846, 5950, 5942, 5947, 5952, 5954, 5931, 5946, 5939, 5930, 5951, 5949, 5945, 5953, 5901, 5902, 5928, 5843, 5937, 5920, 5944, 5863, 5936, 5926, 5923, 5935, 5886, 5925, 5883, 5884, -2]
ACATACTTTAACTTACTTCTTACGCCATAATGTAAGGACGCGAAGCAGATGACAAAGAGAGGAAAAAACATAG
TTGATCTAAAATATTATGAGGCAATGTTGTATATCTAGCTTTCTTTCGTAGATCCTCTGCCGTAAAGCTTTCA
GGTTCCCTTGTGGTACTAACCAATTCTCACACAATCAAATTATGACTCCACTTGTTTCCCTCTAACTTTCAAA
TTTAGGGATCTTCCTTTCCTTTGTCTCAGCATGTACATGTGGCATAGCGTTGCTCATTTCTAACATCTTAATT
TATATTCGCGCCATTCTGTCCCCATCCATATTCTGGCAGTGCTGGATTATCATCATCTCCTCCTAGGCTGTCA
CTAGTAAAATTGTGACCACCATTACCACCAGCCACACCCTGTTGGTGTTGTTGAAACTCGTTTCCATCAAAAG
GAGGTCGAGGCATCATTCCTGAATAATTGTTGCCAGCACCACCACCACTATTCCTCATCATCATCATGTGCGC
CATCCTTTCCTGGTCAGGATAATTGGCAATATCACCATTATTACTCATCATCATCATTTGCGCCATCATTTCC
TGGTTAGGATGATTGGCAGTATTACCACCACTGTTCCTCATCATCATTTGTGCCATCATTTCCTGGTTAGGTT
GATTGGCAGCATTACGATTGATATTGCCACTGCCATCATTCTTCACCATTTGTGTTACCATTTCCCGGTGAGG
TTGATTGGCAGCATTATGATTGATATTGCCAGTGCCACGATTCTTCATCATATCTGCCATCATTTCTGCACGT
GTACCCATCGGCATGCCCATCGGCATACCCATCGGCATTTGATTGGGCACTCCTGCTGACGATGCTCCCATTC
CCATTCCTGCTCCCGTTCCTGCGTTGCTAAAACTTCCCCCAAAGCCAGTGGCAACGTTACTTGCCAAATCATC
AGATGAAGGAGCAGAAGTATCACCTCTTCCCCCATGATTACGACGATGTTTTTCTTTGATTTCCCGTCCAAGA
CAAGAGAGAAATCTCTCCACCTCCTTATCGAACTGATCTTCTTGGGCATTTGTATCTGTTGTAGTAGGTGCAC
CATTATTTGGCAGAGGAGGATGTCTGGGCAAACCTTGCGGCATATTTCCTGCTCCCGGCATGTCTGCTCCAAA
TCTTTCCATAAACTGTCTTCGCGTGGGAGATGGCAGACCACTGGCACCAGCGGTACCTCCAGGCAGAAATGTG
CCTTCGCCCTTGACATCAAACTTGCTATCGTGAGGCGGAAATAAGTCAGAAGAATCACCGCTGCCACCGACAC
TACGACCAACTTGTGGCATCCTTTGCATGTTATCTCTAGTGAATGCATCCCTTGGCATTCCAAATCCATGCCA
ATCTTGTTGTTGACCCTCGTGCAACGCTCGGCTGCTCATCATGCCACCTTGCATTTCCATCTGCATACGATGC
CTCGCCTCTGCCTCTTTCAGTGCCGCAAATTTCTTTTTAATGTCGCCATTCGGCGCAGCATTATGCGCTTCAG
CACGAGTGTCCCTGCGAGAGTTTTCTTGCATGAAATTCGCCCCAGCAGCAGCCATGCGGGCCTGAAGCTGAGC
TTCCTTCATCGCCACAAACTGAGATTCTTTCTTGCAGTGTTCCGCCATTCGAGCTCCATTAAAATTACCACCG
TTTGAGGCGAAGGGCGGAGGTGGTAAGCCTGGGCCATTGCCGTTAGGGTCTGCCCTGCCATCACCAGCGTCGC
CACAGATTCCAGACCAAAATCCGTCAGTGCGGGGTGTATCAAGAGCTCCATCAAAAGGGCCATTCATTGCTCC
ATGATAAGAACCACTCATTCGTGAAAAGGACAAATCTGTGAGGCGACTCATATCGCCACCACCACCGAAGTCA
GAGTTTTGAGGCTTGGCCTGGGCTTCAGTGGGAGTCGACGACCGGTATTGTGATGCTGCAGCCTTAGCCTCTT
CCTTCTTGAATCTCTTCTTCTTCTGGCCCACAATAGTTCCTCCTTCCACAGCCTCGTCAATGAGTCTCTTCTT
CATCTGTGATTTTTTAAACTCATCAGCCTCAGCCTCAGCCTCCATCAGCACTTTTGTCTTCGCCTCCAACTCA
GCCTTCTTCACACACGACTTGTATTTGGTCATGGCCCTCTTGTATGTTTCCAATTCCAAATCTGCCAGCTGTT
GACAATATCGTTTGATGTTGGGATCAGTCTCATCGATCATCTTCCAGTGCGCGGAAACCATTTTAGTCAATTC
GATAAAGCCGCAGGAACCTTTTGTCTTGCGATGTTTGCGCTTTTGCTTGTGACCCACAGAGTACCAGTGCTTG
TCCATGCGAAGGTGTGCGTATCGCGGAGGGATGTTAGGATCGAAGAAGTACTTGGAATCATCACCAGAGTTGG
GCTGTGGTTTCACACAGGCATTCGAGGGAGGGTGTAAAGTAATCTTATCAACGCCTTCTTCCTTGATACTTGG
AGCTGTCGCATTTACTGCCTTTTTATCATAAGAGTATTTGGAATCATCGCCAGAGTTGGGTTGAGGTTTCACA
CAGGCATTCGAGGGAGGGTGTAATGTAAACTTGTCAACGTCTTCATCCTTGGTACTTGGAGCTGTCGCACTTT
CTGCTTTGTCCTCATCTCCAGCCTTGCTTGCCTTGACCTTCGCCATTGCAGTTTTTATACTTGGGTCCTCCGT
TGGTTCCCGACCGTCACTAATCTCTTGCATGATAAATTCCCGCTCCATTTGAAAGAAGAGGTTGTACGCGGAG
AATGGCCGCTTTGGCTTATCGGGCAAGACAACGGAGGCCATTGCGAGGGCTGTGAGGGCCTCTTTTGCCATCT
TTTTGTCAAAGGCTGCCTGGGCCTTTTTCGATTTTTTTTGAGCCTCCAGAGGAGTTGATGTCCTTGGTCTCTT
TCTGCTTGGACTTCTTGGAAGCCTGCTTGCCTCTATTGTTTGACTTGGACGACGCCTTGGTGGATGAATTTTT
AGATTGTTTCGCAGAGGTTTCGGTCTTGGCATGGCTGCTTTTCGTCATCTTCAGTGGCAAAGAATCGTCATCT
GAAGTGTTGGCATTGTTAGGGCCTACTGGGAAATTTTTTACAGTCTCCGTATTTCCAGTGATGGTGTCTTCTG
TGGGAGTTGTCAACGAAGGTAACTTCATGTGCTTCATATTCGCCATCATTGCGCCAGCTTGGCCCATTTGAGA
CACCATCATGTCCCTCTCGTAGTTCTCCATAGGAGTTTGGTGAGATGCCATGGACATATTCGGATGAAACGGT
GGAGCCCGCGACGACACATCATTACCGCCACTCATCCCGCTGTCCAACCTTGGCATGTCGCCGCCAATTGCCG
ATCCAGGACTAGCTCGACGATGATGCTTCTTGAACCCCGCCATCGCATCATTCATATGATTGCGCATTTCAGC
ATCCATAATTCGCTCCCGATGCAAAAATGATTTCTTGGGCAAGGGACTTCGCAGATCGCCACCCGGGTCTCCA
CCACTGTCCCCCATCATGGAGAACTGCTGATCCATGAAGAACTTTTGTTTGGCAGGTAAGGAGTTCGCTGCGT
CATAACTGCTCGCGGTATTTAGACCATGACGATCAACTTTGTCGCCGAAAGTGGGTTTCATAGAAGTTACTGG
AGTGTTCATCATACTACCGGCTTGACTCCCCATTCCCCCTCCACCCATACCCCCCATCGGTCCACGATTCATC
GTTTGCAACATCGCCTTCCTCTGCCTCAAAAAAATCAGGATTATTCAGCCTCAAGTCAGGATTGTTCATCATA
TCAGAATTACTATTCATCATACGATCCCGCAAATCAGCTTTATTGATGTTGCCACTCGGCGAACTCAGCCCGG
AAGTCTTGTCAAAAAAACTCTCCTTCGCCGCCGTGGGCGTAGGACGTCCCATCGAACTACCTCCCATGCCACT

AAGCGAACATGCGCCCAGAGACATCCCGCCCACGGATACGGTATCACCAAAGCTGCCCCAGCTGTTGGATCGG
TGGTGCTGGGTCATATTCCCCGTACCAAAATTCCTGTCGCGGAGGGGGGCGCCAGTGAGGACCATTCGAGTTG
ACTTGTCGTTGCTCCCCCCGCTGATGTTGCGGCTGTTGGGTCGTTCCGTCTCATGGTTCCCCGACCCGTCTCT
CATTTTCGCTGGTAAAGAATCTTGGCAGGTCATCGCTTTGTCGAAGGGTATGGATGGGAAGCAATTATTATTA
TTGGGTGCTCCTCGATGATTATTGGCATCACTACCGCCGCCAATGTTCATTCGGTTACTGGAGTTATTCGAGT
GAGGAGTGGGATTGTTCGAGTTGTTCAAAGATGGTCGATTGAAATCTGAGTCGGGGGTGGGATAGTTGTTGGA
GCGTCGAGGACCCGCATCGCCGTTCATTTGATTCATCATTTGGTTCATCATTGATTCAGTCATACCCTTCATG
GCTCCGCCACCACTAGCATTGCCGCCTCTGCCATTCATCATATTCTCCCCCATTGACTGGTTCATCATTCCAT
TCCACATACCCTGCATGACTCCACCGCCACTGACATAGCCACCTTCATTCATCTTTTTCTCCCTCATCATCTG
GTTCATCATTTCACTGCTCACGCCCGTCATGCCTTCGGTGCCACTGCCGCTGCCATCTATTTTCATTGAGTTC
TTGACAGAGTGTCGTTGATTTGTCATCGTCTCCTCCTTCATCAGCTGGTTCATCATTTCCGACTCCGTTTCCT
CCATGCTGCCGCCGCCCATGATCCGGCTCATCATTTCCAACTTCATATCCTTCGTGACTCCTCTGACATCACT
GTTCATGATTCGGCTCATCATTTCCATCCTAAGATCGTTGGTCATTCGATTGTTGCTGCCGCTGTCGCCGAAA
CTGTTGTTGGTAGAAATCATAGAACTCTTGTTGAAATTTTGACGATTATTGAGGCTTCTATTGTTGTCCATAG
CATGCATGCCACCACTCCCCACAGTGTGAGGGAAGTTCATCCGACCTCCTCCTCCTCCCCCCTGCATATTGTT
ATTATTATTACTCATCATCTGACCTCCTCCTCCCACCGGCATATTACCATTATTCGTCTCACCACCTCCTCCC
CCCATCATATCATTGTTGCCATGACCCATGAGGGTTTTCTCCATCATCATGAGCTGCATCTTGTGCCGAGCTT
GCCTGTGTTTCCGGGGTAGACAAACTCGGAGGTGGTACTCTTGCGGACTAATCAGAGGGTTTTCTTGCGCCTG
GCCGCTCGGGAGAGTCATCCCGTTATTCATGGGTGCATCAACGGCTTGCTGGTGTTGCTCCCCGAGATAGACG
CGCTTGGACAGGGGAGATTCATAATCTTGTCTAGGGGGAGGGGGAGGCGGCGGGGGAGGGTAGAGGGTGTCTC
CGTCATATTCCTTGAGCGGAGGGCGGAGGGTGATTCCGTCATCTTGTTTCGGGGGAGGGTAGAGGGTGTAGAT
CGG
>TR14|c0_g1_i1 len=1581 path=[2226:0-104 2227:105-128 2469:129-169
2490:170-173 2482:174-197 2468:198-396 2503:397-420 2508:421-426
2512:427-458 2466:459-467 2509:468-482 2504:483-490 2514:491-492
2516:493-516 2487:517-593 2505:594-622 2510:623-623 2462:624-647
2444:648-655 2421:656-735 2182:736-759 2183:760-776 2184:777-800
2474:801-959 2506:960-971 2511:972-995 2515:996-1017 2492:1018-1061
2485:1062-1121 2473:1122-1136 2332:1137-1145 2472:1146-1160 @2458@!:1161-
1325 2416:1326-1345 2471:1346-1384 2456:1385-1399 2507:1400-1400
2491:1401-1408 2484:1409-1409 2470:1410-1433 2455:1434-1472 2341:1473-
1507 2342:1508-1580] [-1, 2226, 2227, 2469, 2490, 2482, 2468, 2503, 2508,
2512, 2466, 2509, 2504, 2514, 2516, 2487, 2505, 2510, 2462, 2444, 2421,
2182, 2183, 2184, 2474, 2506, 2511, 2515, 2492, 2485, 2473, 2332, 2472,
2458, 2416, 2471, 2456, 2507, 2491, 2484, 2470, 2455, 2341, 2342, -2]
GTGTGCTCTTCCGATCTTGGGCCACGCAGTTACAATGCGTAAGCCGGCATGAAAGCGTGAATCAATGCTCGAG
CAGTGCTATTAGAAAAATATCAGCATGCGCAAGAGGAAGAGAAAAGAATGGGAGCCAAAGATAGAGACCATAA
ACTTGCGGCACATATCTCGTTCGCATATATTTCAGCTTTCTCACTCAAGTCCTACCATGCTCAAGTTTTGTCT
CAACACTGCAATCACAACTCTTCTCCTCGGCTATGCCCAAGCACGCAGCTTGACCATCACGGCTGATGGCATC
GCAATTATCACCGCGGCAAATGGAGGCAACAGTCTGGTCACCTATGATGCCGTGAGGTCCAAACAAAAGACTC
ATCGTTTCTCTAATCTACCAAATAATGTCGATGGCTTTGATGACGTTGCTGTCGATCCAAACACGTTGGCACG
CGATTCGACGGTGGTGTTTGCGATTGATGCTGAGTCGGGTATTGTCTGTTCCTGTAAGTTTGCTTACACTTAC
ACAATACATGCTGAAATTACTTTGTTCACACACGCTGTGCTTACTTTTCTTACCTTCAATTCACAATCTATTA
TCATTATTTTGCTGTGTATCTGCCAAGTTGATCTATCATTTCGATTTCGACGTCAAAACAATACCAATAACCG
CAACAGGGTATCAGGCGTATCTTTGGACCTCATTGGCTGCACTACACAGTCCGTAAATACAAGTCCATTCGTA
GGTATAGCAGCCATGGGTGGGACGCTCGTAGTCTCGGGAGGGACCGGTGGGGCCTCCGTCTTTTCATACAATA
CCAACACGGGCGTGCTATCGAATCGTGCCACCATCCGTAATGAAGACCTCGGAGATGTTGGTCATCCTGACGT
CACCATGGTGACTCGCAACATTGCTGCCCTATCGACGGATGCCCAACGTGGATTTGGCATAGTTGTTTCAAAC
ATCAATATGAAGTCTAGATCTCTCGATCGTACACGCTTCTTCCGCGTTCAAAATACTGTTGGGTTCGATCATG
CCATTCGACCGGCCAACTTCCCCTGTGGCAGTTCAATGTACTTTCGAAAGGGTGGCAGGAATCTTCTCTATGT
TGCCAATGGACCCATTACTGTCCAGGATCCAAGAAAGAATGGTTCGCCTACAGTTGTTACGACTGCCCCTGAT
GGATTCGAGGCATTGGCTGTCGATGTCAACTCAGAGCGATCCGTGGCGGTCTTCGGAGGGGTGATTGATGATG
GTGAATCATCAGCATATGTCGTGTTGGACATATCTGATCCATTGGCACCATCGTTTGTCAATATGCAAGTCGT
TGGGAGGAATGGGAGGAATGGCAGTGGACGGATTACAAGTGTTGCCTCGGCTGGGCGCAACATTGTCTATGCC
ATGGATGGTTCATCAAAGATTGGACATGATCGTTTGCAATCACTTGCGAATTCCGATGAAAAAAATATTCAAA
GCATCGCGTAGGTATATTTCTATACATAGATGGTTACATTATTAAGGAACATTGTATTGAAAATTCTATCCAT
TTCAAGTGTCCAACGCAAGTATTTGTTAATGGTAATGGAGGGTGCTAC
>TR14|c0_g1_i5 len=1461 path=[2226:0-104 2227:105-128 2469:129-169
2490:170-173 2482:174-197 2468:198-396 2503:397-420 2508:421-426
2512:427-458 2466:459-467 2509:468-482 2504:483-490 2513:491-511

2443:512-535 2421:536-615 2182:616-639 2183:640-656 2184:657-680
2474:681-839 2506:840-851 2511:852-875 2515:876-897 2492:898-941
2485:942-1001 2473:1002-1016 2332:1017-1025 2472:1026-1040 @2458@!:1041-
1205 2416:1206-1225 2471:1226-1264 2456:1265-1279 2507:1280-1280
2491:1281-1288 2484:1289-1289 2470:1290-1313 2455:1314-1352 2411:1353-
1387 2406:1388-1460] [-1, 2226, 2227, 2469, 2490, 2482, 2468, 2503, 2508,
2512, 2466, 2509, 2504, 2513, 2443, 2421, 2182, 2183, 2184, 2474, 2506,
2511, 2515, 2492, 2485, 2473, 2332, 2472, 2458, 2416, 2471, 2456, 2507,
2491, 2484, 2470, 2455, 2411, 2406, -2]
GTGTGCTCTTCCGATCTTGGGCCACGCAGTTACAATGCGTAAGCCGGCATGAAAGCGTGAATCAATGCTCGAG
CAGTGCTATTAGAAAAATATCAGCATGCGCAAGAGGAAGAGAAAAGAATGGGAGCCAAAGATAGAGACCATAA
ACTTGCGGCACATATCTCGTTCGCATATATTTCAGCTTTCTCACTCAAGTCCTACCATGCTCAAGTTTTGTCT
CAACACTGCAATCACAACTCTTCTCCTCGGCTATGCCCAAGCACGCAGCTTGACCATCACGGCTGATGGCATC
GCAATTATCACCGCGGCAAATGGAGGCAACAGTCTGGTCACCTATGATGCCGTGAGGTCCAAACAAAAGACTC
ATCGTTTCTCTAATCTACCAAATAATGTCGATGGCTTTGATGACGTTGCTGTCGATCCAAACACGTTGGCACG
CGATTCGACGGTGGTGTTTGCGATTGATGCTGAGTCGGGTATTGTCTGTTCCTTTGATCTATCATTCCGATTT
CAACGTCAAAACAATACCAATAACCGCAACAGGGTATCAGGCGTATCTTTGGACCTCATTGGCTGCACTACAC
AGTCCGTAAATACAAGTCCATTCGTAGGTATAGCAGCCATGGGTGGACGCTCGTAGTCTCGGGAGGGACCGG
TGGGGCCTCCGTCTTTTCATACAATACCAACACGGGCGTGCTATCGAATCGTGCCACCATCCGTAATGAAGAC
CTCGGAGATGTTGGTCATCCTGACGTCACCATGGTGACTCGCAACATTGCTGCCCTATCGACGGATGCCCAAC
GTGGATTTGGCATAGTTGTTTCAAACATCAATATGAAGTCTAGATCTCTCGATCGTACACGCTTCTTCCGCGT
TCAAAATACTGTTGGGTTCGATCATGCCATTCGACCGGCCAACTTCCCCTGTGGCAGTTCAATGTACTTTCGA
AAGGGTGGCAGGAATCTTCTCTATGTTGCCAATGGACCCATTACTGTCCAGGATCCAAGAAAGAATGGTTCGC
CTACAGTTGTTACGACTGCCCCTGATGGATTCGAGGCATTGGCTGTCGATGTCAACTCAGAGCGATCCGTGGC
GGTCTTCGGAGGGGTGATTGATGATGGTGAATCATCAGCATATGTCGTGTTGGACATATCTGATCCATTGGCA
CCATCGTTTGTCAATATGCAAGTCGTTGGGAGGAATGGGAGGAATGGCAGTGGACGGATTACAAGTGTTGCCT
CGGCTGGGCGCAACATTGTCTATGCCATGGATGGTTCATCAAAGATTGGACATGATCGTTTGCAATCACTTGC
GAATTCCGATGAAAAAAATATTCAAAGCATCGCGTAGGTGTATTTCTATATATAGATGGTTACATTATTAAGG
AACATTGTATTGAAAATTCTATCCATTTCAAGTGTCCAACGCAAGTATTTGTTAATGGTAATGGAGGGTGCTA
C
>TR15|c0_g2_i1 len=1070 path=[10538:0-61 10539:62-85 10599:86-196
10571:197-226 10593:227-277 10582:278-301 10567:302-565 10439:566-589
10591:590-652 10474:653-676 10580:677-832 10402:833-856 10403:857-857
10404:858-875 10405:876-886 10406:887-1069] [-1, 10538, 10539, 10599,
10571, 10593, 10582, 10567, 10439, 10591, 10474, 10580, 10402, 10403,
10404, 10405, 10406, -2]
GTCTTCGGCGCCGCGCGAGCCGTCGGCGATATCCAACCCCCGGGAGTTGAGGCCCACTTCGTTGTGGCCGCAT
GCGAGAACATGATCAATGCCAAGGCGATGGTTCCCAGCGACATTCTCACCGCGAGCAACGGTAAGACGATTGA
GGTACTCAACACCGACGCCGAGGGAAGGCTGACGCTCGCGGACGCATTGGTGTTTGCAGACAAGGAGTGCGGC
TGTGAAAGCATCATCGAACTGTCGACATTGACGGGCGCGTGCATGGTCTCGCTGGGGAAGAAAATTTGTGGTG
TGTGGACAGATGATGATGATCTGGCGAAAGATCTTGAGGAAATTTCGAAAACGACTGGGGATAAGTCCTGGCG
AATGCCCATGGCAAAGGAGTATGCCGAGCTGTTAAAGAGCAAGGTTGCTGATATGTCAAATCTTGGTGGTCCA
TACGGTGGTGCCATCACGGCCGCCCTGTTTTTGACTGAGTTCGTGGATAAGAAGAAGCCGTATGCGCACATTG
ATATTGCGGGTCCAGTATGGGATGATACGAGTGGTGCTACTGGGTTTGGAGCGAAGCTTGTGACTGAATGGGT
GCGTAAGTCAGGCGAGTAAAGAAATAGTGAGAGACAGGAGATATTCAAAACTGATTGAAACATGCATTGACAC
TAGTGTGACCACTGAGTTTGAAACTCTCCTGCCAGTACTAGTCTATGCAGAAAAATAGAGCAGTACCATTTCA
GTGCTATCAAGCGGGTGCAAAGAGGTACAAAAAATAAGCACAACTCTGCGATCAACAGTCATGGCTTATCACT
TTTTAGAACAAAAGAAGTCATTGTTTGACACAGATTGTCATATACCGGTCTTACCTTTAGCTCTATTTAGATC
CATTTAGATCATTGATCATCTAACTGTCGCACCTTAGTCATGTCAATAGACAAGGACTCCTGATAGCTGTACT
TTAGCACGAAAGATACCTCTAAAGGTATCCATTCGAAGTACACATCTGAATATAAATATACCGGATATACAAT
CCGGATATAAACATCATCATTGCACAAACACGTGCGTCAGCACGTGCG
>TR15|c0_g4_i1 len=8482 path=[10505:0-221 10506:222-245 10507:246-246
10583:247-650 10399:651-674 10592:675-942 10542:943-959 10581:960-1319
10532:1320-1343 10566:1344-2097 10484:2098-2121 10579:2122-2247
10503:2248-2271 10564:2272-2454 10545:2455-2478 10578:2479-2883
10563:2884-3540 10418:3541-3564 10590:3565-3636 10453:3637-3660
10603:3661-4456 10589:4457-4702 10576:4703-4777 10408:4778-4780
10597:4781-4793 10560:4794-4922 @10588@!:4923-5191 10421:5192-5226
10559:5227-5449 @10595@!:5450-5632 10527:5633-5650 @10574@!:5651-5856
10522:5857-5873 10602:5874-5887 10600:5888-5911 10594:5912-5981

10557:5982-6291 10573:6292-7172 10471:7173-7196 10556:7197-7665 10459:7666-7689 10572:7690-8247 10492:8248-8271 10555:8272-8477 10520:8478-8481] [-1, 10505, 10506, 10507, 10583, 10399, 10592, 10542, 10581, 10532, 10566, 10484, 10579, 10503, 10564, 10545, 10578, 10563, 10418, 10590, 10453, 10603, 10589, 10576, 10408, 10597, 10560, 10588, 10421, 10559, 10595, 10527, 10574, 10522, 10602, 10600, 10594, 10557, 10573, 10471, 10556, 10459, 10572, 10492, 10555, 10520, -2]
ACCGAAGCGCTTGTCCTCGTTGGTCCGGAGGTCAATTCCTCCGTCGAAGCATCCCCCGGCGGCGTCGTCGTCG
ACCAAGGGCGCCGCCGCGGGGTCGTCTCATCTCTTCAACACCACCGACTCCGCCCGACCCATCCCTCGTAAGA
TGCCTTCGGACGGCGCTCCGATCGGATCCGGCCGCATTCCCAAGCGAGCCATTTCGGAGAAGGGATCGTCCGT
CGTTGCTCCCAAGAAGGGAAAGTTTCGACACCCGCAGAATCAACGCCCGCCGCAGCAGCACCTTCACGTACAA
GCGCGGCAGGAGCGGCATTGCCCCGAGATTGCCCCGTCTCCGGCCGATGCGGACCTCAGCGAGAACGTCAACG
TTCACGGAATTTGGAGGCGTCGCACCCGCGCTCCCTCGGGCACCGACGCGATTCCCTTGCAGAAACACACTTC
GACGAGGATGGTGCGGAAGGGCAACCAGAGCGCGTTCGGCACGGAGAGTCCCGAGGGGAGGGCCACGACGTTC
GGCCCTGGGAGGGATGGGAATGGGATGGCGTCGTCGAAATCTCCGGGGGAGATCGACGAGTCGGGATTGCACG
ATACGGAGAGCGAGGATGGGAGCATGTCGGAGGGCACTGTCATGGAGGAGGACGATGGATTCAGCGAGAGCGC
GTCGGTGGGGAACGAAAATCCACCAGTGCAGAAGCAGGCTGTGGAGTTGATTGTGCTGGACGACTCCTCGGAC
GAGGACGATGCTGGTGTCGGCGAAGACGGGGAGGGTGCGAACAGTGGGATCGACTACTCTCCAGATGGGGAAG
ACTCCTCGAACGAAGATGACAAAGATGAGAGGTCGAAGGAGCAGGATTTAGAAGACGACCCATTATTGTATAG
AAGCTTGAATAGAAGAAATTCCTTGCAGCAGGATCACTACCAGCTGAAGCCTGAGCAGATCAAAAAAGCTCCT
GAGGACCAGAATATCCCAGAACATGGCCACAATGATACGATGAGTGATGATGTAGAAGACGATGGTGTAGAAG
ACGAGGAGGTGAATGAGGAGGAAGAAAATGGCAAGGAAGAGAATGCCCAAAAAATTGCTGTTCCTGTGGCAGA
TGAAAGCGACCAGGAATCAAGTCAGGTAGGAGCAGATAGCGGCGAAGATGAACAGGAAGGCAAAGCCATGGAG
CTGGACAATGAAGGCACTGCTGTGATTCTGGGTGAGGACGCTGTGGCCGCCGAATGTGAAAAGGATTTGTTCT
ATCTCATTGATCATGGAATTGAACTCCACGCTCGATGGTGTAGAAGAATTTCATCTAAATTTTCCAATCTGCA
TCATCCAGAGGAAGCATATCACGAGAATAAGCAGCAGGAGGTGGAGCCACAGGAGAACAGTGGCAGGGAAGGA
AATGAAGGCGTGTGTACAGACCGCGCCAGTGATAGCTCCAACATGAGTATTTCCGAGGCTGATCACAGTGCGC
GGCACTGTGAGAGCGAAATGGTGGATGAGGAAGAGGTGAATGATTCCGTGAGCGGCACCAACGATGGCAATTT
AAATGGCCATGGCCATGACAAAAATATGCCCAATACAGATGATGATGGTCTGCCACATTTAGAGACCGAATTG
GGTGAATCAATGGATTTGTCAAATGAAGATGAGGCTGCCAATGAGGAACGTCCAAATATTGTTTCAAATGATA
AGGACGGAAGCATGTCCACTCCTGCTGCTGATAGAAAACAGGTCAGTGACTCGCCGACGTCCACAAATGAGCT
GCGGATAGACCCCATCCTGGATTCTATAGCGAGCGAGGGGAATGTGACGAGGAAAAATTCTGAGTCTGCGAGC
GAGACCAAAGACGGCGGTGGCAATAGCCATGGCAAAAATGCACTCACTGCACATGATGATGGTCTGCACCATA
TGGAGAGTGATTTGGGTGAATCAATGGATATATCAGATGAAGATGAGGCTGTCAATGAGGAACGTCCAAAGAT
TGTTTCAAATGATAAGGGCGGGAGTATGTCCAATACTGCTGCTGATAGAGAACATGGAAGTGACTCATCTTCC
TCCCCAAATGAGCTGCGGACAGACCCCACTACGGTTTCCCTTGCACACAAGGGGAATGTGATGACAAAGAATT
TCGATGAGACCAACTGTGCAGCTTCGCACACCAAGGCGAATCCTCGTCGTGATTTGGAATGGCCAGAGATGTG
GAATCTGATGAAAGACGAGGGCTGGTCCCTCATGCCAGGGAGTGGCTTGATCTCGTGGTATTACCTCGACCCC
ACCTGCGCCGGCATGAAGAAAGTGGAGTTGCGGAGGACCAAGGAGGAGGGCGTTGATTACTTCTCGAACATAG
AATGTCTGAAGCAGCATGCAAGGATCAATCTTGGATGGGAGTCAGATCGCATTCCAAAGGATTCGGTGAGATC
AGGAAACACTCTAAGTACTGACCCTCTCCATGACGCAGACTTCAATATTGTGCCAAAAAGCGCAAGTGACGAC
CCCGAAAAATCCAGCAACGAGGAATGTCCTCAAATTGAATCAAGTGGTGGTAAGAGTGAATCTAGTGCAGATG
TACATCCTGGGACGATCAAATCCAAGAATCCTCTGACAGTAGGCGGGGAAATATTGTCACCTCTGTCGTCTAC
CATGAGGGGCAAGGAGTCGTTGAGCGAAGTCGACAATGGTACTATGATGCGCAGCGATCAAGATATTATTGTT
GGACGGAATGAGGACGCCAACCCTCTGGACAATGAATCATCGACAGCTGTCAATGAAGCCGACGTTGTCAGTG
CTGATGTTGGTCGCACTGATGATAAGGTGCACTCTCGTAACGAATCATTTGACAAGTTGAATGTAGATGAGAT
AGAGCCCCTTACTGCATCAGCAGTGCCAGACAAGAAAGAGCATGGCGGCGTTGATAAACTCGTCGGAAACAAA
TGTGCGCAGGTTTTGAATGATACATGCGAGTCAGCAGTAGGCAAGGAAAATAATGATGGCATAGGTATGCAAA
TTGACGAGAAAGATGTGCGAAGCGTCGAAGAGACTGTCAATGGTCATGACTCGGCATCTTCTGCACCGTGTGC
TGCTGTTTCCACATCTCAAGGCGATACAGCCAAGAAGGGAAGCTCAGCCACATCCTCCAAAGCCAAGACAGCA
GAGAGCAACAAAGAGAAGAATAAGCTTAAAAAGGAAAAAAAGAGGCAGAAAAGGGAAAGAAGAAAGGCTAAGA
AAAAGCGCAAACTCTCCAAAGAAGGCGAGAGCTGCCATGATGCCAAGAAGCAAAAGCAGCGAGCAGCTGGCGC
TGGTCAGGGTGTGGATAAGCCATGGACTGAAGAGGAACAATGTCTCTTTCATGAGGGCGTTGAAAAGTGTGGA
GGGAACAACTGGGGCAGAATTGCAAGTGATTATGTGACGTCCCGTACTCCCCTTCAAATTCGGCAATATGCAC
GGCATAGTCAACTCTCCAATGCGGACGAGAACTGCAATATTGGTCAGTCACAAACACAAGGAGGTGATATTAG
CCATGATGTTGACAAACCATGGACTGAAGAGGAGACTTGCCTTTATCTTGAGGGCGTTGACAAGTGCGGTAGG
AATAACTGGGGCAAAATTGCGACTGAGCATGTGACCACTCGCACTCCCCAACAAGTTAGAAAGTATGCAAGGT
GTGGCTCTAACTTACTTTTGGACCAAGGGCAGCAGCAGCAACATGCAGAATCAAAAGAGTCACACATTCTAGT
GAATGCTGAACAAGAATTGGACTCTGCTCTGTTGAATGATGTGCATGCAGCTGAAGTCAACATTGACACCAGC
AACATCAATGAACCTAAGCAGGACAAGGATCAGTTAAAACCCGGCCTTCAGGATGAAGATATTCTATCTGGTG

AAGACAGTGATTCAGTTAGCAGTGAGGACGTAGCTGTCGCGGCTTTCCTCACATGCACCAAGTGTGACATGGT
ATTTATGGATACTGATGAGGCTTGGAAGCATGAAATCAATTGCAATATCACAGCCGGTCAAGCTGAAGAAGAA
GGAACAATTTTTGAAGACAAGCTATCGTTGATAAAATATATCTTAGAACTCAACTCGCGTGAGGGGAAGATGG
TGAATGAGGATGATATGGTTTTCTTCAGGCCAGATCTTCTGGCAGGGAAGAGACCAGTATCTTCAGATGAGAC
AGATGAGGATCAGCAGCTGGATGATACAGATGATGACTACCAGCAGGACAATGAAGCCAGTGATTCCAGTGGT
GATTCAGAAACTGAACGGACATTTGTTGAAGATTCTCACATGCACAATAATGAGGAGGCTGAAGAAGGGGATG
ATAGTGGATATGCATATTGGAAGGATGATGAGGGTGAAACTTCCTCTGAGGACAATGAAATGAACCAAGTGAA
GCATCTATGTTTCACAAGAACAGATCCCGTAACAGGTTTTCCTATCCAGAAGCTGACTGTCATGTGCAGCGAG
TCAGGTGAGATTATGTCCTTTGTGGTTCAATGAAAGGAATTGTGTACGGGGGTAGACCTCTTTTGTTTTGATG
CGAATCAAATGCAGTCAGAAATTTCTTTTCAGATGGAGTAGCGTCTGGCACATTTGCCTATCAGCTCCTTCCC
AATCATGTTTTACATAGAGGTTGAACATTGCTAAGCGGTCGCCCTGCTTGCTTTGCTGTGATCATTCTCCAAA
CATTTGATAGACACACCCGACAGATGAAAGCCTAGCACTAAGGCCTTACACCCCCCCTCACTCCAGCCACACC
AATTTTCATGCTTTCAGAGCCTCAGGAAAAAAATCCACCAGTACCCCCACAGACTGTTTCGCCATCTTGTTTT
TCCCCTCTACCTGAAGAAAATTTTGTCAACAAGATTCACACTTCAACTCTCACACCATGTTTGTCTGAAATTG
TGTGGATCTAGAAGCTGAGCAGCAACTGATCCTCAATGAAATCTGGATGACTCACACACAGTCCGGACACAAA
CTTGTATCCTAACAGTTATGATCACAAGAACAAGCTTTGTCATGGCCACCCTTAACACAAGGAATACCTAAAG
AAAGTGTTGAACCTATGTTAGCCCTGTGTTGAGTGCCCCGTCCCAATAATGCCAATGCAAGCTCACGTCTCAA
AGCCAATCATTCACTATTGTTAGTCTCAGAACTGTTGCAAAGACACCAATCATTCATCCCATTGCAGCTACAG
TTCCTGCTCTGTGTACCGAAGAGTTTGTGTCTGGTTATTTTGTTTTATTTCATTGTGATTGTTAGATCAGCTG
TGAATGATGTACACACCATGAGTGGCTCGTGATCATTCACCTTGTTTATGTCAATCACTACTGGCAAATCTGC
ATCGAAACTACCACAAATGCCTTGTAATCATATCCTTGCTGCAATGTACCATACTATGACTCACTTGGTCAGC
TTACATTTATTTACCTCTGATCCAACAGAAACCTTATATGACTTCCAAGTAGACATAAAACAATCCACTATTG
AAAATGCTGGACATGGTGCATTTTTGACCTATCTTGGAGCCAGAGTCTTAAAGAAGAGTGCTGCTGCGCGATC
CACAAGGCTGTTAAGAGGACATTGTATTGAAAATGACATTTGCACACACAATCCCTTGGAAGCAGAGACACTA
AGTGGGAAAAAAAGTCAAAGTGACATTGGCTGGGAACAATCTCCACTACAACGACAACAACATTTACTGGTCA
AAGAAAAGGGCTGACAAATTTGAAGAATTTCATATCAATCAAAACCCTTCTCATTCGAAGTCAACACATGGTA
GCTGCAGAAGTGCGGTTGAATCCTTTACTTTTGATGAGAATGAGGTAAAATGTAAAGTCCACGAGGAAGTCAA
AAAATTGAGGCAAAAAATCCCAGATGGGAATGGGATTGGTTTCTTGGGAATTCACCAAGAGTCTGACTATGAG
CCTGATGATGATGAATTATTCTGCAACCATCCAAATGCGTTTGAACTTGGCCGATATGGACCACACAGGTAAG
TTTTTCTTATTTAGCTCCCACGCGGTTTGGTTAGCTCATTTTCACCATTGAGATTGAGTTCCTGAGGGTGCCC
TACTATCTAGGCATTGCTTGTACCTCACATCTGCGGAGGCTTAGCTGAATATCTATGTGTCACCAAGTTTCTT
GATTTTGCCAGAAACACTGTTTGTGCAAACGTTGGTTTCTCAGCCAGTGCTTCTAATATCCCCTGGGTATTCT
TCAGAGAACTTAATTGTACTTTACCCCATTCAACCTCACATCATTTCATCTTTTCATTTCTATAAGGCCTGGT
GATCGTAAGACCAACTTGCACTTTGATTTCAAGAATTTTGTTCACAATTTTGAGCCTTCAGTATGGTCATACG
AGGTTCCTGAGCCTATGTTAAAGAAAAGACAAATTATTGACATTACTGATGACCATACAGGAGAAGTGCATTC
ACTTGCAAAGAACCATACTCCAATCTATGTCAATGAAATTGGTCATAATCCAGAGCTGCGAAGTAATGTTCTC
ATTCGTGACAAGGATGACCGATCCATTTACTATTATGTGTGCATTACCAAAGAATGCAAAATGAAGAAGGGAG
ACACAGTTGAGCTATTTGCCAACTATGGCGACCATTATGAACAGGTGCGTGAGCGAAAATGCTATGGGCGAGT
AAACAAATCAGAGCATTTGGGAGGTGATGAGGATGATGGTGCAAAACTTCAGCGTAACTTCCATGAAAGGGAA
GAAGTGGAGAAGGACATATCTTCTCTTGAAGTGTTTGCTATGTTCCATTTGGTGGAGTTCTTAACAGAAAATG
TTTTCAAACCAGTCAACGAATCAATTCGTAGTGTGGCCCATGGGAATCTTGATCTGCGTCAACAAAGACATTT
AATTGCGAGGAGGAGATTGCATTGGGTTGGGGAAAAACTTACAGGGCGAGTCCAAGAGGTTATAGCTCATCCT
TCTGTGGACAGCAGTTCTGAGTGCATTTGTAGTATTAGAGAGTCCCTTCGTCAGTGGCGCTTTGTATCAATGC
CAACGCTTTTCAACAAGCTGAATCCTGATCTTGCCAAAATATTACTGGGTGAAATTAGTGAAGAATTGCTGTA
TCAAACACGATCTTGCATACCAAATCCACTTGATGAGACTGTGTGGAGCAATATTGCCATTGATCTCACAAGA
AAAACAACACACAGTATTGGACAACATTTGTTTTGTTCTTCAGAGCCCATTGGTGTACGAAAACAGCACCTAG
GAAAGGATCTATTGAATCATGCCAAGCATGCTTCTATTGCACTGTTTGATGGGTCAAATGCTATCAACAACAT
CGAAGATCAAGACTCTTCCAATCACTCAAACAAGTATCCCAGGATCAAAAGCATGGATGATCACATGAACCTA
CTGTCTTTCAAGGCTCCTGGGCGTAAACTCAAAATAGAAGATCTTCTTGATGGTACAAAAGAACGAATGATCA
GTCTCTACAACTCACATCAAACCATTCGCTTTGGCACAGCTGCAATTATGGCGGACATCCAAGCTTATTATGA
TGCGGTTGACCTTTGTAGTATTGATCAATACTACCTACCTGGGGTAAACAAAGGAATATCTGAATGTGATAGC
TCAATTGTATCACAATATGAGCCAAAAAGTGCAAATTGCTCTGCTAACCACAGTCCACAATTTGATTGGATTG
CGCGCAGTGTTGATGCATTCAAGAAAGGTTGTGCCCGTGTGAATGAGCAGTGGTATCTAGAAAACCAAGTCCT
AGCAATTACTCACAATTTGGCAACAACTTGTGTTGATTGGACAGGTGGACCGGTTGAAAATGGTTGGTATTCT
TTGAGATTATTGTGCAAAAATATCTGTTTAGATATGGACGATGCCAAGAGAGCTTTAGCTCGGGGTACTCTGA
AACCAACTTGGCCTGTTCCAAAAGTAAAGTCAGCTTATTCCCCTGATCTGTGTGATGTTAGCCCTGCAAAAGA
ATCAGGGCGTAAACGTCGTAGGCCCAGGAAAACGCCAGGAGTTCCTTCTGACTCACAGAAGCACCCCAGAAAT
GCTGTTGAGATTTCTAATTGAAAGGCCACTAAATTGAAGAGAGCAAAATGTATGATAAATATGGAAATGAAAA
CCCATTAGATGAATCACTACTCATCCATAATGGTTTCAAAATTAGCTTTTTTGCTAATTTTGAATTTTTCCGT
TTCATATTGGACCTACATTGCAGTGTAAATGTCACAGATAGCATGAATGATGTGCATGGCAACACAGTGCGGC

GAGGCAAACATTTTCATCAATGCTATCATAGCATTTTATTTCACAATTTCAACACAATCATTGCAGAATTTGA
CGTATAATTTATAAAATATAATAATCCATTGGTTAAATGCGTGGTGAAAATTAGGAGGGTTGCATCACAAGCA
AAGCGCCATGTACAGACAGTTGTGGGCGACGGGGTTTAATTTGACAATTTTTTGTTGGTAACATAGATTGTCA
TATACCGGTCTTAC
>TR15|c0_g4_i2 len=8257 path=[10505:0-221 10506:222-245 10507:246-246
10583:247-650 10399:651-674 10592:675-942 10515:943-960 10581:961-1320
10532:1321-1344 10566:1345-2098 10484:2099-2122 10579:2123-2248
10503:2249-2272 10564:2273-2455 10545:2456-2479 10578:2480-2884
10563:2885-3541 10418:3542-3565 10590:3566-3637 10453:3638-3661
10603:3662-4457 10604:4458-4458 10468:4459-4478 10576:4479-4553
10408:4554-4556 10597:4557-4569 10560:4570-4698 @10588@!:4699-4967
10421:4968-5002 10559:5003-5225 @10595@!:5226-5408 10415:5409-5425
@10574@!:5426-5631 10522:5632-5648 10602:5649-5662 10600:5663-5686
10594:5687-5756 10557:5757-6066 10573:6067-6947 10471:6948-6971
10556:6972-7440 10459:7441-7464 10572:7465-8022 10492:8023-8046
10555:8047-8252 10520:8253-8256] [-1, 10505, 10506, 10507, 10583, 10399,
10592, 10515, 10581, 10532, 10566, 10484, 10579, 10503, 10564, 10545,
10578, 10563, 10418, 10590, 10453, 10603, 10604, 10468, 10576, 10408,
10597, 10560, 10588, 10421, 10559, 10595, 10415, 10574, 10522, 10602,
10600, 10594, 10557, 10573, 10471, 10556, 10459, 10572, 10492, 10555,
10520, -2]
ACCGAAGCGCTTGTCCTCGTTGGTCCGGAGGTCAATTCCTCCGTCGAAGCATCCCCCGGCGGCGTCGTCGTCG
ACCAAGGGCGCCGCCGCGGGGTCGTCTCATCTCTTCAACACCACCGACTCCGCCCGACCCATCCCTCGTAAGA
TGCCTTCGGACGGCGCTCCGATCGGATCCGGCCGCATTCCCAAGCGAGCCATTTCGGAGAAGGGATCGTCCGT
CGTTGCTCCCAAGAAGGGAAAGTTTCGACACCCGCAGAATCAACGCCCGCCGCAGCAGCACCTTCACGTACAA
GCGCGGCAGGAGCGGCATTGCCCCGAGATTGCCCCGTCTCCGGCCGATGCGGACCTCAGCGAGAACGTCAACG
TTCACGGAATTTGGAGGCGTCGCACCCGCGCTCCCTCGGGCACCGACGCGATTCCCTTGCAGAAACACACTTC
GACGAGGATGGTGCGGAAGGGCAACCAGAGCGCGTTCGGCACGGAGAGTCCCGAGGGGAGGGCCACGACGTTC
GGCCCTGGGAGGGATGGGAATGGGATGGCGTCGTCGAAATCTCCGGGGGAGATCGACGAGTCGGGATTGCACG
ATACGGAGAGCGAGGATGGGAGCATGTCGGAGGGCACTGTCATGGAGGAGGACGATGGATTCAGCGAGAGCGC
GTCGGTGGGGAACGAAAATCCACCAGTGCAGAAGCAGGCTGTGGAGTTGATTGTGCTGGACGACTCCTCGGAC
GAGGACGATGCTGGTGTCGGCGAAGACGGGGAGGGTGCGAACAGTGGGATCGACTACTCTCCAGATGGGGAAG
ACTCCTCGAACGAAGATGACAAAGATGAGAGGTCGAAGGAGCAGGATTTAGAAGACGACCCATTATTGTATAG
AAGCTTGAATAGAAGAAATTCCTTGCAGCAGGATCACTACCAGCTGAAGCCTGAGCAGATCAAAAAAAGCTCC
TGAGGACCAGAATATCCCAGAACATGGCCACAATGATACGATGAGTGATGATGTAGAAGACGATGGTGTAGAA
GACGAGGAGGTGAATGAGGAGGAAGAAAATGGCAAGGAAGAGAATGCCCAAAAAATTGCTGTTCCTGTGGCAG
ATGAAAGCGACCAGGAATCAAGTCAGGTAGGAGCAGATAGCGGCGAAGATGAACAGGAAGGCAAAGCCATGGA
GCTGGACAATGAAGGCACTGCTGTGATTCTGGGTGAGGACGCTGTGGCCGCCGAATGTGAAAAGGATTTGTTC
TATCTCATTGATCATGGAATTGAACTCCACGCTCGATGGTGTAGAAGAATTTCATCTAAATTTTCCAATCTGC
ATCATCCAGAGGAAGCATATCACGAGAATAAGCAGCAGGAGGTGGAGCCACAGGAGAACAGTGGCAGGGAAGG
AAATGAAGGCGTGTGTACAGACCGCGCCAGTGATAGCTCCAACATGAGTATTTCCGAGGCTGATCACAGTGCG
CGGCACTGTGAGAGCGAAATGGTGGATGAGGAAGAGGTGAATGATTCCGTGAGCGGCACCAACGATGGCAATT
TAAATGGCCATGGCCATGACAAAAATATGCCCAATACAGATGATGATGGTCTGCCACATTTAGAGACCGAATT
GGGTGAATCAATGGATTTGTCAAATGAAGATGAGGCTGCCAATGAGGAACGTCCAAATATTGTTTCAAATGAT
AAGGACGGAAGCATGTCCACTCCTGCTGCTGATAGAAAACAGGTCAGTGACTCGCCGACGTCCACAAATGAGC
TGCGGATAGACCCCATCCTGGATTCTATAGCGAGCGAGGGGAATGTGACGAGGAAAAATTCTGAGTCTGCGAG
CGAGACCAAAGACGGCGGTGGCAATAGCCATGGCAAAAATGCACTCACTGCACATGATGATGGTCTGCACCAT
ATGGAGAGTGATTTGGGTGAATCAATGGATATATCAGATGAAGATGAGGCTGTCAATGAGGAACGTCCAAAGA
TTGTTTCAAATGATAAGGGCGGGAGTATGTCCAATACTGCTGCTGATAGAGAACATGGAAGTGACTCATCTTC
CTCCCCAAATGAGCTGCGGACAGACCCCACTACGGTTTCCCTTGCACACAAGGGGAATGTGATGACAAAGAAT
TTCGATGAGACCAACTGTGCAGCTTCGCACACCAAGGCGAATCCTCGTCGTGATTTGGAATGGCCAGAGATGT
GGAATCTGATGAAAGACGAGGGCTGGTCCCTCATGCCAGGGAGTGGCTTGATCTCGTGGTATTACCTCGACCC
CACCTGCGCCGGCATGAAGAAAGTGGAGTTGCGGAGGACCAAGGAGGAGGGCGTTGATTACTTCTCGAACATA
GAATGTCTGAAGCAGCATGCAAGGATCAATCTTGGATGGGAGTCAGATCGCATTCCAAAGGATTCGGTGAGAT
CAGGAAACACTCTAAGTACTGACCCTCTCCATGACGCAGACTTCAATATTGTGCCAAAAAGCGCAAGTGACGA
CCCCGAAAAATCCAGCAACGAGGAATGTCCTCAAATTGAATCAAGTGGTGGTAAGAGTGAATCTAGTGCAGAT
GTACATCCTGGGACGATCAAATCCAAGAATCCTCTGACAGTAGGCGGGGAAATATTGTCACCTCTGTCGTCTA
CCATGAGGGGCAAGGAGTCGTTGAGCGAAGTCGACAATGGTACTATGATGCGCAGCGATCAAGATATTATTGT
TGGACGGAATGAGGACGCCAACCCTCTGGACAATGAATCATCGACAGCTGTCAATGAAGCCGACGTTGTCAGT
GCTGATGTTGGTCGCACTGATGATAAGGTGCACTCTCGTAACGAATCATTTGACAAGTTGAATGTAGATGAGA

TAGAGCCCCTTACTGCATCAGCAGTGCCAGACAAGAAAGAGCATGGCGGCGTTGATAAACTCGTCGGAAACAA
ATGTGCGCAGGTTTTGAATGATACATGCGAGTCAGCAGTAGGCAAGGAAAATAATGATGGCATAGGTATGCAA
ATTGACGAGAAAGATGTGCGAAGCGTCGAAGAGACTGTCAATGGTCATGACTCGGCATCTTCTGCACCGTGTG
CTGCTGTTTCCACATCTCAAGGCGATACAGCCAAGAAGGGAAGCTCAGCCACATCCTCCAAAGCCAAGACAGC
AGAGAGCAACAAAGAGAAGAATAAGCTTAAAAAGGAAAAAAAGAGGCAGAAAAGGGAAAGAAGAAAGGCTAAG
AAAAAGCGCAAACTCTCCAAAGAAGGCGAGAGCTGCCATGATGCCAAGAAGCAAAAGCAGCGAGCAGCTGGCG
CTGGTCAGGGTGTGGATAAGCCATGGACTGAAGAGGAACAATGTCTCTTTCATGAGGGCGTTGAAAAGTGTGG
AGGGAACAACTGGGGCAGAATTGCAAGTGATTATGTGACGTCCCGTACTCCCCTTCAAATTCGGCAATATGCA
CGGCATAGTCAACTCTCCAATGCGGACGAGAACTGCAATATTGGTCAGTCACAAACACAAGGAGGTGATATTA
GCCATGATGTTGACAAACCATGGACTGAAGAGGAGACTTGCCTTTATCTTGAGGGCGTTGACAAGTGCGGTAG
GAATAACTGGGGCAAAATTGCGACTGAGCATGTGACCACTCGCACTCCCCAACAAGTTAGAAAGTATGCAAGG
TGTGGCTCTAACTTACTTTTGGACCAAGGGCAGCAGCAGCAACATGCAGAATCAAAAGAGTCACACATTCTAG
TGAATGCTGAACAAGAATTGGACTCTGCTCTGTTGAATGATGTGCATGCAGCTGAAGTCAACATTGACACCAG
CAACATCAATGAACCTAAGCAGGACAAGGATCAGTTAAAACCCGGCCTTCAGGATGAAGATATTCTATCTGGT
GAAGACAGTGATTCAGTTAGCAGTGAGGACGTAGCTGTCGCGGCTTTCCTCACATGCACCAAGTGTGACATGG
TATTTATGGATACTGATGAGGCTTGGAAGCATGAAATCAATTGCAATATCACAGCCGGTCAAGCTGAAGAAGA
AGGAACAATTTTTGAAGACAAGCTATCGTTGATAAAATATATCTTAGAACTCAACTCGCGTGAGGGGAAGATG
GTGAATGAGGATGATATGGTTTTCTTCAGGCCAGATCTTCTGGCAGGGAAGAGACCAGTATCTTCAGATGAGA
CAGATGAGGATCAGCAGCTGGATGATACAGATGATGACTACCAGCAGGACAATGAAGCCAGTGATTCCAGTGG
TGATTCAGAAACTGAACGGACATTTGTTGAAGATTCTCACATGCACAATAATGAGGAGGCTGAAGAAGGGGAT
GATAGTGGATATGCATATTGGAAGGATGATGAGGGTGAAACTTCCTCTGAGGACAATGAAATGAACCAAGTGA
AGCATCTATGTTTCACAAGAACAGATCCCGTAACAGGTTTTCCTATCCAGAAGCTGACTGTCATGTGCAGCGA
GTCAGACACACCCGACAGATGAAAGCCTAGCACTAAGGCCTTACACCCCCCCTCACTCCAGCCACACCAATTT
TCATGCTTTCAGAGCCTCAGGAAAAAAATCCACCAGTACCCCCACAGACTGTTTCGCCATCTTGTTTTTCCCC
TCTACCTGAAGAAAATTTTGTCAACAAGATTCACACTTCAACTCTCACACCATGTTTGTCTGAAATTGTGTGG
ATCTAGAAGCTGAGCAGCAACTGATCCTCAATGAAATCTGGATGACTCACACACAGTCCGGACACAAACTTGT
ATCCTAACAGTTATGATCACAAGAACAAGCTTTGTCATGGCCACCCTTAACACAAGGAATACCTAAAGAAAGT
GTTGAACCTATGTTAGCCCTGTGTTGAGTGCCCCGTCCCAATAATGCCAATGCAAGCTCACGTCTCAAAGCCA
ATCATTCACTATTGTTAGTCTCAGAACTGTTGCAAAGACACCAATCATTCATCCCATTGCAGCTACAGTTCCT
GCTCTGTGTACCGAAGAGTTTGTGTCTGGTTATTTTGTTTTATTTCATTGTGATTGTTAGATCAGCTGTGAAT
GATGTACACACCATGAGTGGCTCGTGATCATTCACCTTGTTTATGTCAATCACTACTGGCAAATCTGCATCGA
AACTACCACAAATGCCTTGTAATCATATCCTTGCTGCAATGTACCATACTATGACTCACTTGGTCAGCTTACA
TTTATTTACCTCTGATCCAACAGAAACCTTATATGACTTCCAAGTAGACATAAAACAATCCACTATTGAAAAT
GCTGGACATGGTGCATTTTTGACCTATCTTGGAGCCAGAGTCTTAAAGAAGAGTGCTGCTGCGCGATCCACAA
GGCTGTTAAGAGGACATTGTATTGAAAATGACATTTGCACACACAATCCCTTGGAAGCAGAGACACTAAGTGG
GAAAAAAGTCAAAGTGACATTGGCTGGGAACAATCTCCACTACAACGACAACAACATTTACTGGTCAAAGAAA
AGGGCTGACAAATTTGAAGAATTTCATATCAATCAAAACCCTTCTCATTCGAAGTCAACACATGGTAGCTGCA
GAAGTGCGGTTGAATCCTTTACTTTTGATGAGAATGAGGTAAAATGTAAAGTCCACGAGGAAGTCAAAAAATT
GAGGCAAAAAATCCCAGATGGGAATGGGATTGGTTTCTTGGGAATTCACCAAGAGTCTGACTATGAGCCTGAT
GATGATGAATTATTCTGCAACCATCCAAATGCGTTTGAACTTGGCCGATATGGACCACACAGGTAAGTTTTTC
TTATTTAGCTCCCACGCGGTTTGGTTAGCTCATTTTCACCATTGAGATTGAGTTCCTGAGGGTGCCCTACTAT
CTAGGCATTGCTTGTACCTCACATCTGCGGAGGCTTAGCTGAATATCTATGTGTCACCAAGTTTCTTGATTTT
GCCAGAAACACTGTTTGTGCAAACGTTGGTTTCTCAGCCAGTGCTTCTAATATCCCCTGGGTATTCTTCAGAG
AACTTAATTGTACTTTACCCCATTCAACCTCACATCATTTCATCTTTTCATTTCTATAAGGCCTGGTGATCGT
AAGACCAACTTGCACTTTGATTTCAAGAATTTTGTTCACAATTTTGAGCCTTCAGTATGGTCATACGAGGTTC
CTGAGCCTATGTTAAAGAAAAGACAAATTATTGACATTACTGATGACCATACAGGAGAAGTGCATTCACTTGC
AAAGAACCATACTCCAATCTATGTCAATGAAATTGGTCATAATCCAGAGCTGCGAAGTAATGTTCTCATTCGT
GACAAGGATGACCGATCCATTTACTATTATGTGTGCATTACCAAAGAATGCAAAATGAAGAAGGGAGACACAG
TTGAGCTATTTGCCAACTATGGCGACCATTATGAACAGGTGCGTGAGCGAAAATGCTATGGGCGAGTAAACAA
ATCAGAGCATTTGGGAGGTGATGAGGATGATGGTGCAAAACTTCAGCGTAACTTCCATGAAAGGGAAGAAGTG
GAGAAGGACATATCTTCTCTTGAAGTGTTTGCTATGTTCCATTTGGTGGAGTTCTTAACAGAAAATGTTTTCA
AACCAGTCAACGAATCAATTCGTAGTGTGGCCCATGGGAATCTTGATCTGCGTCAACAAAGACATTTAATTGC
GAGGAGGAGATTGCATTGGGTTGGGGAAAAACTTACAGGGCGAGTCCAAGAGGTTATAGCTCATCCTTCTGTG
GACAGCAGTTCTGAGTGCATTTGTAGTATTAGAGAGTCCCTTCGTCAGTGGCGCTTTGTATCAATGCCAACGC
TTTTCAACAAGCTGAATCCTGATCTTGCCAAAATATTACTGGGTGAAATTAGTGAAGAATTGCTGTATCAAAC
ACGATCTTGCATACCAAATCCACTTGATGAGACTGTGTGGAGCAATATTGCCATTGATCTCACAAGAAAAACA
ACACACAGTATTGGACAACATTTGTTTTGTTCTTCAGAGCCCATTGGTGTACGAAAACAGCACCTAGGAAAGG
ATCTATTGAATCATGCCAAGCATGCTTCTATTGCACTGTTTGATGGGTCAAATGCTATCAACAACATCGAAGA
TCAAGACTCTTCCAATCACTCAAACAAGTATCCCAGGATCAAAAGCATGGATGATCACATGAACCTACTGTCT
TTCAAGGCTCCTGGGCGTAAACTCAAAATAGAAGATCTTCTTGATGGTACAAAAGAACGAATGATCAGTCTCT

ACAACTCACATCAAACCATTCGCTTTGGCACAGCTGCAATTATGGCGGACATCCAAGCTTATTATGATGCGGT
TGACCTTTGTAGTATTGATCAATACTACCTACCTGGGGTAAACAAAGGAATATCTGAATGTGATAGCTCAATT
GTATCACAATATGAGCCAAAAAGTGCAAATTGCTCTGCTAACCACAGTCCACAATTTGATTGGATTGCGCGCA
GTGTTGATGCATTCAAGAAAGGTTGTGCCCGTGTGAATGAGCAGTGGTATCTAGAAAACCAAGTCCTAGCAAT
TACTCACAATTTGGCAACAACTTGTGTTGATTGGACAGGTGGACCGGTTGAAAATGGTTGGTATTCTTTGAGA
TTATTGTGCAAAAAATATCTGTTTAGATATGGACGATGCCAAGAGAGCTTTAGCTCGGGGTACTCTGAAACCAA
CTTGGCCTGTTCCAAAAGTAAAGTCAGCTTATTCCCCTGATCTGTGTGATGTTAGCCCTGCAAAAGAATCAGG
GCGTAAACGTCGTAGGCCCAGGAAAACGCCAGGAGTTCCTTCTGACTCACAGAAGCACCCCAGAAATGCTGTT
GAGATTTCTAATTGAAAGGCCACTAAATTGAAGAGAGCAAAATGTATGATAAATATGGAAATGAAAACCCATT
AGATGAATCACTACTCATCCATAATGGTTTCAAAATTAGCTTTTTTGCTAATTTTGAATTTTTCCGTTTCATA
TTGGACCTACATTGCAGTGTAAATGTCACAGATAGCATGAATGATGTGCATGGCAACACAGTGCGGCGAGGCA
AACATTTTCATCAATGCTATCATAGCATTTTATTTCACAATTTCAACACAATCATTGCAGAATTTGACGTATA
ATTTATAAAATATAATAATCCATTGGTTAAATGCGTGGTGAAAATTAGGAGGGTTGCATCACAAGCAAAGCGC
CATGTACAGACAGTTGTGGGCGACGGGGTTTAATTTGACAATTTTTTGTTGGTAACATAGATTGTCATATACC
GGTCTTAC
>TR15|c0_g4_i3 len=8192 path=[10505:0-221 10506:222-245 10507:246-246
10583:247-650 10399:651-674 10592:675-942 10542:943-959 10581:960-1319
10532:1320-1343 10566:1344-2097 10484:2098-2121 10579:2122-2247
10503:2248-2271 10564:2272-2454 10545:2455-2478 10578:2479-2883
10563:2884-3540 10418:3541-3564 10590:3565-3636 10453:3637-3660
10603:3661-4456 10589:4457-4702 10576:4703-4777 10408:4778-4780
10597:4781-4793 10560:4794-4922 @10588@!:4923-5191 10421:5192-5226
10559:5227-5449 @10595@!:5450-5632 10527:5633-5650 @10574@!:5651-5856
10522:5857-5873 10602:5874-5887 10600:5888-5911 10594:5912-5981
10396:5982-6001 10573:6002-6882 10471:6883-6906 10556:6907-7375
10459:7376-7399 10572:7400-7957 10492:7958-7981 10555:7982-8187
10520:8188-8191] [-1, 10505, 10506, 10507, 10583, 10399, 10592, 10542,
10581, 10532, 10566, 10484, 10579, 10503, 10564, 10545, 10578, 10563,
10418, 10590, 10453, 10603, 10589, 10576, 10408, 10597, 10560, 10588,
10421, 10559, 10595, 10527, 10574, 10522, 10602, 10600, 10594, 10396,
10573, 10471, 10556, 10459, 10572, 10492, 10555, 10520, -2]
ACCGAAGCGCTTGTCCTCGTTGGTCCGGAGGTCAATTCCTCCGTCGAAGCATCCCCCGGCGGCGTCGTCGTCG
ACCAAGGGCGCCGCCGCGGGGTCGTCTCATCTCTTCAACACCACCGACTCCGCCCGACCCATCCCTCGTAAGA
TGCCTTCGGACGGCGCTCCGATCGGATCCGGCCGCATTCCCAAGCGAGCCATTTCGGAGAAGGGATCGTCCGT
CGTTGCTCCCAAGAAGGGAAAGTTTCGACACCCGCAGAATCAACGCCCGCCGCAGCAGCACCTTCACGTACAA
GCGCGGCAGGAGCGGCATTGCCCCGAGATTGCCCCGTCTCCGGCCGATGCGGACCTCAGCGAGAACGTCAACG
TTCACGGAATTTGGAGGCGTCGCACCCGCGCTCCCTCGGGCACCGACGCGATTCCCTTGCAGAAACACACTTC
GACGAGGATGGTGCGGAAGGGCAACCAGAGCGCGTTCGGCACGGAGAGTCCCGAGGGGAGGGCCACGACGTTC
GGCCCTGGGAGGGATGGGAATGGGATGGCGTCGTCGAAATCTCCGGGGGAGATCGACGAGTCGGGATTGCACG
ATACGGAGAGCGAGGATGGGAGCATGTCGGAGGGCACTGTCATGGAGGAGGACGATGGATTCAGCGAGAGCGC
GTCGGTGGGGAACGAAAATCCACCAGTGCAGAAGCAGGCTGTGGAGTTGATTGTGCTGGACGACTCCTCGGAC
GAGGACGATGCTGGTGTCGGCGAAGACGGGGAGGGTGCGAACAGTGGGATCGACTACTCTCCAGATGGGGAAG
ACTCCTCGAACGAAGATGACAAAGATGAGAGGTCGAAGGAGCAGGATTTAGAAGACGACCCATTATTGTATAG
AAGCTTGAATAGAAGAAATTCCTTGCAGCAGGATCACTACCAGCTGAAGCCTGAGCAGATCAAAAAAGCTCCT
GAGGACCAGAATATCCCAGAACATGGCCACAATGATACGATGAGTGATGATGTAGAAGACGATGGTGTAGAAG
ACGAGGAGGTGAATGAGGAGGAAGAAAATGGCAAGGAAGAGAATGCCCAAAAAATTGCTGTTCCTGTGGCAGA
TGAAAGCGACCAGGAATCAAGTCAGGTAGGAGCAGATAGCGGCGAAGATGAACAGGAAGGCAAAGCCATGGAG
CTGGACAATGAAGGCACTGCTGTGATTCTGGGTGAGGACGCTGTGGCCGCCGAATGTGAAAAGGATTTGTTCT
ATCTCATTGATCATGGAATTGAACTCCACGCTCGATGGTGTAGAAGAATTTCATCTAAATTTTCCAATCTGCA
TCATCCAGAGGAAGCATATCACGAGAATAAGCAGCAGGAGGTGGAGCCACAGGAGAACAGTGGCAGGGAAGGA
AATGAAGGCGTGTGTACAGACCGCGCCAGTGATAGCTCCAACATGAGTATTTCCGAGGCTGATCACAGTGCGC
GGCACTGTGAGAGCGAAATGGTGGATGAGGAAGAGGTGAATGATTCCGTGAGCGGCACCAACGATGGCAATTT
AAATGGCCATGGCCATGACAAAAATATGCCCAATACAGATGATGATGGTCTGCCACATTTAGAGACCGAATTG
GGTGAATCAATGGATTTGTCAAATGAAGATGAGGCTGCCAATGAGGAACGTCCAAATATTGTTTCAAATGATA
AGGACGGAAGCATGTCCACTCCTGCTGCTGATAGAAAACAGGTCAGTGACTCGCCGACGTCCACAAATGAGCT
GCGGATAGACCCCATCCTGGATTCTATAGCGAGCGAGGGGAATGTGACGAGGAAAAATTCTGAGTCTGCGAGC
GAGACCAAAGACGGCGGTGGCAATAGCCATGGCAAAAATGCACTCACTGCACATGATGATGGTCTGCACCATA
TGGAGAGTGATTTGGGTGAATCAATGGATATATCAGATGAAGATGAGGCTGTCAATGAGGAACGTCCAAAGAT
TGTTTCAAATGATAAGGGCGGGAGTATGTCCAATACTGCTGCTGATAGAGAACATGGAAGTGACTCATCTTCC
TCCCCAAATGAGCTGCGGACAGACCCCACTACGGTTTCCCTTGCACACAAGGGGAATGTGATGACAAAGAATT

TCGATGAGACCAACTGTGCAGCTTCGCACACCAAGGCGAATCCTCGTCGTGATTTGGAATGGCCAGAGATGTG
GAATCTGATGAAAGACGAGGGCTGGTCCCTCATGCCAGGGAGTGGCTTGATCTCGTGGTATTACCTCGACCCC
ACCTGCGCCGGCATGAAGAAAGTGGAGTTGCGGAGGACCAAGGAGGAGGGCGTTGATTACTTCTCGAACATAG
AATGTCTGAAGCAGCATGCAAGGATCAATCTTGGATGGGAGTCAGATCGCATTCCAAAGGATTCGGTGAGATC
AGGAAACACTCTAAGTACTGACCCTCTCCATGACGCAGACTTCAATATTGTGCCAAAAAGCGCAAGTGACGAC
CCCGAAAAAATCCAGCAACGAGGAATGTCCTCAAATTGAATCAAGTGGTGGTAAGAGTGAATCTAGTGCAGATG
TACATCCTGGGACGATCAAATCCAAGAATCCTCTGACAGTAGGCGGGGAAATATTGTCACCTCTGTCGTCTAC
CATGAGGGGCAAGGAGTCGTTGAGCGAAGTCGACAATGGTACTATGATGCGCAGCGATCAAGATATTATTGTT
GGACGGAATGAGGACGCCAACCCTCTGGACAATGAATCATCGACAGCTGTCAATGAAGCCGACGTTGTCAGTG
CTGATGTTGGTCGCACTGATGATAAGGTGCACTCTCGTAACGAATCATTTGACAAGTTGAATGTAGATGAGAT
AGAGCCCCTTACTGCATCAGCAGTGCCAGACAAGAAAGAGCATGGCGGCGTTGATAAACTCGTCGGAAACAAA
TGTGCGCAGGTTTTGAATGATACATGCGAGTCAGCAGTAGGCAAGGAAAATAATGATGGCATAGGTATGCAAA
TTGACGAGAAAGATGTGCGAAGCGTCGAAGAGACTGTCAATGGTCATGACTCGGCATCTTCTGCACCGTGTGC
TGCTGTTTCCACATCTCAAGGCGATACAGCCAAGAAGGGAAGCTCAGCCACATCCTCCAAAGCCAAGACAGCA
GAGAGCAACAAAGAGAAGAATAAGCTTAAAAAGGAAAAAAAGAGGCAGAAAAGGGAAAGAAGAAAGGCTAAGA
AAAAGCGCAAACTCTCCAAAGAAGGCGAGAGCTGCCATGATGCCAAGAAGCAAAAGCAGCGAGCAGCTGGCGC
TGGTCAGGGTGTGGATAAGCCATGGACTGAAGAGGAACAATGTCTCTTTCATGAGGGCGTTGAAAAGTGTGGA
GGGAACAACTGGGGCAGAATTGCAAGTGATTATGTGACGTCCCGTACTCCCCTTCAAATTCGGCAATATGCAC
GGCATAGTCAACTCTCCAATGCGGACGAGAACTGCAATATTGGTCAGTCACAAACACAAGGAGGTGATATTAG
CCATGATGTTGACAAACCATGGACTGAAGAGGAGACTTGCCTTTATCTTGAGGGCGTTGACAAGTGCGGTAGG
AATAACTGGGGCAAAATTGCGACTGAGCATGTGACCACTCGCACTCCCCAACAAGTTAGAAAGTATGCAAGGT
GTGGCTCTAACTTACTTTTGGACCAAGGGCAGCAGCAGCAACATGCAGAATCAAAAGAGTCACACATTCTAGT
GAATGCTGAACAAGAATTGGACTCTGCTCTGTTGAATGATGTGCATGCAGCTGAAGTCAACATTGACACCAGC
AACATCAATGAACCTAAGCAGGACAAGGATCAGTTAAAACCCGGCCTTCAGGATGAAGATATTCTATCTGGTG
AAGACAGTGATTCAGTTAGCAGTGAGGACGTAGCTGTCGCGGCTTTCCTCACATGCACCAAGTGTGACATGGT
ATTTATGGATACTGATGAGGCTTGGAAGCATGAAATCAATTGCAATATCACAGCCGGTCAAGCTGAAGAAGAA
GGAACAATTTTTGAAGACAAGCTATCGTTGATAAAATATATCTTAGAACTCAACTCGCGTGAGGGGAAGATGG
TGAATGAGGATGATATGGTTTTCTTCAGGCCAGATCTTCTGGCAGGGAAGAGACCAGTATCTTCAGATGAGAC
AGATGAGGATCAGCAGCTGGATGATACAGATGATGACTACCAGCAGGACAATGAAGCCAGTGATTCCAGTGGT
GATTCAGAAACTGAACGGACATTTGTTGAAGATTCTCACATGCACAATAATGAGGAGGCTGAAGAAGGGGATG
ATAGTGGATATGCATATTGGAAGGATGATGAGGGTGAAACTTCCTCTGAGGACAATGAAATGAACCAAGTGAA
GCATCTATGTTTCACAAGAACAGATCCCGTAACAGGTTTTCCTATCCAGAAGCTGACTGTCATGTGCAGCGAG
TCAGGTGAGATTATGTCCTTTGTGGTTCAATGAAAGGAATTGTGTACGGGGGTAGACCTCTTTTGTTTTGATG
CGAATCAAATGCAGTCAGAAATTTCTTTTCAGATGGAGTAGCGTCTGGCACATTTGCCTATCAGCTCCTTCCC
AATCATGTTTTACATAGAGGTTGAACATTGCTAAGCGGTCGCCCTGCTTGCTTTGCTGTGATCATTCTCCAAA
CATTTGATAGACACACCCGACAGATGAAAGCCTAGCACTAAGGCCTTACACCCCCCCTCACTCCAGCCACACC
AATTTTCATGCTTTCAGAGCCTCAGGAAAAAAATCCACCAGTACCCCCACAGACTGTTTCGCCATCTTGTTTT
TCCCCTCTACCTGAAGAAAATTTTGTCAACAAGATTCACACTTCAACTCTCACACCATGTTTGTCTGAAATTG
TGTGGATCTAGAAGCTGAGCAGCAACTGATCCTCAATGAAATCTGGATGACTCACACACAGTCCGGACACAAA
CTTGTATCCTAACAGTTATGATCACAAGAACAAGCTTTGTCATGGCCACCCTTAACACAAGGAATACCTAAAG
AAAGTGTTGAACCTATGTTAGCCCTGTGTTGAGTGCCCCGTCCCAATAATGCCAATGCAAGCTCACGTCTCAA
AGCCAATCATTCACTATTGTTAGTCTCAGAACTGTTGCAAAGACACCAATCATTCATCCCATTGCAGCTACAG
TTCCTGCTCTGTGTACCGAAGAGTTTGTGTCTGGTTATTTTGTTTTATTTCATTGTGATTGTTAGATCAGCTG
TGAATGATGTACACACCATGAGTGGCTCGTGATCATTCACCTTGTTTATGTCAATCACTACTGGCAAATCTGC
ATCGAAACTACCACAAATGCCTTGTAATCATATCCTTGCTGCAATGTACCATACTATGACTCACTTGGTCAGC
TTACATTTATTTACCTCTGATCCAACAGAAACCTTATATGACTTCCAAGTAGACATAAAACAATCCACTATTG
AAAATGCTGGACATGGTGCATTTTTGACCTATCTTGGAGCCAGAGTCTTAAAGAAGAGTGCTGCTGCGCGATC
CACAAGGCTGTTAAGAGGACATTGTATTGAAAATGACATTTGCACACACAATCCCTTGGAAGCAGAGACACTA
AGTGGGAAAAAAAGTCAAAGTGACATTGGCTGGGAACAATCTCCACTACAACGACAACAACATTTACTGGTCA
AAGAAAAGGGCTGACAAATTTGAAGAATTTCATATCAATCAAAACCCTTCTCATTCGAAGTCAACACATGGTA
GCTGCAGAAGTGCGGTTGAATCCTTTACTTTTGATGAGAATGAGGTAAAATGTAAAGTCCACGAGGAAGTCAA
AAAATTGAGGCAAAAAATCCCAGATGGGAATGGGATTGGTTTCTTGGGAATTCACCAAGAGTCTGACTATGAG
CCTGATGATGATGAATTATTCTGCAACCATCCAAATGCGTTTGAACTTGGCCGATATGGACCACACAGGCCTG
GTGATCGTAAGACCAACTTGCACTTTGATTTCAAGAATTTTGTTCACAATTTTGAGCCTTCAGTATGGTCATA
CGAGGTTCCTGAGCCTATGTTAAAGAAAAGACAAATTATTGACATTACTGATGACCATACAGGAGAAGTGCAT
TCACTTGCAAAGAACCATACTCCAATCTATGTCAATGAAATTGGTCATAATCCAGAGCTGCGAAGTAATGTTC
TCATTCGTGACAAGGATGACCGATCCATTTACTATTATGTGTGCATTACCAAAGAATGCAAAATGAAGAAGGG
AGACACAGTTGAGCTATTTGCCAACTATGGCGACCATTATGAACAGGTGCGTGAGCGAAAATGCTATGGGCGA
GTAAACAAATCAGAGCATTTGGGAGGTGATGAGGATGATGGTGCAAAACTTCAGCGTAACTTCCATGAAAGGG
AAGAAGTGGAGAAGGACATATCTTCTCTTGAAGTGTTTGCTATGTTCCATTTGGTGGAGTTCTTAACAGAAAA

TGTTTTCAAACCAGTCAACGAATCAATTCGTAGTGTGGCCCATGGGAATCTTGATCTGCGTCAACAAAGACAT
TTAATTGCGAGGAGGAGATTGCATTGGGTTGGGGAAAAACTTACAGGGCGAGTCCAAGAGGTTATAGCTCATC
CTTCTGTGGACAGCAGTTCTGAGTGCATTTGTAGTATTAGAGAGTCCCTTCGTCAGTGGCGCTTTGTATCAAT
GCCAACGCTTTTCAACAAGCTGAATCCTGATCTTGCCAAAATATTACTGGGTGAAATTAGTGAAGAATTGCTG
TATCAAACACGATCTTGCATACCAAATCCACTTGATGAGACTGTGTGGAGCAATATTGCCATTGATCTCACAA
GAAAAACAACACACAGTATTGGACAACATTTGTTTTGTTCTTCAGAGCCCATTGGTGTACGAAAACAGCACCT
AGGAAAGGATCTATTGAATCATGCCAAGCATGCTTCTATTGCACTGTTTGATGGGTCAAATGCTATCAACAAC
ATCGAAGATCAAGACTCTTCCAATCACTCAAACAAGTATCCCAGGATCAAAAGCATGGATGATCACATGAACC
TACTGTCTTTCAAGGCTCCTGGGCGTAAACTCAAAATAGAAGATCTTCTTGATGGTACAAAAGAACGAATGAT
CAGTCTCTACAACTCACATCAAACCATTCGCTTTGGCACAGCTGCAATTATGGCGGACATCCAAGCTTATTAT
GATGCGGTTGACCTTTGTAGTATTGATCAATACTACCTACCTGGGGTAAACAAAGGAATATCTGAATGTGATA
GCTCAATTGTATCACAATATGAGCCAAAAAGTGCAAATTGCTCTGCTAACCACAGTCCACAATTTGATTGGAT
TGCGCGCAGTGTTGATGCATTCAAGAAAGGTTGTGCCCGTGTGAATGAGCAGTGGTATCTAGAAAACCAAGTC
CTAGCAATTACTCACAATTTGGCAACAACTTGTGTTGATTGGACAGGTGGACCGGTTGAAAATGGTTGGTATT
CTTTGAGATTATTGTGCAAAAATATCTGTTTAGATATGGACGATGCCAAGAGAGCTTTAGCTCGGGGTACTCT
GAAACCAACTTGGCCTGTTCCAAAAGTAAAGTCAGCTTATTCCCCTGATCTGTGTGATGTTAGCCCTGCAAAA
GAATCAGGGCGTAAACGTCGTAGGCCCAGGAAAACGCCAGGAGTTCCTTCTGACTCACAGAAGCACCCCAGAA
ATGCTGTTGAGATTTCTAATTGAAAGGCCACTAAATTGAAGAGAGCAAAATGTATGATAAATATGGAAATGAA
AACCCATTAGATGAATCACTACTCATCCATAATGGTTTCAAAATTAGCTTTTTTGCTAATTTTGAATTTTTCC
GTTTCATATTGGACCTACATTGCAGTGTAAATGTCACAGATAGCATGAATGATGTGCATGGCAACACAGTGCG
GCGAGGCAAACATTTTCATCAATGCTATCATAGCATTTTATTTCACAATTTCAACACAATCATTGCAGAATTT
GACGTATAATTTATAAAATATAATAATCCATTGGTTAAATGCGTGGTGAAAATTAGGAGGGTTGCATCACAAG
CAAAGCGCCATGTACAGACAGTTGTGGGCGACGGGGTTTAATTTGACAATTTTTTGTTGGTAACATAGATTGT
CATATACCGGTCTTAC
>TR17|c0_g1_i1 len=290 path=[268:0-289] [-1, 268, -2]
AGGACATGGCTGGTGTGCTGGGGGTGTCGTGTGTGTTTGTGTCGATGCCAAGAATATTTTATGATCCACAGCT
GCTTGTAGACAGCTCCGACCCATGTGATCGAACAAACACCGGAGCCCGCATCCTTTGCCAAAATATGCATGAG
TACTCATAAACTTCTTCCGGATACAATACTTACTTATGTTGGGGGCGGTTTCCTTTTCTCATGTGTAGAGTTG
GTCGGTACGTAGTTGTTAGGAGATACTGTCTCAAAGGAAAGAGATCTTGTACAGTCTCAAAGAGATCGGAA
>TR19|c0_g1_i1 len=290 path=[268:0-289] [-1, 268, -2]
AGAACACCACGTGGTCCAGGTGGAAGGCCGCTTCCCTGAGCGAGGCATTGAGTGGAACACGAGTAAGTTGTTT
AAGTCAATTTTATCCAGCACGTAAGTCATGTAAAGCTATCACAGACACATGTATCACAAGATAAATCTGACAA
CTCTACCTCCATAAAACAATATCGCGCATGATATCCGTTTCACTACAGGCTCACTACACAACGTTGCAGTGTT
GCATTGTTTCCACTCACCGTAGTAATCTTTGTAAACAAAGAAAACCCCACCAAACCCACAACGCCAAGGTA
>TR20|c0_g1_i1 len=228 path=[206:0-227] [-1, 206, -2]
TGTGCTCTTCCGATCTCTTCACTATAGACCAGAATGATCACATTTGTGTGCCATACCGTATAGGTCCCGCGTG
AGCCACATGTTGAAGATAAGATAGACACCCGCAGCTTGCCTGCAGCATCCTAGCGGCCAGATGGCATGGATAT
GTGAGGAGTAACAAATATTTTACGTGAATTAGACGAACAGCACATTTCATTTGCAGCAGGTCCCATGAGCCAT
TTATTGCAA
>TR21|c0_g1_i1 len=249 path=[221:0-25 222:26-28 223:29-31 224:32-248] [-
1, 221, 222, 223, 224, -2]
CCACCACCACCACCACCACCACCACCACCACCTTCCCCAACAATCCAAACGGCCGACTCCACGAATACGATCC
TCCAATCGCGCGAAAAACGACGCGCTATCGGAAATCAGCACGACAGCGGCCATTACTTCCACGCCGCCGCGAA
CAACGACGGCATCGAAACGAAAAACACCGTCGTCTCCCGAGAAGGAGGAGAAGGGGAGGGGAAACGCGACGGG
CGCGCCGCCGCCGCCATCATCATCGTCGTC
>TR22|c0_g1_i1 len=225 path=[203:0-224] [-1, 203, -2]
CGACTTTGATAGCGTGAGTGAATGGCAAATAGGGTCTGAAGAATGGACACCCGAGTGTAGTCTCTGCCTCCCG
AGTCGCTGCCTTCCACCAGATTCTTGGTGGAGGACAACGACAGCAAGTGTTCGAAGAGCTTGGAAGAAAGCTT
GGAAATGTCAAGAGCACCACTAATGCTGTTACCCAACACATCCAACTTCCCGCGCACGCAATCGGTGACGAGA
TCGGAA
>TR23|c0_g1_i1 len=796 path=[774:0-795] [-1, 774, -2]
CACCATGTGGTCAAAGAACGGTGGATGTGATCAATGTTAGTTGTGATATCTATCCATGTCTGGCAATAAAATC
TACAAACAAACAGCATCATGCTTTTTGCAGCATTTTGATGAAAGAGATTGAATTCGCAAAAAGTTGCTTTTGA
TTTGATCTTTTAGTCTGGGGAGAACCTCTAGAAATTCCTCTTGGCTCCAACACATTTGAGTGGCAGGTGGCAA
AAGTGCTATTTCGAAGCATAACAAAGTCTCCCGAGGTAGCCAACGTCTCCTGTCAATACGCCAACTAACCATT
TCCTGAAGGATGATTCAGAGTCAAAGTGTGTCTACTATCTATTTTCACCACTAACAATTCTCTGTATGCTCTT
CCTCGACCAGTCCTGCCAGTATACTGTCCATTTTTTCTAATGACACACTCAAGTCAGAAATCCTTTTCTCTTC
CGCCGTAACGACAATTTTTTCGCCCGTTATCGTACACCGAGGTGACCCTTGTTCACATTTAACAGCATCTTGC
TGTGAAGGCACAGCAATTTCCTCTACACCAAAACCGTCTTCGTCTGTTTGGGCGGTCGAGTTGCTAGAAGATC
CGTCTCTTGCTACTCCATCCACCACTTGGAATGCAGAACTCTTGGTATTTTCGGAGGCACTGTCTTGACAGCA

CTTGAAGATAGAGATAACTCCACCCATATTGTTATTACGTGCTACTCTTCGACCTGAGAGAAGGGTCTATACC
CGGTTAAACTTAAACGATGGCAGACGCTTACGGTACTGCTAGATAGTGTGATGTGCAGATCGGAAG
>TR24|c0_g1_i1 len=289 path=[267:0-288] [-1, 267, -2]
TTGTGTGACGGTGTCTCCTACCAATCTAATTATGGTGATAAGTATTACACAATACCTTTTAGTTAGTATGGCA
ACTAAGCACCATGTGTTTTTGTAAGATTCATGTACCGGCATGCTCCTAGAGAGGACACACATTTTATTGTATT
TTTTATTCATTCACACACATTACCGATTGATCTCAGTTCATACCTTAATGTATATTATTCAGTCTCACATGAT
TTTCCAAAATAGCAAATTATCATTTATCATGTTCTCTATTGTATCTCTGAAGTCAGTGGTCATTGTACTA
>TR25|c0_g1_i1 len=354 path=[1004:0-332 1005:333-353] [-1, 1004, 1005, -
2]
TTAAATAAATTTACATTACATGAATATTAATGATGATTTAGAAAAACTTCTAAATAATCTACCATTTTTTATT
TATCAACATTTATCTACTTATTCAAATAAAGAGAAATTAATTGAAATTGTTTTGGATTTGGGCCGAAGACCTG
AAGCTCGATTTACTACAGGACCTGAATATTTATCACAAAAAGTCATCTCGTGGCAAGATATAGATTATATAAC
TAAACGTATAAGTAAGTTTAGTGGAGAAAACCGTGCAGGGATTGAACGAACACTTCATCGGATAAGTTGTATT
AGGAATCGGCAATTTTTGATTAATGGTTTAACCTGTCGCGTAGAAGAAGAAAAAAAGAATTA
>TR25|c0_g2_i1 len=1005 path=[1006:0-332 1007:333-1004] [-1, 1006, 1007,
-2]
TTAAATAAATTTACATTACATGAATATTAATGATGATTTAGAAAAACTTCTAAATAATCTACCATTTTTTATT
TATCAACATTTATCTACTTATTCAAATAAAGAGAAATTAATTGAAATTGTTTTGGATTTGGGCCGAAGACCTG
AAGCTCGATTTACTACAGGACCTGAATATTTATCACAAAAAGTCATCTCGTGGCAAGATATAGATTATATAAC
TAAACGTATAAGTAAGTTTAGTGGAGAAAACCGTGCAGGGATTGAACGAACACTTCATCGGATAAGTTGTATT
AGGAATCGGCAATTTTTGATTAATGGTTTAACCTGTCGCGTTGGACGTGCGATTTTTGGTACAATTTCAATTA
TTAGAGACCTACTTGAGTCTGGAGAATCTATTTTAATATTAGGAAAACCAGGTGTTGGAAAAACAACTATTAT
TCGCGAAATTACTCGTGTACTTTCGGATGAAATGGAAAAACGGGTTATTATTATCGATACGTCTAATGAAATT
GCAGGAGATAGTGATGTACCACACAGCGGTATTGGGAGAGCGCGACGTATGCAAGTACCAAAAACTGAACTTC
AATACAAAATAATGTTAGAAGCAATTGAAAATCATATGCCTCAAGTAATTGTTATTGATGAAATTGGAACAAA
ACTTGAAGCTTTAGCTACAAGAACTATTGCAGAAAAAGGTGTTCAATTAGTAGGTACAACTCATGGAAATTGT
TTAGAAAATTTAATTAAAAACCCATCTCTATCTGATTTAATAGGTGGAGTTCAATATGTAACAATAAGTGATG
AAGAAGCTAAACGAAGAGGTACTCAAAAAAGTATTTTAGAACGAAAATCATACTCTGCTTTTCAGCTTGCAAT
TGAAGTTAATAATACATATTCTTGGACAATTCATGAGAATGTAGAGAATTCTGTTGATCTAATCTTACGTAAA
AATTTTAATATTTCACAAATTCGTAATAAAAAAAAAAAATCAAAAATTATTTATTAA
>TR26|c0_g2_i1 len=2061 path=[2060:0-804 2061:805-2060] [-1, 2060, 2061,
-2]
TGTTTAATCTAAGCTTTTATATGAGATCGATTTACACTTGAATATATCTAATTGCCAATGTATCTGGAGCCTC
TCAATCAATCATTACCCACCATTTCAACAAATTAATACGATATGGCACTGCTTACGTGTTGTTCCCATTTTTA
CTTTTCAGATACACGTCATAGGCTGATTCTGTCGTCGACGTAAATTGTAATGGATCGTAATTCAAAATATCAT
CTGGGAAAACCTGCTTCGCCACCTCTGCTCCCTTCCCGCGCTGCTCGGGATCTTCTACCAAAGCATCGTAGCA
ACGAGCGAAGCCTCTTCGCACACCTTCAAATATTGGTTCCCTGCCCGTTTCCAACCGCGTCACCTCGGCCGAT
CCGGGAGGAGCAGCAATGGGGAGCAAATGAGGCTTGTCCGTATCCGTATAAGGTCTGTCCATATCATTGGCCC
CCTCGTTCAGTTCCAGGACCTGTTCCGCGTATATCCTTTTATGGAATCCCTTAATCATATATTCGTACACCTT
TCTGAATCTATTCTTGCGCAGGAAGAATCTCCGAACGATCAGCCTTTCGAAGATGGAACTCTTCCCCGTCACG
TCCTTGCCATCGGGTACCGAACGAAGGCAAACGACGTGCGTCGCCCCCTCCGACACGGCCGCTCTGTAAGGCA
AGGGTTCGTACAACAACGCGTCCGCCAACGGCTCGTAATCCTCGACCTCGTAATTATTCCTCGGTTTCATATC
GTACTGTGGCTGTGTGCCGTCCTCTTGGGGAGATTTCATGTTCATCACTGGACCCGCCACGCCGGGGAGGAGA
CAGCTCGCTCGCATGCAGCATGCTAATTCTTCAATATTGGAAAATGAACCCTTTTCCATATCCATGATGAATG
CCCTTTCACTTTTGAGACCGGATGCCATCACCTTTAGGGGTTGTACTTTTTGCATTTTTTCGAATGTTTCCCA
GTCCAGACGTTTGTTCTCTTGCATTGTCGTATTCAGCAAATAAGTCAAGTCTAGTACCGGTTTCCCATTATTA
CGACGGAAGATGACATCCTTTAACAACCGCGGATTCAAAAGCCCAAGCCCAAGCGCTCGAAACATTCGCTTGC
TATTAATAAATCGATTTCCCGCCGTAGTAAGAGCATCGTAATATAATTCTGGCCCAAACCAAGGAAGCTGCCG
CGTAATAAAGTAAGCCCCAATCACCGTGCCCGCACTCGATCCGTAGACTACGTCGACGGTGTCTTCCAATCCC
AAATGATGAATGGCTGTTACCATTCCTGCGGTGACGCATCCCCGCATACCTCCCCCTTCCACTGCAAGTGCCA
CACGAAATCCATCGTCCGGCGGCATTGTACGATGAATATTACGTTTATTGTTCCCGTTCGCAGGCGTAGATCC
CTCGTTCTCGGTTGATTCATTTTGTGCCTCTTGAGATTTTTTTACCTCTACAATTCGACGTTTTCTTTCGTGC
AATAACTGCACCACTGGGTGCACTGCATCCTTCTTATCAGGTGATCCTATTATTTGAGTATCGCCTTCCACGG
AAAGAGACTTCTGAAGGTTGATATAGTCAATATCGCCATTCTTCTTACGAAATCGTTCCTTTATATCATCGTA
GTAATCATTCAATTCCTCGATGGAATTGATGTGGAAAGTACTTGATTTGATCGCCTCGCCTTCGTTGGACTTT
TCGTCGGCTGTTTCGAATAAATGAACTGGCGCCCTGCATGTCGTCGCGGGAGATGGTACTTTTCTTTGATATC
GAGATGACTGAGCAACCAAACCTGATGCAGGTCCATTCTGAAATGCGGTTACTGTGTGCCTTTGGAGTTGGAG
GATTGCCACCGCCACTACCAGCACCAGGGCACCCAAGGAAAGAAACTTAGGGGTGGCATACCTCGCCACCATT
TTTCGTGTTCTCATTTCGGTGCTCAGTCGACTGGCGTATGGTCATGAATCGTTGAGCGTGTCTGCTCTATCTA

GTGTGGAGTTATTGAGGAAACGATTGCAGGATAGTAAAGCTAGCTGCTGCTGGTTCCGGCCGGTTCGGGTTGT
GCCATCGACATCGCCAT
>TR27|c0_g1_i1 len=1418 path=[1421:0-836 1422:837-860 1423:861-1417] [-1, 1421, 1422, 1423, -2]
GTGCAATATGTGTTTTTACGTATCATAATACTGTATGCTATCCTCTATTGAATTTGTGTTTCTGTGCTTGTTT
CCGAATGCTGTTGCTCATAAGTTCTGAGAGGCTCATGCGGTTGATGATGATACCTGGAAGGACCGGCCGCCAG
GCAACTTTCCAACAGGAATGAGATCCACATATTGACGTGACACCACCCGGTCAATGTGCCTCATCAACCAGTT
GATTGTACCATTAATTCCTCGAGACATATGCGATAGCTCGTTTACCCGACCACCGTCATGTGCGATCTTCGAC
CATTCTCTTCATAATTTTTCTAGTTTATTGAAATGATGGAGGTTTGTGATGAGGATTGTGCACTCTCCTGTGG
TTTCTTATTGTAATTGTGCTCTTCCTGATATTGTTATCTTTTGAGACGAAACAAAGAATATTTTACATGCAAT
TATTTACTGTGTTCCAATACTCATTACACCATGAACATAAATTATACCTTCATACTCCCCACAATCATTCATC
TAACCCCTCCTGACTAATCCTACGAACATGACAAGTACCGAAGAAAACACGACCACATTGGACGAAAATACCC
CTCCGACCAAAGCACTGGCCGCAGAAGAGCTTTCCATAGCTGACTGATCCAACCCGCACTGATAAGCCGCACC
ATCATTACAAAGGCATGTTTCCACCGGATCATAAAGCGGCTGCGTCTTGGATCCATCTGCAAACGAGACAGTG
CCAACACTATCCGGAATGACAAAGGGCCCACTCCAATCCATATATCTTGTATTCCCTGCCCCGCAAGTCTCAC
ACGAGCCACACTCACACGCGTGAGCAACGTCTGCACCCCCCGCCACATCGGGATAAAACATGGTGCTTCCTGA
AATGCAGTGATCGGGTGCCGAAGAAATCGCCGGTTGACGGTAGGTGTACACGGCGGATGCAAAGTTCTCTGAT
TCCACTGTTGTGACCTGAAGGCTGGAGCAGCATCCGCAGGCGCTGGAATCTTCGTCAAAAACAAACATGTTGG
TGGGCGTGTGAGCGGAGCATCCTCCCGCGGCGATGATGCGGCGGAAACAAGCGTCGAGTCCGGATTCTCCTTC
TACCCCGAGAATGTCGGTAGAGCTATTCGTAGCCGCCGCTCCGGTAGAGACAGGCGACGTGGTATTCACATCT
GTGTCCTCCGGAAGTGGAACGGCGCTGCAAAAGCTTTTATCGAACGACACATCCACCTGAGGCGGTGCGCTGC
TTCCCAACGCCCACCTGCTCTTCACCGACGTAGGGATCTGGCACAGCGGAATGCTCGTCTTGCACGTATGCGA
CTGCGAGGACGTGCCCCGTGGACATCGTCCGCTCACGAATCGATCCTGGTCATTGCCGCGACAGTACGGACTA
GAGTGACGGCCGCCGTAGAGAAAGTAACCGG
>TR28|c0_g1_i1 len=406 path=[409:0-117 410:118-141 411:142-405] [-1, 409, 410, 411, -2]
CGCTCTTCCGATCTAGCTGGTCGATGGTCGCGCGACTTCCCTCCCTGTTCTCGTGTTGATCCATGACCTGTTG
GAGTACGGTGGACTTGTAGTCCTTTTTTACGCATTCCCATTTGCTCCCGGCGGAGTTGAATTGGCAGACGAAG
TTTTGGGAGTCGTCATCGCTGCACTGGTTGGCTGGCGCGGAGTGAGGAGAGGTGAGTCCATTGGTTTGCGATT
GCGATGCCATGATGGATTGAGGGATGACGCCGGTGCTGATGCTGGCGGCTCCGTGTGGAGACGACATTGCTTT
TGGTTGTTGTTGGGAGAATAACAACTGTCTGTTACCCTGTCTCTTGTCTCTTGCCGCACTTCGAATATTGTAG
TCGAGTGGTTTAATGAGACGTGACTGTTGACGCGGGAAGTG
>TR29|c0_g1_i1 len=357 path=[367:0-47 368:48-111 369:112-356] [-1, 367, 368, 369, -2]
GAAGCAAAAAGCACTGAAGCATTATTGGAAAGTATTAGTCAAGTAGAATGACCCATCTCTACCATCCAATCGC
CCTCTCATTCCTCATCTTACGGGCAGCAAGCATCTGCCATTCCTTCTCCCATGATACTTCGTTAATTCCACGC
TCTCGCATTCCTTCCAACCTCTTGCGTCATCGTCGACATCCACCATCATGCACCTCAACAATATCTAGCAGAG
GAACCTACTTTCCTTTATGCAAGGCAGTATCACCACCAACGTTGACGTTGCGATGGAACTCTCCAACAGACTC
AGACGTCTCACCGACGACGACGACACCCCCACCAGAAAGGCAGGAAAAGAAGTCAACATCACCAC
>TR30|c0_g1_i1 len=331 path=[1067:0-38 1068:39-330] [-1, 1067, 1068, -2]
TGTGCTCTTCCGATCTGGAGAGTTGAGGAGTTTGAGTTGCCATGTTGTGATTACCTTGGAGAAGGTTGTTGGG
GATAAGAAAGAGTGAATCAAAGTTGGAAATGAATATGGAGTGAGGTCTGGAATGGAGAATTGTTGCTGAAAAG
CTTATTGAGACGTTGAATGCTTTGGGGTTTTAGTAGATGACATATCGTAGGAGAGTGAATTGTAATTTCCTGC
CTCTTGATGGCGGTATTGAAAGTTGCAATATCTTTTCATTCTTTATACTTGACCGTCACTCTACCTATGTGAA
TTCTGGAAAATAGCAACTTAATCGTCAGTTCTACCTTAC
>TR30|c0_g2_i1 len=1059 path=[1065:0-766 1066:767-1058] [-1, 1065, 1066, -2]
ATCTCAACAATCAACACGCCAATGGCCAAGACAATGCGTTGCCTTTTCTCCCTCCTCCTCGCCCTATCCTCAG
GCACACGCCTCTGCGATGCCTTCGCCACCCTCCCTCCCGCAGTATCCGCGGCCAGCATCACCAGTATTACTAA
TGCTAGAATATCATCCCTCTCCTCCACCACCACCACGGAGGACGAAGAAGCCGAAATCTCTTCCCTCCTCCGC
CCCTCCTACACCATCGAACCAATACCCCTCCGCATAGGCCACGGCTTCGACATACATAAAATGGTCCCCCTCT
CCGAAGCAGGACAGCCCGTAGTTATCGGTGGAGTCGTCATCGATCACGTCGATCAAAAGTGGACCGACAAGGA
GGGCAAGTACACCGACGCGCCCAACTCGATTTACGAAACGCAATTAGGAGTATTGGCGCATTCCGATGGTGAT
GTCATCTACCACTCTCTTGTCGATGCCATCTTCGGGGCATTGACATTGCCGGACATCGGTCAGGTATTTCAGG
ATACCGATCCTCGGTGGAAGGGATGTGATTCTTCCAAGTTTATGGATCACGCGTACGAGGTGATGAAGTACTA
CGGATATCAGATTGGGAATATCGATGTGACGTTGATTTTGGAGAGGCCCAAGGTGGCGGGGTTTAAGCCGAGG
ATGAAGGAGAATATTGTGAGGTTGTTGCATACTACTGAGGGGAGGGTGAATATAAAGGCGAGGACGCATGAGA
AAGTGGATAGCGTGGGAGAGTTGAGGAGTTTGAGTTGCCATGTTGTGATTACCTTGGAGAAGGTTGTTGGGGA
TAAGAAAGAGTGAATCAAAGTTGGAAATGAATATGGAGTGAGGTCTGGAATGGAGAATTGTTGCTGAAAAGCT
TATTGAGACGTTGAATGCTTTGGGGTTTTAGTAGATGACATATCGTAGGAGAGTGAATTGTAATTTCCTGCCT

CTTGATGGCGGTATTGAAAGTTGCAATATCTTTTCATTCTTTATACTTGACCGTCACTCTACCTATGTGAATT
CTGGAAAATAGCAACTTAATCGTCAGTTCTACCTTAC
>TR31|c0_g1_i1 len=319 path=[347:0-76 348:77-100 353:101-187 351:188-211 352:212-318] [-1, 347, 348, 353, 351, 352, -2]
GATCTGGCACTCTCCAGCTTCGTCTTGCCATCTGAACCCCGTGCTGTACTTTCAATGGCCACGCCCCCCAACC
CGATTCCAAAATCATTTGCGAATCGAATGGCATTCTCGGCCTCGTCGCGGTAATCAATCTCGCGATATAATAT
CGTCTCGGCATCATTGAAAATGCCCTCCCAGCTTTGTTCTATTCCGCCGACCTGGCCGGCCTTGCCAAAGAAA
TCGACGACCTTGGCAATTTTCTTCATCAAAAGAAGATCCTGATGAAAGAAGTTGGCATTCCATGCATCGCCAT
TTCCATCCTGTCAGAACACAATGCTTC
>TR32|c0_g2_i1 len=1529 path=[1569:0-136 1570:137-1528] [-1, 1569, 1570, -2]
ACGTCGCCACGCTGTCCAAGATGGTGACGAGCGGCTGCGACGCGGTTCTCTATACTATACTGTTGGAGGAAGA
CGCCGAGCAGATTCCATCCGCGACGACGACTATAGGTGCCGATTCGTTGCCGTACGTTGGCGCAGGTCTTGCA
GAGGACGTGGAAAAACGGACACAGTCGCGGTGCAACATCGCCTTTGTTTCTGATGAGAGCCCCTTGGTGCAAC
GCTTACGGTCAGAGGGCGAAGAATATTTGGAGGACGAAAATGCCAACGAAGGCAGTGAGAGCGGCGGGGGTTT
GCCTCCGTTGCTGCGTCACGGATTTTGGACGGTTCTGCCCGTGCCCACCCCGTCGTCTAATACCAATGCCAAG
TCGTCGGCTTCTTGGATGCACGCCTTCGACGGCACCTTTGTGCTTGAGTACCTCCCAAAGGTGTCTCCTGGCC
ATTTCTTCGGCTCCTCCGTACGATATGCTGTCTACGCCGCGCCGTCCATCCTCCTGGCCAATATTCCAAAACT
TCTCCGAAGGATGGAGGAGGGTCCGCCAGCAACGGCCGACTCCGGCAGCGGTGGATTCCCCGAAAACGCCAGC
ACGGCCATCATGCTCTCCGCTAAACGCCCTGCGTGCGATCCCGCCCAGCGTGGCTCTACATGCGCATCCACGT
GGACCCGCCTAGAGAAGAATGACGCGCTCCAGTCGAGCATGTACAACATGATCCGCGTGGCGCTCCGTGGCGA
CATGCTGGGAGGGGGGCTTGACCCTGTCATTGACTCGTCAGTCTTAGTGCACTCACTCCGCGAGGAGGATTCG
CGTCTATTTCGATGCGACTTTTACGGAGAGGCGGCGCAGTGGGGGGCCAGTTCAGACGAACGTGCTTTGGAAT
TTATTGTTAGTTTGCACGACTTGTGGAGTCGAGCGGTGAGTCATTGGTCGGGAGGTAAAGTTTGGTGGAATGC
TGACGGTGGTAGTTGGACACCAGGAGAGGAAGTCGAGGGCGACGGTGGCGAAGAGGAAACTGTAGAAAACCAG
AGACGGCGTCTTGCAGAAAGTATCGGCAATGCTGAGAAGAAACAACAGGAAGAGAAGTGGATGGGAATATTGT
CAGCGACGGAGACACAATTGTTCACTCATATTCTTCCTTCAGAAGTTATGGGTATCATTCATTTGGACGATCA
CCGTCGCTAAGTTGAAGGCTACAATGCCATTTTATGCACAAAGAGAGTGTTAGTTAGGGAGCAGGATTTCGTT
GGACGATTCTACCTCACCTCTACTTTGAAGATTTTGTGTAAATATCTGTTCTTAGAAAGAAGTTATCGAATTA
GCGGGGAAAGTTCTATCTTGACTAGTTGTCAGTTGCCAGCATATTTCCTTTTGTGTCTTCTTTGGTCGAGGAC
CATAGTAAACTATTCCATATCAGTCTAAGAACGTACGATCTTGCGACCACACCTCCTTGCATTATTTGTGACG
TCGTATGGCATTGGCTGATGTGGATAAAGTTTAGATTCAGTGTGGATGAAGTAGGAAAAACATCCGTGC
>TR33|c0_g1_i2 len=602 path=[776:0-49 777:50-73 800:74-224 801:225-248 803:249-252 795:253-306 790:307-324 791:325-325 798:326-390 802:391-391 763:392-601] [-1, 776, 777, 800, 801, 803, 795, 790, 791, 798, 802, 763, -2]
CTGTATCCATTCCGCTCTATATATCTCGAGACAATAGCATTTACTCTCTTCCTCCGCCTCCCTCTAAAATAGC
GAAACACGTCGCAGAAATTCGATTGCATCCGATTTCGACGACGTCTTTTCTCTTTGTTTCTGATTCATCTCTT
TCGTGTTGTAGCAGCATGTAATAGCCTGGCTCTCCCTGAAAAATCGGAATCGTCCATTCATTTCCATCTCAAG
GCCATCTCATCTGCGATCCGTTGGCGGTTTTTCGGATGGACGCAAGAGGCAGAAAAATAGGCTTTTACACACT
CCGGAATCTCATTTGCCTTTTTCAGCGTCGGACATAATGTTGCATCCGGATTTATACCGAGAGAAGGAGTCTC
CTCGATCATATATTCCCTCATTTTCTCCTCCCCCAGGCATTTTTTCATTCACAACCCTAACCATCCAAACTGC
TAGGATGTTGATGATGGACGTCCCGTATTCTTCCATCGCACCGTCGTCGAAGAAGAACGTCGATATCCACATT
TGAATTTTTGTCGCGAATCTTCCCGTTCCGAGGCTACGATGGAATCGCGATCGAATGAATTGTCGTCGGCGTC
GTCGTCGTCGTCGAAATT
>TR34|c0_g1_i1 len=465 path=[608:0-103 609:104-104 628:105-133 635:134-176 636:177-180 626:181-201 632:202-202 629:203-267 623:268-291 625:292-377 613:378-401 614:402-464] [-1, 608, 609, 628, 635, 636, 626, 632, 629, 623, 625, 613, 614, -2]
TGGTGTTTCTTTAAGTACTCCACGTGGAGGGTTTCTGTCGTCGACGCAATTTGGCCCTTCAATCCAAAAGCCA
ACCAAGGCGACGACACAACAACTTCTTCTCCTCTAGAGAGAACCACAAAAATCACCACAAAAAAAACAATACG
AATCAAACCAAAGACACAAATAATACACCTCTAAAAACATGCCATCCCTCATACTCACGGGTCATCCCTGCGT
AGGCAAAACGACCTTCGCCCATCTCCTCGCCGCCCGCGCCCTCCGCCACTCCTCCAACATCATCACTCGCGTC
GTGCACATTCGCGAATCCACGTCCCGTCCGGATCGGACGAAGGCCGAATGCTATTCCGACTCGCATGCGGAGA
AGACGACGCGCGCCGCGTTGAAGTCGGAGTTCGATCGAGCGGTCATGTCCAAATCCACCAAGGACGGCGGCGG
TTGCGGCGGCGGTTGCGGCGGGAGTGG
>TR35|c0_g1_i1 len=1577 path=[1726:0-884 1727:885-908 1750:909-944 1713:945-968 1758:969-1029 1724:1030-1047 @1756@!:1048-1224 1744:1225-1242 @1757@!:1243-1341 1755:1342-1444 1759:1445-1468 1760:1469-1479

1747:1480-1512 1742:1513-1576] [-1, 1726, 1727, 1750, 1713, 1758, 1724, 1756, 1744, 1757, 1755, 1759, 1760, 1747, 1742, -2]
ATGGAATGCAGTTCCAGAGGTGGATGATGCATAATCTGTTCATCAAAATGGGTTAACGTAAATCAACTGCCAA
GAACAGATATCTACTCCTCGCATTTTCATTGTTATTATTAGTCTATCCCACACCATCAGCGAAAAATCTTCAT
TTATTGACCTCCCCCAAAGCTATGGAACCGCATGAAATGTTTAGTCTTTCTTTTCCTCTTCAGATTCAGCGCC
ATTCTGTGAATCTCCTTCCTCTCCATCGCCCTCTTCATCCACATCATCTTCATTGGATTCATCACCTTCCACA
TCTCCCTCCTCGGCCTCTTTCGCAGCTTCAGCGGCTTCCAAAGCTTTCTGCTGCTCCAACTCCATCGCTGCAC
CTGCCTCAATCATTTGAAGAACAGATGGATCCACAGTCTTATGCTTGGTCCACCAAGCCTTCTCCCACGGAGC
AATAATGCTTTCTTGCACAACGCCACTTCCCCCAATCAATATCGAGATGTGAGTCGCCAACTGCATCGTACTC
GCGTACGGACCGCCGCTAATTTGCAATTTCTTCAGATACACTCTCCTCGCTCTCTTCTTAAAGTCCAAATGCG
GACGAGATTTTCTTTTGACGAGAAATCTCACCAGTTCACCCGTTACCCGAAGGGCATCGCTTAACATTTTGTC
CTCAGCACTCTTGGCACTCGATTCTTCCCCTTCTCCAGCCTCAGCAGCATCCTCGCCCGCATTATTCTCAACT
CTTAAATTTAGACCATTCTCTTCCATTTCGAAAAGACCCTTTTGCAACTCCTCTAGCACTTTATCCACCATTA
CCGTTTCCACCGCAAAGTACATTCCAGTCTTCTCATCCAGAATCTGTTCCGCCACAAACACGGCCGCCTGAAC
CCTCGGTTTACCATCCTGGATTTTCCAAGTAGGGGTCATGCCACGCAACATCCCCTCTACAGCAGCGCGACAT
CCCGACATCTGAAGGACGAACCGATTGTACTGTTTGAACTCTTTCAACGCGGTAGCGATCACCTTAGCATCCT
TCTTTTTTTCCCGGCCGGGCGGACTCGCATAACGCGACAAAGTCCGATTGACGGATCATTGCGGCGCCGTTAG
CCGCGTGCTTTTGCAGGACGGATATGCAGTGGCCGGGGGTGACGTTCTCGAGGTTGGCTTGTTCGAGGGCGCT
GGAGGCGGGTCGTTGGTAGTGGCCATCAACGCGTCGGTGTCTGGTTTCATCTTTTTTTGGCAACGCTCACTGA
CGAAAAATTGCTACTCAAAATGGTATTGTCCTTCTCCAAGATCCGATGATCGCTGATATTCCCTAAAGCAATT
GGCATAGAAGCAATGGAATGGCTTCGACGTTTATTTGGATCCGACTGCTGAGCGATCAAGTTTTGTGCACAAA
TCCCCGACGCCGATTCGCTTGTGGAGAATGTATGCCTACTCGCCCGCAGGTAGCACCTGGACCTTTCAACTCC
ACGGAATAATTGATTCATTGTAAATGATACAGGTGATGGCAAGAGTTGCCGCCCGTCTATTTCGGAGAAGCAC
AGCTGTGGAGACAAAAGGAAACGCCGCGTTGTCGCAGGGTAGCG
>TR36|c0_g1_i1 len=889 path=[1323:0-46 1324:47-70 1351:71-218 1355:219-242 1356:243-262 1350:263-312 @1354@!:313-464 1330:465-481 @1352@!:482-747 1337:748-769 1338:770-888] [-1, 1323, 1324, 1351, 1355, 1356, 1350, 1354, 1330, 1352, 1337, 1338, -2]
CTTCCGATCTTTTTTTTTATGTATTGCTGATGTTTCCTCTATTTCTTTGATTAGATCTGTGTTGCAGATTTTT
GAATGATAGCCGCCCGTGCAAAAATAGCGGAGAATGCTTACTTCAGCAAGAATAAATCTCGCTCTCGGCAATG
CTTCCCACATCGTGTTCAGTAGGTATTTGGCTTTACATAAGGGATTTGTGTGTCATCTCAATCATGCAGCATT
TTAATCCTACTACTACACACTCCAACATGCCACAACCACTCATATCGTAGTACCATCTCCTAAAACGCCTATT
TTAACCCTCTCCAACAATCTTAACATCAAACTTACTCTCCTTCAAATCCAACATCGGAGTCCACACCCCCTGA
ATACTCGCAACATCCCCCCTCACCGCCAACCCACCCAACCGAACAATCTTCCTCCCGCTCGTATTCCGCAGCA
TTTCCATCACCTCCATAATCCTTTTTTCGGTCGTGTTCTTGACGCAAATTACCTTGGCCTGGCCGGTGACGTA
ATCTCCCTGAATGTACGGATGGTGGCCGTTGCGCGGTTTGACGATGATTGTTATGTGTGGGTTGGACTTGGCG
AGGTTGATTAGGTGGGGGGATTGTTGGAGGTAATTTCGGACGGTGGTGGATGATCCTCCGATTTCGCAGTAGC
GGAGGGTTAATTTTTTTAGTTGGTTTATTCCTCGTGTTGCCATTTTGGATATATATGTTGCTGTTATTGTTGA
ATATATTATTATTATTACGATGGAGTTATAGAACGATGATAGCTCCTCCTCTTTCCTCTTGAGCTTGTGAGAT
TGAAGATTAATCAACCACCGGGTGATAGAACTTGATTGCTTTTGTGATGGACTTTTGTTGGAGCCGCTCTCGC
ACCGCTTTACCGT
>TR36|c0_g1_i2 len=1198 path=[1323:0-46 1324:47-70 1351:71-218 1355:219-242 1356:243-262 1350:263-312 @1354@!:313-464 1330:465-481 @1352@!:482-747 1345:748-1078 1319:1079-1197] [-1, 1323, 1324, 1351, 1355, 1356, 1350, 1354, 1330, 1352, 1345, 1319, -2]
CTTCCGATCTTTTTTTTTATGTATTGCTGATGTTTCCTCTATTTCTTTGATTAGATCTGTGTTGCAGATTTTT
GAATGATAGCCGCCCGTGCAAAAATAGCGGAGAATGCTTACTTCAGCAAGAATAAATCTCGCTCTCGGCAATG
CTTCCCACATCGTGTTCAGTAGGTATTTGGCTTTACATAAGGGATTTGTGTGTCATCTCAATCATGCAGCATT
TTAATCCTACTACTACACACTCCAACATGCCACAACCACTCATATCGTAGTACCATCTCCTAAAACGCCTATT
TTAACCCTCTCCAACAATCTTAACATCAAACTTACTCTCCTTCAAATCCAACATCGGAGTCCACACCCCCTGA
ATACTCGCAACATCCCCCCTCACCGCCAACCCACCCAACCGAACAATCTTCCTCCCGCTCGTATTCCGCAGCA
TTTCCATCACCTCCATAATCCTTTTTTCGGTCGTGTTCTTGACGCAAATTACCTTGGCCTGGCCGGTGACGTA
ATCTCCCTGAATGTACGGATGGTGGCCGTTGCGCGGTTTGACGATGATTGTTATGTGTGGGTTGGACTTGGCG
AGGTTGATTAGGTGGGGGGATTGTTGGAGGTAATTTCGGACGGTGGTGGATGATCCTCCGATTTCGCAGTAGC
GGAGGGTTAATTTTTTTAGTTGGTTTATTCCTCGTGTTGCCATTTTGGATATATATGTTGCTGTTATTGTTGA
ATATATTATTATTATTACTGAGGATGTATTTGTGGTCCAATCACAGAGTCGATAGAAAGAGGACGGAGAGGAG
ACGGGTGGATTACATGAGGCAAACGCGTTCGAATTTGGTCCAGGGTGACGGTAGGTAAGTATCATCCCATGAA
GAACTAGTGCGCTTCACAAGATGTCATTCTGAACACTGGACCTGAACTGTATTCTGAATAAGCAGCCAGACGA
CCGGGCGTCAATCTATCGCCGGCAATCGTAAACAACGTGACGGCGTTATGCACGTCCATCATCTGGTCCATCC
GCCGCGCACCCTCCACAATAGCGTATAAACTTACCGATGGAGTTATAGAACGATGATAGCTCCTCCTCTTTCC

TCTTGAGCTTGTGAGATTGAAGATTAATCAACCACCGGGTGATAGAACTTGATTGCTTTTGTGATGGACTTTT
GTTGGAGCCGCTCTCGCACCGCTTTACCGT
>TR36|c1_g1_i1 len=934 path=[1486:0-51 1551:52-106 1544:107-107 1556:108-150 1548:151-163 @1553@!:164-429 1524:430-446 @1555@!:447-598 1540:599-648 1552:649-668 1546:669-692 1539:693-840 1494:841-864 1495:865-933] [-1, 1486, 1551, 1544, 1556, 1548, 1553, 1524, 1555, 1540, 1552, 1546, 1539, 1494, 1495, -2]
ACGGTAAAGCGGTGCGAGAGCGGCTCCAACAAAAGTCCATCACAAAAGCAATTAAGTTCTATCATCCGGTGGT
TGATTAATCTCCAATCTCACAAGCTCAAGAGGAAAAAGGAGGAGCTCTCATCGTCCTATAACTCCATCGTAAT
AATAATAATATATTCAACAATAACAGCAACATATATATCCAAAATGGCAACACGAGGAATAAACCAACTAAAA
AAATTAACCCTCCGCTACTGCGAAATCGGAGGATCATCCACCACCGTCCGAAATTACCTCCAACAATCCCCCC
ACCTAATCAACCTCGCCAAGTCCAACCCACACATAACAATCATCGTCAAACCGCGCAACGGCCACCATCCGTA
CATTCAGGGAGATTACGTCACCGGCCAGGCCAAGGTAATTTGCGTCAAGAACACGACCGAAAAAAGGATTATG
GAGGTGATGGAAATGCTGCGGAATACGAGCGGGAGGAAGATTGTTCGGTTGGGTGGGTTGGCGGTGAGGGGGG
ATGTTGCGAGTATTCAGGGGGTGTGGACTCCGATGTTGGATTTGAAGGAGAGTAAGTTTGATGTTAAGATTGT
TGGAGAGGGTTAAAATAGGCGTTTTAGGAGATGGTACTACGATATGAGTGGTTGTGGCATGTTGGAGTGTGTA
GTAGTAGGATTATAATGCTGCATGATTGAGATGACACACAAATCCCTTATGTAAAGCCAAATACCTACTGAAC
ACGATGTGGGAAGCATTGCCGAGAGCGAGATTTATTCTTGCTGAAGTAAGCATTCTCCGCTATTTTTGCACGG
GCGGCTATCATTCAAAAATCTGCAACACAGATCTAATCGAAGAAATAGAGGAAACATCAGCAATACATAACAT
TTCCAATTTTGTTCAACTCGATGCTTAAAAGCTCACAATCACTTTTCAATTGAGCAGA
>TR36|c1_g1_i2 len=1243 path=[1535:0-51 1559:52-106 1558:107-107 1557:108-141 1549:142-459 1491:460-472 @1553@!:473-738 1524:739-755 @1555@!:756-907 1540:908-957 1552:958-977 1546:978-1001 1539:1002-1149 1494:1150-1173 1495:1174-1242] [-1, 1535, 1559, 1558, 1557, 1549, 1491, 1553, 1524, 1555, 1540, 1552, 1546, 1539, 1494, 1495, -2]
ACGGTAAAGCGGTGCGAGAGCGGCTCCAACAAAAGTCCATCACAAAAGCAATCAAGTTCTATCACCCGGTGGT
TGATTAATCTTCAATCTCACAAGCTCAAGAGGAAAGAGGAGGAGCTATCATCGTTCTATAACTCCATCGGTAA
GTTTATACGCTATTGTGGAGGGTGCGCGGCGGATGGACCAGATGATGGACGTGCATAACGCCGTCACGTTGTT
TACGATTGCCGGCGATAGATTGACGCCCGGTCGTCTGGCTGCTTATTCAGAATACAGTTCAGGTCCAGTGTTC
AGAATGACATCTTGTGAAGCGCACTAGTTCTTCATGGGATGATACTTACCTACCGTCACCCTGGACCAAATTC
GAACGCGTTTGCCTCATGTAATCCACCCGTCTCCTCTCCGTCCTCTTTCTATCGACTCTGTGATTGGACCACA
AATACATCCTCAGTAATAATAATAATATATTCAACAATAACAGCAACATATATATCCAAAATGGCAACACGAG
GAATAAACCAACTAAAAAAATTAACCCTCCGCTACTGCGAAATCGGAGGATCATCCACCACCGTCCGAAATTA
CCTCCAACAATCCCCCCACCTAATCAACCTCGCCAAGTCCAACCCACACATAACAATCATCGTCAAACCGCGC
AACGGCCACCATCCGTACATTCAGGGAGATTACGTCACCGGCCAGGCCAAGGTAATTTGCGTCAAGAACACGA
CCGAAAAAAGGATTATGGAGGTGATGGAAATGCTGCGGAATACGAGCGGGAGGAAGATTGTTCGGTTGGGTGG
GTTGGCGGTGAGGGGGGATGTTGCGAGTATTCAGGGGGTGTGGACTCCGATGTTGGATTTGAAGGAGAGTAAG
TTTGATGTTAAGATTGTTGGAGAGGGTTAAAATAGGCGTTTTAGGAGATGGTACTACGATATGAGTGGTTGTG
GCATGTTGGAGTGTGTAGTAGTAGGATTATAATGCTGCATGATTGAGATGACACACAAATCCCTTATGTAAAG
CCAAATACCTACTGAACACGATGTGGGAAGCATTGCCGAGAGCGAGATTTATTCTTGCTGAAGTAAGCATTCT
CCGCTATTTTTGCACGGGCGGCTATCATTCAAAAATCTGCAACACAGATCTAATCGAAGAAATAGAGGAAACA
TCAGCAATACATAACATTTCCAATTTTGTTCAACTCGATGCTTAAAAGCTCACAATCACTTTTCAATTGAGCA
GA
>TR37|c0_g1_i1 len=1604 path=[1910:0-558 1911:559-582 1954:583-608 1964:609-633 1967:634-681 1952:682-739 1963:740-822 1947:823-876 1951:877-1068 1931:1069-1092 1962:1093-1135 1939:1136-1184 1940:1185-1185 1960:1186-1251 1955:1252-1344 1898:1345-1369 1899:1370-1603] [-1, 1910, 1911, 1954, 1964, 1967, 1952, 1963, 1947, 1951, 1931, 1962, 1939, 1940, 1960, 1955, 1898, 1899, -2]
ATCATCATCATCATCATCATCATGTATCGCCATCATCATCGTTTATTGCATCCACTTCTACATCCAAATCCAC
ATCCACATTCACAACAACGGCATTGTACGCAAAGAAAAAAAAGAAAAAACCCATAAAAAAGAACAGCAACAAC
AACAATGGAAAGGGATTCGCACCAAAGTATTCCATCGCCGATAAATCGTACGGCATCATCACCCCTTCGATTA
CCGACAATAGCGTTTCGTTTTCCGCGGAGGAAGAACAACCAACTTCGGAAGAGACGCAGTCGTCATCCTCCAT
GTCCGAATTCTTTGCAACGTACGAGGAATGGAAGCCTCTCTTTCGTCACATCATGATGTCCCCCAGTAGTGGT
GCTGATGATTTTGTGGATACTGCATCATCTTCAACCTCACCACTGGCCTCCTCCTACCTAACCGACGTCGTTC
CATCATCCTCCGAATCGCTATGGGGCATATCCACCCTCGAACACAAGAATCCATGGCGCCTATTGGAATCGAA
ACCGACCACCAATTCCTCCCTCGAGGTGCTGAGCGAATTCCTGGACGAATGGCAACGCTCGTTGTTGGATATA
CCGTTGGACGAAATCGTGACGGGGGTTAATGACGCTCATTTTTTGGAGGAGGGGAGGAGGGTTTTGGCGGTGA
CGAGATTTCACGTTTTGGATGTTGGTGATGATGGTGATGATGCCGTGGAGGGTGATGGTAATGATGCGGGGAA
GTGCGATTGGGAAACGGAATTATTTCGCACGGTCTGGTCCGAATTGGCACACCTGACATCCCAAAACGAGATC

GACACCGGATCGCTGGTACTATTGCCCAAGTCCATCACCACCGCATCATCATCATCCTCCGGAGAGGACGACG
AGGAGGAGGAGGGTGGTAGCAGCAGCAGGGATTTGGAATACGTCCGAACCTTCGTCGAGAGGAAGGTACTTCG
ACCGATTCGATGGTTGGGACGACACGAGGATTGGGAGATTGTTGCCATGGAACGGGGGAGCATCGGCGTTAGG
ATTTTGTATAAGTTGAGTGATATTCCCGATTTGAAGGATAGGGATGCTGGTGGGGACGAGAGTGTTCTGTTTT
AAATCGTGCGTAAAATGTATAGTGTTTTATGGATGAATATATACGAAAAAAGGCGAGACGGCCATTCGTATGT
AAGATGCACTATATTACGAAAGATTTTTTTGTTGAGACACTGTAAGAATTCTATATTGATGTGTGTGATCCTC
TCTCTCTCTTCGTGGCAGGCCTTGCGGGAAACGGGTGTTCGCTACATGCACCCACGAGAGAATCACAATAATT
CACTACATGTGCAGTATGCAACCGCATAACACAATGTCACGACCTGCATCCATTTTTATCATACATCGCATCA
CTACACCTTCGATATATATTTGACAGTTGTAATGTGTATGTACGTTTCTTAGCAACGAAAAAATGAGTAGGCA
CTAAATTACTAAATATTGCCCCCATCCCCCCCCCAGTTATATGGGTGAAGCATGTTTGCAGAGACACACCTT
GCGTTGAGAGAAACAGAGAGTGAACTAAGTAAGAAATATATTTACCGACCCGACCCCAGATCGGAAGAGCA
>TR38|c0_g1_i1 len=2740 path=[3054:0-1099 3055:1100-1120 3084:1121-1334
3058:1335-1358 3080:1359-1538 3061:1539-1562 3083:1563-2037 3085:2038-
2096 3079:2097-2120 3082:2121-2551 3052:2552-2575 3078:2576-2623
3068:2624-2647 3069:2648-2739] [-1, 3054, 3055, 3084, 3058, 3080, 3061,
3083, 3085, 3079, 3082, 3052, 3078, 3068, 3069, -2]
TTGCAAAAAATAGGTTTCTTATATTAGAATATACTTCGCTACCAAATCACGAAGTATCACAAGGACAGGGGAC
GTCACTTCCAAACAGAACTTCTCCCCTGCTCTCTTTCTTTAGCTGTAGAGATCCTCCTCGTCATCTTCATCTG
CTGCATCTCCACCACCGGCAGCAGCCCCTGCCATACCCTCAGACTGTCCTGGGAATGCAAATGCTGCCAAGCT
ACCTCCTGTTGACCCACTCACTGCCGCGCGAGACTGTTGCAGTGTCTGCGCAAAACTCGCATACTGCGCAAGA
TCCCTGTCACTCACACTCCTCCTGGCATTTCTCACAGCGTGTTCAAAGTGACGCGGCAGAATCTCCGGCATGG
AATCCTCGAGCTCATCTTCGTCATCCTCCTCCATCTCCTCCCCAGCCTCCTCGCGCAGGCGGCCGCGTTCAAT
GTCGCGCTCGATCTCTTCACGAATGGCAAGCTTACATGCACTCTGGCAAATTTCCGTAAGATCAGCTCCAGTG
AACTTATCCGTCTGCGTGGCCATGTAGGATAGGTCAACATCCTTAGAGACGGGGGATTTGCGAAGCGTGGCGC
GAAGGATGCCGAGGCGGGATTCAAAGTCAGGCATGGGGATGTAGATGAGCTGATCAAGACGTCCTGGTCGCAT
GAGCGCCGTATCGATAATGTCGGGACGGTTGGTGGCTCCAACAATGAAGACATTCTTCTTCGCGCCAACACCG
TCCATCTCCGTTAGGAGTTGGTTCATGACGCGATCTGCTGCTCCGCCTCCATCCCCGCTAGAACCACCACGTT
GCTGGGCGATGGAATCCAACTCGTCAAAGAAGAGGACGCACGGAGAGGCCTGGCGAGCTTTCTCGAAAACGTC
ACGAACATTAGCCTCGGACTCTCCGAACCACATGGTGAGGAGCTCAGGTCCCTTCACAGAAATGAAGTTAGCC
TGACATTCGTTGGCAACTGCCTTGGCCATAAGGGTCTTACCACACCCAGGGGGACCATAGAAAAGGACTCCCT
TGCTGGGAGACATGCCAAACTTTTCAAATTTCTCAGGGTGCTCAACAGGATATTGAACAAGCTCCTTGAGATC
GCGCTTGACATCTTCCAAACCACCAATATCATCCCACGAAATATTGGGCACCTCAACAACAGTCTCCCTCAAA
CTACTGGGGTTACTCACACCCAATGCATGCCTGAAATGATCCTGGTTCACAGCCATGCTGTCCAAAATCTCCG
CATCAATCTCCTCATCTTCAATATCAATCAAATCCATCTTCTCACGAATACATTGCATCGCCGCCTCGGTACA
CAAGGCGGCAATATCAGCTCCTACGAAACCGTGGGTCTCCCTCGCAATTGCCTCTGGATCCACATCCTCATCG
AGCTTCATGTTACGGGTGTGAATGCGGAAAACCTCTAATCGGCCGTTCTCATCAGGGACTCCAATATCAATTT
CCCTATCGAAACGTCCGAACCTACGCAGGGCAGGATCCATCGAGTTGGGACGGTTGGTAGCGCCAATGACCAC
CACAGATGCACGTTGATTCAAACCATCCATGAGGGTGAGCATCTGACTGACAATGCGCCTCTCTACCTCACCG
TTTGTCTTCTCACGCTTAGGGGCGATTGAATCAATCTCATCGATGAAGATGATAGCGGGGGCATTCTTCTCTG
CCTCCTCGAACGCCTTGCGAAGATTGCTCTCACTTTCTCCCGCCATTTTAGACATAATCTCAGGCCCATTGAT
GAGGAAGAAGAACGCACCCGTCTCGTTGGCCACAGCACGAGCAATGAGGGTCTTACCGCTACCGGGAGGACCA
TACAGCAACACACCACGGGGAGGCTTCACGCCAAGGGTCTTGAAGAGGGCGGGGTGGCGGAGGGGCAATTCAA
TCATCTCGCGAATTTGGGCCATCTGCTTGCGACATCCTCCGACGTCGTCGTAGCCGACATCATCAAGCTTTTC
CTCGTCCTCGCGCTTCACGGGTTCGCCCTCGCAATGGATGACCGTGTCGGGGGCGACAATGCAGTAAGGCGCG
GGATCGGTCTCCACCACCTTGAATTCAACTGGGTGCATAGCCGACCTCACCAAGAAGAGATCCCCCTTCTTGA
CGGGACGGTAAGCCTCAAGGAAGTATGGCTTGAGGTATACGTCGAAAAGGTTGCCAGACACGCCCTCAATGGT
GTCGTCCAAAGGGAGGATATGAATACGTTTTCCGTATGGAACGTCGCCGCAATTCGCGACGCTTACAATGTCC
GCGAGTCGCACGCGGAGGTTCTTGCGCACTACCTTGTTCATTCGCACGCTGGAGTCGTCGCAGGTTTCGTCGG
CGAGCACGATGCAGACTGTGTCACGTCCCTTCTTTCCCTTGATGAGAACAGTGTCGCCGCGAAAGAGGGAGAG
CTCTTCCATCTTGGCGGGGGAGAGGGAGATGACGGAATTGTCGTCGTTCGTGGCATCGTCGACGAGGAGGCGA
TTGGGGGAGCGCTTTTTGCCAAGGATAGCATCCTTCATGTCGGTGTCTTTATCTTCGGATTTCACCATTTCGG
AGAAGGGATGTTGATTGTGTTGATGAGATCTTTGGTCGGCTCTACTGCGGCTCTGAGGTTTCGTTGCTTGGGA
GTCAATGCGATGAGCGCAGAATTGATTGAGAGCGTGATGTTACTTGCTTTTCGGCGGCGGTGGCAACGCGGAA
TGGGAATGTGAAATGAAAGTGTAAGGCGGCTGCCAACCA
>TR39|c0_g1_i1 len=1029 path=[1334:0-68 1375:69-109 1373:110-133
1369:134-343 1363:344-367 1372:368-400 1368:401-420 1347:421-430
1348:431-454 1367:455-526 1360:527-550 1376:551-872 1341:873-912
1371:913-980 1332:981-1014 1333:1015-1028] [-1, 1334, 1375, 1373, 1369,
1363, 1372, 1368, 1347, 1348, 1367, 1360, 1376, 1341, 1371, 1332, 1333, -
2]

GCTCTTCCGATCTGATTGCTCCGCGTAGTGATCGCGGCATCGGCCACAGTGGCCATGGCTGAGGCCTACGTGC
CGCCACACATGGAGCTACCGTCAACTTGGACTGATGTGAATACTGGAACTCTCATCCCGTTGGAATTCAGATC
CGAGGCAGCCGCAGTGCTCGAAGGAGATGCCGAATCATCCATGGTGTCGGAGTCGGCCTGCATGTCTGCTGAA
GATGTAATTAGAAACCATTCTGGTAAAAACGGGTGTATTGCCTTTGCTGTTCGACGGCCAGGGTGAGCCCTAT
GCCGCGAGGAAGGCAGAGCAATTGCTAACCTTGCGGCAAGGGAGGACAAACCGCTGGATGGATTTGGAATCTT
TGGTCTTGTCAAGGAGGTGGGGGTCGATGATGAAGGATTGGCTGAATTTGAGTCAAACTTTTTCCCGTACCCG
CTTTATCGCGATGAATCAAAAACATTCTACAACGCCTTGGGCTTGAGAAAGATGTCGTTGAAACCAAAATCAT
GGAATCCCCTCAAAATCTACAGGGGGATGAGGGATATGCTCAAAAGATTATCGGACAAGAATATAAGCGGAAA
TATGGTTGGAGAAGGATTAATACAAGGTGGAATTATCATTTTTGATAGAAACGGGAAAGCCCGGTATGCATAC
AGAGAGGAAACCGGATTTGAAGTTCCAATCAATGATGTTATTGCCGCAGTGAGGACAGTAAAATCAAAAGACG
AATAGAGCTTTAGCTAGGTCAGAAGGATACTCTCATGCAGAATGACTGTGATTCGAGTTGTTTTGTAACAATC
ATCAAAACAAAAAAATCACCTGAAAATGCATGCGGCTATTTTCATCCAAGCGAGCCACATCTTTTTATGACTA
AAAACCTCGACCCTACAACAATAATTTGGGAGACTTATCTGGCATGAAAAATATAAGCCATACAATAAAAGTA
ATAAGCGTTGTGTCTGTCTGGTGCGCGTCATTTACGAATAAGGTATATAATAGTAACCCACGCTGAAAACTTT
GTGAAAA
>TR40|c0_g1_i1 len=1878 path=[2043:0-357 2044:358-381 2064:382-477
2041:478-501 2062:502-905 2037:906-929 2065:930-951 2063:952-1194
2057:1195-1218 2061:1219-1803 2033:1804-1805 2034:1806-1829 2035:1830-
1877] [-1, 2043, 2044, 2064, 2041, 2062, 2037, 2065, 2063, 2057, 2061,
2033, 2034, 2035, -2]
TCTTCCGATCTGTAAAGATTGAGTCTAAATAAGGCTTCAAACTAGTTAAATACTGGTACCTACAACTCAATGA
GGTGAAAGATGAAACGTGATTTTGTTTAAATTTTCTCAGACCTTTCAATTTTTGAATAGTATCCGCTTCAATC
TTTCATCCAAACTTTCTCGACTTCTTTCTCTCTAAATTCCTTTCTTCCTCAACTCCTTGATACGTGCCTTCTC
AGCATCGAACACTCCCAAATCCATCTCCTGATCGGGATAAAACATAGCCCAGTACATCAAACCGGGATCCTCC
TTGGTACCCGCACACTCCTTCAGTGTCTTAACGTATCTTCGCACGGCATCTCGATAGTGCGTTCCCACCTGCC
GCTCCTGGTACAATCTCTCGCCCGAAATGGCCTGATCCGCTGCGGCAAAAGTCCGGTTGCTGTGCCGCAATGT
CATCATGGCCTCGTCCAACGCGCCCTTCTCGATACCGAGCGGTTCGAGCTCCTTCAAGTCCTCGGGATCGACG
TGACGTTCCCAATCGCTCAGATCGCTGCGCAGTTCCTCGATCGAACGTGCCGGCCGGGCCGTCTCCCGCACGC
CTCTGCGCAGTAGCTTGTCCCAGACCGTGCGGAACGTGACCTCGGCTCCGCCGATGGTGGCCCTGCCGCTCTC
GTACGCCTCCCTGACCCGATCGATCTCGCCGGGCAGCTCAAACCCTACGTAGGCGCACCCCGTGGGTAGGCCC
TGCGAGCAACGCACGATGTGACTCGAACCGTACACGTCCCGTGAATCCGAGCTGAATGGCTGGAAAAACGACG
CCAGGTCCTCCAGGGTTGTGTCCGGGGCCAATCCGGAAACGTGCATCATGTCGTGATGGCTGAGGTACATTTC
ATTGGGAGGAACATCTCCCGTGACGATCAGCCGGGTGTCCAACTTGGACGCCGCATACGTTCGGTGAACGCCT
TGCCACCCATCAAATACGCGTTCGCGCTTGGCACGAAACACCTGGACGGAACGCTTCCCCACAACAGACGTGT
TGTTGGCAATGTTGTTCGACTTCAACGCCTTAGTGACAAGATCGGCGGAGGCGAGATTGAGCAATGCCGTCGT
GGGTGAGACGCGGAAGTAATCGGCTTCGGACAGAGGGCCGAACATGGCCTCGAGCCTCGATCCCGGAGGGAAC
ATCGACCGCAACAATTCCACGGAAGTGGCGTTCGCGGGAAGGCCCGTCACCTTCAGTATGGACGACGGTCCGC
CTGGCGAGGCTGCCACGAATTCCTTCTCCTCTTCCGACATGCCATCCTCCTTGTCAACGCTTCGCTCGTATAC
TTGCAATGCCGCCGGATGCGAAACCCGCAAACGATCATCATTGGCACTATCGACCATGGCCTTCTTGGCCGCC
AACTTCGTGGCAAATCCCACGAACGCTGAGGTAGTCGTGGCCGGCCACACGTCGAGCGAACTCACGCCGAACA
ACTTCGCCACGTCCTCCGATATCGACTGTTTGTGTTGCACGTCGTCGAAATCCTTGATTTCGCCACTGCTTTC
CAAGTGAGGCACCAACCCGCTCACGAACAGGTATCCCTTGTACTTCTTCACGAACTCCTCGTCGGGCAACTTC
CGGTCCTTCGGGAAGGTGGGTCTCGGCTCGCGCTTGCTGTCGGCGTGCTTCACCTTCGATTCCATCACGGCGT
ACTTCGACGGGTGGTCCACCGTAGCGTACAGCCAATCGTCCAACTCCGGTCCGTGCGGCTTCAGCGGCAGGAT
GGCATTGGAATCCGGCTTGGAGGGCTTGAGCAAAAACGGCTTGTCGACGGGGTCCGTGATGTCCCTGAAGTAG
TAATCCCACTCGAACGTTTTCGTGATGGGATCGACGTCCGCGTCGTCCATCGC
>TR41|c0_g1_i1 len=1932 path=[2380:0-149 2432:150-150 2431:151-174
2427:175-395 2420:396-446 2414:447-470 2426:471-856 2358:857-880
2430:881-1059 2433:1060-1103 2417:1104-1130 @2429@!:1131-1258 2422:1259-
1292 2416:1293-1339 2411:1340-1423 2390:1424-1447 2391:1448-1931] [-1,
2380, 2432, 2431, 2427, 2420, 2414, 2426, 2358, 2430, 2433, 2417, 2429,
2422, 2416, 2411, 2390, 2391, -2]
TTTTGTCCCGTACCATAATTTCTGGTCTCCGACTAATTCAGAGTGTTATGATTGTGCATAAATGGTCACAAAC
GAGCAAATTGTGCATAAGTGAGACCAGTGGGACACTAACTACTATTTTTTTTAAGCCTCTTTGTCCGCATCCG
ACTGCAAAGAAGGCTTCCTGGTGGTCCTCTTCCTGCTGCCCCTGCTCCTCTCGGAGGATCTCCTCGCTCCCGC
ACCCCTCGGTCCCGGACCCGATGCTTCCGCAGACGCCACCGCCGGAGGAGCCGCAACCTCCTTCGCAACCACG
GTCGTAACCGGCTTCTTCGCCGCAACCTCCTTCTTCGCCAACTTCGGCTTCGCCGCCAATCCCTTCTCCACCG
CCTGCGACGCCGCATCCGCCAGCCTCCTCACCAAAATATCCTCCCCCGGCAGAGGCTTGTTGCTCACGCCCCC
GTATCCTTTACTCGCATCGCCCCCTCCACGCACCGCCGTCATTCCGCTTTCGTCCACCTCGATTATCATCTTA
CCGCTCGTAACGCTCTGGAAGTCCACGAGTTCCTGCCACACGATCTCGATTTTGGTACCCTCCTCCCCGGTTT

GGATGGCGTACACGGGACGAGCGCCCACGCGCGTTCCGGCAATGTCGTACGAGCGCAATATGGCGGGAGCCCA
CTTGACGGCAACCCACTCGGCGAGACGTTTGGCCGAGGGCACGCTGGAGAATTCGGCGCGGAAGAGGACGGGC
GCGGATCCTCGTGCGTTGGATGGGGAAGATGCCATCATCTCCGCGACGCGTGTGAATTCGGCGTTCCACTCTT
CGTCCGAGGGGGGTGCGGTTCCGACGAAGGAGGAGAGGGATACGTCCATATTCGCGGTTGCGGACTTTACTTT
GCCGAGACCGAGACGGGATGTCGCCTCGCCGACTGTGAGGCGGTTGGATCTGGTGGTTCCGTCTACGCCGGCA
ACGTTTCCGGATGTGGAGACGGTGGGATCGATTCGGACGGAGCGGAGGGTGTACATTTCACGGACTGTGGGGT
CGCGTTTGGGACCTCCCATGAAGGGAGATGACTCAGGCGAAGATTCCGCAGCACCAAACAATTCGCCATGACG
AATCACCGACGCAGCACCACCCACAGCCTCCGCCATGCTACCAGGCACCTGAATCTCATTCCCACCAAACAAC
CCCGCGAAAAATCCCTTACCGCCATCGCTTTCGCCATCCTCGTCATCACTCGGCATCTCCTCTCCGGCCGCCA
ACACCACGATCTTACTCTGACTCCTCCCAGCAGCCTCCCTCGCCGCCACTCGAATACCACTGCGAATCAACTG
TTCCTCGTCCACGCCCATGGTATTCTCGATCTGTTCCGGAGATTTCGTGCGATCGACGCGTCGCTTCTCCTCC
AATGCTCCGTCGTACGTGCAGATTAGCGATCCGGGGGTGCCGCACCATCCCGCAATGGCCGATAACGCATCGT
TGGTGGCAGTGCCTTCGGCGTCGGAAGGAGGGGCGAGGAGACTGCCGGCGTCCGCACCGGCGAGTTCCATCGA
TCCGCCTGCCTTTTCGATGGCGGCGAGTGTTTCGGCGGTCAGGGCCATGTTGGGAGTGTTGCTTGCGGCGGAG
ACGACGAACCACTTGACCGAGCCGCCGAGACGTGCCGTTTCGATGGAGGTGATTTCGCCGATGCCGCCCGGGG
GGGAGATGACGACTACATCGGGTTGTGATGTTGCGGAGAGGGCAGAGAATGAGGTGGATTGGGTGGAGGGTAG
CGATGTTGATGGAGTGATGGATGTGAAGGCGCTGGTCGAGCCGAGCAGGGTGGCCAATAGGCCTGCTCTTGCT
ATTGATGTAATCTTTTGTTGAACCATCCTGTTTTGTACCCTTACTTGAAGTATTGTTCGTGATGGTTGCGAGA
GGTTGCGGTTGCGGGTCATCTAATAGATCGGAAG
>TR41|c0_g1_i2 len=1291 path=[2380:0-149 2432:150-150 2431:151-174
2427:175-395 2420:396-446 2414:447-470 2426:471-856 2358:857-880
2430:881-1059 2364:1060-1290] [-1, 2380, 2432, 2431, 2427, 2420, 2414,
2426, 2358, 2430, 2364, -2]
TTTTGTCCCGTACCATAATTTCTGGTCTCCGACTAATTCAGAGTGTTATGATTGTGCATAAATGGTCACAAAC
GAGCAAATTGTGCATAAGTGAGACCAGTGGGACACTAACTACTATTTTTTTTAAGCCTCTTTGTCCGCATCCG
ACTGCAAAGAAGGCTTCCTGGTGGTCCTCTTCCTGCTGCCCCTGCTCCTCTCGGAGGATCTCCTCGCTCCCGC
ACCCCTCGGTCCCGGACCCGATGCTTCCGCAGACGCCACCGCCGGAGGAGCCGCAACCTCCTTCGCAACCACG
GTCGTAACCGGCTTCTTCGCCGCAACCTCCTTCTTCGCCAACTTCGGCTTCGCCGCCAATCCCTTCTCCACCG
CCTGCGACGCCGCATCCGCCAGCCTCCTCACCAAAATATCCTCCCCCGGCAGAGGCTTGTTGCTCACGCCCCC
GTATCCTTTACTCGCATCGCCCCCTCCACGCACCGCCGTCATTCCGCTTTCGTCCACCTCGATTATCATCTTA
CCGCTCGTAACGCTCTGGAAGTCCACGAGTTCCTGCCACACGATCTCGATTTTGGTACCCTCCTCCCCGGTTT
GGATGGCGTACACGGGACGAGCGCCCACGCGCGTTCCGGCAATGTCGTACGAGCGCAATATGGCGGGAGCCCA
CTTGACGGCAACCCACTCGGCGAGACGTTTGGCCGAGGGCACGCTGGAGAATTCGGCGCGGAAGAGGACGGGC
GCGGATCCTCGTGCGTTGGATGGGGAAGATGCCATCATCTCCGCGACGCGTGTGAATTCGGCGTTCCACTCTT
CGTCCGAGGGGGGTGCGGTTCCGACGAAGGAGGAGAGGGATACGTCCATATTCGCGGTTGCGGACTTTACTTT
GCCGAGACCGAGACGGGATGTCGCCTCGCCGACTGTGAGGCGGTTGGATCTGGTGGTTCCGTCTACGCCGGCA
ACGTTTCCGGATGTGGAGACGGTGGGATCGATTCGGACGGAGCGGAGGGTGTACATTTCACGGACTGTGGGGT
CGCGTTTGGGACCTCCCATGAAGGGAGATGACTCAGGCTGTGTGTTGTTGTTTGTAGTGTCATATTTATGGTG
TTGTTGGATGTACATGGCAAGTGATCACGTCGGTCGGCACCATGAGAAAGGAGGTGAACAGTGGATCAATACA
AAGATATATTTGTCGTGGTATGAAAAAGCCAAACAACCACCTAATTCCCAACATAGAAAAAGATAATGACGAA
AATTAGCAACAAAAAAGCAGGTCTCACCGAAGATTCCGCAGCACCAAACA
>TR42|c0_g1_i1 len=2394 path=[2680:0-93 2681:94-117 2710:118-267
2694:268-284 2702:285-468 2659:469-492 2706:493-584 2662:585-608
2701:609-874 2656:875-898 2705:899-1112 2678:1113-1136 2700:1137-1251
2687:1252-1275 2688:1276-1283 2689:1284-1307 2709:1308-1383 2672:1384-
1407 2704:1408-1626 2684:1627-1658 @2711@!:1659-1755 2703:1756-1959
2667:1960-2393] [-1, 2680, 2681, 2710, 2694, 2702, 2659, 2706, 2662,
2701, 2656, 2705, 2678, 2700, 2687, 2688, 2689, 2709, 2672, 2704, 2684,
2711, 2703, 2667, -2]
TTAGTTTATTAAACGAGAACATGGAATGTTCAATGAATGAGACACACCCGCTAGTGATCTAACGTCGCCTTCT
AAAGCTGAGAGATGGAGTTGGTAGAGAACTTGAGGGGACACGCGAAGAGAGAACTTTCAATGATCCTCCCATT
TTGGCAAGGGCAGCCTGTTTCTGTTTCTCCAATTCTGCCAGCTCCGCCTTCAATCGTTCAGCGTCCGATGCAT
AAGAACTACTCTTCTTCAGCACTGCAGCTGCGCTTCCTGCTGCTTTTTTGTGCTTGGCAAGTGGGTCTGGCAT
GCTACTTCTCAAGTGTTCTGGAATATTGATTGCCCTGTGCCTTTTGGCGTCGATGTCTTCCTGTGTCGGCTCA
TCGAAAGTCCTAGTTTTGAAGTTCACAAACCCACCCTTTTTCCCTTGGCCCAGCTCTTTTGCCTCGTATTTGT
TTAGAACAGATGATGCTTTGACAAAACCTTTCTTTGGGTCCGATTTCGTCTTACTCTCTGCTGCATAGTCGAA
CCCAAGAAGGCCTTCGTCGCTGCCAAGAAAATCGTCGGATCCATAACCATCAAAGCTATCCTCATCAGCCAGT
GAATTGTGGTGAAATTTCTTTCCCTTGTTCTGTTCTACTCTTCCAGCATGCATCAAACGATGCGAGTTCACAA
CCGTGGACATGCATTCGGACGCTTGAATTTCCCTCTCCACTTTTGCCGGGGAGATGCAGTAGTCACATGTCTT
TTTGCAAACGACACTTGCGTCAATCTGCTCTCCAAAATGTTGAAGAACATACTTTCGTTTACACATGGGCTTG

GTACAGTAATTGACCATTCCTTCCAACTCCAGAATTGAATGGTCAACCTTCTGCGATGTGAAGCCATTTTGTG
TCCCCTTCTTTTTCGCGGCACGTTCCGCGTTCAGTTTAGCTAGATATGCAAACTTTGACGCATCGTCTTTTGA
ATAATACAAAAGTGATAATGCTGGCTTCCCGTCCCGTCCACCTCTTCCAGACTCTTGATAGAACCCCTCGAGC
GACTTCGCCATGGTCCAATGGATCACATACCGAACGTGCGGCAGATCGATTCCCATTCCAAAAGCAACGGTAG
CAACTGCAATGTCGCATGCTCCGTTGGTCCACTTTTGTTGAGTCTCCGAACGCAAAGCATCCTTGAGCCCAGC
ATGGTAAGCCAAACACACCAAACCTGTTGCCTTGCCAATCTTGGCAGCCAAAGAACTACAATCTTCTCGTTTG
TGGACATAAACAATGCCACTACAAGGCTCATTCTTCTTCTTGAAAACATTATGCTGTGCCTTCACAAAAGCCA
CGAGGTCCCCCATAGCCCCTTCAGACTTCATCGCATCCAATGAATCTTTGAACCGTACCTCATACGAAATGTT
CAAGCGATTGAATGTCCCCATGATGCAAGGCACATTTTTATCAAATTGGAGAATACTCCTAATGTCCTGGACG
ACTTTTGCGGTTGCAGTTCCTGTACAGGCCATGACAGGAACCTCCGGGAATGCATCGCGAACCCATGTTAGCT
TAGTGAAAGCGGGCCTAAAGTCGTGTCCCCACGTGCTCAAGCAATGTGCCTCGTCGATGGCGAACATGTAGAG
CCGGTTGGATTCGTAAAGTTTCGTCAAAATTGAACGAAATTTATCCGTCTGAATGAGTTCAGGAGTGCAATAA
AGAAGCTGGATTGGAGCAGGCTTCGCATCCGATTGGTTTCTCTTCGTCTCTGTTTGCAGGCGATGAAAGATCT
CCTGTTTCTCCTTCGCTGTATTAGTCGAGCTCAAGCTTGCCGCGGTATGAACTCCCTTTCTGTGCAAATTTTC
GACTTGATCGATCATCAAAGCGATGAGGGGGCAGACGACAATTGTCACGCTTGAATTATCAGCACGCTCGTTT
ATGTTGGTATTTCCTCCCGCAAGTGCTGGAAGTTGGTAACAGATGCTTTTCCCTCCCCCGGTAGCCATTATCA
CAATTTGGCTAGTCTTTTTTAGGGCACCCTTGACAGCAGTATCTTGCAGTGGCCGGAGGGAGTTATGTTTGAA
TGTTTTTCGAAGGATGGAGTTGGCTCGCTCCAGAAGATCTTCTTGCGATTCACAAGGTTGTTCCCTTTGAATG
AAATACTTCGCCAGAGATGGTCCAGTTTGGTTCCTATTACCTTTTCCAGTCTTATTGTCTTTGGTGGAAGAAT
TTTTCCATCGTTTAGAGTCATCGCTGTGTACAGCAGATTTCTTTTTGTTGTCATTTTCAAGAGAATTTGCAGC
TTCCCTAAATTGTCTCTGATTGTCTGGTCTTTGTTTATTACTATTGGAGGGTGCAACCGATCGAGTTGTAGTA
TTGTCAATTGTGTCCTGCGAATTCAATTTCTTATCAATTTTTCTTCTCTTTAGCTTTG
>TR43|c0_g1_i1 len=1231 path=[2929:0-69 2932:70-92 2936:93-124 2904:125-201 @2930@!:202-337 2916:338-372 2903:373-760 2820:761-784 2915:785-881 2863:882-905 2924:906-1000 2870:1001-1012 2871:1013-1036 2913:1037-1099 2836:1100-1133 2837:1134-1230] [2929, 2932, 2936, 2904, 2930, 2916, 2903, 2820, 2915, 2863, 2924, 2870, 2871, 2913, 2836, 2837, -2]
TCTAGGTAGACATGAAAGTAGCTCACCATTTTATGGTTTCTGTTTGCATTTTCATCAACACCTGATGACTGAC
AACTTGAAAAATTACAGTCCCGAATCCGTCGGGAGTGGGATTCATTTCTGATATTTTTTCTTACAAGACCCTT
GCCATTCACGTTGAAGAATCTTGCCATGGCGGAAAAAGCTCTTGACACTGTGATTTACTTTGGGTGTGGCGGT
TCAAGGAGAGGAAAAGCAGCATTTTACCCCTGCGGTATAATATTCGATACTGTCTTAACATAGTTTGTCATAA
TACGAACAAACTTCTCATCGTAATGGAACCTCCCAATCAGACGATAGAACATTGCCAACGAATCTGCCAAATT
TTGTTGCGTATACGGCCGTTCCGTTGGTCCCTCCATTCTCATAGTCGATGATGCTGCCCCTTTATAATTCACG
ACCGTTTGCCAAATGCACGGCACGGATTCCGGTTCGATCACGTTCACTCCTTCGCCTTGCCGCAAAAAGTCCG
CAATTTGCCCTGCCACATTAGGTCCGAAAGTACCATCCGTCAAGGCCTCGTAGGATAAAAACAAGAGATTATT
TTGGCCATGATCGCTAGAAGAGTAGTAGTACTTTTCCATCCAGTAGATTGCATGCTGCTCGTATTTTAAAAGA
CGGCTCGCAAAATCGGCATCTCTGTATTTGACCCATTCTTCATTGGGTGCCCTGACAGTGTGCGTACTGAGAT
GATTCAACTCCTCGTACTGCGCATTGAAGTAGCTGGGTATTGCATGCACTGGATTCCTTAAAATGAGCATGGC
CCTGTGGAAGTACTTGGGGTCTAATTCCTCAAACATTGTATCGCGAATGAATGGATAATGCGTCTTGGCAGCC
ACGACACTCGTGTATCCCCATCCATTTGAATTGCGGTCGTTGTTCGTTTTGATGCCCGTGAGCGCTTGGACTA
AATGCCACGTCATGCGAGAACCGCAGCCTGGGAAGGAGGCTTGCCAGATGGGTGAGATGGGATGTTCCGTGGG
AATTGCTTTGGTTATCTCGCATCCTCCGTTGGGAAGGGAGATTGATAAAGATCTGTGAGATTGTATTGTTGTT
GCTGTTTCAATCTCTGCGTTTGATGATTCTACGGGATAAGTTGTTGCAGATGGAAATGCATGGTGTAGTAGCG
TTGCAACGCCTGCAGTGAAGAAAATTAGGGTTCCCGAAACGAAGTGCGCTTGACGGCGGCGTC
>TR43|c0_g1_i2 len=1945 path=[2873:0-86 2874:87-115 2875:116-148 2942:149-209 2929:210-256 2940:257-269 2910:270-295 2934:296-335 2921:336-378 2908:379-414 2867:415-438 2933:439-493 2939:494-516 2931:517-526 2936:527-558 2905:559-636 @2930@!:637-772 2916:773-807 2903:808-1195 2820:1196-1219 2915:1220-1316 2863:1317-1340 2924:1341-1435 2914:1436-1715 2827:1716-1739 2900:1740-1790 2880:1791-1814 2881:1815-1944] [-1, 2873, 2874, 2875, 2942, 2929, 2940, 2910, 2934, 2921, 2908, 2867, 2933, 2939, 2931, 2936, 2905, 2930, 2916, 2903, 2820, 2915, 2863, 2924, 2914, 2827, 2900, 2880, 2881, -2]
TCTATTTGCAACACATTCATTTTTCTTTATGACATTTGCTGCTACTTACTCATCATGTCATAAATAAGGGAGG
ATATGAGGTAAACATGGGGTTCCCGGGTTTTCCTCATATTCTTAGGCTAACCGCACATAATTGTATTGACCTT
TTGGTCACCAATTTATACCACAAACAATACCAAAATGTCTCTCTAGGTAGACATGAAAGTAGCTCACCATTTT
ATGGTTTCTGTTTGCATTTTCATCAACACCTGATGACTCTAGTATGAATATGAATATTATTTATGGCGTGAGC
AATGTGTGTGAGCATTTAGTTTTCTAATCATGCATGATGTGAGTAATGATATTAAGCGTGCATCTATTTGAAC
ACATATTCTGCGCATAAACTTGGATAGTTACTGGTGAGTTGCAAGCACCAGAGAAGCTGCAACTAACTGTGGT
GTCTCCAATCTTTTCCAAATGTGAAAATACCTGATTGTACCTTGCCTAACTCCGAAATCGACTTACGACAACT

TGAAAAATTACAGTCCCGAATCCGTCGGGAGTGGGATTCATTTCTGATGTTTTTTCTTACAAGACCTTTGCCA
TCAACGCTGAAGAATCTTGCCATGGCGGAGAAAAGCTCTTGACACTGTGATTTACTTTGGGTGTGGCGGTTCA
AGGAGAGGAAAAGCAGCATTTTACCCCTGCGGTATAATATTCGATACTGTCTTAACATAGTTTGTCATAATAC
GAACAAACTTCTCATCGTAATGGAACCTCCCAATCAGACGATAGAACATTGCCAACGAATCTGCCAAATTTTG
TTGCGTATACGGCCGTTCCGTTGGTCCCTCCATTCTCATAGTCGATGATGCTGCCCCTTTATAATTCACGACC
GTTTGCCAAATGCACGGCACGGATTCCGGTTCGATCACGTTCACTCCTTCGCCTTGCCGCAAAAAGTCCGCAA
TTTGCCCTGCCACATTAGGTCCGAAAGTACCATCCGTCAAGGCCTCGTAGGATAAAAACAAGAGATTATTTTG
GCCATGATCGCTAGAAGAGTAGTAGTACTTTTCCATCCAGTAGATTGCATGCTGCTCGTATTTTAAAAGACGG
CTCGCAAAATCGGCATCTCTGTATTTGACCCATTCTTCATTGGGTGCCCTGACAGTGTGCGTACTGAGATGAT
TCAACTCCTCGTACTGCGCATTGAAGTAGCTGGGTATTGCATGCACTGGATTCCTTAAAATGAGCATGGCCCT
GTGGAAGTACTTGGGGTCTAATTCCTCAAACATTGTATCGCGAATGAATGGATAATGCGTCTTGGCAGCCACG
ACACTCGTGTATCCCCATCCATTTGAATTGCGGTCGTTGTTCGTTTTGATGCCCGTGAGCGCTTGGACTAAAT
GCCACGTCATGCGAGAACCGCAGCCTGGGAAGGAGGCTTGCCAGATGGGCGCGATGATGTGCCCGCCTCTCGT
GGGTTTTGGTTCGGTTATCTCGCATCCTCCGTCGGGAAGGGCGTTTGATAGAGCTCTGCGGCCAATGTCAGTT
TGAGGAGAGGATGGTATCATTGATTTTGCATCGTGTATGATGTCTGACGAGTCTACGGTTGGGGCGTCATATG
GAAAGGCATGGCGTAGCGTTGCAACGCCCGCAGTGAAGAAAATTAGGGTTGCCGAACCAAAGAGCGCTCGGCG
GCGGCGCGTTGCATTTGTTTGATGATCATCCATGGCTATCGAATGAAGGCAAGACGGTTAGCAATGAGATGGT
CCGTGGTCACAGAAATGGCGATTTATAAGACCGACGATGAGGAATTATGGTGATGCTGTTGACAGTCGGTCGC
TGAGTAGAGGGAGTGGAAGTGAGGTGCGGCGTTGGTGCAGTGAGCTAGGAGTATTTTTTATACTTAATAAACG
CCGTAGGTAGCATCGGCGGTTATGTTTCATGTTCTCTGTTGTGAATC
>TR43|c0_g1_i3 len=1510 path=[2929:0-69 2932:70-92 2936:93-124 2904:125-201 @2930@!:202-337 2916:338-372 2903:373-760 2820:761-784 2915:785-881 2863:882-905 2924:906-1000 2914:1001-1280 2827:1281-1304 2900:1305-1355 2880:1356-1379 2881:1380-1509] [2929, 2932, 2936, 2904, 2930, 2916, 2903, 2820, 2915, 2863, 2924, 2914, 2827, 2900, 2880, 2881, -2]
TCTAGGTAGACATGAAAGTAGCTCACCATTTTATGGTTTCTGTTTGCATTTTCATCAACACCTGATGACTGAC
AACTTGAAAAATTACAGTCCCGAATCCGTCGGGAGTGGGATTCATTTCTGATATTTTTTCTTACAAGACCCTT
GCCATTCACGTTGAAGAATCTTGCCATGGCGGAAAAAGCTCTTGACACTGTGATTTACTTTGGGTGTGGCGGT
TCAAGGAGAGGAAAAGCAGCATTTTACCCCTGCGGTATAATATTCGATACTGTCTTAACATAGTTTGTCATAA
TACGAACAAACTTCTCATCGTAATGGAACCTCCCAATCAGACGATAGAACATTGCCAACGAATCTGCCAAATT
TTGTTGCGTATACGGCCGTTCCGTTGGTCCCTCCATTCTCATAGTCGATGATGCTGCCCCTTTATAATTCACG
ACCGTTTGCCAAATGCACGGCACGGATTCCGGTTCGATCACGTTCACTCCTTCGCCTTGCCGCAAAAAGTCCG
CAATTTGCCCTGCCACATTAGGTCCGAAAGTACCATCCGTCAAGGCCTCGTAGGATAAAAACAAGAGATTATT
TTGGCCATGATCGCTAGAAGAGTAGTAGTACTTTTCCATCCAGTAGATTGCATGCTGCTCGTATTTTAAAAGA
CGGCTCGCAAAATCGGCATCTCTGTATTTGACCCATTCTTCATTGGGTGCCCTGACAGTGTGCGTACTGAGAT
GATTCAACTCCTCGTACTGCGCATTGAAGTAGCTGGGTATTGCATGCACTGGATTCCTTAAAATGAGCATGGC
CCTGTGGAAGTACTTGGGGTCTAATTCCTCAAACATTGTATCGCGAATGAATGGATAATGCGTCTTGGCAGCC
ACGACACTCGTGTATCCCCATCCATTTGAATTGCGGTCGTTGTTCGTTTTGATGCCCGTGAGCGCTTGGACTA
AATGCCACGTCATGCGAGAACCGCAGCCTGGGAAGGAGGCTTGCCAGATGGGCGCGATGATGTGCCCGCCTCT
CGTGGGTTTTGGTTCGGTTATCTCGCATCCTCCGTCGGGAAGGGCGTTTGATAGAGCTCTGCGGCCAATGTCA
GTTTGAGGAGAGGATGGTATCATTGATTTTGCATCGTGTATGATGTCTGACGAGTCTACGGTTGGGGCGTCAT
ATGGAAAGGCATGGCGTAGCGTTGCAACGCCCGCAGTGAAGAAAATTAGGGTTGCCGAACCAAAGAGCGCTCG
GCGGCGGCGCGTTGCATTTGTTTGATGATCATCCATGGCTATCGAATGAAGGCAAGACGGTTAGCAATGAGAT
GGTCCGTGGTCACAGAAATGGCGATTTATAAGACCGACGATGAGGAATTATGGTGATGCTGTTGACAGTCGGT
CGCTGAGTAGAGGGAGTGGAAGTGAGGTGCGGCGTTGGTGCAGTGAGCTAGGAGTATTTTTTATACTTAATAA
ACGCCGTAGGTAGCATCGGCGGTTATGTTTCATGTTCTCTGTTGTGAATC
>TR43|c0_g1_i5 len=1666 path=[2873:0-86 2874:87-115 2875:116-148 2942:149-209 2929:210-256 2940:257-269 2910:270-295 2934:296-335 2921:336-378 2908:379-414 2867:415-438 2933:439-493 2939:494-516 2931:517-526 2936:527-558 2905:559-636 @2930@!:637-772 2916:773-807 2903:808-1195 2820:1196-1219 2915:1220-1316 2863:1317-1340 2924:1341-1435 2870:1436-1447 2871:1448-1471 2913:1472-1534 2836:1535-1568 2837:1569-1665] [-1, 2873, 2874, 2875, 2942, 2929, 2940, 2910, 2934, 2921, 2908, 2867, 2933, 2939, 2931, 2936, 2905, 2930, 2916, 2903, 2820, 2915, 2863, 2924, 2870, 2871, 2913, 2836, 2837, -2]
TCTATTTGCAACACATTCATTTTTCTTTATGACATTTGCTGCTACTTACTCATCATGTCATAAATAAGGGAGG
ATATGAGGTAAACATGGGGTTCCCGGGTTTTCCTCATATTCTTAGGCTAACCGCACATAATTGTATTGACCTT
TTGGTCACCAATTTATACCACAAACAATACCAAAATGTCTCTCTAGGTAGACATGAAAGTAGCTCACCATTTT
ATGGTTTCTGTTTGCATTTTCATCAACACCTGATGACTCTAGTATGAATATGAATATTATTTATGGCGTGAGC
AATGTGTGTGAGCATTTAGTTTTCTAATCATGCATGATGTGAGTAATGATATTAAGCGTGCATCTATTTGAAC

ACATATTCTGCGCATAAACTTGGATAGTTACTGGTGAGTTGCAAGCACCAGAGAAGCTGCAACTAACTGTGGT
GTCTCCAATCTTTTCCAAATGTGAAAATACCTGATTGTACCTTGCCTAACTCCGAAATCGACTTACGACAACT
TGAAAAATTACAGTCCCGAATCCGTCGGGAGTGGGATTCATTTCTGATGTTTTTTCTTACAAGACCTTTGCCA
TCAACGCTGAAGAATCTTGCCATGGCGGAGAAAAGCTCTTGACACTGTGATTTACTTTGGGTGTGGCGGTTCA
AGGAGAGGAAAAGCAGCATTTTACCCCTGCGGTATAATATTCGATACTGTCTTAACATAGTTTGTCATAATAC
GAACAAACTTCTCATCGTAATGGAACCTCCCAATCAGACGATAGAACATTGCCAACGAATCTGCCAAATTTTG
TTGCGTATACGGCCGTTCCGTTGGTCCCTCCATTCTCATAGTCGATGATGCTGCCCCTTTATAATTCACGACC
GTTTGCCAAATGCACGGCACGGATTCCGGTTCGATCACGTTCACTCCTTCGCCTTGCCGCAAAAAGTCCGCAA
TTTGCCCTGCCACATTAGGTCCGAAAGTACCATCCGTCAAGGCCTCGTAGGATAAAAACAAGAGATTATTTTG
GCCATGATCGCTAGAAGAGTAGTAGTACTTTTCCATCCAGTAGATTGCATGCTGCTCGTATTTTAAAAGACGG
CTCGCAAAATCGGCATCTCTGTATTTGACCCATTCTTCATTGGGTGCCCTGACAGTGTGCGTACTGAGATGAT
TCAACTCCTCGTACTGCGCATTGAAGTAGCTGGGTATTGCATGCACTGGATTCCTTAAAATGAGCATGGCCCT
GTGGAAGTACTTGGGGTCTAATTCCTCAAACATTGTATCGCGAATGAATGGATAATGCGTCTTGGCAGCCACG
ACACTCGTGTATCCCCATCCATTTGAATTGCGGTCGTTGTTCGTTTTGATGCCCGTGAGCGCTTGGACTAAAT
GCCACGTCATGCGAGAACCGCAGCCTGGGAAGGAGGCTTGCCAGATGGGTGAGATGGGATGTTCCGTGGGAAT
TGCTTTGGTTATCTCGCATCCTCCGTTGGGAAGGGAGATTGATAAAGATCTGTGAGATTGTATTGTTGTTGCT
GTTTCAATCTCTGCGTTTGATGATTCTACGGGATAAGTTGTTGCAGATGGAAATGCATGGTGTAGTAGCGTTG
CAACGCCTGCAGTGAAGAAAATTAGGGTTCCCGAAACGAAGTGCGCTTGACGGCGGCGTC
>TR44|c0_g1_i1 len=749 path=[1693:0-37 1694:38-50 1695:51-61 1696:62-74
1697:75-85 1871:86-148 1790:149-172 1905:173-560 1939:561-562 1865:563-
568 1902:569-583 1966:584-598 1862:599-614 2010:615-626 1989:627-628
1965:629-634 1933:635-649 1810:650-676 1896:677-682 1856:683-700
1615:701-715 1616:716-742 1617:743-748] [-1, 1693, 1694, 1695, 1696,
1697, 1871, 1790, 1905, 1939, 1865, 1902, 1966, 1862, 2010, 1989, 1965,
1933, 1810, 1896, 1856, 1615, 1616, 1617]
CCCCTCCATTGAGCTTGAATTTTGGTGGCCGCAACATACTGAATGTACTGCTCTTCTGCCAGAAAGCCCCTCC
ATTGAGCTTGAATCTTGGTGGCCGCAATGTACTGAATGTACTGATCTTCCACAATGAAACGTCTCCATTGTGA
TTGAATTTTGGTGGCTGAGTTGTATTTCACATACTGATCCCGCGCTCTGTAACATCTCCACTTAGCTTGAATC
CGCCTTGCCGTCATGTACAACAGATATTGTTCCTGTGCCAAAAATCCTCTCCAATGTGCCTGGATCGTCCTGG
ACGCTTTGAATTCTCTGTACTTCTTCCACTCTGTGCGGCACCTCCACCACGCCTGAATCTTCTTTACTAACGT
GTAGATCAGGTACTGTTCCTGAGAAACGAAGCCTCTCCAATGTGCCTGGATTTTCGCGGCAGACTTGTAAATC
AGGTACTGCTGCTCTGCTGCGTGACATCTCCACCGCGCTTGGATCTTCGTGACCAACGTGTAAATCAGGTACC
GTTCCTGCGCCACAAAGCACCTCCATTGCGCCTGGATCCTTGTTGCGGCGATGTACTCTAATAACGACATCTG
GGCCGTGTGGGACCTCCAACGCGCCTGGATCCTTGTTGCGGCGATGTACTCTAATAACGACATCTGCGCGCTG
TGGCTCCTCCATCTTGCCTGAATCCTTGTTGCGGCGATGTACTCTAATAACGACATCTGCGCGCTGTGGCTCC
TCCATCTTGCCTGAATCCT
>TR44|c0_g2_i1 len=613 path=[1670:0-39 1671:40-41 1672:42-45 1673:46-50
1674:51-96 1675:97-97 1951:98-387 1884:388-420 1993:421-432 1948:433-441
2056:442-456 1842:457-469 1915:470-480 1879:481-493 1840:494-504
1913:505-507 1971:508-522 1946:523-535 1912:536-540 1877:541-546
1838:547-559 1911:560-570 1876:571-573 1837:574-588 1668:589-601
1669:602-612] [-1, 1670, 1671, 1672, 1673, 1674, 1675, 1951, 1884, 1993,
1948, 2056, 1842, 1915, 1879, 1840, 1913, 1971, 1946, 1912, 1877, 1838,
1911, 1876, 1837, 1668, 1669, -2]
AATACTGTTCCTGCGCCAAAAAACCTCTCCACTTGGCTTGGATCTTTGTTGACGACTTGTAAAATAGGTATTG
CTCGTGTGCCACAAATCCTCTCCACAATGCTTGAATCTTCATAACTGATTTGATAAGTAGATATTGTTCCTGC
ACCACAAACCCTCGCCACTTTGATTGAATTTTTGTGGCGGAAGTGTGCTGCAGGTAATTCTTGCGCAGAGAAT
GGCATCTCCACCATGTTTGAATCTTCCTTCCCGCCATGAAAAGAAGGTACTGCTCTTCCGCCAAAAATCCTCT
CCATCTTGATTGAATCTTGGTTGCAGATTTGTATTGCATGAGCTGCTCTTGCGCAAACAACCCTCTGCATATT
GATTGAATCTTGGTGGCCGCAACATACTGAATAAACTGCTCTTCTGCCAGAAACCCCCTCCATTGAGCTTGAA
TTTTGGTGGCCGCAACATATTGAATGTACTGCCCTTCTGCCAGAAAGCCCCTCCATTGAGCTTGAATTTTGGT
GGCCGCAACATATTGAATGTACTGCTCTTCTGCCAGAAAGCCCCTCCATTGAGCTTGAATTTTGGTGGCCGCA
ACATACTGAATGTACTGCTCTTCTGCCAG
>TR45|c0_g1_i1 len=3606 path=[5501:0-470 5502:471-485 5598:486-651
5540:652-664 5606:665-679 @5603@!:680-1099 5589:1100-1154 5576:1155-1178
5602:1179-1423 5493:1424-1447 5596:1448-1745 5610:1746-1748 5612:1749-
1759 5613:1760-1781 5499:1782-1798 5594:1799-1914 @5584@!:1915-2090
5446:2091-2092 5600:2093-2167 5582:2168-2191 5607:2192-2221 5605:2222-
2240 @5599@!:2241-2545 5568:2546-2763 5451:2764-2766 5580:2767-2913
5566:2914-3049 5537:3050-3073 5591:3074-3243 5459:3244-3267 5579:3268-

3344 5544:3345-3605] [-1, 5501, 5502, 5598, 5540, 5606, 5603, 5589, 5576, 5602, 5493, 5596, 5610, 5612, 5613, 5499, 5594, 5584, 5446, 5600, 5582, 5607, 5605, 5599, 5568, 5451, 5580, 5566, 5537, 5591, 5459, 5579, 5544, -2]
CACGCGGTCAAAGACAGACCAAAAGATTTTGTTGACGTCAACCAGAAGCCCTTAAAGGGAACATGGTGCATGT
GACAACCTCTGAGTGTCACCATGGTGCACCATGATCACTTCTGACCGGCACTATGACCGTAGGGACACCCCCC
CATGATAACTACAAGCTGCCATCCTCCAAAAACGCGTAATGAACACGCTGTTATCCTCATTCTGTTGATGTTT
CGTCCAAAGAAATGGCGTCCTCAACAAGCCAGCAATCGTCAAATCCAAGACTGCCGCTTGCCCAAGCCATCAT
CATCAAACCACTTCCGTCATCACCCTCCTCTTCCGATACCTTCCATCACCGTCCTCTCAAGTAAACAATTCAG
AAAACCAATCCTCTGAATGGATCAGAATCTTGTTCTGGCTCACCATAGCTGTCAGAACGGGTTTATATATCCG
GCTGATGACGGATGATTTGATAATGAAAAAAAACATCATGCGCTCCCACACACAAAAATAACCGGAAGAAGAA
GGCTAGGCAGCAGAAGAAGAAGACAAATAGGGTGCTGAGGATGGCTGGGAGTAAGGGGGGGGCGTTTAAAGAA
AGAGGACGAGGTGGTGTTGAATTTGGAGGATGAATCTGATCGTACATTTTATCTCGTGAAAAAAAAAACACAA
TTTTTTACTTACCGTACACTTTCACAAATCCGTTTTCGTTTCCATGTCACCATGTACCTCACCTCTCTCCTCC
TCCAAATTGTCCCTCCTCTTCCCAAACTGTAGGTATACAACCATCGAGTGCCTATCCCCTGGGCACTCCAGTT
CACCAATGCCAGTATCACCTACCAATTCCTCTCCAAAAGGGTAAGAAAACAGCAACAGAAGGACTCTGGGAAT
ACAAAGGCTGTGACGATGGCCCTTGTCCTTGGGAGCATGGGTGCCTCCCAGTCTCCTACTTCCAAGTCAACCA
CTCCTCTTCCTCAAAGGTCTCTCACTCCTCCAAGGACATCCCTCACTCCTTGGAGGACATCCATCACTCCTCC
ATGAACAGCTGCACAGAACTGCATCAATGAGACATTTGTTGCCATGGCTGCAGCCATTGAGACTCTCCCCAAG
TGGAATGATTGTAAGCTTGTGGGGGTAGCTATGAGAAAGAAGAAGAGGAGCAGTGGTGACATGTCCACCCGGG
ATACATTCGCCAACAAATCAGTGGTTGCCCTCAGTGCTGCCAGATACTCAAAGGCCCTCAAGGATGCCACCAT
ATATTACTCAAGGATCAAGAGTGGTCCGAATGCGGGAAAGAATGGATATGGGCCAGTGTCCATTGTTGAGAAG
TGGAACAGATAGGTTCTCGTCCTACTAAGCGACCAGAAGCTGTCAAAATCCGCGCTCTCTGCTGCAGTGCAGG
CAGGCCAAGTTGGTCAGTCCCCACCTAAAAGAGGAAGTCCTAGAACGATACCGCTTGTGCTCACCAAGACTTT
GGCTGCCCATGCGACGATGATGCAGGTCTCTGGAGAGGGTGAGGCAAATCACCAGAAGATGGTGATGGCGGGG
AAGGCGCTAATGATGGGCACAGAGTGGGAAAATAAGATTTGTCCCGACTACCTCTGGCACAAAGTCCGGGAGG
ATCATCCGGCATTGCTCAACCCTGTGAAGCAGAAGCACAATGAAGACCAGCGGGTGGATTTGGCTGACATATA
ATAATGTCAATGATTGGACAGACCGCACAAAAGCGTATCTGGTTGAGTTGGGCATGGTCAAAAATGAACCGGG
ATTCATAAGTGAGTTGCTTCTTTGTTTTTTACGTTTCCACTTTACAATTCACAGTTTCTAGCACACGTTTTGT
CCAGATGGGGTTTGGTCTGAGGTCTCTCTCTTTCATGACAATGACGCTCGTTTCTTTCTAAACACAGACAAAA
CTCAGCACAAGTTCTCAACGACGAGGAACAAGGGAGGGTCAACGACGATTTCCTATGTGAATGTTTTATTCCC
ACGGTCGGGTGAGCGCGTTGTCGAGAGTTCGAGGCACACCACAGGGTGTTACACCGTGAATCTTGCCGGAGAG
GTACTCCCATCCCTTTATATATTCGACAGTAAAGCTAAAGAAGAAGATAAGTTTTCTGTTGACCCTAGTATTG
GTATTGGTTTGCCTGAAGTTGAAGGAGTCTTTGGACTGTACGAGAAAACTTATTTTGCTCTGTATTTTGCTGT
TTGCCGCAAAGGGTTGATGGACACAAGTCTGTGGTCTCAATTCAATGAGACTGTCATCCTCAATTGTTACCCA
AGATGCAACAAAACGATAGTGCGCTGCCCACTGACAACCAAGTTACTTCAAGGACCTATGGTCATCAAGACCG
ATGAAGGTCCAGGCCGTCTTGCCAAGGAAGCCGTCAGCTGGGAATTTTGAGAGAGGATACACGACCGAGGGCT
CGTCATCACGCTGGGCCTTCCAAATGGTACCTCTGCCATCCAGGAGATGGACCAAGGATACACAGACTTCAAA
TAAGGGTGCAAGAAGAGCACCCTACGTGTAGTATCCATCAAGTTGGCAGCCTGCAATGCTGCACAAAATCGAC
CTCAAGATGAAGTTGATGAATCAGTGGATTCTGACAATGAGAATGCCAACCATGGGAAGAGTGTATGCAATGT
GACTCTTGGTAACAAAGACCTTGCAAACATCATCAATGGATTTCCAGGTGATTCAATTGATCTGCACCCATTT
GACAAGGTCTTTACGCAAGACAATATCAAGTGTTGGTGGGAGAATGTTGGCTTTCTCCCAATGACATGGAATA
CCGCCCAGGACCCAAAGACTCGCTGGGAGCTAGGCAAAGGTGGTACCCCAGAAGAAGGAATAGAAAGGCTCAG
TATGCTTGTCAACCCTTACAAGGTGGGAGCAAAGAGACTAGAAGAGATGGGGTTCAACAAGAACATCCTCTCT
CTACAACCACCAGTAGTATCAACACCAAAAATACCAGAAGGAGAGGAGGCGCAAATTCAAGCCCTCGTGGATG
GCAAGGCAATCAACAAGGCAGGGGCTGTGTTTAAGCTTGGGCTTCAAGTTGCCAACAACAGGGTCATGCTTGA
GACCAACAAGAGGATGGTCAAGTTGGAAGAAGAGGCCAAGGCAAACAAGGAGCGCAAGAAGAAGAAGGAGTCG
GAAGGGAAGGACCAGAAGGCTATTGAAGCATTCAACGCTTGGATCACCAAGGGTAAGTGCATTGATACGAAAG
GGCATCCAGTCCTTAATAAGGATGACTCCCTCGCTATCATTAAGTTATTGATGCCAAAGATAGCTCCTACAGA
AAAGGTTGAGGATTACAAGACTATGAAGAAATGTAATGAGTGGCTGGGTAACTTGGCAGGTGGATCTACATGG
GAGGAGGAGATAAAAGCGATCAAAACCCAGTTTACCGAATCGATTCTAGCGCGTAGTGTGTAACCTTAATCGT
CACCAAACTCATGCATTCTACTCTCCTACATGTTAGTCAAGTGTGACCATCAATATTTGCCATGTTGGATGGT
TACTGGCACCGTACGGGACCATTGAAGTTGACGTCTTTGATTTTTGAAGGCTTAATTGGCCATATTTTAGGTT
AATTCCTACGTACATATGTACCATAGTTC
>TR45|c0_g1_i2 len=2821 path=[5501:0-470 5502:471-485 5598:486-651 5540:652-664 5606:665-679 @5603@!:680-1099 5589:1100-1154 5576:1155-1178 5602:1179-1423 5493:1424-1447 5596:1448-1745 5610:1746-1748 5612:1749-1759 5456:1760-1782 @5584@!:1783-1958 5476:1959-1980 5605:1981-1999 @5599@!:2000-2304 5581:2305-2534 5563:2535-2537 5567:2538-2638 5548:2639-2662 5549:2663-2684 5566:2685-2820] [-1, 5501, 5502, 5598, 5540, 5606,

5603, 5589, 5576, 5602, 5493, 5596, 5610, 5612, 5456, 5584, 5476, 5605, 5599, 5581, 5563, 5567, 5548, 5549, 5566]
CACGCGGTCAAAGACAGACCAAAAGATTTTGTTGACGTCAACCAGAAGCCCTTAAAGGGAACATGGTGCATGT
GACAACCTCTGAGTGTCACCATGGTGCACCATGATCACTTCTGACCGGCACTATGACCGTAGGGACACCCCCC
CATGATAACTACAAGCTGCCATCCTCCAAAAACGCGTAATGAACACGCTGTTATCCTCATTCTGTTGATGTTT
CGTCCAAAGAAATGGCGTCCTCAACAAGCCAGCAATCGTCAAATCCAAGACTGCCGCTTGCCCAAGCCATCAT
CATCAAACCACTTCCGTCATCACCCTCCTCTTCCGATACCTTCCATCACCGTCCTCTCAAGTAAACAATTCAG
AAAACCAATCCTCTGAATGGATCAGAATCTTGTTCTGGCTCACCATAGCTGTCAGAACGGGTTTATATATCCG
GCTGATGACGGATGATTTGATAATGAAAAAAAACATCATGCGCTCCCACACACAAAAATAACCGGAAGAAGAA
GGCTAGGCAGCAGAAGAAGAAGACAAATAGGGTGCTGAGGATGGCTGGGAGTAAGGGGGGGGCGTTTAAAGAA
AGAGGACGAGGTGGTGTTGAATTTGGAGGATGAATCTGATCGTACATTTTATCTCGTGAAAAAAAAAACACAA
TTTTTTACTTACCGTACACTTTCACAAATCCGTTTTCGTTTCCATGTCACCATGTACCTCACCTCTCTCCTCC
TCCAAATTGTCCCTCCTCTTCCCAAACTGTAGGTATACAACCATCGAGTGCCTATCCCCTGGGCACTCCAGTT
CACCAATGCCAGTATCACCTACCAATTCCTCTCCAAAAGGGTAAGAAAACAGCAACAGAAGGACTCTGGGAAT
ACAAAGGCTGTGACGATGGCCCTTGTCCTTGGGAGCATGGGTGCCTCCCAGTCTCCTACTTCCAAGTCAACCA
CTCCTCTTCCTCAAAGGTCTCTCACTCCTCCAAGGACATCCCTCACTCCTTGGAGGACATCCATCACTCCTCC
ATGAACAGCTGCACAGAACTGCATCAATGAGACATTTGTTGCCATGGCTGCAGCCATTGAGACTCTCCCCAAG
TGGAATGATTGTAAGCTTGTGGGGGTAGCTATGAGAAAGAAGAAGAGGAGCAGTGGTGACATGTCCACCCGGG
ATACATTCGCCAACAAATCAGTGGTTGCCCTCAGTGCTGCCAGATACTCAAAGGCCCTCAAGGATGCCACCAT
ATATTACTCAAGGATCAAGAGTGGTCCGAATGCGGGAAAGAATGGATATGGGCCAGTGTCCATTGTTGAGAAG
TGGAACAGATAGGTTCTCGTCCTACTAAGCGACCAGAAGCTGTCAAAATCCGCGCTCTCTGCTGCAGTGCAGG
CAGGCCAAGTTGGTCAGTCCCCACCTAAAAGAGGAAGTCCTAGAACGATACCGCTTGTGCTCACCAAGACTTT
GGCTGCCCATGCGACGATGATGCAGGTCTCTGGAGAGGGTGAGGCAAATCACCAGAAGATGGTGATGGCGGGG
AAGGCGCTAATGATGGGCACAGAGTGGGAAAATAAGATTTGTCCCGACTACCTCTGGCACAAAGTCCGGGAGG
ATCATCCGGCATTGCTCAACCCTGTGAAGCAGAAGCACAATGAAGACCAGCGGGTGGATTTGGCTGACATATA
ATAATGTCAATGATTGGACAGACCGCACAAAAGCGTATCTGGTTGAGTTGGGCATGGTCAAAAATGAACCGGG
ATTCATAAACAAAACTCAGCACAAGTTCTCAACGACGAGGAACAAGGGAGGGTCAACGACGATTTCCTATGTG
AATGTTTTATTCCCACGGTCGGGTGAGCGCGTTGTCGAGAGTTCGAGGCACACCACAGGGTGTTACACCGTGA
ATCTTGCCGGAGAGGTACTCCCATCCCTTTATATATTCGACAGTAAAGCTAAAGAAGAAGAGGGTTGATGGAC
ACAAGTCTGTGGTCTCAATTCAATGAGACTGTCATCCTCAATTGTTACCCAAGATGCAACAAAACGATAGTGC
GCTGCCCACTGACAACCAAGTTACTTCAAGGACCTATGGTCATCAAGACCGATGAAGGTCCAGGCCGTCTTGC
CAAGGAAGCCGTCAGCTGGGAATTTTGAGAGAGGATACACGACCGAGGGCTCGTCATCACGCTGGGCCTTCCA
AATGGTACCTCTGCCATCCAGGAGATGGACCAAGGATACACAGACTTCAAATAAGGGTGCAAGAAGAGCACCC
TACGTGTAGTATCCATCAAGTTGGCAGCCTGCAATGCTGCACGACAATCTCGAGATGAAGTCGTTGTATCAGT
TGATGGTGCCGATGATAACGCCAACCATGTCAGCACCCGGTCAAGGAAGAGTGTATGTAATGTGACTCTTGGC
AATGAAGACCTTGCCAACATCATCAATGGGTTTCCAGGTGATCCAATTGATCTGCGTCCGTTTGACAAGGTCT
TTACTCAAGACAACATCAAGAGTTGGTGGAAGAATGTTGGCTTTCTCCCAATGACACGGAATGCCGTCAACGA
TCCAAAGACTCGGTGGGAGCTAGGCAAAGGCAGTGCCCCAGAAGAAGGTACAGCAAGGCTTGTTATACTCATA
GATGACTACAATGTGGGAGCAAAGAAAGTAGAACAGATGGGGTTCAACAAGAACATCCTCTCTCTACAACCAC
CAGTAGTATCAACACCAAAAATACCAGAAGGAGAGGAGGCGCAAATTCAAGCCCTCGTGGATGGCAAGGCAAT
CAACAAGGCAGGGGCTGTGTTTAAGCTTGGGCTTCAAGTTGCCAACA
>TR45|c0_g1_i3 len=3486 path=[5501:0-470 5502:471-485 5598:486-651 5540:652-664 5606:665-679 @5603@!:680-1099 5589:1100-1154 5576:1155-1178 5602:1179-1423 5493:1424-1447 5596:1448-1745 5610:1746-1748 5612:1749-1759 5456:1760-1782 @5584@!:1783-1958 5446:1959-1960 5600:1961-2035 5582:2036-2059 5607:2060-2089 5605:2090-2108 @5599@!:2109-2413 5581:2414-2643 5563:2644-2646 5567:2647-2747 5548:2748-2771 5549:2772-2793 5566:2794-2929 5537:2930-2953 5591:2954-3123 5459:3124-3147 5579:3148-3224 5544:3225-3485] [-1, 5501, 5502, 5598, 5540, 5606, 5603, 5589, 5576, 5602, 5493, 5596, 5610, 5612, 5456, 5584, 5446, 5600, 5582, 5607, 5605, 5599, 5581, 5563, 5567, 5548, 5549, 5566, 5537, 5591, 5459, 5579, 5544, -2]
CACGCGGTCAAAGACAGACCAAAAGATTTTGTTGACGTCAACCAGAAGCCCTTAAAGGGAACATGGTGCATGT
GACAACCTCTGAGTGTCACCATGGTGCACCATGATCACTTCTGACCGGCACTATGACCGTAGGGACACCCCCC
CATGATAACTACAAGCTGCCATCCTCCAAAAACGCGTAATGAACACGCTGTTATCCTCATTCTGTTGATGTTT
CGTCCAAAGAAATGGCGTCCTCAACAAGCCAGCAATCGTCAAATCCAAGACTGCCGCTTGCCCAAGCCATCAT
CATCAAACCACTTCCGTCATCACCCTCCTCTTCCGATACCTTCCATCACCGTCCTCTCAAGTAAACAATTCAG
AAAACCAATCCTCTGAATGGATCAGAATCTTGTTCTGGCTCACCATAGCTGTCAGAACGGGTTTATATATCCG
GCTGATGACGGATGATTTGATAATGAAAAAAAACATCATGCGCTCCCACACACAAAAATAACCGGAAGAAGAA
GGCTAGGCAGCAGAAGAAGAAGACAAATAGGGTGCTGAGGATGGCTGGGAGTAAGGGGGGGGCGTTTAAAGAA

AGAGGACGAGGTGGTGTTGAATTTGGAGGATGAATCTGATCGTACATTTTATCTCGTGAAAAAAAAAACACAA
TTTTTTACTTACCGTACACTTTCACAAATCCGTTTTCGTTTCCATGTCACCATGTACCTCACCTCTCTCCTCC
TCCAAATTGTCCCTCCTCTTCCCAAACTGTAGGTATACAACCATCGAGTGCCTATCCCCTGGGCACTCCAGTT
CACCAATGCCAGTATCACCTACCAATTCCTCTCCAAAAGGGTAAGAAAACAGCAACAGAAGGACTCTGGGAAT
ACAAAGGCTGTGACGATGGCCCTTGTCCTTGGGAGCATGGGTGCCTCCCAGTCTCCTACTTCCAAGTCAACCA
CTCCTCTTCCTCAAAGGTCTCTCACTCCTCCAAGGACATCCCTCACTCCTTGGAGGACATCCATCACTCCTCC
ATGAACAGCTGCACAGAACTGCATCAATGAGACATTTGTTGCCATGGCTGCAGCCATTGAGACTCTCCCCAAG
TGGAATGATTGTAAGCTTGTGGGGGTAGCTATGAGAAAGAAGAAGAGGAGCAGTGGTGACATGTCCACCCGGG
ATACATTCGCCAACAAATCAGTGGTTGCCCTCAGTGCTGCCAGATACTCAAAGGCCCTCAAGGATGCCACCAT
ATATTACTCAAGGATCAAGAGTGGTCCGAATGCGGGAAAGAATGGATATGGGCCAGTGTCCATTGTTGAGAAG
TGGAACAGATAGGTTCTCGTCCTACTAAGCGACCAGAAGCTGTCAAAATCCGCGCTCTCTGCTGCAGTGCAGG
CAGGCCAAGTTGGTCAGTCCCCACCTAAAAGAGGAAGTCCTAGAACGATACCGCTTGTGCTCACCAAGACTTT
GGCTGCCCATGCGACGATGATGCAGGTCTCTGGAGAGGGTGAGGCAAATCACCAGAAGATGGTGATGGCGGGG
AAGGCGCTAATGATGGGCACAGAGTGGGAAAATAAGATTTGTCCCGACTACCTCTGGCACAAAGTCCGGGAGG
ATCATCCGGCATTGCTCAACCCTGTGAAGCAGAAGCACAATGAAGACCAGCGGGTGGATTTGGCTGACATATA
ATAATGTCAATGATTGGACAGACCGCACAAAAGCGTATCTGGTTGAGTTGGGCATGGTCAAAAATGAACCGGG
ATTCATAAACAAAACTCAGCACAAGTTCTCAACGACGAGGAACAAGGGAGGGTCAACGACGATTTCCTATGTG
AATGTTTTATTCCCACGGTCGGGTGAGCGCGTTGTCGAGAGTTCGAGGCACACCACAGGGTGTTACACCGTGA
ATCTTGCCGGAGAGGTACTCCCATCCCTTTATATATTCGACAGTAAAGCTAAAGAAGAAGATAAGTTTTCTGT
TGACCCTAGTATTGGTATTGGTTTGCCTGAAGTTGAAGGAGTCTTTGGACTGTACGAGAAAACTTATTTTGCT
CTGTATTTTGCTGTTTGCCGCAAAGGGTTGATGGACACAAGTCTGTGGTCTCAATTCAATGAGACTGTCATCC
TCAATTGTTACCCAAGATGCAACAAAACGATAGTGCGCTGCCCACTGACAACCAAGTTACTTCAAGGACCTAT
GGTCATCAAGACCGATGAAGGTCCAGGCCGTCTTGCCAAGGAAGCCGTCAGCTGGGAATTTTGAGAGAGGATA
CACGACCGAGGGCTCGTCATCACGCTGGGCCTTCCAAATGGTACCTCTGCCATCCAGGAGATGGACCAAGGAT
ACACAGACTTCAAATAAGGGTGCAAGAAGAGCACCCTACGTGTAGTATCCATCAAGTTGGCAGCCTGCAATGC
TGCACGACAATCTCGAGATGAAGTCGTTGTATCAGTTGATGGTGCCGATGATAACGCCAACCATGTCAGCACC
CGGTCAAGGAAGAGTGTATGTAATGTGACTCTTGGCAATGAAGACCTTGCCAACATCATCAATGGGTTTCCAG
GTGATCCAATTGATCTGCGTCCGTTTGACAAGGTCTTTACTCAAGACAACATCAAGAGTTGGTGGAAGAATGT
TGGCTTTCTCCCAATGACACGGAATGCCGTCAACGATCCAAAGACTCGGTGGGAGCTAGGCAAAGGCAGTGCC
CCAGAAGAAGGTACAGCAAGGCTTGTTATACTCATAGATGACTACAATGTGGGAGCAAAGAAAGTAGAACAGA
TGGGGTTCAACAAGAACATCCTCTCTCTACAACCACCAGTAGTATCAACACCAAAAATACCAGAAGGAGAGGA
GGCGCAAATTCAAGCCCTCGTGGATGGCAAGGCAATCAACAAGGCAGGGGCTGTGTTTAAGCTTGGGCTTCAA
GTTGCCAACAACAGGGTCATGCTTGAGACCAACAAGAGGATGGTCAAGTTGGAAGAAGAGGCCAAGGCAAACA
AGGAGCGCAAGAAGAAGAAGGAGTCGGAAGGGAAGGACCAGAAGGCTATTGAAGCATTCAACGCTTGGATCAC
CAAGGGTAAGTGCATTGATACGAAAGGGCATCCAGTCCTTAATAAGGATGACTCCCTCGCTATCATTAAGTTA
TTGATGCCAAAGATAGCTCCTACAGAAAAGGTTGAGGATTACAAGACTATGAAGAAATGTAATGAGTGGCTGG
GTAACTTGGCAGGTGGATCTACATGGGAGGAGGAGATAAAAGCGATCAAAACCCAGTTTACCGAATCGATTCT
AGCGCGTAGTGTGTAACCTTAATCGTCACCAAACTCATGCATTCTACTCTCCTACATGTTAGTCAAGTGTGAC
CATCAATATTTGCCATGTTGGATGGTTACTGGCACCGTACGGGACCATTGAAGTTGACGTCTTTGATTTTTGA
AGGCTTAATTGGCCATATTTTAGGTTAATTCCTACGTACATATGTACCATAGTTC
>TR45|c0_g1_i4 len=2809 path=[5501:0-470 5502:471-485 5598:486-651 5540:652-664 5606:665-679 @5603@!:680-1099 5589:1100-1154 5576:1155-1178 5602:1179-1423 5493:1424-1447 5596:1448-1745 5610:1746-1748 5612:1749-1759 5456:1760-1782 @5584@!:1783-1958 5476:1959-1980 5605:1981-1999 @5599@!:2000-2304 5568:2305-2522 5451:2523-2525 5580:2526-2672 5566:2673-2808] [-1, 5501, 5502, 5598, 5540, 5606, 5603, 5589, 5576, 5602, 5493, 5596, 5610, 5612, 5456, 5584, 5476, 5605, 5599, 5568, 5451, 5580, 5566]
CACGCGGTCAAAGACAGACCAAAAGATTTTGTTGACGTCAACCAGAAGCCCTTAAAGGGAACATGGTGCATGT
GACAACCTCTGAGTGTCACCATGGTGCACCATGATCACTTCTGACCGGCACTATGACCGTAGGGACACCCCCC
CATGATAACTACAAGCTGCCATCCTCCAAAAACGCGTAATGAACACGCTGTTATCCTCATTCTGTTGATGTTT
CGTCCAAAGAAATGGCGTCCTCAACAAGCCAGCAATCGTCAAATCCAAGACTGCCGCTTGCCCAAGCCATCAT
CATCAAACCACTTCCGTCATCACCCTCCTCTTCCGATACCTTCCATCACCGTCCTCTCAAGTAAACAATTCAG
AAAACCAATCCTCTGAATGGATCAGAATCTTGTTCTGGCTCACCATAGCTGTCAGAACGGGTTTATATATCCG
GCTGATGACGGATGATTTGATAATGAAAAAAAACATCATGCGCTCCCACACACAAAAATAACCGGAAGAAGAA
GGCTAGGCAGCAGAAGAAGAAGACAAATAGGGTGCTGAGGATGGCTGGGAGTAAGGGGGGGGCGTTTAAAGAA
AGAGGACGAGGTGGTGTTGAATTTGGAGGATGAATCTGATCGTACATTTTATCTCGTGAAAAAAAAAACACAA
TTTTTTACTTACCGTACACTTTCACAAATCCGTTTTCGTTTCCATGTCACCATGTACCTCACCTCTCTCCTCC
TCCAAATTGTCCCTCCTCTTCCCAAACTGTAGGTATACAACCATCGAGTGCCTATCCCCTGGGCACTCCAGTT
CACCAATGCCAGTATCACCTACCAATTCCTCTCCAAAAGGGTAAGAAAACAGCAACAGAAGGACTCTGGGAAT
ACAAAGGCTGTGACGATGGCCCTTGTCCTTGGGAGCATGGGTGCCTCCCAGTCTCCTACTTCCAAGTCAACCA

CTCCTCTTCCTCAAAGGTCTCTCACTCCTCCAAGGACATCCCTCACTCCTTGGAGGACATCCATCACTCCTCC
ATGAACAGCTGCACAGAACTGCATCAATGAGACATTTGTTGCCATGGCTGCAGCCATTGAGACTCTCCCCAAG
TGGAATGATTGTAAGCTTGTGGGGGTAGCTATGAGAAAGAAGAAGAGGAGCAGTGGTGACATGTCCACCCGGG
ATACATTCGCCAACAAATCAGTGGTTGCCCTCAGTGCTGCCAGATACTCAAAGGCCCTCAAGGATGCCACCAT
ATATTACTCAAGGATCAAGAGTGGTCCGAATGCGGGAAAGAATGGATATGGGCCAGTGTCCATTGTTGAGAAG
TGGAACAGATAGGTTCTCGTCCTACTAAGCGACCAGAAGCTGTCAAAATCCGCGCTCTCTGCTGCAGTGCAGG
CAGGCCAAGTTGGTCAGTCCCCACCTAAAAGAGGAAGTCCTAGAACGATACCGCTTGTGCTCACCAAGACTTT
GGCTGCCCATGCGACGATGATGCAGGTCTCTGGAGAGGGTGAGGCAAATCACCAGAAGATGGTGATGGCGGGG
AAGGCGCTAATGATGGGCACAGAGTGGGAAAATAAGATTTGTCCCGACTACCTCTGGCACAAAGTCCGGGAGG
ATCATCCGGCATTGCTCAACCCTGTGAAGCAGAAGCACAATGAAGACCAGCGGGTGGATTTGGCTGACATATA
ATAATGTCAATGATTGGACAGACCGCACAAAAGCGTATCTGGTTGAGTTGGGCATGGTCAAAAATGAACCGGG
ATTCATAAACAAAACTCAGCACAAGTTCTCAACGACGAGGAACAAGGGAGGGTCAACGACGATTTCCTATGTG
AATGTTTTATTCCCACGGTCGGGTGAGCGCGTTGTCGAGAGTTCGAGGCACACCACAGGGTGTTACACCGTGA
ATCTTGCCGGAGAGGTACTCCCATCCCTTTATATATTCGACAGTAAAGCTAAAGAAGAAGAGGGTTGATGGAC
ACAAGTCTGTGGTCTCAATTCAATGAGACTGTCATCCTCAATTGTTACCCAAGATGCAACAAAACGATAGTGC
GCTGCCCACTGACAACCAAGTTACTTCAAGGACCTATGGTCATCAAGACCGATGAAGGTCCAGGCCGTCTTGC
CAAGGAAGCCGTCAGCTGGGAATTTTGAGAGAGGATACACGACCGAGGGCTCGTCATCACGCTGGGCCTTCCA
AATGGTACCTCTGCCATCCAGGAGATGGACCAAGGATACACAGACTTCAAATAAGGGTGCAAGAAGAGCACCC
TACGTGTAGTATCCATCAAGTTGGCAGCCTGCAATGCTGCACAAAATCGACCTCAAGATGAAGTTGATGAATC
AGTGGATTCTGACAATGAGAATGCCAACCATGGGAAGAGTGTATGCAATGTGACTCTTGGTAACAAAGACCTT
GCAAACATCATCAATGGATTTCCAGGTGATTCAATTGATCTGCACCCATTTGACAAGGTCTTTACGCAAGACA
ATATCAAGTGTTGGTGGGAGAATGTTGGCTTTCTCCCAATGACATGGAATACCGCCCAGGACCCAAAGACTCG
CTGGGAGCTAGGCAAAGGTGGTACCCCAGAAGAAGGAATAGAAAGGCTCAGTATGCTTGTCAACCCTTACAAG
GTGGGAGCAAAGAGACTAGAAGAGATGGGGTTCAACAAGAACATCCTCTCTCTACAACCACCAGTAGTATCAA
CACCAAAAATACCAGAAGGAGAGGAGGCGCAAATTCAAGCCCTCGTGGATGGCAAGGCAATCAACAAGGCAGG
GGCTGTGTTTAAGCTTGGGCTTCAAGTTGCCAACA
>TR45|c0_g1_i5 len=3535 path=[5501:0-470 5502:471-485 5598:486-651
5540:652-664 5606:665-679 @5603@!:680-1099 5588:1100-1744 5609:1745-1747
5611:1748-1758 5507:1759-1785 5595:1786-1907 5586:1908-2019 5552:2020-
2021 5573:2022-2084 5604:2085-2169 @5599@!:2170-2474 5568:2475-2692
5451:2693-2695 5580:2696-2842 5566:2843-2978 5537:2979-3002 5591:3003-
3172 5459:3173-3196 5579:3197-3273 5544:3274-3534] [-1, 5501, 5502, 5598,
5540, 5606, 5603, 5588, 5609, 5611, 5507, 5595, 5586, 5552, 5573, 5604,
5599, 5568, 5451, 5580, 5566, 5537, 5591, 5459, 5579, 5544, -2]
CACGCGGTCAAAGACAGACCAAAAGATTTTGTTGACGTCAACCAGAAGCCCTTAAAGGGAACATGGTGCATGT
GACAACCTCTGAGTGTCACCATGGTGCACCATGATCACTTCTGACCGGCACTATGACCGTAGGGACACCCCCC
CATGATAACTACAAGCTGCCATCCTCCAAAAACGCGTAATGAACACGCTGTTATCCTCATTCTGTTGATGTTT
CGTCCAAAGAAATGGCGTCCTCAACAAGCCAGCAATCGTCAAATCCAAGACTGCCGCTTGCCCAAGCCATCAT
CATCAAACCACTTCCGTCATCACCCTCCTCTTCCGATACCTTCCATCACCGTCCTCTCAAGTAAACAATTCAG
AAAACCAATCCTCTGAATGGATCAGAATCTTGTTCTGGCTCACCATAGCTGTCAGAACGGGTTTATATATCCG
GCTGATGACGGATGATTTGATAATGAAAAAAAACATCATGCGCTCCCACACACAAAAATAACCGGAAGAAGAA
GGCTAGGCAGCAGAAGAAGAAGACAAATAGGGTGCTGAGGATGGCTGGGAGTAAGGGGGGGGCGTTTAAAGAA
AGAGGACGAGGTGGTGTTGAATTTGGAGGATGAATCTGATCGTACATTTTATCTCGTGAAAAAAAAAACACAA
TTTTTTACTTACCGTACACTTTCACAAATCCGTTTTCGTTTCCATGTCACCATGTACCTCACCTCTCTCCTCC
TCCAAATTGTCCCTCCTCTTCCCAAACTGTAGGTATACAACCATCGAGTGCCTATCCCCTGGGCACTCCAGTT
CACCAATGCCAGTATCACCTACCAATTCCTCTCCAAAAGGGTAAGAAAACAGCAACAGAAGGACTCTGGGAAT
ACAAAGGCTGTGACGATGGCCCTTGTCCTTGGGAGCATGGGTGCCTCCCAGTCTCCTACTTCCAAGTCAACCA
CTCCTCTTCCTCAAAGGTCTCTCACTCCTCCAAGGACATCCCTCACTCCTTGGAGGACATCCATCACTCCTCC
ATGAACAGCTGCACAGAACTGCATCAATGAGACATTTGTTGCCATGGCTGCAGCCATTGAGACTCTCCCCAAG
TGGAACAACAACAAACGTGTGGGGACCGCCATGACCAAGAAGAAGAGGAACAGGGGTGACCAGTCCTCCCGTG
AGACATTTGCAAACAAAGCAGTGTGTGCCCTCAGTGCTGCCTAATATTCAAAGGCCCTCAAGGACGCAACCAT
CTACTATGCTTGGATCAAGAGTGGCCCAAATGCAGGCAAGATGGGATATGGGCCAGTGTCCATCGTTAACAAG
TGGAACAGAGAGGTTCTGAACCTCTCCAGCAGTTGGAAGCTGTCAAAATCTGCGCTCTCTGCCGCAGTGAAGG
CAGGGCGAGTTGGTTCCTCCCCGCCAAAAAGAGGTAGGCCACAAATGATACCCCTTGTGCTCACTGAGACTTT
AGCTGCCCATGCAACAATGATGCAGTGTCCGGAGAGGGTGAGGCAAATAGACAGAAGATGGTGCTCGTGGCAA
AGGTGCTGATGATGGGCATGGAGTGGGAGAAGAAGATTTCCCCAGACTACCTCTGGCGCAAAGTACGGGAGGA
TCATCCAAGGATACTCAACCCCGTGAAACAGAAACACAACAAAGACCAACGGGTAGATTGGCTGACATATTAA
AAATGTCAAAGATTGGACAGGCCAAGCGAAGGCGTATCTGGTAGAGTTGGGCATGGTCAAAAATGAACCAGGG
TAGATAAATAGGGTTTGGTTCGAGGTCTCTCTCTTTCATGACGACAACGCTTGTTACTTTTAAAAACAGATGA
AACGCAGCACAAGTTCTTGACTGCATGCAACAAGGGTGGTTCAACAACCATCTCGTATGCGAATGTTTCCTTC

CCACGGTCAGGTGAGCATATTGTTGAAAGTTTGAGGCACATCACAGGGTGTTACACCATCAATCTTGCTGGGG
AGGTGCTCCCACCCCTTAATATATTTGACAGTAAAGCTAAAGAAGAAGATATGTTTTGTGTCAAACCGAATAT
TGACATTGGTCTACCTGAAGTTCCTGGAGTCTATGGACTCTATGAAACAACTTGTTATGCTCCATATTTTGCT
GTTTGTTGCAAAGGGTCAATAGACACAAGCCTGTGGTCTCAATTCAATGAGACTGTCATCCTCAATTGTTACC
CAAGATGCAACAAAACGATAGTGCGCTGCCCACTGACAACCAAGTTACTTCAAGGACCTATGGTCATCAAGAC
CGATGAAGGTCCAGGCCGTCTTGCCAAGGAAGCCGTCAGCTGGGAATTTTGAGAGAGGATACACGACCGAGGG
CTCGTCATCACGCTGGGCCTTCCAAATGGTACCTCTGCCATCCAGGAGATGGACCAAGGATACACAGACTTCA
AATAAGGGTGCAAGAAGAGCACCCTACGTGTAGTATCCATCAAGTTGGCAGCCTGCAATGCTGCACAAAATCG
ACCTCAAGATGAAGTTGATGAATCAGTGGATTCTGACAATGAGAATGCCAACCATGGGAAGAGTGTATGCAAT
GTGACTCTTGGTAACAAAGACCTTGCAAACATCATCAATGGATTTCCAGGTGATTCAATTGATCTGCACCCAT
TTGACAAGGTCTTTACGCAAGACAATATCAAGTGTTGGTGGGAGAATGTTGGCTTTCTCCCAATGACATGGAA
TACCGCCCAGGACCCAAAGACTCGCTGGGAGCTAGGCAAAGGTGGTACCCCAGAAGAAGGAATAGAAAGGCTC
AGTATGCTTGTCAACCCTTACAAGGTGGGAGCAAAGAGACTAGAAGAGATGGGGTTCAACAAGAACATCCTCT
CTCTACAACCACCAGTAGTATCAACACCAAAAATACCAGAAGGAGAGGAGGCGCAAATTCAAGCCCTCGTGGA
TGGCAAGGCAATCAACAAGGCAGGGGCTGTGTTTAAGCTTGGGCTTCAAGTTGCCAACAACAGGGTCATGCTT
GAGACCAACAAGAGGATGGTCAAGTTGGAAGAAGAGGCCAAGGCAAACAAGGAGCGCAAGAAGAAGAAGGAGT
CGGAAGGGAAGGACCAGAAGGCTATTGAAGCATTCAACGCTTGGATCACCAAGGGTAAGTGCATTGATACGAA
AGGGCATCCAGTCCTTAATAAGGATGACTCCCTCGCTATCATTAAGTTATTGATGCCAAAGATAGCTCCTACA
GAAAAGGTTGAGGATTACAAGACTATGAAGAAATGTAATGAGTGGCTGGGTAACTTGGCAGGTGGATCTACAT
GGGAGGAGGAGATAAAAGCGATCAAAACCCAGTTTACCGAATCGATTCTAGCGCGTAGTGTGTAACCTTAATC
GTCACCAAACTCATGCATTCTACTCTCCTACATGTTAGTCAAGTGTGACCATCAATATTTGCCATGTTGGATG
GTTACTGGCACCGTACGGGACCATTGAAGTTGACGTCTTTGATTTTTGAAGGCTTAATTGGCCATATTTTAGG
TTAATTCCTACGTACATATGTACCATAGTTC
>TR45|c0_g1_i6 len=2941 path=[5501:0-470 5502:471-485 5598:486-651 5540:652-664 5606:665-679 @5603@!:680-1099 5589:1100-1154 5576:1155-1178 5602:1179-1423 5493:1424-1447 5596:1448-1745 5610:1746-1748 5612:1749-1759 5613:1760-1781 5499:1782-1798 5594:1799-1914 @5584@!:1915-2090 5476:2091-2112 5605:2113-2131 @5599@!:2132-2436 5568:2437-2654 5451:2655-2657 5580:2658-2804 5566:2805-2940] [-1, 5501, 5502, 5598, 5540, 5606, 5603, 5589, 5576, 5602, 5493, 5596, 5610, 5612, 5613, 5499, 5594, 5584, 5476, 5605, 5599, 5568, 5451, 5580, 5566]
CACGCGGTCAAAGACAGACCAAAAGATTTTGTTGACGTCAACCAGAAGCCCTTAAAGGGAACATGGTGCATGT
GACAACCTCTGAGTGTCACCATGGTGCACCATGATCACTTCTGACCGGCACTATGACCGTAGGGACACCCCCC
CATGATAACTACAAGCTGCCATCCTCCAAAAACGCGTAATGAACACGCTGTTATCCTCATTCTGTTGATGTTT
CGTCCAAAGAAATGGCGTCCTCAACAAGCCAGCAATCGTCAAATCCAAGACTGCCGCTTGCCCAAGCCATCAT
CATCAAACCACTTCCGTCATCACCCTCCTCTTCCGATACCTTCCATCACCGTCCTCTCAAGTAAACAATTCAG
AAAACCAATCCTCTGAATGGATCAGAATCTTGTTCTGGCTCACCATAGCTGTCAGAACGGGTTTATATATCCG
GCTGATGACGGATGATTTGATAATGAAAAAAAACATCATGCGCTCCCACACACAAAAATAACCGGAAGAAGAA
GGCTAGGCAGCAGAAGAAGAAGACAAATAGGGTGCTGAGGATGGCTGGGAGTAAGGGGGGGGCGTTTAAAGAA
AGAGGACGAGGTGGTGTTGAATTTGGAGGATGAATCTGATCGTACATTTTATCTCGTGAAAAAAAAAACACAA
TTTTTTACTTACCGTACACTTTCACAAATCCGTTTTCGTTTCCATGTCACCATGTACCTCACCTCTCTCCTCC
TCCAAATTGTCCCTCCTCTTCCCAAACTGTAGGTATACAACCATCGAGTGCCTATCCCCTGGGCACTCCAGTT
CACCAATGCCAGTATCACCTACCAATTCCTCTCCAAAAGGGTAAGAAAACAGCAACAGAAGGACTCTGGGAAT
ACAAAGGCTGTGACGATGGCCCTTGTCCTTGGGAGCATGGGTGCCTCCCAGTCTCCTACTTCCAAGTCAACCA
CTCCTCTTCCTCAAAGGTCTCTCACTCCTCCAAGGACATCCCTCACTCCTTGGAGGACATCCATCACTCCTCC
ATGAACAGCTGCACAGAACTGCATCAATGAGACATTTGTTGCCATGGCTGCAGCCATTGAGACTCTCCCCAAG
TGGAATGATTGTAAGCTTGTGGGGGTAGCTATGAGAAAGAAGAAGAGGAGCAGTGGTGACATGTCCACCCGGG
ATACATTCGCCAACAAATCAGTGGTTGCCCTCAGTGCTGCCAGATACTCAAAGGCCCTCAAGGATGCCACCAT
ATATTACTCAAGGATCAAGAGTGGTCCGAATGCGGGAAAGAATGGATATGGGCCAGTGTCCATTGTTGAGAAG
TGGAACAGATAGGTTCTCGTCCTACTAAGCGACCAGAAGCTGTCAAAATCCGCGCTCTCTGCTGCAGTGCAGG
CAGGCCAAGTTGGTCAGTCCCCACCTAAAAGAGGAAGTCCTAGAACGATACCGCTTGTGCTCACCAAGACTTT
GGCTGCCCATGCGACGATGATGCAGGTCTCTGGAGAGGGTGAGGCAAATCACCAGAAGATGGTGATGGCGGGG
AAGGCGCTAATGATGGGCACAGAGTGGGAAAATAAGATTTGTCCCGACTACCTCTGGCACAAAGTCCGGGAGG
ATCATCCGGCATTGCTCAACCCTGTGAAGCAGAAGCACAATGAAGACCAGCGGGTGGATTTGGCTGACATATA
ATAATGTCAATGATTGGACAGACCGCACAAAAGCGTATCTGGTTGAGTTGGGCATGGTCAAAAATGAACCGGG
ATTCATAAGTGAGTTGCTTCTTTGTTTTTTACGTTTCCACTTTACAATTCACAGTTTCTAGCACACGTTTTGT
CCAGATGGGGTTTGGTCTGAGGTCTCTCTCTTTCATGACAATGACGCTCGTTTCTTTCTAAACACAGACAAAA
CTCAGCACAAGTTCTCAACGACGAGGAACAAGGGAGGGTCAACGACGATTTCCTATGTGAATGTTTTATTCCC
ACGGTCGGGTGAGCGCGTTGTCGAGAGTTCGAGGCACACCACAGGGTGTTACACCGTGAATCTTGCCGGAGAG
GTACTCCCATCCCTTTATATATTCGACAGTAAAGCTAAAGAAGAAGAGGGTTGATGGACACAAGTCTGTGGTC

TCAATTCAATGAGACTGTCATCCTCAATTGTTACCCAAGATGCAACAAAACGATAGTGCGCTGCCCACTGACA
ACCAAGTTACTTCAAGGACCTATGGTCATCAAGACCGATGAAGGTCCAGGCCGTCTTGCCAAGGAAGCCGTCA
GCTGGGAATTTTGAGAGAGGATACACGACCGAGGGCTCGTCATCACGCTGGGCCTTCCAAATGGTACCTCTGC
CATCCAGGAGATGGACCAAGGATACACAGACTTCAAATAAGGGTGCAAGAAGAGCACCCTACGTGTAGTATCC
ATCAAGTTGGCAGCCTGCAATGCTGCACAAAATCGACCTCAAGATGAAGTTGATGAATCAGTGGATTCTGACA
ATGAGAATGCCAACCATGGGAAGAGTGTATGCAATGTGACTCTTGGTAACAAAGACCTTGCAAACATCATCAA
TGGATTTCCAGGTGATTCAATTGATCTGCACCCATTTGACAAGGTCTTTACGCAAGACAATATCAAGTGTTGG
TGGGAGAATGTTGGCTTTCTCCCAATGACATGGAATACCGCCCAGGACCCAAAGACTCGCTGGGAGCTAGGCA
AAGGTGGTACCCCAGAAGAAGGAATAGAAAGGCTCAGTATGCTTGTCAACCCTTACAAGGTGGGAGCAAAGAG
ACTAGAAGAGATGGGGTTCAACAAGAACATCCTCTCTCTACAACCACCAGTAGTATCAACACCAAAAATACCA
GAAGGAGAGGAGGCGCAAATTCAAGCCCTCGTGGATGGCAAGGCAATCAACAAGGCAGGGGCTGTGTTTAAGC
TTGGGCTTCAAGTTGCCAACA
>TR45|c0_g1_i7 len=556 path=[5595:0-144 5480:145-165 5604:166-250
@5599@!:251-555] [5595, 5480, 5604, 5599]
GGTTTGGTTCGAGGTCTCTCTCTTTCATGACGACAACGCTTGTTACTTTTAAAAACAGATGAAACGCAGCACA
AGTTCTTGACTGCATGCAACAAGGGTGGTTCAACAACCATCTCGTATGCGAATGTTTCCTTCCCACGGTCAGT
TCCTGGAGTCTATGGACTCTATGAAACAACTTGTTATGCTCCATATTTTGCTGTTTGTTGCAAAGGGTCAATA
GACACAAGCCTGTGGTCTCAATTCAATGAGACTGTCATCCTCAATTGTTACCCAAGATGCAACAAAACGATAG
TGCGCTGCCCACTGACAACCAAGTTACTTCAAGGACCTATGGTCATCAAGACCGATGAAGGTCCAGGCCGTCT
TGCCAAGGAAGCCGTCAGCTGGGAATTTTGAGAGAGGATACACGACCGAGGGCTCGTCATCACGCTGGGCCTT
CCAAATGGTACCTCTGCCATCCAGGAGATGGACCAAGGATACACAGACTTCAAATAAGGGTGCAAGAAGAGCA
CCCTACGTGTAGTATCCATCAAGTTGGCAGCCTGCAATGCTGCAC
>TR45|c0_g1_i8 len=3547 path=[5501:0-470 5502:471-485 5598:486-651
5540:652-664 5606:665-679 @5603@!:680-1099 5588:1100-1744 5609:1745-1747
5611:1748-1758 5507:1759-1785 5595:1786-1907 5586:1908-2019 5552:2020-
2021 5573:2022-2084 5604:2085-2169 @5599@!:2170-2474 5581:2475-2704
5563:2705-2707 5567:2708-2808 5548:2809-2832 5549:2833-2854 5566:2855-
2990 5537:2991-3014 5591:3015-3184 5459:3185-3208 5579:3209-3285
5544:3286-3546] [-1, 5501, 5502, 5598, 5540, 5606, 5603, 5588, 5609,
5611, 5507, 5595, 5586, 5552, 5573, 5604, 5599, 5581, 5563, 5567, 5548,
5549, 5566, 5537, 5591, 5459, 5579, 5544, -2]
CACGCGGTCAAAGACAGACCAAAAGATTTTGTTGACGTCAACCAGAAGCCCTTAAAGGGAACATGGTGCATGT
GACAACCTCTGAGTGTCACCATGGTGCACCATGATCACTTCTGACCGGCACTATGACCGTAGGGACACCCCCC
CATGATAACTACAAGCTGCCATCCTCCAAAAACGCGTAATGAACACGCTGTTATCCTCATTCTGTTGATGTTT
CGTCCAAAGAAATGGCGTCCTCAACAAGCCAGCAATCGTCAAATCCAAGACTGCCGCTTGCCCAAGCCATCAT
CATCAAACCACTTCCGTCATCACCCTCCTCTTCCGATACCTTCCATCACCGTCCTCTCAAGTAAACAATTCAG
AAAACCAATCCTCTGAATGGATCAGAATCTTGTTCTGGCTCACCATAGCTGTCAGAACGGGTTTATATATCCG
GCTGATGACGGATGATTTGATAATGAAAAAAAACATCATGCGCTCCCACACACAAAAATAACCGGAAGAAGAA
GGCTAGGCAGCAGAAGAAGAAGACAAATAGGGTGCTGAGGATGGCTGGGAGTAAGGGGGGGGCGTTTAAAGAA
AGAGGACGAGGTGGTGTTGAATTTGGAGGATGAATCTGATCGTACATTTTATCTCGTGAAAAAAAAAACACAA
TTTTTTACTTACCGTACACTTTCACAAATCCGTTTTCGTTTCCATGTCACCATGTACCTCACCTCTCTCCTCC
TCCAAATTGTCCCTCCTCTTCCCAAACTGTAGGTATACAACCATCGAGTGCCTATCCCCTGGGCACTCCAGTT
CACCAATGCCAGTATCACCTACCAATTCCTCTCCAAAAGGGTAAGAAAACAGCAACAGAAGGACTCTGGGAAT
ACAAAGGCTGTGACGATGGCCCTTGTCCTTGGGAGCATGGGTGCCTCCCAGTCTCCTACTTCCAAGTCAACCA
CTCCTCTTCCTCAAAGGTCTCTCACTCCTCCAAGGACATCCCTCACTCCTTGGAGGACATCCATCACTCCTCC
ATGAACAGCTGCACAGAACTGCATCAATGAGACATTTGTTGCCATGGCTGCAGCCATTGAGACTCTCCCCAAG
TGGAACAACAACAAACGTGTGGGGACCGCCATGACCAAGAAGAAGAGGAACAGGGGTGACCAGTCCTCCCGTG
AGACATTTGCAAACAAAGCAGTGTGTGCCCTCAGTGCTGCCTAATATTCAAAGGCCCTCAAGGACGCAACCAT
CTACTATGCTTGGATCAAGAGTGGCCCAAATGCAGGCAAGATGGGATATGGGCCAGTGTCCATCGTTAACAAG
TGGAACAGAGAGGTTCTGAACCTCTCCAGCAGTTGGAAGCTGTCAAAATCTGCGCTCTCTGCCGCAGTGAAGG
CAGGGCGAGTTGGTTCCTCCCCGCCAAAAAGAGGTAGGCCACAAATGATACCCCTTGTGCTCACTGAGACTTT
AGCTGCCCATGCAACAATGATGCAGTGTCCGGAGAGGGTGAGGCAAATAGACAGAAGATGGTGCTCGTGGCAA
AGGTGCTGATGATGGGCATGGAGTGGGAGAAGAAGATTTCCCCAGACTACCTCTGGCGCAAAGTACGGGAGGA
TCATCCAAGGATACTCAACCCCGTGAAACAGAAACACAACAAAGACCAACGGGTAGATTGGCTGACATATTAA
AAATGTCAAAGATTGGACAGGCCAAGCGAAGGCGTATCTGGTAGAGTTGGGCATGGTCAAAAATGAACCAGGG
TAGATAAATAGGGTTTGGTTCGAGGTCTCTCTCTTTCATGACGACAACGCTTGTTACTTTTAAAAACAGATGA
AACGCAGCACAAGTTCTTGACTGCATGCAACAAGGGTGGTTCAACAACCATCTCGTATGCGAATGTTTCCTTC
CCACGGTCAGGTGAGCATATTGTTGAAAGTTTGAGGCACATCACAGGGTGTTACACCATCAATCTTGCTGGGG
AGGTGCTCCCACCCCTTAATATATTTGACAGTAAAGCTAAAGAAGAAGATATGTTTTGTGTCAAACCGAATAT
TGACATTGGTCTACCTGAAGTTCCTGGAGTCTATGGACTCTATGAAACAACTTGTTATGCTCCATATTTTGCT

GTTTGTTGCAAAGGGTCAATAGACACAAGCCTGTGGTCTCAATTCAATGAGACTGTCATCCTCAATTGTTACC
CAAGATGCAACAAAACGATAGTGCGCTGCCCACTGACAACCAAGTTACTTCAAGGACCTATGGTCATCAAGAC
CGATGAAGGTCCAGGCCGTCTTGCCAAGGAAGCCGTCAGCTGGGAATTTTGAGAGAGGATACACGACCGAGGG
CTCGTCATCACGCTGGGCCTTCCAAATGGTACCTCTGCCATCCAGGAGATGGACCAAGGATACACAGACTTCA
AATAAGGGTGCAAGAAGAGCACCCTACGTGTAGTATCCATCAAGTTGGCAGCCTGCAATGCTGCACGACAATC
TCGAGATGAAGTCGTTGTATCAGTTGATGGTGCCGATGATAACGCCAACCATGTCAGCACCCGGTCAAGGAAG
AGTGTATGTAATGTGACTCTTGGCAATGAAGACCTTGCCAACATCATCAATGGGTTTCCAGGTGATCCAATTG
ATCTGCGTCCGTTTGACAAGGTCTTTACTCAAGACAACATCAAGAGTTGGTGGAAGAATGTTGGCTTTCTCCC
AATGACACGGAATGCCGTCAACGATCCAAAGACTCGGTGGGAGCTAGGCAAAGGCAGTGCCCCAGAAGAAGGT
ACAGCAAGGCTTGTTATACTCATAGATGACTACAATGTGGGAGCAAAGAAAGTAGAACAGATGGGGTTCAACA
AGAACATCCTCTCTCTACAACCACCAGTAGTATCAACACCAAAAATACCAGAAGGAGAGGAGGCGCAAATTCA
AGCCCTCGTGGATGGCAAGGCAATCAACAAGGCAGGGGCTGTGTTTAAGCTTGGGCTTCAAGTTGCCAACAAC
AGGGTCATGCTTGAGACCAACAAGAGGATGGTCAAGTTGGAAGAAGAGGCCAAGGCAAACAAGGAGCGCAAGA
AGAAGAAGGAGTCGGAAGGGAAGGACCAGAAGGCTATTGAAGCATTCAACGCTTGGATCACCAAGGGTAAGTG
CATTGATACGAAAGGGCATCCAGTCCTTAATAAGGATGACTCCCTCGCTATCATTAAGTTATTGATGCCAAAG
ATAGCTCCTACAGAAAAGGTTGAGGATTACAAGACTATGAAGAAATGTAATGAGTGGCTGGGTAACTTGGCAG
GTGGATCTACATGGGAGGAGGAGATAAAAGCGATCAAAACCCAGTTTACCGAATCGATTCTAGCGCGTAGTGT
GTAACCTTAATCGTCACCAAACTCATGCATTCTACTCTCCTACATGTTAGTCAAGTGTGACCATCAATATTTG
CCATGTTGGATGGTTACTGGCACCGTACGGGACCATTGAAGTTGACGTCTTTGATTTTTGAAGGCTTAATTGG
CCATATTTTAGGTTAATTCCTACGTACATATGTACCATAGTTC
>TR45|c0_g1_i9 len=3474 path=[5501:0-470 5502:471-485 5598:486-651
5540:652-664 5606:665-679 @5603@!:680-1099 5589:1100-1154 5576:1155-1178
5602:1179-1423 5493:1424-1447 5596:1448-1745 5610:1746-1748 5612:1749-
1759 5456:1760-1782 @5584@!:1783-1958 5446:1959-1960 5600:1961-2035
5582:2036-2059 5607:2060-2089 5605:2090-2108 @5599@!:2109-2413 5568:2414-
2631 5451:2632-2634 5580:2635-2781 5566:2782-2917 5537:2918-2941
5591:2942-3111 5459:3112-3135 5579:3136-3212 5544:3213-3473] [-1, 5501,
5502, 5598, 5540, 5606, 5603, 5589, 5576, 5602, 5493, 5596, 5610, 5612,
5456, 5584, 5446, 5600, 5582, 5607, 5605, 5599, 5568, 5451, 5580, 5566,
5537, 5591, 5459, 5579, 5544, -2]
CACGCGGTCAAAGACAGACCAAAAGATTTTGTTGACGTCAACCAGAAGCCCTTAAAGGGAACATGGTGCATGT
GACAACCTCTGAGTGTCACCATGGTGCACCATGATCACTTCTGACCGGCACTATGACCGTAGGGACACCCCCC
CATGATAACTACAAGCTGCCATCCTCCAAAAACGCGTAATGAACACGCTGTTATCCTCATTCTGTTGATGTTT
CGTCCAAAGAAATGGCGTCCTCAACAAGCCAGCAATCGTCAAATCCAAGACTGCCGCTTGCCCAAGCCATCAT
CATCAAACCACTTCCGTCATCACCCTCCTCTTCCGATACCTTCCATCACCGTCCTCTCAAGTAAACAATTCAG
AAAACCAATCCTCTGAATGGATCAGAATCTTGTTCTGGCTCACCATAGCTGTCAGAACGGGTTTATATATCCG
GCTGATGACGGATGATTTGATAATGAAAAAAAACATCATGCGCTCCCACACACAAAAATAACCGGAAGAAGAA
GGCTAGGCAGCAGAAGAAGAAGACAAATAGGGTGCTGAGGATGGCTGGGAGTAAGGGGGGGGCGTTTAAAGAA
AGAGGACGAGGTGGTGTTGAATTTGGAGGATGAATCTGATCGTACATTTTATCTCGTGAAAAAAAAAACACAA
TTTTTTACTTACCGTACACTTTCACAAATCCGTTTTCGTTTCCATGTCACCATGTACCTCACCTCTCTCCTCC
TCCAAATTGTCCCTCCTCTTCCCAAACTGTAGGTATACAACCATCGAGTGCCTATCCCCTGGGCACTCCAGTT
CACCAATGCCAGTATCACCTACCAATTCCTCTCCAAAAGGGTAAGAAAACAGCAACAGAAGGACTCTGGGAAT
ACAAAGGCTGTGACGATGGCCCTTGTCCTTGGGAGCATGGGTGCCTCCCAGTCTCCTACTTCCAAGTCAACCA
CTCCTCTTCCTCAAAGGTCTCTCACTCCTCCAAGGACATCCCTCACTCCTTGGAGGACATCCATCACTCCTCC
ATGAACAGCTGCACAGAACTGCATCAATGAGACATTTGTTGCCATGGCTGCAGCCATTGAGACTCTCCCCAAG
TGGAATGATTGTAAGCTTGTGGGGGTAGCTATGAGAAAGAAGAAGAGGAGCAGTGGTGACATGTCCACCCGGG
ATACATTCGCCAACAAATCAGTGGTTGCCCTCAGTGCTGCCAGATACTCAAAGGCCCTCAAGGATGCCACCAT
ATATTACTCAAGGATCAAGAGTGGTCCGAATGCGGGAAAGAATGGATATGGGCCAGTGTCCATTGTTGAGAAG
TGGAACAGATAGGTTCTCGTCCTACTAAGCGACCAGAAGCTGTCAAAATCCGCGCTCTCTGCTGCAGTGCAGG
CAGGCCAAGTTGGTCAGTCCCCACCTAAAAGAGGAAGTCCTAGAACGATACCGCTTGTGCTCACCAAGACTTT
GGCTGCCCATGCGACGATGATGCAGGTCTCTGGAGAGGGTGAGGCAAATCACCAGAAGATGGTGATGGCGGGG
AAGGCGCTAATGATGGGCACAGAGTGGGAAAATAAGATTTGTCCCGACTACCTCTGGCACAAAGTCCGGGAGG
ATCATCCGGCATTGCTCAACCCTGTGAAGCAGAAGCACAATGAAGACCAGCGGGTGGATTTGGCTGACATATA
ATAATGTCAATGATTGGACAGACCGCACAAAAGCGTATCTGGTTGAGTTGGGCATGGTCAAAAATGAACCGGG
ATTCATAAACAAAACTCAGCACAAGTTCTCAACGACGAGGAACAAGGGAGGGTCAACGACGATTTCCTATGTG
AATGTTTTATTCCCACGGTCGGGTGAGCGCGTTGTCGAGAGTTCGAGGCACACCACAGGGTGTTACACCGTGA
ATCTTGCCGGAGAGGTACTCCCATCCCTTTATATATTCGACAGTAAAGCTAAAGAAGAAGATAAGTTTTCTGT
TGACCCTAGTATTGGTATTGGTTTGCCTGAAGTTGAAGGAGTCTTTGGACTGTACGAGAAAACTTATTTTGCT
CTGTATTTTGCTGTTTGCCGCAAAGGGTTGATGGACACAAGTCTGTGGTCTCAATTCAATGAGACTGTCATCC
TCAATTGTTACCCAAGATGCAACAAAACGATAGTGCGCTGCCCACTGACAACCAAGTTACTTCAAGGACCTAT

GGTCATCAAGACCGATGAAGGTCCAGGCCGTCTTGCCAAGGAAGCCGTCAGCTGGGAATTTTGAGAGAGGATA
CACGACCGAGGGCTCGTCATCACGCTGGGCCTTCCAAATGGTACCTCTGCCATCCAGGAGATGGACCAAGGAT
ACACAGACTTCAAATAAGGGTGCAAGAAGAGCACCCTACGTGTAGTATCCATCAAGTTGGCAGCCTGCAATGC
TGCACAAAATCGACCTCAAGATGAAGTTGATGAATCAGTGGATTCTGACAATGAGAATGCCAACCATGGGAAG
AGTGTATGCAATGTGACTCTTGGTAACAAAGACCTTGCAAACATCATCAATGGATTTCCAGGTGATTCAATTG
ATCTGCACCCATTTGACAAGGTCTTTACGCAAGACAATATCAAGTGTTGGTGGGAGAATGTTGGCTTTCTCCC
AATGACATGGAATACCGCCCAGGACCCAAAGACTCGCTGGGAGCTAGGCAAAGGTGGTACCCCAGAAGAAGGA
ATAGAAAGGCTCAGTATGCTTGTCAACCCTTACAAGGTGGGAGCAAAGAGACTAGAAGAGATGGGGTTCAACA
AGAACATCCTCTCTCTACAACCACCAGTAGTATCAACACCAAAAATACCAGAAGGAGAGGAGGCGCAAATTCA
AGCCCTCGTGGATGGCAAGGCAATCAACAAGGCAGGGGCTGTGTTTAAGCTTGGGCTTCAAGTTGCCAACAAC
AGGGTCATGCTTGAGACCAACAAGAGGATGGTCAAGTTGGAAGAAGAGGCCAAGGCAAACAAGGAGCGCAAGA
AGAAGAAGGAGTCGGAAGGGAAGGACCAGAAGGCTATTGAAGCATTCAACGCTTGGATCACCAAGGGTAAGTG
CATTGATACGAAAGGGCATCCAGTCCTTAATAAGGATGACTCCCTCGCTATCATTAAGTTATTGATGCCAAAG
ATAGCTCCTACAGAAAAGGTTGAGGATTACAAGACTATGAAGAAATGTAATGAGTGGCTGGGTAACTTGGCAG
GTGGATCTACATGGGAGGAGGAGATAAAAGCGATCAAAACCCAGTTTACCGAATCGATTCTAGCGCGTAGTGT
GTAACCTTAATCGTCACCAAACTCATGCATTCTACTCTCCTACATGTTAGTCAAGTGTGACCATCAATATTTG
CCATGTTGGATGGTTACTGGCACCGTACGGGACCATTGAAGTTGACGTCTTTGATTTTTGAAGGCTTAATTGG
CCATATTTTAGGTTAATTCCTACGTACATATGTACCATAGTTC
>TR45|c0_g1_i10 len=2953 path=[5501:0-470 5502:471-485 5598:486-651
5540:652-664 5606:665-679 @5603@!:680-1099 5589:1100-1154 5576:1155-1178
5602:1179-1423 5493:1424-1447 5596:1448-1745 5610:1746-1748 5612:1749-
1759 5613:1760-1781 5499:1782-1798 5594:1799-1914 @5584@!:1915-2090
5476:2091-2112 5605:2113-2131 @5599@!:2132-2436 5581:2437-2666 5563:2667-
2669 5567:2670-2770 5548:2771-2794 5549:2795-2816 5566:2817-2952] [-1,
5501, 5502, 5598, 5540, 5606, 5603, 5589, 5576, 5602, 5493, 5596, 5610,
5612, 5613, 5499, 5594, 5584, 5476, 5605, 5599, 5581, 5563, 5567, 5548,
5549, 5566]
CACGCGGTCAAAGACAGACCAAAAGATTTTGTTGACGTCAACCAGAAGCCCTTAAAGGGAACATGGTGCATGT
GACAACCTCTGAGTGTCACCATGGTGCACCATGATCACTTCTGACCGGCACTATGACCGTAGGGACACCCCCC
CATGATAACTACAAGCTGCCATCCTCCAAAAACGCGTAATGAACACGCTGTTATCCTCATTCTGTTGATGTTT
CGTCCAAAGAAATGGCGTCCTCAACAAGCCAGCAATCGTCAAATCCAAGACTGCCGCTTGCCCAAGCCATCAT
CATCAAACCACTTCCGTCATCACCCTCCTCTTCCGATACCTTCCATCACCGTCCTCTCAAGTAAACAATTCAG
AAAACCAATCCTCTGAATGGATCAGAATCTTGTTCTGGCTCACCATAGCTGTCAGAACGGGTTTATATATCCG
GCTGATGACGGATGATTTGATAATGAAAAAAAACATCATGCGCTCCCACACACAAAAATAACCGGAAGAAGAA
GGCTAGGCAGCAGAAGAAGAAGACAAATAGGGTGCTGAGGATGGCTGGGAGTAAGGGGGGGGCGTTTAAAGAA
AGAGGACGAGGTGGTGTTGAATTTGGAGGATGAATCTGATCGTACATTTTATCTCGTGAAAAAAAAAACACAA
TTTTTTACTTACCGTACACTTTCACAAATCCGTTTTCGTTTCCATGTCACCATGTACCTCACCTCTCTCCTCC
TCCAAATTGTCCCTCCTCTTCCCAAACTGTAGGTATACAACCATCGAGTGCCTATCCCCTGGGCACTCCAGTT
CACCAATGCCAGTATCACCTACCAATTCCTCTCCAAAAGGGTAAGAAAACAGCAACAGAAGGACTCTGGGAAT
ACAAAGGCTGTGACGATGGCCCTTGTCCTTGGGAGCATGGGTGCCTCCCAGTCTCCTACTTCCAAGTCAACCA
CTCCTCTTCCTCAAAGGTCTCTCACTCCTCCAAGGACATCCCTCACTCCTTGGAGGACATCCATCACTCCTCC
ATGAACAGCTGCACAGAACTGCATCAATGAGACATTTGTTGCCATGGCTGCAGCCATTGAGACTCTCCCCAAG
TGGAATGATTGTAAGCTTGTGGGGGTAGCTATGAGAAAGAAGAAGAGGAGCAGTGGTGACATGTCCACCCGGG
ATACATTCGCCAACAAATCAGTGGTTGCCCTCAGTGCTGCCAGATACTCAAAGGCCCTCAAGGATGCCACCAT
ATATTACTCAAGGATCAAGAGTGGTCCGAATGCGGGAAAGAATGGATATGGGCCAGTGTCCATTGTTGAGAAG
TGGAACAGATAGGTTCTCGTCCTACTAAGCGACCAGAAGCTGTCAAAATCCGCGCTCTCTGCTGCAGTGCAGG
CAGGCCAAGTTGGTCAGTCCCCACCTAAAAGAGGAAGTCCTAGAACGATACCGCTTGTGCTCACCAAGACTTT
GGCTGCCCATGCGACGATGATGCAGGTCTCTGGAGAGGGTGAGGCAAATCACCAGAAGATGGTGATGGCGGGG
AAGGCGCTAATGATGGGCACAGAGTGGGAAAATAAGATTTGTCCCGACTACCTCTGGCACAAAGTCCGGGAGG
ATCATCCGGCATTGCTCAACCCTGTGAAGCAGAAGCACAATGAAGACCAGCGGGTGGATTTGGCTGACATATA
ATAATGTCAATGATTGGACAGACCGCACAAAAGCGTATCTGGTTGAGTTGGGCATGGTCAAAAATGAACCGGG
ATTCATAAGTGAGTTGCTTCTTTGTTTTTTACGTTTCCACTTTACAATTCACAGTTTCTAGCACACGTTTTGT
CCAGATGGGGTTTGGTCTGAGGTCTCTCTCTTTCATGACAATGACGCTCGTTTCTTTCTAAACACAGACAAAA
CTCAGCACAAGTTCTCAACGACGAGGAACAAGGGAGGGTCAACGACGATTTCCTATGTGAATGTTTTATTCCC
ACGGTCGGGTGAGCGCGTTGTCGAGAGTTCGAGGCACACCACAGGGTGTTACACCGTGAATCTTGCCGGAGAG
GTACTCCCATCCCTTTATATATTCGACAGTAAAGCTAAAGAAGAAGAGGGTTGATGGACACAAGTCTGTGGTC
TCAATTCAATGAGACTGTCATCCTCAATTGTTACCCAAGATGCAACAAAACGATAGTGCGCTGCCCACTGACA
ACCAAGTTACTTCAAGGACCTATGGTCATCAAGACCGATGAAGGTCCAGGCCGTCTTGCCAAGGAAGCCGTCA
GCTGGGAATTTTGAGAGAGGATACACGACCGAGGGCTCGTCATCACGCTGGGCCTTCCAAATGGTACCTCTGC
CATCCAGGAGATGGACCAAGGATACACAGACTTCAAATAAGGGTGCAAGAAGAGCACCCTACGTGTAGTATCC

ATCAAGTTGGCAGCCTGCAATGCTGCACGACAATCTCGAGATGAAGTCGTTGTATCAGTTGATGGTGCCGATG
ATAACGCCAACCATGTCAGCACCCGGTCAAGGAAGAGTGTATGTAATGTGACTCTTGGCAATGAAGACCTTGC
CAACATCATCAATGGGTTTCCAGGTGATCCAATTGATCTGCGTCCGTTTGACAAGGTCTTTACTCAAGACAAC
ATCAAGAGTTGGTGGAAGAATGTTGGCTTTCTCCCAATGACACGGAATGCCGTCAACGATCCAAAGACTCGGT
GGGAGCTAGGCAAAGGCAGTGCCCCAGAAGAAGGTACAGCAAGGCTTGTTATACTCATAGATGACTACAATGT
GGGAGCAAAGAAAGTAGAACAGATGGGGTTCAACAAGAACATCCTCTCTCTACAACCACCAGTAGTATCAACA
CCAAAAATACCAGAAGGAGAGGAGGCGCAAATTCAAGCCCTCGTGGATGGCAAGGCAATCAACAAGGCAGGGG
CTGTGTTTAAGCTTGGGCTTCAAGTTGCCAACA
>TR45|c0_g1_i11 len=3618 path=[5501:0-470 5502:471-485 5598:486-651 5540:652-664 5606:665-679 @5603@!:680-1099 5589:1100-1154 5576:1155-1178 5602:1179-1423 5493:1424-1447 5596:1448-1745 5610:1746-1748 5612:1749-1759 5613:1760-1781 5499:1782-1798 5594:1799-1914 @5584@!:1915-2090 5446:2091-2092 5600:2093-2167 5582:2168-2191 5607:2192-2221 5605:2222-2240 @5599@!:2241-2545 5581:2546-2775 5563:2776-2778 5567:2779-2879 5548:2880-2903 5549:2904-2925 5566:2926-3061 5537:3062-3085 5591:3086-3255 5459:3256-3279 5579:3280-3356 5544:3357-3617] [-1, 5501, 5502, 5598, 5540, 5606, 5603, 5589, 5576, 5602, 5493, 5596, 5610, 5612, 5613, 5499, 5594, 5584, 5446, 5600, 5582, 5607, 5605, 5599, 5581, 5563, 5567, 5548, 5549, 5566, 5537, 5591, 5459, 5579, 5544, -2]
CACGCGGTCAAAGACAGACCAAAAGATTTTGTTGACGTCAACCAGAAGCCCTTAAAGGGAACATGGTGCATGT
GACAACCTCTGAGTGTCACCATGGTGCACCATGATCACTTCTGACCGGCACTATGACCGTAGGGACACCCCCC
CATGATAACTACAAGCTGCCATCCTCCAAAAACGCGTAATGAACACGCTGTTATCCTCATTCTGTTGATGTTT
CGTCCAAAGAAATGGCGTCCTCAACAAGCCAGCAATCGTCAAATCCAAGACTGCCGCTTGCCCAAGCCATCAT
CATCAAACCACTTCCGTCATCACCCTCCTCTTCCGATACCTTCCATCACCGTCCTCTCAAGTAAACAATTCAG
AAAACCAATCCTCTGAATGGATCAGAATCTTGTTCTGGCTCACCATAGCTGTCAGAACGGGTTTATATATCCG
GCTGATGACGGATGATTTGATAATGAAAAAAAACATCATGCGCTCCCACACACAAAAATAACCGGAAGAAGAA
GGCTAGGCAGCAGAAGAAGAAGACAAATAGGGTGCTGAGGATGGCTGGGAGTAAGGGGGGGGCGTTTAAAGAA
AGAGGACGAGGTGGTGTTGAATTTGGAGGATGAATCTGATCGTACATTTTATCTCGTGAAAAAAAAAACACAA
TTTTTTACTTACCGTACACTTTCACAAATCCGTTTTCGTTTCCATGTCACCATGTACCTCACCTCTCTCCTCC
TCCAAATTGTCCCTCCTCTTCCCAAACTGTAGGTATACAACCATCGAGTGCCTATCCCCTGGGCACTCCAGTT
CACCAATGCCAGTATCACCTACCAATTCCTCTCCAAAAGGGTAAGAAAACAGCAACAGAAGGACTCTGGGAAT
ACAAAGGCTGTGACGATGGCCCTTGTCCTTGGGAGCATGGGTGCCTCCCAGTCTCCTACTTCCAAGTCAACCA
CTCCTCTTCCTCAAAGGTCTCTCACTCCTCCAAGGACATCCCTCACTCCTTGGAGGACATCCATCACTCCTCC
ATGAACAGCTGCACAGAACTGCATCAATGAGACATTTGTTGCCATGGCTGCAGCCATTGAGACTCTCCCCAAG
TGGAATGATTGTAAGCTTGTGGGGGTAGCTATGAGAAAGAAGAAGAGGAGCAGTGGTGACATGTCCACCCGGG
ATACATTCGCCAACAAATCAGTGGTTGCCCTCAGTGCTGCCAGATACTCAAAGGCCCTCAAGGATGCCACCAT
ATATTACTCAAGGATCAAGAGTGGTCCGAATGCGGGAAAGAATGGATATGGGCCAGTGTCCATTGTTGAGAAG
TGGAACAGATAGGTTCTCGTCCTACTAAGCGACCAGAAGCTGTCAAAATCCGCGCTCTCTGCTGCAGTGCAGG
CAGGCCAAGTTGGTCAGTCCCCACCTAAAAGAGGAAGTCCTAGAACGATACCGCTTGTGCTCACCAAGACTTT
GGCTGCCCATGCGACGATGATGCAGGTCTCTGGAGAGGGTGAGGCAAATCACCAGAAGATGGTGATGGCGGGG
AAGGCGCTAATGATGGGCACAGAGTGGGAAAATAAGATTTGTCCCGACTACCTCTGGCACAAAGTCCGGGAGG
ATCATCCGGCATTGCTCAACCCTGTGAAGCAGAAGCACAATGAAGACCAGCGGGTGGATTTGGCTGACATATA
ATAATGTCAATGATTGGACAGACCGCACAAAAGCGTATCTGGTTGAGTTGGGCATGGTCAAAAATGAACCGGG
ATTCATAAGTGAGTTGCTTCTTTGTTTTTTACGTTTCCACTTTACAATTCACAGTTTCTAGCACACGTTTTGT
CCAGATGGGGTTTGGTCTGAGGTCTCTCTCTTTCATGACAATGACGCTCGTTTCTTTCTAAACACAGACAAAA
CTCAGCACAAGTTCTCAACGACGAGGAACAAGGGAGGGTCAACGACGATTTCCTATGTGAATGTTTTATTCCC
ACGGTCGGGTGAGCGCGTTGTCGAGAGTTCGAGGCACACCACAGGGTGTTACACCGTGAATCTTGCCGGAGAG
GTACTCCCATCCCTTTATATATTCGACAGTAAAGCTAAAGAAGAAGATAAGTTTTCTGTTGACCCTAGTATTG
GTATTGGTTTGCCTGAAGTTGAAGGAGTCTTTGGACTGTACGAGAAAACTTATTTTGCTCTGTATTTTGCTGT
TTGCCGCAAAGGGTTGATGGACACAAGTCTGTGGTCTCAATTCAATGAGACTGTCATCCTCAATTGTTACCCA
AGATGCAACAAAACGATAGTGCGCTGCCCACTGACAACCAAGTTACTTCAAGGACCTATGGTCATCAAGACCG
ATGAAGGTCCAGGCCGTCTTGCCAAGGAAGCCGTCAGCTGGGAATTTTGAGAGAGGATACACGACCGAGGGCT
CGTCATCACGCTGGGCCTTCCAAATGGTACCTCTGCCATCCAGGAGATGGACCAAGGATACACAGACTTCAAA
TAAGGGTGCAAGAAGAGCACCCTACGTGTAGTATCCATCAAGTTGGCAGCCTGCAATGCTGCACGACAATCTC
GAGATGAAGTCGTTGTATCAGTTGATGGTGCCGATGATAACGCCAACCATGTCAGCACCCGGTCAAGGAAGAG
TGTATGTAATGTGACTCTTGGCAATGAAGACCTTGCCAACATCATCAATGGGTTTCCAGGTGATCCAATTGAT
CTGCGTCCGTTTGACAAGGTCTTTACTCAAGACAACATCAAGAGTTGGTGGAAGAATGTTGGCTTTCTCCCAA
TGACACGGAATGCCGTCAACGATCCAAAGACTCGGTGGGAGCTAGGCAAAGGCAGTGCCCCAGAAGAAGGTAC
AGCAAGGCTTGTTATACTCATAGATGACTACAATGTGGGAGCAAAGAAAGTAGAACAGATGGGGTTCAACAAG
AACATCCTCTCTCTACAACCACCAGTAGTATCAACACCAAAAATACCAGAAGGAGAGGAGGCGCAAATTCAAG

CCCTCGTGGATGGCAAGGCAATCAACAAGGCAGGGGCTGTGTTTAAGCTTGGGCTTCAAGTTGCCAACAACAG
GGTCATGCTTGAGACCAACAAGAGGATGGTCAAGTTGGAAGAAGAGGCCAAGGCAAACAAGGAGCGCAAGAAG
AAGAAGGAGTCGGAAGGGAAGGACCAGAAGGCTATTGAAGCATTCAACGCTTGGATCACCAAGGGTAAGTGCA
TTGATACGAAAGGGCATCCAGTCCTTAATAAGGATGACTCCCTCGCTATCATTAAGTTATTGATGCCAAAGAT
AGCTCCTACAGAAAAGGTTGAGGATTACAAGACTATGAAGAAATGTAATGAGTGGCTGGGTAACTTGGCAGGT
GGATCTACATGGGAGGAGGAGATAAAAGCGATCAAAACCCAGTTTACCGAATCGATTCTAGCGCGTAGTGTGT
AACCTTAATCGTCACCAAACTCATGCATTCTACTCTCCTACATGTTAGTCAAGTGTGACCATCAATATTTGCC
ATGTTGGATGGTTACTGGCACCGTACGGGACCATTGAAGTTGACGTCTTTGATTTTTGAAGGCTTAATTGGCC
ATATTTTAGGTTAATTCCTACGTACATATGTACCATAGTTC
>TR47|c0_g1_i1 len=2060 path=[2679:0-164 2680:165-188 2723:189-250
2643:251-278 @2734@!:279-487 2592:488-504 @2732@!:505-614 2624:615-638
2625:639-647 2731:648-677 2733:678-707 2721:708-746 2728:747-759
2712:760-788 2720:789-875 2736:876-899 2738:900-914 2698:915-946
2737:947-970 2708:971-1097 2620:1098-1121 2696:1122-1153 2726:1154-1177
2718:1178-1237 2694:1238-1283 2725:1284-1284 2717:1285-1308 2706:1309-
1424 2662:1425-1456 @2724@!:1457-1676 2603:1677-1709 @2705@!:1710-1984
2683:1985-2059] [-1, 2679, 2680, 2723, 2643, 2734, 2592, 2732, 2624,
2625, 2731, 2733, 2721, 2728, 2712, 2720, 2736, 2738, 2698, 2737, 2708,
2620, 2696, 2726, 2718, 2694, 2725, 2717, 2706, 2662, 2724, 2603, 2705,
2683, -2]
CTCGGATCTTGATACCAATCAAGCCGAATTGATTTCGTAGAAGAGTTCACAAACACCAAGACCCCCCAGGACA
ATGGCGCTCTACCAATCATCGAAGTCGGCCACATCGCTCATCAGGCGATGTTTCTCCTCCACCCTCACCCAAC
GCTGTGCCCAAACACCAGCAAGCAGCAGCGTCTCCAGCATCCGATTTCCAACAACGCCCACCTTCATCAAAAA
CAATAACTACACATCATGTGCCCAGCACAGCAGCAATCACAGCATCATCATCCGCTCTTTCTCCGCAATGCCC
CCTCCCCCCACAGCCACCCCTCCCAACGCAAACGCAATGTACACACCCACCTCCCTCTCCCCCGAAGCAGGCC
TAGGCGCACAAGACGCCATGAAACTATTCATCGAACACGGACTCGGGAAACAAAAGCTACAACTAATCGCTGC
CGAAAAGGCCAATGACAATAACTCGCCCCTAGTATCCCGATGGCAAAAAATGATTGCCACCTACCTCGAAACG
CAATGCCACGTTATTTCCCTCTTGGGATACCAACCGAACGAACAGGGGATTGCATTGTACACCCAACAACTGA
CCGAATCGCTACGTCTCGCTTCTCCCGATGTACAAGATCAGCTTCGCGTGGCGGGTCGCGATACATACCGTAT
GGTATTGTCCGCGGCATTCAATTTGCCGTCGTTGATCGAGGAGTCGAAGACTAAGGGGGAATTGAGCGTGGTC
GATGCACGGAACATGATGCACAAGGTGTCTCTGCGCATGCAGGATCCCGAGGTGTTGGAGAAGGTGGCCAAGT
TGTGCAGTGTGTCGGTGGCGATGAATGATAGTCCTGAGGCCAAGCAGATTGATTTGGCGAGGAAGCATACAGT
GGTGCAGAATGTGATGGTGGAAGATGTGTATCTGACAGAGAAGAAGGATAGTGTTAGTTTGGTCCAGGAGCTG
GGATTCGGGGAGGGGGAGGAGGGGTACATACGGATGCAGAGTGCGTTGGCGGAGCATCAGGGTGATCCGTTGA
TTACGCAGTATGTGGGTGCGGCGATGATGAAGTTGTTGACGAGTGCGGGGATTGATATGGAGGCGTTGCAGAA
GGGAGAGGCGTAGGCGCGTGAGGAATTGAGATTCTGGAGAGGGAAGAAAGAAGAGACTACTCTCTGCAGAGTG
TGTCGTTACTTTTACAATCCGCCTTAGAGAAAGAGACTTCATTCATTGCTTTTTAGTGGTTTTGCATTGTTGT
GAAGTGGGTGGCTTGTGCTCTCCTCCTGCTGTTGAATACGCAGTAAGATTGCCCTTTCGAACATGTTTTTATT
ATGTTGTTCATTGTTTTGATGATTTGATAAATAACGGATTAAGATACGGTACAAAACGAAGAAGAATGTCGAG
TCCCCAACAACATTATGTTATGTTGTCCCAATTTACTCATCGGCCCCAGAGAGGGTAAAATAATGCAGCAAGA
GACTTCTTTCACAATTATTGGTTATCTCAAGAAATTTTGTTGCGTCCGCATATTGAACAATTTGGACAAAATG
CAATCACAAGTGGATTTCTTTTACCCACTCTTCACTACTGAATGGTCCCACGCATCCCAAATTTTTGTCTTCG
ACACAATTATAACACAGCAATTTCGACAAAATGTTATCACAAGTGGGTTTCTTTTTCCCAGTCTTTCGTACTG
AATGGTCCCACATATCCCAAACTTTTGTCTTCGACACAATTAAAACACAGCAATAACCACTGTCCAAAATTAT
TGGAATTCCTGACCTAATATTTAATTTAATTTAACCTATTGACGCGACATTGGATCTGTTCATTCGCCACGTA
GCAATATATTTCATCATTCAGAGGGGCGGGGAACACTACCAAACTTTTCTCCACCAGACTAAAAGATTCTACT
CGGTGTAAGAAAGGGAAAGGTGAACATAGGCATCCTCTCCCATAATCCTGTTGTACACCTTCTCCTTCACCGG
CGTGGGGGTGTCAGAAAGACCAAGTTTCTCGAGCTTAATTTTGCATTTGCCCATCTTATCGTCTTTGAGAGTA
TCGTCATCCATGACCT
>TR48|c0_g1_i1 len=517 path=[495:0-516] [-1, 495, -2]
TTCTGCTTCTGATGAACTACAAATGGCGACCGCGGAACCGACGTCAGCGCCGTGGTTGAACTGCTGCGTCGTA
GATGGAGGAGAGGTCGCCGACGGTGGTGGAGTCCTAGAGCGGGTGTTTGCAGAAGGTGTGCTCATGGCCGAAA
AAGGCTTAGTGCTGAAAGTGAGGAACGATTGTGGAAGAAGAAATAAATGGAAAGGAGACCGCGTTTGTGGACG
ACGCTTTTCGAAAGGTCACTAGTAGTTTTTCACTCTTGTTTCTTGTCTACTCTTTACGCCATGGGTTGCGACA
GGTTGCGACTGGATAGAGCTTAAGGTTCTTGAAACTTATCGACAGGTCGCGCCGTCTGCGTGTTTTCTTTTTT
CGTAGTTCCAGCCGTTTTTTATTAAACATTCCAGTCACCAGGTATCATACTCATCTACCAAGTCTTACGACGG
AATCCAGCGAAAGATCTGAAAGAGTCAATAATGCCGATGTCCAGAAAAGCACGGCGTTGGATGGTTGGTCGGG
CGACCT
>TR49|c0_g1_i1 len=296 path=[274:0-295] [-1, 274, -2]

GACATTTTTGAATTCAACATCCCTCGATGAAATCATTTATAGCGTGAATGCTTGAATACTAGATGGAAATGTC
AGCTCCATGTTTTCGATTCATGTCAAAGAGGGCAAGGAAGCAACGAAATAATGGACGAAGATTCCGACGAAGA
TTCTTCGTCCATTTCGTTACAACTCAATTTTTAAATTTCGAAAAATAATTTTTTTTTCAATAAAATAGGCAGA
CCCCGGCAGTAAACAAAGATGTCCGGGAGAATCAGCCGGGGAACTCAAATCGGAAAATATTCAGACTCGGGAG
ATCG
>TR53|c0_g1_i1 len=376 path=[354:0-375] [-1, 354, -2]
GATGTGCAGCATCGATATTCTACGTCTTCAAAGCGGCGTTGATACAGAGTTTTTGCAAAAGGGCAACATCCTT
TTTCCAATTCCTTTTGGAACCATGCTTCGTCGCGCACCCACTGCACTTCTTGTATTACCTCAAGTCTTTGATG
TGCATGCGCTGTTGTATTTGATTTGATAGTGATGTTGCTGATGGCGATGTTGAGTACTGTGATTGTGATTATG
CACATATAAATAATCCGATGACCTTTCACCAGTGCACCGATGGTGTAATGTTGTTGGGTTGGAGTATGTTCGA
CCAGCCCTTGCATGATGGTGATGGGTGCTATCGTCCTCTGTGAATAATAACGTGCCGTTGCGTTTGTTGAGGG
TGGAGAAAGGA
>TR54|c0_g1_i1 len=415 path=[392:0-408 393:409-409 394:410-411 395:412-
412 396:413-414] [-1, 392, 393, 394, 395, 396, -2]
TTCCGATCTCGAACCATCAGCATCGTCGTCGTCTCAGCATTGTAGTGTTTGTGGCGGTGACCGGTGTTCTAGG
GCTCGCAACGCCAACCCGCAATTGCAATACCGCTGTCACTGCGAACGCGAATGCCAATGGCCGCAACGGTGTC
TTGCGATCCGCAATACCAACAACAATAACTACAACCGCATTGTCGTTATTCTTTGCTCCCGTGGACGGTTTTC
TCCTCCGTGGCCAAATTATTACCACCAAAGACCTTTGCCCTGGGCGGACAGTTTCGCCTCTGCTGGCGAAAGC
CTCGGATGCGCTCGATCCGAGCAATAGCAACAGCTCAGAAGTATTGGAAAGCATCACGCCTTTTTGTTCCCTG
CCATCGTCGCCAGCAACAACAACGACGACGACGACGACGACGACGACGGG
>TR55|c0_g1_i1 len=532 path=[525:0-121 526:122-531] [-1, 525, 526, -2]
GGTGACAAACCTCTTCAACTAAGAGTATGGATACATGATCAATGATGCAGCATGGTTACAGTCAGAACACTGT
ATTTGCTATGTTGGTAGTAGAGGAGAGGTGACAAACCTCTTTGACTCAGAGTATGTATGTGTGGTCAAGATGT
AGGATGGTTACAGTTAGGGTACTATAGTGTATTTGCAGTGGGGGTGGTGGAAGAGGGATGACAAACCTTTTTA
ACTCAGAGTATGGATGTGTGATGAAGGACATGGGATGGTTGCAGTTCAATTGCTGCATTTGTCATGGTGTTAG
CAGGGGAAGGATGGTGACCCTCTTTGACTCAGAGTGTAGATGTGTGATCAATGATGTGGGGAGTCAGAGCACT
GTATTTGCAGTGGTGCTGGTAGAGGAGGGGTTGCAAAGCTCTTTGACTCAAAGTATGGACACATGGTCAATGA
TACAAGATGGTTCCAGTTAGAATACTGTATTTGAAATAGTGGTAGTAGAGGAGGGGTGATGAACCTTTTTGAC
TCAGATTATGGTTGTTTGAGC
>TR56|c0_g1_i1 len=1015 path=[1008:0-100 1009:101-1014] [-1, 1008, 1009,
-2]
GCTCTTCCGATCTCTGCTGCGGTGTCTGCTCACTTACTTCTGCCGGCTTCTCTTCACTCTGCAGCGTGTGATC
AACTGCGGCATTGTCGTCATTCTGCGTGTCACCATCGGGCATGACCGTAGTGGTTGCCGTTGCCGTTGACGTT
GCCACTGTTGGAGGTAGCATTTGTTTTCCCGCTGGCTTCGGTTGATTTTCAGCTGCTGGCTTCGTCGTCGGCC
GTCGTTCCGACAGCGGTCCCGGCGGCATTTCGTCAGAGATTTCGTCGCCGTCTGTCAAGTCAACAATTTTCTG
GACAGCGGCGTTTGTCGTTCCTCGCATTTGCGCGGCGGCGGTGGTCGTCGGAGGTTTCTTAGAGCCGTTCGTG
GATTGAATGGCACCTCGAGGGAGATTGGCATGTTGCAGTTGCTGTTGTTGGGTTCGCATTTTCTCTTTTGATT
GTTGCGGTTGGTCGTGGTGCTGTTGGTGCTGGAGCGGATGCTTCCACGACGGTGGTAGATTGTCACCACTACC
CATTAATTTCGTCAGTTCTAATTGCTTCAACAACGACGTCTCCCCCTCGCGAATGTTGTGAATTCTCACAACC
TCGAACAGGAGCGGCCTACTGTTCACTCTTCCCTCATCACCGCCAACTTGACGATTCTGACTGGCCACCGCCT
CCATCTTGGTGCGAAACCACCCACGAATACCATGCATAATTCGTCCTGTGGTGGTGGGTCGACAAATGATATC
CTTCGCCTGCAGGCCATCCTTCGCAGCGAGGGAATAGGGACGCACGGCCCCCACCAACGTGAGCGGGTACGAA
CCCACCACCTTCTCTTCAATGCCAAAGCCGAGCGTGTTCTCGTCAACGGTTGTCGACTTGTCGTCGCGCATGG
CGAATCGATGTATCGAATAGTGAATCCTGCGCAACACCTTCGGACTGGGGCATATATCCCCCACGAAGCGATC
CACTGCGTCGTCCCCACCCATCTTTCGAAACACGTGGAACGTCAGCGGCAATTGATGGCACTTCTC
>TR57|c0_g1_i1 len=957 path=[954:0-717 955:718-738 956:739-956] [-1, 954,
955, 956, -2]
ACCGGAGCCGCCGCCATGATTGGAGCGGCGGGAGCCAGCTTGGGCTGTTGCTGCGGTGACGCGTGACGCATCC
CCATCATTACCGGCTCGACGGGCTGCTGCTGCTGGGTCATCATGGGAGTGACTGCCATTTGAGTTTGAGGTTG
GGCCAAGGGAGGCTGGGGAGCAGCGACGATGCCGACGTCCGTAGCCTGCTGTTGTTGTTGTTGCGTCGCGGCG
ATCATTTGCTGCACCTGCGACAATTCTCCCGCCAGGTCCCCCCCGGTCGGGAGGGGGGCCGCGGGGATATCGG
CATTGGCTTGAGGCTGTTGCAGCTGGGTCGTCACATCGGTCAAGGGGTCGATGAACGCGGGACCATCTTGATT
AGGATTGGGCGTGACTATTCCGGGGTCTGGCAAAACTGACGTTGGTTGAGTTTTCAACGTGTCGATAATTGGA
GGGGACACTGCTTCGGCGCTTGCGCCAAAATCGAACCCCCCCGCACCCATATCCTCCAACAGATATTCTGCCA
ACAAATCGAAATCGAGAGCGCTGTCCCCCATATTGTTCCATTCGGAGGATTCCCTCAAATGCGTTTCGTTCGA
TGTTAATTTGTTGTTGGTGGCGGCGTATTGAGGGATTGTTACTAGTACGGCGGCGGGAGCCATATCGCGGGAA
CAATCGGGAACACACTTTATAAGAAGATGAAAATATGTATATTTGCAGAAGGTCCAAATATATTCTAAGACAG
AGAGAGAGATATAAGCAGAGAGAGAGTTGTACTGTATAAATAGAGATTATTTGCCGCTAGAATACAGCCCAAT
AATATATATCGTCCGATATATCGATCCAGTCTTGGCAATGGCGGTGTGTAGGAATGCCGGCGGCAAATGCAGT

ACTCGAATCCAAAAGACAAAAATTGCCTCAACAATCCAACAACACAAAAGCGGAAAGAAAGTTTGGTTGATGT
TGATGGGT
>TR58|c0_g1_i1 len=305 path=[327:0-242 328:243-286 329:287-304] [-1, 327, 328, 329, -2]
CGTTTGTCGGCAAAGCAACGGGTTTGCATTTGTACTAAATCTATAGGTATCGATGGGTAAGTGTAGCGGTCGG
CACATGGACACGATGCAACGGCCGTTGAGGTTGCTGGCAGCAGCCTTCCATTCAGAAAACTGGCTATTGTTTT
TGATGCAACGAGCTGTAATATGGACATCTTCCCCGGTTCCGTTATAGGATTGGGTGGTAGAGCTAAGAGAGGA
GAAAGATATTGTTGCGATTTATGAATATTGTTTTGGTGCGCCCTGGAACTTGACACTTAAGTAGAAGAACTCA
TATTCTTTTAGAC
>TR59|c0_g2_i1 len=1226 path=[1221:0-626 1222:627-641 1223:642-1225] [-1, 1221, 1222, 1223, -2]
ATTTTTAAATCTAATACGCTATTCAAGTGAATACGGTAAATCTAATTTTATGTCTTCTTTTGTTATTAAAACA
ATAAGATGAGGAAAAAATTATTTATTATCTATTTTAGATGGTCATTTAATCCATTACCCTAGCCCATTAAATT
TAACATACGCTTGAAGTTTTGGATCTTCAGCAGGAATATGTTTAGTTATTCAAATTTTGTCCGGAATTTTTCT
AGCCATGCACTATACTCCACATATTGATCTTGCATTTAGTAGTGTAGAGCATATCATGCGAGATGTAAATAAT
GGTTGATTAATACGTTATATTCATGCAAACGGTGCTTCAATGTTCTTCATAGTAGTGTATTGTCATATCTTTC
GAGGTTTATATTATGGTTCTTATATGCAACCAAGACAATTACTTTGGTGTTCTGGTGTTCTTATATTTATCTT
GATGATGGGTACTGCTTTCATGGGATACGTACTTCCTTGGGGCCAAATGAGTTTCTGGGGTGCTACTGTAATT
ACCAGTTTAGTAACTGCAGTTCCAATTGTTGGTCATTCCATCGTGGATTGATTGTGGGGAGGATTTACAGTAA
ATAACGCAACATTAAATAGATTTTTTAGTTTACATTTTTTTTTTGCCATTTGTTATTGCAGCTTTAACTTTAC
TACATTTAGCGCTTTTACACAAAGACGGATCAAATAACCCATTAGGGATAGATAGCAAAGGGGATAAAATACC
TTTTTACCCTTATTTTGTCATAAAAGATTTATTTGCATTTTTTTGTTTTCTAACTTTTTTTGGTGTTTTCGTC
TTTTATTTCCCAAATACGCTTGGTCACCCTGATAATTACATACCAGCAGATCCTATGCAAACACCTGCTCATA
TTGTACCTGAGTGGTATTTTTTACCATTTTATGCAATTTTACGGTCTATTCCTGATAAGCTTGGTGGTGTAGC
TGCAATGGGTGGTGCTCTTGTTGTTTTATTTTTAATTCCTTTCATAAATACGTCTGAAATTAGAAGTACGACT
TTCAGACCTATTTTTAAAATTTTTTATTGGTTACTTGTTGCTGATTTCTTATTGCTAGGTTGAATTGGTCAAA
AGCCTGTTAAAGACATTTATGTTGTGGTAGGCCAAGCTGGTACAGTATTTTATTTCTTATTTTTTGTTGTTTT
AATTCCGGCAATAGGTACTTTAGAAGCTAAATTATTACTTATTAATAAAACAGATCGG
>TR60|c0_g1_i1 len=1437 path=[1440:0-155 1441:156-179 1442:180-1436] [-1, 1440, 1441, 1442, -2]
ACAGGGACCGGTCTCTCGTCAATGACAATCCCAAAATGCACACCAAACAAGCTGGTAGTATGTAGACACTAGT
AGTAGTGCAACCAATTCGTTGTTTCCTACGTTTCTTCCGTTATCACTACTGAATGCATCCATCCGCGATGAAC
CGAGCAGACCTTTTCTTGCGCTTCTTGATTTTCCAACCAATCCAGCCACGATGGACGAACCGCCCCATTCTGC
CTGAATTCTTCGTTACTCCACAACTGTATTTCAATCACTTGCCTCCTCCCGCTTTTGGTGCCGAAAACAACTA
CCGTTCGACCAAACCCTCCTGTAAAACTGACCAACTCTTTCATTTGTACGAGCCCCGGGGCCAGTATTTCGAC
CGCCTCGCCCCACAATGGCAATGGTCGATCGGTCGCTACACCGACGGCCCATATTGATCCGTCTTCGAGCAGC
ATTGCCGCGTGATGAGTGCCACATGCAACCCTCTCTATGGAAAGATTCGGCGGTAACCCTGGGATAAAGCGTG
GGTATGCCGAGTCTTGCACAGGTTTGGATCGCAGATTAACGCCCGATTCCGAGTAGTTTGGATTTAGGCAGAT
GTTTTTACCCCAGATGTAGGGAATATTCGACTCTGTTACTGCCAATGTGAAATTCATTCCAGCCGAGACCATC
CGAACTTTGGAATCTTGCTGCGACAGGACTGGAGCATAAATCTCATCGGGAGATGGGGATATCGTCGAATAAG
GATCGTAAAAACTGGCAACCTGGACGGCGTATTCAAATGTACGATTCTGGTTGGGCTCCTCGTAAACTTCATT
ATTGGGTGCTTCTTCTTCGCGTCCTTCGTGTGCCATTCTCCTTCCTAGCCCTAGCTGACCCCTTTCACCCTTG
CCCCAACAAAACACCTGTCCTTCACTGTCCAATGCCACTCCGTGCTGCAACCCCAATGCCACGCTCACAATCG
GATTCTCCTGTTCTTTAAACTGTTTGAACAGTTCATCTCCAGAGCCGGTGCCATGATCTAATATAAATCGGGT
GGATGCTAGACCAACTACTCGGCTTGGAATCCATACATTTGGAAGGAAGGAGCCTGTTCCGCATTGACCTCGA
TGGTTCAATCCAAAGGTGTATAATGTGCCTGTTTTAGAAATGACAGCTGTAACACCTGCATTGGCGACCAGTG
TGTTTTGGAAGCGTGTATGCAGCCTTGAATTATTCGATTCACCATTCTCTCGAGCAATCCTTCCAGCAGCATG
CATATCAAACGCAGTGTTGTCGATTATACCACCTTCCAATGCGGGAATGTCTCCATCTGGCAAATCTATCTCC
AACAAAGTAGGTACATTGTTGTAACGACGGCACTCGTCCTCGTTAAAAGTGACTTCATTTTCTCCCGCAAAAT
AAGTTGCTATTCTTTGCAGTGTCGATGGAGACTGATCCGATGATTGCTCA
>TR61|c0_g2_i1 len=1035 path=[1031:0-438 1032:439-1034] [-1, 1031, 1032, -2]
GGGGGGGGGGGGTTGCTCTGCGAAGCCCAACTTCTATTTCTCTATTTTTATCGTTCATTTTTTACAAGCTACT
GGGCATTCTCCTCTGCTGTTCGTTTAGACTTCTTCCTTGCGATCGCATAGTATAATCACAAAATAGGACAGGA
GATGTAAAAGTAGGCACCCTATATTACACACAATACTTCCTACAGTTGGTTATCTGTACGTGGGATACATGAT
ACATTTCCCCCCCTTCCCTGCAGTTGGGAATCACACACATCTTTATCGGCTCAGATCGTTTTTGACATATCAA
GAGTGCGTATGGATGTATGCATGCCATGGTAAGGCAAAGTGCTTTGTGTAGAACTCAGCCCATTTCTGCAAGC
CCCACCAATGAGTGTATGGTGGCATGTAAAAATCTTTGTCTTCGATTAATCTTGACTCCACTCTGATGTTTTT
TCTTTACATCTCTTGGGCTGCGCTATATGTTCACGTAAATATATTCAGAATGATATTCTCTCTCCACACATAA
TGTAACGTTCATAACCAGAACTCGACCTAGAGTGAGAGAGAGATAGAGTAGTCCAGTTCTAAATCAATACCAT

ACTCGATTGTGAGAAAAGCTGTATCCGCATCGATCTATTTATCGCCCACGCCCATCCATTCCTATCAACTGCA
CCAACAGCGACAACTGATCGGGTGTAATTGCTCGCAGATCCAGGCCGTGTTTCTGCGCCACCTCCAGTAGAAT
CTCCGGCGATGTGCCTGCCCCCAGCGGCTGAGACACTTCCAGTGTCATCGCCTGTTGCAGCACGATCTCCTGC
ATTTGCATTTGTTGTTCAGCCCTCGATGAATTGATTTCGGCCACGAGGATGGCCTTCTGGAGCTCGTCATTAG
ACATCCTAGTGGCTGTGCGCTCCCGCTCAAGTATGAGTTCGGTATTAGGGTTGAGGCTGTTGAGCTGGCTGTG
GCCGCCATCATTGAAGTTAAAGCGACCATAGCTACCGCCACCGCGAGCTCTTCCTTCTAACATCTGAGTCTGA
GTCACCAGATCCT
>TR62|c0_g1_i1 len=384 path=[574:0-133 575:134-383] [-1, 574, 575, -2]
GCTTTCCTCAAATGTAACCTCGTGGCCTCCAAATGAATCGCACTGGTGACTTTCCCCTGCTGTTTAAGATATC
GTTGAAGGTAATCGTCATTGAAAGCGGTAGTCGTACCATCCTTGGAAGTGTATCCGAATGGGACCAACGAAGA
AGAAGTTGAAGTTTGACTATCGTCCAGGCATTTGTACGATCCCTTGGTAACGTCGGGAAAGGTCAAGTATTGA
ATCACCGTCGTTTTATTCTTCCCATTCGTTTCGTTTGCAGCGTTGGTATTATCTCGTAGATAGCGGATGGACT
CCCCTCGACTTAGTACGCGACACGGCTTGAGAGTTTCGCGTTTTCGTCCTACCGCTCCATCCTTCGCCGGGAG
ACGTATGCGATGCCAGATC
>TR63|c0_g1_i1 len=294 path=[293:0-113 294:114-135 295:136-293] [-1, 293, 294, 295, -2]
CGATCTATGTGTACAAGTGGAGCATTTCACGTCAGTGGTCACGACTCTTGGGAACAGTGGGTACATAGGTGCC
ATCACCTTGTTCCTGACAAAGAAAGAATCATTCACAGGCAAAGGGGTTTCACCCTGTCAAGGTGTGAAAGCAA
AACTTGCGTCATGTCAGACCAAAAATGCATTTAGGTTCACGCCCCAGCCGACTGCGTTGGCGAAGGAGAACTC
TTGTTCATATTCATCAACAAATTCGCATCGCTCAACGATGCCATTCTCTGAAACGAAGGAGACATTCTCGGGG
CA
>TR63|c1_g1_i1 len=577 path=[580:0-70 581:71-94 582:95-576] [-1, 580, 581, 582, -2]
GCCGCCCTCCAACACAACTCTCGCCTCTGGGTACAATGCATCTGGCTTATCGCTTAGCCAGCAACACGGCTCG
AATACTAGAGGAACTTAAGTTGATCCCGCCTCCCAATAGACGCTGCAGAACCCCCAGCTAGCTTCTAAATTAT
CAATGCCACATAAATTAGTGCAATTCAGATTCGATAAGAGATTCCAAAGGACACCAACGTCCACGTGGGTTTT
AGTGGCATCACCCTGACCCCGCGGCAGGTTTGGAAACTCAGTTCGCAGTTGGTATCTACAATGGCGTTTGGCT
GAGTCCTTGAGTATCTACATCTATGTTGTAAAAGAAGAAGGAACCAAAGAAGAATATGTGTAATTATTTCATG
TTATTGTCATTATCATTATGGTATGAATTATTTAATAAGACCTTAACCCAACTTTTATGAGTCAAATCAAGCC
CAATCATTATATATATATATACATATATCGCCACACTTTGTTACAGTGCCACAAAGTCATAGAGCAGTAAGACGA
CACTATTCGTGGAAACGAACCGTTCCCAGTAAGGCAGGATGAATGTGTACAAGTGGAGCATTTCAC
>TR64|c0_g1_i1 len=2261 path=[2421:0-149 2422:150-2260] [-1, 2421, 2422, -2]
ATCGTCATTTGTTTTCTCTGATTTCTCAGCATCACCAAACAACTTGCGTATCCCATCCGACGCCATTCCTACT
TCTTTTCGGAAGCATTCTATTGAACGTAAAGGACAAGCTAGCGACCATACCATTGATACTACTAGCATTTGAC
TCTTGAACGATATTATATAACTACCAGCTACCATGTCTCTCACACAGAATTCCGTGCAGACCATGTTCAGTAT
GAACGCGTCAGGAGACAACCCATCCTTCGCACCCACCGTGCAGGTCATCCACTTGAAGAAGATCGACAACAAG
GGAGGTGGAGGAGAAGAGCGGTGGAAGGTCATCCTGTCTGACGGCAAATACTTCCTCTCGGGCATGTGCGCCA
CTCAACTCAACAACCTTGTCCACACTGGCGTCATTGCACTAAATTCCATTCTGCGCGTGAACGACTTCATTGT
CAACACAATGGGATCTGGCCAAAAGATAGTCATCGTACTGGGAGCAGATCAGATGGGGGCCAACCCTGGGGAG
CGGATAGGTTCCCCGGTCGACATCGCTAAGGCCGGGGCGGCTGCTCTACCGCTGCCTCAGGCCAACAACGCAG
GAGGGGCGCAGCCCATGTATGGCAATATGCAGTCGCCGTCGGGTAGGAATAGCAACCCATACGGCAAAGCTTC
TCCCCGCGGCGGCGGCAATCCCTACGGGGGAGGAAATAACAGTAATAGTTATGGAGGCGGAAGTAGCGGTGGC
TCTTCTGCTCCCATCGTCCGCACCTCTGCCACAGGACAGCAGATCACACCGATCAATGGTCTCAACATGTATT
CAAACCGTTGGGTTATCCGCGCAAAGGTCTGTAACAAGTCTGCCATTCGCCACTGGAGTAATGCAAAGGGCGA
GGGATCGCTATTCAGCATCGAGCTACTAGACTCAAGCGGGTTCGACGTTAAGTGTACCTTCTTCAAGGAGGCG
GTTGACAAATTCTACAGCTTTTTGGAGGAGGGGAGCGTGTACACCTTCTCGGGTGGAAGATTAAAGGTAGCCA
ACATACAATACAATAAGTGCAAGTCGCAGTTCGAGATTACATTCGATCAAAATTCGGAGATCCACCTTGACAA
GACGGGGGGACCTGAGATCACGGAGTCGTACGAGTTTATGAAGATTGCAGGCCTGGAGGGCGTCGACCCGAAC
AGCTACGTCGACATCCTGGCAGTAGTAAAGCACGTGGAGGAGCCATCAACCATCGTGTCGAAAAAGAGCGGGA
AGGAATTGATAAAGTGCGAAATGATTCTCGAGGATGACTCCGCGGCGGAGGTCAAGCTGACCGTGTGGGGGGA
CGCGGCGCAGACGGCCCAGCAGAAGTTCGCAAACGCCCCGATCGTAGCGTTCAAGAGGGCGCGGGTGAGCGAC
TATGGCGGCAGGACATTGAGCGGCCAATCCTTCAATGTGAACCCCAACATTCCCCAGGCGAATGGGCTCCGGC
AATGGTGGTCCGTCAACGGCAATAAAACGCAGACGCGTAGCCTCTCCTCCGCCATGGGAGGGAATCGCGGCCC
TGACCCTTTCCCCCAGCGGAAGGATGTCTCGTCCATCAAGGGTGAGGGCCTTGGGCACGGCGAGAAGCCCGAC
TGGCTTACATTCAAGGCCACCATCACCTTTCTCAAGAAGGAGAAACAGGGCGACGAGGGGGCGTGGTACACGG
CGTGCGCCAGGTCCGAAGATCCTTGCAGGAATATGTTCAAGGCCACGCAAACATCTGACGGAAACTGGCACTG
CGACAAGTGTCAGCAGACCTATGAGACGTGCGTCCGGAGATTCATCTTCTCGGGGACTGTGGCGGACGACACG
TGCACGTCGTGGGTGTCGGTTTTCAATGAGCAGGCCGAGGCGTTGTTCAACGGGGAGCCAACGGCGAAGGCAG
ACTACCTTTACGAACAGTTGATCAACGGGGATGGGAGAGATCTTTATGATTCTACGTTCATGAAGGCGACGTA

TACTGATTGGATTATCAAGTGTAAGGTGAAGCAGGAAATGGTGGGAGACGAGCAGAGGATTAAGACGAGTGTT
GTGAGCGTGGCTCCGATCGATTATGTGGTGGAGAGTCGGAATCTTTTAACGAGCCTGTGCGCACAGTGAAAGA
GGCTCTGATCGACTTTGGGAACCGGTCGGCACCATGAACGAGTGCAATATATTTCTACCTGTTTTGCATTCAA
GAAGATAATTATGTAACATATCACACAAAAATGAATGAATAAGTAGATATCCAGATTCCCGATAAAAAAAA
>TR64|c0_g2_i1 len=2290 path=[2416:0-78 2417:79-95 2423:96-178 2420:179-2289] [-1, 2416, 2417, 2423, 2420, -2]
GCAATGATGCATGTGTGGTGGCGTAAAGACGGCTTTGTTTTTGATCGTATCAGAACAAAAGGGTTGACTAGCC
TTTTTTCTGAATATTTGGTACATTTCACGGTCTCTTTCAAATTTAACATTCAGCATTGTCTTCCTCTCACTCT
TCATTCTTCAGATACTACTAGCATTTGACTCTTGAACGATATTATATAACTACCAGCTACCATGTCTCTCACA
CAGAATTCCGTGCAGACCATGTTCAGTATGAACGCGTCAGGAGACAACCCATCCTTCGCACCCACCGTGCAGG
TCATCCACTTGAAGAAGATCGACAACAAGGGAGGTGGAGGAGAAGAGCGGTGGAAGGTCATCCTGTCTGACGG
CAAATACTTCCTCTCGGGCATGTGCGCCACTCAACTCAACAACCTTGTCCACACTGGCGTCATTGCACTAAAT
TCCATTCTGCGCGTGAACGACTTCATTGTCAACACAATGGGATCTGGCCAAAAGATAGTCATCGTACTGGGAG
CAGATCAGATGGGGGCCAACCCTGGGGAGCGGATAGGTTCCCCGGTCGACATCGCTAAGGCCGGGGCGGCTGC
TCTACCGCTGCCTCAGGCCAACAACGCAGGAGGGGCGCAGCCCATGTATGGCAATATGCAGTCGCCGTCGGGT
AGGAATAGCAACCCATACGGCAAAGCTTCTCCCCGCGGCGGCGGCAATCCCTACGGGGGAGGAAATAACAGTA
ATAGTTATGGAGGCGGAAGTAGCGGTGGCTCTTCTGCTCCCATCGTCCGCACCTCTGCCACAGGACAGCAGAT
CACACCGATCAATGGTCTCAACATGTATTCAAACCGTTGGGTTATCCGCGCAAAGGTCTGTAACAAGTCTGCC
ATTCGCCACTGGAGTAATGCAAAGGGCGAGGGATCGCTATTCAGCATCGAGCTACTAGACTCAAGCGGGTTCG
ACGTTAAGTGTACCTTCTTCAAGGAGGCGGTTGACAAATTCTACAGCTTTTTGGAGGAGGGGAGCGTGTACAC
CTTCTCGGGTGGAAGATTAAAGGTAGCCAACATACAATACAATAAGTGCAAGTCGCAGTTCGAGATTACATTC
GATCAAAATTCGGAGATCCACCTTGACAAGACGGGGGGACCTGAGATCACGGAGTCGTACGAGTTTATGAAGA
TTGCAGGCCTGGAGGGCGTCGACCCGAACAGCTACGTCGACATCCTGGCAGTAGTAAAGCACGTGGAGGAGCC
ATCAACCATCGTGTCGAAAAAGAGCGGGAAGGAATTGATAAAGTGCGAAATGATTCTCGAGGATGACTCCGCG
GCGGAGGTCAAGCTGACCGTGTGGGGGGACGCGGCGCAGACGGCCCAGCAGAAGTTCGCAAACGCCCCGATCG
TAGCGTTCAAGAGGGCGCGGGTGAGCGACTATGGCGGCAGGACATTGAGCGGCCAATCCTTCAATGTGAACCC
CAACATTCCCCAGGCGAATGGGCTCCGGCAATGGTGGTCCGTCAACGGCAATAAAACGCAGACGCGTAGCCTC
TCCTCCGCCATGGGAGGGAATCGCGGCCCTGACCCTTTCCCCCAGCGGAAGGATGTCTCGTCCATCAAGGGTG
AGGGCCTTGGGCACGGCGAGAAGCCCGACTGGCTTACATTCAAGGCCACCATCACCTTTCTCAAGAAGGAGAA
ACAGGGCGACGAGGGGGCGTGGTACACGGCGTGCGCCAGGTCCGAAGATCCTTGCAGGAATATGTTCAAGGCC
ACGCAAACATCTGACGGAAACTGGCACTGCGACAAGTGTCAGCAGACCTATGAGACGTGCGTCCGGAGATTCA
TCTTCTCGGGGACTGTGGCGGACGACACGTGCACGTCGTGGGTGTCGGTTTTCAATGAGCAGGCCGAGGCGTT
GTTCAACGGGGAGCCAACGGCGAAGGCAGACTACCTTTACGAACAGTTGATCAACGGGGATGGGAGAGATCTT
TATGATTCTACGTTCATGAAGGCGACGTATACTGATTGGATTATCAAGTGTAAGGTGAAGCAGGAAATGGTGG
GAGACGAGCAGAGGATTAAGACGAGTGTTGTGAGCGTGGCTCCGATCGATTATGTGGTGGAGAGTCGGAATCT
TTTAACGAGCCTGTGCGCACAGTGAAAGAGGCTCTGATCGACTTTGGGAACCGGTCGGCACCATGAACGAGTG
CAATATATTTCTACCTGTTTTGCATTCAAGAAGATAATTATGTAACATATCACACAAAAATGAATGAATAAGT
AGATATCCAGATTCCCGATAAAAAAAA
>TR65|c0_g2_i1 len=2890 path=[3157:0-167 3158:168-191 3179:192-531 3181:532-938 @3180@!:939-1425 3178:1426-1512 3176:1513-2367 3156:2368-2889] [-1, 3157, 3158, 3179, 3181, 3180, 3178, 3176, 3156, -2]
GATCTGTGACTTTGGGAGGGCCGCCGACTCCCGTTTCGGCGACGGTGGTCCAAGGAGGAGTGATTGCGCACCC
ACCGATGAATGGTATTCGCCACGAGAATCGAAGGCCAGCGTTGTTACCCAGTCATCCTGCAGCGAAGAAGCAA
AAGCTGAACAATGGTGTGGCTTGTCCTCTCATTGATCCAAATACCGCTACAATCACTGTGCAGGCCCACACGC
CATTGTCGCCATTGCCAATCGCATCTACGCATCATCACCATCTACAACAACAACCATGCCCAGTGCTATTCGC
CGCCGTCAACGACACCCTCAAAACACCCTATCTTCTGAAAAATATCGCCTTGTTTCTTGACCCAATGTCCCTC
CTTCGATGTACCCGCGTCTCGAGGCGATGGAATTCTTGGGAGAACATATTTCAACATCCGAAATTGTGGTTTC
ATTTATGTTTACGACGCTTTGGCCCGAATACCGTGCGGGCGTGGGAAGATGCTCACGATGAGGACGAGGACGA
GGACGTTCGAACCAAAGCGAAGAGTCAGCTTTGCACTAAAAGTGGTTCAGCAACTAATACTGCCACTACCACT
ACGAATTACATGAATATCTACCGTCGAATGTCGGAACGAAACGCAAAGCCATCACATTGTCCGACGATTGAAA
AGTCGGTGCCTTTGGGAGGATCGACGTTGGACGGACTCGTTGGTGGCTGGGCTACGTTGGTGGATCGGAGCAA
TGGCGAGACGTCTCGAAGTGTACTGCGTCGAAAAGCAGTTCACCCTGGTGAAGGAACTGCAGCAACGCCATGC
TTCTACGGACCCATGCCAGTGGTGGAAATACGCCTGCTCGTTCAAAACACGGGTTTCTCGAAGGGAGCAATAA
TAGTGCCTGCACAGACGTTTTGCGTGGATGCTAGCACAAGGCGTAAAGGCGAAAAGTTCTTGGAGGTGGGAGA
TGGGGACGAGCGGTTCCGACGGCGGGTCATGCATGTCGAGAGCAACGGCAGCAGCAGCAGCAGTAGTAGTAAG
CTTACAGAGTTTGGCAAACAACAACCACAAGCTTCATCGCTGACCCGTAATGAAATGTGTCGTCTCCGCCTCT
ATGAATCGGCCGTGGTCTCGCTCCACATCCACGCTCGGGGGTGTTCCACCACGGCGAAATTTCGAAGCAAGTC
GAAGAATATTCAGATTTTGGTTTGCGTGGATGGGACAACGCGACCGTTGGTCATTCCGCTCCATGCTAACAAG
TGTTTGCAGTTGAAATAATGAAAGACATTCATACAGTTCATAGAAAATGAAAAATGGAGTTGGACCGAGACGA
GAGATATTCTATGTTTTGTCTACGATATGATTTGACCGCTGCTATTCCCGTGTCGCGGCTTCGCAAACAGGAA

AATGGTTAATTAAATAGCCCGATTCAGGAAACGCACGTCGTAAGTCCCACTTGCCATCATTCCGCAAGTCACT
TCACCTTATCACCTCTGAATATTTTCCCAGTGTACCATGCACCCTCCTGTCTTTGATTTCGAAGTCCCCTGCG
TAGTACCTACCACCGAATTGTTGGCCACAATTTCAACATAAAAAATCACACGTGCCAAATATATCAGAATCGT
ACACATAAAACATCTCATTCAGCGGAGAAAATAGAGCTTTCACTCAATTAAGCCATCTACTGGTCATTTATTT
CGGGTGTTCACAAATAGTAATCATCTCATTTAATAGTTTTATTTGCCAGATTCAAACCCTGGCGAATTAACCT
CGGAGCCGGTGGTAGTGTAAACGAAGTATCCGCATCTCACGTCATCGTCATGTCTCTTTAATTACTGTGACAT
CTTCTCATTTTTCAAAGTATTGTAGGTACCCTCCGTACCATAGCACCATTGACCAAATTGTATACCTATCACT
TCCTTCTCAATCGACTCTTCAATTGTTTCTTCAATTTCATCTTTTCATCCATCTTAGCAAAGTCCTGTATCGA
ATCGTCCACCAGTTTCACGTGCTCCTCGCATTGCTGCATCCCGCTCTTCATGAACCAGAATCCGATCGTCACG
ATGGTGTGATAGATGACCACAACGGGAAATTGATGCCTATCCTCTACTTCGGCGTCTACACTCTCGATATCGT
CCTCCCCTCCACCAGGGAAACATCCAAGATGTTTGGAGTGAAAGTACAAACCCAGGGTAATGGGGACCAGAGC
GCTCGAGAGCATATACGACCAATTGTCCAATTCCAGCATTCCTACGTTCACCTTACTCAGGGCCAAAAAGTAG
GACAGCAGCGACGAGATGACAACCCCCAGAATCTCGCAGTAAGAATCCCCAAAAATGAGTTTCACAGCTTCCG
CACCAGTCACCGCCGCCGTAACGGATCCATCATCTTTGCCGTCCCCTTCTTTAATCTCGCGAATGCAAGAGGA
TATCGAGGTCTGGAGCTGATGCATCGTAACGAGCACTACGAGGAGGGACAGCAGGAATAATTGGCCCTTCCAC
GCAGCGTGGAGGTCGGATGTGCGATCGCGGGAGATGACGAGTCGGGCCCGTGCGCTTCGAAGCTGTTCGATGG
GTGAGAGTTCTGGTTGTTCATCATTTTCATTATTTTTGGCTGCATCGGTGGTGGTGGTGGTTGCCGATGGTGG
TGTTGTTGTTTCTGATGCGGGGGAAGTGGCGGTGCTGCTGCTCTTTTTGCCGCGACGTTGTCTCAAGTTGTCG
GACATGGCTATCGGACGGAGGAGGTGTATTATTTCTGTGTTTGGCTCGGTGGTTAGTGTTAGTCTGCGGGTCT
CGGTGGGTGCATACGGTCAATATTATTGGGAGGGAGTTTCTGTTGCTTCACTCTGTTACTCTAATAATTGTGC
GGAGCGCTTGCTTGCATTCCCGACGCGGTTTTGGATCTTTTTG
>TR65|c0_g2_i2 len=2826 path=[3157:0-167 3158:168-191 3179:192-531
3181:532-938 @3180@!:939-1425 3161:1426-1426 3162:1427-1448 3176:1449-
2303 3156:2304-2825] [-1, 3157, 3158, 3179, 3181, 3180, 3161, 3162, 3176,
3156, -2]
GATCTGTGACTTTGGGAGGGCCGCCGACTCCCGTTTCGGCGACGGTGGTCCAAGGAGGAGTGATTGCGCACCC
ACCGATGAATGGTATTCGCCACGAGAATCGAAGGCCAGCGTTGTTACCCAGTCATCCTGCAGCGAAGAAGCAA
AAGCTGAACAATGGTGTGGCTTGTCCTCTCATTGATCCAAATACCGCTACAATCACTGTGCAGGCCCACACGC
CATTGTCGCCATTGCCAATCGCATCTACGCATCATCACCATCTACAACAACAACCATGCCCAGTGCTATTCGC
CGCCGTCAACGACACCCTCAAAACACCCTATCTTCTGAAAAATATCGCCTTGTTTCTTGACCCAATGTCCCTC
CTTCGATGTACCCGCGTCTCGAGGCGATGGAATTCTTGGGAGAACATATTTCAACATCCGAAATTGTGGTTTC
ATTTATGTTTACGACGCTTTGGCCCGAATACCGTGCGGGCGTGGGAAGATGCTCACGATGAGGACGAGGACGA
GGACGTTCGAACCAAAGCGAAGAGTCAGCTTTGCACTAAAAGTGGTTCAGCAACTAATACTGCCACTACCACT
ACGAATTACATGAATATCTACCGTCGAATGTCGGAACGAAACGCAAAGCCATCACATTGTCCGACGATTGAAA
AGTCGGTGCCTTTGGGAGGATCGACGTTGGACGGACTCGTTGGTGGCTGGGCTACGTTGGTGGATCGGAGCAA
TGGCGAGACGTCTCGAAGTGTACTGCGTCGAAAAGCAGTTCACCCTGGTGAAGGAACTGCAGCAACGCCATGC
TTCTACGGACCCATGCCAGTGGTGGAAATACGCCTGCTCGTTCAAAACACGGGTTTCTCGAAGGGAGCAATAA
TAGTGCCTGCACAGACGTTTTGCGTGGATGCTAGCACAAGGCGTAAAGGCGAAAAGTTCTTGGAGGTGGGAGA
TGGGGACGAGCGGTTCCGACGGCGGGTCATGCATGTCGAGAGCAACGGCAGCAGCAGCAGCAGTAGTAGTAAG
CTTACAGAGTTTGGCAAACAACAACCACAAGCTTCATCGCTGACCCGTAATGAAATGTGTCGTCTCCGCCTCT
ATGAATCGGCCGTGGTCTCGCTCCACATCCACGCTCGGGGGTGTTCCACCACGGCGAAATTTCGAAGCAAGTC
GAAGAATATTCAGATTTTGGTTTGCGTGGATGGGACAACGCGACCGTTGGTCATTCCGCTCCATGCTAACAAG
TGTTTGCAGTTGAAATAATGAAAGACATTCATACAGTTCATAGAAAATGAAAAATGGAGTTGGACCGAGACGA
GAGATATTCTATGTTTTGTCTACGATATGATTTGACCGCTGCTATTCCCGTGTCGCGGCTTCGCAAACAGGAA
AATGGTTAATTAAATAGCCCGATTCAGGAAACGCACGTCTGTACCATGCACCCTCCTGTCTTTGATTTCGAAG
TCCCCTGCGTAGTACCTACCACCGAATTGTTGGCCACAATTTCAACATAAAAAATCACACGTGCCAAATATAT
CAGAATCGTACACATAAAACATCTCATTCAGCGGAGAAAATAGAGCTTTCACTCAATTAAGCCATCTACTGGT
CATTTATTTCGGGTGTTCACAAATAGTAATCATCTCATTTAATAGTTTTATTTGCCAGATTCAAACCCTGGCG
AATTAACCTCGGAGCCGGTGGTAGTGTAAACGAAGTATCCGCATCTCACGTCATCGTCATGTCTCTTTAATTA
CTGTGACATCTTCTCATTTTTCAAAGTATTGTAGGTACCCTCCGTACCATAGCACCATTGACCAAATTGTATA
CCTATCACTTCCTTCTCAATCGACTCTTCAATTGTTTCTTCAATTTCATCTTTTCATCCATCTTAGCAAAGTC
CTGTATCGAATCGTCCACCAGTTTCACGTGCTCCTCGCATTGCTGCATCCCGCTCTTCATGAACCAGAATCCG
ATCGTCACGATGGTGTGATAGATGACCACAACGGGAAATTGATGCCTATCCTCTACTTCGGCGTCTACACTCT
CGATATCGTCCTCCCCTCCACCAGGGAAACATCCAAGATGTTTGGAGTGAAAGTACAAACCCAGGGTAATGGG
GACCAGAGCGCTCGAGAGCATATACGACCAATTGTCCAATTCCAGCATTCCTACGTTCACCTTACTCAGGGCC
AAAAAGTAGGACAGCAGCGACGAGATGACAACCCCCAGAATCTCGCAGTAAGAATCCCCAAAAATGAGTTTCA
CAGCTTCCGCACCAGTCACCGCCGCCGTAACGGATCCATCATCTTTGCCGTCCCCTTCTTTAATCTCGCGAAT
GCAAGAGGATATCGAGGTCTGGAGCTGATGCATCGTAACGAGCACTACGAGGAGGGACAGCAGGAATAATTGG
CCCTTCCACGCAGCGTGGAGGTCGGATGTGCGATCGCGGGAGATGACGAGTCGGGCCCGTGCGCTTCGAAGCT
GTTCGATGGGTGAGAGTTCTGGTTGTTCATCATTTTCATTATTTTTGGCTGCATCGGTGGTGGTGGTGGTTGC

CGATGGTGGTGTTGTTGTTTCTGATGCGGGGGAAGTGGCGGTGCTGCTGCTCTTTTTGCCGCGACGTTGTCTC
AAGTTGTCGGACATGGCTATCGGACGGAGGAGGTGTATTATTTCTGTGTTTGGCTCGGTGGTTAGTGTTAGTC
TGCGGGTCTCGGTGGGTGCATACGGTCAATATTATTGGGAGGGAGTTTCTGTTGCTTCACTCTGTTACTCTAA
TAATTGTGCGGAGCGCTTGCTTGCATTCCCGACGCGGTTTTGGATCTTTTTG
>TR66|c0_g1_i1 len=568 path=[665:0-79 666:80-121 669:122-267 663:268-291 671:292-377 657:378-416 658:417-567] [-1, 665, 666, 669, 663, 671, 657, 658, -2]
TGATTTCAGTAAAAGATCTGCTTAAGAAAGCCCCAAACAAAAACTCAGGAGACATACCATGGCGTAACGCCAC
TCATTCTCTTCTAGGTGCTTGATTACAGAAACACCAATCGATGCAATAACCGGCCAATGAGGCGTTCAGCCCG
TGGCCGATTCATCAGACACAATGAAAGCATTGTCCATCAAGCCTTCTAACCTTACGATCTTGTGGTCTTTTTG
CAAAATGAGAGGACGAACCATATAGTGTATTTTATATTTTGATGGGAATGCATTGTACATTAAGAAAACCAAG
CAATACATTCCTCTGGTACACCCTATTTCTGATGAAATATTCAAAACGATCGCCTGACGGTACTGAAAAATGT
ACATCGTACCATGTGATTGCCACACGGGGGTTGGTTCGTCTAGAGTCAGTCTCTCGAATCATGCGTATTCGGC
GTTGTTTTTTCAGTAGCTATACGAATAAAGCCAATTCTGCGCAAGCTGAAGCGCCTTCTTGCTCGCCCTATCC
GCCCGAGACGAGAGCCTCCGCTGAAATATCTCCAACCCGGCAATATCGACGGCGAGG
>TR67|c0_g1_i1 len=4174 path=[4212:0-1153 4213:1154-1171 4224:1172-1546 4223:1547-2502 4218:2503-4173] [-1, 4212, 4213, 4224, 4223, 4218, -2]
TCAGGAAAAATATTACGGGTGAAACACATACTGCCCCCTATCCGGATGCCTACATTCTCGATGAATGCTCCCG
CGCCGCCATTCGTATCACATTCCACAAGATCCCACATCCCACAAGATCCCACATCCCCGCTTCGAGCTGCTAC
GAGCGCAGAGGAGAACCGAGCGAGGGTCGTGTGCACAAGCCTCCCGATCATAGAATTTGATAGTTGCAGCCGT
GCCACTGCGCTAAATAAGGCAAGAGGACCCACCATTGCCGTCTCGTCGGACAATAAAAAATGATTTCGCGATG
GAGACAACGGAAACCGCCGTCAACTCCACAAAAAGGCAACAACAATGGCGCTGCGAGCGCCAGTGGTCTGCCA
TCAGCGATGCGCACTCCTACTACAGCACAACCCAGGAAAATGTACGGTGGAGGGCCCGCCGGCCAATCTCAAT
ACGGCAATAATGGTGGTCATCTCCAATCACCATGTAACAGCTTTTTGCTCGGTGGTAACTTCCACGCAGGAAA
AGATAAACGAAAAAGAAGAGCATACCACCCTCGTCACAAATCTCTTTGGTATCGAGTGTTCTTCTCTACACCA
GGAAGAGCAATCGCTTCGACACTGATGATCCTGTATTTATCGTGGCGATATGCAATCGTGCCCGGTGTGGGAA
TTGTTTTGAAATGGGGGCGTTATTTAAGTCAGGATGGAGACGCTGCATTTCCCCCCGTGATGTCGGCAGCAGA
TGACAAACATATCAGAGACGAGCTCTGGAAGCCTATCAAAAGTCTAAGGAGCAATGCAAAGCTACTCAATGAT
CGTATCGAAACATTACGAAGGGTGAACACAAAGAAGAAGACGGGCACAAAACGACTAGAGGCTATCAAGCGGA
TTGTTCCAAAATGGTTCGATAGAAATCTGGCTCCTCCGATTGATAAAACTACGCCACAACGGAAACAAAAGTC
GAACGTAACGTCGGCGAAGCCAAAGAAGCACCAGAAAGCATCAGAGAAAAACAACAATAGAGCCAAGAGAGGT
TCAAAGGCAGTTGCAAGTAAAAAATCTGCAGTTGCGAATAAGAAATCTCAGTCACTTTCAGTGGTTGAGAAAA
TGCATCCTCAATATCACGATGCATTACTACTTGACATCCAAAAACAACTTGAGAAAAAAATCCGAATCTTCAC
CAAGGACCCTCCAGACACTGGAGAAAGATTTGAAGCACTCTCCCAACTCATGTCCCAAGGAAGGATTATCGTT
GCCATTAGATATCTCTGTATCTCTTGTCATTCAATGCAGTTTGGATAGGATATGGCTATTGTCAGAAACTTGT
CTACGATGGCCCGACCCTATAGTGCTCGTCGTTTATCTCCCCTCCCAGACGGTTCTTAATTCCTCTGAACTGT
CCACCGCCCTTGATTCCATTTCCAACGTCATGGCAGAGTGTCCGCAAATGACAGTATTGCCACATGTGCACGG
GCATGATGAAAAGAAAGACGGATCCTCTACATATCCTGTAAATGTCATGAGGAATAAGGGGTTAGACGCAGTG
AAAACCTCGCATGTCCTCATCATGGATGTGGATCTCATCCCCTCCGCCGATCTAAGCCATGTGGCGAAAGACA
ATGTAATGGACCAAATAACAATGGCAAAGCGATCCAACAACGACATTGGAATCCCTGTGAATGCAATAGTAGT
TCCGGCATTTGAACGAAAGGTTGACTCGCCCTGTTCCGACGTTGAGTCTTGTCGCAGGTATTTACAAAATGAT
TCCAAGTTTCTTCCGCTCCTATTTCAGGATTTGAAAGAGTGTACTGAAGATAAAGACTGCATCGTGTTTCAGG
CGGATATGAATTGGGAAGGTCACCACACAACACAATCAGAGAAATGGCTAAAAAAGCATTGGTATGAGAGTTC
GTCGAAGGAGAAGGAGGGAGAGAAAACCAGAACAATTAGACAAATCAAATGCTTTGACTCATTACGCTATGAA
CCGTACATTGTCATCCCGTGGTGTCCCTCTTCCAAATCATCCAACCCACAACCACTCACCCCTTACTACGATG
AAAGATTCTATGGATATGGCAAGAACAAAATCCAACACATTTCTCATTTGAGATTCCGGGGAGTTCCTTTCTC
TGTGCTCCCGCAATCATTTGTGGTGCATCATCCACACCCAGAATCAAGCGTCAAACAGGTTTGGAATAATCGC
AAAAAGAACTCATTGCATCGGAATATGGATCTGTTATATCGAGAGTACATCAATGATTTGACTGAAAAATATT
CAGATGTAGACAATGCCGTTCCTCAGTGTGACGGCTAACCGAAAACAGTATGTCATGTGAATGTAAGGTACCG
AATTAATCGGGTAACTGTTGCCTGGAACTTGAAGAAGGAGAAAAAAGTTACTTATCAGCAGACCGCTTCATTT
TCTGTTCACAAATGATACGAAATCGAAACCGGAGCTGACTATAATATATTCAATAGCAATGACAAATACGCCA
CTGGGCACCTGTCAATTTTTTAAAGAAGGCATCCAAAAGAGTACACTTAACATGTTTTGATGTTCAGATTCTC
GTCACCATAAATCTTGTAACAGCGACTGCTGTGTTTGGCTGGAATTTGCCCTGGCCATGTACCCATTGTTGAT
GTTGGCGCAATGCCCAATACATTTTGCTCAAGCGCGACCATCTATTATTGCACCGACTGACTTAGACTAACCA
CGAACTTTCAAGGTGAAGTATGGCACACGACCAGACTAGAATAGGCTGCAGGCAATGAGACGATATTAAAGAG
ATATTTAGCTAAAAATTTGCATTGCATTGTCAACTGCTATGATATTGAATGGCATCATAAATGCTATTGTACA
CGCTTGATAAACTCAACGAGATCGCCCAACAGCAAATCTGGATTTTCCAATGCAGCAAAGTGCCCGCCTGTCT
CATGCTTTTGAAAAAGAACAATGTTCCGGAACCTCGTCCTGATTTGTGCATCTGCCGGCGGAAAAATCTCGTG
CGGAAACAGCGACAGCGCCACGGGCAACGTAACCCTGAGGTTATACACGGCCATGGCCTCGTCCGAATGAAAT
TGCTCCTTGTACAATCGCATCGACGACGTTATGCTATTTGTCGCCCAGTAAATCATGCAGTTCGTGAGTATGT

CTTGCGTGTTGAACTTGTCTCCCAGCGGGAGGGACGGGTCAGACCACGATTGGAACTTTTCCGATATCCACGC
GCACATGCCCGCCGGGGAGTCAGAGAGGGCATGCCCAGTCGTTTCTGGCTTTGTCGCCTGTTGATGCAGGTAC
CCAAGTTGTGATAAAATAGCGGCGATGTCACGAGACTTCCTAGCTCTCTCCCGACTACCCTCATCGTCCCAAA
ATCGAATCTGGAATTCCTCCCGCAAAAAGTGTCCCGGTGAAAAAGTGCACGGGAAGAAATTGAGATGCAATCC
AATCAACGGCGAGGGATCGAGGTACGCCATCGACTGGGCAACCATACTACCCCAATCCCCTCCCTGGACTACG
TAGCTTTCGTACCCGAGCTTCTCCATCAGCCTCTTCATCGTGCCGGCCATAGCGACGGTGTTGTACCCCTCAA
TATTCGGCGCGCCGCTGAATCCGTACCCAGGAAGACTCGGGCAAACGATGCCGTACTCATCGAGGGTCGTTTG
ATTCTGCATAAGTGGTATGAATTTGTTGAATTCCCATATGCTGCCGGGCCATCCGTGGAGGAGCAAAATGGCC
TTCTTGCCGGAGCCGCGTTGGATGTGATGCATCCTGATGATCAGGCCGTCAACCACCGTCTCGCTCAATCCCA
CTATATTAAGTTGTGCCTCGTACTTTCCCCAGTCATAATCATCTCTCCATTCGTCCACCAACTTTGCCATGAA
GTTGGAGTTAATTCCATAGCTCCAGCCATCGTCGTGCTCCATCGGCGCACCTGGCGCTCTCCACCGAACGAGG
CGCTCTTTTAAATCAGCGAGTACTTCATCTTCTACGTGGAGCTTGTATTCGTCCGATGCTGCCCCCTCTACGC
TGCATTCCTCGTCCTTGTGTGGACCCCATTGCCTGGGCTGCCCGCTCGATGTTTCTGGAGATGCAGGGCGTGT
CAATAGCCAGTTATCGATGCTATCTGGGCATAGCGTGCAGGCCAATAGGATGAATGTGATGGCACCAACTGAT
ACAACGATGGGTC
>TR69|c0_g2_i1 len=1018 path=[1099:0-691 1100:692-695 1121:696-708
1120:709-717 1119:718-741 1117:742-760 1093:761-1017] [-1, 1099, 1100,
1121, 1120, 1119, 1117, 1093, -2]
ATCCATGACATTGAATTCTTCAGCGATGCGTGGTGGCGGCGGCGGTGTTAGTGGTAGTGGTGGTGGTGGTGCA
TTTCAAGGGAGTTGGTATGGCATGTCCGGTCCCTTTCTAGTACGTCAAGCATTCGAAGAATTGTGCGAACGAT
GCGATGGAGGCGTGGTATTCGTTTCGTTGATTGGTACGAGTGGTGTTTTGGATTTGGACGAGTTGGACTTGGA
CTACGAAGGAGGAGGAGGAGAATCCAATAATTGGAAGGATGGTGATTATTCAGGTGACAATGGGGATGATGAT
GATGAGGATGATCGCGAAGGTCAACAGCATACCAATTTGAAATCTTTTAGGAAACGAAACAATGCATTGCGGA
AGTTTCGTCGAAATGGGGCGTGGGTGGACCTATCGTCCGATCCCTTTGGCTGGGATGAAGACACCGAAAATCA
CAATGACGGCATCAGCAACAAAAACAACAACTCGTTCGTGCAAAAATCTACGATGAATAAATTACAATCCATT
GCTATGGCAATTCGTCGTGCCGCAGCGACCGTCGAAACACGCCACCCACCATTATTTCGAGGAAAGGAACAAA
ACAATCAAGGGAAGAAGAAGGAACAAAAAAACAACATGATGCAAAAACCAATACCAATCGTGTTTGAAACTTT
AACCCCCCTTCTTCAATTGCATGGTGTGGAAAAAATATGTCTGTTGATTAAAAGTTTGGGAAGATGTATTGCG
TCCTCCTCGTCGTTAATGGCAGTACCTTCTGGCATGGCTAATGTGCCAATTCTATCGCCAATTGTCGCACCCA
TCCTCTACGAATCGTTGCGCCCATCGGAACACCGATGTCTTGAGGACATTGCAGATGCAATGCTTCATTTGAA
TTTGATGGAGGATAATCCAAGGGTCAGTGATCATCCCAACTCTACACCCCTTATTTCGGGAGTGATGGACTTG
GTGCGACGGGGTGGAGGCGGAGGCGGAGGAGGAGGAAAGGCTGATATGGGAGGGAAATTAATAGATCGG
>TR70|c0_g1_i1 len=978 path=[1102:0-392 1103:393-416 1127:417-475
1109:476-499 1129:500-570 1112:571-609 @1126@!:610-797 1106:798-836
1107:837-977] [-1, 1102, 1103, 1127, 1109, 1129, 1112, 1126, 1106, 1107,
-2]
TCCGATCTTTTTTCGGACAGCAGAGGGGAGGCTTTCACGACGGCGATTCAAAACAGGTTGGAGCAATGCGTAA
TCCAAGATGATATCGACTACAAGGTGGCGATCGAGAAGAAGTCCAAGGGAGACCCGAAACAGGTGAAACAAAA
ACGACGCAAGAAGAAGGATATGGTAGGACCGTTGTTGGAGGGTGCGCGAAAGGCATTCTCGGCCTTTGTGAGG
GCGTATCCCGCGAAGGAGAAGGCGGTGCGGCACATTTTCAACGCGCGGGCGCTCCACTTGGGCCACATTGCAA
GGTCGCTGGCGTTGAAGGAAACTCCCAAGATGGTGTCCAAGGGAAACAATAGCAAGGACAAGAATGCAACTCG
CGACAAGTCACAAACAAGCGGGGGCAAAGGAGGAGAGAAACGAAAGTCTAGACTGACGTTTGGGAACGATGAT
GATGAAGACCAAGAGGATGACTCTGCGATGGCAATCATCGAGGATGAGGAACGGGATCGTAATCTCAGCTTGT
TGGGCGATGCAATCGGTTCAAGCAGTAGGAAGTCCCATGCGAACAAAAAGATAAAAACGTTGGCGGGAGGCAG
TGGAGGTGGGAAGAATGTTCAAAAGAGAATGATGGAAGCGGCGATGATGATGCAAGCTCAGGAGTTCATGTAG
ACATTATGAAATTGTTCTAATCCCATAAGCGAGTTCAACTTGTTTGAAGCTGGGACAGTCTTGGAGAAAGTAC
TGACCAGCGTAATTATGTGCGGTGGCATTCCCCACCTTACAGTCTACGAAACGATAGGAACACACAAGGCTTT
GCTCTTTCTCTACTTTCCCCCCTAAGTATTTATTACTTAGCTGCCTATTCTTGTGCTGCCCATTACCCTCTTT
AAAGGAGGATGTATATCCATAACTCACACGCATTGGACAACTATCATCTTCTCACAATAGATACAAAGGTACA
TCAGGCAAGTAAACCCAACAAATGACATC
>TR71|c0_g1_i1 len=1638 path=[1699:0-366 1700:367-385 1701:386-390
1720:391-403 1719:404-523 1718:524-1534 1713:1535-1558 1714:1559-1637] [-
1, 1699, 1700, 1701, 1720, 1719, 1718, 1713, 1714, -2]
TTATGTATCACCTAGATCGTTGTATTTGACCACGAAGATCAAACTCTATCTCAATATATGCAGTCGAAATGGT
ACAATGATTGAAGAAGGGATGAATTAATAAGGAGTCGACTCAAATGCTCGTAATGACTGTTTTATTTTCTGGA
TTGTGAGCCCGTGTAAATGGATCAAATGATGTTCAAATGTCGTGTTAGAGGCGAATGTTATTGTCTGAAACCG
ATAAGGTATGAACTTGTGACCTTAGTGTAGACACCCCATCATGGCTGTGGAACACGACACCCATCTGAATGCA
TATGGATCATAGCTTTGGTTAAAATACGGAGACGCAATAAAACCACCCAACTCGAAACCAATTCTATTTTTTC
GTGAATTCGTTCTGTCTTTTTCTCAGTCTTGTGCACAATTTTACATCTACATCATGGCATCCTGCAACTCCTT
CAATATCGGCCCCACCTCCGGATCGCTCATGTACTTCGTCATCGCCATCGGATTCCCCATACACTCCTGCACC

GCTGCCATAACCTTGGGATTCTTCTGCGCCTTTTGAATGACCGCCATGACTTTGGGATTGGACATGGCCTGTT
GTGCGGCCTTCATCGCATCGTCCATTCCTCCTCCTCCTCCGAAGGGGTTTCCACCCATTCCTCCGAATGG
GTTTCCACCGCCACCTGCGGCGGCACCTCCGAAGGGATTTCCTCCTGCGGCTCCTCCGGGCGCACCGCCCATT
CCTCCCATGCCCTTCAACATGTCGGCAATGTTTCCCATGTCCATTCCTCCCATTCCACCTGGTCCTCCCCCTC
CTCCGAAGGGATTGCCGCCTGCTCCTCCAAAGGGATTGCCCCCTGCTCCGCCAAATGGATTGCCTGCCCCTGC
TCCGGCATCATTCGCATCCCTCGTCTTCTTCGTCTTCGGTCCCACATTATCGTTGGTAATCACCTCGACCTTC
GCTGCCGTAACCTCCTCGACATCATCATCATCGACTGCCGCAGCCCGCCTCTTTTTCTTCTTGGACTTTTTCA
CTCCATCGACCCTCGCCTCCGCCAGGGCTTTTACGAATTCGCTCTTTTCGAAAAACGTGTTGGTGGATACGCC
GTAGGATTCTAATTCGGATTTGAGTTCAGATACTTTGAGGGATTTGCAGTTTTCAAGCTCTGTTTGGAGGCGT
TGGTTCCTGTCTTCAGTGGTGGCTGTCGTCGTCGACGTCGTTGTTGTTGTTGTTGTCGTTTTTTCAATTTCTT
CTTCTGGAGTTTCCTCTGTTTTTTAATGCTGGAATGATTCTTCTTGGCGAGTATTCCGTGGAGGCTCAATACC
GTTACGGTAACTTCCATCGTCTTGCTCTTGCTGTTGTTGTTTTGATGGGAGGCGGGATCTGAATTGTTGTCAT
TGTTGCTGGAGTTAATGCTGTCGGCGTAGGGATTGCTATTGCTATTGATGCTATCGGCGTACGGATTCGAATT
GGTGGAGTAGCCATCGGAGGATGCCATTTTAGTATTGATATTGGAGATGGAATGATTGCTCGTTACCGATTGA
GGTGTGGCGGGTCGATGCTGCTGATGCTGATGATGTTGTTGTTGGGTCTTGCTTGGAGTAACGGTTGGCGACG
CGAGTGATTTCGTAAGCGAGGTTGTGAGATCG
>TR72|c0_g1_i1 len=1781 path=[1901:0-535 1902:536-559 1925:560-1117
1927:1118-1141 1928:1142-1161 1922:1162-1200 1924:1201-1494 1899:1495-
1518 1920:1519-1712 1905:1713-1736 1906:1737-1780] [-1, 1901, 1902, 1925,
1927, 1928, 1922, 1924, 1899, 1920, 1905, 1906, -2]
TCTTCCGATCTCGCATCAGCAATACGGCACAAAGCAAGCAAAAAAGTATCTATCATTTAGTTCGCGATCTAGT
CACAATAGATAACACAGCTCGTTGTTCCAGGCAACACAACATCAGTAGAGCGAGGTCGCTTCACCTGAAAATC
GATGTCACATCTCCAACAGCAGTGCCAAAAGAAAGGCACAGAAGTATGAGGAGAGCACCTGCATTGATACCCT
CCTGTACCCGTTGGTCAATGCGACGACCCGCCGCAGCTTCTGCAAGCACAAACGCCAGCTGGCCACCATCCAA
GGCAGGCAGCGGCAACGCGTTCACAACCGCCAGATTAACGCTGATAGATGCAGCGAACGCCACAACGGCAGCA
AAATCGGACGTGCTCACGACTTCCGCTCCCGACTTGACTACTCCAATAGGGCCGCTCATACTGGTTCCAGCAG
GAAGGCCTTTTCCTGAAATGAACCCCAACAGTAGACCGAAAATGGAACGAGAGGTTTGTGATGTGATATCGTA
TACGGCCGATCCCGCCTTGGAAATGGCATCCGTTACAGAGGCGGCTTGAACCATATCCGTTCGCAAATAGTTT
GGGCCCAACATCACTCCAATAGACGTCACTCCATCATCGTTCACCTTGGGAGCTACGGAAACTACATCTGTGG
CCTCACTATCCTTCCCATGAATAATTGTCAATTTGACGCTCTCTCCCACTGGAGTCTCGCGGATGGTGGAAAT
GACATCTGAAATTTGCTTTTGAGACGCCCAAATACCGCCCTGGGAATTTCCCGACGTGACCACGTCATTCACG
CCGAGAATGACATCGCCCCGCTTCAACAGGCCGAACGATGGACTGTCCTCGCTGGGCATTCGGCTTATAACTA
CTCCAGCGTCAAATACGGGCATGGGAAGACCTTTCCCTCCAGTCAGTTCACCAAAGTAGCATGCAAATGCCAG
GAGTATGTTGAACACCACTCCACCCGATAGGACGATGGCGCGTTGTTGCCAGGGTCGATTTTGGAGAAGGTTG
GGATCATCGAAGTAGTCAATGTCAGGGACTTTTGCGGCTTTCAGAATTGCAAGCTTGTCATTTCCTTCCAGCT
CTTCCTTTTCATCCTTCTTGCTGGCCCAGGGTAATGCACTCAACCAGGATCCATTCTTGTTTCCATTAGATTC
ATTTGCCATGAATAATTTCGCATCTTCTTCCGCCTGCTTGAGCTGTTCCGCTACCCGATTGGACTGCCATTTC
TTCAACACTCCCAACGTAAGTATATTGAACGCAAGGGATTTGGCTGCTACTGCCTCCAAACCCTTTTCCCATG
GCGACGCATTCTCTATGCGCATTAATCGGGCCTCATCCCTCGCCTTCCTGGCAATATCCTCTTGCTCAAATGC
CAGAGTTCGATTGTAATTCTCTGGGAAACGAACGTATCCACCCAACGGAATCGCCCTCAAACTAAATTCAATC
CCATCATCCGACAGTAATTCCGAACCTTCATCATTTCCCCCATCCTCCTTCGACTTCGATGCTGCATCATCAA
TCTTCTCGAAGACGAACTTGTCATCCACCAACCGACGACGTACCCCCACAATACGCGGTCCAACACCCACGCT
AAACTCATCGACGTTCATTCCGAGTGAACGCGCTGCGATGAAGTGACCAGCCTCGTGGACCAAAATGACGAAT
GCCAGGACAGCGATGCTCCCCAGAGGAGATCCGATGGCAGATAGTGCCGCGGCGGGTGTCATAGACTCGAGGG
ATGTAGTTGTTACGTCATGACGTGACAGT
>TR73|c0_g1_i1 len=1520 path=[1606:0-39 1607:40-61 1628:62-144 1629:145-
229 1621:230-246 1627:247-475 1618:476-499 1625:500-1454 1604:1455-1478
1605:1479-1519] [-1, 1606, 1607, 1628, 1629, 1621, 1627, 1618, 1625,
1604, 1605, -2]
CTGTTCACCATGACCATGTGAGTTGTTCTGTTCCACCCGCCCGCTCTGCCTACTTGTCCACCTGACAGATTCG
CCCACTCCCGCCATCATTCAGCACTACGATATCACCTGGCCACTCACAGCCAAGCAAATCCTTTCCTGAACAA
CAACCATGCCCGCATCAGTCTATCTCGTAACCGGCGGCGCCCGCTCCGGTAAATCATCTTACGCTCAGAGCCT
CTGCGAAAAAACATGTCCCAATCCTATCTACCTCGCCACCTCCAAGGTATGGGATGATGATTTCAAGGATAGA
GTCAAACGGCATCAAAATGAACGCGGTGACAATTGGACGACAATCGAAGAGCCACTACACCCAAGTAAACACT
GCGATAAATTTGGCGGCAAAGCAATCCTCGTCGATTGCCTCACATTATGGTTGACCAATTACTTCATGGAGCT
CGGTGCATTTACCGAACCCGATGGCGACAAAGATGCAATGGCGAATGAAAACAACGACAAAATTGGCGACGCA
TCGGAGCAGGCGTTGGAAAAAACGAAGGCTGAGTTTAACAAAATGATTACCCAATGGGATGCAACCTTCGTCT
TCGTCACGAACGAGATAGGGTCGGGATTGCATGCTGACAGTGCAGCATCGCGCAAGTTCGTGGATGCACAAGG
ATGGCTCAACCAATACGTATCATCCAAGGCAGACATGGTCATTCACATGGTCGCTGGTGTTCCAAACATTATC
AAACAATTTCCACCAGAGAGCCGTAATCCGCTCAAGGCCCCTGCCTCTCACGATCTGACGGAGCGTGCAGTGC

TCGATCAATTTTTATCAACACGCAGTCTAGCTATGGACGACAAGGGATACTTCATGATGAAACTCGATCACGC
CAAAGGCGTTATACGCGTCACGTATCATTCGTGTATTAAAAACGACAAGGGGGAGATATGCGATGTCCACGGG
AAGAAAATTAGCTGTTGCGAAGGGAACAATAATCGTCCGGAACCGATGGCGACGTTCGAGGCACGGACGGCGA
AGGAGCTCACGGTGATGGTGTTCGAGCGATGGGAGCATGCGAAAGATTTAGTGAGTGTGGGACACGCTGCGTA
TATTGGTAGGGAGGCGCAGAAGGCGGAGGAGTGTTTGTTTGCTGGAAGGTTTTATCAGCAGGATTGATGGAGG
AATGTGTCGTACAACATACAAAGAATGCTGCGCTGTCGATTTCATCTCATCTATTTGTGCAAACCATGAACCC
GAACACCATGAACCCCGTGTCCATTTTCCTGAAGTGCTCATAAGTTTAAGGCATGGAAGATGTGATTTGAAGG
AAGGGGAAGAAACAGAGACAAAAAGAAACATAATGAAAAATGCTACCAACTACCTGGTTTTGTTCAACGTGCT
CCGATGTGTCTAGCATTCTCAGAATCAAACTGGGTTTTTCGTAGCTAGATATTTGGCTTAAAATTCTGCATCC
GCTGGGTGGATCACGATTAAGGTGCATGTTACTACCCTGATTGTTTTAGAATTTGTCTCC
>TR74|c0_g1_i1 len=446 path=[647:0-121 648:122-145 685:146-259 692:260-283 695:284-316 688:317-340 694:341-358 696:359-371 681:372-394 662:395-445] [-1, 647, 648, 685, 692, 695, 688, 694, 696, 681, 662, -2]
CCCTTCGGCCGATCGGATCGCCTCGGTCGCTGCGCCGACTTCACCACCCAATCCTTCGCGCGCACGGGAGACG
CCACCGCGGGATACTTCCGCAACGCACGTCACCACAACCGCAATCGGGATGCCGAGGCGAACAAAAATTTGGA
ATTTCAGTACAAGGTGGATGCCGAGGAGGCGAAGGAGTTTCAGCTGGTGGATTCGAGCAAGGTTGGGCCGACG
AACGGGGGTGGGCCGAATGGCGCTCCCGGCGGCGGGAAGAGATTCGTGCCGCAGGCGAGGAGGAGGGCGAATG
CGGCGAGGTTGAGGCAGTTGAATGCTAGGAGGAACGATGGGAACCAGCAGGGGGGAAATGTTGGGAGGTACAA
TCAGCCACAGAGGACGAGGGCAGGTGGGAGATTCGGAGGAGGAAGAGGCGGACCCGGAGGTCGCGGTGGCAGA
GGGCGCGG
>TR74|c0_g1_i2 len=439 path=[647:0-121 648:122-145 685:146-259 692:260-283 695:284-316 688:317-340 694:341-358 696:359-371 681:372-394 666:395-438] [-1, 647, 648, 685, 692, 695, 688, 694, 696, 681, 666, -2]
CCCTTCGGCCGATCGGATCGCCTCGGTCGCTGCGCCGACTTCACCACCCAATCCTTCGCGCGCACGGGAGACG
CCACCGCGGGATACTTCCGCAACGCACGTCACCACAACCGCAATCGGGATGCCGAGGCGAACAAAAATTTGGA
ATTTCAGTACAAGGTGGATGCCGAGGAGGCGAAGGAGTTTCAGCTGGTGGATTCGAGCAAGGTTGGGCCGACG
AACGGGGGTGGGCCGAATGGCGCTCCCGGCGGCGGGAAGAGATTCGTGCCGCAGGCGAGGAGGAGGGCGAATG
CGGCGAGGTTGAGGCAGTTGAATGCTAGGAGGAACGATGGGAACCAGCAGGGGGGAAATGTTGGGAGGTACAA
TCAGCCACAGAGGACGAGGGCAGGTGGGAGGTGAGTACCTTGTTACGGCTTTTGTCAGTATGGTTGGTTGTTT
T
>TR75|c0_g1_i1 len=1667 path=[2552:0-156 2553:157-175 2615:176-184 2607:185-199 @2611@!:200-476 2569:477-496 2606:497-1198 2618:1199-1203 2614:1204-1226 @2609@!:1227-1368 2543:1369-1389 2544:1390-1666] [-1, 2552, 2553, 2615, 2607, 2611, 2569, 2606, 2618, 2614, 2609, 2543, 2544, -2]
TTGTCAACTGACATGACGCTTGTTGAACGTAAAACGGAACATAGAAAAGGCGATACATCATCATACCGTACTC
TAGCGCAACGCGTAGCCTTTGCGACCAGCATCACAATTATTATTGGCCTCCCACAGTTCCTGCACTGTTCCCA
ATAGGCTGAAGACACCCTCATCCGTGCGTACAAAAAAAACGTAAACACCAAAACTATATCATGTCGGCAGTAA
CAGTAACGTCCAACCCCTTCTCCATTACCATTCCATCGTCACTCCTCCTCCTCCTCCTCCTTGCATCAGTCGC
CGTTACCACTACTCGAGCACAAGATCATCCGATCCCCTATCCGCCTCCCGTCGATCACGTCGTCCATCCTCTC
CCACACACGTACCTCGCTTCGACGACATTGCCCCGAGAATTCACGTGGCAAGATGTGAATGGTCATTCGTATT
TGACGCGGGTGCGAAATCAACATATTCCACAGTATTGTGGGAGCTGTTGGGCACACGCAGCACTAAGTTCATT
GGCGGATCGTGTCAAGATCATGAGGAGTTACCGTGGTCCGAGAGAGAATTGTCATCAACAAGCGGAAGCAGCA
GCACAAGTGCAAGAAATGAACAGTGAGATGCATTTTCATAAGGAGACGGAGGATGATGTTCCCCAACCTATTC
TGGGAGAATTGGGTCCTCCTCCTGGACCGGATATTGATTTGTCAGTGCAATACATTTTGAACTGCGGACCCAA
ATCGGCCGAACCACACCATCATCTATCCTGCCACGGAGGAAGCACTCTCCGCGCGTACGAATACATCCATTCC
ACATTGAAATTCGTACCCGAAGATACCTGCCTGCAGTACATTGCGTGTTCGGACGATAGCGAAGAGGGGTGGT
GTCCTCACGTGCGGAGTTTGACCTCGTGTGATACGTGGAATGTCTGTCGTACGTGCGATGGTTTTTCGACATC
GAAGAGGGGAAGGAGCAACGACAACAAATTGTTGAGAGGGGAGGGTCACCGGGGTGGTGGGGAGGTTAGTCTT
AGTGTGGAAGGCGAGATTGGGGGAGGGGAGGAGGAGAGGGATTCGTACGGATGTCGGGCTATTCCTCATGGGT
CCATTCCCAATGTGACCATTTCGGAGTTTGGAAGTATTGAACCGGGGAATATTCATGCCATTCAAGCTGAAAT
TTACGCTCGAGGGCCAATCAAGACAGCCATTAATGCCGAACTACTCAAAACCTATACCGGTGGCATCCTCTGC
TCCGACGACGATCCCGCGTGCTTGGACAGGAATCACAACCACGGCGTGAGCATCGTGGGATGGGGTTACGATA
GTAGCAGGGACAAACAACACTGGATCATTCGGAATAGTTGGGGCCACTATTGGGGCGAGATGGGATTTTTCCG
AATTGAACTGGGAAGTAACTTTCTTGGGGTGGAAGAAAATTTTGCATGGGCCACGCCGGGCACATGGATGGGG
AGAAATGATGGCGATTGTCTCAATACGTACATTGATCCTTCGTTGGATGTTTCTGCTGTCAGCCTTCGCTAGA
GAAAGTGATCATACAACAGATGATAGATCGAGATGTCCTGTTTAGTTATCGTTGTTAAATACTGTTGTTGCGA
GAGAGATAAAAATGTATAATATCAAGTTTTAGTTCTAAAAAAAAAAGATCGGAAGAGCGTC
>TR75|c0_g1_i2 len=1795 path=[2552:0-156 2553:157-175 2615:176-184 2607:185-199 @2611@!:200-476 2569:477-496 2606:497-1198 2618:1199-1203

2613:1204-1354 @2609@!:1355-1496 2543:1497-1517 2544:1518-1794] [-1, 2552, 2553, 2615, 2607, 2611, 2569, 2606, 2618, 2613, 2609, 2543, 2544, -2]
TTGTCAACTGACATGACGCTTGTTGAACGTAAAACGGAACATAGAAAAGGCGATACATCATCATACCGTACTC
TAGCGCAACGCGTAGCCTTTGCGACCAGCATCACAATTATTATTGGCCTCCCACAGTTCCTGCACTGTTCCCA
ATAGGCTGAAGACACCCTCATCCGTGCGTACAAAAAAAACGTAAACACCAAAACTATATCATGTCGGCAGTAA
CAGTAACGTCCAACCCCTTCTCCATTACCATTCCATCGTCACTCCTCCTCCTCCTCCTCCTTGCATCAGTCGC
CGTTACCACTACTCGAGCACAAGATCATCCGATCCCCTATCCGCCTCCCGTCGATCACGTCGTCCATCCTCTC
CCACACACGTACCTCGCTTCGACGACATTGCCCCGAGAATTCACGTGGCAAGATGTGAATGGTCATTCGTATT
TGACGCGGGTGCGAAATCAACATATTCCACAGTATTGTGGGAGCTGTTGGGCACACGCAGCACTAAGTTCATT
GGCGGATCGTGTCAAGATCATGAGGAGTTACCGTGGTCCGAGAGAGAATTGTCATCAACAAGCGGAAGCAGCA
GCACAAGTGCAAGAAATGAACAGTGAGATGCATTTTCATAAGGAGACGGAGGATGATGTTCCCCAACCTATTC
TGGGAGAATTGGGTCCTCCTCCTGGACCGGATATTGATTTGTCAGTGCAATACATTTTGAACTGCGGACCCAA
ATCGGCCGAACCACACCATCATCTATCCTGCCACGGAGGAAGCACTCTCCGCGCGTACGAATACATCCATTCC
ACATTGAAATTCGTACCCGAAGATACCTGCCTGCAGTACATTGCGTGTTCGGACGATAGCGAAGAGGGGTGGT
GTCCTCACGTGCGGAGTTTGACCTCGTGTGATACGTGGAATGTCTGTCGTACGTGCGATGGTTTTTCGACATC
GAAGAGGGGAAGGAGCAACGACAACAAATTGTTGAGAGGGGAGGGTCACCGGGGTGGTGGGGAGGTTAGTCTT
AGTGTGGAAGGCGAGATTGGGGGAGGGGAGGAGGAGAGGGATTCGTACGGATGTCGGGCTATTCCTCATGGGT
CCATTCCCAATGTGACCATTTCGGAGTTTGGAAGTATTGAACCGGGGAATATTCATGCCATTCAAGCTGAAAT
TTACGCTCGAGGGCCAATCAAGACAGCCATTAATGCGTAAGTCGAATGCCGAGGGCAATTGTCAAGGTCACAC
TTCACGCGACAGTATATTGATGCCGTCCTTTCATGTTATTCTCAAACCCGCCTTTGTTCTTCGCGCGCTCAAA
TATTTACGACAACAACAGCGAACTACTCAAAACCTATACCGGTGGCATCCTCTGCTCCGACGACGATCCCGCG
TGCTTGGACAGGAATCACAACCACGGCGTGAGCATCGTGGGATGGGGTTACGATAGTAGCAGGGACAAACAAC
ACTGGATCATTCGGAATAGTTGGGGCCACTATTGGGGCGAGATGGGATTTTTCCGAATTGAACTGGGAAGTAA
CTTTCTTGGGGTGGAAGAAAATTTTGCATGGGCCACGCCGGGCACATGGATGGGGAGAAATGATGGCGATTGT
CTCAATACGTACATTGATCCTTCGTTGGATGTTTCTGCTGTCAGCCTTCGCTAGAGAAAGTGATCATACAACA
GATGATAGATCGAGATGTCCTGTTTAGTTATCGTTGTTAAATACTGTTGTTGCGAGAGAGATAAAAATGTATA
ATATCAAGTTTTAGTTCTAAAAAAAAAAGATCGGAAGAGCGTC
>TR75|c0_g1_i3 len=627 path=[2619:0-158 2618:159-163 2614:164-186 @2609@!:187-328 2543:329-349 2544:350-626] [-1, 2619, 2618, 2614, 2609, 2543, 2544, -2]
GCTGAAATTTACGCTCGGTATGTATTTATGATGTAGATTGTCCTTGTTTTGGTGTCGCTGAAATGTATATTCC
ACCCCCCTCCCCTCTCTCCACAGTTTTTCTTTGCGTGTGTTTCAATCCATGATATATGCAACAGAGGGCCAAT
CAAGACAGCCATTAATGCCGAACTACTCAAAACCTATACCGGTGGCATCCTCTGCTCCGACGACGATCCCGCG
TGCTTGGACAGGAATCACAACCACGGCGTGAGCATCGTGGGATGGGGTTACGATAGTAGCAGGGACAAACAAC
ACTGGATCATTCGGAATAGTTGGGGCCACTATTGGGGCGAGATGGGATTTTTCCGAATTGAACTGGGAAGTAA
CTTTCTTGGGGTGGAAGAAAATTTTGCATGGGCCACGCCGGGCACATGGATGGGGAGAAATGATGGCGATTGT
CTCAATACGTACATTGATCCTTCGTTGGATGTTTCTGCTGTCAGCCTTCGCTAGAGAAAGTGATCATACAACA
GATGATAGATCGAGATGTCCTGTTTAGTTATCGTTGTTAAATACTGTTGTTGCGAGAGAGATAAAAATGTATA
ATATCAAGTTTTAGTTCTAAAAAAAAAAGATCGGAAGAGCGTC
>TR75|c0_g1_i4 len=1893 path=[2598:0-156 2612:157-367 2608:368-384 2603:385-401 2615:402-410 2607:411-425 @2611@!:426-702 2569:703-722 2606:723-1424 2618:1425-1429 2614:1430-1452 @2609@!:1453-1594 2543:1595-1615 2544:1616-1892] [-1, 2598, 2612, 2608, 2603, 2615, 2607, 2611, 2569, 2606, 2618, 2614, 2609, 2543, 2544, -2]
TTGTCAACTGACATGACGCTTGTTGAACGTAAAACGGAACATAGAAAAGGCGATACATCATCATACCGTACTC
TAGCGCAACGCGTAGCCTTTGCGACCAGCATCACAATTATTATTGGCCTCCCACAGTTCCTGCACTGTTCCCA
ATAGGCTGAAGGTGAGGGTTTGCAATTTTTTGAACGAGAGTCTCTCCGTAGAGTTCCCAAGGGGTGGTGTTTT
TGCTCGCTGCGCTGTGTATATCGTATGATGTGTGCAGTGTGTTTTATCTGTTCTCTGTTCATGTTCATGTTCA
GACATTGTGTCGGTAGAAAAGATTAGGACGTGATATTATATCTGACATAACATATACATCTGCCCTTGTCTTT
TTTCTTCCCACCTCGAAGACACCCTCATCCGTGCGTACAAAAAAAACGTAAACACCAAAACTATATCATGTCG
GCAGTAACAGTAACGTCCAACCCCTTCTCCATTACCATTCCATCGTCACTCCTCCTCCTCCTCCTCCTTGCAT
CAGTCGCCGTTACCACTACTCGAGCACAAGATCATCCGATCCCCTATCCGCCTCCCGTCGATCACGTCGTCCA
TCCTCTCCCACACACGTACCTCGCTTCGACGACATTGCCCCGAGAATTCACGTGGCAAGATGTGAATGGTCAT
TCGTATTTGACGCGGGTGCGAAATCAACATATTCCACAGTATTGTGGGAGCTGTTGGGCACACGCAGCACTAA
GTTCATTGGCGGATCGTGTCAAGATCATGAGGAGTTACCGTGGTCCGAGAGAGAATTGTCATCAACAAGCGGA
AGCAGCAGCACAAGTGCAAGAAATGAACAGTGAGATGCATTTTCATAAGGAGACGGAGGATGATGTTCCCCAA
CCTATTCTGGGAGAATTGGGTCCTCCTCCTGGACCGGATATTGATTTGTCAGTGCAATACATTTTGAACTGCG
GACCCAAATCGGCCGAACCACACCATCATCTATCCTGCCACGGAGGAAGCACTCTCCGCGCGTACGAATACAT
CCATTCCACATTGAAATTCGTACCCGAAGATACCTGCCTGCAGTACATTGCGTGTTCGGACGATAGCGAAGAG

GGGTGGTGTCCTCACGTGCGGAGTTTGACCTCGTGTGATACGTGGAATGTCTGTCGTACGTGCGATGGTTTTT
CGACATCGAAGAGGGGAAGGAGCAACGACAACAAATTGTTGAGAGGGGAGGGTCACCGGGGTGGTGGGGAGGT
TAGTCTTAGTGTGGAAGGCGAGATTGGGGGAGGGGAGGAGGAGAGGGATTCGTACGGATGTCGGGCTATTCCT
CATGGGTCCATTCCCAATGTGACCATTTCGGAGTTTGGAAGTATTGAACCGGGGAATATTCATGCCATTCAAG
CTGAAATTTACGCTCGAGGGCCAATCAAGACAGCCATTAATGCCGAACTACTCAAAACCTATACCGGTGGCAT
CCTCTGCTCCGACGACGATCCCGCGTGCTTGGACAGGAATCACAACCACGGCGTGAGCATCGTGGGATGGGGT
TACGATAGTAGCAGGGACAAACAACACTGGATCATTCGGAATAGTTGGGGCCACTATTGGGGCGAGATGGGAT
TTTTCCGAATTGAACTGGGAAGTAACTTTCTTGGGGTGGAAGAAAATTTTGCATGGGCCACGCCGGGCACATG
GATGGGGAGAAATGATGGCGATTGTCTCAATACGTACATTGATCCTTCGTTGGATGTTTCTGCTGTCAGCCTT
CGCTAGAGAAAGTGATCATACAACAGATGATAGATCGAGATGTCCTGTTTAGTTATCGTTGTTAAATACTGTT
GTTGCGAGAGAGATAAAAATGTATAATATCAAGTTTTAGTTCTAAAAAAAAAAGATCGGAAGAGCGTC
>TR75|c0_g1_i5 len=1826 path=[2552:0-156 2553:157-175 2615:176-184
2607:185-199 @2611@!:200-476 2561:477-526 2610:527-655 2606:656-1357
2618:1358-1362 2614:1363-1385 @2609@!:1386-1527 2543:1528-1548 2544:1549-
1825] [-1, 2552, 2553, 2615, 2607, 2611, 2561, 2610, 2606, 2618, 2614,
2609, 2543, 2544, -2]
TTGTCAACTGACATGACGCTTGTTGAACGTAAAACGGAACATAGAAAAGGCGATACATCATCATACCGTACTC
TAGCGCAACGCGTAGCCTTTGCGACCAGCATCACAATTATTATTGGCCTCCCACAGTTCCTGCACTGTTCCCA
ATAGGCTGAAGACACCCTCATCCGTGCGTACAAAAAAAACGTAAACACCAAAACTATATCATGTCGGCAGTAA
CAGTAACGTCCAACCCCTTCTCCATTACCATTCCATCGTCACTCCTCCTCCTCCTCCTTGCATCAGTCGC
CGTTACCACTACTCGAGCACAAGATCATCCGATCCCCTATCCGCCTCCCGTCGATCACGTCGTCCATCCTCTC
CCACACACGTACCTCGCTTCGACGACATTGCCCCGAGAATTCACGTGGCAAGATGTGAATGGTCATTCGTATT
TGACGCGGGTGCGAAATCAACATATTCCACAGTATTGTGAGTGTCGTTGTGTCGTTTTGTGTAGGGTGTAGTT
TCATTTTATGTCGTATCGCATTATATTTGTGAGAGGAACCATATATTGTTGTGTATATTATTTCTTACATCTC
CACGCGGTCAATAACACTATCATGACGATGACTTGCATATATAAAAAAGGTGGGAGCTGTTGGGCACACGCAG
CACTAAGTTCATTGGCGGATCGTGTCAAGATCATGAGGAGTTACCGTGGTCCGAGAGAGAATTGTCATCAACA
AGCGGAAGCAGCAGCACAAGTGCAAGAAATGAACAGTGAGATGCATTTTCATAAGGAGACGGAGGATGATGTT
CCCCAACCTATTCTGGGAGAATTGGGTCCTCCTCCTGGACCGGATATTGATTTGTCAGTGCAATACATTTTGA
ACTGCGGACCCAAATCGGCCGAACCACACCATCATCTATCCTGCCACGGAGGAAGCACTCTCCGCGCGTACGA
ATACATCCATTCCACATTGAAATTCGTACCCGAAGATACCTGCCTGCAGTACATTGCGTGTTCGGACGATAGC
GAAGAGGGGTGGTGTCCTCACGTGCGGAGTTTGACCTCGTGTGATACGTGGAATGTCTGTCGTACGTGCGATG
GTTTTTCGACATCGAAGAGGGGAAGGAGCAACGACAACAAATTGTTGAGAGGGGAGGGTCACCGGGGTGGTGG
GGAGGTTAGTCTTAGTGTGGAAGGCGAGATTGGGGGAGGGGAGGAGGAGAGGGATTCGTACGGATGTCGGGCT
ATTCCTCATGGGTCCATTCCCAATGTGACCATTTCGGAGTTTGGAAGTATTGAACCGGGGAATATTCATGCCA
TTCAAGCTGAAATTTACGCTCGAGGGCCAATCAAGACAGCCATTAATGCCGAACTACTCAAAACCTATACCGG
TGGCATCCTCTGCTCCGACGACGATCCCGCGTGCTTGGACAGGAATCACAACCACGGCGTGAGCATCGTGGGA
TGGGGTTACGATAGTAGCAGGGACAAACAACACTGGATCATTCGGAATAGTTGGGGCCACTATTGGGGCGAGA
TGGGATTTTTCCGAATTGAACTGGGAAGTAACTTTCTTGGGGTGGAAGAAAATTTTGCATGGGCCACGCCGGG
CACATGGATGGGGAGAAATGATGGCGATTGTCTCAATACGTACATTGATCCTTCGTTGGATGTTTCTGCTGTC
AGCCTTCGCTAGAGAAAGTGATCATACAACAGATGATAGATCGAGATGTCCTGTTTAGTTATCGTTGTTAAAT
ACTGTTGTTGCGAGAGAGATAAAAATGTATAATATCAAGTTTTAGTTCTAAAAAAAAAAGATCGGAAGAGCGT
C
>TR75|c0_g1_i6 len=755 path=[2619:0-158 2618:159-163 2613:164-314
@2609@!:315-456 2543:457-477 2544:478-754] [-1, 2619, 2618, 2613, 2609,
2543, 2544, -2]
GCTGAAATTTACGCTCGGTATGTATTTATGATGTAGATTGTCCTTGTTTTGGTGTCGCTGAAATGTATATTCC
ACCCCCCTCCCCTCTCTCCACAGTTTTTCTTTGCGTGTGTTTCAATCCATGATATATGCAACAGAGGGCCAAT
CAAGACAGCCATTAATGCGTAAGTCGAATGCCGAGGGCAATTGTCAAGGTCACACTTCACGCGACAGTATATT
GATGCCGTCCTTTCATGTTATTCTCAAACCCGCCTTTGTTCTTCGCGCGCTCAAATATTTACGACAACAACAG
CGAACTACTCAAAACCTATACCGGTGGCATCCTCTGCTCCGACGACGATCCCGCGTGCTTGGACAGGAATCAC
AACCACGGCGTGAGCATCGTGGGATGGGGTTACGATAGTAGCAGGGACAAACAACACTGGATCATTCGGAATA
GTTGGGGCCACTATTGGGGCGAGATGGGATTTTTCCGAATTGAACTGGGAAGTAACTTTCTTGGGGTGGAAGA
AAATTTTGCATGGGCCACGCCGGGCACATGGATGGGGAGAAATGATGGCGATTGTCTCAATACGTACATTGAT
CCTTCGTTGGATGTTTCTGCTGTCAGCCTTCGCTAGAGAAAGTGATCATACAACAGATGATAGATCGAGATGT
CCTGTTTAGTTATCGTTGTTAAATACTGTTGTTGCGAGAGAGATAAAAATGTATAATATCAAGTTTTAGTTCT
AAAAAAAAAAGATCGGAAGAGCGTC
>TR76|c0_g1_i1 len=2062 path=[2460:0-342 2474:343-343 2471:344-367
2467:368-418 2438:419-442 2470:443-867 2457:868-891 2466:892-919
2473:920-943 2469:944-1668 2465:1669-1740 2441:1741-1764 2475:1765-1805
2454:1806-1824 2464:1825-1980 2444:1981-2004 2445:2005-2061] [-1, 2460,

2474, 2471, 2467, 2438, 2470, 2457, 2466, 2473, 2469, 2465, 2441, 2475, 2454, 2464, 2444, 2445, -2]
CAATCATCATCATCATCACCCACAACAACAAACAGACAACAACAACAACGTCCAAATTTATTCGAGAGGATAT
TCACCACGCGTCGTTTCCTCGCCAATCATCCTGACAATAACAACAACAACAACAAAACTGCCATCCTACAATT
GCACAATCACCTCCGAAAAGAAAACTGGAGCCTCGCGACGGTATTACTCGAATCGAATCCGAACTTGGCACGA
ACGTGGCATCCCGTCGATCGGTTGTACGGTGGACGGTACGATGGCGAAGCGCTGCCGATACATGCCGCGTGCG
CATTGCGTCCGCCTCCATCTTTTATCGAATTGTTGGGCCGGTTGTATCCGGAGGGGTTGGTGGAGAAGGATAA
GGCGTTTGGGAGGGTGCCGCTGCATGTGGCTTGTAGGAGTTTGGCGGAGAGTGGTGTGATTCGAGTGCTTTGC
GAGATGGATCCAAAATGTGTGGAGGAGAGAGATACTTTGAAGAGAGTTCCTTTGCATTATTTGATTAAGAATT
ATTCCACTTTTGGCGATGATAATGATGACGACGACGACAGCATTGACGAAGACAACGTCGCTGATACTACTAT
CGACGACAACAATACAAATGGCGAAAAAAACAACAACAACGACGACGGAATGATCGCATTGAAACTATTCATC
GAAACCAATCCCGCCTGCGTCCGCGCCACGGACCATCGAGGGTGGCTCCCGATACATGTCGCATGCAGCTGCT
CTTCCCGCAAGGGTATGATCCGCGTGATGAGGGTCCTTCTGAAAGCTTGGCCGGAGAGCGTAAATTGTAAAAC
GGACAAAAACAGCGATGCCTTTGCTTGTGTCGATATGGCGGGGAAAATTCATCCCACTAAGGATACAGTTCTT
GCGTTGTTGCATGAGGTGAGGAGTAAGATAGAGCGGAGGGGAATTAGTGCGGTGGAGGAGAATTCTGAGGGGT
GTGATGGTGATGACGCGGCGATTGGAAATGGTGATGAAGATGATGTTGAGCACGAAGATCAATCCTTGTCTGA
CAAAAGCGTGGAAGAAAGTAATTCTTCACTGCCGCAATGCGAAACGACATTAGAGGAGCAGAAGGAGCATTTG
CACGAGGAAGAAGAGTGTACTCGTTCAACATTGTCAAGCGGAGAAACCCATCCAATAATACCATGCCCTGAGG
AAGGAATTTTGATTGATTTGGAGGAGGCAAGAAGCAAGTTAGAACGGAGCGGAAATACCGGGGATGATAATTC
TGAGGGCTGTGATGATGAAGCGGCTGTTGAAAATAGATATGAGGATGTGGTTAATCAAGAAGATCAATCCCTG
TCTGAAAGAAGTGTTGAAGAAGGCAAATCCTCACTGCCGCAATGTGAAACCACGCCAGAGGAACAAAGTAAGC
AGTTGCACAAGGTAGAAGAGTGCATCACAACTTTGTCTAGTGGAGAAAAGTATCCAGCAATGGCATACCCAGA
GGAAGGTGTTTTAATTAATTTGTAAATACAGATATGCGAAACGAAAGTATCATCTAGCACATTCCCAAGGTTT
GATAGGTGGTATCGCAGTACAAGCGACAAGGGTTCCCTCAATTATCTATTTCCCTGGTTAAGTCTCCACAACA
ACATTTGCCAAGTGCCTTCCCGCTTGCCATCGGACTTAAAAGCCAACTTTAGAGCTAGCAAGACTGCCATATT
TTACTGAATCTCATGTAGAGTCCATCGAGCACCTACATCCTGTCGTTCAACACTACTGTCGGGGTAGATGTCT
AATGACAAGGTCATTGTGGTGTACACAATAAATGTTTTGCTCAAATAGATGGTATCTGCATTCATCCATGTAA
ATTGATTCTTCTCACACAACTGCTCTACACTGCTACCTATCATGATGTTTATAGCATTCCAGTAGTCATATGT
ATGATGATGAGAGTGTAGATGACTTATGTTCATCATTTACCACATGTCAGTGTCTAAAGGTCCACTAGTAATC
TGAATCTCTCATGTCATTTGAAGTGTATTTAGGCCAGGGTTGTCAATAGACTTAAATTACAAATAAACTCATA
AAAAAGACTGTGAAAAAA
>TR76|c0_g1_i2 len=2152 path=[2460:0-342 2474:343-343 2471:344-367 2467:368-418 2438:419-442 2470:443-867 2457:868-891 2466:892-919 2473:920-943 2469:944-1668 2465:1669-1740 2441:1741-1764 2475:1765-1805 2468:1806-1914 2464:1915-2070 2444:2071-2094 2445:2095-2151] [-1, 2460, 2474, 2471, 2467, 2438, 2470, 2457, 2466, 2473, 2469, 2465, 2441, 2475, 2468, 2464, 2444, 2445, -2]
CAATCATCATCATCATCACCCACAACAACAAACAGACAACAACAACAACGTCCAAATTTATTCGAGAGGATAT
TCACCACGCGTCGTTTCCTCGCCAATCATCCTGACAATAACAACAACAACAACAAAACTGCCATCCTACAATT
GCACAATCACCTCCGAAAAGAAAACTGGAGCCTCGCGACGGTATTACTCGAATCGAATCCGAACTTGGCACGA
ACGTGGCATCCCGTCGATCGGTTGTACGGTGGACGGTACGATGGCGAAGCGCTGCCGATACATGCCGCGTGCG
CATTGCGTCCGCCTCCATCTTTTATCGAATTGTTGGGCCGGTTGTATCCGGAGGGGTTGGTGGAGAAGGATAA
GGCGTTTGGGAGGGTGCCGCTGCATGTGGCTTGTAGGAGTTTGGCGGAGAGTGGTGTGATTCGAGTGCTTTGC
GAGATGGATCCAAAATGTGTGGAGGAGAGAGATACTTTGAAGAGAGTTCCTTTGCATTATTTGATTAAGAATT
ATTCCACTTTTGGCGATGATAATGATGACGACGACGACAGCATTGACGAAGACAACGTCGCTGATACTACTAT
CGACGACAACAATACAAATGGCGAAAAAAACAACAACAACGACGACGGAATGATCGCATTGAAACTATTCATC
GAAACCAATCCCGCCTGCGTCCGCGCCACGGACCATCGAGGGTGGCTCCCGATACATGTCGCATGCAGCTGCT
CTTCCCGCAAGGGTATGATCCGCGTGATGAGGGTCCTTCTGAAAGCTTGGCCGGAGAGCGTAAATTGTAAAAC
GGACAAAAACAGCGATGCCTTTGCTTGTGTCGATATGGCGGGGAAAATTCATCCCACTAAGGATACAGTTCTT
GCGTTGTTGCATGAGGTGAGGAGTAAGATAGAGCGGAGGGGAATTAGTGCGGTGGAGGAGAATTCTGAGGGGT
GTGATGGTGATGACGCGGCGATTGGAAATGGTGATGAAGATGATGTTGAGCACGAAGATCAATCCTTGTCTGA
CAAAAGCGTGGAAGAAAGTAATTCTTCACTGCCGCAATGCGAAACGACATTAGAGGAGCAGAAGGAGCATTTG
CACGAGGAAGAAGAGTGTACTCGTTCAACATTGTCAAGCGGAGAAACCCATCCAATAATACCATGCCCTGAGG
AAGGAATTTTGATTGATTTGGAGGAGGCAAGAAGCAAGTTAGAACGGAGCGGAAATACCGGGGATGATAATTC
TGAGGGCTGTGATGATGAAGCGGCTGTTGAAAATAGATATGAGGATGTGGTTAATCAAGAAGATCAATCCCTG
TCTGAAAGAAGTGTTGAAGAAGGCAAATCCTCACTGCCGCAATGTGAAACCACGCCAGAGGAACAAAGTAAGC
AGTTGCACAAGGTAGAAGAGTGCATCACAACTTTGTCTAGTGGAGAAAAGTATCCAGCAATGGCATACCCAGA
GGAAGGTGTTTTAATTAATTTGTAAATACAGATATGCGAAACGAAAGTATCATCTAGCACATTCCCAAGGTTT
GATAGGTGGTATCGCAGTACAAGCGACAAGGGTTCCCTCAATTATCTATTTCCCTGGTTAAGTCTCCACAACA
ACATTTGCCAAGTGCCTTCCCGCTTGCCATCGGACTTAAAAGCCAACTTTAGAGCTAGCAAGACTGCCATATT

TTACTGAATCTCATGTAGAGTCCATCGAGCACCTACATCCTGTCGTTCAACACTACTGTCGGGGTAGATGTCT
AATGACAAGGTCATTGTGGTGTACACAATAAATGTTTTGCTCAAATAGATGGTACGAAGACTAAAAGATCCAA
AGTGTTCCTTTTTGTAAATAGACAATGGTACATTCATATTTCCCTTCATTTTGACACAAAACCAATAGGTATC
TGCATTCATCCATGTAAATTGATTCTTCTCACACAACTGCTCTACACTGCTACCTATCATGATGTTTATAGCA
TTCCAGTAGTCATATGTATGATGATGAGAGTGTAGATGACTTATGTTCATCATTTACCACATGTCAGTGTCTA
AAGGTCCACTAGTAATCTGAATCTCTCATGTCATTTGAAGTGTATTTAGGCCAGGGTTGTCAATAGACTTAAA
TTACAAATAAACTCATAAAAAAGACTGTGAAAAAA
>TR77|c0_g1_i1 len=1370 path=[2527:0-165 2685:166-225 2681:226-232
2668:233-274 2651:275-314 2547:315-326 2645:327-330 2626:331-491
2518:492-515 2658:516-542 2675:543-563 @2641@!:564-715 2657:716-739
2640:740-763 2673:764-775 2656:776-799 2639:800-842 2619:843-991
2469:992-1009 2470:1010-1033 2471:1034-1055 2472:1056-1369] [-1, 2527,
2685, 2681, 2668, 2651, 2547, 2645, 2626, 2518, 2658, 2675, 2641, 2657,
2640, 2673, 2656, 2639, 2619, 2469, 2470, 2471, 2472, -2]
AAAGGGCGGACAAGATGTAGATATATTTACGTATATCAACATGCCCCCAAATCTACTTCAAAGGCCAAAAATA
ATCAGTCTTCCCCTCCATCCGTCTTCGCATCGCTCGTCGCTCGCAATGGCCAATCTCTCTAACGCCTGTGACT
GACTTGATTTTTACTCAAAAAAAGAGCGAATCATTATTCAACCGACCGCCTCTCTCTAGAACAATATCAAAAT
GAAGTCAATCATCGCCCTTATCTCAATTGTGTGCCTGGCACTCATCCATGGCTCTGTTGCGTCTGCTGGCGAG
GAAGTGACCACAAAGGTTTTTTTCGACATTGACATCGAGGGAGGAGAAGGAGGACGCATCGTCCTCGGCCTTT
TCGGCGCCACCGTCCCAAAGACGGTCGAAAACTTCCGTGCCCTATGCACGGGCGAGAAGGGAGTGGGCAAACG
CGGAAAGCCCCTCCATTACAAGGGATCAACCTTCCACCGCGTCATTCCCAACTTTATGTTGCAGGGTGGTGAT
TTCACGGACGGCAATGGACGTGGTGGCGAGAGTATCTACGGAGAAAAATTTGCTGATGAGAACTTCGACCTGA
AGCACGAGGGTCCCTACTACCTTTCCATGGCCAATGCTGGACCCAACACGAACGGATCCCAATTTTTTATCAC
CACCGTCACGACCTCCTGGCTTGATGGACGTCACGTCGTTTTTGGAAAGGTTCTCGAGGTAAGTCGTTTAGTT
TTGTGTCGGGATTGGTGTGAATAATCTCCTCGCAAGTGAACATGACGAGGCCTGATCAACTTTTTCCATCTCG
TATTCCATGTTGGTTGCAGGGACAGGATGTCGTAAAGCGTGTTGAGGGCGTTGGCTCAAGCGGAGGCAAGACG
ACGAAGACGGTAACCATTGCCGACTCTGGAGAGCTCAAGGACTAAGTGGCTGCCCCTGCCCTGCACGCAAGCT
ACCAAGTGGACGGTGACATACGTTGATTGAGCTTCAATTTGCTTACAACAAGCGCTCATGTTATCCAGAATTT
GACAGAGACAATTTAGCTCAGACATCAAGTCCCATTGAATTTATTATAAATTAGATGAATCTAAATGACGTGT
GTACGTGATCCTTCATCGAGGGGAATCGTTTACCCAAGATGGAGGATTGCATTTTCTTTTATCTCCTAAATGA
AGTCCTTCATTGCCGTGTTTTTTAGGAGTAGCACGTAGTATACTATCATTCCAACAGGCTGCGTATGACATGT
AAATCGAAGTCGTGCCACAACGGCCAAGTCCAAGAGAAGAGGGATATAATTTGAAGCAATCAAATAAAACTCC
TATCGTGTGACTCTTGATGTAAGGATGATTCACTGTTATGAATTTGAATGTATTCA
>TR77|c0_g1_i2 len=1017 path=[2500:0-49 2672:50-152 2689:153-156
2582:157-180 2674:181-210 @2641@!:211-362 2657:363-386 2640:387-410
2673:411-422 2656:423-446 2639:447-489 2619:490-638 2469:639-656
2470:657-680 2471:681-702 2472:703-1016] [-1, 2500, 2672, 2689, 2582,
2674, 2641, 2657, 2640, 2673, 2656, 2639, 2619, 2469, 2470, 2471, 2472, -
2]
ATGTTGCAGGGTGGTGATTTCACGGACGGCAATGGACGTGGTGGCGAGAGGTGAGTGTTGAACATAGCACTTG
TTTGGGAAGGTGATGTTTGGAGATGTGTCTGTGTTGGGATAGAATTACACCAATGGCATCATGGCCAGATTCA
TGGTAATACTCACCGTTCACCAACACACGTCACCTCCACCACAGTATCTACGGAGAAAAATTTGCTGATGAGA
ACTTCGACCTGAAGCACGAGGGTCCCTACTACCTTTCCATGGCCAATGCTGGACCCAACACGAACGGATCCCA
ATTTTTTATCACCACCGTCACGACCTCCTGGCTTGATGGACGTCACGTCGTTTTTGGAAAGGTTCTCGAGGTA
AGTCGTTTAGTTTTGTGTCGGGATTGGTGTGAATAATCTCCTCGCAAGTGAACATGACGAGGCCTGATCAACT
TTTTCCATCTCGTATTCCATGTTGGTTGCAGGGACAGGATGTCGTAAAGCGTGTTGAGGGCGTTGGCTCAAGC
GGAGGCAAGACGACGAAGACGGTAACCATTGCCGACTCTGGAGAGCTCAAGGACTAAGTGGCTGCCCCTGCCC
TGCACGCAAGCTACCAAGTGGACGGTGACATACGTTGATTGAGCTTCAATTTGCTTACAACAAGCGCTCATGT
TATCCAGAATTTGACAGAGACAATTTAGCTCAGACATCAAGTCCCATTGAATTTATTATAAATTAGATGAATC
TAAATGACGTGTGTACGTGATCCTTCATCGAGGGGAATCGTTTACCCAAGATGGAGGATTGCATTTTCTTTTA
TCTCCTAAATGAAGTCCTTCATTGCCGTGTTTTTTAGGAGTAGCACGTAGTATACTATCATTCCAACAGGCTG
CGTATGACATGTAAATCGAAGTCGTGCCACAACGGCCAAGTCCAAGAGAAGAGGGATATAATTTGAAGCAATC
AAATAAAACTCCTATCGTGTGACTCTTGATGTAAGGATGATTCACTGTTATGAATTTGAATGTATTCA
>TR77|c0_g1_i3 len=726 path=[2577:0-165 2684:166-224 2680:225-231
2652:232-273 2683:274-305 2666:306-356 2650:357-484 2690:485-508
2693:509-531 2688:532-555 2687:556-591 2682:592-621 2629:622-645
2661:646-649 2644:650-673 2624:674-685 2598:686-725] [-1, 2577, 2684,
2680, 2652, 2683, 2666, 2650, 2690, 2693, 2688, 2687, 2682, 2629, 2661,
2644, 2624, 2598, -2]

AAAGGGCGGACAAGATGTAGATATATTTACGTATATCAACATGCCCCCAAATCTACTTCAAAGGCCAAAAATA
ATCAGTCTTCCCCTCCATCCGTCTTCGCATCGCTCGTCGCTCGCAATGGCCAATCTCTCTAACGCCTGTGACT
GACTTGATTTTTACTCAAAACGAAGCGAATCATTATTCAAACGACCGCCTCTCTCCAGAACAATATCAAAATG
AAGTCAATCATCGCCCTTATCTCAATTGTGTGCCTGGCACTCATCCATGGCTCTGCTGCGTCGTGAGTTGAAT
CAAGATCGATCATTTTAGCGGACACCTCGGGCGCCGGAGCGTCGTCGAACGTATGCGCGTCCGACAACCGCAG
ACAGCTGTTACTTTATCATCGACCACATCCGCGGCGTGGAGCTACGGATCGTATCAAGTAACATGACGACGTC
CCCCGTAGCAGAAGGAACCCTCTAGTCATCCTGGCCTATTCATGAGTCAGAGTTTCATCTCATTTGACGACTA
ATCCAATTTCTAATTTTGTTTGCGCCCCTATTCCCACCAGTGCTGGCGAGGAAGTGACCACCAAGGTTTTTTT
CGACATTGTGAGTATAATATATCCATAACATCGTTTTCAAAGGAAAAAATCGCGCACGTGTAACGATGAATAA
TTCTCATCGAATCGCGCAACACACATCTCAAATAACAATTAACACAACAAATACAGGACATCGAGGGAG
>TR77|c0_g1_i4 len=1639 path=[2577:0-165 2684:166-224 2680:225-231
2652:232-273 2683:274-305 2666:306-356 2650:357-484 2690:485-508
2693:509-531 2688:532-555 2687:556-591 2662:592-595 2645:596-599
2626:600-760 2518:761-784 2658:785-811 2675:812-832 @2641@!:833-984
2657:985-1008 2640:1009-1032 2673:1033-1044 2656:1045-1068 2639:1069-1111
2619:1112-1260 2469:1261-1278 2470:1279-1302 2471:1303-1324 2472:1325-
1638] [-1, 2577, 2684, 2680, 2652, 2683, 2666, 2650, 2690, 2693, 2688,
2687, 2662, 2645, 2626, 2518, 2658, 2675, 2641, 2657, 2640, 2673, 2656,
2639, 2619, 2469, 2470, 2471, 2472, -2]
AAAGGGCGGACAAGATGTAGATATATTTACGTATATCAACATGCCCCCAAATCTACTTCAAAGGCCAAAAATA
ATCAGTCTTCCCCTCCATCCGTCTTCGCATCGCTCGTCGCTCGCAATGGCCAATCTCTCTAACGCCTGTGACT
GACTTGATTTTTACTCAAAACGAAGCGAATCATTATTCAAACGACCGCCTCTCTCCAGAACAATATCAAAATG
AAGTCAATCATCGCCCTTATCTCAATTGTGTGCCTGGCACTCATCCATGGCTCTGCTGCGTCGTGAGTTGAAT
CAAGATCGATCATTTTAGCGGACACCTCGGGCGCCGGAGCGTCGTCGAACGTATGCGCGTCCGACAACCGCAG
ACAGCTGTTACTTTATCATCGACCACATCCGCGGCGTGGAGCTACGGATCGTATCAAGTAACATGACGACGTC
CCCCGTAGCAGAAGGAACCCTCTAGTCATCCTGGCCTATTCATGAGTCAGAGTTTCATCTCATTTGACGACTA
ATCCAATTTCTAATTTTGTTTGCGCCCCTATTCCCACCAGTGCTGGCGAGGAAGTGACCACCAAGGTTTTTTT
CGACATTGACATCGAGGGAGGAGAAGGAGGACGCATCGTCCTCGGCCTTTTCGGCGCCACCGTCCCAAAGACG
GTCGAAAACTTCCGTGCCCTATGCACGGGCGAGAAGGGAGTGGGCAAACGCGGAAAGCCCCTCCATTACAAGG
GATCAACCTTCCACCGCGTCATTCCCAACTTTATGTTGCAGGGTGGTGATTTCACGGACGGCAATGGACGTGG
TGGCGAGAGTATCTACGGAGAAAAATTTGCTGATGAGAACTTCGACCTGAAGCACGAGGGTCCCTACTACCTT
TCCATGGCCAATGCTGGACCCAACACGAACGGATCCCAATTTTTTATCACCACCGTCACGACCTCCTGGCTTG
ATGGACGTCACGTCGTTTTTGGAAAGGTTCTCGAGGTAAGTCGTTTAGTTTTGTGTCGGGATTGGTGTGAATA
ATCTCCTCGCAAGTGAACATGACGAGGCCTGATCAACTTTTTCCATCTCGTATTCCATGTTGGTTGCAGGGAC
AGGATGTCGTAAAGCGTGTTGAGGGCGTTGGCTCAAGCGGAGGCAAGACGACGAAGACGGTAACCATTGCCGA
CTCTGGAGAGCTCAAGGACTAAGTGGCTGCCCCTGCCCTGCACGCAAGCTACCAAGTGGACGGTGACATACGT
TGATTGAGCTTCAATTTGCTTACAACAAGCGCTCATGTTATCCAGAATTTGACAGAGACAATTTAGCTCAGAC
ATCAAGTCCCATTGAATTTATTATAAATTAGATGAATCTAAATGACGTGTGTACGTGATCCTTCATCGAGGGG
AATCGTTTACCCAAGATGGAGGATTGCATTTTCTTTTATCTCCTAAATGAAGTCCTTCATTGCCGTGTTTTTT
AGGAGTAGCACGTAGTATACTATCATTCCAACAGGCTGCGTATGACATGTAAATCGAAGTCGTGCCACAACGG
CCAAGTCCAAGAGAAGAGGGATATAATTTGAAGCAATCAAATAAAACTCCTATCGTGTGACTCTTGATGTAAG
GATGATTCACTGTTATGAATTTGAATGTATTCA
>TR77|c0_g1_i5 len=1532 path=[2577:0-165 2684:166-224 2680:225-231
2652:232-273 2683:274-305 2667:306-356 2650:357-484 2690:485-508
2693:509-531 2688:532-555 2687:556-591 2662:592-595 2645:596-599
2626:600-760 2518:761-784 2658:785-811 2675:812-832 @2641@!:833-984
2454:985-1004 2619:1005-1153 2469:1154-1171 2470:1172-1195 2471:1196-1217
2472:1218-1531] [-1, 2577, 2684, 2680, 2652, 2683, 2667, 2650, 2690,
2693, 2688, 2687, 2662, 2645, 2626, 2518, 2658, 2675, 2641, 2454, 2619,
2469, 2470, 2471, 2472, -2]
AAAGGGCGGACAAGATGTAGATATATTTACGTATATCAACATGCCCCCAAATCTACTTCAAAGGCCAAAAATA
ATCAGTCTTCCCCTCCATCCGTCTTCGCATCGCTCGTCGCTCGCAATGGCCAATCTCTCTAACGCCTGTGACT
GACTTGATTTTTACTCAAAACGAAGCGAATCATTATTCAAACGACCGCCTCTCTCCAGAACAATATCAAAATG
AAGTCAATCATCGCCCTTATCTCAATTGTGTGCCTGGCACTCATCCATGGCTCTGCTGCGTCGTGAGTTGAAT
CAAGATCGATCATTCTAGCGGACACCTCGGGCGCGGGAGCGGCGTCGAACGTATGCGCGTCCGACAACCGCAG
ACAGCTGTTACTTTATCATCGACCACATCCGCGGCGTGGAGCTACGGATCGTATCAAGTAACATGACGACGTC
CCCCGTAGCAGAAGGAACCCTCTAGTCATCCTGGCCTATTCATGAGTCAGAGTTTCATCTCATTTGACGACTA
ATCCAATTTCTAATTTTGTTTGCGCCCCTATTCCCACCAGTGCTGGCGAGGAAGTGACCACCAAGGTTTTTTT
CGACATTGACATCGAGGGAGGAGAAGGAGGACGCATCGTCCTCGGCCTTTTCGGCGCCACCGTCCCAAAGACG
GTCGAAAACTTCCGTGCCCTATGCACGGGCGAGAAGGGAGTGGGCAAACGCGGAAAGCCCCTCCATTACAAGG

GATCAACCTTCCACCGCGTCATTCCCAACTTTATGTTGCAGGGTGGTGATTTCACGGACGGCAATGGACGTGG
TGGCGAGAGTATCTACGGAGAAAAATTTGCTGATGAGAACTTCGACCTGAAGCACGAGGGTCCCTACTACCTT
TCCATGGCCAATGCTGGACCCAACACGAACGGATCCCAATTTTTTATCACCACCGTCACGACCTCCTGGCTTG
ATGGACGTCACGTCGTTTTTGGAAAGGTTCTCGAGGGACAGGATGTCGTAAAGCGTGTTGAGGGCGTTGGCTC
AAGCGGAGGCAAGACGACGAAGACGGTAACCATTGCCGACTCTGGAGAGCTCAAGGACTAAGTGGCTGCCCCT
GCCCTGCACGCAAGCTACCAAGTGGACGGTGACATACGTTGATTGAGCTTCAATTTGCTTACAACAAGCGCTC
ATGTTATCCAGAATTTGACAGAGACAATTTAGCTCAGACATCAAGTCCCATTGAATTTATTATAAATTAGATG
AATCTAAATGACGTGTGTACGTGATCCTTCATCGAGGGGAATCGTTTACCCAAGATGGAGGATTGCATTTTCT
TTTATCTCCTAAATGAAGTCCTTCATTGCCGTGTTTTTTAGGAGTAGCACGTAGTATACTATCATTCCAACAG
GCTGCGTATGACATGTAAATCGAAGTCGTGCCACAACGGCCAAGTCCAAGAGAAGAGGGATATAATTTGAAGC
AATCAAATAAAACTCCTATCGTGTGACTCTTGATGTAAGGATGATTCACTGTTATGAATTTGAATGTATTCA
>TR77|c0_g1_i6 len=910 path=[2500:0-49 2672:50-152 2689:153-156 2582:157-180 2674:181-210 @2641@!:211-362 2454:363-382 2619:383-531 2469:532-549 2470:550-573 2471:574-595 2472:596-909] [-1, 2500, 2672, 2689, 2582, 2674, 2641, 2454, 2619, 2469, 2470, 2471, 2472, -2]
ATGTTGCAGGGTGGTGATTTCACGGACGGCAATGGACGTGGTGGCGAGAGGTGAGTGTTGAACATAGCACTTG
TTTGGGAAGGTGATGTTTGGAGATGTGTCTGTGTTGGGATAGAATTACACCAATGGCATCATGGCCAGATTCA
TGGTAATACTCACCGTTCACCAACACACGTCACCTCCACCACAGTATCTACGGAGAAAAATTTGCTGATGAGA
ACTTCGACCTGAAGCACGAGGGTCCCTACTACCTTTCCATGGCCAATGCTGGACCCAACACGAACGGATCCCA
ATTTTTTATCACCACCGTCACGACCTCCTGGCTTGATGGACGTCACGTCGTTTTTGGAAAGGTTCTCGAGGGA
CAGGATGTCGTAAAGCGTGTTGAGGGCGTTGGCTCAAGCGGAGGCAAGACGACGAAGACGGTAACCATTGCCG
ACTCTGGAGAGCTCAAGGACTAAGTGGCTGCCCCTGCCCTGCACGCAAGCTACCAAGTGGACGGTGACATACG
TTGATTGAGCTTCAATTTGCTTACAACAAGCGCTCATGTTATCCAGAATTTGACAGAGACAATTTAGCTCAGA
CATCAAGTCCCATTGAATTTATTATAAATTAGATGAATCTAAATGACGTGTGTACGTGATCCTTCATCGAGGG
GAATCGTTTACCCAAGATGGAGGATTGCATTTTCTTTTATCTCCTAAATGAAGTCCTTCATTGCCGTGTTTTT
TAGGAGTAGCACGTAGTATACTATCATTCCAACAGGCTGCGTATGACATGTAAATCGAAGTCGTGCCACAACG
GCCAAGTCCAAGAGAAGAGGGATATAATTTGAAGCAATCAAATAAAACTCCTATCGTGTGACTCTTGATGTAA
GGATGATTCACTGTTATGAATTTGAATGTATTCA
>TR77|c0_g1_i7 len=249 path=[2527:0-165 2685:166-225 2681:226-232 2508:233-248] [-1, 2527, 2685, 2681, 2508, -2]
AAAGGGCGGACAAGATGTAGATATATTTACGTATATCAACATGCCCCCAAATCTACTTCAAAGGCCAAAAATA
ATCAGTCTTCCCCTCCATCCGTCTTCGCATCGCTCGTCGCTCGCAATGGCCAATCTCTCTAACGCCTGTGACT
GACTTGATTTTTACTCAAAAAAAGAGCGAATCATTATTCAACCGACCGCCTCTCTCTAGAACAATATCAAAAT
GAAGTCAATCATCGAGATCGGAAGAGCGTC
>TR77|c0_g1_i8 len=1263 path=[2527:0-165 2685:166-225 2681:226-232 2668:233-274 2651:275-314 2547:315-326 2645:327-330 2626:331-491 2518:492-515 2658:516-542 2675:543-563 @2641@!:564-715 2454:716-735 2619:736-884 2469:885-902 2470:903-926 2471:927-948 2472:949-1262] [-1, 2527, 2685, 2681, 2668, 2651, 2547, 2645, 2626, 2518, 2658, 2675, 2641, 2454, 2619, 2469, 2470, 2471, 2472, -2]
AAAGGGCGGACAAGATGTAGATATATTTACGTATATCAACATGCCCCCAAATCTACTTCAAAGGCCAAAAATA
ATCAGTCTTCCCCTCCATCCGTCTTCGCATCGCTCGTCGCTCGCAATGGCCAATCTCTCTAACGCCTGTGACT
GACTTGATTTTTACTCAAAAAAAGAGCGAATCATTATTCAACCGACCGCCTCTCTCTAGAACAATATCAAAAT
GAAGTCAATCATCGCCCTTATCTCAATTGTGTGCCTGGCACTCATCCATGGCTCTGTTGCGTCTGCTGGCGAG
GAAGTGACCACAAAGGTTTTTTTCGACATTGACATCGAGGGAGGAGAAGGAGGACGCATCGTCCTCGGCCTTT
TCGGCGCCACCGTCCCAAAGACGGTCGAAAACTTCCGTGCCCTATGCACGGGCGAGAAGGGAGTGGGCAAACG
CGGAAAGCCCCTCCATTACAAGGGATCAACCTTCCACCGCGTCATTCCCAACTTTATGTTGCAGGGTGGTGAT
TTCACGGACGGCAATGGACGTGGTGGCGAGAGTATCTACGGAGAAAAATTTGCTGATGAGAACTTCGACCTGA
AGCACGAGGGTCCCTACTACCTTTCCATGGCCAATGCTGGACCCAACACGAACGGATCCCAATTTTTTATCAC
CACCGTCACGACCTCCTGGCTTGATGGACGTCACGTCGTTTTTGGAAAGGTTCTCGAGGGACAGGATGTCGTA
AAGCGTGTTGAGGGCGTTGGCTCAAGCGGAGGCAAGACGACGAAGACGGTAACCATTGCCGACTCTGGAGAGC
TCAAGGACTAAGTGGCTGCCCCTGCCCTGCACGCAAGCTACCAAGTGGACGGTGACATACGTTGATTGAGCTT
CAATTTGCTTACAACAAGCGCTCATGTTATCCAGAATTTGACAGAGACAATTTAGCTCAGACATCAAGTCCCA
TTGAATTTATTATAAATTAGATGAATCTAAATGACGTGTGTACGTGATCCTTCATCGAGGGGAATCGTTTACC
CAAGATGGAGGATTGCATTTTCTTTTATCTCCTAAATGAAGTCCTTCATTGCCGTGTTTTTTAGGAGTAGCAC
GTAGTATACTATCATTCCAACAGGCTGCGTATGACATGTAAATCGAAGTCGTGCCACAACGGCCAAGTCCAAG
AGAAGAGGGATATAATTTGAAGCAATCAAATAAAACTCCTATCGTGTGACTCTTGATGTAAGGATGATTCACT
GTTATGAATTTGAATGTATTCA
>TR78|c0_g1_i1 len=2819 path=[3441:0-168 3486:169-253 3444:254-270 3491:271-282 3485:283-671 3495:672-695 3498:696-713 3414:714-733

3493:734-801 3496:802-821 3463:822-845 3474:846-1163 3448:1164-1187
3482:1188-1310 3405:1311-1334 3488:1335-1598 3497:1599-1622 3499:1623-
1640 3471:1641-1662 3452:1663-1664 3487:1665-1665 3479:1666-1683
3470:1684-2336 3423:2337-2360 3478:2361-2459 3434:2460-2483 3469:2484-
2666 3457:2667-2690 3458:2691-2818] [-1, 3441, 3486, 3444, 3491, 3485,
3495, 3498, 3414, 3493, 3496, 3463, 3474, 3448, 3482, 3405, 3488, 3497,
3499, 3471, 3452, 3487, 3479, 3470, 3423, 3478, 3434, 3469, 3457, 3458, -
2]
CCGATGAGATGGAAAGAGGGTACTTCTTGCTCGAGAAGGCAGAGTTGAGCATCCCAACGATCACAGGGATTCC
GACGGACGCAAGTCCCACTTCATCCAAAGTAGAGTCCATCTCGGGCTCAATACCAGTCATTTTCTCGATGAGG
CCACACAAAGTCACACCAATATCTAAAAGAAAAACAACATAGAGTGTTCAATGAAGTGGCCATTTTGAACAGG
ATTACTTAGTGTCTTGCATTCTAACCATAAAAAAAACTCACCAATTTCCACCTCCTCTTCTTCACCCTTGAAT
AGACCTGTGATCCAATCCCACACATCCTTAATGACAGGGGTGAAAGTGTTGTCCATAAAATTGGAAAAGGCGA
CAACGATCCCTACCACAAAGAAGTATTCATACCATTCAACAGGGCAAGGGCCCGGGCTGAACCAGTAGAAGGA
CTTCCTGTACGACCACCAGTTCCAGAACTCTCCAGGCCTAGTTGCTGCATAATACCATTGGCCAATCATTTGG
TGAAAGAGGAAACAGTTATAGCTGTTGGGGCCCAAAGTGTGAACTAGGAAATCGCCACGAAGAATCTTAGCGG
TGTATCCTTCGCCAGTAGCCATCGCAAATATCCAAAGAGTGGTCAAAGGACACATGATCCTGCCGCACAGATT
GTCCCAGAGGCGTTGGGCCGAGGAAGCATCGCCGATGTAGTCAGCGTCACTTGGCCTGAACCATCTAGTCGCA
TTTGGATCGTTGTACAACGGTTGACAGATGATGGTAACAGTCAGGCCAAGTTGGACAAGCGTGCATGCATCTG
CAACATATCCCCAAACATGGGCATTGTGGCGCTCTGCGGGCTTGTACGCGTCATAGATGAATGCGAGCACTCC
ACCTATGACGAAGTAAAGGGCCCAAAATGGAGCAAATAAGTAGAAGGACAGAATAACTGCATTATACACCACT
GGGTCAGAGCCGTAGGCATTATCAAACTCCGAAACATCGTTTTTCTCTCCCGTTTCTGCATCATAGTGATTGT
AGGATGGAGCCGATTTCATGGAAAACCACGCTGCGACCAAAATTATCGCATTTAATACTTGTAGAAAAATCAT
GACCTTCATCAAATATCCAGTTCGCTTCCTAGTCTTTAGGAAAAGCCAATTATACATCGCTGGAAAGACGGCC
AAGCACTGGTAATACATACTGGAGAACCAAAGGTAATACCCCATCCACCAGTTGAGCGGCCAAATTGGTGTAA
TCGCCAGGCCAAAGATATACGTGATGATGGTAGTGATCAAGCTCCCCCACCAACTGTTCTTTGTGGCAGGTGT
TCCTTCACAAAAAAAACCACGTGACTTGTCATCTGGCTGCCCATCCCAATGAAAATTTGAATCAAACGTCGAA
GGACGGCAGACGATCAACAGGTTGATTAACCCGAAAATCATTGACATGGTGTACATGGGATACATATTCCCAA
TACGCGCCAAGAAATACGAAAACTTCTTCTTGATGGCAGGATTCATGGGAGAAGCCATGCTGAAGCCACCCAG
AGTGTAAAAACAATGGACGTGCCAAGGCCATCCTCGAAGGTTGTTAAAGTGTGACCACGATGTGTTCGAACCA
ATGTGCATGAACATTACATAGCACGCGAGGAAGAACCGAAATCCAGTAATGACTCCCCAGTCGATCGCAGCCT
TAGTATCTTTGGATTCAGCGTTGGCATCGCCTGCATCTTCTTCGCCAAACCCAAGTTTTTCTGCGAGGCCAAC
ACGGATGTACTTCTTCGTCTTCGTCTTGGGGAGTTCTTCATCAGGACCGATCCACCATTTGGTTGGCCACTTC
ACTGGTGCAAATTTCTTCTGTTTCATCCATTTCCGGAGGCTCTTGATCACTTCATTATCCGGGACGTCCTTTG
GCGCGTCGTCATTGAGTACAATGGCACATCCAACTTCCTCGCCATACACCTTGGACGGAACAGAAAAACACAC
GGGCAACCTTACCCAGGGATGGTCCAAAAGTTCTGCCTCCACTTCGTAAGGAGATACTTGCTCTCCGCCCTTC
TTGATCAATTCCTTCGCTCGCCCCTTCAGGGACAAAAAGCCTTCCTTGTCGAGAACACCGACATCTCCAGTGA
GGAAGTAGCGACCCTTGGAAGGAGTTGCGTCTTCGGGGTCACCAGGTAAGGTCAAGTAGAAGTAATTCTTTGC
GTCAGCTTCTGGATTCTCCAGGTAGCTGCTCATGACGGTATCACCCGAAATGGCAATCTCACCCTCTTGTCCA
TAAGGCTGGGGGCGAAGATGAGAACGACTGACAATGGCCAAGGACGTCGCTACAGGCACACCAACCGATCCTG
GTTTACTAGTCACTGTGTCTCCCTTGCCAGCAGGTGGCTGAGAAATAGGCATCTGTTCACTCATCGAATAGGT
TGGATAGATGGGCAACTCGCCATAGGTTGCTGCAAGGGCCACAGCGTCTGGGGGAAGAAGCGCTGCCGCACCG
GAACGAATCATCCTCAACGAATGCCCACTGTGCCATATCCCATCCTTAATACCATACCCCGCAAATTTGGGAT
CGTCCTTCGCGTGCTCCTTCAAGAAAGCCACCGTGGCATTGTGAATCGTGGGCACGCTCGAATACCATGTGGG
CTGCGGATTCGACACCGCGAGTGCATCCACCATGCGACTGGGATCGAACGGCGCGTCGTCGCAACACACACTT
CCGCCCGTGGATAGGGTGCAGAGGATGGAGGCGGAAATGCCACCGATGTGGAAGAGCGGCATGACACTGTAGC
ACACATCCGCGTCAGTCAGCTGCATGCTCGCCGCGATGATGGCGC
>TR78|c0_g1_i2 len=2890 path=[3401:0-168 3402:169-190 3485:191-579
3495:580-603 3498:604-621 3494:622-728 3493:729-796 3490:797-893
3483:894-916 3474:917-1234 3448:1235-1258 3482:1259-1381 3405:1382-1405
3488:1406-1669 3497:1670-1693 3499:1694-1711 3471:1712-1733 3452:1734-
1735 3487:1736-1736 3479:1737-1754 3470:1755-2407 3423:2408-2431
3478:2432-2530 3434:2531-2554 3469:2555-2737 3457:2738-2761 3458:2762-
2889] [-1, 3401, 3402, 3485, 3495, 3498, 3494, 3493, 3490, 3483, 3474,
3448, 3482, 3405, 3488, 3497, 3499, 3471, 3452, 3487, 3479, 3470, 3423,
3478, 3434, 3469, 3457, 3458, -2]
CCGATGAGATGGAAAGAGGGTACTTCTTGCTCGAGAAGGCAGAGTTGAGCATCCCAACGATCACAGGGATTCC
GACGGACGCAAGTCCCACTTCATCCAAAGTAGAGTCCATCTCGGGCTCAATACCAGTCATTTTCTCGATGAGG
CCACACAAAGTCACACCAATATCAATTTCCACCTCCTCTTCTTCACCCTTGAATAGACCTGTGATCCAATCCC
ACACATCCTTAATGACAGGGGTGAAAGTGTTGTCCATAAAATTGGAAAAGGCGACAACGATCCCTACCACAAA

GAAGTATTCATACCATTCAACAGGGCAAGGGCCCGGGCTGAACCAGTAGAAGGACTTCCTGTACGACCACCAG
TTCCAGAACTCTCCAGGCCTAGTTGCTGCATAATACCATTGGCCAATCATTTGGTGAAAGAGGAAACAGTTAT
AGCTGTTGGGGCCCAAAGTGTGAACTAGGAAATCGCCACGAAGAATCTTAGCGGTGTATCCTTCGCCAGTAGC
CATCGCAAATATCCAAAGAGTGGTCAAAGGACACATGATCCTGCCGCACAGATTGTCCCAGAGGCGTTGGGCC
GAGGAAGCATCGCCGATGTAGTCAGCGTCACTTGGCCTATTTCGGGATAGGTCAGTTCAAGACCATTTTAGTT
GAAGCATCGAACGAGTTAAAACTCTGAGACAACTAGTTTGCTCGACTCACCTGAACCATCTAGTCGCATTTGG
ATCGTTGTACAACGGTTGACAGATGATGGTAACAGTCAGGCCAAGTTGGACAAGCGTGCATGCATCTAAAAAG
TCAAGAGACGGTGAGTAAGTGAGTAAGAAACGAGCAAGAAACTATATACTAATGTGATAACATCCTACCTGCA
ACATATCCCCAAACATGAGCATTGTGGCGCTCTGCGGGCTTGTACGCGTCATAGATGAATGCGAGCACTCCAC
CTATGACGAAGTAAAGGGCCCAAAATGGAGCAAATAAGTAGAAGGACAGAATAACTGCATTATACACCACTGG
GTCAGAGCCGTAGGCATTATCAAACTCCGAAACATCGTTTTTCTCTCCCGTTTCTGCATCATAGTGATTGTAG
GATGGAGCCGATTTCATGGAAAACCACGCTGCGACCAAAATTATCGCATTTAATACTTGTAGAAAAATCATGA
CCTTCATCAAATATCCAGTTCGCTTCCTAGTCTTTAGGAAAAGCCAATTATACATCGCTGGAAAGACGGCCAA
GCACTGGTAATACATACTGGAGAACCAAAGGTAATACCCCATCCACCAGTTGAGCGGCCAAATTGGTGTAATC
GCCAGGCCAAAGATATACGTGATGATGGTAGTGATCAAGCTCCCCCACCAACTGTTCTTTGTGGCAGGTGTTC
CTTCACAAAAAAAACCACGTGACTTGTCATCTGGCTGCCCATCCCAATGAAAATTTGAATCAAACGTCGAAGG
ACGGCAGACGATCAACAGGTTGATTAACCCGAAAATCATTGACATGGTGTACATGGGATACATATTCCCAATA
CGCGCCAAGAAATACGAAAACTTCTTCTTGATGGCAGGATTCATGGGAGAAGCCATGCTGAAGCCACCCAGAG
TGTAAAAACAATGGACGTGCCAAGGCCATCCTCGAAGGTTGTTAAAGTGTGACCACGATGTGTTCGAACCAAT
GTGCATGAACATTACATAGCACGCGAGGAAGAACCGAAATCCAGTAATGACTCCCCAGTCGATCGCAGCCTTA
GTATCTTTGGATTCAGCGTTGGCATCGCCTGCATCTTCTTCGCCAAACCCAAGTTTTTCTGCGAGGCCAACAC
GGATGTACTTCTTCGTCTTCGTCTTGGGGAGTTCTTCATCAGGACCGATCCACCATTTGGTTGGCCACTTCAC
TGGTGCAAATTTCTTCTGTTTCATCCATTTCCGGAGGCTCTTGATCACTTCATTATCCGGGACGTCCTTTGGC
GCGTCGTCATTGAGTACAATGGCACATCCAACTTCCTCGCCATACACCTTGGACGGAACAGAAAAACACACGG
GCAACCTTACCCAGGGATGGTCCAAAAGTTCTGCCTCCACTTCGTAAGGAGATACTTGCTCTCCGCCCTTCTT
GATCAATTCCTTCGCTCGCCCCTTCAGGGACAAAAAGCCTTCCTTGTCGAGAACACCGACATCTCCAGTGAGG
AAGTAGCGACCCTTGGAAGGAGTTGCGTCTTCGGGGTCACCAGGTAAGGTCAAGTAGAAGTAATTCTTTGCGT
CAGCTTCTGGATTCTCCAGGTAGCTGCTCATGACGGTATCACCCGAAATGGCAATCTCACCCTCTTGTCCATA
AGGCTGGGGGCGAAGATGAGAACGACTGACAATGGCCAAGGACGTCGCTACAGGCACACCAACCGATCCTGGT
TTACTAGTCACTGTGTCTCCCTTGCCAGCAGGTGGCTGAGAAATAGGCATCTGTTCACTCATCGAATAGGTTG
GATAGATGGGCAACTCGCCATAGGTTGCTGCAAGGGCCACAGCGTCTGGGGGAAGAAGCGCTGCCGCACCGGA
ACGAATCATCCTCAACGAATGCCCACTGTGCCATATCCCATCCTTAATACCATACCCCGCAAATTTGGGATCG
TCCTTCGCGTGCTCCTTCAAGAAAGCCACCGTGGCATTGTGAATCGTGGGCACGCTCGAATACCATGTGGGCT
GCGGATTCGACACCGCGAGTGCATCCACCATGCGACTGGGATCGAACGGCGCGTCGTCGCAACACACACTTCC
GCCCGTGGATAGGGTGCAGAGGATGGAGGCGGAAATGCCACCGATGTGGAAGAGCGGCATGACACTGTAGCAC
ACATCCGCGTCAGTCAGCTGCATGCTCGCCGCGATGATGGCGC
>TR78|c0_g1_i3 len=2727 path=[3401:0-168 3402:169-190 3485:191-579
3495:580-603 3498:604-621 3414:622-641 3493:642-709 3496:710-729
3463:730-753 3474:754-1071 3448:1072-1095 3482:1096-1218 3405:1219-1242
3488:1243-1506 3497:1507-1530 3499:1531-1548 3471:1549-1570 3452:1571-
1572 3487:1573-1573 3479:1574-1591 3470:1592-2244 3423:2245-2268
3478:2269-2367 3434:2368-2391 3469:2392-2574 3457:2575-2598 3458:2599-
2726] [-1, 3401, 3402, 3485, 3495, 3498, 3414, 3493, 3496, 3463, 3474,
3448, 3482, 3405, 3488, 3497, 3499, 3471, 3452, 3487, 3479, 3470, 3423,
3478, 3434, 3469, 3457, 3458, -2]
CCGATGAGATGGAAAGAGGGTACTTCTTGCTCGAGAAGGCAGAGTTGAGCATCCCAACGATCACAGGGATTCC
GACGGACGCAAGTCCCACTTCATCCAAAGTAGAGTCCATCTCGGGCTCAATACCAGTCATTTTCTCGATGAGG
CCACACAAAGTCACACCAATATCAATTTCCACCTCCTCTTCTTCACCCTTGAATAGACCTGTGATCCAATCCC
ACACATCCTTAATGACAGGGGTGAAAGTGTTGTCCATAAAATTGGAAAAGGCGACAACGATCCCTACCACAAA
GAAGTATTCATACCATTCAACAGGGCAAGGGCCCGGGCTGAACCAGTAGAAGGACTTCCTGTACGACCACCAG
TTCCAGAACTCTCCAGGCCTAGTTGCTGCATAATACCATTGGCCAATCATTTGGTGAAAGAGGAAACAGTTAT
AGCTGTTGGGGCCCAAAGTGTGAACTAGGAAATCGCCACGAAGAATCTTAGCGGTGTATCCTTCGCCAGTAGC
CATCGCAAATATCCAAAGAGTGGTCAAAGGACACATGATCCTGCCGCACAGATTGTCCCAGAGGCGTTGGGCC
GAGGAAGCATCGCCGATGTAGTCAGCGTCACTTGGCCTGAACCATCTAGTCGCATTTGGATCGTTGTACAACG
GTTGACAGATGATGGTAACAGTCAGGCCAAGTTGGACAAGCGTGCATGCATCTGCAACATATCCCCAAACATG
GGCATTGTGGCGCTCTGCGGGCTTGTACGCGTCATAGATGAATGCGAGCACTCCACCTATGACGAAGTAAAGG
GCCCAAAATGGAGCAAATAAGTAGAAGGACAGAATAACTGCATTATACACCACTGGGTCAGAGCCGTAGGCAT
TATCAAACTCCGAAACATCGTTTTTCTCTCCCGTTTCTGCATCATAGTGATTGTAGGATGGAGCCGATTTCAT
GGAAAACCACGCTGCGACCAAAATTATCGCATTTAATACTTGTAGAAAAATCATGACCTTCATCAAATATCCA
GTTCGCTTCCTAGTCTTTAGGAAAAGCCAATTATACATCGCTGGAAAGACGGCCAAGCACTGGTAATACATAC

TGGAGAACCAAAGGTAATACCCCATCCACCAGTTGAGCGGCCAAATTGGTGTAATCGCCAGGCCAAAGATATA
CGTGATGATGGTAGTGATCAAGCTCCCCCACCAACTGTTCTTTGTGGCAGGTGTTCCTTCACAAAAAAAACCA
CGTGACTTGTCATCTGGCTGCCCATCCCAATGAAAATTTGAATCAAACGTCGAAGGACGGCAGACGATCAACA
GGTTGATTAACCCGAAAATCATTGACATGGTGTACATGGGATACATATTCCCAATACGCGCCAAGAAATACGA
AAACTTCTTCTTGATGGCAGGATTCATGGGAGAAGCCATGCTGAAGCCACCCAGAGTGTAAAAACAATGGACG
TGCCAAGGCCATCCTCGAAGGTTGTTAAAGTGTGACCACGATGTGTTCGAACCAATGTGCATGAACATTACAT
AGCACGCGAGGAAGAACCGAAATCCAGTAATGACTCCCCAGTCGATCGCAGCCTTAGTATCTTTGGATTCAGC
GTTGGCATCGCCTGCATCTTCTTCGCCAAACCCAAGTTTTTCTGCGAGGCCAACACGGATGTACTTCTTCGTC
TTCGTCTTGGGGAGTTCTTCATCAGGACCGATCCACCATTTGGTTGGCCACTTCACTGGTGCAAATTTCTTCT
GTTTCATCCATTTCCGGAGGCTCTTGATCACTTCATTATCCGGGACGTCCTTTGGCGCGTCGTCATTGAGTAC
AATGGCACATCCAACTTCCTCGCCATACACCTTGGACGGAACAGAAAAACACACGGGCAACCTTACCCAGGGA
TGGTCCAAAAGTTCTGCCTCCACTTCGTAAGGAGATACTTGCTCTCCGCCCTTCTTGATCAATTCCTTCGCTC
GCCCCTTCAGGGACAAAAAGCCTTCCTTGTCGAGAACACCGACATCTCCAGTGAGGAAGTAGCGACCCTTGGA
AGGAGTTGCGTCTTCGGGGTCACCAGGTAAGGTCAAGTAGAAGTAATTCTTTGCGTCAGCTTCTGGATTCTCC
AGGTAGCTGCTCATGACGGTATCACCCGAAATGGCAATCTCACCCTCTTGTCCATAAGGCTGGGGGCGAAGAT
GAGAACGACTGACAATGGCCAAGGACGTCGCTACAGGCACACCAACCGATCCTGGTTTACTAGTCACTGTGTC
TCCCTTGCCAGCAGGTGGCTGAGAAATAGGCATCTGTTCACTCATCGAATAGGTTGGATAGATGGGCAACTCG
CCATAGGTTGCTGCAAGGGCCACAGCGTCTGGGGGAAGAAGCGCTGCCGCACCGGAACGAATCATCCTCAACG
AATGCCCACTGTGCCATATCCCATCCTTAATACCATACCCCGCAAATTTGGGATCGTCCTTCGCGTGCTCCTT
CAAGAAAGCCACCGTGGCATTGTGAATCGTGGGCACGCTCGAATACCATGTGGGCTGCGGATTCGACACCGCG
AGTGCATCCACCATGCGACTGGGATCGAACGGCGCGTCGTCGCAACACACACTTCCGCCCGTGGATAGGGTGC
AGAGGATGGAGGCGGAAATGCCACCGATGTGGAAGAGCGGCATGACACTGTAGCACACATCCGCGTCAGTCAG
CTGCATGCTCGCCGCGATGATGGCGC
>TR78|c0_g1_i4 len=2814 path=[3401:0-168 3402:169-190 3485:191-579
3495:580-603 3498:604-621 3494:622-728 3493:729-796 3496:797-816
3463:817-840 3474:841-1158 3448:1159-1182 3482:1183-1305 3405:1306-1329
3488:1330-1593 3497:1594-1617 3499:1618-1635 3471:1636-1657 3452:1658-
1659 3487:1660-1660 3479:1661-1678 3470:1679-2331 3423:2332-2355
3478:2356-2454 3434:2455-2478 3469:2479-2661 3457:2662-2685 3458:2686-
2813] [-1, 3401, 3402, 3485, 3495, 3498, 3494, 3493, 3496, 3463, 3474,
3448, 3482, 3405, 3488, 3497, 3499, 3471, 3452, 3487, 3479, 3470, 3423,
3478, 3434, 3469, 3457, 3458, -2]
CCGATGAGATGGAAAGAGGGTACTTCTTGCTCGAGAAGGCAGAGTTGAGCATCCCAACGATCACAGGGATTCC
GACGGACGCAAGTCCCACTTCATCCAAAGTAGAGTCCATCTCGGGCTCAATACCAGTCATTTTCTCGATGAGG
CCACACAAAGTCACACCAATATCAATTTCCACCTCCTCTTCTTCACCCTTGAATAGACCTGTGATCCAATCCC
ACACATCCTTAATGACAGGGGTGAAAGTGTTGTCCATAAAATTGGAAAAGGCGACAACGATCCCTACCACAAA
GAAGTATTCATACCATTCAACAGGGCAAGGGCCCGGGCTGAACCAGTAGAAGGACTTCCTGTACGACCACCAG
TTCCAGAACTCTCCAGGCCTAGTTGCTGCATAATACCATTGGCCAATCATTTGGTGAAAGAGGAAACAGTTAT
AGCTGTTGGGGCCCAAAGTGTGAACTAGGAAATCGCCACGAAGAATCTTAGCGGTGTATCCTTCGCCAGTAGC
CATCGCAAATATCCAAAGAGTGGTCAAAGGACACATGATCCTGCCGCACAGATTGTCCCAGAGGCGTTGGGCC
GAGGAAGCATCGCCGATGTAGTCAGCGTCACTTGGCCTATTTCGGGATAGGTCAGTTCAAGACCATTTTAGTT
GAAGCATCGAACGAGTTAAAACTCTGAGACAACTAGTTTGCTCGACTCACCTGAACCATCTAGTCGCATTTGG
ATCGTTGTACAACGGTTGACAGATGATGGTAACAGTCAGGCCAAGTTGGACAAGCGTGCATGCATCTGCAACA
TATCCCCAAACATGGGCATTGTGGCGCTCTGCGGGCTTGTACGCGTCATAGATGAATGCGAGCACTCCACCTA
TGACGAAGTAAAGGGCCCAAAATGGAGCAAATAAGTAGAAGGACAGAATAACTGCATTATACACCACTGGGTC
AGAGCCGTAGGCATTATCAAACTCCGAAACATCGTTTTTCTCTCCCGTTTCTGCATCATAGTGATTGTAGGAT
GGAGCCGATTTCATGGAAAACCACGCTGCGACCAAAATTATCGCATTTAATACTTGTAGAAAAATCATGACCT
TCATCAAATATCCAGTTCGCTTCCTAGTCTTTAGGAAAAGCCAATTATACATCGCTGGAAAGACGGCCAAGCA
CTGGTAATACATACTGGAGAACCAAAGGTAATACCCCATCCACCAGTTGAGCGGCCAAATTGGTGTAATCGCC
AGGCCAAAGATATACGTGATGATGGTAGTGATCAAGCTCCCCCACCAACTGTTCTTTGTGGCAGGTGTTCCTT
CACAAAAAAAACCACGTGACTTGTCATCTGGCTGCCCATCCCAATGAAAATTTGAATCAAACGTCGAAGGACG
GCAGACGATCAACAGGTTGATTAACCCGAAAATCATTGACATGGTGTACATGGGATACATATTCCCAATACGC
GCCAAGAAATACGAAAACTTCTTCTTGATGGCAGGATTCATGGGAGAAGCCATGCTGAAGCCACCCAGAGTGT
AAAAACAATGGACGTGCCAAGGCCATCCTCGAAGGTTGTTAAAGTGTGACCACGATGTGTTCGAACCAATGTG
CATGAACATTACATAGCACGCGAGGAAGAACCGAAATCCAGTAATGACTCCCCAGTCGATCGCAGCCTTAGTA
TCTTTGGATTCAGCGTTGGCATCGCCTGCATCTTCTTCGCCAAACCCAAGTTTTTCTGCGAGGCCAACACGGA
TGTACTTCTTCGTCTTCGTCTTGGGGAGTTCTTCATCAGGACCGATCCACCATTTGGTTGGCCACTTCACTGG
TGCAAATTTCTTCTGTTTCATCCATTTCCGGAGGCTCTTGATCACTTCATTATCCGGGACGTCCTTTGGCGCG
TCGTCATTGAGTACAATGGCACATCCAACTTCCTCGCCATACACCTTGGACGGAACAGAAAAACACACGGGCA
ACCTTACCCAGGGATGGTCCAAAAGTTCTGCCTCCACTTCGTAAGGAGATACTTGCTCTCCGCCCTTCTTGAT

CAATTCCTTCGCTCGCCCCTTCAGGGACAAAAAGCCTTCCTTGTCGAGAACACCGACATCTCCAGTGAGGAAG
TAGCGACCCTTGGAAGGAGTTGCGTCTTCGGGGTCACCAGGTAAGGTCAAGTAGAAGTAATTCTTTGCGTCAG
CTTCTGGATTCTCCAGGTAGCTGCTCATGACGGTATCACCCGAAATGGCAATCTCACCCTCTTGTCCATAAGG
CTGGGGGCGAAGATGAGAACGACTGACAATGGCCAAGGACGTCGCTACAGGCACACCAACCGATCCTGGTTTA
CTAGTCACTGTGTCTCCCTTGCCAGCAGGTGGCTGAGAAATAGGCATCTGTTCACTCATCGAATAGGTTGGAT
AGATGGGCAACTCGCCATAGGTTGCTGCAAGGGCCACAGCGTCTGGGGGAAGAAGCGCTGCCGCACCGGAACG
AATCATCCTCAACGAATGCCCACTGTGCCATATCCCATCCTTAATACCATACCCCGCAAATTTGGGATCGTCC
TTCGCGTGCTCCTTCAAGAAAGCCACCGTGGCATTGTGAATCGTGGGCACGCTCGAATACCATGTGGGCTGCG
GATTCGACACCGCGAGTGCATCCACCATGCGACTGGGATCGAACGGCGCGTCGTCGCAACACACACTTCCGCC
CGTGGATAGGGTGCAGAGGATGGAGGCGGAAATGCCACCGATGTGGAAGAGCGGCATGACACTGTAGCACACA
TCCGCGTCAGTCAGCTGCATGCTCGCCGCGATGATGGCGC
>TR78|c0_g1_i5 len=771 path=[3459:0-117 3470:118-770] [-1, 3459, 3470]
ACCGAAATCCAGTAATGACTCCCTACAAAAGGGATAGAGTTAGAATAAATGGTAAAATAAATACAGAGATGAT
ACAAACCTCACTAGCTTGCTCACCCAGTCGATCGCAGCCTTAGTATCTTTGGATTCAGCGTTGGCATCGCCTG
CATCTTCTTCGCCAAACCCAAGTTTTTCTGCGAGGCCAACACGGATGTACTTCTTCGTCTTCGTCTTGGGGAG
TTCTTCATCAGGACCGATCCACCATTTGGTTGGCCACTTCACTGGTGCAAATTTCTTCTGTTTCATCCATTTC
CGGAGGCTCTTGATCACTTCATTATCCGGGACGTCCTTTGGCGCGTCGTCATTGAGTACAATGGCACATCCAA
CTTCCTCGCCATACACCTTGGACGGAACAGAAAAACACACGGGCAACCTTACCCAGGGATGGTCCAAAAGTTC
TGCCTCCACTTCGTAAGGAGATACTTGCTCTCCGCCCTTCTTGATCAATTCCTTCGCTCGCCCCTTCAGGGAC
AAAAAGCCTTCCTTGTCGAGAACACCGACATCTCCAGTGAGGAAGTAGCGACCCTTGGAAGGAGTTGCGTCTT
CGGGGTCACCAGGTAAGGTCAAGTAGAAGTAATTCTTTGCGTCAGCTTCTGGATTCTCCAGGTAGCTGCTCAT
GACGGTATCACCCGAAATGGCAATCTCACCCTCTTGTCCATAAGGCTGGGGGCGAAGATGAGAACGACTGACA
ATGGCCAAGGACGTCGCTACAGGCACACCAACCGATCCTGG
>TR79|c0_g1_i1 len=3180 path=[4039:0-102 4040:103-126 4159:127-262
4084:263-286 4188:287-442 4135:443-475 4158:476-595 4099:596-619
4174:620-694 4025:695-718 4157:719-778 4015:779-802 4187:803-1170
4009:1171-1194 4173:1195-1235 4198:1236-1259 4199:1260-1291 4192:1292-
1323 4202:1324-1324 4204:1325-1337 4153:1338-1415 @4197@!:1416-1483
4069:1484-1528 4194:1529-1588 4200:1589-1591 4190:1592-1640 4117:1641-
1654 4167:1655-1659 4148:1660-1691 4181:1692-1719 4165:1720-1725
4146:1726-1813 4179:1814-1846 4163:1847-1875 4145:1876-1885 4177:1886-
1929 4162:1930-2011 4144:2012-2244 3999:2245-2268 4161:2269-2582
4047:2583-2606 4143:2607-2902 4002:2903-2926 4189:2927-2961 4176:2962-
2977 @4160@!:2978-3091 4138:3092-3135 4139:3136-3179] [-1, 4039, 4040,
4159, 4084, 4188, 4135, 4158, 4099, 4174, 4025, 4157, 4015, 4187, 4009,
4173, 4198, 4199, 4192, 4202, 4204, 4153, 4197, 4069, 4194, 4200, 4190,
4117, 4167, 4148, 4181, 4165, 4146, 4179, 4163, 4145, 4177, 4162, 4144,
3999, 4161, 4047, 4143, 4002, 4189, 4176, 4160, 4138, 4139, -2]
GACAAACACCATGGCAAATTTTACATAGCTGTGTAATAAAAAGAATAGATGTAACCTGAGGTAACTATCTAAT
TATTGAAAACCATGGTAGCCGAAGAGGCATATGAGCATAGTCAATACGGCGAGTCTGAGTCCGAAGCTGACTT
CGACATCGAGACCGTCGATGGACCTGCCCTCACCCACCAAATCGCGGGCATCGTGGGACTGTGCATCACATCA
CTTCTCTGGATCATCGCATCATGGCGATTGCTCTATCATTATTCTGGATGGTGCAGCACATGCTGCACCAAGC
GCACGAAGGAAGACGAAGACATTAATCACCTGCGAGCGGACGGTCTGACGACAAAGCGATTGCTTCACGGATT
GCTGTGGACGGCCATGGTGGTGGAGGGGGTGGGATACGCGGATATGGTGGGAACGAACTCGAGTAACAAGCTT
AATTATGTCCTACTCGATATAATAGGGAGGGGCATTCTCGAGTTTTCCACATTTGTGATAGGGACCGTTCATT
GGTTTAATATCACATCTCAGGCGCGGGCGGGGGATGAGAAGCTGGCGTTTACCATCTTCCCGGCCATACTCAT
GATTATAACTATCGCGGTAACGATCTCGTCTACGTTCGAAGCCGTGGCCCTTTTGCACGGAGGGTTTGAAACG
GTCCACGAATTCAGGTCGCAGTCGGATGTTCATAGAATACAATTGTTGGTCGAGTCGACGGGGTGGGGAATTC
ACGCCGTAATTGTCGCAATATGTGGCAAAATGGTCTACGAGCGCATATCCTCCCTCCCGACGTTCTCCCAAGT
GCGAAGTCAAGCCAAACGAAACATCATCACCAAAATGATCATTCCCATGATATTTTGTTCCCTGTGCTACCTC
CTGCGAGCGGGGTGGATGGCGGCCGATTTCGTTAGTAGGATAATTTATCCGGAAACTACATTCGAATCGGGGG
TGGGGTGGTGGGTGGGAAATTGTTGGGTGCCCACGTTCATACCCTCCATCATGCTCCTTTACTCCATTAGGAA
ACGGGATAGGGCGCCAGGGTCTATTGATGGGGTGCCCGAGTCGTTGCTGCGGACGTCCGGCGGGGAAGGGCCG
ACGGATCCCTTTCTGTCGTTCCAGCAAACTTTTCAAGATTTCGAAGATGAAGATGCGAGTATGTCAAGCAATA
AATGAGATGAGATAACATCAAGGTCCCGTGACGGATGATAAGGGCTAGGGGATGGCATACTCAGGAAATATAC
CATCTTGAAGAAGTTTCACTGGGTTGTTATTTTCAGATGGCATAGAGAGTGTCATTCAATAATTGTAAATATA
ATGAATTCAATTGTGTGTGTTTAACTTTTTTGTAACATGGTATATGTATTGGTTCCAAACAACAGAGATCATC
AAACATATAAGTATCACACAAGTAGATGGAAGTATAGAGTGGTGCATACATTTCTCCTTGAAGTATTTCATAT
CAAATACTAGTTCGTCAAAAAGACAAGTACCCAGTTGATTTAGATAAGTACGTTTACTTATCGCCTCAGAGGT
TGCCTAGTTCGAGCGCATTACCGTACGGAAATCGTCCTCGAATTCGGGGAATATCGTAGGCGTGGCTGACAAG

AAACCATTTATATCAAGTGTCAATTGGGGGGGGGGGAAGCCATGTCACCTTCTAATCTGATCCAATTTTCGAT
CGGAGGAAGGCGTTAATACCCAAGCATATGCAGTCATAATTAAAATAATAGCTTGAAACAAAGTAGTTGTGCA
GCATTCTGCACAAACGATCAGCTGCACATATTGTGCTAATTTTGTCCCCTTGGCAACTTTACCAAAATCATGT
GTTACTAATAAATAGCTCTTCACAGCACTAGCGAGATTCCACGGTGTCGCACAGCACCTTCTCTCGTAAAAAA
ATCATGAACCCAATAAGTAACCCCGCAATGAAACGGCGAGATTCCACATCCCACCTGGTCGCACAGTGTCACA
AAAATCTGATCCCCCTCACTCAAGAACAGTGAACCCGCCTCCTTCTCCCTCCATATCATCATCATCCTCAAGT
TCTCTCTTCCTCTTTTCGGCTTCCACCCTAACCTTGTACCTCTTTACTTTCAATATCATTTCTTCGTCCTCTT
CCACCTGGGATAGACTCAAACTTTTGTTGGAACACCCAGGAATAGGACAATTATATTTGCGATTGTTCAGTAA
TTGCGCCAATCCTGCCTTATCATAGGTGTGACTGCACACCTTATTCCTCACGGGGTTCTCGAATAACAATCCC
GTAACAGGGCACTTCAACGACGTGATATCATCTGTTCGATTGTGAACAATTTCCAAATCATCTTCATCGTCAT
CCCCGCCCCTGGAGGGGCGAGACTTCTTCTTCGGAACCTTCTCCCCCAACGCCTCGCGGAGTTCCCTCGACAG
ATCGTGATCCTCGGGAGCCACGTGTGATGGATCGCTCTCCCGCTCATGGCGAATCCTATCCATGATGTCGTGG
ATGGAACGCTCGTAATCGGGAGCGTCATTGTTATTGCCCTCCTCCGCGTCACCCTGGTTCTCGTTCTCAACTT
TGGTCCGCACTTCCTTCACGGAATCGACGAAATATTCGATTTCGTACATGCGTTTCGCATTCCCCTCGGCGAT
GGCCTTCAAGCGATTGTGAGTCGAGGATAGGAAGTTGTCTGCTACGCCGTCTTCGGATTCGGCATTTAGAACC
TCGGCGATTCTGCCGTCGGACCGCAGGGCCTCGGCGAGTTGTTTCGTCCGTATGTGAAGGGTTTTGATGTTTT
TCTTGTAATCGTCTTGGCGTTCGATGGCTCTGTTGCAGGCTAGGAGAGCGCCGCCTCCGCCTCCGTGGCTGGT
CCGTCCTCGTGCCATGGTGAAGTTGTTGTATTTGGTGCTGTTGGTGTTGTTCTGAAAAAGACACCGCCTTATC
TACCGTACGTTTGCGTTGCTAGATATGCATTGAGATTTTTTTCTAAGTAACATACCTACTCTACCTATCTCGC
GGGAAAGGTTTCAAGAAAATTTGGTTGGTTCACAAGCATAGAAAGCCCGAGCCCAATTGGAAAAATATATAAC
AACACAAACGTACGGTGTCTGTGTTCAGTCGCCCGCGCCATTTTCGTTCCATAAATTACATCGATAATTTATG
TCGGGTCTCAATTTCATTCCCAAAAATGCTGCATAAATTTTT
>TR80|c0_g1_i2 len=2230 path=[2857:0-116 2858:117-141 2969:142-231 2950:232-336 2906:337-360 2968:361-516 2949:517-740 2914:741-764 2975:765-783 2967:784-807 2980:808-811 2958:812-843 2978:844-898 2973:899-939 2966:940-1022 2946:1023-1086 2979:1087-1107 2977:1108-1136 2971:1137-1185 2944:1186-1273 @2964@!:1274-1397 2887:1398-1430 2943:1431-1505 2875:1506-1529 2976:1530-1565 2939:1566-1613 @2970@!:1614-2026 2962:2027-2068 2951:2069-2114 2941:2115-2138 2927:2139-2229] [-1, 2857, 2858, 2969, 2950, 2906, 2968, 2949, 2914, 2975, 2967, 2980, 2958, 2978, 2973, 2966, 2946, 2979, 2977, 2971, 2944, 2964, 2887, 2943, 2875, 2976, 2939, 2970, 2962, 2951, 2941, 2927, -2]
GCCTGCCGAAGTGCCGGCGGCGCTTCGCGAGTCTCGATTCCTACGCCGAGCGACGCGATGTGATCAAGAGAGA
GAGAGTGGCCGGAACCGTGTCGGCTTCGGTGAGCAGGGGGAAGGATGGCCTGCGGTATGCGATGGGAGAGAAT
CGCATCCGCTGGCACAGGGAGGTCGTTCGGAGTGTGACTCAAGAGGAGGCTGGCGGTGGCAAACTGACCCTCT
GGATGCCGTCGGGTGACAGCATCACTGTGGATCAAATTATCCTTGCTACCGGATTTGGCAAAGATCCACCTGC
TAGGCACCTTATTAACAGTATTGCGCAGAGAAATGGCCTCAAGCTTTGTGACTGTGGGTATCCAGCTCCGGAT
GAGTCATTACGATGGCATAAACGAATATTCTTGGCGGGCGCCTTGGCCGAGCTGGAACTCGGACCGTCGGCGA
GGAATATTGCTGGAGCGAGGCTGGCATCGGAACGAATTGTTGCTGCTGCGTAAAGAGCCATCAATTAGGAGGA
AGTAGGGGGAATTTTTACAGCGTTTGGTGTGTAATGCCCGCTGAGATTTTGCAAGTGATCCAAAACAACACAT
ATGCAATCATCATTTGCGACGAAGTAACTCTTGAGATTTTTAAGGTCGAAAGTATCGCAACATCTTATTTATT
ATTTTGGTGGAACTGTGCATGGTAGTACAGCAGGGCGCATTTAATTATTTGCAGCTTCGAGCCGGTAATCTCC
AACCTTGTGTTGCCAAAAGGTAGGTAAGCCCCTGAATTCCTCTGCCCACAAAACAGGAAAGATTTCTTACCAT
GGCATTGCGCGACCTAGGTAGGAGTAGACAATAATGATGGTGTACCTAGAAGTACTACTGCCAAAGTCCCCAT
AATTCCAATTACGTCTGAGGCATTGGGCACATGACCGCCAGTGGTTTCCAATTTCCAGTGTTTTCCTACATTC
TAAGTTGATAACATCATGGGAAAAGAAGAAAGTTTCGAGTTTTCATTTCTAATATTATTGTGCCAGACATCAT
TCTGTAGTTGTCCATGGAGGATCAGATTTCAATTATACTTGACCTGTATTGAGCTCTTGGGCAAATGAAGTCG
TAGCGACCAGCACCCTAATGTGCAGTACTTGCAATAACGTATTCAGCATTTTGCAGAGGCATCAGCAGTCATC
GTGACCGCAGCCTAATGAATTGAACTTTGCTGGGAGATTGCAAGTGTAATGGAACCTCCCAAAACAACGAAAA
ACAATCAATTCTCCGTCCTAAATGCCAGGTAGCTTTCCAACGATACCCATCAAAACGGACCAATAGCATAATC
AGCAGCACCACTAATGAAAGGTATCTGATCCGGCCTCTTCCCTCTCAAATTGCTCACCACGCAGTAAATCACC
ATGCTAAACAAACCAAACCACACGAAATTACTACACGGTTCCATCACCGCCCTCGGCAAATTGGCATCGGCAT
CCGCCACGGCCCCCCCGATCAACTGCGGAATGATCAGCGAAACATCGATCAAAATCGCCTGCTGGGCATTGAA
TCGCACCTCTCGCGATTGATCGGTAAGTCGCGGCCCAAGCGCGAACGCCACGAAGAGAAGAATGCTGAGGATG
GGCACGGCCTCGAATCCCTCCACTATCGGACGGAGGAGGACGTAATCGAGCATTCCGAGGGGGGGTATGCGGT
CGTAGATGTACGCGCCGAAGGAGTCGCCGTCGATGAGGGGGAGGACGTAGGGTATGCAGCTCTTGATGCGATC
GGGTCCTTGGATTTCCGTCTCTTCGAAGGCTTTTTTGTTTTTCCGTTTCGTTATGCTGTCGTCGTCGTTGGGT
TGGCCGAATATGTCCAGTTTGTTGGTTCGTCGGTCTTTGTTGTCCTCTCCTTCGTCATCTTGTTTGCTGAGTA
AGCTCGTCGTCGTGCTGGGTCTATTTAATTGACTGTCGACGTGGATCATGGATGCTAATGATCCTTTGTTTCG
CGGTTGTGAGAGTCCGTGATTCGCACCCCTGAATGCAGAACAATCACTGCATCTTATGAATAGATGGGAAAAC

GGGATGAACGCTGAAGTGTAGAATCTTTGAAGCCTCATTTTTTGATTCAATGGAAGAGATGGAGTAAAAATGT
TTTGAAATTATGACGGCGCGTTGATGCTACGAAACGCCTAATCTGCACCTCATGGCGGCCTTTTCGTTCCCTT
GCAAAAATCGAAACAACATGATTCCCATTGTCGTCGGTTT
>TR81|c0_g1_i1 len=2011 path=[2623:0-400 2624:401-459 2697:460-525
2694:526-549 2701:550-579 2688:580-670 2648:671-691 2681:692-758
2665:759-760 2698:761-929 @2696@!:930-1154 2692:1155-1200 2685:1201-1217
2677:1218-1248 2695:1249-1556 2614:1557-1580 2691:1581-1661 2702:1662-
1667 2690:1668-1801 2683:1802-1802 2675:1803-1895 2611:1896-1919
2612:1920-2010] [-1, 2623, 2624, 2697, 2694, 2701, 2688, 2648, 2681,
2665, 2698, 2696, 2692, 2685, 2677, 2695, 2614, 2691, 2702, 2690, 2683,
2675, 2611, 2612, -2]
ACATCCATAGTGACAACAGAAACAACAAAAACCTCCACCACCACCAACAACAACAACAACAACAACATGCCCA
CAATTATTGACGATCCCCTACTACTAGCCGATCTCCAATCCCGTGTATCAGATCTCGAACTATCCAAAACACA
CAATGACGTATCCGCCATTCTCCAAAAGGAACGTGCCGATATGTTACTTCATTTACGAAAGATAAAGGAGGCA
ATGGTAAAAGAAGCTAGTGGTGATGGTGGAAGTGGGAGTGGAAGTGGACGTGGAGGAGCGAGTAGTAAGGAAT
TGGAACAATTGAGGGCGGAGAATAAAGAGTTGAAGAAGGTGAATGCTAAGCAACAATATCGGATTGAACATTT
GGTTAGTAATTTGAGGGGGGCGTTGGAGGGTGATAAGCAGTGAATGAATTAAAACATAGCACCATCATCACGT
GGAAAAATTGAGAAAATTTAGTGATGGAAGAAAATTTTAAGAGTCGGTGAAGACATTATTGCCATGCAACAAC
ATTGAGTTGGATGGTTGTCACATCATGTGTGGGCGCTGCAAAATTGGAATTGGAGCAGTAAGGTTGATAACCA
AGTGTTTTATTTCAGAGTAATATGACTTGGGTCTATGAAGTTTGGAAACAGAGGCCATAAATTCTGACATGCA
TCAAGTGGATTCATCATACATTAATGAAATAGATGTGTGATATTTTCCACAAAGAGATACCAGGTTGCCAAGA
CCTGAGTGATTGCCTCTACTTGTGACCAAAATCCATTACGAGCCTAAACATTGACCGCACTGCCTATCGCAGG
TGGCAATGGATTTATCACATTCAACTAAGCGTGATCGTTCATGGAAATAGTTATTATCCAGCCGCCATTACCT
CTCGTTTCCCAACCAATTCACCGCGTATCCCAAACCTTACAGAGCAGACAAAACCTCCGCCAACATCCCCGAA
TTCGGGCACCAATCTCTATCAAAACGCATCGGCTTATTCGCAGGCGTCTTCCCTCCAAACCCGCTCGAAACAG
TCGACTCGGGATTGGACGACACTTCCAAAATCCTACTCTCCCCCCAATCCATCGACATCAGGGATTGCACGAT
CAACGCCGCGAAATCTTCCCGCATCACCGCCGGTTCCTTCTCCGACTTTTCCGCATCAAAAGGGATCAAACTC
GATCCATCTTGTACCTTTTTCACATTCAATTGATTCGTAATCCCCTTCTCAAACGAAAACGTTTTATCCTTCT
TCAACGGCGACATGGTTCTCAAATACGTGTACGTCATCCCCCCCTCCTCTTCCAAAATGGACACGAAGTCCGA
CGGGTTAACGGTACGAGGAGTTTCCACCACGACGAGATGCGACAGTCCCGCCTCCCTGCACGCCCTCACCGAA
TTTCGAAAAAATGGATTCGTGAACGTCGGCATCGTTTTCCGGTACGTTATTCTGCCACGCGCCGTACCGTTCGT
CCAAGTACAGTTCGTAGGTTTTGTCATTGACATTCTTTTCGTAGGTGTTGAGCGTGACGGCGCGTTGTTCGAG
TTGGTAGGTCGTGCCGAGGATGCAGGCGTCGAATGTTTTGAGACGTTCGGTCATGTGGGACGTGGAGGAGGTG
TCGACGAGGCGGGCTAGGTCTTCTCCCGCGTAGGCGAGTTTGAAGCACGGTCCGAGCACCTTTTATTGCGTGA
TCGTAGGTGGGATGAATGCACACATCAGATGACCTCCGAGAGTGAGAATAAAAAGACGCCCAACGAATGTGTA
CATGCATCACAGTATGATGCATACTCCTACCTTATTGAGCCCTTCCAATCCACCACTGGTTGCACTGATCGAA
CGAGGGCTGGAACTTGCCGATATTCCTGTTCCGTATATTGACGCTGCGCGCTGCAGGAAACCAAAGGCAGTAG
CACCAAGATAACCGCCAGCTCCGAATATTGCAATTCTCTTCTTTTCGAATGGTTTTGCAGCACAGCCCTTGGG
TGGCTCCATGTTCAGCAGAGATGACGACGACGACGACGAT
>TR81|c0_g1_i2 len=1894 path=[2623:0-400 2624:401-459 2697:460-525
2694:526-549 2701:550-579 2688:580-670 2648:671-691 2681:692-758
2665:759-760 2698:761-929 @2696@!:930-1154 2692:1155-1200 2685:1201-1217
2677:1218-1248 2695:1249-1556 2614:1557-1580 2691:1581-1661 2702:1662-
1667 2656:1668-1685 2675:1686-1778 2611:1779-1802 2612:1803-1893] [-1,
2623, 2624, 2697, 2694, 2701, 2688, 2648, 2681, 2665, 2698, 2696, 2692,
2685, 2677, 2695, 2614, 2691, 2702, 2656, 2675, 2611, 2612, -2]
ACATCCATAGTGACAACAGAAACAACAAAAACCTCCACCACCACCAACAACAACAACAACAACAACATGCCCA
CAATTATTGACGATCCCCTACTACTAGCCGATCTCCAATCCCGTGTATCAGATCTCGAACTATCCAAAACACA
CAATGACGTATCCGCCATTCTCCAAAAGGAACGTGCCGATATGTTACTTCATTTACGAAAGATAAAGGAGGCA
ATGGTAAAAGAAGCTAGTGGTGATGGTGGAAGTGGGAGTGGAAGTGGACGTGGAGGAGCGAGTAGTAAGGAAT
TGGAACAATTGAGGGCGGAGAATAAAGAGTTGAAGAAGGTGAATGCTAAGCAACAATATCGGATTGAACATTT
GGTTAGTAATTTGAGGGGGGCGTTGGAGGGTGATAAGCAGTGAATGAATTAAAACATAGCACCATCATCACGT
GGAAAAATTGAGAAAATTTAGTGATGGAAGAAAATTTTAAGAGTCGGTGAAGACATTATTGCCATGCAACAAC
ATTGAGTTGGATGGTTGTCACATCATGTGTGGGCGCTGCAAAATTGGAATTGGAGCAGTAAGGTTGATAACCA
AGTGTTTTATTTCAGAGTAATATGACTTGGGTCTATGAAGTTTGGAAACAGAGGCCATAAATTCTGACATGCA
TCAAGTGGATTCATCATACATTAATGAAATAGATGTGTGATATTTTCCACAAAGAGATACCAGGTTGCCAAGA
CCTGAGTGATTGCCTCTACTTGTGACCAAAATCCATTACGAGCCTAAACATTGACCGCACTGCCTATCGCAGG
TGGCAATGGATTTATCACATTCAACTAAGCGTGATCGTTCATGGAAATAGTTATTATCCAGCCGCCATTACCT
CTCGTTTCCCAACCAATTCACCGCGTATCCCAAACCTTACAGAGCAGACAAAACCTCCGCCAACATCCCCGAA
TTCGGGCACCAATCTCTATCAAAACGCATCGGCTTATTCGCAGGCGTCTTCCCTCCAAACCCGCTCGAAACAG

TCGACTCGGGATTGGACGACACTTCCAAAATCCTACTCTCCCCCAATCCATCGACATCAGGGATTGCACGAT
CAACGCCGCGAAATCTTCCCGCATCACCGCCGGTTCCTTCTCCGACTTTTCCGCATCAAAAGGGATCAAACTC
GATCCATCTTGTACCTTTTTCACATTCAATTGATTCGTAATCCCCTTCTCAAACGAAAACGTTTTATCCTTCT
TCAACGGCGACATGGTTCTCAAATACGTGTACGTCATCCCCCCCTCCTCTTCCAAAATGGACACGAAGTCCGA
CGGGTTAACGGTACGAGGAGTTTCCACCACGACGAGATGCGACAGTCCCGCCTCCCTGCACGCCCTCACCGAA
TTTCGAAAAATGGATTCGTGAACGTCGGCATCGTTTTCCGGTACGTTATTCTGCCACGCGCCGTACCGTTCGT
CCAAGTACAGTTCGTAGGTTTTGTCATTGACATTCTTTTCGTAGGTGTTGAGCGTGACGGCGCGTTGTTCGAG
TTGGTAGGTCGTGCCGAGGATGCAGGCGTCGAATGTTTTGAGACGTTCGGTCATGTGGGACGTGGAGGAGGTG
TCGACGAGGCGGGCTAGGTCTTCTCCCGCGTAGGCGAGTTTGAAGCACGGTCCGAGCACCTTATTGAGCCCTT
CCAATCCACCACTGGTTGCACTGATCGAACGAGGGCTGGAACTTGCCGATATTCCTGTTCCGTATATTGACGC
TGCGCGCTGCAGGAAACCAAAGGCAGTAGCACCAAGATAACCGCCAGCTCCGAATATTGCAATTCTCTTCTTT
TCGAATGGTTTTGCAGCACAGCCCTTGGGTGGCTCCATGTTCAGCAGAGATGACGACGACGACGACGAT
>TR81|c1_g1_i1 len=1219 path=[2374:0-214 2375:215-238 2418:239-279
2427:280-282 2348:283-303 @2434@!:304-492 2322:493-514 @2441@!:515-603
2416:604-626 2432:627-652 2424:653-676 2440:677-706 2437:707-730
2431:731-796 2413:797-891 2351:892-915 2422:916-961 2397:962-985
2430:986-1000 2442:1001-1001 2436:1002-1014 2444:1015-1015 2445:1016-1024
2428:1025-1137 2409:1138-1201 2345:1202-1218] [-1, 2374, 2375, 2418,
2427, 2348, 2434, 2322, 2441, 2416, 2432, 2424, 2440, 2437, 2431, 2413,
2351, 2422, 2397, 2430, 2442, 2436, 2444, 2445, 2428, 2409, 2345, -2]
GATCTCGACGCCGCCCCGCGCGCAATCGCCTCCCTCAGCGGAATCGAACCGGGCGAAGTCGCCCCCAAAATAA
AAGGATGATCCCTCCCGTCCACGTTCGAATCCAACAGCGTCGCCGCCTCGGCGTCCGTCCTCGCGACGATGAC
CAACTCCACGCCCAGCACGTCCGCCGCCAACCTCGCCGCGACCAATCGATCGGCGTGTTCCCGCGTCGATACC
AAAACCTTCCCGCCCATGTGGCCGCATTTTTTCGTGCCGGGTTTCTGATCCTCGAAGTGTATCCCGGCGGCGC
CCGCCTCGACGAAGAGCTTGACGAGTTTCATTACGGCGGAGAGGCCTCCGTGGCCGGTGTCGCCGTCGGCGAC
CACGGGGGTGAGGTAGTCGACCTTGGGAGGGAGGGGGGTTGTTGTCGGGTCGTTGGAGAGGATGGCGGAGGCG
CGTTCTTCGTTCTGGCGGCGATCGTGGTGGAGTTGGGCGCGTACGAGCTGATCGCACTTATTTGGCACCGTGT
TCATGGGATAATCGGCGAAGTCAGGACCCGGTTCATTGGTGCTGCTCGCCGTGCTCGAACATTGCCATCCGCT
CACATAGATACTACTCAGATGAGGAGCCATCTGCACGACTTGGACCGGATCCAATGCCCCGAAGGTATGAGAA
TACCCTCCCTTCGAATGGAGTCGGGTTAACAGCGAGTACAACTTGCGAGAAGACGCGTTGGAGTAAGAGCATT
TGGGCGACATGATTCCATTGCCGGGACGTGCCGACGGAGAGGGACGGAGGGAGGCTACATCGGCGGCCGAGTA
GGTCCTCTTGGTACCCTTCCATCGTTCCTCGCGCCACCATTGTTCGATTTGTGCAATTTCGGTCAGGAGTGCT
TCTCGCTCCGCCTTGATCTCGCCGCCGCCGCCGTCGTCGCTGGAGGAGGATGAAGACGACTTCTTCGGAAGGA
CGCGAAGTCCTCGACTGACCTTGGGGGTTGATTTGGATGTCATTTTTTTTTTTTGAGGCGTGTATGCTATTTT
TTATGTGTTTTATGTTGTAGAAGACTGGTGCCGTAGTATGAAGGGTGTTGCGTGTGTGAACGTTGTGTGCAGG
AGTAGATGAGACCTGAGACGATGTACGTGCTGTCAACCGTGCGTGACCGTGTCAACCCCGATAGAGATGTCAA
CGATGCGCTTTGGATGGGGCGGGTTGGAGGATGGGAAATTGGACGTCGAGG
>TR81|c1_g1_i2 len=1204 path=[2374:0-214 2375:215-238 2418:239-279
2427:280-282 2348:283-303 @2434@!:304-492 2322:493-514 @2441@!:515-603
2416:604-626 2432:627-652 2424:653-676 2440:677-706 2437:707-730
2431:731-796 2413:797-891 2351:892-915 2422:916-961 2397:962-985
2430:986-1000 2442:1001-1001 2436:1002-1014 2444:1015-1015 2445:1016-1024
2410:1025-1186 2320:1187-1203] [-1, 2374, 2375, 2418, 2427, 2348, 2434,
2322, 2441, 2416, 2432, 2424, 2440, 2437, 2431, 2413, 2351, 2422, 2397,
2430, 2442, 2436, 2444, 2445, 2410, 2320, -2]
GATCTCGACGCCGCCCCGCGCGCAATCGCCTCCCTCAGCGGAATCGAACCGGGCGAAGTCGCCCCCAAAATAA
AAGGATGATCCCTCCCGTCCACGTTCGAATCCAACAGCGTCGCCGCCTCGGCGTCCGTCCTCGCGACGATGAC
CAACTCCACGCCCAGCACGTCCGCCGCCAACCTCGCCGCGACCAATCGATCGGCGTGTTCCCGCGTCGATACC
AAAACCTTCCCGCCCATGTGGCCGCATTTTTTCGTGCCGGGTTTCTGATCCTCGAAGTGTATCCCGGCGGCGC
CCGCCTCGACGAAGAGCTTGACGAGTTTCATTACGGCGGAGAGGCCTCCGTGGCCGGTGTCGCCGTCGGCGAC
CACGGGGGTGAGGTAGTCGACCTTGGGAGGGAGGGGGGTTGTTGTCGGGTCGTTGGAGAGGATGGCGGAGGCG
CGTTCTTCGTTCTGGCGGCGATCGTGGTGGAGTTGGGCGCGTACGAGCTGATCGCACTTATTTGGCACCGTGT
TCATGGGATAATCGGCGAAGTCAGGACCCGGTTCATTGGTGCTGCTCGCCGTGCTCGAACATTGCCATCCGCT
CACATAGATACTACTCAGATGAGGAGCCATCTGCACGACTTGGACCGGATCCAATGCCCCGAAGGTATGAGAA
TACCCTCCCTTCGAATGGAGTCGGGTTAACAGCGAGTACAACTTGCGAGAAGACGCGTTGGAGTAAGAGCATT
TGGGCGACATGATTCCATTGCCGGGACGTGCCGACGGAGAGGGACGGAGGGAGGCTACATCGGCGGCCGAGTA
GGTCCTCTTGGTACCCTTCCATCGTTCCTCGCGCCACCATTGTTCGATTTGTGCAATTTCGGTCAGGAGTGCT
TCTCGCTCCGCCTTGATCTCGCCGCCGCCGCCGTCGTCGCTGGAGGAGGATGAAGACGACTTCTTCGGAAGGA
CGCGAAGTCCTCGACTGACCTTGGGGGTTGATTTGGATGTCATTTTTTTTTTTTGAGGCGTGTATGCTATTTT
TTACGTGTTTTATGTTGTAGAAGACTGCTGCGTAGTATGAAGTGTGAACGTTGTGTGCAAGGGTAGCTGAGAC

CTGAGACGATGTACTACGTGCTGACAACCGTGACCGTGTCAACCCAGATAGATGTCAACGATGCGCTTGGGAT
GGGGCGGGTTGGAGGATGGGAAATTGGACGTCGAGG
>TR81|c1_g1_i3 len=1617 path=[2374:0-214 2375:215-238 2418:239-279 2427:280-282 2348:283-303 @2434@!:304-492 2443:493-685 @2441@!:686-774 2438:775-869 2330:870-904 2415:905-1024 2432:1025-1050 2424:1051-1074 2440:1075-1104 2437:1105-1128 2431:1129-1194 2413:1195-1289 2351:1290-1313 2422:1314-1359 2397:1360-1383 2430:1384-1398 2442:1399-1399 2436:1400-1412 2444:1413-1413 2445:1414-1422 2428:1423-1535 2409:1536-1599 2345:1600-1616] [-1, 2374, 2375, 2418, 2427, 2348, 2434, 2443, 2441, 2438, 2330, 2415, 2432, 2424, 2440, 2437, 2431, 2413, 2351, 2422, 2397, 2430, 2442, 2436, 2444, 2445, 2428, 2409, 2345, -2]
GATCTCGACGCCGCCCCGCGCGCAATCGCCTCCCTCAGCGGAATCGAACCGGGCGAAGTCGCCCCCAAAATAA
AAGGATGATCCCTCCCGTCCACGTTCGAATCCAACAGCGTCGCCGCCTCGGCGTCCGTCCTCGCGACGATGAC
CAACTCCACGCCCAGCACGTCCGCCGCCAACCTCGCCGCGACCAATCGATCGGCGTGTTCCCGCGTCGATACC
AAAACCTTCCCGCCCATGTGGCCGCATTTTTTCGTGCCGGGTTTCTGATCCTCGAAGTGTATCCCGGCGGCGC
CCGCCTCGACGAAGAGCTTGACGAGTTTCATTACGGCGGAGAGGCCTCCGTGGCCGGTGTCGCCGTCGGCGAC
CACGGGGGTGAGGTAGTCGACCTTGGGAGGGAGGGGGGTTGTTGTCGGGTCGTTGGAGAGGATGGCGGAGGCG
CGTTCTTCGTTCTGGCGGCGATCGTGGTGGAGTTGGGCGCGTACGAGCTGATCGCCTTTTGTTGTTGTTGTTG
TTGTTGTGTTATCATTGGGCGAATTGTGTTCGAAAGAGAGAGAGTGTGTGTGTGTGTATAGTGAAAATGTAGA
ATAGCATTCAGTATTGCTGTGTTAGTTTCGTGTATTGTGATACGATGTTGTATTACAAGACAATATCATTGAA
CGCTTACACTTATTTGGCACCGTGTTCATGGGATAATCGGCGAAGTCAGGACCCGGTTCATTGGTGCTGCTCG
CCGTGCTCGAACATTGCCATCCGCTCACATAGATACTACTCAGATCTAGAGAGAGAGAGATGACGACGACGAC
GACGATGTTGTAATATGCACATGATGCAAAATTGAATAGAACCAACGAAATTAGGAGAAGGACATATATGAGA
AAGAAGAAAGAGTCCAATATTCCTACGCAAACTCTGACATCGACATGACACGCGAACGAATAATAATAACAAT
AATAACAATGACAACAATAACACATGCATTCATTCCAAGACCAAACTACTTACGAGGAGCCATCTGCACGACT
TGGACCGGATCCAATGCCCCGAAGGTATGAGAATACCCTCCCTTCGAATGGAGTCGGGTTAACAGCGAGTACA
ACTTGCGAGAAGACGCGTTGGAGTAAGAGCATTTGGGCGACATGATTCCATTGCCGGGACGTGCCGACGGAGA
GGGACGGAGGGAGGCTACATCGGCGGCCGAGTAGGTCCTCTTGGTACCCTTCCATCGTTCCTCGCGCCACCAT
TGTTCGATTTGTGCAATTTCGGTCAGGAGTGCTTCTCGCTCCGCCTTGATCTCGCCGCCGCCGCCGTCGTCGC
TGGAGGAGGATGAAGACGACTTCTTCGGAAGGACGCGAAGTCCTCGACTGACCTTGGGGGTTGATTTGGATGT
CATTTTTTTTTTTTTGAGGCGTGTATGCTATTTTTTATGTGTTTTATGTTGTAGAAGACTGGTGCCGTAGTATG
AAGGGTGTTGCGTGTGTGAACGTTGTGTGCAGGAGTAGATGAGACCTGAGACGATGTACGTGCTGTCAACCGT
GCGTGACCGTGTCAACCCCGATAGAGATGTCAACGATGCGCTTTGGATGGGGCGGGTTGGAGGATGGGAAATT
GGACGTCGAGG
>TR81|c1_g1_i4 len=1260 path=[2374:0-214 2375:215-238 2418:239-279 2427:280-282 2348:283-303 @2434@!:304-492 2322:493-514 @2441@!:515-603 2438:604-698 2330:699-733 2415:734-853 2432:854-879 2424:880-903 2440:904-933 2437:934-957 2431:958-1023 2369:1024-1259] [-1, 2374, 2375, 2418, 2427, 2348, 2434, 2322, 2441, 2438, 2330, 2415, 2432, 2424, 2440, 2437, 2431, 2369, -2]
GATCTCGACGCCGCCCCGCGCGCAATCGCCTCCCTCAGCGGAATCGAACCGGGCGAAGTCGCCCCCAAAATAA
AAGGATGATCCCTCCCGTCCACGTTCGAATCCAACAGCGTCGCCGCCTCGGCGTCCGTCCTCGCGACGATGAC
CAACTCCACGCCCAGCACGTCCGCCGCCAACCTCGCCGCGACCAATCGATCGGCGTGTTCCCGCGTCGATACC
AAAACCTTCCCGCCCATGTGGCCGCATTTTTTCGTGCCGGGTTTCTGATCCTCGAAGTGTATCCCGGCGGCGC
CCGCCTCGACGAAGAGCTTGACGAGTTTCATTACGGCGGAGAGGCCTCCGTGGCCGGTGTCGCCGTCGGCGAC
CACGGGGGTGAGGTAGTCGACCTTGGGAGGGAGGGGGGTTGTTGTCGGGTCGTTGGAGAGGATGGCGGAGGCG
CGTTCTTCGTTCTGGCGGCGATCGTGGTGGAGTTGGGCGCGTACGAGCTGATCGCACTTATTTGGCACCGTGT
TCATGGGATAATCGGCGAAGTCAGGACCCGGTTCATTGGTGCTGCTCGCCGTGCTCGAACATTGCCATCCGCT
CACATAGATACTACTCAGATCTAGAGAGAGAGAGATGACGACGACGACGACGATGTTGTAATATGCACATGAT
GCAAAATTGAATAGAACCAACGAAATTAGGAGAAGGACATATATGAGAAAGAAGAAAGAGTCCAATATTCCTA
CGCAAACTCTGACATCGACATGACACGCGAACGAATAATAATAACAATAATAACAATGACAACAATAACACAT
GCATTCATTCCAAGACCAAACTACTTACGAGGAGCCATCTGCACGACTTGGACCGGATCCAATGCCCCGAAGG
TATGAGAATACCCTCCCTTCGAATGGAGTCGGGTTAACAGCGAGTACAACTTGCGAGAAGACGCGTTGGAGTA
AGAGCATTTGGGCGACATGATTCCATTGCCGGGACGTGCCGACGGAGAGGGACGGAGGGAGGCTACATCGGCG
GCTGGGTGTGAAAACAAAGAGTATGTGTATAATGACGGTGAGTTTTTGGGGGTGTGCGTGTTTGTGTGTGCAT
ATGTAGTATATATATCATGTCATGGGGATATACATGATTTATTTTTGTTCGTGTGTACAAGATGTATATGAGT
AGTATAGATGGGATAGAAGCAGGAGATGATATATCCAACAAATGAATGAACAGACTGCAAGAAGTGTCATGCA
AATAATACATCATCGAGAT
>TR81|c1_g1_i5 len=1375 path=[2374:0-214 2375:215-238 2418:239-279 2427:280-282 2348:283-303 @2434@!:304-492 2443:493-685 @2441@!:686-774

2416:775-797 2432:798-823 2424:824-847 2440:848-877 2437:878-901
2431:902-967 2413:968-1062 2351:1063-1086 2422:1087-1132 2397:1133-1156
2430:1157-1171 2442:1172-1172 2436:1173-1185 2444:1186-1186 2445:1187-
1195 2410:1196-1357 2320:1358-1374] [-1, 2374, 2375, 2418, 2427, 2348,
2434, 2443, 2441, 2416, 2432, 2424, 2440, 2437, 2431, 2413, 2351, 2422,
2397, 2430, 2442, 2436, 2444, 2445, 2410, 2320, -2]
GATCTCGACGCCGCCCCGCGCGCAATCGCCTCCCTCAGCGGAATCGAACCGGGCGAAGTCGCCCCCAAAATAA
AAGGATGATCCCTCCCGTCCACGTTCGAATCCAACAGCGTCGCCGCCTCGGCGTCCGTCCTCGCGACGATGAC
CAACTCCACGCCCAGCACGTCCGCCGCCAACCTCGCCGCGACCAATCGATCGGCGTGTTCCCGCGTCGATACC
AAAACCTTCCCGCCCATGTGGCCGCATTTTTTCGTGCCGGGTTTCTGATCCTCGAAGTGTATCCCGGCGGCGC
CCGCCTCGACGAAGAGCTTGACGAGTTTCATTACGGCGGAGAGGCCTCCGTGGCCGGTGTCGCCGTCGGCGAC
CACGGGGGTGAGGTAGTCGACCTTGGGAGGGAGGGGGGTTGTTGTCGGGTCGTTGGAGAGGATGGCGGAGGCG
CGTTCTTCGTTCTGGCGGCGATCGTGGTGGAGTTGGGCGCGTACGAGCTGATCGCCTTTTGTTGTTGTTGTTG
TTGTTGTGTTATCATTGGGCGAATTGTGTTCGAAAGAGAGAGAGTGTGTGTGTGTGTATAGTGAAAATGTAGA
ATAGCATTCAGTATTGCTGTGTTAGTTTCGTGTATTGTGATACGATGTTGTATTACAAGACAATATCATTGAA
CGCTTACACTTATTTGGCACCGTGTTCATGGGATAATCGGCGAAGTCAGGACCCGGTTCATTGGTGCTGCTCG
CCGTGCTCGAACATTGCCATCCGCTCACATAGATACTACTCAGATGAGGAGCCATCTGCACGACTTGGACCGG
ATCCAATGCCCCGAAGGTATGAGAATACCCTCCCTTCGAATGGAGTCGGGTTAACAGCGAGTACAACTTGCGA
GAAGACGCGTTGGAGTAAGAGCATTTGGGCGACATGATTCCATTGCCGGGACGTGCCGACGGAGAGGGACGGA
GGGAGGCTACATCGGCGGCCGAGTAGGTCCTCTTGGTACCCTTCCATCGTTCCTCGCGCCACCATTGTTCGAT
TTGTGCAATTTCGGTCAGGAGTGCTTCTCGCTCCGCCTTGATCTCGCCGCCGCCGCCGTCGTCGCTGGAGGAG
GATGAAGACGACTTCTTCGGAAGGACGCGAAGTCCTCGACTGACCTTGGGGGTTGATTTGGATGTCATTTTTT
TTTTTTGAGGCGTGTATGCTATTTTTTACGTGTTTTATGTTGTAGAAGACTGCTGCGTAGTATGAAGTGTGAA
CGTTGTGTGCAAGGGTAGCTGAGACCTGAGACGATGTACTACGTGCTGACAACCGTGACCGTGTCAACCCAGA
TAGATGTCAACGATGCGCTTGGGATGGGGCGGGTTGGAGGATGGGAAATTGGACGTCGAGG
>TR81|c1_g1_i7 len=1033 path=[2374:0-214 2375:215-238 2418:239-279
2427:280-282 2348:283-303 @2434@!:304-492 2322:493-514 @2441@!:515-603
2416:604-626 2432:627-652 2424:653-676 2440:677-706 2437:707-730
2431:731-796 2369:797-1032] [-1, 2374, 2375, 2418, 2427, 2348, 2434,
2322, 2441, 2416, 2432, 2424, 2440, 2437, 2431, 2369, -2]
GATCTCGACGCCGCCCCGCGCGCAATCGCCTCCCTCAGCGGAATCGAACCGGGCGAAGTCGCCCCCAAAATAA
AAGGATGATCCCTCCCGTCCACGTTCGAATCCAACAGCGTCGCCGCCTCGGCGTCCGTCCTCGCGACGATGAC
CAACTCCACGCCCAGCACGTCCGCCGCCAACCTCGCCGCGACCAATCGATCGGCGTGTTCCCGCGTCGATACC
AAAACCTTCCCGCCCATGTGGCCGCATTTTTTCGTGCCGGGTTTCTGATCCTCGAAGTGTATCCCGGCGGCGC
CCGCCTCGACGAAGAGCTTGACGAGTTTCATTACGGCGGAGAGGCCTCCGTGGCCGGTGTCGCCGTCGGCGAC
CACGGGGGTGAGGTAGTCGACCTTGGGAGGGAGGGGGGTTGTTGTCGGGTCGTTGGAGAGGATGGCGGAGGCG
CGTTCTTCGTTCTGGCGGCGATCGTGGTGGAGTTGGGCGCGTACGAGCTGATCGCACTTATTTGGCACCGTGT
TCATGGGATAATCGGCGAAGTCAGGACCCGGTTCATTGGTGCTGCTCGCCGTGCTCGAACATTGCCATCCGCT
CACATAGATACTACTCAGATGAGGAGCCATCTGCACGACTTGGACCGGATCCAATGCCCCGAAGGTATGAGAA
TACCCTCCCTTCGAATGGAGTCGGGTTAACAGCGAGTACAACTTGCGAGAAGACGCGTTGGAGTAAGAGCATT
TGGGCGACATGATTCCATTGCCGGGACGTGCCGACGGAGAGGGACGGAGGGAGGCTACATCGGCGGCTGGGTG
TGAAAACAAAGAGTATGTGTATAATGACGGTGAGTTTTTGGGGGTGTGCGTGTTTGTGTGTGCATATGTAGTA
TATATATCATGTCATGGGGATATACATGATTTATTTTTGTTCGTGTGTACAAGATGTATATGAGTAGTATAGA
TGGGATAGAAGCAGGAGATGATATATCCAACAAATGAATGAACAGACTGCAAGAAGTGTCATGCAAATAATAC
ATCATCGAGAT
>TR81|c1_g1_i8 len=1204 path=[2374:0-214 2375:215-238 2418:239-279
2427:280-282 2348:283-303 @2434@!:304-492 2443:493-685 @2441@!:686-774
2416:775-797 2432:798-823 2424:824-847 2440:848-877 2437:878-901
2431:902-967 2369:968-1203] [-1, 2374, 2375, 2418, 2427, 2348, 2434,
2443, 2441, 2416, 2432, 2424, 2440, 2437, 2431, 2369, -2]
GATCTCGACGCCGCCCCGCGCGCAATCGCCTCCCTCAGCGGAATCGAACCGGGCGAAGTCGCCCCCAAAATAA
AAGGATGATCCCTCCCGTCCACGTTCGAATCCAACAGCGTCGCCGCCTCGGCGTCCGTCCTCGCGACGATGAC
CAACTCCACGCCCAGCACGTCCGCCGCCAACCTCGCCGCGACCAATCGATCGGCGTGTTCCCGCGTCGATACC
AAAACCTTCCCGCCCATGTGGCCGCATTTTTTCGTGCCGGGTTTCTGATCCTCGAAGTGTATCCCGGCGGCGC
CCGCCTCGACGAAGAGCTTGACGAGTTTCATTACGGCGGAGAGGCCTCCGTGGCCGGTGTCGCCGTCGGCGAC
CACGGGGGTGAGGTAGTCGACCTTGGGAGGGAGGGGGGTTGTTGTCGGGTCGTTGGAGAGGATGGCGGAGGCG
CGTTCTTCGTTCTGGCGGCGATCGTGGTGGAGTTGGGCGCGTACGAGCTGATCGCCTTTTGTTGTTGTTGTTG
TTGTTGTGTTATCATTGGGCGAATTGTGTTCGAAAGAGAGAGAGTGTGTGTGTGTGTATAGTGAAAATGTAGA
ATAGCATTCAGTATTGCTGTGTTAGTTTCGTGTATTGTGATACGATGTTGTATTACAAGACAATATCATTGAA
CGCTTACACTTATTTGGCACCGTGTTCATGGGATAATCGGCGAAGTCAGGACCCGGTTCATTGGTGCTGCTCG

CCGTGCTCGAACATTGCCATCCGCTCACATAGATACTACTCAGATGAGGAGCCATCTGCACGACTTGGACCGG
ATCCAATGCCCCGAAGGTATGAGAATACCCTCCCTTCGAATGGAGTCGGGTTAACAGCGAGTACAACTTGCGA
GAAGACGCGTTGGAGTAAGAGCATTTGGGCGACATGATTCCATTGCCGGGACGTGCCGACGGAGAGGGACGGA
GGGAGGCTACATCGGCGGCTGGGTGTGAAAACAAAGAGTATGTGTATAATGACGGTGAGTTTTTGGGGGTGTG
CGTGTTTGTGTGTGCATATGTAGTATATATATCATGTCATGGGGATATACATGATTTATTTTTGTTCGTGTGT
ACAAGATGTATATGAGTAGTATAGATGGGATAGAAGCAGGAGATGATATATCCAACAAATGAATGAACAGACT
GCAAGAAGTGTCATGCAAATAATACATCATCGAGAT
>TR81|c1_g1_i9 len=1390 path=[2374:0-214 2375:215-238 2418:239-279
2427:280-282 2348:283-303 @2434@!:304-492 2443:493-685 @2441@!:686-774
2416:775-797 2432:798-823 2424:824-847 2440:848-877 2437:878-901
2431:902-967 2413:968-1062 2351:1063-1086 2422:1087-1132 2397:1133-1156
2430:1157-1171 2442:1172-1172 2436:1173-1185 2444:1186-1186 2445:1187-
1195 2428:1196-1308 2409:1309-1372 2345:1373-1389] [-1, 2374, 2375, 2418,
2427, 2348, 2434, 2443, 2441, 2416, 2432, 2424, 2440, 2437, 2431, 2413,
2351, 2422, 2397, 2430, 2442, 2436, 2444, 2445, 2428, 2409, 2345, -2]
GATCTCGACGCCGCCCCGCGCGCAATCGCCTCCCTCAGCGGAATCGAACCGGGCGAAGTCGCCCCCAAAATAA
AAGGATGATCCCTCCCGTCCACGTTCGAATCCAACAGCGTCGCCGCCTCGGCGTCCGTCCTCGCGACGATGAC
CAACTCCACGCCCAGCACGTCCGCCGCCAACCTCGCCGCGACCAATCGATCGGCGTGTTCCCGCGTCGATACC
AAAACCTTCCCGCCCATGTGGCCGCATTTTTTCGTGCCGGGTTTCTGATCCTCGAAGTGTATCCCGGCGGCGC
CCGCCTCGACGAAGAGCTTGACGAGTTTCATTACGGCGGAGAGGCCTCCGTGGCCGGTGTCGCCGTCGGCGAC
CACGGGGGTGAGGTAGTCGACCTTGGGAGGGAGGGGGGTTGTTGTCGGGTCGTTGGAGAGGATGGCGGAGGCG
CGTTCTTCGTTCTGGCGGCGATCGTGGTGGAGTTGGGCGCGTACGAGCTGATCGCCTTTTGTTGTTGTTGTTG
TTGTTGTGTTATCATTGGGCGAATTGTGTTCGAAAGAGAGAGAGTGTGTGTGTGTGTATAGTGAAAATGTAGA
ATAGCATTCAGTATTGCTGTGTTAGTTTCGTGTATTGTGATACGATGTTGTATTACAAGACAATATCATTGAA
CGCTTACACTTATTTGGCACCGTGTTCATGGGATAATCGGCGAAGTCAGGACCCGGTTCATTGGTGCTGCTCG
CCGTGCTCGAACATTGCCATCCGCTCACATAGATACTACTCAGATGAGGAGCCATCTGCACGACTTGGACCGG
ATCCAATGCCCCGAAGGTATGAGAATACCCTCCCTTCGAATGGAGTCGGGTTAACAGCGAGTACAACTTGCGA
GAAGACGCGTTGGAGTAAGAGCATTTGGGCGACATGATTCCATTGCCGGGACGTGCCGACGGAGAGGGACGGA
GGGAGGCTACATCGGCGGCCGAGTAGGTCCTCTTGGTACCCTTCCATCGTTCCTCGCGCCACCATTGTTCGAT
TTGTGCAATTTCGGTCAGGAGTGCTTCTCGCTCCGCCTTGATCTCGCCGCCGCCGCCGTCGTCGCTGGAGGAG
GATGAAGACGACTTCTTCGGAAGGACGCGAAGTCCTCGACTGACCTTGGGGGTTGATTTGGATGTCATTTTTT
TTTTTTGAGGCGTGTATGCTATTTTTTATGTGTTTTATGTTGTAGAAGACTGGTGCCGTAGTATGAAGGGTGT
TGCGTGTGTGAACGTTGTGTGCAGGAGTAGATGAGACCTGAGACGATGTACGTGCTGTCAACCGTGCGTGACC
GTGTCAACCCCGATAGAGATGTCAACGATGCGCTTTGGATGGGGCGGGTTGGAGGATGGGAAATTGGACGTCG
AGG
>TR81|c1_g1_i10 len=1446 path=[2374:0-214 2375:215-238 2418:239-279
2427:280-282 2348:283-303 @2434@!:304-492 2322:493-514 @2441@!:515-603
2438:604-698 2330:699-733 2415:734-853 2432:854-879 2424:880-903
2440:904-933 2437:934-957 2431:958-1023 2413:1024-1118 2351:1119-1142
2422:1143-1188 2397:1189-1212 2430:1213-1227 2442:1228-1228 2436:1229-
1241 2444:1242-1242 2445:1243-1251 2428:1252-1364 2409:1365-1428
2345:1429-1445] [-1, 2374, 2375, 2418, 2427, 2348, 2434, 2322, 2441,
2438, 2330, 2415, 2432, 2424, 2440, 2437, 2431, 2413, 2351, 2422, 2397,
2430, 2442, 2436, 2444, 2445, 2428, 2409, 2345, -2]
GATCTCGACGCCGCCCCGCGCGCAATCGCCTCCCTCAGCGGAATCGAACCGGGCGAAGTCGCCCCCAAAATAA
AAGGATGATCCCTCCCGTCCACGTTCGAATCCAACAGCGTCGCCGCCTCGGCGTCCGTCCTCGCGACGATGAC
CAACTCCACGCCCAGCACGTCCGCCGCCAACCTCGCCGCGACCAATCGATCGGCGTGTTCCCGCGTCGATACC
AAAACCTTCCCGCCCATGTGGCCGCATTTTTTCGTGCCGGGTTTCTGATCCTCGAAGTGTATCCCGGCGGCGC
CCGCCTCGACGAAGAGCTTGACGAGTTTCATTACGGCGGAGAGGCCTCCGTGGCCGGTGTCGCCGTCGGCGAC
CACGGGGGTGAGGTAGTCGACCTTGGGAGGGAGGGGGGTTGTTGTCGGGTCGTTGGAGAGGATGGCGGAGGCG
CGTTCTTCGTTCTGGCGGCGATCGTGGTGGAGTTGGGCGCGTACGAGCTGATCGCACTTATTTGGCACCGTGT
TCATGGGATAATCGGCGAAGTCAGGACCCGGTTCATTGGTGCTGCTCGCCGTGCTCGAACATTGCCATCCGCT
CACATAGATACTACTCAGATCTAGAGAGAGAGAGATGACGACGACGACGATGTTGTAATATGCACATGAT
GCAAAATTGAATAGAACCAACGAAATTAGGAGAAGGACATATATGAGAAAGAAGAAAGAGTCCAATATTCCTA
CGCAAACTCTGACATCGACATGACACGCGAACGAATAATAATAACAATAATAACAATGACAACAATAACACAT
GCATTCATTCCAAGACCAAACTACTTACGAGGAGCCATCTGCACGACTTGGACCGGATCCAATGCCCCGAAGG
TATGAGAATACCCTCCCTTCGAATGGAGTCGGGTTAACAGCGAGTACAACTTGCGAGAAGACGCGTTGGAGTA
AGAGCATTTGGGCGACATGATTCCATTGCCGGGACGTGCCGACGGAGAGGGACGGAGGGAGGCTACATCGGCG
GCCGAGTAGGTCCTCTTGGTACCCTTCCATCGTTCCTCGCGCCACCATTGTTCGATTTGTGCAATTTCGGTCA
GGAGTGCTTCTCGCTCCGCCTTGATCTCGCCGCCGCCGCCGTCGTCGCTGGAGGAGGATGAAGACGACTTCTT

CGGAAGGACGCGAAGTCCTCGACTGACCTTGGGGGTTGATTTGGATGTCATTTTTTTTTTTTGAGGCGTGTAT
GCTATTTTTTATGTGTTTTATGTTGTAGAAGACTGGTGCCGTAGTATGAAGGGTGTTGCGTGTGTGAACGTTG
TGTGCAGGAGTAGATGAGACCTGAGACGATGTACGTGCTGTCAACCGTGCGTGACCGTGTCAACCCCGATAGA
GATGTCAACGATGCGCTTTGGATGGGGCGGGTTGGAGGATGGGAAATTGGACGTCGAGG
>TR82|c1_g1_i1 len=1324 path=[1377:0-289 1378:290-313 1379:314-323 1380:324-347 1385:348-675 1383:676-699 1384:700-1323] [-1, 1377, 1378, 1379, 1380, 1385, 1383, 1384, -2]
GAGACTTGGTCGCGTATGGAGTTGATGCTGCATTGTTGTTGTTGTTGTTGTTGTCGTCGTCGTCGGGTTTGAC
AGTGCAATCCTCCTCCTCTGGAGCCTCCATCGAGGATAGGAAATAAAAGTCAGTTTGGGCCTCCTTCTCCACG
TCATTCGCCTTGATTGGAAGGTTGACAGATATTTTTACATTGTATTCGCTGCCTGCATTCTCATGACAACGAG
GAGGTAGATGCTCCTTTCCTCCTCCGCCAGTCATTATTTCGGGCATCCTTCCAAAACTTCCGACAATGCTTTC
ATCGCCATTGACACTCATATCCACACCATCAGGATAACTCCAGCAATCTCCATCGATTTCAGAGACTGGCGAC
GCTAAATCCGCCGCTGCACATCGAAACCTCTCCCTCAAATCCCCCCGCACCAAAGCCACGTACAACTTCGTCG
CATCCTTCCTCCTCAATCGCCCGTGCAATCGTCCCGCGGTCCGGGAATCGAATCCCATGATGACCGCGCCGCT
CGTGCGGTGATCCAATCGATGTACCAAGTAGGGTTTGCGCGATAATTGTTTGTGTATCGTGGTTTGAAGGACG
GGGCCTTTGGATCGACCCCATCGCGTGTTGGAGTTGTGATGCATCGTCATGCCCGGTGGTTTGGAGACGATGA
CGTGATCGTTTTGGTAGGAGAGGATGGGTATTCGAGGTCGATTCGATCGCTTTTTGAGTTTGGTTGGTTGTTC
GGTGGGTTGACTTGATTTGGTCATGTTGACATTTTGGTCATTGTTGATGTTGAATTCTTCCTTCGTGATTTCA
ATGGTTGAACCTTCCCGCAATGATTCTGAAAGTGAATGTGCCGTTGTGATTCCCGAATATCTTCCACTGATAC
GATCCGATGAAGATATACCACGTCGGCCGAGCCGTTCTGGGAATCTTTGCGAATTCGAGCAGAACACAACTAC
GCTACTCCCGCAATTATCACGCGCGGCGACGCCATCCCACCTCGTTCTACCCCGCGATGATGACGACGCCGGA
TCCAACGAAAAACCCAACACGCCCTTCGCACCCCGCAATCCCAAGAGTTCCTCCCTCGATGGCGGTATGCCGC
CGTTGCTTCCTTCGTCGAGGAGGAAAAAGGATGCGAATTTGAGCAAGAAATGGACTGCGACGATGTACACGAG
CAGGATGGAGAGGATTTCGAACGGTCGATTGTGGTCGGGGGGGCGGCGTAGGGCGAGGCGTCTTCTTTGGGGG
GGGATGGTGTTGGGTGGAGCGTTGGCGGTGGAGGTTGATGGTGTCGTGTCGGTGCCCATGGTCACGTCGTTAG
ATCGGAAGAG
>TR82|c2_g1_i1 len=647 path=[6749:0-145 6815:146-451 6770:452-452 6804:453-469 6821:470-493 6812:494-506 6801:507-530 6688:531-646] [-1, 6749, 6815, 6770, 6804, 6821, 6812, 6801, 6688, -2]
GGGTATCAACTCCTGCCACTTTAGAGGGAGGTCCATCCTTGCTATTTTGGACAACAAAGATGCGACATTAGTT
TGCAACGCGCGGTCTTGGATTAGTTCGACTTGTTCCCTCGTTATACTATTGGGATTGGCGATCATAAAGTCAG
TCTGGTGTTGATTAGATCCTCCTTCCGCTGCTTCTTTTGCTGCTACTACTCTCCCGGCGAGTACCAAATTCAA
CATCGATGTCCTCAACAAAGTTTTGGCACCTTCGCTCAACAAATTCTTGGTGGCCCCGACTTTGCCGCGTCCC
TTGTCTTTCCATCGGCGTGTGATCGCTGCCTTCAGGGACAATACTGCGGCTAGACGCATGGTTGCTGCTGCTG
ATTCCTTGTGCGTAGCCGACGACGATTCATTTTCCTGATCCACCACAGTGGCAGTTGTGAACGGTGTTGATGT
TGATGTTGATGTTGTGTGTCCAATCAATGATGTGAGAGTGATCGAGTACTGATCGGTGTGTGATTCTTCGCAT
TGTAACAGTGCCCGCTGGGCTTCTTGAATGATTTCGGGTGATGAGGGTGAAGTGATGCATTGGAGAGCCGATT
GGATGTGAGCGCACAGTTCTGTACTGGATGCCATGGCCGAGTGCTCCATGGCCATGGTCATGG
>TR82|c2_g2_i1 len=2102 path=[6673:0-79 6823:80-84 6814:85-97 6826:98-531 6757:532-548 @6820@!:549-651 6828:652-828 6776:829-850 @6818@!:851-1082 6798:1083-1171 6707:1172-2101] [-1, 6673, 6823, 6814, 6826, 6757, 6820, 6828, 6776, 6818, 6798, 6707, -2]
CTTGATACCATTGTGACCATGATTGGCCAACCGGTTGAAGGGTTATTGATCAATGATGGTTGATTTTAGGGGT
ACTTTTTCCTTGGCGGAGTTGAAAATGCGCGCCGTCCACCAAAGTTTGATTTTTTTCTTCGCGCAGTTGCGTG
TACGGGATAATAGCTCTGCTTCACAGGACTAAAAAGCAGCACTAAACATCGTTTGAACCTGACGAAGCTTGGA
TTTATGATTCACGATCCTAGAATATTTTGTATGGACAGGCCCTAATATCCTTCTCGAATATGTCCAGCATCTC
CTTCGCCATATCCCCCACGTCGCTATCCTCCCCATTAAACAAAATGGCATTTTCGAACACCAACTTTGTATCG
CTCACGAACCCCGCCATATCCTTGTATGCTCCCTCCTCAAGCTTGTTCACAACCAATGTCAAATCCATGGGCT
GCTCAACGACGTCGAAGTACTCCGGAATGCCCAGTTCGACAGGGTCAATAGGATCCTTGAACACCCAGCCATG
TTCATGGTGGAGAAGTTTTTTGGCGAGGGGGAGGCATTTGGTGGCAATTTGTCGTTCGATGGTGGTTGGCGAT
GCATCGTTGAGTTGGAGAGATGGAGCAGTGGAAGAGGAATCCCCCAGCTTGTCGCTGGCGGAAGGCGCTGCTC
CGTTGGAATCGGTGGGACGCATGCCTCCTGCGGAAGTGCTACCGCTTGCAACACCGCCACTACTTTGTCTGGG
CATCTTGCTGAGTCGAAGCGTATCTTTGCCTTCCTCCTCCTGCCCCTCTGTTGTGGAAACTAGATCTGCTGAC
CGTTTCGAAGCCCCTGAGACAGTCGCAGCACCACTGTTGTTGATGACCTGCTGCTGGACATTACCTAATTGGC
CAACGCCTGTTTGCTGCTGCTGCTGTTGTTGGAGCTGGAACGTGTGCTGAAGAATCTCCCCCTGGAGTATTGA
TTGTTGTTGTTCGAATTGTTGGCTCAGTTGCTGGAGAATGGCATGTTGCTTTTGGAGCTGCTGCGCCTGCTGT
GGGTTTGGGGTTGCTATGAGTTGTTGCTGCAGCCTTTGGCTTTGTCCAAAAAGGTTTTCCTACCAAAAATGAA
ACAAATGGTGAGAAAATTAGGCGAATGGGGAAAGATCAACCTAGGACAACCTCACCTTCTGCTGCTGAATCTG
CTGCATAATGAGCTGCTTCTTCTGAATTTCCTGTTGGAGCTCCTGCATATTGCTCAGAAATTGTTGAGGCATA
CCGCCCCCCGCTGTCACTACCGACGCCGCTTGAGCTTGCTGGCGTGGGACGGAGGTGCTGTTGGAATCATTAT

TGTCGACGGGCATGAAAGCGCCAAATGCTTCCAAACCCTGTCCGCTGACGCTGGCCTGACCGGCCGCCCCAAT
GCCCATATCAATCCCACCGTCCCCTGTGGCGTTGGCGGTGGGGGCGGTATCCAACCCATCGAGACCGACACTG
TTGAGATCCAAATCGTCGCCGCCGCCAAACGGAACGCCGGCGTCACCGCCGCCCGTTTGCCTTATGTGGTTCA
TAACCGGCGCACATATGGCACAACTGGCTGTCTGGTTCTCCGCCTTGCATAATCTATAGTGATTTAGAACACA
TCTGCTACTAAGACAGTGGGAGACCTTGCAATGCTTGTCGCGACACTTCTTCATGTGTTTCCATAGCGTGACC
ATCTGGGTACAGAATTTAGTGGGGCAGGTGGGGCCCTTGTCGCACTTGGAAGCGTGGCGGAGGAGGAGGAGTC
GCCGTTGCTGTTGAATGAGAATGGCCTTCTTTTTGTCGGATCCGCTTTGACTGTTGTTGTTGTTCCCCGGGTT
TTTTAGTTGGGTGGCTTTCGCAGCGGTCAGGTCCTGTGGTATTTCCGGAATGGCAGGAAGCTGATCGTCCGTC
ATTTGCTGCTGCTGCTGCTGGACAAGAATGTTGGCCGGCTGCACGTGTGGGACCTGCTGCTGCAGCTGCTGCA
TTTGTTGTTGAAGCTGCTGCTGACGCTGTAGAAGCATATGCTGCGGCTGCTCTTGCTGGTTCGTTTGCTGCGG
CGACGTATTCGATAACAATCCAATAGCCGCGGACAAGCCGCCGCCATTGTCAGCGGCG
>TR82|c2_g2_i2 len=2177 path=[6673:0-79 6823:80-84 6814:85-97 6826:98-531
6757:532-548 @6820@!:549-651 6828:652-828 6825:829-925 @6818@!:926-1157
6798:1158-1246 6707:1247-2176] [-1, 6673, 6823, 6814, 6826, 6757, 6820,
6828, 6825, 6818, 6798, 6707, -2]
CTTGATACCATTGTGACCATGATTGGCCAACCGGTTGAAGGGTTATTGATCAATGATGGTTGATTTTAGGGGT
ACTTTTTCCTTGGCGGAGTTGAAAATGCGCGCCGTCCACCAAAGTTTGATTTTTTTCTTCGCGCAGTTGCGTG
TACGGGATAATAGCTCTGCTTCACAGGACTAAAAAGCAGCACTAAACATCGTTTGAACCTGACGAAGCTTGGA
TTTATGATTCACGATCCTAGAATATTTTGTATGGACAGGCCCTAATATCCTTCTCGAATATGTCCAGCATCTC
CTTCGCCATATCCCCCACGTCGCTATCCTCCCCATTAAACAAAATGGCATTTTCGAACACCAACTTTGTATCG
CTCACGAACCCCGCCATATCCTTGTATGCTCCCTCCTCAAGCTTGTTCACAACCAATGTCAAATCCATGGGCT
GCTCAACGACGTCGAAGTACTCCGGAATGCCCAGTTCGACAGGGTCAATAGGATCCTTGAACACCCAGCCATG
TTCATGGTGGAGAAGTTTTTTGGCGAGGGGGAGGCATTTGGTGGCAATTTGTCGTTCGATGGTGGTTGGCGAT
GCATCGTTGAGTTGGAGAGATGGAGCAGTGGAAGAGGAATCCCCCAGCTTGTCGCTGGCGGAAGGCGCTGCTC
CGTTGGAATCGGTGGGACGCATGCCTCCTGCGGAAGTGCTACCGCTTGCAACACCGCCACTACTTTGTCTGGG
CATCTTGCTGAGTCGAAGCGTATCTTTGCCTTCCTCCTCCTGCCCCTCTGTTGTGGAAACTAGATCTGCTGAC
CGTTTCGAAGCCCCTGAGACAGTCGCTGGATTTAAATTGTGAGTGTTTTCTTCCGACCCGCTAGCACTAGCTG
AACAAAATTTATCTGCTTTGCTCTCACCAGCACCACTGTTGTTGATGACCTGCTGCTGGACATTACCTAATTG
GCCAACGCCTGTTTGCTGCTGCTGCTGTTGTTGGAGCTGGAACGTGTGCTGAAGAATCTCCCCCTGGAGTATT
GATTGTTGTTGTTCGAATTGTTGGCTCAGTTGCTGGAGAATGGCATGTTGCTTTTGGAGCTGCTGCGCCTGCT
GTGGGTTTGGGGTTGCTATGAGTTGTTGCTGCAGCCTTTGGCTTTGTCCAAAAAGGTTTTCCTACCAAAAATG
AAACAAATGGTGAGAAAATTAGGCGAATGGGGAAAGATCAACCTAGGACAACCTCACCTTCTGCTGCTGAATC
TGCTGCATAATGAGCTGCTTCTTCTGAATTTCCTGTTGGAGCTCCTGCATATTGCTCAGAAATTGTTGAGGCA
TACCGCCCCCCGCTGTCACTACCGACGCCGCTTGAGCTTGCTGGCGTGGGACGGAGGTGCTGTTGGAATCATT
ATTGTCGACGGGCATGAAAGCGCCAAATGCTTCCAAACCCTGTCCGCTGACGCTGGCCTGACCGGCCGCCCCA
ATGCCCATATCAATCCCACCGTCCCCTGTGGCGTTGGCGGTGGGGGCGGTATCCAACCCATCGAGACCGACAC
TGTTGAGATCCAAATCGTCGCCGCCGCCAAACGGAACGCCGGCGTCACCGCCGCCCGTTTGCCTTATGTGGTT
CATAACCGGCGCACATATGGCACAACTGGCTGTCTGGTTCTCCGCCTTGCATAATCTATAGTGATTTAGAACA
CATCTGCTACTAAGACAGTGGGAGACCTTGCAATGCTTGTCGCGACACTTCTTCATGTGTTTCCATAGCGTGA
CCATCTGGGTACAGAATTTAGTGGGGCAGGTGGGGCCCTTGTCGCACTTGGAAGCGTGGCGGAGGAGGAGGAG
TCGCCGTTGCTGTTGAATGAGAATGGCCTTCTTTTTGTCGGATCCGCTTTGACTGTTGTTGTTGTTCCCCGGG
TTTTTTAGTTGGGTGGCTTTCGCAGCGGTCAGGTCCTGTGGTATTTCCGGAATGGCAGGAAGCTGATCGTCCG
TCATTTGCTGCTGCTGCTGCTGGACAAGAATGTTGGCCGGCTGCACGTGTGGGACCTGCTGCTGCAGCTGCTG
CATTTGTTGTTGAAGCTGCTGCTGACGCTGTAGAAGCATATGCTGCGGCTGCTCTTGCTGGTTCGTTTGCTGC
GGCGACGTATTCGATAACAATCCAATAGCCGCGGACAAGCCGCCGCCATTGTCAGCGGCG
>TR82|c2_g2_i3 len=2108 path=[6673:0-79 6823:80-84 6814:85-97 6826:98-531
6757:532-548 @6820@!:549-651 6828:652-828 6825:829-925 @6818@!:926-1157
6725:1158-1177 6726:1178-2107] [-1, 6673, 6823, 6814, 6826, 6757, 6820,
6828, 6825, 6818, 6725, 6726, -2]
CTTGATACCATTGTGACCATGATTGGCCAACCGGTTGAAGGGTTATTGATCAATGATGGTTGATTTTAGGGGT
ACTTTTTCCTTGGCGGAGTTGAAAATGCGCGCCGTCCACCAAAGTTTGATTTTTTTCTTCGCGCAGTTGCGTG
TACGGGATAATAGCTCTGCTTCACAGGACTAAAAAGCAGCACTAAACATCGTTTGAACCTGACGAAGCTTGGA
TTTATGATTCACGATCCTAGAATATTTTGTATGGACAGGCCCTAATATCCTTCTCGAATATGTCCAGCATCTC
CTTCGCCATATCCCCCACGTCGCTATCCTCCCCATTAAACAAAATGGCATTTTCGAACACCAACTTTGTATCG
CTCACGAACCCCGCCATATCCTTGTATGCTCCCTCCTCAAGCTTGTTCACAACCAATGTCAAATCCATGGGCT
GCTCAACGACGTCGAAGTACTCCGGAATGCCCAGTTCGACAGGGTCAATAGGATCCTTGAACACCCAGCCATG
TTCATGGTGGAGAAGTTTTTTGGCGAGGGGGAGGCATTTGGTGGCAATTTGTCGTTCGATGGTGGTTGGCGAT
GCATCGTTGAGTTGGAGAGATGGAGCAGTGGAAGAGGAATCCCCCAGCTTGTCGCTGGCGGAAGGCGCTGCTC
CGTTGGAATCGGTGGGACGCATGCCTCCTGCGGAAGTGCTACCGCTTGCAACACCGCCACTACTTTGTCTGGG
CATCTTGCTGAGTCGAAGCGTATCTTTGCCTTCCTCCTCCTGCCCCTCTGTTGTGGAAACTAGATCTGCTGAC

CGTTTCGAAGCCCCTGAGACAGTCGCTGGATTTAAATTGTGAGTGTTTTCTTCCGACCCGCTAGCACTAGCTG
AACAAAATTTATCTGCTTTGCTCTCACCAGCACCACTGTTGTTGATGACCTGCTGCTGGACATTACCTAATTG
GCCAACGCCTGTTTGCTGCTGCTGCTGTTGTTGGAGCTGGAACGTGTGCTGAAGAATCTCCCCCTGGAGTATT
GATTGTTGTTGTTCGAATTGTTGGCTCAGTTGCTGGAGAATGGCATGTTGCTTTTGGAGCTGCTGCGCCTGCT
GTGGGTTTGGGGTTGCTATGAGTTGTTGCTGCAGCCTTTGGCTTTGTCCAAAAAGGTTTTCCTTCTGCTGCTG
AATCTGCTGCATAATGAGCTGCTTCTTCTGAATTTCCTGTTGGAGCTCCTGCATATTGCTCAGAAATTGTTGA
GGCATACCGCCCCCCGCTGTCACTACCGACGCCGCTTGAGCTTGCTGGCGTGGGACGGAGGTGCTGTTGGAAT
CATTATTGTCGACGGGCATGAAAGCGCCAAATGCTTCCAAACCCTGTCCGCTGACGCTGGCCTGACCGGCCGC
CCCAATGCCCATATCAATCCCACCGTCCCCTGTGGCGTTGGCGGTGGGGGCGGTATCCAACCCATCGAGACCG
ACACTGTTGAGATCCAAATCGTCGCCGCCGCCAAACGGAACGCCGGCGTCACCGCCGCCCGTTTGCCTTATGT
GGTTCATAACCGGCGCACATATGGCACAACTGGCTGTCTGGTTCTCCGCCTTGCATAATCTATAGTGATTTAG
AACACATCTGCTACTAAGACAGTGGGAGACCTTGCAATGCTTGTCGCGACACTTCTTCATGTGTTTCCATAGC
GTGACCATCTGGGTACAGAATTTAGTGGGGCAGGTGGGGCCCTTGTCGCACTTGGAAGCGTGGCGGAGGAGGA
GGAGTCGCCGTTGCTGTTGAATGAGAATGGCCTTCTTTTTGTCGGATCCGCTTTGACTGTTGTTGTTGTTCCC
CGGGTTTTTTAGTTGGGTGGCTTTCGCAGCGGTCAGGTCCTGTGGTATTTCCGGAATGGCAGGAAGCTGATCG
TCCGTCATTTGCTGCTGCTGCTGCTGGACAAGAATGTTGGCCGGCTGCACGTGTGGGACCTGCTGCTGCAGCT
GCTGCATTTGTTGTTGAAGCTGCTGCTGACGCTGTAGAAGCATATGCTGCGGCTGCTCTTGCTGGTTCGTTTG
CTGCGGCGACGTATTCGATAACAATCCAATAGCCGCGGACAAGCCGCCGCCATTGTCAGCGGCG
>TR82|c2_g3_i1 len=292 path=[6670:0-145 6671:146-168 6672:169-291] [-1, 6670, 6671, 6672, -2]
GGGTATCAACTCCTGCCACTTTAGAGGGAGGTCCATCCTTGCTATTTTGGACAACAAAGATGCGACATTAGTT
TGCAACGCGCGGTCTTGGATTAGTTCGACTTGTTCCCTCGTTATACTATTGGGATTGGCGATCATAAAGTCAG
GGGTATCAACTCCTGCCACTTTAGAGGGAGGTCCATCCTTGCTATTTTGGACAACAAAGATGCGACATTAGTT
TGCAACGCGCGGTCTTGGATTAGTTCGACTTGTTCCCTCGTTATACTATTGGGATTGGCGATCATAAAGTCAG
>TR82|c2_g4_i1 len=3420 path=[6727:0-43 6728:44-56 6822:57-73 6813:74-96 6802:97-196 6741:197-220 6819:221-257 6799:258-281 6827:282-357 6824:358-401 6817:402-464 6714:465-485 6807:486-571 6796:572-711 6719:712-735 6806:736-871 6784:872-895 6795:896-922 6816:923-946 6805:947-949 6794:950-1437 6765:1438-1461 6766:1462-1462 6767:1463-1486 6793:1487-1809 6690:1810-1833 6691:1834-1854 6792:1855-3296 6748:3297-3419] [-1, 6727, 6728, 6822, 6813, 6802, 6741, 6819, 6799, 6827, 6824, 6817, 6714, 6807, 6796, 6719, 6806, 6784, 6795, 6816, 6805, 6794, 6765, 6766, 6767, 6793, 6690, 6691, 6792, 6748, -2]
AATATGAGTCATTAACTATCAATTTTAGGGGTACTTTTTCCTTGGCGGAGTTGAAAAATAGGTCGTCGTCTAG
AATTTTAAAAATTCGAACCCTTGCGCAGTTGCGTGCACAGGATAATATCTAGTGTTCTTGGTCTCAGGGCCAA
ATAAATGGCAAGAGATATTTCTGCGCGCTAAGGCAAGGTAGCTCAGTTCATCAAAACCGGAAAAATCTCTAGC
TCACCTGATGAAGCTTAGATTATATTATTTGTTGCGCTTGTGCTTTCAATATATTGCACCAAGCAAGCATGCT
GATAGCGATAATTTGCACGATCAACCCTCAATATTCAAAATGACTTCTTTCAATTGCTGTAAGGCACCATTTT
CAGCAAGGCAAAAATCTTGGTAATTTTGACCAAGTTCATTAGGAAGGATGTTCAGTTTTGTAGAAACAGCTTC
ATGAAGTGATGTGGAGATGATAATGTCCTGCAACAAAGGAAATCAAAAGACAATAAATTCATTAGCACAAGTG
TAAACAAAACAAACAGCACGACCATAAAATTATATTCACATTTGACCTCTGTCCCTGTAACAGTGCTTCGTAC
AAATCTTCACCAATGTAAATAGACTCCTCGTCGTCGCCGCAACCTACTTCATAAGAGAGAAGATTGGTCCCAT
TGGGATGTACGTTCTTCAACACACCAATGAAGATGTTGAATATTGTCTTGGACTTCCTGAGGACTGTGTTGCG
ACAGGATGCCAGTTGGCACGGTGGGTAGAACGAAAGCAGAAGCAATGCCCAAAGCTTTTGGTACAGCAGTCCG
TGAGCACCATTCCCTGCAACCTGGAACTTTCCCATGTACAGAGAAATAAATTCTTCCTCCCCAAAAACAGCAC
CTGATGGGAGCTTTAGAGGGAGTTGCAGCGTGTCTGGCCTAGACAATGTCACCCTAGCCAATGCAGCCAAATA
CAGCACAATCACTCTATCGGGTTCACATTTTTCATCGTCAAAATAACTACTGGCACACGCCTCCATGAAAGTC
GTTAACACTCCACAGCTGTTCAGCAATACGCCGCCTTCGACAGGAAAGGAGCGAAGGAGAGCCTCGATAACCA
AAAATATGTACGCCGTACCACGAGGGCGGACTTCTCCAACAACATTACTAAGAACTGATTGAATTGTGGTGGC
ATGGTCGTTTAGGAAGGTAGCTCCGCCAAGTATGATGTAGTGTTCAGTGACTCGCATCAGTATCATCAGGTGA
TCAAAATCTTTACTCAACTCCACAGTATGAATGAACAATTTTCCCAGCAACGAATCATACTTTCGTGACAATC
GAAGGAACACCCACCATATTTTCAACGCCTCCTCAATGAGAAAAACGTTTTCCTCCCTTGCAAAACTCTCGTC
GATCATGGGCAATACCAAGGGATACAGTACGCTACAGCTTTCAGGCCCAACAAATGATGCTATGCACGAGAGA
ATAGTTAAAACACTCCGTTTCAATCGCAGATTCTGATCAATTGCATTGTCCCAGATAGACGAAAGCGGAGCAA
CGATGGTGTTAAGTATTTCATTCGTGAGCGAACCTCCAGTCTGCATACAAATAAGGTTGGAGAGAAGATCGAG
GCAAGATGATCGAGATTCGACCTCCGCGCATTCGTTTACTAACTGATACAAAGCGGGAGCTGTTGGTTCAACG
ATAGAGTGGAGCAGTGACGGAGCGTCTTCGCATTGTGGAAGCAATGCATCCATGGCTTGGACTGCGGTGAGTC
GGACACATACATCTTGATCCGAAATCGAAGAAGATAGCATACCAAAAGGATTGAACGGCGATGATATTGGTAC
CTGATGGGCATTGCAGGAAAGGAGCCAAATCATCCGCCGTTGCAGGATGGGAAGTTGTGTGCGCTGTTGTTTA
CTCTGTCGCAACAACATGGACAGACAATCACCAACCGAAACACGAAACCAAGTTTGAAAGGAGTATCCAGGAT

AATTTTCCAAAATGCTCCCCATCAACCCGGCGGCCGTGTAGATTGCGTCCCATTGCAATAACAGGTCCATCGA
CGCGTTCTCCACGCCACTGGATAGCTTTGCGCACGGAGCCCCCAATGGCAACGCCGAATATATAATTTGAGGA
TCAACGCCGAAAGAAATGCCTCCCTCCAATTCCACCGCCAATCGCTGCGATACGACATTGGTCACGAACCCCA
TCAGCCAGGGCAGCACAACGTCGCCGGAAAAGGAAGACTCCGCCAGTGCCAGGAATAGATTCTGTGCCGCGCA
ACCGACATCTTCCTCACTAGACCGATTCATTTCCCAATGGTAAAATGCTTCCGGATCGTCTTGCCACAGAACT
GCATCTTCTTTGCTGTCATCGTCATCGTCGTCATGGACAATCTGGATGGGAGAAATGTGGATCGAGAATAACT
GAAGTAGAGTCCGCGATAGAGATTGGACCAGCGACGGAGTGAAGAAGCTCGCCATCTCGCTTTTGGCGTTTAC
CAAGTCCGCATACTTGGATGTTCCCGCCACATTGGCGAGGAAGGAGAGGAATGCAATGGCAAATCGGTCTGTA
CTACCCCGCAGGTTGCTGTTATTCGCACCGAGGTCATTATTATTCGCGACATTTCTCCTTTGACATCTCTCCT
GCTCCGGAACGCCCACAATGCACATTAACGACGAATGAAACAAAGTGAGAAAGGGTGTCAAATATCTCGCAAA
CGATTCCGGATGAGAGGATTGCAGATCAACAATAAGGTCGCAATGAACGCGAAGGAGTTGCTTCAGGGGTTTG
CGGAGGGCAACATGCACAGCATCGTCGTTGTCGCCCAAGATGCACGGGAGCCACCGAGAGAGAAACGAGTGGG
TCAGCGTAAGGATCTGATCCACCAATGGCGAATTTTCTTCCACCAATTTAGAAAAGGAAGAAATCATCATGTG
AGAAACAACTCGCGATGTGAGGGTGGCATTTCGCAGCGTGAAAGCGGTACATTCTCCTCCTCCCCTCATTATC
TCATTGTTGTTGGATGCAGTATTCACCAAGGATTCCAACTCCTGGAAGGGAGGCATCAGCCCACTCTCCACGA
TCGATCCCAGATATTGCAATGCAATGGAGTTCCTGTACTTTTTGTTCACCAATAATCGTTGTGTGGATATTTC
CGACAAGATGGCTTCCAGCGAATTCATTGCGTTGTAAATTACAGCCTGAAATAATTGCTGCATTTGTTTCGAT
TGCGGTTGATGTTTTGTTTGATACACTGCCCCTTGGGCACATCTCACTGCTTCCACCAAGGAGGGTATCAACT
CCTGCCACTTTAGAGGGAGGTCCATCCTTGCTATTTTGGACAACAAAGATGCGACATTAGTTTGCAACGCGCG
GTCTTGGATTAGTTCGACTTGTTCCCTCGTTATACTATTGGGATTGGCGATCATAAAGTCAG
>TR82|c2_g4_i2 len=3335 path=[6727:0-43 6728:44-56 6822:57-73 6813:74-96 6802:97-196 6741:197-220 6819:221-257 6799:258-281 6827:282-357 6824:358-401 6817:402-464 6808:465-486 6796:487-626 6719:627-650 6806:651-786 6784:787-810 6795:811-837 6816:838-861 6805:862-864 6794:865-1352 6765:1353-1376 6766:1377-1377 6767:1378-1401 6793:1402-1724 6690:1725-1748 6691:1749-1769 6792:1770-3211 6748:3212-3334] [-1, 6727, 6728, 6822, 6813, 6802, 6741, 6819, 6799, 6827, 6824, 6817, 6808, 6796, 6719, 6806, 6784, 6795, 6816, 6805, 6794, 6765, 6766, 6767, 6793, 6690, 6691, 6792, 6748, -2]
AATATGAGTCATTAACTATCAATTTTAGGGGTACTTTTTCCTTGGCGGAGTTGAAAAATAGGTCGTCGTCTAG
AATTTTAAAAATTCGAACCCTTGCGCAGTTGCGTGCACAGGATAATATCTAGTGTTCTTGGTCTCAGGGCCAA
ATAAATGGCAAGAGATATTTCTGCGCGCTAAGGCAAGGTAGCTCAGTTCATCAAAACCGGAAAAATCTCTAGC
TCACCTGATGAAGCTTAGATTATATTATTTGTTGCGCTTGTGCTTTCAATATATTGCACCAAGCAAGCATGCT
GATAGCGATAATTTGCACGATCAACCCTCAATATTCAAAATGACTTCTTTCAATTGCTGTAAGGCACCATTTT
CAGCAAGGCAAAAATCTTGGTAATTTTGACCAAGTTCATTAGGAAGGATGTTCAGTTTTGTAGAAACAGCTTC
ATGAAGTGATGTGGAGATGATAATGTCATTTGACCTCTGTCCCTGTAACAGTGCTTCGTACAAATCTTCACCA
ATGTAAATAGACTCCTCGTCGTCGCCGCAACCTACTTCATAAGAGAGAAGATTGGTCCCATTGGGATGTACGT
TCTTCAACACACCAATGAAGATGTTGAATATTGTCTTGGACTTCCTGAGGACTGTGTTGCGACAGGATGCCAG
TTGGCACGGTGGGTAGAACGAAAGCAGAAGCAATGCCCAAAGCTTTTGGTACAGCAGTCCGTGAGCACCATTC
CCTGCAACCTGGAACTTTCCCATGTACAGAGAAATAAATTCTTCCTCCCCAAAAACAGCACCTGATGGGAGCT
TTAGAGGGAGTTGCAGCGTGTCTGGCCTAGACAATGTCACCCTAGCCAATGCAGCCAAATACAGCACAATCAC
TCTATCGGGTTCACATTTTTCATCGTCAAAATAACTACTGGCACACGCCTCCATGAAAGTCGTTAACACTCCA
CAGCTGTTCAGCAATACGCCGCCTTCGACAGGAAAGGAGCGAAGGAGAGCCTCGATAACCAAAAATATGTACG
CCGTACCACGAGGGCGGACTTCTCCAACAACATTACTAAGAACTGATTGAATTGTGGTGGCATGGTCGTTTAG
GAAGGTAGCTCCGCCAAGTATGATGTAGTGTTCAGTGACTCGCATCAGTATCATCAGGTGATCAAAATCTTTA
CTCAACTCCACAGTATGAATGAACAATTTTCCCAGCAACGAATCATACTTTCGTGACAATCGAAGGAACACCC
ACCATATTTTCAACGCCTCCTCAATGAGAAAAACGTTTTCCTCCCTTGCAAAACTCTCGTCGATCATGGGCAA
TACCAAGGGATACAGTACGCTACAGCTTTCAGGCCCAACAAATGATGCTATGCACGAGAGAATAGTTAAAACA
CTCCGTTTCAATCGCAGATTCTGATCAATTGCATTGTCCCAGATAGACGAAAGCGGAGCAACGATGGTGTTAA
GTATTTCATTCGTGAGCGAACCTCCAGTCTGCATACAAATAAGGTTGGAGAGAAGATCGAGGCAAGATGATCG
AGATTCGACCTCCGCGCATTCGTTTACTAACTGATACAAAGCGGGAGCTGTTGGTTCAACGATAGAGTGGAGC
AGTGACGGAGCGTCTTCGCATTGTGGAAGCAATGCATCCATGGCTTGGACTGCGGTGAGTCGGACACATACAT
CTTGATCCGAAATCGAAGAAGATAGCATACCAAAAGGATTGAACGGCGATGATATTGGTACCTGATGGGCATT
GCAGGAAAGGAGCCAAATCATCCGCCGTTGCAGGATGGGAAGTTGTGTGCGCTGTTGTTTACTCTGTCGCAAC
AACATGGACAGACAATCACCAACCGAAACACGAAACCAAGTTTGAAAGGAGTATCCAGGATAATTTTCCAAAA
TGCTCCCCATCAACCCGGCGGCCGTGTAGATTGCGTCCCATTGCAATAACAGGTCCATCGACGCGTTCTCCAC
GCCACTGGATAGCTTTGCGCACGGAGCCCCCAATGGCAACGCCGAATATATAATTTGAGGATCAACGCCGAAA
GAAATGCCTCCCTCCAATTCCACCGCCAATCGCTGCGATACGACATTGGTCACGAACCCCATCAGCCAGGGCA
GCACAACGTCGCCGGAAAAGGAAGACTCCGCCAGTGCCAGGAATAGATTCTGTGCCGCGCAACCGACATCTTC
CTCACTAGACCGATTCATTTCCCAATGGTAAAATGCTTCCGGATCGTCTTGCCACAGAACTGCATCTTCTTTG

CTGTCATCGTCATCGTCGTCATGGACAATCTGGATGGGAGAAATGTGGATCGAGAATAACTGAAGTAGAGTCC
GCGATAGAGATTGGACCAGCGACGGAGTGAAGAAGCTCGCCATCTCGCTTTTGGCGTTTACCAAGTCCGCATA
CTTGGATGTTCCCGCCACATTGGCGAGGAAGGAGAGGAATGCAATGGCAAATCGGTCTGTACTACCCCGCAGG
TTGCTGTTATTCGCACCGAGGTCATTATTATTCGCGACATTTCTCCTTTGACATCTCTCCTGCTCCGGAACGC
CCACAATGCACATTAACGACGAATGAAACAAAGTGAGAAAGGGTGTCAAATATCTCGCAAACGATTCCGGATG
AGAGGATTGCAGATCAACAATAAGGTCGCAATGAACGCGAAGGAGTTGCTTCAGGGGTTTGCGGAGGGCAACA
TGCACAGCATCGTCGTTGTCGCCCAAGATGCACGGGAGCCACCGAGAGAGAAACGAGTGGGTCAGCGTAAGGA
TCTGATCCACCAATGGCGAATTTTCTTCCACCAATTTAGAAAAGGAAGAAATCATCATGTGAGAAACAACTCG
CGATGTGAGGGTGGCATTTCGCAGCGTGAAAGCGGTACATTCTCCTCCTCCCCTCATTATCTCATTGTTGTTG
GATGCAGTATTCACCAAGGATTCCAACTCCTGGAAGGGAGGCATCAGCCCACTCTCCACGATCGATCCCAGAT
ATTGCAATGCAATGGAGTTCCTGTACTTTTTGTTCACCAATAATCGTTGTGTGGATATTTCCGACAAGATGGC
TTCCAGCGAATTCATTGCGTTGTAAATTACAGCCTGAAATAATTGCTGCATTTGTTTCGATTGCGGTTGATGT
TTTGTTTGATACACTGCCCCTTGGGCACATCTCACTGCTTCCACCAAGGAGGGTATCAACTCCTGCCACTTTA
GAGGGAGGTCCATCCTTGCTATTTTGGACAACAAAGATGCGACATTAGTTTGCAACGCGCGGTCTTGGATTAG
TTCGACTTGTTCCCTCGTTATACTATTGGGATTGGCGATCATAAAGTCAG
>TR82|c3_g2_i1 len=243 path=[7881:0-27 7882:28-49 8631:50-53 8627:54-55 8620:56-121 8611:122-131 8636:132-134 8642:135-138 8648:139-139 8651:140-140 8562:141-187 8522:188-190 8468:191-214 7909:215-242] [-1, 7881, 7882, 8631, 8627, 8620, 8611, 8636, 8642, 8648, 8651, 8562, 8522, 8468, 7909, -2]
TTATGCTGTGCACGCAACTGCGCGAAGAAAAAAATCGAACTTTTGCGGACGGCGCGCATTTTCAACTCCGCTG
AGGAAACAGTACCCCTAAAATCAACTGTCATTGGTCAATAATCCTTCAACCGGTTGGCCAATCATGGCCGCAA
TGGTATCGATAGACGGGGATTTCTTAGACGAATCCATGGATAATCCAATTGTCCTACTTTCCCAGCTATTTTT
CTTATTAAGTATATTGACTTTCAA
>TR82|c3_g3_i1 len=909 path=[8285:0-63 8286:64-64 8287:65-72 8599:73-180 8635:181-183 8641:184-199 8647:200-207 8650:208-235 8609:236-249 8524:250-259 8563:260-266 8594:267-406 7968:407-442 8560:443-462 8465:463-509 8519:510-520 8634:521-548 8630:549-639 8637:640-642 8645:643-684 8607:685-696 8591:697-711 8554:712-725 8615:726-755 8604:756-764 8551:765-788 8504:789-840 8585:841-848 8547:849-866 8411:867-908] [-1, 8285, 8286, 8287, 8599, 8635, 8641, 8647, 8650, 8609, 8524, 8563, 8594, 7968, 8560, 8465, 8519, 8634, 8630, 8637, 8645, 8607, 8591, 8554, 8615, 8604, 8551, 8504, 8585, 8547, 8411, -2]
CTGCGCAAAGAAAAAAAATCGAACTTTTGCAAACGGCGCATATTTTCAACTCCGCCGAGGAAAAAGTACCCCT
TAAATCAACCCTCATTGGTCAATAATCCTTCCACCGGTTAGTTGGCCAATCATGGCCGCAATGATATCGTATC
AATAGACGGGGTTTTCTTAGACGAATCCATGGATAATCTAATTGTCCTACTTTCCCAGCTATTTTTCTTATTA
AGTATATTGACTTTCAACAGGGAACTTTTTTGGTGTACACACGACATTGTCAATCCTTCTCAAGCGTCAGTTT
TGCACGCGCAATCTTCTGTGTGCATTCGACCGTCATCTGCACAGGCCCGAATTCATTCATCTTCACCGACGTC
GTGCCCCGCCGGCCTCCGCCCATCCGCAGACTCAAGCATCCTTCTCGTCTCCACGGCCGTCATATCGATGTTC
ACTACTCTTAGGTACAGAGATGACAGCGGCTGTGATATGGAGATGTGACGAGGAGGAGGATGGTTAGTTGTCA
GGTGACCAGTCGATCTTCGGTCGGCCGGGCTCGTCGTCAGTAAACAGCAAGCGGGCGACTTGGGGGCCACTCA
CTAATCGCGCGTGAACATACACACGTGAGAAGATGATCAACACAGACACCAAAAAAGTTCGGTATTGAAGTCA
ATACAAGGAGGAAAAAAAGAAGGGAAAGTAAGACAATTAGATTATCCATGGCATTGTCTAAGAAACCCCGTCT
ATAGATACCATTTAACCCATGATCAACCTACCTCAGGAGCCAATTCATAAGTCATTAACCATCAATTTTTGGG
GTACTTTTCCCTCCGAGGAGTTGAAAAAACATGTCATTGTCTAGAAATTTGGAAATTCGAACGTTCGTGCAGT
TGCGTGCACAGCATAACAGGGTAGGGCACGCCA
>TR82|c3_g3_i2 len=1327 path=[8285:0-63 8286:64-64 8287:65-72 8599:73-180 8635:181-183 8641:184-199 8647:200-207 8650:208-235 8609:236-249 8524:250-259 8563:260-266 8594:267-406 7968:407-442 8560:443-462 8465:463-509 8519:510-520 8634:521-548 8630:549-639 8637:640-642 8644:643-656 8649:657-683 8555:684-695 8512:696-710 8616:711-754 8604:755-763 8550:764-797 8622:798-804 8601:805-813 8585:814-821 8547:822-839 8498:840-881 @8584@!:882-982 8546:983-1009 8497:1010-1017 8437:1018-1041 8327:1042-1326] [-1, 8285, 8286, 8287, 8599, 8635, 8641, 8647, 8650, 8609, 8524, 8563, 8594, 7968, 8560, 8465, 8519, 8634, 8630, 8637, 8644, 8649, 8555, 8512, 8616, 8604, 8550, 8622, 8601, 8585, 8547, 8498, 8584, 8546, 8497, 8437, 8327, -2]
CTGCGCAAAGAAAAAAAATCGAACTTTTGCAAACGGCGCATATTTTCAACTCCGCCGAGGAAAAAGTACCCCT
TAAATCAACCCTCATTGGTCAATAATCCTTCCACCGGTTAGTTGGCCAATCATGGCCGCAATGATATCGTATC
AATAGACGGGGTTTTCTTAGACGAATCCATGGATAATCTAATTGTCCTACTTTCCCAGCTATTTTTCTTATTA

AGTATATTGACTTTCAACAGGGAACTTTTTTGGTGTACACACGACATTGTCAATCCTTCTCAAGCGTCAGTTT
TGCACGCGCAATCTTCTGTGTGCATTCGACCGTCATCTGCACAGGCCCGAATTCATTCATCTTCACCGACGTC
GTGCCCCGCCGGCCTCCGCCCATCCGCAGACTCAAGCATCCTTCTCGTCTCCACGGCCGTCATATCGATGTTC
ACTACTCTTAGGTACAGAGATGACAGCGGCTGTGATATGGAGATGTGACGAGGAGGAGGATGGTTAGTTGTCA
GGTGACCAGTCGATCTTCGGTCGGCCGGGCTCGTCGTCAGTAAACAGCAAGCGGGCGACTTGGGGGCCACTCA
CTAATCGCGCGTGAACATACACACGTGAGAAGATGATCAACACAGACACCAAAAAAGTTTGGTATTGAAGTCA
ATACAAGGAGAAAAATAGAAGGGAAAGTAAGACAATTAGATTATCCATGGCATTTTCTAAGAAACCCCGTCTA
TCGATACCATTTAACCCATGATCAACCTACCTCATGAGTCATTAGGGTACTTTTCCCTCGAAGGAGTTGAAAA
AACATGTCATTGTCTAGAAATTTGGAAATTCGAACGTATGTGCAGTTGCGTGCACCGCATAACAGGGTAGGGC
ACGCCATCAATGCGTAAAAAGAACAAAATGTAAAACTCAAGATCAGAAACAAAAATTCTTCGATTACTTCTTT
CGACCGGCGTAGGTAAATATTCTGCTACTTCACGCGCCCCCTCATCCAAAAACAAATGCTGGATCAAGTTGAA
ATGTATGTAGTCATGGCAGTTCTTCGACAGCTAATGCCTTTCCTTACAACTTCGTCATTCCATTGACAAAATC
GCCGAGAGCATTCAGGAATCCCATCCCCTCCTGCGGGGCATTCATGGCACCATTCTCGATATCGTAAATATCC
TCCACCTCCGGGTTGCTGGGCAAGCGATCCATCGCCTTGATCATCCTGTATCCCCCCTCGGCCCCCTCGTCCT
CCTGCCACACCGTGAACAGCTGCGGCCAAACCCGCGTCAGCGCCCCCCACGGCAGGGTGCGCAAGTAGTACGC
CGGCGCCAACCCG
>TR82|c3_g3_i3 len=827 path=[8285:0-63 8286:64-64 8287:65-72 8599:73-180
8635:181-183 8641:184-199 8647:200-207 8650:208-235 8609:236-249
8524:250-259 8563:260-266 8594:267-406 7968:407-442 8560:443-462
8465:463-509 8519:510-520 8634:521-548 8630:549-639 8637:640-642
8645:643-684 8458:685-706 8504:707-758 8585:759-766 8547:767-784
8411:785-826] [-1, 8285, 8286, 8287, 8599, 8635, 8641, 8647, 8650, 8609,
8524, 8563, 8594, 7968, 8560, 8465, 8519, 8634, 8630, 8637, 8645, 8458,
8504, 8585, 8547, 8411, -2]
CTGCGCAAAGAAAAAAAAATCGAACTTTTGCAAACGGCGCATATTTTCAACTCCGCCGAGGAAAAAGTACCCCT
TAAATCAACCCTCATTGGTCAATAATCCTTCCACCGGTTAGTTGGCCAATCATGGCCGCAATGATATCGTATC
AATAGACGGGGTTTTCTTAGACGAATCCATGGATAATCTAATTGTCCTACTTTCCCAGCTATTTTTCTTATTA
AGTATATTGACTTTCAACAGGGAACTTTTTTGGTGTACACACGACATTGTCAATCCTTCTCAAGCGTCAGTTT
TGCACGCGCAATCTTCTGTGTGCATTCGACCGTCATCTGCACAGGCCCGAATTCATTCATCTTCACCGACGTC
GTGCCCCGCCGGCCTCCGCCCATCCGCAGACTCAAGCATCCTTCTCGTCTCCACGGCCGTCATATCGATGTTC
ACTACTCTTAGGTACAGAGATGACAGCGGCTGTGATATGGAGATGTGACGAGGAGGAGGATGGTTAGTTGTCA
GGTGACCAGTCGATCTTCGGTCGGCCGGGCTCGTCGTCAGTAAACAGCAAGCGGGCGACTTGGGGGCCACTCA
CTAATCGCGCGTGAACATACACACGTGAGAAGATGATCAACACAGACACCAAAAAAGTTCGGTATTGAAGTCA
ATACAAGGAGGAAAAAAAGAAGGGAAAGAGCCAATTCATAAGTCATTAACCATCAATTTTTGGGGTACTTTTC
CCTCCGAGGAGTTGAAAAAACATGTCATTGTCTAGAAATTTGGAAATTCGAACGTTCGTGCAGTTGCGTGCAC
AGCATAACAGGGTAGGGCACGCCA
>TR82|c3_g3_i4 len=889 path=[8285:0-63 8286:64-64 8287:65-72 8599:73-180
8635:181-183 8641:184-199 8647:200-207 8650:208-235 8527:236-259
8563:260-266 8594:267-406 8521:407-442 8561:443-462 8466:463-507
8520:508-518 8633:519-546 8630:547-637 8637:638-640 8644:641-654
8649:655-681 8632:682-697 8629:698-703 8453:704-726 8548:727-727
8499:728-745 7895:746-787 @8584@!:788-888] [-1, 8285, 8286, 8287, 8599,
8635, 8641, 8647, 8650, 8527, 8563, 8594, 8521, 8561, 8466, 8520, 8633,
8630, 8637, 8644, 8649, 8632, 8629, 8453, 8548, 8499, 7895, 8584]
CTGCGCAAAGAAAAAAAAATCGAACTTTTGCAAACGGCGCATATTTTCAACTCCGCCGAGGAAAAAGTACCCCT
TAAATCAACCCTCATTGGTCAATAATCCTTCCACCGGTTAGTTGGCCAATCATGGCCGCAATGATATCGTATC
AATAGACGGGGTTTTCTTAGACGAATCCATGGATAATCTAATTGTCCTACTTTCCCAGCTATTTTTCTTATTA
AGTATATTGACTTTCAATAGGGAACTTTTTTGGTGTACACACGACATTGTCAATCCTTCTCAAGCGTCAGTTT
TGCACGCGCAATCTTCTGTGTGCATTCGACCGTCATCTGCACAGGCCCGAATTCATTCATCTTCACCGACGTC
GTGCCCCGCCGGCCTCCGCCCATCCGCAGACTCAAGCATCCTCCTCGTCTCCACAGCCGTCATATCGATGTTC
ACTACTCTTAGGTACAGAGATGACAACGGCTGTGATGAAGATGTGATGAGGAGGAGGATGGTTAGTTGTCAGG
TGACCAGTTGATGTTCGGTCGGCCGGGCTCGTCGTCAGTAAACAGCAAGCGGGCGACTTGGGGGCCACTCACT
AATCGCGCGTGAACATACACACGTGAGAAGATGATCAACACAGACACCAAAAAAGTTTGGTATTGAAGTCAAT
ACAAGGAGAAAAATAGAAGGGAAAGGTACTTTTCCCTCGAAGGAGTTAAAAAAACATGTCATTGTCTAGAAAT
TTGGAAATTCGAACGTTCGTGCAGTTGCGTGCACAGCATAACAGGGTAGGGCACGCCATCAATGCGTAAAAAG
AACAAAATGTAAAACTCAAGATCAGAAACAAAAATTCTTCGATTACTTCTTTCGACCGGCGTAGGTAAATATT
CTGCTACTTCACG
>TR82|c3_g4_i1 len=3902 path=[8204:0-149 8205:150-173 8206:174-186
8207:187-210 8537:211-997 8023:998-1021 8482:1022-1264 8111:1265-1288
8565:1289-1323 8523:1324-1347 8471:1348-2120 7887:2121-2144 7888:2145-

2181 8467:2182-2467 8396:2468-2491 8397:2492-2492 8398:2493-2516
8463:2517-3007 7998:3008-3031 8557:3032-3058 8513:3059-3082 8456:3083-
3218 8393:3219-3242 8605:3243-3382 8639:3383-3383 8506:3384-3404
8602:3405-3407 8586:3408-3467 8501:3468-3511 8440:3512-3587 8210:3588-
3611 8600:3612-3648 8583:3649-3672 8545:3673-3772 8640:3773-3794
8646:3795-3802 8436:3803-3826 8401:3827-3829 8402:3830-3835 8403:3836-
3841 8404:3842-3870 8405:3871-3871 8406:3872-3872 8407:3873-3901] [-1,
8204, 8205, 8206, 8207, 8537, 8023, 8482, 8111, 8565, 8523, 8471, 7887,
7888, 8467, 8396, 8397, 8398, 8463, 7998, 8557, 8513, 8456, 8393, 8605,
8639, 8506, 8602, 8586, 8501, 8440, 8210, 8600, 8583, 8545, 8640, 8646,
8436, 8401, 8402, 8403, 8404, 8405, 8406, 8407, -2]
TCAACATCAGACCATGACCATGGCCATGGAGCACTCGGCCATGGCATCCAGTACAGAACTGTGCGCTCACATC
CAATCGGCTCTCCAATGCATCACTTCACCCTCATCACCCGAAATCATTCAAGAAGCCCAGCGGGCACTGTTAC
AATGGGAAGAATCACACACCGATCAGTACTCGATCACTCTCTCATCATTGATTGGACACACAACATCAACATC
AACATCAACACCGTTCACAACTGCCACTGTGGTGGATCAGGAAAATGAATCGTCGTCGGCTACGCACAAGGAA
TCAGCAGCAGCAACCATGCGTCTAGCCGCAGTATTGTCCCTGAAGGCAGCGATCACACGCCGATGGAAAGACA
AGGGACGCGGCAAAGTCGGGGCCACCAAGAATTTGTTGAGCGAAGGTGCCAAAACTTTGTTGAGGACATCGAT
GTTGAATTTGGTACTCGCCGGGAGAGTAGTAGCAGCAAAAGAAGCAGCGGAAGGAGGATCTAATCAACACCAG
ACTGACTTTATGATCGCCAATCCCAATAGTATAACGAGGGAACAAGTCGAACTAATCCAAGACCGCGCGTTGC
AAACTAATGTCGCATCTTTGTTGTCCAAAATAGCAAGGATGGACCTCCCTCTAAAGTGGCAGGAGTTGATACC
CTCCTTGGTGGAAGCAGTGAGATGTGCCCAAGGGGCAGTGTATCAAACAAAACATCAACCGCAATCGAAACAA
ATGCAGCAATTATTTCAGGCTGTAATTTACAACGCAATGAATTCGCTGGAAGCCATCTTGTCGGAAATATCCA
CACAACGATTATTGGTGAACAAAAAGTACAGGAACTCCATTGCATTGCAATATCTGGGATCGATCGTGGAGAG
TGGGCTGATGCCTCCCTTCCAGGAGTTGGAATCCTTGGTGAATACTGCATCCAACAACAATGAGATAATGAGG
GGAGGAGGAGAATGTACCGCTTTCACGCTGCGAAATGCCACCCTCACATCGCGAGTTGTTTCTCACATGATGA
TTTCTTCCTTTTCTAAATTGGTGGAAGAAAATTCGCCATTGGTGGATCAGATCCTTACGCTGACCCACTCGTT
TCTCTCTCGGTGGCTCCCGTGCATCTTGGGCGACAACGACGATGCTGTGCATGTTGCCCTCCGCAAACCCCTG
AAGCAACTCCTTCGCGTTCATTGCGACCTTATTGTTGATCTGCAATCCTCTCATCCGGAATCGTTTGCGAGAT
ATTTGACACCCTTTCTCACTTTGTTTCATTCGTCGTTAATGTGCATTGTGGGCGTTCCGGAGCAGGAGAGATG
TCAAAGGAGAAATGTCGCGAATAATAATGACCTCGGTGCGAATAACAGCAACCTGCGGGGTAGTACAGACCGA
TTTGCCATTGCATTCCTCTCCTTCCTCGCCAATGTGGCGGGAACATCCAAGTATGCGGACTTGGTAAACGCCA
AAAGCGAGATGGCGAGCTTCTTCACTCCGTCGCTGGTCCAATCTCTATCGCGGACTCTACTTCAGTTATTCTC
GATCCACATTTCTCCCATCCAGATTGTCCATGACGACGATGACGATGACAGCAAAGAAGATGCAGTTCTGTGG
CAAGACGATCCGGAAGCATTTTACCATTGGGAAATGAATCGGTCTAGTGAGGAAGATGTCGGTTGCGCGGCAC
AGAATCTATTCCTGGCACTGGCGGAGTCTTCCTTTTCCGGCGACGTTGTGCTGCCCTGGCTGATGGGGTTCGT
GACCAATGTCGTATCGCAGCGATTGGCGGTGGAATTGGAGGGAGGCATTTCTTTCGGCGTTGATCCTCAAATT
ATATATTCGGCGTTGCCATTGGGGGCTCCGTGCGCAAAGCTATCCAGTGGCGTGGAGAACGCGTCGATGGACC
TGTTATTGCAATGGGACGCAATCTACACGGCCGCCGGGTTGATGGGGAGCATTTTGGAAAATTATCCTGGATA
CTCCTTTCAAACTTGGTTTCGTGTTTCGGTTGGTGATTGTCTGTCCATGTTGTTGCGACAGAGTAAACAACAG
CGCACACAACTTCCCATCCTGCAACGGCGGATGATTTGGCTCCTTTCCTGCAATGCCCATCAGGTACCAATAT
CATCTCCGTTCAATCCTTTTGGTATGCTATCTTCTTCGATTTCGGATCAAGATGTATGTGTCCGACTCACCGC
AGTCCAAGCCATGGATGCATTGCTTCCACAATGCGAAGACGCTCCGTCACTGCTCCACTCTATCGTTGAACCA
ACAGCTCCCGCTTTGTATCAGTTAGTAAACGAATGCGCGGAGGTCGAATCTCGATCATCTTGCCTCGATCTTC
TCTCCAACCTTATTTGTATGCAGACTGGAGGTTCGCTCACGAATGAAATACTTAACACCATCGTTGCTCCGCT
TTCGTCTATCTGGGACAATGCAATTGATCAGAATCTGCGATTGAAACGGAGTGTTTTAACTATTCTCTCGTGC
ATAGCATCATTTGTTGGGCCTGAAAGCTGTAGCGTACTGTATCCCTTGGTATTGCCCATGATCGACGAGAGTT
TTGCAAGGGAGGAAAACGTTTTTCTCATTGAGGAGGCGTTGAAAATATGGTGGGTGTTCCTTCGATTGTCACG
AAAGTATGATTCGTTGCTGGGAAAATTGTTCATTCATACTGTGGAGTTGAGTAAAGATTTTGATCACCTGATG
ATACTGATGCGAGTCACTGAACACTACATCATACTTGGCGGAGCTACCTTCCTAAACGACCATGCCACCACAA
TTCAATCAGTTCTTAGTAATGTTGTTGGAGAAGTCCGCCCTCGTGGTACGGCGTACATATTTTTGGTTATCGA
GGCTCTCCTTCGCTCCTTTCCTGTCGAAGGCGGCGTATTGCTGAACAGCTGTGGAGTGTTAACGACTTTCATG
GAGGCGTGTGCCAGTAGTTATTTTGACGATGAAAAATGTGAACCCGATAGAGTGATTGTGCTGTATTTGGCTG
CATTGGCTAGGGTGATATTGTCTAGGCCAGACACGCTGCAACTCCCTCTAAAGCTCCCATCAGGTGCTGTTTT
TGGGGAGGAAGAATTTATTTCTCTGTACATGGGAAAGTTCCAGGTTGCAGGGAATGGTGCTCACGGACTGCTG
TACCAAAAGCTTTGGGCATTGCTTCTGCTTTCGTTCTACCCACCGTGCCAACTGGCATCCTGTCGCAACACAG
TCCTCAGAAAGTCCAAGACAATATTCAACATCTTCATTGGTGTGTTGAAGAACGTACATCCCAATGGGACCAA
TCTTCTCTCTTATGAAGTAGGTTGCGGCGACGACGAGGAGTCTATTTACATTGGTGAAGATTTGTACGAAGCA
CTGTTACAGGGACAGAGGTCAAATGACATTATCATCTCCACATCACTTCATGAAGCTGTTTCTACAAAACTGA
ACATCCTTCCTAATGAACTTGGTCAAAATTACCAAGATTTTTGCCTTGCTGAAAATGGTGCCTTACAGCAATT
GAAAGAAGTCATTTTGAATATTGAGGGTTGATCGTGCAAATTATCGCTATCAGCATGCTTGCTTGGTGCAATA

TATTGAAAGCACAAGCGCAACAAATAATATAATCTAAGCTTCATCAGGTGAGCTAGAGATTTTTCCGGTTTTG
ATGAACTGAGCTACCTTGCCTTAGCGCGCAGAAATATCTCTTGCCATTTATTTGGCCCTGAGACCAAGAACAC
TAGATATTATCCTGTGCACGCAACTGCGCAAGGGTTCGAATTTTTAAAATCTAGACGACGACCTATTTTTCAA
CTCCGCCAAGGAAAAAGTACCCCTAAAATCAACCGTCATTGGTCAATAATCTTTCAACCGGTTGGCCAATCAT
GGCAGCAATGGTATCAATTGACGGGGGTTTCTT
>TR82|c3_g4_i2 len=3987 path=[8204:0-149 8205:150-173 8206:174-186
8207:187-210 8537:211-997 8023:998-1021 8482:1022-1264 8111:1265-1288
8565:1289-1323 8523:1324-1347 8471:1348-2120 7887:2121-2144 7888:2145-
2181 8467:2182-2467 8396:2468-2491 8397:2492-2492 8398:2493-2516
8463:2517-3007 7998:3008-3031 8557:3032-3058 8513:3059-3082 8456:3083-
3218 8393:3219-3242 8605:3243-3382 8508:3383-3468 8334:3469-3489
8602:3490-3492 8586:3493-3552 8501:3553-3596 8440:3597-3672 8210:3673-
3696 8600:3697-3733 8583:3734-3757 8545:3758-3857 8640:3858-3879
8646:3880-3887 8436:3888-3911 8401:3912-3914 8402:3915-3920 8403:3921-
3926 8404:3927-3955 8405:3956-3956 8406:3957-3957 8407:3958-3986] [-1,
8204, 8205, 8206, 8207, 8537, 8023, 8482, 8111, 8565, 8523, 8471, 7887,
7888, 8467, 8396, 8397, 8398, 8463, 7998, 8557, 8513, 8456, 8393, 8605,
8508, 8334, 8602, 8586, 8501, 8440, 8210, 8600, 8583, 8545, 8640, 8646,
8436, 8401, 8402, 8403, 8404, 8405, 8406, 8407, -2]
TCAACATCAGACCATGACCATGGCCATGGAGCACTCGGCCATGGCATCCAGTACAGAACTGTGCGCTCACATC
CAATCGGCTCTCCAATGCATCACTTCACCCTCATCACCCGAAATCATTCAAGAAGCCCAGCGGGCACTGTTAC
AATGGGAAGAATCACACACCGATCAGTACTCGATCACTCTCTCATCATTGATTGGACACACAACATCAACATC
AACATCAACACCGTTCACAACTGCCACTGTGGTGGATCAGGAAAATGAATCGTCGTCGGCTACGCACAAGGAA
TCAGCAGCAGCAACCATGCGTCTAGCCGCAGTATTGTCCCTGAAGGCAGCGATCACACGCCGATGGAAAGACA
AGGGACGCGGCAAAGTCGGGGCCACCAAGAATTTGTTGAGCGAAGGTGCCAAAACTTTGTTGAGGACATCGAT
GTTGAATTTGGTACTCGCCGGGAGAGTAGTAGCAGCAAAAGAAGCAGCGGAAGGAGGATCTAATCAACACCAG
ACTGACTTTATGATCGCCAATCCCAATAGTATAACGAGGGAACAAGTCGAACTAATCCAAGACCGCGCGTTGC
AAACTAATGTCGCATCTTTGTTGTCCAAAATAGCAAGGATGGACCTCCCTCTAAAGTGGCAGGAGTTGATACC
CTCCTTGGTGGAAGCAGTGAGATGTGCCCAAGGGGCAGTGTATCAAACAAAACATCAACCGCAATCGAAACAA
ATGCAGCAATTATTTCAGGCTGTAATTTACAACGCAATGAATTCGCTGGAAGCCATCTTGTCGGAAATATCCA
CACAACGATTATTGGTGAACAAAAAGTACAGGAACTCCATTGCATTGCAATATCTGGGATCGATCGTGGAGAG
TGGGCTGATGCCTCCCTTCCAGGAGTTGGAATCCTTGGTGAATACTGCATCCAACAACAATGAGATAATGAGG
GGAGGAGGAGAATGTACCGCTTTCACGCTGCGAAATGCCACCCTCACATCGCGAGTTGTTTCTCACATGATGA
TTTCTTCCTTTTCTAAATTGGTGGAAGAAAATTCGCCATTGGTGGATCAGATCCTTACGCTGACCCACTCGTT
TCTCTCTCGGTGGCTCCCGTGCATCTTGGGCGACAACGACGATGCTGTGCATGTTGCCCTCCGCAAACCCCTG
AAGCAACTCCTTCGCGTTCATTGCGACCTTATTGTTGATCTGCAATCCTCTCATCCGGAATCGTTTGCGAGAT
ATTTGACACCCTTTCTCACTTTGTTTCATTCGTCGTTAATGTGCATTGTGGGCGTTCCGGAGCAGGAGAGATG
TCAAAGGAGAAATGTCGCGAATAATAATGACCTCGGTGCGAATAACAGCAACCTGCGGGGTAGTACAGACCGA
TTTGCCATTGCATTCCTCTCCTTCCTCGCCAATGTGGCGGGAACATCCAAGTATGCGGACTTGGTAAACGCCA
AAAGCGAGATGGCGAGCTTCTTCACTCCGTCGCTGGTCCAATCTCTATCGCGGACTCTACTTCAGTTATTCTC
GATCCACATTTCTCCCATCCAGATTGTCCATGACGACGATGACGATGACAGCAAAGAAGATGCAGTTCTGTGG
CAAGACGATCCGGAAGCATTTTACCATTGGGAAATGAATCGGTCTAGTGAGGAAGATGTCGGTTGCGCGGCAC
AGAATCTATTCCTGGCACTGGCGGAGTCTTCCTTTTCCGGCGACGTTGTGCTGCCCTGGCTGATGGGGTTCGT
GACCAATGTCGTATCGCAGCGATTGGCGGTGGAATTGGAGGGAGGCATTTCTTTCGGCGTTGATCCTCAAATT
ATATATTCGGCGTTGCCATTGGGGGCTCCGTGCGCAAAGCTATCCAGTGGCGTGGAGAACGCGTCGATGGACC
TGTTATTGCAATGGGACGCAATCTACACGGCCGCCGGGTTGATGGGGAGCATTTTGGAAAATTATCCTGGATA
CTCCTTTCAAACTTGGTTTCGTGTTTCGGTTGGTGATTGTCTGTCCATGTTGTTGCGACAGAGTAAACAACAG
CGCACACAACTTCCCATCCTGCAACGGCGGATGATTTGGCTCCTTTCCTGCAATGCCCATCAGGTACCAATAT
CATCTCCGTTCAATCCTTTTGGTATGCTATCTTCTTCGATTTCGGATCAAGATGTATGTGTCCGACTCACCGC
AGTCCAAGCCATGGATGCATTGCTTCCACAATGCGAAGACGCTCCGTCACTGCTCCACTCTATCGTTGAACCA
ACAGCTCCCGCTTTGTATCAGTTAGTAAACGAATGCGCGGAGGTCGAATCTCGATCATCTTGCCTCGATCTTC
TCTCCAACCTTATTTGTATGCAGACTGGAGGTTCGCTCACGAATGAAATACTTAACACCATCGTTGCTCCGCT
TTCGTCTATCTGGGACAATGCAATTGATCAGAATCTGCGATTGAAACGGAGTGTTTTAACTATTCTCTCGTGC
ATAGCATCATTTGTTGGGCCTGAAAGCTGTAGCGTACTGTATCCCTTGGTATTGCCCATGATCGACGAGAGTT
TTGCAAGGGAGGAAAACGTTTTTCTCATTGAGGAGGCGTTGAAAATATGGTGGGTGTTCCTTCGATTGTCACG
AAAGTATGATTCGTTGCTGGGAAAATTGTTCATTCATACTGTGGAGTTGAGTAAAGATTTTGATCACCTGATG
ATACTGATGCGAGTCACTGAACACTACATCATACTTGGCGGAGCTACCTTCCTAAACGACCATGCCACCACAA
TTCAATCAGTTCTTAGTAATGTTGTTGGAGAAGTCCGCCCTCGTGGTACGGCGTACATATTTTTGGTTATCGA
GGCTCTCCTTCGCTCCTTTCCTGTCGAAGGCGGCGTATTGCTGAACAGCTGTGGAGTGTTAACGACTTTCATG
GAGGCGTGTGCCAGTAGTTATTTTGACGATGAAAAATGTGAACCCGATAGAGTGATTGTGCTGTATTTGGCTG

CATTGGCTAGGGTGATATTGTCTAGGCCAGACACGCTGCAACTCCCTCTAAAGCTCCCATCAGGTGCTGTTTT
TGGGGAGGAAGAATTTATTTCTCTGTACATGGGAAAGTTCCAGGTTGCAGGGAATGGTGCTCACGGACTGCTG
TACCAAAAGCTTTGGGCATTGCTTCTGCTTTCGTTCTACCCACCGTGCCAACTGGCATCCTGTCGCAACACAG
TCCTCAGAAAGTCCAAGACAATATTCAACATCTTCATTGGTGTGTTGAAGAACGTACATCCCAATGGGACCAA
TCTTCTCTCTTATGAAGTAGGTTGCGGCGACGACGAGGAGTCTATTTACATTGGTGAAGATTTGTACGAAGCA
CTGTTACAGGGACAGAGGTCAAATGTGAATATAATTTTATGGTCGTGCTGTTTGTTTTGTTTACACTTGTGCT
AATGAATTTATTGTCTTTTGATTTCCTTTGTTGCAGGACATTATCATCTCCACATCACTTCATGAAGCTGTTT
CTACAAAACTGAACATCCTTCCTAATGAACTTGGTCAAAATTACCAAGATTTTTGCCTTGCTGAAAATGGTGC
CTTACAGCAATTGAAAGAAGTCATTTTGAATATTGAGGGTTGATCGTGCAAATTATCGCTATCAGCATGCTTG
CTTGGTGCAATATATTGAAAGCACAAGCGCAACAAATAATATAATCTAAGCTTCATCAGGTGAGCTAGAGATT
TTTCCGGTTTTGATGAACTGAGCTACCTTGCCTTAGCGCGCAGAAATATCTCTTGCCATTTATTTGGCCCTGA
GACCAAGAACACTAGATATTATCCTGTGCACGCAACTGCGCAAGGGTTCGAATTTTTAAAATCTAGACGACGA
CCTATTTTTCAACTCCGCCAAGGAAAAAGTACCCCTAAAATCAACCGTCATTGGTCAATAATCTTTCAACCGG
TTGGCCAATCATGGCAGCAATGGTATCAATTGACGGGGGTTTCTT
>TR83|c0_g1_i2 len=2483 path=[4044:0-69 4045:70-93 4126:94-261 4136:262-285 4139:286-298 4125:299-320 4131:321-354 4116:355-378 4124:379-695 4075:696-730 4115:731-846 3985:847-870 4123:871-880 4130:881-904 4135:905-913 4099:914-955 4129:956-970 4121:971-994 4134:995-1049 4000:1050-1070 4128:1071-1110 4137:1111-1122 4095:1123-1902 4052:1903-1926 4119:1927-2092 4094:2093-2125 4127:2126-2142 4118:2143-2166 4108:2167-2308 4138:2309-2332 4140:2333-2351 4106:2352-2413 4091:2414-2482] [-1, 4044, 4045, 4126, 4136, 4139, 4125, 4131, 4116, 4124, 4075, 4115, 3985, 4123, 4130, 4135, 4099, 4129, 4121, 4134, 4000, 4128, 4137, 4095, 4052, 4119, 4094, 4127, 4118, 4108, 4138, 4140, 4106, 4091]
AGAGAGGCGCGAGAACGAGGAACATCATCATACTCACAATATTTTTTCACTCATTTTATTCTCAACAGATCCA
GTCATCTGAAGCACACACACAAAGACTCAAGTTCACTGGTAATAAACACAACAATCATCAATAAAATTTCTTC
CTTCTTTATCTCCACAATATGAACCCCTACGCTAGCTTCACTATTGTCATCTTCGCTGCGGCGTTGAGTAGCG
TTTCTTCCTTCGTTAACCCCGCAACAACTTCTTCTCGTGTGACCACTCACCTTAACCTCGAGAATCACATTGC
AGACATGATCGACAGTGAAGTCAAAAGACTCCAAAACATTGCAGGCTGGCGACGCGAACAAACCGAAAAACTC
AAAAAGGGACGGGATCCCTCCATCCCCGCCGGCTTCGACTTCAACTCCGTCAGCGACTTCACGCCCAACGCCG
GAGCCGCCCAAAAGATACAAATGCGCAAGGACAAACGCATGGCACGAGACGATCCGGCACGGTACTGCGCCGA
TCGATGCGTCGCTACCGGCAATTGCGAAATTTGGGAGGAGATGTTCGAGATGGCGGCGGATGAGGTTCAGGCC
TTTTGTACCGGGTGCGTGTTGAGCGAGGAGGAGGAGCCGTGCGATGTTCCCGAGAAGTTTATGGAGAATGCGG
GGAAGAATTCGTGGGAGCTGCTCACGCCCTGAGTTTTTGGGACGATGTATATAGGTTCAGGTTTGTTTAGGAT
GAAATACTGAAGCACGGCGATGCTGCAACATGCATCTCGGGACGGGAACATGAGACCGCTGCAAGCCATGATG
GGTTAGGATATGATTTTGTTGAACTGCAATGTACAATGGTCCCAAACGTACATTCAAAATTGTTCTTCGAGGC
AAACGCGGCATCTCGAGTTAGTAGGGTACACGGCGGATGGTTTATACGCACGGAAATGATATGATTTTGATCG
ACAAGCGCAGCTACAATTCCATTATGGTAAACACGCCACAGTTGATGCTCCAGAAGATTCTTTTCAGCACTTT
TCCACAAGACTCCAGAAGATAAAAAGATGTCACGAACAAAATGCATTGGAGTTGTAATTTGGCTGATTTATAA
ATTAAATAGAAACGCTGTTTGAATTGTAGAAACACGTTTCTGGTCTATGCCTCAGCTGGCGTCGGTATCACGT
TTGGAGCATGGCAATACTTTACACTTTGTGTAAGTTTGCAGTTTATTCGATCAGTGCTACGTGTACGCATATA
ATCTTTTCTGCTATTGCATGGCAGTGAGATCAGTTGACTATCGTTTAAAATTATTTCTGCCAACATTATTTAT
GTACCTACAACGCTCAAGACTTGGTACTCAGGCATAAGGAGAAGCTTCTTAAGGTAGATGCTCCTAGTTACCT
TATTCCCTACTCCAATCTCATAATGGTAGCATGGCAAAGTACACTTTCTAAGGTCAACTGGTATGCCTATGTA
TCACAAGGGAAGAGAATGAATCACCACTGGCAGGTCTATTGTCGGTTGGGTAATATTATTTCAGTGATTTATG
ATGCCAAGGGTAGTCACACGGGCCCCGACTGGCATGTGACCGTTGCGCTTGCTTCCCATACACTTGTTTATCG
AAAAATTATCACAGCCGGTTAGTACGAATGTGCATACTCCACTCCACCGCCTGTTCTCTTATTCTCTCTACCC
AAATACTTCATAACATTTCCCTATAAAGGCGAGAACAAAAGAACGATCCAGCACTAGCACCATCATGTTGTTC
TACAAGCCTCTCCTTTCTTGTCTCTAATTCTCGTAAGCATACTTCGGATTGGGCACATCCCACCCCTTCTCTT
TCAACATACCACCCAAATAAATCAACCTCTTCCTATTCACGAATAAATCACCGGACCCCATACGACTAGAACT
CTTCACCTCCACCGTAATACTACCTTTCGCATCATCCTTTCCTAATTCGAGCTTTAAATCGTCAATGAAGGGT
TGACCCAAATTGATCGCCAATGCTGCCGGTCCCACGCACGATTTGTATTCCAACTTGATCGTATTACTACTAC
TACTGCTTCCCGCCTCGGCACCAAGTTCGTCTTTGACGATTTTCCATCCACTGCAATCGACGCCCGCTTGTCC
CTTTTGAGGATAGGAATTGAGCACGTGGCGGATGGTGTTGGCAGCTTCGGATGATGACGTTACCGAGGAGGGG
GCAGTCCATGTGGTGCGAATGCAATTGTGGGCATTGGGTCTGCATGCCTCGAGGGCATTGGCTGGAGGAGTTG
GTTGTAGTACGGACGTAGTAGTAATGATGGGTGTGAGGAGAAATGCGTATTTCAATAGTGCATCGCGTCGTGA
TAATGATGACGCCGATACCTCTTTGATTTGGTTGGTAGGTTGATGGTGGTTGTTGTTGATGTTCCTTTGAGCT
TTCACTTTCACCTGAGGTTGAGGTACCAATGCATTTGTTGCATCGAAGAAGCACAGTAAAGACAATGATCCAT
A

>TR83|c0_g1_i3 len=2596 path=[4044:0-69 4045:70-93 4126:94-261 4136:262-285 4139:286-298 4102:299-433 4131:434-467 4116:468-491 4124:492-808 4075:809-843 4115:844-959 3985:960-983 4123:984-993 4130:994-1017 4135:1018-1026 4099:1027-1068 4129:1069-1083 4121:1084-1107 4134:1108-1162 4000:1163-1183 4128:1184-1223 4137:1224-1235 4095:1236-2015 4052:2016-2039 4119:2040-2205 4094:2206-2238 4127:2239-2255 4118:2256-2279 4108:2280-2421 4138:2422-2445 4140:2446-2464 4107:2465-2526 4091:2527-2595] [-1, 4044, 4045, 4126, 4136, 4139, 4102, 4131, 4116, 4124, 4075, 4115, 3985, 4123, 4130, 4135, 4099, 4129, 4121, 4134, 4000, 4128, 4137, 4095, 4052, 4119, 4094, 4127, 4118, 4108, 4138, 4140, 4107, 4091]
AGAGAGGCGCGAGAACGAGGAACATCATCATACTCACAATATTTTTTCACTCATTTTATTCTCAACAGATCCA
GTCATCTGAAGCACACACACAAAGACTCAAGTTCACTGGTAATAAACACAACAATCATCAATAAAATTTCTTC
CTTCTTTATCTCCACAATATGAACCCCTACGCTAGCTTCACTATTGTCATCTTCGCTGCGGCGTTGAGTAGCG
TTTCTTCCTTCGTTAACCCCGCAACAACTTCTTCTCGTGTGACCACTCACCTTAACCTCGAGAATCACATTGC
AGACATGTAAGTGCAACCAAAACCCTGATTCATTCACACCATTTAGTTCCTTCTAAAGTGCCATTTGCGTACT
GACGATATTATATGCCATACTCTCCTTTTCTACACATCTCCCCCAGGATCGACAGTGAAGTCAAAAGACTCCA
AAACATTGCAGGCTGGCGACGCGAACAAACCGAAAAACTCAAAAAGGGACGGGATCCCTCCATCCCCGCCGGC
TTCGACTTCAACTCCGTCAGCGACTTCACGCCCAACGCCGGAGCCGCCCAAAAGATACAAATGCGCAAGGACA
AACGCATGGCACGAGACGATCCGGCACGGTACTGCGCCGATCGATGCGTCGCTACCGGCAATTGCGAAATTTG
GGAGGAGATGTTCGAGATGGCGGCGGATGAGGTTCAGGCCTTTTGTACCGGGTGCGTGTTGAGCGAGGAGGAG
GAGCCGTGCGATGTTCCCGAGAAGTTTATGGAGAATGCGGGGAAGAATTCGTGGGAGCTGCTCACGCCCTGAG
TTTTTGGGACGATGTATATAGGTTCAGGTTTGTTTAGGATGAAATACTGAAGCACGGCGATGCTGCAACATGC
ATCTCGGGACGGGAACATGAGACCGCTGCAAGCCATGATGGGTTAGGATATGATTTTGTTGAACTGCAATGTA
CAATGGTCCCAAACGTACATTCAAAATTGTTCTTCGAGGCAAACGCGGCATCTCGAGTTAGTAGGGTACACGG
CGGATGGTTTATACGCACGGAAATGATATGATTTTGATCGACAAGCGCAGCTACAATTCCATTATGGTAAACA
CGCCACAGTTGATGCTCCAGAAGATTCTTTTCAGCACTTTTCCACAAGACTCCAGAAGATAAAAAGATGTCAC
GAACAAAATGCATTGGAGTTGTAATTTGGCTGATTTATAAATTAAATAGAAACGCTGTTTGAATTGTAGAAAC
ACGTTTCTGGTCTATGCCTCAGCTGGCGTCGGTATCACGTTTGGAGCATGGCAATACTTTACACTTTGTGTAA
GTTTGCAGTTTATTCGATCAGTGCTACGTGTACGCATATAATCTTTTCTGCTATTGCATGGCAGTGAGATCAG
TTGACTATCGTTTAAAATTATTTCTGCCAACATTATTTATGTACCTACAACGCTCAAGACTTGGTACTCAGGC
ATAAGGAGAAGCTTCTTAAGGTAGATGCTCCTAGTTACCTTATTCCCTACTCCAATCTCATAATGGTAGCATG
GCAAAGTACACTTTCTAAGGTCAACTGGTATGCCTATGTATCACAAGGGAAGAGAATGAATCACCACTGGCAG
GTCTATTGTCGGTTGGGTAATATTATTTCAGTGATTTATGATGCCAAGGGTAGTCACACGGGCCCCGACTGGC
ATGTGACCGTTGCGCTTGCTTCCCATACACTTGTTTATCGAAAAATTATCACAGCCGGTTAGTACGAATGTGC
ATACTCCACTCCACCGCCTGTTCTCTTATTCTCTCTACCCAAATACTTCATAACATTTCCCTATAAAGGCGAG
AACAAAAGAACGATCCAGCACTAGCACCATCATGTTGTTCTACAAGCCTCTCCTTTCTTGTCTCTAATTCTCG
TAAGCATACTTCGGATTGGGCACATCCCACCCCTTCTCTTTCAACATACCACCCAAATAAATCAACCTCTTCC
TATTCACGAATAAATCACCGGACCCCATACGACTAGAACTCTTCACCTCCACCGTAATACTACCTTTCGCATC
ATCCTTTCCTAATTCGAGCTTTAAATCGTCAATGAAGGGTTGACCCAAATTGATCGCCAATGCTGCCGGTCCC
ACGCACGATTTGTATTCCAACTTGATCGTATTACTACTACTACTGCTTCCCGCCTCGGCACCAAGTTCGTCTT
TGACGATTTTCCATCCACTGCAATCGACGCCCGCTTGTCCCTTTTGAGGATAGGAATTGAGCACGTGGCGGAT
GGTGTTGGCAGCTTCGGATGATGACGTTACCGAGGAGGGGGCAGTCCATGTGGTGCGAATGCAATTGTGGGCA
TTGGGTCTGCATGCCTCGAGGGCATTGGCTGGAGGAGTTGGTTGTAGTACGGACGTAGTAGTAATGATGGGTG
TGAGGAGAAATGCGTATTTCAATAGTGCATCGCGTCGTGATAATGATGACGCCGATGCCTCTTTGATTTGATT
GTTAGGTTGATGGTGGTTGTTATTGATGTTCCTTTGAGCTTTCACTTTCACCTGAGGTTGAGGTACCAATGCA
TTTGTTGCATCGAAGAAGCACAGTAAAGACAATGATCCATA
>TR83|c0_g1_i6 len=1145 path=[4044:0-69 4045:70-93 4126:94-261 4136:262-285 4139:286-298 4125:299-320 4131:321-354 4116:355-378 4124:379-695 4075:696-730 4115:731-846 3985:847-870 4123:871-880 4130:881-904 4135:905-913 4133:914-955 4129:956-970 4121:971-994 4134:995-1049 4132:1050-1072 4128:1073-1112 4137:1113-1124 4050:1125-1144] [-1, 4044, 4045, 4126, 4136, 4139, 4125, 4131, 4116, 4124, 4075, 4115, 3985, 4123, 4130, 4135, 4133, 4129, 4121, 4134, 4132, 4128, 4137, 4050, -2]
AGAGAGGCGCGAGAACGAGGAACATCATCATACTCACAATATTTTTTCACTCATTTTATTCTCAACAGATCCA
GTCATCTGAAGCACACACACAAAGACTCAAGTTCACTGGTAATAAACACAACAATCATCAATAAAATTTCTTC
CTTCTTTATCTCCACAATATGAACCCCTACGCTAGCTTCACTATTGTCATCTTCGCTGCGGCGTTGAGTAGCG
TTTCTTCCTTCGTTAACCCCGCAACAACTTCTTCTCGTGTGACCACTCACCTTAACCTCGAGAATCACATTGC
AGACATGATCGACAGTGAAGTCAAAAGACTCCAAAACATTGCAGGCTGGCGACGCGAACAAACCGAAAAACTC
AAAAAGGGACGGGATCCCTCCATCCCCGCCGGCTTCGACTTCAACTCCGTCAGCGACTTCACGCCCAACGCCG

GAGCCGCCCAAAAGATACAAATGCGCAAGGACAAACGCATGGCACGAGACGATCCGGCACGGTACTGCGCCGA
TCGATGCGTCGCTACCGGCAATTGCGAAATTTGGGAGGAGATGTTCGAGATGGCGGCGGATGAGGTTCAGGCC
TTTTGTACCGGGTGCGTGTTGAGCGAGGAGGAGGAGCCGTGCGATGTTCCCGAGAAGTTTATGGAGAATGCGG
GGAAGAATTCGTGGGAGCTGCTCACGCCCTGAGTTTTTGGGACGATGTATATAGGTTCAGGTTTGTTTAGGAT
GAAATACTGAAGCACGGCGATGCTGCAACATGCATCTCGGGACGGGAACATGAGACCGCTGCAAGCCATGATG
GGTTAGGATATGATTTTGTTGAACTGCAATGTACAATGGTCCCAAACGTACATTCAAAATTGTTCTTCGAGGC
AAACGCGGCATCTCGAGTTAGTAGGGTACACGGCGGATAGTTTACACGCACGGAAACGATATGATTTTGATCG
ACAAGCGCAGCTACAATTCCATTATGGTAAACACGCCACAGTTGATGCTCCAGAAGATTCTTTTCAGCACTTT
TCCACAAGACTCCAGAAGATAAAAAGATATGTCACGAACAAAATGCATTGGAGTTGTAATTTGGCTGATTTAT
AAATTAAATAGAAACGCTGTTTGAATTGTAAAAAAAAAAGATCGGAAGAGC
>TR83|c0_g1_i8 len=1258 path=[4044:0-69 4045:70-93 4126:94-261 4136:262-285 4139:286-298 4102:299-433 4131:434-467 4116:468-491 4124:492-808 4075:809-843 4115:844-959 3985:960-983 4123:984-993 4130:994-1017 4135:1018-1026 4133:1027-1068 4129:1069-1083 4121:1084-1107 4134:1108-1162 4132:1163-1185 4128:1186-1225 4137:1226-1237 4050:1238-1257] [-1, 4044, 4045, 4126, 4136, 4139, 4102, 4131, 4116, 4124, 4075, 4115, 3985, 4123, 4130, 4135, 4133, 4129, 4121, 4134, 4132, 4128, 4137, 4050, -2]
AGAGAGGCGCGAGAACGAGGAACATCATCATACTCACAATATTTTTTCACTCATTTTATTCTCAACAGATCCA
GTCATCTGAAGCACACACACAAAGACTCAAGTTCACTGGTAATAAACACAACAATCATCAATAAAATTTCTTC
CTTCTTTATCTCCACAATATGAACCCCTACGCTAGCTTCACTATTGTCATCTTCGCTGCGGCGTTGAGTAGCG
TTTCTTCCTTCGTTAACCCCGCAACAACTTCTTCTCGTGTGACCACTCACCTTAACCTCGAGAATCACATTGC
AGACATGTAAGTGCAACCAAAACCCTGATTCATTCACACCATTTAGTTCCTTCTAAAGTGCCATTTGCGTACT
GACGATATTATATGCCATACTCTCCTTTTCTACACATCTCCCCCAGGATCGACAGTGAAGTCAAAAGACTCCA
AAACATTGCAGGCTGGCGACGCGAACAAACCGAAAAACTCAAAAAGGGACGGGATCCCTCCATCCCCGCCGGC
TTCGACTTCAACTCCGTCAGCGACTTCACGCCCAACGCCGGAGCCGCCCAAAAGATACAAATGCGCAAGGACA
AACGCATGGCACGAGACGATCCGGCACGGTACTGCGCCGATCGATGCGTCGCTACCGGCAATTGCGAAATTTG
GGAGGAGATGTTCGAGATGGCGGCGGATGAGGTTCAGGCCTTTTGTACCGGGTGCGTGTTGAGCGAGGAGGAG
GAGCCGTGCGATGTTCCCGAGAAGTTTATGGAGAATGCGGGGAAGAATTCGTGGGAGCTGCTCACGCCCTGAG
TTTTTGGGACGATGTATATAGGTTCAGGTTTGTTTAGGATGAAATACTGAAGCACGGCGATGCTGCAACATGC
ATCTCGGGACGGGAACATGAGACCGCTGCAAGCCATGATGGGTTAGGATATGATTTTGTTGAACTGCAATGTA
CAATGGTCCCAAACGTACATTCAAAATTGTTCTTCGAGGCAAACGCGGCATCTCGAGTTAGTAGGGTACACGG
CGGATAGTTTACACGCACGGAAACGATATGATTTTGATCGACAAGCGCAGCTACAATTCCATTATGGTAAACA
CGCCACAGTTGATGCTCCAGAAGATTCTTTTCAGCACTTTTCCACAAGACTCCAGAAGATAAAAAGATATGTC
ACGAACAAAATGCATTGGAGTTGTAATTTGGCTGATTTATAAATTAAATAGAAACGCTGTTTGAATTGTAAAA
AAAAAGATCGGAAGAGC
>TR84|c1_g1_i1 len=3432 path=[7284:0-48 7732:49-86 7700:87-113 7777:114-117 7356:118-132 7699:133-141 7656:142-206 7342:207-233 7343:234-257 7608:258-315 7524:316-339 7525:340-349 7730:350-419 7778:420-431 7606:432-443 7753:444-458 7729:459-491 7654:492-515 7764:516-554 7604:555-617 7652:618-683 7220:684-707 7601:708-812 7179:813-836 7751:837-870 7726:871-894 7779:895-896 7691:897-938 7725:939-1004 7597:1005-1013 7724:1014-1028 7689:1029-1061 7649:1062-1085 7723:1086-1100 7763:1101-1124 7688:1125-1154 7721:1155-1193 7686:1194-1197 7646:1198-1208 7768:1209-1238 7762:1239-1254 7719:1255-1288 7683:1289-1294 7642:1295-1318 7718:1319-1355 7767:1356-1384 7781:1385-1415 7681:1416-1424 7745:1425-1473 7715:1474-1495 7679:1496-1536 7743:1537-1542 7632:1543-1543 7713:1544-1570 7790:1571-1578 7766:1579-1587 7756:1588-1603 7738:1604-1622 7672:1623-1646 7625:1647-1693 7709:1694-1706 7671:1707-1730 7765:1731-1744 7736:1745-1779 7708:1780-1810 7564:1811-1817 7669:1818-1831 7622:1832-1855 7563:1856-1873 7707:1874-1876 7668:1877-1900 7621:1901-1933 7784:1934-1957 7791:1958-1992 7560:1993-2101 7735:2102-2114 7706:2115-2118 7666:2119-2224 7011:2225-2248 7618:2249-2324 7042:2325-2349 7705:2350-2415 7404:2416-2458 7617:2459-2573 7785:2574-2574 7704:2575-2764 7663:2765-2824 7554:2825-2852 7703:2853-2878 7661:2879-2902 7614:2903-3105 7786:3106-3109 7551:3110-3144 7702:3145-3156 7659:3157-3184 7612:3185-3252 7787:3253-3276 7792:3277-3277 7191:3278-3431] [-1, 7284, 7732, 7700, 7777, 7356, 7699, 7656, 7342, 7343, 7608, 7524, 7525, 7730, 7778, 7606, 7753, 7729, 7654, 7764, 7604, 7652, 7220, 7601, 7179, 7751, 7726, 7779, 7691, 7725, 7597, 7724, 7689, 7649, 7723, 7763, 7688, 7721, 7686, 7646, 7768, 7762,

7719, 7683, 7642, 7718, 7767, 7781, 7681, 7745, 7715, 7679, 7743, 7632, 7713, 7790, 7766, 7756, 7738, 7672, 7625, 7709, 7671, 7765, 7736, 7708, 7564, 7669, 7622, 7563, 7707, 7668, 7621, 7784, 7791, 7560, 7735, 7706, 7666, 7011, 7618, 7042, 7705, 7404, 7617, 7785, 7704, 7663, 7554, 7703, 7661, 7614, 7786, 7551, 7702, 7659, 7612, 7787, 7792, 7191, -2]
CTTTATTATCTTTCAGTTTCAGAGTAGTCAAATCATGGCCAGCACCTCCACCCCGTTCGCTCCCTCGAGCAGT
CCGCTGCAGGTCAAGGTAGCTCAAGAGGGCAAATATTCGCCTGTCCCCGATGTATGTGTCGTCACCGGCGGCA
CCGGGTTTGTCGGCAACCGTCTAGTAGAAATGCTCGTCGAGCGTGGTGCGAAGAAAGTGATTTCGTTCGATGT
CGTGCCTCCTCCACCAGATGCCTGGAAGCATCCCAACATCGAATGGCGCATCGGCGATATCACCGATAAGGCG
GCCGTGGACGATCTCATGTCCTCCGTGCCAAACATCGGCTGTGTCTGGCACAATGCAGCGGCAGTCGGACCGT
TCCATCCCACACCTCTCTACTACAAGGTGAACTACGAAGGAACAATCAACATCATCGAGTCGGCAAAGAAACA
CGGAATTAACAAAATCGTCTTTAGCTCTTCGCCGTCCACTCGCTTCACGGGCGTCGACGTCGACGGGTTAACC
GAAGACGAGATGCCCAAACTGCCCTTGAAAAAGTACATGCAAACGTATGCCGGAACAAAAGCCATGGCCGAAT
TGGAAATATCTAAAGCCTGCACCAACGAATTCATGACGGTCGCCGTTGCACCACACCAAGTGTACGGTCCGCG
CGACAATCTATTCCTGCCCAATTTACTGGAGGCGTCCATGACGGGAAAGCTGCGGGTTTTCGGCGAGGGATAC
AATCGCATCTGCTTCACGCACGTGGACAATTACTGCCACGGTTTGATCATTTCGGAAAAGGCGCTGTATCCCG
ATAGCCCCGCGTTGGGGAAATTTTACATTGTTACCGATGGAAAAACTCATCCGAACGGCGAACAATACTTAAT
TTTTTGGGAAATTCTCGACGAAGCCGTGAAGGCGATGGGGTTCACCTCGCTCTGGGATAAGGCCCATCTTCCG
AAGGGTCTTCTCATGTTTGTTGCCACTATTTGCGAGTTCATTGGGAATTTGCTTGGGATGACAATGAAACTCA
ACAGGTTCAATGTCATTGTCCTGACAATGCATAGATGGTTTGATATCAAAGGGGCAGAAAAAGACTTGGGATT
TCAGCCAATTATTGGATTCCGGGAAGGATGGGACGAAACGATAGAGTGGTTTAAACAGCACTGGCTCCCCAAA
CAGCCCAAATCACGCAGTATATTTGGCATTGCGCGCACCACCCAAGAAAAGATTGATATACAGGACCAGAGTG
CAAAGAAAGTACAGTAAGATCGAGAAACTGCACAAATTTCCTTTTCGTTCGTAGTTAATTTTTCGGCTAAAGG
TTTTGATGGGACTGCTTTGTTGATATGCAGGGAGGGATAGACAATTAAACAAGTAACAGTATCTCGCACAAGT
GAGGCGATGGCCGGAAGTGCCACAAAACCCAAGAGATGGTATTGATGTTTGAATGCGAATTCAGTTTATACAT
TACATGGACCAGATATTTAATCTTCCTTCTTGTAAACCACTCTTTCGTAAATTTCATCCAGTGTTGGTGTCAA
CTTATTTGTCCTTACCGCTGCCCGCGCGATTCTTTTTTGGAGTTCTGGCAAGCCATGTTGATCTTGAATCCGC
CATGGAAGAGCCATGTTAAGGAGACTATTCATTTTAAGAGCTCTCCAATAGGCAGCCATCGGAGTCTGACGGT
CATTGTGACGAATGCGAGGTTCTCTGGTACCCCATAAACTCGCCTTTCCAGGGACTGGAAGTAATTTTACGTC
AACGCCAACATGAACAAAATTGCTTCCTCTCTTCGTCGGCTTCATGTTGAAAGGGAGGTGTGCAACTGTAATG
ATTTTAACAGAATTGTAAAGGTTTCTGTTTGATGCTTGTGCTCCAAGAATAACGGTTACTGCGAGCATATGAT
GGGACGTACTTACCAATATCATTACTATTATAAAAACGTGCATGCTGCACTTTCTTCTTCCTCTCTTGATGTC
TGAATGCATCTGCAAAGGCATGTCGGGCAGCGTACGGCGATCCGTTCGAAAATATTTTCACCGGACCATTTTG
AGTAAAGCGGTCATCCGTTGATGCATAAAACCCGAAGAGGATCGAATTAGCCCCGCCGAGGCTGTACCCATTT
ACAGTCACAGTAAAGTCGCTGCCTATGATCTTATTTTGAATCCACCAAGAACGTCTTTGTCAAGAAAACTTCA
CTTGTGATGAGGTTAAAGTGAGAAATGCATTCACTTGGGAGAGTGAGATTGATATTCTTGGACAGATGATTGT
TCATAGAGAAGAGTTGCTCGCAAAAGTCAAAGATACCTTTCTCGTCTTGAATAGCATTTCCATTCAAGATTGG
AATGAATAGATTTGAATGCCACGTATCATGCATAGCATTCTCATCAGTAACTAGCTTGAACGAGAACCGGTAG
GTAGGAACAGCACAACTTGGGGGAACTGTCGACTGAATTGTGATAGCCTTGAGGATGTCATTAGCATAGCGCC
ACATCGCCATGACCCCAGGTACAAATGTAACATGATTCATCTGGTACTTGTGACCAGAGAAGGACATGATCTT
GATGTCGGATGTTACAAAAAGCCAGAATGGAGGCATAGACCTTGTCTTCCCATAAGAGTACTTGGATGAAGCA
TTATGTTGATGGTGTGAGTCACAAGAATACATACTATGGGATACGCGATCAGAGTGACAAGGATAAGAGTCAC
AATAATCATTGCTGCAATCAACAAAGTCTGCCTCCTGCAACATCAATGAGCAGCTGTCACCCTTGTGAGGTAC
GGCAAGCTTGTTGACAAGAGTCTTGTCATCAACATTGTTGTCATCAACGGTCAGAGACTCAGCCTTGCACTCC
AAATTGTCAATAACTTCATTCTGACGCTTGGGACGCAAAGTAGCAGACATGTGTTGAATCCTCTCCTTCTTTG
TGGCATTTTCATGCTCGTGGCAATGACGTTCGGCCTCATCACAAAGACACACAACGTGAGACTTGTGTGAGGC
CGAACTTTGGGCAGTGCTAACCCCATGATTGCTCAATGTCCTACAATGAGTGGTGCTAACCTCATGGTTGCTT
GATGTCCTGTTTGATACAGTGGCATTGTTGTCATTGCCGTTCTTGTCAGCAATGGTAGGAAGACCATGATCTG
AATTTTGAAAGGAAGGTTCAACAACATTATCAACAGATGGACTTGGAGGAGATGAAGTAACAGCAGAATCCTC
TGACAATTGCTTAGATCCTGTGAGAGATCAAAACTTATAGTTCTTTTGCATTGGAATCAGATGGAGACAATCC
AGAAGAATCAGTAAGAGGGCGGTTAGCAGGCTTGCATACCAGGTATGGCTTAGAGTGCATAGACTTCTTCTTT
GGAGGTATCATGTTAATATACTAGTTTTGTATGACCGTGCATACGACCTATAAATACGTAGTATAAGAGATCG
G
>TR84|c1_g1_i3 len=1629 path=[7284:0-48 7732:49-86 7700:87-113 7777:114-117 7356:118-132 7699:133-141 7656:142-206 7342:207-233 7343:234-257 7608:258-315 7524:316-339 7525:340-349 7730:350-419 7778:420-431 7696:432-458 7729:459-491 7654:492-515 7764:516-554 7604:555-617 7652:618-683 7220:684-707 7601:708-812 7179:813-836 7751:837-870 7726:871-894 7779:895-896 7691:897-938 7725:939-1004 7597:1005-1013 7724:1014-1028 7689:1029-1061 7649:1062-1085 7723:1086-1100 7763:1101-

1124 7688:1125-1154 7721:1155-1193 7686:1194-1197 7646:1198-1208
7768:1209-1238 7762:1239-1254 7719:1255-1288 7683:1289-1294 7642:1295-
1318 7718:1319-1355 7767:1356-1384 7781:1385-1415 7681:1416-1424
7309:1425-1439 7759:1440-1446 7744:1447-1447 7714:1448-1474 7577:1475-
1482 7633:1483-1507 7738:1508-1526 7674:1527-1543 7626:1544-1567
7737:1568-1591 7709:1592-1604 7671:1605-1628] [-1, 7284, 7732, 7700,
7777, 7356, 7699, 7656, 7342, 7343, 7608, 7524, 7525, 7730, 7778, 7696,
7729, 7654, 7764, 7604, 7652, 7220, 7601, 7179, 7751, 7726, 7779, 7691,
7725, 7597, 7724, 7689, 7649, 7723, 7763, 7688, 7721, 7686, 7646, 7768,
7762, 7719, 7683, 7642, 7718, 7767, 7781, 7681, 7309, 7759, 7744, 7714,
7577, 7633, 7738, 7674, 7626, 7737, 7709, 7671]
CTTTATTATCTTTCAGTTTCAGAGTAGTCAAATCATGGCCAGCACCTCCACCCCGTTCGCTCCCTCGAGCAGT
CCGCTGCAGGTCAAGGTAGCTCAAGAGGGCAAATATTCGCCTGTCCCCGATGTATGTGTCGTCACCGGCGGCA
CCGGGTTTGTCGGCAACCGTCTAGTAGAAATGCTCGTCGAGCGTGGTGCGAAGAAAGTGATTTCGTTCGATGT
CGTGCCTCCTCCACCAGATGCCTGGAAGCATCCCAACATCGAATGGCGCATCGGCGATATCACCGATAAGGCG
GCCGTGGACGATCTCATGTCCTCCGTGCCAAACATCGGCTGTGTCTGGCACAATGCAGCGGCAGTCGGACCGT
TCCATCCCACACCTCTCTACTACAAGGTGAACTACGAAGGAACAATCAACATCATCGAGTCGGCAAAAAAGCA
CGGAATTAACAAAATCGTCTTTAGCTCTTCGCCGTCCACTCGCTTCACGGGCGTCGACGTCGACGGGTTAACC
GAAGACGAGATGCCCAAACTGCCCTTGAAAAAGTACATGCAAACGTATGCCGGAACAAAAGCCATGGCCGAAT
TGGAAATATCTAAAGCCTGCACCAACGAATTCATGACGGTCGCCGTTGCACCACACCAAGTGTACGGTCCGCG
CGACAATCTATTCCTGCCCAATTTACTGGAGGCGTCCATGACGGGAAAGCTGCGGGTTTTCGGCGAGGGATAC
AATCGCATCTGCTTCACGCACGTGGACAATTACTGCCACGGTTTGATCATTTCGGAAAAGGCGCTGTATCCCG
ATAGCCCCGCGTTGGGGAAATTTTACATTGTTACCGATGGAAAAACTCATCCGAACGGCGAACAATACTTAAT
TTTTTGGGAAATTCTCGACGAAGCCGTGAAGGCGATGGGGTTCACCTCGCTCTGGGATAAGGCCCATCTTCCG
AAGGGTCTTCTCATGTTTGTTGCCACTATTTGCGAGTTCATTGGGAATTTGCTTGGGATGACAATGAAACTCA
ACAGGTTCAATGTCATTGTCCTGACAATGCATAGATGGTTTGATATCAAAGGGGCAGAAAAAGACTTGGGATT
TCAGCCAATTATTGGATTCCGGGAAGGATGGGACGAAACGATAGAGTGGTTTAAACAGCACTGGCTCCCCAAA
CAGCCCAAATCACGCAGTATATTTGGCATTGCGCGCACCACCCAAGAAAAGATTGATATACAGGACCAGAGTG
CAAAGAAAGTACAGTAAGATCGAGAAACTGCACAAATTTCCTTTTCGTTCGTAGTTAATTTTTCGGCTAAAGG
TTTTGATGGGACTGCTTTGTTGATATGCAGGGAGGGATAGACAATTAAACAAGTAACAGTATCTCGCACAAGT
GAGGCGATGGCCGGAAGTGCCACAAAACCCAAGAGATGTGTTGGTGTCAACTTATTTGTCCTTACCGCTGCCC
GCGCGATTCTTTTTTGGAGTTCTAACAAGCCATGTTGATCTTGAATCCGCCATGGAAGAGCCATGTTTAGGAG
ACCATTCATTTAAAGAGCTCTCCAATACGATGGACACTGACGGTCATTGTGACGAATGCGAGGTTCTCTGGTA
CCCCATAAACTCGCCTTTCCAGG
>TR84|c1_g1_i4 len=3533 path=[7284:0-48 7732:49-86 7700:87-113 7777:114-
117 7356:118-132 7699:133-141 7656:142-206 7342:207-233 7343:234-257
7608:258-315 7524:316-339 7525:340-349 7730:350-419 7778:420-431
7606:432-443 7753:444-458 7729:459-491 7654:492-515 7764:516-554
7604:555-617 7652:618-683 7220:684-707 7601:708-812 7179:813-836
7751:837-870 7726:871-894 7779:895-896 7691:897-938 7725:939-1004
7597:1005-1013 7724:1014-1028 7689:1029-1061 7649:1062-1085 7723:1086-
1100 7763:1101-1124 7688:1125-1154 7721:1155-1193 7686:1194-1197
7646:1198-1208 7768:1209-1238 7762:1239-1254 7719:1255-1288 7683:1289-
1294 7642:1295-1318 7718:1319-1355 7767:1356-1384 7781:1385-1415
7681:1416-1424 7745:1425-1473 7715:1474-1495 7679:1496-1536 7743:1537-
1542 7632:1543-1543 7713:1544-1570 7628:1571-1588 7756:1589-1604
7738:1605-1623 7673:1624-1647 7625:1648-1694 7709:1695-1707 7671:1708-
1731 7765:1732-1745 7736:1746-1780 7708:1781-1811 7564:1812-1818
7669:1819-1832 7622:1833-1856 7563:1857-1874 7707:1875-1877 7668:1878-
1901 7621:1902-1934 7784:1935-1958 7791:1959-1993 7561:1994-2017
7620:2018-2115 7446:2116-2119 7559:2120-2842 7383:2843-2866 7665:2867-
3091 7144:3092-3124 7145:3125-3532] [-1, 7284, 7732, 7700, 7777, 7356,
7699, 7656, 7342, 7343, 7608, 7524, 7525, 7730, 7778, 7606, 7753, 7729,
7654, 7764, 7604, 7652, 7220, 7601, 7179, 7751, 7726, 7779, 7691, 7725,
7597, 7724, 7689, 7649, 7723, 7763, 7688, 7721, 7686, 7646, 7768, 7762,
7719, 7683, 7642, 7718, 7767, 7781, 7681, 7745, 7715, 7679, 7743, 7632,
7713, 7628, 7756, 7738, 7673, 7625, 7709, 7671, 7765, 7736, 7708, 7564,
7669, 7622, 7563, 7707, 7668, 7621, 7784, 7791, 7561, 7620, 7446, 7559,
7383, 7665, 7144, 7145, -2]

CTTTATTATCTTTCAGTTTCAGAGTAGTCAAATCATGGCCAGCACCTCCACCCCGTTCGCTCCCTCGAGCAGT
CCGCTGCAGGTCAAGGTAGCTCAAGAGGGCAAATATTCGCCTGTCCCCGATGTATGTGTCGTCACCGGCGGCA
CCGGGTTTGTCGGCAACCGTCTAGTAGAAATGCTCGTCGAGCGTGGTGCGAAGAAAGTGATTTCGTTCGATGT
CGTGCCTCCTCCACCAGATGCCTGGAAGCATCCCAACATCGAATGGCGCATCGGCGATATCACCGATAAGGCG
GCCGTGGACGATCTCATGTCCTCCGTGCCAAACATCGGCTGTGTCTGGCACAATGCAGCGGCAGTCGGACCGT
TCCATCCCACACCTCTCTACTACAAGGTGAACTACGAAGGAACAATCAACATCATCGAGTCGGCAAAGAAACA
CGGAATTAACAAAATCGTCTTTAGCTCTTCGCCGTCCACTCGCTTCACGGGCGTCGACGTCGACGGGTTAACC
GAAGACGAGATGCCCAAACTGCCCTTGAAAAAGTACATGCAAACGTATGCCGGAACAAAAGCCATGGCCGAAT
TGGAAATATCTAAAGCCTGCACCAACGAATTCATGACGGTCGCCGTTGCACCACACCAAGTGTACGGTCCGCG
CGACAATCTATTCCTGCCCAATTTACTGGAGGCGTCCATGACGGGAAAGCTGCGGGTTTTCGGCGAGGGATAC
AATCGCATCTGCTTCACGCACGTGGACAATTACTGCCACGGTTTGATCATTTCGGAAAAGGCGCTGTATCCCG
ATAGCCCCGCGTTGGGGAAATTTTACATTGTTACCGATGGAAAAACTCATCCGAACGGCGAACAATACTTAAT
TTTTTGGGAAATTCTCGACGAAGCCGTGAAGGCGATGGGGTTCACCTCGCTCTGGGATAAGGCCCATCTTCCG
AAGGGTCTTCTCATGTTTGTTGCCACTATTTGCGAGTTCATTGGGAATTTGCTTGGGATGACAATGAAACTCA
ACAGGTTCAATGTCATTGTCCTGACAATGCATAGATGGTTTGATATCAAAGGGGCAGAAAAAGACTTGGGATT
TCAGCCAATTATTGGATTCCGGGAAGGATGGGACGAAACGATAGAGTGGTTTAAACAGCACTGGCTCCCCAAA
CAGCCCAAATCACGCAGTATATTTGGCATTGCGCGCACCACCCAAGAAAAGATTGATATACAGGACCAGAGTG
CAAAGAAAGTACAGTAAGATCGAGAAACTGCACAAATTTCCTTTTCGTTCGTAGTTAATTTTTCGGCTAAAGG
TTTTGATGGGACTGCTTTGTTGATATGCAGGGAGGGATAGACAATTAAACAAGTAACAGTATCTCGCACAAGT
GAGGCGATGGCCGGAAGTGCCACAAAACCCAAGAGATGGTATTGATGTTTGAATGCGAATTCAGTTTATACAT
TACATGGACCAGATATTTAATCTTCCTTCTTGTAAACCACTCTTTCGTAAATTTCATCCAGTGTTGGTGTCAA
CTTATTTGTCCTTACCGCTGCCCGCGCGATTCTTTTTTTGGAGTTCTGGCAAGCCATGTTGATCTTGAATCCG
CCATGGAAGAGCCATGTTGAGGAGACTATTCATTTTAAGAGCTCTCCAATAGGCAGCCATCGGAGTCTGACGG
TCATTGTGACGAATGCGAGGTTCTCTGGTACCCCATAAACTCGCCTTTCCAGGGACTGGAAGTAATTTTACGT
CAACGCCAACATGAACAAAATTGCTTCCTCTCTTCGTCGGCTTCATGTTGAAAGGGAGGTGTGCAACTGTAAT
GATTTTAACAGAATTGTAAAGGTTTCTGTTTGATGCTTGTGCTCCAAGAATAACGGTTACTGCGAGCATATGA
TGGGACGTACTTACCAATATCATTACTATTATAAAAACGTGCATGCTGCACTTTCTTCTTCCTCTCTTGATGT
CTGAATGCATCTGCAAAGGCATGACCGGCAGCGTACGGCGATCCGTACGAAAATATTTTCACCGGACCGTTTT
GAGTAAAGCGGTCATCGGTCGATGCGTAAAACCCGAAGAGGATCGAAAGAGCCCCGCCGAGGCTGTACCCATT
TACTGTCACCGTAAAGTCACTGCCTATGATCTTATTCCCGTACTCGTACACCTTGTTCGCAATTTCGTCATAC
TTTCGTGTGGAGGTGTCTTTCCTCGTTCGGAAGAGGTAGGTGTGGAAGCCTCGGTGTATGGTGACGAATTGTT
TCTTTCCTTCGTATGTATCCTTAACTGGATTTTGGGTTTTCTTCATCGCAGCGTCAAACCCGTGATTCCAGTC
AGCCCGAGTCGTTGCGCCTCTGAAAACTACCAATACTCTTTTCTTCTTCTCGTCGACTGAAATAGCATAAATA
CAATCTTTGATCGGGAACCAATCTTGCAGCCAAACAACCTGCGCTTTGCTCTTGACCATTGTTTGCGAAATGG
TATTCGGTCTGAAAAAATCTGCCATAAAGTCCAACGAATCGACATTCCTGGATTCGACACATCTTTTCATGAA
ATCGAGCAATTCCTTCTCTGACATATTCCGCTCTGGATCTCCGCCCTGAGCTAGCGTTTGGGGCACGCCCGAC
ATCGACGCCATCGTCCCCAAATTAAAACTCGACGCCAATCTCACCATGTTGTTTCCCACGTCTTCCGCCCCAG
CGAACGTTTCGTAATCATCCGCGGGAATGATCAGCTTCTTGTGCAACACGTGATCCTCCCGGTACCTCTCGAC
GCGGCTCGTCAAATGTTGCGTGAGATCCCTCTTCAGAGTGCTGGCCCTCTTGCTCATCGGGGGCGGAGGTGGC
CGGCTCCTCGCATCCGAGAGTTCAGGAACGATGCCGTCCCCGGGCCAACTCTGATTGGGGCCGGGGTCCGATT
TGAGCGAGTGCAAGTCGTCGTCTGGAGCACGAGCGGCCCTAATCCCTTTCATCGACTCGATTATTTCGAAGAG
TCTGGATTGGTTCATCTTCCCGCCACCGCTGTTAGTGATTTTGGGTTGGGCAGCAGCTTCGAGTCCATCCATA
ACTTCCAGATCTTGACTTGCGCTTGGGTCAAGAGTGATTGATGAATTCCGTTTGTGCTCTTCGCCGTCTTCTA
TTATTATAGATAACGCAGACTGATCCCGTTTGTCCTCGTCCCCTTCTTCTATTAGAGATAACGCAGACTGTGG
CTTCGATCTCCCCTTAGTAGCCGATCTCCCCGCACTAACTGTTCCCAAATTATCTTCATCATTTTCATTATTT
GCAACCCCTTCCGATGGTGGCGCCGATGTCGCTTCCACCGTTAAGCCGGTCGGTTCCTTCGGCGATTTGTTCC
GCATCAACGACCCGGTAATTCTGGCAGAAAGCTTCATCTTTCTCATGCCACTCCCCTCGTCATCCGATTCTTG
CCCCGCGGTAGGCGACTGTTGATCTGGATTCTGATTCTGATCGACGTCATCATACGAGAACAATCGCTGGGTC
AATTGCCCTCTCATCCACGGCATCATCGG
>TR84|c1_g1_i5 len=1521 path=[7284:0-48 7732:49-86 7700:87-113 7777:114-117 7356:118-132 7699:133-141 7656:142-206 7342:207-233 7343:234-257 7608:258-315 7524:316-339 7525:340-349 7730:350-419 7778:420-431 7606:432-443 7753:444-458 7729:459-491 7654:492-515 7764:516-554 7604:555-617 7652:618-683 7220:684-707 7601:708-812 7179:813-836 7751:837-870 7726:871-894 7779:895-896 7691:897-938 7725:939-1004 7597:1005-1013 7724:1014-1028 7689:1029-1061 7649:1062-1085 7723:1086-1100 7763:1101-1124 7688:1125-1154 7750:1155-1184 7687:1185-1193 7592:1194-1197 7769:1198-1238 7762:1239-1254 7719:1255-1288 7773:1289-1355 7412:1356-1366 7640:1367-1379 7585:1380-1462 7151:1463-1474 7152:1475-1496 7153:1497-1513 7154:1514-1520] [-1, 7284, 7732, 7700,

7777, 7356, 7699, 7656, 7342, 7343, 7608, 7524, 7525, 7730, 7778, 7606, 7753, 7729, 7654, 7764, 7604, 7652, 7220, 7601, 7179, 7751, 7726, 7779, 7691, 7725, 7597, 7724, 7689, 7649, 7723, 7763, 7688, 7750, 7687, 7592, 7769, 7762, 7719, 7773, 7412, 7640, 7585, 7151, 7152, 7153, 7154, -2]
CTTTATTATCTTTCAGTTTCAGAGTAGTCAAATCATGGCCAGCACCTCCACCCCGTTCGCTCCCTCGAGCAGT
CCGCTGCAGGTCAAGGTAGCTCAAGAGGGCAAATATTCGCCTGTCCCCGATGTATGTGTCGTCACCGGCGGCA
CCGGGTTTGTCGGCAACCGTCTAGTAGAAATGCTCGTCGAGCGTGGTGCGAAGAAAGTGATTTCGTTCGATGT
CGTGCCTCCTCCACCAGATGCCTGGAAGCATCCCAACATCGAATGGCGCATCGGCGATATCACCGATAAGGCG
GCCGTGGACGATCTCATGTCCTCCGTGCCAAACATCGGCTGTGTCTGGCACAATGCAGCGGCAGTCGGACCGT
TCCATCCCACACCTCTCTACTACAAGGTGAACTACGAAGGAACAATCAACATCATCGAGTCGGCAAAGAAACA
CGGAATTAACAAAATCGTCTTTAGCTCTTCGCCGTCCACTCGCTTCACGGGCGTCGACGTCGACGGGTTAACC
GAAGACGAGATGCCCAAACTGCCCTTGAAAAAGTACATGCAAACGTATGCCGGAACAAAAGCCATGGCCGAAT
TGGAAATATCTAAAGCCTGCACCAACGAATTCATGACGGTCGCCGTTGCACCACACCAAGTGTACGGTCCGCG
CGACAATCTATTCCTGCCCAATTTACTGGAGGCGTCCATGACGGGAAAGCTGCGGGTTTTCGGCGAGGGATAC
AATCGCATCTGCTTCACGCACGTGGACAATTACTGCCACGGTTTGATCATTTCGGAAAAGGCGCTGTATCCCG
ATAGCCCCGCGTTGGGGAAATTTTACATTGTTACCGATGGAAAAACTCATCCGAACGGCGAACAATACTTAAT
TTTTTGGGAAATTCTCGACGAAGCCGTGAAGGCGATGGGGTTCACCTCGCTCTGGGATAAGGCCCATCTTCCG
AAGGGTCTTCTCATGTTTGTTGCCACTATTTGCGAGTTCATTGGGAATTTGCTTGGGATGACAATGAAACTCA
ACAGGTTCAATGTCATTGTCCTGACAATGCATAGATGGTTTGATATCAAAGGGGCAGAAAAAGACTTGGGATT
TCAGCCAATTATTGGATTCCGGGAAGGATGGGACGAAACGATAGAGTGGTTTAAACAGCATTGGCTCCCCAAA
CAACCCAAATCACGCAGTATATTTGGCATTTCACGCACCACCCAAGACAAGATTGATATACAGGACCAGAGTG
CAAAGAAAGTACAGTAAGATCGAGAAACTGCACAAATTTCCTTTTCGTACATAGTAAATTTTTCGGCTAAAGA
TTTTGATGGGACTGCTTTATTGATATGCAGGGAGGGATAGACGATTAAACAACTAACATTATCTCGCACAAGT
GAGGCCTATGGCCGGAAGTGCCACAAGACCCAAGAGACGGTATTAATGATTGAATGCGAATTCAATTTATACA
TTACATGGACCAGATATTTAATCTTCCTTCTTGTAAATCACTCTTTCGTAAATTTCATCCA
>TR84|c1_g1_i6 len=248 path=[7442:0-38 7443:39-76 7731:77-122 7699:123-131 7656:132-196 7407:197-247] [-1, 7442, 7443, 7731, 7699, 7656, 7407, -2]
TGTGCTCTTCCGATCTGTCAAATCATGGCCAGCACCTCCACCCCGTTCGCTCCCTCGAGCAGTCCGCTGCAGG
TCAAAGCAGCTCAAGAGGGCAAATATCCGCCTGTCCCCGATGTATGTGTCGTCACCGGCGGCACCGGGTTTGT
CGGCAACCGTCTAGTAGAAATGCTCGTCGAGCGTGGTGCGAAGAAAGTGATATCGTTCGATGTCGTGCCTCCT
CCGCCAGATGCCTGGAAGCACCCCAACAT
>TR84|c1_g1_i7 len=1442 path=[7007:0-38 7732:39-76 7700:77-103 7777:104-107 7356:108-122 7699:123-131 7656:132-196 7342:197-223 7343:224-247 7608:248-305 7524:306-329 7525:330-339 7730:340-409 7607:410-433 7753:434-448 7729:449-481 7654:482-505 7764:506-544 7604:545-607 7652:608-673 7220:674-697 7601:698-802 7179:803-826 7751:827-860 7726:861-884 7779:885-886 7691:887-928 7725:929-994 7597:995-1003 7724:1004-1018 7689:1019-1051 7649:1052-1075 7723:1076-1090 7763:1091-1114 7688:1115-1144 7750:1145-1174 7647:1175-1198 7768:1199-1228 7762:1229-1244 7719:1245-1278 7683:1279-1284 7642:1285-1308 7718:1309-1345 7767:1346-1374 7639:1375-1397 7583:1398-1424 7790:1425-1432 7766:1433-1441] [-1, 7007, 7732, 7700, 7777, 7356, 7699, 7656, 7342, 7343, 7608, 7524, 7525, 7730, 7607, 7753, 7729, 7654, 7764, 7604, 7652, 7220, 7601, 7179, 7751, 7726, 7779, 7691, 7725, 7597, 7724, 7689, 7649, 7723, 7763, 7688, 7750, 7647, 7768, 7762, 7719, 7683, 7642, 7718, 7767, 7639, 7583, 7790, 7766]
TGTGCTCTTCCGATCTGTCAAATCATGGCCAGCACCTCCACCCCGTTCGCTCCCTCGAGCAGTCCGCTGCAGG
TCAAGGTAGCTCAAGAGGGCAAATATTCGCCTGTCCCCGATGTATGTGTCGTCACCGGCGGCACCGGGTTTGT
CGGCAACCGTCTAGTAGAAATGCTCGTCGAGCGTGGTGCGAAGAAAGTGATTTCGTTCGATGTCGTGCCTCCT
CCACCAGATGCCTGGAAGCATCCCAACATCGAATGGCGCATCGGCGATATCACCGATAAGGCGGCCGTGGACG
ATCTCATGTCCTCCGTGCCAAACATCGGCTGTGTCTGGCACAATGCAGCGGCAGTCGGACCGTTCCATCCCAC
ACCTCTCTACTACAAGGTGAACTACGAAGGAACAATCAACATCATTGAGTCGGCAAAGAAACACGGAATTAAC
AAAATCGTCTTTAGCTCTTCGCCGTCCACTCGCTTCACGGGCGTCGACGTCGACGGGTTAACCGAAGACGAGA
TGCCCAAACTGCCCTTGAAAAAGTACATGCAAACGTATGCCGGAACAAAAGCCATGGCCGAATTGGAAATATC
TAAAGCCTGCACCAACGAATTCATGACGGTCGCCGTTGCACCACACCAAGTGTACGGTCCGCGCGACAATCTA
TTCCTGCCCAATTTACTGGAGGCGTCCATGACGGGAAAGCTGCGGGTTTTCGGCGAGGGATACAATCGCATCT
GCTTCACGCACGTGGACAATTACTGCCACGGTTTGATCATTTCGGAAAAGGCGCTGTATCCCGATAGCCCCGC
GTTGGGGAAATTTTACATTGTTACCGATGGAAAAACTCATCCGAACGGCGAACAATACTTAATTTTTTGGGAA
ATTCTCGACGAAGCCGTGAAGGCGATGGGGTTCACCTCGCTCTGGGATAAGGCCCATCTTCCGAAGGGTCTTC

TCATGTTTGTTGCCACTATTTGCGAGTTCATTGGGAATTTGCTTGGGATGACAATGAAACTCAACAGGTTCAA
TGTCATTGTCCTGACAATGCATAGATGGTTTGATATCAAAGGGGCAGAAAAAGACTTGGGATTTCAGCCAATT
ATTGGATTCCGGGAAGGATGGGACGAAACGATAGAGTGGTTTAAACAGCATTGGCTCCCCAAACAACCCAAAT
CACGCAGCATATTTGGCATTGCGCGCACCACCCAAGAAAAGATTGATATACAGGACCAGAGTGCAAAGAAAGT
ACAGTAAGATCGAGAAACTGCACAAATTTCCTTTTCGTTCGTAGTTAATTTTTCGGCTAAAGGTTTTGATGGG
ACTGCTTTGTTGATATGCAGGGAGGGATAGACAATTAAACAAGTAACAGTATCTCGCACAATGTTGGTGTCAA
CTTATTTGTCCTTACCGCTGCCCGCGCGATTCTTTTTTGGAGTTCTGGCAAGCCA
>TR84|c1_g1_i8 len=1421 path=[7007:0-38 7732:39-76 7700:77-103 7777:104-107 7356:108-122 7699:123-131 7656:132-196 7342:197-223 7343:224-247 7608:248-305 7524:306-329 7525:330-339 7730:340-409 7778:410-421 7606:422-433 7753:434-448 7729:449-481 7654:482-505 7764:506-544 7604:545-607 7652:608-673 7220:674-697 7601:698-802 7179:803-826 7751:827-860 7726:861-884 7779:885-886 7691:887-928 7725:929-994 7597:995-1003 7724:1004-1018 7689:1019-1051 7649:1052-1075 7723:1076-1090 7763:1091-1114 7688:1115-1144 7721:1145-1183 7686:1184-1187 7646:1188-1198 7768:1199-1228 7762:1229-1244 7780:1245-1255 7788:1256-1279 7585:1280-1362 7151:1363-1374 7152:1375-1396 7153:1397-1413 7154:1414-1420] [-1, 7007, 7732, 7700, 7777, 7356, 7699, 7656, 7342, 7343, 7608, 7524, 7525, 7730, 7778, 7606, 7753, 7729, 7654, 7764, 7604, 7652, 7220, 7601, 7179, 7751, 7726, 7779, 7691, 7725, 7597, 7724, 7689, 7649, 7723, 7763, 7688, 7721, 7686, 7646, 7768, 7762, 7780, 7788, 7585, 7151, 7152, 7153, 7154, -2]
TGTGCTCTTCCGATCTGTCAAATCATGGCCAGCACCTCCACCCCGTTCGCTCCCTCGAGCAGTCCGCTGCAGG
TCAAGGTAGCTCAAGAGGGCAAATATTCGCCTGTCCCCGATGTATGTGTCGTCACCGGCGGCACCGGGTTTGT
CGGCAACCGTCTAGTAGAAATGCTCGTCGAGCGTGGTGCGAAGAAAGTGATTTCGTTCGATGTCGTGCCTCCT
CCACCAGATGCCTGGAAGCATCCCAACATCGAATGGCGCATCGGCGATATCACCGATAAGGCGGCCGTGGACG
ATCTCATGTCCTCCGTGCCAAACATCGGCTGTGTCTGGCACAATGCAGCGGCAGTCGGACCGTTCCATCCCAC
ACCTCTCTACTACAAGGTGAACTACGAAGGAACAATCAACATCATCGAGTCGGCAAAGAAACACGGAATTAAC
AAAATCGTCTTTAGCTCTTCGCCGTCCACTCGCTTCACGGGCGTCGACGTCGACGGGTTAACCGAAGACGAGA
TGCCCAAACTGCCCTTGAAAAAGTACATGCAAACGTATGCCGGAACAAAAGCCATGGCCGAATTGGAAATATC
TAAAGCCTGCACCAACGAATTCATGACGGTCGCCGTTGCACCACACCAAGTGTACGGTCCGCGCGACAATCTA
TTCCTGCCCAATTTACTGGAGGCGTCCATGACGGGAAAGCTGCGGGTTTTCGGCGAGGGATACAATCGCATCT
GCTTCACGCACGTGGACAATTACTGCCACGGTTTGATCATTTCGGAAAAGGCGCTGTATCCCGATAGCCCCGC
GTTGGGGAAATTTTACATTGTTACCGATGGAAAAACTCATCCGAACGGCGAACAATACTTAATTTTTTGGGAA
ATTCTCGACGAAGCCGTGAAGGCGATGGGGTTCACCTCGCTCTGGGATAAGGCCCATCTTCCGAAGGGTCTTC
TCATGTTTGTTGCCACTATTTGCGAGTTCATTGGGAATTTGCTTGGGATGACAATGAAACTCAACAGGTTCAA
TGTCATTGTCCTGACAATGCATAGATGGTTTGATATCAAAGGGGCAGAAAAAGACTTGGGATTTCAGCCAATT
ATTGGATTCCGGGAAGGATGGGACGAAACGATAGAGTGGTTTAAACAGCACTGGCTCCCCAAACAGCCCAAAT
CACGCAGTATATTTGGCATTGCGCGCACCACCCAAGAAAAGATTGATATACAGGACCAGAGTGCAAAGAAAGT
ACAGGAGGGATAGACAATTAAACAACTAACATTATCTCGCACAAGTGAGGCCTATGGCCGGAAGTGCCACAAG
ACCCAAGAGACGGTATTAATGATTGAATGCGAATTCAATTTATACATTACATGGACCAGATATTTAATCTTCC
TTCTTGTAAATCACTCTTTCGTAAATTTCATCCA
>TR85|c0_g1_i1 len=281 path=[259:0-280] [-1, 259, -2]
TGCTGTTGTTGTTGTTGGAGTCTCCCATCATCGTCGTCGTCGGATGAACAATGCTGCTATCGCTGTTACTAAC
AAGGACGCTGACATTTCCTCCCTTCAATGATTCCTTAACGGTATTATTAGCGTTGGTGATTCTTTTGGTGTTG
TTGGTGTCAGTTGTTGTTGTAGTTGCTTCGTGACGCTGTTGCTTTTCATGCCATTTTTGTCGTCTTAGTTGTC
TTTGTTCCTCCTCGTCTTCTACCATTGTCTCTTCGTTTCCTTTTCGTTTTCCTCTCTCGTTG
>TR86|c0_g1_i1 len=809 path=[787:0-808] [-1, 787, -2]
TGGACTTCTGCGACATCACAAACAGCGCGTACTGCGACCGTTACGTCCCCTCTTTATTTGTTTCTACTCAAGC
CAACCCTTTGTCTGCCCTTTCGTCTCTCCTTTGAAGAACATGGATCCCGCTTGTCTTTGACGGACATTATTTT
GAATTACAGTGCTTTTGGTGCGAGGGCGATCGCTTCGTTTGACTTTGGACGATGACTGATCAACTCGGGTAGC
CTTTCGAGTACGTCTTGCACGTATACGCTGTAAGTCCACGGCGACATTGGATGGGAGGTCAGCGGAAGTTGGT
CGATTGGGTTGGTGCGCCGGTGCGTTGTGGCCGTTAGTGTTTGCTTTGGAAGCCTTTTGAGCTCGCGTCTCAC
GGATACGTTTCAGGTCATCGGCAACATTGGTCGGAAGATTGGCTGAGGTCGGTCGGTTATTGGGCCTGTTGTC
GCTTTTGTCCTTTGTCTCCACAGCCGGCCGCTGGCTATCTTCGGAAGCTTTCTCTTTGCGCTTCTCTCTGATA
CGTTTCAATTCCATAGCAACGTTGGCTGGAATATTGGCCGATGTTGGTTGATCGTTTGGTGTGCTATTTTCGT
TGGCAATAATTGCCTCGGATCCAACGTCATCATTGTTCGCGTCCTTTTTCTTAGACCTTGATTTGAATTCTCT
TATCCGCGCAACTGCATTTTGCTTCGTAACTTTCCCATCTAATATGTTTCTCTTGACACTACCCCATTTTTGA
GTAGCGTTTGCCTTGCCGTTCACCCGTTCTCTAATTAAATTCCATCCCTGAAGTCCAGTCGTTTTGCCGTCTC
CATAGA

>TR87|c0_g1_i1 len=1104 path=[1082:0-1103] [-1, 1082, -2]
ATTTAGCGGGCGCGGGCGCCACCCACGCGCCAAACTTCCATATCATCCAACAATCAGCATCCTCATGAAAGAT
TGCACACGGTTTCCGTAGCACAAAACCTCAACCACGAAGTTCGTTGATCTTACGAATATTCATAATTGAAATT
AACTCAACTTGAAAATATGGTACCATACACTGTAAAAACTGCGAAAGGTACAGTATTTCGATTTGTACCTATG
CATACCAAGCGGAAACACTGAGCTCAACCCTGATCCCACCTTCAAGCATTCTTCTCATCGAGCTTATTTTATC
TCGTCGAAAGTACTCGAACCCCAAGAATATGTCAATGATTTGTGTTTCTATCCATTCCATCTCAGATAACCCC
TTTTAAATAATATTAAGCATTCAATTTCAATCATTCCGGGCCAGTTTCTCCTCTACTTGATCTCTATCGACAC
TCCTCTCGGTTAAAAGTATAATATGCATCCCGAAATCAGTAACCACCGGCCCTAACAACGCTCCCACAGGAGG
AGCCTCCCCCACATCCGGAAAAAGCAATTCATCAAACTCAGGTGCCATCTTCCCTCGTCCAAACGAACCCAAA
TCTCCCCCCTCCGACCGCGACGGACACTGAGATTCCCGCTTGGCCAACTCGGAGAATATAGGTTGCACCAGCG
CAATCTTTGACATGGCGCCCTCCTCGGCGTTGCCATCATCGACCTCTCGCACACGCTCGTTGAGTTCCCCCAT
GATGGTTTTTATTTTCGTCATGACGTAGCCGACATCATCGCCCTTGATGAGAATGTGTGAGGCGCGGACGCGT
TGGTCTTGGGCTTCGTAGTCGTTGTTTGCGAAGGCTGCCGAGATGCTGTCGAAGAAACCCATTTGGAGTGCGG
ATAGTGTTGGAGAGGTGAGGCATTTTACAGAGGGGTGCTGGTGGTGAGATCCGTTGTTGTTCCTATATCCGGG
TCTTTGTAGCGAAGTTTTTACGGTCGACGATGGGGAGAACCCATGCGAGAGCTGCACCAATATGATGAGCAAA
AGGATAGAGATTAGGTGTCTGGTGTTATTCATGGTGCTCTTCTTCGAGTTGTGATCTTGTGAGTTCGTACTTA
CGAAATCAC
>TR89|c0_g1_i1 len=564 path=[536:0-352 537:353-358 542:359-529 540:530-
535 541:536-563] [-1, 536, 537, 542, 540, 541, -2]
TTTTTTTTTAAAATAAAATATCTTCATTTTACTGAAAGGATCCAAATCAATCAATAGTGTTGAATGTGGCATA
GTTTTCTATCATTTAGTGCAGAGCCATATAAGCTTAAAGCAGGACTTGATAACATTTCATCACAAATGGAATA
TCGAGTCAAACATTAGTATCTGCGTCCTCCTCGTCCTCCACGCCCACCACGTCCACCGCGGCCTCTTCCGCCG
CCGCGCCCACCTCCGCGCCCACCTCCACGACCTCCTTTTCTCTCCTCGGGGCAGTCTCTACTGATGTGACCAC
TGTTGCCGCAGTTGTAACATGATCTGTCTCCACCTCCTCCACCTTCCTTTCTGGGCTCAGTGCAGTCTCTGCT
GATGTGGCCGCTGCCACCACAATTGTAACAGGACCTATCTCCTCCATAACCTCCGCCACCTTCTTTTTTCTCC
TCGGGGCAGTCTCTGCTCATGTGGCCACTTTTACCACAGTTGTAGCACGACCTGTCTCCACCTCCTCCACCAC
CTTCCTTTCTGGGCTCAGTGCAGTCCCTGCTGATGTGGCCATCGCCACCGCAG
>TR90|c0_g1_i1 len=266 path=[244:0-265] [-1, 244, -2]
TGTCACATATGGCTCTTTCGTCATCATAGGAAACCATACCTGTTCTCTGATACAAATATTGAGTCATTGTCTG
GTCGTCTCGCACACTTGTGAACTGATCACTCATTTTATCCAAAATATTGTAATACTGGGGTACGCCTTCATCC
ATATGCCAAGTTATTTCGTCATTGCCTTCGTGAGGTTCGTACAAGTGCTCTGCATTGAGGAACGAGCGTCCAT
AAAATGGAAATCCAATATTTATTTTTTCCCTTGGTGCTCCGCCTTTC
>TR92|c0_g1_i1 len=260 path=[238:0-259] [-1, 238, -2]
CTTTCAAAACAAACACAGGCAAGTATTTATCGGTTTCCTGGTATTGGGGTGTTCACGCTAACGGAAACGCAGT
TGGAGATGAGGAAAAGTGGTCGAAGGAAGAAACCGAAGACCCCAAACTTGTGTGTCTCATGTCGTCCTACGGC
AGCTACCTTTCTTGCGACGAACGAGGTAGAGTCGTTCATAAAGAGGGAAAACAGCCAGAGCGTATGGAAAAGT
TTGCAATTGTCGACGATCCGCCTACAATGGAGGAGGTGGAT
>TR93|c0_g1_i1 len=231 path=[209:0-230] [-1, 209, -2]
TGATGTGCATCACAATCCTACTTCCTTGGATGATACCGTAGACAGCAGTGGAGTTATTGCATTCTCGCCAGTG
GAATTTCGAAGGGTGTTGTGGAGAGGTGGAGGAGGGGATACTCCTTCTAGCACTAGGAGTGGAAATGGTATGT
CATCTCATCATCAGAATCATGCCACAGTTGACGGGAACAAGACTACTTGTTTTACACCCCTGGGAACAACAAC
ATCATTGCATAT
>TR94|c0_g1_i1 len=409 path=[387:0-408] [-1, 387, -2]
ATCTGATTAGATGCAGCAGGGGGCTCTGTCTCCGCCGTGACGGGAGGACGGGAAATGGGGGGGCAGGTTCTTC
TATCACATCATTCGATCGTGCCTTCATTTGTTTTTCCGCCGCGCCACCGCAGGAACCTTTTTTGGTGAAGGTG
TCTTCCGTTGTGACACAACAATCATACTCGAGGAGGGGACGGGAGTAGAACACCAGTCTTTCTATTCACAAAC
GCATTAGCGAGAAGAGGAGAACATGATGGATTGTTCGCTGCCGTCATTAGGTAGACGAAGATGCCGGAAGTGA
CGTAGGAGGCGTGAGGAGAACACCGGTCGGTGTGTCGTTAGTGAAGGATGTGCCATGGGTGAGGATAGCTTTG
ACAGTCATTGATGTCTTTGGCATTGTTGTTGGTGTTGTTGTGGG
>TR95|c0_g1_i1 len=229 path=[257:0-164 258:165-228] [-1, 257, 258, -2]
CTTCCGATCTGCTGAAAAATGTGCCTTGCAAGGAAAGGAAGAGGATGCCGCAATCCCCCGCTGCGCAGGCCGG
TCGTAAATCCAGCCGGATCCAAGTGCGCAACCGCAAGCGGATTCTGGAGGCGGCGCTTGAGGTGTTTTCCACC
CATGGCTATCGCGGCGCGAAGAAAATAAAAAGTATGAGGAAAACGAAAAAAAGAAGAAAGGACCTAAAGAGAT
CGGAAGAGCG
>TR97|c0_g1_i1 len=1534 path=[1528:0-551 1529:552-568 1530:569-1533] [-1,
1528, 1529, 1530, -2]
ATTCTATCACGCACTAACTAAGATTCAGTGGATATACCGAGAAGTCAAGTAAAAAATATATCAATCGCTATCG
CTATCGCTATCCATCATCATGGCGGAAAAGGCACTCCCGCCATTCGACCTAGAATTCTTCTTCGTCGCCTTCT
TCCCCCTCGCCGCCTGCCTCGACGCCAATCCACCGGGAGTGACCCAAGTGGGCGCACCGCTCGCCGACGATCG
AGGTGCCGCCTTTTTCTTTCCCGCCGACGACGATCCCTTGCCCGCGGATCCTCCTACGGCCGTCGAAATCCTC

TTCCGACTGGCCTCCAATCGAATGCGAAAGTTCTCGTCGTCCTCGAACCAACCCCGGGATGCCTCCTCCAGCT
CCTCGATCTGCTCCTGCATCTCTTCCATTTCGGTCAATTGCTTGGTCTCCTTCACGCTCAGCAATCGTCCCTT
GGAGGATGCTTCCAATTTCAACAGCGGTTTGAGCATCTTTTTCAATTTGAGAATCTGGGGTTCGTGTTTCAAT
TTGTGCGGGACCACCGCCGCGTGTCCCTCCAGCATTTTTTTTGCGCGCCTCCGCCTTCTCCTTTTGCGTGTTC
AATTTGGACGCCTTCTTCTCCTGAGACTTTTGCAATGCCGCGTCGATGTCCTCGTCGAAGGAATCGATTTTCT
CGCATACTTGCGCCAATAGTCGATCGATTTCGTGTCGCGTGAGGGAACCCTTTTCCACCTGTTGTTGCATGAG
CTTTTTGAGACAGTCCGACTTGGTCGTGATGGATGTCGCTCCTCCCACGGCGTCGTAGAATGCATTTTCGTCG
TCGGAGAGTGGTGTGCCCGACATGAGTTTGGCATCCATCTTCTCCACAGTCTCCACGGCAGCCTTCAAAACCT
CCGTACACATCTTCTTCTCCGACCACGAAGGACACATTCCCAGAGCATTCTTCGCAAACACTTCCTCGGACGC
CACTAGAATGATATGTTCGGGAGTACCCTCCGCCAATGGAAAATACATAGGATACTCCTCCGCCGGTGTTGAC
GACGAGGCTCCTCCAGCACCACCATCCTCTCCGTCTTGCACCAATTTAGGAAATACAACAACGGTGAATAGAT
TCTTACCGGTATCGTGTTGGGTTTTGTAACATCCTCCGCATTCGTTGACGCAGCTCGCTCCGCGATCTTCGAC
GTAGTCTAATTTTCGCAGTGCCGAGGTCAAGGTTTGCATGGCCTTGTCATCGTATTTTTGACGTGCTAATTTG
CCGGCCCCGGTGACGAAGGTCACTCGTATCATTCGATCAAGGTGGAATGGTTGGGAGAAGGCATCGCTTATTG
TTTGGTTGATGTTGCTGGCATCGTGACCGGCCAAATCAAACGTTTCGTTATCCGTTGCGGCACGGATAGATGC
TGCCATTCCGCTTACGGCATTCGTCATGGTAGAGGTGTTGGTGTTTCGTCTGGTCGAGGGGTAGTTCGAGGAG
GAGTTGTGATCGGATTGGCAGTTGTATCGAGTTCGATCGGGCGAGGCCGAAGCACGTCCGAGGTGTGGGTGTG
A
>TR98|c0_g1_i1 len=1812 path=[1836:0-703 1841:704-886 1835:887-1811] [-1, 1836, 1841, 1835, -2]
CTAGCATTGTAGGCACTTGTAGATTTTAATATCATCTGATCTATTTCCCACAGCATGAGAATGTTAATTGGTG
TCCTAACCAAACTTGATTTTTCTCGAAGATGATTTATAAATTTCTACACTGTGTGCGAAAAATTATATATCAT
TGACCTCTACTTTTGCAACAAAAGAAAGTAACCTCCCCACATGAAACTATGTTATTGCAACAAATTCTATTGC
TTTTCAACCCGATGATTGATATTGTTATGTCTACCAGTCGAAGCCTTTTTGAAAGATTATGATTTCAAGTTTA
TGCATTCCTTTGACAAAATTTCGGACGCACCAACTCTGTGTTAAATAATGTTAAACTCATCACAGGTCGCATC
GACATCACGACGATTGGATCGCCGTTGTCTTAATCTCTGCCTCAAGATAATCCTGTGAAATGAATGAAATTGG
AATGGATGACGGATCCACAACCTGCGGATCCAAACTTGATGTGCTATCACCGTCACCAGCGTCACCACCCTCC
GGAGGAGCGTACACCACGTTCAACGACCTCAATACCGGATTGGGAGCCACAGTTTTACTCCTGCCACGCTTTT
CATTCTTCAAAAGTAATATCCCCATGGAGTTGGCCGAGGGCGCGACGTACACCGATTTGTCCGGTTGTGGCGG
TTCGAATTGTCCCTGACCCTGATTGAGTCGGCAATAGGGGTATTCCTTCGTTATTATGGGTCACGAGATGTGT
GTATTTTGTTGCATCGACGAGAAAGGTACCACAATAGTTTTGAAATGATCCATCAGTATACCACGACGCATGA
GACACTAACAATCAGAGATGCCCATTGCAAAAAAATCACACCCTCTCATCCAACACACTTACCTGCCGTACGA
ATCCTAACTTGGCATAAAACTCCTCGCTCTTCTTCACATCAGCGCAATTCACCGCCAAAAACATCATCGGATC
GGGGCTCACCCCCACGATGGCTCGAATCGGCAATCCCGCCGGACTCACGACGTTCACCCACCCGTACGCATCC
AGCACGTTTCCCCCGTTCTTGACCATTTGACTTATGCGGTACTGCGCCACGCCGATTTGCAAATAGGCCACAT
TATCCCCCGGGGCGGGTTCATTGTTGAACTCACCCGATGACGGTTTGTAGAGTATGGCGGTAGACTTGGGATC
GTAGCGGACGTGGATGGCGGCGTGTCCTCCGTATTGGGAGAAGGATGAGACGGGGAGTTCGAAGGTGGGTGGG
ATGGAGAGAGTTTCGGGACCGAATCCTAGCCACGTATCCTTCACCGCGGCACCACCACCCCCAGTTCGTTCCC
TCAACACCTTCATACCATCAAACCCATCCAAAAAGAACTGGATCGTCTGCGCGTATTGATCCTTGTCTGTGAC
GTCAATGGTGAGGGATTGGACGATGGCATTAGCATCACTGAACTCGGGGAGGGTGCCGAGGGCAGCGTTGGCC
CGTTGGGGGAATGCGATTGTGGTCGTGGCCGCTAATGCCGTAGTGGTAACTATCGACGTCGCCAGCAGTCCTC
CTTGGTGGAGGATAGACCGTCGCGTTTCGCCGATGGACGATGATGGTGATGGTTCGTTGTCGTCAATGCCATC
ATCGCCGTCGCTAGTCCTAATCCGTGATTGAAGATGCGATGCGTGCGGCTTCAGCGGCGTTAAGGCTGACCGC
ACTGGCGCCACGGAGAAGGATGCCGCATCATTGGGAAGACAGGCGAATAGGAATGCTGCTAGAGTTGCTCTGG
CCATGGTGTGATAGTGTGCTGTCGTGAGCCTTCAGCGTTTGACAGGTTGTGCAGATCGGA
>TR98|c0_g2_i1 len=1649 path=[1838:0-703 1839:704-723 1840:724-1648] [-1, 1838, 1839, 1840, -2]
CTAGCATTGTAGGCACTTGTAGATTTTAATATCATCTGATCTATTTCCCACAGCATGAGAATGTTAATTGGTG
TCCTAACCAAACTTGATTTTTCTCGAAGATGATTTATAAATTTCTACACTGTGTGCGAAAAATTATATATCAT
TGACCTCTACTTTTGCAACAAAAGAAAGTAACCTCCCCACATGAAACTATGTTATTGCAACAAATTCTATTGC
TTTTCAACCCGATGATTGATATTGTTATGTCTACCAGTCGAAGCCTTTTTGAAAGATTATGATTTCAAGTTTA
TGCATTCCTTTGACAAAATTTCGGACGCACCAACTCTGTGTTAAATAATGTTAAACTCATCACAGGTCGCATC
GACATCACGACGATTGGATCGCCGTTGTCTTAATCTCTGCCTCAAGATAATCCTGTGAAATGAATGAAATTGG
AATGGATGACGGATCCACAACCTGCGGATCCAAACTTGATGTGCTATCACCGTCACCAGCGTCACCACCCTCC
GGAGGAGCGTACACCACGTTCAACGACCTCAATACCGGATTGGGAGCCACAGTTTTACTCCTGCCACGCTTTT
CATTCTTCAAAAGTAATATCCCCATGGAGTTGGCCGAGGGCGCGACGTACACCGATTTGTCCGGTTGTGGCGG
TTCGAATTGTCCCTGACCCTGATTGAGTCGGCAATAGGGGTATTCCTGCCGTACGAATCCTAACTTGGCATAA
AACTCCTCGCTCTTCTTCACATCAGCGCAATTCACCGCCAAAAACATCATCGGATCGGGGCTCACCCCCACGA
TGGCTCGAATCGGCAATCCCGCCGGACTCACGACGTTCACCCACCCGTACGCATCCAGCACGTTTCCCCCGTT
CTTGACCATTTGACTTATGCGGTACTGCGCCACGCCGATTTGCAAATAGGCCACATTATCCCCCGGGGCGGGT

TCATTGTTGAACTCACCCGATGACGGTTTGTAGAGTATGGCGGTAGACTTGGGATCGTAGCGGACGTGGATGG
CGGCGTGTCCTCCGTATTGGGAGAAGGATGAGACGGGGAGTTCGAAGGTGGGTGGGATGGAGAGAGTTTCGGG
ACCGAATCCTAGCCACGTATCCTTCACCGCGGCACCACCACCCCCAGTTCGTTCCCTCAACACCTTCATACCA
TCAAACCCATCCAAAAAGAACTGGATCGTCTGCGCGTATTGATCCTTGTCTGTGACGTCAATGGTGAGGGATT
GGACGATGGCATTAGCATCACTGAACTCGGGGAGGGTGCCGAGGGCAGCGTTGGCCCGTTGGGGGAATGCGAT
TGTGGTCGTGGCCGCTAATGCCGTAGTGGTAACTATCGACGTCGCCAGCAGTCCTCCTTGGTGGAGGATAGAC
CGTCGCGTTTCGCCGATGGACGATGATGGTGATGGTTCGTTGTCGTCAATGCCATCATCGCCGTCGCTAGTCC
TAATCCGTGATTGAAGATGCGATGCGTGCGGCTTCAGCGGCGTTAAGGCTGACCGCACTGGCGCCACGGAGAA
GGATGCCGCATCATTGGGAAGACAGGCGAATAGGAATGCTGCTAGAGTTGCTCTGGCCATGGTGTGATAGTGT
GCTGTCGTGAGCCTTCAGCGTTTGACAGGTTGTGCAGATCGGA
>TR99|c0_g1_i1 len=701 path=[732:0-550 733:551-574 735:575-615 730:616-
639 731:640-700] [-1, 732, 733, 735, 730, 731, -2]
CATCGCTTATCTTGCAGGTGACCAACCACTTGAACTTACTGTTCTGGCATTAACTGTGAACATAACCAACCCA
ATAACTTTCTATTGATTGAAAAGCCAAGGTTGGGGAGGGATGTTACGGCAGTCATTAGTCCCGAAGATACAAA
GCACAGATTGATGATGACGTATAAGAATTCAGTACTTGTACGGAGGCAGGTTCCTCCAGTTAAATCATAATTT
TGTTAGAAAAACAATAGTTGGGTACCGCCTGTTCTCCTTGCACCCTTTCCTAGAAAGAGACATACATTCCATC
TTCCAATGTCAACGCCATACATGATGCAAAATGAAACTCATTCTCACATGACCATGGAAACTGCAGCCATACC
ACCCTCCTCCCGTTCCTCCCTCCACGCAGCACTCCCAAACAACTCCCTCCTCACCTCCACCGTAATCTTCGGA
TGACTGTGCACCTTCATCAAATCCCTCAATGCGGCCTTCCCCTCCACCCCAACCTCGTTCTTATACCTTTCCA
CGAACGCCAACAGCGCCTGATGCCACAACACCGGCAGCTCGGCCCCCTCGTCCTCCCGAAACCTCACAAAGTG
CCGTATCAAACTCTCGATCACCGGCGCCGGCAACGAGTACTTCTTGTTCAAAAGCGTCTTGATGAATATCGAC
GCGGCGCCATTGTACTCCGTCATGCACGCCAGCTTGTGAATCGC
>TR100|c0_g1_i1 len=1235 path=[1263:0-96 1264:97-120 1269:121-498
1267:499-522 1268:523-1234] [-1, 1263, 1264, 1269, 1267, 1268, -2]
AACGGAGTCAAATGGTGTTTTATACAAGTTTGACTAGTATGTGCAATATATGAACCCTATCAGCCACACAAAT
CCCTTCGCCTCCAAAAAATAACCTTAAGTCTCCTTCCAATAACTCTCGTTAGACTCTCAAAGCTGACACTACT
TTACATGTACCACATCAACATTCACATTCTGTACAAGTCAAACCGTTTAGATCATGGTGCTCATAGAGCGCTA
CTTTTCTTTTTCTTCTTCTTGCTGTTCTTCTCCTTCGTTTTCTTCTCCTTCGTTTTCTTCTCTTTCGTTTTCT
TCTTCTTAGTGTTTGTCTCCTTAGTTTTCGACTCCTTGATTTTCAGCTCCTTGGTCTTCGACTCCTTACTCAA
CGCCTCTTTCATCCCCTTCCTTTTCATCGCCTTACCTTTCTTCATTTTGATATTGATCTTTTTGGCTTTCGTC
TCTTTGCTCTTGTTCTCGTTGTTTCTTTGGTCATTGTCACTGTAGGGATCACATTTATTGCGATAAGAGGACG
AAGACGGCGGTTTCGAATTTTGCTTCTGTTCGGCACCTTTTTTCTGGTTCTTAATTTCTGTCTCGAACTTGGC
CAGGTCACCGCTGCTTTCCTTCTTGTTCTTCTTCTTTTTGCTTTTCTCCGGACGAGGTGGACGGCCTTGACGC
TCGATTCTTTTCTCACCCCTCTTCCTCGAAGGAGAACTGGCAGCCGCTCCTCGACAAGCATAATCACTGCGTT
CACTCCTGCCCCGCACCACCCCTGCTAGCATCGTCCCCTCAGCTAAGGTATCAAAATCGAATGATTTAATCAA
CGGAGACCTCGACTTGATCATCTTTATCTGTGCAGTGACGTGCGCCGTGGTATCCTCAAAGGCATCCTTTAGA
TCTTCCATAATTTCCTCCTGGGGGATGCCAAAGGGCTTGAAGCTGGAAACTTTACCTAGCGGTTCTGGGGCAT
TCCTACTGACACCGCGGCGGTCGCCCTTGTCCCATTCGTCATCGGATTCCTCTTCTTCGCTCTGACTGGACTC
GGTGTAGATCGATCTTCTGCCGGAGGAACCACTACTGCTGTCGCCATCATCCTCGCTGACAGAAGATTCGTCA
TCAGTATCATCATCTTTGGTGCCTTTATGATAGTACGTGCTCTCGCCGTCACTTTCGTAGTGCGAATCCTCCT
CACTATTGGAGGTGTAATCACTTTCGAACGATCTCGTCACAGATCTCGATCGTCTGCCACGACAGAT
>TR101|c0_g1_i1 len=1679 path=[1717:0-1678] [-1, 1717, -2]
GATAGTCTTATTCGCAATAATGATGGTGTGAACGACGACGACGACGACGACGAAGACATGACCGATTGGACAG
AGGATGCTGCATCATCGTCGGTAACTTCATCCTCTGCATTGCAATGCACATGCAACCTATGTCATCAAACTTA
CATCGATTGGGAAATATCCTACGAGCAATGGGGGTTGATTGACTCGGTATTACAGCCGCTTAACTTGTGTACG
AAATGTTACATAAAGAGTCTTCCGGGAGATAGTGTCGATGCGTCCACGATTGATATTCGACAGAAGTGCAAAT
TATTGCCCGACTGTCTTTATATATTGATGGAATATGCAGGGAATACTTTGAATGAGGAAATGAATAGTCTGAA
TGATGATGATGATGCCGCCGAAACAGCGGATGATACTGCTGTCAAACGTAGATGGTCGTTATTTGCTCAATGC
GTTCAAGGTTTGTATTCAATACACGAAGAAGGATACGTGCATCGTGATATCAAACCCACCAATATACTTGTCC
AGGATGGTGTTGTAAAAATTGCGGACTTGGGCCTCGCGAGTTACCAAATCAAATCTTCCATCATTGCGTCGCC
GCCGTCATCTGATTCCGCCAATACACATCACGGGTCGGAAGGCAAGGAAAGTGCCGGCATTGGAACGCTGCTG
TACAGCGCTCCCGAGGTAGACGAAAGCCGCTACGGAAAAGCGAACGACATATACTCCATGGGGATAGTACTCA
TCGAGCTATTCAGCCACTTTACCACGGGCATGGAGAGGGTGGCAGTATTGAGTAAAATAAGAGAGGGGAAATT
CCCCTCCTCCGACGAGTGGGGAAGTCCTCGCAGTAGTACTACCGACATAGATCAAGCGATGGAGTTGGCGCGG
TCGATGGTCGATCTCGATCCGGGCCGACGGCCTTCGGCGCGGCGGATACTGGAAATCCTCGTGCGGAAGGGAT
TCATCGTGGATCCGGACTCGACGGTGTTGGCGGAGATCATCGCGCGGTTGCGGGATCGAGTCGGGACGTTGGA
ACGGAAGGTGCTTGAGAAGGAAAGGGAGTCGGAGGGGCTGCGGCGACTGTTGCGAGAGAATGGCATACATATA
CCGCAGAGTTTTTCTGTGCAGGGAGTATAGATCGACTTAAGATGGAGGTGCACGAAAGGGCGACGCTTGTTGA
CTAGATGACTTGGAATGACAAATAGGCTGTGGTTGAGAGACGAGGAATAAATCATGTCAACCTCGTCAAATGA
TTAAAAAAAAGCACAAATAGACTTTCAGGTTGACAAGTGATGCTGGATTAGGGCTTTTGTGGTAGCAATGTAA

AACGAACGGGAAAAATGATCATCAGATGGATGGAATGAAGTTGAGGGATTTTTAACCTTTTTAGTTTTTTAAA
AGTGAAAGGATGATAGGATCTCGCTCTCTCGGACATCGCGATCTCGCCTCCAAGCCCTCCGATGGCAGTATCA
ATTCTTACAGGAACCTACGTAGGAATGAAATTAAGTAACTCTCTCGGACATTGCTATCTCGGGGTTTCCTTTG
CCTGGATTTTTTGTGGCTTGTGGCGGGTTTGTGCCACCATATCGACAAGTCCATGCCCCAATTTGTATACAAA
ATCACACCTGCCTATTAAGTGACAAATTTAAAAAATAGGAGTAATCAAAATGATATCTACCCAGTTATCGTTC
>TR102|c0_g1_i1 len=988 path=[1076:0-87 1077:88-111 1082:112-155 1071:156-179 1080:180-533 1067:534-557 1081:558-572 1079:573-596 1075:597-987] [-1, 1076, 1077, 1082, 1071, 1080, 1067, 1081, 1079, 1075, -2]
TGGGAGGAGGAAATGCTGCTGCTGCTGCTGTTTTTGTTGTTGTTTCCGTCGACGATGATGATGGACTCCTACG
TCCTCTATCAAGTCGGAAAAGAGAGGATTGGTAGAATCCAATAGGAATGCAAAGGAAGATCGAACTGATTTTT
GTATGGTGTGGTGGAGAGATGTGGCGAGTTGATATCCGAGATGTGAATTTCCCGTTGCCAATATTAGGCAGTT
GCAGTGCTCTACTTCGTCTCGACCTTGGATTGTCAATTGAAAGCCAGCATTCGTGACATCATCATCATCATCA
TCATTCTTGGATATGGATGTTATCTTAGTCGATGTTTCCACGAGTTCAGCCACGTCCTCTGCTAATATCGCAT
CACATATCTTACTGCGATTACACACTGCCTGATTACTACCATCAGCATCATTAGTAGCAGCGCTCATATCCAC
AACCACGGCTCCATCCCTCTCCACACGAAATACCACACCCCGCTCTTCAAACCACGCTTGCTGCTCTACAGGT
GGAAAGTCTTTCGTCAACAACGAAGTAATCTCCTTCCTCCCTCGAGGATATCCTTCGTTTAACAATTCCCGTA
TCTTTTTCGAAGTGTCGTGTAGGATGCCCCCATTGCTACTACTATAGGTACTGCTGCTGCTACTACTGCGAAT
GGTGCGTAATACTTTGTGGGTGGATTCGTACACTTTTACTTCGATCATGGAGTTTGACAGTGATGTCGATGAC
TTTTCCAAGTTTTTGTGAAGGGTATTGACGATGGAGGAGGCGGCGAACATGCCACTCGTTCCACCGCCGATAA
TTGCGATGCGATATTTTTCATTAGATAAACACGGCGATGACGCTGGAGGTGGTGATGGCGATGGTGATGGCGG
AACGCTAAAGGCGCATACTGGATTGGTCGCCGTAAGAGCTACCAGCAGAATGAAGCCAAAATATTTGGGAGCA
ATGGTGTGCCAGCCTGCCGCGGCTGGAAGGTGCCGCTGC
>TR104|c0_g1_i1 len=1544 path=[1739:0-1094 1764:1095-1122 1768:1123-1147 1767:1148-1207 @1766@!:1208-1423 1769:1424-1430 1754:1431-1447 1755:1448-1448 1756:1449-1543] [-1, 1739, 1764, 1768, 1767, 1766, 1769, 1754, 1755, 1756, -2]
GAATGAAAGCAATGATGAAACAATCTGATTCCGATGATGATGGTGATGATGATGATGAATGGGGCGGGAAGAA
AAAGAAGGCAAAGACAACAACAACAACAACTGCAGCAGACTACATAACATCCACCATCAAATCCAATCCTGAC
GCTATCGATTCCACAAAGAACTTTGGTGCCAAAACCCAACAATTGAGTCTTGCGGAATTGAAAAGCAATACGA
CTACCATGTCTGCAGATGGGAAGAAGAAAATTGTGGAAGTGGTGGAAATGAATAGCACGGAGGTTATTGAGGG
TATTGAGAATGTGGCTGTGGTGGTGGCACATCAGGTTTTGGCGAAGCAGGGGTTTCAGCTTGATATTCCATCT
CGTGCCGCTTCGAATCAAATCTACGTTCCAGAATTGGATAGGATTGTATTGGGGGATAAACGTGGGACGAGGA
GTTTTCTCAATGTCAAGGAATCCAGAAAGGCCGCCATCACAACACGAGTCCTCCAACTCCTACACGCCGTCCT
CCAAAAACGAATCCACATTACCAAACGTGATTTATTTTATACGGATGTTAAACTATTCGTCGATCAGACCGAT
AGCGATGGAGTGCTAGATGATGTGGCCACCATGATTGGCTGCACGAGGAGTAATCTGCATGTTGTGGCGAGTG
ATAAGGGATTAGTGGTTGGACGGATTTCTTTTTTTGAGGATGGAGATTTTATTGATTGTACTAAAATGGGTGT
TGGTGGCAAGGCCATTCCGCCGTATATTGACAAGATTGAGAACATCCAGAGTGATGCTGAGTTTATTTTGTTG
GTAGAAAAAGAGGCTGCTTACATGAGAATGGCTGAAGATAGATTCTACCAAAAATATCCATGCATCGTCATAA
CCGCCAAGGGACAACCCGACGTGGCCTCGCGCATGTTCCTCTCTCGCATCGCGTCCGAGTTGCAAATCCCCGT
GTTGGCATTGGTGGATTCGGATCCATACGGGCTCAAAATTCTCTCGGTTTACATGTCGGGGAGCAAAAATATG
AGTTACGATAGTGCCAGTTTGACTACTCCAGATATTAAGTGGTTGGGGTTGCGTCCTAGTGATTTGAATAGGT
GAGAGTGGTGGATGGTCGTTTGCAAAATAGGTGGTTTGAGTATTATATATATATGTTGGTGTGTCTTAATCCT
CTCCTTTTGTTCTTTGCAGGTACGATCTTCCCGAGCAGTGCCGTTTGAAGATGACGGATAACGATATCAAAAC
TGGCAAGGAATTGCTTCAGGAATCTTTCATCATGAAAAATCCCGAATGGATGAAGGAGCTTCAGAAAATGGTG
AAGACGAAGGAGAAGGCTGAGATTCAAGCTCTTTCGTCGTTTGGCTTTCAGTATGTCACGGAGGAGTATCTTC
CTCGAAAGTTGAGGGAAGGGGATTGGATCTGAAGGATCTTTTTTTAAGAAGGGTTTAGATTCGAACATTCGTA
ATATTTGACAATTCTATGAGGAGCCACACTTTATTAATTGTTAATCTTTATAGTTTTTAGTATATACTGTGTG
AAAAAAAAAAA
>TR105|c0_g1_i1 len=545 path=[658:0-75 688:76-107 692:108-113 690:114-137 695:138-216 694:217-258 691:259-291 685:292-327 681:328-544] [-1, 658, 688, 692, 690, 695, 694, 691, 685, 681, -2]
CGTGTGGCGACCAACGCGAAGGTTCATTGCCATTGTACCAACTCACCCTTTGTAGTGAACCAGATAATGTCCA
TTTGCCCAACTGCAGATCAGCCAAAATGATAACCAGACAAAACATTATAACGCTCCTGAGCGCAACAGCCCTC
CTGTCCATCGAAATCGAAGCATTTTCACCATCACTCGTAATCAACAGACATGAACACCAACATGACTCCACCA
ACGCCTTCGGCTTCGGGTACCAATCACCAGCATCCTCCACTGTTACTACTCGACGACATACACCCACCAGCAC
ATCCATCAGCACGTCCATCATCACATCGACATCCCTCTTTGCATTCAAAAAGAAACCAAACATGCGAACCCTC
CAACGTTTCCTCGAGGTAGAATGCTGGAAACTCCCCCACCTCAAATCCATGGATCGGACCCTCAACGCCGCCG
CCGACGCCTGCAAACAAATCAATCGCATCGTCCAACGTGCCGCCACCGACGACCTCTACGGAGCGGCGGTCGA
TTCCACCACCGGGGAACAACTCGAGGAGAACGTG

>TR106|c0_g1_i1 len=768 path=[854:0-173 855:174-197 858:198-316 847:317-340 848:341-354 849:355-378 857:379-648 852:649-672 853:673-767] [-1, 854, 855, 858, 847, 848, 849, 857, 852, 853, -2]
ATAGTAAGAATAATCCATCTAGACTGCATTTTCAATTCAAGAGATTTGAAGTCTCATGTGCTTATCAAATACA
ATCTCATCTTTTGTTGTCATAAAAGGTTAACGTTGGATATTATTTGTTTCTCTCCAACCACACCACTGGTTTT
CTTGTTTATCTCGTGTCATAACTAGAGTGGCATGTTACCTACAACTCTGAGTGATGCTCAATTTCCTCCCCCT
TCCGTCTTTTCAATCCACCCACAACTGCTTCGACAACCGATTCCAACTCTCCTTCTCTCAACGTCACTCCCAC
AGCACCGAGAGACCTGGCATCCTTTACTAGAAATACATCCACGTGATGCAAGTCCAATGCCTGAGATAAATGC
CTGTAATGACTCGCTTGCGATAAGCCTTCCAATCCCATTTCGACTACTGTGGTGCCTACGTTGGCAAATGCAA
TATTTGCAAGGGACGCGCCGTGGACACCTACGACGACATCGGCGGATTGAAATAAACGAATGGTATCCATAAT
GAATTCGAGCGGAAAGCGACTTTTCCCATTCTTATCCGTCTTCTTTTCGAACAAGATCACTTCGGCATGAAGA
CCTTCCTCTCGTATCCCGTCCTTGATCCGCAACAAAAGCTCTTCCTCTTCCATCAGACTCCTCGTCGGACTCG
TCGATCTCGAGCAATATACGACTTTCAAATTCTTATTGATCGTTTCGTCCACCCCTCTCAACGCATTGACTGC
GTTCCACACACCCCGACGCATGGCACGAAGCAGCGGAG
>TR107|c0_g1_i1 len=528 path=[696:0-276 712:277-335 685:336-354 714:355-398 710:399-431 707:432-455 708:456-527] [-1, 696, 712, 685, 714, 710, 707, 708, -2]
AGGATGAGGAGGAGGATGAGTATGGAATGTCGGATAGGGAGATTAATGAGTTTGAAAGTTCGTTTGGTGTGGA
GTATGATCCTTACTATGACGAACCATACGAAGAATCAGAACTCCCAGTCGGTAAATACAAGGAAGATAAATCC
TACGGAGATCGACGATATGCCAATGGAGAAGTGTTTTACAAGGATGAAAGTACGGGAATGTTTTACAGACAGG
GTAGTCGTCCTAGACAGAAGAAGTTTTGGGATTTGAATTCTTCGTGAAGAGGAGAAAGGTAGGTTGGATGTGT
AGTGGTGGTGGTGATGGTGTGGTGTGTATTGCTAGATTGGGATATGTGCATGAATCACATTTTACCTGTTTGG
CGGCATATATATGTGTGTATTGAGTGGTTGTGAAAAACAAAGGGGTGGTGTGTGTTACCATACAGGAAATATA
TCCTTTGGGGAAAGTAATGAGTAAAAATGGCAGCTATAACCGCAATAGATTAAGAGTACACTATATTATAGGG
TGGCAGCTATATCCAAA
>TR107|c0_g2_i1 len=533 path=[702:0-276 717:277-336 715:337-355 718:356-366 713:367-403 709:404-436 690:437-460 691:461-532] [-1, 702, 717, 715, 718, 713, 709, 690, 691, -2]
AGGATGAGGAGGAGGATGAGTATGGAATGTCGGATAGGGAGATTAATGAGTTTGAAAGTTCGTTTGGTGTGGA
GTATGATCCTTACTATGACGAACCATACGAAGAATCAGAACTCCCAGTCGGTAAATACAAGGAAGATAAATCC
TACGGAGATCGACGATATGCCAATGGAGAAGTGTTTTACAAGGATGAAAGTACGGGAATGTTTTACAGACAGG
GTAGTCGTCCTAGACAGAAGAAGTTTTGGGATTTGAATTCTTCGTGAAGAGGAGAAAGATAGATTGGATGTGT
AGTGGTGGTGTGGTGTGTGGTTGTGTGTATTGCTAGATTGGGATATGTGCATGAATCACATTTTGCCTGTTTG
GGGGTATATATATATGTGTGTGTATTGAGTGGTTGTGAAAAACAAAGGGGTGGTGTGTGTTACCATACAGGAA
ATATATCCTTTGGGGAAAGTAATGAGTAAAAATGGCAGCTATAACCGCAATAGATTAAGAGTACACTATATTA
TAGGGTGGCAGCTATATCCAAA
>TR108|c0_g1_i1 len=1107 path=[1180:0-206 1181:207-230 1189:231-386 1184:387-410 1185:411-432 1186:433-448 1188:449-974 1178:975-998 1179:999-1106] [-1, 1180, 1181, 1189, 1184, 1185, 1186, 1188, 1178, 1179, -2]
AATAAATATTGCCAATGATCAATTGTTTTACGATCTATTGCGACTTCCATAGGATTCGGAGGAGGTAGCGAGA
GAAATAATCTTACTTTAGTACCAAAAAATCTCCCCGTGTGCCACCTTGCAATTATTCCTAAGTATGCCTCACT
GTTATTTTGTTTGGGCTTTTGATCTAGCATTTTGAAACGCATTCGTTTGCCTGGGCGGATGTGATTTTCGGTT
CCCTTTTCGTTTCCTCCTAATCTTGACATTGCAAGTCCACTCCGGCGGTAAATCGTGAACCGTCAGCTGATGC
ACGTCCTCTTTCGTTTTCAGCTTTTTTTGCAAATATTGCGGAGGAAGGAAACCTCTCATGTTATTGAACAGCA
ATTCGTTGACGGATCCGCTCTTTATGGTAAGTTCGAAGATGCGGTTGATCCACGCGAAATCTTTTTTTACGTC
GAGCAGGTTCTGGCATGCTTTCCATACGGCAGGCCACGTGTAGGCGTCTGGGAGCAAGTGGTTGCTGTCCTGC
AGTTCTATCAGGGCATTGACTACCAAAGAGAAGGCAGCTTGTCTGTTCTCACTGTTGCCTTCGGCCATGGCAC
ATGCAGAGAAAAAAATGTTGTATGTCCGTATGTTGGGCTTGATCCTTGACTCGCCATCTTCATAAAGTTGCTT
CATTCGATTCAAAAGTTGTTCTGCCTTTAAATGCTTCGTCGGATGCGAACTATTCTGAATGGCTTTCATGACA
ACATTAAACGTTGTAGTCGTGGGTGCGGAATCTGAATAACCCGATTCAGAATATTCACATTCTCTCTTGGATC
GTTCAATCATACGCTCTAAAATGGCCTCTACTTTCTCCAGAGAATTGGACTGGCGGCTGCTGATCCAAGCTTC
CATGGCATTCGTGTATGCGATGGCATTCGGCTTCAAATCAGTCAACCCTTTCTCTTGGTAGCCTTCCATCTTC
TCCAGCAATGCATCCGCGCGTTCTCCCGCATCGCCTCGCCTCGAATTTCGCCAAGCCAGGATACAAGTCGAAT
AGGCGATGGTATTCGGCGAAACGTTACCATCCCCCGATTCCTCCAACTCGTGCATCGTCCGCAGCAGTTTCTC
GGCCTTCGTCGC
>TR109|c0_g1_i1 len=1885 path=[1962:0-346 2003:347-649 1991:650-666 1998:667-839 1952:840-848 1953:849-881 1954:882-887 1997:888-1006 1978:1007-1007 1979:1008-1013 1980:1014-1014 1996:1015-1111 1965:1112-1120 1966:1121-1152 1967:1153-1153 1968:1154-1159 1969:1160-1160

1995:1161-1268 @2005@!:1269-1741 2002:1742-1758 1999:1759-1813 1958:1814-1884] [-1, 1962, 2003, 1991, 1998, 1952, 1953, 1954, 1997, 1978, 1979, 1980, 1996, 1965, 1966, 1967, 1968, 1969, 1995, 2005, 2002, 1999, 1958, -2]
GAGAGCTAAGCTCCCCCCAAACTTTGCCATGCTTTTCATACCAAGTGGACAGTACGGAAGATTATAAACTCGA
GTTGGGAATAGTATAAAGAGTCGTCTCGTCAACTCCCCTATACCATCAACTTCATCTTTCCACAAAACACTCT
CTCTTGTCGGCGTTCCACTCAAACTGAACATCACCACCACCAATGAAGATCCAAGCCATCCTATCCGCCGCCC
TCGTAGCCTCGTGCGAGGCCTTCGTGTCCCTTAATAATCAGATTCCTCAAACTAGTACTCAACTCCGAGCAGA
GATCGGTGAAACTGGTGTTGCGTTCGAGAATGTAGCGCGGGAATGGCGATGCAAGGTGCGTGTACTTGCTGCT
TGAACGAAGACGATGATGGTGTGATGTTGAGCGACGCTACATTATTATTATTACTCCGATTGTAAGGGAATGT
GATAAATGTCTCAAAGGACGTATCATTATTACGCAACGTATTGCGTTACCTCGGATGCCTGAAAAAGAGACCT
ACAAAACATCGTCTACAACCGAGCTGGCAGGTCTTCATATGGTTATATATCTTCGTATCATTTACCTCGTCTT
TGATCACACGCACACTATTTACTCGATAAGGGAAACCATACTGATGAAACACATTTTCCAAAAAAAATTACAT
GGATTTTCGCCTGGGAGATGAAAGATAGTTTCAAAATTGATTTTTATACTCTGTACTTGAACTGGAGAGAATG
GTCTAGTGGGTGGTAATGCGAGGTGACTATTATACTGCCAACTGAAACATTCAAATAAACAATGATGCTGTGC
TACCGTATGCCCTGATGAGGAACAAATTTACTGCTGAAACTTGTTCTAAACAAGTGATGTGGCTTTAGAAACT
TGACCTGAAGGACGTTATATTTTGCTGCCCGCTGTCAAGTGTAGCTGCTTTCCAACTGTGGTTTTGAGATATA
TCTAACGAGGAACAAATTTACTGCAGGAAACGAGAAATGTGGCTTTAGAAACTTGACCTGAAGGATATCCGTA
TTGTGCTGCCTGCTGTTGAGTAGAACTGCTTTCCCAGCGAAGCAACTATGTTTTTGTGACATATCTAATGAGG
AACAAATTTACTGCTGAAACTTGTTCCAAACAAGTAATGTGGCTTTAGAAACTTGACCTGAAGGATGTCAATA
TTATTTCCATGCTTTCTCTGTTCATTTTGCTGTTCCTTCTGACTCGTACCGTGCTACTCCTACATCAAAAAAA
CAAACAGTACTCCCCTGGCGAATCCGGAGGACCGGGTGACTCCGAATCACTCAAAGCCTGCCAATCCCTCCTC
GACGAATACCTACCGAAACTAAAGGCACTTCCGGGCGCCCAGGTGACGCGCCAAGTTTGCGGCGGCTGTTTGG
ACTTCAAGGTCAGCATCACCCAACCGCTCGAGGAGCACGGGGCGTGGGCCGAGGCGAATTACGAACCGTTGGA
GAGCGAGTTTATGGAGAAGTTGAAGGGGATTGAGGGGACGAGTTTGCATGAGACGCAGGAGATTACGTTTGAG
GCCCTGTGAGATGGATGGTATGCAATTATTGTGTTGAGATGTGGAGTTTAGGACGGGGGGTGAATAGGCCGGA
GAAGAAATTCATATCAATGATTAACGTTTCTCCCAAGGAAGAAGAGCAATGGATTTGTCTAGGGATCGGTTGA
TCAGACCGCCATAATGAAAATAACAGCTACGAAGAAATCTAGATGTTCATGTGGAGCAAAAAATAATTAATGC
CATGTTTCAGTTGCTGCGGGAAACACTCTGTACTAGGAGTAGATAGCATCGTTTTATTTTCACTCATGAAAAA
CCTTCTAATGTATCTAGGAGTGTGCAATTATGATATTATTACGAGATAATAATATGACAC
>TR109|c0_g1_i2 len=984 path=[1971:0-346 1972:347-367 @2005@!:368-840 2002:841-857 1999:858-912 1958:913-983] [-1, 1971, 1972, 2005, 2002, 1999, 1958, -2]
GAGAGCTAAGCTCCCCCCAAACTTTGCCATGCTTTTCATACCAAGTGGACAGTACGGAAGATTATAAACTCGA
GTTGGGAATAGTATAAAGAGTCGTCTCGTCAACTCCCCTATACCATCAACTTCATCTTTCCACAAAACACTCT
CTCTTGTCGGCGTTCCACTCAAACTGAACATCACCACCACCAATGAAGATCCAAGCCATCCTATCCGCCGCCC
TCGTAGCCTCGTGCGAGGCCTTCGTGTCCCTTAATAATCAGATTCCTCAAACTAGTACTCAACTCCGAGCAGA
GATCGGTGAAACTGGTGTTGCGTTCGAGAATGTAGCGCGGGAATGGCGATGCAAGTACTCCCCTGGCGAATCC
GGAGGACCGGGTGACTCCGAATCACTCAAAGCCTGCCAATCCCTCCTCGACGAATACCTACCGAAACTAAAGG
CACTTCCGGGCGCCCAGGTGACGCGCCAAGTTTGCGGCGGCTGTTTGGACTTCAAGGTCAGCATCACCCAACC
GCTCGAGGAGCACGGGGCGTGGGCCGAGGCGAATTACGAACCGTTGGAGAGCGAGTTTATGGAGAAGTTGAAG
GGGATTGAGGGGACGAGTTTGCATGAGACGCAGGAGATTACGTTTGAGGCCCTGTGAGATGGATGGTATGCAA
TTATTGTGTTGAGATGTGGAGTTTAGGACGGGGGGTGAATAGGCCGGAGAAGAAATTCATATCAATGATTAAC
GTTTCTCCCAAGGAAGAAGAGCAATGGATTTGTCTAGGGATCGGTTGATCAGACCGCCATAATGAAAATAACA
GCTACGAAGAAATCTAGATGTTCATGTGGAGCAAAAAATAATTAATGCCATGTTTCAGTTGCTGCGGGAAACA
CTCTGTACTAGGAGTAGATAGCATCGTTTTATTTTCACTCATGAAAAACCTTCTAATGTATCTAGGAGTGTGC
AATTATGATATTATTACGAGATAATAATATGACAC
>TR110|c0_g1_i1 len=1769 path=[1903:0-206 1904:207-230 1913:231-356 1893:357-380 1916:381-586 1912:587-677 1909:678-701 1914:702-760 1896:761-784 1911:785-1431 1899:1432-1455 1900:1456-1768] [-1, 1903, 1904, 1913, 1893, 1916, 1912, 1909, 1914, 1896, 1911, 1899, 1900, -2]
AAGAAAGAGATGCGTTGCTAAAGTAAATTTTATCTTCCTACATTCTTTTTCAACTGTCCTCACCATCAATTTG
TACGAGCAAACCCAAAGCTTTCTTTCTAAGCTCTAACAAGCAAGCTCTTTCCTCGAGAGAAATTAACATCCCA
TACTTGAACTTTTGAAGAATGCCAGCAAATTTACCTTCATGACATGTCATTATGTTCGACATCACATTGGCAT
CTGCCACTTCGTTATCACCCCAGTAAAACAACTCGCTCTTTTGACTATCGCACTCCGACCGGTAGAAACCTCC
CAAGAAATCCAAAAATTCTCGAGTTTGTCCAGGCCGTTTGATATCCGTCGGCAGATGTGCCGAAGACACTCTA
TACGTATTCAATTTCTCCTTCAAAGCATCGACTATAGCTCGCAGCACAAGCTTGCCGAGGAAGTAATCTTCCA
GTACTTCCTTCCCCAGCAACACCACCTCCGTGGTCGATTCATCGAGGAGTTCGCCAATGGCATCATCGGGCAG
GAAATGCATGCAGCACACGGTGATCAATTCGTCCGAGAGAGATCCACCAAAGGGAATTACAAGTTCTTCCGAA
AATGGCTTCAACGACTCCCATCTCGAACCGGAATCCTCGTCTAATTTGGAAATCTCCCAATGTTCCCATCCGT

CGTCTACCATCCCAGACTGGTCCATGAATTTACGTAGCGCGTCGGGGTAGGAAGAGCTGGCAAGTCGCTTGCA
TGCGCGGGAAATTTCTTTCTTTGTGAGTATCGCCGGCGTAATGTTGTTACAGAGATTGGCAAAATGACGACCT
CCTTGATTGTTGTTTCCAATCGCACCGTCGTTCTTTCGGTAGTAGCCCAATATCCTCTTCCCAGCCTCATTGA
CATCTACAAATCCAAATGTTTGCAACAATTGAGCAGAGTTCAAGGAACTTGATTCTTCCTTCGCAACTTCTTC
CCGACACGCTGCAGTGGATGCACCACCATCGGCTGTAGGTGTAGCTGCTGCCGTAGAAGACGACGCCGATCCT
GAATCGTAGGAGTGACAAACCTCCTCCTCCATCCCAATATCTCGCTCCGCCACCATCAGGAACGATCCATCCC
CGCCATAATCTCGACGCAACGTCGTCACGTGTTTCTCAGAGCCTCTTGGCGAGTGGTTCAGAAGATCAATGTA
GGGAAGGAGATATGGTCCTTGGTAGTCCTCGTTGTTGTTTGCGTTTGTATCATCCTCATTTTTGTCGTTTTTT
GTCGTTGCAGATGGCGGAGACTGCATGTGGAAGGCTCTCGTCGAGATGCACATGCATCCCTCGCGGAAGAGAG
GATACATCCTTTGGGCCTCGTGATAATTTTTGACATTCTCGTCGTCCTTCTTGTTGCTGTGGGATGTCGTTGT
CGTCGTCGTGTCCATCTTTCGCCGTTTTGGACGTTGAGCGTCGTAATTGCCTTCTTCGCCATTGTCCCTGCAA
TTCGATTCGTCCATTGTAGCCATTGTCGTCGCTGCATCCTTATCATACTTATCATCACCATCATCCCCATTCC
TCTCATTATTGTCATCACCTCCCAAGAAGAATCCATTACCTTTCAAGAATGTCAAATAGGGAACAACGGTTTG
CAAGAACCTTTCGTGCAATGCCCTTTCGTGTTGGTCCTCCTCCGTGATGGTATTCTTTATCGCAACACTGCTG
CCACCACCACCATCACGATGATCACAGCCATCAACAACAACATCATCATCATCATCATCATTATCAAAACCGA
CTGCATTGGAATTGGCC
>TR111|c0_g1_i1 len=691 path=[2075:0-89 2076:90-113 2099:114-225
2070:226-248 @2097@!:249-600 2081:601-690] [-1, 2075, 2076, 2099, 2070,
2097, 2081, -2]
TTTCCTCAGTTGATGGAGGAGATGTGGTAGACTCAATTGGGCTTTCAGTAGAGTCTGGGCTCTGCGTTTCCTC
CCCAAAGGCTGGCTGAATAGTTGGAGTCTGTGATGCAGTTGGCCCTGGTCCCAAAACAAATGTGGTTGACTCA
GATATATTAAAGTCTAGACTTGATTGCACAAGGATTGTGTTCCATTCAATGCCTATGTAATAACCCGAAGCAC
CTGCGAGTTGGCTCGCATCCTCTCCTGAGTTGTATAATGTGAATGTGAATTGTCCATCTGGTAAGCACGCAAA
CTCTCTATAGAGACCGTAGACCTTGTCATAAGGTCCACCGTCGACCACAGTCTCGCCATCTTCGTTAGTCACA
TTCCACGTGATTCCCTGGGGGTTCTCATCTGTGACAAGAATCAAGAAAAAGTCACCAGAACAAACAGGGTGTC
CGTCAATGACACTCATTTCATCATTAAGTTCTCGTTGATAGAGAGGCAAGCGAAACTTCACGATGTCAACATC
CCAGAAGTCAGTCATGAACCCTATTGGGAGCCCATCGATAAAAATGAAGTATCCGCAAGTTGTGCATTCCCCA
AACCCATTGCCTCCACTGAAACGAATTGCAAAAAATATTGAGAATGAAGAAAATATCTGGTGGAGAAGTGTTT
ACCAAATATAGCGGCTCACCTATTGGTTATATTG
>TR111|c0_g1_i2 len=1763 path=[2075:0-89 2076:90-113 2099:114-225
2070:226-248 @2097@!:249-600 2093:601-1183 2073:1184-1207 2095:1208-1318
2086:1319-1342 2092:1343-1503 2083:1504-1527 2084:1528-1762] [-1, 2075,
2076, 2099, 2070, 2097, 2093, 2073, 2095, 2086, 2092, 2083, 2084, -2]
TTTCCTCAGTTGATGGAGGAGATGTGGTAGACTCAATTGGGCTTTCAGTAGAGTCTGGGCTCTGCGTTTCCTC
CCCAAAGGCTGGCTGAATAGTTGGAGTCTGTGATGCAGTTGGCCCTGGTCCCAAAACAAATGTGGTTGACTCA
GATATATTAAAGTCTAGACTTGATTGCACAAGGATTGTGTTCCATTCAATGCCTATGTAATAACCCGAAGCAC
CTGCGAGTTGGCTCGCATCCTCTCCTGAGTTGTATAATGTGAATGTGAATTGTCCATCTGGTAAGCACGCAAA
CTCTCTATAGAGACCGTAGACCTTGTCATAAGGTCCACCGTCGACCACAGTCTCGCCATCTTCGTTAGTCACA
TTCCACGTGATTCCCTGGGGGTTCTCATCTGTGACAAGAATCAAGAAAAAGTCACCAGAACAAACAGGGTGTC
CGTCAATGACACTCATTTCATCATTAAGTTCTCGTTGATAGAGAGGCAAGCGAAACTTCACGATGTCAACATC
CCAGAAGTCAGTCATGAACCCTATTGGGAGCCCATCGATAAAAATGAAGTATCCGCAAGTTGTGCATTCCCCA
AACCCATTGCCTCCACTATTGGTTATATTGAATACGTAGTCATCTTGTTCGATACATTCTGCATCATACTGTA
CAACGGCATTTTCCTTAAACTGACCACCCTGTAGAATCACCTCCCGAGATCCTTCCTTGTATAGCTCCCAAGA
CGTTACATCATCCTCCAACCCTTCCGTGTTTGCACCTGTGGTCATTATTACAACCCGGATGTCATAGCAGATC
GTTGGGGCTGAGCTTGAGGTAGGAGTATCACTCGGAGCGACTGACGCCGATGGTACTGAACTGGATGTGGGGA
CTGGTGAAGGAGCAAATGTTGACGTGGGTGCTGATGACGTAGGGATTACAATGGTGGGTCGAACCGAAGCGCT
TGGTGTAGCGCTGGGTGCAATAGACGAGGACGGCACCTGCGTCGGCATTTGTTGCATGCAATCGGCGAAGTCC
ATCGAGTACACCTCGTTGTCATACCTGATGGCAATCGCATTCCGGTCAGACATTTGGAAGCTATTGAACGAAT
CCTCCAACTGAAATATTTCTTCAAAATAACCATTTTCTCTTTGAGTAAACACGAAGGTGTGGTCACCTCCACC
AATGACTAGTACATCCTGGTGCATAGCGAGTTCCCAACCAAAATTATCCTCGTAATCAGAAGCATTAAAATGC
TGCAGAAAAGCGAATGTTTGGTTGATCTCGTGACCGCCACCACTTCTTTGGTAGACAACAACCTCTTTAAAGG
TCCAAATATTATCATCACTGCTGAATGTTCTAAGCGAGTAAACGAGGTGCTCTTCGCTCATGTGCATATCGAA
AACTTGCGACGTGGTCCCAGTCGCCATGATTTGCTTCATGGTAGAACCATACTCTTTTTTCTCGGGATCGATC
ACCAACGCATCTTGCATCGGCACGACTTGATTTCCACCCCGATCATATCGGTAGAAATGAATCTTAGAAAACA
CCGGATCCAACGCGACGATGGTATCGCCCCTCAAAAAGCAACTGCTCATCTCGTAGCCTACATCAAACCGAAG
CCGCTGGCGCCACAATGACTCGCCACCGGCGTTGGTATATTTCCGAAAGACGTAAAGGGCCTTCTCGTTGAAC
GCGCCGATCACCGCCAGATCGCCGTACGCGTCCATCCTGTGACCAAACGAAGCCCCTCGACGGCCCCCGTCGA
GATCGGAAGAG
>TR111|c0_g1_i3 len=1878 path=[2075:0-89 2076:90-113 2099:114-225
2098:226-363 @2097@!:364-715 2093:716-1298 2073:1299-1322 2095:1323-1433

2086:1434-1457 2092:1458-1618 2083:1619-1642 2084:1643-1877] [-1, 2075, 2076, 2099, 2098, 2097, 2093, 2073, 2095, 2086, 2092, 2083, 2084, -2]
TTTCCTCAGTTGATGGAGGAGATGTGGTAGACTCAATTGGGCTTTCAGTAGAGTCTGGGCTCTGCGTTTCCTC
CCCAAAGGCTGGCTGAATAGTTGGAGTCTGTGATGCAGTTGGCCCTGGTCCCAAAACAAATGTGGTTGACTCA
GATATATTAAAGTCTAGACTTGATTGCACAAGGATTGTGTTCCATTCAATGCCTATGTAATAACCCGAAGCAC
CTGCGAGCTAGAGACGCATTGAAGGTGAAAGGGCATACGTGGTTTTCAAAGAAAGTCAGCACGGGGAGGGTTG
GCAATCGATGATTATTTCCGACATAGTATTGCTACAATATGAAACTCACTTGGCTCGCATCCTCTCCTGAGTT
GTATAATGTGAATGTGAATTGTCCATCTGGTAAGCACGCAAACTCTCTATAGAGACCGTAGACCTTGTCATAA
GGTCCACCGTCGACCACAGTCTCGCCATCTTCGTTAGTCACATTCCACGTGATTCCCTGGGGGTTCTCATCTG
TGACAAGAATCAAGAAAAAGTCACCAGAACAAACAGGGTGTCCGTCAATGACACTCATTTCATCATTAAGTTC
TCGTTGATAGAGAGGCAAGCGAAACTTCACGATGTCAACATCCCAGAAGTCAGTCATGAACCCTATTGGGAGC
CCATCGATAAAAATGAAGTATCCGCAAGTTGTGCATTCCCCAAACCCATTGCCTCCACTATTGGTTATATTGA
ATACGTAGTCATCTTGTTCGATACATTCTGCATCATACTGTACAACGGCATTTTCCTTAAACTGACCACCCTG
TAGAATCACCTCCCGAGATCCTTCCTTGTATAGCTCCCAAGACGTTACATCATCCTCCAACCCTTCCGTGTTT
GCACCTGTGGTCATTATTACAACCCGGATGTCATAGCAGATCGTTGGGGCTGAGCTTGAGGTAGGAGTATCAC
TCGGAGCGACTGACGCCGATGGTACTGAACTGGATGTGGGGACTGGTGAAGGAGCAAATGTTGACGTGGGTGC
TGATGACGTAGGGATTACAATGGTGGGTCGAACCGAAGCGCTTGGTGTAGCGCTGGGTGCAATAGACGAGGAC
GGCACCTGCGTCGGCATTTGTTGCATGCAATCGGCGAAGTCCATCGAGTACACCTCGTTGTCATACCTGATGG
CAATCGCATTCCGGTCAGACATTTGGAAGCTATTGAACGAATCCTCCAACTGAAATATTTCTTCAAAATAACC
ATTTTCTCTTTGAGTAAACACGAAGGTGTGGTCACCTCCACCAATGACTAGTACATCCTGGTGCATAGCGAGT
TCCCAACCAAAATTATCCTCGTAATCAGAAGCATTAAAATGCTGCAGAAAAGCGAATGTTTGGTTGATCTCGT
GACCGCCACCACTTCTTTGGTAGACAACAACCTCTTTAAAGGTCCAAATATTATCATCACTGCTGAATGTTCT
AAGCGAGTAAACGAGGTGCTCTTCGCTCATGTGCATATCGAAAACTTGCGACGTGGTCCCAGTCGCCATGATT
TGCTTCATGGTAGAACCATACTCTTTTTTCTCGGGATCGATCACCAACGCATCTTGCATCGGCACGACTTGAT
TTCCACCCCGATCATATCGGTAGAAATGAATCTTAGAAAACACCGGATCCAACGCGACGATGGTATCGCCCCT
CAAAAAGCAACTGCTCATCTCGTAGCCTACATCAAACCGAAGCCGCTGGCGCCACAATGACTCGCCACCGGCG
TTGGTATATTTCCGAAAGACGTAAAGGGCCTTCTCGTTGAACGCGCCGATCACCGCCAGATCGCCGTACGCGT
CCATCCTGTGACCAAACGAAGCCCCTCGACGGCCCCCGTCGAGATCGGAAGAG
>TR111|c0_g1_i4 len=806 path=[2075:0-89 2076:90-113 2099:114-225 2098:226-363 @2097@!:364-715 2081:716-805] [-1, 2075, 2076, 2099, 2098, 2097, 2081, -2]
TTTCCTCAGTTGATGGAGGAGATGTGGTAGACTCAATTGGGCTTTCAGTAGAGTCTGGGCTCTGCGTTTCCTC
CCCAAAGGCTGGCTGAATAGTTGGAGTCTGTGATGCAGTTGGCCCTGGTCCCAAAACAAATGTGGTTGACTCA
GATATATTAAAGTCTAGACTTGATTGCACAAGGATTGTGTTCCATTCAATGCCTATGTAATAACCCGAAGCAC
CTGCGAGCTAGAGACGCATTGAAGGTGAAAGGGCATACGTGGTTTTCAAAGAAAGTCAGCACGGGGAGGGTTG
GCAATCGATGATTATTTCCGACATAGTATTGCTACAATATGAAACTCACTTGGCTCGCATCCTCTCCTGAGTT
GTATAATGTGAATGTGAATTGTCCATCTGGTAAGCACGCAAACTCTCTATAGAGACCGTAGACCTTGTCATAA
GGTCCACCGTCGACCACAGTCTCGCCATCTTCGTTAGTCACATTCCACGTGATTCCCTGGGGGTTCTCATCTG
TGACAAGAATCAAGAAAAAGTCACCAGAACAAACAGGGTGTCCGTCAATGACACTCATTTCATCATTAAGTTC
TCGTTGATAGAGAGGCAAGCGAAACTTCACGATGTCAACATCCCAGAAGTCAGTCATGAACCCTATTGGGAGC
CCATCGATAAAAATGAAGTATCCGCAAGTTGTGCATTCCCCAAACCCATTGCCTCCACTGAAACGAATTGCAA
AAAATATTGAGAATGAAGAAAATATCTGGTGGAGAAGTGTTTACCAAATATAGCGGCTCACCTATTGGTTATA
TTG
>TR112|c0_g1_i1 len=1021 path=[1085:0-698 1086:699-717 @1100@!:718-812 1098:813-878 1094:879-1020] [-1, 1085, 1086, 1100, 1098, 1094, -2]
CTTCCGATCTGGGGCGTTCGGCGGCGGGTTTCTTTTTGCCCTTCTTTTTCTTCTTCTTTTTGGAGGACGTCGC
GGCGGCGGTCGTCGCCTTGGCGTCGACGTCGTCGTCCCCCTTCTTCTCCTCCTCCTTCTCGTTGGCGGTTCCC
TGGTCGTCGGCCTGCTCGTCCCCTTTCAAACCGAACTCTCCCAGCAAATCGTCCAAATCGTCCAATGCCGCCT
CCGCCCTCTTCTTCTCCAGAGCCTTCTTCTCCTTTTTGCTCATGGTGGCTAATTTATGCTGTTCGGCCTTGCG
TCGTTTTTCCTCTTCCTCTTTGGCGAGTCGGAGGGATTCGAGTCGGGCGGCTTTTTGAGCGGGCGTTTCGATT
TCGACGTCGTCGGAGGAGCGTTCGGATTCGCTGGAACTGGAGGAGGAGGAGGAGGCGATGGATTGGGCGTCGT
CCTCTTCGGTTAAGGCGGGTGCTGGTGCTTCATCACGCGACAGATCGATCATTGACGTGTTGATCACACGAGA
GGGATGCGTCTGAAACTCATCATCAGAGTCCTCAGAGTCCGTATCGGCCCACCTCATACTTTTCTATTTCGAC
AATTTTTAATATAGAATATGATGTATATATCCTCGTCTCTCTCTCTCTCTTTCAATGTCCGTCTTTATTGTGT
TGTTCGATGTGGGGGGATAGAGAGTCGTTGAATTGACAAAAAAGTTTGAAAACGAAATCCGAGGAGGAGGGGG
GAGAGAGAGTGTGTCGATATTTATTTGGAAAGGGTCGTGGTACGTGTTGTTGCGCTGTGACAATGTGAAATGT
TCGAAGGCACTGACTACTTCTATTTTGATTCAGATGGGAAGATGAGCGCAGAGATGAAACGGGGGGTTGGTAA
GTAGTTGCGTTGGGTGTGGATGGCAGTGAGGACCGCGCGGATGAGACGAACAGGGGAGTGTGTGTGTTTTGAT
TGAACTCCTTTCGATTTTTGATTCAGAGGTGTCGGGAGATAGATGGAGGTGAATGCTGTCGAGAGGAGAGGA

>TR112|c1_g1_i1 len=1751 path=[1891:0-373 1892:374-397 1897:398-622 1885:623-646 1901:647-1081 1876:1082-1105 1899:1106-1136 1902:1137-1160 1903:1161-1177 1894:1178-1219 1883:1220-1750] [-1, 1891, 1892, 1897, 1885, 1901, 1876, 1899, 1902, 1903, 1894, 1883, -2]
TTTTTTTTTTTCGCGTTACTGAATGAATGCTACTAAATTTAATCATGTGTTGTATGTTGTACTTTAAAACACA
GAAGCTCTCACAAAGCAACAACATGGCTAACCTTCACATGGGGTTTAGATTCATACGCCATATCAGGGTCCTG
ATCTGCCATCTCCAGCTTCAACAACATCATATCCTCCTTTTCGTACTTGCCCTCTCCAAACTGCTTCAGCACA
CGAACGCACTTCTTATTCGGATAGACATCCATCGTCGACTTGTCCAACACCCGATTGTTCAAACTGACCGTCA
CGTTCTGATTCTGGAGGAACATGGCCTCGGCAATGCTCAACCCGCCAGGCTGCTTCCCACAGCAAAAGCACAA
CATTACCAATCCCGCCGTCATTTTCGAGGACGGCAGTTCAAAGACCAACGTCCCCTGGGTGGAATCGGACGCA
AAAATCCCCCCGCAGGCATCGAGGTGGGTGCACTTTCGTTCCCATTCGGGATCGTGCATTGCTTTGATGATGG
CGGTATCCATTTTACCTACCATTTTGTTCCATTTGTTTTTTTCGGTGAATATTTCCCAGCCGGCTTGGTTTTG
GACCGCGATTCCCGGCGTTCCGAAGGTGGGCTTCTCGTAATTGAGGCAATTGGGCGGATGGGGGGTGGAGCAG
TAGAGGGGGTCGCAGAAGAGAGGTTCTCGCATGTTGAGCGCCGGGAGAGCTCGATGCAGCGGGCGATGCCGGG
AAGGTTCGGATGGAGGTGAAATGGCCAATGATCTCAATGCTGGCGAGTTTCCATTCTCCTTATCTGTCGAACC
CAACTGCCTTCCATCCGTATCGAACCAGCGATCCAAAATATCCACGCCGGCGTTCATATCTTCCTCGATGATA
TCCAATGCCATCAACAATCCCGTCACGTAAACGTAAGCAAATGCATCCGACGCAATCTGAAACCCCAACGGCC
CCGGGTGCCAGTTCCTGAACACCGTTCCCAAGCTACCCCTCCTCGCATCGCCCTCCGGCAGCTCCTCTCCGTA
CCTAGTCGTATTGTGATATCCATCCCCCTGAAACTTCCATCCGAAGCGATTGACCCCAGCTTCCACCTCGGCA
TCGTAATCGTGTCCCCCAAAGTACAACCCCGTCTGAATGCAAAACGCATTATAACCATACACGGCATACGCCT
TGTCCAAATCAACATTGGCCCGATTGTCCCCATTGCACGTATTCTTCTTCCCCCGTGCCACCAAGTGATGCAC
CATCGGTCGACGTTCCATCCGTTGCGCCCACCGTATCAACTGCTCCCTCTGCTCCTCCGCACCATCATTCTCA
AAGTACGTCCACGAGTAATGCACCACGTCCACATCAGGACTTACATTGTGCGTAATGCAAAACACCTGGTTCT
GATGACTATCCCCGCATCCTCCCCCCTCGCCTGCATTCTGCACGATGGCGAACATGTTCGCCGCGGCGAAGAC
GGGGGATAACGTGCGTTCGAGCTGACTCTCGTAGGAATCGTAGTGACAGTTGTCGTGTCCCGCAGCGACGGAC
GAGCCGATGGTGCCGATGACGAAACGGTCTTGGGTTGGGTTGAGGATGGATCGGGCCATGGTGGTAACGAGTT
TGGTCGTGCGTTGACGTCGGTGGTGGTGGTGGGTTTTGAGTTCGGCGGTGGACATTCGGGTTGGGTCTTCGAT
TAGTTTGCCTTCTTTAACGCCCTTCTTTTTCTTCTTCTTTTTATTGTCTTTGTTGTCATTGTCCTTGTTGTC
>TR113|c0_g1_i1 len=909 path=[1137:0-31 1138:32-55 1149:56-78 1156:79-102 1157:103-181 1147:182-214 1154:215-226 1151:227-266 1146:267-688 1131:689-712 1150:713-780 1120:781-804 1145:805-852 1115:853-908] [-1, 1137, 1138, 1149, 1156, 1157, 1147, 1154, 1151, 1146, 1131, 1150, 1120, 1145, 1115, -2]
AGTAGTGCCGCAGCTAGTGGTGGCGGATCCACCGGAGTCACCTCTACCGGAGGTGCCACCGCCGTTGGCGGAT
CGAACGTATCCGCATCCACCCCGGTGGACGCCCACGACATCACTCTCACCACCACCACCACCAAGCCCGGCCA
ATCCAAGTCGATCATGACCCTCCTCTCCTCCGCACTGGGGATCGGAATCGTTGCCGCGGGCCTCGCCGTTTCC
CGACCGCGGACCGAACCCTCGAACACCAAGGAGAAAGAGCTCAACAACAAAGATCATCGACTGCAGGGGAGCA
TCCAGAAGCGCATGGATATGTTCAATCACATGATCGAGATGAGTTCTCGATCGGAGGGGAGCGGATTGAGCGG
AGTGTCGGGGGCCGTCGTTGCTCCGGAGGAGCGATCGGTTTCGTCCTCCTCTCCACGGTCAGTGGGAAGCAGC
AAGGGTGATAGGAGCGTCTCTACGAGTAAATCTGGACGCAGCGGTAGAGGGTTCGGCAGCAAGCTGAGCATTG
GTGCCAAGAGTAGATTGGCGAAAAAGCTCAGTAAGAGCATGAATAGAAGCAAGCAGAGTCATGAGCAGGAGAA
TGATAGTGTTACTGCGACATCGTCCATGTATGTGAAGACAGAGGAGGATGGCACTGCTTTGAGACCGGGGATG
ATGGTGGTGTGATGATAGGGATGATGATGATGGATGAAGTTTCTTCATCAAATATATGTTACACGGAGAAGAG
CTATACTTTGGATTTGTTGTCGTGTTTCTTTAGGTCGCTGTTAGCTTTCTTAGGGGCCATTGGATTGGCTTAC
TCGAACTTCGTAAGAGGAGTTGAGTTAGTTGACAAAATACATTAATAAAGTGAGTTATTGAGTATCTACAATA
AAGAAACCCCGAAATAAATATGTGTTTTTGTTT
>TR114|c0_g1_i1 len=1770 path=[2451:0-163 2623:164-207 2672:208-249 2668:250-273 2655:274-730 2675:731-743 2620:744-767 2654:768-787 2660:788-814 2673:815-823 2670:824-840 2666:841-864 2658:865-1011 2650:1012-1046 2633:1047-1082 2614:1083-1152 2648:1153-1165 2631:1166-1178 2612:1179-1226 2647:1227-1358 2597:1359-1382 2630:1383-1458 2669:1459-1504 2665:1505-1506 2657:1507-1530 2676:1531-1545 2677:1546-1569 2607:1570-1602 2641:1603-1603 2624:1604-1627 2604:1628-1684 2438:1685-1708 2439:1709-1769] [-1, 2451, 2623, 2672, 2668, 2655, 2675, 2620, 2654, 2660, 2673, 2670, 2666, 2658, 2650, 2633, 2614, 2648, 2631, 2612, 2647, 2597, 2630, 2669, 2665, 2657, 2676, 2677, 2607, 2641, 2624, 2604, 2438, 2439, -2]
TCTTCCGTTCTCGAATACCTTCACGCTGCTCGCTTCTTCCCGCCCATCGCCCATACAAGAGGTAGGAAGTACC
TAACAATCTAAGTAGGTTGCAGCTCGAATCTCAAATCACCCTTTTCCATTTGTTGATGGGTCCTTCGACAATG
TTGTCTTGGCATTTACAACCAAAGAACCCTATAATGGCTAGGAGGATATTGCTCGGCGTCTTCTTGCTTTTGC

TGCCATGGGTATATCTGAAATCTTTGCATAGTCAAATTATTGCTGTCACGCATAATGTTCCAGTGGGAATCAA
TGGAACAACATCAACAATACACTACAGAAGCAAAGAAGTGGCTCATTTACATGTGCAAAGTGATGTTGTTGTC
AAGCAGGGGACATCGTCAGCATCAGTATCAGCTGCAGTGAATGCAACACAAACATTTGACCAGAAGAAGAATG
GAGATTCGATAAATAATTGTAGGAGAACGTGTCCCCACTACAATAATAGAATCATCCTCCAATCAAGACAACA
AGCTGGAATGAAGGATCGCACATACATCATGCTAGCCTGGGCAAACCTGGGCATGTTCTTATGTGCCCACGTC
AAGATTCCCACCCCCAGCGTTTGGCTCGTTCCAAAGCATAACATAAATGGATCAAAAGTCCACGAGAATGTAA
CTTTTGAGAAAGATTTGCTTTATTTTGTGGATTCCAACAACCCAAACGAAATAGTCCTCAGCGATCTGATGGA
TATTGGAGGGCCAACGGAAGGATGGGGTATTGAGTTTGTGTCAAATGTTGGGAGAACGAAAGAGAAAAAGAAA
AAGTTTGACGATGGCTTTTTTCGTGCGTATGCACATACACTACAACAGCAGGAAAAGGGATCAGATGTCAAAG
CATTTCAGTGGACGATCAAAGGCAGTCATAATAATTTCTTGGGGAGGATTCATAGCGTGATCAACGAATTGCG
GGAGGCGAATATGATTGTTGATGACACTGCCCCCTTGACTTCCATTACACCCAAACAACTTCCAAAGTGGACT
AGATGGCGAGATGATACAGGATGCGTTTATGCCTCACGTGTTGAGGCAAAAAGTATTCTCACTACTGTCGATC
GCGTGATGGATCAATTGAGGCACCGTTTTGGTGCACTTGAGGAAACAGCAGGACGATTTGTTTATGGTTACCT
CCATGTGCGTCATGGAGATTCTCACCGTTGTGATAAAAATCTAGTTCAGATGGGAAAATATATTGAATGTACA
TTTGGTACGTTCTGCCAAAATAACGAGGGGCACGATTGCAAGAAAGACTTTCCGCTACTAATTGGCAGCGACG
AACGGAATGAAACATACCGCCGGGGTCTTTCCTCCTTGATAAATTCTCACCCAAACCTTCAAGCTGTCAATCT
GGACGAGGAGGTTACAAAAGAAATCAAAAGAGAAATCTCAGAAGGAGAGTCACCTCCTTACAAACTGAATAAC
TTCTGCATCTTCTTGGTTGAAAAGGTTATACTTCAAGATGCAGCCTTCTATTTGCAGCAGAATAGACAATACT
GCCCTGATTGTGAATCGGGTAATGTTGTGATCGATCCGGATGAAAGGAGGAGGTGGTGGTCTTCGTGGCTACC
CGTGAATATTAGAGAAAACGAGGACGAAGACTCAGAGCATGCGTATGATAATCTCCGTATTGTGAAAAGTTCA
AATTAAGTCTCTCAATTAATGGTTTGCTTTATTTTTATGTATTCAAGTACATTCAGAAACAAATCACTGCTAA
GATTCATTTTTGTGGCTC
>TR114|c0_g1_i2 len=512 path=[2507:0-36 2648:37-49 2631:50-62 2612:63-110 2647:111-242 2597:243-266 2630:267-342 2645:343-388 2629:389-390 2609:391-414 2643:415-429 2626:430-478 2656:479-486 2641:487-487 2624:488-511] [-1, 2507, 2648, 2631, 2612, 2647, 2597, 2630, 2645, 2629, 2609, 2643, 2626, 2656, 2641, 2624]
TGCTCTTCCGATCTCTTGAGGAAACAGCAGGACGATTTGTTTATGGTTACCTCCATGTGCGTCATGGAGATTC
TCACCGTTGTGATAAAAATCTAGTTCAGATGGGAAAATATATTGAATGTACATTTGGTACGTTCTGCCAAAAT
AACGAGGGGCACGATTGCAAGAAAGACTTTCCGCTACTAATTGGCAGCGACGAACGGAATGAAACATACCGCC
GGGGTCTTTCCTCCTTGATAAATTCTCACCCAAACCTTCAAGCTGTCAATCTGGACGAGGAGGTTACAAAAGA
AATCAAAAGAGAAATCTCAGAAGGAGAGTCACCTCCTTACAAACTGAATAATTTCTACATCTTCTTGGTTGAA
CAGGTTATACTTCAAGATGCAGCCTTCTATTTGCAGCAGAATAGACAATACTGCCCTGATTGTGATTCGGAAA
ACGTTGTGATCGATCAGAATGAAAGTAGGAGGTGGTGGTCTTCGTGGCTACCCGTGAATATTAGAGAAAACGA
G
>TR115|c0_g1_i1 len=2401 path=[2811:0-908 2812:909-927 2908:928-997 2809:998-1021 2927:1022-1087 2918:1088-1135 2929:1136-1138 2928:1139-1162 2926:1163-1207 2905:1208-1249 2925:1250-1288 2894:1289-1312 2922:1313-1627 2930:1628-1640 2831:1641-1651 2921:1652-1656 2914:1657-1867 2856:1868-1891 2902:1892-2031 2841:2032-2055 2913:2056-2084 2901:2085-2108 2870:2109-2117 2871:2118-2141 2920:2142-2207 2865:2208-2231 2911:2232-2239 2898:2240-2293 2828:2294-2400] [-1, 2811, 2812, 2908, 2809, 2927, 2918, 2929, 2928, 2926, 2905, 2925, 2894, 2922, 2930, 2831, 2921, 2914, 2856, 2902, 2841, 2913, 2901, 2870, 2871, 2920, 2865, 2911, 2898, 2828, -2]
TCGTCGAGTCCTTCCGAGTCGCCGTCGGCCGCGCCCGTCACGTCGTGCACGGAATCGATACGCTCGCAATACA
GCACGCTGGGGGTGGGGAACAGTCCCGGTGAACAGGCGCCCAATATCGCCATGGATGGAACGAATGCCGTCGT
GGTGAACAGATCAGGAGGAGTGCGATTTTATTCATTGGTCAACAGCGATGGCACTGGTGAATGGGTCGAGGTG
GATTATTTTCCCAATCTTAATCGATACCCGGTGACTCCACACGTAGCGATTTCCGGCAACGTTGCTGTAGTGG
GTTTTGTATACCAGGTGCTGGGTTTTGATATAGGAACGGGCGGCGCGTACGTGTACGAGCAGGACACTGCCAC
CGGTCGATGGAAGAGAGAGGACGTGCTGAAACCCGAACCTGGCGACAACATCGACAACTCAGCCAAATTCGGC
TGGTCTGTCGACGTTGATGCCGACCAGAAGGAACCTCTGATCGTCGTGGGCGCATACGGCGAAAACGACAAGG
CGGGGAGCGTGTACGTCTTCCAAAGGATCCGTCTCCGGCAACGTACTTACTGGTCCCGGGTCGGCAAGGTCTT
ACCAAAATTTTGCCCCTCTGGTGACTTTGAATTTAATTTTGGATATTCCGTCGCGGTCTCTGGCAATGTCATT
GCGGCGAGTACCGACTGCGATTTCATCGTGCAGCTTAGTTCGTACAATGGAACCTTGAATGGAATCGAGACAT
ACCAGAATATTCGTTACATCGATTTTAAGTTGGGTGCCATTAGCACCATTATCATGGATGAACGGTATTTAGC
ATATTCTACTGTGTTTGGTGGTGTTGCCATCTACGAACGAACGATGGGAAGTAATGATACTGATTACGAGTTC
CTCGAGCAATTAGACTTTTCCGGAAACGAAAAAATACTGCATTATCCGTTGGCAATCGATAGAGATATTTTAG
TGGTGGGCGTTGGCAATGATTATTGGATCTTCCCAAAGAAGGATAGCTTATGGAAGGATGGTGCCTTCTTCAT
CCAAAATGGAGATGTGGACGCTTTTGACATACCGTCCGTGGCAGTCAGTGGTCGGAATGTCCTGGCCGGTTCT

GGAAATCCTCACGAAGTGTATCATTACGACATTACGAACTGTGCCCATGCCATGCCGACACAGACTCCGACGT
GGTCAAGTGCGCCGACTCCATTTACGACCTGGCCTCCCTCGCTAAAACCGACGACGGAAAAGCCTACAATATC
TCCGACCACGGAACGGCCTTCAACATCTCCAACACGTTCAAATGTTCCAACGCCGTCGACTCCAATACCGACA
GCCACTTTGTCATTGAGACCCTCAACCTCTTCAGCCCCGACGCCAAACCCAACAATATCACCAACTCTGAGAC
CTACAGATTCGCCTAGTGTGAGTCCAGTCGCCTCAAGTTTAAGCCCAAGCCTCAGTCCTATAAAAAATCCAAC
ACAGTCACCTTCAGCGCCACCAACCCAGCAGGCTATATCATCGTCATCAACAGTTACACCGGCCTTATCGTGT
TATCCACTCGAAATTAGCATATTATATGACAGCTCTCCCTTTGGTGTGGGTTATGTCCTCACCAAGGTAGACA
CCACTGGTGCGCAAAGCAACATAGACACTTTTTTTGCTCCTTTTGTTTCAGAATTGGCCAATCAACTCTCTAC
TAAGTCACTTTGTCTGGAGGAGGGAAGCTACAAGTTTACTATGTATGATAGTTACGGAGATGGTCTTTGCTGT
GTCAATGGTAACGGCGGGTATGTTATTAAAAGCAACGGGGTCACTCTTGGACAGGGTGGGGAATTTAGATACA
ACGACGAGAGGTTATTTGAACTACCCGCTTCAGTCTGAGGTGGGAATTATCTATACTGCTATTATACGTTGAT
GTTATTATTTTTGGAGAAATATTTTGTATCATCTGCAGTGTCCAATATCGGAGGTACCCAAACAAACAATCGC
ATCTCTGAGCATACACGAGAACTATCCCAGAGTAACAAAAGAAGTGAAAAACATTGAGCCTGGGAATGAGACT
GTTTGGAAGCCAGAGGATGAGCTTTAGGTTCCAACTGTTATGGATGAATAGTATCAAGCTTGAGCTAGATATG
TCCTTAAATACTCAACCCAAAGCTAGCGCAGCACTGTGGATGACTTCTTCTCCTTCCTTGGTCTGTGCAATAT
TGATACCTACTACGAGTATTCCCTCTGTCTCAAAAAAGTGGGAAATCTCACGTTACATGTTAAAATAAGGTCG
ATTTCAGGGTTAATTTGGAGGATTCAGGTACCGGTTGGAAAGCTCTCATCAAGCGACGTCAAGTGATATGAGC
CTTCCATAGGAATTCATTCATTATCATATAGTGTTAAGTACACCAATGTAATAACAATTAGATCG
>TR116|c0_g1_i1 len=1636 path=[2187:0-71 2188:72-95 2274:96-408 2183:409-433 2184:434-440 2260:441-460 2287:461-510 2271:511-529 2280:530-607 2288:608-631 2289:632-641 2279:642-665 2283:666-722 2256:723-771 2277:772-780 2267:781-804 2255:805-862 2241:863-886 2242:887-920 2254:921-1007 2231:1008-1008 2232:1009-1033 2286:1034-1055 2282:1056-1079 2276:1080-1088 2265:1089-1127 2285:1128-1150 2281:1151-1174 2275:1175-1221 2204:1222-1248 2251:1249-1343 2166:1344-1367 2167:1368-1635] [-1, 2187, 2188, 2274, 2183, 2184, 2260, 2287, 2271, 2280, 2288, 2289, 2279, 2283, 2256, 2277, 2267, 2255, 2241, 2242, 2254, 2231, 2232, 2286, 2282, 2276, 2265, 2285, 2281, 2275, 2204, 2251, 2166, 2167, -2]
ATGTTTTAGACTTGCACCAGCGTCACTATGATATTTGATGACGCTGTTTTTACTATTGTGTTACTGTCACAGC
TCACAATATATTTTCGGATTGTGACATTTTTGAGACATTTTTTATCTTTGCCTTCTGGTATTGTCTTCGATCA
CCTTCGATGTGTCAGTGACGCGGTGATTCGTACTCATATATCTACCTAGACGTACCTGTCTAAAACTATGGCA
CGTACAATACACGATACCGGGTGATTTTCATTTTGCGCAATCTGCATAATAATAATATTAAGGCATGCGACAT
GGGGTATTGTACTCCTCGAGCCAAAGAACCAGCAAAGAACCAGCAAAGAACAACAGCAAAGTCAAATAACGCA
AGAGGTGAGAATGCGTGGGTTTGTTTGGATGGACCTGGTTCTGTTCGTACGGTATCGTACAGTACCTCTCTCG
GAGTAGAATAGAACAAAACTCTCTTACGGAACGCACGGGTCCGGTCTGCGCGTATTACAATATCTCTAGAGAG
AGGGTAAAATAACAACATGTCGCTCGATGATGCACGCACCGCCACCTACCTCTGCTCTTTCTTTCTGTGGAAA
ATCACGATCATCTTGCGTGTTTCAATGAAACTGCGCAGGCAAGCTCTGGCCATGACCATGCCTATGCCTCTGA
GGAGATTCCGATGACGAATGAGGAGGAAGATTTATCGTACTATTGTAGGAGTGTCTCTAGTATCACGATTTAC
CCGGGATAGAGAATGGCGCTTGCGTCTGACCCAGAGATACATTGAGTTGTAGGAGAGGAACAATACGATGGCA
TGGTAGGTACGTTTAGGAAGAGCGAGGGAGATGGAGCTTGATTCTGATTTGATCGTTGTGGTTCAAACCTCTC
TAGGTATCGTAGAAGTTCTCGTACTCCAGTGCATACTTACGATATACTTACATGATAGCGGCGAGCGTCGTCG
GATCTCCGCGTGCTTTCCAGGCTTCGTCATGGTATCTACATCAGATAGAGAGAGATGATTACACAGGTAAAGC
AATGTGTGCACTTTAGACGAAACAGATCATGGCCGAGCAACGGCACTAGCTACTACCTTGCTGCAGTACTATG
TTACTCTCTCTCTCTGAATGATGTGCATCTAATTGTGAAATGATAACCATGATATTCTTCATTAATATCTGGG
CCGGTGACGCCCAGAGCTTTTGTGCGACCTGCCCGCATGTTTCCCAGAACCATTGCGACGACCGTGCTCTCTA
TGATGACGCTCTCCATCACCATTCTTCCTTCCCTTACCAGATCGTTTCTCATCCAAAACGACGTAGTCATCCA
TGCGCCTACCATCCCCGCCCACATCGTCGATACCGTCGCCGATCCTGTCCAACGCCGACCTCCTGGAATCGTG
GTCGACGTGCCGCTTGCATTTGAACTTCGTCCCCAGCCAATCCCCGCCGCGTTCGCATTCGCGTTCGTCCTCG
TCGACCCGGCCGCTGTAACCCGGCATGGCGGCGAACGCCGCCTCCCTCTCGTCGCGCAAGTCCTCCTCGTCCT
CCGTCCGTTTCGCTCGCTTCGCCATTCGCGACGCGAGGGAATCGTCGGCGCGGCATCGCTTCGCCTCGCCGTG
GGTACCGCCTCCGTCGTGGTCGTCGTGATC
>TR116|c1_g1_i1 len=1639 path=[2223:0-187 2299:188-252 2187:253-276 2326:277-371 2310:372-398 2317:399-445 2325:446-469 2327:470-492 2297:493-531 2322:532-540 2315:541-564 2306:565-670 2321:671-710 2305:711-749 2314:750-821 2328:822-845 2329:846-854 2320:855-903 2313:904-960 2303:961-984 2291:985-994 2197:995-1018 2198:1019-1031 2302:1032-1053 2290:1054-1098 2319:1099-1115 2312:1116-1165 2288:1166-1187 2266:1188-1194 2267:1195-1215 @2311@!:1216-1374 2176:1375-1398 2300:1399-1411 2286:1412-1508 2255:1509-1528 2256:1529-1552 2257:1553-1638] [-1, 2223, 2299, 2187, 2326, 2310, 2317, 2325, 2327, 2297, 2322,

2315, 2306, 2321, 2305, 2314, 2328, 2329, 2320, 2313, 2303, 2291, 2197, 2198, 2302, 2290, 2319, 2312, 2288, 2266, 2267, 2311, 2176, 2300, 2286, 2255, 2256, 2257, -2]
GGCGAAGAGATGCCGCGCCGACGATTCCCTCGCGTCGCGAATGGCGAAGCGAGCGAAACGGACGGAGGACGAG
GAGGACTTGCGCGACGAGAGGGAGGCGGCGTTCGCCGCCATGCCGGGTTACAGCGGCCGGGTCGACGAGGACG
AACGCGAATGCGAACGCGGCGGGGATTGGCTGGGGACGAAGTTCAAATGCAAGCGGCACGTCGACCACGATTC
CAGGAGGTCGGCGTTGGACAGGATCGGCGACGGTATCGACGATGTGGGCGGGGATGGTAGGCGCATGGATGAC
TACGTCGTTTTGGATGAGAAACGATCTGGTAAGGGAAGGAAGAATGGTGATGGAGAGCGTCATCATAGAGAGC
ACGGTCGTCGCAATGGTTCTGGGAAACATGCGGGCAGGTCGCACAAAAGCTCTGGGCGTCACCGGCCCAGATA
TTAATGAAGAATATCATGGTTATCATTTCACAATTAGATGCACATCATTCAGAGAGAGAGAGTAACATAGTAC
TGCAGCAAGGTAGTAGCTAGTGCCGTTGCTTGGCCATGATCTGTTTCGTCTAAAGTGCACACATTGCTTTACC
TGTCAAATCATCTCTCTCTATCTGATGTAAGTAGATACCATGGCGAAGCCTGGAAAGCACACAGAGATCCGAC
GACTCTCGCCGCTATCATGTAAGAATATCGTAAGTATGCACTGGAGTACGAGAAATTCTACGATACCTAGAGA
GAGGTTTGAACCACAACGATCAAATCAGAATCAAGCTCCATCTCCCTCGCTCTTCCTAAACGTACCTACCATG
CCATCGTATTGTTCCTCTCCTACAACTCAATGTATCTCTGGGTCAGACGCAAGCGCCATTCTCTATCCCGGGT
AAATCGTGATACTAGAGACACTCCTACAATAGTACGATAAATCTTCCTCCTCATTCGTCATCGGAATCTCCTC
AGAGGCATAGGCATGGTCATGGCCAGAGCTTGCCTGCGCAGTTTCAGTGAAACACGCAAGATGATCGTGATTT
TCCACAGAAAAAGAAAGAGCAGAGGTAGGTGGCGGTGCGTGCATCATCGAGCGACATGTTGTTATTTTACCCT
CTCTAGAGATATTGTAATACGCGCAGACCGGACCCGTGCGTTCCGTAAGAGAGTTTTGTTCTATTCTACTCTC
CGAGAGAGGTACTGTACGATACCGTACAGAACCAGGTCCATCCAAACAAACCCACGCATTCTCACCTCTTGCG
TTATTTGACTTTGCTGTTGTTCTTTGCTGGTTCTTTGCTGGTTCTTTGGCTCGAGGAGTACAATACCCCATGT
CGCATGCCTTAATATTATTATTATGCAGATTGCGCAAAATGAAAATCACCCGGTATCGTGTATTGTACGTGCC
ATAGTTTTAGACAGGTACGTCTAGGTAGATATATGAGTACGAATCACCGCGTCACTGACACATCGAAGGTGAT
CGAAGACAATACCAGAAGGCAAAGATAAAAAATGTCTCAAAAATGTCACAATCCGAAAATATATTGTGAACTG
TGACAGTAACACAATAGTAAAAACAGCGTCATCAAATATCATAGTGACGCTGGTGCAAGTCTAAAACATTGAA
GTTTCTACGCTCACTTACTACATTCTTGACTCG
>TR116|c1_g1_i2 len=571 path=[2262:0-72 2299:73-137 2187:138-161 2326:162-256 2309:257-283 2317:284-330 2325:331-354 2327:355-377 2307:378-416 2322:417-425 2315:426-449 2306:450-555 2270:556-570] [-1, 2262, 2299, 2187, 2326, 2309, 2317, 2325, 2327, 2307, 2322, 2315, 2306, 2270, -2]
TGCTCTTCCGATCTCGAGGACGAACGCGAACGCGAACGCGGCGAGCGCGCCGGGGATTGGCTGGGGACGAAGT
TCAAATGCAAGCGGCACGTCGACCACGATTCCAGGAGGTCGGCGTTGGACAGGATCGGCGACGGTATCGACGA
TGTGGGCGGGGATGGTAGGCGCATGGATGACTACGTCGTTTTGGATGAGAAACGATCTGGTAAGGGAAGGAAG
AATGGTGATGGAGAGCGTCATCATAGAGAGCACGGTCGGCGTAATGGTTCTGGGAAACATGCGGGCAGGTCGC
ACAAAAGCTCTGGGCGTCACCGGCCCAGATATTAATGAAGAATATCATGGTTATCATTTCACAATTAGATGCA
CATCATTCAGAGACAGAGAGTAACATAGCACTGCAGCAAGGTAGTAGCTAGTGCCGTTGCTTGGCCATGATCT
GTTTCGTCTAAAGTGCACACATTGCTTTACCTGTCAAATCATCTCTCTCTATCTGATGTAAGTAGATACCATG
GCGAAGCCTGGAAAGCACACAGAGATCCGACGACTCTCGCCGCTACCATGCCATCGTATT
>TR117|c0_g1_i1 len=284 path=[262:0-283] [-1, 262, -2]
CGATTGCCGACGATAATCCACCGCCGCTACTCTGTTTTGAAATTATATCCTTGAAATCCTTCAAAAGTTTATC
ATGGGTATAGTCATCATCTCGACTGTCAGTCTTATCATCGTCGTCACCGTCGCCATCGTCATTCTCATCAAAG
CTCTCCTCCTGAATAAATCCTAAGTTGTGCACCTCATCGTCCGCATATGTGTTGATATCCTCCTCACTCAAGT
TATGTCTGGGACGGTATAGAGGACTCACTCCCCGCCTCTTGTGATCGTCCTCCTCATCGAAATGC
>TR119|c0_g1_i1 len=504 path=[482:0-503] [-1, 482, -2]
GACGCCAAACACATAAGCGTGAAGCACGCCGCCTCGTGCACCTCCAACGAGTCAGGATGGTTCAGTATCCCAT
TAATCACGGCATCGACCTCCCGTTCACCCACAGGGGCCACGAACGACGTGTCGACCGACGCGGCCAGGTTGCC
AAGGATGACGCATCCCTCCGATTGGATGGTCGGGTTCTTAAGATGGTTTTGCATCACATTCGCGACATGGGTC
AGTGTCCCTCGCGAGCGCATTGTGGATTTTGCTATGTTGTCGAACGACAGTACCTTTAGCGCCCCCAACGCCA
TGACCTGTAGCGCGTCGTCGTCCGAATGCACCAGCATGGCGTTCAGTATTGCGTCGCATCCCCCTGCTTGCGC
CACGTGCTTTCGGTCGTTCGGGTCCATGGACAGCGACCAGAGCACGCCACACGCCCGATTCTGGATTTCGGCC
GAACGCTCCCGGTAGATCTGCATCGCCTCGATGATGGCGTCGAACGTCGATTCCTCAAAAATAATC
>TR120|c0_g1_i1 len=315 path=[293:0-314] [-1, 293, -2]
TCCGATCTGTAGCGTCGTCTTACAGACATCATTTGCCAATCTTTCTAGAGACGATGATAAGTATCCATTCTTG
GCCATTTCAAACGCAGCGTTATCAATATCTTGCAAAAATTGGAGGGCGGCAAAATTCAAAAACATGCCTAATA
CGTCCACCGCATACAACATCAGCGTGACAATGATGAATAGCCAGGTGACTCCGTCGAGTGCACGGAACGTAGT
TGCCATCCAAAATTTGACCTGCGTCGCTTGAGGATGGCTTTCGATTTTCTGCCAACTCCTCTCACCCAAAACG
TCCATGGTGATCATAAGTTCTTC
>TR121|c0_g1_i1 len=565 path=[543:0-564] [-1, 543, -2]

CTCCAACTGCGGCTGCAGCTCCGACAACCGAATTATTCACCAAGGACCTCAATATACTTCGCAACCAAAATCG
TAATACGGAGGACTGCGCGACGTTATCTTTCGCAACAGAAAATGAATAGTTGATTGTAGACGGTCGTGCGCCC
AACACGTGACATATTCGTTCTTTGCTCGTTCGAAAAAGTTTAAGTAAAGAACCTGATTGTAGATATCCGGCAA
AGTGAAGTACCTGTGCACTAAATATTGTGAACCAAATATACCCCCTCAATCGCCAAATTATCCATGGCAAACA
TGGTACTATAAGGGGTGTGTAACCCAAACGCACATTCGTGCCAGTGATATCCCACCGTTGGAAGCTCAATAGG
TTGAAGCGAAAGGTGCAGTCTCGAGCTGATATGGAGATATTGGCAAGTTTCCCTTGCAAGAGTGCACTGTTGG
ACGATGCGTCAATGTTAACATCAATAGAAGAGAATTGACGAACGAGAAGATACTTTAGTACCCATTGGATGGT
TCTTTCGAGTGCTGACGATAGACGTGGCTTCTTCGCGACCTGACGATGGTCGTC
>TR122|c0_g1_i1 len=1572 path=[1550:0-1571] [-1, 1550, -2]
CCCCGTTCCCACTTAAGTACTTCCCTCTTACTCCCACTCTCCATCCTCCGCCCTGCTTGCTTGGGGTACGAAT
CCGCGTTGATATTGACAAGACGCAAGAACCAACTCTGAACCCTGAAATTGAACGAAGCATTCTTGGAACGAAG
ACCATGCCACCCCCTCCGCCCAACTTTCTCCCTGCCAACCGCCGCCGTCGACACCTCGACCCCACACGGCATT
TGAAATCTCGAGAATTATGGAATCAAGTGCGCTTCCATGCCTCAGCCTCGCAACATCCTACCAACGGTAGGGC
GTCATCGTCGTCGGACAACTCAAAGTGGGCTTACTCGTGTGCACTTTTCCACGCAATTGCTTATGAAATCCAG
CCCTTTCGGATACGACCATGCCTCCAGCGCGCCAAGATCTTCGTCTGCGAGGAAGAGGGTCTAAAACATCTCT
CTGCATCTATTTTGGAATATGCGAAATCTGTATTATCTGTTCTGAAAGAATTGTTCCAAATCTTACACAAACA
ATTCCAACAAATGGATGTGGACTCAGTCAAATCATTTTTTGTATCGAACAAATGTTTCCTCGCCAAATTCACC
ACCATACTATTCGCATTGAGAATTTATTATCACGTCCTACTTTACCTCCACGATCTCCTCCACGCTGGTCCTA
TAATTGTAATCCTCACCCTAATGACTTTACTCTACACCATCGGTCTGGGGGATAATACCGGTGCCGGAAGTGG
GATACCCTCGGCGTACAGCGTGTTCAACCGGGGCATGCGGAAGATTATGGGCACCGTCGACGGGGAGGAGCTG
GCGCGGCAATATGCTGGGGGAGCCATGATGGCCGGGATGAATAATGACAATGCACATCGTCACAATAGGAACG
ATGAGTTTGAAGATGGGAATGATATCTGGTTGGCGAACGAGGAAGAGGATACGGAGGGGGAGGAGGGAGAACG
TAATTTGGTGAATGAACGTAGAAGACGGAGGCGTTTGGAGCGGCTACAGCAACAACAGCGGAATGCTAATGAG
GGAGGGAATGGAGGTAACGATAATAATCTACCAGAGCAACCGGGGGACGAAAATGATGGCGGAGCGCCGCACC
CTAATGAAAACGAAGAAATGGGAGAAGATGGTGCACCGGAATTAGCAGAAGAAGCTCGCCCACCAACGAATGG
AACTGCCGAAGCAACGGCGACAGCGGGTGGGAGAAAATCGGGTAAGAAGGCCCGTCGACGAAACTTGGAATTG
CGTCGCGAGATCCAACGACAGCGACAGGCCGCGGCGGCCATGGGTTTTGTTGGAGGTGACATGGAGAATGCAA
ACGATGGAAGAGAGTTCGAAGCAAGAATGGCGTTGGAGTGAATTTATCAAGAGCGGGCATGTGGGTACATTGG
AACGAGGCATATTGGCATAACGAGAGGAGAACAGCCTGTTCTGATAAGTGCAACAGAATTTATCTCTTCTCGG
GTACCCCAAATATTTTTGTATGGTTGAGAACTTTCCCCTTCGTATTATCAAGAGTGGATGTGTTTTAACAATG
TAAGATAAACATCTGCTCTTGTTTTTTCTCGAAAAAAAA
>TR123|c0_g1_i1 len=639 path=[619:0-121 620:122-638] [-1, 619, 620, -2]
GGCGATTTATTCTACACCTCCACCGGATTCATGCCGGCGGGCATCGGCCCGAGGGCGAGCAGGGGAGGATCCA
GCGTACCGGGAGGAGATGCGCTCGGCGGCGATTTATTCTACACCTCCACGCTGGCAGCGTCGATCCCATTCCC
TTCGTACTTTGCCAAGTTGCGTCATAATGGAGCTCGATTGTTTGGATTTGCCAATGCTGGTACGTGTGTCGCT
ACAGGTGCTAGTTTGCTGGGAGGGCAGCAATCTTCTTCTCCCTTGCTTAGCTTGCCAGTGTGGAGCCAAGTAT
TGCAGAGTTCTCGGTTGTCTGTTGGGGGTGGCGTGTCGGCGGGCACTCCCATGGGTCGCTTTGAGGCCACGTA
TGCTGTTCCCATTCGGTATGGTCCACGAGATGCGAGAAAGCCAGTTCAGTTTGGGTTTGGTTTTTCTTTTGGA
TGATTATATAATGATAAGAAGAATTGCCAAATGCATCGGATTGAATACACGTAAAAACACAATCATGTGTATG
CAAATAAGCAGGATTAGTTTACTTCAACCTATTTCTGCTACCTAAGTAGGTGCGAAAATTAGTTTCGGTATAG
GGGGGTATATTCTCAATTGATTTAAAATAATCATCGTTTTTTGTAATTTAAAAAA
>TR124|c0_g1_i1 len=363 path=[341:0-362] [-1, 341, -2]
GAGCTTGATGTTTCAAATTTTAAGAAGCTCGCAGCATCGCTTTCGGTAGTAGTAGTAGAGGCCATACTATCCT
CTTTTTCACTGCCACTACTGGCATCAACACTAACATCTTCACTCTTGATTTCGGCGGCAAACTCCTCCAGCAA
GCCACCCAGAGACGTCGTACTAGAATCAGTCGAATCACTGTCGGTTCCCAATAGCACGACACCACCGCCGACC
AGGCGTTGTTGTTGTCTAGAATTCGCATGGGATGCTCTCGGATGTTGTGATGGTAGCAAAAACTGTTGTCGTG
TGTTTATGTTGTTTTTGTGTAGTCTGTTGTACGGTAGATGATGTCCGGCGAATCCGATTTGTGGTTGTGAT
>TR125|c0_g1_i1 len=230 path=[208:0-229] [-1, 208, -2]
CACACACCACGAGTTCATAGGCCATCCGTACTTGACCTTGGCATTGGCGCATTCGTCGGCGTCTGTTTCGGTT
TGAGTGTGACAGTCTCTGTCTCCGACGCCTCCTTCGAAACAAACATCGATTGCGTATCTGAGTTCTTCGCGGT
TGGCGAAGCATTGGTTCTCAGTAGTATCCTCCTCGTCTTTCAATTCCTGCTCGACAGATTCAGAGCCGAGACA
ATAGATCGGAA
>TR126|c0_g1_i1 len=229 path=[232:0-91 233:92-115 234:116-228] [-1, 232, 233, 234, -2]
TCCGAGGCTGCATCTGCGCCGAATTCATCATTGTCGTCGTCGTCCTCCTCATCGGATGACATGAGCTTCATCT
TTTTGGTTTGTTTCGGTTTGAGCCAGTTAGAGTTGTCATCGGTGAAGCCTGATTTGTTCTCCTCTGCGTCACT
GGAACCATCTTCTTCATCGCTGTCATCATCATCCTCGTCGGAGCTCTCGTCCTTCGTGGCGGGGGGGACAGAA
TTCTTAAGCG
>TR127|c0_g1_i1 len=253 path=[255:0-215 256:216-252] [-1, 255, 256, -2]

ACTCTATGGGAGGGAGAACGAGGTCTCCTTGATTACCGATGCATTCTGCAGAGTTTCCGCCGGAGGAGTGAAG
CCTTCTTCATTGGAGGATTCTCGGGAAGCGGGAAGAGCAGGCTTGTCAACAGCCTGACTGCTCGAGTAGATAT
CAACGAGGGATACGTCATCACGCACAAATTCGATCAAATATCGAAGGAAATGCCGCTGACGGGAGTCATCCCC
GTATTTAACGATTTGTGCCTCCTGATAGATCGGA
>TR128|c0_g1_i1 len=1336 path=[1336:0-397 1337:398-1335] [-1, 1336, 1337, -2]
AAAAACATACATTAATTGTTACGGAAACTATAGTACTAACAAGGAACTACGCTTGGACGTTTTAGCTCCATGG
TACTGCGGTTCCGTCGCTCGTTTGGGTCATTTCTGTTTGGGTCATTTCTGTTTGAGCCTCATCGTCCATTGTC
TTGTAATCGGTCATTTCTTCAATCTCGTCAAGTCCCAGCTCGCTACGAAGCACATCCTGAGTGCAATCACCTT
GCTTGATTCGGGTGTCCAAGTAAAACATTGCTTGAAACACCGACCCCATGGCAGCAAGGAAAACCACAAAGGG
AACGGATATCCAGGATGTCCCAATCTCCCATAGATCGGAATTGGTGCTCCAAAGGATTCGAATGGGGTGCAGT
GGAATGCCTGGGTATTCCCAGACAACCCAAAAACGCATCTCGTGCAGGTAGTACATCCAATTGATTGCCTCTA
CGAACTTGGTTAGTGCAATCTTGATGAGGAACCAGAGAACCGTCACCGCTCCAATGTGCGACCAAGACTTTTT
GAATGACTCTGACGATTCTTTCAAACTCGCAATCGCCTGTCCTCCTTTGACAGTTATAATCGAATAACTCATG
CAAGTCGCCATCATGAACGTGCTTGACGCGACCAAATACGGAAGTCCGAAAACCAGTTTCGACAGCCACGGGT
CATAGTAGATGGCGATGCCATACTGTTGCTCTTGCGTTTGGCCTTCGGGAGCCATGCACAGCGCGACCACAAT
CAGCGGCAGCATGAGAACCGCGCACCAAATTGCGCAGACGAGTCCGAGAGAAGGGAGATTGGGTTTAATCGCG
CGGAGAGTCTGCTGAATGCTTTCGGCGTCTCGTCCGGCATAAAGACCGGCGACGGTTCGAATGTTTGCGGCGT
TGGCGGGCAAAAAGAGAAAAGAGAAGAGGAAAAACTCCGCGACGAGACCTAGCCTCGAGAGCGTCGTGTGAAG
GCTCTGCACCTGATACGAATAGTAATCGCCGCCCATTTCGAACGACAAGTTCTCGAGGTGGGATGCGCTGATT
CCGGCGAAGACATTGCGGATACCGATGGCGGTTATGTACCAAAGAAAGATAGGGATCGCGGAGATGCCTAGCA
AATGCTTCCAACGCTGGCGAAACACGTGGATTGCCCTCTCGAGGGACGCCTTGATTCCCAGAGGCTCCAAAGG
CTCAAAGTAGGCATCTGGGACCTCAGTAGTGCTTGGCTCTGACTCCATTGCTTGTGCTGGTGGTGTTGAAGTG
AATGCAATCTTTTCGGTGTACGAAAATGTATGAACCGTACGAAACGATGTTGTGAATTTGTGAGGTGTTCGGC
GATTAATAAATCTCAATGGTGC
>TR129|c0_g1_i1 len=245 path=[248:0-174 249:175-198 250:199-244] [-1, 248, 249, 250, -2]
GCTGGTTATTGCTACCGCTGCTGCTGCTGTTGCCGTTGCCGATGCGAGGATTGCTACGGATGCTAGTGCCAAT
CGATTTGTGAATGTTTTGGCATTGTCTGATTTGAGGAACGTGTTCCATTGTCGTTGGATTGCCGCTTTGGCTG
CCATGGTGTTGCGATAAGTATATATTTGCGATCTGTTGTTTGCTTCGATCGATTTGATTGGTATCGTAGTTGG
TAGAATTGCCTGTGGGGTTCGTTGGC
>TR130|c0_g1_i1 len=1375 path=[1392:0-1179 1393:1180-1203 1397:1204-1252 1396:1253-1374] [-1, 1392, 1393, 1397, 1396, -2]
CCAATGCCAACATCGCCCATCGCCCATCGCAATCAGTTAGGCTTAGGGATCCCTGGCCCACCGCCCATCGCCA
TCGGATGCCATCGGTGGCGAAAACAACAACGAAACAATCTACAAAAATGCCTCGACGGTTCCCCCATTACTAT
CTAATCTTTGCATGTGTCCTCTTCGCTTTAACGTCCCATCGCGTATACATTGCTACAGTTGGACTCTCAAAAC
TACTCCAAAGTGCATCACCCGATGATATCTACAAGCTGGAATTCTATCCTAGATTGTCGGAGCGGATGACCTT
GGAGACCAAAATCGCATCATACCCACGAGTTGCACCGACGCTGTCCAATTTGCGCACCCTCGATGAATCGAAG
CTGCCTTATCAGTGTGGCATCATATTTTTCTACCACATTGCATGTACAGGCGGCTCTTCCATGAATAAATGGC
TGGGCAAAGAAAAAGATAACAACGAGAACGTCACGTATTGGACGCATTGGGGACGGCATGATGGAGTGCAAGA
TAAATTTATTATAGGCATGGAAAAGCAGGTCAAAGATCTTGCTCCTCAACAATGGAGGATTGTTCATGCGCAT
GGATTTAGTCTATTCCCGAACACGAGTGAACCTTATTTGTACCAGTGGAGAGAGGAGGTCGAAAGGCAAGGGT
GTGGTTTCGTGATGACCACAATGCTTCGTGATGCCATCGGACATACCATAAGTCAATCGAAAGGAATGATAAA
TCCCAATTATACTTTGGATACGTTCGTCAATCATCTGGAACCAGAAAACTACAACCAACGTGGGATCTTCAAT
ACGCAAATTGATTACCTCGTTTACAATCGTGGTCCGAGGAATCAACACAATGCTACAAAAGAAGAAAAAGTGA
GACGAGCCATGGAGTTACTACAAAGGCACTTTGATGTTGTGTTATTGAGTGACTACAAGAGATTCACGGAAAT
TATCCTCAAGGTCACTGGGTGGAATCCTGTTGGTATGCCACATGCCAACGTTTTCAAAGGGGAATTGAATTTT
ACAGAAAGGGAATTGAACAAGATAAAAACCTTGACAGAGGCCAACGGGGACGTGCTGTTTATTGATGCAGTAA
AACACATTTATTATGGGCATTTGGATTATTTGCTTGTTTGATATTGGCATACAAAATCTGTGATATCTGAATG
AAATTGCAGTGAGAGTAAATTATTCTGGGCACCTGGGTCATGTGTTTCATATGAGAGTGAGAATTGGATTCTT
GCATAGACAGATATAATGCTTTTGGACTAAATAGTTTTTCACATCGTATTCATGTCTTCATTGGAGGATGGGA
TCGGCATTATGATGACATGTATTGCTCACAAACCCACACCATGCCCAAGCGCCAAAAACAT
>TR131|c0_g1_i1 len=781 path=[809:0-350 810:351-374 815:375-664 813:665-688 814:689-780] [-1, 809, 810, 815, 813, 814, -2]
CGCGCTCCCTCACCGCCTCCACCACCCTCGCCGCGGCGCACACGATCCCGTCCACGTACCGCAGGTGATCCCT
CACGAACCGCTTGCTCCATAGATCCTGCCTCCAATCCGCGAAGAAGAGGAAGGCGTAAAAGTGCGTGAGGAGC
CTCACGCCCTTCTCCACCTTGAGGTGGATGAGCGTAGACGTTTGGAGGGTCCTGTCGTAGATGCAGAGCTTGT
TCCGATCGGCGAGCATCTCTCGCATGCGGTCGTGCATGGTGGCGTTGACGGGGGTGGGACGGCCGTTGAATTC
CTCGAGGGTGGGTTTGAACCTGCCGTCGGAGCCCTGCATGATGAATTGGAGGGTGCGGTTGAGATCGTCGACG
GATTTTTGTTCGGTGGAGGAGGGTATGGCGAGGGCGCACTCCCACGGATCCCACTCGGGGGTGTTCCCGACCG

ATCGGAGGTACTGGAAGAGATCCCGACGATTGCCGTTGCCACCGGCGCCGCCCCAGCTGGTCTGATTCCCGGG
AGGATAGACGGGTCGTCGAGTGTTAGGGTGTTTCAACCTCCCCTCCCTGGCCACCCTGACCAAAAACTCCTCC
ATGGTAATGACCTTGAACCCCCTGTGCTCGATGGAAATGGCATCCAAGTGAAAGAAATCGTTGAACGAGAAGT
GATCGTCCCTCTTCCCCTTGTCCAGCAGGTACATGCGCTGCTCGGGAGGCAAGACCAGCGTCCTTCCCATCGC
GTGGGACATGACCATCGCCGTCTCCATCGCCATGCGTATGTTGTTCCACCC
>TR133|c0_g1_i1 len=260 path=[306:0-109 323:110-136 321:137-156 319:157-
193 313:194-259] [-1, 306, 323, 321, 319, 313, -2]
AGGATAACATAGCTTAATTTTACATACTTATGAAGACTACAGTATCCGAATACAATAGCATACGAGCCTCTAG
AGTTCTACTTTGCCATACTCTCCCAGCCATGTATCGTATATCATCCATCACCACCACACTTCACTCGCATTCA
CTTATTTTCATTCCGTCTCCACTTGGCCTCCCCTCCATGAGAACTAACGCCGAAATGACCATTCGTACTCCCC
GTCAACCCCGTCATGCTGAAGCTGCTCGAACTCTTCTTGCC
>TR133|c0_g2_i1 len=260 path=[302:0-109 322:110-136 320:137-156 318:157-
193 317:194-259] [-1, 302, 322, 320, 318, 317, -2]
AGGATAACATAGCTTAATTTTACATACTTATGAAGACTACAGTATCCGAATACAATAGCATACGAGCCTCTAG
AGTTCTACTTTGCCATACTCTCCCAGCCATGTATCGTGTAACATCCATCACCACCACACTTCACTCGCATTCA
CTTATTTTCATACCGTCTCCACTTAGCCTCCCCTCCATGAGAACTAACGCCGAAATGACCATTCGTACTCCCC
GTCAACCCCGTCATGCTGAAGCTGCTCGAACTCTTCTTGCC
>TR134|c0_g2_i1 len=1590 path=[1610:0-307 1622:308-778 1621:779-1363
1615:1364-1589] [-1, 1610, 1622, 1621, 1615, -2]
CGCTGCGACGAGGTACCGTCCCGGTTCGGCGATGATGCAAACGCCGCTGGAGGGAGGGAACATTTCGTCGAGC
ATGGGGGCGATGGCCTCCGCGATTTCATTGAAGTACATGGACTGATGCTCTCTTTTGTCCTCTTGGGACACAC
CATCCTCGCTAGGTTCTTTACCCTCCGGGTCATCTTCTTCGTCGATTTCTTCGTCATCATTGTCCCCTTCCCC
TTCTTCACTGATAGATGGAATGTCTTCCAGAGCAGTAGGTTTTTTCGGTGGAAGATCTCTCTCCTCAGCTGTG
GGGTCCCCAAATGCCTGTATCAAAGAAATAATGGTTGACATGCATGAATCTACTCATCACACAAATGTTTCGA
ATTGTACCATGGCCCAGACAACTAGAAGTAAAAAACCAGCCAGCCAACAAGTGGGTAATTCAAAGTTACACCC
CAAAAAACCAAGGAGAAAACCAATCCCTGTGCCTCAGAAGTGCAACAGATATGCACATCAAGATGGGGAATGT
ATAGTATTTGAGTAGGAAAATACTTCTGAGGAGGCTCGTATCTGCATTCATTATTGGCACAGAAAAATTTTCT
CTCTCTCTCTCTCTCTACCCGCCTGCCTTTTCTCCGTTGGCAGGAGGACACAAAATACTCCGATCATCCAATC
CAAAGCGTGTGCACAACTGGTGCGCTTTGTTCAAACAAACCGGACATGCACACATCTAGTTCGATGACAAATC
AGATGCCAGCCCAACATAGAGTCTCACCTTAGCCGGATTCCACAAACTGTGCGTCTCTCCCGGAAATCCTCCC
CCAATATCCAAAATAGTCATATTAAACCCATAATCCCTCTTGGCCATTTCAAAAAGCTTCTTGCAATCCCTCA
ACGCCATCTCATACCTCGTCGCATCCCTACATCCACTCCCCACGTGAAAACTCACTCCCACGACATCCAACCC
ACAACTCTTCGCCTCGGCCAACAAAACGGGCCAATGCTGCGGAGGCGCCCCAAACTTACTCGAAAGCCTGCAC
TGACTCCCCGAATCATCCGTGATAATCCTCATCACCAATTGTATCTTCTTGCTCACGGCCGCGCACTTGCGCA
CCTCCGCGACATTATCGAACGTCATCATGCGCACGCCGCGACACACCGCATCGATCACGTGTCCCCGCGCCTT
GGTCGGGTTGGCGTACACGATCTCCGGTTTGGCGATTTCCTTCGGCAAGGAGGCGCTGATTCTCTGGACCAAT
TCGATTTCGGCGCGGGAGGCGCAGTCGAAGTTGCATCCGAGGCTCATGAGGGTGCGCACGATGGCGGGGTCGG
GGTTGCATTTGACGGCGTAGTAGGGTGCGACGCGGGGGAAGGCGGCGCGCCAGCGGGCTAGTTGGGTGGAGAC
GATGCCGAGGTCGACGATGTAGAAGGGATCTTCGATGGTGTGTTGTACTCCTGGTTCGAGGACCGAGTCGTCC
CATGGAGTGCAGTTGGATACAGCTGCCTTTTTGGTGGCGTTGGCGTTGTCGATGGAGAGGTTGCCGAGGAGGG
CTAGTGATTGTGGAGCGCGTTTGTGTAGGTGGTAGCGTGCAGCGAGGCGGAAGCCCT
>TR134|c0_g2_i2 len=1139 path=[1618:0-307 1619:308-327 1621:328-912
1615:913-1138] [-1, 1618, 1619, 1621, 1615, -2]
CGCTGCGACGAGGTACCGTCCCGGTTCGGCGATGATGCAAACGCCGCTGGAGGGAGGGAACATTTCGTCGAGC
ATGGGGGCGATGGCCTCCGCGATTTCATTGAAGTACATGGACTGATGCTCTCTTTTGTCCTCTTGGGACACAC
CATCCTCGCTAGGTTCTTTACCCTCCGGGTCATCTTCTTCGTCGATTTCTTCGTCATCATTGTCCCCTTCCCC
TTCTTCACTGATAGATGGAATGTCTTCCAGAGCAGTAGGTTTTTTCGGTGGAAGATCTCTCTCCTCAGCTGTG
GGGTCCCCAAATGCCTTAGCCGGATTCCACAAACTGTGCGTCTCTCCCGGAAATCCTCCCCCAATATCCAAAA
TAGTCATATTAAACCCATAATCCCTCTTGGCCATTTCAAAAAGCTTCTTGCAATCCCTCAACGCCATCTCATA
CCTCGTCGCATCCCTACATCCACTCCCCACGTGAAAACTCACTCCCACGACATCCAACCCACAACTCTTCGCC
TCGGCCAACAAAACGGGCCAATGCTGCGGAGGCGCCCCAAACTTACTCGAAAGCCTGCACTGACTCCCCGAAT
CATCCGTGATAATCCTCATCACCAATTGTATCTTCTTGCTCACGGCCGCGCACTTGCGCACCTCCGCGACATT
ATCGAACGTCATCATGCGCACGCCGCGACACACCGCATCGATCACGTGTCCCCGCGCCTTGGTCGGGTTGGCG
TACACGATCTCCGGTTTGGCGATTTCCTTCGGCAAGGAGGCGCTGATTCTCTGGACCAATTCGATTTCGGCGC
GGGAGGCGCAGTCGAAGTTGCATCCGAGGCTCATGAGGGTGCGCACGATGGCGGGGTCGGGGTTGCATTTGAC
GGCGTAGTAGGGTGCGACGCGGGGGAAGGCGGCGCGCCAGCGGGCTAGTTGGGTGGAGACGATGCCGAGGTCG
ACGATGTAGAAGGGATCTTCGATGGTGTGTTGTACTCCTGGTTCGAGGACCGAGTCGTCCCATGGAGTGCAGT
TGGATACAGCTGCCTTTTTGGTGGCGTTGGCGTTGTCGATGGAGAGGTTGCCGAGGAGGGCTAGTGATTGTGG
AGCGCGTTTGTGTAGGTGGTAGCGTGCAGCGAGGCGGAAGCCCT

>TR135|c0_g1_i1 len=319 path=[419:0-117 420:118-141 428:142-158 435:159-175 439:176-203 440:204-204 430:205-250 425:251-279 372:280-280 373:281-289 374:290-318] [-1, 419, 420, 428, 435, 439, 440, 430, 425, 372, 373, 374, -2]
CTGCCACATGCATATCTAATAACAGTTTTTTACCTCTCGAAAGCTACAGAACAACAGGCAGGCTCTCCAGTTA
AGTATGCTTAGTGCCTATGAACATCTCTATTTTGGTAACTACACATATACACCCCACCTCACGCACCCTTGCA
CCGTCTCCCCCCCCTCCCCCCACAGCCTTTACCCGGACGGTTACGCAGTGATCTCTTCAGCAGGGACTTCTGG
AATCTCTTCAGCAGGGAATTCTTCAGCAGGGACTTCTTGAATCTCTTGAGCAGGGAATTCTTCAGCAGGGACT
TCTTGAATCTCTTGAGCAGGGAATTCT
>TR137|c0_g1_i1 len=1014 path=[1083:0-160 1092:161-256 1081:257-272 1090:273-360 1093:361-361 1072:362-377 1073:378-1013] [-1, 1083, 1092, 1081, 1090, 1093, 1072, 1073, -2]
CTCGACTCTCCAAAATCAACCTGGAAGCGTATGCATAATATTTTCAGGTAGGGAAGGAAGATGGTACCAACGA
CAAACAAAAAAGCAACACACATACTATGCATCACATCCATCAGCAACATTTTTGTTGTTGATTCCTGTTCTCA
CTATCCTCTCTGCGTATAACAGCTCGAAAACTTCGTACCTACACACACGCCTCAAAAAACCTCTGAATTACGA
AGAACAACAACATCATGATTTTCGCAATCCATTTTTTTCGCATTTGTCATTCAAAGTATATATAATATAGTAC
AGACCTGGATTATAATCGCACAAAACCAAAACGCTCTGCACAACCAAATCAACGCTACATACAAAAAAAAAGT
GTACCACAATCAATATCCAACCCATCATCATCATCATCATCAAAAAATGATGTAAACTATATATATATATATA
CTCACTTTGAATTGGGCGCAATCGTGGGAATCGAAAACGGATTCTCATTCGGATTAATCGGAGAATCATTCGA
TGGGATCATGGAAGGTACCGTCGTGATCGGGTTACGTGTTGGCTGTATAATATGTGATAATGTGAATGATGAG
TGATGTGATATATAATGTGTGAACATGTGATTTTTCAAGTGTGTGTGTGCGTGTATGCATTGGAGAAATGTGA
TAGATGTGTTAGAATCCGAGTGTGTGTTTGCTTATTACACTAAATTGCAATGGTAGTAGGTAGAATGATCATA
TTATCCTCATAGCAAACACAAGTGTGTGGTTTAATTCTTACCTCTCCATCAATGATGATACCTTTGGTTGGAC
CAAGCGTTGGAATATCCGTTTCCTCCTCCGTACTAATTGTTGGCCCCGTAGTCGGCGTACTACTTGTTGGTCC
TGCCGTAGTTGGCTGCCCAGTTGTAGGACTTGAACTTGTGGGCTTTTTTCCCCATTCATTTGGTTTTAAAAAA
AGAAGCAAAAGACGAGTATAAAAATGTATATATCAGTTTTTCCTCTTCGTTCATTTTTAATTTGA
>TR138|c0_g1_i1 len=786 path=[873:0-96 874:97-121 880:122-208 871:209-232 881:233-371 868:372-395 879:396-568 865:569-592 866:593-785] [-1, 873, 874, 880, 871, 881, 868, 879, 865, 866, -2]
CGCTCTTCCGATCTGGCGTTCGTGCGCTCGACGTCTTCGCCGTCCACCGAAACCACCACCCAGCCGGGCCGTA
TGCCCAATTTTTCAGCGAGGGAATTCGGCGCCACGGATTTGACGACGACTCGAAAGTTGGTGCGAAAGGTTAC
CTGACCCAATTCGAGACCGAAGCCTCCTTTCATTTTCAGGATGTCCGATTTGTAAATGGTGAATAGGATCTCG
TCCGAGAGGGCCGTCGATGTTGCCGACACTCCCGTTTGCGTGGTGGTGACGCCTTGGGAGGTGGGCGGGGACT
GTTGCCTGTAGGACGCGTCCGTAAATTGATTCGCGAAGGATGCGGTTTCGTCGGTCATGGCCGCTTCGGCATT
TGGAATCCGGGACGGAGATGGGATGACGAGCGACGCCAAGGATGCGCCGGCGACTTGTTGCATGATTTTTCGC
CTCGATGTGTAAAATGAGTCGTTGTCGGCATCATTCGACGTAGAATACAGTGCATTCTGGCAATAATTTGACT
CGGCGATAGCGTGACTGGTGACGCCGCCAGCACGGAGGCAGCGTTGCGTAAGTGATGGATTTCTCACTGTCAA
GCCCAACGTAAAAACGACCGGGAGTACGGCACAGAGGAGTATTGCAACGGGATGAATCATCCCTGCTATTGTA
CGAAAGGTGAAGGTGCGGCTCAGACACAGAACAAATGACATCGAGTTCACACTAGCTCGTCTCTATTTGAGCT
TGAGCTTGGTGGTGAATAAGAAAACGGAATTCATTACGAAAGGTGAAAGATCCAAA
>TR139|c0_g1_i1 len=766 path=[1549:0-466 1586:467-601 1594:602-625 1595:626-626 1593:627-672 1590:673-688 1584:689-718 1578:719-741 1545:742-765] [-1, 1549, 1586, 1594, 1595, 1593, 1590, 1584, 1578, 1545, -2]
ACCCCCCGCCGCAGCCGCGCCCCCCACGCCGACCAAAACGGGAAAGCTCATCATCATGATCGCTCCGAGTATC
CAGAGCATGATGAGGTGTATTCCCGGCACCTTGCGCTGGAGGGCGCCGAGGCGTCGTGCCCGGGCCTGACGGA
GGGAGCGGACGGTGTCGTATACCTGGCCGGGGATGCCGACGCTGGTGAGGTAGAGTATGGCCTCGAGGGGGTC
GTCGGCGGGACGGGCGCTCAGGATGAGGGAGGGCGTTACGGATCCGTATTTGGTGTCGGATTCGTTGGAGGGG
TTGGAATCGGTTTTGAGGTAGGAGAGCGTGCCGGCGAGGAGGTCGTCCTTGACGTAGCGGGCGATGGAGTTGA
GACAGGTGGAGTACATGGTTCGGCCTTGGGAGACGAGGGCTACTTGTTCTAGCAATGACTTTGCCTCGGTCAC
CTCGTCGAACAGTGCGTAGTAGAGTGCCTTATTGTTGTATATGGTATGATTGTTGTGTATTTGGTTGATTGCA
TTGTTCATTGAATGCATTGCGCATTATATTGTAAGGTATTGGGTGCACCGAGGATTGTAATTGAGATTTGTAT
TGAAATATTTGCTCAGCTGATACTGCGACACATGATATGTCCACACGACCCATAGTAACAACGAAATCCTTCC
CACAAAACTAAAAAAAGGCAGAAATCATACCCCCACTCACCTCCTGCTGACTATACAAAAATCCAAACACCTG
CCCCATCAAAATGGAATACAACAATCCCACCAATCC
>TR139|c0_g2_i1 len=1134 path=[1573:0-466 1587:467-486 1591:487-509 1581:510-533 1585:534-677 1530:678-701 1592:702-779 1589:780-825 1583:826-873 1577:874-897 1541:898-1133] [-1, 1573, 1587, 1591, 1581, 1585, 1530, 1592, 1589, 1583, 1577, 1541, -2]

ACCCCCCGCCGCAGCCGCGCCCCCCACGCCGACCAAAACGGGAAAGCTCATCATCATGATCGCTCCGAGTATC
CAGAGCATGATGAGGTGTATTCCCGGCACCTTGCGCTGGAGGGCGCCGAGGCGTCGTGCCCGGGCCTGACGGA
GGGAGCGGACGGTGTCGTATACCTGGCCGGGGATGCCGACGCTGGTGAGGTAGAGTATGGCCTCGAGGGGGTC
GTCGGCGGGACGGGCGCTCAGGATGAGGGAGGGCGTTACGGATCCGTATTTGGTGTCGGATTCGTTGGAGGGG
TTGGAATCGGTTTTGAGGTAGGAGAGCGTGCCGGCGAGGAGGTCGTCCTTGACGTAGCGGGCGATGGAGTTGA
GACAGGTGGAGTACATGGTTCGGCCTTGGGAGACGAGGGCTACTTGTTCTAGCAATGACTTTGCCTCGGTCAC
CTCGTCGAACAGTGCGTAGTAGAGTGCCTCCTGCTGACTATACAAAAATCCAAACACCTGCCCCATCAAAATG
GAATACAACAATCCCACCAATCCAATAGCACTCGACACCACCTCGGTCAACATGCCAATCTTGGCCGGCTCGG
TCACACGCGTCACGAAGATGGCGACCCTCACGCACATGGCCGGCAATGCCACATACGCGGCAATGCCCGCGAG
CAAGGCGGGAAGGAGGACGCGTACCAGAGTTTGCAATTCGAAGAGGAAACGTTCTCGACGTGCTAGGAATGGA
TTATTGTCGCGGTCGTTGTTGTTGTTGTAGGAGGATGATGTGGCTTGGAGACGAGTGGGACTGGCATGGCCGT
TTCTCCCGACTGTCGTTGTTGTTGGTGACGTAGCTCGTCTTCGACGCGATGATGATGTTATTGATGTCGCTGA
CGTTGATGTTGATCGAATTGCGCCGTGATGACGACATGGCGACGATGGTGAAATTCCGACGGTGGGAGGCGAA
GTGATGGCCGCTGGTGAAAATGCCGCCACTCCTATAATAACGTTGGCAATTGAAAACAGGTGTAGAAAATTGA
GGAGGACCATTGCGCCATGTATGGGGTTATTTGGAGGTCCCTCTTCGATGGTTGCTGCCTACTCCTACTCCTC
TATTTGTATTTGCTATTGCAGCCGTGTGGATGTGGGGAC
>TR140|c0_g1_i1 len=1442 path=[1537:0-279 1538:280-303 1555:304-545
1556:546-561 1532:562-562 1554:563-585 1552:586-915 1550:916-1018
1528:1019-1042 1529:1043-1441] [-1, 1537, 1538, 1555, 1556, 1532, 1554,
1552, 1550, 1528, 1529, -2]
TATGTTATTATGTTGTTGATGTGCCCGCATATCATAAGTACACCAAATGAACTATCAAGCTCGAGGACCAAGC
TAATTACTCCTACGCAGCAGCAAGAAAGCCGCCGCTCAAAGCTCTTATGTACGGTATCGTAGTGGCGGCCAGC
GTCGCAGGCAGTATGAAACTATTTATGCATTTCATCCTCCAGCATTAGTGTTACAATAGTAAGTACAACTTCT
GACAAAATTAAATGTTCCAACTTAATCCATGATCATGATGAGATCCTTCGTTTTCGGCGCTGCCCTGGCGTCC
CCAATATCTACCTCGCTGGCCTTCGTTTCTCCCAGTGGCATTAATGTCAATGCCGCCGTCGAGAGATCTGCCG
TCGTATGCAACAACGGCATCCTCGACTCCGTCCGAGCGATATTCAGCAAGGACGAAACATCATCCACATCCTC
GCAACCCGATGGAAAAAGTGGAGGGGAGAAGTTTCTCATGGCACAATCCATCCTCCTTTCGCTAATTAACGAC
GAAAAGTGCTTCACCACCGAGGAGGGAGCAAAAAAGTTCGCAGCCTCCTGTACCCCTGACATCGTCTACGAGG
ATTGCTACGAACCCCAACCCATCGTCGGTCGCGGAAAGGTTGAGGAACACTTGATCAACCGAGCCTTGGCACG
TGGCGACGGCGATGTCGGATTCCGCATCGATAAAATATCCGATGGATCCACCGCCTGCGGCTTTGCGTGGACG
TGGACCTCGTCGGATATGGAGGGACTGCGCGGAACGACTTTTGTGGAATTGAATAGCGCGAATCAAATTGCTT
ACGTGCGGGAAATTCCAGAGCCGTTATATAAGCCTGGGGATTTGATTGTGGAAGTGCTGAAGGCGGCTACTGC
CGACGCAACGCCCAAGCCTCCGCCAGAGTATACCCCCAGGACTCCGAAGAAGGCGAATGAGGTTGCGAAGTAT
TTATTTAATGATGTGCAGGGGGCTGATTCTGAGGAGGGGATGCGATTCTTTGATGATTCCATCATTTACAGGG
ATTTCAACTATGAAGAAGTGCTTCGAGGAAAAGCGGAAGTGAAGAAGTTTATTGATGATTTTAGTTTCCCTGG
AATCACTTTTGCTACGCAACGATTTGATGATGGAGCCGTGTCCACCTGTTTCACCTGGGAAGTTGTGATTGAG
GGTGCACCAGAAGGAAGTGCAATTAAAGGCATAAGTTTTTATGAGGTAGATCCCGACACTGGACTGATTACAT
ATGTGAGAGATGTGCCGGAGAGCGGAATCAAACCCCCACCATTGGGAAAACTAGCCAGACAGTTCCGTCCTGC
TGTAGGAGTATTTCAGCCGGTTGCCATTGGCAGCAGAGAGGGTGGTCTTTAGGCTCCAATTTTTGTTGGCCCC
GCCCGAACAATTAATCTCGGCGCTGCTTTAAAATAAGAAAATTGAGATCGGAAGA
>TR140|c0_g1_i2 len=564 path=[1535:0-37 1550:38-140 1528:141-164
1529:165-563] [-1, 1535, 1550, 1528, 1529, -2]
GTGCTCTTCCGATCTCTCCGCCAGAGTATACCCCCAGGACTCCGAAGAAGGCGAATGAGGTTGCGAAGTATTT
ATTTAATGATGTGCAGGGGGCTGATTCTGAGGAGGGGATGCGATTCTTTGATGATTCCATCATTTACAGGGAT
TTCAACTATGAAGAAGTGCTTCGAGGAAAAGCGGAAGTGAAGAAGTTTATTGATGATTTTAGTTTCCCTGGAA
TCACTTTTGCTACGCAACGATTTGATGATGGAGCCGTGTCCACCTGTTTCACCTGGGAAGTTGTGATTGAGGG
TGCACCAGAAGGAAGTGCAATTAAAGGCATAAGTTTTTATGAGGTAGATCCCGACACTGGACTGATTACATAT
GTGAGAGATGTGCCGGAGAGCGGAATCAAACCCCCACCATTGGGAAAACTAGCCAGACAGTTCCGTCCTGCTG
TAGGAGTATTTCAGCCGGTTGCCATTGGCAGCAGAGAGGGTGGTCTTTAGGCTCCAATTTTTGTTGGCCCCGC
CCGAACAATTAATCTCGGCGCTGCTTTAAAATAAGAAAATTGAGATCGGAAGA
>TR141|c0_g1_i1 len=1187 path=[1328:0-88 1361:89-89 1359:90-113 1356:114-
203 1352:204-227 1358:228-500 1349:501-524 1360:525-669 1343:670-686
1357:687-839 1346:840-863 1354:864-923 1337:924-947 1338:948-1186] [-1,
1328, 1361, 1359, 1356, 1352, 1358, 1349, 1360, 1343, 1357, 1346, 1354,
1337, 1338, -2]
GATGACCGCGTCGTTGCGTTCCTTGAGTCCCTCGTTGGTGAATTTTTCGGATTTTACGAGGGAATACCTTCTC
TTTCGACGGCTGCCCCCGGTGCCGCCGTTGTTCGTTTCCCCGAATCCTGCATACTCCTGATTTTTCCATTGCC
GCAGCATGTCTGTGAGTAGCTGTGCGGATGCCGAAACCGACGCGTACAAGTGGGTGGTATCGTCCAATTGCGC
TTTGAGTACCACGGTTTCGTTTGCGGATCGAACGTGACGCTGTAGGGAGGCGATGAGATCGTGCGATTTTTCG
AGGTGATTGAGTTTGAGTTTGGACCATTCCGAGAGCGGAGGGTCGGGAATGGATGGGACTTTCATCGAAGAGG

ACAAGGACGTGGATTGCTTTTGTTCCGTTAAGTTATTACGTTGCTGATTTTGTTGCTCTTCGATTATTGCTGC
CTCTGCCCCCTCCTTTATTTGCTGCAATAAGTCAGGATTGATACCTGCTAACCTCTCCAAAGAAACCTTGTCT
AACTCATCAACGCCAGAGCTCTGAACCTCATCCAATAAAAGTTGCATTTGTTTTCGGAGGGATGGGCTTAATT
GCAGCGTCCCGTCCGCGTTTCTACCAATGCTCGGAGCCGCAGTCAGATCCACAGTGGGCTTTTCCTTTTCGAC
CGGCTTCAAAAAACACTGGCCGATCCGACGCCACTTCTCCTCATCCTTGATAACTCCAAATTCTCGCCACGTG
TTCCATACCTTTTTCAACCGCGTTTTCTCGTTTTCCTTATTCGCCTTATCGAACATTTCGTACACCGTTGACA
TCCAATTGGCAGCATCGTCCTGTATAATGTCAATGTAAACGCCCTTCACATTCTTGAGCAGCGAATCGAGGAC
ATACACGAGCGGAAGCTTGCGGTCGGGGGAGGAGATTCCGGGAGAGGTCCGGTCTACGAGGCGGCTGCGTATG
CAGTCGTAGATGGTGGATGCCGACGTGGGAGAGGAGGCGAAGTCTTCGGCCGCCATGGAGAGGCTGTTGATGG
CGACTTTTTCGGGGAAGTTTCCCAGACCGTCGAGCATTGCGCAGTAGTCTTCCACTTGCTCTTCGTCGATCTC
CTCTTCCTCCTCGTCCATGTTAGCATCATATTGATTGGACATTGTTGAAGAGAGAGTGTTGTCGCGTCGCTGG
TTGATATTTTTAGATCGGA
>TR142|c0_g1_i1 len=725 path=[1591:0-148 1592:149-169 1621:170-448
1567:449-472 1617:473-596 1583:597-613 1584:614-724] [-1, 1591, 1592,
1621, 1567, 1617, 1583, 1584, -2]
GTGTGCGCTTCCGATCTCTAATACTCCAACCATTCCGATTCCATCCCAATAACATCCAATAATACCTTCATCA
TGGCTGATCAACTTACCGAAGAGCAGATTGCTGAATTTAAGGAGGCCTTCTCCCTCTTTGACAAGGATGGCGA
TGGCACCATCACCACCAAGGAACTTGGAACCGTCATGCGATCTTTGGGACAGAACCCCACGGAGGCAGAGCTT
ATGGACATGATTAACGAGATCGACTCCGACGGAAACGGGACCATTGACTTCCCTGAATTCCTTACAATGATGG
CGCGTAAAATGAAGGACACGGACAGTGAGGAGGAGATCCTTGAGGCATTCAAGGTCTTCGACAAGGACGGAAA
TGGTTTCATCTCGGCCGCCGAATTGCGTCACATCATGACCAACCTGGGAGAGAAGCTTACGGATGAAGAAGTT
GATGAGATGATTCGTGAGGCTGATATTGATGGGGACGGCCAAATCAACTATGAAGAGTTCGTCAAGATGATGA
TGTCGAAATAAATCCCGTAATCCCCCTCTGTGAAAACCTTTTTGTTTTTGGTCTAGGCTGGTAAACACTTTAG
AGCAGTCTTTTTTGTCGTTGGCGCCTTAAGCACCTGCGAGGGATCTGAGAAGATTCTCGAATGGAATTGGCTG
CCCACCCTTTATATAGAACATATATTTAAACTGGACTTTGTCTACTTGAAAAAAAAAAAAAGATCGGAA
>TR142|c0_g1_i2 len=1270 path=[1603:0-148 1616:149-256 1595:257-280
1620:281-349 1570:350-373 1615:374-446 1606:447-470 1625:471-568
1614:569-592 1622:593-639 1618:640-663 1612:664-714 1621:715-993
1567:994-1017 1617:1018-1141 1583:1142-1158 1584:1159-1269] [-1, 1603,
1616, 1595, 1620, 1570, 1615, 1606, 1625, 1614, 1622, 1618, 1612, 1621,
1567, 1617, 1583, 1584, -2]
GTGTGCGCTTCCGATCTCTAATACTCCAACCATTCCGATTCCATCCCAATAACATCCAATAATACCTTCATCA
TGGCTGATCAACTTACCGAAGAGCAGATTGCTGAATTTAAGGAGGCCTTCTCCCTCTTTGACAAGGATGGCGA
TGGTGAGTAGGGCGGGACGCAGCTGTAGATTTTAAATTTTGGGGCGCGCCCTGCTTCGTTGTCGGCGGCTTAG
GTGGAAGGGCCTGTTGGCTGTCAGGGTTTGTCTCGAGAGGGAGTCGAGTGATGGAGTTCAAATATCATTCCAG
CGGTGTGTCACCTAAGGGGAAATGCCGGTCAGGAAACCATGCATCGCCACAACCACCAACGCGGAGGCGGGAA
TGACCGTATCCAATGACTTTCGTGCCGCCGCACGACCGCGTGGCCAACCCCCACCATGACTTTCCCCCGCGCC
ATTACAATCATCCATCAACTCCCGGCGGCCCCGCCCTCACATTCAAACTCCACTATGAAAATTTGAGGGGAGA
TCGGGGCAGGAATGATGGAATGGAGAGTTGGCGTAATGGTTTTAAAATCGCCCGGCAATTTCGCACCCCCGAT
ACAGATTTCCACGTGATGTCAACCTCTTTTATATTCACGGTTGACCAATGAGCGCCTTCTTCTTGAGCTAACT
TCACCTGATTGTTGCCGTCATCCCTCCCCCCTTCAGGCACCATCACCACCAAGGAACTTGGAACCGTCATGCG
ATCTTTGGGACAGAACCCCACGGAGGCAGAGCTTATGGACATGATTAACGAGATCGACTCCGACGGAAACGGG
ACCATTGACTTCCCTGAATTCCTTACAATGATGGCGCGTAAAATGAAGGACACGGACAGTGAGGAGGAGATCC
TTGAGGCATTCAAGGTCTTCGACAAGGACGGAAATGGTTTCATCTCGGCCGCCGAATTGCGTCACATCATGAC
CAACCTGGGAGAGAAGCTTACGGATGAAGAAGTTGATGAGATGATTCGTGAGGCTGATATTGATGGGGACGGC
CAAATCAACTATGAAGAGTTCGTCAAGATGATGATGTCGAAATAAATCCCGTAATCCCCCTCTGTGAAAACCT
TTTTGTTTTTGGTCTAGGCTGGTAAACACTTTAGAGCAGTCTTTTTTGTCGTTGGCGCCTTAAGCACCTGCGA
GGGATCTGAGAAGATTCTCGAATGGAATTGGCTGCCCACCCTTTATATAGAACATATATTTAAACTGGACTTT
GTCTACTTGAAAAAAAAAAAAAGATCGGAA
>TR143|c0_g1_i1 len=1312 path=[1541:0-192 1542:193-216 1555:217-402
1548:403-437 1556:438-484 1554:485-508 1546:509-604 1550:605-675
1524:676-699 1545:700-1015 1530:1016-1039 1549:1040-1066 1544:1067-1090
1512:1091-1311] [-1, 1541, 1542, 1555, 1548, 1556, 1554, 1546, 1550,
1524, 1545, 1530, 1549, 1544, 1512, -2]
CGATCTTGGAAATCTAATTACGCTACGATACCGCTAGTTTTACACTATTTGATACATGTCAATACAAAGAAAA
CAAATACACAACAGACAATCGATCGTTGTGAATCGTCGCCGACAACAGCAGCGAGTGCGATGATTTACTCATG
AGAGAACATGTGGATCTTCGTATGTTGTAATCGCTCAAGTGCTGATTCATTTACTGTTTTGGTAAATAAAACT
GCAATAGTGCGATTACATCAAAGACGGCTCATCAATCGAGGTTGATTGGTCTCCACGGACGGAATCTCCCTCT
CCGATGCTGTCCCCGAATTCTCCACCAACAACCCCTTCGCAGCCTCCCATTCTTCCAACAGCTCACTCTCCGT
CACCTTCAATTTCTCCTGTGCAAACTCGCTCAACTCCAGACCCTCCTTCACGAACCACTCTCCACCTCTGCAT

TCGCACGGAAAGGAAAACACAATGCCCTCCTTCACTCCGTAATACCCCCTATCGTTGTAAACCGCCATGGAAA
TTGTTTCTCCATCCTTCGTCCCGGTAACGAGCCACGTCTTGAGACAGTCTGCCGTACCCATGGCCGCCGACAA
CGCGCTCGAATTCCCACGCACTTCGATCACCGCCTTTCCGCGATTTTGAACCGTGGTTACGAAATCGTTGTTC
GCCCATTCCAGATCGTTGGACAGCAATGTTCTCAAAGGAACCTTCTCTCCGCTATCGCCTAGAATATACCCAT
CCGTCAAAGCATCGGGATATTGAGTCTCCGAGTGATTTCCCCATATGATCACATTCTTCACACGATCGGCAGA
CACTCCTCCTCCTCCTCCGAGCCGTTGGGCAATCTGACCCTTGGCACGCTGATAATCCAAATACGTAAGAGCA
CAAAAATTCTTCGACGGTATGCGACCATTCGCAGAAGCCTCCTTCAATGCCACGAGACAATTCGTATTCGCCG
GATTTGCCACCACCAACACTTTCGCTTCAGGTGATGCCACCTCATTTATCGCCCGGCCCATTGTAGCGAATAT
TCTAGTATTGGCTCGAATTAAATCTTTGCGCGCCATGCCTTTTTTGCGAGGATATCCTCCGACGAGTATGATT
ACGTCGGCGTGGTCGAATGCAACGCGTGGGTCGGTGGTGCATATTACATCGTTGAGGGAGGGGAAGGCACAGT
CCAAGAGTTCCATGCGGGTCCCGTCGAGGGATCTTATGGCGGGTGGTATTTCGAGGAGGCGGAGGGATATGGG
TCGATTTTGGAAGACGTAGCCGGAGGCGAGGAGGGGGAGGAGGGCGTAGCCGATGTTTCCGGCGGCGCCGG
>TR144|c0_g1_i1 len=817 path=[809:0-159 810:160-816] [-1, 809, 810, -2]
ATCCACACAAACGAAAGGAAGAATGAAGCATAGCAGTACTTTCCAAAACCTGGCCGTCTCACGTCCGGAATAG
TGAAGGTCAGTACAGCCACAAGAGGCAACACGAGCAAATACTTCAACCACGCGAAACCCCCCTCGCCTGATGG
AGGCGACAGCGCCTCGCAGACCCCCTGAGCCTCTTCCTCGATCTGCTTCTCAATAAGAGACTGCTGGCGGGTA
TAAATGATGGAGTGGACATACCAATCAGCAAATTCCTCATAAGTAATTTCTTCTGTGGAGCCACTCTTGTACA
TAGCGTGGATAGCATCGTCAACATCTGATTCGGTGACTTTTGGTTCCAATGTTTTGAGAAGTTCTCTAACCTC
ATTGCGATCGATGGTACCAGATTTGTCAGCGTCAAATGTATCGAAGATGGGTTTCACTTTCGAGAGGATACGC
TCTTCGGAACTTATGTACCATTTAGAGAATTCTTCCTCAGAGATCGTTCCATCACCATCCAAATCTAAATTAC
GAAATACACTCCCCAACTCCTCCTCAGTAATTTCGTGCTCCAACTTAGCGAACAATTTCCCCAGCTCCTCCCT
GTCAATAGTCCCGTCCCCATTAACATCTACCTGTTTAAATACATGATCCACATCACCCTGAATTTTTGCTACT
ATACCAATGCCACCCTTTTCTTCCCAAGATTGGGGGTGAAGTAAGAGTTTGAGGACTCCAGCTCGGAAGGTGC
CTGGCCAACGAAAGTTAGCGTTACCTTTTTTTGGACTGTTCGGTGGACCCCCCCCCCCCACTGTTGCAGCCAC
ACTATTCCCATTGT
>TR144|c0_g2_i1 len=694 path=[811:0-36 812:37-693] [-1, 811, 812, -2]
CGCTCTTCCGATCTGCCTGATGGAGGCGACAGCGCCTCGCAGACCCCCTGAGCCTCTTCCTCGATCTGCTTCT
CAATAAGAGACTGCTGGCGGGTATAAATGATGGAGTGGACATACCAATCAGCAAATTCCTCATAAGTAATTTC
TTCTGTGGAGCCACTCTTGTACATAGCGTGGATAGCATCGTCAACATCTGATTCGGTGACTTTTGGTTCCAAT
GTTTTGAGAAGTTCTCTAACCTCATTGCGATCGATGGTACCAGATTTGTCAGCGTCAAATGTATCGAAGATGG
GTTTCACTTTCGAGAGGATACGCTCTTCGGAACTTATGTACCATTTAGAGAATTCTTCCTCAGAGATCGTTCC
ATCACCATCCAAATCTAAATTACGAAATACACTCCCCAACTCCTCCTCAGTAATTTCGTGCTCCAACTTAGCG
AACAATTTCCCCAGCTCCTCCCTGTCAATAGTCCCGTCCCCATTAACATCTACCTGTTTAAATACATGATCCA
CATCACCCTGAATTTTTGCTACTATACCAATGCCACCCTTTTCTTCCCAAGATTGGGGGTGAAGTAAGAGTTT
GAGGACTCCAGCTCGGAAGGTGCCTGGCCAACGAAAGTTAGCGTTACCTTTTTTTGGACTGTTCGGTGGACCC
CCCCCCCCCACTGTTGCAGCCACACTATTCCCATTGT
>TR144|c1_g1_i1 len=1597 path=[1723:0-45 1724:46-61 @1740@!:62-1065
1727:1066-1087 1737:1088-1305 1730:1306-1329 1731:1330-1596] [-1, 1723,
1724, 1740, 1727, 1737, 1730, 1731, -2]
GGCTGCGACAGTGGGGGGGGGGGGTCCACCGAACAGTCCAAAAAAAGGTAACGCTAACTTTCGTTGGCCAGGC
ACCTTCCGAGCTGGAGTCCTCAAACTCTTACTTCACCCCCAATCTTGGGAAGAAAAGGGTGGCATTGGTATAG
TAGCAAAAATTCAGGGTGATGTGGATCATGTATTTAAACAGGTAGATGTTAATGGGGACGGGACTATTGACAG
GGAGGAGCTGGGGAAATTGTTCGCTAAGTTGGAGCACGAAATTACTGAGGAGGAGTTGGGGAGTGTATTTCGT
AATTTAGATTTGGATGGTGATGGAACGATCTCTGAGGAAGAATTCTCTAAATGGTACATAAGTTCCGAAGAGC
GTATCCTCTCGAAAGTGAAACCCATCTTCGATACATTTGACGCTGACAAATCTGGTACCATCGATCGCAATGA
GGTTAGAGAACTTCTCAAAACATTGGAACCAAAAGTCACCGAATCAGATGTTGACGATGCTATCCACGCTATG
TACAAGAGTGGCTCCACAGAAGAAATTACTTATGAGGAATTTGCTGATTGGTATGTCCACTCCATCATTTATA
CCCGCCAGCAGTCTCTTATTGAGAAGCAGATCGAGGAAGAGGCTCAGGGGGTCTGCGAGGCGCTGTCGCCTCC
ATCAGGCGAGGGGGGTTTCGCGTGGTTGAAGTATTTGCTCGTGTTGCCTCTTGTGGCTGTACTGACCTTCACT
ATTCCGGACGTGAGACGGCCAGGTTTTGGAAAGTACTGCTATGCTTCATTCTTCCTTTCGATTGTGTGGATTG
CACTTTTCTCTCTCGCTATGGTTGATTGGGTAGAAATTGTAGGAAATACCATTGGTATCCCTTCTGTGGTAAT
GGGGTACACAGTACTGGCAGCAGGGACATCGGTCCCTGACTTGCTCTCGAGTGTGATTGTAGCAAGGATGGGC
GAGGGAGACATGGCTGTCTCCAGCTCCATTGGTAGCAATATTTTTGATATTCTCGTGGGGTTGCCACTTCCTT
GGTTGATTTTCACACTTTGGCCTACAACACCTGACGTAGTAATGATACAAGCCAAAGGAATTTGGCAGTCTAT
ATTCATTTTGGTCGGAATGCTGTTCGCTATAATAGCACTTGTTCATTGCCAGGGGTGGAAAATGACAAGGACA
CTGGGTGGGCTCATGTTCCTATTGTACTTCGCATTTCTTGTGCAAGCCATTTTGACTGAATATTATAGAAACC
CCTGCTTTGGGTAGGATTCTGGTGTGACAGGAAGAGAAAAGTTCTGTTTCTTGGTAGTGGCAGGGAGAAGAGA
AATATTTACTCAACTGCCATCTTAGCGACATTCATACTGGCCTGTAAACCCCTTTGTAGAATCGAATCAAATC
CATTTAGTCGAAATATGTGTGCTGGGGGCGAGGGAGGGAGCCAAGAAGTTGTTGGTTTTTGATTTTGAATGGT

GCAGGTACAAATGATCCATAATTTAAAGAGGAGGATGCATGAGACTTCGCACACTTTTAAGTCACTGAAATTC
TCCCGCTACTTGTCCTAAAATAATATCTGCCTATTTCAATTTCTGAAAAAAAAAAAGATCGGAA
>TR144|c1_g1_i2 len=1679 path=[1723:0-45 1724:46-61 @1740@!:62-1065 1738:1066-1169 1737:1170-1387 1730:1388-1411 1731:1412-1678] [-1, 1723, 1724, 1740, 1738, 1737, 1730, 1731, -2]
GGCTGCGACAGTGGGGGGGGGGGTCCACCGAACAGTCCAAAAAAAGGTAACGCTAACTTTCGTTGGCCAGGC
ACCTTCCGAGCTGGAGTCCTCAAACTCTTACTTCACCCCCAATCTTGGGAAGAAAAGGGTGGCATTGGTATAG
TAGCAAAAATTCAGGGTGATGTGGATCATGTATTTAAACAGGTAGATGTTAATGGGGACGGGACTATTGACAG
GGAGGAGCTGGGGAAATTGTTCGCTAAGTTGGAGCACGAAATTACTGAGGAGGAGTTGGGGAGTGTATTTCGT
AATTTAGATTTGGATGGTGATGGAACGATCTCTGAGGAAGAATTCTCTAAATGGTACATAAGTTCCGAAGAGC
GTATCCTCTCGAAAGTGAAACCCATCTTCGATACATTTGACGCTGACAAATCTGGTACCATCGATCGCAATGA
GGTTAGAGAACTTCTCAAAACATTGGAACCAAAAGTCACCGAATCAGATGTTGACGATGCTATCCACGCTATG
TACAAGAGTGGCTCCACAGAAGAAATTACTTATGAGGAATTTGCTGATTGGTATGTCCACTCCATCATTTATA
CCCGCCAGCAGTCTCTTATTGAGAAGCAGATCGAGGAAGAGGCTCAGGGGGTCTGCGAGGCGCTGTCGCCTCC
ATCAGGCGAGGGGGGTTTCGCGTGGTTGAAGTATTTGCTCGTGTTGCCTCTTGTGGCTGTACTGACCTTCACT
ATTCCGGACGTGAGACGGCCAGGTTTTGGAAAGTACTGCTATGCTTCATTCTTCCTTTCGATTGTGTGGATTG
CACTTTTCTCTCTCGCTATGGTTGATTGGGTAGAAATTGTAGGAAATACCATTGGTATCCCTTCTGTGGTAAT
GGGGTACACAGTACTGGCAGCAGGGACATCGGTCCCTGACTTGCTCTCGAGTGTGATTGTAGCAAGGATGGGC
GAGGGAGACATGGCTGTCTCCAGCTCCATTGGTAGCAATATTTTTGATATTCTCGTGGGGTTGCCACTTCCTT
GGTTGATTTTCACACTTTGGCCTACAACACCTGACGTAGTAATGGTAAGTTTTTTGGGGGGGAAAGTGATTTT
CCTCGTTCGACAGTCACTAAATCATATTTTGTATGTTTCTGTAATATTATCAGATACAAGCCAAAGGAATTTG
GCAGTCTATATTCATTTTGGTCGGAATGCTGTTCGCTATAATAGCACTTGTTCATTGCCAGGGGTGGAAAATG
ACAAGGACACTGGGTGGGCTCATGTTCCTATTGTACTTCGCATTTCTTGTGCAAGCCATTTTGACTGAATATT
ATAGAAACCCCTGCTTTGGGTAGGATTCTGGTGTGACAGGAAGAGAAAAGTTCTGTTTCTTGGTAGTGGCAGG
GAGAAGAGAAATATTTACTCAACTGCCATCTTAGCGACATTCATACTGGCCTGTAAACCCCTTTGTAGAATCG
AATCAAATCCATTTAGTCGAAATATGTGTGCTGGGGGCGAGGGAGGGAGCCAAGAAGTTGTTGGTTTTTGATT
TTGAATGGTGCAGGTACAAATGATCCATAATTTAAAGAGGAGGATGCATGAGACTTCGCACACTTTTAAGTCA
CTGAAATTCTCCCGCTACTTGTCCTAAAATAATATCTGCCTATTTCAATTTCTGAAAAAAAAAAAGATCGGAA
>TR144|c2_g1_i1 len=1103 path=[2030:0-57 2031:58-60 2047:61-173 2044:174-1102] [-1, 2030, 2031, 2047, 2044, -2]
GCTCTTCCGATCTCACACTCGAGAACAAGTCAGGAACCGATGTCCCTGCTGCCAGTACTGTTAATCCCATGAT
CACAGACGGAATGCCAATGGTATTCCCCACAATTTCTACCCAAGTAACCATAGCAAAGGAGAAAAGTGCAATC
CACAAAATCGAAAGGAAGAATGAAGCATAGCACCAGTTGCCCCAACCTTGTCGCCGCACGTCTGGAATAGTAA
AAGTCAGTGCTGTCACGAGAGGCAACACGATCAAATACTTCGACCAAGCAAAAAACCCTTCTCCTCTTGGAGG
CGTCAACGCGGCGCAAATTCCCTGCGATTCTCCCTCGATCTGCATCCCATAGGAATGCTGGTGAGAAGTGGCG
ATGAAGTTCATGTACCAATTGGCAAACTCCTCAAACGTAATTTCTTCTGTGGAGCCACTTTTGTACATGGCAT
TGATTGCATCGTCAACATCTGACTCTTTGACGTTAGGTTCTAACTTTTTGAGAAGTTCGGAAACCTCATAACG
ATCAATGGCACCAGATTTGTCAGCGTCGAATGTATCAAAGATAGGTTTCACTTTCGAGAGGACATGTTTTTCA
GATTTCAAATACCAGTTCATGAATTCATCTTCGCTAATAGTTCCATCACTATCCAAATCCAAACTACTAAATA
CCTCCGAGAGTTCCTCATCGCTAATGTCATGCTCCAACTTTGCAAACAGTTTCCTCAACTCCTCCTTGTCAAT
ACTCCCATCTCCATTAATATCAACTTGTTTAAATGTAGTATCGACATCGCCCTCAATTTTAGTAACAATTCCG
ATACCAGCCTTTTGCTCCCAGGCTTCAGGATGGAGTAAGAGTTGGAGGACCCCAGCACGAAAGGTGCCTGGCC
AGCGAAAGTCAGTGGTGGTGCATGGATTATCGTTTGTTGGCATGGCCGTTTGTGGCGCTGAGGACTTGTGATC
GCTTGATGATTTCAAACCATCATCGCTGAGAAGAGGCGTGTGGTTTCCTCCCATATCCCCCGGTTTCGTTGAC
ACCGCTTGCCCCTCATCCTCTGCTAATGTATCATTATCGCTCTCCCCAGGCAAGACCAACTCCTTTCCAGTCA
ACTTCGTG
>TR144|c2_g2_i1 len=1756 path=[2024:0-263 2025:264-297 @2050@!:298-531 2041:532-553 2048:554-710 2036:711-713 2045:714-826 2039:827-1755] [-1, 2024, 2025, 2050, 2041, 2048, 2036, 2045, 2039, -2]
TTCCGATCTTTTTTTTTTTCAGAAATTGAAATAGGCAGATATTATTTTAGGACAAGTAGCGGGAGAATTTCAG
TGACTTAAAAGTGTGCGAAGTCTCATGCATCCTCCTCTTTAAATTATGGATCATTTGTACCTGCACCATTCAA
AATCAAAAACCAACAACTTCTTGGCTCCCTCCCTCGCCCCCAGCACACATATTTCGACTAAATGGATTTGATT
CGATTCTACAAAGGGGTTTACAGGCCAGTATGAATGTCGCTAAGATGGCAGTTGAGTAAATATTTCTCTTCTC
CCTGCCACTACCAAGAAACAGAACTTTTCTCTTCCTGTCACACCAGAATCCTACCCAAAGCAGGGGTTTCTAT
AATATTCAGTCAAAATGGCTTGCACAAGAAATGCGAAGTACAATAGGAACATGAGCCCACCCAGTGTCCTTGT
CATTTTCCACCCCTGGCAATGAACAAGTGCTATTATAGCGAACAGCATTCCGACCAAAATGAATATAGACTGC
CAAATTCCTTTGGCTTGTATCATTACTACGTCAGGTGTTGTAGGCCAAAGTGTGAAAATCAACCAAGGAAGTG
GCAACCCCACGAGAATATCAAAAATATTGCTACCAATGGAGCTGGAGACAGCCATGTCTCCCTCGCCCATCCT
TGCTACAATCACACTCGAGAGCAAGTCAGGGACCGATGTCCCTGCTGCCAGTACTGTGTACCCCATTACCACA
GAAGGGATACCAATGGTATTTCCTACAATTTCTACCCAATCAACCATAGCGAGAGAGAAAAGTGCAATCCACA

CAATCGAAAGGAAGAATGAAGCATAGCACCAGTTGCCCCAACCTTGTCGCCGCACGTCTGGAATAGTAAAAGT
CAGTGCTGTCACGAGAGGCAACACGATCAAATACTTCGACCAAGCAAAAAACCCTTCTCCTCTTGGAGGCGTC
AACGCGGCGCAAATTCCCTGCGATTCTCCCTCGATCTGCATCCCATAGGAATGCTGGTGAGAAGTGGCGATGA
AGTTCATGTACCAATTGGCAAACTCCTCAAACGTAATTTCTTCTGTGGAGCCACTTTTGTACATGGCATTGAT
TGCATCGTCAACATCTGACTCTTTGACGTTAGGTTCTAACTTTTTGAGAAGTTCGGAAACCTCATAACGATCA
ATGGCACCAGATTTGTCAGCGTCGAATGTATCAAAGATAGGTTTCACTTTCGAGAGGACATGTTTTTCAGATT
TCAAATACCAGTTCATGAATTCATCTTCGCTAATAGTTCCATCACTATCCAAATCCAAACTACTAAATACCTC
CGAGAGTTCCTCATCGCTAATGTCATGCTCCAACTTTGCAAACAGTTTCCTCAACTCCTCCTTGTCAATACTC
CCATCTCCATTAATATCAACTTGTTTAAATGTAGTATCGACATCGCCCTCAATTTTAGTAACAATTCCGATAC
CAGCCTTTTGCTCCCAGGCTTCAGGATGGAGTAAGAGTTGGAGGACCCCAGCACGAAAGGTGCCTGGCCAGCG
AAAGTCAGTGGTGGTGCATGGATTATCGTTTGTTGGCATGGCCGTTTGTGGCGCTGAGGACTTGTGATCGCTT
GATGATTTCAAACCATCATCGCTGAGAAGAGGCGTGTGGTTTCCTCCCATATCCCCCGGTTTCGTTGACACCG
CTTGCCCCTCATCCTCTGCTAATGTATCATTATCGCTCTCCCCAGGCAAGACCAACTCCTTTCCAGTCAACTT
CGTG
>TR144|c2_g2_i2 len=1838 path=[2024:0-263 2025:264-297 @2050@!:298-531
2046:532-635 2048:636-792 2036:793-795 2045:796-908 2039:909-1837] [-1,
2024, 2025, 2050, 2046, 2048, 2036, 2045, 2039, -2]
TTCCGATCTTTTTTTTTTTCAGAAATTGAAATAGGCAGATATTATTTAGGACAAGTAGCGGGAGAATTTCAG
TGACTTAAAAGTGTGCGAAGTCTCATGCATCCTCCTCTTTAAATTATGGATCATTTGTACCTGCACCATTCAA
AATCAAAAACCAACAACTTCTTGGCTCCCTCCCTCGCCCCCAGCACACATATTTCGACTAAATGGATTTGATT
CGATTCTACAAAGGGGTTTACAGGCCAGTATGAATGTCGCTAAGATGGCAGTTGAGTAAATATTTCTCTTCTC
CCTGCCACTACCAAGAAACAGAACTTTTCTCTTCCTGTCACACCAGAATCCTACCCAAAGCAGGGGTTTCTAT
AATATTCAGTCAAAATGGCTTGCACAAGAAATGCGAAGTACAATAGGAACATGAGCCCACCCAGTGTCCTTGT
CATTTTCCACCCCTGGCAATGAACAAGTGCTATTATAGCGAACAGCATTCCGACCAAAATGAATATAGACTGC
CAAATTCCTTTGGCTTGTATCTGATAATATTACAGAAACATACAAAATATGATTTAGTGACTGTCGAACGAGG
AAAATCACTTTCCCCCCCAAAAAACTTACCATTACTACGTCAGGTGTTGTAGGCCAAAGTGTGAAAATCAACC
AAGGAAGTGGCAACCCCACGAGAATATCAAAAATATTGCTACCAATGGAGCTGGAGACAGCCATGTCTCCCTC
GCCCATCCTTGCTACAATCACACTCGAGAGCAAGTCAGGGACCGATGTCCCTGCTGCCAGTACTGTGTACCCC
ATTACCACAGAAGGGATACCAATGGTATTTCCTACAATTTCTACCCAATCAACCATAGCGAGAGAGAAAAGTG
CAATCCACACAATCGAAAGGAAGAATGAAGCATAGCACCAGTTGCCCCAACCTTGTCGCCGCACGTCTGGAAT
AGTAAAAGTCAGTGCTGTCACGAGAGGCAACACGATCAAATACTTCGACCAAGCAAAAAACCCTTCTCCTCTT
GGAGGCGTCAACGCGGCGCAAATTCCCTGCGATTCTCCCTCGATCTGCATCCCATAGGAATGCTGGTGAGAAG
TGGCGATGAAGTTCATGTACCAATTGGCAAACTCCTCAAACGTAATTTCTTCTGTGGAGCCACTTTTGTACAT
GGCATTGATTGCATCGTCAACATCTGACTCTTTGACGTTAGGTTCTAACTTTTTGAGAAGTTCGGAAACCTCA
TAACGATCAATGGCACCAGATTTGTCAGCGTCGAATGTATCAAAGATAGGTTTCACTTTCGAGAGGACATGTT
TTTCAGATTTCAAATACCAGTTCATGAATTCATCTTCGCTAATAGTTCCATCACTATCCAAATCCAAACTACT
AAATACCTCCGAGAGTTCCTCATCGCTAATGTCATGCTCCAACTTTGCAAACAGTTTCCTCAACTCCTCCTTG
TCAATACTCCCATCTCCATTAATATCAACTTGTTTAAATGTAGTATCGACATCGCCCTCAATTTTAGTAACAA
TTCCGATACCAGCCTTTTGCTCCCAGGCTTCAGGATGGAGTAAGAGTTGGAGGACCCCAGCACGAAAGGTGCC
TGGCCAGCGAAAGTCAGTGGTGGTGCATGGATTATCGTTTGTTGGCATGGCCGTTTGTGGCGCTGAGGACTTG
TGATCGCTTGATGATTTCAAACCATCATCGCTGAGAAGAGGCGTGTGGTTTCCTCCCATATCCCCCGGTTTCG
TTGACACCGCTTGCCCCTCATCCTCTGCTAATGTATCATTATCGCTCTCCCCAGGCAAGACCAACTCCTTTCC
AGTCAACTTCGTG
>TR144|c3_g1_i1 len=1121 path=[1848:0-210 1849:211-234 1915:235-240
1910:241-289 1904:290-301 1922:302-334 1918:335-355 1908:356-394
1902:395-413 1891:414-414 1892:415-439 1916:440-508 @1911@!:509-613
1923:614-643 1874:644-656 1875:657-674 1876:675-1120] [-1, 1848, 1849,
1915, 1910, 1904, 1922, 1918, 1908, 1902, 1891, 1892, 1916, 1911, 1923,
1874, 1875, 1876, -2]
TTTTTTTGGGTTTTTGGGGGGCCCCCCCCCCCACTGTCGCAGCCACACTATTCCCATTGTGAACTACTACCG
CCATTCCATTTGTCGCCGCCGTCCCTTCCTCTTCCTGCGTATCCTCACTCTCCCCAGGCAACACTAACTCCTT
TCCAGTCAACTTCGTGTACAATTTTCGGTTGTAATACATCAACAGCACATACCCAAAGTACATAAACAGCAAA
ATCAATGCCTCCCACCAGTGAACCTCCCCCACGGTGAGCACGCCAATAAACACACTCAACACGATTAGTCCCA
CGGCGTAATACGATGAATCACGAAAGAGTGGCCACCAGGTCAAGGTCAATACCTCCTTCGAAAGTAAGGAACA
CATTCCGATGACGAACAAAACATTGAAAACCGCACTCCCCACAATGGTACCGATGCCGATATCACTCTGCTTG
AATGTTCCCACGAAGGATGTGAAGAGCTCGGGGGCGCTCCCCCCCGCCGCCATGAGGGTGGCGCCGGCCACGT
CCATACTGAGGTTAAGGTGGCGTTCGCTGCTCATTTCTTCGAGGGCGGGGACGAAGAATTCATCGCACACGAT
GGCTAATGCGAGGAACATGTAGAACGTTCCAAGGGCGTAGAGCGCCGCAGACCATTTGGGGTCGGCAACCTCG
AGCACCGGGCATGGCGGATCGTCGGCTTGGGAGGATGGCGTCGCTTCTTGTTTGTCACTATCACCTCCTTGTT
CGTCTTTATCGTCTTTTAACAGCCTAGAAAAGTTGTTGATATTCCCCTCGCGACGGGTGTCCCCTGCAATGTT

TCTTAAATTGGACGTCCCAGCGTCACCTTTCGAGCGCTGCGACGGCGGGATCAAGCTGCCAGTGCCATCTCCC
CCGAAGTGATTGTATAACGCGTATCCTCCAACCAGCAATACTAAAGACGCGCTTCGAAAAATGGCACGGAGGG
TGTGTTTCCGACGGTAGACTCTGCCGAAGCAGCGAGAAGGAGGAGGAATGATGCTACAGTCGCCGCTGATATC
AGCCATGGCGTAGAAATTGTCGACTGCTTATTTGTCCGGGTAGTATAATAACATAAGAATTCGAGTAAGCGAG
GATTAGTGAAGGGCGTAGTGGTAGCA
>TR144|c3_g1_i2 len=1121 path=[1865:0-210 1866:211-234 1919:235-240
1914:241-289 1909:290-301 1921:302-334 1918:335-355 1912:356-394
1907:395-413 1901:414-414 1920:415-439 1917:440-508 @1911@!:509-613
1923:614-643 1874:644-656 1875:657-674 1876:675-1120] [-1, 1865, 1866,
1919, 1914, 1909, 1921, 1918, 1912, 1907, 1901, 1920, 1917, 1911, 1923,
1874, 1875, 1876, -2]
TTTTTTTGGGTTTTTGGGGGGCCCCCCCCCCCACTGTCGCAGCCACACTATTCCCATTGTGAACTACTACCG
CCATTCCATTTGTCGCCGCCGTCCCTTCCTCTTCCTGCGTATCCTCACTCTCCCCAGGCAACACTAACTCCTT
TCCAGTCAACTTCGTGTACAATTTTCGGTTGTAATACATCAACAGCACATACCCAAAGTACATAAACAGCAAA
ATCAATGCCTCCCACCAGTGAATAATACCCTTGCTAACAACGCCGGCCAACACACTCAACACGATTAGTCCCA
CGGCGTAATAAGAGGTGTCGCGAAAGAGTGGCCACCAGGTCAAGGTCAATACCTCCTTCGAAAGGATGGAACA
CATCCCAATGACGAACAAAACATTGAAAACCGCACTCCCCACAATGGTACCGATGCCGATATCACTCTGCTTG
AACGTCCCCACGAGGGACGTGAAGAGCTCGGGCGCGCTCCCCCCCGCGGCCATGAGGGTGGCGCCGGCCACGT
CCATACTGAGGTTAAGGTGGCGTTCGCTGCTCATTTCTTCGAGGGCGGGGACGAAGAATTCATCGCACACGAT
GGCTAATGCGAGGAACATGTAGAACGTTCCAAGGGCGTAGAGCGCCGCAGACCATTTGGGGTCGGCAACCTCG
AGCACCGGGCATGGCGGATCGTCGGCTTGGGAGGATGGCGTCGCTTCTTGTTTGTCACTATCACCTCCTTGTT
CGTCTTTATCGTCTTTTAACAGCCTAGAAAAGTTGTTGATATTCCCCTCGCGACGGGTGTCCCCTGCAATGTT
TCTTAAATTGGACGTCCCAGCGTCACCTTTCGAGCGCTGCGACGGCGGGATCAAGCTGCCAGTGCCATCTCCC
CCGAAGTGATTGTATAACGCGTATCCTCCAACCAGCAATACTAAAGACGCGCTTCGAAAAATGGCACGGAGGG
TGTGTTTCCGACGGTAGACTCTGCCGAAGCAGCGAGAAGGAGGAGGAATGATGCTACAGTCGCCGCTGATATC
AGCCATGGCGTAGAAATTGTCGACTGCTTATTTGTCCGGGTAGTATAATAACATAAGAATTCGAGTAAGCGAG
GATTAGTGAAGGGCGTAGTGGTAGCA
>TR144|c3_g1_i3 len=1058 path=[1848:0-210 1849:211-234 1915:235-240
1910:241-289 1904:290-301 1922:302-334 1918:335-355 1908:356-394
1902:395-413 1891:414-414 1892:415-439 1916:440-508 @1911@!:509-613
1853:614-656 1854:657-674 1899:675-820 1870:821-844 1871:845-1057] [-1,
1848, 1849, 1915, 1910, 1904, 1922, 1918, 1908, 1902, 1891, 1892, 1916,
1911, 1853, 1854, 1899, 1870, 1871, -2]
TTTTTTTGGGTTTTTGGGGGGCCCCCCCCCCCACTGTCGCAGCCACACTATTCCCATTGTGAACTACTACCG
CCATTCCATTTGTCGCCGCCGTCCCTTCCTCTTCCTGCGTATCCTCACTCTCCCCAGGCAACACTAACTCCTT
TCCAGTCAACTTCGTGTACAATTTTCGGTTGTAATACATCAACAGCACATACCCAAAGTACATAAACAGCAAA
ATCAATGCCTCCCACCAGTGAACCTCCCCCACGGTGAGCACGCCAATAAACACACTCAACACGATTAGTCCCA
CGGCGTAATACGATGAATCACGAAAGAGTGGCCACCAGGTCAAGGTCAATACCTCCTTCGAAAGTAAGGAACA
CATTCCGATGACGAACAAAACATTGAAAACCGCACTCCCCACAATGGTACCGATGCCGATATCACTCTGCTTG
AATGTTCCCACGAAGGATGTGAAGAGCTCGGGGGCGCTCCCCCCCGCCGCCATGAGGGTGGCGCCGGCCACGT
CCATACTGAGGTTAAGGTGGCGTTCGCTGCTCATTTCTTCGAGGGCGGGGACGAAGAATTCATCGCACACGAT
GGCTAATGCGAGGAACATGTAGAACGTTCCGAGGGCGTAGAGCACCGCAAACCATTTGGGGTCGGCAACCTCG
AGCACCGGGCATGGCGGAGGCTCCTCTTCGGCTAGGTCTGAAACGAGTTCCTGTGCTCCCTCCACCTCTCCAT
CCACTAATAACCGTGAAAAGTTAATAAAATTCTTGTGGCGGTTGTCATCTATGCTATTTCTTAAATTGGAGGT
CCCGGCGCCGATGGGGTCACTCTTCAAATGCTGCGACGGGGGCACCATCCCGCCATTGCTACCTTCCCCGAAG
TGATTGTACAAGGCGTATCCCCCCACCAATAGTACTAAAGACGAACTTCGAAATATTGCGCGGAGAGTGTGTT
TCCGACGATAGATTCCGAAAACGCTGCGGGAGGGGGGGCTAAGGCCTTCGCCATAATCGCCGCCGTTGTCGTC
GGCCATGGCGTTGGCGATGCGAGATCGGAAGAGCAC
>TR144|c3_g1_i4 len=1058 path=[1865:0-210 1866:211-234 1919:235-240
1914:241-289 1909:290-301 1921:302-334 1918:335-355 1912:356-394
1907:395-413 1901:414-414 1920:415-439 1917:440-508 @1911@!:509-613
1853:614-656 1854:657-674 1899:675-820 1870:821-844 1871:845-1057] [-1,
1865, 1866, 1919, 1914, 1909, 1921, 1918, 1912, 1907, 1901, 1920, 1917,
1911, 1853, 1854, 1899, 1870, 1871, -2]
TTTTTTTGGGTTTTTGGGGGGCCCCCCCCCCCACTGTCGCAGCCACACTATTCCCATTGTGAACTACTACCG
CCATTCCATTTGTCGCCGCCGTCCCTTCCTCTTCCTGCGTATCCTCACTCTCCCCAGGCAACACTAACTCCTT
TCCAGTCAACTTCGTGTACAATTTTCGGTTGTAATACATCAACAGCACATACCCAAAGTACATAAACAGCAAA
ATCAATGCCTCCCACCAGTGAATAATACCCTTGCTAACAACGCCGGCCAACACACTCAACACGATTAGTCCCA
CGGCGTAATAAGAGGTGTCGCGAAAGAGTGGCCACCAGGTCAAGGTCAATACCTCCTTCGAAAGGATGGAACA
CATCCCAATGACGAACAAAACATTGAAAACCGCACTCCCCACAATGGTACCGATGCCGATATCACTCTGCTTG

AACGTCCCCACGAGGGACGTGAAGAGCTCGGGCGCGCTCCCCCCCGCGGCCATGAGGGTGGCGCCGGCCACGT
CCATACTGAGGTTAAGGTGGCGTTCGCTGCTCATTTCTTCGAGGGCGGGGACGAAGAATTCATCGCACACGAT
GGCTAATGCGAGGAACATGTAGAACGTTCCGAGGGCGTAGAGCACCGCAAACCATTTGGGGTCGGCAACCTCG
AGCACCGGGCATGGCGGAGGCTCCTCTTCGGCTAGGTCTGAAACGAGTTCCTGTGCTCCCTCCACCTCTCCAT
CCACTAATAACCGTGAAAAGTTAATAAAATTCTTGTGGCGGTTGTCATCTATGCTATTTCTTAAATTGGAGGT
CCCGGCGCCGATGGGGTCACTCTTCAAATGCTGCGACGGGGGCACCATCCCGCCATTGCTACCTTCCCCGAAG
TGATTGTACAAGGCGTATCCCCCCACCAATAGTACTAAAGACGAACTTCGAAATATTGCGCGGAGAGTGTGTT
TCCGACGATAGATTCCGAAAACGCTGCGGGAGGGGGGGCTAAGGCCTTCGCCATAATCGCCGCCGTTGTCGTC
GGCCATGGCGTTGGCGATGCGAGATCGGAAGAGCAC
>TR144|c4_g1_i1 len=1162 path=[3350:0-509 3351:510-527 3352:528-570
@3381@!:571-675 3370:676-744 3361:745-789 3380:790-828 3375:829-849
3368:850-882 3383:883-894 3372:895-943 3366:944-949 3382:950-973
3384:974-984 3355:985-1014 3339:1015-1023 3340:1024-1161] [-1, 3350,
3351, 3352, 3381, 3370, 3361, 3380, 3375, 3368, 3383, 3372, 3366, 3382,
3384, 3355, 3339, 3340, -2]
AGTTGTCTACATCTTCTTCTCTCGAGTCATACTTACCTCTTTGCTACCACTACGCCCTTCACTAATCCTCGCT
TACTCGAATTCTTATGTTATTATACTACCCGGACAAATAAGCAGTCGACAATTTCTACGCCATGGCTGATATC
AGCGGCGACTGTAGCATCATTCCTCCTCCTTCTCGCTGCTTCGGCAGAGTCTACCGTCGGAAACACACCCTCC
GTGCCATTTTTCGAAGCGCGTCTTTAGTATTGCTGGTTGGAGGATACGCGTTATACAATCACTTCGGGGGAGA
TGGCACTGGCAGCTTGATCCCGCCGTCGCAGCGCTCGAAAGGTGACGCTGGGACGTCCAATTTAAGAAACATT
GCAGGGGACACCCGTCGCGAGGGGAATATCAACAACTTTTCTAGGCTGTTAAAAGACGATAAAGACGAACAAG
GAGGTGATAGTGACAAACAAGAAGCGACGCCATCCTCCCAAGCCGACGATCCGCCATGCCCGGTGCTCGAGGT
TGCCGACCCCAAATGGTCTGCGGCGCTCTACGCCCTTGGAACGTTCTACATGTTCCTCGCATTAGCCATCGTG
TGCGATGAATTCTTCGTCCCCGCCCTCGAAGAAATGAGCAGCGAACGCCACCTTAACCTCAGTATGGACGTGG
CCGGCGCCACCCTCATGGCCGCGGGGGGGAGCGCGCCCGAGCTCTTCACGTCCCTCGTGGGGACGTTCAAGCA
GAGTGATATCGGCATCGGTACCATTGTGGGGAGTGCGGTTTTCAATGTTTTGTTCGTCATTGGGATGTGTTCC
ATCCTTTCGAAGGAGGTATTGACCTTGACCTGGTGGCCACTCTTTCGCGACACCTCTTATTACGCCGTGGGAC
TAATCGTGTTGAGTGTGTTGGCCGGCGTTGTTAGCAAGGGTATTATTCACTGGTGGGAGGCATTGATTTTGCT
GTTTATGTACTTTGGGTATGTGCTGTTGATGTATTACAACCGAAAATTGTACACGAAGTTGACTGGAAAGGAG
TTAGTGTTGCCTGGGGAGAGTGAGGATACGCAGGAAGAGGAAGGGACGGCGGCGACAAATGGAATGGCGGTAG
TAGTTCACAATGGGAATAGTGTGGCTGCGACAGTGGGGGGGGGGGGCCCCCCAAAAACCCAAAAAAA
>TR144|c4_g1_i2 len=2327 path=[3278:0-365 3279:366-381 3363:382-887
3286:888-905 3287:906-948 @3381@!:949-1053 3376:1054-1122 3369:1123-1167
3379:1168-1206 3374:1207-1227 3367:1228-1260 3383:1261-1272 3373:1273-
1321 3366:1322-1327 3382:1328-1351 3384:1352-1362 3364:1363-1392
3354:1393-1401 3313:1402-2326] [-1, 3278, 3279, 3363, 3286, 3287, 3381,
3376, 3369, 3379, 3374, 3367, 3383, 3373, 3366, 3382, 3384, 3364, 3354,
3313, -2]
TAAAATTCACTATTTTCCTTGGCCCGTGGTAATATCTACCTAAAAAGGTCAAACCACAAAATGTGTATGTTTT
GCCAACAGTTTGTATGATACGAATAAACAACCCATTACGATATCGACAGGGATAATAATAACAGCTTCTGCTC
ATTGTTGCACAGGCTCAGAGGCACATCAAAACCTTCTTTATCTAATCCCATAGAGAAATATTTTTCAGCCGTG
GATGTTCCCATCGTCTTCGTCGACTACCAACTGTGAGCATTAAACGGGAGGGGCTGATCAGCTCAACGCCAAT
CGTCATTATTATTATTAATTATCAATCCGCCATAGCCACCACGACCACCACATAAATCTCAAAATTGAAAAAA
ATCATTTCAATTTTCATTTTCAAACAAGCAAAGGGCAAACCAACCATGGAGGAGCCCTCCACGCCGCCAAACC
CGCTAGCGAGATCCAAGGAAAATATTAACCAAAATCAACATCGCGGCTGCGGCAGCATCCGCATCGCCAACGC
CATGGCCGACGACAACGGCGGCGATTATGGCGAAGGCCTTAGCCCCCCCTCCCGCAGCGTTTTCGGAATCTAT
CGTCGGAAACACACTCTCCGCGCAATATTTCGAAGTTCGTCTTTAGTACTATTGGTGGGGGGATACGCCTTGT
ACAATCACTTCGGGGAAGGTAGCAATGGCGGGATGGTGCCCCCGTCGCAGCATTTGAAGAGTGACCCCATCGG
CGCCGGGACCTCCAATTTAAGAAATAGCATAGATGACAACCGCCACAAGAATTTTATTAACTTTTCACGGTTA
TTAGTGGATGGAGAGGTGGAGGGAGCACAGGAACTCGTTTCAGACCTAGCCGAAGAGGAGCCTCCGCCATGCC
CGGTGCTCGAGGTTGCCGACCCCAAATGGTTTGCGGTGCTCTACGCCCTCGGAACGTTCTACATGTTCCTCGC
ATTAGCCATCGTGTGCGATGAATTCTTCGTCCCCGCCCTCGAAGAAATGAGCAGCGAACGCCACCTTAACCTC
AGTATGGACGTGGCCGGCGCCACCCTCATGGCGGCGGGGGGGAGCGCCCCCGAGCTCTTCACATCCTTCGTGG
GAACATTCAAGCAGAGTGATATCGGCATCGGTACCATTGTGGGGAGTGCGGTTTTCAATGTTTTGTTCGTCAT
CGGAATGTGTTCCTTACTTTCGAAGGAGGTATTGACCTTGACCTGGTGGCCACTCTTTCGTGATTCATCGTAT
TACGCCGTGGGACTAATCGTGTTGAGTGTGTTTATTGGCGTGCTCACCGTGGGGGAGGTTCACTGGTGGGAGG
CATTGATTTTGCTGTTTATGTACTTTGGGTATGTGCTGTTGATGTATTATAACCGGAAATTGTACACGAAGTT
GACTGGAAAGGAGTTGGTCTTGCCTGGGGAGAGCGATAATGATACATTAGCAGAGGATGAGGGGCAAGCGGTG
TCAACGAAACCGGGGGATATGGGAGGAAACCACACGCCTCTTCTCAGCGATGATGGTTTGAAATCATCAAGCG
ATCACAAGTCCTCAGCGCCACAAACGGCCATGCCAACAAACGATAATCCATGCACCACCACTGACTTTCGCTG

GCCAGGCACCTTTCGTGCTGGGGTCCTCCAACTCTTACTCCATCCTGAAGCCTGGGAGCAAAAGGCTGGTATC
GGAATTGTTACTAAAATTGAGGGCGATGTCGATACTACATTTAAACAAGTTGATATTAATGGAGATGGGAGTA
TTGACAAGGAGGAGTTGAGGAAACTGTTTGCAAAGTTGGAGCATGACATTAGCGATGAGGAACTCTCGGAGGT
ATTTAGTAGTTTGGATTTGGATAGTGATGGAACTATTAGCGAAGATGAATTCATGAACTGGTATTTGAAATCT
GAAAAACATGTCCTCTCGAAAGTGAAACCTATCTTTGATACATTCGACGCTGACAAATCTGGTGCCATTGATC
GTTATGAGGTTTCCGAACTTCTCAAAAAGTTAGAACCTAACGTCAAAGAGTCAGATGTTGACGATGCAATCAA
TGCCATGTACAAAAGTGGCTCCACAGAAGAAATTACGTTTGAGGAGTTTGCCAATTGGTACATGAACTTCATC
GCCACTTCTCACCAGCATTCCTATGGGATGCAGATCGAGGGAGAATCGCAGGGAATTTGCGCCGCGTTGACGC
CTCCAAGAGGAGAAGGGTTTTTTGCTTGGTCGAAGTATTTGATCGTGTTGCCTCTCGTGACAGCACTGACTTT
TACTATTCCAGACGTGCGGCGACAAGGTTGGGGCAACTGGTGCTATGCTTCATTCTTCCTTTCG
>TR144|c4_g1_i3 len=1540 path=[3278:0-365 3279:366-381 3363:382-887
3286:888-905 3287:906-948 @3381@!:949-1053 3370:1054-1122 3361:1123-1167
3380:1168-1206 3375:1207-1227 3368:1228-1260 3383:1261-1272 3372:1273-
1321 3366:1322-1327 3382:1328-1351 3384:1352-1362 3355:1363-1392
3339:1393-1401 3340:1402-1539] [-1, 3278, 3279, 3363, 3286, 3287, 3381,
3370, 3361, 3380, 3375, 3368, 3383, 3372, 3366, 3382, 3384, 3355, 3339,
3340, -2]
TAAAATTCACTATTTTCCTTGGCCCGTGGTAATATCTACCTAAAAAGGTCAAACCACAAAATGTGTATGTTTT
GCCAACAGTTTGTATGATACGAATAAACAACCCATTACGATATCGACAGGGATAATAATAACAGCTTCTGCTC
ATTGTTGCACAGGCTCAGAGGCACATCAAAACCTTCTTTATCTAATCCCATAGAGAAATATTTTTCAGCCGTG
GATGTTCCCATCGTCTTCGTCGACTACCAACTGTGAGCATTAAACGGGAGGGGCTGATCAGCTCAACGCCAAT
CGTCATTATTATTATTAATTATCAATCCGCCATAGCCACCACGACCACCACATAAATCTCAAAATTGAAAAAA
ATCATTTCAATTTTCATTTTCAAACAAGCAAAGGGCAAACCAACCATGGAGGAGCCCTCCACGCCGCCAAACC
CGCTAGCGAGATCCAAGGAAAATATTAACCAAAATCAACATCGCGGCTGCGGCAGCATCCGCATCGCCAACGC
CATGGCCGACGACAACGGCGGCGATTATGGCGAAGGCCTTAGCCCCCCCTCCCGCAGCGTTTTCGGAATCTAT
CGTCGGAAACACACTCTCCGCGCAATATTTCGAAGTTCGTCTTTAGTACTATTGGTGGGGGGATACGCCTTGT
ACAATCACTTCGGGGAAGGTAGCAATGGCGGGATGGTGCCCCCGTCGCAGCATTTGAAGAGTGACCCCATCGG
CGCCGGGACCTCCAATTTAAGAAATAGCATAGATGACAACCGCCACAAGAATTTTATTAACTTTTCACGGTTA
TTAGTGGATGGAGAGGTGGAGGGAGCACAGGAACTCGTTTCAGACCTAGCCGAAGAGGAGCCTCCGCCATGCC
CGGTGCTCGAGGTTGCCGACCCCAAATGGTTTGCGGTGCTCTACGCCCTCGGAACGTTCTACATGTTCCTCGC
ATTAGCCATCGTGTGCGATGAATTCTTCGTCCCCGCCCTCGAAGAAATGAGCAGCGAACGCCACCTTAACCTC
AGTATGGACGTGGCCGGCGCCACCCTCATGGCCGCGGGGGGGAGCGCGCCCGAGCTCTTCACGTCCCTCGTGG
GGACGTTCAAGCAGAGTGATATCGGCATCGGTACCATTGTGGGGAGTGCGGTTTTCAATGTTTTGTTCGTCAT
TGGGATGTGTTCCATCCTTTCGAAGGAGGTATTGACCTTGACCTGGTGGCCACTCTTTCGCGACACCTCTTAT
TACGCCGTGGGACTAATCGTGTTGAGTGTGTTGGCCGGCGTTGTTAGCAAGGGTATTATTCACTGGTGGGAGG
CATTGATTTTGCTGTTTATGTACTTTGGGTATGTGCTGTTGATGTATTACAACCGAAAATTGTACACGAAGTT
GACTGGAAAGGAGTTAGTGTTGCCTGGGGAGAGTGAGGATACGCAGGAAGAGGAAGGGACGGCGGCGACAAAT
GGAATGGCGGTAGTAGTTCACAATGGGAATAGTGTGGCTGCGACAGTGGGGGGGGGGGGCCCCCAAAAACCC
AAAAAAA
>TR144|c4_g1_i4 len=1949 path=[3350:0-509 3351:510-527 3352:528-570
@3381@!:571-675 3376:676-744 3369:745-789 3379:790-828 3374:829-849
3367:850-882 3383:883-894 3373:895-943 3366:944-949 3382:950-973
3384:974-984 3364:985-1014 3354:1015-1023 3313:1024-1948] [-1, 3350,
3351, 3352, 3381, 3376, 3369, 3379, 3374, 3367, 3383, 3373, 3366, 3382,
3384, 3364, 3354, 3313, -2]
AGTTGTCTACATCTTCTTCTCTCGAGTCATACTTACCTCTTTGCTACCACTACGCCCTTCACTAATCCTCGCT
TACTCGAATTCTTATGTTATTATACTACCCGGACAAATAAGCAGTCGACAATTTCTACGCCATGGCTGATATC
AGCGGCGACTGTAGCATCATTCCTCCTCCTTCTCGCTGCTTCGGCAGAGTCTACCGTCGGAAACACACCCTCC
GTGCCATTTTTCGAAGCGCGTCTTTAGTATTGCTGGTTGGAGGATACGCGTTATACAATCACTTCGGGGGAGA
TGGCACTGGCAGCTTGATCCCGCCGTCGCAGCGCTCGAAAGGTGACGCTGGGACGTCCAATTTAAGAAACATT
GCAGGGGACACCCGTCGCGAGGGGAATATCAACAACTTTTCTAGGCTGTTAAAAGACGATAAAGACGAACAAG
GAGGTGATAGTGACAAACAAGAAGCGACGCCATCCTCCCAAGCCGACGATCCGCCATGCCCGGTGCTCGAGGT
TGCCGACCCCAAATGGTCTGCGGCGCTCTACGCCCTTGGAACGTTCTACATGTTCCTCGCATTAGCCATCGTG
TGCGATGAATTCTTCGTCCCCGCCCTCGAAGAAATGAGCAGCGAACGCCACCTTAACCTCAGTATGGACGTGG
CCGGCGCCACCCTCATGGCGGCGGGGGGGAGCGCCCCCGAGCTCTTCACATCCTTCGTGGGAACATTCAAGCA
GAGTGATATCGGCATCGGTACCATTGTGGGGAGTGCGGTTTTCAATGTTTTGTTCGTCATCGGAATGTGTTCC
TTACTTTCGAAGGAGGTATTGACCTTGACCTGGTGGCCACTCTTTCGTGATTCATCGTATTACGCCGTGGGAC
TAATCGTGTTGAGTGTGTTTATTGGCGTGCTCACCGTGGGGGAGGTTCACTGGTGGGAGGCATTGATTTTGCT
GTTTATGTACTTTGGGTATGTGCTGTTGATGTATTATAACCGGAAATTGTACACGAAGTTGACTGGAAAGGAG
TTGGTCTTGCCTGGGGAGAGCGATAATGATACATTAGCAGAGGATGAGGGGCAAGCGGTGTCAACGAAACCGG

GGGATATGGGAGGAAACCACACGCCTCTTCTCAGCGATGATGGTTTGAAATCATCAAGCGATCACAAGTCCTC
AGCGCCACAAACGGCCATGCCAACAAACGATAATCCATGCACCACCACTGACTTTCGCTGGCCAGGCACCTTT
CGTGCTGGGGTCCTCCAACTCTTACTCCATCCTGAAGCCTGGGAGCAAAAGGCTGGTATCGGAATTGTTACTA
AAATTGAGGGCGATGTCGATACTACATTTAAACAAGTTGATATTAATGGAGATGGGAGTATTGACAAGGAGGA
GTTGAGGAAACTGTTTGCAAAGTTGGAGCATGACATTAGCGATGAGGAACTCTCGGAGGTATTTAGTAGTTTG
GATTTGGATAGTGATGGAACTATTAGCGAAGATGAATTCATGAACTGGTATTTGAAATCTGAAAAACATGTCC
TCTCGAAAGTGAAACCTATCTTTGATACATTCGACGCTGACAAATCTGGTGCCATTGATCGTTATGAGGTTTC
CGAACTTCTCAAAAAGTTAGAACCTAACGTCAAAGAGTCAGATGTTGACGATGCAATCAATGCCATGTACAAA
AGTGGCTCCACAGAAGAAATTACGTTTGAGGAGTTTGCCAATTGGTACATGAACTTCATCGCCACTTCTCACC
AGCATTCCTATGGGATGCAGATCGAGGGAGAATCGCAGGGAATTTGCGCCGCGTTGACGCCTCCAAGAGGAGA
AGGGTTTTTTGCTTGGTCGAAGTATTTGATCGTGTTGCCTCTCGTGACAGCACTGACTTTTACTATTCCAGAC
GTGCGGCGACAAGGTTGGGGCAACTGGTGCTATGCTTCATTCTTCCTTTCG
>TR145|c0_g1_i1 len=5732 path=[6132:0-265 6133:266-289 6148:290-997
@6145@!:998-1443 6147:1444-2048 6117:2049-2072 6144:2073-4019 6122:4020-
4035 6146:4036-4615 @6143@!:4616-4769 6130:4770-4786 6131:4787-5731] [-1,
6132, 6133, 6148, 6145, 6147, 6117, 6144, 6122, 6146, 6143, 6130, 6131, -
2]
TTCCGATCTGTAAGGCATTCTTGACAAGCATGATGGTATGCCGAAGATGACCGAAAATAATTTCAATCAAACG
AACACCATGGGTTATCGAATAAACTTACAGCGGACTGTTGATCAAAACAACAAGTTAGCTTTCCAACAACAAC
CCCGGCGAACTTCCACCGGCGACATCCCCATCACCGAAGATAAGCCTCACATAATGCAACCACCAACGATAAA
TGAAAAGGTGCTTGACAGCGACGACGCGATGACCAGTGGCATGATGGACACATACATGGCGAGCGAGGAGGGG
TACGACAGCGGGGATGGCAGCGGTTCCACGAAATCTTTCGAAGCGCTGCTACAGGATAGCACGCCACTCGAGG
TGGACGACCTCGTTGCCGCCGTCCTGTCATCTACGCCGGCGCCGGCGAGGAACAGCAGGACGAAATCCAAAAA
ATTTAACATCGTCGAGGGGGGGGGAGGCAGGTAGACCCAACAATTATTCAGTCCGAGCAGGTCCGTATGGTAAG
AAGGAAATGGCGACAATTCACGACGAGCCGCAGTTTCCCAGTGATAGGGAGAGACCTCACGTCAAATTTTCAG
GCCCGCCGTCCAACACGCCGTACAAGAAATCAAGGAGTTTTGACAACTTGATGTTGAAAGATGAGAAAACCCG
GCGCGGTCGACTGGGCTCTTCGGGAGATCTTGCGGCCCTCGATGACGATCCTATGCCACCTGCGAGGTCGAAG
AGTTTTCACGCCCAAGAGAAACCCCGACAGGATGAGAAACCCCGGCGCGTTCGATTGGGCTCTTTGGGAGATC
GTGCGGCCCTTGACTACGACCTGTCTCACGACTTCCTGGACGTGAGGGGGGATGGAGGGTCGGGCAGGTTGAC
CGCTGACAAGCCACCCGCGAAGTCGATGAATTTTCACGTCGCGAATTCGATGGGTTTTCATGCTAAGGAGGAA
CCCCAGAACTTAGTCCGAGGTTTTTCCACCCTCTCAAACGAGAGTGAAAGTGTGCCCCAGCATCATAATGTGT
CAGGCAAAAAGAATTTGCCTCCTTCTATAATAGCCCAGAAGTCAACGAGTTTTCACGATGATTCTAAAAGAAA
CCAGCATGCCCTTCCTCCCCCAAAACGTGATGCCGACCCATTCTCGAACCTCTCAAATGATAGGCAAAGACTT
GACTATTTAGTAAACAAGTTGAACCAGGCTGGTATATCACATGAAGAGCTAACGATTCGTAGCGGCCGTTCAA
AATCATCTACGAATAGTGGCAAGTCCAAGGCGACATCTTTCCACTCCGCCTCACCAGTAAAATCAATACGTGT
ATCCAATTCCGCGACAGGGGGACAGCAGCAGAACAATCAAAAAAAGGTTGACGAGCTGGCCAGTCGTCTCATC
AACTTAAAAAAAATGTCTAAATCCACAGCGAAAACAGGCAATACCAGTGCGTTAAGGTCACAGCCATATCGAA
ACGATCGAGGGGCACAAACAGGCTCTAATTTAGCAACTCCCAATGGTACAAAGGTTACCAGTACCCTCGGCAC
TGACAAGGCTGATGCCGTTCCGACACCGAGACGCCACCATAATCATGAGAATGGCTCTTCTGGTTCTGGTTCT
CTACCCAGTGTGCCATCACTGCCTTCAGCCGATTCGTACAGCAAAAAGAACAGTAGAAAACCCCCTCCGCCCC
CTCCGCGTCGTCCTCATCGAGATGTAGGACCGCCGTCGGCCAAAAGCAGACCTCCTCCACCGCCACCATCAGT
AACTGTGATATCAACGAATAGGAACAACAATGGAGAGTGGAATGCGTCAGCATCCTCGACTTCCCGTTTATCC
ATAGATTCTGAAGTCATCAATAGCATAATGAATGATCATCAATGCAGTAATAATGAACAGGAGTACGGTAGCT
TCAAAAATATGCTGCAGCGGCGCTCTAGCGATGGAGGTGGGTTCAGCGAAAGGCTGTCTCCCAGGAAAAATTA
CGATTCTGGGAACACTTCAAAGAGTCCGAACCACAAGGGTGGTAGCTTTGATCATCACAACGGTAGCAGCATT
AAGAGCAGTGGCAGTAATATCCGCAAGGGCGGCAGCTTTGATCATCACAACGGTGGCAGCAGCAAGAATAAGA
ATAAAAGTGAGCCAGCATTGGAGGTCCTATCTGAGTACGCCAAAATGTCGACGGAACAGCTCGCCGAAGTCGA
GGCCTCCAACTTCCAGAAATTAGGCTCGCAATCCTTTCCAGAGTTGGACCCCTCGGCCCTTTATCGTCCCATT
CATGGTGACAGTAGTGACGGCAACGAGAGAAGCGGCAGCTACAACAACAGCAACAGCAAACATAATCATCACG
CCAGCCTGGCTGGTCATGAGTTCAACCATTCTTCCGCATCCTTCGCCCCTCGCCCTGTCCGCGTTACTCAGTT
TGAGGACAATGGGAAAAATATGGATACTCCGTCCACTCCAAAAAGTCGGCCGACGGCAGTGGCGACCAAAACC
TTTTTCAATTCCATGCGTAAACCTGACAATGACGGAGCAAATGATTCCGTGAATAATACTCCCATGGAAATAC
CCAATACCTACTACGCAACTCCAAGTTCGGCCTCCAAAAATAAGACCCCGTCGGTATCCTCTGGGATGACCTC
CAACTCCGGCAGGACTTACCCCTCCAAGTGCACCGCTGACAACCTACCAGATAATGTCGACAAGAAGGGAAGG
TGCATCCGCCATCCGAGAGTAAAAATCTTTAAGAAGAAATTAATGGGGGGGTACGAGCAGGTTCGCGACATGT
GCCCAAAATGCCATGAGGAGGCTCCCTACGAGAATTTGTGGAGGGGCCGAGCACGGGTCAAGCCGATGAAGAA
TTCGCAACACTCAAGCAGGGGACCGCAGTCAAGAGACCAGTCTAAGGAGCGGAAGCCAATGTTAAAAGGACGG
ACGTCGCGATCCAGGGAGAGAAAATGCGGCAAGCTGGATGACACTGCTTCGACGCACCCTGGCGTTCCATCGT
TGTTACATGGTGGAGAAGCGGAGCTGCCCATGCAGCAGGAGGAGCCCATGCTGAGGACAGGATCAGGACCGTC
AATGGAGCCCAACATCCAGAATAATCTGATTCCTCCTACACCGCTGATGTCAGCCCCGACGCTAACACCACCT

CCTTTGAATCAGGATAATATTCCTGTCCCATCACCTCGTCGCATGGATAGGAGACCCGAGAGGGGAAGGTCCA
ATCTGCGAGGGCGTACTCGGTCGTCGGCATCATCGAACAACGGTCGGTCGACGCCCGTCCCGGAAAGCAAGAA
GCTCAGGGCGAGGTCTTCGTCGCGAACCCCCGCTCACGAAGAATCAGCGACTGTATTAGCTCGCGATGGAAGA
ACCATGGGCAACCGCAGCGCATCCCCCTACTCGAACAAGAAATCGCATAAGAACAAATTTGACCAGAAGACAG
GACGATGTAAGATTCACCCTTCCATCTTAATGGCTAGAAAGTCCAAGTTTAACAAGGGATGGGATATACTTCG
CGAATGTCCGATTTGTGACGAAGAGAGAAAGGCCACCAATCCACATCATAGAAGGGGGGGAGACGTGGATGAA
TTTGATGAAATATCCAAGAAGAAGATGAATGCCTTGCTTAGCGGCAGTGATACCACGGATGTTGACGGGGGCG
AATGCCCGCAGCATTCATTAGATAAGACTCCACCCCCCTCTGGTCCAATGAATTATAGTTCTCGAACAACTCC
ACGCAAAAATTCCAGTTTACACGAAACCAACCAAACGAACAAACAAATCAGCACCAAAGGCTCCCGCCGCAGT
CTTACGCCCGATCACCTTGACTTCATGGACAATGCAAATCCCTCCAATGATCTCGAAGCGGGCTACGTGTCAC
GAATGCCCTATATCACCCCCTGGGGCGAGTCTGGATGGTACACTGGTGAGATAAACAAGGCGGGTAAACCCCA
TGGGAAGGGTCGAATGAGGGCCAATAATGGGAATTCAATCGATGGCAAGTGGGTCAATGGTTACTCGAGCGTT
TTTTTGGAGAAGCAAGGCAGAATGAAGAGCGGATTCGGGACTAATATGGCCAAGTGGGAGGAGAAGTATCTTG
CGGATCCTAATGGAGTTCGAAGATCTCGCTCGGGGAGCCGAGGTCGATCGCCATCGAGGCAATCCCATGCCCA
ATCTACTCATTCTATGGCTCCTGTTCGACGATCTCGCTCGAGGAGCCGGGGTCAATCGCCATCAGAACAACCT
CATGCCCAATCTTCTCATTCCGTGGCTCAATCCTCTCATTCCATGGCTCAGTATCAAGGGCACTATTTGGCGC
CACAAATCGCCAGTCCATATGAATATCCAGTCCCACCTTCACCACAGGCAACGTACCATCCATCTCTTTATGG
AATGGCCCCTGCTCACTCGCAACCAATGGAAATATGGGACCACCCTCCTGGTGGCTATCAGAGTTATCAGTCG
CAATTTTATAGTGGGGGCCATGACAACAGGCCTTCTCCTCCTGCCTACCATCCCGGCTATCATTAGTAGGTTG
CTTCGGACTCGCTTCTGGCCGTCGACCTTCAGATGGAATGCTTGACTACAGGAGTATACACTTTTCCTTTTTT
GTGTAGAAGAAACAGCTCAGAACTGAACTGAGTGGTGTAGAGAAGCGGAGCGTTTTAATCAACACTTCTTCGC
GTCATTATGGGGAACCGTGGGAATGCTAATGTAAAGTAAATGCATGCTACAAACCAAGCAAGATCTTAGAAAA
AGCTTCAGATTGTCAAAGCTTTTTTAAGAAACCAATAAGTAACCCTTTCCAGCCTACAGACCACTTTATTTTG
CAAAATTTACAGAAAGTGGATTCTGCCAAACCCAATAGCCTACCCTGTTCTGCTGGGGTTTCTTATCTCAGGG
AAGTTCGCGGTGTACTAGCATAAGATCTTTTGATGGATGGTTTGATTACAAGGAAGCTCTTCATGATGTTCTC
ACCGGTGAACGTTCGATCAAAAGGGTGATCTTCAATCGGATCTCCTCGTTTTCCATTTATCATTTCTGGCAAG
TCCTCACTGTTGAGATAGGCAACTGGTATCCTAACTGATTTGTTTGTCTTGTGGCATTCGGACGCCTCTGTTT
GTTCACAAGTATTCTGTGCAAACACCGACTGCACCTTCTTGTCAGTCCAATCCTTGTACGTTATGTACAAGAA
GTCAAGCTCTTGAGATACTGACATGGAGTTTGGAAGATCCACCATGATGTGGAAGCCCTCGGTATTCATATCA
GCGCGTAACTTTATGCTCCTCTCACATTTGTACCGGCGGCCAACACCGCTGTGCCCTTGGACAAGCTTGCTCC
CTTCCGACTTGAATTTTGGACAACAATTGGAGAAAGAGAACCATAAAGCAAGCAGGTTTCAATAAATAGATCC
TCCTGCATCACCAAGCCACTTATTAACATTTGGATAAGTAATCCAAAACGTGCACCGCCCACTCCTCCATCTC
CACCGATCATGTGGAACTTACCATGTCTGAATACTTCTGTTGTGCACAGGTTACTAAGTTAGCTTGGACCAGT
ACCACTTCATGCTAGAAATCTGCTAGGTTAGCTTTATAGTTGTACCTGCTTGCTTGTGCACTGCAGTATTGCT
GCAGCACACAGCCAAACCCTAGGGTATATGTACATACATACTTACTTCAGCAAGTGATAAATTGATACAGTGT
TTTTACCTTATAGTTAGTGCGGAGATCGGAAGAGCGTC
>TR146|c0_g1_i1 len=2319 path=[4242:0-37 4317:38-80 4309:81-248 4323:249-608 4324:609-612 4308:613-632 4295:633-852 4272:853-876 4320:877-964 4294:965-1063 4262:1064-1087 4263:1088-1106 4314:1107-1364 4325:1365-1388 4329:1389-1396 4218:1397-1420 4304:1421-1424 4291:1425-1512 4213:1513-1536 4319:1537-1774 4232:1775-1798 4312:1799-2009 4326:2010-2012 4171:2013-2029 4322:2030-2033 @4318@!:2034-2288 4327:2289-2306 4330:2307-2307 4180:2308-2318] [-1, 4242, 4317, 4309, 4323, 4324, 4308, 4295, 4272, 4320, 4294, 4262, 4263, 4314, 4325, 4329, 4218, 4304, 4291, 4213, 4319, 4232, 4312, 4326, 4171, 4322, 4318, 4327, 4330, 4180, -2]
CCCCAATAAGATAGCCCCGCCGCGCTGGCCATTATTTTGTTTGTACGACTGCAGCCTACCGCATCACATTTTC
TACTGCCCGTAGCACTGGGGTGACTGGGCTACTACCATCAACATCACCTGTCTATCGTGTCGATCCAATCATC
ACACACCACCAAACAAACCCACCATGTCCGACGACGAAGAATCCAACGCCCCGCCCTCCGGCGCCGTCCACCA
AGTAGTACCCCGCCTCCAATCCGGCCCGGCCCAAACTACCACCTCCGAACTCGCCGCGGGACTCTCCCGCTGG
ATGGACGCCTCCAACAGCTTCATCTACGTGGAACTCGCCTCCCTCGTCCTCCTCTTCGCATGCGTCGCTGATT
GGTATCCCACCGTATGGCACAAATACGCGCTGAGCGTGGCGTGCGTTTCGTTGTTGGTGTGTCTGATATTGCA
GACGGCGGAGTTTTTGGTCCCGGGGTTTTTGGAGTGGTCGGTGGTGAAGCAGAGGGATGATGGGACGGGTGGG
CATTCGATTCAGAAGGTTTGTTCGGTGTTGATGTTGCTTTGGTGGATTTTTGGGACAGGGATTATTACCTTTC
ATGCTCCATTCGTCGTGACATCCAACGGTTGGTTCGGCGCTTGGGGAGGTCTCCTCGCCACAGTAAAATGGTG
CATCGGACTCAAATTTTCGGTCTACGATGATCAACCACCGGGACTCAAACAACTATACTACATTTTTACGTGC
TCCATCATCTTGCTGCTCGCTTCCATCCCCCCGCTCATTCAAAAATGGGAACATTATGGCGGAGCCGGATTTT
CGATAGCTGGCTCCGCGCTCACAATCATTGCCTGCGCTTACATGGTGACCATGTACAGTGACATTCCGAAAAG
CGTGTTGAAGATCAACGTGGTTGTGCTTTTCGTGCTTTGGACTCTTGTTGCGGGAGTTTGCACATTTCACGGA
CCTTTTCTGATCACGAGTAAGTTTTTTTACGCTGTTGGATTTATGAATATGTTTTGTTTGTTCGTACATATCA
CTGTTTATTGACACGTCATTCTATCCATACGTCCGTACGCTCCAATCATTTGCGTGGAATAGACAATGGGTTC

TTTGCATGTTGGTTGGGCTGCCTTTGTTCACTGAACCTAATGGTGATTGAAATGAAAGATGCCTCTCCAGAAA
ACTACACGTAACATGCCGCCAGTGCAGCGAAAGCCACCGATTTTTGTACAAATTAGGCTCAACAATTAACTTT
GGTTATTGGGATGTTTTAACTCAGCACTTCAATTCCGTAAAAACACTGTCTACGGGATGTTTGCGCGGAATGT
GTTTAGTGTCTTCTTGTTTCAGTCGAGGAGAAGATGTTATAATGAAGGTGTGTCCGTGGCGAATCATGTATGG
TGTAGGCGGGTAGGTAATTGGGAGCATATCATGCCGTATGTAGGCACAACCAACAAATTCATAAAATTAAGCT
AATGCCACGTTGATAGCATTACCAATAACCAACCCTCACAGGTGAGAGGGGGTTCCTCGAATGCAGCCAAAGC
TACAGGTGTGTATTCAGCACCACTGAAAAATCATCAAGTGGAATCTAAGCCCGGAAAACCAGCCACCTCCAAA
TGCATCACAATTCCGGGGACGACTGTGGTTACAACGGGCCACCAACATCTCACCAAGTGTATAATCACCCCTG
TGAGGAGCATCACCACCCCATAGACCAGATGAGCCACCTATCCCTGGGTCAATTTAAAATGTCGCTTCCCAGT
GAATTGTCGAATGGTATGGTATCATTACTGAGAATGCCAGAGAATATGGTAGGACACACTACGTACTTAGAGA
GGTGAAATGCCATTGAAAATAAAACTCTACCCAAGTACATGGGATGTAATTTTAAATTTGAGATATACTCCTA
CTTAGATTCTTTTCATAAGCTGGTTGCACGTTACTTCGCCACTAACACAAGAGATTTTCAATACAAGTCACCA
TAAAATTCCAGCTTCTCTCATCATGATGAATTGGGGAAAAAACACTTTTTCCTCAAAAAAAATTATTACTTTG
ACCTCTTGTCAAGAGCATGGTATCTTTACTGGGATTACACCCGACTGACATTGGAATTGAAACTCTACCAACA
GTAGAGACATGGATTCTTTTCATAAAACTGATTGTTCTTTACTTTGTCGCTCACAGATAGTATGTTTCGACCT
CAATCATCATACAATTTCATCCTCTCTCGTTATGATGAATTGAAGAAAGAACGTAGACAGAAGAAAGCCGCCC
CAACATGCAAAGTATCCATTTCCTGCTTGAAGGAATGGCCCATCAGATCGGAAGAG
>TR146|c0_g1_i2 len=1416 path=[4201:0-48 4323:49-408 4324:409-412
4307:413-535 4295:536-755 4272:756-779 4320:780-867 4294:868-966
4262:967-990 4263:991-1009 4314:1010-1267 4325:1268-1291 4329:1292-1299
4218:1300-1323 4304:1324-1327 4291:1328-1415] [-1, 4201, 4323, 4324,
4307, 4295, 4272, 4320, 4294, 4262, 4263, 4314, 4325, 4329, 4218, 4304,
4291]
CCTCCGGCCCCGTCCACCAAGTAGTCCCCCGCCTCCAATCCGGCCCGGCCCAAACTACCACCTCCGAACTCGC
CGCGGGACTCTCCCGCTGGATGGACGCCTCCAACAGCTTCATCTACGTGGAACTCGCCTCCCTCGTCCTCCTC
TTCGCATGCGTCGCTGATTGGTATCCCACCGTATGGCACAAATACGCGCTGAGCGTGGCGTGCGTTTCGTTGT
TGGTGTGTCTGATATTGCAGACGGCGGAGTTTTTGGTCCCGGGGTTTTTGGAGTGGTCGGTGGTGAAGCAGAG
GGATGATGGGACGGGTGGGCATTCGATTCAGAAGGTTTGTTCGGTGTTGATGTTGCTTTGGTGGATTTTTGGG
ACAGGGATTATTACCTTTCATGCTCCATTCGTCGTGACATCCAACGGTACGACAATCATGTATTAATTTATTA
TATGGACTGTATCATAATTTTTATCAACACCTCGTTCCATAACCTCAACACGACACCTATTCCTACCAACGCA
CAGGTTGGTTCGGCGCTTGGGGAGGTCTCCTCGCCACAGTAAAATGGTGCATCGGACTCAAATTTTCGGTCTA
CGATGATCAACCACCGGGACTCAAACAACTATACTACATTTTTACGTGCTCCATCATCTTGCTGCTCGCTTCC
ATCCCCCCGCTCATTCAAAAATGGGAACATTATGGCGGAGCCGGATTTTCGATAGCTGGCTCCGCGCTCACAA
TCATTGCCTGCGCTTACATGGTGACCATGTACAGTGACATTCCGAAAAGCGTGTTGAAGATCAACGTGGTTGT
GCTTTTCGTGCTTTGGACTCTTGTTGCGGGAGTTTGCACATTTCACGGACCTTTTCTGATCACGAGTAAGTTT
TTTTACGCTGTTGGATTTATGAATATGTTTTGTTTGTTCGTACATATCACTGTTTATTGACACGTCATTCTAT
CCATACGTCCGTACGCTCCAATCATTTGCGTGGAATAGACAATGGGTTCTTTGCATGTTGGTTGGGCTGCCTT
TGTTCACTGAACCTAATGGTGATTGAAATGAAAGATGCCTCTCCAGAAAACTACACGTAACATGCCGCCAGTG
CAGCGAAAGCCACCGATTTTTGTACAAATTAGGCTCAACAATTAACTTTGGTTATTGGGATGTTTTAACTCAG
CACTTCAATTCCGTAAAAACACTGTCTACGGGATGTTTGCGCGGAATGTGTTTAGTGTCTTCTTGTTTCAGTC
GAGGAGAAGATGTTATAATGAAGGTGTGTCCGTGGCGAATCATGTATGGTGTAGGCGGGTAGGTAATTGGGAG
CATATCATGCCGTATGTAGGCACAACCAACAAATTCATAAAATTAAGCTAATGCCACGTTGATAGCATTACCA
ATAACCAACCCTCACAGGTGAGAGGGGGT
>TR146|c0_g1_i3 len=3168 path=[4245:0-108 4317:109-151 4309:152-319
4323:320-679 4324:680-683 4308:684-703 4295:704-923 4272:924-947
4320:948-1035 4210:1036-1058 4314:1059-1316 4325:1317-1340 4329:1341-1348
4218:1349-1372 4304:1373-1376 4291:1377-1464 4213:1465-1488 4319:1489-
1726 4232:1727-1750 4312:1751-1961 4326:1962-1964 4171:1965-1981
4322:1982-1985 @4318@!:1986-2240 4327:2241-2258 4330:2259-2259 4288:2260-
2984 4249:2985-3008 4250:3009-3009 4310:3010-3052 4328:3053-3076
4331:3077-3080 4286:3081-3119 4205:3120-3167] [-1, 4245, 4317, 4309,
4323, 4324, 4308, 4295, 4272, 4320, 4210, 4314, 4325, 4329, 4218, 4304,
4291, 4213, 4319, 4232, 4312, 4326, 4171, 4322, 4318, 4327, 4330, 4288,
4249, 4250, 4310, 4328, 4331, 4286, 4205, -2]
ACACGAGCGAACCCACATCGAACAGCCCCACCCACAAGTTCGGCAAGCAACACAGCCAGTGGCAACATCTGCC
CCAATAAGATAGACCCGCCGCGCTGGCCATTATTTTGTTTGTACGACTGCAGCCTACCGCATCACATTTTCTA
CTGCCCGTAGCACTGGGGTGACTGGGCTACTACCATCAACATCACCTGTCTATCGTGTCGATCCAATCATCAC
ACACCACCAAACAAACCCACCATGTCCGACGACGAAGAATCCAACGCCCCGCCCTCCGGCGCCGTCCACCAAG
TAGTACCCCGCCTCCAATCCGGCCCGGCCCAAACTACCACCTCCGAACTCGCCGCGGGACTCTCCCGCTGGAT
GGACGCCTCCAACAGCTTCATCTACGTGGAACTCGCCTCCCTCGTCCTCCTCTTCGCATGCGTCGCTGATTGG

TATCCCACCGTATGGCACAAATACGCGCTGAGCGTGGCGTGCGTTTCGTTGTTGGTGTGTCTGATATTGCAGA
CGGCGGAGTTTTTGGTCCCGGGGTTTTTGGAGTGGTCGGTGGTGAAGCAGAGGGATGATGGGACGGGTGGGCA
TTCGATTCAGAAGGTTTGTTCGGTGTTGATGTTGCTTTGGTGGATTTTTGGGACAGGGATTATTACCTTTCAT
GCTCCATTCGTCGTGACATCCAACGGTTGGTTCGGCGCTTGGGGAGGTCTCCTCGCCACAGTAAAATGGTGCA
TCGGACTCAAATTTTCGGTCTACGATGATCAACCACCGGGACTCAAACAACTATACTACATTTTTACGTGCTC
CATCATCTTGCTGCTCGCTTCCATCCCCCCGCTCATTCAAAAATGGGAACATTATGGCGGAGCCGGATTTTCG
ATAGCTGGCTCCGCGCTCACAATCATTGCCTGCGCTTACATGGTGACCATGTACAGTGACATTCCGAAAAGCG
TGTTGAAGATCAACGTGGTTGTGCTTTTCGTGCTTTGGACTCTTGTTGCGGGAGTTTGCACATTTCACGGACC
TTTTCTGATCACGAACAATGGGTTCTTTGCATGTTGGTTGGGCTGCCTTTGTTCACTGAACCTAATGGTGATT
GAAATGAAAGATGCCTCTCCAGAAAACTACACGTAACATGCCGCCAGTGCAGCGAAAGCCACCGATTTTTGTA
CAAATTAGGCTCAACAATTAACTTTGGTTATTGGGATGTTTTAACTCAGCACTTCAATTCCGTAAAAACACTG
TCTACGGGATGTTTGCGCGGAATGTGTTTAGTGTCTTCTTGTTTCAGTCGAGGAGAAGATGTTATAATGAAGG
TGTGTCCGTGGCGAATCATGTATGGTGTAGGCGGGTAGGTAATTGGGAGCATATCATGCCGTATGTAGGCACA
ACCAACAAATTCATAAAATTAAGCTAATGCCACGTTGATAGCATTACCAATAACCAACCCTCACAGGTGAGAG
GGGGTTCCTCGAATGCAGCCAAAGCTACAGGTGTGTATTCAGCACCACTGAAAAATCATCAAGTGGAATCTAA
GCCCGGAAAACCAGCCACCTCCAAATGCATCACAATTCCGGGGACGACTGTGGTTACAACGGGCCACCAACAT
CTCACCAAGTGTATAATCACCCCTGTGAGGAGCATCACCACCCCATAGACCAGATGAGCCACCTATCCCTGGG
TCAATTTAAAATGTCGCTTCCCAGTGAATTGTCGAATGGTATGGTATCATTACTGAGAATGCCAGAGAATATG
GTAGGACACACTACGTACTTAGAGAGGTGAAATGCCATTGAAAATAAAACTCTACCCAAGTACATGGGATGTA
ATTTTAAATTTGAGATATACTCCTACTTAGATTCTTTTCATAAGCTGGTTGCACGTTACTTCGCCACTAACAC
AAGAGATTTTCAATACAAGTCACCATAAAATTCCAGCTTCTCTCATCATGATGAATTGGGGAAAAAACACTTT
TTCCTCAAAAAAAATTATTACTTTGACCTCTTGTCAAGAGCATGGTATCTTTACTGGGATTACACCCGACTGA
CATTGGAATTGAAACTCTACCAACAGTAGAGACATGGATTCTTTTCATAAAACTGATTGTTCTTTACTTTGTC
GCTCACAGATAGTATGTTTCGACCTCAATCATCATACAATTTCATCCTCTCTCGTTATGATGAATTGAAGAAA
GAACGTAGACAGAAGAAAGCCGCCCCAACATGCAAAGTATCCATTTCCTGCTTGAAGGAATGGCCCATCAAAA
GTGCACACTCCAGCTACAGTCGCCCAAATCACAAACAAGCCTATAGCTGTAACCTTCACCAACTGCGCAGGAA
TATCATTAAATGCCTTGAAAAACACACCAGTAATAATCATGGTAATCAACCCCGCCGACAAACCCCACGCCGA
CTGACCGACGAAAACCTTATACCTCAAAAACGGAATGCACGCAAATAGCAGCACGGCCGCGGCCGTAAACATT
CCCGCCAATATCTTCCTCCCCTTCTCCAAATCCGTAAATCGCGCCGTATCAATATTTAATGCCCAATGAGTCG
TGAAGATCAAACCCGCCCACGCGGAAAAGTACCCATTTCCCACCCCGGTAAAAGGAGCCCTAAATGTAATAAC
GCCGGTGCCTACCACCCACCACACGAATAAGAAGAGACTAACCGGCTTCTCGACTCGTTCCAACATACCCGGC
CTCATAAATTCGCCAGTTTGAATGATGAGGCATGCCGCTAGGGAGATGACCGATACGGATAGAGCGTAGGCGA
GTTCGGGATATCCCTTGGTCCATACGCCTATGCAGGCGAACATTAGGACGAGGGAGGAGACTTCGACGTACAT
GAAGGATTTGGCGACTGCGGACCATGCTTTGGTTGCTTCGACTATTTCGGCGGGGGAGGTTTGGGCGCTGGTT
GGCGTGCCGGGTGCCGGGGTGGTGGGGGCGTGGTCTTCTCCGTCGCTGCTCATTTTGTTGTTGGTGTGGTGGA
TGTAGTGCTAGACGGATCGTCTTCGATTTGATGAGAAGAGAAGCACTACTGTCTGCTGTAGTGCTGTATTGTT
TGTATCTCTCGAGTCTCTTGAGGGTACTTTTCAGGCTTGTATTTTATTTGCGACTCTCTCTTGGTTCTTCTGG
CTGCGCGTTTCGTGTAGATCGGAAGAGCA
>TR146|c0_g1_i4 len=991 path=[4194:0-62 4195:63-86 4321:87-205 4324:206-209 4308:210-229 4295:230-449 4272:450-473 4320:474-561 4210:562-584 4314:585-842 4325:843-866 4329:867-874 4218:875-898 4304:899-902 4291:903-990] [-1, 4194, 4195, 4321, 4324, 4308, 4295, 4272, 4320, 4210, 4314, 4325, 4329, 4218, 4304, 4291]
GACAGGGATTATTACCTTTCATGGTGAGATAATATGAGATGTATTTGTGGAGCATATCACCGAGTATGGTGAT
GTGGTCATGGCGTCGCATGCACCTCATGCATATCATTTCATCTCTTTGCTCAACGAAATATCTTTCATCTCGT
TAATTTGACACACATGCGCACATTTTGCAAACAAACAGCTCCATTCGTCGTGACATCCAACGGTTGGTTCGGC
GCTTGGGGAGGTCTCCTCGCCACAGTAAAATGGTGCATCGGACTCAAATTTTCGGTCTACGATGATCAACCAC
CGGGACTCAAACAACTATACTACATTTTTACGTGCTCCATCATCTTGCTGCTCGCTTCCATCCCCCCGCTCAT
TCAAAAATGGGAACATTATGGCGGAGCCGGATTTTCGATAGCTGGCTCCGCGCTCACAATCATTGCCTGCGCT
TACATGGTGACCATGTACAGTGACATTCCGAAAAGCGTGTTGAAGATCAACGTGGTTGTGCTTTTCGTGCTTT
GGACTCTTGTTGCGGGAGTTTGCACATTTCACGGACCTTTTCTGATCACGAACAATGGGTTCTTTGCATGTTG
GTTGGGCTGCCTTTGTTCACTGAACCTAATGGTGATTGAAATGAAAGATGCCTCTCCAGAAAACTACACGTAA
CATGCCGCCAGTGCAGCGAAAGCCACCGATTTTTGTACAAATTAGGCTCAACAATTAACTTTGGTTATTGGGA
TGTTTTAACTCAGCACTTCAATTCCGTAAAAACACTGTCTACGGGATGTTTGCGCGGAATGTGTTTAGTGTCT
TCTTGTTTCAGTCGAGGAGAAGATGTTATAATGAAGGTGTGTCCGTGGCGAATCATGTATGGTGTAGGCGGGT
AGGTAATTGGGAGCATATCATGCCGTATGTAGGCACAACCAACAAATTCATAAAATTAAGCTAATGCCACGTT
GATAGCATTACCAATAACCAACCCTCACAGGTGAGAGGGGGT
>TR146|c0_g1_i5 len=3352 path=[4245:0-108 4317:109-151 4309:152-319 4323:320-679 4324:680-683 4307:684-806 4295:807-1026 4272:1027-1050 4320:1051-1138 4210:1139-1161 4314:1162-1419 4325:1420-1443 4329:1444-

1451 4218:1452-1475 4304:1476-1479 4291:1480-1567 4213:1568-1591
4319:1592-1829 4232:1830-1853 4312:1854-2064 4326:2065-2067 4171:2068-
2084 4322:2085-2088 @4318@!:2089-2343 4311:2344-2442 4330:2443-2443
4288:2444-3168 4249:3169-3192 4250:3193-3193 4310:3194-3236 4328:3237-
3260 4331:3261-3264 4285:3265-3303 4270:3304-3351] [-1, 4245, 4317, 4309,
4323, 4324, 4307, 4295, 4272, 4320, 4210, 4314, 4325, 4329, 4218, 4304,
4291, 4213, 4319, 4232, 4312, 4326, 4171, 4322, 4318, 4311, 4330, 4288,
4249, 4250, 4310, 4328, 4331, 4285, 4270, -2]
ACACGAGCGAACCCACATCGAACAGCCCCACCCACAAGTTCGGCAAGCAACACAGCCAGTGGCAACATCTGCC
CCAATAAGATAGACCCGCCGCGCTGGCCATTATTTTGTTTGTACGACTGCAGCCTACCGCATCACATTTTCTA
CTGCCCGTAGCACTGGGGTGACTGGGCTACTACCATCAACATCACCTGTCTATCGTGTCGATCCAATCATCAC
ACACCACCAAACAAACCCACCATGTCCGACGACGAAGAATCCAACGCCCCGCCCTCCGGCGCCGTCCACCAAG
TAGTACCCCGCCTCCAATCCGGCCCGGCCCAAACTACCACCTCCGAACTCGCCGCGGGACTCTCCCGCTGGAT
GGACGCCTCCAACAGCTTCATCTACGTGGAACTCGCCTCCCTCGTCCTCCTCTTCGCATGCGTCGCTGATTGG
TATCCCACCGTATGGCACAAATACGCGCTGAGCGTGGCGTGCGTTTCGTTGTTGGTGTGTCTGATATTGCAGA
CGGCGGAGTTTTTGGTCCCGGGGTTTTTGGAGTGGTCGGTGGTGAAGCAGAGGGATGATGGGACGGGTGGGCA
TTCGATTCAGAAGGTTTGTTCGGTGTTGATGTTGCTTTGGTGGATTTTTGGGACAGGGATTATTACCTTTCAT
GCTCCATTCGTCGTGACATCCAACGGTACGACAATCATGTATTAATTTATTATATGGACTGTATCATAATTTT
TATCAACACCTCGTTCCATAACCTCAACACGACACCTATTCCTACCAACGCACAGGTTGGTTCGGCGCTTGGG
GAGGTCTCCTCGCCACAGTAAAATGGTGCATCGGACTCAAATTTTCGGTCTACGATGATCAACCACCGGGACT
CAAACAACTATACTACATTTTTACGTGCTCCATCATCTTGCTGCTCGCTTCCATCCCCCCGCTCATTCAAAAA
TGGGAACATTATGGCGGAGCCGGATTTTCGATAGCTGGCTCCGCGCTCACAATCATTGCCTGCGCTTACATGG
TGACCATGTACAGTGACATTCCGAAAAGCGTGTTGAAGATCAACGTGGTTGTGCTTTTCGTGCTTTGGACTCT
TGTTGCGGGAGTTTGCACATTTCACGGACCTTTTCTGATCACGAACAATGGGTTCTTTGCATGTTGGTTGGGC
TGCCTTTGTTCACTGAACCTAATGGTGATTGAAATGAAAGATGCCTCTCCAGAAAACTACACGTAACATGCCG
CCAGTGCAGCGAAAGCCACCGATTTTTGTACAAATTAGGCTCAACAATTAACTTTGGTTATTGGGATGTTTTA
ACTCAGCACTTCAATTCCGTAAAAACACTGTCTACGGGATGTTTGCGCGGAATGTGTTTAGTGTCTTCTTGTT
TCAGTCGAGGAGAAGATGTTATAATGAAGGTGTGTCCGTGGCGAATCATGTATGGTGTAGGCGGGTAGGTAAT
TGGGAGCATATCATGCCGTATGTAGGCACAACCAACAAATTCATAAAATTAAGCTAATGCCACGTTGATAGCA
TTACCAATAACCAACCCTCACAGGTGAGAGGGGGTTCCTCGAATGCAGCCAAAGCTACAGGTGTGTATTCAGC
ACCACTGAAAAATCATCAAGTGGAATCTAAGCCCGGAAAACCAGCCACCTCCAAATGCATCACAATTCCGGGG
ACGACTGTGGTTACAACGGGCCACCAACATCTCACCAAGTGTATAATCACCCCTGTGAGGAGCATCACCACCC
CATAGACCAGATGAGCCACCTATCCCTGGGTCAATTTAAAATGTCGCTTCCCAGTGAATTGTCGAATGGTATG
GTATCATTACTGAGAATGCCAGAGAATATGGTAGGACACACTACGTACTTAGAGAGGTGAAATGCCATTGAAA
ATAAAACTCTACCCAAGTACATGGGATGTAATTTTAAATTTGAGATATACTCCTACTTAGATTCTTTTCATAA
GCTGGTTGCACGTTACTTCGCCACTAACACAAGAGATTTTCAATACAAGTCACCATAAAATTCCAGCTTCTCT
CATCATGATGAATTGGGGAAAAAACACTTTTTCCTCAAAAAAAATTATTACTTTGACCTCTTGTCAAGAGCAT
GGTATCTTTACTGGGATTACACCCGACTGACATTGGAATTGAAACTCTACCAACAGTAGAGACATGGATTCTT
TTCATAAAACTGATTGTTCTTTACTTTGTCGCTCACAGATAGTATGTTTCGACCTCAATCATCATACAATTTC
ATCCTCTCTCGTTATGATGAATTGAAGAAAGAACGTAGACAGAAGAAAGCCGCCCCAACATGCAAAGTATCCA
TTTCCTGCACACCAAATGAGTTTTTTAGAACATCGAACGACAAATAAGGAAACAATGAACATGGATAATTTTT
GTTTTGTCTCACCTGCTTGAAGGAATGGCCCATCAAAAGTGCACACTCCAGCTACAGTCGCCCAAATCACAAA
CAAGCCTATAGCTGTAACCTTCACCAACTGCGCAGGAATATCATTAAATGCCTTGAAAAACACACCAGTAATA
ATCATGGTAATCAACCCCGCCGACAAACCCCACGCCGACTGACCGACGAAAACCTTATACCTCAAAAACGGAA
TGCACGCAAATAGCAGCACGGCCGCGGCCGTAAACATTCCCGCCAATATCTTCCTCCCCTTCTCCAAATCCGT
AAATCGCGCCGTATCAATATTTAATGCCCAATGAGTCGTGAAGATCAAACCCGCCCACGCGGAAAAGTACCCA
TTTCCCACCCCGGTAAAAGGAGCCCTAAATGTAATAACGCCGGTGCCTACCACCCACCACACGAATAAGAAGA
GACTAACCGGCTTCTCGACTCGTTCCAACATACCCGGCCTCATAAATTCGCCAGTTTGAATGATGAGGCATGC
CGCTAGGGAGATGACCGATACGGATAGAGCGTAGGCGAGTTCGGGATATCCCTTGGTCCATACGCCTATGCAG
GCGAACATTAGGACGAGGGAGGAGACTTCGACGTACATGAAGGATTTGGCGACTGCGGACCATGCTTTGGTTG
CTTCGACTATTTCGGCGGGGGAGGTTTGGGCGCTGGTTGGCGTGCCGGGTGCCGGGGTGGTGGGGGCGTGGTC
TTCTCCGTCGCTGCTCATTTTGTTGTTGGTGTGGTGGATGTAGTGCTAGACGGATCGTCTTCGATTTGATGAG
AAGAGAAGCACTACTGTCTGCTGTAGTGCTGTATTGTTTGTATCTCTCGAGTCGATTGAGGGTACTTTGCAGG
CTTGTATTTTATTTGCGACTCTCTCTTGGTTCTTCTGGCTGCGCGTTTCGTGTAGATCGGAAGAGCA
>TR146|c0_g1_i6 len=3249 path=[4245:0-108 4317:109-151 4309:152-319
4323:320-679 4324:680-683 4308:684-703 4295:704-923 4272:924-947
4320:948-1035 4210:1036-1058 4314:1059-1316 4325:1317-1340 4329:1341-1348
4218:1349-1372 4304:1373-1376 4291:1377-1464 4213:1465-1488 4319:1489-
1726 4232:1727-1750 4312:1751-1961 4326:1962-1964 4171:1965-1981
4322:1982-1985 @4318@!:1986-2240 4311:2241-2339 4330:2340-2340 4288:2341-

3065 4249:3066-3089 4250:3090-3090 4310:3091-3133 4328:3134-3157
4331:3158-3161 4285:3162-3200 4270:3201-3248] [-1, 4245, 4317, 4309,
4323, 4324, 4308, 4295, 4272, 4320, 4210, 4314, 4325, 4329, 4218, 4304,
4291, 4213, 4319, 4232, 4312, 4326, 4171, 4322, 4318, 4311, 4330, 4288,
4249, 4250, 4310, 4328, 4331, 4285, 4270, -2]
ACACGAGCGAACCCACATCGAACAGCCCCACCCACAAGTTCGGCAAGCAACACAGCCAGTGGCAACATCTGCC
CCAATAAGATAGACCCGCCGCGCTGGCCATTATTTTGTTTGTACGACTGCAGCCTACCGCATCACATTTTCTA
CTGCCCGTAGCACTGGGGTGACTGGGCTACTACCATCAACATCACCTGTCTATCGTGTCGATCCAATCATCAC
ACACCACCAAACAAACCCACCATGTCCGACGACGAAGAATCCAACGCCCCGCCCTCCGGCGCCGTCCACCAAG
TAGTACCCCGCCTCCAATCCGGCCCGGCCCAAACTACCACCTCCGAACTCGCCGCGGGACTCTCCCGCTGGAT
GGACGCCTCCAACAGCTTCATCTACGTGGAACTCGCCTCCCTCGTCCTCCTCTTCGCATGCGTCGCTGATTGG
TATCCCACCGTATGGCACAAATACGCGCTGAGCGTGGCGTGCGTTTCGTTGTTGGTGTGTCTGATATTGCAGA
CGGCGGAGTTTTTGGTCCCGGGGTTTTTGGAGTGGTCGGTGGTGAAGCAGAGGGATGATGGGACGGGTGGGCA
TTCGATTCAGAAGGTTTGTTCGGTGTTGATGTTGCTTTGGTGGATTTTTGGGACAGGGATTATTACCTTTCAT
GCTCCATTCGTCGTGACATCCAACGGTTGGTTCGGCGCTTGGGGAGGTCTCCTCGCCACAGTAAAATGGTGCA
TCGGACTCAAATTTTCGGTCTACGATGATCAACCACCGGGACTCAAACAACTATACTACATTTTTACGTGCTC
CATCATCTTGCTGCTCGCTTCCATCCCCCCGCTCATTCAAAAATGGGAACATTATGGCGGAGCCGGATTTTCG
ATAGCTGGCTCCGCGCTCACAATCATTGCCTGCGCTTACATGGTGACCATGTACAGTGACATTCCGAAAAGCG
TGTTGAAGATCAACGTGGTTGTGCTTTTCGTGCTTTGGACTCTTGTTGCGGGAGTTTGCACATTTCACGGACC
TTTTCTGATCACGAACAATGGGTTCTTTGCATGTTGGTTGGGCTGCCTTTGTTCACTGAACCTAATGGTGATT
GAAATGAAAGATGCCTCTCCAGAAAACTACACGTAACATGCCGCCAGTGCAGCGAAAGCCACCGATTTTTGTA
CAAATTAGGCTCAACAATTAACTTTGGTTATTGGGATGTTTTAACTCAGCACTTCAATTCCGTAAAAACACTG
TCTACGGGATGTTTGCGCGGAATGTGTTTAGTGTCTTCTTGTTTCAGTCGAGGAGAAGATGTTATAATGAAGG
TGTGTCCGTGGCGAATCATGTATGGTGTAGGCGGGTAGGTAATTGGGAGCATATCATGCCGTATGTAGGCACA
ACCAACAAATTCATAAAATTAAGCTAATGCCACGTTGATAGCATTACCAATAACCAACCCTCACAGGTGAGAG
GGGGTTCCTCGAATGCAGCCAAAGCTACAGGTGTGTATTCAGCACCACTGAAAAATCATCAAGTGGAATCTAA
GCCCGGAAAACCAGCCACCTCCAAATGCATCACAATTCCGGGGACGACTGTGGTTACAACGGGCCACCAACAT
CTCACCAAGTGTATAATCACCCCTGTGAGGAGCATCACCACCCCATAGACCAGATGAGCCACCTATCCCTGGG
TCAATTTAAAATGTCGCTTCCCAGTGAATTGTCGAATGGTATGGTATCATTACTGAGAATGCCAGAGAATATG
GTAGGACACACTACGTACTTAGAGAGGTGAAATGCCATTGAAAATAAAACTCTACCCAAGTACATGGGATGTA
ATTTTAAATTTGAGATATACTCCTACTTAGATTCTTTTCATAAGCTGGTTGCACGTTACTTCGCCACTAACAC
AAGAGATTTTCAATACAAGTCACCATAAAATTCCAGCTTCTCTCATCATGATGAATTGGGGAAAAAACACTTT
TTCCTCAAAAAAAATTATTACTTTGACCTCTTGTCAAGAGCATGGTATCTTTACTGGGATTACACCCGACTGA
CATTGGAATTGAAACTCTACCAACAGTAGAGACATGGATTCTTTTCATAAAACTGATTGTTCTTTACTTTGTC
GCTCACAGATAGTATGTTTCGACCTCAATCATCATACAATTTCATCCTCTCTCGTTATGATGAATTGAAGAAA
GAACGTAGACAGAAGAAAGCCGCCCCAACATGCAAAGTATCCATTTCCTGCACACCAAATGAGTTTTTTAGAA
CATCGAACGACAAATAAGGAAACAATGAACATGGATAATTTTTGTTTTGTCTCACCTGCTTGAAGGAATGGCC
CATCAAAAGTGCACACTCCAGCTACAGTCGCCCAAATCACAAACAAGCCTATAGCTGTAACCTTCACCAACTG
CGCAGGAATATCATTAAATGCCTTGAAAAACACACCAGTAATAATCATGGTAATCAACCCCGCCGACAAACCC
CACGCCGACTGACCGACGAAAACCTTATACCTCAAAAACGGAATGCACGCAAATAGCAGCACGGCCGCGGCCG
TAAACATTCCCGCCAATATCTTCCTCCCCTTCTCCAAATCCGTAAATCGCGCCGTATCAATATTTAATGCCCA
ATGAGTCGTGAAGATCAAACCCGCCCACGCGGAAAAGTACCCATTTCCCACCCCGGTAAAAGGAGCCCTAAAT
GTAATAACGCCGGTGCCTACCACCCACCACACGAATAAGAAGAGACTAACCGGCTTCTCGACTCGTTCCAACA
TACCCGGCCTCATAAATTCGCCAGTTTGAATGATGAGGCATGCCGCTAGGGAGATGACCGATACGGATAGAGC
GTAGGCGAGTTCGGGATATCCCTTGGTCCATACGCCTATGCAGGCGAACATTAGGACGAGGGAGGAGACTTCG
ACGTACATGAAGGATTTGGCGACTGCGGACCATGCTTTGGTTGCTTCGACTATTTCGGCGGGGGAGGTTTGGG
CGCTGGTTGGCGTGCCGGGTGCCGGGGTGGTGGGGGCGTGGTCTTCTCCGTCGCTGCTCATTTTGTTGTTGGT
GTGGTGGATGTAGTGCTAGACGGATCGTCTTCGATTTGATGAGAAGAGAAGCACTACTGTCTGCTGTAGTGCT
GTATTGTTTGTATCTCTCGAGTCGATTGAGGGTACTTTGCAGGCTTGTATTTTATTTGCGACTCTCTCTTGGT
TCTTCTGGCTGCGCGTTTCGTGTAGATCGGAAGAGCA
>TR146|c0_g1_i7 len=1094 path=[4194:0-62 4195:63-86 4321:87-205 4324:206-
209 4307:210-332 4295:333-552 4272:553-576 4320:577-664 4210:665-687
4314:688-945 4325:946-969 4329:970-977 4218:978-1001 4304:1002-1005
4291:1006-1093] [-1, 4194, 4195, 4321, 4324, 4307, 4295, 4272, 4320,
4210, 4314, 4325, 4329, 4218, 4304, 4291]
GACAGGGATTATTACCTTTCATGGTGAGATAATATGAGATGTATTTGTGGAGCATATCACCGAGTATGGTGAT
GTGGTCATGGCGTCGCATGCACCTCATGCATATCATTTCATCTCTTTGCTCAACGAAATATCTTTCATCTCGT
TAATTTGACACACATGCGCACATTTTGCAAACAAACAGCTCCATTCGTCGTGACATCCAACGGTACGACAATC
ATGTATTAATTTATTATATGGACTGTATCATAATTTTTATCAACACCTCGTTCCATAACCTCAACACGACACC
TATTCCTACCAACGCACAGGTTGGTTCGGCGCTTGGGGAGGTCTCCTCGCCACAGTAAAATGGTGCATCGGAC

TCAAATTTTCGGTCTACGATGATCAACCACCGGGACTCAAACAACTATACTACATTTTTACGTGCTCCATCAT
CTTGCTGCTCGCTTCCATCCCCCCGCTCATTCAAAAATGGGAACATTATGGCGGAGCCGGATTTTCGATAGCT
GGCTCCGCGCTCACAATCATTGCCTGCGCTTACATGGTGACCATGTACAGTGACATTCCGAAAAGCGTGTTGA
AGATCAACGTGGTTGTGCTTTTCGTGCTTTGGACTCTTGTTGCGGGAGTTTGCACATTTCACGGACCTTTTCT
GATCACGAACAATGGGTTCTTTGCATGTTGGTTGGGCTGCCTTTGTTCACTGAACCTAATGGTGATTGAAATG
AAAGATGCCTCTCCAGAAAACTACACGTAACATGCCGCCAGTGCAGCGAAAGCCACCGATTTTTGTACAAATT
AGGCTCAACAATTAACTTTGGTTATTGGGATGTTTTAACTCAGCACTTCAATTCCGTAAAAACACTGTCTACG
GGATGTTTGCGCGGAATGTGTTTAGTGTCTTCTTGTTTCAGTCGAGGAGAAGATGTTATAATGAAGGTGTGTC
CGTGGCGAATCATGTATGGTGTAGGCGGGTAGGTAATTGGGAGCATATCATGCCGTATGTAGGCACAACCAAC
AAATTCATAAAATTAAGCTAATGCCACGTTGATAGCATTACCAATAACCAACCCTCACAGGTGAGAGGGGGT
>TR146|c0_g1_i8 len=3271 path=[4245:0-108 4317:109-151 4309:152-319 4323:320-679 4324:680-683 4307:684-806 4295:807-1026 4272:1027-1050 4320:1051-1138 4210:1139-1161 4314:1162-1419 4325:1420-1443 4329:1444-1451 4218:1452-1475 4304:1476-1479 4291:1480-1567 4213:1568-1591 4319:1592-1829 4232:1830-1853 4312:1854-2064 4326:2065-2067 4171:2068-2084 4322:2085-2088 @4318@!:2089-2343 4327:2344-2361 4330:2362-2362 4288:2363-3087 4249:3088-3111 4250:3112-3112 4310:3113-3155 4328:3156-3179 4331:3180-3183 4285:3184-3222 4270:3223-3270] [-1, 4245, 4317, 4309, 4323, 4324, 4307, 4295, 4272, 4320, 4210, 4314, 4325, 4329, 4218, 4304, 4291, 4213, 4319, 4232, 4312, 4326, 4171, 4322, 4318, 4327, 4330, 4288, 4249, 4250, 4310, 4328, 4331, 4285, 4270, -2]
ACACGAGCGAACCCACATCGAACAGCCCCACCCACAAGTTCGGCAAGCAACACAGCCAGTGGCAACATCTGCC
CCAATAAGATAGACCCGCCGCGCTGGCCATTATTTTGTTTGTACGACTGCAGCCTACCGCATCACATTTTCTA
CTGCCCGTAGCACTGGGGTGACTGGGCTACTACCATCAACATCACCTGTCTATCGTGTCGATCCAATCATCAC
ACACCACCAAACAAACCCACCATGTCCGACGACGAAGAATCCAACGCCCCGCCCTCCGGCGCCGTCCACCAAG
TAGTACCCCGCCTCCAATCCGGCCCGGCCCAAACTACCACCTCCGAACTCGCCGCGGGACTCTCCCGCTGGAT
GGACGCCTCCAACAGCTTCATCTACGTGGAACTCGCCTCCCTCGTCCTCCTCTTCGCATGCGTCGCTGATTGG
TATCCCACCGTATGGCACAAATACGCGCTGAGCGTGGCGTGCGTTTCGTTGTTGGTGTGTCTGATATTGCAGA
CGGCGGAGTTTTTGGTCCCGGGGTTTTTGGAGTGGTCGGTGGTGAAGCAGAGGGATGATGGGACGGGTGGGCA
TTCGATTCAGAAGGTTTGTTCGGTGTTGATGTTGCTTTGGTGGATTTTTGGGACAGGGATTATTACCTTTCAT
GCTCCATTCGTCGTGACATCCAACGGTACGACAATCATGTATTAATTTATTATATGGACTGTATCATAATTTT
TATCAACACCTCGTTCCATAACCTCAACACGACACCTATTCCTACCAACGCACAGGTTGGTTCGGCGCTTGGG
GAGGTCTCCTCGCCACAGTAAAATGGTGCATCGGACTCAAATTTTCGGTCTACGATGATCAACCACCGGGACT
CAAACAACTATACTACATTTTTACGTGCTCCATCATCTTGCTGCTCGCTTCCATCCCCCCGCTCATTCAAAAA
TGGGAACATTATGGCGGAGCCGGATTTTCGATAGCTGGCTCCGCGCTCACAATCATTGCCTGCGCTTACATGG
TGACCATGTACAGTGACATTCCGAAAAGCGTGTTGAAGATCAACGTGGTTGTGCTTTTCGTGCTTTGGACTCT
TGTTGCGGGAGTTTGCACATTTCACGGACCTTTTCTGATCACGAACAATGGGTTCTTTGCATGTTGGTTGGGC
TGCCTTTGTTCACTGAACCTAATGGTGATTGAAATGAAAGATGCCTCTCCAGAAAACTACACGTAACATGCCG
CCAGTGCAGCGAAAGCCACCGATTTTTGTACAAATTAGGCTCAACAATTAACTTTGGTTATTGGGATGTTTTA
ACTCAGCACTTCAATTCCGTAAAAACACTGTCTACGGGATGTTTGCGCGGAATGTGTTTAGTGTCTTCTTGTT
TCAGTCGAGGAGAAGATGTTATAATGAAGGTGTGTCCGTGGCGAATCATGTATGGTGTAGGCGGGTAGGTAAT
TGGGAGCATATCATGCCGTATGTAGGCACAACCAACAAATTCATAAAATTAAGCTAATGCCACGTTGATAGCA
TTACCAATAACCAACCCTCACAGGTGAGAGGGGGTTCCTCGAATGCAGCCAAAGCTACAGGTGTGTATTCAGC
ACCACTGAAAAATCATCAAGTGGAATCTAAGCCCGGAAAACCAGCCACCTCCAAATGCATCACAATTCCGGGG
ACGACTGTGGTTACAACGGGCCACCAACATCTCACCAAGTGTATAATCACCCCTGTGAGGAGCATCACCACCC
CATAGACCAGATGAGCCACCTATCCCTGGGTCAATTTAAAATGTCGCTTCCCAGTGAATTGTCGAATGGTATG
GTATCATTACTGAGAATGCCAGAGAATATGGTAGGACACACTACGTACTTAGAGAGGTGAAATGCCATTGAAA
ATAAAACTCTACCCAAGTACATGGGATGTAATTTTAAATTTGAGATATACTCCTACTTAGATTCTTTTCATAA
GCTGGTTGCACGTTACTTCGCCACTAACACAAGAGATTTTCAATACAAGTCACCATAAAATTCCAGCTTCTCT
CATCATGATGAATTGGGGAAAAAACACTTTTTCCTCAAAAAAAATTATTACTTTGACCTCTTGTCAAGAGCAT
GGTATCTTTACTGGGATTACACCCGACTGACATTGGAATTGAAACTCTACCAACAGTAGAGACATGGATTCTT
TTCATAAAACTGATTGTTCTTTACTTTGTCGCTCACAGATAGTATGTTTCGACCTCAATCATCATACAATTTC
ATCCTCTCTCGTTATGATGAATTGAAGAAAGAACGTAGACAGAAGAAAGCCGCCCCAACATGCAAAGTATCCA
TTTCCTGCTTGAAGGAATGGCCCATCAAAAGTGCACACTCCAGCTACAGTCGCCCAAATCACAAACAAGCCTA
TAGCTGTAACCTTCACCAACTGCGCAGGAATATCATTAAATGCCTTGAAAAACACACCAGTAATAATCATGGT
AATCAACCCCGCCGACAAACCCCACGCCGACTGACCGACGAAAACCTTATACCTCAAAAACGGAATGCACGCA
AATAGCAGCACGGCCGCGGCCGTAAACATTCCCGCCAATATCTTCCTCCCCTTCTCCAAATCCGTAAATCGCG
CCGTATCAATATTTAATGCCCAATGAGTCGTGAAGATCAAACCCGCCCACGCGGAAAAGTACCCATTTCCCAC
CCCGGTAAAAGGAGCCCTAAATGTAATAACGCCGGTGCCTACCACCCACCACACGAATAAGAAGAGACTAACC
GGCTTCTCGACTCGTTCCAACATACCCGGCCTCATAAATTCGCCAGTTTGAATGATGAGGCATGCCGCTAGGG

AGATGACCGATACGGATAGAGCGTAGGCGAGTTCGGGATATCCCTTGGTCCATACGCCTATGCAGGCGAACAT
TAGGACGAGGGAGGAGACTTCGACGTACATGAAGGATTTGGCGACTGCGGACCATGCTTTGGTTGCTTCGACT
ATTTCGGCGGGGGAGGTTTGGGCGCTGGTTGGCGTGCCGGGTGCCGGGGTGGTGGGGGCGTGGTCTTCTCCGT
CGCTGCTCATTTTGTTGTTGGTGTGGTGGATGTAGTGCTAGACGGATCGTCTTCGATTTGATGAGAAGAGAAG
CACTACTGTCTGCTGTAGTGCTGTATTGTTTGTATCTCTCGAGTCGATTGAGGGTACTTTGCAGGCTTGTATT
TTATTTGCGACTCTCTCTTGGTTCTTCTGGCTGCGCGTTTCGTGTAGATCGGAAGAGCA
>TR147|c1_g1_i1 len=1734 path=[2928:0-198 2929:199-249 3165:250-261
3138:262-315 3172:316-319 3164:320-373 @3153@!:374-464 3116:465-519
3168:520-549 3152:550-585 3115:586-590 3135:591-614 3029:615-633
3113:634-897 2993:898-918 3134:919-954 3151:955-959 3111:960-1014
3132:1015-1022 3161:1023-1056 3149:1057-1110 3130:1111-1134 3046:1135-
1165 3107:1166-1236 3171:1237-1272 3167:1273-1296 3159:1297-1318
3146:1319-1364 3173:1365-1383 3158:1384-1392 3144:1393-1419 3125:1420-
1422 3099:1423-1452 3142:1453-1476 3123:1477-1497 3156:1498-1530
3141:1531-1554 3122:1555-1578 3174:1579-1579 3096:1580-1606 2956:1607-
1630 2957:1631-1733] [-1, 2928, 2929, 3165, 3138, 3172, 3164, 3153, 3116,
3168, 3152, 3115, 3135, 3029, 3113, 2993, 3134, 3151, 3111, 3132, 3161,
3149, 3130, 3046, 3107, 3171, 3167, 3159, 3146, 3173, 3158, 3144, 3125,
3099, 3142, 3123, 3156, 3141, 3122, 3174, 3096, 2956, 2957, -2]
ATACATCATGACATTCCTCCTAATTTACATTAAACAATCATCTACACCATCCTTCTGGAAGGAGGGGCAAAAG
TTCTGCAATCACATCAGGTCGCGATTGATCTAATGGCTCAAAATCATCTGGATCTCTTATTGTATCATAAAGC
ATGATGTGCCAAGGTGCACCGCTAGGGAAGGGTGTGGCTGAGCGAGGTGCACCTTTCTTCCATGCCTTCCCGC
TCTTCCAGCAAATTAGTTTGTATGACGTAATCGTTGGATCTTCCGGCGATGTTGGCATTTCCTTGATGACAGC
CCTCCAATCCCCGTATCCAGACGAGACAAATGGTCGACGGGTGTCAGTCATATAACCAGCTAGGATGGAGCGA
AGACTCGCAGTGGTCATCCCAGGTCCCAAGTTACTAACACCGGCATAGTCCAGCCAGGTGCCACCTAAATCCA
ATGTCGATACCGGACCTTCATGCACTTTCCCTTCTCCAACTCCAGGGCCCATGATAAAAAGCGGTGTAGATAT
CGAAGCATGCCACGGCATACCCTTCCCACTAGCTCCATTATCACCGAGATTGTCACCATGATCGCCCGCCACA
ATCACAATAGTATTGTCAAGTTCTCCCATTTCTTCCACGTGTTTGACTATAGAAAACATCAGTGCGTCGAGGT
GCTCAAGCTCAGCACCGTAGTTGCATCGCCCATCCATCTGGGGCGGCAGTATTTGTCCGCGATTCTTTCGCTG
GAAACGTTTATTCTGCTGTGGACACGCCAGAAGACCAGGACTATCGATTGGATCTGGCCATTGCCTGTCCATG
ACACTTGCGGCCATTCTCGATGTAGAGACAATGGGAAAGTGCGGTCCTGGAAAATTCACCTGCATGAACCATG
GCTTGTCTGCTGGTTTACGGTCAAGCAATTCAATTGCCCTGTCTCTCACAAAGTCGTCAGGGTAAATTTCTTC
CTGAAACGACGATGCATCACAACCATCCTTAACACCACGCCCTATCATACATCCCTTGTAGGCCTCCCTTGCA
CTGATTTCAGTTCCATCATCTGTTTTGACAGTTATACCATTAAGAAATGTTCGATAAGGCTCGTCCTTACATC
TTTCATCTGAAAGCTTTGCGACACGAACCTTCCCATCCGACCGTATTGCATCTGCAAATCCAAGATCCACGGA
TGGCGTATCGTGATGCTTTTGAGTGGCATATTTCGGAAACTTGATGTCCCATTCATAGAAGTCATCCTTTCCA
CAAGTCATGGTGTGATATCCCCCCTCATCCCGTAGCAGTTTGTACGTTGTTGTCCGCTGAAAAATCCCTGGTG
CGCGTTTCTCGCTCCATTCTGAGTTAGTCGGCACACCAGTGGCATCATACTCCATACCAAATGCCATGCAGGA
TCGAACAGGTCCACAGAATGGACTAGGAACATATGCTTGTGTGAATCGTGTTCCGCGTTGACCCATTTCGTTG
AGGAGAGGCATGTTCAGTGGGAGAGGCCCCGTTCGCGTGTCTGGATGATGACCATCCCAATCTGGACGCCACT
GATCTAGAAGGAGATAGAGAATGTTGGGACGAGAGGATGTGGATGGTATACCACCTTCTGATTGGTGATGCAC
ACCAGCTTCAGTTTGTTTTATAAATTTAGATTGAGGATCCGCATATTGGTTTAGATCGATTTGCAGCCGTCGT
CGTTGTTGGCGGCGACCACGGCGGCGCCGGTCGCTGTCTTCATGGGTAGAGGATG
>TR147|c1_g1_i2 len=1704 path=[3093:0-198 3170:199-249 3165:250-261
3139:262-315 3172:316-319 2960:320-373 @3153@!:374-464 3116:465-519
3168:520-549 3152:550-585 3115:586-590 3135:591-614 3029:615-633
3113:634-897 2993:898-918 3134:919-954 3151:955-959 3111:960-1014
3132:1015-1022 3161:1023-1056 3149:1057-1110 3130:1111-1134 3046:1135-
1165 3107:1166-1236 3171:1237-1272 3167:1273-1296 3159:1297-1318
3145:1319-1364 3173:1365-1383 3101:1384-1407 3157:1408-1419 3143:1420-
1422 3124:1423-1497 3156:1498-1530 3141:1531-1554 3122:1555-1578
3174:1579-1579 2975:1580-1703] [-1, 3093, 3170, 3165, 3139, 3172, 2960,
3153, 3116, 3168, 3152, 3115, 3135, 3029, 3113, 2993, 3134, 3151, 3111,
3132, 3161, 3149, 3130, 3046, 3107, 3171, 3167, 3159, 3145, 3173, 3101,
3157, 3143, 3124, 3156, 3141, 3122, 3174, 2975, -2]
ATACATCATGACATTCCTCCTAATTTACATTAAACAATCATCTACACCATCCTTCTGGAAGGAGGGGCAAAAG
TTCTGCAATCACATCAGGTCGCGATTGATCTAATGGCTCAAAATCATCTGGATCTCTTATTGTATCATAAAGC
ATGATGTGCCAAGGTGCACCGCTAGGGAAGGGTGTGGCTGAGCGAGGTGCACCGTACTTCCATGTCTTTCCGC
TCTTCCAACAAATTAGTTTGTATGACGTAATCGTTGGATCTTCAGGTGATGTTGGCATCTCCTTGATGACAGC
TCTCCAATCCCCGTATCCAGACGAGACATATGGACGACGGGGGATGGTAATATCACCAACTAGGATGGAGCGA

AGACTCGCAGTGGTCATCCCAGGTCCCAAGTTACTAACACCGGCATAGTCCAGCCAGGTGCCACCTAAATCCA
ATGTCGATACCGGACCTTCATGCACTTTCCCTTCTCCAACTCCAGGGCCCATGATAAAAAGCGGTGTAGATAT
CGAAGCATGCCACGGCATACCCTTCCCACTAGCTCCATTATCACCGAGATTGTCACCATGATCGCCCGCCACA
ATCACAATAGTATTGTCAAGTTCTCCCATTTCTTCCACGTGTTTGACTATAGAAAACATCAGTGCGTCGAGGT
GCTCAAGCTCAGCACCGTAGTTGCATCGCCCATCCATCTGGGGCGGCAGTATTTGTCCGCGATTCTTTCGCTG
GAAACGTTTATTCTGCTGTGGACACGCCAGAAGACCAGGACTATCGATTGGATCTGGCCATTGCCTGTCCATG
ACACTTGCGGCCATTCTCGATGTAGAGACAATGGGAAAGTGCGGTCCTGGAAAATTCACCTGCATGAACCATG
GCTTGTCTGCTGGTTTACGGTCAAGCAATTCAATTGCCCTGTCTCTCACAAAGTCGTCAGGGTAAATTTCTTC
CTGAAACGACGATGCATCACAACCATCCTTAACACCACGCCCTATCATACATCCCTTGTAGGCCTCCCTTGCA
CTGATTTCAGTTCCATCATCTGTTTTGACAGTTATACCATTAAGAAATGTTCGATAAGGCTCGTCCTTACATC
TTTCATCTGAAAGCTTTGCGACACGAACCTTCCCATCCGACCGTATTGCATCTGCAAATCCAAGATCCACGGA
TGGCGTATCGTGATGCTTTTGAGTGGCATATTTCGGAAACTTGATGTCCCATTCATAGAAGTCATCCTTTCCA
CAAGTCATGGTGTGATATCCCCCCTCATCCCGTAGCAGTTTGTACGTTGTTGTCCGCTGAAAAATCCCTGGTG
CGCGTATCTCGCTCCATCCTGAGTTCGCCGGCACACCAGTGGCATCATACTCCATACCAAATGCCATGCATGA
TCGAACTGGACCACAGAATGGACTAGGAACATATGCCTGCGTGAATCGTGTTCCGCGTTTACCCATGTCGTTG
AGGAAGGGCATGTTTAGTGGGAGAGGCCCCGTTCGCGTGTCTGGATGATGACCATCCCAATCTGGACGCCACT
GATCTAGAAGGAGATAGAGAATGTTGGGACGAGAGGATGTGGATGGTGTATCATCAGATTGGTGATGTACAGC
AGCTTCAGCTTGTTTATTAAATTTGGATTGAGGATCCGCGTATTGGCCTAGATCCATTTGCATCTGTCGTCGT
TGCTGCCGGCGATCACGGCGGCGCC
>TR147|c1_g1_i3 len=1737 path=[2928:0-198 2929:199-249 3165:250-261
3138:262-315 3172:316-319 3164:320-373 @3153@!:374-464 3117:465-519
3168:520-549 3136:550-585 3114:586-590 2946:591-614 2947:615-633
3112:634-897 3081:898-918 3133:919-954 3162:955-959 3150:960-1017
3131:1018-1025 3160:1026-1059 3149:1060-1113 3130:1114-1137 3046:1138-
1168 3107:1169-1239 3171:1240-1275 3167:1276-1299 3159:1300-1321
3146:1322-1367 3173:1368-1386 3158:1387-1395 3144:1396-1422 3125:1423-
1425 3099:1426-1455 3142:1456-1479 3123:1480-1500 3156:1501-1533
3141:1534-1557 3122:1558-1581 3174:1582-1582 3096:1583-1609 2956:1610-
1633 2957:1634-1736] [-1, 2928, 2929, 3165, 3138, 3172, 3164, 3153, 3117,
3168, 3136, 3114, 2946, 2947, 3112, 3081, 3133, 3162, 3150, 3131, 3160,
3149, 3130, 3046, 3107, 3171, 3167, 3159, 3146, 3173, 3158, 3144, 3125,
3099, 3142, 3123, 3156, 3141, 3122, 3174, 3096, 2956, 2957, -2]
ATACATCATGACATTCCTCCTAATTTACATTAAACAATCATCTACACCATCCTTCTGGAAGGAGGGGCAAAAG
TTCTGCAATCACATCAGGTCGCGATTGATCTAATGGCTCAAAATCATCTGGATCTCTTATTGTATCATAAAGC
ATGATGTGCCAAGGTGCACCGCTAGGGAAGGGTGTGGCTGAGCGAGGTGCACCTTTCTTCCATGCCTTCCCGC
TCTTCCAGCAAATTAGTTTGTATGACGTAATCGTTGGATCTTCCGGCGATGTTGGCATTTCCTTGATGACAGC
CCTCCAATCCCCGTATCCAGACGAGACAAATGGTCGACGGGTGTCAGTCATATAACCAGCTAGGATGGAGCGA
AGACTCGCAGTGGTCATCCCAGGTCCCAAGTTACTAACACCGGCATAGTCCAGCCAGGTGCCACCTAAATCCA
ATGTCGATACCGGACCTTCATGCACTTGCCCTTCTCCAATTCCAGGGCCCATGATGAATAGCGGTGTAGATAT
CGAAGCATGCCACGGCATACCCTTCCCACTAGCTCCATTGTCACCAAGGTTTTCACCATGATCGCCCGCCACA
ATCACAATAGTATTGTCAAGTTCTCCCATTTCTTCCACGTGTTTGACTATTGAAAACATGAGCTCGTCGAGGT
GCTCAAGCTCAGCAGCGTAGTTGCATCGTCCACCTATCTGAGGCGGCAGTACTTTTCCGCGATTCATTATTTG
GAACTTTCTCTTTTGCTGTGGACACGCCAGATAACCAGGACGATCAATTGGATCCGGCCATTCTCTGTCCATG
ACACTTGCAGCCATTTTCGATGTAGAGACGATGGGTTCGTGCGGTCCTGGAAAGTTCACCTGCATGAACCACG
GCTTGTCTGCTGGTTTACGGTCAAGCAATTCAATTGCCCTGTCCCTCACAAAGTCATCAGGGTAAATTTCTTC
CTGAAACGACGTTGCGTCACAACGATTCTTAGCACCAAGATGCCACATCATACATCCCTTGTAGGCCTCCCTT
GCACCGATCTCAGTCCCATCATCTGTTTTGACAGTTATACCATTAAGAAATGTTCGATAAGGCTCGTCCTTAC
ATCTTTCATCTGAAAGCTTTGCGACACGAACCTTCCCATCCGACCGTATTGCATCTGCAAATCCAAGATCCAC
GGATGGCGTATCGTGATGCTTTTGAGTGGCATATTTCGGAAACTTGATGTCCCATTCATAGAAGTCATCCTTT
CCACAAGTCATGGTGTGATATCCCCCCTCATCCCGTAGCAGTTTGTACGTTGTTGTCCGCTGAAAAATCCCTG
GTGCGCGTTTCTCGCTCCATTCTGAGTTAGTCGGCACACCAGTGGCATCATACTCCATACCAAATGCCATGCA
GGATCGAACAGGTCCACAGAATGGACTAGGAACATATGCTTGTGTGAATCGTGTTCCGCGTTGACCCATTTCG
TTGAGGAGAGGCATGTTCAGTGGGAGAGGCCCCGTTCGCGTGTCTGGATGATGACCATCCCAATCTGGACGCC
ACTGATCTAGAAGGAGATAGAGAATGTTGGGACGAGAGGATGTGGATGGTATACCACCTTCTGATTGGTGATG
CACACCAGCTTCAGTTTGTTTTATAAATTTAGATTGAGGATCCGCATATTGGTTTAGATCGATTTGCAGCCGT
CGTCGTTGTTGGCGGCGACCACGGCGGCGCCGGTCGCTGTCTTCATGGGTAGAGGATG
>TR147|c1_g1_i4 len=1707 path=[3093:0-198 3170:199-249 3165:250-261
3139:262-315 3172:316-319 2960:320-373 @3153@!:374-464 3117:465-519
3168:520-549 3136:550-585 3114:586-590 2946:591-614 2947:615-633
3112:634-897 3081:898-918 3133:919-954 3162:955-959 3150:960-1017

3131:1018-1025 3160:1026-1059 3149:1060-1113 3130:1114-1137 3046:1138-1168 3107:1169-1239 3171:1240-1275 3167:1276-1299 3159:1300-1321 3146:1322-1367 3173:1368-1386 3158:1387-1395 3144:1396-1422 3125:1423-1425 3099:1426-1455 3142:1456-1479 3123:1480-1500 3156:1501-1533 3141:1534-1557 3122:1558-1581 3174:1582-1582 2975:1583-1706] [-1, 3093, 3170, 3165, 3139, 3172, 2960, 3153, 3117, 3168, 3136, 3114, 2946, 2947, 3112, 3081, 3133, 3162, 3150, 3131, 3160, 3149, 3130, 3046, 3107, 3171, 3167, 3159, 3146, 3173, 3158, 3144, 3125, 3099, 3142, 3123, 3156, 3141, 3122, 3174, 2975, -2]
ATACATCATGACATTCCTCCTAATTTACATTAAACAATCATCTACACCATCCTTCTGGAAGGAGGGGCAAAAG
TTCTGCAATCACATCAGGTCGCGATTGATCTAATGGCTCAAAATCATCTGGATCTCTTATTGTATCATAAAGC
ATGATGTGCCAAGGTGCACCGCTAGGGAAGGGTGTGGCTGAGCGAGGTGCACCGTACTTCCATGTCTTTCCGC
TCTTCCAACAAATTAGTTTGTATGACGTAATCGTTGGATCTTCAGGTGATGTTGGCATCTCCTTGATGACAGC
TCTCCAATCCCCGTATCCAGACGAGACATATGGACGACGGGGGATGGTAATATCACCAACTAGGATGGAGCGA
AGACTCGCAGTGGTCATCCCAGGTCCCAAGTTACTAACACCGGCATAGTCCAGCCAGGTGCCACCTAAATCCA
ATGTCGATACCGGACCTTCATGCACTTGCCCTTCTCCAATTCCAGGGCCCATGATGAATAGCGGTGTAGATAT
CGAAGCATGCCACGGCATACCCTTCCCACTAGCTCCATTGTCACCAAGGTTTTCACCATGATCGCCCGCCACA
ATCACAATAGTATTGTCAAGTTCTCCCATTTCTTCCACGTGTTTGACTATTGAAAACATGAGCTCGTCGAGGT
GCTCAAGCTCAGCAGCGTAGTTGCATCGTCCACCTATCTGAGGCGGCAGTACTTTTCCGCGATTCATTATTTG
GAACTTTCTCTTTTGCTGTGGACACGCCAGATAACCAGGACGATCAATTGGATCCGGCCATTCTCTGTCCATG
ACACTTGCAGCCATTTTCGATGTAGAGACGATGGGTTCGTGCGGTCCTGGAAAGTTCACCTGCATGAACCACG
GCTTGTCTGCTGGTTTACGGTCAAGCAATTCAATTGCCCTGTCCCTCACAAAGTCATCAGGGTAAATTTCTTC
CTGAAACGACGTTGCGTCACAACGATTCTTAGCACCAAGATGCCACATCATACATCCCTTGTAGGCCTCCCTT
GCACCGATCTCAGTCCCATCATCTGTTTTGACAGTTATACCATTAAGAAATGTTCGATAAGGCTCGTCCTTAC
ATCTTTCATCTGAAAGCTTTGCGACACGAACCTTCCCATCCGACCGTATTGCATCTGCAAATCCAAGATCCAC
GGATGGCGTATCGTGATGCTTTTGAGTGGCATATTTCGGAAACTTGATGTCCCATTCATAGAAGTCATCCTTT
CCACAAGTCATGGTGTGATATCCCCCCTCATCCCGTAGCAGTTTGTACGTTGTTGTCCGCTGAAAAATCCCTG
GTGCGCGTTTCTCGCTCCATTCTGAGTTAGTCGGCACACCAGTGGCATCATACTCCATACCAAATGCCATGCA
GGATCGAACAGGTCCACAGAATGGACTAGGAACATATGCTTGTGTGAATCGTGTTCCGCGTTGACCCATTTCG
TTGAGGAGAGGCATGTTCAGTGGGAGAGGCCCCGTTCGCGTGTCTGGATGATGACCATCCCAATCTGGACGCC
ACTGATCTAGAAGGAGATAGAGAATGTTGGGACGAGAGGATGTGGATGGTGTATCATCAGATTGGTGATGTAC
AGCAGCTTCAGCTTGTTTATTAAATTTGGATTGAGGATCCGCGTATTGGCCTAGATCCATTTGCATCTGTCGT
CGTTGCTGCCGGCGATCACGGCGGCGCC
>TR148|c0_g1_i1 len=481 path=[1033:0-101 1254:102-112 1248:113-114 1241:115-154 1260:155-157 1257:158-176 1247:177-209 1265:210-210 1252:211-270 1246:271-306 1225:307-325 1263:326-330 1262:331-350 1266:351-368 1258:369-369 1255:370-375 1249:376-392 1242:393-414 1229:415-419 1215:420-423 1195:424-447 1105:448-480] [-1, 1033, 1254, 1248, 1241, 1260, 1257, 1247, 1265, 1252, 1246, 1225, 1263, 1262, 1266, 1258, 1255, 1249, 1242, 1229, 1215, 1195, 1105, -2]
TGTGCTCTTCCGATCTATTAAATACTACAATAGAAGTAGGAGGTCAATGACCTCCTACTTCTATTGTTGTGTT
TAACCCATTCCAAATTCCTTTACTAAACACAGCTATCTTATTAGCCTCCGGTGTTACTGTCACATGAAGACAC
CACGCTATCGTAGAAAATAATCATTCTCAAGCCTTCAAAGCCCTTATACTTACTATTACTTTAGGTGTTTATT
TCACAGCTCTTCAAGCCCTGGAGTATTACGAAGCTCCTTTTACTATTGCAGACTCTGTTTATGGTTCAACTTT
CTTTGTAGCCACAGGATTCCATGGTTTACATGTCATTATTGGCTCAACTTTTCTTGCCGTTTGTTTATTACGA
ATAATCTTAGGACATTTCTCCAGAAAACATCACACAGGTTATGAATCCGCTATTTGATACTGACATTTTGTCG
ATGTTGTATGATTATTTTTGTATGTATCAATTTATTCCTGAGA
>TR148|c0_g1_i2 len=229 path=[1033:0-101 1254:102-112 1248:113-114 1241:115-154 1260:155-157 1257:158-176 1247:177-209 1090:210-228] [-1, 1033, 1254, 1248, 1241, 1260, 1257, 1247, 1090, -2]
TGTGCTCTTCCGATCTATTAAATACTACAATAGAAGTAGGAGGTCAATGACCTCCTACTTCTATTGTTGTGTT
TAACCCATTCCAAATTCCTTTACTAAACACAGCTATCTTATTAGCCTCCGGTGTTACTGTCACATGAAGACAC
CACGCTATCGTAGAAAATAATCATTCTCAAGCCTTCAAAGCCCTTATACTTACTATTACTTTAGAGATCGGAA
GAGCGTCGTG
>TR148|c0_g1_i3 len=434 path=[1033:0-101 1254:102-112 1248:113-114 1241:115-154 1260:155-157 1257:158-176 1247:177-209 1265:210-210 1252:211-270 1246:271-306 1225:307-325 1263:326-330 1262:331-350 1266:351-368 1250:369-388 1243:389-392 1217:393-414 1110:415-433] [-1, 1033, 1254, 1248, 1241, 1260, 1257, 1247, 1265, 1252, 1246, 1225, 1263, 1262, 1266, 1250, 1243, 1217, 1110, -2]

TGTGCTCTTCCGATCTATTAAATACTACAATAGAAGTAGGAGGTCAATGACCTCCTACTTCTATTGTTGTGTT
TAACCCATTCCAAATTCCTTTACTAAACACAGCTATCTTATTAGCCTCCGGTGTTACTGTCACATGAAGACAC
CACGCTATCGTAGAAAATAATCATTCTCAAGCCTTCAAAGCCCTTATACTTACTATTACTTTAGGTGTTTATT
TCACAGCTCTTCAAGCCCTGGAGTATTACGAAGCTCCTTTTACTATTGCAGACTCTGTTTATGGTTCAACTTT
CTTTGTAGCCACAGGATTCCATGGTTTACATGTCATTATTGGCTCAACTTTTCTTGCCGTTTGTTTATTACGA
ATAACCTTAGGACATTTCTCCAGAAAACATCACACAGGTTATGAATCCGCAGATCGGAAGAGCGTCGTG
>TR149|c0_g1_i1 len=228 path=[206:0-227] [-1, 206, -2]
AGCTTTATGTTCCTCTTCCGTGCTACTGCTACTGCTACTGCTGGTGTCGCTGCTGATGGAAATTATTGAATCA
TCGTCATCATCATTACCCATCGGTTGAGTCAAGGGTTGAGTATGAAGCCTCGAATCATCGGTCCCGGCATCGA
CATAGTTGCTATCTTCTGTGGAGGTGTTCGAGTCATCATCGTCATCGCTGGAGGAAGAGGAAGATTGTTGATT
GTCGCTGTT
>TR150|c0_g1_i1 len=475 path=[453:0-474] [-1, 453, -2]
GGAGGATGCCATGGAAGAAGAAGAAGAAGATGATGATGATGATGAATTGGAGACTGCAGTAGAACGAACGTCG
TCGTCGTCGTCGTTATCGACGAATGACGATCCTTCGTCGGTAGTACTGTAGGCTGGTGGATCCATCGAATGCG
AATCACGCATTTTGTGTGTGTGTATGTGTGTGTGTGTGCGTGTATGTGCGTGTATGTGCGTATAATATGGAGT
GTGGAGAGCCCTTTGATTATCGGTGTGTGTGTGTGTGTTGCGGGGTTGGTTGTGTGTGCTGTGTGCTATATTA
TATATATGGCGTGGAAGAAAATATAGTTCGTGCGTTCCCGGCAAAATATTTTCCCGGGTTTGTGTGTGATTTT
TTTTGCTTGGCAAGGAGTGTAGTGTGTGTAATGTGTGCTTAGTGTGTGTTTGTGTGTTTGATTATGCTCGAGA
GAGGTTTAGAGATTTCAGAGTTCAGAGATCGGAAGAG
>TR154|c0_g1_i1 len=323 path=[301:0-322] [-1, 301, -2]
AAATGACTGGGGGCTGGAGCAACGGCCCCGCCATTCCATCGTCCGGTGGTTCTAACTCAGCATCAACTGCCAC
GGCATTGCCGCCATCGTCGCCATCGTCCTCCTCTTCCCGTCTGTCATCGACATCGCTGTGAAGAGTGCCCGTC
ACAACACCCTCCCCCTCGGTATCCTCCGCCGGGGCCAAAGTAAGGTCGCGTTCGTTATCGTCCGTCCCCGATT
GTTCGCCAATATCGTCCGAACGCACAGGCGCATCCACGCGAGGGGTTCGAACCTGTATCGTTACAGGATCGTC
TTCGAGTTCACCCCTCGAATCGGAGTCGTCA
>TR155|c0_g1_i1 len=250 path=[228:0-249] [-1, 228, -2]
GGGGACAAACTCCCAAAGAACACGTGATATAGAAACGCCGATGGTTGATGGACGACAACTGTGCTGAATCTTG
TGATGGAGTACACACACCAAACGGCCGGCCGGGCGTCCAATTATTTTTGTCAAAAGACAAACACCCAGGGACC
ACTTGATGTGGCGCAAATGCAATCTAAATGCATTGAACTGCCATACAACTGAAGAAGTGCCACCATGATCATC
AGAACTTGTGAGAGAGTTACATCGTTCTCTC
>TR156|c0_g1_i1 len=1277 path=[1255:0-1276] [-1, 1255, -2]
CCGATCTATACATTACAAAGTCAGTTTGGATATATTATGCACCCAATTAAATATTGATCTTGCATCATAATAT
TATAATACACATTCTCAGACCAGCAATAATATATGATCATTGTCCGTCTGTTTCTCCCTTCCATCCTCAAGCC
GAATCGTATAATACGGTTCGAGCTCATCGTCCAGGTGAACATCGAGAATACGCGCTGGCGTGATGGTACTACC
AGACGATTGATAAAATACATTCATTCCTTTTTGGTGAAGAGGACCGCTAGCGCTAGTTTTACGACCACGACCA
CCACTACCCGAATCCACGGAATCATTGGGCGAAGAAGGAGGAAGCAATGGCTGTGTTCTATCGTTATTGCGAG
TATTCGTGCTTCCGAGTTGTTGATTTTGACTGTTGTTGTTGTAACTATCATCATCTGCTCTTCCTTGCTGCGA
CGACGATTGAGAAGAAGGTATGATGGTGCGTTGGGGAATACCACCTTGAAATATCACCTCCAATTCACGTTGA
AAACGTCGTTGACACCACATATTCGTACATGATAAAAGAACCAACAATACAGCACATACAGCAGTCCACTGTA
GGATGGATTCTTTTTGATCATCACCTCCTTCCATGGCATACAGCTCTACCATGGTAAGGTATGCAAATATACC
AACAAGGCACACCCCCGGTACAAGTATTAAGCACGTTCGACAATGAGAATGATTACGAATATTCGTGTCGATG
ACTTCTCGTATGCATCCCGATCGTGGCATTCCGTGTAAGACATATAAGTCGATTCTCGTTCCCCGTGGTATTG
GTTCGTTCACTGTAAATTTTCTCAAATAGCGTTGGTGTTTGATTGCATTTGGGTATCGAAATTTGCTACGCGA
ATCAATTTCGTATTTGTGAATGGTTGTTGTGTAAAGCACTGACACATTGTAGTATTTGCCATTATTATGCTGT
ATTGTAGTTGTAGTGGAAGATGATGATGGTTCGGAGGAGAGTACATCACCAATTACTTTCACGGCGGAATTTT
CATTGGTGTATTTTTTTCCAAATCTTGTGTGTTGCAAGATGATTGTAAAGTAACAAAGAAACACCGGCATACAC
GAAGAAAATACCAAGAAGGCATTTTATCACACCGCTCAAATCGTATACCAATTCGATCCAATCATCGATTTTA
TTTTGTTCTGCTCTTGCTGATGTGGTGACACAGGCTAGTAGAATGCCAGCTGTTGGGAATGGTGGTATTATTG
TCCGACTCATATCAAGATGATGGTCCCCTTGATGTG
>TR157|c0_g1_i1 len=274 path=[252:0-273] [-1, 252, -2]
GACACACTGACTTCTTACCACACCACCAACAACAACAACAACATCACCAACAACAACAGCAGCAATATCCCAC
AACATCAAAAAATTCACCTCCATCAATGTCCAAACATGGCCCCCGGAATACACTGTCTCCAACCCAATGCACT
CACGTCCTACGCGCGGTCTTCGTCGTTGATGAGATGCCTACACTCGATCGTGGGCGATAATATTTTGAGAGAG
ATGTTGGTGAGTTCCGTGGTCCTCGTTCCGGCCGTGGTAGTTTCGTCGTCGCCGT
>TR158|c0_g1_i1 len=521 path=[499:0-520] [-1, 499, -2]
TCACTGGCGCTACTGAATCTGTTAGCGTCCTCGAACTGCTCCTTGGAAGTTCCCGCCATGCGAGTCTTGCAGT
GTCGCCCATATATCAGGTCGCATTCAAATAAATGACCGTGAACGAGGACCAGAGCGGCATGGGCGAATGTGTC
GCCTTCGACGTAGTGTAACTCCGCCCCTCCATCATTGGCAAGTGCCGCATTCAATGATTTTAGATCCTCCTTC
TTGGTGTACACAAAGTCGGCATTTCTCATACCGATGGCGGTGCACTTCATTTCTGCCAGTTCGGCACAAACAA
TCTTGGCTTTCGGAAAGGCTTCCGACGCCTGACAAAGTTGATTTGTATGAGCGGACGATCCGCTTACGATGTA

CGAAACCGTCCCGATCCCGTCTAGCCACTCCATAATGCTCGGGCGCATCCTACAGGGATTATAAACCAGTAGC
TTCTCCTTACCGCTCTCCTCATCCTTTGCGTCCAATCTCGCCACCAGCATATTTAGGCTAGACAAATATTTCT
TCTCCACACG
>TR159|c0_g1_i1 len=1006 path=[984:0-1005] [-1, 984, -2]
TTCAAGGTCACCTTCACATTTGACAAGCTTTGGAGAACGCGTGATGCGCTACGCAGACCGTCCTCGGATTTGA
GGATAGGGCTAACAAGTTCAAATTGGTTGCACTTGGGATCCGAAATGCTGCACTTGATGCTTCCATCATGAAC
TAGCTTCCAGTTGCCGTGGGTTCTCTTAGACTCGTTCCAATTCTCCTCAATATTTTCAACGTAGATACCAGAG
CGCGATAACTCATTGGCAATGTGGCCGCGGGTCCAATGACCTGCAGATGTCATTTCCAACTCAATGCCAAACC
GGGCCCCCTGTCGAATTTCCATCATTTTAGGGGTTGGGTTATACAGGACATTCCTCTTTAGACGGAAGACATA
AAATTTAGCGTATTGAAATTCTATTTCTTCATTGTTGGCATAGCGAGACATGCAGTTGGGAAACATGGCACAA
TCAAAGTCTTCATACCACTCCAAGCAAAAACCTGCATGCTGTGATATCTGCATTACATTCCATTTGGAGAATT
GATTGCCTTGTAATGCAATACCGACTTCTATATGCGACATAGTCCGCACTTTGGACTGGAAGTAAGCTGCAGC
ACTCATGAAAAATCCTCGAAGGAGTGATTCGTTCTCTTCGTCAGTCGCTGCATTACTGGCAGAAGGGAAAGTC
CAAGCACACTTTGAAACTTGCGCGTTCAGGTTACGATGCAAGAGGAGATTTCTATTTTTGTGGAAATGAGCGA
GGTCAAAAATTGATCCATCTTCAGCCTGGCTGATGAAAAATTTCGGTGGTGTTCCCATCGCTTGAAGCGAATG
GCAAAAATCACAACTCCCAAGTAACAACATTGAACCTGAATTCTCAACAAACCATGCCAACGCACCACCATGA
AATTTCCCATGCGTAGGCGCAATCATTTCTTCACTGGAAATATTTGCCGGTTCATCAATGTGAGAATATTTAC
AATCATCGTTTGAACACCCTCCTCTGAGGTGAAACCTGCAAGGAGGCGCTGGTGCGG
>TR160|c0_g1_i1 len=1505 path=[1483:0-1504] [-1, 1483, -2]
TGATGTTAGATGCATGACCTATTCTCTTTCCTTTTACGGATGGGACAATGCAGAGATGGGTGTCAATGATGAT
GGTGCTACTCTGGGTGATAATTCTATTGCATTGCCTTCACTGGTTGCTGCATCGGGTACAGTCTTTGGGGAAA
TATTGGTTTGGGGTGTTGCGAATAAAGTCGAGGAGAAGGCTACGTCGCCAATCAAACAGGGCATCCATTTGGA
TGCCATTGCGGAGAGGTGGCGTTCAGATACCATCAACAAAAATAATTCTCCAACTCAGCCCGCAAACGCAATC
AAAACTTCGAGGATACGAGTCACTCCTTCATACTGTCTAAAGGGTCATCTTGGAAGTGTATTTTGTGTCAAAT
TTTGTGGGAATTATATTGCATCGACATCAGATGATAGATCAGTGAGGCTGTGGGCTCTGACGCCAGGAAACCA
AGACACCAACAAGGGGCATATTCATGCATTTGCTGGCAGAACCAGTGCAAAGAAAATCCTTGAAGGAGAGTCA
ACACACACCTATTCAAATATATGGACAGGTTGGGGCCATACTGCGCGTGTATGGGATGTAACATTCGCTTCTT
CGTCTTCTAATAAGCAAATAGACTCGTCGTTCCCACTCCTAGTGTCAGCAGGAGAAGACAGCACGGCGAGAAT
ATGGAGTCCATTGGCATCTAACACTGAGATTTCTTACCCACTCCGTGGGCATCGCTGTGAATCACTATGGACG
GTAGATGTTTGCGAAGGCATCATCGTAACTGGAGGTAATGATGGATGCGTGAAGTTATTTGACCTAGAGAGTC
GTGTTAGATCAAAGGAAGAATCCGTTAAAACATTTTGTGTTCCAAAAGACCCTATTCCAATTGCACCTGTGAC
TACCAACGGAGAAGCAAAATTGAATCAACCACTATTGGGAAGAGACGCGGAGAACTCCTCTGCCAGCACTATG
CCACGCAAAAAGCGCAAAAAGAAATCCAAACCGAATGGGCAGGCCATTTTTGGCATGGAATTTTACGGCAGTA
AAGGCAAAGTACGAAGCAATCTTCTGGTCGCAACAAGGGCTGGCGGACTTTTCTCTCTCGATTTGATAAGCCA
TATCTGGACTGATCACAGTAGCTGGTCTGGACAAGTTGTTTCCTCTTCGAAAAATGGAAATATGATTAGCATC
AATTCATCATCAGGCAGTTGTATAGGTGTGCATCCAAGTGGAGAAGGTGTAGTAGTCGGGACGTCAGAAGGCT
GGCTCGTCACTGTATCTCTATCCAAGTTTCCCTCAACCGAACACCATCATAAAAAGATTGCATTCCACGCAAC
ATCCCACCGGTCTGTTCAATCGGTTTCTTTCATAAACGATGAGAGTTTACTAGTATTCTACGCAAGAGGTTCA
GTCATATGGTTTAAGTTTGGCGAGTCCCCTATACCATTGCATGTGATGAAATTGAGCGCTGCAGGTATTCCTC
TGTCGTTTGCGTACGACGAGGCAGAGCAGGCCATGTACATTGGTG
>TR161|c0_g1_i1 len=335 path=[313:0-334] [-1, 313, -2]
GCTGAAAGAGACAAAACAGGACGAATTGTTCTCCGCACAGCTGCGACCTGAGTGCCGAGATTTAGAACTGACT
GAGTAGATGCAATGCCGATCAATCTGACAGCCACAAACTCTATCTGTCTCAGATCGAGGAAATTTGACAAATA
TTCATCTGCTGTGTATTGTGATTTGATGTGACATGGTATCAAATGTTCGCGACAGAGGTATCCTACGGAATAT
ACAAGATATGCGAAAGAAACGGCCAACCCCTCCTAAATATTTGGAGGAGAGTCTGATTCAGTAGATACACGGT
ACGAATTGAATCACTTAAGGGATAAGAGTTAGATAGGGCACAT
>TR162|c0_g1_i1 len=1151 path=[1129:0-1150] [-1, 1129, -2]
TATTATACTACACTAAAAATTATCGAAGCAAAATAAGATGGGATTTGGTGCTTAGTCGAGTGCGAATCTCCCC
CCCCCCAAAGAAATAAACCGATCTACTGCTACACGTTGCAATTAGGGGGCATGTAGTCTGCCAGTTTTGATGT
AAAACAGTATTCACATGTGTGACCCACTGCAGCTGATGATGGTCTACCCAAGATGAGGACGTAACCAGAAATT
ATTGTTTTAATGATATAACACGAACAGTAAAATAATCTAGTGGATCTCATTGACTATCGTGGGATTACTTTAA
TCAAATATTTATGTGCATGGAATGGTCGTGTCCTAGGATGAGATGTGCAAGAACGTTAGGATGCCAATTCAAA
TCAAAATGAGACATCCTTGTGTGATTGGCGTTGTAAACAACTTGATAGCGATGCAACGTGCTAAGTTTGGTCA
ATTGCAATAAAAATGTCGCTCGAGCTTCGATTTACACAATTTGATAACGAAACTCGACAGACGCCTTCAAGCG
ACGTGCCTCATATCTTCAAAGTGGTCCAGTACTGGTTAGCAATGTTACGAGCTCTCGTGATTGTCCATTCAGA
ATTCCGTTCCGATATCCTCGAAAGGTGCTCGTCATTAGGCATATTCAACAGCCTCAGGCGATTCTGCGCCGCG
AGTATCGTGCGAAGGTGTTGGCGCCTGCGTTCGACAGTGTGAATGTAGACTTCTTGAGAGAGGAACCTTTCCA
TGCCTACGCAATCGTAGAGATCTTCTTGTCCGATCAATGTGGTGGGCACTGTTGAGAGTTTGTAACGCGTTGC
GCTCACGTGGCGAGAGAAATCAGCCCGCAAGTAGCTTCGCTCTGCTTTGGAGTACCAACGGTGCTGCAGCTCT
TCTTTCGTTGCACGGTCGGTTACGATTAATACGCTGGTCTCTGAGAATCGCACCGAGCGTCGGGCGGGCGTAG

TAGTGGACAACATTAGTTTGCTCGGGCGACTTTCAAGCATGGGTCTTAGGTTGCGGGGAAAGTCTACTATCAA
TTGAGACATGGTAACAAACTTGATAGGAGTTTCTTTTTGCGCGTGCAGATTTTATGGTAGTAAGTAATACTTT
ATGTTGTGGAGTATCGCTGTTCACTTAAATTTTAATTTGAAGTATGTAGATCGGAA
>TR163|c0_g1_i1 len=381 path=[359:0-380] [-1, 359, -2]
TCAACCACTTTATCAATCATCAATATACTTCTAGCAATCGTGAAGGCGAACATCACATGCCAAGAATAACACA
ATCTCATACGTCTTGACCCTCCTTGTAATATTAATTAATACTACAAAATACTACAACAATGGCCATGTCACCA
GGAAAAGACAAGCCGAAGATGGATTCCTTCAGCATTGAAACGCCGTCAAAAACGGAATCCACCAACGCGTCCC
AATCCCCAGAATCACCATCGTCATCACCAACACCATCGAGAAACTGCTTCTACTCCATGTGGCAATATGGAGG
CATTCCGGAGGCGAAGGGTTACGCTCTCGTCGCGATGGGGCGTGGCGTTGGGGTGATGAGCAACGTCGTTGTC
AATGCGGCTTTGCTCG
>TR165|c0_g1_i1 len=263 path=[241:0-262] [-1, 241, -2]
CATGAGCAGTGCGCAGCGGCAAAGCTCACTCATGATTCCAGTATTTTGCCTCAGCATTTGATCGAAATGCTCA
ACCATTCTGTAGAAGTTTTTTGGTGGTGCTCGTGAAAGGGAATCCAAAGTCTTACTTCTACTTTGGATTCCCG
TTCACCATGGTCAACCTCTCGCCGACCGACGGACGCTTTGTACTCTAGAGAGAGAAAGATTGTAGATCCCGAG
TTCACAAAACGCCAGACTATGACCAAAGCCTCAATAATAATATG
>TR166|c0_g1_i1 len=278 path=[256:0-277] [-1, 256, -2]
GGCGTGCCGTCGCCCTTCCATGATCCATATACTTGAATCGTCATTCTGAGCATTGTCGTCGCTCTGCTTTGGC
GCATTGGTTGTTGAATGATTTTTATTGTCATTCCAAAACATGTTCAGACGAAATCGCATCTGGACTCGCCCGG
TCTGGACGTCTATCGTTCCTATATGCCATATGATCACTTGAAATCGAATAGATTCATGAAGCAATTGCGAACG
AGTCATGCTCGCCATCCGAAACATCGTCCGAATGCTATGCCGCGGTTTGAATTCACTGC
>TR167|c0_g1_i1 len=296 path=[282:0-159 283:160-167 284:168-196 291:197-225 290:226-260 289:261-295] [-1, 282, 283, 284, 291, 290, 289, -2]
ATGGAATGGAATCAACTCGATTGGAATGGAATGGAATGGAATCCAGCCGAGTACAGAGGAATGGAATGGAATG
GAATGCAATGGAATGGAATCATCCGTAATGGAATGGAAAGGAATGGAATGGAATGGAATCGAATGGAATGGAA
TGGAATGGAATCAACTCGATTGCAATCGAATGGAATGGAATGGAATTAACCCGAATAGAATGGAATGGAATGG
AATGGAACGGAACGGAACGGAATGGAATGGAATGGAATGGAACGGAACGGAACGGAATGGAATGGAATGGAAT
GGAA
>TR167|c0_g2_i1 len=305 path=[276:0-159 277:160-167 278:168-304] [-1, 276, 277, 278, -2]
ATGGAATGGAATCAACTCGATTGGAATGGAATGGAATGGAATCCAGCCGAGTACAGAGGAATGGAATGGAATG
GAATGCAATGGAATGGAATCATCCGTAATGGAATGGAAAGGAATGGAATGGAATGGAATCGAATGGAATGGAA
TGGAATGGAATCAACTCGATTGGAATGGAATGGAATGGAATCCAGCCGAGTACAGAGGAATGGAATGGAATGG
AATGCAATGGAATGGAATCATCCGTAATGGAATGGAAAGGAATGGAATGGAATGGAATCGAATGGAATGGAAT
GGAATGGAATCAA
>TR169|c0_g1_i1 len=387 path=[883:0-372 884:373-386] [-1, 883, 884, -2]
GATAAATGTGGTCATCGCTTCTTCTTCTTTTTCTTCTTCTTCTTTTTCTTACCACCACCACCCCCCGCCATCA
CCCCATTCTCTTCCTCCTCCGCCCTAACCCTCTCCATCATCTCCTGCCTTGCATTCCGTCCACTTTCTCTCTT
CTCCATCTGCTCCAACCTCTCTCGTTCCTGCCTTTCCAAATTCAATTTATATATCGCATCGCATTTCTCCGCC
ATCGTCATTCCAGTTTTACCACCCTCCTCCCCATCGCCATCACCATCATCCCCACCACCATCATTCTCATCAC
AACCATCAATCTCATCCACAAAACAAACCCGTTCCGCATCCACCCCCAACTCCTTATAATAATCCTCCAAAAT
AATACAAGAGATCGGAAGAGCA
>TR169|c0_g2_i1 len=891 path=[885:0-372 886:373-890] [-1, 885, 886, -2]
GATAAATGTGGTCATCGCTTCTTCTTCTTTTTCTTCTTCTTCTTTTTCTTACCACCACCACCCCCCGCCATCA
CCCCATTCTCTTCCTCCTCCGCCCTAACCCTCTCCATCATCTCCTGCCTTGCATTCCGTCCACTTTCTCTCTT
CTCCATCTGCTCCAACCTCTCTCGTTCCTGCCTTTCCAAATTCAATTTATATATCGCATCGCATTTCTCCGCC
ATCGTCATTCCAGTTTTACCACCCTCCTCCCCATCGCCATCACCATCATCCCCACCACCATCATTCTCATCAC
AACCATCAATCTCATCCACAAAACAAACCCGTTCCGCATCCACCCCCAACTCCTTATAATAATCCTCCAAAAT
AATACAAGCCGCATCGGCATCCAACTCCCCCCCCAAATCGCTCTTGCTCGGTATCCTCTCCCCCCTCGCCATC
GCCTCCGCTGATATCCTCGCCACCGCCTCCTTACTCGTATACCTCTCATCCCACATCTCCACGCTCACGCCCT
TCCCACATGCACATCGTACCTCCCAAAGTAAAGCTCTCGCGAACATGCGTGTTATATCGGATTGTTCGCTCTC
CGTTCCATTTTTATGAAGTGGTAATCCTATAACGATGTTGGTAGGACGTTCGGGGATTATGATTTGGGTGATG
ATGGTGGTGATGAGGAGCGTGGTGTTTGCGGCGGGGAGGATGGCGAGGGGACGCGGGCGGTATCCTCCCGTTG
TGATGGCGATGCCGGTGCGGGCTAGTCCGTAGTCTATTCCGATGGAACGCAGGGTGGAGAAACGTTGTTGTAG
TTGTAGTTGTTGCTGTTGTTGTAACAGTTGTGATTGTTGGTGTTGGGTTGTTGTTGTTGTTGTTGTTGCGGAT
GATGATGTTAATGTT
>TR170|c0_g1_i1 len=245 path=[247:0-136 248:137-163 249:164-244] [-1, 247, 248, 249, -2]
ATCTCGAGGAACCATGGTTCGATGATGTAGTCGAGGGAGGCGGATGGACAGCTGTGTTACGTTGATTTGGTAT
TTTGTTGGTTTTGTGTGGTATTGCGTATCGTTGTACCAAAGAGCGAGTTTGTTAGATATCTCCACATAATATA

TAACAATCACAACTCTCTCGTATGTAACGATATATATATCAATCATACATATCGAATAAAAACACCCTCTCTT
ACCGTCCCTCCAACATCTGCACGGCG
>TR171|c0_g1_i1 len=692 path=[703:0-394 704:395-424 705:425-691] [-1, 703, 704, 705, -2]
CTCCCTCCTCTTCCTCCCGGGCAGCCACCCCACACTCGCATTGTACAATGCCGTATAGAAATTGGACGCTCTG
CGTTTCGCATCGAGCAATTTGATGAACACGTCGTACTCCTCCGCGGTGGGTCCGTTTTCGCCCAATGCAAACT
CCTTCGCGAATCCGTCCTCTACCGCGTTCGTCGACCCTCCGTTATCATTGTTGTCGTTGGTACTACTACTTCC
AAAATTTTTCGATGATCCGTTCATTCGTTTCTTCTGTCTCTCCATATCCCTCTTCAATTCGACCACCAACTTC
TCCCCCTTCAATTCTTCCATCACCAATACGCGCTTGGTGCATAACTCCAAATACGGCTTGGGAATCGCACACA
TCTTCTTCGACTTGGACGAATTATCGTCGTCGTTTCCTCCGGATATATCCGCCTTCATCATATTCCCACGGAC
TCGATCGAGCTGCTCCGATTCCAACTTGTAATCGAACTCTGTCATGAACTGTTTTTCTATTTCGATGAGAGGA
GGTACGTGTACCGGTTGGGCTACCTGACAGAACATTTTGATGGTGCGTACGTCCCCGCGAAATACGTCCTCGA
CTCCCGGATACATGACTTTCACAACTACTCTATTCCCATTCTCTTTCAATGTGGCACGGTGCACCTGACCGAT
GGAGGCGGCGCCAATCGGAGTTTCGTCGAAGGAAG
>TR172|c0_g1_i1 len=247 path=[237:0-90 244:91-168 240:169-246] [-1, 237, 244, 240, -2]
CTACTTCTCGAAGGATCATTTTAGCTTCCGGTCTGAGATTGAGTAGCTGCGACTTCAGTTGCGGCTCTCGTGG
TGATGTCGAAGCTTCGCTTAGACTGAGGGATGACAATGATGGCGATGGCTTGCGAGCTTGATAAATGGGAGTT
GAAGCTTCGCTAAGACTGAGGGATGAAAATGATGGCGATGGCTTGCGATCTTGATAAACGGGAGACAATGAAA
AATCTGGATTACTCCCATTAGTGGAAGA
>TR173|c0_g1_i1 len=286 path=[289:0-171 290:172-195 291:196-285] [-1, 289, 290, 291, -2]
TCTACTCCCCTTCGTAGTGCGTCAGTAGATGTAGGCGAATCTAAGGGACTCAACACTTCGCTACATGATGTCA
ATCCGCGTCGAAAGGTACACTTTTCTCCCACCGAATGCGGCCATGAAAACCACGGAAAAGGCGCATCGTCATT
CTTTGCCGAAACTAAAGTCGACAGCAGCATGTCATGGATGGACCAAAATGGTATCATTGGGAGATACACCGGT
CAAGTCAATGATTATAATCAACCCCATGGGCCAGGATTGTTAGTCTACGATGATGGCACGGTCCAGA
>TR174|c0_g1_i1 len=1939 path=[1942:0-1812 1943:1813-1836 1944:1837-1938] [-1, 1942, 1943, 1944, -2]
GAACAGATCCAACTACAATCCAACCAAATAAATCCACCCCCTATCTCCACCGCAAACCGAGCAACAGCATCAA
CAAAACAGGAAGCGTACCTCGAACAGTAACCCGGCATCATGAATGTAGCAGCAACAGCAGCTCGAGCGGCGCG
CCTTTACTCCCCACGGCGTCCGCTGCATACCATCAGAGCGCCAAGTAGATCATTCGCCGCCGCCCAAACACAA
GCGTCACCATCCGAACCGGCGAAGACTTACTCCGAAAATGAATTCCCCGATCGACAGGAGCCTCCCATCATCA
AGCGAACAAATGCCGCCGATGTAAGTGTAACAAAAGCAGCCAAGCCTCCGGCCAATCCAACGTACACAATTTC
TCCAACGCGTTGCATCGGTACCGAACGCCCCGCCCATATTCCTCCCAACGTCCCACTTGAGTCACTGGAAATC
CCGGACACCAAAATCACCACACTGTCCAATGGCGTACGAGTCGGGTCGATGGATACATTCTCCCAAGTCTCCT
CCGTGGGGGTCTTGATAGATTTTGGATCACGTCACGAATTAGACCAATATACAATCCCCTCTACGAACGAAAC
CGTGTCAACAGCGGGAGTGAACCATTTGATGGAACTGCTGGCATTCCAATCCACCAAAAATCACACTGGGGAC
CAAATTCGCAACATCATGGAGAACTTGGGAGGGGCGTCCTTCGCCACGTCCAGCCGCGAGCAAATGCTGTATT
GTGTCGATGTCCTGCGCCCCAATGTCAAAGAGGCATTTAATTTGTTGGGGGACGTTATCAAGTGCCCCATGAT
TGAAACCAACGAAGTGGAAGAGATGAAGCAAGTCATTGAATTTCAAATGATGGACATGATGCCTCAGATTTCG
ATGGGGGAGGGGTTGCAAATGGCGGGGTACGGGCCGTTGGATGGAAAGGTACAGCAGCTTGGACGGCCACATT
TTTGCACCGCGGAAGCTCTTGCAAATCTGACGGTGGATTCGGTCCACGCTTTCCGAGAACAGCATCTGCTCCA
CAAACCGGAGGGAATCGTGGTTTCGGGGACGGGCATCTCTCACGATGCACTGGTGGAGCTTGCGGAAGCCAAT
TTTGGCCATATTTCTCCGGTTGATACGAACAACAATGATGACTCTCGTACCATTCCCTCCGTCTACACTGGAG
GAGAGTATCGCCTTCAGAGCCCGCCCGGTCTCAACCCGGCGAAGGAGGAGTTTACGCATGTAGCCATTGCTTT
TGAGGTCGGCGGCTGGCACTCCTCTGACCTCGTCCCAACGTGCGTCCTTCAAACACTTTTGGGAGGCGGAAGT
TCCTTCTCGGCCGGCGGACCTGGTAAAGGGATGTATTCGCGCCTGTATCGAGAAATATTAAATAAGCACCACT
GGACGGAGTCCTCGGAGGCATTCTCGTCGTTTCACTCGGAAAGCGGCCTTTGGGGCATTTCAGGTTCGTGTAG
GCCGGAGAAAACTCGCGAGATGACGTTTGCGTTGGTGGAGCAGTTTCACAAGTTGAAAGACGATTTCGTGACC
GACGAGGAGCTGGATCGGGCGCGGAACATGCTCAAGTGCAACGTTCTGACGCAGTTGGAGTCTCGCTTGGTCC
TTTTTGAGGACATCGGCAGGCAAATATTAACGTATGGTAAACGAGAGGATGCGGCTACTATGTGTGCTAAGAT
TGATGCGGTATCAAAAGAGGATATTCGAGAAGTCGTGAGGAAGGCGATCAAGCAGCCGCCGACTTTCTCGACT
GTGGGCATTGATATAAAGAATGTTCCCAGGGTAGAGGAGGTGACGCAGTGGCTTGGGGGGGATCTTCCTAGGA
AAAAGTCTACGAAGTCTTGGTTTTAGATAGAGCACAAGAGTAGATTGTGTCAGCAACTTACTTTTCAATTATT
TAAAATGCAAGACATAAAATCGCATATTGTTTGATTTAAAA
>TR175|c0_g1_i1 len=920 path=[922:0-919] [-1, 922, -2]
TCCGATCTTATATGTCTAAGAAAAATGCAGATCCGTTGCGCCCAGAAGCACAGCCCCCAGCTCTGCCTAAAGT
AGTTGGTGGGTTCTCAACGGTGTTAGCCGACCCTCCTTGGCGTTTTACTAACCGTACCGGCAAAGTAGCCCCG
GAGCATCGTCGTCTCGACCGCTATTCGACAATGGACCTCGAGGGGATTAAAGCCCTCCCTGTTTCAGAAGTTT
GCGCAAAGAACGCACACCTTTATCTGTGGGTTCCGAACGCACTGTTGCCAGACGGCCTCGCCGTTATGGAGGC

TTGGGGGTTCCGTTATATATCAAACATCATATGGGCAAAGCGCCGCAAAGATGGAGGTCCAGATGGTCGCGGA
GTGGGGTTCTATTTTCGAAACGTAACTGAGATCCTATTGTTTGGGGTCAAAGGCTCCATGCGGACCCTCGCTC
CAGCGAGGTCACAGGTAAATATGATTGAAACCCGCAAGCGTGAGCACTCACGTAAACCAGATGAACAGTACGG
CTTGATAGAAGCCTGTTCGCCAGGTCCTTATCTGGAGATGTTTGCCCGCCACCCGCAGAAGGGGTGGACGCTA
TGGGGAGATGAGGCTTCGGAGGAGGTGGCACCTCGCGGTCAAGTTCATAAAGGGTACGCTGGTGGCGAGATAT
TCCCTCCGTTACATACCAATGAGCAAATTCCTGAGACAGTAGCGGCTGAAATCGGGCGGGAGATAGGCATTCG
TTATGAAGCTGGCGCAAGCATTAGGGATCTCGCCGTAGAAACGGGTTATCCCATTCAAAGGGTAAGATCGTTG
CTAGAATTGGTGGAGACACCGATGAGGAAACGCGGACGGCCCGCGGCCGAGTAGTTAAGTAGCCTGATAAGGT
GTCCCATCCCATGTGTTCTCGTTCGCAGCCACAATCAATACAGG
>TR176|c0_g2_i1 len=577 path=[578:0-513 579:514-536 580:537-576] [-1, 578, 579, 580, -2]
GTCGTATGTCCGTGATTCGCTGTTGCTGCTGCTGCTGCTGCTGTTTCTGGCCAAGGTCAGCTATGCAGAGTTG
TTGGCAGAACATGTCCTCCTTCATTTTAAGACGATAGGGCGAAGATTGGATGCGATCTCCAGCGAGGTCTTGG
CCGAGATTGTTTTTGATGTTGTCGTTGTCCATCTCCCAGCCGCCTTCGGGTTGACAGAATGGGAGAGATTTGT
AATCGATGGGAAGGAAAGTTGCACGGGAGGTCATTTTGTTTACTTTTAGGTCGACAGGTTCGCCCACAGCATA
CGCATGCGGCTTGTCTTCCGAAAAATAAGAGCATTGTACTACCCCCAGTATTGGGAGGAGAATTATTGTGACG
TTGAAGAACCTCATTATATTCCGCGTATATTGTTTGATTTTCACAACATGTTCAGACGGATCATGTACGGGCA
ACTTCATCAAGTTCTACGCGGTGTAAAAAGGATACATAACACAACATAGAAAGTACGATACGATAGGGTTCGT
ACAACATTATGATACCATCAGAAGTGCAAACACTGAGTTTTGAAACTTCAAGAAAAAAGGGGGAAA
>TR177|c0_g1_i1 len=1204 path=[1197:0-266 1198:267-283 1199:284-1203] [-1, 1197, 1198, 1199, -2]
GGAGTAGGTAGCCATCCAATGACGAAAAAATCTTTTCTCATTGTCCTAGTGAGATCAAGAGAGCCTGGTGAAA
TAATAAGACCTGGCTGAGGATGCCACCCCAATTTCTCACTCATCTCGCTCTTATCATCGTATCAATAATTTGC
CTATTTCCGACAACAAGTGCAACCACCGAGCAAATCGTAGAAAAGAGCTGTCTTGAAACCCCTCCATCCGAAA
GCTGCAACACCAAACTTGATTCATCCACCATGTCCGCCACAAAAAAAAAACGTACGAACAAGATGGCGACGAA
GCCCGTCAAATCGCACAATACCTGCCCTACTTCCCATTCAAGGGCATCCCCCGCTTCTACGACATTGGCGGTT
TCTTAGAAAAACCCGAGGTATTCCAACAGATCGTAGACATCTTCGTGGAACGGTACAACGAAATCGGGATCGA
TTCCGTCGCCGGATTGGACGCACGCGGTTTCATCCTCGGTCCGCCCATCGCACTCGCACTGAAGAAACCATTC
ATCATGATGCGAAAGCAGGGTAAAATGCCCAATGCCATCTCTTCCGACGCGTACGATACCGAATACGGGAAAC
GAACGGGACTTACCGTTCAGAAGGATAAGGTGAAGAAGGGGGACAGAGTGCTCATCATTGACGACCTCGTGGC
GACGGGTGGAACTTTGAGTTCGGCAATTAATTTGGTGCATATGCTCGGTGGGGAAGTGGTGGAATGCGCGTGT
GTCGTGGAGCTCAAAATGTTCATCGATCCATCGGAGGAGAGTGGATTGCCGAGCAGGACGAAACTGTTCAAGT
CGTCCAATATTAATCACGTCCCTGTGTGGGGGTTAATTTCAGAGGACGTGTTGACCAATGAAGCAACATTACC
AGAGGATTATGTCGATGATGGGGAAGAACATTGAGTGCATTGAAATGTTGGCGTGTGATCATAATGGGGAAGA
ACATTGAGGGGGGGATCGTTTGACTTATTGCTACGAGCAGGATGAAGTTTTAATTTTTAACCTGTTGTAATAGT
GAAGTACGTGACAAATCACACCACGACCAGCGGATAAAATGATGACAATAAAGAGCTTGAGCTTATCTTTGTT
TCTCGGGCTAGCAACCAAATTCATCCGAAAGAGCTATCTACGCGAATCACATTGATTGGATCAAAGTAAACCT
AAATCTATCTTCTTTTTCCAAAAAAAAAAAGATCGGA
>TR178|c0_g1_i1 len=271 path=[310:0-132 311:133-156 322:157-231 323:232-255 324:256-256 320:257-270] [-1, 310, 311, 322, 323, 324, 320, -2]
CCTTCCAAGCTCCATCCGCTGTCCCCACAAGCACAAGTTGTTGTTTCGCAATCCACAAAGACCGCCACAAAAT
GAAAAGCGACGACGTCCCAGACGTTCCCATCCTTCCTGATATTCCCGACCAGTTGCCACTACCACAATTTGTT
TTGAATGCATTGGCGGCGATTCGGGAAGTGGACACAGTCAAACACGGAGCACCAGAATTAAATTCCGAGGTGC
AAACACTACCGCGCAGCAGCAACCGCGAACCGCCCCCACATGACGATGATGG
>TR178|c0_g1_i2 len=268 path=[310:0-132 311:133-156 322:157-231 323:232-255 324:256-256 316:257-267] [-1, 310, 311, 322, 323, 324, 316, -2]
CCTTCCAAGCTCCATCCGCTGTCCCCACAAGCACAAGTTGTTGTTTCGCAATCCACAAAGACCGCCACAAAAT
GAAAAGCGACGACGTCCCAGACGTTCCCATCCTTCCTGATATTCCCGACCAGTTGCCACTACCACAATTTGTT
TTGAATGCATTGGCGGCGATTCGGGAAGTGGACACAGTCAAACACGGAGCACCAGAATTAAATTCCGAGGTGC
AAACACTACCGCGCAGCAGCAACCGCGAACCGCCCCCAAGATCGGAAGA
>TR179|c0_g1_i1 len=752 path=[782:0-444 785:445-534 781:535-751] [-1, 782, 785, 781, -2]
TGTTTAAAATGCGTGCATAAGTTATGATCATAAATTCCCGTACTTCTATTACCAACAGTTCCACCATGGCGGT
AGGTGTCATGTCCCATAGGGTAGCTCCGTCGCCGGTACGATAGCTTCCTGCCTCGTCACTCGCCCATCTTGCT
TCCTCCCCCACTTCATCACCAGCTACGTTACTTTATAACAACTATTTACTCCTAGTCAGAAGTCAACCAGCAG
TGGTATGGTATCGTATTAGCAGACCTAGCAGCGTCAAATTAATTGACGGTTCTACTTCCACCCGAGAGTATCC
TTCCTCGCAACACTTGAACACACTATTCTTCACACGCGATTCATAACATCTTCACAATATTATCAAAAAACCA
TGGTAGCCCATCAAATACCGCTGGCACTCCTGCTCATCAGCCAAAATGTGCACGCATTTCAAATTCCAGTCAC
TAGCATTAATATCGGACAGCAGCACGCGATTCACGGCCGAAATCTGTCTTCGTCAACGTCACCATTATCGTCA
TCGCCATTGCAACGATCGCGAAATATCGCACCGCAATTAGCATCGACAGCGAACACTGACGTCGATGAAGTGT

TGGATACAATCGACAGAGCTCCCTGCTTAGATGGCGTCTGTGCCTCGTCACCCGCGAGTAGTGAAACCGACAA
TCTAACTGGAGATTCACCGTCCGCAGACGCATCCAACGGCGACGAATCATCATCATCAACATCAACATCAACA
TCAACATCGGGCGAGGCAATCC
>TR182|c0_g1_i1 len=485 path=[536:0-100 537:101-124 553:125-125 552:126-239 550:240-263 551:264-484] [-1, 536, 537, 553, 552, 550, 551, -2]
TCACACTTACAATTACAGTTACTTGTTTATAAAATGAGTTACAAAAAATCAGTTGTATAAATGAGCACAGATT
TTATTTGATTTTTTCACAGATTCCCTTCTGCTCATCTCTGGTCCGTTAGCATTCATCATCAGAGGTCGAAAAT
GGATCAAACGAGGTCGCAAAAGGACCAAACGCTTCCTGGTAGTATTGATCTTGGGACGTGGAAGAAAGCATAG
CTATACTTGGATCATCCTCTACCACTGCCTCCTCTTTCGGTTCTTCCTCATTCTCGTTTGTCACGATCGACGC
GAGGCTCGTGCTCGTAACCCCGGAATTATCGTTCGCACTCCCCTCTTCCTCTTCAGGAATCCCATCGACCTCT
GGCACGTGGATCCGTTGCCGCTGCTGATGATACCGCGTCTTTTGCTGCTGCCTCTGCCTCTCCACCATCTCCT
TCCTCCTCTTCAACCTCTCCTTCTCCTCCTCCTCCTTCTTCCGTAGA
>TR183|c0_g2_i1 len=1098 path=[1249:0-362 1250:363-1097] [-1, 1249, 1250, -2]
CTTCCGATCTCTCTCTAATACGACTCAAATACAAATCCACCATGCTTCGCGTATTGATCTCCGTCCTCTTGGC
ATGTGCCGTCCTCCTCACCCCATCATCAGCCTTTGTCGCACCATCCTCATCCAACATTGTTAGAACCGCAAAC
GCCAACCAACCACTCGCAATGGGATTCTTCGATGGCATTTTCGGCGGCGCCAAGACTGCTGACGCATCGCACA
TTCTACTCAAGGGTCCGAATGCGAACAAGCAATGCGAGAAGTTGAAGACGGATATTTACAAAAAGGCGATTGG
AAGGGGTGATCCAGCGGCGGGTGTGGCCCCGGCGAAGCTTATGGATGCGTTTGCAGCACAGGCAAAGAGGAGC
AGCACATGTCCCAGCAAGAACAACGGCGGATCATTGGGATCCTTTGGTCCTGGAGAAATGGTACCGGAATTCG
ATGCGGTTGTATTCAAGGAGCAAGTGGGAGTGATTCATGGACCGGTGGAGACACAGTTTGGCAGCCATCTCAT
TCTTGTTACGGACCGCAGTTAAAACGATTTAGAGATTTGATTCAGAGGACGACTCAGTCGCAGTTGAATGGGG
TTGTCTGCTTTAATGGAAAAAGGAATCGATAACAATTGAATTACCAGTATGCTTCATCAATAATGAATTCAAT
ACATACGCCATAGCAGGAAATATAGATAGATTTGAATCGAATATTACTCTTTCTGATTTGCGAGCTCAAATGT
GGAGAACAAATCTGTACACAAGGGTTAGACTAAAATCCAACCGAGCCAGGCACACCTATCGCATCACCCTCTC
GACACATGAAATAAAAAACAATAAAGAATACATTCATGCAAACAAAGTGCTTAAGCCAACACTCGAGGACAAG
AGGATTGTGTCTCTCCCACTTCCACACCACCACCAAAATGACCAGTGTTACTGCCAGCAATAACAACCTCGAC
AAAGTCAATACACTGGCAGTAAAGACCAGGTACGCTGAGTCCTGTTATCTTAAGATAATGTAATTGACTTTCA
TTCAGGACTTCATTGTAAAAAGTGAGCTTATCTTTCTTGGATGAACGATTTTTAAAACATTATGTGAAACAGA
TCG
>TR183|c0_g3_i1 len=876 path=[1239:0-91 1240:92-115 1241:116-129 1242:130-140 1243:141-875] [-1, 1239, 1240, 1241, 1242, 1243, -2]
TGCTCTTCCGATCTCCCGGCGAAGCTTATGGATGCGGTACGTTAAATTTCCTTTCCGTGGTACTATCAAACTT
TCGAATCGCCAAATCGCTAATATTACTTATCTAATCATGGGAGCAGTTTGCAGCACAGGCAAAGAGGAGCAGC
ACATGTCCCAGCAAGAACAACGGCGGATCATTGGGATCCTTTGGTCCTGGAGAAATGGTACCGGAATTCGATG
CGGTTGTATTCAAGGAGCAAGTGGGAGTGATTCATGGACCGGTGGAGACACAGTTTGGCAGCCATCTCATTCT
TGTTACGGACCGCAGTTAAAACGATTTAGAGATTTGATTCAGAGGACGACTCAGTCGCAGTTGAATGGGGTTG
TCTGCTTTAATGGAAAAAGGAATCGATAACAATTGAATTACCAGTATGCTTCATCAATAATGAATTCAATACA
TACGCCATAGCAGGAAATATAGATAGATTTGAATCGAATATTACTCTTTCTGATTTGCGAGCTCAAATGTGGA
GAACAAATCTGTACACAAGGGTTAGACTAAAATCCAACCGAGCCAGGCACACCTATCGCATCACCCTCTCGAC
ACATGAAATAAAAAACAATAAAGAATACATTCATGCAAACAAAGTGCTTAAGCCAACACTCGAGGACAAGAGG
ATTGTGTCTCTCCCACTTCCACACCACCACCAAAATGACCAGTGTTACTGCCAGCAATAACAACCTCGACAAA
GTCAATACACTGGCAGTAAAGACCAGGTACGCTGAGTCCTGTTATCTTAAGATAATGTAATTGACTTTCATTC
AGGACTTCATTGTAAAAAGTGAGCTTATCTTTCTTGGATGAACGATTTTTAAAACATTATGTGAAACAGATCG
>TR184|c0_g1_i1 len=941 path=[980:0-84 993:85-293 994:294-317 995:318-338 987:339-940] [-1, 980, 993, 994, 995, 987, -2]
CCGATCTCAACACGACGCTACACTCGTCGTCGACAAGCAACGAAGACAACGCGGATCGTTGGTCGATTGGATG
ATCACCGCGCATTACAAACTCGAACTCGATCCACAGACGCTTTACCTCGCGGTGAATATATTCGATCGGTACT
TCCACGCTACCACCGCCGAGGAAGAAGTTTCGGGCGAGTACGAGATGCGACTCGCTGGCGTAACGGCGCTTTT
GATCGCGTCCAAGTACGAGGAGATATACCCGCCCGAATTCTGCGATCTATTGTACGTTAGCGACGTGGAGCTG
TCAAAAAAGGAGATTATCTGCTACGAAGAAACCATGCTGTCGAAGCTAGAGTATCGGATCACCGTCCCGACCG
CACGTAACTTTCTCGTGCGATACCTGAAGGCATCCGCCGATCGACCTGCCGACGAGAGGATCGTGCAGCTGTC
GTGCTTCGTACTTGACGGTACGCTCGTTAGCTCGGGTCTGTTGTTGCGGTACCTCCCGAGTCAGCTCGCGGCA
GCTGCGGTCTATGTTGCGCGAAAAACCGCGGGTCGGAAGAAGGGGTGGAGTCCGACTCTGACCGAGTGCACGA
GGTATGGAGAGGAGGATGTGTTGCCGGTGGCGCGTGCCGTTTTGGCCGACAGGGCTTTGATGGCAGTGGAGTG
GCGTGCCGTTGACAAGAAGTACATGAAGCATAGATACGGTTGTGTCGCCAACATTAGATTATGCTCGGACTTT
TGATCTTGTGACTACTTGTGTCGTCAATGAGACATGATTGCTGGCTTTGAAAGCCCCCTATACAATGTCTCTT
CTAGGCGTCATGTTTTCATATTGTTAACTTAGTGCGAGGTAGTACTGCAGTGAATGAATAGTAATGGGTCCAT
TCGGTGCATAGATTTTATCCACACTTTGCATACTTTAAAAGCTATCAATGCAAGATCGGAAGAGC

>TR185|c0_g1_i1 len=2222 path=[2305:0-857 2306:858-879 2307:880-903 2325:904-1503 2303:1504-1527 2322:1528-2061 2316:2062-2085 2317:2086-2221] [-1, 2305, 2306, 2307, 2325, 2303, 2322, 2316, 2317, -2]
AAGTCAAGATCAGGATGTTAAATCTTAATAGTATCATTCCTTTCTATATCTATTTCGATTTGCACTGCAACTC
AATCTCAGTCGCAATGTTTCTCATCCCGTAAAGACTCCTCTCAGTCGAATGCGTAATCGCGTACCCGCCCACC
AAACTCGCGGCAAAGCACAACAACAGCGGGTGATAATTCCACCCAGCATTTCTCCAAATCAACATGGCGCTCA
AAAGCATGCTCGCCAACACAACGCATCCCATGCTCAAACACGGCATCATGTTGGAGGAGCTGGGATGACCCAA
CTCGTCGCAAAACAGATCGTGCGCCGCGCGTAAAAAGGGATCAAAAGGCGGTCCCTCTGGGGAGTTGGATGCT
GCCTCGTATACGGCGTCGTCGTAAGCGATACGATCTCCCTTTTGGTAGCGACGTTTGGGATCCCATTGCATCA
AGGTAGCGTTGCTGTTGTTGTTGCCTCCGTTGTTCGATCCTCGACCGTGACCGCCTCGGGACGACCCTCCATG
GTGGTGGGAGGGAGGATTGCGCGAAGTGGAAGAGGAACCATTCTCGATGCCAAGGACGTTGTACAGCCACGAC
TTCGGGCGGAACCGCTGGACCGGCGAGACACCGGAAGACGAGGACGACGAAGTGTTGTTGTTGTCGCCATCGG
CATCGTCAGAAGTCTTTTCAGTCGCCAGTTCCCATTCCCCGCTGATGTATCCCATGATGGCAAAATACGTCCG
ATTCATCAATGTCATCTGCGCCACTAGTAACACAGCTGTGATGAGGACCGTCGAGGTAATAATCAGCCGCGTC
GCAAAGTCATCCCCCATCAGCGCGTCATATGAGGTCGTGAGAAATGTTGTTCCCTTCCCGAATCGTCGGCCAG
CAATACCAGCCACACCAGATGGTCCCCACAGTAAAGGCCCTGGTTCGCAGTGCTCCGTGATGCGGCACACAAT
CTCCGTCTGGGGCAATATGGCGCACACTATGATGTTTTCAAGGATGCGTAGTATGGCAACCTTGTAGTACAAA
CGAAGGCGATTTTCGGTCGTGATGGACTTGTAGCTCTCCGCCCACGGTAGGTCCTTGTCCTTCCATTTCGAAG
ATTTGCTACCGCCCGCACCGCCTCCCCCTCCGGATCCACTGCCGCCGCCCAATATCCTCGTTTCAGCGCGAAT
CGCCTTCAACACGTTTGTTCCCGCCACGAACTCCGGTATGGGGAGCATGACCCTCCCGTAGAAGAGCAAGCTT
GACGCTGTACCAAATGAAATGACCAAGAACATCAAACTCACCCACGAAGGAGGCTCAGCGTCGAACGTTGCCG
GCGCCGGAAACGGAACCTGATTCCTCCAGTAATGCGCCTGATTCACCGCATCGTGGGGCTGGTTGTGATTCGG
CATGTTGTGATTGGGATTCCCACCAAAGTTGGGTATGTTGCTTTCGACCGTCTGATAATCCGCTCCGTTGGTG
CCGGTGCCATTCCCAATGAGCCATACATATCCCCCCAAAGTGAACAGCCATCCGGAAATTAAAAAGACGGTGG
CAGCCATGGCGATGACGAGCTGCGAGGAGGGGAGGACCTCCCACAGCCGTTCGAGGGCACGGTACATGTCTGA
GGTACGCATCTGTTGGACGTGGAGGGATTGCGATCGCACGGCGGTGGGAAAGAGTCGGAAGGTCAGCTTGCCG
GTAACGAAATCAAGGGATCGGTCCGGGTCGGACGAGCTGCCTGAGGCGTTGCGGCCCTGCGACGCGTGGTGAT
GATGAGAATCGCTGGAGTGATGACCCTGATTGGACTGCTGGTGTTTCCGACGGTTCTTTTTGCTGCTGCCGCC
TCCTCCGCCAGAACCGCGATCGTGGCTCCCCATGGACGATTCCGAACCGAATGCCGATCGCATCTCCGATGGG
GAGGATAGGCCTACCAACGATAGGCACGATTTTGTCCAACTCCAGATCACTCCCCCGACGAGTCCCACTGTTT
CTCCGACCGCGCCTCTTACGCCGCCCCCCTCTCCGCGGAGGAGGGACCAAATTGCAAAGGATAAACCGCATAA
CGTTCCCCATGATCTCGCGAGCGACAAAATAACAGCGGTATGCACGTCATTCGACTGAGGATACGGCCCAAAC
AACTGCTGTCCCGAATGCTGCGTCCCTTTCCCGGAACTCTTCGACGACGAATGCAAATACTGATCGGAATCTC
CAAACCCTCCTCCACCACCTGCCATCATCATG
>TR186|c0_g1_i1 len=4663 path=[4757:0-1869 4758:1870-1893 4764:1894-1911 4762:1912-1935 4765:1936-2281 4755:2282-2305 4763:2306-3138 4748:3139-4662] [-1, 4757, 4758, 4764, 4762, 4765, 4755, 4763, 4748, -2]
TTCATCTAAGCTTACCCAACTATTCAATTCCTTACAATCCCTTGGCCCTATTCTTATTCCAAAGTGAGGATAT
AACATATAATACACAGAGGTAACACTCAAGTGATGAACTCTTTTGTGCGACACCACCGACACAAAACGACGAA
AACTGCGCCTCGATTGACACAATACTTAGTAGAAACAGCATTATGCAAATACATTTCACAATCAACACTAGCC
AAAATTGATGAGTGTGAAGTGTTGAGGAGCTCCATTTCAGAATGATACAAATCCCCGTCAGACATTCCGCACC
CAGTCTGAACCCGAAGTAATGGATATCTTATTTTGACTATACATCGATCAGACTAATGGTCGCTCATATTGAC
CATTTACTATTACAAATTTTATGACTCTAAACACTCGTTGGTCTGCAGCATGAAGAAAGCTCTGCAGCAGGCG
GATTTGGACTCTTTCAGATACGCTCCTGTAACCCAACTCGGAGCTATGCCATCAACACAGCCAATGGATCCAT
CAGTTCCCCACACAGGGAAGAAGAGTCCAGTATTAGAACTCATTGGGTCTTGACATGGTACAAAGTTGCAATC
GTTTGCTGCATCCCTTGACACAAATGATCCATCGGAACATTGTAAGAGATCTGCAGTGCAGATTCCACTTGGT
TGTGATTCCATTTCATCTACCATTTCATCTTCCATTTCGAGTAGTAGTTCCGGCGGTTCTTGCCCTTCCTTGC
TACTATACCGGGCACGATTCCATGAATTTGAAGTTATTCGAAGGCTTACTTCATTTTTGTTCTCGCCAGAGAA
TAGGCCAATGACATCTACCTCGTACCAAGTGGCTCTTTCGACGGTGCCAAGACTGTTTATTGGAGCACCAAAT
GCAGAAGGGGCACTGCTCCATGTCACCGATCCTTCATTCCAGCTGGAAACCCCCGTTTTCCCGATTATCCCTC
CGGAATCAGATCCATCAGTACAGTACAGGCGCAGTGTCGCAGATTTCACACTTCTAGTATCAATATTTGACAA
ATCGAACCGTATTAGTGAGTCGAATACTCCAGAATCGTCATCAACCTGAAGCGTTTCTTCAAAGCCAAAATTC
TGATCTGGGCGATTAGACACAATGGTTGCATCATCTGATGGAGTTAAGACAAGTACGCTAGGGTCGGATGCAA
CGGTAGATGATATTGGTACAGTGGCAGACATAATTGGTCTTCGCGTGGGTCTGGCGGTGAGTCTTCGCGTTGG
ATTAGGAGTGACTGGATTGGGCTTCGACTCTGCCATACCTGAAGAAGTCAGCAATCCATATGCCACAATTATT
TCCGGTGGGTGGTGCCCCTCTTTTGAGCTATACTTGACCATTTGGGGGCTTCGTGAACGGATTCTAATAGTGA
CCGCACCTTCATTCCCGTTGAGACTGCTGACGGCCGCGGACACGTCCATAGTATACCATTGGTCTTTTTTAAC
GGAGCCAATATTGCCGAGACGGGTGTCCCAGTCAGGGGCACTATTCCACGTCACACTTCGCTCAGACCACGGT
CTGGTTTCAGGTGCAATGTGGAAGATTCCTCCGTGCTGTGCATCGGTGAGGGAGTATATTTTTAGGAAAGCGT
CAACAGGCGTCGACGCACTGAGCCCGGATGCAGGAATGTCAAACCTTATGATTGTATCATGGGCATTCGGACC

GGTGGAAAGGCCTTTGACTTTCAAGAAAGGTTCTTGGCCGTGGTTTGAGTCGGGAGAACCTTTCATGATAGTG
GCATCTTCCGATGCTATAACGGATACCACGGTTGCCTGAGATCCAAATGATTCCATCAGTTCTACAGGTGGCA
GTGTTGGAGACGTGGTTGGGTCTTTGACTGGCCTTCTTGATGGGGCAATTGTTGAGATTGGTGTAACCAAATC
AGGGTTTTGACAATCATACGGGTGGTCAAGATCCCCACACACAAAGTGAGCATATGTCGGGTCACAAGTTTCC
TGGCAGCAGTCACCACCATCCCAATTGCAAGCCTCGGTATTGTACATTTCAATATCGGCGTCACATCGCCCAT
CTCCAACCCTCGTACGATCTGACACTACACATAATTGAGGATCAATATATTCATCCAATTTGGGATTGACGCA
GAAGTAACCAAAAGGTCCATAGCCGAAGCTCTCACGGCTACAGCCATAATTTGTTGAAAGATCACACGTTGCT
TGGCAACAGTCACCCCCGTCAAAATTGCACTCAGGTGAATTGTATGGTGGATCAGGATCGCAAGCTCCGTCTC
CATGGTATGCAGGCTCAGGAACGCAGCAGCCTGGTAATCCATCTACAGGTCGCAATTCCCCCGGGCAAGAAAA
TGTCACGATTTCTTCTTTGTTCTCTGACATTGCGGCTTCTACAGGCTGGCGCGTGGGATTACGCGTAGGTTTG
GTAGTAGGATTCGCAGTGTCCCATTGAATTTGCCATGATGGGGTGCTCAACGGAGTTGGATCCATTGGCTGCG
ATGTGGGCTTACGAGTAGGATTCGGAGTTGCCACAGGGATAGGAGGAGTAGGTGGCGTTAGAGCGGTGACAGC
ATTATGTGACACTGTATGCGACACCCCATCACTGAACTGTTGACAGTCTGGACAAGTGCCGTCACCAAGACAT
TCTGCGAAGCTATTGTTGAGACCTTTGCACCCTTCGAAGACAAGGATGGATTCCAAACCAAGCGCATGAGATG
CCTTACAATACCTGTTGATACGAAGCTTGATCGCCCCACCATGCCATCCACCATGGTAGGCAGGACACTCCAA
CCCGCCGTTAATGTTATTCAATGTGCCACCAAAGTCATGATTTCTCAAGGCTTCAATATGGCATGTTGTTTCT
TCCTTGAGATTCTCCATCCAAAAGAAAACGCCGGCGCCCCACGCATATGCAGGAGTGGTGGCGACAAGATCGG
GATCTTCGCAGAACGTCATTGGATCTCCCGTCAACGCTTCCGATGCTGCGCGGTAGTTATAGTTCCATGATAG
CTGAATCGCTCCTCGACCAAAGAAAACCTTGCTGGCCGGAATGTATCCATCCAGGGGCGCAGGCTCGGACTTG
AGGGTGTCCTCGCACGCACACGCGGCGGGAGGTTCGACGACGCGATTGCAATATCCGTTGAAGGTGAGTCCGT
CGCCAACCAAACCGGCACCGTTGCATACGAAATTGTCAAAATCGAACGATTCCGAGTTGCAGGGCTTGCAGTA
CGTTTCCGTCCCAACCACCTTCTTATCCCCACACATCAAATACTCTCTAGAGGCTTGCCACTCATCACTCTCG
TGTAGAGTGTGGCCAAGAAATGATGCCAGTTCCGACTTCCGTTCGTGTTCCGTGCCCATCATGAAGATCTTTT
CGGCGTGACCATCGTTGTAACCATCGATGGCTTCGCACAATCCTTCATACGTATACGGATATTGAGCCTTTGG
CGCCAACATATCGAATGTCGCCTTCGTGAAGTAAGTGCAAGGCCGCTCTTCGGTTAACTGTGCCAACCCGCTG
CCTGGCGCGGGGGGTATACGTGGCGTGACGGGATCCCAGCAGAAGCAAAGACCGTCGACATCGCAGGGCCAAT
ACGTTTGACCGGACTGACAGGGTCGGCAGTCGGAATCGGCGATGTTTTGGGGATGGTTCTGGTTGCCCACGCA
TTTCTCGTTGGCATTCTTGCATCCACCCAAACACCAAGTTTCCGGCGCTTGAGGGCTAACTCCGTCACGAGAG
TTCTTCGTGCTGACAACTTGTGCTGCGCCGAACCTAAGAGTTGGTCCAAGGGTGGGCGGATCCGTAATGATTG
GCGACAAGGTGGGTTCTCTTGTGGGCTTCTTCGTTGGTTCCTCCGTTGGTGGCCCGACGCAAGCGTCGAATTT
ATATTCAAACTTGTAACCACAACATTCTTCCTTGCGTCGGAACAAGTTGTCTTGTGTTTCCCAGGCGGGTTGT
TTCCCATCATTGACGCATGTGCCACTTTCCCAGTCAGAATACCAAGGATTGGGTTCGGCATTAGCTTGTACCG
CAGCTGCTCCATTCCCAGTTTCCATTCGAAACTGCTCACACGCAAATCCATCTCCGTGCTCCAACTTGTAATT
GACCACATCCAACAACGGCGTCAATAAATCTTCCGTCAAATCACCACTCAATTCCCATATAATGTACCCGTTC
AATTGCTTCTCCCTTGCATACTCCACCTTATCGCATATGGCCTGGCTATCATCAAATGAGACAATACCCCCTT
TCTCGAAGTATGCATACTGCGTCTTCGTTACGTCGTCCCTCAGCGATATCATGTCCGGCAACTTCTCCAGTAT
ATTATAGTATTGAGGCTGGCCCTGATCAGCCCACCAATTCATTCCATCCGGGCCGTCAAAGTCCCCATACAAC
TCGGTCGCCCCGCCGAACGATCGGCCATAGAAGGGCAAACCAATATTAATCTTGGCTCGATCGGCACCCTCCT
CCACCCACCGCTCCACACAGCCATCCACGCTGTAACCCGGCGAGTAAAATCTGTCCTCGGGCTGATCCATCAA
CGGAGCGTTGATCCCCACCTTGTCGTCCCACGTCCCGTGAAAGTCGTACGTCATGAGGTTCAACTCGGTGAGT
GCCGCGGAGACGCGGGGCACGTCCTGGTGGTCGATGATTTGAGGACCGCATGGCAATGCGGCCG
>TR187|c0_g1_i1 len=500 path=[1211:0-66 1212:67-499] [-1, 1211, 1212, -2]
CTCGAAAACGAAATGAATCGCGTGTTCCGACGTTCTTTACTCACCGCACCTCCGATTCGGGACGAGTCTCGGG
TCCATTGGCATGCGCCCACCATCCGTTCTCCTCTCCACTCGCAAACCCCTCGAAGGATGCATTGGTCACACCC
GCCGGATTCAAAAGGGAAGCGTTGATCTGCTGTGCATTCATGCCCGGCTCGACCTTCTCACGGGTGTGACACC
CTTCGCTCTCGTGGTATAATCCATTGATCATGACGCTGCACGCTGCTCCTCCATCGTCGCTTCCACTGTTACC
GTTGTCATTCAACGCCACCAACGTGGGCAAAATAGTCTCGATCGTCCTCCTCATCGGACTCACCACGAACCTC
ACCGGACGACTCGCCTGATTCGCCAAGTGCGGCTCCAAATACCGCGCCAGCCTCCTCGCTTGAATCCGTCCGA
TGTCGGACAATCCCGGATCGGCCGAACGGCGTTCGTCGACGTAGCGGAGCCATCCCCGCAGG
>TR187|c0_g2_i1 len=594 path=[1225:0-201 1238:202-222 1235:223-228
1232:229-252 1237:253-254 1234:255-278 1231:279-498 1223:499-522
1224:523-593] [-1, 1225, 1238, 1235, 1232, 1237, 1234, 1231, 1223, 1224,
-2]
TTGGATACGGCGACAACAAATCAATCGAATCTGGATCAAGCAGTTCTTCGCACCCATGTGGGATTTTTCTCGT
AAAGTTGGATGCTGTACCCATTTTGATGTTTCGATCTAAATGAGAATAAATTTATGACCAACGTTTGTCAAAT
AGAAACAAAGTGCCCTTGTCGTCCTGTTCCGAGGCCTCGCGCAGTTGTTGGTCGAGGTATTCCATCATCCAAA
CGTAGAATTTCGCAGCTCTGGAAGAACACAGGAAACGTGGTAAACTGGGCACCAAAAATTGCACAAACGAAGG
CAAGAAGTGACACCAGCAAAACACCTGTCGCGTTGGCATCAGGAATTGAAACTATGTAAAACGATTCATAAAC
TGGCCAAACTTCTGGAGTATTTCAGTACTACCACTACGAATTCGATCTCATTCATGCACTTGTATACCAGAAA
TGCTTCAACACAACTCATGATATATGTTGTGCTACCTCGTCCTCAATCTCATTACTCGTACCCCACTATCATC

ACATCTGTAGTTCACGCAAATCGATGAATACGCTCCGTTGATTTCCTCTTTCTTTCCCACATTCCCACCACCC
CCACTACCGC
>TR187|c0_g3_i1 len=561 path=[1213:0-70 1236:71-91 1233:92-127 1210:128-560] [-1, 1213, 1236, 1233, 1210, -2]
CGCTCTTCCGATCTGTCGAAGATGTCGCGGACGTCGTCCTGTTCCGACGCCTCGCGCAGTTGTTGGTCGAGGT
ATTCCATCATCCAAACGTAAAATTTGGCGGCTCGCACCTCCGATTCGGGACGAGTCTCGGGTCCATTGGCATG
CGCCCACCATCCGTTCTCCTCTCCACTCGCAAACCCCTCGAAGGATGCATTGGTCACACCCGCCGGATTCAAA
AGGGAAGCGTTGATCTGCTGTGCATTCATGCCCGGCTCGACCTTCTCACGGGTGTGACACCCTTCGCTCTCGT
GGTATAATCCATTGATCATGACGCTGCACGCTGCTCCTCCATCGTCGCTTCCACTGTTACCGTTGTCATTCAA
CGCCACCAACGTGGGCAAAATAGTCTCGATCGTCCTCCTCATCGGACTCACCACGAACCTCACCGGACGACTC
GCCTGATTCGCCAAGTGCGGCTCCAAATACCGCGCCAGCCTCCTCGCTTGAATCCGTCCGATGTCGGACAATC
CCGGATCGGCCGAACGGCGTTCGTCGACGTAGCGGAGCCATCCCCGCAGG
>TR188|c0_g1_i1 len=1985 path=[2154:0-621 2181:622-941 2178:942-1045 @2179@!:1046-1437 2168:1438-1453 2177:1454-1573 2159:1574-1597 2175:1598-1645 2151:1646-1646 2152:1647-1670 2153:1671-1984] [-1, 2154, 2181, 2178, 2179, 2168, 2177, 2159, 2175, 2151, 2152, 2153, -2]
TCATCATCATCACCATCAGCATCTCCTTCATCACAAGAGGCGTATGACAAAGAATTCAATCAGCCGAATAATA
TTCGGGAGGAAATGTATAGATTGGAGAAAAATAAAATGAGATCGCGTACGGAGGGTAAGGCGTGTTTGGATAG
GTACCGACTTGCTTCCTCTCGTATATTTTCGCTTATCGATGAGACATTGGCCGAAAATCTAGGTCGGAAAAAT
TTCATATTGGAAAGAGCATCCATAGATGAACTATTCATTGATGTCACTGCCTTCTGCTGTTCTTCATCAGAAG
ATGGCGATGACGATGGCAACGACGACAATGATGATGATGATACCAAAGAAAAAGTTACAACAACAGCAGCAGC
AGCAATGCAGTTTCGTTCCGACTGTGGTACCGCTGCAGTACAATCATTACGTAAAACAGCTTTATGTCATGGA
GAATATGTAGATTCGGCCGAGAGGAATGACCAAATTGGCCAAGCTCTTCGCCTTGGTTGTCACGTCGCTTTGA
CTGCTCGTCGTGCCGTGTTTGAGAAACTTGGATTCACTCTTTCGGCTGGTATTTCGACGTCGAAGCTGGTATC
CAAACTTGCTGCTACATATGGGAAGCCAGATGGACAGGCTGTAGTGTTCCCAGGAGCCATGGCAAAACTAATG
GAAGAAACTCCAATCAAAAAGACCCGTTTGCTGGGAGGGAAGCTCGGGAAGAAAGTGCAATCGCTCTTGCCGG
ACTTCGAAACGACGTTGGGATCCATCTCGCGATTGTTGCCGCTGGATGTTCTGGAGAAGGCGATCGGTGAAGA
ATCTGGAAGATGGGTGTTCGATGCTTGCCGTGGGATTGACTTTGAAGAAGTGAGGCCTACTCTTAAGGTCTTA
CCAAAGTCCATCACTGCGTTCAAGTCATTTTCAAAGGTGTCATATCCAGAGCTCGAAAAGTGGACGGTAAGAT
ATTTCTTTGGATACATGGGCAGAAATTTTCTCCCTCGATGATCATGACAGTTTTCGCTACACGTTTCGATCTC
AGACATTACTAATACGGGATGTCATGAAAAGGGTGGAGACGGACAATGCCCGTAACAATCGAATTCCAAAATC
CATCACAGTTGGCTATACCATGGTTCCAGGAGGTGCATGGATTGCCCATACCTTCCGCCTCCCATTCCCAACA
GATAAAGACTTCGATTCACGAGTGCAAAGACTAGTTGACAGCACACGGAAAAGTTTGAGAGAAACAGGACATT
CATCTTTTATCAGGATAGGTTTCAGTGCCATAGATTTTGTCCACAGACCGAAGATAGGGATTGATTCGTTTTT
CAGCAAGGACAACACCCAACCAACAATTTCTTCGAAGCGTCTAGTTAGTGGGGCAAATCATGTCAGAAAGGAA
ACTCATTCAAAATCAGGGGATATTGGTTCCTTCTTTTCCGCCAGAAAAAAACAACCTGCTACTCCTAAATCGA
AGACAATAACTTCTCCCCAACTCGTGGGTCATGGCGACAATGAATTGCACGATGACATTCCTTCCCACCAATC
AAACCAGAAGCAAGACGGATCACAATTGTTGCATTTAAAGGGTAAAGGAACCGATGTACGCAATTGGTTAGCA
GACGATTCGACAGTATCAAAAAAGAATTCTTCGCTTGCATCTCAACACCCTCACATCATGACTGACGAGGAGA
TTGCAAGGCAACTACAAAACTCCTATGACAACGAAACATGGAAGGAGAGCAAACATAATGATGCATTTTCAGA
CAAGGACGAAGCTTTCGCTTTCCAACTTCAATCATCTTACGATAGAGAGCACTCTGTTCTCTCACACGTGGAG
AGATTCTCTGGGAAGAGGAACAATAATTCAAATACCAAGTCTCATACAAAAGGAAACAGCAAAAAGAGAAGCA
AAATAGATTTTTTTCTGAAAAAGTGATTACACATATCGCATATCATCTCATTGCAACAAATTAAAAATTGTAT
CATGCAGATCGGAA
>TR188|c0_g1_i2 len=1903 path=[2154:0-621 2181:622-941 2171:942-963 @2179@!:964-1355 2168:1356-1371 2177:1372-1491 2159:1492-1515 2175:1516-1563 2151:1564-1564 2152:1565-1588 2153:1589-1902] [-1, 2154, 2181, 2171, 2179, 2168, 2177, 2159, 2175, 2151, 2152, 2153, -2]
TCATCATCATCACCATCAGCATCTCCTTCATCACAAGAGGCGTATGACAAAGAATTCAATCAGCCGAATAATA
TTCGGGAGGAAATGTATAGATTGGAGAAAAATAAAATGAGATCGCGTACGGAGGGTAAGGCGTGTTTGGATAG
GTACCGACTTGCTTCCTCTCGTATATTTTCGCTTATCGATGAGACATTGGCCGAAAATCTAGGTCGGAAAAAT
TTCATATTGGAAAGAGCATCCATAGATGAACTATTCATTGATGTCACTGCCTTCTGCTGTTCTTCATCAGAAG
ATGGCGATGACGATGGCAACGACGACAATGATGATGATGATACCAAAGAAAAAGTTACAACAACAGCAGCAGC
AGCAATGCAGTTTCGTTCCGACTGTGGTACCGCTGCAGTACAATCATTACGTAAAACAGCTTTATGTCATGGA
GAATATGTAGATTCGGCCGAGAGGAATGACCAAATTGGCCAAGCTCTTCGCCTTGGTTGTCACGTCGCTTTGA
CTGCTCGTCGTGCCGTGTTTGAGAAACTTGGATTCACTCTTTCGGCTGGTATTTCGACGTCGAAGCTGGTATC
CAAACTTGCTGCTACATATGGGAAGCCAGATGGACAGGCTGTAGTGTTCCCAGGAGCCATGGCAAAACTAATG
GAAGAAACTCCAATCAAAAAGACCCGTTTGCTGGGAGGGAAGCTCGGGAAGAAAGTGCAATCGCTCTTGCCGG
ACTTCGAAACGACGTTGGGATCCATCTCGCGATTGTTGCCGCTGGATGTTCTGGAGAAGGCGATCGGTGAAGA
ATCTGGAAGATGGGTGTTCGATGCTTGCCGTGGGATTGACTTTGAAGAAGTGAGGCCTACTCTTAAGGTCTTA

CCAAAGTCCATCACTGCGTTCAAGTCATTTTCAAAGGTGTCATATCCAGAGCTCGAAAAGTGGACGACATTAC
TAATACGGGATGTCATGAAAAGGGTGGAGACGGACAATGCCCGTAACAATCGAATTCCAAAATCCATCACAGT
TGGCTATACCATGGTTCCAGGAGGTGCATGGATTGCCCATACCTTCCGCCTCCCATTCCCAACAGATAAAGAC
TTCGATTCACGAGTGCAAAGACTAGTTGACAGCACACGGAAAAGTTTGAGAGAAACAGGACATTCATCTTTTA
TCAGGATAGGTTTCAGTGCCATAGATTTTGTCCACAGACCGAAGATAGGGATTGATTCGTTTTTCAGCAAGGA
CAACACCCAACCAACAATTTCTTCGAAGCGTCTAGTTAGTGGGGCAAATCATGTCAGAAAGGAAACTCATTCA
AAATCAGGGGATATTGGTTCCTTCTTTTCCGCCAGAAAAAAACAACCTGCTACTCCTAAATCGAAGACAATAA
CTTCTCCCCAACTCGTGGGTCATGGCGACAATGAATTGCACGATGACATTCCTTCCCACCAATCAAACCAGAA
GCAAGACGGATCACAATTGTTGCATTTAAAGGGTAAAGGAACCGATGTACGCAATTGGTTAGCAGACGATTCG
ACAGTATCAAAAAAGAATTCTTCGCTTGCATCTCAACACCCTCACATCATGACTGACGAGGAGATTGCAAGGC
AACTACAAAACTCCTATGACAACGAAACATGGAAGGAGAGCAAACATAATGATGCATTTTCAGACAAGGACGA
AGCTTTCGCTTTCCAACTTCAATCATCTTACGATAGAGAGCACTCTGTTCTCTCACACGTGGAGAGATTCTCT
GGGAAGAGGAACAATAATTCAAATACCAAGTCTCATACAAAAGGAAACAGCAAAAAGAGAAGCAAAATAGATT
TTTTTCTGAAAAAGTGATTACACATATCGCATATCATCTCATTGCAACAAATTAAAAATTGTATCATGCAGAT
CGGAA
>TR188|c0_g2_i1 len=699 path=[2165:0-621 2166:622-698] [-1, 2165, 2166, -2]
TCATCATCATCACCATCAGCATCTCCTTCATCACAAGAGGCGTATGACAAAGAATTCAATCAGCCGAATAATA
TTCGGGAGGAAATGTATAGATTGGAGAAAAATAAAATGAGATCGCGTACGGAGGGTAAGGCGTGTTTGGATAG
GTACCGACTTGCTTCCTCTCGTATATTTTCGCTTATCGATGAGACATTGGCCGAAAATCTAGGTCGGAAAAAT
TTCATATTGGAAAGAGCATCCATAGATGAACTATTCATTGATGTCACTGCCTTCTGCTGTTCTTCATCAGAAG
ATGGCGATGACGATGGCAACGACGACAATGATGATGATGATACCAAAGAAAAAGTTACAACAACAGCAGCAGC
AGCAATGCAGTTTCGTTCCGACTGTGGTACCGCTGCAGTACAATCATTACGTAAAACAGCTTTATGTCATGGA
GAATATGTAGATTCGGCCGAGAGGAATGACCAAATTGGCCAAGCTCTTCGCCTTGGTTGTCACGTCGCTTTGA
CTGCTCGTCGTGCCGTGTTTGAGAAACTTGGATTCACTCTTTCGGCTGGTATTTCGACGTCGAAGCTGGTATC
CAAACTTGCTGCTACATATGGGAAGCCAGATGGACAGGTAAAAAGAAATGGTTTTTGTCGTCTCGTTATACTT
ATCGATGTGGTCTGTGTGCGCTTTTTTTTCGCTAGAAACTCA
>TR189|c0_g1_i1 len=432 path=[636:0-45 637:46-69 651:70-73 649:74-124 647:125-148 632:149-172 652:173-256 648:257-358 629:359-431] [-1, 636, 637, 651, 649, 647, 632, 652, 648, 629, -2]
TCTCAATTCGGCGTGCTGCCCCGCATGTGACCGGTGATCTGATGACCATTTGGCGGGACAGTTTGTGGTGAAA
ATGTGGCGTGATCTGGTCGCAGACGGACAAATTGGCTCAGGCGCATTGCGGCAAAACAAGGGCTATTGCCGGA
TGGCCTGTCGCTTTTGAACGGAAGTGTTGGAAGTGTAAAACACCCCTAAGTTGTATTTTTTTGGGGTTATTGA
CTTGGTTCTGGGCACGGTGCATCAACGTTTAAGTTAGCTTAATACTTAGTAAATCTGGAAACGACAGGCAGTG
GGGGCATTGGCCGGTTGTATCCTTGGGGTAAGTTGCCCGACCGAAGTCATATGCGAAAAGTGACGCATTACGT
GTGCCTAAGCCTGCCGAACTCTGGCGACAGTTACGCCATCTTTCGGACATAATTGCGGAGACGTGCC
>TR189|c0_g1_i2 len=302 path=[636:0-45 637:46-69 651:70-73 649:74-124 647:125-148 632:149-172 652:173-256 635:257-301] [-1, 636, 637, 651, 649, 647, 632, 652, 635, -2]
TCTCAATTCGGCGTGCTGCCCCGCATGTGACCGGTGATCTGATGACCATTTGGCGGGACAGTTTGTGGTGAAA
ATGTGGCGTGATCTGGTCGCAGACGGACAAATTGGCTCAGGCGCATTGCGGCAAAACAAGGGCTATTGCCGGA
TGGCCTGTCGCTTTTGAACGGAAGTGTTGGAAGTGTAAAACACCCCTAAGTTGTATTTTTTTGGGGTTATTGA
CTTGGTTCTGGGCACGGTGCATCAACGTTTAAGTTAGCCAAAAAAGAAAAAAAATTAAAAAAAAAAAAAACAT
AACCAAAAAA
>TR189|c0_g1_i3 len=402 path=[636:0-45 637:46-69 651:70-73 649:74-124 647:125-148 632:149-172 652:173-256 648:257-358 645:359-401] [-1, 636, 637, 651, 649, 647, 632, 652, 648, 645, -2]
TCTCAATTCGGCGTGCTGCCCCGCATGTGACCGGTGATCTGATGACCATTTGGCGGGACAGTTTGTGGTGAAA
ATGTGGCGTGATCTGGTCGCAGACGGACAAATTGGCTCAGGCGCATTGCGGCAAAACAAGGGCTATTGCCGGA
TGGCCTGTCGCTTTTGAACGGAAGTGTTGGAAGTGTAAAACACCCCTAAGTTGTATTTTTTTGGGGTTATTGA
CTTGGTTCTGGGCACGGTGCATCAACGTTTAAGTTAGCTTAATACTTAGTAAATCTGGAAACGACAGGCAGTG
GGGGCATTGGCCGGTTGTATCCTTGGGGTAAGTTGCCCGACCGAAGTCATATGCGAAAAGTGACGCAGGAAAG
GAAAGTTATCAAAAAAAAAAGATATAGAAGAAAAAGA
>TR190|c0_g1_i1 len=1274 path=[1798:0-96 @1827@!:97-780 1821:781-928 1825:929-992 1823:993-1016 1819:1017-1210 1804:1211-1234 1805:1235-1273] [-1, 1798, 1827, 1821, 1825, 1823, 1819, 1804, 1805, -2]
CTCTTCCGATCTCAATAATTCAATTCTCAACCATCTTCTTTACTCTCCTGTCGTGTATGAATCATATGAATCA
GGTATACAACGTAAAAAATGGCTTCCCATTCGCAGCCTTCAGCGAAACATTCGTAATAACACTGGAATCAGCA
ATCGTCCTCGGATTAATCCTCTACCATCAAAAACGCATCAACGACTCCACGCTCCTAGCGTGCCTAGCCTACC
TCGCAATCACATCGTGGGCACTATTCTCCCCCTCGCACACCTTCCCCTACGGACCACCCAACGAATGGATCGA

CGCCGCTCAAATTCTCTCCATCGCACTCAGCGCTTCGGCATTGGTACCGCAATTAAAGCAGAATTTCGATCGA
AGGTCTTCCGGGGATTACAGTCCGATCACTGCCTCGTTGGCTTCGGTCGGGTGTGCCATTCGATTGTTTACCA
CGATGGAATTGGCCGATGGGGATGTGTTGTTGTTGTGTAATTACGGCGTGGCATTGGTGTTGAATTTGTGTGT
TTTGTTTCAGATTGTTCTTTTTGGGATGCAGGTGGAGGGTAAGAGTTTGGTCGATTTGTTTTTGGCAGATGTT
AAATCTACGGATGTGGAGATATAATACACACACGAACAATCAACACACACACACCGACACGTACGTGCGTGGG
AAAATGTTGGAGGAAGGAATAGAGTTTGAATCAAAACAGCAATGACACAGACAGTGGAGCAATATGAGGAAGA
TGTGGAGGAGAAATGTCACTCTGTGGGGTCGTGCAATTCAGATGAGAACAGGTAGGTGTGCGTGGCGAGGGGC
CCAAATATTTGTGGTTGAATTAAAAAATCTATTTTGGAATGGATGAAAAAGTCTCCAGATCTGACATATACCA
AATTATGAAAACGACTCACTTGATTGAAAAAGACATCCATTGCATGGAAGTACCCTATGCAGCAAATACGGAT
GTGGAACAATAAGATGTATTATGGGAAACACTGGGTAGGGATTTTTTTTGCAGCAGCATCAGCATCTCTTGTC
AAGTTCATTCATGCAACGACGAAGTGCGGATATAACCTTTTTGGAAATTTAATGGCAATCAATTTTCAGGGCC
AATCAGTTTTGACAGGCTCGATCTATGCATTTTCATCATATCTCTTAGGATATGATTCATTCCTATGCAGTAC
AGCTGAGTTGGGATATTTTTTACATAAGGAAGAAAAATGTCAGTTCTGGAATATTTATCTCCATTTTCTAGGA
GTAATTATTTATTAATGAAATCATTTATATGCA
>TR190|c0_g1_i2 len=1595 path=[1795:0-226 1796:227-250 1822:251-311
1810:312-335 1826:336-417 @1827@!:418-1101 1821:1102-1249 1825:1250-1313
1823:1314-1337 1819:1338-1531 1804:1532-1555 1805:1556-1594] [-1, 1795,
1796, 1822, 1810, 1826, 1827, 1821, 1825, 1823, 1819, 1804, 1805, -2]
CGATCTAACAACAACAACAACATGCTGTCGGGGTTGGAGCAATTGGCGTCCGCATTCGAGATAAAAACATAAT
ACACTCTCCTCGATATCATGCATCACCATCTTCTTCTTCTCTATCGATGTTCCCCTCCTCCGATGCTGCATCG
ATATTATTATCCAGCGGTAGCAGTAGCATCAACAGTATCATCAGTAGCAGTAGCGCAGTTGTTGCGGAGGGAG
GTAGCACGGATGCAGTAGCGCTCAAATCAATCGCGGAGGCACTGGGGTATCTCATCGGCGCTGCGTCCCTCCT
CCTCTACACTCCCATTGCCGTACGAATTATTCGAACAAAGTCCGCGGATGGGTTGGCGGTGAGCACGTGGTGG
CTCAAGTTGACGAGCTTTACATGCACGGATGTATACAACGTAAAAAATGGCTTCCCATTCGCAGCCTTCAGCG
AAACATTCGTAATAACACTGGAATCAGCAATCGTCCTCGGATTAATCCTCTACCATCAAAAACGCATCAACGA
CTCCACGCTCCTAGCGTGCCTAGCCTACCTCGCAATCACATCGTGGGCACTATTCTCCCCCTCGCACACCTTC
CCCTACGGACCACCCAACGAATGGATCGACGCCGCTCAAATTCTCTCCATCGCACTCAGCGCTTCGGCATTGG
TACCGCAATTAAAGCAGAATTTCGATCGAAGGTCTTCCGGGGATTACAGTCCGATCACTGCCTCGTTGGCTTC
GGTCGGGTGTGCCATTCGATTGTTTACCACGATGGAATTGGCCGATGGGGATGTGTTGTTGTTGTGTAATTAC
GGCGTGGCATTGGTGTTGAATTTGTGTGTTTTGTTTCAGATTGTTCTTTTTGGGATGCAGGTGGAGGGTAAGA
GTTTGGTCGATTTGTTTTTGGCAGATGTTAAATCTACGGATGTGGAGATATAATACACACACGAACAATCAAC
ACACACACACCGACACGTACGTGCGTGGGAAAATGTTGGAGGAAGGAATAGAGTTTGAATCAAAACAGCAATG
ACACAGACAGTGGAGCAATATGAGGAAGATGTGGAGGAGAAATGTCACTCTGTGGGGTCGTGCAATTCAGATG
AGAACAGGTAGGTGTGCGTGGCGAGGGGCCCAAATATTTGTGGTTGAATTAAAAAATCTATTTTGGAATGGAT
GAAAAAGTCTCCAGATCTGACATATACCAAATTATGAAAACGACTCACTTGATTGAAAAAGACATCCATTGCA
TGGAAGTACCCTATGCAGCAAATACGGATGTGGAACAATAAGATGTATTATGGGAAACACTGGGTAGGGATTT
TTTTTGCAGCAGCATCAGCATCTCTTGTCAAGTTCATTCATGCAACGACGAAGTGCGGATATAACCTTTTTGG
AAATTTAATGGCAATCAATTTTCAGGGCCAATCAGTTTTGACAGGCTCGATCTATGCATTTTCATCATATCTC
TTAGGATATGATTCATTCCTATGCAGTACAGCTGAGTTGGGATATTTTTTACATAAGGAAGAAAAATGTCAGT
TCTGGAATATTTATCTCCATTTTCTAGGAGTAATTATTTATTAATGAAATCATTTATATGCA
>TR190|c0_g1_i3 len=1468 path=[1795:0-226 1796:227-250 1822:251-311
1810:312-335 1826:336-417 @1827@!:418-1101 1820:1102-1122 1825:1123-1186
1823:1187-1210 1819:1211-1404 1804:1405-1428 1805:1429-1467] [-1, 1795,
1796, 1822, 1810, 1826, 1827, 1820, 1825, 1823, 1819, 1804, 1805, -2]
CGATCTAACAACAACAACAACATGCTGTCGGGGTTGGAGCAATTGGCGTCCGCATTCGAGATAAAAACATAAT
ACACTCTCCTCGATATCATGCATCACCATCTTCTTCTTCTCTATCGATGTTCCCCTCCTCCGATGCTGCATCG
ATATTATTATCCAGCGGTAGCAGTAGCATCAACAGTATCATCAGTAGCAGTAGCGCAGTTGTTGCGGAGGGAG
GTAGCACGGATGCAGTAGCGCTCAAATCAATCGCGGAGGCACTGGGGTATCTCATCGGCGCTGCGTCCCTCCT
CCTCTACACTCCCATTGCCGTACGAATTATTCGAACAAAGTCCGCGGATGGGTTGGCGGTGAGCACGTGGTGG
CTCAAGTTGACGAGCTTTACATGCACGGATGTATACAACGTAAAAAATGGCTTCCCATTCGCAGCCTTCAGCG
AAACATTCGTAATAACACTGGAATCAGCAATCGTCCTCGGATTAATCCTCTACCATCAAAAACGCATCAACGA
CTCCACGCTCCTAGCGTGCCTAGCCTACCTCGCAATCACATCGTGGGCACTATTCTCCCCCTCGCACACCTTC
CCCTACGGACCACCCAACGAATGGATCGACGCCGCTCAAATTCTCTCCATCGCACTCAGCGCTTCGGCATTGG
TACCGCAATTAAAGCAGAATTTCGATCGAAGGTCTTCCGGGGATTACAGTCCGATCACTGCCTCGTTGGCTTC
GGTCGGGTGTGCCATTCGATTGTTTACCACGATGGAATTGGCCGATGGGGATGTGTTGTTGTTGTGTAATTAC
GGCGTGGCATTGGTGTTGAATTTGTGTGTTTTGTTTCAGATTGTTCTTTTTGGGATGCAGGTGGAGGGTAAGA
GTTTGGTCGATTTGTTTTTGGCAGATGTTAAATCTACGGATGTGGAGATATAATACACACACGAACAATCAAC
ACACACACACCGACACGTACGTGCGTGGGAAAATGTTGGAGGAAGGAATAGAGTTTGAATCAAAACAGCAATG
ACACAGACAGTGGAGCAATATGAGGAAGATGTGGAGGAGAAATGTCACTCTGTGGGGTCGTGCAATTCAGATG
AGAACAGACATCCATTGCATGGAAGTACCCTATGCAGCAAATACGGATGTGGAACAATAAGATGTATTATGGG

AAACACTGGGTAGGGATTTTTTTTGCAGCAGCATCAGCATCTCTTGTCAAGTTCATTCATGCAACGACGAAGT
GCGGATATAACCTTTTTGGAAATTTAATGGCAATCAATTTTCAGGGCCAATCAGTTTTGACAGGCTCGATCTA
TGCATTTTCATCATATCTCTTAGGATATGATTCATTCCTATGCAGTACAGCTGAGTTGGGATATTTTTTACAT
AAGGAAGAAAAATGTCAGTTCTGGAATATTTATCTCCATTTTCTAGGAGTAATTATTTATTAATGAAATCATT
TATATGCA
>TR191|c0_g1_i1 len=1125 path=[1795:0-112 @1846@!:113-796 1835:797-812
1837:813-1022 1812:1023-1046 1813:1047-1124] [-1, 1795, 1846, 1835, 1837,
1812, 1813, -2]
TGATTGAATCCTTTAAAATAATTAATGGGATATTGAGTGAATTACATGTATTCATTGAGAATAGGTAGAGATA
CACGCACTTGTGACTCACCTCTATCAATGGTCCAAATCCTGCCAGTCGCTTCAAATTCTCTTCTTCGCTCCTC
CACGAATACCGAGCTACTGTTCTTACCCTCGTCTTCAACGTACCCCTCTGGGTCCGGGACTCCTCCTCTAGAC
GTCGCATATTTCTCAGAAGAGAGCGTCGTCGGAGAAAGAGTTCATTGGATTCGAAGCTAAATTCGAAACAGTC
AACGTCATCAATCGTGTATACGCTCGACAGATAAATCGTGTCGAGTATGTGATCTTTCATGGCATCGCATAAT
TTTTCTTGAAGTTCGACATCAATTTCTATTATGGACGACCCCACTGCATCTACCGTTGCATTGCGAAACTCGA
TGTCTTGGTCGGATCGGACCCCGGAGGCCGATATTATGTCGCCGGGATCGAAAGTCGGCGATGACGACGGCGT
GGCTGTGGGACTGCCCGTTATTATCGCACCACGGCTCGGTGGCTCCGTCGGGGCAATACTCGGCGGGATCGTC
GTCGTTACCGTTATGTTATCGTTGAATAATCCTTGCTGTTCTTCGTTGAGGAGGGGGCGGCCATGATTTTCCG
GGGGAATGTATTGCGGTGGCGGACCAGCGCCGGTGCACTCGCGCGATGTCGGGTTGAAGCATTCATCGTAGAA
TTCGACATAACAGTATCCAGGATTGTCGCAAGCGTTGGACCAAGAGATGATGTCCATGAGTTTTTTTCTTCTG
TAGACTCTGACTTGGAAGTAGGGGACCAGTTGATTGATGACCAGAGGGAGTCTGGGGGTGCGTGTGACGTGAT
TAATACAAAATATCAATGGCAGCCTCCGTTCTCGGTCGAGGCGCGTGATGTGAGTCTATCGAGGGTGCTGCAT
GCAGATGCACGATTGATGACCAGGTATGGACATTGAGGAGGAAGCAATGAGATGTTCCTGTGTTCCCGTGGCT
CGTGTATTCATGTTACGGAGAAAAGAACTCGTGACTAGATTAGAGTTCATTATATCGGCCAGTTCAAAAATGC
TAGTAACTCACTAGTAAAATGCATTTTTAT
>TR191|c0_g1_i2 len=1675 path=[1823:0-55 1824:56-58 1825:59-59 1841:60-79
1849:80-85 1851:86-118 1850:119-142 1848:143-148 1844:149-184 1839:185-
185 1847:186-195 1843:196-219 1838:220-628 1831:629-652 1832:653-662
@1846@!:663-1346 1835:1347-1362 1837:1363-1572 1812:1573-1596 1813:1597-
1674] [-1, 1823, 1824, 1825, 1841, 1849, 1851, 1850, 1848, 1844, 1839,
1847, 1843, 1838, 1831, 1832, 1846, 1835, 1837, 1812, 1813, -2]
TGTGGTTGAGCGTTTCTCTTCCATGTGTTCCAGTCGAATGGCACTATTTCTGTTGAATTGCTCTGTGGTTGAG
CGTTTCTCTTCCATGTGTTCCAGTCGAATGGCACTATTTCTGTTGAGTTGCTCTGTGGTTGAGCGTTTCTCTT
CCACGTGTTCCAGTCAAATGGCACTATTTCTGTTGAATTGGTCTGTGTCCTGTTTCTTTGCCCCCGTTCTTCA
AGGCGATCGAACTGGCTTAGAGATCCTTTAGAAGAAGCAGAGAGAGTGTGCTTGTCATCATTATCGGCATGCA
GATTCCTTGTTGCTTTGGTTTGAAAGGCATCGGTTAGGTCAATTACTACTTCATCTTCCCATTGTTTATCTTC
ACCTTTGCCTTTCTGCATACCCTTGTAAAACAAGAATGCCGCGAAGATAAGTGCAATCACCGAGCAAGAGATA
ATTATCGACATCCCCACCGCAGTAGAGGCAAAGGACAAAAACATAAATTCGTGTTCCCTTAAAGGCGCGGGAT
CAGTTGGGATTGGGACTGACACTGGTGCTGATGTCTGATTGGCAACTACTTCTGTATCTCTTAACACGGATAT
GTCTCTGTCTTCTAGTTTCCTTTTCAATCGAACACCACCATCTCGGTTGATTGAATCCTCTATCAATGGTCCA
AATCCTGCCAGTCGCTTCAAATTCTCTTCTTCGCTCCTCCACGAATACCGAGCTACTGTTCTTACCCTCGTCT
TCAACGTACCCCTCTGGGTCCGGGACTCCTCCTCTAGACGTCGCATATTTCTCAGAAGAGAGCGTCGTCGGAG
AAAGAGTTCATTGGATTCGAAGCTAAATTCGAAACAGTCAACGTCATCAATCGTGTATACGCTCGACAGATAA
ATCGTGTCGAGTATGTGATCTTTCATGGCATCGCATAATTTTTCTTGAAGTTCGACATCAATTTCTATTATGG
ACGACCCCACTGCATCTACCGTTGCATTGCGAAACTCGATGTCTTGGTCGGATCGGACCCCGGAGGCCGATAT
TATGTCGCCGGGATCGAAAGTCGGCGATGACGACGGCGTGGCTGTGGGACTGCCCGTTATTATCGCACCACGG
CTCGGTGGCTCCGTCGGGGCAATACTCGGCGGGATCGTCGTCGTTACCGTTATGTTATCGTTGAATAATCCTT
GCTGTTCTTCGTTGAGGAGGGGGCGGCCATGATTTTCCGGGGGAATGTATTGCGGTGGCGGACCAGCGCCGGT
GCACTCGCGCGATGTCGGGTTGAAGCATTCATCGTAGAATTCGACATAACAGTATCCAGGATTGTCGCAAGCG
TTGGACCAAGAGATGATGTCCATGAGTTTTTTTCTTCTGTAGACTCTGACTTGGAAGTAGGGGACCAGTTGAT
TGATGACCAGAGGGAGTCTGGGGGTGCGTGTGACGTGATTAATACAAAATATCAATGGCAGCCTCCGTTCTCG
GTCGAGGCGCGTGATGTGAGTCTATCGAGGGTGCTGCATGCAGATGCACGATTGATGACCAGGTATGGACATT
GAGGAGGAAGCAATGAGATGTTCCTGTGTTCCCGTGGCTCGTGTATTCATGTTACGGAGAAAAGAACTCGTGA
CTAGATTAGAGTTCATTATATCGGCCAGTTCAAAAATGCTAGTAACTCACTAGTAAAATGCATTTTTAT
>TR192|c0_g1_i1 len=423 path=[3989:0-346 3990:347-387 3991:388-422] [-1,
3989, 3990, 3991, -2]
TAACAATAAAGAATAACATTTTTATTTAATAAAAATGAGAAAGGAAATGATATGTTTGAAAAATTTACTGAAG
GTGCGATTAAAGTAATTATGCTAGCACAAGAAGAAGCTAGACGAATGGGTCATAATTTTGTAGGCACTGAACA
ATTATTACTTGGTGTAATTGGACAAAGACATGGAATAGGTGGGCGTGCATTAGCACAATTAAATATAACATTA
AAGAAAACACGAAAAGAAATTGAAAAATATATTGGTCGTGGAACTGGTTTTGTTGCTTCTGAAATACCATTTA

CTCCGCGTGCAAAACGTGTATTAGAAATGTCAATTCATGAAGGTCAAGATCTTGGTGTTAATTATGTTGGTAC
TGAACATGTTTTATTATCTTTAAAAGAAAAGAAAAAAAAAAGAGAAAAAAATCGAAAGG
>TR192|c0_g2_i1 len=396 path=[3987:0-346 3988:347-395] [-1, 3987, 3988, -2]
TAACAATAAAGAATAACATTTTTATTTAATAAAAATGAGAAAGGAAATGATATGTTTGAAAAATTTACTGAAG
GTGCGATTAAAGTAATTATGCTAGCACAAGAAGAAGCTAGACGAATGGGTCATAATTTTGTAGGCACTGAACA
ATTATTACTTGGTGTAATTGGACAAAGACATGGAATAGGTGGGCGTGCATTAGCACAATTAAATATAACATTA
AAGAAAACACGAAAAGAAATTGAAAAATATATTGGTCGTGGAACTGGTTTTGTTGCTTCTGAAATACCATTTA
CTCCGCGTGCAAAACGTGTATTAGAAATGTCAATTCATGAAGGTCAAGATCTTGGAAAAGAACACGCTGGAGA
TTACAAGGACCAAGGCAAAAAGAAAAGGGCT
>TR192|c0_g3_i1 len=3728 path=[3980:0-346 3981:347-387 3982:388-406 4003:407-688 3972:689-705 @3999@!:706-2139 3975:2140-2156 @4002@!:2157-2580 3998:2581-2996 3995:2997-3727] [-1, 3980, 3981, 3982, 4003, 3972, 3999, 3975, 4002, 3998, 3995, -2]
TAACAATAAAGAATAACATTTTTATTTAATAAAAATGAGAAAGGAAATGATATGTTTGAAAAATTTACTGAAG
GTGCGATTAAAGTAATTATGCTAGCACAAGAAGAAGCTAGACGAATGGGTCATAATTTTGTAGGCACTGAACA
ATTATTACTTGGTGTAATTGGACAAAGACATGGAATAGGTGGGCGTGCATTAGCACAATTAAATATAACATTA
AAGAAAACACGAAAAGAAATTGAAAAATATATTGGTCGTGGAACTGGTTTTGTTGCTTCTGAAATACCATTTA
CTCCGCGTGCAAAACGTGTATTAGAAATGTCAATTCATGAAGGTCAAGATCTTGGTGTTAATTATGTTGGTAC
TGAACATGTTTTATTATCTTTAATAAATGAAGCAGATGGAATTGCAATGCGAACTCTAGATAAACTAAGAGTA
AACATACCTAAGTTACGACATCTAATTTTAACTTATATTGAAGAACAACAAGAAGATATTTTAAAACCACCTT
TAACTGATCAAGAAAAATGGGCGATTGCTAGAGAGAAGTCTGGATTAAAAACACCTGCTTTAGATAGTTATAC
CGAAAATATCACAAAAGATGTTATGAAGCAGCGTTTAGATCCAGTTATTGGTCGTGATGAAGAAATAAATTCT
TTAGTTAAAGTTTTAGGTCGTCGTCGAAAAAATAATCCTGTTCTAGTTGGAGAAGCAGGGGTTGGTAAAACAG
CTTGTGCTGAAGGACTTGCTATATTAATGCAAACTATGGATTGTCCAGAATTTTTATTAGAACATGATATACG
ATCTCTTGATTTAGGTTCAGTTTTAGCGGGAACAAAATATCGAGGTGAATTTGAAGAACGTATGAAACATATT
ATTGATGAAGTTAACCAACATGGAAAAACAATTTTAGTAATTGATGAAATTCATACATTAGTAGGGGCAGGTG
CAGCCGAAGGTGCTGTTGATGCTGCAAATCTTTTAAAACCTTCTTTAGCTCGAGGTACTTTACGTGTTATTGG
TGCTACGACTATAGATGAATACCGTCGATATATTGAAAAAGACCCAGCATTAGAACGAAGATTTCATTCAATT
CTTGTAGAAGAACCATCTGTAGAAACAACAATTGATATTTTAAAAGGTTTAAAATCTGAATTTCAACGTCACC
ATGCTTTAACGTATAATGACGCTGCTCTTGAGGAAGCTGCAAAACTTGCCAGTAGATTTATTGCAGACCGTAA
TTTACCAGATAAAGCTATTGATGTGATTGACGAGGCAGGTTCGCGTGTTAGATTACGGAATAATTCATTACCT
ACAGGTCTACAAAAGTTACTTGTTGAATTACATGATACGATAAAAGATAAAAATGATGCGATCGATGAAAATG
ATTTTGTTACTGCAAAATTATTAGTTGAACATGAAATAGAAGTCCGTACACATTTACGAATAATGCGATTTGC
ATTAACTAGTAAAGAAAGATATGCGAAATATGCAAGTCCAAATGGACCACGTTTTGATGAAGTTACTGAGTCA
GATATTTCTAAAGTTATTTCTGCATGGACGGGTATTCCAGTAACAAAAATTAATCAGTCGGAAAATGAACGTT
TAAAAGTCATGGAAAGTATTCTTCATAAACGACTTATTGGTCAACATCATGCAATCGTTGCTGTTTCAAAAGC
AGTACGTCGAGCACGAGTTGGTATTCAAGATCCTAAACGTCCGATTGCAAGTTTTATCTTTGCAGGTCCAACA
GGTGTTGGAAAAACGGAATTAACAAAAGCTTTAGCCGAATACATGTTTGATGATGAAAAAAGTCTTATTCGAT
TTGATATGTCGGAATTTATGGAAAGACATACGGTTGCAAAATTAATTGGATCACCACCGGGTTATGTAGGTTT
CCAAGATGGAGGGCAGTTAACAGAAGCAGTAAGACAAAAGCCATATTCAATTGTTTTATTTGATGAAGTTGAG
AAAGCACACCCAGATGTTTTTAACTTATTTTTACAAATATTAGATGATGGACATTTAACAGACTCAAGTGGTA
AAGAAGTTAGTTTTAAAAACTGTATTATTGTAATGACAACTAACTTAGGTTCTAGAGTTATTGAAAAAGAGTC
TCCAGTTCTTTCGAAAGAAAAAATTGGATTTGGGCGTAGTCTTGATTCAACAGCATTAGAAAGAGAAAGTAAA
AAAATTGAATTACTCGTAAATTCAGATGGTAGTTTCTCATTAAAACCTCCGGTTGAAAGAGAAATAACAGCAG
AAGATGAAGAAAAATTTGTTAAAATAACAGGTTTAGTTCAAGAAGAATTGAAAAAATTCTTCCGCCCAGAATT
CTTGAATCGAATAGATGATATTATTGTCTTTAACCATTTATCTAAACATGATATTTGGGAAATTTGTGGACTA
ATGTTAGGTGGGCTTAGTAAGCGACTTAAAGAACAAGGAGCAACTTTAAATGTAGATAATGCTGTAAGATTCT
TCCTTACGGACGAAGGTTATGACCCAATTTACGGAGCTCGTCCACTACGTCGTTCAATTACAAAATTCTTGGA
AGATAAATTAGCTGAAACTTGTTTAAGTAATGTGATACATGAAGGTACAGAAATAAATGTGACACAAAAAATA
AAACCAAATGTTTATGGGAATAGACCAATCTATTCACCAATATTTGATGACATTTATACATCCGAAATTTTAG
TTGAATTTGATAATAGTGGTGTAGATTTTAGTAAAATTCAAAAAACTGAAGAACCAAAAAATGTCGGTGAAGA
AGCGAAAAGTAAATCTAAAGTACCAGTAGAAGAGTAGGAGATATAAGTGTAAAATAAAATAATACTAAATATT
ATTATATTTACATATAATAATATTTAGTTTTTATATTTATTGTATTTGACTTATTTGATTTATTGGAGAACTA
GGGGTCGCATAATATTTTTTTTCCATTCGACCAGCTAAATACCCTAACCGTCCTGCTTCAACACCTAATTTCA
TGGCTAACGCCATTTGTTCAGGATTTTTAGCTTGAGCAACTGCTGTATTTAATAAAATTCCATCAGCCCCTAA
TTCCATTGCCATTGTAGCTTCACTTGGAGCCCCAATTCCAGCATCTATAATAACAGGGATATTTGCATTTTCA
ATAATGATTTGAAGATTAGCCAAATTATTTAATCCTTGTCCAGACCCAATTGGCGAACCAAGTGGCATAACAG
TAGCACATCCTAAATCTTCAAGATGTAATGCTAACATAGGATCAGCATTGATATAAGGTAAAACAGTAAATCC
TTTTTTTACTAAAAATTCGGCTGCTTTTAAAGTTCCTATTGGATCGGGTAATAAATATTTTGGGTCAGAAATA

ACCTCTAATTTAACAAAAAAATTATCTTCTTGACCTAATTGACAAGCTAATTCATGACCTAAAAATGCCATTC
TAATAGCATCTTCAGCTGTTTGTGAACCCGCTGTGTTTGGTAATAACCATAACTTTTCCCAATCTAGAGATTT
AAGAAAACTAATATTATCTTGATTTATATTTGTAGGTAATCGTCGAATAGCGACTGTAACAATCTCACATTTG
CTTGCTTTAATGCTTTTTATCGCATCTTGACTAGTTCTATATTTACCAGTTCCTAACATCAAATGCGAAGAGA
ATGATTTATCCCCAATTCGTAAAGGATCAAACATATTGTTCATAATTTTTTTAACTTTAAAAAAAGCAGCGAA
AGGGA
>TR193|c0_g1_i1 len=296 path=[287:0-295] [-1, 287, -2]
TTCCGATCTAAAAACAACGCGATCCCTTCCCATCCTTCCGAACGCGTCGCGGGTGCATCCTGCCAGGCTCATT
ACAATATGAGATTCTTTGTATCTCTATGAAACGACCAAGAGACTCACACACACCTTGAGACGTGCGTACTGAG
TACTCCTATTCAATAATAATGTCATGGGGAATTGCACTAGTCAAAACCAAACCCAAGGTGATCAACATTACCA
TCATGGAATAACCGATCCCACCAACGACAACGACGACGACGACGAAAATAACGAGGATAACAACAATGAAGAT
ATAC
>TR193|c1_g1_i1 len=1115 path=[1196:0-556 1197:557-580 1211:581-1006
1212:1007-1030 1213:1031-1051 1206:1052-1097 1195:1098-1114] [-1, 1196,
1197, 1211, 1212, 1213, 1206, 1195, -2]
CGTACCTCAATCTCAATACTCATAACCACGATAGGCAATCAATCCATGACCACCATGAAGCTAACCGCAGTGG
CATTGCATGTCACTGCAACGATGACATCCAAAATTGTTGCCGAGGATCATGCTTCGTCATCCGTGAATAGATG
GAGGACTCATGATCTCCATCCGGTGAGAAGGAAACAGCAGCAGCAGCAGCAGCGCCTTCGTCGTCGATCGAGT
AATACTGCAAACGACTCATACTACCGCTCTAATGACGAAGAAGTTGTTCGAACGAAAGAAAAAATATTACATG
AGCGGCGGCGTGATCTTCTGGGCGAACTATTGGTAGAGTCCGGAGCCGCGTTTTTATACACTGTTGAAAATAA
TTATGATGATTATGTAGCGCCCGTTGTCTTACCGACTCAGCCATTAGAAGAAGAGAACGACAATAACAGCAAG
AGCGACGATAATGACGACTGTCTCAATAATCGTGCATGGCATCCAGATCTCACCTCTTTGAACATGGCCTGCA
GCAACAACCTAAGTTATCCATCATTTTGGGACGAGGATCCCTCCATGTACGAAGTGGCCATATTTGATTCCTC
GTTAGATTGCTGCGAGGCACTGCTAGATGTCATAACTACTACGGGGAAATGCTCGGTAATAAATGACTGCGAA
CGCATCTCAAACAGCAACAACAACGACGACGACGAATCATCAACAGTAACAGCACAACCACCAGCAGAAATTA
TAGCAGCCTCCAAAACAAGAGAACGGAGAACAGTTGGCTGCAGCAGCAGTGGATGGCACATGGACCTCACATA
TAAAAATGGATGTACCAACGACGACAATTATCCCGAAAGATGGGTCAATAATCCGACCGCAAAAAGTATCATG
TTTCACGATACGGCGCAAAAATGCTGTGATAAAGTCTATGCTGGACGAGAATGTCAGATTTATCATGTTGATG
GATGTAGTAGCCAGTTCGAAGCATCGCCTGCAGGAGGAGCAAATTCAGCGACCGCAAGACCACCAACAAGGCT
ACCAACACCCCCACCAGCAAAATCCAACAATGAATCCACTGAGGACGTAGGATCGACCTGCCCATGGCACAGG
GACATCACACATGAGGACGG
>TR193|c2_g1_i1 len=3096 path=[4152:0-1224 4153:1225-1241 @4171@!:1242-
2924 4169:2925-3082 4164:3083-3095] [-1, 4152, 4153, 4171, 4169, 4164, -
2]
TGATTCTCGTCATCTACCTACTACCTATCCTTCGACAGTCTTCCACTCCCATCCACTGGATCAAAACTGGCGA
CAATTCGGTACGATAGTACTATTCCTTGGAAACCTCGACCTGATCCTCAACAAAACAGTGCTCAACAATGATG
GATATCCAACCACTATCTTCCCCAGGAAAAAGTTTTGCAATGGAAGAAACGACCACATCAACAACAGTCAGCA
GTACGACGACGACGTTGTCGTCATCGTCGGAAACAACAACACGCAGCAATACAGACAGTGTGGATGATGACAT
CAACAACGCTGATGAAGATGTAGAAATGTCGTCTACTACGAAAGACAACAATGATCAGAATCAGGATGGAAAA
GCAATCGCAGAGGATGACGCGCCACCGAACAATATCAACGCGATCACAGCAGCTGCAGCAACGTTTCCCAAAC
AGATTGGTAATGCATGGGGAACCAACAAAACCCCAACAACCACAACCAATCCCACAACTAATCCCCAAGATGA
TATGGACAATAGAAGTTCTACTCCCAAAAATAGTAAAAAGCGACTCAAATTACTACCAGGCGGAAAAGAAAAC
AACAACAAAGACGAAAGTCAAACCAAACTTAATTTCGCCGACATTATGGCAGAGCAAGAACACGAAAAGAAGA
AGAAGGAAGAATCATCCACTCGCGGTGGTGGTGGTGGTGGTGGTGGTGCGAAAGGCAGCGTCAGCTTTTGCGAGAA
TCGAATACTAGTGGAAAGCGAGGAAGATCGTATGATGCGCCTTGCTATTGAGGCTTCGTTGTTGGATCAACAG
CAGGGGCAGCAATATCATCAGAAAGGTGGCCATCCACTGAATGATACGAAGATGCCAGCGTCGGTACTGAAGG
GAAAGAGTAATAGCGGTGGTGATGATGATGATGATGATGTCAAAATGTCGTCATTCGGCACGAAAGGGGATTA
TTATGATTACGATGATCCGAAAAAGGAAATTGACAATTATTTGAATGATGATGAGGATGATGGTATGGATGAG
GATATGAGAATGGCCATTGCATTGTCTCTACAAGAAGCCAATGGAGGAGGTGGAGGAGGAGATAATGGAGGAT
TTGTAGCTAGCTCGGTAGCAGCAGCAACGGCGACAGGAGGAGGAGGAACCAACAGCCATGACACCATTGCCCT
CAAAGAAATGTCCAAAAATGCATTCTCGGAAGATCCGACAGAGTTTTACGAAAAAAAAGGGAGACTGGGCAGA
AGGAGGAATCTCCCATTTCACATCGTCGGCATTCGATGTTGCGGCGAAGGCTTCTTCTCCCTCAGTAGTTTCC
ACCTTTGACGCGTTCCCTGCTGTTGTCGCTGCATCCAAAAAACCGCCGCCTTCCTTCGATGCAACATCCGCCG
TGGCTTCGAAACCCCCACCTTCCTTCGATGATGCATCATCGTCCGCATCATCGAAACTCCCCCCATCGTGCGC
TGCATCATCGTCCGCATCCGCCACTACCGCATCATCGAAACCACCACCACCCTCCATCGTCGACGGATCCGAA
TCCCTCGCTCGCGCATTGCACGAAGCCGAACTCGCCGAATACGATGCTTCCAAATCCGCGAGTGCCGCCGCCG
AAATGGCATCGTTGAAGTTGGCCATGGCGCTCCAGGAGGAGGAGGATGCGGCGAGGAATCGCCTTTCCAATAA
TCGAGATTGCGTGGCAGGTCCATTGAATGTTGGGGTGAGGACTGTGGGGAGGGAGGAGTTTCATTCTCTCAAG
ATGGGTGGCGGCAAGGATGATTATGGAAATAATATAGGGGGAAGTAGTAGTAGGATGGTGGGGAGGAGGAAAC
AAGAGGAGAGGGACGTGGGGAAATTATTGCTCTCGAGGAATAGTAATAGTTTTGGGAAGGAAGAAGAAGATGC

CACGACGGGCGATGTTCCCATGGGCGACGATGATGACACCGATAATTACTACTACTACGCAGACGATCGACTC
AAATCAGATGATGCGCAACACGATCACGATCTCGACGAGGTGGGCGATAACGAAGACGACGGTATACGAATGA
ATTCCCAAACGTCTTCCTCCGCCTGGAAGCGGTTCGACAAGGATACCTTCATCGGACCCAACAACGAAATCCG
AACGAAGCACGATCCCGAACTCAAACACCGTTCCAACGCCGCGAATCTCCTCGGCAGTCACGGCGCGAAGCTC
AAGGCCGCCGAAAAAAAGTCCGTCGCCGTGAGCGATCGCGCGTACAACGCCTTCAAACGGGCCGAATCGCGAC
AGTCCGGATTCAAGAAGGGAGTGGCGAAGCAGGGACACGGACGGGCCGAGGATGCCGGCGCGGGTAAGACGAG
GGGAGGTGCGATGGACGGGAATGTGAGGATGCATATTGCCGCGGCGATTAATGCGGGGTTGATCGATCGTTGC
AACGGGGTCGTGAAGGAGGGGAAGGAGGCGATGGTGTACCATGCGGATGGAGGTTGGAAGGGAAGGAGGAGTC
GACAGCAGGCGGAGGGTGATGATGGTGGAGGTGCGGAAGATGAAATATCGTCCGTGGTTCATTATTCTGGAGA
GGGGGCATTGGATACAGTAGGGAGCGATGGGTACGATGTCGCTGTCAAGGTGTTTAAGAGAATTTCGGAGTTC
AAGGGAAGAGGCACCTACGTCGACGGCGATCCCCGCTATCACAAGCAAAAGTTCAAAACCAACGATCAACGCG
ACCAAGTCGTATTGTGGACCGAGAAGGAATACCGCAATCTAATCCGTGCATACAGAGCAGGCGTTCCAGTCCC
AAAACCGCTCTATCGAAAGGAAAACATTTTATTTCTGCGTTTCTTGGGCGAGAATGGATGGCCGTCGCCGCAG
ATCAAAGAGATAGATATGAAGAAGGGGAGTAAGAAGTGGACTACGTTATACGCGCAGACGCTTGTGGCTGTCC
GTAGGTGAGTGTCGATTACCACATCATTTTCTCTGGAAGCTTATGTTTGTTTGTTCATATTCGTGCCCGGAAT
GTTCACACTTGTGCTCAAATCGCAAACCTTGATTACGTTGAAATAACATCTCCTCTTTTCTCTCGCTCGATCA
GGCTCTACCACTGTGCAGATCGGAAGAGCG
>TR193|c2_g1_i2 len=3943 path=[4152:0-1224 4153:1225-1241 @4171@!:1242-2924 4156:2925-2944 4157:2945-3942] [-1, 4152, 4153, 4171, 4156, 4157, -2]
TGATTCTCGTCATCTACCTACTACCTATCCTTCGACAGTCTTCCACTCCCATCCACTGGATCAAAACTGGCGA
CAATTCGGTACGATAGTACTATTCCTTGGAAACCTCGACCTGATCCTCAACAAAACAGTGCTCAACAATGATG
GATATCCAACCACTATCTTCCCCAGGAAAAAGTTTTGCAATGGAAGAAACGACCACATCAACAACAGTCAGCA
GTACGACGACGACGTTGTCGTCATCGTCGGAAACAACAACACGCAGCAATACAGACAGTGTGGATGATGACAT
CAACAACGCTGATGAAGATGTAGAAATGTCGTCTACTACGAAAGACAACAATGATCAGAATCAGGATGGAAAA
GCAATCGCAGAGGATGACGCGCCACCGAACAATATCAACGCGATCACAGCAGCTGCAGCAACGTTTCCCAAAC
AGATTGGTAATGCATGGGGAACCAACAAAACCCCAACAACCACAACCAATCCCACAACTAATCCCCAAGATGA
TATGGACAATAGAAGTTCTACTCCCAAAAATAGTAAAAAGCGACTCAAATTACTACCAGGCGGAAAAGAAAAC
AACAACAAAGACGAAAGTCAAACCAAACTTAATTTCGCCGACATTATGGCAGAGCAAGAACACGAAAAGAAGA
AGAAGGAAGAATCATCCACTCGCGGTGGTGGTGGTGGTGGTGGTGCGAAAGGCAGCGTCAGCTTTTGCGAGAA
TCGAATACTAGTGGAAAGCGAGGAAGATCGTATGATGCGCCTTGCTATTGAGGCTTCGTTGTTGGATCAACAG
CAGGGGCAGCAATATCATCAGAAAGGTGGCCATCCACTGAATGATACGAAGATGCCAGCGTCGGTACTGAAGG
GAAAGAGTAATAGCGGTGGTGATGATGATGATGATGATGATGTCAAAATGTCGTCATTCGGCACGAAAGGGGATTA
TTATGATTACGATGATCCGAAAAAGGAAATTGACAATTATTTGAATGATGATGAGGATGATGGTATGGATGAG
GATATGAGAATGGCCATTGCATTGTCTCTACAAGAAGCCAATGGAGGAGGTGGAGGAGGAGATAATGGAGGAT
TTGTAGCTAGCTCGGTAGCAGCAGCAACGGCGACAGGAGGAGGAGGAACCAACAGCCATGACACCATTGCCCT
CAAAGAAATGTCCAAAAATGCATTCTCGGAAGATCCGACAGAGTTTTACGAAAAAAAAGGGAGACTGGGCAGA
AGGAGGAATCTCCCATTTCACATCGTCGGCATTCGATGTTGCGGCGAAGGCTTCTTCTCCCTCAGTAGTTTCC
ACCTTTGACGCGTTCCCTGCTGTTGTCGCTGCATCCAAAAAACCGCCGCCTTCCTTCGATGCAACATCCGCCG
TGGCTTCGAAACCCCCACCTTCCTTCGATGATGCATCATCGTCCGCATCATCGAAACTCCCCCCATCGTGCGC
TGCATCATCGTCCGCATCCGCCACTACCGCATCATCGAAACCACCACCACCCTCCATCGTCGACGGATCCGAA
TCCCTCGCTCGCGCATTGCACGAAGCCGAACTCGCCGAATACGATGCTTCCAAATCCGCGAGTGCCGCCGCCG
AAATGGCATCGTTGAAGTTGGCCATGGCGCTCCAGGAGGAGGAGGATGCGGCGAGGAATCGCCTTTCCAATAA
TCGAGATTGCGTGGCAGGTCCATTGAATGTTGGGGTGAGGACTGTGGGGAGGGAGGAGTTTCATTCTCTCAAG
ATGGGTGGCGGCAAGGATGATTATGGAAATAATATAGGGGGAAGTAGTAGTAGGATGGTGGGGAGGAGGAAAC
AAGAGGAGAGGGACGTGGGGAAATTATTGCTCTCGAGGAATAGTAATAGTTTTGGGAAGGAAGAAGAAGATGC
CACGACGGGCGATGTTCCCATGGGCGACGATGATGACACCGATAATTACTACTACTACGCAGACGATCGACTC
AAATCAGATGATGCGCAACACGATCACGATCTCGACGAGGTGGGCGATAACGAAGACGACGGTATACGAATGA
ATTCCCAAACGTCTTCCTCCGCCTGGAAGCGGTTCGACAAGGATACCTTCATCGGACCCAACAACGAAATCCG
AACGAAGCACGATCCCGAACTCAAACACCGTTCCAACGCCGCGAATCTCCTCGGCAGTCACGGCGCGAAGCTC
AAGGCCGCCGAAAAAAAGTCCGTCGCCGTGAGCGATCGCGCGTACAACGCCTTCAAACGGGCCGAATCGCGAC
AGTCCGGATTCAAGAAGGGAGTGGCGAAGCAGGGACACGGACGGGCCGAGGATGCCGGCGCGGGTAAGACGAG
GGGAGGTGCGATGGACGGGAATGTGAGGATGCATATTGCCGCGGCGATTAATGCGGGGTTGATCGATCGTTGC
AACGGGGTCGTGAAGGAGGGGAAGGAGGCGATGGTGTACCATGCGGATGGAGGTTGGAAGGGAAGGAGGAGTC
GACAGCAGGCGGAGGGTGATGATGGTGGAGGTGCGGAAGATGAAATATCGTCCGTGGTTCATTATTCTGGAGA
GGGGGCATTGGATACAGTAGGGAGCGATGGGTACGATGTCGCTGTCAAGGTGTTTAAGAGAATTTCGGAGTTC
AAGGGAAGAGGCACCTACGTCGACGGCGATCCCCGCTATCACAAGCAAAAGTTCAAAACCAACGATCAACGCG
ACCAAGTCGTATTGTGGACCGAGAAGGAATACCGCAATCTAATCCGTGCATACAGAGCAGGCGTTCCAGTCCC
AAAACCGCTCTATCGAAAGGAAAACATTTTATTTCTGCGTTTCTTGGGCGAGAATGGATGGCCGTCGCCGCAG
ATCAAAGAGATAGATATGAAGAAGGGGAGTAAGAAGTGGACTACGTTATACGCGCAGACGCTTGTGGCTGTCC

GTAGGCTCTACCACTGTGCAAGGTTGATCCACGCTGATTTGAGCGAGTACAACATCCTAATCTGTCCCACCTG
GCAAGCTTCGCATGGCCATTTAATCCCGTCGGATGAACGCACCGCCGACGACGAAACACTGCAAGTGGTCCTG
ATCGATTTCGGACAGTCTGTTGAGATTGGACATCCTTCCGCGGAGAAATGGCTCGGGAGGGATCTCTCCACGG
TACGTGACTTCTTTGTGAAGCAGGGTATCAAGGCGCTGTCGAATGAGGACGCCGAGCGGTATGTGACTGATCC
ATTTGAAGAAGTAGATGATGCTGAGAATGGTGAAACTGGTATAACTGAGATAGAAGTAGATTCGGCGAAGACG
GAAGATAAAGATAACATTTGGAGACACACCAAACGAGGTTGGGACGACAAGAAAGAGATGGAACTCCTGCTTG
AAAAGCTGAAAGGAACCAGCCGCTAAGATATTCGTCTCGAGATAATATCAAGTTGTTGCTCTACTAATAAATT
TACCATGCGTACAATTCAAATATTTGATACGGCAAACGTGTCTCCGTGTAAATTTGTTAGGACCTTTGCAATC
GACATCGTGTCTTTCGCATCTTGATCCCGGGCAACGTCATGCCTGTGGGTGTCTCTATGCTCGTCAACTTTGG
GATCATTGGCGCCATTTATTGGGTGTGAAATCTCACCATGCTCTTCTCGAATCTTGGTTGCGTCTTGATCCAT
GTAAGTACTTGTTCTTCCATCACTCTCACTATTGGCACCACTTGTTTGATGCGAAATCATGCTATTTTGTTTT
TGAAAGGCAAGGGTATCTCGATCAATGTCGGCACTGGTTTTTCCATCAACCCCATGGTCGGCACCACTCTTTT
GATGAGAAATCATGTCACCTTCCTTTTGAAAAACGGTGGCATCTTGATCAGTACCTGTTTTTCCATCAGCCTT
GGGATCGGCACTTTTATTTTGATGCGGATCAATGTTATTCTCTTTTTGAAATGCCCTGGCGTCTTGATCACAC
G
>TR193|c3_g2_i1 len=490 path=[605:0-88 667:89-89 665:90-113 661:114-116 656:117-132 664:133-140 659:141-156 @666@!:157-289 662:290-327 657:328-348 653:349-365 636:366-489] [-1, 605, 667, 665, 661, 656, 664, 659, 666, 662, 657, 653, 636, -2]
AGCCCAAGAATTGTTGGGGTGTAGGGGGTGGCAAGATACAACAACAACAACAACAACATAGGACAATGGACAA
TTATGGTCATCATACTCGCGACGGTATGATTTCGCGCATTTCGACTTTTTCGGATGAGAGCACGGGGATGTCC
GAGAGCTTTGATACGATGGATGACGATGAGCGTATGCTGAGGCTCGCGTTCACGGGGACCATTTCGGAAGGGA
CGGGTTTTAGCAAAGATAGAATGTATCAGTCGCATTCTATTTCTGAAACCCACAGCGACGAGAAGGAAGACGG
ATCTGACAATAACATTGATGATTATGACAAAGATGTCAACAACGATGACGGAAAAAATGAAAATGACGACGAC
GATAACAGCAACAACAACGACGACGACCAATCAAACGCGTACATGTGCTCCGACGACGACGATGACAACAGCA
TCCCATCCGAAAGCGGAATCGAAATGCAAATCTCCTCCCAAAAGAAAACGAA
>TR193|c4_g2_i1 len=2400 path=[2939:0-753 @2985@!:754-934 2965:935-955 2988:956-1112 2960:1113-1134 @2986@!:1135-1698 2980:1699-1824 2975:1825-1933 2942:1934-2399] [-1, 2939, 2985, 2965, 2988, 2960, 2986, 2980, 2975, 2942, -2]
CGCAACTGCAGCGATACTACACAACAGAACCAAGACGACGACGACGACAACGACGACAACGACGACACGAAAT
ACACAACAAGATGGCTACTACTGCCAAGGCCCCAACGCTCATCCGAGCACACATCCTCCCATCCGCACCCACC
ACCAACGAAGAAGACGGCGACTCCAATGCCAACAATCTAGGCATACTCATCAAACGAATCGACGATCCTCACC
CCGCCTCCATCGTCATCTACCACGTCATCCCCTCCTCGTACGCCGCCACCCACACCGCCCTCAGGGCGGGGTG
CGAAATCCTCGCCGTGAACAAACACCGCGTGCGCGGGTCGGCCATTCGCACGATGGAAATGATGGAACACAGC
CTCGCCAAATACGGCAAGGTCAATCTACTGTGCAGTCACGGAGAACGTCGAAGCGGGAGCTGTTACGTCATTG
GCAAAATGCACGTTGCCACGGCGGGAACGTCGATATCGGTGGAACCTCAAACTCGTATTGCTCAGGATGTCTT
CGAGGCTGGTGGTGGTGGCGGCGATGCCGAGGGGAAGGGCGCGGGCGATTACGACACTATCGTGGATGGATTG
GAATTGGAAGAAACCTCCCGTACGGGACGCGTGCGAATCGTGTCCAATGTTGGCGCGACGAAGCATTCGGTGT
TCGGTGGACTCAAATTGAACAGGGGGGATAATCTACATGCCATCGACGGACGCGTGATTACTTCCATTGCCGA
CGTGAGGGGTGCATTGATGGATGCGGCGAGTGGGAATCACGTCATGGTGCCGATATTGACGTATAATGTGTTT
CGCAAGTTGAAGACGGCGGTGATGGGACTGAGCATGAGTAGGGCGTTGGGGGAAGAGGTTGGTGTGGAGTGGA
CTAATAAGGAGAATAATATGGGGAGGAGTATTCAGGATGAGTATGATGTGCAGGAAAAGCTTGGCGAGGGAGC
ATTTGCAGTGGTCAAGAAGGCGACGCACAAGAAATCGGGACAAGTCTACGCCATTAAAATTATTAACCGTTCG
AGCTTGAACAAGGATATGGAAGTTGCACTGAAGGATGAGATCTCGATATTGAAGGAGCTCGATCACGAGCACA
TTTATAAGTTGAACGATGTGATCACATCCATCAACCAGCATTATCTCGTCACCGAGTATCTCGACGGCGGAGA
GCTCTTCGATCGTATCGTAGAAAAATCCACCTACAACGAATTCGAGGCGCGGGATGTATGCAACGTCATCTTC
GGCGCCCTAAAGTACATGCACTCCAAGGGAATCGCCCACCGCGATCTCAAACCCGAGAACCTCCTGTTGGAAA
ACAGACACGACGATTCGCAGATCAAGATTGCCGACTTTGGATTTGCCAAGAAGATCAGGGCGGATGATTCAGA
TAGAGATCGGTCGTTGAAGACCATGTGCGGCACACCGGGATACGTCGCACCGGAGGTTTTGAGAAAGGAGTGG
TACGGGACCAAGGCGGACATGTTCTCGATGGGAGTCATTGTCTTTATTTTGCTGGGAGGGTATCCTCCATTTT
ATGCGGATAGTCAGAAAGATTTGCTGCTAATGTGTAGACGTGGTCAGTTTGAGTTTGATCGCGAGTACTGGGG
TAAAATTTCCATTGAAGTGAAGAATATGATTCGCCATCTCCTTGTGGTAAATCCTGACAAGCGCCTCTCTGCA
GAAGATGTTCTATCTCATCCGTAAGTTGATATTATCTCTTGTCTGACTTTCCCGCGTGCTTTTGTCTTTTTGG
CTTCTCCAATTTATGTTTGCGGTTTCGTTATTTGTGTAACTTGTTACTAGATGGATGACGACAGACAAGAATG
TGCTTCAAGCCGCAGATCTCTCCAAGTCGCAAGCCGCATTGAAGAAGTACAATGCTAGGAAAAGGTTCAAGAA
GGCAATCAATTCCATTCTCTTTGTCAACACCTTTACTGGTGAGAATGCAGTATTCTCTGGTGCGAATAAGCGT
AAGAATGTTCAGGCTCTTACAAAAGGAATTGATGAGATTGGGGTAGAGGTGTAATGCTGTGAAGAATGATACT
CCTAGAGAACTCAATGGATCGTTTCTCATGGCGAAAATTTATAATACCTGTTAGCACGAATAAAAATAATAGA
CAAGGATTTTTTTCTTCCAATGATTTTGAAGGGTTTTGTTTGATGCCTTATGATGTTAGTCTCGTACCACAAG

ATTTTGTTGATCGTGGAAGGGGAGATGGTAAGAGGTGACGGTTTGATTGCGGATGGTCTCTTGATGACTTTAT
GGTGTATGGCCAGGAAGCACTTGCATCATTGTCTCTGGAGTTTTGTGTGGTTAGGCTCCATGGTTGCCTTATG
ATTACTTTGTCTTTTGAGATATTACTGCAGCGTATTTTCCATTCCTAACTTTACATCATGTGAC
>TR193|c4_g2_i2 len=2588 path=[2939:0-753 @2985@!:754-934 2987:935-1068
2978:1069-1085 2984:1086-1143 2988:1144-1300 2960:1301-1322 @2986@!:1323-
1886 2980:1887-2012 2975:2013-2121 2942:2122-2587] [-1, 2939, 2985, 2987,
2978, 2984, 2988, 2960, 2986, 2980, 2975, 2942, -2]
CGCAACTGCAGCGATACTACACAACAGAACCAAGACGACGACGACGACAACGACGACAACGACGACACGAAAT
ACACAACAAGATGGCTACTACTGCCAAGGCCCCAACGCTCATCCGAGCACACATCCTCCCATCCGCACCCACC
ACCAACGAAGAAGACGGCGACTCCAATGCCAACAATCTAGGCATACTCATCAAACGAATCGACGATCCTCACC
CCGCCTCCATCGTCATCTACCACGTCATCCCCTCCTCGTACGCCGCCACCCACACCGCCCTCAGGGCGGGGTG
CGAAATCCTCGCCGTGAACAAACACCGCGTGCGCGGGTCGGCCATTCGCACGATGGAAATGATGGAACACAGC
CTCGCCAAATACGGCAAGGTCAATCTACTGTGCAGTCACGGAGAACGTCGAAGCGGGAGCTGTTACGTCATTG
GCAAAATGCACGTTGCCACGGCGGGAACGTCGATATCGGTGGAACCTCAAACTCGTATTGCTCAGGATGTCTT
CGAGGCTGGTGGTGGTGGCGGCGATGCCGAGGGGAAGGGCGCGGGCGATTACGACACTATCGTGGATGGATTG
GAATTGGAAGAAACCTCCCGTACGGGACGCGTGCGAATCGTGTCCAATGTTGGCGCGACGAAGCATTCGGTGT
TCGGTGGACTCAAATTGAACAGGGGGGATAATCTACATGCCATCGACGGACGCGTGATTACTTCCATTGCCGA
CGTGAGGGGTGCATTGATGGATGCGGCGAGTGGGAATCACGTCATGGTGCCGATATTGACGTATAATGTGTTT
CGCAAGTTGAAGACGGCGGTGATGGGACTGAGCATGAGTAGGGCGTTGGGGGAAGAGGTTGGTGTGGAGTGGA
CTAATAAGGAGAATAATATGGGGAGGAGTATTCAGGATGAGTATGATGTGCAGGAAAAGGTGCGTTTGATGTT
ATGTGTGTGTGTTTTAGAATTACTCTTTTTTATTTTGTTCTCCATCCTATACTTATTGTGTGTAAAAGTGGTT
GTTTGAGGAGTGCGGATTTATGTAACTCAGAACGTGTACACTTTTTTCCATTTTTGTTTGTACACATACACGC
ATATATGTACACGCATATATGCACACAGCTTGGCGAGGGAGCATTTGCAGTGGTCAAGAAGGCGACGCACAAG
AAATCGGGACAAGTCTACGCCATTAAAATTATTAACCGTTCGAGCTTGAACAAGGATATGGAAGTTGCACTGA
AGGATGAGATCTCGATATTGAAGGAGCTCGATCACGAGCACATTTATAAGTTGAACGATGTGATCACATCCAT
CAACCAGCATTATCTCGTCACCGAGTATCTCGACGGCGGAGAGCTCTTCGATCGTATCGTAGAAAAATCCACC
TACAACGAATTCGAGGCGCGGGATGTATGCAACGTCATCTTCGGCGCCCTAAAGTACATGCACTCCAAGGGAA
TCGCCCACCGCGATCTCAAACCCGAGAACCTCCTGTTGGAAAACAGACACGACGATTCGCAGATCAAGATTGC
CGACTTTGGATTTGCCAAGAAGATCAGGGCGGATGATTCAGATAGAGATCGGTCGTTGAAGACCATGTGCGGC
ACACCGGGATACGTCGCACCGGAGGTTTTGAGAAAGGAGTGGTACGGGACCAAGGCGGACATGTTCTCGATGG
GAGTCATTGTCTTTATTTTGCTGGGAGGGTATCCTCCATTTTATGCGGATAGTCAGAAAGATTTGCTGCTAAT
GTGTAGACGTGGTCAGTTTGAGTTTGATCGCGAGTACTGGGGTAAAATTTCCATTGAAGTGAAGAATATGATT
CGCCATCTCCTTGTGGTAAATCCTGACAAGCGCCTCTCTGCAGAAGATGTTCTATCTCATCCGTAAGTTGATA
TTATCTCTTGTCTGACTTTCCCGCGTGCTTTTGTCTTTTTGGCTTCTCCAATTTATGTTTGCGGTTTCGTTAT
TTGTGTAACTTGTTACTAGATGGATGACGACAGACAAGAATGTGCTTCAAGCCGCAGATCTCTCCAAGTCGCA
AGCCGCATTGAAGAAGTACAATGCTAGGAAAAGGTTCAAGAAGGCAATCAATTCCATTCTCTTTGTCAACACC
TTTACTGGTGAGAATGCAGTATTCTCTGGTGCGAATAAGCGTAAGAATGTTCAGGCTCTTACAAAAGGAATTG
ATGAGATTGGGGTAGAGGTGTAATGCTGTGAAGAATGATACTCCTAGAGAACTCAATGGATCGTTTCTCATGG
CGAAAATTTATAATACCTGTTAGCACGAATAAAAATAATAGACAAGGATTTTTTTCTTCCAATGATTTTGAAG
GGTTTTGTTTGATGCCTTATGATGTTAGTCTCGTACCACAAGATTTTGTTGATCGTGGAAGGGGAGATGGTAA
GAGGTGACGGTTTGATTGCGGATGGTCTCTTGATGACTTTATGGTGTATGGCCAGGAAGCACTTGCATCATTG
TCTCTGGAGTTTTGTGTGGTTAGGCTCCATGGTTGCCTTATGATTACTTTGTCTTTTGAGATATTACTGCAGC
GTATTTTCCATTCCTAACTTTACATCATGTGAC
>TR193|c4_g2_i3 len=2485 path=[2939:0-753 @2985@!:754-934 2987:935-1068
2978:1069-1085 2984:1086-1143 2988:1144-1300 2960:1301-1322 @2986@!:1323-
1886 2937:1887-1909 2975:1910-2018 2942:2019-2484] [-1, 2939, 2985, 2987,
2978, 2984, 2988, 2960, 2986, 2937, 2975, 2942, -2]
CGCAACTGCAGCGATACTACACAACAGAACCAAGACGACGACGACGACAACGACGACAACGACGACACGAAAT
ACACAACAAGATGGCTACTACTGCCAAGGCCCCAACGCTCATCCGAGCACACATCCTCCCATCCGCACCCACC
ACCAACGAAGAAGACGGCGACTCCAATGCCAACAATCTAGGCATACTCATCAAACGAATCGACGATCCTCACC
CCGCCTCCATCGTCATCTACCACGTCATCCCCTCCTCGTACGCCGCCACCCACACCGCCCTCAGGGCGGGGTG
CGAAATCCTCGCCGTGAACAAACACCGCGTGCGCGGGTCGGCCATTCGCACGATGGAAATGATGGAACACAGC
CTCGCCAAATACGGCAAGGTCAATCTACTGTGCAGTCACGGAGAACGTCGAAGCGGGAGCTGTTACGTCATTG
GCAAAATGCACGTTGCCACGGCGGGAACGTCGATATCGGTGGAACCTCAAACTCGTATTGCTCAGGATGTCTT
CGAGGCTGGTGGTGGTGGCGGCGATGCCGAGGGGAAGGGCGCGGGCGATTACGACACTATCGTGGATGGATTG
GAATTGGAAGAAACCTCCCGTACGGGACGCGTGCGAATCGTGTCCAATGTTGGCGCGACGAAGCATTCGGTGT
TCGGTGGACTCAAATTGAACAGGGGGGATAATCTACATGCCATCGACGGACGCGTGATTACTTCCATTGCCGA
CGTGAGGGGTGCATTGATGGATGCGGCGAGTGGGAATCACGTCATGGTGCCGATATTGACGTATAATGTGTTT
CGCAAGTTGAAGACGGCGGTGATGGGACTGAGCATGAGTAGGGCGTTGGGGGAAGAGGTTGGTGTGGAGTGGA
CTAATAAGGAGAATAATATGGGGAGGAGTATTCAGGATGAGTATGATGTGCAGGAAAAGGTGCGTTTGATGTT

ATGTGTGTGTGTTTTAGAATTACTCTTTTTTATTTTGTTCTCCATCCTATACTTATTGTGTGTAAAAGTGGTT
GTTTGAGGAGTGCGGATTTATGTAACTCAGAACGTGTACACTTTTTTCCATTTTTGTTTGTACACATACACGC
ATATATGTACACGCATATATGCACACAGCTTGGCGAGGGAGCATTTGCAGTGGTCAAGAAGGCGACGCACAAG
AAATCGGGACAAGTCTACGCCATTAAAATTATTAACCGTTCGAGCTTGAACAAGGATATGGAAGTTGCACTGA
AGGATGAGATCTCGATATTGAAGGAGCTCGATCACGAGCACATTTATAAGTTGAACGATGTGATCACATCCAT
CAACCAGCATTATCTCGTCACCGAGTATCTCGACGGCGGAGAGCTCTTCGATCGTATCGTAGAAAAATCCACC
TACAACGAATTCGAGGCGCGGGATGTATGCAACGTCATCTTCGGCGCCCTAAAGTACATGCACTCCAAGGGAA
TCGCCCACCGCGATCTCAAACCCGAGAACCTCCTGTTGGAAAACAGACACGACGATTCGCAGATCAAGATTGC
CGACTTTGGATTTGCCAAGAAGATCAGGGCGGATGATTCAGATAGAGATCGGTCGTTGAAGACCATGTGCGGC
ACACCGGGATACGTCGCACCGGAGGTTTTGAGAAAGGAGTGGTACGGGACCAAGGCGGACATGTTCTCGATGG
GAGTCATTGTCTTTATTTTGCTGGGAGGGTATCCTCCATTTTATGCGGATAGTCAGAAAGATTTGCTGCTAAT
GTGTAGACGTGGTCAGTTTGAGTTTGATCGCGAGTACTGGGGTAAAATTTCCATTGAAGTGAAGAATATGATT
CGCCATCTCCTTGTGGTAAATCCTGACAAGCGCCTCTCTGCAGAAGATGTTCTATCTCATCCATGGATGACGA
CAGACAAGAATGTGCTTCAAGCCGCAGATCTCTCCAAGTCGCAAGCCGCATTGAAGAAGTACAATGCTAGGAA
AAGGTTCAAGAAGGCAATCAATTCCATTCTCTTTGTCAACACCTTTACTGGTGAGAATGCAGTATTCTCTGGT
GCGAATAAGCGTAAGAATGTTCAGGCTCTTACAAAAGGAATTGATGAGATTGGGGTAGAGGTGTAATGCTGTG
AAGAATGATACTCCTAGAGAACTCAATGGATCGTTTCTCATGGCGAAAATTTATAATACCTGTTAGCACGAAT
AAAAATAATAGACAAGGATTTTTTTCTTCCAATGATTTTGAAGGGTTTTGTTTGATGCCTTATGATGTTAGTC
TCGTACCACAAGATTTTGTTGATCGTGGAAGGGGAGATGGTAAGAGGTGACGGTTTGATTGCGGATGGTCTCT
TGATGACTTTATGGTGTATGGCCAGGAAGCACTTGCATCATTGTCTCTGGAGTTTTGTGTGGTTAGGCTCCAT
GGTTGCCTTATGATTACTTTGTCTTTTGAGATATTACTGCAGCGTATTTTCCATTCCTAACTTTACATCATGT
GAC
>TR193|c4_g2_i4 len=2297 path=[2939:0-753 @2985@!:754-934 2965:935-955
2988:956-1112 2960:1113-1134 @2986@!:1135-1698 2937:1699-1721 2975:1722-
1830 2942:1831-2296] [-1, 2939, 2985, 2965, 2988, 2960, 2986, 2937, 2975,
2942, -2]
CGCAACTGCAGCGATACTACACAACAGAACCAAGACGACGACGACGACAACGACGACAACGACGACACGAAAT
ACACAACAAGATGGCTACTACTGCCAAGGCCCCAACGCTCATCCGAGCACACATCCTCCCATCCGCACCCACC
ACCAACGAAGAAGACGGCGACTCCAATGCCAACAATCTAGGCATACTCATCAAACGAATCGACGATCCTCACC
CCGCCTCCATCGTCATCTACCACGTCATCCCCTCCTCGTACGCCGCCACCCACACCGCCCTCAGGGCGGGGTG
CGAAATCCTCGCCGTGAACAAACACCGCGTGCGCGGGTCGGCCATTCGCACGATGGAAATGATGGAACACAGC
CTCGCCAAATACGGCAAGGTCAATCTACTGTGCAGTCACGGAGAACGTCGAAGCGGGAGCTGTTACGTCATTG
GCAAAATGCACGTTGCCACGGCGGGAACGTCGATATCGGTGGAACCTCAAACTCGTATTGCTCAGGATGTCTT
CGAGGCTGGTGGTGGTGGCGGCGATGCCGAGGGGAAGGGCGCGGGCGATTACGACACTATCGTGGATGGATTG
GAATTGGAAGAAACCTCCCGTACGGGACGCGTGCGAATCGTGTCCAATGTTGGCGCGACGAAGCATTCGGTGT
TCGGTGGACTCAAATTGAACAGGGGGGATAATCTACATGCCATCGACGGACGCGTGATTACTTCCATTGCCGA
CGTGAGGGGTGCATTGATGGATGCGGCGAGTGGGAATCACGTCATGGTGCCGATATTGACGTATAATGTGTTT
CGCAAGTTGAAGACGGCGGTGATGGGACTGAGCATGAGTAGGGCGTTGGGGGAAGAGGTTGGTGTGGAGTGGA
CTAATAAGGAGAATAATATGGGGAGGAGTATTCAGGATGAGTATGATGTGCAGGAAAAGCTTGGCGAGGGAGC
ATTTGCAGTGGTCAAGAAGGCGACGCACAAGAAATCGGGACAAGTCTACGCCATTAAAATTATTAACCGTTCG
AGCTTGAACAAGGATATGGAAGTTGCACTGAAGGATGAGATCTCGATATTGAAGGAGCTCGATCACGAGCACA
TTTATAAGTTGAACGATGTGATCACATCCATCAACCAGCATTATCTCGTCACCGAGTATCTCGACGGCGGAGA
GCTCTTCGATCGTATCGTAGAAAAATCCACCTACAACGAATTCGAGGCGCGGGATGTATGCAACGTCATCTTC
GGCGCCCTAAAGTACATGCACTCCAAGGGAATCGCCCACCGCGATCTCAAACCCGAGAACCTCCTGTTGGAAA
ACAGACACGACGATTCGCAGATCAAGATTGCCGACTTTGGATTTGCCAAGAAGATCAGGGCGGATGATTCAGA
TAGAGATCGGTCGTTGAAGACCATGTGCGGCACACCGGGATACGTCGCACCGGAGGTTTTGAGAAAGGAGTGG
TACGGGACCAAGGCGGACATGTTCTCGATGGGAGTCATTGTCTTTATTTTGCTGGGAGGGTATCCTCCATTTT
ATGCGGATAGTCAGAAAGATTTGCTGCTAATGTGTAGACGTGGTCAGTTTGAGTTTGATCGCGAGTACTGGGG
TAAAATTTCCATTGAAGTGAAGAATATGATTCGCCATCTCCTTGTGGTAAATCCTGACAAGCGCCTCTCTGCA
GAAGATGTTCTATCTCATCCATGGATGACGACAGACAAGAATGTGCTTCAAGCCGCAGATCTCTCCAAGTCGC
AAGCCGCATTGAAGAAGTACAATGCTAGGAAAAGGTTCAAGAAGGCAATCAATTCCATTCTCTTTGTCAACAC
CTTTACTGGTGAGAATGCAGTATTCTCTGGTGCGAATAAGCGTAAGAATGTTCAGGCTCTTACAAAAGGAATT
GATGAGATTGGGGTAGAGGTGTAATGCTGTGAAGAATGATACTCCTAGAGAACTCAATGGATCGTTTCTCATG
GCGAAAATTTATAATACCTGTTAGCACGAATAAAAATAATAGACAAGGATTTTTTTCTTCCAATGATTTTGAA
GGGTTTTGTTTGATGCCTTATGATGTTAGTCTCGTACCACAAGATTTTGTTGATCGTGGAAGGGGAGATGGTA
AGAGGTGACGGTTTGATTGCGGATGGTCTCTTGATGACTTTATGGTGTATGGCCAGGAAGCACTTGCATCATT
GTCTCTGGAGTTTTGTGTGGTTAGGCTCCATGGTTGCCTTATGATTACTTTGTCTTTTGAGATATTACTGCAG
CGTATTTTCCATTCCTAACTTTACATCATGTGAC
>TR193|c4_g2_i5 len=2615 path=[2939:0-753 @2985@!:754-934 2987:935-1068
2978:1069-1085 2984:1086-1143 2988:1144-1300 2976:1301-1452 @2986@!:1453-

2016 2937:2017-2039 2975:2040-2148 2942:2149-2614] [-1, 2939, 2985, 2987, 2978, 2984, 2988, 2976, 2986, 2937, 2975, 2942, -2]
CGCAACTGCAGCGATACTACACAACAGAACCAAGACGACGACGACGACAACGACGACAACGACGACACGAAAT
ACACAACAAGATGGCTACTACTGCCAAGGCCCCAACGCTCATCCGAGCACACATCCTCCCATCCGCACCCACC
ACCAACGAAGAAGACGGCGACTCCAATGCCAACAATCTAGGCATACTCATCAAACGAATCGACGATCCTCACC
CCGCCTCCATCGTCATCTACCACGTCATCCCCTCCTCGTACGCCGCCACCCACACCGCCCTCAGGGCGGGGTG
CGAAATCCTCGCCGTGAACAAACACCGCGTGCGCGGGTCGGCCATTCGCACGATGGAAATGATGGAACACAGC
CTCGCCAAATACGGCAAGGTCAATCTACTGTGCAGTCACGGAGAACGTCGAAGCGGGAGCTGTTACGTCATTG
GCAAAATGCACGTTGCCACGGCGGGAACGTCGATATCGGTGGAACCTCAAACTCGTATTGCTCAGGATGTCTT
CGAGGCTGGTGGTGGTGGCGGCGATGCCGAGGGGAAGGGCGCGGGCGATTACGACACTATCGTGGATGGATTG
GAATTGGAAGAAACCTCCCGTACGGGACGCGTGCGAATCGTGTCCAATGTTGGCGCGACGAAGCATTCGGTGT
TCGGTGGACTCAAATTGAACAGGGGGGATAATCTACATGCCATCGACGGACGCGTGATTACTTCCATTGCCGA
CGTGAGGGGTGCATTGATGGATGCGGCGAGTGGGAATCACGTCATGGTGCCGATATTGACGTATAATGTGTTT
CGCAAGTTGAAGACGGCGGTGATGGGACTGAGCATGAGTAGGGCGTTGGGGGAAGAGGTTGGTGTGGAGTGGA
CTAATAAGGAGAATAATATGGGGAGGAGTATTCAGGATGAGTATGATGTGCAGGAAAAGGTGCGTTTGATGTT
ATGTGTGTGTGTTTTAGAATTACTCTTTTTTATTTTGTTCTCCATCCTATACTTATTGTGTGTAAAAGTGGTT
GTTTGAGGAGTGCGGATTTATGTAACTCAGAACGTGTACACTTTTTTCCATTTTTGTTTGTACACATACACGC
ATATATGTACACGCATATATGCACACAGCTTGGCGAGGGAGCATTTGCAGTGGTCAAGAAGGCGACGCACAAG
AAATCGGGACAAGTCTACGCCATTAAAATTATTAACCGTTCGAGCTTGAACAAGGATATGGAAGTTGCACTGA
AGGATGAGATCTCGATATTGAAGGAGCTCGATCACGAGCACATTTATAAGTTGAACGATGGTATGATTTACAC
CGACACACCGAGGGATTCTGTGTGTGTTTCTCTAATTTCTATATGCTACCTGTATGCATGCACTGACACAACA
TTCTTGAAAATGTATATTATACCAATATCATGAAAAAACAACAGTGATCACATCCATCAACCAGCATTATCTC
GTCACCGAGTATCTCGACGGCGGAGAGCTCTTCGATCGTATCGTAGAAAAATCCACCTACAACGAATTCGAGG
CGCGGGATGTATGCAACGTCATCTTCGGCGCCCTAAAGTACATGCACTCCAAGGGAATCGCCCACCGCGATCT
CAAACCCGAGAACCTCCTGTTGGAAAACAGACACGACGATTCGCAGATCAAGATTGCCGACTTTGGATTTGCC
AAGAAGATCAGGGCGGATGATTCAGATAGAGATCGGTCGTTGAAGACCATGTGCGGCACACCGGGATACGTCG
CACCGGAGGTTTTGAGAAAGGAGTGGTACGGGACCAAGGCGGACATGTTCTCGATGGGAGTCATTGTCTTTAT
TTTGCTGGGAGGGTATCCTCCATTTTATGCGGATAGTCAGAAAGATTTGCTGCTAATGTGTAGACGTGGTCAG
TTTGAGTTTGATCGCGAGTACTGGGGTAAAATTTCCATTGAAGTGAAGAATATGATTCGCCATCTCCTTGTGG
TAAATCCTGACAAGCGCCTCTCTGCAGAAGATGTTCTATCTCATCCATGGATGACGACAGACAAGAATGTGCT
TCAAGCCGCAGATCTCTCCAAGTCGCAAGCCGCATTGAAGAAGTACAATGCTAGGAAAAGGTTCAAGAAGGCA
ATCAATTCCATTCTCTTTGTCAACACCTTTACTGGTGAGAATGCAGTATTCTCTGGTGCGAATAAGCGTAAGA
ATGTTCAGGCTCTTACAAAAGGAATTGATGAGATTGGGGTAGAGGTGTAATGCTGTGAAGAATGATACTCCTA
GAGAACTCAATGGATCGTTTCTCATGGCGAAAATTTATAATACCTGTTAGCACGAATAAAAATAATAGACAAG
GATTTTTTTCTTCCAATGATTTTGAAGGGTTTTGTTTGATGCCTTATGATGTTAGTCTCGTACCACAAGATTT
TGTTGATCGTGGAAGGGGAGATGGTAAGAGGTGACGGTTTGATTGCGGATGGTCTCTTGATGACTTTATGGTG
TATGGCCAGGAAGCACTTGCATCATTGTCTCTGGAGTTTTGTGTGGTTAGGCTCCATGGTTGCCTTATGATTA
CTTTGTCTTTTGAGATATTACTGCAGCGTATTTTCCATTCCTAACTTTACATCATGTGAC
>TR193|c5_g1_i1 len=889 path=[1109:0-193 1110:194-217 1137:218-515 1139:516-517 1141:518-541 1142:542-572 1136:573-620 1140:621-622 1126:623-636 1096:637-646 1130:647-660 1125:661-787 1107:788-811 1108:812-888] [-1, 1109, 1110, 1137, 1139, 1141, 1142, 1136, 1140, 1126, 1096, 1130, 1125, 1107, 1108, -2]
ACATCTACCTACCACATCTCCCTACCCAACAGGAGGCACAACGAGCAAAACACACATGAGATCCCTCCGCGAC
ACTGCCGCCCAGTGCTGCAAGAAGGTATACATCGGCGCGGCCGTTCTTCCCAAGCCCCTTTTTGTGCCCGATC
ACGACGACGACGACAACGACGACATAATAACTGATGGCGCCAACTGCGGAACCAACGACAACGACGACGACGA
CATTATCAGCAGCAAAGTCAAACAGAAACGGAAACGACCTCAAATCAAATGCGCCACCATCTCCACAGTCGAA
CCATCCGACAATCCCTCCCTCTCCCAAAATGCCGCATCCCGACCGCTCACACGCTACGCCCATCACCTCACAC
GCGAATTCAACTCCATCGTCATCGAACATCACCTCAAATGGTCTCCCCTCGTCCACACCTACCCCGGCCCAAT
CCACGAATCGGCTCCTCCCTCGACGCACCTGGGCAGGTACGATTTCCACCACGTCGATTCCATCGTGGACTTT
GCCCTCCGTCGGAATATGCACGTCAAGGGACACGTATTGGTGTGGCACGTCACCTCCCCCCATTTCTCGAGG
ACATGTCCCCCGCGGAAGTTCGGGAGGCGGTCAAACGACACATCTTCGCGACCGTGGGATACTTTCGAGGACG
GATCCGAATGTGGGACGTGGTGAACGAATCGCTGGCGAGCGATGGAACGCTGGTCGAGAATGTATTTTACAGG
AAGATGGGACCGAATTACATAGAGGATTGTTTTCGATGGGCGCACGAGGCGGATCCGAGTGCTTTTTTGATTT
ACAATGATAATAAGGTGGAGGGATGCGGGTATTACGATGGAAGCGAAGGAGGCCGTGGTGGTGGTGGTGATGA
TGATGATGATGAT
>TR193|c6_g1_i1 len=3404 path=[4104:0-226 4105:227-250 4179:251-263 4172:264-295 4162:296-424 4080:425-448 4170:449-480 4120:481-504 4178:505-921 4077:922-947 4169:948-1836 4092:1837-1860 4177:1861-1988 4130:1989-2020 4168:2021-2487 4136:2488-2511 4159:2512-2707 4151:2708-

2731 4180:2732-2782 4158:2783-2841 4176:2842-2861 4166:2862-3030
4156:3031-3051 4174:3052-3075 4164:3076-3098 4155:3099-3122 4084:3123-
3131 4085:3132-3155 4173:3156-3233 4153:3234-3314 4123:3315-3333
4124:3334-3366 4125:3367-3403] [-1, 4104, 4105, 4179, 4172, 4162, 4080,
4170, 4120, 4178, 4077, 4169, 4092, 4177, 4130, 4168, 4136, 4159, 4151,
4180, 4158, 4176, 4166, 4156, 4174, 4164, 4155, 4084, 4085, 4173, 4153,
4123, 4124, 4125, -2]
CTCGGTCCAGTTTTCACAACAACATCCCAACGAAGACGACGACGACGACGACGGCGAAACAAAAAATCAT
GCTATGCACCCGCTTGCACATCCAATCGGCAACGTACGGACCTGCCGAAGGACGACGATTGTTCAATGGGATG
TTGGTCGATTATCACGAGAAGGAGACGTATGTTCCGTATACGAGAGATGTGCTTCCTTTTTTGAAGGCGCTCA
TGAGCCTCATGTCCGATGATGAGGAAGACTTGGAGGAGGAGGAAGAGGAGGAGAAAGGTGGCGACTTTGAAAA
GAAGGAAAGTGATGCTTTCGGTTTGCGTCCAGGGGAGGAGAACGCTCACGAGAAGGATGATGATGAAGTGGAT
AATCATCATGAAGGAGATCTCTACGGGTGTTCACGCACAGATGATGACACTCGATTTAGAGTACTGCAGCAAG
GGAATAATAGCACAAATGCCTTCGCACTGATGGATGGACGTCCCATGAACGCCGTATTCGGCGATCCTTGTCC
TGGAACTACCAAATTGTTGAGGGTGGAATATTCATTCCGAGATTACTTCTACGATGGCGGAGAGATTGACAAC
GCAACAATAGAGGGTGAATGCGGCATTGGTATTGACGAACTCGGTAAGAGAAGAGACCATGATGATGGCGGTA
GGGACAATAGTTTGAAAGCCGTTCGAGAAGAAGTAGATTTGAATAATGGAAAGATGATGCGACAAAATCAAAG
AAGGAGGCAGCAACGACAACGCCGCCGCTATCATTGCAAAACATCTCGCCTATTTCAGTCGACATTTCGCGAA
CACGAACGGGTGTTGTTGAAACGTCAGGATCCGTTGTTCCGGCTGACGATGGATGACACTCATGGTGACTGTT
CAAACGAGGCAGATCCAGAGAATGCAGCGAAGATAGATGATGAATCTGGAACGATACCCATTTCATCATCATC
GACGATACCACAATCATCTGCACCATCCACCGCCTCTCCCTCCTCGTCCTCGTCGCCATCCAAGCAATGGAAA
CTAGCACCCACCACCTCCGAAATCACCCTCCCCATAATCCTACCATTCCTCACAATACACCAACGCGCCAAAT
GTCAAATGGTATGCCGTTCCTGGAAAAGTATCGTCCTCGATAGGGGCATTGCCGTGGTCGTGGACGTAAACGA
CGTGGGTTTGTTTCCATTGTCGAAAGATCGAATGATGATGGACGTTGCTCCAACTACCACTGGTGCGACCGGG
GCGGCCACCGCGACCATTGCGCGTTTGCGCATTCCGCACTATCCTCCGTTCGGCGGCAGGTCTTTCGGTGCTA
CCGATAACGATGCGGTAGATGCTGCATCATCATCCTATTCTCCTAATAATGTTCATATTCAACAACAACAACA
TCAACATCCATCCCGCTCGCTCCTCCGAGGACTACTGAACCACTCCCATTCCTCATTGGAAGCCCTCGTCCTC
AACGACTTCTGCCCTCTCCATCCCGTCATCGATCTGCATCCCGCGTTGCCGCACTTGCGCAAACTCAAACGAT
TGGACATCAGTCGCGTCCCCTCCATCACCGACGACACATTGCACCTATTATCGTCCTTCGTGGGCAAACGGTT
GGAAGTACTGTACATGAAGGGATTACGGCACGTTACCAACGAGGGAATAGTACGATTGGTAAAATCGTGTAGT
AATTTGCGTGTGTTGGACGTGAGTCAAGTCAATCAGCTCGATGATACGGCGGGCGTCGCGATAGGTACGCATT
TGACCAAGTTGGAAGTCTTCCATTCGAGGGATTGTCACAAGATGACGAATCGGAGCGTGGATTTGATTACGAG
GAATTGTTGCAGTTTGATTCAGGGGACGTTTTGGGGGTGTATTCGATTGACTCATGTTTGCTTTGGTGGTAGT
GGTGATGATGATGATGATGATCGACGACAACAACAGCAACAACAGTGTGCGGAGAATGATGGCGATGCTGATG
CTGCTACTGCCATGGATGGGGGTACATTTGCCAATTCGACGTTTCAGAGTGCTACCACCACTACAGCAACAAT
GCCTCGACCATCACCCTCCTTTACCAACATTCGCAACACATCGACCGCGAAAAATCTAACCTTGCTCAACCTA
TGGGGATGTCACAATCTAACGGACGCTGCCGCGTTGCGAATGACGAACCTAACTCGTCTACGCTCGCTCTGCG
TTAGCGAATGTCATCGTCTCACAGACAAATTCGTTACGGGAGTTACCGAGTCCTTGAATCAATTATTACATCT
CCAGCTTCGATACCTTCGGAGGATCACGGATGCGAGTCTGGAGTACATATCGCATAGGATGTCGGGTTTGTAT
TCGTTGGACGTTAGCTTTTGCACCAAGCTTACCGTTGGGGGATTGGCGCGATTGTTGACGGAGAAGTGTTCCT
CGTTGTCGGAATTGAGACTTTATTCGTGTCGACAGCTGGACGTGGAAGGCGGGTCGCTGATGATGATGAACAA
CGGTGGTGGTGGTGGTAGAAATAATAATAATGGTGGCAATAATGGACACGGAGGAGTGGGTGGTGGTCGACGT
TTCGTCCAAGCACTGAGATCTCTCCGCGGTCGGAGCATACTCTCGTTACTTGATTTGAGAGAGTGTCAACATC
ACGAGCCATTCGCGAGAGATGATGTCTTCTTGGATGGGATGGCGGGGTTAGGTTTCGACGAGACGTTGCATGG
ATTGTTTGTTAGGCCTGCTCGTTGGAATGGAGAAGTGATGAGACAATTGGTGGCAAATTTAAGCGATGCTGGT
GGAACTTAAGGTGTGAAAGGAGATGAGACAGGACGAAAGTCTAGGTATCCGCACTGTTTCTCGGACGGCCAAA
TATCTTATTGTGTCATCTTTCTGCCTTCTAGCTCAACGTTGTCATTTTGAAAGGTTTCGGACATGTTACGGAC
CACATGAGAATTTCTTGCGCAATAGAAATATGGCATGCAATTGCACATCTCTGCAAACTGTCATTTTTCACAG
AATGGGTACGAAATGAGCCTCTGCTTGATCGATCAAGAGATGGAGAATCCACGAAGGAATATGGATGTTGAGG
GCTTCCCATTACCTCCACTTCCTACCGAGAATCGTCATCAGTAGTGCTTTCTACTGCCATGGGCATAGGAGGG
TGAAAGAAGAAATAAGATCCACAGAAACAACTCGTCAAGAGAAACAATCCATATCTAATGCTTTGACCAAAAC
ACTGCATTATTTTACAGTGGTACCAGGAAATGGAGAAATTAACTGTTGGTTCTTCTCGTCAATCATTTTAAAG
AAACTGCCATCTGTGGCACTTACCTACCTATTGTTGTAGGGACAAAACTGAAATCTTTGTAGGTGAAATGAAC
TCACCATCTTAAAATAGCCCGCACTCGGTGTCAAAAAAAAAGATCGG
>TR193|c7_g1_i1 len=3224 path=[3965:0-414 3966:415-438 4092:439-456
4083:457-480 4068:481-545 3980:546-569 4082:570-778 3938:779-802
4067:803-888 4034:889-912 4035:913-916 4036:917-940 4081:941-996
4046:997-1020 4066:1021-1201 4004:1202-1225 4005:1226-1226 4080:1227-1267
3963:1268-1291 4065:1292-1524 4049:1525-1548 4098:1549-1655 4079:1656-
1695 4064:1696-1733 3996:1734-1757 4097:1758-1818 4061:1819-1846

4089:1847-1860 4075:1861-1901 4101:1902-1925 4096:1926-1949 4100:1950-2031 4074:2032-2051 4095:2052-2110 4087:2111-2133 4099:2134-2134 4094:2135-2169 4085:2170-2176 4070:2177-2200 4093:2201-2248 4052:2249-2286 @4084@!:2287-3097 4054:3098-3141 3986:3142-3223] [-1, 3965, 3966, 4092, 4083, 4068, 3980, 4082, 3938, 4067, 4034, 4035, 4036, 4081, 4046, 4066, 4004, 4005, 4080, 3963, 4065, 4049, 4098, 4079, 4064, 3996, 4097, 4061, 4089, 4075, 4101, 4096, 4100, 4074, 4095, 4087, 4099, 4094, 4085, 4070, 4093, 4052, 4084, 4054, 3986, -2]
ATCTCGACGACGACGATCCCGTAATCCGCACCATCGACGTGTACATCTCCCCGCCCTCTCCTCCACCCTCCA
CCTCCTCCAATTCCCCATCCAACCCGCCGTGAACAAACGGAACGCTCATCATCACCATCATCGATCGAACGGT
CAGGATAATCGCGCCAACACTCCCGCCGACGCGCGATTCCGTCCCAAACACAACATGCTCGAACTCGACTATC
CCATCCCGCCGTCGGCCCAGGCGGCGCAACGTCAGCTTCCCGACACCATGTGCCTCAGCCAACGCACCTTCGC
CTCCAATGCCATCGCGCCGGTCACGCACATGGCCCTCGCGAGATTGAACCGCAGCGGAGAACGACTCAACGTC
GTCCCGTTGACCAAATCGGTCATGCAAATGAGACCGTCGTTTCGACACCTCCACGATCCGGACGACGATGGCG
ATCCGAATGCGGCGGGACCGAATAACGACGCTATGCCCTCCGGCACCGGCAAGGCGGGGAGGGTGCGTCCGGT
CATGTTTCAGAAGAAGGAGACGGAACGTACCGTGGCCCAGCGTCGGAATTCCTACGCGTACAAACGCGCCGCC
GAGGAGGGAGAGGAATGGATCGAATTGGACGTGCACGGTTCGGTCGGGAAGTGGACGCCGTTGCGGAAGGAGG
TCATGGCCAAGATCAAATGCACGGATCGGGAGAACGAATTGAGATTCAAGACCAATTTGAAACACGAGGATTG
CGAGGATGGGGGATACGTCAAGAGTTTGAATTACCTGGATGCTCTCTCGTTGCAGGCGAGGTCAGGCGTGAGC
GGGGAGGGTTTCGTGGAGAATTTATCGGAGTGGTCTCCGTCGTCCGCGGCGGAGGATGCCGCCGTGGGGGATG
ATGGCGGAGAGAATGCGGAGGAGCAGGAGGATGCCGACGATATCATGGATGCGGACGATATGCGATCGCTCTC
CTCCAACACCTCCGCGGCACCGCCCGCCGTGGGCGCCATCGAAAGGGCCACGGCGGAATTAGCATCCAAGCTC
GCCGTCCTACTCCAGACGGGCAACGGCACCATGATCCCGTACCGCGTACTGCGATCGAGATTCAACCCCGCCA
AGGTGTCGGACGAATTGCTCACCATGGCCCTATCCTCCTGCGCGGTACTCGTGCGCGGGAACTTCTCGCTCAA
GTCCGACCTGTGCAAGTTCCTCCACCTCGCGGGCGCGGGACTGGGCACCGGCGGAGCCGAGGAGCGCAAGAAG
CGCCTTCGAGAATTGCGAGATCTGATACTGCTCCTGTTGAACATGCACGGCGCCGTCCAACGCGAGAGGCTGA
CGTGGGCGTACGCAGTGCGGGCCGAGGCGTATCGGAGTTACGAGGCGATACATCCCGACGTCATCACGTTCGT
GCTGCGGACCGTGGCGAAGCGATCGGAGGATTGCTGGGTGGCCAAGGTGGAGGACGACGAGCAGTTCGGGGCG
GATTTTCCGGAGGTCGCGGCGAGTCACGGCGCGTACTGGTTTCAGAAGAAGGATATGCTGATGGATTTGATCG
AGTTGTACGAGAATGCGGAGGTGGGGGATCACGGGGCCGGGGATCACTTGTGTCCGAGCATTTTGGACAATTC
GGTGTGATGATGTGTGGTTTTGTGGTCATGTTTTTTCCAGTTCTAGGAACAATCTCGCAGACGTTTCGGGTAT
TTCAGGTAGGATAGCATCATGGTAGCTTGGGAGGATTGAGAAAGGAAAGCACTTGGCCATGCTGGCATGAGTG
AAAGAAAGGTGAACCACCAATTCGTCGTCGGATTCTTTTTCTGATAGTTTCGCCCAGCGCAAGTGGTACTGGT
GGTACCTATGTGAAGGAAATCTCAACTGGAGTCACAGGTACTTGGTTATATGCCAACCTTGTACCATCGAGTG
ATCACAAAAATCTAATCAAACATATTACCTCTTATCACGCATGATCATGGTACACACATCTTTTGTAAAATGC
AATGAATAGAGCCGATTTGTATTTTATGGATCTGAATAAATGCAGAATTTCCTTACAATTTATCCTTCAGATT
TTTCCTGTTTGTATCAACAAATATGAACGAAAAATATTCAATCAACATCACCTGTAGCAAGTCATGAATCATA
CCGTACGTGGTTGTTGCATCTAGCCTGCAGGAATTGTTGGCATTTCCAGTAATAATGTCCACGAAAACGATTT
TGTACTGTTGATTGACGCGATGATGCCTCTTCCGCATAGGGTGGCAAGCTCCCAAAAAAAGGCGTAACTCTGC
ATAAATCCGCTTGTTGCCATGACAATCATCTCCCTTCCCTGAAAATCCCTCCGAAATGATTTCGTTTCTCATT
GATCTCTCAACACCACTCCTCTGCAGATGGTACTATACCATATCCCAAGTAGATTCAAACAACCTCTCCCAAA
ACAAACAAAACCGCATAATCCTGCTTCTCGCATCCATCAATCGCCGGCATCGCCGCCCCGGGTTTCACGGGAA
AGTCTCTTAAATTCGCATTCTTCGGCGCGCATCCCTCCCCCCGATACGCGGCGCTCCGACACGGCAAATGCGT
AACGTCGTACAGCGTTCCCCTCTCCAACTCCCAACTCCCATTTTCCCGCACCGTCAACACGGTGGTCACCTCC
CTGTCCCCCGTCACGACGTACTTTTTCTCAGGCACTATCGCCCTGGTCTCCCTGTCGAGGCTCTTCCGCGGCA
ACGGTTCCGGAGTGGACATTATGAGACCGTGCTCCTCGACCCACCAATCGGACGGATCGAATTTCCATCCGGC
GGGTGCCGCCAGGTCGTCGTTCGATCCGATTCGACGATCGTAGTCTCGAAGGTACACGCCTCGAGGGCCGGGA
TCGTCGCGCCAGAGACCCCAGGATTCGGCGCCGCTGTCGCCGCTGGATGCTGTTGGGTCGCCTAGTGCGGAGA
TGAAGCGGATGGATGGGTAGCGCTGCAATGTTCGTCGTTGTTTGGCACCACCATCCTCCGCGAGAGCCGAAGA
GGTCGGCGTCAATAATGCGAGAGCCGCCGTGGAAAGGGAAGCGAGGGCGAGACGACGCGATTGCATTGGTGAA
GAAGGGCGGACGGATGATGATTCGCCTCGAGACGGGGGGAGGGTCGTGAGTCCGAGTCCGCAGACTGTTGGTA
GATGGTGTGTCGATGCTGCGAGGAGGAGCGTGACGATGCCAGCGGATTTCATGGTTGGCATATGATCTGAGTT
CTTCTTTGAGAT
>TR194|c0_g1_i1 len=6961 path=[7910:0-44 7911:45-68 7912:69-69 7913:70-93 7961:94-495 7843:496-519 7969:520-611 7920:612-644 7960:645-815 7935:816-839 7949:840-887 7836:888-911 7959:912-1010 7878:1011-1034 7968:1035-1425 7958:1426-1840 7900:1841-1864 7947:1865-2277 7927:2278-2301 7973:2302-3490 7967:3491-3555 7946:3556-4104 7956:4105-4492 7850:4493-4516 7974:4517-4637 7873:4638-4659 @7972@!:4660-4917 7944:4918-4939 7965:4940-4977 7954:4978-5001 7942:5002-5555 7870:5556-5579 7971:5580-5677

7883:5678-5733 7953:5734-5819 7888:5820-5843 7963:5844-5878 7975:5879-5902 7976:5903-5907 7938:5908-5959 7962:5960-5970 7951:5971-5994 7937:5995-6068 7930:6069-6092 7931:6093-6093 7932:6094-6117 7933:6118-6960] [-1, 7910, 7911, 7912, 7913, 7961, 7843, 7969, 7920, 7960, 7935, 7949, 7836, 7959, 7878, 7968, 7958, 7900, 7947, 7927, 7973, 7967, 7946, 7956, 7850, 7974, 7873, 7972, 7944, 7965, 7954, 7942, 7870, 7971, 7883, 7953, 7888, 7963, 7975, 7976, 7938, 7962, 7951, 7937, 7930, 7931, 7932, 7933, -2]
AACAAACAGCCAAAACAGCGACCGGCGAGGAGGGAGCAATAGCACCGGACACGATGGCGTCTACCCCGCCGTC
GCTCTCCCCACCCTCGGATCCTCCTACGCATCATCGTCGTCCTCTCCCTACTACAGCAGCGGTGGTGGGCTTA
CCGGCAGCAGCAGGAGTGCGAATAATCTAAACGGCGGTACCAGTTCCGCCACCAACGGCCGCCTTTCCTCCCC
AAACACAAACCTCGGCCTGCGAAAACGAGATGCCGTTGCAATACTCACGTGTCTCCTCCTCCTTCGATTGGCG
TTTACTCCTTCCAACAATGCTGGAGGTGGTGGGGGGCTTGTCCATTTGTCCGATTCGAGTATAAGTGCATCGA
TGGAATTGGCATTTTATTCTCCATTTCAATCCCATCCTCCCAGACGTGGCAAGAATATGAGCGAAAGGACGGC
GAGGCCAGTGCCTCTGGATGTCGATGGGGACGGCGTCGTCGAGGCGATGGTGGTGCCTATATTCCTAAAACGA
AAAGAAGTCACGGAAGAGAAGAAGATGGATTTATTGTTGGAGGCGGTGAAGAATAAACGACCGTTTGCGAAAA
AGAAAGTCGAGGATGTCGTTGTCGATGAAGCGGTGTTGGAAGGATGGCATGATGATGGAACATGGGGACTACG
CGTCTTGAATCTGAAACCATTGCGGATCAATGCCGATAGCGACAAGCAGGAGGGGGGTTCGACGAATGCCGGT
CCATTTGCTCCTAGGAGTCTGTTCCTCTCGCCTCTCTTGCCGTACGCATCCAAGCAAAGTGACGATCAAACAA
TACCAAATGAAAAAGAACGTAGAAAGGTCTCTCCTGACACCTATCCCATCAAACTCCTTTCCGTCCAAATACC
TATCCAGCGAACTCGCCTCGGAGAAGAAGAAAAATCGCGGCAGCGGCACAAACCGTCCACCTCCGGTACTGGC
TCTTACGGCAAGAACTCGGAGATTCCGCCGAAGGATGATCCGAATTTTGCAGACTACGACCGAAAGCGACATT
ACTTTTGCGGGAAGGATTGGCACCACGCCGCGCAGAGTTGTCACAAGCACTGTCCTGGGGGACAGTCGAGCGA
GTGCGGGGAGGGTGAGACTTGTTATGCCGATACGCCGTGTGATGCTCATCTTGATCTGAGCTCTGAATCAGCC
TCTACCTCGAAAGATGATGCAAACAGTAATAAAGAATCTAACACTTCGATGGCATTGACAAAAAGTGGCACGC
TACCGGGCGTTGCAACCGTCTGGTCTGACGGCAGCGTCAGCCTCCACGTTATAACGGCGGACATTAGTCCATC
CATGAGCGGGGACGGGCAACAGCATCGGCCGAAGAGAGTGAAGCAAAAGAGCTTGAAGCCGGAGTTGGAATTT
CGACAGTTGTGGAGGGTGCACCCATTGGAAAAAGAAAATGGCGGTAGAGAGGGCGGTACTACTATTTTGGGGG
TTGATTTCGACGAACTCGGAATTACTTATGAATCGGGAACAATTTACGGACCCGCTTCGACTTCAGAGGGTGA
CAAGGAAACGAAACGATTTGGTGACAACGGAGCCATTCTACTCGGAGGGCGCTACACGCTCCGATCTAACGAT
GATGAGTTCCATCAGTTGAAAGTTTCGTTTCACGCCCTTGATGCCATGACGGGGACGTCTCTGTGGGAGCTAA
AGGGAAATCACCACGGGGACATTGATTCAACATCCAATACGACAAACGACAGCACAACCGATGTACCACCAAT
ACCAATTATCCACACCACCTCCTCGGCACGCAGACGCTCACACCTCCCCACCATCGACGTTCTCGATCCCGAA
CTCGACAACGAGAACGTCTTCATAGAAGGAGATGACGTCATGACCTCCGAGGAGTGCATGACGCACTTCCGCG
CGTCCGTGTTGGATCCGGAGGGTGGGGCATTGCCGCACGAATTTTGGGACGATGGAGAGTACGGGAGCATTGG
GGTCGGACGGTTCGATCGAGGGAAGAAGGTCGGAGGGAGGAAGTGGAAGGCGCTTGGGAAGAAAGGAAGGAAT
TACTTGCTGAACAACAAGAAGGGTGGTGCAGGAAGTGGGGGCCGTGGTGGAAGTGCTGCTGCCGTGGGCGGAC
TCGGAGGCATTGGACAGGGAAGAACGCGCTCCGCAGGCAGCAAGGGTGGTGGCAGTGGAGGTAAAGGAGCTTC
AGGCGGTCAGAGCGGCAGCTGGCAGTCAGACATCTTACACCGTGCTGTTCCTCGTCGACTCATCCAACAACAA
AAATATAACGCCAGGTACCCACGCACAGGCAGGCCCAATGTTGTCCTCTTCCACGGCCGTGACGGACTAGCCG
TCCTTTCTCTCAAAAACGGCCGGCCCGTTTGCCACGTCTCCCTCGTGGATCACTCATTGTATGCAGACATGGA
TGGCGATGGTGTCGTTGATGTGGTGCAGGTTGTCACCTCGCCCTCTGGCTTTGAAAGATCCAGCGGCGTCCAA
AGTTTGATCCATCGACTTGCTAAGGAGGCCAGCCAGTGGCACAGGCTGCCAGACGCACCCGTAATCTGCCACG
CGTTGGTAACGAGTGGACTTCCACCGCGGGAGGAAGTTTTCACTGCACCGCTTTGTCTGGGCGGGCCGGTGGA
TATTCGTCGTCCCCAGCACGTTAGTTCGGCCCCGCCGCTGTTACTAGAAGGATCGATGGGGTACGGCAACGAC
GTAGTGTTTGCGATGAACAACGGCGTTGTGGTCCGGTATGATTTCAACGGGAGAGAAATCTGGAGGAAGAAGG
GGGGTTTGAAGGACGGGACGCCCTCCTGGGACATGTCAGGAAATCGTGGTGGTCATTCCTCCGCCTCGTTTCT
GGGACGTATCCAATTCGGATCGGTCCGCGAATCTCATTCTTCGGTGTCGGCATCATCTCACAGAAAGAACCAG
CACCGTCCTGGATCGCCTGCGCGTCCCATCCTCCTCTCTGGAGAGGACGGCGCGGCGCTATTATCGCCAGCGT
CGGGGAAAGTCCTCTCCTCCGTCGTCTATCCCCAACGTGTGGTGAGCCAGCCGCTGCTGGCCGACCTGGATGG
AGATGGCACGGACGACCTGTTGGTCACGAGCGACGACGCCGTGTGGGGATATCGCGTTGTGGTTGAGACAGGA
CGAAGCGGGGGATTTACCATCGTTGTTGTGACGTTGTTGATCGGTGTGGCGTTGGCGGCTTTGGTGAACAGGA
CCAGTTATCAACTAGGACAAACTCCGAAGAGGAGTACGGATGCATAGTACTGAGTATACCGTCTATAACCTTC
GAGCTCATTGAAAACATTCATTCTACAGACTACCCATTATGCGACAATTTAGTTACACTGCGGATACCTGTTC
GTCCAATAAATAGACGGCAGTACCTTACGATTGAGACACAATTCTCCCGTTCATCATAAAAGGGCAAGAAAAT
TTGGCCATCTACGTTTGTTATATCTATTCGTTGGCCACTTGAATTCAGTTGATCATGCTTGCATGAGTCAAAG
CACCGCCAAAAACCATTTTCTCGTAATAATCTTTAGACAACGATCCTGCACAATCACAAGTTGGAAATATATC
GCATTGGCGTCCTAGTCATTTAGCGGCTCGTCAAAAGAACTCGCTCGATTATCAAAGCAGCGGGCGCTCCGCT
CGCCAGATAATAAACTTCCACAAGCCTACATGCTACTTACAACTCTCCACGAATGTCTACGAAAAAGAGTCAT
CCCGTTGAACCTGCAAGGCATTCTCTGTTCTCATCACATGCACCAACGTGACCCCGCATACAGTAGGCGACCC

ATGTGAGCGGAGCCCCAGTAACTTTAGGTGCAAGGTTGGCCACCAGCTGAATCACCCCTCTTCGATTTTTTGT
ATCCACCAAACAAATCTTAATGCTCGAATCCTTTAGACCTTCCCACTTCAATGCATTCAGCTGTGAATTGATT
TCGTTGCAGATCTCATCAGATGGGTTTCGTTTCATCCTTTGAATATCGCATTCAATCTTCACACCTCACCGCA
CTCACATTTCAATTCGAGCCAAGGGGTGCACCTTAGCCAAGTCATCTTAGACCCTCTGAAACTGGTCCAAAGC
GAAAATTGTCTTACTTGTCCTGAGGATGATAGGATCTCTACAGAAGTTTAAGTGTATAATAGCTTCTCCATCT
CGCACAAGGGAATCGTAGTCCATCGCTGTCTCCAGAGCGAGTATTGCTAGATGCCCATGCATACTTTTATCGA
AGATAAAGCGGCGAACAAACTCGGCCTACCAGTTTTGGTTGTTTTCGACAGCAAAGATTTCGCCACAGGGCCC
TCGAGAACATGATACATCAATTCTGAACCGCTTCCGTCTTAGAGCAGCGCTTACTGTAGAATGCTTCTCAAAG
AGTTCCATTGTTGAGCACAGGCCTGCGAAGAGGAGAGCAAGCCGTGCAAAGACGAACTAACAATACACGGTCC
AGAAACTCAGCAATGCGATCGTTCATTTCTGCCGGTAAATTACTGAGCGAGGCCACCATAGATATGACACAGG
GGAATGAAGCAAGAGCTTCCGATGAGCAAACAGAGGTGGATGCATGGTTCATTACGCTTGATGGGTAGATAAG
TTAGTTCAAGTGGGGCTGAGATCAAGCGCGAGGACACTGAAATCAACAGTCACATGGCTCAGTTTTTCGGTCA
CGAATGTTGGTTTGCCTGCTTCGCCTAAACACTGCCTGATCTCCGCTTCGACAGTTTTTGATCTACAAAAGTA
ACTCTCTTTGGAAGGCCCTCCACAACAAGTGCTGGTATAGTGGCAAGCGGTTATGATCTGATGGACGACTTCT
CGAAGGCCTCGTGGCGGAATCATTGTTGATAATGACAACCTGAACTGGAAGGTCGACTTTGCTCGCCGCTTCA
CACTCGATCAGTGCGTGCCTGAGCGTTTAGCAAGAACCGCATCAGGAGGTATCTCCTCTGACTTTGAGAGTTC
AATTGATTTACTGACCACTAACAAGAACAGTACAGGAAGAGGCGATGAAGTAGAAGTAACTCTTGTGCACGAT
AGAGCAGATATTCATGATAAGCTCACACTTCTCTGTGGGGTTCAATGCAGTATTGTTTTGGATCAATATGGTC
GAGTCTTCGTGTGATTGGGATAGGCTGCATGACTTGGCTCAAGTTGGGAACGAAAATACTGTAGGGTATGAAG
ATGGAATGATGGCAATATCCAAACTATTCCATAGCCACAGAGATCTCACACGTCGGCAATGACCGGATGAGAT
CTATGATGAAATCAATCTGCAGCCGGATTGCTTGACATTGGGGGATATGGACGACTTCGAATCTGTAATAGTA
TTCGACGGGGATGTTAACTTCTTCTTGATAAAGTATCGTAGAATCGAAAAAGGATGGTTCAATTGGTGGCCGT
TATCAAAGAAGAATAGTTATTGCATCTATAGTGGATGGGATCCCACCACCAGATCCTGCTGTAAAATATTAGC
ACATTATGGGGGATCAGGTTTCTGATTGGGTTGAGGAGTTCTAACTTCTTGAGGAGAATTCGTATAGCCTGTT
ATCCAACAACAGTTTGAATCGGACCAAGAATCTGGCATAGTTCTAATGACAAACGCAATTGCACACAGATTGC
ACCAGGCAGTTCAGCGTTCTTTGTCCAATAGATGGACGAAAGCAACGCGAGAGTCCAACGTAACAGAGACCAG
GCAGACCGAGATTTGGCTACCTGTGTAGACTAGACCATTCTAAATAATAGGTAAAATTTCGGCCGTATTGAGC
GTCGATAGTCCAGATGAAATAATTGATCTGTTATGGAGACATAGCAAACACTCTGCACATCATGGTCCATAAT
CTGTAAAGCTGGCCTCGTTTTGGCACCATGGGTATGAACCGGCGAAGTCTCAAGAGAGAAGTCAGACGGCAGT
ACTATGGGCAATGTCTCTAGGGGAAGGGGTCACTCCTAATATCCACATGCAACCTCAATGTGACTGTCTCCGT
CAAAATCCTCATGTGAAAACACAAGGTCTTTAGGAGTATGATGTAAACGACTACGCCCCCCTTTCATGATGCT
TTCCAAATATCGTGATTCCGATGAGATATTGAGATATATTCTTCCGATTGTAGATTGTTTCTGATAATGCAAG
ATCAAACAGCATCACCATCTTCCATTTCCGACCTCAACACCCAATCACCCTCCGTTATCTCCCTCCCCCTCAA
ACACGACCCATCCCTCACATTCCTCTCCACGAAGAACATCGGCTCTCCCCCTTGTTCATCATACACCTTGCCC
ACAAACCCAACAAATGACGCCTTCACATCATCCCCGTTGTACATAGTCAGGTAATTGAACGCTTTCGTGAATT
GGATGTTGGGATTGTTTCGTGCCGCTTCCAGTTCCGAGTTACCGGGAGCGTACCATACCTCGAAGGGAGTGCT
CAGGTCGGAGAGCGAGGGGCGTACGGGGAGTCGGAGGGCCCATACGTGTCTTTCGATGAGGGGCCATTGGCGT
TCTATGGCCTGTTCTGGGGAGACATCCGATATACGCGCAACTTTACCAACAAGCCACCACTCGATTTTCCCCG
GAGCTCTGACATAGACATCATTCACCGGTCCTCCAATGGATTCGAGTTGATCGAAGGTGTTGGCCTGTCGTCG
AATGTTTGATTCGATATACATCATATCCGATGGCATATTCTCGATTTTGTTTGCGGGAATACTGTCGTTGGTG
GATATCTCATCGTCATCATCTTCGTCAACGAGATCGTCCCATTTGTCCTTTGGTCGGGCATGGAGCGATAAGA
ATGCATCGCCATTGTTGTTCGGTGACGGGTGCAATTGCCATTGCTGTGGCGAGGGGAGAATATTAGGTCGGTG
TCGTTGATGCGTTGCTTGTAGAAACGCCTTCGCTGTGGTGGCGCAGAGCAAAATAATAACAATTGATGTAGGA
GTATTCATTTTTCATTTGTCGTTGGG
>TR194|c0_g1_i2 len=7044 path=[7910:0-44 7911:45-68 7912:69-69 7913:70-93 7961:94-495 7843:496-519 7969:520-611 7920:612-644 7960:645-815 7935:816-839 7949:840-887 7836:888-911 7959:912-1010 7878:1011-1034 7968:1035-1425 7958:1426-1840 7900:1841-1864 7947:1865-2277 7927:2278-2301 7973:2302-3490 7967:3491-3555 7946:3556-4104 7956:4105-4492 7850:4493-4516 7974:4517-4637 7966:4638-4742 @7972@!:4743-5000 7944:5001-5022 7965:5023-5060 7954:5061-5084 7942:5085-5638 7870:5639-5662 7971:5663-5760 7883:5761-5816 7953:5817-5902 7888:5903-5926 7963:5927-5961 7975:5962-5985 7976:5986-5990 7970:5991-6042 7962:6043-6053 7951:6054-6077 7937:6078-6151 7930:6152-6175 7931:6176-6176 7932:6177-6200 7933:6201-7043] [-1, 7910, 7911, 7912, 7913, 7961, 7843, 7969, 7920, 7960, 7935, 7949, 7836, 7959, 7878, 7968, 7958, 7900, 7947, 7927, 7973, 7967, 7946, 7956, 7850, 7974, 7966, 7972, 7944, 7965, 7954, 7942, 7870, 7971, 7883, 7953, 7888, 7963, 7975, 7976, 7970, 7962, 7951, 7937, 7930, 7931, 7932, 7933, -2]

AACAAACAGCCAAAACAGCGACCGGCGAGGAGGGAGCAATAGCACCGGACACGATGGCGTCTACCCCGCCGTC
GCTCTCCCCACCCTCGGATCCTCCTACGCATCATCGTCGTCCTCTCCCTACTACAGCAGCGGTGGTGGGCTTA
CCGGCAGCAGCAGGAGTGCGAATAATCTAAACGGCGGTACCAGTTCCGCCACCAACGGCCGCCTTTCCTCCCC
AAACACAAACCTCGGCCTGCGAAAACGAGATGCCGTTGCAATACTCACGTGTCTCCTCCTCCTTCGATTGGCG
TTTACTCCTTCCAACAATGCTGGAGGTGGTGGGGGGCTTGTCCATTTGTCCGATTCGAGTATAAGTGCATCGA
TGGAATTGGCATTTTATTCTCCATTTCAATCCCATCCTCCCAGACGTGGCAAGAATATGAGCGAAAGGACGGC
GAGGCCAGTGCCTCTGGATGTCGATGGGGACGGCGTCGTCGAGGCGATGGTGGTGCCTATATTCCTAAAACGA
AAAGAAGTCACGGAAGAGAAGAAGATGGATTTATTGTTGGAGGCGGTGAAGAATAAACGACCGTTTGCGAAAA
AGAAAGTCGAGGATGTCGTTGTCGATGAAGCGGTGTTGGAAGGATGGCATGATGATGGAACATGGGGACTACG
CGTCTTGAATCTGAAACCATTGCGGATCAATGCCGATAGCGACAAGCAGGAGGGGGGTTCGACGAATGCCGGT
CCATTTGCTCCTAGGAGTCTGTTCCTCTCGCCTCTCTTGCCGTACGCATCCAAGCAAAGTGACGATCAAACAA
TACCAAATGAAAAAGAACGTAGAAAGGTCTCTCCTGACACCTATCCCATCAAACTCCTTTCCGTCCAAATACC
TATCCAGCGAACTCGCCTCGGAGAAGAAGAAAAATCGCGGCAGCGGCACAAACCGTCCACCTCCGGTACTGGC
TCTTACGGCAAGAACTCGGAGATTCCGCCGAAGGATGATCCGAATTTTGCAGACTACGACCGAAAGCGACATT
ACTTTTGCGGGAAGGATTGGCACCACGCCGCGCAGAGTTGTCACAAGCACTGTCCTGGGGGACAGTCGAGCGA
GTGCGGGGAGGGTGAGACTTGTTATGCCGATACGCCGTGTGATGCTCATCTTGATCTGAGCTCTGAATCAGCC
TCTACCTCGAAAGATGATGCAAACAGTAATAAAGAATCTAACACTTCGATGGCATTGACAAAAAGTGGCACGC
TACCGGGCGTTGCAACCGTCTGGTCTGACGGCAGCGTCAGCCTCCACGTTATAACGGCGGACATTAGTCCATC
CATGAGCGGGGACGGGCAACAGCATCGGCCGAAGAGAGTGAAGCAAAAGAGCTTGAAGCCGGAGTTGGAATTT
CGACAGTTGTGGAGGGTGCACCCATTGGAAAAAGAAAATGGCGGTAGAGAGGGCGGTACTACTATTTTGGGGG
TTGATTTCGACGAACTCGGAATTACTTATGAATCGGGAACAATTTACGGACCCGCTTCGACTTCAGAGGGTGA
CAAGGAAACGAAACGATTTGGTGACAACGGAGCCATTCTACTCGGAGGGCGCTACACGCTCCGATCTAACGAT
GATGAGTTCCATCAGTTGAAAGTTTCGTTTCACGCCCTTGATGCCATGACGGGGACGTCTCTGTGGGAGCTAA
AGGGAAATCACCACGGGGACATTGATTCAACATCCAATACGACAAACGACAGCACAACCGATGTACCACCAAT
ACCAATTATCCACACCACCTCCTCGGCACGCAGACGCTCACACCTCCCCACCATCGACGTTCTCGATCCCGAA
CTCGACAACGAGAACGTCTTCATAGAAGGAGATGACGTCATGACCTCCGAGGAGTGCATGACGCACTTCCGCG
CGTCCGTGTTGGATCCGGAGGGTGGGGCATTGCCGCACGAATTTTGGGACGATGGAGAGTACGGGAGCATTGG
GGTCGGACGGTTCGATCGAGGGAAGAAGGTCGGAGGGAGGAAGTGGAAGGCGCTTGGGAAGAAAGGAAGGAAT
TACTTGCTGAACAACAAGAAGGGTGGTGCAGGAAGTGGGGGCCGTGGTGGAAGTGCTGCTGCCGTGGGCGGAC
TCGGAGGCATTGGACAGGGAAGAACGCGCTCCGCAGGCAGCAAGGGTGGTGGCAGTGGAGGTAAAGGAGCTTC
AGGCGGTCAGAGCGGCAGCTGGCAGTCAGACATCTTACACCGTGCTGTTCCTCGTCGACTCATCCAACAACAA
AAATATAACGCCAGGTACCCACGCACAGGCAGGCCCAATGTTGTCCTCTTCCACGGCCGTGACGGACTAGCCG
TCCTTTCTCTCAAAAACGGCCGGCCCGTTTGCCACGTCTCCCTCGTGGATCACTCATTGTATGCAGACATGGA
TGGCGATGGTGTCGTTGATGTGGTGCAGGTTGTCACCTCGCCCTCTGGCTTTGAAAGATCCAGCGGCGTCCAA
AGTTTGATCCATCGACTTGCTAAGGAGGCCAGCCAGTGGCACAGGCTGCCAGACGCACCCGTAATCTGCCACG
CGTTGGTAACGAGTGGACTTCCACCGCGGGAGGAAGTTTTCACTGCACCGCTTTGTCTGGGCGGGCCGGTGGA
TATTCGTCGTCCCCAGCACGTTAGTTCGGCCCCGCCGCTGTTACTAGAAGGATCGATGGGGTACGGCAACGAC
GTAGTGTTTGCGATGAACAACGGCGTTGTGGTCCGGTATGATTTCAACGGGAGAGAAATCTGGAGGAAGAAGG
GGGGTTTGAAGGACGGGACGCCCTCCTGGGACATGTCAGGAAATCGTGGTGGTCATTCCTCCGCCTCGTTTCT
GGGACGTATCCAATTCGGATCGGTCCGCGAATCTCATTCTTCGGTGTCGGCATCATCTCACAGAAAGAACCAG
CACCGTCCTGGATCGCCTGCGCGTCCCATCCTCCTCTCTGGAGAGGACGGCGCGGCGCTATTATCGCCAGCGT
CGGGGAAAGTCCTCTCCTCCGTCGTCTATCCCCAACGTGTGGTGAGCCAGCCGCTGCTGGCCGACCTGGATGG
AGATGGCACGGACGACCTGTTGGTCACGAGCGACGACGCCGTGTGGGGATATCGCGTTGTGGTTGAGACAGGA
CGAAGCGGGGGATTTACCATCGTTGTTGTGACGTTGTTGATCGGTGTGGCGTTGGCGGCTTTGGTGAACAGGA
CCAGTTATCAACTAGGACAAACTCCGAAGAGGAGTACGGATGCATAGTACTGAGTATACCGTCTATAACCTTC
GAGCTCATTGAAAACATTCATTCTACAGACTACCCATTATGCGACAATTTAGTTACACTGCGGATACCTGTTC
GTCCAATAAATAGACGGCAGTACCTTACGATTGAGACACAATTCTCCCGTTCATCATAAAAGGGCAAGAAAAT
TTGGCCATCTACGTTTGTTATATCTATTCGTTGGCCACTTGAATTCAGTTGATCATGCTTGCATGAGTCAAAG
CACCGCCAAAAACCATTTTCTCGTAATAATCTTTAGACAACGATCCTGCACAATCACAAGTTGGAAATATATC
GCATTGGCGTCCTAGTCATTTAGCGGCTCGTCAAAAGAACTCGCTCGATTATCAAAGCAGCGGGCGCTCCGCT
CGCCAGATAATAAACTTCCACAAGCCTACATGCTACTTACAACTCTCCACGAATGTCTACGAAAAAGAGTCAT
CCCGTTGAACCTGCAAGGCATTCTCTGTTCTCATCACATGCACCAACGTGACCCCGCATACAGTAGGCGACCC
ATGTGAGCGGAGCCCCAGTAACTTTAGGTGCAAGGTTGGCCACCAGCTGAATCACCCCTCTTCGATTTTTTGT
ATCCACCAAACAAATCTTAATGCTCGAATCCTTTAGACCTTCCCACTTCAATGCATTCAGCTGTGAATTGATT
TCGTTGCAGATCTCATCAGATGGGTTTCGTTTCATCCTTTGAATATCGCATTCAATCTTCACACCTCACCGCA
CTCACATTTCAATTCGAGCCAAGGGGTGCACCTTAGCCAAGTCATCTTAGACCCTCTGAAACTGGTCCAAAGC
GAAAATTGTCTTACTTGTCCTGAGGATGATAGGATCTCTACAGAAGTTTAAGTGTATAATAGCTTCTCCATCT
CGCACAAGGGAATCGTAGTCCATCGCTGTCTCCAGAGCGAGTATTGCTAGATGCCCATGCATACTTTTATCGA
AGATAAAGCGGCGAACAAACTCGGCCTACCAGTTTTGGTTGTTTTCGACAGCAAAGATTTCGCCACAGGGCCC
TCGAGAACATGATACATCAATTCTGAACCGCTTCCGTCTTAGAGCAGCGCTTACTGTAGAATGCTTCTCAAAG

AGTTCCATTGTTGAGCACAGGCCTGCGAAGAGGAGAGCAAGCCGTGCAAAGACGAACTAACAATACACGGTCC
AGAAACTCAGCAATGCGATCGTTCATTTCTGCCGGTAAATTACTGAGCGAGGCCACCATAGATATGACACAGG
GGAATGAAGCAAGAGCTTCCGATGAGCAAACAGAGGTGGATGCATGGTTCATTACGCTTGATGGGTAGATAAG
TTAGTTCAAGTGGGGCTGAGATCAAGCGCGAGGACACTGGTGCGTCCAATGTCAAGCTCTTGCTTTACTTTCT
ACTGACGCGCCTCGTCAGTCAGTGTTCTCGCTCACCCATATTTCAGCAGAAATCAACAGTCACATGGCTCAGT
TTTTCGGTCACGAATGTTGGTTTGCCTGCTTCGCCTAAACACTGCCTGATCTCCGCTTCGACAGTTTTTGATC
TACAAAAGTAACTCTCTTTGGAAGGCCCTCCACAACAAGTGCTGGTATAGTGGCAAGCGGTTATGATCTGATG
GACGACTTCTCGAAGGCCTCGTGGCGGAATCATTGTTGATAATGACAACCTGAACTGGAAGGTCGACTTTGCT
CGCCGCTTCACACTCGATCAGTGCGTGCCTGAGCGTTTAGCAAGAACCGCATCAGGAGGTATCTCCTCTGACT
TTGAGAGTTCAATTGATTTACTGACCACTAACAAGAACAGTACAGGAAGAGGCGATGAAGTAGAAGTAACTCT
TGTGCACGATAGAGCAGATATTCATGATAAGCTCACACTTCTCTGTGGGGTTCAATGCAGTATTGTTTTGGAT
CAATATGGTCGAGTCTTCGTGTGATTGGGATAGGCTGCATGACTTGGCTCAAGTTGGGAACGAAAATACTGTA
GGGTATGAAGATGGAATGATGGCAATATCCAAACTATTCCATAGCCACAGAGATCTCACACGTCGGCAATGAC
CGGATGAGATCTATGATGAAATCAATCTGCAGCCGGATTGCTTGACATTGGGGGATATGGACGACTTCGAATC
TGTAATAGTATTCGACGGGGATGTTAACTTCTTCTTGATAAAGTATCGTAGAATCGAAAAAGGATGGTTCAAT
TGGTGGCCGTTATCAAAGAAGAATAGTTATTGCATCTATAGTGGATGGGATCCCACCACCAGATCCTGCTGTA
AAATATTAGCACATTATGGGGGATCAGGTTTCTGATTGGGTTGAGGAGTTCTAACTTCTTGAGGAGAATTCGT
ATAGCCTGTTATCCAACAACAGTTTGAATCGGACCAAGAATCTGGCATAGTTCTAATGACAAACGCAATTGCA
CACAGATTGCACCAGGCAGTTCAGCGTTCTTTGTCCAATAGATGGACGAAAGCAACGCGAGAGTCCAACGTAA
CAGAGACCAGGCAGACCGAGATTTGGCTACCTGTGTAGACTAGACCATTCTAAATAATAGGTAAAATTTCGGC
CGTATTGAGCGTCGATAGTCCAGATGAAATAATTGATCTGTTATGGAGACATAGCAAACACTCTGCACATCAT
GGTCCATAATCTGTAAAGCTGGCCTCGTTTTGGCACCATGGGTATGAACCGGCGAAGTCTCAAGAGAGAAGTC
AGACGACAGTACTATGGGTAATGTCTCTAGGGGGAGGGGTCACTCCTAATATCCACATGCAACCTCAATGTGA
CTGTCTCCGTCAAAATCCTCATGTGAAAACACAAGGTCTTTAGGAGTATGATGTAAACGACTACGCCCCCCTT
TCATGATGCTTTCCAAATATCGTGATTCCGATGAGATATTGAGATATATTCTTCCGATTGTAGATTGTTTCTG
ATAATGCAAGATCAAACAGCATCACCATCTTCCATTTCCGACCTCAACACCCAATCACCCTCCGTTATCTCCC
TCCCCCTCAAACACGACCCATCCCTCACATTCCTCTCCACGAAGAACATCGGCTCTCCCCCTTGTTCATCATA
CACCTTGCCCACAAACCCAACAAATGACGCCTTCACATCATCCCCGTTGTACATAGTCAGGTAATTGAACGCT
TTCGTGAATTGGATGTTGGGATTGTTTCGTGCCGCTTCCAGTTCCGAGTTACCGGGAGCGTACCATACCTCGA
AGGGAGTGCTCAGGTCGGAGAGCGAGGGGCGTACGGGGAGTCGGAGGGCCCATACGTGTCTTTCGATGAGGGG
CCATTGGCGTTCTATGGCCTGTTCTGGGGAGACATCCGATATACGCGCAACTTTACCAACAAGCCACCACTCG
ATTTTCCCCGGAGCTCTGACATAGACATCATTCACCGGTCCTCCAATGGATTCGAGTTGATCGAAGGTGTTGG
CCTGTCGTCGAATGTTTGATTCGATATACATCATATCCGATGGCATATTCTCGATTTTGTTTGCGGGAATACT
GTCGTTGGTGGATATCTCATCGTCATCATCTTCGTCAACGAGATCGTCCCATTTGTCCTTTGGTCGGGCATGG
AGCGATAAGAATGCATCGCCATTGTTGTTCGGTGACGGGTGCAATTGCCATTGCTGTGGCGAGGGGAGAATAT
TAGGTCGGTGTCGTTGATGCGTTGCTTGTAGAAACGCCTTCGCTGTGGTGGCGCAGAGCAAAATAATAACAAT
TGATGTAGGAGTATTCATTTTTCATTTGTCGTTGGG
>TR195|c0_g1_i1 len=470 path=[448:0-469] [-1, 448, -2]
GGTTATAATAATATTGTCCCGGCGGAGCGGTATGCCGACGTGACAATAATCCTGGCTGCGGCCGTGCCTCTGC
TTGTGCCCGTGCGACGTTATTGGTGTCGTCGACGGCATTGCTGGCATTACTGGAGTTGATGCTTGTGTTAGGA
TCGTAGAGTGCTAGCCGTGGCGGTGCCGGTCTTGGTAAGGGCGAACCATAATAACCGTGGTAAGATGAGATGC
TGCTGCTGCTATTATTATTTTCGCTGTCGTGGTGATGGCTATTACTACTTGTTGTTCGACGATGAAGTACATC
ATTATTATTGTGATCAGCAGGAGACGATTCTCGGTCCTTGGTCATGAGGATGTTGTGGCTGCTGCGTCTGTTA
CTGTCGGATGGAACTAGCGATGAGGTGAGGAGAGGCGGCATGATGGATGAATCCTTTTATGTTGCATGTTGCA
TGATGTCCATAATTATGGTTGCGATGTTGCTT
>TR197|c0_g1_i1 len=272 path=[250:0-271] [-1, 250, -2]
CTCCGGTGACCTCATTGCCGAGGAATAATGCAACGCCCCTACTACTACTACTACTACTGCTACTTGAATCATC
ATAATCACCACCACTAAAATCCTCACCAGTGGATATCTCAGTAGTCGTGGATTCCACCTCCCCATCCTTCGAA
ACTATAGGACCGGGATACGCAAACTCCGTATAAACCCTACTCCGTTCCGTAGTCTCCATCCCCACCAAACACA
ATCCCGGCCAACATTCCCTCAAATACTCCAAAGCATCCATAGTCGTCGTAAAA
>TR198|c0_g1_i1 len=855 path=[833:0-854] [-1, 833, -2]
CCTCGCCGCAAAACGATCCATGTCCTCTTCCACTCCCGACGGCGGCGGCGGTGGTGGTGGTGGACACCACGAA
GCGATAAATCTCCTGTGCAACACGACGGCTGACACCTTCAAAGATGGATTTACCAACCAACCCGGCCTCCTCT
CCGCGCTCGGATCGGCCATTTGCTACTTCCGGGATGCATTTCGCACCCCCGCGAATCTTGCGGCGTGCGAACG
GGTCGGCCTCTCGGATTACGATGTCGCGCATCACATTACGGATATGTACCACCATAGGAACTTTCGGGGCGCG
TATACCAAAACTTTGAGTCCCAGTGGGACGGGCGAGATCGCGGCGAGGGAAGCTGTTGGTTTGGGTGCGGATG
GAGATGTTGGAGTAGATGCTAGTACTGCGGACGATGATGGAGTGGCATTGCTTGAGGAGGACGATGAGGATGA
ATGCATCCTTTGGTCTCCGACGACATCTGGGGTTTGCATTCATTGGAAATCGGATGATGAGGCTTGGAGGAAG
AAGAGGTTCGAAAGAATACAGATGGAGGGAACGAAAGATCCCAGACGCGGGGGTAGCGGCGGGACAAGGTGAT
CTGCGTCAGTGCTATCGAATGCGCACCTATTGTGGATTCGCTACGTCCATGGAGATATTTTGCAATTTTGACG

AGCATTAAGGAAGGCATGGTAGAAGACTTTACTCGTTGCTATGATACAATTCAAGTCAGATAAAATGGGAATT
TTGGTGTGTGAGGTATTATTTGGAAGATAGTATAAGAAAGAGTCTAATACTACTTCAGCACCTCTTCAATAAT
GAACGCTGTGTTGAGACCTGTGATATGGTCCACTAGGGAGTCAATGGGGAAC
>TR199|c0_g1_i1 len=904 path=[882:0-903] [-1, 882, -2]
TACTGATTTGGACCTCGATTTGGATTTGCATCGTCGTGTCCTAACTGATTTGGACCCCGATATTGATTTGGAT
CGGCGTGTCCGGACTGATTTGGACCCGGAGACGGATTTGGATCTGGATCGGCTTGTCCTGACTGATTTGGACC
CCGAGACGGATTTAGTAGATCGGGTATCACTCCTACCTCGAACTGGTTTGGAGGAACCTGAGTTCTCTGTGGT
GGATTTCTGGGTTCTTCTTCGCACTGATTTGGATCGTCGCATAGTTCGTACTGATTTGGGGGTGCTGACGTTG
TTGAATCTGTCGGTTACCATCGTGGCAGCGTCCGACGAGTTGTTGGTATTGACGGGTATCATGGTGCGAGAAT
GAAGTTACCGTCGTGTCTTACTCGTTGACGGGGTGTTCACGGATGGAAGCTAGAGTTCTCGAAAAGAGAGAGG
TATGATGGTGTGCGATGGAGCGAGAGAGGTCGCGTGCGCTCTAGAGTGCTAGTAGGTTTTTAGTACGTGATCT
AGATAAAGTCCCGCGATGGCAATGATTAATGATGTTTGGACTCGGGCATGGGCTTCGGTGCCATTATTTTATC
GTTGTTCGATATTGAATCGTTTTCATCGATGGGTGCATGCATGCATCCGCCTGACCCGAAGGCCGAAAGTGAT
GGAGCCTTAACCGAAGAAATGCCTCGACGATCGACGATGCGTGAGATGAGACGTCTTGAAAAAAATGGCGGGA
ACTTAAATTGCTCACGACTTTCTCATTTTGAAATCAACAACTCATATTGGAATTCATATGTTTTACGTATGTA
TGTGGAAAATGTTGGTATTGAATATGAACAACTGAGTTGTCCTCGAATGGGGAATGATTTTACGCGAGTCTGG
GCGATACTCGCTCATATTGGTGATATTT
>TR200|c0_g1_i1 len=840 path=[818:0-839] [-1, 818, -2]
CTTCCGATCTATTTATTTATCCTTTGTGGATTTGGCAATGACATTCTTTGACAAGGACCCGTATTTATTTTAT
CGTACTGTACCATGCCCAAACCGATCTCGCTCCGACGGCAATAAATCATGATGTCGTCAACGCCATACCAAGG
AAGTGAATCCGACGCACCTGAAGCGTCGCTCTCTCCCATCTTATCGACAGCGACATCGGCTTCACCATCGCCA
CATGCTTTTACACGACGACGCAAATCACCAACATCATCACTCCCGCCGACCACTAGGGAGAACAATTTGACAC
TTTCGCTTTCAAAGGAAGACCTAACCAATGAGGATGACGACGAGCCTACCATGAGTTCATCGCCAACGCATGT
CGACGGGGTAACGTCGCCTTCTTTCGAAAACGACGAAAGGCATTATGCATCGATGCGCGAAATGATTTGTAAT
TTTATCTTGGGAAAATCAAAAGCATTGGTCTTTGGTCAATTTCTTTCACTGATGTTGGCAGGCACCGGAGCCA
TACAATCAGCATTATACCTAGACTGTCATTTATCTGCCCCCACATTCTCGATGCTTTCGTTCTACTTACCTCT
GAGCATCGCATGTCTCGCGCGCTTATTTTGGAGAGAAAGGAAACATGTATTATTTTCTCGGGTACAGTCCTCT
TGTTCCATAAATCAGGTGAATGAAAATGGACAGAAGAGTAAGGCAGGAGAGGTATCTGAATTGCAAAATGTAA
CACAACAACAGTCACAGCAACAGCAGTCATCCGAGTCCACGAACAGTGAAATAACAACAAAAGCAACAACAAC
AACAATACGCGCCAAACCAGAAAAGAGAAATCATTTA
>TR201|c0_g1_i1 len=484 path=[462:0-483] [-1, 462, -2]
GCAAGGTGGGAGGAGGACGACGATCCACGACCGACTGGGACGCCATTCTCCAACGTGCCCGAAGTCATCCTCA
CGAGGCTTGCTTTTTCGATCCTAATGCGGGGGGACACGTTTACGCGTTGCATCGATTGTTGAGGAGAGGGGAG
TCCTGCTGCGAGGATGGGGACTGCTGCGACTCGTCGTCGTCGTCGAACTCGTCCACGTGTCGTCCTCCGGTGG
CGGTCGTCGAGGCCGTGATACAGGCGTGTCCTCGGGCGGTCACGAGGAAACAGGCTGTGATCGACGAGGATGG
GATTCTCACGGGCATGGATAACGAGGGAGGTTCGAACGTCGGGATCATGGGAGGGGGGAACGGTGCCGTCGTC
AACGATGGGAATAATAATCCCATGGAATGGCAGGCTCAGGCACAACACCATCAGCAGCAGCAACAACAACAAC
AACAACAAGGCCAATTATTTCCACCCCCCAACGAAAATTTGCTCCT
>TR204|c0_g1_i1 len=262 path=[240:0-261] [-1, 240, -2]
TAGGCGGGTGCAGACGCGACGACTGCAACGCCAACCGCAAGTGCGGCGACAATCGATTTCAAGCTGTTCATTG
CTGTTCCCTTTCGGTTGATATTGGCGCCCTTCGGGCAGTGTCAAAGCCATTACTGTGGGTTGCTTTCATCCGG
CCGCGAGGCCGGATGCGCACCTTCTTCTTCGTGCCTTCGTCGAGGAGTTTTGGGTCTCAACCCATCCGCTCGC
TGGCGTAGGAGCCCGGCGATGCCGTGAAAACGACGGTCTTGTT
>TR207|c0_g1_i1 len=243 path=[221:0-242] [-1, 221, -2]
TAAGGTTTTTTACTGGAAAGAGGCCTTCACAACTAGCCATAGCACCGAGTAGAATCATTATATCATCCTCCTC
AAATTCGTTATTAATCTCATCCCTTATTTCTGCACACGATCATTTGCCACAACGAATTTTGTAAGCATCGCC
ATGGCGTCAGCAAAAGCTGATAATGGCAGTCCTGTGTTGTTGAGATCAAATTTACCACCTGCGAACACCATCC
CCATCTTGTGTGCCGGATCCCTAA
>TR209|c0_g1_i1 len=523 path=[501:0-522] [-1, 501, -2]
GAATAAAACTCTGCTCCCCGCTCCTTCATCCTGCAATAATGGTTCCTTCCCCATCTGGCCCCGAAGCGAAACA
TTCAACTCAAGATAACTTCATCCATCCCCGTCGATCAGTGCAATTTTCTCCAACCAGTCAATTAGCCTTGTAC
ATAAAGGCGGAAGGCGCAAGTAGCTCCTGGTCCACCGACGACGACATGGTCCGTTTCAAACGAGATCTTGTAA
GAGACCTACAGAGAGTTGGAATGCTACTGACGACGCGATTGCCTGAAGATGCATCTCGAGATGATATATGTAG
ATGCGTCGGGATAGAGAATATGATGTCATTGGACAAGGCAAGGCGTGCAGTGGAACATAGGCGCAGCCACGTG
AAGCTTGTTACTGAATCGCAGAGTATATGTAGCGAGGAAGACCTGCGATCTATTTCTATCCAAAGTTCTAGGT
CTACCTGCAAGAGAGCGCATAGGCTCGCGGCCGGTTATTGGGGTATGCGAGATGATTAGACGTATGACTTGCC
CGAGAGAGAGAG
>TR210|c0_g1_i1 len=237 path=[215:0-236] [-1, 215, -2]
GGCGATGATGGCACCTCCTCCCATGCCATCGTACGCTTCTTCCACCGGAACGTCGAATCGGTCGAATGCAGCA
TTGCCCAAACATCCGAGGAAGCGTGTTAGCAGTCACCATACCGCCCATTTTCGAAAAAGCACGAGGGAGGTAG

CCCAGGAAAAAGAGAGAATAACTCGGAACAACGATCACTTCGACGATATCAATGGGAAAGAGGATCATCATCA
CCGTGCCGGCACAAAGGT
>TR211|c0_g1_i1 len=407 path=[385:0-406] [-1, 385, -2]
GAGTTGGTCACGCCGCGTTCCTTCGCGTACGCCACGAGGGCCTTCGCCTGTTGGACGTGGGATGCGTCGTTGT
TCATGTCGGAGATCATGTCGACTATTTGGATTTTATCCAATGGGTCCATTGCCACATTTGTTGCTGTTGCTGC
TGGGGTTGTAGCGGAAGATGGTTCCACACGAGGATCATCCTCCCGGAACAAAGCGTCCACCTTCTCTTTCCTC
GGATGTAACTCTTCCATACACCTCACGATACTCTGTGCATCGCCACCACCACCAGCATCACCATTGTCATCAT
CATCACCACCTTCCCTCTCCATCTCCAGCAGATATCGAATCGATTCCAATCCCAGGCGTTTTCCAACTTGCTC
CTCGCTCCATCCCTTGACGCACGGCTCCTCGATAAGAACGCA
>TR212|c0_g1_i1 len=580 path=[583:0-73 584:74-97 585:98-579] [-1, 583,
584, 585, -2]
TCCGGATCAAAGTTTAGATTACTAGTGTAGTAATTCAACGCTGCTCTAATTTCGTCCTTTTCCTCCCTCGCCT
TTTCGTCCTTCTTGAGATCTCTCCAGGCGTTGATTTCTCCGTTTTGTACTAACATGGCCCCGGGCTTTTCGAG
GATGGTCACTCGATTACGATATTGGGCGTAGGCGTCTAGGTAGGACTGGGCCGGTTTTTGAGGTATGGAGAGG
GGTGTTGTATCGCGATTGTAGTTTTGGGGAGTGGACAGCAGGCGTTCGAGATTGTGTAGCATTTCTGTTCGAG
TAGATTCGTTGGTCTTCTCCTCGGCGACTAAATTCGAAGCAATATCAACAGCCCGATCGATTGTTATCTTGAC
TCTCGTCGCGGGCAATAGAGCATCGATGGGAGCGAATGGAGCCACGGCCAAAGCAGGTTGTTTATTTGTTGTG
CAGATTATCAGTGCAGCAGCGGTACTGGACGTTGTTGTTGTTAATAATTCACGAATCGCGGATCTCCTATCGG
TTGTCATTATGCCAAATGCTGACACATCCGTGGGAGCAGACATTAGCGCCATGGAGAAGATGCCGATGG
>TR213|c0_g1_i1 len=956 path=[959:0-438 960:439-462 961:463-955] [-1,
959, 960, 961, -2]
AGATTGATTGAGCAAGGCTAACATTTTACACACTAAATATCTCTCCAAGACTAATTCCCTCTGAGCTATCACT
TGTGGCCAAAGTGGCGGGTGAAGATCTGGCACTACTGCCCGGCACACGCTTTCCAACGGTAGTCTCCACAATC
GCATTGTTTGCGGTTGATTTGTCGTGAGTATCAAAAGAAGTGATAAGTGTGGTTGATTCGTCATCACCTGCTC
CACCACAAGTTGTGAACCAGTTGATCAGTTTTTCACCACCAGAGGCTCCATGTTCTCTGGCAGTGAAACTGTG
GTCCGCTCCCTTCGAAAAAGTTGAGTCACCTTCCATTTCTTCCTCGGACATCGACTGCCCTTCTTCTGTCTCA
TCATCGGTCAATCCTCCGGAGATATCATCAATCTCCTGCAGAAGGTCAGCATGCTCCCAACTCACGATTCCCG
GCGTAGTACCATTTTTTCCATCCTCCTTCATCGCCGTGAGATTTGCCTCCATTTCTTTGAGCTTCTTATTCTG
ATCTTCTTTAGTTTTCCGCACCTTTTTGTCGGCATCGGAAATACCCTTCCGCAGCCCGGCGATCTTCGACCGA
AGTTTGGTATTCTGCCGAATTTCCTTCTTGAGCTTGGCTTCCATTTTATGCATTTCGTCTTCCTGAATCTTTC
TCTCCATTTCTGCGAATTGCCTCGTGGCGAATATCTTCTCGCTGAGCTGCAATATTTGATGATTGAGCAATTC
GTTCTCCTCGCTTTCATGCGCGTAGTTCGCTTCCGACGTCGTGAATACCTTCGGTTGGTCCACAGACGACTCA
TAGAGTGTATCCATTCGATTCTTTTGAGCTTCAATATTCGATGACGTTTGTATGAACTGCTTTTTTTCCAACT
TGGCATTCGCGAGCTCCAAATCTTTTTGCAACGCAAGCACCATCTTCACCTTTTCGTTGAAATTGCTCTGGAT
ATGGCTC
>TR214|c0_g1_i1 len=362 path=[366:0-140 367:141-361] [-1, 366, 367, -2]
AGGATGCGCCGTGTTTTAGTTTTGGGTGTGGTGGTGGTGGTGGTGGTAGTAGTAGTCGGGGTTGTTGGGATTG
TGCTTGTTTCGGTGATGGGTGTATTGATGGCATAATCATTGATATCGCGATGGATGGTCATGCTTTTTGTTGG
TTCCAAATGGCAAGAAGAAAAGATGTCTAGGGATGAGGAGCCGTGGCAAGGGCCGGCATGCATGCAAGTCGGG
GTGGCTACTTCTAGTTAGGCCGGTGTTGTCATTGTCACGTTTTTCCCAAAACTCTCCAGGTCTCAGGTCCCAA
GCCCCATCTACTTAGGTACGGTAATCAGGTATGAGTACAACCTAAGTATAGTACCAGTACTTTACTAGTT
>TR215|c0_g1_i1 len=527 path=[530:0-409 531:410-433 532:434-526] [-1,
530, 531, 532, -2]
ATGATGACGACGATGACGATGATGATAACGTCGATGATGATGACAACGAGAGCGTCGTCCTCTTGCTAACAAC
CGATGTTCTTGCAGATAAAAAGGCACAGCTATTATTCGATGAAAACGAAGATGTTTTCATTGGCGTCGTCAAT
CCCAACTTGTTACTGCCAATGCCAATGCGCGACAACGACGTCGTTGGGTGTATTGTTAAAGAAGAGGATGCGG
TGGCAAGGAGCGCCACTGTGAGTGATGATAGGGTAGGAGGGATCGATGATCGCGCCATGGTCGAGAGAGAAGA
TTTTTTTATTTTCCGTAGTTCCCTTACAATGATTTTTTGACGACGTTTGGACTCTCTAGACGCGCTGAGAATA
TAACTGGATACTGTGTTTAATTGAAGATTGAAATGTGACACGATGTTTTGTGTTCTTTGCTGTCATTGAAGAG
GGGATCTTTGAAGCACTTTTGAAGCACTTTGAAGCGTGCCGCCGATCGAGAGATGGTGTTCTTTGTTCTTTGT
CCTTTTTGTCATTGTT
>TR216|c0_g1_i1 len=279 path=[305:0-79 306:80-278] [-1, 305, 306, -2]
AAATGAAAATAATCAACACAATCAATAAATAGCAACGAGAATTACTCCGTTATCATTGCAAAAGTTAACCAAC
TTCTCCCAGAGCAACCGAGACTTCTTACCGAGACAACCAAACCGTCACACAACAACCACTACTTTTTGATTCT
AACCAAGACAACCAACCCCTCACACAACAAACACGAAGATGAAGGCTCCCATCAACACCGCCATCCTCTACCT
GCTCTCCTCCGCGATTGGACCTGCCGACGTCAACGCCCAAGTCGCCAAGATATGCGAATC
>TR216|c0_g2_i1 len=270 path=[307:0-70 308:71-269] [-1, 307, 308, -2]
ATCTCTTGGATTGTATCATGCCAATTGATCCTCATAATTCTTACAAAAGCAAAAGTTAACCAACTTCTCCCAG
AGCAACCGAGACTTCTTACCGAGACAACCAAACCGTCACACAACAACCACTACTTTTTGATTCTAACCAAGAC

AACCAACCCCTCACACAACAAACACGAAGATGAAGGCTCCCATCAACACCGCCATCCTCTACCTGCTCTCCTC
CGCGATTGGACCTGCCGACGTCAACGCCCAAGTCGCCAAGATATGCGAATC
>TR218|c0_g1_i1 len=860 path=[863:0-467 864:468-491 865:492-859] [-1, 863, 864, 865, -2]
TTCCGATCTTTTTTTTTTGATTCATATGCTTCTAATTTTTAATTTATGCAGCTATATTCTCAGCCCTCTTCTC
CATTGTTCCCATTTGATCCACTGGCAAAATATCTCACAAACTTGATAACTGACCTCTAATCTAACAACTAGAG
AAGCATCAATAATGTATCATGCCGACAAATTTGGCCATGGTTACCAAATGTAATCTGATATCGGCATAAATAA
CACTTTTCCTTTCTTTGCTATACCCTGAACTGAAAGAAGGGTGGAGCTGTTGTCATGATAATGTCCCAGTCCT
TTCTTTGGTATGTGATACAAGACTTCTCAGGATTTAACACAATGTAGAATCTGTTCACAAATTGTAACTGTTC
ACATCCAAGGATCTGCAAGCAGTACATGTATCAATATCCACATATCCGTAAAATTACCCCCTACATCTCTTTG
CATGCCGTGCATGCAATCAATGATATCTCGAAAACATTGCTCTGCTCATTTCACCAGTATCCTAAGGATTGAA
AGAAAACTGCTCATCGGACCCTCTCTTGAGAAAGAAAGAAGATTGTAGATGGAGGGCACCAACAGTGTGAGTT
CTTGTTGGCTTTCTTATCTTTACCCGTCCACGATGTCAAATTCGGATATCGATACGCTGTTGTCCGAAACTTT
GTCTGGCGGCGTTTATGTGAACGACATCACCTTCCAGAAGTCTCCTCCTCATCTTCCATTCGGAGGCATCGGC
ATGAGCGGCATGCGAGTCCATACAAGCTCGGAGTTCTTTGCCAACTCATGAGCACATGGTACAGTGGCAAGTG
CGGTGGTCACGAAGGCTACCTTGAGCTGTCGCGCACGCATCCCGTTATTCACCGCTC
>TR220|c0_g1_i1 len=430 path=[458:0-155 459:156-179 466:180-209 465:210-233 464:234-429] [-1, 458, 459, 466, 465, 464, -2]
GCGAGGACGTCGCTGAATGCTTCGTTCGATGCCGCCGCCGCCGCTTCTGCCACCGTTACCGCTGGCGCGACCA
CCACCCCTCTGTCGCGTTCGAATAGCTCCCAGCAATTGACGATGATCGCGTCATCGTCATCGGCAACGTCTTC
CGGATCGAAGGCAAATCCAAAGTACACATCGCCGAAACGTCGAAGGCAGAGTTCGGACAAACAATACCCCGCG
CAAAAACACCATTCGTCGACCGGACAGGCATACACCCCCCTCCGACCGCCCCGAACGCAACAAAAACGATATC
GAAAAAAGGAATCCGAAGGACGATCCTCCTCGTCGTCGAACGTGTTATTCTCGCCCAAACCCGTCTACAGCGC
CGAGAAGAAGGGCGGTCGATCGAAGCGTCCGCCCCGTCCCTCTCCTTCCGGAAGCGGAAGCGCTT
>TR221|c0_g1_i1 len=1276 path=[1300:0-246 1301:247-270 1307:271-584 1298:585-601 1299:602-1275] [-1, 1300, 1301, 1307, 1298, 1299, -2]
TTTTTGTTTGTTAAATCATCAATTAGTATTTTTAATACAACCTACCTCCCATCAAGATATCGAATTCATGGGT
ATACATTTCCAAGATGCATACATCATCAAACTCTTTTTTCGCATGCATCGCTTAGTAAATTTGGATCTCACCC
AGATAAGAAATTCCTCCGCGCCCCATTCAAGATTTCTCGTATTTGTTGCTTTTGTCTTGGCGACATCTGGTGT
TTGATCCCGCTACACATAGACCCACCACTATCCTTTTTTTTTCTTCTGTCGGTCTTGCGAGTGCCTGTCCCTTC
CGCGCCACCTACGTCTTTCGGAAGTCCTCTTGTGTCCATTTCTGGGTTTGAGCAATTCTGGCTTTTTTGATGA
AGCAGAAACATCGTCTCTGCTCTCTTCGCTCTTAATGTCGACCTCATTTTGACTGACTGATGAGTTTGATGTG
CTTTCTTGCGTACCGTTTGTGGAATGAGATGTTTCGTCATTGAGCTGTTGCCGTCTTTGTCGTGCGCGTCGCC
TGCGGGAATTTCTGCCGCGTCCCTCTCCTGGTGGTGATTGCTTGATTTTCGGGTTTTGCGCATGGGTGTTTTT
TGCTTCAGCCTTACGGTTTCGTTCTCTGCGTTCCTTAGTCTCTCTTCGTTGTACATTCCTACCAACGCGCCCC
TCTCCCCGCGGCCGATGTATCATTTGTTTCCGCTCAGACATCTTACGAGCATCTATGGCCTGCGGAAGTCCAT
TCTTCGTTGTTCCCGGCGTGCCCTCTTTCCTACCGATCTTTCTTGTGACAACTTTATGACTTTTATTATCATT
CCCCCCTTTTGCAGCAACTTTTTCGTTGGCATAGCTGCTGGGAGTTTGATTGGGGGGGCAGCTCTTTTCATTA
TCAGGTTTGTCCTCTCCACCATAAATGTCATTTGCATATCTTAGTTTCAGTGGTGTCAGCAACGAGGAGGTAA
TTCCCAACTCAATCAATTCCTGCACCTCAGTATCCAAAGATTTCTCGAATTCATCAAGTTGCCCGTAAATGTC
ACCGCTGGAATTTTCATGATCACGTTCTGCCTCGCTAATAGTTGCGGGATATGGTTCGCCTCCCATCGTCACT
TTACAGTTGCCATCGTCGAGGATAACGTTTGAGCTCGGTATTTCGCGTTGAGACGACATTGTTGAAAGGCTGG
TTAGATGGTTGTGTTTACGGTGCTGTCTGGGATACAATGCGAGAGGTGTTATGATCATGGTGTCGTCGCATGA
ATATTATGCTACTGTACGTTTGACTTCGAGAGATC
>TR222|c0_g1_i1 len=666 path=[719:0-176 720:177-200 721:201-201 722:202-225 727:226-444 725:445-468 726:469-665] [-1, 719, 720, 721, 722, 727, 725, 726, -2]
TTCCGATCTAAGCTAGATCTCACATAACCATGGTTACGACCTCGGCGCGATGCACAAGTTCACAAGCGAGCAT
GGTCTTAATGCGATATGAAACTACCACGGCAGGGGGATAACATGTTCCAAACAGATGATTCTGACGATCAAGC
GAGACGAATTATTTTTGGCAATTAACAAATCGACGTGATAGACTAAGAAGTGCCTATCCTATAAGCTCAACAA
CATCATCTAGATACGCGGATACAAGCTTGGCGCCATGCGATGTACCACCTTGCCGACAAGTGACAATACCATC
AGCATGGGACAAGCATCGCCTTGTCAGGTAAAGAACAAGATTACCATTAGTGAGGTCGGGGAGGTCGTTGGTT
TCTCCTGGTACTAGCAATGAGTATCAAAAAGACTTGAACTTTTCCTACGTTTGAATTTCGTACCCGCAAGCAG
CACATCTAAAAAAGAGCCTATTATCCAAACATAAGTACGGCGATTAGTGAGCGGTAAATAGCCGAGATATGAC
GATCTACGAACCGTAAAAAATGGCACCCTTTTGTACGATTCGCATGAAATCAGGGAGGATTGCAACCTGCCTT
ACCCTTCGCATGAAATCGGGGTATTACAACCTACTCAGTCCGTCCCGTTATTATTTATCTATTTTTTATAATT
TTAAATAAC
>TR223|c0_g1_i1 len=4258 path=[4462:0-823 4463:824-846 4475:847-847 4474:848-871 4473:872-2188 4455:2189-2212 4472:2213-3730 4458:3731-3758

4459:3759-4257] [-1, 4462, 4463, 4475, 4474, 4473, 4455, 4472, 4458, 4459, -2]
CAAGTCTCTATTTCGATATTTATCATAAGTCGTATCATAGGCATGTCCGTCCAATGTTAAGACAGACACGCTC
TAACACCCACATCCTCCATCTTTCAATTTCCGTCCAGCCACTTCAGTTCTAGTAAATTGATTACCGCCTTGGC
CTTTCTTGCTGCTCCCCATTCCATGTAAGCATCATGGGATCTCTTGATCATTTCCCGTGTCTTCTCCTTCAGT
CCAACATTTCCAAAGTAATGTGCAGCTAATTCACAAGCAAGAGCTGAATGATGAAAGAAACGGTGTTTCTGCG
CAGCTTCAGCTGAGAGGTAATAGGCTTGGGCAGCTTCGTTCGTCTCTCCTCTTGTATGATGCATCTCTGCCTT
CAAAAGTAAATACATATGTTCAAAATTCCACTCATTGATCTTTGAAAATTTCTCGAATGCAGCTACTACATTT
GTCCCCTTTTCCATCCAATGTGCCTCTCTTGTTTCCCGCGCCATGTGAAACGATACCAGTCCTGCATTAAACG
CTACCATGAATTGGTTTATTCTTACTTGGCCATTTTCTAGCATTTTGAAGAAGTCTGGATATTTGAGTATAAT
TTGAGAGGCTTGGGCAAAATTCCTCAAAGAGAGTGATTCTTCCAAAGATAACGCGATAGCAGTGGGGACGTAA
AGCATTTCTTTTTTAATACTGGCCGTTTCGACAATATCTTCGCTGCAAGTAGCTACTAGCTGAGGAAGAAGCT
CCGAAGAATCCAATGAGATACCACTTAAGGCGGATACAATTTTATAAATTGGTGCAAGGAAAAGCTGCAACAT
GGCGAAGTGACTTTGTTGTTTATGCTGATGGGCGAATGCATTAATTTCGTTCATGACCAGGGGAAGCTTTTTC
CCAGCTTGCCAAGACCTAGAAATGTAAGTGATTGTATTGATAGTTGAACTCTCAAAATGGCCGTTTGAAAATG
ACAGACGACATGCTCTGAGAAGAGGCTCGACAAGCGATTGAAGAGGTTTTTCCGAAACAAGGACATTTCCATA
AATAAATCCATATACACGTTGGATCATCTGATTGGCATCGTAGAACTCCGCCAATGACAGCGCGGTACGTGCC
AAAGCGCGGCCTTCATCTATATCTTGCAAGTGGTTTAGGAGAGTATTGCTAAATACAGACAAGGCAAATACCG
TATCATCGCAAAGACCGTATTGCAGAGAAATGCGTATCATTTTACAAGCCAAATACCCACTCATGAATGCATG
CTGCTGTAGGTAATACGTCACCCGCATTGCCAATAGCTTCATCGTCATCAACTTTGTACGATCCGTCATCTGG
TAATGATTGAGTAATATGTCCTTTTGACCGCCAGCTAGAAGGCCCCGGATGGACGCGAATTCTGTTATGGCAA
CTTGAGCACTGATCAAATTGGGGTCTATGTTTTCGCCAAGATGTTTTAGAAGGTCGTAGATCTTTTCGGTGGC
CCTTTCCATCGATCCAATTGCAACGACATTTATGAATACACAATGCGATTTGAACTTGTCCTCAAAAGATTTG
GCATTCGAGAGGACATGGTTGACCTGGATAACCACCTGTTCGTGATTTCCTTCTGAATAATTCGCCAACGCAG
AATTCTCAAACAGATTCAGACTTAGATCGTATTCATCTTTCCAATAATTTGTTCGAAGGAACGAAATTCCAGA
TTTGAAGTGCATCACCGCAGAGGAGAACTCCATCGCGGCCATGGACTTCTTTCCCGCCTCGAGATTCAGCCTG
GCAAATAGTTGACTCTGAGTTGGATTCTCGGATACTGCCTCGCTTCCAATCTTGTTGATTAGGTCCACCGCGA
CAAAGAGAAAGGAGTCCGATCCCCCCTCCAGCATGCAACAGGGGATCAGGCATGCTACTAGCTTCTGCAAGAG
TGGGACGCGATTCGCCACTGGGACAAGATCGAACGCCGCTTGTTGAATCAAGTCGTGAGAGAATGTGTATGTT
GGACCGGCTTTCTCGAGAAGACCCTCTTTTTGAGCTGTATCGAGAGCTGGCATCAGCGTTGTACCAGAAGTCC
CATCGTAATTCTTAACGGCTTCGAGGATTTCGACATCCACGTGAGACCCAAAGCAAGATAGAACCTGGACTCC
CGAGAGAGCGTCGTTAGATAAATTTTTGAGTTTAATGGCCAAAAGCTCTGCGACACTCTTGGATATGCCCTTC
AAATCAATAGCATCTACATCCCATTCCCATCCATCGACTGGGTTGTGGATGAGCAATTTCTCCGTCCACAATG
CATCCAGAAACTCGACCACAAAGAGAGGGACACCTTGGGTCTTGATATGGATAATTTCGGACAAGGATCTTGT
TTTTCGACGAGGAAGACACAGTGCTTCGGACACCATTTCATTTAGGGATTCCAGTTGGAATCCACAGAGGGAA
ATCTGGGTGGTGTTAATGGCATCAGATGCCTCAAATTCGCGTATCATGCCCTTCAGCTTGTTTTCTTCCACAA
TATTATCTGAACGATAAGCACCCGCAAAGAGAATTTTATTGGTACTGCTGTTATCCCCATCAAAGTTGGTGAC
TGCGCGATTGATGAGATCAGTTAGCAATTCTAAAGATGCAGCATCTGCCCATTGTAAATCGTCCACGAAAAAC
AAAACTGGATTAAACGAAGCCAACGTATCCAACAGCGTCCCAAAAAGATGATGCGTATGGAATTTCCCCTGCA
GCCCTCCGCTAATATGATCCAAAGAATCCCTCCTGTGGGAATACATGCTCTCGACAAGGCTGTGCAAACTCGG
CATTTGCTGCGACAGACACTTCAGCCCGTCCAATCCAATCAATGTTTCCAATCGCTCGCACACTTTCCTAGGA
CAGCTGGGTGGTCTCATGGAGGAATTCACAGCCTGTTCCTGAGTGGATCCCATGGGCATGGGGAAATTGCAAA
CCGCGTTGGACGAAAAGTACTCGTCCAACCCCAGGGCGATGACCGACAGAGGTTCTGGCTGGACCATCTTCCC
GAATTTACAGCGTAAAAATAGCCACCCCCGCGCCTGTAGCGGTTTGCGTATCTCCCTGACCAATCGACTCTTT
CCCGATCCCGCGTGTCCGTTGACGGTGATGACCTCCTTCTTCCTTCGGGCTACGGGATTGTTGCCACCATCGC
CATCGTTCGCCGTAGCACCAGTGGTAGTCTTGTTGTTATTGCTGTTCCCAACCCCATCCGAATTCTGAATCCG
ATCCGCCGCATCCATGAGCAACTGCAACTCCGTCTTCCTCCCAAACATGTGATTATTCCCAAATACCAATTCC
CACCTCGAATTAATCGATCCGAACAAAAACGCATCGGGAAAATCTACCATCTGCTGTATGTCAGACCGCACGT
CCGACAGCGAATCAAAGCAGAATCCGCCGCGATCATCACTCTCTCCTCCTCCTCCCATGATACTATTCGGCGA
TGCGTATACAACTTCGTTGAAATCTTTACTCGTTTTCATGCTTACAGAGGTGTGGAATATATCGGTTACGAGA
CGACATAATGGCATGGGGAGGCGATTGTTGTTGTTGTTGCTGTTACTACTACCACTACCAGTGCCACTCCCCT
CTCCCCGCATGGCGGATATGATGTGATCCATTTTACCATTCGATTCGCAACCGCCGGCGGTGGGGAGATTACG
GAGCATGTCCAATATGTCATCCTCATTGGGGATGGCGATGGATGACGAGTCGATAATGTTGCCGATAGAGGAG
GAGGTTGCATTGATCGAAGATGACGTGGCGTTGTTGGTTTCTGTGGGAACATCCTCCCCATCCATAAATAACT
TAAAGAGTAATTTCCCCAACGCTTCGAATATCAATCCCAACTGTTTCACCTTGGGATTGGACGCGAATAAGCT
ACTGCTACTGTCTTCATCCTCCTCGGTTAGATTTTGATCCCAATTAAAGTTTTTGTAGATGTCGAAGCCGACA
TTGATGATTTCGCCCGTGAGGGCATTTTCGGAGACGGTGATGCATTCCTCGTCGATGTCCTGGGGTTTGGGTT
CCCAATCGTCCTCAGTTTCGGCTGCATCGTCGAAGCTGTCGGCAGCACTATTGGTATTGTCGGATTTGGCGGT
GGTTGGGGTTGGAACTCGTTCGAAGCTTTTGGCTAAGTTGGTGACTAAAAAGAGAGCGATTTTGACGCATTTT

TGGATGTGCTGTTTCTTGCTGGGACTCATTGCCGTCGTCCCGTTGTTGTTGGCACCACCACCCCCAGCAAATG
TAGCAGTGGCAGCCGACGCAACCG
>TR224|c0_g1_i1 len=579 path=[687:0-156 700:157-216 702:217-282 693:283-306 701:307-385 703:386-409 704:410-410 698:411-578] [-1, 687, 700, 702, 693, 701, 703, 704, 698, -2]
ACTAACATTCAACACTTGACAACATTTATGCATTATAAATAAACTCCTATTGTATTGTATTCCCATATCCTCA
CTCCATCAACGTATATCAAGCAATCAATCCCTTCTCCTTCAATTTACCAAGCAATGCATCCACGCTCTCGACC
GTCTCCCCTTCTTTCCTCGGAGGAGGTTCATACACTTCCACCACTTCGTTCCTCGGATTACACACATCGTCCA
ATTCCAAACCTAATTCGGTCACCATATCCTCCAGAGAGATGGTTTGTACTGGCTTCTTCTTGGCCTTCATAAT
GTTTGGCATGGTGGGATAACGTGGGGTGTTGAGTCGTAGATCGCAGGTGATGATGGCGGGTTGCTGTGGATTA
TTGGTTGGATTCAGTTTGATGATTTCGGTCCCAGCGTCGGTCTCGCGTTCTACAGTAAAGGCTCCATCAGCAT
CTCTTTCAATTTTGGCAGCAAAGGTTGCCTGTGGAGCATTCAAAAAGCCAGCTAAAAGTGGGCCAGTTTGACC
ACAATCCGAATCAATACTCTGCTTCCCCAACAAAACCAAATCAACACCCCCCTCCTGCGTTGGCGATA
>TR224|c0_g1_i2 len=425 path=[687:0-156 700:157-216 702:217-282 693:283-306 701:307-385 703:386-409 704:410-410 684:411-424] [-1, 687, 700, 702, 693, 701, 703, 704, 684, -2]
ACTAACATTCAACACTTGACAACATTTATGCATTATAAATAAACTCCTATTGTATTGTATTCCCATATCCTCA
CTCCATCAACGTATATCAAGCAATCAATCCCTTCTCCTTCAATTTACCAAGCAATGCATCCACGCTCTCGACC
GTCTCCCCTTCTTTCCTCGGAGGAGGTTCATACACTTCCACCACTTCGTTCCTCGGATTACACACATCGTCCA
ATTCCAAACCTAATTCGGTCACCATATCCTCCAGAGAGATGGTTTGTACTGGCTTCTTCTTGGCCTTCATAAT
GTTTGGCATGGTGGGATAACGTGGGGTGTTGAGTCGTAGATCGCAGGTGATGATGGCGGGTTGCTGTGGATTA
TTGGTTGGATTCAGTTTGATGATTTCGGTCCCAGCGTCGGTCTCGCAGATCGGAAGAGCA
>TR225|c0_g1_i2 len=1210 path=[1267:0-48 @1273@!:49-172 1265:173-205 1271:206-828 1259:829-852 1260:853-1209] [-1, 1267, 1273, 1265, 1271, 1259, 1260, -2]
CGCTCTTCCGATCTTTTTTTTTTCAGCTGTTATCAAATAACTTAGATTTATGAGAGTACCATAGTAACATAGT
GCCTGATAAAATGCCTGGATTAGATACATCTGGGTCTCTAAGACATTGTTCATTTTTGAAAAAATTGAAGCAC
CAGACTACTCAGAACAGGTGTGATCTGTGACTGTTGTCTGTGATTTGTACAACAATGCGCTGAAAGGGACGCT
CTATGGGTTTGTGCCCGATTCTAGACTCCTGCAAACAAGATGGTCAGCTTCCTCCTAGAATTAGAATGCCAAT
CCTGCGATACTATTTCCTGTCGAACTACCAAATAAATAAATGGATGTACAAGGTGTGCCGACTGCCGACTCGT
ATCGGTTCATGGCGAGGTCATATGGAACCATAACCACCTATGAACTGAGAGATCATAGTTGGACCAATCAATG
CTTTAATGCTTTGATGGGGTAGTAAAGAATTGGACCTTCTGAATAGACGTAGTCGATCGCCTAACGCCATCTC
GGGTCGGACGGGAGCTACTTTGATGATTGTGTTGGCTCGAGGAACGAAAGGCCTTCTGAGCTAGATCTGGTGG
GACTGGTACCACGAAGAATGATCGAATCCTTCCATTGCAAGTTTTGTACAGCCACTTCGATCTTGGAGGTACG
GCTGTTCGTAGAGTGGTGCCATTGGGATAGCGTACCTTCACTCTCTGAGCAGGTTCCAATTCTGGTGGGATGA
AGACTCTCATCATTTCCTTGCTTTTTTCCCCTCGGGTTTGACAACTTGGATGGAATCGTTTGCTCTTGTTCTT
AAGGTCTGAATGTTCAGCCGATTGTGAATAAGGCAGAACAGATTCTTCGTCGGAGGGCACGATGCATGGAATG
CAAGCTGCTCCATTTACTTTGAGAAATGCATCTTCAGGCATAACGGGAGTACACGAAGGACGGATGGACACAT
CGTCTGAAGTAACATTGGTCGATTTATCAGTCTTTTCACTCTGGAGAAGGACAGTCCAGTTGCTATCGTGACG
GCTTCCTACGATCTTTTCCTGGCCGACGAATTTACTTTTCAATTGCTCGATCATGGTGATTTTTATTTGACGA
AGAGCGTTTTGAGTTTACTGAGTTGTGTGTTGTGGAAGCTAGAATTAATTCTTTGGAGAGACTTTGGTTTTGG
ACTGAAATAATGTGAAGGTTCATTTATGTTTTTTATGACAAG
>TR226|c0_g1_i1 len=1483 path=[1535:0-38 1549:39-39 1548:40-63 1547:64-555 1533:556-579 1546:580-726 1540:727-1482] [-1, 1535, 1549, 1548, 1547, 1533, 1546, 1540, -2]
TGTGCTCTTCCGATCTCTTATCTCAAAAGTGTGTCCATACCTAAAAGTAAGTAGGTGCAACCTACAACGTTGA
TTCTTCTCCAGCCTGCAACGATGCCAACCATCAAGTTCGCCTACAAAGGAAAAACGGCATACGTCGACGACAT
ATCCCACGAAACAACATCCGCCGACCTCCTCGACGAGGTCCGCAGCTGCTACAAAGTCAAGAAGGGCTACACC
CTCCACCTCCTCTTCAAGGGCAAGAAGATCGCCGAGGAGACGCTCAACAACGACGGCGACATTCTCATCCGCA
ATCATCCCGCCTTCGACGACTCGGTGGACCTGACGCTGGGCGCCACAGTGAACGTCGTTGGGACCAACGCCGC
CAAGCAAAAGTCCAAGCCGAAGAAGGACCCCAACAGATTTCTGGATGATGGCATATGGGGCACCTACGATGGA
GATCTCGACGACCATGGTCAGCGTCATGGACAGGGTAAGATATCCTGCGAGGACGGCGAGGTATACGAAGGCG
AGTGGTCGAACAACAAGCGTCACGGTAGAGGTACGTGTCATATGACCAATGGCGCTATCTACGAGGGCGAATT
CAATAAGGGCAAGAGGGAGGGATGGGGTAGATATAGCTGCACCAACGGAGACATATACGAGGGCGACTACAAG
GGACATTTACGGCACGGTTATGGGTCTTACACGAGGAGGGACGGGAGCAGGTTCGAGGGTGAGTACGCCATGG
GGACGGCGAATGGGAGGGGTACGTTTCGCTGGCAGGATGGGATGGTCGACGTGGGCAAATATAAGGGGGAGAA
GGACGTTGTGACGGCCACGGGCGTGGGGGTGCGATGGAGTATGGACCGTGGGACCGCGTGGTCCTTGATGAAC
GGGTCGGTCGTGAAGGAGATTGGGCTACTGGAGGCGAGGAAGGTCGCGGCTCAAATGGGGTTTTGCGATGTGC
CTCCGTGAAGAGACTACATGTTTAGCATTCTGAATATTCAAATCTTCTTCTAGGTGTTGCGATGGATGGCAGG
AGATGTTAAACTATGGGTGGTGTTGCCTTCTCGTTGCATGCACGCATTCAGCAAAACTCGCCCTCGATAAATA

AGTATTCAAGCGAGGTGCTCGGAGTTTTCTCTCTCTCTCGCACTCTCTTATTATTTGTCAAACACAGTGCCTA
GTGTCAAACAGAAACTATGTATGAGTATAATTTTGTGAAGTAAAGATATTGTACATTTTTATAGTGGCGTGAT
GTTGTTTAGCAAATAATGGCGCCCAGATGAACATATTTTTCCATTTTGCACGTCACTCCTAACTCTTTAGTGC
CTGGGACAGTCATGTGCAAATAAATGTATAAACATAGCTATGGTCGGATGATTTGGCCAACTCAAGGTACATT
GGTAAGTGCGCTGAATTAGTCATATATGATGGGTTGGCAATGTGTTTTGATGGTTGGACCATAATACAAATCC
CGTTTCAGGCCAAGATCTAGGTG
>TR227|c0_g1_i1 len=1114 path=[1173:0-61 1174:62-85 1177:86-147 1168:148-171 1176:172-924 1171:925-948 1172:949-1113] [-1, 1173, 1174, 1177, 1168, 1176, 1171, 1172, -2]
TCTTTTTTTTAAACACCCAACTCTTTTGTTTAGATTAGATCTCTCTTATACCGTTTTGATTGAATAGCCATGT
TTAAATCTTTGACTTCGGAATAGTTGCACTTGAATCTTTGATTATTTGACTCAAATCTGCCATGAATTCTTCT
CGTGCCGAATTGTGAATGCTATGAGTACCTTCCGAGTATGTCTTTATTGACAGAGGAACGTCCTTTCCTTCCA
TGATTTGTCGCATACTTTCGATATTTTCTTCATCGCAAACAGTTCCAATGCCAGCCACCATCAAATGTATTTT
CGTACTGCCAACCTCTTTGTCAATGTTCATCTTTTGTTGTAGACTTCTAGCAATGGCAGCCCAAGAATCATCG
CCTACATTTGAATTAAAAATTGTACGGTAACACAATGCTCTAAATGCAGGATTCACATCACTCCAAAAATTCC
CCTCATCCTCGTATATTCGCCCTTCACCAATCCATTTGTTGTACATCTCAGAAGGATAGCCAATATTCCCTAA
TTCTTTCTTAACTCCATCCAAAGTATCATGACGCCTCGAGAATGCAATCGCCCTCTTCTCGTCAAAATTTCGT
ATAAAGTTTTCACTCCTACGCCTAATATCCATATCCTCAACCACCAACGCCGATAAATCCTCTGGATATTTCG
CCGCGTAGCACATGGCCACTCGTCCACCCATGCTGTGACCCACAAGTACGAAAGGTTTGTCACGTAGCAAGGG
ATGGGATTTGGAAAGACTTTTCACATCTTCGACTAATGCATCGGGAGAGAAATTGTCGGGATCGCCTAGGTCG
GACCGACCGCAGCCTCGGCAGTCGATGGCGACGGATGGCGTGGCAAGCGACTGTTGTACGCCTTGCCATGTCT
GAGAAGAAGAGTCTAGTCCATGGATAAATAGGGTCGTGGGACCATTGTCGTTTTTGCCTTGGCTTTCAACGTA
TGATATTGATAGATGGGCCGAGGAAATGATGCTACCGGACATTAGGTGAAAGGATGAGGTGGATGGTAAATAT
GGTATTGTCATTATTGCTGCGGAAAGGCAGGCAACAAATGCTTTCATCCTTTGATATGGGGTGAGAGGTGAGA
GGTCGAAGGGACACAGAAG
>TR228|c0_g1_i1 len=1101 path=[1476:0-55 1493:56-193 1464:194-236 @1489@!:237-457 1449:458-490 1488:491-505 1484:506-511 1494:512-529 1492:530-580 1482:581-827 1442:828-846 1443:847-885 1444:886-1100] [-1, 1476, 1493, 1464, 1489, 1449, 1488, 1484, 1494, 1492, 1482, 1442, 1443, 1444, -2]
TCGCGAGTGGGAGTGGAATGTTGTTGATGGTGTTGTTGTTGTTGTTGTTGATGATGTAGCTGTTGCTCCGCTG
TCGAGGAGGATGACCCGCGATGACCCTCGGTAAGAAGGTCCCCTATCGACGAGTTGTTGGAGAGAGTGCCGAA
GGCTCCGAACGATGCAATGGGACCCGACACTCCGAATTGAGCAAAGCTGGATAGGCTATTATTGTACATGAGC
CGCGACCGTTTGCCCTCCTCGTTATTGTCCTTACAGTCGTCGACGACGCTCCCCATCGCCGACGAGACGTCGC
TCTTGCGCTTCCTCGCAACGACGTCGCCGCTCGCTCCGGTGGAAACGGAAGAAGTCTCGTTGAAGAAGTGATC
CATGTTGCCGTAGTCCCAATCCACGGGAGATGCGTTGTACGCGATGGGGGAGGTGCTGTATCCGTGCTGGTCG
CGAGTGGGAGTGGAATGTTGTTGATGGTGTTGTTGTTGTTGTTGTTGATGATGATGGTTGGCATTGTCTCCCG
TGGTGCCCCCCGGGGTGATGGCAAAGCTGTGACTGTGGGAGAGTTCGTAGCTTCGTCTTCGTGGATCTAATAG
TGAATCCTATGGGAAGAATACATGAGCACACGTGATGGTGTTTGTGTTTCAAGTTATTCATCAGTTTGTCAAG
AGTTCAATATATAAAGTGCAATCACGTGACATGCATGTCAACGAAAAATGGACCATGGAGAGGCAAAGCATGC
AACGCACAAGGCGGAACAGAGCATGCAAACATGTGTGAGTGCATTGATTATACGCGTTTGAATCAATGAGAGA
ATACCCGTCCGTGGGCATTCAGCAAATACATATATGGCGGATAATTCGTAACCTTTGCTCGTGTGCAGTTTCG
TATTTACTCAAAAAGTGGTTTCAATAAAGCGATGTAACACTTCACCAGCAATGATACACGCGTAAAAACCTTT
TGCCGAATGCGAGTACAAAAATCATCGCACTCTCGCTATACCGGAACAAAATGATCGGAAGGCATTCGAGAGA
GAGTGCGTGCATTAGGTTTTCGTAACAGGAAGCATATACATGCCACTGCCTAACACCGCCGAAACTGGAGAAT
CGATTT
>TR228|c0_g1_i2 len=1086 path=[1476:0-55 1493:56-193 1464:194-236 @1489@!:237-457 1473:458-496 1494:497-514 1492:515-565 1482:566-812 1442:813-831 1443:832-870 1444:871-1085] [-1, 1476, 1493, 1464, 1489, 1473, 1494, 1492, 1482, 1442, 1443, 1444, -2]
TCGCGAGTGGGAGTGGAATGTTGTTGATGGTGTTGTTGTTGTTGTTGTTGATGATGTAGCTGTTGCTCCGCTG
TCGAGGAGGATGACCCGCGATGACCCTCGGTAAGAAGGTCCCCTATCGACGAGTTGTTGGAGAGAGTGCCGAA
GGCTCCGAACGATGCAATGGGACCCGACACTCCGAATTGAGCAAAGCTGGATAGGCTATTATTGTACATGAGC
CGCGACCGTTTGCCCTCCTCGTTATTGTCCTTACAGTCGTCGACGACGCTCCCCATCGCCGACGAGACGTCGC
TCTTGCGCTTCCTCGCAACGACGTCGCCGCTCGCTCCGGTGGAAACGGAAGAAGTCTCGTTGAAGAAGTGATC
CATGTTGCCGTAGTCCCAATCCACGGGAGATGCGTTGTACGCGATGGGGGAGGTGCTGTATCCGTGCTGGTCG
CGAGTGGGAGTGGAATGTTGATGATGATGTTGTTGTTGATGGTTGGCATTGTCTCCCGTGGTGCCCCCCGGGG
TGATGGCAAAGCTGTGACTGTGGGAGAGTTCGTAGCTTCGTCTTCGTGGATCTAATAGTGAATCCTATGGGAA
GAATACATGAGCACACGTGATGGTGTTTGTGTTTCAAGTTATTCATCAGTTTGTCAAGAGTTCAATATATAAA
GTGCAATCACGTGACATGCATGTCAACGAAAAATGGACCATGGAGAGGCAAAGCATGCAACGCACAAGGCGGA

ACAGAGCATGCAAACATGTGTGAGTGCATTGATTATACGCGTTTGAATCAATGAGAGAATACCCGTCCGTGGG
CATTCAGCAAATACATATATGGCGGATAATTCGTAACCTTTGCTCGTGTGCAGTTTCGTATTTACTCAAAAAG
TGGTTTCAATAAAGCGATGTAACACTTCACCAGCAATGATACACGCGTAAAAACCTTTTGCCGAATGCGAGTA
CAAAAATCATCGCACTCTCGCTATACCGGAACAAAATGATCGGAAGGCATTCGAGAGAGAGTGCGTGCATTAG
GTTTTCGTAACAGGAAGCATATACATGCCACTGCCTAACACCGCCGAAACTGGAGAATCGATTT
>TR228|c0_g1_i3 len=1104 path=[1476:0-55 1493:56-193 1464:194-236 @1489@!:237-457 1449:458-490 1488:491-505 1484:506-511 1494:512-529 1492:530-580 1490:581-826 1486:827-845 1481:846-888 1462:889-1103] [-1, 1476, 1493, 1464, 1489, 1449, 1488, 1484, 1494, 1492, 1490, 1486, 1481, 1462, -2]
TCGCGAGTGGGAGTGGAATGTTGTTGATGGTGTTGTTGTTGTTGTTGTTGATGATGTAGCTGTTGCTCCGCTG
TCGAGGAGGATGACCCGCGATGACCCTCGGTAAGAAGGTCCCCTATCGACGAGTTGTTGGAGAGAGTGCCGAA
GGCTCCGAACGATGCAATGGGACCCGACACTCCGAATTGAGCAAAGCTGGATAGGCTATTATTGTACATGAGC
CGCGACCGTTTGCCCTCCTCGTTATTGTCCTTACAGTCGTCGACGACGCTCCCCATCGCCGACGAGACGTCGC
TCTTGCGCTTCCTCGCAACGACGTCGCCGCTCGCTCCGGTGGAAACGGAAGAAGTCTCGTTGAAGAAGTGATC
CATGTTGCCGTAGTCCCAATCCACGGGAGATGCGTTGTACGCGATGGGGGAGGTGCTGTATCCGTGCTGGTCG
CGAGTGGGAGTGGAATGTTGTTGATGGTGTTGTTGTTGTTGTTGTTGATGATGATGGTTGGCATTGTCTCCCG
TGGTGCCCCCCGGGGTGATGGCAAAGCTGTGACTGTGGGAGAGTTCGTAGCTTCGTCTTCGTGGATCTAAGAG
TGAATCCTATGGGAAGAATGCATGAGCATACGTGATGGTGTGTGTGTTTCAAGTTATTCGCCAGTTTGTCAAG
AGTTCAATATGTAAAGTGCAATCATGTGATATGCATGTCAACGAAAATTGGACCATAGAGAGGCAGAGCATAC
AACACAAAGCGGAAACAGAGCATGCAAACATGTGCCAGTTCATTGATCATTCGCATTCGAATCAACGAGAGAA
CACCCGTCCGTGGGCATTCAGCAAATACATATATGGCGGATAAAATTCGTAACCTTTGCTCGTGTGTGCAGTT
TCGTATTTACTCAAAAAGTGGTTTCAATAAAGCGATGTAACACTTCACCAGCAATGATACACGCGTAAAAACC
TTTTGCCGAATGCGAGTACAAAAATCATCGCACTCTCGCTATACCGGAACAAAATGATCGGAAGGCATTCGAG
AGAGAGTGCGTGCATTAGGTTTTCGTAACAGGAAGCATATACATGCCACTGCCTAACACCGCCGAAACTGGAG
AATCGATTT
>TR228|c0_g1_i4 len=1089 path=[1476:0-55 1493:56-193 1464:194-236 @1489@!:237-457 1473:458-496 1494:497-514 1492:515-565 1490:566-811 1486:812-830 1481:831-873 1462:874-1088] [-1, 1476, 1493, 1464, 1489, 1473, 1494, 1492, 1490, 1486, 1481, 1462, -2]
TCGCGAGTGGGAGTGGAATGTTGTTGATGGTGTTGTTGTTGTTGTTGTTGATGATGTAGCTGTTGCTCCGCTG
TCGAGGAGGATGACCCGCGATGACCCTCGGTAAGAAGGTCCCCTATCGACGAGTTGTTGGAGAGAGTGCCGAA
GGCTCCGAACGATGCAATGGGACCCGACACTCCGAATTGAGCAAAGCTGGATAGGCTATTATTGTACATGAGC
CGCGACCGTTTGCCCTCCTCGTTATTGTCCTTACAGTCGTCGACGACGCTCCCCATCGCCGACGAGACGTCGC
TCTTGCGCTTCCTCGCAACGACGTCGCCGCTCGCTCCGGTGGAAACGGAAGAAGTCTCGTTGAAGAAGTGATC
CATGTTGCCGTAGTCCCAATCCACGGGAGATGCGTTGTACGCGATGGGGGAGGTGCTGTATCCGTGCTGGTCG
CGAGTGGGAGTGGAATGTTGATGATGATGTTGTTGTTGATGGTTGGCATTGTCTCCCGTGGTGCCCCCCGGGG
TGATGGCAAAGCTGTGACTGTGGGAGAGTTCGTAGCTTCGTCTTCGTGGATCTAAGAGTGAATCCTATGGGAA
GAATGCATGAGCATACGTGATGGTGTGTGTGTTTCAAGTTATTCGCCAGTTTGTCAAGAGTTCAATATGTAAA
GTGCAATCATGTGATATGCATGTCAACGAAAATTGGACCATAGAGAGGCAGAGCATACAACACAAAGCGGAAA
CAGAGCATGCAAACATGTGCCAGTTCATTGATCATTCGCATTCGAATCAACGAGAGAACACCCGTCCGTGGGC
ATTCAGCAAATACATATATGGCGGATAAAATTCGTAACCTTTGCTCGTGTGTGCAGTTTCGTATTTACTCAAA
AAGTGGTTTCAATAAAGCGATGTAACACTTCACCAGCAATGATACACGCGTAAAAACCTTTTGCCGAATGCGA
GTACAAAAATCATCGCACTCTCGCTATACCGGAACAAAATGATCGGAAGGCATTCGAGAGAGAGTGCGTGCAT
TAGGTTTTCGTAACAGGAAGCATATACATGCCACTGCCTAACACCGCCGAAACTGGAGAATCGATTT
>TR229|c0_g1_i1 len=1349 path=[1547:0-82 1548:83-106 1557:107-527 1551:528-548 1556:549-878 1538:879-902 1553:903-1032 1545:1033-1056 1546:1057-1348] [-1, 1547, 1548, 1557, 1551, 1556, 1538, 1553, 1545, 1546, -2]
GCAGAGAGAGCACGATTTGATCCAGCTGACAAAAATATTGTATTCACTAAATTATGTATTTTTGATCTTTAAC
CTGTTAAGAAGTAGTTCTTGCCCCAGCTCTCATTCATTACTCACATATTGGTCATATTTGTTTGCCCACACCC
TGAGTTTGGTGATACGGAACTCTATATAGTATTTGCAATGCACCTGAGTTGAAAGCACAATCTACCACCTATA
GTGAGGCATTCGTCATACTACCACCAGGATTCAAGGCATATTTCATGTCAGGTCCGTTACTAGGAGGCTTAGG
ATCATGCCCATAACTGGGAGGCATCGGATAATCTCCACCCATTTGAACCATAGGACCACCGGGCATCTGACCC
CCTCCCGACTGGACCCACTGCGCACCAGCTCTGAAAGCATCCCACAATTGGTACTGCATATTTGGATTACTCG
GCGCGGCAGGGGGCATCATGTAGCCACCACTTGACGGGGATTGGATGGGCTTCGCTGATGCATCTGGATATCG
AATTTGTTCGCACCACTTGACGGTGATCCTCTTGGATGAAATTGTAGCAAAGAATGTTTTTGACTCATGAATT
ACAGTTATATCATCACTTTTGTCATTATCATCCCCAACCGCATCATCAGGGCCTCGCCTCGGTTTTCCATCTG
CATTTTCCTCTGGCTTCCTTTTCTTCTTCTTGGAAGGCCGTAAATTATTATCATCCTCTTGCTTCAACACTAA
TTTCACATAGATAGATTGATACGGGGGCGTCGAATGTTTCAACGTATTCCTACAATGATCCCTGGTGTAATTT

TTCTCCTTGCAGACTGGGCAGATCGGTTCCATCTTCATGGAACCATCCGCCGGCCCACGGCAAAGTTCCGGCA
TATGTTGCAGCTCGGCGACCACCGGAACGTCGGCCAGGATGAGAGCGCCGTCTTCTCCCTTTATCAAAACCTC
GTCCGTGAGTGTCACAACAACGTAAGTCATCTGCCACGGCAGAGCCTTGTGTCCATCCCGCTGACGGCAAAGT
TCGCGACTTCGATAATTTTTCTTGCATCGATCGCACACGGGCAGTCTTTTCTCTGGGAACGGCACATCAAAAG
GCTGGGGCATTCCGTCGGGGTGCACCGTGCAAACTTGCGTCACGGACTTCTTCGGGGAAGGCGCTACCCCCCG
AGGCATGGCGTACGGTTGAGGTTGAGGTGGTGCCATCATGGGCATTGTGCCGCCTCCTCCTGGCATTGGCATG
GGGAACATCATCATGGGCTGAGGGGGGTAGGAAAATGCGGGTGGCATCTGATAGCCGTAGCCCATACCCGGGG
CTGGGGCGGACATGGCGCTCGGACCCATCGGGGCC
>TR229|c0_g1_i2 len=1436 path=[1547:0-82 1548:83-106 1557:107-527
1541:528-542 1542:543-566 1554:567-635 1556:636-965 1538:966-989
1553:990-1119 1545:1120-1143 1546:1144-1435] [-1, 1547, 1548, 1557, 1541,
1542, 1554, 1556, 1538, 1553, 1545, 1546, -2]
GCAGAGAGAGCACGATTTGATCCAGCTGACAAAAATATTGTATTCACTAAATTATGTATTTTTGATCTTTAAC
CTGTTAAGAAGTAGTTCTTGCCCCAGCTCTCATTCATTACTCACATATTGGTCATATTTGTTTGCCCACACCC
TGAGTTTGGTGATACGGAACTCTATATAGTATTTGCAATGCACCTGAGTTGAAAGCACAATCTACCACCTATA
GTGAGGCATTCGTCATACTACCACCAGGATTCAAGGCATATTTCATGTCAGGTCCGTTACTAGGAGGCTTAGG
ATCATGCCCATAACTGGGAGGCATCGGATAATCTCCACCCATTTGAACCATAGGACCACCGGGCATCTGACCC
CCTCCCGACTGGACCCACTGCGCACCAGCTCTGAAAGCATCCCACAATTGGTACTGCATATTTGGATTACTCG
GCGCGGCAGGGGGCATCATGTAGCCACCACTTGACGGGGATTGGATGGGCTTCGCTGATGCATCTGGATATCG
AATTTGTTCGCACCACTGATCGAAAGTAAGAAAAGAATAGCAGAGAGGTTTAAAAGTGCACCAGAGATTAGCC
CCTCTACAACTTGGTCTTCCTGGACTTACCTTGACGGTGATCCTCTTGGATGAAATTGTAGCAAAGAATGTTT
TTGACTCATGAATTACAGTTATATCATCACTTTTGTCATTATCATCCCCAACCGCATCATCAGGGCCTCGCCT
CGGTTTTCCATCTGCATTTTCCTCTGGCTTCCTTTTCTTCTTCTTGGAAGGCCGTAAATTATTATCATCCTCT
TGCTTCAACACTAATTTCACATAGATAGATTGATACGGGGGCGTCGAATGTTTCAACGTATTCCTACAATGAT
CCCTGGTGTAATTTTTCTCCTTGCAGACTGGGCAGATCGGTTCCATCTTCATGGAACCATCCGCCGGCCCACG
GCAAAGTTCCGGCATATGTTGCAGCTCGGCGACCACCGGAACGTCGGCCAGGATGAGAGCGCCGTCTTCTCCC
TTTATCAAAACCTCGTCCGTGAGTGTCACAACAACGTAAGTCATCTGCCACGGCAGAGCCTTGTGTCCATCCC
GCTGACGGCAAAGTTCGCGACTTCGATAATTTTTCTTGCATCGATCGCACACGGGCAGTCTTTTCTCTGGGAA
CGGCACATCAAAAGGCTGGGGCATTCCGTCGGGGTGCACCGTGCAAACTTGCGTCACGGACTTCTTCGGGGAA
GGCGCTACCCCCCGAGGCATGGCGTACGGTTGAGGTTGAGGTGGTGCCATCATGGGCATTGTGCCGCCTCCTC
CTGGCATTGGCATGGGGAACATCATCATGGGCTGAGGGGGGTAGGAAAATGCGGGTGGCATCTGATAGCCGTA
GCCCATACCCGGGGCTGGGGCGGACATGGCGCTCGGACCCATCGGGGCC
>TR231|c0_g1_i1 len=1727 path=[1878:0-323 1879:324-347 1893:348-804
1876:805-828 1894:829-1039 1882:1040-1056 1892:1057-1468 1888:1469-1492
1890:1493-1607 1873:1608-1631 1874:1632-1726] [-1, 1878, 1879, 1893,
1876, 1894, 1882, 1892, 1888, 1890, 1873, 1874, -2]
CAGATGATGGCCAATTTTGTTCGTGTTTGAGAAGCCTAGCCGCCAGCCGACGAAATAATAGAGTCAATCAAGC
AGTTTCAGTTTTAGAGAACACCACCACGCTCTCTAGAGATCAATATGATGGAGTAGATAGTAGGTAGAGTAGA
TTTTACTGTTCCGAGGATCCAACACCAACAGCAACTCTCGAATTCGATTCGAGAAGTTTAGACTCCATCACCT
CATATTTCTGGTATTTATATCATTCGTAAAGATAGAAGATAATCAAAACTTCTTGTTGCCTGCCTTCTCGCTT
CCCACTTTCCAAAACATAACCACAAACTAGATTGAAATCATGAATTCTTCCAGCGCTGCTCCCCTCTACGACC
TCGGCCTCAACCCTCCTTACCAAATCAATTACCGCGCCAAACGCGCCGGGAAACGCATTTCGGCTTCCAAGCG
TCGCATCACCTTCCAATTCGGCTTCTCGTCCGCCTCCGCCATTGCCCGTGGTCTCGCCGAGAAAGAATGCCGC
GGCTGCGAACACGAGATCGTCCTCGTCTGGAGCCACATGACCGGGAAGCGCCAGCTCTTCATGGACTCGAAGG
AGATTCACATGTCCAAGGCGGCTCGTGGGAACACGAGGTTTGAGTACTCGTGGAGTACTTTTGGGAGCCACAC
GATGAAGATTATCGCCAATGGTACGCCGCCAAAGCAGAATAGCGGAGAGAGGCAGTTTGATCTGGAGTTGGAT
GGTATGAGCTTCTTTGAGTTCAGCAAGATCTACCAGTTGGGTCGCAGTAAGAGTGCGACGAAAAGGGCAAGCA
TCGAACCTTCTCGTGCCTATTCCTATCGTGGTGTGGCATATGACGCACGTGACGACGATGAAGAGGACGACGA
ACTTCCCATGCCGGAGGCCAAGATAGACCTCTTTTCACAGCCATCACCAATGCTCGTCTCTCCCATGTCGAAT
TACAGCTCCGTGCCATCACTCCTGGATTCCTCCACGTCGTCCACTGGTTCCTACGATGAGTTCATGCCAGTTG
AGTACAATAGCCAAAAAACGTTTGAATCTGTCTCGAGTCAAATTATGCACGCCTACAATACCAAAGCTCCCGA
TGCAGACATGCCTCGTTCGGCCCCAAGCAGCTCTCGCGCACTGATCCCCGCAAGCGAAGAGGGCATGGATCAA
ATCTCCAAGTCCATGAAGAATCTCGTCAATCTGGACGACATTACCACCACGGCGTTCCAACCAATCTCGCCGA
AGTCTCCTTCTCTCGTCGCCATGAAGAGAGGAGAGGCCAACTGGGGCCTTGCTGGCAGGGCTCCCACGCTTTC
GGAGATGCGAGATTCCGTCGCGCAGAGTCCTACTGCTTTGGTGAGGGAGGCGATGAACGCTCGACCCATGCAG
CCTATTGCACATCTACCGTATCAGGTCGCAGTTTCTCCAGAACAAGATGGTTATTATGGAGCACCCCGTACGA
ATATGGGATGTGCACCTCATTACTAGACTAGAGAATCGCGACAAAAGGTACCCACCAGAAAAGACTTGCTGGA
CATAATAAAGAAGATACACCTGTGGCAAGAGCTATCTACACAATTAGAACACTTATATGAGACTGCACAGAGA
TCTTCGGAGCGACTGGGAATATTGCCAACAGATAGAGATGTACCAAATGCTCCGAGAGATATAATATTATTAG
AGAGTTAAAATAAGACAGGTAAAACTGAAAAAAAAAAAAAAGATCGGAA

>TR232|c0_g1_i1 len=985 path=[1156:0-80 1157:81-104 1161:105-143 1153:144-167 1164:168-187 1163:188-211 1160:212-545 1139:546-569 1140:570-572 1141:573-596 1162:597-762 1150:763-786 1159:787-882 1147:883-906 1148:907-984] [-1, 1156, 1157, 1161, 1153, 1164, 1163, 1160, 1139, 1140, 1141, 1162, 1150, 1159, 1147, 1148, -2]
CGATCTTTTTTTTTTAATGTGAATAATGGTGTATTTATAGATGGATATAGTGCACTTCTTGACAGAATGGGAA
CCGACTATTATAATTAAATAGAATGGAGGAGTATGAACAGGAGAATCGATTGAATGCATTGTGGTTTCCTCCT
GCATCTTCAAATGGGTCTCTGGGAGCATGCTCCTTCCCCTTTCCCCCATCCCACACCACTTGCCAGTCACTTG
TAATAGTACTATGAAACACTTTATGCACAACATTTGCATCTTCTATTGTATCAACTAAAACATACTAATGATG
ATTGCTACACCCATCAAAATTTGAAGGGGCATTGTATAATGTAATCTATTTGAGATAGAAAGGTTGTGGATAT
CTTGTATGAAAGCAGATTGCCGCATATCATGTGCATGTTGTGGGCTCGGTGTGATCGGATTCCCAGTTGGCAC
ACCAGACTGTATTTCTTTCGTTGGACTTGCAGTAGGAGATATTGTACTATCATATTCATCCACCGTTTCATAC
TTCGCCTTATCTACACACCATGGATCAAAAAAGGAATATTCAAATCCTTTATCGTCCAAATCCGACGGTCGAA
AGTTCACGGGAACAGTACACTCTTGCGTAAAAAGGCACATCTTCGCATCGCAGCTACCAGTGGTCAATCCACC
ATTCGTATGATTGTAACGACAAACGACTAGCGAATTTCCGCTCGTTTTTACGTGGTTTTCTTCACACTCGCAC
AGGACAAATGATGCGATGTCTATGCCATCATCACGTGGGTATGATGGGAGGACCGTCGCGCAGAAGCCTTCGT
GGTCGAAATGGGCGAAGGCGTGAGGGATTATGATGGATGGAAGGGCGAGTAGGAAGGAAATCGAGATGCGGTT
CATATTATTTCTAAGTTACTTAATTGGAAATTTTGACCGTCGGCAGTGTTATTGGGCTGCCTGTCTTGCTACT
TGCCAAGTCTTTGCTTTCTGCCAAGAGATCGGAAGA
>TR233|c1_g1_i1 len=570 path=[705:0-204 722:205-223 728:224-228 730:229-280 729:281-304 731:305-315 724:316-342 720:343-405 689:406-429 690:430-569] [-1, 705, 722, 728, 730, 729, 731, 724, 720, 689, 690, -2]
AAATAAGATACATTCAATTTAAGTGAGCCTAAATGACCTCTTCAATGAAGCATGCTCGGCACGCGCTGCTGCC
AACTTACTCCCTCCTCCCCACATCTCTGGTCTCTGAATATCTTCTGTCAAATCGCTTGAAACTTTTGGAATCC
ATCACAGCGAAGAAGATGGTCAAGCCGGCCATTCAAAGGAAGAGGATATCCTCCTCTGCGTTAGACCAGCTCA
TCTCGCTGTACGGATTCTCGACCGCGGCCGGACTGTCGCCGACGAACCGCATCCTCTCCAACATCACGCCACT
GGCCGGTGGATTCAGCGGAACCAATTACCGAATCGACTTCTCCGACGGCACAAAGTTCTGTCTGAAGATTTGC
CACGGGTACGACCGACCGTTCGTCGAGTCCCAGGCCAGAGTACAGGATCATCTGCGTCAGAGTGGTTTCGAAC
GGGCGTGCTTTGCCATTCCGCTACTACTTGTCAACAATGACGGCATCCAAAGCGGCAGAGATGACGATTATGG
CGACGACGACGACGACGACGACGATGATGATGATGAATTTCGTTTCGCCGCAATGGATC
>TR233|c2_g2_i1 len=1524 path=[2202:0-95 2203:96-122 2237:123-275 2171:276-299 2227:300-315 2251:316-339 2248:340-621 2253:622-624 2234:625-648 2250:649-674 2252:675-687 2247:688-744 2249:745-762 2239:763-831 @2245@!:832-996 2221:997-1053 2238:1054-1074 2229:1075-1098 2220:1099-1320 2219:1321-1523] [-1, 2202, 2203, 2237, 2171, 2227, 2251, 2248, 2253, 2234, 2250, 2252, 2247, 2249, 2239, 2245, 2221, 2238, 2229, 2220, 2219, -2]
CATCTCACATCTAAACCCCGCCCCCCCACTTACTACCCACAAGCCACGGCACACAGCAGACAAAGCCAATAAT
AATACTCATAGTAACCTGCTGCCATCACTGCCGTCACCAATCACTAGGCAGCCTCAGCAGCATGTGACCAAAA
AATGATCATCTCTGATTCGAAGAGGGAGATGAACGCATTTTTGCGGCCTCCCTACGCAGGGGTTTGGTTGGTG
CGGTATCGGTATCGGCATTCGATTCGGGTTCGCGAGACTCGCGCGACGCATCTCGCTTCTGCTCGATGGTTCT
CATTCCAGTGTACGGATACAGCGGGGCCTTGCTCCTACCAGTGGGGCGTTCGCGTTGCGACCGCCTAATTTCT
ACCTTCATTACCTCATATCACGTCCTGGTCTTCATGTTTCATGAACCAGAACAGTTCATAAATCCCCCAATCC
CTGACAGCACTTCTCATTGCTATCGTATCCAGCTATCATCCTATCATCATCTCACATCATACTCACATACTAT
GAGCACTATGGTTGTCACATCCGCTGTCATTTACATTGGAGGCTATATATCCGACTCCCCAGCGACTGACAGC
ATCATCACCGTCAACGCTCCCACGAAAGAGTCGTTATCCAGTTGCCTCTCGTCTGGCAAAGCGAACTTCGCAT
CTCTCCAATCCATCGATATCTTCCTCCAATCCTCCGAGTTATTAAGCCACTACGATTCTTTAGCGCTGGCATC
ATTAGTTCCATTTCTTGGTTCTAATGGCACACTTTCGGTCCATGTCGTATCTGATGGAAATGGAGCCGCTCAA
GACATAACAGATTGGGGTGTCATCATGACATCGTTTGTTCTAGCTGGTCTCAAGACCGAATCGGAACAAAGAG
TAGGGGGTGGAGGACGCGTATTCAATGCTCGCAAGCCGGCTAAAACCAAAGCGGCTTCGGCTCCTATGCGTTT
GAATCTCGGTGGCAATAAGATTAAGGTCACACTGAACTTGGATGACGACGACGACGACGACGATGATCAAATT
GACGAGGACGATCTATTGGCCGGAGGAGGGGATGGCATGCTGGCCCCTCCACCAAAAATTGATATGGATGCAC
GTGCCAAAGCTGCCACAGATGATTGCGGGGGGCGCAAAGCGTGCGATAACTGCTCTTGCGGCCGGGCCGAAAT
GGAAGCTGCCGAAGCGCGGGGAGAAGAGAAAAAGGAGGAGCCGCACAAAAGCGATTGCGGAAATTGTTCGAAG
GGAGATGCCTTCCGGTGTGCTGGATGCCCTTATCTGGGGAAACCAGCGTTTAAGGAGGGAGAAGAGCATTTGG
TGTTGAACTTGAGTGATGATATTTGATTTCGCTTGGAACATTTCGTGATGCAATTTTGAAGATCTACCTTGGT
GGGGTGTTCACGTAGGGAGGGGGGATGGGGTGGTCTCGGTAAGAGAATTCTCGGTCACTTGGATATGACATGA
TGTCTTCTTTAGGTGATAGATCATTAGATAAAATACTGTTATGAAAAGAGTGAAGACCTTTTTT
>TR233|c2_g2_i2 len=1166 path=[2161:0-269 2244:270-272 2236:273-284 2228:285-322 2252:323-335 2246:336-392 2249:393-410 2240:411-473

@2245@!:474-638 2221:639-695 2238:696-716 2229:717-740 2220:741-962 2219:963-1165] [-1, 2161, 2244, 2236, 2228, 2252, 2246, 2249, 2240, 2245, 2221, 2238, 2229, 2220, 2219, -2]
CTCTTCCGATCTCGATACTCCTACCTATACTTACGAACCAAAAGAGAATCATTGTACCTCTTGACTTGAGGGT
ACCAATCATATAATATCGCAACCTCTTCAGGCTGCCGCAACCTCTCATCATCCCTACATCTACTATTACATTT
TCCCCTTACCTCTCGTTACGATGACCGCCACCTCCGCCGCCATTTACATTGGAGCCTACGTCTCCGACTCCCC
AGCGGCTGACAGCATCATCACCGTCAAAGCTCCCACGAAAGAGTCGTTATCCAGCTGCCTCTCGTCAAGCAAA
ATGAACTCCGCATCTCTCCAATCCATCGATATCTTCCTCCAATCTTCCGAGTTGTTAAGCCACTACGATTCTT
TAGCACTGGCATCATTAGTTCCATTTCTTGGTTCTAATGGCACACTGTCGATCCATGTCGTATCTGATGGGAC
CGCGCAAGACAATACAGATTGGGGTGTCATCATGACATCGTTTGTTCTAGCTGGTCTCAAGACCGAATCGGAA
CAAAGAGTAGGGGGTGGAGGACGCGTATTCAATGCTCGCAAGCCGGCTAAAACCAAAGCGGCTTCGGCTCCTA
TGCGTTTGAATCTCGGTGGCAATAAGATTAAGGTCACACTGAACTTGGATGACGACGACGACGACGACGATGA
TCAAATTGACGAGGACGATCTATTGGCCGGAGGAGGGGATGGCATGCTGGCCCCTCCACCAAAAATTGATATG
GATGCACGTGCCAAAGCTGCCACAGATGATTGCGGGGGGCGCAAAGCGTGCGATAACTGCTCTTGCGGCCGGG
CCGAAATGGAAGCTGCCGAAGCGCGGGGAGAAGAGAAAAAGGAGGAGCCGCACAAAAGCGATTGCGGAAATTG
TTCGAAGGGAGATGCCTTCCGGTGTGCTGGATGCCCTTATCTGGGGAAACCAGCGTTTAAGGAGGGAGAAGAG
CATTTGGTGTTGAACTTGAGTGATGATATTTGATTTCGCTTGGAACATTTCGTGATGCAATTTTGAAGATCTA
CCTTGGTGGGGTGTTCACGTAGGGAGGGGGGATGGGGTGGTCTCGGTAAGAGAATTCTCGGTCACTTGGATAT
GACATGATGTCTTCTTTAGGTGATAGATCATTAGATAAAATACTGTTATGAAAAGAGTGAAGACCTTTTTT
>TR234|c0_g1_i1 len=1693 path=[1951:0-64 1952:65-65 1953:66-1692] [-1, 1951, 1952, 1953, -2]
GATCTCTTTAAGATTGTGATTATTTCCGTTTTATAAAGCTATCTAAAAGGCTTGGAATGAAGCATCGAATGCG
TCAAACACACCAAGCCTGCATTGCGTCAAATTGACACATTGACACAAAGGCTGCTGCCATTTAATTTCATCAC
AGCTACGTCACGCGTGGCCTTTTGTTCTCTGCAATAACTCTTATGTTGTCTTCGATTGTCAAATACGCCAACA
GTGGTACAGAATCAATTGTTCAAAAAGTAATCTTTTGCATAATTTCTTTGAAATAATATAAGATACTGTCCAA
GAGAAATTATCCAAGTTATTGCAAACTCCACTGAAACAAAGAGACGGTAAATTTACTTTGTTCGGGAATATAT
GACGGAGTAGTAAGGAATCCAGCCTCTTTAAGTCGGGTGCTGCTTCCGAATATCATGGTAAACTGATGTTCCT
CCCGCCGAAGCTCTGGCGGTGCCATTACCCTGTTTTTCAGCCCTATTCAAGGCAGAAACTCCAGTTGGAATCG
TCGTCCTCGGCGGAGGAACATTCACCTCTGGTGTTTCAGTATACCTTCCACTCTTTCGTTGGAAGTGTAACGG
CACATGACCCAACGTCCCATCGAATCGATTCTTCTTCACATCCACAAATTTCCGTTTCCCGTCGCTCTGCAAG
ATGATGACCGTGTCGGCTTCTTGGGTCGCCTTGGCGCTTCCGTAGAAGGACGACATTCCAAGCTTCGCCCCTT
CGTCTTCCTTCCGAGGGTGGACCACAAGGGTGACGTGCACGTTGTACTCCGTCGCAAACTTACGAAATTTCTC
AACGGCAATATCCTGCATGTCGAACTTATCGTACGACGATCCCTTCCCTACCGACTGTCGGCTGATCATGAAT
TGCATGTTGTCCAGTATGATGTGCTCCACGTCGTGAACATAGGCGGCGTACTCCATGGCATCCAACACATCAT
CGACGTCGGAACCTCCGTGGAATTTCATGAAGTGCATGGGCAGGGTTTCGAATTTGTCGGCGAGAGCAGTGAG
TCCCTCCATATGCTGCCGTCGTTCCTCGGCGGGCAGGTCTTTGTTGGCGAGTCCCACGGGAAGCACGTCCTTC
ATGTATTGTTGTAGAAGTTTGTGCATCAGTCTCGTATTTTTGATTTCAAAGCTGCCCCAGAGAACGTTGATGC
CTTGCTCGGCAAGGTCCAAGCTTGTTTGGCCCAGGAATGTGGTCTTACCAGAGCCAGTAGGTCCTGTGAGTAC
AGTCATTTCACCCCGACGGAACCCTTTGATAAGAGAGGTGAAGCCAGGAAGGCTTGGTATGGGAGCGCCTCGG
TATTTTTCTGGATTCACTATCTCATGAATTACCTGATCTCTCAAATCCGAAAATTTCAATATTCTCTCGTGCG
GAAGCTCGCTGGCCTCGTCCAACAATTCACCCAGGTCCCATCCCTGCAACAAAGCTTCGTTGGCATCTTTTGG
CGGATGAGATGGCCTCGCCTTGTCGTCTACAGCATCATCCAAAGTACATGCCGGCGAAAGATCACTGCTGTTA
GTCCCAGTTTCAACGTGCTGGCCAGCGTTTCCAACATCAACATCTCCAGCAGCATCATCCATCCCATTCTTCT
TCGTTCCACCACCATCATTCCAACCACGCTTCCCGCTCGGCTGCACCACCAAGCACCTCTCCACGCCCAGTTT
CCTCGCGAACAAGA
>TR234|c1_g2_i1 len=2697 path=[3671:0-116 3672:117-140 3729:141-259 3710:260-283 3721:284-426 3655:427-450 3741:451-978 3732:979-1792 3742:1793-1817 3743:1818-1841 3744:1842-1846 3739:1847-1875 3735:1876-1896 @3730@!:1897-2047 3717:2048-2572 3675:2573-2578 3676:2579-2696] [-1, 3671, 3672, 3729, 3710, 3721, 3655, 3741, 3732, 3742, 3743, 3744, 3739, 3735, 3730, 3717, 3675, 3676, -2]
CCGGGTGGCGGAATCGGGGGAGCCGCGGCGAGGGATGGATTCTACACCATTCGCAGTCGCGATCACGAGGCCG
ATGACGAATTTCTCGATACTGCTCCCCCGCCACAAGCCACTCCCACCGTTGCGGTGGTGGACGAAAGCAAGGA
ATTGGAAGCCCTCCGTGCCGTGACGGATCAGGCCGGATCCATGTACGGACCCGGTGGATCGTCCTCCATGATG
CCCGCGCAACGCGGGAGGGATCCTCGTGTGGCGGGACAGGGTGCTCCTCCGCAGTTTGCCAAGCCTCCGCCAT
TTAATAATAATAACAATAGGCATCGTCCCAACGCCGATCCAGAATTGAGGATGCAGGAGATGATGAACATGCC
TCAGACCAAGAAAAGGGCGACTGGAATTCCTCGTACCTTTTTGAATTTGAGCGCTCCCTCGAACCCTCCCCCT
GCGGAGGGGGGGGATGGCAACTCGGGGGGAGAAGCTGTCGCCGATCAGGAGGGAAATTTGGCGGCGCAGCTCC
AACCCTCCGCGCAGGCCTTCCAGGCTCTGGTGCGTTCGGGCGGGGGACAATCCACCTCCTCCGCGTCCAAGCG
ACGCGATCTCGACTATGCTCTCAAATTGACCGCCACGTCAATACCGGAACATTTGCAGTGCGGTATTTGCCAC

GGCATGGTCAGGAATGCCATGCTAATTCCCTGGGACACGGAGGGACGGCCTACTTGCGAATCTTGCATTCGGG
ACGGTCTCACGAAAAATGGATTCACTTGTCCCTTGACAGGGACGGAGGGGGTGAGTCCCGACGATCTGTTTCC
CAACGTCGGATTGCGTAAGGCGGCTGAGGCGTTCATAAAGGGTGTTATGGAAAAGTTGGATAGTATCGAACAG
CTGCTTGAGGCCGAGGAGGAAGAGGAGGCACGGAAACGAGCCGCCGATGCGAGGATGAAGGAGAGTAATAACG
AGTTTGAGGATGCCGGGGATGGAATAATTATGGGTAATAAATCAAATACGAGTCAAAAGAAAAGTAATGGCGA
TGATGATGATTTGTTTGGGGGGGACGATGAGTTTGGTGGTGATGTCTTCGATGTTGCTGAGGATGAGCCCGAA
GAGCCTGATGATAATGACGACACAATAATTGAACCGAAATCTGCTTCCTTGGCAATAGATGATAATAGTAGAA
AGGACGAGGAATCGAAGGGAACCTCATCAAAAGGAGAAAATGGAAACGACAATAAGGTTGATAGCGACGGAAA
AACTACGAATAACGATCAACAAACCAATAATGAAACTAACTTTCATTCACAAAATGAAAATCCTGCCAATGAC
AACCAAGAGAGCAAGGGTGGTAATGGAGGCGGCCCTGCATCCTCAAGTCCGACCAAGATGGTGGAGTCTGGAT
CGACCACTTCTTTGACGCAAAAGGCCAATGTGAGAAAGGAGGCAATGAAACGTAGGGGTCCGCCCGTTGGTTA
TGTCCTTGGACCAGCGGGTGGCGGCATGGCATCGCCAGCCGTCGTGGGCATACAGCACCAGCAGCAGTTCCCT
CCTCCGCCGCCGCCCATGATGATGGGTGGGGGCGGTCCGCCACCACATGGTGGAAGGGGAGGGAGCGGGCCGC
ACAACATGTCGGGTCCTCAGTTTGGTGACATGAGTAGAGGCGGAGGAGGACATGGCGGACCACCTGGTGGTGG
TGGTGGCGGCGGCAGTGGTAGGTTCTATCCAAATCAGTTTGGAGGGAGAGGCGGAGGTGGTGGACGGGGATAC
CATCCGAATCAACAACCCTATCCCAGTGGTCGTGGCGGCGGTGGTCCTCCCCCGTCCCAGCAGGGGTACAATC
CGAATCAAGGAGGCCGTGGCGGTGGTGGGTTCCATAGAAATCAACAATGGGGCGACAATAATAATGGCAATAA
TGATTGGAACAGGAAGCGCCCTCGAGAAGCCATGCTGGAACAACAGCAACACAATGGTAGCCCAGCCAACATG
TACCAAGGAGAGCCAAACCAGCAGCATCATCATCAGGGTGGATATCAACAACAGGGAGGCTGGAACGGCGGCG
GAGGTCGAGGACGAGGAGGGCCTCCTGGGGGCAACTATCAAGGAAGAGGAGGAGGATACAATCCTCATCAGTT
TCAGGGTGGACGTGGTGGCGGCGGACGTTTCCAAGGAGGAAGAGGAGGTGGTGGTGGCAGGGGATACAACGAT
TTTCGTGGGAGGGGTGGCTGGGGCGGACGGGGTCGGTTCTGAAACTTGGGGTGAATAGATAGACATTGGACAC
CGTTGCAGGTATAAAGAATTGAAATAACAAGAATTTTACCTCGGGGTGGAGCTATTCGTTGAACTATTTGGAG
AAGTGCCATTACAAAAAGCTTTAATGACAGTAAATCCAATTTATCCATGTCGGCAAACATGAGGATGATATGA
TTTTACTTACCCACATGTCAATGTGCCCCAACAAAGGGAGTGTAAGTTCGTACACTCCCAAAATCGTGGCAGG
AGGAGCATACAGTCCACGTGAGGATCAGTATGCTCCACTCTTTCTACAGATTAGGGCTGAGCTATCCAAATTT
TGGATTGCTGTTCATCAGCCCGAAGTTTCTGTTTGATGAAAAACAAGAAGCTTGGCTTGCCAAGCTTTCTTAT
TTGTCAAAGTATTTGTGCAGCTCAAGGTTTGGCTTATTTGTTAGCATGCAATTCGAGCATGCACCTTTG
>TR234|c1_g2_i2 len=2836 path=[3671:0-116 3672:117-140 3729:141-259
3710:260-283 3721:284-426 3655:427-450 3741:451-978 3732:979-1792
3742:1793-1817 3743:1818-1841 3744:1842-1846 3739:1847-1875 3734:1876-
2035 @3730@!:2036-2186 3717:2187-2711 3675:2712-2717 3676:2718-2835] [-1,
3671, 3672, 3729, 3710, 3721, 3655, 3741, 3732, 3742, 3743, 3744, 3739,
3734, 3730, 3717, 3675, 3676, -2]
CCGGGTGGCGGAATCGGGGGAGCCGCGGCGAGGGATGGATTCTACACCATTCGCAGTCGCGATCACGAGGCCG
ATGACGAATTTCTCGATACTGCTCCCCCGCCACAAGCCACTCCCACCGTTGCGGTGGTGGACGAAAGCAAGGA
ATTGGAAGCCCTCCGTGCCGTGACGGATCAGGCCGGATCCATGTACGGACCCGGTGGATCGTCCTCCATGATG
CCCGCGCAACGCGGGAGGGATCCTCGTGTGGCGGGACAGGGTGCTCCTCCGCAGTTTGCCAAGCCTCCGCCAT
TTAATAATAATAACAATAGGCATCGTCCCAACGCCGATCCAGAATTGAGGATGCAGGAGATGATGAACATGCC
TCAGACCAAGAAAAGGGCGACTGGAATTCCTCGTACCTTTTTGAATTTGAGCGCTCCCTCGAACCCTCCCCCT
GCGGAGGGGGGGGATGGCAACTCGGGGGGAGAAGCTGTCGCCGATCAGGAGGGAAATTTGGCGGCGCAGCTCC
AACCCTCCGCGCAGGCCTTCCAGGCTCTGGTGCGTTCGGGCGGGGGACAATCCACCTCCTCCGCGTCCAAGCG
ACGCGATCTCGACTATGCTCTCAAATTGACCGCCACGTCAATACCGGAACATTTGCAGTGCGGTATTTGCCAC
GGCATGGTCAGGAATGCCATGCTAATTCCCTGGGACACGGAGGGACGGCCTACTTGCGAATCTTGCATTCGGG
ACGGTCTCACGAAAAATGGATTCACTTGTCCCTTGACAGGGACGGAGGGGGTGAGTCCCGACGATCTGTTTCC
CAACGTCGGATTGCGTAAGGCGGCTGAGGCGTTCATAAAGGGTGTTATGGAAAAGTTGGATAGTATCGAACAG
CTGCTTGAGGCCGAGGAGGAAGAGGAGGCACGGAAACGAGCCGCCGATGCGAGGATGAAGGAGAGTAATAACG
AGTTTGAGGATGCCGGGGATGGAATAATTATGGGTAATAAATCAAATACGAGTCAAAAGAAAAGTAATGGCGA
TGATGATGATTTGTTTGGGGGGGACGATGAGTTTGGTGGTGATGTCTTCGATGTTGCTGAGGATGAGCCCGAA
GAGCCTGATGATAATGACGACACAATAATTGAACCGAAATCTGCTTCCTTGGCAATAGATGATAATAGTAGAA
AGGACGAGGAATCGAAGGGAACCTCATCAAAAGGAGAAAATGGAAACGACAATAAGGTTGATAGCGACGGAAA
AACTACGAATAACGATCAACAAACCAATAATGAAACTAACTTTCATTCACAAAATGAAAATCCTGCCAATGAC
AACCAAGAGAGCAAGGGTGGTAATGGAGGCGGCCCTGCATCCTCAAGTCCGACCAAGATGGTGGAGTCTGGAT
CGACCACTTCTTTGACGCAAAAGGCCAATGTGAGAAAGGAGGCAATGAAACGTAGGGGTCCGCCCGTTGGTTA
TGTCCTTGGACCAGCGGGTGGCGGCATGGCATCGCCAGCCGTCGTGGGCATACAGCACCAGCAGCAGTTCCCT
CCTCCGCCGCCGCCCATGATGATGGGTGGGGGCGGTCCGCCACCACATGGTGGAAGGGGAGGGAGCGGGCCGC
ACAACATGTCGGGTCCTCAGTTTGGTGACATGAGTAGAGGCGGAGGAGGACATGGCGGACCACCTGGTGGTGG
TGGTGGCGGCGGCAGTGGTAGGTTCTATCCAAATCAGTTTGGAGGGAGAGGCGGAGGTGGTGGACGGGGATAC
CATCCGAATCAACAACCCTATCCCAGTGGTCGTGGCGGCGGTGGTCCTCCCCCGTCCCAGCAGGGGTACAATC
CGAATCAAGGAGGCCGTGGCGGTGGTGGGTTCCATAGAAATCAACAATGGGTAAGTAGATTGTATGCATGTCT

GACTGCCCACAGGACTTGCGTAGTACAGAACAAAACTAGTATTGTTTCATTGCGTATTTACAAGTAGTTTCAT
TCAACAATTGATTTTTGGTCTTCGTGTCTCCCTTTCGTATTAGGGCGACAATAATAATGGCAATAATGATTGG
AACAGGAAGCGCCCTCGAGAAGCCATGCTGGAACAACAGCAACACAATGGTAGCCCAGCCAACATGTACCAAG
GAGAGCCAAACCAGCAGCATCATCATCAGGGTGGATATCAACAACAGGGAGGCTGGAACGGCGGCGGAGGTCG
AGGACGAGGAGGGCCTCCTGGGGGCAACTATCAAGGAAGAGGAGGAGGATACAATCCTCATCAGTTTCAGGGT
GGACGTGGTGGCGGCGGACGTTTCCAAGGAGGAAGAGGAGGTGGTGGTGGCAGGGGATACAACGATTTTCGTG
GGAGGGGTGGCTGGGGCGGACGGGGTCGGTTCTGAAACTTGGGGTGAATAGATAGACATTGGACACCGTTGCA
GGTATAAAGAATTGAAATAACAAGAATTTTACCTCGGGGTGGAGCTATTCGTTGAACTATTTGGAGAAGTGCC
ATTACAAAAAGCTTTAATGACAGTAAATCCAATTTATCCATGTCGGCAAACATGAGGATGATATGATTTTACT
TACCCACATGTCAATGTGCCCCAACAAAGGGAGTGTAAGTTCGTACACTCCCAAAATCGTGGCAGGAGGAGCA
TACAGTCCACGTGAGGATCAGTATGCTCCACTCTTTCTACAGATTAGGGCTGAGCTATCCAAATTTTGGATTG
CTGTTCATCAGCCCGAAGTTTCTGTTTGATGAAAAACAAGAAGCTTGGCTTGCCAAGCTTTCTTATTTGTCAA
AGTATTTGTGCAGCTCAAGGTTTGGCTTATTTGTTAGCATGCAATTCGAGCATGCACCTTTG
>TR234|c1_g3_i1 len=314 path=[3687:0-86 3688:87-110 3689:111-112
3690:113-118 3722:119-238 3715:239-241 3716:242-313] [-1, 3687, 3688,
3689, 3690, 3722, 3715, 3716, -2]
GTACACTCCAAAGTTTGGGACAGAGGGAGTATACCTTCTCGTAAGAATGGCATACTCGCCCAAACCACAATAT
TCGAGGCTGAGCAACCCAATTTTGGATTGCTGTTCATCAGCCCGAAATTTCTGTTCAAGAACAATAAGAAGCT
TGGCGTACCAAGGACTTCTTATCTCGTTTCTTATTCAAGTGTCTGTACGCCAAAATTTGGCTTATTTGTTAGC
ATGCGAGTCGAGCATGCACCTTTGTACAGCAGGTTGGAATGTTTGATGCATTCGATGCTTCATTCCAACCCTT
TTAGATAGCTATAATTCGGAAA
>TR235|c0_g1_i1 len=328 path=[3429:0-227 3430:228-228 3533:229-278
3521:279-302 3467:303-303 3468:304-327] [-1, 3429, 3430, 3533, 3521,
3467, 3468, -2]
TTACGTGGAGCGATGGAGCGGCAGAGGGAGCTGGGGAAGCATGTTATTTTGGTGGGCGATTTGAATTTGAAGA
TTGATAAGCGAGACGTTTTTTGGAGATGGCGGAATGTTTACGTGGATGTAGTATTGAATCAAATGAAAGAAAA
TAGGAAAGATGAAAATCATGGATGGAAGAAGGACATTGCCAATAATTGGGATGAGATTGTACGAGTCCTCCAT
ACAATTGAGGTGAGTCAATAATGTATCTATGGCATTGGTGCGCAAGCAACTGAATATCAATAAGACTTCAAAA
CAAGACCCAACAAAGCTATGCACGCAACGAGAGCGA
>TR235|c0_g2_i1 len=471 path=[3367:0-35 3534:36-61 @3524@!:62-457
3464:458-470] [-1, 3367, 3534, 3524, 3464, -2]
GCTCTTCCGATCTTATCGTTGTTGATGAAGAACAGCTTCGGGGTAGAGTGTGCTGGTAATGGTGAGTTGTCGT
TGGGTGGAGATGCATCAACGAGGAAAGCACAGCCGCACAAGAAGCAACGATCGGTGGCGTCCTTTTTCTCTGC
TGCAGGTTCTAAATCGTCTTCTACAAGTAGTGGTAGCGTCTCTTCTTCTCTGTCGTCCTCGTGTTCTGGCGAG
AAGCGGGCCATCATTTCGCAGGATATCAATAATTACAAATCTTCCAAAAAGAAATCTATACACTCTTATTTTG
GTGAACCAAAAGGAAAGTCAACAAAGGAGAGTTCTGGCAATCCTAATTCTACAAAATCTGCGTCATCCAACAA
AAAGAGTTACACACCAAGGAATAGTGTTCTGAAGCACTTTGGTAAAAAGTAAGGCAATATGATAGGCCATTTT
ATACAATACATTAAAAAACAGATCGGAAGAGCG
>TR235|c0_g2_i2 len=1525 path=[3370:0-227 3371:228-248 3542:249-251
3532:252-289 3552:290-301 3550:302-323 3548:324-350 3537:351-405
3528:406-450 3514:451-515 3385:516-557 3386:558-581 3526:582-643
3425:644-667 3512:668-734 3407:735-758 3543:759-840 3535:841-1035
3491:1036-1059 3534:1060-1085 @3524@!:1086-1481 3495:1482-1524] [-1,
3370, 3371, 3542, 3532, 3552, 3550, 3548, 3537, 3528, 3514, 3385, 3386,
3526, 3425, 3512, 3407, 3543, 3535, 3491, 3534, 3524, 3495, -2]
TTACGTGGAGCGATGGAGCGGCAGAGGGAGCTGGGGAAGCATGTTATTTTGGTGGGCGATTTGAATTTGAAGA
TTGATAAGCGAGACGTTTTTTGGAGATGGCGGAATGTTTACGTGGATGTAGTATTGAATCAAATGAAAGAAAA
TAGGAAAGATGAAAATCATGGATGGAAGAAGGACATTGCCAATAATTGGGATGAGATTGTACGAGTCCTCCAT
ACAATTGAGTAAATTCCCTGACATGAGAACCATTGCATGCAAAACCGCAGAACTCCATGAGTAAATCACGCAC
CACATTCGAGCATTGCCACGCCAGACCAAAAATCCTTTGACGAAAAAAACACATGATAGGTATATTGCCCGTG
TCAAGCTAGCAGAAAACAAATATATCACACTTGGCAATTACGAAGATACCGCTGAAGATGCATTGGGTTGCTA
TGACATCAATGCAAAATACTACATAAACCCGTACAATGAGACTTCCACCATCTATCGAAAGGCAAACTGCTTG
AGCATCGATACACTGGCGGAGCTGATGGCCAAAATTGTGAATGTAACATGGGACAATAAGGTGCTACGACAAA
TATCCGAGTCCAGCGAGTCTGGTTTGAATCCAGATTTGCCCTCCTACATGTGGATGAAAAAGCTGTTGGATGA
TGATGGGATGGTGGACGTCTTTCGTCACTTCTACCCAGCTGCAGAAGCACGGTTCACTTGTTGGCATCAAATG
AAAAATATGAGATACTCAAATGCAGGAGGAAGGATAGACTTTACCCTCGTGGACAAGGCTCTCTTAACCCACG
TCGAACTGAACAACAATGTACTATTGCGGTGTGGCAGGTCAAACCCACACGACAATCCGCTCGGAGAAGAAGC
CGCTCTCTTGGCGGCCACCGCCTCGGGTCTTTTCGTGCCCGGTACATTTGCAGGAGGAGGAATCGCCGACGCC
ACGAAACGGGCGTTGGACACGCAGTTCGCAGGGAAGGCTCACAGCGGCATGATATATACACCTCCTTCGTACA
GTGACCACATAGCCATATCGTTGTTGATGAAGAACAGCTTCGGGGTAGAGTGTGCTGGTAATGGTGAGTTGTC

GTTGGGTGGAGATGCATCAACGAGGAAAGCACAGCCGCACAAGAAGCAACGATCGGTGGCGTCCTTTTTCTCT
GCTGCAGGTTCTAAATCGTCTTCTACAAGTAGTGGTAGCGTCTCTTCTTCTCTGTCGTCCTCGTGTTCTGGCG
AGAAGCGGGCCATCATTTCGCAGGATATCAATAATTACAAATCTTCCAAAAAGAAATCTATACACTCTTATTT
TGGTGAACCAAAAGGAAAGTCAACAAAGGAGAGTTCTGGCAATCCTAATTCTACAAAATCTGCGTCATCCAAC
AAAAAGAGTTACACACCAAGGAATAGTGTTCTGAAGCACTTTGGTAAAAAGTAAGGCAATATGATAGGCCATT
TTATACAATACATTAAAAAACACTGTTGACAATCACCTCCATGTTTCAATCGTAGAATGGGCGAT
>TR235|c0_g2_i3 len=1477 path=[3455:0-180 3547:181-201 3541:202-203
3532:204-241 3552:242-253 3550:254-275 3548:276-302 3537:303-357
3528:358-402 3514:403-467 3385:468-509 3386:510-533 3526:534-595
3425:596-619 3512:620-686 3407:687-710 3543:711-792 3535:793-987
3491:988-1011 3534:1012-1037 @3524@!:1038-1433 3495:1434-1476] [-1, 3455,
3547, 3541, 3532, 3552, 3550, 3548, 3537, 3528, 3514, 3385, 3386, 3526,
3425, 3512, 3407, 3543, 3535, 3491, 3534, 3524, 3495, -2]
AGCTATGCACGCAACGAGAGCGAGCTCTATCTATTGATTGGAGAGCATTGTGGTTTTGCACGAAGAACCACTC
AATTTTTCTCGGAAGAAGATACCTGTCATGTGAATGGATTTTAGCCTTAATGGCAATCATCCAAAACATGATG
AAACCTCACGGATGCATTTTCGTGTTTTCAAAAAGAAATTCCCTGACATGAGAACCATTGCATGCAAAACCGC
AGAACTCCATGAGTAAATCACGCACCACATTCGAGCATTGCCACGCCAGACCAAAAATCCTTTGACGAAAAAA
ACACATGATAGGTATATTGCCCGTGTCAAGCTAGCAGAAAACAAATATATCACACTTGGCAATTACGAAGATA
CCGCTGAAGATGCATTGGGTTGCTATGACATCAATGCAAAATACTACATAAACCCGTACAATGAGACTTCCAC
CATCTATCGAAAGGCAAACTGCTTGAGCATCGATACACTGGCGGAGCTGATGGCCAAAATTGTGAATGTAACA
TGGGACAATAAGGTGCTACGACAAATATCCGAGTCCAGCGAGTCTGGTTTGAATCCAGATTTGCCCTCCTACA
TGTGGATGAAAAAGCTGTTGGATGATGATGGGATGGTGGACGTCTTTCGTCACTTCTACCCAGCTGCAGAAGC
ACGGTTCACTTGTTGGCATCAAATGAAAAATATGAGATACTCAAATGCAGGAGGAAGGATAGACTTTACCCTC
GTGGACAAGGCTCTCTTAACCCACGTCGAACTGAACAACAATGTACTATTGCGGTGTGGCAGGTCAAACCCAC
ACGACAATCCGCTCGGAGAAGAAGCCGCTCTCTTGGCGGCCACCGCCTCGGGTCTTTTCGTGCCCGGTACATT
TGCAGGAGGAGGAATCGCCGACGCCACGAAACGGGCGTTGGACACGCAGTTCGCAGGGAAGGCTCACAGCGGC
ATGATATATACACCTCCTTCGTACAGTGACCACATAGCCATATCGTTGTTGATGAAGAACAGCTTCGGGGTAG
AGTGTGCTGGTAATGGTGAGTTGTCGTTGGGTGGAGATGCATCAACGAGGAAAGCACAGCCGCACAAGAAGCA
ACGATCGGTGGCGTCCTTTTTCTCTGCTGCAGGTTCTAAATCGTCTTCTACAAGTAGTGGTAGCGTCTCTTCT
TCTCTGTCGTCCTCGTGTTCTGGCGAGAAGCGGGCCATCATTTCGCAGGATATCAATAATTACAAATCTTCCA
AAAAGAAATCTATACACTCTTATTTTGGTGAACCAAAAGGAAAGTCAACAAAGGAGAGTTCTGGCAATCCTAA
TTCTACAAAATCTGCGTCATCCAACAAAAAGAGTTACACACCAAGGAATAGTGTTCTGAAGCACTTTGGTAAA
AAGTAAGGCAATATGATAGGCCATTTTATACAATACATTAAAAAACACTGTTGACAATCACCTCCATGTTTCA
ATCGTAGAATGGGCGAT
>TR235|c0_g2_i4 len=1452 path=[3554:0-227 3553:228-228 3551:229-248
3549:249-250 3548:251-277 3537:278-332 3528:333-377 3514:378-442
3385:443-484 3386:485-508 3526:509-570 3425:571-594 3512:595-661
3407:662-685 3543:686-767 3535:768-962 3491:963-986 3534:987-1012
@3524@!:1013-1408 3495:1409-1451] [-1, 3554, 3553, 3551, 3549, 3548,
3537, 3528, 3514, 3385, 3386, 3526, 3425, 3512, 3407, 3543, 3535, 3491,
3534, 3524, 3495, -2]
TTACGTGGAGCGATGGAGCGGCAGAGGGAGCTGGGGAAGCATGTTATTTTGGTGGGCGATTTGAATTTGAAGA
TTGATAAGCGAGACGTTTTTTGGAGATGGCGGAATGTTTACGTGGATGTAGTATTGAATCAAATGAAAGAAAA
TAGGAAAGATGAAAATCATGGATGGAAGAAGGACATTGCCAATAATTGGGATGAGATTGTACGAGTCCTCCAT
ACAATTGAGGCATTGCCACGCCAGACCAAAAATCCTTTGACGAAAAAAACACATGATAGGTATATTGCCCGTG
TCAAGCTAGCAGAAAACAAATATATCACACTTGGCAATTACGAAGATACCGCTGAAGATGCATTGGGTTGCTA
TGACATCAATGCAAAATACTACATAAACCCGTACAATGAGACTTCCACCATCTATCGAAAGGCAAACTGCTTG
AGCATCGATACACTGGCGGAGCTGATGGCCAAAATTGTGAATGTAACATGGGACAATAAGGTGCTACGACAAA
TATCCGAGTCCAGCGAGTCTGGTTTGAATCCAGATTTGCCCTCCTACATGTGGATGAAAAAGCTGTTGGATGA
TGATGGGATGGTGGACGTCTTTCGTCACTTCTACCCAGCTGCAGAAGCACGGTTCACTTGTTGGCATCAAATG
AAAAATATGAGATACTCAAATGCAGGAGGAAGGATAGACTTTACCCTCGTGGACAAGGCTCTCTTAACCCACG
TCGAACTGAACAACAATGTACTATTGCGGTGTGGCAGGTCAAACCCACACGACAATCCGCTCGGAGAAGAAGC
CGCTCTCTTGGCGGCCACCGCCTCGGGTCTTTTCGTGCCCGGTACATTTGCAGGAGGAGGAATCGCCGACGCC
ACGAAACGGGCGTTGGACACGCAGTTCGCAGGGAAGGCTCACAGCGGCATGATATATACACCTCCTTCGTACA
GTGACCACATAGCCATATCGTTGTTGATGAAGAACAGCTTCGGGGTAGAGTGTGCTGGTAATGGTGAGTTGTC
GTTGGGTGGAGATGCATCAACGAGGAAAGCACAGCCGCACAAGAAGCAACGATCGGTGGCGTCCTTTTTCTCT
GCTGCAGGTTCTAAATCGTCTTCTACAAGTAGTGGTAGCGTCTCTTCTTCTCTGTCGTCCTCGTGTTCTGGCG
AGAAGCGGGCCATCATTTCGCAGGATATCAATAATTACAAATCTTCCAAAAAGAAATCTATACACTCTTATTT
TGGTGAACCAAAAGGAAAGTCAACAAAGGAGAGTTCTGGCAATCCTAATTCTACAAAATCTGCGTCATCCAAC

AAAAAGAGTTACACACCAAGGAATAGTGTTCTGAAGCACTTTGGTAAAAAGTAAGGCAATATGATAGGCCATT
TTATACAATACATTAAAAAACACTGTTGACAATCACCTCCATGTTTCAATCGTAGAATGGGCGAT
>TR235|c0_g2_i5 len=806 path=[3401:0-227 3402:228-229 3403:230-248
3404:249-260 3545:261-428 3531:429-545 3549:546-547 3548:548-574
3555:575-585 3536:586-629 3527:630-674 3513:675-739 3410:740-781
3411:782-805] [-1, 3401, 3402, 3403, 3404, 3545, 3531, 3549, 3548, 3555,
3536, 3527, 3513, 3410, 3411, -2]
TTACGTGGAGCGATGGAGCGGCAGAGGGAGCTGGGGAAGCATGTTATTTTGGTGGGCGATTTGAATTTGAAGA
TTGATAAGCGAGACGTTTTTTGGAGATGGCGGAATGTTTACGTGGATGTAGTATTGAATCAAATGAAAGAAAA
TAGGAAAGATGAAAATCATGGATGGAAGAAGGACATTGCCAATAATTGGGATGAGATTGTACGAGTCCTCCAT
ACAATTGAGAACTCCATGAGTAAATCACGCACCACATTCGAGTGAGTGTGGTTTGATGTGCATTGATGTTTGC
ATTATTGACACTGAAGTTTACTACCTCAAACAACAAATAAAGCGACACTTGAGCAACAACATGCAACGCAGTA
CATGATCGCACTCGCTATTGCCAATGACAACTCCTTGCTTTTTCCAGCAAGCGTGGTTATTTTTTCACCTATG
CAGCTACCAATTCATTAATGAAAACTTGCAATCCCATCCACCCTGTCATCTGACATTCTTCATTTTGCTCATT
TTTTGGTCATGTAGGCATTGCCACGCCAGACCAAAAATCCTTTGACGAAAAAAACACATGATAGATTTATTGC
CCGTGTCAAACTAGCAGAAAACGAATATATCACACTTGGCAATTACGAAGATACCGCTGAAGATGCATTGGGT
TGCTATGACATCAATGCACAATACTACATGAACCCGTCCAATGAGACTTCCACCATCTACCGAAAGGCAAACT
GCTTGAGCATCGATACACTGGCGGAGCTGATGGCCAAAATTGTGAATGTAACATGGGACAATAAGGTGCTACG
ACA
>TR235|c0_g2_i6 len=541 path=[3388:0-227 3389:228-229 3390:230-248
3552:249-260 3550:261-282 3548:283-309 3555:310-320 3536:321-364
3527:365-409 3513:410-474 3410:475-516 3411:517-540] [-1, 3388, 3389,
3390, 3552, 3550, 3548, 3555, 3536, 3527, 3513, 3410, 3411, -2]
TTACGTGGAGCGATGGAGCGGCAGAGGGAGCTGGGGAAGCATGTTATTTTGGTGGGCGATTTGAATTTGAAGA
TTGATAAGCGAGACGTTTTTTGGAGATGGCGGAATGTTTACGTGGATGTAGTATTGAATCAAATGAAAGAAAA
TAGGAAAGATGAAAATCATGGATGGAAGAAGGACATTGCCAATAATTGGGATGAGATTGTACGAGTCCTCCAT
ACAATTGAGAACTCCATGAGTAAATCACGCACCACATTCGAGCATTGCCACGCCAGACCAAAAATCCTTTGAC
GAAAAAAACACATGATAGATTTATTGCCCGTGTCAAACTAGCAGAAAACGAATATATCACACTTGGCAATTAC
GAAGATACCGCTGAAGATGCATTGGGTTGCTATGACATCAATGCACAATACTACATGAACCCGTCCAATGAGA
CTTCCACCATCTACCGAAAGGCAAACTGCTTGAGCATCGATACACTGGCGGAGCTGATGGCCAAAATTGTGAA
TGTAACATGGGACAATAAGGTGCTACGACA
>TR235|c0_g3_i1 len=882 path=[3483:0-320 3484:321-344 3523:345-583
3395:584-607 3546:608-646 3539:647-652 3530:653-670 3517:671-676
3462:677-881] [-1, 3483, 3484, 3523, 3395, 3546, 3539, 3530, 3517, 3462,
-2]
AGCAGTGGACACTGCTTCTCCAATATCCAATAGGAGTAGGAGGAATAAAAAATAAGACAAAAGACAGATCAAC
CTCTCGTTCTCTCGCTCTCTCAAACTCTTAAACTCTCAAACGTTCAAACATTGGCAAACATCATGTTGATTGT
GAGCTGGAACGTCGCCGGTCTGAAGCCAGCGCTCCAACGAATCCAGAGCGACTACGGCACACCAACATCCAGC
GACAGCAGCAGCATTGGTAGTAGCAATGCCAAATGTTCAAAACCAAAACCAAAACCAGACCCATCCCGCGCAT
TCGCCAACTACCTCCACCTCCACGGCTCCGTCGACATCCTCTGCCTCCAAGAACACAAAATACCCCTGAGCCA
GCTCTCCTCCCGCTCCGAACCGGATCGCTGTTCGACCATCGACGGCTACGAATCATTTTGGTCATGTGTAACG
ACAGATCAAAAATCGCGCGGATTTAACGGGGTGGTCACCTACGCCAAGGTGGGGTTGGTGCAAGCAGCGGACT
GTACGCCGTTGAAGGATGCGGAGTTGGACGGGCAGGGACGATGTATTTTGACGGATCATGGGAAGTTCGTGGT
GTTCAATGTGTACGTTCCTTATTCGAGGGATGCGGAGACATTGGCCAAGAAGATGAAGTTTCTAAATGCTTTA
CGTGGAGCGATGGAGCGGCAGAGGGAGCTGGGGAAGCATGTTATTTTGGTGGGCGATTTGAATTTGAAGATTG
ATAAGCGAGACGTTTTTTGGAGATGGCGGAATGTTTACGTGGATGTAGTATTGAATCAAATGAAAGAAAATAG
GAAAGATGAAAATCATGGATGGAAGAAGGACATTGCCAATAATTGGGATGAGATTGTACGAGTCCTCCATACA
ATTGAG
>TR236|c0_g1_i1 len=3634 path=[5542:0-276 5543:277-300 5579:301-610
5572:611-638 5562:639-662 5578:663-665 5571:666-689 5561:690-829
5534:830-853 5570:854-926 5479:927-950 5560:951-1016 5537:1017-1040
5569:1041-1310 5476:1311-1334 5559:1335-1771 5516:1772-1795 5568:1796-
1810 5558:1811-1827 5584:1828-1852 5582:1853-1867 5577:1868-2486
5587:2487-2491 5556:2492-2515 5576:2516-2567 5566:2568-2591 5555:2592-
2785 5586:2786-2786 5585:2787-2810 5583:2811-3093 5580:3094-3185
5588:3186-3202 5472:3203-3207 5564:3208-3226 5553:3227-3528 5551:3529-
3552 5552:3553-3633] [-1, 5542, 5543, 5579, 5572, 5562, 5578, 5571, 5561,
5534, 5570, 5479, 5560, 5537, 5569, 5476, 5559, 5516, 5568, 5558, 5584,
5582, 5577, 5587, 5556, 5576, 5566, 5555, 5586, 5585, 5583, 5580, 5588,
5472, 5564, 5553, 5551, 5552, -2]

AAGTGAACGGCGGTATCGTATTTGGCACTCTCCACATCAGGCTCTCATCGTTGTCATCATAGACTGCCAAAAA
AAAGAAAACAGCACCATGTTTCTTCTCGCAACGTTGCTGCTCTCATCCAAATATTATGCCAATGGATTCGGAC
AACCGTTAACAGCGTCACCAAGATGGCTGCATCATCCGCATTCGTATTCACGATCACGGCAGACATCAACATC
AGCATCAACAACACCTTGCCAGAAAAATCCTGCCAACTATCGACGTAGAAGAGATCCAGTCGTTGTCGTTCTT
TTTGGTGCCGCAACCGATACGAATAAAATAGATGGTAGGAGCAGGGAGCAAATAATGAGTGATGCAATTGCAT
CAGCGGGTTTAACGCAAGTCAAGAAGAAAAAGAAGAAAACAACGGCTACTGCTACTGATAGAACCTCAGGGAA
GAGCACCGGTGGTAGGTCAAATAATTCAAACGCAAATAATGGAAAGAAGAGGAACAATAACGAGAAGAATAAC
AAAAAGCCAGTACCAAAACCAAAACCAAAACAATCCACCTCAAAATCAAAACAACAAACAATAACCTCAACAA
CCTCGAAAATCTCTCTCCAATCCCAACTATCCTACTCACGAGAAGGACACGCTTCTCTCCGCTCCTTAATCCC
ACCATCCATCATACACGAACTCTACACCGACCTTCAAGCCTACTCACACGCCTCGTCGGTACATCGTAAAAAG
GGCGAAGATTCCGCACCCTTTTTGCAATTTTACAATACATGGAGAGATTTACCGAGTGTCAAGGCGTTGGCGA
CGAGTCCTTTGCTTTGTTCTGCTGCGGCAACTCTCATGGGAATGCGGAGTGTTACTACTACCGGCAGTAGTGG
CAGTGGAGCTGGATCGTCATCGTCACCATCATTGCCAAAACTGCGACTCTATCAAGATTCCGTATTCATCAAA
CGTAAAAACAAGGATGGGATAACACCCTGGCACATCGATGGAAGGATGATGCCATTCGATACCTCCAAAATAA
TCACGTTTTGGATTCCATTGCATTCGATACCCGCGATTGAGGATGGGGGTACTGGTTTGTTGTTTATTAGTAA
GAGTCATTCGGATTTTAGTTTGCCTTATTGGAATGGACGGGGACTCGATGGGAAGGCTGGCGATAATGATGGT
GGTGATAGTTATGGCAATGGTGGTGGTGGTGGTGATGCTGACATTGAGGATAGTGCATATAACAATCTGACCG
GACGATATGGTATTGAAAATGTCGGAGATGATCGCATAGTGGATAATAATCCGGGTGTGATTGGTCATCATAT
GCCTTTGGATGTTGGGGATTGTACGGTTCACAATGGATGGACCATGCATTGTGCGAATGGCAACATGAGGAAT
ATGACGAAGAAGAAAAACAACAAAAATAAAAAGAATGGTGGGCAGCAGCAGCAGAGCGATGAAAATAGCTTGT
TCCAGACGCGGTATGCTATTGCCATTTCGTATATTGACAGCCATGCCGAAGTTCGGGGAGATGTACCTGGCGT
CGGAAAGAATGTTGTAGGGAATCGTGGTGGTGGTGGAAAGAGGAAGGGAGGTAACACCAACAAGAAAAGCAGG
GTATCGTACAAAGGTGATGCCGAGGACAGGGCGAGTTATGCACAATGGATTGGGGATGTTTTGCCGAGAACAC
GGTTTGAGCATGAACTCATGCCTGATGTTTGGCCACCATCAGCATGAGGGTTATCATTTGTAGAGTCATTCAC
AGTTGCCCCTGCAAAGTCGTACGTCGGGGAATGAGGACCGCTGAAAATGGCACAAAAAAGTGAGCGATGCATT
CACATTCGAGTATGTGTACAAACAAGAGCGAGAAGTCAACAATAAGCTAAATAAATTCTCGAGGAAAGTTTTT
GTGTCTCTACCCACTTGCTGCCATAATCAACTCGAACTATTCATTTTTGATCAGCGTATCACCTTTGCCTGCA
AGCAGGTATCATTTACCCTCTATTCCAATTAACGATGTGGTTTTAAATAGTTCTGTCTTGGTTCCGTCCGCAT
GAATTGAACACCTTCGATCCTTCTGTTGTTTGCCAGTCGGATCCAAGATGGTCACACTACCATACCGTTTGCG
GCCATTTTATGGGTAAGCATGTTTGGGATCATTGTGGTGGCGCATCCTATTTCCACTTGATCAGCACAACATC
TTCACCATTAAGCAAGTACTCCTTACCCTCCATTCTGATTTTGCCATTCTCTTTTGCCGCGTTGTAATTGTCG
TATTGCACCAACTCCCGTGCTTCTATTGCTTCTGCATTCATAAAACCTTTTCTTATATCCCCATGCAGTTTAC
CAGCAAGATCCAGGACTGTCATACCATTTGATCCCAAAATGTGTGTCTTGGTTGTTTGGGATCGCTCCGATGG
CACGCCCGGGCCAGTGTATACAATGCCAAAGTCCAGAACGTCCTTCACACGTAACGGCAGTGCGTGATAACTC
AATTGGTCCGTGTAGGTCGTCCCCGAAGCATCATCCGAGTCGCCGCATTCCACTCCCATGGATTCCAAATATT
CATGACGCTCTTCTGCACTCAACGAACCCATTTCCGACTCCAGCTTGGCGCTCACCACCATGCACGAATCTGT
CTCCAGATCGCAATACTGAATTTGTTTCATGTAGCCTTCCGCCAATTTGATCGATTCATCCCTATTCAATGCA
AAATCGACCTCGTCCAAATTAAAGGCATAAATCACAGGTTTCAACGTCAACAACCCCATGGATTTGATTGCGA
AGCACTCCTCGGATGATAGTTCCATCGTCCTTGCTGGCAATCCATTCTCTAATCCTTTTTCAAGTTTCATTAA
CACATCCCTCTCTTCTCCTACACATGTGGTCCTCTCCAATCGTCGTTGGACATGGGCTAGATCCGCCAGGAGT
AATTCCAAATTGACGACTTCTGCATCGGCCACAGGATCCACCTTCCCGTCGACGTGGATGACATCTTCGTCGA
TGTAGTTTCGGACGACGTGGATTATTAGATCGCATTCTCGGATGGTTGCCAGGAATTTATTTCCTAATCCTTC
TCCTCGAGAGGCACCTTTGACTAATCCCGCGACATCGATCAAGAATATGCTTGCAGGAAGGGCCTTTTTGCTC
TTGGCCAATTTTGCCAGCTTTTCCAAATTGGCATCGGGTATCGGAACAGGCGTGATATTTGGCTCGATAGTGC
AAAAGGGGAAGTTTTTGGCATCGGCAATTGCTTTTTGGGCAATGGCATTGAATAGCGTGCTTTTTCCGACATT
AGGAAGTCCAACTAGACCAACTTTTATCTTCATGGATAGCAGCGTGTTTCGGGGTGATGTAGATTTCAATTTA
GCTGGCGCATGATGCGAGGTGGTGTGGCTGTAAATCGTGCTGTCGATTCTTATGGATTGCGTGAATGATGATG
TTGAAGACCAGCATAGCCCTTTTCTTGTCAAGAGAGTTATGCATATTAAGAATGCCCGCATGACCTCCATGAC
CATTTTCAGTAAAATCCTAGTTGACAGCGAAGCAGGTAGACCTCCAGATGCATGCGGAGAGGACGGGATGTTG
AGGGGATAGATAAACGGAGGATAGAGGAGATGGCATGGGAGATGGGAGACGGAGATG
>TR236|c0_g1_i3 len=3719 path=[5542:0-276 5543:277-300 5579:301-610
5572:611-638 5562:639-662 5578:663-665 5571:666-689 5561:690-829
5534:830-853 5570:854-926 5479:927-950 5560:951-1016 5537:1017-1040
5569:1041-1310 5476:1311-1334 5559:1335-1771 5516:1772-1795 5568:1796-
1810 5558:1811-1827 5584:1828-1852 5582:1853-1867 5577:1868-2486
5587:2487-2491 5505:2492-2510 5581:2511-2515 5576:2516-2567 5566:2568-
2591 5555:2592-2785 5586:2786-2786 5585:2787-2810 5583:2811-3093
5580:3094-3185 5574:3186-3292 5564:3293-3311 5553:3312-3613 5551:3614-
3637 5552:3638-3718] [-1, 5542, 5543, 5579, 5572, 5562, 5578, 5571, 5561,
5534, 5570, 5479, 5560, 5537, 5569, 5476, 5559, 5516, 5568, 5558, 5584,

5582, 5577, 5587, 5505, 5581, 5576, 5566, 5555, 5586, 5585, 5583, 5580, 5574, 5564, 5553, 5551, 5552, -2]
AAGTGAACGGCGGTATCGTATTTGGCACTCTCCACATCAGGCTCTCATCGTTGTCATCATAGACTGCCAAAAA
AAAGAAAACAGCACCATGTTTCTTCTCGCAACGTTGCTGCTCTCATCCAAATATTATGCCAATGGATTCGGAC
AACCGTTAACAGCGTCACCAAGATGGCTGCATCATCCGCATTCGTATTCACGATCACGGCAGACATCAACATC
AGCATCAACAACACCTTGCCAGAAAAATCCTGCCAACTATCGACGTAGAAGAGATCCAGTCGTTGTCGTTCTT
TTTGGTGCCGCAACCGATACGAATAAAATAGATGGTAGGAGCAGGGAGCAAATAATGAGTGATGCAATTGCAT
CAGCGGGTTTAACGCAAGTCAAGAAGAAAAAGAAGAAAACAACGGCTACTGCTACTGATAGAACCTCAGGGAA
GAGCACCGGTGGTAGGTCAAATAATTCAAACGCAAATAATGGAAAGAAGAGGAACAATAACGAGAAGAATAAC
AAAAAGCCAGTACCAAAACCAAAACCAAAACAATCCACCTCAAAATCAAAACAACAAACAATAACCTCAACAA
CCTCGAAAATCTCTCTCCAATCCCAACTATCCTACTCACGAGAAGGACACGCTTCTCTCCGCTCCTTAATCCC
ACCATCCATCATACACGAACTCTACACCGACCTTCAAGCCTACTCACACGCCTCGTCGGTACATCGTAAAAAG
GGCGAAGATTCCGCACCCTTTTTGCAATTTTACAATACATGGAGAGATTTACCGAGTGTCAAGGCGTTGGCGA
CGAGTCCTTTGCTTTGTTCTGCTGCGGCAACTCTCATGGGAATGCGGAGTGTTACTACTACCGGCAGTAGTGG
CAGTGGAGCTGGATCGTCATCGTCACCATCATTGCCAAAACTGCGACTCTATCAAGATTCCGTATTCATCAAA
CGTAAAAACAAGGATGGGATAACACCCTGGCACATCGATGGAAGGATGATGCCATTCGATACCTCCAAAATAA
TCACGTTTTGGATTCCATTGCATTCGATACCCGCGATTGAGGATGGGGGTACTGGTTTGTTGTTTATTAGTAA
GAGTCATTCGGATTTTAGTTTGCCTTATTGGAATGGACGGGGACTCGATGGGAAGGCTGGCGATAATGATGGT
GGTGATAGTTATGGCAATGGTGGTGGTGGTGGTGATGCTGACATTGAGGATAGTGCATATAACAATCTGACCG
GACGATATGGTATTGAAAATGTCGGAGATGATCGCATAGTGGATAATAATCCGGGTGTGATTGGTCATCATAT
GCCTTTGGATGTTGGGGATTGTACGGTTCACAATGGATGGACCATGCATTGTGCGAATGGCAACATGAGGAAT
ATGACGAAGAAGAAAAACAACAAAAATAAAAAGAATGGTGGGCAGCAGCAGCAGAGCGATGAAAATAGCTTGT
TCCAGACGCGGTATGCTATTGCCATTTCGTATATTGACAGCCATGCCGAAGTTCGGGGAGATGTACCTGGCGT
CGGAAAGAATGTTGTAGGGAATCGTGGTGGTGGTGGAAAGAGGAAGGGAGGTAACACCAACAAGAAAAGCAGG
GTATCGTACAAAGGTGATGCCGAGGACAGGGCGAGTTATGCACAATGGATTGGGGATGTTTTGCCGAGAACAC
GGTTTGAGCATGAACTCATGCCTGATGTTTGGCCACCATCAGCATGAGGGTTATCATTTGTAGAGTCATTCAC
AGTTGCCCCTGCAAAGTCGTACGTCGGGGAATGAGGACCGCTGAAAATGGCACAAAAAAGTGAGCGATGCATT
CACATTCGAGTATGTGTACAAACAAGAGCGAGAAGTCAACAATAAGCTAAATAAATTCTCGAGGAAAGTTTTT
GTGTCTCTACCCACTTGCTGCCATAATCAACTCGAACTATTCATTTTTGATCAGCGTATCACCTTTGCCTGCA
AGCAGGTATCATTTACCCTCTATTCCAATTAACGATGTGGTTTTAAATAGTTCTGTCTTGGTTCCGTCCGCAT
GAATTGAACACCTTCGATCCTTCTGTTGTTTGCCAGTCGGATCCAAGATGGTCACACTACCATACCGTTTGCG
GCCATTTTATGGGTAAGCATGTTTGGGATCATTGTGGTGGCGCATCCTATTTCCACTTGATCAGCACAACATC
TTCACCATTAAGCAAGTACTCCTTACCCTCCATTCTGATTTTGCCATTCTCTTTTGCCGCGTTGTAATTGTCG
TATTGCACCAACTCCCGTGCTTCTATTGCTTCTGCATTCATAAAACCTTTTCTTATATCCCCATGCAGTTTAC
CAGCAAGATCCAGGACTGTCATACCATTTGATCCCAAAATGTGTGTCTTGGTTGTTTGGGATCGCTCCGATGG
CACGCCCGGGCCAGTGTATACAATGCCAAAGTCCAGAACGTCCTTCACACGTAACGGCAGTGCGTGATAACTC
AATTGGTCCGCGTAGGTCGTCCCCGAAGCATCATCCGAGTCGCCGCATTCCACTCCCATGGATTCCAAATATT
CATGACGCTCTTCTGCACTCAACGAACCCATTTCCGACTCCAGCTTGGCGCTCACCACCATGCACGAATCTGT
CTCCAGATCGCAATACTGAATTTGTTTCATGTAGCCTTCCGCCAATTTGATCGATTCATCCCTATTCAATGCA
AAATCGACCTCGTCCAAATTAAAGGCATAAATCACAGGTTTCAACGTCAACAACCCCATGGATTTGATTGCGA
AGCACTCCTCGGATGATAGTTCCATCGTCCTTGCTGGCAATCCATTCTCTAATCCTTTTTCAAGTTTCATTAA
CACATCCCTCTCTTCTCCTACACATGTGGTCCTCTCCAATCGTCGTTGGACATGGGCTAGATCCGCCAGGAGT
AATTCCAAATTGACGACTTCTGCATCGGCCACAGGATCCACCTTCCCGTCGACGTGGATGACATCTTCGTCGA
TGTAGTTTCGGACGACGTGGATTATTAGATCGCATTCTCGGATGGTTGCCAGGAATTTATTTCCTAATCCTTC
TCCTCGAGAGGCACCTTTGACTAATCCCGCGACATCGATCAAGAATATGCTTGCAGGAAGGGCCTTTTTGCTC
TTGGCCAATTTTGCCAGCTTTTCCAAATTGGCATCGGGTATCGGAACTGCGAAACAAAGGTATGTGAGTAAAG
AATGAAGAATCGTCGATAATATTTTTATCTGAAAATTTATATTCATTTTCGTACGAACCAGGCGTGATATTTG
GCTCGATAGTGCAAAAGGGGAAGTTTTTGGCATCGGCAATTGCTTTTTGGGCAATGGCATTGAATAGCGTGCT
TTTTCCGACATTAGGAAGTCCAACTAGACCAACTTTTATCTTCATGGATAGCAGCGTGTTTCGGGGTGATGTA
GATTTCAATTTAGCTGGCGCATGATGCGAGGTGGTGTGGCTGTAAATCGTGCTGTCGATTCTTATGGATTGCG
TGAATGATGATGTTGAAGACCAGCATAGCCCTTTTCTTGTCAAGAGAGTTATGCATATTAAGAATGCCCGCAT
GACCTCCATGACCATTTTCAGTAAAATCCTAGTTGACAGCGAAGCAGGTAGACCTCCAGATGCATGCGGAGAG
GACGGGATGTTGAGGGGATAGATAAACGGAGGATAGAGGAGATGGCATGGGAGATGGGAGACGGAGATG
>TR236|c0_g2_i1 len=1234 path=[5508:0-370 5573:371-425 5563:426-1134 5497:1135-1158 5498:1159-1172 5499:1173-1187 5500:1188-1233] [-1, 5508, 5573, 5563, 5497, 5498, 5499, 5500, -2]
TTCGAACCATAATTTCAATCGACATCAGTTCGTCAGTTTCATTATCAATCCCAAAAAATTTCAAAGATGGTAT
TCGGTTCAGAAACGCAAGTGCCTCTCGGCCAGCCTCTTAGCTCCGCATCTGGAGTGTCCCACGGATCGGGAAC
AAACCTCAATCTCTCATCACATGGAGACGAGAATACAACAGAATACCGTATCAAGGCGGCTGACAAGAGCGGA
AAGAAGACTATTTCTCTGTGGCATGATGTTACCCTTGTTCACGTGGACCCCGCGACCGATCGGCCAACTCCCT

ACCTTAACTTTGTTTGTGAGATTCCCAAATTTTCAAGGAAAAAGTACGAAATTGCTACTGATGAAGTGGGTAA
CTTTATCAAGCAAGATGAAAAGAAAGGTGTTCTGCGCGAATTCAAAAAGGGTGACATTTTCTTCAACTATGGC
TGTCTTCCGCGTACTTGGGAGGATCCGACGTTTGTCCACCCCGATGCGGAGGGATGCAGAGGTGACAATGATC
CAGTTGATGTTTGCGAAATCGGATCGAGAATTATCGGAACCGGCGAAATCCGTCCCACCAAAGTCCTTGGTAT
CCTCTGCATGATCGACGAAGGCGAAGCCGACTGGAAAGTTGTCACCATCGATGCCGAGGACAAATGGGCACCC
TTCCTCAATGACATTGATGACGTAGAGAAGCTCCTCCCTGGAACTCTGTCCGCGATCCGAGAATGGTTCCGCA
CGTACAAAATTCCAGATGGCAAGCCACCGAATGTCTTTGGATTGGACGAGAAATTCATGGACAAGCATTATGC
GCTTGAAATCATCAAGGAGTGCAACCATGCGTGGAAGGATTTGATTACCGGAGAGAAGGAGCGAAGTTTGGAG
AATTGCGATGCTGAGGTTAAGGGATTGGTGAGGAAGCTTTCCAAGTCGAATCTTGTGGATTTGGCCATCAATG
ATGACGAAGACGAGGCGCCGAATTTCTAAACTGGTGATTATTGACTGAGATACCTGGGGAAGGTTGAGGTATT
TTGTGGATCTGCTATGTAAGTTTCATTCTAGGTACTCCCAGTTTTCAAGACACACGAAGTGAAGTGTCATAGG
GAATGCTAGTACAAAAGCTCAACAGGAGATACAAGAAAGTGAGATGCTCACAGCGTCGAGTATGTGTACAAAC
AAGAGCGAGAAGTCAACAATTAGCTAAATAAAATCTATAGGAAAGTTTCTGCCATAAAAAAAAGAT
>TR236|c0_g2_i2 len=964 path=[5530:0-100 5573:101-155 5563:156-864
5497:865-888 5498:889-902 5499:903-917 5500:918-963] [-1, 5530, 5573,
5563, 5497, 5498, 5499, 5500, -2]
TGCTGAGATAACAAATGACAAATTTGACATTGAACCACACGCCATCATTCCTCGTCCAGGAAAAAGTACGAAA
TTGCCACTGATGAAGTGGGTAACTTTATCAAGCAAGATGAAAAGAAAGGTGTTCTGCGCGAATTCAAAAAGGG
TGACATTTTCTTCAACTATGGCTGTCTTCCGCGTACTTGGGAGGATCCGACGTTTGTCCACCCCGATGCGGAG
GGATGCAGAGGTGACAATGATCCAGTTGATGTTTGCGAAATCGGATCGAGAATTATCGGAACCGGCGAAATCC
GTCCCACCAAAGTCCTTGGTATCCTCTGCATGATCGACGAAGGCGAAGCCGACTGGAAAGTTGTCACCATCGA
TGCCGAGGACAAATGGGCACCCTTCCTCAATGACATTGATGACGTAGAGAAGCTCCTCCCTGGAACTCTGTCC
GCGATCCGAGAATGGTTCCGCACGTACAAAATTCCAGATGGCAAGCCACCGAATGTCTTTGGATTGGACGAGA
AATTCATGGACAAGCATTATGCGCTTGAAATCATCAAGGAGTGCAACCATGCGTGGAAGGATTTGATTACCGG
AGAGAAGGAGCGAAGTTTGGAGAATTGCGATGCTGAGGTTAAGGGATTGGTGAGGAAGCTTTCCAAGTCGAAT
CTTGTGGATTTGGCCATCAATGATGACGAAGACGAGGCGCCGAATTTCTAAACTGGTGATTATTGACTGAGAT
ACCTGGGGAAGGTTGAGGTATTTTGTGGATCTGCTATGTAAGTTTCATTCTAGGTACTCCCAGTTTTCAAGAC
ACACGAAGTGAAGTGTCATAGGGAATGCTAGTACAAAAGCTCAACAGGAGATACAAGAAAGTGAGATGCTCAC
AGCGTCGAGTATGTGTACAAACAAGAGCGAGAAGTCAACAATTAGCTAAATAAAATCTATAGGAAAGTTTCTG
CCATAAAAAAAAGAT
>TR237|c0_g1_i1 len=3053 path=[3763:0-378 3764:379-395 3891:396-554
3907:555-578 3909:579-587 3910:588-602 3875:603-626 3896:627-680
3887:681-704 3873:705-884 3784:885-947 3903:948-1033 3793:1034-1088
3895:1089-1202 3767:1203-1226 3885:1227-1571 3862:1572-1588 3905:1589-
1817 3902:1818-1899 3908:1900-1943 3869:1944-2000 3883:2001-2170
3807:2171-2194 3867:2195-2472 3770:2473-2485 3771:2486-2509 3772:2510-
2536 3900:2537-2591 3892:2592-2594 3881:2595-2618 3865:2619-2674
3800:2675-2698 3801:2699-2700 3802:2701-2724 3880:2725-2786 3829:2787-
2810 3864:2811-2973 3859:2974-2997 3860:2998-3052] [-1, 3763, 3764, 3891,
3907, 3909, 3910, 3875, 3896, 3887, 3873, 3784, 3903, 3793, 3895, 3767,
3885, 3862, 3905, 3902, 3908, 3869, 3883, 3807, 3867, 3770, 3771, 3772,
3900, 3892, 3881, 3865, 3800, 3801, 3802, 3880, 3829, 3864, 3859, 3860, -
2]
GAACAAATGCACAATATCACAACCAATCATCTGAATCGTCGTCACCATGGAAGACACTAATGGAGATTCTACA
TCCCAGCATTGCACTGCCAAAAATATTGGTCCATCGCAACAAAATAATGACGATGGAGTTCTCGTTCATCTAG
AACCTAGTAGGGGTAAATCGGACGGGAACGATGACGACAGCGATTGCAGCGAAAATGGTCCTATCGCTCTCCT
TCCTCCATCATGGTCCATCGAATATGTCCAAACCATCGAAAAGAAAGCGCCCATAGTAGTATCGGAAAAAATCG
AATGATGATACGAACAATATTATACCATCGGTCATGGATGTGGGTATGCAAATGGTACGGGGCACTCTCGATG
GAATATCCAAAAAACTAAAGAAGAAGAACAAAGGATCGGCAAATAATAATAAATCAGTATTGGTGAATCCTAC
ACCCATTCAATTGAAATTGTGGCCAGCATTGCTTCATTCCTTCCAATCGAAAGTTAGCGCAGATGATGACGAT
GAGCCTTCTGCTACTGTAGCATTGAATGTTGTTGGGATTGCTCCCACTGGAACAGGAAAGACCATGGCCTACG
CAACTCCCATTTTATCACATTGCGTTCGAACCCTCCTTTGCCAGTTACATCCTGCACATGCACCACCATCGTC
ATCGTCTTCAAAACAATTATGGAACCATTCAACAATGGTACACGGATTAGTACTCGTTCCAACCCGTGAATTG
GCCATACAGGTATCGAAGGAATTTAAAGTGGGTGCTAAGATTGCCAATAAGTATTTGTCGAAGTACTGTGCTG
CCTACGCGGGGATGGAGGGCAAGAGTGAGACTGATGGAGCAGATGCGCCATCATTGAAGGTTAAATCAATTGC
CGTGTATGGAGGTGTTGATATCGGAGAACAAATTACTACACTTTTGGGAAAAGGAATGGAATCATCCTCCTCC
TCTGGTCAGATCAGTCGGAGGTCTTTGGTCGTGGCAGCAACAGCAGGTCGGTTATTAGACATTCTCAAAAAGA
CCGATGCAAACGGCCAGCCAATAACAGTAGCATCTGCCTTTGCCAACCTCCAGGCGATCGTCTTCGACGAAGC
GGATCGTATAGCGGTGAATGCCGACATGGCGAGCCAAGTGGATGAGATTATCTCCATTGTGAATACGGAAAAA
AGGAATCGTTCCAAGGATGATGATGATGGTGGAGTGGTGGTGGCCAACTGCGTGGATGATGTTGTATCTTGCC

TGGTATCGGCGACGCTGCCCGAGAAGGCGAAAGAAATGTGTGACAAATGGGTACCGCGCAGTCGTATCATCGT
CAAGGTCGACAATGTGAAAGTGGGAGGAAGGCAAGACATGAAAATGGATTCCCCCGACAATGACACAACAACA
ACAAAGGACAGAAAATGCAATGGTGGTGATGACGGCGAAGGCTCTCCCAACGAATCATCCTCAAAGACAGCAG
AGAAGGAAGGCAAGAAAAGGAGTTCCATTCCAGCAAATCTAGATCTGGGATCGATACCATCCAACATAGTCCA
AACATTACACGTATGCTCCCCTCACAAAAAGCCAAAAAAGCTCCTTCTCACACTACAAAGAATTTACTCGCCG
AAGAAGGGTGCTCAGCAGAACAACGGACGATATTCGGTCAATAATCGACTGTGCATCGTGTTCTTCGCCCAGA
TCAAAACGGTCAAGTTCGCTTCCAAGTTTTTGATCAAGGAAGGATTGAGGTGTGTGGAGTTGTATGGGAGTTT
GAATCAGAGCGAGCGCGATAAGCGACTGCTTGAGTTTAAGTGCGGCAAAACTCCCATCCTTCTCGCGACGGAT
GTGGCTGCGAGAGGTCTTCATATTTCCAATGTTCACTTTGTTGTAAATTATGACTTTCCCGGATCACTGGATC
AGTATGTGCATAGATGCGGACGAGCAGGAAGAAAGCACGCTCTCAGCGGAGGAGATGCAGGGTCGTATCCACC
AACCGTATACAGCTTCTTCCCAAGAGAGTTCCCCAGCGAATATGCAGATGCTGTGGTAGACTTGTTGAAGGCT
TCGAGTGCCTGGGTCGATCCCAATCTACTGGCGCTTACGAAGAGGAATAAGACATTGGATAACGGTACGAGTA
AGACGCGAAGAAAGAGAAAGAGGGGGAAGAAGGATAAACCAGCGGATGATGCCGAAAACAGCAAAACTGCGGA
AGGTCAAGATAAAAATAATGATGATGATGATGATGACAGGGAATTTTCCTTCCTCGGTCAAATTAAATTGAAG
CGTGCATCTCATGTTAGTGACGCGGAAGACTCGGATGATAATGATAGCAGCTAATTATTTTAAATATTGCTAC
CTTTTCCAGATCTCTCGATTATTGCCAGTCACTGAGGGGATATTCCCACCGGCTGAGGGGGATTTCCCATTTG
CGAGGAGCAATTTCCTACCCGTTCCCACTGGCCTAATATGCAGCTTTTTAATTCGAAAGGAGTGCCATCGAAC
AAACCATTATGACACCTAAATTATCAATACCATGCGATTATGAAAATTGTGACCCGTCTGTAGACCACGCTAA
TGATGTCATAGATCTTTCTGGTGGGCGGGAAAAGGTGGGAAGTTCATCGGGCAAAAAATACTTGATCTGGAAA
TTGTACCGTGACAACACTGAATAAACAAGCAGGTACAATAATATGGTAACAAATTGAAATTCTGGAGGGATCC
GCGGACAATGTTCCCGCAAAGCTGCAATCTAGCATTGGAGCGGTGAGCATCATAATATGTCCCAACCTTGGTT
TCTATCATATTTTCATGCTGCTGGACAGTGTTTCGTGGCTTTGGCGCCCTGTAAAGTAGTGTTTTCAACATTT
CGAGGATGGTGTTGAAACAATGGACCTGACTTGCTGTTAGCCTTCCAATGGTATCCTGGGGCCCTTTCAAGAC
CCCTTTTCACTATTTATTGCATGTCCAACCAAAGCAAATGTGCATTACTGTCATGCAGTAAAGTTTAACATGG
ACCTTACGATACTATCACCAGCAGGTTAACTATGGCCTGTAAGTACAATTAGCCTCCAGC
>TR237|c0_g1_i2 len=431 path=[3751:0-77 3902:78-159 3908:160-203
3869:204-260 3883:261-430] [-1, 3751, 3902, 3908, 3869, 3883]
GTGCTCTTCCGATCTGAAGTTTGAATGCGTCTGCATGTGTCAATTTGTATGGATAGGCAAAACTCCCATCCTT
CTCGCGACGGATGTGGCTGCGAGAGGTCTTCATATTTCCAATGTTCACTTTGTTGTAAATTATGACTTTCCCG
GATCACTGGATCAGTATGTGCATAGATGCGGACGAGCAGGAAGAAAGCACGCTCTCAGCGGAGGAGATGCAGG
GTCGTATCCACCAACCGTATACAGCTTCTTCCCAAGAGAGTTCCCCAGCGAATATGCAGATGCTGTGGTAGAC
TTGTTGAAGGCTTCGAGTGCCTGGGTCGATCCCAATCTACTGGCGCTTACGAAGAGGAATAAGACATTGGATA
ACGGTACGAGTAAGACGCGAAGAAAGAGAAAGAGGGGGAAGAAGGATAAACCAGCGGATGATGCCG
>TR238|c0_g2_i1 len=3227 path=[4197:0-471 4198:472-495 4356:496-823
4336:824-876 4355:877-877 4349:878-901 4334:902-902 4317:903-926
4354:927-928 4348:929-952 4333:953-1037 4267:1038-1063 4347:1064-1066
4332:1067-1090 4315:1091-1176 4234:1177-1200 4346:1201-1249 4299:1250-
1273 4331:1274-1395 4357:1396-1419 4359:1420-1441 4345:1442-1463
@4353@!:1464-2077 4310:2078-2146 4344:2147-2165 4328:2166-2189 4309:2190-
2194 4343:2195-2218 4327:2219-2257 4308:2258-2281 4342:2282-2308
4326:2309-2332 4307:2333-2585 4272:2586-2609 4341:2610-2632 4325:2633-
2656 4306:2657-2663 4352:2664-2687 4340:2688-2718 4324:2719-2742
4358:2743-2780 4351:2781-3043 4323:3044-3077 4304:3078-3086 4337:3087-
3110 4321:3111-3135 4302:3136-3174 4265:3175-3226] [-1, 4197, 4198, 4356,
4336, 4355, 4349, 4334, 4317, 4354, 4348, 4333, 4267, 4347, 4332, 4315,
4234, 4346, 4299, 4331, 4357, 4359, 4345, 4353, 4310, 4344, 4328, 4309,
4343, 4327, 4308, 4342, 4326, 4307, 4272, 4341, 4325, 4306, 4352, 4340,
4324, 4358, 4351, 4323, 4304, 4337, 4321, 4302, 4265, -2]
GCAGCGGCGGCGACGCAGTACCATACAGCCAGTTCAGGAGAAGACGGACCCCAGCTATCTACGATACTTATCT
ATCCGTTCAATCGAGCGGGGGATAAGGCACCAAACAAGAGCAAAGGCAGCGGTGTCGATCGTCTCGGGGGGTG
CATGCACGCACTGAAGGCAACGCGATATCTACTTCTCTAGGTAGATACTTACGATCGGCGGTCGCGGATCAAT
AATTGATAATTTGTGGGACCAGTTTCGGTGCGCAGAGTGATTTTCGCGTCTCCGCACGAGGTACTATTCCTAT
CTACGTCTCCACCAGACTCTTGAACCAAGTTGAACGAGGAACACGAAAACGAAGACCATGATGATGTGCCGCC
AATCAATGTGTGCCACATCATAATATTAGAGCCTTTTAATAAAATGTCATCAAAAAGCCCCGGCCAAGGAAAC
AGCCGGGGCTCGCCTGACCGTCCCAGCGCAGGCACAAATCCTGATCTAAGTATTGACGAATCAAATTTACTGC
CAGAAGAAGACAGAATAGATCAAATCATACATCAACATCGATATAGACCAACAAGCATTCAAACACAGCTAAA
CCTGCTTCAGCAGCATATCGCACCAAGAGATCAGGGAGACGCCTCAATTGTACGTGGAGGCACACACTCTCCA
CATCAATCAAGATTAATAGGCTACACTGCATCATTCTCTACTGGACATCAGTCGCTACCTCAATCTTACCCAG
CTGGCCAACCTTCCGCGCAATCATTTGCACAGAGTTATTCCCCTCCATTTTCGCAAAATTATTCGCCTCCATT
ACCGCCAATTCATTTGACAAACGAAGCAAGCCTATCTGGGAATATCGCAGGAAGTTCATCTGGGAATATCGGC

GAAGTAACTCATGAATTATTGGGTTCTTCATCACAATGGTCTGTAACCCAATCGCGAATACCGTCCTTACCTT
CCTCGGCAGTCAGTCCTCCCCAACATACCCCTCGAGCTCGGGGCAGACGATTCAGCGCTTCAACGATTCCTCC
CGCCCAGGCATCATTATCACAAGCAACTTCTCACCCACAAATACCCCGCACATTACTCCCGTCTATAACCAAT
CTTAGCGTGGACACAGACACTCAGCGCAGCATATCGGGACAATTACCATCACCTATTTCCCCCTCGACATCAT
CTGTTCAATTACCATTTCTTGCGTCGCCAGACGATCTAAAACCCCTTCCTCAGAATCTGCGGGGAGATCCGTT
CCGTTCCGCGAAGGTCAAGACTGAGCTTTGCCGCTTTTACAAAACTCCCAAAGGTTGCATTTTTGGTGATAAA
TGCAACTATGCACATGGAGAGAATGAGCTGAAATTCAATAAGCTGATGGATTTGGAGACGGCGGGATTGGTAG
ATGTGGAGGTGTTTATGTGTCATGTTTGTTTGACCTGGGTGGCGACTGGCGCTTGCCCTTTCGACCAAAGATG
TACGAGATTACACGACCCTCGAGTTATTGGCATAGAACCGTCGTGGCTACCACATGCTGAAGTATTAGTCAAT
AATACGAAGAAAGGGAGGGAGGTTGACAAATTGTACCACCAACAGTACTGTTCAATTTACAGCTGCTCGCCCA
TCTATGGCTTTGCCCCCAAGAAGAGATGGAAAGCGGATGAGACGAGTACCAATGCGGCATGGAAAGAATTATA
TTCCTTTTGCTGCAATACAGATGCAAATCAACCATACTCCCAATGTAAGCGATGGCAGCTATCTCCACAGAAG
AATGCGCAAGGTGCCGAAATCACCGAAATGCATTGGCTTGCTATGGCACTCATGATGAGAGAAGGACGAAAGG
CACAGTGCTTCACGTATTTACCATCTCATGTATTTTGGGGCGAGCTTTGCTTGCTCCTTCAAACGAGCTATTT
TGAGTTGAAAGTAGTCGAGTCTTACCAGGACATTCAAAGATATGAAGTTGTACAAGTGTCTGAAGAAGAGGCC
ACCAAAATATCTGATTCGGGAAAAGGTAGCCGCATAATCGTTGCTAGACAGATTGCGTTTGGGCCAGTTGCTG
ATGCTAGCGTCCGCCAAGTATCTATTTGGTTCGACATCAAGCAAGAGGACATTGTTCCGTGTACACGTCAACA
GGCGCGCAGACACAGGCGTTCCCGACATCGAATTCGAGCGAAACGGAATTATGAGAATCAGCCCGGCGAAGCG
AAAATTGCTGCGTCTTACTCTATTCCACCATTTACCTGCCACGAGCCCACGGATGAGGCCGCATTTAACTTGA
TTACTGGAATTCAAACTTATCGGTATCGCGTGCTCAGGTATCTTTCATCCAGTCCTGACGAACGTGCACTTCG
TTCTTTGGCATTGGAAGAAGAGAAGCTCCGAAATAGCTTTGAAAGTCAGAGACGATTTTGGATGACCTGGACG
TGGCCCAAAAAGATTGGGAGTTGTGATATCGGAAACGAAACTGAAGTTCCAGATGTTGATGGGACTTATAACT
TTGTGATACACGGCGATCCAAAATACAGGGAAGACTCTCTCTTCTTTGGATCTGATGTACAAGATGGAAATGC
CGATGATAGCCTCGAAATAGTTTCAAAACAGACCAAGCTAGCTACTGGTCTTGTTTGGAAATCGTTTGTGATG
AATTTGCAACTATTTTCTTACCAAGGAGCTATTGCATTGCCAAAGGAAGAAGACGAAATGCCAATACACGATC
CTATTATTCCGACGATACATCGTATCCCCACTTTGCGGAGCTTATCATTAGGATTTTCAGCTCTTCGACACTC
TACCAGGAATATTCCAAGAATTAATTCTCCTGTGTGCACAACAGCTGCCCCCGTGGTCACTTCATTGGATGTT
TTGATACACGAGTGGAAGATGATGCAACGCCAATACGAGGAAAATTCATCGTACCCCTTGAAAGATGCAAAAC
AAGGGAACGGACGCCATCCTGATCCAGCGAAACGCAGGCCCCTCTCTTATTCGTTCGAGAAAGCAGCACGTAG
TTGGACTCGAGAACTGCCCAAATTGTGACGGAACTGATTTCATACAAAACACTTTCATTTCATGGGAGCCAGA
GTTCATGGTCTGGTTCCACTGTATCATCCCCGAAGTTCACTCTCTCATGTTGAACTTTGGCGCGGTAAAATCA
TTGCCTGACAAGCAGTCGATACATTGATAGATAAATTTGATACATATTTTAAGAACAAATATCAAAAGCTAAA
AAAAAAAGATCGGAA
>TR238|c0_g2_i2 len=1992 path=[4175:0-169 4176:170-228 @4353@!:229-842
4311:843-911 4344:912-930 4328:931-954 4309:955-959 4343:960-983
4327:984-1022 4308:1023-1046 4342:1047-1073 4326:1074-1097 4307:1098-1350
4272:1351-1374 4341:1375-1397 4325:1398-1421 4306:1422-1428 4352:1429-
1452 4340:1453-1483 4324:1484-1507 4358:1508-1545 4351:1546-1808
4323:1809-1842 4304:1843-1851 4337:1852-1875 4321:1876-1900 4302:1901-
1939 4265:1940-1991] [-1, 4175, 4176, 4353, 4311, 4344, 4328, 4309, 4343,
4327, 4308, 4342, 4326, 4307, 4272, 4341, 4325, 4306, 4352, 4340, 4324,
4358, 4351, 4323, 4304, 4337, 4321, 4302, 4265, -2]
GTGAGTAATCATGTTGTTGCAGGAGGTGCCATTAGAATGGATCGTTGACCGGAGATTCGAGTATTGGCATCAT
ATTTTACACATGGGCCGAAAAATTAATTTCAGTTGATATCGGATAAATGGCGGCAGCGCTGAATTGCAAGATT
TTCTTCTCTATGGGAAAACGACTCATCCATCACTTCTATTTTTCTGTGTACGTAAAAATAGCCCTTTCGACCA
AAGATGTACGAGATTACACGACCCTCGAGTTATTGGCATAGAACCGTCGTGGCTACCACATGCTGAAGTATTA
GTCAATAATACGAAGAAAGGGAGGGAGGTTGACAAATTGTACCACCAACAGTACTGTTCAATTTACAGCTGCT
CGCCCATCTATGGCTTTGCCCCCAAGAAGAGATGGAAAGCGGATGAGACGAGTACCAATGCGGCATGGAAAGA
ATTATATTCCTTTTGCTGCAATACAGATGCAAATCAACCATACTCCCAATGTAAGCGATGGCAGCTATCTCCA
CAGAAGAATGCGCAAGGTGCCGAAATCACCGAAATGCATTGGCTTGCTATGGCACTCATGATGAGAGAAGGAC
GAAAGGCACAGTGCTTCACGTATTTACCATCTCATGTATTTTGGGGCGAGCTTTGCTTGCTCCTTCAAACGAG
CTATTTTGAGTTGAAAGTAGTCGAGTCTTACCAGGACATTCAAAGATATGAAGTTGTACAAGTGTCTGAAGAA
GAGGCCACCAAAATATCTGATTCGGGAAAAGGTAGCCGCATAATCGTTGCTAGACAGATTGCGTTTGGGCCAG
TTGCTGATGCTAGCGTCCGCCAAGTATCTATTTGGTTCGATATCAAGCAAGAGGACATTGTCCCGTGTACACG
TCAACAGGCACGTAGACACAGGCGTTCCCGACATCGAATTCGAGCGAAACGGAATTATGAGAATCAGCCCGGC
GAAGCGAAAATTGCTGCGTCTTACTCTATTCCACCATTTACCTGCCACGAGCCCACGGATGAGGCCGCATTTA
ACTTGATTACTGGAATTCAAACTTATCGGTATCGCGTGCTCAGGTATCTTTCATCCAGTCCTGACGAACGTGC
ACTTCGTTCTTTGGCATTGGAAGAAGAGAAGCTCCGAAATAGCTTTGAAAGTCAGAGACGATTTTGGATGACC
TGGACGTGGCCCAAAAAGATTGGGAGTTGTGATATCGGAAACGAAACTGAAGTTCCAGATGTTGATGGGACTT
ATAACTTTGTGATACACGGCGATCCAAAATACAGGGAAGACTCTCTCTTCTTTGGATCTGATGTACAAGATGG

AAATGCCGATGATAGCCTCGAAATAGTTTCAAAACAGACCAAGCTAGCTACTGGTCTTGTTTGGAAATCGTTT
GTGATGAATTTGCAACTATTTTCTTACCAAGGAGCTATTGCATTGCCAAAGGAAGAAGACGAAATGCCAATAC
ACGATCCTATTATTCCGACGATACATCGTATCCCCACTTTGCGGAGCTTATCATTAGGATTTTCAGCTCTTCG
ACACTCTACCAGGAATATTCCAAGAATTAATTCTCCTGTGTGCACAACAGCTGCCCCCGTGGTCACTTCATTG
GATGTTTTGATACACGAGTGGAAGATGATGCAACGCCAATACGAGGAAAATTCATCGTACCCCTTGAAAGATG
CAAAACAAGGGAACGGACGCCATCCTGATCCAGCGAAACGCAGGCCCCTCTCTTATTCGTTCGAGAAAGCAGC
ACGTAGTTGGACTCGAGAACTGCCCAAATTGTGACGGAACTGATTTCATACAAAACACTTTCATTTCATGGGA
GCCAGAGTTCATGGTCTGGTTCCACTGTATCATCCCCGAAGTTCACTCTCTCATGTTGAACTTTGGCGCGGTA
AAATCATTGCCTGACAAGCAGTCGATACATTGATAGATAAATTTGATACATATTTTAAGAACAAATATCAAAA
GCTAAAAAAAAAAAGATCGGAA
>TR238|c0_g2_i3 len=1612 path=[4197:0-471 4198:472-495 4356:496-823
4336:824-876 4355:877-877 4349:878-901 4334:902-902 4317:903-926
4354:927-928 4348:929-952 4333:953-1037 4221:1038-1063 4347:1064-1066
4332:1067-1090 4315:1091-1176 4234:1177-1200 4346:1201-1249 4299:1250-
1273 4331:1274-1395 4357:1396-1419 4359:1420-1441 4312:1442-1464
4162:1465-1611] [-1, 4197, 4198, 4356, 4336, 4355, 4349, 4334, 4317,
4354, 4348, 4333, 4221, 4347, 4332, 4315, 4234, 4346, 4299, 4331, 4357,
4359, 4312, 4162, -2]
GCAGCGGCGGCGACGCAGTACCATACAGCCAGTTCAGGAGAAGACGGACCCCAGCTATCTACGATACTTATCT
ATCCGTTCAATCGAGCGGGGGATAAGGCACCAAACAAGAGCAAAGGCAGCGGTGTCGATCGTCTCGGGGGGTG
CATGCACGCACTGAAGGCAACGCGATATCTACTTCTCTAGGTAGATACTTACGATCGGCGGTCGCGGATCAAT
AATTGATAATTTGTGGGACCAGTTTCGGTGCGCAGAGTGATTTTCGCGTCTCCGCACGAGGTACTATTCCTAT
CTACGTCTCCACCAGACTCTTGAACCAAGTTGAACGAGGAACACGAAAACGAAGACCATGATGATGTGCCGCC
AATCAATGTGTGCCACATCATAATATTAGAGCCTTTTAATAAAATGTCATCAAAAAGCCCCGGCCAAGGAAAC
AGCCGGGGCTCGCCTGACCGTCCCAGCGCAGGCACAAATCCTGATCTAAGTATTGACGAATCAAATTTACTGC
CAGAAGAAGACAGAATAGATCAAATCATACATCAACATCGATATAGACCAACAAGCATTCAAACACAGCTAAA
CCTGCTTCAGCAGCATATCGCACCAAGAGATCAGGGAGACGCCTCAATTGTACGTGGAGGCACACACTCTCCA
CATCAATCAAGATTAATAGGCTACACTGCATCATTCTCTACTGGACATCAGTCGCTACCTCAATCTTACCCAG
CTGGCCAACCTTCCGCGCAATCATTTGCACAGAGTTATTCCCCTCCATTTTCGCAAAATTATTCGCCTCCATT
ACCGCCAATTCATTTGACAAACGAAGCAAGCCTATCTGGGAATATCGCAGGAAGTTCATCTGGGAATATCGGC
GAAGTAACTCATGAATTATTGGGTTCTTCATCACAATGGTCTGTAACCCAATCGCGAATACCGTCCTTACCTT
CCTCGGCAGTCAGTCCTCCCCAACATACCCCTCGAGCTCGGGGCAGACGATTCAGCGCTTCAACGATTCCTCC
CGCCCAGGCATCATTACCGCAAGCAACTTCTCACCCACAAATACCCCGCACATTACTCCCGTCTATAACCAAT
CTTAGCGTGGACACAGACACTCAGCGCAGCATATCGGGACAATTACCATCACCTATTTCCCCCTCGACATCAT
CTGTTCAATTACCATTTCTTGCGTCGCCAGACGATCTAAAACCCCTTCCTCAGAATCTGCGGGGAGATCCGTT
CCGTTCCGCGAAGGTCAAGACTGAGCTTTGCCGCTTTTACAAAACTCCCAAAGGTTGCATTTTTGGTGATAAA
TGCAACTATGCACATGGAGAGAATGAGCTGAAATTCAATAAGCTGATGGATTTGGAGACGGCGGGATTGGTAG
ATGTGGAGGTGTTTATGTGTCATGTTTGTTTGACCTGGGTGGCGACTGGCGCTTGGTGAGTAATCATGTTGTT
GCAGGAGGTGCCATTAGAATGGATCGTTGACCGGAGATTCGAGTATTGGCATCATATTTTACACATGGGCCGA
AAAATTAATTTCAGTTGATATCGGATAAATGGCGGCAGCGCTGAATTGCAAGATTTTCTTCTCTATGGGAAAA
CGACTC
>TR239|c0_g1_i1 len=2135 path=[2849:0-207 2974:208-246 2994:247-282
2993:283-306 2989:307-386 2866:387-403 2972:404-628 2995:629-652
2999:653-677 2970:678-708 2996:709-741 2981:742-858 3000:859-862
2987:863-1009 2979:1010-1057 2992:1058-1065 2986:1066-1089 2978:1090-1198
2891:1199-1222 2966:1223-1267 2997:1268-1291 3001:1292-1292 2965:1293-
1380 2899:1381-1404 2949:1405-1591 2977:1592-1694 2838:1695-1718
2964:1719-1737 2991:1738-1761 2998:1762-1781 2962:1782-1814 2990:1815-
1852 2975:1853-1865 2843:1866-1889 2844:1890-2134] [-1, 2849, 2974, 2994,
2993, 2989, 2866, 2972, 2995, 2999, 2970, 2996, 2981, 3000, 2987, 2979,
2992, 2986, 2978, 2891, 2966, 2997, 3001, 2965, 2899, 2949, 2977, 2838,
2964, 2991, 2998, 2962, 2990, 2975, 2843, 2844, -2]
TATCTGCCGTTGTTCTTATTGTCGCCGTGGAGGAGAGAGAGAGAGAGAGAGAGAGCATACACCTATTCTCCGT
TGTCCCTCGGCTGATATCCCAAGGTTGTTCTCGGCCGGTGGTATACATTGCGAATTCTTGAGCAGTCAAAATC
AACACGGGGTCAATTGGAATCGCAATCACTGACAATTGGTAGATAACCCCCCTTTGAGACATAGTCGACTTCT
TCAGCCTTCGGACGTAACTTTTTATCACCGTGGAGAGGGGGTAGGAGGGCATCCCCATCCCTTTGCGTGGGCC
TCATTCCACCGTTGGACAGTGCATTCTAGATAGTACTACTATTTGATAATAGCGTGGGAGAGAATTGCGTTAT
TGTTCGAGAGGAAGAGAGAGAGAGAGGGAGAGATTGGCATTCGCCCTATTGACGTGATTTATTTTCGAATAGC
AGAGGCGTCGAGATTCGTCATTAGATTCAGGTTGACAGGTAGTACGCTTTTTCCTTAAACGACAAACATACAG
GGAGATTCTACGCAATGATTCGACAACACAACGCCAAAGTTCACGACAATCGTCACGACGGAAATAATGGCAA

AAGCAGATCACTGGGCCCATCGGCACAACCGCTATCCCAAATCAAGAACAGCCCTTCCGCACCACCTCCGCCA
ACGTCGAAATTCCAACAAATGTTCTGCCTAAAACACCGTCGTTGTCGAACCAGCGACCTCCTCCTCGTCCTCA
TCGGATTCGCGGCCTGTCTCCTCTCGGTGACGCACATCCTCTGGCATTCGGAAAGTCTGCCCCACGTCCATCG
ACACGACAATGGCAAATCGTCCTCATGGCGGGAACACAACAAAATGAGCGCGCTGAAAACCCATTTGGACGGC
TTCGTGAATGCCAAACGGATGAAGAAAGCCATGAGGGGCAGGAGGGTTCCATCTCGCGAGGACGGAGCACGAA
AACAACAAAATGATTTGACGGTGGGAGCACACAACGAAAGCATCAACGATGAACCTCACCCGGTGAAAACTGC
AAACAACAATCCTAGTCTCCCCTTGCCCTCAAAACAACCTCACCTTGAACAACAACCTCACCCGGTAGCGCAC
CTCAACTGCGCCGATCACGGCGGACCCACCGATCCTCGCATCATCGACGAAATGGTATTCTGGTCCGACATCC
CCGCCGACGCCTCCTACCTGAGTCCCATGCATCCCCTCAACGACCCCCACTCTCCCGACGACGCGGAGCGATT
CCTCACGTTCGAACCCGACCACGGAGGCTGGAACAACATACGCATGGCGATGGAGACGGCGATGGTCATGTCC
CACGCGATGGGAAGGACGCTGGTCTTGCCTCCCGAGCAGCGCATGTACCTGCTGGACAAGGGGAAGAGGGACG
ATCACTTCTCGTTCAACGATTTCTTTCACTTGGATGCCATTTCCATCGAGCACAGGGGGTTCAAGGTCATTAC
CATGGAGGAGTTTTTGGTCAGGGTGGCCAGGGAGGGGAGGTTGAAACACCCTAACACTCGACGACCCGTCTAT
CCTCCCGGGAATCAGACCAGCTGGGGCGGCGCCGGTGGCAACGGCAATCGTCGGGATCTCTTCCAGTACCTCC
GATCGGTCGGGAACACCCCCGAGTGGGATCCGTGGGAGTGCGCCCTCGCCATACCCTCCTCCACCGAACAAAA
ATCCGTCGACGATCTCAACCGCACCCTCCAATTCATCATGCAGGGCTCCGACGGCAGGTTCAAACCCACCCTC
GAGGAATTCAACGGCCGTCCCACCCCCGTCAACGCCACCATGCACGACCGCATGCGAGAGATGCTCGCCGATC
GGAACAAGCTCTGCATCTACGACAGGACCCTCCAAACGTCTACGCTCATCCACCTCAAGGTGGAGAAGGGCGT
GAGGCTCCTCACGCACTTTTACGCCTTCCTCTTCTTCGCGGATTGGAGGCAGGATCTATGGAGCAAGCGGTTC
GTGAGGGATCACCTGCGGTACGTGGACGGGATCGTGTGCGCCGCGGCGAGGGTGGTGGAGGCGGTGAGGGAGC
GCGCGAGGAAGAACATTCGTTACGACAACAACAATAACAATAACAATAACAACAACAACAACAATAACGCGAG
TCGCAGTCGTGCCGACGA
>TR239|c0_g1_i2 len=761 path=[2849:0-207 2974:208-246 2994:247-282 2993:283-306 2989:307-386 2866:387-403 2972:404-628 2995:629-652 2999:653-677 2971:678-708 2996:709-741 2936:742-760] [-1, 2849, 2974, 2994, 2993, 2989, 2866, 2972, 2995, 2999, 2971, 2996, 2936, -2]
TATCTGCCGTTGTTCTTATTGTCGCCGTGGAGGAGAGAGAGAGAGAGAGAGAGAGCATACACCTATTCTCCGT
TGTCCCTCGGCTGATATCCCAAGGTTGTTCTCGGCCGGTGGTATACATTGCGAATTCTTGAGCAGTCAAAATC
AACACGGGGTCAATTGGAATCGCAATCACTGACAATTGGTAGATAACCCCCCTTTGAGACATAGTCGACTTCT
TCAGCCTTCGGACGTAACTTTTTATCACCGTGGAGAGGGGGTAGGAGGGCATCCCCATCCCTTTGCGTGGGCC
TCATTCCACCGTTGGACAGTGCATTCTAGATAGTACTACTATTTGATAATAGCGTGGGAGAGAATTGCGTTAT
TGTTCGAGAGGAAGAGAGAGAGAGAGGGAGAGATTGGCATTCGCCCTATTGACGTGATTTATTTTCGAATAGC
AGAGGCGTCGAGATTCGTCATTAGATTCAGGTTGACAGGTAGTACGCTTTTTCCTTAAACGACAAACATACAG
GGAGATTCTACGCAATGATTCGACAACACAACGCCAAAGTTCACGACAATCGTCACGACGGAAATAATGGCAA
AAGCAGATCACTGGGCCCATCGGCACAACCGCTATCCCAAATCAAGAACAGCCCTTCCGCACCACCTCCGCCA
ACGTCGAAATTCCAACAAATGCTCTGCCGAAAACACCGTCGTTGTCGAACCAGCGACCTCCTCCTCGTCCTCA
TCGGATTCGCGGAGATCGGAAGAGCGTCGTG
>TR240|c0_g1_i1 len=3514 path=[4237:0-51 4365:52-247 4215:248-271 4380:272-357 4328:358-381 4364:382-463 4256:464-487 4379:488-778 4306:779-802 4363:803-872 4277:873-896 4397:897-1024 4361:1025-1058 4390:1059-1069 4377:1070-1093 4360:1094-1114 4389:1115-1138 4396:1139-1263 4359:1264-1297 4401:1298-1326 4394:1327-1367 4375:1368-1394 4400:1395-1418 4393:1419-1439 4373:1440-1464 4385:1465-1486 4355:1487-1528 4372:1529-1588 4221:1589-1612 4354:1613-1672 4316:1673-1696 4384:1697-1710 4371:1711-1734 4353:1735-1914 4331:1915-1938 4399:1939-2139 4352:2140-2205 @4392@!:2206-2309 4368:2310-2346 4398:2347-2353 4382:2354-2377 4391:2378-2480 4297:2481-2498 4381:2499-2519 4366:2520-2543 4347:2544-2621 4338:2622-2645 4339:2646-3513] [-1, 4237, 4365, 4215, 4380, 4328, 4364, 4256, 4379, 4306, 4363, 4277, 4397, 4361, 4390, 4377, 4360, 4389, 4396, 4359, 4401, 4394, 4375, 4400, 4393, 4373, 4385, 4355, 4372, 4221, 4354, 4316, 4384, 4371, 4353, 4331, 4399, 4352, 4392, 4368, 4398, 4382, 4391, 4297, 4381, 4366, 4347, 4338, 4339, -2]
GGGACATACAGCTGGTTATTACTGTTATCATATAACAGTTCTATTGTGCGGATTTGTTAAAGTGGGCCAACAT
GGGAATGCCAAAATGGTTGCCCTGATTACAACTAGTTATCTTGAATTGTCAGGAATAAATTTCAAAGTTGAGG
TATTTTTCTAATTTGCAGTATAGCTCCTATGATGAAACACCAGTCAGTATCTTGTATATTGTTGAGCGTGGTT
GGACCTAGATACCATACTCATGGCTTGAATTAGAATAGGCATGATGTAAAAGTACCACATTGTTATGAAACCT
GCTAAGGAATTGGCTGTTCAGGAAGTCTTTCGATGAAGTCAAGTAGATCACCCGCCCTTAGGGATAATGTTCG
ATCGTGATATTTCAACCGCTGGCTACTGGCAACACAGAGATCTTTTACGTCATTGAAGGCCGGTACCGCCTTA
TTCGTTATCGTTATCACTGAGCTTCGCATCATGGGGTGGCACGGAAATGCCCTCCCGGAGAGCGTCAGCAATC
CCCGCCATAGAGCATCGCGGTCTACGAGCAGCATTGCTTTCAATGCTACTAAGGCCGCATCGCCAATGGGACC

AGTATCCGAGAGCAATGGAAAGAGCATGGTTCCGGCAGAAAGGATGAGGTTCGTCAAGCCATAGCGGCAGCTG
GACTCGAAGGTACACGTGAGGCAATGGACGATGGGCAACACTAGCGAATGCTTTGATTTGGCAAACAATGAGG
ATGGTGGCTGCTGGCCAGGGAAATGGCGGCCCCCGCTACCATTGCCATCATCAGGCAGAATGTCTTGCATCAG
TCCAGCGACAATTGGGTATGCATCGTTCTCGAATCGATCGGCAAAGAAGCCATCCTCGGAGCTACAGAGTTCC
GAGAGGATGAGAAGTAACTTGGAAATCAAAACTTCAAGGCTGGACTTGGATGGCGCCCCATGTTCTGGCTGAT
CAGTGGCCATCACGTGGCGTATTGATAGCAGGTCGGATCGAGATAGTCGATTCGCCGAAGTGAGGGACGCTGA
TGCCGATCGTAGTTGGGCTGCGATGGAGGGCCAAAACTCGGCGATTGCTGGCAACAGTGGATTGATGCTGCCA
AATTTTCCATGCAAGTTCTTCCGGAACGAACCGACCTTGCCGAGGGAGCGAAATCCAGAGAGTATGGTTTCGC
AGCACAGCACTTGTATCCGTAGATCTGCGTTGCAGAGGAGATAGCAGCACCGAGATAGGATGCCGTTGATGGC
ACAAATCTCTTGTACAAAATCTGACGTCTCGTCCGCTTCGTCGTTGTTTGAGGAATGATCACTGTTAGGAAAA
GGCGGAGCTTGTTCGGTGGGGTCACCTTCCTCATATGGCGCTTGCTGGCCAAAAATATCATCGTTTGAATCAA
TATCCTCCGAACATCCAGTCGATTCGATACTCAACTCAAAGTCAAGCCTCCTCAGAAAACAACCGTCGTCATT
ATCCTCCTCTGGAATTATCGGCGCAGCCCCATCTTTATTATTGCTTATAAAAACGTCGATGGATGCATCCATG
AACGTATTCATCGATCGAAAGACACTAACCGTGTCAAATAAACTCAAATTGTCCATGTTGTAAGACTGGCGAT
CAAAGTGACCCAGTAAACAATTTAACATGTGTTCCACCATAGCAACATGCCCCATGAAGAGAGGCTCGTCTCC
ATTTTCTACGTCAGAACTTGCTGGACGAACACGAGAAATGGCTGACTGGAGAATGACATCCACCACGCCAAAA
AGCGATCGTTCCAAGGCACTCTGCTCTCTTGCATATTTGCATAGTCTTAACGATAGTATATCAACCAAATAGT
CGAAATTTGATGCCAACAAACACGAAATATTTCTGTAGCCGACCGATCGAGATATTTTCCAAAGACATGCAAA
GGCAGCTCTCTGGACATACGAGTGATTGCCGATGGGAGATGCCCTTTCGAGAAGCGGGAAGAGAATGGTCGGC
AGCTGTAGCTTCACGCTGTCGCCAAGAGCACGTGCGAGTTGGCAGAGGAATCCCATTAACGTTGAAATTAAGA
TGGCATTGCTGTTCATGATGGTAATAGGCAGATTGGGTGCGTTGACTTCATTACTTGGCAACACCTGCGCATC
GCTGATACTTTCCTCCGTAGGAACGTCCAAAGTTGTAGGTAACATCCATACCGGGCCGCTGACAACGACAGGT
AGAATTGACGACGCCAACGAAGAGAGGACGTGCAGGTTCGACTTTGATAATTTGAAAGATTCCTTCCTACCGC
CATCGTCCCCAAATCCGCATATCCCTCTCATTAATTCCGTGGCGAAGGCGCACACTCCAGACCAGCATCGTCG
TCGATTAAGGAGTTCTTCATTGGTCAAAAGATGCTGACCGTCACATCGAGAACAAGCCTCAAACATGTCCGCA
AAGCAGGCATCGATCACGAACGCACACCTCTTCGTTCCAAGGGCTCTCGCAAATAAACGAACAGTGTTTTTTT
GCAGCTTCGATTACCTGCTCGTCCCTAAGATAGAGAAAACGACATCCGTTGCTTTCTCCGTATAATGCAATCT
CATCAGAGGAAGAACCTGTTTCAATCATGGGACCACATGTAATAGAGTCGATATCTGGGGCGAAAATCACCGA
AAAAGCGTCTTTGACGACGTCCACAACCTCCGTACACGAAAGCGCAGACCCAATATCACTCTTTCTCTTCCCC
GTCGACAAACACTCTTGCAAATCGAAATCATTTGTCATGCCCCTAAACGATAGCACGATATACCCATCCACCA
GGCGCAAATGATGTCGAACCTGCGATTCCCGGCCGCTCTTCGCCAACGCCGGAAGCGCTTCCACCAATCCGAG
TATCGTGGGAACGGCGGAGCGGCTAAGCTGTCGCTTCCAGTGATTCGTCGCCCCGAGGTGCGCCTTGTAAGAT
TGCAAGATTGAACGAGAGCAGTGGGCAATTTCGTCGTCTTCGTCGCCCAGCATCATCAAGCAGTATTCCAATG
CCTTTCGTGCGAGATCTTTTATGTTGGGTGCCGTCCAGACGGATCGGATATCCATCAAAATGATTCGACATAA
ATATAGTCCGCTTTTTCGCACTGCGGCGGAGCGGTTTGTGGGTAAAATACTGAGTAATACGGAAAGAGGACCT
CGTAGCCGTTCATTCACTTCTATTCTCAATTCTTGCATTTGATTTTCTGGAGGTGGCAATGGCTCTGTGTCTG
AGGTGTCAACTGTTTTATATTGTTGGTTTGATTGTTGCACAGCAGCCATGAGAGATGCCGTGATGGATTGCAT
TTTGTCATTGCCGTCCATGCTACCAGTTGGGTCTGCACATGTATTCAACGGTTTACGACTCAATGACTGCTTC
AACAATATCGTGAGAACATGAACACACGAAGATGCAACCTTGTAAGAAGACGCACATGACGAATATCGAAGTT
TTGATAGAGCCGTCCGGTACAAACCCGCGAAGCATCCCGGCAAAATAGATGCCCACAACTCCATCACTGGAAC
ACCCAGATCG
>TR241|c0_g1_i1 len=2236 path=[4262:0-151 4263:152-181 4620:182-248 4358:249-261 4664:262-361 4785:362-395 4736:396-453 4661:454-477 4767:478-489 4734:490-493 4754:494-511 4702:512-545 4733:546-548 4766:549-560 4701:561-584 4753:585-600 4657:601-624 4232:625-638 4610:639-725 4280:726-749 4730:750-771 4699:772-795 4786:796-806 4608:807-813 4698:814-820 4654:821-840 4607:841-915 4498:916-939 4752:940-1008 4787:1009-1034 4793:1035-1044 4652:1045-1068 4728:1069-1106 4695:1107-1122 4765:1123-1136 4751:1137-1160 4727:1161-1180 4694:1181-1204 4788:1205-1219 4794:1220-1238 4747:1239-1265 4722:1266-1282 4641:1283-1322 4778:1323-1350 4685:1351-1418 4789:1419-1433 4795:1434-1439 4720:1440-1481 4782:1482-1494 4777:1495-1529 4744:1530-1549 4677:1550-1577 4634:1578-1578 4715:1579-1597 4673:1598-1598 4630:1599-1603 4714:1604-1639 4758:1640-1651 4741:1652-1654 4711:1655-1656 4668:1657-1661 4625:1662-1685 4709:1686-1720 4666:1721-1729 4623:1730-1783 4570:1784-1803 4792:1804-1844 4665:1845-1853 4621:1854-1877 4566:1878-2047 4332:2048-2071 4333:2072-2235] [-1, 4262, 4263, 4620, 4358, 4664, 4785, 4736, 4661, 4767, 4734, 4754, 4702, 4733, 4766, 4701, 4753, 4657, 4232, 4610, 4280, 4730, 4699, 4786, 4608, 4698, 4654, 4607, 4498, 4752, 4787, 4793, 4652, 4728, 4695, 4765, 4751, 4727, 4694, 4788,

4794, 4747, 4722, 4641, 4778, 4685, 4789, 4795, 4720, 4782, 4777, 4744, 4677, 4634, 4715, 4673, 4630, 4714, 4758, 4741, 4711, 4668, 4625, 4709, 4666, 4623, 4570, 4792, 4665, 4621, 4566, 4332, 4333, -2]
ATGATCAAGATAACATAATTTTACCCAGAGGATAGATGGAATAAATGAATTATGGCATTCTGTGCTGCTCCTG
TGGTATGGATTTGGTCTGTGGAATGAAGGAATGAACTTCCCTCTCTCTCTCTCTGTTCCAATTTCCCCAATTA
GTCAAACAACACAAGGATATTACTTCAGCTCATACTTTAGTTGATGTGGATACCATATGCTGTATCCCTGGGT
TGTCCCCAGGTTGGTGGACAAATGAGCCAGAAGTTATTCCATTATTTAGAGTATTGGGAGAAAAATGTGCTCT
TCGTACAAAGTACTACTCCTTAATAAACCGCACCAACGCAACATAGTCATGAGAATGTTATGCGCGTAGCAGG
CAATGCCATCTGGTCTCTTCTTCCATTAGATAGTCTAGATGACATTCGCACCAATAGACCGGGTAAACAAACT
CACTCATGCATGTGACCTAATATCAGAAATTTAAACATGCATCTAAATCACAATCATTATCTAACGTGGTGCC
AGCGCTGTCACAACACCAGTCATAATATTGTTCCCCTTCTTGTCCATCTTATTCATATTCATTCGCTGTCGGT
ACTGCAATCTCTGTCCCTTAGCATCGTCGCGTCGCTGCTTCTTCACCACAGCACGATAACGATACAACGACAG
AGACGTAAATCCCCCCTTGCCCTCGCCAGCGACGAGAATTCCCTTGCCACTCCGTCCCGTAGGAATGGACCCC
GCCATCCGCCCCATGGCCGCCAAAGCCGTGGTCTGCTCTCCACCTCCCTCCGCCTCTGCCTCCGCTCTCATTT
GATACAAATTCACCACCCGCCCTTGGTACAAGCGATCTCCCGCCGCATCCTTCGCATGTCGGACGGCGACCCG
CTCGGTCCGATCGGGTGCGAATCGCTGCTTGTAATACCGCGACAGCGCGCGGTGGCCGACGATGCGTCCGTCG
GGGAAGATGAGCTCCCCGAGGGGGGTGATGTCGAACCCATGCGAGGCGATTTCGTTTTCGCATGCGGCGTGAA
GATCGTCGTCGTCCTCTTCCTTCGTGGAGGCGTCTTCCCATCCTTCGCCGTCATCGTCTTCTACGTCCTCCCA
CCCTTCGTCATCCTCCTCCATCGCATCAGTGATATCTCTATTAATCTTCGTAGCGCTGGTGCCCAAAAACTCA
CGATTCGCCTCGCGAAAGTCGTAAAACACGTCGAATTCCTCCTAATCGACACCCCTCTCGTACAGAATCTTGC
AATGTTCCTTGTCCTTCATGTGCTTTAGCAGAGCCCCCGTGCTCGCGAACGATTTTTGGCAGTACAGACACAT
ATTGCCCAAGCGGACCTTCTCATTGGCATATCCCAGAAATCCCTCGAGGTCGGTAATGTATTCGGCATCAGGT
AGGTAGAAGGAGAATTTGGATTGCATGTACTCGAGGTTGAGCTCAGGAGTGTCAGAGATGTGGTTGTCGAAAA
GGGATTGGCAAGGGTTGATTTCCGGCTGGCTTTCATCCATGGAAGAAGCGTCGTCATCGTCCGCGTCATCAAC
AACATCGGCTTGCATGGATTCTGCTACCGGAGACGTTGTTTCCTCGCTCTCCTCCACGGAATCATTCGTCAGA
TTATTTTCACACTCCGCTCTGTTCTTTGCGAACGCCGGCTTTCTCTTGTGACTCTTCCCCTGCTGTTTCTTAT
TCTTTTTCTCCTTTTTGCCCTTGCTTTTATCCTTGCGATGGTCCACCCCCGTCCTCTCTTCCCTCCCCTCTCT
CTCCTTTCTCAGTGCGACGGCCGCTTCCAACCTCGCATTGAAGTCGGTTTCGTTCAGCATGGGCAGGCCCGCC
TCGCGTCGTTTGAGGTTGTAGCGGTGCCAATCCGATTTGTAGTGTTGTTGGAGGTCGTTACGGGAGGTGAAGG
TTTTTCCGGGGGCGGTAGTGGAGCTTAGTGTTGGTGCGGAGGTGAAGGCGGTGGCTCCCGCAGAGGGTATGGG
TGTTGGGAGTGGTAGTTGCATCATGATTCTTTGTTGGTGTGAAGGGAGATTAATGGGGAGAGATATCACGATT
GGATAACACGATAGGGATGGCAATAAGACGGCGGAGTAAGGCGTTTGCTCTTGCTTGCTACCAATCTTGGATG
GCCTCAGATGCTAGAAGCTTCCGGCTGCTTGTGTTGCACTGAATGTTGTATCGTATCTGCTCAGTGGTGGTGG
TGGTGGTTGTAAGGTTTGGTACGTAGAGCGAGATTGGTGATCGGGT
>TR241|c0_g1_i2 len=963 path=[4461:0-131 4462:132-144 4463:145-148 4464:149-153 4465:154-174 4768:175-241 4785:242-275 4736:276-333 4661:334-357 4767:358-369 4734:370-373 4754:374-391 4614:392-425 4733:426-428 4766:429-440 4701:441-464 4753:465-480 4700:481-494 4656:495-518 4610:519-605 4280:606-629 4730:630-651 4699:652-675 4786:676-686 4608:687-693 4698:694-700 4654:701-720 4607:721-795 4498:796-819 4752:820-888 4787:889-914 4793:915-924 4696:925-962] [-1, 4461, 4462, 4463, 4464, 4465, 4768, 4785, 4736, 4661, 4767, 4734, 4754, 4614, 4733, 4766, 4701, 4753, 4700, 4656, 4610, 4280, 4730, 4699, 4786, 4608, 4698, 4654, 4607, 4498, 4752, 4787, 4793, 4696]
CTCTCTGTTCCTACTTATTTCCCCAATTAGTCAACCAACACAAGGATATTATTTTGGCTCATAATTTATTTTA
TGTGGATAATGTATGCTGTATCCCTGAGTTGTCACTAGGTTGGTGGACAAATGAGCCAGAAGTTATTCCATTT
TTTAGAGTATTGGGAGAAAAATGCGCTGTTCGTACAAAACACTACTTTACAAACTGCACCAACGCAACATAGC
CATGAGAATGTTATGCGCGTAGCAGGCAATGCCATCTGGTCTCTTCTTCCATTAGATAGTCTAGATGACATTC
GCACCAATAGACCGGGTAAACAAACTCACTCATGCATGTGACCTAATATCAGAAATTTAAACATGCATCTAAA
TCACAATCATTATCTAACGTGGTGCCAACGCTGTCACCACACCAGTCATAATATTGTTCCCCTTCTTGTCCAT
CTTATTCATATTCATTCGCTGTCGGTACTGCAATCTCTGTCCCCTAGCATCGTCGCGCCGCTGCTTCTTCACC
ACAGCACGATAACGATACAACGACAGAGACGTAAATCCCCCCTTGCCCTCGCCAGCGACGAGAATTCCCTTGC
CACTCCGTCCCGTAGGAATGGACCCCGCCATCCGCCCCATGGCCGCCAAAGCCGTGGTCTGCTCTCCACCTCC
CTCCGCCTCTGCCTCCGCTCTCATTTGATACAAATTCACCACCCGCCCTTGGTACAAGCGATCTCCCGCCGCA
TCCTTCGCATGTCGGACGGCGACCCGCTCGGTCCGATCGGGTGCGAATCGCTGCTTGTAATACCGCGACAGCG
CGCGGTGGCCGACGATGCGTCCGTCGGGGAAGATGAGCTCCCCGAGGGGGGTGATGTCGAACCCATGCGAGGC
GATTTCGTTTTCGCATGCGGCGTGAAGATCGTCGTCGTCCTCTTCCTTCATGGAGGCGTCCTCCCATCCTTCG
CCGTCATCGTCGTC
>TR241|c0_g1_i3 len=2101 path=[4315:0-131 4316:132-144 4317:145-148 4318:149-153 4319:154-165 4737:166-176 4768:177-243 4785:244-277

4735:278-326 4703:327-350 4660:351-354 4615:355-358 4732:359-413
4766:414-425 4701:426-449 4753:450-465 4657:466-489 4232:490-503
4610:504-590 4280:591-614 4730:615-636 4699:637-660 4786:661-671
4608:672-678 4698:679-685 4654:686-705 4607:706-780 4498:781-804
4752:805-873 4787:874-899 4793:900-909 4652:910-933 4728:934-971
4695:972-987 4765:988-1001 4751:1002-1025 4727:1026-1045 4694:1046-1069
4788:1070-1084 4794:1085-1103 4747:1104-1130 4722:1131-1147 4641:1148-
1187 4778:1188-1215 4685:1216-1283 4789:1284-1298 4591:1299-1330
4790:1331-1335 4718:1336-1346 4782:1347-1359 4777:1360-1394 4744:1395-
1414 4635:1415-1442 4251:1443-1443 4584:1444-1462 4674:1463-1463
4631:1464-1468 4713:1469-1504 4758:1505-1516 4741:1517-1519 4711:1520-
1521 4668:1522-1526 4625:1527-1550 4709:1551-1585 4666:1586-1594
4623:1595-1648 4570:1649-1668 4792:1669-1709 4665:1710-1718 4621:1719-
1742 4566:1743-1912 4332:1913-1936 4333:1937-2100] [-1, 4315, 4316, 4317,
4318, 4319, 4737, 4768, 4785, 4735, 4703, 4660, 4615, 4732, 4766, 4701,
4753, 4657, 4232, 4610, 4280, 4730, 4699, 4786, 4608, 4698, 4654, 4607,
4498, 4752, 4787, 4793, 4652, 4728, 4695, 4765, 4751, 4727, 4694, 4788,
4794, 4747, 4722, 4641, 4778, 4685, 4789, 4591, 4790, 4718, 4782, 4777,
4744, 4635, 4251, 4584, 4674, 4631, 4713, 4758, 4741, 4711, 4668, 4625,
4709, 4666, 4623, 4570, 4792, 4665, 4621, 4566, 4332, 4333, -2]
CTCTCTGTTCCTACTTATTTCCCCAATTAGTCAACCAACACAAGGATATTATTTTGGCTCATAATTTATTTTA
TGTGGATAATGTATGCTGTATCCCTGAGTTGTCACTAGGTTGGTGGACAAATGAGCCAGAAGTTATTCCATTT
TTTAGAGTGTATTGGGAGAAAAATGCGCTGTTCGTACAAAACACTACTTTACAAACTGCACCAACGCAACATA
GCCATGAGAATGTTATGCGCGTAGCAGGCAATGCCATCTGGTCTCTTCTTCCATTAGATCACTAATAGACCGG
GTAAAGAAACGCACTCATGCATGGGACTTAATATCCGAACTTTAAACATGCATCTAAATCACAATCACTGTCT
AACGTGGTGCCAATGCTGTCACCACTCCAGTCATAATATTGTTCCCCTTCTTGTCCATCTTATTCATATTCAT
TCGCTGTCGGTACTGCAATCTCTGTCCCTTAGCATCGTCGCGTCGCTGCTTCTTCACCACAGCACGATAACGA
TACAACGACAGAGACGTAAATCCCCCCTTGCCCTCGCCAGCGACGAGAATTCCCTTGCCACTCCGTCCCGTAG
GAATGGACCCCGCCATCCGCCCCATGGCCGCCAAAGCCGTGGTCTGCTCTCCACCTCCCTCCGCCTCTGCCTC
CGCTCTCATTTGATACAAATTCACCACCCGCCCTTGGTACAAGCGATCTCCCGCCGCATCCTTCGCATGTCGG
ACGGCGACCCGCTCGGTCCGATCGGGTGCGAATCGCTGCTTGTAATACCGCGACAGCGCGCGGTGGCCGACGA
TGCGTCCGTCGGGGAAGATGAGCTCCCCGAGGGGGGTGATGTCGAACCCATGCGAGGCGATTTCGTTTTCGCA
TGCGGCGTGAAGATCGTCGTCGTCCTCTTCCTTCGTGGAGGCGTCTTCCCATCCTTCGCCGTCATCGTCTTCT
ACGTCCTCCCACCCTTCGTCATCCTCCTCCATCGCATCAGTGATATCTCTATTAATCTTCGTAGCGCTGGTGC
CCAAAAACTCACGATTCGCCTCGCGAAAGTCGTAAAACACGTCGAATTCCTCCTAATCGACACCCCTCTCGTA
CAGAATCTTGCAATGTTCCTTGTCCTTCATGTGCTTTAGCAGAGCCCCCGTGCTCGCGAACGATTTTTGGCAG
TACAGACACATATTGCCCAAGCGGACCTTCTCATTGGCATATCCCAGAAATCCCTCGAGGTCGGTAATGTATT
CGGCATCAGGTAGGTAGAAGGAGAATTTGGATTGCATGTACTCGAGGTTGAGCTCAGGGGTGTCGGAGACGTG
GTTGTCGAAGAGGGATTGGCAAGGGTTGATTTCCGGCTGGCTTTCATCCATGGAAGAAGCGTCGTCATCGTCC
GCGTCATCAACAACATCGGCTTGCATGGCTTCCGCTACCGGAGACGTTGTTTCCTCGCTCTCCTCCACGGAAT
CATTCGTCAAATTAGTTTCACCCTCCGCTCTGTTCTTTGCGAACGCCGGCTTTCTCTTGTGACTCTTCCCCTG
CTGTTTCTTATTCTTTTTCTCCTTTTTGCCCTTGCTTTTATCCTTGCGATGGTCCACCCCCGTCCTCTCTTCC
CTCCCCTCTCTCTCCTTTCTCAGTGCGACGGCCGCTTCCAACCTCGCATTGAAGTCGGTTTCGTTCAGCATGG
GCAGGCCCGCCTCGCGTCGTTTGAGGTTGTAGCGGTGCCAATCCGATTTGTAGTGTTGTTGGAGGTCGTTACG
GGAGGTGAAGGTTTTTCCGGGGGCGGTAGTGGAGCTTAGTGTTGGTGCGGAGGTGAAGGCGGTGGCTCCCGCA
GAGGGTATGGGTGTTGGGAGTGGTAGTTGCATCATGATTCTTTGTTGGTGTGAAGGGAGATTAATGGGGAGAG
ATATCACGATTGGATAACACGATAGGGATGGCAATAAGACGGCGGAGTAAGGCGTTTGCTCTTGCTTGCTACC
AATCTTGGATGGCCTCAGATGCTAGAAGCTTCCGGCTGCTTGTGTTGCACTGAATGTTGTATCGTATCTGCTC
AGTGGTGGTGGTGGTGGTTGTAAGGTTTGGTACGTAGAGCGAGATTGGTGATCGGGT
>TR241|c0_g1_i4 len=821 path=[4485:0-151 4486:152-181 4620:182-248
4358:249-261 4664:262-361 4785:362-395 4736:396-453 4661:454-477
4767:478-489 4734:490-493 4754:494-511 4614:512-545 4733:546-548
4766:549-560 4701:561-584 4753:585-600 4700:601-614 4656:615-638
4610:639-725 4280:726-749 4730:750-771 4699:772-795 4786:796-806
4353:807-820] [-1, 4485, 4486, 4620, 4358, 4664, 4785, 4736, 4661, 4767,
4734, 4754, 4614, 4733, 4766, 4701, 4753, 4700, 4656, 4610, 4280, 4730,
4699, 4786, 4353, -2]
ATGATCAAGATAACATAATTTTACCCAGAGGATAGATGGAATAAATGAATTATGGCATTCTGTGCTGCTCCTG
TGGTATGGATTTGGTCTGTGGAATGAAGGAATGAACTTCCCTCTCTCTCTCTCTGTTCCAATTTCCCCAATTA
GTCAAAGAACACCAGGATATTACTTCAGCTCATACTTTAGTTGATGTGGATACCATATGCTGTATCCCTGGGT

TGTCCCCAGGTTGGTGGACAAATGAGCCAGAAGTTATTCCATTATTTAGAGTATTGGGAGAAAAATGTGCTCT
TCGTACAAAGTACTACTCCTTAATAAACCGCACCAACGCAACATAGTCATGAGAATGTTATGCGCGTAGCAGG
CAATGCCATCTGGTCTCTTCTTCCATTAGATAGTCTAGATGACATTCGCACCAATAGACCGGGTAAACAAACT
CACTCATGCATGTGACCTAATATCAGAAATTTAAACATGCATCTAAATCACAATCATTATCTAACGTGGTGCC
AACGCTGTCACCACACCAGTCATAATATTGTTCCCCTTCTTGTCCATCTTATTCATATTCATTCGCTGTCGGT
ACTGCAATCTCTGTCCCCTAGCATCGTCGCGCCGCTGCTTCTTCACCACAGCACGATAACGATACAACGACAG
AGACGTAAATCCCCCCTTGCCCTCGCCAGCGACGAGAATTCCCTTGCCACTCCGTCCCGTAGGAATGGACCCC
GCCATCCGCCCCATGGCCGCCAAAGCCGTGGTCTGCTCTCCACCTCCCTCCGCCTCTGCCTCCGCTCTCATTT
GATAAGATCGGAAGAGCA
>TR241|c0_g1_i5 len=2001 path=[4461:0-131 4462:132-144 4463:145-148
4464:149-153 4465:154-174 4768:175-241 4785:242-275 4735:276-324
4703:325-348 4660:349-352 4615:353-356 4732:357-411 4766:412-423
4701:424-447 4753:448-463 4657:464-487 4232:488-501 4610:502-588
4280:589-612 4730:613-634 4699:635-658 4786:659-669 4608:670-676
4698:677-683 4654:684-703 4607:704-778 4498:779-802 4752:803-871
4787:872-897 4793:898-907 4652:908-931 4728:932-969 4695:970-985
4765:986-999 4751:1000-1023 4727:1024-1043 4694:1044-1067 4788:1068-1082
4794:1083-1101 4747:1102-1128 4722:1129-1145 4641:1146-1185 4778:1186-
1213 4685:1214-1281 4789:1282-1296 4591:1297-1328 4790:1329-1333
4718:1334-1344 4782:1345-1357 4777:1358-1392 4760:1393-1441 4743:1442-
1460 4676:1461-1461 4633:1462-1501 4581:1502-1504 4771:1505-1506
4759:1507-1513 4742:1514-1537 4712:1538-1549 4626:1550-1558 4740:1559-
1611 4710:1612-1631 4624:1632-1672 4571:1673-1745 4384:1746-2000] [-1,
4461, 4462, 4463, 4464, 4465, 4768, 4785, 4735, 4703, 4660, 4615, 4732,
4766, 4701, 4753, 4657, 4232, 4610, 4280, 4730, 4699, 4786, 4608, 4698,
4654, 4607, 4498, 4752, 4787, 4793, 4652, 4728, 4695, 4765, 4751, 4727,
4694, 4788, 4794, 4747, 4722, 4641, 4778, 4685, 4789, 4591, 4790, 4718,
4782, 4777, 4760, 4743, 4676, 4633, 4581, 4771, 4759, 4742, 4712, 4626,
4740, 4710, 4624, 4571, 4384, -2]
CTCTCTGTTCCTACTTATTTCCCCAATTAGTCAACCAACACAAGGATATTATTTTGGCTCATAATTTATTTTA
TGTGGATAATGTATGCTGTATCCCTGAGTTGTCACTAGGTTGGTGGACAAATGAGCCAGAAGTTATTCCATTT
TTTAGAGTATTGGGAGAAAAATGCGCTGTTCGTACAAAACACTACTTTACAAACTGCACCAACGCAACATAGC
CATGAGAATGTTATGCGCGTAGCAGGCAATGCCATCTGGTCTCTTCTTCCATTAGATCACTAATAGACCGGGT
AAAGAAACGCACTCATGCATGGGACTTAATATCCGAACTTTAAACATGCATCTAAATCACAATCACTGTCTAA
CGTGGTGCCAATGCTGTCACCACTCCAGTCATAATATTGTTCCCCTTCTTGTCCATCTTATTCATATTCATTC
GCTGTCGGTACTGCAATCTCTGTCCCTTAGCATCGTCGCGTCGCTGCTTCTTCACCACAGCACGATAACGATA
CAACGACAGAGACGTAAATCCCCCCTTGCCCTCGCCAGCGACGAGAATTCCCTTGCCACTCCGTCCCGTAGGA
ATGGACCCCGCCATCCGCCCCATGGCCGCCAAAGCCGTGGTCTGCTCTCCACCTCCCTCCGCCTCTGCCTCCG
CTCTCATTTGATACAAATTCACCACCCGCCCTTGGTACAAGCGATCTCCCGCCGCATCCTTCGCATGTCGGAC
GGCGACCCGCTCGGTCCGATCGGGTGCGAATCGCTGCTTGTAATACCGCGACAGCGCGCGGTGGCCGACGATG
CGTCCGTCGGGGAAGATGAGCTCCCCGAGGGGGGTGATGTCGAACCCATGCGAGGCGATTTCGTTTTCGCATG
CGGCGTGAAGATCGTCGTCGTCCTCTTCCTTCGTGGAGGCGTCTTCCCATCCTTCGCCGTCATCGTCTTCTAC
GTCCTCCCACCCTTCGTCATCCTCCTCCATCGCATCAGTGATATCTCTATTAATCTTCGTAGCGCTGGTGCCC
AAAAACTCACGATTCGCCTCGCGAAAGTCGTAAAACACGTCGAATTCCTCCTAATCGACACCCCTCTCGTACA
GAATCTTGCAATGTTCCTTGTCCTTCATGTGCTTTAGCAGAGCCCCGTGCTCGCGAACGATTTTTGGCAGTA
CAGACACATATTGCCCAAGCGGACCTTCTCATTGGCATATCCCAGAAATCCCTCGAGGTCGGTAATGTATTCG
GCATCAGGTAGGTAGAAGGAGAATTTGGATTGCATGTACTCGAGGTTGAGCTCAGGGGTGTCGGAGACGTGGT
TGTCGAAGAGGGATTGGCAAGGGTTGATTTCCGGCTGGCTTTCATCCATGGAAGAAGCGTCGTCATCGTCCGC
GTCATCGACAACATCGGATCGCATGGGTTCCACTACCGGAGACGTTGTTTCCTCGCTCTCCTCCACGGAATCA
TTTGTCAGATTATTTTCACCTTTGCGAACGCCGGCTTTCTCTTGTGATTCTTTTTCTCCTTTTTGCTCTTGCT
TTTATCCTTGCGATGGTCCACCCCCGACCTCTCTTCCCTCCCCTCTATCTCCTTTCCAGTGCGACGGCCGCTT
CCAACCTCGCATTGAAGTCGGTTTCGTTCAGCATGGGCAGGCCCGCCTCGCGTCGTTTGAGGTTGTACCATTT
TCGATGACATCAAATCGCGCACCGCCGTAGGGTATGCATCGGATATCGCCTCAGGAACGGGCTCTGACCCTGC
AACTGCCACTGTTTCGCTCTCCATGCTCTTGGCTTTAATATGTGCCATCGCTCGATTTGTAGAAGCGCTTGGC
GGCGGAGTCCGATCTGCCCCATTCATGACGGCATTACTACGATGGGGGTAAATGAAATGGCCGTTGATGTGTA
ATGTAGGTAGTAATGCTCAAAAACTAGAATGGTACCATAGTGGTTAAAAGAGTCCCTATGACCAAATCGAGGG
TGCTGAACATGATGGTGTTGTCTAAAGTTT
>TR241|c0_g1_i6 len=2138 path=[4262:0-151 4263:152-181 4620:182-248
4358:249-261 4664:262-361 4785:362-395 4736:396-453 4661:454-477
4767:478-489 4734:490-493 4754:494-511 4702:512-545 4733:546-548

4766:549-560 4701:561-584 4753:585-600 4657:601-624 4232:625-638
4610:639-725 4280:726-749 4730:750-771 4699:772-795 4786:796-806
4608:807-813 4698:814-820 4654:821-840 4607:841-915 4498:916-939
4752:940-1008 4606:1009-1034 4793:1035-1044 4696:1045-1082 4604:1083-1122
4765:1123-1136 4751:1137-1160 4727:1161-1180 4694:1181-1204 4788:1205-1219 4794:1220-1238 4747:1239-1265 4722:1266-1282 4641:1283-1322
4778:1323-1350 4685:1351-1418 4789:1419-1433 4591:1434-1465 4790:1466-1470 4718:1471-1481 4782:1482-1494 4777:1495-1529 4760:1530-1578
4743:1579-1597 4676:1598-1598 4633:1599-1638 4581:1639-1641 4771:1642-1643 4759:1644-1650 4742:1651-1674 4712:1675-1686 4626:1687-1695
4740:1696-1748 4710:1749-1768 4624:1769-1809 4571:1810-1882 4384:1883-2137] [-1, 4262, 4263, 4620, 4358, 4664, 4785, 4736, 4661, 4767, 4734, 4754, 4702, 4733, 4766, 4701, 4753, 4657, 4232, 4610, 4280, 4730, 4699, 4786, 4608, 4698, 4654, 4607, 4498, 4752, 4606, 4793, 4696, 4604, 4765, 4751, 4727, 4694, 4788, 4794, 4747, 4722, 4641, 4778, 4685, 4789, 4591, 4790, 4718, 4782, 4777, 4760, 4743, 4676, 4633, 4581, 4771, 4759, 4742, 4712, 4626, 4740, 4710, 4624, 4571, 4384, -2]
ATGATCAAGATAACATAATTTTACCCAGAGGATAGATGGAATAAATGAATTATGGCATTCTGTGCTGCTCCTG
TGGTATGGATTTGGTCTGTGGAATGAAGGAATGAACTTCCCTCTCTCTCTCTCTGTTCCAATTTCCCCAATTA
GTCAAACAACACAAGGATATTACTTCAGCTCATACTTTAGTTGATGTGGATACCATATGCTGTATCCCTGGGT
TGTCCCCAGGTTGGTGGACAAATGAGCCAGAAGTTATTCCATTATTTAGAGTATTGGGAGAAAAATGTGCTCT
TCGTACAAAGTACTACTCCTTAATAAACCGCACCAACGCAACATAGTCATGAGAATGTTATGCGCGTAGCAGG
CAATGCCATCTGGTCTCTTCTTCCATTAGATAGTCTAGATGACATTCGCACCAATAGACCGGGTAAACAAACT
CACTCATGCATGTGACCTAATATCAGAAATTTAAACATGCATCTAAATCACAATCATTATCTAACGTGGTGCC
AGCGCTGTCACAACACCAGTCATAATATTGTTCCCCTTCTTGTCCATCTTATTCATATTCATTCGCTGTCGGT
ACTGCAATCTCTGTCCCTTAGCATCGTCGCGTCGCTGCTTCTTCACCACAGCACGATAACGATACAACGACAG
AGACGTAAATCCCCCCTTGCCCTCGCCAGCGACGAGAATTCCCTTGCCACTCCGTCCCGTAGGAATGGACCCC
GCCATCCGCCCCATGGCCGCCAAAGCCGTGGTCTGCTCTCCACCTCCCTCCGCCTCTGCCTCCGCTCTCATTT
GATACAAATTCACCACCCGCCCTTGGTACAAGCGATCTCCCGCCGCATCCTTCGCATGTCGGACGGCGACCCG
CTCGGTCCGATCGGGTGCGAATCGCTGCTTGTAATACCGCGACAGCGCGCGGTGGCCGACGATGCGTCCGTCG
GGGAAGATGAGCTCCCCGAGGGGGGTGATGTCGAACCCATGCGAGGCGATTTCGTTTTCGTACGCGGCGTGAA
GATCGTCGTCGTCCTCTTCCTTCATGGAGGCGTCCTCCCATCCTTCGCCGTCATCGTCGTCTACGTCCTCCCA
CCCTCCGTCATCCTCCTCCATCGCATCAGTGATATCTCTATTAATCTTCGTAGCGCTGGTGCCCAAAAACTCA
CGATTCGCCTCGCGAAAGTCGTAAAACACGTCGAATTCCTCCTAATCGACACCCCTCTCGTACAGAATCTTGC
AATGTTCCTTGTCCTTCATGTGCTTTAGCAGAGCCCCCGTGCTCGCGAACGATTTTTGGCAGTACAGACACAT
ATTGCCCAAGCGGACCTTCTCATTGGCATATCCCAGAAATCCCTCGAGGTCGGTAATGTATTCGGCATCAGGT
AGGTAGAAGGAGAATTTGGATTGCATGTACTCGAGGTTGAGCTCAGGGGTGTCGGAGACGTGGTTGTCGAAGA
GGGATTGGCAAGGGTTGATTTCCGGCTGGCTTTCATCCATGGAAGAAGCGTCGTCATCGTCCGCGTCATCGAC
AACATCGGATCGCATGGGTTCCACTACCGGAGACGTTGTTTCCTCGCTCTCCTCCACGGAATCATTTGTCAGA
TTATTTTCACCTTTGCGAACGCCGGCTTTCTCTTGTGATTCTTTTTCTCCTTTTTGCTCTTGCTTTTATCCTT
GCGATGGTCCACCCCCGACCTCTCTTCCCTCCCCTCTATCTCCTTTCCAGTGCGACGGCCGCTTCCAACCTCG
CATTGAAGTCGGTTTCGTTCAGCATGGGCAGGCCCGCCTCGCGTCGTTTGAGGTTGTACCATTTTCGATGACA
TCAAATCGCGCACCGCCGTAGGGTATGCATCGGATATCGCCTCAGGAACGGGCTCTGACCCTGCAACTGCCAC
TGTTTCGCTCTCCATGCTCTTGGCTTTAATATGTGCCATCGCTCGATTTGTAGAAGCGCTTGGCGGCGGAGTC
CGATCTGCCCCATTCATGACGGCATTACTACGATGGGGGTAAATGAAATGGCCGTTGATGTGTAATGTAGGTA
GTAATGCTCAAAAACTAGAATGGTACCATAGTGGTTAAAAGAGTCCCTATGACCAAATCGAGGGTGCTGAACA
TGATGGTGTTGTCTAAAGTTT
>TR241|c0_g1_i7 len=1409 path=[4315:0-131 4316:132-144 4317:145-148
4318:149-153 4319:154-165 4737:166-176 4768:177-243 4785:244-277
4735:278-326 4617:327-337 4704:338-350 4660:351-354 4615:355-358
4732:359-413 4766:414-425 4701:426-449 4753:450-465 4657:466-489
4232:490-503 4610:504-590 4280:591-614 4730:615-636 4699:637-660
4786:661-671 4608:672-678 4698:679-685 4654:686-705 4607:706-780
4498:781-804 4752:805-873 4787:874-899 4793:900-909 4652:910-933
4728:934-971 4695:972-987 4765:988-1001 4751:1002-1025 4727:1026-1045
4694:1046-1069 4788:1070-1084 4794:1085-1103 4774:1104-1105 4764:1106-1129 4748:1130-1136 4722:1137-1153 4642:1154-1193 4778:1194-1221
4721:1222-1242 4789:1243-1257 4795:1258-1263 4719:1264-1295 4790:1296-1300 4588:1301-1324 4777:1325-1359 4760:1360-1408] [-1, 4315, 4316, 4317, 4318, 4319, 4737, 4768, 4785, 4735, 4617, 4704, 4660, 4615, 4732, 4766,

4701, 4753, 4657, 4232, 4610, 4280, 4730, 4699, 4786, 4608, 4698, 4654, 4607, 4498, 4752, 4787, 4793, 4652, 4728, 4695, 4765, 4751, 4727, 4694, 4788, 4794, 4774, 4764, 4748, 4722, 4642, 4778, 4721, 4789, 4795, 4719, 4790, 4588, 4777, 4760]
CTCTCTGTTCCTACTTATTTCCCCAATTAGTCAACCAACACAAGGATATTATTTTGGCTCATAATTTATTTTA
TGTGGATAATGTATGCTGTATCCCTGAGTTGTCACTAGGTTGGTGGACAAATGAGCCAGAAGTTATTCCATTT
TTTAGAGTGTATTGGGAGAAAAATGCGCTGTTCGTACAAAACACTACTTTACAAACTGCACCAACGCAACATA
GCCATGAGAATGTTATGCGCGTAGCAGGCAATGCCATCTGGTCTCTTCTTCCATTAGATCACTAATAGACCGG
GTAAAGAAACGCACTCATGCATGGGACTTAATATCAGAACTTTAAACATGCATCTAAATCACAATCACTGTCT
AACGTGGTGCCAATGCTGTCACCACTCCAGTCATAATATTGTTCCCCTTCTTGTCCATCTTATTCATATTCAT
TCGCTGTCGGTACTGCAATCTCTGTCCCTTAGCATCGTCGCGTCGCTGCTTCTTCACCACAGCACGATAACGA
TACAACGACAGAGACGTAAATCCCCCCTTGCCCTCGCCAGCGACGAGAATTCCCTTGCCACTCCGTCCCGTAG
GAATGGACCCCGCCATCCGCCCCATGGCCGCCAAAGCCGTGGTCTGCTCTCCACCTCCCTCCGCCTCTGCCTC
CGCTCTCATTTGATACAAATTCACCACCCGCCCTTGGTACAAGCGATCTCCCGCCGCATCCTTCGCATGTCGG
ACGGCGACCCGCTCGGTCCGATCGGGTGCGAATCGCTGCTTGTAATACCGCGACAGCGCGCGGTGGCCGACGA
TGCGTCCGTCGGGGAAGATGAGCTCCCCGAGGGGGGTGATGTCGAACCCATGCGAGGCGATTTCGTTTTCGCA
TGCGGCGTGAAGATCGTCGTCGTCCTCTTCCTTCGTGGAGGCGTCTTCCCATCCTTCGCCGTCATCGTCTTCT
ACGTCCTCCCACCCTTCGTCATCCTCCTCCATCGCATCAGTGATATCTCTATTAATCTTCGTAGCGCTGGTGC
CCAAAAACTCACGATTCGCCTCGCGAAAGTCGTAAAACACGTCGAATTCCTCCTAATCGACACCCCTCTCGTA
CAGAATCTTTGTCCTTCGTTGTTCCTTGTCCTTCATGTGCTTTAGCAGAGCCCCCGTGCCCGCGAACGATTTT
TGTCAGTACAGACACATATTGCCCAAGCGGACCTTCTCATTGGCATATCCCAGAGAATTTGGATTGCATGTAC
TCGAGGTTGAGCTCAGGAGTGTCGGAGACGTGGACGTGGTTGTCGAAGAGGGATTGGCAACGGTTGATTTCCG
GCTGGCTTTCATCCATGGAAGAAGCGTCGTCATCGTCCGCGTCATCGACAACATCGGATCGCATGGGTTCCAC
TACCGGAGACGTTGTTTCCTCG
>TR241|c0_g1_i8 len=2012 path=[4262:0-151 4263:152-181 4620:182-248 4358:249-261 4664:262-361 4785:362-395 4736:396-453 4661:454-477 4767:478-489 4734:490-493 4754:494-511 4702:512-545 4733:546-548 4766:549-560 4701:561-584 4753:585-600 4657:601-624 4232:625-638 4610:639-725 4280:726-749 4730:750-771 4699:772-795 4786:796-806 4608:807-813 4698:814-820 4654:821-840 4607:841-915 4498:916-939 4752:940-1008 4787:1009-1034 4793:1035-1044 4652:1045-1068 4728:1069-1106 4695:1107-1122 4765:1123-1136 4751:1137-1160 4727:1161-1180 4694:1181-1204 4788:1205-1219 4794:1220-1238 4774:1239-1240 4764:1241-1264 4748:1265-1271 4722:1272-1288 4642:1289-1328 4778:1329-1356 4721:1357-1377 4789:1378-1392 4795:1393-1398 4719:1399-1430 4790:1431-1435 4718:1436-1446 4782:1447-1459 4777:1460-1494 4760:1495-1543 4743:1544-1562 4676:1563-1563 4633:1564-1603 4581:1604-1606 4771:1607-1608 4759:1609-1615 4742:1616-1639 4712:1640-1651 4626:1652-1660 4740:1661-1713 4710:1714-1733 4301:1734-1756 4302:1757-2011] [-1, 4262, 4263, 4620, 4358, 4664, 4785, 4736, 4661, 4767, 4734, 4754, 4702, 4733, 4766, 4701, 4753, 4657, 4232, 4610, 4280, 4730, 4699, 4786, 4608, 4698, 4654, 4607, 4498, 4752, 4787, 4793, 4652, 4728, 4695, 4765, 4751, 4727, 4694, 4788, 4794, 4774, 4764, 4748, 4722, 4642, 4778, 4721, 4789, 4795, 4719, 4790, 4718, 4782, 4777, 4760, 4743, 4676, 4633, 4581, 4771, 4759, 4742, 4712, 4626, 4740, 4710, 4301, 4302, -2]
ATGATCAAGATAACATAATTTTACCCAGAGGATAGATGGAATAAATGAATTATGGCATTCTGTGCTGCTCCTG
TGGTATGGATTTGGTCTGTGGAATGAAGGAATGAACTTCCCTCTCTCTCTCTCTGTTCCAATTTCCCCAATTA
GTCAAACAACACAAGGATATTACTTCAGCTCATACTTTAGTTGATGTGGATACCATATGCTGTATCCCTGGGT
TGTCCCCAGGTTGGTGGACAAATGAGCCAGAAGTTATTCCATTATTTAGAGTATTGGGAGAAAAATGTGCTCT
TCGTACAAAGTACTACTCCTTAATAAACCGCACCAACGCAACATAGTCATGAGAATGTTATGCGCGTAGCAGG
CAATGCCATCTGGTCTCTTCTTCCATTAGATAGTCTAGATGACATTCGCACCAATAGACCGGGTAAACAAACT
CACTCATGCATGTGACCTAATATCAGAAATTTAAACATGCATCTAAATCACAATCATTATCTAACGTGGTGCC
AGCGCTGTCACAACACCAGTCATAATATTGTTCCCCTTCTTGTCCATCTTATTCATATTCATTCGCTGTCGGT
ACTGCAATCTCTGTCCCTTAGCATCGTCGCGTCGCTGCTTCTTCACCACAGCACGATAACGATACAACGACAG
AGACGTAAATCCCCCCTTGCCCTCGCCAGCGACGAGAATTCCCTTGCCACTCCGTCCCGTAGGAATGGACCCC
GCCATCCGCCCCATGGCCGCCAAAGCCGTGGTCTGCTCTCCACCTCCCTCCGCCTCTGCCTCCGCTCTCATTT
GATACAAATTCACCACCCGCCCTTGGTACAAGCGATCTCCCGCCGCATCCTTCGCATGTCGGACGGCGACCCG
CTCGGTCCGATCGGGTGCGAATCGCTGCTTGTAATACCGCGACAGCGCGCGGTGGCCGACGATGCGTCCGTCG
GGGAAGATGAGCTCCCCGAGGGGGGTGATGTCGAACCCATGCGAGGCGATTTCGTTTTCGCATGCGGCGTGAA
GATCGTCGTCGTCCTCTTCCTTCGTGGAGGCGTCTTCCCATCCTTCGCCGTCATCGTCTTCTACGTCCTCCCA

CCCTTCGTCATCCTCCTCCATCGCATCAGTGATATCTCTATTAATCTTCGTAGCGCTGGTGCCCAAAAACTCA
CGATTCGCCTCGCGAAAGTCGTAAAACACGTCGAATTCCTCCTAATCGACACCCCTCTCGTACAGAATCTTTG
TCCTTCGTTGTTCCTTGTCCTTCATGTGCTTTAGCAGAGCCCCCGTGCCCGCGAACGATTTTTGTCAGTACAG
ACACATATTGCCCAAGCGGACCTTCTCATTGGCATATCCCAGAGAATTTGGATTGCATGTACTCGAGGTTGAG
CTCAGGAGTGTCGGAGACGTGGACGTGGTTGTCGAAGAGGGATTGGCAAGGGTTGATTTCCGGCTGGCTTTCA
TCCATGGAAGAAGCGTCGTCATCGTCCGCGTCATCGACAACATCGGATCGCATGGGTTCCACTACCGGAGACG
TTGTTTCCTCGCTCTCCTCCACGGAATCATTTGTCAGATTATTTTCACCTTTGCGAACGCCGGCTTTCTCTTG
TGATTCTTTTTCTCCTTTTTGCTCTTGCTTTTATCCTTGCGATGGTCCACCCCCGACCTCTCTTCCCTCCCCT
CTATCTCCTTTCCAGTGCGACGGCCGCTTCCAACCTCGCATTGAAGTCGGTTTCGATCGCCTCAGGAACGGGC
TCTGACCCTGCAACTGCCACTGTTTCGCTCTCCATGCTCTTGGCTTTAATATGTGCCATCGCTCGATTTGTAG
AAGCGCTTGGCGGCGGAGTCCGATCTGCCCCATTCATGACGGCATTACTACGATGGGGGTAAATGAAATGGCC
GTTGATGTGTAATGTAGGTAGTAATGCTCAAAAACTAGAATGGTACCATAGTGGTTAAAAGAGTCCCTATGAC
CAAATCGAGGGTGCTGAACATGATGGTGTTGTCTAAAGTTT
>TR241|c0_g1_i9 len=1981 path=[4315:0-131 4316:132-144 4317:145-148
4318:149-153 4319:154-165 4737:166-176 4768:177-243 4785:244-277
4735:278-326 4703:327-350 4660:351-354 4615:355-358 4732:359-413
4766:414-425 4701:426-449 4753:450-465 4657:466-489 4232:490-503
4610:504-590 4280:591-614 4730:615-636 4699:637-660 4786:661-671
4608:672-678 4698:679-685 4654:686-705 4607:706-780 4498:781-804
4752:805-873 4787:874-899 4793:900-909 4652:910-933 4728:934-971
4695:972-987 4765:988-1001 4751:1002-1025 4727:1026-1045 4694:1046-1069
4788:1070-1084 4794:1085-1103 4747:1104-1130 4722:1131-1147 4641:1148-
1187 4778:1188-1215 4685:1216-1283 4789:1284-1298 4591:1299-1330
4790:1331-1335 4718:1336-1346 4782:1347-1359 4777:1360-1394 4760:1395-
1443 4743:1444-1462 4675:1463-1484 4771:1485-1486 4759:1487-1493
4742:1494-1517 4712:1518-1529 4626:1530-1538 4740:1539-1591 4710:1592-
1611 4624:1612-1652 4571:1653-1725 4384:1726-1980] [-1, 4315, 4316, 4317,
4318, 4319, 4737, 4768, 4785, 4735, 4703, 4660, 4615, 4732, 4766, 4701,
4753, 4657, 4232, 4610, 4280, 4730, 4699, 4786, 4608, 4698, 4654, 4607,
4498, 4752, 4787, 4793, 4652, 4728, 4695, 4765, 4751, 4727, 4694, 4788,
4794, 4747, 4722, 4641, 4778, 4685, 4789, 4591, 4790, 4718, 4782, 4777,
4760, 4743, 4675, 4771, 4759, 4742, 4712, 4626, 4740, 4710, 4624, 4571,
4384, -2]
CTCTCTGTTCCTACTTATTTCCCCAATTAGTCAACCAACACAAGGATATTATTTTGGCTCATAATTTATTTTA
TGTGGATAATGTATGCTGTATCCCTGAGTTGTCACTAGGTTGGTGGACAAATGAGCCAGAAGTTATTCCATTT
TTTAGAGTGTATTGGGAGAAAAATGCGCTGTTCGTACAAAACACTACTTTACAAACTGCACCAACGCAACATA
GCCATGAGAATGTTATGCGCGTAGCAGGCAATGCCATCTGGTCTCTTCTTCCATTAGATCACTAATAGACCGG
GTAAAGAAACGCACTCATGCATGGGACTTAATATCCGAACTTTAAACATGCATCTAAATCACAATCACTGTCT
AACGTGGTGCCAATGCTGTCACCACTCCAGTCATAATATTGTTCCCCTTCTTGTCCATCTTATTCATATTCAT
TCGCTGTCGGTACTGCAATCTCTGTCCCTTAGCATCGTCGCGTCGCTGCTTCTTCACCACAGCACGATAACGA
TACAACGACAGAGACGTAAATCCCCCCTTGCCCTCGCCAGCGACGAGAATTCCCTTGCCACTCCGTCCCGTAG
GAATGGACCCCGCCATCCGCCCCATGGCCGCCAAAGCCGTGGTCTGCTCTCCACCTCCCTCCGCCTCTGCCTC
CGCTCTCATTTGATACAAATTCACCACCCGCCCTTGGTACAAGCGATCTCCCGCCGCATCCTTCGCATGTCGG
ACGGCGACCCGCTCGGTCCGATCGGGTGCGAATCGCTGCTTGTAATACCGCGACAGCGCGCGGTGGCCGACGA
TGCGTCCGTCGGGGAAGATGAGCTCCCCGAGGGGGGTGATGTCGAACCCATGCGAGGCGATTTCGTTTTCGCA
TGCGGCGTGAAGATCGTCGTCGTCCTCTTCCTTCGTGGAGGCGTCTTCCCATCCTTCGCCGTCATCGTCTTCT
ACGTCCTCCCACCCTTCGTCATCCTCCTCCATCGCATCAGTGATATCTCTATTAATCTTCGTAGCGCTGGTGC
CCAAAAACTCACGATTCGCCTCGCGAAAGTCGTAAAACACGTCGAATTCCTCCTAATCGACACCCCTCTCGTA
CAGAATCTTGCAATGTTCCTTGTCCTTCATGTGCTTTAGCAGAGCCCCCGTGCTCGCGAACGATTTTTGGCAG
TACAGACACATATTGCCCAAGCGGACCTTCTCATTGGCATATCCCAGAAATCCCTCGAGGTCGGTAATGTATT
CGGCATCAGGTAGGTAGAAGGAGAATTTGGATTGCATGTACTCGAGGTTGAGCTCAGGGGTGTCGGAGACGTG
GTTGTCGAAGAGGGATTGGCAAGGGTTGATTTCCGGCTGGCTTTCATCCATGGAAGAAGCGTCGTCATCGTCC
GCGTCATCGACAACATCGGATCGCATGGGTTCCACTACCGGAGACGTTGTTTCCTCGCTCTCCTCCACGGAAT
CATGCGAACGCCGGCTTTCTCTTGTGATTCTTTTTCTCCTTTTTGCTCTTGCTTTTATCCTTGCGATGGTCCA
CCCCCGACCTCTCTTCCCTCCCCTCTATCTCCTTTCCAGTGCGACGGCCGCTTCCAACCTCGCATTGAAGTCG
GTTTCGTTCAGCATGGGCAGGCCCGCCTCGCGTCGTTTGAGGTTGTACCATTTTCGATGACATCAAATCGCGC
ACCGCCGTAGGGTATGCATCGGATATCGCCTCAGGAACGGGCTCTGACCCTGCAACTGCCACTGTTTCGCTCT
CCATGCTCTTGGCTTTAATATGTGCCATCGCTCGATTTGTAGAAGCGCTTGGCGGCGGAGTCCGATCTGCCCC
ATTCATGACGGCATTACTACGATGGGGGTAAATGAAATGGCCGTTGATGTGTAATGTAGGTAGTAATGCTCAA

AAACTAGAATGGTACCATAGTGGTTAAAAGAGTCCCTATGACCAAATCGAGGGTGCTGAACATGATGGTGTTG
TCTAAAGTTT
>TR241|c0_g1_i10 len=1890 path=[4315:0-131 4316:132-144 4317:145-148 4318:149-153 4319:154-165 4737:166-176 4768:177-243 4785:244-277 4735:278-326 4703:327-350 4660:351-354 4615:355-358 4732:359-413 4766:414-425 4701:426-449 4753:450-465 4657:466-489 4232:490-503 4610:504-590 4280:591-614 4730:615-636 4699:637-660 4786:661-671 4608:672-678 4698:679-685 4654:686-705 4607:706-780 4498:781-804 4752:805-873 4787:874-899 4793:900-909 4652:910-933 4728:934-971 4695:972-987 4765:988-1001 4751:1002-1025 4727:1026-1045 4694:1046-1069 4788:1070-1084 4794:1085-1103 4747:1104-1130 4722:1131-1147 4641:1148-1187 4778:1188-1215 4685:1216-1283 4789:1284-1298 4591:1299-1330 4790:1331-1335 4718:1336-1346 4782:1347-1359 4777:1360-1394 4760:1395-1443 4743:1444-1462 4675:1463-1484 4771:1485-1486 4759:1487-1493 4742:1494-1517 4712:1518-1529 4626:1530-1538 4740:1539-1591 4710:1592-1611 4301:1612-1634 4302:1635-1889] [-1, 4315, 4316, 4317, 4318, 4319, 4737, 4768, 4785, 4735, 4703, 4660, 4615, 4732, 4766, 4701, 4753, 4657, 4232, 4610, 4280, 4730, 4699, 4786, 4608, 4698, 4654, 4607, 4498, 4752, 4787, 4793, 4652, 4728, 4695, 4765, 4751, 4727, 4694, 4788, 4794, 4747, 4722, 4641, 4778, 4685, 4789, 4591, 4790, 4718, 4782, 4777, 4760, 4743, 4675, 4771, 4759, 4742, 4712, 4626, 4740, 4710, 4301, 4302, -2]
CTCTCTGTTCCTACTTATTTCCCCAATTAGTCAACCAACACAAGGATATTATTTTGGCTCATAATTTATTTTA
TGTGGATAATGTATGCTGTATCCCTGAGTTGTCACTAGGTTGGTGGACAAATGAGCCAGAAGTTATTCCATTT
TTTAGAGTGTATTGGGAGAAAAATGCGCTGTTCGTACAAAACACTACTTTACAAACTGCACCAACGCAACATA
GCCATGAGAATGTTATGCGCGTAGCAGGCAATGCCATCTGGTCTCTTCTTCCATTAGATCACTAATAGACCGG
GTAAAGAAACGCACTCATGCATGGGACTTAATATCCGAACTTTAAACATGCATCTAAATCACAATCACTGTCT
AACGTGGTGCCAATGCTGTCACCACTCCAGTCATAATATTGTTCCCCTTCTTGTCCATCTTATTCATATTCAT
TCGCTGTCGGTACTGCAATCTCTGTCCCTTAGCATCGTCGCGTCGCTGCTTCTTCACCACAGCACGATAACGA
TACAACGACAGAGACGTAAATCCCCCCTTGCCCTCGCCAGCGACGAGAATTCCCTTGCCACTCCGTCCCGTAG
GAATGGACCCCGCCATCCGCCCCATGGCCGCCAAAGCCGTGGTCTGCTCTCCACCTCCCTCCGCCTCTGCCTC
CGCTCTCATTTGATACAAATTCACCACCCGCCCTTGGTACAAGCGATCTCCCGCCGCATCCTTCGCATGTCGG
ACGGCGACCCGCTCGGTCCGATCGGGTGCGAATCGCTGCTTGTAATACCGCGACAGCGCGCGGTGGCCGACGA
TGCGTCCGTCGGGGAAGATGAGCTCCCCGAGGGGGGTGATGTCGAACCCATGCGAGGCGATTTCGTTTTCGCA
TGCGGCGTGAAGATCGTCGTCGTCCTCTTCCTTCGTGGAGGCGTCTTCCCATCCTTCGCCGTCATCGTCTTCT
ACGTCCTCCCACCCTTCGTCATCCTCCTCCATCGCATCAGTGATATCTCTATTAATCTTCGTAGCGCTGGTGC
CCAAAAACTCACGATTCGCCTCGCGAAAGTCGTAAAACACGTCGAATTCCTCCTAATCGACACCCCTCTCGTA
CAGAATCTTGCAATGTTCCTTGTCCTTCATGTGCTTTAGCAGAGCCCCCGTGCTCGCGAACGATTTTTGGCAG
TACAGACACATATTGCCCAAGCGGACCTTCTCATTGGCATATCCCAGAAATCCCTCGAGGTCGGTAATGTATT
CGGCATCAGGTAGGTAGAAGGAGAATTTGGATTGCATGTACTCGAGGTTGAGCTCAGGGGTGTCGGAGACGTG
GTTGTCGAAGAGGGATTGGCAAGGGTTGATTTCCGGCTGGCTTTCATCCATGGAAGAAGCGTCGTCATCGTCC
GCGTCATCGACAACATCGGATCGCATGGGTTCCACTACCGGAGACGTTGTTTCCTCGCTCTCCTCCACGGAAT
CATGCGAACGCCGGCTTTCTCTTGTGATTCTTTTTCTCCTTTTTGCTCTTGCTTTTATCCTTGCGATGGTCCA
CCCCCGACCTCTCTTCCCTCCCCTCTATCTCCTTTCCAGTGCGACGGCCGCTTCCAACCTCGCATTGAAGTCG
GTTTCGATCGCCTCAGGAACGGGCTCTGACCCTGCAACTGCCACTGTTTCGCTCTCCATGCTCTTGGCTTTAA
TATGTGCCATCGCTCGATTTGTAGAAGCGCTTGGCGGCGGAGTCCGATCTGCCCCATTCATGACGGCATTACT
ACGATGGGGGTAAATGAAATGGCCGTTGATGTGTAATGTAGGTAGTAATGCTCAAAAACTAGAATGGTACCAT
AGTGGTTAAAAGAGTCCCTATGACCAAATCGAGGGTGCTGAACATGATGGTGTTGTCTAAAGTTT
>TR241|c0_g1_i11 len=2103 path=[4262:0-151 4263:152-181 4620:182-248 4358:249-261 4664:262-361 4785:362-395 4736:396-453 4661:454-477 4767:478-489 4734:490-493 4754:494-511 4702:512-545 4733:546-548 4766:549-560 4701:561-584 4753:585-600 4657:601-624 4232:625-638 4610:639-725 4280:726-749 4730:750-771 4699:772-795 4786:796-806 4608:807-813 4698:814-820 4654:821-840 4607:841-915 4498:916-939 4752:940-1008 4787:1009-1034 4793:1035-1044 4652:1045-1068 4728:1069-1106 4695:1107-1122 4765:1123-1136 4751:1137-1160 4727:1161-1180 4694:1181-1204 4788:1205-1219 4794:1220-1238 4774:1239-1240 4764:1241-1264 4748:1265-1271 4722:1272-1288 4642:1289-1328 4778:1329-1356 4721:1357-1377 4789:1378-1392 4795:1393-1398 4719:1399-1430 4790:1431-1435 4718:1436-1446 4782:1447-1459 4777:1460-1494 4760:1495-1543 4743:1544-1562 4676:1563-1563 4633:1564-1603 4581:1604-1606 4771:1607-1608

4759:1609-1615 4742:1616-1639 4712:1640-1651 4626:1652-1660 4740:1661-1713 4710:1714-1733 4624:1734-1774 4571:1775-1847 4384:1848-2102] [-1, 4262, 4263, 4620, 4358, 4664, 4785, 4736, 4661, 4767, 4734, 4754, 4702, 4733, 4766, 4701, 4753, 4657, 4232, 4610, 4280, 4730, 4699, 4786, 4608, 4698, 4654, 4607, 4498, 4752, 4787, 4793, 4652, 4728, 4695, 4765, 4751, 4727, 4694, 4788, 4794, 4774, 4764, 4748, 4722, 4642, 4778, 4721, 4789, 4795, 4719, 4790, 4718, 4782, 4777, 4760, 4743, 4676, 4633, 4581, 4771, 4759, 4742, 4712, 4626, 4740, 4710, 4624, 4571, 4384, -2]
ATGATCAAGATAACATAATTTTACCCAGAGGATAGATGGAATAAATGAATTATGGCATTCTGTGCTGCTCCTG
TGGTATGGATTTGGTCTGTGGAATGAAGGAATGAACTTCCCTCTCTCTCTCTCTGTTCCAATTTCCCCAATTA
GTCAAACAACACAAGGATATTACTTCAGCTCATACTTTAGTTGATGTGGATACCATATGCTGTATCCCTGGGT
TGTCCCCAGGTTGGTGGACAAATGAGCCAGAAGTTATTCCATTATTTAGAGTATTGGGAGAAAAATGTGCTCT
TCGTACAAAGTACTACTCCTTAATAAACCGCACCAACGCAACATAGTCATGAGAATGTTATGCGCGTAGCAGG
CAATGCCATCTGGTCTCTTCTTCCATTAGATAGTCTAGATGACATTCGCACCAATAGACCGGGTAAACAAACT
CACTCATGCATGTGACCTAATATCAGAAATTTAAACATGCATCTAAATCACAATCATTATCTAACGTGGTGCC
AGCGCTGTCACAACACCAGTCATAATATTGTTCCCCTTCTTGTCCATCTTATTCATATTCATTCGCTGTCGGT
ACTGCAATCTCTGTCCCTTAGCATCGTCGCGTCGCTGCTTCTTCACCACAGCACGATAACGATACAACGACAG
AGACGTAAATCCCCCCTTGCCCTCGCCAGCGACGAGAATTCCCTTGCCACTCCGTCCCGTAGGAATGGACCCC
GCCATCCGCCCCATGGCCGCCAAAGCCGTGGTCTGCTCTCCACCTCCCTCCGCCTCTGCCTCCGCTCTCATTT
GATACAAATTCACCACCCGCCCTTGGTACAAGCGATCTCCCGCCGCATCCTTCGCATGTCGGACGGCGACCCG
CTCGGTCCGATCGGGTGCGAATCGCTGCTTGTAATACCGCGACAGCGCGCGGTGGCCGACGATGCGTCCGTCG
GGGAAGATGAGCTCCCCGAGGGGGGTGATGTCGAACCCATGCGAGGCGATTTCGTTTTCGCATGCGGCGTGAA
GATCGTCGTCGTCCTCTTCCTTCGTGGAGGCGTCTTCCCATCCTTCGCCGTCATCGTCTTCTACGTCCTCCCA
CCCTTCGTCATCCTCCTCCATCGCATCAGTGATATCTCTATTAATCTTCGTAGCGCTGGTGCCCAAAAACTCA
CGATTCGCCTCGCGAAAGTCGTAAAACACGTCGAATTCCTCCTAATCGACACCCCTCTCGTACAGAATCTTTG
TCCTTCGTTGTTCCTTGTCCTTCATGTGCTTTAGCAGAGCCCCCGTGCCCGCGAACGATTTTTGTCAGTACAG
ACACATATTGCCCAAGCGGACCTTCTCATTGGCATATCCCAGAGAATTTGGATTGCATGTACTCGAGGTTGAG
CTCAGGAGTGTCGGAGACGTGGACGTGGTTGTCGAAGAGGGATTGGCAAGGGTTGATTTCCGGCTGGCTTTCA
TCCATGGAAGAAGCGTCGTCATCGTCCGCGTCATCGACAACATCGGATCGCATGGGTTCCACTACCGGAGACG
TTGTTTCCTCGCTCTCCTCCACGGAATCATTTGTCAGATTATTTTCACCTTTGCGAACGCCGGCTTTCTCTTG
TGATTCTTTTTCTCCTTTTTGCTCTTGCTTTTATCCTTGCGATGGTCCACCCCCGACCTCTCTTCCCTCCCCT
CTATCTCCTTTCCAGTGCGACGGCCGCTTCCAACCTCGCATTGAAGTCGGTTTCGTTCAGCATGGGCAGGCCC
GCCTCGCGTCGTTTGAGGTTGTACCATTTTCGATGACATCAAATCGCGCACCGCCGTAGGGTATGCATCGGAT
ATCGCCTCAGGAACGGGCTCTGACCCTGCAACTGCCACTGTTTCGCTCTCCATGCTCTTGGCTTTAATATGTG
CCATCGCTCGATTTGTAGAAGCGCTTGGCGGCGGAGTCCGATCTGCCCCATTCATGACGGCATTACTACGATG
GGGGTAAATGAAATGGCCGTTGATGTGTAATGTAGGTAGTAATGCTCAAAAACTAGAATGGTACCATAGTGGT
TAAAAGAGTCCCTATGACCAAATCGAGGGTGCTGAACATGATGGTGTTGTCTAAAGTTT
>TR241|c1_g1_i1 len=813 path=[4639:0-287 5161:288-360 5327:361-401 5314:402-421 5265:422-474 5335:475-518 5326:519-542 5342:543-549 5352:550-552 5357:553-564 5262:565-600 5153:601-605 5212:606-606 5259:607-625 5150:626-626 5290:627-654 5341:655-674 5339:675-709 5334:710-722 5324:723-733 5310:734-738 5197:739-770 5287:771-785 5249:786-812] [-1, 4639, 5161, 5327, 5314, 5265, 5335, 5326, 5342, 5352, 5357, 5262, 5153, 5212, 5259, 5150, 5290, 5341, 5339, 5334, 5324, 5310, 5197, 5287, 5249]
ACATTTTAATAAACTTTAGACAACACCATCATGTTCAGCACCCTCGATTTGGTCATAGGGACTCTTTTAACCA
CTATGGTACCATTCTAGTTTTTGAGCATTACTACCTACATTACACATCAACGGCCATTTCATTTACCCCCATC
GTAGTAATGCCGTCATGAATGGGGCAGATCGGACTCCGCCGCCAAGCGCTTCTACAAATCGAGCGATGGCACA
TATTAAAGCCAAGAGCATGGAGAGCGAAACAGTGGCAGTTGCAGGGTCAGAGCCCGTTCCTGAGGCGATATCC
GATGCATACCCTACGGCGGTGCGCGATTTGATGTCATCGAAAATGGTACAACCTCAAACGACGCGAGGCGGGC
CTGCCCATGCTGAACGAAACCGACTTCAATGCGAGGTTGGAAGCGGCCGTCGCACTGGAAAGGAGATAGAGGG
GAGGGAAGAGAGGTCGGGGGTGGACCATCGCAAGGATAAAAGCAAGGGCAAAAAGGAGAAAAAGAATAAGAAA
CAGCAGGGAAAGAGTCACAAGAGAAAGCCGGCGTTCGCAAAGAACAGAGCGGAGGGTGAAACTAATTTGACGA
ATGATTCCGTGGAGGAGAGCGAGGAAACAACGTCTCCGGTAGCGGAAGCCATGCAAGCCGATGTTGTTGATGA
CGCGGACGATGACGACGCTTCTTCCATGGATGAAAGCCAGCCGGAAATCAACCCTTGCCAATCCCTCTTCGAC
AACCACGTCTCCGACACCCCTGAGCTCAACCTCGAGTACATGCAATCCAAATTCTCCTTCTACCTACCTGATG
CCGAATACAT
>TR241|c1_g1_i2 len=2556 path=[4669:0-200 4670:201-224 5160:225-394 5050:395-418 5220:419-427 5327:428-468 5314:469-488 5266:489-542 5335:543-586 5326:587-610 5342:611-617 5352:618-620 5357:621-632

5262:633-668 5153:669-673 5212:674-674 5259:675-693 5150:694-694
5290:695-722 5341:723-742 5339:743-777 5334:778-790 5324:791-801
5310:802-806 5197:807-838 5287:839-853 5249:854-880 5195:881-904
5332:905-921 5286:922-949 5308:950-989 5343:990-1006 5193:1007-1033
5322:1034-1037 5306:1038-1061 5283:1062-1067 5244:1068-1091 5331:1092-
1111 5321:1112-1135 5344:1136-1149 5184:1150-1189 5129:1190-1216
5240:1217-1227 5182:1228-1237 5126:1238-1263 5280:1264-1332 4835:1333-
1356 5239:1357-1360 5340:1361-1384 5338:1385-1408 5345:1409-1418
5300:1419-1431 5234:1432-1442 5174:1443-1455 5118:1456-1458 5275:1459-
1476 5173:1477-1500 5232:1501-1522 5317:1523-1546 5299:1547-1633
5347:1634-1647 5171:1648-1671 5115:1672-1687 5228:1688-1711 5169:1712-
1723 5112:1724-1726 5226:1727-1760 5349:1761-1778 5356:1779-1782
5223:1783-1794 5164:1795-1818 5268:1819-1876 5350:1877-1910 5267:1911-
2010 5036:2011-2023 5221:2024-2090 4873:2091-2120 4874:2121-2555] [-1,
4669, 4670, 5160, 5050, 5220, 5327, 5314, 5266, 5335, 5326, 5342, 5352,
5357, 5262, 5153, 5212, 5259, 5150, 5290, 5341, 5339, 5334, 5324, 5310,
5197, 5287, 5249, 5195, 5332, 5286, 5308, 5343, 5193, 5322, 5306, 5283,
5244, 5331, 5321, 5344, 5184, 5129, 5240, 5182, 5126, 5280, 4835, 5239,
5340, 5338, 5345, 5300, 5234, 5174, 5118, 5275, 5173, 5232, 5317, 5299,
5347, 5171, 5115, 5228, 5169, 5112, 5226, 5349, 5356, 5223, 5164, 5268,
5350, 5267, 5036, 5221, 4873, 4874, -2]
TCTTCCGATCTCTGACCCGATCACCAATCTCGCTCTACGTACCAAACCTTACAACCACCACCACCACCACTGA
GCAGATACGATACAACATTCAGTGCAACACAAGCAGCCGGAAGCTTCTAGCATCTGAGGCCATCCAAGATTGG
TAGCAAGCAAGAGCAAACGCCTTACTCCGCCGTCTTATTGCCATCCCTATCGTGTCATCCAATCGTGATATCT
CTCCCCATTAATCTCCCTTCACACCAACAAAGAATCATGATGCAACTACCACTCCCAACACCCATACCCTCTG
CGGGAGCCACCGCCTTCACCTCCGCACCAACACTAAGCTCCACTACCGCCCCCGGAAAAACCTTCACCTCCCG
TAACGACCTCCAACAACACTACAAATCGGACTGGCACCGCTACAACCTCAAACGACGCGAGGCGGGCCTGCCC
ATGCTGAACGAAACCGACTTCAATGCGAGGTTGGAAGCGGCCGTCGCACTGAGAAAGGAGAGAGAGGGGAGGG
AAGAGAGGACGGGGGTGGACCATCGCAAGGATAAAAGCAAGGGCAAAAAGGAGAAAAAGAATAAGAAACAGCA
GGGAAAGAGTCACAAGAGAAAGCCGGCGTTCGCAAAGAACAGAGCGGAGGGTGAAACTAATTTGACGAATGAT
TCCGTGGAGGAGAGCGAGGAAACAACGTCTCCGGTAGCGGAAGCCATGCAAGCCGATGTTGTTGATGACGCGG
ACGATGACGACGCTTCTTCCATGGATGAAAGCCAGCCGGAAATCAACCCTTGCCAATCCCTCTTCGACAACCA
CGTCTCCGACACCCCTGAGCTCAACCTCGAGTACATGCAATCCAAATTCTCCTTCTACCTACCTGATGCCGAA
TACATTACCGACCTCGAGGGATTTCTGGGATATGCCAATGAGAAGGTCCGCTTGGGCAATATGTGTCTGTACT
GCCAAAAATCGTTCGCGAGCACGGGGGCTCTGCTAAAGCACATGAAGGACAAGGAACATTGCAAGATTCTGTA
CGAGAGGGGTGTCGATCAGGAGGAATTCGACGTGTTTTACGACTTTAGCGAGGCGAATCGTGAGTTTTTGGGC
ACCAGCGCTACGAAGATCAATAGAGATATCACTGATGCGATGGAGGAGGATGACGGAGGGTGGGAGGACGTAG
ACGACGATGACGGCGAAGGATGGGAGGACGCCTCCATGAAGGAAGAGGACGACGACGATCTTCACGCCGCGTA
CGAAAACGAAATCGCCTCGCATGGGTTCGACATCACCCCCCTCGGGGAGCTCATCTTCCCCGACGGACGCATC
GTCGGCCACCGCGCGCTGTCGCGGTATTACAAGCAGCGATTCGCACCCGATCGGACCGAGCGGGTCGCCGTCC
GACATGCGAAGGATGCGGCGGGAGATCGCTTGTACCAAGGGCGGGTGGTGAATTTGTATCAAATGAGAGCGGA
GGCAGAGGCGGAGGGAGGTGGAGAGCAGACCACGGCTTTGGCGGCCATGGGGCGGATGGCGGGATCCATTCCT
ACGGGACGGAGTGGCAAGGGAATTCTCGTCGCTGGCGAGGGCAAGGGGGGATTTACGTCTCTGTCGTTGTATC
GTTATCGTGCTGTGGTGAAGAAGCAGCGACGCGACGATGCTAAGGGACAGAGATTGCAGTACCGACAGCGAAT
GAATATGAATAAGATGGACAAGAAGGGGAACAATATTATGACTGGTGTTGTGACAGCGCTGGCACCACGTTAG
ATAATGATTGTGATTTAGATGCATGTTTAAATTTCTGATATTAGGTCACATGCATGAGTGAGTTTGTTTACCC
GGTCTATTGGTGCGAATGTCATCTAGACTATCTAATGGAAGAAGAGACCAGATGGCATTGCCTGCTACGCGCA
TAACATTCTCATGACTATGTTGCGTTGGTGCGGTTTATTAAGGAGTAGTACTTTGTACGAAGAGCACATTTTT
CTCCCAATACTCTAAATAATGGAATAACTTCTGGCTCATTTGTCCACCAACCTGGGGACAACCCAGGGATACA
GCATATGGTATCCACATCAACTAAAGTATGAGCTGAAGTAATATCCTGGTGTTCTTTGACTAATTGGGGAAAT
TGGAACAGAGAGAGAGAGAGAGGGAAGTTCATTCCTTCATTCCACAGACCAAATCCATACCACAGGAGCAGCACA
GAATGCCATAATTCATTTATTCCATCTATCCTCTGGGTAAAATTATGTTATCTTGATCATCATATTTAACATG
CAAGAAAGTAAGATTGCTAGTAGGCTTGGTTTTGCCAGGGCACCAGCAGTTTACAATGGTGGGGTGCTGCCCC
TATCAAATGGTGACAGAAGCCGCCTCGCATTTCACCTCATAGTTTAGAGCTCTGGCTGAGTTCAAGGTGCAAC
AGTGCAGTCTGCACAGCAACAATCTCAACAGCATTATAACCCAATGATGAATACTGGCTGATGCTGCCTTTTA
AGCACCTTTGACTGACATTCCCAACCTTTACATCAACCAAATTACACACATATAATACAAACACACATTGTAT
T
>TR241|c1_g1_i3 len=736 path=[4639:0-287 5161:288-360 5327:361-401
5314:402-421 5265:422-474 5335:475-518 5326:519-542 5342:543-549
5155:550-571 5292:572-590 5261:591-639 5339:640-674 5325:675-698

5310:699-703 5250:704-735] [-1, 4639, 5161, 5327, 5314, 5265, 5335, 5326, 5342, 5155, 5292, 5261, 5339, 5325, 5310, 5250]
ACATTTTAATAAACTTTAGACAACACCATCATGTTCAGCACCCTCGATTTGGTCATAGGGACTCTTTTAACCA
CTATGGTACCATTCTAGTTTTTGAGCATTACTACCTACATTACACATCAACGGCCATTTCATTTACCCCCATC
GTAGTAATGCCGTCATGAATGGGGCAGATCGGACTCCGCCGCCAAGCGCTTCTACAAATCGAGCGATGGCACA
TATTAAAGCCAAGAGCATGGAGAGCGAAACAGTGGCAGTTGCAGGGTCAGAGCCCGTTCCTGAGGCGATATCC
GATGCATACCCTACGGCGGTGCGCGATTTGATGTCATCGAAAATGGTACAACCTCAAACGACGCGAGGCGGGC
CTGCCCATGCTGAACGAAACCGACTTCAATGCGAGGTTGGAAGCGGCCGTCGCACTGGAAAGGAGATAGAGGG
GAGGGAAGAGAGGTCGGGGGTGGACCATCGCAAGGATAAAAGCAAGGGCAAAAAGGAGAAAAAGAATAAGAAA
CAGCAGGGAAAGAGTCACAAGAGAAAGCCGGCGTTCGCATGATTCCGTGGAGGAGAGCGAGGAAACAACGTCT
CCGGTAGTGGAACCCATGCGATCCGATGTTGTCGATGACGCGGACGATGACGACGCTTCTTCCATGGATGAAA
GCCAGCCGGAAATCAACCGTTGCCAATCCCTCTTCGACAACCACGTCCACGTCTCCGACACTCCTGAGCTCAA
CCTCGA
>TR241|c1_g1_i4 len=238 path=[4793:0-29 4794:30-32 4795:33-72 4796:73-73 5292:74-92 5261:93-141 5339:142-176 5334:177-189 5251:190-231 5309:232-237] [-1, 4793, 4794, 4795, 4796, 5292, 5261, 5339, 5334, 5251, 5309]
AAGAGTCACAAGAGAAAGCCGGCGTTCGCAAAGGTGAAAATAATCTGACAAATGATTCCGTGGAGGAGAGCGA
GGAAACAACGTCTCCGGTAGTGGAACCCATGCGATCCGATGTTGTCGATGACGCGGACGATGACGACGCTTCT
TCCATGGATGAAAGCCAGCCGGAAATCAACCCTTGCCAATCCCTTTTCGACAACCACATCTCTGACACTCCTG
AGCTCAACCTCGAGTACAT
>TR241|c1_g1_i5 len=2046 path=[4669:0-200 4670:201-224 5160:225-394 5050:395-418 5220:419-427 5327:428-468 5314:469-488 5266:489-542 5335:543-586 5326:587-610 5342:611-617 5352:618-620 5357:621-632 5262:633-668 5153:669-673 5212:674-674 5259:675-693 5150:694-694 5290:695-722 5341:723-742 5339:743-777 5334:778-790 5324:791-801 5310:802-806 5197:807-838 5287:839-853 5249:854-880 5195:881-904 5332:905-921 5286:922-949 5308:950-989 5343:990-1006 5193:1007-1033 5322:1034-1037 5306:1038-1061 5283:1062-1067 5244:1068-1091 5331:1092-1111 5321:1112-1135 5344:1136-1149 5353:1150-1165 5185:1166-1189 5129:1190-1216 5240:1217-1227 5182:1228-1237 5126:1238-1263 5280:1264-1332 4835:1333-1356 5239:1357-1360 5340:1361-1384 5338:1385-1408 5345:1409-1418 5300:1419-1431 5234:1432-1442 5174:1443-1455 5118:1456-1458 5275:1459-1476 5173:1477-1500 5232:1501-1522 5317:1523-1546 5299:1547-1633 5347:1634-1647 5229:1648-1657 5273:1658-1671 5170:1672-1687 5329:1688-1711 5316:1712-1723 5271:1724-1778 5356:1779-1782 5315:1783-1786 5296:1787-1810 5269:1811-1859 5350:1860-1893 5163:1894-1960 5016:1961-1983 5017:1984-1992 5018:1993-2005 5019:2006-2045] [-1, 4669, 4670, 5160, 5050, 5220, 5327, 5314, 5266, 5335, 5326, 5342, 5352, 5357, 5262, 5153, 5212, 5259, 5150, 5290, 5341, 5339, 5334, 5324, 5310, 5197, 5287, 5249, 5195, 5332, 5286, 5308, 5343, 5193, 5322, 5306, 5283, 5244, 5331, 5321, 5344, 5353, 5185, 5129, 5240, 5182, 5126, 5280, 4835, 5239, 5340, 5338, 5345, 5300, 5234, 5174, 5118, 5275, 5173, 5232, 5317, 5299, 5347, 5229, 5273, 5170, 5329, 5316, 5271, 5356, 5315, 5296, 5269, 5350, 5163, 5016, 5017, 5018, 5019, -2]
TCTTCCGATCTCTGACCCGATCACCAATCTCGCTCTACGTACCAAACCTTACAACCACCACCACCACCACTGA
GCAGATACGATACAACATTCAGTGCAACACAAGCAGCCGGAAGCTTCTAGCATCTGAGGCCATCCAAGATTGG
TAGCAAGCAAGAGCAAACGCCTTACTCCGCCGTCTTATTGCCATCCCTATCGTGTCATCCAATCGTGATATCT
CTCCCCATTAATCTCCCTTCACACCAACAAAGAATCATGATGCAACTACCACTCCCAACACCCATACCCTCTG
CGGGAGCCACCGCCTTCACCTCCGCACCAACACTAAGCTCCACTACCGCCCCCGGAAAAACCTTCACCTCCCG
TAACGACCTCCAACAACACTACAAATCGGACTGGCACCGCTACAACCTCAAACGACGCGAGGCGGGCCTGCCC
ATGCTGAACGAAACCGACTTCAATGCGAGGTTGGAAGCGGCCGTCGCACTGAGAAAGGAGAGAGAGGGGAGGG
AAGAGAGGACGGGGGTGGACCATCGCAAGGATAAAAGCAAGGGCAAAAAGGAGAAAAAGAATAAGAAACAGCA
GGGAAAGAGTCACAAGAGAAAGCCGGCGTTCGCAAAGAACAGAGCGGAGGGTGAAACTAATTTGACGAATGAT
TCCGTGGAGGAGAGCGAGGAAACAACGTCTCCGGTAGCGGAAGCCATGCAAGCCGATGTTGTTGATGACGCGG
ACGATGACGACGCTTCTTCCATGGATGAAAGCCAGCCGGAAATCAACCCTTGCCAATCCCTCTTCGACAACCA
CGTCTCCGACACCCCTGAGCTCAACCTCGAGTACATGCAATCCAAATTCTCCTTCTACCTACCTGATGCCGAA
TACATTACCGACCTCGAGGGATTTCTGGGATATGCCAATGAGAAGGTCCGCTTGGGCAATATGTGTCTGTACT
GCCAAAAATCGTTCGCGAGCACGGGGGCTCTGCTAAAGCACATGAAGGACAAGGAACATTGCAAGATTCTGTA
CGAGAGGGGTGTCGATCAGGAGGAATTCGACGTGTTTTACGACTTTAGCGAGGCGAATCGTGAGTTTTTGGGC
ACCAGCGCTACGAAGATCAATAGAGATATCACTGATGCGATGGAGGAGGATGACGAAGGGTGGGAGGACGTGG

ACGACGATGACGGCGAAGGATGGGAGGACGCCTCCATGAAGGAAGAGGACGACGACGATCTTCACGCCGCGTA
CGAAAACGAAATCGCCTCGCATGGGTTCGACATCACCCCCCTCGGGGAGCTCATCTTCCCCGACGGACGCATC
GTCGGCCACCGCGCGCTGTCGCGGTATTACAAGCAGCGATTCGCACCCGATCGGACCGAGCGGGTCGCCGTCC
GACATGCGAAGGATGCGGCGGGAGATCGCTTGTACCAAGGGCGGGTGGTGAATTTGTATCAAATGAGAGCGGA
GGCAGAGGCGGAGGGAGGTGGAGAGCAGACCACGGCTTTGGCGGCCATGGGGCGGATGGCGGGATCCATTCCT
ACGGGACGGAGTGGCAAGGGAATTCTCGTCGCTGGCGAGGGCAAGGGGGGATTTACGTCTCTGTCGTTGTATC
GTTATCGTGCTGTGGTGAAGAAGCAGCGACGCGACGATGCTAGGGGACAGAGATTGCAGTACCGACAGCGAAT
GAATATGAATAAGATGGACAAGAAGGGGAACAATATTATGACTGGAGTGGTGACAGCATTGGCACCACGTTAG
ACAGTGATTGTGATTTAGATGCATGTTTAAAGTTCGGATATTAAGTCCCATGCATGAGTGCGTTTCTTTACCC
GGTCTATTAGTGATCTAATGGAAGAAGAGACCAGATGGCATTGCCTGCTACGCGCATAACATTCTCATGGCTA
TGTTGCGTTGGTGCAGTTTGTAAAGTAGTGTTTTGTACGAACAGCGCATTTTTCTCCCAATACACTCTAAAAA
ATGGAATAACTTCTGGCTCATTTGTCCACCAACCTAGTGACAACTCAGGGATACAGCATACATTATCCACATA
AA
>TR241|c1_g1_i6 len=1229 path=[4669:0-200 4670:201-224 5160:225-394
5050:395-418 5220:419-427 5327:428-468 5314:469-488 5266:489-542
5335:543-586 5326:587-610 5342:611-617 5352:618-620 5357:621-632
5154:633-668 5214:669-673 5260:674-674 5312:675-693 5291:694-694
5258:695-722 5341:723-742 5339:743-777 5325:778-801 5310:802-806
5250:807-838 5309:839-844 5287:845-859 5333:860-880 5286:881-908
5323:909-948 5343:949-965 5307:966-972 5284:973-996 5245:997-998
5322:999-1002 5306:1003-1026 5283:1027-1032 5244:1033-1056 5331:1057-1076
5321:1077-1100 5344:1101-1114 5353:1115-1130 5281:1131-1168 5128:1169-
1192 5320:1193-1202 5303:1203-1228] [-1, 4669, 4670, 5160, 5050, 5220,
5327, 5314, 5266, 5335, 5326, 5342, 5352, 5357, 5154, 5214, 5260, 5312,
5291, 5258, 5341, 5339, 5325, 5310, 5250, 5309, 5287, 5333, 5286, 5323,
5343, 5307, 5284, 5245, 5322, 5306, 5283, 5244, 5331, 5321, 5344, 5353,
5281, 5128, 5320, 5303]
TCTTCCGATCTCTGACCCGATCACCAATCTCGCTCTACGTACCAAACCTTACAACCACCACCACCACCACTGA
GCAGATACGATACAACATTCAGTGCAACACAAGCAGCCGGAAGCTTCTAGCATCTGAGGCCATCCAAGATTGG
TAGCAAGCAAGAGCAAACGCCTTACTCCGCCGTCTTATTGCCATCCCTATCGTGTCATCCAATCGTGATATCT
CTCCCCATTAATCTCCCTTCACACCAACAAAGAATCATGATGCAACTACCACTCCCAACACCCATACCCTCTG
CGGGAGCCACCGCCTTCACCTCCGCACCAACACTAAGCTCCACTACCGCCCCCGGAAAAACCTTCACCTCCCG
TAACGACCTCCAACAACACTACAAATCGGACTGGCACCGCTACAACCTCAAACGACGCGAGGCGGGCCTGCCC
ATGCTGAACGAAACCGACTTCAATGCGAGGTTGGAAGCGGCCGTCGCACTGAGAAAGGAGAGAGAGGGGAGGG
AAGAGAGGACGGGGGTGGACCATCGCAAGGATAAAAGCAAGGGCAAAAAGGAGAAAAAGAATAAGAAACAGCA
GGGAAAGAGTCACAAGAGAAAGCCGGCGTTCGCAAAGAACAGAGCGGAGTGTGAAAATAATCTGACGAATGAT
TCCGTGGAGGAGAGCGAGGAAACAACGTCTCCGGTAGCAGAATCCATGCAAGCCGATGTTGTTGATGACGCGG
ACGATGACGACGCTTCTTCCATGGATGAAAGCCAGCCGGAAATCAACCGTTGCCAATCCCTCTTCGACAACCA
CGTCCACGTCTCCGACACTCCTGAGCTCAACCTCGAGTACATGCAATCCAAATTCTCTGGGATATGCCAATGA
GAAGGTCCGCTTGGGCAATATGTGTCTGTACTGACAAAAATCGTTCGCGGGCACGGGGGCTCTGCTAAAGCAC
ATGAAGGACAAGGAACAACGAAGGACAAAGATTCTGTACGAGAGGGGTGTCGATCAGGAGGAATTCGACGTGT
TTTACGACTTTAGCGAGGCGAATCGTGAGTTTTTGGGCACCAGCGCTACGAAGATCAATAGAGATATCACTGA
TGCGATGGAGGAGGATGACGAAGGGTGGGAGGACGTAGAAGACGATGACGGCGAAGGATGGGAAGACGCCTCC
ACGAAGGAAGAGGACGACGACGATCTTCACGCCGCATGCGAAAACGAAATCGCCTCGCATG
>TR241|c1_g1_i7 len=992 path=[4669:0-200 4670:201-224 5160:225-394
5050:395-418 5220:419-427 5327:428-468 5314:469-488 5266:489-542
5335:543-586 5326:587-610 5342:611-617 5155:618-639 5292:640-658
5261:659-707 5339:708-742 5334:743-755 5324:756-766 5310:767-771
5250:772-803 5309:804-809 5287:810-824 5333:825-845 5286:846-873
5323:874-913 5343:914-930 5307:931-937 5284:938-961 5245:962-963
5322:964-967 5306:968-991] [-1, 4669, 4670, 5160, 5050, 5220, 5327, 5314,
5266, 5335, 5326, 5342, 5155, 5292, 5261, 5339, 5334, 5324, 5310, 5250,
5309, 5287, 5333, 5286, 5323, 5343, 5307, 5284, 5245, 5322, 5306]
TCTTCCGATCTCTGACCCGATCACCAATCTCGCTCTACGTACCAAACCTTACAACCACCACCACCACCACTGA
GCAGATACGATACAACATTCAGTGCAACACAAGCAGCCGGAAGCTTCTAGCATCTGAGGCCATCCAAGATTGG
TAGCAAGCAAGAGCAAACGCCTTACTCCGCCGTCTTATTGCCATCCCTATCGTGTCATCCAATCGTGATATCT
CTCCCCATTAATCTCCCTTCACACCAACAAAGAATCATGATGCAACTACCACTCCCAACACCCATACCCTCTG
CGGGAGCCACCGCCTTCACCTCCGCACCAACACTAAGCTCCACTACCGCCCCCGGAAAAACCTTCACCTCCCG
TAACGACCTCCAACAACACTACAAATCGGACTGGCACCGCTACAACCTCAAACGACGCGAGGCGGGCCTGCCC
ATGCTGAACGAAACCGACTTCAATGCGAGGTTGGAAGCGGCCGTCGCACTGAGAAAGGAGAGAGAGGGGAGGG

AAGAGAGGACGGGGGTGGACCATCGCAAGGATAAAAGCAAGGGCAAAAAGGAGAAAAAGAATAAGAAACAGCA
GGGAAAGAGTCACAAGAGAAAGCCGGCGTTCGCATGATTCCGTGGAGGAGAGCGAGGAAACAACGTCTCCGGT
AGTGGAACCCATGCGATCCGATGTTGTCGATGACGCGGACGATGACGACGCTTCTTCCATGGATGAAAGCCAG
CCGGAAATCAACCCTTGCCAATCCCTCTTCGACAACCACGTCCACGTCTCCGACACTCCTGAGCTCAACCTCG
AGTACATGCAATCCAAATTCTCTGGGATATGCCAATGAGAAGGTCCGCTTGGGCAATATGTGTCTGTACTGAC
AAAAATCGTTCGCGGGCACGGGGGCTCTGCTAAAGCACATGAAGGACAAGGAACAACGAAGGACAAAGATTCT
GTACGAGAGGGGTGTCGATCAGGAGGAATTCGACGTGTTTTAC
>TR241|c1_g1_i8 len=779 path=[4528:0-287 4529:288-310 5314:311-330
5265:331-383 5335:384-427 5326:428-451 5342:452-458 5155:459-480
5292:481-499 5261:500-548 5339:549-583 5334:584-596 5324:597-607
5310:608-612 5250:613-644 5309:645-650 5287:651-665 5333:666-686
5286:687-714 5323:715-754 5343:755-771 5307:772-778] [-1, 4528, 4529,
5314, 5265, 5335, 5326, 5342, 5155, 5292, 5261, 5339, 5334, 5324, 5310,
5250, 5309, 5287, 5333, 5286, 5323, 5343, 5307]
ACATTTTAATAAACTTTAGACAACACCATCATGTTCAGCACCCTCGATTTGGTCATAGGGACTCTTTTAACCA
CTATGGTACCATTCTAGTTTTTGAGCATTACTACCTACATTACACATCAACGGCCATTTCATTTACCCCCATC
GTAGTAATGCCGTCATGAATGGGGCAGATCGGACTCCGCCGCCAAGCGCTTCTACAAATCGAGCGATGGCACA
TATTAAAGCCAAGAGCATGGAGAGCGAAACAGTGGCAGTTGCAGGGTCAGAGCCCGTTCCTGAGGCGATCGAA
ACCGACTTCAATGCGAGGTTGGAAGCGGCCGTCGCACTGGAAAGGAGATAGAGGGGAGGGAAGAGAGGTCGGG
GGTGGACCATCGCAAGGATAAAAGCAAGGGCAAAAAGGAGAAAAAGAATAAGAAACAGCAGGGAAAGAGTCAC
AAGAGAAAGCCGGCGTTCGCATGATTCCGTGGAGGAGAGCGAGGAAACAACGTCTCCGGTAGTGGAACCCATG
CGATCCGATGTTGTCGATGACGCGGACGATGACGACGCTTCTTCCATGGATGAAAGCCAGCCGGAAATCAACC
CTTGCCAATCCCTCTTCGACAACCACGTCCACGTCTCCGACACTCCTGAGCTCAACCTCGAGTACATGCAATC
CAAATTCTCTGGGATATGCCAATGAGAAGGTCCGCTTGGGCAATATGTGTCTGTACTGACAAAAATCGTTCGC
GGGCACGGGGGCTCTGCTAAAGCACATGAAGGACAAGGAACAACGAAGG
>TR242|c0_g1_i1 len=505 path=[483:0-504] [-1, 483, -2]
CTCTTCCGATCTCCTCAGCATCGATGGCATCGTGCATTGAACCTGCGCCTCCCTCAGGGCCTAACGCGCCCTG
CATGATGCGCAGGAGTTCGGCGTTATCCATTCCTCCGGGTCCGGCGGCAGCTGCGTTGCCAGAGGCCGCAGCG
GTGGAAGGGGTGGCGACACCGGCAGCAGATGCAGCCTCGGAATAATCGCTCATATAGAGATTGACCTTAACAC
AAAGGTCTTCGTCTGGTGTGATATCCTTGTCTTGCATCCAAAAGAAGTGCCTCGAATCCGCATTACTGCCACA
CTGCAACAAATATACGCGATCACCCTCCTTCCCCGTCTCAACCCGTTCGAACGTAGCATCGTCCGTGGGAAAA
ATGGGAATCGTGTTGACCGTGGTCTTGGTGCGGCGATCCTTCCATTCCAATTTCAGATGCCCACTAGGTCCAG
CAGTACTGGCCACAGCGGCTGCGGCCGTGGCGGTCCAGACAACGTGAAGCTCGCCACGACAGATCGG
>TR243|c0_g1_i1 len=238 path=[216:0-237] [-1, 216, -2]
TACGTATCTTACGCCTTGCTGACAACTTGGTAACCAGCTAAGATTTAAACATTAACCCAAGTGCTGCATGAAA
ATAAAAGCTTTCGTACCTCGCGCCTCGCTTGCAGAAGTAGAGTTACTTATCAAGGGGCAGGATAAGGAGCAGA
CTGTCCAAGGCACGATTCTCGTTCTGTGGATAGTATACCAATTGCCAAAATCATCATGATCTGCTTTCGATTG
TTGAGGGATAGTAAAAGTA
>TR244|c0_g1_i1 len=226 path=[204:0-225] [-1, 204, -2]
CGCAGACGTCGCCCGATTTGCACGATCCCTCCGGAAGATTGTTGAACTGGCCACCGGACAGTTCCAGCATTCC
TCCAATTTTCGAGACGGGAAATTTACGCTTTTTGTTCTTATCATTGGTATTGTCGCCGTCACCGTTGCTGGCA
CCACTCAAACTGTTGGGAGAAATCACGCGGACGCTATCACGCGCTTTCAAGGTACCCGAGTAGACACGAGCGA
AGGCGAC
>TR245|c0_g1_i1 len=3432 path=[3410:0-3431] [-1, 3410, -2]
CGCAGTAGCATATTACCTATGAGCATTATGTTAACGTGTATTTTACATCGCAGACTTGGGTACCATACAATGG
TTAATCTCGCTCCATGGTGCAATGGATGGGGTGTCAAGGGCGACATAGCCTCATTTTATCCCCCTTCCATGAT
AACTTAGATGTGTACAATAATTGTACACCCTAACGTTGTTGGCGTTTAAGTTAGAGTGCATGAAATGCACGAT
TAACCCTTGATTAATCAGTAGCCATGTGCGATGCATAGTTCTCATTGCCACATTTGTCGTCTTTGCCAATATG
CATCCAACCTGTCCATCCGGGCTGAATCGAATCATATTGCAGCAAGATACACAAGCATTGTGGCTATACCTAG
AAAATCATTCTCGTTGTAAAACCTTCCATATCTGAATCAAAGTTTAGCAAGATGAATCAATTTGCTCTTCGAT
CAAGTCGTCTATGAGTTTCCAGCCAACATATAATGCGCAATAGCATTCGTCGGCTATATATCCAGAGGCGAAG
ATACGGCAACCTAGACGTAAGGAGGAGTACTTTCACTTGTCGTCCAGGAGTATTCTGGATATTACATGGGAGC
ACCATGAGATCAAGATCTCGACTATTCACAGACAGGAGATGTGTTTTGGATCCAATCCTTGTCGAGTATGTTC
AATTAATGACTCCAGAGCAGCCTCAAATGCATGCACCCAAAGCTGACAATCAAAAAGAGTGTTACTATTCCGA
GCATCATCCAACAGGTGGATTACATCCAAGAATTTCTCCTCGTCTTCGCCCAGGGAAATTGCCAGCTCCTTAT
ACTCTGTCAAATTTGACGCAATGAACTCTTCTACCCCTAGAGAACGAAGCATACTAGCGCCAATTCTATTACA
CATACGGTCACCAGCAATCGTGATCAAGGGAGCGCCAGACAGCAGTGCATCGCACCCAGTAGCAATCCCACTG
CACACTCGAGTATCGAGAAATACGTCAACCATGCAACATCGTGCCAGATGTTCTCGTCTTTGTACTAAATTCA
AGAACACTATTCGGTCGTTTGCAACGTGGTGTTTGATAGCTTCTTTTCTGAGATTTGCTTCCATGGCAGCAGA
AAATCGAAGCAGCACGAGAAGCGAATTAGGTTGACGCACGAGGATTTCCATCCATACTTCAAACAGCTCAGGA

TCGATTTTGTATGGTTGCGAAAATGACGCATATACAAATGCATCGTCGGAAATACCATATTTTGTCCGCAATC
TGGAGCTGTCGGCATCTAAATCGGCTACACCGTTGCATAAAATATTTGGCATGTAGATGATCTTCTCGCTGAA
ATGTGAACGGTGATGTTCAGGGATTGTAAAGTCGTCTCCGATAACATAATCAATTCCACTCCCGGAGCCCATC
GTTCCACAGTTCATTGTTATTTGGATTGGTGCCGGGCGAAGAGTAAAAATTTCAGGGCAAGCTCCTTGGCAAT
AGCCATCTAAATCAACCAAAATATGAATATCATGTGTGTTAATTGTATCTGCAGCATCCACGCAGTTGAGTCT
GCTCAAATCTACTAAAAGATCGACGCTTTCTTGAACATGCTTCCATTCAGCACTGCCGTCTGAAGCTGTAAGT
GCAAAACAAACTACTTGAAATTTCTGCCGGTTGTGAAAATCACATATTCCGCGGAAAAGGGAATTGTTCGTAT
GATTTCCAAAGGTCGATGACACGTACCCTATTCTAAGAATATCACCATTTTCGGAGAGTGTGTGCTTGTAGGG
ACGATTTCCCTTTTTGTAAAGAGCTCGTTGGTGGAATCGCTGAGACACTTTATCAGAGATGGCACGAGCCATT
TCTGGTGTGATCTGAGGAATGATTTGGGCACAAAATGGCTGGATGACAGCGTCACATAAGTTGACACTCGCAA
TTTGCTCGAAGCTTTGCTCACGATTGGACCATTGGCAGACAAACGCATCAAAAATAGCCAATGTAGATTCCTT
ACTATTTGGATCCAGTTTTTGTTTTAGATGGAGGCATGTGTTCGATGCTTTCTCGACTTCGTTTTGAGAAAGA
TTCAAAAGCGCAAGATTGTTCAGAGCCACAACGCTGTATTCGGATTTGGAATCGATTGAAGTTTGATAGCATT
TTTCTGCATATTCTTTTTGCCTACTAGTAAAGTATGCTGACGATGGCATACCATGTAACACGTGAGGTGGGAA
GAAAGTTTGACGTCTAGAAACAGCACAAGAGCAGAAACTGACCATTTCCCATGTTTAGAAAGGCAGGAATGTT
GAAAGGGTTCAATGCAATAGCCTTCTTTAGAAGTTTTGATGCAGCTAGAGTGTCTCCAAAGTCGTTTAAGACG
GATCCAAGGTTAGAACACGTGGTGGGATTAGGTTCCAGTTTCTCTGCAATACGAAGGCATTCAAGTGCGTCGT
CTACGGATCCCAGTCGTAACAAAGCCAAGCCGAGAGTGCCAAATCCTCGTGCATTGTTGGGATCAAGCTGCAA
TGCCCGAAGGCAAAGTTTTACAGATTGCTCATTTTCCCCAGTTTCAATATGTAGAGCTGCGAGGTTGCACACT
GCATCCGTGCTTGTCGGATCAAGTGTGACCGCATGCCACAAATAGTGATGAGCTAATTTGTGCCGGCCTTCGT
GCCTGTGACACATACCTAGCCTACAATTGGCTGATTGAGAGTTCTTAAACGAGACAACTATTCGGTCGAGGCG
TGACGTGGCTTCTTGCAACAGTATTCGGTTATTTGTGGCCTTGAAAGACTCTATTTGAGAACCGCACAAATTG
GTGGCAGCGTCAATGAATGATGGCTCCAAACGGAGAGCATGCGAGTAATGATCAATTGCTGTATCAAAATCAC
TCTCTTCGTGTGCTAAGTTTCCTAGACTGAGGCGAGCGATGGCACTCGATTTTGATTGCGATAGATGAACTTT
CCCACCAGCTTTATCACCAGCTGCGACTAAAATCAGACCAAGATTTTTCTTGAAGGAAGGATCGAGTGATACA
GCCATCTCGAAGCAGTCATTCGCCTCTTTCAGACGATTTTTCTGAAAGTGGATACAACCTAATGCCGCATAAG
CATGAGCAAATCTTGGCTGAATTCTTAATGCTTTGTTGTATAGTTCAGCCGCATCATCTTCATTTCCAAGCTC
TTGAAGAGCACTGCCAACTGCTACGTGAGCCTCCACGAATGTAGGATCAACTCCGATCGCCTGTTGCCCGTAA
AAAATGCTTTCTCGCATATGATTCACCTGAAAATGACAAGCTGCAAGGAGTAACAAATTTTCTGCGCTGGAAG
GATTTTTGCTGTAGAAGTACTGGCATTGTCGTAGGCACTCTTGATAGCGGCCTTCTTCAAATTTGAGACGCGC
ACTGGTCAAATGGATCCTAGTGTCGTCCATTTTTTATTTAGCCTTTCCCTTGGATTCAAACTGTCTCTCGTGA
C
>TR248|c0_g1_i1 len=514 path=[492:0-513] [-1, 492, -2]
CGATCCTTCATTACCATCAAGACTAGATAGCTCGGGATGTTGTAGAACAATGGAAAGGAGAAGAAGCCCCATC
AAAGGAGACAATTATTTTTTCTCGTCACAGACTGAATTGTCACACCAGTCGCTCAGTCGGGTAGCCTAGTGGG
TCCATTCTCTTCTAATTTAGACGCCGTTCCATATGAAGCGAATATAAGGGCCAGAAGATCGACATTCGACGTT
GCTAAAAGGAGGTCATCTCTTAGATCTATAACTCCCGGCAGCTGAGTTGACCCAATCTCTCCTCTATTTCGGA
TGAAGTTCCATGCCAATACGAACCTGACGTAAGAGTAAGAGGTAGAAGGTCGATACCTGCCCATCCTAGACGG
AGAGCATTTCTTACACTTGCATCTGCAAGTGCAGCTAACAGAGCAATCAATGAATACCGTTTTTGCTCGTCTG
AGACCGACTTCTTAGGCCAGTCAGGCAGTCTAGTTGACCCATTCTCCTCTAACTTGGACGAGGTTCCATGTCA
ACG
>TR249|c0_g1_i1 len=242 path=[220:0-241] [-1, 220, -2]
TTTGTGGGCGGCACGATATGTCGCATTCAAGAGTCATCTTTCATCCCCATCTGACACTTAGCCATGAATGGAG
TGAAAGATGAGACCCGAAAGGCAATTTGAGACCGACGAGACCGCAACACTGGAGGTTCACCATAGTCCGCGCG
ATATCCATGATCGGATCGCGCTGCGGATTGTGAAAATCATGCGGGTTTTTGCCGACGCCTTTTTCTCAAAGCG
CTACGGGCATCGTGCTGTGGTGC
>TR251|c0_g1_i1 len=508 path=[486:0-507] [-1, 486, -2]
GTCCACGCACTCTTCAATTCCCGCCACTCCTCCGAACACTATGAGCAGGTGGTCGAACTGTTCGTCGTTACCG
TCGCCATCGGTCGCATCTGCTTTGGACTTTGATTTTTTCGTTGTACGTTTCTTACGGAGGGTGAACTTTGGAT
CGTCGATGGAGACATCGCCGCGCTCACTCGTTCCGACCTTCAGATCGTATCCGCCTTCATAGGGGCACGCCTC
GAAAATGGCATTGATGGAGGGTGCCATCCTTGTTGTGTATCCCCAATATATGCCGTCTTCGTCACGGGGTGTG
GTGGGACTTACCACTTTACCTTTCATGACGCCCTTTTTGGAGGGTGATTTTTGTTTTGATTCGTAGGCTTTGA
TATCGAGTTGGACGGTGCATCGTATTCCTGGTGTAAGAACACGGTCAATCTGCACCAATCGATTGGGAACACC
ACAATCCACAAACGACCCATCCTCCTCCTCTTTCCCACCACCACCCTCTTTTCTCTCCACAACAACGCCC
>TR252|c0_g1_i1 len=305 path=[268:0-25 269:26-28 270:29-31 271:32-34 272:35-37 273:38-40 274:41-304] [-1, 268, 269, 270, 271, 272, 273, 274, -2]
ACAACAACAACAACAACAACAACAACAACAACAACAACAACGAAAGAGATTCGCTCCGCCTCCGATGATGCCA
CCTCCTATGTCGAGATCGGCGGGAACCACCGGCGGAAGCGGAAGCGGAATAGGAGGCGGATACAGAAGCCATC
AAGGCCTCCATTCCAATTCCAGTTCTCACCACGAGGACGGCAGTGGGAGCGGGAGCAGGCAACATCAGCAACA

GCATCAGCAACAGCAGCAACATCAGCAACAGCAGCGCCCTGCTCGTGGCAGCCTCACCATGTCCGCTACCGCC
GAGATCGGAAGAG
>TR253|c0_g1_i1 len=315 path=[315:0-247 316:248-314] [-1, 315, 316, -2]
TCATTATTGCATTCTCTGCTACTAGTTTGAAGGTCTGACTTACGCAGTTGCTTCACTTTTGATTGCTTTCTCG
CTCCTTATGCAGAATGTCCAGTTCTGCATTAGAAGAACATCGAATCGAAGAGTGGAATTACGTCAGTTCTTGG
TAATGGATATCTTCCTCCTCAGCCAATGATATTGTATTACTTCTGACATTTCTCATTGATGCTACTTCCACTT
CTAGTTTGGACGAGTGTTGTGCAGCAGCTCCTCCTCTCAGGTGATAGATTGTCCAAATGGTTGTAACTAAAGC
GCCCATGGTAGCACCAAACCAGG
>TR255|c0_g1_i1 len=625 path=[628:0-62 629:63-86 630:87-624] [-1, 628, 629, 630, -2]
AATGCCAGCACAGAAGAGGGTGGAGGTGGGACGGTACGCCACCGCGCCCAACGACGCGGCGAAACGACTCTTG
AACCGCGAGTCGGTCAACAGACTCAAAAACAATCGGTGAAGACTACTCCTCAATTGCTTCGGAGAGTATGGAT
CCAACAACAATAATAGCAGAACGGGCGAACTCGGCGCCACAAACGAACTACATGACGCAATCTTTTTCGAATG
CTCTCTAGTAGTCACCCCCACATTGGATTTGTTGCCAAAGTAATTAGTGCGGGAAGAGCCATGCTCGTCAACA
TGAGAGACTCCGGCTACAATATTGAGACCCGCCATCTTATGCGGAAACAAAGAGGAGGTGAGGTGTTCACCCT
CCTCGTGCTTGCGGAGATCGGTGTCCAAGACAAAGACTCTGGACTGCAGTCGTACGGATTCATCATCGGCGAT
GGGAAGTTGTGAAGTCGTTTTGTAGGCGTCTCTTTCCAATTTTCTCAATAAAGGATGGGTGAATGCTTCTTCA
TCGGCGTTGGACTGGGAGAGATGGGATGCAGGCAGCGTTCCCCATAAAGCGTGCGGAGATTTACGCAGGCGTG
AATCGGGAAGGCTGTATGGCACATTGGCGTAGAAGTCAGGG
>TR256|c0_g1_i1 len=329 path=[332:0-98 333:99-122 334:123-328] [-1, 332, 333, 334, -2]
TGCCGCTGCTGCTGCTGCTGCGGACATCATCGCGTTGTTGTCTCCTCCTCCTCCTCTTCTCCATCGCACCATC
CTGCACCAACGGATATCATCGAGCACCCTGCAAAAAGTTATCCATACGACGTCAATACCAATTTTCCGACTGT
TACGTCCCCAACCTAAACCCGAATATCTTTTTAAATCAAGCATCCTTCGTCATGGCACACGATGCCGCCACCG
GTTATCTCAACATGAACAATCAAAACGGCGATGACGACGACGATACCAATAAAAATTCCAACAACAACAATAA
CAACAACCGCAACAATTACAACAACAACAACAACAAC
>TR257|c0_g2_i1 len=301 path=[319:0-206 320:207-223 321:224-300] [-1, 319, 320, 321, -2]
GTGCTCTTCCGATCTGACGACGATGCGACGAAGAAGGGCGGCGGATTGCATCCTCCTCCGCAGTTCGTGGGGA
GGGTGGATGTGCCCGGTGCGACGACGACGACTTCGACGCGGAATGGAAATTTGAAACGTTCGGCGTAGGGCCG
TGTAGCGCCCGGAGAGACGGGTAACAGCACCCAGAGAGAGGAAGAGGGATGAAGTTTTTTTTAAGTTTCTACT
TGAATTCGAGTGCAGCTGGAAAACGAATATCTTAGAATATATAAGCATGTTATAATTTTTGTACATTAAATTG
GAAAAAAAA
>TR259|c0_g1_i1 len=843 path=[900:0-254 901:255-278 915:279-600 904:601-617 913:618-750 910:751-779 911:780-842] [-1, 900, 901, 915, 904, 913, 910, 911, -2]
CCATCACCATGAACATGAAAATGAAACCAACGCATGATAACTAAGTACTGTACGTACCGTACCCTTCACACTT
GGCAACTATTGGTTGTAACCATACTAGATACTCCTACCCAGTTTTCAAAAAGTTCTCAAGAATAACCATGTTT
TCTCCCTCGTTGATACTTTTGACCCTGGCCCCAGCGGCGTTGGACGTTGGAGCCTTCTCACCTCAGACGCAAC
ATCATGTCCTTCCAATTAGCCGAACTCGACTGTCACAACATCAATCCCGGCTAAATGCTCGCGGAGACGGATT
CAATTTGGACGACGACGATGAGGACGATCGCGACTTCGTTTACGCCGGGCGCGGATCGGGACGCCGCGGAAGG
GAAGAAGGAGGATATAACGACCAAGCGTCATCCGGGTCGTCTAGACGGTATCAGGATAATGTCGAATTCTTTG
ACCTCGACGACGATGACATATTCGAAGGTGATGGCAATAGAGGGGGTGATGTTTACGAGGCGACCTACAATGG
AATCATTCCTAACCCTCTGTTGGATGCGATGGATCCAGATGGTGTTTATGAACGGCTGGGGCCGGAATTGTTC
AAGGATTGGACTTTTTTCAGGGATTTGGCCTTGTTCGCTGCTTTCTTGACGTTCTTTACAAGGGATACGCATC
ATTATGGCACGTTTGATTCTGTGGTAGAGGGTTTGGAAAGATTGCCTGCCGATTTTATCCATTGAATAAAAGA
GAGACGCGAGATTGTGTACGTCCATAGGGGCGATTGATGGCTTGAAATGTAGATTTTTCAGTGTAAACTCAAT
AGAAATATAACTATAGAAGTGGAATAGGATAGATCGGAAG
>TR260|c0_g1_i1 len=2310 path=[2428:0-305 2439:306-586 2434:587-610 2438:611-2115 2431:2116-2139 2432:2140-2309] [-1, 2428, 2439, 2434, 2438, 2431, 2432, -2]
ATGACGTGGGCATCGCTGGCGGTGGCAGGGGTTTGCCATCTTCTTCGTGGGCCGCGGAGGCATTGAGATGGTT
GTCGTTGTCCTCTACCAACTATGGATTCCTGCAATCTATCCCTCGTTTGCACAACAGTCGAGATGGGGAGAAC
GAGCCACTTCAATTTTCCAAGTTTGCGATGGCATGCCAACCGAGCAGTGGTGGAGAGAGCGACGACCGAGTGT
ATCTAACGAAAATTAAACGGAGTCGATTGCCAATTCTGAAAAGGATCTTTCGTCCTCTGCTTCAAGATCTGTT
CGTATTCAAGGTTGTGAACTCTTCATTTGTACAACTTGGCATAAACGAGGATTTCGATACTCTCCAAAAATAC
TTCGGAGAATGGGCCAGTACTCTATCATCGAATGATTTAGCAAAATCAAACATGTATGCCACGGCAACGTGGC
GTCCCATAATTCGATGGCTTCACGACCTCATCCTCACTGCTTACGACGTGAACCAACGGTATCAGGGCCCATC
ACAATCACAAGCTAAGTTAGAGGATGATTCATTGAGAAAAAATGCCAAGTTGGAAACCCTGATTAAGTTCTGC
ACCGAAATCGAAGACCTCCCAAAAGCCTTCCTCCTTGCAGCCATTTGCACAGACGCCATTTCCTCTGCCTCTT

TACAACTCGAGCAAAAGACGTACGGGAAGATCACTCAGGTCGAATGCATTCGGCCATGGGAAGAGTTGCTGCG
CAAATTGAGAACGTGCCTCCTCGTTTCGCTGCGGCTTGGAGGGGACGTTTGTCCGCCGGGCGGAGGGAACGAT
CCCATGACGGTGAGCAACGTAGAGCGTCCGGACATGTTCTCCGTTTATTCTTGGATTGCGCGCGACGAACTGT
ACCTCAGCCACGATAACCGAGTGATCATGGCTTTGGAGAGCGCGTGCGTATCGAGCGCGGATGCGTTCTACCC
TTCCACCGTCGATGGGGATGGGGATCGACAGAAGACGATTATTTTTCAGAGTTGTCGCAGTGACATCAGGGAC
CTTCGCAACCCCACGGGACTGACAGACGAATCGCATTCGCGTTCTTTGCTGTTCTACTTGAAAGATCATGCAC
ACTTCCCCACCCGTCTAGCCGCAAATCGTGCGTTGATACTGGCGAATCTGTGGTCTCAAACTCCCGATCAGTT
GCACCTCCTCTACCACTCCATATCCGCGCTTCGTACGGTGGTGGACAGCGTCGAGAGATTCAGCCTGGCGGTG
GTGGCTGAAATATACCAATCGCGAATACGACCTGTGTGTCGCGCCATGCTGTTCGGATTCGGCGAACAGGAGC
TTAGCGAGGAGGTGGTCAGTCCTCTGTTCGGTGATGCTGTCTGGTTGAGCGAATTCATAAGTGTCGCGAAACT
GATCCTATCCATGATGGCTGAGTGCATGTCAAAAGTACCACCGCCGAGCGAAGACCATGAACAACTTGAGCCC
AGCAGCGATATGTGGCCACCGCTACGACAATGTCACGTACTCGACGGTCTCGTTCGAAAACTCAGAGGAGACG
TGCGGCCTTCCTCGGTCGCGTTGCATCATACGATCATATTTACCTTTGAAATGACTCACAGCATTGACTCCTT
CCAGGCCATTGTGCCATCCTTTTCCAACCTGTTTTTGACTGGGACTCTGTTTACTGAACTGCCCCCCGTGCCC
AAAGGATCTGCCCGGGAGAGGGACCTCCTTGACAAGGCCATAGGCGAACACGCCGAGAAAAGGAACGCCCCGG
TCATCGATAGCTTTGCTGGGATTAAGGATGTTGAGCTGTTTGGCGAGGCCATGGGATTGGAATCGAATTACGT
AAAAACGAAATTCCTTATCAAAATGATTCGACGTGGAAAAGAATCGAGCATCAATGATCTTTTGGGTGCAAGC
AATTCTTTGCTAGATAAGGAATTGTTCACATTCAAAGTCACGCGGATCATGTGCGCGAGGTTGGACGCCACCA
TTACATCGCTGAAACGAACGAAGAACTATCGGGGCATACTGAGTCTCTTGGATGCCGACGCGAGCCGATGGGT
ACGGGAGGAGGCTGCCGCAGAGTGTGCTTCATGGAAAACTAATAACTTTGCCACGGCGTCGTTGGTCGCTACA
CACTCGATGGTGATGCGACTTAAGAGCATATCGGAAAGTTTAGATGATGCATCGATGAAGGAGAAAGTCGATG
TGATTTGTCTCATGAGCGGGACATTGCTGAAAGCGGTTCAGGCGCAGGAAGAAGAGGACATACAGAATGTGTG
ATTAAAAGTAAATAATCCTGTACAAATTTCTCGAAAAAAAAAAAAGAT
>TR261|c0_g1_i1 len=1481 path=[1682:0-148 1683:149-169 1704:170-352
1686:353-376 1706:377-400 1703:401-424 1708:425-601 1680:602-625
1705:626-1180 1697:1181-1216 1698:1217-1480] [-1, 1682, 1683, 1704, 1686,
1706, 1703, 1708, 1680, 1705, 1697, 1698, -2]
CCTAACAAAGATATGAATTTAATGTATTATCATGTTTACTATTTGTCATCCCTATCTACATATTTATGCGTAA
CCATAATATCTCCTAGATCTGGTGGCGCTTAATCGAAGCACCACCATTCTGTTGCAAGCGCCTCACATAGGCA
GCTGATTTTTTGCTTTTTTCGGGTTTATTTACCTGATTTCCTTTTTCCAGCACGAAGCTTGGCAATAGTGTAG
AGGATGCCTCCCATCAAACTGATAGAAATGAAAGTCCACGTCTCCTCAGAAATGGCATTATCAAAAATGAGGT
AACCCATGATGGCTATAGGGATCTTGTTGAGAGATCCAATCATGGCATACGTCGTTGGACCTGTCTGTGCCAC
GCAGTTAAGACTGGCAAAATTGAGGAAGAATCCCACAAACCCTGCAAATGCATTCTTCACCGCATAATCAGGA
GTGTGAAGCGCAGATGTTCGCATAAAGGTGGCAAATTCGCCATTCATCACCGAAAAAGGGAACAAGAAGAGGG
TGCAAAGCACATTGTTGTAGAACACCATTCCAAATTTCGAGAGCTTCACCGACTTGGTGGCAAATTTGAGATA
CAAAACGTATCCAGAAGTGCACAAGCAGTTGGCGAGCATCCAGAACAATCCCGTCTGTGTGACGTCGGCATCG
TTCCTCGCTGCCATGACGGCTCCCATCAACATGATGCCAAATGCGGCAATCACCAAAAGTTCAACGCTGGCGC
CGTAAATAATACAGTCTCCGATGGTGGTCATGATATTGGTAATGTTTTTGAAGACGGTGACCATGGGAACGGA
GTTGTGTTCGAGACTGGCCATTCCGGTGAAGAGCATGCCACAGAAGAGGATGTTGACGGGGGCCCATAGTTGA
GCGGTTCGGATGTTGAAACTGGGGTAGTCGACCCATCCCATGCGCTTGGAGGTTTCGACGCAGATAACGGCGG
TTATGGCTTGGAATACCACGAGGAGAATATTGAGATCGCCCTGGATAAGGTGATTGTAACTCGAGGCAAGACT
CTTATTTACGAGAACCATCCCAACGCTGCATCCACTGTACATAGTACTAGCAATGATGCTCTTCGACAACGAT
GCTGCGTCCGCCTCTTTCTGCATAGTCCGCGGCGTTCCGGGCATTTTCAAACTGTTCGCATCGCCATTCCCAA
CGGTCAACGCTGCTGACTTTTCGACGGGATCGGACTCCTTCGCCTTGAAATTGCGCCTGTGTCCCGTCGTTCC
TCCGTGTGATGATGATCCCAGTTCGACATCTTCCATGCTCCGCAGATTGTTGGGGTTGCTTCCTTTCGCGGGA
GGGGATCGGACGGTCATTCCAGTGTTCATGGTCATGGTTGTAGAGCCGATAGACATTGTATCTAGGTAGATGT
TACTTTTTCCTCTTCGGGTGGGCGGGCGAACGGCGGAAGGGCGGTGAAAACGCGGTTCACACGGGGACGTGAA
ATACGGTTGCGGCGGGCGGGG
>TR261|c0_g1_i2 len=1569 path=[1689:0-148 1707:149-257 1704:258-440
1686:441-464 1706:465-488 1703:489-512 1708:513-689 1680:690-713
1705:714-1268 1697:1269-1304 1698:1305-1568] [-1, 1689, 1707, 1704, 1686,
1706, 1703, 1708, 1680, 1705, 1697, 1698, -2]
CCTAACAAAGATATGAATTTAATGTATTATCATGTTTACTATTTGTCATCCCTATCTACATATTTATGCGTAA
CCATAATATCTCCTAGATCTGGTGGCGCTTAATCGAAGCACCACCATTCTGTTGCAAGCGCCTCACATAGGCA
GCTAAGAGTTAAACAAAAACCCGGATTCAACAGAATGAGTATGCATCCCCCTGAGCAAATTAAACACAGAAGG
AAGGCATATAAATTACCTGATTTTTTGCTTTTTTCGGGTTTATTTACCTGATTTCCTTTTTCCAGCACGAAGC
TTGGCAATAGTGTAGAGGATGCCTCCCATCAAACTGATAGAAATGAAAGTCCACGTCTCCTCAGAAATGGCAT
TATCAAAAATGAGGTAACCCATGATGGCTATAGGGATCTTGTTGAGAGATCCAATCATGGCATACGTCGTTGG
ACCTGTCTGTGCCACGCAGTTAAGACTGGCAAAATTGAGGAAGAATCCCACAAACCCTGCAAATGCATTCTTC
ACCGCATAATCAGGAGTGTGAAGCGCAGATGTTCGCATAAAGGTGGCAAATTCGCCATTCATCACCGAAAAAG

GGAACAAGAAGAGGGTGCAAAGCACATTGTTGTAGAACACCATTCCAAATTTCGAGAGCTTCACCGACTTGGT
GGCAAATTTGAGATACAAAACGTATCCAGAAGTGCACAAGCAGTTGGCGAGCATCCAGAACAATCCCGTCTGT
GTGACGTCGGCATCGTTCCTCGCTGCCATGACGGCTCCCATCAACATGATGCCAAATGCGGCAATCACCAAAA
GTTCAACGCTGGCGCCGTAAATAATACAGTCTCCGATGGTGGTCATGATATTGGTAATGTTTTTGAAGACGGT
GACCATGGGAACGGAGTTGTGTTCGAGACTGGCCATTCCGGTGAAGAGCATGCCACAGAAGAGGATGTTGACG
GGGGCCCATAGTTGAGCGGTTCGGATGTTGAAACTGGGGTAGTCGACCCATCCCATGCGCTTGGAGGTTTCGA
CGCAGATAACGGCGGTTATGGCTTGGAATACCACGAGGAGAATATTGAGATCGCCCTGGATAAGGTGATTGTA
ACTCGAGGCAAGACTCTTATTTACGAGAACCATCCCAACGCTGCATCCACTGTACATAGTACTAGCAATGATG
CTCTTCGACAACGATGCTGCGTCCGCCTCTTTCTGCATAGTCCGCGGCGTTCCGGGCATTTTCAAACTGTTCG
CATCGCCATTCCCAACGGTCAACGCTGCTGACTTTTCGACGGGATCGGACTCCTTCGCCTTGAAATTGCGCCT
GTGTCCCGTCGTTCCTCCGTGTGATGATGATCCCAGTTCGACATCTTCCATGCTCCGCAGATTGTTGGGGTTG
CTTCCTTTCGCGGGAGGGGATCGGACGGTCATTCCAGTGTTCATGGTCATGGTTGTAGAGCCGATAGACATTG
TATCTAGGTAGATGTTACTTTTTCCTCTTCGGGTGGGCGGGCGAACGGCGGAAGGGCGGTGAAAACGCGGTTC
ACACGGGGACGTGAAATACGGTTGCGGCGGGCGGGG
>TR262|c0_g1_i1 len=2655 path=[2754:0-2042 2755:2043-2059 2815:2060-2094
2811:2095-2199 2820:2200-2226 2816:2227-2243 2823:2244-2259 2809:2260-
2349 2759:2350-2654] [-1, 2754, 2755, 2815, 2811, 2820, 2816, 2823, 2809,
2759, -2]
TACTGCTGACAACGACAACCGACAACACAACGGTGGCAAGAATTAGAGGCTACACGCAACATGAACTGTAGAG
ATAATAACAGCAGCAATGCGCAAGCGAGTATCATTGGGAAACTTGCGTTCCATGTCGCTCATTGATTCTACGA
AGATTCCTGACAATGACACTTACACTACTATTGAGACGAGTACATTGAATATGGAGTGGCAGGAGCAGGAACC
TCTGGTCGTGCCTCCTCTTCCGTTTTCTTCGAACGAAAAACTATCATCAACGATGGATTCTCCTGCCGAGGAG
CGGGTGCGGGATCGACCATCACAGCGGACGAACAAGGAGACACCGAGGAAGAAGCGAGAAAAGTCAACAAAGG
TCATCTCTCTCCCCTGGACGGACTCTCGCGGCATCCGAGGCGAATACTCGGGGGAGATAAATTCCTCCATTCA
GCCGCACGGCAAGGGCACATTTCGTGCCGATGATGGATCAATAGTAGGGTTCCCCTCCTCTACCTGGATAAAT
GGGAAGCTGGTCGCAGCCAAGATTCCCGAGGATCACAGTCTCAACAACAACAATTCGTCATCGGATTCATTAT
TGCGCAGTGGGACAAAACTTACACGTCCAAAGTTGAACTCTCCCGAACACAGACGATCCACGACGCTGACAGG
TTACAAGCTGGGCGATACGGCCAAGCATCCCTCTCACATGTACAAACTACCGCGCAAAATTCACCCTAAAGTC
GCAATAATGAATGCCGATAGTCTAAAACCACTCGACTTTGCCTTTGTACGAAGAAACAACAGAAAGTGGGTCT
ATAGCATAGTTGCCGGTCGTAAATTTATCGAGGGCGAGCTGGCAATTATATTCGTGCTGAATGAAAAGGGCAG
CATCAAGACTATTGGGAAGAAGTATTGGGCCAAGGTGATACGGTTGGTGAATACCACTCACATGAGTGGTATA
GTCAGTGCTGAAGCTGGTGGAAATGATCCCATGTCCAAAGAAGAGTCTCGCGATTGCAATGAAACGAAGAAAA
GGCGAAAAGAAAGAGTAGCGAATATAACAGGGAGCAAAATTTCCAGGAAATTGGATGGAAGAAGATCTGCCAA
AATTAAACCAGAACTCATGTCGGCGAACTCTTGTTCCACCAATGTGGCTGTAGTGGTGCCTAATACCAAGATC
TCCAATATTAAGTATGGTAAATATCATGTTCATCCAGAGACAAAAGTGAATAGTTCAGCGGATAGACATATGT
TGGGAAGATTTGACAAGCATCATCCACAGGGGGAGGACCGAGCACAACATTCATCTCATGGGGAACTTAAGTA
TAATGCTTCTACAGTGCATAATTCATTGCAAACTGTCAACCGGCATCCCACGGATGACCAGGCCGTCACAACG
AGAAGAGACAGCTACGAGTCTAACGAACGGGGTGACACTTCAACGAAGAAAAACATCTCTTCGAGTCCTATCA
ACAGGGGTAACAACGAGGAGATCCCAAAAAGAATAGGTAGGACTGGTGCCAGCTTTCATGGGATTGTCGCAGA
AGGGCCCAGGAAAAGAAGTAGAAGCAGGGAGAGCATAGCAAGCGGTGTGTCGGGTCTCAGCACCTTGTTCCGT
GAAATGTACTCGTCGTATGAAAGTTTGGAGATGAAGAAGTCACGTACGACACACTACCGTCCGACATCGAACC
AAAAATGACCATGTGAATACTAGACGTCTACAACCACTTCAGTTCTACCTTTGCATTAAGAAGTAACAGAATG
TGAGTCTATTTCACAGTTGGCGATTATCCGTTTCCATACAACGCGATGTGGACTCAATTCATGGGGAAAGCAC
AACTTCAAGACTACTTTGAACAAGTTTAGGCTGGGTCGATATGGCTATCGATGCCACTGACAAAACTCCCATT
GCATTGCCATTGCCGAAGAACTGGCTGCGTGTTCGACCAATAGAGAAGAATACATCGACGAAGAAAATAACTC
ATTCAATTATACTGACTCGGGCAAAAAGGATAATGTTCCACAGAGGCGAGGTAGGTCTGATACCAGTTTTTTG
TTGGACTGTCAGGAGGGCCGGGAAAAGAAGCAGAAGCAGGGAGAGCATAGTAAGTGATGTGCCAAGTTGCGAC
TCAGTGTTTTGTTAAATATAGATACTTACTCCTTAGAAAATGCGGCACGGAAATATTAAGGTTTTGTGATGAG
GAAATCATAAGCTTGATTATTTTTACAGAATTTTTTTTGTAGATTATATCAGTTCAGGCCAACATGAGAGGGA
AATGGCCAATTGAGAGCTCAGAATAGAGCACTGGCATACCCATATAGGTGGCACCTTCCCTATCGAATTGGAT
GGAAATCGTTTTTTGCATCAGCTCCTTAGAAAGATGTGTTTAGTATTCATTAACCCAGGAGGTCATGGCACAA
TGTGTGATCCCCACCATTTGCTTAAGGCACAAAACAAGTAAAGGCACATTGTGCATCCTACAATGCCTACAAT
ATTTGTGATGGTCAACAGTAAGCCACACACAAACAACTTCATAAATAATAACACCAACATGACATCAATGAGT
AGGATTCATATATTGCGGTTGCAATAGCTTGTTCATTGGAGGACATGCCCACAGTTGTGCTGAATAGTGAAAA
CATGATATCAATCCCCAATGCCTCAAT
>TR263|c0_g1_i1 len=743 path=[1078:0-109 1079:110-133 1095:134-135
1091:136-212 1086:213-244 1094:245-313 1089:314-379 1074:380-403
1075:404-407 1076:408-428 1096:429-496 1063:497-535 1088:536-624
1048:625-648 1049:649-742] [-1, 1078, 1079, 1095, 1091, 1086, 1094, 1089,
1074, 1075, 1076, 1096, 1063, 1088, 1048, 1049, -2]

ATCTCAATCATTTTTTAGACAAACTCCCACAACAACAATTTCCCTCCCCCCGACAAGAGAGATAGATGCCATG
TCAATAATAATAATTGACCACCTTCGATAGCACACATCGATCACCACTCTATCAATATCATCATCGTCTGTTG
CTATTGTACAGCCCTGATAGACCACACAAGCACAAGGCCATTTCCATTTCCAAAAGGAAGTTACAGACCGAAG
AAAAGAAAAAGCTCCACACCTAAGTAGTATATATAAAAAACAAAAAACAAAATACAAAATAATATGGCACCAC
CCCCCCTCTGCGGCACCCTAATAACATCCCGCCGTTCCTTCCTCGCATTAGCATGGACGTTAACCACCGTCCT
CTCCTTCTTCTCCTTTGGCGTTTCCCTTTTCCTGGGTTCCAAAATAACCAATCAATACAATTCCATGCAAACG
GTAGAATACGCGGAATGGTATCGAGATACGTATGGACAATATAATAATAATTGCAACAATAACCGTGACAGGT
ACAGTCGTAGGTATCGACGGTTAGATGATACCGACGACGATAGCGACGACGATAGCGACGACAACAACAACAA
CCATAGCAACAATAACAACAATCGGTATATCAACCGAAATATCAACAACAATTACATTCGACATTTAAGTAGC
AATGATAACGATGGAAGCGGAGACAATAACGACAACAACAACAACAACAACGGAAGCGGCGATAACAACAACA
ACAACAACAACGG
>TR264|c0_g1_i1 len=2018 path=[3108:0-36 @3156@!:37-418 3158:419-420
3143:421-443 3150:444-544 3146:545-1056 3153:1057-1762 3149:1763-1788
3145:1789-1811 3137:1812-1837 3138:1838-2017] [-1, 3108, 3156, 3158,
3143, 3150, 3146, 3153, 3149, 3145, 3137, 3138, -2]
CGCTCTTCCGATCTGGCGGTCATTGTCAAAGGCTTGGATATGACTGCACTTGGTGGAGTGGAGCAACTTGATA
GGATGACGGTTTGCAGCAAGATTGCCTACTTCATCTTAGGCGAGCACATTGGTACAGTGGATGCTACCGCCCG
TGGGAAACTTTTCGATGTCAAAAGGTACGTTTTGCATGCAAATAAACTTCAACGTTCCACTCTTGCTCGTCTT
CATTTAGGACTCTATTTCACTAACATGTTTTTCGCTTCACTCACCCCCCACTTCCATCTGTCCAGTGCCAATA
TTGATGCCATGTCTAAATCGAATGACAATGTCCTCTTCTCGGTGTCTGACACTGAAGCTGGATGGCACACTGA
TGGTGCCTCAAAAGATCGTGTGTATGACGTTGTATCTCTCTTGTGCATCTCTCCTGTCTCTCTCTCACGCAAA
CACATTATTTGTTCTTAATGTGAATAGGTGATGTTTATGACGAGCTGAACAGGGCGTTGCCCAAGTTCCTTCT
GTATGAGCTCACCCGGTAAGTTGTGACCAGTGGCAGGTAGCACACCCTCTAGTGGTCCCACTCTCCTAACTCA
CACTCTCCTTTTTGTCATTTCCTTTTCACAGTCCCATTCCTCGCGATATTCTCGAAAATGGTGGTGGTAAAGG
AACTGTTGGAGCTGCTACTTCATTGAGCCGTTCAGCAGCAATTCTTGCTATGCGCATCAAATACAACTCTTAC
CCAATCTATCTCGTCGAAGGAGACCGCATGCGGTAAGATAGCTGTGTCACATCCTTGGCTCTCTTCATCTCCT
TTTTGGTGCATTTATCAACTGTGGATTTTTGTTTGTGCTAACTGACCACATTCTCTCTCTCTTCGCCATCCTC
AGTTTCCGTTACATGCGCCATTGGATCGAAACAGGTCACGAGAAGACTTTTTGGAAGGTGCCTACCCTCCTCA
AGATTGCTATGGATGTATTGGATGATCATCTTGATGATGCATGCTGCTTCCATCAAGCCCTTGAGAGGGGAGA
CATGATTTTTGCCAACAATGCAATGCTCGCACATGCACGGGATCAGTTCAAGAATGATCCCGATGCACCCCCT
CGCCACAAGGTTCGTGCGTGGATTCAAGTGCAAAAGGCTGCAATTCTTAACGACGAAGAAACTTCATTCACCA
CCAGTGATGAGAAGGTAAGTGCTGATTCCAACCTTAATGCCTACAGAAACAGAAAGTTATTCTCATTCTCATC
ATTTTCTTTGATCTTTCCTGAATGAACCTCTAGTTGCACATGTCTCGAGTTGATACTATCAACTCCCTTGCTC
GAGTCGAGTCTCTCACCAATATGATATTGGACGACTCGAATCGTAATGGTGCTCCCACTTGGTGAACGAACCA
CACCATAAGTAGTATTTACTTCCTAGGATGCAACCCCTCTGTCGGCCCCAACAGCCGATTAGGATTAAGTGAA
GTAGTATCTTGAACAATTCACTACTTTTTACTTAAGAAGTTCAAGTAAAATCATGTTTTCATTTTGTTCCTTC
CTACTTGCTGTCTATGTAGTTTCTATGTGAATGGGGATAGTAGCTTATATCATTGCGTTGAAGGAGGCGCAGT
TTATTGGAGACACTGTGATGAAGCTCCTTTTACCCGTGTTGCTCTCAGTGTAGTAGACATTGCAATAGCCAAA
TGCGATGACGGCCACAGCCTATGGGGATCTTACCGCCTCTCTCACATCATCATGTGAGAGAGGGATGAGTATT
GATTGCGTGCGTCGTCCACGACTTGACGGGCATTTTGGGGATGAGTATTGATTGCGTGCGTCGTCCACGACTT
GACGGGCATTTTGTCATATTGCCTCAAGGTTCATGGTACTGGTGCTCGTCGGAGGGGATGAGTGCTGCTGACG
ACGGAGGCAATGGGCAACGGAATTGTGTTGTGCGATGGAGGCTTCATTGTTGGCGGGGTCATTTGGTTTGAAC
GAGCAAACTTCGGTCGAAATGAGAATGATTTTGGCAGTGCCCAACAA
>TR264|c0_g1_i2 len=3037 path=[3129:0-189 3130:190-213 3152:214-417
3157:418-949 @3156@!:950-1331 3158:1332-1333 3154:1334-1462 3150:1463-
1563 3146:1564-2075 3153:2076-2781 3149:2782-2807 3145:2808-2830
3137:2831-2856 3138:2857-3036] [-1, 3129, 3130, 3152, 3157, 3156, 3158,
3154, 3150, 3146, 3153, 3149, 3145, 3137, 3138, -2]
CTCTTCCGATCTGGGTGATAATATCTGGTTTAGATTTATTATAAGAAAAGGTAGGATCACACGAGGATCACAC
AAGGATCACACGGGGAACACACGAGAGATTACTTGTACGTAGGAACTGATAAGGTTCTCTAGTGACGTGGTAA
ACGACTTCAATATGAGATCATTGAGATTTGGGTATGGATCCGATTGCTAGATCGAAAGGTGTTCGTCATCAGT
GACGTGGTATGGAGGCCACGGCACAATTGGGAGTCCTAGTCCAATGGGATTAGGGCTCAGGAAATAGCTGGGA
CTCCTTGATACAAGGGAACAAGGGAACTGCTTATGTCACATAGATGGGGAGAAATTGAATGTCAGGTATGTTT
CAGCCCCTTTTGACGGTTTGTACTGGGCTGCATAGTAATTCATAGACTGACTCCACCTGCTCTTTTTTGTCTT
CAACAGAATGCATTTCAAGACGATTGAAGACTACATCCATGCTGCTACTTCGCATGGTTTTGAAATTGTTGAT
TTTACTGAAGCTAGGGTGAGGGCAGAAGATATGGCCGGTAACATACCCTTTTTCAGCAGTGTCAATGGATACC
CTCTCCACCTTGTCATCAAGATTCGTAAACCCATGGCCACCAGTGATGGTGCTTCTCTTGGCTCGATGAACAC
ACTTGACATCCTCCCAAAGAAGCTGAACTGGACCCGTACAGTCACCAGCAACATAGAAAACTCTCTTGTGATG
TGGATTCCAACAGATGTAAATGCTGAACTTATTGATGCTTCCCTCCAAGTGTACGAAAAAGGAATTTCTGTGG
ACGATCTTGATATTGGGAAGGATATCAACTTACCTACCAAGCGACGCTCGAGAAGGATGGTCCTATTCAGCGC

CACGAAGGAGTTTGCCAGAGCTATTCGTAGTAGGTTACTACATGAGACAGGGGCGGTCATTGTCAAAGGCTTG
GATATGACTGCACTTGGTGGAGTGGAGCAACTTGATAGGATGACGGTTTGCAGCAAGATTGCCTACTTCATCT
TAGGCGAGCACATTGGTACAGTGGATGCTACCGCCCGTGGGAAACTTTTCGATGTCAAAAGGTACGTTTTGCA
TGCAAATAAACTTCAACGTTCCACTCTTGCTCGTCTTCATTTAGGACTCTATTTCACTAACATGTTTTTCGCT
TCACTCACCCCCCACTTCCATCTGTCCAGTGCCAATATTGATGCCATGTCTAAATCGAATGACAATGTCCTCT
TCTCGGTGTCTGACACTGAAGCTGGATGGCACACTGATGGTGCCTCAAAAGATCGTGTGTATGACGTTGTATC
TCTCTTGTGCATCTCTCCTGCTACAGAGGGTGGAAAATTTAGAATTTCAAATGCATGTGAGTCGATATTGACC
AGTGCTCCATTTGAGCTGTGTGGATGAAATTGTTTTAGACCTTTTTCTCTCACTCTCTCTCTCACGCAAACAC
ATTATTTGTTCTTAATGTGAATAGGTGATGTTTATGACGAGCTGAACAGGGCGTTGCCCAAGTTCCTTCTGTA
TGAGCTCACCCGGTAAGTTGTGACCAGTGGCAGGTAGCACACCCTCTAGTGGTCCCACTCTCCTAACTCACAC
TCTCCTTTTTGTCATTTCCTTTTCACAGTCCCATTCCTCGCGATATTCTCGAAAATGGTGGTGGTAAAGGAAC
TGTTGGAGCTGCTACTTCATTGAGCCGTTCAGCAGCAATTCTTGCTATGCGCATCAAATACAACTCTTACCCA
ATCTATCTCGTCGAAGGAGACCGCATGCGGTAAGATAGCTGTGTCACATCCTTGGCTCTCTTCATCTCCTTTT
TGGTGCATTTATCAACTGTGGATTTTTGTTTGTGCTAACTGACCACATTCTCTCTCTCTTCGCCATCCTCAGT
TTCCGTTACATGCGCCATTGGATCGAAACAGGTCACGAGAAGACTTTTTGGAAGGTGCCTACCCTCCTCAAGA
TTGCTATGGATGTATTGGATGATCATCTTGATGATGCATGCTGCTTCCATCAAGCCCTTGAGAGGGGAGACAT
GATTTTTGCCAACAATGCAATGCTCGCACATGCACGGGATCAGTTCAAGAATGATCCCGATGCACCCCCTCGC
CACAAGGTTCGTGCGTGGATTCAAGTGCAAAAGGCTGCAATTCTTAACGACGAAGAAACTTCATTCACCACCA
GTGATGAGAAGGTAAGTGCTGATTCCAACCTTAATGCCTACAGAAACAGAAAGTTATTCTCATTCTCATCATT
TTCTTTGATCTTTCCTGAATGAACCTCTAGTTGCACATGTCTCGAGTTGATACTATCAACTCCCTTGCTCGAG
TCGAGTCTCTCACCAATATGATATTGGACGACTCGAATCGTAATGGTGCTCCCACTTGGTGAACGAACCACAC
CATAAGTAGTATTTACTTCCTAGGATGCAACCCCTCTGTCGGCCCCAACAGCCGATTAGGATTAAGTGAAGTA
GTATCTTGAACAATTCACTACTTTTTACTTAAGAAGTTCAAGTAAAATCATGTTTTCATTTTGTTCCTTCCTA
CTTGCTGTCTATGTAGTTTCTATGTGAATGGGGATAGTAGCTTATATCATTGCGTTGAAGGAGGCGCAGTTTA
TTGGAGACACTGTGATGAAGCTCCTTTTACCCGTGTTGCTCTCAGTGTAGTAGACATTGCAATAGCCAAATGC
GATGACGGCCACAGCCTATGGGGATCTTACCGCCTCTCTCACATCATCATGTGAGAGAGGGATGAGTATTGAT
TGCGTGCGTCGTCCACGACTTGACGGGCATTTTGGGGATGAGTATTGATTGCGTGCGTCGTCCACGACTTGAC
GGGCATTTTGTCATATTGCCTCAAGGTTCATGGTACTGGTGCTCGTCGGAGGGGATGAGTGCTGCTGACGACG
GAGGCAATGGGCAACGGAATTGTGTTGTGCGATGGAGGCTTCATTGTTGGCGGGGTCATTTGGTTTGAACGAG
CAAACTTCGGTCGAAATGAGAATGATTTTGGCAGTGCCCAACAA
>TR265|c0_g1_i1 len=451 path=[1080:0-68 1112:69-205 1109:206-219
1105:220-238 1111:239-239 1108:240-263 1103:264-280 1095:281-304
1065:305-450] [-1, 1080, 1112, 1109, 1105, 1111, 1108, 1103, 1095, 1065,
-2]
ATGTCGGATATTCCCAGGGCGACATATGATTCAATCAAAGACTACTTTGTCTCGTTCTTGAAGAACAAGTGAG
ATACATTTTTTAACATTATTGTCGTCATTTATGATGCTGGCGAAGGCGATGCACAAAAATGTTTAGTTTCTCC
CGTTTGAATTATACTCATTCGCGCCATACATTTTTTATTGAACATACACAGGCCACAGGGTGAAATCTTTGAG
GGCCACGTCACAATTGGACACAACTGGTGCCAAGACGCAGGGATATATGAGTTTACCATGGGTGTATCTGGCG
CGAAGGTCAAGGGCCGGTATTCCTTTGTCTATGTTTGGGAGAATGGTCAGTGGATGATCAGTCATCACCATTC
GTCTGTCATGCCCGGCCAAAATTGAGGATGATTTTCGAAGAATCTTTGTGCTCGTGCGTTATCGAGACTAGCT
ACTTAAACAGATC
>TR265|c0_g2_i1 len=533 path=[1038:0-72 1039:73-97 1040:98-120 1100:121-
150 1104:151-291 1050:292-315 1107:316-420 1102:421-466 1094:467-488
1058:489-507 1059:508-508 1060:509-532] [-1, 1038, 1039, 1040, 1100,
1104, 1050, 1107, 1102, 1094, 1058, 1059, 1060, -2]
TGCTTTTGCCCACGGTTAGCGATACGCCGCGAACGGACTTTGCTGGAATTGAGGACTACTTTGTCAACTTTTT
GAAGGGTCATCCCACGGGTGAAATCTTGGAAAGTCACGTCACCATCGGGCGTAACTGGTGCCAAGACGTGGGA
CTTTACGAGTTCACCATGGGGACAACGGGAAAGAAGGTGAAAGGTCGCTATTCATTCGTCTACGTTCAAGAGG
ATGGCAAATGGATGATCAATCACCACCATTCCTCGGTCATGCCTGAGGGCACCATGTCACAGCCCATCGAAAC
CGATGAGGTCAGAAACCTGTTTCAATTATGGAACTCAGCGCTGGCCACCGAAGATCCCGATGCAGTGGCCAAG
AGATATGCCTCTAAGGCGGTCCTTCTTCCTACTATGTCGGATATTCCCAGGGCGACATATGATTCAATCAAAG
ACTACTTTGTCTCGTTCTTGAAGAACAAGCCACAGGGTGAAATCTTTGAGGGCCACGTCACAATTGGACACAA
CTGGTGCCAAGACGCAGGGATA
>TR265|c0_g2_i2 len=514 path=[1052:0-53 1053:54-78 1110:79-101 1099:102-
131 1104:132-272 1050:273-296 1107:297-401 1102:402-447 1094:448-469
1058:470-488 1059:489-489 1060:490-513] [-1, 1052, 1053, 1110, 1099,
1104, 1050, 1107, 1102, 1094, 1058, 1059, 1060, -2]
AGGGTCATCCCACGGGTGAAATCCTGGAATCGAGGACTACTTTGTCAACTTTTTGAAGGGTCATCCCACGGGT
GAAATCTTGGAAAGTCACGTCACCATCGGACATAAATGGTGCCAAGACGTGGGACTTTACGAGTTCACCATGG
GGACAACGGGAAAGAAGGTGAAAGGTCGCTATTCATTCGTCTACGTTCAAGAGGATGGCAAATGGATGATCAA

TCACCACCATTCCTCGGTCATGCCTGAGGGCACCATGTCACAGCCCATCGAAACCGATGAGGTCAGAAACCTG
TTTCAATTATGGAACTCAGCGCTGGCCACCGAAGATCCCGATGCAGTGGCCAAGAGATATGCCTCTAAGGCGG
TCCTTCTTCCTACTATGTCGGATATTCCCAGGGCGACATATGATTCAATCAAAGACTACTTTGTCTCGTTCTT
GAAGAACAAGCCACAGGGTGAAATCTTTGAGGGCCACGTCACAATTGGACACAACTGGTGCCAAGACGCAGGG
ATA
>TR266|c0_g1_i1 len=1328 path=[1559:0-183 1560:184-207 1598:208-419
1596:420-509 1591:510-776 1594:777-953 1563:954-977 1590:978-1107
1585:1108-1131 1586:1132-1142 1587:1143-1166 1593:1167-1266 1575:1267-
1308 1576:1309-1327] [-1, 1559, 1560, 1598, 1596, 1591, 1594, 1563, 1590,
1585, 1586, 1587, 1593, 1575, 1576, -2]
GGGAGGATCAGGACTGAGGCGACAGAAAGTGCAAGCTAAATCCTTAGCAATGCTGTATGGAGTGGCCATGCGC
TACATCTGCAGAGGGAGAGCAATGAACAAAGACTTGTGAAGGAATAATAGAATTGAAGTTACGAACCTACACA
ACATAATGTTGGACAGTATTGATTGACTGCCAAGCAGATTTTTTTCCCCCAACACTAAACATTTTCGACCATG
GTGTTTTCATCACTGAAGTAATCCTTTTCGATCAAATGATTTCGTATCCTGAGAATATACCATATGATCTTAT
TTTCCCTCGCAACCATTCTACAACCACAACTCATAAACTACCGGAATCAACGGCTGCGCCCTATCAAAGAAGG
GAGAATTGATGTAAAACTCCCAAAGCACCAAAAGACCAATGCCAACCCACGCTCCGTTTTCAAGAAGCTTCAA
AATATTCAAATCCTCAAAGGGTGACTTCTCCGCCTCAATCTCCGCCTCACGCTGCAACGCGGCAGCCTTGCGT
TCGGCATTCCTCGCGGCCTTCTTCCCCATCCCCTGCGAGTCCATCAACATGCCAGCATCTTCCACCCTCCCTT
CCTGCATCGCCTCTCGCTGCTTGCGACGGGATATGACATCGTCAAAATCGGGAAGTTCAACCGCGTTTCCGCC
GCCATCCTGCTCGATCATTTTATCGAAATCCATGAATTTTTCGAGACGTCGATCGCTGAGCGTGTCTTGAATG
GTTTGGGGAGGCTGCCAGTTTGTACCAGCTTTTCCGCGCATGGCTCCGAGGATGACTTCTCCGTCGAGACCAT
CGACGTTGCCTCCCACTTCTTCCGCGAGTTGGGCGTCGAGACCGGCTCCTCGTTTGATGGGGGTGGCGTTTAT
CGGCGGAGACGATGATGATGCGGAGGATGCGACGGCGGCCCTCATGGGGCTACCGGTAGGTTCAACGATGGGC
GCTTGTTCAACAATAGCATCCTCTCCATCATCAAAGTCCGGCAGTTCGTCAGATTGTGTCGTCGGAGCAGGAG
AGGCCTTTCTTCGACGTCTCTTTTTCGCCATGAGAAACGACGATGATGTGCGACTGCTGCTTATCACAGCCGT
CGATCTGGAGAAAGTACTTCGCACCCCATTAGATGTGAGAGCGAGCCGTGCTGCCGGAGAAAAAGAGCATGAC
TGGAGTGGCAGTGAGAGCAATAAAGCGAGGATGATGTTCATGGTTTGCCCAGTGGTTTGCCTATCTGCTTCGT
ATTGGCCTCTCTAATCTCTTGCACACGCGGCTACTCCTACTTCTTAAATTAATTTTGACAGGCTCTCTAGAGA
GGCGGCCGACGTCA
>TR266|c0_g1_i2 len=495 path=[1559:0-183 1560:184-207 1598:208-419
1595:420-481 1568:482-494] [-1, 1559, 1560, 1598, 1595, 1568, -2]
GGGAGGATCAGGACTGAGGCGACAGAAAGTGCAAGCTAAATCCTTAGCAATGCTGTATGGAGTGGCCATGCGC
TACATCTGCAGAGGGAGAGCAATGAACAAAGACTTGTGAAGGAATAATAGAATTGAAGTTACGAACCTACACA
ACATAATGTTGGACAGTATTGATTGACTGCCAAGCAGATTTTTTTCCCCCAACACTAAACATTTTCGACCATG
GTGTTTTCATCACTGAAGTAATCCTTTTCGATCAAATGATTTCGTATCCTGAGAATATACCATATGATCTTAT
TTTCCCTCGCAACCATTCTACAACCACAACTCATAAACTACCGGAATCAACGGCTGCGCCCTATCAAAGAAGG
GAGAATTGATGTAAAACTCCCAAAGCACCAAAAGACCAATGCCAACCCACGCTCCATTTTCAAGAAGCTTCAA
AATGTTCAAATCCTCAAAGGGTGATTTCTCCGCCTCAATCTCCGAGATCGGAAGAGC
>TR267|c0_g1_i1 len=1230 path=[1459:0-130 1460:131-154 1474:155-333
1472:334-363 1465:364-386 1468:387-410 1471:411-434 1464:435-458
1467:459-513 1450:514-537 1463:538-603 1428:604-627 1470:628-909
1453:910-937 1462:938-1033 1425:1034-1057 1426:1058-1229] [-1, 1459,
1460, 1474, 1472, 1465, 1468, 1471, 1464, 1467, 1450, 1463, 1428, 1470,
1453, 1462, 1425, 1426, -2]
GTCCCTAAACAAAAATAGATTATCATCCCGCAATCCCGCGTTTCCTCTCTTCGTTCGTTCGATACATACATCA
TCATTCCCTCTCTTTTCGCCAGAACACTGTGTACTTTTTCTGAGAAATATAAATACATAATATCATGAGCCTC
TCTCCAGGATGTTATCGGAAACTACCATATCCCCATCGAAACTCTCTCCTCGCTGGTGGCGCAGAATGGAAAG
ATATGGTCATGGCAAAAAACGCTCACACCATCATAGGCTCATTTTGTCCGCAATAGTTTCAGCGATTTTTCAT
CCTCCACCCTCCCTATGGATGATATTCCGATCTCGTCAGCGCTTGTCAAAACGACGCATCATCGAATACTTTT
TGGAATGGCAATCGGAGACATATTATTCTCATTGAGTATAGCATGCTTTAACGCTTTGATACCCAGCGATATT
TTGATTATCTCGCGTGGAATGTGCCGTGGTAGGCAACCAAGCAAACGTGCAATGCCGATGGGATTCACGACGA
TGACGGGCGTGTGGGTATTTCGATGATGTACAGCTCTGCTCTTTGAATATTCATTCACTGGCGATTGTGAAAA
GTACAACAAGACACGGATTCCTACATTCGTACCAAGATGGAGAGCCATTTCTTCTTGGCGTTTTCCGATTGTG
ATTGCACTTCTATTCGGTGTGCCCCTCGGCTATTCACTCGGGTTGTTACAATTCTGATTTGGGAGGGACCTGC
CTGCCTTGCTCCCGTCTATGAACCACCTCATTTCATAGGGATCGAGGATGGTCAGGTTCGCGAAGGGTTCGAG
ATCACCATGCGGACGTGGGCGGGATGGGCCATCGGCAATCATTTTCCATGTGGCATTCTTGCTGTATTTCTTT
GGGTTGCCAACCATTGTTGGCGTAACTCTTGGTATAATGCATAGGCATGTATCTTGTCAGGAGAGAAGGATGC
GAAGGTATGGGGCAGGTGCTCTCAACCACAACGTCCCCAATGAAAATAATGATACATCACCTGATGCTACTGA
TGTTGGCGAGGGTAATAGCATCCACAGCACAATGAGATCGATTGTCTCTTCTGTGAAGAGCACATTTGGGAGA

TCGTCGTCCCGGTCAAATTCAAATCAAAACAATTCTACGAGCCGCGTTGTAATGGATCGGGCACGGGCCTACA
CCGCTGCATTTCTCTTGACATGGTCATGGTACATTGCTATCGTTGTGTTACGAGGGGCAGGC
>TR268|c0_g1_i1 len=2978 path=[3159:0-202 3160:203-226 3172:227-298 3157:299-322 3176:323-469 3163:470-493 3177:494-664 3175:665-892 3166:893-916 3170:917-1006 3174:1007-1405 3151:1406-1429 3169:1430-1613 3138:1614-1637 3173:1638-2262 3154:2263-2286 3168:2287-2887 3145:2888-2917 3146:2918-2947 3147:2948-2977] [-1, 3159, 3160, 3172, 3157, 3176, 3163, 3177, 3175, 3166, 3170, 3174, 3151, 3169, 3138, 3173, 3154, 3168, 3145, 3146, 3147, -2]
CCGCCGGCGCCCGTGCCGGCGCCGGCACCCGTCCTTCCCATCACATATCCAATCGTGACGACGAATCATCCAA
TCAAGATAAAGTTGGTCAACCTTCGTCAGGGATATTCCATGACAGCCGGGGTTCGTGCGATGATTATTAGATA
CATTCTGGAATGGATGCGACGAAATCTTAGTCCTGATTTGGAATTGGTCGAGGTTGCATTTGCGGGATATCTC
CCCACAAATGGCAACACTGGAAACGGGAGAGATCACAACTTGGCACTCAGAGTTACCGTCCGGCATCCATCCA
ATTTATCAGACGTTGCCCTATCCTACGTCCTCGACGCTATGCCAGACTTCGGCGACCTCAAGATAGAACTGAA
AAACCTAGACTTTAACGCCTTCAAGAATGTGGGTTTCTCCGCATCGTCGTTTAACGTTAACGAATTATCCGCC
ACGACATACCGACCGACACGCAGGCCCACGACTCGTCGTCCCACTCCCAGACCGGTTCAGAAGTTTGCGACGA
CGTTCCACGGGCAAAAGCTGGTGTTGCAGAATTTACGACCGGGATACACTCTGACCACCCGGGATCGCAATCG
CGTTATCGATTGGACGAGGGAGTGGCTGGAAACGAAACTCGACGGCACCGATATGGAATTGGTCAATGTGGTA
TCGGGGGGATTTCTGCAAACCAACATGTTGAACAGTGTGGCCATCCCCCTTCGCATTACACTGAGAGGTCCCG
AGGATTCATCACAGTTCGCATCGTCGCTTCTGATTAACCTTATGAATGAGTTCAGGGTGGAGCTTGGAAATAA
ATTAGCGTCATTGGATTGGAACTCGTTCATGAATGTGGATGTTTCGACGGGGACTTATGATTTTGAACAGGTT
CTGGATACAACGGTACGACCGACGCGAGAGCCGACGAGGAGGCCGACGCCAATACCGACGAAGAAGCCGACGC
GAAGACCGACGCCCTCCAGGAGACAGAGTACCCATGGCGTGCAGTTCCTCGCCAAAAAGGTCGCTCCCAGCTT
CACTCTCTCCTCCAGAGATCGGACATCCATCATTGGATTCATCAAGGTTGCATTGAGGAGAGATTTGAATCCC
TCACTAGAACTGATCGATGTTGACTACCGAGGCGGCGGCGGTAGGAATGGTCTGAAGCCATTGCCGCTAGATA
TCACCGTCAACGGACCTGTCAACACTTCGGACGACATCAAGTTAGGTTACATTCTGGAAGCGCTCGAGGATAA
TAGGCAAGACATTTCACAGAAGATCAAGAACCTCAATCGGAATGCATTCCAGGGGTTTGAGTTGTCTGTGGGA
CCGCATGATCCTACCTTATCGGTCCATGATGTGCAGTTGTCATTCAAGAATGTCGCCCCCAGCTACACTTTGT
CCTCCAGCGATCGGGCGTCAATTATCGTAATTGTCCAGGATGTATTGAAGAAAAACTTGGATCCCTCGTTGGA
ACTGATTGACGTTGAATACGAAGGCGGTGGGGATAACTTATCGCCGTTGCCGCTTGCGGTCACCGTCAAGGGA
CCTGTCGAATCTTCACCTGAAGTTGCGTTGGGTTACATTCTGGAAGGTCTCGAGGAGAGCAAGCAAGAGATTT
TGCGTAGGGTCAAGGCCCTCAATCGGAATGCGTTCAAGGAAGTCTCCGTGTCCTCAGGACAGTTTGATCCTAC
GCTTAAGATGGAACGATCGGAACACATGGTGCAACTGACCTTTACAGAGAGTTATCGTCTCTCTCAAAAAGAC
AGAACATCTATTATTGATTACATCAAGAATTTAATGCAGGATAACCTCGATGAATCATTGGAGGTGGTTCAAG
TCATTGATGCAGGTGGGGGGAATAATGGCAATTCGCCTATTCGCAGATTACCCCTCCGCATCACGGTGAAAGG
ACCTGACACTGAATCCGACCTCGCATTGGCCCACGTCATGACAGTTCTAGAAGAAAAGGACAAAGAGATAGAA
ATATATGTCAAATCGTTAGATTGGAACGAACTCAAGAATGTTGGGATTTCCGTGGAAGCGTTCGATCCTGAAG
CGGTGGAGATGGAGATGGAGAGGACGGAGCACCCGGTGCAGTTGACCTTCGGGAACGTCCCTTCTACATATAG
ACTCAATCCTGTCGATCGGGCGTCTATTATTCTCTTCGTCAAAGAGTTGATGGAGGAGCACCTTGACGACTCG
CTTGAGTTAATTTCAGTAACATATGCTGGCAACGGCAACAGACGATCGGATGCTCAATCGCTACCTCTTCTAA
TAAAGGTCGATGGGCCGGCGGATGAATCGGACTACGCTCTGGTGTACGTCATGAAGGTTCTCGAAGATCACAA
CGAAGAAATCGAGATGATGTTCAAATCTTTAGATTGGGATGAACTAAAAAACGCCAGCGTCTCCCCGGAAACG
TCTGATTCTAACTCCTTCACCTTCCCAGTGGGAGAGGTGGAAATGAAAGTGACGGAACTTCCAGTGCAGTTGA
ATCTCAACCAAATTGCCAGCGGATACCGCCTTTCTCCCGCCGATCGGGCATCCATCATTCGCTTCGTCAAAGA
GGAGTTGTTGGACAAGCACCTCGACGAGTCGTTAGAGTTAATATCAGTTAGATTTGCTGGCGATTATACGAGA
AACCTTCTCTCTTCAACGCTCCCTCACACTCGACGTTTGGGCACTCTTTCCATACCCCTTACTGTCGCAGTGA
AAGGACCAGTCTCTGAAAAAGACTTCGCCTTGTCGTACGTGATGGACGTTCTTGAAGATAACGAGAAGGAAAT
TGAGAGGTTTGTCGAGACTTTGGATTGGAACTCATTCAAGGACGTTACTGTTGATGTGGCATCCTATCATTCC
GACCTTGTGTCTACTGGACCTGGAGGAGGATCTGGAGGAGGATCTGGGCCTGGAGGAGGATCTGGAGGAGGAT
CTGGGCCTGGAGGAGGATCTGGAGGAGGATCTGGGCCTGGAGGAGGATCTGGAGGAGG
>TR269|c0_g1_i1 len=7481 path=[7804:0-652 7805:653-676 7863:677-739 7852:740-763 7872:764-1744 7840:1745-1764 7862:1765-2631 7808:2632-2655 7877:2656-2848 7794:2849-2866 @7871@!:2867-3663 7879:3664-3684 7881:3685-3685 7834:3686-3703 7874:3704-4446 7837:4447-4470 7870:4471-4689 7822:4690-4713 @7873@!:4714-6235 7869:6236-6251 7864:6252-6284 7857:6285-6308 7830:6309-7480] [-1, 7804, 7805, 7863, 7852, 7872, 7840, 7862, 7808, 7877, 7794, 7871, 7879, 7881, 7834, 7874, 7837, 7870, 7822, 7873, 7869, 7864, 7857, 7830, -2]
GCCAATGACGGTGGCGATGACGGCGGTGATGACGCAGCTGGCCTCAGCAATGGTGGCAACAGTAAGTCTCGCA
GTGTCGGCCGCTCCGCCATGGGCATCGCAGCTGCTGCAGCTGTGGGCGTGGCTGCTGCCTCATCACGTTCTTC

AGCCGCAAGGGAAGACGATGGCACCGCTGCCATGGAGCAAGATCAAGTTAGCACCGTCAATGAAGCTGCTGAC
GATCAACAAGTTTATGACGAAACACTTAGAGAATCTACCACTGGCACTAGCACCATCGAGCCATCGTCATCGC
AAATGTCATACAATGCTGATTACAACAGCTTCTCGCCGACAGAGAAGAGGCAGTTTTTGAGATTACTCAATTC
TGGACTGAGTCCTCAGGATGCTGCGCAGAATATTGTATTGGAGCGAAGCGGTGACAACGGGGAGATTATGGAT
AGTGACACTGCTCCTGCTGTAGTAGAAGAGGAACTGGAGGAAGAGTACGAAGAGGATAGTGATGAGAATGAAC
TGGAGGAGGAGGTCATGTATGCTAATGATGGGCCAGGGAAGGGACTTGCTGTAAGAACATCAAGTGACCCTGA
CGACGAGAGCAATGACATGCTCTCGTTAATTTTAGATCCACCAGGCGATACCATGCAGGGTGCCTCTGGTGAG
AGTGCCGAATTATCCACCCAATTTGAGCAGGTGGTAATGGAAGACCAAGAAGAAGTGGCGTCGAGGTCGGTGA
TGGCTCAAGCAAACAGCAAGTCTATTTCTCGCATGGGATCAATTGCTGCCATTGCGGCATTACCATTGGTCCC
AGCCATTGCCATTGTTCGCGCTAAGAGATCGAGGTCCAAGTCGAAAGAACGGAAACGACAGGCTTTGCAGGAA
GAACAGGAACAGGACGACGATGATGAGAAAGATGTGCAACAACAGCAGGAGGAGGACGACACAGGACAATATC
TAGCTACTCCAGGTGCCGATGGTCTGATATCCAGCGGCATTGGCTACTATGATGCCCACGATCGTGGAATTGG
TCCCGGAAACGAGGCGAACAGCAACATCGATGATGGTTACATTTATAGCGACTCTAGCCTTTTGGGGGACAAT
GACGAAGTTACTACTTCAGGTTCTATCACTCGCGGTAGATCACGAAGGGGCAAGCTCCTCGGAGCCGCAGTGG
CTTCCAGTTTGGCCGCGAAGGTTGCTTCACGGCGCCATCGTGATTCATTTAGGGGACATCAGCTGTCGCGTGG
CACCAAGTACAAGAATGTGTCATCATCAGTGCCTGCCTTGACGATTCGAGACGATTCACATATCGTCGCTGCA
TCCTACAGTTCTCCAGTTGCCAGAGACTACGAATATGAACAGCGCGCGACCAAGTCGGACTTCGCAACCAAAT
CACCTAAGTCGTTATCTTCAGCATTCAACTTCATGAATATCAGTAAGCCCATCAACATTAGCAAGAAGGGTAA
GAGTTCCAAGTGGGGTAAACTTGATTCGGATCTTGCGTTGGATCAGGTGGCGCCTTCAAGGTCAGCTGAGGTG
GAGGGCATGCCGTCGTGGATGTTACCCCGCGATGATAGCGAGACTGACGAAGAAGAGGAAAAGGAAGAAACAG
AAGAAGTGGAACCAAGCGGGGATCAAGCAGGACGAGACGAAGATGGAGAAGGACTATCTGTAGATGACGCTGC
GTCTGAGTCCATAGTTGAGTCAACGGTTCAAGAGCAAGAGGACGAAGAAAAAGAAGAAGGGGAGGAGGAATAC
GAAGATGAATCTGAAGCACCAGTTCTTTCTTCTGCATCTGCAACACACAGGGCGAATCGCCCACTAAACGTTG
GCACGAGTAGTCCTGGGATGGCATCCAAATTCACGAGTAAGAGGAATGTTGTCACCCCAATGGCATCTGAGAG
TACGGGCAACATCAGTGATGGTTACGATATTATGTCCCTGCAAGAATCCACGGCTGAGTCGTTGGCCCTTTCT
CCCGACGACTCTTTGGTCTTGTCTCCCGGCAGCACTGTGTTCTCACCGGGCACTTTGTCGTCACCCTCGTCTC
AAGCTTACGCGATGTCTGAAGATGAAGTTATATCACCCGAAGAACCTACCATTACAGGAGTAGAGAGACATCG
GCAGGAACAGTTGATCGAAGAGGATGAGGGCGGCGACGAGGGTTCTGTGGACAGAGCCGCGAGCGCCTTTCCG
ACAATTCCTTCATCTTCTACAGAATCATTAGTAAATCGCACTAGTCGTGATAGTATGTCAGCGGCACCCTCGC
GCACTGATGTATCCCTCACAAGCCCTGGAGAAGACGCTGCAAGCTTGATGTCTTATGCAACGAATACGACAGG
CACTGGATCCTGGACGACATCATCTAAGTCCCACCGAATACACCACAAGGGTGCCGCGCCCAAGCGAATAACA
AAACAAGTTGAATTTTCTCCCAGCCTTTTGTCCCCAGCCCCGCCTCAAGCAGACTCTCCTACCTCAGAAAATT
ATCAGCAGGAAAAACAGCATAGGCAGAGGGACAAGGTCGAGGGTGGGGAGAAGGAGGATGAAGATTCTGTTCG
TTTTACGAGTAGTGTCAAAAGCTCCCATCCTCGGCCAATTCCTTCGTCTTCTGCAGTATCATTGCCGGGTGAT
AGTCGTAATGATACTGCAGCAGCATCCTCGCCCCCAATTGAATCTCCCACCAGCCCCGATGAAGACACCGCAA
GCATAATGTCTTATGCCACGGGGATGACGGGCACAGGCTCCACGTGGACAGCGGCGTCAAAATCTCAACGCAA
ACGCCACAAGGGTGCCGCCTCCAAGCGATTGCAACAAGCTAAGCAGGCTGAAAATGACAACGCCGGCGGAAAA
AAACAAGGGATGGCTTGATACTATTCGGAATGCAGCCTCGACGCAAGGGCAGGTATGGGATCCCGAGTTGGGA
TGGGTTAACTACACCGAGCCAGAAGAAGTGACCTACGAGCATGATTACAAGCCCATCGGGAATTTGCACCTTG
CGATCCCTTCCAAGAGAAAATCATCTCCGGCAACGTCGCCGTCGCCAGTCAAGGCGCATCTGCCTGTGCACAA
CTATGTCGACCACCCCGAGCCCGTCGCATTTCCGGAAAAATGGGCTGATGAGCGAGAGACCATGATCAAATCG
GACGAACAACCAGCTAGTGCCCTTCAAAGAACTGTCATTGATTCCGATGGCGGCGAAATGAAGCCGGGTGTCC
TTCTTGATGACGAATCTATTTTTGACGCAACGACAACGCACACGATAAACACAGTGGTAGAGACTTCCAGTGT
GGTTAGCACCTCGACTGCGCCTACGCATCGATCAGCCCCTAGGCACCGTTCGAATGACTGGAGAAAGACGTCC
GCAGCTGCCGCTCACCACTTAGACCAGAAGCCCACCGGGTGGAAGAAATCGATGGAAAATGCCACCAACCACA
TGATGGAGATGAACGACGATAGCATTGGACGTCGTTGGGAATACGAGAAGGGGTGGGTCCGATCGGCTCCAAA
GCACCGTTCAACTGACCAGAGGAAGATTGGAGTGTTGCATACTGGCACCACTTCAGACAATAAACCGGTTGAA
TGGAAGGAACCAACGGAAAATGTCACTGCACAAACAGTGGCGAGAAACCGTACGGCCCCAAGGCACCGCCCCA
CAGACCAGAGGAAAATTGGAGCGGCAGAAGTTTTTTTCTGTTGCTGCTTCAAATGTTTCAAATGACGATGAAA
TGCCATCCGCTGTCCCCTATGACGACTCTGTCAATGATATGGTGACCGTGCACACCATCAGCACGACCGCGAA
GGGTTCCAGTATATTTAGTAACTCGACTGCACCCATGCACCGATCAGCAGCCCCTACGCACCGCTCCACCGAT
CAAAGGAAGACTGCCGCAGTTGCCGCTCACCTCTCAGATAATAATTCCATCGGGTCGAAGAAATTGATGGAAA
ATGCCACCGCCCAAATGGTGGAAACGAATAATGAGGGTAATGGCCGTCATTCGGCACCTATACGTCGCATCAA
AGACCGAAGGAAGATTTCCCAGCAGCACTCGGAAAAGAAACCCCTGGGATGGAAGGAATCAATGGAAAATGCC
ACTGTCCACATGGTGAATGGCGACCGTCTTTGGGACTACGAGAAGGGGTGGGTACGGGCAAACGGGGCTGTGG
ATGATGACATTGTGAGTGAATTGACTAGATCGGTTTTTGAACCAAGTCCTGAACGAGATCATGCAGTGAAGAA
TAACACGGCATCGGGTCATGATACCAGTTTCATGGCGGCGGAAGACTCTGCTGCGCCGTCGCAACATCTCCCG
TCCGTTGCGGAAGAGATGGAAGAGTTACTTGATTCCAAATCTGAGGAAGAACAAAAGGACGATGCGGTCACGA
GCAAAGTTGCGAAAGAAGGCAAAGATAAAACAGGTGGCGCTAAGCATAATGATGTTACGTTGGACAATGTCCC
ACCTCAAAAACGGCGCATTGGTCTACCTTCCCTCATGACGGCTGACAGTGGCAGCGACAAAGAAAACCAGGAG

AGCGAAAACGATATCCCCAGCACCTTCGCATATGAGCAATATAACGACCGAAAACCTCGATACGACCAAAAGC
CCCCCCTTCCGACGAAGAAACGGTCCCTCAATCAATGGTTGGAGAAGCATGTCAGCGACAACAGTAGTGCCCC
ACCAGAGCCCCTGGACGAATCGGCAGATGCCGTCGTATCTCGCGAAAGTAACGACTCGAGCGCTACTGCCACT
CACAGCAGTCATCTTCTTTTGAGCCCACCTAAATTATCTCCGTCTAATTTCTCAAATACTGATCCTTTCTTCG
GCGTAACTGACGAGAGAAAATTATCTCTATCGAATCTTTCGAATTTGGACCCTTTCCTTGGGGTAACCAACGA
TGACAATGGTGGAGACTCCACGCCGCGGACGGGGGAAGTGCAGGTCACGAAGGAGAAGTTCGACGATGCCGAC
AGCGATTTGTTCGAAACTACAAATATCACAGAGAGCTTTGAAAAAGCTATGGGCAGCAATTCTGATTCACTCG
ATAGTGGCCGTGCCAGGCAATGGGAAGTGCAGGTTGTGAAGGAAAATCTTGATGATACCGACAGCGATTTATT
CGAACCTTCTAAAATCACTGAGAGCTTTGTGAACAGGATATGTCGCAACCGGGTGTCATGTGATGATGACGGC
GCCAGACAATCCTTGATGAAAACATCCAATTCTCACGATTCCCATTACGGTAGCACGACGATTTCGCCTCCTG
CACTTTCTCCGATAGAGCCAAAGCACTCTCTCCTGATTGAAAAATCAGGTGGTGGTGCCAGGCCTGCTTCTTC
GAGAGTGAGGGAGTTAATGAGCTCTGCGGAGAAGAATACTCAGGCAGAACAGGCCGAGTCCGACGCGGGAGAC
GACAGCGATGTTCCTCAAATAAACAAGGTCACCGCGGTCTCTGACGTCAGCACCGCTCGTGATTTGGGTCAGA
AGCCAGGCCCTGTGAGTAGTTCGATAATTGGCAAGAATTCAAATAAGACCTCTATCTCGGGGATTCGAGGAAG
GAACTATGTGAAAAAAGATGAAGAAGAAAATCATTCCTCAAGGAATGCTCCTGACTCCTGGATAAAGTCGAGA
AATGACCTAGAGAAAGAAGAATATTCCAATTCCTCCTCTAGCAAGCCTGCGCAAGCAAAACCCAATGTGGCTA
ATCTACGATCCCAGTTCGAGACCAAGGGCAAACTTCCTGTCGAAAAAGAGGCAAATCCAGTGTTTATCCCCAC
TAAAAGTTCATCAGAAAATGATGTGTTCTTCCGCTCGACTGCAATGGGTATTAAATTGAAGCGCGGCGAGGAC
GGCCTTGTTCGAGTGGTTTCCGTCACAGAATCTATACCTGGATCTTCCATAATTCGAGATGGAATGATTGAGC
CAGAGGATTTGGTGATGGAGGCCGCTGGAGTGGACCTACGGTCACCTATCACGAATAGCCAGTGGGGAGAAGC
CGTCACGGTGATTAGGAATGCGCCGCGACCGATGAAATTCGTTGTTGCCGGCGGACCAAGGAGAAGGAATGAG
GAAACCAAACCATTGGTAGCATCCCAGCCCCCTAATCCTCCTCCTCCAGCGAAGTATGTGATTGAGTCCCGTT
TGTCCAAATCACCTGAAGGGCAACAAGTACTCGAATCACTTTCTGCATCTCGCCCTTATGAGCATTCCCAAGT
GGCATCACCGTCAGCGATTCCTCCAAAAAAATCCAAACCATCCCAAGCATCTGCCGGGGGGGTTGGCGGCGCA
GCATCAAGCTCATTCGGTGAAGACGAGACAAGCCACCGAACTAAGGAATCCCTCTTCAAACGCGTCGTTGCTT
CTTGTGCATCTCCCAGCCCATCTAAATCGTCAGCAGACCAACATCATGACGATGGAAGTCAGGATGTGCCTAT
GGCACATTTGCAGTTTTTGCGAACGAACCCAACAATAGCAAGAGTTACGAGTGCTGCATCTCGCCGTTATCCT
GTCTTTTGTGGAAGACCCGACACTATATTTGAAGAACCAGATGATGATGAACGAGAGACTAAGGGCTCAAGAA
CAAAAGAAAGGTCATCTTCTCTTCCCCGGGCATATGGCCTGGGGCAATCTAGATCTGCGGCATCATCAATGCA
TGATGGCTCACAAACGTACGACGGCTCACAAACGGACGACGGCTCACAGACGTATGATGGCTCACGGACTTAC
CTTTCGCAAACGGCAACACTGTCAACGTTTGGTGGTACGAAAACTATCACACCCATTAGCATTACGTCTACTA
ATGGCTCCATGGGTTCTGGTGCTGGCGGTGGCAGTGGCGACAATACAGCCTTCCTAGATAAGCTTGCCCTCAA
TCCTGCAGTAGCTAGTAAACCGGTTACACAAAGAAGAAATGGCGGCGCAAACATCGCCACACCGAAACTGGGT
ACGGAATCTTACAGGGACCATCCATCTTACCACGGATCCCAAGGAAACGATGCATTGGGATGGCCCGAGAGTG
ATGGTCCAGAGTACGAAAATGACATTGATAATGCGTCAACCTACAGCTCAAACATGAGCCACTCGTCTTATCA
CAGCAATAAGAAAAAGAAGGACACCGTGAGGCAAGCTGAGTTGCTCGCTGCGGCCAAAGTAGAAGACATGATG
AATGAGCTTCATAATGCAGACTCAGACGATCAATGTGAAATTTAACGTTCATACTGGAATGAGGCCATTAGAC
TGTCATCGCAGACGATGGATGTTTCACAACACTTCAATATCCATAAAATAAATGTATTTATGTATCTGGTATT
TTGCTCATAAAGGCGAAGCACAGCTTTCAAGGTGATCATACCGGCAGATTCATTTCCCTTGTTTTGCCTAAAA
AAATCGAACTGAGAAACCTGTACACAGGTAGAGGCAGAAAGTAATGAAACCAGACAACTGGATCAGTGTCTCT
TCACACAACAATTAAAGGAGGGAGGAAGGGCATCG
>TR270|c0_g1_i1 len=690 path=[2955:0-198 2956:199-200 3053:201-361
2976:362-362 3033:363-446 2995:447-457 2996:458-461 3030:462-515
2881:516-521 2882:522-522 2883:523-535 3025:536-643 2924:644-662
2925:663-689] [-1, 2955, 2956, 3053, 2976, 3033, 2995, 2996, 3030, 2881,
2882, 2883, 3025, 2924, 2925, -2]
TCAAAACTATTTATGAGTTATAATAGTGCTTTCATATACTACACACGCACAAACGCACACCCTGTTCTACCAA
CTAAAACATATCACTCAAGACATAAATGCAATACTTCACAAGCAAAAAAGGACTTGAGAAATGGCCCCACTTG
TTCTCCAACCCTATTTCATTTTCTATACAAAGCATGCAGTGCAGCAGCTGCACACGAGTTGCGGCGCCCCACC
TCCAGTGGGTGGCTGGCAATCGGCATGAAAGACGCCCAAATTCCCACCGGGTACGGTATCATTTCGGATCAGA
CACAAGGATTCGTCCACTTCACCCGTCATTTCGTTGTTATTTAACAGTAACTCCTCCAAGAACAAAAATTCTC
CCTCTGCAATGATGGGCAAGGTCCCAGTGAGGACATTATCGTTCATCCAAAGATCCTTTAATCTTGGACTTTG
TCCATAATTCTCCGGCACCGGACCGCTCAACTGATTATCATTCAGGTACAATATCCTCAAGCCTGGAATATTC
TGCCCAAAGATTCTCGGGGGTATAGACCCAGTCAGCTGATTGCTCTCGATCGACAAGAACTCTGCCGATTCCA
TGTTCCCAAACTCCTCCGGTATGGACCCGGTCAAATCGTTATTCTGCAATTCGAGCTCCTCGAGGTTGAGACA
CTCGCCGAACGTGGTGGGAATCTCCCCCGAGAG
>TR270|c0_g2_i1 len=567 path=[2964:0-107 3065:108-150 3052:151-166
3035:167-190 2961:191-202 2962:203-204 3070:205-300 3028:301-342
3083:343-348 3079:349-353 3081:354-354 3082:355-366 3045:367-369
3023:370-448 3074:449-451 3071:452-453 3067:454-464 3056:465-468

3042:469-522 3020:523-528 2861:529-529 2862:530-557 2863:558-566] [-1, 2964, 3065, 3052, 3035, 2961, 2962, 3070, 3028, 3083, 3079, 3081, 3082, 3045, 3023, 3074, 3071, 3067, 3056, 3042, 3020, 2861, 2862, 2863, -2]
TCTCTACTATGGTATATTATCATTTTGGACTAAATTGTTGATGATTCACCACAAACAAACAAACAAACACACG
AACACAACGATCACCTAATAGATCACTCTAGACATTACTACTGTAAAATGTCTTAAAAAGTAGACCTGAAATA
GCTCCACCTGTTCTCCGCCTAAATTTCTACACATAGCATGCAGTGCAGCAGCTGCACACTATTTGCGGTGGTG
CTCCGCTTCGGAACGACGGCTGGCAATCGGCATGGAATACGCCCAAATTCCCGCCGGGTATGATATCGTTTCG
GATTATACACAAGGAATCGTCCACTTCCCCGTCATTTCGTTGTTGTTTATTATTAACTCCTCCAAGAACAAA
AACTCTCCTTCTGCAATGGCGGCAACGTCCCAGTGAGCAAATTATCATTCATATAAAGATCCTTCAATCTTG
GACTTTGTCCATAATTCTCCGGCACCGGACCACTCAACTCGTTGTCACCCAAGTATAATAGTCTCAAGCCTGG
AATATTCTGCCCAAAGATTGTTGAGGGTATAGTCCCAGTCAGCTGATTGCTCTCCA
>TR270|c0_g2_i2 len=1227 path=[2937:0-293 3054:294-317 3083:318-323 3079:324-328 3081:329-329 3082:330-341 3075:342-343 3072:344-344 3068:345-365 3057:366-377 3044:378-423 3074:424-426 3071:427-428 3067:429-439 3021:440-503 2910:504-504 2911:505-517 2912:518-532 2913:533-541 2914:542-565 3041:566-625 3080:626-644 3040:645-790 3066:791-817 3055:818-841 3039:842-867 2974:868-1226] [-1, 2937, 3054, 3083, 3079, 3081, 3082, 3075, 3072, 3068, 3057, 3044, 3074, 3071, 3067, 3021, 2910, 2911, 2912, 2913, 2914, 3041, 3080, 3040, 3066, 3055, 3039, 2974, -2]
GTACCGGCCCAGCCGGTACTAATTCCTACGCGTTCTCCTCACGCCTGCCTCCCAGCAATAATTTCAGATGGAC
CAGGCGATGACCAGACAGAAGGGTGATGCGAAGCTGACAGTGCATGATCTCCAGATGCCGATATCAGCTCAAC
CTTGACGTACCCAGCAAGCAGTGCAGCAACTGCACACGATTTGTGGTGCCCTGCTTCCTATGGATGGCTGGCA
ATCAGCATGGAAGGCACCCAAGTTCCCGCCGGGTATGGTATCGTTTCGGATTAAACACAATGAATCATCCACT
TCTCCCGTCATTTCGTTGTTGTTTATTATTAACTCCTCCAAGAACAAAAATTCCCCCTCTGCAATGATGGGCA
ATGTCCCAGTGAGCAAATTATCGTTCATCCAAAGAACCTTCAATCTTGGACTTTGTCCATAATTCTCCGGCAC
CGAACCGCTCAACTCGTTATCATTCAGGTATAATATTTGCAACCCTGGAATATTCTGCCCAAAGATTCTCGGG
GGTATAGTCCCAGTCAGCTGATTGCTCTCCACGGACAAGAACTCGGCCGATTTTATATTCCCAAACTCCTCTG
GTATAGAACCAGTCAGTTTGTTATTCTGCAATTCGAGCTCCTCGAGGTTGAGACACTCGCCAAACGTGGTGGG
TATCTCCCCTGAGAGCTGGTTGAAGCTTGCCCAGAACGTGACGAGCTTGGACAGATCACCGACTCTGTCGGAG
ATGGTTCCCGATAGGGAGTTGTTCTTGAAGTAAACTTTCTCGAGATCGAGGTTGGAGTAGAAGGACTGGGGGA
TGGTGCCGACGATTTTGTTGTCTTCCATGTCGATCCACGTGAGCTTGTCGAGGTTGCCGATTTCGGTGGGGAT
GGTGCCTGCAATTTTGTTTTTGTAGAAGAGGAATGCATTCATGTCGACCAAGTTCCCAATCTGCCTGGGAATT
GTCCCGGTAATCTCGTTATCGGCAAACCAGAGATTTTGCAACTTGGTCCAGCGTCCGATCTCCGTGGGAATGT
TGCCCCGAAAGTTGTTGAGGCTCGCTCGAAAGTTGACCACCTCCGTGAGCCCGTTGGGTCCGCTGTACTCGGG
CTGGAAGAGCTCGCCCTCCATGTTGTTGGATTCGACGTCGAAGGTCTGGAGCGGCGCCACGAGGCCGAGGGAG
GTGGGGATGGGACCTGACTGGGCGTTGAAGAAGGATATGTAGGATGTCATGTAGACGAG
>TR270|c0_g3_i1 len=430 path=[2978:0-293 2979:294-317 2980:318-323 2981:324-328 2982:329-329 2983:330-429] [-1, 2978, 2979, 2980, 2981, 2982, 2983, -2]
GTACCGGCCCAGCCGGTACTAATTCCTACGCGTTCTCCTCACGCCTGCCTCCCAGCAATAATTTCAGATGGAC
CAGGCGATGACCAGACAGAAGGGTGATGCGAAGCTGACAGTGCATGATCTCCAGATGCCGATATCAGCTCAAC
CTTGACGTACCCAGCAAGCAGTGCAGCAACTGCACACGATTTGTGGTGCCCTGCTTCCTATGGATGGCTGGCA
ATCAGCATGGAAGGCACCCAAGTTCCCGCCGGGTATGGTATCGTTTCGGATTAAACACAATGAATCATCCACT
TCTCCCGTCATTTCGTTGTTGTTTATTATTAACTCCTCTGAGTTGCAAACATAAATTAGAGGCTGCATAGCAA
GAACAAAATTCACACTCTCACAAAGTAGAATACAATCAAGTACTCACCCAAGAACAAAAATTCCC
>TR271|c0_g1_i1 len=1362 path=[2229:0-45 2230:46-71 2231:72-88 2296:89-154 2255:155-182 2288:183-332 2219:333-351 2278:352-360 2184:361-382 @2300@!:383-494 2240:495-529 2294:530-530 2285:531-554 2299:555-618 2293:619-666 2301:667-678 2274:679-704 2298:705-729 2283:730-774 2272:775-909 2245:910-933 2297:934-1001 2271:1002-1036 2236:1037-1052 2237:1053-1076 2238:1077-1361] [-1, 2229, 2230, 2231, 2296, 2255, 2288, 2219, 2278, 2184, 2300, 2240, 2294, 2285, 2299, 2293, 2301, 2274, 2298, 2283, 2272, 2245, 2297, 2271, 2236, 2237, 2238, -2]
AATTACACGTGTCATTATTGATCGATTACAATGCCCCTCCTGATCTTATGATGAATGCTCTTTCTCACCCTAT
GCAGAATAAAATGAGACATTTGTCCAGAATTTTTGAAAGTTTCTCCATGCCGCCACCCGAGGAGACCCTAGCA
CACAACCCCATTTCAAAATGCTTCTACTGAGCACCTGGGTACTGGCTGATTTTGCATTTATTTTCAAGGTTGG
ATGTCCGAAACTCCATTCCACATCCTAAATTGCTTCCATCACAATCAACATCATGTTTTCAACTCCCGTGGTT
GAGGCAAAAATGCTTCGAATCTTTTTACGCTATCCTGCCACTAGCATCATGCACAAATGAAAAATCATCCACC
CCCTTGTAAGGATTCAGGATTCTCGCTATGATCTGTAGTTACTTGCTAGAAATCCACTCAATATTTGCCAAGT

GTCGCTCCAAAAATATTGTTTGTATCCACTTTACTCCTATCTACTTTGACGGGAACGACGGTGCAGTAAAATA
AAGCTTTGGAAACATTGTTTATTATTCTCGAGTAGAAAAACTGGAGAATATTCTTCACTGGTGGAGAGAGCAA
TTGCATTCATAGTCTTGAACCCTTCTGGAGTACTTTTTGGAACTATATCGAATGCCCTGAACTGAGCGTTATT
GTATTGAATAAGCAAATACATGCGCATTTACTTAAGAGCCATCAGACTAAATCCAGTCCTCGTAGATTTGCTT
AGGCGCGCCCTTCCACAAATGAGATATTTGCATTTCGCCCTTTCATTGTCCATACAAACGAAAAGACACATCC
ATTGGTATTCTGAGATAATATACGTATCAGTTTAAATAAAATCGATACGCCCCGGCAGCAGCCCACGCGGTGC
TGCACGCAAACAGAAATGGTCGGTGTCTCTTCGGGGCTGGATTACCCTGCATGAATTTCATCAGATTCGTTTG
GAAACCTCGCCATGTTGCTGGTATTATTCGGCGTGACTTCGCATGTTGCCCATAAATTTTAGGAGATTGACGA
GATGACGATGACGTGTGTTTATTGTTCGTTCTGCCGATCGAGTCCTCTCGTTTGTGTTGTGGTTTCGTCATCG
TTTGATTTTTTGGATGCTCCTCCCGTTCCATGGCCAGGGAGAGGAAGTAGCCCGAGAGTGCCATGCACGTTCC
CACTCCGGTGTACAAGCATGACCGGCAGGATTCTTTTTCTTGTGTGTTGCGGCCTCCTCCGGCGTTGTCGTCC
TCGTTGGTCATGGTCAGTTGTTGAGTTGTTTGTACGAATAGATTTTGATTCAATTTCTATCGTATCGTACCAC
AAGCCGGTTCAAGCTCTTTTGTTGGAGCTTTTGTGTTGAATTCGCGGC
>TR271|c0_g1_i2 len=1587 path=[2211:0-87 2212:88-111 2289:112-134
2280:135-160 2252:161-177 2287:178-243 2264:244-271 2277:272-441
2207:442-450 2295:451-607 @2300@!:608-719 2240:720-754 2294:755-755
2285:756-779 2299:780-843 2292:844-891 2301:892-903 2273:904-929
2298:930-954 2282:955-999 2272:1000-1134 2245:1135-1158 2297:1159-1226
2271:1227-1261 2236:1262-1277 2237:1278-1301 2238:1302-1586] [-1, 2211,
2212, 2289, 2280, 2252, 2287, 2264, 2277, 2207, 2295, 2300, 2240, 2294,
2285, 2299, 2292, 2301, 2273, 2298, 2282, 2272, 2245, 2297, 2271, 2236,
2237, 2238, -2]
GAAGTTTCACGTTCCATATTAGAGCGTATTTAAGTGTAGAACTAAAATGTCAATATGTCTCTATCCAAATGAA
CCCATGGTAGCTAATGAATTACACGTGTCATTATTGATCGATTACAATGCCCCTCCTGATCTAACGATGAATG
CTCTTTCTCACCCTATGCAGAATAAAATGAGATATCTGTCCAGAATTTTTGGAAGTTTCTCCATGCCGCCACC
CAAGGAGACCCTAGCACACAACCCCATTTCAAAATGCTTCTACTGAGCACCTGCGTACTGGCTGATTTTGCCT
TTATTTTCAAGGTTGGATGTCCCAAACTCCATTCCACACCCTTAATTGCTTCCATTACAATCAACATCAGGTT
TTCAACTCCCGTGGTTGGAGGCAAAAATGCTTTGAATCTTTTCACGCTACCTTTCCACTAGCATCATGCACAA
ATGAAAAATCATCTTGATTGTAGTACATGAGATCATCAATGCAAAGTTAGAGGTCCTGCACACCATGTCTGTG
ATACAATTGAGTTCTATGAGGTGCACCCACCCGAGTAGGTTGTGCCACACAAAGCCTAGCCGTTTATTACTTA
CCCACCCCCTTGTAAGGATTCAGGATTCTCGCTATGATCTGTAGTTACTTGCTAGAAATCCACTCAATATTTG
CCAAGTGTCGCTCCAAAAATATTGTTTGTATCCACTTTACTCCTATCTACTTTGACGGGAACGACGGTGCAGT
AAAATAAAGCTTTGGAAACATTGTTTATTATTCTCGAGTAGAAAAACTGGAGAATATTCTTCACTGGTGGAGA
GAGCAATTGCATTCATAGTCTTGAACCCTTCTGGAGTACTTATTGGAGCTATATCGAATGCACTGGACTGAGC
GTTATTGTATTGAATAAGCAAATACATGTGTATTTACTTAAGAGCCATCAGACTAAATCCAGTCCTCGTAGAT
TTGCTTCGGCATGCCCTTCCACGAATGCGATATTTGCATTTCGCCCTTTCATTGTCCATACAAACGAAAAGAC
ACATCCATTGGTATTCTGAGATAATATACGTATCAGTTTAAATAAAATCGATACGCCCCGGCAGCAGCCCACG
CGGTGCTGCACGCAAACAGAAATGGTCGGTGTCTCTTCGGGGCTGGATTACCCTGCATGAATTTCATCAGATT
CGTTTGGAAACCTCGCCATGTTGCTGGTATTATTCGGCGTGACTTCGCATGTTGCCCATAAATTTTAGGAGAT
TGACGAGATGACGATGACGTGTGTTTATTGTTCGTTCTGCCGATCGAGTCCTCTCGTTTGTGTTGTGGTTTCG
TCATCGTTTGATTTTTTGGATGCTCCTCCCGTTCCATGGCCAGGGAGAGGAAGTAGCCCGAGAGTGCCATGCA
CGTTCCCACTCCGGTGTACAAGCATGACCGGCAGGATTCTTTTTCTTGTGTGTTGCGGCCTCCTCCGGCGTTG
TCGTCCTCGTTGGTCATGGTCAGTTGTTGAGTTGTTTGTACGAATAGATTTTGATTCAATTTCTATCGTATCG
TACCACAAGCCGGTTCAAGCTCTTTTGTTGGAGCTTTTGTGTTGAATTCGCGGC
>TR271|c0_g1_i3 len=1334 path=[2229:0-45 2230:46-71 2231:72-88 2296:89-
154 2255:155-182 2288:183-332 2258:333-354 @2300@!:355-466 2240:467-501
2294:502-502 2285:503-526 2299:527-590 2293:591-638 2301:639-650
2274:651-676 2298:677-701 2283:702-746 2272:747-881 2245:882-905
2297:906-973 2271:974-1008 2236:1009-1024 2237:1025-1048 2238:1049-1333]
[-1, 2229, 2230, 2231, 2296, 2255, 2288, 2258, 2300, 2240, 2294, 2285,
2299, 2293, 2301, 2274, 2298, 2283, 2272, 2245, 2297, 2271, 2236, 2237,
2238, -2]
AATTACACGTGTCATTATTGATCGATTACAATGCCCCTCCTGATCTTATGATGAATGCTCTTTCTCACCCTAT
GCAGAATAAAATGAGACATTTGTCCAGAATTTTTGAAAGTTTCTCCATGCCGCCACCCGAGGAGACCCTAGCA
CACAACCCCATTTCAAAATGCTTCTACTGAGCACCTGGGTACTGGCTGATTTTGCATTTATTTTCAAGGTTGG
ATGTCCGAAACTCCATTCCACATCCTAAATTGCTTCCATCACAATCAACATCATGTTTTCAACTCCCGTGGTT
GAGGCAAAAATGCTTCGAATCTTTTTACGCTATCCTGCCACCACCCCCTTGTAAGGATTCAGGATTCTCGCTA
TGATCTGTAGTTACTTGCTAGAAATCCACTCAATATTTGCCAAGTGTCGCTCCAAAAATATTGTTTGTATCCA
CTTTACTCCTATCTACTTTGACGGGAACGACGGTGCAGTAAAATAAAGCTTTGGAAACATTGTTTATTATTCT
CGAGTAGAAAAACTGGAGAATATTCTTCACTGGTGGAGAGAGCAATTGCATTCATAGTCTTGAACCCTTCTGG

AGTACTTTTTGGAACTATATCGAATGCCCTGAACTGAGCGTTATTGTATTGAATAAGCAAATACATGCGCATT
TACTTAAGAGCCATCAGACTAAATCCAGTCCTCGTAGATTTGCTTAGGCGCGCCCTTCCACAAATGAGATATT
TGCATTTCGCCCTTTCATTGTCCATACAAACGAAAAGACACATCCATTGGTATTCTGAGATAATATACGTATC
AGTTTAAATAAAATCGATACGCCCCGGCAGCAGCCCACGCGGTGCTGCACGCAAACAGAAATGGTCGGTGTCT
CTTCGGGGCTGGATTACCCTGCATGAATTTCATCAGATTCGTTTGGAAACCTCGCCATGTTGCTGGTATTATT
CGGCGTGACTTCGCATGTTGCCCATAAATTTTAGGAGATTGACGAGATGACGATGACGTGTGTTTATTGTTCG
TTCTGCCGATCGAGTCCTCTCGTTTGTGTTGTGGTTTCGTCATCGTTTGATTTTTTGGATGCTCCTCCCGTTC
CATGGCCAGGGAGAGGAAGTAGCCCGAGAGTGCCATGCACGTTCCCACTCCGGTGTACAAGCATGACCGGCAG
GATTCTTTTTCTTGTGTGTTGCGGCCTCCTCCGGCGTTGTCGTCCTCGTTGGTCATGGTCAGTTGTTGAGTTG
TTTGTACGAATAGATTTTGATTCAATTTCTATCGTATCGTACCACAAGCCGGTTCAAGCTCTTTTGTTGGAGC
TTTTGTGTTGAATTCGCGGC
>TR272|c0_g1_i1 len=2337 path=[4966:0-80 4967:81-104 4968:105-105 4969:106-129 4990:130-765 4916:766-789 5009:790-1038 4998:1039-1139 4989:1140-1391 4940:1392-1398 4941:1399-1442 @5012@!:1443-1747 4996:1748-1788 5011:1789-1830 5003:1831-1863 4994:1864-1878 4985:1879-1917 5002:1918-2019 4959:2020-2043 4993:2044-2122 4972:2123-2147 5001:2148-2151 4992:2152-2175 4983:2176-2253 4919:2254-2277 4920:2278-2278 4921:2279-2336] [-1, 4966, 4967, 4968, 4969, 4990, 4916, 5009, 4998, 4989, 4940, 4941, 5012, 4996, 5011, 5003, 4994, 4985, 5002, 4959, 4993, 4972, 5001, 4992, 4983, 4919, 4920, 4921, -2]
AGCAACCTCCTACCATGTTCAACCACCCATTCTCAAAGAATACTTTATTCGCTTCAGCCATATCAGTAGGTGA
CTCATCATACCCTCCCATCGCATTCGGCAGACCAGCATTCGAGTACACATGCAAAAAGCACTCAACACACTTA
GCAAGCCTCTCTACAAATGGAGTCATGTGCTTCGCACCAAGGGCGCAGTTCAAACCCACACACATTGGCTTCG
CATGCCGGATCGATGCATAGAATGCCTCCCCTGTCTGACCTGACAATGTACGACCTGATTGATCTACCAATGT
ACCTGATACAAATACTGGAATGTCCAAACCTGTAAACTCCAAATACTCTCCTACAGCATACAGGGCTGCCTTC
GCATTCAGTGTATCAAAAATCGTCTCAACCATCAAAATGTCTGATCCACCATCCATCAGACCCACAATCTGCT
CAAAGTATGCATCCACAAGCTCGTCAAATGTCACGTTACGAGCAGCAGGATCCTCCACCGATGGGGAAATCGA
TCCAGTACGATTCGTCGGACCAATCGCTCCCACCACAAACCGTGGCTTAGTAGGGTCCTTCGCTGTCACCTCA
TCACACACCTCACGTGCAAGGCGTGCACCCTCGTAGTTAAGCTCATACACATAATCCTCCATCTCGTAGTCTG
CCATCGCAATCGTAGTCGACGAAAATGTATTCGTCCCAATCATATCAGACCCTCCAACCTCAAGGTACTCCCT
GTAAATACCCTGTATAATATGAGGTTGTGAGATCGACAGCTGATCATTGTTCCCCTTTGTGTTGCATGTCCAG
TCCTTAAACCTATCACCTCTGTACTCCTCCTCCTCCAGTCTGTTCCTCTTCGCATAGTTCTGAATCATGGTGC
CCATCGCTCCATCGTACATTGCAATACGACTTGTGAACAGGGATGTAATGTGCTCACGGAGGCTGTCACGACC
GCGAACGAAGTGGGGGAAGGACTCGGGATACATGCCGCTCTCCTGGACATAGTCGAGATTGGGCTTGGTTGGG
TTAGTTGCCTTCGCGCGAATGGCGGCGATCTTCTCATGAGTTAGTTTGGAGTTGAGGTAGGGATTGGGAACGT
GGTTTTGGGCCGCTTCGGAGACTGGGAGGGGAGGCTCCACGGCGGGTTTCGGCTCGACCTTATCGTAGTCAAA
TTGCAATCTTGGAAGGTTGGGCATCTTACCCCTGGGCTTGCGAGGCAAAGGAAGTTTCTTCTTTCTTGCATCA
ACACAGGCCCTTTCGTAATTGGTCCTCTCAAGCATGTCATCTGTGGCTTCTTCATTCTTGTTGAACACCAAGT
TGTCACAAAGAATTCGCATGTCATCTTCAAGATCATCATATGCTATCATCTCTTTCGGGTTGACAATGCCAAC
ATCCATTCCAGATTCCAAAATGGCACTTTGAAGGAACACAGAGTGGATTGACTCGCGAATCTTCATCACACCA
CGGAACCCAAACGACAAGTTCGAGATACCACCACTAATCTTCACGTAAGGACACTGCTCCTTGATTGTTTTCG
TGGCGTTGATGAAATCAACACCATAGTTCGCATGCTCTTCCATTCCTGTTCCAATCGTCAACACATTCGGGTC
AAATATGATGTCTTCAGGAGGGAAATGGACTTCATTCACCAAAATGTCATACGAGCGCTTGCAGATTCGTACT
TTCTCTGATTCAGTCGCTGCCTGACCTTCCTCATCGAAAGCCATCACTACCACAGCACATCCATGCTTCCTCA
GTAGGTTAGCCTGCTCCTTGAACAGCTCCTCGCCAACCTTCAATGAAATTGAGTTCACAATGCACTTTCCTTG
GCACCATTTAAGTCCTGCAAGGACAATCTCAAACTTCGATGCGTCAAGCATGAAAGGAACCTTCGAAACTTCA
GGCTCTGTAATTGCAATCTTCACGAATTTCTGCATTGCAGCAAGACCGTCAAGCATACCATCATCCACATTGA
TATCAATCACATGCGCTCCATCCACTACTTGCTTCTTCGCAATGTCCATCGCAGTTCCATAATCACCTGCCAT
CATCAGCTTCTTGAAACGGATTGACCCTGAAATATTACAGCGCTCACCCACATTCAAAAATGGCATTCCAAGA
TGATTGTGCACATCATCAACAACCAAGTCCTCTAAACCTGACAGCCACATCTTGGGACGACCTACATCTGGAA
GCTTGCGCGGCTTGAAACTCTCAGACATCTCTCTGATCGCCTTGATGTGTGGTGGTGTTGAACCACAGCAACC
TCCTACCATGTTCAACCACCCATTCTCAAAGAATACTTTATTCGCTTCAGCCATATCAGTAGGTGACTCATCA
T
>TR272|c0_g1_i2 len=3029 path=[4922:0-1120 5010:1121-1553 4933:1554-1574 4991:1575-2083 4936:2084-2090 4937:2091-2134 @5012@!:2135-2439 4996:2440-2480 5011:2481-2522 5003:2523-2555 4994:2556-2570 4985:2571-2609 5002:2610-2711 4959:2712-2735 4993:2736-2814 4972:2815-2839 5001:2840-2843 4992:2844-2867 4983:2868-2945 4919:2946-2969 4920:2970-2970 4921:2971-3028] [-1, 4922, 5010, 4933, 4991, 4936, 4937, 5012, 4996,

5011, 5003, 4994, 4985, 5002, 4959, 4993, 4972, 5001, 4992, 4983, 4919, 4920, 4921, -2]
TTCCGATCTTTTTTTTTTACACTGGCAAGTAAATTTTATCTTATGTTTTCCCCCGAAACACTATTACTCAACT
TAATCCCTTTCGTAGTTGAGGATAGGAGACAGCCACCTTTCACATGGAGACAACTCCATATTTTTGCGCTTGG
CATAACTTTCTACTTGATCCTTGCCGACCTGTCCCACCGCAAAATAACAGGACTCAGGATGGGCAAACACCAA
AGCCGATACAGATGCAGCAGGCATCATTGAGAGAGATTCCGATAGCTGGATGCCAGCAAGCTGTTCTGCCTTG
ATGACATTCCACATGGTGCTTTTTTCAGTATGGTCAGGTTGCGATGGGTAACCTGGTGCTGGACGAATTCCAA
CGTACTTAATTTTGAGTAGGTCTTCCTCGTTCAGACTCTCATCCTTGGCATATCCCCACGTGTCTGTACGCAT
CACCCGATGCAGATATTCTGCAAAAGCTTCAACAAAGCGGTCTGCTAAAGCCTGGGACATAATCTTGTTGTAG
TCATCGTGCTCAGCTTCATACTTCTGTACTAGGGCGTCACACCCGAAGCAAGATACAGCAAACATACCAATGT
GGTCTTTGTAACCGGCTGGGGCAATGAAATCTGCTTGGCTCAAATAAGGATCATCACTCTCTTTCTCTGCTTG
TTGCCGAAGCATGCAGAATTTGTCAAGGGGTTTGCCAGCTTCACGATCTTCTTCGCTAGAGTAGACAAGAACA
TCTTCACCATCATCTGTGCGGTTTGCTGCGAACATGCCAACGACACCCTTCAGGTTCATGCTTTTGTCGGCAA
TAATCTTTTTCATCAGCTCCTGGGCATCATTAAACAATTTCTTGGCCTCAGATCCGACAGCTTCGTCGTTGAA
GATTTTTGGATATCCACGATTGGGGTATCGGCCTCTAAGCTCCCACGTTTGGAAAAACGGATTCCAGTCAATG
TATGGTACAACATCTTCAAGCTGCACATCCTCAATGAGAGTAACACCAAGCTTCTTCGGCGCTGGTGCTGGGG
GGACAGCATCAAAGTCAATGACAAGTTTCTGCTGTTTCGCTTGATCAAATGTGAGGAAGTATCTGTCCTCCAA
ACCTGCGTAGTAATCCTCTCTCATCTCGTCATACTCTTCCATAATGTCTGTCGTGTAGTCTTCCTTATTCTCG
CCAAGCAGTGATGACACCACTGTCACGGATCTGGAGGCATCCAATACATGAATCACTGGGTGATCAACTGTAA
AGTAATTGGGAGAAACTTTCACGGCTGTGTGCATTTTGGATGTAGTGGCACCTCCAATCAGTAGAGGCTGCGT
GAAGCCACCCTTGGACATCTCTTTCGCGACGACAACCATTTCATCCAATGATGGTGTAATCAACCCAGAAAGG
CCAATAACATCAACATTATATTCCTTGGCTTTCTCAAGAATTTTCTCACACGAGCACATGACACCGATATCAT
ACACTTTGTAATTGTTACAGCCAAGTACAACCGCAACAATGTTCTTTCCAATATCGTGCACATCACCTTTGAC
AGTGGCCATGAGTACTTTTCCAGCAAAGTTTGAATCATCGTTTTCGTCGAAATCATCAGGGTTCAACCCGTCC
GCAATCATCTTGTCTATCTTTTCTTGTTCCATGAAAGGAATCAAATACGCAACTGCTTTCTTCATCACACGGG
CCGACTTGATAACTTGAGGCAAAAACATCTTTCCCGCCCCAAACAAATCCCCAATAATGTTCATTCCATCCAT
GAGAGGACCTTCGATGATATGGAGTGGTTTTTCTGCTTCTTGGCGAGCTTCTTCCGTATCCTTTTCAACGTAT
TGAGAGATTCCGTTAATAAGAGCATGCTCTAGCCTCTTGATCACAGGCAAGTCACGCCATGATGATTTTTTAA
CTTCAACAGCCCCTGGACCATTCTTCAAGGCTTCACGACGGGCTCTCTCTCTCTTCGTCAATTCAAGCATGAC
ATCAGTCGCATCCTCCGTCTTGTTGAACACCAAGTTATCGCAAGCAACCTTGACGTCCGGCTCGATTTCAGAT
AGTGCTAACATCTCATGCGGGTTGACAATGCCAACATCCATTCCAGACTCCAAAATGGCATGTTGCAAGAACA
CAGAGTGGATTGACTCGCGAATCTTCATCACACCACGGAACCCAAACGACAAGTTCGAGATACCACCACTAAT
CTTCACGTAAGGACACTGCTCCTTGATTGTTTTCGTGGCGTTGATGAAATCAACACCATAGTTCGCATGCTCT
TCCATTCCTGTTCCAATCGTCAACACATTCGGGTCAAATATGATGTCTTCAGGAGGGAAATGGACTTCATTCA
CCAAAATGTCATACGAGCGCTTGCAGATTCGTACTTTCTCTGATTCAGTCGCTGCCTGACCTTCCTCATCGAA
AGCCATCACTACCACAGCACATCCATGCTTCCTCAGTAGGTTAGCCTGCTCCTTGAACAGCTCCTCGCCAACC
TTCAATGAAATTGAGTTCACAATGCACTTTCCTTGGCACCATTTAAGTCCTGCAAGGACAATCTCAAACTTCG
ATGCGTCAAGCATGAAAGGAACCTTCGAAACTTCAGGCTCTGTAATTGCAATCTTCACGAATTTCTGCATTGC
AGCAAGACCGTCAAGCATACCATCATCCACATTGATATCAATCACATGCGCTCCATCCACTACTTGCTTCTTC
GCAATGTCCATCGCAGTTCCATAATCACCTGCCATCATCAGCTTCTTGAAACGGATTGACCCTGAAATATTAC
AGCGCTCACCCACATTCAAAAATGGCATTCCAAGATGATTGTGCACATCATCAACAACCAAGTCCTCTAAACC
TGACAGCCACATCTTGGGACGACCTACATCTGGAAGCTTGCGCGGCTTGAAACTCTCAGACATCTCTCTGATC
GCCTTGATGTGTGGTGGTGTTGAACCACAGCAACCTCCTACCATGTTCAACCACCCATTCTCAAAGAATACTT
TATTCGCTTCAGCCATATCAGTAGGTGACTCATCAT
>TR272|c0_g1_i3 len=3106 path=[4922:0-1120 5010:1121-1553 5000:1554-1651 4991:1652-2160 4936:2161-2167 4937:2168-2211 @5012@!:2212-2516 4996:2517-2557 5011:2558-2599 5003:2600-2632 4994:2633-2647 4985:2648-2686 5002:2687-2788 4959:2789-2812 4993:2813-2891 4972:2892-2916 5001:2917-2920 4992:2921-2944 4983:2945-3022 4919:3023-3046 4920:3047-3047 4921:3048-3105] [-1, 4922, 5010, 5000, 4991, 4936, 4937, 5012, 4996, 5011, 5003, 4994, 4985, 5002, 4959, 4993, 4972, 5001, 4992, 4983, 4919, 4920, 4921, -2]
TTCCGATCTTTTTTTTTTACACTGGCAAGTAAATTTTATCTTATGTTTTCCCCCGAAACACTATTACTCAACT
TAATCCCTTTCGTAGTTGAGGATAGGAGACAGCCACCTTTCACATGGAGACAACTCCATATTTTTGCGCTTGG
CATAACTTTCTACTTGATCCTTGCCGACCTGTCCCACCGCAAAATAACAGGACTCAGGATGGGCAAACACCAA
AGCCGATACAGATGCAGCAGGCATCATTGAGAGAGATTCCGATAGCTGGATGCCAGCAAGCTGTTCTGCCTTG
ATGACATTCCACATGGTGCTTTTTTCAGTATGGTCAGGTTGCGATGGGTAACCTGGTGCTGGACGAATTCCAA
CGTACTTAATTTTGAGTAGGTCTTCCTCGTTCAGACTCTCATCCTTGGCATATCCCCACGTGTCTGTACGCAT
CACCCGATGCAGATATTCTGCAAAAGCTTCAACAAAGCGGTCTGCTAAAGCCTGGGACATAATCTTGTTGTAG
TCATCGTGCTCAGCTTCATACTTCTGTACTAGGGCGTCACACCCGAAGCAAGATACAGCAAACATACCAATGT

GGTCTTTGTAACCGGCTGGGGCAATGAAATCTGCTTGGCTCAAATAAGGATCATCACTCTCTTTCTCTGCTTG
TTGCCGAAGCATGCAGAATTTGTCAAGGGGTTTGCCAGCTTCACGATCTTCTTCGCTAGAGTAGACAAGAACA
TCTTCACCATCATCTGTGCGGTTTGCTGCGAACATGCCAACGACACCCTTCAGGTTCATGCTTTTGTCGGCAA
TAATCTTTTTCATCAGCTCCTGGGCATCATTAAACAATTTCTTGGCCTCAGATCCGACAGCTTCGTCGTTGAA
GATTTTTGGATATCCACGATTGGGGTATCGGCCTCTAAGCTCCCACGTTTGGAAAAACGGATTCCAGTCAATG
TATGGTACAACATCTTCAAGCTGCACATCCTCAATGAGAGTAACACCAAGCTTCTTCGGCGCTGGTGCTGGGG
GGACAGCATCAAAGTCAATGACAAGTTTCTGCTGTTTCGCTTGATCAAATGTGAGGAAGTATCTGTCCTCCAA
ACCTGCGTAGTAATCCTCTCTCATCTCGTCATACTCTTCCATAATGTCTGTCGTGTAGTCTTCCTTATTCTCG
CCAAGCAGTGATGACACCACTGTCACGGATCTGGAGGCATCCAATACATGAATCACTGGGTGATCAACTGTAA
AGTAATTGGGAGAAACTTTCACGGCTGTGTGCATTTTGGATGTAGTGGCACCTCCAATCAGTAGAGGCTGCGT
GAAGCCACCCTTGGACATCTCTTTCGCGACGACAACCATTTCATCCAATGATGGTGTAATCAACCCAGAAAGG
CCAATAACATCAACATTATATTCCTTGGCTTTCTCAAGAATTTTCTCACACGAGCACATGACACCGATATCAT
ACACTTTGTAATTGTTACAGCCAAGTACAACCGCAACAATGTTCTTTCCAATATCGTGCACATCACCTTTGAC
AGTGGCCATGAGTACTTTTCCTGCAATTGACACAGTAATAATTCGTCGCCATTAGACATGAGAAATGGAAATG
CATTGTAGTAGAAGAAACGCTTACCAGCAAAGTTTGAATCATCGTTTTCGTCGAAATCATCAGGGTTCAACCC
GTCCGCAATCATCTTGTCTATCTTTTCTTGTTCCATGAAAGGAATCAAATACGCAACTGCTTTCTTCATCACA
CGGGCCGACTTGATAACTTGAGGCAAAAACATCTTTCCCGCCCCAAACAAATCCCCAATAATGTTCATTCCAT
CCATGAGAGGACCTTCGATGATATGGAGTGGTTTTTCTGCTTCTTGGCGAGCTTCTTCCGTATCCTTTTCAAC
GTATTGAGAGATTCCGTTAATAAGAGCATGCTCTAGCCTCTTGATCACAGGCAAGTCACGCCATGATGATTTT
TTAACTTCAACAGCCCCTGGACCATTCTTCAAGGCTTCACGACGGGCTCTCTCTCTCTTCGTCAATTCAAGCA
TGACATCAGTCGCATCCTCCGTCTTGTTGAACACCAAGTTATCGCAAGCAACCTTGACGTCCGGCTCGATTTC
AGATAGTGCTAACATCTCATGCGGGTTGACAATGCCAACATCCATTCCAGACTCCAAAATGGCATGTTGCAAG
AACACAGAGTGGATTGACTCGCGAATCTTCATCACACCACGGAACCCAAACGACAAGTTCGAGATACCACCAC
TAATCTTCACGTAAGGACACTGCTCCTTGATTGTTTTCGTGGCGTTGATGAAATCAACACCATAGTTCGCATG
CTCTTCCATTCCTGTTCCAATCGTCAACACATTCGGGTCAAATATGATGTCTTCAGGAGGGAAATGGACTTCA
TTCACCAAAATGTCATACGAGCGCTTGCAGATTCGTACTTTCTCTGATTCAGTCGCTGCCTGACCTTCCTCAT
CGAAAGCCATCACTACCACAGCACATCCATGCTTCCTCAGTAGGTTAGCCTGCTCCTTGAACAGCTCCTCGCC
AACCTTCAATGAAATTGAGTTCACAATGCACTTTCCTTGGCACCATTTAAGTCCTGCAAGGACAATCTCAAAC
TTCGATGCGTCAAGCATGAAAGGAACCTTCGAAACTTCAGGCTCTGTAATTGCAATCTTCACGAATTTCTGCA
TTGCAGCAAGACCGTCAAGCATACCATCATCCACATTGATATCAATCACATGCGCTCCATCCACTACTTGCTT
CTTCGCAATGTCCATCGCAGTTCCATAATCACCTGCCATCATCAGCTTCTTGAAACGGATTGACCCTGAAATA
TTACAGCGCTCACCCACATTCAAAAATGGCATTCCAAGATGATTGTGCACATCATCAACAACCAAGTCCTCTA
AACCTGACAGCCACATCTTGGGACGACCTACATCTGGAAGCTTGCGCGGCTTGAAACTCTCAGACATCTCTCT
GATCGCCTTGATGTGTGGTGGTGTTGAACCACAGCAACCTCCTACCATGTTCAACCACCCATTCTCAAAGAAT
ACTTTATTCGCTTCAGCCATATCAGTAGGTGACTCATCAT
>TR272|c0_g2_i1 len=1151 path=[4943:0-1120 4944:1121-1150] [-1, 4943, 4944, -2]
TTCCGATCTTTTTTTTTTACACTGGCAAGTAAATTTTATCTTATGTTTTCCCCCGAAACACTATTACTCAACT
TAATCCCTTTCGTAGTTGAGGATAGGAGACAGCCACCTTTCACATGGAGACAACTCCATATTTTTGCGCTTGG
CATAACTTTCTACTTGATCCTTGCCGACCTGTCCCACCGCAAAATAACAGGACTCAGGATGGGCAAACACCAA
AGCCGATACAGATGCAGCAGGCATCATTGAGAGAGATTCCGATAGCTGGATGCCAGCAAGCTGTTCTGCCTTG
ATGACATTCCACATGGTGCTTTTTTCAGTATGGTCAGGTTGCGATGGGTAACCTGGTGCTGGACGAATTCCAA
CGTACTTAATTTTGAGTAGGTCTTCCTCGTTCAGACTCTCATCCTTGGCATATCCCCACGTGTCTGTACGCAT
CACCCGATGCAGATATTCTGCAAAAGCTTCAACAAAGCGGTCTGCTAAAGCCTGGGACATAATCTTGTTGTAG
TCATCGTGCTCAGCTTCATACTTCTGTACTAGGGCGTCACACCCGAAGCAAGATACAGCAAACATACCAATGT
GGTCTTTGTAACCGGCTGGGGCAATGAAATCTGCTTGGCTCAAATAAGGATCATCACTCTCTTTCTCTGCTTG
TTGCCGAAGCATGCAGAATTTGTCAAGGGGTTTGCCAGCTTCACGATCTTCTTCGCTAGAGTAGACAAGAACA
TCTTCACCATCATCTGTGCGGTTTGCTGCGAACATGCCAACGACACCCTTCAGGTTCATGCTTTTGTCGGCAA
TAATCTTTTTCATCAGCTCCTGGGCATCATTAAACAATTTCTTGGCCTCAGATCCGACAGCTTCGTCGTTGAA
GATTTTTGGATATCCACGATTGGGGTATCGGCCTCTAAGCTCCCACGTTTGGAAAAACGGATTCCAGTCAATG
TATGGTACAACATCTTCAAGCTGCACATCCTCAATGAGAGTAACACCAAGCTTCTTCGGCGCTGGTGCTGGGG
GGACAGCATCAAAGTCAATGACAAGTTTCTGCTGTTTCGCTTGATCAAATGTGAGGAAGTATCTGTCCTCCAA
ACCTGCGTAGTAATCCTCTCTCATCTGTGTTGCGAGAAAAGCAAAGTTTAATGTTG
>TR273|c0_g1_i1 len=2533 path=[3035:0-59 3036:60-108 3099:109-250 3085:251-309 3091:310-312 3098:313-333 3102:334-357 3101:358-381 3097:382-387 3083:388-411 3089:412-714 3059:715-738 3100:739-1330 3096:1331-1462 3056:1463-1478 3081:1479-2114 3017:2115-2138 3095:2139-2159 3087:2160-2183 3103:2184-2203 3094:2204-2300 3104:2301-2301 3078:2302-2388 3028:2389-2412 3029:2413-2532] [-1, 3035, 3036, 3099,

3085, 3091, 3098, 3102, 3101, 3097, 3083, 3089, 3059, 3100, 3096, 3056, 3081, 3017, 3095, 3087, 3103, 3094, 3104, 3078, 3028, 3029, -2]
CACCATTCCAGAACCTTCAGCTGCAGCTAGATCAAAATCCGACACTTAGAGGTATTTATCTCGCGGAAAGATT
TCTCCAATCCGTGTCTTTCAAGAGAAAAGAACGCATACGCCGCCACGCAACATGAAGATCAAATCAAATCGCC
AACAACGCATGCCGCACATGTCGTATTCCAATCCAATGATGATGGCGTTGGCTATCTGCTTTCTGATTCTGGT
TCTCTCAATACTCTTGTTTGGTGGGGAGGATACTCATACACATCAAATAATTCGAGGAGAGAGGAATCACCAC
ACCAAAACTCTTGAATACGCTGACAAACCAAGCAGATACCCCTTCCATCAGTCATCATCTAGCACTATCGAAG
AAGCAACATCCGGAAAAGGACAGGTAATACAGCAGCACCATAATCATTTTTCACTCTCTCCTGGAGAGTTACC
TGGATATACAGGATGGGCTAGACCAGAACAAACATTGGCAGGATACTTTGACATCGATTCTTTATCTCATCGC
CCTCTACTGACAGAATTGCATGGGGCGGCAGTAGCTCCTAAATATCACTCCATTGTAAAGAGTGGTGACAGAT
TCTCCCTCCTCCTAACTTGCAACCACAACCGTATCAACAACATAGATCCAGAATTCGAATTACCGTATGAATG
TCCACCCAATGGAGGGACTCTTTTTTACGCTCGAGCATATGGGCCGTCGGTCATCACTGGACTCGTCACTGAC
TATCACAATGCTTCTTATTCTTTGGAGATGCAATTTACAGATCCTGGCGAGTACACTCTGGAAGTGGTGGTGA
CTTTTTCGGTACCGCTAGATTTTGATGAGTTTCCATTGAATATGGAAACGATGAGTACGGACGAAGAAGAGAA
AATAGAGCCGGGGTATGAGGGCTACATGGCTTCAGGATTTCCTCTGATGATACGTGTGGATCCGGTGCACGAG
CCGGCCTCGTCCCTGACCGAAGATACTGGGAAACAATGGTGCACACTATCCCAACTCACAGAATCGTCTCCCC
AATCGGCGCTTTATAAAGGTCGTTGGCGAGTCATCGATAACGTTGCCCACTCATCTCATAATCAAATTACTCC
AGATGGAACTATGGTAACGTTGGATGGGTATCGAATCGGACTGAACTCAGTAGGGGTACGTATGATGTATGAA
TACAAAGGTTGCGAGCTGATTCACATACGAGAAATTCTGTCTATAATCTCTGATGGGATGGATGAATGCTTTC
CGGAACAATTGGGGTTCAGCATTCACCGTGGCGATGGTAATGGTAGCTTGGATGATACAAGTGGCGATCAGAC
TGGAGCTGGTCAAAAAAGAACTGCAAATAGCACCAACGACAAAACTGAGATTAAGTCTCTCGAAAATAGCAGC
GAGGACAGCAGTTTTGAAGGAATCCATGTTATTTTCATTGGAGATAGTGTCATGAAACTCATTGAGGGATTTT
TTTCGAAACTCGTCCGCAATTTTTTTGGCATCAAAGTGTCATTTTTAGAGACCAATGGAGGAATTCATCTTAC
TATGCCCAACATAACTTCTACCTTGAATAAAATTCGGCGAGAAGAGCAGGGCGCAAATATCAAAAGAGCGATT
TTGTTCAACACAGGTCTCCATGATATCGATGTTCTTTGCAGTTCCAAGAGAAAGCACACTCGAATAAAAACAA
AAATTGCGAACAAAGTTAAGTCTTGTACCGATGCATACCGTGACGCCATGTTGCATTTGGTTCAAGTGTTAGA
GGATTATCCAGCAGAGTTAAAGGTATTCCAATCAACTACTGCAGGCTGGCAAAAGTACGGAAACTACGGATTC
TCATGGAGTGCCAAAGAAATGCAACCGATGTCACGTTCACCCCACCTCGCCCATCATTTCAACACAATTGCCT
ACGATATCATACAGAACCAGAATAGAAATATTCTCATTGCCGATGGATATTGGATGACGTTACCCCGTCCAGA
TCACACACAAATTAGTACGCAGAATAAAGTGGGAAAGCATTTGGTTCATCCTGGATTTGAAGTTTTGAGTGTT
TTTGCTCGAAGGTGGTTCATGATGATTTTACATGGAATTTGTGGTGAATAATTGGTTGAGGAGATTCCCCCGT
ATGATTGGATGGGATCGACTCTAACTTCAAAAGCATTTCCCTAGGTTTCCAATGAATAAATGCCAATGGTCCA
CGCTGTGACCAAATTCTCTCCCCATGTTGACAGATTGGACCTTGGACTGATTGCTCAGCATTGAGTCTGTGTA
TTTTGCAATAATGGTAAAATATAGTATTCTAAATAATGGCAGAAAGCAGCATCCTTTTCGCGGGTGGTAATAG
GCAAATCGTTTGGTCCATGCCTTTGGGATACCTTGCAGATAATGGTACCATCACTAACTAATACTTCCCGTTG
TGATATTTACCAATTGGTCATTGGGGATGTATGGAATGGATTCTGGGCCATCATTCACATTGATAGCGGGTCT
CACATCGTGATGCGGACAGCACTACGTTACCTTAAACTGGTCTAACAATAA
>TR274|c0_g1_i1 len=3348 path=[8199:0-363 8200:364-387 8320:388-475 8310:476-550 8327:551-574 8330:575-575 8297:576-752 8185:753-776 8316:777-842 8193:843-866 8307:867-1815 8323:1816-1875 8315:1876-1899 8305:1900-1952 8190:1953-1968 8314:1969-2047 8223:2048-2071 8303:2072-2550 8328:2551-2574 8331:2575-2600 8313:2601-2637 8322:2638-2967 8265:2968-2987 8312:2988-2988 8301:2989-3112 8226:3113-3136 8288:3137-3225 8252:3226-3347] [-1, 8199, 8200, 8320, 8310, 8327, 8330, 8297, 8185, 8316, 8193, 8307, 8323, 8315, 8305, 8190, 8314, 8223, 8303, 8328, 8331, 8313, 8322, 8265, 8312, 8301, 8226, 8288, 8252, -2]
AGCATGATTTAGGGGGTGCGACAGGAGTGTGTTTAAACTCGTACTTATTTCTCTCTCCGGCTCACTTTACCTA
GCAAAAAATCAAGAAAAGTGGCTCTTCCGACAATCTCTGGCTCTGGTAAATGATCGCTTTGTGAAAATCAACA
ACGACGCTTTGCTCCCTACTCCAGCCGTCAACACTGGTCAACACTTCATTGGCAGGCGCAAATCTTTGGCATT
CGCTTCATGGGGAACCCGCCTCGCTCCCACCTCCGCTTGATCGCCCTCGTGTGCGTTCGTATCGTAGGTTTTC
ATCCACCGTTACAGCTTCAGCTAAACGACGACGGACGTCTAAATCCCCTCAAACAGGATCAATGAAAATCATT
CACAACAGGGAATTAACTCCCACGGCGAGCCTCAACCATTTGTGCCAAGCAAGCATGAGATAAATTGCTAGTG
GTTACGCTCAGTTACGATAGTTCCCACTCCTTCATCCCATGGCGATCCTCCCATCCCTCCGACGTCGACGTCG
ACTTCCTGACAAAGCTGCCGCGCCATCGGGGCCATCGGCGTGGTACACGTTTGCTGCTTCAAGGGCTCACTGC
TCCGGCATGTCCAAGTGGCTGATTATCCTCCCTGCATATGTTATCTGTATCAGCTGCTGTCTCTTCCTCCTTG
ACCTTCATCCAACAATCCCAACTACTGATTCCCAAACCGGGGCAGGCCAGAGCAATCACAATAATATGAATAT
GCCATTCCTCGAGCTTCGACCCAGCACCCATCATACCACTTCCCAAACTCGAAATGTAAATAAAAATCGAACT
TTGTTACCAGATCAAGATCCACAACCTCCAGCGAATCACTTTCACAATAATCATTGGGTATCAGAGCTGCACC
CATCCAAAATTGCCATTGTCCAATTCGACGGAAGGCCACTGTGGCAAGAATGGCATATTGGTCCCACCACGAA
AAAGATTCATCCAACGGTCTGGAACACTGCGGCAATCTGGAACTGGCGATACAGCCAGAAACACGGGCACGAC

TACATTTATTACCACTGGCCCAACAATTTGGCCAAAGCGTGCCGCAGCAGAAACAACAAAGAATTGGCCGAAG
CATGGTGCAAGGTCAAAGTATTTCTGCAGGTTCAAGAAGACTTGCCGCACATTGACTATTTTCTCTTTATGGA
CACTGATGCCGTGGTGGATTATCGATTCCGGGACCAATCCATATTGCAAGTTCTGCACAACATGACAACGCAA
TGGCTGCCGAGTTGGAACATTGACGAAAGGCCGTTTGTTTTGAACAAGGAAGGTCCGTCGTATTGGTGCTCCC
TCGTCCACGATGCTCTGTTCTCTCACTGTCTTAATACGGGGACAGTGTTTTGGAAAAGATCGGATAAAGCGAC
GGCGGTCTTGGAGCAGTGGTGGACGTCTGCAGATGATCCCTATGAGTCCGAGAATGTTAAAAGGAGTCATCAC
CCGAATCTTGGGATCAAGTTTCGCACTGACACGCCGTGGGAGCAGGTCCAGGCACAGTCTCTACTGCGTAATC
CCTCGGTGAATGAGTCTATTCAAATTGCCTCCCAGCCTGAGGCCTTTGTGCAATTGCAAGGTCCGTGCTTGTC
AGACTGTTGGCAACCTCCAATCTTTGAGCAGTTGGGCTGTTTCATTTTACACTTTTGCATGAATAAGACAGAG
TTGGTGGAGGTGTACGGGAATTATTTGAAGGGGTTGGCGAACGAGGAGGAGAGAGATATGGTGGTAGATGATG
GTCATGGTGGTTGCTATCCAAGGGCCGTTTTGTCAATGAACAATGAAAATACCAATGTGCAGCATCATCAGCA
CGGTGAAACTAAGTTTGACACAGCTCCATGTAGGTTTGATTGGATCGTGGAGCGTGCGTTGAGTGGTTTTAAT
GTTGAGGAGGAGGGGAGTTAACAGTTGTTGCCCCTGTCAAGAAGGGTGAAAAAAAGCAATACCTTGTTGAAAT
TGCAATACGACGGATGATGCGCCCGTGTGATGTAACGGAGCGCATCACTTGGCGGTTAAGAATTTAGGGATAC
CGCGCGAGTAAACGCTAAAAGTCTAGTGCTGAGTAGATATTAACAAGGGTTTCGAAACATGTCATACCATCAT
CTCGAGATATCTTATCATGGGACTGCTGCAATGCCATGGTCGAGGTCGGCACATGAGAGAAGCGTAACCTTAG
TAGATCCCTGGACTCAGGCGAGTCATGTGCATTAAAGCTATGCACATCAGACAGCGCACCCAGCACCACCACA
ATATTTATCCACCATGAAGTACCATACATTTTCAATTCCTCCATTGCCAACATTGTTATCATCACTCCTATCT
CTGTCAATTTCCTAGAGATTAAAAGCATTATTGGGGGCACGACGGGCAGCACACAGTCACCTGAAAGAGCAGT
ACCAACCAAACCCGTGTCAGGAGGCATCAGCTAGGTACCGTTATCGCCCCTAAAGATGGTAACTTCTCCGTAA
TACTACCATTGCCAAAAACATCTGTTGTGATGATCTACACCCCACAAGCATAGTCAGCTTGAATATACTAGTG
TAAATCAAAGGCAACTGAAGCCAATTTCCAGAAAAGAAAATCACCGAAATAAATTGATATTGCTACTGGTTTT
ACTCTTGTCTATGATGAAAATAGGGAAAGATGATTTGATTAACAAAACTAATAGTCAGCAAACACAGCTGGAA
TTGCCATCCGGGCGTACAAATGTGGTCACCACCATACGATCGTATGGCAATGTTCTAAATCAATATAATTGCA
CTTTGGAAAGTGAAGAGAAGAACCCTAAGGAAAACATTGCAAGATGCAACACGCATGGTGCTCCCGCCTTGTT
GCCCGTCACTGCGGCCGGTTCGTTGTTCTGCATCAATGCCAATAGAATGCTGCTGCTGTTTCTCTCCGCGTTT
TTTCTCAATGATTGTTTTTAGCCATGTTTCATATTCTATTACTTTCCTTTCCAGCTAATTTCTATGTAGTATT
GACTTTTCAGTAGTGAAGTTTCGTTATTCAACATCTTCAAGCCTTCTCAAGTGTCAGTCTGCACACGCAAACT
GTGTGCATTCACCTACCATCTGCACACGACCAAATTCATTCATCATCACCAACGTCGAGCACCACAAGCTGCC
AGCCCCATCCACAGACGCCAACATCCTCCTGGGCATCTTGCTCTAGATAGAGATATCACAGCTGTCATATCTA
TGTTTACTACTCCTACAACAATGACAACGGCTGTGATAGATGTGATGATGAGGAGGGTGGTTGTCCAGTGACC
AGTTGACCTTCGGTCGGCCAGGCTCATCTTCAGTGAGTAGCAAACAGTCAACTTGTGTTGGCA
>TR274|c0_g1_i2 len=3018 path=[8199:0-363 8200:364-387 8320:388-475 8310:476-550 8327:551-574 8330:575-575 8297:576-752 8185:753-776 8316:777-842 8193:843-866 8307:867-1815 8323:1816-1875 8315:1876-1899 8305:1900-1952 8190:1953-1968 8314:1969-2047 8223:2048-2071 8303:2072-2550 8328:2551-2574 8331:2575-2600 8313:2601-2637 8272:2638-2658 8301:2659-2782 8226:2783-2806 8288:2807-2895 8252:2896-3017] [-1, 8199, 8200, 8320, 8310, 8327, 8330, 8297, 8185, 8316, 8193, 8307, 8323, 8315, 8305, 8190, 8314, 8223, 8303, 8328, 8331, 8313, 8272, 8301, 8226, 8288, 8252, -2]
AGCATGATTTAGGGGGTGCGACAGGAGTGTGTTTAAACTCGTACTTATTTCTCTCTCCGGCTCACTTTACCTA
GCAAAAAATCAAGAAAAGTGGCTCTTCCGACAATCTCTGGCTCTGGTAAATGATCGCTTTGTGAAAATCAACA
ACGACGCTTTGCTCCCTACTCCAGCCGTCAACACTGGTCAACACTTCATTGGCAGGCGCAAATCTTTGGCATT
CGCTTCATGGGGAACCCGCCTCGCTCCCACCTCCGCTTGATCGCCCTCGTGTGCGTTCGTATCGTAGGTTTTC
ATCCACCGTTACAGCTTCAGCTAAACGACGACGGACGTCTAAATCCCCTCAAACAGGATCAATGAAAATCATT
CACAACAGGGAATTAACTCCCACGGCGAGCCTCAACCATTTGTGCCAAGCAAGCATGAGATAAATTGCTAGTG
GTTACGCTCAGTTACGATAGTTCCCACTCCTTCATCCCATGGCGATCCTCCCATCCCTCCGACGTCGACGTCG
ACTTCCTGACAAAGCTGCCGCGCCATCGGGGCCATCGGCGTGGTACACGTTTGCTGCTTCAAGGGCTCACTGC
TCCGGCATGTCCAAGTGGCTGATTATCCTCCCTGCATATGTTATCTGTATCAGCTGCTGTCTCTTCCTCCTTG
ACCTTCATCCAACAATCCCAACTACTGATTCCCAAACCGGGGCAGGCCAGAGCAATCACAATAATATGAATAT
GCCATTCCTCGAGCTTCGACCCAGCACCCATCATACCACTTCCCAAACTCGAAATGTAAATAAAAATCGAACT
TTGTTACCAGATCAAGATCCACAACCTCCAGCGAATCACTTTCACAATAATCATTGGGTATCAGAGCTGCACC
CATCCAAAATTGCCATTGTCCAATTCGACGGAAGGCCACTGTGGCAAGAATGGCATATTGGTCCCACCACGAA
AAAGATTCATCCAACGGTCTGGAACACTGCGGCAATCTGGAACTGGCGATACAGCCAGAAACACGGGCACGAC
TACATTTATTACCACTGGCCCAACAATTTGGCCAAAGCGTGCCGCAGCAGAAACAACAAAGAATTGGCCGAAG
CATGGTGCAAGGTCAAAGTATTTCTGCAGGTTCAAGAAGACTTGCCGCACATTGACTATTTTCTCTTTATGGA
CACTGATGCCGTGGTGGATTATCGATTCCGGGACCAATCCATATTGCAAGTTCTGCACAACATGACAACGCAA
TGGCTGCCGAGTTGGAACATTGACGAAAGGCCGTTTGTTTTGAACAAGGAAGGTCCGTCGTATTGGTGCTCCC
TCGTCCACGATGCTCTGTTCTCTCACTGTCTTAATACGGGGACAGTGTTTTGGAAAAGATCGGATAAAGCGAC

GGCGGTCTTGGAGCAGTGGTGGACGTCTGCAGATGATCCCTATGAGTCCGAGAATGTTAAAAGGAGTCATCAC
CCGAATCTTGGGATCAAGTTTCGCACTGACACGCCGTGGGAGCAGGTCCAGGCACAGTCTCTACTGCGTAATC
CCTCGGTGAATGAGTCTATTCAAATTGCCTCCCAGCCTGAGGCCTTTGTGCAATTGCAAGGTCCGTGCTTGTC
AGACTGTTGGCAACCTCCAATCTTTGAGCAGTTGGGCTGTTTCATTTTACACTTTTGCATGAATAAGACAGAG
TTGGTGGAGGTGTACGGGAATTATTTGAAGGGGTTGGCGAACGAGGAGGAGAGAGATATGGTGGTAGATGATG
GTCATGGTGGTTGCTATCCAAGGGCCGTTTTGTCAATGAACAATGAAAATACCAATGTGCAGCATCATCAGCA
CGGTGAAACTAAGTTTGACACAGCTCCATGTAGGTTTGATTGGATCGTGGAGCGTGCGTTGAGTGGTTTTAAT
GTTGAGGAGGAGGGGAGTTAACAGTTGTTGCCCCTGTCAAGAAGGGTGAAAAAAAGCAATACCTTGTTGAAAT
TGCAATACGACGGATGATGCGCCCGTGTGATGTAACGGAGCGCATCACTTGGCGGTTAAGAATTTAGGGATAC
CGCGCGAGTAAACGCTAAAAGTCTAGTGCTGAGTAGATATTAACAAGGGTTTCGAAACATGTCATACCATCAT
CTCGAGATATCTTATCATGGGACTGCTGCAATGCCATGGTCGAGGTCGGCACATGAGAGAAGCGTAACCTTAG
TAGATCCCTGGACTCAGGCGAGTCATGTGCATTAAAGCTATGCACATCAGACAGCGCACCCAGCACCACCACA
ATATTTATCCACCATGAAGTACCATACATTTTCAATTCCTCCATTGCCAACATTGTTATCATCACTCCTATCT
CTGTCAATTTCCTAGAGATTAAAAGCATTATTGGGGGCACGACGGGCAGCACACAGTCACCTGAAAGAGCAGT
ACCAACCAAACCCGTGTCAGGAGGCATCAGCTAGGTACCGTTATCGCCCCTAAAGATGGTAACTTCTCCGTAA
TACTACCATTGCCAAAAACATCTGTTGTGATGATCTACACCCCACAAGCATAGTCAGCTTGAATATACTAGTG
TAAATCAAAGGCAACTGAAGCCAATTTCCAGAAAAGAAAATCACCGAAATAAATTGATATTGCTACTGGTTTT
ACTCTTGTCTTTCCAGCTAATTTCTATGTAGTATTGACTTTTCAGTAGTGAAGTTTCGTTATTCAACATCTTC
AAGCCTTCTCAAGTGTCAGTCTGCACACGCAAACTGTGTGCATTCACCTACCATCTGCACACGACCAAATTCA
TTCATCATCACCAACGTCGAGCACCACAAGCTGCCAGCCCCATCCACAGACGCCAACATCCTCCTGGGCATCT
TGCTCTAGATAGAGATATCACAGCTGTCATATCTATGTTTACTACTCCTACAACAATGACAACGGCTGTGATA
GATGTGATGATGAGGAGGGTGGTTGTCCAGTGACCAGTTGACCTTCGGTCGGCCAGGCTCATCTTCAGTGAGT
AGCAAACAGTCAACTTGTGTTGGCA
>TR274|c0_g1_i3 len=3684 path=[8199:0-363 8200:364-387 8320:388-475
8310:476-550 8327:551-574 8330:575-575 8297:576-752 8185:753-776
8316:777-842 8193:843-866 8307:867-1815 8323:1816-1875 8315:1876-1899
8305:1900-1952 8190:1953-1968 8314:1969-2047 8223:2048-2071 8303:2072-
2550 8328:2551-2574 8331:2575-2600 8313:2601-2637 8322:2638-2967
8289:2968-3323 8312:3324-3324 8301:3325-3448 8226:3449-3472 8288:3473-
3561 8252:3562-3683] [-1, 8199, 8200, 8320, 8310, 8327, 8330, 8297, 8185,
8316, 8193, 8307, 8323, 8315, 8305, 8190, 8314, 8223, 8303, 8328, 8331,
8313, 8322, 8289, 8312, 8301, 8226, 8288, 8252, -2]
AGCATGATTTAGGGGGTGCGACAGGAGTGTGTTTAAACTCGTACTTATTTCTCTCTCCGGCTCACTTTACCTA
GCAAAAAATCAAGAAAAGTGGCTCTTCCGACAATCTCTGGCTCTGGTAAATGATCGCTTTGTGAAAATCAACA
ACGACGCTTTGCTCCCTACTCCAGCCGTCAACACTGGTCAACACTTCATTGGCAGGCGCAAATCTTTGGCATT
CGCTTCATGGGGAACCCGCCTCGCTCCCACCTCCGCTTGATCGCCCTCGTGTGCGTTCGTATCGTAGGTTTTC
ATCCACCGTTACAGCTTCAGCTAAACGACGACGGACGTCTAAATCCCCTCAAACAGGATCAATGAAAATCATT
CACAACAGGGAATTAACTCCCACGGCGAGCCTCAACCATTTGTGCCAAGCAAGCATGAGATAAATTGCTAGTG
GTTACGCTCAGTTACGATAGTTCCCACTCCTTCATCCCATGGCGATCCTCCCATCCCTCCGACGTCGACGTCG
ACTTCCTGACAAAGCTGCCGCGCCATCGGGGCCATCGGCGTGGTACACGTTTGCTGCTTCAAGGGCTCACTGC
TCCGGCATGTCCAAGTGGCTGATTATCCTCCCTGCATATGTTATCTGTATCAGCTGCTGTCTCTTCCTCCTTG
ACCTTCATCCAACAATCCCAACTACTGATTCCCAAACCGGGGCAGGCCAGAGCAATCACAATAATATGAATAT
GCCATTCCTCGAGCTTCGACCCAGCACCCATCATACCACTTCCCAAACTCGAAATGTAAATAAAAATCGAACT
TTGTTACCAGATCAAGATCCACAACCTCCAGCGAATCACTTTCACAATAATCATTGGGTATCAGAGCTGCACC
CATCCAAAATTGCCATTGTCCAATTCGACGGAAGGCCACTGTGGCAAGAATGGCATATTGGTCCCACCACGAA
AAAGATTCATCCAACGGTCTGGAACACTGCGGCAATCTGGAACTGGCGATACAGCCAGAAACACGGGCACGAC
TACATTTATTACCACTGGCCCAACAATTTGGCCAAAGCGTGCCGCAGCAGAAACAACAAAGAATTGGCCGAAG
CATGGTGCAAGGTCAAAGTATTTCTGCAGGTTCAAGAAGACTTGCCGCACATTGACTATTTTCTCTTTATGGA
CACTGATGCCGTGGTGGATTATCGATTCCGGGACCAATCCATATTGCAAGTTCTGCACAACATGACAACGCAA
TGGCTGCCGAGTTGGAACATTGACGAAAGGCCGTTTGTTTTGAACAAGGAAGGTCCGTCGTATTGGTGCTCCC
TCGTCCACGATGCTCTGTTCTCTCACTGTCTTAATACGGGGACAGTGTTTTGGAAAAGATCGGATAAAGCGAC
GGCGGTCTTGGAGCAGTGGTGGACGTCTGCAGATGATCCCTATGAGTCCGAGAATGTTAAAAGGAGTCATCAC
CCGAATCTTGGGATCAAGTTTCGCACTGACACGCCGTGGGAGCAGGTCCAGGCACAGTCTCTACTGCGTAATC
CCTCGGTGAATGAGTCTATTCAAATTGCCTCCCAGCCTGAGGCCTTTGTGCAATTGCAAGGTCCGTGCTTGTC
AGACTGTTGGCAACCTCCAATCTTTGAGCAGTTGGGCTGTTTCATTTTACACTTTTGCATGAATAAGACAGAG
TTGGTGGAGGTGTACGGGAATTATTTGAAGGGGTTGGCGAACGAGGAGGAGAGAGATATGGTGGTAGATGATG
GTCATGGTGGTTGCTATCCAAGGGCCGTTTTGTCAATGAACAATGAAAATACCAATGTGCAGCATCATCAGCA
CGGTGAAACTAAGTTTGACACAGCTCCATGTAGGTTTGATTGGATCGTGGAGCGTGCGTTGAGTGGTTTTAAT
GTTGAGGAGGAGGGGAGTTAACAGTTGTTGCCCCTGTCAAGAAGGGTGAAAAAAAGCAATACCTTGTTGAAAT
TGCAATACGACGGATGATGCGCCCGTGTGATGTAACGGAGCGCATCACTTGGCGGTTAAGAATTTAGGGATAC

CGCGCGAGTAAACGCTAAAAGTCTAGTGCTGAGTAGATATTAACAAGGGTTTCGAAACATGTCATACCATCAT
CTCGAGATATCTTATCATGGGACTGCTGCAATGCCATGGTCGAGGTCGGCACATGAGAGAAGCGTAACCTTAG
TAGATCCCTGGACTCAGGCGAGTCATGTGCATTAAAGCTATGCACATCAGACAGCGCACCCAGCACCACCACA
ATATTTATCCACCATGAAGTACCATACATTTTCAATTCCTCCATTGCCAACATTGTTATCATCACTCCTATCT
CTGTCAATTTCCTAGAGATTAAAAGCATTATTGGGGGCACGACGGGCAGCACACAGTCACCTGAAAGAGCAGT
ACCAACCAAACCCGTGTCAGGAGGCATCAGCTAGGTACCGTTATCGCCCCTAAAGATGGTAACTTCTCCGTAA
TACTACCATTGCCAAAAACATCTGTTGTGATGATCTACACCCCACAAGCATAGTCAGCTTGAATATACTAGTG
TAAATCAAAGGCAACTGAAGCCAATTTCCAGAAAAGAAAATCACCGAAATAAATTGATATTGCTACTGGTTTT
ACTCTTGTCTATGATGAAAATAGGGAAAGATGATTTGATTAACAAAACTAATAGTCAGCAAACACAGCTGGAA
TTGCCATCCGGGCGTACAAATGTGGTCACCACCATACGATCGTATGGCAATGTTCTAAATCAATATAATTGCA
CTTTGGAAAGTGAAGAGAAGAACCCTAAGGAAAACATTGCAAGATGCAACACGCATGGTGCTCCCGCCTTGTT
GCCCGTCACTGCGGCCGGTTCGTTGTTCTGCATCAATGCCAATAGAATGCTGCTGCTGTTTCTCTCCGCGTTT
TTTCTCAATGATTGTTTTTAGCCATGTTTCATATTCTATTACTTTCCTAGGATAACCCAATATTCGTTGAGTC
AAAAACAGCAAGTGCTTAATTAGTGTTGCAGCTGTTTGTTGTCTCAATTTCCCCTACAATGATTCCTATGATT
CTGATTATTCCTGGTATTGTCCCTGGCTGTTGCTGTACAATAATATCCTGTGCACGCAATTGCACGAAGAAAA
AAAGCAAACATTGGCAGACAACATGCAGTTTCAACTTCACCATTATCTCCAAAATTGATCGGCATTGATCAAT
AACCCTTCAACCAGTTGGTCAATCATGGCAGCAATGGTATCTATAGTTGGGTTTCCATTGATGGTATCATGGA
TAATCTAATTGTCTTACCTTTCCAGCTAATTTCTATGTAGTATTGACTTTTCAGTAGTGAAGTTTCGTTATTC
AACATCTTCAAGCCTTCTCAAGTGTCAGTCTGCACACGCAAACTGTGTGCATTCACCTACCATCTGCACACGA
CCAAATTCATTCATCATCACCAACGTCGAGCACCACAAGCTGCCAGCCCCATCCACAGACGCCAACATCCTCC
TGGGCATCTTGCTCTAGATAGAGATATCACAGCTGTCATATCTATGTTTACTACTCCTACAACAATGACAACG
GCTGTGATAGATGTGATGATGAGGAGGGTGGTTGTCCAGTGACCAGTTGACCTTCGGTCGGCCAGGCTCATCT
TCAGTGAGTAGCAAACAGTCAACTTGTGTTGGCA
>TR274|c0_g2_i1 len=3736 path=[8253:0-265 8321:266-374 8326:375-381
8329:382-382 8319:383-504 8298:505-738 8318:739-1068 8308:1069-1069
8296:1070-1634 8229:1635-1658 8306:1659-1806 8249:1807-1830 8294:1831-
1890 8304:1891-2839 8275:2840-2863 8292:2864-2929 8269:2930-2953
8270:2954-3735] [-1, 8253, 8321, 8326, 8329, 8319, 8298, 8318, 8308,
8296, 8229, 8306, 8249, 8294, 8304, 8275, 8292, 8269, 8270, -2]
TGCCAACACAAGTTGACTGTTTGCTACTCACTGAAGATGAGCCTGGCCGACCGAAGGTCAACTGGTCACTGGA
CAACCACCCTCCTCATCATCACATCTATCACAGCCGTTGTCATTGTTGTAGGAGTAGTAAACATAGATATGAC
AGCTGTGATATCTCTATCTAGAGCAAGATGCCCAGGAGGATGTTGGCGTCTGTGGATGGGGCTGGCAGCTTGT
GGTGCTCGACGTTGGTGATGATGAATGAATTTGGTCGTGTGCAGATGGTAGGTGAATGCACACAGTTTGCGTG
TGCAGACTGACACTTGAGAAGGCTTGAAGATGTTGAATAACGAAACTTCACTACTGAAAAGTCAATACTACAT
AGAAATTAGCTGGAAAGGTAAGACAATTAGATTATCCATGATACCATCAATGGAAACCCAACTATAGATACCA
TTGCTGCCATGATTGACCAACTGGTTGAAGGGTTATTGATCAATGCCGATCAATTTTGGAGATAATGGTGAAG
TTGAAACTGCATGTTGTCTGCCAATGTTTGCTTTTTTTCTTCGTGCAATTGCGTGCACAGGATATTATTGTAC
AGCAACAGCCAGGGACAATACCAGGAATAATCAGAATCATAGGAATCATTGTAGGGGAAATTGAGACAACAAA
CAGCTGCAACACTAATTAAGCACTTGCTGTTTTTGACTCAACGAATATTGGGTTATCCTAGGAAAGTAATAGA
ATATGAAACATGGCTAAAAACAATCATTGAGAAAAAACGCGGAGAGAAACAGCAGCAGCATTCTATTGGCATT
GATGCAGAACAACGAACCGGCCGCAGTGACGGGCAACAAGGCGGGAGCACCATGCGTGTTGCATCTTGCAATG
TTTTCCTTAGGGTTCTTCTCTTCACTTTCCAAAGTGCAATTATATTGATTTAGAACATTGCCATACGATCGTA
TGGTGGTGACCACATTTGTACGCCCGGATGGCAATTCCAGCTGTGTTTGCTGACTATTAGTTTTGTTAATCAA
ATCATCTTTCCCTATTTTCATCATAGACAAGAGTAAAACCAGTAGCAATATCAATTTATTTCGGTGATTTTCT
TTTCTGGAAATTGGCTTCAGTTGCCTTTGATTTACACTAGTATATTCAAGCTGACTATGCTTGTGGGGTGTAG
ATCATCACAACAGATGTTTTTGGCAATGGTAGTATTACGGAGAAGTTACCATCTTTAGGGGCGATAACGGTAC
CTAGCTGATGCCTCCTGACACGGGTTTGGTTGGTACTGCTCTTTCAGGTGACTGTGTGCTGCCCGTCGTGCCC
CCAATAATGCTTTTAATCTCTAGGAAATTGACAGAGATAGGAGTGATGATAACAATGTTGGCAATGGAGGAAT
TGAAAATGTATGGTACTTCATGGTGGATAAATATTGTGGTGGTGCTGGGTGCGCTGTCTGATGTGCATAGCTT
TAATGCACATGACTCGCCTGAGTCCAGGGATCTACTAAGGTTACGCTTCTCTCATGTGCCGACCTCGACCATG
GCATTGCAGCAGTCCCATGATAAGATATCTCGAGATGATGGTATGACATGTTTCGAAACCCTTGTTAATATCT
ACTCAGCACTAGACTTTTAGCGTTTACTCGCGCGGTATCCCTAAATTCTTAACCGCCAAGTGATGCGCTCCGT
TACATCACACGGGCGCATCATCCGTCGTATTGCAATTTCAACAAGGTATTGCTTTTTTTCACCCTTCTTGACA
GGGGCAACAACTGTTAACTCCCCTCCTCCTCAACATTAAAACCACTCAACGCACGCTCCACGATCCAATCAAA
CCTACATGGAGCTGTGTCAAACTTAGTTTCACCGTGCTGATGATGCTGCACATTGGTATTTTCATTGTTCATT
GACAAAACGGCCCTTGGATAGCAACCACCATGACCATCATCTACCACCATATCTCTCTCCTCCTCGTTCGCCA
ACCCCTTCAAATAATTCCCGTACACCTCCACCAACTCTGTCTTATTCATGCAAAAGTGTAAAATGAAACAGCC
CAACTGCTCAAAGATTGGAGGTTGCCAACAGTCTGACAAGCACGGACCTTGCAATTGCACAAAGGCCTCAGGC
TGGGAGGCAATTTGAATAGACTCATTCACCGAGGGATTACGCAGTAGAGACTGTGCCTGGACCTGCTCCCACG
GCGTGTCAGTGCGAAACTTGATCCCAAGATTCGGGTGATGACTCCTTTTAACATTCTCGGACTCATAGGGATC

ATCTGCAGACGTCCACCACTGCTCCAAGACCGCCGTCGCTTTATCCGATCTTTTCCAAAACACTGTCCCCGTA
TTAAGACAGTGAGAGAACAGAGCATCGTGGACGAGGGAGCACCAATACGACGGACCTTCCTTGTTCAAAACAA
ACGGCCTTTCGTCAATGTTCCAACTCGGCAGCCATTGCGTTGTCATGTTGTGCAGAACTTGCAATATGGATTG
GTCCCGGAATCGATAATCCACCACGGCATCAGTGTCCATAAAGAGAAAATAGTCAATGTGCGGCAAGTCTTCT
TGAACCTGCAGAAATACTTTGACCTTGCACCATGCTTCGGCCAATTCTTTGTTGTTTCTGCTGCGGCACGCTT
TGGCCAAATTGTTGGGCCAGTGGTAATAAATGTAGTCGTGCCCGTGTTTCTGGCTGTATCGCCAGTTCCAGAT
TGCCGCAGTGTTCCAGACCGTTGGATGAATCTTTTTCGTGGTGGGACCAATATGCCATTCTTGCCACAGTGGC
CTTCCGTCGAATTGGACAATGGCAATTTTGGATGGGTGCAGCTCTGATACCCAATGATTATTGTGAAAGTGAT
TCGCTGGAGGTTGTGGATCTTGATCTGGTAACAAAGTTCGATTTTTATTTACATTTCGAGTTTGGGAAGTGGT
ATGATGGGTGTTGGGTCGAAGCTCGAGGAATGGCATATTCATATTATTGTGATTGCTCTGGCCTGCCCCGGTT
TGGGAATCAGTAGTTGGGATTGTTGGATGAAGGTCAAGGAGGAAGAGACAGCAGCTGATACAGATAACATATG
CAGGGAGGATAATCAGCCACTTGGACATGCCGGAGCAGTGAGCCCTTGAAGCAGCAAACGTGTACCGCGCCGA
TGGCCCCGATGGCGCGGCAGCTTTGTCAGGAAGTCGACGTCGACGTCGGAGGGATGGGAGGATCGCCATGGGA
TGAAGGAGTGGGAACTATCGTAACTGAGCGTAACCACTAGCAATTTATCTCATGCTTGCTTGGCACAAATGGT
TGAGGCTCGCCGTGGGAGTTAATTCCCTGTTGTGAATGATTTTCATTGATCCTGTTTGAGGGGATTTAGACGT
CCGTCGTCGTTTAGCTGAAGCTGTAACGGTGGATGAAAACCTACGATACGAACGCACACGAGGGCGATCAAGC
GGAGGTGGGAGCGAGGCGGGTTCCCCATGAAGCGAATGCCAAAGATTTGCGCCTGCCAATGAAGTGTTGACCA
GTGTTGACGGCTGGAGTAGGGAGCAAAGCGTCGTTGTTGATTTTCACAAAGCGATCATTTACCAGAGCCAGAG
ATTGTCGGAAGAGCCACTTTTCTTGATTTTTTGCTAGGTAAAGTGAGCCGGAGAGAGAAATAAGTACGAGTTT
AAACACACTCCTGTCGCACCCCCTAAATCATGCTGATGTGTCACTCCCGACGCAGTATCAGTAAAGTAGGTAA
ATAATCGCGACAT
>TR274|c0_g2_i2 len=3400 path=[8253:0-265 8321:266-374 8326:375-381
8329:382-382 8240:383-402 8318:403-732 8308:733-733 8296:734-1298
8229:1299-1322 8306:1323-1470 8249:1471-1494 8294:1495-1554 8304:1555-
2503 8275:2504-2527 8292:2528-2593 8269:2594-2617 8270:2618-3399] [-1,
8253, 8321, 8326, 8329, 8240, 8318, 8308, 8296, 8229, 8306, 8249, 8294,
8304, 8275, 8292, 8269, 8270, -2]
TGCCAACACAAGTTGACTGTTTGCTACTCACTGAAGATGAGCCTGGCCGACCGAAGGTCAACTGGTCACTGGA
CAACCACCCTCCTCATCATCACATCTATCACAGCCGTTGTCATTGTTGTAGGAGTAGTAAACATAGATATGAC
AGCTGTGATATCTCTATCTAGAGCAAGATGCCCAGGAGGATGTTGGCGTCTGTGGATGGGGCTGGCAGCTTGT
GGTGCTCGACGTTGGTGATGATGAATGAATTTGGTCGTGTGCAGATGGTAGGTGAATGCACACAGTTTGCGTG
TGCAGACTGACACTTGAGAAGGCTTGAAGATGTTGAATAACGAAACTTCACTACTGAAAAGTCAATACTACAT
AGAAATTAGCTGGAAAGGAAAGTAATAGAATATGAAACATGGCTAAAAACAATCATTGAGAAAAAACGCGGAG
AGAAACAGCAGCAGCATTCTATTGGCATTGATGCAGAACAACGAACCGGCCGCAGTGACGGGCAACAAGGCGG
GAGCACCATGCGTGTTGCATCTTGCAATGTTTTCCTTAGGGTTCTTCTCTTCACTTTCCAAAGTGCAATTATA
TTGATTTAGAACATTGCCATACGATCGTATGGTGGTGACCACATTTGTACGCCCGGATGGCAATTCCAGCTGT
GTTTGCTGACTATTAGTTTTGTTAATCAAATCATCTTTCCCTATTTTCATCATAGACAAGAGTAAAACCAGTA
GCAATATCAATTTATTTCGGTGATTTTCTTTTCTGGAAATTGGCTTCAGTTGCCTTTGATTTACACTAGTATA
TTCAAGCTGACTATGCTTGTGGGGTGTAGATCATCACAACAGATGTTTTTGGCAATGGTAGTATTACGGAGAA
GTTACCATCTTTAGGGGCGATAACGGTACCTAGCTGATGCCTCCTGACACGGGTTTGGTTGGTACTGCTCTTT
CAGGTGACTGTGTGCTGCCCGTCGTGCCCCCAATAATGCTTTTAATCTCTAGGAAATTGACAGAGATAGGAGT
GATGATAACAATGTTGGCAATGGAGGAATTGAAAATGTATGGTACTTCATGGTGGATAAATATTGTGGTGGTG
CTGGGTGCGCTGTCTGATGTGCATAGCTTTAATGCACATGACTCGCCTGAGTCCAGGGATCTACTAAGGTTAC
GCTTCTCTCATGTGCCGACCTCGACCATGGCATTGCAGCAGTCCCATGATAAGATATCTCGAGATGATGGTAT
GACATGTTTCGAAACCCTTGTTAATATCTACTCAGCACTAGACTTTTAGCGTTTACTCGCGCGGTATCCCTAA
ATTCTTAACCGCCAAGTGATGCGCTCCGTTACATCACACGGGCGCATCATCCGTCGTATTGCAATTTCAACAA
GGTATTGCTTTTTTTCACCCTTCTTGACAGGGGCAACAACTGTTAACTCCCCTCCTCCTCAACATTAAAACCA
CTCAACGCACGCTCCACGATCCAATCAAACCTACATGGAGCTGTGTCAAACTTAGTTTCACCGTGCTGATGAT
GCTGCACATTGGTATTTTCATTGTTCATTGACAAAACGGCCCTTGGATAGCAACCACCATGACCATCATCTAC
CACCATATCTCTCTCCTCCTCGTTCGCCAACCCCTTCAAATAATTCCCGTACACCTCCACCAACTCTGTCTTA
TTCATGCAAAAGTGTAAAATGAAACAGCCCAACTGCTCAAAGATTGGAGGTTGCCAACAGTCTGACAAGCACG
GACCTTGCAATTGCACAAAGGCCTCAGGCTGGGAGGCAATTTGAATAGACTCATTCACCGAGGGATTACGCAG
TAGAGACTGTGCCTGGACCTGCTCCCACGGCGTGTCAGTGCGAAACTTGATCCCAAGATTCGGGTGATGACTC
CTTTTAACATTCTCGGACTCATAGGGATCATCTGCAGACGTCCACCACTGCTCCAAGACCGCCGTCGCTTTAT
CCGATCTTTTCCAAAACACTGTCCCCGTATTAAGACAGTGAGAGAACAGAGCATCGTGGACGAGGGAGCACCA
ATACGACGGACCTTCCTTGTTCAAAACAAACGGCCTTTCGTCAATGTTCCAACTCGGCAGCCATTGCGTTGTC
ATGTTGTGCAGAACTTGCAATATGGATTGGTCCCGGAATCGATAATCCACCACGGCATCAGTGTCCATAAAGA
GAAAATAGTCAATGTGCGGCAAGTCTTCTTGAACCTGCAGAAATACTTTGACCTTGCACCATGCTTCGGCCAA
TTCTTTGTTGTTTCTGCTGCGGCACGCTTTGGCCAAATTGTTGGGCCAGTGGTAATAAATGTAGTCGTGCCCG
TGTTTCTGGCTGTATCGCCAGTTCCAGATTGCCGCAGTGTTCCAGACCGTTGGATGAATCTTTTTCGTGGTGG

GACCAATATGCCATTCTTGCCACAGTGGCCTTCCGTCGAATTGGACAATGGCAATTTTGGATGGGTGCAGCTC
TGATACCCAATGATTATTGTGAAAGTGATTCGCTGGAGGTTGTGGATCTTGATCTGGTAACAAAGTTCGATTT
TTATTTACATTTCGAGTTTGGGAAGTGGTATGATGGGTGTTGGGTCGAAGCTCGAGGAATGGCATATTCATAT
TATTGTGATTGCTCTGGCCTGCCCCGGTTTGGGAATCAGTAGTTGGGATTGTTGGATGAAGGTCAAGGAGGAA
GAGACAGCAGCTGATACAGATAACATATGCAGGGAGGATAATCAGCCACTTGGACATGCCGGAGCAGTGAGCC
CTTGAAGCAGCAAACGTGTACCGCGCCGATGGCCCCGATGGCGCGGCAGCTTTGTCAGGAAGTCGACGTCGAC
GTCGGAGGGATGGGAGGATCGCCATGGGATGAAGGAGTGGGAACTATCGTAACTGAGCGTAACCACTAGCAAT
TTATCTCATGCTTGCTTGGCACAAATGGTTGAGGCTCGCCGTGGGAGTTAATTCCCTGTTGTGAATGATTTTC
ATTGATCCTGTTTGAGGGGATTTAGACGTCCGTCGTCGTTTAGCTGAAGCTGTAACGGTGGATGAAAACCTAC
GATACGAACGCACACGAGGGCGATCAAGCGGAGGTGGGAGCGAGGCGGGTTCCCCATGAAGCGAATGCCAAAG
ATTTGCGCCTGCCAATGAAGTGTTGACCAGTGTTGACGGCTGGAGTAGGGAGCAAAGCGTCGTTGTTGATTTT
CACAAAGCGATCATTTACCAGAGCCAGAGATTGTCGGAAGAGCCACTTTTCTTGATTTTTTGCTAGGTAAAGT
GAGCCGGAGAGAGAAATAAGTACGAGTTTAAACACACTCCTGTCGCACCCCCTAAATCATGCTGATGTGTCAC
TCCCGACGCAGTATCAGTAAAGTAGGTAAATAATCGCGACAT
>TR274|c0_g2_i3 len=3193 path=[8253:0-265 8321:266-374 8326:375-381 8329:382-382 8319:383-504 8196:505-524 8197:525-526 8296:527-1091 8229:1092-1115 8306:1116-1263 8249:1264-1287 8294:1288-1347 8304:1348-2296 8275:2297-2320 8292:2321-2386 8269:2387-2410 8270:2411-3192] [-1, 8253, 8321, 8326, 8329, 8319, 8196, 8197, 8296, 8229, 8306, 8249, 8294, 8304, 8275, 8292, 8269, 8270, -2]
TGCCAACACAAGTTGACTGTTTGCTACTCACTGAAGATGAGCCTGGCCGACCGAAGGTCAACTGGTCACTGGA
CAACCACCCTCCTCATCATCACATCTATCACAGCCGTTGTCATTGTTGTAGGAGTAGTAAACATAGATATGAC
AGCTGTGATATCTCTATCTAGAGCAAGATGCCCAGGAGGATGTTGGCGTCTGTGGATGGGGCTGGCAGCTTGT
GGTGCTCGACGTTGGTGATGATGAATGAATTTGGTCGTGTGCAGATGGTAGGTGAATGCACACAGTTTGCGTG
TGCAGACTGACACTTGAGAAGGCTTGAAGATGTTGAATAACGAAACTTCACTACTGAAAAGTCAATACTACAT
AGAAATTAGCTGGAAAGGTAAGACAATTAGATTATCCATGATACCATCAATGGAAACCCAACTATAGATACCA
TTGCTGCCATGATTGACCAACTGGTTGAAGGGTTATTGATCAATGCCGATCAATTTTGGAGATAATGACAAGA
GTAAAACCAGTAGCAATATCAATTTATTTCGGTGATTTTCTTTTCTGGAAATTGGCTTCAGTTGCCTTTGATT
TACACTAGTATATTCAAGCTGACTATGCTTGTGGGGTGTAGATCATCACAACAGATGTTTTTGGCAATGGTAG
TATTACGGAGAAGTTACCATCTTTAGGGGCGATAACGGTACCTAGCTGATGCCTCCTGACACGGGTTTGGTTG
GTACTGCTCTTTCAGGTGACTGTGTGCTGCCCGTCGTGCCCCCAATAATGCTTTTAATCTCTAGGAAATTGAC
AGAGATAGGAGTGATGATAACAATGTTGGCAATGGAGGAATTGAAAATGTATGGTACTTCATGGTGGATAAAT
ATTGTGGTGGTGCTGGGTGCGCTGTCTGATGTGCATAGCTTTAATGCACATGACTCGCCTGAGTCCAGGGATC
TACTAAGGTTACGCTTCTCTCATGTGCCGACCTCGACCATGGCATTGCAGCAGTCCCATGATAAGATATCTCG
AGATGATGGTATGACATGTTTCGAAACCCTTGTTAATATCTACTCAGCACTAGACTTTTAGCGTTTACTCGCG
CGGTATCCCTAAATTCTTAACCGCCAAGTGATGCGCTCCGTTACATCACACGGGCGCATCATCCGTCGTATTG
CAATTTCAACAAGGTATTGCTTTTTTTTCACCCTTCTTGACAGGGGCAACAACTGTTAACTCCCCTCCTCCTCA
ACATTAAAACCACTCAACGCACGCTCCACGATCCAATCAAACCTACATGGAGCTGTGTCAAACTTAGTTTCAC
CGTGCTGATGATGCTGCACATTGGTATTTTCATTGTTCATTGACAAAACGGCCCTTGGATAGCAACCACCATG
ACCATCATCTACCACCATATCTCTCTCCTCCTCGTTCGCCAACCCCTTCAAATAATTCCCGTACACCTCCACC
AACTCTGTCTTATTCATGCAAAAGTGTAAAATGAAACAGCCCAACTGCTCAAAGATTGGAGGTTGCCAACAGT
CTGACAAGCACGGACCTTGCAATTGCACAAAGGCCTCAGGCTGGGAGGCAATTTGAATAGACTCATTCACCGA
GGGATTACGCAGTAGAGACTGTGCCTGGACCTGCTCCCACGGCGTGTCAGTGCGAAACTTGATCCCAAGATTC
GGGTGATGACTCCTTTTAACATTCTCGGACTCATAGGGATCATCTGCAGACGTCCACCACTGCTCCAAGACCG
CCGTCGCTTTATCCGATCTTTTCCAAAACACTGTCCCCGTATTAAGACAGTGAGAGAACAGAGCATCGTGGAC
GAGGGAGCACCAATACGACGGACCTTCCTTGTTCAAAACAAACGGCCTTTCGTCAATGTTCCAACTCGGCAGC
CATTGCGTTGTCATGTTGTGCAGAACTTGCAATATGGATTGGTCCCGGAATCGATAATCCACCACGGCATCAG
TGTCCATAAAGAGAAAATAGTCAATGTGCGGCAAGTCTTCTTGAACCTGCAGAAATACTTTGACCTTGCACCA
TGCTTCGGCCAATTCTTTGTTGTTTCTGCTGCGGCACGCTTTGGCCAAATTGTTGGGCCAGTGGTAATAAATG
TAGTCGTGCCCGTGTTTCTGGCTGTATCGCCAGTTCCAGATTGCCGCAGTGTTCCAGACCGTTGGATGAATCT
TTTTCGTGGTGGGACCAATATGCCATTCTTGCCACAGTGGCCTTCCGTCGAATTGGACAATGGCAATTTTGGA
TGGGTGCAGCTCTGATACCCAATGATTATTGTGAAAGTGATTCGCTGGAGGTTGTGGATCTTGATCTGGTAAC
AAAGTTCGATTTTTATTTACATTTCGAGTTTGGGAAGTGGTATGATGGGTGTTGGGTCGAAGCTCGAGGAATG
GCATATTCATATTATTGTGATTGCTCTGGCCTGCCCCGGTTTGGGAATCAGTAGTTGGGATTGTTGGATGAAG
GTCAAGGAGGAAGAGACAGCAGCTGATACAGATAACATATGCAGGGAGGATAATCAGCCACTTGGACATGCCG
GAGCAGTGAGCCCTTGAAGCAGCAAACGTGTACCGCGCCGATGGCCCCGATGGCGCGGCAGCTTTGTCAGGAA
GTCGACGTCGACGTCGGAGGGATGGGAGGATCGCCATGGGATGAAGGAGTGGGAACTATCGTAACTGAGCGTA
ACCACTAGCAATTTATCTCATGCTTGCTTGGCACAAATGGTTGAGGCTCGCCGTGGGAGTTAATTCCCTGTTG
TGAATGATTTTCATTGATCCTGTTTGAGGGGATTTAGACGTCCGTCGTCGTTTAGCTGAAGCTGTAACGGTGG
ATGAAAACCTACGATACGAACGCACACGAGGGCGATCAAGCGGAGGTGGGAGCGAGGCGGGTTCCCCATGAAG

CGAATGCCAAAGATTTGCGCCTGCCAATGAAGTGTTGACCAGTGTTGACGGCTGGAGTAGGGAGCAAAGCGTC
GTTGTTGATTTTCACAAAGCGATCATTTACCAGAGCCAGAGATTGTCGGAAGAGCCACTTTTCTTGATTTTTT
GCTAGGTAAAGTGAGCCGGAGAGAGAAATAAGTACGAGTTTAAACACACTCCTGTCGCACCCCCTAAATCATG
CTGATGTGTCACTCCCGACGCAGTATCAGTAAAGTAGGTAAATAATCGCGACAT
>TR274|c0_g2_i4 len=3070 path=[8253:0-265 8321:266-374 8326:375-381
8317:382-402 8308:403-403 8296:404-968 8229:969-992 8306:993-1140
8249:1141-1164 8294:1165-1224 8304:1225-2173 8275:2174-2197 8292:2198-
2263 8269:2264-2287 8270:2288-3069] [-1, 8253, 8321, 8326, 8317, 8308,
8296, 8229, 8306, 8249, 8294, 8304, 8275, 8292, 8269, 8270, -2]
TGCCAACACAAGTTGACTGTTTGCTACTCACTGAAGATGAGCCTGGCCGACCGAAGGTCAACTGGTCACTGGA
CAACCACCCTCCTCATCATCACATCTATCACAGCCGTTGTCATTGTTGTAGGAGTAGTAAACATAGATATGAC
AGCTGTGATATCTCTATCTAGAGCAAGATGCCCAGGAGGATGTTGGCGTCTGTGGATGGGGCTGGCAGCTTGT
GGTGCTCGACGTTGGTGATGATGAATGAATTTGGTCGTGTGCAGATGGTAGGTGAATGCACACAGTTTGCGTG
TGCAGACTGACACTTGAGAAGGCTTGAAGATGTTGAATAACGAAACTTCACTACTGAAAAGTCAATACTACAT
AGAAATTAGCTGGAAAGACAAGAGTAAAACCAGTAGCAATATCAATTTATTTCGGTGATTTTCTTTTCTGGAA
ATTGGCTTCAGTTGCCTTTGATTTACACTAGTATATTCAAGCTGACTATGCTTGTGGGGTGTAGATCATCACA
ACAGATGTTTTTGGCAATGGTAGTATTACGGAGAAGTTACCATCTTTAGGGGCGATAACGGTACCTAGCTGAT
GCCTCCTGACACGGGTTTGGTTGGTACTGCTCTTTCAGGTGACTGTGTGCTGCCCGTCGTGCCCCCAATAATG
CTTTTAATCTCTAGGAAATTGACAGAGATAGGAGTGATGATAACAATGTTGGCAATGGAGGAATTGAAAATGT
ATGGTACTTCATGGTGGATAAATATTGTGGTGGTGCTGGGTGCGCTGTCTGATGTGCATAGCTTTAATGCACA
TGACTCGCCTGAGTCCAGGGATCTACTAAGGTTACGCTTCTCTCATGTGCCGACCTCGACCATGGCATTGCAG
CAGTCCCATGATAAGATATCTCGAGATGATGGTATGACATGTTTCGAAACCCTTGTTAATATCTACTCAGCAC
TAGACTTTTAGCGTTTACTCGCGCGGTATCCCTAAATTCTTAACCGCCAAGTGATGCGCTCCGTTACATCACA
CGGGCGCATCATCCGTCGTATTGCAATTTCAACAAGGTATTGCTTTTTTTCACCCTTCTTGACAGGGGCAACA
ACTGTTAACTCCCCTCCTCCTCAACATTAAAACCACTCAACGCACGCTCCACGATCCAATCAAACCTACATGG
AGCTGTGTCAAACTTAGTTTCACCGTGCTGATGATGCTGCACATTGGTATTTTCATTGTTCATTGACAAAACG
GCCCTTGGATAGCAACCACCATGACCATCATCTACCACCATATCTCTCTCCTCCTCGTTCGCCAACCCCTTCA
AATAATTCCCGTACACCTCCACCAACTCTGTCTTATTCATGCAAAAGTGTAAAATGAAACAGCCCAACTGCTC
AAAGATTGGAGGTTGCCAACAGTCTGACAAGCACGGACCTTGCAATTGCACAAAGGCCTCAGGCTGGGAGGCA
ATTTGAATAGACTCATTCACCGAGGGATTACGCAGTAGAGACTGTGCCTGGACCTGCTCCCACGGCGTGTCAG
TGCGAAACTTGATCCCAAGATTCGGGTGATGACTCCTTTTAACATTCTCGGACTCATAGGGATCATCTGCAGA
CGTCCACCACTGCTCCAAGACCGCCGTCGCTTTATCCGATCTTTTCCAAAACACTGTCCCCGTATTAAGACAG
TGAGAGAACAGAGCATCGTGGACGAGGGAGCACCAATACGACGGACCTTCCTTGTTCAAAACAAACGGCCTTT
CGTCAATGTTCCAACTCGGCAGCCATTGCGTTGTCATGTTGTGCAGAACTTGCAATATGGATTGGTCCCGGAA
TCGATAATCCACCACGGCATCAGTGTCCATAAAGAGAAAATAGTCAATGTGCGGCAAGTCTTCTTGAACCTGC
AGAAATACTTTGACCTTGCACCATGCTTCGGCCAATTCTTTGTTGTTTCTGCTGCGGCACGCTTTGGCCAAAT
TGTTGGGCCAGTGGTAATAAATGTAGTCGTGCCCGTGTTTCTGGCTGTATCGCCAGTTCCAGATTGCCGCAGT
GTTCCAGACCGTTGGATGAATCTTTTTCGTGGTGGGACCAATATGCCATTCTTGCCACAGTGGCCTTCCGTCG
AATTGGACAATGGCAATTTTGGATGGGTGCAGCTCTGATACCCAATGATTATTGTGAAAGTGATTCGCTGGAG
GTTGTGGATCTTGATCTGGTAACAAAGTTCGATTTTTATTTACATTTCGAGTTTGGGAAGTGGTATGATGGGT
GTTGGGTCGAAGCTCGAGGAATGGCATATTCATATTATTGTGATTGCTCTGGCCTGCCCCGGTTTGGGAATCA
GTAGTTGGGATTGTTGGATGAAGGTCAAGGAGGAAGAGACAGCAGCTGATACAGATAACATATGCAGGGAGGA
TAATCAGCCACTTGGACATGCCGGAGCAGTGAGCCCTTGAAGCAGCAAACGTGTACCGCGCCGATGGCCCCGA
TGGCGCGGCAGCTTTGTCAGGAAGTCGACGTCGACGTCGGAGGGATGGGAGGATCGCCATGGGATGAAGGAGT
GGGAACTATCGTAACTGAGCGTAACCACTAGCAATTTATCTCATGCTTGCTTGGCACAAATGGTTGAGGCTCG
CCGTGGGAGTTAATTCCCTGTTGTGAATGATTTTCATTGATCCTGTTTGAGGGGATTTAGACGTCCGTCGTCG
TTTAGCTGAAGCTGTAACGGTGGATGAAAACCTACGATACGAACGCACACGAGGGCGATCAAGCGGAGGTGGG
AGCGAGGCGGGTTCCCCATGAAGCGAATGCCAAAGATTTGCGCCTGCCAATGAAGTGTTGACCAGTGTTGACG
GCTGGAGTAGGGAGCAAAGCGTCGTTGTTGATTTTCACAAAGCGATCATTTACCAGAGCCAGAGATTGTCGGA
AGAGCCACTTTTCTTGATTTTTTGCTAGGTAAAGTGAGCCGGAGAGAGAAATAAGTACGAGTTTAAACACACT
CCTGTCGCACCCCCTAAATCATGCTGATGTGTCACTCCCGACGCAGTATCAGTAAAGTAGGTAAATAATCGCG
ACAT
>TR275|c0_g1_i1 len=6209 path=[6985:0-194 7023:195-211 7009:212-445
6951:446-469 7019:470-572 7008:573-1002 7000:1003-1026 7022:1027-1231
6962:1232-1265 @7018@!:1266-1551 7013:1552-1617 6989:1618-1641 7006:1642-
3020 6957:3021-3044 7012:3045-3140 6995:3141-3164 7005:3165-3499
6992:3500-3523 7021:3524-4151 7011:4152-4266 7004:4267-5309 6979:5310-
5333 6980:5334-6208] [-1, 6985, 7023, 7009, 6951, 7019, 7008, 7000, 7022,
6962, 7018, 7013, 6989, 7006, 6957, 7012, 6995, 7005, 6992, 7021, 7011,
7004, 6979, 6980, -2]

ATCCAGTGTCAACTGGTAAGTCGAAGAATTCACCTATGAACGCCATAAGTCGTTATTAATTTGTGTGGTTATT
TACGCCGTTAACCGGTCGAGAAATGCGGAAATTATCTCCTAACGCCCGTCTATGACGTTGCGCCTCATTGCGC
ACACCACACCCCTTCCCCATTACCCAGGTCCGCCACAGTCTTCAAGGGAGACCCCGAACTCTCCGCCATGGTC
GCCGGCTTCCGCGCCTGGACGGCCCAAGCCGACGCCATGGCCGAGAAGTACTCAGCCCCTCCCGCCCCCATCG
ACTTCGCCACGGCCAAATCCTCCGTCCGCGACGTCGCCCTCGTCGACGCCCTCGAATCCATGTACAAATCGTC
CAGCCCTCCCGCCATCACTTACGAGTGGAGCGAGGAGGAGCAGGCAAAGAAGGCGGAGTTGATCAAGGGAGCC
GAGGCGGGTTTGGCGTTCAACCAGGAGATGATCGATGATACGGAGAAGGAGATTGCGTTGCTTCGCACCAACA
GGCTGTCTCACGATAGTTCCCCGAGTCTTATGGCTGAGGTTTATCCTGATATTGCGGAGGAGATTGAGGATGA
GATTGAGAACAGAGAGTGGTTTAAGGATACTTTGAATAAGTAGAGGTTGAATAGTCTCTGACTTTAGGTTAGG
GGGTTGGGTGCTTTGCGGAATGTTGTTGGAGTGAATGGGTATTAGTTAGAGTGCATTTGGATCGTGTGGGGAG
GTTGAGTAATTCCCAATGAACCCTCATTGGAATTCGACAAACATCCAACCTACTGAGTCATGCTACAGAATTG
CATCTAATGGATACTAACAGTGACATTGTCAATGAAATCATCGGCATGTCAATGGGGGGTCATGATTATCTGT
CATTTTGGCAAAACAATAATAGATTTGGGTATCTAAATATCCCACTCCTGTTATCTGACGGGGAATAAATCAG
CAAATCCTCTTGGAAAAGCATGGTTACAATTACAGCTTTTGTGAATTTCCCACTTTGTGTTGACTCAATGACA
ACCAATGCATGGATTCAATCAAGACATCTTCCACCCTTGAAATGCCCACTGTTGAAATCCGTGGGCATGCTTA
GAGTAGGCATTCACATTTTAGCGTGGAAGACTGGGGTCGCTCTGAGCTTTCTTTCTTTCCAAGCAGATGGGGG
TGGAACTTGGCTCTTTCTTCCGTCCTTTCGAGTGTACTAAAATATTTTGAACCATTGTGGTCTTCGGGGGGCC
AAATATATTCCGACCGACTGTGCAGTGAACCAGAGCAAGTGGTCTTAGTTTTATGAAACCAGTCTCTAGCTAC
CCTGCCATTTCTCGTCATTCATATGAATTGTCCCCTTCGAATCCAAGTGAACCCTGTATATTTCGTTTCGTTT
TGGATGACACAGTAGAGGCAGCAGAAGAAGAACAGTAGCATGGCTTGATGGCACAAACAATACCGAAACCTCC
ATCCAACCGAAATAGTAATTTTTCCCTATATTCTTGTTAACCAGACATGAACAAAGACTGTTTAATTTACCTA
ACAAAACCCTTGAGGCTGATCTTCTTCTTCGTTACCCGCTTGGGGGCCGCATACCCGCTCATGTCCGCCCGCA
CGGCCGACTCTAAAACTGCTCTACTCTCGGGGGAGATGGCAACCATGGTTGATTTGAACAACATGGGGGCCGT
TCGAACAACGTGAACGAAGACCTGTGCACACATTCTCCCAATAGTTTCGTCTGCCCCCTTTTTGCCACTGGGA
TGGTTTGGCAACCCATTGAATGAAATGCTCTCGACGAGAATTTCAAACAGTGCTGATATGAAAGAGACACTTT
TGTCTTCTTCTTTTGACGCAGAAATCAGGTATTGGAATGCCATGAGATTCACTTTTACTGACTCAGCACATAC
AGTAACTCGATTTTCGTCAAAGCCTGGCCCAGATAAAACTCGAACTCCATGAGCTCTCAATAACTGAAGGATC
TCAAACCCGAGAGATTGCAGCACATAATTGAGTACAGATGAGTCTTCGGCAATAGCAGAATGCAACGCAGATC
GCAAGGCAACAAGGGCGGCAGCATGACGAGGGCCTGACTGAAGATCTTGTAGCTGTATCTTGATTGCATCAAT
GCTACACTTGATCCCATATCCTGAAGGGAGCGACAAGCATAGAACAGCCCAAGCATCCCACAATGCATGAGTC
GCAGAATAATTCGCAATTTGTCCCCATTCTTCATGTGACAAGGTTGTCTCTTGACAGCAGGCTTGAAGGAGTG
ACAAACATGCAGCCTTTATTTCTTCTTCTGGCTCATCTATCAGCAGTGTCAAGACTAGTTGTACCAAGGCTTT
CTCGAGTTCTGCGCGGCCTTCTTCTGGGTGAGATTGAAGTAATGATTGGCTGGATCTGATGCATGAAGAAATG
ATGATTCCGTGATTGGGCTCCATTTCCCGCTTTTCTTGCAAAGTTACAAGAGGGGTCACAACTGATCGAGTAA
GTATTGCCGCATCTACCCCTATGGCATAACATTCTTTGCACAACTGTGCGATCAAACCACACGACTGTTGTAC
CAGGATTTCATCGTCCTGAGACAAAGATCGTTTGCTTCCCAGAACACGAAATATGACAGATTCAGTCACGATG
TTGGCAACATCGCCAAGTTCATTTGGACATAATCCCTCCACTACGCACTCGTTGTCCCTCACAAGAAGGCGGA
CGGCATAAACGCACAACGCTGCAGTTTGTGAGGCAGATGGATGTTCTGAGGAAGGTTCCTTGTCACCGAGGTG
AAGGCTAGAAAGTGAATTGCGTAACCCAGTAACCATCAAGGGAGTAAGCTTGCTGAGCCACACCTGCAAAGAC
TTCGTTTCTTCACTTTCACTGTCTCCAGCTTTTATTGCCTTAATAATCGACGAAATAGCAGAGGCGCTAAGCA
TAGGCCAATTCTCGATCAGCGCTTCTTGAACAGATCCATCCAAATCAGTCGTATTCTTGTACGTCAAACCGGG
ACGACTAATGGTATTATTAGACTGACTCTGTTGAGAATCTTGCAGTAAGAAACCGTCAATAGCGGCGGCGGCG
CAGTGAATCGCCCTCCCCGTTTCTTCCTTTTCTAGCTCACTGGCAACTATGGAGAGTTTGGACTGTTCGATAT
GATTTTCTCCCAGTGCAATCAGCATGGATACCTTGGACAAAAAGCACAGCTTCGACAGTCGAAAGAGGGGGTA
GGAGCGTGAATCATCTGTTATATGAGAAGAACTCATCCAAAGGGCACTCTCATTTTCATCTCCTGTCGGAAAT
GGAACAAAAGGAGTTTCTGTGGCACCCAATAGCACTGGTTGCAATAGACGCCTCATAGCAATGGGGTCAGAAA
TCAGCTTTTCAGATATCATCACAAATAGTGCCTCACACCCTGTTGAAAACAGGCGATGAAACGCAGTTCCTGG
GACGGACTCATTGAGTAAACTCTCAGAATTTAGTGCATGTTTCACTGCCGAAACTATCTGTGACGAGTATTGT
TCGAGCACCGATGTGCCATCATTTATGGTCGCATCCAATTGGCTACCAAAGGCGCGAAACAAAGAAATCAAAA
GCCGAAGCCCAGATATCTGAACTGAGGGAAGCTCAGAGTGGTTTGATGTTGATGTGGATGTGCTGCATGCTGT
TGTAACCAGTTCTTCTAAGTGAAAAATTGGTTGTGAGTGTAGTTCAACACCGTATTCTTTGTTCTCTTTCCTT
AACATAGCAGTGCATCGTGACTGGGCAGATTTCGAGTTGAAGAGGAATCCACCACCGTCAGTTCCATCGTCCA
TAGAGAGAAGCATCATCATTGCGACTGATGCAACATTAGCCGATACACATCTCAGTTGCCACCGGGGTGGATT
TGAATATTGCAGCGCGTCCGAAGCACAAAGCCGCGCGATATACCTTGTTCTTTCGATGAGACCTGGAACCGAA
CCGTCGGAATCAACATCATTAAGAGTTTTGGCTCCATCTCCAGAAGCCAGACACCTGGAGAACAGTAACCATT
GAATGATAATGAAAGATTTTTCATTCTCATCTCTCCCCATGATTTCTGAGCTGAGAATTGCTTGGATCAGGGA
AATCACTTCAGACTCTAACATCTGACAATCTTCAAAGAAGTTAACTACTGCGCGAGAGAGAGCTGTAGTGCCA
CGAGAAATAAATTGGAATGGTGAAACAAGAAAAGTTAGCGAATGAAAAACGCACCAACGTTGATAAGTTACGA
TGATCATCACCTAATGAGCGAAAGTCCGAAAAGTGTTGGAAACGTCGACGCCCGATTCTGTCATGTAGAAATG
AGAATAAGGCCTTCCCTACGCCAAACGTGAATGTATCGTCGGGACTTTTTGACGATGCACACATCTCTTTCAT

GCACAAGATAGCTGAGCGCTGCGCATCAATAGGGCCTTGACAGTTGGAAAAGTCTAGCTCACTATCGGCCACA
ACATTTGGATAAGGCGGCTGGAGTACAGAAATCAAGAACGGCGTTGCTGTGGCTGAAAGGTTGTGGGTTGTGA
CAACTGAACAAGAGTTCGGCGTAATAAGGTCCCTATGTGCACTCAAACGCTCGAAGAAAACAGAGCCTTCAAA
CAGAATGACTGGATGATTTGAGCTGTATTCCAAAATTGTGGATGTAATGCATGAGAATCGACTAGCCAACCAA
CGGTCCAAGGACTTCAGCTCCCTGAGCCACGGGAGTAGCGTCGTCATGAGTCGTAGAATGACTGCAAGAAATG
CAGGCCCTGACGCAAACTTCTCACGCTCTCGACCTCTGCAGGTGTCAGAAACAACCGCGCTCCATGCACCGAG
GAAACTTGACTCAACAGCTTCAGCTGCTTTCCATATGATTTGAACTTGGTCAGGTGTTAGGACTGTGTCCGTC
GATATAGAATTTGATATCAAAACACCGAACGAGTGCAAAGCATATGTTCTGGCGAGGAGAGAATCCCCTTCAG
GGTTTTGGGTGGCAAGGGAAGGCAAAACTCGAGTCATCATAATCATAACTGGTGTTCTGTCAATCTCATTCTT
ATCTGACTCTCGTGAAACTGATCTTGCGATCGCTCGTTCTTCTTCATTTTTCTTCATTCTTTTCGCTGCTCGT
TGAATGCATGCCAAGGCAAGTAACGTGCCTGCTTTCGCAAATGCGAGAGTTTCAACGTGATTTTTTGGGTTTG
CCATGACGTTGCTCCCTTTCATAACTTCATCAAGAGAATGAAGGATGGTGCTCTGCAGCGTGTGTGCATCTTC
GCTTACTCCAAGTGTTGCTAAAAGAGAAAGGCCTTCAGCAGCACCACGCCGAATAATGCCTGATGGCGATGGA
AGGAGGCGCAACAACAGAGAGGTTGCCCTCTCCATCCAGGGAGGCCCTCTATCGATAAGAGTGTCGTACGTCG
ATTCGTGTAAGGGAAGCGCCTGAAGACAAGCCAAGACAGTGGCAATGACATTTGAAGCTGGCACATCTTCCTC
TTGCGGCTTCACTTTCCCTTGACTTTCACTAATAAGGGAGGTACTGACATTAAATCGATTAGACTTCTCAGTT
TGTGTCAATAGATAGAGATTTTCAAGCATCTTCAAGGCGTCAGACTGCGTGTGGCCATCCTGAAGGCCGAAGG
TCGCCGCGAAGACATGTATACTGGAGTCAAGAAGTCTCACTTTGGAAGTGCCCGTTGGGTTTAGAGGAACTCG
CCAAGTGCCTACCTCGTGAAGTGGCGTTGGAATTGAGTTCTCGTCCCCTTCACGTAGATATAAGCTGAGAATA
TCATCATGGAAACGCATGCGAAACTTCTTTCTCCATGCAAGAAGGTGTAAGACGGCCTCCCTTTCATTGTGAT
GAACTATGTCTGATGAACGGATAGATATGTTATTGTCTAGTGTAACTGAGCCACCAACCTGGCCCGGACCAAC
AGCGCGTTCGATTGAATGAGCTTCTATGAGTTTGTCTTCACGGCTCACAAGGCCATCAAGAATAGAACACAGG
ACATCGTCTGCACTCAAATCCTGGATCTGGGCCGCGGCGAATGAAAATATCCTATCTGCAGACAACGGAAAAG
ACCCTGGTGGCAACCAAGAATAGGCCTCCATGACAGAAGATCTTGTGCTGGAGAGCCGAGCGCTACCAACAGG
ATCGTTTCCTTCTCGCTCAAATCTACCCCCACTCGATATTAGTGGGAATACTTTTTCGAGAAGCGCCGTCAGG
CGGG
>TR275|c0_g1_i2 len=6115 path=[6985:0-194 7023:195-211 7009:212-445 6951:446-469 7019:470-572 7008:573-1002 7000:1003-1026 7022:1027-1231 6962:1232-1265 @7018@!:1266-1551 7013:1552-1617 6989:1618-1641 7006:1642-3020 6957:3021-3044 7012:3045-3140 6995:3141-3164 7005:3165-3499 6992:3500-3523 7021:3524-4151 6970:4152-4172 7004:4173-5215 6979:5216-5239 6980:5240-6114] [-1, 6985, 7023, 7009, 6951, 7019, 7008, 7000, 7022, 6962, 7018, 7013, 6989, 7006, 6957, 7012, 6995, 7005, 6992, 7021, 6970, 7004, 6979, 6980, -2]
ATCCAGTGTCAACTGGTAAGTCGAAGAATTCACCTATGAACGCCATAAGTCGTTATTAATTTGTGTGGTTATT
TACGCCGTTAACCGGTCGAGAAATGCGGAAATTATCTCCTAACGCCCGTCTATGACGTTGCGCCTCATTGCGC
ACACCACACCCCTTCCCCATTACCCAGGTCCGCCACAGTCTTCAAGGGAGACCCCGAACTCTCCGCCATGGTC
GCCGGCTTCCGCGCCTGGACGGCCCAAGCCGACGCCATGGCCGAGAAGTACTCAGCCCCTCCCGCCCCCATCG
ACTTCGCCACGGCCAAATCCTCCGTCCGCGACGTCGCCCTCGTCGACGCCCTCGAATCCATGTACAAATCGTC
CAGCCCTCCCGCCATCACTTACGAGTGGAGCGAGGAGGAGCAGGCAAAGAAGGCGGAGTTGATCAAGGGAGCC
GAGGCGGGTTTGGCGTTCAACCAGGAGATGATCGATGATACGGAGAAGGAGATTGCGTTGCTTCGCACCAACA
GGCTGTCTCACGATAGTTCCCCGAGTCTTATGGCTGAGGTTTATCCTGATATTGCGGAGGAGATTGAGGATGA
GATTGAGAACAGAGAGTGGTTTAAGGATACTTTGAATAAGTAGAGGTTGAATAGTCTCTGACTTTAGGTTAGG
GGGTTGGGTGCTTTGCGGAATGTTGTTGGAGTGAATGGGTATTAGTTAGAGTGCATTTGGATCGTGTGGGGAG
GTTGAGTAATTCCCAATGAACCCTCATTGGAATTCGACAAACATCCAACCTACTGAGTCATGCTACAGAATTG
CATCTAATGGATACTAACAGTGACATTGTCAATGAAATCATCGGCATGTCAATGGGGGGTCATGATTATCTGT
CATTTTGGCAAAACAATAATAGATTTGGGTATCTAAATATCCCACTCCTGTTATCTGACGGGGAATAAATCAG
CAAATCCTCTTGGAAAAGCATGGTTACAATTACAGCTTTTGTGAATTTCCCACTTTGTGTTGACTCAATGACA
ACCAATGCATGGATTCAATCAAGACATCTTCCACCCTTGAAATGCCCACTGTTGAAATCCGTGGGCATGCTTA
GAGTAGGCATTCACATTTTAGCGTGGAAGACTGGGGTCGCTCTGAGCTTTCTTTCTTTCCAAGCAGATGGGGG
TGGAACTTGGCTCTTTCTTCCGTCCTTTCGAGTGTACTAAAATATTTTGAACCATTGTGGTCTTCGGGGGGCC
AAATATATTCCGACCGACTGTGCAGTGAACCAGAGCAAGTGGTCTTAGTTTTATGAAACCAGTCTCTAGCTAC
CCTGCCATTTCTCGTCATTCATATGAATTGTCCCCTTCGAATCCAAGTGAACCCTGTATATTTCGTTTCGTTT
TGGATGACACAGTAGAGGCAGCAGAAGAAGAACAGTAGCATGGCTTGATGGCACAAACAATACCGAAACCTCC
ATCCAACCGAAATAGTAATTTTTCCCTATATTCTTGTTAACCAGACATGAACAAAGACTGTTTAATTTACCTA
ACAAAACCCTTGAGGCTGATCTTCTTCTTCGTTACCCGCTTGGGGGCCGCATACCCGCTCATGTCCGCCCGCA
CGGCCGACTCTAAAACTGCTCTACTCTCGGGGGAGATGGCAACCATGGTTGATTTGAACAACATGGGGGCCGT
TCGAACAACGTGAACGAAGACCTGTGCACACATTCTCCCAATAGTTTCGTCTGCCCCCTTTTTGCCACTGGGA
TGGTTTGGCAACCCATTGAATGAAATGCTCTCGACGAGAATTTCAAACAGTGCTGATATGAAAGAGACACTTT
TGTCTTCTTCTTTTGACGCAGAAATCAGGTATTGGAATGCCATGAGATTCACTTTTACTGACTCAGCACATAC

AGTAACTCGATTTTCGTCAAAGCCTGGCCCAGATAAAACTCGAACTCCATGAGCTCTCAATAACTGAAGGATC
TCAAACCCGAGAGATTGCAGCACATAATTGAGTACAGATGAGTCTTCGGCAATAGCAGAATGCAACGCAGATC
GCAAGGCAACAAGGGCGGCAGCATGACGAGGGCCTGACTGAAGATCTTGTAGCTGTATCTTGATTGCATCAAT
GCTACACTTGATCCCATATCCTGAAGGGAGCGACAAGCATAGAACAGCCCAAGCATCCCACAATGCATGAGTC
GCAGAATAATTCGCAATTTGTCCCCATTCTTCATGTGACAAGGTTGTCTCTTGACAGCAGGCTTGAAGGAGTG
ACAAACATGCAGCCTTTATTTCTTCTTCTGGCTCATCTATCAGCAGTGTCAAGACTAGTTGTACCAAGGCTTT
CTCGAGTTCTGCGCGGCCTTCTTCTGGGTGAGATTGAAGTAATGATTGGCTGGATCTGATGCATGAAGAAATG
ATGATTCCGTGATTGGGCTCCATTTCCCGCTTTTCTTGCAAAGTTACAAGAGGGGTCACAACTGATCGAGTAA
GTATTGCCGCATCTACCCCTATGGCATAACATTCTTTGCACAACTGTGCGATCAAACCACACGACTGTTGTAC
CAGGATTTCATCGTCCTGAGACAAAGATCGTTTGCTTCCCAGAACACGAAATATGACAGATTCAGTCACGATG
TTGGCAACATCGCCAAGTTCATTTGGACATAATCCCTCCACTACGCACTCGTTGTCCCTCACAAGAAGGCGGA
CGGCATAAACGCACAACGCTGCAGTTTGTGAGGCAGATGGATGTTCTGAGGAAGGTTCCTTGTCACCGAGGTG
AAGGCTAGAAAGTGAATTGCGTAACCCAGTAACCATCAAGGGAGTAAGCTTGCTGAGCCACACCTGCAAAGAC
TTCGTTTCTTCACTTTCACTGTCTCCAGCTTTTATTGCCTTAATAATCGACGAAATAGCAGAGGCGCTAAGCA
TAGGCCAATTCTCGATCAGCGCTTCTTGAACAGATCCATCCAAATCAGTCGTATTCTTGTACGTCAAACCGGG
ACGACTAATGGTATTATTAGACTGACTCTGTTGAGAATCTTGCAGTAAGAAACCGTCAATAGCGGCGGCGGCG
CAGTGAATCGCCCTCCCCGTTTCTTCCTTTTCTAGCTCACTGGCAACTATGGAGAGTTTGGACTGTTCGATAT
GATTTTCTCCCAGTGCAATCAGCATGGATACCTTGGACAAAAAGCACAGCTTCGACAGTCGAAAGAGGGGGTA
GGAGCGTGAATCATCTGTTATATGAGAAGAACTCATCCAAAGGGCACTCTCATTTTCATCTCCTGTCGGAAAT
GGAACAAAAGGAGTTTCTGTGGCACCCAATAGCACTGGTTGCAATAGACGCCTCATAGCAATGGGGTCAGAAA
TCAGCTTTTCAGATATCATCACAAATAGTGCCTCACACCCTGTTGAAAACAGGCGATGAAACGCAGTTCCTGG
GACGGACTCATTGAGTAAACTCTCAGAATTTAGTGCATGTTTCACTGCCGAAACTATCTGTGACGAGTATTGT
TCGAGCACCGATGTGCCATCATTTATGGTCGCATCCAATTGGCTACCAAAGGCGCGAAACAAAGAAATCAAAA
GCCGAAGCCCAGATATCTGAACTGAGGGAAGCTCAGAGTGGTTTGATGTTGATGTGGATGTGCTGCATGCTGT
TGTAACCAGTTCTTCTAAGTGAAAAATTGGTTGTGAGTGTAGTTCAACACCGTATTCTTTGTTCTCTTTCCTT
AACATAGCAGTGCATCGTGACTGGGCAGATTTCGAGTTGAAGAGGAATCCACCACCGTCAGTTCCATCGTCCA
TAGAGAGAAGCATCATCATTGCGACTGATGCAACATTAGCCGATACACATCTCAGTTGCCACCGGGGTGGATT
TGAATATTGCAGCGCGTCCGAAGCACAAAGCCGCGCGATATACCTTGTTCTTTCGATGAGACCTGGAACCGAA
CCGTCGGAATCAACATCATTAAGAGTTTTGGCTCCATCTCCAGAAGCCAGACACCTGGAGAACAGTAACCATT
GAATGATAATGAAAGATTTTTCATTCTCATCTCTCCCCATGATTTCTGAGCTGAGAATTGCTTGGATCAGGGA
AATCACTTCAGACTCTAACATCTGACAATCTTCAAAGAAGTTAACTACTGCGCGAGAGAGAGCTAATGAGCGA
AAGTCCGAAAAGTGTTGGAAACGTCGACGCCCGATTCTGTCATGTAGAAATGAGAATAAGGCCTTCCCTACGC
CAAACGTGAATGTATCGTCGGGACTTTTTGACGATGCACACATCTCTTTCATGCACAAGATAGCTGAGCGCTG
CGCATCAATAGGGCCTTGACAGTTGGAAAAGTCTAGCTCACTATCGGCCACAACATTTGGATAAGGCGGCTGG
AGTACAGAAATCAAGAACGGCGTTGCTGTGGCTGAAAGGTTGTGGGTTGTGACAACTGAACAAGAGTTCGGCG
TAATAAGGTCCCTATGTGCACTCAAACGCTCGAAGAAAACAGAGCCTTCAAACAGAATGACTGGATGATTTGA
GCTGTATTCCAAAATTGTGGATGTAATGCATGAGAATCGACTAGCCAACCAACGGTCCAAGGACTTCAGCTCC
CTGAGCCACGGGAGTAGCGTCGTCATGAGTCGTAGAATGACTGCAAGAAATGCAGGCCCTGACGCAAACTTCT
CACGCTCTCGACCTCTGCAGGTGTCAGAAACAACCGCGCTCCATGCACCGAGGAAACTTGACTCAACAGCTTC
AGCTGCTTTCCATATGATTTGAACTTGGTCAGGTGTTAGGACTGTGTCCGTCGATATAGAATTTGATATCAAA
ACACCGAACGAGTGCAAAGCATATGTTCTGGCGAGGAGAGAATCCCCTTCAGGGTTTTGGGTGGCAAGGGAAG
GCAAAACTCGAGTCATCATAATCATAACTGGTGTTCTGTCAATCTCATTCTTATCTGACTCTCGTGAAACTGA
TCTTGCGATCGCTCGTTCTTCTTCATTTTTCTTCATTCTTTTCGCTGCTCGTTGAATGCATGCCAAGGCAAGT
AACGTGCCTGCTTTCGCAAATGCGAGAGTTTCAACGTGATTTTTTGGGTTTGCCATGACGTTGCTCCCTTTCA
TAACTTCATCAAGAGAATGAAGGATGGTGCTCTGCAGCGTGTGTGCATCTTCGCTTACTCCAAGTGTTGCTAA
AAGAGAAAGGCCTTCAGCAGCACCACGCCGAATAATGCCTGATGGCGATGGAAGGAGGCGCAACAACAGAGAG
GTTGCCCTCTCCATCCAGGGAGGCCCTCTATCGATAAGAGTGTCGTACGTCGATTCGTGTAAGGGAAGCGCCT
GAAGACAAGCCAAGACAGTGGCAATGACATTTGAAGCTGGCACATCTTCCTCTTGCGGCTTCACTTTCCCTTG
ACTTTCACTAATAAGGGAGGTACTGACATTAAATCGATTAGACTTCTCAGTTTGTGTCAATAGATAGAGATTT
TCAAGCATCTTCAAGGCGTCAGACTGCGTGTGGCCATCCTGAAGGCCGAAGGTCGCCGCGAAGACATGTATAC
TGGAGTCAAGAAGTCTCACTTTGGAAGTGCCCGTTGGGTTTAGAGGAACTCGCCAAGTGCCTACCTCGTGAAG
TGGCGTTGGAATTGAGTTCTCGTCCCCTTCACGTAGATATAAGCTGAGAATATCATCATGGAAACGCATGCGA
AACTTCTTTCTCCATGCAAGAAGGTGTAAGACGGCCTCCCTTTCATTGTGATGAACTATGTCTGATGAACGGA
TAGATATGTTATTGTCTAGTGTAACTGAGCCACCAACCTGGCCCGGACCAACAGCGCGTTCGATTGAATGAGC
TTCTATGAGTTTGTCTTCACGGCTCACAAGGCCATCAAGAATAGAACACAGGACATCGTCTGCACTCAAATCC
TGGATCTGGGCCGCGGCGAATGAAAATATCCTATCTGCAGACAACGGAAAAGACCCTGGTGGCAACCAAGAAT
AGGCCTCCATGACAGAAGATCTTGTGCTGGAGAGCCGAGCGCTACCAACAGGATCGTTTCCTTCTCGCTCAAA
TCTACCCCCACTCGATATTAGTGGGAATACTTTTTCGAGAAGCGCCGTCAGGCGGG
>TR275|c0_g1_i3 len=6192 path=[6964:0-87 6965:88-111 7020:112-177 7023:178-194 7009:195-428 6951:429-452 7019:453-555 7008:556-985

7000:986-1009 7022:1010-1214 6962:1215-1248 @7018@!:1249-1534 7013:1535-1600 6989:1601-1624 7006:1625-3003 6957:3004-3027 7012:3028-3123 6995:3124-3147 7005:3148-3482 6992:3483-3506 7021:3507-4134 7011:4135-4249 7004:4250-5292 6979:5293-5316 6980:5317-6191] [-1, 6964, 6965, 7020, 7023, 7009, 6951, 7019, 7008, 7000, 7022, 6962, 7018, 7013, 6989, 7006, 6957, 7012, 6995, 7005, 6992, 7021, 7011, 7004, 6979, 6980, -2]
TTGCCTGTTGCACCCACCGCCCACGCCCACCCCACGCAACCCCACCCCACCTTGCTTGCACGTGGACAACCTC
TTACCTCCGACGCCAATAACAACGTCCACCATGAGCATGTCCGCCTTCCGCCGATCGGCCCAGCGCCTATCCA
GTGTCAACTGGTCCGCCACAGTCTTCAAGGGAGACCCCGAACTCTCCGCCATGGTCGCCGGCTTCCGCGCCTG
GACGGCCCAAGCCGACGCCATGGCCGAGAAGTACTCAGCCCCTCCCGCCCCCATCGACTTCGCCACGGCCAAA
TCCTCCGTCCGCGACGTCGCCCTCGTCGACGCCCTCGAATCCATGTACAAATCGTCCAGCCCTCCCGCCATCA
CTTACGAGTGGAGCGAGGAGGAGCAGGCAAAGAAGGCGGAGTTGATCAAGGGAGCCGAGGCGGGTTTGGCGTT
CAACCAGGAGATGATCGATGATACGGAGAAGGAGATTGCGTTGCTTCGCACCAACAGGCTGTCTCACGATAGT
TCCCCGAGTCTTATGGCTGAGGTTTATCCTGATATTGCGGAGGAGATTGAGGATGAGATTGAGAACAGAGAGT
GGTTTAAGGATACTTTGAATAAGTAGAGGTTGAATAGTCTCTGACTTTAGGTTAGGGGGTTGGGTGCTTTGCG
GAATGTTGTTGGAGTGAATGGGTATTAGTTAGAGTGCATTTGGATCGTGTGGGGAGGTTGAGTAATTCCCAAT
GAACCCTCATTGGAATTCGACAAACATCCAACCTACTGAGTCATGCTACAGAATTGCATCTAATGGATACTAA
CAGTGACATTGTCAATGAAATCATCGGCATGTCAATGGGGGGTCATGATTATCTGTCATTTTGGCAAAACAAT
AATAGATTTGGGTATCTAAATATCCCACTCCTGTTATCTGACGGGGAATAAATCAGCAAATCCTCTTGGAAAA
GCATGGTTACAATTACAGCTTTTGTGAATTTCCCACTTTGTGTTGACTCAATGACAACCAATGCATGGATTCA
ATCAAGACATCTTCCACCCTTGAAATGCCCACTGTTGAAATCCGTGGGCATGCTTAGAGTAGGCATTCACATT
TTAGCGTGGAAGACTGGGGTCGCTCTGAGCTTTCTTTCTTTCCAAGCAGATGGGGGTGGAACTTGGCTCTTTC
TTCCGTCCTTTCGAGTGTACTAAAATATTTTGAACCATTGTGGTCTTCGGGGGGCCAAATATATTCCGACCGA
CTGTGCAGTGAACCAGAGCAAGTGGTCTTAGTTTTATGAAACCAGTCTCTAGCTACCCTGCCATTTCTCGTCA
TTCATATGAATTGTCCCCTTCGAATCCAAGTGAACCCTGTATATTTCGTTTCGTTTTGGATGACACAGTAGAG
GCAGCAGAAGAAGAACAGTAGCATGGCTTGATGGCACAAACAATACCGAAACCTCCATCCAACCGAAATAGTA
ATTTTTCCCTATATTCTTGTTAACCAGACATGAACAAAGACTGTTTAATTTACCTAACAAAACCCTTGAGGCT
GATCTTCTTCTTCGTTACCCGCTTGGGGGCCGCATACCCGCTCATGTCCGCCCGCACGGCCGACTCTAAAACT
GCTCTACTCTCGGGGGAGATGGCAACCATGGTTGATTTGAACAACATGGGGGCCGTTCGAACAACGTGAACGA
AGACCTGTGCACACATTCTCCCAATAGTTTCGTCTGCCCCCTTTTTGCCACTGGGATGGTTTGGCAACCCATT
GAATGAAATGCTCTCGACGAGAATTTCAAACAGTGCTGATATGAAAGAGACACTTTTGTCTTCTTCTTTTGAC
GCAGAAATCAGGTATTGGAATGCCATGAGATTCACTTTTACTGACTCAGCACATACAGTAACTCGATTTTCGT
CAAAGCCTGGCCCAGATAAAACTCGAACTCCATGAGCTCTCAATAACTGAAGGATCTCAAACCCGAGAGATTG
CAGCACATAATTGAGTACAGATGAGTCTTCGGCAATAGCAGAATGCAACGCAGATCGCAAGGCAACAAGGGCG
GCAGCATGACGAGGGCCTGACTGAAGATCTTGTAGCTGTATCTTGATTGCATCAATGCTACACTTGATCCCAT
ATCCTGAAGGGAGCGACAAGCATAGAACAGCCCAAGCATCCCACAATGCATGAGTCGCAGAATAATTCGCAAT
TTGTCCCCATTCTTCATGTGACAAGGTTGTCTCTTGACAGCAGGCTTGAAGGAGTGACAAACATGCAGCCTTT
ATTTCTTCTTCTGGCTCATCTATCAGCAGTGTCAAGACTAGTTGTACCAAGGCTTTCTCGAGTTCTGCGCGGC
CTTCTTCTGGGTGAGATTGAAGTAATGATTGGCTGGATCTGATGCATGAAGAAATGATGATTCCGTGATTGGG
CTCCATTTCCCGCTTTTCTTGCAAAGTTACAAGAGGGGTCACAACTGATCGAGTAAGTATTGCCGCATCTACC
CCTATGGCATAACATTCTTTGCACAACTGTGCGATCAAACCACACGACTGTTGTACCAGGATTTCATCGTCCT
GAGACAAAGATCGTTTGCTTCCCAGAACACGAAATATGACAGATTCAGTCACGATGTTGGCAACATCGCCAAG
TTCATTTGGACATAATCCCTCCACTACGCACTCGTTGTCCCTCACAAGAAGGCGGACGGCATAAACGCACAAC
GCTGCAGTTTGTGAGGCAGATGGATGTTCTGAGGAAGGTTCCTTGTCACCGAGGTGAAGGCTAGAAAGTGAAT
TGCGTAACCCAGTAACCATCAAGGGAGTAAGCTTGCTGAGCCACACCTGCAAAGACTTCGTTTCTTCACTTTC
ACTGTCTCCAGCTTTTATTGCCTTAATAATCGACGAAATAGCAGAGGCGCTAAGCATAGGCCAATTCTCGATC
AGCGCTTCTTGAACAGATCCATCCAAATCAGTCGTATTCTTGTACGTCAAACCGGGACGACTAATGGTATTAT
TAGACTGACTCTGTTGAGAATCTTGCAGTAAGAAACCGTCAATAGCGGCGGCGGCGCAGTGAATCGCCCTCCC
CGTTTCTTCCTTTTCTAGCTCACTGGCAACTATGGAGAGTTTGGACTGTTCGATATGATTTTCTCCCAGTGCA
ATCAGCATGGATACCTTGGACAAAAAGCACAGCTTCGACAGTCGAAAGAGGGGGTAGGAGCGTGAATCATCTG
TTATATGAGAAGAACTCATCCAAAGGGCACTCTCATTTTCATCTCCTGTCGGAAATGGAACAAAAGGAGTTTC
TGTGGCACCCAATAGCACTGGTTGCAATAGACGCCTCATAGCAATGGGGTCAGAAATCAGCTTTTCAGATATC
ATCACAAATAGTGCCTCACACCCTGTTGAAAACAGGCGATGAAACGCAGTTCCTGGGACGGACTCATTGAGTA
AACTCTCAGAATTTAGTGCATGTTTCACTGCCGAAACTATCTGTGACGAGTATTGTTCGAGCACCGATGTGCC
ATCATTTATGGTCGCATCCAATTGGCTACCAAAGGCGCGAAACAAAGAAATCAAAAGCCGAAGCCCAGATATC
TGAACTGAGGGAAGCTCAGAGTGGTTTGATGTTGATGTGGATGTGCTGCATGCTGTTGTAACCAGTTCTTCTA
AGTGAAAAATTGGTTGTGAGTGTAGTTCAACACCGTATTCTTTGTTCTCTTTCCTTAACATAGCAGTGCATCG
TGACTGGGCAGATTTCGAGTTGAAGAGGAATCCACCACCGTCAGTTCCATCGTCCATAGAGAGAAGCATCATC
ATTGCGACTGATGCAACATTAGCCGATACACATCTCAGTTGCCACCGGGGTGGATTTGAATATTGCAGCGCGT
CCGAAGCACAAAGCCGCGCGATATACCTTGTTCTTTCGATGAGACCTGGAACCGAACCGTCGGAATCAACATC

ATTAAGAGTTTTGGCTCCATCTCCAGAAGCCAGACACCTGGAGAACAGTAACCATTGAATGATAATGAAAGAT
TTTTCATTCTCATCTCTCCCCATGATTTCTGAGCTGAGAATTGCTTGGATCAGGGAAATCACTTCAGACTCTA
ACATCTGACAATCTTCAAAGAAGTTAACTACTGCGCGAGAGAGAGCTGTAGTGCCACGAGAAATAAATTGGAA
TGGTGAAACAAGAAAAGTTAGCGAATGAAAAACGCACCAACGTTGATAAGTTACGATGATCATCACCTAATGA
GCGAAAGTCCGAAAAGTGTTGGAAACGTCGACGCCCGATTCTGTCATGTAGAAATGAGAATAAGGCCTTCCCT
ACGCCAAACGTGAATGTATCGTCGGGACTTTTTGACGATGCACACATCTCTTTCATGCACAAGATAGCTGAGC
GCTGCGCATCAATAGGGCCTTGACAGTTGGAAAAGTCTAGCTCACTATCGGCCACAACATTTGGATAAGGCGG
CTGGAGTACAGAAATCAAGAACGGCGTTGCTGTGGCTGAAAGGTTGTGGGTTGTGACAACTGAACAAGAGTTC
GGCGTAATAAGGTCCCTATGTGCACTCAAACGCTCGAAGAAAACAGAGCCTTCAAACAGAATGACTGGATGAT
TTGAGCTGTATTCCAAAATTGTGGATGTAATGCATGAGAATCGACTAGCCAACCAACGGTCCAAGGACTTCAG
CTCCCTGAGCCACGGGAGTAGCGTCGTCATGAGTCGTAGAATGACTGCAAGAAATGCAGGCCCTGACGCAAAC
TTCTCACGCTCTCGACCTCTGCAGGTGTCAGAAACAACCGCGCTCCATGCACCGAGGAAACTTGACTCAACAG
CTTCAGCTGCTTTCCATATGATTTGAACTTGGTCAGGTGTTAGGACTGTGTCCGTCGATATAGAATTTGATAT
CAAAACACCGAACGAGTGCAAAGCATATGTTCTGGCGAGGAGAGAATCCCCTTCAGGGTTTTGGGTGGCAAGG
GAAGGCAAAACTCGAGTCATCATAATCATAACTGGTGTTCTGTCAATCTCATTCTTATCTGACTCTCGTGAAA
CTGATCTTGCGATCGCTCGTTCTTCTTCATTTTTCTTCATTCTTTTCGCTGCTCGTTGAATGCATGCCAAGGC
AAGTAACGTGCCTGCTTTCGCAAATGCGAGAGTTTCAACGTGATTTTTTGGGTTTGCCATGACGTTGCTCCCT
TTCATAACTTCATCAAGAGAATGAAGGATGGTGCTCTGCAGCGTGTGTGCATCTTCGCTTACTCCAAGTGTTG
CTAAAAGAGAAAGGCCTTCAGCAGCACCACGCCGAATAATGCCTGATGGCGATGGAAGGAGGCGCAACAACAG
AGAGGTTGCCCTCTCCATCCAGGGAGGCCCTCTATCGATAAGAGTGTCGTACGTCGATTCGTGTAAGGGAAGC
GCCTGAAGACAAGCCAAGACAGTGGCAATGACATTTGAAGCTGGCACATCTTCCTCTTGCGGCTTCACTTTCC
CTTGACTTTCACTAATAAGGGAGGTACTGACATTAAATCGATTAGACTTCTCAGTTTGTGTCAATAGATAGAG
ATTTTCAAGCATCTTCAAGGCGTCAGACTGCGTGTGGCCATCCTGAAGGCCGAAGGTCGCCGCGAAGACATGT
ATACTGGAGTCAAGAAGTCTCACTTTGGAAGTGCCCGTTGGGTTTAGAGGAACTCGCCAAGTGCCTACCTCGT
GAAGTGGCGTTGGAATTGAGTTCTCGTCCCCTTCACGTAGATATAAGCTGAGAATATCATCATGGAAACGCAT
GCGAAACTTCTTTCTCCATGCAAGAAGGTGTAAGACGGCCTCCCTTTCATTGTGATGAACTATGTCTGATGAA
CGGATAGATATGTTATTGTCTAGTGTAACTGAGCCACCAACCTGGCCCGGACCAACAGCGCGTTCGATTGAAT
GAGCTTCTATGAGTTTGTCTTCACGGCTCACAAGGCCATCAAGAATAGAACACAGGACATCGTCTGCACTCAA
ATCCTGGATCTGGGCCGCGGCGAATGAAAATATCCTATCTGCAGACAACGGAAAAGACCCTGGTGGCAACCAA
GAATAGGCCTCCATGACAGAAGATCTTGTGCTGGAGAGCCGAGCGCTACCAACAGGATCGTTTCCTTCTCGCT
CAAATCTACCCCCACTCGATATTAGTGGGAATACTTTTTCGAGAAGCGCCGTCAGGCGGG
>TR275|c0_g1_i4 len=6098 path=[6964:0-87 6965:88-111 7020:112-177 7023:178-194 7009:195-428 6951:429-452 7019:453-555 7008:556-985 7000:986-1009 7022:1010-1214 6954:1215-1248 @7018@!:1249-1534 7013:1535-1600 6989:1601-1624 7006:1625-3003 6957:3004-3027 7012:3028-3123 6995:3124-3147 7005:3148-3482 6992:3483-3506 7021:3507-4134 6970:4135-4155 7004:4156-5198 6979:5199-5222 6980:5223-6097] [-1, 6964, 6965, 7020, 7023, 7009, 6951, 7019, 7008, 7000, 7022, 6954, 7018, 7013, 6989, 7006, 6957, 7012, 6995, 7005, 6992, 7021, 6970, 7004, 6979, 6980, -2]
TTGCCTGTTGCACCCACCGCCCACGCCCACCCCACGCAACCCCACCCCACCTTGCTTGCACGTGGACAACCTC
TTACCTCCGACGCCAATAACAACGTCCACCATGAGCATGTCCGCCTTCCGCCGATCGGCCCAGCGCCTATCCA
GTGTCAACTGGTCCGCCACAGTCTTCAAGGGAGACCCCGAACTCTCCGCCATGGTCGCCGGCTTCCGCGCCTG
GACGGCCCAAGCCGACGCCATGGCCGAGAAGTACTCAGCCCCTCCCGCCCCCATCGACTTCGCCACGGCCAAA
TCCTCCGTCCGCGACGTCGCCCTCGTCGACGCCCTCGAATCCATGTACAAATCGTCCAGCCCTCCCGCCATCA
CTTACGAGTGGAGCGAGGAGGAGCAGGCAAAGAAGGCGGAGTTGATCAAGGGAGCCGAGGCGGGTTTGGCGTT
CAACCAGGAGATGATCGATGATACGGAGAAGGAGATTGCGTTGCTTCGCACCAACAGGCTGTCTCACGATAGT
TCCCCGAGTCTTATGGCTGAGGTTTATCCTGATATTGCGGAGGAGATTGAGGATGAGATTGAGAACAGAGAGT
GGTTTAAGGATACTTTGAATAAGTAGAGGTTGAATAGTCTCTGACTTTAGGTTAGGGGGTTGGGTGCTTTGCG
GAATGTTGTTGGAGTGAATGGGTATTAGTTAGAGTGCATTTGGATCGTGTGGGGAGGTTGAGTAATTCCCAAT
GAACCCTCATTGGAATTCGACAAACATCCAACCTACTGAGTCATGCTACAGAATTGCATCTAATGGATACTAA
CAGTGACATTGTCAATGAAATCATCGGCATGTCAATGGGGGGTCATGATTATCTGTCATTTTGGCAAAACAAT
AATAGATTTGGGTATCTAAATATCCCACTCCTGTTATCTGACGGGGAATAAATCAGCAAATCCTCTTGGAAAA
GCATGGTTACAATTACAGCTTTTGTGAATTTCCCACTTTGTGTTGACTCAATGACAACCAATGCATGGATTCA
ATCAAGACATCTTCCACCCTTGAAATGCCCACTGTTGAAATCCGTGGGCATGCTTAGAGTAGGCATTCACATT
TTAGCGTGGAAGACTGGGGTCGCTCTGAGCTTTCTTTCTTTCCAAGCAGATGGGGGTGGAACTTGGCTCTTTC
TTCCGTCCTTTCGAGTGTACTAAAATATTTTGAACCATTGTGGTCTTTGGGGGGCCAGATATATTCCGACCGA
CTGTGCAGTGAACCAGAGCAAGTGGTCTTAGTTTTATGAAACCAGTCTCTAGCTACCCTGCCATTTCTCGTCA
TTCATATGAATTGTCCCCTTCGAATCCAAGTGAACCCTGTATATTTCGTTTCGTTTTGGATGACACAGTAGAG
GCAGCAGAAGAAGAACAGTAGCATGGCTTGATGGCACAAACAATACCGAAACCTCCATCCAACCGAAATAGTA
ATTTTTCCCTATATTCTTGTTAACCAGACATGAACAAAGACTGTTTAATTTACCTAACAAAACCCTTGAGGCT

GATCTTCTTCTTCGTTACCCGCTTGGGGGCCGCATACCCGCTCATGTCCGCCCGCACGGCCGACTCTAAAACT
GCTCTACTCTCGGGGGAGATGGCAACCATGGTTGATTTGAACAACATGGGGGCCGTTCGAACAACGTGAACGA
AGACCTGTGCACACATTCTCCCAATAGTTTCGTCTGCCCCCTTTTTGCCACTGGGATGGTTTGGCAACCCATT
GAATGAAATGCTCTCGACGAGAATTTCAAACAGTGCTGATATGAAAGAGACACTTTTGTCTTCTTCTTTTGAC
GCAGAAATCAGGTATTGGAATGCCATGAGATTCACTTTTACTGACTCAGCACATACAGTAACTCGATTTTCGT
CAAAGCCTGGCCCAGATAAAACTCGAACTCCATGAGCTCTCAATAACTGAAGGATCTCAAACCCGAGAGATTG
CAGCACATAATTGAGTACAGATGAGTCTTCGGCAATAGCAGAATGCAACGCAGATCGCAAGGCAACAAGGGCG
GCAGCATGACGAGGGCCTGACTGAAGATCTTGTAGCTGTATCTTGATTGCATCAATGCTACACTTGATCCCAT
ATCCTGAAGGGAGCGACAAGCATAGAACAGCCCAAGCATCCCACAATGCATGAGTCGCAGAATAATTCGCAAT
TTGTCCCCATTCTTCATGTGACAAGGTTGTCTCTTGACAGCAGGCTTGAAGGAGTGACAAACATGCAGCCTTT
ATTTCTTCTTCTGGCTCATCTATCAGCAGTGTCAAGACTAGTTGTACCAAGGCTTTCTCGAGTTCTGCGCGGC
CTTCTTCTGGGTGAGATTGAAGTAATGATTGGCTGGATCTGATGCATGAAGAAATGATGATTCCGTGATTGGG
CTCCATTTCCCGCTTTTCTTGCAAAGTTACAAGAGGGGTCACAACTGATCGAGTAAGTATTGCCGCATCTACC
CCTATGGCATAACATTCTTTGCACAACTGTGCGATCAAACCACACGACTGTTGTACCAGGATTTCATCGTCCT
GAGACAAAGATCGTTTGCTTCCCAGAACACGAAATATGACAGATTCAGTCACGATGTTGGCAACATCGCCAAG
TTCATTTGGACATAATCCCTCCACTACGCACTCGTTGTCCCTCACAAGAAGGCGGACGGCATAAACGCACAAC
GCTGCAGTTTGTGAGGCAGATGGATGTTCTGAGGAAGGTTCCTTGTCACCGAGGTGAAGGCTAGAAAGTGAAT
TGCGTAACCCAGTAACCATCAAGGGAGTAAGCTTGCTGAGCCACACCTGCAAAGACTTCGTTTCTTCACTTTC
ACTGTCTCCAGCTTTTATTGCCTTAATAATCGACGAAATAGCAGAGGCGCTAAGCATAGGCCAATTCTCGATC
AGCGCTTCTTGAACAGATCCATCCAAATCAGTCGTATTCTTGTACGTCAAACCGGGACGACTAATGGTATTAT
TAGACTGACTCTGTTGAGAATCTTGCAGTAAGAAACCGTCAATAGCGGCGGCGGCGCAGTGAATCGCCCTCCC
CGTTTCTTCCTTTTCTAGCTCACTGGCAACTATGGAGAGTTTGGACTGTTCGATATGATTTTCTCCCAGTGCA
ATCAGCATGGATACCTTGGACAAAAAGCACAGCTTCGACAGTCGAAAGAGGGGGTAGGAGCGTGAATCATCTG
TTATATGAGAAGAACTCATCCAAAGGGCACTCTCATTTTCATCTCCTGTCGGAAATGGAACAAAAGGAGTTTC
TGTGGCACCCAATAGCACTGGTTGCAATAGACGCCTCATAGCAATGGGGTCAGAAATCAGCTTTTCAGATATC
ATCACAAATAGTGCCTCACACCCTGTTGAAAACAGGCGATGAAACGCAGTTCCTGGGACGGACTCATTGAGTA
AACTCTCAGAATTTAGTGCATGTTTCACTGCCGAAACTATCTGTGACGAGTATTGTTCGAGCACCGATGTGCC
ATCATTTATGGTCGCATCCAATTGGCTACCAAAGGCGCGAAACAAAGAAATCAAAAGCCGAAGCCCAGATATC
TGAACTGAGGGAAGCTCAGAGTGGTTTGATGTTGATGTGGATGTGCTGCATGCTGTTGTAACCAGTTCTTCTA
AGTGAAAAATTGGTTGTGAGTGTAGTTCAACACCGTATTCTTTGTTCTCTTTCCTTAACATAGCAGTGCATCG
TGACTGGGCAGATTTCGAGTTGAAGAGGAATCCACCACCGTCAGTTCCATCGTCCATAGAGAGAAGCATCATC
ATTGCGACTGATGCAACATTAGCCGATACACATCTCAGTTGCCACCGGGGTGGATTTGAATATTGCAGCGCGT
CCGAAGCACAAAGCCGCGCGATATACCTTGTTCTTTCGATGAGACCTGGAACCGAACCGTCGGAATCAACATC
ATTAAGAGTTTTGGCTCCATCTCCAGAAGCCAGACACCTGGAGAACAGTAACCATTGAATGATAATGAAAGAT
TTTTCATTCTCATCTCTCCCCATGATTTCTGAGCTGAGAATTGCTTGGATCAGGGAAATCACTTCAGACTCTA
ACATCTGACAATCTTCAAAGAAGTTAACTACTGCGCGAGAGAGAGCTAATGAGCGAAAGTCCGAAAAGTGTTG
GAAACGTCGACGCCCGATTCTGTCATGTAGAAATGAGAATAAGGCCTTCCCTACGCCAAACGTGAATGTATCG
TCGGGACTTTTTGACGATGCACACATCTCTTTCATGCACAAGATAGCTGAGCGCTGCGCATCAATAGGGCCTT
GACAGTTGGAAAAGTCTAGCTCACTATCGGCCACAACATTTGGATAAGGCGGCTGGAGTACAGAAATCAAGAA
CGGCGTTGCTGTGGCTGAAAGGTTGTGGGTTGTGACAACTGAACAAGAGTTCGGCGTAATAAGGTCCCTATGT
GCACTCAAACGCTCGAAGAAAACAGAGCCTTCAAACAGAATGACTGGATGATTTGAGCTGTATTCCAAAATTG
TGGATGTAATGCATGAGAATCGACTAGCCAACCAACGGTCCAAGGACTTCAGCTCCCTGAGCCACGGGAGTAG
CGTCGTCATGAGTCGTAGAATGACTGCAAGAAATGCAGGCCCTGACGCAAACTTCTCACGCTCTCGACCTCTG
CAGGTGTCAGAAACAACCGCGCTCCATGCACCGAGGAAACTTGACTCAACAGCTTCAGCTGCTTTCCATATGA
TTTGAACTTGGTCAGGTGTTAGGACTGTGTCCGTCGATATAGAATTTGATATCAAAACACCGAACGAGTGCAA
AGCATATGTTCTGGCGAGGAGAGAATCCCCTTCAGGGTTTTGGGTGGCAAGGGAAGGCAAAACTCGAGTCATC
ATAATCATAACTGGTGTTCTGTCAATCTCATTCTTATCTGACTCTCGTGAAACTGATCTTGCGATCGCTCGTT
CTTCTTCATTTTTCTTCATTCTTTTCGCTGCTCGTTGAATGCATGCCAAGGCAAGTAACGTGCCTGCTTTCGC
AAATGCGAGAGTTTCAACGTGATTTTTTGGGTTTGCCATGACGTTGCTCCCTTTCATAACTTCATCAAGAGAA
TGAAGGATGGTGCTCTGCAGCGTGTGTGCATCTTCGCTTACTCCAAGTGTTGCTAAAAGAGAAAGGCCTTCAG
CAGCACCACGCCGAATAATGCCTGATGGCGATGGAAGGAGGCGCAACAACAGAGAGGTTGCCCTCTCCATCCA
GGGAGGCCCTCTATCGATAAGAGTGTCGTACGTCGATTCGTGTAAGGGAAGCGCCTGAAGACAAGCCAAGACA
GTGGCAATGACATTTGAAGCTGGCACATCTTCCTCTTGCGGCTTCACTTTCCCTTGACTTTCACTAATAAGGG
AGGTACTGACATTAAATCGATTAGACTTCTCAGTTTGTGTCAATAGATAGAGATTTTCAAGCATCTTCAAGGC
GTCAGACTGCGTGTGGCCATCCTGAAGGCCGAAGGTCGCCGCGAAGACATGTATACTGGAGTCAAGAAGTCTC
ACTTTGGAAGTGCCCGTTGGGTTTAGAGGAACTCGCCAAGTGCCTACCTCGTGAAGTGGCGTTGGAATTGAGT
TCTCGTCCCCTTCACGTAGATATAAGCTGAGAATATCATCATGGAAACGCATGCGAAACTTCTTTCTCCATGC
AAGAAGGTGTAAGACGGCCTCCCTTTCATTGTGATGAACTATGTCTGATGAACGGATAGATATGTTATTGTCT
AGTGTAACTGAGCCACCAACCTGGCCCGGACCAACAGCGCGTTCGATTGAATGAGCTTCTATGAGTTTGTCTT
CACGGCTCACAAGGCCATCAAGAATAGAACACAGGACATCGTCTGCACTCAAATCCTGGATCTGGGCCGCGGC

GAATGAAAATATCCTATCTGCAGACAACGGAAAAGACCCTGGTGGCAACCAAGAATAGGCCTCCATGACAGAA
GATCTTGTGCTGGAGAGCCGAGCGCTACCAACAGGATCGTTTCCTTCTCGCTCAAATCTACCCCCACTCGATA
TTAGTGGGAATACTTTTTCGAGAAGCGCCGTCAGGCGGG
>TR277|c0_g1_i1 len=302 path=[280:0-301] [-1, 280, -2]
GCGTTTGGACCTGGTGTAGTTTCGAACGATGTCAGGATATGTATGAGACACGAGTGATGGTTTCATTTGTCCA
AATGGAAATTAAGGCGACAGTCGAGCGATGACACATGAAACCAAACTTCAATGTGCAAATCATTGCACGCTAG
CCATCGATTGTGACGCTGTTTGACGCATGGGAGCGCGTGTGGTTTCTCGTAGAAAGCAGACCCCTGGCAGTCA
CTCCAGCTAAAGTGCCGAGAGCACGGCAAAAGATGGAAATAAAACTACTCACAATATCCAATGCATCGTGACA
CTTCTTCTGC
>TR279|c0_g1_i1 len=890 path=[868:0-889] [-1, 868, -2]
GCGCTGCGCAGCCTTCGCGGGGTCGGAGGCGCGCATCCGCCGAATCCGGCCGGTTGGAAGGGAGCTTTCGTCG
GTTCCCACGAAGCGTTCTCCTCGGGCGCTTCCACTTCGCTGGCGGACAGCACGTCTCCCCGGAGGAGCTTTTG
ACGAAACCAAGCCAATCCCTCTGCTTCGGTTACCTTGGGACATTTCGCCCATTGCCCCGTTTCTCGTTTTGAT
TGGGTTACAGATTCTACTCCTTCTGAAATGCCATCCTGATGCTGCAACTCGTCCAAAGCAGACTTGGCCGACG
CAATGCACAGTCGATACCTCATTATAGCCTCCTGTACCCGATTGGAACGCGCTAATACCGTACCAATGTCGTT
TTGCAGTGCTGCTATGGACATGGTCGTAAATGACGAAAACGGATGATACAATTCATTGCTGGGCATCGGCTGG
AGTGCAAAGGAATTATTTTTGGGTGAGGAATCGTAGTCGTTGGGTGAATAATGATGGAAGGAACGAGAACGTT
GCCTCGGTGGATGTTGCTGCTGACTGTGTATCAAGAGGGGCTGTATCGTAAGCGAAGTGGTCGGCTCGGCAAT
GCTGTCTCCCTCACCCGACGATAACGTTTCCACCTCCGTGTCTATGTAACTATCATCCGCATTCACCTCCACC
GAACGAGTGCCGTTGCACAGTGGCAAAGGAGGGATTCCCACAATGGGATCGAACGAATCGGACTCCGTTTTTT
CCGTTATCGCTACCGTAGTATCTGCCGATGAAATAATTGCCGACGTTGATGGTGATGACGCCGTTGTTGTTGT
GACATCCCTCACGCTGTTCCCGGTAACCTTGGCCGACCACGACGCCTCCCTTCCCTCGACGGTCGGTCGCTTG
GGAGATCGGAAGAG
>TR280|c0_g1_i1 len=881 path=[859:0-880] [-1, 859, -2]
CGCACTCGTCGATGTTGTCGATCACCACGGTGCGCTTCGCCCTCACGAAGTCGGGAGACGTCCAGGGAGTGGC
CACCGTGTAATTCACGTGGAATTCGCCGACTTCCAAGAGGCATCCCTGCGGCAGCGGTCGACTGTAGGCAATC
TTCAGACTGCGGTAGTATTCCTCGGCGTTGTCGTCGATGACGTCGACGCCGTATTCCTCGTAGCGATCTCCTT
GGGAGAGGTGGGTGGTGCCCGTTTTGGGATCGGTGCCGTGGGTGGGATCGGGACGGAGGTGGAGTAGAGGTGG
AGATTCGTCGACGCAACCGTAGTCGGGATCGTGGACGTAGCCGTTTTGGCAGGTGCAGCCGGGGGTGTTGGAG
GAGGATACAGCGCACATTTCGTGTTTTCCAGGACACTTAGGAAAACCATCACAAGCATCCACCGTAGCCTCCA
CGCATCCGCAAATCAATCCCTTCTCCAACGCCCTCCTCGTCTCCTCGGTAGGCGTCTTCCCATCGTACTTGAC
TTTCGGTTCCACCTTCCTCCCGTTCCTCGTACACGGTTTTCCATTCCCCATGAGGCCGGAGGGACACTGGCAC
GTGTAATTGGGACGATGGAGGGGAGATGTCGCCCGTTGGCATATCGCATTGGACGCACACGCCGACGATGCTG
CCGATCTGGTTTTGGAAGCGTTGTTGTTGTTTTTCGTCGTCGTCGACGTTCGGGCACACGGATCCACCGGACA
GCGGAACGACGACCTGCAACCCTCGCCATCGGCGGAATGTCCGTCCTCGTCGCAACATCCGCGAGTCGACGGA
GAATCGGGACAGGAACTGTCGGACGAACTCCCCAGCGACAATTCCCACGCGTTTCGCGATTGGGAGTCGAGCG
TTTGC
>TR282|c0_g1_i1 len=342 path=[320:0-341] [-1, 320, -2]
GATCTGCTGCTGCTGCTGCTGCTGGCGACGTCATCGGCACCAACGGAGCAGGTGCCTCCATCAACGGCACCCC
GCACTCCTCCCACGAAATCAAGTGCCTCTTATCCTCCTTGATTTCCACCTGAAACGACAGCGCCAACGTGTCC
GGATGCCCAACGCTCTTCACGCAGTGCACCCAAAACTTGGGCAAAAATATCACAGCTCCGGGGTACATCATCA
CAGTCTCCCACGTGAATCCCCCATGCATTTTCTCGTCCTCACTGAACGGATCTATCCCGTGCAGTATCCCGTC
CTCGGGTCGATCCACCGGTCGCATCGGCCGCGCGATCTTCACCTCCTTGA
>TR284|c0_g1_i1 len=831 path=[809:0-830] [-1, 809, -2]
TCTCTTTTCTGGTTTGGCGCGTATTGTTGTTGTTGTTGCTTTTGTTGTTATTTCACTGTTCGTGGACTCGGAT
GACTGCTGTTGCTGTGACTGTTGTTGTGTTACATTTTGCAATTCAGATACCTCTCCTGCCTTACTCTTCTGTC
CATTTTCATTCACCTGATTTATGGAACAAGAGGACTGTACCCGAGAAAATAATACATGTTTCCTTTCTCTCCA
AAATAAGCGCGCGAGACATGCGATGCTCAGAGGTAAGTAGAACGAAAGCATCGAGAATGTGGGGGCAGATAAA
TGACAGTCTAGGTATAATGCTGATTGTATGGCTCCGGTGCCTGCCAACATCAGTGAAAGAAATTGACCAAAGA
CCAATGCTTTTGATTTTCCCAAGATAAAATTACAAATCATTTCGCGCATCGATGCATAATGCCTTTCGTCGTT
TTCGAAAGAAGGCGACGTTACCCCGTCGACATGCGTTGGCGATGAACTCATGGTAGGCTCGTCGTCATCCTCA
TTGGTTAGGTCTTCCTTTGAAAGCGAAAGTGTCAAATTGTTCTCCCTAGTGGTCGGCGGGAGTGATGATGTTG
GTGATTTGCGTCGTCGTGTAAAAGCATGTGGCGATGGTGAAGCCGATGTCGCTGTCGATAAGATGGGAGAGAG
CGACGCTTCAGGTGCGTCGGATTCACTTCCTTGGTATGGCGTTGACGACATCATGATTTATTGCCGTCGGAGC
GAGATCGGTTTGGGCATGGTACAGTACGATAAAATAAATACGGGTCCTTGTCAAAGAATGTCATTGCCAAATC
CACAAAGGATAAATAAATAGATCGGAAG
>TR285|c0_g1_i1 len=414 path=[392:0-413] [-1, 392, -2]
CTTCCGATCTTAACGACCCCAAGCTCACCCTCGACGATACGCGCGACCATATCAAGATGCTCGGTAATGCAAC
TGCCTTCGCGGATGCGGCCACCGCCGCCAGGCAGCGCGAGAGGAAGGAGAGCGGGGAATTGGATGATTCTACT
ACGCCTGCGCCCCCCCCTGAACCTATCGAAGGGGACACCAAACGCAGGAGAGAGGACGGTAGGAAGTAGAAAA

CAGAGTTTTGACACGGATGAAATCTTGTCACTGTAGTCGTAGCATTGTCATTGCTTAGTTTTATATGGCTCGC
AAGCCCATGTTACTCTTTTGGAAACGAGGAACCCTAGCATAACCTCAAGCTTATCTCGGTTAGTTTTGATCAA
TGTGGCAATTAGGGGCCTTAAATTAACTTTATGTTTTTCTTCTCAAAAA
>TR286|c0_g1_i1 len=326 path=[304:0-325] [-1, 304, -2]
AGGGACGATGATGGCAACAATGATCGACGAAGCCTCCTCCCAGTATTGAATTCCTTTGCCAAAGCCAGAAACA
CGAATTCAGCTTCGATAGGCTTTTTAGGTTTCTTGGCGATGAGTAGTTTGGCATGTCTATCATCCAGAGCAGT
GGCGTTGACGTTACATGAACCATTTCATCATCGCGCGAGATTAGGCAAGCATTCAACAATGATAGATTCAACG
GTGCCGCGGTATTGCAGAATTCACTCGGCAAGAGCAACTAGCAACACTGGCATTAGAATGATACTCACAGCGG
AAGAGACTGGAAACAGCAATGGAACCAACGGAAA
>TR287|c0_g1_i1 len=266 path=[244:0-265] [-1, 244, -2]
AACAACAACTGTGCCTTGCTCCAACCAATCATTTAAAACTCTCAAGAATTCCATCTCTTTGTGGAACGAAAGT
ACAAATGCTTTCTCCAACGTTCTGAAGACGCAATAATGAAATCCCTCCTACAAGAATTGGCCACAAAGCAAAA
CAAACGTAAACAAATCAAATTTGTGCTGACCAATGCCAGATACGGTGATGTGCCTGAAATCATATGGCAGGAA
CGTCTCAGTCAAGAACGCGACGTGGAAAGATTGCACGGGGAGGAATG
>TR288|c0_g1_i1 len=239 path=[217:0-238] [-1, 217, -2]
ATCCTTGATTTTCGCAGGCGACTCCTCCGTTTATTACATTGTTGTTGATGGTGTTGTTCTCGTCGTCATCGTT
CGTCTCACATGTGATGATGCCGATTGTGGAGAGTAGAATACTGATGGTGATGAGCACCGCCGGCGGCACAGGA
AGATGCCCCATTACACTCATAAGTACTTACAATTAGAGGAGTAAGTAGTTTGTACTGTAGTATCGTACTTAGC
GGACAAAGCGATCGGACAGA
>TR289|c0_g1_i1 len=1431 path=[1434:0-986 1435:987-1010 1436:1011-1430]
[-1, 1434, 1435, 1436, -2]
AGTGCAAGGAGCTAGAATCTAAGCTTGAACATGACCGCTGTGCGTTGAAGGTTCAATCAGAACAAAACACAAT
TTTGGAGAAGGAGCTTGACCAGTTGCGTCAACAAGCACACAGTGTACAGGGTAAAATGAAGGCGGCATTTGAC
GCTGATAGGAAGGCCCAGGATAAATCAGTTGAAGAGGTTATCATAGCCAAGGAAGAAGCTTTGTCACGACAGG
AGGAATTAGGGCAAAGAGTAGATGCACTTCTAAAAGAAATTGCCAATCTGAATGAGCGGATAGCTGTTCTTCA
AGGCGCGACTGACGAATCCAGACAAGAATGCCAGGATGCTACATCAAAGTACGAAGATGTCGCCTCAAAATTT
GAAGAATGTCAGGGACAGCTGGCTTCCCTTCTCGAAAAGTCAGACGCATCTAAAAACATAGTAGAAGAGTTGA
CGAATCACTACATGAAGATTGAAGACGACCTTAGAGAACAGCTTCAATTGGTTCAGTGCGAAAAAGATGATTT
GGATCAAAAACTCAATGCTGCGCAGAGGGAATTAGAGAGTAAAATGGCCGATTCGATCCAAGAGATTGAAAGG
CTAACAGAAGAACTGAAGAGGAACAAAATTGAGTTACATCAGTTTGATTCTTTATCCAGCGAAATTGATAACT
TGAAGCAAGGCTTCTACAGGGAAAAGGATGAGTTAATGTCCACCCTTGTGTTGAAAGAAGAAGTGATTCAAGA
GCTTGAAACAAAATCAAATGTACTGGCGAAGGACAAATGCGAGTTGATGTCTGCTCTTGGAATGAAAGAAGAA
TCGATACAGAGGCTGGAGAAAGAAACAGAGGAACTGGAGGAGAAGATAGACCAGGCTCAGACAAAGTGTGACA
CTCTATCAAATGGAATCAGCGACTTAAACAAAGAGAACGCGGAGTTAAAGAAAGAACACGATCAAGTCTATGG
CGAGCGAGGCATTCTTAGTTTGAAGGCAGAGAAGCTGACATTGGCTTTAGCAGAGTTGGAATCATTGAAATCT
GAAAATTCGAAGGAGCTGAATTCAACGATAAATTCTTTACGTGTAAACCATGCTGCTACTGTCGAATCTCTGC
AAAACGACATTCACAGCATTAAGATGGAGCGAGACTCTTTATCACAACAGGTCCATCACTTATCTTCAGCCCT
AGCGGCATCAAAATCATCCCAGGAAGCAAAGGACTTTACTTGTAAAGAGTTGAAGTCTAACTTGGAGGAAGAA
CGTATGGCACATACAGATGCTTTGAATACATTGCAGAATGATATTCGCATTATCACACTGGATAGAGATTCCT
TGACTCGACGTGCAGAAGGCTTGGTTTCGACCCTCGCGGCGTTACAATCATCTCATGATGCTCAAGACGCTGC
AAGTAAGGAGTTGGCATCGACTCTTGAGGAGGAACGCGAAGCGC
>TR290|c0_g1_i1 len=260 path=[238:0-259] [-1, 238, -2]
TGCATGTCGTCATCGTCATCGCCATCGCCATCGCCATCATCATCATCAATATCAACATAGTGATCGTGATCGT
TGTTGTCGTCCTCGACAATGCTGGAGGATGGGGATATAATTCGTAGGAGTTCATGATTGTACGCCATATTGGA
AGAGGAGGACGATGTCGAAGAAGAAGACGACGAGGACGAAGACGAGCTGGCATTATCTTCTCCATTGGACGAA
GAGAGTAGTAGCGAAGATGTTTGTACTATCGGATAGGCATG
>TR291|c0_g1_i1 len=260 path=[301:0-209 302:210-259] [-1, 301, 302, -2]
GGGTCTATCGCTCAGTTTGAACGGGAAATGATGCTGGAACGTCAGCGCGAAGGCATCGCCAAGGCAAAGGCTC
TGGGCAAATACAAGGGGAGAAAGCCCACTGCAATCCTGAAGGCCGATGCAGTTCAAAGCCTCTTGCGTGCTGG
AGAGACGAAACGAGGAATTTCAAAAAAGCTTGGAATTTCAGAGCGCTCAGTTCACCGCATCTGTATAAGAAGG
AGGAAGGAAAAGAACAGGAAAAGTCGACAGGCGAGATCGGA
>TR291|c0_g2_i1 len=273 path=[303:0-209 304:210-272] [-1, 303, 304, -2]
GGGTCTATCGCTCAGTTTGAACGGGAAATGATGCTGGAACGTCAGCGCGAAGGCATCGCCAAGGCAAAGGCTC
TGGGCAAATACAAGGGGAGAAAGCCCACTGCAATCCTGAAGGCCGATGCAGTTCAAAGCCTCTTGCGTGCTGG
AGAGACGAAACGAGGAATTTCAAAAAAGCTTGGAATTTCAGAGCGCTCAGTTCACCGCATCTGTTCGATGAAG
TGAAGTTTGAGCGTCTTTCAAGGGCAAAACCTACAAAGCTGCACACCATCAAGG
>TR292|c0_g1_i1 len=913 path=[908:0-254 909:255-272 910:273-912] [-1,
908, 909, 910, -2]
AAATAACCTAACTTCACCCCATTGCTTCAGTTTATCACTACCTATTCATGTCCGTGGTCCTCCAGTATCACAA
TGCAATCATATTATCAATTATGAGCACATAGAGGGGCATGCACTTCCTATCCAAATCGCCTCGCCATGTCTAC

TTTGTTCCATATACTTCATCCAATCTTCCTCTTGTACTGATGTCCAGCCACAAGTGGCTTTGGGATTGTTTGC
TGCCTTGCGTTGCTGGGTCAGGGCTAAAGCTTTTTTTGTGGTTGTGTTGAAATAGCAATAGAATGGGTGTGTG
ATGGGGCACACGGTCTACCAGTGCCTTCATAATCTCATCTAATTTGATTACTTCATTCTTCCATCCGCAGTTG
CCGTCCGTACTTCGATCTTCACAACGTAAGTTGGCCCAAAGAAGACCAGCTCTTTTAGAGGACTTATCACTCG
ATAGCCATGAAGGAACCGCCATGGACATGGAGTTCAAAATTGACTCACAACTAAGCACATGATGAGCCCCATC
GGAAGCATATTTCGATATCCAATCGTTGGGGCCGATGCAAACACAGGATCCGTCGTTTCTTTCCGCAAAATTT
ACAAACTCCTTACTCTTGCCATTTGCCGCACGATTCACATTTTGGAACACAATAGGAATCGCTTGTCGCCGAG
CGCCCTCCTTCAACCCAAACGAATTGCCACGGCGTGCCCGTCTCAGCGCTAACACCAGGGCGGCATTTGCATC
CTCTGTGCTACAATGGCCACCACATCCCTGCTGAATTTTCTTTTGCAATAATACATTCGATAGATGCCGAAGG
CTAAATTTTCTTCCATTGACCCCGCGAAAGACGACAGAGGTATCGACGACCTTTTTATGGACCAACCGAAGTG
CTCGCAGATCGTTTTCCAATGAATGCCCCACGATGAT
>TR293|c0_g1_i1 len=293 path=[296:0-139 297:140-163 298:164-292] [-1, 296, 297, 298, -2]
ACGGATGGCGGTACGATTCGCAGGGATGATTCGATCATTACGGACGCCTTTCACAGCACCCTCACGCTGGATA
TGGAAGAAACGGATCTTCGTTCGATCTACACCGAAAAATGTGCAGGGGCGAAAACTGCACAGGAGGAAGTCAA
GACACGATCGAGCTGCGATGGCAGTACCATCTCGAAAGAAGAAAAGGAACAAGCCGAAAGTTTGTCGGAAGAA
GAAATGAACGAAGCAGAAGAGAGCAGTTGCCTCATGTCCATGTTCTCATGCTACTTTGGCTTGGGCAAGAGAT
C
>TR294|c0_g1_i1 len=1258 path=[1286:0-435 1287:436-459 1292:460-1186 1290:1187-1210 1291:1211-1257] [-1, 1286, 1287, 1292, 1290, 1291, -2]
TTCTTATTTTATCTGGAGCAATGGCAATCATAACCATATTTCTAACAATAATGAAAACAAAAATTGTCAAGTA
AAAATGCCCACTCTGACTAATTTTTTATTGTTGATATTCTTTGTTTGACATCAAGATCACCTCACCTGGCCCA
CTCCAAACTATTAGATTGCCTATAATACATAATCATGAACGTAGTTTTCTATGATTTGCCCTTGGAGCAACCC
CCCTCCAGCCAATCTGACAAGGAAGGCCTTTCTTTGTACATTACCTGCTGATTATTTTCAAGATATTAGATCC
ATCTCTATCTTGAAAAAGATAATTGAGAATAAGAATGCACGGCATGACACACGGTCAGATATTAAGATAAATC
ATACTGAACAAATACTCATACAAGGCAATCGAAGTACGGATGTTATCAATGATGCCCAAGATAACGAGCTTTG
ACTCCGTTCATGACGAGTGAATTGGTTATAGGCAATCTTCTCCAAGAGAGAAAGGTGGCAAATTGCGAGGCAG
TTGCACGGTTTGCGTCCATCGATATGGGGTAAATCTTCGATGGCATCTGATGTGGAGGCAAATTACTCGTTCC
CTGACTATAATATAAGGCATTTTGTACAAATTCTCTGTGTAGAAGCAAGAAATCGAAGGAGGATAGAGCATAT
GCTCTAACCCCCGAATGACCGGAATTCTTTCATTTTGCCATTAAATTTATGATGATTTAATGATTGTCAAAAA
TTACAACACCAAATACAGTTCATCTTTATTTCCCCCCATCTTAGTACTTGGCGAGGTCGACTCCGAACTTCTT
GGCGGCGGCATCAATTCCTCCGTATTTATTGACTGTCTTGATTCCCTTCGTGCTCAATCGGAGGCGCACCCGG
CGTCCCAAATCATCCGAGTAAAACCTCTTCCACTGCAAATTGACCTGCTGGACCTTGTGAATACGCTTGTGCG
AGAATGTAACGACGCGGGCCTTGCGGTTGGCGGTCTTGCCGAGGAGGTCGCACTTTCGCTCCCTCATGACGAG
GGTGGAGCGACTGCTGGATTGGGCAGCGGCGGAGACACGGTTGATCTGGAGGGGGGTGCCGGTGAAGGCGGAG
GCTGCGGCGATGACCAAGGCCAGGAGGAGGGCGGTGATAGCGGATCTCATCATCTTCAATTAGGGTGAGGTGC
TGCAATAGTGGAGAGAGTTGGAGGCGCGGCGGTGACTTGATTGATGACAGATGCCGAAACAGAAGTGCATGGG
GCGGCAATGGCGGCCGG
>TR296|c0_g1_i1 len=265 path=[324:0-54 349:55-91 352:92-115 354:116-139 353:140-163 355:164-195 356:196-197 346:198-198 342:199-237 323:238-264] [-1, 324, 349, 352, 354, 353, 355, 356, 346, 342, 323, -2]
AGACGATGGCCACGGAGACAAGGACGATGATGCGCGGCCGACCGATGGTCACGGCGACGACGATGCGGATCGC
GACGGCGATGGCCATGGCGTAGACAAGGATGACGATGAAAACGGTCACGATGGGGACGCCGTGTGGGAAGACG
ATGGCCACGGAGACAAGGACGATGATGCGCGGCCGACCGATGGTCACGGCAACGATAACAACAACAACAACGA
CGACGACGAAAATGACGAAAACAGCGTCTACGGGGAAGCCCTCGTT
>TR297|c0_g2_i1 len=5284 path=[5586:0-864 5587:865-885 @5616@!:886-1062 5612:1063-1168 @5615@!:1169-1366 5574:1367-1389 5611:1390-1671 5608:1672-2453 5597:2454-5283] [-1, 5586, 5587, 5616, 5612, 5615, 5574, 5611, 5608, 5597, -2]
GGGAATACCGTAATCATCCATACGCCAATGTACGGAAAACCTTCCAAAGGAATCATAACCTGTAGGAACGGAA
GGTATCTACAACTTCATATATTAGGATTTTTGCGACTATCTTGAGTGTTTTCGATTTTGAACTTCAAATATAT
CCATGTCCAAATTGTGCTTCCTCAAAATTCTCTCCGCTATTGCAGTCGCTCCCCATTGAGTATAATACAGGTA
TCCATTTTTGAAGCATTCAATTGAGACGAGATGACATCCACGGTCTGAATAATAATCACCCAGCAATTCATGA
GCTAGAGCAAGTTCTTGGATGTTGCCATGATCTTGAGCAGATTTGATTGAATCTTTATACATTTGCAGGGCTT
CGTCAAACTTCTCAACGGAAGCAGCATATTCTGCCTTCAGAAGAAGCCACTTGTTATTGAAAACATCCTTCGA
CAAAAGAGACCATCTCCCCATTAAATCCATCATTTCCTTTCCATCCTTCAATTTGTTATTAGCACTTCCAACA
ATGCTTCCCGAGATACGATGCCGCTTGAAAAGAATAAGTGCCCGGTAGAAGGTGTGATATATCAAGTATATCG
TTGGTTCTTTGCACGCAGGGTACATCATTGAAGCAAGACGAGAGTGTTCCTCAGCTGCCATATAGTTTCCTCT
CCAATAATTAATCAAAACATTAAACACATGACAGCTTTGGAGTAAGCTGAAATCTTTCGTTGCCTTCGCCTCA
TCTTTGAGGTCATCGATGTTGCAAATTGATCCACTAAAAACAGAGAAATAATCTCCAGATTTGCCCGTCAATT

CAGTAAGGAGAATACTATCTAATGCCATATATTTTGCCACAAACGATTTGCTGTGTTTGGCCACGAGACGAAA
CTTGTCATCGTATAATTGTTGTAACAAGGATAGGTTTTCTCCTCCCAAGAGCAAATAACGGCACGAGAGGCCC
AGCGCAGCCACTGCAATATGAACTTGACCCATTTTCATAGCGTTGTGGTGATACGTGATCATATTTTGGGAGA
TTTCTTTCGGAGGTTGAAACCAAATGTCAACGGCAACATGCTTGAACAATTGTGAACAACAGGAAGAATATGA
GATGAGTCTCGCAGGAGAAATGACGAAACTACTTGCAAGTGTTTTTCTCACCAATAGCAAATGGGCATGGGTT
TCCATGTTCATATTTGACTTGGCCATTCCCCGTAGTTTCTTTGTCATTGCTCTGACAATGTTAGCCCAGTATC
GCCCTCCGTCGTAATCCCCTGCGAGAAAAATCTTCATCGTTGCAAAGGCAGCAAATGCTGCCAGTGCTTCATG
ACATATGCCATGTTGAAGCGAAAACTGGATTACAGCACAAGATATCAATGGCAAAAAGGAGACGACGCGACA
TAGCAGGAGGTGTAGAAAGCGTCGATAATCATAACGGCGCGATGAACTGTATCATCAACAGTCTTTCCGCATA
GATCCATCATCTGGTCAGTGCTAAACTTGGACGTTATCTCATTCGTTGATGCCATGCCTTTCATCACGCTTTC
GGGTGTTGGGGCAAGAGGAATGTCGAACTTGAGTCGATGGAGAATTTGAATGCCCTTAGAAATAGATTCTTCG
TGCTGCCCCGATTGAGATAATGACCTCAGAACGAATGGCTGGATGTCGAGACTGTCGTCAAATGCCACATTCT
TCGTAATCTGTTCGGCGTACTGAGCGACATCGTCAGGCTCGCCAAGCGTAAACGATGCCGTAACTGCAGCCTT
GTGCAACGAGAGGCACAAATTATTATAACTCGCGTGCCAACAATCCCGTCCAAGAAATGAAATTCCTTTGGAG
AAATAGTAAAGAGCTGCGCCTGAATTTGACTGACGATGCGCCTGTCGCCCGCTGAATAAATTCCACGTAGCAA
ATCTAACCCTTTCGATATCGTCGCCGATAGCGTCGGGACCTGCAAAATCAATCTGATCGCATGCGATGGAGAT
CAAAGACGACTCCGGGGTATCTAGCTGAAGGTGATCAAGATTGGATGTTTCTGGATCACTTGTTGCGGAATCA
TCGATATTAGCTCGTTCACCGAGAAACTGCCCAATATCAAGATGCAGTGATTTCCGCTCATTCAAAGACATGC
TCTCGTAAACAGCCTGCTGGATTAAGTCATGAGTGAATATTACTATTGGCCCTGCTCGATCGAGAATCCCTTG
TTCAATGAAATCATCAGTTGAGCAGACAATACCATTCTGAAAGTTTTCCAAGTATTGCAATAAGGTTGTGTCT
GTCTGTATACCAAAGCACGATAAGATTTGCAGCACTCGTCTCTGGCTCGGCGGCAACGACTTGAGATTCGACG
CGATCAACCCTGCCACCCCGGCTCCCGTCTGGATTATATTGATCGTATCCTGGTCCCATGCAAAGCGTCGGAT
TTGCGTACTATACGCAACGGTGGAGGAGCGTACCAGCGAGTTCAGCAGTTCCACGGCGAATAAGGCGTGGCCG
GACGTCTTCTTGTGGACTACGTCGGCCAGCGGTGCGACGTATCGTCGGGGTAGCCCGAGCTCTGCCATCAACA
CGTCTCCAATATCTTCCTTCAATAGGCTGGAAAGCTCGATCTTGGTGGTACTGATCTTCTCCGTCATCATTTG
AAGGTAGGTATATTGAACTGAAAATGGGTGATTATCGTCGATTTCGTCGTGACGAAACAATCCTACAAACAGG
CATCGTGGAGAGACACGCCCGAAGCTCGCAACGTTGACGAGGATTTCTACTATTAACGCAAGCGAAGTTTTAT
CCGCCCACTGCAGATCATCGCAGCAGATCATCATGAGCCGGTCAGAGGATGATTCCAAAAGGGCCTCGATGAT
CTTCGATATAGAATAGATCAATCGAAAAGCATTCTCTTCTGCTTGCACCTTGATTGATTTATCGCCATCGAGA
CCAGGAAATAAGGAGCGAAGACTGGGCAGAAAGTCTACTAGCAATGCCAGCCTAGAATATCCAATGGCATCAA
GGATCAACTTCGAAGCTTGTCGGGAATAGGCAACATCGGATGGTCTCTCACTCCCTTTCATTTTGACCAGATT
GACAATCAGCTGATCGAACATGAACGAAACAATACTTTTTGAGATGTGCTCCTTCCCTCGTTCGAACTTTGCC
TTGATGACAATCCATCCTGGGCTGCGCAACATTTGTATGACAGTCTGGATGAGATGAGACTTGCCCGATCCGG
CAATACCAGACACAAATATCGCATCGAGGCAGCCTGTGTCGTTAGGAAACCCCATCGTATTTGTTTGCTGCAG
GGATTCTTCCATTTGGTATGCAACTTTTCTGAGAGTTGCTACGTCTTTCCCCCTTCCGTGGTAGGTATGGCCA
AACATTGGCGGAGGTGGCATGCCCCGAGAGAAGAGTGAATTGTGCAAGAAAATCTCGGGCTGGTGCACCATGC
ATTCCAAGTCTTGAATCACATCATCGTACAATCGAATTGGATGATTAACCTGGGATTTTGGACCGGTGTCCAC
CATGTCAGAAATCAATCTGCATATCGGATCAGGGAGAGAATTGCTTTCAATCAGTTTGGAGAATACTGTTGAC
TTTTGGGTGCGAAACATTTTGGATGATCGACCAGTGTTGTCTTCTTCGCCTTGATCATTGACACGAGACACCG
CTTCTCCTCGACTTGATGTGGTCGATCCTGTCAGCAGAGCAGAAGGGAGTAATCCGCCCAGAGAAAAGACAGA
ATATAGAAGCTTCCCAAGGACGGTCATCCCTGCTGAAACTTCCAGTGTATTCTGCTCTTCACATATATCGGGA
GTAATTATTGAGACGGACAAATCGATCCAGAGGAGAATCCATTTCGAAACCTCGCTTGATGTAATGCATGAAG
GGTCGTTGACGTGCTCGCCCCCAACTTCTTGTTTAAGATGAATTGAGAAATCATCCAATTGAATTGATTCCAA
ATTGATACCCGGAAGACGGCCGACGAATGATCGAGCGAGCAGTAATGCCATTCGGGCAGCATTTTCCATTACC
AAGCGGTCCTTGCTACCATCGCCATTGCTCCTTTCCGGCGAGGGTGGTCCAACTGCTTGAGTATTTGCATTCA
CAAGAGCATCATTGTCCTCTAGTAACACCATGCGATTTTTCAAACAACCCGCCTTGAGGAACTCTCCCGCCGA
CACTGGAAAGCTCGTGATTGAATTACTGGCCTCTAAGAAAAAGAGGTTTCTATTTGTAATGTTAGAAGACTCG
ATCAGACGCTCCACCATGGTAGTGGAGGTATCATTCAACTTTCCAAGTGGTTCATTTGCAGAGGGATGCTTTG
AGTTTGACGCCAGAATTGAAGAAGTCTGCACCACTGACTGAGGTGGCAGAAAGTTAAGCTGGGGGGGTATTCC
ATTTTGCTGATGAGTGTTGTGGGCAGTCTTCATCGCCTCCCCCGGTACATAGGAAGACAGGTTCGCTGGGGGG
CAGCGTATCTTAGCTTGTTCTGTTGCCTGATTCAAACCAGAAATGCCATCGCTTCTGTTTACACTGCTCATGA
CATTCGTGGCATTGAATTGTTGATTCACCACTTGATTCAAATCAAACAGTTCCATCTGCTGCATGTAGTGCAA
AAAGCTGGACATGAATTCAGAAGAGCCAACAGCTGGTGAAGCTACTCCCCCTTGAGAAGTTGATAAACCAGTC
GGTCCTTGCAAGAAGCCGGCGGACATGGTGCTCGAGGGAATTTGGCCTGTAAAGCCACCTCCTGCGCCAGTTG
CTGCAACGGCCACCCTTTCATTATCAAGAGTAGAAAAACATCCATTGGAGCGTGCGTTCTGTTGTCTCAGGAG
ATGAATCTGCTGCAATTGATGTTCCATCTGCTGCAGTAATAGTTCAGGATCCTCGCCTTCTTTGTTCTCCATA
GAGTTCTTGTCATTCTCGCGCAGGCGATGCATGACCTTCGACACGGCCTTTTCGGTATCGACGCATATCCAAG
CTTTTGCATATATCCACTCCTTATCTTTCTTCCTCGCTTTTTGATCACTTCCATTTTGCGATGCTACATGATC
ATCGGGGTCATCCGTTAAAAATCTTCCTCCCAATTTATATTGAATATCACCCACGATCTTTCTCGCAATGTCT
CGTTTTTCGGTTTTTTTTGTTTTTAGAAAGAGTGGTTTGTGTGCGCGGACCATGCAGCGTAGGATTTGATTTC

CGGGGTGGGCGCTGATGGCGTAGCCACGCCCGAATAGTACATCGTTATTACGGGGAGTTACGTCCGCCATGTC
GTTGGATCATAGCTCAAAGCATGCATTG
>TR297|c0_g2_i2 len=5308 path=[5598:0-864 5599:865-888 5600:889-913 5610:914-993 @5616@!:994-1170 5592:1171-1192 @5615@!:1193-1390 5574:1391-1413 5611:1414-1695 5608:1696-2477 5597:2478-5307] [-1, 5598, 5599, 5600, 5610, 5616, 5592, 5615, 5574, 5611, 5608, 5597, -2]
GGGAATACCGTAATCATCCATACGCCAATGTACGGAAAACCTTCCAAAGGAATCATAACCTGTAGGAACGGAA
GGTATCTACAACTTCATATATTAGGATTTTTGCGACTATCTTGAGTGTTTTCGATTTTGAACTTCAAATATAT
CCATGTCCAAATTGTGCTTCCTCAAAATTCTCTCCGCTATTGCAGTCGCTCCCCATTGAGTATAATACAGGTA
TCCATTTTTGAAGCATTCAATTGAGACGAGATGACATCCACGGTCTGAATAATAATCACCCAGCAATTCATGA
GCTAGAGCAAGTTCTTGGATGTTGCCATGATCTTGAGCAGATTTGATTGAATCTTTATACATTTGCAGGGCTT
CGTCAAACTTCTCAACGGAAGCAGCATATTCTGCCTTCAGAAGAAGCCACTTGTTATTGAAAACATCCTTCGA
CAAAAGAGACCATCTCCCCATTAAATCCATCATTTCCTTTCCATCCTTCAATTTGTTATTAGCACTTCCAACA
ATGCTTCCCGAGATACGATGCCGCTTGAAAAGAATAAGTGCCCGGTAGAAGGTGTGATATATCAAGTATATCG
TTGGTTCTTTGCACGCAGGGTACATCATTGAAGCAAGACGAGAGTGTTCCTCAGCTGCCATATAGTTTCCTCT
CCAATAATTAATCAAAACATTAAACACATGACAGCTTTGGAGTAAGCTGAAATCTTTCGTTGCCTTCGCCTCA
TCTTTGAGGTCATCGATGTTGCAAATTGATCCACTAAAAACAGAGAAATAATCTCCAGATTTGCCCGTCAATT
CAGTAAGGAGAATACTATCTAATGCCATATATTTTGCCACAAACGATTTGCTGTGTTTGGCCTGAATGAAATA
AAGGCAACACAAATTAGCATAAAAGTTTAAGGCATGCAGATAACCAAGATTAGGAACAAGAAACCAGGAACTG
CAAATCAGTGCATGTTTACATACCACGAGACGAAACTTGTCATCGTATAATTGTTGTAACAAGGATAGGTTTT
CTCCTCCCAAGAGCAAATAACGGCACGAGAGGCCCAGCGCAGCCACTGCAATATGAACTTGACCCATTTTCAT
AGCGTTGTGGTGATACGTGATCATATTTTGGGAGATTTCTTTCGGAGGTTGAAACCAAATGTCAACGGCAACA
TGCAATAGCAAATGGGCATGGGTTTCCATGTTCATATTTGACTTGGCCATTCCCCGTAGTTTCTTTGTCATTG
CTCTGACAATGTTAGCCCAGTATCGCCCTCCGTCGTAATCCCCTGCGAGAAAAATCTTCATCGTTGCAAAGGC
AGCAAATGCTGCCAGTGCTTCATGACATATGCCATGTTGAAGCGAAAACTGGATTACAGCACAAGATATCAAT
GGCAAAAAAGGAGACGACGCGACATAGCAGGAGGTGTAGAAAGCGTCGATAATCATAACGGCGCGATGAACTG
TATCATCAACAGTCTTTCCGCATAGATCCATCATCTGGTCAGTGCTAAACTTGGACGTTATCTCATTCGTTGA
TGCCATGCCTTTCATCACGCTTTCGGGTGTTGGGGCAAGAGGAATGTCGAACTTGAGTCGATGGAGAATTTGA
ATGCCCTTAGAAATAGATTCTTCGTGCTGCCCCGATTGAGATAATGACCTCAGAACGAATGGCTGGATGTCGA
GACTGTCGTCAAATGCCACATTCTTCGTAATCTGTTCGGCGTACTGAGCGACATCGTCAGGCTCGCCAAGCGT
AAACGATGCCGTAACTGCAGCCTTGTGCAACGAGAGGCACAAATTATTATAACTCGCGTGCCAACAATCCCGT
CCAAGAAATGAAATTCCTTTGGAGAAATAGTAAAGAGCTGCGCCTGAATTTGACTGACGATGCGCCTGTCGCC
CGCTGAATAAATTCCACGTAGCAAATCTAACCCTTTCGATATCGTCGCCGATAGCGTCGGGACCTGCAAAATC
AATCTGATCGCATGCGATGGAGATCAAAGACGACTCCGGGGTATCTAGCTGAAGGTGATCAAGATTGGATGTT
TCTGGATCACTTGTTGCGGAATCATCGATATTAGCTCGTTCACCGAGAAACTGCCCAATATCAAGATGCAGTG
ATTTCCGCTCATTCAAAGACATGCTCTCGTAAACAGCCTGCTGGATTAAGTCATGAGTGAATATTACTATTGG
CCCTGCTCGATCGAGAATCCCTTGTTCAATGAAATCATCAGTTGAGCAGACAATACCATTCTGAAAGTTTTCC
AAGTATTGCAATAAGGTTGTGTCTGTCTGTATACCAAAGCACGATAAGATTTGCAGCACTCGTCTCTGGCTCG
GCGGCAACGACTTGAGATTCGACGCGATCAACCCTGCCACCCCGGCTCCCGTCTGGATTATATTGATCGTATC
CTGGTCCCATGCAAAGCGTCGGATTTGCGTACTATACGCAACGGTGGAGGAGCGTACCAGCGAGTTCAGCAGT
TCCACGGCGAATAAGGCGTGGCCGGACGTCTTCTTGTGGACTACGTCGGCCAGCGGTGCGACGTATCGTCGGG
GTAGCCCGAGCTCTGCCATCAACACGTCTCCAATATCTTCCTTCAATAGGCTGGAAAGCTCGATCTTGGTGGT
ACTGATCTTCTCCGTCATCATTTGAAGGTAGGTATATTGAACTGAAAATGGGTGATTATCGTCGATTTCGTCG
TGACGAAACAATCCTACAAACAGGCATCGTGGAGAGACACGCCCGAAGCTCGCAACGTTGACGAGGATTTCTA
CTATTAACGCAAGCGAAGTTTTATCCGCCCACTGCAGATCATCGCAGCAGATCATCATGAGCCGGTCAGAGGA
TGATTCCAAAAGGGCCTCGATGATCTTCGATATAGAATAGATCAATCGAAAAGCATTCTCTTCTGCTTGCACC
TTGATTGATTTATCGCCATCGAGACCAGGAAATAAGGAGCGAAGACTGGGCAGAAAGTCTACTAGCAATGCCA
GCCTAGAATATCCAATGGCATCAAGGATCAACTTCGAAGCTTGTCGGGAATAGGCAACATCGGATGGTCTCTC
ACTCCCTTTCATTTTGACCAGATTGACAATCAGCTGATCGAACATGAACGAAACAATACTTTTTGAGATGTGC
TCCTTCCCTCGTTCGAACTTTGCCTTGATGACAATCCATCCTGGGCTGCGCAACATTTGTATGACAGTCTGGA
TGAGATGAGACTTGCCCGATCCGGCAATACCAGACACAAATATCGCATCGAGGCAGCCTGTGTCGTTAGGAAA
CCCCATCGTATTTGTTTGCTGCAGGGATTCTTCCATTTGGTATGCAACTTTTCTGAGAGTTGCTACGTCTTTC
CCCCTTCCGTGGTAGGTATGGCCAAACATTGGCGGAGGTGGCATGCCCCGAGAGAAGAGTGAATTGTGCAAGA
AAATCTCGGGCTGGTGCACCATGCATTCCAAGTCTTGAATCACATCATCGTACAATCGAATTGGATGATTAAC
CTGGGATTTTGGACCGGTGTCCACCATGTCAGAAATCAATCTGCATATCGGATCAGGGAGAGAATTGCTTTCA
ATCAGTTTGGAGAATACTGTTGACTTTTGGGTGCGAAACATTTTGGATGATCGACCAGTGTTGTCTTCTTCGC
CTTGATCATTGACACGAGACACCGCTTCTCCTCGACTTGATGTGGTCGATCCTGTCAGCAGAGCAGAAGGGAG
TAATCCGCCCAGAGAAAAGACAGAATATAGAAGCTTCCCAAGGACGGTCATCCCTGCTGAAACTTCCAGTGTA
TTCTGCTCTTCACATATATCGGGAGTAATTATTGAGACGGACAAATCGATCCAGAGGAGAATCCATTTCGAAA
CCTCGCTTGATGTAATGCATGAAGGGTCGTTGACGTGCTCGCCCCCAACTTCTTGTTTAAGATGAATTGAGAA

ATCATCCAATTGAATTGATTCCAAATTGATACCCGGAAGACGGCCGACGAATGATCGAGCGAGCAGTAATGCC
ATTCGGGCAGCATTTTCCATTACCAAGCGGTCCTTGCTACCATCGCCATTGCTCCTTTCCGGCGAGGGTGGTC
CAACTGCTTGAGTATTTGCATTCACAAGAGCATCATTGTCCTCTAGTAACACCATGCGATTTTTCAAACAACC
CGCCTTGAGGAACTCTCCCGCCGACACTGGAAAGCTCGTGATTGAATTACTGGCCTCTAAGAAAAAGAGGTTT
CTATTTGTAATGTTAGAAGACTCGATCAGACGCTCCACCATGGTAGTGGAGGTATCATTCAACTTTCCAAGTG
GTTCATTTGCAGAGGGATGCTTTGAGTTTGACGCCAGAATTGAAGAAGTCTGCACCACTGACTGAGGTGGCAG
AAAGTTAAGCTGGGGGGGTATTCCATTTTGCTGATGAGTGTTGTGGGCAGTCTTCATCGCCTCCCCCGGTACA
TAGGAAGACAGGTTCGCTGGGGGGCAGCGTATCTTAGCTTGTTCTGTTGCCTGATTCAAACCAGAAATGCCAT
CGCTTCTGTTTACACTGCTCATGACATTCGTGGCATTGAATTGTTGATTCACCACTTGATTCAAATCAAACAG
TTCCATCTGCTGCATGTAGTGCAAAAAGCTGGACATGAATTCAGAAGAGCCAACAGCTGGTGAAGCTACTCCC
CCTTGAGAAGTTGATAAACCAGTCGGTCCTTGCAAGAAGCCGGCGGACATGGTGCTCGAGGGAATTTGGCCTG
TAAAGCCACCTCCTGCGCCAGTTGCTGCAACGGCCACCCTTTCATTATCAAGAGTAGAAAAACATCCATTGGA
GCGTGCGTTCTGTTGTCTCAGGAGATGAATCTGCTGCAATTGATGTTCCATCTGCTGCAGTAATAGTTCAGGA
TCCTCGCCTTCTTTGTTCTCCATAGAGTTCTTGTCATTCTCGCGCAGGCGATGCATGACCTTCGACACGGCCT
TTTCGGTATCGACGCATATCCAAGCTTTTGCATATATCCACTCCTTATCTTTCTTCCTCGCTTTTTGATCACT
TCCATTTTGCGATGCTACATGATCATCGGGGTCATCCGTTAAAAATCTTCCTCCCAATTTATATTGAATATCA
CCCACGATCTTTCTCGCAATGTCTCGTTTTTCGGTTTTTTTTGTTTTTAGAAAGAGTGGTTTGTGTGCGCGGA
CCATGCAGCGTAGGATTTGATTTCCGGGGTGGGCGCTGATGGCGTAGCCACGCCCGAATAGTACATCGTTATT
ACGGGGAGTTACGTCCGCCATGTCGTTGGATCATAGCTCAAAGCATGCATTG
>TR297|c0_g2_i3 len=5200 path=[5586:0-864 5587:865-885 @5616@!:886-1062 5592:1063-1084 @5615@!:1085-1282 5574:1283-1305 5611:1306-1587 5608:1588-2369 5597:2370-5199] [-1, 5586, 5587, 5616, 5592, 5615, 5574, 5611, 5608, 5597, -2]
GGGAATACCGTAATCATCCATACGCCAATGTACGGAAAACCTTCCAAAGGAATCATAACCTGTAGGAACGGAA
GGTATCTACAACTTCATATATTAGGATTTTTGCGACTATCTTGAGTGTTTTCGATTTTGAACTTCAAATATAT
CCATGTCCAAATTGTGCTTCCTCAAAATTCTCTCCGCTATTGCAGTCGCTCCCCATTGAGTATAATACAGGTA
TCCATTTTTGAAGCATTCAATTGAGACGAGATGACATCCACGGTCTGAATAATAATCACCCAGCAATTCATGA
GCTAGAGCAAGTTCTTGGATGTTGCCATGATCTTGAGCAGATTTGATTGAATCTTTATACATTTGCAGGGCTT
CGTCAAACTTCTCAACGGAAGCAGCATATTCTGCCTTCAGAAGAAGCCACTTGTTATTGAAAACATCCTTCGA
CAAAAGAGACCATCTCCCCATTAAATCCATCATTTCCTTTCCATCCTTCAATTTGTTATTAGCACTTCCAACA
ATGCTTCCCGAGATACGATGCCGCTTGAAAAGAATAAGTGCCCGGTAGAAGGTGTGATATATCAAGTATATCG
TTGGTTCTTTGCACGCAGGGTACATCATTGAAGCAAGACGAGAGTGTTCCTCAGCTGCCATATAGTTTCCTCT
CCAATAATTAATCAAAACATTAAACACATGACAGCTTTGGAGTAAGCTGAAATCTTTCGTTGCCTTCGCCTCA
TCTTTGAGGTCATCGATGTTGCAAATTGATCCACTAAAAACAGAGAAATAATCTCCAGATTTGCCCGTCAATT
CAGTAAGGAGAATACTATCTAATGCCATATATTTTGCCACAAACGATTTGCTGTGTTTGGCCACGAGACGAAA
CTTGTCATCGTATAATTGTTGTAACAAGGATAGGTTTTCTCCTCCCAAGAGCAAATAACGGCACGAGAGGCCC
AGCGCAGCCACTGCAATATGAACTTGACCCATTTTCATAGCGTTGTGGTGATACGTGATCATATTTTGGGAGA
TTTCTTTCGGAGGTTGAAACCAAATGTCAACGGCAACATGCAATAGCAAATGGGCATGGGTTTCCATGTTCAT
ATTTGACTTGGCCATTCCCCGTAGTTTCTTTGTCATTGCTCTGACAATGTTAGCCCAGTATCGCCCTCCGTCG
TAATCCCCTGCGAGAAAAATCTTCATCGTTGCAAAGGCAGCAAATGCTGCCAGTGCTTCATGACATATGCCAT
GTTGAAGCGAAAACTGGATTACAGCACAAGATATCAATGGCAAAAAAGGAGACGACGCGACATAGCAGGAGGT
GTAGAAAGCGTCGATAATCATAACGGCGCGATGAACTGTATCATCAACAGTCTTTCCGCATAGATCCATCATC
TGGTCAGTGCTAAACTTGGACGTTATCTCATTCGTTGATGCCATGCCTTTCATCACGCTTTCGGGTGTTGGGG
CAAGAGGAATGTCGAACTTGAGTCGATGGAGAATTTGAATGCCCTTAGAAATAGATTCTTCGTGCTGCCCCGA
TTGAGATAATGACCTCAGAACGAATGGCTGGATGTCGAGACTGTCGTCAAATGCCACATTCTTCGTAATCTGT
TCGGCGTACTGAGCGACATCGTCAGGCTCGCCAAGCGTAAACGATGCCGTAACTGCAGCCTTGTGCAACGAGA
GGCACAAATTATTATAACTCGCGTGCCAACAATCCCGTCCAAGAAATGAAATTCCTTTGGAGAAATAGTAAAG
AGCTGCGCCTGAATTTGACTGACGATGCGCCTGTCGCCCGCTGAATAAATTCCACGTAGCAAATCTAACCCTT
TCGATATCGTCGCCGATAGCGTCGGGACCTGCAAAATCAATCTGATCGCATGCGATGGAGATCAAAGACGACT
CCGGGGTATCTAGCTGAAGGTGATCAAGATTGGATGTTTCTGGATCACTTGTTGCGGAATCATCGATATTAGC
TCGTTCACCGAGAAACTGCCCAATATCAAGATGCAGTGATTTCCGCTCATTCAAAGACATGCTCTCGTAAACA
GCCTGCTGGATTAAGTCATGAGTGAATATTACTATTGGCCCTGCTCGATCGAGAATCCCTTGTTCAATGAAAT
CATCAGTTGAGCAGACAATACCATTCTGAAAGTTTTCCAAGTATTGCAATAAGGTTGTGTCTGTCTGTATACC
AAAGCACGATAAGATTTGCAGCACTCGTCTCTGGCTCGGCGGCAACGACTTGAGATTCGACGCGATCAACCCT
GCCACCCCGGCTCCCGTCTGGATTATATTGATCGTATCCTGGTCCCATGCAAAGCGTCGGATTTGCGTACTAT
ACGCAACGGTGGAGGAGCGTACCAGCGAGTTCAGCAGTTCCACGGCGAATAAGGCGTGGCCGGACGTCTTCTT
GTGGACTACGTCGGCCAGCGGTGCGACGTATCGTCGGGGTAGCCCGAGCTCTGCCATCAACACGTCTCCAATA
TCTTCCTTCAATAGGCTGGAAAGCTCGATCTTGGTGGTACTGATCTTCTCCGTCATCATTTGAAGGTAGGTAT
ATTGAACTGAAAATGGGTGATTATCGTCGATTTCGTCGTGACGAAACAATCCTACAAACAGGCATCGTGGAGA
GACACGCCCGAAGCTCGCAACGTTGACGAGGATTTCTACTATTAACGCAAGCGAAGTTTATCCGCCCACTGC

AGATCATCGCAGCAGATCATCATGAGCCGGTCAGAGGATGATTCCAAAAGGGCCTCGATGATCTTCGATATAG
AATAGATCAATCGAAAAGCATTCTCTTCTGCTTGCACCTTGATTGATTTATCGCCATCGAGACCAGGAAATAA
GGAGCGAAGACTGGGCAGAAAGTCTACTAGCAATGCCAGCCTAGAATATCCAATGGCATCAAGGATCAACTTC
GAAGCTTGTCGGGAATAGGCAACATCGGATGGTCTCTCACTCCCTTTCATTTTGACCAGATTGACAATCAGCT
GATCGAACATGAACGAAACAATACTTTTTGAGATGTGCTCCTTCCCTCGTTCGAACTTTGCCTTGATGACAAT
CCATCCTGGGCTGCGCAACATTTGTATGACAGTCTGGATGAGATGAGACTTGCCCGATCCGGCAATACCAGAC
ACAAATATCGCATCGAGGCAGCCTGTGTCGTTAGGAAACCCCATCGTATTTGTTTGCTGCAGGGATTCTTCCA
TTTGGTATGCAACTTTTCTGAGAGTTGCTACGTCTTTCCCCCTTCCGTGGTAGGTATGGCCAAACATTGGCGG
AGGTGGCATGCCCCGAGAGAAGAGTGAATTGTGCAAGAAAATCTCGGGCTGGTGCACCATGCATTCCAAGTCT
TGAATCACATCATCGTACAATCGAATTGGATGATTAACCTGGGATTTTGGACCGGTGTCCACCATGTCAGAAA
TCAATCTGCATATCGGATCAGGGAGAGAATTGCTTTCAATCAGTTTGGAGAATACTGTTGACTTTTGGGTGCG
AAACATTTTGGATGATCGACCAGTGTTGTCTTCTTCGCCTTGATCATTGACACGAGACACCGCTTCTCCTCGA
CTTGATGTGGTCGATCCTGTCAGCAGAGCAGAAGGGAGTAATCCGCCCAGAGAAAAGACAGAATATAGAAGCT
TCCCAAGGACGGTCATCCCTGCTGAAACTTCCAGTGTATTCTGCTCTTCACATATATCGGGAGTAATTATTGA
GACGGACAAATCGATCCAGAGGAGAATCCATTTCGAAACCTCGCTTGATGTAATGCATGAAGGGTCGTTGACG
TGCTCGCCCCCAACTTCTTGTTTAAGATGAATTGAGAAATCATCCAATTGAATTGATTCCAAATTGATACCCG
GAAGACGGCCGACGAATGATCGAGCGAGCAGTAATGCCATTCGGGCAGCATTTTCCATTACCAAGCGGTCCTT
GCTACCATCGCCATTGCTCCTTTCCGGCGAGGGTGGTCCAACTGCTTGAGTATTTGCATTCACAAGAGCATCA
TTGTCCTCTAGTAACACCATGCGATTTTTCAAACAACCCGCCTTGAGGAACTCTCCCGCCGACACTGGAAAGC
TCGTGATTGAATTACTGGCCTCTAAGAAAAAGAGGTTTCTATTTGTAATGTTAGAAGACTCGATCAGACGCTC
CACCATGGTAGTGGAGGTATCATTCAACTTTCCAAGTGGTTCATTTGCAGAGGGATGCTTTGAGTTTGACGCC
AGAATTGAAGAAGTCTGCACCACTGACTGAGGTGGCAGAAAGTTAAGCTGGGGGGGTATTCCATTTTGCTGAT
GAGTGTTGTGGGCAGTCTTCATCGCCTCCCCCGGTACATAGGAAGACAGGTTCGCTGGGGGGCAGCGTATCTT
AGCTTGTTCTGTTGCCTGATTCAAACCAGAAATGCCATCGCTTCTGTTTACACTGCTCATGACATTCGTGGCA
TTGAATTGTTGATTCACCACTTGATTCAAATCAAACAGTTCCATCTGCTGCATGTAGTGCAAAAAGCTGGACA
TGAATTCAGAAGAGCCAACAGCTGGTGAAGCTACTCCCCCTTGAGAAGTTGATAAACCAGTCGGTCCTTGCAA
GAAGCCGGCGGACATGGTGCTCGAGGGAATTTGGCCTGTAAAGCCACCTCCTGCGCCAGTTGCTGCAACGGCC
ACCCTTTCATTATCAAGAGTAGAAAAACATCCATTGGAGCGTGCGTTCTGTTGTCTCAGGAGATGAATCTGCT
GCAATTGATGTTCCATCTGCTGCAGTAATAGTTCAGGATCCTCGCCTTCTTTGTTCTCCATAGAGTTCTTGTC
ATTCTCGCGCAGGCGATGCATGACCTTCGACACGGCCTTTTCGGTATCGACGCATATCCAAGCTTTTGCATAT
ATCCACTCCTTATCTTTCTTCCTCGCTTTTTGATCACTTCCATTTTGCGATGCTACATGATCATCGGGGTCAT
CCGTTAAAAATCTTCCTCCCAATTTATATTGAATATCACCCACGATCTTTCTCGCAATGTCTCGTTTTTCGGT
TTTTTTTGTTTTTAGAAAGAGTGGTTTGTGTGCGCGGACCATGCAGCGTAGGATTTGATTTCCGGGGTGGGCG
CTGATGGCGTAGCCACGCCCGAATAGTACATCGTTATTACGGGGAGTTACGTCCGCCATGTCGTTGGATCATA
GCTCAAAGCATGCATTG
>TR297|c0_g2_i4 len=5285 path=[5586:0-864 5587:865-885 @5616@!:886-1062
5592:1063-1084 @5615@!:1085-1282 5613:1283-1390 5611:1391-1672 5608:1673-
2454 5597:2455-5284] [-1, 5586, 5587, 5616, 5592, 5615, 5613, 5611, 5608,
5597, -2]
GGGAATACCGTAATCATCCATACGCCAATGTACGGAAAACCTTCCAAAGGAATCATAACCTGTAGGAACGGAA
GGTATCTACAACTTCATATATTAGGATTTTTGCGACTATCTTGAGTGTTTTCGATTTTGAACTTCAAATATAT
CCATGTCCAAATTGTGCTTCCTCAAAATTCTCTCCGCTATTGCAGTCGCTCCCCATTGAGTATAATACAGGTA
TCCATTTTTGAAGCATTCAATTGAGACGAGATGACATCCACGGTCTGAATAATAATCACCCAGCAATTCATGA
GCTAGAGCAAGTTCTTGGATGTTGCCATGATCTTGAGCAGATTTGATTGAATCTTTATACATTTGCAGGGCTT
CGTCAAACTTCTCAACGGAAGCAGCATATTCTGCCTTCAGAAGAAGCCACTTGTTATTGAAAACATCCTTCGA
CAAAAGAGACCATCTCCCCATTAAATCCATCATTTCCTTTCCATCCTTCAATTTGTTATTAGCACTTCCAACA
ATGCTTCCCGAGATACGATGCCGCTTGAAAAGAATAAGTGCCCGGTAGAAGGTGTGATATATCAAGTATATCG
TTGGTTCTTTGCACGCAGGGTACATCATTGAAGCAAGACGAGAGTGTTCCTCAGCTGCCATATAGTTTCCTCT
CCAATAATTAATCAAAACATTAAACACATGACAGCTTTGGAGTAAGCTGAAATCTTTCGTTGCCTTCGCCTCA
TCTTTGAGGTCATCGATGTTGCAAATTGATCCACTAAAAACAGAGAAATAATCTCCAGATTTGCCCGTCAATT
CAGTAAGGAGAATACTATCTAATGCCATATATTTTGCCACAAACGATTTGCTGTGTTTGGCCACGAGACGAAA
CTTGTCATCGTATAATTGTTGTAACAAGGATAGGTTTTCTCCTCCCAAGAGCAAATAACGGCACGAGAGGCCC
AGCGCAGCCACTGCAATATGAACTTGACCCATTTTCATAGCGTTGTGGTGATACGTGATCATATTTTGGGAGA
TTTCTTTCGGAGGTTGAAACCAAATGTCAACGGCAACATGCAATAGCAAATGGGCATGGGTTTCCATGTTCAT
ATTTGACTTGGCCATTCCCCGTAGTTTCTTTGTCATTGCTCTGACAATGTTAGCCCAGTATCGCCCTCCGTCG
TAATCCCCTGCGAGAAAAATCTTCATCGTTGCAAAGGCAGCAAATGCTGCCAGTGCTTCATGACATATGCCAT
GTTGAAGCGAAAACTGGATTACAGCACAAGATATCAATGGCACTATGGTGTGGCAGCAATGTAAACTCTGTTA
GATAAATGGGGGGTAACAGAGGGGTGTCGATTTTGACATTCGCATATGACTCACAAAAAGGAGACGACGCGAC
ATAGCAGGAGGTGTAGAAAGCGTCGATAATCATAACGGCGCGATGAACTGTATCATCAACAGTCTTTCCGCAT
AGATCCATCATCTGGTCAGTGCTAAACTTGGACGTTATCTCATTCGTTGATGCCATGCCTTTCATCACGCTTT

CGGGTGTTGGGGCAAGAGGAATGTCGAACTTGAGTCGATGGAGAATTTGAATGCCCTTAGAAATAGATTCTTC
GTGCTGCCCCGATTGAGATAATGACCTCAGAACGAATGGCTGGATGTCGAGACTGTCGTCAAATGCCACATTC
TTCGTAATCTGTTCGGCGTACTGAGCGACATCGTCAGGCTCGCCAAGCGTAAACGATGCCGTAACTGCAGCCT
TGTGCAACGAGAGGCACAAATTATTATAACTCGCGTGCCAACAATCCCGTCCAAGAAATGAAATTCCTTTGGA
GAAATAGTAAAGAGCTGCGCCTGAATTTGACTGACGATGCGCCTGTCGCCCGCTGAATAAATTCCACGTAGCA
AATCTAACCCTTTCGATATCGTCGCCGATAGCGTCGGGACCTGCAAAATCAATCTGATCGCATGCGATGGAGA
TCAAAGACGACTCCGGGGTATCTAGCTGAAGGTGATCAAGATTGGATGTTTCTGGATCACTTGTTGCGGAATC
ATCGATATTAGCTCGTTCACCGAGAAACTGCCCAATATCAAGATGCAGTGATTTCCGCTCATTCAAAGACATG
CTCTCGTAAACAGCCTGCTGGATTAAGTCATGAGTGAATATTACTATTGGCCCTGCTCGATCGAGAATCCCTT
GTTCAATGAAATCATCAGTTGAGCAGACAATACCATTCTGAAAGTTTTCCAAGTATTGCAATAAGGTTGTGTC
TGTCTGTATACCAAAGCACGATAAGATTTGCAGCACTCGTCTCTGGCTCGGCGGCAACGACTTGAGATTCGAC
GCGATCAACCCTGCCACCCCGGCTCCCGTCTGGATTATATTGATCGTATCCTGGTCCCATGCAAAGCGTCGGA
TTTGCGTACTATACGCAACGGTGGAGGAGCGTACCAGCGAGTTCAGCAGTTCCACGGCGAATAAGGCGTGGCC
GGACGTCTTCTTGTGGACTACGTCGGCCAGCGGTGCGACGTATCGTCGGGGTAGCCCGAGCTCTGCCATCAAC
ACGTCTCCAATATCTTCCTTCAATAGGCTGGAAAGCTCGATCTTGGTGGTACTGATCTTCTCCGTCATCATTT
GAAGGTAGGTATATTGAACTGAAAATGGGTGATTATCGTCGATTTCGTCGTGACGAAACAATCCTACAAACAG
GCATCGTGGAGAGACACGCCCGAAGCTCGCAACGTTGACGAGGATTTCTACTATTAACGCAAGCGAAGTTTTA
TCCGCCCACTGCAGATCATCGCAGCAGATCATCATGAGCCGGTCAGAGGATGATTCCAAAAGGGCCTCGATGA
TCTTCGATATAGAATAGATCAATCGAAAAGCATTCTCTTCTGCTTGCACCTTGATTGATTTATCGCCATCGAG
ACCAGGAAATAAGGAGCGAAGACTGGGCAGAAAGTCTACTAGCAATGCCAGCCTAGAATATCCAATGGCATCA
AGGATCAACTTCGAAGCTTGTCGGGAATAGGCAACATCGGATGGTCTCTCACTCCCTTTCATTTTGACCAGAT
TGACAATCAGCTGATCGAACATGAACGAAACAATACTTTTTGAGATGTGCTCCTTCCCTCGTTCGAACTTTGC
CTTGATGACAATCCATCCTGGGCTGCGCAACATTTGTATGACAGTCTGGATGAGATGAGACTTGCCCGATCCG
GCAATACCAGACACAAATATCGCATCGAGGCAGCCTGTGTCGTTAGGAAACCCCATCGTATTTGTTTGCTGCA
GGGATTCTTCCATTTGGTATGCAACTTTTCTGAGAGTTGCTACGTCTTTCCCCCTTCCGTGGTAGGTATGGCC
AAACATTGGCGGAGGTGGCATGCCCCGAGAGAAGAGTGAATTGTGCAAGAAAATCTCGGGCTGGTGCACCATG
CATTCCAAGTCTTGAATCACATCATCGTACAATCGAATTGGATGATTAACCTGGGATTTTGGACCGGTGTCCA
CCATGTCAGAAATCAATCTGCATATCGGATCAGGGAGAGAATTGCTTTCAATCAGTTTGGAGAATACTGTTGA
CTTTTGGGTGCGAAACATTTTGGATGATCGACCAGTGTTGTCTTCTTCGCCTTGATCATTGACACGAGACACC
GCTTCTCCTCGACTTGATGTGGTCGATCCTGTCAGCAGAGCAGAAGGGAGTAATCCGCCCAGAGAAAAGACAG
AATATAGAAGCTTCCCAAGGACGGTCATCCCTGCTGAAACTTCCAGTGTATTCTGCTCTTCACATATATCGGG
AGTAATTATTGAGACGGACAAATCGATCCAGAGGAGAATCCATTTCGAAACCTCGCTTGATGTAATGCATGAA
GGGTCGTTGACGTGCTCGCCCCCAACTTCTTGTTTAAGATGAATTGAGAAATCATCCAATTGAATTGATTCCA
AATTGATACCCGGAAGACGGCCGACGAATGATCGAGCGAGCAGTAATGCCATTCGGGCAGCATTTTCCATTAC
CAAGCGGTCCTTGCTACCATCGCCATTGCTCCTTTCCGGCGAGGGTGGTCCAACTGCTTGAGTATTTGCATTC
ACAAGAGCATCATTGTCCTCTAGTAACACCATGCGATTTTTCAAACAACCCGCCTTGAGGAACTCTCCCGCCG
ACACTGGAAAGCTCGTGATTGAATTACTGGCCTCTAAGAAAAAGAGGTTTCTATTTGTAATGTTAGAAGACTC
GATCAGACGCTCCACCATGGTAGTGGAGGTATCATTCAACTTTCCAAGTGGTTCATTTGCAGAGGGATGCTTT
GAGTTTGACGCCAGAATTGAAGAAGTCTGCACCACTGACTGAGGTGGCAGAAAGTTAAGCTGGGGGGGTATTC
CATTTTGCTGATGAGTGTTGTGGGCAGTCTTCATCGCCTCCCCCGGTACATAGGAAGACAGGTTCGCTGGGGG
GCAGCGTATCTTAGCTTGTTCTGTTGCCTGATTCAAACCAGAAATGCCATCGCTTCTGTTTACACTGCTCATG
ACATTCGTGGCATTGAATTGTTGATTCACCACTTGATTCAAATCAAACAGTTCCATCTGCTGCATGTAGTGCA
AAAAGCTGGACATGAATTCAGAAGAGCCAACAGCTGGTGAAGCTACTCCCCCTTGAGAAGTTGATAAACCAGT
CGGTCCTTGCAAGAAGCCGGCGGACATGGTGCTCGAGGGAATTTGGCCTGTAAAGCCACCTCCTGCGCCAGTT
GCTGCAACGGCCACCCTTTCATTATCAAGAGTAGAAAAACATCCATTGGAGCGTGCGTTCTGTTGTCTCAGGA
GATGAATCTGCTGCAATTGATGTTCCATCTGCTGCAGTAATAGTTCAGGATCCTCGCCTTCTTTGTTCTCCAT
AGAGTTCTTGTCATTCTCGCGCAGGCGATGCATGACCTTCGACACGGCCTTTTCGGTATCGACGCATATCCAA
GCTTTTGCATATATCCACTCCTTATCTTTCTTCCTCGCTTTTTGATCACTTCCATTTTGCGATGCTACATGAT
CATCGGGGTCATCCGTTAAAAATCTTCCTCCCAATTTATATTGAATATCACCCACGATCTTTCTCGCAATGTC
TCGTTTTTCGGTTTTTTTTGTTTTTAGAAAGAGTGGTTTGTGTGCGCGGACCATGCAGCGTAGGATTTGATTT
CCGGGGTGGGCGCTGATGGCGTAGCCACGCCCGAATAGTACATCGTTATTACGGGGAGTTACGTCCGCCATGT
CGTTGGATCATAGCTCAAAGCATGCATTG
>TR297|c0_g2_i5 len=5369 path=[5586:0-864 5587:865-885 @5616@!:886-1062 5612:1063-1168 @5615@!:1169-1366 5613:1367-1474 5611:1475-1756 5608:1757-2538 5597:2539-5368] [-1, 5586, 5587, 5616, 5612, 5615, 5613, 5611, 5608, 5597, -2]
GGGAATACCGTAATCATCCATACGCCAATGTACGGAAAACCTTCCAAAGGAATCATAACCTGTAGGAACGGAA
GGTATCTACAACTTCATATATTAGGATTTTTGCGACTATCTTGAGTGTTTTCGATTTTGAACTTCAAATATAT
CCATGTCCAAATTGTGCTTCCTCAAAATTCTCTCCGCTATTGCAGTCGCTCCCCATTGAGTATAATACAGGTA
TCCATTTTTGAAGCATTCAATTGAGACGAGATGACATCCACGGTCTGAATAATAATCACCCAGCAATTCATGA

GCTAGAGCAAGTTCTTGGATGTTGCCATGATCTTGAGCAGATTTGATTGAATCTTTATACATTTGCAGGGCTT
CGTCAAACTTCTCAACGGAAGCAGCATATTCTGCCTTCAGAAGAAGCCACTTGTTATTGAAAACATCCTTCGA
CAAAAGAGACCATCTCCCCATTAAATCCATCATTTCCTTTCCATCCTTCAATTTGTTATTAGCACTTCCAACA
ATGCTTCCCGAGATACGATGCCGCTTGAAAAGAATAAGTGCCCGGTAGAAGGTGTGATATATCAAGTATATCG
TTGGTTCTTTGCACGCAGGGTACATCATTGAAGCAAGACGAGAGTGTTCCTCAGCTGCCATATAGTTTCCTCT
CCAATAATTAATCAAAACATTAAACACATGACAGCTTTGGAGTAAGCTGAAATCTTTCGTTGCCTTCGCCTCA
TCTTTGAGGTCATCGATGTTGCAAATTGATCCACTAAAAACAGAGAAATAATCTCCAGATTTGCCCGTCAATT
CAGTAAGGAGAATACTATCTAATGCCATATATTTTGCCACAAACGATTTGCTGTGTTTGGCCACGAGACGAAA
CTTGTCATCGTATAATTGTTGTAACAAGGATAGGTTTTCTCCTCCCAAGAGCAAATAACGGCACGAGAGGCCC
AGCGCAGCCACTGCAATATGAACTTGACCCATTTTCATAGCGTTGTGGTGATACGTGATCATATTTTGGGAGA
TTTCTTTCGGAGGTTGAAACCAAATGTCAACGGCAACATGCTTGAACAATTGTGAACAACAGGAAGAATATGA
GATGAGTCTCGCAGGAGAAATGACGAAACTACTTGCAAGTGTTTTTCTCACCAATAGCAAATGGGCATGGGTT
TCCATGTTCATATTTGACTTGGCCATTCCCCGTAGTTTCTTTGTCATTGCTCTGACAATGTTAGCCCAGTATC
GCCCTCCGTCGTAATCCCCTGCGAGAAAAATCTTCATCGTTGCAAAGGCAGCAAATGCTGCCAGTGCTTCATG
ACATATGCCATGTTGAAGCGAAAACTGGATTACAGCACAAGATATCAATGGCACTATGGTGTGGCAGCAATGT
AAACTCTGTTAGATAAATGGGGGGTAACAGAGGGGTGTCGATTTTGACATTCGCATATGACTCACAAAAAGGA
GACGACGCGACATAGCAGGAGGTGTAGAAAGCGTCGATAATCATAACGGCGCGATGAACTGTATCATCAACAG
TCTTTCCGCATAGATCCATCATCTGGTCAGTGCTAAACTTGGACGTTATCTCATTCGTTGATGCCATGCCTTT
CATCACGCTTTCGGGTGTTGGGGCAAGAGGAATGTCGAACTTGAGTCGATGGAGAATTTGAATGCCCTTAGAA
ATAGATTCTTCGTGCTGCCCCGATTGAGATAATGACCTCAGAACGAATGGCTGGATGTCGAGACTGTCGTCAA
ATGCCACATTCTTCGTAATCTGTTCGGCGTACTGAGCGACATCGTCAGGCTCGCCAAGCGTAAACGATGCCGT
AACTGCAGCCTTGTGCAACGAGAGGCACAAATTATTATAACTCGCGTGCCAACAATCCCGTCCAAGAAATGAA
ATTCCTTTGGAGAAATAGTAAAGAGCTGCGCCTGAATTTGACTGACGATGCGCCTGTCGCCCGCTGAATAAAT
TCCACGTAGCAAATCTAACCCTTTCGATATCGTCGCCGATAGCGTCGGGACCTGCAAAATCAATCTGATCGCA
TGCGATGGAGATCAAAGACGACTCCGGGGTATCTAGCTGAAGGTGATCAAGATTGGATGTTTCTGGATCACTT
GTTGCGGAATCATCGATATTAGCTCGTTCACCGAGAAACTGCCCAATATCAAGATGCAGTGATTTCCGCTCAT
TCAAAGACATGCTCTCGTAAACAGCCTGCTGGATTAAGTCATGAGTGAATATTACTATTGGCCCTGCTCGATC
GAGAATCCCTTGTTCAATGAAATCATCAGTTGAGCAGACAATACCATTCTGAAAGTTTTCCAAGTATTGCAAT
AAGGTTGTGTCTGTCTGTATACCAAAGCACGATAAGATTTGCAGCACTCGTCTCTGGCTCGGCGGCAACGACT
TGAGATTCGACGCGATCAACCCTGCCACCCCGGCTCCCGTCTGGATTATATTGATCGTATCCTGGTCCCATGC
AAAGCGTCGGATTTGCGTACTATACGCAACGGTGGAGGAGCGTACCAGCGAGTTCAGCAGTTCCACGGCGAAT
AAGGCGTGGCCGGACGTCTTCTTGTGGACTACGTCGGCCAGCGGTGCGACGTATCGTCGGGGTAGCCCGAGCT
CTGCCATCAACACGTCTCCAATATCTTCCTTCAATAGGCTGGAAAGCTCGATCTTGGTGGTACTGATCTTCTC
CGTCATCATTTGAAGGTAGGTATATTGAACTGAAAATGGGTGATTATCGTCGATTTCGTCGTGACGAAACAAT
CCTACAAACAGGCATCGTGGAGAGACACGCCCGAAGCTCGCAACGTTGACGAGGATTTCTACTATTAACGCAA
GCGAAGTTTTATCCGCCCACTGCAGATCATCGCAGCAGATCATCATGAGCCGGTCAGAGGATGATTCCAAAAG
GGCCTCGATGATCTTCGATATAGAATAGATCAATCGAAAAGCATTCTCTTCTGCTTGCACCTTGATTGATTTA
TCGCCATCGAGACCAGGAAATAAGGAGCGAAGACTGGGCAGAAAGTCTACTAGCAATGCCAGCCTAGAATATC
CAATGGCATCAAGGATCAACTTCGAAGCTTGTCGGGAATAGGCAACATCGGATGGTCTCTCACTCCCTTTCAT
TTTGACCAGATTGACAATCAGCTGATCGAACATGAACGAAACAATACTTTTTGAGATGTGCTCCTTCCCTCGT
TCGAACTTTGCCTTGATGACAATCCATCCTGGGCTGCGCAACATTTGTATGACAGTCTGGATGAGATGAGACT
TGCCCGATCCGGCAATACCAGACACAAATATCGCATCGAGGCAGCCTGTGTCGTTAGGAAACCCCATCGTATT
TGTTTGCTGCAGGGATTCTTCCATTTGGTATGCAACTTTTCTGAGAGTTGCTACGTCTTTCCCCCTTCCGTGG
TAGGTATGGCCAAACATTGGCGGAGGTGGCATGCCCCGAGAGAAGAGTGAATTGTGCAAGAAAATCTCGGGCT
GGTGCACCATGCATTCCAAGTCTTGAATCACATCATCGTACAATCGAATTGGATGATTAACCTGGGATTTTGG
ACCGGTGTCCACCATGTCAGAAATCAATCTGCATATCGGATCAGGGAGAGAATTGCTTTCAATCAGTTTGGAG
AATACTGTTGACTTTTGGGTGCGAAACATTTTGGATGATCGACCAGTGTTGTCTTCTTCGCCTTGATCATTGA
CACGAGACACCGCTTCTCCTCGACTTGATGTGGTCGATCCTGTCAGCAGAGCAGAAGGGAGTAATCCGCCCAG
AGAAAAGACAGAATATAGAAGCTTCCCAAGGACGGTCATCCCTGCTGAAACTTCCAGTGTATTCTGCTCTTCA
CATATATCGGGAGTAATTATTGAGACGGACAAATCGATCCAGAGGAGAATCCATTTCGAAACCTCGCTTGATG
TAATGCATGAAGGGTCGTTGACGTGCTCGCCCCCAACTTCTTGTTTAAGATGAATTGAGAAATCATCCAATTG
AATTGATTCCAAATTGATACCCGGAAGACGGCCGACGAATGATCGAGCGAGCAGTAATGCCATTCGGGCAGCA
TTTTCCATTACCAAGCGGTCCTTGCTACCATCGCCATTGCTCCTTTCCGGCGAGGGTGGTCCAACTGCTTGAG
TATTTGCATTCACAAGAGCATCATTGTCCTCTAGTAACACCATGCGATTTTTCAAACAACCCGCCTTGAGGAA
CTCTCCCGCCGACACTGGAAAGCTCGTGATTGAATTACTGGCCTCTAAGAAAAAGAGGTTTCTATTTGTAATG
TTAGAAGACTCGATCAGACGCTCCACCATGGTAGTGGAGGTATCATTCAACTTTCCAAGTGGTTCATTTGCAG
AGGGATGCTTTGAGTTTGACGCCAGAATTGAAGAAGTCTGCACCACTGACTGAGGTGGCAGAAAGTTAAGCTG
GGGGGGTATTCCATTTTGCTGATGAGTGTTGTGGGCAGTCTTCATCGCCTCCCCCGGTACATAGGAAGACAGG
TTCGCTGGGGGGCAGCGTATCTTAGCTTGTTCTGTTGCCTGATTCAAACCAGAAATGCCATCGCTTCTGTTTA
CACTGCTCATGACATTCGTGGCATTGAATTGTTGATTCACCACTTGATTCAAATCAAACAGTTCCATCTGCTG

CATGTAGTGCAAAAAGCTGGACATGAATTCAGAAGAGCCAACAGCTGGTGAAGCTACTCCCCCTTGAGAAGTT
GATAAACCAGTCGGTCCTTGCAAGAAGCCGGCGGACATGGTGCTCGAGGGAATTTGGCCTGTAAAGCCACCTC
CTGCGCCAGTTGCTGCAACGGCCACCCTTTCATTATCAAGAGTAGAAAAACATCCATTGGAGCGTGCGTTCTG
TTGTCTCAGGAGATGAATCTGCTGCAATTGATGTTCCATCTGCTGCAGTAATAGTTCAGGATCCTCGCCTTCT
TTGTTCTCCATAGAGTTCTTGTCATTCTCGCGCAGGCGATGCATGACCTTCGACACGGCCTTTTCGGTATCGA
CGCATATCCAAGCTTTTGCATATATCCACTCCTTATCTTTCTTCCTCGCTTTTTGATCACTTCCATTTTGCGA
TGCTACATGATCATCGGGGTCATCCGTTAAAAATCTTCCTCCCAATTTATATTGAATATCACCCACGATCTTT
CTCGCAATGTCTCGTTTTTCGGTTTTTTTTGTTTTTAGAAAGAGTGGTTTGTGTGCGCGGACCATGCAGCGTA
GGATTTGATTTCCGGGGTGGGCGCTGATGGCGTAGCCACGCCCGAATAGTACATCGTTATTACGGGGAGTTAC
GTCCGCCATGTCGTTGGATCATAGCTCAAAGCATGCATTG
>TR298|c0_g1_i1 len=832 path=[970:0-127 971:128-158 @997@!:159-339
965:340-371 996:372-384 992:385-510 998:511-624 974:625-648 995:649-778
977:779-831] [-1, 970, 971, 997, 965, 996, 992, 998, 974, 995, 977, -2]
CAGAGACTTAAAGCCTCAAAATATTGGATTCGATGTACGAGGTGATGTGAAAATCTTTGACTTTGGAATGGCT
CGCATCGTGCCTGAAGGTGGAAATCCATACGATGATAGATTCAAGATGTCAGGAGTTGGTTCGCGAAGGTACA
TGGCACCCGAGTGTCTTATCGGTGAAAACTATAACTTGAAAGCTGACGTCTATACATTTAGCATTGTATTGTG
GGAGATGCTGTCTAGAGAAACCCCTTATTCGTTTGCGAGGACTAAAGAGCAACTAAATTATCATGTTGTTGAA
GAACAAGGTCGGCCAGATATAGATGATGCATGGCCTTCTCAAATCAAATGTATGCTGAGAGCCAGTTTTGATG
GTGACATGGATAAGCGCCCCGTAAGTTCAAGTGCCAGCGTCAATACAAATGCTGCTAATTTGGTTTCTTTCGT
TCTTATAATTCAATGATATCTGTACACATATTTCTTCCATTCAATCAAAGAAAATGATATTCTTTCATAACAG
TATCAAGGAGGCATTGATTATGTTGCGTGGCGGGGACAATACAGGATTGACTCGCTCTCATATTCTCCTCAGA
CGGTCGGATACCAGCATCACAGCCAACATTACCGACGTTTGTGGCACTAGCGCTCATTTTCCACAGGCCCCTT
TTTAAGACGTGGACAAGAAAGGGTATACATGTTGTCGCTCATGGATCGATTTCTACATCGGTCCAAGACAAAG
TGACTATAAAAGGCCTGTGACTAGCACATAGCAAATATGGCAACACAGCATAGCATAATTTTGCGAAAAGTTT
TCCCTCTGATGTTGTCAAGCATATATTAT
>TR298|c0_g1_i2 len=729 path=[970:0-127 971:128-158 @997@!:159-339
988:340-371 989:372-384 993:385-407 998:408-521 974:522-545 995:546-675
977:676-728] [-1, 970, 971, 997, 988, 989, 993, 998, 974, 995, 977, -2]
CAGAGACTTAAAGCCTCAAAATATTGGATTCGATGTACGAGGTGATGTGAAAATCTTTGACTTTGGAATGGCT
CGCATCGTGCCTGAAGGTGGAAATCCATACGATGATAGATTCAAGATGTCAGGAGTTGGTTCGCGAAGGTACA
TGGCACCCGAGTGTCTTATCGGTGAAAACTATAACTTGAAAGCTGACGTCTATACATTTAGCATTGTATTGTG
GGAGATGCTGTCTAGAGAAACCCCTTATTCGTTTGCGAGGACTAAAGAGCAACTAAATTATCATGTTGTTGAA
GAACAAGGTCGGCCAGATATAGATGATGCATGGCCTTCTCAAATCAAACGTATGCTAAGAGCCAGTTTTGATG
GTGACATGGATAAGCGCCCCAAAATGATATTCTTTCATAACAGTATCAAGGAGGCATTGATTATGTTGCGTGG
CGGGGACAATACAGGATTGACTCGCTCTCATATTCTCCTCAGACGGTCGGATACCAGCATCACAGCCAACATT
ACCGACGTTTGTGGCACTAGCGCTCATTTTCCACAGGCCCCTTTTTAAGACGTGGACAAGAAAGGGTATACAT
GTTGTCGCTCATGGATCGATTTCTACATCGGTCCAAGACAAAGTGACTATAAAAGGCCTGTGACTAGCACATA
GCAAATATGGCAACACAGCATAGCATAATTTTGCGAAAAGTTTTCCCTCTGATGTTGTCAAGCATATATTAT
>TR299|c0_g1_i1 len=2348 path=[2602:0-425 2615:426-538 2610:539-761
2572:762-785 2618:786-869 2609:870-905 2617:906-924 2613:925-957
2607:958-1339 2597:1340-1363 2611:1364-1778 2594:1779-1802 2606:1803-2157
2591:2158-2181 2592:2182-2347] [-1, 2602, 2615, 2610, 2572, 2618, 2609,
2617, 2613, 2607, 2597, 2611, 2594, 2606, 2591, 2592, -2]
ATCTATCTCGAGGAAAGGAAACATCAATTCAAGATTAGCCCAAGACTTCAAAATCTTCGCTCTGGTTCTGAGG
CTAATCATATCAGCGCAGAATCAACCAAAACCAGGAGAAGGATCAACCAAGGGAAGGATCAACCGAGGGAAGG
ATCAACCAAGCAAGGGAAGGATCAACCAAGCGAGATACACACCCGCCCAATTGCAATTGCAGTCTACGGATTA
TAATTCTATCCTGCCACTCTCTGCTCTTTGAGAACGCACTCATCTATCGCTCACGATACCCATTCCACAGCAA
CCGGGGGACTCGCATCCAAATTACACCCTTCTCTTTTCTCTTGACTCACATCCAAATTACACCATTCTCTTTT
CTCTTCCAACGCAAAAACTTATCATCTGTGCATCTGTTCTCGGAGGCGGTGGAGGTACCACGTGAGTCGGAAT
TCGGAATGCAAATATTATCTCAATCTTTGTTAATAACTGCAACTGTCGAATGATAATAATACGATTATCACAC
AACAGATTGTAACCATGGACGCGATCCCTCTTCAAGAAGACCGTGTCAATAATGGCACCGGATGCTGCAACAG
CGATCAAGATCGAGACACGAGTCTAGGTTTGCTGCAGGATAATGAAAACAAAAACGCGACACAAAAGGACATT
TCCTTGAGACGTCCTGCTCGAAGGGCACCGCTTTCCTCAAGAATGCACTCCAGTCTCCGCGTGAGATCCAATA
ATTTGACATCAATGTTTACCAATAGTAACGCGGCTGAACGTATTGTCGAGGTTAGCGATCACGAAAACGAAGT
TGCTGGATACGTCTCTCCTACGAATGCGGCGCCATTGGCGGACAAGGAATGTTTTGATGAGGAGCCTTGGACA
GAGCCAATTACCAGGTCGATGCAATTATACGACAAAGAAGAGGGTTCTGCCAACATTCGTAGCAACTCCAAAG
ACGACACTGACTCTATGTGCTCCGAAACCTGCCCCGTAGATGCGTTAGCAATTGGAATCGGTGCTCATGAATA
CCTGGAGGAGTGCTTCTACACTGAAGTCTCGGTTTTAGATGAGGAGAAGTTCAATGCCATCCCAGAGATTCTC
AAGTCAGACCTTACAATTATGGAGCATATTGGAAAGGGAAGCTTTTGCGACGTTTTCGAAGTTGTTCAGAATG
GCGAACGACTTGTTGATGTTGAGACAAGCAATGGCATCTCTGCTGACACGCAGCCTAAACGCAGACGTTTTCC

TCTTTCCGGTTCCATCACTAAGGCGACACTGGCAAGGCCTTCCGCGAGAGGCATGAGAGCTTCTCTCTCGAGG
TCCATCACAACCGCGACATTAGTAACTCATTCGCAGTGTGAGAAGCGCAACCCCGTCTTTGCTATGAAGTGCC
TACGTCCTCAGATTCGATCTGACGCTGACCAATTCACTATCGGAGCTGAAGATCTAATCCACGAAACAGCTAT
CTTATCCACTCTCAACCACCCCAACATTATTAAGTTGCATGGCCATGCCTCTGGGAACCTATCCGATGCATTT
GTTTGTAATGATGGGTATTTCATTCTTCTCGATCGTCTGACAGAGACATTCGTAGAACGCATGATGGTGTGGA
AACAAAACCCCAAGTGTTTCCTTCAAAGACCCACAGCGAATCAGATCAAAGTAGCCCGTTCAATAGCCGATGC
TGTATCTTACCTGCATTCGAAGAACATCGTCTTTCGCGATTTGAAGCCTGACAATATAGGGTTTGACTCGACG
GGGACACTAAAGCTTTTCGACTTCGGGCTTGCGATTGGTCTACCAGAGAAAGATGACTCGAATCCGGATGGTC
TTCTGTTTGATAGGTGTGGCACTCCTCGTTACATGGCACCAGAAGTTGGGCTTTCCCTAGGGTATGATGTGAA
AGCTGATGTGTACTCATTTGGGATCCTGCTCTGGGAAATATGTGCATTGACCAAGCCATTTTCATTCATCACT
TCGGCGATTGAGTTTGAAAGGACCGTTTATGTTGGAGGGAAACGTCCTGTGAATGAGACTTATTGGCCAACTA
CCCTGAAGGAACTCATCATCAGTTGTTGGTCTTGTAGCCCAGACAAAAGACCAACCGATTGGGTGGCCATTAA
GTCCTCCCTGTCATCTATTGAAAAGAAGTTTCAAATGAGAAGTATCCGATCCAGAATAACTAGGAAGTGTTCA
GATCAATCAGATTAGAGGTTGGCCCCAGTGCAATGCAGTGTACAGTGTGTAAGGGGTCCTGGACTAAGTTCCT
ACACAAATCATTTTAATGTAATCAATCATGTATCTACTATTTAGAGGCAGTAAATCTGAACTGTTAATTGAAT
CGACTTTTCCTC
>TR299|c0_g1_i2 len=2258 path=[2599:0-425 2600:426-448 2610:449-671
2572:672-695 2618:696-779 2609:780-815 2617:816-834 2613:835-867
2607:868-1249 2597:1250-1273 2611:1274-1688 2594:1689-1712 2606:1713-2067
2591:2068-2091 2592:2092-2257] [-1, 2599, 2600, 2610, 2572, 2618, 2609,
2617, 2613, 2607, 2597, 2611, 2594, 2606, 2591, 2592, -2]
ATCTATCTCGAGGAAAGGAAACATCAATTCAAGATTAGCCCAAGACTTCAAAATCTTCGCTCTGGTTCTGAGG
CTAATCATATCAGCGCAGAATCAACCAAAACCAGGAGAAGGATCAACCAAGGGAAGGATCAACCGAGGGAAGG
ATCAACCAAGCAAGGGAAGGATCAACCAAGCGAGATACACACCCGCCCAATTGCAATTGCAGTCTACGGATTA
TAATTCTATCCTGCCACTCTCTGCTCTTTGAGAACGCACTCATCTATCGCTCACGATACCCATTCCACAGCAA
CCGGGGGACTCGCATCCAAATTACACCCTTCTCTTTTCTCTTGACTCACATCCAAATTACACCATTCTCTTTT
CTCTTCCAACGCAAAAACTTATCATCTGTGCATCTGTTCTCGGAGGCGGTGGAGGTACCACATTGTAACCATG
GACGCGATCCCTCTTCAAGAAGACCGTGTCAATAATGGCACCGGATGCTGCAACAGCGATCAAGATCGAGACA
CGAGTCTAGGTTTGCTGCAGGATAATGAAAACAAAAACGCGACACAAAAGGACATTTCCTTGAGACGTCCTGC
TCGAAGGGCACCGCTTTCCTCAAGAATGCACTCCAGTCTCCGCGTGAGATCCAATAATTTGACATCAATGTTT
ACCAATAGTAACGCGGCTGAACGTATTGTCGAGGTTAGCGATCACGAAAACGAAGTTGCTGGATACGTCTCTC
CTACGAATGCGGCGCCATTGGCGGACAAGGAATGTTTTGATGAGGAGCCTTGGACAGAGCCAATTACCAGGTC
GATGCAATTATACGACAAAGAAGAGGGTTCTGCCAACATTCGTAGCAACTCCAAAGACGACACTGACTCTATG
TGCTCCGAAACCTGCCCCGTAGATGCGTTAGCAATTGGAATCGGTGCTCATGAATACCTGGAGGAGTGCTTCT
ACACTGAAGTCTCGGTTTTAGATGAGGAGAAGTTCAATGCCATCCCAGAGATTCTCAAGTCAGACCTTACAAT
TATGGAGCATATTGGAAAGGGAAGCTTTTGCGACGTTTTCGAAGTTGTTCAGAATGGCGAACGACTTGTTGAT
GTTGAGACAAGCAATGGCATCTCTGCTGACACGCAGCCTAAACGCAGACGTTTTCCTCTTTCCGGTTCCATCA
CTAAGGCGACACTGGCAAGGCCTTCCGCGAGAGGCATGAGAGCTTCTCTCTCGAGGTCCATCACAACCGCGAC
ATTAGTAACTCATTCGCAGTGTGAGAAGCGCAACCCCGTCTTTGCTATGAAGTGCCTACGTCCTCAGATTCGA
TCTGACGCTGACCAATTCACTATCGGAGCTGAAGATCTAATCCACGAAACAGCTATCTTATCCACTCTCAACC
ACCCCAACATTATTAAGTTGCATGGCCATGCCTCTGGGAACCTATCCGATGCATTTGTTTGTAATGATGGGTA
TTTCATTCTTCTCGATCGTCTGACAGAGACATTCGTAGAACGCATGATGGTGTGGAAACAAAACCCCAAGTGT
TTCCTTCAAAGACCCACAGCGAATCAGATCAAAGTAGCCCGTTCAATAGCCGATGCTGTATCTTACCTGCATT
CGAAGAACATCGTCTTTCGCGATTTGAAGCCTGACAATATAGGGTTTGACTCGACGGGGACACTAAAGCTTTT
CGACTTCGGGCTTGCGATTGGTCTACCAGAGAAAGATGACTCGAATCCGGATGGTCTTCTGTTTGATAGGTGT
GGCACTCCTCGTTACATGGCACCAGAAGTTGGGCTTTCCCTAGGGTATGATGTGAAAGCTGATGTGTACTCAT
TTGGGATCCTGCTCTGGGAAATATGTGCATTGACCAAGCCATTTTCATTCATCACTTCGGCGATTGAGTTTGA
AAGGACCGTTTATGTTGGAGGGAAACGTCCTGTGAATGAGACTTATTGGCCAACTACCCTGAAGGAACTCATC
ATCAGTTGTTGGTCTTGTAGCCCAGACAAAAGACCAACCGATTGGGTGGCCATTAAGTCCTCCCTGTCATCTA
TTGAAAAGAAGTTTCAAATGAGAAGTATCCGATCCAGAATAACTAGGAAGTGTTCAGATCAATCAGATTAGAG
GTTGGCCCCAGTGCAATGCAGTGTACAGTGTGTAAGGGGTCCTGGACTAAGTTCCTACACAAATCATTTTAAT
GTAATCAATCATGTATCTACTATTTAGAGGCAGTAAATCTGAACTGTTAATTGAATCGACTTTTCCTC
>TR300|c0_g1_i1 len=978 path=[2473:0-88 2517:89-928 2488:929-952
2489:953-953 2490:954-977] [-1, 2473, 2517, 2488, 2489, 2490, -2]
TCGTGAATTACGATTGGCCAAGTCGAAGAAAGAACAACAACAACAACAACAGGATGTAGGAGCAGCATCAGTT
ACAGGTTTTAGCAAGGGAAAGAAGAAAAAGAAGACTGGTGCCAAATCGACATTGGGATTGGAATTAGTTGCTT
CTCCTGAATTATTATTGAAGCATAGTGGTGAGTGGTTGAATAAGGAAGATAATGCCAAGGGTGGTCAGAAAGA
GATGGAGGAGAAACATCAACATCAGCAAGGTCCTACACTAGTATCTCCTGAGAAGCAGAGTATTGACCAAAGC
CAACATAGTTCCACAAGGCCTCCCAACGAGGCAAAGGCTGTAGTCGTGACGCCAGAACCGCTGGAGAAATCAA
GTCGCAGTCGTCTCAAGCTGCGCCATCGGCTCGGTGGCGGTAGTATTAAGAACAGCAGCGGGAGCAATGCCGT

GCCCGAGTTGTACACAGCTTTCAATACTCCTCCCAAGGATGAGGAACGAAAGAATGGTGGTCACATTGCTTCG
TCTGCTGCCATGATCGGCAAGGCTGCGGGCAATGATGGTGCGTTAGGTCAAGGACCTGTTGGTGGTAGTAGCA
GTCGAGAACAACAAGGTGGCGTCACACTGTCATCCTACCGTGTTCTCTCCAAAGGACGAAGTCGATCCAGCGC
ATTGCCCAAGATGCGTCGTCTTGGTGCTAGTCAACGTGATACACCGTCCGCATCCCCCAAGGCTATCAAGAAT
AATGCATCGACATCGTCCACAACAGCACTACTATCATCATCACAGCAGCAGCAACCTGATAATAACACAGCAT
CCTCCGCAGCAGCAGCAGCTGCACCTACCAAGAGGGCAACATCATCCCGAGGATCGAGTATGAAACGAATAGC
CAATGCCAAGGCGGCAGCTGCCAAACAAGAGCGTATGCAACAGCTGTTGCGTGCGAGATCGGTAGAACGTAAG
CGGAATCAATCCCTGGAGAGGGATGGTGT
>TR300|c0_g2_i1 len=1171 path=[2493:0-39 2494:40-63 2523:64-491 2500:492-522 @2522@!:523-637 2515:638-786 2505:787-825 2506:826-849 2521:850-911 2513:912-988 2479:989-1012 2518:1013-1046 2512:1047-1070 2477:1071-1170] [-1, 2493, 2494, 2523, 2500, 2522, 2515, 2505, 2506, 2521, 2513, 2479, 2518, 2512, 2477, -2]
CAATCCCTGGAGAGGGATGGTGTTGGTACTGGTCAAAATACTGGTGCTGATACTAGTCGAGAGCAGCAGGTGC
AGTCACAACGGTATCAGCATGAGCAGCAACATCAAGTAGAAATGCCAATACCACAACGAAAGTTGCTGGACAA
GGAGATAGAACGCCGCACCCAATCACAACCGAGGTCAATCAAGCCCAAGGAAGAGGATTTATTCGGCGATCTC
GACGAACCATTCGATGAGAAAGAAGGCCACATTGCTGGCGGCAATGCTGCAATGATTGCGTCAGCTTCTGCGG
CAGCTACAGCACGAATGGGAGAATTACCAGATCAGAATACGCGTGATTATCTCAAGACCAAAGGACTTGAGAG
TGTACGAGCTGCACGATCGGGACGAGTACGTAGTTTCTTCTCCAATAGGAGGAGCATGTCGACTGGCAAGGCT
GGTAGGAAGAGTGCTGCCACTGACAAGCAGATCAAAGTCACTGCAGTGGAGCAAGTTAGTGTGGAGGGTGAAA
ATAACCAGGATAATAATGTTGACGATCATGAGGGTTTGGCAACGCCAGCGAAATCGAATAGTAGCAATGATGT
TGTTGCTAAGAAGGGTGTGGCGGGTTTCCTCAGGGGTCGCAGTTTCAAGAGCAATGATAGCGAGAACAAAAAC
AGTCCTTCCGCGAAAACGAAGACGATGGATGGTACGCCTAGTTCCACTTCCGCGGACTCTTTCACAATCGACG
CTAACAAGAACACTACCGCCGACGTACAACAACAGCAACAACACATAGCCAAGGACGAAGCTTATACCAGTGT
CGAAGTCGATAATTTACAACCCGTTCTCGAAGACGAAGAAGTATCACATCCCATGGACGAAGTACTGGTAACT
CCCATCTCCATAGTCACACCGCCTAACACCACCAGTACTTCCGACAGTGCTTCCAATAATATGAAACTACCCT
CGCCTGCCATGGTACGCGATCGACGTGCCAAGATGGTCGTACAAGAACGGTTGCAAAACATTCAGGAGGAAGC
GAAGCAGAAACAGATTCGACGCGATAATCTTCGTATCAAGCAGCAGGAGTTAGAGCAGCAACGTGCCGAGTTG
GAACGTGTGGACTTGATTCAACATCAACATCAACAGCAACAACGAGTAGTAGTAGAACAGGGAAAACGCGTGA
CGT
>TR301|c0_g1_i2 len=2363 path=[2542:0-227 2566:228-229 2564:230-247 2563:248-855 2561:856-1003 2559:1004-1209 2547:1210-1233 2562:1234-1294 2538:1295-1311 2558:1312-1541 2518:1542-1542 2519:1543-1566 2520:1567-2362] [-1, 2542, 2566, 2564, 2563, 2561, 2559, 2547, 2562, 2538, 2558, 2518, 2519, 2520, -2]
TCTTCCGATCTTCCCTTCAATAAGCATCTCCAACTCCAAATGTACCTAGGGTTGTAGTTTTATGTCTCATGTC
TCCACGCATGCAGAGTCGTGGCCAGGTCGGGGCAGTCGTGGGTAATATTTACTAGGAGTAGCTCGATCTTCTT
AGAACTTTCTCACGCTCGCTTCAATAACAATGCTCATAAAGTATCAAGATAGACATAAACAATGAATCATTCC
ACTACAAAAAAACAAAGCACCGACCATGTCTATCAAGAATATCTTCTCTCCCACGCCACAGAAAATATCTCCT
GATCCATCACATACAAATATGAATAAAGAAAACACCGAAGCCCTGACTTGGCAGCTACCGCTGCATACACCTG
TCTATGATTCTACGATGAAGCCATCCGTCGTGGAAATTGCAGTCCCGGCTCGTCCGCAGAAGAAGCCAGCACA
TCCTCACCGGAAGAGTGTTGCTAGTGCGATAGGAAGACCTGTTGCTGCTGGTGTTGCTGTCGATAGGCGAGCC
TTGTTGCAGAACGAGGAAGATCGGATACTCGTGAGAAAGCTGCGCAAAATCAAGAGAAGATTGTTTCTCGAGG
CGGTGAATCCAGAGCAACCGTTACCTATCAAGTCGCCTCGGAGTGTTGTGCGTCAAGAACTTACAGGCAAGAA
AAGTCGGACGGGTAAAGAATGAAACACGGAATATGACGTAAACGTTGCGCAGAGTGCCAATTCACTCTATTCT
CTGGAGCTGTAAATGCTCTCTTGCACTTGTCCAGGTCGGAAACAGCACGAGTGCTGAGAGCTCCAGAGGCACG
GCTTCTCTCCTGTAGCATGCATTGCACAGGTTTTCTGAAAGTCGGGACAAGACTGCCGAGATGGGATCCATCA
CATTTTTCTAGGAGCACACATTAGCGAAAATCTTTAGTATCAAGTCCTCTCATGGGCGTGGGCCCAGTCTATC
GACATGTATCAAATTGTATAGAGAGATATCACGGAATCTTCTCCAGATACTAGGAGTATACCATAATCTTGGA
AAAATCACTTGAGACAACTTGTTAGAAGTAAATAATGTGCTGAAAATAGGCTGATCTTTCGTAAACAGGATGT
TCATAAATAATATGTAGTTGAGCCAGTGAAGATAAAATAACCGACACGTACCTAGCAACAATTTTCGGTGGGG
TGTTTAGCGTCGATAATTTGCAAACAAGCATCAGCCTGGTATAAAAGTAAACGCTGGGAATAATATGAGTTTT
TGCAGCTACAGTGGCGTCGCTTCTATCCCTGTTGTTAGTTTTGATCAGAAAAAACTGTTCGGGTGATTTTGTA
ACTGAGTAGTTTGTGCCAAGAGGTAATGGTAATACAACGCGTTGGGAGGGTCAACTAGTAATATAACCCAGTT
GATTATATTACTCTAATCGTAAGCATTATGTTCATAAATTGTAGCGTGGTCTTCTGTGTGTGTGCTCTTCCTG
TGTGAATGATTTTCGACAAGTGGCATTTAGCAACGGTGCATCTACCCTGGCGCAGCTTGTCCGGAAGGTTGTC
TAGCAAGTGTTCCCCATATAGCCGCACCGCCCTTACGGTGTACACAAGTATTTTCGCTCTTAGTTGTCCGCCC
GAACAAACCCGGCTCAACCTACTAGCAAGTCATAACTGGACCGATCCCAAGCATAGCCATCTCTCATCAGATC
ATTTACCAACCATCATCCGCCTCCTCCGCAATCCTTTTCTTATCCTCCTCTGACATTTGCAATTGCTTCTGGT
CCGCACCCCCCGTCTCCATCAATGCAGCCTCGAGCTTATCTGTCAAACTGCTCAAGTCAGCAACCGAGTCTAC

CTCGACATCTTCCATTAATCCACCGGTGATTCGTGTGGCCATATCGTCGGATAGGTTGCTGCGCTCTCCCATG
CTTTCGGCGAAGGACACGAGGCCGATTCCTGCCACGAGGCCGGCTACGGCAACTCCTCCTCCCATGAGAATTT
GAGTGTCAACCATGGAGAGGCTGGAGGAAGATGACTTCGTCGGCGAGGCATTTCGCGGCGTATTATTATTGGA
TAGGAGGCTGGATCGGGAAGAGGAGGGTTGGACAAACGCAAAGCCGTGCGCCAAAGGGGCAGAGAGGCAGAGA
AGCGTGGCTACTGATTTCATCATGATAACTTGGTAGAGAAGGCGTGGTATCGTACAGGGATTAAAAAATGGTC
TTAATGTTGTCCCGTTGGATCCCATTGCATTCGTTCAGCATGTGCAGCTCCTCACTGACGCTCTGAGGATGAC
GCGTGTGACACGTGTGCGAAAATAGTACTGTGTGTTTGGAAAGATGAAGGGCAACCAAACCAGGAGATTTTTA
CTTTTCAATTATTCATATTATCAAGAG
>TR302|c0_g1_i1 len=3716 path=[3919:0-1098 3920:1099-1122 3926:1123-1380 3917:1381-1404 3928:1405-1461 3914:1462-1485 3925:1486-1660 3901:1661-1684 3930:1685-1893 3927:1894-2251 3924:2252-2465 3909:2466-2489 3910:2490-2496 3911:2497-2520 3912:2521-3715] [-1, 3919, 3920, 3926, 3917, 3928, 3914, 3925, 3901, 3930, 3927, 3924, 3909, 3910, 3911, 3912, -2]
TCTCTATCCAAAACCACAACTGCTTTCCCCCATGATTTCAGTTCACAGCTGTGGTTTTGGAGATGGTTGAGAT
GTCGGTTGATTCATTTTTCATAAGTGGCACTGTTGTGTCTTCAACTTTTCGACAGGTCTAAGATAACGGAGTA
AAGGGGCAACCAGAAAAGACCACGCCGTGTGACGGGTAATTTGGGAGACATAGCTGGACCAAACTGGACAACA
ACTAATACAACGTGCTGGTGACAATTAGATGTAACGTAGTTATTAGAACACAAGAACTAATATTTAGCAATTG
CCACAAAACCACCAATGCACTTCATTCATGATATATTGCACCATTGTGATTTTATGCAAACTTGAGATACTAA
TAACTTTTCTACCCATGGTGAACGTGTGCGTTTTTCGTCTATACCACAGATGTTTCAATGGTGATTGGACAAA
TGAGTTGTTCTCACGGGTTAGTGTTAGCGGGATTTGTCATCCACGCTGGCTTGTTCACGTGCGCACCACGTCC
CCTTCCACGTCCTCGCCCCCTGCCTCGTCCTTGCCCGTTGGCAGTTACTCCACCAGATGGACCTATTGCATTG
AGTGCGTTCATTTGCGTAATGGCAGTGGCGGGTTGATTCACACTTGGCACAGGAGCCTGTTGTCCCATGCTCG
GCGGCCCATTGCTGGGTGCACTTTCCTGCCTTGTCATCCATGCTGGCATGTTTACGTGCGCGCCCCGTCCCCT
ACCAGCACCACCAGGCGGAACAGCAACAGAGGTATTCGCCGAAAACCTATTGCTGCTAGACAAACTGGAAACT
GATGAGTTAGTTCCATGAGCATGTGGGTTCAAATTTTCAACTGGTGCAGGAGCCTGATGTCCCATTCCTGTTG
GTCCATTGCTTGACGCACTGCCTTGCCTTGTCATCCACGCTGGCTTGTTTACGTGCGCGCCACGTCCCCTACC
CGCACCACCAGCCGGAGCAGCTGCAGAAGCGTTCGATGAAGCAGAGGTGGGATAATTGGCATTCATTCCTGCC
ACATCTGTGCCTGGATTGGTGGCCGAACCGACGCCATTGCCGGACATTCCTCCTGTGGGTCCAGGATTTGTCG
GGCTGTGCCCTGGTTGAGTCATCCAAGCCGGTTTGTTCACATGAGCTCCCCGTCCTCTGCCAGCAGCTCCGGT
GGGAGCAGCGGGGGCGGGCGCTGTCTCATCTGACGCCCTATATCGCTTTTGGGGTCGGACTTCCCCATCGCTA
TCTTGACTTCGACTGCGGGATTCTTGCCCATACCCGCTTCCACCATGGTCTCGGGAGCCGCCGTCCTGACTAC
CACGTGAACGACGAGGCTCATCCCCTCGGTAATTATCACGACTGTAATCTCCACTTCTAGATCTGCCGCGAGA
GTAGTCCTCACGGTCATACGACCTTCCACCATCCCTGCCACGGCCGGAAGATCGACTGTCAAAGCGACTGTCG
CTGCGTCGAGAAGAATATTTGTCAGAGCGGGAGGAGTCGTAGCGGTCCCGACTTTCATACTCACGACCTCGGC
TTTGGTCACGACTAGAATCATCTCGGCCACGGCCACTATTATCTCGGGAGCGCAAATCATTCAGTGCATCCAT
ACCGGCCTTATAATTTTCTTCTCTGGGAGCGGAGCGTTCGTTCCGATCAGCAATCACCGGTGGCTCCCGTTGG
ACAGACTCGACCTTGATATCAAAACTATTAGTCCCCCTCCGATGCGTATACGAACCCTTAGTCTTGAACACTT
TTGAAGAAGAGTCCTCACCACGTTTTCGTCCCCATCCATCTCCTCGTGCGGCAATGGATGTGTTGTGTCGTGC
AGCAGGTGACGGAATACTTGTAATCGATTGCCCGCCGCCGCTAGTTGGCCTAACAGGGGACTTTCTCTCGTAA
GTATCATTACTAGTTGCATGTTGTTCCGGAGAACGGTTCCGACTGTGACCATCCCCAGCAGGGGCTGGATTAT
GCCCTTGAGGTTTGGGGGCATCAAATCTCGACCGTGAGTCAGCCGATCTCGGGGCCTCCCTTCCTCGTGCATA
TGATTCTCTGACAGGAGCTGGGGTTACTGCTTTCGGCAGGGACGTAGCGGCTGCACCATTCGCCCCACTCTGA
AACGTTGAAGGAACTTTTCTCATGGCTGCCCTCATACCTTGCATTCTCTTCTTTCTAGCCTCATCGTTCTTTT
TCTTCATTTCTTCCTCACTGATACGAAATTTTTTCGCAGACTTCTCTGCGGCACCATTTGTAGCAGGTTTAAC
ATCAGTGGCGGCAGCTAGTTTCGGTTTGGCTGTGCTCTTGAGACCCTCCAAATGGGTCTCTACTTCTTTCAGC
TCTTTGTCCAAAAAAATGGGACGGTCCGCACTGATTGATAACCTTGCGTGCCGCTTTCACATTTTCCACCCAAT
CAATTCCATAATCTGTATTTAATTTTTGCAGTGCTTTCCCATTCTTAATCAAATGACTACTGCCTTTGATGAA
GGCGAACAGTGATTTCATGATCTTCACAGCAGTATAATTTTCGAGAGACTCGTCTTTAAGACCTTCGGAGGAT
AATTGCAATATACGTTGAAGGGAATAATTCCACCATGGCCCAAATACGACCGCGCCCCCCATTTCTTTATTCT
TGAACATTCTGTTATACACCGCTTTCTCAGATCGTTGTATGGCTTCAATATCGAGAAGAACAGTTTCCTCCGA
TATACTCGATTCAAATTCCTCACCACCCGTACCATGCACCCGGTACATATCCCTATCGAAGAAAGACTTTTCC
GTGCCCCTTATGACGCGATACTTTTGGTAAAAATGACGGGCATCTCCCGCGTTCTCTGCCTTTTCGAGCGGTG
GTTCACCCAACATGGCAATAGCTCGAAACATTTCATCTTCCGAGTACTTGCCTTGATTTTTATTCATTTTTAT
GTTGCATCTATCGATGGCGTGTTCTTCTTCCGGCGTGAGAAAACTGTTTAGGCCGGTGGGGACGCCGTCGTCG
TCTGAGTCGGAGTCTTCTAGAATGTGACGTTTCGACATGATGGGATTGCTCTGGTGATTGCTCCTCGCCTACG
CGGCTCCCACGCAAATTCAATACTTTTGGTGGGTATGAAGATGCCCTGATGGTGGTGGACGGCTACTGCTGTT
TTATTGGTGTCCGAGGTAGAGGGGAGGAGGTGGGACCCAGCGTAGCCGAAGAAGCGATACTAATAATCTGTGA
TGATGACAATGGGACACTGCTCAGGACCAATCAAAAAGAATCCTATGTCAACGCGCTCCGAAATAATGCGAGA
TCGATGTTAGATTCAGCTATCTTTCTTTAGGGGGGCGGATGAACTTCGGGTGGGGCTACGCTGGGCGCTGATA

ACTAGAATAAAGCGCGCGATTTGAGGGTCAAATTTTCGATTCGGTGTGTCCTTTGTCCATCTTTCGCTTTGTC
TGCCGCTTATATTATGTAGGAGTACTTACGATACTTCGTCGCCGAAGGAGACCATGTCAATTAGCGATGATGT
TTCAAAAGAAAGATATCGAAAATTGAGATTCTACATCCATACATACGAGACATTTATCGCCAACATAGTGAGT
GTCATCCCACGTGCTGATCCCAGCGCGCGCCACGCCCGCGCCATCTTGCCACATCACCACAACATATGATGTC
ACAGCTGGAACATATTAATACTGGTCGAGCGATATTTTAGCAATTAAATTTAGTACAAGTGTACGG
>TR302|c0_g1_i2 len=3381 path=[3919:0-1098 3920:1099-1122 3926:1123-1380
3917:1381-1404 3928:1405-1461 3914:1462-1485 3925:1486-1660 3901:1661-
1684 3930:1685-1893 3906:1894-1916 3924:1917-2130 3909:2131-2154
3910:2155-2161 3911:2162-2185 3912:2186-3380] [-1, 3919, 3920, 3926,
3917, 3928, 3914, 3925, 3901, 3930, 3906, 3924, 3909, 3910, 3911, 3912, -
2]
TCTCTATCCAAAACCACAACTGCTTTCCCCCATGATTTCAGTTCACAGCTGTGGTTTTGGAGATGGTTGAGAT
GTCGGTTGATTCATTTTTCATAAGTGGCACTGTTGTGTCTTCAACTTTTCGACAGGTCTAAGATAACGGAGTA
AAGGGGCAACCAGAAAAGACCACGCCGTGTGACGGGTAATTTGGGAGACATAGCTGGACCAAACTGGACAACA
ACTAATACAACGTGCTGGTGACAATTAGATGTAACGTAGTTATTAGAACACAAGAACTAATATTTAGCAATTG
CCACAAAACCACCAATGCACTTCATTCATGATATATTGCACCATTGTGATTTTATGCAAACTTGAGATACTAA
TAACTTTTCTACCCATGGTGAACGTGTGCGTTTTTCGTCTATACCACAGATGTTTCAATGGTGATTGGACAAA
TGAGTTGTTCTCACGGGTTAGTGTTAGCGGGATTTGTCATCCACGCTGGCTTGTTCACGTGCGCACCACGTCC
CCTTCCACGTCCTCGCCCCCTGCCTCGTCCTTGCCCGTTGGCAGTTACTCCACCAGATGGACCTATTGCATTG
AGTGCGTTCATTTGCGTAATGGCAGTGGCGGGTTGATTCACACTTGGCACAGGAGCCTGTTGTCCCATGCTCG
GCGGCCCATTGCTGGGTGCACTTTCCTGCCTTGTCATCCATGCTGGCATGTTTACGTGCGCGCCCCGTCCCCT
ACCAGCACCACCAGGCGGAACAGCAACAGAGGTATTCGCCGAAAACCTATTGCTGCTAGACAAACTGGAAACT
GATGAGTTAGTTCCATGAGCATGTGGGTTCAAATTTTCAACTGGTGCAGGAGCCTGATGTCCCATTCCTGTTG
GTCCATTGCTTGACGCACTGCCTTGCCTTGTCATCCACGCTGGCTTGTTTACGTGCGCGCCACGTCCCCTACC
CGCACCACCAGCCGGAGCAGCTGCAGAAGCGTTCGATGAAGCAGAGGTGGGATAATTGGCATTCATTCCTGCC
ACATCTGTGCCTGGATTGGTGGCCGAACCGACGCCATTGCCGGACATTCCTCCTGTGGGTCCAGGATTTGTCG
GGCTGTGCCCTGGTTGAGTCATCCAAGCCGGTTTGTTCACATGAGCTCCCCGTCCTCTGCCAGCAGCTCCGGT
GGGAGCAGCGGGGGCGGGCGCTGTCTCATCTGACGCCCTATATCGCTTTTGGGGTCGGACTTCCCCATCGCTA
TCTTGACTTCGACTGCGGGATTCTTGCCCATACCCGCTTCCACCATGGTCTCGGGAGCCGCCGTCCTGACTAC
CACGTGAACGACGAGGCTCATCCCCTCGGTAATTATCACGACTGTAATCTCCACTTCTAGATCTGCCGCGAGA
GTAGTCCTCACGGTCATACGACCTTCCACCATCCCTGCCACGGCCGGAAGATCGACTGTCAAAGCGACTGTCG
CTGCGTCGAGAAGAATATTTGTCAGAGCGGGAGGAGTCGTAGCGGTCCCGACTTTCATACTCACGACCTCGGC
TTTGGTCACGACTAGAATCATCTCGGCCACGGCCACTATTATCTCGGGAGCGCAAATCATTCAGTGCATCCAT
ACCGGCCTTATAATTTTCTTCTCTGGGAGCGGAGCGTTCGTTCCGATCAGCAATCACCGGTGGCTCCCGTTGG
ACAGACTCGACCTTGATATCAAAACTATTAGTCCCCCTCCGATGCGTATACGAACCCTTAGTCTTGAACACTT
TTGAAGAAGAGTCCTCACCACGTTTTCGTCCCCATCCATCTCCTCGTGCGGCAATGGATGTGTTGTGTCGTGC
AGCAGGTGACGGAATACTTGTAATCGATTGCCCGCCGCCGCTAGTTGGCCTAACAGGGGACTTTCTCTCACTT
CTCTGCGGCACCATTTGTAGCAGGTTTAACATCAGTGGCGGCAGCTAGTTTCGGTTTGGCTGTGCTCTTGAGA
CCCTCCAAATGGGTCTCTACTTCTTTCAGCTCTTTGTCCAAAAAATGGGACGGTCCGCACTGATTGATAACCT
TGCGTGCCGCTTTCACATTTTCCACCCAATCAATTCCATAATCTGTATTTAATTTTTGCAGTGCTTTCCCATT
CTTAATCAAATGACTACTGCCTTTGATGAAGGCGAACAGTGATTTCATGATCTTCACAGCAGTATAATTTTCG
AGAGACTCGTCTTTAAGACCTTCGGAGGATAATTGCAATATACGTTGAAGGGAATAATTCCACCATGGCCCAA
ATACGACCGCGCCCCCCATTTCTTTATTCTTGAACATTCTGTTATACACCGCTTTCTCAGATCGTTGTATGGC
TTCAATATCGAGAAGAACAGTTTCCTCCGATATACTCGATTCAAATTCCTCACCACCCGTACCATGCACCCGG
TACATATCCCTATCGAAGAAAGACTTTTCCGTGCCCCTTATGACGCGATACTTTTGGTAAAAATGACGGGCAT
CTCCCGCGTTCTCTGCCTTTTCGAGCGGTGGTTCACCCAACATGGCAATAGCTCGAAACATTTCATCTTCCGA
GTACTTGCCTTGATTTTTATTCATTTTTATGTTGCATCTATCGATGGCGTGTTCTTCTTCCGGCGTGAGAAAA
CTGTTTAGGCCGGTGGGGACGCCGTCGTCGTCTGAGTCGGAGTCTTCTAGAATGTGACGTTTCGACATGATGG
GATTGCTCTGGTGATTGCTCCTCGCCTACGCGGCTCCCACGCAAATTCAATACTTTTGGTGGGTATGAAGATG
CCCTGATGGTGGTGGACGGCTACTGCTGTTTTATTGGTGTCCGAGGTAGAGGGGAGGAGGTGGGACCCAGCGT
AGCCGAAGAAGCGATACTAATAATCTGTGATGATGACAATGGGACACTGCTCAGGACCAATCAAAAAGAATCC
TATGTCAACGCGCTCCGAAATAATGCGAGATCGATGTTAGATTCAGCTATCTTTCTTTAGGGGGGCGGATGAA
CTTCGGGTGGGGCTACGCTGGGCGCTGATAACTAGAATAAAGCGCGCGATTTGAGGGTCAAATTTTCGATTCG
GTGTGTCCTTTGTCCATCTTTCGCTTTGTCTGCCGCTTATATTATGTAGGAGTACTTACGATACTTCGTCGCC
GAAGGAGACCATGTCAATTAGCGATGATGTTTCAAAAGAAAGATATCGAAAATTGAGATTCTACATCCATACA
TACGAGACATTTATCGCCAACATAGTGAGTGTCATCCCACGTGCTGATCCCAGCGCGCGCCACGCCCGCGCCA
TCTTGCCACATCACCACAACATATGATGTCACAGCTGGAACATATTAATACTGGTCGAGCGATATTTTAGCAA
TTAAATTTAGTACAAGTGTACGG
>TR303|c0_g1_i1 len=1324 path=[1775:0-126 1888:127-179 1896:180-196
1894:197-213 1892:214-270 1879:271-323 1886:324-359 1840:360-383

1877:384-628 1897:629-652 1898:653-660 1864:661-689 1891:690-708
1883:709-751 1863:752-788 1875:789-812 1882:813-818 1862:819-842
1874:843-1083 1893:1084-1107 1890:1108-1179 1881:1180-1216 1872:1217-1232
1859:1233-1276 1793:1277-1323] [-1, 1775, 1888, 1896, 1894, 1892, 1879,
1886, 1840, 1877, 1897, 1898, 1864, 1891, 1883, 1863, 1875, 1882, 1862,
1874, 1893, 1890, 1881, 1872, 1859, 1793, -2]
GACGCTCTTCCGATCTATCGTGTCGCGGCAGCGACCGTCTTCGACGACGGATGCAGCGAGAGAGAGAGGATGA
GAGGAAAAAACGAGTTGAGACGCATTGGGATGGTGTGTGTTGATGTGTGTGTGTGTTTTTCGTTTCTCGATTT
TATTTTACTATTGATGGGGCTGATATTTTTACTTTACAACTCTCCTTTTTTCGACCGTTGGACGAAAAAGGAG
GATATTATTTTGTGTGGTGGTTTGTGGTGTGCGGGGCGGGCGGTGTGTGTGTGCGTGATGTGTGTGTGGTGTG
TGTTGTGCCATTTGTCATTTCCGGAAGATCGCGAATCGGCCCTTTTTCTTCTTTTTGGAAGATGGAACACCAC
CATCGTCCATGCTGCCACCTCGTCCGAATTGAGTTATATCATTGTTATTTCGTTCGATATCGTCGGAATCTTT
GGTGGGTGGGATGAACCCCAACCGCAAGATATTGAGCTGGTCGGCGGCCGATAGTTGACGCCTACCGCTACCG
GACTTGGGAACTCGAGACGACGACTGGATTGCGGTGGTGACCGCAATCGGCCACACCAGCAAGGCAGAGAAGA
TGAGGGTTAGAGATGATTTGCAATGCATTGATGTCTACTCTATTGATGTTCGTTGCGTTTCAGTGTGTTTGGG
TTTTTTGGGCCGATTAATTTATATGCAAATAATACGAACGGTATGTTACGATAGAGGGTTGCAGTTTGGCAGT
TGGCAGTTGGCAATTGACAGTCGGAGACAGAGGTGACGGTGACCGGTGAGAAGCGGAATCTCGGAGAATCCAA
CGCCCAGGTGGATGGCGGGGCATGGCTTAATCTTAATGCATGATCGCCATCTGCGTCAATGCGATGGCGATGG
TGTCTTTCATTTTCTTCGGAATTTGGAACCCGAACGAATCTTTCTTCTTTGAGGCTCGTGGATTTAGCGGGCA
TGATATCAATTTTAAGAAGGCTACGTCCGCCTACTCACTAAGTCTAAATTATGAATGGAAGCATTTGTAGGTA
GGTAGGACCGAACAAATAGAGGAGAGTAGTTACCAGAGAGAAATGCCGCTATTCGGGTCAGCCCTCGTACCCG
GAATATTTACGGACCTACTGCTCCGTCAGATCAGTCAGGTGCGGCAGATTGAGTGGCAGCTGGATCTTAGATC
TTGGCGATAGATAGGTAGGTTCGCATCCGCGATGCACCCAAGACCCAAGATACCGGTGGCTTTTCACCTTAGA
TATATGTGAAACTTTCCATGGTGTAAAAATGAAATTGATCTCCATAATAATTCTTATTCTAAGTAATATTGCC
AGTAATGCTT
>TR303|c0_g1_i2 len=1309 path=[1853:0-126 1889:127-179 1896:180-196
1894:197-213 1892:214-270 1879:271-323 1886:324-359 1840:360-383
1877:384-628 1897:629-652 1898:653-660 1864:661-689 1891:690-708
1883:709-751 1863:752-788 1875:789-812 1882:813-818 1862:819-842
1874:843-1083 1893:1084-1107 1890:1108-1179 1873:1180-1222 1860:1223-1238
1762:1239-1261 1763:1262-1308] [-1, 1853, 1889, 1896, 1894, 1892, 1879,
1886, 1840, 1877, 1897, 1898, 1864, 1891, 1883, 1863, 1875, 1882, 1862,
1874, 1893, 1890, 1873, 1860, 1762, 1763, -2]
GACGCTCTTCCGATCTATCGTGTCGCGGCAGCGACCGTCTTCGACGACGGATGCAGCGAGAGAGAGAGGATGA
GAGGAAAAAACGAGTTGAGACGCATTGGGATGGTGTGTGTTGATGTGTGTGTGTGTTTTTCGTTTTTCTCGATTT
TATTTTACTAATGATGGGGCTGATATTTTTACTTTACAACTCTCCTTTTTTCGACCGTTGGACGAAAAAGGAG
GATATTATTTTGTGTGGTGGTTTGTGGTGTGCGGGGCGGGCGGTGTGTGTGTGCGTGATGTGTGTGTGGTGTG
TGTTGTGCCATTTGTCATTTCCGGAAGATCGCGAATCGGCCCTTTTTCTTCTTTTTGGAAGATGGAACACCAC
CATCGTCCATGCTGCCACCTCGTCCGAATTGAGTTATATCATTGTTATTTCGTTCGATATCGTCGGAATCTTT
GGTGGGTGGGATGAACCCCAACCGCAAGATATTGAGCTGGTCGGCGGCCGATAGTTGACGCCTACCGCTACCG
GACTTGGGAACTCGAGACGACGACTGGATTGCGGTGGTGACCGCAATCGGCCACACCAGCAAGGCAGAGAAGA
TGAGGGTTAGAGATGATTTGCAATGCATTGATGTCTACTCTATTGATGTTCGTTGCGTTTCAGTGTGTTTGGG
TTTTTTGGGCCGATTAATTTATATGCAAATAATACGAACGGTATGTTACGATAGAGGGTTGCAGTTTGGCAGT
TGGCAGTTGGCAATTGACAGTCGGAGACAGAGGTGACGGTGACCGGTGAGAAGCGGAATCTCGGAGAATCCAA
CGCCCAGGTGGATGGCGGGGCATGGCTTAATCTTAATGCATGATCGCCATCTGCGTCAATGCGATGGCGATGG
TGTCTTTCATTTTCTTCGGAATTTGGAACCCGAACGAATCTTTCTTCTTTGAGGCTCGTGGATTTAGCGGGCA
TGATATCAATTTTAAGAAGGCTACGTCCGCCTACTCACTAAGTCTAAATTATGAATGGAAGCATTTGTAGGTA
GGTAGGACCGAACAAATAGAGGAGAGTAGTTACCAGAGAGAAATGCCGCTATTCGGGTCAGCCCTCGTACCCG
GAATATTTACGGACCTACTGCTCCGTCAGATCAGTCAGGTGCGGCAGATTGAGTGGCAGCTGGATCTTAGATC
TTGGCGATAGATTGGTACTCCTAGGTTCGCACCCGCGATGCACCCAAGACCCAAGATACCGGTGGCTTTTCTT
CCATGGTGTAAAAATGAAATTGATCTCCATAATAATTCTTATTCTAAGTAATATTGCCAGTAATGCTT
>TR304|c0_g1_i1 len=2217 path=[2622:0-36 2699:37-37 2697:38-61 2695:62-
400 2665:401-424 2690:425-505 2610:506-531 2693:532-563 2698:564-614
2682:615-654 2692:655-656 2688:657-680 2681:681-719 2658:720-743
2691:744-828 2637:829-852 2687:853-991 2640:992-1008 2641:1009-1024
2642:1025-1048 2679:1049-1138 2614:1139-1162 2686:1163-1288 2634:1289-
1312 2678:1313-1415 2655:1416-1439 2685:1440-1720 2672:1721-1744
2677:1745-2080 2675:2081-2104 2676:2105-2216] [-1, 2622, 2699, 2697,
2695, 2665, 2690, 2610, 2693, 2698, 2682, 2692, 2688, 2681, 2658, 2691,

2637, 2687, 2640, 2641, 2642, 2679, 2614, 2686, 2634, 2678, 2655, 2685, 2672, 2677, 2675, 2676, -2]
TCGAGTAGTTATTATGTGCAATTCATTATCTTATTTTCTAAGTGAAAATGCTCTTTTCAGAAGAAACCCAAAA
TTTAAACAGTACCCAACCAGCATCCAATGCCTTGTCGTAGTCCGTGCTTTCCTTTCATTAATTATGAATATTT
GCAACCCAAAACAACAACCAAAAAGATGGCTCCCCATTAGTCGTATCCCATGGTCCGCTTGTACGTGCGCTTC
TCGGCAAGAGTCACATGGTCTTTCGTCCAGTTGCCAGAACTCGTTCTCCTCTGGTACCTAGACTTTTTCTTGT
CAAATATCTCCTCAAGATTGCAGGTGATCACCTCCCCAAATATTTTGTCTGGATCGAGACGTTCAGGTCCATC
TGCGAACTGTCGCTCAAGTGCACGATGAAGGTTAGCTTTTTGTGCCCAATCCGGGATCGATTTTTTCGGCCGC
TGTCTTTCGTACTCGTCATCAGAATCTGATTCCGATTCACTGCTCTCCTCCTCACGGTCTGACATCTCATATG
TTTGTATGGGTGATAATGGCTTCTCCTTTGCCGGCTCTGACTTAGTTGGTGGCGAAACTGCTGGTCTGTTTGA
CGGATCAAGAATGCTCTTTAGAGCTGTCGACTTTGGAGCAGGTTTGTACGTTGCCATTTGAGAAACTTGCTTC
ACTGTGCTCAATGATGATGTCACGCTAATGCTGCTGCTGCTGCTGCTGCCTTGTTTGAGGAGTCCCGCCGGTT
TTGTTGGCGGGGCCACCATGTTCTCAGTGTTTTTATCCGCGGCAGCCTTTTCGCGCATCTTTGCGCGTATGTC
ATTTGACTTGCTCTCACCCAACGTGGGACCGCTCGCCATCTTCTTTAACGTTGACGACACTGCATATTTTGAA
TGAGATGGAGACGGTTTTGTGGCAGAGCTCACATTTGCTGCACTAGGCTTGGCCTTTTGCCTCATCTCTGCCA
TTCGTGCTTTTCGAGCCTCCATGGCTGCCGCATGAGCTTTTTTCTTCTCTAGAAGTTTCGCCGATCCAGTCCC
CCCAACCTTTGCCTTGAATGCAGATCCGCCTGGTGCAGTTGGTGCGAGAGTAGATAATGCCTTCCCTTTTTCT
TCACTCGCCTGCATTAGTTTGCTTGTCGTAGTAGATTTGGAACCCACGGCCACTTCATATGAAGACTCAGAAG
AAGAGTCAGATACCTTGTGCGCTAGCAATTCGTCCTCCGCCGAACTACTAGGTTCAGGGTTCACCGCAGGCTT
TATATCATGGATCTGTGTCGAGCTTTGAACAATGGTAGAAGCGTATTTCGATGGCTTCAAGGCGGATTTGGGA
GCCTGGTTCTTCATCGCGTTATTTTCCATCGTCCCAACTATTGTTGGTTTCACGGGAGCTTTCTTCAATGGTT
TGACCCCCTCCGGTTCAATCTTCGTATTCTCACTCGCCTCACTGGCATCCTCCATGACAACATCCATCTCCCC
CTTATTTTTCAAACTTCTATTCTCGCCATTGCCGATCTTCACATGTTTAATTTGGACTTCCTTCTTTTTCGCG
GCATAGTTCGCAGCCCACTTCGCATCCCGCTCCCTCTTCGCCTCGGCCAGGTGCTGTTTCAATCTCGCCTGGA
GCTCAGCCTTCCTCCCAGTCCCGATCAGTCCTCTCCTCCTCAGCTCCTTTCGCAGGTCGTTAACAAGCATTCT
ATCGACGTTGATTTTGTCGTCCGGATGGGGTGGCATCGTCGCAGGAGCAACTTTCTTCGCTGTAGCTGCTACC
TTGGATTTCGCCACCGAATTCGCCGCAGCCTCTGTCGCCACGCTGTGTTGATTTGGAATGGCATTTTCTTCTC
TTACGGGAACCATATCAGCTTCCGACGTCCGTAGCTTCTTAGGGACGGCTACGCCGGTGAAGCCTTCCCATTT
GCGTTTCATGCCCTCGAAGTTCTGAACATGCGATCTCAATCGTCCTTCGTAAAAACCACTGATATCGTGCTTC
TCCCTTTGCTGCTCTTGGTTAAGTGTGGCAAAGAGGTTGTATATCTCCCTCTCAGGTTTTCCCTTTGCATGAC
GAGTCCCCGTCATGATAGTTTCGTTTTCTTTTTGATTCGCACGGCGCGTAAAAGCAATAGAGAGTAATTCGAA
AAGTGGGCTTCGAAATCAAAGAGAGCTCCGAGGGCGATCAATGACCCGATGGGAATGATGTGGTGGTGGCACG
TCGATCTAAACGATCAAATGATTGCCC
>TR305|c0_g1_i1 len=492 path=[8578:0-114 @8697@!:115-491] [-1, 8578, 8697]
TCCACGCCATGTGGCAGCCTAGCGTCAGAGATTGTGGCAACAAATATTATTATGTGCGGGTACAAACGATTGA
TATCAATGAGTTTGCCTTACCTAGCATTCACATCCCAGTCTTCGTATCCAATCATCCTAAACGAATCATCGGT
TCCCTCCTCTAACGGACGGAGGCTGATGGCACAGTTCCATGCGACATCAATGCCCATCTGACTCGCCAATTCC
TTCGTCCGCCTCATGTTTCGAGGAGAAAAGTAGACGAACCGCACTCCCGCGGCGGCACAGGAGGCGACGAGGG
GCTCGATTTCTTTCCGTGGACGGACAGAACTTCCGAGAAGACCGAGGAATATTTGATTTTTGACGAGAGACCA
AAAGCCGTGGGAAGGGATGTATGCGTTGGGCGCGGCGTTGTCCAAGAGGTAAATATGAGAACCTTTCGCACCG
CCCCATTCTATCTTTTGATCGGCAGTATATGGTAGAGGGGCGTAGCTAAAAGCT
>TR305|c0_g1_i2 len=231 path=[8565:0-112 8701:113-170 8693:171-194 8709:195-199 8713:200-211 8506:212-230] [-1, 8565, 8701, 8693, 8709, 8713, 8506, -2]
TATTTCGTCCGAATTTTTAGTGCAGGTGAATTCACTGCCAAAAAGACATTGGCAACAGCTCTCGGGACCGCTT
ATGCTTAGAAATAGGAGCACTTGTAATAAACCACGGCTTTTGAGTCTAGAGGTGCTATAGCAGAACAATGCAA
TACTGATCCAATAGTTTGAATTTTAACATCCATTACAGGCAATACTGGCGGGGGGGGGCTAAACAAATGTCCT
CTTTTGACCACT
>TR305|c0_g1_i3 len=7337 path=[8640:0-33 8641:34-57 8704:58-104 8701:105-162 8693:163-186 8709:187-191 8622:192-215 8660:216-460 8692:461-471 8679:472-495 8659:496-668 8557:669-692 8700:693-875 8529:876-899 8691:900-1094 8535:1095-1118 8677:1119-1120 @8690@!:1121-1488 8532:1489-1536 8675:1537-1857 8656:1858-2186 8539:2187-2210 8674:2211-2434 8625:2435-2458 8655:2459-2740 8563:2741-2764 8673:2765-2929 8698:2930-2977 8654:2978-3001 8689:3002-3524 8510:3525-3541 8653:3542-3975 8485:3976-3999 8703:4000-4152 @8697@!:4153-4529 8594:4530-4550 8666:4551-4924 8526:4925-4948 8646:4949-5425 8597:5426-5449 8695:5450-5722 8500:5723-5746 8682:5747-5985 8712:5986-6002 8716:6003-6040 8644:6041-6112 8600:6113-6136 8694:6137-6643 8662:6644-6703 8643:6704-7012

8617:7013-7036 8618:7037-7336] [-1, 8640, 8641, 8704, 8701, 8693, 8709, 8622, 8660, 8692, 8679, 8659, 8557, 8700, 8529, 8691, 8535, 8677, 8690, 8532, 8675, 8656, 8539, 8674, 8625, 8655, 8563, 8673, 8698, 8654, 8689, 8510, 8653, 8485, 8703, 8697, 8594, 8666, 8526, 8646, 8597, 8695, 8500, 8682, 8712, 8716, 8644, 8600, 8694, 8662, 8643, 8617, 8618, -2]
TTTCGTCTGAATTTTTAGTGCAGGTGAAGGCACTGCCAAAAAGACAGTGGCAATAGCTCTCGGGACCGCTTGG
AAATAGGACCACTTGTAATAAACCACGGCTTTTGAGTCTAGAGGTGCTATAGCAGAACAATGCAATACTGATC
CAATAGTTTGAATTTTAACATCCATTACAGGCAATACTGGCGGGGGTGGGCTAAACAACTGTCCTCTTTTGAC
CACTGACACTGAGATCGAGAATCGAAGGAAATTCTTTCGCCACAGTATAAAAATAAAGATGAGGTTCGACATC
GCCTTCTTCCTCTGCTGCTGCCGCTCCTTCCTCAGCTCCAACGGACGAGACGATGGCGAGACCTTCTACGAAC
TCCTCGGTGTCTCCCCGAAAGCCAACCCGGACGAACTCAAACGTGCCTACAAACGCCAATCCCTCCTCATGCA
CCCCGACAAACTCGCCCAAAAGGGACAATCCGTCACCCCCGCCGATCGCGATCGATTTACCCGCATGAGACAC
GCCTACGAGGTCCTATCCGATCCCCGTCGGCGGGAAACCTACGACGCCATCGGCGAACGGGGAATGAAGTGGG
TCGAGGAACCTCTGTCGGTCGATCCGCAGGAGTTGGCGCACAACTTTGCCACGTCGAGCATATTGGACCGATC
GAAGATCTTCGCTATATTTGCGGGGATATACATTGCGGTCTTTCTGTTGCCGATATTGGTATGTCTGATGGCT
GATGGGACGTTGGGAATGGGTGTGAAGTGGGTGGCCGTGGTGACCCCTCTTTGGGTGTGGGACGTGTTCATTT
TGTTTTACCACTCGCGCGTGATTTTGATGGGACCGATCAAGAGGCCGGATCATATTCCGGAGGAGGAGTGGGT
TGATCCGCTGCCTATGTGGAAGAGGGTGATGGCCATGGTTCGGTTTGCGTGTTTGGTGTTGTTTCAGGTGCTA
TTGGCGCTTCGGTTGGATGGGTACATTGCCGCTCCGTGGTCGATTGTCTTCATTCCCATGTACCTCTGGGACG
TCATTGCGTTACGACGAAAGATCATGCTCATGAGGATAGTCATTGTCAATCACGAGGAGTTGGAGCTGGCGAT
TGGAAAAAAGATTGGAGAGTGCACTGCAGCAGAGCGGGAGGATATTCACCGTCGTTATATCGTCGTGCCGACC
AAGAGCGGTCCTCTCTATGACGCGGCATGCCGACTACGTGATGAGGCCAAGATGGACATCATTCGAATATTGG
CACGCGTCACTTTCACGGTACTACTGGTGCTGAATTTGGATCTTGATGTGGGTTGGAGTTGGTGGCTCGTCTT
TCTCCCAATGTTCGCCATGGCAGGTTGCATCGTAGGCTCGGCGTTTGGGAACTTTGCGAAGACACAGTCTGAG
GCAGTGAAAAGGGACCCTGCTATGTTTGGACTGGATCCTAATGGGGAAGAGAGTTCATATTCCAAGATGGATG
AAAATAAACCAGCAGAGCTAACAGATGAAAAGAAGGATGAGCTGAAAGCCAAAGTAGCTCAATCGGCCTATAG
AGCAGTGGGCACCTGCTTCTCTCAGTGTTTCTTCATCATCATAACATGCGTCTTGATTGGAAAAATTGAAGGC
GCTGGCTATTCCGCCTTAGTAATCATATCTCCGTTTTTGGCTGCCGGAGGTATCGTCCTCTGCTGTCTCGCAT
GCACCATCTTTTGCATTTCGGAGGTTGACGAAAACGCGGGCATGGCGGAGTTCGACACCGCCGTAAATCAGGC
GACCGCGGCAGCTTCTGGACACGGTGTTGTGGAGAAAGAACAGGCAAACAATAGCACCTATGCGCCGCCGACA
CAACCAGATCAAAAAAGCGGTGGTATATCGGATGCTGGCACGACAGACAGCGGTGGTACGGGGTCGGGTAGCG
TGTCTTCTTCGAAGTCAAAACCTCCGCCTAGTTCAATATGGGATCCTGAATTGGGAGAGGTATGGCAAAACAC
GACTGTGGATGAATTTGATGAGGAAGAGCTTAAAGTCGATAATCGAACAACTGAGAAAGAAACGAATTCTGTG
GATGAACAACAACCGACTTTAATAGAACCGAATCCCTCAATCACTTCTGTAACATCGAGCCAGTGCGATCTCG
ATTAGAAGGGGTCTTCGTTGTGTAGGATATGCTGGTGTGTATTAAGATTTAATGTGACATTGTTAATGAGTGG
ATCATAGGGTTACTACGTATTCTTCATCTTGGTCATCTAAGTTGTTTTGTGCTGTTTTTCGCTCTATGGCATC
ATTTCGTGTTGGGCCACTCACGATGTAAACTTGATGGCAGATGTATATTACTTCCACCCTGCAGTAGGTGCAA
TCATCCTGCAAGTGGCCACATCTTTGAATATATCTTCCACTGGCAAGCTGTAGTGCAATGCGCATGTGTTGCC
ATTTTACTGCTCTACCAAAATCAATCCTAACTCTCACATCGGCATTTATATGAAAATCTTTAATATTGAAGTC
CGGTTTACTTGAATGCTCTAAGAATGGCACTATCCCAAGTTTGAGGCACTGTATTCTGTGGGCCATAAACTCG
TCTTTTATGCCGTTTATGATGGGTTCCAGAGGGCTAAACTTTGTTGGTTACTTTCAAATTTCTCCCCAGGCCA
GTCGTTGAAGTATACACCGCTTTCGTTCATTGGCCTCTTGATAGAGGACCTAATTTAACTTATGGAAGTGTTG
AGATAGAGGCTCGAGCCAAAGTGTCACAATCACGATTTGTGCTTTCTTCGTCTCTTGAGCACAAATTAATTAT
ACGTGAACAGTAATTCGGTACGTCCTTGGGATGCCACAGGCAGTTGGTTTCAACCAATTAGCGCTCTACCAAT
TACACCATAGGAGAAGTATTTTCTCTTCGATTGTTGCTCATACCATCCCCCATGTGAGTACTCTCTTTGGGGC
TCCACATCCCAAGCCTAGTCTCGAACTGCAGCCTTCTCATCATTACTGCACGCTTCTCATGCCTCTGGTCCAT
TTTCTTCACTGCCTCGCAGAAATAAAGACAACCAAATGGAGCCACCAAAAACAATAAGTAAAAATACCAGGAG
AGTGTTGTCATCGAACCTTCCTCTAATACCAATGCCATGTAGATAGCAATAAGTACAAATGATAATGCCAATG
TTCCCAGCCATAAATGATTCCTTTTCCATGGAGGTTCTGATCGAATTGATTCAGTGCGAAATACGTAAGAAGC
CGATGCCACACATGTGCAAATCGCAAGAGCTGCCAATGAGATAGTTCCAGCAGATTGCACGGCAGGACCAGAG
TAGTCACGCAGGGCTTCACAACGAATGATGGATGCTATTGGTGGTTTACGATACTCAGATTTGTCTCCATCAA
GGCAGAAGTCTTTCACGAATGGAGAATCAAATTCCCACATTAATTCACCCAGCACAATGAGATAAAAGAATTG
TGGTATGATTGCTGGAATCGCAGCTCTCAACAGCATACTTAGGTAAGGCCGCATGTTTGATCTTAGAGAGTAT
TTGACCGCGGGGTCATTTTTTGGCGGGACACGTGTCATAAAGTCTTTTGCATCATCTGTCGATGCCATCGAAA
GTCCAATCAAAGGCAGTAGCACTTGAGCATACATAAAGGAACCGAGGGTGGGAACCATCGGGACGGCAGTTGC
TGCCGTACACGGACAAAGCAATATGAAGATGCTGAAGGAGAGATTCCCTGACAAAGCAAAGCTAGCGCCGGAT
GTGGCAGCCTCGAGACATACCCGTCCGATTCGAATAATCTCCACAAAGTGACATGTTGCCGTTCTGTGGAGAT
TGAAAACAGATGAATGCGCAGATATGGAAGTAACAAATGCAATCTCATGGGGCTGTAAATTATTCAAAGCATG
GACGTTTGTGTCAATATTGTTGAAAGAAATATCCTCCGCGAGAACGTCAACCCCAATAGCAATATCAGCTCGT
GAGAAAATCTCCTGGTTGCCCGAGAGATGGGATAAACCAACGGACAATACTGTGTCGTTGTATTCTTGAAACA

CATTAACCATATCCGCTGCCGTTTTTTTATTCACGTCGGTGTAAAGAGAAACCAGCAACGGTACATTGTCCAC
CTCCTCCAAATGCCTTCTCACGTCATCCACGCCATGTGGCAGCCTAGCATTCACATCCCAGTCTTCGTATCCA
ATCATCCTAAACGAATCATCGGTTCCCTCCTCTAACGGACGGAGGCTGATGGCACAGTTCCATGCGACATCAA
TGCCCATCTGACTCGCCAATTCCTTCGTCCGCCTCATGTTTCGAGGAGAAAAGTAGACGAACCGCACTCCCGC
GGCGGCACAGGAGGCGACGAGGGGCTCGATTTCTTTCCGTGGACGGACAGAACTTCCGAGAAGACCGAGGAAT
ATTTGATTTTTGACGAGAGACCAAAAGCCGTGGGAAGGGATGTATGCGTTGGGCGCGGCGTTGTCCAAGAGGT
AAATATGAGAACCTTTCGCACCGCCCCATTCTATCTTTTGATCGGCAGTATATGGTAGAGGGGCGTAGCTAAA
AGCTTGTACATCCATATCAGACAACATCCAATTCTTTTGCGTTTCGTTTATGATCTGCCGATCTGCCGCCGTG
AGAGGGGATATGGTCGAATTTTCGCCCTGCCACCAATCGGGACATAGTTGCGTGACTACCCTAGAATCTCCCA
CTGTCAGCACCAAGTCTCCACCCGACCGTGCATCCCTCACAAAAACCGTGTCCGCATCGGTAAACAATCGACT
CCAATTCCTCGATTCCTCCAATCCCAACGCGTGGGAGTTCAATTGGATTCGTTGCCGGAGGAGATCGGTGGAT
ACGATGTGAAGTCTCCGACGTTCATGAAAGCAGCTCAAATCGCCACGTGGACCGTTACTCGTGTCGAATCCGA
TGCATTGCGCGAGGAGTCGGAGCTGCTTCCGTTCACGTTCGTCGTTGCAAATGTGATCGATGAGGGAAGTTTC
TTCGGGAGTCAGAGGACAGGGCGATGGTTTCGCGCCGTTATTGCTGAAGCGACTGCTGCTCGATAGTGATTTG
CCATTATCGAACGTAACTTTTTGGGATGGAGTATTCGGCGCACTCCTGGTATTTCCGCCGCTTGGCGTTGTTG
CGCAGACTATCTTTCTTCTGGCCCTACCGGTCCTCTTGGTTCTTTCCTCCACGAGGAGACAGCCTAATCCTAT
GCATTTTAAACTTGGCAGATGTTTCCACCACTGCGGATCTTCGAATTGAACTTCCACGTCATCGTCGTCAAAT
CCACCGCAAGGGCCTTGAACGGACCCGCCGCTGCCATCCCGCTGAAATGACACCCCACGCTTCTTCTTTCGTC
CGTAGTTCCTCCTGATATTCCTCAGAGTCCTCGCCGGCGCTGAAAACGAGTGATTGAAATGCACTGCCGTCTC
CTCGTTGGAATCATCGTCCGAGTCGATCGAAACATCCGAGCTCTGCTGTGGGTTGATGTTCGTAGTTTCGTTG
TTGACGTGGGGGTTATTGTTGTTGCCTTCATCATCCGTGCTGCAGTACCCGTCCTCCCCATCAAACACAGGGC
AAATATCGAGGAGAGAGAAGCCTCCCTGACCCGACGGAATCAACAGCTGCTGTGGAGTCGAAAAGGGTTCGCA
CGCGAGTTCGTCATCCACGAGGGCGAACGCCGTCACCACGCCCAGTTTTTCCAATAGGTGCAACGATGCAGGG
GGAATGGAGAGCAGTGCCTCTCCTCTCGGGGACGACGACAACGACGAGGATTTATCTTTTGCTTTGCTCCTCA
TGGCTAAAATCTTTTTCAATAACGATTTCGTAAAGAGGCGGGATGACGCCGTGGCCATCAAATAGCGACACAG
CAACCACGGAGACGGTTTTTCGATCCTTCCATTCTCCGAGGAGGAGCGATTATCGTCCATGGTCAGGATGCTA
TATTGTCGCCGCGACGAATCCTCCGCTGCCATTCCCTTCGGAGGTTCCATACTCTGCTGTTGTTGAGTTTTTT
GGATGGAAGCCAACGGATGCACCGTCGCCAATATCCGCGACGTGCCCATGCATTCAATCCAAAAGAGGAGGAG
CGGGGACATCACCGGCAAAATTCCTAGTGATGCAAGCAGCGGCAACGTCCAATACGCGTTCGGAGTCCGAAAG
AAACTCTGAGCCATGCCCGGACGTACCAGAAGAACGAACAGCGTCCCCACAAACACTATAATGGCACACGGAA
GGAGCGTTTTGCGCACGGCCTCGCCCTGACGAAGCACCTGCGGAGTCGAACTGTGCTTCTCCGTCGAATCTTC
CTTTCGCAAGAACGAATCTAACGGAGTCTCCAACAACACGAATATTCGTACGCCGTTCGCGAGGAGGAGCATC
TCGTCGGAATGATACTTGAGGGTGGATCTCCCGGGGGGTAACAATTTGGGCGACGACGATGTCGACGTCTTTT
TGTTGTCTTTGGATGCTGCAGCGTTGTTTCTTTTGGGCGTGACGTTGGTGGTCGGTACTGTGTTCGCGCTTGC
GATGGAAGCCGTGCTGGGGTGTTTGGTTGATATCATCTTCGATTTTAATTCCAGGGTATTTTGGACCATTGGC
GATAGTGAATTGATGGTAAGCCGTTCTCCCGCTTCGATAACGTTGTTAGTGGCGTCATCATCGTCGCTAGTAT
CATCATCCTTATTACAGTTTTTAACCGCTTTGCATTTTGCAGGAGCCGTATCGCCCACCTTCAATGCGATGAA
ATCGCCGGTTACCAATAACAAGGATGGTATTCGATGCCAATGCCCTCGTCCACTATCATCATCACCGGGTGCA
TTCTTGTTGGAATCATCGCCGCCGCTCCTTTCACTAATCTGATCGACCAGGCGGTATGTGGAATACACATCCT
CAACGTTCTTCCTCGGAATAGTATCCATGTTGTACATTTCTTGATATTCTAGTGGATTCTGCTGCTGTTGAAC
ACCGTCACCATCATCGCCGCTCGTACGTTGCTGAAGATTACGACTCGTTATCGTCTTCATCCCATCCAAAAAT
GCCGACACGCACCGTCGTCGCTCGATGCTCCGATCCGTCTCCCTCGAAACCTTTCGTCTCCTCCGCGTGATCC
ACGCCGACGTTGTGGAAGAAATTAAAAATAAAAGAGCCGCCGCCTGTAGTTGTTGCCTTCCGTAGAAAGACGT
AGACGTACTATTATTGATCCACCCATCCGAATCACTTCTTGGTAGGATGAATGATAATAACACAAATAGAAAA
GAGAGTGGTATGGCCAGAAGCATCATGTCGGGATCATCCACCGGACATGATGAACATGGTCCTCTGTCTCCGT
TGATGTCATCATTATTAGTTTAGTGATGTCGGCCCC
>TR307|c0_g1_i1 len=1016 path=[994:0-1015] [-1, 994, -2]
CGCTCTTCCGATCTTTTTTTTATATCATATTCGTAATTAGGCAATGGTTATGGATATACCAGAATTCAACTAC
AAATAGTATTCAGTAAGCGACATTTTGTTGATTTGGTGATGCGGATCAGCCGTTCACCAGCGTCATGATGATA
TATTCAATCGCAGATCCAGTCCGACGATTTGAAATGGCGGCTATAATATTATCGCCTCAATGCCCTTCGAAGA
ATATGGCCAGTCTGTTTCATCCCAGGCATTTTCAGCGGATAAACTTCCCCCAAACTAGCTTTGTGTTCAAACC
ATGTGCCGAATGCAGTTGGATCCATCAAACATTCCTTCTCTACATGATATGGCGGTCCGAATTCTCTACCCGT
TTCCTTATTCTTCACTTCAATATACAGTGTCCCCCCATCTTTGTGGAATTCTGATAATCGCTCGCAAAATTTG
GGACGCATCTCAAGAGAAAGTGCCCCAAAAGAGTCCTTATCGTAAATGACATCAAAGGTATTCACCAGCTCTG
GCCGTTTCATCAAAATGTCACCCTCGAATAATGTGGCCCTTCCACTCTTGTGCTTCCATACCAGCCCGCTTTT
TTGTTGATTCTCCGACGTCCAATCGTCGACATCATCACCAAATTGACTTCTCATGGCCTTCAATGCATCGGGT
ACAATGTCAATGGCCGTCACATCGTGACCTTGACTCCATGCGTACTGAACGAATGGATCGTCTCCGGCGAGGG
GGCATAGAATGCGCATCGGCTCGCAATCATCGAGCGATGAGCCCCCGGCGTGATGATGTTCTATGATGTGTGC
GAGAGCTTTTCGTTTTGCATCCAGATCGTCGACCTTCCAGCGGGGGGATCCACCATTTAAGATGTCGGCCCAT

GTGTCTTTCCATGCCATGGCGATGATGAATGATTGTACGGGTTGGGTGTTGAGGAAGAGCAGCGTGGTGGCAA
GTTGTAAGGCGATACGGATTATTCCCATTTTTCCAGATGGGCTGTTGGAAGTTGGAAGTCGGACAGT
>TR308|c0_g1_i1 len=559 path=[537:0-558] [-1, 537, -2]
GCAACAGTCATGTATAGTGAAAATAGTTCAATATTCAATGTTGAGAACCCTCATATGCCCTGATTTCTCCTTG
ACATGGGGAGATCGAATGGCCACAGTGTTGCCGTCCTAAGAAAGAGCCACAGACCGCCCCAAGTTATCACTTA
TTGTGTCACCATTGATATCACCACCAACCTCCTTCCAAACCAGACCATCATACACGATGCGATACGCCCTCAC
ATGGCCTTCATTAGAACCATTCCCATCGTTACCATGGGCCTCAAATGTTAACTAAGTCTAATGTCCAGTTTGA
GTTGGAAGAAGATGATGTCGATTGTTGTTCCTATGCCCGTCAAATAGTTTCTGGAAACAAAACTTACCAATGA
GATACTGATGTGAATGAATTATCGAGATCTCTCTTTGCTGTCAAGGGTCATTGTTGGGTTGGGATTCCCCCCA
CAGATGAAGGCAATATGTGGTATTATCTTGATAATGCCTTGGCTGAAGGTTTTAGGTGAATTTCACTATCCTG
ACCTAACACTGGAGGGACAAAGTGGACTCTGGGAGTCAATGCACACCA
>TR309|c0_g1_i1 len=530 path=[508:0-529] [-1, 508, -2]
ATCCAGTATTCTCCTTCTGTTGCCATTTCGATGTCCAGAGAACGGAAAAAAGACTGGTGCTCGCCTTCACACC
ATTTCTCAGTCAGGTCGATACTAGAATAGCCTTGAAATACTTTGCCATAATGTGATGGAATAATCACAGAAAA
AGTATTGCGTTTCACATATTTGGTGCTAGATTGCCGAAGCCCGTGCGTCCCTAGCTTGTCACCTTCTTTTGAA
AACGGATCAATGTGAGTGGCTGGCTGAACCTGAACAATATCCACCGACTGGATCGTTAACGAATTTTGCCAGT
CCAAAGCATTATTCGAAACTGTGTTCATGAGCCCCCCTTTTTTTCGAATGTCCTCTTCTCGGTCGATCTTTGC
TTTCAGATAATCTGGAAGAGGTACGGACGAACCGATCATCTTATCATTTTGTTGTATGGATTCGTTGTCGGTG
CTCGTTCTGTGAGACTCGAATTCTCCAAAGCTCAGCCTTGCTTTCTTCTTTCGCCGACGCCCATTGTATCGCT
GGGCTTGCTCTCGTAGCGC
>TR310|c0_g1_i1 len=357 path=[335:0-356] [-1, 335, -2]
GCAGAACAGTCACCAGAGCTCTCGCGGCTTTCCAGAGGTGAAGAATTTCAGGCAGATCGATCAATTGGTCCAA
GCGGCCTTCGATTACCCAAATCTGTCGCCTGGAAACATGGCAGCTATATGGTATCGCATTTCACACTTGCTCG
CGGCGCGCAGTGGGGAACTAAAAGAACATCAACGTCAGTCGCTGAGCCATGAATTGGGTGTCTTGGTTACCCG
CACGCAGAATAGGATATCCAACTATAAGCCAAGGCATTTGTGTAAAACTGCTTGGAGCATCGCAAATATCATC
AAGGTCGTGCGGAACTATCAGAACAGTAGTGAAGCATTGACACATCACCAGATCCTTCACAATGT
>TR311|c0_g1_i1 len=432 path=[410:0-431] [-1, 410, -2]
TTCCCCTCCGAGGAGCTAACCACGGATACAGATACAATTTGTCAAACTGCCGAAGACGATTGTCTCTGCGGAG
TATGCGTCTCAGAGTGCCCACCTGGCTTCCCCGACACTACCTTTTGCAACAAGCGTTGCCAATACCAAGAAAC
AACAAACGACGGTTCTACTACCAGCGAGCCACCACTCGTTCTCTTTCTCAACCATGCCGATGGATACACGGGT
CAAATTGAAGTTGCATTCGGAACAAAAAATAGTGCATGCACTGGGATATTCGGTCTATCGACCGTTGACGATG
ATGATGATGGAACTGGATTGTACCAAATCCAGAAGCCTCTTGGTGTTCCCAGTTGTTACTCGGATAATGAAAA
ACTCGATGTTAATATATTTATCAAACATTATCCGGATGGGTCGGCGTTCAAGAAGATTCGCACGTCG
>TR312|c0_g1_i1 len=578 path=[556:0-577] [-1, 556, -2]
CGATATCGCTTTTTGGCAAGGATCTCGTACATGTAGCGACAGCGACCAGTCAACGGTCGGTGACACTGGATCT
CTCGAAACATTGATGGATAATTCAAAAGAGAGCAACGCCATCGAGAATCTAGTGACTGATGTATCGTCACTTT
CTATCGCATCAAATGATACGACGGAAGAGTTTTCAACAAAAAGCGAACCACAATCTCCTGGATGTCCGGCAGG
CGCCCTCGTGTCCCAAGGACCGAACATAATCGAGACCGAAGCCTCGACGATTTTGTCAGCTACCAAATCGTCT
CTTTCTGATTTGTCGGATGACGCTACACAAGACTTTTTGGAAAATAGCGAAGCGCCCCCAGTATCGGAGGAAG
AGATGGAGTCCAAAAGCCTAGATGCATTGATAAATCGAAAATCGAGCACAATCGAAATCGAGCCCCCGGAAAA
CCAAATTGCGGATATATCGTCATTATATGTATCCAATGTTATCGCAGTAGCTGCACGAAACGAACTAGCGATA
TCACCCGATGCGACATCTACTCCCACTGACCATCAAATAGAAAATGCACTTGCAACTCGTGCCCGGA
>TR314|c0_g1_i1 len=333 path=[738:0-177 739:178-332] [-1, 738, 739, -2]
AGGATAAGCAGTATTGGCAAGTGTAGAAGAAAATCAAGGGAGCAATAACTTACGCAACAGTTGCCCATTGTGA
CCGAGAAGGAGTCGGTGGACCCACCATGTGGTGATGCGATGGCTTAACATACGCCCTTCCCCCCATGTTGTAT
CTAATAAGTAGGATAAGCAGTATTGGCAAGTGTAGAAGAAAATCAAGGGAGCAATAACTTACGCAACAGTTGC
CCATTGTGACCGAGAAGGAGTCGGTGGACCCACCATGTGGTGATGCGATGGCTTAACATACGCCCTTCCCCCC
ATGTTGTATCTAATAAGTAGGATAAGCAGTATTGGCAAGTG
>TR314|c0_g2_i1 len=734 path=[736:0-177 737:178-733] [-1, 736, 737, -2]
AGGATAAGCAGTATTGGCAAGTGTAGAAGAAAATCAAGGGAGCAATAACTTACGCAACAGTTGCCCATTGTGA
CCGAGAAGGAGTCGGTGGACCCACCATGTGGTGATGCGATGGCTTAACATACGCCCTTCCCCCCATGTTGTAT
CTAATAAGTAGGATAAGCAGTATTGGCAAGTGCAGAAAGAACCTAGGGAGTAACAACTTATACAGCAGTTGCC
CACTGTGACCAAGAAGGAGTCGATGGAACCAACGTGTGTTGCTGTGGGGGCTTAAGATATGCCCTCCCCCTAT
GTTGTAAGTATCTAACAGTTAGGATGAAAGGTGGCAAAGAGAGCACTTATAAATTACTCAGTGTGGGACCTTT
TGTGAATAAATGGCGCCGTTTAGAGTAATTTATTTTAAAAGTTGTTGTGAAGGATCCCCCCAGTCTGCAAATT
TCTTTAGGAAATGCTACATCGCCCATGGAATGATGTGCATTGGAGGTCACCGGGTATCACATCTTTGCCACAG
GAAAAGCTGAATTGCATATAGAATTTGATCATCTGCACAGCACATTCATGCGCTTGGACACAATACATGGAAT
ATCATTCAGTCCCAGATGTTGCCTCTACTCTCACTAATAAACATTTATTTATCTCACTCACCACAAACACTGC
GCATAAGAAGTGCCCCCCCCCCTCCTCCAATGGGTACAATTAATACCCTGAATGTTAGTTTGATAAGTCCTGA
AATA

>TR315|c0_g1_i1 len=447 path=[452:0-78 453:79-117 454:118-446] [-1, 452, 453, 454, -2]
AAAAAGAGCCGTTCAGCAGCCGTCACCCCCCCATTGAGAATTGATATATATTGTATCAAACGTCGACTCTGAA
CAAACGGCAACTCTCTCTCTCTCTCTGAACAAACGGCAACTCCATCAAAGTCGAAACAACGACAGTGTGTGGA
AGCAAACCTTCAATACAAAGGGCAAGGCTAGAAAAACGAAACGACGAAGAGCCAACAGCAATATATATATATA
TATATACACACATATACATCAACAGCCATGGTGTTAAAGATCGAACGACCACCCAACATGATACCCGTCAACA
ACAACTCGCGGGACAATCGCAGTCCGCGAGACGACCGTCATCTCGAAGGCAATCCGCGTCACGCGAAAAAGCC
GAAGATGACGACGCAAGTCAAAGACGATGATGACGATGCGACTGCGAACAACGTCATTATTGCCAATGGCGGA
AGCGGAAGC
>TR315|c0_g2_i1 len=435 path=[455:0-78 456:79-105 457:106-434] [-1, 455, 456, 457, -2]
AAAAAGAGCCGTTCAGCAGCCGTCACCCCCCCATTGAGAATTGATATATATTGTATCAAACGTCGACTCTGAA
CAAACGTCAACTCTGAACAAACGGCAACTCCATCAAAGTCGAAACAACGACAGTGTGTGGAAGCAAACCTTCA
ATACAAAGGGCAAGGCTAGAAAAACGAAACGACGAAGAGCCAACAGCAATATATATATATATATATACACACA
TATACATCAACAGCCATGGTGTTAAAGATCGAACGACCACCCAACATGATACCCGTCAACAACAACTCGCGGG
ACAATCGCAGTCCGCGAGACGACCGTCATCTCGAAGGCAATCCGCGTCACGCGAAAAAGCCGAAGATGACGAC
GCAAGTCAAAGACGATGATGACGATGCGACTGCGAACAACGTCATTATTGCCAATGGCGGAAGCGGAAGC
>TR316|c0_g2_i1 len=731 path=[726:0-127 727:128-145 728:146-730] [-1, 726, 727, 728, -2]
CCGATCTTTTTTTTTTTTTAAACATCTAAACAATGGCATTAAAGAAGATTTTGTATCCTCAACAACATCTTAG
ACTCAATCCTCTACAGCTTGAATACTTCCAAACTCTCAAAGAAATCTCCTTTTTTTCTTCTTCTTCGCCGCGG
AGACATCAACTCCATCATACTCCTCGCTCTCGCTCTCAAAAATGGCATCGTAGTCACCATCGCCACCATTACC
TCCAGAAGACTTCTTCTCCTCCTCCAAGTCGAATGCAGTATCCCTAGCAATCGCATCCGCCAGCACATCCCTC
CGAATGGCATCGCAATATATGCTCAATCGCAAGGTCCCCTGAAACGGATCCTCGATCCTGCTACCTATCTCGT
ATACGCCATACAAAATCCACGAAAGAACCGCCACCACGGGCCCGGTCAGCAGACCCAAATCCTTCACCAACAC
AAACGGCAGCGTCCATAACCACACCGTCAAACTTCTCAACGCATGCCTGGCATAATTCAACGGCACCACCGTC
TGGTGAATCCTCTCGCACGCGCCAATCGACCGAGACAACTTATCGACATATCCAATCATCGCCAACCTTTCCC
GATTCGTAAACTTCGGAGCCATGTCCTCCACCACCGCCAGCTCCTTCGCCATCCGATCGCACACCCACAGCGG
TCGATTCTGCGCCCTGGCGCACAGTTCCAACGCCGAGCCTGGAATGAGCTTCCACGGCAGCGTCCGCTTGAGA
T
>TR317|c0_g1_i1 len=796 path=[938:0-262 939:263-284 946:285-638 942:639-795] [-1, 938, 939, 946, 942, -2]
CTTTTCACTCCACATCGTCACGATGATGGTAACGAGGATTCGACGGCAACAGTACGGACTCAAAATAAAAATA
ACGCATTACGGAGATCCTGTCGCGGTAGTTCAGCTTTGTCGTTGTTGGCATCAGCGCTTGCAACAGTGGCACT
TGCGGCAAAGAAAAGAGCGGTCAATGCTGCGCATGTCGCGAGCGTGACACCATCCTCCACACAGAGATCGATT
ATTCAGAATCGCCCGGCAGCAGCAGTGCGAGTAACGAGGAAGCATAACAATGATGATGACGCAGGAGGATCTG
AAACCGAAGAAGAAGACGAAGCGCGGACCATTCGCGAAGAATGCACCCTCCGCCAAGAAAGCGAACTTGGCAT
CGCCAATTTGGAGAACGCGATGGCAAATAATGGGAAGGAATCTCCGTACTTCAAGGGGTCCAAAAATCGATCA
AAAGTAAAGCGGCCAAAACCGCAGCCGTGCGACAACGCAAATTGATTCTAGCATCCAGCAAAGAGGAGGAGGT
CGACAAAAACGTTGCCAACGATGTCAACGGAATGAAAAAGCCTCCTTTGAAGAATTTGAAGGAGGCAGCTGCC
GCAGCCACCAGGGCAAAGAAACGAACCAACGCGACAACGGTCGCTAAGAAGCCTCCCGCCAAATTGATCACGG
CAACGAGCAAGGAGATAATCATCCCCACCACTACAGCAGTCACCACTTGCTGATTTTGCCCAATTCTCCCGAT
CGCGATGCCGTGGAGGATTGGCAGCTCAGTCTCGTCACGGCCTCCACGAGCGGGATGCCCCCCTTGT
>TR317|c0_g2_i1 len=319 path=[936:0-161 937:162-318] [-1, 936, 937, -2]
AATTGATTCTGGCATCCAGTAAAGAGGAGGAGGTTGACAAAAACGTTGCCAACAATGCCAACGGAACAAAAAA
GCCTCCTTTGAAGAATTCGAAAGAGGCATCTGCTGCAGCCACCAGGGTGAAGAAACAAACCAACGTGACAACG
GTCGCTAAGAAGCCTCCCGCCAAATTGATCACGGCAACGAGCAAGGAGATAATCATCCCCACCACTACAGCAG
TCACCACTTGCTGATTTTGCCCAATTCTCCCGATCGCGATGCCGTGGAGGATTGGCAGCTCAGTCTCGTCACG
GCCTCCACGAGCGGGATGCCCCCCTTGT
>TR318|c0_g1_i1 len=553 path=[584:0-289 585:290-313 587:314-355 582:356-379 583:380-552] [-1, 584, 585, 587, 582, 583, -2]
GATGATGCTGATGCTGATGTCGCCGCCTCCACTCCTCCATGCGCCTGCCCCAGATGTCCCCCCTGCGGCTGTT
GCTGCTGCGGTAAAAAGGACTGGATCGGTGGATCACTGTCACATTCACACGCACAGACCCCCGGTGGTAGATC
CACCTGCTTCGCCTTCAACGCCGGCCAGATGAACTCGTCCTCGGCCGCGGAATGAGCCCGAAAAACACTCCAA
ATGACGGTGAAGCGGCCAGCAACTCGTCGTTCCAGATCGCAAATGCGTCCGTCCCCAGCGTACTTATTGATGT
CCTCCCCGGCGGCGGTAATCTCGCTACATAATTCGGTCACATCCGCCTCAAGATTTTTGAGGGCACCGCGGAA
GCAGTCGTGTAAGTGGATGATTTCTACGAGGGCGCAGTCGCCGAAGATGTCCTTCGCCCTGCGTGGAGGAGAC
GATGGCCGTTGTCCCAAGGTGCTGTTCGCGGTTGTTTCAACGGTATCTCCGCTAGCACCTGTTGGAGTGGTGC
TCATGATGGCAGGGTTCCTCTTGAATTATTTGAATTGAGATT

>TR319|c0_g1_i1 len=694 path=[750:0-203 751:204-251 753:252-323 746:324-347 747:348-693] [-1, 750, 751, 753, 746, 747, -2]
TCCTGTCCCCTCATAATATTCGAAATATTGACAAGATGTCTCAAGTTATCTTGTTACCTTCGGTTATTTACTC
TCGTATTCCAATAACAACAATATTCAAGTAATGACGTTCACATCCCTGATCATACATAGTAAGCAGTTCGTAA
TCATAGTTCGTAATCATAGTTCGTAATGATTCATGGTGTCGTTATTCCTATACGCATGGCGAAGTCGTTAAGT
TTACTCGTGTCGTTTCGTCGTCGCTCTAGTATGCCCAAGTATAATATACATTCCCTAATGTGCGCGTCCCTTT
CATTCCCCTCCATCTCTCACAGCCTTGTACTTGGCAACCAACTCTTGCAACTTTTTCTTCGGCAAAGGCTTCT
GGAAGAATTCCGTAAATCCGATCCCCTCACACTCGCTTCTGATATCCTCGCGCATCTCTCCCGTGAGCGCCAA
TATAGGCAGGTCGTTTATCTTCAGTTCGTTTCGAATCAACTTGGTCGCCTCGAGTCCGTCCATGACGGGCATC
CTCAGGTCCATGAGGACCATGTCGAAGGTGCCAGGCAAGGAATCCTTGAGGAGGTTCACCGCGACTTGACCGT
TCTCGGCCCGTTGGGTGGAGCACTTCATCTTTTTCAGTTGCATGGCCAGCAACCGAGCGCAAATGTTCGTATC
TTCCACGATGAGAACCGAAAATGGCATGGACGCCTTG
>TR320|c0_g1_i1 len=445 path=[472:0-147 473:148-164 475:165-276 467:277-300 468:301-444] [-1, 472, 473, 475, 467, 468, -2]
TTTTACATGTATCATTCTGTTATTACCCTTCTTTTACATTATTTAAAATCAATGACACCAAGCGTGCCCTCTT
TTTAGTAGTATGAGACAACATGTCTGCGCCACACACCCTTGGCTGCCATGCACCCCAGTGAGAGATTGCAAAA
AACCGTTGTCATCTCACAGGACTCAAAGGAAGACTGGGTGCGGCTAGCCCAGATTCGTCTTACCGTGATGGCG
AAGGAAAAGGCTTGTTTATCCTAAAATTGCCTCGATTTTGCTCAATCTCTCACGGAAACCCTCTGCTTCATTT
GTCAGTCTCACTATCTCTTCTTTAGCTGCTCTGAGTTCAACCCTCATTGTAGAAATTTCATTTGCTAGCTCTT
CCTGGGGATTGGCCGTTTGTATTGACATTCTTCTCACCCAGCGAGAGTTGACAACCACCAAAATTCCAAATAT
TAATTCA
>TR321|c0_g1_i1 len=993 path=[1206:0-405 1207:406-429 1211:430-458 1210:459-482 1199:483-992] [-1, 1206, 1207, 1211, 1210, 1199, -2]
CCTACATCTGGTGTAAGTACACATTCAAGATTTTATAATCAGGCCCAGTAGGTTCACACTCAAAACGCCAAGC
ATAGTTGTGCGATTGTGATCTTGAGGGAATGTGTCCAGAGTGTTGGAAAAAAACTCATGGAAGTGAGTGACTG
TCACCCATTTCACTCATGGCAGTCACAGGTGCTTTCCGCTCGTTTTGTCTCCAACACTCACGATCTTATGCGC
ACAAGATCACAAGCACTCCCGCTAGTGGTGTGTCTTCAAATATCCTCTCGTCTGAGAGTGAGTCTCCTTAAAA
TATCTTTGCATACCCCCTCTTCGGTACATCAGCGTCTGGTTGATCGCCTAATCCAGCATGCATTTGATCATTT
TTATGGGGGTTCACGAGGCTGAAAATAATGCAAAATGTCACGGCACTGGAATGCACAACTCAGAAGAACTTTC
CGGCAGATTGCACTTATATATTGTGATGGATCAGGAGAGGGGATGTAAACAATCCATCTCAACTCCTGATTGA
TAAAGCCGCAAATTCGTCCCGTTTGCCCAAATCCTGCGAATCACGCTTGAAGGAAATTTCCTCTTTACATTTG
ACTTTTCCAACATGGTTATTACATATCTTCAGTTGTCACACGTTTGGTCGTCCTCCTCCTCCAGAGTGTCATT
GCTGCAGTGATCGCAAGTTAACATGCATTTCTCACTCATAAATGACGGGTTCTCCTCACACTGATTCTGATTG
GCCCAAAATTGACAATGTCCGCTCAAGTCCAAGCAATCTTTCCTTGGGATATCGATCACCACGCTCCCGTTTT
CTCCAATAGGAGGCATCGAACTACCACTACCCTGTCCTTGCTTCCCCTCCTCTTTCACCTCGACGCCAATCTT
CCAACTGCCAAGGCTGCTATTAGAAGTCAATTTCCATCTCCCGGGACTCCCCGACCACGCATCGACACGTGCA
TCCCGCGCGTAGAATTCGTGCGTGAGCATCGAGTAGTGCACCCA
>TR322|c0_g1_i1 len=458 path=[517:0-32 518:33-56 521:57-205 512:206-229 520:230-386 515:387-410 516:411-457] [-1, 517, 518, 521, 512, 520, 515, 516, -2]
CATCGGCATCGGTAGGCTTTCCTATCCAACTATTGTTAGTTGTTATTGCACTTCTATCAACATCATCATGGCC
CACAGGAAACTTTCTCAGAGCGTTTCAGTCAGTGCAGCCGCGTTCTTGGTAGGAGGAACAGCAGTTGCAGTCG
GATCACGCGGTACGGACTCCACTCTCAATGTCTCCTCGCTGCCGCGAGGGTCTTCTGTTGGATCATCGTATCT
CTCGGATACTACTGCAACAACGACCAAAGACGGACGGTTCATCCCCGTTAACTCATTTCTCTCCACGAAACAC
CCATCGTCTTCCCATGCTTTATGCGAGGGAGAAAATTCAGCAGAGGAGACCGAGTCCCAAAGATTCGAACGTG
CGCTCGCCTGCCACCGCTCACGAATATCGTCCTATCGACAAAAATGGGAGTACACGCCGGACAGCGCCGCAAC
GACATCGACGAAGACTCCGA
>TR323|c0_g1_i1 len=1548 path=[1608:0-378 1609:379-409 1620:410-636 1612:637-660 1619:661-1102 1615:1103-1547] [-1, 1608, 1609, 1620, 1612, 1619, 1615, -2]
CGAACTGGAGCTTATCTGGCCGGCCCGATTTTCGGGCCCCAGGACAAGATCTGTCAGCGGACAGGATTTTGAG
TTTTGATAATTTGGCGCCTTACTATGTTCATTGCCGTTTTTGATTACTTTCATTTGATAGTGATGTCAAAAAT
ATCAAATAGATTGGAAGATGTAGTACCTTCTTACGCTTGATACTGCCTTGATCCGGAAGACGAACGTTGTCTT
TGGCTGGTGCTACTGCTCCGTCTCCCTGATCGTTCACCTTTCCTCGGACGGGAGCCACTACGACTGCCTTCGT
CACTCTTGCCGCCACCATTGCTACCGCGATATTGTCCGTTCTTCCATTCACCATCCTTCACTCTGCCATTGTG
GTCATATTCGAAGGATCCTTTACCATGTGGAACCAAACTGTCATTGACTTGTCCATTGTAAGTGCCACATCGA
CCCTTGATGTCAGTGTATCGTATTTGAACTGCACGCATGGTGCTTCTCCTTTTTTCGTGAGTCTTTTCTAGCT
GTGGGCGCTGCTCTTGTTGGCGGTCCTTCAACTTACACCTAGGTTTCCTTCTTTTCTTGTGTACTTTGCTCTC
CAAGACAGGTTTGTTATCGGCTGGGGGCAACCCTTGATGTTTATCAATGAACGTTTTATTGGCCTCGTCTGCG
CTGGGGGCTCTTGATCTAAGCCTGGCAATTCTGTTCAAGCTCTGGGAGGGACTTTCACCAATCTGTCCGTAAG

ACGATTCACTGGAGCTTCCCGTGAGCGATGCCGTGTCATCAGATGGATGATTAGATGGACGCTTTGACTTGTC
GGCAATGGGGACAGTAATGACACTTTTAGTAATGGAGGCATGTCGAAGGTTCTCTCGGGGTAGAGATATACTT
GGTGATTGTGGCGGCAGTGAGGGTGGGTGGGACATCGTCTTAGGGGAATCCAAACTGTTATTGCTGCCCATGG
TAATGGTGGGTCGAAGCTGAGACGCCGTGTTTCTATCCTCTCCGTCAAAAGACTTCTCCTTCACCTTACTCTC
GATCTTCTCCACAACCATCACCTTCTGCAGCTCCTCCATACAACAGTCCGGGCATGCACTCATGAGGATTGTC
CATCCTCTGCCAAAGAGTTTCCTTTTTCGAAGACGAACATGTGAATGGTGGATGCATCGACCTTTAGCATCAT
GCGCGTGGTACATGGTAGTCAGTGATTGAGATTGCGCAGGGCTGGAGTCTTGGTTTTGAGGTTCTTGATCTTG
GTTATAATTCAGTAGCACGTCGTTGGTATTACTAAAAGAGGTATCAATACCTGGAGACAATTGGACTGCATCT
CCCATTAAGAAATAATCGGATACTTCATCGTCGGTGCTGCCCACCAGTGGTAAAAATGTGGACAACTCATCCT
CGTAGTCAGGAGCGGTCACATCAATGTCGGTGTTGCACTGCGAACGTGTTGCCTGGATAGATTCGATCATGTT
CATCTGTTGAGATGGGGGGGGATGTTTTTGTATAACCTCCCCTCTCTGTTGCTCTGATTGTTGCTCTGATGGC
TGGTTTAGATCTTGA
>TR325|c0_g2_i1 len=2883 path=[3621:0-1551 3622:1552-1570 @3673@!:1571-2211 3662:2212-2229 3665:2230-2372 3637:2373-2396 3667:2397-2584 3664:2585-2673 3648:2674-2696 3649:2697-2718 3650:2719-2882] [-1, 3621, 3622, 3673, 3662, 3665, 3637, 3667, 3664, 3648, 3649, 3650, -2]
TCTGTTCGTTCCATATTATAATGCATGACACTCTGCGTTGCTGCAGGTCGCACGATAGCTGCGCTTGAGGCAG
ATCAAAGAACGAAAGCAAAAGAGAAGAGCACGAAAAGAGACAGCAAAAGAGTGAATACCTATCAACAATAGGC
ACCGGGGGAGTCGTTACCATTACAACTACAATCCCGGCGCAGCGATATTTGATTAAACTTATGATCGAAGACA
CCAACAAATATAATATGCGTTATAATTCACCATTACTCGTCCTACCATCCTCAATCCTCCTCCTCTCCTCTTT
CTCCAACAATAACAACACACTACTTGTACACGCCCAACAACGCTGTGGAGCCGAATTCGGCAACACAATCTGC
TCCTTCAACCTGTGCTGCTCCTCCTTCGGATGGTGCGACTTCGGAGATGCATGGTGCGGCGACGGCTGTCAAA
GCGGTCCATGCTACTCCAACCCCGTACCCCTTCCTCCTCCCCCTCCAACGAGTCTGTCACCCACTCCACCTCC
CGCAAGACCCAAAACTCCCGCTCCATTCAACTCCTGCGATACGAGGAAACGCAACACGGTCAACTTTGGATAT
TACGCATCATGGGCGATATGGCGTGGATCGGATTGCAATAACATAGGACCGGACGATATCGACGTGGAGGGGT
TTGGATATACTCACTTGGCGTATGCCTTTGCGGGGATCAATGAGTTTGGAGAATTAGAAGCGTGGAATGGCAA
TACGTTCGACGAGACTCGTCTGTACGGTAGACTCAATTTGCTCAAGACGGCTTCCCCTGATTTGAAAACGTTG
ATTGCCGTGGGAGGGGCGTCGTTAGATAGTTCGCTGTTTTCGAATGCCGCGAGTACGGCATTGAAGCGTCAGA
ACTTTGCGGAATCGGCGGTGGATTTTTTGGACGCGTACGAATTTGATGGGATCGATTTGGATTGGGAATATCC
CGGAGAGGCAACTTCGAGGGATTTCGTGAATTATCCATTGTTGTGTAAGGCGTTGAGGGATGCGTTTAACGAA
GCGGGACATAGGGACTGGTTGATTACGGTGGCGACACCTATTGATAAAGCCAAGTTGCAGATTGGATATGATT
TACCGGGGATGGCTCCTTATGTGGATTGGTTCAACATGATGGCCTACGACATTTACAGTATCACGGGTGATAG
GGCAAAAGCCCACACGGACTTAAACACCATGCCCTCCATTCGCTCCACCATGGATTTCGTATTCGAGCTGGGC
ATTTCACGCTCCAAATTGGTTCTGGGGCTGGCAACCTACGGCCGTTCGTTTCGCTTGTTGGAACCCGAAACTT
GCACGACAGTTGGTTGTCCCATCAGCGGTCCGGGATTGACAGGGTGTTACGGACAAGCGGGGGAATTGCCGTT
TTTTCAACTTCAAGAGAGTTACTCGTCATCGGCTACTGTTAATCTTGAGACGGGTAGCGTGGAGTTGGTCGTT
GGGGAGACTTGGACGACCTACGACAACGCAGAAACCTTCGAGACGAAATATCAGTATGCGTATAGGCAGTGTA
TGAGGGGCGTTATGTGGTGGACCGTTGATTTCATCAAAGAGCCCCTCCTCGACACCTTCGTCGAAGCCATACC
CACCGCCTCTCCCACCAAGTCTGAAGGGCCCAGCATGTCAGCAAATCCTTCCATTGGTCCTACCGTGACCTTA
GTTCCCAGTGAATCGACAGAACCCAGCCGTGACCCGACGGTTAAGCCGACAGGTCCCCCAACGATACCGCAAG
ACTGCGGGTTTTGTCCCGGCGAATTTACTGGGAATTTACCCACGCTGGACTGCGTTGGATTTATCATGTGCCG
GGATGGGGTAGAAGTTAGCTCTTTCGGATGCCCATCCGGGCTTCTTTTTGACACAAGTACGGGCGTGTGCAAC
TGGAGTTATGCATTTGAATGCGACTGTAAGAGAATCACAGGGCCGCCAACACCACATCCCACCCCCACCGCAC
CCGGTCCTCATGAGCCGCCCGCACCTTCCCTTCCAACTCTGTCGACCCAGTCCCAGTGCAGCCCGTGTCCGGC
CTCTGGCAGCACCCTGGTTGAGGCCGCTGGGTGCATGGGTTTCATCCGCTGTAATGGGGGAGTGAGAGGTCCT
TACCAGAGTTGTCAAGGCGGTTTGCAGTTCTCGTTGCAGGCGCAGAGGTGCGAATGGCCACGAAATGTCCAAT
GCTCCTGTTCGAGCGGGACAGGGCTGACGCAGCCGGGACCCGTTCCGACTACGCCCACCCTTCGATTTCCAAC
GCCTCCCGCATATCCCGCAATCCAGCCTCGAACGTCGCCCGCCGGACTGTGGTACCCCGATTGGCCGCATGGC
ACGTGCGTGGACGATGGGATGGCACCGGAGTGGATGGTTAGACATTATTCATACTTCACTTCAACACAGGCCG
AGTGTTGCGATCAATGGTATTGGTGGGATTATAGTTGTTATTGGACGGGCGCGAGTTGATGCCAAGACAATTT
CGTACCTGAGGGAAACGGCATGCTATGGCCCTACCAGGGCATGATTCTCTCTTTCTATGTTGATGAAATATAG
TTACGACGGTGGGAGATGGCACCAAAATGTAGGGTGACACCTAAACCTGAATTTGTAATTGTAGGCCGTTGGT
TGGATGTAAACACTTTGCAATAAGGAGTATCCACAGTTGTCCCAAAGCTGGACAAATGCACAGTAGACACATT
ATTTGGATAATGGTGATAATACGTGGCCAAATTTGCAGAGAAGTAGTGTACCCATTTAAATGTCTGGATGTGG
GTCTGCATGGAAATTACCATAGAGTAAAGTACATGAGGACATATTAATCCAAAAGTGGCATGCATGTTCGTCC
TAAATTCTGATATTTGGCAGAATCTATCGGATGTGG
>TR325|c0_g2_i2 len=1713 path=[3628:0-400 @3673@!:401-1041 3662:1042-1059 3665:1060-1202 3637:1203-1226 3667:1227-1414 3664:1415-1503 3648:1504-1526 3649:1527-1548 3650:1549-1712] [-1, 3628, 3673, 3662, 3665, 3637, 3667, 3664, 3648, 3649, 3650, -2]

TGCATGACGATGTGTGTAATCACACACGCTCTCGTGTGTGATTTGCGTGAATCATCGCACTCGGTATCCCTCG
CGACGACGATTTATCGCTCCCTAGCGAGTGTGATCGACGAAATAGCGCACCAATCCTGCTTTCTATGACACGT
GGGTCTAGGGCCTTCGGCTAGTCAGTGAGCTTTCCGGGGAGGAATTTTGATCGGACCAAAGTTGATTTGATGG
ATCCCCGAAACTTGCTCTTTTTCCAGCAAACAATTTCATCCAAAGTTTTCATGATAGATCGGCTCGGATATGC
CACATAATCAGCCATAGTTGCTTGAGCTGGAACTATTGCATGGTCTAAACCACAATGCCCCTCTGTGCAACAT
TTTCTCGCTTTCAGGTGGACCGTTGATTTCATCAAAGAGCCCCTCCTCGACACCTTCGTCGAAGCCATACCCA
CCGCCTCTCCCACCAAGTCTGAAGGGCCCAGCATGTCAGCAAATCCTTCCATTGGTCCTACCGTGACCTTAGT
TCCCAGTGAATCGACAGAACCCAGCCGTGACCCGACGGTTAAGCCGACAGGTCCCCCAACGATACCGCAAGAC
TGCGGGTTTTGTCCCGGCGAATTTACTGGGAATTTACCCACGCTGGACTGCGTTGGATTTATCATGTGCCGGG
ATGGGGTAGAAGTTAGCTCTTTCGGATGCCCATCCGGGCTTCTTTTTGACACAAGTACGGGCGTGTGCAACTG
GAGTTATGCATTTGAATGCGACTGTAAGAGAATCACAGGGCCGCCAACACCACATCCCACCCCCACCGCACCC
GGTCCTCATGAGCCGCCCGCACCTTCCCTTCCAACTCTGTCGACCCAGTCCCAGTGCAGCCCGTGTCCGGCCT
CTGGCAGCACCCTGGTTGAGGCCGCTGGGTGCATGGGTTTCATCCGCTGTAATGGGGGAGTGAGAGGTCCTTA
CCAGAGTTGTCAAGGCGGTTTGCAGTTCTCGTTGCAGGCGCAGAGGTGCGAATGGCCACGAAATGTCCAATGC
TCCTGTTCGAGCGGGACAGGGCTGACGCAGCCGGGACCCGTTCCGACTACGCCCACCCTTCGATTTCCAACGC
CTCCCGCATATCCCGCAATCCAGCCTCGAACGTCGCCCGCCGGACTGTGGTACCCCGATTGGCCGCATGGCAC
GTGCGTGGACGATGGGATGGCACCGGAGTGGATGGTTAGACATTATTCATACTTCACTTCAACACAGGCCGAG
TGTTGCGATCAATGGTATTGGTGGGATTATAGTTGTTATTGGACGGGCGCGAGTTGATGCCAAGACAATTTCG
TACCTGAGGGAAACGGCATGCTATGGCCCTACCAGGGCATGATTCTCTCTTTCTATGTTGATGAAATATAGTT
ACGACGGTGGGAGATGGCACCAAAATGTAGGGTGACACCTAAACCTGAATTTGTAATTGTAGGCCGTTGGTTG
GATGTAAACACTTTGCAATAAGGAGTATCCACAGTTGTCCCAAAGCTGGACAAATGCACAGTAGACACATTAT
TTGGATAATGGTGATAATACGTGGCCAAATTTGCAGAGAAGTAGTGTACCCATTTAAATGTCTGGATGTGGGT
CTGCATGGAAATTACCATAGAGTAAAGTACATGAGGACATATTAATCCAAAAGTGGCATGCATGTTCGTCCTA
AATTCTGATATTTGGCAGAATCTATCGGATGTGG
>TR325|c0_g2_i3 len=2344 path=[3621:0-1551 3622:1552-1570 @3673@!:1571-2211 3671:2212-2287 3625:2288-2309 3626:2310-2321 3627:2322-2343] [-1, 3621, 3622, 3673, 3671, 3625, 3626, 3627, -2]
TCTGTTCGTTCCATATTATAATGCATGACACTCTGCGTTGCTGCAGGTCGCACGATAGCTGCGCTTGAGGCAG
ATCAAAGAACGAAAGCAAAAGAGAAGAGCACGAAAAGAGACAGCAAAAGAGTGAATACCTATCAACAATAGGC
ACCGGGGGAGTCGTTACCATTACAACTACAATCCCGGCGCAGCGATATTTGATTAAACTTATGATCGAAGACA
CCAACAAATATAATATGCGTTATAATTCACCATTACTCGTCCTACCATCCTCAATCCTCCTCCTCTCCTCTTT
CTCCAACAATAACAACACACTACTTGTACACGCCCAACAACGCTGTGGAGCCGAATTCGGCAACACAATCTGC
TCCTTCAACCTGTGCTGCTCCTCCTTCGGATGGTGCGACTTCGGAGATGCATGGTGCGGCGACGGCTGTCAAA
GCGGTCCATGCTACTCCAACCCCGTACCCCTTCCTCCTCCCCCTCCAACGAGTCTGTCACCCACTCCACCTCC
CGCAAGACCCAAAACTCCCGCTCCATTCAACTCCTGCGATACGAGGAAACGCAACACGGTCAACTTTGGATAT
TACGCATCATGGGCGATATGGCGTGGATCGGATTGCAATAACATAGGACCGGACGATATCGACGTGGAGGGGT
TTGGATATACTCACTTGGCGTATGCCTTTGCGGGGATCAATGAGTTTGGAGAATTAGAAGCGTGGAATGGCAA
TACGTTCGACGAGACTCGTCTGTACGGTAGACTCAATTTGCTCAAGACGGCTTCCCCTGATTTGAAAACGTTG
ATTGCCGTGGGAGGGGCGTCGTTAGATAGTTCGCTGTTTTCGAATGCCGCGAGTACGGCATTGAAGCGTCAGA
ACTTTGCGGAATCGGCGGTGGATTTTTTGGACGCGTACGAATTTGATGGGATCGATTTGGATTGGGAATATCC
CGGAGAGGCAACTTCGAGGGATTTCGTGAATTATCCATTGTTGTGTAAGGCGTTGAGGGATGCGTTTAACGAA
GCGGGACATAGGGACTGGTTGATTACGGTGGCGACACCTATTGATAAAGCCAAGTTGCAGATTGGATATGATT
TACCGGGGATGGCTCCTTATGTGGATTGGTTCAACATGATGGCCTACGACATTTACAGTATCACGGGTGATAG
GGCAAAAGCCCACACGGACTTAAACACCATGCCCTCCATTCGCTCCACCATGGATTTCGTATTCGAGCTGGGC
ATTTCACGCTCCAAATTGGTTCTGGGGCTGGCAACCTACGGCCGTTCGTTTCGCTTGTTGGAACCCGAAACTT
GCACGACAGTTGGTTGTCCCATCAGCGGTCCGGGATTGACAGGGTGTTACGGACAAGCGGGGGAATTGCCGTT
TTTTCAACTTCAAGAGAGTTACTCGTCATCGGCTACTGTTAATCTTGAGACGGGTAGCGTGGAGTTGGTCGTT
GGGGAGACTTGGACGACCTACGACAACGCAGAAACCTTCGAGACGAAATATCAGTATGCGTATAGGCAGTGTA
TGAGGGGCGTTATGTGGTGGACCGTTGATTTCATCAAAGAGCCCCTCCTCGACACCTTCGTCGAAGCCATACC
CACCGCCTCTCCCACCAAGTCTGAAGGGCCCAGCATGTCAGCAAATCCTTCCATTGGTCCTACCGTGACCTTA
GTTCCCAGTGAATCGACAGAACCCAGCCGTGACCCGACGGTTAAGCCGACAGGTCCCCCAACGATACCGCAAG
ACTGCGGGTTTTGTCCCGGCGAATTTACTGGGAATTTACCCACGCTGGACTGCGTTGGATTTATCATGTGCCG
GGATGGGGTAGAAGTTAGCTCTTTCGGATGCCCATCCGGGCTTCTTTTTGACACAAGTACGGGCGTGTGCAAC
TGGAGTTATGCATTTGAATGCGACTGTAAGAGAATCACAGGGCCGCCAACACCACATCCCACCCCCACCGCAC
CCGGTCCTCATGAGCCGCCCGCACCTTCCCTTCCAACTCTGTCGACCCAGTCCCAGTGCAGCCCGTGTCCGGC
CTCTGGCAGCACCCTGGTTGAGGCCGCTGGGTGCATGGGTTTCATCCGCTGTAATGGGGGAGTGAGAGGTCCT
TACCAGAGTTGTCAAGGCGGTTTGCAGTTCTCGTTGCAGGCGCAGAGGTGCGAATGGCCACGAAATGTCCAAT
GCTCCTGTTCGAGCGGGACAGGTGAGTAGTTTTCACCATTTTGGGTGTCTGGTGTTTTGCATGTTTACGCTGT
GCATCGAGATTTTAGTATGGTTCCTCTGTAATTTTTGTCTTTATATTTTTTGCATCGCACAGGGCTGACGCAG
CCGGGACC

>TR325|c0_g4_i1 len=1654 path=[3634:0-1551 3635:1552-1653] [-1, 3634, 3635, -2]
TCTGTTCGTTCCATATTATAATGCATGACACTCTGCGTTGCTGCAGGTCGCACGATAGCTGCGCTTGAGGCAG
ATCAAAGAACGAAAGCAAAAGAGAAGAGCACGAAAAGAGACAGCAAAAGAGTGAATACCTATCAACAATAGGC
ACCGGGGGAGTCGTTACCATTACAACTACAATCCCGGCGCAGCGATATTTGATTAAACTTATGATCGAAGACA
CCAACAAATATAATATGCGTTATAATTCACCATTACTCGTCCTACCATCCTCAATCCTCCTCCTCTCCTCTTT
CTCCAACAATAACAACACACTACTTGTACACGCCCAACAACGCTGTGGAGCCGAATTCGGCAACACAATCTGC
TCCTTCAACCTGTGCTGCTCCTCCTTCGGATGGTGCGACTTCGGAGATGCATGGTGCGGCGACGGCTGTCAAA
GCGGTCCATGCTACTCCAACCCCGTACCCCTTCCTCCTCCCCCTCCAACGAGTCTGTCACCCACTCCACCTCC
CGCAAGACCCAAAACTCCCGCTCCATTCAACTCCTGCGATACGAGGAAACGCAACACGGTCAACTTTGGATAT
TACGCATCATGGGCGATATGGCGTGGATCGGATTGCAATAACATAGGACCGGACGATATCGACGTGGAGGGGT
TTGGATATACTCACTTGGCGTATGCCTTTGCGGGGATCAATGAGTTTGGAGAATTAGAAGCGTGGAATGGCAA
TACGTTCGACGAGACTCGTCTGTACGGTAGACTCAATTTGCTCAAGACGGCTTCCCCTGATTTGAAAACGTTG
ATTGCCGTGGGAGGGGCGTCGTTAGATAGTTCGCTGTTTTCGAATGCCGCGAGTACGGCATTGAAGCGTCAGA
ACTTTGCGGAATCGGCGGTGGATTTTTTGGACGCGTACGAATTTGATGGGATCGATTTGGATTGGGAATATCC
CGGAGAGGCAACTTCGAGGGATTTCGTGAATTATCCATTGTTGTGTAAGGCGTTGAGGGATGCGTTTAACGAA
GCGGGACATAGGGACTGGTTGATTACGGTGGCGACACCTATTGATAAAGCCAAGTTGCAGATTGGATATGATT
TACCGGGGATGGCTCCTTATGTGGATTGGTTCAACATGATGGCCTACGACATTTACAGTATCACGGGTGATAG
GGCAAAAGCCCACACGGACTTAAACACCATGCCCTCCATTCGCTCCACCATGGATTTCGTATTCGAGCTGGGC
ATTTCACGCTCCAAATTGGTTCTGGGGCTGGCAACCTACGGCCGTTCGTTTCGCTTGTTGGAACCCGAAACTT
GCACGACAGTTGGTTGTCCCATCAGCGGTCCGGGATTGACAGGGTGTTACGGACAAGCGGGGGAATTGCCGTT
TTTTCAACTTCAAGAGAGTTACTCGTCATCGGCTACTGTTAATCTTGAGACGGGTAGCGTGGAGTTGGTCGTT
GGGGAGACTTGGACGACCTACGACAACGCAGAAACCTTCGAGACGAAATATCAGTATGCGTATAGGCAGTGTA
TGAGGGGCGTTATGTGGTGAGTGTTTTTCGTCTGTGCTGTTGACTTTATAATATGTCCTGCATAGTTGGCATG
ATCAATATGATATTGTGATCAAGGGAATAAACCGTGAGGGCACACATG
>TR326|c0_g1_i1 len=253 path=[231:0-252] [-1, 231, -2]
CTTCGACTGGGGCATCGACCAGTTGGCCTTGCAGTTGTCTGTTGGGTTAGCTGGCGAGGATGCAGAATATAGG
CGGCATGTATGCGATGCCATTTCGACGCTGTATGGCATAGGGACTAGCTATGGACTGGATGGCTACCCGTAGT
TGAAGTGTTTGTTGGAAATTGAAGGTCAATGGCGGTGTGAGAGGCGTTGGGGCTGGCCAGGGTACTGTCGGCG
TGGTTGTAATTGTGGTTGAACGGCATCGAAATGG
>TR327|c0_g1_i1 len=352 path=[330:0-351] [-1, 330, -2]
TCCTCCCAATGCATTATCATTATTATCGTCATCGTCGTCGTAAAATGGTATTAACGCCGGCGTACTGTCATCC
CAGGGTTGCTTTGGTATTGGCCGAGCGATGTATTGGCGCATGAGGGCCAGGCGAGATTGGAGGTGTTCCTCGT
CGAATACCATTGCGTCTTCGGCGGCTTCATTCATTCTCTAGATTTTCATTCAGTTCTTGGCCTCCGTTGCACG
CACCTTGCACATTGCATTTCCTTGCACCTAAAATTAATACACACTTGAATATGAAATATGTGTGCTGCTGAAG
CTTCACATTGCCTTCTCATTGAAGTATTGAGGATGCAAAGAACGACGTTCCCTCTCTCTC
>TR328|c0_g1_i1 len=311 path=[289:0-310] [-1, 289, -2]
GCATAATTTCCCCGCCCATCCAAAGCCCAACAACTTACAATACTCCACATTGCCCTCAAGCATTTAACAAATC
CCTACAGTACCAGGAGGGTGATATCGTGACTCGCCGTGACAGCATGTTTAAATGCAAGCATTGGCCGGATTCA
GCCTTCTGTAACCGTTTCGTGCCCGGGAAACGCAGTTCCAATCGAGGGTGGTTGAAAATTGGACATTGCGCTG
GTGGTCCATCGAATAATGTCCCGAAAGTGGAGTCTGTTTCAAGCACTCCAACTCTGTCTCCTGTGGCTGGAGA
TACCGGTGAACCTGTCACG
>TR329|c0_g1_i1 len=401 path=[379:0-400] [-1, 379, -2]
TTTACATATTGATTACTTTTTACGAATAACATTGTATTTCGTAATAATTCCATTTGGATAATTTTCTCCTCAT
TTTGTTGAGGAATTAAAATTAATGATCCTGAATTTTTAGTAACTAAAACGTCATCCCCCCGATTTGTCCGTAA
TATAATTGTCTTTAAAATTTTAGAAAATTTAATAATGCCATTTGTTGGACTAATAATTTGTTGTCGGGCTTCC
GATGTAAAAATACCGCCCGTATGAAAAGTCCGCATTGTTAATTGTGTTCCTGGTTCCCCAATGGATTGTCCAG
CAAGAATTCCAACTGCTTCTCCTAAATCAACCAGATTTTGGTTTGATAAGTCCCATCCATAACACATTTGACA
AATAGAATGATATAAACTACAAGTTAATGGTGAACG
>TR330|c0_g1_i1 len=700 path=[678:0-699] [-1, 678, -2]
GAGAGTCGACGACGGTATGATGGCGGATGGGGGGATGGCAGCAATTCTAATAATAGAAGTGGGCGATCATCGT
CTTCGTCGTCGTTCCACTGGCAGAATCAATTCTTTCAACAACAAAGACAGAGACAACCCATTCATTTAAAAGA
CAAATTTCAAGTGAAACAAGCCCAATCGCGGGTCCTACACATCGTGAGTCTGGATCAACTGCGCACCATCATG
CTAGACGATGACGATCGACTCGAACGTCATTTGCTCCTTTGTTTCTTCACCCCCAATATAGAACGTTTGGTCG
AAGACGAGATCGTATATCCGTATCCGTTCGCGGGCATGTCACCGCAGTCGATATGGTGGGAGGATCTGTTGCA
GACGGTGAGCATACGTTTTCATAGGGGGAATGATCTGACAAGATATTTCAACATTGATGGTGGTGGCGATGGC
ATGACCGCGCCGGTATTCCTGTTCGTGAAGAAAGGTACATTGCTAGGAGAGACCTTTAATAATAATTACCACG
AGGGCAGTACCAATAACTCGTTCGTATACACGACGAAAGATCGCGAAGCATTCGAAACATGGATGTGGTCCAA
GATGGAAGTCCAAGTCACCTTTCGCAACTATCACTCCCATCCCGTGCAACTATACTGGATCTCCGGCACCCGC
GCCCATATCAAGGAGACGATACCGGCGGGACATTCATGGGTGC

>TR331|c0_g1_i1 len=433 path=[411:0-432] [-1, 411, -2]
AACCCACCCCCAGACACCACAACCATGGCCATGACAGGGCAGCAGATGGTAGCATAGAGCAGATCATGTCAAA
GACAGGCATGCGAATGAGTAGACGCAGACAATTTTTCTTCTAAAAAAATACCCACTGTGTAATTTAAATCGGT
TACGATGCGTTACTCTACCGGTCTTTGTCTGGCCTTCAGTCCTTCCATTTCCTCATTCGCTCCATCGAATCCG
AGAATAATACAGCCTGCAACATCTACGAGAATACACGCCTACGGAAAAGGATCCGAAATATGGCCAGAATGCA
ATGAAGAACCAATCGGTCTATCAGCATCATTTCCGGGAGGCGTAATTCCCCAACCTGCGAAAGATCTTTTGGA
TTCCTCCGACCCGACGGTGTCCCAATCCGCCACATCCCAAACAACGTCTGCAGTCGATGCCCTCGCCG
>TR332|c0_g1_i1 len=364 path=[367:0-304 368:305-328 369:329-363] [-1, 367, 368, 369, -2]
ATATTGGCATTGATTGAAAAGGGAATGCGGACCAATTCGTATACCAACGATGTTGTGATCAAGGTTTCTTATA
TTGGTGTACGAGAGAATGTTGCTGCCCAAGAAGGATCCGATTTACAGCATATCAACACAGGCAATGCGGAAAA
TGCTTTGGACAATACTAATGGTAATGTCAACCCCGCTATGGGAAGTCCCAACCTCAACGGGGTCAAGCTAGTC
GAACCTGGCAAACTAGAATCCAACAATCTGCCTTTCGTCGTAGTCATTGTTATCAGCTTCATCTTTCTCTTCG
CATTCGCGCTCGCGATATTCCTTGGAGTGCTTTTTCTTAGAAAGAATAGAGTCCAGCTCGTTCACGACGGAC
>TR334|c0_g1_i1 len=334 path=[330:0-187 331:188-333] [-1, 330, 331, -2]
CGGCCATCAAGCCCACAAGGATGCTATGGTCGTGAAGAATGGACTGCCAGATGGAAGGGGCTTGCTTCACTTC
TGATGGACCCATCTGCTACTAAAGATCTTCAATCAAGCATTTTAGCGTGCCATCATCATGTGCACCCAGCCAG
CCACAGCATCTACACTATAATCTTGAGCAGCATCCCCTAGGTTTTGTTTACTTTGGTGGGCTGACAGACTACA
CAAAATCCTCAACCCATACCTGCCCTATTTGTTGGATTTCGGGGTTTTTCAATGAGCACTTTTACCTAGATAC
ATGCACACTCTTCAAAAATAGAGCTGGTGTCCCACCCATTAT
>TR335|c0_g1_i1 len=850 path=[875:0-45 876:46-69 881:70-369 874:370-849] [-1, 875, 876, 881, 874, -2]
CGGACGATTTGTCGCTTAGCCTGATTGCTCATCGATCGGAGCGTGTTGCGTTACTGTTGAGCGTTTTCCCTAC
AGTACACTGGGGAATCCTCATTGGCCTCAGTCTCAGCATCTGCATGACCTTCCTCCTCGTGTCAAATCAACAG
GTGCTACAGTACCTAAATTCGATCCAACTTCGTTTCTTGTTTGCCACAATCGTAGGGGTTTTGGCAAGTACGG
CCACATTATGTTGGGATCTGAGTGATCCATTCAGTGGCTCATATAGTATTGAGGGTGCCAGCACACAGTTGGC
TGATGTTAGAGTCTGCCTCAGAGAAGATGTGAGGGAAGCCCTCATAGAGGCAAAAGTCTCTTCCAGCACTAGA
AAGTTCTTCCGATCCATACTTGGTGGACCAGATTTTCCCACGACAGAAAGACTTTCCACCAAGTCTGGTGAGA
ATGTAGACAGTGAGGATATCAGTCCTTCGTCGCGGTATGGGTTGATATCAACTCTATACTTCCACCTCCTTAC
AGGACCACTAGGAGACAATGTTCGTGTAATTGGAGATGTCATGGTTTGGTTGACCACCCTTTTCGGGAGGAGG
TTAAAAGCAATAGCAAACTGGCGACAATGGCCATGGTTGTGGAAGAAGGAAAGATCCCGAAGACAGGAACGTC
AATAATTGATAGAATTACAGCATTTGCTTGTGTAGTGGGTAGGATGTATCTGTTCTTATTTGGTCGATGATGA
GCTTCGTAGTTTTCTGTGCCTCTTATTTAGGTCATATCCTTTTCTCGCTTTCTCTTCGATCTCGTTCGTTCGT
CTAGATAATACAATCGCACACAGAGGTCGTCAGTTTCATATTGCCCT
>TR335|c0_g1_i2 len=390 path=[875:0-45 876:46-69 881:70-369 879:370-389] [-1, 875, 876, 881, 879, -2]
CGGACGATTTGTCGCTTAGCCTGATTGCTCATCGATCGGAGCGTGTTGCGTTACTGTTGAGCGTTTTCCCTAC
AGTACACTGGGGAATCCTCATTGGCCTCAGTCTCAGCATCTGCATGACCTTCCTCCTCGTGTCAAATCAACAG
GTGCTACAGTACCTAAATTCGATCCAACTTCGTTTCTTGTTTGCCACAATCGTAGGGGTTTTGGCAAGTACGG
CCACATTATGTTGGGATCTGAGTGATCCATTCAGTGGCTCATATAGTATTGAGGGTGCCAGCACACAGTTGGC
TGATGTTAGAGTCTGCCTCAGAGAAGATGTGAGGGAAGCCCTCATAGAGGCAAAAGTCTCTTCCAGCACTAGA
AAGTTGTGAGTACAAATGAGGTGTG
>TR336|c0_g1_i1 len=1644 path=[2567:0-565 2568:566-586 2569:587-1643] [-1, 2567, 2568, 2569, -2]
CCGATCTGGGGCATTGTAGAAATAGATACAAAACAATATGGCCTACATCTTTTGTCGATTTGAAAGGTTGCGA
CGTTGGACTCGCTGCATTGCAACAAAATCATGGCATCCTTTTCAGTTGTGTGCGGGGTTGAGACGAGGTTGGA
GACGACACATCAACAAACACTCAAATGAGTATCCATACCTCATCTTTATGTGCAGGGATACCATTGCCATCAT
GTACTATCAAATGCATGAACGCCCACGAAGTGTCCTCCTCTCAGAGAGTAACATCATGGGCATACACTTATGC
ATAAATCTGTTTCGGAAGTGACAAGGGCCAAAACAAATATTTCCAGGCACATTAGTATATCCGAAGAGGTTTA
GACAATAAACTGCGGAATAAGTGATATGTTTAGCGAAATTGGCTTTGGGCTCCTAACATCTATTAAAGTAGAT
GTCATTGGCCAGTGCTTGGCTCGCCCAATAGTTACAAGTGCAACGCCACTTTGTTGGCCAAATTATCCACATG
AACATCACGGTGTGGTGATCAAGACGGATCATGACACGCTTACACGTGTGAGTAGCTTATGCGGCCTACAAAT
TGGGAAGTTTTTACTACACGGGACTACATGGGTTTAAGGAAGATCGCAAGAGAGCTAGCAAATGGTTGAAACG
TGCGGAGAGCAAATTGTCTGAATTAAACGAGTCAGAAAGGAGAAATGTTTGCAACTACTTGAGCACTATTTCT
TCTGATTCTGCGCCCTCGACAATGAGCACGTGTCGCCCTTCACCGGTGCCAGAACAAATTGATGTGAAGAATG
ATTGTGAAAGATCTATGTCAACAATTGGTATGGCATCTGCGGGGCGCATCTCGGACACTACTCATCCGTTGAC
ATATCAAACATGATACCAGCATTAGCTTCACGCATCGACGATCCATTTTAGATCACTCCAGTAGTTTTTTCGT
TCCGCATAAGCGGTTTAAATTGTTATCAGACACATGTGCTGGGATGTTATCATTGGTCTCGCACACTGGAACA
GATACATCTCGGTCAAAATGGGTTGTGAACTCGATCCAATAGGCATCGAATGTACATTTGAGGCTCTTTTGCT
TGGTCCGACATAGGACTGGCGACAAATGAATAGTGCGAAGAATGCAGGGGAAAAGGTGGAGGAGAGGGTACGA

TGGTCCAACGAAGTAGAGTGTGTGGTGTATGTCACCGTACACGCCCGATGTATGGACGACATCTGGTTGAAGT
GCTAAGTGAACAATCTCTTACTACCAATCGGACACCATTCTTCCCTACTTTTATACCATTGCGTGAACATATA
CACTCGTGCTTCTAGTAATTGCGTGCCAGCGAAACGCGATGGCATCGTAAAAAGTTCCCGTCGGAAGAATGCA
CAGAGTGCGGAATGTGTATATGAACCAAATGATGGGTGACTATGATCGGCTGCTTCCTGTAAACCTCGTTCTA
GATTCTTGTTGAATCGGCATATATTTCTCGAAACACAAGTGATGAATCATTCGACTCAAGGCTCCAATATGAA
GTATAAAACTAGGCAGCATTGGCAGATAGTTTTAAGCGGCAAAGTTTAGTTGCTAGTTGCCTTGGTTGTGGAC
AAGACTAAATTTAAACTAAAGAATCTAGTGGCGACAGT
>TR336|c0_g2_i1 len=2530 path=[2581:0-565 2590:566-1360 2591:1361-1384 2585:1385-1401 2587:1402-1471 2575:1472-1472 2576:1473-2529] [-1, 2581, 2590, 2591, 2585, 2587, 2575, 2576, -2]
CCGATCTGGGGCATTGTAGAAATAGATACAAAACAATATGGCCTACATCTTTTGTCGATTTGAAAGGTTGCGA
CGTTGGACTCGCTGCATTGCAACAAAATCATGGCATCCTTTTCAGTTGTGTGCGGGGTTGAGACGAGGTTGGA
GACGACACATCAACAAACACTCAAATGAGTATCCATACCTCATCTTTATGTGCAGGGATACCATTGCCATCAT
GTACTATCAAATGCATGAACGCCCACGAAGTGTCCTCCTCTCAGAGAGTAACATCATGGGCATACACTTATGC
ATAAATCTGTTTCGGAAGTGACAAGGGCCAAAACAAATATTTCCAGGCACATTAGTATATCCGAAGAGGTTTA
GACAATAAACTGCGGAATAAGTGATATGTTTAGCGAAATTGGCTTTGGGCTCCTAACATCTATTAAAGTAGAT
GTCATTGGCCAGTGCTTGGCTCGCCCAATAGTTACAAGTGCAACGCCACTTTGTTGGCCAAATTATCCACATG
AACATCACGGTGTGGTGATCAAGACGGATCATGACACGCTTACACGTGTGAGTAGGTGAGTAGTAATTTTCTG
AAATTTTCAGTCAGTACGCAGGACTAGTATAGGTAGACTGGTATGCATTTTGTCATGTGCACATGTATGTGCT
GCATGCACCAACCTCAAGCCGCATGGTTGAACGACGACCAAGTAGGGTATCTCTCTAGAGTGCCATACCACAC
AAAAGCTTTTAATGACAACGACCTATTGCACGTTTGAGGCATCACTCTCTGCCTAGATATTGGTACGAACAAA
ATAAAATGGACGCATATTCTCTAGCCTTGAGGCATGTGTGCAACGTTTGTGGAAAGCTTCCTGAACATCCTTA
CGCCGCAGAAGATGGTTATTTCTATCATAAAGGATGCATTGAGGGAATATTTGAACGCGCAGCTGCTACTGAA
GTGATCAACTCACCTGTCACAGGTCGGCCCATGGGAAAGAGGCTGATTTTCGCTAAAACAGTCCAGTGTGTGA
TTGAAAAGCTAGTTTCCAATAACGAAGAAGTCAAACGAAAACTAGTTCATTCGGACTCTGGAGATGCTATTAT
TACAGTGAGAGCGACCAAAGATGAAGCGCGAGAGGGGTCGGCAAAACACATGGCACTTCTTGGTCGTTGGCAC
TTGTTTGGCGAGCAAGACGGAATGGAATGTAATTTAGCGGAAGGGTATCAATGGTGTCAAAAGGCTGTGGATA
AAGGTGACGTCGACGGAATGGCCTACCAAGCAATTTGTTTATTGCATGGCTATGCCGGGATTAAAAAGGATAG
AAACGAGGGCTTTGAACTCCTCGTTGATGCAGCCAATGAAGGGTCAGGTATAGACTGGATATCTCTTTTTTCG
TTCATTCCCATTGTGATGACAATCTCAACATTGTGAACTCTATCTGCCACGATGAATTAATACAGCTTATGCG
GCCTACAAATTGGGAAGTTTTTACTACACGGGACTACATGGGTTTAAGGAAGATCGCAAGAGAGCTAGCAAAT
GGTTGAAACGTGCGGAGAGCAAATTGTCTGAATTAAACGAGTCAGAAAGGAGAAATGTTTGCAACTACTTGAG
CACTATTTCTTCTGATTCTGCGCCCTCGACAATGAGCACGTGTCGCCCTTCACCGGTGCCAGAACAAATTGAT
GTGAAGAATGATTGTGAAAGATCTATGTCAACAATTGGTATGGCATCTGCGGGGCGCATCTCGGACACTACTC
ATCCGTTGACATATCAAACATGATACCAGCATTAGCTTCACGCATCGACGATCCATTTTAGATCACTCCAGTA
GTTTTTTCGTTCCGCATAAGCGGTTTAAATTGTTATCAGACACATGTGCTGGGATGTTATCATTGGTCTCGCA
CACTGGAACAGATACATCTCGGTCAAAATGGGTTGTGAACTCGATCCAATAGGCATCGAATGTACATTTGAGG
CTCTTTTGCTTGGTCCGACATAGGACTGGCGACAAATGAATAGTGCGAAGAATGCAGGGGAAAAGGTGGAGGA
GAGGGTACGATGGTCCAACGAAGTAGAGTGTGTGGTGTATGTCACCGTACACGCCCGATGTATGGACGACATC
TGGTTGAAGTGCTAAGTGAACAATCTCTTACTACCAATCGGACACCATTCTTCCCTACTTTTATACCATTGCG
TGAACATATACACTCGTGCTTCTAGTAATTGCGTGCCAGCGAAACGCGATGGCATCGTAAAAAGTTCCCGTCG
GAAGAATGCACAGAGTGCGGAATGTGTATATGAACCAAATGATGGGTGACTATGATCGGCTGCTTCCTGTAAA
CCTCGTTCTAGATTCTTGTTGAATCGGCATATATTTCTCGAAACACAAGTGATGAATCATTCGACTCAAGGCT
CCAATATGAAGTATAAAACTAGGCAGCATTGGCAGATAGTTTTAAGCGGCAAAGTTTAGTTGCTAGTTGCCTT
GGTTGTGGACAAGACTAAATTTAAACTAAAGAATCTAGTGGCGACAGT
>TR336|c0_g2_i2 len=2439 path=[2581:0-565 2590:566-1360 2578:1361-1380 2579:1381-1381 2580:1382-2438] [-1, 2581, 2590, 2578, 2579, 2580, -2]
CCGATCTGGGGCATTGTAGAAATAGATACAAAACAATATGGCCTACATCTTTTGTCGATTTGAAAGGTTGCGA
CGTTGGACTCGCTGCATTGCAACAAAATCATGGCATCCTTTTCAGTTGTGTGCGGGGTTGAGACGAGGTTGGA
GACGACACATCAACAAACACTCAAATGAGTATCCATACCTCATCTTTATGTGCAGGGATACCATTGCCATCAT
GTACTATCAAATGCATGAACGCCCACGAAGTGTCCTCCTCTCAGAGAGTAACATCATGGGCATACACTTATGC
ATAAATCTGTTTCGGAAGTGACAAGGGCCAAAACAAATATTTCCAGGCACATTAGTATATCCGAAGAGGTTTA
GACAATAAACTGCGGAATAAGTGATATGTTTAGCGAAATTGGCTTTGGGCTCCTAACATCTATTAAAGTAGAT
GTCATTGGCCAGTGCTTGGCTCGCCCAATAGTTACAAGTGCAACGCCACTTTGTTGGCCAAATTATCCACATG
AACATCACGGTGTGGTGATCAAGACGGATCATGACACGCTTACACGTGTGAGTAGGTGAGTAGTAATTTTCTG
AAATTTTCAGTCAGTACGCAGGACTAGTATAGGTAGACTGGTATGCATTTTGTCATGTGCACATGTATGTGCT
GCATGCACCAACCTCAAGCCGCATGGTTGAACGACGACCAAGTAGGGTATCTCTCTAGAGTGCCATACCACAC
AAAAGCTTTTAATGACAACGACCTATTGCACGTTTGAGGCATCACTCTCTGCCTAGATATTGGTACGAACAAA
ATAAAATGGACGCATATTCTCTAGCCTTGAGGCATGTGTGCAACGTTTGTGGAAAGCTTCCTGAACATCCTTA
CGCCGCAGAAGATGGTTATTTCTATCATAAAGGATGCATTGAGGGAATATTTGAACGCGCAGCTGCTACTGAA

GTGATCAACTCACCTGTCACAGGTCGGCCCATGGGAAAGAGGCTGATTTTCGCTAAAACAGTCCAGTGTGTGA
TTGAAAAGCTAGTTTCCAATAACGAAGAAGTCAAACGAAAACTAGTTCATTCGGACTCTGGAGATGCTATTAT
TACAGTGAGAGCGACCAAAGATGAAGCGCGAGAGGGGTCGGCAAAACACATGGCACTTCTTGGTCGTTGGCAC
TTGTTTGGCGAGCAAGACGGAATGGAATGTAATTTAGCGGAAGGGTATCAATGGTGTCAAAAGGCTGTGGATA
AAGGTGACGTCGACGGAATGGCCTACCAAGCAATTTGTTTATTGCATGGCTATGCCGGGATTAAAAAGGATAG
AAACGAGGGCTTTGAACTCCTCGTTGATGCAGCCAATGAAGGGTCAGCTTATGCGGCCTACAAATTGGGAAGT
TTTTACTACACGGGACTACATGGGTTTAAGGAAGATCGCAAGAGAGCTAGCAAATGGTTGAAACGTGCGGAGA
GCAAATTGTCTGAATTAAACGAGTCAGAAAGGAGAAATGTTTGCAACTACTTGAGCACTATTTCTTCTGATTC
TGCGCCCTCGACAATGAGCACGTGTCGCCCTTCACCGGTGCCAGAACAAATTGATGTGAAGAATGATTGTGAA
AGATCTATGTCAACAATTGGTATGGCATCTGCGGGGCGCATCTCGGACACTACTCATCCGTTGACATATCAAA
CATGATACCAGCATTAGCTTCACGCATCGACGATCCATTTTAGATCACTCCAGTAGTTTTTTCGTTCCGCATA
AGCGGTTTAAATTGTTATCAGACACATGTGCTGGGATGTTATCATTGGTCTCGCACACTGGAACAGATACATC
TCGGTCAAAATGGGTTGTGAACTCGATCCAATAGGCATCGAATGTACATTTGAGGCTCTTTTGCTTGGTCCGA
CATAGGACTGGCGACAAATGAATAGTGCGAAGAATGCAGGGGAAAAGGTGGAGGAGAGGGTACGATGGTCCAA
CGAAGTAGAGTGTGTGGTGTATGTCACCGTACACGCCCGATGTATGGACGACATCTGGTTGAAGTGCTAAGTG
AACAATCTCTTACTACCAATCGGACACCATTCTTCCCTACTTTTATACCATTGCGTGAACATATACACTCGTG
CTTCTAGTAATTGCGTGCCAGCGAAACGCGATGGCATCGTAAAAAGTTCCCGTCGGAAGAATGCACAGAGTGC
GGAATGTGTATATGAACCAAATGATGGGTGACTATGATCGGCTGCTTCCTGTAAACCTCGTTCTAGATTCTTG
TTGAATCGGCATATATTTCTCGAAACACAAGTGATGAATCATTCGACTCAAGGCTCCAATATGAAGTATAAAA
CTAGGCAGCATTGGCAGATAGTTTTAAGCGGCAAAGTTTAGTTGCTAGTTGCCTTGGTTGTGGACAAGACTAA
ATTTAAACTAAAGAATCTAGTGGCGACAGT
>TR337|c0_g1_i1 len=565 path=[711:0-39 712:40-40 742:41-81 740:82-122
739:123-146 726:147-163 727:164-187 728:188-564] [-1, 711, 712, 742, 740,
739, 726, 727, 728, -2]
TATCAATAAATTATAGAAGTATAAGTAGATCAAGGCAATTGCACTTCAATCGCAATGCTTTATAATCTCTCTC
ATCGATTTAAAGCTGCAATAAATACACAAAGGCAAACACCAAAGCACCAGTCCTATATACAAACGAATCAATA
GCAACAGTCGCAACAGTAACAGTCGCACCATTCGCAAACTCTGAATATTGAATCATATTCGAAACCTTACTCA
ACCCACCACTGTTCGCACCTTCAACATTGGACCTATCGTAATCACGACTCTGAGTTGATTTTGATCCACTGCT
CTTGCCACTCTTGCTATTTTTGGAAGATTTGCTGCCACCGCTGCTGCCACCCGATTTACCGCTTTTGCTTCCA
GATTTACCAGATTTGCTCGTCGATGATGACCACGAGGATGACCATGCGCTTTCACCACTGCGGTCAATGGAAC
CACCGCCATTCCAGGATGGACTGGGCTGACTCGTCGTGGGAAATAATGTGGGAGTAATCGAGGGACTGAGTGA
GGGACTGAGTGTGGGACTATCCGTCGGTCGAAGCGTAGGAAATAATGTGGGACT
>TR338|c0_g1_i1 len=1170 path=[1408:0-146 1433:147-359 1415:360-380
1428:381-661 1424:662-685 1431:686-712 1429:713-736 1427:737-1006
1418:1007-1030 1419:1031-1169] [-1, 1408, 1433, 1415, 1428, 1424, 1431,
1429, 1427, 1418, 1419, -2]
CACCGCACGCACTGGTTTCACGGATCGGCTCTCATAATATTCACCCGCCCGTCAATGTTCTCATCATTTCTGA
ACCGAACATTATCACGGCGCGGAGGTATGGGCAATTCTCCCTCTTCTGGCGACGACGGTGGCGGTGAAGGCGA
TGGCAATGACGAAGGCGGGACCGGACGAAGCAGGCCTGGCCTCGCGAGTAGAGGACTCAGTCATCGCATCGTG
GGGGGCGGATCAAATAATAATTCGAATAGGGCGGGATTGCCTCATCTGACGCTGCCGAGTTCTTTGCCCTCCC
CATTTCGTAGCGGGGGTAGCTTGGGGTTGAGTCGGTCCGAGCTGGATGCAAAATGTCAACCTTCCGGGCTGTA
CCCAAGCTGCGAATGGGAACCCAAACAAATCCGCCGGCTGATCTCGAATGGCCAACTAGCCGCACGCCTGAAA
GGTTCCGATGCCCGACTTGCGAAAACGGATCGCGAGTGCCCAATATGCTTCATGTACTATTCCGAAAGCAATG
TTACCAAATGCTGCAATGCAACGGTATGCACCGAGTGCTACCTCCAAATCAAGTCGCAGAAGGATCGATGCAC
GACCTGTCCGTTTTGCAATAATCCCAAGATGACCGTGACTGTCCAACGGTTGATGGACGAGTGTGACGTCGTC
AAGAGAGAAGAGGACGAACAACGCGTCATCGAGGCCACCATTAAGAGCAGGCTGAGTCGATTGCACGGGGAGG
ACGGCCCAGCGTCCGACGATGGCAGCCCCGGTGCTGCTGGCGGCGAACACGGAGATTCGGGGGAGACCGCCTC
CCAGGGCTCCTTCGGCTCCTCCCTCGAAAACTACAATCGCGCGCGAACCCTCAGCGCGTCCTCCAACAACTCG
GGTTCCGAGGCGGGCGCGGTCGACGCATCCGGCAACAATCCCCCCGGAACACCCAACCGACCCGGCGAAAGCG
ACGCCGGATCCGAAATCATGTCCCTCGCCATGTCTCCCGCCGATCGACGCGCTCTGGAAAGCGAAATGCGCGC
CCAACTCTCCCACGAGACGCACCGTCGCATGGAGACCGAGGCGGAGGAGGCGCGGATGCGACACACGCAGGAA
TGGTACGGATCGCAAGCGGGGAGCAGGAGCCGCATGCGGGAGGCGAGGCTGGCGGAGTTGACGGGGCTGTTGG
AA
>TR338|c0_g1_i2 len=1319 path=[1408:0-146 1433:147-359 1430:360-529
1428:530-810 1424:811-834 1431:835-861 1429:862-885 1427:886-1155
1418:1156-1179 1419:1180-1318] [-1, 1408, 1433, 1430, 1428, 1424, 1431,
1429, 1427, 1418, 1419, -2]
CACCGCACGCACTGGTTTCACGGATCGGCTCTCATAATATTCACCCGCCCGTCAATGTTCTCATCATTTCTGA
ACCGAACATTATCACGGCGCGGAGGTATGGGCAATTCTCCCTCTTCTGGCGACGACGGTGGCGGTGAAGGCGA
TGGCAATGACGAAGGCGGGACCGGACGAAGCAGGCCTGGCCTCGCGAGTAGAGGACTCAGTCATCGCATCGTG

GGGGGCGGATCAAATAATAATTCGAATAGGGCGGGATTGCCTCATCTGACGCTGCCGAGTTCTTTGCCCTCCC
CATTTCGTAGCGGGGGTAGCTTGGGGTTGAGTCGGTCCGAGCTGGATGCAAAATGTCAACCTTCCGGGTAAGT
ATGCGTGTTTCTATACAGTGTCTGTTTTGAGTTTGAGAGAGCCATCAAACCATCAATTCTTTCCTAGAGAGGG
CCCATTCGCATTTTGATGATGACGCGCATCCATTTATCTTATCATTTCCATACATCCAAAAAACTCTCAGGCT
GTACCCAAGCTGCGAATGGGAACCCAAACAAATCCGCCGGCTGATCTCGAATGGCCAACTAGCCGCACGCCTG
AAAGGTTCCGATGCCCGACTTGCGAAAACGGATCGCGAGTGCCCAATATGCTTCATGTACTATTCCGAAAGCA
ATGTTACCAAATGCTGCAATGCAACGGTATGCACCGAGTGCTACCTCCAAATCAAGTCGCAGAAGGATCGATG
CACGACCTGTCCGTTTTGCAATAATCCCAAGATGACCGTGACTGTCCAACGGTTGATGGACGAGTGTGACGTC
GTCAAGAGAGAAGAGGACGAACAACGCGTCATCGAGGCCACCATTAAGAGCAGGCTGAGTCGATTGCACGGGG
AGGACGGCCCAGCGTCCGACGATGGCAGCCCCGGTGCTGCTGGCGGCGAACACGGAGATTCGGGGGAGACCGC
CTCCCAGGGCTCCTTCGGCTCCTCCCTCGAAAACTACAATCGCGCGCGAACCCTCAGCGCGTCCTCCAACAAC
TCGGGTTCCGAGGCGGGCGCGGTCGACGCATCCGGCAACAATCCCCCCGGAACACCCAACCGACCCGGCGAAA
GCGACGCCGGATCCGAAATCATGTCCCTCGCCATGTCTCCCGCCGATCGACGCGCTCTGGAAAGCGAAATGCG
CGCCCAACTCTCCCACGAGACGCACCGTCGCATGGAGACCGAGGCGGAGGAGGCGCGGATGCGACACACGCAG
GAATGGTACGGATCGCAAGCGGGGAGCAGGAGCCGCATGCGGGAGGCGAGGCTGGCGGAGTTGACGGGGCTGT
TGGAA
>TR339|c0_g1_i1 len=1235 path=[1445:0-514 1471:515-572 1478:573-596
1476:597-939 1470:940-955 1473:956-983 1477:984-1007 1475:1008-1140
1472:1141-1181 1468:1182-1197 1463:1198-1234] [-1, 1445, 1471, 1478,
1476, 1470, 1473, 1477, 1475, 1472, 1468, 1463, -2]
CGCGCGTCGTAATTTCCCGTGCCGTCGAGCCGGACTATCTCGCTCTCGACGTCGGTCGTGGCGAATCGAGGGC
CGCAGAGACGCGGCGCGGGATCGGCGGATCGCGCCTCGAGGTCGGCGGTGTGGGGACGGGGATAGTGGCTTAT
TATGGGTTTGTCCGACGGGGCCTTCTCCAGCATGTCGACGGAGTGCGCGTCCCAATCTTCGAGGAACGTCATG
TGGGAATCTATTTGCATGTAGTACTGTTCGCCCATCCACATCTTGGCCGCGAGGTACCGCGCCATGTAGGGTC
CGAGCGCCGACGTATCCTTCATTTTCAACAGGCGGAGGTTTCCGCTGTTGCAGTACTTCCTCCCGACGCTCGA
ACTGCAGAAGAGCGCGTAGCAATCCGGATCCGTCGGAACGTCGATCATCTTCCCGCCCTCCAGGATGCCGCTG
CGGCATTTCGCCTCCTCGCAATTCTGCTGCACCAGGCCGACGAACAACTTGTCCGGATGCTTCGCCTTGTCGA
ACGCCTGTATCAGGGTCTCGGAACAGAACTCGTCCCGAAACGATGCCACGCTCAGGTACAGAGTACCGTCCTC
CCTCCTATTCGGCATCCTTCGCATGTACTCTCTATCCCAGGGGTAGAGGTACTTCACCGTCAACTCGTGATAC
GCGCTCCATATCCCGTGGTACGTCGATCGCTTATTCTTCATGTTCGACGAGTTTAATTTATTCAGGAAGGAAT
GAAGGAAATCGATTATTTCCGTGAGGGTCATCGGTGCCGATTTGTGATTGCGGGATGACGATCCGGCGCCGAT
GTGGACGCTCGATCCGATTCCGCCATTGTTGTTGTTGTTGATGTTGATGGTGACGATGATGCCTCTATCGGTT
TGGGAACTGTTGCCATTAAACTCGTGCTGCTCGATGATGATGATGATTCTGATGGCAGCAGCAGCAGTCGGAG
AGGAGGATTCAACGATACTTCACGTTGTTTCGACAAAGTTTTCAACGAAGGAGAAGTTGGGTGGTGATGGATG
TGCAATACCGATTGTCTGTGCGGCATGTTGAATGCAGACGAGAGGAAGGGTGATTGCAATAATAGGGCCAATA
ACGTCGTCGTCCGAATTGCCGGCGTCATCATTGACGGGGTGCCGATTACGGTGCGTCGATCAACCATTTTTCG
TGTAGGAAAAGGTGTCGTTGATGAATCGACAGGGTTGCGATTGAAAAGATTTGGGGACGATTGAGGG
>TR340|c0_g1_i1 len=1167 path=[1477:0-135 1478:136-159 1540:160-242
1551:243-266 1553:267-270 1511:271-300 1539:301-451 1552:452-475
1554:476-483 1548:484-537 1550:538-567 1546:568-591 1547:592-626
1531:627-650 @1545@!:651-809 1527:810-852 @1536@!:853-1128 1525:1129-
1166] [-1, 1477, 1478, 1540, 1551, 1553, 1511, 1539, 1552, 1554, 1548,
1550, 1546, 1547, 1531, 1545, 1527, 1536, 1525, -2]
AGGTCGTCAATGTGACCACCGAGGCGGCGGCGGGAGCGCTCGTGGGCGTGGGGGATGTGGTGGAGGAGGCGGC
GAGTAGTCTGAGGGTGCATCAGGTGAAGGGCAGGCCTTTGTTTGTGATGGGGGGGATGGAGGAGGATGATGAT
TCGTATTACGAGGATGAAGATGAGGAGGAGATTGATTTGGAAGAGGAAGAGGGAGAGGTAGGATCGAGCGGGT
GGAATGCCAATGGTGGAGAGGATACCAAGGAAGAAGAGATCATTCACGACGAGGAAGCAGAGGAGGAGGGAGA
CGAGGAGGTGAATTTTCTAGAGGTGTCCCTCCTCACCGATGATGGCGGGTTGGATGCAGTGAGGTTACGGAAG
AGTTTGATGCACGCCGAGGAAGAGAGGAATACGTTGATGCAAATGGTGGACGAGCGTACTACTGAATTGAGCA
AATTGAGATCTGCACTGTTGAAGCAAGACCAGCAACATTGCCCGCCAGTGTCTGGTCCTGCTAGAATTCATGG
TTTAGAGAGAGAAGTAGAGTGGTTGAAGCATTTGGTTGCTGCTACAGCACCTACCGATAGCTCTGACGCACGA
GATACGAGCGTGAATGCATTGAAATCGATCCTAATTCAGATGAGGAAGGAGCTCCTTTGTCAAAAAACGAAAC
CTACTGACAAGTCAGCTATATCCAAATCCACCCTAGACTCCAAGGCGGTCGTTGTTCGAGATTTGCAATCGAA
GATTCAAGCATTAAAGAACAATACTACACCAGACAATTCTCCCAATATTGAAACCCTGCCCGAAAGCAGTGAA
CAGGATCGGCTTCTACGCGAACAACTCCATCAAACGAACGAAAAATGCAATAAACTTCGTTTAGAAATAGAGG
AGACGAATCAATGCATCGCCCAACGTCAAGCACAGCTTCAATTACTGCGAGAAAAATTAAGTGACAAGGTATA
GAATTAGGCGCTTTGCAATGTAGTGGTGCGATAGTGAATCAAAATCGGACGTGTACAATATAAAGTAGTTGTC
TCTGTGCGGCGTGTCTCTCGCTCTCTCCGACAAAGAATGAGATTTGTACAAGAGATATCTTGGCGAATATATC
GCCTGTTTTGTAGAATAGAGAGTATATAATCGATAAATTGGTATTTATTTTTAGGAAAAAAAAAGATCGGAA

>TR341|c0_g1_i1 len=1128 path=[3187:0-266 3188:267-281 @3245@!:282-868 3251:869-881 3175:882-890 3247:891-901 3243:902-1010 3183:1011-1127] [-1, 3187, 3188, 3245, 3251, 3175, 3247, 3243, 3183, -2]
CGCATGCATGAACCATGGCCATGACCCACGACCCGCAACCTCTTCATATTTTTGACATGACCATGTCAAGGCC
GTGAGACCTAGACCTCCATCATATCACACCTGCAGACCCACACGCACGACGCACGATCGACACTCTAGAATGG
TAATACCGACAGAGGAAGCATCCCGGATAAGACAACAGGGCCCAAACCTGTCATAAACAAAGAATTTGTTCAA
AGACCATTACCAATACCAAATAACAGCAAATAGTAGGAAGAAAAAAAACGCTACAAATCGAGGGAATATTAGC
CATAGAAAGACTAACAACAGCAACAAATAGAATATAATGCAATCATGATGAACTCAGGAAAAATTGAATTCTC
TCATCTCGAACAACCCATAGAAATCTACGAAAACCAACGCATGTGGATAGGACGCGGTTTCTCGACGGTAGGC
CTCCTACCCACCGAACGCGGCCCTTATTCGACCAAGGATGGTTCACTAAGTTGGAAGACTATGCAAGACGCCA
GCTTAGCGTTGTTGAGAGGTGATGTCACCCCTAACAATGCTGGCGGAATGGATAGCAAGAAGGGTTTTGGAAG
AAAGAATGGTAAACTAGTCGCCAGTACAAATATAAAGAGAATACGACGTGGCTGGTCCTTTCACGAAGAGGAT
TCGTCTGACCACCCAACAGATAGAAATGACAATGACAAAGAGAACTTGCGTAGGGAAGATGAATACGAGTGCA
GTTCTAATCATAGCGATGGTGACGTCGCTGTCAATTACTGCGGATTTGTCCCATGCACCGGACCGGAAGACGG
ACGGAAAGATAGTATTGATGGCTGGCAGTACTTTCCGGATTTTACTCCCCAGTCGTTGTTGAGTCCCAATAGG
AAAAGGGGAATCTTGGACTTTGTCCGTCGACGGAAGTTGCGTAGAGTTGCCATATTTCGACCAGATCATTTCT
TACCGCGAGAGGTGTATGCCAAATGTGACTATTGCGATTCCAATATAGTAAATATGCTCTCGGTATGTTGTAG
TCGATGCTTTTATGCCATGCATGCCCTTTCAATACTTACTTTCATTCTGCTGAGCTAACTTCTCTCTTTGTCT
CTACACAATAGAATGCAATGCTCGATGCAATCG
>TR341|c0_g1_i2 len=374 path=[3210:0-40 3211:41-64 3240:65-152 3167:153-176 3250:177-180 3246:181-201 3242:202-260 3237:261-269 3224:270-272 3225:273-296 3226:297-314 3227:315-373] [-1, 3210, 3211, 3240, 3167, 3250, 3246, 3242, 3237, 3224, 3225, 3226, 3227, -2]
TCTCCACATAAGAGTTGGATCTATTTAAAGTTTATCACAATGTTTTGAACTGAGGGTAATGCAATTGACTCAT
CTGTCAAAATCTTAGACAGAAGATTGTTGCAACAGGTTGATACCCGCAGACGTGTAAATTGTGATCAAAACTT
GTTAGTACGCCATACACCCCCCGGATCGGTTGTTTTCGGCCGTCTCAAACAGTCGCGCTGTGGGCAGGGACCC
ACGCACGCAGGGCATCAAGATCTATCTAACGAAATACAGATGTTTTAGAACCGTGCCGGACAAAATGGGACTC
AACACATCCATAAAGTTATTTTTAGCTAAATTTTCAAAATGTTTTTAATATAGAAAGATGTTTTTATTCAAAA
AAGATCGGA
>TR341|c0_g1_i3 len=2516 path=[3187:0-266 3188:267-281 @3245@!:282-868 3251:869-881 3175:882-890 3247:891-901 3243:902-1010 3233:1011-2318 3250:2319-2322 3246:2323-2343 3242:2344-2402 3237:2403-2411 3224:2412-2414 3225:2415-2438 3226:2439-2456 3227:2457-2515] [-1, 3187, 3188, 3245, 3251, 3175, 3247, 3243, 3233, 3250, 3246, 3242, 3237, 3224, 3225, 3226, 3227, -2]
CGCATGCATGAACCATGGCCATGACCCACGACCCGCAACCTCTTCATATTTTTGACATGACCATGTCAAGGCC
GTGAGACCTAGACCTCCATCATATCACACCTGCAGACCCACACGCACGACGCACGATCGACACTCTAGAATGG
TAATACCGACAGAGGAAGCATCCCGGATAAGACAACAGGGCCCAAACCTGTCATAAACAAAGAATTTGTTCAA
AGACCATTACCAATACCAAATAACAGCAAATAGTAGGAAGAAAAAAAACGCTACAAATCGAGGGAATATTAGC
CATAGAAAGACTAACAACAGCAACAAATAGAATATAATGCAATCATGATGAACTCAGGAAAAATTGAATTCTC
TCATCTCGAACAACCCATAGAAATCTACGAAAACCAACGCATGTGGATAGGACGCGGTTTCTCGACGGTAGGC
CTCCTACCCACCGAACGCGGCCCTTATTCGACCAAGGATGGTTCACTAAGTTGGAAGACTATGCAAGACGCCA
GCTTAGCGTTGTTGAGAGGTGATGTCACCCCTAACAATGCTGGCGGAATGGATAGCAAGAAGGGTTTTGGAAG
AAAGAATGGTAAACTAGTCGCCAGTACAAATATAAAGAGAATACGACGTGGCTGGTCCTTTCACGAAGAGGAT
TCGTCTGACCACCCAACAGATAGAAATGACAATGACAAAGAGAACTTGCGTAGGGAAGATGAATACGAGTGCA
GTTCTAATCATAGCGATGGTGACGTCGCTGTCAATTACTGCGGATTTGTCCCATGCACCGGACCGGAAGACGG
ACGGAAAGATAGTATTGATGGCTGGCAGTACTTTCCGGATTTTACTCCCCAGTCGTTGTTGAGTCCCAATAGG
AAAAGGGGAATCTTGGACTTTGTCCGTCGACGGAAGTTGCGTAGAGTTGCCATATTTCGACCAGATCATTTCT
TACCGCGAGAGGTGTATGCCAAATGTGACTATTGCGATTCCAATATAGTAAATATGCTCTCGAATGCAATGCT
CGATGCAATCGCTCTCGCCACGCTATTCGCACACGGACCTAAAAACAATATAACCGACGCTCAAGTTCTCCCC
CTAAAAAGCAAACTGATCGATTCCCTTTCCATCGGAACCAATTGTAGCCACGGTAGCAGGGCCATGGCGCATC
AAAGCAGTAGCAGCAGTGACAACGACAGCGACTACGATCCATTTGTAGACATCCTCAAAGTCAAGGATAGACT
TCGCAACTTTGCCGAAACATGCGTGGGCAAACCGGGGTCGGTGAGGCAGTTGCTCAATCCAGATGTTAGCGAT
CAAAATTTGATCTCGGCCATGCCTGGCAGACGGAAGGTCGCGGAAAGGTACTTGACTGAACGGGAACGCGTGT
GTCTGGCACGGCTATTGATCAAGGATGTCGATCGTCATTCCTTTCAATTTCACTGCAGGAATGAATCATGCTC
CTGTATCATGATGGGGGGATCACAACATAGCAATAATGACGGTTCGTATCGGATTGACGACGACGACGATGAC
ACCAAACTACCCGACTGTGAATTCCGTCTCGTACCATGCCCTAACCCGAACTGCCCAGATCAATTCTCCCACA
AACATCGAATGCAACACGATGAAGAATGCGGATTCAAACTAGTCCCATGTCCCAGCGAATGCGGTGCCAACAT
CCCCAAGAACGAAGTGCATATCCACGTTCGCGACCAATGCATACTACGTTCCGCAGAGTGTCCTTTGTCCATC
GTCGGATGTACCGCCACCGTGCGAGCCAAGGACGTCGCCTCCCACTTGAATGAACACGCCGATCAACACTTTA

TACTGATGGCCAATCGCATGATGGAATACCAAAACGTGATGAAGGATATGAATGCCCGTCTAAAGTCATTGGA
AGAGAAGAATGTGGTGTTGGAGAGTGAGTTAAAGAGGGCTAAGGTGCAGTTGCAGAGTAAGAGCGAGGCAAAG
ACTGTTGCAAATAATGTGAAGAAGTTGACGAAGAGAATCGGGACGTTGGAGACTACGTGTCGCAAGGAGTTCA
AGCTGGTGGAGCATGATAGAAAGAATAGATAGATTGTTAGAGGAACATGACATACATTGCTAAACATTGGCTG
AACAAGTTGCATTGAAAAGGTCGTAGCTAGACCAATGAGAGCATCATCTTCTTTCATTATTTCGATCTTTGGC
CGGAAACCTAGGCCATTGTCAAAAACCTGAACTGTTGTGCTCTTGAGTTTAAACTCGATCTCTTCCCATAAAT
GTGGCTTTCCTGCCATTATTGCCTGGTTAGTATGCCATACACCCCCCGGATCGGTTGTTTTCGGCCGTCTCAA
ACAGTCGCGCTGTGGGCAGGGACCCACGCACGCAGGGCATCAAGATCTATCTAACGAAATACAGATGTTTTAG
AACCGTGCCGGACAAAATGGGACTCAACACATCCATAAAGTTATTTTTAGCTAAATTTTCAAAATGTTTTTAA
TATAGAAAGATGTTTTTATTCAAAAAAGATCGGA
>TR341|c0_g1_i5 len=2629 path=[3187:0-266 3188:267-281 @3245@!:282-868
3251:869-881 3248:882-1014 3243:1015-1123 3233:1124-2431 3250:2432-2435
3246:2436-2456 3242:2457-2515 3237:2516-2524 3224:2525-2527 3225:2528-
2551 3226:2552-2569 3227:2570-2628] [-1, 3187, 3188, 3245, 3251, 3248,
3243, 3233, 3250, 3246, 3242, 3237, 3224, 3225, 3226, 3227, -2]
CGCATGCATGAACCATGGCCATGACCCACGACCCGCAACCTCTTCATATTTTTGACATGACCATGTCAAGGCC
GTGAGACCTAGACCTCCATCATATCACACCTGCAGACCCACACGCACGACGCACGATCGACACTCTAGAATGG
TAATACCGACAGAGGAAGCATCCCGGATAAGACAACAGGGCCCAAACCTGTCATAAACAAAGAATTTGTTCAA
AGACCATTACCAATACCAAATAACAGCAAATAGTAGGAAGAAAAAAAACGCTACAAATCGAGGGAATATTAGC
CATAGAAAGACTAACAACAGCAACAAATAGAATATAATGCAATCATGATGAACTCAGGAAAAATTGAATTCTC
TCATCTCGAACAACCCATAGAAATCTACGAAAACCAACGCATGTGGATAGGACGCGGTTTCTCGACGGTAGGC
CTCCTACCCACCGAACGCGGCCCTTATTCGACCAAGGATGGTTCACTAAGTTGGAAGACTATGCAAGACGCCA
GCTTAGCGTTGTTGAGAGGTGATGTCACCCCTAACAATGCTGGCGGAATGGATAGCAAGAAGGGTTTTGGAAG
AAAGAATGGTAAACTAGTCGCCAGTACAAATATAAAGAGAATACGACGTGGCTGGTCCTTTCACGAAGAGGAT
TCGTCTGACCACCCAACAGATAGAAATGACAATGACAAAGAGAACTTGCGTAGGGAAGATGAATACGAGTGCA
GTTCTAATCATAGCGATGGTGACGTCGCTGTCAATTACTGCGGATTTGTCCCATGCACCGGACCGGAAGACGG
ACGGAAAGATAGTATTGATGGCTGGCAGTACTTTCCGGATTTTACTCCCCAGTCGTTGTTGAGTCCCAATAGG
AAAAGGTAATTATATTGCTGCTCATTTATTTTCCACGAATTATGAATGTTCGCAATCATATCGTGTCATCTGA
TATCATCCATGTTCGATAATGTTCGATGCTATAATGCATCTTTAGGGGAATCTTGGACTTTGTCCGTCGACGG
AAGTTGCGTAGAGTTGCCATATTTCGACCAGATCATTTCTTACCGCGAGAGGTGTATGCCAAATGTGACTATT
GCGATTCCAATATAGTAAATATGCTCTCGAATGCAATGCTCGATGCAATCGCTCTCGCCACGCTATTCGCACA
CGGACCTAAAAACAATATAACCGACGCTCAAGTTCTCCCCCTAAAAAGCAAACTGATCGATTCCCTTTCCATC
GGAACCAATTGTAGCCACGGTAGCAGGGCCATGGCGCATCAAAGCAGTAGCAGCAGTGACAACGACAGCGACT
ACGATCCATTTGTAGACATCCTCAAAGTCAAGGATAGACTTCGCAACTTTGCCGAAACATGCGTGGGCAAACC
GGGGTCGGTGAGGCAGTTGCTCAATCCAGATGTTAGCGATCAAAATTTGATCTCGGCCATGCCTGGCAGACGG
AAGGTCGCGGAAAGGTACTTGACTGAACGGGAACGCGTGTGTCTGGCACGGCTATTGATCAAGGATGTCGATC
GTCATTCCTTTCAATTTCACTGCAGGAATGAATCATGCTCCTGTATCATGATGGGGGGATCACAACATAGCAA
TAATGACGGTTCGTATCGGATTGACGACGACGACGATGACACCAAACTACCCGACTGTGAATTCCGTCTCGTA
CCATGCCCTAACCCGAACTGCCCAGATCAATTCTCCCACAAACATCGAATGCAACACGATGAAGAATGCGGAT
TCAAACTAGTCCCATGTCCCAGCGAATGCGGTGCCAACATCCCCAAGAACGAAGTGCATATCCACGTTCGCGA
CCAATGCATACTACGTTCCGCAGAGTGTCCTTTGTCCATCGTCGGATGTACCGCCACCGTGCGAGCCAAGGAC
GTCGCCTCCCACTTGAATGAACACGCCGATCAACACTTTATACTGATGGCCAATCGCATGATGGAATACCAAA
ACGTGATGAAGGATATGAATGCCCGTCTAAAGTCATTGGAAGAGAAGAATGTGGTGTTGGAGAGTGAGTTAAA
GAGGGCTAAGGTGCAGTTGCAGAGTAAGAGCGAGGCAAAGACTGTTGCAAATAATGTGAAGAAGTTGACGAAG
AGAATCGGGACGTTGGAGACTACGTGTCGCAAGGAGTTCAAGCTGGTGGAGCATGATAGAAAGAATAGATAGA
TTGTTAGAGGAACATGACATACATTGCTAAACATTGGCTGAACAAGTTGCATTGAAAAGGTCGTAGCTAGACC
AATGAGAGCATCATCTTCTTTCATTATTTCGATCTTTGGCCGGAAACCTAGGCCATTGTCAAAAACCTGAACT
GTTGTGCTCTTGAGTTTAAACTCGATCTCTTCCCATAAATGTGGCTTTCCTGCCATTATTGCCTGGTTAGTAT
GCCATACACCCCCCGGATCGGTTGTTTTCGGCCGTCTCAAACAGTCGCGCTGTGGGCAGGGACCCACGCACGC
AGGGCATCAAGATCTATCTAACGAAATACAGATGTTTTAGAACCGTGCCGGACAAAATGGGACTCAACACATC
CATAAAGTTATTTTTAGCTAAATTTTCAAAATGTTTTTAATATAGAAAGATGTTTTTATTCAAAAAAGATCGG
A
>TR342|c0_g2_i1 len=322 path=[358:0-106 359:107-138 360:139-321] [-1,
358, 359, 360, -2]
CTGGAGTATTTATTTAGACCAAGATTTGGATGGTGATCTGGATGATGTTGCACCAGATGCCAATGTTGGCCTT
CTCCTGTGTGGCGCTGCTTCATTCGAAGGGATTCAGTCATAAAAAATATCAAACTCCAACAACACCATGGGCA
GCACCGCCGGACAAGGGACTACCTCAGAATGCGCCGTATCAACCAGGAAGTCGAGGAGTCAAAAAGATGGTAA
CACACTAAATGGCGGCAAGGGATGTCATTCCATTGATGATGATGGTGGCGACGACGACGACGACGACAACACT
TCTTCCTCTTCATCGTCCAGTAGTAGCAAC

>TR342|c1_g1_i1 len=3453 path=[3551:0-349 3585:350-634 3549:635-651 3589:652-730 3568:731-746 3584:747-879 3557:880-894 3588:895-1919 @3586@!:1920-2985 3580:2986-3144 3566:3145-3452] [-1, 3551, 3585, 3549, 3589, 3568, 3584, 3557, 3588, 3586, 3580, 3566, -2]
CCAGCACTGGTAACAGTCCTAGCCCTCTGTTCCCTTCTCATCGCAATATCACTCCAAAACTCGACGACGACGA
CGACAACAACAACAATAACAACAACATCATCGCCGGGGGCAGCGGAAACAGATTCCAAGAAACGACGACGAAG
GCTTTCCGAATCATCATCATCATCGGCTTCATCGGCTTCATCGGCATCGGATCACCATCACGATCTCGAACGG
GTCCTGCATCTCGCCACGCGTCCCATCGAGGATGCGTATACCGAGGACGATACTCCGTTTCGTGTATTATTCA
TCGTGACGACTTTGGCGGAGTACGATAAGGGTACGAGGGGTACGATTCACGGTGCAGAGTGAGTTTATTCTTG
TTGTTGTTGTTGTCGTTGTTGCTCTTGTGTGTGCGTGCGTCGTGTGCGTGCGTGTGTGTGGGGTCCAGATGTA
TATATTTCTTTTGAATATCTATTGAGGTTTTAAAAAACGATGTAATGTACAAATTATTGAAAGCTCTATATTG
GGTAGAATCGCATGTGAATTGTTTGGGGCTGATGGGTTGTGTGCGACAAAAACGATCCCACCCGCCTTCTTCG
TTGTACGCAACAATATCGTCGTCGAGAGTTATCGCCGCCACCGCTTTTTTTTCTCTCATCCGCCAGTTTCGAT
TGATTGATTGTTGTTTTTTTGGCCAGAATATGGGGTAGGAAAGAAATGTCAATGTTAGAATGCATCGAAAAAA
ATGATGTGTTTTTCACCGTCCCTCTCTAGTGTGCCATTTTGGTAAAGACAAGTTGGTGCGTCGTACACCGACG
CGCCGGTCACGCGTTTTTTGTTTTATTGTTTTTCATTTTTAGGATGCATACATACACACGCGGCTTCCAAAAA
AAAAACGCGCTGATATACATCGAGCCGAGAGAGACGACCTCTCTTTTGGGTGGCGTGTCGAAACTTTTTATGG
AAAAGGCTGTGGTGTCTGTGTGATGATGGTCGCAGAATCGGGGGAGGGGTTTTTTTTCCAACGATGAGATATG
TATTCATTTTTCGACTTGTCGGGACTTTAAGTTGGGTAATATATTACGGGTCTTTCTCCTTTTATTGTGTGAC
ATGGGAGTATAGATACAAGTGAAAATGGTAAAAACGGAACGAAGAGATGATTTGAGGCAAGAATATTAAGTAT
ATGATATAGTTGAAACGTACGCATTTTTCACTTGTCGGCTGTGAGTTGGATAATATACAGGCCTTTCTCCTTT
TATTGAATGACATGTGTGAGTACAATTGAAAATGGCAAAATGGAAAGAAGAGAATTTGATGGGTGTGTGATAG
ATGCCCGTGCTCTGCCAAAATTGGTGTGAAAAATTCCATTTTGTCGAGAAGGTGGATATATATGCATATTTCA
AGAGATGAGGATATATCGATTTCAGTTGGCGCTCTCGATGTTTTGATATCGTTGATCCGATTCGTATATTGTC
CACACGTCTCATCGGCACACTTTTGACATTGCAAAAGATCTTTTGACAACTCGTTGATTTTCGAGCAAGATGA
CCACTGTTGAGGTCAATTCGATGATGTGCGGTCGAAATATTTTGACGCGTTGACTGTTGTATGGATGAATCAT
GAGTCAGAGAGGTTTAAATGTTGGTCCACGTTTCATTTTCGTAGGGTCATCACTGTGAGAAATAATGATGAAC
TGGTTTTGTTCGTCTATTGCTGTGTTCATGTTGATCCATGGTCCGAACATGGCGTAGAAAAATCATTCTATGC
TGTGAAGAGTATCTTTTGGTTTGTATTTGGCATGGTTGGTTTCTTTTAACTTTTTATCACTCTTCTCCAAGTC
TGAAGCCTTGTCTGCGAAATCTTTTGACAAAAATATCAATACCCATCATCGATGTTTACACACTCTCTCTAGC
CGGTTGAAAGATTTGGTCCTGCCTCTCCTGGTCGATGGAGTCACCAGCATGATCACAAGGGGATGGCAGGTCG
ACGTCTACCTAATCTGCGGATTCGAAACCCTCACCGACTCCCGAAAGTCAATGATCCAAAACGCCCTCCCTCC
CAACGTCGGACTCGAGATATGGGCCGATGCCATCCCCTTCTACTACGTCAAAAAGTTCAATCAGGAATTGAAA
CGCGTCGATCAGACGATAGAGCACGCTCCGCACGGATTATCGAGGCAGCATCGTTTCGTTGTCAGGGACAAGT
TATTGGAGTACGATTTCTTCACGGCATTCGAGGACGACATGCGCATCACGGCGGATCACGTTATCAACTTCTT
GGAGATGAGCGTGGAGATCGATCGGATGAGGAGGAAGGCGGAGGAGTCTCCCGATGGCAAGGTGCGCGTGGAG
GAGGAGGAGGATGGCATCATCGTCGAGGATCGCAGGACCACAAGGGGGAAGAGCATGGATAAGGCCAATGTGG
GCAACGATCTGGTGCAAGATCCAATGACCGCCGAGGACTTGCGAAGGCTGTGGCCCGGCTTCGTGAGGACGGA
GGTGCTGGACGCTCGAGGAGGGGAACCGCATCAGCTGCTGGGCGGAGCGCCATCGCCGCTGGATCGTTACAAA
TGGATGGAGGACGTGCCGGCCTCGTTAGAGTACGAGCAAACGCACGGTACGATCGATCCGAACGTGTGCTGCG
GCGTTCCACCGGGCAGGGCACGGACGCCGCCCGATCCCCAAAGGACGACCTCCTGCTGTGGGAGACCGACAT
CAGCGCCATGGGAGTGAGGCACTATCCCGGTATCGGTTGGGCCGCGGCCATGACGGTCGAGGACAGGGCCGAC
GTCGGGTCGTACTGGTCCGGGGAAGGGCACAACTACAACGATCCGGGGATGAAACGACCCAGGCGTGTCAATT
CGCTGATTGGTCAACAGGCGGGTTGGATGGCGACGCGGTCGCAGATATTGTACTTTCACGAGCACGCCTGCCC
TGGCGGGTTCTTGCCTCCATTTGATGGAAAGGAATGGGTGAATGATTCATTGCAAACAAGGAATGGGTGAGTG
AATGAAAATGTGCATCATTGTGATTTCTCCATGACATTTGGAGCACATATAAAGTGCATCATGCACAAATATC
CTCTGCTTACACCTCTTGCCACTTCTCCACCTTGGTTTTTGTGTGTGTGTATTTCAGTGCTGTTGAATTCTGG
AGTGGTGGCTACCAACTGTTTGGGAAATGTTACTTCAATCGAATCTTGAGTCTGGATCCCAAACGTTTTTCAA
GGCAACTGCTTTACCATGCCTCGAATAATAAGCAAAGGACTGTGAGCAGTGGCAAATTTGTGAGGTATACTCA
TTTTCTTGGTCAGTTGTACACGGTGAAGGAGAGGGCTTTGCGATCGCTGATGGGCTGAGATGATGATGAGGAA
GGGGGGTCAAATCATCTGATCATATCATTGTCTCTTTACAAAAATAACACTTTACATTAGCTTGCTCGTTATG
CTAGAAAAAAAAAAGATCGGAAG
>TR342|c1_g1_i2 len=3315 path=[3551:0-349 3585:350-634 3554:635-650 3589:651-729 3568:730-745 3584:746-878 3557:879-893 3588:894-1918 @3586@!:1919-2984 3571:2985-3006 3572:3007-3314] [-1, 3551, 3585, 3554, 3589, 3568, 3584, 3557, 3588, 3586, 3571, 3572, -2]
CCAGCACTGGTAACAGTCCTAGCCCTCTGTTCCCTTCTCATCGCAATATCACTCCAAAACTCGACGACGACGA
CGACAACAACAACAATAACAACAACATCATCGCCGGGGGCAGCGGAAACAGATTCCAAGAAACGACGACGAAG
GCTTTCCGAATCATCATCATCATCGGCTTCATCGGCTTCATCGGCATCGGATCACCATCACGATCTCGAACGG
GTCCTGCATCTCGCCACGCGTCCCATCGAGGATGCGTATACCGAGGACGATACTCCGTTTCGTGTATTATTCA

TCGTGACGACTTTGGCGGAGTACGATAAGGGTACGAGGGGTACGATTCACGGTGCAGAGTGAGTTTATTCTTG
TTGTTGTTGTTGTCGTTGTTGCTCTTGTGTGTGCGTGCGTCGTGTGCGTGCGTGTGTGTGGGGTCCAGATGTA
TATATTTCTTTTGAATATCTATTGAGGTTTTAAAAAACGATGTAATGTACAAATTATTGAAAGCTCTATATTG
GGTAGAATCGCATGTGAATTGTTTGGGGCTGATGGGTTGTGTGCGACAAAAACGATCCCACCCGCCTTCTTCG
TTGTACGCAACAATATCGTCGTCGAGAGTTATCGCCGCCACCGCTTTTTTTCTCTCATCCGCCAGTTTCGATT
GATTGATTGTTGTTTTTTTGGCCAGAATATGGGGTAGGAAAGAAATGTCAATGTTAGAATGCATCGAAAAAAA
TGATGTGTTTTTCACCGTCCCTCTCTAGTGTGCCATTTTGGTAAAGACAAGTTGGTGCGTCGTACACCGACGC
GCCGGTCACGCGTTTTTTGTTTTATTGTTTTTCATTTTTAGGATGCATACATACACACGCGGCTTCCAAAAAA
AAAACGCGCTGATATACATCGAGCCGAGAGAGACGACCTCTCTTTTGGGTGGCGTGTCGAAACTTTTTATGGA
AAAGGCTGTGGTGTCTGTGTGATGATGGTCGCAGAATCGGGGGAGGGGTTTTTTTTCCAACGATGAGATATGT
ATTCATTTTTCGACTTGTCGGGACTTTAAGTTGGGTAATATATTACGGGTCTTTCTCCTTTTATTGTGTGACA
TGGGAGTATAGATACAAGTGAAAATGGTAAAAACGGAACGAAGAGATGATTTGAGGCAAGAATATTAAGTATA
TGATATAGTTGAAACGTACGCATTTTTCACTTGTCGGCTGTGAGTTGGATAATATACAGGCCTTTCTCCTTTT
ATTGAATGACATGTGTGAGTACAATTGAAAATGGCAAAATGGAAAGAAGAGAATTTGATGGGTGTGTGATAGA
TGCCCGTGCTCTGCCAAAATTGGTGTGAAAAATTCCATTTTGTCGAGAAGGTGGATATATATGCATATTTCAA
GAGATGAGGATATATCGATTTCAGTTGGCGCTCTCGATGTTTTGATATCGTTGATCCGATTCGTATATTGTCC
ACACGTCTCATCGGCACACTTTTGACATTGCAAAAGATCTTTTGACAACTCGTTGATTTTCGAGCAAGATGAC
CACTGTTGAGGTCAATTCGATGATGTGCGGTCGAAATATTTTGACGCGTTGACTGTTGTATGGATGAATCATG
AGTCAGAGAGGTTTAAATGTTGGTCCACGTTTCATTTTCGTAGGGTCATCACTGTGAGAAATAATGATGAACT
GGTTTTGTTCGTCTATTGCTGTGTTCATGTTGATCCATGGTCCGAACATGGCGTAGAAAAATCATTCTATGCT
GTGAAGAGTATCTTTTGGTTTGTATTTGGCATGGTTGGTTTCTTTTAACTTTTTATCACTCTTCTCCAAGTCT
GAAGCCTTGTCTGCGAAATCTTTTGACAAAAATATCAATACCCATCATCGATGTTTACACACTCTCTCTAGCC
GGTTGAAAGATTTGGTCCTGCCTCTCCTGGTCGATGGAGTCACCAGCATGATCACAAGGGGATGGCAGGTCGA
CGTCTACCTAATCTGCGGATTCGAAACCCTCACCGACTCCCGAAAGTCAATGATCCAAAACGCCCTCCCTCCC
AACGTCGGACTCGAGATATGGGCCGATGCCATCCCCTTCTACTACGTCAAAAAGTTCAATCAGGAATTGAAAC
GCGTCGATCAGACGATAGAGCACGCTCCGCACGGATTATCGAGGCAGCATCGTTTCGTTGTCAGGGACAAGTT
ATTGGAGTACGATTTCTTCACGGCATTCGAGGACGACATGCGCATCACGGCGGATCACGTTATCAACTTCTTG
GAGATGAGCGTGGAGATCGATCGGATGAGGAGGAAGGCGGAGGAGTCTCCCGATGGCAAGGTGCGCGTGGAGG
AGGAGGAGGATGGCATCATCGTCGAGGATCGCAGGACCACAAGGGGGAAGAGCATGGATAAGGCCAATGTGGG
CAACGATCTGGTGCAAGATCCAATGACCGCCGAGGACTTGCGAAGGCTGTGGCCCGGCTTCGTGAGGACGGAG
GTGCTGGACGCTCGAGGAGGGGAACCGCATCAGCTGCTGGGCGGAGCGCCATCGCCGCTGGATCGTTACAAAT
GGATGGAGGACGTGCCGGCCTCGTTAGAGTACGAGCAAACGCACGGTACGATCGATCCGAACGTGTGCTGCGG
CGTTCCACCGGGCAGGGCACGGACGCCGCCCGATCCCCAAAAGGACGACCTCCTGCTGTGGGAGACCGACATC
AGCGCCATGGGAGTGAGGCACTATCCCGGTATCGGTTGGGCCGCGGCCATGACGGTCGAGGACAGGGCCGACG
TCGGGTCGTACTGGTCCGGGGAAGGGCACAACTACAACGATCCGGGGATGAAACGACCCAGGCGTGTCAATTC
GCTGATTGGTCAACAGGCGGGTTGGATGGCGACGCGGTCGCAGATATTGTACTTTCACGAGCACGCCTGCCCT
GGCGGGTTCTTGCCTCCATTTGATGGAAAGGAATGGGTGAATGATTCATTGCAAACAAGGAATGGTGCTGTTG
AATTCTGGAGTGGTGGCTACCAACTGTTTGGGAAATGTTACTTCAATCGAATCTTGAGTCTGGATCCCAAACG
TTTTTCAAGGCAACTGCTTTACCATGCCTCGAATAATAAGCAAAGGACTGTGAGCAGTGGCAAATTTGTGAGG
TATACTCATTTTCTTGGTCAGTTGTACACGGTGAAGGAGAGGGCTTTGCGATCGCTGATGGGCTGAGATGATG
ATGAGGAAGGGGGGTCAAATCATCTGATCATATCATTGTCTCTTTACAAAAATAACACTTTACATTAGCTTGC
TCGTTATGCTAGAAAAAAAAAAGATCGGAAG
>TR342|c1_g1_i3 len=1906 path=[3562:0-349 3563:350-372 @3586@!:373-1438
3580:1439-1597 3566:1598-1905] [-1, 3562, 3563, 3586, 3580, 3566, -2]
CCAGCACTGGTAACAGTCCTAGCCCTCTGTTCCCTTCTCATCGCAATATCACTCCAAAACTCGACGACGACGA
CGACAACAACAACAATAACAACAACATCATCGCCGGGGGCAGCGGAAACAGATTCCAAGAAACGACGACGAAG
GCTTTCCGAATCATCATCATCATCGGCTTCATCGGCTTCATCGGCATCGGATCACCATCACGATCTCGAACGG
GTCCTGCATCTCGCCACGCGTCCCATCGAGGATGCGTATACCGAGGACGATACTCCGTTTCGTGTATTATTCA
TCGTGACGACTTTGGCGGAGTACGATAAGGGTACGAGGGGTACGATTCACGGTGCAGACCGGTTGAAAGATTT
GGTCCTGCCTCTCCTGGTCGATGGAGTCACCAGCATGATCACAAGGGGATGGCAGGTCGACGTCTACCTAATC
TGCGGATTCGAAACCCTCACCGACTCCCGAAAGTCAATGATCCAAAACGCCCTCCCTCCCAACGTCGGACTCG
AGATATGGGCCGATGCCATCCCCTTCTACTACGTCAAAAAGTTCAATCAGGAATTGAAACGCGTCGATCAGAC
GATAGAGCACGCTCCGCACGGATTATCGAGGCAGCATCGTTTCGTTGTCAGGGACAAGTTATTGGAGTACGAT
TTCTTCACGGCATTCGAGGACGACATGCGCATCACGGCGGATCACGTTATCAACTTCTTGGAGATGAGCGTGG
AGATCGATCGGATGAGGAGGAAGGCGGAGGAGTCTCCCGATGGCAAGGTGCGCGTGGAGGAGGAGGAGGATGG
CATCATCGTCGAGGATCGCAGGACCACAAGGGGGAAGAGCATGGATAAGGCCAATGTGGGCAACGATCTGGTG
CAAGATCCAATGACCGCCGAGGACTTGCGAAGGCTGTGGCCCGGCTTCGTGAGGACGGAGGTGCTGGACGCTC
GAGGAGGGGAACCGCATCAGCTGCTGGGCGGAGCGCCATCGCCGCTGGATCGTTACAAATGGATGGAGGACGT
GCCGGCCTCGTTAGAGTACGAGCAAACGCACGGTACGATCGATCCGAACGTGTGCTGCGGCGTTCCACCGGGC
AGGGCACGGACGCCGCCCGATCCCCAAAAGGACGACCTCCTGCTGTGGGAGACCGACATCAGCGCCATGGGAG

TGAGGCACTATCCCGGTATCGGTTGGGCCGCGGCCATGACGGTCGAGGACAGGGCCGACGTCGGGTCGTACTG
GTCCGGGGAAGGGCACAACTACAACGATCCGGGGATGAAACGACCCAGGCGTGTCAATTCGCTGATTGGTCAA
CAGGCGGGTTGGATGGCGACGCGGTCGCAGATATTGTACTTTCACGAGCACGCCTGCCCTGGCGGGTTCTTGC
CTCCATTTGATGGAAAGGAATGGGTGAATGATTCATTGCAAACAAGGAATGGGTGAGTGAATGAAAATGTGCA
TCATTGTGATTTCTCCATGACATTTGGAGCACATATAAAGTGCATCATGCACAAATATCCTCTGCTTACACCT
CTTGCCACTTCTCCACCTTGGTTTTTGTGTGTGTGTATTTCAGTGCTGTTGAATTCTGGAGTGGTGGCTACCA
ACTGTTTGGGAAATGTTACTTCAATCGAATCTTGAGTCTGGATCCCAAACGTTTTTCAAGGCAACTGCTTTAC
CATGCCTCGAATAATAAGCAAAGGACTGTGAGCAGTGGCAAATTTGTGAGGTATACTCATTTTCTTGGTCAGT
TGTACACGGTGAAGGAGAGGGCTTTGCGATCGCTGATGGGCTGAGATGATGATGAGGAAGGGGGGTCAAATCA
TCTGATCATATCATTGTCTCTTTACAAAAATAACACTTTACATTAGCTTGCTCGTTATGCTAGAAAAAAAAAG
ATCGGAAG
>TR342|c2_g1_i1 len=389 path=[4992:0-52 5692:53-75 5686:76-104 5681:105-
111 5676:112-126 5663:127-173 5693:174-178 5627:179-190 5703:191-201
5488:202-214 5680:215-216 5674:217-225 5662:226-226 5649:227-249
5590:250-250 @5626@!:251-388] [-1, 4992, 5692, 5686, 5681, 5676, 5663,
5693, 5627, 5703, 5488, 5680, 5674, 5662, 5649, 5590, 5626]
CGCATCGAACTCCACTATATTCACCCCATCAATGAATCAGAATACACATGGCTTCCTTCAAAGTAATTCTCCA
TTGAACAATGGAAATGCAACACAACATGATGAATTCTCCACAACCCATCAATATACATAGCATTCTTCCCAAT
ATATAGCAGCAGCGTCACGTCTCACATCATCAATCTTCACGCAGTCCCACTCCACTACTTTCCTCTAGACCAC
ATCCGCCTCTCCCTCCAAACCTTGGCGCAAACGCGATGCACCAAAGCATGTCTCCTCCCCACCACCCTCGCAT
CATCATCGTACCCCCCGCCCACCACGCATGCCACGGGTATACCATTCTCCACGCACGTTCGTATCACGTGTTC
GTCCCTTCGTTCCATCCCTTCCCA
>TR342|c2_g2_i1 len=496 path=[5656:0-90 5565:91-304 5044:305-335
5045:336-495] [5656, 5565, 5044, 5045, -2]
TCGCGGACACTTTGGAGCAGGTTGATTGCCAGGTAGAGAATGGATCGCTGTTGCGCTTCGGACAGAGTTGAGT
ACGCACCATCCTCCTTTCCCTTGCCAGAGCCTCTCAAGGCGAGTGCAGCAACTTCAGATACGTCCACTGCCAT
GTTGCCACGGGAGGAATTCTCGCCAGAGAATGATGATAGGCCGCAAATATAGGACATTAACTTCTCGGCATAC
TGCAGAAGTGAAAGAGAAACTTGAATTTTGATCCCTCTGGTAGTGTTGGCGAGCACACGTAGGTCGAGCCTCG
TCTCAGGATTATGCTCCGCATCATCTGACATTTCAGAATCAGTATCATTGCTTGAAGTTGTCTTGGGCTTTTG
AATGTTGAATGCCTGCAGAGCCATCACACACGCTGCCAGAGATGCAACGGCTTCACTCGAACTCTCCTTGGTG
GGAAGCAACTTCAAAGCATCCACCAAGTGATCGAGCATATTGCACCTCCATTTTTCAT
>TR342|c2_g1_i4 len=3135 path=[4932:0-69 4933:70-85 5668:86-113 5656:114-
181 5424:182-203 5512:204-305 5163:306-329 5561:330-630 5258:631-654
5507:655-714 5415:715-738 5603:739-774 5505:775-798 5558:799-898
5240:899-922 5602:923-1032 5692:1033-1055 5500:1056-1084 5630:1085-1091
5595:1092-1115 5675:1116-1157 5693:1158-1162 5593:1163-1200 5702:1201-
1209 5650:1210-1210 5589:1211-1234 @5626@!:1235-1372 5694:1373-1380
5588:1381-1423 5661:1424-1474 5624:1475-1524 5586:1525-1579 5540:1580-
1603 5696:1604-1618 5623:1619-1762 5539:1763-1806 5646:1807-1828
5622:1829-1842 5536:1843-1884 5477:1885-1924 5323:1925-1948 5535:1949-
2252 4937:2253-2276 5476:2277-2442 5116:2443-2466 5117:2467-2471
5118:2472-2495 5685:2496-2541 5582:2542-2697 5699:2698-2722 5705:2723-
2723 5706:2724-2726 5704:2727-2728 5682:2729-2757 5678:2758-2791
5670:2792-2843 5658:2844-2866 5700:2867-2880 5528:2881-2891 5614:2892-
2913 5701:2914-2915 5525:2916-2916 5611:2917-2940 5570:2941-2955
5522:2956-2979 5460:2980-2981 5569:2982-2994 5521:2995-3014 5610:3015-
3054 5568:3055-3057 5520:3058-3065 5458:3066-3089 5155:3090-3092
5156:3093-3093 5157:3094-3134] [-1, 4932, 4933, 5668, 5656, 5424, 5512,
5163, 5561, 5258, 5507, 5415, 5603, 5505, 5558, 5240, 5602, 5692, 5500,
5630, 5595, 5675, 5693, 5593, 5702, 5650, 5589, 5626, 5694, 5588, 5661,
5624, 5586, 5540, 5696, 5623, 5539, 5646, 5622, 5536, 5477, 5323, 5535,
4937, 5476, 5116, 5117, 5118, 5685, 5582, 5699, 5705, 5706, 5704, 5682,
5678, 5670, 5658, 5700, 5528, 5614, 5701, 5525, 5611, 5570, 5522, 5460,
5569, 5521, 5610, 5568, 5520, 5458, 5155, 5156, 5157, -2]
CAAAAAAGAAACAACACACACACACACTTCGAAACAAACTAAACACGTCAAAACAAACAGCACGGAAAAAAACTC
ACTCGCTTCATCATTAATTCGCGGACACTTTGGAGCAGGTTGATTGCCAGGTAGAGAATGGATCGCTGTTGCG
CTTCGGACAGAGTTGAGTACGCACCATCCTCCTTTCTTTCGCCAGAGCCTCTCAAGGCGATGCTGCCCCATTC
CGAGGCGACGATGACGAGGATGCGCAGCGTTCCCTCCTTCGTAGCACGAGGTCGAAGGCAGGCGTGGAGGAAG
ACGGAAAAGAGACCACCGGGTGCGAGATCCCCGTATTTTTTGGTGTCATGGCTGGCCAAAAGGCCTGAAATTG
CGGGCTTGACGATTTCCTCAAATGCCATTGTGGAAGGTGTGGACGCAAGCTTCAGAATAGCTTCTTCGCATGC

GTCTCGTTGTTGGACGATGGCCGGCGAATGAAAATACCGGGAGCAAGTCTCGTGTAGAGAAGACACCTCCGAT
TGAAACGTGCTGCTGGTGGTACTGCTGCTATTCTTGGATTCTTTCCCGTACGAATGCATTTGGTACAAGGCCA
AATCCAAGGTGATTCGAAGAATCCTGCTCATCGATGATGTTTGTTGCTAGTCGCGCGAACCGACCAGATCCGA
ATAGGAACTTAACGCCAGCCAGAGGAAGCGCGATTGTAGCAATTTGGGGCCCGCCATCGGTTCCGACAACCCC
TTCTCTGCTTGTCCACGAGAGAATAAGCGGGACAACACTTGTTGACCGATTGAATTCGTAGTGATAAAGCCGG
TAGCATCAACCTTGCGTGTTTCAACACCTCCCGTCAGCCGTGAAAGTTCATCCAATGCCGCATCGGAAGCTCT
CTCGATTGGTTGCGGAGTGTCCGATTCGCGAACATTGGCAGCGTATCCGATGTGCGCACCCAGGGAAAGGAAG
AGTCGACAGAACAAATCGCTCAGTGGATGGTCCTCCTCCAAATCACCATCATCATTACAGGAATGAATCAGAA
TACACATGGCTTCCTTCAAAGTAATTCTCCATTGGACAAAGGAAATGCAACACAACATGATGAATTCTCCCCA
ACCCATCAATATGTATAGCATTCTTCCCAATATAGCAGTGGCAGCAGCGTCACGTCTCACATCATCAAGTTTC
ACGCAGTCCTACTCCACTACTTTCCTCTAGACCACATCCGCCTATCCCTCCAAACCTTGGCGCAAACGCGATG
CACCAAAGCATGTCTCCTCCCCACCACCCTCGCATCATCATCGTACCCCCCGCCCACCACGCATGCCACGGGT
ATACCATTCTCCACGCACGTTCGTATCACGTGTTCGTCCCTTCGTTCCATCCCTTCCCAGGATAGGTCGAGGC
GTCCCAATTTATCCGCGTGGAAGACGTCGACGCCGGCATTGTACAGTACCAGCTGCGGTTGTACCTCGGTCAA
CGCTTTGGCGAGGGATGTTTTCAGTGCCGTGAGGTAGGTTGCATCATCGCATTCGTCCGGTAAGGGAATATCG
TAGGTGCTAGTTTCCTTCGGATGAGGGTAGTTGTTAGCCGCGTGGAGATCTAGCGTGAAGAGCTTGCCGTGTA
GATTGTTACGAAGAATGCGATCGCGATCGCCGTGGTCGTGGTCGCGCGTTGCAATGCCACCCTCGGTAGCGGG
AGGTTGTTGGGAGAAGGTCGACGTGCCATCTCCCTGGTGAACGTCGCAATCGACCACCAACACGCGATCCATC
GACGTGCCTCCCTCGTCGCCCCTGTAGAATTTGCGCAACAGCAGACGACGACGACGACGACAATCCTCATCAT
CGACCTCCCCATTGTTGTCGTCGTCGTTGGCGGCAACGGCGGCAACGTCACCATCGTCCCCCTCCTTCCAAGT
CATCAACCTCGCCACCACGGCCAAATCATTCAGTATCGTAAATCCCCTCCCCGCATCGCTCGTGGCGTGATGC
GTCCCACCCGCCAAATTACTCGCCAAACCGTACCTCACGGCGAGTTGCGCCGCGAGCATCGTGCCGGCGACCT
CGAGCAGCGTGCGTTCGATGAGTTCGGGACGGGCGGCGTGTTCTCGAAAACCGACGATGCGACGCTGTTCGTA
GGTTAAATCGGAGGATAGGAATGTTCGGAGGAAGGTCGAGTCGATGGGCGGGGATAGGAAGGCGGTCGTGGGG
AAGGTCGATGTCGGGAGGGGTCGATGGAAGTGCGTTGGGGAGAGTACCAGTGGCTTCGTTGGCCTTTCCTTAG
TGTTGTTGCGGTGATCTCTATCAACGTCTTTATTCTCGTTGCGCTCTTTGTCCTCGTAGACATCCTGCTGACT
CTCCAACAATAAGAATTCATCAGGATCGTGAAGTAACGAAAGTGCAGTCTCCTTGAACTTGGCCATGGGAAAG
GTATGCTTCGCGGGCCAATCGGGACAAGAGTATAAATCGTGATATACCATGGGTGCATGACGGGTGTTGTCAT
TTGGGTGAAGGCTCGGTCGACTGTTCGTTACCAATCCGCCATCGAGGAAAATGTCACGTGGTTCGTATTGCTC
GTGTCGTTGTTGTTGTTGTTGTCGTTGTTGTTGTTCGTCGGAAGGAGAGAAAGAAGTGCTACTGGTGATGGTG
CTGGATGTCGATGCGAATTTTTTAGCTTGATGTTGATTCTTCTTATTCTTCGTCTTGTTCTTCTTTGATGCAA
GAAGCTGTTGCTGCACGCGGGCCAGAGTTGGAGCCATCATGATGGAGTAATCCGTATCATCGTCGTTGTTTTT
GTTGGAGGATGATAATAACGATGACGACGAGACTACGGACGAGATCAACGGGATCGACGTCATCCTCCGATGA
TGACGGTTTGATAATTGGTGCATTACGATGGCACTGCATCCTCCTCCTCTGCACATGCGATGATGGACGAATC
TGCTTCTGGCATATATCATGGCCATTATGCTGCAGCAGACTCGCATACTTTGCCACTCGCAATGGTGTCGGGT
CACGCCACCCTCTACTACCTACACGACAACAGTCGAATGATGTGCGCTCGGATCAATAAAATAATGATTAGGG
TTAACCAACATTAAAAAAGAAATTTTATTAAACTACAAAGTCAGTTATAAAAAACATTATACCTGTTAA
>TR344|c0_g1_i1 len=627 path=[605:0-626] [-1, 605, -2]
CCCAATTCCAACAACAACGATGATGACACAACAACAACAATCCGCAGGGATAACGAAAATGAACATCATAATA
AAAGACGAAAGAAAGGCAATCATAATAATCATCATCGCGGTCGTCGTCGCAACAATAAGAACGACACATCTGG
GAAAAACACTGATCCAAGACTGAATGAATCAACCACCGTGGATGACATCTCTGATAACATGGACGCGGCAACG
TATCTCGCTTGGGTGAATCGACAGGCGAATTCTCTGCCCAATGTCTTTGTTTCGAATCAGTGTAATTATAATG
ATGATGATGACACAAAAGAAATAGGAGGAGGAGGAGGAGAGGCGAAAAATGACAGCAACAACACCAGCAACAA
CAATAACGCGTTCAAAAACGCATGCCAAACGAAGGCAACATCATCATCCAAAGAAGATGAAGAAGATGATCCA
ATCCACGGGTCGGCAGCCACATTACAAGTATTGCTATCCAAACGTATGGATATTCTCCCTCCTCCTTCGGAAA
ATCATCTTCCTCCTCGTACTACTGACAATGCCAATGCCAGGTATGCGACTATAGACGAGGAAAACGCACAATC
ATCATCATCATCATCATCTCATGATCATGGTTGGGTGTACGCT
>TR346|c0_g1_i1 len=1288 path=[1266:0-1287] [-1, 1266, -2]
TTATTGCATGCATTCACTGATTTAATTTCCCATGGTATCTATTGCACAACCATCCAGTTCAAAGAAAGGCTGA
TGAGAAAATTCAAACAGGCAAGGTATCAACCTATCCATTGAAATAGGAGAGCTTGTTTGATGAATTCATTTGA
GTATCCCTTCCTATTGCACAGACAGCAAGTTCAAAAGGAAGGAACGATACGAAAATTTATACCAACATGCAAA
GTATCAACGTATTCATCTGAAGGGGTGCCCTTCCTTGATAGAGTCATTACGTGTGAATAATATAAATATCGAA
TGCCAGCCTATGCCTGCTACTTCATCTCCGGACAAACATACACGCACCATGCTCAATTGCATTCATTTTTATT
GCCATTCGCAGCCATTATCTTCTTTGCAGCATACCTATCATCCTTGTGATGAACACTATTCGCACTCATAAAC
GACCCTCCATCCAAATGTCCCGTAAACACCACACCACTATGATCGTCCAACAAATAACAACCAAACTTGGCAT
GCCCGTCCCTCAATCCCAAACGTTCGCAACTTCGCACCATGCACGGACTTAATTTATCCTCGACGAATCCACC
TCGTGCCACCATTTTATATTGAGGATGGAAGATGAAGTCACGGTAGTGGGACGTGGTTACAATGTGGGTATTG
TCGTACCAGAATAGGGTGGGCGTGAGTGATAGTTGGTGATGTCGTCCATTCTCGTTTTTTGTTTTTCCTTCGA
ATGAATTGAGCCATTCGTCGTAGGTGATGCGTTGATGACTTCCGCGATAGGTATCCTTCAATGCCTCGAGATT
TTTACGGCGTTTTTCCGTACGCGGCATTGCCATATTCGCCACGGATGTTCCATCGGGACCATAAATATTGCCA

TCGATGCGCAACTCGTTCGGATATAAAATCATCGTTTTGAACTCATCCACCGCTCGTCTTCCATACTTGCCAC
TGAATATATCGTGATACATCAAGTTGGACCTCATGCTCACACCGACATATTTGATTTGTTGCTTTGCATCGTT
TTCCATGGCGGTCACTACCTCCTTGATGGGAGTGGGTCGCATGAAGGTGCGATCGTGTTGAATGACGCATACG
TACGGAGTATCGACGCAATGGTAGAGGGCATGGCGTAATGCGAATCCGTACCCGTGTCGTTCTTCGAGTTCTA
CCACTCGAGTATTTCGAAAGGGGCTCTTCTTTTCATTCTCATCATTATTATTGGCTTCTTCGCAGAGTTTTCG
CAGTCTCAATTTGAACTCCGCGTAATTCCCCGCCTGATCCACCGTGG
>TR347|c0_g1_i1 len=273 path=[251:0-272] [-1, 251, -2]
TCTTCGATTGGACAGGTGTTTACGTATTACCGTGCGCCCTCGATTACCTCGATCTTTCCGACATACGGATTTT
TGGAGGGAGGGGAAAATGTATTTGTTTCGGGACGTTTTTTTCGACATGACGTCCGATGTGCATTTGGTGATTT
AGTCTTATCCAAAACAGTGTGGCTCTCGGATACATCAATAATTTGCACTTCGCCGGTGTCAGCAAATAAGATG
GCTGTGGCTCTACGTGTGACTAATAATGGCATGGACTATTCTGACAGGGGTGGC
>TR348|c0_g1_i1 len=630 path=[608:0-629] [-1, 608, -2]
TCTTGATGATGATGCTCCTGCTCCTGCGGTAACATCCAGTGGAGGTGGTACCAATCCTCCAAACAAGAATAGT
TTATCATTCCTCTTCTTTTTGTTGTTGTTGTTAGTACTGCCGCCGCTAGTGCTACTGCTGCTGCTGCTGCTGG
TAGTGACGTCGCCGATGTCGACTATGGGGGATGCAACGCAGGCGTGACCGCATACCTGTGGACCAGCGGATAT
GGAATTGTCGTTATTCGTCGAGAGGGACCATGACCATGCGGACACCGACGATGATGGCAGTGATGTCATTTGG
TACAATGACAATGCCAATAGGAATGGATAGCATGATGATGATGGCATTTTTTTTTTTGTCAATTGTGTTATTG
TCCGATTTTTTTACGTCTGTCTCTCGTATCGATGGGGACAAACAAATAATGATGTAGGACGTAGGATCCCCCC
CCCTCTCTCGTTTGCTGTTTTTTAATCGAAATTTTGTTTTCGTGAGATTGCTCTTGATGATAGTGATAGATGC
TGATGCATTGCTGAATTGCTGAGATCTACTACTTATATGCCCGTGATCTGTTGCTATGGTTGTGGTTGTGAAT
GGAATATACTCCTAGTATGAGTACGTCGTTACTACAGATCGGAAGA
>TR349|c0_g1_i1 len=275 path=[253:0-274] [-1, 253, -2]
GGAGAAGAGAACATTGCCATCAGCCAAGTTATTTTTGCTTATGGAGTTGACTGATAGGGCTCTCGGACGAGTC
CTTGGACGCTTCCGTCCAGAGGAAGGCCATAGCGTGTCGTCGTTGTCGTCTTCAATAGCAGACATTTTGACAC
CGCCGCGATCTCCGTCGATAAACTTGATTCCTCTTCGCTCCATAACTGCCTCATTTTGATGTTCCCCCGGGGT
TTGCCGTTCTCTCGATATTGGCTCCCTTTTCTCTACGTTTCGAAATGAAATACGTT
>TR351|c0_g1_i1 len=289 path=[267:0-288] [-1, 267, -2]
CCATCACGACGAAAGCGCTCCTGCTATCCAAACCAGAGGTCGGCTCATCGAGAAATAGAATCTTCGGCTTCTT
CATAAGCTCAAGACCAATATTCACACGCTTTTTCTCCCCTCCACTGACACCACGACGGGTTGCATCACCCACG
AGAGAATTTGCAATTCTAGATAGTCCCAGGGATGCCATAGTGGCGTCGGCGAGTTCGGCAATCTCCTGTTCGG
TAGTGCCCGAGGGCAGTAAGAGCCTCCCCGGGTATAGCAAATTCTCGCGAACAGTCAAATCGGGAAATAC
>TR353|c0_g1_i1 len=422 path=[400:0-421] [-1, 400, -2]
CTCTTCCGATCTCACAGCCGTATTACGGTCTCCGGAGGCTGGTTCTCGACAGTTGCCATGGCCTGCTGTTGTC
GTGAATGTCCGCGCTCCCGCTGCTGTTGCCGCGGGAGTGGCTGCTGCTGTGGTTGCTTGTCCGATGGCAGGGG
TTGAGGTCCGTAAATAATCTGCGCGGTGGGACGCGTCGTCGTGGGCGCGCCATTATTCCCGCTAGTACCGCCG
GATGCGGCCGAAGCGGATGCGGGATTGGAGACATTCTCGTAATTGCCCATTATTTCGTTATTTGTGATCCCTG
ATGCTGGCGGATGAGATGCCTCCGGGTGGCGATGTTTGTTGCCTACTGCAGCTGCGGCTGCGGCTGCTGGTCT
TGGTGGCGGCGGAGGTGGAGCTCCTTCGTTCGTTGCTGCGGCCGCGCCTCGACCAGC
>TR354|c0_g1_i1 len=247 path=[225:0-246] [-1, 225, -2]
TTGGGATCTTTGAGGAGTACGTGAAGTCGCCAAAGAAAACGAAATCCTCTGCATTGTTGGATACAGATAGAGG
AAACGCTTTTTCTTGGATTGAATTGATGTAGTTCTTTCCATGGATTGGTGGCGAGAAACAGAGGACAGCTTCT
CGAGTCAGAAAATGAGCTGGGGTAATTACACTTCCAAATCGACATGTGAGTTGTGTGTTGTTGACGAAATTTG
ACCCACTGATATAAACAGGAGTTCCTCC
>TR355|c0_g1_i1 len=264 path=[242:0-263] [-1, 242, -2]
CTTCTTTTTCTTCTTCTTCTTTTGGCGAGCAGGAGATGATGTCACACTGGTCTCGTCCTTCCTAGGGCGGCCT
CTGCTTCGTTTGATAACAGCGAGGTTGTTTTCGTGTTTGGAGACGCAAGACCCTTCCTCTGCTTTTTTCAAAC
AATCCCGGTTATTCTTCGTCGTTCTCGCCATGTTTGCTGCAGTTGCTGCGTTCTCTCGTTCGATGATTGTATT
TGTTTCTGCACTGCCCCTACTGTCGCCATCTTTTCTAGATCCGAG
>TR356|c0_g1_i1 len=243 path=[238:0-154 239:155-172 240:173-242] [-1,
238, 239, 240, -2]
GCATCGATCAATCCCGCGGGCAATTCGATCGTGTGGGCGTCGACAGGCGGACGGAATTGTTTGACGCATACGA
TTTCGTCCTTCGTCGGGTCGTCGGCGTCGTTTTTGAGTATGGCGAGGATGATTACGGCATCGATGGAATTTTC
GGATTTTTTTCGTGGTGCGTACGGCTCGATCCCATTTGCGTAATGTGTTGTTGTTGTTGTTGTTGTTATCGTC
TTTTTCGGGAGGGACGTGATAGGA
>TR357|c0_g1_i1 len=571 path=[574:0-342 575:343-366 576:367-570] [-1,
574, 575, 576, -2]
GATCTGAACAAAAAGCAAATTCATTGCCATTGGCCATTGCCATTGAGCATCATATCTAGGTACTCTGCAGTCT
CTACCTCACAGCATAGAATAGTGGCCAGGAATTCCAGCGGCAGTATCTACCATTGAAGGGAAAGAAGCAGCAG
CAACAAGCACAGTTATGTTGTAATCTAATAGGAGATCGTAGGATAATACCATTCCAAGCGCGGCGCATCTGAG
ATATAAACTTCAGACGAGATCTGCTATTGCGGCAACGAATCATTTTCATCCCAATTTATCAGGCACATTCATT

GAGACATCGATTCGAAAAAGGTCATATAGCATCATAACATCACTATCACCATCACCATGACAGCCTCCAATTC
AAAGAGCAAATCCAACAATGGCAGCGGAGGGGGGTTCCAATCTCTCAACCTCTCTCCGCCCATCTTCGCGGGC
ATTAAACGGCTGGGATATCGTACACCCACACCCGTGCAGCGAAAATCATTGCCTATACTGTTAACGGGTTCGG
ATGCTTGCGTCATGGCCCGTACCGGAAGTGGTAAGACAGTTGCATTCTTGGCAGATCGGA
>TR358|c0_g1_i1 len=425 path=[459:0-227 460:228-424] [-1, 459, 460, -2]
CTCTTCCGATCTACACAGAATATTTACAAATCTAAATAATGTTCACTTGAGACACACTTATGTTGATCACGTC
CAACCTCCAGAACTATCCATATTCGGCATAGACTTCCACACATCCGAAACATCCACGTTATTCGTAGACTCCT
CCGTGCTCGACCACGTCGTCATCGTATCCTCGCTCGTCCTCCCCGTACTATTCCTCGCGCTCTGATTAAAACT
CACCTCCCCCACCTTCAAAGGCGCGGCCAAAGAAGAAGAACCCTTGCTGGATCCGCTGACCCTGCCGGAATTA
AAGTTGCCCAACTGCGCCCTCAATTCCGAACTCTCCCTCATGAGCTCCCCGTTCCGCTCTTCCAATTCCCTCG
TTTCCCTGCGCAGCGTCCGCTCGCGTTCGAGGGAGTCGTTGAGCTGCATGCGGATTCGCG
>TR358|c0_g2_i1 len=282 path=[457:0-227 458:228-281] [-1, 457, 458, -2]
CTCTTCCGATCTACACAGAATATTTACAAATCTAAATAATGTTCACTTGAGACACACTTATGTTGATCACGTC
CAACCTCCAGAACTATCCATATTCGGCATAGACTTCCACACATCCGAAACATCCACGTTATTCGTAGACTCCT
CCGTGCTCGACCACGTCGTCATCGTATCCTCGCTCGTCCTCCCCGTACTATTCCTCGCGCTCTGATTAAAACT
CACCTCCCCTCGGAATTGAGGGCGCAGTTGGGCAACTTTAATTCCGGCAGGGTCAGCGGATCC
>TR359|c0_g1_i1 len=238 path=[241:0-133 242:134-157 243:158-237] [-1,
241, 242, 243, -2]
GGCCATGTTCACGTCCATCAAGCCGATAACGCTGTCGGTGGGGATGACGCCGCAGGAGAGAGGCGAGAACTTG
TTTCCCATGGCCAAAACGACGTCCGCTTCGAGATCTTGGAGAGTGCCATCGTAAGAGGAGGGGGCGCGGTTCT
TCATATAGCGAGGGAGCATGATGGACAGGAACAGAAGAGAGGCGTGGTTGTACTTATGATATGTCTACCCACC
AACAACTGTTAGTGGAAAT
>TR360|c0_g1_i1 len=476 path=[479:0-112 480:113-136 481:137-475] [-1,
479, 480, 481, -2]
CGTTCCCCGTATCGTAAATTTCCTGTAGTTTCACATCCCTGATTTCGTGGACGAAGCTAAATCCTCCCCGGCC
GATAATTTCACCGAGGACAACTTCTTGGTATGCCAATGCCGGTAGCGGATTCGACGATCCATCCTCGGATCTA
TTCAGTGAGGACGCCATCGCTAGTAGGTCGTAGTAGTGTTGGATCAATGTTGGGTGGAGACGTGTCAGATAGT
CGGAGGTGGAGGACTGGCTCGCCAACGACGAAGATGAGACGCTCTTAGTTTCGAAGACACCGCTGTCTTGCAA
TGACTGATGGATGAGCAACGATTGATTTTCCGGCAAGATGACACTGCTCGGCGTTTGTTGCTGATGTGAAAAT
GACGATGTATTGAGGTTATTCGTTTGTGGTCTTCCTCGAAGACGCAGTCCAGTCGAGTTTGTATTTTTACTTG
GCGAGCTTTTCATGTGCATGTGCCACCAAATCTCCCAT
>TR361|c0_g1_i1 len=2271 path=[2293:0-314 2294:315-337 2306:338-1376
2300:1377-1377 2308:1378-1520 2303:1521-1521 2307:1522-2189 2291:2190-
2206 2292:2207-2270] [-1, 2293, 2294, 2306, 2300, 2308, 2303, 2307, 2291,
2292, -2]
TATTGCATGATTTGCCAATCGGCACCCGACCCCCCGCCCCTATCGCGTGCGCCATTGTAAAATTGTCAATGGC
GTCGTCCAACTTTCGGGGCTTTTCTTATGCTCAATCTCCCCCAAACAGATATCCAAGGACGGTGTTATTACTT
CGTACCCAAGAAAAAATATATTCACCATACTTGCTCACGTCTTCCCATCGCCGTAAATCGAATCTCCAAAGTT
CGATTGATGAAGATCAAGAAGTAGACTGCTTGCAACCACAGCTCAGTGTATCTGTGGCTCGGTGCTTGTCCAA
TTCCGACGCCCGCTCGTCCTAGCGCAACTCCATCCAACACCGAAAAATATTTGCCAAAAACGACAGCTACCGC
AAAATGAAATACAGCTTCTACGAATCGCACGGTGATGACGATATTGGGAAAGACAAAAAGACAGACATCACCG
ACAAGCAAGCGCCGTGGTCGAACAAAAATGTCCATCAATTCTACCAAGTAACAGAAGAGTCAGGGGATAGCAA
AGGAAAGACCGACGCGAGCAAGAATGAGCACCAACATGGAAAAACACCAACGCCGAGGAGTGTGATTCATTTT
CCTTCTGAGAATGCACATGATGTGAAAAACAACAACAACAAGCAAGATCATACGTCTGGAAAGAAGGTGCATC
AATTCTACGAACCATCGGAGGTTTCGGAGGATAAATTCTCCCCTCTTGGTACCTTTCCTAGGAAGGCTGTACA
AGATGACGTGATCGCTGCCAGAGAGCAGGAGCCTTTGTCAAACAAAAATGTGCATCAGTTCTACCAGCCATCG
GATGAACCAAAGAGTACTACCTTGTCACTTCTTGATAGTCTCCCCGTCGATAATACAGAAGACGTTAATGCCA
ATGAGCAAGAGCCTTGGTCAAACAGTAATGTCCAACAGTTCTATCAAACATCAGGAGAACCCAATGGTAGTAA
AGAGAAGACTAACGCACCAGAAGGCAAGAAGGTGCATCAATTCTACGTACCAGCAGAAGCATCAGCGTTTTCT
GTTCCCCTTTTTGGTGGCTTCCCAACGAAGGCTGCACAAGATGATGTGATCGCTGCCAGAGAGAAGGAGTCTT
TGTCAAATGACAAGATTTATCAATTCTACCAGCCATCTGAGGAAGCCACGGAGGGTGCTATTGCCCTTCTTGG
TAGATTTCCCGCGAAGACTGAACAAGATGATGTCATTGCTGCTAGGGAGAAGGAGTCTTCATCAAATGATAAG
ATTCATCAATTCTACCAGCCATCTGGGGAAGCTGGGTGTGATTTTTCACTTTTTGATAGCTTCTCGGTCAAGA
ATACGCAAGATGTCAATGCCAATGCTATCGCCAACAAGCGAGAGTCTCCGCCGAACAAGAATGTACACCAATT
CTATCAGTCAGTGGAAACAGTATCACTTGGCGAGACAGCCGCCGCCGCTGCCCGTTTTGATAGACATTCAGTT
GAGAATACTCAGAACGTGGTAGTCAATGTCAACAAGCAAGAGTCTCCGCCGAACAAGAATGTGCACCAATTCT
ACCTTTCAGAGGAAACAAGAGAGGATAACAATAGCCACCCAGTCGAGAGAATCAACACCGACGACGCGTCTTC
GTTGGACAATGATGTGCACCAGTGCCTTGATAATGTGTTTAATAATATGGACGAAATTTGCCACCAAGAAGCC
GACGCACAAGTAAGCGACACTTCACTGCATGGCGCGCCCTTGTTCGCAATCGGTTCTACGAAAGACAACTTCT
ACGAACAAGGCGGTGATAGTTGTTGTCCAGCGGAGGAGATTGTGCACGATGCCAAGAAAGATTCCAAGGGGCC

AGAATCCTCCCTGTCAATCAAGAGTTTTCGCCAACTCTACCGACCGTCGGAGGAATCAAAGAATACCATTACG
CTGTCTGTGAAAGATATCTCTACCCGCTCGGCTCGGAAGGCTCATCTTACATCCTTGAATTTGAAAACCGTGG
AATCGTTGGCTCTTTTGCAAAGTTATCATGAAAGCAAGAGGTCGGAGGTGAGATTGTTGGATAAGTACGGGCT
GGGGTCGGCTGCTTCAAACTAATCGGAGGGGATTCGGCGAAAAGCCAACTCTATTTTTGTGGAGAGCAACAGA
CAGCAGCAATTTGGCAAAATAGATTGTTTGTGTTCAGATAGATGGTAGTAGATAGAGTTTCTTCAGCAAAAAA
GGATGACGATTTAGTACAATGCATACTCTTCCCTATCTGGAGGGTGAAATAAGATAAATGTTTTTTGTGACAG
TGAAAAAA
>TR362|c0_g1_i1 len=434 path=[490:0-298 500:299-333 498:334-360 485:361-384 486:385-433] [-1, 490, 500, 498, 485, 486, -2]
ACATCTACAATACAGGTGTAGAAGTAAGTTGGATGTGAATACTTACACTTAGGTACACAAAATAACCCTGCAT
AACAACATCATGTTCCCTTCTGAACAGTCGGAAGTTCGTCCCTCTTCGTAGGCACTTCTGGCATCGCATTGCG
TTGATGTCTTCTCAGTCGACACGAATACAAAATGATACTACTACATCATGGTCGGAATAGCTGCTGCATATCG
GGAGTCGCACTAGTCTCTGTGTTGTTACAGCTTTCATCCTTCGCCTTCGGTTTCCTTGCACCGCGCTGTTGTC
GGTTTCAGAACTTTAAGAACGTACCGCCAAGCAGGAAATTTCTTGAATCTGTTGGAAATTGTAAAGATCTAGG
CGAGAATTATGATGACGACGACGACGACGACGACGACTCCACAATCAAGCCATATCGAAATAGGTCGCT
>TR363|c0_g1_i1 len=1264 path=[1295:0-218 1296:219-242 1298:243-1129 1293:1130-1153 1294:1154-1263] [-1, 1295, 1296, 1298, 1293, 1294, -2]
CTCTTCCGATCTTTTTTTTTCTTAAGAGGGTTAAAACTGCTTTATTTGAATAGCTTTAAACTGCTATGACGCA
TATTTACGACACGCAATCTTCAACACTCTTAGTACCTAGTACTAACTTCTGTGGGGGTATCAATTAAATAACT
GGTGCTCGGTGACACATTCTTTCAAATCATAAGAATAGACATTGTCGCTAGAAGAAATCAATACCTTGGTTCC
CGATAATGCAATTTGATCACCAAAGTATGGACTCTCAGAATTTGGAGCATTTATTTTGGCCACCTCCGTCCAA
TTACTCTCTTGGCTCTCGCGCACGAAAACATATGCTAAACCGTTTTCGTTCCTGTACTCCCCAATCGTCATGA
CATTCCCGTCCGCTGATATTTGTTGAGGCCCTCCGAATCTATTTCCATCGGCTCCATCGGATGCGGTGAGTAT
TTGCCGAAGAGAATATGCACCACCATCTTTTGACTGTGTGTAGTGATATACAGCTCCTTTCTTTTTACCGTAA
AAAGGACAGCCGACCATCAAATCGTGCTCATTTGCCAACGCAACGATAACACCGAAACGTCCATCACAAGTTA
TCTCTTGAATAGGATCCGGTAACTGACTCCAATTTCCCAAATTTTCGTTATACTCGTAAAGGTACACGACTCC
TTCAGGTTTATAACCAAACAAGCGATCTCCCACAACTATCCTATTCCCTTTCACTTTTGCAGATTTACCAAAA
TCTTTATTCAATGGTTGGTTTGGAGCAATTTTTGCTTCCTCTACCCATGTGTTATTCACATCTCGGCGATACA
CATACACAGAACCTGTGCGATCGTACGCGCTAACAACTATCACATCGCCGTCGATGTCCACATGCCTCCCGAA
CTGATGAGCGTCAGCGATGTCGCGCGGGATTAAGCGCCCCGCTGGATTCCAAACCCCATCAGATGACGACGAC
GAGCCGTAGCTGTCTTTGTCGTACACGTAAACGGTGCCGGTGAAGCTGTCTTTGTCGTAACTAGTATCTTCTG
GAGTGCCCACCACCGCGACGTTCCCTGATATCGACACTCCGCCATCGGCTCCGCCTACTCTATAATCTATGCT
GAATACCGATGAATTCTCGAATTGACCATAATCAGTGAACGAATAGAAATTCAAGCTTTTCTTCTTGACTGCG
GTCACCGTCGTATTGCCGTATATGTCCACGGGGGGTGAACATCCGTTGCAGTCGACTTCGAACAGGGCCCCCG
TATATTCTACACAAGAACCTGAT
>TR364|c0_g1_i1 len=717 path=[748:0-391 749:392-404 750:405-428 751:429-716] [-1, 748, 749, 750, 751, -2]
AAGGGTGTAGGTAAGACTAGTGCTTGCACCGACGTGCTGATTCCGTGCAAACTGATTCAGGATACACTGCACG
AATATCGACGCGCTATTTTACTTGGATTCAAACTGAAGGAATGCAATCAACCTAGTGCAATCGCTATGTTTAA
GACCTCTCTATTGATTTGATACTGCACAGTAGTTCCCGGTAACTGCACGTATTGGTCACAACATTTGCCCTAT
GCTGCAGAGTTGAACCGCATGATGTTTGATACTGTATACAATGTATACCAAACTTGGGTAGATAGGTAGATCA
GCGCCGGTTTCGCGAGGCACAGACGCAAAGTAATTGGCAGAGTATCAGATGATGCCTAAATTTCCAAACTTTC
CCTTGACTTGTTGAGCTTCACTGTTAAACTTTAGGCTCTCGACATTCCGGACGTCTACTTCTACACATACACC
TGCATACACCACACATGGATCGAGATCACAGCAGACACCCTATCTCCAAGAATCAGGAAATGGCGAACACCTC
CTGCCTCCTCTATTGTAGTTCCACACACAATGGAAGAAATCAAACGTTCTCAAAGCAGCACATGCCTCGTCTT
AGACCGCAGTTTACTCCTTCTCTCTTGCGCCTGTTTCATCAACGTCGTTTTTTTCACGAGGTGATCTGTCACG
TCCGCCATGGCGCGCGGGACCACGCTGCTGCCCTCCAAATCGTTCAAGGAGATCGGAAGA
>TR365|c0_g2_i1 len=1218 path=[1280:0-695 1281:696-1217] [-1, 1280, 1281, -2]
CCTCTTCGGCGCGACCTTCCAACTCATCGACCTCGCCCTCGCGCAACTCGACACCCAACTTCCCCTCCCCGCG
ATTGCCTTCGTCTTCTACGCCGCCTCGCTTAAATCCCGAGTATTCAACCCATTAAACAATCAACGTCCCGACC
GCTCGAAGGCCGCCAACGAGGAGGGCTCTAGTGGATTTCGCGATCGGGTCATGCCCTCGTGGACTCCCCCCGG
CGTCATCTTCCCGATCATGTGGGTGCTCATCATCGGTCCCCTACGGGCGTATTCGGCGGCACTCGTGGTTTCG
TCCACCGGGAGCTTCCTCACACTTCCCACCATGGCATTCATGCTGCATTTGACGGTAGGAGACATTTGGAACA
CCATCAACAATACCGAAAAGCGATATGGGGCTGCAGTCATCGGAGTGTTGTGCGTGGTCATCAGCGCGGCGAA
TGCGGCGTACCAGTACTCGACGGTGGATCCCCTCGCTGGGAAGCTTTTGGGAGGGACGTGTATCTGGCTGGTC
ACGGCGGCGGCTTTGATCACGGATACTTGGAGGTTGAATCCTACGGAGAATGGGGAGAGAGTGCCGCTGTATC
CTGTCAAGGGGGAGGCGGAGACTTCTTTCATGTGGTTTGACAAGGGAGAGGAGTAGAAGTGAAAATAAGTATG
AATCCGCACATCGACGTTTTTTGTCAGCAACGAATAGCAATAGTATTGGGAATTAGTTTTTTAGGCAGACCT
GCAAGGCCATACTGCTTCAAGAATGGAGAGAAGAAAAGAAAGGTCGACGAAAAACAGTTCAGTTAAAGAATCC

ATAATATCGTATCGTGATTTTCGACCCGCGTCGAACTATTCTAATTCATTATATGTTGGCATTCATTTCGAGG
AGCATAGGTAGCGTGATTGGCACCACCACATCTTTCAGAAATTGGATTTGTCCTTCGATTCGAATTCGAGAAA
GTCTCACCATACTTTTGTCGACTCGCAATTTCCGCATCATCACTCTGGCCCTTTACAGAACGAACGAATTTTA
TTTCACCCCATCCCAGCCCCAGTACTGAAACCATAAATTGGCCTGCCTGACCTCTTCAAAGCATGAACCACAT
CCATTGCCGTCACCGTCTTCCTATTGGCATGCTCGGTATAAATGACAGCGTCACCTATCGTTTTTTCGAGAAA
ATTTCTCAGTACCCCACGAGTCTCTTCGTATATCAAGCCAGAGATTCGCT
>TR366|c0_g1_i1 len=1313 path=[1399:0-154 1400:155-178 1408:179-501 1407:502-1071 1405:1072-1095 1406:1096-1312] [-1, 1399, 1400, 1408, 1407, 1405, 1406, -2]
CCGATCTCTCGAGTATCTACCGTCAATATATCGGAGTGCATGCTAGTGTGTATGAAGTGATCATAATATTTCT
TTTTCTTGGCAAGTCATCCACATCCATCCAAATCCAGATCTCAGAATATCCGCTCAAAGAATCTAAAAATACA
TCATGATGACCATGACCACTAACAACAGCCACACCACCGACATCATCGAACGCCTCAACGCCATGACCGTTCA
AGAGTCGACGACACCATCATGCTTCAACTATTTCAACAACAACATCAACAGCAACAATAAATTTACATTAAAC
CAAGATCTAAACGAAACATCCCGCAAATCAATGGTAACATGGATCACCCAAGTTCAAACCACACTCTCCCTTA
GCCCTGAATCGGTATGGATCGGCATGTCCTTCTTCGACCGCTACCTTAGCTCCAACAAAGGACAATCCCAACA
AGTTCTCAATAACAAAGCCAAATTCCAACTAGCCGCCATCACCTCGTTCTATACTGCCGTGAAGATCTACGAG
CCGGTAGTATTGGGTATGGATATCCTGATACAGATATGCCGTGGGACGTATAGTGAAATGGATATTACATCTA
TGGAGAATGAAATATTGAATGCGTTGGATTGGAGAGTTTCGTGTCATACTCCTACGGAGTTTGCGAGACATTT
GTTGGAATTGCTACCTGAAGAAGACGACATTCCTTCGTGTCAGTCGGAAGTCATTCTTGAAGAATGTCAGAAA
AATTTGGATTGCACAATTGCTGATATGTACTTTTCGTCCTTAGCTCCATCTGTTGTGGGCATCGGTTGCCTAG
CAAGCGCTTTGACGAAAAACAATACATTAACTCTATCGGAAAAGCAAACGATTTGGTCTCGATTATCATCGAA
GGAGGAGTGTCAATTTGATCTATCGTCGAAAGAAGTTATTGCAGTGATTCATCGTCTCGATCCTCCTACATCG
TCGCATGATGCATCATCGAAACTTACTACTACACCATCACAGTCGAACAACCACGTGCTGTCTGTTCCATCAT
CTCCAGTCTGTGTGACGCAAGTTGCCCGACAGGCATGAAATGAAATACTTCACACAATATAATTGGTCTTAAT
AATGCAAGCACCTCTACTACATGATCCCACATCCAACCTAAAAATGTAGTACAATAATTTGTTAGATGCATAT
GATATACCCTGTTTGATCAATCATCTACAAATCATGTAGGTTCAAAAATATCAGACTAGACAATATTTGAATT
TGACAACTCTAAAGAAGTGAACGTTTCCCATTTTTTTAATTGAAACTGCCCATCCCTAGAGAGGCAAGGTAT
>TR367|c0_g1_i1 len=348 path=[493:0-91 494:92-120 495:121-121 528:122-145 522:146-146 530:147-147 526:148-198 520:199-229 490:230-253 491:254-254 492:255-347] [-1, 493, 494, 495, 528, 522, 530, 526, 520, 490, 491, 492, -2]
GATTGCGCTGCACCGCCGCCACCTCTGCCCACCTCTTCCACCGCGCTGCTGCTGATGCACTCGCTGACGCGCT
GCCTTCGGCACGCATGCGCTATGATATATGGAGGATCGATGGCTGGTGAGGTGAATGAAAACAATTTCGAAAA
GTAAGGGAACAAACTATACACCAAAGATTCAAAGACAGCATCAATTTCAATAACCATGACATCCAACCTCTTC
CTCTTCCTCGCGGCACTGATTGCCACGGTAACGGTATCGTCCACCAATGCCTTCTCTCCCATCTCCACATCAT
GCCAACAACAACAACAACAACAACAATCTTCCATGCGCATCCCCACCGCCCGCCCC
>TR367|c0_g2_i1 len=262 path=[509:0-91 525:92-120 532:121-121 529:122-145 523:146-168 487:169-261] [-1, 509, 525, 532, 529, 523, 487, -2]
GATTGCGCTGCACCGCCGCCACCTCTGCCCACCTCTTCCACCGCGCTGCTGCTGATGCACTCGCTGACGCGCT
GCCTTCGGCACGCATGCGCTATGATATATGGAGGATCGATGGCTGGTGAGGTGAATGAAAACAATTTCGAAAA
CACTGATTGCCACGGTAACGGTATCGTCCACCAATGCCTTCTCTCCCATCTCCACATCATGCCAACAACAACA
ACAACAACAACAATCTTCCATGCGCATCCCCACCGCCCGCCCC
>TR367|c0_g3_i1 len=347 path=[514:0-91 515:92-118 516:119-119 524:120-144 531:145-145 527:146-197 521:198-228 480:229-252 481:253-253 482:254-346] [-1, 514, 515, 516, 524, 531, 527, 521, 480, 481, 482, -2]
GATTGCGCTGCACCGCCGCCACCTCTGCCCACCTCTTCCACCGCGCTGCTGCTGATGCACTCGCTGACGCGCT
GCCTTCGGCACGCATGCGCGATGATATGGAGGATCGATGGCTGGTGACATGAATGAAAACAATTTCGAAAAGT
GAGGGGACAAACTATTATACCACAGAGTTAAAGACAGCATCAATTTCAATAACCATGACATCCAACCTCTTCC
TCTTCCTCGCGGCACTGATTGCCACGGTAACGGTATCGTCCACCAATGCCTTCTCTCCCATCTCCACATCATG
CCAACAACAACAACAACAACAACAATCTTCCATGCGCATCCCCACCGCCCGCCCC
>TR368|c0_g1_i1 len=1675 path=[1766:0-258 1767:259-282 1776:283-1203 1764:1204-1227 1778:1228-1271 1761:1272-1303 1775:1304-1515 1773:1516-1539 1774:1540-1674] [-1, 1766, 1767, 1776, 1764, 1778, 1761, 1775, 1773, 1774, -2]
GGCGTATCCTCCCGACGAGGTTGTCGGACGTGATGTCGTCGCAAGTTGTACCGTGATGGGAGGTTTCGAAGGA
GACGCCGATGGAATGGAGGGCGTTGACGATGATGGTGGTGCCAGCGTCGGAGCATTCGCGTTGAGCATGGATA
CCGCAGGAGAAGCACTCATCGTGGGAGCGGACGTCGTGAGCGTTGCGGTAATCGACGGATTCGATGGGTAGGA
TATCGGCGCGGCGGTGTTGCGTACTTGGCTCGAAGGAGGATGAGTGGGATCCGTCATTGGCGATTTCGATGGC
ATTCCGGAGGGAGTTGGCAACGTCGATGGGATCGACGCGTCAGCGAGAACCGACGTGGGATCCGCCGTCGGAG
CGGAGGAAGCGATGATGGGAGATTTCGATTTCGATTTCGATGGCGATGCTGTTGCTGCCGCTGCCGATGGCCC

ATTCGTTGGCAAAGACGCTGGATTTGCCATTATCGTTGGTCCCGAAGTGGGAGGGACGACGACATCCAAAGTC
GTTAGCCCGAAACTTACAATAGCGTCCACCCCCTCCTCGAGATCGAAACACTTCATCCGACCCGTTTCGGGAT
CCACGCGTCCTGTATTGGTACTCCCCCGTCGTTCCAAACCTCGCTAAACGCGTACCCCCGTGGTGCCGATGG
AATGAAAACGTAATACTGCCTTCCGCATTCCAAATCCTCGAATTCGTAATATCCCGAAGAATTTGTTTTTGCG
ATTTTGATATTCCCATCGATACACGTGAATAGGAAGATCATAATGTTGGAAACAACGCCGGCGTGATTGTCGT
GTGTGGTATTGGCATCGAGGTCGTTTGATTCGACGAAGGCGTATCCGGAGATGCTGCTGCCGTTAATGGACGA
AGCGTTGATGGGTGATTCTATTGGCGATGCCGTTATTGGCAATTTGGAAGGTGATTTGGTCGATGGCATTTTA
TTCGGAGGAGAGGAGGCAGGAATAGTAGACGCCGACGCGGAATTCGAAGTAGGAGCTTTCGTTGGAGCAGAGG
TGGCAATGACGTTGTCGATGACATTGTCGATGATGTTGTCGATGACATTGTCGATGACATTGTCGATGACGGC
GGGAGATTTCGAAGGCGGTATTGACGACGATGGCACTTTTGTCAGATGTTGGCTCGAATTCATCGTTGGAGCA
GATGTGATAGGATTCGAAGTCGTCGGTTTCAAAGCGAGTGGGACGTCGTGCGTCATTCCAAAGCTGACATTCT
TGTCTTCGCCCTCCTCCAAATAAAAGCACACCGACCGACCAGTCTCGGGATTCACCGCATTGTCCACATTTTG
ATACAATAATGCTCCGGCGTCATCAGTCGCGCCATTCCAAAACTCGCTAAAGACACACCCACTCGGCAGCGAA
GGGATGTAAACGTAATACTGGACATCATCGCCATGCCCCAAATCCTCAAATTTGTAATGTCCGCTCCCATCCG
TTCGGGTCGTCCTCCTAATCTCGTTTGCACAAGTGAAGAGGTAGACCGATATACCATCTACTTCGTACTCTGG
CGCAGCATTCATATCCAAGCCGAGATTGCCAGACGTGTTCGTATCGTCGCCGATTGCGTTTGAGAAGGCATTC
GCGAGCGTATTCAAATCGAGGTCTAGATTGCCATCATTATTCGCGTCGAAGAACGCATGGCCGTAGACG
>TR369|c0_g1_i1 len=1066 path=[1147:0-519 1148:520-543 1168:544-737
1167:738-865 1157:866-914 1158:915-1065] [-1, 1147, 1148, 1168, 1167,
1157, 1158, -2]
ACTAAAGGTGAGTGACATATCATTGGACATCTGCACGAAAAGATTTGCAGCTAGGCAACAGAGCTCACATGCA
TGAGGTACTGTACAGCGCAGAAACGTTCTCTAGTACAGCGACAAGGCCTCAGCGGTTGACCCATCCAACTTCA
TCACGTTATTGTGCCAGCAAAATGTAACGTGGCTGCCTACCTATTGAATACGTAACATGTATCGACGAGGTCA
ATTGATCGAACGCGGTTTTACCACAACACCCAAGTACATGAAGGGGGCTTGTTGAGTTCAAATGGTTTTAGAT
AGAATCTACACGTCACCAATGAAATGAATGCTCCACCCTCCCGGAATATTAGAGGGTGCAGCCAATTATGTAG
AGTTGTGATAATGAGGGTATGGGGACTCTCATTATTGTGACACAATACTTCTAGTAAATTGTAAATTATAAGC
ATTACATGTTTTCCCATCGGTTCTTTTGTTTAGTAGTCGCCGCCATCGTCTGCGCCTCCGAACATGTCTACGG
CAGGAGGGGGCTCTTCAACCTCTTCTTCAACCTCTGCTGCTGCTTCCTCGGCAGCATCTCCTCCTCCTCCACC
GCCGCCTCCTCCTCCACCTCCACCGCCTCCTGGCGCTAAGCAAAAGTCTTCCACTGATTTTGCCTTGCCAACT
GCCGATGCAAAGACCGACGCCCACGCTCCCGAAAGGCTATTTCCACTGGCAGTGGCAACGGCGGAGAGAGACT
CTGCCGATACTTCACCTCCGGACGTTCCGACCACAATGGCCGAAAGTGAGGCAATTAGCTCCTGCTTCTGTGC
GTCAGAAAGACCTTCGAGGGATGAGACAACCATTTTTGTTTATATTTATACTGCGCGTCGGAAATTTGAAATG
ATGGGAATTTTGTGTTAGTTAGCTGTTTGCGGTATATTTCGAGCATGCATGATGCCTCCCCCATCAAGCGATC
GTTGCGAAGTACACAAATCGAGCGCGAGCAGTAGAGCCAATCTACGACGGCAACGCTACTCCCGGCAGGCGAA
CTTTCAACGCAGCCAGATCCATCATGACAGTCATCGCCATGGTC
>TR370|c0_g1_i1 len=265 path=[426:0-54 427:55-78 443:79-113 450:114-140
449:141-200 445:201-202 440:203-226 437:227-231 438:232-264] [-1, 426,
427, 443, 450, 449, 445, 440, 437, 438, -2]
GTGCTGGAAAGTCGACGTTGATGGTAGCCCTCCTTAGGATCGTGGAGCTGTATTCCGGTAGTATCAAAATTGA
TGGCGTTGATATTCGGGCTGTTGGTCTTAAGAAATTGAGGTAAGCACGGCATCTTTTGTTGTCTATTGTTCAT
TACATTGTCAGCAATCTACTCAATGAACCACCCCCCTTGTTCATCTCTACTACTCGCGTCTGTAGGTCTGCAA
TAGCAGTTATTCCTCAGGACCCAGTTTTGTTCTCTGGATCTGTTCG
>TR370|c0_g1_i2 len=265 path=[426:0-54 427:55-78 443:79-113 450:114-140
441:141-176 448:177-200 444:201-202 439:203-226 403:227-231 404:232-264]
[-1, 426, 427, 443, 450, 441, 448, 444, 439, 403, 404, -2]
GTGCTGGAAAGTCGACGTTGATGGTAGCCCTCCTTAGGATCGTGGAGCTGTATTCCGGTAGTATCAAAATTGA
TGGCGTTGATATTCGGGCTGTTGGTCTTAAGAAATTGAGGTAAGCACGGCATCTTTTGTTGTCTATTGCTCAT
TACATTGTCAGCAATTCCCTCAATGAATAACTCCCCTTGTTCATCTCTACTACTCGCGTCTGTAGGTCTGCAA
TAGCAGTTATTCCTCAGGACCCAGTTTTGTTCTCTGGATCTGTTCG
>TR372|c0_g1_i1 len=476 path=[990:0-112 1011:113-191 1009:192-278
993:279-302 1006:303-320 1010:321-344 1008:345-383 1005:384-407 1000:408-
475] [-1, 990, 1011, 1009, 993, 1006, 1010, 1008, 1005, 1000, -2]
GAAAAGGTAGGAATTATTATACGCTGAGAGTCCAGCAGCGTCTATCATGAAACGCGGATCACTAACATTGTCA
CTGTATGTCATCTTCACAGTACATGAATGCAACCATGTGGCGGCACTGGTAGGACGGTTCCCACCAACATCAA
TCAGTACAAGCAGGTTGGTCGCACATCAATTTCATTCGCTGTCGCCCTATTTGCATCGCGACGCTGGCTATGC
TCCATCCGAAGGGATTAGAACTCGACGGGCGGTTATTCATTCAGCCTTGCTTGCCTCCAAGGATGACACGAAC
AATTCATCAACCAACAGCGAAGAGATTGATGACCAAGGCACTTCATCAAAGACAAACAATTCATCGAAACGAG
AAATGCTGAAATTTGCCATACCGGCCCTTGGCATCTACCTCACAAATCCCCTCCTCTCCAACATTGACAACGC
CTTCGTGGGACGGACCGTCGGAGCAGCCGGTCTCGCGG
>TR372|c0_g2_i1 len=497 path=[1011:0-101 987:102-496] [1011, 987, -2]

AGTACATGAATGCAACCATGTGGCGGCACTGGTAGGACGGTTCCCACCAACATCAATCAGTACAAGCAGGTTG
GTCGCACATCAATTTCATTCGCTGTCGCCATATTTGCATCGCGGCTCTCAGTCCCGCCACGCTCTGCATCGAT
CAGGTCCTCTACCTCTTCTCCTTTCTCAGCCGTGCCGCCACCGCCCTCGCCGCCAGGGGATACAACAATGTCA
ACAACAAATCGGAAGCGGAGGCAAAGCAAAGGATGAAGGACGCGGCCTCTCCCGCCCTCTCCATCGCGCTATT
TTGCGGCATCCTCCTGTCGGGCATGTACGCAACCTCGTCACCCTTCATCCTCGAAACCCTCAACGTCGATCCC
CTCCTCCACGCTTCGGCATCGTCTTACATCCACTGGCGCGGCGTGGCATCCTGGGCGGCCATGGTGCAATCCA
TCCTCCTCACCGTCTTCATGGTCACCAAGGATGCCAAGACGCCGCTCAAGATCATCGCC
>TR373|c0_g1_i1 len=3811 path=[4208:0-62 4209:63-86 4233:87-808 4197:809-832 4238:833-892 4212:893-916 4232:917-1472 4182:1473-1496 4237:1497-1558 4206:1559-1582 4241:1583-1753 4185:1754-1797 4236:1798-2136 4176:2137-2160 4243:2161-2365 4203:2366-2394 @4240@!:2395-2623 4188:2624-2646 4229:2647-2768 4179:2769-2792 4242:2793-3250 4221:3251-3265 @4234@!:3266-3482 4194:3483-3506 4227:3507-3692 4173:3693-3716 4174:3717-3810] [-1, 4208, 4209, 4233, 4197, 4238, 4212, 4232, 4182, 4237, 4206, 4241, 4185, 4236, 4176, 4243, 4203, 4240, 4188, 4229, 4179, 4242, 4221, 4234, 4194, 4227, 4173, 4174, -2]
ATCTAACCACTGCCAATGAAAGAGCGTAATCTTTTCGAACCAGCCACCCCAAGCACTCCTAACAAAGGATGTG
ATGCCGCGGTGCCCGAAGATAAATCCCGGAAATCACGTCGGCTCCGCAGCTCGCCCTCGCGCTACATAGCAAG
ACCTTCTCAACAGGGGCTGGGCGTTGAGGAGGGAGATGCTGCTTTTTTGGCATGCGGGCGGTTGGAATCCGTA
CGACAATCTAAGCGATTGATAGAACGTCAAGATGCGGGTCATGCAAACGATGGCGAGGATGCCGACAACGACG
ATCACGCCAAGACGAATAACGAGCGAGTTCATGCTTCCCCGGTGAGGGACACACGCCATTCAATGGATAATAT
CAAAAACACCAAAAAATATGGCGAGAGGAAACAGCCGCGGCCGATGTCATCGCCCGCGTCAAAATCACCGAAG
AAACGTCGAATGCCGTCGATGCCATCGCCAGCGTCGAAGTCACCGAAGAAAAATCCCGTAGTCGGAAGCGGAG
CTCCACGTCGTCCCCGAGTCATAATTATAGGGGCGGGAATCTCGGGGTTGGCGTGTGCGCGCGAGCTATCCGA
ACGACGGCACGACGTGCTGGTCCTCGAGGCGCGCAATAGGATAGGGGGACGCTGTCGAACGGTCGACTTGATG
CTCGACAAGGAGTGGAAAGACAACAGAGATGCGAAAAGCGATTTGTACAAAGTCCAGAAGTGGAGCCCGGTGG
ATGTCGGAGGAGCGTTCATTCACGGCACGGGCGTTTCCACGTCCGATGCCGATCGTCCCCCCTCGACTCACGT
GGGTTCTCATGATTTCGGCACCAGCAGCGGCAGCATGAATGGAAAACGAAAAAGCAAGGTTTCCGAACCCATT
CGCAAATCGGCGAGGATTCACACTGGAAACTTGGGCCGGAAACGATCCTACAAAAATATGGATAGCACAACGG
GCGACAGAAGGGACACCAATATCCGAAGGAGCGACGATGGAATCGGAGGCCTCAATCCGCTGTACGTTCTCGC
TCGTCAAAAATTGCGTCTGCCCGTGCACGCCGCCGAGGGGGCGTACACGTGTCTCGTGGACCACAATGGGAAT
TTAATCGGCGAGGAAGTCGACCGAAGAGTCGCGAAGGAATTCAACGAGGTGTTGGACCTGGCGACGAAATGTT
GCGAGCAGGGGAGGTACGAATGGAAAGATGAAAGCGCCGTGGCGGGGGATTCTAGTTTACATCAAAAGCAAGG
TTTGTCCACAACGGCTGGAATGGGTGAAAATGGAGAGGCAACGGTTCGACCCCCGCTGCCTAATCAGTTGCAG
CCGGTTTTATCCGTGGAAACTGGGGATGTGACGCGTTCGCCAAATTATGTATCGGATGACACGACTCCGAGCA
ATAGTGCATCGGAGATTCTTTCACAAAGTATAACAAATGCGGGCAGCGCAAATATTAGCCCAGAAACTGATTT
CGGGGAAATATTTGCCGAATGCAAAAGGCACCATGATGACATCATTTCCACTGACGGAAAGTGCGGCAGCACT
GGAACAAAAAGCGGCAATGGAAACTCAACCGAACAAGACGTTTGCGATAATTTGTTCCGATGGCACGTGGCCA
ACCTCGAAATGAGCTCGGGGGCACCCATCGGCAAGCTGGGTCAACGGTGGAACGATGACGAACCGTTCGGATA
CGGCGGCGACCATTCCTATCTCGAGGGCGGATTGCGAGACGTCATCGAGACGCTGGCGGAGGGGTTCGAGTGC
AGAGGCATCGGAGATCGATCGATTTCGGGCACCAGCAGGGGGAGCAAGGGCAATATCCGCCGTCGCAGCGGCG
ACTTTGCGAGCACATTCGGTAGACGACCCGCCGCCGCTGGACACTCGGATTTATCGGGAGGGACGGCGAACAG
CAGGGGAATTATTCAGTGTGGAATGGAAGTGAATGGAGTTAAGGTAATCGAAAGCGAAGAAGCGAAACTCCTG
AGACGGAGAAAGCGGAAAAAGCAACCGATTCGAGTTGACAATTCTGCGCTTCGTCGGTCTAATCGCGAGAATA
GAGGCGAGAAGATGGCTACTTTTTTGGATGCACTGACTTCACGGCCGAAGGGGAAGCAAAAACCTGCGGCTGG
GCCTGCAGACCCCATTTCTGGGATTGCGGGAAAAACACCGTCCATTCCAATTTCGTCCTATGGAAACAAACAA
AACAGCAGTGTTCAAGTCACCACCAAATGCGGATTAACGTTCGAGGCGGATGCTGTAATTGTGACGACTCCAC
TTGCCATTTTATCGATTCCATCTGGCTCACCTGGCCACATATCATTTAGCCCTCCCTTACCGCTCACAAAACA
AAATGCATTGAGTCGATTAGGGGTTGGCGCTTATAGCAAGTGCTGCATGTCATTTGAAAAACCGTTTTGGAAC
AGTTTGCCTCGTCACCTGTCGACGGCAACACTGCCGGGCAATTGGGCGGATGAAATGACTAAAAGATTTGATT
TCATAGGACATGCATCAGCAGAACATGGCAAGGATATCCTCTTTTTCAATCTGAGAAATGCACCTGTCTTGGT
TGCCATCTACGGTGGCTCAGATTACTCAGAACAGATTGAGGGTATGCACGATGGCCTTGTCGTCCTGGAATGC
ATGACAGTTTTGAAGAAAATATGCAGCAAGGCAATTAAGGCGAGAGACAATTCCCTTAAGACACGCCGACAGG
TTGATCTTTCTGTCCCGAATTGGCCAATTGATTATTTTGTCTCACGGTGGGGATCTGATCCTTACAGCAGAGG
TGCATTTAGCTATGTGCCTGCAGGAGTCAATGGGTTCGAGGAGTTGAGAGCCATGTCAACACCAATCTGTGAC
TTTGGTCCCAAGCGGACATGCGAATCGAAGGAAAAGCCAGAGCGACCGTTGATAATGTTTGCCGGAGAGGCCA
CAACACCGTATCATCCATCGACAATGCATGGGGCTTTTGAGTCGGGAATCCGAGAGGCATACAGACTGGACCT
GGCGCTAGAGCCTGAGTTAAATGGCATTACATTTGACGAGGCATTTCTCTATCGGCCTACGTTTTCAGTACGA
CGTGCCCAAGCAGATCCAAACTTGACATCGAACAAGGTACCTGAAACTGCTTCGCAAAATAGGAGAAATGGCA
GTAAGAATGATCCAGAAGCAAAGAGCTGGTGGTTTGACCACGACGCTTCAATTTTGAGGGGGGTGGAGACTTT

TGGTTACTCTGCCCAAACGATGTCCATAATTAAAAAAAAGCTGCTAGCACCAGCAAGTGATAATCACAGCGAG
GAGGAGATGCTAGACAGATACAAGTCACTCATGCATATGATAACGAACGAAGTGCAAACACCAGAACCTCATG
AATCAAATACGCTGGCCGGTGTCGACTCTAACGCAACGTACAGCATCGAAAACGAGTGGAGTATTCCTGGGCA
ACGAGGAACGTGGCTCGTAGCTGATGTTGCCAAAAATGCGGAAGGAAATATTCGTGTTGAGCCTGCGCCTGAT
GTTGTTGCGAAAGCCGACCTAAGAAACTTTCAAGGTGAATCAACTGTCAGAACTAATTCCGGAACCCGCCGTT
CTTCGAGAAAGAGTCAGAGGAAACTTGATGAATCGTTTACTTTTTATTAAATGATATTCGCACTTGAAAAATT
ATTTAATTTCTACTCGTTGTGATTTACAAATAGAGAAATTGGCTTCGCACCACAAGTAAAAAGGGCTATGGCA
GGGCAGTCTATCACTTAAGAAACTTTACATCAGTACTCGAATAAATACTTAGTAAGTCCGATTAGGTACTAAA
AAAAAAAAGATCGGAA
>TR374|c0_g1_i1 len=3659 path=[4070:0-301 4071:302-302 4072:303-316
4152:317-317 4150:318-331 4145:332-332 4139:333-398 4047:399-422
4131:423-539 4068:540-563 4138:564-745 4065:746-769 4130:770-978
4149:979-1125 4118:1126-1185 @4144@!:1186-1698 4153:1699-1702 4041:1703-
1726 4128:1727-2216 4044:2217-2240 4148:2241-2713 4135:2714-2799
4105:2800-2817 4142:2818-2823 4151:2824-2847 4147:2848-2885 4141:2886-
2895 4133:2896-2909 4125:2910-3066 4037:3067-3090 4146:3091-3194
4097:3195-3218 4140:3219-3355 4154:3356-3360 4079:3361-3384 4080:3385-
3658] [-1, 4070, 4071, 4072, 4152, 4150, 4145, 4139, 4047, 4131, 4068,
4138, 4065, 4130, 4149, 4118, 4144, 4153, 4041, 4128, 4044, 4148, 4135,
4105, 4142, 4151, 4147, 4141, 4133, 4125, 4037, 4146, 4097, 4140, 4154,
4079, 4080, -2]
AGAACGTTGCGGCGTCATCGCCCTGGGGGATGAATTACTGGCCGTCAACGAAGTGGGACTGGAGGGAATGCCG
TTCGAGGAGGCCATTGCCACTGTGCGTCAGATCGGGTTTGACTCGTACGGCGCGCCTTTACGAATGCGGTTTC
GCCGATGTCGCGGAAAGAGGGGCTCGTTATTGTCTTCCCTCGGATCGGGTGGGAGGCGGAGCAGGACTCACAC
AGTAGATTCCAAAGGGTCTCAAGGGGCTGCATTGGAAATCGCATCCACTATGGCCACACCAGGAGGAGAGTTG
GTAGGAGGAGAGTTGGTAGGAGGAGAGTTGGTAGGAGGAGACGCCGATGTTGGTATTGACGTTGGGGATACTA
CCAGGAGTCTTGCCACCGTCGAGGTCGGCGCCGATGCGGAGATCCAACAGGGATTCGGGCGTATTATAGCCAT
TGTTAAGAATGCCGTGTCTGTCGATTCCGGCATCCACCATCAACATGCAATGGATGTTGCCGGTCCATCCGCA
GTGCTATTGCTCCCGTGGAACTTCGGCAAGGGTGCGACGGTGTCACATAAAATGTATGGTGGTGCGTTGATAT
TGTGGGCAATCCCCACTGCCATCTCGGGCACAGGGAAACGACGAAACGTCATTAAGGCGGCGCGATTGGAGGC
TGTACTGGATATCGATCCCGAGAATGCACGATTCGTGGAGATGGGCTCGATTGTTTTGGACAACGACGAAACA
GGGAGTGCGAACCAATCCAATGTCAAATCGATATCGTTTGTGAGTTCAACCGAGAAAGGTTGGCTAGTTGCTA
TTCACGATTGTGCTGGAAACGTCTCGCTGCTCTTCGTCGAAACCAACCCTGCGGACGGCACCAGCGGCATTGT
CTCTAACGTCGACTCTACTTCGCACTCGGGCGCGTGCCACCTCTGTACAACTTTCCGCCATTATCCTTGCATA
TTCAACTCTTTTGGAAAAAAATATTGGTTCACTTGGGAAAACCCAACAGGACAGTGGTTCTTTCATTCTGCGCT
CTTTTTCTCTGGAACTTTTCGGTGGCATGGAAGAATGCAATGGGGGCTGTAAGGAGCTAACCATATGGTCGGC
ATTGCCACAATCGAAGCACCAGTCTATGGACATAAGTGATGAAGGTACTCAATTTTTTGAGTACCACACTGTT
AAAATTTGCATTGACGATGTTCCCCAGCTTTGTTCGGAAGAGGTGATTCTTGACTTCCGATGGCTTTCCAGCG
GTTTTGTAGATGCTTTCCCTTGGTTGGTTGTATTCACCAAGAGCGCTGCGGTGGTGTACCATCGATCGGCTTT
GCAGCGGAGATGGCAACCCGCTGGCGTATTTTCGTACAAGGAGAAGTTGTGCACCACTGGAGGTTCGGGAGGA
TCGATCAATCCTCAGGATGCATTTCCCCATCTAATAACTGCCCTACGCTGTGTCGATCTATCGAATGATGAAC
AGAACGGTATGGTAAAGTCCGACTGGCATCCCGAATCGATTTTGGCCAGTATTTGCACAGAGGAGGACGGCGT
TGCCATCGCGTTGAGTAGCTACGTGCAGGGATTGTATAGCTGGCTTTCTCGTTGGATGGGCCTAGACGAGTCG
ATGAGGCCAACTTGGGACGGTCACGGTCCTTTGAGCAGTGCTCCGTTCCGGATTGTGAATGACAAGACTGTGC
TGATAGAAGATGATGGCGAAGACGGCAACGAACTAGGTGCAACATCTGCCAATTTGTTCACAACGGTACCTGA
AAAAGACACCGAGAAGCTCCCGTCAGAGGAGGAAGTCCTCCTGTCCAACTTACAAACAGCGCTCTTACCTACT
GTCGATACTGAATTAAATAAAGAGAACCAGCCTGACAAGGCAAATGGCAGTGCTAAACGGAGCAGAGAGTTTC
TACTGGCCATGTCGTCTGCGCGAACCAAAGCGAAGAAAGAAGCGGAGGACGCCAAAAACAAGAAGCCGTTGAG
TCCGTTGCCTCTCCCGCTGCAGAAATTGAACAATGACGAGCGACGCTGCCTCTGGGCCATTGGCGATATTTTG
TCCAACAAACCTCCTTCCTTTAAGAAGCTAGATTCGCTGTCCCAGCTATGTTTATTTAGCGTGACATTGATGC
GACGTTTATTGGATATCGAAACAACTGCAGGCTCGTCAAAAAAAGCCACTGATCTTGTCAATTCAATGCCCGA
TTACGAGGGCGGAAGGCCGGCTATGCCTACGAAACAGGCATCCACCAGAAATATGTTTGGACAGGAACAGTTG
TGCTTCGACCGCGTCGCCTCTGCTGCGCTCTTATCGGCCCTCATGAGTGACAGCCAGGCAAAACTACTTGATT
CTTGCCGCCTGTCCAAGGGCGAAAAGTTCAGCTGGGAATTGACACGTGCCATTGGCGCTCCCTACTGGGTGCG
TTCTGATAAACTTTTGGTCAAAATCGCTGAGGAGATTGCCCAGACCGTGTACAAATCTACGAAAGATGTGATG
GACTGCGCGCTGTTCTACGTTGCCATGCGCAACATGACGAAGCTTAAGGCCATCGCTGCCACAGACCGGTCGG
ACCAGGGCAAGAAGTTCCTAAAGTTTATCATGGATAACGACTTTACATCTAACCGAGGAAGGGTGACGGCGGA
GAAAAATGCTTATAGTTTACTGAGGAAACGAAAATATTCGTCGGCCGCGTGTTTCTTTCTGCTGGCAGAACCC
CCCATGATCAAGACGGCGTTGGACGTGATCAAGTCGCAGTTGCAGGATCCTTCTCTTGCGTTCTTTGTTGCAA
GACTGGTGGAGGGCGCGCCCAAAAAAATTATCTAGTGATGGAGGGAGTCTTACGATTGGGGGCGGTTTTAATC

TCCACAGTATGGGAGGAGGAGGCGGGTTTGCCGGAGGAGGAGGATTTGCTGGCTCGTCTGGAACTATTGGAGG
AGTTGACATAGAGAAGGATACAACTGTCAAGTTCAACTGCTGGAAGCCAAAATTGGGCAACAGCGCTCGTAGC
GTTCTCCTGCCAAAGTACTCCGAGGGGGATAAAGACATTTGCTTCGACTCCCTACAGCTCCTTTGGCTTAACA
GACCGAGGGAGGCGACTTTGCGCCTTTCTCATATGCCCACAAAAAGTCTTCGCGATGATACTGAGGACTGTGT
TGTGGAATCGGGGGATCTTTCCTTCCCTCCCCTCTTTGACGCTCCTCTTGGCGCACGTTTATCTAAAGCCGAA
TCACCTGCCGTGGAAAAATCCCGAGTACTTAGTAAGGCGAACGGAATCATCACTTTTTGCGCAGGTCCATTAT
TGTTGAAAAAAATGAAACCAAAGAAACGCATTTTGATGACTTCGGCATTGCTCGTCTCGAGGGCGCTCAGCAG
GTGCGGGATGGAGATTCCGTCGATGCGTATCTTGTTGCAGGTTGCCGATCCTGAATACGAAGAGGAGGAGGAG
GATAAACCCACAAAATCTAGCAGCCAACGGACAGAGATAAAATCATCCAACAACGCGCCATCATCAATTTTCG
ACTCCTATGATGCGGCGCCCTCGAAGCCCAAGCCTGCAGTCCCTAAACCATCACAGTCTGATGCAATGTCATC
ATCAATCTTTGACTCTTTTGATGCAGCCCCTTCAAAACCCAAGCCTGCACCTTCACAACAACCATCACAGTCT
GATGCAATG
>TR375|c0_g1_i1 len=2582 path=[2560:0-2581] [-1, 2560, -2]
TGCAACCTTAATGGTCCATGGCAACAGTTCTCAGATTGGCATGCCCTGGGAGGGGCAAAAGAACTGAAATAAA
ATAATGCTAAAAAGCTGATTTAAATTTTAACCAACATATTTTGAAGTGGACAAACTATTTCTTGCTCTCTGCC
TAGAAGGACACAATCTTGATACCAGAGTTCACAGTAATCCCTTCCAAGTCCACCCACATAAATACTAGACTAG
AGAAAGACAGCTGGAACGATTCAAGGTGTGTTCAAATCCTATGACGAGCAGTTGCAGTCTGTGCAAAAATTGT
TACTTGCCACTCTGCTAAAGATTGGTCGGACGTGTACCAATCTACGTTTGAACAGGGTGACACCCGTAATACT
TCGGTCTTCACTGACCAAGTGCCAAATATGAACCCCCCTCGTTGACTTGGGTATGCTAAAGCATCTTGAATGA
CCACTGTAATCAGCAAGCTTCAATACTCGGCAACAACTCAAGTTGCAAACGGATTATTAGTCTGCGGCATACC
ATGATGTTGCGATGGTGGGTGCCCCCCTAGATTGGCATAAAGAGGCTGTCCCGCTCCAGACGATGCATCAATA
CTAGGTTCGAATAAAAATTTCATAATGGACTCGGATGCATCCTCTGTCTCATGGTGCTCTGTCGTTGGCGGAG
GTATCAAGAGATCTAGGTCGTTGGATGACCCGAGTAGCTCATTGGGAGCCTGGATTTGGTTGTTATGGTTCAA
AAGGAAATCGAGTGTTGGATCAAGTCCTCCTCTATTCACAGTATGCTGCTGTAGGTTGACACCACCACTTTGA
TTGGGCAGATTCAAAAGGTGGGATTGATTCAACCTTGAAGACGAATTTGCGGCAATGAAATTATTGTTGAACG
ATGGTTGTGGAGCTTGGACAAAAAATGGATTCATCGTTTCAAACATGCCCGATGCCGGGTAGATGCTAGGCCC
TGCTTGTGGCATCATACCTGGAGGAGCAGGCTGGTTCTGTCCAAACGGTGTAACGCTCTCTTCTGCTTGGCGG
GAATGGACCGGGGCTGAGAATTCTGCCAAGGAGATCCCAGTGTTGCTTGGGCCATTGAAGGGGAGTGGTAGTT
GGGGACGTTGTGCTGGTGGATTTGGCATGGAAAAGCCAGGAGGCGGAGGCAATGGTGCTTTCGCCAGTGGCTT
TTGTTGTGGCTTAATTGCAAATGCATCGTCGATGAGAGCATCCACACCATTCGCAGCCGCCGCCGTGTTCAGA
TCTGTTGGCTCAAGATTCATTGGCGCCAAGATGGAATCAACAGGAAGAACATTTTGAGCCTTTGATACTTCCT
TGCTGCTCTTCACAGCAACATCCACCGGCAGATCGATCGGTTGCTGGTCGCCAACACCAGCCAGAAGAGCTGC
TGGAGTGAGCAGCGCCATGCCCATCCCATAATTTGCAGGATTTAGAGGAGCAGTGGAATCCTTCTCCACTGTC
CCCCCAGTCTTCTTGACCACATCCATATTGTTGTTATCAGTGACATTTGGTTGTTGATCACGTGGCGTTGACT
CCTCGTCAGACACAGTCTCTTCTTCATATTCACCATCGGAAACATGGTCTCCCGCCTTTTCTATGCCACTTGT
GACAAATTCCCTCATCTTCGTTTCTGGGTTCTTGGACAAGAAATTATGTCCACAATTGACTTTATTATCACTG
TTCGTACTGCTATCTGCTATTGAAAGAATGAGATTAATTTTCAAGCGTGTCTCCGCCTCCACCCCCTTCGCTC
CCCCCGAGTGTTTCTTTTCAGAAAGGGCCTGGATGACCTCCTCGGTGCTTGCATACTTCGTTGTTGTTTTTGT
GCCTTGTATGGGAGCATCATCGTTGGCAGTATCCAAAAAGGAGGCGAAAGGAATATATCCGTGGAATTCGGCA
AAGTCCTTCACATCTTCTATGACATGGCTTTTCTGCGCACCACGCTGTTTGGTCAGCAAGGTATCAATGCGGT
TCGACATGTTCGCTATGGACTCCCAGAATTCAACATGGGATTGTTTGCATAGCTCGTGGATTGAACTGCTTTC
CATTCCGTCGTTTGCAGATTCCCGGGCACTGGGGAAAAAGGGAAGACCATGAAACCAATCACACCCCTCATAA
AGGGAGGTGGCAAAGCGAAACCCGAGCAGTAGCGGAGAAAGAGATCGAATCACCCCTAACTTGTGCGCTTCCT
TCTTGGAAAGTACCGCATCCACATCCTTCGCCAGCGTGGCGCATACACGCAAGAGGAAAGAAAACGCCAACGC
TTGATTCGTCATCACAATTCCCTCGTTCCACTTCGGATCCTTATTCCTCCTTGCGTAAAATCCATCGGCAGTA
ATTAGCTTTCCGTGGTTGCTTGCCCCTAATGTCGTGAATGCCAAAATAGCCATGACTTTACAGAGCAGAGACT
CGCTGAACGACGCCTGACCGATCAAACTGCTGAGCGTATCGTGCATCCCCGCCATCTTCTTTTTCAGACCTGC
CAGATCGATGCGTCCGTCTGCGTACCCGTCGAGCGAGACTCCCCGAAAGAGCGCCCATTGGAGGTCCACGGTC
CGCGCCAACGCCTTGCGGTTCGCGGCG
>TR376|c0_g1_i1 len=888 path=[866:0-887] [-1, 866, -2]
GGAATGAAATGGAGACTTGACATAAATTATTACTGATTTGTGTCTCAAATTTATGTCACTACATAAATGTATT
TTGAGTTCGCTCCACTGTCACAGCACTACAAACATGACTTTTTTGTTTTTAGTAAGTCCTTACTTACTATCAT
ATTGTGCATCTTGCTAGAAGTAGGAACCACCCTAATCATGACTAATGTGAGGGTCAAAGTAGATTATGGTGCA
GTTATTGAACCATATTTCTGGGTGAAGAATAAAGCAAGCTCTCGTCTGGATAAACAGTTTAATGCCGACCCAA
GCAGAGTGTACTGACGGCATTCCTCGATTCGTCCCACAGAGACTCTTAGGCAATCCATTCAACTCCTGCTAAT
ACCATGGGTGAGAAAACATGGGCGAAATCATTGTGGTCATTGTCTTCGCTCCAGCCTCGACGATTGGCTTGGT
CCTATCAGCATTGCAGATCTCCCAGTTCTTCATGCGTTTGCGCAATCGAATGAGAACCCATGCATGGTCGCTT
GCCGTGTGGCTGTGTACAACATCCCCTGTAATGTATCCTCGCTCATGGTCTTATTTCAAGCTCTAGAGCACAC
CATACTGGAAAGTAGATGGAATGTCGTCGCCATCCGATGCCAGTATATTCTCACAACAATCAAGCATATCACC
AATGGAAAGGGAATCACGTGTCCGTGATATTTCATTCGAACCGCGTTCTTCCGAGATCGAGCTGTAAGGCAAC

TTCTGGATGCTCGGACAAATCATATCCATTGGCTTGTCCAGCATGTGGTCAATAACGGGAGTACTACCCCATG
ATGAACTATTCGAGCATTGGTCCCCCGAGCTCGTACTGCAGAAAGACGACGACGACGATGATGATGATGACTC
GAGGAGGCTAGG
>TR378|c0_g1_i1 len=224 path=[202:0-223] [-1, 202, -2]
CCATGCCGTGGTGATGGTATCAATAATTCTCTTCGCACAAGCAAATCAATATGGCCCCGTTGAACGTGAGATG
CTAGCTGAGTTCCCATGGAGACATACTCAGGTCCAAATGTGGTCTCGCCCCAGGATTCATGGGGAGATTCTTG
TAGCGCGATGATGTCGGGATGATGATGATGCGATCGGAGGCATTCCTCGCGAATGAGACGGGGTGCTTCCTGT
GCCCG
>TR379|c0_g1_i1 len=367 path=[345:0-366] [-1, 345, -2]
CTAGCACCCCAGTCGATACTTCCTCTGCAAGCCTCCCGACGAAATTCTTCACAAACACACCCGTCGCGCACGC
ATCCGCAAACAGCTCTCCAATGGGCAAGTGCACTACCAAATGCCGACTAAATTTCTTGTCCGTGGATGAGTCG
AGATCGACAATGCAAGATCTATCAATCTGAATGGCATGTATAATTCGAATCTCGCTACAAAGCTCCTGGATAA
ACTCTGTCATCAAAAATTCGCTATCCTCCGAAGTAATGTGGGGATTGGCCCGCTTGCAAAATTCCAAGTCGAA
ATACAATCGACAGGGCGCACCTTCGCGTATGAGCTCATAATAATGACGTCCGTGAGGATCGCAGATCGGAAGA
GC
>TR380|c0_g1_i1 len=2251 path=[2229:0-2250] [-1, 2229, -2]
ACCATGGCCTCCACAATGGCGGCAAGGAATGCAATCAAGCGTTGTTTCCCTCCGAAACAAATAACCACAAACT
CACGCCATTTCTCCTCCTCAAGGGCCTCAGCAGTGCCCGCCAGCAGCATCCTCTTTGATCACGATTCACGAAA
GCAAACATGGGCAACGGCACTGTTGACGGCCATCGCCGTGACATCCTCGGTAGCCTTCACCGCTGGAACAACT
ACAGCACTCGCAGAGGAAGACCATGCCGACGATGACAATAATGAAGAAGAAGAAGAATCGACCAAGATTCTCA
ATTGGTCGGGAACGCATTCCGTCGAACTCGCACCTGGAAAGTACCACGAACCCGAAACTATGGACGAATTAAT
ACAACTCGTCTCCAAATCGTACCACGAGGGAACGCACATTCGTCCCGTGGGCTCGGCCCTATCTCCCAACGGA
TTATCATTCGACTCCCGCGGCATGGTATGCCTCTCCAACCTCGACAAAATTATCGAAATTAACAAGGAGAACA
TGACCGTGACCGTGGAGGCGGGAGCACGCGTGTCTCACGTGTTGGATGCGCTGCGCCCCCATGGATTGACATT
GCCCAATCTCGCCTCGATTGCAGAGCAGCAGATTGGAGGGTTCGTTAGTGTGGGAGCGCATGGAACGGGAGCA
GCTATTCCTCCGTGCGATGAGTTTGTCGAGGCGTTGAAAATCGTGACTCCGTCGGAACATGGGGTTGTGAAGA
TGACAGAAGAGAGTCACGGTCCGATGTTTAAGTTGGCGAGGCTTGGTTTGGGAGGTTTGGGGATATTGAGCGA
GGTTACGCTGAAGGTGATTCCCGCTCATCGGTTGGTGGAACAGACGATCGTGTTAACTAGGTCAGAGGCTAAA
GAGCAATTGCCCACGTTGTTGAAACGTCACAAGCATGTGAGATACATGTGGATTCCGTATGAAGACGCGGTGG
TGGTTGTCTCCAACGATCCCGAAAATGATCTTCCGCTCATGGGACCTGGGACGGAAGTGGACGATGGAAATGG
AGGGAAGAAAAAGATCGTGACAGTGGAGGATCTCGAACCAAAATATTCCAAAGACGAAAAGCTTGCGCCATTG
CGGGAATTTCTTCGCAAGTTACTGAAGAGCAAAGGGACACCCATCGACGAAGAGACTATCAGCGGAATGGGAT
TTGGCGATTTGCGCGATTTCATCCTTGCATCGGGAAACATGCTCGATCCCGACCATATCAAACGTTGCAACAA
GGTCGAACACGAATTCTGGGCGAAAGCACAGGGAATAGAGATAGCGCCGTCGGATCAAAAGCTGCAGTTCGAT
TGCGGCGGACAACAATGGGTATACGAAGTGTGCTTTCCGGTGGGAACGTATGGAATTCCCAATTCCAACTCGA
TGGATTTCGTGGAAGAGCTTTTGAAGGAAATTGAATCGTCTGGCATTGCCGCCCCCTCTCCCATCGAGCAGCG
ATGGTCGAGCGCGTCAACGTCGCCGCTCAGTCCCGCGTCGGTCGGACCCAGAGAATACGAAACGTACGAGAGC
CAGAACGCCCTTTTCTCCTGGGTCGGCGTCATCATGTACCTTCCATCGGAAGATTTCGACCCTACGGGATTTC
GACGGGAGTTCATCACTCAATCATTCAAGGAGGAGTACTGCAAATTAGTGCGCAAGGTAGGAGAGAAATATGG
CCTCATGTGTCACTGGGCCAAATTGGAAGTATTGGAGGATCCAAGTGATCCCAGTCGCGGCGTGGCAGAGAGT
GTACGCAATCGCTTTGGACCGGAGGTGGTTGGTGCATTCAATGCTGCTAGAGATTTGTATGACCCTAGACGAT
TGTTATCTTGCTCGCTACTTGACCAAATCTTTGGGCCTCCTTCGCCCGAGAAATGATAAAAATAGAGCCTTTT
GTGGGGTATTGTACTTATGTGTGGAGCTCAGTTTTAACACCCCGACCGCTTTTACTTGACAAGTTGGAGAACG
GAAACCAACGGACAGTTCATACACACAAATCTGACGTTAGATTGATGTGAAGTATTAGCATGATCAGAGAGCT
GGGCACCAGCGTCTCTTCATGCCCACAATTGTGTTCCCTTTCAAAATGAGGCGGTTGAAGGACACAGGGACTT
AAGGTATCACCAACAGATTTTGATGGTACAGCTACTGTATTAAAGTAATAATTCCAAACTATGATGCGATCTT
GGCTAAGGTAACGAATAAGGACTGGTATCTTAGACCGCTATTTGCAAATAAATGCATAAAA
>TR382|c0_g1_i1 len=423 path=[401:0-422] [-1, 401, -2]
TCCGATGGTGGCGAGTACGGTGGAGGCGTGTCCCATTCCGAATTGGTAGTACATGCATCCCGTGACCACCCCC
ACTTCGAGGATGGTGGGCACGGTGTTGAACACCATGGCATTTAACACGAATGAAATACTGCGATTTCCTCGTT
CGATGATTCGGCCCAGAGCGCCGGTGTTTCGATTCAGGTGGAATTGTAAATCGAGGGCGTGGACGTGGTCAAA
GGTGGATCGTCCCACGGAGCGGATGGCGGATTGGGCGACATTCGCAAACACTGCATTTCGAGATTCTTGGAAG
AGGGAGGAGAGGGTGCGTGATGCTCCGTAGGAGAGGAGGAGAACGAATGGCAATGCCGGTACTCCTGCCAATG
TCGATGTTGTGAGAGAGTCGACTAGGATTTCTGGTACAAATGTTGGTAGGTTGGAAGT
>TR383|c0_g1_i1 len=426 path=[426:0-292 427:293-425] [-1, 426, 427, -2]
AGCGATTCCCCTACAGTCGATACTTCGGCACCGCCAAGTTCGAGCGAAACCCCATCTCAAACAAACAAGGATG
GTGCTGCTAATGTCAACCTTGAGTCCCCTACTAGTGATCCCAAGAATGAGGACTCGGTTACAAGACGCTTCTT
TATTGGAATGTCCTCCGTTTTGGCTTTGTCTCTTACAGCCGCATGTATCGTGTATGCTTATCACCGACCTCGT
GATGATTCAGAATCTGTACCTGATAAATACTGCTCGAAGGATGCGAATCATTTTGGCATTGCAGCTCATGAGC

CGTGGGGGAGATCAATTTCATCCCTACCCATGTCTTACATGGGCCTGAATTGCTCGCTGAGCTCGACCTCTTC
ATCTTTCAGAGACTATCGATTCGTCAGCGTTGTTGGGCCAGATGGTACGTCGATAAGATCG
>TR384|c0_g1_i1 len=3515 path=[3515:0-1670 3516:1671-1686 3525:1687-1877 3522:1878-1893 3524:1894-2438 3513:2439-2439 3514:2440-3514] [-1, 3515, 3516, 3525, 3522, 3524, 3513, 3514, -2]
ATACAGAGTGGGCTATTATTGGGATTACCCACAAAAATGTGAGAAAGGATGTGACACACCTCAGAAAGGATAT
GACACACCAACCACGGTCTGACATTCACCAGAAAGAATGTGTGGATCGCAGGCAACCTGGTCTAGGTAAATCA
TATGCCCAAGTAGTTAGTGAGTACTCCTAGATATGAGCTAAACACTCACAATTAACAGATTATTTAGGCACCT
GCTCAACTCTAGTGCACGTCTGAATAATCACCCTCAATTGACCGTAGGTTGAGGTTATGCTCACCATTAGTCG
TACAAGGGGGCTATATCACAATACACACCCTAGGTGTTTCTCACTGGTTCGCCGCCACAGTAGAGTGTTGCAG
TGACGCATCCTGGAAGAGTGATGGCCCAGAGGGTGCACGAGGCATCAATTTTTGAGTCGAAAATGCAATCACA
ATGATTGCCTTGGCTAAGTTTTTGGAACCCACATGAGGAACGTCGCTATGGAGAATAATTGGCACCATGATCT
CGCTAAGATGTATCTGGTTTGTTTTTTGTATTGATTTCCAAGTGAAAAGATGCTTCTCTTCTCCTCTTCTTTG
CTTGGACAAATCAGATGAAAAGATGAAAAATAGTATTACTCGAGATGAAAATGTTTATCGTATCAAGTCGGTA
AAAAAGGAAATAAATCAACAAAGGCATAATTCGTATCTTAGCAGAGATCTGGTGCTGTTGTAATAGTGGAATG
TGAAGACTCCGTTTCTTCGGCACCCAATGTCGGGACTTTCCTGTATTGGACATGTTCCGCCGACACATTATCT
GCCACATGAAATTGATTTCCTTTCTCAACGATCAGAATGGTATAGAAATATGAAAATTTGTTATCAGAAGAGT
GTGAGGCCTGGTCTTTCTCTTCATTGGCAATTCCCTTCCATTTTTCGACTAATGCATGCGCTTGTTTACCCAC
AATATCATTGCTGTGTGATTCGAGCGTTACAATATATTTCCCAATGAAAGTCTCAGATAGGATTTGATGGGAG
ACGGAAACAGAGGCAAATTGGTCTAATATATCAATCAGTTTCTCGAAATCATTTGAGATATTGGATAGACGCG
CTTGTTCCATGAGCTGCATAACAACATTGATGTTGATATCATGTTGTGATTTAGCCATACACATCAATATTTC
ACCTTGAAGGAGATGGGTGTTTGTTCCCATGGTGCTGGATGAAGATTCAGATACGCTCACCGGGCATCTTGGT
GCATAATGAAGTAGATATTCTGGAATCATTTCACTTTGCTCAGTACCCTGCCTGGTGGTCAGACTAACTTTGT
ACATGATATCTCGAGACGACAGATCTATTAAGACAGCTGATACCACTCCTGTGCGGAATGATTGTTTCGGATT
ATCACCGTCATTTGAGTTGTCGACTTCGTCAAATTGGAACCAAACCGAGCAGCCTACTGGATAAAGCAAGTCG
AGCTCTTCAACTTGATTGATGGTAAGCTCCACTATTTCAATTTTCTTTTTTCTCTTGGTATGCATGGCATTTG
CATTCATTGCTGAGAGCAGCGGCTGATCGTGTGACTTTGGGCTGGGGTTTTCGCATGATACTCTTTCAGAAGG
TAACGGGGCGAATGTGCCTTTGGAAAATTTATTTGGTTTTCTCCTCGAAATGTTCAGTTCATTGCCCACTTGA
GACGCTTCTTTGTCGGGGGTTTGAGCTACAATAGCCTTCCCAGTATCAAGAGAATTCACCCCCGGAGTTTCGT
CGCATAATTGATTTAAAAGACGAGCTTTATCCGATAATGCATTGCCTAATGAAACAGTTGATAGCACCGGCTC
GCCGTCTTCGGTGGATTTGTTTCGTTTTCCCCTCGAAATGTTCAGTTCATTGCCCACTTGAGACGCTTCCTCG
TCGGGGTTTCGAGCAGTGATCACCTTGCCGGCATCAAAAGCATTTGATTCCAGACTGTCGTATCTTGATTGGT
TTTGAAGACTATCTTTATTTAACAATGCATTGCCAAATGAAACAGTTAACACTGGCTCTTCAACAGGCACAAT
AGATTCAGGTTTCTTCAAACGTTTGGAATGATCAACTGTAACGTTTGCTGATGACAGTGGGGCCTGATTTTCT
AACTGTGGACGTGGGTTCTTGAATGTTAGCGTATTAGGGGGCGAAGAGAGGCTCGATTTCTGAGTAGATGCAT
TTTTGATGTACTCATTTGGTTTTTCCTTGGCGCGAAGTTTATTGTGATCCTGAGACGACGTGTCACCGTTTTT
TTGAGCTGGAGCCGCCTTGCCAGGATCCGAAGCACTCGTTCCAATATTTTCATCTCTGGATTTGTTTCGAAGA
TGATTTTTACTGATATTGTGAGCTTCATTCGAATCTTTGGAACAGAGGCTCTGATCTGATAATTCATTACCCA
CTGAAACAGTCGATGACATCGGGGTATCAAACGCAATACCTTGAGGTTTCAAATCCTGAAAGATTTCGTCAAT
GCCTTTTTTGTATAACTCAATATACTCTTGTCCTGTCTCTGGTGGCAGATGTGACGCATACTTCTTCGCCATG
GCAGCATTAAGCATAGCGATTTCAGGACAAATTTCTGGAGGAATAGGAGCATCTTTTTCGAGGGCATGGACAG
GATGGCGCCGTGACGTCTTGTCCTTGGAGGTCTTCCATAACTGTCCGCTGACATGGGAGAGGGGGCAGCCGTT
GGTGCCGTTGATGGACTGTTCGCTTGGGCGTTCCTTTACGGAATAGTTGCACTCTGGCGTTCCGGAGAAAGTT
CCGACAGTTTCTTCGTTTGCAGTACCAATTTTCTGGCGTATTCTCTCCTTTACATAGGATTTTATTGCTGCCG
AGGTCAACGTAGCGTCCATACCAAAACCTTTTCTTTCCATCTCGAGCTTCTTTGCTTTCTCATAATCTTGCAA
TGTGAAAAAATTCTTCTCTTTTTTTGATTTTAGTTCCTCCAAGGCCACGTCATGTGACAGTGCTGCATCCGTC
TCAAGAGAGTATAAACCAAGATGGATCCGTACCCCATCGTGCCTAATATTGGACGTATACTTTTGGTGTGGTT
TCGAGTATGAAAAACCAACATAATCGGAGTGTCTTTTCCTCTTGAGGTTAGTCTGAGGCAAATCCAAGCCAAT
TTTGCCAAGTTTAGAAGTCGTTCGGGGATTGCAATCATCCTGGGACGACGCAACGCTAGCGCGTAGAGATCTC
TCCTGCTTTCCTTTTTTCTCGGTTAGTTGTTCTCCATCCTGGCTGCAGCTTGAAGAAGCTATAAACTTCGAAA
TGATGTGACTTACGGTCGATGTTATATACAGTCGAACAGCGTCAAAGTCATCATCTAATCTCGTTTTACCAGT
TTTACTCAACTCCGCAGCCCTCTCATTCTTGTGATCTTGATCCGTGGCAAAGTTGGGCTTAACATTCGAAATC
TTCAATTGTCTCAATGCTTCATCGTGGGCAAGAGCTGCGTCCGAGGCTAGCATATAATATCCTAAATGGCAGG
CCTTTCCTCCG
>TR385|c0_g2_i1 len=463 path=[491:0-368 492:369-386 493:387-406 494:407-462] [-1, 491, 492, 493, 494, -2]
CAAAAGTCTCAATCGTAGCCCCTTCTGGGCTTGGACTTCCATCGGTGTGTCTCGGTGTCAACGTTCCCACATC
AACGGCCACGATCCTAATCTCCCCGACTAAAATCCAATCGGCCATAAATTCGTAGTGCAAACGCTCACACAAC
AACATCACCAAAAGAATCAATATTTTGTTCTGCCGCGCGGCAAATCCAAGAATTGAATTGTAAGAGCATCGGC
TCTTTATGACCAATCCTATCCACCCAACGTCCCAAAGCAGAACGCAACAGCAACCAACAATTTTTAAGAGTCG

GCTGTGGCCTGCAGGGATATCGTATCAGTGTCAAAATAGCGTTCGCATGTCAGCAGAAAAAGTACAAGTAGAA
AAAAATAATTATGGATGCTAAATCAGCTGGATCTCGGTCCCCTTCCCCGTCGGCGGCAGCGTCCCGAGAGCCT
ACCGCTACCCGCCGAGAGATCGGAA
>TR386|c0_g1_i1 len=1800 path=[1873:0-88 1874:89-112 1891:113-756
1892:757-780 1893:781-781 1887:782-800 1889:801-1421 1882:1422-1445
1883:1446-1799] [-1, 1873, 1874, 1891, 1892, 1893, 1887, 1889, 1882,
1883, -2]
CTTCCGATCTTTTTTTTACCGATGCAGTGTCTTTATTTTATCCCTACCATTCTACATTGTGCGTTCGTTTTTA
ACTATTCACAGTATTTGTACACATTATCTCACAAATGAAATATACACTGCATCTTGGAGCTAAACAGAGGATG
GCAAGGGCTGCGAATAAACCAAATTGCCACCATAAACCAAACAACTAGAGTATGCGATGCTAGACGAGACCAA
AGCAGAACAAAACATATGCCCCCGATATATCCTTCTAAGACATACAGTATCATAACAGGAAACAATATGCACT
AAAGTTGAACCTCAGTCAGACGGCGAGCTTTCATTGTCAAGACCGGGCAGAAGATTGTCTAACCCTGCAAAGC
CCATGGCGGCAATGATGCATCTGACTGGCATGATTCGCCTTCAGCCTCTTGGACATACCCATCAAATCCTCCA
CGATGAGACAGTCCCTTCGGTGGGCTTCTCCTACACATGGGACACCCAGAGATTACACAAAAATCACTCGAGT
TCTCTCCCGATACCCATTTATCAATGCAACCGCAATGAAACCGGTGTTGGCATGGCAAAACACGCAAGCATTC
TCCATCCTTAAACGGTTCTTGGCAAATGACGCAATGCTCATAGGTATCGTCATTCATAGTGCATTCCATGTTC
ACTGAAGAGTTGGAGGAAGACCTGCAAATTGATATGGAATCGGGAACTTCTCCAGCCTGAATTACGAAGTCTG
GCATTCGTGTCGTATCTTCTGTGGGATCTTTTGGCACCATCATCCAGGAAAACTCAGGAGGCATAGAATCATT
TGGATTTCCCCCCTCGCTCTCGATGGGCTCATTGTTCTCTTCTGTGTTGTCAAAGTAATCTTCAATGATGTGA
GCATTGCGCAGCAAGAAGTCATCGACATTTTCTTCTTCTTCTTCGTGGCTGGAGCATATCAACGCACTTTCGG
CACGGTAGATAATTGTAGGCAACTGGTGGATGCTTTCGACATTCAAACGTCGAGAAGCTGTCCGAGCCATTCG
AGAAATATCGACTTGGGTGATCACGTAATCGAGTCGCTGAGTGCTTTCTTCCGTGTCGAAATCAGCGTTTTGG
AACACGAGACCACCTTGGTTGTCTTGCGTATTCTGTTCGTAAGAAGACGAGTCTGACATGTCCGAAGTCACCG
GTGGGGGTTCGTCGTCTCGGTCAGGTGTATCAGGAGAATACTTATCTTCAAGAACATTGGCAGGCTGTAATGC
ATTCAATGCTGAATAGAAATAAGGAGATATCGTCGAAGACAAGATGGAATTCGTTAGAGCCTCGACTTCAGCA
AGCGCCGGAGCATCCAATCCTTCAATTATTCCTAGCATATTTTCATCCCAAACAAAGTCCTGAATACCAAAAT
CAAAATCCTGAAGCATTCCCCATACGAGATTTGCTACTTGGGGTATTCCGGATGCTGCTCTTTTCAATAAGTA
TAGAGTTGCCGAATTAACGTGGACATGCGACAAATCAGAAGCATCGCTTCCGTTTTTCAAGGGACATGAATTA
ATTAATTTGCTTTTTTTGTTGTTTATCGAGGCGGACGTGCATTTTGATGCGGTGTCGATCGACGCCATTTTTT
ACTACGAAGACGTAGAGCTATGCTTATTTGGTGCTTCAGTGCTTGGGGATCTCGTGGTGCTTTTTACTGCTGC
AATTGGCCAGATAATAAGGCCTCGAGAGGAGCAGGTCTCAGTCATTAGCAGTCAAATTTAAGTTGGTTCGTCG
AATGGCAATTGTGATAGCAAGAATAAAAATGACAATGAGTTGATAGTA
>TR387|c0_g1_i1 len=1850 path=[2063:0-161 2064:162-185 2102:186-304
2097:305-326 2101:327-385 2098:386-980 2081:981-1004 2095:1005-1640
2055:1641-1655 2056:1656-1849] [-1, 2063, 2064, 2102, 2097, 2101, 2098,
2081, 2095, 2055, 2056, -2]
TTGCGCGTCAGCCCCTTGTTATTTGTAGTATGTACATCTAACAACTGCTCCTATCGCCTCCCCAATTTTAAGC
AACAATATTCCCTCACCTCAATCAACTCTTCACCCGCATACACAAGAACATGTTTGTCCGTTCCATTCTCTCT
CTCGCTGTAGCGGTAGTCTTCTTCGTCTCCACCGCAAATGCGGTATCTTCCATCGACATTGGACCCCGCGCGG
GATATGGATATACCATGCTCCCTGGTTCCTCCGTCCAGACACCAGACGACGGTTGGCAAGTGTCGGGATCCGG
ATCTGACATCTGGATTGATAATTGGCATTTGCGTCCTTTCTTATTCTCAATCATACGTATTATCATAAAAAGG
GACACTGGGATGAATTCCACTATCTGCACTTTAACCGCACCACCGACGTCACCGTAACCTGCCTCGTCAAATC
CTTCGGACCCACCACCCTCGAAAGCTGGCGCAAGGGTGGCATCATGATCCGCAACAAGACACCCGGCATCGAC
GACCAGCGCATGGCGATGAGTATGCTCGAAGTCACCGGTTGGGGCGTCGCACATCAATCCCGTCTAGAGGACA
ATAGCCATTCCGTGAGCAATCACGATCGCTTCGATACCGAGAATGTATGGCTCCGTATGGTAAAGGTGGGGAA
TACGGTAACTAGTTACGTGAAGCGGGATGGCGAATTCGACTACATGCGTTTTTGGGAGGAGGAGGTTCAGTTC
GGACCGGAGTTTCATGTGGGACTTGCGGTGTCCTCTCATGAGAGGACTCTTCTTGCTACGTTGGATGTGAAGC
ATTTTAAAATGTCGGATGAGATTTACACTCAGCCTGGCATTCCTACGGAAATTGGAGAGACTGGGCAGCGTGT
TTCGGTGCAGCAGGTGAAGGAGGGTATCTGGTCTGTCAAGGCTGGGGGGGCGGATATTGGGGGAACCGCAGAC
AGCTTCGGATTTTTCGGCTCCGAACACACCGGTAACACCAACGCGACCCTGCACCTCGAATCTCTCCTCAGGA
ACAACAACGACAGCAAGGGCGGACTAATGATGCGCGCAAGCACCGATCCCGATGCCGCTCACGTCAGTCTCGT
CGTGAAGGCGGGAGACGGCGTCACCATGTACTACCGCGCACAGGCCGGCGGGGAGACTACCACCATCAAGAAT
GTTGGTGTTTGGCGCGATAATATCGAGTTGAGCCTTGTGAAGAATGGTGTTTCGGTGGAGGCCTCTTACAAGC
ATAAGGCTGCTCCGACTTGGTATGTGCTTGGTACTGCGAATGTGCCACTTGGTGCGACCTACCTTATTGGGCA
TGCTGTGACGTCTGCTGACACCACTCAGCCTGGTCAGTTGATGGCTGGCGAGGTGGCTGTTATGACGATTTAA
TTTGCATCTGTTTTATGGGGAACTGCATATGATTAGGGTTGACAATTGCTTTCCATCCAATGGACAGAAAAGG
TGATATGTATTGATATTGTTAAGGGGATTTTTCGCAGTGATGACCACGAGCATGTTTATGAGTAAAAAGTGAA
TTTACGGGCAGTCACATTCAGCAACATAAACGCACACGCTCTCTCATATGGAAATCACGGGTGATTTATCCCA
CTTGTGATAAATAGTTAGGGATGAGACAAAAAAAAGTAAAGTTTGTACAATTTTGACACTTGACAATAACTAA
GAGTTGCCATCTTGCCACGATAGCAACTGATCGTCTCCACACGTTGAACAATCATCCAATAGCTTCACTAAGA

AAGGGATAAATAAATCTCGATTCAATTGGACAGCCATGCACGACATCCCTGTTGCAACTATGCCCCCTCCCCA
AACAAAAAACCACGTTTCCGTAGCC
>TR387|c0_g1_i2 len=1791 path=[2063:0-161 2064:162-185 2102:186-304
2103:305-305 2071:306-326 2098:327-921 2081:922-945 2095:946-1581
2055:1582-1596 2056:1597-1790] [-1, 2063, 2064, 2102, 2103, 2071, 2098,
2081, 2095, 2055, 2056, -2]
TTGCGCGTCAGCCCCTTGTTATTTGTAGTATGTACATCTAACAACTGCTCCTATCGCCTCCCCAATTTTAAGC
AACAATATTCCCTCACCTCAATCAACTCTTCACCCGCATACACAAGAACATGTTTGTCCGTTCCATTCTCTCT
CTCGCTGTAGCGGTAGTCTTCTTCGTCTCCACCGCAAATGCGGTATCTTCCATCGACATTGGACCCCGCGCGG
GATATGGATATACCATGCTCCCTGGTTCCTCCGTCCAGACACCAGACGACGGTTGGCAAGTGTCGGGATCCGG
ATCTGACATCTGGGGACACTGGGATGAATTCCACTATCTGCACTTTAACCGCACCACCGACGTCACCGTAACC
TGCCTCGTCAAATCCTTCGGACCCACCACCCTCGAAAGCTGGCGCAAGGGTGGCATCATGATCCGCAACAAGA
CACCCGGCATCGACGACCAGCGCATGGCGATGAGTATGCTCGAAGTCACCGGTTGGGGCGTCGCACATCAATC
CCGTCTAGAGGACAATAGCCATTCCGTGAGCAATCACGATCGCTTCGATACCGAGAATGTATGGCTCCGTATG
GTAAAGGTGGGAATACGGTAACTAGTTACGTGAAGCGGGATGGCGAATTCGACTACATGCGTTTTTGGGAGG
AGGAGGTTCAGTTCGGACCGGAGTTTCATGTGGGACTTGCGGTGTCCTCTCATGAGAGGACTCTTCTTGCTAC
GTTGGATGTGAAGCATTTTAAAATGTCGGATGAGATTTACACTCAGCCTGGCATTCCTACGGAAATTGGAGAG
ACTGGGCAGCGTGTTTCGGTGCAGCAGGTGAAGGAGGGTATCTGGTCTGTCAAGGCTGGGGGGGCGGATATTG
GGGGAACCGCAGACAGCTTCGGATTTTTCGGCTCCGAACACACCGGTAACACCAACGCGACCCTGCACCTCGA
ATCTCTCCTCAGGAACAACAACGACAGCAAGGGCGGACTAATGATGCGCGCAAGCACCGATCCCGATGCCGCT
CACGTCAGTCTCGTCGTGAAGGCGGGAGACGGCGTCACCATGTACTACCGCGCACAGGCCGGCGGGGAGACTA
CCACCATCAAGAATGTTGGTGTTTGGCGCGATAATATCGAGTTGAGCCTTGTGAAGAATGGTGTTTCGGTGGA
GGCCTCTTACAAGCATAAGGCTGCTCCGACTTGGTATGTGCTTGGTACTGCGAATGTGCCACTTGGTGCGACC
TACCTTATTGGGCATGCTGTGACGTCTGCTGACACCACTCAGCCTGGTCAGTTGATGGCTGGCGAGGTGGCTG
TTATGACGATTTAATTTGCATCTGTTTTATGGGGAACTGCATATGATTAGGGTTGACAATTGCTTTCCATCCA
ATGGACAGAAAAGGTGATATGTATTGATATTGTTAAGGGGATTTTTCGCAGTGATGACCACGAGCATGTTTAT
GAGTAAAAAGTGAATTTACGGGCAGTCACATTCAGCAACATAAACGCACACGCTCTCTCATATGGAAATCACG
GGTGATTTATCCCACTTGTGATAAATAGTTAGGGATGAGACAAAAAAAAGTAAAGTTTGTACAATTTTGACAC
TTGACAATAACTAAGAGTTGCCATCTTGCCACGATAGCAACTGATCGTCTCCACACGTTGAACAATCATCCAA
TAGCTTCACTAAGAAAGGGATAAATAAATCTCGATTCAATTGGACAGCCATGCACGACATCCCTGTTGCAACT
ATGCCCCCTCCCCAAACAAAAAACCACGTTTCCGTAGCC
>TR387|c0_g1_i3 len=1927 path=[2063:0-161 2064:162-185 2102:186-304
2103:305-305 2099:306-370 2074:371-387 2096:388-403 2101:404-462
2098:463-1057 2081:1058-1081 2095:1082-1717 2055:1718-1732 2056:1733-
1926] [-1, 2063, 2064, 2102, 2103, 2099, 2074, 2096, 2101, 2098, 2081,
2095, 2055, 2056, -2]
TTGCGCGTCAGCCCCTTGTTATTTGTAGTATGTACATCTAACAACTGCTCCTATCGCCTCCCCAATTTTAAGC
AACAATATTCCCTCACCTCAATCAACTCTTCACCCGCATACACAAGAACATGTTTGTCCGTTCCATTCTCTCT
CTCGCTGTAGCGGTAGTCTTCTTCGTCTCCACCGCAAATGCGGTATCTTCCATCGACATTGGACCCCGCGCGG
GATATGGATATACCATGCTCCCTGGTTCCTCCGTCCAGACACCAGACGACGGTTGGCAAGTGTCGGGATCCGG
ATCTGACATCTGGGTGAGCGTTATCAAACTGTAAATATCTTCAATCTTCGTGTCTAGTTATTGACAATCTGCC
TTTTTTGTCTCACTCAGATTGATAATTGGCATTTGCGTCCTTTCTTATTCTCAATCATACGTATTATCATAAA
AAGGGACACTGGGATGAATTCCACTATCTGCACTTTAACCGCACCACCGACGTCACCGTAACCTGCCTCGTCA
AATCCTTCGGACCCACCACCCTCGAAAGCTGGCGCAAGGGTGGCATCATGATCCGCAACAAGACACCCGGCAT
CGACGACCAGCGCATGGCGATGAGTATGCTCGAAGTCACCGGTTGGGGCGTCGCACATCAATCCCGTCTAGAG
GACAATAGCCATTCCGTGAGCAATCACGATCGCTTCGATACCGAGAATGTATGGCTCCGTATGGTAAAGGTGG
GGAATACGGTAACTAGTTACGTGAAGCGGGATGGCGAATTCGACTACATGCGTTTTTGGGAGGAGGAGGTTCA
GTTCGGACCGGAGTTTCATGTGGGACTTGCGGTGTCCTCTCATGAGAGGACTCTTCTTGCTACGTTGGATGTG
AAGCATTTTAAAATGTCGGATGAGATTTACACTCAGCCTGGCATTCCTACGGAAATTGGAGAGACTGGGCAGC
GTGTTTCGGTGCAGCAGGTGAAGGAGGGTATCTGGTCTGTCAAGGCTGGGGGGGCGGATATTGGGGGAACCGC
AGACAGCTTCGGATTTTTCGGCTCCGAACACACCGGTAACACCAACGCGACCCTGCACCTCGAATCTCTCCTC
AGGAACAACAACGACAGCAAGGGCGGACTAATGATGCGCGCAAGCACCGATCCCGATGCCGCTCACGTCAGTC
TCGTCGTGAAGGCGGGAGACGGCGTCACCATGTACTACCGCGCACAGGCCGGCGGGGAGACTACCACCATCAA
GAATGTTGGTGTTTGGCGCGATAATATCGAGTTGAGCCTTGTGAAGAATGGTGTTTCGGTGGAGGCCTCTTAC
AAGCATAAGGCTGCTCCGACTTGGTATGTGCTTGGTACTGCGAATGTGCCACTTGGTGCGACCTACCTTATTG
GGCATGCTGTGACGTCTGCTGACACCACTCAGCCTGGTCAGTTGATGGCTGGCGAGGTGGCTGTTATGACGAT
TTAATTTGCATCTGTTTTATGGGGAACTGCATATGATTAGGGTTGACAATTGCTTTCCATCCAATGGACAGAA
AAGGTGATATGTATTGATATTGTTAAGGGGATTTTTCGCAGTGATGACCACGAGCATGTTTATGAGTAAAAAG
TGAATTTACGGGCAGTCACATTCAGCAACATAAACGCACACGCTCTCTCATATGGAAATCACGGGTGATTTAT
CCCACTTGTGATAAATAGTTAGGGATGAGACAAAAAAAAGTAAAGTTTGTACAATTTTGACACTTGACAATAA

CTAAGAGTTGCCATCTTGCCACGATAGCAACTGATCGTCTCCACACGTTGAACAATCATCCAATAGCTTCACT
AAGAAAGGGATAAATAAATCTCGATTCAATTGGACAGCCATGCACGACATCCCTGTTGCAACTATGCCCCCTC
CCCAAACAAAAAACCACGTTTCCGTAGCC
>TR388|c0_g1_i1 len=1803 path=[1966:0-559 1967:560-583 1975:584-712
1952:713-736 1974:737-1536 1976:1537-1560 1977:1561-1568 1957:1569-1593
@1973@!:1594-1703 1970:1704-1802] [-1, 1966, 1967, 1975, 1952, 1974,
1976, 1977, 1957, 1973, 1970, -2]
CCACCATGAGTAAGATTAACAGCCAAAATTATCTCAGGATATTTGGGTCAGCTTTTTCTGTCTTCGCTTCTAA
GAATACTTATCTCTACCCCCCAACCCACACAAAAGCAAGGCACACATTCAACCAAATTTGATTAACATATTAT
CTTTCTTTATAGCCAAGCCTGGTACGCTGATCTACTCCAATCATACATCAGAAACCCTAACTACCTAGTGGTA
AAGATCAGATACCTGGCTCGTCATCTTTCCACTTTCAAACTTGTGCATAAACTGTTCCGGAATCGGTGCTTCT
TTGATTTCTCCTTCGTCATCTGGCCAACTCACATTCATCTCGCTACTCTTGCTCGTCACAAAGGAACTGGTCG
CAATTGCCTCATTTGTCAAATGGACCACAATTCCATCCGTTTTGTCGATCGAAAAGGATGGACACACATTAGT
AGCCTGCACTTGCACACTCTTGCAGTTGACAACCTCGCACGCCGAAATGGCCGTGTCGAATACCACCCCACAT
TTCGTACAGCTGTCAAGAACAACCGACTTCAGTTTGCCCGTGATTTGGATCGTCACGTTTTGACATTTGTAAA
TGTACACCTGCTGCTTCGGATCGGTGACCGTAATCTTGCACACGCCGTTCTCACAGGTATCCTTGGTCTGATT
CTCGACATTCCACTTGCAACCCCGCGTTTGGTATTCGCATTTCGGCGCCCCCAGCGCTTTCTTGGCACCGAAT
TTGGGTGCAGCCGCAGGTTTCGCAGCGGTGGCGGCGGGCTGCGATGGCGCATCGCCGGATTTTTTAAACTCTT
TCCTCCAGGTTTGTTGATCCCTCGTCACCTTTTTGAGACCCGTCGCGGCGGAGTCGCCGGCGCTTGTTTTTTT
CTTCTGCAGTTCAGCGAATACGTCGCCAGCGCCACCTGCTGCTGGCACCTTTGCCGCCGCTGGAGCCGACTTC
GCCTTCGGTGCACTCCCGCCCTCCCCAACTTTAGCCTCCGAAAAGTCCTTCCCGCGAGGATTCCAAGCCAATC
CCGACAAATGATACTCCTTCAAATAAGCACTCAAATCTAACACCAGCGCCTTCATCGAATCACAAAACTTAGT
ATGTTTCGCCCCCTCCTCGCCCTTATTCTTTTGCTCTTTACGCACCTTGTTCGCCCAAAAGTCAGAAGCGCCG
ATGGTATCCTTCACGAAGCTCGACGGCGTTGGAGGCGGGCTCATGACCACCCAGCTCACGCACGCCAGCATCT
CCATTACCGCCTTAATATGCCAATCAAATTTTCGATCAAGACGCGCCGAGCGGATTTTGGACATGGCGTCCTG
ACACGGTTTGATGTGGGGCTGAATGGCGCTCGCCACGGGTTCGGGCGGCTTTTTATACTCGGTGCCGAGTTCG
ACGATGAGTCGTACGCCGTTCCAGACATCGCGTACGGAGTCGCCGATGCCACCCATTTCGGATCCTAGGGCGG
TGCATGAGTCAGTGAAGGGGGTGAGGGCGCGGACCATGTGTTCGTCGTATGCGGCGAGGCGGGGATGGACTTC
ATCCTCATCCTCTACTACGGCGGAAGTGGCGACAGCGTCCGATGGAACAGTGGCGCCCTTGGGAGGAGTGACG
CCGAGCTTGCACTCTACAGCGGCAAGGCGTTGAAGGACATCGTCCAGGATGCCGCAGAGGGGTTTTAGCTGCT
CCGACATTGAAATAATGGATACGACCAGAGTATTATTATGCCGCCCAAATAATGGTATTGATATTGGTAATGC
GTTATGCCAATGACAAACAATAATACTATCGTTGTGATCTAGGAGTATGAG
>TR389|c0_g1_i1 len=372 path=[1196:0-44 1197:45-46 1198:47-47 1208:48-157
1166:158-160 1167:161-371] [-1, 1196, 1197, 1198, 1208, 1166, 1167, -2]
ATACGACGTGGAAATTTGGAACCGGGAGACTTGCCTCCCATACAGGTACGTGCTCTGCTGCACGTTTGCCATT
CTCAATTTTCATGAAAGACACATAATATATGTATCACGTGACAACAATATCATTCTTACTCTAGTAGGTATTA
ATCGGTCCAAACGAAAATGACGGCCTCGGACCATGGTATTTCTCGCTCAACAATCGACGACTATGGGTACTCA
AACAATGTCACAAGGACGGTTTACTGGACAACGAACGATACAACAATAAAATACCGGTGAGGGTGAGAATACC
CAAGAGTCAGGCGGAGAGCGAGAGGTATACCGTTGCGAATTGCGCGTTGGAGGCCAAATTCATGAGGGAGGGT
GGTGGCA
>TR389|c0_g2_i1 len=955 path=[1175:0-129 1216:130-259 1211:260-431
1215:432-623 1214:624-624 1212:625-700 1217:701-722 1181:723-724
1182:725-725 1183:726-743 1184:744-954] [-1, 1175, 1216, 1211, 1215,
1214, 1212, 1217, 1181, 1182, 1183, 1184, -2]
CGCACTGAGTAGATAGTTGTGCCGTACTCATACAAACTAGGAGTAACTCTCTAGGCATAGGCTCCTACTTTTT
CGTTGCTCTATTTGGTTCTATCTCGAATTTCGGTGCGCTGATTGTCACTCGACTCCTCCAACGACCCCGACCT
ACCTCCCAATACAAGCCCAAACTACTCTTCCGAGCACACGCCAAAATCCTCTCTCGTCTCGCTATTCTAGATT
CGCCGTGTGCATAATTTTTCTTGAAGAGAAGTTAAAAAGGTAAGGGGACGGCACAAAGCACAAAAGCACAACA
ATCATGGCAGCGAAGAAAAAGAAGACAAAGAGCAAAAAAGCGAAACAAACAACCCAATCTGGTACAAACGAAG
GCAGTCAAACTACAACGAGCGTCTCCGCCAATGGAGAAGATCTCATTCATATTTCACCATCCAGGGTAAGGAG
GTATATTATATAGTATATAACAACAGCCACGAAGCAACAACACGTATCATGATGAGAACACACATCTCTCTCG
TTACACGGAGTATTTTGAATTATCGAAACTCTCTCATATGTCTTCCTATATATTCCAACGAAAATGCCATAAA
TAAATAAAAACACACGCAGGTCCGATTCCAACATTCCAAAATTCGTCCGGTATTCTCCGGTTGCGGACGAAGT
GTCACAGAAACACTGGACGAGATACGACGTGGAAATTTGGAACCGGGAGACTTGCCTCCCATACAGGTATTAA
TCGGTCCAAACGAAAATGACGGCCTCGGACCATGGTATTTCTCGCTCAACAATCGACGACTATGGGTACTCAA
ACAATGTCACAAGGACGGTTTACTGGACAACGAACGATACAACAATAAAATACCGGTGAGGGTGAGAATACCC
AAGAGTCAGGCGGAGAGCGAGAGGTATACCGTTGCGAATTGCGCGTTGGAGGCCAAATTCATGAGGGAGGGTG
GTGGCA
>TR389|c0_g2_i2 len=610 path=[1175:0-129 1216:130-259 1188:260-278
1214:279-279 1212:280-355 1217:356-377 1181:378-379 1182:380-380

1183:381-398 1184:399-609] [-1, 1175, 1216, 1188, 1214, 1212, 1217, 1181, 1182, 1183, 1184, -2]
CGCACTGAGTAGATAGTTGTGCCGTACTCATACAAACTAGGAGTAACTCTCTAGGCATAGGCTCCTACTTTTT
CGTTGCTCTATTTGGTTCTATCTCGAATTTCGGTGCGCTGATTGTCACTCGACTCCTCCAACGACCCCGACCT
ACCTCCCAATACAAGCCCAAACTACTCTTCCGAGCACACGCCAAAATCCTCTCTCGTCTCGCTATTCTAGATT
CGCCGTGTGCATAATTTTTCTTGAAGAGAAGTTAAAAAGGTCCGATTCCAACATTCCAAAATTCGTCCGGTAT
TCTCCGGTTGCGGACGAAGTGTCACAGAAACACTGGACGAGATACGACGTGGAAATTTGGAACCGGGAGACTT
GCCTCCCATACAGGTATTAATCGGTCCAAACGAAAATGACGGCCTCGGACCATGGTATTTCTCGCTCAACAAT
CGACGACTATGGGTACTCAAACAATGTCACAAGGACGGTTTACTGGACAACGAACGATACAACAATAAAATAC
CGGTGAGGGTGAGAATACCCAAGAGTCAGGCGGAGAGCGAGAGGTATACCGTTGCGAATTGCGCGTTGGAGGC
CAAATTCATGAGGGAGGGTGGTGGCA
>TR389|c0_g2_i3 len=782 path=[1175:0-129 1216:130-259 1211:260-431 1201:432-440 1202:441-451 1212:452-527 1217:528-549 1181:550-551 1182:552-552 1183:553-570 1184:571-781] [-1, 1175, 1216, 1211, 1201, 1202, 1212, 1217, 1181, 1182, 1183, 1184, -2]
CGCACTGAGTAGATAGTTGTGCCGTACTCATACAAACTAGGAGTAACTCTCTAGGCATAGGCTCCTACTTTTT
CGTTGCTCTATTTGGTTCTATCTCGAATTTCGGTGCGCTGATTGTCACTCGACTCCTCCAACGACCCCGACCT
ACCTCCCAATACAAGCCCAAACTACTCTTCCGAGCACACGCCAAAATCCTCTCTCGTCTCGCTATTCTAGATT
CGCCGTGTGCATAATTTTTCTTGAAGAGAAGTTAAAAAGGTAAGGGGACGGCACAAAGCACAAAAGCACAACA
ATCATGGCAGCGAAGAAAAAGAAGACAAAGAGCAAAAAAGCGAAACAAACAACCCAATCTGGTACAAACGAAG
GCAGTCAAACTACAACGAGCGTCTCCGCCAATGGAGAAGATCTCATTCATATTTCACCATCCAGGGTCCGATT
CCAACATTCCAAAATTCGTCCGGTATTCTCCGGTTGCGGACGAAGTGTCACAGAAACACTGGACGAGATACGA
CGTGGAAATTTGGAACCGGGAGACTTGCCTCCCATACAGGTATTAATCGGTCCAAACGAAAATGACGGCCTCG
GACCATGGTATTTCTCGCTCAACAATCGACGACTATGGGTACTCAAACAATGTCACAAGGACGGTTTACTGGA
CAACGAACGATACAACAATAAAATACCGGTGAGGGTGAGAATACCCAAGAGTCAGGCGGAGAGCGAGAGGTAT
ACCGTTGCGAATTGCGCGTTGGAGGCCAAATTCATGAGGGAGGGTGGTGGCA
>TR390|c0_g1_i1 len=290 path=[1203:0-52 1214:53-126 1197:127-150 1217:151-261 1206:262-289] [-1, 1203, 1214, 1197, 1217, 1206, -2]
CGAACACGGTGGGCACCACCGCCGTCAGCTACCACAATCCGAGTGATGATGGTCAACCTGAAATTACATCATC
CCCCCGTCGAGAGCTCTTATTCCTCATTCCAAAAATGGCAGCAGCAACAACCATCGCAACAGCAACAGTCATG
ACGTTGCCCCATCAACGAGCCTTGGCAGCCGGAGGAATGACCGCCGATTCGGCAAGAGACCAGTGGAAAAATG
CCTCACCAGCAATAGACGAATTGTTACAGAATTGGTCCACCGAGAAATGGGCCGAGGAAGTAGATCGGAAG
>TR390|c0_g1_i2 len=1054 path=[1209:0-281 1210:282-305 1218:306-337 1216:338-367 1214:368-441 1197:442-465 1217:466-576 1213:577-968 1200:969-988 1201:989-1012 1202:1013-1053] [-1, 1209, 1210, 1218, 1216, 1214, 1197, 1217, 1213, 1200, 1201, 1202, -2]
ATATCCATTGACAAGCTATGCGTTCGTATTTTACGATATCCACCCGACGTCGTTAGGCAAACGCAATCGAGTA
GGAAAACGATACTAATCCGAACCCGATGCCTGATCGTCTCATATCTCTATGATAGGGTAGTAATCACAGTTTA
AACTTCCTTTTATTGCACTTAGATCGAGATACCACGATGATGGCATCAAGATCAGCAGTTTCCTTTGTGTTAC
TTCACGTATCCATCATTTTATTAATCCTGCGCAACGATAAAGTTGCGGCATGGCAAATCGGTAGTAATAACGG
TAGCAGGGTGGCAGCTAGGACGACGAACACGGTGGGCACCACCGCCATCAGCCACCACAATCCGAGTGATGAT
GGTCAACCTGAAATTACATCATCCCCCCGTCGAGAGCTCTTATTCCTCATTCCAAAAATGGCAGCAGCAACAA
CCATCGCAACAGCAACAGTCATGACGTTGCCCCATCAACGAGCCTTGGCAGCCGGAGGAATGACCGCCGATTC
GGCAAGAGACCAGTGGAAAAATGCCTCACCAGCAATAGACGAATTGTTACAGAATTGGTCCACCGATAAATGG
GCCGAGGAAGTGGGCGGCGGCGATATCATTCGCACGAAATTGGGGCAAATGGGCACGACGTCCCCCTTGTTCC
AGATCGAAAAGGCGCTCAAGGCGCTGGGCGATTCGGACTACGTGGATGATTTCATCGAATTCACCGAAACGGG
GGAAGAATTCATGGACGCGTTGTACCAGGCCGATTCGTTGGCGAATTCGTCGAATCTCAAAACGGGGAGTGGG
AAACAGACCCCGCCGGCCGTCTTTATTGAACAATCTAGGAAAGAAGTTGAGAAAATGCAGAGTATCGCGAAGA
GAATGGATGCCATGGTGAAATAAATACTTGAATAAATAAGAAACCAGTGATTGAATGAGCTGAAGCCTCGTGG
ATAATATATGCACTCTGACAGGTAAAACAGTAGGGTCCCATGCTTTTGATGGGACGCTGAGGTACCATATATT
TGTAATTGGACTAATTGCATCCGTTGTTTCAA
>TR391|c0_g1_i1 len=877 path=[1161:0-192 1162:193-216 1215:217-408 1212:409-432 1208:433-464 1216:465-473 1210:474-489 1158:490-513 1213:514-528 1209:529-647 @1206@!:648-768 1195:769-789 1205:790-792 1200:793-810 1194:811-834 1154:835-876] [-1, 1161, 1162, 1215, 1212, 1208, 1216, 1210, 1158, 1213, 1209, 1206, 1195, 1205, 1200, 1194, 1154, -2]
TGTGCTCTTCCGATCTCTACGCCCAGTTCTCTTCCGCCAGAGCCATGTACAGATTTGAACCTTTCCCTGGTGA
CATCGAGGATGCTGCAGGCGACGCCCCGATCACTGTGGCTGTAGACACGTCGACCACCTACCAGGCTATCCTC
GGCTTCGGTGGTGCCTTCACCGATTCCGTCGGCATCAATATTGACAACCTGTCTCCCCCTGCACGAGAATTAC

TCCTCAGGTCCTTCTGGGCACCCGAAGGAGCTGAGTACTCCATCGGCCGCATCCCCATCGGAGGATGTGACAT
GTCTACCCGCCCCTACACTCTCGATGACGTCGAGGGCGACGTGGAATTGGTCCACTGGTCCCTGGCCCCTGAG
GACTACAACTACAAGATTCCGTACGTCAAGGAAGCCATGGGCATTTCTGAGGAAGAAATTCTCATCTTCGGAT
CTCCCTGGACGCCACCCATCTGGATGAAGAGCAACAACATGTTCAACGGAACAGGCTACCTACTACGGGAATA
CTACCAGCCTTACGCCAACTACATTGTCAAGTGGGTCAGTGCCTACGAGTCCGAAGGTGTCCCCATCTGGGGC
CTCACTCCCCAGAACGAGCCCCTCGACCAAGAAGAGGACTGGGAAAACAATGGCTGCGTGTGGCACCCTGAGG
ATATGAGGGACTGGATCAGAGACTTCCTGGGACCGAGCCTTTCTGCTGCTGGATACGACAGACTCATGATCAT
GGCTGACGATTTCAACCGCTACACTCTGCCTTATTACGTCGAGCCTTGCTTCACGGACCCTGAATGCTCGAAA
TACGTGGACGGCGTCGCTGTTCACTGGTATGCGGACGACACAGAGCAGATCGGAAGAGCGTCGTGCAGGGAAA
G
>TR391|c0_g1_i2 len=488 path=[1122:0-75 1216:76-84 1210:85-100 1158:101-124 1213:125-139 1209:140-258 @1206@!:259-379 1195:380-400 1205:401-403 1200:404-421 1194:422-445 1154:446-487] [-1, 1122, 1216, 1210, 1158, 1213, 1209, 1206, 1195, 1205, 1200, 1194, 1154, -2]
CGGCATACGAGATTGGTCAGTGACTGGAGTTCAGACGTGTGCTCTTCCGATCTCTGGACGCCACCCATCTGGA
TGAAGAGCAACAACATGTTCAACGGAACAGGCTACCTACTACGGGAATACTACCAGCCTTACGCCAACTACAT
TGTCAAGTGGGTCAGTGCCTACGAGTCCGAAGGTGTCCCCATCTGGGGCCTCACTCCCCAGAACGAGCCCCTC
GACCAAGAAGAGGACTGGGAAAACAATGGCTGCGTGTGGCACCCTGAGGATATGAGGGACTGGATCAGAGACT
TCCTGGGACCGAGCCTTTCTGCTGCTGGATACGACAGACTCATGATCATGGCTGACGATTTCAACCGCTACAC
TCTGCCTTATTACGTCGAGCCTTGCTTCACGGACCCTGAATGCTCGAAATACGTGGACGGCGTCGCTGTTCAC
TGGTATGCGGACGACACAGAGCAGATCGGAAGAGCGTCGTGCAGGGAAAG
>TR391|c0_g1_i3 len=268 path=[1169:0-38 @1206@!:39-159 1195:160-180 1205:181-183 1200:184-201 1194:202-225 1154:226-267] [-1, 1169, 1206, 1195, 1205, 1200, 1194, 1154, -2]
TGTGCTCTTCCGATCTGGAAAACAATGGCTGCGTGTGGCACCCTGAGGATATGAGGGACTGGATCAGAGACTT
CCTGGGACCGAGCCTTTCTGCTGCTGGATACGACAGACTCATGATCATGGCTGACGATTTCAACCGCTACACT
CTGCCTTATTACGTCGAGCCTTGCTTCACGGACCCTGAATGCTCGAAATACGTGGACGGCGTCGCTGTTCACT
GGTATGCGGACGACACAGAGCAGATCGGAAGAGCGTCGTGCAGGGAAAG
>TR392|c0_g1_i1 len=1045 path=[1952:0-324 1953:325-348 1987:349-464 1926:465-488 1983:489-793 1994:794-817 1997:818-818 1982:819-833 1978:834-857 1993:858-862 1916:863-886 1992:887-986 1996:987-987 1998:988-1003 2000:1004-1011 1944:1012-1044] [-1, 1952, 1953, 1987, 1926, 1983, 1994, 1997, 1982, 1978, 1993, 1916, 1992, 1996, 1998, 2000, 1944, -2]
TCCCGAGGTCGGCGGTTTCCCGATCACCCAAGGGTGTCTACGGTACCCTCACCTGTTTGGGGTTCCCATTACA
GCCGTGTACGGGTCACTTATACATAGGAATCAGTATTACAGTCATATCGACTCCTCCTGCTCTCTTGACGGCA
TGCATCACGGTGGCGAACACCGTATTACCATTATTTCCTCTCCATGCTGAACAAAAAAATGATAATTCAAAAG
GTTCTCGTGATCGCAATCATTCTATTTGATACAACTGGATTCATGAATTTGACAATGGATAGATCAAGAAGAG
CTATGCTGACCAATCTGGGAATCGTGGCGGGAACCATGCACACCAACGTCGTCCGACTCGGCTTTGTAGGGGA
TGTCATGATTGGTAGAGGGATTGATGCTATACTTCCATCCAGTGTGGATAGCACTCTACACGAGTCATACATG
AAAGATGCAAATGGGTATGTGAGAATTGCCGTAAGGGAAAATGGTCCTTTATCAAAAGCAGAACTTGAAGAAA
AAGGTCACAGTTATATTTGGGGCGACGTGGTGGAGGAGCTCAAGTCTCCAGACTCATTAATCATTAATCTCGA
AACTGCTTTAACTCAGAGCAATGATTGGAATCGTGATAAAGGAATACATTATAAATCACATCCAAACAACGTT
GCGAGTTTATCGGCTATTGGAGTCAAAGTAGCAACACTTGCCAACAATCATGTCCTAGACTGGGGAGAGGAAG
GATTGAAAGAAACGACAAGCGCCTTGGACATGGCCGGGATCAATTATTCTGGCGCAGGCCCCAATGAAGACAT
TGCGAAGAGACCAACTTTCTTCAAAGTTGATGTTAATCCCATGTCTCCTAAAGGTGGATCAGTTACAATTGCT
ATTCAAGCGATTGGGTTTCCATCAGCGGGCGTCCCCATGGAATGGCAAGCGAGGAAGAACAACTGTGGAGTGA
ATGTTGAGGAAGATCTCAGCGAGGAAGTGGCCAAAAAAGCGTTTGATGCTGTACAGACGAGAGATCGGAAGAG
CGTCGTATAGGGAAAGAGTGTAG
>TR392|c0_g1_i2 len=1566 path=[1952:0-324 1953:325-348 1987:349-464 1926:465-488 1983:489-793 1994:794-817 1997:818-818 1982:819-833 1978:834-857 1993:858-862 1916:863-886 1992:887-986 1996:987-987 1999:988-1001 1948:1002-1003 1988:1004-1028 1984:1029-1035 1980:1036-1171 1919:1172-1195 1976:1196-1395 1962:1396-1419 1963:1420-1565] [-1, 1952, 1953, 1987, 1926, 1983, 1994, 1997, 1982, 1978, 1993, 1916, 1992, 1996, 1999, 1948, 1988, 1984, 1980, 1919, 1976, 1962, 1963, -2]
TCCCGAGGTCGGCGGTTTCCCGATCACCCAAGGGTGTCTACGGTACCCTCACCTGTTTGGGGTTCCCATTACA
GCCGTGTACGGGTCACTTATACATAGGAATCAGTATTACAGTCATATCGACTCCTCCTGCTCTCTTGACGGCA
TGCATCACGGTGGCGAACACCGTATTACCATTATTTCCTCTCCATGCTGAACAAAAAAATGATAATTCAAAAG
GTTCTCGTGATCGCAATCATTCTATTTGATACAACTGGATTCATGAATTTGACAATGGATAGATCAAGAAGAG

CTATGCTGACCAATCTGGGAATCGTGGCGGGAACCATGCACACCAACGTCGTCCGACTCGGCTTTGTAGGGGA
TGTCATGATTGGTAGAGGGATTGATGCTATACTTCCATCCAGTGTGGATAGCACTCTACACGAGTCATACATG
AAAGATGCAAATGGGTATGTGAGAATTGCCGTAAGGGAAAATGGTCCTTTATCAAAAGCAGAACTTGAAGAAA
AAGGTCACAGTTATATTTGGGGCGACGTGGTGGAGGAGCTCAAGTCTCCAGACTCATTAATCATTAATCTCGA
AACTGCTTTAACTCAGAGCAATGATTGGAATCGTGATAAAGGAATACATTATAAATCACATCCAAACAACGTT
GCGAGTTTATCGGCTATTGGAGTCAAAGTAGCAACACTTGCCAACAATCATGTCCTAGACTGGGGAGAGGAAG
GATTGAAAGAAACGACAAGCGCCTTGGACATGGCCGGGATCAATTATTCTGGCGCAGGCCCCAATGAAGACAT
TGCGAAGAGACCAACTTTCTTCAAAGTTGATGTTAATCCCATGTCTCCTAAAGGTGGATCAGTTACAATTGCT
ATTCAAGCGATTGGGTTTCCATCAGCGGGCGTCCCCATGGAATGGCAAGCGAGGAAGAACAACTGTGGAGTGA
ATGTTGAGGAAGATCTCAGCGAGGAAGTGGCCAAAAAAGTGTTTGATGCTGTATAGACGAGAGTAGCTGAGCT
TGGGACAGTCCCGGATATCATCATATTCTCACTTCATATTGGGTCCAACTGGAACTGGGGCATTCCTAAAAAA
TGGCGGATGTTTGCTCATAAACTCATTGATCTTGGCGCTGACGTGGTCGTTGGACACTCTTCCCATCATGTGA
AGGGGATGGAGGTATACAAAAACAAGTTCATCTCGTATGGTTTGGGTGATTTTCTCAACGATTATGAAGGCAT
TGTAGGCCAGGGTTATGAAGATTTCAGGAATGATCTAGCGGCGTTGTACCTTCCAGTAATTGATCTTCAGACC
AAACAACTCTTAAAGGTTGATATTATTCCATGCAAAATAAAGCACCTCAAAGTGCAAAGGGCCACGGAATCGT
CTGACATTGTCTGGCTGAAATCAACTTTCAACCAAGAAGGTAAAGGCATTGGAACTTCATGCGAAAGTGAAAA
AACGGCATCAGGAGACTACAACTTGAAACTTGTATGGTAGCAAAATGATTTTAGAGACAAATTATTTACTTTG
TGTTTGTTGGTATTCTTCCATGTAAGATCGGAA
>TR393|c0_g1_i1 len=1879 path=[2252:0-179 2253:180-203 2292:204-655
2232:656-679 2286:680-772 2249:773-796 2291:797-823 2285:824-847
2295:848-877 2290:878-901 2284:902-955 2262:956-979 2263:980-983
2264:984-1007 2289:1008-1039 2229:1040-1063 2283:1064-1114 2226:1115-1138
2294:1139-1196 2275:1197-1220 2288:1221-1285 2246:1286-1310 2297:1311-
1630 2235:1631-1654 2296:1655-1705 2299:1706-1747 2301:1748-1759
2244:1760-1878] [-1, 2252, 2253, 2292, 2232, 2286, 2249, 2291, 2285,
2295, 2290, 2284, 2262, 2263, 2264, 2289, 2229, 2283, 2226, 2294, 2275,
2288, 2246, 2297, 2235, 2296, 2299, 2301, 2244, -2]
TGCTGGGTCCCTACCGTCCCTACCCAGCAGCGCAAACATGATGTTGTGAAGGAGCCGGAGAAAAGACTTGCTT
CGCGGTGCAGGCAGACAAACAAGTTGAATAACTTCTTGTCCACCGCGATGGCATCATTGTTTCTCTACTACTA
TTCGGTATTACAAGTGGATCATGCATGTGCCTGGGGTCATCATCCTTTAGCTTTGGCATATAGAAATAACGCC
GTATTTGGCAATCTCAACCGGCTGAAAGGCAACGACCATCATGGGTTTCGCCGGGATCCATTGATTGCATTCC
AGTCATCCGAATCGGATTCTTCATCGGATTCGGATACGCTGGCTCCACCAATACCACACTTGGTAGATGATAA
TGCTCCCAGCTGGGAGGACCTTCGAAAACAATTGTTGGCCACTTCCACGGGGAAACGTTTACAGCAGGAATCC
ATCGACAGAGAGAAGGGACACGGTCCACCTCATGCCCATGCTAAATTTCGACTCTTTGACGCGAAGGCTGAAT
CCGAAATTCGAGTGACATTTTATCGTGATCAAGCCGCCTGGTGTCCCTATTGTCAAAAGGTTTGGTTGCTCCT
GGAGGAGAAGCGCATTCCATACCGTGTCAAAACTGTCCCGCTTAATGCATACGGAGATAAGCCAGCGTGGTAC
ACGAGGTTAGTGGATGGAGGAAAGCTCCCTACGCTGGAACTGGATGGCACGCTGTACCGAGAAAGTCTGGAAA
TCATGAAGGTATTGGATGACGAGTTCCCCGATTGTGGTCCAAAAATGTTCCCATCCAACGAATCAAATCGAAC
CAACCTGCTCTTCGAACTGGAGCAGGAGCTACAGCGAGACTGGTTTTCGCTCACCTTCTATCCCGTTGAGGGG
GGACGTCTACGCTCCGCTCGAACCGCCCTGCTCCAGACCTTGCAAAAGGTCGACGATGAGCTGGGCAAAATAA
CATCCGGACCGTGGTTTTTGGGAGGCGATGCTCCGTCCGCTGTGGATCTCCAGTACGTCACCACCGTCGAACG
AATCATCGCGTCGGTGCTGTACTATAAAGGACTCGTTATCCGCGGAAAGTTCGTGAATGTCGATGAATGGCTC
GCGGCATTCGAACGACGGTCGTCATACGCGGCTTCGAAGTCGGATTACTACACTCTCATAATGGCGATGCCGT
CCCAGAACGGACCGGGCTACATGACGGCATCGGGGGACGTCGACGACGACAAGAATGCGAACCACGATGCCTC
GTCCATAATATACGGCTTCGACGACGCGTGGAAACTGCCGCTGCATTTCACGGACGACCCGTCGTCGTCGTTC
GAGCCGATCCCTTCCTCGCAGTTTCGACTGGGCGAACCATCGGAATTTCGTCACGAGGCGGCATTTCGCGTCA
TATCGAACCACGATGGCATCGTCGAATTTGCGTGCCGCGGGGCGGGGGAGGCGGGGAGGCCGTCGTACCACGC
GGAGCTCGCCGATCCGTACGCCGAGTCGAACGAGGATTTTATCGCGCCGGTGGATGTTTGCCTACGACACGTT
ACGAACGCTCTGTTGAACGGCGTTGACGATGGCGCGGGCGTTGCCACTGTTGTCGCCGCCGCCGTGATGGATC
TGGAAGGGAAGGGAGGTACGGGCGAGCTGCGGGAGGACTGGGAATCGTACATCGACGAGGGGACGGGCCGTAC
CTACTTTTGGAACTACGAGACGGGCGACGTTTCGTACACGGCTCCGACGAGGCAGCTCGACACGTGTCTGGTC
TATTTGAGGGATAGGGTTGGGGTGCCGCGAGATATGAATTTGCCAGCTGCGATGCAGCTTCGAGCACATTTGA
ATTGGTGCATAGACTTAATGAAGTAAATGATATTGATTGTTTAAACTCAAAAAA
>TR394|c0_g1_i1 len=947 path=[1421:0-92 1422:93-106 1423:107-125
1491:126-158 1494:159-202 1492:203-226 1495:227-240 1496:241-264
1497:265-266 1487:267-296 1481:297-382 1475:383-532 1431:533-556
1480:557-688 1469:689-712 1474:713-724 1485:725-748 1479:749-771
1473:772-795 1457:796-946] [-1, 1421, 1422, 1423, 1491, 1494, 1492, 1495,
1496, 1497, 1487, 1481, 1475, 1431, 1480, 1469, 1474, 1485, 1479, 1473,
1457, -2]

AACATTTTATTATTAGAAGAAGTATTATGATATGATCACGTTTGCGGCCTACGCCTCGGTTTCATAATGCCTT
ATGGACGATGAGGGGGGGGGATAGATGATGGGAGACGTCGTTACTGCTCGCGACTCCCTGGACTGTCTCGATC
CTTTTGTGCTCAAATATTGAAAGTTTCTTGGAAGAGGCAACACATCATGGGCATAAAGGAAGAGGAGCGAATA
GCCACGGATTGGATCAGAATGCGCGTCATCATCTAGCCAAGGGGGCACCGATTAATATTTAGGAGAATGAAAA
GTCTGTCCACTCTATCAATCTGACACATGACGCATAACATCTTTTTGATCCTTCCCCGAACATGAAAATAGAC
ATTTGTAGGCGCGTGTAGCTTTCCCCCTCAACGGCATCGCGACACAACGATAGAACACACCATCACCATGGAA
ACATACGAATACTCCTGGTCCGAGCAATTCTACTACTTCTCCCTCTTCCGCGCCCTCCACGGCCCTGACGCCG
CGCCCAACAAAGACGACGAATCCCCCATCGACGAAGAAACCTACTACACCCAATCCGACCTAGCAACATGGAT
CCGCGAACAGAACAATTGTCGTAAAATGTTCCTGGACGGGAAGGCATCCGACATCACGCCCAATCAAATCAAG
GTGTTGGACCAAGCGTCCTTTCCTTGGATACGAGATAGCAGCGAACGATGGGACGGGCACTTTCGCGAATTGA
TGGCGTTTAAGGAGGAGCACGGGCATTGCAACGTGCCGAGGAAGGCGGGGAAGTTGGGGATATGGGTGAAGAA
TCAACGGGCGGCGTATCACAAATTGTACGATTTGGCTACCGGAGGAGGAGGAGGAGGGAATGGAGGGAACAAT
GAAGGGGACGAACACGACAACAATAACAACAACAACAATAACCATCAGAATAACGTCAAGAAGACCACCGC
>TR394|c0_g1_i2 len=732 path=[1447:0-167 1475:168-317 1431:318-341
1480:342-473 1469:474-497 1474:498-509 1485:510-533 1479:534-556
1473:557-580 1457:581-731] [-1, 1447, 1475, 1431, 1480, 1469, 1474, 1485,
1479, 1473, 1457, -2]
GCACCGTTTGTATTGGAACAGATAAAATTCATCATCAAAATCACAGCGTTTATCGCTTTGGGCCAACCGGGGA
ACACAACATCACACGCGGTTGTGTCGGTTGTGTCAATTCAGTCAAGGATATTCAATCCTCAAAATCCCAAAGT
AGACATTTGTAGGCGCGTGTAGCTTTCCCCCTCAACGGCATCGCGACACAACGATAGAACACACCATCACCAT
GGAAACATACGAATACTCCTGGTCCGAGCAATTCTACTACTTCTCCCTCTTCCGCGCCCTCCACGGCCCTGAC
GCCGCGCCCAACAAAGACGACGAATCCCCCATCGACGAAGAAACCTACTACACCCAATCCGACCTAGCAACAT
GGATCCGCGAACAGAACAATTGTCGTAAAATGTTCCTGGACGGGAAGGCATCCGACATCACGCCCAATCAAAT
CAAGGTGTTGGACCAAGCGTCCTTTCCTTGGATACGAGATAGCAGCGAACGATGGGACGGGCACTTTCGCGAA
TTGATGGCGTTTAAGGAGGAGCACGGGCATTGCAACGTGCCGAGGAAGGCGGGGAAGTTGGGGATATGGGTGA
AGAATCAACGGGCGGCGTATCACAAATTGTACGATTTGGCTACCGGAGGAGGAGGAGGAGGGAATGGAGGGAA
CAATGAAGGGGACGAACACGACAACAATAACAACAACAACAATAACCATCAGAATAACGTCAAGAAGACCACC
GC
>TR394|c1_g1_i1 len=1488 path=[3432:0-270 3433:271-277 3434:278-287
3738:288-301 3712:302-400 3726:401-421 3711:422-451 3685:452-475
3725:476-526 3484:527-550 3710:551-630 3752:631-632 3596:633-655
3597:656-679 3708:680-730 3682:731-754 3723:755-772 3707:773-796
3680:797-816 3754:817-828 3721:829-946 3677:947-970 3743:971-973
3748:974-1028 3719:1029-1075 3742:1076-1076 3731:1077-1100 3718:1101-1108
3741:1109-1132 3729:1133-1145 3716:1146-1195 3747:1196-1211 3694:1212-
1261 3728:1262-1278 3664:1279-1290 3714:1291-1293 3692:1294-1334
3739:1335-1374 3727:1375-1398 3756:1399-1436 3660:1437-1461 3498:1462-
1487] [-1, 3432, 3433, 3434, 3738, 3712, 3726, 3711, 3685, 3725, 3484,
3710, 3752, 3596, 3597, 3708, 3682, 3723, 3707, 3680, 3754, 3721, 3677,
3743, 3748, 3719, 3742, 3731, 3718, 3741, 3729, 3716, 3747, 3694, 3728,
3664, 3714, 3692, 3739, 3727, 3756, 3660, 3498, -2]
TGTTTGAAAATAAAAGTTAATCGTAACTATGGTAATATTTATTTCCACGCTTTCGTATCGCTGATCTTGCAGT
ACCCAAGAACAACAACAAATAACACGATAGACCTCCTCGTCTTCCTCCCCGTTCGAAGGAAGTGGCTATGAGG
GGCAATTCCAACACCGCTGCAGCTTCTTCCGTGGCGTTGGCAAAACAAACGTGACACATAATCTCTCTCAATT
ATGTCAAACCCATCCTATTCTCCCATCGGCAATTCCACCAAACGTCAAAAAACCGACGACGCACTCGACAACG
TCGATCTAAGCGATGACGACCTTGACAACAACAACGACGACGATGGCATGCCCAATGCAGACATCGAACGCCT
AAGCCTCCCAAGTCTCAAATCCGAAACGGTCCGCCTCGAATACGAAGCGGCCCGCATCCAAACTCGTCTCACC
CTCGCCAAGTCGCGTATCGCACGAATAACAGCCTTCCATCGCGGCAATACCACTCCACAACGAGGCACGCGCG
CCGATCGCATGAAGTGTATAGCGGCAGGATGCACATCTCATGCGCAGATGAATGGATTGTGTCAACGCCATGG
GGAATATGTTGGTAAACGATGTCGATATAAGGGATGTACGAATCATGTTCAGAATGATGGATTGTGTTATCGA
CATGGTGCTACTATTACGCTCTGTGGTGTGGAAGGTTGTAGTAATAGAGTGCAGAGGAAGGGAGTTTGTTATA
AGCACGGTGCGAGGTTGGAGGTTGTGACTTGTAGTGTAAGGAGTTGTAGGAATGTTGCCAAGGTTGGTGGATT
GTGTAAGAAGCATAGGGATACGATGGATACCAATACAAATGTCAATGACAATACCAATACAAATACGAATCCA
ATCAATTCAAATCCAACAAATCCTGATCAGATGATGATGATGATGGGAGTGGTGGACGTGCCAAATCCAGAGG
ATGTTTGTCAGGTGGTCCCTACAGACGTGGCTACCACGCATGCTTTGACTACGGGGGGAGCGGATGATCATAA
TCAGCAGTCGCAGCTACCAGAGGGTGACGACTTTGAAACAAAGGGATTTTCTATGCAGCGTGATGATCAATGT
GGATGACAAAACCATCAGGAGGGGCTGGCGTTGGCGCAAATGTGTTTCGTAGGAAACACCCTTTTGGGCGGAA
TTGGTACCCCAATACCCAAGGCTGATTCAAATTTATCAAGTTTTCACTTCTTTCGAGTCAGAATGATTCATAT
GCAAATCATGTCACTGTTTGAGATAGAAAGAGATCACGAAAGGGAACAATACAACAACTCTACACAATTAAG
ACAGGCGTGCAACATGTATCCGCAATGCGATGTGATACCCAGGTTCAGAAAGCATCGTCAGATTGTGGGGAGG

GGGATCATTTTTCCACATTTGACTTCCTTTTTATCTCTAAATATGATTTTTCATTCAATATTGCCTACGGTTT
GGTGGTGGTGGTGGAGATCGGAAGAGCG
>TR394|c1_g1_i2 len=1548 path=[3432:0-270 3433:271-277 3434:278-287 3738:288-301 3712:302-400 3478:401-421 3687:422-684 3752:685-686 3736:687-784 3724:785-808 3709:809-870 3753:871-882 3681:883-982 3517:983-988 3518:989-991 3722:992-1149 3678:1150-1185 3744:1186-1221 3733:1222-1245 3720:1246-1311 3451:1312-1335 3674:1336-1372 3701:1373-1402 3717:1403-1434 3669:1435-1479 3612:1480-1547] [-1, 3432, 3433, 3434, 3738, 3712, 3478, 3687, 3752, 3736, 3724, 3709, 3753, 3681, 3517, 3518, 3722, 3678, 3744, 3733, 3720, 3451, 3674, 3701, 3717, 3669, 3612, -2]
TGTTTGAAAATAAAAGTTAATCGTAACTATGGTAATATTTATTTCCACGCTTTCGTATCGCTGATCTTGCAGT
ACCCAAGAACAACAACAAATAACACGATAGACCTCCTCGTCTTCCTCCCCGTTCGAAGGAAGTGGCTATGAGG
GGCAATTCCAACACCGCTGCAGCTTCTTCCGTGGCGTTGGCAAAACAAACGTGACACATAATCTCTCTCAATT
ATGTCAAACCCATCCTATTCTCCCATCGGCAATTCCACCAAACGTCAAAAAACCGACGACGCACTCGACAACG
TCGATCTAAGCGATGACGACCTTGACAACAACAACGACGACGATGGCATGCCCAATGCAGACATCGAACGCCT
AAGCCTCCCAAGTCTCAAATCCGAAACGGTCCGCCTCGAATACGAAGCGGCCCGCATACAAACTCACCTAACC
CGCATACAAACTCGACTAACCCTCGCCAAGTCACGTATCACTCAAATAACCTCCCTCCATCGAAGCAATGCCA
CCTTTCATCAAGGCTCGACTAACCCCCCGCAACGTCGACAGCGAGGCACGCGTCCCGATCGCATAAGGTGCAT
GGCGGCAGGATGCACATCTAATGCTCAAAAGAAGGGATTGTGTGAGCGCCATGGTGAATACATTGGTAAACGA
TGTTCATATAAGGGATGTACGAATCATGTTAAGAATAATGGATTATGTTATCGACATGGTGCGACAATTAAGC
TCTGTGGCGTGGAAGGTTGTAGTAATAGAGTACAGAGGAAGGGAGTTTGTTATAAGCACGGTGCGAGGTTGGA
GGTTGTAACTTGTTGGATGAGGAGTTGTAGGAATGTCGCCAAGGCTGGTGGATTGTGTAAGAAGCATAGGGAT
ACGATGGGTAACAATACAAATTCAAATACCTATACGAATCCTGAGCAGATGGTGACAATGATGGGAGTGGTGA
ATGTGCCAGCACCAGAAGATGTTTGTCAGGTGGTCCCTACAGATGTGGCTACCACGTGTGCTTTGACTACGGG
GGGAGTGGATGATGATAATCAGCAGCCGGATCTACCAGAGGGTGAAGAATTTGAAACAAAGCGATTTTCTATC
CGACGTGATGATCAATGTAGATGACACCCGGCGGTATGGGCTGAGGGGGGGGGGGCTGACACAAAAGTTTTTC
TTGGATACCCTTTTGGGCGAAATTGGTACCCCAGTACCCAAGGCGTTGATTCGTATTGAGCAAGTTTCACGTT
ATTGAACCAAAAGGATTTGCATCCAAATTATGTCACTGTTTGAGATAGGAAGAGATCACGAAAGGGAAACAGT
ACGACAATGCTACACAATTAATACAGGCGTGCAACATGGATGCAATGGGATGTGTTACCTACCAAGTAAAGAG
AACTCCGACAGGTTTCAGAAAGTTTGGCTAAGTTGTCATCAACCTGTTGCAAGTGGGGACAAGGCACTTAAAT
TTTGTGAGTCAATCCCATACTACGTACATAGATAAATTTTACCTAGAGGCTAGTTAGTCTAAAATTATTGCGG
TCAGTAAAAAAAAAAA
>TR394|c1_g1_i3 len=1253 path=[3555:0-112 3737:113-165 3726:166-186 3711:187-216 3685:217-240 3725:241-291 3484:292-315 3710:316-395 3752:396-397 3596:398-420 3597:421-444 3708:445-495 3682:496-519 3723:520-537 3707:538-561 3680:562-581 3754:582-593 3721:594-711 3677:712-735 3743:736-738 3748:739-793 3719:794-840 3742:841-841 3731:842-865 3718:866-873 3741:874-897 3729:898-910 3716:911-960 3747:961-976 3694:977-1026 3728:1027-1043 3664:1044-1055 3714:1056-1058 3692:1059-1099 3739:1100-1139 3727:1140-1163 3756:1164-1201 3660:1202-1226 3498:1227-1252] [-1, 3555, 3737, 3726, 3711, 3685, 3725, 3484, 3710, 3752, 3596, 3597, 3708, 3682, 3723, 3707, 3680, 3754, 3721, 3677, 3743, 3748, 3719, 3742, 3731, 3718, 3741, 3729, 3716, 3747, 3694, 3728, 3664, 3714, 3692, 3739, 3727, 3756, 3660, 3498, -2]
GCGATGACGACCTTGCCAACATACCCCATAATGACAACAATAACAACAACAACAACACCGAAATGACATCACC
CCCCCATGATGATAACGACGACAACGAAGATATGCCCAATGCAGACATCGAACACCTAACGCTACAACAACTC
AAATCCGAAACGGTCCGCCTCGAATACGAAGCGGCCCGCATCCAAACTCGTCTCACCCTCGCCAAGTCGCGTA
TCGCACGAATAACAGCCTTCCATCGCGGCAATACCACTCCACAACGAGGCACGCGCGCCGATCGCATGAAGTG
TATAGCGGCAGGATGCACATCTCATGCGCAGATGAATGGATTGTGTCAACGCCATGGGGAATATGTTGGTAAA
CGATGTCGATATAAGGGATGTACGAATCATGTTCAGAATGATGGATTGTGTTATCGACATGGTGCTACTATTA
CGCTCTGTGGTGTGGAAGGTTGTAGTAATAGAGTGCAGAGGAAGGGAGTTTGTTATAAGCACGGTGCGAGGTT
GGAGGTTGTGACTTGTAGTGTAAGGAGTTGTAGGAATGTTGCCAAGGTTGGTGGATTGTGTAAGAAGCATAGG
GATACGATGGATACCAATACAAATGTCAATGACAATACCAATACAAATACGAATCCAATCAATTCAAATCCAA
CAAATCCTGATCAGATGATGATGATGATGGGAGTGGTGGACGTGCCAAATCCAGAGGATGTTTGTCAGGTGGT
CCCTACAGACGTGGCTACCACGCATGCTTTGACTACGGGGGGAGCGGATGATCATAATCAGCAGTCGCAGCTA
CCAGAGGGTGACGACTTTGAAACAAAGGGATTTTCTATGCAGCGTGATGATCAATGTGGATGACAAAACCATC
AGGAGGGGCTGGCGTTGGCGCAAATGTGTTTCGTAGGAAACACCCTTTTGGGCGGAATTGGTACCCCAATACC
CAAGGCTGATTCAAATTTATCAAGTTTTCACTTCTTTCGAGTCAGAATGATTCATATGCAAATCATGTCACTG
TTTGAGATAGAAAGAGATCACGAAAGGGAAACAATACAACAACTCTACACAATTAAGACAGGCGTGCAACATG
TATCCGCAATGCGATGTGATACCCAGGTTCAGAAAGCATCGTCAGATTGTGGGGAGGGGGATCATTTTTCCAC

ATTTGACTTCCTTTTTATCTCTAAATATGATTTTTCATTCAATATTGCCTACGGTTTGGTGGTGGTGGTGGAG
ATCGGAAGAGCG
>TR394|c2_g1_i2 len=1558 path=[3490:0-61 3715:62-106 3710:107-138
3683:139-168 3709:169-204 3702:205-205 3681:206-295 3719:296-319
3722:320-355 3678:356-391 3651:392-549 3522:550-552 3523:553-558
3648:559-658 3421:659-670 3696:671-732 3537:733-756 3671:757-854
3720:855-856 3723:857-896 3670:897-1119 3504:1120-1140 3639:1141-1239
3501:1240-1263 3502:1264-1557] [-1, 3490, 3715, 3710, 3683, 3709, 3702,
3681, 3719, 3722, 3678, 3651, 3522, 3523, 3648, 3421, 3696, 3537, 3671,
3720, 3723, 3670, 3504, 3639, 3501, 3502, -2]
GACTAACTAGCCTCTAGGTAAAATTTATCTATGTACGTAGTATGGGATTGACTCACAAAATTTAAGTGCCTTG
TCCCCACTTGCAACAGGTTGATGACAACTTAGCCAAACTTTCTGAAACCTGTCGGAGTTCTCTTTACTTGGTA
GGTAACACATCCCATTGCATCCATGTTGCACGCCTGTATTAATTGTGTAGCATTGTCGTACTGTTTCCCTTTC
GTGATCTCTTCCTATCTCAAACAGTGACATAATTTGGATGCAAATCCTTTTGGTTCAATAACGTGAAACTTGC
TCAATACGAATCAACGCCTTGGGTACTGGGGTACCAATTTCGCCCAAAAGGGTATCCAAGAAAAACTTTTGTG
TCAGCCCCCCCCCCCTCAGCCCATACCGCCGGGTGTCATCTACATTGATCATCACGTCGGATAGAAAATCGCT
TTGTTTCAAATTCTTCACCCTCTGGTAGATCCGGCTGCTGATTATCATCATCCACTCCCCCCGTAGTCAAAGC
ACACGTGGTAGCCACATCTGTAGGGACCACCTGACAAACATCTTCTGGTGCTGGCACATTCACCACTCCCATC
ATTGTCACCATCTGCTCAGGATTCGTATAGGTATTTGAATTTGTATTGTTACCCATCGTATCCCTATGCTTCT
TACACAATCCACCAGCCTTGGCGACATTCCTACAACTCCTCATCCAACAAGTTACAACCTCCAACCTCGCACC
GTGCTTATAACAAACTCCCTTCCTCTGTACTCTATTACTACAACCTTCCACGCCACAGAGCTTAATTGTCGCA
CCATGTCGATAACATAATCCATTATTCTTAACATGATTCGTACATCCCTTATATGAACATCGTTTACCAATGT
ATTCACCATGGCGCTCACACAATCCCTTCTTTTGAGCATTAGATGTGCATCCTGCCGCCATGCACCTTATGCG
ATCGGGACGCGTGCCTCGCTGTCGACGTTGCGGGGGGTTAGTCGAGCCTTGATGAAAGGTGGCATTGCTTCGA
TGGAGGGAGGTTATTTGAGTGATACGTGACTTGGCGAGGGTTAGTCGAGTTTGTATGCGGGTTAGGTGAGTTT
GTATGCGGGCCGCTTCGTATTCGAGGCGGACCGTTTCGGATTTGAGACTTGGGAGGCTTAGGCGTTCGATGTC
TGCATTGGGCATGCCATCGTCGTCGTTGTTGTTGTCAAGGTCGTCATCGCTTAGATCGACGTTGTCGAGTGCA
TCGTCGGTTTTTTGACGTTTGGTGGAATTGCCGATGGGAGAATAGGATGGGTTTGACATAATTGAGAGAGATT
ATGTGTCACGTTTGTTTTGCCAACGCCACGGAAGAAGCTGCAGCGGTGTTGGAATTGCCCCTCATAGCCACTT
CCTTCGAACGGGGAGGAAGACGAGGAGGTCTATCGTGTTATTTGTTGTTGTTCTTGGGTACTGCAAGATCAGC
GATACGAAAGCGTGGAAATAAATATTACCATCTCTTTGTTGTTCAGGGTACGTATCTCCTTACGGGTAGAATT
TCGCTATAGCGAAATTCTAGGTATT
>TR394|c2_g2_i1 len=1234 path=[3512:0-48 3685:49-73 3718:74-111 3714:112-
135 3708:136-175 3701:176-231 3713:232-248 3716:249-297 3707:298-313
3677:314-363 3711:364-376 3705:377-400 3694:401-408 3672:409-432
3457:433-433 3642:434-480 3692:481-535 3668:536-538 3641:539-544
3582:545-562 3667:563-680 3638:681-692 3690:693-712 3666:713-736
3637:737-754 3689:755-778 3665:779-829 3636:830-853 3518:854-876
3519:877-878 3664:879-958 3540:959-982 3635:983-1033 3623:1034-1057
3704:1058-1087 3688:1088-1108 3663:1109-1160 3721:1161-1161 3610:1162-
1233] [-1, 3512, 3685, 3718, 3714, 3708, 3701, 3713, 3716, 3707, 3677,
3711, 3705, 3694, 3672, 3457, 3642, 3692, 3668, 3641, 3582, 3667, 3638,
3690, 3666, 3637, 3689, 3665, 3636, 3518, 3519, 3664, 3540, 3635, 3623,
3704, 3688, 3663, 3721, 3610, -2]
GACGCTCTTCCGATCTACCACCACCACCAAACCGTAGGCAATATTGAATGAAAAATCATATTTAGAGATAAAA
AGGAAGTCAAATGTGGAAAAATGATCCCCCTCCCCACAATCTGACGATGCTTTCTGAACCTGGGTATCACATC
GCATTGCGGATACATGTTGCACGCCTGTCTAAATTGTGTAGAATTGTTGTATTGTTTCCCCTCCGTGATCTCT
TTCTATCTCAAACAGTGACATGATTTGCATGTGAATCATTTTGACTCGAAAGAAGTAAAACTTGATAAATTTG
AATCAGCCTTGGGTATTGGGGTGCCAATTCCACCCAAAAGGGTGTATCGTACGAAACACATTTGCGCCAACGC
CAGCCCCTCCTGCTGGTTTTGTCATCCACATTGATCATCACGCTGCATAGAAAATCCCTTTGTTTCAAAATCG
TCACCCTCTGGTAGCTGCGGCTGCTGATTATGATCATCCGCTCCCCCCGTAGTCAAAGCATGCGTGGTAGCCA
CGTCTGTAGGGACCACCTGACAAACATCTTCTGGATTTGGCACGTCCACCACTCCCATCATCATCATCATCTG
ATCAGGATTTGTTGGATTTGAATTGATTGGATTCGTATTTGTATTGGTATTGTCATTGACATTTGTATTGGTA
TCCATCGTATCCCTATGCTTCTTACACAATCCACCAACCTTGGCAACATTCCTACAACTCCTTACACTACAAG
TCACAACCTCCAACCTCGCACCGTGCTTATAACAAACTCCCTTCCTCTGCACTCTATTACTACAACCTTCCAC
ACCACAGAGCGTAATAGTAGCACCATGTCGATAACACAATCCATCATTCTGAACATGATTCGTACATCCCTTA
TATCGACATCGTTTACCAACATATTCCCCATGGCGTTGACACAATCCATTCATCTGCGCATGAGATGTGCATC
CTGCCGCTATACACTTCATGCGATCGGCGCGCGTGCCTCGTTGTGGAGTGGTATTGCCGCGATGGAAGGCTGT
TATTCGTGCGATGCGCGACTTGGCGAGGGTGAGACGAGTTTGGATGCGGGCCGCTTCGTATTCGAGGCGGACC

GTTTCGGATTTGAGTTGTTGTAGCGTTAGGTGTTCGATGTCTGCATTGGGCATATCTTCGTTGTCGTCGTTAT
CATCATGGGGGGGTGATGTCATTTCGGTGTTGTTGTTGTTGTTATTGTTGTCATTATGGGGTATGT
>TR394|c2_g2_i2 len=948 path=[3444:0-35 3706:36-72 3697:73-77 3711:78-90 3646:91-96 3695:97-114 3673:115-122 3643:123-146 3426:147-147 3693:148-194 3669:195-249 3640:250-252 3691:253-276 3667:277-394 3638:395-406 3690:407-426 3666:427-450 3637:451-468 3689:469-492 3665:493-543 3636:544-567 3518:568-590 3519:591-592 3664:593-672 3540:673-696 3635:697-747 3623:748-771 3704:772-801 3688:802-822 3663:823-874 3721:875-875 3610:876-947] [-1, 3444, 3706, 3697, 3711, 3646, 3695, 3673, 3643, 3426, 3693, 3669, 3640, 3691, 3667, 3638, 3690, 3666, 3637, 3689, 3665, 3636, 3518, 3519, 3664, 3540, 3635, 3623, 3704, 3688, 3663, 3721, 3610, -2]
GCTCTTCCGATCTCTTGGGTATTGGGGTACCAATTCCGCCCAAAAGGGTGTTTCCTACGAAACACATTTGCGC
CAACGCCAGCCCCTCCTGATGGTTTTGTCATCCACATTGATCATCACGCTGCATAGAAAATCCCTTTGTTTCA
AAGTCGTCACCCTCTGGTAGCTGCGACTGCTGATTATGATCATCCGCTCCCCCCGTAGTCAAAGCATGCGTGG
TAGCCACGTCTGTAGGGACCACCTGACAAACATCCTCTGGATTTGGCACGTCCACCACTCCCATCATCATCAT
CATCTGATCAGGATTTGTTGGATTTGAATTGATTGGATTCGTATTTGTATTGGTATTGTCATTGACATTTGTA
TTGGTATCCATCGTATCCCTATGCTTCTTACACAATCCACCAACCTTGGCAACATTCCTACAACTCCTTACAC
TACAAGTCACAACCTCCAACCTCGCACCGTGCTTATAACAAACTCCCTTCCTCTGCACTCTATTACTACAACC
TTCCACACCACAGAGCGTAATAGTAGCACCATGTCGATAACACAATCCATCATTCTGAACATGATTCGTACAT
CCCTTATATCGACATCGTTTACCAACATATTCCCCATGGCGTTGACACAATCCATTCATCTGCGCATGAGATG
TGCATCCTGCCGCTATACACTTCATGCGATCGGCGCGCGTGCCTCGTTGTGGAGTGGTATTGCCGCGATGGAA
GGCTGTTATTCGTGCGATGCGCGACTTGGCGAGGGTGAGACGAGTTTGGATGCGGGCCGCTTCGTATTCGAGG
CGGACCGTTTCGGATTTGAGTTGTTGTAGCGTTAGGTGTTCGATGTCTGCATTGGGCATATCTTCGTTGTCGT
CGTTATCATCATGGGGGGGTGATGTCATTTCGGTGTTGTTGTTGTTGTTATTGTTGTCATTATGGGGTATGT
>TR394|c3_g1_i1 len=453 path=[431:0-452] [-1, 431, -2]
AGCGTGCAGAATCTGATTCCAAATTTCTTCCGCCCCGGCATTGTTGTTGTTGTTGTTATTGTTGCCGTTACCG
TCACGAAGATAACGCACGCATTGATCCAACCAGCAATGGGAGGGACGGACGCCTGCTTGATTTTCGAGTTGTT
CTTGTAAAGATCGATGAGGTGAGGAGGACGAAGACGAGGACGACACGTCGCGCTGCTGCTGCTGCTGCTGGCC
GTTATTTGTCGAATCGACATCCATGTCGGCATTGTCGCTTTCGTCGTCCGAGTCGATAATTCGTCGTCTCGGC
ATTTTATTGGAAGCTGTACGGGAAACGCTACGGTTGGGGGGGTTATGTTGCTTCAAAGGCCATTGCACCGTTG
GTTTCGTTCGTTCCACTTTCCTCTTGCTCTGTTGTCCTCGTGAATTGTGAAATGGACACCAGGAGTTCCGAAA
GGTTCTGAGATCGGA
>TR395|c0_g1_i1 len=4127 path=[5061:0-472 5262:473-519 5233:520-543 5247:544-675 5160:676-699 5260:700-816 5184:817-839 5246:840-920 5134:921-944 5269:945-1047 5053:1048-1071 5245:1072-1374 5278:1375-1398 5279:1399-1407 5257:1408-1430 5227:1431-1431 5268:1432-1473 5256:1474-1497 5226:1498-1644 5242:1645-1949 5153:1950-1973 5255:1974-2058 5210:2059-2073 5241:2074-2143 5224:2144-2238 5201:2239-2262 5273:2263-2541 5266:2542-2569 5240:2570-2586 5275:2587-2610 5272:2611-2636 5264:2637-2652 5252:2653-2679 5238:2680-2703 5220:2704-2781 5079:2782-2805 5251:2806-2839 5237:2840-2856 5219:2857-3183 5070:3184-3207 5271:3208-3227 5263:3228-3269 @5250@!:3270-3388 5277:3389-3394 5066:3395-3412 5249:3413-3430 5235:3431-3560 5150:3561-3584 5217:3585-3707 5098:3708-3731 5234:3732-3758 5216:3759-3782 5168:3783-4126] [-1, 5061, 5262, 5233, 5247, 5160, 5260, 5184, 5246, 5134, 5269, 5053, 5245, 5278, 5279, 5257, 5227, 5268, 5256, 5226, 5242, 5153, 5255, 5210, 5241, 5224, 5201, 5273, 5266, 5240, 5275, 5272, 5264, 5252, 5238, 5220, 5079, 5251, 5237, 5219, 5070, 5271, 5263, 5250, 5277, 5066, 5249, 5235, 5150, 5217, 5098, 5234, 5216, 5168, -2]
ACAATAAGACGAGAATGAATAACACCAGATTCAGAGCCAGAGCCACATCAACACAATCTATATCTGCCATATT
GAACACCCTCGAAACAGACGATGGTCGCGTCAAATCATCCCACTTTCGGAATTCCGGTAGCGACCGTTCGTTG
ACACTTCTCGCCATCGGTCTATGTGCGCTATTGCTGGGCGTCGTCAACATTATTGTGTCGACTCGGGTCCACA
GCGTGGTGATACGACCGCATTATCCGCATCCCTTCGACCCCAATAACAATACCGCCAAGCTACGTAATGATGA
CACTGCTATCGACAATACCGCCGCCGGTGCTCAAACCGACCATGCATTATTATTGCCCCCCATCTATTGGATT
AACCTCCAAACGTCCACAGACAGGAGGGAAGCAATGCTAAAATCATTTGAATCGCTCGGGATACCCTCCAACC
ATCATCATCGCGTCATTGCATCCAACATCCCACAGTTATTGAATGCATCGAAATCCGGCCGTCTGATCTTCCA
TCCCGAAATCCGCCTGGAGGCACATGGCATCCGTCCCAAACCGCACCAGCAAAACATTTATTACTACAGCGAG
GCAGCGTGTCTATTATCTCATCTCAATGCAATCAAACGAGCGTACGAAGATGGCACGACCTCGTCTTATTTC
TCGAAGATGATGCGGTACTGTCCAGAACATTTCTCGAACACTGGAGGGATTACGTGAATCACGAAGCTACTCC

CGATGGATGGAAGATATTGCAATTCGCCACGGTCAATCCTCATGTTATTCGGCAGGGTGTGCTATTGCGCGAG
CATTTCGTTTCGTGGCAGCCTTATCACTGGTCTACGCGTGCGTACGTGATGAATAGGGAGGGTATGCAGACTC
TTATGGAGAGGGCGTACTTTTCTTCGTCTACCGATAACAGCAGGGGCAACGCGTTGGGCGGTTGGCAGATACC
ATTCTCATTGCCGCCCATGGTGGTGGCGGACGAAGTTATTTATTCCATCGTGGGGGATGCATACACCTCCACG
GGTCTTTGGGTTGATTCTTTTGAAGGGCACGGTTCCACGGTCCAGTCGAAGGAGTCGAATGCCGATGTGAACG
AGGGCATCATGCATTCTTATTCCGTTCCGGGGGACTTGGTGGACCACGTTACGATGAAAAAAGAACACCACAA
TTTCTGGTTCGAAGAATCGCTACTGGTTCTGATGAATGTGAGAGTTTCCGACGAGGGGGGAATGGAGCGAGAT
ATTCAATGGATTCGGCAAGACTTCAATGCGGCGTGTCGATTCCATCGAATTTGCGACTGGGAAATCAACGTGG
TGATCGCGAACCCTGCTCTGACGGAAAACTTCCGTGTTTCTTCCTCCGAGTTGCCTCCCAACATTCATTTTCA
CACCACCGTGTCCGCCGAACCATTCAACAAATTTTCCTATGTGCGTACCATCGTCGATCGAATGGACGATTAC
GACCTGGTCCTGCTCAAGGACAACGACCTGCGAATATCCGGCTTCCCGTGGCGCACTTTCGTCGAACGAAAGG
AAAACGCCGTAGTCTCCGGGCCGCTCCGACAATCCCTCCGAGAGGCCATGGCGTACGCCCTCAACTCGCGACG
GATGAGCGATCAAAAGTCCTTCTTCCACGAGGCTCACAACTGGGTCGAGAAGGATTGGAGCGCGGATCTCTTC
GCGAACGTGATGCCGACGGAGGTCCCCATCGTCGAAATGTATTTCGTTCTGTTCGACGGCGCGTTCGCGAAAT
ACTTCTTCGATCGGATACTGACGCCGGAATTCGTGGATCAGAGCAGCGATTGGGGACCCGATCTGCTGTGGTG
TGCCGCCGCGAAGGAATGGGACGATAGTCGTCCTGGTTGCTACTTGGTCCCACTCGTGTCGGGACACGAGGAT
AGTCGTCAGATAGACAAGACGAGGAGGGTTGAGTTTAATGCTCGTGGTGATAGGGTGTTGGCTGCGTTCAGGA
ATGATCCTCACATTGGAAAGTGGATGCGTGTTTCGACGGCATGGCATGGTATCATCCACAGTCAAAGTCTTCA
GAGGATCAAAAAAAACTGCCTCAATTTGCTTGGGATTAAGGAGAAGGAACTCTTCGATCTGCAGGCATGTGCC
ATGAAGCTAATATTTTCGTTTCAGCATTACGGTTACGTGCCACAGGTTTCCCAACAGCAGCATCGGCGGCCTG
AATCAGACTCAGTACCGAGCCATGTGAATGTTGAAAGTGGAACTGGTTCTTGGGCAAATTGCTCGTCGCCAAA
AGAATCAACTCGGGAAGAACGCAAGCATCATCTCACTCGCTGGGAGAACCATCGCGACTCTGCTTATACGAGC
ATTTCATGCACCAATATTCATTACAGGATACCCGGCCGCTCATTGCACGATATTAAATTTTCGGAGCAGACCA
TGGTGATTGGGGTGTTGTCCGGCGCATCAGGAGAGGGGCCGAACCGACGCAGAAACATCCGATCCACGTGGGC
ATCGGGGCGAAGTAATGTGCTCTTCGTTGTTGCTGGGCCGTGGAGCCAGATTGAAGAGGAGTATGCAAAACAC
GGAGACCTTCTATGGCTTGACATGGACGAGATTTACTTGACAGAAATATCGCACCTCACCTTCAAAACCGAAT
CCTTTTTTTCAATATTGTATGAACGCGTTGTGTCAAAATCTGAGTCAGTGCAGTATCTTTTCAAAACAGACGA
TGATTCGTATGTAGCCTTGGAAAAGCTGTACCATGTGCTGCTTAAAGGGAACCGGGGAGATGATGGGCTTGAC
TATTGGGGGAAATGCATGGCAGATGCCAAGCCTCACAGGAATCAAGTGGTAGAATGGCAGAAAAAATGGTTCA
TTCCATATGAAGTGTACGCTTTCGATCATTATCCGACTTATGCCATGGGGGCCGGTTATGCGCTTTCGAGGAA
ATTCTTATCTTGCGCTTTTGGAAACAAGCAACATGCTGCTCGAATCCGGTACATGCCCAATGAAGATGTGGCA
GTGGGCATGCTGGCGGAAAGGTGTGATGTAAAATGCACCAATAACAATGGAGTATGGATCCGTTATGACAGAG
CTGTTGATGGGATAAACATGGATCGAAGGATTGTACAACACTATGTCAAGAGCGAGGAAGAAATGCGTGTATT
TCATAATAGTCTTCCTTCTATAAGTAAATAGTTGTCATGGAGCTTCCCCAGTAGCATTTCTCCGGCAGTGTGT
CCCCGCGCGAGTAGCATTAGGAGAACCATTTTCAAGTGGTGCCCAAGCGAATTTCAAGCATCATCGTGTTAGG
TGCAACCTCTCACTATAGGCTCAGGGTGTATACTTTGTTGCAATTCGCCGTGGGGAGAGCACGAATATGTTTT
CAAATCAATGTCACGTATCGCCTCGGAAGAGCTTCAAACCAATATACTAATCAAGAACGATTCATTTGATTGT
GATTGAATCAAATCATCGGGGCCTCATCCGAACCTCCTCATTTCTTCTTCTCTTCCAAACCAAGCTCCTTCGC
CAAGAAGTTGTAGGTCAACGCCCACATGAAAGCTCGCTGGCTATTATCGGCTGCGCCGCCGTGACCCCCCTCG
GTGTTTTCCCAATACTGGACCTCCGGGGCCAACCCCGCACCAGCCTCCTCTAGCAGAGCCGCGACCATCTTGC
GAGCGTGTCCTGGGTGGACTCGGTCGTCGCGCGTGGATGTGGTGAAGAGCGTCTTGGGGCATTTCCAGTCGGG
GTCGGTGGATTCGGTGGGTTGCCCGAGGACGCCGTCATCGCCGAGTTCTGGCTTGCCGAGGATGTCGTGACGG
AGCAAGTGGTACGGAGAGTGTTTGCGGAGGAATTTCCAATCGGATGTCTCTGGATTACCGTATTCGCCCATCC
ATGAAGCACCGGCAAGTAGCTGAGAATATCGCTTCATATCTAGGAGCGGAACCTGGCATACAGCAGCACCAAA
GAGACTGGATCCGATTGGTCGAGTGATTAAATTCCCCACGAGCAAACCGCCGTTGGACCCTCCGATGCACGCC
AGCTTTTCGCGGCTGGTGAGCTTGCTGCGAATAAGATCCCGGGCGACGCCCTCCACGTCTTCGAAGCACTTGT
GACGATTCTCCTTCAGCGCCATCTGATGCCACCTCGGTC
>TR396|c0_g1_i1 len=828 path=[806:0-827] [-1, 806, -2]
CTTCCGATCTGGCGGCCCTGGCCGCGTCCAAGGGGGCGTCGTATGAGGTCGCCATGGCAGCCGAAGAAGATTT
GGAGGACGGAAGCGAGGACGTTGATTACGACCAGGCCGAGGAGGATGCGGAGAAGGAATTGTGGGTGGATCAG
GAAGAACAATGGGGGGATATGGATAGGAAGGTGTTCGACATGACGCCTCCTAGTTCAAGGTTGACGCCGGACG
ATGTTGAATGGATCGTTGAACAACTGAACGCCAAGGCCAAAAAGACGGAGGAACGACTTGACTTTGAGGACAA
GATCCGAAGGAAGGAGATGGAGAAGATGATGAGTGGGGACATGGGGTCCAAACCCATCGAGAGTCTGGAAGAG
GTGGACAGATACGTAATGGAGGGGCTGGGGTACGACATGGACGCGATTGAGGCGTTGGTGATGGAATTGACGC
CGGAGCAAAGCGCTGCTTTGGAGGAGATTGATTTTGCGAGTAGAGGAGATACGGACATTACTGAGGAGGAGAT
GACGAATGAACTTAAGGTGGTACCGGGATTGACGGACGAACAGATCAGGGCGTTGGTGGGAATGGAGATGAAG
TTGCTGAGGGACGAGAAGCTGAAAAATATTGCGTAGATTGGATGATGGCGATATTGGCGTTTGTGGAGTGATT
ATCTTAAGAACTTCAGGAGGCTATGGTACCCACCCTGAGACTGTAATCAACGCGCAACAATAAAATCAGCCCG
TGCACGCTCGGGTGATATTTTGGATAATGCAATTAAACAAACAATGGTAACCCTTTCTATGTCTGCAAGCCCT
CACACTTACCAAAATAAGACCAATT

>TR400|c0_g1_i1 len=412 path=[390:0-411] [-1, 390, -2]
CGATCTATAGTATCAGCAAGCGACGCTTGTATTATAGAATAGATTTCATATTCCACTTCTACCGACTAGAGAG
AGAGAGGAGTAGAGGGAACTATAGCATAAACTTACTACAAGTGCATTCACACTTATATATATTTCGTGTCGTC
GATGCCTATATATTAGCTCCTGAAGGCTCCGAATCGATTAAGGTGAACTGAGATCTACCTATCTACCCAGAGA
GAGAGAGAGAGAAAATGAAAATAATGTCTACTATGAGTAGGAACAAACGACAACATCCACAAACTGGAAATCA
ATGGCACGGTCATCATCATCGCCATTACAATCATCGCTCGCCATCATCAACACTTCCCCTCCTCCTCCTCCTC
CTCCTCCTACCAACACCAACACTCTCCCAAGAGATCGGAAGAGCGTC
>TR401|c0_g1_i1 len=249 path=[227:0-248] [-1, 227, -2]
GAAAAGAAACAATCCACTCAAGGGAACCATTCAATATCTCTCTCTCGGAATGCATGGTATAAAAACAATGCCA
CACACATACCTCATCGCTATCGTTGCCGCTGCCTCTTCCTTTCTTCTCGTCGACATACACGCACACCGCATCA
TCCGCACACCGGTGGTACTACTAACAACAACAACACCACCACTACTCGAGTCTACCTGCTGGCGACGTTCTTC
GCGATGCCATCATCATCATCGTTATCGCCA
>TR402|c0_g1_i1 len=346 path=[360:0-300 361:301-333 362:334-345] [-1,
360, 361, 362, -2]
TCTCAAACCTTTATTTAAAGCATGTTACTCATCATTGTTGTAAAAGATAAATGATTAACATGTCATCCTAAAT
CTTCCAAGTAATAAATGTTGCCTAAACGTGGAATCCAGACGCCGATCTTGTTAATTTTTAGTTATGAAACATC
TAAGTATGTACTTCAAACAAGAAAGTTGCCAACTGCTTTCAGGCAGCGATAAATCTCCCGTAGCGTCATCGAC
ACGCAGTATCCAACTGCGCCCGGCAATTCGGCGGATAGCCCACACCAAGCTCCATCTTCGACAGGTCTTCTCT
TCTCCATCCGACAGATAGTGGCTTATCTCGAAAACGAAACGCACGGCGCTAAAC
>TR404|c0_g1_i1 len=2580 path=[2558:0-2579] [-1, 2558, -2]
CGCTCTTCCGATCTTTTTTTTTTCATTCTAGTATTCTACTTCTAGCAATAATTTAAGGTCTTATTAAAAGACA
TCGAGTATCTAGGTAAATCAAATGATATAATGAGCCGTCCTCGTTATTGCAATAGCGTCTCAAGAAGAGTCTT
CTAGCGTGTCAATGCTTTTGAGTGTCAAAATAGCATCATTCTTCTCTATGTGGTAGCTACACATAACTCTATT
CAAAACTTTGCTATTGCTTTTTCTTGGCATCCCCCTCCTTGGGTGTTTTTACATGAGAACTTTTCCTCGATGA
TGATGACGACGTAACTCCACCTCCTCCTTTCGCTACCCTATCACAGGCACCCCCAAGGTATCCCCTTGCCACA
TACCGGATGCACAAGCCCAGCGCTATTTTGAACAGCACCAGTCCAACCAAGCCCTGGAACAACCCCGTGTAAA
ACTTCCACTTGTTTCCATCCGCCATCAACTCCACCAGCGCCGCCACGGGGTGGTCCACGTCTCCGTTTCCCTG
AAACGCAATGGCGAATCGCACCGCCTCGTGCAGATAGCGGGCCGTAACGCAAACCAATGGGGTTTGCGCCAGG
CCCAGCCGCTTCACTGCGGCGTGGGTGTAGTCAAGGTTGAGCCCCTCGTGACCGATGCCTGTGACGTCTCCGG
AAAGAATGAGAGCATAATCGAAGTAGATGGTACTCTTTATCAGGTTGAACTTGGTGATGAACGAATGCTTGAT
CCAGTCGCTGACCAATTCTGCCGCGAAGATGATGCCGCACATGGAATAATAATCCTTCACCATGGAACTCGTC
ATGTCTCCCTGAAAGCGATTGAGGAAAAGAATCAAAAGGAGGAATAACCCTAGCTTAAACCGTTCGCATATGT
CGGACGTAGTGATTTTAAAGAGGTTTTGTTTGTTGTATTTCTTGAACACGGTGGATTTAATTTCGGCGAAGTT
GCCACTAATGAGAAGGGTCAGGAGGGCATTGTCGGCCGAGTTGACTGCTACGCTCAGGGTCGCGACGTGTACA
AATAAGAAAAGACTGTGGATAGTCACGTACACTCCAACAACGATAATGCTCACAAACATGCGCGGATGTCGGG
GTCGACGCGTCGTGTTCCAATACAAGCTATCCCAAGCATCCTGACCAAACGAACAAAGAAGTCGGTCAAACAC
TTCGACAATGGCAATGAGAACATACAACTTCAGCATGGCCTGGCCTCGAATCCAATGGTACAGCTTTCCAATG
GAAATGGGACAAAGGACTTGACTGTAGACGAAATAGATGAGGGCACTCTGCATTATTGGGTAGAGATGTCGAC
GGCGAAACCTAATCCCCCGTAACCCCTTCCCAGCACTGCACACCGAACAAGCCAAACACACCCACCCCCAGAG
AAACTTCAACGGCAGCATGGCCCACGCATACAGAAACGAATCTATGCACACCGCCAGGGAGAAGACCATCAAC
CATTCCAGCTTGTGCGGTATGCGAAGGAATTCTTCCATATTTTCGTCAATGTCCTCCGTGGCGGAACCGTCGT
GGTTGCGCGAGTCGATGTTGAGGAGGTCGTTGATGACGTATCCCCCCATGGTTTGGTTGTTTTTGTTGTCGTC
GGCCACGACCGCCGATAACGTGGTATTGGGCGGCTGTTGGGATGGGTGTTGTTTGTGGGGAGACTTTCCCTTC
TTCGATTCGCTCTCGGTGCTTTTGCGAGGTGACTTTCCCCTAGTTTCAGTTTTGTGGATCGATTTCCCCTTGG
CCTCCCTTTGCGTAGACGGCGTAGTAGCGGAAGTCGTATTCACGTCGTCGCTAGGAGCATCCGCAGTAGGACC
ATCCTCTTTTTTCGGTGTCGAACCCTCGTCCGATTTGGAGCTCTCGGCACTTTGGGAGCGTTCCCCTTCGTGG
GGAGTGCTCAGCGAAACACCGATGTCCACGCGGAGCTTCTTCTTTTGTGCGGAAGAGACTTTGGCATTCAAAT
TCCCGCTCTTGAGCGTCTGGGAGGACATGTTCGAAGCGCACGACGTGGAGTGCGCCATGATATCCGCGGTGTT
GAAAGTTGTGTTGGTCCCCACCGATTTCGTCTCCGTCTTCGTTTTGGCATCTATTTTCCCCTCGTTCTCGTGA
CGAATGGTGGTCTGCACGCTTTTCTTGACCTCCACTTCCCGAGGTCTTCTTGAGTCTGGCAACGACAAGTCGG
GCATGGCGATTACATCGACTCCTTTGTTTTCGTCGGCATCCGCAAAAGTACCCACGCTGATAACGCTAATGGC
CGAATCCGGTTCTTGCACACTGCCATCCAGGACGTCCAACGACGGCATCCAATGCTGCTTGGGAGCATCCTCT
TCTCTACCCTTCCTGCGAATCAGATCAGAATCATTGAGGGAGTACGCTCGTCCCTTCCCAGCTTCGCGACGAA
GCCGCAATGCTTCCAAATGGGTATCCGTGAACGCCTGGCATTTGAGATGGTTCTCGGCACCAGGAGGTTCCTT
GAGCGACGACGAGAGATGACGTGGCGGAACGCGTCGAAGATGACGACGGTCGCCACAGTGAAACGGAGGCGTC
GGAGGGCTTTCGTTCGACGAGCTGA
>TR405|c0_g1_i1 len=338 path=[316:0-337] [-1, 316, -2]
CTCTATCGGCAAAAAGATGCTTCTACCATCAACTCATCAAGCATCTTATCATCAGTTGAAAAGAGAGAAAGCA
GGCAGTTGAGCTTGAAGAGTCGAGTTCCCACCACTATTCCTCATTGTCTCTGCGTTATCAGGATGATTTCTCT
GTCTTGGATCTAGATGTGAGATTTTTGGGGCTCTGTCCGGGATGTGTCTGAATACTCGAACCTGAGCATCCTG

ATTGACACATTGTGGCTGAATTGTTCCACTTGCTTTCTGGTGCAAAGTAGAAATGTGTCGCTGGACCATCTTC
CAATCATCTTTTTATTGCGTATACTGGGCATTCACCTAGGTACATG
>TR406|c0_g1_i1 len=384 path=[362:0-383] [-1, 362, -2]
TCTTCCGATCTCAAAGTCAAAGATCTCGACATCACCGCGTACATCAAAGCCAATATTGGCCGGCTTCAAATCT
CTAAATATAATAGAATGCGAGTGCAAATAAATCATTGCTGCGGATATCTGAAGAGCAACACTCAAACGTTCAT
CTAACAATTCATCACTCATTCCAAATTTACCATCTGTCTGATTAAGACTATTCTGGCCATTACATTCCTCTTT
GCATTCATCAATGCCCATCTCGGTTACCATAACGCTATCGGTATCTACTTTTTCTTCTTCTGTGGTACTATTA
CTCCATCTCTGTATCCTTTGATCCAACGTTTCGAATAAACGATCTATGATTAGAAAGTATCCATATGGACCTT
GGCTAAAACCACCAGATCG
>TR407|c0_g1_i1 len=236 path=[214:0-235] [-1, 214, -2]
CGTCCCTCCACCAACGTCCGCAACGTTCGACCGAGCCTGCCGAACAACAGCTGAAAAATAAACTGCAGATTTT
TGGACAGCGTGGGGTATCCCTTTTTATTCTTCAAAAACACGGCCGTGTCCCAACTCACCGCCCTCACGTTTCC
CGCGCCGATGAACCCGTGCAATGCCCTCACCATCGCGGGCATTTCGGCGCCGTCGATCCTGCGCGCAATGTAT
AGATCGGAAGAGCACAC
>TR408|c0_g1_i1 len=618 path=[596:0-617] [-1, 596, -2]
GCCCTTCCGATGGAGCTTGCCCTTTCGCTTGTCCGCGGACGACGCCCCCGCCTCGGTTGGATCGGGATTGTAC
ATGTTATCGTAGCCGTCGTCGCTCCCCAGATCGTCGTCGTTGTCCATGGTGTCGTCGTCCCCGTAGTCCTCTC
CGCTCATGAGACCGCCGTGGCCATCGGCACCCAACGCCCTCGGTCTTAACAACGGGCGATTCGTCGATCGGAC
CAGCACGATGTAACGCAATTTGTTCCTGGATTTGGCGATGGGTATGTGGCGCTTCTTCCCCCCCGAGGTTTTC
GACAGTCCCACGGAAAGATCGAATTGGGAAACGCACATGCTGAAGAGACATTCGTTTGTGAGTCTATTCATGG
GGCGCGACGGTGGCACCAGTGCGTTCTTCCTCTTATCTCCCCCATTTTCCTCCTCCACGGCAGCACCACCGGC
AGCAGCGCCACCGGTGGCGGCATCCTTATTCTCCAACAACTCCTCCCAACCCTCTTTTAATAAGACTTTGCCG
GTTCTCGCCTCGACCTTGCCCTCGCCGCTGAGCATCCTTCCCCCGATGGAACGCACGTCGATCCGCATTTTGG
AGGAAATGCGACACGTCATTTCCCCCACCGAGAT
>TR410|c0_g2_i1 len=1050 path=[1067:0-37 1068:38-61 1072:62-153 1066:154-
1049] [-1, 1067, 1068, 1072, 1066, -2]
GAAGCTTGTGGTTGTTGTTTGCCAAACTCTGTAAGCTTCCTACTACTGCTGCTGCTGCTGCCGTTGCTCTCGA
CATGCATGACCCGCCGTCGGAACCGCTCGTCCCCATCTCCCACCTCCAAGAACTTTTCGCCTTTACGCCTTGT
GCTAGCATCCACGCAAAACGTCTGTGCAGGCACTATTATTGCTCCCTTCGAGAAACCCGTGTTTTGAACGAGC
AGGCGTATTTCCACCACTGGCATGGGTCCGTAGAAGCATGGCGTTGCTGCAGTTCCTTCACCAGGGTGAACTG
CTTTTCGACGCAGTACACTTCGAGACGTCTCGCCATTGCTCCGATCCACCAACGTAGCCCAGCCACCAACGAG
TCCGTCCAACGTCGATCCTCCCAAAGGCACCGACTTTTCAATCGTCGGACAATGTGATGGCTTTGCGTTTCGT
TCCGACATTCGACGGTAGATATTCATGTAATTCGTAGTGGTAGTGGCAGTATTAGTTGCTGAACCACTTTTAG
TGCAAAGCTGACTCTTCGCTTTGGTTCGAACGTCCTCGTCCTCGTCCTCATCGTGAGCATCTTCCCACGCCCG
CACGGTATTCGGGCCAAAGCGTCGTAAACATAAATGAAACCACAATTTCGGATGTTGAAATATGTTCTCCCAA
GAATTCCATCGCCTCGAGACGCGGGTACATCGAAGGAGGGACATTGGGTCAAGAAACAAGGCGATATTTTTCA
GAAGATAGGGTGTTTTGAGGGTGTCGTTGACGGCGGCGAATAGCACTGGGCATGGTTGTTGTTGTAGATGGTG
ATGATGCGTAGATGCGATTGGCAATGGCGACAATGGCGTGTGGGCCTGCACAGTGATTGTAGCGGTATTTGGA
TCAATGAGAGGACAAGCCACACCATTGTTCAGCTTTTGCTTCTTCGCTGCAGGATGACTGGGTAACAACGCTG
GCCTTCGATTCTCGTGGCGAATACCATTCATCGGTGGGTGCGCAATCACTCCTCCTTGGACCACCGTCGCCGA
AACGGGAGTCGGCGGCCCTCCCAAAGTC
>TR411|c0_g1_i1 len=773 path=[776:0-551 777:552-575 778:576-772] [-1,
776, 777, 778, -2]
CCTCATCTCATTCGGAGTCTTCCTCTTTTCCTGAGCGTACACCACCTCCAACTCCTGCCCCTTCATCAGAAAC
CTATCCATCTCATCACGCGCCTCTCTCGCCATCTCTGGCGTGGCATACTCGATGAATGCAAAGCCTTTGGGCT
GCTGCGAGTGAAAATCGCGGGGGATATATACGTCCCTTACTTCACCGATGCGTCCAAACGCCATTTGGAGATC
TTGGGTGGTCATGTCATGGGAGACATTGCGGACAAGGAGGCTTACGCCGGGTGGGCCATCGTCGCGGCGGACA
CGGCCACCACCTCCTCCTCCACCTCCTCCACGGCGATTGTGGTTATTTCCCCCGCCCCCGTAAGGGGGGTGGT
GGTTGTTAGGGCCACCGGGGGGGAAGTCTCCGGGGAAACCGTAAGGGCCTGGGGGCGGAGGACCGTGGCCGAA
GAAGGGTGGAGGTGGGCCGTTGTAGCGATCGTGACGGCCACCACGGTGATCCATTGGGGGGCCGTGAGGGGGG
CCGTGCCTGTCGCGGTAGCGCCTATCATCACGGTCTCGGTTGCGATCCCGATCTCTACGGCGTTCCCGACTGC
GGCTCCTAGGGCTTCTACTCCTACTCCTACTCCTGCTCCCTCCGCCGCGATCCCTGCGGTGATGACGATCATA
CGATCGACTGCGGCTCCTACTCGATCCTCCCCTACGACTACCCCCGCCGCGATCCCTCCGATGATTGCTCCGA
CTGCGCGAACGGGAATGGGTACCGCCGCGATCTCCGAGATCGG
>TR412|c0_g1_i1 len=1314 path=[1317:0-458 1318:459-482 1319:483-1313] [-
1, 1317, 1318, 1319, -2]
CCGATCTCATTCACCTGTGATTGCTCAATCTGCCCCTCGCATTGCTCGAGTCGCTCTGTCGAACAAACCCTGG
TTCACACCCTGAACTCTTCTCTTCCTTTACGCAGGCAATAGCTATAATTGCGTTGCGCTAGACTTGAGAGGTA
TCGAATGCACTTTGTAGTTTCCGTTACTTGAATATTATCTTCACACCACTATGACCGACTTCAGCCAAATTGA
CAATGACGAAACGAGCAACGCCTCATTCAGCTCGGAAGACAGTCAAGAGCCCTACTTTCACGTTCAGACCGGG

AAGGTATTCCCTGCGAGAGAGGCGTGGGGTATTCTAGCTGCGGAGGGTCATAGTCGTCCCTCCAAACATGAAA
AAGAAGTACATCTTGGTGGCGACGATGTAGTCGACTCTGCCAACTCCTCTGCCAACTCTGCACTTCGCACTCC
CAAGGAAACGGAGGAGACAACGGAAATATACGACGACCAACCTCCGGATATTTCTCCCCCGCCACCGCCCACC
TTGTCCAGAGAAGTCTCCGAAACATCGTCCATTCAACAAACGTCCTCCTTCGGACCCACCCAATCTTCCATCG
ATAAGGCTTTACATCCCGAATCCCCCAACTACATCTCCCCCAACAAGAAGGATATCAAGAATCAAACATCCAT
CTCCAGCGAAGCGGAACTCCCACCCAGCGAATCTCAAGACGAACTCGAGGAAGACGACCCCGTCATCCTACGC
GCCAAGGCCATGGCCCTCGCCGCCCAAAACGGACAAAAACTAACCGCCGCACAGATGCAACTCATCGCACAAC
CGGACAAACAACAACAAAAGCTCATCGAGGAGGCAAAACGCGTACAAAAGGCCAAGGATGCGGCAAAGGCGAA
AGACATGCAGGATCAGCTTTTGAAACAGCTGAGCGCTCCCGTGGACAATTTGCAGAGGGTCGGGGCCGACTTT
AAGAAGTTTATACAAACGGAGAAGGAGAAGGGGGCGGCGCCGTTGCAGTGGGGGGCGGATTTGGGTAAGTTTA
TCGAAAGTCAGACTAGCATTCGCATGGGCAAGGATGATGGTTTGCAACAGGATGGTGCAGCGGAGGCAGAAGG
GGGAGCGGGCATCGCGGCCAAGAACGAGGATGATGCGGCATCGTCGAAGCCGAGTTCGTCGTCTCTCCCGCCG
TCGGCCGCGTCGTCGCCGAAACGCGAGAAGAAAAAGATGGAGGGGTTCGGCACCGATCCGCTGGCCCCTCATC
CGATGTCGCCGGGTCCCGGATCGAAGCACGGGCCTCCCGATCCGTCTTCGGCGGAGGTGACGCCCCGTCCATC
>TR413|c0_g1_i1 len=1703 path=[1706:0-1278 1707:1279-1302 1708:1303-1702]
[-1, 1706, 1707, 1708, -2]
TTTTTTTTTGAGCATAATTGGTAGTCTTTATTAATTAAAAAAGTAGTCTTTTGCTGCTTAGCACATGGCAGCT
TTCATTGTTTGAAATATCATCGTGATTGTCATTATTCAAAATCGTATCCTTTTTCGATGAGGTTTATTTTCTC
AAGACACACATGCAAATCAGTTGGAGGTTATAGCAGATAGGCCAACTCGTAATTTAGTATATCATGGCCAAGT
CAACGAGGGAGCTGTCGACGTTGTGGTAGTGATACTTCCCATCGTCGGTTACCCATGCCGGTACACCCTCGTT
ATCTGTTGAATGGACATCGACGTCATCACTGAAAAGAATATGTGACGGTGGTAAGGAGGTACTCAGTAGCTCC
AAGAGAGTTGGCTGGGGTTCTGATGGTTCTGATGCCGACTGATTTGACAATGTGGTTGGTGGTTGACTGACCT
GTAGCTTGTGCGTGAATGTATTCGTAGCTGTCACCGTTTTACTAGAGGTCGTCACGATTGGTTGCGTTTGAAG
CTGATTTGGCTCAATAGCATCAGAGCTTTGGCTCATCATAGGTGGGCCATATTCTTCTGCCTCCACTTGGTGC
GGTATAGGCTTAACTGGAAGTAACTGCTTGCCCTGAGACTTGTGTTTTTTATCTTTTTTATTTCGGTTTTTGT
TGCTGCTGCTCTTCTTCGACTTCTTATCGACCGTTACTCTGCGCTTCTTGGATTTGCTCGGACTTGTTGAGTT
ATCTGCCTCGTCGTGGCTGCTCGCGTTCCCTTCGCAGTCCGCATCCTCGCTCGTTCTCTTCTTCGCCGGAGGC
CTTCTCTTGAGCGTAAACAAGCTACCAACGTCGTCCGTCAAAGACTCGTGAATGTAAGAGCACGGACTGAGCT
TGCCCTTCTTGCCCCCGTGTAATCGCTTCTTGAATCCAAAGTAATTCAGCTGCCTCTGAAAGCTCGCATACTT
GGAATGCCGATAATATTTGCCCAACACAAAGTCTTGGAGCGACTCGGGCTTGTGCACGACGATCTTGCCGATG
CCGCGAATGCCACCGCCCATGTGATCAGGCTCGTCCGAACCGGGGACCGTCCACGAGATGAGATCGATGTAGG
ATGGGTCGCGCAGCATGGAGAAGAGATGGCATAGGAATTCGGGTACAGGTTGGGTGACGGCCTCAGCTCCAAG
GGGTGATTGGGCAAGCATTAAGGTGGATGGGAGGGAGTGTCGTCGTTCAGCGGCGGACGGCCGTTTCTTTTTG
GTGATGGTGGTCGTACTCGTGGTGACAACGTCCAACGGAACCTCGTCGTAAGATGACGCCGTGGAGACTACCG
TGGCGCCCGAATCTTGGGGAACACCGAAGAGTTCGCTCTCCAGTGCCGAGGCGGGGAGCATAAGGTCGGACAT
TTCTATGAAACCTACCGATGGTGAAGATGATTCTGGCGATGATAGGATGTCATCGGTGTCGACTTCAGGTGCC
CCTAATTCTGTCGATGCGGGTGATTTGGATAGGGCATCCATGTAATTGCTCCTTAGAGCACGGGAGGGCGAGG
TCATTTGCGCCAACATGATGATGATAATATTATAATGAGGTGTAGTCGCTGACTGATGTTGTGTGCGTAGTGT
TTGATGGCTCTGAGTAGTGTGACGATCGGAACACCGGTGCAGGTAAGTTTGTTTAAATTCGAATTGTGAGATT
CTTGTTTTATGCTCTTATGCTGTG
>TR414|c0_g4_i1 len=1235 path=[1559:0-548 1560:549-569 1561:570-1234] [-
1, 1559, 1560, 1561, -2]
CAACTCCGAACATCATCTTCTCAATCTCATCTCAATCATGCGATGACTTGGCTCACGGCGATAACATAAATTT
ATCCGCAGCAAATATCACTGATCCCACCAAAAATCTAGCAGCTTACACCGCCTTCAGCTCTAAACCAACGCAA
TATATCACCCATTTGATTGCGCTATCCCTCCATTTTCCAAAGTATACCCAGCTCGTACCGCTTCCGCTTCCAA
ATTTCCAACCACCCCACATCTCGAGGATGATCCAACGCAATTTAATTCGCACGACCTGCACCATCCTCGCCCT
CTCTGGCGGAACATCTGCATGGACCAGTCCTCCCACCACCCTCCATCGGAGTAGTTCCGTCACCACCACTCTC
CTTTCGAGGCCCGACGCTAATAATCCATGCTGGCAAGACAATTACGATTCGGAAGATGATTGCATGTCCACCG
TGTATTCATCGGCGTTCGTCGCCGAAGAGTGGATAAAGTCCATGCCGTGTGGAAAGGATGCAGATTGTTTGCC
TGAGAAGTTGTCTCGTCCGGGGACTATGGGAGGTTCGGGGGTGGAGAAGGTGGATGTCATGGAATATTTGAAC
ATTCGCAAGGCTGCACCAGTTGCTGAGAAAATCGAGAAGAAGGGGGCGGCAGGTCCTTAATAAGGAAGTGTAG
AATTTTGGTTAGAGATACGAGCAAGATATAGGGGTAGGTAGCGAATGAAAAATTCAACGATTGAGGTTCTTAA
ACAGAGTGCGAATATTCTATAGATGCAGCAATAATGGACCTACACATGTAATAAAAGATGTTAAATTTATCGG
CGTAGAGTGGAAGGCATTTGATGTTGGAAGGCGCAATTCATGTGCGCATTGTTGTATTTTACCGAAATGGGGC
ATAGAAGCAATTCTTAAATGCAAATTATAAATGGTAAATTCATATTGCACCTTGCATCATGGATACAGTCTAT
GATGTTACAAAATTAGCATTAGACATGGTGCTACGAGATTAAAGAAAGGCTGGAAAATTATTTTGTTTCACCT
CAACTGTGCAAAAGAAAGAATCCCATGTACAATTAATTTCACATCATGTTTCTATCTATCCATCACAATATTT
GAAGTTCGCAAATCATGGAAAGTTTTGATGGAGTGATTGGTTTCGATTGGGGATCCAAGAAAGAAACCTGAAT
TGACCAGATGACTGAGATAATTTCATAAATATACTCCAGAATCCGAATCAAAAAAAAAAGATCGGAA

>TR414|c0_g5_i1 len=680 path=[1565:0-548 1566:549-570 1567:571-679] [-1, 1565, 1566, 1567, -2]
CAACTCCGAACATCATCTTCTCAATCTCATCTCAATCATGCGATGACTTGGCTCACGGCGATAACATAAATTT
ATCCGCAGCAAATATCACTGATCCCACCAAAAATCTAGCAGCTTACACCGCCTTCAGCTCTAAACCAACGCAA
TATATCACCCATTTGATTGCGCTATCCCTCCATTTTCCAAAGTATACCCAGCTCGTACCGCTTCCGCTTCCAA
ATTTCCAACCACCCCACATCTCGAGGATGATCCAACGCAATTTAATTCGCACGACCTGCACCATCCTCGCCCT
CTCTGGCGGAACATCTGCATGGACCAGTCCTCCCACCACCCTCCATCGGAGTAGTTCCGTCACCACCACTCTC
CTTTCGAGGCCCGACGCTAATAATCCATGCTGGCAAGACAATTACGATTCGGAAGATGATTGCATGTCCACCG
TGTATTCATCGGCGTTCGTCGCCGAAGAGTGGATAAAGTCCATGCCGTGTGGAAAGGATGCAGATTGTTTGCC
TGAGAAGTTGTCTCGTCCGGGGACTATGGGAGGTTCGGTGAGTTTTTGACCTTGATTGGATGATGACGATGAT
GATGCATGTGGACCATCTATCTGCTGTTTGTTTACGATTGGATCTCTATTACCTCTTTCTGTGAAATGAGATG
ACGTCTGTTTTATTCATTGTCTC
>TR415|c0_g1_i1 len=1655 path=[1747:0-126 1748:127-150 1755:151-541 1745:542-565 1757:566-1069 1754:1070-1495 1753:1496-1654] [-1, 1747, 1748, 1755, 1745, 1757, 1754, 1753, -2]
CAACAAAGGGATGTATTCCATCCACTCGAACGATAGATCTCTTATTAGCTTATTGTCAACACTAATCTATCTG
CTAGAACCTTCTAAGTTTGCCTCTTGACATAATATTTTCTGTCTATTAGCCGTACATGATACTTATGGTGTAA
TGAAGTGACACCACCATGATAGCACTTGCCATTGATTTTGATGTGAGTTACTGAGCTTTACTCTTATCAGACG
GCGATGATGACGACTGAATCTTGACCAAAAGGGATTTGACTTTATCTGCCAACAGCGACCCAACAAAGAAACT
TGGCCACCACTTAAGTAAAACATTAGGATATCCAGCAAATGCTAAAAATATGGCAGGCCCGGGACACAAACCT
GCAATACCCCATCCTAATCCAAACATTGCCTCTCCAAGTATGAGCGAGGTGTCAATAACTTTATTGGTGGGAA
CATTGAAGTGACCGACTGTTGATTTCTGGCTTAGTGGACATTCGAGCGCCTTAGGATTCTTGAAAGCATTGAA
CCCCTTCACCCACTGATATGAAACAAAGCTCACTACCAATCCTCCACCCATGACACAAGCAAGGGTAGGATCC
CACGTGCCATCCCTGAGACCCTTCACATTTAAAAATCCATAAATTTTACTGGATATGGTCATCTGGGAAATAG
CCAAACCAACCGAGAAGAATGCAGCACTGAAAATGGCAGGCACGATCTTTCTCTTATTATTGTCATATTCGTC
CTTCTCCATTGCAGTAGCATTTTCATGAAGACCCCTACCAAAAAAGCTGGGCAAAACCGCTCCCGCGGCCAAG
GAGGCGAGCACGGTGCCAATCCCAATGGTAAATTCGGTCGGCAAGTGCTTGCCAACGGATTCGTAGCTGTCGC
GAAGGTACGGGTGGAAGGGACAGGTGGGCGAACAACCGGTGGCCGCGAATATTCCCGTTGCCATGAATGCGAG
TACTGCCGTGAGCGAACGGGTGCTTCGACGTGCCAGGCCGCATATTCCGTGTCCTGTGGTGCATCCATTTCCG
AGCTTGCTACCGAATCCAACAAAGAATCCTCCAAGAATAAATCCAAGAGGTGACACAATAGGAATCCCCTCCG
AATAACCCAACCTCTCATCCTTCAAAGCATCTACATCGACCATCGTAACATAAACTCTCGTCGTCAAACTAAA
TGCCGCCAAAAAGGCAAGTTTCCATTGTTGAGTTGGATCCGTCAATATTTCTCGGGGAGTGAGGATGAAGGAG
GACATCATTCCGGATGCTCCGAGTATATCGCCATTGAAGAGGAGCAGGGTTGCGGCGGAGAGACCGATGAGGG
ATCCGCCGAGGAGACCTGACATGGGTGTGAAGTAGTTGCTGAGGGACATGATGCTTGGAATGCGGTGTTTTAT
TGTGTAGAGTGCATAGGATGAAATGGTTGGTGTTGTGGTTGTAATGAAGATTTAGGATTTCCTTGATTCAAGA
TTGAAAAGTCGGCCTTCTGTTTACTCTGCTCCCACGGTCCTCAGCGAGCGTGCTGTCTGAATTGCCGGCGCAG
CTATTGAGCAGTACGTTTGTCATTCGTGTCATCAGCATCAACATGTCAGCAGAGATCAGCATTCCTTTCTACT
TCCGATGCGATCGGGGGAACGGGCCTATCGGGCGGACGGATTTATTTTC
>TR415|c0_g1_i2 len=1114 path=[1747:0-126 1748:127-150 1755:151-541 1745:542-565 1757:566-1069 1741:1070-1113] [-1, 1747, 1748, 1755, 1745, 1757, 1741, -2]
CAACAAAGGGATGTATTCCATCCACTCGAACGATAGATCTCTTATTAGCTTATTGTCAACACTAATCTATCTG
CTAGAACCTTCTAAGTTTGCCTCTTGACATAATATTTTCTGTCTATTAGCCGTACATGATACTTATGGTGTAA
TGAAGTGACACCACCATGATAGCACTTGCCATTGATTTTGATGTGAGTTACTGAGCTTTACTCTTATCAGACG
GCGATGATGACGACTGAATCTTGACCAAAAGGGATTTGACTTTATCTGCCAACAGCGACCCAACAAAGAAACT
TGGCCACCACTTAAGTAAAACATTAGGATATCCAGCAAATGCTAAAAATATGGCAGGCCCGGGACACAAACCT
GCAATACCCCATCCTAATCCAAACATTGCCTCTCCAAGTATGAGCGAGGTGTCAATAACTTTATTGGTGGGAA
CATTGAAGTGACCGACTGTTGATTTCTGGCTTAGTGGACATTCGAGCGCCTTAGGATTCTTGAAAGCATTGAA
CCCCTTCACCCACTGATATGAAACAAAGCTCACTACCAATCCTCCACCCATGACACAAGCAAGGGTAGGATCC
CACGTGCCATCCCTGAGACCCTTCACATTTAAAAATCCATAAATTTTACTGGATATGGTCATCTGGGAAATAG
CCAAACCAACCGAGAAGAATGCAGCACTGAAAATGGCAGGCACGATCTTTCTCTTATTATTGTCATATTCGTC
CTTCTCCATTGCAGTAGCATTTTCATGAAGACCCCTACCAAAAAAGCTGGGCAAAACCGCTCCCGCGGCCAAG
GAGGCGAGCACGGTGCCAATCCCAATGGTAAATTCGGTCGGCAAGTGCTTGCCAACGGATTCGTAGCTGTCGC
GAAGGTACGGGTGGAAGGGACAGGTGGGCGAACAACCGGTGGCCGCGAATATTCCCGTTGCCATGAATGCGAG
TACTGCCGTGAGCGAACGGGTGCTTCGACGTGCCAGGCCGCATATTCCGTGTCCTGTGGTGCATCCATTTCCG
AGCTTGCTACCGAATCCAACAAAGAATCCTCCAAGAATAAATCCAAGACTAATAAGTCATGGGTGTGCGTAAG
ATGTGTTTGTGCAAAGCGT
>TR416|c0_g1_i1 len=561 path=[622:0-83 623:84-112 641:113-342 640:343-406 638:407-476 631:477-560] [-1, 622, 623, 641, 640, 638, 631, -2]

CCTTCATCGCATCTCGCATCCCTGCCTTCATCTTCGTTTACATCGGTCATTCACCGAGCTACCGAACATCCAC
CCAGCCCCCCCCTTAGTCTCAATCTCAATAGCCGATAGCAGACTCTCCTCTCTCTGCCATAGATCGTAGATCG
CTCAACCATGACATCCCAAGCCGCCGCCAATCTCCTCCACGCCAAGGAATGGTCGAAAGTAGATCCCAACCCA
GAAACATCGAGCTTCGTTCAAAACCTGATTGAACAGGTCGAAGCAGGTGGCAGCAATGCTGACGCCCGCCGTC
CATTACAAACCCTATTTCCCGTTGCGCCAAACAGTGATACGAATAACTACCAACCACAGCTACACCCTCGAAT
CGGATTTGGTACCGCAGGTCTACGTGCGAAGATGGAGCCGGGTCCACTTGGGATGAACGACCTGACCGTCATA
CAAACTGCACAGGGCCTTGCAAGTTACATCCTCCAATGCAATAATAATGATGATGATGATGATATCACAAACA
ACAACAACAACAACAAACGTCCACTCAAAGCTGTAGTAGGATACGATCAT
>TR417|c0_g1_i1 len=1017 path=[1018:0-193 1025:194-757 1021:758-1016] [-1, 1018, 1025, 1021, -2]
TCCGATCTCTCATCTGTCTCTATTGTACTTATCACATCATCATCATCATGGCAACTTTCTGTTCAGATGAATA
GAGTTGTCCCTAATTTGCCTCTCGGATAATATCCATGACATGTACCAGAAACTCCTCTAGAGTAACAATAAAG
AGAGAATTGACTAAATTGTCCGTCCGTACAATAATATTGGGAAATGATGTGAGCGCGCTGCGATAGAGTTGGC
GACTGATAGAATGAATCTGATGAATGACGACTTGCATACCAAAACATCAATACTAATATTGTATGAAGAAGAA
TGAGTACCAATATGAATAAGTATAGAGCAGAGCACTGTTGAGAGTGCATATTGTACGGAATCTTTGCTTGAAT
TATACCTTCATTTCATTTGGGGGATACAAACCAATTATCTTGGCGTGCTATATCTATTACCAATGATATGTAG
CTTTCAACGACATGATCTCTTTCATCAGTTTGTAATTGAAAGAAATAATCCTACCTACTCGCCAAGTCTTGGG
TATTCTTATCCGATTCAGTGAAGAGTAGGTTCGAGCAATCATACTGTTGGTTCGTGTGAATCTTAGAACAATA
CCGATGCATCTCGTGAGCTACCTACATGGATTTTCTTATTGCATCTTCCGCATTTGATGCCTTGACTTCGTCA
TTAGATGATCGCAATCTAGTTGCTTCCTATAAAACGTTCTTTTACCTTGTATCATTTACTCCTCGCATCGATA
TCTAGATCAAATACAACAACTATGTGTGAAATACAGCAATGAAATATGATATCTTCGATAATTCTATTCATTA
ATATTTTACAACAGCCCTTCTCTGAACTCTAGATATTACGACACGAAAGAACTAGTTTCGAACAGTTTCGCAC
CATTCTCTAATCCAATACATTCCATTCACACAAAGCGTCTCAGGGAGAAGGAACACTCTTATTCGTCTTCCCA
GCAGCAGCAGCAGCAGCAGCAGCATCACGAAACTGCTTCCACCTCAACACCGGTCTCACAGATCGGAA
>TR417|c0_g2_i1 len=476 path=[1022:0-193 1023:194-216 1024:217-475] [-1, 1022, 1023, 1024, -2]
TCCGATCTCTCATCTGTCTCTATTGTACTTATCACATCATCATCATCATGGCAACTTTCTGTTCAGATGAATA
GAGTTGTCCCTAATTTGCCTCTCGGATAATATCCATGACATGTACCAGAAACTCCTCTAGAGTAACAATAAAG
AGAGAATTGACTAAATTGTCCGTCCGTACAATAATATTGGGAAATGATATCAAATACAACAACTATGTGTGAA
ATACAGCAATGAAATATGATATCTTCGATAATTCTATTCATTAATATTTTACAACAGCCCTTCTCTGAACTCT
AGATATTACGACACGAAAGAACTAGTTTCGAACAGTTTCGCACCATTCTCTAATCCAATACATTCCATTCACA
CAAAGCGTCTCAGGGAGAAGGAACACTCTTATTCGTCTTCCCAGCAGCAGCAGCAGCAGCAGCAGCATCACGA
AACTGCTTCCACCTCAACACCGGTCTCACAGATCGGAA
>TR417|c1_g2_i1 len=1356 path=[1494:0-979 1495:980-980 1496:981-1004 1508:1005-1085 1492:1086-1120 1506:1121-1279 1504:1280-1303 1505:1304-1355] [-1, 1494, 1495, 1496, 1508, 1492, 1506, 1504, 1505, -2]
GATCTGCAACAGGAGCAGCAGCAGCAGCAGCAGCAATGATAGCATCAATTCCTTTGATACTGTCACTGTCCCA
ATCCTGTCGGAATCGACCAAAAGTGCATGCCACCAAAAGTCAATACTACTCACCGGCGCGTCCCGCGGCCTAG
GCAAATCACTAGCACACGCATTATCCTCATGCCAACCCTCCCTCTTGATATTGAGCGGACGAGACGAGACAGC
ATTGCGAGAGGTACAACAAGAGTGCCTGCAAATTTCGAAAAAACCATCAGACGATGAGAATGATAGTGCAACG
ACAGCACCAACATTCACTGAAGTAGAAATTATTGCGTGCGATTTGGAGGACAAATCTTCCGTGGAGGAGTTGG
CATCTCAATCCCTCAAATTGGCAGAACAATGCAATTCTTCGGGGGGAAGTGTTGACATACTCATTAACAATGG
AGGTATATCCTCTCGATCCTCATTTCTCGAAACAAAGCTGGATGTCGATGAGAAGCTGATGCAGGTTAATTTC
TTTTCAGGTGCCGCTTTGGCCAAACGGTTGGTTCCAGGGATGGTCGATAAAAAGGATGGGATGGTGATTTGGA
TCAGTTCGGTACAGGGGAAATTGGGAACACCTTATCGAACGAGCTATGCTGCATCGAAATTCGCTGTGCAGGG
GTATTGTGAGGCGCTCCGATCGGAACTGGCCTCGAGCAACGTTTCCGTCCACGTCGTTAGTCCGGGATATATT
CGTACCAATTTGAGCGTCTCTGCTGTGATGGGGGATGGGAGGGCTTACAGCAAGATGGATGAGACTACAGCAA
ATGGCGCCGATCCAGACGAGGTGGCAGCGAATATTCTCAACACTGCCGCTGCTGGCACGACCGATTTTGTCGT
GGCAGCCACTCTTTCCGCCAAGGCGGCTTTGTGGTTAAAGGTTCTAGCGCCATCATTTTTGGAGAGCATGTTG
GTGAAGCGATTCGAGAAACAAAAACAAAAAAGTTGAGTCGAAATTTTGAATAAAAACTCCTCGAAACAACAAC
GCTAGATTGCTATTTCCTTCCTTTGATACGATATCTTTTGGCGCACTCTGATCCATAATGAAACTCCATGGTT
TGTTGCTGGTGTATGTGGGTATAAAGAGCTTGGACCCTGCGCAAAAAGTATCTATCATGCATTAGATTCGATT
CCAGGTGAGAGGATACCGCACCTGTCTTCAAGCCGAAATTCTGCTCCTGGCATCTTGCAGATGGGACTGTTCC
ATCCTTTTGCGGTCAGTTCTTTCACCTAGAGTCTGGAAATCGTTTCCCCGTCTCAAACCAAAGCGTTTAAATC
AACGTAATAATTAGGCAAAATTTTACTAGTTTAAAAAAAAAG
>TR418|c0_g1_i2 len=2062 path=[2541:0-291 2542:292-308 2598:309-345 2594:346-387 2591:388-651 2584:652-675 2597:676-1127 2593:1128-1195 2561:1196-1216 2596:1217-1564 @2592@!:1565-1765 2557:1766-1770 2558:1771-1788 2559:1789-2061] [-1, 2541, 2542, 2598, 2594, 2591, 2584, 2597, 2593, 2561, 2596, 2592, 2557, 2558, 2559, -2]

ACAACAACAACAACAATAACAACACGGAAAGATTACTAATCATCTACGCATCGATCCTCACCGCATACCACGC
CTGGACAACACATCAATCATCATCATCGCTGTTGTCGAATACTCCAATCCCATTCTCGTTGGTCGTTGTACTT
TCGTTCCTTTCGGGATATGGCATTGCTCATTACGAACAAGAACTACTACTGCTACGTAACAACAACAACAACA
ACAATCCTACCAATAACTTTCCAACGGGGGGTATTGTTGGATCTGCTTTGGGTGACATTCTTTTAACTTTTTT
GAGACGCGTGGTATCAATGTGCGGCAGTAGCAGTGGTATTGTTCCCAATGCAGGCGATGGTAGGGTAGTACAG
CTGGTACAACATGCGCCTACTGAGACGACGACGACGACGACATCATCCATTGCCGTTACAACGTTGGAGAATC
GAGAACAACAACCAAGAGGCATCATCATCACCGACGACGAGAACGATGGCCTGCGTCAACGCACCAAGAAATT
GGCCCGACGCGGAAAATCGCGTCTCAAACGCTTCGGTCGAAAGTACGTCACGGGACCCATCGAAGACCACCTC
GCGAATAACAACAACAACGACGGAGGAGAGTGGGAGGAGAAACTCGCGGCTCCGTTTTCGCAGGAGGGACCGT
TGATGGATCACCTGTTGAAGTATTCGGATTTTACGAAACACGGCAGGAGGAGGAGGAAATCTGCGATTGCGGC
TGGTGCTACGACAAAGACGACGGCAACCGACGACGATGGTGCGAAGAACGTAGCACGGGACGATGACGACGAT
GACGATCGTCGAAGAGGGGACGACATCGACGATAACGACAACGCGAGCAAGAGAAAACCGTCGTCGTCCGACA
TTTCCATGGGAAATGCCAATTTGAAAAATACCAAGGCGGAATCGCTGGAGAGCGACGAACAGTTTATGGACAA
GGTTCTCGACGTCATGTGCGAATTGCGCGGTATGGATTTATTTCTGACGGATGATCCAGAGGAGGAGATTTGG
CGAGAGCCTCTCTTGATTCAGTCTGGACTGCGAGATGTGCCAACGTTCATTGCAAACATGATGTTTCCTTTTG
GGAATATGACGGCGTATTTTAAGCTGCCAGATTGGTTCGATGATTGGGATAACATCCCGGAAGAGAAAGAGGA
CGACCCTCCGAATGTTAAGGCATTAAAGAGATTCCTCCTGGGAGACGAAGACTACCGAAATGCCCGATGCAAG
GTAATCCCACACGTCGTCGACGGACCCCTCGCCATACGACTCATCAAACCCCGTCCCATGGAAGTGAACATCG
ACGGACCGCGTCATCCCGCGACGTGGCACCAGGTGTCGAAGAAGAAGATCGCGGAGGACCCCCTGACGAACGA
GACGACGAGCGCGGCCGTGTTGGAGTGCGATATCGACGTGGGCATGTCGAACAAGCAGATTCGGAAGATTATC
AATGTTATTCGACCGCACATTGAGTCGGTTACGATAGACGTGGCTTTGATCGTTGGTACGCCCAATTTTTCGG
AGGTGGAGGAGCCGAGTGCCTGCATTGGACTTTGGAGGATTGAAAAGGTCGATTTCGAAAGCGCGGCGGTCTT
TCCCGAGCTGGCGGTGGAAGATGCCGCAGAAAAAGTGAAGATGATCATGTCAGGGATGGAGATGGAGGAAGTA
GTAGGAGGTGTAGCTGCTGCTTAGATGGGTAATTGACGAATTTCATTTTATATGATTATGGGGTAGCATTTGA
CCTTCATGACAACATATAGTATGGACTTCTTTCTGCAAATCATTAACGATGTATAATAGTTGATTGCAGGATT
TATGCTGCTTTTATGAATTCATGCCTATCACTTATTCTCGTTTCATAAAAAAGGTTGAATTTGCCATGGGAAT
ATCATTGTCTTGACTTAACAGAACGAGTCCGAATAGTCCAAGTTACTGCAGTGGACAAACAGTTGCTACTTGG
CTTGGTGTAGATTCTTGAACTGTAACTTTTCCGGGCAGCTCGACATAACCGAAGGAGTCCTCACTCAATATAA
CTGCTGATTTTGGAGGTT
>TR418|c0_g1_i3 len=1199 path=[2563:0-123 2593:124-191 2600:192-329 2596:330-677 @2592@!:678-878 2574:879-925 2575:926-1198] [-1, 2563, 2593, 2600, 2596, 2592, 2574, 2575, -2]
ATCTGAAGAATACACATCTTGCACTGTATATTCCAATGCGTGCGAAGATATATAACAATACATTGTTTTTCAT
TCTCTCTCGTCACGTATATTTTATGGAAGGCGTATTTTAAGCTGCCAGATTGGTTCGATGATTGGGATAACAT
CCCGGAAGAGAAAGAGGACGACCCTCCGAATGTTAAGGCATTAAAGGTAAATATATCAGCACACACGCTCAAA
ATGTATCATTTCTTTTTTCGAAGCAAGCACACCAGAACACACACATGTTAAAACTCCAACATCCCGCTATTGT
ATAAAACGAAAAAACAGAGATTCCTCCTGGGAGACGAAGACTACCGAAATGCCCGATGCAAGGTAATCCCACA
CGTCGTCGACGGACCCCTCGCCATACGACTCATCAAACCCCGTCCCATGGAAGTGAACATCGACGGACCGCGT
CATCCCGCGACGTGGCACCAGGTGTCGAAGAAGAAGATCGCGGAGGACCCCCTGACGAACGAGACGACGAGCG
CGGCCGTGTTGGAGTGCGATATCGACGTGGGCATGTCGAACAAGCAGATTCGGAAGATTATCAATGTTATTCG
ACCGCACATTGAGTCGGTTACGATAGACGTGGCTTTGATCGTTGGTACGCCCAATTTTTCGGAGGTGGAGGAG
CCGAGTGCCTGCATTGGACTTTGGAGGATTGAAAAGGTCGATTTCGAAAGCGCGGCGGTCTTTCCCGAGCTGG
CGGTGGAAGATGCCGCAGAAAAAGTGAAGATGATCATGTCAGGGATGGAGATGGAGGAAGTAGTAGGAGGTGT
AGCTGCTGCTTAGATGGGTAATTGACGAATTTCATTTTATATGATTATGGGGTAGCATTTGACCTTCATGACA
ACAGTCCAGATTACATATGTCCTCTTGTATAGTATGGACTTCTTTCTGCAAATCATTAACGATGTATAATAGT
TGATTGCAGGATTTATGCTGCTTTTATGAATTCATGCCTATCACTTATTCTCGTTTCATAAAAAAGGTTGAAT
TTGCCATGGGAATATCATTGTCTTGACTTAACAGAACGAGTCCGAATAGTCCAAGTTACTGCAGTGGACAAAC
AGTTGCTACTTGGCTTGGTGTAGATTCTTGAACTGTAACTTTTCCGGGCAGCTCGACATAACCGAAGGAGTCC
TCACTCAATATAACTGCTGATTTTGGAGGTT
>TR418|c0_g1_i4 len=2086 path=[2541:0-291 2542:292-308 2598:309-345 2594:346-387 2591:388-651 2584:652-675 2597:676-1127 2593:1128-1195 2561:1196-1216 2596:1217-1564 @2592@!:1565-1765 2574:1766-1812 2575:1813-2085] [-1, 2541, 2542, 2598, 2594, 2591, 2584, 2597, 2593, 2561, 2596, 2592, 2574, 2575, -2]
ACAACAACAACAACAATAACAACACGGAAAGATTACTAATCATCTACGCATCGATCCTCACCGCATACCACGC
CTGGACAACACATCAATCATCATCATCGCTGTTGTCGAATACTCCAATCCCATTCTCGTTGGTCGTTGTACTT
TCGTTCCTTTCGGGATATGGCATTGCTCATTACGAACAAGAACTACTACTGCTACGTAACAACAACAACAACA
ACAATCCTACCAATAACTTTCCAACGGGGGGTATTGTTGGATCTGCTTTGGGTGACATTCTTTTAACTTTTTT
GAGACGCGTGGTATCAATGTGCGGCAGTAGCAGTGGTATTGTTCCCAATGCAGGCGATGGTAGGGTAGTACAG
CTGGTACAACATGCGCCTACTGAGACGACGACGACGACGACATCATCCATTGCCGTTACAACGTTGGAGAATC

GAGAACAACAACCAAGAGGCATCATCATCACCGACGACGAGAACGATGGCCTGCGTCAACGCACCAAGAAATT
GGCCCGACGCGGAAAATCGCGTCTCAAACGCTTCGGTCGAAAGTACGTCACGGGACCCATCGAAGACCACCTC
GCGAATAACAACAACAACGACGGAGGAGAGTGGGAGGAGAAACTCGCGGCTCCGTTTTCGCAGGAGGGACCGT
TGATGGATCACCTGTTGAAGTATTCGGATTTTACGAAACACGGCAGGAGGAGGAGGAAATCTGCGATTGCGGC
TGGTGCTACGACAAAGACGACGGCAACCGACGACGATGGTGCGAAGAACGTAGCACGGGACGATGACGACGAT
GACGATCGTCGAAGAGGGGACGACATCGACGATAACGACAACGCGAGCAAGAGAAAACCGTCGTCGTCCGACA
TTTCCATGGGAAATGCCAATTTGAAAAATACCAAGGCGGAATCGCTGGAGAGCGACGAACAGTTTATGGACAA
GGTTCTCGACGTCATGTGCGAATTGCGCGGTATGGATTTATTTCTGACGGATGATCCAGAGGAGGAGATTTGG
CGAGAGCCTCTCTTGATTCAGTCTGGACTGCGAGATGTGCCAACGTTCATTGCAAACATGATGTTTCCTTTTG
GGAATATGACGGCGTATTTTAAGCTGCCAGATTGGTTCGATGATTGGGATAACATCCCGGAAGAGAAAGAGGA
CGACCCTCCGAATGTTAAGGCATTAAAGAGATTCCTCCTGGGAGACGAAGACTACCGAAATGCCCGATGCAAG
GTAATCCCACACGTCGTCGACGGACCCCTCGCCATACGACTCATCAAACCCCGTCCCATGGAAGTGAACATCG
ACGGACCGCGTCATCCCGCGACGTGGCACCAGGTGTCGAAGAAGAAGATCGCGGAGGACCCCCTGACGAACGA
GACGACGAGCGCGGCCGTGTTGGAGTGCGATATCGACGTGGGCATGTCGAACAAGCAGATTCGGAAGATTATC
AATGTTATTCGACCGCACATTGAGTCGGTTACGATAGACGTGGCTTTGATCGTTGGTACGCCCAATTTTTCGG
AGGTGGAGGAGCCGAGTGCCTGCATTGGACTTTGGAGGATTGAAAAGGTCGATTTCGAAAGCGCGGCGGTCTT
TCCCGAGCTGGCGGTGGAAGATGCCGCAGAAAAAGTGAAGATGATCATGTCAGGGATGGAGATGGAGGAAGTA
GTAGGAGGTGTAGCTGCTGCTTAGATGGGTAATTGACGAATTTCATTTTATATGATTATGGGGTAGCATTTGA
CCTTCATGACAACAGTCCAGATTACATATGTCCTCTTGTATAGTATGGACTTCTTTCTGCAAATCATTAACGA
TGTATAATAGTTGATTGCAGGATTTATGCTGCTTTTATGAATTCATGCCTATCACTTATTCTCGTTTCATAAA
AAAGGTTGAATTTGCCATGGGAATATCATTGTCTTGACTTAACAGAACGAGTCCGAATAGTCCAAGTTACTGC
AGTGGACAAACAGTTGCTACTTGGCTTGGTGTAGATTCTTGAACTGTAACTTTTCCGGGCAGCTCGACATAAC
CGAAGGAGTCCTCACTCAATATAACTGCTGATTTTGGAGGTT
>TR419|c0_g1_i1 len=6581 path=[6843:0-74 6844:75-98 6875:99-934 6868:935-958 6872:959-3131 6852:3132-3155 6878:3156-4982 6865:4983-5004 6871:5005-5223 6860:5224-5247 6876:5248-5853 6873:5854-5976 6847:5977-5992 6848:5993-6580] [-1, 6843, 6844, 6875, 6868, 6872, 6852, 6878, 6865, 6871, 6860, 6876, 6873, 6847, 6848, -2]
GGACGTCGTTTTTCCCGTGATGGATGATCGCTGGGTGGTGGCCGTGTTGTTCTTCTCCCTTACGTTGCTTTTG
TCAACTGCCCTTGCCTTGAGGGGTAGTAGTAGTAGTGGTAGCAGTAGTAGTAGTAGCAGTATCAGTGCCAGTG
TCAGTGGGGAGAGGAAGACGACGGGTTCTTCGTCCTCGTCCTTTTTTCGTTCTTTGCCGGTCGACGACGACGA
CCATGTGGCGAGGATGGGCCTCGGAAATAAAGAATCCACCCGCCCGGAGAATACTCCGCTCGTGGATGTCGAG
GTAGGGAACGATTACGATTACGATGTTCTTCTCAACGGAAAAACGGACGAGCGAGATCATCATCATCACGTCG
AGAGTATCACCACCGACGACGATGATGCGCTTCTTCGGCCTGTGATGATGACGATGACTTTGGAAGATGGGGA
GGATATCGCGGCGATGGCGAAGCGCGACGAGAGGGAATTGCGCAAGGGAGAATCCGCCCTTCCGGCCTCGCTT
CGCGGTGGTGACGATGACACGGAGGGAGGGGAACATCATCGTCGTCATCATCGTCGCGATTCCGACGGAAAAA
TAACGGGCGAACAACCCGACGGAAGAGTAGAACGGATCGACGACGTTTACGAAAAAACAACCGACGACGACCA
TCGTCATCCTCCTCCATTCGGCGAAGGAGAGTTCGATCCGACTGCCGCGGAGGGAGGGGATGATCTCGAGCGC
GGGAGGAATTTGGAGGGGGACGAAGGCGAACAACAACATGCATCGAGCCCGCACGCTGGAGCTACTGCTTCTC
GCGGTGGCATCCTCGACGACGACGCAGCGACCCTCTACTACGTCGATTGGTCGAATCTCGTTTGCACGAATGA
TCCGGAGCGGCGTCCTCCCTATGCCACCGAAGTCGGGACGACTCTGTTCGAAACCGCCGTAGAATGCTGTGAA
ATGTGGCTTTCGCATCAACCCGACATTGATTGTTCGGAACTCTTGGCCGCATCGCCTTCCCTTTCATCGCAAT
CCAAACGCACCGATAACGCGACGGAAGCAACAACAACAACTTCCGTGACGAATCGCCCGACCGACGGAAGCAC
CGATGCTTCTCCTCCTCCAACCCCGGCAGCATGGGCCGACGCTGCTGCTACGTATTGGCCGACTTACTCTCCG
ATTTCCTTCTGCGAGTTCCACAACCCCGAAATATGCGGGTGCGAGGATGTCAAACAATCCGATTATCGGGGGA
CGATAAACACGACTAAATCGGGCGTCACTTGCGACCAATGGAGCAATTCGCCTCTCACCCCGGAATCCTACCC
CGATAGCGGTCTCGAGAACAATTACTGTCGCAACCCGGTTGGCTTTGGCGAAGTCGCATTTTGTTACTCGATG
GATAGTAAATTCGAGGATTGCGACGTTCCGTACTGCAAAGACGTGCCGCCCGATTGCGAGTATCCCAACCCCG
AATCATGCGGATGCGACAACGTCCGTCAGGCCGATTACCGAGGAACGACGAACGTCACCGCTTCGGGAAGGGA
ATGCCGACGATGGAGTTCCCCGGAAGTGCTCGGTCGCGGATACACCGCGGAAAATTACCCGTACGGCGGGCTG
GACGAGAATTACTGCCGCAACCCCACTCCCGACGAGGATCACCAGGACGCTTGGTGCTTTACCACGGACCCGA
GCGTGGAATGGGAGCACTGCGACGTCCCTCTGTGTGCGGCGCCCACGATTTCTCCGCAACCCACGCTCTCCTC
GCCGCCGACTCCGAATCCGACGTCCTCCTCCGATCCCACTTTGGCATTGATCTCTCCGTCCGAAACGCCATCT
GCTTCTTCTTCTCCGACGCATCCGGCCAGTTGCATGGACCTAACTATCGCTCTCCTCATGCCCCCACGGACAA
ATGACTGGAATGACGACAAGTCGGCTTTTTGGTGGCGTTTGCGCGACGGCACAGATATTAAGACTGTTGATTG
GATAATTGAGTACGATGTAAACTCCTTAATTGAAGGTGATCTTCGTTATGGAGAGTACAACGTAATTGTGATA
GAACATGCCAGAAGCACAAATTCAAGGAATGAGAAAATTTGCCTTCCAGAAGGGAACTACTCCTTCATTGGAC
AAATGAAATCTCTTTCTGGGAACGAAGATGCTGGGAAAAGTGTGTGGTATGTTTTATATTTTAATGGGAGGAA
GATTGGGCGTGGCGATCTGATTCGGGAGAAATTTGAGTTTCAGCTCCCATTCGAACTGATTGGTGGGGACAAG
GAAGGACCAGGCTCGACCACAAATGAAGAATCTCTGGGTGGCGAGTTGGAGCCCTTGCAGTGCTACTTTGACG

CCTTCAATTACGAAAATGTTGACCGGAGGGAGGTATGGTTCAGCGACAAGTGTGGGCCAATGCTAGAGGATGC
ATGCCAAAATGATCATGCTTTGGGTTTCACAAACGACGTAGACTTTCCCGAGATTGATGATTTTTGCGCTTAC
CACAGATGTGCATACGATGCCATCGGCACTGACAGTACCTTCCAAGAGTGGGCCTCGAGCGGAGAATTTCTCA
ACTGCGAATGTCTCTACCATAGTTGGGACTGCGAGCATGGAAGTGGTGGTTGTGAAAGCGATCTCAGAAAAAA
TGCAATAGAGTGTTGCGACAATGACACAGGCTGCGATTGCCTGGGCGTGCTGAGTTGTTATGAAGGGAACTTA
GCAAGTTGTGGTGATGTTGCTGATTATTGCTGCGGCGATGAGGACGGAGGGTGCCAATGTGACTTTGAAAAAA
CAGCGTGTGAAGGAAGCCTTCAATATCTAACAGAAGAGATAGTGTTGAAGTGGCAGTATTCAAGTGAATGCGC
AATTGCTGATGATGTTTGTTCGTCAGGTCGCCCATTGTTTGAGAAGAAACTAGACGAAGTTTGCCCAGAAATC
AATCGACGATGCGGCCGAGGAGATGGTCAAAGCTGCCTTGGCTTTGCGAATAATTGTTGCAGCGATGTTGATG
GCGATTGTAAATGTGAATACATGAAAAAATCATGTACCGCAAGCCTCAAAGGTTTATCAATCGACGAGAAATA
TCTTTCTCAAGAGTGCCTTGAGGCCGAAGAGATTTGCTGCGAAGATTGCAAGCCAGTCTGGCAATTTGACGAC
GTGAGGATTGAGACTTGCCGATGCGGCTGCGATGTTTGGGAACCAATTTGCGCTACCCATCGGGGTCAAGCTT
GCGAGTTCTTTGCTGGAAGGTGCTGCGGCTCGCCTACAGTTGATATCCACAACCCCCAATGCTACTGCGAGGC
GTATACTGAGTTGGAGCAACACATGAACTATGAACTTAGCCACAAAGAGTCTTGCTCAAATGCGGACGAAGCA
GTTTTCAAGTCAGATTATGTCGACGAACGCGAAAGTCTTAGCAACATCTACAGTGAATTAGGAGGAAAATATT
GGAATGATAGAAGCATGTGGCTGGAGGGTGATACGGAAACGGCGCATTGCATGTGGCATGGTGTCAAGTGTGA
CGCTACTGGTTTCATCTCTGAAATTGACCTAACAGGCAACAATCTTGTAGGCAATATGCAGCCATCACGATTC
TTCTCGTCAATGCATGCACTCAAGTCACTCAGACTTGCGAGCAACAAGCTTGAAGGCATAATAGGGCCCAATT
CTTTTTATGGACTACGGGCCCTTGAGCATGTCGACTTTTCCCAGAATAATCTCAATGGAGAAGTGGATGTCTT
ACTTTCGCCATCAATAAAGGAAGTCAACATGAGTCATAATAGTTTCACATACATGGCTCCTTTTAAGAAGTTC
AGAGGCGCACAACAAACTTTGAGGCATTTGGACTTGAGCTATAACAGCATCCATGAGGACATCTCCGATATTT
TGAAAGATATTCCATCTAATCTCAAGGAGTTAGTGCTTACAATGAATCACATCTTTGGTTCTCTACCTGATGC
ACTCCCAGAATTGGATGACATGAATCGGTTTGTTTTGAACAACAATCGCATGACAGGGCCAATTCCTGATATC
TCACGTTCTTTCCCACGTCTGAAAGAACTGGATTTGTCAAGTCAGAAGCAAGCCAACAATAATTATACAGGGT
TGTCGGGATTAATACCAGCAGGTTGGACATTATCTCCAGAACTTGCTGTATTGGATCTGTCAAATAATAGACT
AACAGGTGATATACCTCCAGGCATTGGAAACCTTCTTCACCTGAAAAAGCTCAATGTATCGAACAATGTTTTG
AGCCGAAACAGTGAGTTGGATGGAATATCTGCCGACCTTGGAAACCTTGCAGGTATTTGTGAAGTTATGGATT
TGTCAAATAACAAGTTGTTAGGAAAAATCCCTTCAACATTCAAAAGATTTAAGAGTTCGCCGAAACCTACGTT
GATTCACCTTAATGGCAACGAAGACTTATTCGCTCCAGCGCCTCTAGACCTATGTAATGTGACGAAGTTCGAC
CTTTTGAATCATACCAAATGGTGCCCGACTGAGAGAAATATTTTAAACGAATTTTATGACTTTACCAAGGGCG
GAGAGTGGACAGAGAGTACTTACTGGAATACACCATATACACCTCATTGTCAATGGTTAGGTGTAACTTGCAA
TGGTAATGGTGCTGTTATCGGGATTAACTTGACAAGTAATGGCTTATCTGGAAAGTTGACCCCAAGGATTTCA
GAGTTTCATTCACTTCGAGTTCTCGATCTTGGCGACAATGATATTAAGGACGAAATTCCTTCAACAATCGGAG
ATATTGAATATCTCACCCATTTGAGATTGAGCTTTAACTCATTTGGTGGCACAATACCAACCGAGTTGGAAAA
ATTACAACACTTGGAGATGGTGCATCTTCATGGTAACAGATTGCAGGGTATAATGCCATTATTGAATACTCAG
CATTTGGGAAATGAATCTTCTTTCATATCTGACTGTGGCTACCCCACCAACTTTGATCAGCCGATTTCCTGTA
AAGAGTGCACTATTTGCTGCAACTCTAATGGAATTTGTGAATCAACAGTGGAAAATGCACTGCAGCAACAAGG
GTTTGCGAGTTACATCTACTTTGTGCCTGTCTTTGCCCTAATAATTCTTGGATTCACTTTGACTGTGGCCATT
CTATCCTCCTACTATGAACGATGGTACAACCGAAAATATCCCAATGAGAGTGATGGACAAGACGGCCGTCTGG
AAGCAAATGGAGCAGCTGATACGCTCTCTATCATCGAGATGAGCCCAATTAAAGAAGATGAAGAATATGCATT
GGAGTCCATCGGTGAAGACTCGGTGTACTCTTACATCTTCTCGAAGAACAAATTTTCTTGGCTTATTACATTG
TTTGTGGTAGTTCTCCAAACCGGCTGCCTTTTATACTTCATTGATGCCGCAATGAGAGATACTGAAAATGAAG
CAAGTGACTGGATTTATTCATGGAAATGCCCTCGGGATAATCTCGAGTGTTTGAATGAAAATGATGTCAGTCC
ATTGGGTTGGTTAATCTTTACCTTTCTCATGCTTGCCCATCTATCGAAGGATTTTATCAATGGGTTAAAGTTG
ACTGTTCTTTGTGGCAAGCTTAGGCATGAAAGAAGTGCAATGATCAAATACTTTAGTGGTGGATTGATCCTCC
TTTGGATATCCTCACTTGCTATATTGTCTTCATTTCTTTACAACCTTGCAATTGCAAGAACCGAGACTCAACT
TATTGAGAATGCAGTCATCGTCCTTTTCGTCTGTGACGTGGATGAATTTAGTCACATGGCAGTCAGAGCTGCA
CAATCATTACGGACGGGTACCCGAGAGTGTGTTGATGAAATAGCCACACTATCTGCTGCTGTCGAAAAAATGT
CTGCGGATTTTACCAAGTTACAAAAGAAATTGAAAAATCAAGAGGAAGAAATAGAATGCCTTTCAGAAGAGAG
GGATGAAAAATTGAAACTTTTGCAAAAGCAGTATGAGGAGATGGAGATGTGTTTGCAAAAAAAAAGATGAAGA
ATTAAAGAAGGTTTTACAATCCAATGTGCAATTGGAGTTGCGCATGAAAGCCCTGGAGGGAAGGGTAATGTTG
TGGATACAGTCCCACCCCACAGAGAGGCCCATAGATAGTCCTGACATCGGGGACTATGAGACACCATCAGCAA
GAGGTGTTGATTTAGAACCAATGAACCTATGAAGACTAAACTAAAAATCATAGTATCGCACAAGGGCCGAATC
ACGAACCTACCAGGGGGAGGGAAGTACTAGAAAATTGGGTTAGGGCACACTAAAATTATGAGACAGGATGGGG
CATCTAATAGCGAACCATAAGTTAGTTGCCATCTGAAGGTGCACAGGTAATGAAACCGTGCGGGAAAAAATAA
GACCTCAAAAAGTTAATTTATGCCCATTCAATTCTCTGAATGATTGACGACATCTGTACAGAGGAGGATTTGT
CTTCTATCTCACCCAGAAAGTCTGAGTGGCATTAGTAGGAGATACTTGTGCTTCCGGCACGGAAAAGGAAAGA
ACAAGGAAAACAGGAAGAAAAGAAGTGCAATGCCACACGGGCATTGCACTGGGCTTACTTCCGTTTAATATGA
GCATAACAACC

>TR419|c0_g1_i2 len=6662 path=[6843:0-74 6844:75-98 6875:99-934 6868:935-958 6872:959-3131 6852:3132-3155 6878:3156-4982 6874:4983-5085 6871:5086-5304 6860:5305-5328 6876:5329-5934 6873:5935-6057 6847:6058-6073 6848:6074-6661] [-1, 6843, 6844, 6875, 6868, 6872, 6852, 6878, 6874, 6871, 6860, 6876, 6873, 6847, 6848, -2]
GGACGTCGTTTTTCCCGTGATGGATGATCGCTGGGTGGTGGCCGTGTTGTTCTTCTCCCTTACGTTGCTTTTG
TCAACTGCCCTTGCCTTGAGGGGTAGTAGTAGTAGTGGTAGCAGTAGTAGTAGTAGCAGTATCAGTGCCAGTG
TCAGTGGGGAGAGGAAGACGACGGGTTCTTCGTCCTCGTCCTTTTTTCGTTCTTTGCCGGTCGACGACGACGA
CCATGTGGCGAGGATGGGCCTCGGAAATAAAGAATCCACCCGCCCGGAGAATACTCCGCTCGTGGATGTCGAG
GTAGGGAACGATTACGATTACGATGTTCTTCTCAACGGAAAAACGGACGAGCGAGATCATCATCATCACGTCG
AGAGTATCACCACCGACGACGATGATGCGCTTCTTCGGCCTGTGATGATGACGATGACTTTGGAAGATGGGGA
GGATATCGCGGCGATGGCGAAGCGCGACGAGAGGGAATTGCGCAAGGGAGAATCCGCCCTTCCGGCCTCGCTT
CGCGGTGGTGACGATGACACGGAGGGAGGGGAACATCATCGTCGTCATCATCGTCGCGATTCCGACGGAAAAA
TAACGGGCGAACAACCCGACGGAAGAGTAGAACGGATCGACGACGTTTACGAAAAAACAACCGACGACGACCA
TCGTCATCCTCCTCCATTCGGCGAAGGAGAGTTCGATCCGACTGCCGCGGAGGGAGGGGATGATCTCGAGCGC
GGGAGGAATTTGGAGGGGGACGAAGGCGAACAACAACATGCATCGAGCCCGCACGCTGGAGCTACTGCTTCTC
GCGGTGGCATCCTCGACGACGACGCAGCGACCCTCTACTACGTCGATTGGTCGAATCTCGTTTGCACGAATGA
TCCGGAGCGGCGTCCTCCCTATGCCACCGAAGTCGGGACGACTCTGTTCGAAACCGCCGTAGAATGCTGTGAA
ATGTGGCTTTCGCATCAACCCGACATTGATTGTTCGGAACTCTTGGCCGCATCGCCTTCCCTTTCATCGCAAT
CCAAACGCACCGATAACGCGACGGAAGCAACAACAACAACTTCCGTGACGAATCGCCCGACCGACGGAAGCAC
CGATGCTTCTCCTCCTCCAACCCCGGCAGCATGGGCCGACGCTGCTGCTACGTATTGGCCGACTTACTCTCCG
ATTTCCTTCTGCGAGTTCCACAACCCCGAAATATGCGGGTGCGAGGATGTCAAACAATCCGATTATCGGGGGA
CGATAAACACGACTAAATCGGGCGTCACTTGCGACCAATGGAGCAATTCGCCTCTCACCCCGGAATCCTACCC
CGATAGCGGTCTCGAGAACAATTACTGTCGCAACCCGGTTGGCTTTGGCGAAGTCGCATTTTGTTACTCGATG
GATAGTAAATTCGAGGATTGCGACGTTCCGTACTGCAAAGACGTGCCGCCCGATTGCGAGTATCCCAACCCCG
AATCATGCGGATGCGACAACGTCCGTCAGGCCGATTACCGAGGAACGACGAACGTCACCGCTTCGGGAAGGGA
ATGCCGACGATGGAGTTCCCCGGAAGTGCTCGGTCGCGGATACACCGCGGAAAATTACCCGTACGGCGGGCTG
GACGAGAATTACTGCCGCAACCCCACTCCCGACGAGGATCACCAGGACGCTTGGTGCTTTACCACGGACCCGA
GCGTGGAATGGGAGCACTGCGACGTCCCTCTGTGTGCGGCGCCCACGATTTCTCCGCAACCCACGCTCTCCTC
GCCGCCGACTCCGAATCCGACGTCCTCCTCCGATCCCACTTTGGCATTGATCTCTCCGTCCGAAACGCCATCT
GCTTCTTCTTCTCCGACGCATCCGGCCAGTTGCATGGACCTAACTATCGCTCTCCTCATGCCCCCACGGACAA
ATGACTGGAATGACGACAAGTCGGCTTTTTGGTGGCGTTTGCGCGACGGCACAGATATTAAGACTGTTGATTG
GATAATTGAGTACGATGTAAACTCCTTAATTGAAGGTGATCTTCGTTATGGAGAGTACAACGTAATTGTGATA
GAACATGCCAGAAGCACAAATTCAAGGAATGAGAAAATTTGCCTTCCAGAAGGGAACTACTCCTTCATTGGAC
AAATGAAATCTCTTTCTGGGAACGAAGATGCTGGGAAAAGTGTGTGGTATGTTTTATATTTTAATGGGAGGAA
GATTGGGCGTGGCGATCTGATTCGGGAGAAATTTGAGTTTCAGCTCCCATTCGAACTGATTGGTGGGGACAAG
GAAGGACCAGGCTCGACCACAAATGAAGAATCTCTGGGTGGCGAGTTGGAGCCCTTGCAGTGCTACTTTGACG
CCTTCAATTACGAAAATGTTGACCGGAGGGAGGTATGGTTCAGCGACAAGTGTGGGCCAATGCTAGAGGATGC
ATGCCAAAATGATCATGCTTTGGGTTTCACAAACGACGTAGACTTTCCCGAGATTGATGATTTTTGCGCTTAC
CACAGATGTGCATACGATGCCATCGGCACTGACAGTACCTTCCAAGAGTGGGCCTCGAGCGGAGAATTTCTCA
ACTGCGAATGTCTCTACCATAGTTGGGACTGCGAGCATGGAAGTGGTGGTTGTGAAAGCGATCTCAGAAAAAA
TGCAATAGAGTGTTGCGACAATGACACAGGCTGCGATTGCCTGGGCGTGCTGAGTTGTTATGAAGGGAACTTA
GCAAGTTGTGGTGATGTTGCTGATTATTGCTGCGGCGATGAGGACGGAGGGTGCCAATGTGACTTTGAAAAAA
CAGCGTGTGAAGGAAGCCTTCAATATCTAACAGAAGAGATAGTGTTGAAGTGGCAGTATTCAAGTGAATGCGC
AATTGCTGATGATGTTTGTTCGTCAGGTCGCCCATTGTTTGAGAAGAAACTAGACGAAGTTTGCCCAGAAATC
AATCGACGATGCGGCCGAGGAGATGGTCAAAGCTGCCTTGGCTTTGCGAATAATTGTTGCAGCGATGTTGATG
GCGATTGTAAATGTGAATACATGAAAAAATCATGTACCGCAAGCCTCAAAGGTTTATCAATCGACGAGAAATA
TCTTTCTCAAGAGTGCCTTGAGGCCGAAGAGATTTGCTGCGAAGATTGCAAGCCAGTCTGGCAATTTGACGAC
GTGAGGATTGAGACTTGCCGATGCGGCTGCGATGTTTGGGAACCAATTTGCGCTACCCATCGGGGTCAAGCTT
GCGAGTTCTTTGCTGGAAGGTGCTGCGGCTCGCCTACAGTTGATATCCACAACCCCCAATGCTACTGCGAGGC
GTATACTGAGTTGGAGCAACACATGAACTATGAACTTAGCCACAAAGAGTCTTGCTCAAATGCGGACGAAGCA
GTTTTCAAGTCAGATTATGTCGACGAACGCGAAAGTCTTAGCAACATCTACAGTGAATTAGGAGGAAAATATT
GGAATGATAGAAGCATGTGGCTGGAGGGTGATACGGAAACGGCGCATTGCATGTGGCATGGTGTCAAGTGTGA
CGCTACTGGTTTCATCTCTGAAATTGACCTAACAGGCAACAATCTTGTAGGCAATATGCAGCCATCACGATTC
TTCTCGTCAATGCATGCACTCAAGTCACTCAGACTTGCGAGCAACAAGCTTGAAGGCATAATAGGGCCCAATT
CTTTTTATGGACTACGGGCCCTTGAGCATGTCGACTTTTCCCAGAATAATCTCAATGGAGAAGTGGATGTCTT
ACTTTCGCCATCAATAAAGGAAGTCAACATGAGTCATAATAGTTTCACATACATGGCTCCTTTTAAGAAGTTC
AGAGGCGCACAACAAACTTTGAGGCATTTGGACTTGAGCTATAACAGCATCCATGAGGACATCTCCGATATTT
TGAAAGATATTCCATCTAATCTCAAGGAGTTAGTGCTTACAATGAATCACATCTTTGGTTCTCTACCTGATGC
ACTCCCAGAATTGGATGACATGAATCGGTTTGTTTTGAACAACAATCGCATGACAGGGCCAATTCCTGATATC

TCACGTTCTTTCCCACGTCTGAAAGAACTGGATTTGTCAAGTCAGAAGCAAGCCAACAATAATTATACAGGGT
TGTCGGGATTAATACCAGCAGGTTGGACATTATCTCCAGAACTTGCTGTATTGGATCTGTCAAATAATAGACT
AACAGGTGATATACCTCCAGGCATTGGAAACCTTCTTCACCTGAAAAAGCTCAATGTATCGAACAATGTTTTG
AGCCGAAACAGTGAGTTGGATGGAATATCTGCCGACCTTGGAAACCTTGCAGGTATTTGTGAAGTTATGGATT
TGTCAAATAACAAGTTGTTAGGAAAAATCCCTTCAACATTCAAAAGATTTAAGAGTTCGCCGAAACCTACGTT
GATTCACCTTAATGGCAACGAAGACTTATTCGCTCCAGCGCCTCTAGACCTATGTAATGTGACGAAGTTCGAC
CTTTTGAATCATACCAAATGGTGCCCGACTGAGAGAAATATTTTAAACGAATTTTATGACTTTACCAAGGGCG
GAGAGTGGACAGAGAGTACTTACTGGAATACACCATATACACCTCATTGTCAATGGTTAGGTGTAACTTGCAA
TGGTAATGGTGCTGTTATCGGGATTAACTTGACAAGTAATGGCTTATCTGGAAAGTTGACCCCAAGGATTTCA
GAGTTTCATTCACTTCGAGTTCTCGATCTTGGCGACAATGATATTAAGGACGAAATTCCTTCAACAATCGGAG
ATATTGAATATCTCACCCATTTGAGATTGAGCTTTAACTCATTTGGTGGCACAATACCAACCGAGTTGGAAAA
ATTACAACACTTGGAGATGGTGCATCTTCATGGTAACAGATTGCAGGGTATAATGCCATTATTGAATACTCAG
CATTTGGGAAATGAATCTTCTTTCATATCTGACTGTGGCTACCCCACCAACTTTGATCAGCCGATTTCCTGTA
AAGAGTGCACTATTTGCTGTAAGTGTATGAGAATGATCCAATATTCAATCGGTTTTTTGCTAACAATGAATTT
TGGATGAAACTTGACCCTTTTTTCAGGCAACTCTAATGGAATTTGTGAATCAACAGTGGAAAATGCACTGCAG
CAACAAGGGTTTGCGAGTTACATCTACTTTGTGCCTGTCTTTGCCCTAATAATTCTTGGATTCACTTTGACTG
TGGCCATTCTATCCTCCTACTATGAACGATGGTACAACCGAAAATATCCCAATGAGAGTGATGGACAAGACGG
CCGTCTGGAAGCAAATGGAGCAGCTGATACGCTCTCTATCATCGAGATGAGCCCAATTAAAGAAGATGAAGAA
TATGCATTGGAGTCCATCGGTGAAGACTCGGTGTACTCTTACATCTTCTCGAAGAACAAATTTTCTTGGCTTA
TTACATTGTTTGTGGTAGTTCTCCAAACCGGCTGCCTTTTATACTTCATTGATGCCGCAATGAGAGATACTGA
AAATGAAGCAAGTGACTGGATTTATTCATGGAAATGCCCTCGGGATAATCTCGAGTGTTTGAATGAAAATGAT
GTCAGTCCATTGGGTTGGTTAATCTTTACCTTTCTCATGCTTGCCCATCTATCGAAGGATTTTATCAATGGGT
TAAAGTTGACTGTTCTTTGTGGCAAGCTTAGGCATGAAAGAAGTGCAATGATCAAATACTTTAGTGGTGGATT
GATCCTCCTTTGGATATCCTCACTTGCTATATTGTCTTCATTTCTTTACAACCTTGCAATTGCAAGAACCGAG
ACTCAACTTATTGAGAATGCAGTCATCGTCCTTTTCGTCTGTGACGTGGATGAATTTAGTCACATGGCAGTCA
GAGCTGCACAATCATTACGGACGGGTACCCGAGAGTGTGTTGATGAAATAGCCACACTATCTGCTGCTGTCGA
AAAAATGTCTGCGGATTTTACCAAGTTACAAAAGAAATTGAAAAATCAAGAGGAAGAAATAGAATGCCTTTCA
GAAGAGAGGGATGAAAAATTGAAACTTTTGCAAAAGCAGTATGAGGAGATGGAGATGTGTTTGCAAAAAAAAA
GATGAAGAATTAAAGAAGGTTTTACAATCCAATGTGCAATTGGAGTTGCGCATGAAAGCCCTGGAGGGAAGGG
TAATGTTGTGGATACAGTCCCACCCCACAGAGAGGCCCATAGATAGTCCTGACATCGGGGACTATGAGACACC
ATCAGCAAGAGGTGTTGATTTAGAACCAATGAACCTATGAAGACTAAACTAAAAATCATAGTATCGCACAAGG
GCCGAATCACGAACCTACCAGGGGGAGGGAAGTACTAGAAAATTGGGTTAGGGCACACTAAAATTATGAGACA
GGATGGGGCATCTAATAGCGAACCATAAGTTAGTTGCCATCTGAAGGTGCACAGGTAATGAAACCGTGCGGGA
AAAAATAAGACCTCAAAAAGTTAATTTATGCCCATTCAATTCTCTGAATGATTGACGACATCTGTACAGAGGA
GGATTTGTCTTCTATCTCACCCAGAAAGTCTGAGTGGCATTAGTAGGAGATACTTGTGCTTCCGGCACGGAAA
AGGAAAGAACAAGGAAAACAGGAAGAAAAGAAGTGCAATGCCACACGGGCATTGCACTGGGCTTACTTCCGTT
TAATATGAGCATAACAACC
>TR420|c0_g1_i1 len=1241 path=[1541:0-179 1542:180-203 1563:204-366
1545:367-390 1554:391-522 1527:523-546 1558:547-654 1530:655-678
1565:679-873 1562:874-920 1557:921-963 1552:964-987 1564:988-1056
1511:1057-1094 @1556@!:1095-1178 1520:1179-1240] [-1, 1541, 1542, 1563,
1545, 1554, 1527, 1558, 1530, 1565, 1562, 1557, 1552, 1564, 1511, 1556,
1520, -2]
GTCTGTCCTATCGCAGAAGACCTCCTCTGCAGAGTCACCACTACTCCTGATTGTTGTTCGAAACAGACTTATA
TATATATTAGAAGACACTGTAAAATCGCAGTCCTCCCATGGCCAAATAAACATCCATCTCTCTCCCAATCCCC
ACAATACCAATACGTCGTAATTCCGAAGCATCCAGTACAGGTGGCATATCGGGACCTCCTCCATACGCCTCGA
AGCACGAAAATGGAACTCGAATCAGCTCCCAGGTGTTGGGTTGTTCAATTCGAAAGGTATGCCGATAGGATGC
CTGCTGCAACGTCCCCGGAGTGCGTATGTGCATATTGAACGATCCGACATTCTTCTCGTCATTGTCGTTGTCG
TCCACCACGCGATAGAGAATATCAAATTCAATTCCATCGTAGTCCGAAGCATCCACGCTTTGATCGGAAGCAT
CCAACGGCAAATCCGTCACCATCTGCAAGAACCCTCCGTTGTTCGCAAGCGACACGCGTCCCTCCAACACGTT
CGCGGCCCTCCCTTCCAAATCCAATTCGCGACGCAACATCCCATTGCTGAGGCCGCCCATGACGCGATCGGTA
ATTCCCCTCCATTCGTTGCCGGAACTCGTCATCGGGAATACGTTGGTCAAGTCGTCGATGATTCCCGTGGTCG
TGCATTCTCGATAGGATGCTTTCGATTTGGCAATGTTGGTGGTACCACCACCACTGCCGATGCCCATGCCACC
CACGCCGGCGCTGTCGCGTTGGGCGGGCGGTTGACGTCCCGTCGAACCCTCATCGGATCCCACGGCATTCACG
AAGTTCTTGTTGGCGGCGCCACCGACTCGTCCGATCCTTTTGAGGAAGGCCATGCTGTTATTGTCTTTTTTTT
TGGGTGTTGTTGTTGACTTTTCGTTGGAAGAAGCGGCGGCGGAGGATTGTTCGAGTTTCTTTGTTAACGATTT
TAAAGTGTCGTCGGAGATCGAGGAGGGATCTTCTTCGTTGTCGTCATCTCGCAATCCTTCGTCGGGAAGACCG
AAGGCCATGGATAATTGGAATTGCGATTTTTTGGCGGTGGAGGTGGTGATATGGCGGCGAGAAGAAGCGATGA
TGATGGGAGGGCTGGAATAAGCCGTCGTCGTCGTTGGTGTGAGAATTGCAGCAGCAGCTGTGATGGCTACTAT
TGCAACGTTGGATTGGTACGCTTTCATATTTGCTTGCAGTCTATTGTTACTTCTACTATGCGTTCATTGTGGC

>TR420|c1_g1_i1 len=1547 path=[2109:0-81 2188:82-110 2197:111-137 2204:138-171 2185:172-209 2195:210-278 2157:279-302 2200:303-345 2097:346-392 2194:393-587 2127:588-611 2183:612-679 2140:680-703 2141:704-719 2142:720-743 2193:744-875 2160:876-892 2192:893-899 2181:900-1062 2170:1063-1086 2203:1087-1225 2137:1226-1261 2180:1262-1314 2130:1315-1338 2205:1339-1385 2190:1386-1441 2121:1442-1444 2122:1445-1448 2123:1449-1462 2124:1463-1472 2125:1473-1546] [-1, 2109, 2188, 2197, 2204, 2185, 2195, 2157, 2200, 2097, 2194, 2127, 2183, 2140, 2141, 2142, 2193, 2160, 2192, 2181, 2170, 2203, 2137, 2180, 2130, 2205, 2190, 2121, 2122, 2123, 2124, 2125, -2]
ACAATGAGCGCATAGTAGAAGTAACGATTAACTGCAGGCAAATATGAAAGCGTACCAACCCAACGTTGCAATA
GTAGCCATCACAGCTGCTGCTGCAATTCTCACACCAACAACGACGACGACGACGGCTTATTCCAGCCCTCCCA
TCATCATCGCTTCTTCTCGCCGCCATATCACCACCTCCACCGCCAAAAAATCGCAATTCCAATTATCCATGGC
CTTCGGTCTTCCCGACGAAGGATTGCGAGATGACGACAACGAAGAAGATCCCTCCTCGATTTCCGACGACACT
TTAAAATCGTTAACAAAGAAACTCGAACAATCCTCCGCCGCCGCTTCTTCCAACAAAAAGTCAACAACAACAC
CCCCCAAAAAAGACAATAACAGCATGGCCTTCCTCAAAAGGATCGGACGAGTCGGTGGCGCCGCCAACAAGAA
CTTCGTGAATGCCGTGGGATCCGATGAGGGTTCGACGGGACGTCAACCGCCCGCCCAACGCGACAGCGCCGGC
GTGGGTGGCATGGGCATCGGCAGTGGTGGTGGTACCACCAACATTGCCAAATCGAAAGCATCCTATCGAGAAT
GCACTACCACGGGAATCATCGACGACTTGACCAACGTATTCCCGATGACGAGTTCCGGCAACGAATGGAGGGG
AATTACCGATCGCGTCATGGGCGGCCTCAGCAATGGGATGTTGCGTCGCGAATTGGATTTGGAAGGGAGGGCC
GCGAACGTGTTGGAGGGACGCGTGTCGCTTGCGAACAACGGAGGGTTCTTGCAGATGGTGACGGATTTGCCGT
TGGATGCTTCCGATCAAAGCGTGGATGCTTCGGACTACGATGGAATTGAATTTGATATTCTCTATCGCGTGGT
AGACGACAACGACAATGACGAGAAGAATGTCGGATCGTTCAATATGCACATACGCACTCCGGGGACGTTGCAG
CAGGCATCCTATCGGCATACCTTTCGAATTGAACAACCCAACACCTGGGAGCTGATTCGAGTTCCATTTTCGT
GCTTCGAGGCGTATGGAGGAGGTCCCGATATGCCACCTGTACTGGATGCTTCGGAATTACGACGTATTGGTAT
TGTGGGGATTGGGAGAGAGATGGATGTTTATTTGGCCATGGGAGGACTGCGATTTTACAGTGTCTTCTAATAT
ATATATAAGTCTGTTTCGAACAACAATCAGGAGTAGTGGTGACTCTGCAGAGGAGGTCATCTGTGACAGGGCA
GACGCCGCTCGCTCCAACTCCAAAAGCCGACAAATCGTCGTCTCTTTTATTTTTCACAACAAATCTGCATGCA
CCATGGCGGCGCCGAACTAGTACACGCTCCACTGGTCCTCGCGGATCCTGAATCCCACCAAACTTCCATGGCC
GGAGGATCTTTATGGAAATCACCATTAGTTGTTTGAGGATACAACCTGACAGAAAATTGATGTGATATAGCAT
CCCTCCCCCTCGTCCGAAACACATGTCCGCAAATGCCGTCGTCAAAATAGTTGCAGCATTAATTAATTAATTA
AAAATACTTGAGAT
>TR420|c1_g1_i2 len=1563 path=[2144:0-103 2187:104-132 2196:133-153 2204:154-187 2186:188-225 2195:226-294 2157:295-318 2200:319-361 2173:362-408 2194:409-603 2127:604-627 2183:628-695 2140:696-719 2141:720-735 2142:736-759 2193:760-891 2160:892-908 2192:909-915 2181:916-1078 2170:1079-1102 2203:1103-1241 2191:1242-1277 2180:1278-1330 2130:1331-1354 2205:1355-1401 2198:1402-1460 2189:1461-1464 2178:1465-1488 2155:1489-1562] [-1, 2144, 2187, 2196, 2204, 2186, 2195, 2157, 2200, 2173, 2194, 2127, 2183, 2140, 2141, 2142, 2193, 2160, 2192, 2181, 2170, 2203, 2191, 2180, 2130, 2205, 2198, 2189, 2178, 2155, -2]
TCGCTTTTAGCGGTTTGTCGCCACAATGAACGCATAGTAGAAGTAACAATAGACTGCAAGCAAATATGAAAGC
GTACCAATCCAACGTTGCAATAGTAGCCATCACAGCTGCTGCTGCAATTCTCACACCAACGACGACGACGGCT
TATTCCAGCCCTCCCATCATCATCGCTTCTTCTCGCCGCCATGTCACCACCTCCACTGCCAAAAAATCGCAAT
TCCAATTATCCATGGCCTTCGGTCTTCCCGACGAAGGATTGCGAGATGACGACAACGAAGAAGATCCCTCCTC
GATTTCCGACGACACTTTAAAATCGTTAACAAAGAAACTCGAACAATCCTCCGCCGCCGCTTCTTCCAACGAA
AAGTCAACAACAACACCCAAAAAAAAAGACAATAACAGCATGGCCTTCCTCAAAAGGATCGGACGAGTCGGTG
GCGCCGCCAACAAGAACTTCGTGAATGCCGTGGGATCCGATGAGGGTTCGACGGGACGTCAACCGCCCGCCCA
ACGCGACAGCGCCGGCGTGGGTGGCATGGGCATCGGCAGTGGTGGTGGTACCACCAACATTGCCAAATCGAAA
GCATCCTATCGAGAATGCACTACCACGGGAATCATCGACGACTTGACCAACGTATTCCCGATGACGAGTTCCG
GCAACGAATGGAGGGGAATTACCGATCGCGTCATGGGCGGCCTCAGCAATGGGATGTTGCGTCGCGAATTGGA
TTTGGAAGGGAGGGCCGCGAACGTGTTGGAGGGACGCGTGTCGCTTGCGAACAACGGAGGGTTCTTGCAGATG
GTGACGGATTTGCCGTTGGATGCTTCCGATCAAAGCGTGGATGCTTCGGACTACGATGGAATTGAATTTGATA
TTCTCTATCGCGTGGTAGACGACAACGACAATGACGAGAAGAATGTCGGATCGTTCAATATGCACATACGCAC
TCCGGGGACGTTGCAGCAGGCATCCTATCGGCATACCTTTCGAATTGAACAACCCAACACCTGGGAGCTGATT
CGAGTTCCATTTTCGTGCTTCGAGGCGTATGGAGGAGGTCCCGATATGCCACCTGTACTGGATGCTTCGGAAT
TACGACGTATTGGTATTGTGGGGATTGGGAGAGAGATGGATGTTTATTTGGCCATGGGAGGACTGCGATTTTA
CAGTGTCTTCTAATATATATATAAGTCTGTTTCGAACAACAATCAGGAGTAGTGGTGACTCTGCAGAGGAGGT
CTTCTGCGATAGGACAGACGCCGCTCGCTCCAACTCCAAAAGCCGACAAATCGTCGTCTCTTTTATTTTTCAC
AACAAATCTGCATGCACCATGGCGGCGCCGAACTAGTACACGCTCCACTGGTCCTCGCGGATCCTGAATCCCA

CCAAACTTCCATGGCATCAGGATCCTTATGAGGAGCGCCATTCTCTCTCTAAGGATACAACCTGACAGAAAAT
TGATGGGATATAGCATCCCTCCCCCTCGTCCGAAACACATGTCCGCAAATGCCGTCGTCAAAATAGTTGCAGC
ATTAATTAATTAATTAAAAATACTTGAGAT
>TR421|c0_g1_i1 len=345 path=[4383:0-197 5550:198-202 5669:203-211
5665:212-219 5657:220-221 5644:222-234 5621:235-258 5588:259-280
5530:281-286 5450:287-287 5356:288-311 5102:312-344] [-1, 4383, 5550,
5669, 5665, 5657, 5644, 5621, 5588, 5530, 5450, 5356, 5102, -2]
GATCTATTGGCTTGACAGCTCCAGAATCCAGACCTCTCTGTGTAGAGATAGACTCCAACCATTACATAGCAAG
AGAGCAATCCGGCGTATCATAAGAGGTGCTTTCATCACTTCCATTTGATCAATGCAGTCTTATCATGGCGATG
GCAAGTTCTCACAAAATGACAATTGCGGACAAGCATAATTCTACAGCTACAGCAAGATACCAACGAATTAAAG
CAAGGCATCACATTTTTTGACAGGGTGAAATCTACACATAGAATAAAATCATGACAGACAAACATACAGAAGC
AGATGTTCTCCTTGGCCGAGGTGTTGCGACCACTCGCCATCCAGGCAATCAAC
>TR421|c0_g2_i2 len=890 path=[4602:0-39 4603:40-60 5642:61-77 5619:78-96
5585:97-103 5526:104-127 5444:128-157 5655:158-177 5673:178-197 5679:198-
204 5663:205-221 5653:222-258 5637:259-278 5576:279-292 5512:293-322
5670:323-335 5667:336-351 5633:352-356 5607:357-380 5568:381-386
5630:387-405 5602:406-433 5564:434-469 5308:470-540 5598:541-574
5677:575-580 5401:581-605 4714:606-627 5556:628-694 5483:695-728
5398:729-774 5553:775-788 5479:789-812 5394:813-836 5284:837-842
4970:843-843 4971:844-889] [-1, 4602, 4603, 5642, 5619, 5585, 5526, 5444,
5655, 5673, 5679, 5663, 5653, 5637, 5576, 5512, 5670, 5667, 5633, 5607,
5568, 5630, 5602, 5564, 5308, 5598, 5677, 5401, 4714, 5556, 5483, 5398,
5553, 5479, 5394, 5284, 4970, 4971, -2]
TAGCTCTGTCAAACCATCGTTGAGCCAACCTCGTAAGCAGATGTGTGATGTCAAACAGGAAAAGAAACACAGA
CGAACGAAATCAGATCCATTGAAAACCAATATTGCCTCATCTCCTGACACTGTAGACCAACCTCATTCAAAGA
AACAACGCGTCGTCCTAGACGTTGAACGAAGCACAAGTGCTCCCATTCAGGGGAAAAAAGTGTATATGAATGA
GATACAGCATAGTCCATTAGTTGCATCTCGTCGCCAGCAACACGAATCGGCAGAGCAGCAACACCTGCCATAT
TCGCAGCAACAACCCTCGCTACAATTGTTTGAAACTTCAGAAGACAGCATTGCATTCTCCGAGGCTTTGGCGC
CAGGCCTAGCTGATGACTACGAGCCTCTTCCATTTAATCCGCTGCACGGAGATACAGAACACAGCGGTTTCTA
TGGGTGATATCTTGCCATAAATGGAACTTCTTCATGACACATGAATTCTACCCCCAGTGCGTCAGCAAACAAT
GGTGGAGCAATGTGCGCAAATGAAGGTTATATGGAGCAGCAAAGCCTTCTTGAAAATGGTTAGTGTAGATTTT
GGCTGGCGAGATGTTCTCAGTGAGTGGCAGTCAGACTCCAACCTTGACCAGAGACAGTAATCGGGGTGACGAT
AATATGAATGATACTGCAGCTATGCTTGTATTGAGAGAAATTGTTGAGTGAAGAAATGTACACAATATATTGG
TTAGGAAAACATAGTTTTGTATCAAATCATCATGAAAACCGCTCATGCTATCTTTGATGCAAGCTCTACCATG
ATAAATTGTGATGATGTCAGAAGAAAGAAAGAATAGAAATTTACACGGACTATTTAATAATACCTAACATATT
GAATTTAAAAGATC
>TR421|c0_g2_i3 len=889 path=[4602:0-39 4603:40-60 5642:61-77 5619:78-96
5585:97-103 5526:104-127 5444:128-157 5655:158-177 5673:178-197 5679:198-
204 5663:205-221 5653:222-258 5637:259-278 5513:279-302 5652:303-309
5635:310-311 5508:312-335 5667:336-351 5675:352-360 5649:361-395
5604:396-429 5563:430-454 5659:455-497 5648:498-520 5559:521-523
5488:524-573 5677:574-579 5597:580-594 5557:595-604 5626:605-626
5596:627-670 5554:671-694 5647:695-751 5625:752-776 5678:777-800
5682:801-817 5395:818-835 5551:836-841 5476:842-842 5392:843-852
5280:853-876 5170:877-888] [-1, 4602, 4603, 5642, 5619, 5585, 5526, 5444,
5655, 5673, 5679, 5663, 5653, 5637, 5513, 5652, 5635, 5508, 5667, 5675,
5649, 5604, 5563, 5659, 5648, 5559, 5488, 5677, 5597, 5557, 5626, 5596,
5554, 5647, 5625, 5678, 5682, 5395, 5551, 5476, 5392, 5280, 5170, -2]
TAGCTCTGTCAAACCATCGTTGAGCCAACCTCGTAAGCAGATGTGTGATGTCAAACAGGAAAAGAAACACAGA
CGAACGAAATCAGATCCATTGAAAACCAATATTGCCTCATCTCCTGACACTGTAGACCAACCTCATTCAAAGA
AACAACGCGTCGTCCTAGACGTTGAACGAAGCACAAGTGCTCCCATTCAGGGGAAAAAAGTGTATATGAATGA
GATACAGCATAGTCCATTAGTTGCATCTCGTCGCCAGCAACACGAATCGGCAGAGCAGCATCACCTAACATAT
TTACGGCGACAACCCTCGCTGCAATTGTTTGAAACTTCAGAAGACAGCATTGCATTCTCCAAGGCTTTGGCAC
CAGGCCTAGCTGATGACTATGAGCCTCTTCCATTGAAGGCGTTGCACGGAGAGACAGAAGCAGCGGATTCTAT
GGATGATATCTTGCAAGCAATGGAACTTCTTTATGATACAGGAATTCTACCCCCGGTGCGTCAGCAAACAATG
GCGGATCAATATGTACAAATGAAGGTTACATCGAGCAGCGAAGCCTTCTTGAAAATGGTTAGTGTAGATGATG
GCTGGCGAGATGTTCTCAGTGAGTGGCAGTCAGACTCCAACCTCGACCGGTGACAGTAATCGGGGTGCCGATG
ATATGAATGATACTGCAGCTGTGCTTGTATTATGAAATATGGTGGAGGGAAGAAATGCACACGAGAGTTTGGT
TAGACAAACATAGTTTTGTACCCTATCATCCTCAAAACCGCTAATGCTATCTTTGATGTAAACTCTACCATGA

TAAATTGTGTTGATGTCAGAAGAAAGAAAGAATAGAAATTCACACCTACTGTTTAATAATGTAACACTTGATG
ATATCAACATTGT
>TR421|c0_g2_i5 len=835 path=[4616:0-41 5549:42-95 5473:96-154 5668:155-169 5664:170-193 5654:194-203 5637:204-223 5576:224-237 5511:238-254 5429:255-256 5330:257-267 5670:268-280 5667:281-296 5633:297-301 5607:302-325 5568:326-331 5630:332-350 5602:351-378 5564:379-414 5308:415-485 5598:486-519 5677:520-525 5401:526-550 4714:551-572 5556:573-639 5483:640-673 5398:674-719 5553:720-733 5479:734-757 5394:758-781 5284:782-787 4970:788-788 4971:789-834] [-1, 4616, 5549, 5473, 5668, 5664, 5654, 5637, 5576, 5511, 5429, 5330, 5670, 5667, 5633, 5607, 5568, 5630, 5602, 5564, 5308, 5598, 5677, 5401, 4714, 5556, 5483, 5398, 5553, 5479, 5394, 5284, 4970, 4971, -2]
CAGGAAAAGAAACACAGACGAACGAAATCAGATCCATTGAAAGCCAATATTGCCCCATCTCCTGACAATGTAG
ACCAACATCATTCAAAGAAACAATCCGACGTCCTAGACGTTGAACGAAGCATTAGTGCCCCCATTCAGGGGAA
CAAAGTGTATATGAATGAAATACAGCATAGTCCAATAGTTGCATCTCGTCGCCAGCAACACGAATCGGCAGAG
CAGCAACACCTGCCATATTTACGGCGACAACCCTCGCTACAATTGTTTGAAACTTCAGAAGACAGCATTGCAT
TCTCCGAGGCTTTGGCGCCAGGCCTAGCTGATGACTACGAGCCTCTTCCATTTAATCCGCTGCACGGAGATAC
AGAACACAGCGGTTTCTATGGGTGATATCTTGCCATAAATGGAACTTCTTCATGACACATGAATTCTACCCCC
AGTGCGTCAGCAAACAATGGTGGAGCAATGTGCGCAAATGAAGGTTATATGGAGCAGCAAAGCCTTCTTGAAA
ATGGTTAGTGTAGATTTTGGCTGGCGAGATGTTCTCAGTGAGTGGCAGTCAGACTCCAACCTTGACCAGAGAC
AGTAATCGGGGTGACGATAATATGAATGATACTGCAGCTATGCTTGTATTGAGAGAAATTGTTGAGTGAAGAA
ATGTACACAATATATTGGTTAGGAAAACATAGTTTTGTATCAAATCATCATGAAAACCGCTCATGCTATCTTT
GATGCAAGCTCTACCATGATAAATTGTGATGATGTCAGAAGAAAGAAAGAATAGAAATTTACACGGACTATTT
AATAATACCTAACATATTGAATTTAAAAGATC
>TR421|c0_g2_i6 len=888 path=[4901:0-39 4902:40-56 4903:57-57 4904:58-60 5465:61-76 5619:77-95 5585:96-102 5526:103-126 5444:127-156 5655:157-176 5673:177-196 5679:197-203 5663:204-220 5653:221-257 5637:258-277 5513:278-301 5652:302-308 5635:309-310 5508:311-334 5667:335-350 5675:351-359 5418:360-385 5630:386-404 5601:405-446 5562:447-453 5659:454-496 5648:497-519 5559:520-522 5404:523-547 5486:548-593 5557:594-603 5626:604-625 5596:626-669 5554:670-693 5647:694-750 5625:751-775 5678:776-799 5682:800-816 5395:817-834 5551:835-840 5476:841-841 5392:842-851 5280:852-875 5170:876-887] [-1, 4901, 4902, 4903, 4904, 5465, 5619, 5585, 5526, 5444, 5655, 5673, 5679, 5663, 5653, 5637, 5513, 5652, 5635, 5508, 5667, 5675, 5418, 5630, 5601, 5562, 5659, 5648, 5559, 5404, 5486, 5557, 5626, 5596, 5554, 5647, 5625, 5678, 5682, 5395, 5551, 5476, 5392, 5280, 5170, -2]
ACCTTATTAGATACTAATAGCTCTGTCAAACCATCGTTGAGCCAACCTCGTAAGCAGGAAAAGAAACACAGAC
GAACGAAATCAGATCCATTGAAAACCAATATTGCCTCATCTCCTGACACTGTAGACCAACCTCATTCAAAGAA
ACAACGCGTCGTCCTAGACGTTGAACGAAGCACAAGTGCTCCCATTCAGGGGAAAAAAGTGTATATGAATGAG
ATACAGCATAGTCCATTAGTTGCATCTCGTCGCCAGCAACACGAATCGGCAGAGCAGCATCACCTAACATATT
TACGGCGACAACCCTCGCTGCAATTGTTTGAAACTTCAGAAGACAGCATTGCATTCTCCAAGGCTTTGACACC
AGGCCTAGCTGATGACTACGAGCCTCTTCCATTTAATCCGTTGCACGGAGAGACAGAAACAGCGATTTCTATG
GATGATATCTTGCAAGCAATGGAACTTCTTTATGATACAGGAATTCTACCCCCGGTGCGTCAGCAAACAATGG
CGGATCAATATGCACAAATGAAAGTTACATCGAGCAGCGAAACCTTCTTGAAAATGGTTGGTGTAGATGATGG
CTGGCGAGATGTTCTCAGTGAGTGGCAGTCAGACTCCAACCTCGACCGGTGACAGTAATCGGGGTGCCGATGA
TATGAATGATACTGCAGCTGTGCTTGTATTATGAAATATGGTGGAGGGAAGAAATGCACACGAGAGTTTGGTT
AGACAAACATAGTTTTGTACCCTATCATCCTCAAAACCGCTAATGCTATCTTTGATGTAAACTCTACCATGAT
AAATTGTGTTGATGTCAGAAGAAAGAAAGAATAGAAATTCACACCTACTGTTTAATAATGTAACACTTGATGA
TATCAACATTGT
>TR421|c0_g2_i7 len=890 path=[4602:0-39 4603:40-60 5642:61-77 5619:78-96 5585:97-103 5526:104-127 5444:128-157 5655:158-177 5673:178-197 5679:198-204 5663:205-221 5653:222-258 5637:259-278 5513:279-302 5652:303-309 5635:310-311 5508:312-335 5667:336-351 5675:352-360 5649:361-395 5417:396-433 5564:434-469 5491:470-498 5648:499-521 5628:522-534 5487:535-540 5598:541-574 5677:575-580 5401:581-605 4714:606-627 5556:628-694 5483:695-728 5398:729-774 5553:775-788 5479:789-812 5394:813-836 5284:837-842 4970:843-843 4971:844-889] [-1, 4602, 4603, 5642, 5619, 5585, 5526, 5444, 5655, 5673, 5679, 5663, 5653, 5637, 5513, 5652, 5635, 5508, 5667, 5675, 5649, 5417, 5564, 5491, 5648, 5628, 5487,

5598, 5677, 5401, 4714, 5556, 5483, 5398, 5553, 5479, 5394, 5284, 4970, 4971, -2]
TAGCTCTGTCAAACCATCGTTGAGCCAACCTCGTAAGCAGATGTGTGATGTCAAACAGGAAAAGAAACACAGA
CGAACGAAATCAGATCCATTGAAAACCAATATTGCCTCATCTCCTGACACTGTAGACCAACCTCATTCAAAGA
AACAACGCGTCGTCCTAGACGTTGAACGAAGCACAAGTGCTCCCATTCAGGGGAAAAAAGTGTATATGAATGA
GATACAGCATAGTCCATTAGTTGCATCTCGTCGCCAGCAACACGAATCGGCAGAGCAGCATCACCTAACATAT
TTACGGCGACAACCCTCGCTGCAATTGTTTGAAACTTCAGAAGACAGCATTGCATTCTCCAAGGCTTTGGCAC
CAGGCCTAGCTGATGACTATGAGCCTCTTCCGTTTAATCCGCTGCGCGGAGATACAGAACACAGCGGTTTCTA
TGGGTGATATCTTGCCATAAATGGAACTTCTTTATGACACAGGAATTCTACCCCCGGTGCGTCAGCAAACAAT
GGCGGATCAATGTGCGCAAATGAAGGTTATATGGAGCAGCAAAGCCTTCTTGAAAATGGTTAGTGTAGATTTT
GGCTGGCGAGATGTTCTCAGTGAGTGGCAGTCAGACTCCAACCTTGACCAGAGACAGTAATCGGGGTGACGAT
AATATGAATGATACTGCAGCTATGCTTGTATTGAGAGAAATTGTTGAGTGAAGAAATGTACACAATATATTGG
TTAGGAAAACATAGTTTTGTATCAAATCATCATGAAAACCGCTCATGCTATCTTTGATGCAAGCTCTACCATG
ATAAATTGTGATGATGTCAGAAGAAAGAAAGAATAGAAATTTACACGGACTATTTAATAATACCTAACATATT
GAATTTAAAAGATC
>TR421|c0_g2_i8 len=572 path=[4835:0-41 5548:42-95 5472:96-102 5655:103-122 5523:123-154 5668:155-169 5664:170-193 5654:194-203 5637:204-223 5514:224-245 5432:246-247 5652:248-254 5635:255-256 5508:257-280 5667:281-296 5675:297-305 5418:306-331 5630:332-350 5601:351-392 5562:393-399 5659:400-442 5648:443-465 5628:466-478 5405:479-493 5486:494-539 5557:540-549 5626:550-571] [-1, 4835, 5548, 5472, 5655, 5523, 5668, 5664, 5654, 5637, 5514, 5432, 5652, 5635, 5508, 5667, 5675, 5418, 5630, 5601, 5562, 5659, 5648, 5628, 5405, 5486, 5557, 5626]
CAGGAAAAGAAACACAGACGAACGAAATCAGATCCATTGAAAGCCAATATTGCCCCATCTCCTGACAATGTAG
ACCAACATCATTCAAAGAAACAACGCGTCGTCCTAGACGTTGAACGAAGCGCAAGTGCTCCCATTCAGGGGAA
CAAAGTGTATATGAATGAAATACAGCATAGTCCAATAGTTGCATCTCGTCGCCAGCAACACGAATCGGCAGAG
CAGCAGCACCTAACATATTTACGGCGACAACCCTCGCTGCAATTGTTTGAAACTTCAGAAGACAGCATTGCAT
TCTCCAAGGCTTTGACACCAGGCCTAGCTGATGACTACGAGCCTCTTCCATTTAATCCGTTGCACGGAGAGAC
AGAAACAGCGATTTCTATGGATGATATCTTGCAAGCAATGGAACTTCTTTATGATACAGGAATTCTACCCCCG
GTGCGTCAGCAAACAATGGCGGATCAATGTGCGCAAATGAAAGTTACATCGAGCAGCGAAACCTTCTTGAAAA
TGGTTGGTGTAGATGATGGCTGGCGAGATGTTCTCAGTGAGTGGCAGTCAGACTCCAACCT
>TR421|c0_g2_i9 len=889 path=[4901:0-39 4902:40-56 4903:57-57 4904:58-60 5465:61-76 5619:77-95 5585:96-102 5526:103-126 5444:127-156 5655:157-176 5673:177-196 5679:197-203 5663:204-220 5653:221-257 5637:258-277 5513:278-301 5652:302-308 5635:309-310 5508:311-334 5667:335-350 5675:351-359 5649:360-394 5417:395-432 5564:433-468 5491:469-497 5648:498-520 5628:521-533 5487:534-539 5598:540-573 5677:574-579 5401:580-604 4714:605-626 5556:627-693 5483:694-727 5398:728-773 5553:774-787 5479:788-811 5394:812-835 5284:836-841 4970:842-842 4971:843-888] [-1, 4901, 4902, 4903, 4904, 5465, 5619, 5585, 5526, 5444, 5655, 5673, 5679, 5663, 5653, 5637, 5513, 5652, 5635, 5508, 5667, 5675, 5649, 5417, 5564, 5491, 5648, 5628, 5487, 5598, 5677, 5401, 4714, 5556, 5483, 5398, 5553, 5479, 5394, 5284, 4970, 4971, -2]
ACCTTATTAGATACTAATAGCTCTGTCAAACCATCGTTGAGCCAACCTCGTAAGCAGGAAAAGAAACACAGAC
GAACGAAATCAGATCCATTGAAAACCAATATTGCCTCATCTCCTGACACTGTAGACCAACCTCATTCAAAGAA
ACAACGCGTCGTCCTAGACGTTGAACGAAGCACAAGTGCTCCCATTCAGGGGAAAAAAGTGTATATGAATGAG
ATACAGCATAGTCCATTAGTTGCATCTCGTCGCCAGCAACACGAATCGGCAGAGCAGCATCACCTAACATATT
TACGGCGACAACCCTCGCTGCAATTGTTTGAAACTTCAGAAGACAGCATTGCATTCTCCAAGGCTTTGGCACC
AGGCCTAGCTGATGACTATGAGCCTCTTCCGTTTAATCCGCTGCGCGGAGATACAGAACACAGCGGTTTCTAT
GGGTGATATCTTGCCATAAATGGAACTTCTTTATGACACAGGAATTCTACCCCCGGTGCGTCAGCAAACAATG
GCGGATCAATGTGCGCAAATGAAGGTTATATGGAGCAGCAAAGCCTTCTTGAAAATGGTTAGTGTAGATTTTG
GCTGGCGAGATGTTCTCAGTGAGTGGCAGTCAGACTCCAACCTTGACCAGAGACAGTAATCGGGGTGACGATA
ATATGAATGATACTGCAGCTATGCTTGTATTGAGAGAAATTGTTGAGTGAAGAAATGTACACAATATATTGGT
TAGGAAAACATAGTTTTGTATCAAATCATCATGAAAACCGCTCATGCTATCTTTGATGCAAGCTCTACCATGA
TAAATTGTGATGATGTCAGAAGAAAGAAAGAATAGAAATTTACACGGACTATTTAATAATACCTAACATATTG
AATTTAAAAGATC
>TR421|c0_g3_i1 len=345 path=[4741:0-197 4742:198-202 4743:203-211 5540:212-219 5377:220-236 5622:237-258 5589:259-280 5531:281-286 5451:287-287 5358:288-311 4541:312-344] [-1, 4741, 4742, 4743, 5540, 5377, 5622, 5589, 5531, 5451, 5358, 4541, -2]

GATCTATTGGCTTGACAGCTCCAGAATCCAGACCTCTCTGTGTAGAGATAGACTCCAACCATTACATAGCAAG
AGAGCAATCCGGCGTATCATAAGAGGTGCTTTCATCACTTCCATTTGATCAATGCAGTCTTATCATGGCGATG
GCAAGTTCTCACAAAATGACAATTGCGGACAAGCATAATTCTACAGCTACAGCAAGATACCAACGAATTAAAG
CGAGGCATTACATGTTCTGACAGGGTGAAATCTACACATAGAATAAAATCATGACAGACAAACATACAGAAGC
AGATGTTCTCCTTGGCCGAGGTGTTGCGACCACTCGCCATCCAGGCAATCAAC
>TR421|c0_g4_i1 len=440 path=[4531:0-28 4532:29-29 5546:30-53 5641:54-86
5618:87-110 5672:111-116 5616:117-138 5580:139-140 5516:141-152 5435:153-
176 5334:177-203 5636:204-227 5611:228-267 5574:268-291 5674:292-294
5680:295-318 5683:319-324 5684:325-327 5660:328-342 5631:343-366
5603:367-383 5565:384-400 5492:401-401 5409:402-404 5302:405-420
5092:421-439] [-1, 4531, 4532, 5546, 5641, 5618, 5672, 5616, 5580, 5516,
5435, 5334, 5636, 5611, 5574, 5674, 5680, 5683, 5684, 5660, 5631, 5603,
5565, 5492, 5409, 5302, 5092, -2]
AGAATAAAATCATGACAGACAAACATACAGAAGCAGATGTTCTCCTTGGCCGAGGTGTTGCGACCACTCGCCA
TCCAGGCAATCAACATTTTAGGTCAATTGTAAGCCAGCATTGCGAGCTCTACGCAACAAGTACTAAGAAACAA
AAAATGAACATATCGCGCTCCATTGTTGCCAAGATCCAAACCGAAGGCAAATTTCTAGAAAAGGAAGCCAATA
CTGGACTCTGGCACGAGGTCGACGATAGAAGAGCGTTGGAAAAAACGGCCCAGGCGTTGCGTGATGGTGCGGC
CCCACTTAGGAAACGGCTATTGGAGGATATGAGCGATCCTGCCTTTTTGGACACCTTATTAGATACTAATAGC
TCTGTCAAACCATCGTTGAGCCAACCTCGTAAGCAGGAAAAGAAACACAGACGAACGAAATCAGATCCATTGA
AA
>TR421|c0_g4_i2 len=431 path=[4754:0-70 5646:71-88 5624:89-107 5462:108-
129 5579:130-131 5516:132-143 5435:144-167 5334:168-194 5636:195-218
5611:219-258 5574:259-282 5674:283-285 5680:286-309 5683:310-315
5684:316-318 5660:319-333 5676:334-339 5681:340-350 5629:351-357
5600:358-374 5561:375-391 5489:392-392 5406:393-395 5296:396-411
4805:412-430] [-1, 4754, 5646, 5624, 5462, 5579, 5516, 5435, 5334, 5636,
5611, 5574, 5674, 5680, 5683, 5684, 5660, 5676, 5681, 5629, 5600, 5561,
5489, 5406, 5296, 4805, -2]
TCTTCCGATCTCGTGAGTAAAACATTGCCACTGCCATGCTCCTATGATACGTCGCTCAAAGATCTCACGTAAC
GTGTTTGTATCTGATGACGATGAACGACGATCAGGCGCTCTACGCAACAAGTACTAAGAAACAAAAAATGAAC
ATATCGCGCTCCATTGTTGCCAAGATCCAAACCGAAGGCAAATTTCTAGAAAAGGAAGCCAATACTGGACTCT
GGCACGAGGTCGACGATAGAAGAGCGTTGGAAAAAACGGCCCAGGCGTTGCGTGATGGTGCGGCCCCACTTAG
GAAACGGCTATTGGAGGATATGAGCGATCCTGCCTTTTTGGATACCTTATTAGATACTAATAGCTCTGTCAAA
CCATCGTTGAGCCAACCTCGTAAGCAGGAAAAGAAACACAGACGAACGAAATCAGATCCATTGAAA
>TR421|c0_g4_i3 len=721 path=[4403:0-171 4404:172-180 5547:181-212
5643:213-227 5620:228-251 5586:252-255 5671:256-256 5527:257-266
5656:267-280 5641:281-313 5618:314-337 5672:338-343 5442:344-365
5579:366-367 5516:368-379 5435:380-403 5334:404-430 5636:431-454
5611:455-494 5574:495-518 5674:519-521 5680:522-545 5683:546-551
5684:552-554 5319:555-631 5026:632-647 5027:648-666 5028:667-720] [-1,
4403, 4404, 5547, 5643, 5620, 5586, 5671, 5527, 5656, 5641, 5618, 5672,
5442, 5579, 5516, 5435, 5334, 5636, 5611, 5574, 5674, 5680, 5683, 5684,
5319, 5026, 5027, 5028, -2]
GCAAACTTTACAATATCGTAGGAGTACGTACCATACCTCATGAACCACACAGAATATCCGACATGTCATACGA
GTACGAGGTACTTTCATCACACTCCCATCTGATAATGTCAATGCAATCTCATCATGGCGATGGCAAGTTTAGA
TGAATAATTCTACAGCTACAGCAAGATACCAACGAGTTAAAGCGAGGCATCACATTTTCTGACAGGGCGAAAT
CTACACGAAAAATGAAATCATGACAGACAAACATACAGTAGCAGATGTTCTCCTTGGCCGAGGTGTTGCGACC
ACTCGCCATCCAGGCAATCAACATTTTAGGTCAATTGTAAGCCAGCATTGCGCGCTCTACGCAACAAGTACTA
AGAAACAAAAAATGAACATATCGCGCTCCATTGTTGCCAAGATCCAAACCGAAGGCAAATTTCTAGAAAAGGA
AGCCAATACTGGACTCTGGCACGAGGTCGACGATAGAAGAGCGTTGGAAAAAACGGCCCAGGCGTTGCGTGAT
GGTGCGGCCCCACTTAGGAAACGGCTATTGGAGGATATGAGCGACCCTGCCTTTTTGGATACCTTATTGGATA
CTAATAGCTCTGCCAAACCATCGTCGAGCCAACCTCGTAAGCAGGAAAAGAAACACAGACGAACGAAATCAGA
TCCATTGAAAGCCAATATTGCCCCATCTCCTGACAATGTAGACCAACATCATTCAAAGAAACAA
>TR421|c0_g4_i4 len=495 path=[4473:0-53 5391:54-79 5658:80-80 5646:81-98
5624:99-117 5536:118-122 5458:123-139 5580:140-141 5516:142-153 5435:154-
177 5334:178-204 5636:205-228 5611:229-268 5574:269-292 5674:293-295
5680:296-319 5683:320-325 5684:326-328 5319:329-405 5026:406-421
5027:422-440 5028:441-494] [-1, 4473, 5391, 5658, 5646, 5624, 5536, 5458,
5580, 5516, 5435, 5334, 5636, 5611, 5574, 5674, 5680, 5683, 5684, 5319,
5026, 5027, 5028, -2]

GTCAATTGTAAGCCAGCATTGCGTGAGTAAAACATTGACACTGCCATGCTCCTATGACACGTCGCTCAAAGAT
CTCACGTAACGTGTTTGTATCTGATGACGATGAACGACGATCAGGAGCTCTACGCAACAAGTACTAAGAAACA
AAAAATGAACATATCGCGCTCCATTGTTGCCAAGATCCAAACCGAAGGCAAATTTCTAGAAAAGGAAGCCAAT
ACTGGACTCTGGCACGAGGTCGACGATAGAAGAGCGTTGGAAAAAACGGCCCAGGCGTTGCGTGATGGTGCGG
CCCCACTTAGGAAACGGCTATTGGAGGATATGAGCGACCCTGCCTTTTTGGATACCTTATTGGATACTAATAG
CTCTGCCAAACCATCGTCGAGCCAACCTCGTAAGCAGGAAAAGAAACACAGACGAACGAAATCAGATCCATTG
AAAGCCAATATTGCCCCATCTCCTGACAATGTAGACCAACATCATTCAAAGAAACAA
>TR421|c0_g4_i5 len=666 path=[4403:0-171 4404:172-180 5547:181-212
5643:213-227 5620:228-251 5586:252-255 5671:256-256 5527:257-266
5656:267-280 5641:281-313 5618:314-337 5672:338-343 5442:344-365
5579:366-367 5516:368-379 5435:380-403 5334:404-430 5636:431-454
5611:455-494 5574:495-518 5674:519-521 5680:522-545 5683:546-551
5684:552-554 5660:555-569 5676:570-575 5681:576-586 5629:587-593
5600:594-610 5561:611-627 5301:628-648 4793:649-665] [-1, 4403, 4404,
5547, 5643, 5620, 5586, 5671, 5527, 5656, 5641, 5618, 5672, 5442, 5579,
5516, 5435, 5334, 5636, 5611, 5574, 5674, 5680, 5683, 5684, 5660, 5676,
5681, 5629, 5600, 5561, 5301, 4793, -2]
GCAAACTTTACAATATCGTAGGAGTACGTACCATACCTCATGAACCACACAGAATATCCGACATGTCATACGA
GTACGAGGTACTTTCATCACACTCCCATCTGATAATGTCAATGCAATCTCATCATGGCGATGGCAAGTTTAGA
TGAATAATTCTACAGCTACAGCAAGATACCAACGAGTTAAAGCGAGGCATCACATTTTCTGACAGGGCGAAAT
CTACACGAAAAATGAAATCATGACAGACAAACATACAGTAGCAGATGTTCTCCTTGGCCGAGGTGTTGCGACC
ACTCGCCATCCAGGCAATCAACATTTTAGGTCAATTGTAAGCCAGCATTGCGCGCTCTACGCAACAAGTACTA
AGAAACAAAAAATGAACATATCGCGCTCCATTGTTGCCAAGATCCAAACCGAAGGCAAATTTCTAGAAAAGGA
AGCCAATACTGGACTCTGGCACGAGGTCGACGATAGAAGAGCGTTGGAAAAAACGGCCCAGGCGTTGCGTGAT
GGTGCGGCCCCACTTAGGAAACGGCTATTGGAGGATATGAGCGATCCTGCCTTTTTGGATACCTTATTAGATA
CTAATAGCTCTGTCAAACCATCGTTGAGCCAACCTCGTAAGCAGATGTGTGATGTCAAACAGGAAAAGAAACA
CAGACGAAC
>TR421|c0_g4_i6 len=440 path=[4473:0-53 5391:54-79 5658:80-80 5646:81-98
5624:99-117 5536:118-122 5458:123-139 5580:140-141 5516:142-153 5435:154-
177 5334:178-204 5636:205-228 5611:229-268 5574:269-292 5674:293-295
5680:296-319 5683:320-325 5684:326-328 5660:329-343 5676:344-349
5681:350-360 5629:361-367 5600:368-384 5561:385-401 5301:402-422
4793:423-439] [-1, 4473, 5391, 5658, 5646, 5624, 5536, 5458, 5580, 5516,
5435, 5334, 5636, 5611, 5574, 5674, 5680, 5683, 5684, 5660, 5676, 5681,
5629, 5600, 5561, 5301, 4793, -2]
GTCAATTGTAAGCCAGCATTGCGTGAGTAAAACATTGACACTGCCATGCTCCTATGACACGTCGCTCAAAGAT
CTCACGTAACGTGTTTGTATCTGATGACGATGAACGACGATCAGGAGCTCTACGCAACAAGTACTAAGAAACA
AAAAATGAACATATCGCGCTCCATTGTTGCCAAGATCCAAACCGAAGGCAAATTTCTAGAAAAGGAAGCCAAT
ACTGGACTCTGGCACGAGGTCGACGATAGAAGAGCGTTGGAAAAAACGGCCCAGGCGTTGCGTGATGGTGCGG
CCCCACTTAGGAAACGGCTATTGGAGGATATGAGCGATCCTGCCTTTTTGGATACCTTATTAGATACTAATAG
CTCTGTCAAACCATCGTTGAGCCAACCTCGTAAGCAGATGTGTGATGTCAAACAGGAAAAGAAACACAGACGA
AC
>TR421|c0_g4_i7 len=417 path=[4778:0-35 5656:36-49 5641:50-82 5618:83-106
5672:107-112 5616:113-134 5580:135-136 5516:137-148 5435:149-172
5334:173-199 5636:200-223 5611:224-263 5574:264-287 5674:288-290
5680:291-314 5683:315-320 5608:321-344 5569:345-355 5497:356-379
5414:380-396 4599:397-397 4600:398-400 4601:401-416] [-1, 4778, 5656,
5641, 5618, 5672, 5616, 5580, 5516, 5435, 5334, 5636, 5611, 5574, 5674,
5680, 5683, 5608, 5569, 5497, 5414, 4599, 4600, 4601, -2]
GCTCTTCCGATCTGACAAACATACAGTAGCAGATGTTCTCCTTGGCCGAGGTGTTGCGACCACTCGCCATCCA
GGCAATCAACATTTTAGGTCAATTGTAAGCCAGCATTGCGAGCTCTACGCAACAAGTACTAAGAAACAAAAAA
TGAACATATCGCGCTCCATTGTTGCCAAGATCCAAACCGAAGGCAAATTTCTAGAAAAGGAAGCCAATACTGG
ACTCTGGCACGAGGTCGACGATAGAAGAGCGTTGGAAAAAACGGCCCAGGCGTTGCGTGATGGTGCGGCCCCA
CTTAGGAAACGGCTATTGGAGGATATGAGTGATCCTGCCTTTTTGGATACCTTATTAGATACTACTAGCTCTG
TCAAACCATCGTTGAGCCAACCTCGTAAGCAGGAAAAGAAACACAGACGAAC
>TR422|c0_g1_i1 len=227 path=[205:0-226] [-1, 205, -2]
TTGAGGACGGTGCAGTGACTTCGTCGACATCCGTAACAGGAGTGATGTCTACTTTCAGATGTGGAAACCTCGG
CGTTTTTCTCAGTGCCTGCAACGACAATGATACACCGTCTGTCGATTGCGATTCGACATCGTTGTCATCTTCT
GGCACAACATGGGATGGACGAGGAAATCGAACCGCAGGAACGCATTTCTCCTTCCTTCCTCCTCCACTCACAC
CAGACGAT

>TR423|c0_g1_i1 len=260 path=[238:0-259] [-1, 238, -2]
CCGAGATCTACACTCTTTCCCTACACGACGCTCTTCCGATCTCTTTTTCAATGTGAGGAGTAGTGTATTGCGT
TTTGATGCCTCTAACCATACTGTTTTTTCCAAGCCGTCAAAGGTGAACCCAAGTATTTCTTTCTTTAGAGCCC
AAATGGATTCCAGCTTCTTCATTTTCTTTACCGACACTGCATCTTCATCGGCCTTTTCTTTGGCCAGGAACAC
ATCATGGATTCCCATCATCACCACATTTGCGACGTGAACAA
>TR424|c0_g1_i1 len=287 path=[265:0-286] [-1, 265, -2]
CCTTCTGACGACGGCGTTTAGCAGCGGTGCAAGACGATTGATTGTTATTGTCGTTGTGGGCGTAGAAGGAGCT
GTTGTTATCAGGATGCTGACGTTTCTTGGTGGCGGATGTCATTATTTTAGTTGGCGGCGGATGGATGTGATTC
TGATTGTGGTTGGCGACGGCGTTGTACTCTTCGCGACTAGGATGATGACTCTCAATATACTTTCGTTTGTTTT
CGTTGACAATGATGGGGTTAAACCTGGTGGTCAAATTGATTAATCTCGTGCTGTATGTTGTGACCATG
>TR427|c0_g1_i1 len=422 path=[400:0-421] [-1, 400, -2]
TGGAGGAGGAGGAGGGCGGTGCAGAAGAGAGCGGCGAATGTGAATCCGCGGGCTAGGTCGTCTAGGATTGTGT
CGTCTTCGAGTATCGAGTCATCGTCGTTCTTGTCTTCTGCGTCGTTTGGTATTTGAGTTGACGACGATTGTGT
ACTGTTGGTTTGGTTGTGCTTGTGGGTGGTATTACCAAACAAATGGGTGTGGGCACTGTCCTCTTTTTTCTCG
CCCTTCTTTTCATTGCCCTTCTTTGTGACATTTGTTTCTTTCTCCATAATACCCTTTTCTGAATTCTCAGCGG
TAGGAGGAGGAGGAGGAGGTGGCGGAGGTGGTGGTATTTTCTTATGAGATTCTTTGGCTCCGACATCGCCCGA
GTATATGATGCCCTTGTCTTTATTGCTGTTCTTTTTCTTTGCTTTCTTTTTTTCCTT
>TR428|c0_g1_i1 len=296 path=[274:0-295] [-1, 274, -2]
CGCTCTTCCGATCTTGATGATTTTGCCGACTGCGAAGCCAATGCCGAGGATGAAGGAGTTCATGAGCATCTTT
GCGCCGGTGATTTCGCCGGTAAAGCCGCCTTGCGTGAACAGCGCCTTGTCGAAATTCTCGGCCATGTGCGGCC
CGAGCGAGAGGTGAAGCCCGTCGCGGTAGATATCGACATCGCTCACGCTGGTGGACAGGAAGACCGCGAACAC
CGGCATGATCATCAGAAGTGAACCCGCGATCAGAACCGCATGGTCGATCACGGCCGACCACCGGATCGGTCGT
CCGG
>TR429|c0_g1_i1 len=263 path=[235:0-250 236:251-256 237:257-262] [-1,
235, 236, 237, -2]
TCGTCGTCATCATCATCATCCTCTTTACCATCCGTACAAGACGCACGAGGATCCTCGCTCTCGTCCAAACTGA
TCACCGGCCTCGCCGTCGGAGGAAGGAACGGAAGTCCGTAGGCATTGACCTTCCTCACGATCTCTCTCATTTT
CTTGGCATAAAGAGCTTGTTCCTTTTTCTCTGCACTCTCCCCCTCTTCCTTTTTGTTTTCGTTTTCGTCGTCG
TCGTCGTCATCATCGTCATCGTCATCGTCATCGTCATCGTCATC
>TR430|c0_g1_i1 len=258 path=[236:0-257] [-1, 236, -2]
AGGCAATTGATATTTACTGAAGTATTATTTCCGCCATCAAGCAGCCTTTTGGTTGTGCGTAATCCGTGCAGGC
ACTGCGTCATCTCTTACATGAGCTAAGATGACATCTTCGGGCAGCCGCAAAAGGGGAAGTCAGTGCAACAGCC
AGACGCACTTGGCATTTCTTTGGGAGGACTAGCGGGCACTTCCAGCGCAGGAGCAACTCTTTCGTGAGACGTG
GACATTCCCAATTTTGGACAGCCGCAAAATGGATAATCA
>TR431|c0_g2_i1 len=406 path=[408:0-374 409:375-405] [-1, 408, 409, -2]
TCACATCGCGTCCACGTCGTACAACTCCATCCGAACGGACTGATGCAGCAGCTCGACTTCCAATGAAAACCAA
GTTGCAAGGGCCAAGTCCACAGGAGACGAGATTGGTAGAAAAGCACGATCTAGTGGTCCGAATGCGAACCGCG
ATTCGAGCACGGTACATCCGTAGCGCCATGGATGCAAAACACGACAACGAAACAATTTTTCGCCAATTTGGGT
GGTGATTCGATCAACCAAAAAAATTGACGGCATACCCTAGCTGGCCTGTGCCGTATTTAGCCAAATTCTCGCC
CACGTCTCCTTCCCACGCTCCGCAAACGACGAGTAAAATCCCGAAACTCGCACATCCCACTCGAAAAAGGCGT
CCACCCAAAAGTATTAAAAAAGAAACACTCTCACCTCCTCG
>TR433|c0_g1_i1 len=243 path=[234:0-176 235:177-242] [-1, 234, 235, -2]
CATATATAGGCGTCAAAAGTCGTGATATATATTGTTGTTGTTGTTGTCGTTGCTGCACCCCATTTTGTCCTTT
CTTTCCTTTCTCTCTCGCCATTGTGTTTTTATTCCATGTTTCGTTCTCTGTCTCTCTACACTGCAGAAAAAAT
CTCGTATTTTATTACCGGCACACACGCATTTTTTCATACGCATATTGTATTCACGGTAAAACACGCAACATAC
CAAATAACAACAATAACAACAACA
>TR434|c0_g1_i1 len=1524 path=[1708:0-963 1713:964-1499 1711:1500-1511
1712:1512-1523] [-1, 1708, 1713, 1711, 1712, -2]
ACTCTCTGTCGCTCTGCCAGTTTTTCATCAGCGGTGCCTATTTCATGTTCATGTTCTTTGTTATTCCTCGCAC
ATGAAAAATATTGTTCCAAAAGCTGAATACGATTTTGGGCCTTGTGTGAATTAACGCGAAACGTCTCAGGCGG
CGAAGAAATCCATTCGTTTCTGGCGTGGAGGAGGCCGCGTATATATGTTGCTATTTTTGGTTTGAACGCTGTG
GTAACCCCACCGCGCGCGGTAGGTTGCAGTTCATGATGTGTTGCGTTGTTTATTTTTCTGTCGCTTTTTCAGT
CGTCCGATGGGAAGATAATTTCTTCGGCTCGCTTTATGTTATATTATTGGAATTGATGATGATTACATCATAT
GCTTATTTTGGAAGTTGTTTGAGGTTGTCGTCGTGTCTCAATTATGGTTTGCTTCACCCTCACCGCCAAAAAT
CGAGAGGCGAAGGGATCAACATCGAGTTTCGATCCCGCCAAATTGATTTCTTCTATGATTCTTTTCCGTGTTT
GTTGACAAATATTGTATTTGCCGTTCTTTATCGGGCTGGTCCTTGTAGCAGACGATATATTATTCGGCTTACT
CTGATAGTTTTGGATTTGATGATGACTGCACCATGCTTGTTTTGGAGGTTGTGTAGGCGGTCATCGATTCTCA
ATATTTTCCATTTATAGGCATTCATATCATCTCCATGTCCTTTAAAATTATTCGCAACGCCGTCCAACGCCGC
AATATCTCCATCGGCATGCGGAAAAATTGAGAGGTGAAGCGAGCATCACTAAACACTGGGCGTTTTTGAATCC
TGACTGTTTTGGTTTTGCCATTTTTGAATCGTGCTGGTTTTGGGGCAGGTCATCCGACTGTATTAACTATTTT

TGTTTACAAGTCTAACATTATATTATGCATTCAATACTTTCTTTTTAATCCTCTTCACATTTTCAGACATGAT
AACAGTATGCAGCAGATAAACAGTGGGTTCTCTTCCCTATCCTTTACCCCTTCCATGACTAGCATGACCGCTA
TCTCCAGCATGAGCAAGGGGGGAGTAATCCAAAAGCCAGACGTGCCGATTTCACCCATGTCATCCACCATGAA
CATGAACAACGGGAGCTCCTTTTCTAGCTTTGTTCCACAAGAATCTGACGGACCATCGCCATACTATCCCTCC
TCTCCCATGTCTCCCGTCTTTCGCCAACATCCGACCATACCGCACATGATCAACCGTGCGCAAAGTTTGGGAG
GAGAAGACAATACTCAGGGTCAGGGTAGCCAGTTAGGAAGAGGAGGAGGTGGAGGAGGTAATAATAACTTTGC
AGCATCTCCCGTCATACATTCCAAGCACACCTTCTCCCCCATTCTTAATAATAATGCTAATGGCCCAGCCTCG
ATGATGTCGTCTTCGTTCGATGGGAGATCGTCCTCTCAATATTTGTCATCGGGGAGTAATAACAACAACAACA
ACAATCAGAACCAACAACAACAGCAGCAGCAACAGCAGCAGCAACAGCAGCAGCAACAGCAGCA
>TR434|c0_g1_i2 len=799 path=[1706:0-238 1713:239-774 1711:775-786 1712:787-798] [-1, 1706, 1713, 1711, 1712, -2]
CGCTATGCACTTTCCAGATTCGGGCACCTAGAAACATCTCGCAAAAAACGAACACATGACACATGCTCTATGC
ATAGCATGATGAACATCGGAAAAAAGATTTCTCGAAAACGCACCCACCACTGCGGCCACGGATAGAACAATTA
TCATCACCAACTCTCATGATGGCCAACCCGCCCTTTAGTAGCGGGGCATCCCTGCTAGAAGACCTCAAGGGAC
ATGATAACAGTATGCAGCAGATAAACAGTGGGTTCTCTTCCCTATCCTTTACCCCTTCCATGACTAGCATGAC
CGCTATCTCCAGCATGAGCAAGGGGGGAGTAATCCAAAAGCCAGACGTGCCGATTTCACCCATGTCATCCACC
ATGAACATGAACAACGGGAGCTCCTTTTCTAGCTTTGTTCCACAAGAATCTGACGGACCATCGCCATACTATC
CCTCCTCTCCCATGTCTCCCGTCTTTCGCCAACATCCGACCATACCGCACATGATCAACCGTGCGCAAAGTTT
GGGAGGAGAAGACAATACTCAGGGTCAGGGTAGCCAGTTAGGAAGAGGAGGAGGTGGAGGAGGTAATAATAAC
TTTGCAGCATCTCCCGTCATACATTCCAAGCACACCTTCTCCCCCATTCTTAATAATAATGCTAATGGCCCAG
CCTCGATGATGTCGTCTTCGTTCGATGGGAGATCGTCCTCTCAATATTTGTCATCGGGGAGTAATAACAACAA
CAACAACAATCAGAACCAACAACAACAGCAGCAGCAACAGCAGCAGCAACAGCAGCAGCAACAGCAGCA
>TR435|c0_g1_i1 len=374 path=[372:0-98 373:99-116 374:117-373] [-1, 372, 373, 374, -2]
CTATTGCACATCCACCTATCCATCATTTTTCTCACCAACAAAATGCCGAAAATGAAGAAGTTCGGGCTGGTCG
CCATGGCGTCCTTAAAGGGCAAAAAAACTAACTAAGGCTACAGGCAGTGCCATGCAAAGTTTTCGCACCAGTG
TGCAAGGATCCTATCAGGGCAACAGCGTCGACAGTTACAGCAGTGGATCGTCCTATTACAGCAGCAGCGGCAG
CGAGGAGGGGGATTCAGAGAGTGACAGTGGTTCTGATTACAGCGATGATAGCAGCAGTGATGAATCATTAAAA
AAGAAGGGGGGCGATAAGAATAGTAATAATGGTAGTTCGAACGGGAACGGGACCGCTGGGACTGGGGGCGGGG
GGATAAAAC
>TR436|c0_g1_i1 len=282 path=[326:0-160 332:161-190 330:191-219 321:220-243 322:244-281] [-1, 326, 332, 330, 321, 322, -2]
TTGGCACAACATATTACATTAATGACTGGTGGTGAAAGCCTTGTTTAAGCTTGGACATGCGCTTGAGTTTGTG
GATTTTGGAGAGAGAGACAAATGAATGTGCACTATCTCTTCCTGCACCCAAAGCGACATCTTCCGCGCACTAC
CCACTTTCCGGACATAACAAAGTGCACCACTGGATTGTAGGAGTGAAGAAGGGTGATGGGACAATTGTTTGAT
TCGGATCTCCTTCGGTGATGGAACTGTACAGTTTGGTATTCGCACATAGTGCGTTTTGTGCCT
>TR436|c0_g2_i1 len=282 path=[323:0-160 331:161-190 329:191-219 317:220-243 318:244-281] [-1, 323, 331, 329, 317, 318, -2]
TTGGCACAACATATTACATTAATGACTGGTGGTGAAAGCCTTGTTTAAGCTTGGACATGCGCTTGAGTTTGTG
GATTTTGGAGAGAGAGACAAATGAATGTGCACTATCTCTTCCTGCACCCAAAGCGACATCTTCCGCGCACTAC
CCACTTTCCGGACATGACATAATGCACCACTGGATTGTAGGAGTGAAGAAGGGTGATGGGACAATTGTTTGAT
TCGGATCTCCTTCGGTGATGGAACTGTACAGTTTGGTATTCGCACATAGTGCGTTTTGTGCCT
>TR437|c0_g1_i1 len=347 path=[650:0-172 651:173-346] [-1, 650, 651, -2]
CGTTTCCCGTGCCGTCGGATTTGCGACGGCGGATTACGGCCGATACCGCGGGGGTGGACATGATGCCACTTTG
AGGGATCCAAATGTTTTTGATTCCATTTGCGGCAAGTAGACGAGCTATCGTCTGAATGGCTTCGTTGTTGTAG
TACCGTCCATCACCTGCAACTACAATCGTATCCAACATCTTCCCCCCATTCGCATCCACCGCCGTATCGATCA
AACTCTGAATAAAATTCTCCACGTAATTCTCACCCATCCACACCTCCACCTTCTTTCGCAATCCGGACGTGCC
GGGACGCATCCCCTCGATGACAGTGGTGGGGACTACGGCGACGGCCACGCCCTCG
>TR437|c0_g2_i1 len=435 path=[643:0-172 654:173-350 652:351-407 649:408-434] [-1, 643, 654, 652, 649, -2]
CGTTTCCCGTGCCGTCGGATTTGCGACGGCGGATTACGGCCGATACCGCGGGGGTGGACATGATGCCACTTTG
AGGGATCCAAATGTTTTTGATTCCATTTGCGGCAAGTAGACGAGCTATCGTCTGAATGGCTTCGTTGTTGTAG
TACCGTCCATCACCTGCAACTACAATCCTAAGAGATGGATAGAATTCGTTGTATTTGATTGTATTGAACGATG
ATATGAATATCATTGTGGATGAATGAGGTAGTGAAATGCAATAATGTAAATGCATGAATACAAATTGTACAAA
ATTGAGATTCCCAGAAAGCAATGTTCGCAAGTTTGGTATATTACTGAACGATCAAGGATATGTGCGAATATGG
CAACAACGAAGAGGGGGGACAGTGCACACATTATAACCGTGAAATTAACTCAATCTCATGCTGCCATTTT
>TR438|c0_g1_i1 len=369 path=[490:0-146 491:147-147 512:148-160 515:161-164 513:165-214 510:215-231 504:232-368] [-1, 490, 491, 512, 515, 513, 510, 504, -2]

GAGCGACCTCGAATTAGTATAGTTGCATACTCCTAGATCATTTGACAAGGTGTATCAACCCCCATATTGAGTA
ACAAAGCGAAGTCATTTTTTCATTTTTTGAAACCTACCCAGATTGACCTCGAAAGTCCACCTCCTACACTTTT
TTCCACACGTCACCCATGACGTCGTGAGGCAATGATTAGTGACCGTAAGAAGCAGGGAATATAGCTGGTAACC
TCGGCCAGGGGTGCAATGGTCGGTGGGGCTGAGGAAACCACCAGAACTACACAGCGCGTCACATCGTCGCCAC
TAATAGGGTGTTTTTGGGTTGTTGTGGGCCGCTACAGCGACCCAAGTGACGCAACAACCGCCATGTATTATAC
CCAT
>TR439|c0_g1_i1 len=906 path=[1133:0-75 1158:76-179 1156:180-310
1155:311-385 1149:386-401 1150:402-905] [-1, 1133, 1158, 1156, 1155,
1149, 1150, -2]
TCTTCCGATCTCTTTCCCATTTTGTGGTGTGTGTGTGTGCGTGCGACGCACAGGATCCCAATGCTCCCAAGAG
ATCACCCCCCGCCTTCTTTCTGTTCGTGAACCATTGTCGTCCAGAGTTGAAAATGCAGTATCCCGAATTGCGT
CATACTGAATTAGTCAAAATGCTTGGGCAAAAGTAAGTTGTTATTGTTGTTGAATACACATTTGGTTTATATA
ATATTGTAACGCGAAAAAAATTGGTACCTGTCCTCATATACGATCATCTGAAAATTCGATGTATTACAGGTGG
TCGCAAATGTCAGAGGAAGAGAAGAGACCATTTCGCGACCACGAGGAACAGTTGCGACAACAATACCACATTG
ATATCAAGCATTACAAAAAAAGAGCGGTGCCGGAGCAGGAATACACGAATTTGGGATCGCTTCATGTTGCCGT
GATGAAACCTCAACAGCAACATGTTGTTGGAGTCGACAACAGAGAAGACAAGATGTCGGTTCCTCCGTCGGGT
ATGCATACGATGCATGGGGAAGTGGATTATCATCAACAACCTGGGGTGCAGAATCATGAACCTCATTATCAAC
AATCTTTGAAGGAAGGAGAGAATGAATTGTTACTGCTCTAAATTCCATTATGTATAATCATATGTATTAATCA
TTGTATTATCTACACATCCATACCTGTAGGGACATCTTGTCGTGAATACAAGCACTTCCTTTTGTATGACTGC
TACTTAATTCTCCCCAGCCCAAACCAGATAGTTCAAGTGGTACAATATTTATAAGTAATATTACGAATGCCAC
GCCATAATCTAAACAACAGATAAACCACAGTAGTATCATCCAACAACAACGACGACAACAACAACAACAACAT
TCATCAACTCATCAACAAAGCGCCCGAATA
>TR439|c0_g1_i2 len=936 path=[1139:0-200 1159:201-215 1158:216-319
1143:320-340 1155:341-415 1149:416-431 1150:432-935] [-1, 1139, 1159,
1158, 1143, 1155, 1149, 1150, -2]
TGGCGGTGGTGGGGGAACAAGAGCAGCAGCAGCAACAACAACAACAACAACGAGTGGAGGAGTGGAAAGGTTG
CCTCCCGAACAACTTACCATCCCGCAATCGGGTCACAATGCATACATGAATCCACCTGGATACGCTCCTCCTT
TTGTAAAAGCTCCTGCTGCTACTAATCGTGGTAAAAAACGCAAACGAGATCCCAATGCTCCCAAGAGATCACC
CCCCGCCTTCTTTCTGTTCGTGAACCATTGTCGTCCAGAGTTGAAAATGCAGTATCCCGAATTGCGTCATACT
GAATTAGTCAAAATGCTTGGGCAAAAGTGGTCGCAAATGTCAGAGGAAGAGAAGAGACCATTTCGCGACCACG
AGGAACAGTTGCGACAACAATACCACATTGATATCAAGCATTACAAAAAAAGAGCGGTGCCGGAGCAGGAATA
CACGAATTTGGGATCGCTTCATGTTGCCGTGATGAAACCTCAACAGCAACATGTTGTTGGAGTCGACAACAGA
GAAGACAAGATGTCGGTTCCTCCGTCGGGTATGCATACGATGCATGGGGAAGTGGATTATCATCAACAACCTG
GGGTGCAGAATCATGAACCTCATTATCAACAATCTTTGAAGGAAGGAGAGAATGAATTGTTACTGCTCTAAAT
TCCATTATGTATAATCATATGTATTAATCATTGTATTATCTACACATCCATACCTGTAGGGACATCTTGTCGT
GAATACAAGCACTTCCTTTTGTATGACTGCTACTTAATTCTCCCCAGCCCAAACCAGATAGTTCAAGTGGTAC
AATATTTATAAGTAATATTACGAATGCCACGCCATAATCTAAACAACAGATAAACCACAGTAGTATCATCCAA
CAACAACGACGACAACAACAACAACAACATTCATCAACTCATCAACAAAGCGCCCGAATA
>TR439|c1_g1_i1 len=520 path=[498:0-519] [-1, 498, -2]
AGAAGAGGATACTGGGATGGTCTTCCGCGAGGGACGCGGCGATGGATTTGTCGACTGTGGAGGGAGCGGGCAA
GACGGATTTGTTCGCGTCGCTTTCGAACGGGGAGAAGAAGGCGTTGTTGAAGCGATTTCAATCGGACGAGAGT
TACGAGAGCCTTTGTGGTGGACAGGGTGGGATGACATTTCCTCAATTGCGACAGTATAACGGGTTCGATGATG
AACTCACGTTGCGACCCGTGGCCACTCGCCTCTCCGAATTGCTCAACGACGACAACAAAAACAACAACAATAA
CGATGCCAGTTCAGCACAACAATCACAACCACACATAGAAGTCAAATTCGCACCAGACTGCCTGGACGCCACG
TCGATAATATCCACCCTCCAACCATCCCAAGTGCTCCTCCTCGAAAACGTCCGATTCTACTCCGACGAAAACT
CCCGCAACCCCGCCGAACGCCTCGCCATGGCACGACAAATATCGACCTACGGCGATTACTTCGTAAGTGACGC
CTTCGGAGA
>TR440|c0_g1_i1 len=599 path=[1168:0-132 1169:133-156 1188:157-210
1184:211-342 1187:343-411 1174:412-435 1185:436-547 1167:548-598] [-1,
1168, 1169, 1188, 1184, 1187, 1174, 1185, 1167, -2]
ACGATGAGGACTTTGGCAATTTCAATAACAATTTCAACAACAACAACAACAACAACTATGCACGACCCCCAGA
GGAGGGAACTAATCGACCGCAGACGGATGGATATTGCGTCGCCGCCGGCATACCGTGTCCGAACAGGAACATC
GCGCTGTGCTGCAGCGATATTTGCATCAAGGATAGGGATACGGGAGAACAGTTGTGCGCACCGATGCCGGAGG
AGGAGGATACTACGCCTGCTTCTACCAACACTTCGCCAGAGGAGGAGGAGGAGGAACCGGTGCTACCACTGGC
AGTAGCGGATGAGGAGGAAGATGCTGCCTATTGCGTCCAAGAAGGAAATCCGTGTCCGGATAGGAAGGTTGCA
TTGTGCTGTAGCGACATTTGCGAGAAGAATAAGGAGACGAAGCAGTATGAGTGTGCGGCGGTGGTAACATCTT
CGCAAAAGGATAAGGACAAGAATAAAGACAAGAACAAGGACAAGGATAAGAACAAGGATCGCTGAAAGTGAAG
ATGCGAATGTTCATATTATAATGGCAGTACTTTAATGATAGTAATTTAATGTATTTGTCCAAATGAAAAAAAA
AAGATCGGAAGAGCG

>TR440|c0_g1_i2 len=930 path=[1168:0-132 1169:133-156 1188:157-210 1183:211-339 1187:340-408 1174:409-432 1185:433-544 1181:545-929] [-1, 1168, 1169, 1188, 1183, 1187, 1174, 1185, 1181, -2]
ACGATGAGGACTTTGGCAATTTCAATAACAATTTCAACAACAACAACAACAACAACTATGCACGACCCCCAGA
GGAGGGAACTAATCGACCGCAGACGGATGGATATTGCGTCGCCGCCGGCATACCGTGTCCGAACAGGAACATC
GCGCTGTGCTGCAGCGATATTTGCATCAAGGATAGGGATACGGGAGAACAGTTGTGCGCACCGATACCGGAGG
TGGAGGATACTACGCCTGCGTTTACCAACACTTCGCCAAAGGAGGAGGAGGAGTTGCCGCTACCAACGGTAGT
AGCGGATGTGGAGGAAAATGCTGCGTATTGCGTCCAAGAAGGAAATCCGTGTCCGGATAGGAAGGTTGCATTG
TGCTGTAGCGACATTTGCGAGAAGAATAAGGAGACGAAGCAGTATGAGTGTGCGGCGGTGGTAACATCTTCGC
AAAAGGATAAGGACAAGAATAAAGACAAGAACAAGGACAAGGATAAGAACAAGGATCGCTGAAAGTGAAGATG
CGAATGTTCATATTATAATGGCAGTACTTTAATGTATTTGTCCAAATGATATAAATATGGACAGTGGTTTGTA
AATTCGAGGGGGTATAAGTGGCGATGATGTCTTCATGAAATGCATACAAATATAAACACAATGCAAACCCTAG
ATACCAATCACTTATGAGTAGTGGGAGTAGTTGATAATGTGATCTAATATCCGAAGAATCAATGCACGAAAAG
AGGAGCACAAGGGGAGAAACACGTATATGGTATTCAATAACAGCGAGATGGACACTTACACCATGTATATATG
TTGAACATTTTTGATATGTGTTGTTCAAATAATACTCCAACTGAGAAATGTCTCGGCATACCGCCTCTGCGGA
TAAACCTGTCTTCTGAAACTACTCAGGTTATAGTATTGTTTAACCCTCTCCTAC
>TR440|c0_g1_i3 len=596 path=[1168:0-132 1169:133-156 1188:157-210 1183:211-339 1187:340-408 1174:409-432 1185:433-544 1167:545-595] [-1, 1168, 1169, 1188, 1183, 1187, 1174, 1185, 1167, -2]
ACGATGAGGACTTTGGCAATTTCAATAACAATTTCAACAACAACAACAACAACAACTATGCACGACCCCCAGA
GGAGGGAACTAATCGACCGCAGACGGATGGATATTGCGTCGCCGCCGGCATACCGTGTCCGAACAGGAACATC
GCGCTGTGCTGCAGCGATATTTGCATCAAGGATAGGGATACGGGAGAACAGTTGTGCGCACCGATACCGGAGG
TGGAGGATACTACGCCTGCGTTTACCAACACTTCGCCAAAGGAGGAGGAGGAGTTGCCGCTACCAACGGTAGT
AGCGGATGTGGAGGAAAATGCTGCGTATTGCGTCCAAGAAGGAAATCCGTGTCCGGATAGGAAGGTTGCATTG
TGCTGTAGCGACATTTGCGAGAAGAATAAGGAGACGAAGCAGTATGAGTGTGCGGCGGTGGTAACATCTTCGC
AAAAGGATAAGGACAAGAATAAAGACAAGAACAAGGACAAGGATAAGAACAAGGATCGCTGAAAGTGAAGATG
CGAATGTTCATATTATAATGGCAGTACTTTAATGATAGTAATTTAATGTATTTGTCCAAATGAAAAAAAAAAG
ATCGGAAGAGCG
>TR441|c0_g1_i1 len=1108 path=[1340:0-313 1341:314-337 1369:338-429 1373:430-443 1362:444-467 1371:468-512 1370:513-536 1368:537-761 1374:762-784 1348:785-808 1364:809-877 1323:878-901 1359:902-966 1320:967-990 1321:991-1107] [-1, 1340, 1341, 1369, 1373, 1362, 1371, 1370, 1368, 1374, 1348, 1364, 1323, 1359, 1320, 1321, -2]
GGCGGCTTCGGATGCGCTCGCCGTAAGGAGATCCAAGCATTCCAGGAAGATTGCCAAGCGTCAGTTGAGGGGG
ACGGGCCGTTCCGTCAAGATCACTGGCAGCGGGAACAACAATGGTAACAAAAGGTTGGGGGATAGCTTTGGGA
ATAGCTTCGGAAGTTCGGTAGAGGCGAGTTCCTCCTGTCTTTCGCAGCGTCGAAATTCGCCCGTGACTGCGGA
GACCGCCACCAATCATCATCGCCGGAGAAGTGATCGCAATGGTTTCGAAGGTGCTGATCTGGACGGTTCCGAG
AATGCCAACGCGGTGACCAGCGCTTTTGAAAGCGAAACAATCAGCCAAAACACCGACGATAAAGTTTTGAACA
GCATGTTGGGCATGGCGAGCCGGTTGGAAAGATCTCACACGCTGGAGGAACCGTCCATCGTCGAAGGGACCAC
CGACGCTCAGTACTGGAAGAACGTCAGGGGGGAATCGAATAGCAGACGTCAAATAATGCGCGGGAGCGGCCGT
GCGATCGGCTTTTCACGGCAACGAGGGAGCAGCGCTAAAATCGAGAATTACGGAGCTCGAAGCGGCGGCGGCG
GCGGCGGCAAAACCGCCAAGGAAAAGAAACTGACGCCCAGCGAACATCGGAAATTGCATCACGAGAAACGAAA
GCTGAAGCGAATACAGGAGAAGAAGAATCAAGAACAGCGACAAGAGTCGATGAATCATGGCAATCGACAAAGT
CGCAGTGGCAAGAACACCGAGGAACAAACACTGTCGCTCAGCGAGCATAGGAAACCGCACCACGAGAATCGAA
AGATAAAACGGATGCAGGAGTGGAATGATCAACGGCAGATACAACAGCAGGAACCATCTAGCAGTGCCACTTC
TGCTTTGGCGAACACGGCTCGTGACAGCGGCGGTAGCAGCGCGGCACCCATGCACAATCACCAACGTGGTCGA
CGGCGGCAGCAGCCTCAAAATCAACAATTCCAACAACGGCACCACCAGCACCCGCCGAAAACAAGCCTATTAG
GCCGATTGAAACATCGGGGCAAGAATAAGAAGAAAGCTAACGACAACTTCGACGATTCCAATCCCTCGCTGCG
ATAGATCGGAAGA
>TR442|c0_g2_i1 len=2946 path=[3162:0-360 3199:361-1123 3208:1124-1142 3207:1143-1166 3205:1167-1248 3185:1249-1272 3206:1273-1282 3204:1283-1284 3201:1285-1636 3154:1637-1660 3203:1661-1816 3182:1817-1833 3200:1834-2340 3194:2341-2364 3196:2365-2723 3175:2724-2747 3176:2748-2945] [-1, 3162, 3199, 3208, 3207, 3205, 3185, 3206, 3204, 3201, 3154, 3203, 3182, 3200, 3194, 3196, 3175, 3176, -2]
CACAATCCATGCAGATCCATCTCAAAAGGACATTTTACTGCAAATGTACTTAAATGAGATTAATTTATATTTC
TGCTGGTTCGTTAGTGCTACGCGAATGCAAGCTCCGTATGACTTTGGTACTTATCATCGATCATCGCTCAAGA
CGTATTTATGAACATTGCGTTGAAGTTTGTTCTCTCAAGTGGATGTCGGAGGGGTTTTCGTTGGAATCGACAA
AATACCGTGGACCATTCATAAGACGGAAGGGTTCTCAGCAGTATTGGATCAAACCGTCGAAAAGCTATGAGAC
CTTTTTACCATAAAAAAGAACGAGAACACCTTCCGCATCGATATTTTGCTGCGTGTTCTCTTCCTGCCAATAA

TTTCGATGTAATCAGCTACAAGGCCCGCTAGTACAACGATGCGCACATCTGGGAGCTTCAGAAGCATAAAGAG
TTATATATATTCCTCATGTTGTACGTCTTACGCGAACATTTTTAGCATCAACATTGCGCATTTTCTACCAGTC
GTTCTCTTTTACATACAGCAGTCCACGTGCCATTGCACATCTTGTTTTTCTGGTTCGTGCGCCAAAGGATCCT
TCCAACACTTGCATCAAACGAAGTAGATATATTACGGAGATAGAAGAAACAACAACACCTTCATGTTGGCATT
AATACTAAACTGAATCCCAACTCTTTATGGCAGAGAGACTCTCCTGTTGACATCATAAGCCGCACATAAACTT
CTTTTCCCATCCTACCTGAAAGAAAAGGTTAAACTTACTAGTTCGATGTGGTTGAGCACTAGCGCTGCAATAG
CCTGTGCTGCGATTTATGTGCGGAAGTTAATATGGCGTTTTCGAATTTGCTCGATATGGAAGCTCGCGTTCAC
ATTTGGTGCTTGCCTCGCCGGTGATCAATTCGTGGAGAGAGATATTGACGATACAGACGATGCTACGAATACT
TTGCGCATCCACCGCACAATATTTCGAACGGACGAGTACATAAAATTTTTTGTTATTGAGCACGGAGGTATCG
AAACCACCGTCAAAGCAATGAAGAGATACGATGGCGATCACGAGGGCGTAGCAGCTAGCGGAGTAGGGTTGAT
CCAAGTTATTTACGGATGCAATTGCGCCACAGATAAAAAAACTTGTCGAATGCAGTGCAATCCGCGTTGTGGT
CCAGACCACGAGATGTTGGCCTGAAAAGGAAGAGGTGCAAGCAAACGCATCCAATGCACTGAACCGTCTTGCA
TCGAATAAGGACAGTGCCATCCGGAATAAGATCATTGATGTTGGCGGGCTGATAGCTCTTGCAGAAACTCAGA
CAAAACACCAGAACGATGCAAGAGTGAATGGCCCAGCTACCCACGCCATTCTCCTTCTCGTCACTAAGGGAAA
AGACCACTGTGGGGACCCCATCATTATTTGGCGAAGCTACATTTGAGGTATTAGGTACTATCACACTAATTCA
CTAACCCCCCCCCTAATGTTATAATAGTTCACATACGACATGACCAAACCAGGGTTCGTTCCGCATTATGACA
TCGCATGAGATAACAGGCAAGCTCTTCGAGACGATCAAATCACATGGAACTGCTACAACCGTCCTGTATTGTA
GATTGTGGTAAAATGTGTAGATTACCATACTCTTCTAATATTCACAGACTTACGCCTCAACTATTGCTATTCC
TATTGCAGATGGTTGTCACTTGTTTTCATAGCCCAGGGATCATTTCATCTACAGCCTGGCGCCACTTTGGCAA
CAGCGGATTGGACAAATAATCCGTTGTTTTCGGGCCCACTTTAAGGCCACTTGATTGAATTTTTTGCGCAAGA
TGATACAAACGCTCCAAATTGTCCAGCCCAAATTTGACCACCCCCTTTGTTTGTTCAGTAGGGGGAAAGATGC
CACCCAGATCACTGTATTGGAATTGTAGAATCCGGCACTTATTCTCTCCAAGCGGAACAACTCGCAAAAAGTA
GCAAAGGGGAGAGCGCAGAGTTCGGGATGGCCTCTTTGAACACGTCCCAATTAGCCCGTGCAAGCATGTCGGA
ACCGGAGTCTGGGGTCTGCCGGGATGTAGACACGACCTACTGACCAGCATGAGATTCTCCTCATCGACAAAGT
CAAATGTGACAAAATCTCGATGGGCAAACAATGGGATGATCTTGTCAGCTGTGTATGCAACGTGGGCCCAACT
AAATTTGCCGGCGGTATCAATTTCTTCTAACATCTCCACTAAGTATGTGTAGCTGTCAATGGCCTCAGTGTAG
TTCAGACGAACATACATGTCGAATAAAGTTTTGGCATCTACATCAAATTCAGCTGTATGACGGACACATTCAA
GGCCAGGAAAAGGGACGCCAACAACTGGCGCCTTTTCCGCTTGGATGGTCAAGCTGCCCCTTGTGAGACTCGT
CAAATATTCCATAGGAGTTATTTCTGCATCGGTCAATCGTAGACCAGCCTGAACCATGTTCAACATGTAATGA
AGGCAAACTCCAGCATAATCTTTTCCATTAGTTTCCTCAGATTTAAGCTTGTTAAAATCGAGAGCATTCGGCC
AGGAGACAAGCGAGATGGAGGAATATGGCATTGTCGTTGATTTTTGATCCTCGTCATATTCTGTTGTCCTCTT
CTCTACAGGGTCAAATCCTGGCTCTTCTGGGACGCGGCAGATGCCCATTCCTACTCAAACTATCAGAGAGACA
CAATTATGCACCCTCCACTCTGCACGCTGCTATTGCAAGTATCTAGTATGGGTCTCTTGTGATTAAGAGCGCG
TGATGACTACTCCTATCTATCAATGGCCGCGGAAAGTATACAACGCAGCAGTACGATAGTAAATATGTACGAC
CTCGTATGGTCTTTCAACCATTTCGCGTTACCAGATTGCGTCCTTTCAGATGCTCACTGCGAGCTCAATTTTT
GACTTCATGATTATCTAAGACTATCG
>TR443|c0_g1_i1 len=1624 path=[2307:0-36 2364:37-93 @2369@!:94-220
2275:221-257 2363:258-461 2315:462-485 2356:486-604 2372:605-605
2368:606-629 2362:630-655 2355:656-679 2319:680-680 2376:681-915
2374:916-979 2371:980-1035 2366:1036-1056 2359:1057-1059 2375:1060-1097
2373:1098-1121 2370:1122-1222 2322:1223-1246 2365:1247-1310 2377:1311-
1313 2350:1314-1535 2265:1536-1536 2266:1537-1623] [-1, 2307, 2364, 2369,
2275, 2363, 2315, 2356, 2372, 2368, 2362, 2355, 2319, 2376, 2374, 2371,
2366, 2359, 2375, 2373, 2370, 2322, 2365, 2377, 2350, 2265, 2266, -2]
CGATCGAGAGCAGCAGCAACAACAGCAGCAGCAGCAGCAGATTGCGTCAAATACGAATACAAAATTTCGCATA
GTCCTCGGCCTCGGTCCGGGGCGATCCGGCACCAAAAGCCTATCCGAACTTCTGGCGTTACAACATGACGTGG
TGAAATGCGAGCACGAAATGATCGTTCCGAGAGTTTACTCTCGCACTCCCTCCGGACGCATTGACGCGACCAT
GACAATGGAAACAACAACTACTACTACAACAGCAACACCCACAAAGGCGAAGAAGGGTTCCTGGGGCGCAGAT
CGGAGGCTCGAATGGGACGCACCAAATCTCGCGAGGGGGGAGCCGCGTCGTACCGAAAAAGAGGAGGCGACGT
GGAGGATGTTGCGATTGATCGAACAGCGCGCCGCCTTCGAAGGATGGATAGCTGAGGTAGGAGGATGGAGGGA
GCAGCGCACTCATAATCAAACGAATTGCAGTGCGGCGCGACGCCTTTCGAAATGCTGTGGGGGAGAAGTCAAT
TCCAATTCCATCGCGGACGAAAATAAACACGTACCACCGACGATCGTACTGCCCGTGGTGGCGGCCGTGAGCT
CCGTCGGACTGGCCTACGCGCACGAATACATCGCCTTGTATCCATCCGCGAAAATCGTCGTGATGCTACGACA
GCGCGAGGAGGTTGTCGATTCCTTCTTGGCCAAGTCGAAGGGACGGAATCACTGGCAGGCGCACGTCCATCAT
CATCATCATCGTCGGAGAAAGAGCAGTGGCAGTGGCAATGATGGCAATGGTGCCGAACGTCGCTTGGACGATG
AGATTGAATTGGTCCAACGCGATAAAACTTGGGATGGCGCATTTCCCAACATGTCGGATGAGGAATGCAGACT
GGTCATGATGGGGGCACGGAATAATCACGAGGATGATGGTGCGACTGCTGCTGCTGCTGCTACAACAATCATC
CCCGGGAAGAAGGAGGAGGAGGAGATCCAACGACTACGAGGACGACGAGGAGCATGTCGGAAACCCGACAAGG
TGTGGGCATTGCGATCGTACTGCGAGCTTTACGAAGATGTCGCTCGGGAGCTATCGGAGCGGTATCCGAACAA
TGTTCGAATTTACGATATGCGGGATGCTTTGAACGAGGCGTCGGTGCAGGAAAATATGCTGCGTTGGTGTGGG

TTCGAGGAGCCGGTGTTGGATACTCGTTTGCATTTGAATAAAAAGAAGTGACAAAGACTATGAAGCGAGACAC
TCTGCTGTACACTATTTTATTCTCGGCGTAGTGAAATTGCATTGACAAGTATGTAGTCATTACACCTTTCTTA
CGGGACTTTTGAAACCGTGTACTAAAAACATGTAGCCAATACGATTTTATCTTGCTGGCTAAACATCTGGTGC
TTGTGAAGCCTTGAGTGTGTGTTGCAACTCTAGGCAAGACGTGGACGGAAACTCTTGTCCCTACAAAATTTCT
CACTTCACAGGTAAGAGAGAAGTCGAAAAAAACCATCTTGGGTAGAAGAATATTGGTACCCACCATTTTCGTT
TTTTCCGTTCGATGTCCTCACTTCATTTTTTGTTCACTTATTACGTCTCGTGTGCGACCCAGCCCGCGCCTGC
GCCGACGTTGGAGCCAAC
>TR443|c0_g1_i2 len=747 path=[2277:0-24 2372:25-25 2368:26-49 2362:50-75
2355:76-99 2319:100-100 2376:101-335 2367:336-399 2371:400-455 2360:456-
479 2375:480-517 2373:518-541 2370:542-642 2322:643-666 2365:667-730
2377:731-733 2273:734-746] [-1, 2277, 2372, 2368, 2362, 2355, 2319, 2376,
2367, 2371, 2360, 2375, 2373, 2370, 2322, 2365, 2377, 2273, -2]
CTCTCCGTCGGACTGGCCTACGCGCACGAATACATCGCCTTGTATCCATCCGCGAAAATCGTCGTGATGCTAC
GACAGCGCGAGGAGGTTGTCGATTCCTTCTTGGCCAAGTCGAAGGGACGGAATCACTGGCAGGCGCACGTCCA
TCATCATCATCATCGTCGGAGAAAGAGCAGTGGCAGTGGCAATGATGGCAATGGTGCCGAACGTCGCTTGGAC
GATGAGATTGAATTGGTCCAACGCGATAAAACTTGGGATGGCGCATTTCCCAACATGTCGGATGAGGAATGCA
GACTGGTCATGATGGGGGCACGGAATAATCACGAGGATGATGGTACGACTGCTGCTGCTGCTGCTGCTACATC
AATCATCCCCGGGAAGGAGGAGGAGGAGATCCAACGACTACGAGGACGACGAGGAGCATGTCGGAAACCCGAC
AAGGTGTGGGCATTGCGAGCGTACTGCGAGCTTTACGAAGATGTCGCTCGGGAGCTATCGGAGCGGTATCCGA
ACAATGTTCGAATTTACGATATGCGGGATGCTTTGAACGAGGCGTCGGTGCAGGAAAATATGCTGCGTTGGTG
TGGGTTCGAGGAGCCGGTGTTGGATACTCGTTTGCATTTGAATAAAAAGAAGTGACAAAGACTATGAAGCGAG
ACACTCTGCTGTACACTATTTTATTCTCGGCGTAGTGAAATTGCATTGACAAGTATGTAGTCATTACACCTTT
CTTAGATCGGAAGAGCG
>TR444|c0_g1_i1 len=1632 path=[3475:0-332 4141:333-391 4133:392-415
4146:416-420 4151:421-424 4155:425-432 4119:433-456 4095:457-458
4053:459-462 4132:463-490 4118:491-526 3673:527-550 4092:551-649
4147:650-650 3746:651-674 4131:675-739 4091:740-752 4048:753-763
4116:764-782 4130:783-791 4090:792-815 4115:816-816 4139:817-839
4089:840-853 4128:854-863 4114:864-877 4138:878-887 4127:888-911
4112:912-923 4087:924-941 4043:942-947 4143:948-965 4137:966-989
4126:990-1033 4086:1034-1082 4110:1083-1091 4040:1092-1103 4084:1104-1115
4125:1116-1116 4109:1117-1140 4083:1141-1141 4038:1142-1144 3982:1145-
1228 3822:1229-1236 3823:1237-1246 3824:1247-1251 3825:1252-1262
3826:1263-1269 4106:1270-1361 4149:1362-1366 4153:1367-1377 4124:1378-
1380 4104:1381-1382 4071:1383-1407 4123:1408-1410 4102:1411-1434
4068:1435-1438 4020:1439-1441 4122:1442-1449 4065:1450-1480 3683:1481-
1490 3684:1491-1493 3685:1494-1497 3955:1498-1576 3926:1577-1579
3927:1580-1585 3928:1586-1631] [-1, 3475, 4141, 4133, 4146, 4151, 4155,
4119, 4095, 4053, 4132, 4118, 3673, 4092, 4147, 3746, 4131, 4091, 4048,
4116, 4130, 4090, 4115, 4139, 4089, 4128, 4114, 4138, 4127, 4112, 4087,
4043, 4143, 4137, 4126, 4086, 4110, 4040, 4084, 4125, 4109, 4083, 4038,
3982, 3822, 3823, 3824, 3825, 3826, 4106, 4149, 4153, 4124, 4104, 4071,
4123, 4102, 4068, 4020, 4122, 4065, 3683, 3684, 3685, 3955, 3926, 3927,
3928, -2]
TTTGGTTGGGCAGAGGCTGCAGCATTGTGGGGAGGAGATGTGGATGTCATCGTTCATCGGCAACTATCTAACT
ATCTAATAAATGAGCAATTTAATCTCCCTTACTCAGTTCCAGTTGCCTTGGCCCCTGTCCCCCCCCTCACAAA
CTCTAGGCAGAATGGGATCTTACTAATCACCAATCAAGGAGAGAACAATGCTCAGGATGCAGAAATTATATTT
GAGAGATGGCAACCCCTGATTGCTATCCTTGCCCTGCCCTCTACTTTGTCAAGATCCAAGAGGAATACATGGT
GGTTCTTAGACTATGTGCCAAGAATTTTCCTTGAGAAGTGCAATCATCGCTTCCCTCGAGCCATCAACATCCA
ACCCCTTCTCACTAAGCATTCTCCTCAGAGCAGCCACCCTCATGGTATTAAAGCCACCAGTAGCGTCACTGCT
GTCATCATCTTTTCTTTTGCTCCTTCCCACTGCTGCCAAAAGGTCCGTCACAACAGATCCAGGGACATTGTCG
AAAGATACCTTGCCCATAATATCATCTCCGTTTTTTACAATAAAATCCATCACCGCCTCCTTCAGAAGTGCAC
AATTTTTCGATTCGGCATAAAGTAAATTGTCTAGAATGTTGTCAATATCAAGTTTGGTTGATTCCACATAGTA
TGCCTCCGCCTTCAATTTTAGGCTCACGATTCCATATTTATCAGCAGCATCAATGAAGTCTTTCGCATGAGAC
CTTAAGGATTTATCAGACAGTTTCCCCCCGTACACGTAGTACAAGGCCTGCTGGAAAATGTCAGGACCGACAT
CAGTGATTGAAACTTGACACGGGTCTCTCCCCGGCTTGCAAAATTGGGCTAAAATGGGTGCGCCATCTTGTAA
AATCAAACGATGAACGTAGAACATGGTGGGAGATATCCTGGCCTTCTTGGAAGGATTCCCCCCTTGATCACTC
TTATGTTCCACCTCAAAGATAACATCCGCCGACTCTTCATCCATGAATTTGTTAAGAATATTTCTGCAGAGCG
GATTTTCAGGAACGAATGCCAAAGAAGAAGATTTTGTGGGTCCAATTCTCATCATCCGTACTTCAATGGTCAA
TGTTCCTTTTACCAGGGCATCGGTGATCGTCGAGCGCCTGGCGAAATTAGAGGTTATATAGGCCCTATTGGGC

CGGTAAGGATCAATTCTTACTTTTGTTGAAGAAAAACGTACAATTGGTTTGTCGGCTGAGTCTCTGACGATGA
ATCTATACCGTAACTTAATGCTCTCAGCAAATAAATTTTGAAGACCCACAGCAACATTTCCATCATCGCTTCT
TTCTTCGCCTCTGGGATAGATACTCAAGCGCCAACGATTCCCAACACACGTGAACTCCGGCGATATAATGTTC
TCTCCTCTCGCTGAAGAGAGATCTCCAAACCCATGGAAATTCACAATGGCTGAATCCCAATTTCGAAAACCGC
TGGGCAATGTTCCGACATCAACTGTGGTTGTATTGGGATGTATTTGACTACTCATAATTATTTAGGAGTTGGT
ATCGTTCTTCGATTTTGACTAAAGTACAACTTGAGAACATTGTAATCCAACTTGTGCTGATTTATTTACCCAA
AATAAAAAATCATTGATATTCGAATG
>TR444|c0_g1_i2 len=1625 path=[3475:0-332 4141:333-391 4133:392-415
4146:416-420 4151:421-424 4155:425-432 4119:433-456 4095:457-458
4053:459-462 4132:463-490 4118:491-526 3673:527-550 4092:551-649
4147:650-650 3746:651-674 4131:675-739 4091:740-752 4048:753-763
4116:764-782 4130:783-791 4090:792-815 4115:816-816 4139:817-839
4089:840-853 4113:854-877 4138:878-887 4127:888-911 4112:912-923
4087:924-941 4043:942-947 4143:948-965 4137:966-989 4126:990-1033
4086:1034-1082 4110:1083-1091 4040:1092-1103 3690:1104-1127 3691:1128-
1129 3985:1130-1233 3805:1234-1243 3806:1244-1248 4037:1249-1273
3981:1274-1275 4081:1276-1318 4034:1319-1327 4107:1328-1363 4077:1364-
1374 4031:1375-1377 3970:1378-1379 4073:1380-1403 4025:1404-1404
3965:1405-1407 4069:1408-1416 4021:1417-1423 3960:1424-1431 3863:1432-
1435 4066:1436-1459 4019:1460-1490 4145:1491-1494 4144:1495-1501
4142:1502-1503 4134:1504-1528 4150:1529-1531 4154:1532-1544 3951:1545-
1578 3849:1579-1624] [-1, 3475, 4141, 4133, 4146, 4151, 4155, 4119, 4095,
4053, 4132, 4118, 3673, 4092, 4147, 3746, 4131, 4091, 4048, 4116, 4130,
4090, 4115, 4139, 4089, 4113, 4138, 4127, 4112, 4087, 4043, 4143, 4137,
4126, 4086, 4110, 4040, 3690, 3691, 3985, 3805, 3806, 4037, 3981, 4081,
4034, 4107, 4077, 4031, 3970, 4073, 4025, 3965, 4069, 4021, 3960, 3863,
4066, 4019, 4145, 4144, 4142, 4134, 4150, 4154, 3951, 3849, -2]
TTTGGTTGGGCAGAGGCTGCAGCATTGTGGGGAGGAGATGTGGATGTCATCGTTCATCGGCAACTATCTAACT
ATCTAATAAATGAGCAATTTAATCTCCCTTACTCAGTTCCAGTTGCCTTGGCCCCTGTCCCCCCCCTCACAAA
CTCTAGGCAGAATGGGATCTTACTAATCACCAATCAAGGAGAGAACAATGCTCAGGATGCAGAAATTATATTT
GAGAGATGGCAACCCCTGATTGCTATCCTTGCCCTGCCCTCTACTTTGTCAAGATCCAAGAGGAATACATGGT
GGTTCTTAGACTATGTGCCAAGAATTTTCCTTGAGAAGTGCAATCATCGCTTCCCTCGAGCCATCAACATCCA
ACCCCTTCTCACTAAGCATTCTCCTCAGAGCAGCCACCCTCATGGTATTAAAGCCACCAGTAGCGTCACTGCT
GTCATCATCTTTTCTTTTGCTCCTTCCCACTGCTGCCAAAAGGTCCGTCACAACAGATCCAGGGACATTGTCG
AAAGATACCTTGCCCATAATATCATCTCCGTTTTTACAATAAAATCCATCACCGCCTCCTTCAGAAGTGCAC
AATTTTTCGATTCGGCATAAAGTAAATTGTCTAGAATGTTGTCAATATCAAGTTTGGTTGATTCCACATAGTA
TGCCTCCGCCTTCAATTTTAGGCTCACGATTCCATATTTATCAGCAGCATCAATGAAGTCTTTCGCATGAGAC
CTTAAGGATTTATCAGACAGTTTCCCCCCGTACACGTAGTACAAGGCCTGCTGGAAAATGTCAGGACCGACAT
CAGTGATTGAAACTTGACACGGGTCTCTCCCCGGCTTGCAAAATTGGGCTAGAATGGGTGCGCCATCTTGTAA
AATCAAACGATGAACGTAGAACATGGTGGGAGATATCCTGGCCTTCTTGGAAGGATTCCCCCCTTGATCACTC
TTATGTTCCACCTCAAAGATAACATCCGCCGACTCTTCATCCATGAATTTGTTAAGAATATTTCTGCAGAGCG
GATTTTCAGGAACGAATGCCAAAGAAGAAGATTTTGTGGGTCCAATTCTCATCATCCGTACTTCAATGGTCAA
TGTTCCTTTAACCAGGGCATCGATGATCGTCGAGCTCCTGAAAAAATTAGGGGCTATCCAGGCCTCATTGGGA
TCAAGTGTTGTGCCTTCTTCTTCTGATGAAAAAGATGCAACTAGTTTGTCGGCTGAGTCTCTGACGATGAATC
TATACCGTTTCTTAATGCTCTCATCACTAAAATTTTGAAGACTCAAAGCAACTTTTCCATCATTGGATCTTTC
ATGGCCACCGGGATAGATACTCAAGCACCAACGATTCCCAACACACGTGAACTCCGGCGATATAATTCTCTCT
CCTCTCGCTGAAGAGAGATCTTCAAACCCATGGAAATTCACAATGGCTGTATCCCAATTTTGAAAACCGCTGG
GCAATGTGCCGACATCAACTGTGGTTGTATTGGGACGTATTTGACTGCTCATAATTATTTAGGAGTAGGTATT
GTTCTTCGATTTCGACCTACAATTTGAGAACATTGTAATCCAACTTGTGCTGATTTATTTACCCAAAATAAAA
AATCATTGATATTCGAATG
>TR444|c0_g1_i3 len=1623 path=[3475:0-332 4141:333-391 4133:392-415
4146:416-420 4151:421-424 4155:425-432 4119:433-456 4095:457-458
4053:459-462 4132:463-490 4118:491-526 3673:527-550 4092:551-649
4147:650-650 4000:651-673 4140:674-674 4131:675-739 4091:740-752
4048:753-763 4116:764-782 4130:783-791 4090:792-815 4115:816-816
4139:817-839 4089:840-853 4128:854-863 4114:864-877 4138:878-887
4127:888-911 4112:912-923 3991:924-947 4143:948-965 4137:966-989
4126:990-1033 4111:1034-1082 4085:1083-1091 4041:1092-1103 4148:1104-1115
4152:1116-1116 3546:1117-1140 3547:1141-1141 3983:1142-1219 3466:1220-
1227 3467:1228-1237 3980:1238-1267 4082:1268-1269 4035:1270-1321

4108:1322-1357 4079:1358-1368 4032:1369-1371 3971:1372-1399 4074:1400-1408 4026:1409-1410 3966:1411-1417 4103:1418-1425 4068:1426-1429 4020:1430-1432 4122:1433-1440 4065:1441-1471 3683:1472-1481 3684:1482-1484 3685:1485-1488 3955:1489-1567 3926:1568-1570 3927:1571-1576 3928:1577-1622] [-1, 3475, 4141, 4133, 4146, 4151, 4155, 4119, 4095, 4053, 4132, 4118, 3673, 4092, 4147, 4000, 4140, 4131, 4091, 4048, 4116, 4130, 4090, 4115, 4139, 4089, 4128, 4114, 4138, 4127, 4112, 3991, 4143, 4137, 4126, 4111, 4085, 4041, 4148, 4152, 3546, 3547, 3983, 3466, 3467, 3980, 4082, 4035, 4108, 4079, 4032, 3971, 4074, 4026, 3966, 4103, 4068, 4020, 4122, 4065, 3683, 3684, 3685, 3955, 3926, 3927, 3928, -2]
TTTGGTTGGGCAGAGGCTGCAGCATTGTGGGGAGGAGATGTGGATGTCATCGTTCATCGGCAACTATCTAACT
ATCTAATAAATGAGCAATTTAATCTCCCTTACTCAGTTCCAGTTGCCTTGGCCCCTGTCCCCCCCCTCACAAA
CTCTAGGCAGAATGGGATCTTACTAATCACCAATCAAGGAGAGAACAATGCTCAGGATGCAGAAATTATATTT
GAGAGATGGCAACCCCTGATTGCTATCCTTGCCCTGCCCTCTACTTTGTCAAGATCCAAGAGGAATACATGGT
GGTTCTTAGACTATGTGCCAAGAATTTTCCTTGAGAAGTGCAATCATCGCTTCCCTCGAGCCATCAACATCCA
ACCCCTTCTCACTAAGCATTCTCCTCAGAGCAGCCACCCTCATGGTATTAAAGCCACCAGTAGCGTCACTGCT
GTCATCATCTTTTCTTTTGCTCCTTCCCACTGCTGCCAAAAGGTCCGTCACAACAGATCCAGGGACATTGTCG
AAAGATACCTTGCCCATAATATCATCTCCGTTTTTTACAATAAAATCCATCACCGCCTCCTTCAGAAGTGCAC
AATTTTTCGATTCGGCATAAAGTAAATTGTCTAGAATGTTGTCAATATCAAGTTTGGTTGATTCCACGTAGTA
TGCCTCCGCCTTCAATTTTAGGCTCACGATTCCATATTTATCAGCAGCATCAATGAAGTCTTTCGCATGAGAC
CTTAAGGATTTATCAGACAGTTTCCCCCCGTACACGTAGTACAAGGCCTGCTGGAAAATGTCAGGACCGACAT
CAGTGATTGAAACTTGACACGGGTCTCTCCCCGGCTTGCAAAATTGGGCTAAAATGGGTGCGCCATCTTGTAA
AATCAAACGATGAACGTAGAACATGGTGGGAGATATCCTGGCCTTCTTTGAAGGATTCCCCCCTTGATCACTC
TTATGTTCCACCTCAAAGATAACATCCGCCGACTCTTCATCCATGAATTTGTTAAGAATATTTCTGCAGAGCG
GATTTTCAGGAATGAATGCCAAAGAAGAAGATTTTGCTGGTCCAATTCTCATCATCCGTACTTCAATGGTCAA
TGTTCCTTTTACCAGGGCATCGGTGATCGTCGAGCGCCTGGCGAAATCAGGGAATATCCAGCCTTCATTGGGC
TCCATTCTTGCTTGTGTTGAAGAAAAAGATACAATTGGTTTGTCGGCTGAGTCTCTGACGATGAATCTATGCC
GTAACTTAATGCTCTCATCACTAAAATTTAGAAGACCCACAGCAACATTTTCATCATCGGATCTTTCATGGCC
ACCGGGATAGATACTCAAGCACCAACGATTCCCAACACACGTGAACTCCGGCGATATAGTGTGCTCTCCTCTC
ACTGAAGAGAGATCTTCAAACCCATGGAAATTCACAATGGCTGAATCCCAATTTCGAAAACCGCTGGGCAATG
TTCCGACATCAACTGTGGTTGTATTGGGATGTATTTGACTACTCATAATTATTTAGGAGTTGGTATCGTTCTT
CGATTTTGACTAAAGTACAACTTGAGAACATTGTAATCCAACTTGTGCTGATTTATTTACCCAAAATAAAAAA
TCATTGATATTCGAATG
>TR444|c0_g1_i6 len=271 path=[3764:0-76 4141:77-135 4133:136-159 4146:160-164 4007:165-200 3556:201-212 3557:213-234 3558:235-270] [-1, 3764, 4141, 4133, 4146, 4007, 3556, 3557, 3558, -2]
GATCTTTTTTTTTTAGATTTAAAATCCCAATTATTTTAGCTTATTAAAGGAGTTCAAGAATTTTCCTTGAGAA
GTGCAATCATCGCTTCCCTCGAGCCATCAACATCCAACCCCTTCTCACTAAGCATTCTCCTCAGAGCAGCCAC
CCTCATGGTATTAAAGCCAACAGTAGCGTCACTGCTGGCATCTACTTTTCTTTTGCTTCTTCCCATTGCTGCC
AAAAGGTCCGTCACAACAGATCCAGGGACATTGTCGAAAGATACCTTGCCCA
>TR444|c0_g1_i7 len=1382 path=[3475:0-332 4141:333-391 4133:392-415 4146:416-420 4151:421-424 4155:425-432 4119:433-456 4095:457-458 4053:459-462 4132:463-490 4118:491-526 3673:527-550 4092:551-649 4147:650-650 3746:651-674 4131:675-739 4091:740-752 4048:753-763 4116:764-782 4130:783-791 4090:792-815 4115:816-816 4139:817-839 4089:840-853 4128:854-863 4114:864-877 4138:878-887 4127:888-911 4112:912-923 3991:924-947 4143:948-965 4137:966-989 4126:990-1033 4111:1034-1082 4085:1083-1091 4041:1092-1103 4148:1104-1115 4152:1116-1116 3758:1117-1127 3759:1128-1129 3760:1130-1140 3761:1141-1141 3762:1142-1144 4036:1145-1277 3979:1278-1284 4080:1285-1336 4034:1337-1345 4107:1346-1381] [-1, 3475, 4141, 4133, 4146, 4151, 4155, 4119, 4095, 4053, 4132, 4118, 3673, 4092, 4147, 3746, 4131, 4091, 4048, 4116, 4130, 4090, 4115, 4139, 4089, 4128, 4114, 4138, 4127, 4112, 3991, 4143, 4137, 4126, 4111, 4085, 4041, 4148, 4152, 3758, 3759, 3760, 3761, 3762, 4036, 3979, 4080, 4034, 4107]
TTTGGTTGGGCAGAGGCTGCAGCATTGTGGGGAGGAGATGTGGATGTCATCGTTCATCGGCAACTATCTAACT
ATCTAATAAATGAGCAATTTAATCTCCCTTACTCAGTTCCAGTTGCCTTGGCCCCTGTCCCCCCCCTCACAAA
CTCTAGGCAGAATGGGATCTTACTAATCACCAATCAAGGAGAGAACAATGCTCAGGATGCAGAAATTATATTT
GAGAGATGGCAACCCCTGATTGCTATCCTTGCCCTGCCCTCTACTTTGTCAAGATCCAAGAGGAATACATGGT
GGTTCTTAGACTATGTGCCAAGAATTTTCCTTGAGAAGTGCAATCATCGCTTCCCTCGAGCCATCAACATCCA

ACCCCTTCTCACTAAGCATTCTCCTCAGAGCAGCCACCCTCATGGTATTAAAGCCACCAGTAGCGTCACTGCT
GTCATCATCTTTTCTTTTGCTCCTTCCCACTGCTGCCAAAAGGTCCGTCACAACAGATCCAGGGACATTGTCG
AAAGATACCTTGCCCATAATATCATCTCCGTTTTTACAATAAAATCCATCACCGCCTCCTTCAGAAGTGCAC
AATTTTTCGATTCGGCATAAAGTAAATTGTCTAGAATGTTGTCAATATCAAGTTTGGTTGATTCCACATAGTA
TGCCTCCGCCTTCAATTTTAGGCTCACGATTCCATATTTATCAGCAGCATCAATGAAGTCTTTCGCATGAGAC
CTTAAGGATTTATCAGACAGTTTCCCCCCGTACACGTAGTACAAGGCCTGCTGGAAAATGTCAGGACCGACAT
CAGTGATTGAAACTTGACACGGGTCTCTCCCCGGCTTGCAAAATTGGGCTAAAATGGGTGCGCCATCTTGTAA
AATCAAACGATGAACGTAGAACATGGTGGGAGATATCCTGGCCTTCTTTGAAGGATTCCCCCCTTGATCACTC
TTATGTTCCACCTCAAAGATAACATCCGCCGACTCTTCATCCATGAATTTGTTAAGAATATTTCTGCAGAGCG
GATTTTCAGGAATGAATGCCAAAGAAGAAGATTTTGCTGGTCCAATTCTCATCATCCGTACTTCAATGGTCAA
TGTTCCTTTTACCAGGGCATCGATGATCGTCGAGCGCCTGGCGAAATTAGGGACTGTCTTGCCTAAATTGGGC
GCACATCTGCGGTAAGGATGAAATCTTTTGCCTTCTTCTTTTGAAGAAAAAGATGCAATTGGTTTGTCGGCCG
AGTCTCTGATGAAAAATCTATACCGTAACTTAATGCTCTCAGCATTATTACTGTGAAGCCTCACAGCAACATA
TCCATCATTGGATCTTTCATGGCCACCGGGATAGATACTCAAGCACCAACGATTCCCAACACACGTGA
>TR445|c0_g1_i1 len=921 path=[4633:0-74 4634:75-78 4704:79-98 4770:99-145
4731:146-178 4700:179-181 4780:182-240 @4768@!:241-533 4788:534-534
4449:535-554 4724:555-556 4749:557-716 4670:717-726 4671:727-740
4776:741-816 4790:817-824 4499:825-840 4743:841-845 4718:846-920] [-1,
4633, 4634, 4704, 4770, 4731, 4700, 4780, 4768, 4788, 4449, 4724, 4749,
4670, 4671, 4776, 4790, 4499, 4743, 4718]
CCGATCTCATCGTACAACCTTGTACAATAAATGGCAACCCATAAAATAAATCTCCTCCACTCTACTCAGAAGC
CATCGCATCAGCGTTCTCTCATTTGTTAACCCCTCGCTTTCCCTCTAATCTGCATTAGTTTTCCTCGCCAACA
CCAGTATTGAACCGCGAGCTATTTTCCGACGAATAACAGGTCTATATTTAAAATGTCTGATACGGGGTGCAAC
TGGCGATTCTTCTCCCGCCTCTGTACATTTTTTATACCTGACTCTTTACTCTGGTCCATTGGCCGCGATATAG
AAGACAAAGCCTCCCGAAAAGAGGCTAAACAAGCTTGGAGGGAGAAAATCGCGCTCTGTGTCATCATGTTGAG
TTCCTGCGCAGCGTTTATTGGATTTGGTGGACTTTTATTACTACTTGAGTGTGGAGTTGAATCGATGCAGTCT
GGGGCACGTCTTGGCATTGACTCAGCACCTCTTGGCACTTGTGGCAACGGGAAACGAGGTAACGGACTTTGCT
TTGATGGCAGCTGCTGCTCTCAGCGACGGAATTTGTCAGGCTCTCAAAGGTACATCATATGCTGTCATGGGAA
TTGTTGTCGCAGTCATGGCAATCCAAGGTCTTTGTTCGATATACTACCTTGTCCGAGGGATCGTCCTTCAACA
AAGTCGTAAAATCAAAAGCGAGCATACAAAACAAACTGTTATGATAATGGTCCCTTGCTACAACGAAGGAGAC
AAGGAACTCAGGAAGACGATTGATAGCATCATGGACACCTCTTATCCAGACTCAAACAAAGTTTTGATTGTGG
TTGCAGATGGTGATGTCACTGGAAAAGGTGAAACTAAGTCCACTCCAGAGGTTATATCCAGCATTCTTCAGTA
CCCCAGATCCCCACAGGACATCACATACGATTGTACAAGCGTTGG
>TR445|c0_g1_i2 len=2134 path=[4456:0-74 4705:75-98 4770:99-145 4732:146-
172 4701:173-175 4779:176-234 @4768@!:235-527 4788:528-528 4646:529-547
4784:548-625 4789:626-649 4794:650-677 4796:678-684 4726:685-700
4777:701-701 4766:702-761 4724:762-763 4749:764-923 4670:924-933
4671:934-947 4776:948-1023 4790:1024-1031 4499:1032-1047 4743:1048-1052
4718:1053-1127 4538:1128-1151 4687:1152-1356 4426:1357-1380 4763:1381-
1421 4741:1422-1444 4717:1445-1557 4638:1558-1574 4684:1575-1790
4413:1791-1814 4762:1815-1935 4663:1936-1957 4738:1958-2133] [-1, 4456,
4705, 4770, 4732, 4701, 4779, 4768, 4788, 4646, 4784, 4789, 4794, 4796,
4726, 4777, 4766, 4724, 4749, 4670, 4671, 4776, 4790, 4499, 4743, 4718,
4538, 4687, 4426, 4763, 4741, 4717, 4638, 4684, 4413, 4762, 4663, 4738]
CCGATCTCATCGTACAACCTTGTACAATAAATGGCAACCCATAAAATAAATCTCCTCCACTCTACTCAGAAGC
CACCGCATCAGCGTTCTCTCATTTGTTAACCCCTCGCTTTCCCTCTAATCTGCATTAGTTTTCCTCGCCAACA
ACAGACCGCGAGCTATTTTCCGACGAATAAGAGGTCCATATTTAAAATGCCTGATACGGGGTGCATCTGGCGA
TTCTTCTCCCGCCTCTGTACATTTTTTATACCTGACTCTTTACTCTGGTCCATTGGCCGCGATATAGAAGACA
AAGCCTCCCGAAAAGAGGCTAAACAAGCTTGGAGGGAGAAAATCGCGCTCTGTGTCATCATGTTGAGTTCCTG
CGCAGCGTTTATTGGATTTGGTGGACTTTTATTACTACTTGAGTGTGGAGTTGAATCGATGCAGTCTGGGGCA
CGTCTTGGCATTGACTCAGCACCTCTTGGCACTTGTGGCAACGGGAAACGAGGTAACGGACTTTGCTTTGATG
GCAGCTGCTGCTCTCAGCATGGATTTTGTGGATCTTCGGTCGAGCATTGTTCTGGAAGGTCGCTCGATAGTGA
TAACCTTTTTGATATTAAAGACGATATCCAGGCTGATATTAAAGATTCTTTTGATGATGAAGATATGTGGGTA
ACTAAACATCGTATCAGCTTCCTTTTTTCATGCCTCTTCTCTTATATTTAACATTGTGGTCTTTCACTTCTTT
ATTCTTTAAAGCGACGGAATTTGTCAGGCTCTCAAAGGTACATCATATGCTGTCATGGGAATTGTTGTCGCAG
TCATGGCAATCCAAGGTCTTTGTTCGATATACTACCTTGTCCGAGGGATCGTCCTTCAACAAAGTCGTAAAAT
CAAAAGCGAGCATACAAAACAAACTGTTATGATAATGGTCCCTTGCTACAACGAAGGAGACAAGGAACTCAGG
AAGACGATTGATAGCATCATGGACACCTCTTATCCAGACTCAAACAAAGTTTTGATTGTGGTTGCAGATGGTG
ATGTCACTGGAAAAGGTGAAACTAAGTCCACTCCAGAGGTTATATCCAGCATTCTTCAGTACCCCAGATCCCC
ACAGGACATCACATACGATTGTACAAGCGTTGGCCCTGACGAAAGAAGTCACAACCGGGCAAAGGTTTACTAC

GGCACAAAAAAAAAGTGAAAAAAATCCAGGCAAGATGTTACATTATATTGTAATCGAGAAATGTGGTATACCAA
TAGAGAAAGGGAGCCCAAAAGCTGGAAACCGTGGCAAACGTGACTCACAGCTCCTTATTACTGGTCTTCTCAA
CAAAATTGGGTGTCATCCTGATGATCCTTTGAACGGCCTTGAATCGACCATCGTTCATGCACTTGCCGATTTA
CAGCTCGGACCAATGAACGACATCAAATACCTCTTAGCCATTGACGCAGATACCGAGATTAGCGAAGATAGCA
TAACGCAAATGTGTTACAATATGGAAGAACAACCTTTTGTTCTAGCACTTTGCGGAGAAACTCAGGTAGCCAA
CCCGTCAGATAGCTGGGTCACCAGGATACAAGTATTTGAATACTACACGAATCACCACTTGAAGAAAGCGTTC
GAGTCTGTTTTTAACACTGTGACATGTCTCCCTGGATGTTTCACAATGTACCGGCTGTTTGATTACGAAAACG
AACCTCTTCTTGCCTGCGATTCTGTCTACATACCTTACGCTATTAATGACGCTGAGATAAATTCACTACACGC
AAAGAACTTGTACCATCTTGGAGAAGACCGCATGCTGACGACTCTTCTACTGCGGCACTTCCCCGAAATGAAT
CTCTCCTTCGTACCTGAAGCAACTTGCCAGACCGTCGTGCCAGACACATTCTCTGTTCTTCTTAGTCAAAGAA
GAAGATGGGTCAATTCGACGTTCCATAATTTACTTGAGCTTTGGAAAGTGAAAACGTGTGGAATATGCTGCTT
CTCTATGAAGGTCATTATCTTACTTGATTTGGTAGGAACTCTTATTCTTCCCGTAGCATTCATATACGTGGGC
ATTATGCTATACTTTGCACTTAATGGAAATGGGGACACTTTAGCACAGATAATATTGGGGTTTTCTGTTGCTG
TTCAACTTGTTGTCCCT
>TR445|c0_g1_i3 len=1891 path=[4633:0-74 4634:75-78 4704:79-98 4770:99-145 4731:146-178 4700:179-181 4780:182-240 @4768@!:241-533 4788:534-534 4646:535-553 4784:554-631 4789:632-655 4725:656-678 4749:679-838 4670:839-848 4671:849-862 4776:863-938 4790:939-946 4721:947-962 4690:963-969 4745:970-1008 4775:1009-1031 4764:1032-1055 4744:1056-1062 4718:1063-1137 4538:1138-1161 4687:1162-1366 4426:1367-1390 4763:1391-1431 4741:1432-1454 4717:1455-1567 4483:1568-1572 4484:1573-1596 4685:1597-1617 4716:1618-1641 4410:1642-1674 4684:1675-1890] [-1, 4633, 4634, 4704, 4770, 4731, 4700, 4780, 4768, 4788, 4646, 4784, 4789, 4725, 4749, 4670, 4671, 4776, 4790, 4721, 4690, 4745, 4775, 4764, 4744, 4718, 4538, 4687, 4426, 4763, 4741, 4717, 4483, 4484, 4685, 4716, 4410, 4684]
CCGATCTCATCGTACAACCTTGTACAATAAATGGCAACCCATAAAATAAATCTCCTCCACTCTACTCAGAAGC
CATCGCATCAGCGTTCTCTCATTTGTTAACCCCTCGCTTTCCCTCTAATCTGCATTAGTTTTCCTCGCCAACA
CCAGTATTGAACCGCGAGCTATTTTCCGACGAATAACAGGTCTATATTTAAAATGTCTGATACGGGGTGCAAC
TGGCGATTCTTCTCCCGCCTCTGTACATTTTTTATACCTGACTCTTTACTCTGGTCCATTGGCCGCGATATAG
AAGACAAAGCCTCCCGAAAAGAGGCTAAACAAGCTTGGAGGGAGAAAATCGCGCTCTGTGTCATCATGTTGAG
TTCCTGCGCAGCGTTTATTGGATTTGGTGGACTTTTATTACTACTTGAGTGTGGAGTTGAATCGATGCAGTCT
GGGGCACGTCTTGGCATTGACTCAGCACCTCTTGGCACTTGTGGCAACGGGAAACGAGGTAACGGACTTTGCT
TTGATGGCAGCTGCTGCTCTCAGCATGGATTTTGTGGATCTTCGGTCGAGCATTGTTCTGGAAGGTCGCTCGA
TAGTGATAACCTTTTTGATATTAAAGACGATATCCAGGCTGATATTAAAGATTCTTTTGATGATGAAGATATC
GACGGAATTTGTCAGGCTCTCAAAGGTACATCATATGCTGTCATGGGAATTGTTGTCGCAGTCATGGCAATCC
AAGGTCTTTGTTCGATATACTACCTTGTCCGAGGGATCGTCCTTCAACAAAGTCGTAAAATCAAAAGCGAGCA
TACAAAACAAACTGTTATGATAATGGTCCCTTGCTACAACGAAGGAGACAAGGAACTCAGGAAGACGATTGAT
AGCATCATGGACACCTCTTATCCAGACTCAAACAAAGTTTTGATTGTGGTTGCAGATGGTGATGTCACTGGTA
AGAGCTGTTCCTTGTTGTAAAATAATAATTACTGGCTGTCTGTCAATTCCCGCTCCTTTTCAAACATTATTCC
GTTGTTTTACGAATCAAAGGAAAAGGTGAAACTAAGTCCACTCCAGAGGTTATATCCAGCATTCTTCAGTACC
CCAGATCCCCACAGGACATCACATACGATTGTACAAGCGTTGGCCCTGACGAAAGAAGTCACAACCGGGCAAA
GGTTTACTACGGCACAAAAAAAAAGTGAAAAAAATCCAGGCAAGATGTTACATTATATTGTAATCGAGAAATGT
GGTATACCAATAGAGAAAGGGAGCCCAAAAGCTGGAAACCGTGGCAAACGTGACTCACAGCTCCTTATTACTG
GTCTTCTCAACAAAATTGGGTGTCATCCTGATGATCCTTTGAACGGCCTTGAATCGACCATCGTTCATGCACT
TGCCGATTTACAGCTCGGACCAATGAACGACATCAAATACCTCTTAGCCATTGACGCAGATACCGAGATTAGC
GAAGATAGCATAACGCAAATGTGTTACAATATGGAAGAACAACCTTTTGTTCTAGCACTTTGCGGAGAAACTC
AGGTAGCCAACCCGTCAGATAGCTGGGTCACCAGGTAGGTCAAATCCACACTGTTGAACCAATACGCAAGCGA
TCACTAAGCTGACCTCACCTGCTGCTGCCTCTTTCCCCTATCATTTACCAGGATACAAGTATTTGAATACTAC
ACGAATCACCACTTGAAGAAAGCGTTCGAGTCTGTTTTTAACACTGTGACATGTCTCCCTGGATGTTTCACAA
TGTACCGGCTGTTTGATTACGAAAACGAACCTCTTCTTGCCTGCGATTCTGTCTACATACCTTACGCTATTAA
TGACGCTGAGATAAATTCACTACACGCAAAGAACTTGTACCATCTTGGAGAAGACCGCATGCTGAC
>TR445|c0_g1_i4 len=859 path=[4633:0-74 4634:75-78 4704:79-98 4770:99-145 4731:146-178 4700:179-181 4780:182-240 @4768@!:241-533 4699:534-612 4787:613-643 4784:644-721 4789:722-745 4794:746-773 4796:774-780 4696:781-798 4399:799-858] [-1, 4633, 4634, 4704, 4770, 4731, 4700, 4780, 4768, 4699, 4787, 4784, 4789, 4794, 4796, 4696, 4399, -2]
CCGATCTCATCGTACAACCTTGTACAATAAATGGCAACCCATAAAATAAATCTCCTCCACTCTACTCAGAAGC
CATCGCATCAGCGTTCTCTCATTTGTTAACCCCTCGCTTTCCCTCTAATCTGCATTAGTTTTCCTCGCCAACA
CCAGTATTGAACCGCGAGCTATTTTCCGACGAATAACAGGTCTATATTTAAAATGTCTGATACGGGGTGCAAC
TGGCGATTCTTCTCCCGCCTCTGTACATTTTTTATACCTGACTCTTTACTCTGGTCCATTGGCCGCGATATAG

AAGACAAAGCCTCCCGAAAAGAGGCTAAACAAGCTTGGAGGGAGAAAATCGCGCTCTGTGTCATCATGTTGAG
TTCCTGCGCAGCGTTTATTGGATTTGGTGGACTTTTATTACTACTTGAGTGTGGAGTTGAATCGATGCAGTCT
GGGGCACGTCTTGGCATTGACTCAGCACCTCTTGGCACTTGTGGCAACGGGAAACGAGGTAACGGACTTTGCT
TTGATGGCAGCTGCTGCTCTCAGGTACGTGAAATAAAGGGAAAGAAGAAGCTCTCCTGCATATTCATTCATTC
CTAAAAAGTTCCAATAATTGTATTTTGTGATTTCATACAGCATGGATTTTGTGGATCTTCGGTCGAGCATTGT
TCTGGAAGGTCGCTCGATAGTGATAACCTTTTTGATATTAAAGACGATATCCAGGCTGATATTAAAGATTCTT
TTGATGATGAAGATATGTGGGTAACTAAACATCGTATCAGCTTCCTTTTTTTCATGCCTCTTCTCTTATATTT
AACATTGTGGTCTTTCACTTCTTTATTCTTTAAAGCGACGGAATTTGTCAGGCTCT
>TR445|c0_g1_i5 len=2992 path=[4456:0-74 4705:75-98 4770:99-145 4732:146-172 4701:173-175 4779:176-234 @4768@!:235-527 4788:528-528 4646:529-547 4784:548-625 4789:626-649 4794:650-677 4727:678-699 4777:700-700 4766:701-760 4724:761-762 4749:763-922 4670:923-932 4671:933-946 4776:947-1022 4790:1023-1030 4721:1031-1046 4690:1047-1053 4745:1054-1092 4775:1093-1115 4764:1116-1139 4744:1140-1146 4718:1147-1221 4538:1222-1245 4687:1246-1450 4426:1451-1474 4763:1475-1515 4741:1516-1538 4717:1539-1651 4638:1652-1668 4684:1669-1884 4413:1885-1908 4762:1909-2029 4740:2030-2060 4786:2061-2084 4783:2085-2092 4774:2093-2094 4681:2095-2101 4760:2102-2127 4712:2128-2149 4738:2150-2325 4792:2326-2349 4795:2350-2364 4677:2365-2400 4758:2401-2490 4708:2491-2538 4782:2539-2549 4772:2550-2573 4793:2574-2595 4673:2596-2619 4587:2620-2638 4588:2639-2991] [-1, 4456, 4705, 4770, 4732, 4701, 4779, 4768, 4788, 4646, 4784, 4789, 4794, 4727, 4777, 4766, 4724, 4749, 4670, 4671, 4776, 4790, 4721, 4690, 4745, 4775, 4764, 4744, 4718, 4538, 4687, 4426, 4763, 4741, 4717, 4638, 4684, 4413, 4762, 4740, 4786, 4783, 4774, 4681, 4760, 4712, 4738, 4792, 4795, 4677, 4758, 4708, 4782, 4772, 4793, 4673, 4587, 4588, -2]
CCGATCTCATCGTACAACCTTGTACAATAAATGGCAACCCATAAAATAAATCTCCTCCACTCTACTCAGAAGC
CACCGCATCAGCGTTCTCTCATTTGTTAACCCCTCGCTTTCCCTCTAATCTGCATTAGTTTTCCTCGCCAACA
ACAGACCGCGAGCTATTTTCCGACGAATAAGAGGTCCATATTTAAAATGCCTGATACGGGGTGCATCTGGCGA
TTCTTCTCCCGCCTCTGTACATTTTTTATACCTGACTCTTTACTCTGGTCCATTGGCCGCGATATAGAAGACA
AAGCCTCCCGAAAAGAGGCTAAACAAGCTTGGAGGGAGAAAATCGCGCTCTGTGTCATCATGTTGAGTTCCTG
CGCAGCGTTTATTGGATTTGGTGGACTTTTATTACTACTTGAGTGTGGAGTTGAATCGATGCAGTCTGGGGCA
CGTCTTGGCATTGACTCAGCACCTCTTGGCACTTGTGGCAACGGGAAACGAGGTAACGGACTTTGCTTTGATG
GCAGCTGCTGCTCTCAGCATGGATTTTGTGGATCTTCGGTCGAGCATTGTTCTGGAAGGTCGCTCGATAGTGA
TAACCTTTTTGATATTAAAGACGATATCCAGGCTGATATTAAAGATTCTTTTGATGATGAAGATATGTGGGTA
ACTAAACATCGTATCAGCTTCTTTTTTTCATGCCTCTTCTCTTATATTTAACATTGTGGTCTTTCACTTCTTTA
TTCTTTAAAGCGACGGAATTTGTCAGGCTCTCAAAGGTACATCATATGCTGTCATGGGAATTGTTGTCGCAGT
CATGGCAATCCAAGGTCTTTGTTCGATATACTACCTTGTCCGAGGGATCGTCCTTCAACAAAGTCGTAAAATC
AAAAGCGAGCATACAAAACAAACTGTTATGATAATGGTCCCTTGCTACAACGAAGGAGACAAGGAACTCAGGA
AGACGATTGATAGCATCATGGACACCTCTTATCCAGACTCAAACAAAGTTTTGATTGTGGTTGCAGATGGTGA
TGTCACTGGTAAGAGCTGTTCCTTGTTGTAAAATAATAATTACTGGCTGTCTGTCAATTCCCGCTCCTTTTCA
AACATTATTCCGTTGTTTTACGAATCAAAGGAAAAGGTGAAACTAAGTCCACTCCAGAGGTTATATCCAGCAT
TCTTCAGTACCCCAGATCCCCACAGGACATCACATACGATTGTACAAGCGTTGGCCCTGACGAAAGAAGTCAC
AACCGGGCAAAGGTTTACTACGGCACAAAAAAAAAGTGAAAAAAATCCAGGCAAGATGTTACATTATATTGTAA
TCGAGAAATGTGGTATACCAATAGAGAAAGGGAGCCCAAAAGCTGGAAACCGTGGCAAACGTGACTCACAGCT
CCTTATTACTGGTCTTCTCAACAAAATTGGGTGTCATCCTGATGATCCTTTGAACGGCCTTGAATCGACCATC
GTTCATGCACTTGCCGATTTACAGCTCGGACCAATGAACGACATCAAATACCTCTTAGCCATTGACGCAGATA
CCGAGATTAGCGAAGATAGCATAACGCAAATGTGTTACAATATGGAAGAACAACCTTTTGTTCTAGCACTTTG
CGGAGAAACTCAGGTAGCCAACCCGTCAGATAGCTGGGTCACCAGGATACAAGTATTTGAATACTACACGAAT
CACCACTTGAAGAAAGCGTTCGAGTCTGTTTTTAACACTGTGACATGTCTCCCTGGATGTTTCACAATGTACC
GGCTGTTTGATTACGAAAACGAACCTCTTCTTGCCTGCGATTCTGTCTACATACCTTACGCTATTAATGACGC
TGAGATAAATTCACTACACGCAAAGAACTTGTACCATCTTGGAGAAGACCGCATGCTGACGACTCTTCTACTG
CGGCACTTCCCCGAAATGAATCTCTCCTTCGTACCTGAAGCAACTTGCCAGACCGTCGTGCCAGACACATTCT
CTGTTCTTCTTAGTCAAAGAAGAAGATGGGTCAATTCGACGTTCCATAATTTACTTGAGTAAGAATTGAACAA
ACATTTTGAACATATCTTCCAATTGTAAAGACTCATCCTTGTTTTTTTTCTGCACTCGATGTTATGAAACCCA
TGGCCGCCAGGCTTTGGAAAGTGAAAACGTGTGGAATATGCTGCTTCTCTATGAAGGTCATTATCTTACTTGA
TTTGGTAGGAACTCTTATTCTTCCCGTAGCATTCATATACGTGGGCATTATGCTATACTTTGCACTTAATGGA
AATGGGGACACTTTAGCACAGATAATATTGGGGTTTTCTGTTGCTGTTCAACTTGTTGTCCCTACTCTACGCT
TACACTGGAATATCTTGTTTTGGTCCATTGCTTTTTATGTGGTCGGTGTGCCCATATTTTACTTCATTTTGCC
AATTTATGCCTTCTGGCATATGGACGATGTATCGTGGGGATCTACTCGGTTAGTAGCCACCTAGAGAGGGACT

TAGTTGAGTGGATACAAGAGTTGTTGATGCTGGGGCTGCTGCTGGGGAGGATGGTGTGGCGATGCAGATGAGG
CAGCGCGCTATATGAAACCAATGGTGCATCGGAGTGGCTGGTAATAATGATGGCAAGGAGTAAAATGCAGCTA
ATTATTTGTTCAACTGCCAAGTAATGAGATATTAAGAGTTTTAATACCCAAATATATGGAATTGAGTTATTCC
ATCCTCTTGTATCTACTTTGGCAATCAATAAGTGTTCTTAGGAGGGCTTGGTCAATGCATCAAATTGTAGACT
GACGTGTACTATCTAGAATGGTAAGTCAGATAATTGGTTCTGAGAGAGCAATGGCAAGTTGATGCAATACAGA
ATAAATGGTAACTGTGTTTTAACCCTCAAGTGTCCAGAAAGCATCAGAAAGTCATTTACAAATAAATACATAA
AAGAACTGTAGCTGTCTGATTTGGCTGATTCCAAGTACCGTGCAGTTGGGATCATTCAGTCAACCCTAAATT
>TR445|c0_g1_i6 len=298 path=[4443:0-63 4771:64-84 4756:85-108 4781:109-130 4769:131-154 4742:155-184 4717:185-297] [-1, 4443, 4771, 4756, 4781, 4769, 4742, 4717]
CGACCATCGTTCATGCACTTGCCGATTTACAGCTCGGACCAATGAACGACATCAAATACCTCTTGTGAGTGGC
AATCATTGATTTGCGTTGTTTATTCTCTACCAGCTATCATCTTGGCTGTATATTACTCACTTCCTCCTTGCTG
TATTTGATTCCAACAGAGCCATTGACGCAGATACCGAGATTAGCGAAGATAGCATAACGCAAATGTGTTACAA
TATGGAAGAACAACCTTTTGTTCTAGCACTTTGCGGAGAAACTCAGGTAGCCAACCCGTCAGATAGCTGGGTC
ACCAGG
>TR445|c0_g1_i7 len=1886 path=[4633:0-74 4634:75-78 4704:79-98 4770:99-145 4731:146-178 4700:179-181 4780:182-240 @4768@!:241-533 4788:534-534 4646:535-553 4784:554-631 4789:632-655 4794:656-683 4727:684-705 4777:706-706 4766:707-766 4724:767-768 4749:769-928 4670:929-938 4671:939-952 4776:953-1028 4790:1029-1036 4499:1037-1052 4743:1053-1057 4718:1058-1132 4538:1133-1156 4687:1157-1361 4426:1362-1385 4763:1386-1426 4741:1427-1449 4717:1450-1562 4483:1563-1567 4484:1568-1591 4685:1592-1612 4716:1613-1636 4410:1637-1669 4684:1670-1885] [-1, 4633, 4634, 4704, 4770, 4731, 4700, 4780, 4768, 4788, 4646, 4784, 4789, 4794, 4727, 4777, 4766, 4724, 4749, 4670, 4671, 4776, 4790, 4499, 4743, 4718, 4538, 4687, 4426, 4763, 4741, 4717, 4483, 4484, 4685, 4716, 4410, 4684]
CCGATCTCATCGTACAACCTTGTACAATAAATGGCAACCCATAAAATAAATCTCCTCCACTCTACTCAGAAGC
CATCGCATCAGCGTTCTCTCATTTGTTAACCCCTCGCTTTCCCTCTAATCTGCATTAGTTTTCCTCGCCAACA
CCAGTATTGAACCGCGAGCTATTTTCCGACGAATAACAGGTCTATATTTAAAATGTCTGATACGGGGTGCAAC
TGGCGATTCTTCTCCCGCCTCTGTACATTTTTTATACCTGACTCTTTACTCTGGTCCATTGGCCGCGATATAG
AAGACAAAGCCTCCCGAAAAGAGGCTAAACAAGCTTGGAGGGAGAAAATCGCGCTCTGTGTCATCATGTTGAG
TTCCTGCGCAGCGTTTATTGGATTTGGTGGACTTTTATTACTACTTGAGTGTGGAGTTGAATCGATGCAGTCT
GGGGCACGTCTTGGCATTGACTCAGCACCTCTTGGCACTTGTGGCAACGGGAAACGAGGTAACGGACTTTGCT
TTGATGGCAGCTGCTGCTCTCAGCATGGATTTTGTGGATCTTCGGTCGAGCATTGTTCTGGAAGGTCGCTCGA
TAGTGATAACCTTTTTGATATTAAAGACGATATCCAGGCTGATATTAAAGATTCTTTTGATGATGAAGATATG
TGGGTAACTAAACATCGTATCAGCTTCTTTTTTCATGCCTCTTCTCTTATATTTAACATTGTGGTCTTTCACT
TCTTTATTCTTTAAAGCGACGGAATTTGTCAGGCTCTCAAAGGTACATCATATGCTGTCATGGGAATTGTTGT
CGCAGTCATGGCAATCCAAGGTCTTTGTTCGATATACTACCTTGTCCGAGGGATCGTCCTTCAACAAAGTCGT
AAAATCAAAAGCGAGCATACAAAACAAACTGTTATGATAATGGTCCCTTGCTACAACGAAGGAGACAAGGAAC
TCAGGAAGACGATTGATAGCATCATGGACACCTCTTATCCAGACTCAAACAAAGTTTTGATTGTGGTTGCAGA
TGGTGATGTCACTGGAAAAGGTGAAACTAAGTCCACTCCAGAGGTTATATCCAGCATTCTTCAGTACCCCAGA
TCCCCACAGGACATCACATACGATTGTACAAGCGTTGGCCCTGACGAAAGAAGTCACAACCGGGCAAAGGTTT
ACTACGGCACAAAAAAAAGTGAAAAAAATCCAGGCAAGATGTTACATTATATTGTAATCGAGAAATGTGGTAT
ACCAATAGAGAAAGGGAGCCCAAAAGCTGGAAACCGTGGCAAACGTGACTCACAGCTCCTTATTACTGGTCTT
CTCAACAAAATTGGGTGTCATCCTGATGATCCTTTGAACGGCCTTGAATCGACCATCGTTCATGCACTTGCCG
ATTTACAGCTCGGACCAATGAACGACATCAAATACCTCTTAGCCATTGACGCAGATACCGAGATTAGCGAAGA
TAGCATAACGCAAATGTGTTACAATATGGAAGAACAACCTTTTGTTCTAGCACTTTGCGGAGAAACTCAGGTA
GCCAACCCGTCAGATAGCTGGGTCACCAGGTAGGTCAAATCCACACTGTTGAACCAATACGCAAGCGATCACT
AAGCTGACCTCACCTGCTGCTGCCTCTTTCCCCTATCATTTACCAGGATACAAGTATTTGAATACTACACGAA
TCACCACTTGAAGAAAGCGTTCGAGTCTGTTTTTAACACTGTGACATGTCTCCCTGGATGTTTCACAATGTAC
CGGCTGTTTGATTACGAAAACGAACCTCTTCTTGCCTGCGATTCTGTCTACATACCTTACGCTATTAATGACG
CTGAGATAAATTCACTACACGCAAAGAACTTGTACCATCTTGGAGAAGACCGCATGCTGAC
>TR445|c0_g1_i8 len=2144 path=[4633:0-74 4634:75-78 4704:79-98 4770:99-145 4731:146-178 4700:179-181 4780:182-240 @4768@!:241-533 4788:534-534 4646:535-553 4784:554-631 4789:632-655 4725:656-678 4749:679-838 4670:839-848 4671:849-862 4776:863-938 4790:939-946 4721:947-962 4690:963-969 4745:970-1008 4775:1009-1031 4764:1032-1055 4744:1056-1062 4718:1063-1137 4538:1138-1161 4687:1162-1366 4426:1367-1390 4763:1391-1431 4741:1432-1454 4717:1455-1567 4638:1568-1584 4684:1585-1800 4413:1801-1824 4762:1825-1945 4663:1946-1967 4738:1968-2143] [-1, 4633,

4634, 4704, 4770, 4731, 4700, 4780, 4768, 4788, 4646, 4784, 4789, 4725, 4749, 4670, 4671, 4776, 4790, 4721, 4690, 4745, 4775, 4764, 4744, 4718, 4538, 4687, 4426, 4763, 4741, 4717, 4638, 4684, 4413, 4762, 4663, 4738]
CCGATCTCATCGTACAACCTTGTACAATAAATGGCAACCCATAAAATAAATCTCCTCCACTCTACTCAGAAGC
CATCGCATCAGCGTTCTCTCATTTGTTAACCCCTCGCTTTCCCTCTAATCTGCATTAGTTTTCCTCGCCAACA
CCAGTATTGAACCGCGAGCTATTTTCCGACGAATAACAGGTCTATATTTAAAATGTCTGATACGGGGTGCAAC
TGGCGATTCTTCTCCCGCCTCTGTACATTTTTTATACCTGACTCTTTACTCTGGTCCATTGGCCGCGATATAG
AAGACAAAGCCTCCCGAAAAGAGGCTAAACAAGCTTGGAGGGAGAAAATCGCGCTCTGTGTCATCATGTTGAG
TTCCTGCGCAGCGTTTATTGGATTTGGTGGACTTTTATTACTACTTGAGTGTGGAGTTGAATCGATGCAGTCT
GGGGCACGTCTTGGCATTGACTCAGCACCTCTTGGCACTTGTGGCAACGGGAAACGAGGTAACGGACTTTGCT
TTGATGGCAGCTGCTGCTCTCAGCATGGATTTTGTGGATCTTCGGTCGAGCATTGTTCTGGAAGGTCGCTCGA
TAGTGATAACCTTTTTGATATTAAAGACGATATCCAGGCTGATATTAAAGATTCTTTTGATGATGAAGATATC
GACGGAATTTGTCAGGCTCTCAAAGGTACATCATATGCTGTCATGGGAATTGTTGTCGCAGTCATGGCAATCC
AAGGTCTTTGTTCGATATACTACCTTGTCCGAGGGATCGTCCTTCAACAAAGTCGTAAAATCAAAAGCGAGCA
TACAAAACAAACTGTTATGATAATGGTCCCTTGCTACAACGAAGGAGACAAGGAACTCAGGAAGACGATTGAT
AGCATCATGGACACCTCTTATCCAGACTCAAACAAAGTTTTGATTGTGGTTGCAGATGGTGATGTCACTGGTA
AGAGCTGTTCCTTGTTGTAAAATAATAATTACTGGCTGTCTGTCAATTCCCGCTCCTTTTCAAACATTATTCC
GTTGTTTTACGAATCAAAGGAAAAGGTGAAACTAAGTCCACTCCAGAGGTTATATCCAGCATTCTTCAGTACC
CCAGATCCCCACAGGACATCACATACGATTGTACAAGCGTTGGCCCTGACGAAAGAAGTCACAACCGGGCAAA
GGTTTACTACGGCACAAAAAAAAGTGAAAAAAATCCAGGCAAGATGTTACATTATATTGTAATCGAGAAATGT
GGTATACCAATAGAGAAAGGGAGCCCAAAAGCTGGAAACCGTGGCAAACGTGACTCACAGCTCCTTATTACTG
GTCTTCTCAACAAAATTGGGTGTCATCCTGATGATCCTTTGAACGGCCTTGAATCGACCATCGTTCATGCACT
TGCCGATTTACAGCTCGGACCAATGAACGACATCAAATACCTCTTAGCCATTGACGCAGATACCGAGATTAGC
GAAGATAGCATAACGCAAATGTGTTACAATATGGAAGAACAACCTTTTGTTCTAGCACTTTGCGGAGAAACTC
AGGTAGCCAACCCGTCAGATAGCTGGGTCACCAGGATACAAGTATTTGAATACTACACGAATCACCACTTGAA
GAAAGCGTTCGAGTCTGTTTTTAACACTGTGACATGTCTCCCTGGATGTTTCACAATGTACCGGCTGTTTGAT
TACGAAAACGAACCTCTTCTTGCCTGCGATTCTGTCTACATACCTTACGCTATTAATGACGCTGAGATAAATT
CACTACACGCAAAGAACTTGTACCATCTTGGAGAAGACCGCATGCTGACGACTCTTCTACTGCGGCACTTCCC
CGAAATGAATCTCTCCTTCGTACCTGAAGCAACTTGCCAGACCGTCGTGCCAGACACATTCTCTGTTCTTCTT
AGTCAAAGAAGAAGATGGGTCAATTCGACGTTCCATAATTTACTTGAGCTTTGGAAAGTGAAAACGTGTGGAA
TATGCTGCTTCTCTATGAAGGTCATTATCTTACTTGATTTGGTAGGAACTCTTATTCTTCCCGTAGCATTCAT
ATACGTGGGCATTATGCTATACTTTGCACTTAATGGAAATGGGGACACTTTAGCACAGATAATATTGGGGTTT
TCTGTTGCTGTTCAACTTGTTGTCCCT
>TR445|c0_g1_i9 len=1133 path=[4633:0-74 4634:75-78 4704:79-98 4770:99-145 4731:146-178 4700:179-181 4780:182-240 @4768@!:241-533 4699:534-612 4787:613-643 4784:644-721 4578:722-745 4725:746-768 4749:769-928 4670:929-938 4671:939-952 4776:953-1028 4790:1029-1036 4499:1037-1052 4743:1053-1057 4718:1058-1132] [-1, 4633, 4634, 4704, 4770, 4731, 4700, 4780, 4768, 4699, 4787, 4784, 4578, 4725, 4749, 4670, 4671, 4776, 4790, 4499, 4743, 4718]
CCGATCTCATCGTACAACCTTGTACAATAAATGGCAACCCATAAAATAAATCTCCTCCACTCTACTCAGAAGC
CATCGCATCAGCGTTCTCTCATTTGTTAACCCCTCGCTTTCCCTCTAATCTGCATTAGTTTTCCTCGCCAACA
CCAGTATTGAACCGCGAGCTATTTTCCGACGAATAACAGGTCTATATTTAAAATGTCTGATACGGGGTGCAAC
TGGCGATTCTTCTCCCGCCTCTGTACATTTTTTATACCTGACTCTTTACTCTGGTCCATTGGCCGCGATATAG
AAGACAAAGCCTCCCGAAAAGAGGCTAAACAAGCTTGGAGGGAGAAAATCGCGCTCTGTGTCATCATGTTGAG
TTCCTGCGCAGCGTTTATTGGATTTGGTGGACTTTTATTACTACTTGAGTGTGGAGTTGAATCGATGCAGTCT
GGGGCACGTCTTGGCATTGACTCAGCACCTCTTGGCACTTGTGGCAACGGGAAACGAGGTAACGGACTTTGCT
TTGATGGCAGCTGCTGCTCTCAGGTACGTGAAATAAAGGGAAAGAAGAAGCTCTCCTGCATATTCATTCATTC
CTAAAAAGTTCCAATAATTGTATTTTGTGATTTCATACAGCATGGATTTTGTGGATCTTCGGTCGAGCATTGT
TCTGGAAGGTCGCTCGATAGTGATAACCTTTTTGATATTAAAGACGATATCCAGGCTGATATTAAGGATTCTT
TTGATGATGAAGATATCGACGGAATTTGTCAGGCTCTCAAAGGTACATCATATGCTGTCATGGGAATTGTTGT
CGCAGTCATGGCAATCCAAGGTCTTTGTTCGATATACTACCTTGTCCGAGGGATCGTCCTTCAACAAAGTCGT
AAAATCAAAAGCGAGCATACAAAACAAACTGTTATGATAATGGTCCCTTGCTACAACGAAGGAGACAAGGAAC
TCAGGAAGACGATTGATAGCATCATGGACACCTCTTATCCAGACTCAAACAAAGTTTTGATTGTGGTTGCAGA
TGGTGATGTCACTGGAAAAGGTGAAACTAAGTCCACTCCAGAGGTTATATCCAGCATTCTTCAGTACCCCAGA
TCCCCACAGGACATCACATACGATTGTACAAGCGTTGG
>TR445|c0_g1_i10 len=2897 path=[4456:0-74 4705:75-98 4770:99-145 4732:146-172 4701:173-175 4779:176-234 @4768@!:235-527 4788:528-528 4646:529-547 4784:548-625 4789:626-649 4794:650-677 4727:678-699 4777:700-700 4766:701-760 4724:761-762 4749:763-922 4670:923-932

4671:933-946 4776:947-1022 4790:1023-1030 4499:1031-1046 4743:1047-1051
4718:1052-1126 4538:1127-1150 4687:1151-1355 4426:1356-1379 4763:1380-
1420 4741:1421-1443 4717:1444-1556 4638:1557-1573 4684:1574-1789
4413:1790-1813 4762:1814-1934 4740:1935-1965 4786:1966-1989 4783:1990-
1997 4774:1998-1999 4681:2000-2006 4760:2007-2032 4712:2033-2054
4738:2055-2230 4792:2231-2254 4795:2255-2269 4678:2270-2305 4758:2306-
2395 4785:2396-2443 4782:2444-2454 4772:2455-2478 4793:2479-2500
4673:2501-2524 4587:2525-2543 4588:2544-2896] [-1, 4456, 4705, 4770,
4732, 4701, 4779, 4768, 4788, 4646, 4784, 4789, 4794, 4727, 4777, 4766,
4724, 4749, 4670, 4671, 4776, 4790, 4499, 4743, 4718, 4538, 4687, 4426,
4763, 4741, 4717, 4638, 4684, 4413, 4762, 4740, 4786, 4783, 4774, 4681,
4760, 4712, 4738, 4792, 4795, 4678, 4758, 4785, 4782, 4772, 4793, 4673,
4587, 4588, -2]
CCGATCTCATCGTACAACCTTGTACAATAAATGGCAACCCATAAAATAAATCTCCTCCACTCTACTCAGAAGC
CACCGCATCAGCGTTCTCTCATTTGTTAACCCCTCGCTTTCCCTCTAATCTGCATTAGTTTTCCTCGCCAACA
ACAGACCGCGAGCTATTTTCCGACGAATAAGAGGTCCATATTTAAAATGCCTGATACGGGGTGCATCTGGCGA
TTCTTCTCCCGCCTCTGTACATTTTTTATACCTGACTCTTTACTCTGGTCCATTGGCCGCGATATAGAAGACA
AAGCCTCCCGAAAAGAGGCTAAACAAGCTTGGAGGGAGAAAATCGCGCTCTGTGTCATCATGTTGAGTTCCTG
CGCAGCGTTTATTGGATTTGGTGGACTTTTATTACTACTTGAGTGTGGAGTTGAATCGATGCAGTCTGGGGCA
CGTCTTGGCATTGACTCAGCACCTCTTGGCACTTGTGGCAACGGGAAACGAGGTAACGGACTTTGCTTTGATG
GCAGCTGCTGCTCTCAGCATGGATTTTGTGGATCTTCGGTCGAGCATTGTTCTGGAAGGTCGCTCGATAGTGA
TAACCTTTTTGATATTAAAGACGATATCCAGGCTGATATTAAAGATTCTTTTGATGATGAAGATATGTGGGTA
ACTAAACATCGTATCAGCTTCTTTTTTCATGCCTCTTCTCTTATATTTAACATTGTGGTCTTTCACTTCTTTA
TTCTTTAAAGCGACGGAATTTGTCAGGCTCTCAAAGGTACATCATATGCTGTCATGGGAATTGTTGTCGCAGT
CATGGCAATCCAAGGTCTTTGTTCGATATACTACCTTGTCCGAGGGATCGTCCTTCAACAAAGTCGTAAAATC
AAAAGCGAGCATACAAAACAAACTGTTATGATAATGGTCCCTTGCTACAACGAAGGAGACAAGGAACTCAGGA
AGACGATTGATAGCATCATGGACACCTCTTATCCAGACTCAAACAAAGTTTTGATTGTGGTTGCAGATGGTGA
TGTCACTGGAAAAGGTGAAACTAAGTCCACTCCAGAGGTTATATCCAGCATTCTTCAGTACCCCAGATCCCCA
CAGGACATCACATACGATTGTACAAGCGTTGGCCCTGACGAAAGAAGTCACAACCGGGCAAAGGTTTACTACG
GCACAAAAAAAAAGTGAAAAAAATCCAGGCAAGATGTTACATTATATTGTAATCGAGAAATGTGGTATACCAAT
AGAGAAAGGGAGCCCAAAAGCTGGAAACCGTGGCAAACGTGACTCACAGCTCCTTATTACTGGTCTTCTCAAC
AAAATTGGGTGTCATCCTGATGATCCTTTGAACGGCCTTGAATCGACCATCGTTCATGCACTTGCCGATTTAC
AGCTCGGACCAATGAACGACATCAAATACCTCTTAGCCATTGACGCAGATACCGAGATTAGCGAAGATAGCAT
AACGCAAATGTGTTACAATATGGAAGAACAACCTTTTGTTCTAGCACTTTGCGGAGAAACTCAGGTAGCCAAC
CCGTCAGATAGCTGGGTCACCAGGATACAAGTATTTGAATACTACACGAATCACCACTTGAAGAAAGCGTTCG
AGTCTGTTTTTAACACTGTGACATGTCTCCCTGGATGTTTCACAATGTACCGGCTGTTTGATTACGAAAACGA
ACCTCTTCTTGCCTGCGATTCTGTCTACATACCTTACGCTATTAATGACGCTGAGATAAATTCACTACACGCA
AAGAACTTGTACCATCTTGGAGAAGACCGCATGCTGACGACTCTTCTACTGCGGCACTTCCCCGAAATGAATC
TCTCCTTCGTACCTGAAGCAACTTGCCAGACCGTCGTGCCAGACACATTCTCTGTTCTTCTTAGTCAAAGAAG
AAGATGGGTCAATTCGACGTTCCATAATTTACTTGAGTAAGAATTGAACAAACATTTTGAACATATCTTCCAA
TTGTAAAGACTCATCCTTGTTTTTTTTCTGCACTCGATGTTATGAAACCCATGGCCGCCAGGCTTTGGAAAGT
GAAAACGTGTGGAATATGCTGCTTCTCTATGAAGGTCATTATCTTACTTGATTTGGTAGGAACTCTTATTCTT
CCCGTAGCATTCATATACGTGGGCATTATGCTATACTTTGCACTTAATGGAAATGGGGACACTTTAGCACAGA
TAATATTGGGGTTTTCTGTTGCTGTTCAACTTGTTGTCCCTACTCTACGCTTACACTGGAATATCTTGTTTTG
GTCCATTCCTTTTTATGTGATCGGTGTGCCCATATTTTACTTCATTTTGCCAATTTATGCCTTCTGGCATATG
GACGATGTATCGTGGGGATCTACTCGGTTAGTAGCCACCTAGAGAGGGACTTAGTTGAGTCGATACAAGAGTT
GTTGATGCTGGAGCTGCTGCTGGGGAGGATGGTGTGGCGATGCAGATGAGGCAGCGCGCTATATGAAACCAAT
GGTGCATCGGAGTGGCTGGTAATAATGATGGCAAGGAGTAAAATGCAGCTAATTATTTGTTCAACTGCCAAGT
AATGAGATATTAAGAGTTTTAATACCCAAATATATGGAATTGAGTTATTCCATCCTCTTGTATCTACTTTGGC
AATCAATAAGTGTTCTTAGGAGGGCTTGGTCAATGCATCAAATTGTAGACTGACGTGTACTATCTAGAATGGT
AAGTCAGATAATTGGTTCTGAGAGAGCAATGGCAAGTTGATGCAATACAGAATAAATGGTAACTGTGTTTTAA
CCCTCAAGTGTCCAGAAAGCATCAGAAAGTCATTTACAAATAAATACATAAAAGAACTGTAGCTGTCTGATTT
GGCTGATTCCAAGTACCGTGCAGTTGGGATCATTCAGTCAACCCTAAATT
>TR445|c0_g1_i12 len=2894 path=[4456:0-74 4705:75-98 4770:99-145
4732:146-172 4701:173-175 4779:176-234 @4768@!:235-527 4788:528-528
4646:529-547 4784:548-625 4789:626-649 4794:650-677 4727:678-699
4777:700-700 4766:701-760 4724:761-762 4749:763-922 4670:923-932
4671:933-946 4776:947-1022 4790:1023-1030 4721:1031-1046 4690:1047-1053
4745:1054-1092 4775:1093-1115 4764:1116-1139 4744:1140-1146 4718:1147-
1221 4538:1222-1245 4687:1246-1450 4426:1451-1474 4763:1475-1515

4741:1516-1538 4717:1539-1651 4638:1652-1668 4684:1669-1884 4413:1885-1908 4762:1909-2029 4663:2030-2051 4738:2052-2227 4792:2228-2251 4795:2252-2266 4678:2267-2302 4758:2303-2392 4785:2393-2440 4782:2441-2451 4772:2452-2475 4793:2476-2497 4673:2498-2521 4587:2522-2540 4588:2541-2893] [-1, 4456, 4705, 4770, 4732, 4701, 4779, 4768, 4788, 4646, 4784, 4789, 4794, 4727, 4777, 4766, 4724, 4749, 4670, 4671, 4776, 4790, 4721, 4690, 4745, 4775, 4764, 4744, 4718, 4538, 4687, 4426, 4763, 4741, 4717, 4638, 4684, 4413, 4762, 4663, 4738, 4792, 4795, 4678, 4758, 4785, 4782, 4772, 4793, 4673, 4587, 4588, -2]
CCGATCTCATCGTACAACCTTGTACAATAAATGGCAACCCATAAAATAAATCTCCTCCACTCTACTCAGAAGC
CACCGCATCAGCGTTCTCTCATTTGTTAACCCCTCGCTTTCCCTCTAATCTGCATTAGTTTTCCTCGCCAACA
ACAGACCGCGAGCTATTTTCCGACGAATAAGAGGTCCATATTTAAAATGCCTGATACGGGGTGCATCTGGCGA
TTCTTCTCCCGCCTCTGTACATTTTTTATACCTGACTCTTTACTCTGGTCCATTGGCCGCGATATAGAAGACA
AAGCCTCCCGAAAAGAGGCTAAACAAGCTTGGAGGGAGAAAATCGCGCTCTGTGTCATCATGTTGAGTTCCTG
CGCAGCGTTTATTGGATTTGGTGGACTTTTATTACTACTTGAGTGTGGAGTTGAATCGATGCAGTCTGGGGCA
CGTCTTGGCATTGACTCAGCACCTCTTGGCACTTGTGGCAACGGGAAACGAGGTAACGGACTTTGCTTTGATG
GCAGCTGCTGCTCTCAGCATGGATTTTGTGGATCTTCGGTCGAGCATTGTTCTGGAAGGTCGCTCGATAGTGA
TAACCTTTTTGATATTAAAGACGATATCCAGGCTGATATTAAAGATTCTTTTGATGATGAAGATATGTGGGTA
ACTAAACATCGTATCAGCTTCTTTTTTCATGCCTCTTCTCTTATATTTAACATTGTGGTCTTTCACTTCTTTA
TTCTTTAAAGCGACGGAATTTGTCAGGCTCTCAAAGGTACATCATATGCTGTCATGGGAATTGTTGTCGCAGT
CATGGCAATCCAAGGTCTTTGTTCGATATACTACCTTGTCCGAGGGATCGTCCTTCAACAAAGTCGTAAAATC
AAAAGCGAGCATACAAAACAAACTGTTATGATAATGGTCCCTTGCTACAACGAAGGAGACAAGGAACTCAGGA
AGACGATTGATAGCATCATGGACACCTCTTATCCAGACTCAAACAAAGTTTTGATTGTGGTTGCAGATGGTGA
TGTCACTGGTAAGAGCTGTTCCTTGTTGTAAAATAATAATTACTGGCTGTCTGTCAATTCCCGCTCCTTTTCA
AACATTATTCCGTTGTTTTACGAATCAAAGGAAAAGGTGAAACTAAGTCCACTCCAGAGGTTATATCCAGCAT
TCTTCAGTACCCCAGATCCCCACAGGACATCACATACGATTGTACAAGCGTTGGCCCTGACGAAAGAAGTCAC
AACCGGGCAAAGGTTTACTACGGCACAAAAAAAAGTGAAAAAAATCCAGGCAAGATGTTACATTATATTGTAA
TCGAGAAATGTGGTATACCAATAGAGAAAGGGAGCCCAAAAGCTGGAAACCGTGGCAAACGTGACTCACAGCT
CCTTATTACTGGTCTTCTCAACAAAATTGGGTGTCATCCTGATGATCCTTTGAACGGCCTTGAATCGACCATC
GTTCATGCACTTGCCGATTTACAGCTCGGACCAATGAACGACATCAAATACCTCTTAGCCATTGACGCAGATA
CCGAGATTAGCGAAGATAGCATAACGCAAATGTGTTACAATATGGAAGAACAACCTTTTGTTCTAGCACTTTG
CGGAGAAACTCAGGTAGCCAACCCGTCAGATAGCTGGGTCACCAGGATACAAGTATTTGAATACTACACGAAT
CACCACTTGAAGAAAGCGTTCGAGTCTGTTTTTAACACTGTGACATGTCTCCCTGGATGTTTCACAATGTACC
GGCTGTTTGATTACGAAAACGAACCTCTTCTTGCCTGCGATTCTGTCTACATACCTTACGCTATTAATGACGC
TGAGATAAATTCACTACACGCAAAGAACTTGTACCATCTTGGAGAAGACCGCATGCTGACGACTCTTCTACTG
CGGCACTTCCCCGAAATGAATCTCTCCTTCGTACCTGAAGCAACTTGCCAGACCGTCGTGCCAGACACATTCT
CTGTTCTTCTTAGTCAAAGAAGAAGATGGGTCAATTCGACGTTCCATAATTTACTTGAGCTTTGGAAAGTGAA
AACGTGTGGAATATGCTGCTTCTCTATGAAGGTCATTATCTTACTTGATTTGGTAGGAACTCTTATTCTTCCC
GTAGCATTCATATACGTGGGCATTATGCTATACTTTGCACTTAATGGAAATGGGGACACTTTAGCACAGATAA
TATTGGGGTTTTCTGTTGCTGTTCAACTTGTTGTCCCTACTCTACGCTTACACTGGAATATCTTGTTTTGGTC
CATTCCTTTTTATGTGATCGGTGTGCCCATATTTTACTTCATTTTGCCAATTTATGCCTTCTGGCATATGGAC
GATGTATCGTGGGGATCTACTCGGTTAGTAGCCACCTAGAGAGGGACTTAGTTGAGTCGATACAAGAGTTGTT
GATGCTGGAGCTGCTGCTGGGGAGGATGGTGTGGCGATGCAGATGAGGCAGCGCGCTATATGAAACCAATGGT
GCATCGGAGTGGCTGGTAATAATGATGGCAAGGAGTAAAATGCAGCTAATTATTTGTTCAACTGCCAAGTAAT
GAGATATTAAGAGTTTTAATACCCAAATATATGGAATTGAGTTATTCCATCCTCTTGTATCTACTTTGGCAAT
CAATAAGTGTTCTTAGGAGGGCTTGGTCAATGCATCAAATTGTAGACTGACGTGTACTATCTAGAATGGTAAG
TCAGATAATTGGTTCTGAGAGAGCAATGGCAAGTTGATGCAATACAGAATAAATGGTAACTGTGTTTTAACCC
TCAAGTGTCCAGAAAGCATCAGAAAGTCATTTACAAATAAATACATAAAAGAACTGTAGCTGTCTGATTTGGC
TGATTCCAAGTACCGTGCAGTTGGGATCATTCAGTCAACCCTAAATT
>TR445|c0_g1_i13 len=2902 path=[4456:0-74 4705:75-98 4770:99-145 4732:146-172 4701:173-175 4779:176-234 @4768@!:235-527 4788:528-528 4646:529-547 4784:548-625 4789:626-649 4725:650-672 4749:673-832 4670:833-842 4671:843-856 4776:857-932 4790:933-940 4721:941-956 4690:957-963 4745:964-1002 4775:1003-1025 4764:1026-1049 4744:1050-1056 4718:1057-1131 4538:1132-1155 4687:1156-1360 4426:1361-1384 4763:1385-1425 4741:1426-1448 4717:1449-1561 4638:1562-1578 4684:1579-1794 4413:1795-1818 4762:1819-1939 4740:1940-1970 4786:1971-1994 4783:1995-2002 4774:2003-2004 4681:2005-2011 4760:2012-2037 4712:2038-2059 4738:2060-2235 4792:2236-2259 4795:2260-2274 4678:2275-2310 4758:2311-2400 4785:2401-2448 4782:2449-2459 4772:2460-2483 4793:2484-2505

4673:2506-2529 4587:2530-2548 4588:2549-2901] [-1, 4456, 4705, 4770, 4732, 4701, 4779, 4768, 4788, 4646, 4784, 4789, 4725, 4749, 4670, 4671, 4776, 4790, 4721, 4690, 4745, 4775, 4764, 4744, 4718, 4538, 4687, 4426, 4763, 4741, 4717, 4638, 4684, 4413, 4762, 4740, 4786, 4783, 4774, 4681, 4760, 4712, 4738, 4792, 4795, 4678, 4758, 4785, 4782, 4772, 4793, 4673, 4587, 4588, -2]
CCGATCTCATCGTACAACCTTGTACAATAAATGGCAACCCATAAAATAAATCTCCTCCACTCTACTCAGAAGC
CACCGCATCAGCGTTCTCTCATTTGTTAACCCCTCGCTTTCCCTCTAATCTGCATTAGTTTTCCTCGCCAACA
ACAGACCGCGAGCTATTTTCCGACGAATAAGAGGTCCATATTTAAAATGCCTGATACGGGGTGCATCTGGCGA
TTCTTCTCCCGCCTCTGTACATTTTTTATACCTGACTCTTTACTCTGGTCCATTGGCCGCGATATAGAAGACA
AAGCCTCCCGAAAAGAGGCTAAACAAGCTTGGAGGGAGAAAATCGCGCTCTGTGTCATCATGTTGAGTTCCTG
CGCAGCGTTTATTGGATTTGGTGGACTTTTATTACTACTTGAGTGTGGAGTTGAATCGATGCAGTCTGGGGCA
CGTCTTGGCATTGACTCAGCACCTCTTGGCACTTGTGGCAACGGGAAACGAGGTAACGGACTTTGCTTTGATG
GCAGCTGCTGCTCTCAGCATGGATTTTGTGGATCTTCGGTCGAGCATTGTTCTGGAAGGTCGCTCGATAGTGA
TAACCTTTTTGATATTAAAGACGATATCCAGGCTGATATTAAAGATTCTTTTGATGATGAAGATATCGACGGA
ATTTGTCAGGCTCTCAAAGGTACATCATATGCTGTCATGGGAATTGTTGTCGCAGTCATGGCAATCCAAGGTC
TTTGTTCGATATACTACCTTGTCCGAGGGATCGTCCTTCAACAAAGTCGTAAAATCAAAAGCGAGCATACAAA
ACAAACTGTTATGATAATGGTCCCTTGCTACAACGAAGGAGACAAGGAACTCAGGAAGACGATTGATAGCATC
ATGGACACCTCTTATCCAGACTCAAACAAAGTTTTGATTGTGGTTGCAGATGGTGATGTCACTGGTAAGAGCT
GTTCCTTGTTGTAAAATAATAATTACTGGCTGTCTGTCAATTCCCGCTCCTTTTCAAACATTATTCCGTTGTT
TTACGAATCAAAGGAAAAGGTGAAACTAAGTCCACTCCAGAGGTTATATCCAGCATTCTTCAGTACCCCAGAT
CCCCACAGGACATCACATACGATTGTACAAGCGTTGGCCCTGACGAAAGAAGTCACAACCGGGCAAAGGTTTA
CTACGGCACAAAAAAAAAGTGAAAAAAATCCAGGCAAGATGTTACATTATATTGTAATCGAGAAATGTGGTATA
CCAATAGAGAAAGGGAGCCCAAAAGCTGGAAACCGTGGCAAACGTGACTCACAGCTCCTTATTACTGGTCTTC
TCAACAAAATTGGGTGTCATCCTGATGATCCTTTGAACGGCCTTGAATCGACCATCGTTCATGCACTTGCCGA
TTTACAGCTCGGACCAATGAACGACATCAAATACCTCTTAGCCATTGACGCAGATACCGAGATTAGCGAAGAT
AGCATAACGCAAATGTGTTACAATATGGAAGAACAACCTTTTGTTCTAGCACTTTGCGGAGAAACTCAGGTAG
CCAACCCGTCAGATAGCTGGGTCACCAGGATACAAGTATTTGAATACTACACGAATCACCACTTGAAGAAAGC
GTTCGAGTCTGTTTTTAACACTGTGACATGTCTCCCTGGATGTTTCACAATGTACCGGCTGTTTGATTACGAA
AACGAACCTCTTCTTGCCTGCGATTCTGTCTACATACCTTACGCTATTAATGACGCTGAGATAAATTCACTAC
ACGCAAAGAACTTGTACCATCTTGGAGAAGACCGCATGCTGACGACTCTTCTACTGCGGCACTTCCCCGAAAT
GAATCTCTCCTTCGTACCTGAAGCAACTTGCCAGACCGTCGTGCCAGACACATTCTCTGTTCTTCTTAGTCAA
AGAAGAAGATGGGTCAATTCGACGTTCCATAATTTACTTGAGTAAGAATTGAACAAACATTTTGAACATATCT
TCCAATTGTAAAGACTCATCCTTGTTTTTTTTCTGCACTCGATGTTATGAAACCCATGGCCGCCAGGCTTTGG
AAAGTGAAAACGTGTGGAATATGCTGCTTCTCTATGAAGGTCATTATCTTACTTGATTTGGTAGGAACTCTTA
TTCTTCCCGTAGCATTCATATACGTGGGCATTATGCTATACTTTGCACTTAATGGAAATGGGGACACTTTAGC
ACAGATAATATTGGGGTTTTCTGTTGCTGTTCAACTTGTTGTCCCTACTCTACGCTTACACTGGAATATCTTG
TTTTGGTCCATTCCTTTTTATGTGATCGGTGTGCCCATATTTTACTTCATTTTGCCAATTTATGCCTTCTGGC
ATATGGACGATGTATCGTGGGGATCTACTCGGTTAGTAGCCACCTAGAGAGGGACTTAGTTGAGTCGATACAA
GAGTTGTTGATGCTGGAGCTGCTGCTGGGGAGGATGGTGTGGCGATGCAGATGAGGCAGCGCGCTATATGAAA
CCAATGGTGCATCGGAGTGGCTGGTAATAATGATGGCAAGGAGTAAAATGCAGCTAATTATTTGTTCAACTGC
CAAGTAATGAGATATTAAGAGTTTTAATACCCAAATATATGGAATTGAGTTATTCCATCCTCTTGTATCTACT
TTGGCAATCAATAAGTGTTCTTAGGAGGGCTTGGTCAATGCATCAAATTGTAGACTGACGTGTACTATCTAGA
ATGGTAAGTCAGATAATTGGTTCTGAGAGAGCAATGGCAAGTTGATGCAATACAGAATAAATGGTAACTGTGT
TTTAACCCTCAAGTGTCCAGAAAGCATCAGAAAGTCATTTACAAATAAATACATAAAAGAACTGTAGCTGTCT
GATTTGGCTGATTCCAAGTACCGTGCAGTTGGGATCATTCAGTCAACCCTAAATT
>TR445|c1_g1_i1 len=2964 path=[4410:0-368 4508:369-369 4461:370-409 4535:410-412 4530:413-434 4521:435-458 4506:459-460 4534:461-469 4520:470-517 @4505@!:518-607 4456:608-643 4533:644-658 4528:659-682 4518:683-858 4540:859-880 4480:881-909 4517:910-919 4502:920-943 4478:944-974 4452:975-1095 4293:1096-1119 4527:1120-1335 4541:1336-1352 4547:1353-1353 4500:1354-1465 4214:1466-1488 4449:1489-1529 4499:1530-1553 4515:1554-1714 4447:1715-1729 4537:1730-1758 4532:1759-1782 4526:1783-1857 4542:1858-1864 4548:1865-1888 4549:1889-1911 4493:1912-1950 4470:1951-1957 4441:1958-1973 4539:1974-1981 4536:1982-2018 4525:2019-2056 4544:2057-2057 4512:2058-2071 4394:2072-2081 4511:2082-2095 @4489@!:2096-2241 4545:2242-2243 4488:2244-2304 4465:2305-2326 4546:2327-2327 4487:2328-2378 @4464@!:2379-2456 4250:2457-2475 4523:2476-2476 @4509@!:2477-2769 4486:2770-2828 4462:2829-2831 4431:2832-2864 4291:2865-2963] [-1, 4410, 4508, 4461, 4535, 4530, 4521, 4506, 4534,

4520, 4505, 4456, 4533, 4528, 4518, 4540, 4480, 4517, 4502, 4478, 4452, 4293, 4527, 4541, 4547, 4500, 4214, 4449, 4499, 4515, 4447, 4537, 4532, 4526, 4542, 4548, 4549, 4493, 4470, 4441, 4539, 4536, 4525, 4544, 4512, 4394, 4511, 4489, 4545, 4488, 4465, 4546, 4487, 4464, 4250, 4523, 4509, 4486, 4462, 4431, 4291, -2]
GGGTTGACTGAATGATCCCAACTGCACGGTACTTGGAATCAGCCAAATCAGACAGCTACAGTTCTTTTATGTA
TTTATTTGTAAATGACTTTCTGATGCTTTCTGGACACTTGAGGGTTAAAACACAGTTACCATTTATTCTGTAT
TGCATCAACTTGCCATTGCTCTCTCAGAACCAATTATCTGACTTACCATTCTAGATAGTACACGTCAGTCTAC
AATTTGATGCATTGACCAAGCCCTCCTAAGAACACTTATTGATTGCCAAAGTAGATACAAGAGGATGGAATAA
CTCAATTCCATATATTTGGGTATTAAAACTCTTAATATCTCATTACTTGGCAGTTGAACAAATAATTAGCTGC
ATTTTACTCCTTGCCATCATTATTACCAGCCACTCCGATGCACCATTGGTTTCATATAGCGCGCTGCCTCGTC
TGCATCGCCACACCATCCTCCCCAGCAGCAGCCCCAGCATCAACAACTCTTGTATCCACTCAACTAAGTCCCT
CTCTAGGTGGCTACTAACCGAGTAGATCCCCACGATACATCGTCCATATGCCAGAAGGCATAAATTGGCAAAA
TGAAGTAAAATATGGGCACACCGATCACATAAAAAGGAATGGACCAAAACAAGATATTCCAGTGTAAGCGTAG
AGTAGGGACAACAAGTTGAACAGCAACAGAAAACCCCAATATTATCTGTGCTAAAGTGTCCCCATTTCCATTA
AGTGCAAAGTATAGCATAATGCCCACGTATATGAATGCTACGGGAAGAATAAGAGTTCCTACCAAATCAAGTA
AGATAATGACCTTCATAGAGAAGCAGCATATTCCACACGTTTTCACTTTCCAAAGCCTGGCGGCCATGGGTTT
CATAAGAGTGTAGAAAAAAAAACAAGGATGAGTCTTTACAATTGGAAGATATGTTCAAAATGTTTGTTCAATTC
TTACTCAAGTAAATTATGGAACGTCGAATTGACCCATCTTCTTCTTTGACTAAGAAGAACAGAGAATGTGTCT
GGCACGACGGTCTGGCAAGTTGCTTCAGGTACGAAGGAGAGATTCATTTCGGGGAAGTGCCGCAGTAGAAGAG
TTGTCAGCATGCGGTCTTCTCCAAGATGGTACAAGTTCTTTGCGTGTAGTGAATTTATCTCAGCGTCATTAAT
AGCGTAAGGTATGTAGACAGAATCGCAGGCAAGAAGAGGTTCGTTTTCGTAATCAAACAGCCGGTACATTGTG
AAACATCCAGGGAGACATGTCACAGTGTTAAAAACAGACTCGAACGCTTTCTTCAAGTGGTGATTCGTGTAGT
ATTCAAATACTTGTATCCTGGTGACCCAGCTATCTGACGGGTTGGCTACCTGAGTTTCTCCGCAAAGTGCTAG
AACAAAAGGTTGTTCTTCCATATTGTAACACATTTGCGTTATGCTATCTTCGCTAATCTCGGTATCTGCGTCA
ATGGCTAAGAGGTATTTGATGTCGTTCATTGGTCCGAGCTGTAAATCGGCAAGTGCATGAACGATGGTCGTTT
CAAGGCCGTTCAAAGGATCATCAGGATGACACCCAATTTTGTTGAGAAGACCAGTAATAAGGAGCTGTGAGTC
ACGTTTGCCACGGTTTCCAGCTTTTGGGCTCCCTTTCTCTATTGGTATACCACATTTCTCGATTACAATATAA
TGTAACATCTTGCCTGGATTTTTTTCACTTTTTTTTGTGCCGTAGTAAACCTTTGCCCGGTTGTGACTTCTTT
CGTCAGGACCAACGCTTGTACAATCGTATGTGATGTCCTGTGGGGATCTGGGGTACTGAAGAATGCTGGATAT
AACCTCTGGAGTGGACTTAGTTTCACCTTTTCCTTTGATTCGTAAAACAACGGAATAATGTTTGAAAAGGAGC
GGGAATTGACAGACAGCCAGTAATTATTATTTTACAACAAGGAACAGCTCTTACCAGTGACATCACCATCTGC
AACCACAATCAAAACTTTGTTTGAGTCTGGATAAGAGGTGTCCATGATGCTATCAATCGTCTTCCTGAGTTCC
TTGTCTCCTTCGTTGTAGCAAGGGACCATTATCATAACAGTTTGTTTTGTATGCTCGCTTTTGATTTTACGAC
TTTGTTGAAGGACGATCCCTCGGACAAGGTAGTATATCGAACAAAGACCTTGGATTGCCATGACTGCGACAAC
AATTCCCATGACAGCATATGATGTACCTTTGAGAGCCTGACAAATTCCGTCGCTTTAAAGAATAAAGAAGTGA
AAGACCACAATGTTAAATATAAGAGAAGAGGCATGAAAAAAGAAGCTGATACGATGTTTAGTTACCCACATAT
CTTCATCATCAAAAGAATCCTTAATATCAGCCTGGATATCGTCTTTAATATCAAAAAGGTTATCACTATCGAG
CGACCTTCCAGAACAATGCTCGACCGAAGATCCACAAAATCCATGCTGAGAGCAGCAGCTGCCATCAAAGCAA
AGTCCGTTACCTCGTTTCCCGTTGCCACAAGTGCCAAGAGGTGCTGAGTCAATGCCAAGACGTGCCCCAGACT
GCATCGATTCAACTCCACACTCAAGTAGTAATAAAAGTCCACCAAATCCAATAAACGCTGCGCAGGAACTCAA
CATGATGACACAGAGCGCGATTTTCTCCCTCCAAGCTTGTTTAGCCTCTTTTCGGGAGGCTTTGTCTTCTATA
TCGCGGCCAATGGACCAGAGTAAAGAGTCAGGTATAAAAAATGTACAGAGGCGGGAGAAGAATCGCCAGTTGC
ACCCCGTATCAGACATTTTAAATATAGACCTGTTATTCGTCGGAAAATAGCTCGCGGTTCAATACTGGTGTTG
GCGAGGAAAACTAATGCAGATTAGAGGGAAAGCGAGGGGTTAACAAATGAGAGAACGCTGATGCGGTGGCTTC
TGAGTAGAGTGGAGGAGATTTATTTTATGGGTTGCCATTTATTG
>TR445|c1_g1_i2 len=2272 path=[4410:0-368 4508:369-369 4461:370-409 4535:410-412 4530:413-434 4521:435-458 4506:459-460 4534:461-469 4520:470-517 @4505@!:518-607 4456:608-643 4533:644-658 4528:659-682 4518:683-858 4540:859-880 4480:881-909 4517:910-919 4502:920-943 4478:944-974 4452:975-1095 4293:1096-1119 4527:1120-1335 4541:1336-1352 4547:1353-1353 4500:1354-1465 4214:1466-1488 4449:1489-1529 4499:1530-1553 4515:1554-1714 4447:1715-1729 4537:1730-1758 4532:1759-1782 4526:1783-1857 4543:1858-1862 4472:1863-1878 4539:1879-1886 4536:1887-1923 4525:1924-1961 4544:1962-1962 4512:1963-1976 4394:1977-1986 4511:1987-2000 @4489@!:2001-2146 4177:2147-2193 @4464@!:2194-2271] [-1, 4410, 4508, 4461, 4535, 4530, 4521, 4506, 4534, 4520, 4505, 4456, 4533, 4528, 4518, 4540, 4480, 4517, 4502, 4478, 4452, 4293, 4527, 4541, 4547, 4500, 4214, 4449, 4499, 4515, 4447, 4537, 4532, 4526, 4543, 4472, 4539, 4536, 4525, 4544, 4512, 4394, 4511, 4489, 4177, 4464]

GGGTTGACTGAATGATCCCAACTGCACGGTACTTGGAATCAGCCAAATCAGACAGCTACAGTTCTTTTATGTA
TTTATTTGTAAATGACTTTCTGATGCTTTCTGGACACTTGAGGGTTAAAACACAGTTACCATTTATTCTGTAT
TGCATCAACTTGCCATTGCTCTCTCAGAACCAATTATCTGACTTACCATTCTAGATAGTACACGTCAGTCTAC
AATTTGATGCATTGACCAAGCCCTCCTAAGAACACTTATTGATTGCCAAAGTAGATACAAGAGGATGGAATAA
CTCAATTCCATATATTTGGGTATTAAAACTCTTAATATCTCATTACTTGGCAGTTGAACAAATAATTAGCTGC
ATTTTACTCCTTGCCATCATTATTACCAGCCACTCCGATGCACCATTGGTTTCATATAGCGCGCTGCCTCGTC
TGCATCGCCACACCATCCTCCCCAGCAGCAGCCCCAGCATCAACAACTCTTGTATCCACTCAACTAAGTCCCT
CTCTAGGTGGCTACTAACCGAGTAGATCCCCACGATACATCGTCCATATGCCAGAAGGCATAAATTGGCAAAA
TGAAGTAAAATATGGGCACACCGATCACATAAAAAGGAATGGACCAAAACAAGATATTCCAGTGTAAGCGTAG
AGTAGGGACAACAAGTTGAACAGCAACAGAAAACCCCAATATTATCTGTGCTAAAGTGTCCCCATTTCCATTA
AGTGCAAAGTATAGCATAATGCCCACGTATATGAATGCTACGGGAAGAATAAGAGTTCCTACCAAATCAAGTA
AGATAATGACCTTCATAGAGAAGCAGCATATTCCACACGTTTTCACTTTCCAAAGCCTGGCGGCCATGGGTTT
CATAAGAGTGTAGAAAAAAAAACAAGGATGAGTCTTTACAATTGGAAGATATGTTCAAAATGTTTGTTCAATTC
TTACTCAAGTAAATTATGGAACGTCGAATTGACCCATCTTCTTCTTTGACTAAGAAGAACAGAGAATGTGTCT
GGCACGACGGTCTGGCAAGTTGCTTCAGGTACGAAGGAGAGATTCATTTCGGGGAAGTGCCGCAGTAGAAGAG
TTGTCAGCATGCGGTCTTCTCCAAGATGGTACAAGTTCTTTGCGTGTAGTGAATTTATCTCAGCGTCATTAAT
AGCGTAAGGTATGTAGACAGAATCGCAGGCAAGAAGAGGTTCGTTTTCGTAATCAAACAGCCGGTACATTGTG
AAACATCCAGGGAGACATGTCACAGTGTTAAAAACAGACTCGAACGCTTTCTTCAAGTGGTGATTCGTGTAGT
ATTCAAATACTTGTATCCTGGTGACCCAGCTATCTGACGGGTTGGCTACCTGAGTTTCTCCGCAAAGTGCTAG
AACAAAAGGTTGTTCTTCCATATTGTAACACATTTGCGTTATGCTATCTTCGCTAATCTCGGTATCTGCGTCA
ATGGCTAAGAGGTATTTGATGTCGTTCATTGGTCCGAGCTGTAAATCGGCAAGTGCATGAACGATGGTCGTTT
CAAGGCCGTTCAAAGGATCATCAGGATGACACCCAATTTTGTTGAGAAGACCAGTAATAAGGAGCTGTGAGTC
ACGTTTGCCACGGTTTCCAGCTTTTGGGCTCCCTTTCTCTATTGGTATACCACATTTCTCGATTACAATATAA
TGTAACATCTTGCCTGGATTTTTTTCACTTTTTTTTGTGCCGTAGTAAACCTTTGCCCGGTTGTGACTTCTTT
CGTCAGGACCAACGCTTGTACAATCGTATGTGATGTCCTGTGGGGATCTGGGGTACTGAAGAATGCTGGATAT
AACCTCTGGAGTGGACTTAGTTTCACCTTTTCCAGTGACATCACCATCTGCAACCACAATCAAAACTTTGTTT
GAGTCTGGATAAGAGGTGTCCATGATGCTATCAATCGTCTTCCTGAGTTCCTTGTCTCCTTCGTTGTAGCAAG
GGACCATTATCATAACAGTTTGTTTTGTATGCTCGCTTTTGATTTTACGACTTTGTTGAAGGACGATCCCTCG
GACAAGGTAGTATATCGAACAAAGACCTTGGATTGCCATGACTGCGACAACAATTCCCATGACAGCATATGAT
GTACCTTTGAGAGCCTGACAAATTCCGTCGATATCTTCATCATCAAAAGAATCTTTAATATCAGCCTGGATAT
CGTCTTTAATATCAAAAAGGTTATCACTATCGAGCGACCTTCCAGAACAATGCTCGACCGAAGATCCACAAAA
TCCATGCTG
>TR445|c1_g1_i3 len=1681 path=[4179:0-368 4180:369-369 4485:370-412
4530:413-434 4521:435-458 4506:459-460 4534:461-469 4519:470-517
@4505@!:518-607 4456:608-643 4533:644-658 4528:659-682 4518:683-858
4540:859-880 4479:881-913 4517:914-923 4502:924-947 4478:948-978
4452:979-1099 4293:1100-1123 4527:1124-1339 4451:1340-1446 4547:1447-1447
4500:1448-1559 4450:1560-1635 4258:1636-1659 4259:1660-1680] [-1, 4179,
4180, 4485, 4530, 4521, 4506, 4534, 4519, 4505, 4456, 4533, 4528, 4518,
4540, 4479, 4517, 4502, 4478, 4452, 4293, 4527, 4451, 4547, 4500, 4450,
4258, 4259, -2]
GGGTTGACTGAATGATCCCAACTGCACGGTACTTGGAATCAGCCAAATCAGACAGCTACAGTTCTTTTATGTA
TTTATTTGTAAATGACTTTCTGATGCTTTCTGGACACTTGAGGGTTAAAACACAGTTACCATTTATTCTGTAT
TGCATCAACTTGCCATTGCTCTCTCAGAACCAATTATCTGACTTACCATTCTAGATAGTACACGTCAGTCTAC
AATTTGATGCATTGACCAAGCCCTCCTAAGAACACTTATTGATTGCCAAAGTAGATACAAGAGGATGGAATAA
CTCAATTCCATATATTTGGGTATTAAAACTCTTAATATCTCATTACTTGGCAGTTGAACAAATAATTAGCTGC
ATTTTCCTCCTTGCCATCATTATTCCCAGCCACTCCGATGCACCATTGGTTTCATATAGCGCGCTGCCTCGTC
TGCATCGCCACACCATCCTCCCCAGCAGCAGCTCCAGCATCAACAACTCTTGTATCGACTCAACTAAGTCCCT
CTCTAGGTGGCTACTAACCGAGTAGATCCCCACGATACATCGTCCATATGCCAGAAGGCATAAATTGGCAAAA
TGAAGTAAAATATGGGCACACCGATCACATAAAAAGGAATGGACCAAAACAAGATATTCCAGTGTAAGCGTAG
AGTAGGGACAACAAGTTGAACAGCAACAGAAAACCCCAATATTATCTGTGCTAAAGTGTCCCCATTTCCATTA
AGTGCAAAGTATAGCATAATGCCCACGTATATGAATGCTACGGGAAGAATAAGAGTTCCTACCAAATCAAGTA
AGATAATGACCTTCATAGAGAAGCAGCATATTCCACACGTTTTCACTTTCCAAAGCCTGGCGGCCATGGGTTT
CATAACATCGAGTGCAGAAAAAAAAACAAGGATGAGTCTTTACAATTGGAAGATATGTTCAAAATGTTTGTTCA
ATTCTTACTCAAGTAAATTATGGAACGTCGAATTGACCCATCTTCTTCTTTGACTAAGAAGAACAGAGAATGT
GTCTGGCACGACGGTCTGGCAAGTTGCTTCAGGTACGAAGGAGAGATTCATTTCGGGGAAGTGCCGCAGTAGA
AGAGTTGTCAGCATGCGGTCTTCTCCAAGATGGTACAAGTTCTTTGCGTGTAGTGAATTTATCTCAGCGTCAT
TAATAGCGTAAGGTATGTAGACAGAATCGCAGGCAAGAAGAGGTTCGTTTTCGTAATCAAACAGCCGGTACAT
TGTGAAACATCCAGGGAGACATGTCACAGTGTTAAAAACAGACTCGAACGCTTTCTTCAAGTGGTGATTCGTG
TAGTATTCAAATACTTGTATCCTGGTAAATGATAGGGGAAAGAGGCAGCAGCAGGTGAGGTCAGCTTAGTGAT

CGCTTGCGTATTGGTTCAACAGTGTGGATTTGACCTACCTGGTGACCCAGCTATCTGACGGGTTGGCTACCTG
AGTTTCTCCGCAAAGTGCTAGAACAAAAGGTTGTTCTTCCATATTGTAACACATTTGCGTTATGCTATCTTCG
CTAATCTCGGTATCTGCGTCAATGGCTCTGTTGGAATCAAATACAGCAAGGAGGAAGTGAGTAATATACAGCC
AAGATGATAGCTGGTAGAGAATAAACAACGTAAATCAATGATTGCCACTCACAAGAGGTATTTGATGTCGTTC
AT
>TR445|c1_g1_i4 len=3048 path=[4179:0-368 4180:369-369 4485:370-412
4530:413-434 4521:435-458 4506:459-460 4534:461-469 4520:470-517
@4505@!:518-607 4456:608-643 4533:644-658 4528:659-682 4518:683-858
4540:859-880 4480:881-909 4517:910-919 4502:920-943 4478:944-974
4452:975-1095 4293:1096-1119 4527:1120-1335 4541:1336-1352 4547:1353-1353
4500:1354-1465 4214:1466-1488 4449:1489-1529 4499:1530-1553 4515:1554-
1714 4448:1715-1730 4537:1731-1759 4532:1760-1783 4526:1784-1858
4542:1859-1865 4548:1866-1889 4549:1890-1912 4471:1913-1950 4442:1951-
1957 4384:1958-1973 4538:1974-1981 4536:1982-2018 4525:2019-2056
4544:2057-2057 4512:2058-2071 4394:2072-2081 4511:2082-2095 @4489@!:2096-
2241 4545:2242-2243 4488:2244-2304 4465:2305-2326 4546:2327-2327
4487:2328-2378 @4464@!:2379-2456 4522:2457-2566 @4509@!:2567-2859
4463:2860-2918 4432:2919-2921 4389:2922-2948 4390:2949-3047] [-1, 4179,
4180, 4485, 4530, 4521, 4506, 4534, 4520, 4505, 4456, 4533, 4528, 4518,
4540, 4480, 4517, 4502, 4478, 4452, 4293, 4527, 4541, 4547, 4500, 4214,
4449, 4499, 4515, 4448, 4537, 4532, 4526, 4542, 4548, 4549, 4471, 4442,
4384, 4538, 4536, 4525, 4544, 4512, 4394, 4511, 4489, 4545, 4488, 4465,
4546, 4487, 4464, 4522, 4509, 4463, 4432, 4389, 4390, -2]
GGGTTGACTGAATGATCCCAACTGCACGGTACTTGGAATCAGCCAAATCAGACAGCTACAGTTCTTTTATGTA
TTTATTTGTAAATGACTTTCTGATGCTTTCTGGACACTTGAGGGTTAAAACACAGTTACCATTTATTCTGTAT
TGCATCAACTTGCCATTGCTCTCTCAGAACCAATTATCTGACTTACCATTCTAGATAGTACACGTCAGTCTAC
AATTTGATGCATTGACCAAGCCCTCCTAAGAACACTTATTGATTGCCAAAGTAGATACAAGAGGATGGAATAA
CTCAATTCCATATATTTGGGTATTAAAACTCTTAATATCTCATTACTTGGCAGTTGAACAAATAATTAGCTGC
ATTTTCCTCCTTGCCATCATTATTCCCAGCCACTCCGATGCACCATTGGTTTCATATAGCGCGCTGCCTCGTC
TGCATCGCCACACCATCCTCCCCAGCAGCAGCCCCAGCATCAACAACTCTTGTATCCACTCAACTAAGTCCCT
CTCTAGGTGGCTACTAACCGAGTAGATCCCCACGATACATCGTCCATATGCCAGAAGGCATAAATTGGCAAAA
TGAAGTAAAATATGGGCACACCGATCACATAAAAAGGAATGGACCAAAACAAGATATTCCAGTGTAAGCGTAG
AGTAGGGACAACAAGTTGAACAGCAACAGAAAACCCCAATATTATCTGTGCTAAAGTGTCCCCATTTCCATTA
AGTGCAAAGTATAGCATAATGCCCACGTATATGAATGCTACGGGAAGAATAAGAGTTCCTACCAAATCAAGTA
AGATAATGACCTTCATAGAGAAGCAGCATATTCCACACGTTTTCACTTTCCAAAGCCTGGCGGCCATGGGTTT
CATAAGAGTGTAGAAAAAAAACAAGGATGAGTCTTTACAATTGGAAGATATGTTCAAAATGTTTGTTCAATTC
TTACTCAAGTAAATTATGGAACGTCGAATTGACCCATCTTCTTCTTTGACTAAGAAGAACAGAGAATGTGTCT
GGCACGACGGTCTGGCAAGTTGCTTCAGGTACGAAGGAGAGATTCATTTCGGGGAAGTGCCGCAGTAGAAGAG
TTGTCAGCATGCGGTCTTCTCCAAGATGGTACAAGTTCTTTGCGTGTAGTGAATTTATCTCAGCGTCATTAAT
AGCGTAAGGTATGTAGACAGAATCGCAGGCAAGAAGAGGTTCGTTTTCGTAATCAAACAGCCGGTACATTGTG
AAACATCCAGGGAGACATGTCACAGTGTTAAAAACAGACTCGAACGCTTTCTTCAAGTGGTGATTCGTGTAGT
ATTCAAATACTTGTATCCTGGTGACCCAGCTATCTGACGGGTTGGCTACCTGAGTTTCTCCGCAAAGTGCTAG
AACAAAAGGTTGTTCTTCCATATTGTAACACATTTGCGTTATGCTATCTTCGCTAATCTCGGTATCTGCGTCA
ATGGCTAAGAGGTATTTGATGTCGTTCATTGGTCCGAGCTGTAAATCGGCAAGTGCATGAACGATGGTCGTTT
CAAGGCCGTTCAAAGGATCATCAGGATGACACCCAATTTTGTTGAGAAGACCAGTAATAAGGAGCTGTGAGTC
ACGTTTGCCACGGTTTCCAGCTTTTGGGCTCCCTTTCTCTATTGGTATACCACATTTCTCGATTACAATATAA
TGTAACATCTTGCCTGGATTTTTTTCACTTTTTTTTTGTGCCGTAGTAAACCTTTGCCCGGTTGTGACTTCTT
TCGTCAGGACCAACGCTTGTACAATCGTATGTGATGTCCTGTGGGGATCTGGGGTACTGAAGAATGCTGGATA
TAACCTCTGGAGTGGACTTAGTTTCACCTTTTCCTTTGATTCGTAAAACAACGGAATAATGTTTGAAAAGGAG
CGGGAATTGACAGACGGCCAGTAATTATTATTTACAACAAGGAACAGCTCTTACCAGTGATATCACCATCTGC
AACCACAATCAAAACTTTGTTTGAGTCTGGATAAGAGGTGTCCATGATGCTATCAATCGTCTTCCTGAGTTCC
TTGTCTCCTTCGTTGTAGCAAGGGACCATTATCATAACAGTTTGTTTTGTATGCTCGCTTTTGATTTTACGAC
TTTGTTGAAGGACGATCCCTCGGACAAGGTAGTATATCGAACAAAGACCTTGGATTGCCATGACTGCGACAAC
AATTCCCATGACAGCATATGATGTACCTTTGAGAGCCTGACAAATTCCGTCGCTTTAAAGAATAAAGAAGTGA
AAGACCACAATGTTAAATATAAGAGAAGAGGCATGAAAAAAGAAGCTGATACGATGTTTAGTTACCCACATAT
CTTCATCATCAAAAGAATCCTTAATATCAGCCTGGATATCGTCTTTAATATCAAAAAGGTTATCACTATCGAG
CGACCTTCCAGAACAATGCTCGACCGAAGATCCACAAAATCCATGCTGTATGAAATCACAAAATACAATTATT
GGAACTTTTTAGGAATGAATGAATATGCAGGAGAGCTTCTTCTTTCCCTTTATTTCACGTACCTGAGAGCAGC
AGCTGCCATCAAAGCAAAGTCCGTTACCTCGTTTCCCGTTGCCACAAGTGCCAAGAGGTGCTGAGTCAATGCC
AAGACGTGCCCCAGACTGCATCGATTCAACTCCACACTCAAGTAGTAATAAAAGTCCACCAAATCCAATAAAC

GCTGCGCAGGAACTCAACATGATGACACAGAGCGCGATTTTCTCCCTCCAAGCTTGTTTAGCCTCTTTTCGGG
AGGCTTTGTCTTCTATATCGCGGCCAATGGACCAGAGTAAAGAGTCAGGTATAAAAAATGTACAGAGGCGGGA
GAAGAATCGCCAGATGCACCCCGTATCAGGCATTTTAAATATGGACCTCTTATTCGTCGGAAAATAGCTCGCG
GTCTGTTGTTGGCGAGGAAAACTAATGCAGATTAGAGGGAAAGCGAGGGGTTAACAAATGAGAGAACGCTGAT
GCGGTGGCTTCTGAGTAGAGTGGAGGAGATTTATTTTATGGGTTGCCATTTATTG
>TR445|c1_g1_i5 len=2367 path=[4179:0-368 4180:369-369 4485:370-412
4530:413-434 4521:435-458 4506:459-460 4534:461-469 4519:470-517
@4505@!:518-607 4456:608-643 4533:644-658 4528:659-682 4518:683-858
4540:859-880 4480:881-909 4517:910-919 4502:920-943 4478:944-974
4452:975-1095 4293:1096-1119 4527:1120-1335 4541:1336-1352 4547:1353-1353
4500:1354-1465 4214:1466-1488 4449:1489-1529 4499:1530-1553 4515:1554-
1714 4447:1715-1729 4537:1730-1758 4532:1759-1782 4526:1783-1857
4542:1858-1864 4548:1865-1888 4549:1889-1911 4493:1912-1950 4470:1951-
1957 4441:1958-1973 4539:1974-1981 4536:1982-2018 4525:2019-2056
4544:2057-2057 4524:2058-2081 4511:2082-2095 @4489@!:2096-2241 4177:2242-
2288 @4464@!:2289-2366] [-1, 4179, 4180, 4485, 4530, 4521, 4506, 4534,
4519, 4505, 4456, 4533, 4528, 4518, 4540, 4480, 4517, 4502, 4478, 4452,
4293, 4527, 4541, 4547, 4500, 4214, 4449, 4499, 4515, 4447, 4537, 4532,
4526, 4542, 4548, 4549, 4493, 4470, 4441, 4539, 4536, 4525, 4544, 4524,
4511, 4489, 4177, 4464]
GGGTTGACTGAATGATCCCAACTGCACGGTACTTGGAATCAGCCAAATCAGACAGCTACAGTTCTTTTATGTA
TTTATTTGTAAATGACTTTCTGATGCTTTCTGGACACTTGAGGGTTAAAACACAGTTACCATTTATTCTGTAT
TGCATCAACTTGCCATTGCTCTCTCAGAACCAATTATCTGACTTACCATTCTAGATAGTACACGTCAGTCTAC
AATTTGATGCATTGACCAAGCCCTCCTAAGAACACTTATTGATTGCCAAAGTAGATACAAGAGGATGGAATAA
CTCAATTCCATATATTTGGGTATTAAAACTCTTAATATCTCATTACTTGGCAGTTGAACAAATAATTAGCTGC
ATTTTCCTCCTTGCCATCATTATTCCCAGCCACTCCGATGCACCATTGGTTTCATATAGCGCGCTGCCTCGTC
TGCATCGCCACACCATCCTCCCCAGCAGCAGCTCCAGCATCAACAACTCTTGTATCGACTCAACTAAGTCCCT
CTCTAGGTGGCTACTAACCGAGTAGATCCCCACGATACATCGTCCATATGCCAGAAGGCATAAATTGGCAAAA
TGAAGTAAAATATGGGCACACCGATCACATAAAAAGGAATGGACCAAAACAAGATATTCCAGTGTAAGCGTAG
AGTAGGGACAACAAGTTGAACAGCAACAGAAAACCCCAATATTATCTGTGCTAAAGTGTCCCCATTTCCATTA
AGTGCAAAGTATAGCATAATGCCCACGTATATGAATGCTACGGGAAGAATAAGAGTTCCTACCAAATCAAGTA
AGATAATGACCTTCATAGAGAAGCAGCATATTCCACACGTTTTCACTTTCCAAAGCCTGGCGGCCATGGGTTT
CATAAGAGTGTAGAAAAAAAACAAGGATGAGTCTTTACAATTGGAAGATATGTTCAAAATGTTTGTTCAATTC
TTACTCAAGTAAATTATGGAACGTCGAATTGACCCATCTTCTTCTTTGACTAAGAAGAACAGAGAATGTGTCT
GGCACGACGGTCTGGCAAGTTGCTTCAGGTACGAAGGAGAGATTCATTTCGGGGAAGTGCCGCAGTAGAAGAG
TTGTCAGCATGCGGTCTTCTCCAAGATGGTACAAGTTCTTTGCGTGTAGTGAATTTATCTCAGCGTCATTAAT
AGCGTAAGGTATGTAGACAGAATCGCAGGCAAGAAGAGGTTCGTTTTCGTAATCAAACAGCCGGTACATTGTG
AAACATCCAGGGAGACATGTCACAGTGTTAAAAACAGACTCGAACGCTTTCTTCAAGTGGTGATTCGTGTAGT
ATTCAAATACTTGTATCCTGGTGACCCAGCTATCTGACGGGTTGGCTACCTGAGTTTCTCCGCAAAGTGCTAG
AACAAAAGGTTGTTCTTCCATATTGTAACACATTTGCGTTATGCTATCTTCGCTAATCTCGGTATCTGCGTCA
ATGGCTAAGAGGTATTTGATGTCGTTCATTGGTCCGAGCTGTAAATCGGCAAGTGCATGAACGATGGTCGTTT
CAAGGCCGTTCAAAGGATCATCAGGATGACACCCAATTTTGTTGAGAAGACCAGTAATAAGGAGCTGTGAGTC
ACGTTTGCCACGGTTTCCAGCTTTTGGGCTCCCTTTCTCTATTGGTATACCACATTTCTCGATTACAATATAA
TGTAACATCTTGCCTGGATTTTTTTCACTTTTTTTTGTGCCGTAGTAAACCTTTGCCCGGTTGTGACTTCTTT
CGTCAGGACCAACGCTTGTACAATCGTATGTGATGTCCTGTGGGGATCTGGGGTACTGAAGAATGCTGGATAT
AACCTCTGGAGTGGACTTAGTTTCACCTTTTCCTTTGATTCGTAAAACAACGGAATAATGTTTGAAAAGGAGC
GGGAATTGACAGACAGCCAGTAATTATTATTTTACAACAAGGAACAGCTCTTACCAGTGACATCACCATCTGC
AACCACAATCAAAACTTTGTTTGAGTCTGGATAAGAGGTGTCCATGATGCTATCAATCGTCTTCCTGAGTTCC
TTGTCTCCTTCGTTATAGCAAGGGACCATTATCATAACAGTTTGTTTTGTATGCTCGCTTTTGATTTTACGAC
TTTGTTGAAGGACGATCCCTCGGACAAGGTAGTATATCGAACAAAGACCTTGGATTGCCATGACTGCGACAAC
AATTCCCATGACAGCATATGATGTACCTTTGAGAGCCTGACAAATTCCGTCGATATCTTCATCATCAAAAGAA
TCTTTAATATCAGCCTGGATATCGTCTTTAATATCAAAAAGGTTATCACTATCGAGCGACCTTCCAGAACAAT
GCTCGACCGAAGATCCACAAAATCCATGCTG
>TR445|c1_g1_i6 len=2959 path=[4179:0-368 4180:369-369 4485:370-412
4530:413-434 4521:435-458 4506:459-460 4534:461-469 4520:470-517
@4505@!:518-607 4456:608-643 4533:644-658 4528:659-682 4518:683-858
4540:859-880 4480:881-909 4517:910-919 4502:920-943 4478:944-974
4452:975-1095 4293:1096-1119 4527:1120-1335 4541:1336-1352 4547:1353-1353
4500:1354-1465 4214:1466-1488 4449:1489-1529 4499:1530-1553 4515:1554-
1714 4447:1715-1729 4537:1730-1758 4532:1759-1782 4526:1783-1857

4543:1858-1862 4472:1863-1878 4539:1879-1886 4536:1887-1923 4525:1924-1961 4544:1962-1962 4512:1963-1976 4394:1977-1986 4511:1987-2000 @4489@!:2001-2146 4545:2147-2148 4488:2149-2209 4465:2210-2231 4546:2232-2232 4487:2233-2283 @4464@!:2284-2361 4522:2362-2471 @4509@!:2472-2764 4486:2765-2823 4462:2824-2826 4431:2827-2859 4291:2860-2958] [-1, 4179, 4180, 4485, 4530, 4521, 4506, 4534, 4520, 4505, 4456, 4533, 4528, 4518, 4540, 4480, 4517, 4502, 4478, 4452, 4293, 4527, 4541, 4547, 4500, 4214, 4449, 4499, 4515, 4447, 4537, 4532, 4526, 4543, 4472, 4539, 4536, 4525, 4544, 4512, 4394, 4511, 4489, 4545, 4488, 4465, 4546, 4487, 4464, 4522, 4509, 4486, 4462, 4431, 4291, -2]
GGGTTGACTGAATGATCCCAACTGCACGGTACTTGGAATCAGCCAAATCAGACAGCTACAGTTCTTTTATGTA
TTTATTTGTAAATGACTTTCTGATGCTTTCTGGACACTTGAGGGTTAAAACACAGTTACCATTTATTCTGTAT
TGCATCAACTTGCCATTGCTCTCTCAGAACCAATTATCTGACTTACCATTCTAGATAGTACACGTCAGTCTAC
AATTTGATGCATTGACCAAGCCCTCCTAAGAACACTTATTGATTGCCAAAGTAGATACAAGAGGATGGAATAA
CTCAATTCCATATATTTGGGTATTAAAACTCTTAATATCTCATTACTTGGCAGTTGAACAAATAATTAGCTGC
ATTTTCCTCCTTGCCATCATTATTCCCAGCCACTCCGATGCACCATTGGTTTCATATAGCGCGCTGCCTCGTC
TGCATCGCCACACCATCCTCCCCAGCAGCAGCCCCAGCATCAACAACTCTTGTATCCACTCAACTAAGTCCCT
CTCTAGGTGGCTACTAACCGAGTAGATCCCCACGATACATCGTCCATATGCCAGAAGGCATAAATTGGCAAAA
TGAAGTAAAATATGGGCACACCGATCACATAAAAAGGAATGGACCAAAACAAGATATTCCAGTGTAAGCGTAG
AGTAGGGACAACAAGTTGAACAGCAACAGAAAACCCCAATATTATCTGTGCTAAAGTGTCCCCATTTCCATTA
AGTGCAAAGTATAGCATAATGCCCACGTATATGAATGCTACGGGAAGAATAAGAGTTCCTACCAAATCAAGTA
AGATAATGACCTTCATAGAGAAGCAGCATATTCCACACGTTTTCACTTTCCAAAGCCTGGCGGCCATGGGTTT
CATAAGAGTGTAGAAAAAAAACAAGGATGAGTCTTTACAATTGGAAGATATGTTCAAAATGTTTGTTCAATTC
TTACTCAAGTAAATTATGGAACGTCGAATTGACCCATCTTCTTCTTTGACTAAGAAGAACAGAGAATGTGTCT
GGCACGACGGTCTGGCAAGTTGCTTCAGGTACGAAGGAGAGATTCATTTCGGGGAAGTGCCGCAGTAGAAGAG
TTGTCAGCATGCGGTCTTCTCCAAGATGGTACAAGTTCTTTGCGTGTAGTGAATTTATCTCAGCGTCATTAAT
AGCGTAAGGTATGTAGACAGAATCGCAGGCAAGAAGAGGTTCGTTTTCGTAATCAAACAGCCGGTACATTGTG
AAACATCCAGGGAGACATGTCACAGTGTTAAAAACAGACTCGAACGCTTTCTTCAAGTGGTGATTCGTGTAGT
ATTCAAATACTTGTATCCTGGTGACCCAGCTATCTGACGGGTTGGCTACCTGAGTTTCTCCGCAAAGTGCTAG
AACAAAAGGTTGTTCTTCCATATTGTAACACATTTGCGTTATGCTATCTTCGCTAATCTCGGTATCTGCGTCA
ATGGCTAAGAGGTATTTGATGTCGTTCATTGGTCCGAGCTGTAAATCGGCAAGTGCATGAACGATGGTCGTTT
CAAGGCCGTTCAAAGGATCATCAGGATGACACCCAATTTTGTTGAGAAGACCAGTAATAAGGAGCTGTGAGTC
ACGTTTGCCACGGTTTCCAGCTTTTGGGCTCCCTTTCTCTATTGGTATACCACATTTCTCGATTACAATATAA
TGTAACATCTTGCCTGGATTTTTTTCACTTTTTTTTGTGCCGTAGTAAACCTTTGCCCGGTTGTGACTTCTTT
CGTCAGGACCAACGCTTGTACAATCGTATGTGATGTCCTGTGGGGATCTGGGGTACTGAAGAATGCTGGATAT
AACCTCTGGAGTGGACTTAGTTTCACCTTTTCCAGTGACATCACCATCTGCAACCACAATCAAAACTTTGTTT
GAGTCTGGATAAGAGGTGTCCATGATGCTATCAATCGTCTTCCTGAGTTCCTTGTCTCCTTCGTTGTAGCAAG
GGACCATTATCATAACAGTTTGTTTTGTATGCTCGCTTTTGATTTTACGACTTTGTTGAAGGACGATCCCTCG
GACAAGGTAGTATATCGAACAAAGACCTTGGATTGCCATGACTGCGACAACAATTCCCATGACAGCATATGAT
GTACCTTTGAGAGCCTGACAAATTCCGTCGCTTTAAAGAATAAAGAAGTGAAAGACCACAATGTTAAATATAA
GAGAAGAGGCATGAAAAAAGAAGCTGATACGATGTTTAGTTACCCACATATCTTCATCATCAAAAGAATCCTT
AATATCAGCCTGGATATCGTCTTTAATATCAAAAAGGTTATCACTATCGAGCGACCTTCCAGAACAATGCTCG
ACCGAAGATCCACAAAATCCATGCTGTATGAAATCACAAAATACAATTATTGGAACTTTTTAGGAATGAATGA
ATATGCAGGAGAGCTTCTTCTTTCCCTTTATTTCACGTACCTGAGAGCAGCAGCTGCCATCAAAGCAAAGTCC
GTTACCTCGTTTCCCGTTGCCACAAGTGCCAAGAGGTGCTGAGTCAATGCCAAGACGTGCCCCAGACTGCATC
GATTCAACTCCACACTCAAGTAGTAATAAAAGTCCACCAAATCCAATAAACGCTGCGCAGGAACTCAACATGA
TGACACAGAGCGCGATTTTCTCCCTCCAAGCTTGTTTAGCCTCTTTTCGGGAGGCTTTGTCTTCTATATCGCG
GCCAATGGACCAGAGTAAAGAGTCAGGTATAAAAAATGTACAGAGGCGGGAGAAGAATCGCCAGTTGCACCCC
GTATCAGACATTTTAAATATAGACCTGTTATTCGTCGGAAAATAGCTCGCGGTTCAATACTGGTGTTGGCGAG
GAAAACTAATGCAGATTAGAGGGAAAGCGAGGGGTTAACAAATGAGAGAACGCTGATGCGGTGGCTTCTGAGT
AGAGTGGAGGAGATTTATTTTATGGGTTGCCATTTATTG
>TR445|c1_g1_i7 len=1244 path=[4235:0-25 4508:26-26 4461:27-66 4535:67-69 4530:70-91 4521:92-115 4506:116-117 4534:118-126 4520:127-174 @4505@!:175-264 4456:265-300 4533:301-315 4528:316-339 4518:340-515 4540:516-537 4480:538-566 4517:567-576 4502:577-600 4478:601-631 4452:632-752 4293:753-776 4527:777-992 4541:993-1009 4547:1010-1010 4500:1011-1122 4450:1123-1198 4258:1199-1222 4259:1223-1243] [-1, 4235, 4508, 4461, 4535, 4530, 4521, 4506, 4534, 4520, 4505, 4456, 4533, 4528, 4518, 4540, 4480, 4517, 4502, 4478, 4452, 4293, 4527, 4541, 4547, 4500, 4450, 4258, 4259, -2]

AGGTGAACAAATAATTAGCTGCATTTTACTCCTTGCCATCATTATTACCAGCCACTCCGATGCACCATTGGTT
TCATATAGCGCGCTGCCTCGTCTGCATCGCCACACCATCCTCCCCAGCAGCAGCCCCAGCATCAACAACTCTT
GTATCCACTCAACTAAGTCCCTCTCTAGGTGGCTACTAACCGAGTAGATCCCCACGATACATCGTCCATATGC
CAGAAGGCATAAATTGGCAAAATGAAGTAAAATATGGGCACACCGATCACATAAAAAGGAATGGACCAAAACA
AGATATTCCAGTGTAAGCGTAGAGTAGGGACAACAAGTTGAACAGCAACAGAAAACCCCAATATTATCTGTGC
TAAAGTGTCCCCATTTCCATTAAGTGCAAAGTATAGCATAATGCCCACGTATATGAATGCTACGGGAAGAATA
AGAGTTCCTACCAAATCAAGTAAGATAATGACCTTCATAGAGAAGCAGCATATTCCACACGTTTTCACTTTCC
AAAGCCTGGCGGCCATGGGTTTCATAAGAGTGTAGAAAAAAAACAAGGATGAGTCTTTACAATTGGAAGATAT
GTTCAAAATGTTTGTTCAATTCTTACTCAAGTAAATTATGGAACGTCGAATTGACCCATCTTCTTCTTTGACT
AAGAAGAACAGAGAATGTGTCTGGCACGACGGTCTGGCAAGTTGCTTCAGGTACGAAGGAGAGATTCATTTCG
GGGAAGTGCCGCAGTAGAAGAGTTGTCAGCATGCGGTCTTCTCCAAGATGGTACAAGTTCTTTGCGTGTAGTG
AATTTATCTCAGCGTCATTAATAGCGTAAGGTATGTAGACAGAATCGCAGGCAAGAAGAGGTTCGTTTTCGTA
ATCAAACAGCCGGTACATTGTGAAACATCCAGGGAGACATGTCACAGTGTTAAAAACAGACTCGAACGCTTTC
TTCAAGTGGTGATTCGTGTAGTATTCAAATACTTGTATCCTGGTGACCCAGCTATCTGACGGGTTGGCTACCT
GAGTTTCTCCGCAAAGTGCTAGAACAAAAGGTTGTTCTTCCATATTGTAACACATTTGCGTTATGCTATCTTC
GCTAATCTCGGTATCTGCGTCAATGGCTCTGTTGGAATCAAATACAGCAAGGAGGAAGTGAGTAATATACAGC
CAAGATGATAGCTGGTAGAGAATAAACAACGTAAATCAATGATTGCCACTCACAAGAGGTATTTGATGTCGTT
CAT
>TR445|c1_g1_i8 len=659 path=[4235:0-25 4508:26-26 4461:27-66 4535:67-69
4530:70-91 4521:92-115 4506:116-117 4534:118-126 4519:127-174
@4505@!:175-264 4457:265-300 4533:301-315 4528:316-339 4518:340-515
4321:516-537 4452:538-658] [-1, 4235, 4508, 4461, 4535, 4530, 4521, 4506,
4534, 4519, 4505, 4457, 4533, 4528, 4518, 4321, 4452]
AGGTGAACAAATAATTAGCTGCATTTTACTCCTTGCCATCATTATTACCAGCCACTCCGATGCACCATTGGTT
TCATATAGCGCGCTGCCTCGTCTGCATCGCCACACCATCCTCCCCAGCAGCAGCTCCAGCATCAACAACTCTT
GTATCGACTCAACTAAGTCCCTCTCTAGGTGGCTACTAACCGAGTAGATCCCCACGATACATCGTCCATATGC
CAGAAGGCATAAATTGGCAAAATGAAGTAAAATATGGGCACACCGACCACATAAAAAGCAATGGACCAAAACA
AGATATTCCAGTGTAAGCGTAGAGTAGGGACAACAAGTTGAACAGCAACAGAAAACCCCAATATTATCTGTGC
TAAAGTGTCCCCATTTCCATTAAGTGCAAAGTATAGCATAATGCCCACGTATATGAATGCTACGGGAAGAATA
AGAGTTCCTACCAAATCAAGTAAGATAATGACCTTCATAGAGAAGCAGCATATTCCACACGTTTTCACTTTCC
AAAGCTCAAGTAAATTATGGAACGTCGAATTGACCCATCTTCTTCTTTGACTAAGAAGAACAGAGAATGTGTC
TGGCACGACGGTCTGGCAAGTTGCTTCAGGTACGAAGGAGAGATTCATTTCGGGGAAGTGCCGCAGTAGAAGA
GT
>TR446|c0_g1_i1 len=751 path=[1185:0-171 1239:172-197 1252:198-224
1244:225-248 1249:249-398 1216:399-422 1243:423-458 1248:459-482
1257:483-486 1256:487-510 1255:511-525 1204:526-547 1233:548-650
1190:651-674 1191:675-692 1192:693-716 1193:717-750] [-1, 1185, 1239,
1252, 1244, 1249, 1216, 1243, 1248, 1257, 1256, 1255, 1204, 1233, 1190,
1191, 1192, 1193, -2]
TAACATTCTCAGCCATGGAAAATCGTTAAAAGACAAGTTAGATGCATACTATCTCAAGGTTGCACTGCTACAT
GCTCGCCAAGAGACAAATCCCGCTTATGAGACGTGCATAGAAGTTCTCCAGCAACTGGGAGAAACGATCCCGT
CATCAGTCGAACCAGAAATTATTGTGGAGAAGGTGGACAAGATCCAATCAAAAATGAAGAGACTAACGGAGAG
TGATTTCATGAATATCAAAACAATGGACAGTCCATTTCACTGCTCTCTGATGCAGTTCTACAATCAAGTTTGT
TATATATCTTATTTTGTCCACACTCCTATGCTAAATTGGTTTGCCTGTAAGTTAGTTGAAATCTCTTTTGAGC
ATGGCTTCAGCAAATATTCGGCATTGGGTATTTTACGATATTCAATGGCACTTGGAGGCAAACTCATTGACGA
TGCAGAGGAGGGTTATAGAGTCGGCAAGTTGGCATTGAAATTGTTGGATCGATTTGATGCCCCAGATTTATTG
CCAATGGCATACCTGTGTTATTATGGTTACATTGCGGTTCAAATCGAGCCATTTCAATCCTGTGGAGACATGT
TAAAACGAGGTTTTGAGATAGGACTTTCGATTGGGGATACACAAGTCGCCTTACTGAATGGGGTTCATTATAT
CCAAAAATGTTTTCTTAGTGGTACAAATCTTTCAATACTGAAGAAGGAATGCGATTATCAAATGCGACTCATT
GAAGCTCATTCTCAGCCGGTG
>TR446|c0_g1_i2 len=846 path=[1185:0-171 1239:172-197 1252:198-224
1244:225-248 1249:249-398 1216:399-422 1243:423-458 1248:459-482
1257:483-486 1256:487-510 1255:511-525 1258:526-539 1246:540-601
1240:602-642 1233:643-745 1190:746-769 1191:770-787 1192:788-811
1193:812-845] [-1, 1185, 1239, 1252, 1244, 1249, 1216, 1243, 1248, 1257,
1256, 1255, 1258, 1246, 1240, 1233, 1190, 1191, 1192, 1193, -2]
TAACATTCTCAGCCATGGAAAATCGTTAAAAGACAAGTTAGATGCATACTATCTCAAGGTTGCACTGCTACAT
GCTCGCCAAGAGACAAATCCCGCTTATGAGACGTGCATAGAAGTTCTCCAGCAACTGGGAGAAACGATCCCGT
CATCAGTCGAACCAGAAATTATTGTGGAGAAGGTGGACAAGATCCAATCAAAAATGAAGAGACTAACGGAGAG
TGATTTCATGAATATCAAAACAATGGACAGTCCATTTCACTGCTCTCTGATGCAGTTCTACAATCAAGTTTGT

TATATATCTTATTTTGTCCACACTCCTATGCTAAATTGGTTTGCCTGTAAGTTAGTTGAAATCTCTTTTGAGC
ATGGCTTCAGCAAATATTCGGCATTGGGTATTTTACGATATTCAATGGCACTTGGAGGCAAACTCATTGACGA
TGCAGAGGAGGGTTATAGAGTCGGCAAGTTGGCATTGAAATTGTTGGATCGATTTGATGCCCCAGATTTATTG
CCAATGGCATACCTGGTAAGCCAGTTTTTTTGCAAATAATGACATTTTATCTTGAACAACTAAAAATAATCAA
CCTTTTTGCATCTTCCAATTGGCGGATCGCCTAATAGTGTTATTATGGTTACATTGCGGTTCAAATCGAGCCA
TTTCAATCCTGTGGAGACATGTTAAAACGAGGTTTTGAGATAGGACTTTCGATTGGGGATACACAAGTCGCCT
TACTGAATGGGGTTCATTATATCCAAAAATGTTTTCTTAGTGGTACAAATCTTTCAATACTGAAGAAGGAATG
CGATTATCAAATGCGACTCATTGAAGCTCATTCTCAGCCGGTG
>TR446|c1_g1_i1 len=726 path=[6723:0-39 7715:40-173 7782:174-213
7827:214-234 7889:235-241 7861:242-265 7826:266-280 7609:281-289
7778:290-305 7954:306-321 7707:322-329 7860:330-331 7824:332-343
7777:344-345 7888:346-347 7823:348-382 7702:383-398 7956:399-403
7470:404-445 7820:446-448 7597:449-465 7698:466-470 7957:471-471
7296:472-483 7819:484-495 7773:496-507 7958:508-514 7922:515-523
7912:524-538 7901:539-539 7883:540-547 7981:548-558 7816:559-562
7686:563-586 7853:587-610 7815:611-619 7765:620-631 7682:632-638
7575:639-647 7432:648-662 7262:663-687 6992:688-725] [-1, 6723, 7715,
7782, 7827, 7889, 7861, 7826, 7609, 7778, 7954, 7707, 7860, 7824, 7777,
7888, 7823, 7702, 7956, 7470, 7820, 7597, 7698, 7957, 7296, 7819, 7773,
7958, 7922, 7912, 7901, 7883, 7981, 7816, 7686, 7853, 7815, 7765, 7682,
7575, 7432, 7262, 6992, -2]
TCACGCAAAAAGAGGACTTTTTTATCGGCACTGTCTTTTCATGAAGCAATTGAAAGGAGTAGTACTATGGAGG
AGGATCACAACAAAGCTGTTGATCAAACTGACAATATCGTTCAAAACAAAAACAAAAACAACAAGAAATTTAC
TTATGCAACACAACAAGTTTCGAGGGATAGCCTATCGTCAAACGACAAACCGTCCAAATACGTACCATTGAAG
GAATTAGGATTCCCATCCTCCCTTTCGTTACTCGTTACAAATCTACTCGATTGCGGATGGGGAGAATTTCGCC
CAGACAATGCCTTGCCTTCGTTGGAGGCAGCTAGTAAGGATTTGCATTTGCTCCTTCTCGATCCAGAACGCTT
TTTGTTTGATCGTATGATTCCTGGTCATAATCAAGGAGGCGGAGGAGGAGGTAAGGGGACAGGTTTGGATTTA
GATTACAAACAAAACAAGGTGTATGGGCGAGAGACGGAGAGGTCGCTTATTTCGGATGTTTTCTGTCGTGTGT
CGTTTACTGGGGAGAGCGAGGCGTTGTTTGTTGAAGGCTTCTCTGGGTCGGGAAAGTCATCTCTGGTCCAGAG
CGTTGTTGAATATATCGACGTTGCAGGCGCATACATTATACAAAAAAAGTTTGACGAGATTTCTCAAGAACGG
CCCATGTCAGTTGTGATTGCTGCATTCGATGATCTGTGCCACTCGATGAGCAAGAATAACTCTCCTCAA
>TR446|c1_g1_i2 len=2191 path=[5901:0-46 7715:47-180 7782:181-220
7827:221-241 7889:242-248 7323:249-287 7609:288-296 7778:297-312
7318:313-338 7605:339-350 7479:351-386 7313:387-389 7706:390-405
7477:406-469 7308:470-474 6547:475-475 6548:476-487 7819:488-499
7773:500-511 7958:512-518 7291:519-542 7690:543-543 7817:544-567
7770:568-582 7687:583-590 7444:591-614 7984:615-623 7678:624-665
7850:666-666 7811:667-691 7759:692-729 7919:730-738 7909:739-771
7564:772-783 7876:784-795 7846:796-821 7806:822-850 7670:851-874
7964:875-876 7986:877-877 7995:878-878 7240:879-924 7805:925-928
7750:929-962 7667:963-986 8015:987-990 8009:991-1014 7965:1015-1021
7874:1022-1028 7842:1029-1052 7801:1053-1061 7744:1062-1084 7872:1085-
1091 7798:1092-1132 7966:1133-1145 7797:1146-1154 7391:1155-1178
7796:1179-1196 7740:1197-1224 7539:1225-1265 7386:1266-1302 7870:1303-
1326 7839:1327-1361 7967:1362-1364 7738:1365-1373 7987:1374-1385
7968:1386-1388 7996:1389-1394 8002:1395-1413 7736:1414-1418 7941:1419-
1437 7934:1438-1441 7926:1442-1442 7916:1443-1466 7905:1467-1475
7891:1476-1499 7864:1500-1544 7524:1545-1609 7970:1610-1610 7787:1611-
1664 7641:1665-1678 7727:1679-1721 7863:1722-1736 7638:1737-1737
7786:1738-1743 7972:1744-1750 7635:1751-1790 7511:1791-1794 7355:1795-
1821 7169:1822-1842 7508:1843-1843 7632:1844-1877 7352:1878-1878
7784:1879-1903 7720:1904-1910 7629:1911-1934 7503:1935-1939 7718:1940-
1951 7344:1952-1952 7621:1953-1976 7156:1977-2007 6965:2008-2010
7336:2011-2019 7150:2020-2021 7618:2022-2034 7492:2035-2077 7332:2078-
2105 7977:2106-2120 5876:2121-2190] [-1, 5901, 7715, 7782, 7827, 7889,
7323, 7609, 7778, 7318, 7605, 7479, 7313, 7706, 7477, 7308, 6547, 6548,
7819, 7773, 7958, 7291, 7690, 7817, 7770, 7687, 7444, 7984, 7678, 7850,
7811, 7759, 7919, 7909, 7564, 7876, 7846, 7806, 7670, 7964, 7986, 7995,
7240, 7805, 7750, 7667, 8015, 8009, 7965, 7874, 7842, 7801, 7744, 7872,
7798, 7966, 7797, 7391, 7796, 7740, 7539, 7386, 7870, 7839, 7967, 7738,

7987, 7968, 7996, 8002, 7736, 7941, 7934, 7926, 7916, 7905, 7891, 7864, 7524, 7970, 7787, 7641, 7727, 7863, 7638, 7786, 7972, 7635, 7511, 7355, 7169, 7508, 7632, 7352, 7784, 7720, 7629, 7503, 7718, 7344, 7621, 7156, 6965, 7336, 7150, 7618, 7492, 7332, 7977, 5876, -2]
TCATTATCACGCAAAAAGAGGACTTTTTTTATCGGCACTGTCTTTTCATGAAGCAATTGAAAGGAGTAGTACT
ATGGAGGAGGATCACAACAAAGCTGTTGATCAAACTGACAATATCGTTCAAAACAAAAACAAAAACAACAAGA
AATTTACTTATGCAACACAACAAGTTTCGAGGGATAGCCTATCGTCAAACGACAAACCGTCCAAATACGTACC
ATTGAAGGAATTAGGATTCCCATCCTCCCTCTCGTTATTCGTTACGAATCTACTCGATTGCGGATGGGGAGAA
TTTCGCCCAGACAATGCCTTGTCATCGTTGGAGGCAGCTAGTAAGGATTTGCATTTGCTTCTTCTCGATCCGG
ATCGCTTTTTGTTTGATCGTATGATTCCTGGTCATAATCAAAGAGGAGGAGGAGGTAGGGGGGCGGGTTTGGA
TTTAGATTTCAAACAAAACAAGGTGTATGGGCGAGAGACGGAGAGGTCGCTTATTTCGGATGTTTTCTGTCGT
GTGTCGTTCACTGGGGAGAGCGAGGCGTTGTTTATTGAAGGCTTCTCTGGGTCGGGGAAGTCATCTCTGGTCC
AGAGCGTTGTTGAATATATCGACGTTGCAGGCGCATACATGATACAAAAAAAGTTTGATCAGATTTCTCAAGA
ACGGCCCATGTCAGTTGTGATTGCTGCATTCGATGGTCTGTGCCACTCGTTGAGCAAGAATAACTCTCCTCAA
GAATTGCATGCGATTGTAGTGGAACTCATGGAGGTGTTTGGGACTCAGCTTTCGGCACTGGTTCGATTGCTTC
CCAGTGTTGCGATGATAATGCCCAATATGAATGCCCAATCCGGTGATTATGGTCAAGATGCAGAGATCAACAT
GAGCAGTGTCTACTTCATTCTTCAACTGTTTGCACGGGTAATCTCGAGTAAATTACACCCTATTATGATCTTC
CTCGACGATCTACAATGGGCAGACAGTGCTTCGTTGGATGTGTTGCAGCACATCCTGTCCGACATCAAGGGTG
AAAGCTGCGTTTTCATGATTGGATCTTTCAGATCAAATGAGGTGAACGAGGATCATCCCGTGACCACCCTTAT
GAATTCCTTGGAGAGGTGCAACGTCCGATTGAGCAGATTGCATTTGGATGGCATGGATATTGAAGGCGTGAAC
CACATCATTTCGGATGCTCTGGGGACCTTCCCACGCATCTGCAAGCCCCTATCCCAAATTGTGCTGCGCAAAA
CAAAGGGCAATCCTCTATTTGTGCTGGAATGTTTGAGGTCATTGGTAGACCGTGACTTGCTGCACTACAGTAT
CCGGGAGCGACGTTGGGTTTGGGATACTGACATTATTAGTAGGGTAGATATCACGGATGATGTTTGCGAGCTT
CTCTCTGTAAAGATGATTGGTCTTCTGGAAAAAACTCAGGCAGCTCTGAAAATCTCATCTTGCTTTGGGACAA
GTGTGAATTCAACAATTATCAAAACTTTGATGTCGGCATCGCCTTTACTTCAAACCTTTCAGGAGGAACTTGA
CAAGGTCGTTGATGATGGCTTCATGGATAAGGACGACTTAGGCTCCACATATACATTTGTTCATGACAAGGTT
CGAGAAGCTGCATACAGACTTATTCCAGAGGAAGACAGGAATCGGTTCCACTACGATATTGGGATGCTTCTAT
TCAATACAATAGATGGGGACAGCATTGATGGTATCCTATTTTTTATGGTTGATCAAATAAACCACGGTACCAT
TTATCTCAGCAGCAAGCAGCGTATCACTGTCGCAGAGTTGAACCATGGAGCAGCTTCTAAGGCAGTGGGAAAC
TCAAACTTCGCAGCAGCATTCTTCTATTCCAATGCAGCAATGAAGCTCCTCCCCGCAAATCACTGGGAAAGTC
ATTACGATCTGAGTAGGAAGATCTTTTTGGTGCTTGGGAATGCTGCGCGCTCATATGGGCGTATCACAGAGGC
GACAAGGTAAATACTTATGTTGTCCCTTTATCACACAAGGATCCAAAAGTTTTGTTTTCATCTCCTTTCTTTT
ACTCTATTGAAAAGTGCATTGGATGAGATTCTCAAGCAAGGAAAATCGTTGAAAGATAAGTTAGATGCATACT
ATCTTAAGATAGCATTGTTGAACATACACCAAGAACTAAAGAAATCATTTGACACGTGCGTCGAAGTCCTTAA
A
>TR446|c1_g1_i3 len=1816 path=[6723:0-39 7715:40-173 7782:174-213 7827:214-234 7889:235-241 7323:242-280 7609:281-289 7778:290-305 7318:306-331 7605:332-343 7479:344-379 7313:380-382 7706:383-398 7477:399-462 7308:463-467 6547:468-468 6548:469-480 7819:481-492 7773:493-504 7958:505-511 7291:512-535 7690:536-536 7817:537-560 7770:561-575 7687:576-583 7444:584-607 7984:608-616 7678:617-658 7850:659-659 7811:660-684 7759:685-722 7919:723-731 7909:732-764 7564:765-776 7876:777-788 7846:789-814 7806:815-843 7670:844-867 7964:868-869 7986:870-870 7995:871-871 7240:872-917 7805:918-921 7750:922-955 7667:956-979 8015:980-983 8009:984-1007 7965:1008-1014 7874:1015-1021 7842:1022-1045 7801:1046-1054 7744:1055-1077 7872:1078-1084 7798:1085-1125 7966:1126-1138 7742:1139-1162 7871:1163-1165 7840:1166-1171 7796:1172-1189 7740:1190-1217 7538:1218-1258 7386:1259-1295 7870:1296-1319 7839:1320-1354 7967:1355-1357 7738:1358-1366 7204:1367-1434 7926:1435-1435 7916:1436-1459 7905:1460-1468 7891:1469-1492 7864:1493-1537 7969:1538-1597 7788:1598-1643 7187:1644-1737 7522:1738-1777 7728:1778-1808 7640:1809-1815] [-1, 6723, 7715, 7782, 7827, 7889, 7323, 7609, 7778, 7318, 7605, 7479, 7313, 7706, 7477, 7308, 6547, 6548, 7819, 7773, 7958, 7291, 7690, 7817, 7770, 7687, 7444, 7984, 7678, 7850, 7811, 7759, 7919, 7909, 7564, 7876, 7846, 7806, 7670, 7964, 7986, 7995, 7240, 7805, 7750, 7667, 8015, 8009, 7965, 7874, 7842, 7801, 7744, 7872, 7798, 7966, 7742, 7871, 7840, 7796, 7740, 7538, 7386, 7870, 7839, 7967, 7738, 7204, 7926, 7916, 7905, 7891, 7864, 7969, 7788, 7187, 7522, 7728, 7640]

TCACGCAAAAAGAGGACTTTTTTATCGGCACTGTCTTTTCATGAAGCAATTGAAAGGAGTAGTACTATGGAGG
AGGATCACAACAAAGCTGTTGATCAAACTGACAATATCGTTCAAAACAAAAACAAAAACAACAAGAAATTTAC
TTATGCAACACAACAAGTTTCGAGGGATAGCCTATCGTCAAACGACAAACCGTCCAAATACGTACCATTGAAG
GAATTAGGATTCCCATCCTCCCTCTCGTTATTCGTTACGAATCTACTCGATTGCGGATGGGGAGAATTTCGCC
CAGACAATGCCTTGTCATCGTTGGAGGCAGCTAGTAAGGATTTGCATTTGCTTCTTCTCGATCCGGATCGCTT
TTTGTTTGATCGTATGATTCCTGGTCATAATCAAAGAGGAGGAGGAGGTAGGGGGGCGGGTTTGGATTTAGAT
TTCAAACAAAACAAGGTGTATGGGCGAGAGACGGAGAGGTCGCTTATTTCGGATGTTTTCTGTCGTGTGTCGT
TCACTGGGGAGAGCGAGGCGTTGTTTATTGAAGGCTTCTCTGGGTCGGGGAAGTCATCTCTGGTCCAGAGCGT
TGTTGAATATATCGACGTTGCAGGCGCATACATGATACAAAAAAAGTTTGATCAGATTTCTCAAGAACGGCCC
ATGTCAGTTGTGATTGCTGCATTCGATGGTCTGTGCCACTCGTTGAGCAAGAATAACTCTCCTCAAGAATTGC
ATGCGATTGTAGTGGAACTCATGGAGGTGTTTGGGACTCAGCTTTCGGCACTGGTTCGATTGCTTCCCAGTGT
TGCGATGATAATGCCCAATATGAATGCCCAATCCGGTGATTATGGTCAAGATGCAGAGATCAACATGAGCAGT
GTCTACTTCATTCTTCAACTGTTTGCACGGGTAATCTCGAGTAAATTACACCCTATTATGATCTTCCTCGACG
ATCTACAATGGGCAGACAGTGCTTCGTTGGATGTGTTGCAGCACATCCTGTCCGACATCAAGGGTGAAAGCTG
CGTTTTCATGATTGGATCTTTCAGATCAAATGAGGTGAACGAGGATCATCCCGTGACCACCCTTATGAATTCC
TTGGAGAGGTGCAACGTCCGATTGAGCAGATTGCATTTGGATGGAATGGATATCGAAGGCGTGAACCACATCA
TTTCGGATGCTCTGGGGACCTTCCCACGCATCTGCAAGCCCCTATCCCAACTTGTGCTGCGCAAAACTAAGGG
CAATCCTCTATTTGTGCTGGAATGTTTGAGGTCATTGGTAGACCGTGACTTGCTGCACTACAGTATCCGGGAG
CGACGTTGGGTTTGGGATACTGACATTATTAGTAGGGTAGATATCACGGATGACGTTTGCGAGCTTCTCTCTA
TAAAGATGATTGGTCTTGTGGAAACAACTCAGGCAGCTCTGAAAATCTCATCTTGCTTTGGGACAAGTGTGAA
TTCAACAATTATCAAAACTTTGATGTCGGCATCGCCTTTACTTCAAACCTTTCAGGAGGAACTTGACAAGGTC
GTTGACGATGGCTTCATGGATACGGACGACTTAGGCTCCACATATAAATTTGTTCATGACAAGGTCAGAGAAG
CTGCGTATGGACTTATTCCAGAGGAAGATAGGAATCGGGTGAGGAATTTTGTGTATTTTCATCTAGTACTTAG
TGCATTGAATATCCATATCATTCATTCCATTTTTCAGTTCCACTACACTGTTGGGATGCTTCTATTCAATGCA
ATAGATGGGAAAAACATTGATAGCATCATATTTCTTATGGTTGATCAAATAAACCACGGTACCA
>TR446|c1_g1_i4 len=2190 path=[6723:0-39 7715:40-173 7782:174-213
7827:214-234 7889:235-241 7861:242-265 7826:266-280 7609:281-289
7778:290-305 7954:306-321 7707:322-329 7860:330-331 7824:332-343
7777:344-345 7888:346-347 7776:348-379 7703:380-382 7980:383-398
7956:399-403 7857:404-407 7301:408-445 7820:446-448 7597:449-465
7698:466-470 7957:471-471 7463:472-472 7884:473-495 7855:496-507
7818:508-514 7959:515-523 7771:524-547 7981:548-558 7816:559-562
7685:563-563 7581:564-578 7961:579-586 7983:587-596 7577:597-638
7879:639-661 7850:662-662 7811:663-687 7759:688-725 7919:726-734
7909:735-767 7564:768-779 7876:780-791 7846:792-817 7806:818-846
7670:847-870 7964:871-872 7986:873-873 7995:874-874 7240:875-920
7805:921-924 7750:925-958 7667:959-982 8015:983-986 8009:987-1010
7965:1011-1017 7874:1018-1024 7842:1025-1048 7801:1049-1057 7744:1058-
1080 7872:1081-1087 7798:1088-1128 7966:1129-1141 7797:1142-1150
7391:1151-1174 7796:1175-1192 7740:1193-1220 7539:1221-1261 7386:1262-
1298 7870:1299-1322 7839:1323-1357 7967:1358-1360 7738:1361-1369
7204:1370-1437 7926:1438-1438 7916:1439-1462 7905:1463-1471 7891:1472-
1495 7864:1496-1540 7969:1541-1600 7788:1601-1646 7642:1647-1668
7522:1669-1708 7728:1709-1739 7640:1740-1746 7518:1747-1756 7725:1757-
1760 7636:1761-1786 7513:1787-1790 7357:1791-1839 7634:1840-1873
7510:1874-1921 7890:1922-1930 7862:1931-1935 7829:1936-1947 7973:1948-
1948 7989:1949-1963 7998:1964-1972 7495:1973-2018 7335:2019-2020
7149:2021-2076 7617:2077-2104 7976:2105-2119 6091:2120-2189] [-1, 6723,
7715, 7782, 7827, 7889, 7861, 7826, 7609, 7778, 7954, 7707, 7860, 7824,
7777, 7888, 7776, 7703, 7980, 7956, 7857, 7301, 7820, 7597, 7698, 7957,
7463, 7884, 7855, 7818, 7959, 7771, 7981, 7816, 7685, 7581, 7961, 7983,
7577, 7879, 7850, 7811, 7759, 7919, 7909, 7564, 7876, 7846, 7806, 7670,
7964, 7986, 7995, 7240, 7805, 7750, 7667, 8015, 8009, 7965, 7874, 7842,
7801, 7744, 7872, 7798, 7966, 7797, 7391, 7796, 7740, 7539, 7386, 7870,
7839, 7967, 7738, 7204, 7926, 7916, 7905, 7891, 7864, 7969, 7788, 7642,
7522, 7728, 7640, 7518, 7725, 7636, 7513, 7357, 7634, 7510, 7890, 7862,
7829, 7973, 7989, 7998, 7495, 7335, 7149, 7617, 7976, 6091]
TCACGCAAAAAGAGGACTTTTTTATCGGCACTGTCTTTTCATGAAGCAATTGAAAGGAGTAGTACTATGGAGG
AGGATCACAACAAAGCTGTTGATCAAACTGACAATATCGTTCAAAACAAAAACAAAAACAACAAGAAATTTAC
TTATGCAACACAACAAGTTTCGAGGGATAGCCTATCGTCAAACGACAAACCGTCCAAATACGTACCATTGAAG

GAATTAGGATTCCCATCCTCCCTTTCGTTACTCGTTACAAATCTACTCGATTGCGGATGGGGAGAATTTCGCC
CAGACAATGCCTTGCCTTCGTTGGAGGCAGCTAGTAAGGATTTGCATTTGCTCCTTTTCGATCCAGATCGCTT
TTTGTTTGATCGTATGATTCCTGGTCATAATCAAGGAGGAGGAGGAGGAGGTAAGGGTACAGGTTTGGATTTA
GATTACAAACAAAACAAGGTGTATGGGCGAGAGAGGGAGAGGTCGCTTATTTCGGATGTTTTCTGTCGTGTGT
CGTTTACTGGGGAAAGCGAGGCGTTGTTTGTTGAAGGCTTCTCTGGGTCGGGGAAGTCATCTCTGGTCCAGAG
CGTCGTTGAATATCTCGATGTTGCAGGCGCTCACATTATACAAAAAAAGTTTGACCAGATTTCTCAAGAACGG
CCCATGTCAGTTGTGATTGCTGCATTCGATGGTCTGTGCCACTCGTTGAGCAAGAATAACTCTCCTCAAGAAT
TGCATGCGATTGTAGTGGAACTCATGGAGGTGTTTGGGACTCAGCTTTCGGCACTGGTTCGATTGCTTCCCAG
TGTTGCGATGATAATGCCCAATATGAATGCCCAATCCGGTGATTATGGTCAAGATGCAGAGATCAACATGAGC
AGTGTCTACTTCATTCTTCAACTGTTTGCACGGGTAATCTCGAGTAAATTACACCCTATTATGATCTTCCTCG
ACGATCTACAATGGGCAGACAGTGCTTCGTTGGATGTGTTGCAGCACATCCTGTCCGACATCAAGGGTGAAAG
CTGCGTTTTCATGATTGGATCTTTCAGATCAAATGAGGTGAACGAGGATCATCCCGTGACCACCCTTATGAAT
TCCTTGGAGAGGTGCAACGTCCGATTGAGCAGATTGCATTTGGATGGCATGGATATTGAAGGCGTGAACCACA
TCATTTCGGATGCTCTGGGGACCTTCCCACGCATCTGCAAGCCCCTATCCCAAATTGTGCTGCGCAAAACAAA
GGGCAATCCTCTATTTGTGCTGGAATGTTTGAGGTCATTGGTAGACCGTGACTTGCTGCACTACAGTATCCGG
GAGCGACGTTGGGTTTGGGATACTGACATTATTAGTAGGGTAGATATCACGGATGACGTTTGCGAGCTTCTCT
CTATAAAGATGATTGGTCTTGTGGAAACAACTCAGGCAGCTCTGAAAATCTCATCTTGCTTTGGGACAAGTGT
GAATTCAACAATTATCAAAACTTTGATGTCGGCATCGCCTTTACTTCAAACCTTTCAGGAGGAACTTGACAAG
GTCGTTGACGATGGCTTCATGGATACGGACGACTTAGGCTCCACATATAAATTTGTTCATGACAAGGTCAGAG
AAGCTGCGTATGGACTTATTCCAGAGGAAGATAGGAATCGGTTCCACTACACTGTTGGGATGCTTCTATTCAA
TGCAATAGATGGGAAAAACATTGATAGCATCATATTTCTTATGGTTGATCAAATAAACCACGGTACCACTTAT
CTCAGCAGCGATCAGCGTATCACTGTCGCAGAGTTGAACTACGGAGCAGCTTCTAAGGCAGTGGAAAACTCAA
ACTTCGCAGCAGCATTCTTCTATTCCAATGCAGCAATGAAGCTCCTCCCTGAAAATCACTGGGAAAGTCATTA
TGATCTGAGCAGGAAGATCTTTTTGGTGCTTGGGAATGCTGCGCGCTCATACGGGCGTATCGCAGAAGCGACA
AGGTAAATGACTTATGTTGTCCCTATTTATCACACAAGGATCCAAAAGTTCTGTTTTCATCTCCTTTCTCTAA
CTCTATTGAAAAGTGCGTTGGATGAGATTCTCAAGCATGGAAAATCGTTGAAAGATAAGTTAGATGCATACTA
TCTTAAGATAGCATTGTTGAACATACACCAAGAACTAAAGAAATCATTTGACACGTGCGTCGAAGTCCTTAAA
>TR446|c1_g1_i5 len=2172 path=[6723:0-39 7715:40-173 7782:174-213
7827:214-234 7889:235-241 7861:242-265 7826:266-280 7609:281-289
7778:290-305 7954:306-321 7707:322-329 7860:330-331 7824:332-343
7777:344-345 7888:346-347 7776:348-379 7703:380-382 7980:383-398
7956:399-403 7857:404-407 7301:408-445 7820:446-448 7597:449-465
7698:466-470 7957:471-471 7463:472-472 7884:473-495 7855:496-507
7818:508-514 7959:515-523 7771:524-547 7981:548-558 7688:559-602
7448:603-632 7278:633-633 7768:634-640 7684:641-671 7578:672-677
7900:678-685 7882:686-695 7852:696-709 7813:710-718 7762:719-730
7571:731-737 7879:738-760 7850:761-761 7811:762-786 7759:787-824
7919:825-833 7909:834-866 7564:867-878 7876:879-890 7846:891-916
7806:917-945 7670:946-969 7964:970-971 7986:972-972 7995:973-973
7240:974-1019 7805:1020-1023 7750:1024-1057 7667:1058-1081 8015:1082-1085
8009:1086-1109 7965:1110-1116 7874:1117-1123 7842:1124-1147 7801:1148-
1156 7744:1157-1179 7872:1180-1186 7798:1187-1227 7966:1228-1240
7797:1241-1249 7391:1250-1273 7796:1274-1291 7740:1292-1319 7538:1320-
1360 7386:1361-1397 7870:1398-1421 7839:1422-1456 7967:1457-1459
7738:1460-1468 7204:1469-1536 7926:1537-1537 7916:1538-1561 7905:1562-
1570 7891:1571-1594 7864:1595-1639 7969:1640-1699 7788:1700-1745
7642:1746-1767 7522:1768-1807 7728:1808-1838 7640:1839-1845 7518:1846-
1855 7725:1856-1859 7636:1860-1885 7513:1886-1889 7357:1890-1938
7634:1939-1972 7510:1973-2020 7890:2021-2029 7862:2030-2034 7829:2035-
2046 7973:2047-2047 7989:2048-2062 7998:2063-2071 7153:2072-2091
7617:2092-2119 7976:2120-2134 7991:2135-2171] [-1, 6723, 7715, 7782,
7827, 7889, 7861, 7826, 7609, 7778, 7954, 7707, 7860, 7824, 7777, 7888,
7776, 7703, 7980, 7956, 7857, 7301, 7820, 7597, 7698, 7957, 7463, 7884,
7855, 7818, 7959, 7771, 7981, 7688, 7448, 7278, 7768, 7684, 7578, 7900,
7882, 7852, 7813, 7762, 7571, 7879, 7850, 7811, 7759, 7919, 7909, 7564,
7876, 7846, 7806, 7670, 7964, 7986, 7995, 7240, 7805, 7750, 7667, 8015,
8009, 7965, 7874, 7842, 7801, 7744, 7872, 7798, 7966, 7797, 7391, 7796,
7740, 7538, 7386, 7870, 7839, 7967, 7738, 7204, 7926, 7916, 7905, 7891,
7864, 7969, 7788, 7642, 7522, 7728, 7640, 7518, 7725, 7636, 7513, 7357,

7634, 7510, 7890, 7862, 7829, 7973, 7989, 7998, 7153, 7617, 7976, 7991, -2]
TCACGCAAAAAGAGGACTTTTTTATCGGCACTGTCTTTTCATGAAGCAATTGAAAGGAGTAGTACTATGGAGG
AGGATCACAACAAAGCTGTTGATCAAACTGACAATATCGTTCAAAACAAAAACAAAAACAACAAGAAATTTAC
TTATGCAACACAACAAGTTTCGAGGGATAGCCTATCGTCAAACGACAAACCGTCCAAATACGTACCATTGAAG
GAATTAGGATTCCCATCCTCCCTTTCGTTACTCGTTACAAATCTACTCGATTGCGGATGGGGAGAATTTCGCC
CAGACAATGCCTTGCCTTCGTTGGAGGCAGCTAGTAAGGATTTGCATTTGCTCCTTTTCGATCCAGATCGCTT
TTTGTTTGATCGTATGATTCCTGGTCATAATCAAGGAGGAGGAGGAGGAGGTAAGGGTACAGGTTTGGATTTA
GATTACAAACAAAACAAGGTGTATGGGCGAGAGAGGGAGAGGTCGCTTATTTCGGATGTTTTCTGTCGTGTGT
CGTTTACTGGGGAAAGCGAGGCGTTGTTTGTTGAAGGCTTCTCTGGGTGAGAGATAGAGCCTTTATTTTTTAT
TCGTTTTGTTTTATTCGACTCGTTCGTTGCAGAAGTGGTGGATACTAATAATATCGTAATTTCCCCCCTCAGG
TCGGGGAAGTCATCTCTGGTCCAGAGCGTCGTTGAATATATCGACGTTGCAGGCGCATACATTATACAAAAAA
AGTTTGACCAGATTTCTCAAGAACGGCCCATGTCAGTTGTGATTGCTGCATTCGATGGTCTGTGCCACTCGTT
GAGCAAGAATAACTCTCCTCAAGAATTGCATGCGATTGTAGTGGAACTCATGGAGGTGTTTGGGACTCAGCTT
TCGGCACTGGTTCGATTGCTTCCCAGTGTTGCGATGATAATGCCCAATATGAATGCCCAATCCGGTGATTATG
GTCAAGATGCAGAGATCAACATGAGCAGTGTCTACTTCATTCTTCAACTGTTTGCACGGGTAATCTCGAGTAA
ATTACACCCTATTATGATCTTCCTCGACGATCTACAATGGGCAGACAGTGCTTCGTTGGATGTGTTGCAGCAC
ATCCTGTCCGACATCAAGGGTGAAAGCTGCGTTTTCATGATTGGATCTTTCAGATCAAATGAGGTGAACGAGG
ATCATCCCGTGACCACCCTTATGAATTCCTTGGAGAGGTGCAACGTCCGATTGAGCAGATTGCATTTGGATGG
CATGGATATTGAAGGCGTGAACCACATCATTTCGGATGCTCTGGGGACCTTCCCACGCATCTGCAAGCCCCTA
TCCCAACTTGTGCTGCGCAAAACTAAGGGCAATCCTCTATTTGTGCTGGAATGTTTGAGGTCATTGGTAGACC
GTGACTTGCTGCACTACAGTATCCGGGAGCGACGTTGGGTTTGGGATACTGACATTATTAGTAGGGTAGATAT
CACGGATGACGTTTGCGAGCTTCTCTCTATAAAGATGATTGGTCTTGTGGAAACAACTCAGGCAGCTCTGAAA
ATCTCATCTTGCTTTGGGACAAGTGTGAATTCAACAATTATCAAAACTTTGATGTCGGCATCGCCTTTACTTC
AAACCTTTCAGGAGGAACTTGACAAGGTCGTTGACGATGGCTTCATGGATACGGACGACTTAGGCTCCACATA
TAAATTTGTTCATGACAAGGTCAGAGAAGCTGCGTATGGACTTATTCCAGAGGAAGATAGGAATCGGTTCCAC
TACACTGTTGGGATGCTTCTATTCAATGCAATAGATGGGAAAAACATTGATAGCATCATATTTCTTATGGTTG
ATCAAATAAACCACGGTACCACTTATCTCAGCAGCGATCAGCGTATCACTGTCGCAGAGTTGAACTACGGAGC
AGCTTCTAAGGCAGTGGAAAACTCAAACTTCGCAGCAGCATTCTTCTATTCCAATGCAGCAATGAAGCTCCTC
CCTGAAAATCACTGGGAAAGTCATTATGATCTGAGCAGGAAGATCTTTTTGGTGCTTGGGAATGCTGCGCGCT
CATACGGGCGTATCGCAGAAGCGACAAGTGCGTTGGATGAGATTCTCAAGCATGGAAAATCGTTGAAAGATAA
GTTAGATGCATACTATCTCAAGATAGCATTGTTGCACACACACCAAGAACTGAAG
>TR446|c1_g1_i6 len=992 path=[6723:0-39 7715:40-173 7782:174-213 7827:214-234 7889:235-241 7861:242-265 7826:266-280 7609:281-289 7778:290-305 7954:306-321 7707:322-329 7860:330-331 7824:332-343 7777:344-345 7888:346-347 7776:348-379 7703:380-382 7980:383-398 7956:399-403 7857:404-407 7301:408-445 7820:446-448 7597:449-465 7698:466-470 7957:471-471 7463:472-472 7884:473-495 7855:496-507 7818:508-514 7959:515-523 7771:524-547 7981:548-558 7816:559-562 7685:563-563 7581:564-578 7961:579-586 7983:587-596 7577:597-638 7879:639-661 7850:662-662 7811:663-687 7759:688-725 7919:726-734 7909:735-767 7564:768-779 7876:780-791 7846:792-817 7806:818-846 7963:847-848 7242:849-870 7558:871-872 7752:873-873 7668:874-875 7557:876-899 7751:900-920 7666:921-924 7804:925-931 7875:932-938 6506:939-991] [-1, 6723, 7715, 7782, 7827, 7889, 7861, 7826, 7609, 7778, 7954, 7707, 7860, 7824, 7777, 7888, 7776, 7703, 7980, 7956, 7857, 7301, 7820, 7597, 7698, 7957, 7463, 7884, 7855, 7818, 7959, 7771, 7981, 7816, 7685, 7581, 7961, 7983, 7577, 7879, 7850, 7811, 7759, 7919, 7909, 7564, 7876, 7846, 7806, 7963, 7242, 7558, 7752, 7668, 7557, 7751, 7666, 7804, 7875, 6506, -2]
TCACGCAAAAAGAGGACTTTTTTATCGGCACTGTCTTTTCATGAAGCAATTGAAAGGAGTAGTACTATGGAGG
AGGATCACAACAAAGCTGTTGATCAAACTGACAATATCGTTCAAAACAAAAACAAAAACAACAAGAAATTTAC
TTATGCAACACAACAAGTTTCGAGGGATAGCCTATCGTCAAACGACAAACCGTCCAAATACGTACCATTGAAG
GAATTAGGATTCCCATCCTCCCTTTCGTTACTCGTTACAAATCTACTCGATTGCGGATGGGGAGAATTTCGCC
CAGACAATGCCTTGCCTTCGTTGGAGGCAGCTAGTAAGGATTTGCATTTGCTCCTTTTCGATCCAGATCGCTT
TTTGTTTGATCGTATGATTCCTGGTCATAATCAAGGAGGAGGAGGAGGAGGTAAGGGTACAGGTTTGGATTTA
GATTACAAACAAAACAAGGTGTATGGGCGAGAGAGGGAGAGGTCGCTTATTTCGGATGTTTTCTGTCGTGTGT
CGTTTACTGGGGAAAGCGAGGCGTTGTTTGTTGAAGGCTTCTCTGGGTCGGGGAAGTCATCTCTGGTCCAGAG
CGTCGTTGAATATCTCGATGTTGCAGGCGCTCACATTATACAAAAAAAGTTTGACCAGATTTCTCAAGAACGG
CCCATGTCAGTTGTGATTGCTGCATTCGATGGTCTGTGCCACTCGTTGAGCAAGAATAACTCTCCTCAAGAAT

TGCATGCGATTGTAGTGGAACTCATGGAGGTGTTTGGGACTCAGCTTTCGGCACTGGTTCGATTGCTTCCCAG
TGTTGCGATGATAATGCCCAATATGAATGCCCAATCCGGTGATTGTGGTCAAGATGCAGAGATCAACATGACT
AGTGTCTACTTCATTCTTCAATTGTTTGCACGGGTAATCTCGAGTAAATCACACCCTATTATGGTGAGAGCGC
GCCACACTGTGCTTGTATATGGATATAAGTTCGAGAGATCGGA
>TR446|c1_g1_i7 len=231 path=[6098:0-47 7615:48-85 7489:86-94 7828:95-127
7612:128-139 7876:140-151 7846:152-177 7806:178-206 7963:207-208
7242:209-230] [-1, 6098, 7615, 7489, 7828, 7612, 7876, 7846, 7806, 7963,
7242]
AGATTTCTCAAGAACGGCCCATGTCAGTTGTGATTGCTGCATTCGATGATCTGTGCCACTCGATGAGCAAGAA
TAACTCTCCTCAAGAATTGCATACGATTATAGTGGAACTCATGCAGGTGTTTGGGACTCAGCTTTCGGCACTG
GTTCGATTGCTTCCCAGTGTTGCGATGATAATGCCCAATATGAATGCCCAATCCGGTGATTGTGGTCAAGATG
CAGAGATCAACA
>TR446|c1_g1_i8 len=2205 path=[6723:0-39 7715:40-173 7782:174-213
7827:214-234 7889:235-241 7861:242-265 7826:266-280 7609:281-289
7778:290-305 7954:306-321 7707:322-329 7860:330-331 7824:332-343
7777:344-345 7888:346-347 7776:348-379 7703:380-382 7980:383-398
7956:399-403 7857:404-407 7301:408-445 7820:446-448 7597:449-465
7698:466-470 7957:471-471 7463:472-472 7884:473-495 7855:496-507
7818:508-514 7959:515-523 7771:524-547 7981:548-558 7688:559-602
7448:603-632 7278:633-633 7768:634-640 7684:641-671 7578:672-677
7900:678-685 7882:686-695 7852:696-709 7813:710-718 7762:719-730
7571:731-737 7879:738-760 7850:761-761 7811:762-786 7759:787-824
7919:825-833 7909:834-866 7564:867-878 7876:879-890 7846:891-916
7806:917-945 7670:946-969 7964:970-971 7986:972-972 7995:973-973
7240:974-1019 7805:1020-1023 7750:1024-1057 7667:1058-1081 8015:1082-1085
8009:1086-1109 7965:1110-1116 7874:1117-1123 7842:1124-1147 7801:1148-
1156 7744:1157-1179 7872:1180-1186 7798:1187-1227 7966:1228-1240
7797:1241-1249 7391:1250-1273 7796:1274-1291 7740:1292-1319 7539:1320-
1360 7386:1361-1397 7870:1398-1421 7839:1422-1456 7967:1457-1459
7738:1460-1468 7987:1469-1480 7968:1481-1483 7996:1484-1489 8002:1490-
1508 7736:1509-1513 7941:1514-1532 7934:1533-1536 7926:1537-1537
7916:1538-1561 7905:1562-1570 7891:1571-1594 7864:1595-1639 7524:1640-
1704 7970:1705-1705 7787:1706-1759 7641:1760-1773 7727:1774-1816
7863:1817-1831 7638:1832-1832 7786:1833-1838 7972:1839-1845 7635:1846-
1885 7511:1886-1889 7355:1890-1916 7169:1917-1937 7508:1938-1938
7632:1939-1972 7352:1973-1973 7784:1974-1998 7720:1999-2005 7629:2006-
2029 7503:2030-2034 7718:2035-2046 7344:2047-2047 7621:2048-2071
7340:2072-2078 7716:2079-2081 7620:2082-2091 7494:2092-2119 7975:2120-
2134 6079:2135-2204] [-1, 6723, 7715, 7782, 7827, 7889, 7861, 7826, 7609,
7778, 7954, 7707, 7860, 7824, 7777, 7888, 7776, 7703, 7980, 7956, 7857,
7301, 7820, 7597, 7698, 7957, 7463, 7884, 7855, 7818, 7959, 7771, 7981,
7688, 7448, 7278, 7768, 7684, 7578, 7900, 7882, 7852, 7813, 7762, 7571,
7879, 7850, 7811, 7759, 7919, 7909, 7564, 7876, 7846, 7806, 7670, 7964,
7986, 7995, 7240, 7805, 7750, 7667, 8015, 8009, 7965, 7874, 7842, 7801,
7744, 7872, 7798, 7966, 7797, 7391, 7796, 7740, 7539, 7386, 7870, 7839,
7967, 7738, 7987, 7968, 7996, 8002, 7736, 7941, 7934, 7926, 7916, 7905,
7891, 7864, 7524, 7970, 7787, 7641, 7727, 7863, 7638, 7786, 7972, 7635,
7511, 7355, 7169, 7508, 7632, 7352, 7784, 7720, 7629, 7503, 7718, 7344,
7621, 7340, 7716, 7620, 7494, 7975, 6079, -2]
TCACGCAAAAAGAGGACTTTTTTATCGGCACTGTCTTTTCATGAAGCAATTGAAAGGAGTAGTACTATGGAGG
AGGATCACAACAAAGCTGTTGATCAAACTGACAATATCGTTCAAAACAAAAACAAAAACAACAAGAAATTTAC
TTATGCAACACAACAAGTTTCGAGGGATAGCCTATCGTCAAACGACAAACCGTCCAAATACGTACCATTGAAG
GAATTAGGATTCCCATCCTCCCTTTCGTTACTCGTTACAAATCTACTCGATTGCGGATGGGGAGAATTTCGCC
CAGACAATGCCTTGCCTTCGTTGGAGGCAGCTAGTAAGGATTTGCATTTGCTCCTTTTCGATCCAGATCGCTT
TTTGTTTGATCGTATGATTCCTGGTCATAATCAAGGAGGAGGAGGAGGAGGTAAGGGTACAGGTTTGGATTTA
GATTACAAACAAAACAAGGTGTATGGGCGAGAGAGGGAGAGGTCGCTTATTTCGGATGTTTTCTGTCGTGTGT
CGTTTACTGGGGAAAGCGAGGCGTTGTTTGTTGAAGGCTTCTCTGGGTGAGAGATAGAGCCTTTATTTTTTAT
TCGTTTTGTTTTATTCGACTCGTTCGTTGCAGAAGTGGTGGATACTAATAATATCGTAATTTCCCCCCTCAGG
TCGGGGAAGTCATCTCTGGTCCAGAGCGTCGTTGAATATATCGACGTTGCAGGCGCATACATTATACAAAAAA
AGTTTGACCAGATTTCTCAAGAACGGCCCATGTCAGTTGTGATTGCTGCATTCGATGGTCTGTGCCACTCGTT

GAGCAAGAATAACTCTCCTCAAGAATTGCATGCGATTGTAGTGGAACTCATGGAGGTGTTTGGGACTCAGCTT
TCGGCACTGGTTCGATTGCTTCCCAGTGTTGCGATGATAATGCCCAATATGAATGCCCAATCCGGTGATTATG
GTCAAGATGCAGAGATCAACATGAGCAGTGTCTACTTCATTCTTCAACTGTTTGCACGGGTAATCTCGAGTAA
ATTACACCCTATTATGATCTTCCTCGACGATCTACAATGGGCAGACAGTGCTTCGTTGGATGTGTTGCAGCAC
ATCCTGTCCGACATCAAGGGTGAAAGCTGCGTTTTCATGATTGGATCTTTCAGATCAAATGAGGTGAACGAGG
ATCATCCCGTGACCACCCTTATGAATTCCTTGGAGAGGTGCAACGTCCGATTGAGCAGATTGCATTTGGATGG
CATGGATATTGAAGGCGTGAACCACATCATTTCGGATGCTCTGGGGACCTTCCCACGCATCTGCAAGCCCCTA
TCCCAAATTGTGCTGCGCAAAACAAAGGGCAATCCTCTATTTGTGCTGGAATGTTTGAGGTCATTGGTAGACC
GTGACTTGCTGCACTACAGTATCCGGGAGCGACGTTGGGTTTGGGATACTGACATTATTAGTAGGGTAGATAT
CACGGATGATGTTTGCGAGCTTCTCTCTGTAAAGATGATTGGTCTTCTGGAAAAAACTCAGGCAGCTCTGAAA
ATCTCATCTTGCTTTGGGACAAGTGTGAATTCAACAATTATCAAAACTTTGATGTCGGCATCGCCTTTACTTC
AAACCTTTCAGGAGGAACTTGACAAGGTCGTTGATGATGGCTTCATGGATAAGGACGACTTAGGCTCCACATA
TACATTTGTTCATGACAAGGTTCGAGAAGCTGCATACAGACTTATTCCAGAGGAAGACAGGAATCGGTTCCAC
TACGATATTGGGATGCTTCTATTCAATACAATAGATGGGGACAGCATTGATGGTATCCTATTTTTTATGGTTG
ATCAAATAAACCACGGTACCATTTATCTCAGCAGCAAGCAGCGTATCACTGTCGCAGAGTTGAACCATGGAGC
AGCTTCTAAGGCAGTGGGAAACTCAAACTTCGCAGCAGCATTCTTCTATTCCAATGCAGCAATGAAGCTCCTC
CCCGCAAATCACTGGGAAAGTCATTACGATCTGAGTAGGAAGATCTTTTTGGTGCTTGGGAATGCTGCGCGCT
CATATGGGCGTATCACAGAGGCGACAAGTGCATTGGATGAGATTCTCAAGCAAGGAAAATCGTTGAAAGATAA
GTTAGATGCATACTATCTTAAGATAGCATTGTTGAACATACACCAAGAACTAAAGAAATCATTTGACACGTGC
GTCGAAGTCCTTAAA
>TR446|c1_g1_i9 len=2287 path=[6723:0-39 7715:40-173 7782:174-213
7827:214-234 7889:235-241 7861:242-265 7826:266-280 7609:281-289
7778:290-305 7954:306-321 7707:322-329 7860:330-331 7824:332-343
7777:344-345 7888:346-347 7776:348-379 7703:380-382 7980:383-398
7956:399-403 7857:404-407 7301:408-445 7820:446-448 7597:449-465
7698:466-470 7957:471-471 7463:472-472 7884:473-495 7855:496-507
7818:508-514 7959:515-523 7771:524-547 7981:548-558 7688:559-602
7448:603-632 7278:633-633 7768:634-640 7684:641-671 7578:672-677
7900:678-685 7882:686-695 7852:696-709 7813:710-718 7762:719-730
7571:731-737 7879:738-760 7850:761-761 7811:762-786 7759:787-824
7919:825-833 7909:834-866 7564:867-878 7876:879-890 7846:891-916
7806:917-945 7670:946-969 7964:970-971 7986:972-972 7995:973-973
7240:974-1019 7805:1020-1023 7750:1024-1057 7667:1058-1081 8015:1082-1085
8009:1086-1109 7965:1110-1116 7874:1117-1123 7842:1124-1147 7801:1148-
1156 7744:1157-1179 7872:1180-1186 7798:1187-1227 7966:1228-1240
7797:1241-1249 7391:1250-1273 7796:1274-1291 7740:1292-1319 7539:1320-
1360 7386:1361-1397 7870:1398-1421 7839:1422-1456 7967:1457-1459
7738:1460-1468 7204:1469-1536 7926:1537-1537 7916:1538-1561 7905:1562-
1570 7891:1571-1594 7864:1595-1639 7524:1640-1704 7970:1705-1705
7787:1706-1759 7641:1760-1773 7183:1774-1804 7831:1805-1832 7786:1833-
1838 7972:1839-1845 7359:1846-1855 7785:1856-1859 7723:1860-1894
7633:1895-1937 7170:1938-1938 7509:1939-1972 7722:1973-1973 7631:1974-
1998 7507:1999-2005 7721:2006-2010 7630:2011-2020 7890:2021-2029
7862:2030-2034 7829:2035-2046 7974:2047-2050 7499:2051-2103 7990:2104-
2106 7619:2107-2130 7492:2131-2173 7332:2174-2201 7977:2202-2216
5876:2217-2286] [-1, 6723, 7715, 7782, 7827, 7889, 7861, 7826, 7609,
7778, 7954, 7707, 7860, 7824, 7777, 7888, 7776, 7703, 7980, 7956, 7857,
7301, 7820, 7597, 7698, 7957, 7463, 7884, 7855, 7818, 7959, 7771, 7981,
7688, 7448, 7278, 7768, 7684, 7578, 7900, 7882, 7852, 7813, 7762, 7571,
7879, 7850, 7811, 7759, 7919, 7909, 7564, 7876, 7846, 7806, 7670, 7964,
7986, 7995, 7240, 7805, 7750, 7667, 8015, 8009, 7965, 7874, 7842, 7801,
7744, 7872, 7798, 7966, 7797, 7391, 7796, 7740, 7539, 7386, 7870, 7839,
7967, 7738, 7204, 7926, 7916, 7905, 7891, 7864, 7524, 7970, 7787, 7641,
7183, 7831, 7786, 7972, 7359, 7785, 7723, 7633, 7170, 7509, 7722, 7631,
7507, 7721, 7630, 7890, 7862, 7829, 7974, 7499, 7990, 7619, 7492, 7332,
7977, 5876, -2]
TCACGCAAAAAGAGGACTTTTTTATCGGCACTGTCTTTTCATGAAGCAATTGAAAGGAGTAGTACTATGGAGG
AGGATCACAACAAAGCTGTTGATCAAACTGACAATATCGTTCAAAACAAAAACAAAAACAACAAGAAATTTAC
TTATGCAACACAACAAGTTTCGAGGGATAGCCTATCGTCAAACGACAAACCGTCCAAATACGTACCATTGAAG
GAATTAGGATTCCCATCCTCCCTTTCGTTACTCGTTACAAATCTACTCGATTGCGGATGGGGAGAATTTCGCC

CAGACAATGCCTTGCCTTCGTTGGAGGCAGCTAGTAAGGATTTGCATTTGCTCCTTTTCGATCCAGATCGCTT
TTTGTTTGATCGTATGATTCCTGGTCATAATCAAGGAGGAGGAGGAGGAGGTAAGGGTACAGGTTTGGATTTA
GATTACAAACAAAACAAGGTGTATGGGCGAGAGAGGGAGAGGTCGCTTATTTCGGATGTTTTCTGTCGTGTGT
CGTTTACTGGGGAAAGCGAGGCGTTGTTTGTTGAAGGCTTCTCTGGGTGAGAGATAGAGCCTTTATTTTTTAT
TCGTTTTGTTTTATTCGACTCGTTCGTTGCAGAAGTGGTGGATACTAATAATATCGTAATTTCCCCCCTCAGG
TCGGGGAAGTCATCTCTGGTCCAGAGCGTCGTTGAATATATCGACGTTGCAGGCGCATACATTATACAAAAAA
AGTTTGACCAGATTTCTCAAGAACGGCCCATGTCAGTTGTGATTGCTGCATTCGATGGTCTGTGCCACTCGTT
GAGCAAGAATAACTCTCCTCAAGAATTGCATGCGATTGTAGTGGAACTCATGGAGGTGTTTGGGACTCAGCTT
TCGGCACTGGTTCGATTGCTTCCCAGTGTTGCGATGATAATGCCCAATATGAATGCCCAATCCGGTGATTATG
GTCAAGATGCAGAGATCAACATGAGCAGTGTCTACTTCATTCTTCAACTGTTTGCACGGGTAATCTCGAGTAA
ATTACACCCTATTATGATCTTCCTCGACGATCTACAATGGGCAGACAGTGCTTCGTTGGATGTGTTGCAGCAC
ATCCTGTCCGACATCAAGGGTGAAAGCTGCGTTTTCATGATTGGATCTTTCAGATCAAATGAGGTGAACGAGG
ATCATCCCGTGACCACCCTTATGAATTCCTTGGAGAGGTGCAACGTCCGATTGAGCAGATTGCATTTGGATGG
CATGGATATTGAAGGCGTGAACCACATCATTTCGGATGCTCTGGGGACCTTCCCACGCATCTGCAAGCCCCTA
TCCCAAATTGTGCTGCGCAAAACAAAGGGCAATCCTCTATTTGTGCTGGAATGTTTGAGGTCATTGGTAGACC
GTGACTTGCTGCACTACAGTATCCGGGAGCGACGTTGGGTTTGGGATACTGACATTATTAGTAGGGTAGATAT
CACGGATGACGTTTGCGAGCTTCTCTCTATAAAGATGATTGGTCTTGTGGAAACAACTCAGGCAGCTCTGAAA
ATCTCATCTTGCTTTGGGACAAGTGTGAATTCAACAATTATCAAAACTTTGATGTCGGCATCGCCTTTACTTC
AAACCTTTCAGGAGGAACTTGACAAGGTCGTTGATGATGGCTTCATGGATAAGGACGACTTAGGCTCCACATA
TACATTTGTTCATGACAAGGTTCGAGAAGCTGCATACAGACTTATTCCAGAGGAAGACAGGAATCGGTTCCAC
TACGATATTGGGATGCTTCTATATAATACAATAGATGGGGAAAGCATTGATGGCATCATATTTTTTATGGTTG
ATCAAATAAACCACGGTACCACTTATCTCAGCAGCAAGCAACGTATCACTATGGCAGAGTTGAACCATCGCGC
AGCTTCTAAGGCAGTGAGAAACTCAAACTTCGCAGCAGCATTCTTCTATTCCAATGCAGCAATGAAGCTCCTC
CCCATAAATCACTGGGAAAGTCATTACGATCTGAGCAGGAAGATCTTTTTGGTGCTTGGGAATGCTGCGCGCT
CATGCGGACGTGTCACAGAGGCGACAAGGTAAATTAGTTATGTTGTCCCTTTATCACACAAGGGTCCAAAAGT
TTTGTTTTCATCTCCTTTCTTTTACTCTATTGAAAAGTGCATTGGATGAGATTCTCAAGCAAGGAAAATCGTT
GAAAGATAAGTTAGATGCATACTATCTTAAGATAGCATTGTTGAACATACACCAAGAACTAAAGAAATCATTT
GACACGTGCGTCGAAGTCCTTAAA
>TR446|c1_g1_i10 len=2106 path=[6723:0-39 7715:40-173 7782:174-213
7827:214-234 7889:235-241 7861:242-265 7826:266-280 7609:281-289
7778:290-305 7954:306-321 7707:322-329 7860:330-331 7824:332-343
7777:344-345 7888:346-347 7776:348-379 7703:380-382 7980:383-398
7956:399-403 7857:404-407 7301:408-445 7820:446-448 7597:449-465
7698:466-470 7957:471-471 7463:472-472 7884:473-495 7855:496-507
7818:508-514 7959:515-523 7771:524-547 7981:548-558 7816:559-562
7685:563-563 7581:564-578 7961:579-586 7983:587-596 7577:597-638
7879:639-661 7850:662-662 7811:663-687 7759:688-725 7919:726-734
7909:735-767 7564:768-779 7876:780-791 7846:792-817 7806:818-846
7670:847-870 7964:871-872 7986:873-873 7995:874-874 7240:875-920
7805:921-924 7750:925-958 7667:959-982 8015:983-986 8009:987-1010
7965:1011-1017 7874:1018-1024 7842:1025-1048 7801:1049-1057 7744:1058-
1080 7872:1081-1087 7798:1088-1128 7966:1129-1141 7797:1142-1150
7391:1151-1174 7796:1175-1192 7740:1193-1220 7539:1221-1261 7386:1262-
1298 7870:1299-1322 7839:1323-1357 7967:1358-1360 7738:1361-1369
7204:1370-1437 7926:1438-1438 7916:1439-1462 7905:1463-1471 7891:1472-
1495 7864:1496-1540 7524:1541-1605 7970:1606-1606 7787:1607-1660
7641:1661-1674 7727:1675-1717 7863:1718-1732 7638:1733-1733 7786:1734-
1739 7972:1740-1746 7635:1747-1786 7511:1787-1790 7355:1791-1817
7169:1818-1838 7508:1839-1839 7632:1840-1873 7352:1874-1874 7784:1875-
1899 7720:1900-1906 7629:1907-1930 7503:1931-1935 7718:1936-1947
7344:1948-1948 7621:1949-1972 7340:1973-1979 7716:1980-1982 7620:1983-
1992 7494:1993-2020 7975:2021-2035 6079:2036-2105] [-1, 6723, 7715, 7782,
7827, 7889, 7861, 7826, 7609, 7778, 7954, 7707, 7860, 7824, 7777, 7888,
7776, 7703, 7980, 7956, 7857, 7301, 7820, 7597, 7698, 7957, 7463, 7884,
7855, 7818, 7959, 7771, 7981, 7816, 7685, 7581, 7961, 7983, 7577, 7879,
7850, 7811, 7759, 7919, 7909, 7564, 7876, 7846, 7806, 7670, 7964, 7986,
7995, 7240, 7805, 7750, 7667, 8015, 8009, 7965, 7874, 7842, 7801, 7744,
7872, 7798, 7966, 7797, 7391, 7796, 7740, 7539, 7386, 7870, 7839, 7967,
7738, 7204, 7926, 7916, 7905, 7891, 7864, 7524, 7970, 7787, 7641, 7727,
7863, 7638, 7786, 7972, 7635, 7511, 7355, 7169, 7508, 7632, 7352, 7784,

7720, 7629, 7503, 7718, 7344, 7621, 7340, 7716, 7620, 7494, 7975, 6079, -2]
TCACGCAAAAAGAGGACTTTTTATCGGCACTGTCTTTTCATGAAGCAATTGAAAGGAGTAGTACTATGGAGG
AGGATCACAACAAAGCTGTTGATCAAACTGACAATATCGTTCAAAACAAAAACAAAAACAACAAGAAATTTAC
TTATGCAACACAACAAGTTTCGAGGGATAGCCTATCGTCAAACGACAAACCGTCCAAATACGTACCATTGAAG
GAATTAGGATTCCCATCCTCCCTTTCGTTACTCGTTACAAATCTACTCGATTGCGGATGGGGAGAATTTCGCC
CAGACAATGCCTTGCCTTCGTTGGAGGCAGCTAGTAAGGATTTGCATTTGCTCCTTTTCGATCCAGATCGCTT
TTTGTTTGATCGTATGATTCCTGGTCATAATCAAGGAGGAGGAGGAGGAGGTAAGGGTACAGGTTTGGATTTA
GATTACAAACAAAACAAGGTGTATGGGCGAGAGAGGGAGAGGTCGCTTATTTCGGATGTTTTCTGTCGTGTGT
CGTTTACTGGGGAAAGCGAGGCGTTGTTTGTTGAAGGCTTCTCTGGGTCGGGGAAGTCATCTCTGGTCCAGAG
CGTCGTTGAATATCTCGATGTTGCAGGCGCTCACATTATACAAAAAAAGTTTGACCAGATTTCTCAAGAACGG
CCCATGTCAGTTGTGATTGCTGCATTCGATGGTCTGTGCCACTCGTTGAGCAAGAATAACTCTCCTCAAGAAT
TGCATGCGATTGTAGTGGAACTCATGGAGGTGTTTGGGACTCAGCTTTCGGCACTGGTTCGATTGCTTCCCAG
TGTTGCGATGATAATGCCCAATATGAATGCCCAATCCGGTGATTATGGTCAAGATGCAGAGATCAACATGAGC
AGTGTCTACTTCATTCTTCAACTGTTTGCACGGGTAATCTCGAGTAAATTACACCCTATTATGATCTTCCTCG
ACGATCTACAATGGGCAGACAGTGCTTCGTTGGATGTGTTGCAGCACATCCTGTCCGACATCAAGGGTGAAAG
CTGCGTTTTCATGATTGGATCTTTCAGATCAAATGAGGTGAACGAGGATCATCCCGTGACCACCCTTATGAAT
TCCTTGGAGAGGTGCAACGTCCGATTGAGCAGATTGCATTTGGATGGCATGGATATTGAAGGCGTGAACCACA
TCATTTCGGATGCTCTGGGGACCTTCCCACGCATCTGCAAGCCCCTATCCCAAATTGTGCTGCGCAAAACAAA
GGGCAATCCTCTATTTGTGCTGGAATGTTTGAGGTCATTGGTAGACCGTGACTTGCTGCACTACAGTATCCGG
GAGCGACGTTGGGTTTGGGATACTGACATTATTAGTAGGGTAGATATCACGGATGACGTTTGCGAGCTTCTCT
CTATAAAGATGATTGGTCTTGTGGAAACAACTCAGGCAGCTCTGAAAATCTCATCTTGCTTTGGGACAAGTGT
GAATTCAACAATTATCAAAACTTTGATGTCGGCATCGCCTTTACTTCAAACCTTTCAGGAGGAACTTGACAAG
GTCGTTGATGATGGCTTCATGGATAAGGACGACTTAGGCTCCACATATACATTTGTTCATGACAAGGTTCGAG
AAGCTGCATACAGACTTATTCCAGAGGAAGACAGGAATCGGTTCCACTACGATATTGGGATGCTTCTATTCAA
TACAATAGATGGGGACAGCATTGATGGTATCCTATTTTTTATGGTTGATCAAATAAACCACGGTACCATTTAT
CTCAGCAGCAAGCAGCGTATCACTGTCGCAGAGTTGAACCATGGAGCAGCTTCTAAGGCAGTGGGAAACTCAA
ACTTCGCAGCAGCATTCTTCTATTCCAATGCAGCAATGAAGCTCCTCCCCGCAAATCACTGGGAAAGTCATTA
CGATCTGAGTAGGAAGATCTTTTTGGTGCTTGGGAATGCTGCGCGCTCATATGGGCGTATCACAGAGGCGACA
AGTGCATTGGATGAGATTCTCAAGCAAGGAAAATCGTTGAAAGATAAGTTAGATGCATACTATCTTAAGATAG
CATTGTTGAACATACACCAAGAACTAAAGAAATCATTTGACACGTGCGTCGAAGTCCTTAAA
>TR446|c1_g1_i12 len=996 path=[5901:0-46 7715:47-180 7782:181-220 7827:221-241 7889:242-248 7323:249-287 7609:288-296 7778:297-312 7318:313-338 7605:339-350 7479:351-386 7313:387-389 7706:390-405 7477:406-469 7308:470-474 6547:475-475 6548:476-487 7819:488-499 7773:500-511 7958:512-518 7291:519-542 7690:543-543 7817:544-567 7770:568-582 7687:583-590 7444:591-614 7984:615-623 7678:624-665 7850:666-666 7811:667-691 7759:692-729 7919:730-738 7909:739-771 7877:772-783 7847:784-793 7671:794-821 7806:822-850 7963:851-852 7242:853-874 7558:875-876 7752:877-877 7668:878-879 7557:880-903 7751:904-924 7666:925-928 7804:929-935 7875:936-942 6506:943-995] [-1, 5901, 7715, 7782, 7827, 7889, 7323, 7609, 7778, 7318, 7605, 7479, 7313, 7706, 7477, 7308, 6547, 6548, 7819, 7773, 7958, 7291, 7690, 7817, 7770, 7687, 7444, 7984, 7678, 7850, 7811, 7759, 7919, 7909, 7877, 7847, 7671, 7806, 7963, 7242, 7558, 7752, 7668, 7557, 7751, 7666, 7804, 7875, 6506, -2]
TCATTATCACGCAAAAAGAGGACTTTTTTATCGGCACTGTCTTTTCATGAAGCAATTGAAAGGAGTAGTACT
ATGGAGGAGGATCACAACAAAGCTGTTGATCAAACTGACAATATCGTTCAAAACAAAAACAAAAACAACAAGA
AATTTACTTATGCAACACAACAAGTTTCGAGGGATAGCCTATCGTCAAACGACAAACCGTCCAAATACGTACC
ATTGAAGGAATTAGGATTCCCATCCTCCCTCTCGTTATTCGTTACGAATCTACTCGATTGCGGATGGGGAGAA
TTTCGCCCAGACAATGCCTTGTCATCGTTGGAGGCAGCTAGTAAGGATTTGCATTTGCTTCTTCTCGATCCGG
ATCGCTTTTTGTTTGATCGTATGATTCCTGGTCATAATCAAAGAGGAGGAGGAGGTAGGGGGGCGGGTTTGGA
TTTAGATTTCAAACAAAACAAGGTGTATGGGCGAGAGACGGAGAGGTCGCTTATTTCGGATGTTTTCTGTCGT
GTGTCGTTCACTGGGGAGAGCGAGGCGTTGTTTATTGAAGGCTTCTCTGGGTCGGGGAAGTCATCTCTGGTCC
AGAGCGTTGTTGAATATATCGACGTTGCAGGCGCATACATGATACAAAAAAAGTTTGATCAGATTTCTCAAGA
ACGGCCCATGTCAGTTGTGATTGCTGCATTCGATGGTCTGTGCCACTCGTTGAGCAAGAATAACTCTCCTCAA
GAATTGCATGCGATTGTAGTGGAACTCATGGAGGTGTTTGGGGCTCAGCTTTCGGCACTGGTTCAATTACTTC
CCAGTGTTGCGATGATAATGCCCAATATGAATGCCCAATCCGGTGATTGTGGTCAAGATGCAGAGATCAACAT
GACTAGTGTCTACTTCATTCTTCAATTGTTTGCACGGGTAATCTCGAGTAAATCACACCCTATTATGGTGAGA
GCGCGCCACACTGTGCTTGTATATGGATATAAGTTCGAGAGATCGGA

>TR446|c1_g1_i13 len=2110 path=[5901:0-46 7715:47-180 7782:181-220 7827:221-241 7889:242-248 7323:249-287 7609:288-296 7778:297-312 7318:313-338 7605:339-350 7479:351-386 7313:387-389 7706:390-405 7477:406-469 7308:470-474 6547:475-475 6548:476-487 7819:488-499 7773:500-511 7958:512-518 7291:519-542 7690:543-543 7817:544-567 7770:568-582 7687:583-590 7444:591-614 7984:615-623 7678:624-665 7850:666-666 7811:667-691 7759:692-729 7919:730-738 7909:739-771 7564:772-783 7876:784-795 7846:796-821 7806:822-850 7670:851-874 7964:875-876 7986:877-877 7995:878-878 7240:879-924 7805:925-928 7750:929-962 7667:963-986 8015:987-990 8009:991-1014 7965:1015-1021 7874:1022-1028 7842:1029-1052 7801:1053-1061 7744:1062-1084 7872:1085-1091 7798:1092-1132 7966:1133-1145 7797:1146-1154 7391:1155-1178 7796:1179-1196 7740:1197-1224 7539:1225-1265 7386:1266-1302 7870:1303-1326 7839:1327-1361 7967:1362-1364 7738:1365-1373 7987:1374-1385 7968:1386-1388 7996:1389-1394 8002:1395-1413 7736:1414-1418 7941:1419-1437 7934:1438-1441 7926:1442-1442 7916:1443-1466 7905:1467-1475 7891:1476-1499 7864:1500-1544 7969:1545-1604 7788:1605-1650 7642:1651-1672 7522:1673-1712 7728:1713-1743 7640:1744-1750 7518:1751-1760 7725:1761-1764 7636:1765-1790 7513:1791-1794 7357:1795-1843 7634:1844-1877 7510:1878-1925 7890:1926-1934 7862:1935-1939 7829:1940-1951 7973:1952-1952 7989:1953-1967 7998:1968-1976 7153:1977-1996 7617:1997-2024 7976:2025-2039 6091:2040-2109] [-1, 5901, 7715, 7782, 7827, 7889, 7323, 7609, 7778, 7318, 7605, 7479, 7313, 7706, 7477, 7308, 6547, 6548, 7819, 7773, 7958, 7291, 7690, 7817, 7770, 7687, 7444, 7984, 7678, 7850, 7811, 7759, 7919, 7909, 7564, 7876, 7846, 7806, 7670, 7964, 7986, 7995, 7240, 7805, 7750, 7667, 8015, 8009, 7965, 7874, 7842, 7801, 7744, 7872, 7798, 7966, 7797, 7391, 7796, 7740, 7539, 7386, 7870, 7839, 7967, 7738, 7987, 7968, 7996, 8002, 7736, 7941, 7934, 7926, 7916, 7905, 7891, 7864, 7969, 7788, 7642, 7522, 7728, 7640, 7518, 7725, 7636, 7513, 7357, 7634, 7510, 7890, 7862, 7829, 7973, 7989, 7998, 7153, 7617, 7976, 6091, -2]
TCATTATCACGCAAAAAGAGGACTTTTTTTATCGGCACTGTCTTTTCATGAAGCAATTGAAAGGAGTAGTACT
ATGGAGGAGGATCACAACAAAGCTGTTGATCAAACTGACAATATCGTTCAAAACAAAAACAAAAACAACAAGA
AATTTACTTATGCAACACAACAAGTTTCGAGGGATAGCCTATCGTCAAACGACAAACCGTCCAAATACGTACC
ATTGAAGGAATTAGGATTCCCATCCTCCCTCTCGTTATTCGTTACGAATCTACTCGATTGCGGATGGGGAGAA
TTTCGCCCAGACAATGCCTTGTCATCGTTGGAGGCAGCTAGTAAGGATTTGCATTTGCTTCTTCTCGATCCGG
ATCGCTTTTTGTTTGATCGTATGATTCCTGGTCATAATCAAAGAGGAGGAGGAGGTAGGGGGGCGGGTTTGGA
TTTAGATTTCAAACAAAACAAGGTGTATGGGCGAGAGACGGAGAGGTCGCTTATTTCGGATGTTTTCTGTCGT
GTGTCGTTCACTGGGGAGAGCGAGGCGTTGTTTATTGAAGGCTTCTCTGGGTCGGGGAAGTCATCTCTGGTCC
AGAGCGTTGTTGAATATATCGACGTTGCAGGCGCATACATGATACAAAAAAAGTTTGATCAGATTTCTCAAGA
ACGGCCCATGTCAGTTGTGATTGCTGCATTCGATGGTCTGTGCCACTCGTTGAGCAAGAATAACTCTCCTCAA
GAATTGCATGCGATTGTAGTGGAACTCATGGAGGTGTTTGGGACTCAGCTTTCGGCACTGGTTCGATTGCTTC
CCAGTGTTGCGATGATAATGCCCAATATGAATGCCCAATCCGGTGATTATGGTCAAGATGCAGAGATCAACAT
GAGCAGTGTCTACTTCATTCTTCAACTGTTTGCACGGGTAATCTCGAGTAAATTACACCCTATTATGATCTTC
CTCGACGATCTACAATGGGCAGACAGTGCTTCGTTGGATGTGTTGCAGCACATCCTGTCCGACATCAAGGGTG
AAAGCTGCGTTTTCATGATTGGATCTTTCAGATCAAATGAGGTGAACGAGGATCATCCCGTGACCACCCTTAT
GAATTCCTTGGAGAGGTGCAACGTCCGATTGAGCAGATTGCATTTGGATGGCATGGATATTGAAGGCGTGAAC
CACATCATTTCGGATGCTCTGGGGACCTTCCCACGCATCTGCAAGCCCCTATCCCAAATTGTGCTGCGCAAAA
CAAAGGGCAATCCTCTATTTGTGCTGGAATGTTTGAGGTCATTGGTAGACCGTGACTTGCTGCACTACAGTAT
CCGGGAGCGACGTTGGGTTTGGGATACTGACATTATTAGTAGGGTAGATATCACGGATGATGTTTGCGAGCTT
CTCTCTGTAAAGATGATTGGTCTTCTGGAAAAAACTCAGGCAGCTCTGAAAATCTCATCTTGCTTTGGGACAA
GTGTGAATTCAACAATTATCAAAACTTTGATGTCGGCATCGCCTTTACTTCAAACCTTTCAGGAGGAACTTGA
CAAGGTCGTTGACGATGGCTTCATGGATACGGACGACTTAGGCTCCACATATAAATTTGTTCATGACAAGGTC
AGAGAAGCTGCGTATGGACTTATTCCAGAGGAAGATAGGAATCGGTTCCACTACACTGTTGGGATGCTTCTAT
TCAATGCAATAGATGGGAAAAACATTGATAGCATCATATTTCTTATGGTTGATCAAATAAACCACGGTACCAC
TTATCTCAGCAGCGATCAGCGTATCACTGTCGCAGAGTTGAACTACGGAGCAGCTTCTAAGGCAGTGGAAAAC
TCAAACTTCGCAGCAGCATTCTTCTATTCCAATGCAGCAATGAAGCTCCTCCCTGAAAATCACTGGGAAAGTC
ATTATGATCTGAGCAGGAAGATCTTTTTGGTGCTTGGGAATGCTGCGCGCTCATACGGGCGTATCGCAGAAGC
GACAAGTGCGTTGGATGAGATTCTCAAGCATGGAAAATCGTTGAAAGATAAGTTAGATGCATACTATCTTAAG
ATAGCATTGTTGAACATACACCAAGAACTAAAGAAATCATTTGACACGTGCGTCGAAGTCCTTAAA
>TR447|c0_g1_i1 len=239 path=[217:0-238] [-1, 217, -2]

TTAATAATGATGGAAGAGAAATCCCCGAAGAAGAAGAGAAACCAGAGATCCCAGACAGTCAGCAACAGCAAGG
CGATGCCGAATCGGTAATTAGTAGCCTGTCGCGTGGTGTTTGTCCCGCGGTTGAAGAAGAAGAAACAAAGATG
AACTCCAAACAGCAAGGCGACACTAGAAATAACCAAGCTGTGTAAATTAAAAATTGATATGTGTTCCACTTAA
AAAAAAAAAGATCGGAAGAGC
>TR449|c0_g1_i1 len=320 path=[298:0-319] [-1, 298, -2]
CCTTGCACACACAGCGTTTAAACATTTCCTCTTTATACTTCTATCGTCCCGCTTCGAACAAAACATTTCCACT
ATGCCCGAACAAAAGAAGCCAGGATTCGACACCATTGCGATACATGGGGGCTACACTCCAGACCCAAGTGTTA
CCCTCGGTTTAGGCCAGGGTGCTCCAAGTGGCATTGCAGTATATCGTACGTCGCCTTTCGTATTCAAGGATAC
CGAACATGCTGCTCGCTTATTCAAGCTAAAAGAGCTGGGAAATATTTATACTCGACTCATGAATCCCACCTGC
AATATACTTGAATCGCGTTATGCCCAAC
>TR451|c0_g1_i1 len=535 path=[513:0-534] [-1, 513, -2]
TTCCGATCTGGAGCTACTATGAATAATATTATTGGGTCTAATTATGATTTAATCCTCAATCACGGTGAAGCTT
CGTCCGAGCCACGTTTGTACAATCTCGTAAGTCAAGAACATGATGGCCGAGTTGGGAACAACTTTCAGCAAGT
GAACACCCATGCCACCATACAGGCCCTTTCTTCCCTCTTCCTTTCCAATGACGCCTATGGTTTGCCACATCCC
CTTGTACTTGAAGACACCATTGCGTGCCTGCTCTCGTAAGCGAGTTCTCGCAACTTCATGTGGGTACGTAATA
ATAGACGCCGTGCCTTTCGCCAGGGCCGCTGTGAAGAAGTAATAAAGTTTGGGAAGCTTGTCCGTCGGGTGAA
TGCCCTGATCTTCTCTTATCTGGTTCTGCTGCAAAAGAAGCTTCGTTTTGATCTGCTCGTATATGACAAATTG
TATCGATCCCTCGATGCAGCCCCAGTAACTGGCGGAAATACCCTTGTAGAATCCCCCAATGCCCTCCTCCCTG
AAAATCGTAGCGACGGCATCGCGA
>TR453|c0_g1_i1 len=390 path=[368:0-389] [-1, 368, -2]
CGCTGAAGCGTCAAGGAAAGACCCTCTATGGCTTCGGTGGATAATTACCTGCCGAGACACTGATTGAGGTATT
ATGGCCACAAACCGGATTTTCTAAAATCCATCCGTATATCAAAGAACTACTTTATGCCTCATATCGTATTGAA
TCATCTGACAATACGAGAGCTTGTTAGCTGTGAACATTATCGATATCCTTAAATGCAGGCAATACATGGACTG
GTCCAGCGGTCAGCGGAACCAGATTATTTTTGGCCACGTTTTCAGATATGCATGATGAAGCAGATAATCCTCG
TCATTTATTCCATGTACAACAAAGTAGTTGGGAAAGGCGTAACAGCTATTCAGCTGACATACTTTGGATCCTC
ATGATATTCTGACAACATAGTCTAC
>TR454|c0_g1_i1 len=452 path=[430:0-451] [-1, 430, -2]
GGCGCGAAGATCCTTTCGTATCCCGACGATGAAGACTCTTTCTCTCTGTTGCGCCACGTAATGCCTGCTGTTG
CATATATTCCAAGTAACGTCGTACTCGCAATCTTCGAACGCACGCAACATGATTCGAAACACCGGACCGACAT
CCCTCTCCTCCCTCCCGAGCCCGTGCTCTCCGAATCTCCCCCCGACTTCCGACCTTTCCGACCCCATGCCGAC
CAGACCCGAAACGTTCTCGAAAATGAACGACTTGGGATGAGACGCTCGCAGCACCCGCACAATCTCGCGATAC
AATTGTCCCTTCTCGTCGTTCAGCCCTCGTTGCTCCCCCCTCGTCGAGAATGGCTGGCATGGAAATCCGGCGG
TGAGGATGTCGGTATCGGGGGGTATGTCCTTCCGTGCGCACACGTCGAGTATATCGGCCTCGAGGAGTAGTGG
AGGAGGGCACTTCT
>TR455|c0_g1_i1 len=320 path=[298:0-319] [-1, 298, -2]
GCCTTATTTTTTAAATTGTAGTTGATAGGCGCAAGTCCCTAATGTGTTCGATTCCTCTCGCGAGTCGCGTCGT
CATATTCCTCAAAAATGGCTATCCGATGGATTACTGTGTTGTTCTTGTGACTCGTTCAAGCCAAAATACCTTG
CCATTGACGAGTTTGGCAATACCACAAAACAGCAGACATAAAATATCTGCATACTCAAAATATGTTGATGTCC
AAGTCAAAACTCCCCCCAGAAGCAGATATTGCATCATCAAAGCGAACAGCACGGATCTCTGCTCTTCATGTGC
ACCTCGTCAATCTTGGCAAGAACATCCT
>TR456|c0_g1_i1 len=323 path=[301:0-322] [-1, 301, -2]
CACCAGAACCACAACCTCCGTCGCCACACCCACCGCCGCCGCTACCACCGCCATTGCCACACCCTCCTCCTCC
CCCTTTGGTCATTCCCTCGAGCCGTCTGGTTGCACTCCCGCTCAGCTGTTGTTTCGGTATCGATCCCACCACG
ACTTGAAATCGTCCCGTATTCTCAAACTCCATCCTCAATTCCTCCATGGCATGGTGATACTTCACCTCGTACA
ATATCAGCAACTTCTTGCTTTGCTCCTCGTTACTACTAGATGTGCTCATTTGAGCCATCGCTTCGTCCAACGA
TTTGTCAACTTCCATTGCAGACTGATCCCCT
>TR458|c0_g1_i1 len=850 path=[853:0-161 854:162-185 855:186-849] [-1,
853, 854, 855, -2]
GCTACGCAGACATTCGTCGCAGTAGCAGCATCAATAGTTCCAGTTCTAGCCAATCTCGAGTGCGTTTTGATCT
TGAAGTGTTCCAATGTGTAGATCCGAAATCATCAGTAACAACTCAATCGTCTACATCTACATTTACATCGAAT
GACGAAGATTCTGAAATCCAACTTCATGAGCAGTATCTTCACACACAGTCAGGACGACAGAGAGGAAGAGTCA
GCAGACCACTCGCTTCCTCCTTGAACTTTGCCCGAGCGCGGTTCGACATCGGGAAGCCTCCATCTTTTAGCCA
ATCTGAAAGTCAAGACGAACAGCTGTCTCTTCATCGACAGTCAAGAGAAAATGCGAGGACAGAGGAGGGGACG
GACTTGGTTGGTGTGGAGGAGGAGTGGATTCACATGGACGAAGACGAGGCGGAGGAACTACAGCTCTTACCAT
TTCCGGATATGAAGGAATATGAAGGATCTCCATTGATGGAGAAGAGCAAAACAGGGGAGCACATCAATAAGGA
GAACCACAGCCGCCTAGATCAGCTTGGTGATAATAAAATCAAGAACCGCAGGTCGAGCTGGAGTGCTGATACT
AGCCGTCACTGTATGATCAATAGTTTCGGCCAAGGTCAACCGCGTTTGTACCCAGAAGTGCTTCCCTATGTCC
GCCAAGCTCCCGAGAGTCAAGAGCATCTGCAGCCCCTTCAACAACATTCGGGACGGCATGGAGGAGGAGTTAG
TAGACCGCTCGCTTCCTCCTTGAACCTGGCTAAAGTGCAGTTCGACCTCGAAGAATACTTATGTGCTAGCCAA
GTTTCTGAAAGTGAAGATCAACAGCAGTCTCTACAGCGACATTACGG

>TR459|c0_g1_i1 len=627 path=[630:0-349 631:350-373 632:374-626] [-1,
630, 631, 632, -2]
GCTGTATTAGTTTCGTTACTCATTGGAGGAATGGCAACGGCCATCGCAATGCTACTTGTTGGCAATAGTAGTA
ACAATGGAGCTGAGGTGGATACGCAGCCCATCGAACAAATTCCTGTTGTAACAGTGCCAGAGGGAAATGACAC
TGCGTCCGATGTGCTAGTTATTGATGGCAATAGCGGATCTCGGGTTGACACTACACAGCCCATCAAACAAACT
CCCGTAACAACGACCCCGCAGGGTACAGAAGAAAGGGGGCCAATATCCTCAACAACACTCAATACAACGATCC
AATCTCCGCCGCCTCCACCATTCAATTTCAATCCACCTATTACAAATGAAACAACTATCGCTCCCACGACAAC
CGTAACATTGCCGCCAGTGGTTCGACCTACGGACATGCCAATCCCCGCCATGTTGGCTCCCACTCCACGGCCT
GCTCTAAGTCCAATGGTACAGCTACAATCATCCGCCTTGGAGCTAGTTCCAAACGAAGAAATCGTTTCCCATT
CGAGCAGCAGAAATGTTGCCATTCACGGCGATACTGCTATTGTCGGGGCACCCTGGAATAATAACAAGAGAGG
ATTCGCGCTCGTGTACATCAGAGACAACAACCGTGGGGAATGG
>TR461|c0_g1_i1 len=502 path=[490:0-224 491:225-249 503:250-483 494:484-
486 495:487-489 496:490-492 497:493-495 498:496-498 499:499-501] [-1,
490, 491, 503, 494, 495, 496, 497, 498, 499, -2]
TACAATAATACATCATGCCATACAGTGCAAAACACCGACCCAACACCGACCACCGACCGAACACCAACTCACC
CAACGAGGGGAGAGAGATCACAACCACATCATAGCGCCTGCATGCAGCATACACTCTCTAGATCTACTCTTAA
CACGATCCCTACCTACAATTATCGCCATACTACAATTAGTTGTCATTGTTCTACAATGAATAACTCTAACAAC
CAAAGACCAAGTGTATTGCGAACGATGATGGCGTCACCGCTATTCCTCCCTTCGATAGACGGCGCCGATCGTA
TCAACTCTAACGAAAACAACACTCCGCTGACAGTCGACAGTACAGGCCACAATGATTGCAATCACACGAAAAT
CTCATCAAACAAAAATGCAGAAAATGGCCAATGTAATTTATCAGCCTGCGTTATGAGCGGACCACGCGATCGT
CACCACAAACGAAAACTGCCAATGACGACGACGACGACGACGACGACGACGACGACGACGA
>TR462|c0_g1_i1 len=501 path=[524:0-87 525:88-500] [-1, 524, 525, -2]
ATCATGGTATTTAGCAGGCTTTGTGGCCCTGCTTCTACGTTTTTTCTCAGCGTTGCTCAGTGCCATGTTGAAT
CACCTTGGGTATAGTCTGTTCGAAGAAAATCAACGCAATGTGTGAGAAAGAGATGTTCCAGTGGTCGACAGTG
AAGGAAACAAAATGCGCGGTCATCCATGCTCGATATTAATGTGATCAATCCAATATGTACATTCACAACCATT
GCCAATACAACAAGGAGAATCGGGCTCGAAATCATCACTCATGAAGATAATATCGATTTTAAGTATAAGTCGG
ACAGGCAGAGCGTTGATCGACAAGAACAAATTGCCCCAAATGAATAGCAAATCCTCTTCGAACAAGAGAGAAT
GTTAGGAAGCACACGAGAACCGTTGCAAGACAACGCCTACGTTAGAGCGACGCGAACTGCGGGCAATATAAAT
GAACGAACTCTAAACTCCTCTTCTACAGAAAAAGTCTAACGAACAACAATTGCATCATGGCAG
>TR465|c0_g1_i1 len=412 path=[412:0-92 413:93-114 414:115-411] [-1, 412,
413, 414, -2]
CGATCTGTTTTTATAGACACATCAGTTGCAGATGAAGTCGCATCTCTGAGTCACCGGGAAAGTCGTATTCAGA
TGAATCCAGCACTTGGTTATTATCATTTGATCTCCTTTCTCTCGTAGATGACTCTATCAGTGGCATTTTACAT
GTGGCACCAGGTACTAGTATTGCATGTATTCATTGATCTTGAGTGTCAAAATCATGGTCATTTTCGCTTGTAT
TGTATTGCATTGTATATTGTAGGGTACCATGTCAGGAATTCTGATACGTCATGTTTGCCACAACACCATGACA
TGGGGTACCAAATAACATGCTTACATCTCGCCGACAACAGGAAGGACGCCTCCCAGCTGCTCGTGGACCATGA
GTCCAAGGATTCCCATCATGGCCGCACGGCCGTTGTTCAGCTCGATC
>TR466|c0_g1_i1 len=680 path=[658:0-679] [-1, 658, -2]
ATGAGTAGTATAAATTTCAATAAATTGAATTTAAAATTATAACTAAAATTAAAAAAAGGAATGAATATACTCA
AACTCGGAAAATCAAAATGCGGAATTGAGCAAATTGCGAAATTTAGCACAAATCTGAATAAAGCTAAAAACTG
AAAACTTCAAGAGTAAGTTTACCAAGGGGAAAGATTTTTAGAAAACGAAATAATATAAACTGTAGGAAACTTA
AAAAGGAAAAATTAAACACGCATTTTACAACAACGAGTATAAAAAATTGCTTAAATTTAGATTAAACAAAGTT
AGTTGCTTTTTTGCAATCTTATATTTTCCTTCGGAAAATAACGATGGCACAAAATCGCAACTTTCCATAACAC
AAATACGTTGCCCACAATAAAAAATGAATAACTTCAAACAAAATTTAAGAAATAAACCAACTCTATTTTTAAT
AATTGGAATTCTTGTTTTGTTTATTGGAATTCCTTTCGGAATTTATGGATTGACTTTAAATGGAGGAGCAAGT
TTAGGTGGAGTTTTAGTTTTACTTGGAGTTTTTATTTGCATTCCATTTATAGTAATTGACCGAATTTTGGTTA
ATAAAATAAAGCCTACTAAACTCTCAATTTATGAACTGATTTTTTTAGGAATTGTATTGATTTTCTATTTGTC
TGATAATAAAAAACTTTATGTTG
>TR467|c0_g1_i1 len=1452 path=[1448:0-1083 1449:1084-1125 1470:1126-1421
1452:1422-1423 1453:1424-1425 1454:1426-1427 1455:1428-1429 1456:1430-
1431 1457:1432-1433 1458:1434-1435 1459:1436-1437 1460:1438-1439
1461:1440-1441 1462:1442-1443 1463:1444-1445 1464:1446-1447 1465:1448-
1449 1466:1450-1451] [-1, 1448, 1449, 1470, 1452, 1453, 1454, 1455, 1456,
1457, 1458, 1459, 1460, 1461, 1462, 1463, 1464, 1465, 1466, -2]
CTGATCGTTTGTTGGTTTTGTGGTGTGGTATGAACAGTAAAAAATATGGGCAGCGTGGAAGATCAAGGTCACA
TCTGTGTTCGGTTGACGCTGATTTGGACTCGTAGCTGATGTTTCGTTGGCATCGCGTGACAGAACGGACAGTC
GTGCACCTTCAAACAAGATCAAGATCGCATCACCGTCGGGCTTCTGATAGCATATTGAATATGAACAAAGCCT
CCTTCCTTCAGGCCAGTTCACCTACTTACTTACCCCGAAGAAGCTGACACGAGAACCCCTCACCCTGGGACCA
GACCATGACCAATGAAGATGTTTCGCTTAACCCGAATAAGCAAATGAGAACCCCTCATTTAAGCACGACCAGA
CCACGACCAATCACCATTGCTGCCAGCAGGATCTCACTTCTCATAAAAACAATTAAACACACACAGAATACGA

AATACGAACTTGCAACAAGTCAATACAAGTCTCAATTGCCAACAACTCAATCCTTCAATTGGCAGAAAATGAA
CAACAGCACGATGTCCACCACCCCAGCAAGAGCTAGACTATGCTGTGGCTGTTGCTGCGACTTCCGTCGCGCC
GTCATAATCGTCGACCTTGTCATCGTTATACTCGAGGCAATCGTTCTCATATCCTTGCCACTCGGAGCTACGG
ATCAAGTATTATTTGTCCCAGACGAGGACTTAATCAAGACAGAAATGATCTTCAGCGGCGTTTCTATCGCTAC
TGGCCTTCTTTCTATCTTCGGAGCGTACATGTTCAACGTATGGCCTGTATTGATCAATGTGGCGTGGCTTCTC
ATTGGATATATCGCTGCGATCATTTATGCTATCCAATGGTGTAATGACTATATAGAGAAGTACACATCAAAAT
ATCTCTACGCTACTTGTTCCATCAGCCCAGGGTCGGTTATTGTCCAAGGTATCCTCATGCTGCTATGGATATA
TCCTCATGTCGGATTTATTGTTCAGGTAGCAAAAGGCACATTAAAGAAAGATGATTATGTACCTTCTTCTTGC
TGTTGCGTCGTCGGCAACAGTACTGTCGCTGGAGTTGAACAGCAAGTATCACCAGTGGTTGACACCAGTGCAA
CTCCATCAAAGATGGAGCAAGGTGTCATGCAGTAAGCTACAATATAGAAGCATGAATTGTAGGGAATATTGTG
TTTCGTCAAAAGTAAAGCATTATTTAGGACAAAGAAGTGTTATTGTTGTAAAATGAGGTGACGACCAAATATA
AGCAGTATGCGTTGGTACAAAATTCAACGGGAGCGGAATTGAGGATCAATATCAATCCTCTCTTTTACGGCCT
CATGACGCGATGCATAACATAGGCATACTGGAGGCGGGGGCAGGAACACAACACACATAAGGTAGAGCAGGCA
GAGTAATATACCAGAGAGAGAGAGAGAGAGAGAGAGAGAGAGAGAGAGAGAGAGAGAGAGAGAGAGAGA
>TR468|c0_g1_i1 len=481 path=[483:0-480] [-1, 483, -2]
TCGTTAACGAAAAAATAGTCCTACATTTGTGGACCGGACTAAGTATGATCATGTATCATGCATAAGCATGACA
ATAGAAATAGAGCAAATAGAGCCAATGCCTACTATCATGCATCCGCCCAACCTCTCTACTAACCACACCGAAG
AATAGACCATCAACAAAAACAACCAACCGAAACCGCTTACACTCTACCATGGACTTTGACGCAGACAGAATAT
ATTACTCACATCAAAATTTGCAACAGCAGCAACCGCTGGGAGAGGATGGTAGCGCCAATGCTGCCGACCCCGT
CAATACTGCTACAGGTGTAGAGAGTCGATTAGAAGATGAAGAAGAGCGGGCCGTGGATGTCGATGCGTTGCGT
CGTCACTTTCGCGAGTTTTTGAGAAATTACCGCCAAGGAGCCAATCGATACATCTACCGCGATCGCCTGCTCC
GCATGCATCGCCGTCATCACGCAGCCAATAACGACGCCACGCA
>TR469|c0_g1_i1 len=1031 path=[1034:0-199 1035:200-223 1036:224-1030] [-
1, 1034, 1035, 1036, -2]
GGGCGCGGTAGGCCTCGAGTCCCTCGAGGCGGTAGAGACACGCCGCGGATCGGATGGCCAGGGCCCTCCAGTC
CCCTTCCTCGTTGACCGTCAACAGCAGGCTCCTCAGGTTGCGCGCGTCGGCGGAGCTGAGCCTGCCGAGCAGC
TTCCCGTTCTTCCTGACCACCTTGGCCGCGAGCGTTTCCATCGACTTGAGCCTCGCCGCGTCGAGGGCGATCT
TGACGGCGTGCGTCCCGAACTCCTCGATGCCCGATCCTGCGAGGGAGCAGAGGTACTTGGCATCGGACCCCGC
CGCCTTGAGGCATTCCCCCTCCTCCACGGAGCAGGATTCCGCGGTCGAGATGCCCAGCTCGCGTTCTCGTACC
GAGACGAGGGAGCTGTAGTGAAAGGCGGCCGCGCAGAGGGTGGGCGCGTCGGTCATTTCGAGGGAGTTGACGA
GGATGGAGCAGAAGTCGGCGGCGCCGGCCACCTTTTTGGTGTCGTCGCCGGCGGCGAGGTAGATGGCATTGAT
GACCTGGTAAATATCCTTCTGACATAAGTGGGAGGTGAGATCTTTTTGGAGCTTGCGAAGTTTGATGGGTGCG
GAACGTGCTTGGTTGAGCGCGAGCCATGGTGGCAGCGTTGGCGGTTCCGGTTGTTCCATGGGCGTGTCCATTG
ATGTAGTATCTTGATGCGACGCAAAGAAAAACGGTCCAGCAGATTCTGCCTCGTTTAAAATGATGGGTGGTTG
AAAATGACCAATTTCCACTTCTTCAAACTCTTCAAATAGCTGTGACGATGTTGGTGATGATCCCACAACACTG
TGGCCACTAGAAGAAGACCCATCCGACCCTGATCTTTTCTTTTTCGAAAATAATTCTTGTAAATCCAACTCAT
TAAATTCCAAATGAGGCCTGAGCAACGAAAATTCACCTTCTAAATTAGTTTGATCCGTAGCGCACACCACCAC
ATCATTATCCTCCATCGATCCCGATGATGATGACGACGACGATGACGACGACGACTCGGGCGAAGAACCATCG
AGGTAATAA
>TR470|c0_g1_i1 len=1591 path=[1595:0-392 1597:393-1022 1591:1023-1590]
[-1, 1595, 1597, 1591, -2]
TTATGTATCTTGTCATCTAGATAGATAAGCGCTCTGAGCTCATAAATACTTAAATTGACCTACCCATCACAGT
CCCGAGGTCAGCAAATTACCGATCGCCCAAGGGCCCAAGGATATCTTCCCTCACCTAGTACTGCTACAGTTTA
CAATGCACTGCAAATTAAAATACTGATCCTGTTGCACTATTGTTTTTATTAGCATGTTTCCAAACAAAACGCA
AACAACGATGACAATCCAATATCGGCACCTATCAATCCCTAATAGAGATAGAATCTGGATAGAACGGGATCCT
CCCTCAATGCAAGACAGGAAGCAAGCAGCAACTTCCTGCCCAAGGTTAAGGACTGAGAAGATTCATATCATTA
TCATCACTTTTATTGCCACTGAAGGGGTCAAAATTAACCCCCCCTGCAAAGTATTTCCTAGCCACACTCACAT
GATACCCTTCTTTACTAAGACCATCTAGGTCATAACTCCACTCCATCCCTATCATCTCTCCCATGGCATCCAA
ATATTGTTTCGCATAGTCAGGCACCTCTTCATTTGTAATACTCACATAGATACCAAGTTCTCGAATGGCTTTA
CTTGCCTCGTTAAGCAATTTTACCAAATCGAGTAGAGCCGGTGGGCCTGTCCGAGAATGCCACGTTACTGTTG
ATGGGAGCGTAGAAAGAATGCCATTCATTACACCTCCATCAATGATGCAGTCATAACCAGTATTGATAGCCTT
GTCTATGGAACCGCTTCCATCTGTTGCAAGTATTTGACTGAGTTGGTTACTTGCCACATTTGTAATACTGCTT
GATGCCACGACATCATCAAATCCATCTCCATATTGCCAGGGTAGGCGGGTAGTACTTTCTATCTCAGATAATG
GATCGTTTCCAGAATCATCAACACCATCAAATATTCCAGAGACAGTTTGATCACCATCATCATTCAGATCAAT
GGCCATGCACGAAATGTGGTCGTTGAATTGAGGCAAAAAGTCGGTGAATCCGTTCATACTGAAAGAGTAATGC
CAATCTTTCCATTCTTGCTCGGTGGATAGGAGATTGGATATATGAGTCGAGGGTTCTTGTTGCAATGGCAATG
GTTTCACATGATATAATAGATCTGTCATAAAGTGAGCGTAATCTGAATTCTCATCGGTGATGCTGCTGCTGTC
GTCATCGTTGGCCCAAATCATGGAGGATGAAGCCGACGAGAAAGCATAGGACCCCAGTGGTCTCAGCACATAG
CCTCCTTGCGGCGCGAATGATGCGACCAGCGGTAGGATTGCAAATATTAAGGATAATTTTGCCATGATGAGTA
GATCTCTCGCCTATGGATAATGTGTTTGGACAACCAATGTAAGATTTAGGAAGAGATGGTAGCGTGTTGTTTG

TTACTGTTGCGTGCGATGCCAGAGCGCAATGCCAGAGCGCAATGCCATACTATTTTCATTTATGTAGGATTCA
TCACATGGTTCGATCGATCCCGTGGGCGATGTGAGTAGGGCAATACACTACCCTAATGCGATGGATGGGAGAT
TAGATGGGACGATTCGCATTTATCGCATTTACTTACCAAGTAATTTTTCCTTCTGCGT
>TR470|c0_g2_i1 len=982 path=[1592:0-392 1593:393-413 1594:414-981] [-1, 1592, 1593, 1594, -2]
TTATGTATCTTGTCATCTAGATAGATAAGCGCTCTGAGCTCATAAATACTTAAATTGACCTACCCATCACAGT
CCCGAGGTCAGCAAATTACCGATCGCCCAAGGGCCCAAGGATATCTTCCCTCACCTAGTACTGCTACAGTTTA
CAATGCACTGCAAATTAAAATACTGATCCTGTTGCACTATTGTTTTTATTAGCATGTTTCCAAACAAAACGCA
AACAACGATGACAATCCAATATCGGCACCTATCAATCCCTAATAGAGATAGAATCTGGATAGAACGGGATCCT
CCCTCAATGCAAGACAGGAAGCAAGCAGCAACTTCCTGCCCAAGGTTAAGGACTGAGAAGATTCATATCATTA
TCATCACTTTTATTGCCACTGAAGGGGTTCATACTGAAAGAGTAATGCCAATCTTTCCATTCTTGCTCGGTGG
ATAGGAGATTGGATATATGAGTCGAGGGTTCTTGTTGCAATGGCAATGGTTTCACATGATATAATAGATCTGT
CATAAAGTGAGCGTAATCTGAATTCTCATCGGTGATGCTGCTGCTGTCGTCATCGTTGGCCCAAATCATGGAG
GATGAAGCCGACGAGAAAGCATAGGACCCCAGTGGTCTCAGCACATAGCCTCCTTGCGGCGCGAATGATGCGA
CCAGCGGTAGGATTGCAAATATTAAGGATAATTTTGCCATGATGAGTAGATCTCTCGCCTATGGATAATGTGT
TTGGACAACCAATGTAAGATTTAGGAAGAGATGGTAGCGTGTTGTTTGTTACTGTTGCGTGCGATGCCAGAGC
GCAATGCCAGAGCGCAATGCCATACTATTTTCATTTATGTAGGATTCATCACATGGTTCGATCGATCCCGTGG
GCGATGTGAGTAGGGCAATACACTACCCTAATGCGATGGATGGGAGATTAGATGGGACGATTCGCATTTATCG
CATTTACTTACCAAGTAATTTTTCCTTCTGCGT
>TR471|c0_g1_i1 len=286 path=[408:0-43 409:44-67 419:68-108 421:109-132 422:133-144 416:145-197 397:198-203 398:204-285] [-1, 408, 409, 419, 421, 422, 416, 397, 398, -2]
TTCTTAATTTCCTTACTAGACATTGATCAAGTGTTTTCTCAAGGGTACCAACCGGATTTTCTAAAATCCACCC
ATAAATCAAAGAATTCCCTTCATGATATGCAACTGGCCATTAACATCTTTTCAAATTTCTGCATGAGAATATG
CATGCTTGACCTCACTAAATATTGCATACTAATTTGAATCATGTCTTCAAATCCAGCGGAAACGCATTAACAT
GAGTTGTGTTTATTAATTCTCTTAACCAAAACTATCTAACCGCGACCTCACTGATTGTGCAGATCGG
>TR471|c0_g1_i2 len=263 path=[408:0-43 409:44-67 419:68-108 421:109-132 422:133-144 418:145-156 415:157-174 394:175-180 395:181-262] [-1, 408, 409, 419, 421, 422, 418, 415, 394, 395, -2]
TTCTTAATTTCCTTACTAGACATTGATCAAGTGTTTTCTCAAGGGTACCAACCGGATTTTCTAAAATCCACCC
ATAAATCAAAGAATTCCCTTCATGATATGCAACTGGCCATTAACATCTTTTCAAATTTCTGCATGAGAATATT
GCATAGTAATTTGAATCATGTCTTCAAATCCAGCGGAAACGCATTAACATGAGTTGTGTTTATTAATTCTCTT
AACCAAAACTATCTAACCGCGACCTCACTGATTGTGCAGATCGG
>TR472|c0_g1_i1 len=696 path=[763:0-75 764:76-99 774:100-441 770:442-465 773:466-584 761:585-620 762:621-695] [-1, 763, 764, 774, 770, 773, 761, 762, -2]
ACCGAGGAGCGGAGTCGGGGCGGCGAACGTGCCGCACTACGTGACTTACGGGGCGGTGCTGGATCCCCACCAC
GGTGCTCAGGGTGATGTGGGACGGCAGCAGCAGAGTAACAGTAATAACAATTCCAGCAATAGCAACGGTAACA
ACAACAGCGGGGTGAAAGCGGGCAGCAGCACGCCCGCCTCGACCAAAAAAAAACTGGCCCAATTCCAATCCAA
AACTCAATCCACGCCCATCATCTTTTCGTCTCCCTCTTCCTCTTCCCTCCTGGGAGGAGGCACATTGTTGGCC
TCGGGGAGTTTGGCTTCGTTATCTCCCAGCGCGTGCGGACTGGGCAGCGCTGCGGAGGGCGACACGGGCAACA
TGCTGTTCCAAACTCCATCCCATGCGAACGGGGAGGGAAACTTCGCTCGGTACACAGTGGAGAACACCCCGGC
GTCATCCAGGATCCCAGCACACAACAATTACAACGATGGCAACGCGCATCAACAACAGTGGAGGCAGCCCCAG
CCCAAGCTGGGTATCACAATATCTCGCATCCCCCTGGGCCTGTATGTTCAATCCGTCTCACTCGATTCCGAGG
CGTACGGCGTGGGGATATCCCCCGGGAGCATTCTGGTGGATGTCAACGGCATGGGCATGCTTGGGGAGAGGAG
CGATCGGGCGCTGGAGCGTTTATGGACGTACGCAGGGGT
>TR474|c0_g1_i1 len=1598 path=[1650:0-830 1661:831-1131 1653:1132-1155 1654:1156-1156 1655:1157-1180 1660:1181-1573 1658:1574-1585 1659:1586-1597] [-1, 1650, 1661, 1653, 1654, 1655, 1660, 1658, 1659, -2]
CCCAAGGCCGAGCGGGTGCGGATCGTGAACGAGGTCCGGCTGTTGGAACGGCTGCATCACCCGAACATCATCA
GCTTTCACGGATCGTGGGTGAATCGGGAGACGGAGCGGGTCATCTTCGTGACGGAGATACTGAGCAGCGGGAC
GCTCAAGAGTTTCGTGCAGAAGGTGCAGTTGATTCGGTGGAAGATTTTCAAGAGGTGGGCGATACAGATATTG
AAGGGGTTGGAGTATTTGCATTCGCAGGATCCTCCGATTATACATCGGGATTTGAAGTGCGATAATATTTTTA
TTAATGGGACGAGCGGGGATTTGAGGATTGGGGATTTTGGTTTGAGTACGGTGATTAGCAAGACGAATCAGCC
GCTCAGCGTCCTGGGCACCCCGGAATTCATGGCCCCCGAACTATACGACGAAAACTACAATGAAAAGGTGGAT
ATTTATGCCTTCGGAATGCTCCTCCTCGAAATCATCACTCGCGACGTCCCATATCATGAGTGTACCAACCCGG
CCCAAATTTACAAAAAGGTGACGCAGGGGATACCGCCCGCGAGCCTGCGACGGGTCAAGAGCGAGGACGCGAG
GAAGTTCATTCTGTTTTGTTTGGGGAATGGGGAGAATGCCTGTGACAGGCCATCAGCTACCGATTTGTTGAAG
CATCAGTTTCTTACTAATAAATCCGATGACGAGGCCCCAATCGAGGTGGAGCCTGCGGTGGAGGAAATGGTGA
TCGAGGAGGCGGTGGTACCGAACAGCATGACTTTCTTGGAGAAGGGATCGTCGGAAGACTCACACAAATCTAG

GAAGAATGACGAAACTCCAACGGTGAATAACAAGAAGTTTGCGTCGTCCATCGATGGGCATGAAATTCCATCG
CATGGGGCCGCGAGTTCGGCGAGTGCGAAGGACCAACAGCGGAAGCCGCCGAGGAATGACAAAAATGCCGGAG
CTCCACAAAATAACAAAATGACGGATTCCAAAGATTCTGAAATGGAGGATGACCACTTTGGCGAAATGCCCGA
GAACGAAGCCAACATGAAAAAGGTCACGGTCCTCATGGGCCGTGGCACCACGCTGGACGAACACGACGACGAT
GTGGATCCCCCGGTTCAGGAGCTGGGGGTCATTTCGCTCTCGAGCATCCCGCCCCCGGCCAAGGCGGCGGCGA
CGATGACCCGGAGTAACAGCGATACCGAGACCAGCAGCCTGGGGAGCAAGACTTCCCTGCAATATAAAGTGTC
GGCTGTGCCAAGGGAAGGTGTCGTGGACGGAGGGAATACAGGGGCATTCAAACCATTCCCCAACAACCTCATC
AACCTTGCCCTCACCCTCCCAGATGAAGGCCGGACTACCATCGAATTTGACTTTGATCTCGTCGACGATGATC
CTGTGCAGGTCGGGAGGGAGATGGTCACGGAATTAGAAGAGGTCCCGGACGATGCAGTGTTGGATATCAGCGA
GGCCATTAGCGGAGTTGCGCGTCAAGCGAGGATGAAACAGAATCAGTGGACCAAGATACAGCAGCAGCAGATT
GCACAACAGCAGGCGTTGCAGCAACAGGCTCAGCAACAGGCTCAGCAACAGGCTCAGCAACAGGC
>TR475|c0_g1_i1 len=1776 path=[1874:0-369 1875:370-393 1876:394-394
1877:395-418 1892:419-628 1880:629-652 1894:653-1022 1883:1023-1046
1893:1047-1620 1886:1621-1644 1887:1645-1775] [-1, 1874, 1875, 1876,
1877, 1892, 1880, 1894, 1883, 1893, 1886, 1887, -2]
AGGTATAGTTCGCGTGTGGCCTGAGAGGGAATACAGCACCTGAGGATAACAATGACAGAACAAAAATCGAGGC
AAAGTATAATTTGTTCATTAAGTCTGAGTATCCAAGCCTAACGGAACACAACTCGACACTATGGTTTTCGCTC
GACGATTTCAAATAATGAATTTCTGGGGCTTCCATTGAGTGAAACCGAAAATATTGCATGAAGGCGCATTCTC
CATTTCAAGATGATTTGTCCATCTTATCTCTTTAACTGACACTAAGAGGGGATAATCCAAACAAGTTCTATCA
CTATTCCTTTCTCCCAAGATTCTGCGGCAGGAGCATCCGCATGCATAATAGCAGTATTCTCCACAAGAGCACT
GCGCTAAGAGGAAGCTTGCTATGAAACAACACTTCACGTCCATGCTTAGTTCCGCTTCAATTTCCCTTTTCAC
TCTTTCTATTTCAGACTCTTGCTGATACAATCACCATGACTTTCCTGGCAGATAATCCACACCGCACTTTTCA
TTCGCCCAATGATCCGCATTCAACTTCACATTCGTCGTCAATTTCGTAACCTTACTCTCTCCCCCATCCTCCC
TATCCATCCTCTTAATCATCTGTATATCATAACTCAAATCCTTCAGTCCATTCGTCATCCACGTAGTCTTATG
TTCCTCCAACGCCTCCCACTTCACCATACACTTCCACTCCTTATTCTGCTTCAAAAAACCCAGCTTGGTATGC
AAATCCATCTCCTCCAAATCCACAATCGTACCGTGATCCGGCGCATCCCACTTGATACCCAGCGTTTCCAACC
GATTCTGCATCTCGTCATCCTCTCCACCCCATCCCCAGAACGTATTGGGATATCCGTTGATGCGCTTCATATC
CTCCTCCGAGAAGGAAACGACTCCGCCAAAGTACTTGGGATTATTGGCGTACCTGTCCCACACGCGTGCAATA
TGGAGCGGCCGTTGCGGGAATTTGGAATAGGAATCTCCCACGTCGGGTTGGGGGAGCAAATCGACATCGTGGA
AGATGAAAACGTTGTGACGAGGAGGATGCTTTTCGGATCGTTTTAATGCAAAGTCGAATCCGATGTTGAGGAG
CTTTCCTCGGTTGAATTTTCGATTGTCGTTCGATTGCTCGATGATGTATATGTGATAATCTTCGATTTTGTGG
GAGTTTACTAAATTTTGGAGGAAGTTTTTCATGTGGGGTATGAATGATTGTAAGTGTTTGGCTCGATTCTGGG
CGACGTGTAAGTCGCGATAGGGTACGATGATGGCGACCGAGTTGAGTTGTGGCTTCTTCTTTTTGGGGGATTG
TTGTTGCGTAGCGGCCATTCGTCGTTTGGTACCAACAGGATCGCTCGCCTCTAAGGGCGTGGGAAAGTGCCAT
TGAGTGTGTCGCGAGGCACGGTGGAAGTAGTACGGACGATTGTTGCGTGTTCGGGAGTTGCCGCGGGTCCAGC
CCTCGGGGATGGGGACGCTGGTTTGACCGAAATCGTTCATGTGGTTTTTTTATTGCGGTGTGATATTATTGTG
TGATGGATGTGCAAAAGTGGAAAGGTGTTGGTGTAGATGGCAAATCAGATATGAGAGCCACCAAGTCTGCGAG
GAGAGGTGTGGAGAATGAAGGGAGACTATCTAGATAGAAGTATTGAGGAGGAAGTTTTGAGGTTGGCGTTCTA
TTAGGTTGGTGAATGAGACAGCGACAGATTCGCAGAGATTGAATCAAACAGAATCAAGCAGAATGATGTATGA
GCAAGGTTGAAGATGTCTGGGGTC
>TR476|c0_g1_i1 len=270 path=[693:0-35 690:36-36 687:37-119 685:120-269]
[693, 690, 687, 685, -2]
GTAGGGTAAGGTGCCAGTATCCATGGTGCCAGTATCTCAGATAGGTACCATACCCTACCCTACAATCTACGAT
GCCATCTGTAAGTAGTGTCTTCTCAGTTCCCAGGGATCAAACTAACCTAAAACAAGAGGTTCAATGATACGAG
GGTACAACCTTTGACAGGCAGAAAAAGCCGTGGTTTTTTTTTTCCATAGTTAGGAGAGATTTTGTAATGGTTC
CTAGGTCAAAAATAATGGCGCGCTCGGACAAGTCTCAGCCCAAAACCTCAA
>TR477|c0_g1_i1 len=312 path=[1285:0-113 1337:114-114 1324:115-289
1169:290-311] [-1, 1285, 1337, 1324, 1169, -2]
CACGCGACAGGCTGCTAGGTAGCAATTCCATGTCGCCTTGCTGTTGTTGACCGGCTGGGATCTCTGGTTTCTC
ATCACCTTCATGGGGGATTTTCTCTTTCATCATTTTTCGCAGCTCTTCTCTCACGTCATCTGTTAATCGCAGG
GCAGAAAGGCTTTCCATGTTGCCTTGCTGTTGCTCACCGACTGGGATTTCTTGGGGGATTTCTCTTTTGTCCT
TTTTTGCAGCTCTTCTCTCACACCATCTATTAATCAAAGGACAGAATACTTCTGTAGTTGCCTGGCACCAGGA
TAGATTTCCTCCTTGTGATC
>TR477|c0_g1_i2 len=303 path=[1312:0-56 1345:57-74 1364:75-84 1358:85-91
1370:92-97 1366:98-99 1363:100-101 1357:102-123 1369:124-125 1367:126-150
1356:151-181 1348:182-193 1360:194-201 1355:202-214 1372:215-216
1336:217-244 1323:245-280 1311:281-302] [-1, 1312, 1345, 1364, 1358,
1370, 1366, 1363, 1357, 1369, 1367, 1356, 1348, 1360, 1355, 1372, 1336,
1323, 1311, -2]

CATATCAATTTATTATTTACACAACTAGATTGATTTCAGTTTCGCCTTGCGCTTAGGCCTCGATCTCTGTTTT
TTCTTCCTCGCTCTCGGGACGAACGTCACGCGACAGGTTGCTAGCTACCAATTCCGTGTCCTCTTGTTGTTCC
TGACCAACTGGGATCTCTGGTTTCTCTTCTTCTTCTTCTTGGGGGATTTCTCTTTCATCATTTTTCGCGG
CTATTCTCTCACGCCATCTGTTAATCGCAGGACAGAATGCTTCTGTAGTTGCCTGGCACCAGGATAGATTTCC
TCCTTGTGATC
>TR477|c0_g1_i3 len=410 path=[1312:0-56 1345:57-74 1364:75-84 1358:85-91
1370:92-97 1366:98-99 1363:100-101 1357:102-123 1369:124-125 1367:126-150
1356:151-181 1371:182-208 1373:209-210 1304:211-211 1337:212-212
1324:213-387 1169:388-409] [-1, 1312, 1345, 1364, 1358, 1370, 1366, 1363,
1357, 1369, 1367, 1356, 1371, 1373, 1304, 1337, 1324, 1169, -2]
CATATCAATTTATTATTTACACAACTAGATTGATTTCAGTTTCGCCTTGCGCTTAGGCCTCGATCTCTGTTTT
TTCTTCCTCGCTCTCGGGACGAACGTCACGCGACAGGTTGCTAGCTACCAATTCCGTGTCCTCTTGTTGTTCC
TGACCAACTGGGATCTCTGGTTTCTCTTCTTCTTCTATGCGGATTTCTCTTTCATCATTTTTCGCAGCTCTTC
TCTCACGTCATCTGTTAATCGCAGGGCAGAAAGGCTTTCCATGTTGCCTTGCTGTTGCTCACCGACTGGGATT
TCTTGGGGGATTTCTCTTTTGTCCTTTTTTGCAGCTCTTCTCTCACACCATCTATTAATCAAAGGACAGAATA
CTTCTGTAGTTGCCTGGCACCAGGATAGATTTCCTCCTTGTGATC
>TR478|c0_g1_i1 len=611 path=[1258:0-124 1300:125-292 1285:293-316
1298:317-539 1301:540-540 1292:541-563 1293:564-566 1294:567-590
1295:591-610] [-1, 1258, 1300, 1285, 1298, 1301, 1292, 1293, 1294, 1295,
-2]
GGTTAATAAAAAAATTGAAATAATACACCAATAAAAATTGAAAACACTCTCCTTCCATGAACAAAAACGACCC
CCCTCCTCCTCAAAATAACTAGTACTCACCCCTTGGTACTAACATGCCTACTCTTCAACAAATACTGATCCAA
CCCACCCATCTTATCAATACTCCTCAACGCACTCGTCGTAATATGAAACGGTATCATCTCGTCCAAAATCTCG
GAATACACTCGCTTCTCCTGCACATTCGGATTCCATCGACGTCGCGTCTTCTTCATGGAGAAGGGGACGTTGT
TACCGAAACGAACATCCTTTCCGTCGTAGAGGCCACGGCGGGAGCGGGAAGAGCGGGTTCGGATTGCGGTTTG
CGTTGGTTGTTGTTGGGAGTTGTTGATGATGCTGGTTGGTAGAGATGATGATGGAGTCGACGTTGCCGTCGTC
GCTGACATCCATGTTGGGGAGGAGGCGACGGTGGAACAGGTGGTGAGCCAGGATGGAGCCGATTGGCGGACCG
AGGAGAGGGCGAATCGCTGTAACATGGTTGACCCTGGGAGACGGATTGGCTGATTTACCCCGCTACTTTGTTC
AATGGCGTCGGCGTCAGCGACGGGCAG
>TR478|c0_g1_i2 len=1014 path=[1268:0-455 1269:456-479 1299:480-505
1297:506-527 1300:528-695 1285:696-719 1298:720-942 1301:943-943
1292:944-966 1293:967-969 1294:970-993 1295:994-1013] [-1, 1268, 1269,
1299, 1297, 1300, 1285, 1298, 1301, 1292, 1293, 1294, 1295, -2]
CACACACACACACATCATTGCCGGGCTATTCTGACACAGAATCTTTTTCCAAAGACTTGCATGACCATTGCAT
GACTGTCTCTAAGTCTATTATTTGCAATCAATATCGCTGTTGGAACTTGTTTTCTCTACTTTACCTATATCAG
GTGCATCAATAGGAACATCCCACCTCTAGCCATTTCGTATTATCAACAGCCCCCTTTTAAGAATGTTGTGTCG
GTGTACCTCTGTCTAAAGTCATGACGGCACCATAAAACCTGTGAGTTGTATCTCTGCGATTCGACTTGCACAA
AATCATGTGAAGCGGCCCTTGACAAATGCGGGCAATCCAACTCACAACTACTCCTCTTCCGACGCTGAGGGTT
GTTTAATCTTTTTCCCAACCAGAACTATCTTGTCCCAATCCTCCACGCACTCCCCCCTCGCAATCGCTTGCTT
CTTCAATTCCTTTTGATGCCTCATCTTTTGCACGACTCGGTTTCTGATTCGTTGTCCTTCTCCTTCGCCCTTG
GTACTAACATGCCTACTCTTCAACAAATACTGATCCAACCCACCCATCTTATCAATACTCCTCAACGCACTCG
TCGTAATATGAAACGGTATCATCTCGTCCAAAATCTCGGAATACACTCGCTTCTCCTGCACATTCGGATTCCA
TCGACGTCGCGTCTTCTTCATGGAGAAGGGGACGTTGTTACCGAAACGAACATCCTTTCCGTCGTAGAGGCCA
CGGCGGGAGCGGGAAGAGCGGGTTCGGATTGCGGTTTGCGTTGGTTGTTGTTGGGAGTTGTTGATGATGCTGG
TTGGTAGAGATGATGATGGAGTCGACGTTGCCGTCGTCGCTGACATCCATGTTGGGGAGGAGGCGACGGTGGA
ACAGGTGGTGAGCCAGGATGGAGCCGATTGGCGGACCGAGGAGAGGGCGAATCGCTGTAACATGGTTGACCCT
GGGAGACGGATTGGCTGATTTACCCCGCTACTTTGTTCAATGGCGTCGGCGTCAGCGACGGGCAG
>TR479|c0_g1_i2 len=1099 path=[1854:0-177 1855:178-198 1885:199-313
1840:314-337 1878:338-377 1897:378-401 1895:402-614 1884:615-740
1862:741-764 1890:765-916 1850:917-940 1883:941-945 1899:946-969
1898:970-1005 1893:1006-1024 1901:1025-1040 1903:1041-1046 1905:1047-1047
1906:1048-1048 1835:1049-1098] [-1, 1854, 1855, 1885, 1840, 1878, 1897,
1895, 1884, 1862, 1890, 1850, 1883, 1899, 1898, 1893, 1901, 1903, 1905,
1906, 1835, -2]
ACAATATCTTGTAAATTGTGCGAATGCGCACTATCAACAAGCCTAGCCGCAGCTCGCCTCGCCGGATCATTCA
GCTGGGAGTTGTCATAATTCATATTCTCCTGCTTCGACGCTCGGCAAAGATTCCATTGTCATATATCTACCCA
CGCTGCAACGTCTATCTACCATGACATTCAAGCTCAACCCCACCGAAACGGCCCTGGTCCTCATCGAATACCA
AAATGAATTCACAACCGAGGGTGGCAAGCTCTACGAAGCCGTGAAACCCTGCATGGAGTCGAACAACATGCTC
GAAAATTCCATTACGCTCATGGCCGAGATGCGCTCCAAGGGATGCACCGTCTTTCACGTCCCCATCGCGTTCG
AGAAGGGACACAACGAGATCGGGGCCAGGGCGGGCATCCTCGCGGGAATTGCTGAGGGTAAATCGTTCGAATC

CGATTCGTGGAACTCCAAATACCATCCCTCGATGGAGCCGGCCGCGGCTGATATCAACGTGCAAGGCAAGCTT
GGCCTGTGCGGATTCCATTCGACCAACTTGGACTTTCTCCTGCGCCAGAAGGGGATTAAGAATGTGGTGATTG
GTGGATTTTTGACGAACTGTTGTGTTGAGAGTACTATGAGGGCGGCGTACGAGCGTGGTTTCCAAGTTTATAC
GTTGAAGGATTGTACCGCGGCGACGAGTGTGGAGGCACATGAGGCTGCTTTTGAGCACAATTTTGGTATGTTC
TCGACGCCAACAAATTCTGCGGAGGTGACAGCTGCCCTGGCATAGTGTTGCGATCAGCGTTACTGGTTACTAT
TATTGGGCATGGGATATCAGGGTGTCGAGGTGTGCCAATGATCTCCAAATTGATGCATCAGTGGTTGGATACA
TGTGCCTATGGTGGATATGGAACACCACATGCAATCTTTTGAGCTATTCTTTGAAAATCCTCTCAGCCATATA
GTACTTTGAATCTTGTGAAGAGTGCAACCGCACTAGTGCTATTAGTCGGAGATAATTGTCATCGTTAGAGTAT
ATAAAAGTTATGATGGTTATCATTATATTTTCAGGACTAAACTTATCCACAACAAAAAATAAAAAAAAAGATC
GGAA
>TR479|c0_g1_i3 len=1344 path=[1803:0-177 1887:178-197 1892:198-242
1880:243-266 1886:267-319 1785:320-343 1879:344-383 1800:384-407
1801:408-443 1885:444-558 1840:559-582 1878:583-622 1897:623-646
1895:647-859 1884:860-985 1862:986-1009 1890:1010-1161 1850:1162-1185
1883:1186-1190 1899:1191-1214 1898:1215-1250 1893:1251-1269 1901:1270-
1285 1903:1286-1291 1905:1292-1292 1906:1293-1293 1835:1294-1343] [-1,
1803, 1887, 1892, 1880, 1886, 1785, 1879, 1800, 1801, 1885, 1840, 1878,
1897, 1895, 1884, 1862, 1890, 1850, 1883, 1899, 1898, 1893, 1901, 1903,
1905, 1906, 1835, -2]
ACAATATCTTGTAAATTGTGCGAATGCGCACTATCAACAAGCCTAGCCGCAGCTCGCCTCGCCGGATCATTCA
GCTGGGAGTTGTCATAATTCATATTCTCCTGCTTCGACGCTCGGCAAAGATTCCATTGTCATATATCTACCCA
CGCTGCAACGTCTATCTACCATGACATTCAAGGTGAGTGAACATGACTATGGTATCGATCACTAGATGGTCCT
CTCATGAGACGCTGTGGATCTTTGTCGATAGTTTGAGATATCATGTGGGCCGAAAGATGCCTATGCTGACCGT
GCTGCCCTCGCATTGGCTATGTCGATTGTCGGCGCTCGATCGCTCTCGCGAGTCTCACCACGATCGTTCACTG
GCATTGCCACCCTACATCACCATTTGGCCTCTAACCTGTAACCTTTCATACCATGCAGCTCAACCCCACCGAA
ACGGCCCTGGTCCTCATCGAATACCAAAATGAATTCACAACCGAGGGTGGCAAGCTCTACGAAGCCGTGAAAC
CCTGCATGGAGTCGAACAACATGCTCGAAAATTCCATTACGCTCATGGCCGAGATGCGCTCCAAGGGATGCAC
CGTCTTTCACGTCCCCATCGCGTTCGAGAAGGGACACAACGAGATCGGGGCCAGGGCGGGCATCCTCGCGGGA
ATTGCTGAGGGTAAATCGTTCGAATCCGATTCGTGGAACTCCAAATACCATCCCTCGATGGAGCCGGCCGCGG
CTGATATCAACGTGCAAGGCAAGCTTGGCCTGTGCGGATTCCATTCGACCAACTTGGACTTTCTCCTGCGCCA
GAAGGGGATTAAGAATGTGGTGATTGGTGGATTTTTGACGAACTGTTGTGTTGAGAGTACTATGAGGGCGGCG
TACGAGCGTGGTTTCCAAGTTTATACGTTGAAGGATTGTACCGCGGCGACGAGTGTGGAGGCACATGAGGCTG
CTTTTGAGCACAATTTTGGTATGTTCTCGACGCCAACAAATTCTGCGGAGGTGACAGCTGCCCTGGCATAGTG
TTGCGATCAGCGTTACTGGTTACTATTATTGGGCATGGGATATCAGGGTGTCGAGGTGTGCCAATGATCTCCA
AATTGATGCATCAGTGGTTGGATACATGTGCCTATGGTGGATATGGAACACCACATGCAATCTTTTGAGCTAT
TCTTTGAAAATCCTCTCAGCCATATAGTACTTTGAATCTTGTGAAGAGTGCAACCGCACTAGTGCTATTAGTC
GGAGATAATTGTCATCGTTAGAGTATATAAAAGTTATGATGGTTATCATTATATTTTCAGGACTAAACTTATC
CACAACAAAAAATAAAAAAAAAGATCGGAA
>TR479|c0_g1_i4 len=1071 path=[1854:0-177 1855:178-198 1885:199-313
1840:314-337 1878:338-377 1897:378-401 1895:402-614 1884:615-740
1862:741-764 1890:765-916 1850:917-940 1883:941-945 1899:946-969
1898:970-1005 1893:1006-1024 1871:1025-1048 1872:1049-1049 1873:1050-
1070] [-1, 1854, 1855, 1885, 1840, 1878, 1897, 1895, 1884, 1862, 1890,
1850, 1883, 1899, 1898, 1893, 1871, 1872, 1873, -2]
ACAATATCTTGTAAATTGTGCGAATGCGCACTATCAACAAGCCTAGCCGCAGCTCGCCTCGCCGGATCATTCA
GCTGGGAGTTGTCATAATTCATATTCTCCTGCTTCGACGCTCGGCAAAGATTCCATTGTCATATATCTACCCA
CGCTGCAACGTCTATCTACCATGACATTCAAGCTCAACCCCACCGAAACGGCCCTGGTCCTCATCGAATACCA
AAATGAATTCACAACCGAGGGTGGCAAGCTCTACGAAGCCGTGAAACCCTGCATGGAGTCGAACAACATGCTC
GAAAATTCCATTACGCTCATGGCCGAGATGCGCTCCAAGGGATGCACCGTCTTTCACGTCCCCATCGCGTTCG
AGAAGGGACACAACGAGATCGGGGCCAGGGCGGGCATCCTCGCGGGAATTGCTGAGGGTAAATCGTTCGAATC
CGATTCGTGGAACTCCAAATACCATCCCTCGATGGAGCCGGCCGCGGCTGATATCAACGTGCAAGGCAAGCTT
GGCCTGTGCGGATTCCATTCGACCAACTTGGACTTTCTCCTGCGCCAGAAGGGGATTAAGAATGTGGTGATTG
GTGGATTTTTGACGAACTGTTGTGTTGAGAGTACTATGAGGGCGGCGTACGAGCGTGGTTTCCAAGTTTATAC
GTTGAAGGATTGTACCGCGGCGACGAGTGTGGAGGCACATGAGGCTGCTTTTGAGCACAATTTTGGTATGTTC
TCGACGCCAACAAATTCTGCGGAGGTGACAGCTGCCCTGGCATAGTGTTGCGATCAGCGTTACTGGTTACTAT
TATTGGGCATGGGATATCAGGGTGTCGAGGTGTGCCAATGATCTCCAAATTGATGCATCAGTGGTTGGATACA
TGTGCCTATGGTGGATATGGAACACCACATGCAATCTTTTGAGCTATTCTTTGAAAATCCTCTCAGCCATATA
GTACTTTGAATCTTGTGAAGAGTGCAACCGCACTAGTGCTATTAGTCGGAGATAATTGTCATCGTTAGAGTAT
ATATAAAGTTATGATGGTTATCATTATAAAAAAAAAAGATCGGAAGAGC
>TR480|c0_g1_i1 len=330 path=[308:0-329] [-1, 308, -2]

GATCTCTTCCTTTTTTTTAGACCGTTCAGCCCGTCCAACTGTGCCATCTGGTCCACCGGCATCTGGCCGGTCA
GGTCGCGCGCGTGTTTCATTTGGTCAGGATCAGTTTGCCCGCGCGGGTGATGCGCAGGGTGTAGATTTGCCCG
TCGAGCACGATAAGCGCGGTATTGGCACCATTGGTGAGGGCGCGCGCATCGTGGCAGGGGGCCTCGGCCACCA
AAGCGGGATCGGATTTGGTCTGAGCGCGGATCATGCGAAGGCCCTCGCCGGGCTGGTATGAAGAAGTGACATG
GTGTTTCGGTCCTTTGTTACAGGGTGGCCGGGCTGACC
>TR481|c0_g1_i1 len=231 path=[209:0-230] [-1, 209, -2]
ATTTTCTCACGTGGTTCCTTTTATGCTTGAATCAATTTGAGCTTCAAGGTCTTCCTATCCCCTTTCTCATTCT
TCACCAGCGCTCGGATTTTCTCTGATGGTTCTGGACAGGTTATTGTCGCATCAGCTGCATCAAGGCATGGTCC
AACCTTGTCATGTAGCTCCACTTGTGTGTATGGGTTTATCCCTGGCGGGGTCAGAATAGGAAGCATGTTTGGA
ATTGTATCCCTC
>TR482|c0_g1_i1 len=372 path=[350:0-371] [-1, 350, -2]
CTTATTACTAATACATGCCAAATGCCAAAGGCGAAAGGGATGAGGTAAATCATTGACCATGTGATGTATCAGT
ATCGTCCTATGAGTAACACTAAACAAAATATTATCCCGAGCCAAACAGCCATACCCATATCCTTCCTTTGCCC
TTTGAAACAATCTAACGTTCGATCTTAAAATGAAATCTAACCATCATTATTCAGTGGCGATAATATTAAGTAT
CGCCGCTTCCATCGTCGTCGCCCAATCTACATCATCCAGCTGCCACGATGGCGCCGACGGCGACAGCTCCCCT
AGCTACACCAAATTACCCGCTGGTGTCACAACCTACGGCTTCGACGACGGCGGATGTCACTTCTGCGCCGCCA
CCGTCAC
>TR483|c0_g1_i1 len=233 path=[211:0-232] [-1, 211, -2]
TCCGATCTCAGCGGCAACGCGGGGGTTCGAATTACGACGATGGTGGTAAACAACAGCGCGATGAGTCGTATTA
CGACATCGACGATGCTAGGAGGAATAGCAGGGTGTTGGATTTCACATCGCCCAAATTCGATGCGATGCATGCA
TTGAATGTACCCGAACGAATTGTGATGGAGGCGAATAACAACAACAACAACAACGGTAACAATGACCACGCGA
CACTATGTTCCGGA
>TR488|c0_g1_i1 len=237 path=[215:0-236] [-1, 215, -2]
TTTGGCTGCTCTCCTCCTTTGCTTTGTCCTCCCCTCTTCTCTTTTCCTACTTTCTCTGTCTTTTTCCTCCCCT
CCTCTTTTTATCGTTCTCTGTTACTCTTTTCCCTTGTACTTTGCTTTTTTTCCTTCTCCTTCTCCCTCATCCT
CTCCCTTCTCTTCCTTCCTCTTTTCCCTCTCTCCTTTCTTTTGCACGTCCCGCTCTCTTTTCTTTCCCTTTCC
TTTTCTCTTTCTCCCTTT
>TR490|c0_g1_i1 len=329 path=[307:0-328] [-1, 307, -2]
TTATTCGTTTCTGCTTCTGTGAGGGCCTTCTTCTTCGGCTTCTTGTAAGATTTTTGTTTCTCCTCCTCCTCGT
CGTAGTCATCGTGTTCGCTGTCCGTCGACATCGCAGCCATCTCGTTCGACTCCAGTTCCTCCAGCAACTTGCG
CGCCTCCTCCTTCGCTCGCGTACGTTGCGTCTCCATCCCTCGCTTCATGAATTCCAATTGAAACAATCCCTTC
TTCTTCTTACCGTCCCCATTCTCATCACCGCCAACAGTATCATCCTCCATCAATATGTCACGCGCTTTCTTGA
GCAAACCCTCCTCCGTATCGCTCTCGCTCCCGCTATC
>TR492|c0_g1_i1 len=324 path=[327:0-91 328:92-115 329:116-323] [-1, 327, 328, 329, -2]
ACCTGCCCAAGTCTAGGGAGTCCAAGTATCATTATTACAAGAATCTACGCACCAACTCTGAGGTTGGGCATGC
TAGACTCCATGCCAGCCTAGGTGATGATTTGAAGACCCTTGAGAGCGAGGAATGCGCCCCGTTACTGTCGGAG
GTGGAGTCTTCTGTGAAGGAATTCCTGGAGGAGTTCACGGATGATGATATCATCGATAATGCTTGCAATGGTA
TTGAGCTTAAGAAGACCCTTGAAAAACTGGATGCGATGGAGCGTGATATGGTAGAACAAAAGGATCCTTGTAT
CAAGTCAGCTGAAGAAGTTGATAGATCGGAAG
>TR493|c0_g2_i1 len=842 path=[833:0-647 834:648-841] [-1, 833, 834, -2]
CATTTAGATCAATCCTAAAAATATTATGTATGATATCGCCATAGAAGCGATGAAATGCGTAATAATTGTCGCA
AGTCAAAATATTGGATTCTGTCGAAACATTTTTACTTGTTCTCCATCAACTTTTCTGCCCATTCGACTTCTTC
TTCCGTTGCCTCACGGAACTCTCCAACTTTCAAGTCTCCGAGCTCGATGTTGCCATGACGCAATCTCTTCAAC
TCTTCAACTGGATGTCCACAATTAGCCAAAATTCGTCGAACCATTCGATATTTACCCTCACTGACGACTAGTC
TGACATCTGAATATGGTCCTGAGTAGTCCGCCTGTCTCTCGTCCTCCTCCTCACCTTCGTCTATCCCCACAAG
ATCCTCCAATTCATCATCATCCATGGCCGTGGGGCCATCTGAGGGACTGACTGCAATCAGTTTAGCTTTGTGT
ACGCCCTCAGATGTTTTTACCCCATCTGTCAACTTTCGAGCTAACACCGCTTCGTCTACAAGACCCTGCACTG
TCGCTACATACTCCTTCTCCACGCCCCTCCGTGGATGCAGTAATCGTTGCGTCAACTTCCCATCTAAAGAAAA
TAGTATCAACCCCGTCGTATCGTAATCTAACCTGCCAACGGGGTGCATTCCAGACTTGACAAACCTCGGTTCC
AACACCTGTCCCAAATGTTTGCGATTTTGATATTCATATTTCTTCTCATCCTCCATCACCGACAATACGTACT
TCGGCTTGTGATACGCGAGCAACAACGGAGGCGGACCCGGGAGCAAATTCCCATCCAAAAACAGGGACGCAGC
GTACGGCACCTTTTCTTTCGGACCTTTAAGGCGCGTGCG
>TR494|c0_g1_i1 len=1110 path=[1123:0-236 1124:237-252 @1136@!:253-333 1127:334-350 1128:351-1109] [-1, 1123, 1124, 1136, 1127, 1128, -2]
TATTGTACCTGCAATTAATTTAAAGGTCCGCCTTGAAGTGCAGGGATATTATTGCTACCCAGGTTTTATTTGG
GGAACAGCAAAATTTTCCACGTCATAAAATGGGTGAAAAATATGTTTCTTTGCATATTAATTCTAATTGCAAC
ACCATAGTGTATTTTGCTTGTTATCCTTTCACTCATGTGTTGTGTACAAGAACATGGTCAGACGTAAATGTAT
CAAACTCCTCATTTTTTTCATTTCAGTGCTAATCTACCACGAAGGAAGTCTCCTGCCTCATTATATCTATCTC
CCTCTCCTTTATTTCTGTAATGTCATTCCAAATTCGAAAAAATCTATTTCCTGTAACACGCACTCTACCACAT

CTCTCATAGAGACAGTGTACAATATCAACCAACAAATCCACTACCAAGACCATACATGATGTGTCTAACCAGA
CTCGGGTCCACCACCGTCTCCACCACTATCACCACCCCCCTCAAACTTATTATCCGGGGCAATACTCGCCGTC
AATCCCTGCCTTTGCAACCCCAAACAATACTTCATCGCCTGCTCCTTCCAACACGTGTCCACCGTCGCCTGTC
CCTGCCCGTGCGTCACGGTACAAATATCGAATGCCTTCTTCTTGTCCAACGTTCCCATGACCTTTTGTAGGGA
ACGGATGACGTAGTTGAAGGTGTGGACCTCGTCGTTGTGGAGGACGACGCGCCATTGTTCTTCGGGGGTCTCG
TCCTCTTCCATTTCGGTGTCGGTCTTGTCGATGACGATGGTGGACAGTGACTTTTTCTTGTCTTTGGATTTTT
CCATGCCGCCGCCGCCTCCTCCTCCTGAGTCGGACATATGGAGTACCGATGTGCTGATGATTCTGTTGCTGCT
GGAGGAAATTGTTCGTTGATGGAGGGATGATGCTCGGTGTTGTGCTCGTTGGGAGGCTGGCGAGAATGCCGCC
GTTTGAGAGAGGCACATGGCCAGGGCAAGCAGTATTGTTGAGATGTGCATGATGTTCAGTGGGAACTTTTATC
AACTAATGGGCGTGTCGTCTAGTCTATTAAGTTCTACTTATCACGTTGTATTGTCGCGTCGAATAAAGGAAGA
TTGGAGTGAGAGAGC
>TR495|c0_g1_i1 len=1352 path=[1375:0-413 1376:414-437 1382:438-1201
1379:1202-1217 1380:1218-1351] [-1, 1375, 1376, 1382, 1379, 1380, -2]
CTCCAAAACGCGGCGCTCAGCGTCAGAAATTATCGACGGACGCTCCGCATCGCCAAGGCGTGTTACCTCGACG
CGCAGCACCCGCAGCTGCTCCTTTCGATGCTCGGCATGCCGCCCGCGAACCTACTGCCGATGGAAACGAGGCG
GGATATGTTTCGAAGATTGGTCGGCGTGGCCATGATGCCTTTGGGGAGTTTGGTTTCGTCGTCTTCGATCGAG
CACCGCGCGGGAGTGCAGGGTGGAGGTCGCGCGTTGCATGCAGATCACATCGAGAGGAATGTCAGGTCGATTT
TGGAGGGTGGAGGGTCGCTGCCGTTGGTGTTTCCGAGGTATGAATTGGGATTTTCGTACGAGCAGAATGGCGT
GAACGAAGGAAGCAATGCTGCGAACGATGACTCTGGCGATTCGGCGGGGTATTTCCCGAGACTCAAAGTTGGA
CATAGAATGCCTCACGTTTTGGTCGAAGTGTTGGCATCGTCTGCCGACAAAAATGATAAAGGTTGGGATGTTA
TGGAAACTCTCAACCATTGTCGAACAGCTAGCAGCCATGATTTAGCAGGTGCCAATTCTGACAGTGCGGTGCA
TATAAGCCTCACAGACATATCATCCCAATTGCGACGAGTTCATTCGTACTCTTCTCCTGTCTTCACTCTTCTT
GCATTTGGACCGGTATTGGCAAAATCCACCACCCTCACTGGCGAGGTTGTGGATCATGTTATGAGCAAATGGA
ACGTCCCAACAATGCTTGTCAATGTTCTTCCAGCCGAATCTAATATTGGCGAGTACTCCAGATTATCTGGTAC
TCATGATGGAAGCGAGTTAGAAGGATTCAACTTGCTGCATACTGTCGACACACAACAAGCACTGCTTCGACTA
CTAAATGATGAACATGAACTGGCATCTAATACCAAAGAACGCTCAAAGCGGATACAACATTATGGCAAAGCAG
GCGAAAATGCTGATGATTTAGTCGATGCTCTTATTATGGTTCGCCCAGATGGGCACATAGCAAGTATCTCATA
CATTGACGCTGAGAAAGAAAATGTGGAAACAATGACAATCCGAATTCGAGAAGTGATGGAACGAGGATTTCGA
AATGCCTTGGGTGACCGGGTGTTTGAAGATTGATTGGACATGCTGCCACGCCCCCTGACCAATGAGGACTATG
TGCGGTTTGTTGGACCATCTGGGAACCTTTTTTTCGATGATGTGTCTAACTTGTTGCTGAGAACCATATATCG
TTATTTTAAACTGTCCCTTACTTCACTCAATACATCCTTGATAAGTATGGTATCATTATCATAGTGCTTAATA
CATGCTACAATATATTTAGCTTGATTCGATTTATAGGC
>TR496|c0_g1_i1 len=2745 path=[2877:0-2016 2878:2017-2040 2887:2041-2431
2888:2432-2432 2876:2433-2744] [-1, 2877, 2878, 2887, 2888, 2876, -2]
CTATTACTGAACAATTTTAGTAGCATTACTTGGAATTATTGTTCCCGCGAGTTGCTGCGATCATTGACGTTGC
GCCACCACATGCACCCACCTTTTCTAACTCAAACAGGAGCGTCTCGCCTACCCAAGCGCAAAAGTTTACTCCT
TCACCTTCGTTGAAGCCAGACATGGACATAACATTCACTATGTTCGTCACAGTCTGCACAGTTGTCTCAAACA
GAGGGGTGGCGCGACCAGCTTGGAGGTTATCACTCACTATCGATGCAACGCTTATCGTCCAATCTTCTAGCTG
CGACCAATTATCTTTTTGGTTACTGAGTTCAGGCATTAGCGTGAGAAATAACCGACCAATGAGAGAGAGGGAA
TATACTCGCGCTTCTTGTGAGGTGATGCCAGGCGGGTTAGTGGCAACTAGGTCCAAAAAGGTGAACCAATTCG
CCACTGGAATGGATTCAACTTTGGTAGAAAGTACCAGTCCTTGCAGAGATTCAAAACCCTTCTTTACAGTGGC
ACCATCTGTTGCCAAACAAGCATCGTTGTAAATTGCAGTCACCATTGTCACGAACTCATTCTCCTGGAGATGA
GCATTGGCATTGGCAGACCCATTACCAACAAGTGCCCTATCGGAGAAGTGCTGCGAAAAAGAGTACACCTTGT
TGATATATGTCATCGACGGGACCTTAAACGACACATCGTCCTCATAGGAACCATCCATAAACTTCAGGAGCAA
TGAAGCCATCACCTCCACGCCACACTTTGACAATTGGACATCATTTTTGTTTTCGTCGCAGTTCTGCATGTCT
CCATCTCCAGCGGATGGTATCGCATAATCAATTGAATCTATAACAGATGCAATCCCATCAAACACAAAACCAC
GGCCAGATTTGTCTTGAGCAGCAAGGTCCAAAAGGCCCTTGATTGTCGACCAATCATCTGAACTGTCATCAAA
TAAGTAGAAGCAACCACGAAGAATGATAGCTGCACCACAACCGATGCGCTCGTTTATTGCCTTGGTGTATGAT
GAAGGAAGCGTAGCGATGAGATTCAAAGAGCGATTAAGTTGATCTCGAAGGCAAACCTCGGGAACGTCGAACA
AATGAATGCATGCGCGTAATATGGTAACCACGATTCTTTCAAGAATGTAAGGGAACTTGAGCCCCAAGACGAT
ACTTTTCGATGCACCATTTGTTTCCACTATTTGAGGAGAAATCAACTGTTGAAACTTGGTGAGGAAGATCGGG
TAAAGCTCTGCCGCATGGGCTCGATTCGAAATCAGCAACCTCGCTGCCAGTTCCAGCACGAAGACAGAATTAT
GTTCAAATAATGGATCAGATGTGATATCCTCGTCGTCTTCATCAGGAAACATTGATCCAAGCATGGCAAATAA
AATCCGCTTCGCCATGGCGACATCATTCGTCTTCATAATGATTTTATCAAGTTGTGCACATTGGGCAACTTGC
TGCAGTGTCTCCGAAAGAGGAAATAGTTTTTCAGTACTGCCGTCGTCAACTTCTTTTTCCTGGCTCGATGGTG
ACTGTCCAAATCCAAAAAACGATGAAAAGAACCCAGCGTTGGATGATTTTTCCGATGGAGCTATCGACTCCAT
GTATTCATTGACACGTTGATGGCTTCTATTGGCGAATACAGATCTAGGCAATGGGTTACCGCGGCTGTCTGCG
AAATCGTCCAGATCCGAAAGCCGAGGCAGAGCATTCACGTCTCGCAACGCGAAAATCACGTCGAGTAACATCG
GCCATGCTTCTCTGACAAACGAAAGGTGTTGCTTTGTCAACGTGAGTGCGCAATTCAAACTTAGCAATCCTCT
GTGTGCAGCACTCCCGGTCATGTCACTAATATCCATGTGAACACTCTGGCTTCCGACATCTGTCGTCAATGTG

GATAGAAAAGATTCTGGAAGAGGAGGAATGTCTCGTGCCATAAGCTCTGCTTCAAGGTTCGACATCGCCTGGC
CATTGTCATTACGAATACCATTCGATTGCGCCCCAGAAGTTTCCACACCTGCCTCTACGTCTACCGGCTTCGT
CGTGTACATCAACGTAGTCACACTTCCAGCATAATCAAGTCCAAACCCAAGGAGTAACTCTAATATTTGATTG
AACTGTTGATCCAACCCAAAATACGAGCAAATGTAGGCCGAGTTTTGCATGCCATCGAGAGCCTTGGTGACGA
GGACATCGTCCCATGATTCGACGAATACTGCCGATATCGCGTTTATGGCTGCGGAGCAAATGCTAACGAACAT
ATCGCGTTCGTGAACCCCCGCCTGTAGAAATTTGTGGTGTGATGATTGGGTGAAAAAGGGTGTTGCGACGTCG
TTGGCTTTTGCCAAGAGCCCGTCGAACGCGTCTGCGCCGCCGTCCTGCAGCAGGTCTTGATTCACTTGAATCT
CGTCGTTTTTTATTTCGTAATAGAGATTCGTCAGAAATTCAATTGGTAGATCAGCCCCATCATTAATCCCTCT
ATTATTTCTTATGAACTGATCCTGCGTCATTCTTTTTTCGTCCTTCATGTTAGGATTGTGCAAATCAGTATTG
AGCATAATCGTCGAGAAACTAAGTATGAAAGCAGCATCTGAACTCTTGAATGGAAACACAAGATCAACTTCGT
CCGATGCCGCCGGTGGATCAAGCGTCCCAGAACGACCGCTACCGAACTCTCCATCCGAATTACCCCTCCGCGG
AGGATCCAACATGGTCTTCTCCACCGATTGATCGCGAGTAGAGA
>TR496|c0_g2_i1 len=439 path=[2880:0-125 2881:126-126 2882:127-438] [-1, 2880, 2881, 2882, -2]
GGTTGATTGGATACAAAAACGAATCACGAAAAGCATTAGACGGTGAAGATTACATTGTGTGAGGCGTCGTGGT
TCCCCACCAACATCCGCCCAATTGACTCACCGTCGTTTTTTATTTCGTAATAGAGATTCGTCAGAAATTCAAT
TGGTAGATCAGCCCCATCATTAATCCCTCTATTATTTCTTATGAACTGATCCTGCGTCATTCTTTTTTCGTCC
TTCATGTTAGGATTGTGCAAATCAGTATTGAGCATAATCGTCGAGAAACTAAGTATGAAAGCAGCATCTGAAC
TCTTGAATGGAAACACAAGATCAACTTCGTCCGATGCCGCCGGTGGATCAAGCGTCCCAGAACGACCGCTACC
GAACTCTCCATCCGAATTACCCCTCCGCGGAGGATCCAACATGGTCTTCTCCACCGATTGATCGCGAGTAGAG
A
>TR497|c0_g2_i1 len=923 path=[955:0-830 956:831-922] [-1, 955, 956, -2]
AATACCAAAAAAAACGTCATCGCTTACTTTGCCTCCTCCCAAAATAATGCATCCGTAATGTCCGTACGTCCCA
CGGTACTCGACGTGGGATGCGGCTTCGGCGGACTCACCATGGCCCTCTCCCCGGCACTGCCCGATCAAACGAT
ACTCGGACTCGAAATACGCGCCAAGGTCACCGAATACGTGCGACTGCGCATCGTCGCCCAACGGAAGGAAAAC
CCGACCCAATTCCGAAACTGTTCCGTCATGCGCACCAACGCGATGAAGTACCTGCCCAACTTCTTCGCGAAGG
GGAGTTTGACGAAGATTTTCTTCTGCTTTCCCGATCCGCATTTCAAGAAGAAGAATTGGCCGCGTCGTATCGT
ATCGGAACGATTATTGACGGAATACTGTTATTTACTTCAACCTGATACGGGAAGGTTGTATTGTATTACTGAT
GTGAAGGATTTGCACGATTGGCACGTGAGTAAGTGCGATAACCATCCGTTGTTGGAGGAGTTAACCCCAAAAG
AGATGGAGGATGATCCGTGTGTTCATTTGATGAAGACGGAGACGGAGGAGGGGAAGAAGGTCAAGAGGGAGGG
GAAGTTTGGTCATGTCATGTACTATAAAGTCTATCGGAGACGAGGAGTGGTGGATGGTAGTGGTGAGGTAAGC
GGTGTGGGGGCGGAGAACTTTTTTGAGGAGGGGCAGTTTGGTGTGGAAGCGATTAAGAAGTGAAAGAGCGTAT
GGTTTGGTTTTGTTCACTATGGTGATATATTCAACACAGTTTGATTGTTGGATACTGCTCTGTAGAGAAGAGG
GAAGAATATCCGAAGTTTGTGTTCCACATGCTAGTTTTCACGAATTGCTTTGGTAGACATGTTAATGATTAAT
CAAGGCACAAGATAACTGCGCAATAATACCCCATGGCACAGGACTGT
>TR498|c0_g1_i1 len=320 path=[370:0-70 371:71-94 373:95-161 366:162-185 367:186-207 368:208-223 369:224-319] [-1, 370, 371, 373, 366, 367, 368, 369, -2]
CGATCTAACGTGAGCCATGAGGAACAAAGCGAGAGGAACGAAAATGAAAAATGGCAAATAGATCAACGACGGC
ATCATTTATAATTCATACAGCGATCAACCTCAACACATTCAATAATGCCTAGATCAGCTGCTACCATCGCAAC
AATAATATGCTGCTGCCTCGCACCCCTGCATCTTCCCCGCACCGACGCGTTCCAACCCCCCCGTAGTAGCAGC
GGCAACACCGTCTTTTCGCCACCAACAACATCCAACACTGCAGCATCATCATCATCGTTATCATCATCATCAT
CGCTCCAAGCTTTCAAATGGCCAACCCT
>TR499|c0_g1_i1 len=276 path=[367:0-47 377:48-76 376:77-100 375:101-161 361:162-217 362:218-275] [-1, 367, 377, 376, 375, 361, 362, -2]
CGACGCAACTGTTGTCTAGTTTTATATTAATTGTATGTTTCAAAATCAATATACAATATGTGTTGCATCTTCG
CCTAATCCGATGAGTCCAGCACTTGGTTAACGTCATCTTCTTTGGGCTCTCTATATGGCAGCGCTCTTGGACA
ATTGACTTCAGCCATCTTATTCCGACTTCGCTTCAGCGGCGCTATTACGCACACTCCGCTTAATAAGAAGGGA
GGATGGCATCAATGTTTCCAAGCTTCTCATGGACCATGAGCCCAAGGATGCCCATCT
>TR499|c0_g1_i2 len=276 path=[363:0-47 377:48-76 376:77-100 375:101-161 372:162-217 373:218-275] [-1, 363, 377, 376, 375, 372, 373, -2]
CGATGCAACTGTTGTCTAGTTTTACATTAATTGTATGTTTCAAAATCAATATACAATATGTGTTGCATCTTCG
CCTAATCCGATGAGTCCAGCACTTGGTTAACGTCATCTTCTTTGGGCTCTCTATATGGCAGCGCTCTTGGACA
ATTGACTTCAGCCATCGTGTTCCGCCTTCGCTTCAGCGGCGCTATTACACACACTCCGCTTAATAAGAAGGGA
GGATGGCATCAATGTTTCCAAGCTTCTCATGGACCATGAGCCCAAGGATGCCCATCT
>TR500|c0_g2_i1 len=2004 path=[2047:0-357 2048:358-381 2050:382-1039 2046:1040-2003] [-1, 2047, 2048, 2050, 2046, -2]
CTCTCTAGAGAGCGTGATTTGATTTACTGGGACAACCTTCTTCGGGGCCAGGCTTATCGTACTTCTCGCTCTG
CAGTCTCCTCTCTCCCCTGCAATTACAGTTACAACTACCTACTGTTAAGTACTCATACAACTTAGGAAATTGG
GGGGAATACAGAAGGTTCGATATGGGTAGTTGCATCTTGAGCTTCAATTGCTTCATTTATACGAATATCATGC

TGTCGACCCTGCTTAGTTCAGCAAGTGCCTTTACGAAGAGGCATACATATGTAGCTGTAGTGAGGTCTAGACT
CAGCAACCGACTATCTTCTACGAAAGCATCGGAAGGAGTAGATCAAGTCATCGTTGTCGGCGGGGGTATCGCC
GGCCTTGCAACAGCAGCCGCCCTACAAAACATAGCGAACGTACCCAAGATTCAAGTTTTGGAGAAGTCTTCCC
ATGACTCCTTTTTCCAGAAGGAGGGCATTGGCGCCGGAGCTCAACTGGGTCCTAACGGTTTGCGTGCCCTCCG
TGCCATTGGCGGCGAGGAACTCATGCAACGTGTGATTGATTCTGGATCGATCTTGAAGGGGAATGCCATTTAT
CTTCCTGGAATGAGCGATCCTATGATGCAACCTGACACAACAGAAGAAGATTCTGGTCTTCCCCAAGTTTTTG
TCAGATGGAGCGTCTTGAGGAGCAACTTGAATGATCTACTCGCTCCTGCAGTAGACATACAATTTAATGCTGG
GTCAGATATCTGCGGGTATGAGGTCGCTGGTGATGGAAGTTTAAATATTCTTTCCGAGTCAGGGGCTGCCATT
GCATCGACACCACCGGCCTCAGCGCCAACATCTTTGATCGTGGGCGCCGATGGGGCCGTTTCGAAATGCCGCT
ATTGGATCCAAACAGGACAGTCGGACATACCTGCCACGGAGCGTGAAAAAGTGGATACTTTCGACCTTGTTGA
CACGGGCAGAATCAACCTCAAGGCAGTTGTCCCAAAGTCTTTGGGTGATTCATTTGAAGAAGGTCATACGTAT
TCGTACTTTGCGCCGGGCGGTGGAGTTGCCTGCTTTGCTGGACCTGCTGGTGACGGGCATACATATTGGGCCA
TATCAATTGCGGATGAAAAGGATGAGGAGACTGGTCAGGTCACTCAATTTATGGCCAACAAGGGCCAGTCTGA
CCTGAATGAAGTCAAGCTTGCTCTCTTGGATAAAATTTCAAGCTTAGACACTGATACCGTCAAATTTGCTTCC
GATATGATCGAAGCGACCGACCCCGAAACTATCTATGCAACAAGATCAAAAGAAGCGCAGAATATAGGGCCGT
CTCTGGCAACAAATGATGGAAAGGTCGTGCTCGTCGGCGATGCAGCCCATGCAATGAGCGGGGCGTATGGTCA
AAACCCAAACTTCGCGCTGGAAGGAGCGGCGGAACTGGCGACATGCATTCGAAACAACGGGAGCATCGAATCG
GCATTGCAGAAGTACTCGTCGATGCGCGTGGGTAGGTGTTTCGAAATGCAGAAACGCAGCGCGGAACGTGCCG
CAAAAGCAATGAAGGGCGAAAAGGATTTGGAAGATGTTTCTGCCTGGATTCATAAGTGGGATATAGAGAAGAA
TGAGTAAGAGAAGTCCAATGAGTCATGACTGTTAATGATAGAATGAAATGTGCCATTCTCTGTCCACAATAGA
GCTGTATGTGCCTACAACAAGACGAACTGAGAGTAGCTGCTTGCTGATTGGGATACTACGTTAAAGTTAAAAG
GAGCTATGATTCAATAGTACATCTCGGCCTCTTTTATCTAAGTTCAGATGAATCGGTTGAAGTAAGTTTTGTT
AGGGTTGTGCTTGTCACCAAAGTCAAGCACCCCTTAGCTTAATATGACACTGCAGGGTGCGATTCATCTCACC
GTGGCATGATCAAGTACGATGTGTTACATGTCTTCAATTGTGCCGCCTTCGGACACAAGTGATTGGCGAGTGA
TTAGTTTAGTACGATGGCATTCCTACTCTTCAA
>TR501|c0_g1_i1 len=2161 path=[2245:0-106 2246:107-130 2252:131-590
2240:591-614 2253:615-1064 2249:1065-1088 2251:1089-1426 2243:1427-1450
2244:1451-2160] [-1, 2245, 2246, 2252, 2240, 2253, 2249, 2251, 2243,
2244, -2]
TGCAATTCAGTTTCTTCCAAGTTTAGAGTTACAAGTTATTGCACCTAATACTCTTAGATATTTGAAGCTCAAA
CATCAAACAATAATTCATCGGAAATTCAATCGTCAGCCGCTAGCATCCATTTCTCAAAGCTTCGATACCGGTT
CATCTTCAGGAATGACATCTCCTTCATACTTCGTCGTAAGAATGTGGGGATAACATACAGTTGTCAAAAAGTC
ATCAAGTTTATCTTTTGTTAACATGTCCTCCACCAATTTACCAGCCAAGAACCACTTTCCTCTGTCCTCTCCA
TCAGTGCGGCGCTTTACTTCATCGTGGACCAACTTCGAAATCCTTTTCGCCGTAATAATCACTCCATCGTCCT
CGGTTTGGACTCCGTGGTACTTCCACTGCCAAATCTGTGCGCGACTAATTTCCGCGGTGGCAGCATCTTCCAT
GTGATGGTTTAGTGGGATGCATCCAATGCCACGAAGCCATGCCTCTGTGTATGCAAGAACAATGAATACTCCC
TTTTGTAGACCCTCACTTGTAATTGCTTTCCCTTTCGGTACCACTTGTAGCTTCAACAGGTCTATTTCAGTTA
CATCTTTCCCAGAAAGACCTGGATTCTTATCAATTTGATTAAATGTAGGCATGTAGCTGTCAAACACAGATTT
AGCAACAGAAACCAGAGCAGGGTGTGCAACCCACGTTCCATCATGTCCTGCCAGTACTTCCCTAACTTTGTCC
TTCTTTATTCTTGCCATAGCCGCATCATTGGCTTCTGGGTCTCCTTTTATGGGAATCTGAGCCGCCATTCCTC
CCATTGCGAACGTTCCTCTCTTATGGCAAATATAAATCAATCTATCCACGTAACTCCTAAGCAGAGGAGTGTC
CGTCATGGTCAAATGCGATCGATCCGGCGCAATCTTATCATCGTGACATTTGAATTTCTTAATGTACGAGAAC
AAATAATCCCACCGTCCGCAATTCAACCCTAACGAATGCGCTCTCAACTCGTACAATATCTCCTCCATCTGAA
ACGCCGCCGTAATAGTCTCCAACAACACCGTCGCCCGAATCGTGCCCAACGGCACCCCAAAATAGCTCTGCGC
CGCCCGAAAGACATCGTTCCACAACCGCGCCTCCAGATAGTCCTCCAACTTTGGCAGGTAGAAGTACGGCCGC
GATCCCTTCTGCAGCAAGGAGTGCACGTTGTGGTAAAAGTATAGTCCGAAATCGAAGAGACTGCCGGAGGCTA
CGCGACCGTTGACGGTGACGTGCGATTCGTCGAGATGCCACCCGCGGGGACGGACGAAGAGTACGGCGGTCTT
GTCTTTGAGGGCGTAGACTCGTCCGGTGGCGGGGTTGGTGTAGGTGATTTTGCCGCCTACTGCATCGCGGAGG
TTGCGTTGGCCTTCGGTGAGGTTGGACCATGTGGGTGAGGTGGAGTCTTCAAAATCAGCCATGTAGATATTTG
CTCCTGAGTTGAGACCATTAATAACCATTTTGCGATCTACTGGTCCGGTAATTTCCACACGACGATCCTTGAC
ATCGTCCGGAATAGGAGCACATTGCCAATGAGGATCCTCGGTAGCATGCGAATGCGGCTCCCTCCTCATATTA
CCACCACCACCACCATTCTTCCCAGCAACACCTTCAAAATGAGGCACTGCTCCGCTATCGAACTCCATCGCTC
TGCTCGCACGTGCATTCAACAACGCACGACGACGATCCTCGAACTTGTGGCATAAGTATCCGACGAAACGCAA
TGAGTCGGGAGTTAATATCTCGGATGCGGCGGCGCAGATTGGTGCATGGACTTCGACGTGAACGGAGGTCGAT
CGGGCTTCCATGGTGGATATCTTGCGTATTGTTTTCTATGTGGTGGGTTTGTTAGTGAAGTTTTTGATGTTGA
TGTGATTTTTTTATGACGAGGCGATTCTTGTGGCGTTCTGTTGTGAGAAATGGTCAATGATGTCAATGATGTC
AATGATGCTTAGGTTCTTGATTCTTGATGCTCGGCTCAATAAAAGAAGGTGTCAATGATGCTTCCTCGGGTTG
TTCGGTTGTTAGTGGTACAGTGATGTTATACACTACTTATATATGAAGTGACTGAGGTCGGCGGGGAGTGGGC
TGATTATCTGTTTCATCTGGCGTTTCATTTTCATGGTCATTGTC

>TR502|c0_g1_i1 len=1619 path=[1750:0-171 1751:172-195 1756:196-402
1743:403-426 1754:427-639 1738:640-663 1757:664-844 1753:845-1469
1746:1470-1493 1747:1494-1618] [-1, 1750, 1751, 1756, 1743, 1754, 1738,
1757, 1753, 1746, 1747, -2]
CGATGAAACTGAAACAGAGAACAAAGCCAAAGCCAGAGAAACGCCAATATCATCAGTGGCTCAGATATTTCGC
ACATCATCTTCCACTGTGCGCAATATTCTTGCTATCGAATCATTTTCTCTTAGTCCTCTCCTTTCCACTCTGC
TGCTACGCATTTGTCGCTCCAAATAAGGACGAAGATGCCACTCCTCCTGACACACACGTAAATAATGACAACG
GCGACAACGATTATGACGACGATTTCTTTCCTCCCCTCATCGAAAGCGATGATATATCCTTCTTCTTCTCCAC
CTACGATCTCGACGGATTGCATTGGGCGCTCACGAATATCTTCCACCGAAGTGCTCGCAATCCAAACAACAAC
AGCAACAACAAGAAGGCGGTGGTGGAACTTCCACAAACTCTCGTGGCACGAGGAGGAGGAGACACATACACGA
CGGAATACAAACTCAAGCACGACTTGGATCAAGCGAGGTATCTCGCGGCGCAGTTGGAGCATTCCGATCCTAC
CACGTCGGAATACTTTACATCGCGAGTGATACCGATTTACGAAGGTGTGTTGCGAAATATTCCGCCGTTGACG
AAATTGACGCGTACCAAGGGATTGTACGCATTTAGGGAAGAAGACTACGCACTGGGGATTGGAAGCGTGTACA
ACAAGGCGTTATACATGACGACTGCAGATGAGTTGGATCCCGATTGGAAGGAGCGAGGCGGTAGTAGTGGTGG
TGGCCTTCTGAACGATGACTTGGATTGGGATGGGATTCAAAGAGATTGGTTCGGAGAAGGCAGCGAAACATCT
GCACGAACGGAATCGACAAACGATGAGGCAACAACGACAACAAAAGCCACTACCACGCCATCATCATCCATAC
CCGGCGTAATCGTAATCGACGACCTCCTGACGCCCCAAACCCTATCCCTCCTCCGCCGCCTCCTCCTCCACAA
CACCCATTGGTACCAGACCAAAACCCCCCTCGAATTCGGAAAGTACGTCGGATCCTATATCGACGACGGATTG
CACGATCCAATATTCCTCGAGCTCGCGACGCAGTTGCACAAAGCCATGCCTCGAGTGATGTCAGGTCATGATT
TGAAGTACATGTGGGCATACAAGTACGATAGCGAATTCGAGAGCGGGATCAACCTGCACGCCGATCAGGCCGC
GGTCAACGTCAACATTTGGCTGAGCATGGAGGGGAGCGATCTGGAGGAAGAGGGGTACGGCGGGGGAATGGTG
GTTTATACTGCCAAGCCTCCTTCGAGTTGGGACTTCGAGGCTTTCAATACCGATACGGAGTTTGTGGTGGAGG
AGCTGCTTCGACCGAACGACTTTGCGAATGTTACGGTGAAGCACACGACGAATCGCGCTGTTATTTTCGATTC
GGCGTTGTTCCATCAGACGGATAGATATAGATTCAAGAAGGGGTATGAGAATGGGAGGATAAATTTAACCTTG
TTGTTTGGTGAGATGCAAAAGGGCGATGATGACGACATCAGTGGTAGCTCAACATCATCACCTGGCGATGGTA
CGAAACAAGAGCTTTAGTTATTGGGTTATCTGGAAGAAGAGTCCAATAAAGTAGCTTAATAACCAAACTACGT
GTCAGCAAAGCTT
>TR503|c0_g1_i1 len=1172 path=[1397:0-413 1398:414-437 1408:438-478
1406:479-502 1410:503-701 @1409@!:702-865 1395:866-886 1396:887-1171] [-
1, 1397, 1398, 1408, 1406, 1410, 1409, 1395, 1396, -2]
GACGCAGGCGCTGCCGCGGCGGGAGGGGTTACCCTCATCCGTCCCAAAGCGGCTCCGATGCGCGATGGGGCGG
GAGTCCATGAGGAGGGAGCTGTGCCTACGGAGAAGACGGCCATGCCGTTTTTGTCAATGGTGCCATCGTTGTC
ATTGCTGTGGCCTAGGGAGGAGATGCCACCGTTGGTCATTCCTCCCATGCCATTTAAAGAACGGCCTGGAGCT
GCGGCGAACTTGCCATCGTCGTTGCCATCGTCGCTCTCGACGTCCCATTCGTCTTCGAGTTCGGAGATCAGGG
ATCCCCATCGGGATTGACGGGCCTCGAGATCTTCGTTGATCAAGGTTTCGGAGACTCTCTCTGTAATCATGTT
ATTATTGTTGTATTGTTAAGTTGTGAGAGTGTTGAGATGATGATGCCGGTGTCAAATGAATTGAGTGATCGAA
GACATGCAAACTACCGTTCGAGACAGATACCTATCGATAGGTAGAGTTAGTACTACTCAACAACATATCGTAT
CGTAGCAATCACGAACCTATGAGACTTATTTATGAGTACCCTATTTTATGAAGAGGCTGATTGCTACCCTTAC
ATAGGAGTACGAATCTATTTCTGTATCTCTGAGGACGTTTGGTGAAACCGACGCGCATATTTCTTTTCTCAGA
GGCTTTGAGATTGTGTATATGACGTGGTTCGCGCGAACCGATTTCTGAGAGTGGTATGCTTGTGTGTATCGGT
TGAATTTTAATCGACCTTAGTACCGTACCAGTTTCAGCATTTGGCTAGGAGCGATTAAAAAAGAGAGAGATAT
CTAGAGATAGTCGGTTATCGATTTAGTCTACGACCTACAAGAAGTAACAATTTTACAAGCAAGATGCTACCTA
CTTACGATATTGTACAGTCAAAGAACGGTCAACGTCGGATCTCGAAGGCCATTTTCTCGTTTCATACTTATCT
TTCCACATCAGGTCTTTGATAGTGGCACACAGATAAGTACGCATACAATAATCCGACGTCTCATATTTCGTTC
AATCGCAGAGGGTTGTCAATGAAATTGTGATAGGTATCTGATCTAAGTTAGATAAAAGTAGTGCTTTGGGAGG
CTTTTATTTCAATGAGCTCCACAATATGAGTACTCAGTGCGATCCCTAGAAATAGATCTATTTCTTCGATAAC
TATA
>TR503|c0_g1_i2 len=1245 path=[1397:0-413 1398:414-437 1408:438-478
1406:479-502 1410:503-701 @1409@!:702-865 1405:866-959 1404:960-1244] [-
1, 1397, 1398, 1408, 1406, 1410, 1409, 1405, 1404, -2]
GACGCAGGCGCTGCCGCGGCGGGAGGGGTTACCCTCATCCGTCCCAAAGCGGCTCCGATGCGCGATGGGGCGG
GAGTCCATGAGGAGGGAGCTGTGCCTACGGAGAAGACGGCCATGCCGTTTTTGTCAATGGTGCCATCGTTGTC
ATTGCTGTGGCCTAGGGAGGAGATGCCACCGTTGGTCATTCCTCCCATGCCATTTAAAGAACGGCCTGGAGCT
GCGGCGAACTTGCCATCGTCGTTGCCATCGTCGCTCTCGACGTCCCATTCGTCTTCGAGTTCGGAGATCAGGG
ATCCCCATCGGGATTGACGGGCCTCGAGATCTTCGTTGATCAAGGTTTCGGAGACTCTCTCTGTAATCATGTT
ATTATTGTTGTATTGTTAAGTTGTGAGAGTGTTGAGATGATGATGCCGGTGTCAAATGAATTGAGTGATCGAA
GACATGCAAACTACCGTTCGAGACAGATACCTATCGATAGGTAGAGTTAGTACTACTCAACAACATATCGTAT
CGTAGCAATCACGAACCTATGAGACTTATTTATGAGTACCCTATTTTATGAAGAGGCTGATTGCTACCCTTAC
ATAGGAGTACGAATCTATTTCTGTATCTCTGAGGACGTTTGGTGAAACCGACGCGCATATTTCTTTTCTCAGA
GGCTTTGAGATTGTGTATATGACGTGGTTCGCGCGAACCGATTTCTGAGAGTGGTATGCTTGTGTGTATCGGT

TGAATTTTAATCGACCTTAGTACCGTACCAGTTTCAGCATTTGGCTAGGAGCGATTAAAAAAGAGAGAGATAT
CTAGAGATAGTCGGTTATCGATTTAGTCTACGACCTACAAGAAGTAACAATTTTACAAGCAAGGTAGGTAACT
ACTTTCAAGCAATCATTGGCATGACTAAAGTTCTCATTCGAAGCCTATTTTCCGTTCCCGTAGATGCTACCTA
CTTACGATATTGTACAGTCAAAGAACGGTCAACGTCGGATCTCGAAGGCCATTTTCTCGTTTCATACTTATCT
TTCCACATCAGGTCTTTGATAGTGGCACACAGATAAGTACGCATACAATAATCCGACGTCTCATATTTCGTTC
AATCGCAGAGGGTTGTCAATGAAATTGTGATAGGTATCTGATCTAAGTTAGATAAAAGTAGTGCTTTGGGAGG
CTTTTATTTCAATGAGCTCCACAATATGAGTACTCAGTGCGATCCCTAGAAATAGATCTATTTCTTCGATAAC
TATA
>TR504|c0_g1_i1 len=300 path=[2311:0-44 2312:45-299] [-1, 2311, 2312, -2]
AGCACAGACACACAGACACACACCTGGTAAAAATATCAAGATAGAGCCCTCGGACATATCCGCCCCATCAGGA
GCCAACAAAGTGTCACTGCCAGATTTTGAGTTCGTCACAAGCTTCAGAACATCTTCAATAAGATCATAATTAA
TAACCTCCTCGTTCACGCGTTCCATCGATCTACGTGTCGACATCTTATACCCAGCATACGCATCCGACACTCC
CCCCGGCGTAAATCCCTGCGAGTTCAGATCCACCGTCTCCTTCCGCTTCTCTCCACCACGCGTAGTTACCCAA
AGCGATGC
>TR504|c0_g2_i1 len=1688 path=[2318:0-105 2319:106-129 2325:130-1029
2309:1030-1053 2327:1054-1179 2306:1180-1203 2330:1204-1442 2329:1443-
1622 2302:1623-1687] [-1, 2318, 2319, 2325, 2309, 2327, 2306, 2330, 2329,
2302, -2]
TCTTCCGATCTGTGAAAGTTTAGCTATGTTGGCTTACTCTTAGATGAATCAGATCGGCATCAGACTGGCAAGA
GAGTAATGGTGGGAACCTCATCTATTCAATGAAAGACACATTTACGCTGGGGAAAGACCTGGGGATTAATGTT
TTTCAACATTTGCCTGACACCTTCTCTACCTTATTTGTTTACTCAAGTTGAATAAAGTTAAACCTTTCACCTG
CATTTCGCCTCATTCCGTGGAATTAGATCGACATTTAGAAAGCTGTTCATCACGAAGCGAGAATTTTTATGAT
TCCGTCAATCATGCTGGAATCCAACTGTTCAATTTCGCTGTTGTCGGCGCATTCGATCCTTCTTTGCAAAAAT
ATCTCAACTTTCTTTCTCAATTCTCGCAGTATTACACCAGTTTGTGCGGACATACCGATGCTCATCCAGTTGT
CAACAATCACTGTTCGCTCTGTGTGTTTGACGAAGACAGACCCACCAAAAAGAAGCAACGCGAATGGATGAAC
GAAGGCAGTTGTTGACACAGATGTACGATTCGGTGTGCCAAACTTTTCATGGAAGAGCACCCAATTCTCTGAG
CTCAAGAAACGTTTTTTGGCGGCATTCACTGAAGCGCCATGAAAGTAGATGCGTTCATCCTTGTGCCACATGG
TATAATTCATCGAAGGGGATTGCTCTAAACGGAGCACGTTGGGGTATAAGCCAGCGCATATGACAGCATGGAC
CACTTCTGTCTTCTTTGCATGTCGATTGAAATGGCACGACTTCAATTGTTCCGGGTCAACTCTGGATCCATTC
CCCATTACTGCGTTGCGATCCAAAAATCCTATACCGCACAGTAAATCCAGAAATTGTCGACGAGCATCTCCTA
TCTCACGTAGGGCAATATGGTTGAGATAATTCTCTTGGCAAAATCTCCTGCTTGCGATTGTGGATGTTTCATA
TGCATCCCATACATTGCAATACGTCATGAAGTCGCTGTCGGGGTCGGCAAAGACTCTCTGTTTTGCTTTGGCA
ACGGCTGCATCATGGACGAATGCAGCAAAGGGCGACTTCGAAGATAGACTCGCGGCAATGGTGAGGATGGGAT
CAATGCACCGAAACAGAGCGCCAAATATTAGCATCTTGCCTAACTTCACGTCCACGGGCAGTTTAGCCAGATG
AAAACCCAGTGGGGTGAGATGTTCCGATTGTTGCATGATATCACCGGTGCCGGTTCTTTCAATGGCCCCTACA
TCATATAAAACATCCATTGCGGCCTGCACAGATTCCGTTGATGGCGGCTGGGGCGCCTGACCCAAAAAATCCA
TGCAACTCTTCGCAAATCCACTGGCAAGAATTGACATGCATATTTCTTCCAGGGGCACACGTCGAAGTTCTGG
CTCGTTCGTAGCTTTCATAACTCTCTCCGCTGTCGCGAATGAGTAAAGCTTAAGGCAAATCCCCGACTGTACG
CGGCCAGCTCGCCCCTGTCTCTGCTTGGCTGACGATTGTGGGCACCAATCTGTCACAAGCATCGATGTCGAAG
TCTTCTTATTTCGACGGACCTCCCTCACCCGTCCAGTGTCGATCACATAGACAACATCTGGAATTGTCACGCT
GGTCTCAGCAATGTTGGCTTTCAAGATGAAGGTTTGTATATTGACAGTGGGAGGTATTAGTATGGAGATACCT
TCATCGCGA
>TR504|c0_g2_i2 len=1488 path=[2318:0-105 2319:106-129 2325:130-1029
2309:1030-1053 2327:1054-1179 2306:1180-1203 2330:1204-1442 2304:1443-
1487] [-1, 2318, 2319, 2325, 2309, 2327, 2306, 2330, 2304, -2]
TCTTCCGATCTGTGAAAGTTTAGCTATGTTGGCTTACTCTTAGATGAATCAGATCGGCATCAGACTGGCAAGA
GAGTAATGGTGGGAACCTCATCTATTCAATGAAAGACACATTTACGCTGGGGAAAGACCTGGGGATTAATGTT
TTTCAACATTTGCCTGACACCTTCTCTACCTTATTTGTTTACTCAAGTTGAATAAAGTTAAACCTTTCACCTG
CATTTCGCCTCATTCCGTGGAATTAGATCGACATTTAGAAAGCTGTTCATCACGAAGCGAGAATTTTTATGAT
TCCGTCAATCATGCTGGAATCCAACTGTTCAATTTCGCTGTTGTCGGCGCATTCGATCCTTCTTTGCAAAAAT
ATCTCAACTTTCTTTCTCAATTCTCGCAGTATTACACCAGTTTGTGCGGACATACCGATGCTCATCCAGTTGT
CAACAATCACTGTTCGCTCTGTGTGTTTGACGAAGACAGACCCACCAAAAAGAAGCAACGCGAATGGATGAAC
GAAGGCAGTTGTTGACACAGATGTACGATTCGGTGTGCCAAACTTTTCATGGAAGAGCACCCAATTCTCTGAG
CTCAAGAAACGTTTTTTGGCGGCATTCACTGAAGCGCCATGAAAGTAGATGCGTTCATCCTTGTGCCACATGG
TATAATTCATCGAAGGGGATTGCTCTAAACGGAGCACGTTGGGGTATAAGCCAGCGCATATGACAGCATGGAC
CACTTCTGTCTTCTTTGCATGTCGATTGAAATGGCACGACTTCAATTGTTCCGGGTCAACTCTGGATCCATTC
CCCATTACTGCGTTGCGATCCAAAAATCCTATACCGCACAGTAAATCCAGAAATTGTCGACGAGCATCTCCTA
TCTCACGTAGGGCAATATGGTTGAGATAATTCTCTTGGCAAAATCTCCTGCTTGCGATTGTGGATGTTTCATA
TGCATCCCATACATTGCAATACGTCATGAAGTCGCTGTCGGGGTCGGCAAAGACTCTCTGTTTTGCTTTGGCA
ACGGCTGCATCATGGACGAATGCAGCAAAGGGCGACTTCGAAGATAGACTCGCGGCAATGGTGAGGATGGGAT

CAATGCACCGAAACAGAGCGCCAAATATTAGCATCTTGCCTAACTTCACGTCCACGGGCAGTTTAGCCAGATG
AAAACCCAGTGGGGTGAGATGTTCCGATTGTTGCATGATATCACCGGTGCCGGTTCTTTCAATGGCCCCTACA
TCATATAAAACATCCATTGCGGCCTGCACAGATTCCGTTGATGGCGGCTGGGGCGCCTGACCCAAAAAATCCA
TGCAACTCTTCGCAAATCCACTGGCAAGAATTGACATGCATATTTCTTCCAGGGGCACACGTCGAAGTTCTGG
CTCGTTCGTAGCTTTCATAACTCTCTCCGCTGTCGCGAATGAGTAAAGCTTAAGGCCTGGATGTATTTTTTAA
TACCAAGAAAATATTTCGGGTAGTGTGA
>TR504|c0_g2_i3 len=2065 path=[2318:0-105 2319:106-129 2325:130-1029
2309:1030-1053 2327:1054-1179 2306:1180-1203 2330:1204-1442 2329:1443-
1622 2326:1623-1789 2316:1790-1809 2317:1810-2064] [-1, 2318, 2319, 2325,
2309, 2327, 2306, 2330, 2329, 2326, 2316, 2317, -2]
TCTTCCGATCTGTGAAAGTTTAGCTATGTTGGCTTACTCTTAGATGAATCAGATCGGCATCAGACTGGCAAGA
GAGTAATGGTGGGAACCTCATCTATTCAATGAAAGACACATTTACGCTGGGGAAAGACCTGGGGATTAATGTT
TTTCAACATTTGCCTGACACCTTCTCTACCTTATTTGTTTACTCAAGTTGAATAAAGTTAAACCTTTCACCTG
CATTTCGCCTCATTCCGTGGAATTAGATCGACATTTAGAAAGCTGTTCATCACGAAGCGAGAATTTTTATGAT
TCCGTCAATCATGCTGGAATCCAACTGTTCAATTTCGCTGTTGTCGGCGCATTCGATCCTTCTTTGCAAAAAT
ATCTCAACTTTCTTTCTCAATTCTCGCAGTATTACACCAGTTTGTGCGGACATACCGATGCTCATCCAGTTGT
CAACAATCACTGTTCGCTCTGTGTGTTTGACGAAGACAGACCCACCAAAAAGAAGCAACGCGAATGGATGAAC
GAAGGCAGTTGTTGACACAGATGTACGATTCGGTGTGCCAAACTTTTCATGGAAGAGCACCCAATTCTCTGAG
CTCAAGAAACGTTTTTTGGCGGCATTCACTGAAGCGCCATGAAAGTAGATGCGTTCATCCTTGTGCCACATGG
TATAATTCATCGAAGGGGATTGCTCTAAACGGAGCACGTTGGGGTATAAGCCAGCGCATATGACAGCATGGAC
CACTTCTGTCTTCTTTGCATGTCGATTGAAATGGCACGACTTCAATTGTTCCGGGTCAACTCTGGATCCATTC
CCCATTACTGCGTTGCGATCCAAAAATCCTATACCGCACAGTAAATCCAGAAATTGTCGACGAGCATCTCCTA
TCTCACGTAGGGCAATATGGTTGAGATAATTCTCTTGGCAAAATCTCCTGCTTGCGATTGTGGATGTTTCATA
TGCATCCCATACATTGCAATACGTCATGAAGTCGCTGTCGGGGTCGGCAAAGACTCTCTGTTTTGCTTTGGCA
ACGGCTGCATCATGGACGAATGCAGCAAAGGGCGACTTCGAAGATAGACTCGCGGCAATGGTGAGGATGGGAT
CAATGCACCGAAACAGAGCGCCAAATATTAGCATCTTGCCTAACTTCACGTCCACGGGCAGTTTAGCCAGATG
AAAACCCAGTGGGGTGAGATGTTCCGATTGTTGCATGATATCACCGGTGCCGGTTCTTTCAATGGCCCCTACA
TCATATAAAACATCCATTGCGGCCTGCACAGATTCCGTTGATGGCGGCTGGGGCGCCTGACCCAAAAAATCCA
TGCAACTCTTCGCAAATCCACTGGCAAGAATTGACATGCATATTTCTTCCAGGGGCACACGTCGAAGTTCTGG
CTCGTTCGTAGCTTTCATAACTCTCTCCGCTGTCGCGAATGAGTAAAGCTTAAGGCAAATCCCCGACTGTACG
CGGCCAGCTCGCCCCTGTCTCTGCTTGGCTGACGATTGTGGGCACCAATCTGTCACAAGCATCGATGTCGAAG
TCTTCTTATTTCGACGGACCTCCCTCACCCGTCCAGTGTCGATCACATAGACAACATCTGGAATTGTCACGCT
GGTCTCAGCAATGTTGGTTGCACAGATAATTTTTCTACATCCAGGTTTGGACGGGAGGAATGCTCGTCGTTGG
TCTCGGGAGGATAGCGTCGAGTGCAGTGGAATTAAATCAAAACGTTGCCTATCTCTGAATATTCGACTGCTGT
CTAAACGTTCCGACAAAGCTTTGATCTCGCCCAGTCCTGGTAAAAATATCAAGATAGAGCCCTCGGACATATC
CGCCCCATCAGGAGCCAACAAAGTGTCACTGCCAGATTTTGAGTTCGTCACAAGCTTCAGAACATCTTCAATA
AGATCATAATTAATAACCTCCTCGTTCACGCGTTCCATCGATCTACGTGTCGACATCTTATACCCAGCATACG
CATCCGACACTCCCCCCGGCGTAAATCCCTGCGAGTTCAGATCCACCGTCTCCTTCCGCTTCTCTCCACCACG
CGTAGTTACCCAAAGCGATGC
>TR505|c0_g1_i1 len=2749 path=[2950:0-241 2951:242-265 2966:266-722
2940:723-746 2964:747-899 2969:900-923 2970:924-952 2968:953-1220
2934:1221-1244 @2965@!:1245-1352 2937:1353-1394 2960:1395-2129 2931:2130-
2153 2932:2154-2748] [-1, 2950, 2951, 2966, 2940, 2964, 2969, 2970, 2968,
2934, 2965, 2937, 2960, 2931, 2932, -2]
CCGATCTGGAATCTTCAATCTACACCTATTTCATTACTGCGGCCTAGTTTTTCCTTCTCTCATACTACTCGAG
CTATACCCAATTCATGTCTTCCATCCCACTTAATTTGCATTTACTTATCGATTCAAACCAAACCAAAACATTT
AATTTACTACCTGATGCCAAGAAAATATTCCTTCTCCACACCCTGAAATCCCCATTTTGTAATACCCCATGTA
TGAACACTTTAGCGGGCCATTTGATTTTACCCTGTAGCAAATAACTTACGAGTTCTTGATCTCATTCAAAGCA
TTATTCGTAACACTCGCAATGGCACTACCATCTGCCTTGGATATCTGATGATACTTACCCTGTGCTGCATCCG
CAATATCCTTTGCCACGCCAGTACTGATGAACTTGTTCTCTGTATCAATGGCAAGCAAGTTGAATCCAGGAAG
GACTCCCAGTTGCTTCGCGCACGCCAATACCTCATCCTTGAGATACTGCCTGGAAGGTTTGCCATCCTTGGAA
TCCTCATCTGCGTCGGGGTCAAACTCCTCCCCCTTCGAAATGCATAATGGAACATTGGCCCTTCCATCTGAGA
TCAAGACCACAACAACCTTGCCCACGTCTCCCGTCTTCTGGGCATTCAATCCCGTCAGACTAGCCGCCTGCAA
CGCATCCGCCAACGGAGAACCTCCACCGCACGGCATGATCTCCAACCTCTTCTTCGCCTGCGCGATGGATCGG
GTTGGCGGCAGAAGCACATCAGCCCTGTCCCCCTGGAAGGGAATCAGACAAATCTGATCACGGCTCTGGTAAG
CCTCCGTCAGGAGACTCATGGCCGCTCCCTTAGCGGCGTTCATTCGATTGAGAGCCATGGACCCCGAAGCGTC
CACGACGAAGATGATGAGTGATCCGGCCTTCCTCGCCATCTTCTTCGTTCGGACGTCAGACTGCTCGATGAAC
ACGCCTCGTCCCTCGTCCTTTGTCCCGACCGCCCGCCCCCGGCGAGCCCTTTGATACGGAGCCGATGCACGCA
AAGTGGCATCGACTGCGAGACGAACGACCTTGCCCTTAGGGAGCATGGGCTTGATGTACCGTCCGCGATCCTG
GGAGAAGATGAGGCCGCGTCCACCACCCTTGCCGGAACGTTCGCGGGAAGTGAAGTCAATTAGGTCAGGGTCC

ATGGGGGTAGCGTCAACGTCGAACATGAACTCTTGTGGCACATCAGGGACATCCGGTTGTTCTTCTTGTTCAT
TGTCCTCGTCGGGCTCGTCCTCCTCGTCTTGCTCTTCATCCTCCTGATCCTCATCATTTTCACTGTCTTCGTC
TTGCTGATCCATCGGCGGCGGCGGAGGAGGAGGCGGGGGGGGCATCATCTCATCCTCGTCCATGGGAGTGTTC
ACGAACGTTCCACGGGGAGCGATGGCGAGCTTGACAGCCAGCCTCAAGTCTTCACTGGTAATCTGCCGTCCCT
CCAACGCGGCGGCGGCACGGGCCACCTCGCAGGCGAAGATCTCGGCGCGGTGTCCCTGACATCCCGCCCTAAT
GGCCTCCTCACACAGATATTGGATCTGACTGGGAGCGATCTTGACGTCCTTGATGAATTCCCGGGCAAAAACA
ATGGCGGTCTTGAGATCGTCCTCATTCTCGATGGCTTCCTCCAGGGCTAATTCGGCCTTGGCGTTGGCCTGTT
TTCCACCGGCGGAGAAGCCCAGGACGGCGTCCACGGCTTCAACACGGGATTTGATCTCCATCACCTCAGCGTC
CGCGGAGAGGGCGACGGCGATACGATCGAGAAGGTGGTCGCGGAGTTCGCCCTCGTCGGGGTTGAAGGTGGCG
ATGAGGAGGGGAACGCAGGGGTAGGTGAGCGAGATGCCCTCACGTTCGACAACGACCTTGCCCTCGGTGACGA
CGTTCAGAAGAATGTTGGCTGTCTCCTCGTCGAGGAGGTTGATGTCGTCCACGTAAAGAACACCGCGATGAGC
CTTGGCCAACAGTCCAGGGCTGAAGACACTCTTGCCCGTCTTGACACTCTCCTCCAAATCGGCACTACCGATG
AGGCGATCTTCCATCACGTTGAGCGGAACCTGCACGAAGGGAGCGGGGATGACCTCCGTCTCTCTCTCTCCCA
AAGGTACTCCACCTGCGTCAAGATCGGTACGCAAAAAGTCGTCAATTCCAAACTCGCCATCAGGATCGATGTT
GAATGCATTACCCTTAATGACCTCGATGGGAGGCAAAAGCTGATGAAGGGCACGGGCCATGATGGACTTCGCC
GTACCCTTACCTCCCGAAATCACTACGCCTCCCATGCGATTGTTGATGGATGAAAGGAGCAACGCCTGCTTGA
TGGGTTCCTGTCCCACCACCATGGACAATGGGAAGGGCCGTCCGCCAGACACCATTTGATCGGCCTGTCTGCC
GATCTGGACGTCGGGTTTTGTGTCAGCTGCTTCTTCGGCCAACATGGCTGCCTTTGCGCGATCCTCTGCTGTT
TCCGCTACCTGGAGATAGGAAGATCGTTGTATTCTCGACCCTGATTGAAATGCCGGTGGCGCGAAGGCCATGG
CAACGCCGCTGATAGCGGCAGCGATGGTGATAGCTGCGGCTGTTCGTCTTGGTCGCGGCATATTTATTTGGTA
TTTTATTTGATGACTATTCTCCTTTCCTACTCAGAATTTACTGGTTGACAATTTGACAATAAGAAGGATTGTA
TTGTTCAAACAAAAAGATCCACCCCTCTATTGTGAGAGCGACGCAGGT
>TR506|c0_g1_i1 len=1635 path=[1951:0-178 1952:179-202 2002:203-208
1997:209-254 @2000@!:255-633 1975:634-655 1995:656-771 1972:772-797
2001:798-895 1966:896-928 @1994@!:929-1233 1956:1234-1263 1957:1264-1265
1958:1266-1289 1959:1290-1634] [-1, 1951, 1952, 2002, 1997, 2000, 1975,
1995, 1972, 2001, 1966, 1994, 1956, 1957, 1958, 1959, -2]
TACCACACCTGGCACAGCCGCATTCGCAATTCGCAGGTTTGCAGTCGCTGCCGCTACAATCGCCGCCACAATC
AGAGTGCAACAAACCGCAGCCGCGCAACGCCCCCTCCAAACTTCCAGTCTCGCCAACGCCAAAGCGATATTCG
CAGTCCTCCTCCCGGGTCGCCTCCGACTCGCCGTAAAAAGCAGAGCGCACGGGGCAACGGGGGCAAAATCTCT
GGTCGTTTTCTCCTATATATTAGAATCAAACCAAAAATATTCATTCGGTCGTCGCCGTCCCCCGTTGGGTGAC
CTCGCACGAGAGAGAGAGAGAGAGAGGACTAGATTAAAAATCACCCCCCCTTCTTTTCGCTGGGTGTAGCGAGTG
CATAATATTTTTATAGTCGACGAGATTTTGATTACACGCCGCGAGAGGCAAGGATGAAACAAAATAGGATGGA
GGTGGAGGGAAGCAACTGTGATGACGATTTCAATCCCGATTTTTCATCCTGTTCCATCGAATCGCTCCTAAAT
GTTCCCATGCAAATCATCTTTCGGGAGACTAAGACATCCGATCGAGGAAGGGGGAGCGGGAGCGGTGGGAAGA
ACTTTATTTTGTCCAAGAGTGAGGTGTCAACTCTTCTTCGGGTGTCGATGTTATCCTCCGAACTCGACGCACT
CCTTTCCTTGGCATCGGATGCACTCGACAAATCACAAAAATGCAAAACGTATCGCAACCTAATAATAAACGCC
AACAACAACGGAGATCCATCGTCATCCTCCCCCTCCCCAAACATTGCACTTCAACTCCGCGCCCTGCTCCCCA
TCCTGCACGTCCATTCCATCACCGCCAACGCGGCCTTGCAACACGCCACATCCGAAACATTTGGCATCAGTCG
AATACTCGAACAACTCGAAACTTGCGCCAACGATTTATCCAAATTCGGCGAACAGAATCACAACATGGCGGAT
CAACTCGAGTGTTACACCGCGATTGGAGACGATAATAACGAGATGAATTTGTTATCGAACGAAAAGAGAGCGA
TTGCCGAATTGGAGGAGGCGATGGTGGAACGCATTGAGAGTTTGAATGGGATGGCGATGGGGAGTTCTACCGG
AGTTGCTGCGAGTGGAAAAGGTGACGATGCGTCTGATGCGGCCAACGGCGGTGCGTTATTTGAGAGTGGGGCG
GAGGAATTCGTTTCCATGAACGAGCTTTGTGAAAGCTTGTTTTCCAAGTATAAATTATCTTCCAATATCAATA
GTGGGGCACCTCCTCCTCGGCCTGTAGCGGTGAAGGGGGAATTCGGTGATATTACCCAATCGCAGGCAAATGC
AGCAGTGGCTTTGCAATGCATGTTCTCAGATTCTGGAGAAAAATGATGGTTACTCTGGGTCGAGATTATTCTC
CCTTTGTTCTGTAAACTTTATTAACTCCTACAAATGACACAAATAATATCACCATTTTTATCCCTTCCCGAGA
AGGCAATAGACTGTTCTCATAAACAATGTCACACGGTGTCTTCACTGAACCAATCATCATAGTACAAATCATC
CCCATCACCGTCTCGAAACGCATTATCATCATCATTGCCCTCCCCACCACCACCATCCTCCAAAGGACTACCA
CTACCACCACCACCCTTCTTCTTCTCCTC
>TR506|c0_g1_i2 len=1742 path=[1951:0-178 1952:179-202 2002:203-208
1997:209-254 @2000@!:255-633 1983:634-684 1999:685-762 1995:763-878
1949:879-904 2001:905-1002 1969:1003-1035 @1994@!:1036-1340 1956:1341-
1370 1957:1371-1372 1958:1373-1396 1959:1397-1741] [-1, 1951, 1952, 2002,
1997, 2000, 1983, 1999, 1995, 1949, 2001, 1969, 1994, 1956, 1957, 1958,
1959, -2]
TACCACACCTGGCACAGCCGCATTCGCAATTCGCAGGTTTGCAGTCGCTGCCGCTACAATCGCCGCCACAATC
AGAGTGCAACAAACCGCAGCCGCGCAACGCCCCCTCCAAACTTCCAGTCTCGCCAACGCCAAAGCGATATTCG
CAGTCCTCCTCCCGGGTCGCCTCCGACTCGCCGTAAAAAGCAGAGCGCACGGGGCAACGGGGGCAAAATCTCT
GGTCGTTTTCTCCTATATATTAGAATCAAACCAAAAATATTCATTCGGTCGTCGCCGTCCCCCGTTGGGTGAC

CTCGCACGAGAGAGAGAGAGAGAGAGGACTAGATTAAAAATCACCCCCCCTTCTTTTCGCTGGGTGTAGCGAGTG
CATAATATTTTTATAGTCGACGAGATTTTGATTACACGCCGCGAGAGGCAAGGATGAAACAAAATAGGATGGA
GGTGGAGGGAAGCAACTGTGATGACGATTTCAATCCCGATTTTTCATCCTGTTCCATCGAATCGCTCCTAAAT
GTTCCCATGCAAATCATCTTTCGGGAGACTAAGACATCCGATCGAGGAAGGGGGAGCGGGAGCGGTGGGAAGA
ACTTTATTTTGTCCAAGAGTGAGGTGTCAACTCTTCTTCGGGTGTCGATGGTAAGAGCCGATGGTACTATGTA
TTATACAAGTGTCAATCTCCTGGAAAAATGACGAGCATGTTCAACTTTCATCATCTTATTCGACTCACACAAT
CATAATGACAGTTATCCTCCGAACTCGACGCACTCCTTTCCTTGGCATCGGATGCACTCGACAAATCACAAAA
ATGCAAAACGTATCGCAACCTAATAATAAACGCCAACAACAACGGAGATCCATCGTCATCCTCCCCCTCCCCA
AACGCCGCACTTCAACTCCGCGCCCTGCTCCCCATCCTGCACGTCCATTCCATCACCGCCAACGCGGCCTTGC
AACACGCCACATCCGAAACATTTGGCATCAGTCGAATACTCGAACAACTCGAAAATTGCGCCAGCGATTTATC
CAAATTCGGCGAACAGAATCACAACATGGCGGATCAACTCGAGTGTTACACCGCGATTGGAGACGATAATAAC
GAGATGAATTTGTTATCGAACGAAAAGAGAGCGATTGCCGAATTGGAGGAGGCGATGGTGGAACGCATTGAGA
GTTTGAATGGGATGGCGATGGGGAGTTCTACCGGAGTTGCTGCGAGTGGAAAAGGTGACGATGCGTCTGATGC
GGCCAACGGCGGTGCGTTATTTGAGAGTGGGGCGGAGGAATTCGTTTCCATGAACGAGCTTTGTGAAAGCTTG
TTTTCCAAGTATAAATTATCTTCCAATATCAATAGTGGGGCACCTCCTCCTCGGCCTGTAGCGGTGAAGGGGG
AATTCGGTGATATTACCCAATCGCAGGCAAATGCAGCAGTGGCTTTGCAATGCATGTTCTCAGATTCTGGAGA
AAAATGATGGTTACTCTGGGTCGAGATTATTCTCCCTTTGTTCTGTAAACTTTATTAACTCCTACAAATGACA
CAAATAATATCACCATTTTTATCCCTTCCCGAGAAGGCAATAGACTGTTCTCATAAACAATGTCACACGGTGT
CTTCACTGAACCAATCATCATAGTACAAATCATCCCCATCACCGTCTCGAAACGCATTATCATCATCATTGCC
CTCCCCACCACCACCATCCTCCAAAGGACTACCACTACCACCACCACCCTTCTTCTTCTCCTC
>TR507|c0_g1_i1 len=704 path=[971:0-24 972:25-26 973:27-28 974:29-30
1017:31-99 1029:100-126 1025:127-178 1020:179-449 1032:450-472 1022:473-
504 1031:505-525 1013:526-550 1021:551-574 1018:575-598 1033:599-608
985:609-637 986:638-703] [-1, 971, 972, 973, 974, 1017, 1029, 1025, 1020,
1032, 1022, 1031, 1013, 1021, 1018, 1033, 985, 986, -2]
TCTCTCTCTCTCTCTCTCTCTCTCTCTCTCTCTGGATATAGACATGGCGCGCTCTCACTTCCTCTTGCCATGCAG
AGCTCATTCACTACAACTCGGTATTTTGTACATTGACCGGACCGGACACACCTTTATGGGAAGTTCTTGCCAC
GAAGGTGAATGCACTCGATCAAGCACGCACAGCCTTGTGCCTCAAATGTTTTGAATCAAACCAATCATCCGAG
CACTTTCCCTCGACCAGTGCGAGAGTTCTTTCGCGATCTCTGCCATTGCATCAGGGTCATTGATGTCCGCGCG
AGACTGGCGCTCCTGCTCGCCTAGAACAGCCCTTCGAAGCGCCCGCCGGTGGTTCGTGATCTCTCTGGACGTA
GTGTTAGACATCAAATAGTTCTCTAAACCAAGGAATTCGCTTCTTTCTTCGGCGTTCAGTTCCGAAAGCTGTG
CCGTGGTCATTCTCGAAGCAATTGTTCTCAATAGAAGGGTGTGGTAAGCGATGGACCGAAGCTCGATGGCAGA
GAACCATACGTCCTCCCTGTACTTTTCTGGGATGTTTTTGATAACTTTCACCTCAGCGTAGCCTTCAAATGAT
ACGCTGCGCTCATCTTTAGCTTTAGGCAAGACTCGGTGTGGTCGAGGGAGGTTGACGGTGAACTCCATAGGGT
ACCTAGTATTACTTGTTACAATCGTTGATCTAAGTAGCAGATCGGAA
>TR507|c0_g1_i2 len=689 path=[987:0-84 1030:85-111 1024:112-163 1020:164-
434 1032:435-457 1023:458-489 1031:490-510 1026:511-535 1021:536-559
1018:560-583 1033:584-593 1011:594-622 991:623-688] [-1, 987, 1030, 1024,
1020, 1032, 1023, 1031, 1026, 1021, 1018, 1033, 1011, 991, -2]
CTCTCTATCTCTCTCTGGATATAGACATGGTGCGCTCTCACTTCCTCTTGCAATGCAGAGCACATTCACTACA
ACTCGGTATTTTGTACATTGACCGGACCGGACACACCTTCATGACAAGTTCTTGCCACGAAGGTGAAGGCACT
CGATCAAGCACGCACAGCCTTGTGCCTCAAATGTTTTGAATCAAACCAATCATCCGAGCACTTTCCCTCGACC
AGTGCGAGAGTTCTTTCGCGATCTCTGCCATTGCATCAGGGTCATTGATGTCCGCGCGAGACTGGCGCTCCTG
CTCGCCTAGAACAGCCCTTCGAAGCGCCCGCCGGTGGTTCGTGATCTCTCTGGACGTAGTGTTAGACATCAAA
TAGTTCTCTAAACCAAGGAATTCGCTTCTTTCTTCGGCGTTCAGTTCCGAAAGCTGTGCCGTGGTCATTCTCG
AAGCAATTGTTCTCAATAGATTGGTGTGATAAGCGATGGACCGAAGCTCGATGGCAGAGAACCATACGTCCTC
ATTGTACTTTTCTGGGATGTTTTTGATAACTTTCACCTCAGCGTAGCCTTCAAATGATACGCTGCGCTCATCT
TTAGCTTTAGACGAGGCTCGGTGTGGTCGAGGGAGGTTGACGGTGAACTCCATAGGGTACCTAGTATTACTTG
TTACAATCGTTGATCTAAGTAGCAGATCGGAA
>TR508|c0_g1_i1 len=489 path=[540:0-285 544:286-332 543:333-356 539:357-
488] [-1, 540, 544, 543, 539, -2]
CGATGGCCATTCATTGTTATTATTTGGCGATGAAGATGATGATGATGATGATGATGCGGTCGATGTGGATGTC
AACTTATCGGAGAGAGTGGACAGTTGATCTTTCATGCTGTCGATGGTTGGCAGAGTTTGAAATAGTTGCGATG
ATGACCTGCTGATGCTGATGCTGCTGCTGATGCTATCGATGCCAATGTTGGCAGTCGATCGTCCTGGGAGGAA
GGCGGCATTGCTGCTCCCTGCCAAAAGGATTGTCGGAATGAGAAGGGAGGCGACCATCATCCTATTAGTCGTT
GATGGATATTACCTCACTGCCTATATTTATAGACTCGGCCTGCTTAAGTACAGAAGTTGCTCTTATATTCGCC
CGTACGTGCTTCTCGTGGCACACCTCTCGTACAATTATATATATCGCAAAACTCTCTAACAAGAGATGGCGTG
TGTGCTTCCGTGTATGCCGATGAACGATCATAGCACGTCTCTTCGAGATCG
>TR508|c1_g1_i1 len=1939 path=[2468:0-321 2469:322-339 2499:340-470
2475:471-494 2476:495-500 2477:501-524 2508:525-698 2512:699-739

2497:740-779 2510:780-821 2507:822-855 2495:856-880 2466:881-920
2502:921-1125 2437:1126-1149 2494:1150-1235 2509:1236-1280 2506:1281-1304
2501:1305-1396 2513:1397-1420 2514:1421-1448 2492:1449-1518 2443:1519-
1542 2444:1543-1543 2445:1544-1567 2505:1568-1617 2431:1618-1642
2491:1643-1875 2428:1876-1899 2429:1900-1938] [-1, 2468, 2469, 2499,
2475, 2476, 2477, 2508, 2512, 2497, 2510, 2507, 2495, 2466, 2502, 2437,
2494, 2509, 2506, 2501, 2513, 2514, 2492, 2443, 2444, 2445, 2505, 2431,
2491, 2428, 2429, -2]
GTCGTGAGAGATATAACAACATCTCTCTCTCGTACACTGAATACAAAACATCACTCTCTGTCTCTCTCTCTCT
CTCTCTCTCATCAACACCATGTGGTCCTACCCACTACAACCCCTACTACTACCAATCACAACAACACTCACCA
TCGTCGCCCACACCACCCAAGCCGCAGGCACGTACAACGACCAAAGCTTCCACGACCTCCAACAAAAACTCTC
GCCGGCACCCGCTCAAATCCTCGAAGATCTCGATCAGTACGATAAATTCTGGATCGAAGTCATACGTACTCCC
AAGCCCAAATCATCCTCCTCTACAGATAATGACGACAACAACAATAACAAGGTAGACTACAGCACCACGACCA
CCGAACGAAAAAAGGGAAGCAGCTGCGTCTGGTCCGAATGCGGCATCGACGAAACCGACGATGCCTACATGGG
CGATTACCGCGACGGCGACGAACAGTGGTACCAGTACCGCACGCAGAACTTTTGCGCGAACGCTGCGTACTCC
TTGTACGGACGGAGGAAGGATGAGAGCGGATGGTGGGGACCGGGAAGTTGGGGAGGATGCACGAGGAGGCATT
TTATTAATTCGTTTTTCACGTACGGTGGGGCGGATAATTTGTTGAAGGCGATTGGGGTGGAGCCTGTTGTTTA
TTATGATGGGAGCGAGGGTGATGATGATGATGATGATGCGGGACGTCGTCGTCGTCGTCTTGAAGATGTCGCT
GATGATGCTGCTGCTGGGGATGATGCCGCTGCTGGGGATGACGCCGCGGATGACGACGATGATGCATACTCAC
TTTCCAATGCGCAATGTGTCGAAATCGAGTACGAATGGGAAGAAGACAACGACAACGAGGACGGAAGCAACGA
CGGAAGCGGCGACAACAACAACGACGAACGTCACCGTCATCTCTCCGGGAGCAACGACCAAAACGACGGTTTC
GCGAGCACCCTGGGCTGCAGCGCCAACGGCGACTACATCATCGGCGCCTTCCAAAGCAGCTCCTGCGACGGCA
ATTACTTCCGCGAGTCCATCGCGACCTTCGACGAGTACAACGCCCAACACGCGAGCATCGGATGTCGCAACAT
TTGGAAGAGCGGGAACGAGGCCACGTACGATCAGATATACTCCCTGTTGGGGAATTCGTGGAGCTGCGACGTG
CGGTTGTATCCGAACAGTTGTCCGGATCCGTTCGGGGAGAAGGGACGGTACGAGTACGCGATTCGAGTGGCGG
CGCACGGGGGCAATCCCATGTGGGCGTACAATAACATGAGATGGAAACATTCGCTGTGGATGGTGAGCTGGGC
GCTGTTGTGGTTGACCGTCGCCGTCTTCGCGGTTACGTATGTCGTCAAGAAGAGGAAACGGACGAAGGCCAAT
GGTGGGGGGGTGAAAGGGTTCGGATTGAGCGTGAAGGAGGATGCTTTGGCGGGATGGGCGTGGATTACCGGGG
GAGCTCTCGTTGCGTATGTTGCAGCGGCGGCTCTCGTCGAGAAGGCTCGTGTGGCGGCTGTGGAGGGTGTTGA
CAAGGGCAAAGCGAGGATGGCGAGGCGCAGAGGTCGAGGGAAGGCTGGTAAAGCGAAGCGGAAGAAGGCAGTG
GATAGTACCCTTAATGAAGGTGATAGTTACGGAAAGATGACGGATGAAGACAAGGACGAAAAGGTTGAGCAGC
GGAGTACACCGGATAGTGACTTTCAAGTGGTGGACATAGTGAGTAAGAGCAACGGAGACAAAAATGATGACGA
TGGTGGAGTGATGGCCTGAAATAATTATGTCGCCCAACAATGTGCACATGTTGAACTAGGTAGGTGCTACGTG
ACATGAGTGTGGTGAGGCATATGCTGCTGGATAACACGGGATTTTTGAACAGACAAAGGTGAACCTATGAATA
ATAGCTATATGTATTTTTTGTAAGAACTTGTTAGAACCACC
>TR508|c2_g1_i1 len=2236 path=[2976:0-73 2977:74-101 2978:102-133
3143:134-236 3126:237-251 3109:252-277 3149:278-286 3148:287-310
3147:311-400 3134:401-443 3100:444-447 3116:448-471 3132:472-485
3098:486-541 3114:542-642 3077:643-666 3096:667-745 2914:746-769
2915:770-772 2916:773-796 3130:797-1049 3113:1050-1123 3074:1124-1147
3094:1148-1201 3129:1202-1383 3071:1384-1407 3112:1408-1626 3150:1627-
1650 3151:1651-1675 3128:1676-1720 @3111@!:1721-2133 3083:2134-2167
3084:2168-2170 3085:2171-2194 3086:2195-2235] [-1, 2976, 2977, 2978,
3143, 3126, 3109, 3149, 3148, 3147, 3134, 3100, 3116, 3132, 3098, 3114,
3077, 3096, 2914, 2915, 2916, 3130, 3113, 3074, 3094, 3129, 3071, 3112,
3150, 3151, 3128, 3111, 3083, 3084, 3085, 3086, -2]
AATGTCGATCGTAGCGGTGCGTCCTCGGTTGTGTATGCAATCGGCTTTTTCATGTTGGCTGTTTGGCGACGAT
AACGCACTCTCTCTCTCTCTCTCTATTCGCCGATCTAAATATGGAAAACATGGTGCAGAGTATGTTCGATACT
TATATTTAGACACGGATAGCCGATGTGTTTGATATACGTGTTCTTGTTGTACTTGAGTGGGGAGAGGGTGTCA
CGTTTTTATGGCGGTCGGATTTTTAATTTGGTGACCATTTAGTGAAGCTCTCTATCAGCCTCTCCGGACATGA
TTATTCGATGCGTGATTTTGCAGGTGTAGTGAGAGTGTTTGATTTTTTTCATAATTGTTCGGTGTTTGAATTC
ATTTTTGGGAGTTCGCGTTACATTATTTATCGAGGGGCTTATAATAGAGGCATAATATTTGATGGTATTCAGC
GCATTCTGCACAGCGCGTCTCTTTGTTGAATGGCCGCGCAAGTCTCTCTCGGTCAAAATTGAATTTATTTGAA
CTGCGTGAGGAATATATTTTGCCCTCTCCTCCTGCACGAGTCATTTTATATAGTCAACCGCTCTCTGCCTTAT
TTTTGCCAGACATGATCTCTCTGTGGTATGTTATTTTAGCGGATGAGGGAGATGAAAATCAGGGACAAGTCTC
TCTCATTGTGGCTCTCTGTGCCACGTCCAGTCAAGTGCACTGTGTTAGACTAACAGTTCTATGTTATGCAGTT
CGTTTTGCAGCTCGATCATGTTTTGGCTAATTATCTTAGCGAATACATGTCTTGTGTATTTTTTGTCGGTGAT
GTCGATACATTACCAGTGGATGAGATAAAGGCTAATATTGATTTTGACGCACTGTAATTTCGAGAACCAAAAG
GCCAAATTTATCTCGTGCCGTGACTCTGTGGCATTGATAAAGACTGGGAGTGATTTTGTCTGAACATCTCAAG
CATTATATATGCAGAGCAAGGACCATCAATACAATTTTGCTCGTGTATTTTTTAAACTGAAAATGCAGGCAGA

GCATTATTTCATTGATGATGATGGCGGGTGCTTTTAACTTCACGGAGGATGTGCCCATATCGGAGAATGCGCT
GGCCTCTATTGAAGTATCAACCTATGAAGGCATCCCCTGAACATGTTGGCCAATCTCTCAAAATACCATCGCT
TTACCAATCCAGGTCTGATCTTACACCAAGGCCCATACTGATGGATGAATCAACCTCTAAACCCGAAGGAAGC
AAGGGCGAACCCCCTGCTTCCCACATTAGTCCACCAGAGACCAATGATCCAAACGTCAAATCAAAAAAGCGTC
GCAACACCAAATGGGACGTCAACCCCGAAGAAGTCCGCTGCTCCGACTGGGCCATGGTCCCCAAGAGCACACC
CCGAAAATTACTCCCCGAGCATAAGGCAGCCTACAAAGAATTACAAATCGCAGACACCGAACGATGCACCGCC
CAGGAAACGTTGGACATGGCCCGGACAGCACTCGAGGACGCAAAGCGGCACCTGCTTTGGGCGGAGGAACGAT
CCGAGGAGGCCAATGCCAGATTCCGCACAGCGCAAGAGGCCGTCGAAGAGATTGACATAAAGAGACCGGACTT
CAAGTGGAACTTCATGTACGATAAGCTCGTGAAGTTCAAGGAACGGCACGGGCACTGTCGCATTCCGGCAAAC
CAACCGGAACTCGCCAAGCTGCGTGTGTTCGTTCATACGAACAGGACCGCGTACCGAGAGTATCAATCCGGCA
AACGCTGTACGAATAAGGCGCATCGCATGATTGCCCTGGAGCGATTGGGGGTGTTCGCGAGCAAATGGGAGGA
AAGGTATAATGAGTTGGTAGAGTTTAAGAAGGTCCATGGACATTGCAACGTTCCGACGAAATCGAAGGAGAAT
CAGAAGTTGGGGTATTGGGTTGGATGGCAGAGGGGGGGTTACAGGAGGTACAAGGCTGGGAAAGAGAATCAGT
TGTCGGAAGAGCGAATTAAGCTGCTGGAAGATATTGGGTTTTGTTTTAATGTGTATGAGGCGAATGGCATTGT
GAGGAAACGTCGGGGAAAGGAAGCGGAGGAAGCTGTGGCTGATTCGTCGGATTAGGGATGTGAAAATAGAGAG
CGCAAACGAATCATGTAAGTATAACTGGGGTGGGTGTGGATTTGCTGTGAACACAATGTAAAATTTAGAATTA
CTATTATAGCACAATACCTTTAAAATTTAAAAAAAAAAGATCGGAAG
>TR508|c2_g1_i2 len=2260 path=[2918:0-73 2919:74-101 3144:102-102
3110:103-103 3125:104-104 3142:105-105 3108:106-106 3124:107-107
3141:108-108 3107:109-109 3123:110-110 3140:111-111 3106:112-112
3122:113-113 3139:114-114 3105:115-115 3121:116-116 3138:117-117
3104:118-118 3120:119-119 3137:120-120 3103:121-125 3119:126-157
3136:158-260 3102:261-275 3118:276-301 3149:302-310 3148:311-334
3147:335-424 3133:425-467 3099:468-471 3115:472-495 3131:496-509
3097:510-565 3114:566-666 3077:667-690 3096:691-769 2914:770-793
2915:794-796 2916:797-820 3130:821-1073 3113:1074-1147 3074:1148-1171
3094:1172-1225 3129:1226-1407 3071:1408-1431 3112:1432-1650 3150:1651-
1674 3151:1675-1699 3127:1700-1744 @3111@!:1745-2157 3083:2158-2191
3084:2192-2194 3085:2195-2218 3086:2219-2259] [-1, 2918, 2919, 3144,
3110, 3125, 3142, 3108, 3124, 3141, 3107, 3123, 3140, 3106, 3122, 3139,
3105, 3121, 3138, 3104, 3120, 3137, 3103, 3119, 3136, 3102, 3118, 3149,
3148, 3147, 3133, 3099, 3115, 3131, 3097, 3114, 3077, 3096, 2914, 2915,
2916, 3130, 3113, 3074, 3094, 3129, 3071, 3112, 3150, 3151, 3127, 3111,
3083, 3084, 3085, 3086, -2]
AATGTCGATCGTAGCGGTGCGTCCTCGGTTGTGTATGCAATCGGCTTTTTCATGTTGGCTGTTTGGCGACGAT
AGCGCACTCTCTCTCTCTCTCTCTCTCTCTCTCTCTCTCTCTCTCTCTATTCGCCGATCTAAATATGGAAAAC
ATGGTGCAGAGTGTGTTCGATATTTATATTTAGACACGGATAGCCAATGTGTTTGATATACATGTTCTTGTTG
TACTTGAGGGGGGAGAGGATGTCACGTTTTTATGGCGGTCGGATTTTTAATTTGGTGGCGATTTAGTGAAGCT
CTCTATCAGCCTCTCCGGACATGATTATTCGATGCGTGATTTTGCAGGTGTAGTGAGAGTGTTTGATTTTTTT
CATAATTGTTCGGTGTTTGAATTCATTTTTGGGAGTTCGCGTTACATTATTTATCGAGGGACTTATAATAGAG
GCATAACATTTGATGGTATTCAGCGCATTCTGCACAGCGCGTCTCTTTGTTGAATGGCCGCGCAAGTCTCTCC
CGGTCAAATTTGAATTTATTTGAACTGCGTGTGGAATATATTTTGCCCTCTCCTCCTGCACGAGTCATTTTAT
ATAGTCAACCGCTCTCTGCCTTATTTTTGCCAGACATGATCTCTCTGTGGTATGTTATTTTAGCGGATGAGGG
AGATGAAAATCAGGGACAAGTCTCTCTCATTGTGGCTCTCTGTGCCACGTCCAGTCAAGTGCACTGTGTTAGA
CTAACAGTTCTATGTTATGCAGTTCGTTTTGCAGCTCGATCATGTTTTGGCTAATTATCTTAGCGAATACATG
TCTTGTGTATTTTTTGTCGGTGATGTCGATACATTACCAGTGGATGAGATAAAGGCTAATATTGATTTTGACG
CACTGTAATTTCGAGAACCAAAAGGCCAAATTTATCTCGTGCCGTGACTCTGTGGCATTGATAAAGACTGGGA
GTGATTTTGTCTGAACATCTCAAGCATTATATATGCAGAGCAAGGACCATCAATACAATTTTGCTCGTGTATT
TTTTAAACTGAAAATGCAGGCAGAGCATTATTTCATTGATGATGATGGCGGGTGCTTTTAACTTCACGGAGGA
TGTGCCCATATCGGAGAATGCGCTGGCCTCTATTGAAGTATCAACCTATGAAGGCATCCCCTGAACATGTTGG
CCAATCTCTCAAAATACCATCGCTTTACCAATCCAGGTCTGATCTTACACCAAGGCCCATACTGATGGATGAA
TCAACCTCTAAACCCGAAGGAAGCAAGGGCGAACCCCCTGCTTCCCACATTAGTCCACCAGAGACCAATGATC
CAAACGTCAAATCAAAAAAGCGTCGCAACACCAAATGGGACGTCAACCCCGAAGAAGTCCGCTGCTCCGACTG
GGCCATGGTCCCCAAGAGCACACCCCGAAAATTACTCCCCGAGCATAAGGCAGCCTACAAAGAATTACAAATC
GCAGACACCGAACGATGCACCGCCCAGGAAACGTTGGACATGGCCCGGACAGCACTCGAGGACGCAAAGCGGC
ACCTGCTTTGGGCGGAGGAACGATCCGAGGAGGCCAATGCCAGATTCCGCACAGCGCAAGAGGCCGTCGAAGA
GATTGACATAAAGAGACCGGACTTCAAGTGGAACTTCATGTACGATAAGCTCGTGAAGTTCAAGGAACGGCAC
GGGCACTGTCGCATTCCGGCATCCCAACCGGAACTCGCCAAGTTGCGTGTGTTCGTTCATACGAACAGGACCG
CGTACCGAGAGTATCAATCCGGCAAACGCTGTACGAATAAGGCGCATCGCATGATTGCCCTGGAGCGATTGGG
GGTGTTCGCGAGCAAATGGGAGGAAAGGTATAATGAGTTGGTAGAGTTTAAGAAGGTCCATGGACATTGCAAC

GTTCCGACGAAATCGAAGGAGAATCAGAAGTTGGGGTATTGGGTTGGATGGCAGAGGGGGGGTTACAGGAGGT
ACAAGGCTGGGAAAGAGAATCAGTTGTCGGAAGAGCGAATTAAGCTGCTGGAAGATATTGGGTTTTGTTTTAA
TGTGTATGAGGCGAATGGCATTGTGAGGAAACGTCGGGGAAAGGAAGCGGAGGAAGCTGTGGCTGATTCGTCG
GATTAGGGATGTGAAAATAGAGAGCGCAAACGAATCATGTAAGTATAACTGGGGTGGGTGTGGATTTGCTGTG
AACACAATGTAAAATTTAGAATTACTATTATAGCACAATACCTTTAAAATTTAAAAAAAAAGATCGGAAG
>TR508|c2_g1_i3 len=1100 path=[2897:0-65 3129:66-247 3071:248-271
3112:272-490 3150:491-514 3151:515-539 3128:540-584 @3111@!:585-997
3083:998-1031 3084:1032-1034 3085:1035-1058 3086:1059-1099] [-1, 2897,
3129, 3071, 3112, 3150, 3151, 3128, 3111, 3083, 3084, 3085, 3086, -2]
CCGATCTTGACAAAGGCCATAAGTACGAAATGTCGATCGTAGCGGTCTGATCTTACACCAAGGCCCATACTGA
TGGATGAATCAACCTCTAAACCCGAAGGAAGCAAGGGCGAACCCCCTGCTTCCCACATTAGTCCACCAGAGAC
CAATGATCCAAACGTCAAATCAAAAAAGCGTCGCAACACCAAATGGGACGTCAACCCCGAAGAAGTCCGCTGC
TCCGACTGGGCCATGGTCCCCAAGAGCACACCCCGAAAATTACTCCCCGAGCATAAGGCAGCCTACAAAGAAT
TACAAATCGCAGACACCGAACGATGCACCGCCCAGGAAACGTTGGACATGGCCCGGACAGCACTCGAGGACGC
AAAGCGGCACCTGCTTTGGGCGGAGGAACGATCCGAGGAGGCCAATGCCAGATTCCGCACAGCGCAAGAGGCC
GTCGAAGAGATTGACATAAAGAGACCGGACTTCAAGTGGAACTTCATGTACGATAAGCTCGTGAAGTTCAAGG
AACGGCACGGGCACTGTCGCATTCCGGCAAACCAACCGGAACTCGCCAAGCTGCGTGTGTTCGTTCATACGAA
CAGGACCGCGTACCGAGAGTATCAATCCGGCAAACGCTGTACGAATAAGGCGCATCGCATGATTGCCCTGGAG
CGATTGGGGGTGTTCGCGAGCAAATGGGAGGAAAGGTATAATGAGTTGGTAGAGTTTAAGAAGGTCCATGGAC
ATTGCAACGTTCCGACGAAATCGAAGGAGAATCAGAAGTTGGGGTATTGGGTTGGATGGCAGAGGGGGGGTTA
CAGGAGGTACAAGGCTGGGAAAGAGAATCAGTTGTCGGAAGAGCGAATTAAGCTGCTGGAAGATATTGGGTTT
TGTTTTAATGTGTATGAGGCGAATGGCATTGTGAGGAAACGTCGGGGAAAGGAAGCGGAGGAAGCTGTGGCTG
ATTCGTCGGATTAGGGATGTGAAAATAGAGAGCGCAAACGAATCATGTAAGTATAACTGGGGTGGGTGTGGAT
TTGCTGTGAACACAATGTAAAATTTAGAATTACTATTATAGCACAATACCTTTAAAATTTAAAAAAAAAGATC
GGAAG
>TR509|c0_g1_i1 len=836 path=[1085:0-43 1115:44-63 1122:64-114 1118:115-
157 1114:158-203 1094:204-227 1117:228-298 1083:299-322 1113:323-377
1120:378-378 1116:379-402 1112:403-703 1075:704-714 1076:715-738
1077:739-835] [-1, 1085, 1115, 1122, 1118, 1114, 1094, 1117, 1083, 1113,
1120, 1116, 1112, 1075, 1076, 1077, -2]
TCTGCGGTGATAATGACTGGATCCTGAAGTTGGGACGAAATTCACGCATGGAAAGAAGTTGGTTAAGTTGCGT
GATCGAAACTCACAAATTTAGAGCATTCGACAACCAAAAAGTTTAAAGTCTCCAACAAAAATTGTACCAAGCA
TACTATGCTCGGGCCCACACTGATAAAAAGAAAGGCCCAAGTTGTTGGTGCAAGGTTTTGAGATTCTTATTCT
CCCATAGGTATGTTGAAATTGGTAGATGTATTGCAATTGCAACCTGTGAATAAACCGGGAGGTCTCAGCTGTT
TGTACTTCCGTCTCAGGCAAAACATGGTGATGATTGATAATGCTGACAGCATCGTTTTCTCTCGTAGAAGACT
CCCAATGAGAAGTGGCGCCTCCCTATTGGCAGCGTCGACGGAATTGACAACAAAAAGAGATCGCCCATCACAT
GCACCAGAAATCGCACATTTCCCTTCCAAATTGGGTGACTTGAGAGTCATCCCAACTCTCCTCATTTCATCGG
GGCCATCCTTGGCTACGGCCATCAGCATGTCCTTTCAGCAACCTACAGCGACGAAAATTTACCAACCGTGCCA
AATCGAGCGGCCCTAAAAATTCGCCATCATGATCAAATCAACCCAAACGTTCCCCCCAGCCGCCCAGCCCCTC
GTTGCTCGGCACACGGTTTCGCCCGAATTTTGAAGAAAATCAATCATGGAGCTCGTGTCAACTCCCGCCATCA
CAGAAGCGCCGCGCATGGTGTTCTCATGCACTGACAATAATCAAAACGCAAATAGCGGCACTACGTTCCGTAG
CAGGATCGGACATGCCACATCTCGCCCCACCAC
>TR509|c1_g1_i1 len=1187 path=[1460:0-63 1475:64-78 1472:79-102 1468:103-
155 1425:156-179 1426:180-185 1427:186-209 1467:210-252 1440:253-276
1471:277-327 1455:328-351 1466:352-483 1449:484-507 1470:508-609
1434:610-633 1476:634-1053 1473:1054-1094 1469:1095-1129 1464:1130-1153
1444:1154-1186] [-1, 1460, 1475, 1472, 1468, 1425, 1426, 1427, 1467,
1440, 1471, 1455, 1466, 1449, 1470, 1434, 1476, 1473, 1469, 1464, 1444, -
2]
CGCTCGCCGCCTCGCCCCGTGCCATCATGGCGCCGATGGGCACTCCTCCTCCCAGAGCCTTGGCGGAGGTGAA
GACGTCGGGCTCGACTCCGAGCTGTTCGTGTCCCCATAGACTGCCGCTGCGTCCCATGCCGACCTGTACTTCA
TCGCAAATTAGCAGGGCCCCGTTGTCGTCGCATAGCTTTCGCGCGAGCTCGAAGTATTCCCGCTTTCCCGGGA
CGATGCCCCCCTCGCCCTGCAGTGGTTCCAGCATAATGGCGGCCACTCCTCGTTTACGTCCCGCCTTGGCATC
CTCCTCGGGTGTGACATTGATCTCCTCGACCAACTTTTCCAAGGCATCGAGGTCGTTGTAGGGTGTGTATTTG
AATCCACGTACCATTTCTCCTCCGTAGGTGAATCCCTTGTGGTACTTGGGTTGGGCCGTGGCGGTCACCGTAG
CGAGGGTGCGGCCATGGAAGGACTGCTCTGCGGTGATAATGACTGGATCCGTAATGCCACGATCATTCGCATG
CTTCCTGGCCAACTTAATCGCCGCCTCATTACTCTCCGCCCCCGAATTGCAAAAGAACGCCTTGTCCGCACAG
CTATTCTCGCAGAGCCACGCGGCCAACAATCCCTGCCCGGGCGTGTAATACAAATTGCTAACATGATGCAATT
GGCCCATTTGATCGCATACCGCCTTGGTGAGCGATGGGTTATTGTGTCCCAATGCGCACGTGGCAATTCCGGA
TACGAAATCGAGGTAGGTCTTGCCATTCTCGTCGGTGAGGGTGGAGCCGGAGCCGGAGACGAATGTGATGGGA

TACCGTCCGTACGTTTTTTGGACGCGGGTGTCGAATAGATCGGAGGTGGAGGATGAGTCGATGGAGGAGGGTG
GGGTTAGGCTCGAGGTGACTGATCCTGGTGCGGGGATGGTGGAGTGGAGTTGTGTGGTTGTGGTGATTCGATG
GTTGTTATTTTGATTTTGATGGGTGGAGGAGGAAAAAGCATTACAGTTTGTGCCGCTGAGGGCGAGGAGGGCG
GCGAGCACCGTTGATCGATGGACGAGCATCATTATATATATATTATTCTATTGGTTTTGGAATGCCGGGAGGT
CTCTATTGTCAATGGCCAGTGGGTTGCTCTGGGTGCATTCGCGATGGCGTCGTAGTGTGGTGATTGTAAGCTC
GGCGAGACAGTAGCTACTT
>TR509|c2_g1_i1 len=991 path=[4233:0-53 4234:54-77 4374:78-83 4324:84-107
4399:108-142 4206:143-183 4323:184-415 4352:416-603 4162:604-627
4398:628-728 4387:729-809 4371:810-871 4350:872-880 4111:881-896
4349:897-904 4319:905-931 4370:932-945 4348:946-965 4318:966-974
4427:975-990] [-1, 4233, 4234, 4374, 4324, 4399, 4206, 4323, 4352, 4162,
4398, 4387, 4371, 4350, 4111, 4349, 4319, 4370, 4348, 4318, 4427]
ATCAATTTCAATTGAACTCGTCATCTCAAAGTAGCTACTGTCTCGCCGAGCTTACAATCACCACACTACGACG
CCATCGCGAATGCACCCAGAGCAACCCACTGGCCATTGACAATAGAGACCTCCCGGCATTCCAAAACCAACAG
AATAATATATATATTATGATGCTCGTCCATCGATCAACGGTGCTCGCCGCCCTCCTCGCCCTCAGCGGCACAA
ACTGTAATGCTTTTTCCTCCTCCACCCATCAAAATCAAAATAACAACCATCGAATCACCACAACCACACAACT
CCACTCCACCATCCCCGCACCAGGATCAGTCACCTCGAGCCTAACCCCACCCTCCTCCATCGACTCATCCTCC
ACCTCCGATCTATTCGACACCCGCGTCCAAAAAACGTACGGACGGTATCCCATCACATTCGTCTCCGGCTCCG
GCTCCACCCTCACCGACGAGAATGGCAAGACCTACCTCGATTTCGTATCCGGAATTGCCACGTGCGCATTGGG
ACACAATAACCCATCGCTCACCAAGGCGGTATGCGATCAAATGGGCCAATTGCATCATGTTAGCAATTTGTAT
TACACGCCCGGGCAGGGATTGTTGGCCGCGTGGCTCTGCGAGAATAGCTGTGCGGACAAGGCGTTCTTTTGCA
ATTCGGGGGCGGAGAGTAATGAGGCGGCGATTAAGTTGGCCAGGAAGCATGCGAATGATCGTGGCATTACGGT
GAGTTGTGTGGATACTTTGTATGGAACGTTTTTGTTGGAGAGATGTCAAGTTTGGAGGAATGGAATCTCCGTC
CTTTCTTATTCTTCGGCCGCGTGCATTTGAAATGATTATGTTGATTCCGGAGGAGGGTACGTGAGGCTACGGT
TGTATCTCGTGGAAGATGAGAAAGTTTGAAATCGACGTTGAATGATTCGGACTTCGCTTTCGTCGCACCGGAG
GAGGGTACGTGAGGCTACGGTTGTATCTCGTGGAAGATGAGA
>TR509|c2_g1_i2 len=2949 path=[4233:0-53 4234:54-77 4374:78-83 4324:84-
107 4399:108-142 4206:143-183 4323:184-415 4352:416-603 4162:604-627
4398:628-728 4387:729-809 4321:810-896 4227:897-904 4228:905-931
4343:932-990 4313:991-1118 4195:1119-1142 4402:1143-1150 4393:1151-1174
4382:1175-1185 4341:1186-1239 4311:1240-1415 4159:1416-1439 4339:1440-
1486 4223:1487-1510 4310:1511-1536 4365:1537-1566 4428:1567-1590
4436:1591-1598 4381:1599-1661 4364:1662-1685 4337:1686-1730 4429:1731-
1731 4407:1732-1774 4401:1775-1789 4334:1790-1825 4390:1826-1845
4361:1846-1865 4331:1866-1869 4301:1870-2001 4282:2002-2025 4330:2026-
2076 4101:2077-2100 4300:2101-2143 4218:2144-2167 4219:2168-2173
4220:2174-2197 4359:2198-2250 4068:2251-2274 4329:2275-2535 4430:2536-
2559 4437:2560-2563 4378:2564-2588 4358:2589-2592 4328:2593-2640
4389:2641-2674 4355:2675-2714 4326:2715-2755 4375:2756-2779 4354:2780-
2814 4325:2815-2838 4431:2839-2839 4263:2840-2948] [-1, 4233, 4234, 4374,
4324, 4399, 4206, 4323, 4352, 4162, 4398, 4387, 4321, 4227, 4228, 4343,
4313, 4195, 4402, 4393, 4382, 4341, 4311, 4159, 4339, 4223, 4310, 4365,
4428, 4436, 4381, 4364, 4337, 4429, 4407, 4401, 4334, 4390, 4361, 4331,
4301, 4282, 4330, 4101, 4300, 4218, 4219, 4220, 4359, 4068, 4329, 4430,
4437, 4378, 4358, 4328, 4389, 4355, 4326, 4375, 4354, 4325, 4431, 4263, -
2]
ATCAATTTCAATTGAACTCGTCATCTCAAAGTAGCTACTGTCTCGCCGAGCTTACAATCACCACACTACGACG
CCATCGCGAATGCACCCAGAGCAACCCACTGGCCATTGACAATAGAGACCTCCCGGCATTCCAAAACCAACAG
AATAATATATATATTATGATGCTCGTCCATCGATCAACGGTGCTCGCCGCCCTCCTCGCCCTCAGCGGCACAA
ACTGTAATGCTTTTTCCTCCTCCACCCATCAAAATCAAAATAACAACCATCGAATCACCACAACCACACAACT
CCACTCCACCATCCCCGCACCAGGATCAGTCACCTCGAGCCTAACCCCACCCTCCTCCATCGACTCATCCTCC
ACCTCCGATCTATTCGACACCCGCGTCCAAAAAACGTACGGACGGTATCCCATCACATTCGTCTCCGGCTCCG
GCTCCACCCTCACCGACGAGAATGGCAAGACCTACCTCGATTTCGTATCCGGAATTGCCACGTGCGCATTGGG
ACACAATAACCCATCGCTCACCAAGGCGGTATGCGATCAAATGGGCCAATTGCATCATGTTAGCAATTTGTAT
TACACGCCCGGGCAGGGATTGTTGGCCGCGTGGCTCTGCGAGAATAGCTGTGCGGACAAGGCGTTCTTTTGCA
ATTCGGGGGCGGAGAGTAATGAGGCGGCGATTAAGTTGGCCAGGAAGCATGCGAATGATCGTGGCATTACGGT
GAGTTGTGTGGATACTTTGTATGGAACGTTTTTGTTGGAGAGATGTCAAGTTTGGAGGAATGGAATCTCCGTC
CTTTCTTCTTTTTCGGCCGCGTGCATTTGAAATGGTTATGTCGATTCTGGAGGAGGGTACGTGAGGCTACAGT
TGTATCTCGTGGAAGATGAGAAAGTTTGAAATCGACGTTGAATGATTCGGACTTCGGTTTCGTCGCACCGTCC
ACTTTCTGGGCAGGACGCTGGGCAGTGAGGAAATGGCCTATTCGAGATATTATTATTTATCCCAATGCTGGCA

GGTCCGTGGGTGGTGGGGCGAGATGTGGCATGTCCGATCCTGCTACGGAACGTAGTGCCGCTATTTGCGTTTT
GATTATTGTCAGTGCATGAGAACACCATGCGCGGCGCTTCTGTGATGGCGGGAGTTGACACGAGCTCCATGAT
TGATTTTCTTCAAAATTCAGGCGAAGCCGTGTGCCGAGCAATGAGGGGTTGGGCGGCTGGGGGGAACGTTTGG
GTTGATTTGATCATGATGGCGAATTTTTAGGGCCGCTCGATTTGGCACGGTTGGTAAATTTTCGTCGCTGTAG
GTTGCTGAAAGGACATGCTGATGGCCGTAGCCAAGGATGGCCCCGATGAAATGAGGAGAGTTGGGATGACTCT
CAAGTCACCCAATTTGGAAGGGAAATGTGCGATTTCTGGTGCATGTGATGGGCGATCTCTTTTTGTTGTCAAT
TCCGTCGACGCTGCCAATAGGGAGGCGTCACTTCTCATTGGGAGTCTTCTACGAGAGAAAACGATGCTGTCAG
CATTATCAATCATCACCATGTTTTGCCTGAGACGGAAGTACAAACAGCTGAGACCTCCCGGTTTATTCACAGG
TTGCAATTGCAATACATCTACCAATTTCAACATACCTATGGGAGAATAAGAATCTCGAAACCTTGCACCAACA
ACTTGGGCCTTTCTTTTTATCAGTGTGGGCCCGAGCATAGTATGCTTGGTACATTTTTTGTTGGAGACTTTAT
ACTTTTTGGTTGTCGAATGCTCTAAATTTGTGAGTTTCCATCACGCAACTCAACCAACTTCTTTCCATGCGTG
AATTTCGTCCCAACTTCAGGACCCAGTCATTATCACCGCAGAGCAGTCCTTCCATGGCCGCACCCTCGCTACG
GTGACCGCCACGGCCCAACCCAAGTACCACAAGGGATTCACCTACGGAGGAGAAATGGTACGTGGATTCAAAT
ACACACCCTACAACGACCTCGATGCCTTGGAGAAGTTGGTCGAGGAGATCAATGTCACACCCGAGGAGGATGC
CAAGGCGGGACGTAAACGAGGAGTGGCCGCCATCATGCTGGAACCACTGCAGGGCGAGGGGGGCATCGTCCCG
GGAAAGCGGGAATACTTCGAGCTCGCGCGAAAGCTATGCGACGACAACGGGGCCCTGCTAATTTGCGATGAAG
TACAGGTCGGCATGGGACGCAGCGGCAGTCTATGGGGACACGAACAGCTCGGAGTCGAGCCCGACGTCTTCAC
CTCCGCCAAGGCTCTGGGAGGAGGAGTGCCCATCGGCGCCATGATGGCACGGGGCGAGGCGGCGAGCGTCTTC
GGCCCCGGCGATCACGCCAGCACGTACGGCGGCAATCCGCTGGCGTGTGCGGCGGGACTGGCCGTGGCGGAGT
ACTTGAGCGAGCACGACGTGTTGGCGAATGTGCAGGCGCGGGGCGAGCAGCTGATGGCAGGGCTGGCGGACAT
CGCCACGCGGTATCCCACTGTGTTGGGCGAGGCGCGAGGATGGGGACTTTTGCGTGGCGTGCCGATCAAGGAG
GAGGCGGGAGTTACGGCCGCTGAGTTGGTGGGTGATGCTATGGCGGAGGGATTACTGTTGGTAGCCGCGGGGC
CGACCGTTGTTAGGTTTGTTCCTCCGTTGGTGGTGACGGAGGAGGAGATTACCGAGGCTATGGTTAGGTTCGA
GAAGGTGATTGCCAAGCGAGTGGAGTCCTCTTCGTAAAGGAAAGAGAGAGAGCGCCTGCATTAAAATAGGGAT
GCATTTGGCTGTGTCAACAAATACCCAATGAGAGGAATCATATTGCGAATAGAATACATAACCTAGCATCAGT
ACGAAAGAACTCTTTTTCGCTTATGACTTATGTTTTGCAGAAACAAATTCATCACAATAAGTACCTTTAAGCA
TAAGGAATGTAGAAATGACATGTCATCTG
>TR509|c2_g1_i3 len=1833 path=[4233:0-53 4234:54-77 4374:78-83 4324:84-107 4399:108-142 4206:143-183 4323:184-415 4352:416-603 4162:604-627 4398:628-728 4422:729-729 4247:730-749 4331:750-753 4301:754-885 4282:886-909 4330:910-960 4101:961-984 4300:985-1027 4218:1028-1051 4219:1052-1057 4220:1058-1081 4359:1082-1134 4068:1135-1158 4329:1159-1419 4430:1420-1443 4437:1444-1447 4377:1448-1472 4357:1473-1476 4327:1477-1524 4389:1525-1558 4356:1559-1598 4326:1599-1639 4375:1640-1663 4354:1664-1698 4325:1699-1722 4431:1723-1723 4263:1724-1832] [-1, 4233, 4234, 4374, 4324, 4399, 4206, 4323, 4352, 4162, 4398, 4422, 4247, 4331, 4301, 4282, 4330, 4101, 4300, 4218, 4219, 4220, 4359, 4068, 4329, 4430, 4437, 4377, 4357, 4327, 4389, 4356, 4326, 4375, 4354, 4325, 4431, 4263, -2]
ATCAATTTCAATTGAACTCGTCATCTCAAAGTAGCTACTGTCTCGCCGAGCTTACAATCACCACACTACGACG
CCATCGCGAATGCACCCAGAGCAACCCACTGGCCATTGACAATAGAGACCTCCCGGCATTCCAAAACCAACAG
AATAATATATATATTATGATGCTCGTCCATCGATCAACGGTGCTCGCCGCCCTCCTCGCCCTCAGCGGCACAA
ACTGTAATGCTTTTTCCTCCTCCACCCATCAAAATCAAAATAACAACCATCGAATCACCACAACCACACAACT
CCACTCCACCATCCCCGCACCAGGATCAGTCACCTCGAGCCTAACCCCACCCTCCTCCATCGACTCATCCTCC
ACCTCCGATCTATTCGACACCCGCGTCCAAAAAACGTACGGACGGTATCCCATCACATTCGTCTCCGGCTCCG
GCTCCACCCTCACCGACGAGAATGGCAAGACCTACCTCGATTTCGTATCCGGAATTGCCACGTGCGCATTGGG
ACACAATAACCCATCGCTCACCAAGGCGGTATGCGATCAAATGGGCCAATTGCATCATGTTAGCAATTTGTAT
TACACGCCCGGGCAGGGATTGTTGGCCGCGTGGCTCTGCGAGAATAGCTGTGCGGACAAGGCGTTCTTTTGCA
ATTCGGGGGCGGAGAGTAATGAGGCGGCGATTAAGTTGGCCAGGAAGCATGCGAATGATCGTGGCATTACGGA
CCCAGTCATTATCACCGCAGAGCAGTCCTTCCATGGCCGCACCCTCGCTACGGTGACCGCCACGGCCCAACCC
AAGTACCACAAGGGATTCACCTACGGAGGAGAAATGGTACGTGGATTCAAATACACACCCTACAACGACCTCG
ATGCCTTGGAGAAGTTGGTCGAGGAGATCAATGTCACACCCGAGGAGGATGCCAAGGCGGGACGTAAACGAGG
AGTGGCCGCCATCATGCTGGAACCACTGCAGGGCGAGGGGGGCATCGTCCCGGGAAAGCGGGAATACTTCGAG
CTCGCGCGAAAGCTATGCGACGACAACGGGGCCCTGCTAATTTGCGATGAAGTACAGGTCGGCATGGGACGCA
GCGGCAGTCTATGGGGACACGAACAGCTCGGAGTCGAGCCCGACGTCTTCACCTCCGCCAAGGCTCTGGGAGG
AGGAGTGCCCATCGGCGCCATGATGGCACGGGGCGAGGCGGCGAGCGTCTTCGGCCCCGGCGATCACGCCAGC
ACGTACGGCGGCAATCCGCTGGCGTGTGCGGCGGGACTGGCCGTGGCGGAGTACTTGAGCGAGCACGACGTGT
TGGCGAATGTGCAGGCGCGGGGCGAGCAGCTGATGGCAGGGCTGGCGGACATCGCCACGCGGTATCCCACTGT
GTTGGGCGAGGCGCGAGGATGGGGACTTTTGCGTGGCGTGCCGATCAAGGAGGAGGCGGGATGTACGGCCGCT
GAGTTGGTGGGTGATGCGATGGCGGAGGGATTACTGTTGGTCGCCGCGGGGCCGACCGTTGTTAGGTTTGTTC

CTCCGTTGGTGGTGACGGAGGAGGAGGTTACCGAGGCTATGGCTAGGTTCGAGAAGGTGATTGCCAAGCGAGT
GGAGTCCTCTTCGTAAAGGAAAGAGAGAGAGCGCCTGCATTAAAATAGGGATGCATTTGGCTGTGTCAACAAA
TACCCAATGAGAGGAATCATATTGCGAATAGAATACATAACCTAGCATCAGTACGAAAGAACTCTTTTTCGCT
TATGACTTATGTTTTGCAGAAACAAATTCATCACAATAAGTACCTTTAAGCATAAGGAATGTAGAAATGACAT
GTCATCTG
>TR509|c2_g1_i4 len=766 path=[4079:0-31 4427:32-47 4081:48-55 4082:56-82
4367:83-96 4344:97-141 4313:142-269 4195:270-293 4402:294-301 4393:302-
325 4382:326-336 4340:337-390 4311:391-566 4159:567-590 4339:591-637
4223:638-661 4310:662-687 4365:688-717 4428:718-741 4436:742-749
4179:750-765] [-1, 4079, 4427, 4081, 4082, 4367, 4344, 4313, 4195, 4402,
4393, 4382, 4340, 4311, 4159, 4339, 4223, 4310, 4365, 4428, 4436, 4179, -
2]
CCGGAGGAGGGTACGTGAGGCTACGGTTGTATCTCGTGGAAGATGAGAAAGTTTGAAATCGACGTTGAATGAT
TCGGACTTCGCTTTCGTCGCACCGTCGACTCTCTGGGCAGGACGCCGGGCAGTGAGGAAATGGCCTATTCGAG
ATATTATTATTTATCCCAATGCTGGCAGGTCCGTGGGTGGTGGGGCGAGATGTGGCATGTCCGATCCTGCTAC
GGAACGTAGTGCCGCTATTTGCGTTTTGATTATTGTCAGTGCATGAGAACACCATGCGCGGCGCTTCTGTGAT
GGCGGGAGTTGACACGAGCTCCATGATTGATTTTCTTCAAAATTCGGGCGAAACCGTGTGCCGAGCAACGAGG
GGCTGGGCGGCTGGGGGGAACGTTTGGGTTGATTTGATCATGATGGCGAATTTTTAGGGCCGCTCGATTTGGC
ACGGTTGGTAAATTTTCGTCGCTGTAGGTTGCTGAAAGGACATGCTGATGGCCGTAGCCAAGGATGGCCCCGA
TGAAATGAGGAGAGTTGGGATGACTCTCAAGTCACCCAATTTGGAAGGGAAATGTGCGATTTCTGGTGCATGT
GATGGGCGATCTCTTTTTGTTGTCAATTCCGTCGACGCTGCCAATAGGGAGGCGTCACTTCTCATTGGGAGTC
TTCTACGAGAGAAAACGATGCTGTCAGCATTATCAATCATCACCATGTTTTGCCTGAGACGGAAGTACAAACA
GCTGAGACCTCCCGGTTTATAGATCGGAAGAGCGTC
>TR510|c0_g1_i1 len=5323 path=[8901:0-139 8902:140-163 9108:164-175
9083:176-199 9045:200-207 8974:208-231 9105:232-269 8890:270-315
@9125@!:316-428 8685:429-452 9103:453-590 9144:591-614 9148:615-631
9150:632-651 9102:652-655 9142:656-675 9138:676-709 9100:710-719
9073:720-743 9033:744-755 9145:756-758 9132:759-805 9140:806-831
9137:832-856 9131:857-874 9117:875-900 9146:901-901 9067:902-929
9139:930-969 9027:970-1114 8703:1115-1138 8704:1139-1141 8705:1142-1155
9024:1156-1238 8698:1239-1262 9092:1263-1275 9063:1276-1291 9022:1292-
1315 8663:1316-1334 9061:1335-1459 9020:1460-1473 9091:1474-1513
9060:1514-1537 9019:1538-1593 8897:1594-1609 8898:1610-1633 8899:1634-
1652 9018:1653-1995 8922:1996-2019 9059:2020-2553 8828:2554-2577
9017:2578-2765 8678:2766-2789 9058:2790-2953 8883:2954-2977 9016:2978-
3118 8936:3119-3142 8937:3143-3143 8938:3144-3167 9057:3168-3369
8752:3370-3393 9015:3394-3491 8649:3492-3515 9090:3516-3707 8666:3708-
3724 9014:3725-3887 8624:3888-3911 9055:3912-4018 8727:4019-4042
9013:4043-4526 8963:4527-4550 8964:4551-4563 8965:4564-4587 9012:4588-
4684 8635:4685-4708 9089:4709-4719 9129:4720-4743 9112:4744-4757
9052:4758-4816 9010:4817-4935 8708:4936-4959 9111:4960-5117 9050:5118-
5152 9128:5153-5163 9110:5164-5187 9086:5188-5228 9007:5229-5266
8739:5267-5270 8740:5271-5322] [-1, 8901, 8902, 9108, 9083, 9045, 8974,
9105, 8890, 9125, 8685, 9103, 9144, 9148, 9150, 9102, 9142, 9138, 9100,
9073, 9033, 9145, 9132, 9140, 9137, 9131, 9117, 9146, 9067, 9139, 9027,
8703, 8704, 8705, 9024, 8698, 9092, 9063, 9022, 8663, 9061, 9020, 9091,
9060, 9019, 8897, 8898, 8899, 9018, 8922, 9059, 8828, 9017, 8678, 9058,
8883, 9016, 8936, 8937, 8938, 9057, 8752, 9015, 8649, 9090, 8666, 9014,
8624, 9055, 8727, 9013, 8963, 8964, 8965, 9012, 8635, 9089, 9129, 9112,
9052, 9010, 8708, 9111, 9050, 9128, 9110, 9086, 9007, 8739, 8740, -2]
CGCTGCCGCAATGCATCCCCCACACCGCATGTCGCCGCCTCCATGACGAGCCTCCGCCCGCCCTCCTTGTGCC
TACTTGTAGCGTATTACATAGCTCTATGAGTCTTCTCCCTTTGATATGAACGAATCGCACGCATCGATTAAGC
ACCGCAGGGAACCAAACCAGTAAAAGAGACGAATTCCAAAGACATTACAACGGAGATATTCAGGCAGGAGTCG
GGAGATTTTTTTCATGCGTGCAAGATAGAGTGATTTCGTTTGGTGAAATATAAGTTGACAATAATAATATGAC
GATGCGCAAATAATAAGAATGCGCATTCGAGTGTCAAACAGCAACATCAAGCGTGGACGTCTTGTCGTTGTTT
CTTTGGGATTGCTGTCAATCGTCCAACCTGCTAATAATAGCGGTGCGTTATCTTTTGTGCATGCGGAGGACTA
CGGGCCCGGGCCACCATCACGTTCACCAACCCCGTCGTCATCATCGGTATTCACGCCTGTTGGAGGTGTTGGC
ATCGACACACTCGCCAATGATACAGATCCTGTAATTACTATTGATGAACCGTTGGCGCCCACGCAACCCTTGC
TCGCTGCTTCGAGAAGCAGCATATCTGGCCACGCCTACTTAACAAACAGCACGCTTGGCTATGGAATGAACAA
TGTTTCAGTCGATCTCTTCACCTGCACTGGTAATATTATCAATACGACAAAAATAAATGCTTCAGGACATTAC

TCATTTGAGGAATTATACGGGGGTCGATACTTCATTAGTTCTGTGCCACCACAGGGGTACGTATTTAGTAATA
TATGGGACGACTATACCAATGAGACTGAATCGTTGGAGTATCTTGGTGGGGAAAGCGCCGTGAATCCCGATAA
CGGGAGTACAGAATGCTTTGATTTGAGGGAGGGAGAAGACAAAACTGTCAGTTTTGGGATGACGTTCAATGTC
TCCTCAAATACAGGGCCAATGACAACTGCCTCACCTTCAGAGTTGGTACCCAGCAATCCTCCGAGTCATGAGC
TCAATGTCTCCTCAAATCCTATGGAATCTGCATCGCCTTCAAAGTTATTGGAAATTACATCAGAACCATCAAA
TAGTCCTATCACAGCGACTTCAATGCCTCCCTCTGAAATGCCTTCATTGTACACATCACGAGTTCCATCGAAC
AGTCCCACAACCCTCACTGAATCTCAATCTCCATCAACGTTACCATCCCGTGAACCTTCACTGAACCCCACAT
CCGTCACATCAAAACTCCTATCAAACAGCCCTACGGCCCCCGTTGAATCTCAATCTCCATCAAAGATACCATC
CCATGAACCTTCGCTGAATCCCACGTCCATCACAGCAGAGCTTCCATCGGTCAAACCCACGGCCCTTGCTAAA
TCTCAATCCCCACCAATCATACCATCCCATGAACCTTCACTGAATCCTACGCCTCCCTCTGAAATGCCTTCAT
TGTACACATCACGAATTCTATCGAACAGCCCCACGGCCCTCGCTGAATCTCAATTCCCATCAACGATACCATC
CTGTGAACCTTCACTGGATCCCACACCCACCATATCACAACTCCTATCAAACAGCCCTACGGCCCCCGTTGAA
TCTCAATCTCCATCAAAGATACCATCCCATGAACCTTCGCTGAATCCTACGTCAGGATTTTATTTAACTGTCG
CACACTTCTATGAATCATCCCAGCCCACGCCTAATGTTCGTGCCAGCAAAATTCCATCTGCTTCACCTAGCCA
GTCAGTAAGTTTTCACGGTCCGACCACAACAGAGCGGTTGTTGATGACCATTCGCGGGATCAGAATGCTGCAA
GATGAGGAGGCGTGGAGCGTGGCCACCAGTGATTACATCATGTCATACTTTAATTTGATCAGTGACGATCCTG
AAGTGCTTGATGTGACAGTGAACATCGAAGTGACGAAGCAAACGAGTGGCGATGACGACGCGTTTCAACGTCG
ACGTCCGCATCAACGTCATCCAGAGTATCAACACCGAAAAATGAGACGACGACGAGGGCTAGAATCAGATTTC
GATATCGTGGAAGTGATGTACTCTCAAACATCTGTTTACAAGACTTCCAATTCATCTCGCTACGACGAGTTCT
ATGTGGCCACGGCGCCATTCTTTCCGAGACAAGGCTCCGAGGGGTATGTTGCAGCATTACGGGATCTATCACC
CTACTACAAGGAGGTTTGGTCCGTTGGGTTGGTACACGTGGATAACCCCCATCGAATGCCAATTGGTGAAGGC
AACGATGTTACCAACAAAGATAGGGTAGGTGAGGTTCAGAACCCTCCTGAGGAGGAAAATCAGCCCTCCACTA
ATGGTAATGAAGGCAAAGGCTTTAACCAACTATACATCATCGTGGCGGTCGGCTGCGCACTCCTCGCGTTTGT
TAGGGCCAGACTTGATAAAAAAAAACCTACAACTCGCACCACTACGCATAAAAAGTTTAATGTCATTGCGCCC
GCCGGTAGACTTGGCATCATTATCGAAACACCTCCCACTGGCAGTTCAGCCTTTGTTTCCGAGATTACGGAGG
CGTGTCCGATCGCTGGCCAGATCAAGTTGGGGGATCGAATCATTGCCGTAGACGACGAGGATGTACAAAATAT
GCTGACTGAGAACGTAAGCAAATTGCTGATAGAAAAGCATCGACAAGAAGAGAGGAAAATAACTGTTCTGAGG
AAGATTTGACCTGCCAAGGATGTCACTAGGAGTGTTTCTAATGGCGTCAAGTTCAACGAGGAAGTCTCCTCAA
GCATCTCTTCTACATATGGTAACACCGGAAGCTATGCCCATGTTAATGTGTATCAGAAGCAAGAGGCTGAACA
TATGATGATACAGAAGATATCACAGAAGCCTCCTCAAACCTCTTCGTAGCTCAAGCAGTCCTGCCAAAATCTA
TGATAGTAGTCCCGCAGCAAGCAAGCTGAAAGCCATTGGTGAGGGCGGTGTGCGCGATGACGATGACATGAGT
ATCATTATAAGGCGGAAAAGATAGACGCGAGATAGACTTGTGATGGCACGCTGGTACTACTGACAAAATTATG
AAAGACAATGCCTATGCCAACATCCGCCTTTCTCCCGCCCCGGCTTTTGTATCGAACGTATGGGGTGGCAGTC
CATTGGGGGGAAACGATACGTGGGGGGAAGGTGATTCCTATGGACGATCAAGTTTTGCGTTTTGAGGGAGGTT
AAAATCGGTGTGTGTGGTGATGTTAGAGAAGGGCCCGAAACTACTGTGTTGTCAACTGCTACCAAGAGTATGC
ATCGGTACCGAGCCCAAGTTGTGGACCTTGCTCGGCAACAAATCTCAGTAGTTTCCTTGTCGATGACGTCTAA
TGATTTGCTGAAGCAAAGACGCGCAAGTACTTTGCTCGACAAGCTGAGTATTTTAATTCATGTCGAGGTCAAG
ATCGTTGCTCATCAAAAATAGTGAAATATTACAACAGCATACATCTCACTAGTGCATAGAACGGACGATGAAG
GAACACATTGATCACCATTTGAAGGCCAGCATTACACAGTTGGAGAGGGAGTTGAAGAAATAATGATCAAACA
TCGATTCATCGTTGGAGCAGCGGGACACGCCCCCTTTCGACCATGTGCACTTACCTGAACAGCCAATTGTAGC
TCAAGGATGAATCACAGATCTAGTGGGATGCCATTGACAACAAGTTTACGGCAAAAAATGTTAGAGCTGAAGA
ACAGGATCAGTACCTTTGACATGGCAGCCGACCAGTCACCATAAAAACGAAGGCCAACTTGGTGGCCAAGCAA
TCAATGTCCCCGATACAGGAAACCAAGCTATATGACCGAAAGCTTACGAAGATGAGAGACTTGCCACATGCTA
CGTGAAAAGAACACTCAGGCCTATCAATTATAGAGAGACACACAACTACAGGAAAAGGCGTTCAGTGAAAAGA
CTTCATCGAAAGTCAAAATGCAATCTAAGCTGGAAACTCTCCAAATAGTCTGCTGAAAGACGAAGGCCCAGGT
ACATGAAAAAACACTAGGTGCAAGGCGTCGCGGACCCCCCAATATGCATGTCCAGTTGGAGGCACTTTGAATA
GACACTCTAACGATCCATTCAACTGCCGGGTTTGGTACGAACAAGCTAAAGGATGAATTGAAGATTGAGCAGC
AGCAAGCTCACCAATCGCAGCGGCTGAACATGGGATGCGCCAAGGCGAAATCGCTCGCGGTGGCTGTGGAGGA
AGAGGCATGTCAACAAGTTGTGGAAGTGCCAGTAAGGCGAGAGAAGATCGAGCAGCAGTAGGCAGCCAAGCAG
GGGTCGAAACGAGGCACAGTAACGAGCAAAGGGTCAGGACAAGACCATCGAGCCAAGCATGAGAGTACAACAC
AGCGGAGGAAAGTTTGCATAGGGAGAGTCGGTAGTCAAAAAGGAAGCAGAGGCCCATAAAATCAAGGAACGGG
CAGAGGCCAGAGGAAAGACAAAGGCGAAGACATTGGTCGCCAAACAGAAGGTTTCGGTATAAGAGCGGTCCAA
GTCAAAGTGATTCGGCAAGCCTATGACGAAAGAGGCAGTCTCTCCAGCGAAGAAGATTCTATTGGAGGAAATC
ATGGCCGCGGGAGATGCGCATGGCATGCGCACAAAGCAAGGCTCCTGACTGCTCATGTTGCAGGCAGAAAAAG
GGAATGCCGCTCCGAAGAAGGAAGTGGCGGATCCCACTACGACTGAGGTTGCGAAGGAAGTCACAGTGGAAAA
GGCCGGGGTTGGGTTGATGGCTGCCAGCGTGAAGGAGCTAGCGACGTTTAAAAGCGAATCAATGTCTGTAGAA
GATACCTCTCTCCATACCAAGAAATCTCGTTAGGCAATTAATCACGCCCTAAGGAAGGAAGGGCGCCAGTGCC
AATGGCGTGGGGTAGCTGGGGAAAAGTGACCAACCAACCTCCTCCTGGCCAAGGATGCACAATAGGAGACAGT
CAATCGGATGAAAGCCACTGGGAAGGGACTGTGGGCGAGGCTGGTAGGCGGTTGACCACGAATTGGTTGTCAT
GTATCCTCATTTTCGATGAGCATTGCTTTTTCGGTATTTAAATCCCACAAATGATGCAATTAGAAAGTCTTC

AGACTCGATATCTACTCCATTAAATATTGCTTAATGATCTACTCCATACAACACATATAATGGCAGATTCGGA
GAGAGGCGGAATGACTGCACTCGTCCAACGGTTGCAATGGTATTAGTTAATAGTACCAAATAAATATTTCTTA
ACATTATTTTTAATGAAGTAAAAATGAGAATTAATCTAACCCAAATTTCCACTAAAAGATCTTTTGA
>TR510|c0_g1_i2 len=5412 path=[8816:0-388 9085:389-444 9109:445-469
9047:470-487 9135:488-530 9124:531-541 9103:542-679 9144:680-703
9148:704-720 9150:721-740 9102:741-744 9142:745-764 9138:765-798
9100:799-808 9073:809-832 9033:833-844 9145:845-847 9132:848-894
9140:895-920 9137:921-945 9131:946-963 9117:964-989 9146:990-990
9067:991-1018 9139:1019-1058 9027:1059-1203 8703:1204-1227 8704:1228-1230
8705:1231-1244 9024:1245-1327 8698:1328-1351 9092:1352-1364 9063:1365-
1380 9022:1381-1404 8663:1405-1423 9061:1424-1548 9020:1549-1562
9091:1563-1602 9060:1603-1626 9019:1627-1682 8897:1683-1698 8898:1699-
1722 8899:1723-1741 9018:1742-2084 8922:2085-2108 9059:2109-2642
8828:2643-2666 9017:2667-2854 8678:2855-2878 9058:2879-3042 8883:3043-
3066 9016:3067-3207 8936:3208-3231 8937:3232-3232 8938:3233-3256
9057:3257-3458 8752:3459-3482 9015:3483-3580 8649:3581-3604 9090:3605-
3796 8666:3797-3813 9014:3814-3976 8624:3977-4000 9055:4001-4107
8727:4108-4131 9013:4132-4615 8963:4616-4639 8964:4640-4652 8965:4653-
4676 9012:4677-4773 8635:4774-4797 9089:4798-4808 9129:4809-4832
9112:4833-4846 9053:4847-4905 9010:4906-5024 8708:5025-5048 9111:5049-
5206 9050:5207-5241 9128:5242-5252 9110:5253-5276 9086:5277-5317
8640:5318-5341 8641:5342-5355 8642:5356-5359 8643:5360-5411] [-1, 8816,
9085, 9109, 9047, 9135, 9124, 9103, 9144, 9148, 9150, 9102, 9142, 9138,
9100, 9073, 9033, 9145, 9132, 9140, 9137, 9131, 9117, 9146, 9067, 9139,
9027, 8703, 8704, 8705, 9024, 8698, 9092, 9063, 9022, 8663, 9061, 9020,
9091, 9060, 9019, 8897, 8898, 8899, 9018, 8922, 9059, 8828, 9017, 8678,
9058, 8883, 9016, 8936, 8937, 8938, 9057, 8752, 9015, 8649, 9090, 8666,
9014, 8624, 9055, 8727, 9013, 8963, 8964, 8965, 9012, 8635, 9089, 9129,
9112, 9053, 9010, 8708, 9111, 9050, 9128, 9110, 9086, 8640, 8641, 8642,
8643, -2]
CATTCTCCTCACCATTCATGTCCATTGTCCATGACCATGTCGCATGGCGCACGGCGGAATAGACCGCATACAC
TTATATGGCATGAACAACATCATGACCAAAGGCGAAACTGGGCAAAAAACAGACACAGCGACCCGCATATCGT
ACTGTACGCTGCACATCCCACCCCTCCATGACGATCCTCCGTCCTCCCGCCCTGTCCGGCGGACAAGTTGTCG
AAAGGAGTATATCTGATGAGTGAAGTCATGTCACTCCTCCGACTACAGCAGGGAACCAAACGACTGAGAGTAA
AAACGTACATATTCAAGCAGGAGACAAGAGATTTTTTAATGTGTGCAATTGATTTCGTTGGGTGAATAGTAGT
TGACAATAATGTCAATATGCAAATTATAAGAAAGCGCATTCAATCAGCGAACAACAGCATCAAACGTCGACAC
CTCGCATCGTGGTGGACAACCGTCGTCTTTTTGGGATTGCCGTCTATCGTCCAACGAGTATCTCTTGTGCTTG
TGCATGCCGAGGACTACGGGCCCGGGCCACCATCACGTTCACCAACCCCGTCGTCATCATCGGTATTCACGCC
TGTTGGAGGTGTTGGCATCGACACACTCGCCAATGATACAGATCCTGTAATTACTATTGATGAACCGTTGGCG
CCCACGCAACCCTTGCTCGCTGCTTCGAGAAGCAGCATATCTGGCCACGCCTACTTAACAAACAGCACGCTTG
GCTATGGAATGAACAATGTTTCAGTCGATCTCTTCACCTGCACTGGTAATATTATCAATACGACAAAAATAAA
TGCTTCAGGACATTACTCATTTGAGGAATTATACGGGGGTCGATACTTCATTAGTTCTGTGCCACCACAGGGG
TACGTATTTAGTAATATATGGGACGACTATACCAATGAGACTGAATCGTTGGAGTATCTTGGTGGGGAAAGCG
CCGTGAATCCCGATAACGGGAGTACAGAATGCTTTGATTTGAGGGAGGGAGAAGACAAAACTGTCAGTTTTGG
GATGACGTTCAATGTCTCCTCAAATACAGGGCCAATGACAACTGCCTCACCTTCAGAGTTGGTACCCAGCAAT
CCTCCGAGTCATGAGCTCAATGTCTCCTCAAATCCTATGGAATCTGCATCGCCTTCAAAGTTATTGGAAATTA
CATCAGAACCATCAAATAGTCCTATCACAGCGACTTCAATGCCTCCCTCTGAAATGCCTTCATTGTACACATC
ACGAGTTCCATCGAACAGTCCCACAACCCTCACTGAATCTCAATCTCCATCAACGTTACCATCCCGTGAACCT
TCACTGAACCCCACATCCGTCACATCAAAACTCCTATCAAACAGCCCTACGGCCCCCGTTGAATCTCAATCTC
CATCAAAGATACCATCCCATGAACCTTCGCTGAATCCCACGTCCATCACAGCAGAGCTTCCATCGGTCAAACC
CACGGCCCTTGCTAAATCTCAATCCCCACCAATCATACCATCCCATGAACCTTCACTGAATCCTACGCCTCCC
TCTGAAATGCCTTCATTGTACACATCACGAATTCTATCGAACAGCCCCACGGCCCTCGCTGAATCTCAATTCC
CATCAACGATACCATCCTGTGAACCTTCACTGGATCCCACACCCACCATATCACAACTCCTATCAAACAGCCC
TACGGCCCCCGTTGAATCTCAATCTCCATCAAAGATACCATCCCATGAACCTTCGCTGAATCCTACGTCAGGA
TTTTATTTAACTGTCGCACACTTCTATGAATCATCCCAGCCCACGCCTAATGTTCGTGCCAGCAAAATTCCAT
CTGCTTCACCTAGCCAGTCAGTAAGTTTTCACGGTCCGACCACAACAGAGCGGTTGTTGATGACCATTCGCGG
GATCAGAATGCTGCAAGATGAGGAGGCGTGGAGCGTGGCCACCAGTGATTACATCATGTCATACTTTAATTTG
ATCAGTGACGATCCTGAAGTGCTTGATGTGACAGTGAACATCGAAGTGACGAAGCAAACGAGTGGCGATGACG
ACGCGTTTCAACGTCGACGTCCGCATCAACGTCATCCAGAGTATCAACACCGAAAAATGAGACGACGACGAGG
GCTAGAATCAGATTTCGATATCGTGGAAGTGATGTACTCTCAAACATCTGTTTACAAGACTTCCAATTCATCT

CGCTACGACGAGTTCTATGTGGCCACGGCGCCATTCTTTCCGAGACAAGGCTCCGAGGGGTATGTTGCAGCAT
TACGGGATCTATCACCCTACTACAAGGAGGTTTGGTCCGTTGGGTTGGTACACGTGGATAACCCCCATCGAAT
GCCAATTGGTGAAGGCAACGATGTTACCAACAAAGATAGGGTAGGTGAGGTTCAGAACCCTCCTGAGGAGGAA
AATCAGCCCTCCACTAATGGTAATGAAGGCAAAGGCTTTAACCAACTATACATCATCGTGGCGGTCGGCTGCG
CACTCCTCGCGTTTGTTAGGGCCAGACTTGATAAAAAAAAACCTACAACTCGCACCACTACGCATAAAAAGTT
TAATGTCATTGCGCCCGCCGGTAGACTTGGCATCATTATCGAAACACCTCCCACTGGCAGTTCAGCCTTTGTT
TCCGAGATTACGGAGGCGTGTCCGATCGCTGGCCAGATCAAGTTGGGGGATCGAATCATTGCCGTAGACGACG
AGGATGTACAAAATATGCTGACTGAGAACGTAAGCAAATTGCTGATAGAAAAGCATCGACAAGAAGAGAGGAA
AATAACTGTTCTGAGGAAGATTTGACCTGCCAAGGATGTCACTAGGAGTGTTTCTAATGGCGTCAAGTTCAAC
GAGGAAGTCTCCTCAAGCATCTCTTCTACATATGGTAACACCGGAAGCTATGCCCATGTTAATGTGTATCAGA
AGCAAGAGGCTGAACATATGATGATACAGAAGATATCACAGAAGCCTCCTCAAACCTCTTCGTAGCTCAAGCA
GTCCTGCCAAAATCTATGATAGTAGTCCCGCAGCAAGCAAGCTGAAAGCCATTGGTGAGGGCGGTGTGCGCGA
TGACGATGACATGAGTATCATTATAAGGCGGAAAAGATAGACGCGAGATAGACTTGTGATGGCACGCTGGTAC
TACTGACAAAATTATGAAAGACAATGCCTATGCCAACATCCGCCTTTCTCCCGCCCCGGCTTTTGTATCGAAC
GTATGGGGTGGCAGTCCATTGGGGGGAAACGATACGTGGGGGGAAGGTGATTCCTATGGACGATCAAGTTTTG
CGTTTTGAGGGAGGTTAAAATCGGTGTGTGTGGTGATGTTAGAGAAGGGCCCGAAACTACTGTGTTGTCAACT
GCTACCAAGAGTATGCATCGGTACCGAGCCCAAGTTGTGGACCTTGCTCGGCAACAAATCTCAGTAGTTTCCT
TGTCGATGACGTCTAATGATTTGCTGAAGCAAAGACGCGCAAGTACTTTGCTCGACAAGCTGAGTATTTTAAT
TCATGTCGAGGTCAAGATCGTTGCTCATCAAAAATAGTGAAATATTACAACAGCATACATCTCACTAGTGCAT
AGAACGGACGATGAAGGAACACATTGATCACCATTTGAAGGCCAGCATTACACAGTTGGAGAGGGAGTTGAAG
AAATAATGATCAAACATCGATTCATCGTTGGAGCAGCGGGACACGCCCCCTTTCGACCATGTGCACTTACCTG
AACAGCCAATTGTAGCTCAAGGATGAATCACAGATCTAGTGGGATGCCATTGACAACAAGTTTACGGCAAAAA
ATGTTAGAGCTGAAGAACAGGATCAGTACCTTTGACATGGCAGCCGACCAGTCACCATAAAAACGAAGGCCAA
CTTGGTGGCCAAGCAATCAATGTCCCCGATACAGGAAACCAAGCTATATGACCGAAAGCTTACGAAGATGAGA
GACTTGCCACATGCTACGTGAAAAGAACACTCAGGCCTATCAATTATAGAGAGACACACAACTACAGGAAAAG
GCGTTCAGTGAAAAGACTTCATCGAAAGTCAAAATGCAATCTAAGCTGGAAACTCTCCAAATAGTCTGCTGAA
AGACGAAGGCCCAGGTACATGAAAAAACACTAGGTGCAAGGCGTCGCGGACCCCCCAATATGCATGTCCAGTT
GGAGGCACTTTGAATAGACACTCTAACGATCCATTCAACTGCCGGGTTTGGTACGAACAAGCTAAAGGATGAA
TTGAAGATTGAGCAGCAGCAAGCTCACCAATCGCAGCGGCTGAACATGGGATGCGCCAAGGCGAAATCGCTCG
CGGTGGCTGTGGAGGAAGAGGCATGTCAACAAGTTGTGGAAGTGCCAGTAAGGCGAGAGAAGATCGAGCAGCA
GTAGGCAGCCAAGCAGGGGTCGAAACGAGGCACAGTAACGAGCAAAGGGTCAGGACAAGACCATCGAGCCAAG
CATGAGAGTACAACACAGCGGAGGAAAGTTTGCATAGGGAGAGTCGGTAGTCAAAAAGGAAGCAGAGGCCCAT
AAAATCAAGGAACGGGCAGAGGCCAGAGGAAAGACAAAGGCGAAGACATTGGTCGCCAAACAGAAGGTTTCGG
TATAAGAGCGGTCCAAGTCAAAGTGATTCGGCAAGCCTATGACGAAAGAGGCAGTCTCTCCAGCGAAGAAGAT
TCTATTGGAGGAAATCATGGCCGCGGGAGATGCGCATGGCATGCGCACAAAGCAAGGCTCCTGACTGCTCATG
TTGCAGGCAGAAAAAGGGAATGCCGCTCCGAAGAAGGAAGTGGCGGATCCCACTACGACTGAGGTTGCGAAGG
AAGTCACAGTGGAAAAGGCCGGGGTTGGGCTGATGGCTGCCAGCGCGAAGGAGCTAGCGACGTTGAAAAGCGA
ATCAATGTCTGTAGAAGATACCTCTCTCCATACCAAGAAATCTCGTTAGGCAATTAATCACGCCCTAAGGAAG
GAAGGGCGCCAGTGCCAATGGCGTGGGGTAGCTGGGGAAAAGTGACCAACCAACCTCCTCCTGGCCAAGGATG
CACAATAGGAGACAGTCAATCGGATGAAAGCCACTGGGAAGGGACTGTGGGCGAGGCTGGTAGGCGGTTGACC
ACGAATTGGTTGTCATGTATCCTCATTTTCGATGAGCATTGCTTTTTCGGTTATTTAAATCCCACAAATGATG
CAATTAGAAAGTCTTCAGACTCGATATCTACTCCATTAAATATTGCTTAATGATCTACTCCATACAACACATA
TAATGGCAGATTCGGAGAGAGGCGGAATGACTGCACTCGTCCAACGGTTGCAATGGTATTAGCTAATAGTACC
AAACAAATATTTCTTAACATTATTTTAATGAAGTAAAAATGAGAATTAATCTAACCCAAATTTCCACTAAAA
GATCTTTTGA
>TR510|c0_g1_i3 len=1480 path=[8816:0-388 9085:389-444 9109:445-469
9047:470-487 9135:488-530 9124:531-541 9103:542-679 9144:680-703
9148:704-720 9150:721-740 9141:741-764 9138:765-798 9100:799-808
9073:809-832 9033:833-844 9145:845-847 9132:848-894 9140:895-920
9096:921-945 9131:946-963 9116:964-989 9146:990-990 9143:991-1018
9139:1019-1058 9147:1059-1069 9149:1070-1083 9114:1084-1097 9093:1098-
1114 9064:1115-1138 9023:1139-1244 8742:1245-1268 8743:1269-1273
8744:1274-1297 9062:1298-1382 8869:1383-1430 8870:1431-1479] [-1, 8816,
9085, 9109, 9047, 9135, 9124, 9103, 9144, 9148, 9150, 9141, 9138, 9100,
9073, 9033, 9145, 9132, 9140, 9096, 9131, 9116, 9146, 9143, 9139, 9147,
9149, 9114, 9093, 9064, 9023, 8742, 8743, 8744, 9062, 8869, 8870, -2]
CATTCTCCTCACCATTCATGTCCATTGTCCATGACCATGTCGCATGGCGCACGGCGGAATAGACCGCATACAC
TTATATGGCATGAACAACATCATGACCAAAGGCGAAACTGGGCAAAAAACAGACACAGCGACCCGCATATCGT
ACTGTACGCTGCACATCCCACCCCTCCATGACGATCCTCCGTCCTCCCGCCCTGTCCGGCGGACAAGTTGTCG
AAAGGAGTATATCTGATGAGTGAAGTCATGTCACTCCTCCGACTACAGCAGGGAACCAAACGACTGAGAGTAA

AAACGTACATATTCAAGCAGGAGACAAGAGATTTTTAATGTGTGCAATTGATTTCGTTGGGTGAATAGTAGT
TGACAATAATGTCAATATGCAAATTATAAGAAAGCGCATTCAATCAGCGAACAACAGCATCAAACGTCGACAC
CTCGCATCGTGGTGGACAACCGTCGTCTTTTTGGGATTGCCGTCTATCGTCCAACGAGTATCTCTTGTGCTTG
TGCATGCCGAGGACTACGGGCCCGGGCCACCATCACGTTCACCAACCCCGTCGTCATCATCGGTATTCACGCC
TGTTGGAGGTGTTGGCATCGACACACTCGCCAATGATACAGATCCTGTAATTACTATTGATGAACCGTTGGCG
CCCACGCAACCCTTGCTCGCTGCTTCGAGAAGCAGCATATCTGGCCACGCCTACTTAACAAACAGCACGCTTG
GCTATGGAATGGACAATGTTTCAGTCGATCTCTTCACCTGCACTGGTAATATTATCAATACGACAAAAATAAA
TGCTTCAGGACATTACTCATTTGAGGAATTATACGGGGGTCGATACTTCATTAGTTCTGTGCCACCACAGGGG
TACGTATTTAGTAATATATGGGACGACTATACCAATGAGACTGAAGAGTTGGAGTATCTTGGTGGGGAAAGCG
CCGTGAATCCCGATACGAGGAGTACAGAATGCTTTGATTTGAAGGATGGAGAAGACAAAACTGTCAGTTTTGG
GATGACGTTCAATGTCTCCTCAAATACAGGGCCAATGGCAACTGCCTCACCTTCAGAGCTAGTGGAACCCACA
TCGGAAACATCAAATAGTTTGATCACAGCGTCTACAATTCCTCCCTTTGACATAACTTCGTTGCACACGTCTC
AACTTAAATCACAATCCCCATCAATGATACCTTCCCACAAAGCTTCTCTCAATCCTACGTCCAGCTTTCATCC
CACTACAATGCCGTCCGTACAGTCCAGCCGGTCAGGAGATCTTTATGGTCCCGCGACGATGGAAGGGTTGGTG
ATGGCCATTCGCGGGATTCAAGTGTTGGAAGATGAGGCAGCGTGGAGCGTGGCCACCACTAATCACATCATGG
AATACTTTAATTTGACCATTCATGATCCAGGAGTGTTTGATGTGACGGTGAGCATCGAAGTGATGAAGCAGAC
GAGTGGCGATGATGCTGTGT
>TR510|c0_g1_i4 len=1391 path=[8901:0-139 8902:140-163 9108:164-175
9083:176-199 9045:200-207 8974:208-231 9105:232-269 8890:270-315
@9125@!:316-428 8685:429-452 9103:453-590 9144:591-614 9148:615-631
9150:632-651 9102:652-655 9142:656-675 9138:676-709 9100:710-719
9073:720-743 9033:744-755 9145:756-758 9132:759-805 9140:806-831
9137:832-856 9131:857-874 9117:875-900 9146:901-901 9067:902-929
9139:930-969 9147:970-980 9113:981-1008 9093:1009-1025 9064:1026-1049
9023:1050-1155 8742:1156-1179 8743:1180-1184 8744:1185-1208 9062:1209-
1293 8869:1294-1341 8870:1342-1390] [-1, 8901, 8902, 9108, 9083, 9045,
8974, 9105, 8890, 9125, 8685, 9103, 9144, 9148, 9150, 9102, 9142, 9138,
9100, 9073, 9033, 9145, 9132, 9140, 9137, 9131, 9117, 9146, 9067, 9139,
9147, 9113, 9093, 9064, 9023, 8742, 8743, 8744, 9062, 8869, 8870, -2]
CGCTGCCGCAATGCATCCCCCACACCGCATGTCGCCGCCTCCATGACGAGCCTCCGCCCGCCCTCCTTGTGCC
TACTTGTAGCGTATTACATAGCTCTATGAGTCTTCTCCCTTTGATATGAACGAATCGCACGCATCGATTAAGC
ACCGCAGGGAACCAAACCAGTAAAAGAGACGAATTCCAAAGACATTACAACGGAGATATTCAGGCAGGAGTCG
GGAGATTTTTTTCATGCGTGCAAGATAGAGTGATTTCGTTTGGTGAAATATAAGTTGACAATAATAATATGAC
GATGCGCAAATAATAAGAATGCGCATTCGAGTGTCAAACAGCAACATCAAGCGTGGACGTCTTGTCGTTGTTT
CTTTGGGATTGCTGTCAATCGTCCAACCTGCTAATAATAGCGGTGCGTTATCTTTTGTGCATGCGGAGGACTA
CGGGCCCGGGCCACCATCACGTTCACCAACCCCGTCGTCATCATCGGTATTCACGCCTGTTGGAGGTGTTGGC
ATCGACACACTCGCCAATGATACAGATCCTGTAATTACTATTGATGAACCGTTGGCGCCCACGCAACCCTTGC
TCGCTGCTTCGAGAAGCAGCATATCTGGCCACGCCTACTTAACAAACAGCACGCTTGGCTATGGAATGAACAA
TGTTTCAGTCGATCTCTTCACCTGCACTGGTAATATTATCAATACGACAAAAATAAATGCTTCAGGACATTAC
TCATTTGAGGAATTATACGGGGGTCGATACTTCATTAGTTCTGTGCCACCACAGGGGTACGTATTTAGTAATA
TATGGGACGACTATACCAATGAGACTGAATCGTTGGAGTATCTTGGTGGGGAAAGCGCCGTGAATCCCGATAA
CGGGAGTACAGAATGCTTTGATTTGAGGGAGGGAGAAGACAAAACTGTCAGTTTTGGGATGACGTTCAATGTC
TCCTCAAATACAGGGCCAATGGCAACTGCCTCTCCTCCAGAGCTAGTGGAACCCACATCGGAAACATCAAATA
GTTTGATCACAGCGTCTACAATTCCTCCCTTTGACATAACTTCGTTGCACACGTCTCAACTTAAATCACAATC
CCCATCAATGATACCTTCCCACAAAGCTTCTCTCAATCCTACGTCCAGCTTTCATCCCACTACAATGCCGTCC
GTACAGTCCAGCCGGTCAGGAGATCTTTATGGTCCCGCGACGATGGAAGGGTTGGTGATGGCCATTCGCGGGA
TTCAAGTGTTGGAAGATGAGGCAGCGTGGAGCGTGGCCACCACTAATCACATCATGGAATACTTTAATTTGAC
CATTCATGATCCAGGAGTGTTTGATGTGACGGTGAGCATCGAAGTGATGAAGCAGACGAGTGGCGATGATGCT
GTGT
>TR510|c0_g2_i1 len=874 path=[8871:0-54 9126:55-55 9107:56-155 9046:156-
169 9082:170-173 9106:174-214 9044:215-238 9080:239-266 9104:267-267
9042:268-301 9079:302-405 8916:406-429 9040:430-545 8734:546-569
8735:570-570 9036:571-653 8862:654-677 8863:678-873] [-1, 8871, 9126,
9107, 9046, 9082, 9106, 9044, 9080, 9104, 9042, 9079, 8916, 9040, 8734,
8735, 9036, 8862, 8863, -2]
ATCTCCAATTTCCTCTCCGCCAGTTACAATATATGAATGAAGTGCAACAAAAGATCGCCCCTATACAGTGACT
ATCTCCATCCAAAACCCTCCGAATCGCCATCATTCATCCAACGGTTGCAATGGTATAAGATAATAGTACCAAA
CAAATATTTCTTAACATTATTTTTAATGCAGTAAACATGAGAATTAATCTAACCCCAATTTCCCCTAAAAGAT
CTTTTGACTTCGCCAATTACAATACATGAATGAAGTGCAACAAAAGATCACCCCTATACAGTGATAATCACCA
TCCAAAATATCCATTGTTTAGCGACGGTTGTACCCATCCGAATCATCATCCGAATCCGTGCTATCGTAATCCT

TCTCGATCGCCTCATGCTGAGGCTTGGGTTCATAGGATGTTCTCATTGCAGCCACCTTACGAGTTTCACCCCA
TGAAAAGTCGTCCATGTGCCAGAACGCATACAGCGGCAACAGGAAGTAAAACACTGGCACACCAATGATCGTG
AAGTACACGAACCAAAAGATGAAGTCGTAACGGGAACGAAGGATGAAGACCACGAGCTGGACACCGATGATCA
TTCCGTACAATACCAAGAGCAGGAGGGAGAACTGGGTCTTGCCCACGGCCACAAGGAAGATGAGGTACACTGC
GTACACGGTGGACGCCGGCAAAATCATACACGCGATGAGATCGAGGTAAACTACCGTCTTCATGGAGCAGCAG
CACACTCCACACATGGTCTTGACCTTCGTGAGTTCCAGGAGGTTGTGGTACGTGGAGTTAATCCAACGACGAC
GCTGGGAGAGCAAAATCCTGAGCGTGTGTGGCACGATGGTGAAGCACTGCGCTTCTGGGACGTAGCTGAGG
>TR511|c0_g1_i1 len=430 path=[4756:0-234 4888:235-310 4863:311-379
4763:380-403 4764:404-429] [-1, 4756, 4888, 4863, 4763, 4764, -2]
ACAACAATCAACTCGGGAGGTTCACAAACAGCGGCAACAATCGAGGATCAAGATCGTTCTATCGCTCTGGAGC
TGGAGAAATAGAGAGAGAAAATCTTCATCACTTCTGGTCACCATCAACGCTAACCCGCATGTTTTAAAGAGAC
AAATAATACAAACTGGTCCGTGACATCCAATCTCAGCTCAAAATAAACTGGCTAGTAATTTCATTGAGCAAGC
ATCACGTAGACAACATGGCGATGTCCCGACGAAAGGATGATGTCGAATATGCTTGGAATGAGTCGACCATGTC
GCAACCTTTGCGCCGCATGGTGAGAATTGAGAATATCGCTGAATTTATTAGAATTAATTGATGTTCCTACGCG
ATACACATAATTTTCATTTCATTGGTTCACATGTTCCTGTATGTACTTAGCGAGATCGGAAGAGC
>TR511|c0_g1_i2 len=1966 path=[4770:0-184 4888:185-260 4885:261-308
4843:309-332 4875:333-392 4889:393-416 4891:417-445 4860:446-471
4884:472-480 4874:481-504 4887:505-538 4882:539-583 4873:584-697
4858:698-763 4886:764-780 4838:781-838 4856:839-861 4754:862-889
4836:890-958 4673:959-982 4674:983-997 4675:998-1021 4854:1022-1084
4728:1085-1121 4834:1122-1187 4681:1188-1198 4682:1199-1227 4870:1228-
1245 4852:1246-1307 4831:1308-1314 4868:1315-1410 4850:1411-1426
4829:1427-1450 4866:1451-1461 4848:1462-1489 4827:1490-1493 4751:1494-
1965] [-1, 4770, 4888, 4885, 4843, 4875, 4889, 4891, 4860, 4884, 4874,
4887, 4882, 4873, 4858, 4886, 4838, 4856, 4754, 4836, 4673, 4674, 4675,
4854, 4728, 4834, 4681, 4682, 4870, 4852, 4831, 4868, 4850, 4829, 4866,
4848, 4827, 4751, -2]
ACTCAAACAGCGGCAATGATCGATGTCACTCAGATACAACATAGAGAGAGAGAGTCTTCATCACTTCTGGCCA
CAACCCGCACGTTCTAAATAGACAAATAATACAAACTGATCCGTGTCTTCCAATCCAAGCACAAAATAAACTG
GCTAGTAATCATTCTCAGCAAGCATCACGTAGACAACATGGCGATGTCCCGACGAAAGGATGATGTCGAATAT
GCTTGGAATGAGTCGACCATGTCGCAACCTTTGCGCCGCATGCGATACGATGTCCGTGGGGAGATTGTAGCGA
GAGCAGAAAGATTACGCGGCGAAGGACGTGATATCATCTTCACTAATATTGGAAATCCACAAGCTGTGGGACA
AAAACCCATCACATATTACCGCCAAGTACTGGCATTGTGCGATCTACCTCCAGAATGTGGCGTCGATCATCCA
GAAGTGACGAGAATGTATCCCCCCGATGCAATCGCCCGTGCAAAAGAATACAAGGCAGCAATAGGTCCAGCAG
GTACTGGAGCGTACTCACACAGTCAAGGTATACTGGCCTTCCGAGAGCATGTTGCCAACTTTATCGAAGCACG
TGATGGTCATACTGCTCCTTGTGCGGATGACATCTTCCTCACCAATGGTGCGAGTGCTGGCATCAATCACATC
CTGCAGGCATTGATTGCGGGGAGCAACGATGCCGTCATGATACCCATCCCACAGTATCCGATTTACTCGGCGT
TAATCACACTTTTGGGAGGGAGGCAGGTGGGGTACGAATTGGACGAGTCCCTGGGATGGGCCGTGACAAAGTC
CGAGCTCGAGAAGCAACTCCGACAGGCTAGAGAAAATGGACTCACCGTCAAGGCGATGGCAATCATAAATCCA
GGCAATCCTACCGGGCAGGTGATGGACCGCGACGTGCTCGAGATCATCTGCCGATTCTGTTCCGACAATCAAA
TCGTCCTCCTTGCCGACGAGGTGTACCAGCGCAACGTCTACGCTCCCAACAAAACCTTCATCTCAGCCAGGAA
GATTGCACTCGAGACACCGGGCTGCGAAAACCTACAGCTGGTCAGCTTCCACTCCACGTCCAACGGTCTGATC
GGAGAGTGTGGACGGAGGGCTGGGTACATGGAGCTGCACAACATTGAGTCACGGGTGCAGGAGAACGTGCACA
AGCTAGCGGCGACGGGATTGTGCAGCGGATTAGCGGATCAGGTCATGACGTCATTAATGGTGAACCCTCCAAG
GGTGGAAGATGAGTCGTATGAGAGTTTTGTTGCCGAGGAAAAGACTATTTACGATGGCCTAGTCCGCCGTTCC
CATGCACTAGTACAAGGCCTTAACGCCATTTCTGGTGTCACCTGCGAACAGGCTGAAGGATCAATATACGCCT
ACCCCAGCATCGCCATTCCGCAATCGGCATGCATCGAGGCCGACAAGCGCAATCAGACTCCAGACCTCATGTA
TGTTATAAGTTTATTGGAAGAGACGGGGATATGCATGGTACCAGCGAGCAGATTCGGTCAGAGGACGGGGACG
GTGGGGTTTCGTACGACGTTTATGCCTCCCGATGATAAGCTGCAGGAGGCCTTTGAGGGTTTTGCGAGGCATC
ATGCATTGTTTTGCGATAGGTACAGTAGTTCAGCGAGACATACACTCTGCAATGATGACATGATTATCAGGTC
TCCTGGGATGAGGGAAAAGCGTTCTAGGGAAGGTGAGGAGTCGGGGCATGAGCTTTGTACTCGCCCTATTTTC
ACCGGAAGGTGGAAAACCAATAACAATGAGTGGAGTGAGGTCAAGACAAGATACGCCAAAATTAAGTGTGCTT
CGTGCAACACAAGGATTAGGACGTATTGCAAATGCGATAAGAAGGTTCCCATGTGCACAGAATGCTATGGGAT
CCATATTTCAAACTGTAGTAACACATATTAGAGAGTAATAATTAGTATTGGCGAAAAAAAAGATCGGA
>TR511|c0_g1_i3 len=2924 path=[4770:0-184 4888:185-260 4885:261-308
4843:309-332 4875:333-392 4889:393-416 4891:417-445 4860:446-471
4884:472-480 4874:481-504 4887:505-538 4882:539-583 4873:584-697
4858:698-763 4886:764-780 4838:781-838 4856:839-861 4754:862-889
4836:890-958 4673:959-982 4674:983-997 4675:998-1021 4854:1022-1084
4816:1085-1118 4817:1119-1142 4880:1143-1187 4890:1188-1198 4892:1199-

1209 4879:1210-1227 4869:1228-1245 4851:1246-1254 4830:1255-1278 4867:1279-1307 4849:1308-1314 4828:1315-1410 4878:1411-1426 4847:1427-1450 4865:1451-1461 4877:1462-1489 4846:1490-1493 4864:1494-1770 4780:1771-1787 4845:1788-2572 4791:2573-2596 4824:2597-2641 4667:2642-2665 4668:2666-2923] [-1, 4770, 4888, 4885, 4843, 4875, 4889, 4891, 4860, 4884, 4874, 4887, 4882, 4873, 4858, 4886, 4838, 4856, 4754, 4836, 4673, 4674, 4675, 4854, 4816, 4817, 4880, 4890, 4892, 4879, 4869, 4851, 4830, 4867, 4849, 4828, 4878, 4847, 4865, 4877, 4846, 4864, 4780, 4845, 4791, 4824, 4667, 4668, -2]
ACTCAAACAGCGGCAATGATCGATGTCACTCAGATACAACATAGAGAGAGAGAGTCTTCATCACTTCTGGCCA
CAACCCGCACGTTCTAAATAGACAAATAATACAAACTGATCCGTGTCTTCCAATCCAAGCACAAAATAAACTG
GCTAGTAATCATTCTCAGCAAGCATCACGTAGACAACATGGCGATGTCCCGACGAAAGGATGATGTCGAATAT
GCTTGGAATGAGTCGACCATGTCGCAACCTTTGCGCCGCATGCGATACGATGTCCGTGGGGAGATTGTAGCGA
GAGCAGAAAGATTACGCGGCGAAGGACGTGATATCATCTTCACTAATATTGGAAATCCACAAGCTGTGGGACA
AAAACCCATCACATATTACCGCCAAGTACTGGCATTGTGCGATCTACCTCCAGAATGTGGCGTCGATCATCCA
GAAGTGACGAGAATGTATCCCCCCGATGCAATCGCCCGTGCAAAAGAATACAAGGCAGCAATAGGTCCAGCAG
GTACTGGAGCGTACTCACACAGTCAAGGTATACTGGCCTTCCGAGAGCATGTTGCCAACTTTATCGAAGCACG
TGATGGTCATACTGCTCCTTGTGCGGATGACATCTTCCTCACCAATGGTGCGAGTGCTGGCATCAATCACATC
CTGCAGGCATTGATTGCGGGGAGCAACGATGCCGTCATGATACCCATCCCACAGTATCCGATTTACTCGGCGT
TAATCACACTTTTGGGAGGGAGGCAGGTGGGGTACGAATTGGACGAGTCCCTGGGATGGGCCGTGACAAAGTC
CGAGCTCGAGAAGCAACTCCGACAGGCTAGAGAAAATGGACTCACCGTCAAGGCGATGGCAATCATAAATCCA
GGCAATCCTACCGGGCAGGTGATGGACCGCGACGTGCTCGAGATCATCTGCCGATTCTGTTCCGACAATCAAA
TCGTCCTCCTTGCCGACGAGGTGTACCAGCGCAACGTCTACGCTCCCAACAAAACCTTCATCTCAGCCAGGAA
GATTGCACTCGAGACACCGGGCTGCGAAAACCTACAGCTGGTCAGCTTCCACTCCACGTCCAAGGGTCTTATC
GGAGAGTGTGGACGGAGGGGCGGGTACATGGAACTGCACAACATCGATCCACTGGTACGGGAGCACGTGTACA
AGCTAGCGGCGACGGGATTGTGCAGCGGATTGGCTGGTCAGGTCATGACGTCATTAATGGTGAACCCTCCAAG
GGTGGGGGATGCATCGTATGAGAGTTTTGTTTCTGAGGAAAAGGCTATTTACGATGGCCTAGTCCGCCGTTCC
CGTGCACTAGTGCAAGGCCTCAACGCGGTCCCCGGCGTCACCTGCGAACAGGCCGAAGGAGCAATGTACGCCT
TCCCCAGCATCGCCATTCCGCAATCGGCATGCATCGAGGCCGACAAGCGCAATCAGACTCCAGACCTCATGTA
TGCCATCAGTTTATTGGAAGAGACGGGGATATGCGTGGTCCCGGCGAGCGGATTCGGCCAGAGGATTGGAAGG
GTGGGGTTTCGCACGACGTTCTTGCCTCCGGATGATAAGCTGCGGGAGGCGATTGAGGGGTTTGCGAGGCATC
ATGCGTTGTTTTGCGATAGGTACGGTGGTTTAGCGAGATTTACACCAGCTATAGTATCAAAGCTGTGAGATAA
TAATGCGGAATATGCAAATAAATGTGTAGCGAGGAAAAGGTGGTGGAGTAAAAAGTGTCAAACTTGTGTTGGC
GACGGCCTGTCCTTTTTTTTACTTTGTTTGAGGTTTTTATCTCTCTTTCACACTCTATTTGGACACATTTTGC
ACTGGCGACCGAAATTTATGACTGCATCTCAAGTTGAAATGTTCTCATCCCTTTCAGCGAAAACAAACATTTT
CCACTTCATAGCTGTCAATAAAAAGAATGACAAGTACAGTTAAATCATCTGAACTGTACCAGGGGGTGGTATA
ATTTTCATGCCAAGTCTGGGACTGCCATACCAGGATGGTATACATCTAACCTATTGTACTAAGTAGGGCCGAA
GAGAAGGGGTGGTATAATTTTTTCGGTCGGTATTGGCCGAATCAGGTGATAGTTCGGGTGATTTAACTTACAT
TCACATATTTACTTTCTTTCATTTCACATCTCCCATGAAGTCAGTCATGTCCAGAGTGTTCATGGTTCTTTTT
TTATGGTTAACTCTCCATTATTGACTTTCTAGCTGTCGGACAGGTCGCTTCTTCCTTTTTTTATTCGTCGGAT
ACGGCACGTGCACCGACATATTATTATTCTCCCCACAGGGACTGGACACCCGACTGCAACCACTGCAAACCTC
GTGACACGTCTCCGTTCCGGTAATATCGATCCCCCGTCCCTTCAGAACCTCCGCAATGGCTTCTCCGATCGGA
TGATCCTTCGCTCGTGCAGTTTCGACGGTCGATGCACGAAAGAGGTAATCGGCGAACCAAGCATGGAGACACT
TCACGTCGACCGTGCTCGCCAACGGATTTGCACCGGCGACTCCCGCATCGAGAAACGAGGATGCGCCTCGCTC
CCCTAATTTCGATCGTAGAACATTCATCTTGTCGTCCACTTCTTTTTTGTTTGACTCCTTCATATCGAAAATC
AATTTGGCACGTGCTGCGGCATGCGCCTCGTGAGCACCATCGAAGCATTCCATCCACATTCCATTTTCATTGT
CTACTAATTTGGAATTGACCTCCTTCATGAAGCCATCATCCTCGAGGACATCAATACTATTGACCAACATGGG
ACACGTCAGCTTCATCAATCCCGAGTTCAATCGATCCGTGACCGATGGCATGGGATCCAACGAGAATGCCTGG
GAGTATCCGTGTTTGCATTTGCAGTCGGATGGTTTGCCCAGTAGGACATTGGCGGGGGATGTTTGTCCTATTT
GATT
>TR511|c0_g1_i4 len=2016 path=[4756:0-234 4888:235-310 4885:311-358 4843:359-382 4875:383-442 4889:443-466 4891:467-495 4860:496-521 4884:522-530 4874:531-554 4887:555-588 4882:589-633 4873:634-747 4858:748-813 4886:814-830 4838:831-888 4856:889-911 4754:912-939 4836:940-1008 4673:1009-1032 4674:1033-1047 4675:1048-1071 4854:1072-1134 4728:1135-1171 4834:1172-1237 4681:1238-1248 4682:1249-1277 4870:1278-1295 4852:1296-1357 4831:1358-1364 4868:1365-1460 4850:1461-1476 4829:1477-1500 4866:1501-1511 4848:1512-1539 4827:1540-1543 4751:1544-2015] [-1, 4756, 4888, 4885, 4843, 4875, 4889, 4891, 4860, 4884, 4874, 4887, 4882, 4873, 4858, 4886, 4838, 4856, 4754, 4836, 4673, 4674, 4675,

4854, 4728, 4834, 4681, 4682, 4870, 4852, 4831, 4868, 4850, 4829, 4866, 4848, 4827, 4751, -2]
ACAACAATCAACTCGGGAGGTTCACAAACAGCGGCAACAATCGAGGATCAAGATCGTTCTATCGCTCTGGAGC
TGGAGAAATAGAGAGAGAAAATCTTCATCACTTCTGGTCACCATCAACGCTAACCCGCATGTTTTAAAGAGAC
AAATAATACAAACTGGTCCGTGACATCCAATCTCAGCTCAAAATAAACTGGCTAGTAATTTCATTGAGCAAGC
ATCACGTAGACAACATGGCGATGTCCCGACGAAAGGATGATGTCGAATATGCTTGGAATGAGTCGACCATGTC
GCAACCTTTGCGCCGCATGCGATACGATGTCCGTGGGGAGATTGTAGCGAGAGCAGAAAGATTACGCGGCGAA
GGACGTGATATCATCTTCACTAATATTGGAAATCCACAAGCTGTGGGACAAAAACCCATCACATATTACCGCC
AAGTACTGGCATTGTGCGATCTACCTCCAGAATGTGGCGTCGATCATCCAGAAGTGACGAGAATGTATCCCCC
CGATGCAATCGCCCGTGCAAAAGAATACAAGGCAGCAATAGGTCCAGCAGGTACTGGAGCGTACTCACACAGT
CAAGGTATACTGGCCTTCCGAGAGCATGTTGCCAACTTTATCGAAGCACGTGATGGTCATACTGCTCCTTGTG
CGGATGACATCTTCCTCACCAATGGTGCGAGTGCTGGCATCAATCACATCCTGCAGGCATTGATTGCGGGGAG
CAACGATGCCGTCATGATACCCATCCCACAGTATCCGATTTACTCGGCGTTAATCACACTTTTGGGAGGGAGG
CAGGTGGGGTACGAATTGGACGAGTCCCTGGGATGGGCCGTGACAAAGTCCGAGCTCGAGAAGCAACTCCGAC
AGGCTAGAGAAAATGGACTCACCGTCAAGGCGATGGCAATCATAAATCCAGGCAATCCTACCGGGCAGGTGAT
GGACCGCGACGTGCTCGAGATCATCTGCCGATTCTGTTCCGACAATCAAATCGTCCTCCTTGCCGACGAGGTG
TACCAGCGCAACGTCTACGCTCCCAACAAAACCTTCATCTCAGCCAGGAAGATTGCACTCGAGACACCGGGCT
GCGAAAACCTACAGCTGGTCAGCTTCCACTCCACGTCCAACGGTCTGATCGGAGAGTGTGGACGGAGGGCTGG
GTACATGGAGCTGCACAACATTGAGTCACGGGTGCAGGAGAACGTGCACAAGCTAGCGGCGACGGGATTGTGC
AGCGGATTAGCGGATCAGGTCATGACGTCATTAATGGTGAACCCTCCAAGGGTGGAAGATGAGTCGTATGAGA
GTTTTGTTGCCGAGGAAAAGACTATTTACGATGGCCTAGTCCGCCGTTCCCATGCACTAGTACAAGGCCTTAA
CGCCATTTCTGGTGTCACCTGCGAACAGGCTGAAGGATCAATATACGCCTACCCCAGCATCGCCATTCCGCAA
TCGGCATGCATCGAGGCCGACAAGCGCAATCAGACTCCAGACCTCATGTATGTTATAAGTTTATTGGAAGAGA
CGGGGATATGCATGGTACCAGCGAGCAGATTCGGTCAGAGGACGGGGACGGTGGGGTTTCGTACGACGTTTAT
GCCTCCCGATGATAAGCTGCAGGAGGCCTTTGAGGGTTTTGCGAGGCATCATGCATTGTTTTGCGATAGGTAC
AGTAGTTCAGCGAGACATACACTCTGCAATGATGACATGATTATCAGGTCTCCTGGGATGAGGGAAAAGCGTT
CTAGGGAAGGTGAGGAGTCGGGGCATGAGCTTTGTACTCGCCCTATTTTCACCGGAAGGTGGAAAACCAATAA
CAATGAGTGGAGTGAGGTCAAGACAAGATACGCCAAAATTAAGTGTGCTTCGTGCAACACAAGGATTAGGACG
TATTGCAAATGCGATAAGAAGGTTCCCATGTGCACAGAATGCTATGGGATCCATATTTCAAACTGTAGTAACA
CATATTAGAGAGTAATAATTAGTATTGGCGAAAAAAAAAGATCGGA
>TR511|c0_g1_i5 len=2974 path=[4756:0-234 4888:235-310 4885:311-358 4843:359-382 4875:383-442 4889:443-466 4891:467-495 4860:496-521 4884:522-530 4874:531-554 4887:555-588 4882:589-633 4873:634-747 4858:748-813 4886:814-830 4838:831-888 4856:889-911 4754:912-939 4836:940-1008 4673:1009-1032 4674:1033-1047 4675:1048-1071 4854:1072-1134 4816:1135-1168 4817:1169-1192 4880:1193-1237 4890:1238-1248 4892:1249-1259 4879:1260-1277 4869:1278-1295 4851:1296-1304 4830:1305-1328 4867:1329-1357 4849:1358-1364 4828:1365-1460 4878:1461-1476 4847:1477-1500 4865:1501-1511 4877:1512-1539 4846:1540-1543 4864:1544-1820 4780:1821-1837 4845:1838-2622 4791:2623-2646 4824:2647-2691 4667:2692-2715 4668:2716-2973] [-1, 4756, 4888, 4885, 4843, 4875, 4889, 4891, 4860, 4884, 4874, 4887, 4882, 4873, 4858, 4886, 4838, 4856, 4754, 4836, 4673, 4674, 4675, 4854, 4816, 4817, 4880, 4890, 4892, 4879, 4869, 4851, 4830, 4867, 4849, 4828, 4878, 4847, 4865, 4877, 4846, 4864, 4780, 4845, 4791, 4824, 4667, 4668, -2]
ACAACAATCAACTCGGGAGGTTCACAAACAGCGGCAACAATCGAGGATCAAGATCGTTCTATCGCTCTGGAGC
TGGAGAAATAGAGAGAGAAAATCTTCATCACTTCTGGTCACCATCAACGCTAACCCGCATGTTTTAAAGAGAC
AAATAATACAAACTGGTCCGTGACATCCAATCTCAGCTCAAAATAAACTGGCTAGTAATTTCATTGAGCAAGC
ATCACGTAGACAACATGGCGATGTCCCGACGAAAGGATGATGTCGAATATGCTTGGAATGAGTCGACCATGTC
GCAACCTTTGCGCCGCATGCGATACGATGTCCGTGGGGAGATTGTAGCGAGAGCAGAAAGATTACGCGGCGAA
GGACGTGATATCATCTTCACTAATATTGGAAATCCACAAGCTGTGGGACAAAAACCCATCACATATTACCGCC
AAGTACTGGCATTGTGCGATCTACCTCCAGAATGTGGCGTCGATCATCCAGAAGTGACGAGAATGTATCCCCC
CGATGCAATCGCCCGTGCAAAAGAATACAAGGCAGCAATAGGTCCAGCAGGTACTGGAGCGTACTCACACAGT
CAAGGTATACTGGCCTTCCGAGAGCATGTTGCCAACTTTATCGAAGCACGTGATGGTCATACTGCTCCTTGTG
CGGATGACATCTTCCTCACCAATGGTGCGAGTGCTGGCATCAATCACATCCTGCAGGCATTGATTGCGGGGAG
CAACGATGCCGTCATGATACCCATCCCACAGTATCCGATTTACTCGGCGTTAATCACACTTTTGGGAGGGAGG
CAGGTGGGGTACGAATTGGACGAGTCCCTGGGATGGGCCGTGACAAAGTCCGAGCTCGAGAAGCAACTCCGAC
AGGCTAGAGAAAATGGACTCACCGTCAAGGCGATGGCAATCATAAATCCAGGCAATCCTACCGGGCAGGTGAT
GGACCGCGACGTGCTCGAGATCATCTGCCGATTCTGTTCCGACAATCAAATCGTCCTCCTTGCCGACGAGGTG
TACCAGCGCAACGTCTACGCTCCCAACAAAACCTTCATCTCAGCCAGGAAGATTGCACTCGAGACACCGGGCT

GCGAAAACCTACAGCTGGTCAGCTTCCACTCCACGTCCAAGGGTCTTATCGGAGAGTGTGGACGGAGGGGCGG
GTACATGGAACTGCACAACATCGATCCACTGGTACGGGAGCACGTGTACAAGCTAGCGGCGACGGGATTGTGC
AGCGGATTGGCTGGTCAGGTCATGACGTCATTAATGGTGAACCCTCCAAGGGTGGGGGATGCATCGTATGAGA
GTTTTGTTTCTGAGGAAAAGGCTATTTACGATGGCCTAGTCCGCCGTTCCCGTGCACTAGTGCAAGGCCTCAA
CGCGGTCCCCGGCGTCACCTGCGAACAGGCCGAAGGAGCAATGTACGCCTTCCCCAGCATCGCCATTCCGCAA
TCGGCATGCATCGAGGCCGACAAGCGCAATCAGACTCCAGACCTCATGTATGCCATCAGTTTATTGGAAGAGA
CGGGGATATGCGTGGTCCCGGCGAGCGGATTCGGCCAGAGGATTGGAAGGGTGGGGTTTCGCACGACGTTCTT
GCCTCCGGATGATAAGCTGCGGGAGGCGATTGAGGGGTTTGCGAGGCATCATGCGTTGTTTTGCGATAGGTAC
GGTGGTTTAGCGAGATTTACACCAGCTATAGTATCAAAGCTGTGAGATAATAATGCGGAATATGCAAATAAAT
GTGTAGCGAGGAAAAAGGTGGTGGAGTAAAAAGTGTCAAACTTGTGTTGGCGACGGCCTGTCCTTTTTTTTACT
TTGTTTGAGGTTTTTATCTCTCTTTCACACTCTATTTGGACACATTTTGCACTGGCGACCGAAATTTATGACT
GCATCTCAAGTTGAAATGTTCTCATCCCTTTCAGCGAAAACAAACATTTTCCACTTCATAGCTGTCAATAAAA
AGAATGACAAGTACAGTTAAATCATCTGAACTGTACCAGGGGGTGGTATAATTTTCATGCCAAGTCTGGGACT
GCCATACCAGGATGGTATACATCTAACCTATTGTACTAAGTAGGGCCGAAGAGAAGGGGTGGTATAATTTTTT
CGGTCGGTATTGGCCGAATCAGGTGATAGTTCGGGTGATTTAACTTACATTCACATATTTACTTTCTTTCATT
TCACATCTCCCATGAAGTCAGTCATGTCCAGAGTGTTCATGGTTCTTTTTTTATGGTTAACTCTCCATTATTG
ACTTTCTAGCTGTCGGACAGGTCGCTTCTTCCTTTTTTTATTCGTCGGATACGGCACGTGCACCGACATATTA
TTATTCTCCCCACAGGGACTGGACACCCGACTGCAACCACTGCAAACCTCGTGACACGTCTCCGTTCCGGTAA
TATCGATCCCCCGTCCCTTCAGAACCTCCGCAATGGCTTCTCCGATCGGATGATCCTTCGCTCGTGCAGTTTC
GACGGTCGATGCACGAAAGAGGTAATCGGCGAACCAAGCATGGAGACACTTCACGTCGACCGTGCTCGCCAAC
GGATTTGCACCGGCGACTCCCGCATCGAGAAACGAGGATGCGCCTCGCTCCCCTAATTTCGATCGTAGAACAT
TCATCTTGTCGTCCACTTCTTTTTTGTTTGACTCCTTCATATCGAAAATCAATTTGGCACGTGCTGCGGCATG
CGCCTCGTGAGCACCATCGAAGCATTCCATCCACATTCCATTTTCATTGTCTACTAATTTGGAATTGACCTCC
TTCATGAAGCCATCATCCTCGAGGACATCAATACTATTGACCAACATGGGACACGTCAGCTTCATCAATCCCG
AGTTCAATCGATCCGTGACCGATGGCATGGGATCCAACGAGAATGCCTGGGAGTATCCGTGTTTGCATTTGCA
GTCGGATGGTTTGCCCAGTAGGACATTGGCGGGGGATGTTTGTCCTATTTGATT
>TR511|c1_g1_i1 len=3359 path=[4651:0-70 4652:71-94 4871:95-129 4885:130-153 4848:154-213 4870:214-281 4801:282-305 4847:306-434 4793:435-458 4794:459-467 4795:468-491 4884:492-695 4649:696-719 4895:720-764 4903:765-765 4752:766-788 4868:789-789 4845:790-822 4902:823-837 4900:838-861 4867:862-1027 4894:1028-1093 4866:1094-1109 4899:1110-1169 @4893@!:1170-1573 4682:1574-1590 4864:1591-1910 4904:1911-1934 4907:1935-1950 4839:1951-2046 4863:2047-2053 4878:2054-2115 4837:2116-2133 4861:2134-2162 4892:2163-2173 4877:2174-2218 4860:2219-2242 4790:2243-2276 4835:2277-2339 4702:2340-2363 4703:2364-2378 4704:2379-2402 4891:2403-2446 4905:2447-2471 4857:2472-2499 4890:2500-2522 4831:2523-2580 4898:2581-2597 4888:2598-2663 @4874@!:2664-2777 4854:2778-2822 4897:2823-2856 4887:2857-2880 4901:2881-2889 4896:2890-2915 4886:2916-2944 4872:2945-2968 4906:2969-2969 4850:2970-3028 4908:3029-3052 4909:3053-3078 4849:3079-3176 4764:3177-3358] [-1, 4651, 4652, 4871, 4885, 4848, 4870, 4801, 4847, 4793, 4794, 4795, 4884, 4649, 4895, 4903, 4752, 4868, 4845, 4902, 4900, 4867, 4894, 4866, 4899, 4893, 4682, 4864, 4904, 4907, 4839, 4863, 4878, 4837, 4861, 4892, 4877, 4860, 4790, 4835, 4702, 4703, 4704, 4891, 4905, 4857, 4890, 4831, 4898, 4888, 4874, 4854, 4897, 4887, 4901, 4896, 4886, 4872, 4906, 4850, 4908, 4909, 4849, 4764, -2]
GATCTTTTCCAAGCTTTAAAAAGAGATAGTCAATAGAAGATAGAATTGCACCAGTTGAGTCAACATCATGAGA
TTGGCGGCACTACTATCCTTCACGCTTTCCAGCAGTGTCGTCAATGCTCTTGGATCACTATCATATTCTGTAT
CCGTGCAAGACTGTTGTGCATTCTCTCCCGTATTCACTCGATGTCGTCCATCACATCAACACGGACAACGACG
AGCGACGCAACGATTATCAGCGCACACATCAACATCAACAGCAACAGCATCAGCGACAACAGATGCGAAACAA
ATAACCTCAAAACAAAGGTGGCAGCATCCATCTCAACCTGAACCTGACTACATCACCAATCGAAAACACCCCC
AATTCCCAGGAGCCATTCCCATCGAAAACGATGACATCACGACATGCATCAATCAAATAGGACAAACATCACC
CGCCAATGTCCTACTGGGCAAACCATCCGAATGCAAATGCAAACACGGATACTCCCAGGCATTCTCGTTGGAT
CCCATGCCATCGGTCACGGATCGATTGAACTCGGGATTGATGAAGCTGACGTGTCCCATGTTGGTCAATAGTA
TTGATGTCCTCGAGGATGATGGCTTCATGAAGGAGGTCAATTCCAAATTAGTAGACAATGAAAATGGAATGTG
GATGGAATGCTTCGATGGTGCTCACGAGGCGCATGCCGCTGCACGTGCCAAATTGATTTTCGATATGAAGGAG
TCAAACAAAAAAGAAGTGGACGACAAGATGAATGTCCTACGATCGAAATTAGGGGAGCGAGGCGCATCCTCGT
TTCTCGATGCGGGAGTCGCCGGTGCAAATCCGTTGGCGAGCACGGTCGACGTGAAGTGTCTCCATGCTTGGTT
CGCCGATTACCTCTTTCGTGCATCGACCGTCGAAACTGCACGAGCGAAGGATCATCCGATCGGAGAAGCCATT
GCGGAGGTTCTGAAGGGACGGGGGATCGATATTACCGGAACGGAGACGTGTCACGAGGTTTGCAGTGGTTGCA

GTCGGGTGTCCAGTCCCTGTGGGGAGAATAATAATATGTCGGTGCACGTGCCGTATCCGACGAATAAAAAAG
GAAGAAGCGACCTGTCCGACAGCTAGAAAGTCAATAATGGAGAGTTAACCATAAAAAAGAACCATGAACACT
CTGGACATGACTGACTTCATGGGAGATGTGAAATGAAAGAAAGTAAATATGTGAATGTAAGTTAAATCACCCG
AACTATCACCTGATTCGGCCAATACCGACCGAAAAAATTATACCACCCCTTCTCTTCGGCCCTACTTAGTACA
ATAGGTTAGATGTATACCATCCTGGTATGGCAGTCCCAGACTTGGCATGAAAATTATACCACCCCCTGGTACA
GTTCAGATGATTTAACTGTACTTGTCATTCTTTTTATTGACAGCTATGAAGTGGAAAATGTTTGTTTTCGCTG
AAAGGGATGAGAACATTTCAACTTGAGATGCAGTCATAAATTTCGGTCGCCAGTGCAAAATGTGTCCAAATAG
AGTGTGAAAGAGAGATAAAAACCTCAAACAAAGTAAAAAAAAGGACAGGCCGTCGCCAACACAAGTTTGACAC
TTTTTACTCCACCACCTTTTCCTCGCTACACATTTATTTGCATATTCCGCATTATTATCTCACAGCTTTGATA
CTATAGCTGGTGTAAATCTCGCTAAACCACCGTACCTATCGCAAAACAACGCATGATGCCTCGCAAACCCCTC
AATCGCCTCCCGCAGCTTATCATCCGGAGGCAAGAACGTCGTGCGAAACCCCACCCTTCCAATCCTCTGGCCG
AATCCGCTCGCCGGGACCACGCATATCCCCGTCTCTTCCAATAAACTGATGGCATACATGAGGTCTGGAGTCT
GATTGCGCTTGTCGGCCTCGATGCATGCCGATTGCGGAATGGCGATGCTGGGGAAGGCGTACATTGCTCCTTC
GGCCTGTTCGCAGGTGACGCCGGGGACCGCGTTGAGGCCTTGCACTAGTGCACGGGAACGGCGGACTAGGCCA
TCGTAAATAGCCTTTTCCTCAGAAACAAAACTCTCATACGATGCATCCCCCACCCTTGGAGGGTTCACCATTA
ATGACGTCATGACCTGACCAGCCAATCCGCTGCACAATCCCGTCGCCGCTAGCTTGTACACGTGCTCCCGTAC
CAGTGGATCGATGTTGTGCAGTTCCATGTACCCGCCCCTCCGTCCACACTCTCCGATAAGACCCTTGGACGTG
GAGTGGAAGCTGACCAGCTGTAGGTTTTCGCAGCCCGGTGTCTCGAGTGCAATCTTCCTGGCTGAGATGAAGG
TTTTGTTGGGAGCGTAGACGTTGCGCTGGTACACCTCGTCGGCAAGGAGGACGATTTGATTGTCGGAACAGAA
TCGGCAGATGATCTCGAGCACGTCGCGGTCCATCACCTGCCCGGTAGGATTGCCTGGATTTATGATTGCCATC
GCCTTGACGGTGAGTCCATTTTCTCTAGCCTGTCGGAGTTGCTTCTCGAGCTCGGACTTTGTCACGGCCCATC
CCAGGGACTCGTCCAATTCGTACCCCACCTGCCTCCCTCCCAAAAGTGTGATTAACGCCGAGTAAATCGGATA
CTGTGGGATGGGTATCATGACGGCATCGTTGCTCCCCGCAATCAATGCCTGCAGGATGTGATTGATGCCAGCA
CTCGCACCATTGGTGAGGAAGATGTCATCCGCACAAGGAGCAGTATGACCATCACGTGCTTCGATAAAGTTGG
CAACATGCTCTCGGAAGGCCAGTATACCTTGACTGTGTGAGTACGCTCCAGTACCTGCTGGACCTATTGCTGC
CTTGTATTCTTTTGCACGGGCGATTGCATCGGGGGGATACATTTTTGTCACTTCTGGATGATCGACGCCACAT
TCTGGAGGTAGATCGCACAATGCCAGTACTTGGCGGTAATATGTGATGGGTTTTTGTCCCACAGCTTGTGGAT
TTCCAATATTAGTGAAGATGATATCACGTCCTTCGCCGCGTAATCTTTCTGCTCTCGCTACAATCTCCCCACG
GACATCGTATCGCATGCGGCGCAAAGGTTGCGACATGGTCGACTCATTCCAAGCATATTCGACATCATCCTTT
CGTCGGGACATCGCCATGTTGTCTACGTGATGCTTGCTCAATGAAATTACTAGCCAGTTTATTTTGAGCTGAG
ATTGGATGTCACGGACCAGTTTGTATTATTTGTCTCTTTAAAACATGCGGGTTAGCGTTGATGGTGACCAGAA
GTGATGAAGATTTTCTCTCTCTATTTCTCCAGCTCCAGAGCGATAGAACGATCTTGATCCTCGAGATCGGAAG
A
>TR515|c0_g1_i1 len=497 path=[475:0-496] [-1, 475, -2]
TTTTTGAAAATATGGTAGCTGTTCTATTACAGCACACAATTGCACTGAAAGGATTTCTATCTATTCCATTTAC
ATCTAAGTTACCTCATTTTATTGCTGGCACAAACATCAAACATCCAATCGAACTAAATCGCTGTGATTGATGA
ATACCACAACATAAACAGTATTCTATGGTCAACATAGCAATGTAGATTCCAATATGAATAGCAGCACCCCTCA
AGCTCATTCATAATCACTAAACATATCTTTCTCCTCAATCAATTGTTGCTTCTTACTCTCATCTTCCTCCGTC
CACTTCCCCGCCTTCTTCATTTTCTTCTCCCTAAAAGACCTCACCATTTCCTCCGTCTCCTTCTTCTCCTTCT
CCCCCAACCCCAGCGAATTCAAGTAACTTTCCTCATCCTCCCCCATGTTCTGTTCGGCATACGTTTCCGCGAC
GAGGTCAGCATATCCCCCTCCTCCGGATTCAATGAGAAATCCTACGAATATCGTGGAGA
>TR516|c0_g1_i1 len=1769 path=[1747:0-1768] [-1, 1747, -2]
CTATTTCATACCTACATAACTTGAACACGCACGACAAGAGTAAAGCCAATGGCACCAACCGTAAGGAATATAG
AAATGGCTGTATCAGAAACACGAAGAAGAAGAGTTATATATTGTAGTACCAAACTCATTGAGACATGAGTTGA
ACGTGCATTGAAGGGAGACATTTGATTGAGTATCTTGCAAATTATTTCGAAAAGAAAATGTGTTGCTGTTATC
GGCACGATAAAAATAGTGCCGAGTGTAAAATCGTCGTGCACAATACAAGTGAGGCATCTGTATTCCTGTAAAA
CCAAATGCACATGGTAGTGTTTGACTTGTGTGTTATCATAGTCCTGCAAAAGATAGTTAACACCACGCGTTTG
TTCGTATCTAGAAAATATACTTTTCATTAACTCGAGATGAGAAGGAAATAGAAGCGACGTGCCAATATGCAGA
ATTTGGTAGAATTCAACTATCTCTTTCCCGTGGACAATCGGATTGATAATGCCGAACAGCTTCCATTGCACGT
AATCCCCATCTCGTGCAAATACATTGCTGGCATTCGTATACATAGATGGGGATGGTTTTAACCATGACTACTG
ATCTCACAGTTCATTCTTCTTACCAATTCCAAGAAGACGATCTGAGGTATCTTCCAACATGCGATAATTCTTC
ACACTCAAGTATATCATGTCTAAAGCAACTTCCGACTTGGAACTTTTCGTACGCGAATTCTTGGCTTCGTTCT
CGCTCCGATCTTTTGCAATCTGCTTGGCGAGACCCTCGAGCTTAGTGTACATGCCAAGTCGCGACTCCATCTC
TTTAATTTGGTTCTCGTAGATTTTCTGCATCTCTTTTCGCTTCGCTTGTTTGTTTTCCCTGTCTTCTATGACC
GCAGAACGGACGTTTTCGTCTGCCAAGGAATCAAGGATTGAATCTAACTTCGAAGATACGTCTATCGCCTCCT
CTTCCGCTGTCATTTCTTTCTCCCTTGTTCCTTCGGGTGGTGGGCATTTCGTGACTGTCATTTCGTTCTCCCT
TTCCCCCTCTGGTGGTGAATTTTCAGGCGACTCGTCAGATTCGTGGCAAAGATCAATGATGTCAGCAGGTTTG
GTGTTGGCAACAGGAGTGCTGAGTACCATTGCTGCTGTTGCCTTCGCTGGAGATTCTTCCTGCGCTTTAGTAT
CCTCAACCGCCAATCTTTTCGATGGTACATATTCATCCCTGTCATCATTTGGTTTACGCTTTCTTTTGTTCAC
TTTCTTCGATGCGACTATTGGTCGGGCAATACCCCCACCCGATTTACCATCAGTTCCAACATTTGATTTCTTA

TTCCTGGTATCCCCCTGCGAGTCCATAAAAAAATCTCTCATTAACTGTGCCTGAACACGTTGCAGGGGATTTG
GCGAAGTCGTAACGGCATCATTTTGTGTATTCCTTACGGCAACATCCCTTGCAACACTACCATGCTCACGAAG
AGGTAAACTTGCAGCATCGATATTATAAGGTATCTCCTCATGCCGAGGTTGTTTCCCCCTCCCCATCGGTTCC
TGTGGCACTTGCGCCACCTGATGATTCTGCGGCTTTAGTTCTTGCATGAGACTTTCCATAGCAAGAGCCTGCT
GCGTCTCGGTGATTTCAGGCTTGAGTCCCCTGAGTTTCATTTGAATCTTTTGCGTAATCTTTTTCTGGAGAGC
CTGATTCATCTTTTCTTGGATTTTTTCGTGGAGAGATTTCGCCGCGATGGATGCGCTGGTAGCCCGGTCTTTA
TTATTTAGTAATTCGTT
>TR517|c0_g1_i1 len=282 path=[260:0-281] [-1, 260, -2]
TGCGGAGGTATTCGAGGGAGTGGCGTTTTTTGGCGAGGGGGTAGGTTTCTCCGGGGCAGGTGCCGACGATGCG
GAGGGAGGTGGCGGAGAGTTCGACGGATTGCTTTCCTCCTTGGGATTTGACTATTTGGCCGATGGCATTGATG
GAGCATCCTGTGGTGAGGTTTTCGATTTCCTTGAGAGTCTCCTCGTCCACTGCATCAAAAGGCAACACACACT
GAATTCCACTCATACTTGATGCATCATTCACCTCCACGAACGCCAACGTCTTTTGCTTTCGTA
>TR520|c0_g1_i1 len=321 path=[299:0-320] [-1, 299, -2]
TTCCGATCTCAACATGGGACGAAGAATTGGTTGGGAAGGACGAAGTGGAAGTATAAGGCAATTATCGGAAGAC
AAAGCCTCGACGAGGAGGAGGAAGGTGGGGAATATAAAGTTAAGAAAGAAGTACTTCATCCGGAGTATTATCG
AGATGGGGACGAGGGTATTGTTCAGCTTAATTCCAAATGGAGTTCGCTGCGTATGGATAATGATTTCATGTTG
GTATTCCTCAAAACATCCGTGGCCAGTGATGATGTCAAACTTGTACAACTTAACTCCAACGTGACTGTCCCCG
ACAGCAATATCAACTTGGCTGTAATGGGA
>TR521|c0_g1_i1 len=246 path=[224:0-245] [-1, 224, -2]
GCTCTTCCGATCTGTGATTTTGTTGGCGCCGAAGTCGACCGCTTCAATGTATTGTAGTCCGGTGAACATGCTG
ATTGGACCTTCGTCAAATGTAGGAATTTGGTCCTGTTGTGTATTTCTCAGACGCACTTGTTTTATCGCGGGAC
TATTCGTAACAAAATCTGGTGATACGATGATTTTATTAGAAGACAAGTTGATAATCGTTAGTCGTTCGAAAGT
CATACTAGGAATAGGACTCTCAACTTC
>TR522|c0_g1_i1 len=294 path=[297:0-74 298:75-98 299:99-293] [-1, 297, 298, 299, -2]
TGATGGAGGAAGCCTGATGTTGTTGTTGTTGTTGTTGTTGATGGGGTAGTGTGCTGGTGGTGATCGTCGAAGT
AACCGGTATCATCATCATCGACTATTTGACCGTTGTGGAATCGAGATCTGTCAATGTTTCGTACGGAGTCTTC
GTCCATGGAAAGGTGGGTGAAGAAATTGCATTTGCGTTCCGATGGAGATGGTGGTGGGTGTTGTGGCGGGGCT
GCTGGTACTGCTACTGGGCTGTCGCATTTGTCATCCTGGTTTCGAGGCGCCATGGTTGCTACTGATGTTAGGA
GT
>TR523|c0_g1_i1 len=290 path=[293:0-171 294:172-195 295:196-289] [-1, 293, 294, 295, -2]
GGAATCAGAATAAGGACGAATAAGTAACGAGGACCATATTGCATCGGTGGTCAGTGACCAAAACAACCTAGAC
AACGCTCAACATCATTTACATCATGCTGTAATCAATAGAAGAAAGCTCCTTATCTATTCTTTTATCATTCGAA
CTAAACATGGGCTGGCTCAACAGTATCCCCGAAACAGGAGGCGAAACCTCCGATTCCCAATCTGACCGCTCGG
CATCTCCCAGCAGCAGCAGCAACGACAACAACAACAACGCTCTACGATCAAAACTCTCCACCATGGAAGCG
>TR523|c1_g3_i1 len=1956 path=[1981:0-348 1982:349-355 1983:356-371 1984:372-373 1985:374-1955] [-1, 1981, 1982, 1983, 1984, 1985, -2]
GGGTGTTGCCAACGGTGGCAATGGCACTGTCAACAACAGCAGCAACGACAACAACAACAAACCCAACAAGGGA
AACAACATCAACAGCATCAAGGGGATGGACGAAACGTGCCCACTCCTACAAGAGCTACACTTCCTAATGATGA
ACGACGACGATTCATCCACTACTGTCCTTTCTCAGCAGCACACGGCCACAGAAAGACAGTCGCCACACCCCAA
TCACCGACAGCAGGTCGTCGAGCATTTATCCACCGTAGCCGAGATGATGACCGCTACCACCTCCACTCCTCAA
CCGCAAATAATACTCGACTACGGCAACAGCAACAACACTACTATCCCTCCAAGTCAGGAAAAAAACCAACAAC
TCACCTACTCTTACCCCATTGGCGCCTCACTTGCTCGACGCACAGCAATCACCAACCCCACAGGACGACGACC
CAGACAATACCGCTGCTGCCTCGGAAAACAATGGAAAAACAACAGAACCACCGCCCAAGGAATTGAGCGCCTT
CCTCACCTCCCCCACGGGCCTACATTGGGAAGATTTGGTAAGATCCATGAAACAGGCTAATAATAATAATGCC
AAGAAGGATGGAATGGAATATTGGTTGAATAAGGTGATTTTTGTTTCGGAGGATGATACTGTGAGCGAATTGG
GGAGTGTTGTGGGGGGCAAGGGTGGTCATAGTGGTGGTGGTGGTGATGACGATGGCGGAAGGAACAGTAGGGG
ATTCGTGGGGGATTTGGAGATGATGGGCGATGTTGTGATTGATCATCATCATCGCCAGAATCCTCCTCCTCGT
AACCTCACGTTGATTGATGATAAGGATGATGATGACGACGATGTGAGCGAGCTGGGAAGCTGTGTTGATATTG
GGAATGAAAATGGATGTGTGCAGAAGAAGAAGCAGCAGCATAATTTGAACAGGATGGAAGATGCAGTGGGGGA
CGCGGTGGTGGTCCTGGGGCATCAAGAACAGCATGGGAGCAGGAAGGAACAAATTCGCCACGGGAAGAGGGAT
AGCGACGTTTCGAGCATCTCCTCCAAGTCACCGTTTAAGGGGTGGGTTAAGCACAAAGAAAATAATGGTGTGC
ATGCGGCGAATAGTCGTTGTCGGACTAGAGAGGAGCATCTGATATTGAGTACAACTGCCAAGGGAGAATTTCA
ACCACAACAAAAACGGCAGAATTCACCATTACCGAAACGAAAAACAACATCGCCCCCCAAGTTCAAATCGTAT
CCTCGTGACAAGTCACCACTGCGAGGAATTGGAAAGTCCCAGAAATTGACGACTCCCTTGCGCTCTAGGGCGC
GGCCGAGAGAAAGCCTGGAAGAGAAGGTGGAACGGGTACTAAGGAGCGGGAAGTCGTCGGAAGAAAGGGGCAA
GGCTACCAGGAGACGCGCACTGCCTCATCCTCCCTCGCGACCGGCGAAGTCCTCGCCGTCGTCATCAAGCAAG
CCCAAGCCGTTGTCACGAATAGGTTCCAGCAGCAGCTCGGCCTTCTCGCCGATCAAACAATCATCGATCATCA
AATCCCCCAATTCTTTCATAATACGCTCTCCGAACACTCCGGCCACCTCCGCTATGGAAACGACATTCGACTC

GTCCTTCGATCGCTCGCCCAGGAGGAGCATGACCGAGGCAATCACGGCGTCCCCTGCCGTGGTGATTCCCAAT
ATGCAGATGAGGAGTTCCAGCGAGACGACGTTCGTTTCCATCGAACGTCCGGAACAGTTGCCGCAGCATCAAT
TGTTTTCGTCTTCCGTAGTGACGCCGCAGCCGATATCGCCTCGCGTGAGCCGCAGAAGAAGAGTGGACATGGG
CGACGAGCCCCGGCAACAACGACGGGGTCGCAGCAGCAGCGGTTTTGGATGGAATGCGATGGATGGATCCAAA
GACGTCGATGTGTCGCCCAGGCTGGGATCGGCGCCTCGGTCGGCATGCTCGTCCGTGG
>TR526|c0_g1_i1 len=687 path=[690:0-601 691:602-625 692:626-686] [-1, 690, 691, 692, -2]
GCAACCCATCATCGCCTGTTACTGTTTGTTGCAGCACCGACGCGGTGTACATCCAATCAACTCTAACATCCAA
TCTAAAATCACTCCTCCAATTGTGCGGAATGAACTCCTCCGACATTCTCTCTGTGCAGCCCATCATCGAAGAA
GAAGAAAAATCTCCTCGTAATCATAATAATAACGACATCAGCAAAGTACGCATCCAACTAACTTCACCATTCG
CAGCACGACAACTAACCTCTCGATTGCGAAGGGGGAATTACGGACCACGAGATTTACTCGCTTCGACCATTAC
TGTCCAAAATGGAATTATACTGCAGAATGAGGGGATTTGCTCTTTGGGATGTGGTGGTCGATATGCCGGACGT
GCGGTGATGGTGACACAAGTCACTCCTATAACCATTTCTAATACCATTGGAGATATTAATGATACTACCGCTG
CTAATGCTGATGCGGACGAGACAATACTCACAAGATGGACTCGTGCAGCACATCCAAAATTTCGACGACTACT
CGTCAATTCCAATTCGAAAAAGTACAAATTGTTGACTGAGGAAGAAAGTGAGATTCGCAGGGCCATGACGCGG
TTTGTGTATCTTGATAATGTTCTTGGGAATGAGTTGACGTTGGATAATTTTAGTACTATCGCGTTTCTCGGAG
ACGACGACGACGACGATGATGGTGACGATG
>TR527|c0_g1_i1 len=348 path=[377:0-94 378:95-118 387:119-178 381:179-203 382:204-347] [-1, 377, 378, 387, 381, 382, -2]
GAGCCTACACAGCTGTAGCTTCGCAGCACATCACGCCCACCAACAATAGAACATCAACAAACAACAGCAACGA
CGACCATGGGAGCCCTCCAATACATTTCCACATCAGCCCTCTCCCTAACCCTCTCGGGAAACACCGGCGTAGC
ACCCTTCCTAACCCTCTTCCTCGTAGGCATAATCGAAAAATCCGACCCATCATTACTCTACATGGATGGATTC
GCCGAAAAGATCATCGCATCCTGGCCTGGCCTCGTCGTCTTCGGCGCCTTGACCATATTGGAATTCGTTGGAA
AATGCGTACCGGTGGTGGATGAGATTATCGATAGTGCCATGGTGTTCGTGGTACCG
>TR528|c0_g1_i1 len=341 path=[391:0-100 411:101-126 409:127-137 407:138-180 406:181-340] [-1, 391, 411, 409, 407, 406, -2]
TTCTCGCCAACATACTATTTTCAGACGAGTCTATCCGATCTCAACCGCGGATGGAGAACGCCCGATCCTTCGC
CCGAAAGCAAGTGGGTCGAAAAGGCGTCCCGACGCTTGGAAGAAGAATGGAAGGCGTCGTTCGGGTCCGAAGG
AGAAGAAGAAGGAGAAGAGCACCACGACGACGTCTTCGAACGGATGGGCCTCACGCGAACTCTGACGATGCCC
CCTTCTCAAAAGCCCCAACAACAACTCCAAATTACGATGCCTCGTACCAAGGAGTACAATGGTCCAAAAGGCG
ATGATGATACAACAGACATGGAAAAAGGTCCAGGCGAGAAGTCGTAGAT
>TR528|c0_g2_i1 len=344 path=[399:0-100 412:101-126 410:127-137 408:138-183 398:184-343] [-1, 399, 412, 410, 408, 398, -2]
TTCTCGCCAACATACTATTTTCAGACGAGTCTATCCGATCTCAACCGCGGATGGAGAACGCCCGATCCTTCGC
CCGAAAGCAAGTGGGTCGAAAAGGCGTCGCAACGCTTGGAAGAAGAATGGAAGGCGTCGTTCGGGGAGTCCGA
AGCAGAAGAAGGAGAAGAAGAGCACCACGACGACGTCTTCGAACGGATGGGCCTCACGCGAACTCTGACGATG
CCCCCTTCTCAAAAGCCCCAACAACAACTCCAAATTACGATGCCTCGTACCAAGGAGTACAATGGTCCAAAAG
GCGATGATGATACAACAGACATGGAAAAAGGTCCAGGCGAGAAGTCGTAGAT
>TR530|c0_g1_i1 len=749 path=[772:0-133 773:134-134 787:135-176 784:177-200 786:201-333 780:334-346 783:347-472 764:473-473 785:474-572 777:573-596 782:597-672 770:673-685 771:686-748] [-1, 772, 773, 787, 784, 786, 780, 783, 764, 785, 777, 782, 770, 771, -2]
GAAACTGGCGGAAGAACGAGGACCAAGGCTAGACGAGAATCGTCGATTGAACAATAGCCTCTGTGCCAAGGAG
AAAGAGTTCAATCAACTCAGCGATAACTTCAAACAAGCAACTGATGATGCTAGAAAGATGCAGGCAAAGATAG
ATTCAATGGACTTAATAATTGAATCACGAAATGTCGAGAGAGAAAATACAGCAGCAGACATAAAGCGCATGCA
GGATCAAGTCAAAGGTGCCAGAGACGTTATCGAAGAGGAAATGGAGGAGATATCCACTCTTACCGCTAGTGTG
AATCGCCTTGAAAATGAACTGCAAGATACCAAGGACTCTTACGAGAAGAAGCTGGCGGAAGAACGAGGACCCA
GACTAGACGAGAATCGCCGATTGAACCATAGCCTCTGCGCCAAGGAGAAAGAGATCGATCAACTCAATGATAG
CCTCAAACATGTAACTGATGATGCTAGAAAGATGCAAGCAAAGGTGGATTCGATGAACCTAATAATTGAAGCA
AAGATGCTTGAAATCGAAACATCCTCAGAACAGATGAGGGACATGCAAGATCAAATCGAAGCTGCTAGAAACG
TGATCGAAGAGAAAAATGATGAGATATCCACTCTTACCATTAGTGTGAAATGTCTAGAAGACGAAGTGCAAGA
TACCAAGGACTCTTACGAGAAGAAGCTGGTGGAAGAACGAGGACCCAGGCTAGACGAGACTCGTCGACTTCAA
ACCTCTCTCGTTGCCAAGG
>TR531|c0_g3_i1 len=1541 path=[1680:0-102 1681:103-124 1682:125-138 1683:139-1540] [-1, 1680, 1681, 1682, 1683, -2]
TTTTTTTTTTAGTTTTCTTAACTTTCAGTTATTAGATATTAGAATCGCACTGGATAGGGAGTGTGTTGCATCC
GCGCGGTACGTTCCATATTATTGTACAAGCAGGGGTGGAATTACCATGATAAGTGAGGGACAATTATAGAGAC
ACGATATTGTCATCACCTCCTACGGTCAGGAGCCGTCTCATCCATGCTCTCGTCCATCATATGACCACCAGCC
TGAGACTGAACATGAGCCGCTTTCACCTCGCGATAATAATTCGATAACGACTGCCTTTGTTTTTCCTGCGCCG
CGGAAGTGAGCGAGTCAGGGGACTCTATACGCGTGGGCGGATTGGTTGCATTGAGTCTCGCGGCGGCGTGAGC

TTGTTGTGCCATCATTATTTGCAGGTTTGTATAGTTCGACGGCAGAGGCGAAGCCATGGCCGCTGGCGATTCG
TGCCGCGGACTGCTATTTTCGCGTCCCAGGAAAGAGACGTGTCTATCAGCTACATATTCCCTTTCTCTAGCCA
TCATTTGCGATAGCAAAAAATGTGATCCAGTAACATCCTGCAGTCTTGCGGCATGGTCCACGGCATCACTGAC
GGGGCAGTCCTCGATGATGCCATCTCCGACTATGGTCCCTCCGACACTCCGCTTCGACCGCGAGAGGGACGCT
CCCTGCGTGGAAAAATCAGATATTTCTCCAATGGACATGGAGGGAAGCGACATGTCCTCCAAACCGTCGATGA
AGGATTCGCTGCTCTGTCGAGGAACCGCGTGAGGATTATGCTGTGTTCTCGAATCCACGTTGGCACGTCTCCA
ATCACCGCTGCGCACTTGAGGATTGTGCTGCGCTCTCGAATCTACGCTGCTGTGCACCTGGGGTAGGCTATGT
GACTGTGACGTGCTGCCGAACATACTTTCGAACGCTTTCATCTGGGAATCGGAGACTGAGTCTCTGGTTTTTG
TATTGAGGCTCGATAGGGGATATTCGTATGGCTCCTTCCTTTGTGGTAATGATGGAGTGTGTCCTCCGAAGCG
AGACATGTCTTGAAAATTTTGATTCATTATTTGTTGCTCTAGATGATACCTAATATACTCAGATTCGAGAGCT
GAGGAAGCCGAAAGATTATTTGGCTGTCCTGAAAGGGTAGCATTTGGTATAGAATGTTGTTGCATGGATTTGT
ATTGCTGCATAGCGATCGCATTTCGGTAGTGATCGAGATCGACTCCTCCCCCAGCGAGTGGAAAATGAGGGTT
TTGCCCTAGCATGGAATTTGCTTGCATGGCAATATTACGCTGTTGCTGGGCGAAATTTTGCATCTGCAGATTT
CGAATTGCTGCTTCAGCAGGGCTCGCTCGGTAATTATCCTGCTGCTGGGGGGCCGCCGCAAATGCTTGGTGCT
GTTTTGGGCGAACCGGTAAATGCGTCACTCCCTCCATCATATCGGATTGGCGTCCAATATCAGCAACATTGCC
CGGTTTGTTCGATGACGGTGATGATCCCGTCATCATCGACCGCGCCGTCCTATTCTGCCGCAACGCCTGCTTC
GCCTTCTC
>TR531|c0_g4_i1 len=1637 path=[1688:0-102 1689:103-103 1691:104-220
1674:221-234 1675:235-1636] [-1, 1688, 1689, 1691, 1674, 1675, -2]
TTTTTTTTTTAGTTTTCTTAACTTTCAGTTATTAGATATTAGAATCGCACTGGATAGGGAGTGTGTTGCATCC
GCGCGGTACGTTCCATATTATTGTACAAGCCTGTGTATAACATGCATCCGCCAGCAGCCAGCAGTGGTGGTTT
AGATCACATTTGTTTGGACACATGATTAGAATTCGACTAACCAAATGACATACAGGGGTGGAATTACCATGAT
AAGTGAGGGACAATTATAGAGACACGATATTGTCATCACCTCCTACGGTCAGGAGCCGTCTCATCCATGCTCT
CGTCCATCATATGACCACCAGCCTGAGACTGAACATGAGCCGCTTTCACCTCGCGATAATAATTCGATAACGA
CTGCCTTTGTTTTTCCTGCGCCGCGGAAGTGAGCGAGTCAGGGGACTCTATACGCGTGGGCGGATTGGTTGCA
TTGAGTCTCGCGGCGGCGTGAGCTTGTTGTGCCATCATTATTTGCAGGTTTGTATAGTTCGACGGCAGAGGCG
AAGCCATGGCCGCTGGCGATTCGTGCCGCGGACTGCTATTTTCGCGTCCCAGGAAAGAGACGTGTCTATCAGC
TACATATTCCCTTTCTCTAGCCATCATTTGCGATAGCAAAAAATGTGATCCAGTAACATCCTGCAGTCTTGCG
GCATGGTCCACGGCATCACTGACGGGGCAGTCCTCGATGATGCCATCTCCGACTATGGTCCCTCCGACACTCC
GCTTCGACCGCGAGAGGGACGCTCCCTGCGTGGAAAAATCAGATATTTCTCCAATGGACATGGAGGGAAGCGA
CATGTCCTCCAAACCGTCGATGAAGGATTCGCTGCTCTGTCGAGGAACCGCGTGAGGATTATGCTGTGTTCTC
GAATCCACGTTGGCACGTCTCCAATCACCGCTGCGCACTTGAGGATTGTGCTGCGCTCTCGAATCTACGCTGC
TGTGCACCTGGGGTAGGCTATGTGACTGTGACGTGCTGCCGAACATACTTTCGAACGCTTTCATCTGGGAATC
GGAGACTGAGTCTCTGGTTTTTGTATTGAGGCTCGATAGGGGATATTCGTATGGCTCCTTCCTTTGTGGTAAT
GATGGAGTGTGTCCTCCGAAGCGAGACATGTCTTGAAAATTTTGATTCATTATTTGTTGCTCTAGATGATACC
TAATATACTCAGATTCGAGAGCTGAGGAAGCCGAAAGATTATTTGGCTGTCCTGAAAGGGTAGCATTTGGTAT
AGAATGTTGTTGCATGGATTTGTATTGCTGCATAGCGATCGCATTTCGGTAGTGATCGAGATCGACTCCTCCC
CCAGCGAGTGGAAAATGAGGGTTTTGCCCTAGCATGGAATTTGCTTGCATGGCAATATTACGCTGTTGCTGGG
CGAAATTTTGCATCTGCAGATTTCGAATTGCTGCTTCAGCAGGGCTCGCTCGGTAATTATCCTGCTGCTGGGG
GGCCGCCGCAAATGCTTGGTGCTGTTTTGGGCGAACCGGTAAATGCGTCACTCCCTCCATCATATCGGATTGG
CGTCCAATATCAGCAACATTGCCCGGTTTGTTCGATGACGGTGATGATCCCGTCATCATCGACCGCGCCGTCC
TATTCTGCCGCAACGCCTGCTTCGCCTTCTC
>TR532|c0_g1_i1 len=464 path=[492:0-145 493:146-169 498:170-397 496:398-
421 497:422-463] [-1, 492, 493, 498, 496, 497, -2]
GAAAGAGCGCGGGTGTGGTAGTAGTGGCACGTGATGATGATGATGATGATGAGTCACCTCCGGTGTGGAACGC
CAGCTCTACGTCTGCGTCGATCCACTCGTCGGCATAGTAACAAACAACTGATTTTTTGTTACCACGACATGCG
TTACGCTTGAGCCTCCGATGATGTGATGCTGGCAATAATTGTTCGTGTGGCAACAAAGTTCTGTGTTGTTGGG
CACAGTGTGACGACAACGAAGAGTGGAGGATGTTCTTCGGAACAGCCAAAAGAGCGGCTGATTGATGGGTTGT
TGAGAGCACTACCGCCGAGGCAAAAACGGCAGCGGCCGTGGTGGCGAGTCGCAGCCTATTCTGCCGATACTGC
CGTATGTTGCTGGAAACGCTTGAAGACCTCATGGATGTGATGGTGATGCGCAGGTTTTGACTTCCTTTTAATG
CACTTGTGCTGTTTGTTTCAGATCGG
>TR533|c0_g1_i1 len=1445 path=[1505:0-299 1511:300-313 1510:314-337
1509:338-1185 1503:1186-1209 1504:1210-1444] [-1, 1505, 1511, 1510, 1509,
1503, 1504, -2]
CCATTGTAGAAGTATGGTACCTAAATGTTCTAGCATTCTAACATGAAATGATTATCATTCCAATAGCTCATCA
CATCCAAAGCATAATCCGATGCCCATCGGCCTAATCCTAACGAGGGGATTGCCGACACAATCCTCCCGATCAG
CGTCGCCAGCGGTCGTGTCACGGCGTTGTACGCGGGCGATCGAAGCAGGTACAATTTGAGCCGGCCTCGTCTC
CAATCCAATTCTTCCAGCTTCGCTTGTTCCGCGGAAGGAGGGGGCACAATACCATTGAGTCCGATTTGCGGAT
GACGACGAGTACCATCCCCGCCGCCGGCAACTCGCAGTAGTTTATCCGATACCAAGTCCATCAATAACGATAT
CCACCATGCCCTCCAAATGGCAAACGAATACGATAATGGATTCCGCGGTATTTTTCTGAAGGAATACGACCTC

CGTCCGTGCCATTCCTTGTTCTGGGCACGGCTCCAGTACAACGGTCGCAGTATGTGAAGCACTTCGCCCACCG
CGTATATGCAGAGTACTTTGCTCTTCGAAGATGCAAGTTGCGACAGCCACTTCGCGAACGATGCGGACGCGTT
TGATACAAACGATGACGATAATCCACCACGAGACCAAGTACGAGAGGAAGATTTCCTGGCAACGACGGAGCGG
CGTCCCGTCCTCCTTCCGACATATTGCACGACGTTCGCCTCTTCCTCCGCAACTACCAAGGGTACAACTTGTT
CGTAAGGGTCCAACTCTCCTCCTCTCCTCAACAACGACGGCAGCAGCGCCGTTCGATCCTCCTCGTTGTTTGC
ACGATGAGACAGTCTCCTCGCATCTCCATCATCTTCCTCCCGAGTGCATTTTCGACGTCGATGACGCTGCGCC
CACCACGAAATTGAGAGCAACGCCAATCTTGCCAAAAATCTCACATGTTCCAACCGATACGAGACCTGCGCAG
CACGACACTGCCTGCCCTCCCATTCGCGACGTCGTTTTTGCAGCGGTAGATCGGTCGTGGTAGCGAACATTGT
TTCGTATTCTTGTTGGTCACTTTGGTGCTGTTGTTCCGATTCTTCGCTGTAAGGCAATAGCGGCGTGCCATAA
TCCGAAGGATGGGAGATACGACGACGAGACCATGCCTCCATTGCCGGGTAGATGCATGTGGTTGCAGTGACAA
TTGCACGAAGCACGGGTACGACCTTGTTCAATAATTGCAAAATTGTAGTTTTGTTGCAGTAGGAGGATGACAA
TCCCATGATCGTTCTCCTCCGAGAAGAAGAAGAGGACGACGACTTTGCTGGTATTTCTAGCAACCATTCCTCC
CTCCTTCCGATGGTCGTTCCGAGACCTTCGCCCCATCCTTCCAACACGACGTCGTTCATCCATCGAATTATGT
TCCATGCGGCGTAATACATTTCGATGCTCGTGCCGCGATGATCGTGTTTGAAGAGGTA
>TR534|c0_g1_i1 len=444 path=[492:0-95 493:96-119 511:120-131 510:132-150 508:151-255 500:256-279 501:280-443] [-1, 492, 493, 511, 510, 508, 500, 501, -2]
CTGTCTGATCCATAAAATAACTCTCTCACAACCCAAAGCATCGTTTTGAGCCGCGTCATCACACGCACCCAAC
ACACCAAGTTTTTCATCCTCTTCTCGTCGATCAACAAAATACTCAGCAGACTCCGCCCCTCAGCAGAAATAAT
GGTCGGGCGAAAGAGTCGACACTATCGCGATGAAAGCGATCTTCTCCTAGGAGTCTACAGCTCCACTCTCAGC
ACTAGCAGTAAAGACAACGGCAACAGCAACAGCAATAACCATTCTCAACAGCACAACGATTACTACACAACAC
CCAACACGCCCCCGCCCTTAAAACAGAGTTCCTTCTCTAAATTACAGCAGTGTCGCACTCCCACTGACAACGA
CGACAACAACCACAATAACTCGCCACCCAAGAAGAGGACGAGGAAGAAGAAGAAGCAAGGCTATCGCGTCCAC
CGCGTA
>TR535|c0_g1_i1 len=1556 path=[1606:0-413 1607:414-437 1615:438-995 1614:996-1275 1612:1276-1299 1613:1300-1555] [-1, 1606, 1607, 1615, 1614, 1612, 1613, -2]
AAGTAGGAGTAGCAGAACACATCAAACGTCTTCACGATGATGCGTTGCGATGATGGCATAATTCTTAGAGAGC
TCTCACTCATTCAACCAGCCAACAATTCGATTAGAACAGAAGTAATAAAAACGCGACTATTACCCACTGCATG
GATGCACGTTGAAAGAATGGACCCAACAGCCAATCGCCGTGAGACGTCAGCAATAACCTTGAAGCGCGTAGAA
CAGAATGATCCTACATTGAAGAGATTGGAAGTAAGCAGCATTGGTTTTCTCAGGGGACGTTTTACTCATGATG
GAACGGCCTACTACTGGCCCAATGATTGTGCCGATCTTGCACGTCTCGGTGAGGCTATCGGAGATAACAAGAA
TTTGCAGAGCTTGAAGATTCCCAACGCTCGGAATTTGGCAGATATGGCTGTTAATAATAAAGCATTTTTCGAC
GGGATCTACCGCAATGCCTCCATCTGTAGTTTCGACATGTCGTATTTTGATCTTTTGGGGGGAAATGAGCTCG
TGGTCGAATGGTCTTCTGGCGATCAACGCCATTTTTACAGGAATCTTGATCGTTCTGCGAAGATGGGACGCGA
AGCTTTGAATGGATTCATCACGAACAACATCAATCTAACACAAATTGCGTTAGATCGGTGTCCGAACCTTCGC
AATGGGGGGGCTGGCATACTCGCGTCAGCTGTAAAAGGATGCGCAAGTGTAAACGAGATCAAGCTGACATCGT
GCAATGTTGATGATGATATCCTCGTTGAATTCGTTTTGGGAATCAGAGCACTAAAACAGCTCCGCACGCTTGA
TTTGTCTGGGAACCATTTTTGGAGGGCTGGCTGCAAAGCGCTTGCAACCCTGCTAGCAGACAATGATGGCAAT
CTGGAATACCTTGGTCTTCGAGACAACAGCAGAATCGACAACAATTGCGCAAACATTCTTGCAAAGTCTTTGA
CATATAATCGCAAATTGAAGCTACTCCTCCTTTCTGACAACAGTGCAATCACTCACAGAGGCTGGGATGCTTT
CTCTGCAGTCCTGTGCAATACTTCAAGTGTCAACAATACATACCTTTCTAACCACACGCTGCATAGTTTTGGA
AACGTCAATCTACCACCCAAACTCTCGGTTCTTCTCGAAGTGAATTTTGTCGCCGGCAATAAAGGGGCTGCAA
TACGAAAGATCCTGCTTCATCACGCCCATCTTGACATGACACCATTTCACGTAGAGTGGGATTTGAAAGCATT
GCCATTCGCTGTCAATTGGTTTGATAAAGCTAGAGGATATGCGCAAAATGAAGACGAATCAAATATTAATTCG
AGGAAATTAACTGCAGTTTATCAATTTGCAAGAGCCATGCCAATGCTGTTTGTCCCACCACCATCAAAGATGA
CTTATAAAAGAAAGATATGTGAGATCAACTAAAGTCAATGGCAGCAAAACAGGTTTAGTGGGTTTAGTGGAAG
AGACTGCTAGTGAACAGCGACATTCATTTTTGTTTTAGTTTTGTCATGGAAGATTGCAGTGTAGTCATATATA
ATACGTAGTTTTGAATCAATGTT
>TR536|c0_g1_i1 len=753 path=[858:0-365 877:366-366 876:367-390 874:391-501 878:502-525 879:526-539 873:540-565 870:566-752] [-1, 858, 877, 876, 874, 878, 879, 873, 870, -2]
TTTTTTTAAGAAGAAATACGACATCTATTTTTACATAGTTTCATATTACTCTTCATTCTTAGTGTTTTCTCTG
GAAATCTTACGAATTCATAACATTGCACAGCACAAGGGTTCATGAAGACCTTGCCCCCCCAGTTGTCTTGCCT
TGGAGCAGATTCGAAATACTTGCTCCCATTGAAATGTTCAAGAACCACAACAGACGACAACCATCTTGAGTGT
TTTGGACCAGACACCTGATTGAAATACAGAACTTCTCCAACACATGCGTCAAGCTCCTGAGGAACAGGATCAA
ACAATGAATGAACAAGGTCCTCCAGAGACTTTTCCTTGATCAAGGTGGGCAAAGACTTTTCCCTACACCTGAA
GTTCCAGGGGTGAACAATCAACTTGAGGGACCCTGAATCATTATGCACACCTTGCATCAGTACCAGCTTAGCT
GGCAGAAATCCAGGTCCGTAGGTCTTGACAGGTTGTGGTAAGACTGCAGCAATCAATCCCGCTGATGTACTGT
TATAGTTTCTGTTTTGCAGAAGACGAATACAGTCTTGACGTTGAAGAATGTCTTGGCGAAGTAGTGTCCCACT

TGGGAATTTGAAGGTTGAGTATTCTCCAGATGGGAGAATAGTAGTTACAACAGACCCTGACTTCACAGAGTCA
TCCAGAATTATGTCACAATTCATGGTGTGGGATAGCGGCGCAGAAGAAATATTCTTTCTTTGCCAGAAATCTC
AAACACCAAGAAATGCAACACAA
>TR536|c0_g1_i2 len=598 path=[858:0-365 877:366-366 876:367-390 874:391-501 878:502-525 879:526-539 873:540-565 867:566-597] [-1, 858, 877, 876, 874, 878, 879, 873, 867, -2]
TTTTTTTAAGAAGAAATACGACATCTATTTTTACATAGTTTCATATTACTCTTCATTCTTAGTGTTTTCTCTG
GAAATCTTACGAATTCATAACATTGCACAGCACAAGGGTTCATGAAGACCTTGCCCCCCCAGTTGTCTTGCCT
TGGAGCAGATTCGAAATACTTGCTCCCATTGAAATGTTCAAGAACCACAACAGACGACAACCATCTTGAGTGT
TTTGGACCAGACACCTGATTGAAATACAGAACTTCTCCAACACATGCGTCAAGCTCCTGAGGAACAGGATCAA
ACAATGAATGAACAAGGTCCTCCAGAGACTTTTCCTTGATCAAGGTGGGCAAAGACTTTTCCCTACACCTGAA
GTTCCAGGGGTGAACAATCAACTTGAGGGACCCTGAATCATTATGCACACCTTGCATCAGTACCAGCTTAGCT
GGCAGAAATCCAGGTCCGTAGGTCTTGACAGGTTGTGGTAAGACTGCAGCAATCAATCCCGCTGATGTACTGT
TATAGTTTCTGTTTTGCAGAAGACGAATACAGTCTTGACGTTGAAGAATGTCTTGCCAATTCCGCCGCGTCCT
CCCCGTACTTCTTC
>TR537|c0_g1_i1 len=978 path=[1177:0-264 1178:265-315 1198:316-383 1188:384-407 1189:408-409 1190:410-433 1202:434-753 1200:754-855 1197:856-945 1193:946-969 1194:970-977] [-1, 1177, 1178, 1198, 1188, 1189, 1190, 1202, 1200, 1197, 1193, 1194, -2]
TGTCCGATCGCACGCCGCGAGGTGGGTACGACAAGTGCCTCTCGGCGTGCCTCCCGTCGCGATGCTGTTTCGT
TCCCGAAGACGAGACGTACGAGGTTTGGACGACCGCCGACAATGCCGTTCCTCCTCCTCCTCCCGTGGTG
CTGCCGCAGCATTCCTCTGCGACGACGACGACGACGTTCGCGATTATGGGCGACGAGATATCATCGTGTTACC
GCGAATACGACGACGCGTGCGTGCAGTACCTCAATCAGTGTTCGGTGCTGGGTTCGTCCGTATTGCTGCCGCG
AAAACCCCCGAACGACGAGGAGGTGGAGAGCATGACCGTCGGCGAGAGGCTGGAGCTTGCAGAGTGGATTCTT
CACAGCTGCAGTCCTTCTTCCAATGGCGGTGGTGGAGAGAGCGTAGTCGGGGAGATGTGCCAGCAGTTATGTC
GAGAGAAGGAATGTTGCTTCGCGGAGGATGAGGAGGAGGAGGAGGAGAGGAGGGCCGTCGAGGACAAAGAAGA
CGTGCTGCCGACCGATCCGATGGAAGGTCCATCGATCGCCGATGGGTCCGTGGGGGTTGGGACCCTCGCGGAC
GAGACGAGTTCGAACCGTGCCAACGACAACGGTGCGGGTCCTTCGGGTTCTCCGTCGAGCGGTCCGCCGAATA
TCGGTTTGGCGTCCGTGCCATCATCCCCGTCGCCCACTACCACGCACCGGCCCACCGCAGCAGTTACCACGAT
CGATTCCTTACGTGGACGACGAAAACGACAACGGGATTTATCAGAGCAGCAACAGCAACGACAACGTCAACAA
TACCACCACCGCCATCATCATCACCATCATCATCGCAATGCTCAGGAATTCAGCCACATGACGATTCCGGGCG
AGGAAGACTTGACGTACGTCGTGTCCGCGGAGGGTCGTTCCTCCACGGAGGGAGAGTACTCGAATCCGTACGC
GGTGCCATCGGATTGGACGGAAACAACAG
>TR538|c0_g1_i1 len=3269 path=[3364:0-556 3365:557-580 3395:581-1043 3368:1044-1067 3399:1068-1943 3401:1944-1954 3402:1955-1998 3398:1999-1999 3400:2000-2027 3393:2028-2038 3397:2039-2082 3362:2083-2083 3392:2084-2279 3384:2280-2303 3396:2304-2531 3378:2532-2555 3391:2556-2666 3375:2667-2690 3376:2691-3268] [-1, 3364, 3365, 3395, 3368, 3399, 3401, 3402, 3398, 3400, 3393, 3397, 3362, 3392, 3384, 3396, 3378, 3391, 3375, 3376, -2]
TCTTCCGATCTTTTTTTTTTTGATAAAACAAAGTATTAACATGGTGGTCTATCGAACATTTTTTGTTCTTTAT
AACGATACAAAGATTAACAATATTCAAAAGAAGGAACTGATAACACTGCCGCCGCTGCCCTTCTTTTTCTTCT
TCCCCGACTTGTGTTTCAATGAGCCCTTCTTGGAAACGCTTGAGTTGCCGTTGAACAGACCCCCCGTCGACTC
CGACACATTTTGCATCGCCTGCACCTGCCCAGGACCCATGACAGCGTTCATCGCATTCCACAAGTTGAACCCA
TCTCGTCCCCGTTCCCGCTGCGCCACGCTGGATTGATCATTCAACGCGGGATGGTCGATACTCTGGCTCGCCA
CCGCCATGGACTCCACTGCCGACATTGCGCTTTGGACGTGTCCCGACGCGTCCGTCCCAGTCAGACCGCCAGG
GGGGACATACCCGTGCTTCTTGATAAAGTGCAGCTCCCGGCTCATATTCTCAGCCGCAATACGCAGGTCGTCA
ACTCCCTTCAATGCCACAGAGAAAGCCTTCATCTCCATCTCTGACCTTGTCCGTTCCTCGCTGAGCCTCGTCT
TGGCGTCATCCGAATCCTTCTTGTACAGCTCGAGCTGCTTGCTCAGCTCATCGTTGAGGCTGCGCAGGCCCGT
GTTCTGCGCAGCCGCTATGTCTTCCTCGCAGTCCGCGTACTCGCGTACGCCGAACGTTAGATCTTCCTCCGTC
ACCTCTTCTCGTAGGGAGTTCAGCTCAGCATCCTGGGTGTTCACCCTCATTTTGAGTTTTTCTATTTCGAGGT
CCTTTTGCATCCCAGATTCTGCAAGGCGTTTGAGCTCGTCGTACTTGCGCATCATATCATCGGCCGAGAAGGT
ATCGGTATTCCTTGCCTCTTCCTTCGCTACGAGCAGCTCAGTGCTGAGGCGCTCAATTTCATATTGAAGTGAG
GCGATTTCGTTAGCTTCGTGTTCACTGATGGGTTGTTGTGACATTAATGTGCGTGAGCTTCCTCCGTTGTTTT
GTTGTTGATTCAAACGCATTTGAGCTATCTCAGTCCGAAGCTTTGCCTCCCTGGCCACTGCCTCTTCAGCACG
AGCCTTCTCAGCCAACATCCTCGCCTGTGCCTTCTCGAGACTGTCCTTAAATGAACCGTCAATTCCTCCCATG
TTATCTCCATCTTGCAAACCCTCAATCACACTTCGAAGCTCGGCATTCTCTTTCTCGAGACTCAAAAGCGATG
AGAATTGCTGCTCGGCCGTATCTAATATCTTTGTATTGGCTTCCTTCAACTTAGCAATCTCCTTCTTCATCTT
CTTTTCTGACTTGGAATTCGCTATTGTCATAGATTCAGAGGGGGGTGGCATGGGGCTCTTTGTGAATGTTTG
AGCTCGATCCGCAGACGTTCATTCTCCTCCCTTAACTCGTTCGCCTCCACCTCCGAAGAGAGGGAGGTGTACT

CCGTGGAAGAATCGGCATTGCTTTTATTTGACATGGACTCGATCTTCGCCCTCTTGACTTCTTTTTTCAGCGC
GTCGATCTCATTTTGCATTTCTGCCTCCCTCAACTTGTTAGCATTATTGCCGGGCACTTGTTCTGCCATTGCT
AGCTTTTCCTTCGTCGATTCCAACTCTGCTTTTAAGGCATCAAATTCTGCAGATGATGCACCTTGATCGGCAG
ACTGAGGTTGAGACACATGGGGCACGCTGTCAGACATCTGACTCAGCTCACTGGTCAGCTTTTTGATCTCCCT
TTCCCGTTGACGAGACAGGGAGGTCTGTTCATCCAGTTTATTCTGCAGCATCATTTCTTTCGCATTCATCGAG
GCAGATTCGTCGTCACTATCTTTTGCAATGGCTTCCTCCAGTTCCGACTCCAGTTGAGTGATTGACTCCCGGG
CTGTTTCCAAATCTTCTCGCCTCGTCTCGGCCAAGTCATTTGCTTCCTGGAGTTCCGATTCCAGTTGAGTGAT
TGACCCTCGAGCCGTTTCCAATTCTTCTAGCCTTGTCTCGGCCAAGTCATTTGCTTCCTGGAGTTCCGGCTCC
ACTTGAGCAACGGTCTCTCGAGCCGTTTCCAATTCTTCTAATCTTGCCTCTGCCAAGTTATTTGCTTCCTGGA
AGCGCAGACGTTCCTCCTCCAGTTCCTTTTGGGTGTTTGCCAGTTCCTCCTTCACACGAGCCAACTCGTCTTC
CGCATCAGCAGCCATGGATAAGTCGCGGAGACTCGCTTCCTTCTTCAACTTCGCTGCCTCTTCAGCCTCCTTC
TGCGCCGCTACCTTCACCCGCTCCATCTCTATTCTCATCTGCTCCATCCGCTCCCTGAATTTGTCTCGCTCTT
CTGCCACATTCGACAGATCTTTCGCCTCCTGCTTTAGTTTCATCATGACAAGGGTCGACTGTCGAACACGAAC
AGCACATTGTATAACAATGGTGGCACGCCGAATCTCATAAACCTTTTTGTAATGTGCGCGCCCAGCTGCTCCG
CGCTGTCTCCGCTGACACCACCTGGCGACGACCAACATAGCAAGGTACATGCTGCGACCTCGATATTGCCTAT
ACGCGCTCTGTATCCTCTGGGCATTAAACTGTTCTCTCGCCGCTTGCACTTTCCGTTCAGCAATTATGACCCG
GATCCAGCACTGTAGCTGAAGGTTGGCTTCGCAATACTCGTTGAAGTCACGTCGGTTGATGTACCCACGCGCG
ATAGCCTGCACTTTAATGGAGGCGGCGGCCATGAAGTCCTTGCGCATTTTCTCCAAAATATCAAATGCCTTAC
GTCTTAGAAACACCTTCGTCTTGCCCACCTGGATCCCAACAGCCAGTAAATCAACCTTCTCACCAGATGAACT
CGACTTTTTATCCTTTTTGCTCGCCTTCGCTGGAGTCGACGGCGCCTTATTCTCGATCTCCCACATTTTTTTC
GCAATGGCATCCACCAGGGCAACCACCGGTTTCACCTTCCTCGAACTCTTGGACGCCTTTTTCATCTCCTTCG
ATCCCAACGTCCTGTATCTGCTCACGAACTGCGAGTGATTGTACCGCTGCGGATAACCCACGCGACTGACCCT
CACGGCTTCAACAACACCTGCACACCGCAGCTGCTCCACGATCATCAGGGGATCGAAATTATCGGGCACCAAC
TGGCCGTTGGGTTTCAGGCATCGCACGTAATGGGGCGAGGTCAAATCGATCTTCTGCCGGAGCGACGTGAGCT
GGCTCGCGAAGTGCCCTCCCACCGTGACGCTCTTCTTCTTCCCCCGACTCGGCTTGG
>TR539|c0_g1_i1 len=1516 path=[2868:0-149 @2884@!:150-1018 2862:1019-1040
2880:1041-1266 2873:1267-1290 2877:1291-1471 2853:1472-1482 2854:1483-
1493 2855:1494-1504 2856:1505-1515] [-1, 2868, 2884, 2862, 2880, 2873,
2877, 2853, 2854, 2855, 2856, -2]
TCCTTGAGTCATGGGAAGGATGGTAAGCTCTCGTCATGATAGATTCCAGTATACTTTAAAATATGAAATTGAT
TTGTTGCCTGACCAACAGACACGCTGTACCCCTACTCATTTGTTGGATGCCAAAGGCCTGGATACAGAGACAA
TGCTCGGGCGAAGTTGGGACAAGATTATAGAGGCGTCGGAGAACATTTTCTATTGTTGCCCGAGCCTATCCGG
TATTTGACTGACCGGCTCGTAGCTGTCAATACTGCAGTACCCACGACCGACGCTGTCGTCCAAATTTCTTCCG
AGGAACTCCTTCCAGCATTGAATGAAGTATTTACGCTTGATACATTTAGAATCAGCTATGTGAGTGTGGCGAT
CCGCAGTTTTGTGCATCATTTCTTTCCTGCGTATGGTTCGCCATTCGTTGATTATGCTGATAAAATTCTGGAA
GAAGAGAAATAAGCATGATTACATGATTGATTGGTCTTTAACTGCCATGAAAGGTGTTCTGATTGATATTGCT
AGTTACATGAATTTTGATCATGCCAAGGCAGGACGAGCTGGCAATGATGACCATTTGTAGACAAGTACTAGGT
CAACTTTTGCTTTCATAAAGCCAAGCTTGGGTAGAAATAAAGATTGGATTGTGATGGGACACCTGGCGAAATA
ACAGTATTATTAGTCCAACTCAAAAAGTCTCAAAATGGTGTGAATTATGGCTACTCATGCAGAATGATGCGAC
CGAAAAAACCATTTTGACTGGGATTTGGCGACGACGGCGGTATATCTACCACTGCCATTGAAGAGTGCGCAGG
TCGTGCTGGACGTAAAACGGGATGCGGTGTTATTACAAATAGATCAGCTGGAATCCATGCTAGGTGCTAGATA
CCATGAAGGAGCAGGAGCAACTCCACAAAGGCCTCCGTTTACACGCGCTATCCTGGAGCAGGCGGCATGTGTC
GGGATGGGCAGCGGCACTAACCTTGTCGCAACATGCGGGTGCACTGAGGGCATACAACAAGGAGCTATTGATT
ACATCATCACAACAGGCCAAATCTCGATCACAGCGTACAATGTGCATGGGCCCATCTTCGAGCTCGCAGGGAA
AGGGGGAGAAAACGTAGGTCCAGGCCTCGTCAGCTTTGGTGGGACCCACCGATGGAGTTGGATGGGACGAGGA
ACGCAGCGGATGTCAAACCAGATTTCGGGGGATGCGAAGCTACTACTTTGTAGTCGCAAATGATGACCCTCGG
GTACTCACGTTGGCTAGGTGAGTAATACAGTGGATGGGTTACACGGGAGTTCAGGATTGTTCTCTCGAATATC
GTAGTTTCTGCGTTTTCTTAGCAGCCCTTTAATCTTATCATAAGCAAGAGGTTAGACAGCGGACAGAGCCTTG
CCCAATCCTGAGGGATCTGAGTATGGGTAAGACTGTACGCCGCCAGTACCCAAGTTAAGACACACACACTAAG
CACACACTAAGCACACACTAAGCACACACTAAGCACACACTAAGCACACACTAAGC
>TR539|c0_g1_i2 len=2578 path=[2850:0-581 2885:582-805 2879:806-966
2881:967-1054 @2884@!:1055-1923 2878:1924-2102 2880:2103-2328 2873:2329-
2352 2877:2353-2533 2853:2534-2544 2854:2545-2555 2855:2556-2566
2856:2567-2577] [-1, 2850, 2885, 2879, 2881, 2884, 2878, 2880, 2873,
2877, 2853, 2854, 2855, 2856, -2]
GAGTTCGCGAACGGCGTGCTCGTCGGCGGGTACGACCCGTTATTCGACGGCTACCCGTCGGTGAGGAGCGTGC
CTTCTGAAGAATCGCGGCAGAAAATAGTCAAGGGTGCGGAATTCTCGTCTCTTTCGGGGGCTATTTACGAAGA
TGCAATTGCTAACACTCGTGGTCTTGGACATAGCATCGTTGCCGAGGGGAAGACTGCAGATATCGGATGGATG
GTCACGGACAGCATACAGTACGAGCGAGATTTTTGGGCAGACTCGGAGCGGAAAGAGCCGACTCTGGTCAGGA
CTTTCGTCATTCGCGGCTATGATGCGAGTGATGAGGGTGTAGACAGAGAGCGTTTGTTGAATGTTATCTGCAC

GGCATCGCCAGTCCCTATATACAAGAGCGAGCAGGCTTTGGTTCAGGTTCACGAAGGGATGCTCTCGATTGCG
CGCGAGCTATTGCAAGAACTCGAGAAATATATTGATTTGACATCTCCTTCGCACAAGTTTGTTTTCACGGGAC
ACTCTATCGGTGGCTCTCTTGCAATCTTACTGACGATTCTCCTGGCTAATGACCGTGGAGCGAGTTTTGTGAG
GGATAATGTCCTTCGTGTCTTTACCTATGGGTCTCCTCCAATTTTTGAGATAGAGTCCACTTCAAGCACTAAA
CGGGATGGATATCTCGATGATTCATGCTCGATTCTTGATGCATTTGATTTACCAACTGACCTTGTATACGGCT
ATTGCCAACCCTGGGATCCAATCGTGAGATTTTATTCAAAGTATGATCCTTTATACCCTTTGATCGATGATCT
TGGTGAGTGCGAACGTTTTAGACTTCTATGGATTCAATCGCATGATGCCAACCGGTACAGGTAGCCACTCTCT
ATTTCCCTGAACTGATTCTGTCAGTTGCTCACCAAATGATAACTTTATTCTGAAACCCGTTTTAAACAGGCGA
GGATGGAATCACTCTATTTGATTCTGGTCCGAGTAGAACTCTCCGGCCAATTATGAAGACTATCCTTGAGTCA
TGGGAAGGATGGCCTGGATACAGAGACAATGCTCGGGCGAAGTTGGGACAAGATTATAGAGGCGTCGGAGAAC
ATTTTCTATTGTTGCCCGAGCCTATCCGGTATTTGACTGACCGGCTCGTAGCTGTCAATACTGCAGTACCCAC
GACCGACGCTGTCGTCCAAATTTCTTCCGAGGAACTCCTTCCAGCATTGAATGAAGTATTTACGCTTGATACA
TTTAGAATCAGCTATGTGAGTGTGGCGATCCGCAGTTTTGTGCATCATTTCTTTCCTGCGTATGGTTCGCCAT
TCGTTGATTATGCTGATAAAATTCTGGAAGAAGAGAAATAAGCATGATTACATGATTGATTGGTCTTTAACTG
CCATGAAAGGTGTTCTGATTGATATTGCTAGTTACATGAATTTTGATCATGCCAAGGCAGGACGAGCTGGCAA
TGATGACCATTTGTAGACAAGTACTAGGTCAACTTTTGCTTTCATAAAGCCAAGCTTGGGTAGAAATAAAGAT
TGGATTGTGATGGGACACCTGGCGAAATAACAGTATTATTAGTCCAACTCAAAAAGTCTCAAAATGGTGTGAA
TTATGGCTACTCATGCAGAATGATGCGACCGAAAAAACCATTTTGACTGGGATTTGGCGACGACGGCGGTATA
TCTACCACTGCCATTGAAGAGTGCGCAGGTCGTGCTGGACGTAAAACGGGATGCGGTGTTATTACAAATAGAT
CAGCTGGAATCCATGCTAGGTGCTAGATACCATGAAGGAGCAGGAGCAACTCCACAAAGGCCTCCGTTTACAC
GCGCTATCCTGGAGCAGGCGGCATGTGTCGGGATGGGCAGCGGCACTAACCTTGTCGCAACATGCGGGTGCAC
TGAGGGCATACAACAAGGAGCTATTGGTGAGTGGCTAGCTTTTTGGGCGTTAATCATTCATCCTGATATCTAA
CCCGTCAACTGGCCTGGTCGAGTTGCCAGCGTGGTGGCCATTATCAACGATAACTACACCTTGGCCAGCCGCA
ATATGTCTTTGTTGCTAACTCACAAAGCCCAACTCAGATTACATCATCACAACAGGCCAAATCTCGATCACAG
CGTACAATGTGCATGGGCCCATCTTCGAGCTCGCAGGGAAAGGGGGAGAAAACGTAGGTCCAGGCCTCGTCAG
CTTTGGTGGGACCCACCGATGGAGTTGGATGGGACGAGGAACGCAGCGGATGTCAAACCAGATTTCGGGGGAT
GCGAAGCTACTACTTTGTAGTCGCAAATGATGACCCTCGGGTACTCACGTTGGCTAGGTGAGTAATACAGTGG
ATGGGTTACACGGGAGTTCAGGATTGTTCTCTCGAATATCGTAGTTTCTGCGTTTTCTTAGCAGCCCTTTAAT
CTTATCATAAGCAAGAGGTTAGACAGCGGACAGAGCCTTGCCCAATCCTGAGGGATCTGAGTATGGTAAGAC
TGTACGCCGCCAGTACCCAAGTTAAGACACACACACTAAGCACACACTAAGCACACACTAAGCACACACTAAG
CACACACTAAGCACACACTAAGC
>TR539|c0_g1_i3 len=2438 path=[2850:0-581 2885:582-805 2848:806-826
2881:827-914 @2884@!:915-1783 2878:1784-1962 2880:1963-2188 2873:2189-
2212 2877:2213-2393 2853:2394-2404 2854:2405-2415 2855:2416-2426
2856:2427-2437] [-1, 2850, 2885, 2848, 2881, 2884, 2878, 2880, 2873,
2877, 2853, 2854, 2855, 2856, -2]
GAGTTCGCGAACGGCGTGCTCGTCGGCGGGTACGACCCGTTATTCGACGGCTACCCGTCGGTGAGGAGCGTGC
CTTCTGAAGAATCGCGGCAGAAAATAGTCAAGGGTGCGGAATTCTCGTCTCTTTCGGGGGCTATTTACGAAGA
TGCAATTGCTAACACTCGTGGTCTTGGACATAGCATCGTTGCCGAGGGGAAGACTGCAGATATCGGATGGATG
GTCACGGACAGCATACAGTACGAGCGAGATTTTTGGGCAGACTCGGAGCGGAAAGAGCCGACTCTGGTCAGGA
CTTTCGTCATTCGCGGCTATGATGCGAGTGATGAGGGTGTAGACAGAGAGCGTTTGTTGAATGTTATCTGCAC
GGCATCGCCAGTCCCTATATACAAGAGCGAGCAGGCTTTGGTTCAGGTTCACGAAGGGATGCTCTCGATTGCG
CGCGAGCTATTGCAAGAACTCGAGAAATATATTGATTTGACATCTCCTTCGCACAAGTTTGTTTTCACGGGAC
ACTCTATCGGTGGCTCTCTTGCAATCTTACTGACGATTCTCCTGGCTAATGACCGTGGAGCGAGTTTTGTGAG
GGATAATGTCCTTCGTGTCTTTACCTATGGGTCTCCTCCAATTTTTGAGATAGAGTCCACTTCAAGCACTAAA
CGGGATGGATATCTCGATGATTCATGCTCGATTCTTGATGCATTTGATTTACCAACTGACCTTGTATACGGCT
ATTGCCAACCCTGGGATCCAATCGTGAGATTTTATTCAAAGTATGATCCTTTATACCCTTTGATCGATGATCT
TGGCGAGGATGGAATCACTCTATTTGATTCTGGTCCGAGTAGAACTCTCCGGCCAATTATGAAGACTATCCTT
GAGTCATGGGAAGGATGGCCTGGATACAGAGACAATGCTCGGGCGAAGTTGGGACAAGATTATAGAGGCGTCG
GAGAACATTTTCTATTGTTGCCCGAGCCTATCCGGTATTTGACTGACCGGCTCGTAGCTGTCAATACTGCAGT
ACCCACGACCGACGCTGTCGTCCAAATTTCTTCCGAGGAACTCCTTCCAGCATTGAATGAAGTATTTACGCTT
GATACATTTAGAATCAGCTATGTGAGTGTGGCGATCCGCAGTTTTGTGCATCATTTCTTTCCTGCGTATGGTT
CGCCATTCGTTGATTATGCTGATAAAATTCTGGAAGAAGAGAAATAAGCATGATTACATGATTGATTGGTCTT
TAACTGCCATGAAAGGTGTTCTGATTGATATTGCTAGTTACATGAATTTTGATCATGCCAAGGCAGGACGAGC
TGGCAATGATGACCATTTGTAGACAAGTACTAGGTCAACTTTTGCTTTCATAAAGCCAAGCTTGGGTAGAAAT
AAAGATTGGATTGTGATGGGACACCTGGCGAAATAACAGTATTATTAGTCCAACTCAAAAAGTCTCAAAATGG
TGTGAATTATGGCTACTCATGCAGAATGATGCGACCGAAAAAACCATTTTGACTGGGATTTGGCGACGACGGC
GGTATATCTACCACTGCCATTGAAGAGTGCGCAGGTCGTGCTGGACGTAAAACGGGATGCGGTGTTATTACAA
ATAGATCAGCTGGAATCCATGCTAGGTGCTAGATACCATGAAGGAGCAGGAGCAACTCCACAAAGGCCTCCGT
TTACACGCGCTATCCTGGAGCAGGCGGCATGTGTCGGGATGGGCAGCGGCACTAACCTTGTCGCAACATGCGG

GTGCACTGAGGGCATACAACAAGGAGCTATTGGTGAGTGGCTAGCTTTTTGGGCGTTAATCATTCATCCTGAT
ATCTAACCCGTCAACTGGCCTGGTCGAGTTGCCAGCGTGGTGGCCATTATCAACGATAACTACACCTTGGCCA
GCCGCAATATGTCTTTGTTGCTAACTCACAAAGCCCAACTCAGATTACATCATCACAACAGGCCAAATCTCGA
TCACAGCGTACAATGTGCATGGGCCCATCTTCGAGCTCGCAGGGAAAGGGGGAGAAAACGTAGGTCCAGGCCT
CGTCAGCTTTGGTGGGACCCACCGATGGAGTTGGATGGGACGAGGAACGCAGCGGATGTCAAACCAGATTTCG
GGGGATGCGAAGCTACTACTTTGTAGTCGCAAATGATGACCCTCGGGTACTCACGTTGGCTAGGTGAGTAATA
CAGTGGATGGGTTACACGGGAGTTCAGGATTGTTCTCTCGAATATCGTAGTTTCTGCGTTTTCTTAGCAGCCC
TTTAATCTTATCATAAGCAAGAGGTTAGACAGCGGACAGAGCCTTGCCCAATCCTGAGGGATCTGAGTATGGG
TAAGACTGTACGCCGCCAGTACCCAAGTTAAGACACACACACTAAGCACACACTAAGCACACACTAAGCACAC
ACTAAGCACACACTAAGCACACACTAAGC
>TR539|c0_g1_i4 len=2281 path=[2850:0-581 2885:582-805 2848:806-826
2881:827-914 @2884@!:915-1783 2862:1784-1805 2880:1806-2031 2873:2032-
2055 2877:2056-2236 2853:2237-2247 2854:2248-2258 2855:2259-2269
2856:2270-2280] [-1, 2850, 2885, 2848, 2881, 2884, 2862, 2880, 2873,
2877, 2853, 2854, 2855, 2856, -2]
GAGTTCGCGAACGGCGTGCTCGTCGGCGGGTACGACCCGTTATTCGACGGCTACCCGTCGGTGAGGAGCGTGC
CTTCTGAAGAATCGCGGCAGAAAATAGTCAAGGGTGCGGAATTCTCGTCTCTTTCGGGGGCTATTTACGAAGA
TGCAATTGCTAACACTCGTGGTCTTGGACATAGCATCGTTGCCGAGGGGAAGACTGCAGATATCGGATGGATG
GTCACGGACAGCATACAGTACGAGCGAGATTTTTGGGCAGACTCGGAGCGGAAAGAGCCGACTCTGGTCAGGA
CTTTCGTCATTCGCGGCTATGATGCGAGTGATGAGGGTGTAGACAGAGAGCGTTTGTTGAATGTTATCTGCAC
GGCATCGCCAGTCCCTATATACAAGAGCGAGCAGGCTTTGGTTCAGGTTCACGAAGGGATGCTCTCGATTGCG
CGCGAGCTATTGCAAGAACTCGAGAAATATATTGATTTGACATCTCCTTCGCACAAGTTTGTTTTCACGGGAC
ACTCTATCGGTGGCTCTCTTGCAATCTTACTGACGATTCTCCTGGCTAATGACCGTGGAGCGAGTTTTGTGAG
GGATAATGTCCTTCGTGTCTTTACCTATGGGTCTCCTCCAATTTTTGAGATAGAGTCCACTTCAAGCACTAAA
CGGGATGGATATCTCGATGATTCATGCTCGATTCTTGATGCATTTGATTTACCAACTGACCTTGTATACGGCT
ATTGCCAACCCTGGGATCCAATCGTGAGATTTTATTCAAAGTATGATCCTTTATACCCTTTGATCGATGATCT
TGGCGAGGATGGAATCACTCTATTTGATTCTGGTCCGAGTAGAACTCTCCGGCCAATTATGAAGACTATCCTT
GAGTCATGGGAAGGATGGCCTGGATACAGAGACAATGCTCGGGCGAAGTTGGGACAAGATTATAGAGGCGTCG
GAGAACATTTTCTATTGTTGCCCGAGCCTATCCGGTATTTGACTGACCGGCTCGTAGCTGTCAATACTGCAGT
ACCCACGACCGACGCTGTCGTCCAAATTTCTTCCGAGGAACTCCTTCCAGCATTGAATGAAGTATTTACGCTT
GATACATTTAGAATCAGCTATGTGAGTGTGGCGATCCGCAGTTTTGTGCATCATTTCTTTCCTGCGTATGGTT
CGCCATTCGTTGATTATGCTGATAAAATTCTGGAAGAAGAGAAATAAGCATGATTACATGATTGATTGGTCTT
TAACTGCCATGAAAGGTGTTCTGATTGATATTGCTAGTTACATGAATTTTGATCATGCCAAGGCAGGACGAGC
TGGCAATGATGACCATTTGTAGACAAGTACTAGGTCAACTTTTGCTTTCATAAAGCCAAGCTTGGGTAGAAAT
AAAGATTGGATTGTGATGGGACACCTGGCGAAATAACAGTATTATTAGTCCAACTCAAAAAGTCTCAAAATGG
TGTGAATTATGGCTACTCATGCAGAATGATGCGACCGAAAAAACCATTTTGACTGGGATTTGGCGACGACGGC
GGTATATCTACCACTGCCATTGAAGAGTGCGCAGGTCGTGCTGGACGTAAAACGGGATGCGGTGTTATTACAA
ATAGATCAGCTGGAATCCATGCTAGGTGCTAGATACCATGAAGGAGCAGGAGCAACTCCACAAAGGCCTCCGT
TTACACGCGCTATCCTGGAGCAGGCGGCATGTGTCGGGATGGGCAGCGGCACTAACCTTGTCGCAACATGCGG
GTGCACTGAGGGCATACAACAAGGAGCTATTGATTACATCATCACAACAGGCCAAATCTCGATCACAGCGTAC
AATGTGCATGGGCCCATCTTCGAGCTCGCAGGGAAAGGGGGAGAAAACGTAGGTCCAGGCCTCGTCAGCTTTG
GTGGGACCCACCGATGGAGTTGGATGGGACGAGGAACGCAGCGGATGTCAAACCAGATTTCGGGGGATGCGAA
GCTACTACTTTGTAGTCGCAAATGATGACCCTCGGGTACTCACGTTGGCTAGGTGAGTAATACAGTGGATGGG
TTACACGGGAGTTCAGGATTGTTCTCTCGAATATCGTAGTTTCTGCGTTTTCTTAGCAGCCCTTTAATCTTAT
CATAAGCAAGAGGTTAGACAGCGGACAGAGCCTTGCCCAATCCTGAGGGATCTGAGTATGGGTAAGACTGTAC
GCCGCCAGTACCCAAGTTAAGACACACACACTAAGCACACACTAAGCACACACTAAGCACACACTAAGCACAC
ACTAAGCACACACTAAGC
>TR539|c0_g1_i5 len=2421 path=[2850:0-581 2885:582-805 2879:806-966
2881:967-1054 @2884@!:1055-1923 2862:1924-1945 2880:1946-2171 2873:2172-
2195 2877:2196-2376 2853:2377-2387 2854:2388-2398 2855:2399-2409
2856:2410-2420] [-1, 2850, 2885, 2879, 2881, 2884, 2862, 2880, 2873,
2877, 2853, 2854, 2855, 2856, -2]
GAGTTCGCGAACGGCGTGCTCGTCGGCGGGTACGACCCGTTATTCGACGGCTACCCGTCGGTGAGGAGCGTGC
CTTCTGAAGAATCGCGGCAGAAAATAGTCAAGGGTGCGGAATTCTCGTCTCTTTCGGGGGCTATTTACGAAGA
TGCAATTGCTAACACTCGTGGTCTTGGACATAGCATCGTTGCCGAGGGGAAGACTGCAGATATCGGATGGATG
GTCACGGACAGCATACAGTACGAGCGAGATTTTTGGGCAGACTCGGAGCGGAAAGAGCCGACTCTGGTCAGGA
CTTTCGTCATTCGCGGCTATGATGCGAGTGATGAGGGTGTAGACAGAGAGCGTTTGTTGAATGTTATCTGCAC
GGCATCGCCAGTCCCTATATACAAGAGCGAGCAGGCTTTGGTTCAGGTTCACGAAGGGATGCTCTCGATTGCG
CGCGAGCTATTGCAAGAACTCGAGAAATATATTGATTTGACATCTCCTTCGCACAAGTTTGTTTTCACGGGAC
ACTCTATCGGTGGCTCTCTTGCAATCTTACTGACGATTCTCCTGGCTAATGACCGTGGAGCGAGTTTTGTGAG

GGATAATGTCCTTCGTGTCTTTACCTATGGGTCTCCTCCAATTTTTGAGATAGAGTCCACTTCAAGCACTAAA
CGGGATGGATATCTCGATGATTCATGCTCGATTCTTGATGCATTTGATTTACCAACTGACCTTGTATACGGCT
ATTGCCAACCCTGGGATCCAATCGTGAGATTTTATTCAAAGTATGATCCTTTATACCCTTTGATCGATGATCT
TGGTGAGTGCGAACGTTTTAGACTTCTATGGATTCAATCGCATGATGCCAACCGGTACAGGTAGCCACTCTCT
ATTTCCCTGAACTGATTCTGTCAGTTGCTCACCAAATGATAACTTTATTCTGAAACCCGTTTTAAACAGGCGA
GGATGGAATCACTCTATTTGATTCTGGTCCGAGTAGAACTCTCCGGCCAATTATGAAGACTATCCTTGAGTCA
TGGGAAGGATGGCCTGGATACAGAGACAATGCTCGGGCGAAGTTGGGACAAGATTATAGAGGCGTCGGAGAAC
ATTTTCTATTGTTGCCCGAGCCTATCCGGTATTTGACTGACCGGCTCGTAGCTGTCAATACTGCAGTACCCAC
GACCGACGCTGTCGTCCAAATTTCTTCCGAGGAACTCCTTCCAGCATTGAATGAAGTATTTACGCTTGATACA
TTTAGAATCAGCTATGTGAGTGTGGCGATCCGCAGTTTTGTGCATCATTTCTTTCCTGCGTATGGTTCGCCAT
TCGTTGATTATGCTGATAAAATTCTGGAAGAAGAGAAATAAGCATGATTACATGATTGATTGGTCTTTAACTG
CCATGAAAGGTGTTCTGATTGATATTGCTAGTTACATGAATTTTGATCATGCCAAGGCAGGACGAGCTGGCAA
TGATGACCATTTGTAGACAAGTACTAGGTCAACTTTTGCTTTCATAAAGCCAAGCTTGGGTAGAAATAAAGAT
TGGATTGTGATGGGACACCTGGCGAAATAACAGTATTATTAGTCCAACTCAAAAAGTCTCAAAATGGTGTGAA
TTATGGCTACTCATGCAGAATGATGCGACCGAAAAAACCATTTTGACTGGGATTTGGCGACGACGGCGGTATA
TCTACCACTGCCATTGAAGAGTGCGCAGGTCGTGCTGGACGTAAAACGGGATGCGGTGTTATTACAAATAGAT
CAGCTGGAATCCATGCTAGGTGCTAGATACCATGAAGGAGCAGGAGCAACTCCACAAAGGCCTCCGTTTACAC
GCGCTATCCTGGAGCAGGCGGCATGTGTCGGGATGGGCAGCGGCACTAACCTTGTCGCAACATGCGGGTGCAC
TGAGGGCATACAACAAGGAGCTATTGATTACATCATCACAACAGGCCAAATCTCGATCACAGCGTACAATGTG
CATGGGCCCATCTTCGAGCTCGCAGGGAAAGGGGGAGAAAACGTAGGTCCAGGCCTCGTCAGCTTTGGTGGGA
CCCACCGATGGAGTTGGATGGGACGAGGAACGCAGCGGATGTCAAACCAGATTTCGGGGGATGCGAAGCTACT
ACTTTGTAGTCGCAAATGATGACCCTCGGGTACTCACGTTGGCTAGGTGAGTAATACAGTGGATGGGTTACAC
GGGAGTTCAGGATTGTTCTCTCGAATATCGTAGTTTCTGCGTTTTCTTAGCAGCCCTTTAATCTTATCATAAG
CAAGAGGTTAGACAGCGGACAGAGCCTTGCCCAATCCTGAGGGATCTGAGTATGGGTAAGACTGTACGCCGCC
AGTACCCAAGTTAAGACACACACACTAAGCACACACTAAGCACACACTAAGCACACACTAAGCACACACTAAG
CACACACTAAGC
>TR539|c0_g2_i1 len=692 path=[2875:0-581 2876:582-691] [-1, 2875, 2876, -2]
GAGTTCGCGAACGGCGTGCTCGTCGGCGGGTACGACCCGTTATTCGACGGCTACCCGTCGGTGAGGAGCGTGC
CTTCTGAAGAATCGCGGCAGAAAATAGTCAAGGGTGCGGAATTCTCGTCTCTTTCGGGGGCTATTTACGAAGA
TGCAATTGCTAACACTCGTGGTCTTGGACATAGCATCGTTGCCGAGGGGAAGACTGCAGATATCGGATGGATG
GTCACGGACAGCATACAGTACGAGCGAGATTTTTGGGCAGACTCGGAGCGGAAAGAGCCGACTCTGGTCAGGA
CTTTCGTCATTCGCGGCTATGATGCGAGTGATGAGGGTGTAGACAGAGAGCGTTTGTTGAATGTTATCTGCAC
GGCATCGCCAGTCCCTATATACAAGAGCGAGCAGGCTTTGGTTCAGGTTCACGAAGGGATGCTCTCGATTGCG
CGCGAGCTATTGCAAGAACTCGAGAAATATATTGATTTGACATCTCCTTCGCACAAGTTTGTTTTCACGGGAC
ACTCTATCGGTGGCTCTCTTGCAATCTTACTGACGATTCTCCTGGCTAATGACCGTGGAGCGAGTTTTGTGGT
AAGATCCATAGATACACTGTTGACATGTGCTCCTATGATTGATGTCAACTAATACAACACGTGTGTTGTTGTT
TTACACACGCAAGAGGGATAATGTCCTTCGTGTCT
>TR540|c0_g1_i1 len=1402 path=[1513:0-344 1554:345-497 1553:498-642 @1551@!:643-852 1555:853-858 1546:859-874 1552:875-881 1550:882-934 @1548@!:935-1126 1516:1127-1401] [-1, 1513, 1554, 1553, 1551, 1555, 1546, 1552, 1550, 1548, 1516, -2]
CTCCTTCCATGGAGAATACTGTTTGCGTAGCGAACCGCGATGATAGTTGTTGTTGCTCTACCAACATGATGAT
GATGATGATGCAACAGCAACAACAACATCATTCCTCTACTACGAAGAATAGTATGAGAATACTCGATGCAGGA
TTCATTCTAACAACTCCAATATATACCTATTTGGCGACGCATCGACACGTGAATGATTACTTGGCCATGGGGA
ATTCGATCTTGTTGACGTTGCCGTTTTTATACTGCATATATGTCACTCTTTGGAAGGGCGATTTTCGACTTTC
GTTTCGATTGATTGCTACTCATTTATTTCGTTCGTTGTGTGGTTGGTTCACGTGAGTCTCTATGGATATTGAG
AATGAATCGTCATGTACATTGCCTATCTTGTACTTGTGTGTAGCTTCGTGCCAATCTAAGGTTACTCATCACT
CTCAAAATGTGTGATTCTCGCTCTCGTCCGAAAATAGGTACCTGCCTCCCGACCCAGAGTTTTTATCGAGTTC
GTACGACTTTCCCGAAATATTCCTGTGTTTGTTTCAAGAATGCTCGAGCAAGAATAATGCAGAAGAAGTCAAT
TTCTTGACATTCTTTTCGGGGCATGTTGCAACCATCGCAATAATAGCAAATCATCTGTACATGTCAAAACACA
CCCACCTCTCCATCGCTCTCCATACTTTCAACTGGCTCCAAGCCATCCGACTACTCGCTACCCGCGGCCACTA
CTCCATCGATCTCATCATCGGATATGTCGTGTCGGTATTCGTATTATCGAGTCCCGCCGAACGTCTCGGTTTG
TATTATTCACGAGGCATACAGCCCACCTTACCCAATATCGTGGAAACGTTTGAGATATTGGTGGGGGTGGGCG
TTGCTGAATCGAGTGGAGGTAGCAATGGCAACAGTAACATCAACAGCAACAACCAAGAAAACAAATCAATAGC
AGTATCACAACAATCCACAGTCATTCGAGCCGATGATAAACCTGAAACGAGTAGTAGTCATCATAATGGTGGG
TTGTATGATGATGTTCATTCAGAAACGAGTGTGAGAATTGCAGTGGATATCGTGACAAATATAGCTCAAATGA
AAATCGCGAGTAAAAATGAGCAGTAGACGAGAGTGAAACAATGATGCAATATCATGTAGGGTGATACAACATA
AAATAATATTGGAGAGAAGAGATGTTTAGGTAACCAAGTGAGATTGACCATATAAAACCAGTGTGAGACCATC
GAGGGAGATATTGAGCTGTTGTCAAGTATGAAAACGTGTTATTCAGAGTAAGTTTGATGGTTCTATTATACGC

TCGCGAATTTATTCTGTTAGATTAAAGTTGCATACAGCAGGCATTCATTTATTCATACAAACACAGCAATGTG
CCCTAGGGTCGTATT
>TR540|c0_g1_i2 len=1270 path=[1517:0-344 1518:345-365 1553:366-510 @1551@!:511-720 1555:721-726 1546:727-742 1552:743-749 1550:750-802 @1548@!:803-994 1516:995-1269] [-1, 1517, 1518, 1553, 1551, 1555, 1546, 1552, 1550, 1548, 1516, -2]
CTCCTTCCATGGAGAATACTGTTTGCGTAGCGAACCGCGATGATAGTTGTTGTTGCTCTACCAACATGATGAT
GATGATGATGCAACAGCAACAACAACATCATTCCTCTACTACGAAGAATAGTATGAGAATACTCGATGCAGGA
TTCATTCTAACAACTCCAATATATACCTATTTGGCGACGCATCGACACGTGAATGATTACTTGGCCATGGGGA
ATTCGATCTTGTTGACGTTGCCGTTTTTATACTGCATATATGTCACTCTTTGGAAGGGCGATTTTCGACTTTC
GTTTCGATTGATTGCTACTCATTTATTTCGTTCGTTGTGTGGTTGGTTCACGTACCTGCCTCCCGACCCAGAG
TTTTTATCGAGTTCGTACGACTTTCCCGAAATATTCCTGTGTTTGTTTCAAGAATGCTCGAGCAAGAATAATG
CAGAAGAAGTCAATTTCTTGACATTCTTTTCGGGGCATGTTGCAACCATCGCAATAATAGCAAATCATCTGTA
CATGTCAAAACACACCCACCTCTCCATCGCTCTCCATACTTTCAACTGGCTCCAAGCCATCCGACTACTCGCT
ACCCGCGGCCACTACTCCATCGATCTCATCATCGGATATGTCGTGTCGGTATTCGTATTATCGAGTCCCGCCG
AACGTCTCGGTTTGTATTATTCACGAGGCATACAGCCCACCTTACCCAATATCGTGGAAACGTTTGAGATATT
GGTGGGGGTGGGCGTTGCTGAATCGAGTGGAGGTAGCAATGGCAACAGTAACATCAACAGCAACAACCAAGAA
AACAAATCAATAGCAGTATCACAACAATCCACAGTCATTCGAGCCGATGATAAACCTGAAACGAGTAGTAGTC
ATCATAATGGTGGGTTGTATGATGATGTTCATTCAGAAACGAGTGTGAGAATTGCAGTGGATATCGTGACAAA
TATAGCTCAAATGAAAATCGCGAGTAAAAATGAGCAGTAGACGAGAGTGAAACAATGATGCAATATCATGTAG
GGTGATACAACATAAAATAATATTGGAGAGAAGAGATGTTTAGGTAACCAAGTGAGATTGACCATATAAAACC
AGTGTGAGACCATCGAGGGAGATATTGAGCTGTTGTCAAGTATGAAAACGTGTTATTCAGAGTAAGTTTGATG
GTTCTATTATACGCTCGCGAATTTATTCTGTTAGATTAAAGTTGCATACAGCAGGCATTCATTTATTCATACA
AACACAGCAATGTGCCCTAGGGTCGTATT
>TR540|c1_g1_i1 len=348 path=[326:0-347] [-1, 326, -2]
GATGATGAGATCGATGGAGTAGTGGCCGCGGGTGGCGAGTAGTCGGATGGCTTGGAGCCAGTTGAAAGTATGG
AGAGCGATGGAGAGGTGGGTGTGTTTTGACATGTACAGATGATTTGCTATTATTGCGATGGTTGCAACATGCC
CCGAAAAGAATGTCAAGAAATTGACTTCTTCTGCATTATTCTTGCTCGAGCATTCTTGAAACAAACACAGGAA
TATTTCGGGAAAGTCGTACGAACTCGATAAAAACTCTGGGTCGGGAGGCAGGTACCTATTTTCGGACGAGAGC
GAGAATCACACATTTTGAGAGTGATGAGTAACCTTAGATTGGCACGAAGCTACACA
>TR541|c0_g2_i1 len=2801 path=[2921:0-721 2939:722-790 2919:791-814 2941:815-1078 2924:1079-1102 2938:1103-1417 2932:1418-1441 2940:1442-2020 2935:2021-2044 2937:2045-2658 2929:2659-2682 2930:2683-2800] [-1, 2921, 2939, 2919, 2941, 2924, 2938, 2932, 2940, 2935, 2937, 2929, 2930, -2]
TTAACGAAGCTCAAGGACAAATTATCGGATATGAAAATGGACGTCGAGGAGGCCAAGGCAGATAGCGCGATGC
TCGAGGAAGATTTGGAGGAAAAGTGCGTCCAAATCGCCGAATTGGAAGTGGCGTTGGGCGAGAAAGACGATCG
AATTGCTAGGTTGAACGAGGAACTGGATGTCTTGATACAGGTGGAGAACGGCGATGCCTCTGCCGCAGGCACT
GGAAGCAGAAGCAGTCGTAGTGCGGATTCTGTGCAATCTGGAAGCAGCGGTGAGCGAGACACGGCATCGATGC
TCGAAGATCGATTGGATGCCGTGGAGGAAACTCTTGAGTACACTGATGGCGAGTTGGTCGAAACCAGGGCGAG
GCTTGCTGACACCCAAGAACTCTTGGACCAAACAGCAGGCGAGCTCGAACGTTCTGAGGAGGAATTGGCCGAT
GTTCGTGACCAGGTACAAGACTATGAGGTCCAGGTGAACAACTTGTCCAGGGACCTTGCGGAAAAGGAAGTGG
AATGCGCCAACATTACGAAGTTCAGCGATTTCCAAACCACGACGTTGGAGACGTTGAAGGAGAAGCTGTCACG
GAGTGATACAGTCAATGTCGAGTTGCGCACACAGCTCAATTCTTGCTTTCAATCTTTGGTGGATCTGGAAAGG
ATCCTCAGAACGTACGAGGACTTGGACGGTCATGTGGGGAAGAAGATGGCCGAGCAGAGCCGCAAGATTGCAC
GTCTGCTGCGAACCATGGAGCAATTTGTGCAGGATCGTTCTGCATCGCCAGCTCGTGATTTGGAATCGAGAGC
AATAGCGCCGATGGCGCACGAGGAGAAGAACATTGCAGGATCGGGCTCGGAAGAGTCTTCCTCAACATCATTA
AGAAGCACACGAAGTGCGCACTACCAGGAGGAACTAAATGGATTGCGTGAGGAGATTGACGCTGCTGAGATGG
AGCGAGATGATGCCAACAAACAGCTTCAAAAAGCGCACGATTTCCTCCAAGAGTTACGCGATGAATTATCGAA
GCAAGAGGATGAACATCTTGAAGAGACAACGTCTCTCAAGAAGCAGTGTGAAGAGCTGGAAGCTAGGTTATCG
GCGGTGGCCTTGGAACTTCCGACCGGCGAGATGGAAAGTCAGAACTTATCGAAAACTGAAGAGAATGAAGTTT
ATCAGCAATTATCAGTGATTCGTAGCAACAATGCTGAGCTCATTCAAGAAAATGAGAAGCTCCGTGTTGATAT
CCAATCCTTCGAGCTGCAGCTCGAGAACGAGAAAGCGTTGTTGCGTGCCACTCGCGAAGAGGCCAACGGCTAC
CGGCAAGATGTCTCGTTAGGCAAGGCCGCGTTTGAATGCTTGACAACCGAAGCGTCCCTGACAAAAATCCAAC
TTGAGAAAGAAGGCAAAGGTTTGCGAGAAATTGTTGCGCAAAAAGAGAAGGAGCTCAAGGGTTACCAGGAAAA
TCTATCCAAGGCACAAACCTCATTTCAGGAACACACTGAATCGATGAAATCCCATTGTATTGACCTTGAGAGG
AAAATATCGGACGAAGAAAAGTCTCGCGTCAGTGCAGAGGCATCATTGGATGATGCTTCCAAGCGCGTTGCCA
TGTTGGAGGATGCGATTAAAGCAAAGGAAGAGGAATGCCAAGAAAAAGAAAATGAATGCCAAGAACTCATCAT
CTCGCTCAAGCGGGTCCAAAATGCCCTGGGCGAGGCTGAGATGGAACGAGGAGAAGCAACAAACACCATTGAG
AGTTTGGAGAATCAAATGCAAGAAAAAACATCTGTCTTATTTGCCTATGAAGAGGCGATCATTGGATACAAGG
ATGACATTCGCAGATTAAATAACGAACTGCAAACAACTGTTTTAGAAAAGAATAATCGCATTCAAATGCTGGA

GCAGGGTTTGACTTCGCGTCAGACGCTATTCACGGAGCAGTTGGATAGGACGAAGGAAGAGCGTGACGCCTCC
ACAGCCGAGCTCACCGACATGATTGAACGTCTCCAGAATGAGCTACAATCCAGTAACAAAACTCACCAAGAAT
CTGGAGAGAGAAATAATCTTGCAATAAAGGACCTCGAGGAAGAAATCTTGAGAAAGAACGAGGCAATAAAAGA
TCTGGAAGAGGAAATCATTAGGAAAGAAGAGAGTCTCGAAGAAAATACCCAGAAGTTGCAACAAGCCCAGAAT
GAGATTGCGCAAGTGATGGACAGACTTAATTCGTCAGAGATGGATTGCGAGCGTATGGTGACGAAGCACCAAG
ATGAAGCGGAGGAATTAGTGGAGGAATTAAATCGTTTGGAGGGTCAGAAGAACCAAGTTGAGAAGCAAATTCA
ACTACTGCGATCTAACATGGAAGAAAACGAGGAAATTAGAGAAAAGGAGAGACATGAGTTGGATAGACTTTCG
GCTCAAAATGCTTCCTTGCAAAAGAAGTGCAACATCTTCAAAGATAAAATCAAATCGCTGAACGAAAAGAATA
AGGCATGGGAAGTTAGCTACAAAGCTCAGAGCGATGACCTTGTCTTCCATGGGATGGAAATATCGCGATTGAA
CGGCCAGGTTTCGGATTTGAAGGCTCGGTTGATACGCCAGGGTTCTGGCCGATATCTTGTAAACCGTAACGAA
TTGGCGCCGTCTGCTCAAAGTACCCCTCAGGGCGGGGCACACTACCTGGGCAACCCAAACACTATGTGATTTC
GAGCGCAGAAGCCAAAACTAGCGCGCTACTTATGGATTGATTGGAAAGTTGTATAATTTACATATTAGTTGTC
AAATCATAGCACAATATTATCTAGATC
>TR542|c0_g1_i1 len=2810 path=[2956:0-60 2957:61-84 2975:85-636 2972:637-878 2946:879-902 2974:903-1402 2954:1403-1426 2971:1427-1564 2951:1565-1588 2976:1589-2266 2962:2267-2290 2973:2291-2398 2965:2399-2415 2966:2416-2809] [-1, 2956, 2957, 2975, 2972, 2946, 2974, 2954, 2971, 2951, 2976, 2962, 2973, 2965, 2966, -2]
TTTTTTTTCTGATATAATCTCGTTCTAAAACACTTATGGCACATAATCAATGGTTCTTTTAAAAAGTCCTTCT
ATTCATTTTTCATAGTTGAAGCTTCAATCCTCATCGTGGTGCTTTCATTTTTTATTTACACTTCTTTCCGTTT
GTCCTTTTGTGGACGACAGTAGACACTGCTAATATTCAACGCTACATAAGAATTATCCTGCTGGATAAAACAA
AGTCTACGATACTATTCATTCCCCGATCACAGTCTAAATGTTTAAGAACCGCTCATTTATTGTAGCCTCCCCA
AAAGCTGCAAGTTGTACCCTCTCTGTTGACAATGACATGGGGCGTTACAAGTTGAGGGCGCAAAGAGTATCCC
TCTGGAAGAATAGTGTTATTGTGCCTTTGCGTTTTATGCTTTAGCAGATTTCGAAACTCTTCCTGACTTGGCC
TTGGACGAGGACTCTTTCAACACACCTGGTCTGATCATCAAGCCAAACTTGTGAACAGATCTGCGGGGCTGGC
TGCCTGCAGGCTTTCCACCATATAACAAACGATCTTCTGACAAATGACGTCCATTGCTTGCACTATCCACGAT
GTTGATCTGAGCCAACTTTGATTCGGCAGCTGAAATTCCATCAATGGAAGAAGTGGGCGAAACAGATTTTTGA
TTCACCCTGGTTTGGTCTTTAGATGAAGTTGAGTTTGAGGAAAGATACGAGCTTTTGTACAATGGCGTCATTG
AAACAGGGACATCGTTTCGAGGTGCAATGGCATCCCTTGAGTTGGCACGAGATGTTTGATGCGTGACTGCCAC
ATCTGTCATGGTCCCTTTTTCACCAGAAGGAATGTAAGTCTGTGTAGAATTATCCCGCTTCTGCACGTTGCTG
TTAGCAATGGTTCGGACCTGTGAAGCTTGTGCTTCTGGACCATCTACCTCATTCTCATACTCGCTTTCATCTT
CGTCCTCCTCCACCACCTCATAAGAACCGACAGATTCTTCGAGGCTTTCCATGCTCTCCACACCGTCAGCATC
ATTTACGTTGTCATTCATGCCACTATCACCACCGCACACAGGCAAACGAATTCTGCTTGCCAGTGTTCTGCCT
GAGACACCACATTCTCCAAACTGCCAGAGTGGGACAGTCATGGCATCTACTAATACTTTCAAGTTATCGAGTT
CTTCTCTCAAGTGGCCTTCAGCTTCCTGTCTTTCAAGGATCATATTGCGGTTTGAGCTCTGAAGTTCAACAAG
ATTTTCCTCGAGATATGTCACGTGCTGCTCTCTGTTCGACATTTTTTCATTGTATGTTTTATTGATGAGGTGG
AGCTCATTCATCTTCCATATGATAGCTTGCAAATCATGGGTCTTTTTGTCCAGCTGCTGCTTCAATTGAATTG
ACTCCTGAGCGAATTTCTTCTTGTTTAAATTACCGGGTCGGTTAGTCAAAATGTCAACATTTGCCTTCTCTTC
GTTCAGGGCAACTTGAAGCTCGATGCATCTTTCTGTTGCTCTGAGAAGATCACGTTCAAGTTCTCTCTCACGC
ATTGTGGTCAATCGTCCTGCAGGAGTGCCTAAGAATTGTTGGCGATTGGATATTGATTGACCATGAGGTGCTG
ACGCTCCTGCCCCAGCATCTGCTCCACCATAGGCGTCATCACCGGCATTAGCCCCAACCATTCTTTTGCCCTC
TAAGGATGAACGATCAAATGATTCTAATTCATCAACCTTACCATGAAGTGCAGCCTCTTTCCATGCCTTGTTG
GTGGCATCTGTACTACGCCCCAAAGCCTGGGCACTTGGGCGATATGCTCCAGTAGCAGTGTCATCGTGCAACT
CTGATTCCTCAATGAGTCCAGCTTCAAGCAAAAGACTATCCACATCTATTAAATCGTTCAAGTCTGGAACACC
GGGAACCCTCTCGATGACTTCTGATGTCCTGCCAGTCCCTTGGTGAGCTTTATTCCTCAAAAGTCTGCGATAA
AAGCGTCGGAATTGTCGAAGAAATACAGTCGCTTCGTATTCACGAAATTGACTTGTCCGCAGATTATGCTCCA
CCTCTTGCTTCCTTTGTGTCAACAATCGTATCTCGTTTCTCATGGCAACCAATTCTTGCGTATCTTTCTTCTT
CTCCGAGGCATAATTATCATTTTGCGTCCTCAAAACTGCCACCTCCGACTGCCGTTCTGCCAAGAGATTCTCC
ATATTTCCACGCCTCTGTTTCAAGCTTTCAAGTTCGCCTTGTGTCCTCGAGAGCCTTTCCTCGAGCGCAGTTA
CATCTACGTTAGAATTCGATCCCCGCCCGCTCCAATTGGATTTGCTACCCACCGTGTATGTGCCATTTACGAG
TATCGACTCAATCGTTTTCCATAGCGGTCCGTCATAATCTGATTCTACAAATTTTTGTTTTTTCGCATCTTGC
TTCAATTGAAAGCACTCGGCAGATAGATCGTTCACCAGACTAATCAGATCCCCTTGCTTCTTTTGCGAATCGT
TCAGTATTTTCCGATAATCAAGCGGTGACATTTCCACACTAGGCTTGACGGAATTCTTCACACTTCGACAAAA
GTGTCCGAATTTCATCGTCCTTATGGATTCTGATATATTGTAGGATGCCGGCGACGCAGTGAGAATGACAGAC
GTACGGCAATTGCCCCCGAATGCGTCCCCAAGAAGATGGGTGAGCTTGGACTGATTGTAGGGAACTGCATGCA
AGCTCCCCGCAGAATCCTTGCCCTTACTTTCCACATCTTGATTCTCCACCAATGTTTTCACGACGGTATTGAG
CAACGAGAGGGATTTGTTTATCATCCTCGCCTCTTG
>TR543|c0_g1_i1 len=3463 path=[3872:0-1340 3873:1341-1364 3906:1365-1386 3902:1387-1409 @3912@!:1410-2037 3849:2038-2058 3909:2059-2139 3913:2140-2140 3907:2141-2243 3883:2244-2267 3904:2268-2360 3914:2361-2362

3887:2363-2399 3888:2400-3462] [-1, 3872, 3873, 3906, 3902, 3912, 3849, 3909, 3913, 3907, 3883, 3904, 3914, 3887, 3888, -2]
GAGGAGGGAGAAGCTGGCGGCGCAGCAGGCGTTCGAGTCCACCGCCTCCGGGAGCGCCGAGTACTACCGCCGC
GCGTGCGCCTTGCAGGATGCGGCATTGGAATTGCTGCGGGTGGGCGTGGCGGATCGGGTCTCCCAAACTTGTG
CTCAGATTCAGGATGCGTTGAATATACCGAAGGTACCTATAGCCGCAGACAAGCTGGAGGCGTCGTTGGTGTA
CACGCGCTTCCACGGGATATCGAAACGGAGTAATGCGTTGATTTCCATTGCGAGGAAGAGGCTGGACGCGCTG
GGGAACGACAGCGTAGCTTCCGCCTCGTACGATGATTTGCTTACATTGTGCCGCAATACCTACTGCGGAGCGA
GAGAAACACTTTTGACTGCCACAGTGCGAAGGCACATGGACATTTTGAAAGAGCGACACGGGTTGGTGGGCAT
GACGAGATTGGCGTCCTTGTTCCTGATTCGATTGTGCACCGTCGAGACGGCATTGTACTTGGACTTCTTTGGG
GACCCGGAAAAACGACGCTATCCTCGGAAGAAAACCAAGGGTGCTTTATCCCAGGTAGACGAGGAGGATGAGA
CGAAGGAAGGTGGCGATGCTGCTGGAGAGAAGAAGGAGGGCGGAGACGAAGGTGAAAAGGTACCCCCGTCACC
TGGATCCCCTCATTTCAAAAGGATGGGATCTGCAGGCAGTCACACTGTTCCTGCTGCGTCCGCTCTTGCCTCC
CAGGTTTTGTCGGAGGATGCCACGTACCGCGATGCCGAGTTCCAATCCGCGCTCGGCAATCTCTGCTCGTCAT
TGCATCGGACCGTCCGCAGGGGACTCGTCTCCGTCTTGGATTTGGACGTATTGTGTCAGGTGGTCAGCGTGTT
GCGGGAGGAGCGTTCCGTGGCGAATAGTTCTCCCACTACTTTGGCGGCGGCACGCGCGGTGAGCGGGGTTATC
AGGGATGCCCAGGAACGGCTCATCTTTTGCGCGAACGGCGCTTTGCACAAGGAGGTGGCGCGGTTCAAACCCA
CCATTAACGATCTGAACTATCCCGGAAAGTTGTTGGGAGAGGAGCCCAAGGTTTCGATTATCATCGAGGATGA
AAGCAATTCCAACCAAAAGAAGGAGGAAACCACTGCGGAGGCGAAGAAGGAGGGAGATCCCAACGACGCAGTG
GCCGCCCAATTGCGCGTGTACGAATCGTGGTTTCCGCCCATGCGCAGCGTGCTACGTATTTTGTCTAAGATCT
TTCGCGTCGTGGAACCCAGAGTCTTCGAGGACATTGCCTTGTCGAGTGTGCAATCTTGCACAAAGAGTCTAAA
GGACGGCAGCGTCCATATTTTGAGGAAGAGCGGTCAGATCCATGCTGATCTGTTTTTGGTGAAGCATTTGCTT
ATCCTTCGCGAGCAGCTTTCTCCCTTCGACATCCAACTTCGTTCGGTTGAACGGCAGCTCGACTTTTCCGAGG
CTGGACGCGCCGTCACTCGTTTCCTCGCCAATCGCAACCGTCGCCTCTTCTCCATGTCTACTGAGAACGCCCT
CATCACGCTGCTGAGAGAAGGCGTTAGCGTCCAGGAGTCGAGCGTGGATAGCAAACGTGATCTTGAGGATTGT
TTGAGAAGCGCTTGCAATGATTTCATCGAACATACCAGTTCGACGCTGTTGGGCCCCATTGGTGGTTTTGTTG
ATCAGTGCAAGAACGTCGCGGGATCAACATCGTCGAGTGCAGATGCACTGGCCAGAGCTCCCTTCACAAACGG
TGTGGTGGTAAAAGGAGTGTTCGCTAGCGCGCTTCAATACTTGGATGGAGAATTGGACAACGTTTCCAAGCAG
ATGAATTTGTACTTGGAGAATGCGGCGACGCAAGGTATTTTATTGAAGCCAGTCGTGAGGAAGGTGACGCGAA
GTTTAGAGGAGGCGAAGCGATTCATTGGGGAGGTTTCTCAGGGGGAGGATAATGGATGGGATTTGGAGGTGAA
AAGTGATGTGTTGAAGTCTATCGAAGAGTACGAGGCCAAGGTGAAGGTTGCTTCATCTAAGAAATAGAGGTAT
AATGTACTCCCTGATTTAAGCACAACATGACGTGGCCAGACAAAATAAGCCCATAGTGGAATGTAGACTTATG
ACGCATCTTATTTGAAATGGAAGGTGAGCATGAGTGATCGTACCATATTAGTTCGGAGTCAATCTACTTTTTG
AACAATCCAAGTATGTAGTCACTTATCGTGGCTAGTTAATGTGAATGTTGGATGATATTTGGACAGAGGTACT
CTGTCTCGATTGATAGGTTGATTCTTTACATCTTAATTTGCCTATTTGTGTGCATACCTCATACCTCACGCCC
TTAGGCATACCAGAACCCATATCACTTCTATTTGGGGTCTTCTATTCAGATTCGAGCCCGAACCAATTAGTTC
TTTCCTCATCGACTCTAGGGATATCGTACCCTTCAAGAGTGCATCGCCAGCGTAATTTTGCCTGTAATGATGC
ACAAATCCACCAATAGCACCCACAAATCTAAGCACTATTCGAGATATTTTTCCAGATGATCGATTTCCCCAAT
CAATTCCAATACTGTGCATAGTACATAATTGATGTTTCAGTGCCACACGCTCAGTGGAATCGCATGTAGTTTC
TCCTCATTTCTTTCCGAACCAACTCTCAGAAGATTTACGGGTAGAACTACTCCTACTGGCAGAGGATGCTCCC
CTCGAAGATCTAGCCGAATACGACCCAGCAGCGGACGACGACGACGATGCTCCTCTCGACCAACTCGATTTCG
TTGGTGCATCCTTGTCCTTCTTCCCCGACTTATTCCAATTAAAACTCGACATCGGCGTAGCCCCTCCTCCCCT
TTTATTCCCTATAAGGGTCCCATTCACATCCCGTAACGAACTCCGATACTCGTTTATTTTGCTAAATCGTTCC
TTCCGTTCCTCCTTCCGCGAATTGTAAGTTTCCTTCCGTTCCTTTTCCTGGCTGGATAGTTTCATGCTTTTCT
TGACGGACTTGGCGGTGGCGACGGCCGCGGCCTTACCGCCCTTGTATGCGGCGACTCCTCCGGCGGTTATGGC
GGCTGCTTCCAAGATGACCATGATTGAATTCTATTTGTGGGTGGGTGGATGTGACGTGCGGAAGTGAAAGGCT
GCCGACCGCCGATACAAGAGGATTCGTCCGAAGTCACGTCTCAAAAATAATGGAAGTGGAAGAAGAACTGTTC
GTTGTTCGCCAACGATTTTCAATAACTCTTTTTGGCTTCAATCTAATACGCTGAATGCTCGCTGGGCGATGAG
AGGGGGTGGCAGGAGGGGAGGAAAGTCTCGCGTCTCGCGTCAACCAGTTGAATAAGTAAGCAATTAAGCGGTC
TATTGCAGAACGGAAAATGTTTTGTATCATACAACAGGCAGCGAAGGTGAAGATGAAGCAGAACAACGGGAAT
GATGAAAAATAAACAAGATTGAGATCGGAAGA
>TR543|c0_g1_i2 len=3376 path=[3872:0-1340 3873:1341-1364 3906:1365-1386 3902:1387-1409 @3912@!:1410-2037 3910:2038-2171 3909:2172-2252 3913:2253-2253 3915:2254-2273 3916:2274-2275 3857:2276-2312 3858:2313-3375] [-1, 3872, 3873, 3906, 3902, 3912, 3910, 3909, 3913, 3915, 3916, 3857, 3858, -2]
GAGGAGGGAGAAGCTGGCGGCGCAGCAGGCGTTCGAGTCCACCGCCTCCGGGAGCGCCGAGTACTACCGCCGC
GCGTGCGCCTTGCAGGATGCGGCATTGGAATTGCTGCGGGTGGGCGTGGCGGATCGGGTCTCCCAAACTTGTG
CTCAGATTCAGGATGCGTTGAATATACCGAAGGTACCTATAGCCGCAGACAAGCTGGAGGCGTCGTTGGTGTA
CACGCGCTTCCACGGGATATCGAAACGGAGTAATGCGTTGATTTCCATTGCGAGGAAGAGGCTGGACGCGCTG
GGGAACGACAGCGTAGCTTCCGCCTCGTACGATGATTTGCTTACATTGTGCCGCAATACCTACTGCGGAGCGA

GAGAAACACTTTTGACTGCCACAGTGCGAAGGCACATGGACATTTTGAAAGAGCGACACGGGTTGGTGGGCAT
GACGAGATTGGCGTCCTTGTTCCTGATTCGATTGTGCACCGTCGAGACGGCATTGTACTTGGACTTCTTTGGG
GACCCGGAAAAACGACGCTATCCTCGGAAGAAAACCAAGGGTGCTTTATCCCAGGTAGACGAGGAGGATGAGA
CGAAGGAAGGTGGCGATGCTGCTGGAGAGAAGAAGGAGGGCGGAGACGAAGGTGAAAAGGTACCCCCGTCACC
TGGATCCCCTCATTTCAAAAGGATGGGATCTGCAGGCAGTCACACTGTTCCTGCTGCGTCCGCTCTTGCCTCC
CAGGTTTTGTCGGAGGATGCCACGTACCGCGATGCCGAGTTCCAATCCGCGCTCGGCAATCTCTGCTCGTCAT
TGCATCGGACCGTCCGCAGGGGACTCGTCTCCGTCTTGGATTTGGACGTATTGTGTCAGGTGGTCAGCGTGTT
GCGGGAGGAGCGTTCCGTGGCGAATAGTTCTCCCACTACTTTGGCGGCGGCACGCGCGGTGAGCGGGGTTATC
AGGGATGCCCAGGAACGGCTCATCTTTTGCGCGAACGGCGCTTTGCACAAGGAGGTGGCGCGGTTCAAACCCA
CCATTAACGATCTGAACTATCCCGGAAAGTTGTTGGGAGAGGAGCCCAAGGTTTCGATTATCATCGAGGATGA
AAGCAATTCCAACCAAAAGAAGGAGGAAACCACTGCGGAGGCGAAGAAGGAGGGAGATCCCAACGACGCAGTG
GCCGCCCAATTGCGCGTGTACGAATCGTGGTTTCCGCCCATGCGCAGCGTGCTACGTATTTTGTCTAAGATCT
TTCGCGTCGTGGAACCCAGAGTCTTCGAGGACATTGCCTTGTCGAGTGTGCAATCTTGCACAAAGAGTCTAAA
GGACGGCAGCGTCCATATTTTGAGGAAGAGCGGTCAGATCCATGCTGATCTGTTTTTGGTGAAGCATTTGCTT
ATCCTTCGCGAGCAGCTTTCTCCCTTCGACATCCAACTTCGTTCGGTTGAACGGCAGCTCGACTTTTCCGAGG
CTGGACGCGCCGTCACTCGTTTCCTCGCCAATCGCAACCGTCGCCTCTTCTCCATGTCTACTGAGAACGCCCT
CATCACGCTGCTGAGAGAAGGCGTTAGCGTCCAGGAGTCGAGCGTGGATAGCAAACGTGATCTTGAGGATTGT
TTGAGAAGCGCTTGCAATGATTTCATCGAACATACCAGTTCGACGCTGTTGGGCCCCATTGGTGGTTTTGTTG
ATCAGTGCAAGAACGTCGCGGGATCAACATCGTCGAGTGCAGATGCACTGGCCAGAGCTCCCTTCACAAACGG
TGTGGTGGTAAAAGGAGTGTTCGCTAGCGCGCTTCAATACTTGGATGGAGAATTGGACAACGTTTCCAAGCAG
ATGAATTTGTACTTGGAGAATGCGGCGACGCAAGGTATTTTATTGAAGCCAGTCGTGAGGAAGGTGACGCGAA
GTTTAGAGGAGGCGAAGCGATTCATTGGGGAGGTTTCTCAGGGGGAGGATAATGGATGGGATTTGGAGGTGAA
AAGTGATGTGTTGAAGTCTATCGAAGAGTACGAGGCCAAGGTGAAGGTTGCTTCATCTAAGAAATAGGTACGA
GTTGTTCAAGAGTTGTGACCACTCTCTCTGCTGAACAGTAAAATCTAAGGCTGTACTGTATCATTTTGCACCG
AACGCAATCATTCGCTGCCCCCTCCCAACCTCAGAGGTATAATGTACTCCCTGATTTAAGCACAACATGACGT
GGCCAGACAAAATAAGCCCATAGTGGAATGTAGACTTATGACGCATCTTATTTGAAATGGAAGGCATACCAGA
ACCCATATCACTTGTATTTGGGGTCTACTATTCAGATTCGAGCCCGAACCAATTAGTTCTTTCCTCATCGACT
CTAGGGATATCGTACCCTTCAAGAGTGCATCGCCAGCGTAATTTTGCCTGTAATGATGCACAAATCCACCAAT
AGCACCCACAAATCTAAGCACTATTCGAGATATTTTTCCAGATGATCGATTTCCCCAATCAATTCCAATACTG
TGCATAGTACATAATTGATGTTTCAGTGCCACACGCTCAGTGGAATCGCATGTAGTTTCTCCTCATTTCTTTC
CGAACCAACTCTCAGAAGATTTACGGGTAGAACTACTCCTACTGGCAGAGGATGCTCCCCTCGAAGATCTAGC
CGAATACGACCCAGCAGCGGACGACGACGACGATGCTCCTCTCGACCAACTCGATTTCGTTGGTGCATCCTTG
TCCTTCTTCCCCGACTTATTCCAATTAAAACTCGACATCGGCGTAGCCCCTCCTCCCCTTTTATTCCCTATAA
GGGTCCCATTCACATCCCGTAACGAACTCCGATACTCGTTTATTTTGCTAAATCGTTCCTTCCGTTCCTCCTT
CCGCGAATTGTAAGTTTCCTTCCGTTCCTTTTCCTGGCTGGATAGTTTCATGCTTTTCTTGACGGACTTGGCG
GTGGCGACGGCCGCGGCCTTACCGCCCTTGTATGCGGCGACTCCTCCGGCGGTTATGGCGGCTGCTTCCAAGA
TGACCATGATTGAATTCTATTTGTGGGTGGGTGGATGTGACGTGCGGAAGTGAAAGGCTGCCGACCGCCGATA
CAAGAGGATTCGTCCGAAGTCACGTCTCAAAAATAATGGAAGTGGAAGAAGAACTGTTCGTTGTTCGCCAACG
ATTTTCAATAACTCTTTTTGGCTTCAATCTAATACGCTGAATGCTCGCTGGGCGATGAGAGGGGGTGGCAGGA
GGGGAGGAAAGTCTCGCGTCTCGCGTCAACCAGTTGAATAAGTAAGCAATTAAGCGGTCTATTGCAGAACGGA
AAATGTTTTGTATCATACAACAGGCAGCGAAGGTGAAGATGAAGCAGAACAACGGGAATGATGAAAAATAAAC
AAGATTGAGATCGGAAGA
>TR543|c0_g1_i3 len=3576 path=[3872:0-1340 3873:1341-1364 3906:1365-1386 3902:1387-1409 @3912@!:1410-2037 3910:2038-2171 3909:2172-2252 3913:2253-2253 3907:2254-2356 3883:2357-2380 3904:2381-2473 3914:2474-2475 3887:2476-2512 3888:2513-3575] [-1, 3872, 3873, 3906, 3902, 3912, 3910, 3909, 3913, 3907, 3883, 3904, 3914, 3887, 3888, -2]
GAGGAGGGAGAAGCTGGCGGCGCAGCAGGCGTTCGAGTCCACCGCCTCCGGGAGCGCCGAGTACTACCGCCGC
GCGTGCGCCTTGCAGGATGCGGCATTGGAATTGCTGCGGGTGGGCGTGGCGGATCGGGTCTCCCAAACTTGTG
CTCAGATTCAGGATGCGTTGAATATACCGAAGGTACCTATAGCCGCAGACAAGCTGGAGGCGTCGTTGGTGTA
CACGCGCTTCCACGGGATATCGAAACGGAGTAATGCGTTGATTTCCATTGCGAGGAAGAGGCTGGACGCGCTG
GGGAACGACAGCGTAGCTTCCGCCTCGTACGATGATTTGCTTACATTGTGCCGCAATACCTACTGCGGAGCGA
GAGAAACACTTTTGACTGCCACAGTGCGAAGGCACATGGACATTTTGAAAGAGCGACACGGGTTGGTGGGCAT
GACGAGATTGGCGTCCTTGTTCCTGATTCGATTGTGCACCGTCGAGACGGCATTGTACTTGGACTTCTTTGGG
GACCCGGAAAAACGACGCTATCCTCGGAAGAAAACCAAGGGTGCTTTATCCCAGGTAGACGAGGAGGATGAGA
CGAAGGAAGGTGGCGATGCTGCTGGAGAGAAGAAGGAGGGCGGAGACGAAGGTGAAAAGGTACCCCCGTCACC
TGGATCCCCTCATTTCAAAAGGATGGGATCTGCAGGCAGTCACACTGTTCCTGCTGCGTCCGCTCTTGCCTCC
CAGGTTTTGTCGGAGGATGCCACGTACCGCGATGCCGAGTTCCAATCCGCGCTCGGCAATCTCTGCTCGTCAT
TGCATCGGACCGTCCGCAGGGGACTCGTCTCCGTCTTGGATTTGGACGTATTGTGTCAGGTGGTCAGCGTGTT
GCGGGAGGAGCGTTCCGTGGCGAATAGTTCTCCCACTACTTTGGCGGCGGCACGCGCGGTGAGCGGGGTTATC

AGGGATGCCCAGGAACGGCTCATCTTTTGCGCGAACGGCGCTTTGCACAAGGAGGTGGCGCGGTTCAAACCCA
CCATTAACGATCTGAACTATCCCGGAAAGTTGTTGGGAGAGGAGCCCAAGGTTTCGATTATCATCGAGGATGA
AAGCAATTCCAACCAAAAGAAGGAGGAAACCACTGCGGAGGCGAAGAAGGAGGGAGATCCCAACGACGCAGTG
GCCGCCCAATTGCGCGTGTACGAATCGTGGTTTCCGCCCATGCGCAGCGTGCTACGTATTTTGTCTAAGATCT
TTCGCGTCGTGGAACCCAGAGTCTTCGAGGACATTGCCTTGTCGAGTGTGCAATCTTGCACAAAGAGTCTAAA
GGACGGCAGCGTCCATATTTTGAGGAAGAGCGGTCAGATCCATGCTGATCTGTTTTTGGTGAAGCATTTGCTT
ATCCTTCGCGAGCAGCTTTCTCCCTTCGACATCCAACTTCGTTCGGTTGAACGGCAGCTCGACTTTTCCGAGG
CTGGACGCGCCGTCACTCGTTTCCTCGCCAATCGCAACCGTCGCCTCTTCTCCATGTCTACTGAGAACGCCCT
CATCACGCTGCTGAGAGAAGGCGTTAGCGTCCAGGAGTCGAGCGTGGATAGCAAACGTGATCTTGAGGATTGT
TTGAGAAGCGCTTGCAATGATTTCATCGAACATACCAGTTCGACGCTGTTGGGCCCCATTGGTGGTTTTGTTG
ATCAGTGCAAGAACGTCGCGGGATCAACATCGTCGAGTGCAGATGCACTGGCCAGAGCTCCCTTCACAAACGG
TGTGGTGGTAAAAGGAGTGTTCGCTAGCGCGCTTCAATACTTGGATGGAGAATTGGACAACGTTTCCAAGCAG
ATGAATTTGTACTTGGAGAATGCGGCGACGCAAGGTATTTTATTGAAGCCAGTCGTGAGGAAGGTGACGCGAA
GTTTAGAGGAGGCGAAGCGATTCATTGGGGAGGTTTCTCAGGGGGAGGATAATGGATGGGATTTGGAGGTGAA
AAGTGATGTGTTGAAGTCTATCGAAGAGTACGAGGCCAAGGTGAAGGTTGCTTCATCTAAGAAATAGGTACGA
GTTGTTCAAGAGTTGTGACCACTCTCTCTGCTGAACAGTAAAATCTAAGGCTGTACTGTATCATTTTGCACCG
AACGCAATCATTCGCTGCCCCCTCCCAACCTCAGAGGTATAATGTACTCCCTGATTTAAGCACAACATGACGT
GGCCAGACAAAATAAGCCCATAGTGGAATGTAGACTTATGACGCATCTTATTTGAAATGGAAGGTGAGCATGA
GTGATCGTACCATATTAGTTCGGAGTCAATCTACTTTTTGAACAATCCAAGTATGTAGTCACTTATCGTGGCT
AGTTAATGTGAATGTTGGATGATATTTGGACAGAGGTACTCTGTCTCGATTGATAGGTTGATTCTTTACATCT
TAATTTGCCTATTTGTGTGCATACCTCATACCTCACGCCCTTAGGCATACCAGAACCCATATCACTTCTATTT
GGGGTCTTCTATTCAGATTCGAGCCCGAACCAATTAGTTCTTTCCTCATCGACTCTAGGGATATCGTACCCTT
CAAGAGTGCATCGCCAGCGTAATTTTGCCTGTAATGATGCACAAATCCACCAATAGCACCCACAAATCTAAGC
ACTATTCGAGATATTTTTCCAGATGATCGATTTCCCCAATCAATTCCAATACTGTGCATAGTACATAATTGAT
GTTTCAGTGCCACACGCTCAGTGGAATCGCATGTAGTTTCTCCTCATTTCTTTCCGAACCAACTCTCAGAAGA
TTTACGGGTAGAACTACTCCTACTGGCAGAGGATGCTCCCCTCGAAGATCTAGCCGAATACGACCCAGCAGCG
GACGACGACGACGATGCTCCTCTCGACCAACTCGATTTCGTTGGTGCATCCTTGTCCTTCTTCCCCGACTTAT
TCCAATTAAAACTCGACATCGGCGTAGCCCCTCCTCCCCTTTTATTCCCTATAAGGGTCCCATTCACATCCCG
TAACGAACTCCGATACTCGTTTATTTTGCTAAATCGTTCCTTCCGTTCCTCCTTCCGCGAATTGTAAGTTTCC
TTCCGTTCCTTTTCCTGGCTGGATAGTTTCATGCTTTTCTTGACGGACTTGGCGGTGGCGACGGCCGCGGCCT
TACCGCCCTTGTATGCGGCGACTCCTCCGGCGGTTATGGCGGCTGCTTCCAAGATGACCATGATTGAATTCTA
TTTGTGGGTGGGTGGATGTGACGTGCGGAAGTGAAAGGCTGCCGACCGCCGATACAAGAGGATTCGTCCGAAG
TCACGTCTCAAAAATAATGGAAGTGGAAGAAGAACTGTTCGTTGTTCGCCAACGATTTTCAATAACTCTTTTT
GGCTTCAATCTAATACGCTGAATGCTCGCTGGGCGATGAGAGGGGGTGGCAGGAGGGGAGGAAAGTCTCGCGT
CTCGCGTCAACCAGTTGAATAAGTAAGCAATTAAGCGGTCTATTGCAGAACGGAAAATGTTTTGTATCATACA
ACAGGCAGCGAAGGTGAAGATGAAGCAGAACAACGGGAATGATGAAAAATAAACAAGATTGAGATCGGAAGA
>TR543|c0_g1_i4 len=3263 path=[3872:0-1340 3873:1341-1364 3906:1365-1386 3902:1387-1409 @3912@!:1410-2037 3849:2038-2058 3909:2059-2139 3913:2140-2140 3915:2141-2160 3916:2161-2162 3857:2163-2199 3858:2200-3262] [-1, 3872, 3873, 3906, 3902, 3912, 3849, 3909, 3913, 3915, 3916, 3857, 3858, -2]
GAGGAGGGAGAAGCTGGCGGCGCAGCAGGCGTTCGAGTCCACCGCCTCCGGGAGCGCCGAGTACTACCGCCGC
GCGTGCGCCTTGCAGGATGCGGCATTGGAATTGCTGCGGGTGGGCGTGGCGGATCGGGTCTCCCAAACTTGTG
CTCAGATTCAGGATGCGTTGAATATACCGAAGGTACCTATAGCCGCAGACAAGCTGGAGGCGTCGTTGGTGTA
CACGCGCTTCCACGGGATATCGAAACGGAGTAATGCGTTGATTTCCATTGCGAGGAAGAGGCTGGACGCGCTG
GGGAACGACAGCGTAGCTTCCGCCTCGTACGATGATTTGCTTACATTGTGCCGCAATACCTACTGCGGAGCGA
GAGAAACACTTTTGACTGCCACAGTGCGAAGGCACATGGACATTTTGAAAGAGCGACACGGGTTGGTGGGCAT
GACGAGATTGGCGTCCTTGTTCCTGATTCGATTGTGCACCGTCGAGACGGCATTGTACTTGGACTTCTTTGGG
GACCCGGAAAAACGACGCTATCCTCGGAAGAAAACCAAGGGTGCTTTATCCCAGGTAGACGAGGAGGATGAGA
CGAAGGAAGGTGGCGATGCTGCTGGAGAGAAGAAGGAGGGCGGAGACGAAGGTGAAAAGGTACCCCCGTCACC
TGGATCCCCTCATTTCAAAAGGATGGGATCTGCAGGCAGTCACACTGTTCCTGCTGCGTCCGCTCTTGCCTCC
CAGGTTTTGTCGGAGGATGCCACGTACCGCGATGCCGAGTTCCAATCCGCGCTCGGCAATCTCTGCTCGTCAT
TGCATCGGACCGTCCGCAGGGGACTCGTCTCCGTCTTGGATTTGGACGTATTGTGTCAGGTGGTCAGCGTGTT
GCGGGAGGAGCGTTCCGTGGCGAATAGTTCTCCCACTACTTTGGCGGCGGCACGCGCGGTGAGCGGGGTTATC
AGGGATGCCCAGGAACGGCTCATCTTTTGCGCGAACGGCGCTTTGCACAAGGAGGTGGCGCGGTTCAAACCCA
CCATTAACGATCTGAACTATCCCGGAAAGTTGTTGGGAGAGGAGCCCAAGGTTTCGATTATCATCGAGGATGA
AAGCAATTCCAACCAAAAGAAGGAGGAAACCACTGCGGAGGCGAAGAAGGAGGGAGATCCCAACGACGCAGTG
GCCGCCCAATTGCGCGTGTACGAATCGTGGTTTCCGCCCATGCGCAGCGTGCTACGTATTTTGTCTAAGATCT
TTCGCGTCGTGGAACCCAGAGTCTTCGAGGACATTGCCTTGTCGAGTGTGCAATCTTGCACAAAGAGTCTAAA
GGACGGCAGCGTCCATATTTTGAGGAAGAGCGGTCAGATCCATGCTGATCTGTTTTTGGTGAAGCATTTGCTT

ATCCTTCGCGAGCAGCTTTCTCCCTTCGACATCCAACTTCGTTCGGTTGAACGGCAGCTCGACTTTTCCGAGG
CTGGACGCGCCGTCACTCGTTTCCTCGCCAATCGCAACCGTCGCCTCTTCTCCATGTCTACTGAGAACGCCCT
CATCACGCTGCTGAGAGAAGGCGTTAGCGTCCAGGAGTCGAGCGTGGATAGCAAACGTGATCTTGAGGATTGT
TTGAGAAGCGCTTGCAATGATTTCATCGAACATACCAGTTCGACGCTGTTGGGCCCCATTGGTGGTTTTGTTG
ATCAGTGCAAGAACGTCGCGGGATCAACATCGTCGAGTGCAGATGCACTGGCCAGAGCTCCCTTCACAAACGG
TGTGGTGGTAAAAGGAGTGTTCGCTAGCGCGCTTCAATACTTGGATGGAGAATTGGACAACGTTTCCAAGCAG
ATGAATTTGTACTTGGAGAATGCGGCGACGCAAGGTATTTTATTGAAGCCAGTCGTGAGGAAGGTGACGCGAA
GTTTAGAGGAGGCGAAGCGATTCATTGGGGAGGTTTCTCAGGGGGAGGATAATGGATGGGATTTGGAGGTGAA
AAGTGATGTGTTGAAGTCTATCGAAGAGTACGAGGCCAAGGTGAAGGTTGCTTCATCTAAGAAATAGAGGTAT
AATGTACTCCCTGATTTAAGCACAACATGACGTGGCCAGACAAAATAAGCCCATAGTGGAATGTAGACTTATG
ACGCATCTTATTTGAAATGGAAGGCATACCAGAACCCATATCACTTGTATTTGGGGTCTACTATTCAGATTCG
AGCCCGAACCAATTAGTTCTTTCCTCATCGACTCTAGGGATATCGTACCCTTCAAGAGTGCATCGCCAGCGTA
ATTTTGCCTGTAATGATGCACAAATCCACCAATAGCACCCACAAATCTAAGCACTATTCGAGATATTTTTCCA
GATGATCGATTTCCCCAATCAATTCCAATACTGTGCATAGTACATAATTGATGTTTCAGTGCCACACGCTCAG
TGGAATCGCATGTAGTTTCTCCTCATTTCTTTCCGAACCAACTCTCAGAAGATTTACGGGTAGAACTACTCCT
ACTGGCAGAGGATGCTCCCCTCGAAGATCTAGCCGAATACGACCCAGCAGCGGACGACGACGACGATGCTCCT
CTCGACCAACTCGATTTCGTTGGTGCATCCTTGTCCTTCTTCCCCGACTTATTCCAATTAAAACTCGACATCG
GCGTAGCCCCTCCTCCCCTTTTATTCCCTATAAGGGTCCCATTCACATCCCGTAACGAACTCCGATACTCGTT
TATTTTGCTAAATCGTTCCTTCCGTTCCTCCTTCCGCGAATTGTAAGTTTCCTTCCGTTCCTTTTCCTGGCTG
GATAGTTTCATGCTTTTCTTGACGGACTTGGCGGTGGCGACGGCCGCGGCCTTACCGCCCTTGTATGCGGCGA
CTCCTCCGGCGGTTATGGCGGCTGCTTCCAAGATGACCATGATTGAATTCTATTTGTGGGTGGGTGGATGTGA
CGTGCGGAAGTGAAAGGCTGCCGACCGCCGATACAAGAGGATTCGTCCGAAGTCACGTCTCAAAAATAATGGA
AGTGGAAGAAGAACTGTTCGTTGTTCGCCAACGATTTTCAATAACTCTTTTTGGCTTCAATCTAATACGCTGA
ATGCTCGCTGGGCGATGAGAGGGGGTGGCAGGAGGGGAGGAAAGTCTCGCGTCTCGCGTCAACCAGTTGAATA
AGTAAGCAATTAAGCGGTCTATTGCAGAACGGAAAATGTTTTGTATCATACAACAGGCAGCGAAGGTGAAGAT
GAAGCAGAACAACGGGAATGATGAAAAATAAACAAGATTGAGATCGGAAGA
>TR544|c0_g1_i1 len=3268 path=[3556:0-56 3557:57-80 3558:81-91 3559:92-115 3576:116-804 3562:805-828 3581:829-1406 @3580@!:1407-1832 3578:1833-2089 3574:2090-2704 3552:2705-2728 3579:2729-3006 3565:3007-3030 3577:3031-3180 3544:3181-3267] [-1, 3556, 3557, 3558, 3559, 3576, 3562, 3581, 3580, 3578, 3574, 3552, 3579, 3565, 3577, 3544, -2]
TTTGAGGTTGTGTTAACCACCTATCTTATAGGAATTAATAAGTATCTTAAAGGAATTGCAGTTGTTGCTGAAA
GTAAAAAATCTTCTTGCAACCATCAAGGTGGAGCGAGGTCCCACACGCAGCAATAAGAGGATTAAGGTTTAAT
CAGTTGAACTAATCCCCCTTTACAGTGCGACGCCTGCTAGACACCTCAGTCGCCACATGCCTGAAGGACCGGC
TGCTCTTCTCGCTTGTTGCGGCGCAACGAGCGAGCTTGCTTATCTTTATTCCGGTCTTGATATCTGTATTGAA
CACCTCCGAATAGAGAGCACCACGGCGAACTCCAATCCTGACCTGGTCCCACTTGGATGCGGCTGTCTCCTCA
TATGCGGCTTCCTCCAACTCTGTAGGCGTGAGCCCCACCTCCTCCTCGTAATCTGAAGGAGAAAATACAACGA
CGATCACGTTGATCAATACTTGCCAAATTAGACCACCTGCTCCAACTGCAACACTGAACAACCACTGGGCTTT
TGTGAGACCACCAGGGATGATGTTCACCGCAACGCCGCCATATATCATGATGAAGACCTGCATACAAAATGTA
AAAATCATGATGGCAACAAAGTACGGGTTTCGGCTGACGCCCCCAAAGACGTTCCATTCTGACCACGTTGTCC
TTAACTTCTGGGCCGTTGGCAGCCGCCTAGAATTAAATTCATTGAAAAGCTGCAGCATCACAAACACATTGAA
GAAAAGTGACCAATGAAGAGAGCCTTGTGTGGGCTCAAACGCCACTGGCGGGTCTAAGGGAAGAGAAGATGGC
GCGAAGAGTACCCAGTTCAAAATCAATGCCTGGTATATTGCATGCCCAATGATGTTGGCGCCCATTGTGACGC
TGATAATTGGCCGGCGCCTCCCGTAGGGCATGCGGTCGAGAAGTTCCTCTGTTGGGGGCTCCGTTGCAAGAGC
CAATGATGCCAAGCTGTCCATGATCATATTCACCCAAAGCATCTGAACCGCACTCAGCGGTGAGTCGTTGAAT
GCAAACGCTCCGATCACCGCGAGGGTGATAGCCACAACATTCACCGTGAGCTGGAATTGGATGAATTTCGAGA
TGGAATCAAAGATGTTCCGGCCCCACATGACAGCCTGGACAATAGAAGAGAAGCTGTCGTCAAGCAATATAAT
GTCTGCCGCCTGCTTGGCAGTTTCCGTGCCAGCAATACCCATGGCAAAACCGACGTTGGCGGACTTTAGGGCC
GGTGCGTCATTGGTCCCGTCGCCTGTCACTGCCACCACCTGGTAGTCTGGGAAGATACGGATGCCATGGTCAT
TTAAGAGAGCGGCACAGCGTGCCTCATCGAGAAATACTCGACTCTTGCGCATTCCTCTGACCAGGGCCAGCTT
ATCCTCGGGCTGACACCGGGCCAGGACGCGGAGGGTCGGCCACAGATCATCGAATACTTTCTGGTCCAACACT
TTGTTCCCCGCTTCATCGAGCAGAAACGGCTCCGCAAGACGGTCGACCGCATCATTTATCTCTGGATCAAAGA
CCCTCCGGACAGTTTTGGGTTTGGAGGGATCCTTCAGATGGACACGATCGTCGAACTCGGCGCCCGTCATGGC
AAATCCCGTCTTTGGTTGACCGCCTTCGCCAAGGTCAGATACTCGGAGAATGCGGCATCCCTTGGCTATGGCC
ACAGCAGTCGTTAGGGCGTCGCCTGTGCACATGCGAACGTCTATTCCGGCCTTGTGGCACTCAGCAATGGCGC
CGGGTACCGATGGCCTGAGCGGGTCTTCGATGCCCACGACAGTGATGAGCGTCATATTTGTCTCGAGATCTCC
GTTGCCACCCCAACCCCCTTTCGGTACGTCGTCGAAGTCGCGGTATGCAAGTGCCAGAGCCCTCAGACCCTTG
CTTTGGCAGGTAGCGACCATATCCATGAATACTTGCCTGTCTCTTGCTTCCAGTGGTTTGGTGCTCGTAGCGC
CTGCAGCCGTGATGTCCGTCACAAATGATGTGCACGCGGCGAGCATCTGCGTTGGCGCGCCCTTGCTGTACAT
ACGGTAACGTCCAGGGTAGGGGAGTGAGACCGATCCATCGGCCCTCCTTATCATCCATGACATGCGCTTCCTT

TGAGACGAGAATGGAATTGAATGCACGCCCCAGTCCAGCATAGACGTTCCTTCTTTGAACGCCACACGTAGGG
AATCAGCATCACAATCCAGCTGTTGAGTGAGTTTGATCAGAGCACAATCGGTCGGGTTCCCAACGTAAAACGT
TGATCCAGTCTCGGGATTTAAAGACACTGTGGATGTGGGTGATACATTAAGCAGTATTGATTCGCAAAGAAGC
CTCGATTTGGCAGCATTCGTTTTGAAGGCAGAGAGCATCTTTGCTCCAAGGCTGTTATCTTGTCCTCCCTGAC
CCACAACGTGATCGTATTGATAACCTGCGTATCGTGCTGACATCACTGTCATCTTGTTTTCCGTCAGAGTTCC
GGTCTTATCGCTACATATGGTCGTGGCGCTGCCCATGGTCTCGCACGAATCCATGTGCTTCACCTGGTTAGCG
TCCTCCGTCATTCGTTTCATCGATACCGCCAAGGCCAAAGTCACTGCAAGAGGCAATCCTTCTGGGACGGCAA
CAACTAAAATCGTGATGGCGACGATGAAATAATCGAGTATCGTGGACACGTCTTCCGCTACAAAGTGGCGGCC
ACACGTTCCACCGCCGCTCGGCGCAGCAGACCAGGTACATACCAACGTCGACAAGCAATCATCTTCGTCGATC
AACTCGGGGCAAGGTCCTCCCACGATGAAGTCCTTCAACAACCATTGGGTCAGCATGATAAGAAACACCCCTA
CGGCAACAATAAGGCCCATCTTGCCAATGTCCACGGCAAGGACATCGAGTTTGTCCATAAGCGTCGCGCCACT
ATCCGAGCCGCCAAAGGTGAAAAAGGCCCTCAAGGCGGAGAAGGTGGATTTGGCTTCCTCCGTTTCTGCCGAG
TGATCGCTGTCATCATCCTCCTGTTCCCTAATCCGTGCCAAAATCTTTCCCTGAATCGAATTCGGTCCAACCG
CCAAGACGAGCATCAAACCGGTGCCCTGTATAACGTTGGTTCCCGACACGATGAACGGGTCATCGCCAGGCCC
TCCCTCACGGTCTCCCCCTTCCGCTGCGGTCTTAGGTATGAGGGCCGTCTCACCTGTTAGTGCGCTCTCGTCC
ATCTTCATATCGTCGCTCCCCTGTATGACGACGCCGTCGGCTGCGGCGATGTCCCC
>TR544|c0_g1_i2 len=2006 path=[3547:0-144 @3580@!:145-570 3578:571-827
3574:828-1442 3552:1443-1466 3579:1467-1744 3565:1745-1768 3577:1769-1918
3544:1919-2005] [-1, 3547, 3580, 3578, 3574, 3552, 3579, 3565, 3577,
3544, -2]
ATTCCTCTGACCAGGGCCAGCTTTGAGAAGGAGAATTGATATGGAAGCCGAGGAAGCGGAGACTCTTGTCAGT
GAGAAGTTAGGCGTATGTAAGACAATATACCATAATAGCGATGGCATACCTTATCCTCGGGCTGACACCGGGC
CAGGACGCGGAGGGTCGGCCACAGATCATCGAATACTTTCTGGTCCAACACTTTGTTCCCCGCTTCATCGAGC
AGAAACGGCTCCGCAAGACGGTCGACCGCATCATTTATCTCTGGATCAAAGACCCTCCGGACAGTTTTGGGTT
TGGAGGGATCCTTCAGATGGACACGATCGTCGAACTCGGCGCCCGTCATGGCAAATCCCGTCTTTGGTTGACC
GCCTTCGCCAAGGTCAGATACTCGGAGAATGCGGCATCCCTTGGCTATGGCCACAGCAGTCGTTAGGGCGTCG
CCTGTGCACATGCGAACGTCTATTCCGGCCTTGTGGCACTCAGCAATGGCGCCGGGTACCGATGGCCTGAGCG
GGTCTTCGATGCCCACGACAGTGATGAGCGTCATATTTGTCTCGAGATCTCCGTTGCCACCCCAACCCCCTTT
CGGTACGTCGTCGAAGTCGCGGTATGCAAGTGCCAGAGCCCTCAGACCCTTGCTTTGGCAGGTAGCGACCATA
TCCATGAATACTTGCCTGTCTCTTGCTTCCAGTGGTTTGGTGCTCGTAGCGCCTGCAGCCGTGATGTCCGTCA
CAAATGATGTGCACGCGGCGAGCATCTGCGTTGGCGCGCCCTTGCTGTACATACGGTAACGTCCAGGGTAGGG
GAGTGAGACCGATCCATCGGCCCTCCTTATCATCCATGACATGCGCTTCCTTTGAGACGAGAATGGAATTGAA
TGCACGCCCCAGTCCAGCATAGACGTTCCTTCTTTGAACGCCACACGTAGGGAATCAGCATCACAATCCAGCT
GTTGAGTGAGTTTGATCAGAGCACAATCGGTCGGGTTCCCAACGTAAAACGTTGATCCAGTCTCGGGATTTAA
AGACACTGTGGATGTGGGTGATACATTAAGCAGTATTGATTCGCAAAGAAGCCTCGATTTGGCAGCATTCGTT
TTGAAGGCAGAGAGCATCTTTGCTCCAAGGCTGTTATCTTGTCCTCCCTGACCCACAACGTGATCGTATTGAT
AACCTGCGTATCGTGCTGACATCACTGTCATCTTGTTTTCCGTCAGAGTTCCGGTCTTATCGCTACATATGGT
CGTGGCGCTGCCCATGGTCTCGCACGAATCCATGTGCTTCACCTGGTTAGCGTCCTCCGTCATTCGTTTCATC
GATACCGCCAAGGCCAAAGTCACTGCAAGAGGCAATCCTTCTGGGACGGCAACAACTAAAATCGTGATGGCGA
CGATGAAATAATCGAGTATCGTGGACACGTCTTCCGCTACAAAGTGGCGGCCACACGTTCCACCGCCGCTCGG
CGCAGCAGACCAGGTACATACCAACGTCGACAAGCAATCATCTTCGTCGATCAACTCGGGGCAAGGTCCTCCC
ACGATGAAGTCCTTCAACAACCATTGGGTCAGCATGATAAGAAACACCCCTACGGCAACAATAAGGCCCATCT
TGCCAATGTCCACGGCAAGGACATCGAGTTTGTCCATAAGCGTCGCGCCACTATCCGAGCCGCCAAAGGTGAA
AAAGGCCCTCAAGGCGGAGAAGGTGGATTTGGCTTCCTCCGTTTCTGCCGAGTGATCGCTGTCATCATCCTCC
TGTTCCCTAATCCGTGCCAAAATCTTTCCCTGAATCGAATTCGGTCCAACCGCCAAGACGAGCATCAAACCGG
TGCCCTGTATAACGTTGGTTCCCGACACGATGAACGGGTCATCGCCAGGCCCTCCCTCACGGTCTCCCCCTTC
CGCTGCGGTCTTAGGTATGAGGGCCGTCTCACCTGTTAGTGCGCTCTCGTCCATCTTCATATCGTCGCTCCCC
TGTATGACGACGCCGTCGGCTGCGGCGATGTCCCC
>TR545|c0_g1_i1 len=1714 path=[2316:0-64 2317:65-98 2334:99-307 2215:308-
331 2356:332-353 2333:354-377 2346:378-728 2247:729-752 2332:753-773
2355:774-797 2359:798-879 2330:880-909 2353:910-921 2343:922-963
2328:964-999 2351:1000-1024 2341:1025-1025 2326:1026-1065 2230:1066-1071
2339:1072-1159 2367:1160-1193 2366:1194-1227 2365:1228-1239 2363:1240-
1258 2369:1259-1264 2371:1265-1265 2347:1266-1271 2335:1272-1295
2319:1296-1369 2244:1370-1393 2245:1394-1713] [-1, 2316, 2317, 2334,
2215, 2356, 2333, 2346, 2247, 2332, 2355, 2359, 2330, 2353, 2343, 2328,
2351, 2341, 2326, 2230, 2339, 2367, 2366, 2365, 2363, 2369, 2371, 2347,
2335, 2319, 2244, 2245, -2]
TCTATTCCTATCTCTATATATTATACATTTCTATCTAAGGGTCGTATTCAAGATTGTTCTTAAACTCTCATGT
GAAAATCATAGCACTTAGCATCTCGACACATCCAATCCCCATCAAAGGAGCGCAGCTTGCAACACTGCTTGCT

CGCTATTGAACTCATTCACCAAATATGTATTAAAGAAAAAATCCTTTTTTTTCATGGTAGAAGCCTTGGCCTT
ATTGCCCATAGCCCAGTGATTCTCGACCCAGTCGTGCAAAACATTTGCCGCAACCATGGATGCATTCGCAGAC
GATTCCCTTCGAATAGAACTCTTAGAACTATAACTCATACCAGGCGTATAATCCCGCAGCTGGGTATAGCACG
TGGATCTCCCCCATTCATCTGGGGGGGAGGAATCATTTGAGGCATTTGCGTAGTTGCACCAAAGCGCAAACGG
CCTCTTGCTCGTGACTACACAGTTAGATTTGTCCATGACGGCGTCGAGAACTTGCAACACCTTACCGCACTGC
TCCCCCGTTCTCCCCTCTTGAATGGGCCAGTTCACAACAAATGCGCAAACGCGATGTTGCTTCACCGCGGCTT
CCAGTTCCGAAACAAGATCCTCATTCACTTGACTCTTTTTGCATAACGCGCCGTTTCGATCTTGTCGGAGCGA
TATTGAATCCAGAGGAAAGGATTCGGGTCCACGGGTGGCGCATGGGCCGCGGAGGTCTGGATGTTCGGCGAGA
GCAACTCCAATCCGTTCTCGTGTGATATCGAGTCCCATTACAACTCCCATGGCTTTGCGCCAGTCTAATATGT
TAGCAACTTTAGATGGGGACACGAGAGCGTTGGATAATTTTGATGCCATCATTTTAGTTGAGACTATTCTTCT
CTGCGTCGATTGAAAATGGGCTTGCCAATAAGGTCAACTGCAAATCTATCAGTGTAGGTGGCGCGATATGAAT
TTTTCACAACAAAGTTCGATTCGTGCTCTCTCTGAGGAATGGTGACAGTAGGAGGAGCTCGTATGATAACATT
ATTGATGCGATGTATGGACTGAATGCTTGGGCAATGTGATCGATATCGATCCTGTGCATGTGGTGTGAATTAT
CTAGGGTAGTACCATACACGATACTCCTCTTTCTCAATACTCCTACTTCTGCTAAAAGCAAGGCCGATAGCAA
GAACAAGAACGTGTCTACTCCATCCGTGGAACTGGTTGAACATGGAACAGCCACGACAGCAACAAGCGACCTT
GAGATACGTACTTACTCACGAGAGAGAGGTACCGTAGATACTTATACTATCTACTCCTAGAGGTACTTACTTA
CCTAGGTAGAAGTAGGTAGGTATGAGTAGATAAATATCTACTCCTAATTCGTACTTTGGTACACAAGGGATCC
ACTTGTAGGCTCCTAGATTTAGCGGGCAAAAACTATACTTGAAAAGGGCTATGAAAGCGTAAGCCCGGTATCT
AGTTAACTTTACTAGCTTTAGCAATATGATGTCAGCATAATATTTATTACTCATACTTGTCACATCTCCATTG
ATTTACGTGCCTTTATGGGCCTAGCTAAATGTCGTTACATGGATATTTGCTCCCATGCCCGCTAAATCCAAGA
GCCTATCCACCATGATGATGCGCTGTTTCGGGGTTGCGGCCTGTTTGGTCTTCTCTGTTCTTCTTCGCGCTGA
GTTGGCTGGGTTCGGTTCCGGTGCCGTGCCTCCGA
>TR545|c0_g1_i2 len=1715 path=[2316:0-64 2317:65-98 2334:99-307 2215:308-331 2356:332-353 2333:354-377 2346:378-728 2247:729-752 2332:753-773 2355:774-797 2359:798-879 2331:880-909 2354:910-921 2344:922-963 2329:964-999 2352:1000-1024 2342:1025-1025 2327:1026-1065 2263:1066-1071 2340:1072-1160 2367:1161-1194 2366:1195-1228 2360:1229-1265 2371:1266-1266 2347:1267-1272 2335:1273-1296 2319:1297-1370 2244:1371-1394 2245:1395-1714] [-1, 2316, 2317, 2334, 2215, 2356, 2333, 2346, 2247, 2332, 2355, 2359, 2331, 2354, 2344, 2329, 2352, 2342, 2327, 2263, 2340, 2367, 2366, 2360, 2371, 2347, 2335, 2319, 2244, 2245, -2]
TCTATTCCTATCTCTATATATTATACATTTCTATCTAAGGGTCGTATTCAAGATTGTTCTTAAACTCTCATGT
GAAAATCATAGCACTTAGCATCTCGACACATCCAATCCCCATCAAAGGAGCGCAGCTTGCAACACTGCTTGCT
CGCTATTGAACTCATTCACCAAATATGTATTAAAGAAAAAATCCTTTTTTTTCATGGTAGAAGCCTTGGCCTT
ATTGCCCATAGCCCAGTGATTCTCGACCCAGTCGTGCAAAACATTTGCCGCAACCATGGATGCATTCGCAGAC
GATTCCCTTCGAATAGAACTCTTAGAACTATAACTCATACCAGGCGTATAATCCCGCAGCTGGGTATAGCACG
TGGATCTCCCCCATTCATCTGGGGGGGAGGAATCATTTGAGGCATTTGCGTAGTTGCACCAAAGCGCAAACGG
CCTCTTGCTCGTGACTACACAGTTAGATTTGTCCATGACGGCGTCGAGAACTTGCAACACCTTACCGCACTGC
TCCCCCGTTCTCCCCTCTTGAATGGGCCAGTTCACAACAAATGCGCAAACGCGATGTTGCTTCACCGCGGCTT
CCAGTTCCGAAACAAGATCCTCATTCACTTGACTCTTTTTGCATAACGCGCCGTTTCGATCTTGTCGGAGCGA
TATTGAATCCAGAGGAAAGGATTCGGGTCCACGGGTGGCGCATGGGCCGCGGAGGTCTGGATGTTCGGCGAGA
GCAACTCCAATCCGTTCTCGTGTGATATCGAGTCCCATTACAACTCCCATGGCTTTGCGCCAGTCTAATATGT
TAGCAACTTTAGATGGGGACACGAGAGCGTTGGATAATTTTGATGCCATCATTTTAGTTGAGACTATTCTTCT
CTGCATCGATAGAAAATGGGCTTGCCAATAAGGTCAACTGCAAATCAATCAGTGTAGGTGGCGCTATATGAAT
TTTTCACAACAAAGTTCGATTCGTGCTCTCTCTGAGGAATGGTGACAGTAGTGGGAGCTCGTATGATAACATT
ATTGTTGCGATGTATGGACTAAATGCTTGGGCAATGTGATCGATATCGATTCTGTGCATGTGGTGTGAGTTAT
TCAGGGTAGCATACACGATACTCCTCTTCCTCAATACTCTTATCTACTTCTGCTAAAAGCAAGGCCGATAGCA
AGAACAAGAACGTGTCTACTCCATCCGTGGAACTGGTTGAACATGGAACAGCCACGACAGCGATAAGCGACCT
TAAGATACGTACTTACTCACGAGAGAGAGGTACCGTAGATACTTATACTATCTACTCCTAGAGGTACTTACTT
ACCTAGGTAGAAGTAGGTAGGTATGAGTAGATAAATATCTACTCCTAATTCGTACTTTGGTACACAAGGGATC
CACTTGTAGGCTCCTAGATTTAGCGGGCAAAAACTATACTTGAAAAGGGCTATGAAAGCGTAAGCCCGGTATC
TAGTTAACTTTACTAGCTTTAGCAATATGATGTCAGCATAATATTTATTACTCATACTTGTCACATCTCCATT
GATTTACGTGCCTTTATGGGCCTAGCTAAATGTCGTTACATGGATATTTGCTCCCATGCCCGCTAAATCCAAG
AGCCTATCCACCATGATGATGCGCTGTTTCGGGGTTGCGGCCTGTTTGGTCTTCTCTGTTCTTCTTCGCGCTG
AGTTGGCTGGGTTCGGTTCCGGTGCCGTGCCTCCGA
>TR546|c0_g1_i1 len=613 path=[641:0-78 642:79-102 643:103-111 644:112-135 645:136-612] [-1, 641, 642, 643, 644, 645, -2]
CGGAACTTCCCGACACCTGCACCACGCCCACGCCGCACGTGAGCACCACCAACGAGGCCCATTTGGTCCTCGA
GAACCGACGTCCGAGCATGGTCGCGCTGAATACCGCCGTCGTGAGTATTTTGAGTTGGTATAATATTTGATAC
GTGGCTGCGTCCAAGTTCGTGAGTGCCAAATAGAGCAGATTGTTTTGTACGGTGTAGAGGAAGGAGGGGACGC

AGAGTTTGAGCATTTCGTGGGGACTCGCCCAGACGTGGTTGTATAGTTCGGTGCTGAGGCCTGCGAGGGATGA
GCCGGATTGGAAGAAAACTACTCCCAGGCATATTAAGAATTTCATAATCTCCATCATAAAAACCGCAGTGCTC
GCCAAATACATTTCTCCATCTGTATGCGTCCTCGAATATCGCATTATCAACACTAATCCAACCATCTGCCCTA
CCAATAATAACAGACTCCCATATTTTATGAGTGCATTGTGTTCTGGTGTAGGGTTATTGGGACGATTCGATAA
AGGAGTTGGTGATATCGATTGCTGCCTTGTGCTCTGTCTTTGTGAAGATGACGACGACGACGACGACGTTCCC
TTTTTCTTTGTTTTTGTTTTCCGTGGGAT
>TR546|c1_g1_i2 len=1601 path=[2042:0-149 2137:150-177 2132:178-221
2112:222-246 @2141@!:247-488 2122:489-544 2131:545-597 2142:598-621
2140:622-668 2143:669-683 2145:684-684 2107:685-711 2128:712-733
2118:734-757 2105:758-1083 2087:1084-1107 2127:1108-1242 2104:1243-1266
2116:1267-1289 2010:1290-1313 2133:1314-1432 2144:1433-1447 2102:1448-
1464 2125:1465-1482 2113:1483-1492 2100:1493-1540 2079:1541-1600] [-1,
2042, 2137, 2132, 2112, 2141, 2122, 2131, 2142, 2140, 2143, 2145, 2107,
2128, 2118, 2105, 2087, 2127, 2104, 2116, 2010, 2133, 2144, 2102, 2125,
2113, 2100, 2079, -2]
GGCGCCCTCCTCCGGGAGCGTTCCTCGCGGAGGAAGAAGAAGCGAGGTTCACCGCCACGGCGGTGGGGGAGGA
GTCGGATCCGGGAGATGCGGTGCCGGAGGACGAGGCATCATCGGTGCAGGTCGCTATTGTCGTTGTCGTCGTC
AACGTCGACGGGGTATGCGGCGGAGGCGCGCTCTCGCTTCGAGGACGCCGTGAGGATCGAAGGATTTCCTCGG
CGTAGCGTGGAGGTTTCGAACGGGTCGTCCCGTTGTTGTTGCGAAAGCTCTCGGGTCTGCGATGATGGCGATG
ATGATCCTCCGATGCTGCGGCTTCGTTCGACGATGGAACGGAGGCGGTCGTGCGGCGAAAGCTCGCCGACTTG
GTGCTGGCGGAGCGAACGGTACCCGACGAAATGGTCGCCGGGCCGTCCTCGCCGCTGTCGATGGCGTTTTCAT
TTTTCTTCGCGAGTTTCCTACCGAGTCGGCCGGTGCTGTTGTTCGCGTTGTTGCCACTGCTGTTGGTGTTGGT
GTGGTTGGTGGTGGTGTTGTTGCCGCCGAAACTGACCGACTTTCTCGTGGTTCGGGATTTTCCACTCGGAGCC
GAGTTCGACACCTCCTCGTCGGGAAGAACGGTCGACGTCCCATCCGGAGACGGATCATTAAGCATCCGCCGCT
CGGGCAAAGCGTCGTCACTTCCGCTCTTGGGTAATTCCTCGTCTTCGGAAGGCGGAACAACGGAAGACGACGA
TGCGTATTCGTTCCGATGATGCTCGACGACGACCAACCTCAAACACCGTTCCCATTTGCTCCTCTCCAAAATC
TTCTTATTCTTCCCGCCGTCCTTCAAGTGAACGACGAGGCTCGGTTTGCCCGAGTCGTCCGTCGCCCTGTCCG
CGAGCACGGCGTAGGTCCATTCTCCGGAGGTGCGCTTGACGAATAACGGAGAGTCCAATGGCAGTTCGGTCAG
GAGGGTCGTCGCTTGCGCGAGGGAGGCGAACGGCGCGGAGTGTCGCTTCTCGGCGAGCGTCTGCCCTATTTCG
TAGCGGTCGGCGGAGTTGTTGTTGACGTCGACGACCTCGCTGCCATTATTGGCGGCATCATCATCGTTCGATC
GGTATTCCTCGTCTTCCGTCACCCTCGGCATGCTGGGGGGAGACAACGTCTGGCTGCTCGGCCTTGAGACGGC
AGATCTGCAACGACGCGCTCCGGCCACTCGATTCCGTCCTGGGGCGTTTTCGTTCGTCGCATTCGAAATGCCA
GAGTTACAGCATGGATCCGACTTTCTCCTGGACATACGAGGAGGAGACAACAACAACGACTCCTCGCCGCTCG
CCCTCGATCCGGAACGACGCGCTCCGGCCGCTTCGTTCTCGTGAATCGAATTCGAAATGCGCGGATCCGACTT
TCTCTTCGCGTGGCCTTCGGAAGAGGATCGTTTTTTCCGACCGACCGATTTTCCATTTTTTCCATCGGGAGAT
GACGACGACGACGACGACGAGCAAGGCCGACAACATTTCGTCGTAGGATTGGCCTTCGTCTCGGCCTCGTTGG
TGGCAGTGGCCAACTCGTTCGTCGTGACCGACGAGGCCACGGCCATATCGCTGAGGAAGGACGCTTGA
>TR547|c0_g1_i1 len=356 path=[1461:0-57 1520:58-133 1413:134-155
1414:156-355] [-1, 1461, 1520, 1413, 1414, -2]
GGAGGCGCACTGCTCCCGTGATAACTTCGATCGAGCCGGACGTGGTCACGTACACGTCGTTGATATCGGCGTT
GCTCCAGCTGGACAGTCGGGCGGCGACCGAGCGCGCGCGTTCGATGTACGGCGAAATGAAGCGGAAGTGGCTC
ATCTCTCCCGACACAGTGCACGTGCCAAGACGCGTCGGCCATGTCTCTACGACTACCAAACAAATGCCGTTGC
GACGACGGTTCCCTGGTTTCACGTGGGCAGGACGAGTTCAGCGCTCGCCGTGATGTTGACCCAGGGGAGCTTG
GTCTTTTCCGCGAGTGATGAGGGCAGTGGCGGTGCTGCTGCCTCAAAAGGTGGAGTGCGGAAGG
>TR547|c0_g2_i1 len=968 path=[1468:0-72 1539:73-105 1518:106-132
1545:133-145 1538:146-169 1515:170-172 1527:173-196 1548:197-217
1526:218-241 1537:242-249 1543:250-279 1524:280-285 1535:286-314
1547:315-366 1523:367-390 1509:391-634 1421:635-669 1422:670-696
1532:697-743 1521:744-745 1507:746-767 1477:768-967] [-1, 1468, 1539,
1518, 1545, 1538, 1515, 1527, 1548, 1526, 1537, 1543, 1524, 1535, 1547,
1523, 1509, 1421, 1422, 1532, 1521, 1507, 1477, -2]
CTCTTCCGATCTCCGCGCTGACGCCGGCGTACAACGCCTTCATTTCGGGATTGTTGGCGGACGGCAAGAATGG
GCACGTCGGCGGCGCGGACGTTTCCCTGGGGCAATCGTCTCACGCGGCGCACATGGTGGAGGTGTTGGCGGTG
CTGCGAGAGATGCAGGAATTCGATATTCGACCCAATGTCGTGACGGTGGCGCTGGTGGTGGACGGGCTGGGAT
GATGCAATCCCCCTCGATGCAGGGAGGCCAGGGAATTGGTGCAGCATTTGCAATCCCAATCAAAAATGAAGCA
GAGCGATGCCACTAATGGAAATCACAACAATCGAGGTGGCGTAATAGAGGACACAGATGCCAGGGGGATATCG
CTCTCCAACAACAAAATCGCCACGGCGTTGATCGCCTCGTACGGCCGCACAAATGACGTCAAATCCGCCACCG
AGGCGTTCGACCGCATACCCAATCCCGACGTCATGGCGTTCAACGCCCTCCTCGACGCGTGCTGCCGCTGCGA
CAAGCTCAAGCTGGCGCTGGAATTGTTTTGATGGCGCGTTGGCGTCGCCCCTCCTTCTTCTGCGGCAGAGGGC
GATGATGCGGGGAAGGAGAACGCAACAAGGAGGCGCACTGCTCCCGTGATAACTTCGATCGAGCCGGACGTGG

TCACGTACACGTCATTGATATCGGTGTTGCTCCAGTTGGGCAGTCGGGCAGCAACAGAGCGCGTGTGTTCGAT
GTACGGCGAAATGAAGCGGAAGTGGCTCATCTCTCCCGACACAGTGCACGTGCCAAGACGCGTCGGCCATGTC
TCTACGACTACCAAACAAATGCCGTTGCGACGACGGTTCCCTGGTTTCACGTGGGCAGGACGAGTTCAGCGCT
CGCCGTGATGTTGACCCAGGGGAGCTTGGTCTTTTCCGCGAGTGATGAGGGCAGTGGCGGTGCTGCTGCCTCA
AAAGGTGGAGTGCGGAAGG
>TR547|c0_g2_i2 len=970 path=[1483:0-74 1540:75-107 1549:108-134
1545:135-147 1538:148-171 1515:172-174 1527:175-198 1548:199-219
1526:220-243 1537:244-251 1543:252-281 1524:282-287 1535:288-316
1547:317-368 1523:369-392 1509:393-636 1421:637-671 1422:672-698
1532:699-745 1521:746-747 1507:748-769 1477:770-969] [-1, 1483, 1540,
1549, 1545, 1538, 1515, 1527, 1548, 1526, 1537, 1543, 1524, 1535, 1547,
1523, 1509, 1421, 1422, 1532, 1521, 1507, 1477, -2]
GGGACGGAGCACGACTGTGCTGACGCCGGAGTACAATGCCTTCATTTCGGGGTTGTTGGCGGACGGCAAGAAT
GGGCACGTCGGCGGCGCGGACGTTTCCCTGGGGCAGTTATCTCACGCGGCGCACATGGTGGAGGTGTTGGCGG
TGCTGCGAGAGATGCAGGAATTCGATATTCGACCCAATGTCGTGACGGTGGCGCTGGTGGTGGACGGGCTGGG
ATGATGCAATCCCCCTCGATGCAGGGAGGCCAGGGAATTGGTGCAGCATTTGCAATCCCAATCAAAAATGAAG
CAGAGCGATGCCACTAATGGAAATCACAACAATCGAGGTGGCGTAATAGAGGACACAGATGCCAGGGGGATAT
CGCTCTCCAACAACAAAATCGCCACGGCGTTGATCGCCTCGTACGGCCGCACAAATGACGTCAAATCCGCCAC
CGAGGCGTTCGACCGCATACCCAATCCCGACGTCATGGCGTTCAACGCCCTCCTCGACGCGTGCTGCCGCTGC
GACAAGCTCAAGCTGGCGCTGGAATTGTTTTGATGGCGCGTTGGCGTCGCCCCTCCTTCTTCTGCGGCAGAGG
GCGATGATGCGGGGAAGGAGAACGCAACAAGGAGGCGCACTGCTCCCGTGATAACTTCGATCGAGCCGGACGT
GGTCACGTACACGTCATTGATATCGGTGTTGCTCCAGTTGGGCAGTCGGGCAGCAACAGAGCGCGTGTGTTCG
ATGTACGGCGAAATGAAGCGGAAGTGGCTCATCTCTCCCGACACAGTGCACGTGCCAAGACGCGTCGGCCATG
TCTCTACGACTACCAAACAAATGCCGTTGCGACGACGGTTCCCTGGTTTCACGTGGGCAGGACGAGTTCAGCG
CTCGCCGTGATGTTGACCCAGGGGAGCTTGGTCTTTTCCGCGAGTGATGAGGGCAGTGGCGGTGCTGCTGCCT
CAAAAGGTGGAGTGCGGAAGG
>TR548|c0_g1_i1 len=688 path=[2128:0-87 2129:88-88 2656:89-127 2689:128-
140 2674:141-163 2721:164-164 2731:165-166 2739:167-176 2701:177-188
2686:189-200 2672:201-208 2722:209-210 2732:211-212 2740:213-225
2546:226-259 2671:260-302 2723:303-312 2543:313-326 2698:327-336
2684:337-441 2724:442-472 2586:473-566 2734:567-575 2741:576-589
2157:590-687] [-1, 2128, 2129, 2656, 2689, 2674, 2721, 2731, 2739, 2701,
2686, 2672, 2722, 2732, 2740, 2546, 2671, 2723, 2543, 2698, 2684, 2724,
2586, 2734, 2741, 2157, -2]
CCGATCTCCCAGCGCCGCCTCCATGACACCTTCACCAAACCGCACCATAACAACCCGCCGCATAAAATTAAAC
ACAGCGCCAAATTCAGCTTATCATTCATATTGCTCTATCCATAGTATCATTCATCCATCATGTCCAACGAAGA
AGACCTTGAGATGGGCATTCCTCATTCGCTGGTTGAAGATGAGAACGGTGACACAACTCTTGCCAGTGATGCA
AATTCCTTCCCTCAGAATATGGCGGATGCCAACGATGCTGATACTCACTCTTCGCCCACGACACTACTTGACT
CCGAGCGGATTGATGACGTTGTCGATGCAGCTCCCTCAACAGCCAACATTGGTCAATTGCCCTCTGATGCTAA
TGCTGATGCTGTTCGTGGAAATAATACGAGTAATAAAATGAGTATGCAAATGCAAGATCTCATCGCTGAATTA
TTGGACACCAACCAAAGTGGCGAAATCAACCTTGGTCAGATAGAGTCCATTATGATGAGAGCTGCTGTGTCTA
GCAATTCAGCGGTGATAAGCGATGGCGGGAGAGGGGAAGATACTGCTCGTGCGCCATCATCAGTCGTGTCTAC
AATCCAGGATGATTTTGGACCGGCCGATGCAGGAGTACCACCAATAATTGCGGACGAACCTCCCCCAGTATCA
ACACACCTCACTGGCCTGGACGGATTAGCTT
>TR548|c0_g1_i2 len=851 path=[2087:0-87 2088:88-88 2559:89-116 2598:117-
119 2688:120-140 2674:141-163 2721:164-164 2702:165-188 2686:189-200
2672:201-208 2722:209-210 2732:211-212 2740:213-225 2546:226-259
2671:260-302 2723:303-312 2543:313-326 2698:327-336 2684:337-441
2724:442-472 2733:473-487 2622:488-520 2537:521-535 2715:536-566
2709:567-575 2697:576-589 2682:590-623 2725:624-625 2664:626-646
2528:647-670 2705:671-675 2692:676-694 2677:695-699 2659:700-718
2730:719-737 2603:738-744 2561:745-752 2513:753-774 2212:775-850] [-1,
2087, 2088, 2559, 2598, 2688, 2674, 2721, 2702, 2686, 2672, 2722, 2732,
2740, 2546, 2671, 2723, 2543, 2698, 2684, 2724, 2733, 2622, 2537, 2715,
2709, 2697, 2682, 2725, 2664, 2528, 2705, 2692, 2677, 2659, 2730, 2603,
2561, 2513, 2212, -2]
CCGATCTCCCAGCGCCGCCTCCATGACACCTTCACCAAACCGCACCATAACAACCCGCCGCATAAAATTAAAC
ACAGCGCCAAATTCAGTTTATCATTCATCATCCTCTATCCATAGCATCATTCATCCATCATGTCCAACGAAGA
AGACCTTGAGATGGGCATTACTCATTCGCTGGTTGAAGATGAGAACGGTGACACAACTCTTGCCAGTGATGCA
AATTCCTTCCCTCAGAATATGGCGGATGCCAACGATGCTGATACTCACTCTTCGCCCACGACACTACTTGACT

CCGAGCGGATTGATGACGTTGTCGATGCAGCTCCCTCAACAGCCAACATTGGTCAATTGCCCTCTGATGCTAA
TGCTGATGCTGTTCGTGGAAATAATACGAGTAATAAAATGAGTATGCAAATGCAAGATCTCATCGCTGAATTA
TTGGACACCAACCAAAGTGGCGAAATCAACCTTGGCCAGATAGAGTCCATTATGATGAGTGCTGCTGTGTCTA
GTAATTCAGCGGTGACAAGCGATGGCGGGAGAAGGGAAGATACTGCTCGTGCGCCATCATCAGTCGTGTCTAC
AATCCAAGATGATTTCGAGCCAGCCGATGCAGGAGTATCGCCGTTGTTTGCGGAAGAACCTTCTACAGTATCA
ACACACCTCACTAGCCTGGACGGGATAACTCGTGAGAATGGACCTCCCGTGACAGCAGAGGATGAAGCAACAT
CGTCATGGACGCTCGAGAATACCGGCATCGCCACTTTCTCATCTGTGGCAGCATTTGAAGGCGCACCACAAAA
CCAGGAAGAGCATTGCGATGTCGAATCGCCATCTTCGTATCACTCTGA
>TR548|c0_g1_i3 len=268 path=[2248:0-27 2249:28-28 2634:29-56 2655:57-67
2689:68-80 2674:81-103 2721:104-104 2731:105-106 2739:107-116 2701:117-
128 2686:129-140 2672:141-148 2722:149-150 2732:151-152 2740:153-165
2241:166-267] [-1, 2248, 2249, 2634, 2655, 2689, 2674, 2721, 2731, 2739,
2701, 2686, 2672, 2722, 2732, 2740, 2241, -2]
GATCTAATTAAACACAGCGCCAAATTCAGTTTATCATTCATCATCCTCTATCCATAGTATCATTCATCCATCA
TGTCCAACGAAGAAGACCTTGAGATGGGCATTCCTCATTCGCTGGTTGAAGATGAGAACGGTGACACAACTCT
TGCCAGTGATGCAAATTCCTACGGTCAAAACATTGCGGGTGCCAACGATGCTGATACGTACTCTTCGCCCACG
ACGCTACTTGACTCCGAGCGGATTCACGACGTTGTCGATTCAGCTAGCT
>TR548|c0_g2_i1 len=374 path=[2684:0-127 2541:128-261 2388:262-275
2389:276-373] [2684, 2541, 2388, 2389, -2]
CGATGCAGCTCCCTCAACAGCCAACATTGGTCAATTGCCCTCTGATGCTAATGCTGATGCTGTTCGTGGAAAT
AATACGAGTAATAAAATGAGTATGCAAATGCAAGATCTCATCGCTGAATTATTGGGCACTAACCAAAGTGGCG
AAGCCACCCTTGGCCAGGTGGAGTTCATTATGATGAGAGATGCTATGTCGAGCAATTCAGTGGTGACAAGTGA
TGGCGGGAGAGGGGAAGATGCTGCTCGTGCGCCATCATCAGTCGTGTCTACAATCCAGGATGATTTTGGACCG
GCCGATGCAGGAGTACCACCAATAATTGCGGACGAACCTCCCCCAGTATCAACACACCTCACTGGCCTGGACG
GATTAGCTT
>TR548|c0_g1_i4 len=693 path=[2248:0-27 2249:28-28 2634:29-56 2655:57-67
2689:68-80 2674:81-103 2721:104-104 2702:105-128 2686:129-140 2672:141-
148 2722:149-150 2732:151-152 2685:153-199 2671:200-242 2723:243-252
2699:253-276 2684:277-381 2724:382-412 2586:413-506 2734:507-515
2741:516-529 2669:530-563 2649:564-587 2621:588-589 2666:590-592
2646:593-610 2618:611-615 2576:616-637 2661:638-639 2639:640-658
2730:659-677 2603:678-684 2561:685-692] [-1, 2248, 2249, 2634, 2655,
2689, 2674, 2721, 2702, 2686, 2672, 2722, 2732, 2685, 2671, 2723, 2699,
2684, 2724, 2586, 2734, 2741, 2669, 2649, 2621, 2666, 2646, 2618, 2576,
2661, 2639, 2730, 2603, 2561]
GATCTAATTAAACACAGCGCCAAATTCAGTTTATCATTCATCATCCTCTATCCATAGTATCATTCATCCATCA
TGTCCAACGAAGAAGACCTTGAGATGGGCATTACTCATTCGCTGGTTGAAGATGAGAACGGTGACACAACTCT
TGCCAGTAATGCAAATTCCTTCCCTCAGAACATGGCGGATGCCAACGATGCTGATACTCACTCTTCGCCCACG
ACACTACTTGACTCCGAGCGGATTGATGACGTTGCCGATGCAGCTCCCTCAACAGCCAACATTGGTCAATTGC
CCTCTGATGCTAATGCTGATGCTGTTCGTGGAAATAATACGAGTAATAAAATGAGTATGCAAATGCAAGATCT
CATCGCTGAATTATTGGACACCAACCAAAGTGGCGAAATCAACCTTGGTCAGATAGAGTCCATTATGATGAGA
GCTGCTGTGTCTAGCAATTCAGCGGTGATAAGCGATGGCGGGAGAGGGGAAGATACTGCTCGTGCGCCATCAT
CAGTCGTGTCTACAATCCAAGATGATTTCGAGCCAGCCGATGCAGGAGTATCGTCGTTGTTTGCGGAAGAACC
TTCCACAGTATCAACACACCTCACTAGCCTGGGCGGGATAACTCGTGAGAATGGACCTCCCGTGACAGCAGAG
GATGAAGCAACATCGTCATGGACGCTCGAGAATACC
>TR549|c0_g1_i1 len=323 path=[301:0-322] [-1, 301, -2]
GGAACTTTTAGTGCTATTGACAACCTCGGCGCCCTTCCCAATTGTCGCATTATGGTATTGGCGGTGTTTGAGT
CCGCGATGTAAGATTGGATCCACCTCCTCACAGTCTAGATCGTCATCTTGGTCTTCATCCTGGTCTTCCGACT
GTTCGAGGTATTCCGAAGAAGAATCATGGTTATCCTCGCCATCTTCATCGTGGCTGTTACCAGTAATTTCACC
ACCGTCAGGAAGAGTTTCATTACCATCGTAATTGAAATCAAACGATCCCACTTCTTCGCTTTCTTCACTTGCG
CCACCCCCTTCTTGCGCGCTATCCATCCCAT
>TR550|c0_g1_i1 len=420 path=[398:0-419] [-1, 398, -2]
TCATCGCGTCGTCGCGGCGGAATATACCAGCTGCTGCATCCGCTGTCTCTGCCTCCTCCTCCTACAATAATAT
TTCTACGCAAAAATGGCATGGAATGGCAGCGGTTTCGAACGAGTGTTCCATCCACAAGCGTATATTGCGACGA
ATCGGATGGATCATCAGATGATGATAGACGACGTTCATAGCTTTGGGTTTGGGTAGTTGAACTTCGTTTCGTA
TCATTGTTGGTGTCGGATGCCGTCGATGATGATGCGGTGGCGGATGATGTACGACGCGAAGGGTCGTGATGTT
CCCAGTGATAAAACCATTTTAATTCTAGTTGTTTGGCGAGGGAAGCGGACGAGCCTACGAGGGACGCAAAGTC
CATGGTGTGGCCCCTCATATTATCTATGATGTGATCCGCTTCTTGGCTTAGTCGA
>TR552|c0_g1_i1 len=242 path=[220:0-241] [-1, 220, -2]

ATGATGATGGCATCAGAAGATGGCATGAGAATATTTCTCTCTTTAAATATAATATCTAGAGAGAGGTTCGTAC
GAATGCGAATGCGATGCCGGTGTCGCGGCCAGTCGCGGCGTCCGGCGTCACTTGCTAGAGTCAAAGGGTACTG
CCAAAGAGAAGGGTACTGTCTAAAACATTCGTAAAATCTTCGTCGGACGATATGTTTTTTTGTTTGCAGTTTT
TGCACAATCGCTTGAGAGAAGAT
>TR553|c0_g1_i1 len=344 path=[322:0-343] [-1, 322, -2]
GGAAGGTAGTACCTGCTGCTGTTGTTGTTGTTTCTCAATGGGCAAGGGGGAGGAGGTGCCGCCTAAGTCGATA
TCGAACAATTCCGCTACATTCGGAGAAGGATTTAGATTTGGAGGGTTGTTGTTATTATCCTCGTTGGAGGGAG
GTGCCTGCTGTTGTTGTTGCTGTTTTTGTTGATGTTGATCGATGGGCGAGGATATGTCGCCGATTATGTCAAA
CGATTGATCGAGGAGGCGTTCCGCTTCCGCTTCCGCTTCTGCTGCTTCGTCCGGAGGATGACCTCTCGATGCG
GAGACGAAATCCACCATTACCAACGGTCCTTCCAACACTGACGGCAGCTGGG
>TR557|c0_g1_i1 len=1123 path=[1101:0-1122] [-1, 1101, -2]
GACGCTCTTCCGATCTCTTAATTACCTACTATTACTTATCTAATACTTATTGGTTCTGGGAAATGCATACCTA
GCACTGTACAAAAATGGTTGAATTCTACCTAAAAACACTCGTCATGTTATGGTTCCAGGGGTGAGCTGTAGAT
TTTCATGGCGAGTGAATGACACACCGCCCCATGACTCTAAGCCTTGAATGAATCCCTTCCTACTTCCTTCTAC
TTCCTCCAAGCACTACGCCGAGAGATACAAAGGCGCCCCCAATAACTGCACCCGCAAATCCAGCCTTCACCAA
AGCATATGCATCATTATTCGCGGCAACTGCAACCATTTTATCTTCAGCGGCGTCATCACCATCATTCGATGCC
ATGACCCTTTCCGCACGAGGCACCACCCCAATCCACTGGACATTCCAGGGCGATCCCCTCGTGTCCAGCAAGA
TTCCCTGCAGCCGCTGAAAGAGAGTGTGACATCCGTCGATCTCCGATTTAAAGATGGAAACAGATTCGCAGCT
AATACCAAGTGGAACGCCAATACCGACGCATTCCGCGAGTATCCTGCGTAAAATGTCGCACAAGAACTCAACG
CTTTTTTCGACCGGCTTGTCGGCGTTCAGTATCAGCTCCTCGGCCTCTTTTGGGACGCGTACGCCCAGCCATT
TTATGAAATTCATCGTCTTCGCCCGGCTCACGGGGGCAAACGTCAACACGACCTTTCGCGGCGTGACGCCGGC
CTGTTTGCATATCGCAGCGTAATCCCTCAAGAGGCGAATTGTGGGCTCGGCATCGTATACGGCCTGACTGATG
AACCACTCGGCGCCGGCCTTGTGCTTTCGCATCATATTGAGATGCTCGGTAGGATATTTCTTTCCTCGAGCCT
TTGCGGCCTCGAGGGTGTGGCGCTCGGCAATACAGACGGAGCCGAAGTGCATGCCTTTGTGGGCATTTACCTT
TTCCATCGCCTCGGTGATAGTCGGGCCCTCGTACTTGCCTCCCGATGATGCCCTGCCAACAACGTTGATTGCT
GAGTGTCCATGATCATCGTGCGCCCTCTGTAACCATCCATCAAAATCCGGATCAGCGACACACTTGTAAACAA
GACACTCCCGTCCAGACGATCGCGCCAA
>TR558|c0_g1_i1 len=552 path=[530:0-551] [-1, 530, -2]
TCCCAAGTTGTGCTAATTGTTAATTGTTAACGAATAATAATATTGACCAGGAGGTCTTTCAGCCCTCCCATCG
TGTGAGTCTTTTGAAGTCTTGAGTAATGGCAATGGACCAACTTGAGCATTGTCAAGCAGAGATAGAATTCGCT
ATATTTCATTGGCCTTGTGGAGCCATTCGTTGAACGAGGAATCTATCGAGAGAGGAGAGAGTGTGAGGATATT
TATTTCGCTTGGGGTCTTGACGTCTCAGAGAGATAACATTATCGCGAGTATTCGCGAAAACGCTTGTCTAAAA
CCATCGCTAAGAAAGAGCCAAGACAGAGCCTAAGACCGATCCAATCGATCGAGTGGTTTTGCCAAGGTCCCCG
CCGCGCCGAAATCAGCGAGTCATATTATCGTGTCTCCGATCCCATCGGCGGATAGTTGTCGGGCCGATTTTGT
GAACCCCCGGGGAGTTGTTGCACCGTTCCAATAACATTTTTAAAAAACTTCAAATTTGAGAAAATTATTCTGA
GAATTCTGATGCAATCCCAATGATAATTGGTTTATCATTAT
>TR559|c0_g1_i1 len=264 path=[242:0-263] [-1, 242, -2]
CGCTCGTACATTTACCTGCGTGGCGCCAACCATTGCCACTGCAGACGAAGAAGATGTTCGTTGTTTTTGCCAT
TCACTAGATGATGTCGTGATACTATCGCCGATATTTTCACCATTGATGTCATCGCCAGTAGCGGTGGTTTCGA
GCCTATCGGTGTTTGATATATTGGAAGCGGCATAATCAATGTTGTTTCGATCCGATGCAATGGCACCCACATT
TTCAGCTGATGATACCACGGATGGGTCCCCCGTATTCAACGCATC
>TR560|c0_g1_i1 len=297 path=[275:0-296] [-1, 275, -2]
ATTAAAATATTACGCGTGCCGAAGGTGGTTAGCATTAAAAATAGTTCCGAAAAATATGCAGTAAGAGTTTTAT
TATTTTTGAACGACTTTTTGATTTAAAGTGCTAGCGATATTCCATCATTTAGTAATGCAATTTATATGCTAAT
GCCTACTAAGGCAACTGACCGTTTATTTTGTGTGGGCCCTTCGTAGTTAATATTTTGCTGAAGATAAATTGGT
CTTCCAAGATCGTATTACCGTAGATGAATGAGAATGAGATCGGAAGAGCGTCGTGTAGGGAAAGAGTGTAGAT
CTCGG
>TR562|c0_g1_i1 len=1238 path=[1241:0-55 1242:56-79 1243:80-1237] [-1,
1241, 1242, 1243, -2]
TCTTCCGATCTATTTTTTCGATCAAGGATATCGTATCGAAGTCATTCAGTTTGTGCTACGATACTGTTTCTGC
CACGATACTGCTCTACTGTCAATCTCCAATAACCATGACTAAAGTAGTGTTAGTAGCGACATCCGCCACCGAA
CTCAAGGGCCACGCCACTGGACTCTGGATTGAGGAACTTGCGGCGCCCTACTATCAGTTCAAAAAGGCGGGTT
ATGACGTGGTCATCGCCAGCCCCGCCGGTGGCGCCGTCCCGATCGATGCCGGTTCCATGGCCGAAGGTTTCTT
CACCGAGGAGGCTAAGAAATTCATGCACGACGCCGAAGCCTTCGGTCAGCTCAGCCATTCCTTGAAATTAGAT
TCCATCGACTTCACTACGGGTGTGGATGCCATTTTCCTCTGTGGAGGGCATGGGACATGCGCGGATTTCGTCG
AGAATGCTGCTCTGAAAAGTGCCATTGAGACCCTGTATGCCTCCGACAAGATCGTTTCGGCCGTGTGCCATGG
TCCGACGGGCCTGCCGCAATGCAACAAACCAGACGGAACATCGTTGGTGAAGGATAAGTTGGTTACTGGATTC
AAAGACTCCGAAGAGATGGCTGTGCAGTTGCAGGATCTCGTGCCTTTTATGCTGGAGGCGAAGTTAATAGAAC
AGGGAGGTAAATATGAATGTGCCGATGACTGGAACTCCAAAGTATGCGTCGATGGGAATTTGATAACCGGTCA
GAATCCGCAGAGCTCGGAGGAATGTGCTGCTGCTGTCATTTCTGCCCTCTCTTCTTGAGAGTTTGTCCTAGAT

GCAACCCTGTTGATTTATCTTTCCCTTTGGTGAAATAACGAAGGGGCGTCAAAGCGCTTACGTCTTGTTTCTA
ATGGATAGATACGCTGGCTAAATCCCAGTTGAGGATCTCGACACGTTCAACTATTAACGTTTAGTTCAGTAAC
ACATAAGTTGTCTGAAGTCTTCTGCCATGTTGGTTCAACAACCTGAACTCGTCCATTAAGTCTAAACAATGGT
CGTGTTTCTTATGAGTCCCTGTGTTGGTGTCAAATATTCGGCGGTTAACAGCAAAGCGTGAATAAGTGATTGT
AAGTTGTACTCATAAATCAAAAAGAAGTAGCTGACAGAGTTTGGAAAATTTGTGCAAAATTAAGATCTATTCT
CCGCGAAATGATAAGAAGATGCACAATTCGTGCATGCATCATGAGATAAGTAATATTGTAGAATTGCCAG
>TR563|c0_g1_i1 len=283 path=[286:0-202 287:203-226 288:227-282] [-1, 286, 287, 288, -2]
ACATTGATAGAGAACAATGCCATCAATATTGCGAACAACAACGACAACAACGCCAACAGTAGTACGGCAGCAT
ATTTATGGGCGGGAGTTTTGGTACTGAGCGGATTCGTGGTCTTGATGGAACACCACCACGTATTCTTCTTCAC
GTGGAGGAAGGGAATGCAGTATCGCATCTCGAGCATCGCGGCCATATACGACAAATCCCTTCGCCTCAATTCT
ACATCTTTCACGGAGCAATTATCATCATCATCATCATCATCGCCTGGCGACAAAAGCAAGGGCA
>TR564|c0_g1_i2 len=1383 path=[1371:0-760 1388:761-1349 1389:1350-1350 1390:1351-1352 1391:1353-1353 1382:1354-1355 1383:1356-1356 1384:1357-1382] [-1, 1371, 1388, 1389, 1390, 1391, 1382, 1383, 1384, -2]
GTTCTTGCATCCTTCATTCATTCATTCACCTTTCATTCACGGCCAAGGCAAAGAACGGAATGATAACACCTTC
TTGGGATGAATTCAATGGATCACAATTTATCGCACTAAAAAAAGTCACGAACAACTCAACTCATCTTATCGAC
AACCGTTCTTCCGAAACAGCTACATACATCATACATCATACATCATGTTCTCTGTCCATCAGCCACTCTACCT
CCGCACTCAAACTCAAACGACGACGCTCTCCGACACGGATGAATTCTGGCTCTCTCCCCTCCCCCAAGAAAAC
ACCACGCGCTCCACCAATGTCAGCGCGTTTGCCACCTTCCGCGGCATTAACGACATCGGCCAAGACCGAGATG
ACCACGACGTCTTCACGACAACGCCGTCCGCCCCGTCGTCGTCGTCGTCGTCGGCGCACCATCAGCTCCTCGC
ACATGACATTCCTTCCTCCGCAGATCTCAAGGGTTACCATGACATCTACGACAAGGATGATGAATTGTCGTCC
TTCTTTCGTCGTCCAACGCTGACGGAAGTGAAACATCAGGGTGGCGGATCATGGCTGTTGGGATTTGTCGGTG
CTGCCACAACAACAACCCCCGAACAACGGGAGGATAATAATGTAGCACCCATACTACCCGGCCTCGTTCGTGT
TCCCAGCTCTGATAGCGAAAGCGACAGCGACGATGACGACGAAAGCATCGCACTCCAATCTTCCTTCTCGACA
ATCGACGACGAAAACGACAGCATCCAACAACCAAAATCAACAACACCTCAGAAACGAAAAAAGGGAGTATCCT
TCTCCCCCGAGATACAAATCCAACCGATTCCACACGTCTCCACCCTCTCCTCCACTCAACGAAGGAGAATGTA
CTCCTCTTCCGACGAGGTTCGAAGGAATAAAATTCGCAATACGAAGGAATATCGGTACGATGGATGCGATTGG
AGGAATCTTACGGAGGAGAGCGAGATGGATGTGGATGATGTTACGGGAGAGTTGATCCATCCCGTGCATGAAT
ATGAATGGAGATGAGAGAGATTTTTTGATGGAGACACACGGAAAAAGATTAGTGTGTGTGAACATTTATCGTG
AATGAAGGTCTCTCTCCTTCCTTCTCTGTGCTGTACGTTGGTGTGTGGTGTTTTGGGTTTTTTTTGTCTGCCT
GTGAGTTTTCATGGTGTGCATAGAATAAATCAATGACTGCCCACGTATCACATTGAGAGGGTGGGGGAACATA
CATTGCGAAATTCCAAGGTACAAATATGCGGAACTCATGACGCGGCTGCTGCGATATCGTATCTGGATGCCAT
ATGCACACCACACACAACAACAACAACAACAACAACAACAACAGATGAAAATATTGGCAGAAATTCATC
>TR566|c0_g1_i1 len=1232 path=[1229:0-866 1230:867-1231] [-1, 1229, 1230, -2]
TGCAGTTATGAATCTTAACGTATAATTGTAAGAACTCGACGTTCGTTTTCATCTTGGACGTATGTAGGTCCAA
GGTTTTCATGTACAAATTATGTTCCTGCTTCCTACAGGCTAACAGCCCGTTGGCCAGTCTTGTCGAGATCTGT
GGATACCACTGCCCGACTTGCAGAGAATGGAGCAAGGTCGACGGGAGCGGCCTCGCCCGTCAGCAAAGATGCC
ATGCAGTCTCCTAGCAGCGGCCCAAATTTGAACGCTCTCCCACTTCCGCCAGAAAATACGGCAACGTTGTCCG
CCACTTTGTCCATGACAAAGTATGAGTCCTCTGTCATGGTGTATGGATGGCAGTACGAATCAATTCTTTTTCC
AACAGAGGGTAGATGTTCTTTCACCCACCCATCTATCAGTTCCAGCACGTCTTGTGATCCTTCGTAGTCGAAA
GAGCTCATGTCGGATAATGCCTCCCCTGTAGGAAAATCGACTCCTACCTTGACAAAGGACTTCTTGTCATTTT
GGATGGATTCAGTGGCACTCGATGGGAACACGTAGTACAACCCTCCGTCAATATCATTTTCCTTGCGAAAGTG
AAATGCCTGGGGAATTGATGCGGCCACGTTGGTGTCAACTTCATAGTGCGCCCACTGCACTTGCCAAATGTCG
AGTTTCAATTGAGGCAAGTTGGCAGATTCGAGGACTTTATTCGTCCATGCTCCGGCGGATACGACAGCATTGT
CCGCGGTGATGGTCTCGCCATTTTCAGTGACGACTACGACCTTTCCCTCCTCGCCTTGGGCATCCAACGAGAC
AATTCTGCTGTTCAGCTTGACGTCACACTTGTCGCCAGCTCCATCCGCCATGGCTTGGCAAGCTTTCGATGCG
CGGATATGACCGGCGGTATCCTCACACACGCCGCTGTAAGGCTTTCCTCTGCAGCCCTCGAGGGCGGGATACG
CATCCGCTATCTCGTCACCCGTCGCATAAAACTTGTGAGGCAGATTCAAATTCTCCATGACCTCCTTGGCGCC
CAAGACAGAACCTTCTACTGTCTCGCCGGTGTCCTCTCCATAAAACAAAAGACCGTTCTCCTGCAACAACTTG
ATACCGCTCTTTCTCTCGACGTCCTTCCATCGGTCCAATGCCTGGGCCTGCATCTTTGAAAAGAATTCCGAGC
TGTACATCTTACGGTACATTCTCGAGTCGCCGTTCGACGATCCTGTCGGCGGACTCAGAGATGA
>TR566|c0_g2_i1 len=884 path=[1227:0-866 1228:867-883] [-1, 1227, 1228, -2]
TGCAGTTATGAATCTTAACGTATAATTGTAAGAACTCGACGTTCGTTTTCATCTTGGACGTATGTAGGTCCAA
GGTTTTCATGTACAAATTATGTTCCTGCTTCCTACAGGCTAACAGCCCGTTGGCCAGTCTTGTCGAGATCTGT
GGATACCACTGCCCGACTTGCAGAGAATGGAGCAAGGTCGACGGGAGCGGCCTCGCCCGTCAGCAAAGATGCC
ATGCAGTCTCCTAGCAGCGGCCCAAATTTGAACGCTCTCCCACTTCCGCCAGAAAATACGGCAACGTTGTCCG
CCACTTTGTCCATGACAAAGTATGAGTCCTCTGTCATGGTGTATGGATGGCAGTACGAATCAATTCTTTTTCC

AACAGAGGGTAGATGTTCTTTCACCCACCCATCTATCAGTTCCAGCACGTCTTGTGATCCTTCGTAGTCGAAA
GAGCTCATGTCGGATAATGCCTCCCCTGTAGGAAAATCGACTCCTACCTTGACAAAGGACTTCTTGTCATTTT
GGATGGATTCAGTGGCACTCGATGGGAACACGTAGTACAACCCTCCGTCAATATCATTTTCCTTGCGAAAGTG
AAATGCCTGGGGAATTGATGCGGCCACGTTGGTGTCAACTTCATAGTGCGCCCACTGCACTTGCCAAATGTCG
AGTTTCAATTGAGGCAAGTTGGCAGATTCGAGGACTTTATTCGTCCATGCTCCGGCGGATACGACAGCATTGT
CCGCGGTGATGGTCTCGCCATTTTCAGTGACGACTACGACCTTTCCCTCCTCGCCTTGGGCATCCAACGAGAC
AATTCTGCTGTTCAGCTTGACGTCACACTTGTCGCCAGCTCCATCCGCCATGGCTTGGCAAGCTGAAATATGA
CGATAAAA
>TR567|c0_g1_i1 len=508 path=[543:0-293 559:294-321 557:322-339 555:340-363 542:364-507] [-1, 543, 559, 557, 555, 542, -2]
ATTGCTGTTTCCGCCGCCACCGCCTTTCGCGACGGCGGGCGCGTCCCCGGCAATCGACTCGAGCATCGTCCT
CCGGGACGGAGTCTCCCCGTGTCGAATACGGTTACCTCGAGCTCGTAGCGAGAGGATGGCGAGGATGAAGGAT
CGACGTACGACTTCTTGTGTTGCGTCAGCAAGCGAGACGCGCAGGAGAGGCCGGCGATGCCTCCTCCCAAGAT
GGCAATGGACAGTTTCGACTTTTCGTGGTGGTGATCGTTTTCGTTCATTGCCAACGAGGAGGAGGAGTAGGAG
GAGGCCGCCGTTTTGGTGCAAGCTTTGGCAGCACGGATAGCGCGTGATAGAGCAAAACCGAGTACAGACTTTG
GCAACGATGTGCCCAGCAAGCAATACATGCAGGTGCGCAACAGCTTCATATTACGATACTTATTGATGTGTTT
TTCATTGTTGTTGTGTTAACAAGTCTAGGTATCGTACTTGTCGTAAATCATCACATCATGTTACAGATCG
>TR568|c0_g1_i1 len=565 path=[596:0-292 597:293-316 599:317-426 594:427-450 595:451-564] [-1, 596, 597, 599, 594, 595, -2]
TGTCGACGTGAAGCGTGATGGGCACATTTGAGTGCAGTTTTTTGCGCAGTGCGAAGGAAGGTCGATTCGCCGG
AGCTGGCTGCCGACGGACCGCTATCATTGGATTGAGATGCGGGAATGATATTCTTGGTGTAGGCGTGACTTCG
AGGATTTCTATTGGTTTTAGTAGGATGACGACGTGGTTGTTGTGACTGTGGCTGCGGCTTTGGGCTGGAATTG
AACAGATGACCCGATGGAGATGTCAACGAAGGAAGTCCAAGAGGATGATAAAGCGGACGTTTCCGCTTAGCAG
AAGAGAGCGGGGACGAACTTGACGCCCATCTTTTGCCCGGCGTTGTGCTGTTGTTGGGTTGATAATTATTAGA
AGGCGCATTTTGAGACGATCGGGCCAGCCGTGAGTCAATTATTCCAGCTGGCCTTTTTTTTTTTTCAGCATGA
TTATGGCGGGGTCTGTCTCTGCCGGCTTGATGATTCAGGGAATTGGGAGCTTCTGATAATGAGGGCATCCATG
TCGACTCTACAGGCGGGGAGAGTCTGCGGTGGGGCCCGTCTCTGCCGGATTGAT
>TR569|c0_g2_i1 len=1300 path=[1352:0-461 1359:462-520 1350:521-544 1358:545-628 1356:629-897 1343:898-1299] [-1, 1352, 1359, 1350, 1358, 1356, 1343, -2]
TTAATATGACTGGTTTGTCCTACTTAAAGTGCTCTGAATCGTGAATCTTCCGTACCACACAATTTGCACAAAC
CAACACAAACACGTGAAGATAAGAAGCCATCATACGATACCATCGTCTCTACAAAACAACATTTACTCGCGCG
GCATCGTCTCTCACACTCCACCCCTCCCCTAGCCCCAACAGATCAACTATGACACGCTCACACCCTTTCGTCA
CACTCCTCGTAGCTGCCTCGTCGGCATCCGCTTTCGTTTCGAATCATCCATCAACCGGAGTAGTGACGACATC
ATGTGTATCAAATCCCGTTGTTCGAATGCACAGGCGCGCATCAAAAAGAGGGATGGAATTGAAAATGAGTGAT
ACAAAGAAGGCAGCGCCACAGGTAAATGGAGAAGAATTAGAAACCATGCTGCAAGAATGGGACCAACCTCTTG
TGGTGGATGCCTACGCTACCTGGTAAGGAATTGACGCCCAGATTTCGCTGGTTACTTCTACTATTCTTTGTAT
TCAACCGCTCATGAATCAGTTTACGCTCGTTTCCCGATCCATGAACTCGGGTCATTCATCACATAATCAAAAA
TGAAAAAAACACAACCACCTTAGGTGCGGACCCTGCCTCCTGATGGCACCTGAGTTCGAGCAAGCAGCAGAAG
AACTCGCTGGAAAAGTCAGATTCGTCAAACTCGACACCGATCTCGAACCGGAAATGGCCACTCGTCTGGGTAT
CATGGGATTGCCAACTCTGCTCTATTTGGACAAAAATGATGACACGGAAGCGGTGGCAGAGGGCAAAGCACCG
GTGGCCGTATTGAAGGAACGAATTGAGGGAGCACTGAGGAAGCAGAGTATAATGGACCTTTGCGACTTCCACT
TTTTTGATGGTCCACAGCCAACCCAGCTGAGTTAAAATGGGTTGCGCCTCTCGGGGGTTTGATTTGTTTGGTG
GAAGGGTGGACATATTATTTTAATTTTGCTAATAGTTGGAGAAACCCATGTTTGTGTTTCATTTGCCTTGTCA
CGAGAAAATACACAGTATTCTTCGACACTGTGAACACGGAGTTTGCTGTTTGCTTCGAGATCTTATTGAAAGA
TTAATGTCTAGGACCAGTGTCAATTTAAGAATACACGCGAAAGGCAGATCTTAAATGTTTGCCTGTCAAAATT
TCGTAAAAGTGCCAAGTTCGTGCTCTTGGCATCGTCATGACATAAGTCCGTGTGGCGCTCTATCTACCTTTAG
ACGAAGTAATACTGTAACGTCTCTATCTCGACATTTAAAAAAAAAAGATCGGAAGAGCG
>TR569|c0_g2_i2 len=1153 path=[1346:0-461 1347:462-481 1356:482-750 1343:751-1152] [-1, 1346, 1347, 1356, 1343, -2]
TTAATATGACTGGTTTGTCCTACTTAAAGTGCTCTGAATCGTGAATCTTCCGTACCACACAATTTGCACAAAC
CAACACAAACACGTGAAGATAAGAAGCCATCATACGATACCATCGTCTCTACAAAACAACATTTACTCGCGCG
GCATCGTCTCTCACACTCCACCCCTCCCCTAGCCCCAACAGATCAACTATGACACGCTCACACCCTTTCGTCA
CACTCCTCGTAGCTGCCTCGTCGGCATCCGCTTTCGTTTCGAATCATCCATCAACCGGAGTAGTGACGACATC
ATGTGTATCAAATCCCGTTGTTCGAATGCACAGGCGCGCATCAAAAAGAGGGATGGAATTGAAAATGAGTGAT
ACAAAGAAGGCAGCGCCACAGGTAAATGGAGAAGAATTAGAAACCATGCTGCAAGAATGGGACCAACCTCTTG
TGGTGGATGCCTACGCTACCTGGTGCGGACCCTGCCTCCTGATGGCACCTGAGTTCGAGCAAGCAGCAGAAGA
ACTCGCTGGAAAAGTCAGATTCGTCAAACTCGACACCGATCTCGAACCGGAAATGGCCACTCGTCTGGGTATC
ATGGGATTGCCAACTCTGCTCTATTTGGACAAAAATGATGACACGGAAGCGGTGGCAGAGGGCAAAGCACCGG
TGGCCGTATTGAAGGAACGAATTGAGGGAGCACTGAGGAAGCAGAGTATAATGGACCTTTGCGACTTCCACTT

TTTTGATGGTCCACAGCCAACCCAGCTGAGTTAAAATGGGTTGCGCCTCTCGGGGGTTTGATTTGTTTGGTGG
AAGGGTGGACATATTATTTTAATTTTGCTAATAGTTGGAGAAACCCATGTTTGTGTTTCATTTGCCTTGTCAC
GAGAAAATACACAGTATTCTTCGACACTGTGAACACGGAGTTTGCTGTTTGCTTCGAGATCTTATTGAAAGAT
TAATGTCTAGGACCAGTGTCAATTTAAGAATACACGCGAAAGGCAGATCTTAAATGTTTGCCTGTCAAAATTT
CGTAAAAGTGCCAAGTTCGTGCTCTTGGCATCGTCATGACATAAGTCCGTGTGGCGCTCTATCTACCTTTAGA
CGAAGTAATACTGTAACGTCTCTATCTCGACATTTAAAAAAAAAAGATCGGAAGAGCG
>TR570|c0_g1_i1 len=900 path=[952:0-601 953:602-625 958:626-703 956:704-727 957:728-899] [-1, 952, 953, 958, 956, 957, -2]
CGATATGTAAACTTCGGATTTTGAAGTTGTTGGGTGAAAACACCGAAGAACTTCTCATCACACTAAAAAAACA
AAACAAAACAGCCTAGGAGTAATTATTATCAGTGTACAACTATGAAGGAAATACAAATAATGCAAGCAGCTCA
TCATCGGCGGAAGTCACCATGCCGTTCATTATTACTCATAGCATTCACGACATTAGTCCTACTTCACGAAACA
CAAATCGTCACCGTATCGTTATCATTATCATCCCATGCCTTCCAAATCGCACCATCGCAGTATGTCAGAACGA
CGAAACAACAACTGCGTAGCTTGTCCACCACCATCATCACTCGCAGCAGTAGCAGAGACGGCGACAACAGCAG
CGACAACGATAACAGCAGCGACAACGACATCTCCAATTCCAACAACGGCAACAAACTCTACTTCGACATTGCA
ATAGCACCATCGCCCACCGAACTCATCCCCCTGGGCCGCCTGACCTTCCAAATCACGCCCCCCGCTCACCCCG
CCTACCTCCCCCTCCACACATCCAACCTCCTATCGCTGGCCTCCGAACTCCGCTCCTCCGTCGATCCAAAGTG
CACGTATCGAAATTGCGTGTTTCAGTATTCGCCATCGACGATAGACGACGGGAGCGCGCGATACAGGTGGGGA
CACGTGTGCGAAGGCCACGGTAGGAATGGGATACGGACGAGAGATGCCGGTACGGGAAGGGAACGGGGGTGGG
AGGATGCGTTCTCGGATCCGGAGAGGGTGAAGGAGTGTGCCCATTCTTGTTTTGGGGGGGTGTATTACGGCAT
GAGTTACGATGAGATTATTTCGGACAGGGAGAAGAAGAAGAAGGAGGAGGAGGGGGAGAGCGACGACGAAGAC
GATGTCGCGGTGTTGTTGACGGTG
>TR573|c0_g1_i1 len=1018 path=[1069:0-569 1070:570-593 1084:594-812 1085:813-842 1076:843-868 1077:869-1017] [-1, 1069, 1070, 1084, 1085, 1076, 1077, -2]
TACGGCACGCATCGCTAATGGTCACTGGCCACCCACTCATGCACTCCATAGATAGTATCGTACCTCCATACAG
ACAAATTGCCACCCCCGATAATATCCAGCGCAGTAAATCCATCAAAAAGCAGTGTAAGCATGGCGCTCCACGG
GGATGATGAGATTGATTCCCTACTCGCCATGATGGGATCACCGGTAGGCCAAACGCCAACTCTCACATCCATC
ACATCATCATCGACTACGAAGTCCACCACTGGTAATGGTGATGACAAACGAAGTAAACAAAAGACCAGGAAAC
GAAAAAAAGAAAAAAGGGCTTAAAACCAAGGAGGGCGATGCATCTTTTAGACGCAACAAGTCAAATTCCAAAA
ATCATAAAACGAAATCTTATGCAGATACGAGACTAAGTCAAAACTGCTTTGAATGGCCCTGTACCAATAATGG
GCAACGTTGCCTCCACTTGAAAGGTGAATTATTAGACAATTGCGAAAAATACGTTTGTAGCAGCAGTGATGGA
CGTTATGGAGCGAGTGCATGCCAATCCTCCGGAAAAGCGGCTGTCAATTTCGAATTATGTCTCTCCTCCTCTT
GGGAAAGTGGAGTTGACAGTAATGCATCATCATCATCATCGTCCATCGCGGATTGTTTTTCACCCGATATATT
ATCGATAGCGAAGATTATTGTGGCGACGAGGAATGCGAGGTGTTTGATTGGTGAATATTATGCAACGTCAAAC
AGCAAACAAAAAATGAAAACACATGAACTATCAAAGAATGTGCTTCTGCCACCGTTGAAATCTACTTTGACCT
CTTCTGACGTGATATCAGACAATGTAGATCGTTTTCTTGGGAATGCTTTGGCCTCTCTGAGAAGTATGCAGAA
TTCTAGTCCCAAAATAATGAAATCTCGCCATGCTCTTAATGGTGGAATGCAAGAAGTGACAATTTCAGCAAAT
GACATTCAACTGTTACAAGAAAAGGTTTCTGCATTGATCAATGATGTTCAAGGTTACAAGGAAGCGATG
>TR574|c0_g1_i1 len=2046 path=[2085:0-361 2086:362-385 2096:386-806 @2095@!:807-881 2091:882-2045] [-1, 2085, 2086, 2096, 2095, 2091, -2]
CCATGGTTAATACGTCTGTACTAGCTCTAGCAATTAGAACTCAAGTGCAGAGGAACAACACAAAAACGATTGG
TGCACCATATTGGAGCTACACTGTAGGATAGACGGTGCATCGAAAGCTACAAGTAACAATTACCAATTGCATA
AACGCAAGTACGAACAATGCCACATCACAAAAGAAGAATATTCTGCCTTAAGAGAGCAACACCAGGTTATGAA
TAGATCAATATACAGAGAAACGAAAGTACGAGCGATGACGTGTAGGAATAATTTGCACAAACACTGGCGTTCT
CAGCATTCAAGATGTCCGATAAGTCCTTGGGATTATCAGTATCGGTATTACTGACATCTCCAAGTTCGCCACC
TGCCGCTCCGACGTTGTCTCCGGCTGCATTGGTTCCTTTTTCTTCGGGCATACCAACAGTCCCAGCTTCACCA
TCTGCTGTTCCGGCGTTGTCTCCTGCTGCGTTGGATGCTTCCTCTGCAGGTACACCAAGAGCTCCAAATTTAT
CACCTGTCGCTCCGATGTTGTCTCCAACTGCATTGGTTCCTTGCACCGCAGGCACATTAATGGCTCCGACTTC
ACCACCTACCACTCCATTTGTACTGTCAAAACTGCCTCCGGCACAATAAAGGTCAACCATGCCGGGGGGCACA
CGTACCTGTAACCGACCCAACCCTTGTCTGCCAACGTCATCGCATTGGTCACTTAATTGGCAATTACTATGTA
GAATCGAGCATAGCTCGCCCGCAAAGTCGTAAGTCAAATCCGACGCTGTGAGATTCGCGTACGAAGTGGCCGT
AATGGGAGAACACTGCTGCGCAATGGTACTCGCATAATTTCGGAATGGAGCGCAAGTTTGCCACATCAACATA
CTGTAGGTAAGGCTGGCCAATTCTGTTCCGAAGCTAGAGCTGGAGCTCGTGCATTGCATACTCTTCGGATCCC
AGTCCACCGATAAGGCCAGCGCTTCTCGAGTCGAACCGCGACACGTTACCTCGAGGGAAACTCCTTGGAACGT
TTGGGCGACGGCATCGGCGAGATCGTTGGCGTCCATGGAGCACTCGAATGATTGCGGAGTGATTCGGTTGCAG
GCGGGAGTTGAGTCCAGGTCGGCAGACACCAGCTCCGAATCCGGTCCGCAATTGACGGAAATATCGGAAGGCG
CGTCGAAGGAACAGGTGTCTCCATATATAGATTCGACATCACGGAGAGCAGTCAAGACAATTTCATAGTTAGC
CTCTAATGGTCCGTCTGGAAGCGTTGATGAGTCATCAATATTATTAATTCCCGAGCTTAGGTCGTTCGGTCGC
CAAAGGCAGTCTGACCATACTGGCGAGGATGCATCCGTCACATCAAGGCTAAATGATTCCAACAAGGTTCCAG
CCACGCAAGGCCCATCGTCCTCAATATATGGCGTAGGGTTATCCGTACAGAGTCCGCTCCTGTAACAGGAATA

GATCAACGACGGCACGGCTCCATCGCTACATTGATTATTTTCGAACTGCATGGGAAATGACACGAATCCGGGA
ACCGGCGGACATACTGCCGCCACGTTAAGTGTGCGACTGTATGACGATATTCCGAATGATACGCCGGTGCTGC
AGTCGAGGGTGTCTTCTTGCAGTGTGACGGTTGCCTCGCGGGAGGCATCGGTCGGTCCTTCGCAAGCGAATAC
TAAAGAGGCGATGGTTGCGCCGGGCAAAGAATACAGGCTGCTATCGGTGCATGTCATGGTGGTTTCATCGATG
GTGGTACAAGCAGCCAAGCTGAGTCCTTCGTCGGAATCCAAGTTGCGAATTACTCCATCGTCGCCGCATTGCA
CGACGTATGATACGGACTGGGGGAGAACGCAATCCGATGTTGTGACTGCGGAGACGGTGTACATAAGGGCCAT
GTCGACCATGGCTTCATCTGCTTTGACCATATTCAAGAGGTAGAGTCAGGTGGATGCTAGAAAAATTAGCCTA
TGCGGAATGTGTGGAAACATGATAGGATTAAGAAGCATGCTACAACTAAAAAGATTTTGTTTATTCACATCAA
AA
>TR576|c0_g1_i1 len=283 path=[368:0-60 369:61-84 383:85-98 381:99-122
384:123-140 382:141-164 380:165-199 378:200-223 363:224-282] [-1, 368,
369, 383, 381, 384, 382, 380, 378, 363, -2]
AACCGCCATGGCACCGACGCCGATGCCGACCAATCCATATACATAGACAAAATACACCATAACATTCTACACA
ATGAGGCTCCTATTCCCACAAATCTCACTCCTATTGGCCCTCGGCGTCCCCTGCGACGTGAACTCTTTCACCC
CTCCCCAACGTAGTAGCATCAATTGGATCAATAATGCAGTTCCATCACAACTCACGCACTCATCATCATCGCA
GACCAGCACTGCACTCTACGCCAAAAAGAAAAAGAAAAACACGGCGAAAGACGCCGCCCTCGCC
>TR579|c0_g1_i1 len=727 path=[805:0-201 806:202-225 818:226-281 809:282-
305 819:306-429 815:430-453 817:454-526 812:527-550 813:551-726] [-1,
805, 806, 818, 809, 819, 815, 817, 812, 813, -2]
CGCTCTTCCGATCTATGAGGTTTAAGTTAAAATTAGCGTTATTTGTGTTTTTAGTGGTCAACGCAAGACCATT
AGGAGCGTGATTTTTACCAAGAAATTTTGCAACATCCAAAACGTGGATGTTTCTTCAAGGCAAAAAAGAAATA
AACTTGTATTCTTCAGAGCCTGTCATTCGCAATTTTGTCTTCGGTTTGTTTACCACATCCAATCTAGTAAATA
GCTTCTGTCATGCTGACATTAGGTAAGAGATTGCGACACCATCGTTAAATCACTCATATCAACTTGAAACACT
TGCTGCATGTGTACTGAAAAATCAATCTGTGAAGTGCAAGTTGTGTGCCGATGCACTCGTAAATTATTCGTCG
TCTTCCTCCTCGTCATCTTTCGGTTCCCTCTTCAACTCAAAAGCATTCCACACTCCCGTCCCCTTGACTTCAT
CCGTCGCACCAAGACTGTACGCGATTGTGTCGATCGCGGTCTTTATGGATCAGTAAATGAACATGGAGGGCGC
GGCGTCGAGAGAGGTGATGGATGGCTGCGTAGTGGAGATGGATGTAATTGCGTTTCCTCGTTGAATTGGAGAT
GAGGGGACAAACGCTCCTGCGGTGGAGAGCGCGGCGGCGAATACGAGTGCTGAGATACGGACCATCGTTGCAG
AGGGATGCTGAGTGGTATTGTGTAAGTAGTAGATATTGCAGAACAAGTGTAGTTATGCTCCTGCCGTGTG
>TR580|c0_g1_i1 len=1732 path=[2558:0-508 @2591@!:509-675 2595:676-688
2583:689-699 2592:700-708 @2590@!:709-876 2586:877-1034 2574:1035-1731]
[-1, 2558, 2591, 2595, 2583, 2592, 2590, 2586, 2574, -2]
CAATTGTTCGCCAGTGCCACTCGTACGATGAGGGGTTGCGAGGTTACGTCCATCGCCGTGTTGTTCCCGCTAT
CCGCACTATCATTATTGCCATGGTCGTCGTCAACAGCAGTAGTGGGGTAATCGTAGGCACGCAGTTGGAATGC
ATTTCTATTGACGCTCGGAGGTTCGGCACCGGGGGGCAACGATACGGTACCCCCCACCTTCTTTTCGTTGATG
TAACATCGCACCAACACGGGTCGAGAGAAGAAGACGAGCCACGTTAGGTCCGATTCGGAGAAGAATAGTTCCA
CGTCTCGCTCGACCAACATGCCACCACCATCGTGCGATTCTGCAAGAATGTTCCCTCGACTGCTATTTGGATC
TAGGGTGCCGCAACATGACATTCGTTTCGTTCCGTCGATTAAGGGAAGGTGGTCGGGAGAGATGGCTATTTCC
GATGCAATGGTGGGTGTTGTGACCGTGACGACGTCGTCGTCGTCATCGTCCCCATTGCTCCTCCTACAGCGCC
ATTATCCATACCAGAAGAACTGTGATGATGAATGACGTCCAGCATGTACTCTGCCATTTTCCTCCCTCCCGAT
GCCGATGGTTCCACTCGCGCTACGTAATCTTCGCTCTTCGTACCATCGAGAGGATGAAAGAGTGGAATTGGAA
TTACTTGTGATCCTGGTATGCGAATGGAGCTTGTCGCCTCTTCGAAAAACTTTCGGAGTAACAGTTGAATTTT
GGCCGGGTTGGTATTGTAACCAAGCGACCCTAGAGCAGCATTGGCCCACGAGGGTGTATTATTCTCATCGGGG
TAATAAATCATACATACTAATATTTTCTTCGGTTTTGTCTTTGCTGTTATTGCTTCAATGTATTTTTGAGTTC
TGAAAAGACAAGAGAGAGAGACAGAGAGAGTTGATGGTGGTTATATATCAAACACTATTTCATAAGGTCTTTC
TCGACTGTTGCATATAGTGTGCGTGCGTGTATGTCAGCCCAATCATAATTGCGTGCGACATACCTAGTGCCAA
AGAGATGACGGAAATATCCAAAGCAGGGTGGGCAGGAACATGTGCATGAAGCCAATGACGGTCCGCATCCACA
GCAGCAGTCATTCAGCGGAACTGCACCAAACGATCTCCCACTCTCCAAACAACTATTGGGCAAACATAGCAAT
CCCGCAACCGCAGCTATGGTAGCCGGAGTCGGATACAATGCAATATCATTCCCCCCAATGGAAACGATGAGCA
CATCCTCCTCACCGATATTATCGCGTAAGAATTTATCCTGCGGTCGTAATTTAGCACATCGTTCATTGAGCGT
AGTGGCCTCGACTGCCGTATTGATTGCGGCATATTTTACATTACCACGACCATCATCACGACCACCATCACGA
CCACTACCACTATTGGAATCAGAACTACCATCAACGTTATTCTTTGCGAATCGATATCGATCATCGCTCTCCG
ACAAAGAATTCAACCAATACGTAACATCGCAAACGCTACGTGGAGGTTGGAGAATATCCGCATATACGCCCAC
GGCAGGACGTTGATCGGCGAACCAGTGTTTGTTATCGAGGGAGGAATCGCCGGCGGTCCATATTAATGCATCG
GAGTTTCTTCGGAGGGGAGGATAGAGGACTTCGAGGTGGCGTAGTTTGTGTCCGTGGTATTCGCTGTAGAAGC
TGGTGCTGGAAATTTTTGCCATCTTTTGCGGGTGGTGTTTGTTGTGGTTGTTG
>TR580|c0_g1_i2 len=1863 path=[2575:0-467 2576:468-491 2594:492-639
@2591@!:640-806 2595:807-819 2593:820-976 @2590@!:977-1144 2579:1145-1165
2580:1166-1862] [-1, 2575, 2576, 2594, 2591, 2595, 2593, 2590, 2579,
2580, -2]

GGCGTTCACGCTGGTCAATTGTCCATGTCGGCGTAAAGAGGTAAGTACTTCCAAATCCACCTCCAAAATCATC
TTGAAAACGCTTTCTTAGATTAGTTTCCAATTCTTATTGAGTCTGAATTTTACGCTCGCAATTGAAGAATCCT
TCCGAGGGACGACGACTTAACAGCTCGTTTATAAGATGTCTCGGTTGTTTTCCCAAACGCAAAGAGAGAGGGA
GTTGAATGGTTTACATTCATACACCCAATCAATATTTTCTTACTCAAGTCGCTCTCTAATCGATACAATACGA
AGTTGTTGACAGTATATAATACCAAAACTGGAGTCATCATTACCTTGCCATGACATCTGCTCCGATATATGAG
TTTGCGTCGCCATCCCGATTCCTCAGGCACCATGAGCATCATACCTCCTCCAAAAACCATGAAGAGTGTCTAA
AACAAGTATACTCCACCATTACCTCCCTTCAGAGGGCGCGCGGCGGTTTATTCCACTCAGACACACACGCCCA
TATACACACAGTATATCAATTCCAATAATGTCTAAGTATTAGGCCCTCCCATCCATAAACGAAGTCGAAGGTG
CCGAAGAAAAACCCGACGTCGTCGTCGTCATCATCCCCATTGCTCCTCCTACAGCGCCATTATCCATACCAGA
AGAACTGTGATGATGAATGACGTCCAGCATGTACTCTGCCATTTTCCTCCCTCCCGATGCCGATGGTTCCACT
CGCGCTACGTAATCTTCGCTCTTCGTACCATCGAGAGGATGAAAGAGTGGAATTGGAATTACTTGTGATCCTG
GTATGCGAATGGAGCTTTGGTGTGTGTGTGGCGTGTGTATTTTTTGTTGTTGTTATTATTCCAATCAAATGTA
CTAAGTACTTAGCATTCAAATGATTCTTGACAAGTATAGTTTAGAGCAACATGCGTGTAGTAAGAGACATAGA
CATACCTTGTCGCCTCTTCGAAAAACTTTCGGAGTAACAGTTGAATTTTGGCCGGGTTGGTATTGTAACCAAG
CGACCCTAGAGCAGCATTGGCCCACGAGGGTGTATTATTCTCATCGGGGTAATAAATCATACATACTAATATT
TTCTTCGGTTTTGTCTTTGCTGTTATTGCTTCAATGTATTTTTGAGTTCTAGTGCCAAAGAGATGACGGAAAT
ATCCAAAGCAGGGTGGGCAGGAACATGTGCATGAAGCCAATGACGGTCCGCATCCACAGCAGCAGTCATTCAG
CGGAACTGCACCAAACGATCTCCCACTCTCCAAACAACTATTGGGCAAACATAGCAATCCCGCAACCGCAGCT
ATGGTAGCCGGAGTCGGATACAATGCAATATCATTCCCCCCAATGGAAACGATGAGCACATCCTCCTCACCGA
TATTATCGCGTAAGAATTTATCCTGCGGTCGTAATTTAGCACATCGTTCATTGAGCGTAGTGGCCTCGACTGC
CGTATTGATTGCGGCATATTTTACATTACCACGACCATCATCACGACCACCATCACGACCACTACCACTATTG
GAATCAGAACTACCATCAACGTTATTCTTTGCGAATCGATATCGATCATCGCTCTCCGACAAAGAATTCAACC
AATACGTAACATCGCAAACGCTACGTGGAGGTTGGAGAATATCCGCATATACGCCCACGGCAGGACGTTGATC
GGCGAACCAGTGTTTGTTATCGAGGGAGGAATCGCCGGCGGTCCATATTAATGCATCGGAGTTTCTTCGGAGG
GGAGGATAGAGGACTTCGAGGTGGCGTAGTTTGTGTCCGTGGTATTCGCTGTAGAAGCTGGTGCTGGAAATTT
TTGCCATCTTTTGCGGGTGGTGTTTGTTGTGGTTGTTG
>TR580|c0_g1_i3 len=1869 path=[2558:0-508 @2591@!:509-675 2595:676-688
2593:689-845 @2590@!:846-1013 2586:1014-1171 2574:1172-1868] [-1, 2558,
2591, 2595, 2593, 2590, 2586, 2574, -2]
CAATTGTTCGCCAGTGCCACTCGTACGATGAGGGGTTGCGAGGTTACGTCCATCGCCGTGTTGTTCCCGCTAT
CCGCACTATCATTATTGCCATGGTCGTCGTCAACAGCAGTAGTGGGGTAATCGTAGGCACGCAGTTGGAATGC
ATTTCTATTGACGCTCGGAGGTTCGGCACCGGGGGGCAACGATACGGTACCCCCCACCTTCTTTTCGTTGATG
TAACATCGCACCAACACGGGTCGAGAGAAGAAGACGAGCCACGTTAGGTCCGATTCGGAGAAGAATAGTTCCA
CGTCTCGCTCGACCAACATGCCACCACCATCGTGCGATTCTGCAAGAATGTTCCCTCGACTGCTATTTGGATC
TAGGGTGCCGCAACATGACATTCGTTTCGTTCCGTCGATTAAGGGAAGGTGGTCGGGAGAGATGGCTATTTCC
GATGCAATGGTGGGTGTTGTGACCGTGACGACGTCGTCGTCGTCATCGTCCCCATTGCTCCTCCTACAGCGCC
ATTATCCATACCAGAAGAACTGTGATGATGAATGACGTCCAGCATGTACTCTGCCATTTTCCTCCCTCCCGAT
GCCGATGGTTCCACTCGCGCTACGTAATCTTCGCTCTTCGTACCATCGAGAGGATGAAAGAGTGGAATTGGAA
TTACTTGTGATCCTGGTATGCGAATGGAGCTTTGGTGTGTGTGTGGCGTGTGTATTTTTTGTTGTTGTTATTA
TTCCAATCAAATGTACTAAGTACTTAGCATTCAAATGATTCTTGACAAGTATAGTTTAGAGCAACATGCGTGT
AGTAAGAGACATAGACATACCTTGTCGCCTCTTCGAAAAACTTTCGGAGTAACAGTTGAATTTTGGCCGGGTT
GGTATTGTAACCAAGCGACCCTAGAGCAGCATTGGCCCACGAGGGTGTATTATTCTCATCGGGGTAATAAATC
ATACATACTAATATTTTCTTCGGTTTTGTCTTTGCTGTTATTGCTTCAATGTATTTTTGAGTTCTGAAAAGAC
AAGAGAGAGAGACAGAGAGAGTTGATGGTGGTTATATATCAAACACTATTTCATAAGGTCTTTCTCGACTGTT
GCATATAGTGTGCGTGCGTGTATGTCAGCCCAATCATAATTGCGTGCGACATACCTAGTGCCAAAGAGATGAC
GGAAATATCCAAAGCAGGGTGGGCAGGAACATGTGCATGAAGCCAATGACGGTCCGCATCCACAGCAGCAGTC
ATTCAGCGGAACTGCACCAAACGATCTCCCACTCTCCAAACAACTATTGGGCAAACATAGCAATCCCGCAACC
GCAGCTATGGTAGCCGGAGTCGGATACAATGCAATATCATTCCCCCCAATGGAAACGATGAGCACATCCTCCT
CACCGATATTATCGCGTAAGAATTTATCCTGCGGTCGTAATTTAGCACATCGTTCATTGAGCGTAGTGGCCTC
GACTGCCGTATTGATTGCGGCATATTTTACATTACCACGACCATCATCACGACCACCATCACGACCACTACCA
CTATTGGAATCAGAACTACCATCAACGTTATTCTTTGCGAATCGATATCGATCATCGCTCTCCGACAAAGAAT
TCAACCAATACGTAACATCGCAAACGCTACGTGGAGGTTGGAGAATATCCGCATATACGCCCACGGCAGGACG
TTGATCGGCGAACCAGTGTTTGTTATCGAGGGAGGAATCGCCGGCGGTCCATATTAATGCATCGGAGTTTCTT
CGGAGGGGAGGATAGAGGACTTCGAGGTGGCGTAGTTTGTGTCCGTGGTATTCGCTGTAGAAGCTGGTGCTGG
AAATTTTTGCCATCTTTTGCGGGTGGTGTTTGTTGTGGTTGTTG
>TR580|c0_g1_i4 len=2000 path=[2575:0-467 2576:468-491 2594:492-639
@2591@!:640-806 2595:807-819 2593:820-976 @2590@!:977-1144 2586:1145-1302
2574:1303-1999] [-1, 2575, 2576, 2594, 2591, 2595, 2593, 2590, 2586,
2574, -2]

GGCGTTCACGCTGGTCAATTGTCCATGTCGGCGTAAAGAGGTAAGTACTTCCAAATCCACCTCCAAAATCATC
TTGAAAACGCTTTCTTAGATTAGTTTCCAATTCTTATTGAGTCTGAATTTTACGCTCGCAATTGAAGAATCCT
TCCGAGGGACGACGACTTAACAGCTCGTTTATAAGATGTCTCGGTTGTTTTCCCAAACGCAAAGAGAGAGGGA
GTTGAATGGTTTACATTCATACACCCAATCAATATTTTCTTACTCAAGTCGCTCTCTAATCGATACAATACGA
AGTTGTTGACAGTATATAATACCAAAACTGGAGTCATCATTACCTTGCCATGACATCTGCTCCGATATATGAG
TTTGCGTCGCCATCCCGATTCCTCAGGCACCATGAGCATCATACCTCCTCCAAAAACCATGAAGAGTGTCTAA
AACAAGTATACTCCACCATTACCTCCCTTCAGAGGGCGCGCGGCGGTTTATTCCACTCAGACACACACGCCCA
TATACACACAGTATATCAATTCCAATAATGTCTAAGTATTAGGCCCTCCCATCCATAAACGAAGTCGAAGGTG
CCGAAGAAAAACCCGACGTCGTCGTCGTCATCATCCCCATTGCTCCTCCTACAGCGCCATTATCCATACCAGA
AGAACTGTGATGATGAATGACGTCCAGCATGTACTCTGCCATTTTCCTCCCTCCCGATGCCGATGGTTCCACT
CGCGCTACGTAATCTTCGCTCTTCGTACCATCGAGAGGATGAAAGAGTGGAATTGGAATTACTTGTGATCCTG
GTATGCGAATGGAGCTTTGGTGTGTGTGTGGCGTGTGTATTTTTTGTTGTTGTTATTATTCCAATCAAATGTA
CTAAGTACTTAGCATTCAAATGATTCTTGACAAGTATAGTTTAGAGCAACATGCGTGTAGTAAGAGACATAGA
CATACCTTGTCGCCTCTTCGAAAAACTTTCGGAGTAACAGTTGAATTTTGGCCGGGTTGGTATTGTAACCAAG
CGACCCTAGAGCAGCATTGGCCCACGAGGGTGTATTATTCTCATCGGGGTAATAAATCATACATACTAATATT
TTCTTCGGTTTTGTCTTTGCTGTTATTGCTTCAATGTATTTTTGAGTTCTGAAAAGACAAGAGAGAGAGACAG
AGAGAGTTGATGGTGGTTATATATCAAACACTATTTCATAAGGTCTTTCTCGACTGTTGCATATAGTGTGCGT
GCGTGTATGTCAGCCCAATCATAATTGCGTGCGACATACCTAGTGCCAAAGAGATGACGGAAATATCCAAAGC
AGGGTGGGCAGGAACATGTGCATGAAGCCAATGACGGTCCGCATCCACAGCAGCAGTCATTCAGCGGAACTGC
ACCAAACGATCTCCCACTCTCCAAACAACTATTGGGCAAACATAGCAATCCCGCAACCGCAGCTATGGTAGCC
GGAGTCGGATACAATGCAATATCATTCCCCCCAATGGAAACGATGAGCACATCCTCCTCACCGATATTATCGC
GTAAGAATTTATCCTGCGGTCGTAATTTAGCACATCGTTCATTGAGCGTAGTGGCCTCGACTGCCGTATTGAT
TGCGGCATATTTTACATTACCACGACCATCATCACGACCACCATCACGACCACTACCACTATTGGAATCAGAA
CTACCATCAACGTTATTCTTTGCGAATCGATATCGATCATCGCTCTCCGACAAAGAATTCAACCAATACGTAA
CATCGCAAACGCTACGTGGAGGTTGGAGAATATCCGCATATACGCCCACGGCAGGACGTTGATCGGCGAACCA
GTGTTTGTTATCGAGGGAGGAATCGCCGGCGGTCCATATTAATGCATCGGAGTTTCTTCGGAGGGGAGGATAG
AGGACTTCGAGGTGGCGTAGTTTGTGTCCGTGGTATTCGCTGTAGAAGCTGGTGCTGGAAATTTTTGCCATCT
TTTGCGGGTGGTGTTTGTTGTGGTTGTTG
>TR580|c0_g1_i5 len=1863 path=[2575:0-467 2576:468-491 2594:492-639
@2591@!:640-806 2595:807-819 2583:820-830 2592:831-839 @2590@!:840-1007
2586:1008-1165 2574:1166-1862] [-1, 2575, 2576, 2594, 2591, 2595, 2583,
2592, 2590, 2586, 2574, -2]
GGCGTTCACGCTGGTCAATTGTCCATGTCGGCGTAAAGAGGTAAGTACTTCCAAATCCACCTCCAAAATCATC
TTGAAAACGCTTTCTTAGATTAGTTTCCAATTCTTATTGAGTCTGAATTTTACGCTCGCAATTGAAGAATCCT
TCCGAGGGACGACGACTTAACAGCTCGTTTATAAGATGTCTCGGTTGTTTTCCCAAACGCAAAGAGAGAGGGA
GTTGAATGGTTTACATTCATACACCCAATCAATATTTTCTTACTCAAGTCGCTCTCTAATCGATACAATACGA
AGTTGTTGACAGTATATAATACCAAAACTGGAGTCATCATTACCTTGCCATGACATCTGCTCCGATATATGAG
TTTGCGTCGCCATCCCGATTCCTCAGGCACCATGAGCATCATACCTCCTCCAAAAACCATGAAGAGTGTCTAA
AACAAGTATACTCCACCATTACCTCCCTTCAGAGGGCGCGCGGCGGTTTATTCCACTCAGACACACACGCCCA
TATACACACAGTATATCAATTCCAATAATGTCTAAGTATTAGGCCCTCCCATCCATAAACGAAGTCGAAGGTG
CCGAAGAAAAACCCGACGTCGTCGTCGTCATCATCCCCATTGCTCCTCCTACAGCGCCATTATCCATACCAGA
AGAACTGTGATGATGAATGACGTCCAGCATGTACTCTGCCATTTTCCTCCCTCCCGATGCCGATGGTTCCACT
CGCGCTACGTAATCTTCGCTCTTCGTACCATCGAGAGGATGAAAGAGTGGAATTGGAATTACTTGTGATCCTG
GTATGCGAATGGAGCTTGTCGCCTCTTCGAAAAACTTTCGGAGTAACAGTTGAATTTTGGCCGGGTTGGTATT
GTAACCAAGCGACCCTAGAGCAGCATTGGCCCACGAGGGTGTATTATTCTCATCGGGGTAATAAATCATACAT
ACTAATATTTTCTTCGGTTTTGTCTTTGCTGTTATTGCTTCAATGTATTTTTGAGTTCTGAAAAGACAAGAGA
GAGAGACAGAGAGAGTTGATGGTGGTTATATATCAAACACTATTTCATAAGGTCTTTCTCGACTGTTGCATAT
AGTGTGCGTGCGTGTATGTCAGCCCAATCATAATTGCGTGCGACATACCTAGTGCCAAAGAGATGACGGAAAT
ATCCAAAGCAGGGTGGGCAGGAACATGTGCATGAAGCCAATGACGGTCCGCATCCACAGCAGCAGTCATTCAG
CGGAACTGCACCAAACGATCTCCCACTCTCCAAACAACTATTGGGCAAACATAGCAATCCCGCAACCGCAGCT
ATGGTAGCCGGAGTCGGATACAATGCAATATCATTCCCCCCAATGGAAACGATGAGCACATCCTCCTCACCGA
TATTATCGCGTAAGAATTTATCCTGCGGTCGTAATTTAGCACATCGTTCATTGAGCGTAGTGGCCTCGACTGC
CGTATTGATTGCGGCATATTTTACATTACCACGACCATCATCACGACCACCATCACGACCACTACCACTATTG
GAATCAGAACTACCATCAACGTTATTCTTTGCGAATCGATATCGATCATCGCTCTCCGACAAAGAATTCAACC
AATACGTAACATCGCAAACGCTACGTGGAGGTTGGAGAATATCCGCATATACGCCCACGGCAGGACGTTGATC
GGCGAACCAGTGTTTGTTATCGAGGGAGGAATCGCCGGCGGTCCATATTAATGCATCGGAGTTTCTTCGGAGG
GGAGGATAGAGGACTTCGAGGTGGCGTAGTTTGTGTCCGTGGTATTCGCTGTAGAAGCTGGTGCTGGAAATTT
TTGCCATCTTTTGCGGGTGGTGTTTGTTGTGGTTGTTG
>TR581|c0_g1_i3 len=1758 path=[1995:0-122 1996:123-139 2018:140-182
2014:183-206 2022:207-485 2021:486-754 1962:755-772 2013:773-907

2023:908-931 2025:932-945 2020:946-959 2016:960-1020 @2012@!:1021-1458 1986:1459-1495 2019:1496-1567 1954:1568-1592 2011:1593-1714 2024:1715-1716 1990:1717-1720 1991:1721-1722 1992:1723-1726 1993:1727-1728 1994:1729-1757] [-1, 1995, 1996, 2018, 2014, 2022, 2021, 1962, 2013, 2023, 2025, 2020, 2016, 2012, 1986, 2019, 1954, 2011, 2024, 1990, 1991, 1992, 1993, 1994, -2]
GTGCTTATACTTATTGATGCTATAATATACAATTGAAACAAAGGTTGTATAAAAAATGAAAAGTGGCATAAAT
ATGTAATCCTAGTGAGCCTCGGGTGAATTTACTCAGGACAAGTATTTTTTGCCATACTTCTCCAGAACGTGCA
CGTACGGCTGGGCCATAGGTCGCGTCTTCTGAATAAGCGCGGTACCCTCCTTCACCCCCATACCCTCGTACTT
CATCAGCCACCCCACGAGGAGCGTGCAGCTGTTGGTCGTGCCGTACCAATCGTGGCTCAAAACTTTTCCTCCC
TTTTTTCGAACGTGCTCCACAAACTCTACACCATTGTCCAACCTTTCCAGACTTTTGTCGTCGTTGTGGAAGC
CGTCTTCTAAGCAGAGGTACTCGAAGTCATCCTTGAATTGGTTGCACTTCGCGGCCGTAGAGAGGTCCACGAT
GTGCGTAATCCCGGCCTTAATCAGCGACCCGTAAGTGAAGGAGGCGCCTGCAACGCAAATGTATGTGATTTAG
GTTCAGTATTTTGTCAAATATAGTCGTGATCGCCAAGCCATCCGTCCTAGCACCAGATTTGCCAGTTGGGGTT
CCGATTGTTGCGTGTAAGAGCGCGAGTGCAGGTTGCTCGCTCTCACAAAAACAATTGTCACAAACATCCAAGA
TTGTTGCTATTGTGATGCCGCTCGACAAAGTAACCACGCAACAAAGCTTGTGCCCAGGCTTGCTGTGTCGTGC
GATTACCCATCCTCTAATCTTTTTTTGTTTTCCAGAAAAATGTGCAACATACTGCCCTTCCGCCATCTGGGAT
GGAAGAAGAGCGCCCATTTTCTTCAGAGGCCAGCTGAACCAAAGTTTAAGGAATAAAAGGATAGAACTAGCTA
AGACCAGTCAAGCATTTCCCCCAAAACTTTCGCTACAGACTCTACCCACCACATGAGTCTCAAAAAAAAATGG
ATACAACGCTTGACGATCATCACTCCATGCAGGCATCCCCCATTACTCACCGACGGATCCCACGTACAAGAAT
GGATGGCCCTTAATCATCACCGGCTCGTAGTCCACCTCGTACAACTCCATGGCTTCCAACGCATAGTCCAATC
GCGAATAGACCTCCCCAGCAGCGCCTTGATATGCCGAACTCTCAGGATTCGTATAATCATCAATGACCAATTC
CACCACAAAGTCTCGGGCAAAAGGATACTTGGCCAAGAACGTGAACAGCAATTTATCCGTGTCGCTCAATTGG
CTAATGTCAATTGTGGACTCAAAATTCGGTATTTCAGATTCGTCCCACTCCGCCGATTCCTTAATAATATCGT
CCAACGTCAACGTCGATTCGCCGATAGACGCCCGAAGGAGCTCCATCCGATCCTCCTCCGACTTGGGCGATTT
GACTTTCTCGGCCGAGGTGAAATCTGCCTCTGGAGAAGACTCGGAGGCGGCGAAGGTGCCGCTTCCCGGAATT
TTGCCCATGACGGAATCTACTTTTTCGTTCGATGATTTGCCCAAGAAGTACCCCGTTGTTAACACGGCCAGCA
ATCCGAAAACGCAGCCCGTATACTTCATGCAGGTCGTGAAGGAGCTATCGGGTAGATAGGGAGAGGAGCGGTG
CATCTCCCGAATCTCATCGGCATCGTCCGAGTAGCCAGTGTTGGATGTGATCATTGCCACCGAGCTTTCGGCT
AGTTCCATGTCCCCATCGTCATCGTCATCGTCATCGTCATCGTCATCGTCGTGGGAGACGTTTCCGAAGGGGC
TAGGAG
>TR581|c0_g1_i4 len=1244 path=[1995:0-122 1996:123-139 2018:140-182 2014:183-206 2022:207-485 1951:486-506 @2012@!:507-944 1986:945-981 2019:982-1053 1954:1054-1078 2011:1079-1200 2024:1201-1202 1990:1203-1206 1991:1207-1208 1992:1209-1212 1993:1213-1214 1994:1215-1243] [-1, 1995, 1996, 2018, 2014, 2022, 1951, 2012, 1986, 2019, 1954, 2011, 2024, 1990, 1991, 1992, 1993, 1994, -2]
GTGCTTATACTTATTGATGCTATAATATACAATTGAAACAAAGGTTGTATAAAAAATGAAAAGTGGCATAAAT
ATGTAATCCTAGTGAGCCTCGGGTGAATTTACTCAGGACAAGTATTTTTTGCCATACTTCTCCAGAACGTGCA
CGTACGGCTGGGCCATAGGTCGCGTCTTCTGAATAAGCGCGGTACCCTCCTTCACCCCCATACCCTCGTACTT
CATCAGCCACCCCACGAGGAGCGTGCAGCTGTTGGTCGTGCCGTACCAATCGTGGCTCAAAACTTTTCCTCCC
TTTTTTCGAACGTGCTCCACAAACTCTACACCATTGTCCAACCTTTCCAGACTTTTGTCGTCGTTGTGGAAGC
CGTCTTCTAAGCAGAGGTACTCGAAGTCATCCTTGAATTGGTTGCACTTCGCGGCCGTAGAGAGGTCCACGAT
GTGCGTAATCCCGGCCTTAATCAGCGACCCGTAAGTGAAGGAGGCGCCGACGGATCCCACGTACAAGAATGGA
TGGCCCTTAATCATCACCGGCTCGTAGTCCACCTCGTACAACTCCATGGCTTCCAACGCATAGTCCAATCGCG
AATAGACCTCCCCAGCAGCGCCTTGATATGCCGAACTCTCAGGATTCGTATAATCATCAATGACCAATTCCAC
CACAAAGTCTCGGGCAAAAGGATACTTGGCCAAGAACGTGAACAGCAATTTATCCGTGTCGCTCAATTGGCTA
ATGTCAATTGTGGACTCAAAATTCGGTATTTCAGATTCGTCCCACTCCGCCGATTCCTTAATAATATCGTCCA
ACGTCAACGTCGATTCGCCGATAGACGCCCGAAGGAGCTCCATCCGATCCTCCTCCGACTTGGGCGATTTGAC
TTTCTCGGCCGAGGTGAAATCTGCCTCTGGAGAAGACTCGGAGGCGGCGAAGGTGCCGCTTCCCGGAATTTTG
CCCATGACGGAATCTACTTTTTCGTTCGATGATTTGCCCAAGAAGTACCCCGTTGTTAACACGGCCAGCAATC
CGAAAACGCAGCCCGTATACTTCATGCAGGTCGTGAAGGAGCTATCGGGTAGATAGGGAGAGGAGCGGTGCAT
CTCCCGAATCTCATCGGCATCGTCCGAGTAGCCAGTGTTGGATGTGATCATTGCCACCGAGCTTTCGGCTAGT
TCCATGTCCCCATCGTCATCGTCATCGTCATCGTCATCGTCATCGTCGTGGGAGACGTTTCCGAAGGGGCTAG
GAG
>TR582|c0_g1_i1 len=343 path=[4236:0-34 4237:35-85 4238:86-86 4239:87-342] [-1, 4236, 4237, 4238, 4239, -2]
CTCTTCCGATCTCCAACTTCTAAACAGTCATTTTTATTTTATATTTATTCCTACGTGAAAGTAGTCGGGGCTT
GAGAAGCGTACGATTATTTACTTCCTCCCTTCATTCCTTCTTGATTGCTTCCCAATAAGTTCCCACCACGGCT
GGTGTGATTGCTTCCCAAACAGTTCCCACCACGGTGGGGACGTGCGACGAAGGTGCGCTGAAGCACAGAAAAG

AACGCACGGAAAAGAACAACATTCGTTCTTCAAACCTGCTTTGTGAGAGAAGAAAGGGGGGACGGGGACATTA
ACCTTTGAATAACTCGAACTAAAATTGAGGTTTTAACGTCTTCAAAAAAAA
>TR582|c0_g2_i1 len=2322 path=[4310:0-422 4422:423-466 4431:467-472 4427:473-496 4420:497-635 4222:636-659 4405:660-1032 4346:1033-1053 4418:1054-1059 4404:1060-1083 4386:1084-1190 4335:1191-1214 4402:1215-1256 4292:1257-1280 4385:1281-1501 4244:1502-1525 4417:1526-1657 4241:1658-1681 4430:1682-1753 4432:1754-1767 4352:1768-1777 4425:1778-1981 4433:1982-2005 4434:2006-2015 4435:2016-2024 4412:2025-2039 4395:2040-2042 4376:2043-2065 4235:2066-2321] [-1, 4310, 4422, 4431, 4427, 4420, 4222, 4405, 4346, 4418, 4404, 4386, 4335, 4402, 4292, 4385, 4244, 4417, 4241, 4430, 4432, 4352, 4425, 4433, 4434, 4435, 4412, 4395, 4376, 4235, -2]
GGAGGCGCCACACTGGCCGCTGGTCGCCGAGGCGACGACGCCGCCGTTCCCTCCCAAAAGGCGCGGATGTCCG
CACTGAAAGTGCACGCCGGGGGCGGAGGCCGGACTCTCCCCCAGGGCGTGGGGCATCGCTCCCCAGTCTCGAG
ACACCATGGCCGCGGTGGCGGTCAGGGACGATTCCGCCGTGGTGGACGTGCCCGCGCTCGACACCGAATCGAC
GTCGTTGAACGCGGACGAATCGAGCAGGACGGACGCGGGCTTGGACAGGGATGCATTGGGCCGCCACAACGAG
AACGGCAACGTGTTGTTGTGCACCGGAGGAGGGGTGGGGACGGCGCACGGCGTCGTTCGTTGGACCTTGCGGA
GTGCCGTGGGGTAGGCCAGCGCCATGGACGTCAGGTGTTGTCCCGCGGCGGTGTCGCCAGACTCCCTGTTGCT
CGCCGTGTTGTTGTTGTTGCCGTTGTCATTGCTCGCCCCGACGCCAGCGACATTGTTGGGAGATACCGAGAGG
GTGCTCTTGGAACAGGGAGAGTGAGCGTCGCAAGTCGCATTGCAAGCGGGCATCGCGAGGCCGTGGGCGCTGC
TGCCGCAATCGTCGCTCTCCATCATGTCCTCGTCGTCCCCCTGCGAATTGGCATCGACGCTCTCGTTGTGCTG
CGAGTGCCCTCCTCGGATCGACGTGATGCTGAAATCGTGAGATTCCATCACGTGGTGTTGTCCTTCCGTCATG
GTGGTGACTCTGTCTTCCGGAAGGGAGGGCAATCCCGAGATGACGACGACGTGGTTGTTGTTCGTGGAGGATG
GATGGTGGGGTGCCGCCGTCAATGGGGCGCATCCTCGTTGGAGTCCGAGTTGATGGAGGTACCGTTCGCGGAT
GCGATTGGCCGATGCGTTCTTGGGGGCGGGTTTGAAGGGTCTCATGGTCTCTCTTTCTCCGTTGCGCGGATGG
TGATAATATGGGTATGATGTGCGACCCAGTGTGCCTTCTATCAAATGATGATTCTTGGTGTCAAGTTGTGAAC
TACGATGATCCAGCTATGTCGCTCTCTCTTGATCTCTCTATACTGAGGTTCACTAGTGTAGAGGTCAGGTGGG
TCCACTGAATTCAGACCAATGATATTGGTTTACTATCTATGCAGTGCTAATCTACTCTCACGTCCTCTCTGTT
ACACCATCGCCAATGCCAACCTGCCACACTATCTCGTGTAATTCCATCAACTAAATTAAAATGAACCTGGAAT
CCCTGTTTCGGATGGTGGATGCTGCGGATTATCTTTGAGTTCGATGTCGTGTTGGTGATGTCATCCAGACTTC
TCTTCGGATTCGCAGAAAGGATGGAGGTAGATAGAAGTGTCATGTCGGTGACAGGTGACATAAACAGCCGCAT
TGCTTCACTTCTCCCTCTGTTGTTTCCTACAGATTATTTCGCCTTAGAGAAGCAGAAGCAGGAGATTCGACAT
GTGTGTGGGGCTGGATGGGCTGGATTCGCTCCCGATCCACGATCCGGTCGAGAGGCAATGCCGCACATTCGAT
TCGTTCTCAGAAATTCAGAAACTGCCCTCGGCATCCGAACATGACAAAAGACGTCGCTTCGGCACCTTTTGGT
CAGATTTTTTGAGTGGATAGGGGTGCACTTATTCCTGTACAAACGCGGTACATATGGTACAAACATACAAATG
CATTTGAATTTCCAGTGAATTTGTGTCTGATTTTGCATTATTCCGTGATTTTGATGCCGGCCGTCTGCCGGCG
GGTGTCAACCTTCTCGGTGCGTTCAGCTGCGTACAACAGTCGCCGAGACAATCTGTCACTGCTATAATACCTG
CTGTTGGAATAGTCATATTGAATTCCATTGTTCAATTGACGTTACGTGAAGTTTCATTGGCTAAAATAGAATC
TAGAGAGTAAGAAATCGGATAAATGGAAGAGATGGGCACTTGAGGAAGATCGAAGTTCCTGCACTTTCACGTG
GTGTGGCTCTCGCGCTCTTGTTGATTGAGGGCATGCATGCGACATGCAAAAAACGTGCGGGTCTTGCATCTTT
CGGGGCTTGAGAAGCGTACGATTATTTACTTCCTCCCTTCATTCCTTCTTGATTGCTTCCCAATAAGTTCCCA
CCACGGCTGGTGTGATTGCTTCCCAAACAGTTCCCACCACGGTGGGGACGTGCGACGAAGGTGCGCTGAAGCA
CAGAAAAGAACGCACGGAAAAGAACAACATTCGTTCTTCAAACCTGCTTTGTGAGAGAAGAAAGGGGGGACGG
GGACATTAACCTTTGAATAACTCGAACTAAAATTGAGGTTTTAACGTCTTCAAAAAAAA
>TR582|c0_g2_i2 len=1842 path=[4266:0-129 4267:130-153 4428:154-414 4392:415-476 4421:477-536 4406:537-560 4389:561-573 4418:574-579 4404:580-603 4386:604-710 4335:711-734 4402:735-776 4292:777-800 4385:801-1021 4244:1022-1045 4417:1046-1177 4241:1178-1201 4430:1202-1273 4264:1274-1297 4425:1298-1501 4433:1502-1525 4434:1526-1535 4436:1536-1559 4437:1560-1562 4378:1563-1585 4253:1586-1841] [-1, 4266, 4267, 4428, 4392, 4421, 4406, 4389, 4418, 4404, 4386, 4335, 4402, 4292, 4385, 4244, 4417, 4241, 4430, 4264, 4425, 4433, 4434, 4436, 4437, 4378, 4253, -2]
CCTGCTGCAAAAACACACACATTACACGCGAATGAATGTTCAGGCCAAGGCCGTCTCGTAAGTAGAGCGCACG
GCAGGTAAGACAAAAAGAGGCCATCCACTCAAGTGAATGCGTGAAAGCGCTGGAACACCACACACATCGTAGC
AAGGTAATTGTGGGCAGGTCTACCTTCTCATGATAGAGAGCGGATCATGTCGACTTCATACAGAGATACAAAT
ACAACGACGCGGCGTATGACTTTTATGCACAGGGAGACATCACGCGTAACGTAAAGGTGTATGGTACTTCTTC
AAGTAGGCGAGAAATAATCAAATTGCAAGCGCGTGACGCGCCTCGCAATTTGGTGATGGACATCAAAACGGAC
ATCACCCGTAAATTCGATCTGAATTTGATGCCAACGCCTCTCACAGCATCACCCATGAATGCACAACATCATC
GTGGAGGCAAAAAAGCAAATAACCCGAACAATGCCACTGTCAAATGTGGCGCGCCAGATATTGATGGGCGCGG
CATGATATTTGATGGGAGACGCATGCCCGTCATGAACTCACCAGCTATGTCGCTCTCTCTTGATCTCTCTATA

CTGAGGTTCACTAGTGTAGAGGTCAGGTGGGTCCACTGAATTCAGACCAATGATATTGGTTTACTATCTATGC
AGTGCTAATCTACTCTCACGTCCTCTCTGTTACACCATCGCCAATGCCAACCTGCCACACTATCTCGTGTAAT
TCCATCAACTAAATTAAAATGAACCTGGAATCCCTGTTTCGGATGGTGGATGCTGCGGATTATCTTTGAGTTC
GATGTCGTGTTGGTGATGTCATCCAGACTTCTCTTCGGATTCGCAGAAAGGATGGAGGTAGATAGAAGTGTCA
TGTCGGTGACAGGTGACATAAACAGCCGCATTGCTTCACTTCTCCCTCTGTTGTTTCCTACAGATTATTTCGC
CTTAGAGAAGCAGAAGCAGGAGATTCGACATGTGTGTGGGGCTGGATGGGCTGGATTCGCTCCCGATCCACGA
TCCGGTCGAGAGGCAATGCCGCACATTCGATTCGTTCTCAGAAATTCAGAAACTGCCCTCGGCATCCGAACAT
GACAAAAGACGTCGCTTCGGCACCTTTTGGTCAGATTTTTTGAGTGGATAGGGGTGCACTTATTCCTGTACAA
ACGCGGTACATATGGTACAAACATACAAATGCATTTGAATTTCCAGTGAATTTGTGTCTGATTTTGCATTATT
CCGTGATTTTGATGCCGGCCGTCTGCCGGCGGGGGTCAACCTTCTCGGTGCGTTCAGCTGCGTACAACAGTCG
CCGAGACAATCTGTCACTGCTATAATACCTGCTGTTGGAATAGTCATATTGAATTCCATTGTTCAATTGACGT
TACGTGAAGTTTCATTGGCTAAAATAGAATCTAGAGAGTAAGAAATCGGATAAATGGAAGAGATGGGCACTTG
AGGAAGATCGAAGTTCCTGCACTTTCACGTGGTGTGGCTCTCGCGCTCTTGTTGATTGAGGGCATGCATGCGA
CATCCAAAAAACGTGCGGGTCTTGCATCTTTCGGGGCTTGAGAAGCGTACGATTATTTACTTCCTCCCTTCAT
TCCTTCTTGATTGCTTCCCAATAAGTTCCCACCACGGCTGGTGTGATTGCTTCCCAAACAGTTCCCACCACGG
TGGGGACGTGCGACGAAGGTGCGCTGAAGCACAGAAAAGAACGCACGGAAAAGAACAACATTCGTTCTTCAAA
CCTGCTTTGTGAGAGAAGAAAGGGGGGACGGGGACATTAACCTTTGAATAACTCGAACTAAAATTGAGGTTTT
AACGTCTTCAAAAAAAA
>TR582|c0_g2_i3 len=1567 path=[4266:0-129 4267:130-153 4428:154-414
4392:415-476 4421:477-536 4406:537-560 4389:561-573 4418:574-579
4404:580-603 4386:604-710 4335:711-734 4402:735-776 4292:777-800
4385:801-1021 4244:1022-1045 4417:1046-1177 4241:1178-1201 4430:1202-1273
4432:1274-1287 4226:1288-1288 4227:1289-1309 4228:1310-1310 4229:1311-
1566] [-1, 4266, 4267, 4428, 4392, 4421, 4406, 4389, 4418, 4404, 4386,
4335, 4402, 4292, 4385, 4244, 4417, 4241, 4430, 4432, 4226, 4227, 4228,
4229, -2]
CCTGCTGCAAAAACACACACATTACACGCGAATGAATGTTCAGGCCAAGGCCGTCTCGTAAGTAGAGCGCACG
GCAGGTAAGACAAAAAGAGGCCATCCACTCAAGTGAATGCGTGAAAGCGCTGGAACACCACACACATCGTAGC
AAGGTAATTGTGGGCAGGTCTACCTTCTCATGATAGAGAGCGGATCATGTCGACTTCATACAGAGATACAAAT
ACAACGACGCGGCGTATGACTTTTATGCACAGGGAGACATCACGCGTAACGTAAAGGTGTATGGTACTTCTTC
AAGTAGGCGAGAAATAATCAAATTGCAAGCGCGTGACGCGCCTCGCAATTTGGTGATGGACATCAAAACGGAC
ATCACCCGTAAATTCGATCTGAATTTGATGCCAACGCCTCTCACAGCATCACCCATGAATGCACAACATCATC
GTGGAGGCAAAAAAGCAAATAACCCGAACAATGCCACTGTCAAATGTGGCGCGCCAGATATTGATGGGCGCGG
CATGATATTTGATGGGAGACGCATGCCCGTCATGAACTCACCAGCTATGTCGCTCTCTCTTGATCTCTCTATA
CTGAGGTTCACTAGTGTAGAGGTCAGGTGGGTCCACTGAATTCAGACCAATGATATTGGTTTACTATCTATGC
AGTGCTAATCTACTCTCACGTCCTCTCTGTTACACCATCGCCAATGCCAACCTGCCACACTATCTCGTGTAAT
TCCATCAACTAAATTAAAATGAACCTGGAATCCCTGTTTCGGATGGTGGATGCTGCGGATTATCTTTGAGTTC
GATGTCGTGTTGGTGATGTCATCCAGACTTCTCTTCGGATTCGCAGAAAGGATGGAGGTAGATAGAAGTGTCA
TGTCGGTGACAGGTGACATAAACAGCCGCATTGCTTCACTTCTCCCTCTGTTGTTTCCTACAGATTATTTCGC
CTTAGAGAAGCAGAAGCAGGAGATTCGACATGTGTGTGGGGCTGGATGGGCTGGATTCGCTCCCGATCCACGA
TCCGGTCGAGAGGCAATGCCGCACATTCGATTCGTTCTCAGAAATTCAGAAACTGCCCTCGGCATCCGAACAT
GACAAAAGACGTCGCTTCGGCACCTTTTGGTCAGATTTTTTGAGTGGATAGGGGTGCACTTATTCCTGTACAA
ACGCGGTACATATGGTACAAACATACAAATGCATTTGAATTTCCAGTGAATTTGTGTCTGATTTTGCATTATT
CCGTGATTTTGATGCCGGCCGTCTGCCGGCGGGTGTCAACCTTCTCGTCGGGGCTTGAGAAGCGTACGATTAT
TTACTTCCTCCCTTCATTCCTTCTTGATTGCTTCCCAATAAGTTCCCACCACGGCTGGTGTGATTGCTTCCCA
AACAGTTCCCACCACGGTGGGGACGTGCGACGAAGGTGCGCTGAAGCACAGAAAAGAACGCACGGAAAAGAAC
AACATTCGTTCTTCAAACCTGCTTTGTGAGAGAAGAAAGGGGGGACGGGGACATTAACCTTTGAATAACTCGA
ACTAAAATTGAGGTTTTAACGTCTTCAAAAAAAA
>TR582|c0_g2_i4 len=1935 path=[4266:0-129 4267:130-153 4428:154-414
4392:415-476 4421:477-536 4406:537-560 4389:561-573 4418:574-579
4404:580-603 4386:604-710 4335:711-734 4402:735-776 4292:777-800
4385:801-1021 4244:1022-1045 4417:1046-1177 4241:1178-1201 4430:1202-1273
4432:1274-1287 4352:1288-1297 4425:1298-1501 4433:1502-1525 4434:1526-
1535 4436:1536-1559 4437:1560-1562 4424:1563-1596 4410:1597-1616
4394:1617-1626 4375:1627-1677 4301:1678-1678 4302:1679-1934] [-1, 4266,
4267, 4428, 4392, 4421, 4406, 4389, 4418, 4404, 4386, 4335, 4402, 4292,
4385, 4244, 4417, 4241, 4430, 4432, 4352, 4425, 4433, 4434, 4436, 4437,
4424, 4410, 4394, 4375, 4301, 4302, -2]
CCTGCTGCAAAAACACACACATTACACGCGAATGAATGTTCAGGCCAAGGCCGTCTCGTAAGTAGAGCGCACG
GCAGGTAAGACAAAAAGAGGCCATCCACTCAAGTGAATGCGTGAAAGCGCTGGAACACCACACACATCGTAGC

AAGGTAATTGTGGGCAGGTCTACCTTCTCATGATAGAGAGCGGATCATGTCGACTTCATACAGAGATACAAAT
ACAACGACGCGGCGTATGACTTTTATGCACAGGGAGACATCACGCGTAACGTAAAGGTGTATGGTACTTCTTC
AAGTAGGCGAGAAATAATCAAATTGCAAGCGCGTGACGCGCCTCGCAATTTGGTGATGGACATCAAAACGGAC
ATCACCCGTAAATTCGATCTGAATTTGATGCCAACGCCTCTCACAGCATCACCCATGAATGCACAACATCATC
GTGGAGGCAAAAAAGCAAATAACCCGAACAATGCCACTGTCAAATGTGGCGCGCCAGATATTGATGGGCGCGG
CATGATATTTGATGGGAGACGCATGCCCGTCATGAACTCACCAGCTATGTCGCTCTCTCTTGATCTCTCTATA
CTGAGGTTCACTAGTGTAGAGGTCAGGTGGGTCCACTGAATTCAGACCAATGATATTGGTTTACTATCTATGC
AGTGCTAATCTACTCTCACGTCCTCTCTGTTACACCATCGCCAATGCCAACCTGCCACACTATCTCGTGTAAT
TCCATCAACTAAATTAAAATGAACCTGGAATCCCTGTTTCGGATGGTGGATGCTGCGGATTATCTTTGAGTTC
GATGTCGTGTTGGTGATGTCATCCAGACTTCTCTTCGGATTCGCAGAAAGGATGGAGGTAGATAGAAGTGTCA
TGTCGGTGACAGGTGACATAAACAGCCGCATTGCTTCACTTCTCCCTCTGTTGTTTCCTACAGATTATTTCGC
CTTAGAGAAGCAGAAGCAGGAGATTCGACATGTGTGTGGGGCTGGATGGGCTGGATTCGCTCCCGATCCACGA
TCCGGTCGAGAGGCAATGCCGCACATTCGATTCGTTCTCAGAAATTCAGAAACTGCCCTCGGCATCCGAACAT
GACAAAAGACGTCGCTTCGGCACCTTTTGGTCAGATTTTTTGAGTGGATAGGGGTGCACTTATTCCTGTACAA
ACGCGGTACATATGGTACAAACATACAAATGCATTTGAATTTCCAGTGAATTTGTGTCTGATTTTGCATTATT
CCGTGATTTTGATGCCGGCCGTCTGCCGGCGGGTGTCAACCTTCTCGGTGCGTTCAGCTGCGTACAACAGTCG
CCGAGACAATCTGTCACTGCTATAATACCTGCTGTTGGAATAGTCATATTGAATTCCATTGTTCAATTGACGT
TACGTGAAGTTTCATTGGCTAAAATAGAATCTAGAGAGTAAGAAATCGGATAAATGGAAGAGATGGGCACTTG
AGGAAGATCGAAGTTCCTGCACTTTCACGTGGTGTGGCTCTCGCGCTCTTGTTGATTGAGGGCATGCATGCGA
CATCCAAAAAACGTGCGGGTCTTGCATCTTGTGCGTGTGTCGTGGAAATTTTGTGTGATTCCCCCTTCCCGCC
AACTTCTAAACAGTCATTTTTATTTTATATTTATTCCTACGTGAAAGTAGTCGGGGCTTGAGAAGCGTACGAT
TATTTACTTCCTCCCTTCATTCCTTCTTGATTGCTTCCCAATAAGTTCCCACCACGGCTGGTGTGATTGCTTC
CCAAACAGTTCCCACCACGGTGGGGACGTGCGACGAAGGTGCGCTGAAGCACAGAAAAGAACGCACGGAAAAG
AACAACATTCGTTCTTCAAACCTGCTTTGTGAGAGAAGAAAGGGGGGACGGGGACATTAACCTTTGAATAACT
CGAACTAAAATTGAGGTTTTAACGTCTTCAAAAAAAA
>TR582|c0_g2_i5 len=1624 path=[4310:0-422 4422:423-466 4431:467-472 4427:473-496 4420:497-635 4222:636-659 4405:660-1032 4426:1033-1224 4343:1225-1266 @4403@!:1267-1494 4247:1495-1521 4248:1522-1623] [-1, 4310, 4422, 4431, 4427, 4420, 4222, 4405, 4426, 4343, 4403, 4247, 4248, -2]
GGAGGCGCCACACTGGCCGCTGGTCGCCGAGGCGACGACGCCGCCGTTCCCTCCCAAAAGGCGCGGATGTCCG
CACTGAAAGTGCACGCCGGGGGCGGAGGCCGGACTCTCCCCCAGGGCGTGGGGCATCGCTCCCCAGTCTCGAG
ACACCATGGCCGCGGTGGCGGTCAGGGACGATTCCGCCGTGGTGGACGTGCCCGCGCTCGACACCGAATCGAC
GTCGTTGAACGCGGACGAATCGAGCAGGACGGACGCGGGCTTGGACAGGGATGCATTGGGCCGCCACAACGAG
AACGGCAACGTGTTGTTGTGCACCGGAGGAGGGGTGGGGACGGCGCACGGCGTCGTTCGTTGGACCTTGCGGA
GTGCCGTGGGGTAGGCCAGCGCCATGGACGTCAGGTGTTGTCCCGCGGCGGTGTCGCCAGACTCCCTGTTGCT
CGCCGTGTTGTTGTTGTTGCCGTTGTCATTGCTCGCCCCGACGCCAGCGACATTGTTGGGAGATACCGAGAGG
GTGCTCTTGGAACAGGGAGAGTGAGCGTCGCAAGTCGCATTGCAAGCGGGCATCGCGAGGCCGTGGGCGCTGC
TGCCGCAATCGTCGCTCTCCATCATGTCCTCGTCGTCCCCCTGCGAATTGGCATCGACGCTCTCGTTGTGCTG
CGAGTGCCCTCCTCGGATCGACGTGATGCTGAAATCGTGAGATTCCATCACGTGGTGTTGTCCTTCCGTCATG
GTGGTGACTCTGTCTTCCGGAAGGGAGGGCAATCCCGAGATGACGACGACGTGGTTGTTGTTCGTGGAGGATG
GATGGTGGGGTGCCGCCGTCAATGGGGCGCATCCTCGTTGGAGTCCGAGTTGATGGAGGTACCGTTCGCGGAT
GCGATTGGCCGATGCGTTCTTGGGGGCGGGTTTGAAGGGTCTCATGGTCTCTCTTTCTCCGTTGCGCGGATGG
TGATAATATGGGTATGATGTGCGACCCAGTGTGCCTTCTATCAAATGATGATTCTTGGTGTCAAGTTGTGAAC
TACGATGATCCTAAATTGAAAAGAGCAAGAAGAGATGATTAATGTCAGAATGGAGATGAGATGTGGACAAGAT
CCGACGCAATAATGCGATGTGGGACAGTATCATAAGTCAGTGTAAACAACTGTTAAGAGAACATCATCGTGCT
CCTGTCGTGTCCTCTCTCTGATGGGCCCCTAAAAGAATAGCATTGGACAGCAAGCTATACGAGCCAGACGCTT
CCAACGAGCCTAACAACAACCTCTGAAACCCCTTCCAAATCCTTCCACAAAAAAATTGGCGCTACCAATGGGA
GACTGTCAAAACTCAGTCTCTTCTATACTCGAGCCAAACAAAGTCGAAATTTACCCAGCGCTCAAAGAGATGG
ATTCAGGAAAACATCAAAACGGCAATGTGCGCGAAGCACGAACGTGGGAATATTTTCATGGAGACGATGCGCG
AATTACAACTGCAATCATCGAATCATGTTCGTATATACAGTGCGGCAAAACAAAACGAACACAAGGCATGCAA
TCCATGCCGGATGGTGATATTGATGCCAGAGGTTATCCGATATTCAAAGTCAAAACAGATGGCACAACACTAA
ATATGACCGTTGGACATC
>TR582|c0_g2_i6 len=2047 path=[4310:0-422 4422:423-466 4431:467-472 4427:473-496 4420:497-635 4222:636-659 4405:660-1032 4346:1033-1053 4418:1054-1059 4404:1060-1083 4386:1084-1190 4335:1191-1214 4402:1215-1256 4292:1257-1280 4385:1281-1501 4244:1502-1525 4417:1526-1657 4241:1658-1681 4430:1682-1753 4432:1754-1767 4226:1768-1768 4227:1769-1789 4228:1790-1790 4229:1791-2046] [-1, 4310, 4422, 4431, 4427, 4420,

4222, 4405, 4346, 4418, 4404, 4386, 4335, 4402, 4292, 4385, 4244, 4417, 4241, 4430, 4432, 4226, 4227, 4228, 4229, -2]
GGAGGCGCCACACTGGCCGCTGGTCGCCGAGGCGACGACGCCGCCGTTCCCTCCCAAAAGGCGCGGATGTCCG
CACTGAAAGTGCACGCCGGGGGCGGAGGCCGGACTCTCCCCCAGGGCGTGGGGCATCGCTCCCCAGTCTCGAG
ACACCATGGCCGCGGTGGCGGTCAGGGACGATTCCGCCGTGGTGGACGTGCCCGCGCTCGACACCGAATCGAC
GTCGTTGAACGCGGACGAATCGAGCAGGACGGACGCGGGCTTGGACAGGGATGCATTGGGCCGCCACAACGAG
AACGGCAACGTGTTGTTGTGCACCGGAGGAGGGGTGGGGACGGCGCACGGCGTCGTTCGTTGGACCTTGCGGA
GTGCCGTGGGGTAGGCCAGCGCCATGGACGTCAGGTGTTGTCCCGCGGCGGTGTCGCCAGACTCCCTGTTGCT
CGCCGTGTTGTTGTTGTTGCCGTTGTCATTGCTCGCCCCGACGCCAGCGACATTGTTGGGAGATACCGAGAGG
GTGCTCTTGGAACAGGGAGAGTGAGCGTCGCAAGTCGCATTGCAAGCGGGCATCGCGAGGCCGTGGGCGCTGC
TGCCGCAATCGTCGCTCTCCATCATGTCCTCGTCGTCCCCCTGCGAATTGGCATCGACGCTCTCGTTGTGCTG
CGAGTGCCCTCCTCGGATCGACGTGATGCTGAAATCGTGAGATTCCATCACGTGGTGTTGTCCTTCCGTCATG
GTGGTGACTCTGTCTTCCGGAAGGGAGGGCAATCCCGAGATGACGACGACGTGGTTGTTGTTCGTGGAGGATG
GATGGTGGGGTGCCGCCGTCAATGGGGCGCATCCTCGTTGGAGTCCGAGTTGATGGAGGTACCGTTCGCGGAT
GCGATTGGCCGATGCGTTCTTGGGGGCGGGTTTGAAGGGTCTCATGGTCTCTCTTTCTCCGTTGCGCGGATGG
TGATAATATGGGTATGATGTGCGACCCAGTGTGCCTTCTATCAAATGATGATTCTTGGTGTCAAGTTGTGAAC
TACGATGATCCAGCTATGTCGCTCTCTCTTGATCTCTCTATACTGAGGTTCACTAGTGTAGAGGTCAGGTGGG
TCCACTGAATTCAGACCAATGATATTGGTTTACTATCTATGCAGTGCTAATCTACTCTCACGTCCTCTCTGTT
ACACCATCGCCAATGCCAACCTGCCACACTATCTCGTGTAATTCCATCAACTAAATTAAAATGAACCTGGAAT
CCCTGTTTCGGATGGTGGATGCTGCGGATTATCTTTGAGTTCGATGTCGTGTTGGTGATGTCATCCAGACTTC
TCTTCGGATTCGCAGAAAGGATGGAGGTAGATAGAAGTGTCATGTCGGTGACAGGTGACATAAACAGCCGCAT
TGCTTCACTTCTCCCTCTGTTGTTTCCTACAGATTATTTCGCCTTAGAGAAGCAGAAGCAGGAGATTCGACAT
GTGTGTGGGGCTGGATGGGCTGGATTCGCTCCCGATCCACGATCCGGTCGAGAGGCAATGCCGCACATTCGAT
TCGTTCTCAGAAATTCAGAAACTGCCCTCGGCATCCGAACATGACAAAAGACGTCGCTTCGGCACCTTTTGGT
CAGATTTTTTGAGTGGATAGGGGTGCACTTATTCCTGTACAAACGCGGTACATATGGTACAAACATACAAATG
CATTTGAATTTCCAGTGAATTTGTGTCTGATTTTGCATTATTCCGTGATTTTGATGCCGGCCGTCTGCCGGCG
GGTGTCAACCTTCTCGTCGGGGCTTGAGAAGCGTACGATTATTTACTTCCTCCCTTCATTCCTTCTTGATTGC
TTCCCAATAAGTTCCCACCACGGCTGGTGTGATTGCTTCCCAAACAGTTCCCACCACGGTGGGGACGTGCGAC
GAAGGTGCGCTGAAGCACAGAAAAGAACGCACGGAAAAGAACAACATTCGTTCTTCAAACCTGCTTTGTGAGA
GAAGAAAGGGGGGACGGGGACATTAACCTTTGAATAACTCGAACTAAAATTGAGGTTTTAACGTCTTCAAAAA
AAA
>TR582|c0_g2_i7 len=2415 path=[4310:0-422 4422:423-466 4431:467-472 4427:473-496 4420:497-635 4222:636-659 4405:660-1032 4346:1033-1053 4418:1054-1059 4404:1060-1083 4386:1084-1190 4335:1191-1214 4402:1215-1256 4292:1257-1280 4385:1281-1501 4244:1502-1525 4417:1526-1657 4241:1658-1681 4430:1682-1753 4432:1754-1767 4352:1768-1777 4425:1778-1981 4433:1982-2005 4434:2006-2015 4436:2016-2039 4437:2040-2042 4424:2043-2076 4410:2077-2096 4394:2097-2106 4375:2107-2157 4301:2158-2158 4302:2159-2414] [-1, 4310, 4422, 4431, 4427, 4420, 4222, 4405, 4346, 4418, 4404, 4386, 4335, 4402, 4292, 4385, 4244, 4417, 4241, 4430, 4432, 4352, 4425, 4433, 4434, 4436, 4437, 4424, 4410, 4394, 4375, 4301, 4302, -2]
GGAGGCGCCACACTGGCCGCTGGTCGCCGAGGCGACGACGCCGCCGTTCCCTCCCAAAAGGCGCGGATGTCCG
CACTGAAAGTGCACGCCGGGGGCGGAGGCCGGACTCTCCCCCAGGGCGTGGGGCATCGCTCCCCAGTCTCGAG
ACACCATGGCCGCGGTGGCGGTCAGGGACGATTCCGCCGTGGTGGACGTGCCCGCGCTCGACACCGAATCGAC
GTCGTTGAACGCGGACGAATCGAGCAGGACGGACGCGGGCTTGGACAGGGATGCATTGGGCCGCCACAACGAG
AACGGCAACGTGTTGTTGTGCACCGGAGGAGGGGTGGGGACGGCGCACGGCGTCGTTCGTTGGACCTTGCGGA
GTGCCGTGGGGTAGGCCAGCGCCATGGACGTCAGGTGTTGTCCCGCGGCGGTGTCGCCAGACTCCCTGTTGCT
CGCCGTGTTGTTGTTGTTGCCGTTGTCATTGCTCGCCCCGACGCCAGCGACATTGTTGGGAGATACCGAGAGG
GTGCTCTTGGAACAGGGAGAGTGAGCGTCGCAAGTCGCATTGCAAGCGGGCATCGCGAGGCCGTGGGCGCTGC
TGCCGCAATCGTCGCTCTCCATCATGTCCTCGTCGTCCCCCTGCGAATTGGCATCGACGCTCTCGTTGTGCTG
CGAGTGCCCTCCTCGGATCGACGTGATGCTGAAATCGTGAGATTCCATCACGTGGTGTTGTCCTTCCGTCATG
GTGGTGACTCTGTCTTCCGGAAGGGAGGGCAATCCCGAGATGACGACGACGTGGTTGTTGTTCGTGGAGGATG
GATGGTGGGGTGCCGCCGTCAATGGGGCGCATCCTCGTTGGAGTCCGAGTTGATGGAGGTACCGTTCGCGGAT
GCGATTGGCCGATGCGTTCTTGGGGGCGGGTTTGAAGGGTCTCATGGTCTCTCTTTCTCCGTTGCGCGGATGG
TGATAATATGGGTATGATGTGCGACCCAGTGTGCCTTCTATCAAATGATGATTCTTGGTGTCAAGTTGTGAAC
TACGATGATCCAGCTATGTCGCTCTCTCTTGATCTCTCTATACTGAGGTTCACTAGTGTAGAGGTCAGGTGGG
TCCACTGAATTCAGACCAATGATATTGGTTTACTATCTATGCAGTGCTAATCTACTCTCACGTCCTCTCTGTT
ACACCATCGCCAATGCCAACCTGCCACACTATCTCGTGTAATTCCATCAACTAAATTAAAATGAACCTGGAAT
CCCTGTTTCGGATGGTGGATGCTGCGGATTATCTTTGAGTTCGATGTCGTGTTGGTGATGTCATCCAGACTTC

TCTTCGGATTCGCAGAAAGGATGGAGGTAGATAGAAGTGTCATGTCGGTGACAGGTGACATAAACAGCCGCAT
TGCTTCACTTCTCCCTCTGTTGTTTCCTACAGATTATTTCGCCTTAGAGAAGCAGAAGCAGGAGATTCGACAT
GTGTGTGGGGCTGGATGGGCTGGATTCGCTCCCGATCCACGATCCGGTCGAGAGGCAATGCCGCACATTCGAT
TCGTTCTCAGAAATTCAGAAACTGCCCTCGGCATCCGAACATGACAAAAGACGTCGCTTCGGCACCTTTTGGT
CAGATTTTTTGAGTGGATAGGGGTGCACTTATTCCTGTACAAACGCGGTACATATGGTACAAACATACAAATG
CATTTGAATTTCCAGTGAATTTGTGTCTGATTTTGCATTATTCCGTGATTTTGATGCCGGCCGTCTGCCGGCG
GGTGTCAACCTTCTCGGTGCGTTCAGCTGCGTACAACAGTCGCCGAGACAATCTGTCACTGCTATAATACCTG
CTGTTGGAATAGTCATATTGAATTCCATTGTTCAATTGACGTTACGTGAAGTTTCATTGGCTAAAATAGAATC
TAGAGAGTAAGAAATCGGATAAATGGAAGAGATGGGCACTTGAGGAAGATCGAAGTTCCTGCACTTTCACGTG
GTGTGGCTCTCGCGCTCTTGTTGATTGAGGGCATGCATGCGACATCCAAAAAACGTGCGGGTCTTGCATCTTG
TGCGTGTGTCGTGGAAATTTTGTGTGATTCCCCCTTCCCGCCAACTTCTAAACAGTCATTTTTATTTTATATT
TATTCCTACGTGAAAGTAGTCGGGGCTTGAGAAGCGTACGATTATTTACTTCCTCCCTTCATTCCTTCTTGAT
TGCTTCCCAATAAGTTCCCACCACGGCTGGTGTGATTGCTTCCCAAACAGTTCCCACCACGGTGGGGACGTGC
GACGAAGGTGCGCTGAAGCACAGAAAAGAACGCACGGAAAAGAACAACATTCGTTCTTCAAACCTGCTTTGTG
AGAGAAGAAAGGGGGGACGGGGACATTAACCTTTGAATAACTCGAACTAAAATTGAGGTTTTAACGTCTTCAA
AAAAAA
>TR583|c0_g1_i1 len=1424 path=[1630:0-46 1655:47-47 1653:48-71 1651:72-160 1646:161-184 1654:185-294 1626:295-317 @1652@!:318-858 1648:859-1332 1635:1333-1356 1636:1357-1363 1637:1364-1387 1638:1388-1423] [-1, 1630, 1655, 1653, 1651, 1646, 1654, 1626, 1652, 1648, 1635, 1636, 1637, 1638, -2]
TCACAACGGCTCACAGCAGCACACATTACACAGAGCTACACGGAGCTACACGCAACAGCAATATACATTGCAA
CACATTGAGATCTAGACAACTAAAACTGGTACACGAGAGAGCGAGAGAAGAAGATGGTGAAGGTGACAATCCA
ACTAGGAGTACAAAAGATCGACAACGACGGCATTGCCAAGAAGAAGGAACAACGGTCGCCAACGAAAAAAGTC
ATTGTGGATATTTCACAAACATTGGATCATGACAAAGCCACTGCCAATACTCATGGTCGGTGTGGCAGCAATG
ACAATGATGATGTCGATGACGGCGATATGAAAAAATACTCCATGTGTTCCCTATTAATTCAAATTGGAAGAAT
GTTAGGTGTCGTTCCACTACATACACCCTTTGTATTCGACAAGATTCTGGAAAAATGGTGCCTGCATCGTCGT
TGTCGTTCATCACGTGGAGATGGAGAGGAGAACACCGACGATTATGGAGTATCAAGAAATAATATTTATCAAT
TATCGGATATGAATGATGGTGACACAATCGAACTCTGTTGTGGAGATTATGTCAAGGATTTATCGTCCGAGAA
GGAAGGTTACTTATTGGCACTCATACCATCCAGATATAATACCATTGTTGTTGATTCGTTGGAAAATACCCCG
TTGGACAAGGAGAGGGCCGCACACGTGAATGATAAGGCGGATGGAACAGAAAAGGGTTGCCATGATTATCATG
CCAATGATGACGACGACGACGATGATTCTGATATTGAAGATGTTACAGATATCTACGTTTCCAAAAAGCGTCG
TGCTTCGCAGGAGGTTATAGAAGTGGAAGATACCAGCAGCGATGATGACTACTACTGCAAAAACAACAATCAT
GATGAATCTTGCTTTTGGGAGTTTGACAAATATGGCTATCTCAAGAAGAATGCAAATCATTCCGCAAAATGGG
AGAATGCAAAAAGTTCGAGAAAAGATTCGAAAAGCAATGACGACGATGACGACGACGATGATGATGTAAATGC
AAATATTGATATTGAAATTGAAAGCATCATCAGCACTAGTGATGATTCCGACGATGACTCCTCCGATATTGAA
GATGTCACAGAAACAATAATGGCCCAAAGAAAAGAACAAGTTCAAAAATTAATCAACAATGCCGAAGAAATAT
GTGACGACTCGGATTCCAATAACGACGACAATTCACAGGAGGGGGGATCTGGAGGAAAGAAGAGAAAAAACGA
AAATACGACAACTCGCTCATCCCGCTCTTCTTCTTCTTCTCGCCCTACCGCCAAAAAAAAACGAAGGAGAACA
ACGAAAAAGAAGATGAAAGAATCACCAATCGAAGTTCTCATTGATGAGAAAGATATACCCACGCGACCGGGTG
CCAAGATCACAAAGGACGACGACGACGACGACGACGA
>TR583|c1_g1_i1 len=3206 path=[3942:0-645 3943:646-669 3979:670-866 3911:867-890 3984:891-966 3899:967-1014 3978:1015-1360 3954:1361-1384 3969:1385-1473 3905:1474-1497 3977:1498-1785 3902:1786-1809 3968:1810-2100 3932:2101-2124 3976:2125-2127 3983:2128-2151 3967:2152-2179 3975:2180-2301 3908:2302-2325 3966:2326-2526 3888:2527-2550 3987:2551-2715 3964:2716-2743 3981:2744-2751 3973:2752-2796 @3986@!:2797-2994 3937:2995-3016 3980:3017-3082 3922:3083-3108 3923:3109-3205] [-1, 3942, 3943, 3979, 3911, 3984, 3899, 3978, 3954, 3969, 3905, 3977, 3902, 3968, 3932, 3976, 3983, 3967, 3975, 3908, 3966, 3888, 3987, 3964, 3981, 3973, 3986, 3937, 3980, 3922, 3923, -2]
TCCCTCCGTCCAGCAAAAATGGGTCCTGTAAAAAGATGGCGAGGCCCATTATATTTATTCCTTTCGCATCACT
GTCATCGTGGCGGCAGGAGCTAGCGCCGTCCGTTGCGAAAGCATAAATCCTATGAGTTGCTACGGTACGATAC
AATAACGGTTGCCAGTTGACACAACCTTTCAACTTGAAACCCGATCTCAAATTTTCACAGCAATATGACAAGA
GGAATGGTTCATGCGTGGCGAAACGGCCTGAAAACTTCAGACTTTTAATCCACAGACACCAGAGTTTGTCTGC
ACTGGCACTGGCATAAAATCTACCAAAGCGAAATCCAATATCAATATTGTATGGATTGTACATAAAATAGTCA
GTGATGGCATCGGACGCAAATGCAAACGCTGACGCTCCAAAAGCTAGGGCCAACCCTCTAGATATTCTAGGAA
AGGAGGGGTGCAAAGTCCAATCATTGACGGTGTCAGACCAGCATGCTACTCTGCTCATTTCTCACCCAGTTAT
TATCGACGACGCTGCTGGCGACCATGATGGCGATAAAAAAGATGAACCTCCCATCTCATCCACAACCACAACT
TCAACAACCATATCCTCCCTCCTAAAACTAACCCTAGTACCATTCCACAAATCTCTACTGGGAAGCAATCCAG

TCCTAACCCCCGAAGACATTCAACGAGGCCAGATGCCCGAAAGAAATCTTCTCGAACACAATCCCAATGCGAG
TGACAGAATCATCTCCTTTCTGCGCGGATACAATTTTGAACTCAAGAGCGAGAGCGGTGCAGAGTACAGTTAT
TATGCGGCGAGGCCTACTTTGGGCCTTTGTGCCCTGGGCAATGGCGGCGGCGGCGGTAGTAGTAATGGGGGTG
GAGGTGAAAATTCTCCCAATGCAACTGCAGAGGAGAACAATGAAAGCACTAATACCAATACGGCCGTCTCCAA
TTTTGGTTCGTTCGACATAGAACTAATCTCGCCCGCCAGCACGTATCAAATATCACGCGCCATGCCATCCCTC
GGCCACGTCTTAATTCGCGAAACACCTCAACTCTACAATACGGTCGTAAAACCGTATATTCAAAGTATTGTCG
ACAATGGCAGTCTCTCTTGGATCAAAAATGTCATCGAAGTAAAGAAGGAAAAAGAACGGCTATTGCTCAACCA
TGATGATTTTGTGATGAATGTCGATACCAAATGGAGGAATCATCCTCCTCCCCTAACTACCCCGCGGGAAGAA
TGGTACAATCACCCTTCCACGGTGGATTTGTACTGTTTGGGAATAGTGAAACAGAGTGGCATAACTTGCATTC
GCGATTTACGAAGGGAACACATTCCACTACTCATAGAAATGGAAAAGAAAGGACTCGCCGCTATACACACAAT
CTACGGCATCGAGATGGATCAGATTCGAGTGTTTGTGCACTATCAACCGCAGTTCTACCATTTTCATGTTCAT
TTTACGCGGTTGGAGAATGAGGTAGGGTGTGCCGTGGAGAGGGGCCATTTGTTGAGTGATGTGATTCAGAATT
TGGAATTCGATGAAGGTTATTATTGCAAGAGGATTATTACGTACAAGTTGAAGAAGGGAAGTCCACTTCAGAA
TTTGGTTGAGACTTATTCCAAGGATAAAGAGGAGGTCATGGTTGATGCGAAATAGTCATTTACTGCTGTGTGG
CAGGACAGGTGGATTGAATCTAATAGTCTCGCGGTACGTCGTAATCCTGTGGCAATTGGGACGCAGAGATTTG
AAGTGTTGCGTTTGGCAAACAACCCAAAGATGCATCAGTCCAGACGGCAGCATTTGCGTGTCCTTCTCGTTTG
CAGTTAAATTGAATACCGCCCAAAGTAAACTTGGTGTTTTGAAATCCAACGTTCACAAAAGACACCTTTGCAG
CTGTGCCAGAGCTCTGCCATTCTCTACTGTGAATACCAAAATGCCAATCAGGTCCCGCCGAGGAGGAGAGAGC
TCTACCGCCGTATGCCTCCCGTCGGTCGTGTTGTTATCTATCACAGCACTACTTTCCCATCAACATCAAACTA
TCTCATCACTCATCCACCCAACTGGATGATCCACAAAGCGACACAATTAATGGGCATGTGTGAATTTTTGATG
TGCACGCTTAGTAGGAGAGAGTGTAACGCTGTGCACTTACAAATCTCACAATGATGGCAAGGTAGGCCAAAAG
AGCCACCGGGCGTCAGTAATGGAACATATTATTTTATCGCCCAATCAAAATGAATCTCCACACAATTACCTTC
CACTTTAATCTCGACCAGGTCTGGAGCGCTCTGGATTCCGAGAAGTGGTGCGCCTGTCAACGAAATATAATAC
AATGATATTGCGATAGTGCAGAAGAATGAAACAGGAGTGCGTTACATGTTCTGTGTGGAGCATTGCCTCCAAT
CGCTGTATCAATCAACTAAGTTACTAACAGGGCAAATCTATTATTCCAAGCTTTGTCACATATGATAGGCCAA
CCCCATCACAATAAGAAACATCACATAGAAAATGTCGAAACTGCTGCTCACTGAGCCCTTGGGTAACAAGAAA
TGGGTGCCATCATTCTGGCACTGGAAGCCCCATACGCTATCCAACGAGGGCACAATGCCAAAATCGAAGCTTT
TTCATCTATACACTTGAAGATGGCTCACAATCGCCGTGAGACAAGCTGGGCATTGCGCCTCACACCTCATTCT
ACACCCTGAGCACATGATGCGACCGCAAGGTATCAATGCATAGTCCTTGTTTCGCACCTCCATGCAAATCAGG
CACTCAAGAGCTTTTAACGCTCAACCTCTGCCTTCAAGCTTTTAATTTCTTCGTCTCTGGCACCAATCTTCGT
GTCCCTCTTCGTGATAACATTCTCCAGCCCCTTTGCATTTCTTTCAGTGCTACCGGGAATGGCTTCAATCCTC
TTCTTTAGGTGGCCACTCTGTTGGAACCCTCGGAAACGGGTACGCTAGACGTTCCCCATCACGAAGGGTTCCC
AATAAACAGAATGCTATGGCAGCTGCTGCCCTGACTAACTTTAGGCAGCACCACGGAATCCCAATGGCTTATA
ACACATTAGGTTATATATTTATTCCTACATATGTAGATACGAGCTCACAGATGATCAACAGCATCACCTACTT
CAGATGAACTAGAGATAGAGCTCATTAATGATAATAAAATTCGATACCTCAATTGTTAAAAAAAAAA
>TR583|c2_g1_i1 len=239 path=[217:0-238] [-1, 217, -2]
TTCTTCTGGGATAGTAGACGGTTGCAATGGCTATGACGACGACGACGACGACGACATTGATGGTGACGAGGAC
CATTGGGAAACCCTAGATTTCGATACCGGTCTCAATTCTCGAATGGCAGGATGCTTTCACTTGAAACGATTGG
AGCTCATCGACGTGCCATTGCGACGACGACGACGACGACAACAACAACAACAACATGGATCGTCGTCACCGTC
ATGGTCGTACTCCTCGGGAA
>TR584|c0_g1_i1 len=364 path=[1762:0-80 1742:81-119 1765:120-122
1749:123-147 1740:148-154 1726:155-178 1708:179-212 1755:213-236
1748:237-257 1739:258-281 1766:282-284 1768:285-288 1754:289-327
1767:328-339 1769:340-348 1770:349-350 1596:351-363] [1762, 1742, 1765,
1749, 1740, 1726, 1708, 1755, 1748, 1739, 1766, 1768, 1754, 1767, 1769,
1770, 1596, -2]
CACGAAATCCCTTCGCTTATATCAATCCATCACGCAATCGCTCCCCGACTCTCTCCAAATCATTTCCCAGTTT
TACTTTGAGGCAACGTTTCAGAACGCAACCATTTCCGCCTTCGGATGGTGATTGATAATGGTGGCACCACATA
TTTGACATTTGTCTTCTTCGAAATTGTATCAACCAAGAATAGTTGTGATGGGAGACGGTGGTACATATCATCC
AGCAACGAGAGAAACAACCCACGTCCTTTCCCTCCCATTTCACCCCAACTTTAAAGTGATGCCGTGTACGCAT
GTCTTCTCCAACTTGGGTTCCTATCACCGACGATGACGACGACATCGCACATTCTCCAAGATCGGAAGAGCA
>TR584|c0_g1_i2 len=1024 path=[1666:0-108 1667:109-132 1752:133-145
1732:146-180 1744:181-191 1763:192-204 1759:205-248 1751:249-272
1764:273-291 1757:292-315 1762:316-373 1741:374-412 1765:413-415
1709:416-440 1740:441-447 1726:448-471 1708:472-505 1755:506-529
1748:530-550 1739:551-574 1766:575-577 1768:578-581 1754:582-620
1767:621-632 1722:633-656 1745:657-667 1734:668-691 1718:692-810
1641:811-834 1698:835-853 1733:854-877 1717:878-908 1697:909-932
1653:933-933 1654:934-957 1655:958-1023] [-1, 1666, 1667, 1752, 1732,
1744, 1763, 1759, 1751, 1764, 1757, 1762, 1741, 1765, 1709, 1740, 1726,

1708, 1755, 1748, 1739, 1766, 1768, 1754, 1767, 1722, 1745, 1734, 1718, 1641, 1698, 1733, 1717, 1697, 1653, 1654, 1655, -2]
AATCAGACGAGCAGGCATAGGATGATGCTAAGCCGTGCTTACTCATTAAATTAGACACCGGCATCGCATGCAT
ACGGTCAAACAGAAAGAACGAATACAAGTGTGGGCCGTAAGAGAGTTGATATTGATCCTCTTTTTTTAGCAGT
ATTTTTTTCGTGCATGCGTCATTTTGAGTCGAATGATCGATCCCATCAAACTCTCGTCGGCGACATCCAGCTG
GAAAGGGTTACACTGGGACGAGAGGGACGCAGACTCCGACCAAGAAGTTGTGCACGCTTTTTGCTACCATTAC
ACACGAAATCCCTTCGCTTATATCAATCCATCACGCAATCGCTCCCCGACTCTCTCCAAATCATTTCCCAGTT
TTACTTTGAAGCAACGTTTCAAAATGCAACCATTTCCGCCTTCGGATGGTGTATGATAATGGTGGCACCACAT
ATTTGACATTTGTCTTCTTCGAAATTGTATCAACCAAGAATAGTTGTGATGGGAGACGGTGGTACATATCATC
CAGCAACGAGAGAAACAACCCACGTCCTTTCCCTCCCATTTCACCCCAACTTTAAAGTGATGCCGTGTACGCA
TGTCTTCTCCAACTTGGGTTCCTATCACCGACGATGACGACGACATCGCGCATTCTCCAAAATATCATCCAAC
GAAACAACCCTAATCTCGCAATTCGAAAATTCGACCTTCTCATTTTCTTCCTTCCGAAGCAACCATTCGAAAA
CACCAAAGTGTCCCACCAAAGCGATCGCATTTTCCTCGCGACGTTCCAACCAAGTGCACAATTCCAACATTCG
ATTTTCGAAATCGTCGTACGGTTCTCCGGGACAGAAGTAAGTTTGTCCTTCTCCCGTGGGACGCCATTCGGAA
TTATTGTCGATTGGCGATGGTGATGAATCGCTTACGTTGTTGTTGGATTTGACATTGTTCGATTGATACCACC
AGGGTGCATCATTATCGTCATCGTCGTCGTCAGCAGCAGCATCAGCAATAGCAGAAATAATAGCAGCAGTATT
CG
>TR584|c0_g1_i3 len=364 path=[1762:0-80 1742:81-119 1765:120-122 1749:123-147 1740:148-154 1726:155-178 1708:179-212 1755:213-236 1748:237-257 1739:258-281 1766:282-284 1706:285-308 1598:309-324 1599:325-327 1767:328-339 1769:340-348 1770:349-350 1753:351-363] [1762, 1742, 1765, 1749, 1740, 1726, 1708, 1755, 1748, 1739, 1766, 1706, 1598, 1599, 1767, 1769, 1770, 1753]
CACGAAATCCCTTCGCTTATATCAATCCATCACGCAATCGCTCCCCGACTCTCTCCAAATCATTTCCCAGTTT
TACTTTGAGGCAACGTTTCAGAACGCAACCATTTCCGCCTTCGGATGGTGATTGATAATGGTGGCACCACATA
TTTGACATTTGTCTTCTTCGAAATTGTATCAACCAAGAATAGTTGTGATGGGAGACGGTGGTACATATCATCC
AGCAACGAGAGAAACAACCCACGTCCTTTCCCTCCCATTTCACCCCAACTTTAAAGTGATGCCGTGCACGTAT
GTCTTCTCCATCATGGGTTCCTATCACCGACGATGACGACGACATCGCACATTCTCCAAAATATCATCCAAC
>TR585|c0_g1_i1 len=1009 path=[1109:0-109 1110:110-133 1116:134-234 1117:235-258 1118:259-261 1113:262-305 1100:306-1008] [-1, 1109, 1110, 1116, 1117, 1118, 1113, 1100, -2]
TGTGCTCTTCCGATCTCGACGGCAACTTCTTCGGCCGGACCATCGTCGTCATCGTCCTCCGTTACTACCGCAT
CGACTCCACCGCGGAGAACGGCGAAAAGGAGGCGACAGAATTCGGACTCGAACAATGGCTGCGACGATTCTTT
TTCTTCCGTCGGTTCTCGATCTCTGGCTTCTTCCTCGATCGGGGCGTTGTCGGCGAGCAGCAGACCCTACAGG
GATTTCTCAACTAACACTGCGGCATCGGCATCCGCATCAGCAGTACCAACAGCATCGGTAGCAGCAGCAGCAC
GTACCACTCCAACAACCACCGCCAGGACCACGCGGAGACAAAGCTCGAGCATGGACGACAACAGCTGCGATTC
CTCCGCCTTCTCTCAAATTTCTTCCCTCGGCGCTTCCAGTCGAACGTCCGACGATCAATTCTCCATCGCGGCG
AGGTTCAAGAATACGTCGTCGACGATACGCGAAACGACGACCGTGAATCATCATCCCGGCATTCCTCTTCGCG
AATGCTACTACGATGGTACTGATAACGAATCGAGCAACAACGACAACATCAGCAACAACATCAGCAACAACAG
CAGCAACAACCACAGCAACAGCAGCAGAGGAAGAATCGAATCTCGACGTGCTCCTCCCTCGAGACGAGATCTC
CGTGCTCCCTGTCCCAGACGACATTTCAACGCTCAGGACGATCCATCCAGCTATCTCGATGAAATGCTATTCG
CAGAGGTCCACAATGCCACCTCCTCTTCCCCACCATCATCACTGGCACACAACAACGACGAGGTCGAAGACGA
AGTAATGAGAATGGAGAATTATCTATTCATGTCGGTTGGAAACGACGATCATCATCGTTTTTACGATGACGAC
GAGGACGACGAGGAGAGCATGATTGCGCAAATCGAAGCATCGTGGAGGGCGGGCGCCGAGGCGCGTCGGGAGG
TGTACAGTTCCGACGATCACGGTTCCAACATCGCGGACGGCAACAACAACGACGGTCACG
>TR585|c0_g1_i2 len=1024 path=[1109:0-109 1110:110-133 1116:134-234 1117:235-258 1118:259-261 1112:262-320 1097:321-1023] [-1, 1109, 1110, 1116, 1117, 1118, 1112, 1097, -2]
TGTGCTCTTCCGATCTCGACGGCAACTTCTTCGGCCGGACCATCGTCGTCATCGTCCTCCGTTACTACCGCAT
CGACTCCACCGCGGAGAACGGCGAAAAGGAGGCGACAGAATTCGGACTCGAACAATGGCTGCGACGATTCTTT
TTCTTCCGTCGGTTCTCGATCTCTGGCTTCTTCCTCGATCGGGGCGTTGTCGGCGAGCAGCAGACCCTACAGG
GATTTCTCAACTAACACTGCGGCATCGGCATCCGCATCAGCAGCACCAACAGCATCGGCAGCAGCAACAGCAG
CAACAACAGCAGCACGTACCACTCCAACAACCACCGCCAGGACCACGCGGAGACAAAGCTCGAGCATGGACGA
CAACAGCTGCGATTCCTCCGCCTTCTCTCAAATTTCTTCCCTCGGCGCTTCCAGTCGAACGTCCGACGATCAA
TTCTCCATCGCGGCGAGGTTCAAGAATACGTCGTCGACGATACGCGAAACGACGACCGTGAATCATCATCCCG
GCATTCCTCTTCGCGAATGCTACTACGATGGTACTGATAACGAATCGAGCAACAACGACAACATCAGCAACAA
CATCAGCAACAACAGCAGCAACAACCACAGCAACAGCAGCAGAGGAAGAATCGAATCTCGACGTGCTCCTCCC
TCGAGACGAGATCTCCGTGCTCCCTGTCCCAGACGACATTTCAACGCTCAGGACGATCCATCCAGCTATCTCG
ATGAAATGCTATTCGCAGAGGTCCACAATGCCACCTCCTCTTCCCCACCATCATCACTGGCACACAACAACGA
CGAGGTCGAAGACGAAGTAATGAGAATGGAGAATTATCTATTCATGTCGGTTGGAAACGACGATCATCATCGT

TTTTACGATGACGACGAGGACGACGAGGAGAGCATGATTGCGCAAATCGAAGCATCGTGGAGGGCGGGCGCCG
AGGCGCGTCGGGAGGTGTACAGTTCCGACGATCACGGTTCCAACATCGCGGACGGCAACAACAACGACGGTCA
CG
>TR585|c1_g1_i1 len=1257 path=[3178:0-48 3179:49-72 3226:73-238 3215:239-305 3208:306-343 3232:344-365 3225:366-618 3233:619-638 3170:639-640 3223:641-655 3217:656-766 3118:767-787 3201:788-995 3190:996-1019 3191:1020-1054 3192:1055-1256] [-1, 3178, 3179, 3226, 3215, 3208, 3232, 3225, 3233, 3170, 3223, 3217, 3118, 3201, 3190, 3191, 3192, -2]
CTCGCCCAAGACGAACGCCGCTCATATTGCCGCCGCCATGGCCATGGTGGTCTATTGAAATAATAATAACAGA
GAGAGATAGAGAGGCAGGCGACGCCCAGCATGCAAAGCATCTGCCGCCCATTGACGGCACCGAAGAATACTCA
ACGATTACTCCAACGAACAACGCTTCCGATATCAAGAACACACCTGACAAAAGGACGATGTGGAGGAAACAAA
ACATGTTATTATTCCTCCTCGAATAGTAATTCGTCACCATCATCGAAGCCGCCGCCGCCGCCGCCCTCCTCAT
CAGTCGCAAACAATAAGAATGCCAACGATGCATCGCAAACATCAAAATCATCATCACCATCATCGTCATCATC
ATCAACGTCAACGTCCGAAGACAACTCAAGCGGCAGCACAATAGTATCCATCGAAAGTTCCGGATGTCTATCC
CAATGTGGCAAAGGTCCAAACATAATGGTAGTGGATGACAATAATGGCGAAATAAATGAAAAAATGTACTTTG
GAGTGGAGGATGTTACCACCGCATCAGCCGTATTGGATGTGGTGACGGGGGAGGAATATCCTATTAATTTACT
TGTCGCTTCTGCTTCCATTGCTGATGCCGAACATGCCACATCTCCATCGAAAAAAGAAACACTCCTAACCTCT
GCAATATCCGCCCTAACCAAAGATGATGATCCCACGCTCGTGAATTCCTTTGCACATGCCCACGCTCTGTATT
TGCGTGCGGATGCACGATTGGACCTGCTAGCATCCTCGTCATCGTCATCGTCATCACCCAGTAGTACCACTCC
GCCCACTGCTAACACCGCCACCCCTAACAACAACAACAACATACTATACGGAGCCGTAAACGATGCCAAACTA
TCCACTCAAATCCATCCCACCGAAGGTCGAGCATGGCGAACATTAGCCCGAGTCCAAGAGGCGAATGGCAGCA
TAAAAGAGGCAATGGATGCCGTGGAAGAATGGGCGAGGTTAAATCCACCCTTTAGGACCAAGGCGAAGAGGGA
GATGGAGAGGCTTGTTGGTTTATTGTGATTTTTATTGTGAGGAATGGATTGAATGAGGAGAGAGTAGGATGTA
GGATGACACGCGATATATATTGTCGATGGGTTTTTAAGCAGATTGAGCACGTTTGAGTGTATGGATGTGGTGT
TTTGTGTTGTATACTCAAGATAAATATTCAATATACCGTGGGCATTGTATAAAATAAATAAAAATTAGGAAAC
GACAGATCGGAAGAGC
>TR585|c1_g1_i2 len=1557 path=[3178:0-48 3179:49-72 3226:73-238 3215:239-305 3208:306-343 3232:344-365 3150:366-372 3207:373-590 3174:591-614 3229:615-684 3204:685-722 3231:723-754 3228:755-817 3222:818-934 3216:935-1076 3200:1077-1298 3194:1299-1322 3195:1323-1556] [-1, 3178, 3179, 3226, 3215, 3208, 3232, 3150, 3207, 3174, 3229, 3204, 3231, 3228, 3222, 3216, 3200, 3194, 3195, -2]
CTCGCCCAAGACGAACGCCGCTCATATTGCCGCCGCCATGGCCATGGTGGTCTATTGAAATAATAATAACAGA
GAGAGATAGAGAGGCAGGCGACGCCCAGCATGCAAAGCATCTGCCGCCCATTGACGGCACCGAAGAATACTCA
ACGATTACTCCAACGAACAACGCTTCCGATATCAAGAACACACCTGACAAAAGGACGATGTGGAGGAAACAAA
ACATGTTATTATTCCTCCTCGAATAGTAATTCGTCACCATCATCGAAGCCGCCGCCGCCGCCGCCCTCCTCAT
CAGTCGCAAACAATAAGAATGCCAACGATGCATCGCAAACATCAAAATCATCATCACCATCATCGTCATCATC
AAAAATAAGCCAATGGATCGATGCCCTAACCACGGCGGCATCCCACACGGCATCCCGCGCAACCCGAACGGCA
ACCCATGCCGCCACCTCCGTCACCGCGAACGCGACCGTCAAAGCCAGGACGGCGGCCACCCGAGCCGAGACGA
AAGTCCGCCGCTCCCTCGAAAATGCCAAGATCGACGCGAAGCAGACGGGAGAGCGATACGCGTGGGAGGCACA
TCGTTCCATGGCCCAGATGGGGAGTAAAGTCAAGCGATCCGCGGAGGAGAGGGCGAGGGAGACGGGGAGGAAG
GCGAGGCGGTCGATGGAGGAGGTTGCGAAATCTACGAGGAGGAGGGTGATGGATGAAATTAGGCAGAGGGTGC
CTGAGATTCCGAAGCTTCCTTTTACTACTCAGTCGCCTTCGATGACGACCTCGACAACAACATCGACCACAGC
GACCTCCGCGACGTCTGCATCATCCAACAACTCGAAGAGAATAACGGACGCCCTGCCGACCAAAGAAACCGTC
CTAAAATCGGCCTCCACCCTGGCCGCCGAAACACTCACCAAAACCTCCGCCAACGTGACCACGCAAGTGCAGG
AAACGCTGCATAAAACGACGCGTTGGCTGTGGTGGTGGGGACTCGCCGCCGTGGGCGTGTACGGGATGAGCAC
GACCCTCACGAAGGAGGGGGTGCAGTTGCTGAAGGACGCGGTGAGCGGTAAGAAGGAGTCTCCTTGGGGGGCG
GGTGGTGTTGGGGGTACTAGTGCTGGGAGTGGTGTGGATAGTAATAGCGATGGTGCGCCCGCCGGTGTTTCTG
TGGAGGGATCTGCCGTAGGCGCTGTTGGTGCCAAGGATGCAGTGAGTGTCGACAACGACGTAAGAAGCAGTGG
TGGCAGATGGATGTCATCTTGGTTCGGTTGGCGGACGAATAATAATAAAATTGGAAAGGCTGGCGTCGAAGAG
AACGACGATTAGGATCCCCACCGAGTAAGGTTTAAATACGCAATAACCAGCAACGCTAATCAGATTACCGAGT
CGCCATCCATCACGATGCCTGCGGACCAAACAACAACAACCGAACGGCCCTCTCGGAATCCTCCTCCGTCAAT
CCCGACTTCGTGTCGGTGTGCACGAACCGATCGAAGAGGGGAGTGTCCGGCTTGGCGGCGCTGGGCCTATCGT
CCAAAATGACGTACTTCCAGCGAT
>TR585|c1_g1_i3 len=1551 path=[3178:0-48 3179:49-72 3226:73-238 3209:239-311 3126:312-349 3127:350-366 3207:367-584 3174:585-608 3229:609-678 3205:679-716 3231:717-748 3227:749-811 3222:812-928 3216:929-1070 3200:1071-1292 3194:1293-1316 3195:1317-1550] [-1, 3178, 3179, 3226, 3209, 3126, 3127, 3207, 3174, 3229, 3205, 3231, 3227, 3222, 3216, 3200, 3194, 3195, -2]

CTCGCCCAAGACGAACGCCGCTCATATTGCCGCCGCCATGGCCATGGTGGTCTATTGAAATAATAATAACAGA
GAGAGATAGAGAGGCAGGCGACGCCCAGCATGCAAAGCATCTGCCGCCCATTGACGGCACCGAAGAATACTCA
ACGATTACTCCAACGAACAACGCTTCCGATATCAAGAACACACCTGACAAAAGGACGATGTGGAGGAAACAAA
ACATGTTATTATTCCTCCTCCAATAGTAATTCGTCACCGTCATCGAAGCCGCCGCCGCCGCCACCGTCGTCCT
CCTCATCAGTCGCAAACAATAAGAATGCCAACGATGCATCGCAAACATCAAAATCATCGTCATCATCAAAAAT
AAGCCAATGGATCGATGCCCTAACCACGGCGGCATCCCACACGGCATCCCGCGCAACCCGAACGGCAACCCAT
GCCGCCACCTCCGTCACCGCGAACGCGACCGTCAAAGCCAGGACGGCGGCCACCCGAGCCGAGACGAAAGTCC
GCCGCTCCCTCGAAAATGCCAAGATCGACGCGAAGCAGACGGGAGAGCGATACGCGTGGGAGGCACATCGTTC
CATGGCCCAGATGGGGAGTAAAGTCAAGCGATCCGCGGAGGAGAGGGCGAGGGAGACGGGGAGGAAGGCGAGG
CGGTCGATGGAGGAGGTTGCGAGATCTACGAGGAGGCGGGTGATGGATGAAATTAGGCAGAGGGTGCCTGAGA
TTCCGAAGCTTCCTTTTACCACTCAGTCACCTTCGATGACAACCTCGACAACAACAACAACCACAGCGACCTC
CGCGACGTCTGCATCATCCAACAACTCGAAGAGAATAACGGACGCCCTGCCGACCAAAGAAACCGTCCTAAAA
TCGGCCTCCACCCTGGCCGCCGAAACACTCACCAAAACCTCCGCCAACGTGACCACGCAAGTGCAGGAAACGC
TGCATAAAACGACGCGTTGGCTGTGGTGGTGGGGACTCGCCGCCGTGGGCGTGTACGGGATGAGCACGACCCT
CACGAAGGAGGGGGTGCAGTTGCTGAAGGACGCGGTGAGCGGTAAGAAGGAGTCTCCTTGGGGGGCGGGTGGT
GTTGGGGGTACTAGTGCTGGGAGTGGTGTGGATAGTAATAGCGATGGTGCGCCCGCCGGTGTTTCTGTGGAGG
GATCTGCCGTAGGCGCTGTTGGTGCCAAGGATGCAGTGAGTGTCGACAACGACGTAAGAAGCAGTGGTGGCAG
ATGGATGTCATCTTGGTTCGGTTGGCGGACGAATAATAATAAAATTGGAAAGGCTGGCGTCGAAGAGAACGAC
GATTAGGATCCCCACCGAGTAAGGTTTAAATACGCAATAACCAGCAACGCTAATCAGATTACCGAGTCGCCAT
CCATCACGATGCCTGCGGACCAAACAACAACAACCGAACGGCCCTCTCGGAATCCTCCTCCGTCAATCCCGAC
TTCGTGTCGGTGTGCACGAACCGATCGAAGAGGGGAGTGTCCGGCTTGGCGGCGCTGGGCCTATCGTCCAAAA
TGACGTACTTCCAGCGAT
>TR585|c1_g1_i4 len=1426 path=[3178:0-48 3179:49-72 3226:73-238 3215:239-305 3208:306-343 3232:344-365 3225:366-618 3206:619-700 3219:701-709 3213:710-733 3203:734-809 3223:810-824 3217:825-935 3118:936-956 3201:957-1164 3190:1165-1188 3191:1189-1223 3192:1224-1425] [-1, 3178, 3179, 3226, 3215, 3208, 3232, 3225, 3206, 3219, 3213, 3203, 3223, 3217, 3118, 3201, 3190, 3191, 3192, -2]
CTCGCCCAAGACGAACGCCGCTCATATTGCCGCCGCCATGGCCATGGTGGTCTATTGAAATAATAATAACAGA
GAGAGATAGAGAGGCAGGCGACGCCCAGCATGCAAAGCATCTGCCGCCCATTGACGGCACCGAAGAATACTCA
ACGATTACTCCAACGAACAACGCTTCCGATATCAAGAACACACCTGACAAAAGGACGATGTGGAGGAAACAAA
ACATGTTATTATTCCTCCTCGAATAGTAATTCGTCACCATCATCGAAGCCGCCGCCGCCGCCGCCCTCCTCAT
CAGTCGCAAACAATAAGAATGCCAACGATGCATCGCAAACATCAAAATCATCATCACCATCATCGTCATCATC
ATCAACGTCAACGTCCGAAGACAACTCAAGCGGCAGCACAATAGTATCCATCGAAAGTTCCGGATGTCTATCC
CAATGTGGCAAAGGTCCAAACATAATGGTAGTGGATGACAATAATGGCGAAATAAATGAAAAAATGTACTTTG
GAGTGGAGGATGTTACCACCGCATCAGCCGTATTGGATGTGGTGACGGGGGAGGAATATCCTATTAATTTACT
TGTCGCTTCTGCTTCCATTGCTGATGCCGAACATGGTGAGTGTGTTTCCGTTTGATTTGATTTTACTTTGAAT
GCATTTCTTCTTTATAACCACACGCAATAAATACCCCAAGGTGTGATTTCTCGACAAATATTATGTCTGATGC
ACAACAACATGCTCACACACACAAAAAATCTATCAACATACGTCGTGTACTTACACAGCCACATCTCCATCGA
AAAAAGAAACACTCCTAACCTCTGCAATATCCGCCCTAACCAAAGATGATGATCCCACGCTCGTGAATTCCTT
TGCACATGCCCACGCTCTGTATTTGCGTGCGGATGCACGATTGGACCTGCTAGCATCCTCGTCATCGTCATCG
TCATCACCCAGTAGTACCACTCCGCCCACTGCTAACACCGCCACCCCTAACAACAACAACAACATACTATACG
GAGCCGTAAACGATGCCAAACTATCCACTCAAATCCATCCCACCGAAGGTCGAGCATGGCGAACATTAGCCCG
AGTCCAAGAGGCGAATGGCAGCATAAAAGAGGCAATGGATGCCGTGGAAGAATGGGCGAGGTTAAATCCACCC
TTTAGGACCAAGGCGAAGAGGGAGATGGAGAGGCTTGTTGGTTTATTGTGATTTTTATTGTGAGGAATGGATT
GAATGAGGAGAGAGTAGGATGTAGGATGACACGCGATATATATTGTCGATGGGTTTTTAAGCAGATTGAGCAC
GTTTGAGTGTATGGATGTGGTGTTTTGTGTTGTATACTCAAGATAAATATTCAATATACCGTGGGCATTGTAT
AAAATAAATAAAAATTAGGAAACGACAGATCGGAAGAGC
>TR585|c2_g1_i1 len=2282 path=[2915:0-205 2939:206-333 2842:334-375 2946:376-467 2908:468-491 2938:492-535 2875:536-565 @2954@!:566-785 2937:786-871 2860:872-888 2951:889-895 2944:896-961 2959:962-979 2958:980-988 2957:989-999 2935:1000-1070 2927:1071-1352 2864:1353-1376 2941:1377-1396 2933:1397-1420 2926:1421-1496 2911:1497-1520 2947:1521-2008 2845:2009-2031 2925:2032-2186 2896:2187-2281] [-1, 2915, 2939, 2842, 2946, 2908, 2938, 2875, 2954, 2937, 2860, 2951, 2944, 2959, 2958, 2957, 2935, 2927, 2864, 2941, 2933, 2926, 2911, 2947, 2845, 2925, 2896, -2]
GCCTTGAAAAATACATATTTGAAAATATTACCACGCATACACACACACACACACACACAACGGCCTATATTCT
CATCACCGATATAACCAACGGACACACACACGCAACAACCACACAAACATACGCATTCCAACACTCAACCCTC
ACACACACAAAACAACAACAACAACAACAAAAGTGAGACAACAACAACAATGGCAGGATCAGTAACAAATCCA
AGAAGAAGGAAAATATTCCCTCATTTATCAAACAACGAGTCAAAGCAAACGGAGGGTGCGTCATCATCAACAT

CAACATCGGGGTTGGGTGGTGCCAGCAGCAACAGCAGCAACAGCAGTAACAACAATACCGCTAGCAACTCGAA
TAACTCCAAAAATAAATCGGATAACAGCAACAACAGCAACAGCAAGAACAACAACAATGATTCATACGACAAT
GCCAAATCCAGAAGAGGTAAAAAGCAATCTTCCAAATCCTCCTCCACAAATAATGATTCTTCCAACAACAACA
ACAAAGCGGCAACTGCAGCTGCTGCGGGAAGTAACTCTACATCATCATCACCATCATCGTCATCGAATGCAAC
AACGACACCAGTCACCACAACCACAACCACCACAACGACCCTCCATCATCACCTCCTACCATTGCCATTGGTA
TTAACGGTATTGGTATGTTCGGGATTGTTTTGGATAGCATCGTTTCGCGATTTGATGGCGACGGGGAAACCTA
TTTTGGATACGTTGGGCGTGCTTTGGGGTCAGGATGATGCCGATGCTAACTTTTTGGTGAGTTGGCTGAGCAT
TGACAAGTGTTATATTATATTGCTATAGTATGCTACATTGCCGTGTGTGTTTCATCCAATGTAGAACCTGTTT
GAATGAATAATTTATTCACCCCCAAATGTGGACCAATCATCCAATCATCACATCTCACAATCCCCACCCCACG
ACAATACAAAAAAATCAGCAATACACAAAATCAACCGCCTGGTTCGAAGACACCCGCGGCTGGAAATCCACCC
AAGGCGGCCTCTCCACCATAATGTCAACCACCACCGACGCCAACAACATGGGAGGACTCTTCGTCCGCAAGCT
CTCCGGAGTCGCCGGCCTGTCCTTCCACACCACCAAACTCATGCCCGTCGTCTTCCACTCTCCCCCCCAGTAT
CACCACGGGAAGTGGACGGGCGCCAGTTGGAGCGCCGGGCACTTCGATCCGGTGTTGATACTGGGGATGATGG
GGGACGTTTGCCTGGGCGTTTTTTACTTGGGGAGGTTGGAGGAGTTGAAGGATGCGGGTTGCGGCGGGCTGGG
GATGGCGTTCGTGTTGACTAGTTTGGTGGAGGCGTTTATTTTGGGGATGTACATGTTGAGTCGGAGGATGAAT
CACAAATCTCAACCGAACGTTCCGAAAGTGACAACCAGCACGGCATCGGCAAGGACTTCAACCGCCAATCCAC
TCGACGAGAATCCAAACTCCCTCCCCTCCCGCATCGTAGCCCGCACCGTCTCCATCGTCTCCACCCTGATATC
CCTCGTGTCCCTCCGCGACCTCCTCCTCCCGGGCGCCATCCTCTCCTTCCTCCCCCGCGACGACATCTACCTC
GAATGGACGGGAGCCTTCCTCCACTCGCCGCCCCCCGACACCGTCGAGGCGGACGAACACGGACTCGAATCGC
CGTTGTTCGCGGGCGATTTGCTCGTCCATCAGTTGTTGGGATTGTACCTGTTTCTGAGTTGCGCCTTCAAGTT
CGCGAGCGCGGTGGGGTGGTCGAAGGGGAACAGGTCCATGGGAAGCGGTGGTGGTGGTGGTGGGGCGAAGGCG
ATGATGATGGAGGAGAATGCGGAGAGGAGCGGCGTGGTGAGTTCGAAGATGATTTGGAAGGCGCAGGCTTTGG
GCGATGTGGGCGTTTTGACGGTGTTGAGGATGTTTACTCCCGCGGCGAAGAGTGCTAGTTTGGATTTGAGGTG
GCATTTGATGTTGGTGGCGTATGAAGCTTTTATTTTATCCTTGTATGGTTTCTGGTAAAGGAGTTGATGTGTG
GTCATTCGATTTGGCTCAGACTGATTTAAAGGACTTCGAAAGGTTACTTGTCTGGCGAAAGCTCATGTGTATT
TTATGTGGTGGTGTCTTTGCGAGACGGACGCTCCGAAAACTTTATACAAAAAAGATGGTATCATCTTGGAGCA
CAGATAACTGAGAAACTACGCAGATACATACAGCATCAATAACATGTATAAGTCAGAAAAATGAAGCTTAAAA
AAAAAAGATCGGAAGAGCG
>TR585|c2_g1_i2 len=2403 path=[2915:0-205 2939:206-333 2842:334-375
2946:376-467 2908:468-491 2938:492-535 2875:536-565 @2954@!:566-785
2937:786-871 2860:872-888 2951:889-895 2944:896-961 2956:962-978
2958:979-987 2957:988-998 2934:999-1069 2927:1070-1351 2864:1352-1375
2941:1376-1395 2933:1396-1419 2926:1420-1495 2911:1496-1519 2947:1520-
2007 2932:2008-2152 2925:2153-2307 2896:2308-2402] [-1, 2915, 2939, 2842,
2946, 2908, 2938, 2875, 2954, 2937, 2860, 2951, 2944, 2956, 2958, 2957,
2934, 2927, 2864, 2941, 2933, 2926, 2911, 2947, 2932, 2925, 2896, -2]
GCCTTGAAAAATACATATTTGAAAATATTACCACGCATACACACACACACACACACACAACGGCCTATATTCT
CATCACCGATATAACCAACGGACACACACACGCAACAACCACACAAACATACGCATTCCAACACTCAACCCTC
ACACACACAAAACAACAACAACAACAACAAAAGTGAGACAACAACAACAATGGCAGGATCAGTAACAAATCCA
AGAAGAAGGAAAATATTCCCTCATTTATCAAACAACGAGTCAAAGCAAACGGAGGGTGCGTCATCATCAACAT
CAACATCGGGGTTGGGTGGTGCCAGCAGCAACAGCAGCAACAGCAGTAACAACAATACCGCTAGCAACTCGAA
TAACTCCAAAAATAAATCGGATAACAGCAACAACAGCAACAGCAAGAACAACAACAATGATTCATACGACAAT
GCCAAATCCAGAAGAGGTAAAAAGCAATCTTCCAAATCCTCCTCCACAAATAATGATTCTTCCAACAACAACA
ACAAAGCGGCAACTGCAGCTGCTGCGGGAAGTAACTCTACATCATCATCACCATCATCGTCATCGAATGCAAC
AACGACACCAGTCACCACAACCACAACCACCACAACGACCCTCCATCATCACCTCCTACCATTGCCATTGGTA
TTAACGGTATTGGTATGTTCGGGATTGTTTTGGATAGCATCGTTTCGCGATTTGATGGCGACGGGGAAACCTA
TTTTGGATACGTTGGGCGTGCTTTGGGGTCAGGATGATGCCGATGCTAACTTTTTGGTGAGTTGGCTGAGCAT
TGACAAGTGTTATATTATATTGCTATAGTATGCTACATTGCCGTGTGTGTTTCATCCAATGTAGAACCTGTTT
GAATGAATAATTTATTCACCCCCAAATGTGGACCAATCATCCAATCATCACATCTCACAATCCCCACCCCACG
ACAATACAAAAAAATCAGCAATACACAAAATCAACCGCCTGGTTCGAAGACTCCCGCGGCTGGAAATCCACTCA
AGGCGGCCTCTCCACAATAATGTCCACCACCACCGACGCCAACAACATGGGAGGACTCTTCGTCCGCAAGCTC
TCCGGAGTCGCCGGCCTGTCCTTCCACACCACCAAACTCATGCCCGTCGTCTTCCACTCTCCCCCCCAGTATC
ACCACGGGAAGTGGACGGGCGCCAGTTGGAGCGCCGGGCACTTCGATCCGGTGTTGATACTGGGGATGATGGG
GGACGTTTGCCTGGGCGTTTTTTACTTGGGGAGGTTGGAGGAGTTGAAGGATGCGGGTTGCGGCGGGCTGGGG
ATGGCGTTCGTGTTGACTAGTTTGGTGGAGGCGTTTATTTTGGGGATGTACATGTTGAGTCGGAGGATGAATC
ACAAATCTCAACCGAACGTTCCGAAAGTGACAACCAGCACGGCATCGGCAAGGACTTCAACCGCCAATCCACT
CGACGAGAATCCAAACTCCCTCCCCTCCCGCATCGTAGCCCGCACCGTCTCCATCGTCTCCACCCTGATATCC
CTCGTGTCCCTCCGCGACCTCCTCCTCCCGGGCGCCATCCTCTCCTTCCTCCCCCGCGACGACATCTACCTCG
AATGGACGGGAGCCTTCCTCCACTCGCCGCCCCCCGACACCGTCGAGGCGGACGAACACGGACTCGAATCGCC
GTTGTTCGCGGGCGATTTGCTCGTCCATCAGTTGTTGGGATTGTACCTGTTTCTGAGTTGCGCCTTCAAGTTC

GCGAGCGCGGTGGGGTGGTCGAAGGGGAACAGGTCCATGGGAAGCGGTGGTGGTGGTGGTGGGGCGAAGGCGA
TGATGATGGAGGAGAATGCGGAGAGGAGCGGCGTGGTGAGTTCGAAGATGATTTGGAAGGCGCAGGCTTTGGG
CGATGTGGGCGTTTTGACGGTGTTGAGGATGTTTACTCCCGCGGCGAAGAGTGCTAGTTTGGATTTGAGGTGG
CATTTGATGTTGGTGGCGTATGAAGCTTTTATTTTATGTGAGTTTCTATTTTGTGTATAGTTTTGGTGATTTG
TGTGTGTTGTGGAAGGGGTGTGTGTTTTATGATTCATGCGTGTGGGAGTTTCTGATGACTTTTTACTGTTTAC
CCCATGATGTCAGCCTTGTATGGTTTCTGGTAAAGGAGTTGATGTGTGGTCATTCGATTTGGCTCAGACTGAT
TTAAAGGACTTCGAAAGGTTACTTGTCTGGCGAAAGCTCATGTGTATTTTATGTGGTGGTGTCTTTGCGAGAC
GGACGCTCCGAAAACTTTATACAAAAAAGATGGTATCATCTTGGAGCACAGATAACTGAGAAACTACGCAGAT
ACATACAGCATCAATAACATGTATAAGTCAGAAAAATGAAGCTTAAAAAAAAAAGATCGGAAGAGCG
>TR585|c2_g1_i3 len=2101 path=[2915:0-205 2939:206-333 2842:334-375
2946:376-467 2908:468-491 2938:492-535 2875:536-565 @2954@!:566-785
2955:786-807 2957:808-818 2935:819-889 2927:890-1171 2864:1172-1195
2941:1196-1215 2933:1216-1239 2926:1240-1315 2911:1316-1339 2947:1340-
1827 2845:1828-1850 2925:1851-2005 2896:2006-2100] [-1, 2915, 2939, 2842,
2946, 2908, 2938, 2875, 2954, 2955, 2957, 2935, 2927, 2864, 2941, 2933,
2926, 2911, 2947, 2845, 2925, 2896, -2]
GCCTTGAAAAATACATATTTGAAAATATTACCACGCATACACACACACACACACACACAACGGCCTATATTCT
CATCACCGATATAACCAACGGACACACACACGCAACAACCACACAAACATACGCATTCCAACACTCAACCCTC
ACACACACAAAACAACAACAACAACAACAAAAGTGAGACAACAACAACAATGGCAGGATCAGTAACAAATCCA
AGAAGAAGGAAAATATTCCCTCATTTATCAAACAACGAGTCAAAGCAAACGGAGGGTGCGTCATCATCAACAT
CAACATCGGGGTTGGGTGGTGCCAGCAGCAACAGCAGCAACAGCAGTAACAACAATACCGCTAGCAACTCGAA
TAACTCCAAAAATAAATCGGATAACAGCAACAACAGCAACAGCAAGAACAACAACAATGATTCATACGACAAT
GCCAAATCCAGAAGAGGTAAAAAGCAATCTTCCAAATCCTCCTCCACAAATAATGATTCTTCCAACAACAACA
ACAAAGCGGCAACTGCAGCTGCTGCGGGAAGTAACTCTACATCATCATCACCATCATCGTCATCGAATGCAAC
AACGACACCAGTCACCACAACCACAACCACCACAACGACCCTCCATCATCACCTCCTACCATTGCCATTGGTA
TTAACGGTATTGGTATGTTCGGGATTGTTTTGGATAGCATCGTTTCGCGATTTGATGGCGACGGGGAAACCTA
TTTTGGATACGTTGGGCGTGCTTTGGGGTCAGGATGATGCCGATGCTAACTTTTTGCAATACACAAAATCAAC
CGCCTGGTTCGAAGACACCCGCGGCTGGAAATCCACCCAAGGCGGCCTCTCCACCATAATGTCAACCACCACC
GACGCCAACAACATGGGAGGACTCTTCGTCCGCAAGCTCTCCGGAGTCGCCGGCCTGTCCTTCCACACCACCA
AACTCATGCCCGTCGTCTTCCACTCTCCCCCCCAGTATCACCACGGGAAGTGGACGGGCGCCAGTTGGAGCGC
CGGGCACTTCGATCCGGTGTTGATACTGGGGATGATGGGGGACGTTTGCCTGGGCGTTTTTTACTTGGGGAGG
TTGGAGGAGTTGAAGGATGCGGGTTGCGGCGGGCTGGGGATGGCGTTCGTGTTGACTAGTTTGGTGGAGGCGT
TTATTTTGGGGATGTACATGTTGAGTCGGAGGATGAATCACAAATCTCAACCGAACGTTCCGAAAGTGACAAC
CAGCACGGCATCGGCAAGGACTTCAACCGCCAATCCACTCGACGAGAATCCAAACTCCCTCCCCTCCCGCATC
GTAGCCCGCACCGTCTCCATCGTCTCCACCCTGATATCCCTCGTGTCCCTCCGCGACCTCCTCCTCCCGGGCG
CCATCCTCTCCTTCCTCCCCCGCGACGACATCTACCTCGAATGGACGGGAGCCTTCCTCCACTCGCCGCCCCC
CGACACCGTCGAGGCGGACGAACACGGACTCGAATCGCCGTTGTTCGCGGGCGATTTGCTCGTCCATCAGTTG
TTGGGATTGTACCTGTTTCTGAGTTGCGCCTTCAAGTTCGCGAGCGCGGTGGGGTGGTCGAAGGGGAACAGGT
CCATGGGAAGCGGTGGTGGTGGTGGTGGGGCGAAGGCGATGATGATGGAGGAGAATGCGGAGAGGAGCGGCGT
GGTGAGTTCGAAGATGATTTGGAAGGCGCAGGCTTTGGGCGATGTGGGCGTTTTGACGGTGTTGAGGATGTTT
ACTCCCGCGGCGAAGAGTGCTAGTTTGGATTTGAGGTGGCATTTGATGTTGGTGGCGTATGAAGCTTTTATTT
TATCCTTGTATGGTTTCTGGTAAAGGAGTTGATGTGTGGTCATTCGATTTGGCTCAGACTGATTTAAAGGACT
TCGAAAGGTTACTTGTCTGGCGAAAGCTCATGTGTATTTTATGTGGTGGTGTCTTTGCGAGACGGACGCTCCG
AAAACTTTATACAAAAAAGATGGTATCATCTTGGAGCACAGATAACTGAGAAACTACGCAGATACATACAGCA
TCAATAACATGTATAAGTCAGAAAAATGAAGCTTAAAAAAAAAAGATCGGAAGAGCG
>TR585|c2_g1_i4 len=2223 path=[2915:0-205 2939:206-333 2842:334-375
2946:376-467 2908:468-491 2938:492-535 2875:536-565 @2954@!:566-785
2955:786-807 2957:808-818 2934:819-889 2927:890-1171 2864:1172-1195
2941:1196-1215 2933:1216-1239 2926:1240-1315 2911:1316-1339 2947:1340-
1827 2932:1828-1972 2925:1973-2127 2896:2128-2222] [-1, 2915, 2939, 2842,
2946, 2908, 2938, 2875, 2954, 2955, 2957, 2934, 2927, 2864, 2941, 2933,
2926, 2911, 2947, 2932, 2925, 2896, -2]
GCCTTGAAAAATACATATTTGAAAATATTACCACGCATACACACACACACACACACACAACGGCCTATATTCT
CATCACCGATATAACCAACGGACACACACACGCAACAACCACACAAACATACGCATTCCAACACTCAACCCTC
ACACACACAAAACAACAACAACAACAACAAAAGTGAGACAACAACAACAATGGCAGGATCAGTAACAAATCCA
AGAAGAAGGAAAATATTCCCTCATTTATCAAACAACGAGTCAAAGCAAACGGAGGGTGCGTCATCATCAACAT
CAACATCGGGGTTGGGTGGTGCCAGCAGCAACAGCAGCAACAGCAGTAACAACAATACCGCTAGCAACTCGAA
TAACTCCAAAAATAAATCGGATAACAGCAACAACAGCAACAGCAAGAACAACAACAATGATTCATACGACAAT
GCCAAATCCAGAAGAGGTAAAAAGCAATCTTCCAAATCCTCCTCCACAAATAATGATTCTTCCAACAACAACA
ACAAAGCGGCAACTGCAGCTGCTGCGGGAAGTAACTCTACATCATCATCACCATCATCGTCATCGAATGCAAC

AACGACACCAGTCACCACAACCACAACCACCACAACGACCCTCCATCATCACCTCCTACCATTGCCATTGGTA
TTAACGGTATTGGTATGTTCGGGATTGTTTTGGATAGCATCGTTTCGCGATTTGATGGCGACGGGGAAACCTA
TTTTGGATACGTTGGGCGTGCTTTGGGGTCAGGATGATGCCGATGCTAACTTTTTGCAATACACAAAATCAAC
CGCCTGGTTCGAAGACTCCCGCGGCTGGAAATCCACTCAAGGCGGCCTCTCCACAATAATGTCCACCACCACC
GACGCCAACAACATGGGAGGACTCTTCGTCCGCAAGCTCTCCGGAGTCGCCGGCCTGTCCTTCCACACCACCA
AACTCATGCCCGTCGTCTTCCACTCTCCCCCCCAGTATCACCACGGGAAGTGGACGGGCGCCAGTTGGAGCGC
CGGGCACTTCGATCCGGTGTTGATACTGGGGATGATGGGGGACGTTTGCCTGGGCGTTTTTTACTTGGGGAGG
TTGGAGGAGTTGAAGGATGCGGGTTGCGGCGGGCTGGGGATGGCGTTCGTGTTGACTAGTTTGGTGGAGGCGT
TTATTTTGGGGATGTACATGTTGAGTCGGAGGATGAATCACAAATCTCAACCGAACGTTCCGAAAGTGACAAC
CAGCACGGCATCGGCAAGGACTTCAACCGCCAATCCACTCGACGAGAATCCAAACTCCCTCCCCTCCCGCATC
GTAGCCCGCACCGTCTCCATCGTCTCCACCCTGATATCCCTCGTGTCCCTCCGCGACCTCCTCCTCCCGGGCG
CCATCCTCTCCTTCCTCCCCCGCGACGACATCTACCTCGAATGGACGGGAGCCTTCCTCCACTCGCCGCCCCC
CGACACCGTCGAGGCGGACGAACACGGACTCGAATCGCCGTTGTTCGCGGGCGATTTGCTCGTCCATCAGTTG
TTGGGATTGTACCTGTTTCTGAGTTGCGCCTTCAAGTTCGCGAGCGCGGTGGGGTGGTCGAAGGGGAACAGGT
CCATGGGAAGCGGTGGTGGTGGTGGTGGGGCGAAGGCGATGATGATGGAGGAGAATGCGGAGAGGAGCGGCGT
GGTGAGTTCGAAGATGATTTGGAAGGCGCAGGCTTTGGGCGATGTGGGCGTTTTGACGGTGTTGAGGATGTTT
ACTCCCGCGGCGAAGAGTGCTAGTTTGGATTTGAGGTGGCATTTGATGTTGGTGGCGTATGAAGCTTTTATTT
TATGTGAGTTTCTATTTTGTGTATAGTTTTGGTGATTTGTGTGTGTTGTGGAAGGGGTGTGTGTTTTATGATT
CATGCGTGTGGGAGTTTCTGATGACTTTTTACTGTTTACCCCATGATGTCAGCCTTGTATGGTTTCTGGTAAA
GGAGTTGATGTGTGGTCATTCGATTTGGCTCAGACTGATTTAAAGGACTTCGAAAGGTTACTTGTCTGGCGAA
AGCTCATGTGTATTTTATGTGGTGGTGTCTTTGCGAGACGGACGCTCCGAAAACTTTATACAAAAAAGATGGT
ATCATCTTGGAGCACAGATAACTGAGAAACTACGCAGATACATACAGCATCAATAACATGTATAAGTCAGAAA
AATGAAGCTTAAAAAAAAAAAGATCGGAAGAGCG
>TR585|c2_g1_i5 len=2200 path=[2915:0-205 2939:206-333 2842:334-375
2946:376-467 2908:468-491 2938:492-535 2850:536-565 @2954@!:566-785
2922:786-807 2950:808-814 2944:815-880 2956:881-897 2958:898-906
2957:907-917 2934:918-988 2927:989-1270 2864:1271-1294 2941:1295-1314
2933:1315-1338 2926:1339-1414 2911:1415-1438 2947:1439-1926 2845:1927-
1949 2925:1950-2104 2896:2105-2199] [-1, 2915, 2939, 2842, 2946, 2908,
2938, 2850, 2954, 2922, 2950, 2944, 2956, 2958, 2957, 2934, 2927, 2864,
2941, 2933, 2926, 2911, 2947, 2845, 2925, 2896, -2]
GCCTTGAAAAATACATATTTGAAAATATTACCACGCATACACACACACACACACACACAACGGCCTATATTCT
CATCACCGATATAACCAACGGACACACACACGCAACAACCACACAAACATACGCATTCCAACACTCAACCCTC
ACACACACAAAACAACAACAACAACAACAAAAGTGAGACAACAACAACAATGGCAGGATCAGTAACAAATCCA
AGAAGAAGGAAAATATTCCCTCATTTATCAAACAACGAGTCAAAGCAAACGGAGGGTGCGTCATCATCAACAT
CAACATCGGGGTTGGGTGGTGCCAGCAGCAACAGCAGCAACAGCAGTAACAACAATACCGCTAGCAACTCGAA
TAACTCCAAAAATAAATCGGATAACAGCAACAACAGCAACAGCAAGAACAACAACAATGATTCATACGACAAT
GCCAAATCCAGAAGAGGTAAAAAGCAATCTTCCAAATCCTCCTCCACAAATAATGATTCTTCCAACAACAACA
ACAAAGCGGCAACTGCAGCTGCTGCAGGAAGCAACTCTACATCATCATCACCATCATCGTCATCGAATGCAAC
AACGACACCAGTCACCACAACCACAACCACCACAACGACCCTCCATCATCACCTCCTACCATTGCCATTGGTA
TTAACGGTATTGGTATGTTCGGGATTGTTTTGGATAGCATCGTTTCGCGATTTGATGGCGACGGGGAAACCTA
TTTTGGATACGTTGGGCGTGCTTTGGGGTCAGGATGATGCCGATGCTAACTTTTTGAACCTTTTTGAATGAAT
AATTTATTCACCCCCAAATGTGGACCAATCATCCAATCATCACATCTCACAATCCCCACCCCACGACAATACA
AAAAATCAGCAATACACAAAATCAACCGCCTGGTTCGAAGACTCCCGCGGCTGGAAATCCACTCAAGGCGGCC
TCTCCACAATAATGTCCACCACCACCGACGCCAACAACATGGGAGGACTCTTCGTCCGCAAGCTCTCCGGAGT
CGCCGGCCTGTCCTTCCACACCACCAAACTCATGCCCGTCGTCTTCCACTCTCCCCCCCAGTATCACCACGGG
AAGTGGACGGGCGCCAGTTGGAGCGCCGGGCACTTCGATCCGGTGTTGATACTGGGGATGATGGGGGACGTTT
GCCTGGGCGTTTTTTACTTGGGGAGGTTGGAGGAGTTGAAGGATGCGGGTTGCGGCGGGCTGGGGATGGCGTT
CGTGTTGACTAGTTTGGTGGAGGCGTTTATTTTGGGGATGTACATGTTGAGTCGGAGGATGAATCACAAATCT
CAACCGAACGTTCCGAAAGTGACAACCAGCACGGCATCGGCAAGGACTTCAACCGCCAATCCACTCGACGAGA
ATCCAAACTCCCTCCCCTCCCGCATCGTAGCCCGCACCGTCTCCATCGTCTCCACCCTGATATCCCTCGTGTC
CCTCCGCGACCTCCTCCTCCCGGGCGCCATCCTCTCCTTCCTCCCCCGCGACGACATCTACCTCGAATGGACG
GGAGCCTTCCTCCACTCGCCGCCCCCCGACACCGTCGAGGCGGACGAACACGGACTCGAATCGCCGTTGTTCG
CGGGCGATTTGCTCGTCCATCAGTTGTTGGGATTGTACCTGTTTCTGAGTTGCGCCTTCAAGTTCGCGAGCGC
GGTGGGGTGGTCGAAGGGGAACAGGTCCATGGGAAGCGGTGGTGGTGGTGGTGGGGCGAAGGCGATGATGATG
GAGGAGAATGCGGAGAGGAGCGGCGTGGTGAGTTCGAAGATGATTTGGAAGGCGCAGGCTTTGGGCGATGTGG
GCGTTTTGACGGTGTTGAGGATGTTTACTCCCGCGGCGAAGAGTGCTAGTTTGGATTTGAGGTGGCATTTGAT
GTTGGTGGCGTATGAAGCTTTTATTTTATCCTTGTATGGTTTCTGGTAAAGGAGTTGATGTGTGGTCATTCGA
TTTGGCTCAGACTGATTTAAAGGACTTCGAAAGGTTACTTGTCTGGCGAAAGCTCATGTGTATTTTATGTGGT
GGTGTCTTTGCGAGACGGACGCTCCGAAAACTTTATACAAAAAAGATGGTATCATCTTGGAGCACAGATAACT

GAGAAACTACGCAGATACATACAGCATCAATAACATGTATAAGTCAGAAAAATGAAGCTTAAAAAAAAAAGAT
CGGAAGAGCG
>TR585|c3_g1_i1 len=647 path=[4984:0-145 4985:146-169 5047:170-402
4935:403-403 5059:404-520 4993:521-544 5044:545-610 4961:611-612
4962:613-646] [-1, 4984, 4985, 5047, 4935, 5059, 4993, 5044, 4961, 4962,
-2]
TTTTATTTGCGATCACCGTATTTTCATTCATATTGCCATCTCTGCGCAAAATTGCTTTTACACAATAATTACC
TCCAAATTCAGTATATCTCTGTAACGCTCTGTCCATTTTTGAGCCGTCCGTCTCATCCATTCTTCACATTGAT
CATTGCCCATCCATCTCGCGCACTACAACTACACGTATAAATAAACAGAGAATACGACATGTCTCAAATACGA
CAACGCAAACGCACCACGAATAACAAAGACGACGATGATGGTGATGATTCTTCTCCTCCCGATCTATTAGAAT
GTGCCAGAGATAGCGACTCGTCTTCTTCTTTCGAAAAATATTTTGATGTTGGTGATGGCACATATCCCAATGG
ATATGCCACCAATAATAACAACAACAACAACAGCAACAACCGATCGCTCCTCCGAGCACTGCGCGACACGCAT
CGCATCCAACCGTACGCCATGGACTTTCGCGGATTCGGCGGCACTCCCAAGGATGAATCGGGATTCGTGGAGC
CGCTTCGGTGTGTTGCGGATGTTGTTTCCGTGTTGAATTGGATTCACGAGAAACATCATCGCCGTGGGGGGAA
GACTAATGATGATGATGATGATGGTGATGACGATTCGACTTCGTCGTCAAGTAGGTCACGTCC
>TR585|c3_g2_i1 len=927 path=[4976:0-65 4977:66-89 5061:90-319 5025:320-
343 5045:344-402 5058:403-486 4945:487-487 5042:488-577 4928:578-601
4929:602-926] [-1, 4976, 4977, 5061, 5025, 5045, 5058, 4945, 5042, 4928,
4929, -2]
GCTCTTCCGATCTTCTACACTCTAGGCAGCCCCGGGAAGCCAACCATGATGGCAACAACGGCAATAGACTGTA
GAGATTACATCAATCAAATAAATTGAACTACGCACAACCCAAGAACCATGACTTTGACCACCACCGCCGCCGC
CGCGCTCCCACGGCGCTCGCTGCTCCTGTTAGTCCTCGCCGCCATCTACATCGCGCCCTCCCCAGCAGTAGTG
ACCGCAGCATCAACCGACGTCGACGCAACGGCACCGAGGAGGAAGAACAGAAGGAGGAAGAATTATCGCCCCC
ACACGGGCAAGAGTAAGCACGAGGAAGAGGATGAGGACGCCCTACGCGAGCGTGACCTATGGCAGAATCCACT
ATGGGCCACACCCAACACTGATCTCTTCTGGAGTGCGAAAGAACAGCCGCATTATAATGATCCCAACTGGTCT
GCCAATAAGCAGACACCCGCAAACAGTAACAACAACAACAACAGCAACAATAACAGTAGACCCTCTAATCCAA
GGCCACCTGGGAACCCATTCATTAAAGACGATCCGCCTGCGCAGTCGCAATCTCGACCCTCGGGGTCCAATAA
TTATTCTTACAGCCGACCTCCTCCCTCTCGTCCGTCGTCGCAGTCACAGCCGCGACCGGGGGGAAATCCGTGG
GACAATCCTACCAAGTATAACTCATCGGTCGGCGCATCACCTTCATCGCCAACGTCGTCTAGTACTGCTAGCG
CTCCTCAGCGAAGTCCAACATCATCGAATTATCAGAATCCGGTGGGTGGGCGAGGAAGACCGCCAACGGCATC
AGCCCTCATTTCGAGTGTCCAGTCTGGAGGTTCGTCAAACGCGGCTTCTTCGTCAAATATGCAGCAGCAGCAA
CAGCAGCAACAAGTGCCATTACAGCCCAAAGTAACAGCTACCTTCACCTCC
>TR585|c3_g3_i1 len=2573 path=[4963:0-60 4964:61-62 5060:63-142 5071:143-
166 5073:167-192 5057:193-222 5043:223-684 4950:685-708 4951:709-711
4952:712-735 5041:736-786 5003:787-810 5055:811-945 5022:946-969
5040:970-1314 4980:1315-1338 5065:1339-1529 5039:1530-1578 5069:1579-1602
5064:1603-1642 5053:1643-1688 5070:1689-1715 5029:1716-1745 5063:1746-
2078 5072:2079-2102 5074:2103-2129 5035:2130-2177 5062:2178-2431
4942:2432-2455 5049:2456-2498 4948:2499-2572] [-1, 4963, 4964, 5060,
5071, 5073, 5057, 5043, 4950, 4951, 4952, 5041, 5003, 5055, 5022, 5040,
4980, 5065, 5039, 5069, 5064, 5053, 5070, 5029, 5063, 5072, 5074, 5035,
5062, 4942, 5049, 4948, -2]
TCTCAGCTGTTTCCACGTCTGCTGGCGATGACGACAAAAATGATGATGATGATGATGGTGATGCGTCGCCTGC
AGATGTAGATGATGCGACTACTGGCAATACTGCTCCGTCGGATGCGGCTGAATCCAACGAGACATCTGATAGT
GTGTCACCCATCACCCCATCAGACAAACCCGCAGATGCACCATCCTCCACTACGACCACCACGAAAAGTACCG
ACCAACCCGACTGGATCAACAGCATCAATCTACGTCCCAATGAGGTACTCACTCAACTCAACCGCCACATTGT
TGGTCAGGCATCTGCAAAGAAAGCCGTCGCCATTGCCATGCGCAACCGATGGCGCCGTCGCCAACTACCCCCC
GCCCTCCTGAAGGAGGTGACCCCCCGCAACGTCCTGATGATCGGCCCCACCGGCTGCGGCAAAACGGAGGTGG
CTCGACGCATGGCCAAGCTCAGCGACGCGCCCTTCCTAAAGGTCGAGGCGACCAAATTTACTGAGGTTGGGTA
TCACGGACGGGACGTCGATCAGATCGTACGGGATTTGATGGACGTCAGCATGGCGCTGACGAAGAAGCGACAG
ATGGAGAAGTTGCGGGAAGAGGCCAAGCTGATAGTCGAGGATCGGATATTGGACATTCTCGTGGGACCCGAGT
TCTCGGGAACGAAAGGACAGCGGGAATCATTCCGAGGCATGCTCGAGGAGGGACTATTGGAAAACCAAGAGCT
GGAGATCGACGTGCCCGAGAATTTGGGCGCCGCCGGCGGGGGGAAGGATGGGGACGGTGCCGTGCTGGCCTTC
GGTACGGATTCTAACAGCATGTCTGCACAAGCCATCGGGGACATGTTGAAGAAGCTGGGAGCTGCTGGAGGAG
GCAATCGAGGAGGACGGCCGGGCATGGAGAAAAAGAAGATGCCCATTTCGGAGGCACGGGAGGTCATTCTAGA
AATTGAGATTGAGAAGATGTTGGAGAAGGTGGATCTGAAGAAGGAGGCAATCAATGCCGCAGAGGAATCGGGT
ATTGTGTTCATTGACGAGATTGACAAGATTTGTTCCAGCAAGGATTTCAATTCGAAGTCGGCGGATGCTAGCG
CCGAGGGAGTGCAACGAGATCTGTTGCCATTGGTGGAGGGGACGACCATCAGCACCAAATATGGAAATGTCAA
CACAGATTACATGCTTTTTATCGGAAGTGGTGCCTTCCATGCAGTCAAACCTAGTGATATGTTGCCGGAACTG
CAGGGTCGACTACCGATCCGAGTTGAGCTCAATGGCTTGACGGAAGATGATCTGTACAAAATTCTTACTGAGC

CTGAGGCAAATCTGGTAATCCAACAAATCGAATTAATAAAAACCGAGGGTGTTGTCCTAACCTTTAATGATGA
TGCTCTTCGAGAAATCGCCAAACTTGCCGCACACCTTAATAGGACGGTGGAAAATATCGGGGCACGCAGACTT
CATACTGTTATGGAGCGCATTATGGAAGAGATTAGTTTTCAGGCTGCGGAAATGGATACGGCTGAGGAGGTGA
CAGTTACTAAGGAATTGGTTCAGGAGAGGTTATCACATGTCATGACGACGAGTGATTTGTCAAAATACATTCT
ATAACTTATTGTTTAGAGGACAATGATTATCTTTAATCATCAAGAAATAGGATTATCTTTATTAACACAGAAG
GAGCAAATCCACCTGGAGGGTTGCTCAATTTACAACACGTATACATAGATGTGATCTAACATCAGCATCCACA
CGCTGACATATGCTCCGATGACTTTCCCAAGTCTAGTCCAGCGTCCTCTATCAAATATTCATCTCCCATATAT
ATTGGTGGCGGCACCTTCTTCGCAATTACGACGAACATATTTTCAACCCCCTCCCCTGCCTTTGCCGACGTTT
CAATGTAAAATGCACCAATATCATCCGCGTATGCCTCCCCATCGAACGTCCTACCAATCTACCTTCTTCCAA
ATCGGATTTGTTCCCACACACCACCAAAGCTAAATCTGCTGGACCATTCTGTCTCAACTCATCAGCCCAATCC
TGCAACGCAGCAAAGCTGCCCGGTTTTGTTATGTCATAAACGAGAATGGCCGCATTGCTTCCTCTGTAATACA
TGGGTGCGAGCGAGCGATACTTTTCTTGCCCCGCCGTATCCCAAATTTTGAAACCTATGTTACGTTGCTGTTG
TTGTTGCGTAGATTTATGTTCTCCGTTTCCTTTCGCGTGATTTTCGCAATTATTATTATTCTCGGATGTGGAA
TCCTCGTCTGTCGCCGCAGGGGATGCAGGGATGGTTATCGACTTGGACATGAAACTTGCACCAATGGTTGCCT
CGGAATAGGGCCGAAATTCATCGGTCACGAATCGCAACGCTATGCTTGATTTGCCTACGCCACTTTCACCGAG
GAGGACCACCTTGATGGATTCAACCCCTGACATGATGCCTCTTCGTCGAGGCGAATGATTGTCACAGTGTCTC
TTTCTCTAGAGCTCTCCTGAAAGTGTTTTCCTCCTTGCTCAACTCTCCTCTGCTCTCGCTCGCCTCGCTCTGC
CGCTTCGCTTCCTCCGTC
>TR585|c4_g1_i1 len=2581 path=[3485:0-49 3540:50-91 3529:92-115 3548:116-117 3539:118-160 3528:161-184 3425:185-213 @3547@!:214-438 3527:439-486 3553:487-513 3546:514-537 3552:538-870 3554:871-893 3503:894-900 3535:901-927 3523:928-973 3551:974-1013 3544:1014-1037 3534:1038-1277 3555:1278-1301 3556:1302-1302 3521:1303-1646 3416:1647-1670 3543:1671-1805 3520:1806-1880 3471:1881-1904 3472:1905-1907 3473:1908-1931 3550:1932-2393 3518:2394-2423 3542:2424-2448 3531:2449-2449 3516:2450-2473 3445:2474-2580] [-1, 3485, 3540, 3529, 3548, 3539, 3528, 3425, 3547, 3527, 3553, 3546, 3552, 3554, 3503, 3535, 3523, 3551, 3544, 3534, 3555, 3556, 3521, 3416, 3543, 3520, 3471, 3472, 3473, 3550, 3518, 3542, 3531, 3516, 3445, -2]
ATCTCGACCCGCGACGGAGTTGACGGAGGAAGCGAAGCGGCAGAGCGAGGCGAGCGAGAGCAGAGGAGAGTTG
AGCAAGGAGGAAAACACTTTCAGGAGAGCTCTAGAGAAAGAGACACTGTGACAATCATTCGCCTCGACGAAGA
GGCATCATGTCAGGGCTTGAATCCATCAAGGTGGTCCTCCTCGGTGAAAGTGGCGTAGGCAAATCAAGCATAG
CGTTGCGATTCGTGACCGATGAATTTCGGCCCTATTCCGAGGCAACCATTGGTGCAAGTTTCATGTCCAAGTC
GATAACCATCCCTGCATCCCCTGCGGCGACAGACGAGGATTCCACATCCGAGAATAATAATAATTGCGAAAAT
CACGCGAAAGGAAACGGAGAACATAAATCTACGCAACAACAACAGCAACGTAACATAGGTTTCAAAATTTGGG
ATACGGCGGGGCAAGAAAAGTATCGCTCGCTCGCACCCATGTATTACAGAGGAAGCAATGCGGCCATTCTCGT
TTACGACATAACAAAACCGGGCAGCTTTGCTGCGTTGCAGGATTGGGCTGATGAGTTGAGACAGAATGGTCCA
GCAGATTTAGCTTTGGTGGTGTGTGGGAACAAATCCGATTTGGAAGAAGGTAGATTGGTAGGACGTTCGATGG
GGGAGGCATACGCGGATGATATTGGTGCATTTTACATTGAAACGTCGGCAAAGGCAGGGGAGGGGGTTGAAAA
TATGTTCGTCGTAATTGCGAAGAAGGTGCCGCCACCAATATATATGGGAGATGAATATTTGATAGAGGACGCT
GGACTAGACTTGGGAAAGTCATCGGAGCATATGTCAGCGTGTGGATGCTGATGTTAGATCACATCTATTTATA
CCTGTTGTAAATTGAGCAACCCTCCAGGTGGATTTGCTCCTTCTGTGTTAATGAAGGTAATCCTATCTCTTGA
TAATTAAAGATAATCATTGTCCTCTAAACAATAAGTTATAGAATGTATTTTGACAAATCACTCGTCGTCATGA
CATGTGATAACCTCTCCTGAACCAATTCCTTAGTAACTGTCACCTCCTCAGCCGTATCCATTTCCGCAGCCTG
AAAACTAATCTCTTCCATAATGCGCTCCATAACAGTATGAAGTCTGCGTGCCCCGATATTTTCCACCGTCCTA
TTAAGGTGTGCGGCAAGTTTGGCGATTTCTCGAAGAGCATCATCATTAAAGGTTAGGACAACACCCTCGGTTT
TTATTAATTCGATTTGTTGGATTACCAGATTTGCCTCAGGCTCAGTAAGAATTTTGTACAGATCATCTTCCGT
CAAGCCATTGAGCTCAACTCGGATCGGTAGTCGACCCTGCAGTTCCGGCAACATATCACTAGGTTTGACTGCA
TGGAAGGCACCACTTCCGATAAAAAGCATGTAATCTGTGTTGACATTTCCATATTTGGTGCTGATGGTCGTCC
CCTCCACCAATGGCAACAGATCTCGTTGCACTCCCTCGGCGCTAGCATCCGCCGACTTCGAATTGAAATCCTT
GCTGGAACAAATCTTGTCAATCTCGTCAATGAACACAATACCCGATTCCTCTGCGGCATTGATTGCCTCCTTC
TTCAGATCCACCTTCTCCAACATCTTCTCAATCTCAATTTCTAGAATGACCTCCCGTGCCTCCGAAATGGGCA
TCTTCTTTTTCTCCATGCCCGGCCGTCCTCCTCGATTGCCTCCTCCAGCAGCTCCCAGCTTCTTCAACATGTC
CCCGATGGCTTGTGCAGACATGCTGTTAGAATCCGTACCGAAGGCCAGCACGGCGCCGTCCCCATCCTTCCCC
CCGCCGGCGGCGCCCAAATTCTCGGGCACGTCGATCTCCAGCTCTTGGTTTTCCAAGAGTCCCTCCTCGAGCA
TGCCTCGGAATGATTCCCGCTGTCCTTTCGTTCCCGAGAACTCGGGTCCCACGAGAATGTCCAATATCCGATC
CTCGACTATCAGCTTGGCCTCTTCCCGCAACTTCTCCATCTGTCGCTTCTTCGTCAGCGCCATGCTGACGTCC
ATCAAATCCCGTACGATCTGATCGACGTCCCGTCCGTGATACCCAACCTCAGTAAATTTGGTCGCCTCGACCT
TTAGGAAGGGCGCGTCGCTGAGCTTGGCCATGCGTCGAGCCACCTCCGTTTTGCCGCAGCCGGTGGGGCCGAT
CATCAGGACGTTGCGGGGGGTCACCTCCTTCAGGAGGGCGGGGGGTAGTTGGCGACGGCGCCATCGGTTGCGC

ATGGCAATGGCGACGGCTTTCTTTGCAGATGCCTGACCAACAATGTGGCGGTTGAGTTGAGTGAGTACCTCAT
TGGGACGTAGATTGATGCTGTTGATCCAGTCGGGTTGGTCGGTACTTTTCGTGGTGGTCGTAGTGGAGGATGG
TGCATCTGCGGGTTTGTCTGATGGGGTGATGGGTGACACACTATCAGATGTCTCGTTGGATTCAGCCGCATCC
GACGGAGCAGTATTGCCAGTAGTCGCATCATCTACATCTGCAGGCGACGCATCACCATCATCATCATCATCAT
TTTTGTCGTCATCGCCAGCAGACGTG
>TR585|c4_g1_i2 len=1337 path=[3510:0-49 3549:50-117 3539:118-160
3528:161-184 3425:185-213 @3547@!:214-438 3527:439-486 3553:487-513
3546:514-537 3552:538-870 3482:871-900 3545:901-927 3536:928-973
3551:974-1013 3544:1014-1037 3534:1038-1277 3555:1278-1301 3556:1302-1302
3435:1303-1336] [-1, 3510, 3549, 3539, 3528, 3425, 3547, 3527, 3553,
3546, 3552, 3482, 3545, 3536, 3551, 3544, 3534, 3555, 3556, 3435, -2]
ATCTCGACCCGCGACGGAGTTGACGGAGGAAGCGAAGCGGCAGAGCGAGGGGAGCGAGAGCAGAGGAGAGTTG
AACAAGGAGGAAAACACTTTCTGGAGAGCTCTAGAGAAAGAGACACTGTGACAATCATTCGCCTCGACGAAGA
GGCATCATGTCAGGGCTTGAATCCATCAAGGTGGTCCTCCTCGGTGAAAGTGGCGTAGGCAAATCAAGCATAG
CGTTGCGATTCGTGACCGATGAATTTCGGCCCTATTCCGAGGCAACCATTGGTGCAAGTTTCATGTCCAAGTC
GATAACCATCCCTGCATCCCCTGCGGCGACAGACGAGGATTCCACATCCGAGAATAATAATAATTGCGAAAAT
CACGCGAAAGGAAACGGAGAACATAAATCTACGCAACAACAACAGCAACGTAACATAGGTTTCAAAATTTGGG
ATACGGCGGGGCAAGAAAAGTATCGCTCGCTCGCACCCATGTATTACAGAGGAAGCAATGCGGCCATTCTCGT
TTACGACATAACAAAACCGGGCAGCTTTGCTGCGTTGCAGGATTGGGCTGATGAGTTGAGACAGAATGGTCCA
GCAGATTTAGCTTTGGTGGTGTGTGGGAACAAATCCGATTTGGAAGAAGGTAGATTGGTAGGACGTTCGATGG
GGGAGGCATACGCGGATGATATTGGTGCATTTTACATTGAAACGTCGGCAAAGGCAGGGGAGGGGGTTGAAAA
TATGTTCGTCGTAATTGCGAAGAAGGTGCCGCCACCAATATATATGGGAGATGAATATTTGATAGAGGACGCT
GGACTAGACTTGGGAAAGTCATCGGAGCATATGTCAGCGTGTGGATGCTGATGTTAGATCACATCTATGTATA
CGTGTTGTAAATTGAGCAACCCTCCAGGTGGATTTGCTCCTTCTGTGTTAATAAAGATAATCCTATTTCTTGA
TGATTAAAGATAATCATTGTCCTCTAAACAATAAGTTATAGAATGTATTTTGACAAATCACTCGTCGTCATGA
CATGTGATAACCTCTCCTGAACCAATTCCTTAGTAACTGTCACCTCCTCAGCCGTATCCATTTCCGCAGCCTG
AAAACTAATCTCTTCCATAATGCGCTCCATAACAGTATGAAGTCTGCGTGCCCCGATATTTTCCACCGTCCTA
TTAAGGTGTGCGGCAAGTTTGGCGATTTCTCGAAGAGCATCATCATTAAAGGTTAGGACAACACCCTCGGTTT
TTATTAATTCGATTTGTTGGATTACCAGATTTGCCTCAGGCTCAGTAAGAATTTTGTACAGAGATCGGAAGAG
CACATGTCTGAACTCCAGTCACA
>TR585|c4_g1_i3 len=2489 path=[3436:0-121 @3547@!:122-346 3526:347-394
3553:395-421 3546:422-445 3552:446-778 3554:779-801 3503:802-808
3535:809-835 3523:836-881 3551:882-921 3544:922-945 3534:946-1185
3555:1186-1209 3556:1210-1210 3521:1211-1554 3416:1555-1578 3543:1579-
1713 3520:1714-1788 3471:1789-1812 3472:1813-1815 3473:1816-1839
3550:1840-2301 3518:2302-2331 3542:2332-2356 3531:2357-2357 3516:2358-
2381 3445:2382-2488] [-1, 3436, 3547, 3526, 3553, 3546, 3552, 3554, 3503,
3535, 3523, 3551, 3544, 3534, 3555, 3556, 3521, 3416, 3543, 3520, 3471,
3472, 3473, 3550, 3518, 3542, 3531, 3516, 3445, -2]
CATCAAGGTGGTCCTCCTCGGTGTAAGTAACTAAATCAATTGTCATTTTATTATGCACTGGAGTGGATTTTGA
TGATTTCTCACACACATCATTTTCAGGAAAGTGGCGTAGGCAAATCAAGCATAGCGTTGCGATTCGTGACCGA
TGAATTTCGGCCCTATTCCGAGGCAACCATTGGTGCAAGTTTCATGTCCAAGTCGATAACCATCCCTGCATCC
CCTGCGGCGACAGACGAGGATTCCACATCCGAGAATAATAATAATTGCGAAAATCACGCGAAAGGAAACGGAG
AACATAAATCTACGCAACAACAACAGCAACGTAACATAGGTTTCAAAATTTGGGACACGGCGGGGCAAGAAAA
GTATCGATCGCTCGCACCCATGTATTACAGAGGAAGCAATGCGGCCATTCTCGTTTACGACATAACAAAACCG
GGCAGCTTTGCTGCGTTGCAGGATTGGGCTGATGAGTTGAGACAGAATGGTCCAGCAGATTTAGCTTTGGTGG
TGTGTGGGAACAAATCCGATTTGGAAGAAGGTAGATTGGTAGGACGTTCGATGGGGGAGGCATACGCGGATGA
TATTGGTGCATTTTACATTGAAACGTCGGCAAAGGCAGGGGAGGGGGTTGAAAATATGTTCGTCGTAATTGCG
AAGAAGGTGCCGCCACCAATATATATGGGAGATGAATATTTGATAGAGGACGCTGGACTAGACTTGGGAAAGT
CATCGGAGCATATGTCAGCGTGTGGATGCTGATGTTAGATCACATCTATTTATACCTGTTGTAAATTGAGCAA
CCCTCCAGGTGGATTTGCTCCTTCTGTGTTAATGAAGGTAATCCTATCTCTTGATAATTAAAGATAATCATTG
TCCTCTAAACAATAAGTTATAGAATGTATTTTGACAAATCACTCGTCGTCATGACATGTGATAACCTCTCCTG
AACCAATTCCTTAGTAACTGTCACCTCCTCAGCCGTATCCATTTCCGCAGCCTGAAAACTAATCTCTTCCATA
ATGCGCTCCATAACAGTATGAAGTCTGCGTGCCCCGATATTTTCCACCGTCCTATTAAGGTGTGCGGCAAGTT
TGGCGATTTCTCGAAGAGCATCATCATTAAAGGTTAGGACAACACCCTCGGTTTTTATTAATTCGATTTGTTG
GATTACCAGATTTGCCTCAGGCTCAGTAAGAATTTTGTACAGATCATCTTCCGTCAAGCCATTGAGCTCAACT
CGGATCGGTAGTCGACCCTGCAGTTCCGGCAACATATCACTAGGTTTGACTGCATGGAAGGCACCACTTCCGA
TAAAAAGCATGTAATCTGTGTTGACATTTCCATATTTGGTGCTGATGGTCGTCCCCTCCACCAATGGCAACAG
ATCTCGTTGCACTCCCTCGGCGCTAGCATCCGCCGACTTCGAATTGAAATCCTTGCTGGAACAAATCTTGTCA
ATCTCGTCAATGAACACAATACCCGATTCCTCTGCGGCATTGATTGCCTCCTTCTTCAGATCCACCTTCTCCA

ACATCTTCTCAATCTCAATTTCTAGAATGACCTCCCGTGCCTCCGAAATGGGCATCTTCTTTTTCTCCATGCC
CGGCCGTCCTCCTCGATTGCCTCCTCCAGCAGCTCCCAGCTTCTTCAACATGTCCCCGATGGCTTGTGCAGAC
ATGCTGTTAGAATCCGTACCGAAGGCCAGCACGGCGCCGTCCCCATCCTTCCCCCCGCCGGCGGCGCCCAAAT
TCTCGGGCACGTCGATCTCCAGCTCTTGGTTTTCCAAGAGTCCCTCCTCGAGCATGCCTCGGAATGATTCCCG
CTGTCCTTTCGTTCCCGAGAACTCGGGTCCCACGAGAATGTCCAATATCCGATCCTCGACTATCAGCTTGGCC
TCTTCCCGCAACTTCTCCATCTGTCGCTTCTTCGTCAGCGCCATGCTGACGTCCATCAAATCCCGTACGATCT
GATCGACGTCCCGTCCGTGATACCCAACCTCAGTAAATTTGGTCGCCTCGACCTTTAGGAAGGGCGCGTCGCT
GAGCTTGGCCATGCGTCGAGCCACCTCCGTTTTGCCGCAGCCGGTGGGGCCGATCATCAGGACGTTGCGGGGG
GTCACCTCCTTCAGGAGGGCGGGGGGTAGTTGGCGACGGCGCCATCGGTTGCGCATGGCAATGGCGACGGCTT
TCTTTGCAGATGCCTGACCAACAATGTGGCGGTTGAGTTGAGTGAGTACCTCATTGGGACGTAGATTGATGCT
GTTGATCCAGTCGGGTTGGTCGGTACTTTTCGTGGTGGTCGTAGTGGAGGATGGTGCATCTGCGGGTTTGTCT
GATGGGGTGATGGGTGACACACTATCAGATGTCTCGTTGGATTCAGCCGCATCCGACGGAGCAGTATTGCCAG
TAGTCGCATCATCTACATCTGCAGGCGACGCATCACCATCATCATCATCATCATTTTTGTCGTCATCGCCAGC
AGACGTG
>TR586|c0_g1_i1 len=304 path=[4951:0-34 4952:35-58 5257:59-73 5110:74-78 5185:79-127 4550:128-130 5103:131-238 4530:239-243 4531:244-250 4532:251-259 4533:260-262 4534:263-267 4535:268-269 4536:270-275 4537:276-277 4538:278-303] [-1, 4951, 4952, 5257, 5110, 5185, 4550, 5103, 4530, 4531, 4532, 4533, 4534, 4535, 4536, 4537, 4538, -2]
GATTTGGCGACTGCCCCCCATTCGTGGTATTTCTCTTGAGCTTGCATAAAATGTTCTACCGCTGTGCCTTTTC
TGTTCAGCTTTAAAAAGAAATGCCCAGCTAACTCATGGGCAAGTGCTTCTTCGTGAATAAATCTGTGTTCCCT
TGAAGATAACAATGACTTCTCGTACAAAGATTCTGCTCTAGCAATGTCGTTGTAACAGAAGGCCTCCTCCGCT
TCCATCAAGTACAATTTGTTCTGGAAATTCCACTTACAATCTGCCCATTTCTTCATCAAAGATTTAGCCTTGC
GTGCCCGGTCAG
>TR586|c0_g2_i1 len=1649 path=[4720:0-121 5189:122-164 5351:165-188 5336:189-201 5320:202-265 5250:266-277 5098:278-327 5293:328-337 5350:338-362 5335:363-363 5359:364-386 5349:387-388 5334:389-399 5318:400-414 5289:415-419 5243:420-438 5370:439-442 5242:443-459 5358:460-468 5348:469-471 5332:472-498 5241:499-504 5169:505-519 4970:520-531 5084:532-538 5312:539-563 5281:564-594 5161:595-600 5373:601-603 5384:604-608 5390:609-610 5228:611-611 5156:612-642 5345:643-653 5329:654-657 5311:658-694 5374:695-696 5277:697-711 5221:712-750 5375:751-751 5343:752-753 5328:754-783 5275:784-831 5148:832-832 5217:833-859 5309:860-865 5146:866-868 5274:869-889 5377:890-895 5308:896-947 5273:948-975 5378:976-994 5385:995-998 5141:999-1022 5366:1023-1032 5363:1033-1044 5138:1045-1097 5342:1098-1110 5050:1111-1134 5340:1135-1148 5326:1149-1151 5306:1152-1176 5380:1177-1178 5387:1179-1179 5391:1180-1191 5203:1192-1202 5305:1203-1215 5266:1216-1221 5339:1222-1229 5325:1230-1245 5303:1246-1251 5199:1252-1253 5034:1254-1257 5124:1258-1285 5264:1286-1286 5198:1287-1303 5352:1304-1310 5338:1311-1311 5301:1312-1348 5263:1349-1353 5196:1354-1377 5323:1378-1401 5381:1402-1402 5195:1403-1467 5388:1468-1481 5392:1482-1490 5395:1491-1496 5024:1497-1514 5298:1515-1516 5337:1517-1520 5322:1521-1521 5297:1522-1569 5191:1570-1596 5114:1597-1599 5018:1600-1623 4281:1624-1648] [-1, 4720, 5189, 5351, 5336, 5320, 5250, 5098, 5293, 5350, 5335, 5359, 5349, 5334, 5318, 5289, 5243, 5370, 5242, 5358, 5348, 5332, 5241, 5169, 4970, 5084, 5312, 5281, 5161, 5373, 5384, 5390, 5228, 5156, 5345, 5329, 5311, 5374, 5277, 5221, 5375, 5343, 5328, 5275, 5148, 5217, 5309, 5146, 5274, 5377, 5308, 5273, 5378, 5385, 5141, 5366, 5363, 5138, 5342, 5050, 5340, 5326, 5306, 5380, 5387, 5391, 5203, 5305, 5266, 5339, 5325, 5303, 5199, 5034, 5124, 5264, 5198, 5352, 5338, 5301, 5263, 5196, 5323, 5381, 5195, 5388, 5392, 5395, 5024, 5298, 5337, 5322, 5297, 5191, 5114, 5018, 4281, -2]
TTCTATGAACATCAAATTACTACAGTTACTTGGTACGTCAAGTGATCCCTTTGGGATTGTAGTCGATGCACCT
GTTTCGATTGCCATGACTTTTACAGGACGTTTTGACCTTTCAGACACCTACTTGCAGGTGTTATTGTCATCAT
CGTCCAGTAGATTTTGTTCGTTACAACGCCTTACTCAACTATTAATCTAAGCAAAACAGAGGCATCCTCTTGC
GCGTGTTCTTGTCTCCATCATCTACGTTTCCATTTGAAGAAGATGTTAAGCCAGGCAAGATGCATGTGGCTAA
ACTAGACACTTTGCCAAGAACTCGCTGGACAAACTCTAACACAGAATTGGATTTGGCGACTGCCCCCCATTCA
AGGTATTTCTCATGAGCTTGCATAAAATGTTCTACCGCTGTGCCTTTTCTGTTCAACTCTAAAAAGAAATGCC
CAGCTAATTCATGGGCAAGTGCTTCTTCGTGAATGAATCTGTGCTCCCTTGCTGATGATAACGCCTTTTCATA

CAAATATTCTGCATTGGCAATGTCATTGCAACAAAAGGCCTCCTCTGCTTCCATCAAGTATAATTTATTCTGG
AAATTCCACTTACAATCTGCCCATTTCGTCATCAACAATTTGGCCTTGCGTGCCCGGTCAGCCCATTTAGCAT
CCTTCGATTTTCGAAATACCCAAGATGAGACAAGACCGCTGTAGAATGTATGAGCCGTATGAATATATAACAA
CGTCCAAGTGTGAAGATTGAAATCATAATACTTCTCTGCAAGGACCTTCATCTCATCATATTCACGAAACATG
AACTTTATGTACATTTCCTGTAGCGCTTGAGTGTATGAGGCGTGAGGATTTCCTTCTCGGACGTTTTTCACCC
CCTCAATTTCTGAAACATTGCAGTCATTTGTGCCCATTAATATGGAAACATTTTCCTCCCATTGAGATATATG
TGCCAAGAAAGTCATATGATCATGTTCTTCCATTAATCTGCGGGCTAAATGATATCGCTCCCTCCAAACAGAG
AGTTTCGTGCCGGCCCAAAATGAGATACCAATGTAGCATGCACTGTTTAACATTGCACCATGAGTGTCCCCTG
CTGCCATAGCAGTTGTATGGCCTTGAACATGGGTCTCAAGCGTTGCTTGCACTGGTTCAACGAAGCACATAAT
CTGAGTTCCAATTGCAATGACCTCACCTGCCACTTCTTTTGACCCTATTCTGTCCAGTAGCTGCCTCCCTATC
CTCACATATCTACAACCTTCTAGAATATCTCCAAGGCGTGCTAGTAACATGCCAAAGTATGTGAATCCAAGCG
GTGAAACAGGATTCATCCCACGCGTAATTGACAGTTGAACCATCTTCATGGTCACAATGGGCTGCGCTTCAAC
TTTTGTCATATGAAATGATATTTCTAATCTTGCATAGAATTTCATCGCCATTATTTTTTTGGGATCTACCATC
CTTCTATATTCTATCAGCTCTTGGTCTGTTGCAAATCCTTTGAGCATGGCCTGGGTCTGCTCAATGTATAGCT
TCGTGTCTGACTCGGAATAGGAATCTGGCAGTTCCTCTCCAAGTTGCGATAAAACTAAGATAGACCTGTTGAC
TGATTCTGGAAGCTGAGAAGCATAGGCCAAAGCACTGACAGTG
>TR586|c0_g2_i2 len=1314 path=[4720:0-121 5189:122-164 5351:165-188
5336:189-201 5320:202-265 5250:266-277 5098:278-327 5293:328-337
5350:338-362 5335:363-363 5359:364-386 5349:387-388 5334:389-399
5318:400-414 5289:415-419 5243:420-438 5370:439-442 5089:443-484
5368:485-496 5365:497-503 5362:504-504 5357:505-544 5167:545-568
5083:569-573 5235:574-575 5164:576-581 5078:582-605 5355:606-607
5345:608-618 5329:619-622 5311:623-659 5374:660-661 5151:662-715
5375:716-716 5343:717-718 5328:719-748 5276:749-796 5376:797-797
5147:798-824 5060:825-830 5216:831-833 5145:834-886 5057:887-890
5214:891-912 5273:913-940 5378:941-959 5385:960-963 5141:964-987
5366:988-997 5363:998-1009 5138:1010-1062 5342:1063-1075 5050:1076-1099
5340:1100-1113 5326:1114-1116 5306:1117-1141 5380:1142-1143 5387:1144-
1144 5391:1145-1156 5130:1157-1168 5304:1169-1180 5266:1181-1186
5339:1187-1194 5036:1195-1218 4771:1219-1222 4772:1223-1250 4773:1251-
1251 4774:1252-1275 5338:1276-1276 5301:1277-1313] [-1, 4720, 5189, 5351,
5336, 5320, 5250, 5098, 5293, 5350, 5335, 5359, 5349, 5334, 5318, 5289,
5243, 5370, 5089, 5368, 5365, 5362, 5357, 5167, 5083, 5235, 5164, 5078,
5355, 5345, 5329, 5311, 5374, 5151, 5375, 5343, 5328, 5276, 5376, 5147,
5060, 5216, 5145, 5057, 5214, 5273, 5378, 5385, 5141, 5366, 5363, 5138,
5342, 5050, 5340, 5326, 5306, 5380, 5387, 5391, 5130, 5304, 5266, 5339,
5036, 4771, 4772, 4773, 4774, 5338, 5301]
TTCTATGAACATCAAATTACTACAGTTACTTGGTACGTCAAGTGATCCCTTTGGGATTGTAGTCGATGCACCT
GTTTCGATTGCCATGACTTTTACAGGACGTTTTGACCTTTCAGACACCTACTTGCAGGTGTTATTGTCATCAT
CGTCCAGTAGATTTTGTTCGTTACAACGCCTTACTCAACTATTAATCTAAGCAAAACAGAGGCATCCTCTTGC
GCGTGTTCTTGTCTCCATCATCTACGTTTCCATTTGAAGAAGATGTTAAGCCAGGCAAGATGCATGTGGCTAA
ACTAGACACTTTGCCAAGAACTCGCTGGACAAACTCTAACACAGAATTGGATTTGGCGACTGCCCCCCATTCA
AGGTATTTCTCATGAGCTTGCATAAAATGTTCTACCGCTGTGCCTTTTCTGTTCAACTCTAAAAAGAAATGCC
CAGCTGTGCTCCCTTGCTGATGACAACGCCTTTTCATACAAATATTCTGCATTGGCAATGTCATTGTAACAAA
AGGCCTCTTCTGCTTCCATCAAGTACAATTTGTTTTGGAAATTCCACTTACAATCTGCCCATTTCTTCATCGA
GAATTTGGCCTTGCGTGCCCGGTCAGCCCATTTAGCATCCTTCGATTTTCGAAATACCCAAGATGAGACAAGA
CCGCTATAGAATGTATGAGCCGTATATGTAAATAATAACGTCCAAGTGTGAAGATTGAAATCATAATACTTCT
CTGCAAGGACCTTCATCTCCTCATATTCACGAAACATGAATTTGATGTACATTTCCTGTAGCGCTTGAATGCA
TGAGGCGTGAGGATTTCCTTCTCGGACGTTTCTCACCCCCTCAATTTCTGGAACATTGTAATCATTTGTGCCC
GTTAATATGGAAAAATTTTCCTCCCATTGAGATATATGTGCCAAGAAAGTCATATGATCATGTTCTTCCATTA
ATCTGCGGGCTAAATGATATCGCTCCCTCCAAACAGAGAGTTTCGTGCCGGCCCAAAATGAGATACCAATGTA
GCATGCACTGTTTAACATTGCACCATGAGTGTCCCCTGCTGCCATAGCAGTTGTATGGCCTTGAACATGGGTC
TCAAGCGTTGCTTGCACTGGTTCAACGAAGCACATAATCTGAGTTCCAATTGCAATGACCTCCCCTGCCACTT
CTTTTGACCCTATTCTGTCCAGTAGCTTCCTCCCTATCCTCACATATCTACAACCCTCTCGAATATCTCCAAG
GCGTGCTAGTAGCATGCCAAAGTATGTGAATCCAAGCGGTGAAACAGGATTCATCCCACGCGTAATTGACAGT
>TR586|c0_g2_i3 len=1064 path=[4745:0-121 5113:122-144 5256:145-145
5187:146-164 5351:165-188 5336:189-201 5320:202-265 5102:266-337
5350:338-362 5335:363-363 5359:364-386 5349:387-388 5334:389-399
5318:400-414 5289:415-419 5243:420-438 5370:439-442 5242:443-459
5358:460-468 5348:469-471 5332:472-498 5286:499-538 5362:539-539

5357:540-579 5167:580-603 5083:604-608 5235:609-610 5164:611-616
5078:617-640 5355:641-642 5345:643-653 5329:654-657 5311:658-694
5374:695-696 5277:697-711 5221:712-750 5375:751-751 5343:752-753
5328:754-783 5275:784-831 5148:832-832 5217:833-859 5309:860-865
5146:866-868 5274:869-889 5377:890-895 5056:896-921 5212:922-925
5142:926-975 5054:976-994 4926:995-1018 4927:1019-1022 4928:1023-1032
4929:1033-1063] [-1, 4745, 5113, 5256, 5187, 5351, 5336, 5320, 5102,
5350, 5335, 5359, 5349, 5334, 5318, 5289, 5243, 5370, 5242, 5358, 5348,
5332, 5286, 5362, 5357, 5167, 5083, 5235, 5164, 5078, 5355, 5345, 5329,
5311, 5374, 5277, 5221, 5375, 5343, 5328, 5275, 5148, 5217, 5309, 5146,
5274, 5377, 5056, 5212, 5142, 5054, 4926, 4927, 4928, 4929, -2]
TTCTATGAACATCAAATTACTACAGTTACTTGGTACGTCAAGTGATCCCTTTGGGATTGTAGTCGATGCACCT
GTTTCGATTGCCATGACTTTTACAGGACGTTTTGACCTTTCAGACACCTGCTTGCAGGTGTTATTGTCTTCAT
CGTCCAGTAGATTTTGTTCGTTACAACGCCTTACTCAACTATTAATCTAAGCAAAACAGAGGCATCCTCTTGC
GCGTGTTCTTGTCTCCATCATCTACGTTTCCATTTGAAGAAGATGTTGAGCCAGGCAAGGTGCATATGCCCAA
ACTAGACATTTTGCCAAGAACTTGCTGGACAAACTCTAACACAGAATTGGATTTGGCGACTGCCCCCCATTCA
AGGTATTTCTCATGAGCTTGCATAAAATGTTCTACCGCTGTGCCTTTTCTGTTCAACTCTAAAAAGAAATGCC
CAGCTAATTCATGGGCAAGTGCTTCTTCGTGAATGAATCTGTGCTCCCTTGCTGATGATAATGCCTTTTCGTA
CAAACATTCTGCATTGGCAATGTCATTGTAACAAAAGGCCTCTTCTGCTTCCATCAAGTACAATTTGTTTTGG
AAATTCCACTTACAATCTGCCCATTTCTTCATCGAGAATTTGGCCTTGCGTGCCCGGTCAGCCCATTTAGCAT
CCTTCGATTTTCGAAATACCCAAGATGAGACAAGACCGCTGTAGAATGTATGAGCCGTATGAATATATAACAA
CGTCCAAGTGTGAAGATTGAAATCATAATACTTCTCTGCAAGGACCTTCATCTCATCATATTCACGAAACATG
AACTTTATGTACATTTCCTGTAGCGCTTGAGTGTATGAGGCGTGAGGATTTCCTTCTCGGACGTTTTTCACCC
CCTCAATTTCTGAAACATTGGAGTCATTTGTGCCCGTTAATATGGAAAAAATTTCCTCCCATTGAGATATGTG
TGCGAAGAAAGTCATATGATCATGTTCTTCCATTAATCTGCGGGCTGAATCATATCGCTCCCTCCAAACAGAG
AGTTTCGTGCCAGCCCAAAATGAGGTACCAACATAGCACACA
>TR586|c0_g2_i5 len=318 path=[4696:0-23 5190:24-35 5296:36-48 5253:49-59
5321:60-74 5295:75-95 5251:96-119 5358:120-128 5348:129-131 5332:132-158
5286:159-198 5362:199-199 5357:200-239 5372:240-244 5081:245-268
5234:269-270 5163:271-276 5077:277-278 5231:279-286 5159:287-295
5073:296-300 5355:301-302 5345:303-313 5329:314-317] [-1, 4696, 5190,
5296, 5253, 5321, 5295, 5251, 5358, 5348, 5332, 5286, 5362, 5357, 5372,
5081, 5234, 5163, 5077, 5231, 5159, 5073, 5355, 5345, 5329]
GGATTTGGCGACTGCCCCCCATTCGTGGTATTTCTCATGAGCTTGCATAAAATGTTCTACCGCTGTGCCTTTT
CTCTTCAACTCTAAAAAGAAATGTCCAGCTAATTCATGGGCAAGTGCTTCTTCGTGAATGAATCTGTGCTCCC
TTGCTGATGATAATGCCTTTTCGTACAAACATTCTGCATTGGCAATGTCATTGTAACAAAAGGCCTCTTCTGC
TTCCATCAAGTACAATTTGTTCTGGATATTCCACTTACAATCTGCCCATTTCTTCATCAAGAATTTGGCCTTG
CGTGCCCGGTCAGCCCATTTAGCATC
>TR586|c0_g2_i6 len=1029 path=[4720:0-121 5189:122-164 5351:165-188
5336:189-201 5320:202-265 5102:266-337 5350:338-362 5335:363-363
5359:364-386 5349:387-388 5334:389-399 5318:400-414 5289:415-419
5243:420-438 5370:439-442 5089:443-484 5368:485-496 5365:497-503
5362:504-504 5357:505-544 5167:545-568 5083:569-573 5235:574-575
5164:576-581 5078:582-605 5355:606-607 5345:608-618 5329:619-622
5311:623-659 5374:660-661 5277:662-676 5221:677-715 5375:716-716
5343:717-718 5328:719-748 5275:749-796 5148:797-797 5217:798-824
5309:825-830 5146:831-833 5274:834-854 5377:855-860 5056:861-886
5212:887-890 5142:891-940 5054:941-959 4926:960-983 4927:984-987
4928:988-997 4929:998-1028] [-1, 4720, 5189, 5351, 5336, 5320, 5102,
5350, 5335, 5359, 5349, 5334, 5318, 5289, 5243, 5370, 5089, 5368, 5365,
5362, 5357, 5167, 5083, 5235, 5164, 5078, 5355, 5345, 5329, 5311, 5374,
5277, 5221, 5375, 5343, 5328, 5275, 5148, 5217, 5309, 5146, 5274, 5377,
5056, 5212, 5142, 5054, 4926, 4927, 4928, 4929, -2]
TTCTATGAACATCAAATTACTACAGTTACTTGGTACGTCAAGTGATCCCTTTGGGATTGTAGTCGATGCACCT
GTTTCGATTGCCATGACTTTTACAGGACGTTTTGACCTTTCAGACACCTACTTGCAGGTGTTATTGTCATCAT
CGTCCAGTAGATTTTGTTCGTTACAACGCCTTACTCAACTATTAATCTAAGCAAAACAGAGGCATCCTCTTGC
GCGTGTTCTTGTCTCCATCATCTACGTTTCCATTTGAAGAAGATGTTGAGCCAGGCAAGGTGCATATGCCCAA
ACTAGACATTTTGCCAAGAACTTGCTGGACAAACTCTAACACAGAATTGGATTTGGCGACTGCCCCCCATTCA
AGGTATTTCTCATGAGCTTGCATAAAATGTTCTACCGCTGTGCCTTTTCTGTTCAACTCTAAAAAGAAATGCC
CAGCTGTGCTCCCTTGCTGATGACAACGCCTTTTCATACAAATATTCTGCATTGGCAATGTCATTGTAACAAA

AGGCCTCTTCTGCTTCCATCAAGTACAATTTGTTTTGGAAATTCCACTTACAATCTGCCCATTTCTTCATCGA
GAATTTGGCCTTGCGTGCCCGGTCAGCCCATTTAGCATCCTTCGATTTTCGAAATACCCAAGATGAGACAAGA
CCGCTGTAGAATGTATGAGCCGTATGAATATATAACAACGTCCAAGTGTGAAGATTGAAATCATAATACTTCT
CTGCAAGGACCTTCATCTCATCATATTCACGAAACATGAACTTTATGTACATTTCCTGTAGCGCTTGAGTGTA
TGAGGCGTGAGGATTTCCTTCTCGGACGTTTTTCACCCCCTCAATTTCTGAAACATTGGAGTCATTTGTGCCC
GTTAATATGGAAAAAATTTCCTCCCATTGAGATATGTGTGCGAAGAAAGTCATATGATCATGTTCTTCCATTA
ATCTGCGGGCTGAATCATATCGCTCCCTCCAAACAGAGAGTTTCGTGCCAGCCCAAAATGAGGTACCAACATA
GCACACA
>TR586|c0_g2_i7 len=312 path=[5350:0-47 5180:48-71 5247:72-73 5290:74-84 5402:85-99 5400:100-120 5398:121-123 5370:124-127 5242:128-144 5358:145-153 5348:154-156 5332:157-183 5241:184-189 4444:190-216 4445:217-223 4446:224-224 4447:225-236 4448:237-248 5281:249-279 5161:280-285 5373:286-288 5384:289-293 5390:294-295 4544:296-301 4545:302-303 4546:304-311] [5350, 5180, 5247, 5290, 5402, 5400, 5398, 5370, 5242, 5358, 5348, 5332, 5241, 4444, 4445, 4446, 4447, 4448, 5281, 5161, 5373, 5384, 5390, 4544, 4545, 4546, -2]
GCTGGACAAACTCTAACACAGAATTGGATTTGGCGACTGCCCCCCATTGAAGGTATTTCTCATGAGCTTGCAT
AAAATGTTCTACCGCTGTGCCTTTTCTCTTCAACTCTAAAAAGAAATGCCCAGCTAATTCATGGGCAAGTGCT
TCTTCGTGAATGAATCTGTGCTCCCTTGCTGATGATAACGCCTTGTCGTACAAATATTCTGCATTGGCAATGT
CATTGTGACAAAAGGCCTCCTCTGCTTCCATCAAGTATAATTTATTCTGGAAATTCCACTTACAATCTGCCCA
TTTCTTCATCAAGAATTTGG
>TR586|c0_g2_i8 len=861 path=[4720:0-121 5189:122-164 5351:165-188 5336:189-201 5320:202-265 5250:266-277 5098:278-327 5293:328-337 5350:338-362 5335:363-363 5359:364-386 5349:387-388 5334:389-399 5318:400-414 5289:415-419 5243:420-438 5370:439-442 5089:443-484 5368:485-496 5365:497-503 5362:504-504 5357:505-544 5372:545-549 5313:550-556 5282:557-565 5233:566-568 5162:569-573 5075:574-575 5229:576-576 5157:577-600 5071:601-605 5227:606-607 5155:608-618 5279:619-659 5224:660-661 5153:662-676 5344:677-718 5328:719-748 5276:749-796 5376:797-797 4306:798-824 4307:825-830 4308:831-854 4309:855-860] [-1, 4720, 5189, 5351, 5336, 5320, 5250, 5098, 5293, 5350, 5335, 5359, 5349, 5334, 5318, 5289, 5243, 5370, 5089, 5368, 5365, 5362, 5357, 5372, 5313, 5282, 5233, 5162, 5075, 5229, 5157, 5071, 5227, 5155, 5279, 5224, 5153, 5344, 5328, 5276, 5376, 4306, 4307, 4308, 4309, -2]
TTCTATGAACATCAAATTACTACAGTTACTTGGTACGTCAAGTGATCCCTTTGGGATTGTAGTCGATGCACCT
GTTTCGATTGCCATGACTTTTACAGGACGTTTTGACCTTTCAGACACCTACTTGCAGGTGTTATTGTCATCAT
CGTCCAGTAGATTTTGTTCGTTACAACGCCTTACTCAACTATTAATCTAAGCAAAACAGAGGCATCCTCTTGC
GCGTGTTCTTGTCTCCATCATCTACGTTTCCATTTGAAGAAGATGTTAAGCCAGGCAAGATGCATGTGGCTAA
ACTAGACACTTTGCCAAGAACTCGCTGGACAAACTCTAACACAGAATTGGATTTGGCGACTGCCCCCCATTCA
AGGTATTTCTCATGAGCTTGCATAAAATGTTCTACCGCTGTGCCTTTTCTGTTCAACTCTAAAAAGAAATGCC
CAGCTGTGCTCCCTTGCTGATGACAACGCCTTTTCATACAAATATTCTGCATTGGCAATGTCATTGTAACAAA
AGGCCTCTTCTGCTTCCATCAAGTACAATTTGTTCTGGAAATTCCACTTACAATCTGCCCATTTCGCCATCAA
GAATTTGGCCTTGCGTGCCCGGTCAGCCCATTTAGTATCCTTCGATTGACGATATACCCAAGATGAGACAAGA
CCGCTGTAGAATGTATGAGCTGTATGAATATATAATAATGTCCAAGTGTGAAGATTGAAATCATAATACTTCT
CTGCAAGGACCTTCATCTCCTCATATTCACGAAACATGAATTTGATGTACATTTCCTGTAGCGCTTGAGTGTA
TGAGGCGTGAGGATTTCCTTCTCGGACGCTTTTCACCCCCTCAATTTCTGAAACATTG
>TR586|c0_g2_i9 len=1649 path=[4720:0-121 5189:122-164 5351:165-188 5336:189-201 5320:202-265 5250:266-277 5098:278-327 5293:328-337 5350:338-362 5335:363-363 5359:364-386 5349:387-388 5334:389-399 5318:400-414 5289:415-419 5243:420-438 5370:439-442 5242:443-459 5358:460-468 5348:469-471 5332:472-498 5241:499-504 5169:505-519 4970:520-531 5084:532-538 5312:539-563 5281:564-594 5161:595-600 5373:601-603 5384:604-608 5390:609-610 5228:611-611 5156:612-642 5345:643-653 5329:654-657 5311:658-694 5374:695-696 5151:697-750 5375:751-751 5343:752-753 5328:754-783 5276:784-831 5376:832-832 5147:833-859 5060:860-865 5216:866-868 5145:869-921 5057:922-925 5214:926-947 5273:948-975 5378:976-994 5385:995-998 5141:999-1022 5366:1023-1032 5363:1033-1044 5138:1045-1097 5342:1098-1110 5050:1111-1134 5340:1135-1148 5326:1149-1151 5306:1152-1176 5380:1177-1178 5387:1179-1179 5391:1180-1191 5130:1192-1203 5304:1204-1215 5266:1216-

1221 5339:1222-1229 5325:1230-1245 5303:1246-1251 5200:1252-1303
5352:1304-1310 5338:1311-1311 5302:1312-1348 5263:1349-1353 5196:1354-
1377 5323:1378-1401 5381:1402-1402 5195:1403-1467 5388:1468-1481
5393:1482-1516 5337:1517-1520 5322:1521-1521 5020:1522-1582 4400:1583-
1596 4401:1597-1599 4402:1600-1623 4403:1624-1648] [-1, 4720, 5189, 5351,
5336, 5320, 5250, 5098, 5293, 5350, 5335, 5359, 5349, 5334, 5318, 5289,
5243, 5370, 5242, 5358, 5348, 5332, 5241, 5169, 4970, 5084, 5312, 5281,
5161, 5373, 5384, 5390, 5228, 5156, 5345, 5329, 5311, 5374, 5151, 5375,
5343, 5328, 5276, 5376, 5147, 5060, 5216, 5145, 5057, 5214, 5273, 5378,
5385, 5141, 5366, 5363, 5138, 5342, 5050, 5340, 5326, 5306, 5380, 5387,
5391, 5130, 5304, 5266, 5339, 5325, 5303, 5200, 5352, 5338, 5302, 5263,
5196, 5323, 5381, 5195, 5388, 5393, 5337, 5322, 5020, 4400, 4401, 4402,
4403, -2]
TTCTATGAACATCAAATTACTACAGTTACTTGGTACGTCAAGTGATCCCTTTGGGATTGTAGTCGATGCACCT
GTTTCGATTGCCATGACTTTTACAGGACGTTTTGACCTTTCAGACACCTACTTGCAGGTGTTATTGTCATCAT
CGTCCAGTAGATTTTGTTCGTTACAACGCCTTACTCAACTATTAATCTAAGCAAAACAGAGGCATCCTCTTGC
GCGTGTTCTTGTCTCCATCATCTACGTTTCCATTTGAAGAAGATGTTAAGCCAGGCAAGATGCATGTGGCTAA
ACTAGACACTTTGCCAAGAACTCGCTGGACAAACTCTAACACAGAATTGGATTTGGCGACTGCCCCCCATTCA
AGGTATTTCTCATGAGCTTGCATAAAATGTTCTACCGCTGTGCCTTTTCTGTTCAACTCTAAAAAGAAATGCC
CAGCTAATTCATGGGCAAGTGCTTCTTCGTGAATGAATCTGTGCTCCCTTGCTGATGATAACGCCTTTTCATA
CAAATATTCTGCATTGGCAATGTCATTGCAACAAAAGGCCTCCTCTGCTTCCATCAAGTATAATTTATTCTGG
AAATTCCACTTACAATCTGCCCATTTCGTCATCAACAATTTGGCCTTGCGTGCCCGGTCAGCCCATTTAGCAT
CCTTCGATTTTCGAAATACCCAAGATGAGACAAGACCGCTATAGAATGTATGAGCCGTATATGTAAATAATAA
CGTCCAAGTGTGAAGATTGAAATCATAATACTTCTCTGCAAGGACCTTCATCTCCTCATATTCACGAAACATG
AATTTGATGTACATTTCCTGTAGCGCTTGAATGCATGAGGCGTGAGGATTTCCTTCTCGGACGTTTCTCACCC
CCTCAATTTCTGGAACATTGTAATCATTTGTGCCCGTTAATATGGAAAAATTTTCCTCCCATTGAGATATATG
TGCCAAGAAAGTCATATGATCATGTTCTTCCATTAATCTGCGGGCTAAATGATATCGCTCCCTCCAAACAGAG
AGTTTCGTGCCGGCCCAAAATGAGATACCAATGTAGCATGCACTGTTTAACATTGCACCATGAGTGTCCCCTG
CTGCCATAGCAGTTGTATGGCCTTGAACATGGGTCTCAAGCGTTGCTTGCACTGGTTCAACGAAGCACATAAT
CTGAGTTCCAATTGCAATGACCTCCCCTGCCACTTCTTTTGACCCTATTCTGTCCAGTAGCTGCCTCCCTATC
CTCACATATCTGCAACCTTCTCGAATATCTCCAAGGCGTCCTAGTAACATGCCAAAGTATGTGAATCCAAGGG
GCGAGACAGGACTCATCCCACGCGTAATTGACAGTTGAACCATCTTCATGGTCACAATGGGCTGCGCTTCAAC
TTTTGTCATATGAAATGATATTTCTAATCTTGCATAGAATTTCATCGCCATTATTTTTTTGGGATCTACCATC
CTTCTATATTCTATCAGCTCTTTGTTTGTTGTATATCCTTTGAGCATGGCCTGGGTCTGCTCGATGTATAGCT
TCGTGTCTGACTCTGAATAGGAATCTCGCAGTTCCTCCCCGAGTTGCGATAAAACTAAGATAGACCTGTTGAC
TGATTCTGGAAGCTGAGAAGCATAGGCCAAAGCACTGACAGTG
>TR586|c1_g1_i1 len=289 path=[7518:0-50 7519:51-74 7520:75-93 7521:94-95
7522:96-101 8184:102-179 7425:180-181 7426:182-200 7427:201-207 7428:208-
288] [-1, 7518, 7519, 7520, 7521, 7522, 8184, 7425, 7426, 7427, 7428, -2]
GAAGAAAAATTGCCTCTTTCCGTATGAAGACTCGGACATCAACAAAATTTGGTTGATGGCACTGATCGATGCT
GGGTCTGCCCACTGCAAATCGTCTAGAAAAAGGGTCACAGGTGACCCAGAACAACTGGATATTACGCTCACAA
ACTGACACAACAAATACTGCAACCTCTTTTGGGCATCGACACAATCTTGATCTTCCCCCGTTACATCATCTAA
GTCACCAAGAATTGTAAGTAAACTTGGGATCACTTTCACCAAGTGAAGAGCCTCCCTCCCCAATGCAACT
>TR586|c1_g2_i1 len=314 path=[7759:0-263 7760:264-275 7761:276-285
7762:286-313] [-1, 7759, 7760, 7761, 7762, -2]
AGACCTATCGACTGATTCTGGAAGCTGAGAGGCATAGGCCAAAGCACTGACAGTGTTGTACAAGACATTCAGC
TTGTCTTCGAAAGATTTGGCAAATGTGAGAATTTGGTCGGAAAGAAGCTTCAGACTGGTGATATCGCCTGACA
CTACTGCACAATTACTTGCAGATTCGAACAGCTGCAAACTCAAATCGTAGTGTTCCTCCCAATGTTTTTTCCT
AAGGAACGATATTCCGTTGTCAAAAAAAGAGTATGCAGCAGAAAAATCAGACATTTCCATCGCTTTAAGCCCT
GCAGTGAGATTCAGGTTCGCAA
>TR586|c1_g3_i1 len=321 path=[7289:0-38 7290:39-56 8362:57-68 8410:69-78
8323:79-95 8391:96-131 8321:132-162 8456:163-179 8470:180-181 8408:182-
269 8442:270-270 8459:271-290 7312:291-320] [-1, 7289, 7290, 8362, 8410,
8323, 8391, 8321, 8456, 8470, 8408, 8442, 8459, 7312, -2]
ATGTCTTCTCCTAAGGAACGATATTCCATTGTCGAAAAAAGAGTATGCAGCAGAAAAATCAGACATTTCCATC
GCTTTACGCCCCGCTGTGAGATTCAGGTTAGCAATTAGTACTGCCTGCTCGGCATCTTCTAAGGCAGCTGGTC
CACCGCGATTGATCTGGCTAGCCGCTGTAAATAACAAGCCGTCATCTTGGAGCTCGAGCGATCGTGGGACAAG
AGCAGCTCCGTACTTGAAATGAAGTACACACCGATCTTCGGGAGGTATCATGTTGTATGCCGCTTCCTGCAGT
CGATCGTGGCCGAAGCTGTAAGTGCCATT

>TR586|c1_g3_i2 len=1041 path=[7831:0-38 7832:39-68 8410:69-78 8323:79-95 8391:96-131 8259:132-162 8456:163-179 8470:180-181 8408:182-269 8441:270-293 8482:294-299 8311:300-313 8405:314-341 8386:342-372 8245:373-465 7615:466-466 8157:467-474 8306:475-517 8240:518-548 8338:549-563 8447:564-587 8463:588-588 8335:589-614 8283:615-623 8213:624-635 7547:636-656 8369:657-659 8330:660-695 8451:696-697 8115:698-780 7272:781-795 8274:796-806 8366:807-815 8201:816-860 8326:861-863 8269:864-893 8455:894-896 8469:897-912 8478:913-913 8325:914-914 8195:915-933 8392:934-935 8363:936-941 8324:942-953 8096:954-1019 7633:1020-1021 7634:1022-1040] [-1, 7831, 7832, 8410, 8323, 8391, 8259, 8456, 8470, 8408, 8441, 8482, 8311, 8405, 8386, 8245, 7615, 8157, 8306, 8240, 8338, 8447, 8463, 8335, 8283, 8213, 7547, 8369, 8330, 8451, 8115, 7272, 8274, 8366, 8201, 8326, 8269, 8455, 8469, 8478, 8325, 8195, 8392, 8363, 8324, 8096, 7633, 7634, -2]
ATGTCTTCTCCTAAGGAACGATATTCCATTGTCGAAAAACGAGTACGCAGCAGAAAAATCAGACATTTCCATC
GCTTTACGCCCCGCTGTGAGATTCAGGTTAGCAATTAGTACTGCCTGCTCGGCATCTTCAACAGCAACTGGTC
CACCGCGATTGATCTGGCTAGCCGCTGTAAATAACAAGCCGTCATCTTGGAGCTCGAGCGATCGTGGGACAAG
AGCAGCTCCGTACTTGAAATGAAGTACACACCGATCTTCGGGAGGTATCATATTGTATGCCGCTTCCTGCAGC
CGATCGTGGCCGAAGCTATAAGAGTCGTTGACTTTGTCCAGAACACCTTCAGTAACAGCAATTTCGAGTGGTC
CAATCAAGGTCTTTCCACATTTGGTTTCAAGTTCTTTGATGAGTGTTGTGTTCGTTGCATGGCCAAAGCATGA
TAGGATACTCAATGCCATCTGTACCTCTTGCGGTAATTTTTCAATAGAAGAAGTTAGAAATGCCGCTACATCA
TCGGGTAGCTTTCTAGATTGGATCTTCTCCTCTTCCCATTCCCATCGACGGCGACTCAGACTGAGCCGTACCA
AGCCATCTTTGCACAATGACATCATCAACTGAGAAAAGAATAGAGGATTCCCTTTGGTCTTATGGAATACAAT
CTCCGACAATGTCCGAGTAAGACGAGGAGACAGACATAACACATCGGATACCATTGCGTTTATCATCTCTTTG
TCCATACAAGCTAACTCGACTTCCATTGCACGCACACCAAAGTGACGAATACTCGAGACCATATCCCCAATCG
GATGGTTGATCATTCCTTCCTCCCGGCTTGATCCGAGGAAGAAAAATTGTCTCTTTCCATAAGAAGACTCAGA
CATCAACAAAATTTGATTGATGGCACTGATCGATGCTGGGTCTGCCCACTGCAAATCGTCTAGAAAGAGGGTC
ACAGGCGCCCCAGAACAGCTGGAAATGACACCCAGAAACTGACACATTAAATACTGCAACCTCTTTTGGGCAT
CGACACAATCTTGATCTTC
>TR586|c1_g3_i3 len=229 path=[7674:0-59 8461:60-67 8475:68-71 8483:72-72 8488:73-78 8401:79-90 8380:91-110 8296:111-134 8445:135-135 8223:136-144 8399:145-153 8422:154-179 8129:180-203 7543:204-228] [-1, 7674, 8461, 8475, 8483, 8488, 8401, 8380, 8296, 8445, 8223, 8399, 8422, 8129, 7543, -2]
GTGTTGCGTTTGTTGCATGTCCAAAGCATGAAAGGGCACTCAATGCCATCTGTACCTCTTGCGGTAATCTTTC
AATAGAAGAAGTTAAAAATGCCGCTACATCATCGGGTATCTTTCTGGATTGGATCTTCTCCTCGTCCCATTCC
CATCGGCGGCGACTTAGGCTAAGCCGCACCAAGCTATCCTTGCACAATGACACCATCAACTGAGAAAAAAATA
GAGGATTCCC
>TR586|c1_g3_i4 len=1129 path=[7831:0-38 7832:39-68 8410:69-78 8323:79-95 8391:96-131 8259:132-162 8456:163-179 8470:180-181 8408:182-269 8441:270-293 8482:294-299 8311:300-313 8405:314-341 8386:342-372 8245:373-465 7615:466-466 8157:467-474 8306:475-517 8240:518-548 8338:549-563 8447:564-587 8463:588-588 8335:589-614 8283:615-623 8213:624-635 8368:636-659 8330:660-695 8206:696-721 7747:722-727 8328:728-795 8452:796-806 8200:807-815 8106:816-830 8365:831-860 8326:861-863 8269:864-893 8455:894-896 8469:897-912 8478:913-913 7842:914-916 8194:917-1021 8392:1022-1023 8363:1024-1029 8324:1030-1041 8266:1042-1043 8192:1044-1078 8095:1079-1109 7974:1110-1128] [-1, 7831, 7832, 8410, 8323, 8391, 8259, 8456, 8470, 8408, 8441, 8482, 8311, 8405, 8386, 8245, 7615, 8157, 8306, 8240, 8338, 8447, 8463, 8335, 8283, 8213, 8368, 8330, 8206, 7747, 8328, 8452, 8200, 8106, 8365, 8326, 8269, 8455, 8469, 8478, 7842, 8194, 8392, 8363, 8324, 8266, 8192, 8095, 7974, -2]
ATGTCTTCTCCTAAGGAACGATATTCCATTGTCGAAAAACGAGTACGCAGCAGAAAAATCAGACATTTCCATC
GCTTTACGCCCCGCTGTGAGATTCAGGTTAGCAATTAGTACTGCCTGCTCGGCATCTTCAACAGCAACTGGTC
CACCGCGATTGATCTGGCTAGCCGCTGTAAATAACAAGCCGTCATCTTGGAGCTCGAGCGATCGTGGGACAAG
AGCAGCTCCGTACTTGAAATGAAGTACACACCGATCTTCGGGAGGTATCATATTGTATGCCGCTTCCTGCAGC
CGATCGTGGCCGAAGCTATAAGAGTCGTTGACTTTGTCCAGAACACCTTCAGTAACAGCAATTTCGAGTGGTC
CAATCAAGGTCTTTCCACATTTGGTTTCAAGTTCTTTGATGAGTGTTGTGTTCGTTGCATGGCCAAAGCATGA
TAGGATACTCAATGCCATCTGTACCTCTTGCGGTAATTTTTCAATAGAAGAAGTTAGAAATGCCGCTACATCA
TCGGGTAGCTTTCTAGATTGGATCTTCTCCTCTTCCCATTCCCATCGACGGCGACTCAGACTGAGCCGTACCA

AGCCATCTTTGCACAATGACATCATCAACTGAGAAAAGAATAGAGGATTCCCCTTGGTCTTATGGAATACAAT
CTCCGACAATGTCCGAGTAAGACGAGGAGACAGACATAAAACATCGGATACCATTGTGTTTGCCATCTCTTCA
TCCATACAAGCTAAATTAACTTCCGTTGCACGCACACCAAAGCGACGAATACTCGAGACCATATCCCCAATCG
GATGGTTGATCATACCTTCCTCCCGGCTGGATCCAAGGAAGAAAAATTGTCTCTTTCCATAAGAAGACTCAGA
CATCAACAAAATTTGATTGATGGCACTGATCGATGCTGTATTGTACAATGGAAATAGTTTGAATTAAATGGCA
AAATTGACAAAACGATAATACAAAATTGAAAGTGCACATGTAAGTGTCACCTGGGTCTGCCCACTGCAAATCG
TCTAGAAAGAGGGTCACAGGTGCACCAGAACAGCTGGAAATTACACCCACAAACTGGCACAGCAGATACTGCA
ACCTCTTTTGGGCATCGACACAATCTTGATCTTC
>TR586|c1_g3_i5 len=318 path=[8014:0-54 8391:55-90 8321:91-121 8456:122-138 8470:139-140 8408:141-228 8442:229-229 8459:230-249 8473:250-252 8482:253-258 7589:259-317] [-1, 8014, 8391, 8321, 8456, 8470, 8408, 8442, 8459, 8473, 8482, 7589, -2]
AGTACGCAGCAGAAAAACCAGACATTTCCATTGCTTTACGCCCCGCTGTGAGATTCAGGTTAGCAATTAGTAC
TGCCTGCTCGGCATCTTCTAAGGCAGCTGGTCCACCGCGATTGATCTGGCTAGCCGCTGTAAATAACAAGCCG
TCATCTTGGAGCTCGAGCGATCGTGGGACAAGAGCAGCTCCGTACTTGAAATGAAGTACACACCGATCTTCGG
GAGGTATCATGTTGTATGCCGCTTCCTGCAGCCGATCGTGACCGAAGCTATAAGTGCCATTGACTTTGTCCAG
AAAACCTTCGGCAACAGCAATTTCAA
>TR586|c1_g3_i6 len=636 path=[7831:0-38 7832:39-68 8410:69-78 8323:79-95 8391:96-131 8259:132-162 8456:163-179 8470:180-181 8408:182-269 8442:270-270 8459:271-290 8473:291-293 8482:294-299 8311:300-313 8163:314-341 8386:342-372 8353:373-413 8243:414-465 8444:466-466 8461:467-474 8475:475-478 8483:479-479 8488:480-485 8343:486-524 8297:525-527 8379:528-541 8445:542-542 8223:543-551 8399:552-560 8422:561-586 8414:587-590 8396:591-604 7794:605-610 7795:611-635] [-1, 7831, 7832, 8410, 8323, 8391, 8259, 8456, 8470, 8408, 8442, 8459, 8473, 8482, 8311, 8163, 8386, 8353, 8243, 8444, 8461, 8475, 8483, 8488, 8343, 8297, 8379, 8445, 8223, 8399, 8422, 8414, 8396, 7794, 7795, -2]
ATGTCTTCTCCTAAGGAACGATATTCCATTGTCGAAAAACGAGTACGCAGCAGAAAAATCAGACATTTCCATC
GCTTTACGCCCCGCTGTGAGATTCAGGTTAGCAATTAGTACTGCCTGCTCGGCATCTTCAACAGCAACTGGTC
CACCGCGATTGATCTGGCTAGCCGCTGTAAATAACAAGCCGTCATCTTGGAGCTCGAGCGATCGTGGGACAAG
AGCAGCTCCGTACTTGAAATGAAGTACACACCGATCTTCGGGAGGTATCATGTTGTATGCCGCTTCCTGCAGC
CGATCGTGGCCGAAGCTATAAGTGTCATTGACTTTGTCCAGAACACCTTCAGTAACAGCAATTTCGAGTGGTC
CAATCAAGTTATTTCCGCATTTGCTTTCAAGTGCTTCGATGAGTGTTGTGTTCATTGCATGGCCAAAGCATGA
TAGGGTACTCAATGCCATCTGTACCTCTTGCGGTAATCTTTCAATAGAGGAAGTTAAAAATTCAACTACATCA
TCGGGTAGCTTTCTGGATTGGATCTTCTCCTCGTCCCATTCCCATCGGCGGCGACTTAGGCTAAGCCGCACCA
AGCCATCCTTGCACAATGACACCATCAACTGAGAAAAAAAATAGAGGATTCCC
>TR586|c1_g3_i7 len=676 path=[8062:0-39 8191:40-129 8442:130-130 8459:131-150 8473:151-153 8482:154-159 8311:160-173 8163:174-201 8386:202-232 8353:233-273 8243:274-325 8444:326-326 8461:327-334 8475:335-338 8483:339-339 8488:340-345 8343:346-384 8297:385-387 8379:388-401 8445:402-402 8376:403-408 8338:409-423 8447:424-447 8463:448-448 8335:449-474 8283:475-483 8213:484-495 8368:496-519 8330:520-555 8206:556-581 7747:582-587 8328:588-655 8452:656-666 8200:667-675] [-1, 8062, 8191, 8442, 8459, 8473, 8482, 8311, 8163, 8386, 8353, 8243, 8444, 8461, 8475, 8483, 8488, 8343, 8297, 8379, 8445, 8376, 8338, 8447, 8463, 8335, 8283, 8213, 8368, 8330, 8206, 7747, 8328, 8452, 8200]
CTGGTCCACCGCGATTGATCTGGCTAGCCGCTGTAAATAATAGATTGTCATCTTGGAGCTCAGGTGATCGTGG
GACAAGAGCAATTCCGTACTTGAAATGAAAGAGGCACCGATCTTCGGGAGGTATCATGTTGTATGCCGCTTCC
TGCAGCCGATCGTGGCCGAAGCTATAAGTGTCATTGACTTTGTCCAGAACACCTTCAGTAACAGCAATTTCGA
GTGGTCCAATCAAGTTATTTCCGCATTTGCTTTCAAGTGCTTCGATGAGTGTTGTGTTCATTGCATGGCCAAA
GCATGATAGGGTACTCAATGCCATCTGTACCTCTTGCGGTAATCTTTCAATAGAGGAAGTTAAAAATTCAACT
ACATCATCGGGTAGCTTTCTGGATTGGATCTTCTCCTCTTCCCATTCCCATCGACGGCGACTCAGACTGAGCC
GTACCAAGCCATCTTTGCACAATGACATCATCAACTGAGAAAAGAATAGAGGATTCCCCTTGGTCTTATGGAA
TACAATCTCCGACAATGTCCGAGTAAGACGAGGAGACAGACATAAAACATCGGATACCATTGTGTTTGCCATC
TCTTCATCCATACAAGCTAAATTAACTTCCGTTGCACGCACACCAAAGCGACGAATACTCGAGACCATATCCC
CAATCGGATGGTTGATCAT
>TR586|c1_g3_i8 len=409 path=[7816:0-59 7817:60-67 7818:68-71 7819:72-72 7820:73-78 7821:79-90 7822:91-110 8240:111-141 8338:142-156 8447:157-180 8463:181-181 8335:182-207 8283:208-216 8213:217-228 8368:229-252

8330:253-288 8206:289-314 7747:315-320 8328:321-388 8452:389-399
8200:400-408] [-1, 7816, 7817, 7818, 7819, 7820, 7821, 7822, 8240, 8338,
8447, 8463, 8335, 8283, 8213, 8368, 8330, 8206, 7747, 8328, 8452, 8200]
GTGTTGCGTTTGTTGCATGTCCAAAGCATGAAAGGGCACTCAATGCCATCTGTACCTCTTGCGGTAATCTTTC
AATAGAAGAAGTTAAAAATGCCGCTACATCATCGGGTAGCTTTCTAGATTGGATCTTCTCCTCTTCCCATTCC
CATCGACGGCGACTCAGACTGAGCCGTACCAAGCCATCTTTGCACAATGACATCATCAACTGAGAAAAGAATA
GAGGATTCCCCTTGGTCTTATGGAATACAATCTCCGACAATGTCCGAGTAAGACGAGGAGACAGACATAAAAC
ATCGGATACCATTGTGTTTGCCATCTCTTCATCCATACAAGCTAAATTAACTTCCGTTGCACGCACACCAAAG
CGACGAATACTCGAGACCATATCCCCAATCGGATGGTTGATCAT
>TR586|c1_g3_i9 len=1000 path=[8014:0-54 8391:55-90 8321:91-121 8456:122-
138 8470:139-140 8408:141-228 8441:229-252 8482:253-258 8311:259-272
8405:273-300 8386:301-331 8245:332-424 7615:425-425 8157:426-433
8306:434-476 8240:477-507 8338:508-522 8447:523-546 8463:547-547
8335:548-573 8283:574-582 8213:583-594 8368:595-618 8330:619-654
8206:655-680 7747:681-686 8328:687-754 8452:755-765 8200:766-774
8106:775-789 8365:790-819 8326:820-822 8269:823-852 8455:853-855
8469:856-871 8478:872-872 8325:873-873 8195:874-892 8392:893-894
8363:895-900 8324:901-912 8096:913-978 7633:979-980 7634:981-999] [-1,
8014, 8391, 8321, 8456, 8470, 8408, 8441, 8482, 8311, 8405, 8386, 8245,
7615, 8157, 8306, 8240, 8338, 8447, 8463, 8335, 8283, 8213, 8368, 8330,
8206, 7747, 8328, 8452, 8200, 8106, 8365, 8326, 8269, 8455, 8469, 8478,
8325, 8195, 8392, 8363, 8324, 8096, 7633, 7634, -2]
AGTACGCAGCAGAAAAACCAGACATTTCCATTGCTTTACGCCCCGCTGTGAGATTCAGGTTAGCAATTAGTAC
TGCCTGCTCGGCATCTTCTAAGGCAGCTGGTCCACCGCGATTGATCTGGCTAGCCGCTGTAAATAACAAGCCG
TCATCTTGGAGCTCGAGCGATCGTGGGACAAGAGCAGCTCCGTACTTGAAATGAAGTACACACCGATCTTCGG
GAGGTATCATATTGTATGCCGCTTCCTGCAGCCGATCGTGGCCGAAGCTATAAGAGTCGTTGACTTTGTCCAG
AACACCTTCAGTAACAGCAATTTCGAGTGGTCCAATCAAGGTCTTTCCACATTTGGTTTCAAGTTCTTTGATG
AGTGTTGTGTTCGTTGCATGGCCAAAGCATGATAGGATACTCAATGCCATCTGTACCTCTTGCGGTAATTTTT
CAATAGAAGAAGTTAGAAATGCCGCTACATCATCGGGTAGCTTTCTAGATTGGATCTTCTCCTCTTCCCATTC
CCATCGACGGCGACTCAGACTGAGCCGTACCAAGCCATCTTTGCACAATGACATCATCAACTGAGAAAAGAAT
AGAGGATTCCCCTTGGTCTTATGGAATACAATCTCCGACAATGTCCGAGTAAGACGAGGAGACAGACATAAAA
CATCGGATACCATTGTGTTTGCCATCTCTTCATCCATACAAGCTAAATTAACTTCCGTTGCACGCACACCAAA
GCGACGAATACTCGAGACCATATCCCCAATCGGATGGTTGATCATACCTTCCTCCCGGCTGGATCCAAGGAAG
AAAAATTGTCTCTTTCCATAAGAAGACTCAGACATCAACAAAATTTGATTGATGGCACTGATCGATGCTGGGT
CTGCCCACTGCAAATCGTCTAGAAAGAGGGTCACAGGCGCCCCAGAACAGCTGGAAATGACACCCAGAAACTG
ACACATTAAATACTGCAACCTCTTTTGGGCATCGACACAATCTTGATCTTC
>TR586|c1_g3_i10 len=483 path=[8062:0-39 8191:40-129 8442:130-130
8459:131-150 8473:151-153 8482:154-159 8311:160-173 8163:174-201
8386:202-232 8353:233-273 8158:274-338 7860:339-339 7861:340-345
8238:346-357 8150:358-384 8346:385-387 8298:388-411 8226:412-420
7957:421-482] [-1, 8062, 8191, 8442, 8459, 8473, 8482, 8311, 8163, 8386,
8353, 8158, 7860, 7861, 8238, 8150, 8346, 8298, 8226, 7957, -2]
CTGGTCCACCGCGATTGATCTGGCTAGCCGCTGTAAATAATAGATTGTCATCTTGGAGCTCAGGTGATCGTGG
GACAAGAGCAATTCCGTACTTGAAATGAAAGAGGCACCGATCTTCGGGAGGTATCATGTTGTATGCCGCTTCC
TGCAGCCGATCGTGGCCGAAGCTATAAGTGTCATTGACTTTGTCCAGAACACCTTCAGTAACAGCAATTTCGA
GTGGTCCAATCAAGTTATTTCCGCATTTGCTTTCAAGTGCTTCGATGAGTGTTGTATTTGTTGCATGGCCAAA
GAATGACAGGGTGCTCAACGCCGCCTGTACCTCTTGCGGTAATCTTTCAATAGAAGAAGTTAAAAACGACACT
ACATCATCGGGTAGCTTTCTGGACTGGATCTTCTCCTCGTCCCATTCCCATCGGCGACGACTTAGGCTAAGTC
GAACCAAGCCATCCTTGCACACTGACATCATCAACTGAGAAAAGA
>TR586|c1_g3_i11 len=325 path=[7289:0-38 7290:39-56 8362:57-68 8410:69-78
8323:79-95 8391:96-131 8321:132-162 8456:163-179 8470:180-181 8480:182-
196 8252:197-220 8167:221-270 7770:271-290 7771:291-293 7772:294-299
7773:300-324] [-1, 7289, 7290, 8362, 8410, 8323, 8391, 8321, 8456, 8470,
8480, 8252, 8167, 7770, 7771, 7772, 7773, -2]
ATGTCTTCTCCTAAGGAACGATATTCCATTGTCGAAAAAAGAGTATGCAGCAGAAAAATCAGACATTTCCATC
GCTTTACGCCCCGCTGTGAGATTCAGGTTAGCAATTAGTACTGCCTGCTCGGCATCTTCTAAGGCAGCTGGTC
CACCGCGATTGATCTGGCTAGCCGCTGTAAATAACAGGCTGTCATCTTGGAGCTCAAGTGATCGTGGCACAAG
AGCAGCTCCGTACTTAAAATGAAGTATATACCGATCTTCGGGAGGTATCATGTTGTATGCCGCTTCCTGCAGC
CGATCGTGTCCGAAGCTATAAGTGTCATCGACT

>TR586|c1_g3_i12 len=1109 path=[7831:0-38 7832:39-68 8410:69-78 8323:79-95 8391:96-131 8259:132-162 8456:163-179 8470:180-181 8408:182-269 8442:270-270 8459:271-290 8473:291-293 8482:294-299 8311:300-313 8163:314-341 8386:342-372 8353:373-413 8158:414-478 7860:479-479 7861:480-485 8238:486-497 8150:498-524 8346:525-527 8298:528-551 8226:552-560 8422:561-586 8414:587-590 8396:591-604 8448:605-612 8334:613-614 8281:615-623 8121:624-656 8369:657-659 8330:660-695 8451:696-697 8115:698-780 7272:781-795 8274:796-806 8366:807-815 8201:816-860 8271:861-863 8199:864-893 8454:894-896 8468:897-912 7537:913-913 7538:914-916 8102:917-1020 7458:1021-1028 7459:1029-1040 7460:1041-1042 7461:1043-1077 7462:1078-1108] [-1, 7831, 7832, 8410, 8323, 8391, 8259, 8456, 8470, 8408, 8442, 8459, 8473, 8482, 8311, 8163, 8386, 8353, 8158, 7860, 7861, 8238, 8150, 8346, 8298, 8226, 8422, 8414, 8396, 8448, 8334, 8281, 8121, 8369, 8330, 8451, 8115, 7272, 8274, 8366, 8201, 8271, 8199, 8454, 8468, 7537, 7538, 8102, 7458, 7459, 7460, 7461, 7462, -2]
ATGTCTTCTCCTAAGGAACGATATTCCATTGTCGAAAAACGAGTACGCAGCAGAAAAATCAGACATTTCCATC
GCTTTACGCCCCGCTGTGAGATTCAGGTTAGCAATTAGTACTGCCTGCTCGGCATCTTCAACAGCAACTGGTC
CACCGCGATTGATCTGGCTAGCCGCTGTAAATAACAAGCCGTCATCTTGGAGCTCGAGCGATCGTGGGACAAG
AGCAGCTCCGTACTTGAAATGAAGTACACACCGATCTTCGGGAGGTATCATGTTGTATGCCGCTTCCTGCAGC
CGATCGTGGCCGAAGCTATAAGTGTCATTGACTTTGTCCAGAACACCTTCAGTAACAGCAATTTCGAGTGGTC
CAATCAAGTTATTTCCGCATTTGCTTTCAAGTGCTTCGATGAGTGTTGTATTTGTTGCATGGCCAAAGAATGA
CAGGGTGCTCAACGCCGCCTGTACCTCTTGCGGTAATCTTTCAATAGAAGAAGTTAAAAACGACACTACATCA
TCGGGTAGCTTTCTGGACTGGATCTTCTCCTCGTCCCATTCCCATCGGCGGCGACTTAGGCTAAGCCGCACCA
AGCCATCCTTGCACAATGACATCATCAACTGAGAAAAGAACAGGGGATTGCCTTTGGTCTTATGGAATACAAT
CTCCGACAATGTCCGAGTAAGACGAGGAGACAGACATAACACATCGGATACCATTGCGTTTATCATCTCTTTG
TCCATACAAGCTAACTCGACTTCCATTGCACGCACACCAAAGTGACGAATACTCGAGACCATATCCCCAATCG
GATGGTTGATCATTCCTTCCTCCCGGCTTGATCCGAGGAAGAAAAATTGTCTCTTTCCATACGAAGATTCAGA
CATCAACAAAATTTGATTGATGGCACTGATCGATGCTGTATCATACAATAGAAATAGTTTGAGTTGAATAGCA
AAATTGACAAAAGGTAGTGCAAAATTGAAAGTGCAAATGTAAATGTCACCTAGGTCAGCCCACTGCAAATCGT
CTAGAAAGAGGGTCACAGGTGACCCAGAACAGATGGAAATTACACCCACAAACTGGCACAGCAGATACTGCAA
CCTCTTTTGGGCAT
>TR586|c1_g3_i13 len=1109 path=[7831:0-38 7832:39-68 8410:69-78 8323:79-95 8391:96-131 8259:132-162 8456:163-179 8470:180-181 8408:182-269 8441:270-293 8482:294-299 8311:300-313 8405:314-341 8386:342-372 8245:373-465 7615:466-466 8157:467-474 8306:475-517 8240:518-548 8338:549-563 8447:564-587 8463:588-588 8335:589-614 8283:615-623 8213:624-635 7547:636-656 8369:657-659 8330:660-695 8451:696-697 8115:698-780 7272:781-795 8274:796-806 8366:807-815 8201:816-860 8271:861-863 8199:864-893 8454:894-896 8468:897-912 7537:913-913 7538:914-916 8102:917-1020 7458:1021-1028 7459:1029-1040 7460:1041-1042 7461:1043-1077 7462:1078-1108] [-1, 7831, 7832, 8410, 8323, 8391, 8259, 8456, 8470, 8408, 8441, 8482, 8311, 8405, 8386, 8245, 7615, 8157, 8306, 8240, 8338, 8447, 8463, 8335, 8283, 8213, 7547, 8369, 8330, 8451, 8115, 7272, 8274, 8366, 8201, 8271, 8199, 8454, 8468, 7537, 7538, 8102, 7458, 7459, 7460, 7461, 7462, -2]
ATGTCTTCTCCTAAGGAACGATATTCCATTGTCGAAAAACGAGTACGCAGCAGAAAAATCAGACATTTCCATC
GCTTTACGCCCCGCTGTGAGATTCAGGTTAGCAATTAGTACTGCCTGCTCGGCATCTTCAACAGCAACTGGTC
CACCGCGATTGATCTGGCTAGCCGCTGTAAATAACAAGCCGTCATCTTGGAGCTCGAGCGATCGTGGGACAAG
AGCAGCTCCGTACTTGAAATGAAGTACACACCGATCTTCGGGAGGTATCATATTGTATGCCGCTTCCTGCAGC
CGATCGTGGCCGAAGCTATAAGAGTCGTTGACTTTGTCCAGAACACCTTCAGTAACAGCAATTTCGAGTGGTC
CAATCAAGGTCTTTCCACATTTGGTTTCAAGTTCTTTGATGAGTGTTGTGTTCGTTGCATGGCCAAAGCATGA
TAGGATACTCAATGCCATCTGTACCTCTTGCGGTAATTTTTCAATAGAAGAAGTTAGAAATGCCGCTACATCA
TCGGGTAGCTTTCTAGATTGGATCTTCTCCTCTTCCCATTCCCATCGACGGCGACTCAGACTGAGCCGTACCA
AGCCATCTTTGCACAATGACATCATCAACTGAGAAAAGAATAGAGGATTCCCTTTGGTCTTATGGAATACAAT
CTCCGACAATGTCCGAGTAAGACGAGGAGACAGACATAACACATCGGATACCATTGCGTTTATCATCTCTTTG
TCCATACAAGCTAACTCGACTTCCATTGCACGCACACCAAAGTGACGAATACTCGAGACCATATCCCCAATCG
GATGGTTGATCATTCCTTCCTCCCGGCTTGATCCGAGGAAGAAAAATTGTCTCTTTCCATACGAAGATTCAGA
CATCAACAAAATTTGATTGATGGCACTGATCGATGCTGTATCATACAATAGAAATAGTTTGAGTTGAATAGCA
AAATTGACAAAAGGTAGTGCAAAATTGAAAGTGCAAATGTAAATGTCACCTAGGTCAGCCCACTGCAAATCGT

CTAGAAAGAGGGTCACAGGTGACCCAGAACAGATGGAAATTACACCCACAAACTGGCACAGCAGATACTGCAA
CCTCTTTTGGGCAT
>TR586|c1_g3_i14 len=496 path=[8062:0-39 8191:40-129 8442:130-130 8459:131-150 8473:151-153 8482:154-159 8311:160-173 8163:174-201 8386:202-232 8353:233-273 8243:274-325 8444:326-326 8461:327-334 8475:335-338 8483:339-339 8488:340-345 8401:346-357 8380:358-377 8296:378-401 8445:402-402 8223:403-411 8399:412-420 8422:421-446 8129:447-470 7543:471-495] [-1, 8062, 8191, 8442, 8459, 8473, 8482, 8311, 8163, 8386, 8353, 8243, 8444, 8461, 8475, 8483, 8488, 8401, 8380, 8296, 8445, 8223, 8399, 8422, 8129, 7543, -2]
CTGGTCCACCGCGATTGATCTGGCTAGCCGCTGTAAATAATAGATTGTCATCTTGGAGCTCAGGTGATCGTGG
GACAAGAGCAATTCCGTACTTGAAATGAAAGAGGCACCGATCTTCGGGAGGTATCATGTTGTATGCCGCTTCC
TGCAGCCGATCGTGGCCGAAGCTATAAGTGTCATTGACTTTGTCCAGAACACCTTCAGTAACAGCAATTTCGA
GTGGTCCAATCAAGTTATTTCCGCATTTGCTTTCAAGTGCTTCGATGAGTGTTGTGTTCATTGCATGGCCAAA
GCATGATAGGGTACTCAATGCCATCTGTACCTCTTGCGGTAATCTTTCAATAGAAGAAGTTAAAAATGCCGCT
ACATCATCGGGTATCTTTCTGGATTGGATCTTCTCCTCGTCCCATTCCCATCGGCGGCGACTTAGGCTAAGCC
GCACCAAGCTATCCTTGCACAATGACACCATCAACTGAGAAAAAAATAGAGGATTCCC
>TR586|c1_g4_i1 len=392 path=[7642:0-56 8417:57-71 8406:72-73 8387:74-89 8355:90-96 8310:97-156 8246:157-157 8404:158-182 8443:183-193 8460:194-204 8307:205-241 8402:242-243 8381:244-272 8415:273-295 8400:296-307 8140:308-333 7830:334-391] [-1, 7642, 8417, 8406, 8387, 8355, 8310, 8246, 8404, 8443, 8460, 8307, 8402, 8381, 8415, 8400, 8140, 7830, -2]
ATCTCTTCATTGAGTTGCATACCATTCAGTGATGAGTTGTCCAAGTCCAGGTCATGAAGGAATCGGCCGGGTC
TGTCCAGCATCAGCTGCAAATCACTACAGACATCTGACATGTTGCGGTAGGCCTCATCTCCGCTAAGATCGCC
ATTGACGCAATCGATCATATTGCTAATCAAGTCGCAAAGTCTCATGGGCAGCCCCTTCAGTATGAGTGGTTCA
ACCGAGACTGAACGCATCATTGTGCTACGTATTTGTGGCCCCTTCTTTTTGTTGCGAAATTTCATATCAAAAT
CGTCACATGGGTCTTCGTCTTCCATCTTATCAAATGGATCGCCAATATCGCCAATGTTATCAAGTATGTTCAG
TTTACCAGCCATATCAAATGCGCAGCC
>TR586|c1_g4_i2 len=1586 path=[7327:0-53 7328:54-54 7329:55-55 8263:56-75 8361:76-87 8322:88-123 8409:124-168 8359:169-184 8176:185-214 8317:215-215 8253:216-223 8417:224-238 8406:239-240 8387:241-256 8355:257-263 8310:264-323 8246:324-324 8404:325-349 8443:350-360 8460:361-371 8474:372-385 8305:386-408 8402:409-410 8348:411-438 8299:439-439 8231:440-462 8341:463-500 8378:501-558 8294:559-617 8446:618-628 8286:629-676 8216:677-708 8425:709-717 8419:718-752 8411:753-773 8449:774-776 8464:777-780 8277:781-800 8204:801-858 8465:859-860 8476:861-884 8484:885-902 8203:903-906 8108:907-926 8327:927-927 8270:928-935 8198:936-954 8104:955-1146 7875:1147-1170 7876:1171-1173 7877:1174-1197 8100:1198-1236 7895:1237-1260 7896:1261-1585] [-1, 7327, 7328, 7329, 8263, 8361, 8322, 8409, 8359, 8176, 8317, 8253, 8417, 8406, 8387, 8355, 8310, 8246, 8404, 8443, 8460, 8474, 8305, 8402, 8348, 8299, 8231, 8341, 8378, 8294, 8446, 8286, 8216, 8425, 8419, 8411, 8449, 8464, 8277, 8204, 8465, 8476, 8484, 8203, 8108, 8327, 8270, 8198, 8104, 7875, 7876, 7877, 8100, 7895, 7896, -2]
CTGATAGGAACAATCCCCCGCTAGAGCTAACATAAGTGCCGAGTCGATTGGCCAGTACAGATTTTCCAATCCC
CGAGGGGCCTACGATGATTGCACATTCATTCGCTCCCGAAATAGAACGCCTGTAGGAGTTCTGCAGAATTGAA
AAATCCATTTGTCTGTCAAACAAAGCTTCATTAAGTTGCAAACCGTTAAGTGATAAGTTGTCCAAGTCCAGGT
CATGAAGGAATCGGCCGGGTCTGTCCAGCATCAGCTGCAAATCACTACAGACATCTGACATGTTGCGGTAGGC
CTCATCTCCGCTAAGATCGCCATTGACGCAATCGATCATATTGCTAATCAAGTCGCAAAGTCTCATGGGCAGC
CCCTTCAATTTGAGTGGTTCCACCGAGACTGAACGCATCATTGTGCGACGCGTTTGTGGCCCCTTCTTTTTGT
TGCGAAATTTCATATCAAAATCGTCGCATGGGTGTTCATTTTCCATCTTATCAAATGGATCGCCAATATCGCC
AATGTTATCAAGTATGTTCAGTTTACCAGCCATATCAAATGCGCAGCCATTGCCGTGCGGGTTGGGTTCATGG
GCATGTCCATTTTGTGACATAGGCTCCCCAGCATCGCCTTTGAAATGATTGCTTTCATTCCTAGCCCGAGGAC
AGGAATTGGTCTGGGGATGAATGGCAGGATGCTTCTCTCCCCCTGAAAATAATTCATAGAAAACTATCCCCAA
AGAATAAATGAACGGCATCTCATCCACGATGCTGCTGTTGAGATCATTATTATTTGGGAAAGGTAGAAAGTCT
GCCCTAGCCACATTGTGGAAAGAAACACCATTTTGCTCAGAGTCACAGCTGGACGCTCCATCTAAAACGTTAT
CCTCTATAGCAAACAGCGACTCATCTGGAATTAAAGAATTCAAGATATCTTGTAATTCTCGGGGATCTGAGCG
AGGGTTTTCATGAGAGAATGGCAGTGGCTGCAAGTCGCCATCGTACACGTCGATTGGATTGTTGGATAAATGC
ACCGTGATCCGATCTGCCCAGTTGGAGCCTGGAGCAGGTAATTGTACCTGTTGGCTGCATTCGCCGCCGCTGC
TGCCATCCTTGTTATTTTTAGCTTGACAAATTTGATTCGCTAGTGATCTAGCAACCTTCAGCGCAGACTTAAG

ATATGTCTCAGACGACAATGCCATCGACCTCCGTATGTCTGCTGGCCAATAGAGGGAGTCAGCACCGAAGGAT
GAGGTGATTGAATCATTGGCCAGGGAAGATACATCGATGGTTTCCAATGCACCGTCTGCCCATTGTCTCAGTT
TGATTGAGTTGATCATTGCAATTCCTATAACGAAGTATGAAATGAAACCGTTGTAAACATAAGTTTAATTATA
ATAGTGCAGGCGAGTAATTGAGAACGAGATGATGTAATTAGAGCGCGAGTGCGTGCGGCTCTCCAGCAATCGT
GCAAAGTTAACCAGGACGGAGCCCCATCATATCGTCGCTACTTAGTGCCTTCGAATTGTCCGAATTGGGACAT
CGACCCACGTTTTGGAGACGGTCATGTTTTGAGCGTCTGTGGTTGGTGGTTAT
>TR586|c1_g4_i3 len=559 path=[7327:0-53 7328:54-54 7329:55-55 8263:56-75
8361:76-87 8322:88-123 8409:124-168 8177:169-238 8406:239-240 8387:241-
256 8355:257-263 8310:264-323 8246:324-324 8404:325-349 8443:350-360
8460:361-371 8307:372-408 8402:409-410 8381:411-439 8415:440-462
8400:463-474 8140:475-500 7830:501-558] [-1, 7327, 7328, 7329, 8263,
8361, 8322, 8409, 8177, 8406, 8387, 8355, 8310, 8246, 8404, 8443, 8460,
8307, 8402, 8381, 8415, 8400, 8140, 7830, -2]
CTGATAGGAACAATCCCCCGCTAGAGCTAACATAAGTGCCGAGTCGATTGGCCAGTACAGATTTTCCAATCCC
CGAGGGGCCTACGATGATTGCACATTCATTCGCTCCCGAAATAGAACGCCTGTAGGAGTTCTGCAGAATTGAA
AAATCCATTTGTCTGTCAAACAACGCTTCGTTAAGTTGCATACCATTTCGTGATAAGTTGTCCAAGTCCGGGT
CATGAAGGAATCGGCCGGGTCTGTCCAGCATCAGCTGCAAATCACTACAGACATCTGACATGTTGCGGTAGGC
CTCATCTCCGCTAAGATCGCCATTGACGCAATCGATCATATTGCTAATCAAGTCGCAAAGTCTCATGGGCAGC
CCCTTCAGTATGAGTGGTTCAACCGAGACTGAACGCATCATTGTGCTACGTATTTGTGGCCCCTTCTTTTTGT
TGCGAAATTTCATATCAAAATCGTCACATGGGTCTTCGTCTTCCATCTTATCAAATGGATCGCCAATATCGCC
AATGTTATCAAGTATGTTCAGTTTACCAGCCATATCAAATGCGCAGCC
>TR586|c1_g4_i4 len=882 path=[7327:0-53 7328:54-54 7329:55-55 8263:56-75
8361:76-87 8322:88-123 8409:124-168 8359:169-184 8176:185-214 8317:215-
215 8253:216-223 8417:224-238 8406:239-240 8387:241-256 8355:257-263
8310:264-323 8246:324-324 8404:325-349 8443:350-360 8460:361-371
8307:372-408 8402:409-410 8381:411-439 8415:440-462 8400:463-474
8377:475-558 8294:559-617 8446:618-628 8462:629-644 8287:645-679
8217:680-703 8125:704-708 8425:709-717 8419:718-752 8211:753-776
8464:777-780 8276:781-797 8204:798-855 8465:856-857 8476:858-881] [-1,
7327, 7328, 7329, 8263, 8361, 8322, 8409, 8359, 8176, 8317, 8253, 8417,
8406, 8387, 8355, 8310, 8246, 8404, 8443, 8460, 8307, 8402, 8381, 8415,
8400, 8377, 8294, 8446, 8462, 8287, 8217, 8125, 8425, 8419, 8211, 8464,
8276, 8204, 8465, 8476]
CTGATAGGAACAATCCCCCGCTAGAGCTAACATAAGTGCCGAGTCGATTGGCCAGTACAGATTTTCCAATCCC
CGAGGGGCCTACGATGATTGCACATTCATTCGCTCCCGAAATAGAACGCCTGTAGGAGTTCTGCAGAATTGAA
AAATCCATTTGTCTGTCAAACAAAGCTTCATTAAGTTGCAAACCGTTAAGTGATAAGTTGTCCAAGTCCAGGT
CATGAAGGAATCGGCCGGGTCTGTCCAGCATCAGCTGCAAATCACTACAGACATCTGACATGTTGCGGTAGGC
CTCATCTCCGCTAAGATCGCCATTGACGCAATCGATCATATTGCTAATCAAGTCGCAAAGTCTCATGGGCAGC
CCCTTCAGTATGAGTGGTTCAACCGAGACTGAACGCATCATTGTGCTACGTATTTGTGGCCCCTTCTTTTTGT
TGCGAAATTTCATATCAAAATCGTCACATGGGTCTTCATCTTCCATTTTATCAAATAGGTCGCCAATATCGCC
AATTTTATCAAGTATACTCAGTTTGCCAGCCATATCAAATGCGCAGCCATTGCCGTGCGGGTTGGGTTCATGG
GCATGTCCATTTTGTGACATAGGCTCCCCAGCATCGCCTTTGAAACCATTGCTTTCATTCCCAGCCGGAGGAC
AGGAATTTGTCTGGGGATGAATGGCAGGGTGCTTCTCTCCCCCTGAAAATAATTCATAGAAAACTATCCCCAA
AGAATAAATGAACGGCATCTCATTCACGATGCTGCTGTTGAGATCATTATTTGGGAAAGGTAGAAAGTCTGCC
CTAGCCACATTGTGGAAAGAAACACCATTTTGCTCAGAGTCACAGCTGGACGCTCCATCTAAAACGTTATCCT
CTATAG
>TR586|c1_g4_i5 len=876 path=[7964:0-72 7965:73-76 8264:77-152 8438:153-
153 7303:154-154 7304:155-198 @8320@!:199-297 7581:298-313 7582:314-314
8357:315-355 8313:356-362 8249:363-522 7591:523-537 7592:538-538
8415:539-561 8400:562-573 8377:574-657 8294:658-716 8446:717-727
8462:728-743 8287:744-778 8217:779-802 8125:803-807 8425:808-816
8419:817-851 8211:852-875] [-1, 7964, 7965, 8264, 8438, 7303, 7304, 8320,
7581, 7582, 8357, 8313, 8249, 7591, 7592, 8415, 8400, 8377, 8294, 8446,
8462, 8287, 8217, 8125, 8425, 8419, 8211]
CTCTTAATCTTCCCTCCTTAGACATTTTATTGCAGTAATCATTGAAAGCCGAAGCGAGAGCTGAGAATGGCTT
GGCACTTGGAAGTTGATCGAACTTTCCAGATAGGAACAATCCCCCGCTAGATCTAATATAAGTGCCGAGTCGA
TTGGCCAGTGCAGATTTTCCAATCCCCGAAGGGCCTACAATGATTGCACATTCATTCGCCCCCGAAATAGAGC
GCCTATAGGCGTTTTGCAGAGTTGAAAAATCCATTTCTCTGTGAAACAACGCTTCATTGAGTTGCAAACCGGT
AAGTGATAAGTTGTCCAAGTCCATATCATGTAGGAATCGGTCGGGTCTGTCCAGCATCAGCTGCAAATCACAA
CATACATCTGACATGTTGCGGTAAGCCTCGTCTCCGCAAAGATCGCCATTGATGCAATCGATCATATTGTTAA

TCAAGTCACAAAGTCTCACTGGCAGCCCCTTCAGTTTGAGTGGTTCCACAGAGACAGAACGCATCATTGTGCT
ACGTGTTTGTGGCCCCTTCTTTTTGTTGCGAAATTTCATATCAAAATCGTCACATGGGTCTTCATCTTCCATT
TTATCAAATAGGTCGCCAATATCGCCAATTTTATCAAGTATACTCAGTTTGCCAGCCATATCAAATGCGCAGC
CATTGCCGTGCGGGTTGGGTTCATGGGCATGTCCATTTTGTGACATAGGCTCCCCAGCATCGCCTTTGAAACC
ATTGCTTTCATTCCCAGCCGGAGGACAGGAATTTGTCTGGGGATGAATGGCAGGGTGCTTCTCTCCCCCTGAA
AATAATTCATAGAAAACTATCCCCAAAGAATAAATGAACGGCATCTCATTCACGATGCTGCTGTTGAGATCAT
>TR586|c1_g4_i6 len=478 path=[8034:0-53 8035:54-54 8036:55-55 8037:56-75
8038:76-99 @8320@!:100-198 7581:199-214 7582:215-215 8357:216-256
8313:257-263 8249:264-423 8024:424-477] [-1, 8034, 8035, 8036, 8037,
8038, 8320, 7581, 7582, 8357, 8313, 8249, 8024, -2]
CTGATAGGAACAATCCCCCGCTAGAGCTAACATAAGTGCCGAGTCGATTGGCCAGTACAGATTTTCCAATCCC
CGATGGGCCTACAATGATTGCACATTCATTCGCCCCCGAAATAGAGCGCCTATAGGCGTTTTGCAGAGTTGAA
AAATCCATTTCTCTGTGAAACAACGCTTCATTGAGTTGCAAACCGGTAAGTGATAAGTTGTCCAAGTCCATAT
CATGTAGGAATCGGTCGGGTCTGTCCAGCATCAGCTGCAAATCACAACATACATCTGACATGTTGCGGTAAGC
CTCGTCTCCGCAAAGATCGCCATTGATGCAATCGATCATATTGTTAATCAAGTCACAAAGTCTCACTGGCAGC
CCCTTCAGTTTGAGTGGTTCCACAGAGACAGAACGCATCATTGTGCTACGTGTTTGTGGGCCCTTCTTTTTA
TAGGAAATTTGACATCAAAATCGTCACATGGATGTTCATC
>TR586|c1_g4_i7 len=530 path=[7964:0-72 7965:73-76 8264:77-152 8438:153-
153 7303:154-154 7304:155-198 @8320@!:199-297 8257:298-422 7785:423-423
8171:424-448 8314:449-459 8250:460-492 8164:493-516 7655:517-529] [-1,
7964, 7965, 8264, 8438, 7303, 7304, 8320, 8257, 7785, 8171, 8314, 8250,
8164, 7655, -2]
CTCTTAATCTTCCCTCCTTAGACATTTTATTGCAGTAATCATTGAAAGCCGAAGCGAGAGCTGAGAATGGCTT
GGCACTTGGAAGTTGATCGAACTTTCCAGATAGGAACAATCCCCCGCTAGATCTAATATAAGTGCCGAGTCGA
TTGGCCAGTGCAGATTTTCCAATCCCCGAAGGGCCTACAATGATTGCACATTCATTCGCCCCCGAAATAGAGC
GCCTATAGGCGTTTTGCAGAGTTGAAAAATCCATTTCTCTGTGAAACAACGCTTCATTGAGTTGCAAACCGGT
AAGTGACAAGCTGTCCAAGTCCAGGTCATGAAGGTATCGGCCGGGTTTGTCCAGCATCAGCTGCAAATCATTA
CATACATCCGATATGTTGATATAGGCCTCATCTCTGCTAAGATCGCCATTGACGCAATCCGTCATATTGCTAA
TCAAGTCGCAAAGTCTCATGGGGAGTCCCTTCAGTTTGAGTGGTTCCATCGAGACTGTACGCCTCAGTGTGCG
ACGTGTTTGTGGCCCCTTC
>TR586|c1_g4_i8 len=282 path=[7964:0-72 7965:73-76 8264:77-152 8438:153-
153 8186:154-186 7376:187-222 7377:223-281] [-1, 7964, 7965, 8264, 8438,
8186, 7376, 7377, -2]
CTCTTAATCTTCCCTCCTTAGACATTTTATTGCAGTAATCATTGAAAGCCGAAGCGAGAGCTGAGAATGGCTT
GGCACTTGGAAGTTGATCGAACTTTCCAGATAGGAACAATCCCCCGCTAGATCTAATATAAGTGCCGAGTCGA
TTGGCCAGAACAGATTTCCCAATCCCCGAGGGGCCTACGATGATTGCACATTCATTCGCTCCCGAAATAGAAC
GCCTATAGGAGTTCTGCAGAGTTGAAAAATCCATTCCTCTGTCAAACAACGCTTCATTGAGTT
>TR586|c1_g5_i1 len=280 path=[7378:0-51 7379:52-58 7380:59-115 8188:116-
240 7885:241-244 7886:245-279] [-1, 7378, 7379, 7380, 8188, 7885, 7886, -
2]
GACACAGCAAATACTGCAACCTCTTTTGTGCATCGACACAATCTTGATCTTCCCCCGTTTTATTATCTAAGTC
ACCAAGAATTGTAATTAAACTTGGAATCACTTTCACCAAGTGAAGAGCTTCCCCCCCCAATGCAATTCTCAGC
TTCGAAGCCACGATCTGCAAGTGGCTTAATCTTCCTTCCTTAGACATTTTGTTGCAGTAATCATTGAAAGCTG
AAGCGAGAGCTGAGAATGGCTTGGCATGTGGAAGTTGATCGAACTTTCCTGATAGAAACAA
>TR587|c0_g1_i1 len=406 path=[384:0-405] [-1, 384, -2]
CGCCCCAATGGCGTGGCCCGACCTTCTTCGTCGATCCATCGTCCGAGACGATGAATGTGATGGAGGGCGCTGG
AACGTCGAGAGAGACTTCGCACACTTTAGCATAGCGCCAGACCTTGTCGCTGCGACGGATGAATGCGCCATCG
CCCACCTTGAACGAAGGTGCGGCTCGTTTGGCATCGGCGTCTGATGCGTGTGATGCATTGATGTCGCTACTCA
GCGCGACTTCATTGAGCTTGTAGATGTGGGTGTGGTCGACCATGATGAACAGTCAGGATTAGTCTTCAGGTAT
CGTAGTTTCAGAATGAGCAATTAGAATGCTGATGGGTTCCAGGAGGTGGCAATGTGTACAACTGATGTTTCGT
TTCGAGGTAATAATAATAATATTATGAGCCCTGTAACCTGC
>TR588|c0_g1_i1 len=273 path=[251:0-272] [-1, 251, -2]
CTCTTCCGATCTAAAACATGTCCCAAGAAGTCGTCCTATCCTTAACAAAAATCCGAACAGAGGTGGGAACGTC
CACCCTCGAACAACACATTGCCGACGCGCTATTCCTTCCGCGATCCAAAGTGACGGCCCTCATCGACGAAGGC
CTTGCGACACTCATTATCTACCTCCCAGAGCGCGATGATGTTTCGTCAGATGATTATGATGATGCCGACGACA
TCGCCACAAAGCTCAACGATGTCATACAAAGGGGCGAGGCGGACAAATGGACCA
>TR589|c0_g1_i1 len=428 path=[406:0-427] [-1, 406, -2]
CCATACAGCATCCCGTTAGCATGCACGCCCATTGCTCCCCCTGCGCTTCCTTCAAAACCGTCACCTTTTGCAG
GATCTCCTCGATACGGCTTCTTCGAAACGCTGTCACTTTCTCCCGTAAGAGAAGCTTCGTCCACGGTGAACGT
CGACGTGGACACAGATACGAGTCGAATGTCCGCTGGTACTTTTTGCCCCACCGACAATAGCACGACATCTCCT

GGAATGAGTGACGATGCATCCACTTCCACTTCTTCTACCGCAGCTGCATTGTTGGAAAGACTACCGTCGCTGA
CATCGTTACCCACATCGTTGATTCGGACCACCGCCTTCTGCGCTTGCATCTGCTTCAACGCTGAAATACCCTT
GGAGGCATCCAAGGCTTGGTACCCCCCGACCAAGGCATTGATCACTAATATGGTAGTGATGAC
>TR590|c0_g1_i1 len=450 path=[428:0-449] [-1, 428, -2]
CTTTGGCTGGGCATCATGGAACTGTTGGGAACATCTCTGAAGGCAGTTGTACTGGTGATGATGCATGTCACTA
TCTTGGTTCTTCTCATGGAGAGGTTGGGGATGTTTCTGAGGGCAGTTGTGTTGGTGATGATGTATGCCAAGAT
ATTGGATATAATGGAGGAAGTGTCGGAGATATTATAACTGTCAACCTACCAGCAACATCTACCACTGAGTTGG
TGCATGGAATTTGTGTTAAGGACTCTCCATTTGCTTCCGCCACTACCTCTCGTTCCAACCTTGATTACACGCC
AGTTCCTTTTACAGGAGAGCCTCAGGCTTGGGATGATCGTAACTATGTCCTAAGGGGAGTTGAGACTGTCACA
AAATGTAATGGTGGGATCTTCTTGAGGCCATCTTTGGCAAAATCAATTCCATCTGGTACTGAGATATCAATCT
ATGTCACCAAAG
>TR592|c0_g1_i1 len=250 path=[228:0-249] [-1, 228, -2]
TCTCTTTTTAAATGTTAATCCTTGTCTTGTGTGGATTTGATTGTTGTCACTTTGTGGTTGTTGTTGTTTTTCG
TGGTTGTTGTTGTACCGCGCCCACATGCACAACCCAGGTTCCGAGAAAGGTTATATTGTTACACATTTTTGC
TTCTTGTGAAAGGGAGTGTGTTTGTGTTGTTGTTTTTGTGATGTGTGTGTGTGTGTGCGTGGTTTTGTGTGAG
TCTGTGTGTGCGTGATAATTTTATCGTGCAT
>TR594|c0_g1_i1 len=597 path=[575:0-596] [-1, 575, -2]
TTTTTTGCATCTGAGGCATCTGCCTAATCTACCTACTAAAAATCAGATCAGGTTGTATGGTCCCCAAGTGAGT
ACCATACTATTTAACCGGTCATCTCCCGGCAAATAAGTACGAACGGTTGAAACAAGTGACGCCCTCCCTAGAC
TGATCTCTGGTGTGTATCTGTATGAAAGCAGGTCATCGTCTTTCATATTTTATATGTATACGATTGTTAAATA
AAGTTATTTAAGTCTGGTCCCCAATATATGTAGATGTGAATATGAACAAGGCTAATTTGACCTCAGCATGCAG
ATTTCTTCCCCTCCCAGCATTCCCTGCAAGTTTCTGCCTCCATCGTTACACAATCATTGCAATACACCTTTTC
ACACTCAACACAACGCCCCACACAAGTCCCAGTGTGGCAAAAATGCTGCGTGCAAATGTAGCACGTAAAATTC
TGTAACCCATAAAATGGACTCTCCTCGTCGTCTATGAACCACGGAATACCATTTATGTTTTCGTTGCCCCAGT
ACGCATCGTCGCCCTTGCACTTGGTACAAGTCCCGCCAAAATGTTCAAGGAAGGAATCGTATTTGAGGATGAA
GTCGTCGACGAGC
>TR595|c0_g1_i1 len=419 path=[397:0-418] [-1, 397, -2]
CTGCTGTTGTTGTTGTTGTTGTTGTTGTTGCTGTTTCTTCCACAAAGCATCACGTACACCCTCCATCCTATGT
TTCTCCGCCATCAATGCATTTGCTGAAAATTCCAAATAAGTATGACTGTCATCGTCCGTGTCCTCCACTTGAA
ATCCGGCTCGCCGCATGGATCGTTTGAGTAAAGCTTTTGATTCGATGGAGTATGCGAGCAGTGCATCGTCGAG
TTTGAAGTCTTTGGGGTAGGGTATGGGTTGTTGACGGAGGCCTTGGGCTGCGATGGGGGTGGGTAGTGTAAAG
CAGAGCAGCGCTGCTGCCATGGCTGTGGCTAGGGTGATCATGGGTGATATCCTTGACGTTGACGGCATGTTTC
AGTTTTATGGGTCACGGCCGTGTGGTCTTTATGTGGTGCACCTTTCATTTTCGC
>TR596|c0_g1_i1 len=229 path=[207:0-228] [-1, 207, -2]
GTTGCTAAAGTCGACAGCGCATTTACAGAGACAGCACGTCACGTCATTGTAATTTGATCCATCACTGTCGGAT
CCCGGCGTAGCAGCTGCAAGAAGCCCAGGGAAACCCCATTTGACAGTTTTTGTTTTGTTGGTTTTCCCGGTAC
TGCCGCTTTGTTTGGTTTTGGTACCTGTTTTGTTTCCAAAGATTTTCAATGGAATTTGCGACAGCAGCAGAAG
CAGTGGTAAC
>TR598|c0_g2_i1 len=325 path=[321:0-135 322:136-324] [-1, 321, 322, -2]
CGACGTGTGTTCCCACCGCCGCTGATATGTTGGCATCTACGCTCCCGACGAATGCATTTACCGGATTGGCATC
CTCATTTTTCCTCGGATGCCAATTCTTCGGGTGGTATTTCGATGTCGGAAATTGTTTTGTTTTTGTCCAGTCA
GTAGAAGATCCGCCGTATGTTTCGGAGTACTTCGAGGCATACGACGGTTTTGATGATTTGCCATGGGTGTTTC
TTCGAGTGATTCACAGATCGTTAGCATGTCATTGTAAAAAATTGGGATTGGGGGAAGAATGCTGCGAGAGCGA
CGATGCAGAAATTAGACTCCTCAGTTCGAATGC
>TR599|c0_g1_i1 len=639 path=[637:0-119 638:120-137 639:138-638] [-1,
637, 638, 639, -2]
ATCACAACTTTATGGACGCAATGGCTGATAGAAATACTCATCTACTCATTATTCTAAAGTCTAAACTTGTACA
CTATAATGAATGTCTTGGAACTTGGTGATACGCCTAGAAAGTTTTTTTGATTCACCTGACCCTGTACATGCCA
ATCTTCTTATTGCTCTGTTCCATTTTATCAGCAATCGATGTCCTTGTCGAGCTACGGAAAAAGGATGTCACCA
CGGGGTGGTTCAGAACGGTGGATGATAATTGCTGCTGACGTTGTTTTACCTGTTGCTTCTGTTGAGATTGCTG
GTTCGGAAGTCGTTCCTTTGCCAGGTAGAGCGCGCGCTGTTGCTGCAAAGCGAGCATTTTGCGTTGCATCATG
ACGTGACGCCGTGTCATCTCGGATCGTTTCATAGATTCAGCAAGATTATGGAGCGCCTCGATGTATTGATTCT
TGGAGTATGGATGGATTGAAGCAGATTCCTCGACAACTTGTTGGTTGGCAGATGTAGCCTCCGAGGAGCTACG
CACTGATTTCATGGATAAATCGCTGTAGGTGAATCTCTTCGCGGGTGGGTCGAAGTTGACTTGCTGATTGTGC
TGGGGGCGCGGACTTGGCGAGCGTTTTCGTTCACATCGACGGGAATTTTGACAAT
>TR600|c0_g1_i1 len=571 path=[574:0-174 575:175-198 576:199-570] [-1,
574, 575, 576, -2]
GCTAGAAAGTCTGGAGTCGCGTTCTTTACGTTGGTTGTCACCACGAAGCCCCTTGTGCTTTTCTATCTCTCTC
TTCATCTCCGATAAAATTTTGCTCAAGGCCATATTCTCTCGTTGCTCATCAATGACTTTCTGCCGCGTTTCGG
AAACCTGTTTAGAGAGTTTGTCCACTTCGAAAAATTGATTCTCGTCTAGACTGTTACTGCTACCATCTGAACT

GCCCGAATCGAATAATATTCCTTTCAACACGGCGTTCTCATCCGTCAGCGCTTCTTTCTGTCTGAGAAATGAA
TGAGCCGTCGTCTCGAAGTCACTTCTCATCTCTTGAATCTGCTCCTCTAATTGATGAATGCGTTCTTTGCCCC
ATCTTAGTTCGTTTTGGTGCTCGACGTGCTGCTTCTCCAGATAATCCAATCCGACTAGGTCGTGATCGAGTTG
GGATAGTTGTTCTAACAGCGCATGCCTCGTGTGTGAATGACGTAGTATGAGCAGAGTGATGCTGCAATATGTC
ACTAATTTGACTATGCCATTGGTTGTGATTTTCTTCGTCGCGTGTACTGTTGCCGAGATC
>TR601|c0_g1_i1 len=335 path=[395:0-220 398:221-296 394:297-334] [-1, 395, 398, 394, -2]
TTAGCAATTGGGAATTGCTGTTGTTGTTGTTGTTGTTATGCAATGGCAAATCTTGAGGATCGGGAAGCTCCGA
TGAAAGAAGATGAAAGGTTGTCAAGAAGATGAATAGTAGTGAAATACTCAAAAGTAATGGAAGCCCAATTACT
AATACTGCATTTTTCTTGTTGCGAGTGCTGCTGCTGCTGCTGCTTCGATGACAAGGAATTACCATTGTCTGTA
TTTCGATGTGATATGGGAAAAGAGAGATCAGCTCTAGAGAGACGCGAATATGAAGCAAAGAGACACGTCCTAC
GAGAGCGCCGCCAGGATTCGACGGCTTCCTGGGCGACACGGAA
>TR601|c0_g2_i1 len=335 path=[389:0-220 397:221-296 392:297-334] [-1, 389, 397, 392, -2]
TTAGCAATTGGGAATTGCTGTTGTTGTTGTTGTTGTTATGCAATGGCAAATCTTGAGGATCGGGAAGCTCCGA
TGAAAGAAGATGAAAGGTTGTCAAGAAGATGAATAGTAGTGAAATACTCAAAAGTAATGGAAGCCCAATTACT
AATACTGCATTTTTCTTGTTGCGAGTGCTGCTGCTGCTGCTGCTTCGATGACAAGGAATTACCATTGTCTGTA
TTCCGATGTGATATGAGAAAAGAGAGATCAGTTCTAGAGAGGCGCGAATATGAAACAAAGAGACACGTCCTAC
GAGAGCGCCGCCAGGATTCGACGGCTTCCTGGGCGACACGGAA
>TR601|c1_g1_i1 len=672 path=[650:0-671] [-1, 650, -2]
AAGCCAGCAGAAGTGTCCTATTAAAATTATTACACCTGCTCAATCCATGGCCCTCGATAGATATGTATGTACA
TAAATCATGAATCATTCATCCAAATCTTAATTCACACCATCCACGCCACCAAACTCCACCAAATTCTTATGAA
ATTCATCCATCGCGTCTCGACCATCCGCCTCCAAAATCCGTAACTTTTCCAACCACTCATTACGTTCCTCCCT
AGTCAAGACCAAATAATCGTCATTATTCCCCCCCCCTTCCACCACCATCGGTCCAAAAAGACCCGAAACCACT
TCCTCATCATCATCGGCCAGCGCCATTCGTCCCTTATCCTCTCGCGCTTTTTCCATTTCATGCACCCTCCCTC
GTAGAATATTCACCTCGTCGGTGCTCGCGGCAAGTTCCTCTGTCAGTTTGTTCAATTCAAAATTCCGCTGTAA
TGCAATCACGCTCTGTCGAGATTGTTCGGATACAATGTATGTTACTGCTGCCATGTTTAGGAGGAATAAGAGG
AATACCATTAGGGGCAGCAGGATGGTTTTGAGCCATTCGTTTCGTTCGAGGGGTAGATGTGGGAAAGGGACGA
TGGGATGATCGACGGAAGGGATTGTTGCGCTGCTGTTGTTGTTGTTGTTGTTATTGTTCACCACGGATTCTGC
ATTGGATTCTTTTTT
>TR602|c0_g1_i1 len=1304 path=[1316:0-325 @1323@!:326-1068 1321:1069-1303] [-1, 1316, 1323, 1321, -2]
ATTGATATCACTAGGTAGCCCTAAAAAGTACAATCATGCAATATGTCTCCTAATTGACGTACAAATACTCAAA
CATCACCAACATTTGTCACTTTTCTCCAAATTGATCATTTATCCATGATGTTTCATAGTTTGCTACACAGTGA
TAAGGGATTTGTGTTCATCCAAAATACTCATAGTTTGTTTTCGTTGTATTTTTATATCTGTTGGTCATTAAGA
TCAATTTGGTGTGTCAACCATAGAACAACTCAATGTTTTGACTGAGTAATAATTACTCATATATGTATTTTTA
CTAGTAGTCTACTAGAGGTGAGTGTCAAGGTAGGGTGGCAAAATTTGATCAGGTGGCGGAAGTGATTCAGGTC
TCTTCTACTAAGAGCAGTGTATATCCACTCACAGGATACCTTAGTTATCATGTTAACAATTGCTGGTGCGATT
TCCATTGATTCGAAGGATGTGACACTATTTGATCCAATTTGACTTAGGAGTGGGTACTTTACCTTCGAGTTTT
TGGCACTAGAAATGAATATGGTCCAATCCAATATTCGATGTCTGGAGACATTCATTTTCAATTGTTTACTATC
AATCCTGATCTCTAATTATTGACTCTAGTGATTGCCCCACTGTATATTTGATGCATATTTGATAACGTGAATG
TTTATGGAGGAACGATGTGTAGATGCACTCCCTACTCCCTGCATGAGCGGATGACAACTGTGGTGGTGTTACG
TTGTTGGGAGGAATCGTGCAAAGCAACTACTAGGTGGAAGCTGCACGAGATTGGTAGGAACAGTAGAGACGCC
GACATTGTTATGTTAGTGTGTTGTAGATGGTGAATTACTGATTATTGGGATAATAACGATGTCGGTGCCCGGT
GGCTAGACTGCAGTCGTGCTGGTTTTTTATGGTGATTGGAATGATAGGATTTCGGGGCCAAGGATGGTACCGA
CAGACTGTTTGCCCCGGATTGGAACCACCATCGCCGGGGGGTGCAGAATTTAGTGGCGGCCACGCTTCCTAAC
AAGATCCGACATGGATCATCGGGAGGAAGCTAGCAAGAAGAGGAGGGTTGTGGGAAAGCTGCGCCCGTTGTTG
TTCATGGAATGTGACGATCAGAGCGTTCGTTCGGGCTCCCACCCCGGCTCCGGGTCCATTGTTGGACGGCGCC
GTCAGTACCGGAGGCTAATTTGGTAGGAAGGGTAGGCTGGTTGGGAATCGGAAGCGATCGTAGAATCGGTAGA
GGATGGGCTTCTGTGATAAGAAGGTCAGTATGGTGGCAACGTAGGCGTACTTAGTCTATCTAA
>TR604|c0_g1_i1 len=692 path=[843:0-296 844:297-317 @847@!:318-554 836:555-578 837:579-691] [-1, 843, 844, 847, 836, 837, -2]
AGAATTTTGACGCTGGCGATCACAGGTATGGCTTAATCACCAAAGTATGTGTTTGCCGCGATCCATGTTTTGT
CCGTTTTTACCATTTTTTTCAGGTGATCTCTCCCTCTCACCAAATATGTCACTAGTATATATTTTCCATGTAA
ATTTGGACTTTGTCTGAGTCGCTGATGGGGACGCCCATTTTGAACCATGTTGCATGTGCATTGCTGAGGGCAC
GTTTGTATACAGTGATATCGTCAACAAGGTTCCATGGTTGGCAGAGGGCACGCTGGACAATTCCCTTCTTGTA
AGTGGGTGTCATCTGTCTCTTCTTGTGTGGTGTGGTCCACAATGTACCTGTCTTGTTCTGATGCCCATCATAG
GCAATTGAAGTTGGATGAGTTTTTGTTGATGTGGGGGTCTACTTGTGCTGGTTTGTTGCTTTTCTTTGTTACA
GCAACAGGGACACCACTCAGTGCTGCAAACTTGGTCATTGGTCTGTTTTGACAGCAGATGCACTCACGGCAAG
TTGGCCAAGGAGGATTTGGTAGGAGACAAGGGTTAGCTTGCCCACTTTTTTATCTATCATGATAGCATGCATA

TTTCTAGATACATAATTGAGTATGACTGTGGATGAATGGTCAGATGGTTGTGTTGTCTTGGGCAATATTGTTG
CCTACATTGTTATTGTTTGTTTGGGTAGATCTTAC
>TR604|c0_g2_i1 len=405 path=[838:0-296 839:297-404] [-1, 838, 839, -2]
AGAATTTTGACGCTGGCGATCACAGGTATGGCTTAATCACCAAAGTATGTGTTTGCCGCGATCCATGTTTTGT
CCGTTTTTACCATTTTTTTCAGGTGATCTCTCCCTCTCACCAAATATGTCACTAGTATATATTTTCCATGTAA
ATTTGGACTTTGTCTGAGTCGCTGATGGGGACGCCCATTTTGAACCATGTTGCATGTGCATTGCTGAGGGCAC
GTTTGTATACAGTGATATCGTCAACAAGGTTCCATGGTTGGCAGAGGGCACGCTGGACAATTCCCTTCTTGTA
AGTGGTGAGTTTGCACCGTGTGCTCTTGATATGTGCAAGCATCATTTTTGCAATTTTTTGGTGTAACCAACAT
ATTCATATTTGAGTTCTTTGGTGTATTGTTGGTCCACCGC
>TR605|c0_g1_i1 len=259 path=[394:0-183 395:184-258] [-1, 394, 395, -2]
ATGGCAGCTTATGACTAATTCATGGCATCGCTGATGGGGCAGTAGAGAGTGGAAGGGAAAATGATGTTGTTTC
ACTATCTTTGAACCCCCTTCCTTCGACGATCAATTCTCCATACGATGTTACATTGTAATCATCAGTTGCCAAA
AGGCCATCTCCCCACGAATCATAGATGGTGAATTCATACTGCCCTTCTTCCAAGCACATGGAGTGTTTTTGCA
GCGTGTTTCTGTCATCGCGAGTGCCTTGGTATGTTTTCAC
>TR606|c0_g1_i1 len=1483 path=[1515:0-113 1516:114-137 1519:138-718
1510:719-740 1511:741-1482] [-1, 1515, 1516, 1519, 1510, 1511, -2]
TATATCACACGTACATAATTAAGTAAGGCAATTAAGTGTTCATAGTTCGATATTTTTGCAATTCCGTGGCTGA
ATGACGATACGCATCTTCAACGCGTTGCAGGACATCGTACGCAGACTCATAAGTTGTTAGGCATGGAGTATAA
TGGCGATAGACCTGCGCTTGATGTTCGATCTGATCTTTTGGAGTGACAAGGTGTCCATTTATGTCGACAGGGG
CTTCCAAGATTTGATGTGTGACCATATGGACGATATCATGATCCATTCTGGTTCCCTTTACGCCTCGTTCGTC
CAGCGCTTGTAGAATTTCGTAAATGGTATGATCACTGGCTGGGACCATCCATCCGATCAATGCAAATTTCAGC
ATTTCCTGTTCATCCTCAGTCAATCCAAGATAGTTTGCAATCAACATGAATCGTTCGGCCGTACCAGATGGTC
CTGCGAGCATTGGCAAACCTTGTTGAGATGGAATTTTCACTACAGGGTTAGTTGTATATCTTTCATCTATTGT
TTCACGATCAAGATATTCCTTGATCTTCCATCGTCCATTTTTACCACCACCTGGCAACCAATGAATGTATGGA
TCACCATGGAACTGTTCACTGTGCAGTTTAGCTTCGTATTTTCCCAATACAGGCAGTACATCACTAACCAGCA
CGGCTTCGCGAAGATTCCTGTTGAGATATCGTATATCATTTCTAACACGAGCTTCAAAAACGAACCCCCCATT
GGAGGTACACCAAATGGTAGAAAGACTGGATCTTGACTAGTCTTTGAGTGTAGAGCACCGCTATCTTGTTCTC
TCGTTTGTTGTCGTAGAATAAACTCTAGATGGATGAAATCCAATCCTTGTCTCTCTAGCCATTGGTTAGTGCG
TGTGGAATCACTATCAAAGGTTTCGTGATGAAGTTTGGCAATTTGGCTCATATAGTGATACACGTGGGGACTC
CCCAACTCTGAATTGTAGAATTTAGCTGCTGCATAAATGGTTGTCATAGTTTCTCGTAAATTTCCATCTCCAT
GGATGATTTGAAGAATAGTTGTTTCGTCAGTTCCAACCGAACCTGTGCCGGTTGGATCTTTTGTACCAAATAC
GCGACTGCGTATACCGCTGATCGACCGGCCTTCCATTCGTTCCATCGTTTGAGCCAACGCATCGACTCGTTGT
AGCAAAGAAACAGCTATTGCATGCGCAGGAGCATAAGATGCATACCCCAGTCCAATTAAGGTTTCTATATCGT
TTAAATGGACACCGAGATGTTCAATTTTGTCATATAGACTTACTGGCAGAGTGCCGAGTTCAAGTGAAAACAT
TTGCGTTCCCTTTGAGAAACCCATGAACAAACTTCAAACGCCGGCGCTATCATACAATACAATGAACCACACT
GAGAACCAGAAAAGATTGTAATTTCATTCTATTCTCTCGGTCGTCGTTTCACTATTCTGCAGAGTAATTTATG
AGAGAGTCGATGAGTGCGTGCGA
>TR607|c0_g1_i1 len=505 path=[537:0-136 549:137-416 540:417-432 541:433-
453 542:454-504] [-1, 537, 549, 540, 541, 542, -2]
GATCTGGTGTATGCAAAGGAAGCGGGCATTTTGTTTCCTCCAATTGGCGAGGGCGCCCCAGACTCTGCCAAAA
ACTCCGACAGAGAGAGCTCTCCTCCCCATTTCATCATTTCGTAAAGAGGCAAATATAGCATACATGATAGAAC
ACGCGCGTATCCTGAATGTCTGCCACTTCACTCTAGAGAGAGAGAGAGAGATCCTCTATTGTGTATTGCGAGA
GTGATCTACGGAATCGCAAAAAAATCATTGGATGTATCCTCCCGAGACGACCCCATGGACAACTTCATGCGAC
CTATACGACCTGCGACCATAAAGTCTAACTTCTTCTCCAATATTCGCATGTATATATGCGATGTAGACCGTTG
AAAAGCTAAAGAGCCCCAAGTCTGCGATGTTGAAATTTTGCACGAGAGATGCGCGCGATCCAAGGAAAACGCC
GACGGCTGTGAAAAAATATCGAAAAAATCGAAAACACAAACACACCCCCAACTCACACCGAAAAGCG
>TR607|c0_g1_i2 len=404 path=[543:0-35 549:36-315 540:316-331 541:332-352
542:353-403] [-1, 543, 549, 540, 541, 542, -2]
GCTCTTCCGATCTTAAAGAGGCAAATATAGCATACATGATAGAACACGCGCGTATCCTGAATGTCTGCCACTT
CACTCTAGAGAGAGAGAGAGAGATCCTCTATTGTGTATTGCGAGAGTGATCTACGGAATCGCAAAAAAATCAT
TGGATGTATCCTCCCGAGACGACCCCATGGACAACTTCATGCGACCTATACGACCTGCGACCATAAAGTCTAA
CTTCTTCTCCAATATTCGCATGTATATATGCGATGTAGACCGTTGAAAAGCTAAAGAGCCCCAAGTCTGCGAT
GTTGAAATTTTGCACGAGAGATGCGCGCGATCCAAGGAAAACGCCGACGGCTGTGAAAAAATATCGAAAAAAT
CGAAAACACAAACACACCCCCAACTCACACCGAAAAGCG
>TR608|c0_g1_i1 len=733 path=[795:0-143 796:144-167 806:168-229 802:230-
260 804:261-395 793:396-419 794:420-732] [-1, 795, 796, 806, 802, 804,
793, 794, -2]
TCTTCCGATCTCTTCACATCAAGCTATCAAATCATAATAAGTACGTAAAAATATAATGATCGTCAAGTCTTTG
ATTTACAGTGCTGTATTGCTCGCATTCGGATCATGGGTATTGTCAATAGCCGATGCCGCCCGAGTAACGACTC
AATTGTACGAGAACGAATCCATCGCGACGACTCATACGGTTCATTATTCGGGGGGAGGAGGAGGAGGACCCTC

GTCTTTGGACCTTGAGGAGGAGGGAGACGACGTCGTCGAGGACGACGACGATGAGGACGACGACGACGATGAA
AATGTCTCGAATAAGGGAAAAGCATTACTCCACCAACCATCGGGCCAACACCTACTCATCGACATCAAAAACG
TCAACTACGACTTCCTCCGTTCGCAAGATCGCATCGTCGAAGCAATGATCGAAACAATTTCGCTGGCAAAGCA
AACGCTCCTATCCTACCACTGCCATGATAATCGACGGACGCGTATCGTGGGAGGAGTGAGTTGCGTCGGAATT
CTATTGGAGAGTCACATGGCGGTACACACGTTTCCCGCGGATGGGATTGTTATTATGGATTTATTTACGTGTG
GGAAGGGCGAGTTGGTTGGACCGGTTTTACCCGTGGTGGAGAGATTGTTCGCTATTCCGGAGGAGGAGGAGGA
GGAGGAGCGACATCGACAACAACAACAACAACAATCACCACCACCTACACCACCAAGGATGCTGTGGAGATCG
GAA
>TR609|c0_g1_i1 len=934 path=[969:0-81 974:82-576 973:577-792 963:793-816
964:817-933] [-1, 969, 974, 973, 963, 964, -2]
ATGCGATGATGGCGCGATGGTTTAAATTGCAGCACAGTCAGATACTTCTAGTAGCATAGCCTCTAAGCTTATC
CGGTAACTGGGTAACTGCAAGGTTCGTGGCAACGTACAAAGTATACTCTTCCCAATTCCTGACCTACACTCAA
ACAATTTTATAGAAAAATGAAGCATATTTCGGCCATCACTGCGTTATCATTCGCAGCTTCCTCGCAGTTGTAT
CATCCGTCCAATGCCTTTGTGCCAGCGACATACACGAGAAACTTAAATCAACTATCATATCAAGCCAATAATG
TAGGAAGAGATACAATTTTGACGAGTCGAAGACACGAGAGAAGAATACCACCATCCACTTCGTCGGCCTTATC
TGCATCCGATGACGAAAGCAACTCCAGCACGTCCGCACCCCCACGCCAACTGACCATGTACAACTCACTCACA
CGCACCAAAACACCCTTTGTCCCACTCGCCTCCCCCACGTCACCATGTACACCTGCGGCCCAACCGTCTACG
ACTCCGCCCACGTCGGCAACTTCCGCGCCTTCCTAACCTACGACATCCTAAAACGCGTCTTGCTCTACCTCGG
ATACGACGTCGATCACATCTGCAACCTCACCGACGTCGACGACAAAATCATCAAACGATGTGACCGCGAAAAC
GTATCGCTGCTCGAACTCACGCGCAAATTCGAATCCAAATTCTTCGACGATCTGGAGGCACTCAACATCATCA
AGGCCCGGGGATATCCCCGTGCTACCGATCACATTGACGAAATGGCGCAGTTGATCGTCGATCTAGAACGGAA
TGGATTGGCGTACCAGAGCGAGGAGGGGAGTTGGTACTTCTCCGTTTCGAAGAAGGAGGGGTACGGCACGCGG
TTGGTGCAATTGGATCCGGAACAGTTGAAGAAGGGCGCGAGCTCCGAGACGGGGGGAG
>TR610|c0_g1_i1 len=1062 path=[1124:0-760 1125:761-784 1148:785-802
1147:803-832 @1145@!:833-970 1139:971-999 1140:1000-1061] [-1, 1124,
1125, 1148, 1147, 1145, 1139, 1140, -2]
CTCTTCCGATCTATGCAGCTCGCGCGGCGTCCTTTGGTGTAAGCGACGATTTTCGATACGGCCGTGCGGGCGA
GCCGGCGGGTTTGACCTACGTTGAAATCGCGCGGGTGGATGATGTACTCTGTGGAGGAGGCGCTACCGTCGGT
GATGGCGAATGGTGCGAGGAGCGTGGCAAGTGAGTTTGGATCTTCCGTCATGGAACGATGCTGTGCTGCCATT
CTTCTTTGCATTTTCATCGATGGACTTTCCTCCGGGTCTGATTCATCGATTCCTTCGTCACTTTCGAAGGATG
ATGGGAGGGGATCGCGAAGAACGAGTATGTACGAATCGTAATTCCCATTGGCATCGGGCCCTCCATTTCTATT
GTACGAAGCTCCCTTCTTTTTGTAACTCTTCGCCTTGTGTTTCTTTTTGTGTTCGCTCCAGGGTTTGTTCGGT
CGATTCGGCCCCCCTTCCGCCTCGTTGTCTTCCGTCTCAATCTGCTGACTGGCATAAGTAGGAGAATTCAATC
CATAATTTTCCACAATCGTTTGCGACACAGGATTCCATTTGATATTGTCCGCCCTCACAATCTGATCCCGCTC
CAACTTAATCTTCGTCCTCCGTTTCGACCTGCTTCTCGTCTTTACCGGAATACCAGTGCTAGAATTCCTCGGC
AAAAAGCTGTATGGTGCCTGAAGCGTGAGCGTGCATCCATTAGTATCGGAATTATTTCCACAATCCAATACTA
TCGAGGAACTATTCCGTCGAATCCACGACCATCCGCCCCATATGAAGAGTATGCTGATGAAGAACCATATGTA
AGTGCCCTTCATGAATCCGTCTGCCGATCGGATGGCCTTGGCGAGTGGGGCTTCCATTTTGATGGATTTTCCC
GATCCGTCGGGGAGCTTGGATGGGCCGCCGTTGAAGGTAGTGGGGAGCTCGCCACCGGATCCTCGTTTGCGAA
GCATGTTGGCTACTGGTGTGTGGATTTATTTTGCGTTTAATTGCCTGTCCAGTTCCACCTTCTTGGCTGGTTG
TCGATCGGCTTGCGCTTGATCGGCTCGATGAGGGGATTTT
>TR611|c0_g1_i1 len=2023 path=[2129:0-1511 2138:1512-1769 2139:1770-1771
2135:1772-1854 2116:1855-1875 2117:1876-1909 2118:1910-2022] [-1, 2129,
2138, 2139, 2135, 2116, 2117, 2118, -2]
GAGGTTATGAGCCTTTTTTACCGCTGCGAGAGAGCGCTGACCATAGCATCCACCAGTCTACACATCATCACAC
AACTTCCTCCGCTTCTATCAACTTATCACGCCTTCAGACCTTCATTCCGCCACCATCATCGTCTTACCAAGCG
TCTTCCGCACGCATAGATACCCCGCACGGTGCCTATCTTCCAGTCCGACATCCAGAATTTGCCCCGACAAGTT
CCTCGGGTCAAATCCGATGTTGTTCACCTTTCTTTGACATCATCAATGCTGCCACACCTCCACTTCTGGTCTT
CCGACCTCTCCACGGTCGTGGCAAACACCATATCCTTTGCAAATCAAGGGTACAACGCCATTGAAACAACAAT
CTTTGCCGACAGGGATCTTTGGACTACAAAATACGGCACCATTGCCCCGCTAAGTCCCGGCAAATTCACCACC
CAATTAAGCCGCAACAACACTTTGGATGTGCCATCACCACCGTCTTTCGTACCTCCATCGCAACTCCTTGTGT
CCGCGGACGCGCCTAGCTTCGATGCCAGCCCGATAACATTGTTGGGGCCCTTGCAAAACTCCAACATATCTCC
GCAAATGCCGCGGATTGACTTATCAGTCAATAAGGCTGATCAAAACGCGCCAGCAACTCAACAATTTGCACCA
TCGCCTCAACAATTTGTGCTGCATTCCCAATACAGCCATATACAGCTGCCTCAGTAGCCACATTAGCCAACGT
ACCCCACGCTCCACCAATTTCACGTCCCACCTCCATTGCCGAATCCGGTACTTTCCGCCAGACACTTCCCAAA
TCAACTTCCTTCCATCCACACAAATGCTGCCTCCCATCATCATGGCCAGCCCTCGCTGTACCACAAAATGCCA
CCATTTGGGTCACATTACTTGGTGCCTCCTCTGGGCCAACCAATGCCACCAATGTATGTGCCTCCTCTCTGAC
CACTTCCTAAATCAGTACATCTCCCACCATCTTCGGGCCTTTCCCAACCTTCCAATGCACCATCTTTGCATCA
CGGGGATCAATCTGGCCTATCTTTGTCCACACATTCGCCAGCCACTCACCCCGTTGCTCTGTCGTCCACCATC
GATCCAGCCATGGCTGCTCTCACCATGCACATCATGGCTTTGAATCAGCAGAACACACAAGCGATGCAGATGC

TGACCATGTCCAGTACTCCTTGCGGGCCTTGGAACCTATTCCCTACATGGGACGACTCTAAAGTCAACCTTCC
CCTATTCCTAGTCAGAATAGAGTCGTACAAATCAGACCCCTTCTTTGTGTCCGTCACAAATTGGAATGTGACA
CTTCCCGGTCAAGAGATCTTCAGCTAGCGAGTCTATGCCGCCATGATGGCAAAACTTCTGACAGCGCAACTTC
ACCAGTTCCTCTCCCGCCCCGAATTCATAAACCAGGGATTCGAAATGCTACACACACTCCATTCCCATATGGA
CCCTGCAAATGCCAAGAACAGACTTGCAGATGTAAAACATCTCTCAAGCCTGGTAATGAAGCCTGATGAAAAT
GTTTCAACATACATGCTGAACGTCCGAATCTCATCCAACCGCCTATCTTCTGTATCCATCGATGTCCTGATGC
CTCTTTTTGCAACATTGGGCCTCAACGACTCCTGTTTTCCTGGAGTTATCAATTGATTCTCCCAGTCTGTATT
TTTGATAAAAAATGTGACAGGTTAATCTTTGTCCTCATTGGCACAGTATTGTACTTCTCTAATGTGACCATTC
AAGTCATCCCAATTTTGAGTTGAGTCCTTCACATTTTTGACAGTATGAGCCCTGCCATAGTTTGTACTAATTC
CATTGTACATTGATTTCTTCATTTGCATTGAAAATATTTACCCCTTATTGTACTTTATTAAGTACTAATTAGC
TCTCTTACATAGCTAAATATTGAACCTACACCACACATATGTATGCTATTTACCTCTAAACATTTTACATCAT
TATTTTACCGCTTTAATCTAAAAATCTAAATACTAAGTAGATCGGAAGAGCG
>TR611|c0_g1_i2 len=1941 path=[2129:0-1511 2138:1512-1769 2139:1770-1771
2126:1772-1793 2127:1794-1827 2128:1828-1940] [-1, 2129, 2138, 2139,
2126, 2127, 2128, -2]
GAGGTTATGAGCCTTTTTTACCGCTGCGAGAGAGCGCTGACCATAGCATCCACCAGTCTACACATCATCACAC
AACTTCCTCCGCTTCTATCAACTTATCACGCCTTCAGACCTTCATTCCGCCACCATCATCGTCTTACCAAGCG
TCTTCCGCACGCATAGATACCCCGCACGGTGCCTATCTTCCAGTCCGACATCCAGAATTTGCCCCGACAAGTT
CCTCGGGTCAAATCCGATGTTGTTCACCTTTCTTTGACATCATCAATGCTGCCACACCTCCACTTCTGGTCTT
CCGACCTCTCCACGGTCGTGGCAAACACCATATCCTTTGCAAATCAAGGGTACAACGCCATTGAAACAACAAT
CTTTGCCGACAGGGATCTTTGGACTACAAAATACGGCACCATTGCCCCGCTAAGTCCCGGCAAATTCACCACC
CAATTAAGCCGCAACAACACTTTGGATGTGCCATCACCACCGTCTTTCGTACCTCCATCGCAACTCCTTGTGT
CCGCGGACGCGCCTAGCTTCGATGCCAGCCCGATAACATTGTTGGGGCCCTTGCAAAACTCCAACATATCTCC
GCAAATGCCGCGGATTGACTTATCAGTCAATAAGGCTGATCAAAACGCGCCAGCAACTCAACAATTTGCACCA
TCGCCTCAACAATTTGTGCTGCATTCCCAATACAGCCATATACAGCTGCCTCAGTAGCCACATTAGCCAACGT
ACCCCACGCTCCACCAATTTCACGTCCCACCTCCATTGCCGAATCCGGTACTTTCCGCCAGACACTTCCCAAA
TCAACTTCCTTCCATCCACACAAATGCTGCCTCCCATCATCATGGCCAGCCCTCGCTGTACCACAAAATGCCA
CCATTTGGGTCACATTACTTGGTGCCTCCTCTGGGCCAACCAATGCCACCAATGTATGTGCCTCCTCTCTGAC
CACTTCCTAAATCAGTACATCTCCCACCATCTTCGGGCCTTTCCCAACCTTCCAATGCACCATCTTTGCATCA
CGGGGATCAATCTGGCCTATCTTTGTCCACACATTCGCCAGCCACTCACCCCGTTGCTCTGTCGTCCACCATC
GATCCAGCCATGGCTGCTCTCACCATGCACATCATGGCTTTGAATCAGCAGAACACACAAGCGATGCAGATGC
TGACCATGTCCAGTACTCCTTGCGGGCCTTGGAACCTATTCCCTACATGGGACGACTCTAAAGTCAACCTTCC
CCTATTCCTAGTCAGAATAGAGTCGTACAAATCAGACCCCTTCTTTGTGTCCGTCACAAATTGGAATGTGACA
CTTCCCGGTCAAGAGATCTTCAGCTAGCGAGTCTATGCCGCCATGATGGCAAAACTTCTGACAGCGCAACTTC
ACCAGTTCCTCTCCCGCCCCGAATTCATAAACCAGGGATTCGAAATGCTACACACACTCCATTCCCATATGGA
CCCTGCAAATGCCAAGAACAGACTTGCAGATGTAAAACATCTCTCAAGCCTGGTAATGAAGCCTGATGAAAAT
GTTTCAACATACATGCTGAACGTCCGAATCTCATCCAACCGCCTATCTTCTGTATCCATCGATGTCCTGATGC
CTCTTTTTGCAACATTGGGCCTCAACGACTCCTGTTTTCCTGGAGTTATCAATTGATTCTCCCAGTCTGTATT
TTTGATAAAAAATGTGACAGGTTAATCTTTGTCCTCATTGGCACAGTATTGTACTTCTCTAATGTGACCATTC
AAGTCATCCCAATTTTGAGTGAAAATATTTACCCCTTATTGTACTTTATTAAGTACTAATTAGCTCTCTTACA
TAGCTAAATATTGAACCTACACCACACATATGTATGCTATTTACCTCTAAACATTTTACATCATTATTTTACC
GCTTTAATCTAAAAATCTAAATACTAAGTAGATCGGAAGAGCG
>TR611|c0_g1_i3 len=1906 path=[2129:0-1511 2138:1512-1769 2113:1770-1792
2114:1793-1905] [-1, 2129, 2138, 2113, 2114, -2]
GAGGTTATGAGCCTTTTTTACCGCTGCGAGAGAGCGCTGACCATAGCATCCACCAGTCTACACATCATCACAC
AACTTCCTCCGCTTCTATCAACTTATCACGCCTTCAGACCTTCATTCCGCCACCATCATCGTCTTACCAAGCG
TCTTCCGCACGCATAGATACCCCGCACGGTGCCTATCTTCCAGTCCGACATCCAGAATTTGCCCCGACAAGTT
CCTCGGGTCAAATCCGATGTTGTTCACCTTTCTTTGACATCATCAATGCTGCCACACCTCCACTTCTGGTCTT
CCGACCTCTCCACGGTCGTGGCAAACACCATATCCTTTGCAAATCAAGGGTACAACGCCATTGAAACAACAAT
CTTTGCCGACAGGGATCTTTGGACTACAAAATACGGCACCATTGCCCCGCTAAGTCCCGGCAAATTCACCACC
CAATTAAGCCGCAACAACACTTTGGATGTGCCATCACCACCGTCTTTCGTACCTCCATCGCAACTCCTTGTGT
CCGCGGACGCGCCTAGCTTCGATGCCAGCCCGATAACATTGTTGGGGCCCTTGCAAAACTCCAACATATCTCC
GCAAATGCCGCGGATTGACTTATCAGTCAATAAGGCTGATCAAAACGCGCCAGCAACTCAACAATTTGCACCA
TCGCCTCAACAATTTGTGCTGCATTCCCAATACAGCCATATACAGCTGCCTCAGTAGCCACATTAGCCAACGT
ACCCCACGCTCCACCAATTTCACGTCCCACCTCCATTGCCGAATCCGGTACTTTCCGCCAGACACTTCCCAAA
TCAACTTCCTTCCATCCACACAAATGCTGCCTCCCATCATCATGGCCAGCCCTCGCTGTACCACAAAATGCCA
CCATTTGGGTCACATTACTTGGTGCCTCCTCTGGGCCAACCAATGCCACCAATGTATGTGCCTCCTCTCTGAC
CACTTCCTAAATCAGTACATCTCCCACCATCTTCGGGCCTTTCCCAACCTTCCAATGCACCATCTTTGCATCA
CGGGGATCAATCTGGCCTATCTTTGTCCACACATTCGCCAGCCACTCACCCCGTTGCTCTGTCGTCCACCATC
GATCCAGCCATGGCTGCTCTCACCATGCACATCATGGCTTTGAATCAGCAGAACACACAAGCGATGCAGATGC

TGACCATGTCCAGTACTCCTTGCGGGCCTTGGAACCTATTCCCTACATGGGACGACTCTAAAGTCAACCTTCC
CCTATTCCTAGTCAGAATAGAGTCGTACAAATCAGACCCCTTCTTTGTGTCCGTCACAAATTGGAATGTGACA
CTTCCCGGTCAAGAGATCTTCAGCTAGCGAGTCTATGCCGCCATGATGGCAAAACTTCTGACAGCGCAACTTC
ACCAGTTCCTCTCCCGCCCCGAATTCATAAACCAGGGATTCGAAATGCTACACACACTCCATTCCCATATGGA
CCCTGCAAATGCCAAGAACAGACTTGCAGATGTAAAACATCTCTCAAGCCTGGTAATGAAGCCTGATGAAAAT
GTTTCAACATACATGCTGAACGTCCGAATCTCATCCAACCGCCTATCTTCTGTATCCATCGATGTCCTGATGC
CTCTTTTTGCAACATTGGGCCTCAACGACTCCTGTTTTCCTGGAGTTATCAATTGATTCTCCCAGTCTGTATT
TTTGATAAAAAATGTGACAGGTTAATCTTTGTCCTCATTGGCACAGTATTGTACTTCTCTAATGTGACCATTC
AAGTCATCCCAATTTTGATACTAATTAGCTCTCTTACATAGCTAAATATTGAACCTACACCACACATATGTAT
GCTATTTACCTCTAAACATTTTACATCATTATTTTACCGCTTTAATCTAAAAATCTAAATACTAAGTAGATCG
GAAGAGCG
>TR613|c0_g1_i1 len=828 path=[938:0-155 939:156-194 954:195-301 948:302-325 956:326-508 942:509-532 955:533-673 945:674-713 946:714-827] [-1, 938, 939, 954, 948, 956, 942, 955, 945, 946, -2]
CTCTTCCGATCTCAACAACAACGACTTGTGGATGATATCCGATGTCATTGCGTATCCCGAACGTGCCACGGAA
TTATTGAGCAAATGGTGCGAAATGAAGTGGGCATCGCACATGATCGTGACGATGAAATTTCAAGGGAAGGAAC
CCGATTTGGATGAGTTGGATAAGGCAATTGATGTCGTGAGGAATTTGGGATACGATTGTCGTGTGAAGCATTT
TTTCAATAATAAGAATGAGGTCACGTTTATGGTGTCAGAGAAGAAGATAGTTACAGGGAAAGAGAGGAATAAT
GATTTGGAGGCTGGGATAGTGGGTACTTCCATGTTTTCTACTTTATGAGCGTGTTGTGTATGAATGTGGCGGC
CGCGAATATTCTCTTGACCGGTATTTCCAACAGCGGGAGGTATCACAAGCACCGATGCCAGTGTGACGAAGAA
AAAATGATAATACACCATCTAAATAAGGTGGCTTAACCAACAATCAGCGAATAGTACCTTTGCTTCCTACCTG
GTACCCCCCAGAGCCCAGAGACCTCCATGGACGGTGGTTGTGATCACATGGCAAGTATTACAATGGTATCACC
TACTGATAATCGTAAGAGACAACATACGGATTGGACGGAACGAAAACGAACATTTTACAACCCATACTGTTCT
CTACTTCCCTCTTCCAACACCACATCGTTCTGTGTTGACAAAAATATTATAGAAGTGGAGCCCATGTCCAGTA
GTGTAGCGCAACAAAGAGAAGAATCACAGTTGTTTGGAGTAGTATAAACATGATCATGTAATTGATTTTGATT
CTTGGTATTATCATTGGATTTCTTA
>TR615|c0_g1_i1 len=816 path=[1065:0-331 1066:332-332 1067:333-350 1068:351-351 1069:352-357 1070:358-378 1089:379-766 1081:767-790 1082:791-815] [-1, 1065, 1066, 1067, 1068, 1069, 1070, 1089, 1081, 1082, -2]
GCGGACTTGCGCCTGGCGCGTTGGTGCATCGCCGGCGGTTGGGCCGAGGAGGAACCGTGCGACTTGTAGGTCG
GATCGTTATTGTGGGTGGAGGATGTCATGGTTTCGCTTCCCATTGGATATAATATATATCGCGTGTGCGATGA
GTCACGGTTCTCCGCTCGATATGGATATTGCTATGGCTGCGCGCGCCTTGTTGGTTCTACCTTGGGTGAGGTT
TCCTCGGTTGTGTCGTTGTTGCCGAACTTGGTTTAAGTAGGAGGTGTGTCGATAAAACGATTCTGAATTCGAT
GTCGTTCCGTTTCCATGCGAGGCGCGTGTATTGGCTTTTTTTGACTTGTTGCCGTTTTTGTTTTCTTGTATGT
GATATGACGTCGGGGTTGTATTGCGCCTTTAAAGATAATGTGGGAATGTTGTCAACTGAATAAAGCCTTCCAA
AGAAGATCTTTGATATTATTTATGACAATAGATCCGGGACGGCCGATAAGTAGTTCTTTCGCAAGCGCGACGG
ATGACCTCTTGTGAATGGGAGTAATGGGAGTGAATCAGGGTTGGTGGAGCTGTGTTGTGCGCGTATGTGCGTG
AACCGCCACTTACTTAACCACCCACAAGGCTGTGTGTGAACTGCTTTTTGTTGCGTTAGAGTAGTTGGTAGTG
CGCCATGGGAATTCGAAGGGGATTCTGGATCCGTAGTTTCTAGTGGTGGCGAAATAAATCGGCTGACAGCTGG
AGGGAGCAGGAGGCGAAGTATCCAGCAGCTGTTGCTATGATCGGTCCCGTAAGCCTTCTGTAGGGTTAGGTAA
GTCTTGATGCGAT
>TR615|c0_g1_i2 len=1000 path=[1061:0-331 1062:332-349 1091:350-355 1090:356-562 1089:563-950 1081:951-974 1082:975-999] [-1, 1061, 1062, 1091, 1090, 1089, 1081, 1082, -2]
GCGGACTTGCGCCTGGCGCGTTGGTGCATCGCCGGCGGTTGGGCCGAGGAGGAACCGTGCGACTTGTAGGTCG
GATCGTTATTGTGGGTGGAGGATGTCATGGTTTCGCTTCCCATTGGATATAATATATATCGCGTGTGCGATGA
GTCACGGTTCTCCGCTCGATATGGATATTGCTATGGCTGCGCGCGCCTTGTTGGTTCTACCTTGGGTGAGGTT
TCCTCGGTTGTGTCGTTGTTGCCGAACTTGGTTTAAGTAGGAGGTGTGTCGATAAAACGATTCTGAATTCGAT
GTCGTTCCGTTTCCATGCGAGGCGCGTGTATTGGCTTTTTGACTTGTTGCCGTTTTTGTTTTCTAAAAAGAGA
GACATAAGACATATGCAGCATGATGCGTGTGGCAGCGCTGGTTCGTTAGCACAGCAGATAAAGTGCGGTGCAA
TAGACAGCGGCAATGAACCGACACACTACGCCGACATGCACCGGTCCATGCCAATACGCTGACAATCAAACGG
TGACATTGTCTCCTCTCGACGATACCCACCTTGTATGTGATATGACGTCGGGGTTGTATTGCGCCTTTAAAGA
TAATGTGGGAATGTTGTCAACTGAATAAAGCCTTCCAAAGAAGATCTTTGATATTATTTATGACAATAGATCC
GGGACGGCCGATAAGTAGTTCTTTCGCAAGCGCGACGGATGACCTCTTGTGAATGGGAGTAATGGGAGTGAAT
CAGGGTTGGTGGAGCTGTGTTGTGCGCGTATGTGCGTGAACCGCCACTTACTTAACCACCCACAAGGCTGTGT
GTGAACTGCTTTTTGTTGCGTTAGAGTAGTTGGTAGTGCGCCATGGGAATTCGAAGGGGATTCTGGATCCGTA
GTTTCTAGTGGTGGCGAAATAAATCGGCTGACAGCTGGAGGGAGCAGGAGGCGAAGTATCCAGCAGCTGTTGC
TATGATCGGTCCCGTAAGCCTTCTGTAGGGTTAGGTAAGTCTTGATGCGAT

>TR616|c0_g1_i1 len=1365 path=[2682:0-179 2683:180-203 2806:204-618 2741:619-635 2786:636-700 2686:701-724 2795:725-790 2692:791-795 2783:796-960 2689:961-961 2690:962-1364] [-1, 2682, 2683, 2806, 2741, 2786, 2686, 2795, 2692, 2783, 2689, 2690, -2]
CTAGAAGTATCTTCAACGCACACTTTTTGTTGTTGCTCTATGTTATTTTAGGTCACACAGGGGCTATTTGGAG
CACGATGCAAAATAAGACAATGCGGAAGGTATCAAATCCCATCGCCGCAATAGTCCTCCTGGTCTTAGCCGCA
AGCACCGCGCTCCGCTTCCACTCATGGCATGCCGCGGACACACAGCCGTCGGGATGGGAATATTCCTCGTCCG
AATCACGGCGAAAACTCAAACACGTGCCGGATATTGCACCCCCACATACGTCCAACGGCCTTTACGATCAGAT
ATGGCGCGATCTCAAACGGAAAGGGAAGGAACCGGGCACTAAATCGTTCGAGGCGGTCTTACGCAATCGAATT
TCCAAACAAGAGTATGCTTTGATAGATGAGCTCAGTCGTTTTCACCAATCCGAAAGCACCTTGGACAGGACGT
GGCGGGGACTCGTTAAGGAGGGGAAGTCGCCAGGCACCCCACCCTTCGAAAGATTGATGAAGCATAAAGAGCC
AAGGAGGGAGGAGGTGAGAAACGAAAATAAAAATTTGGGGGAAGCGGCCTTGAAAGCCAAGAAAAATTTGGCA
GAGGCCGAAATTAAGTTTGAGGAGGCTGCAAAAAACTTGAAAGATGCGGAGCGGTCCATTGCTCAAATTATGG
AAGAGTCCCAAGGGAGTCACGAAGCGAAGAGGCAATTGGAGGTTGTAAAGGAGACCCTCGAATCAGCGGCCGA
GGTTAAGCTTCAGGAGTTACTGAAGGAGGGTGACTATCAACCCCTTGCCATCTGTGGCCGTAACGCAGACGTG
GGCTCTGTCGAAGACTTTGCGGCCATGCAAAAATATGCCCAATGCTCAGACATTACCAAGGACAAGCCCATCT
TACTCCTGGAGGGGATGGAATTCCACGGTCGCACTGGAAATACCCTCCTCGAATTCCTGCACGCCATCCAGGA
AGCTCGAGATATGGATATTTCGTTGGGCGTCATGCGCCAATCCTGGGCCATGGATGTGCTCCTGAAGATGTGG
ATGGCAATTGATTCCGATGATTGGCAAGCCCAATTTGAAAGAACCTTTTGTTTAAAAATTTTCAACCACGAAA
AAGAATTAGAAGGATGGAACGTATTTGATCAAGATCCAAAAGAGTTGCTTTACTACAGATCTAAAATTTCCCT
AGAGGATTATGTTGCGAGCCAGGAATACAATATTCGAGCAATGCTTCGCAATTACAATACTGGAGAGGGCAAG
GATATAAGTGGGGCACCAGTCAAGGATATGTGTTCGGGCATCAACGCCATCTTCGGCGAGGACCAAAGAAGTT
CTGCCATGTATTCAGTCATACATAGCCGCTCCCTTGAAGGCGAGCCTGGTT
>TR616|c0_g2_i1 len=818 path=[2743:0-178 2810:179-235 2799:236-240 2805:241-249 2819:250-273 2821:274-291 2809:292-348 2804:349-360 2770:361-390 2818:391-405 2817:406-406 2816:407-481 2820:482-507 2781:508-519 2802:520-523 2814:524-531 2811:532-558 2807:559-577 2775:578-630 2670:631-817] [-1, 2743, 2810, 2799, 2805, 2819, 2821, 2809, 2804, 2770, 2818, 2817, 2816, 2820, 2781, 2802, 2814, 2811, 2807, 2775, 2670, -2]
AGAGACAGATGCCGAATTGGAGGTGGGTTTAATACGCCTGGGTTGTGTTTTTATTTTCGCTGTGTGAGTTATT
CTAACAAGTGCATTCGAATATTATGTTGTTGTTATCCGTTTCCCACACGTGATCACCCATGTTCGGCGATGCT
GGCGCTTACTAGGCGCTTTTAACGAGGCCACTCGCTGTAGAGGCGGTGATGAAGGAGGGGGACTTCGAACCCC
TTGCCATCTGTGGCCGTAACGCGGACGTGGGATCCGTCGAAGATTTTGCAGCCCTGCAAAAATATGCTCAATG
CCCACCCATCAATAGGAACAAGCCCATCTTACTTTTGGAAGGAGATCGAGCCTTCGGTCGAACTGGAAACAAC
CTTATTGAATTCCTACATGCCATCCAGGAAGCTCGAGATATGAATATTTCGTTGGGCATCATGTCCAAGTCCT
GGGCCATGAATCTCCTCCTGAAGATGTGGATGGCCATTGAATCAAATGATTGGCGAATTCAATTTGAACGAGC
TTTTTGCGTCAAACTTTTTAACAATGAAAAACAACTCGAAGGTTGGGACATTGTTCGTCCTGACACGCAAGAG
TTGTTCTTCTACAGGACAGATAGTCCTCTTGATGAATATGTTGCAAGCCAGGGACACGTCCTTCGAACATTGT
TTCGTAACTACAACACTGGAGAGGGCAAAGACGTTAGTGGATTGCCTGTCCAGGATATGTGTTCAGGCATCAA
TGCCATCTTTGGTGAAAAGGAGCGAAGTTCTGTCATGTATTCAGTCATACACAGCCGCGCCATTGAAGGCGAG
TCTGGTTATAGGCTG
>TR616|c0_g2_i2 len=747 path=[2736:0-107 2810:108-164 2799:165-169 2805:170-178 2819:179-202 2821:203-220 2785:221-277 2804:278-289 2794:290-319 2818:320-334 2817:335-335 2816:336-410 2808:411-448 2792:449-452 2812:453-475 2777:476-487 2807:488-506 2788:507-520 2774:521-559 2677:560-746] [-1, 2736, 2810, 2799, 2805, 2819, 2821, 2785, 2804, 2794, 2818, 2817, 2816, 2808, 2792, 2812, 2777, 2807, 2788, 2774, 2677, -2]
GGACAGGGGCAAGGCCAGGGACAACTTCAAGGACAGGGTGTGAGGGGGAAACAGGCGATGATGGAAGAGACAG
ATGCCGAATTGGAGGCGCTTTTAACGAGGCCACTCGCTGTAGAGGCGGTGATGAAGGAGGGGGACTTCGAACC
CCTTGCCATCTGTGGCCGTAACGCGGACGTGGGATCCGTCGAAGATTTTGCAGCCCTGCAAAAATATGCTCAA
TGTCCACGCATAAATAGGAACAAGCCCATCTTACTCTTGGAAGGAGATCGAGCCTTCGGTCGAACTGGAAATA
ACCTCATTGAATTCCTACATGCCATCCAGGAAGCTCGAGATATGAATATTTCGTTGGGCATCATGTCCAAGTC
CTGGGCCATGAATCTCCTCCTGAAGATGTGGATGGCCATTGAATCAGATGATTGGCGAATCCAATTTGAACGA
GCTTTTTGCGTCAAAATTTTCCACAACGAAAAACAACTCGAAGGTTGGGACATTGTTCGTCCTGACACGAAAG
AGTTGTTCTTCTACGGGACAGATAAACCTCTTGATGAATATGTTGCAAGCCAGGGACACGTCCTTCGAACATT
GTTTCGTAACTACAACACTGGAGAGGGCAAAGACGTTAGTGGATTGCCTGTCCAGGATATGTGTTCAGGCATC
AATGCCATCTTTGGTGAAAAGGAGCGAAGTTCTGTCATGTATTCAGTCATACACAGCCGCGCCATTGAAGGCG
AGTCTGGTTATAGGCTG

>TR616|c0_g2_i3 len=747 path=[2736:0-107 2810:108-164 2799:165-169 2805:170-178 2819:179-202 2821:203-220 2809:221-277 2804:278-289 2770:290-319 2818:320-334 2817:335-335 2816:336-410 2820:411-436 2781:437-448 2802:449-452 2814:453-460 2811:461-487 2807:488-506 2775:507-559 2670:560-746] [-1, 2736, 2810, 2799, 2805, 2819, 2821, 2809, 2804, 2770, 2818, 2817, 2816, 2820, 2781, 2802, 2814, 2811, 2807, 2775, 2670, -2]
GGACAGGGGCAAGGCCAGGGACAACTTCAAGGACAGGGTGTGAGGGGGAAACAGGCGATGATGGAAGAGACAG
ATGCCGAATTGGAGGCGCTTTTAACGAGGCCACTCGCTGTAGAGGCGGTGATGAAGGAGGGGGACTTCGAACC
CCTTGCCATCTGTGGCCGTAACGCGGACGTGGGATCCGTCGAAGATTTTGCAGCCCTGCAAAAATATGCTCAA
TGCCCACCCATCAATAGGAACAAGCCCATCTTACTTTTGGAAGGAGATCGAGCCTTCGGTCGAACTGGAAACA
ACCTTATTGAATTCCTACATGCCATCCAGGAAGCTCGAGATATGAATATTTCGTTGGGCATCATGTCCAAGTC
CTGGGCCATGAATCTCCTCCTGAAGATGTGGATGGCCATTGAATCAAATGATTGGCGAATTCAATTTGAACGA
GCTTTTTGCGTCAAACTTTTTAACAATGAAAAACAACTCGAAGGTTGGGACATTGTTCGTCCTGACACGCAAG
AGTTGTTCTTCTACAGGACAGATAGTCCTCTTGATGAATATGTTGCAAGCCAGGGACACGTCCTTCGAACATT
GTTTCGTAACTACAACACTGGAGAGGGCAAAGACGTTAGTGGATTGCCTGTCCAGGATATGTGTTCAGGCATC
AATGCCATCTTTGGTGAAAAGGAGCGAAGTTCTGTCATGTATTCAGTCATACACAGCCGCGCCATTGAAGGCG
AGTCTGGTTATAGGCTG
>TR617|c0_g1_i1 len=1455 path=[1952:0-112 2020:113-225 2010:226-295 1959:296-319 2018:320-332 2026:333-356 2009:357-357 2017:358-381 2030:382-407 2008:408-447 2016:448-459 2029:460-483 2031:484-518 2024:519-542 2032:543-563 2023:564-586 2013:587-607 2027:608-611 2022:612-635 2012:636-1043 1932:1044-1067 2001:1068-1238 1921:1239-1262 2021:1263-1313 1999:1314-1375 1976:1376-1454] [-1, 1952, 2020, 2010, 1959, 2018, 2026, 2009, 2017, 2030, 2008, 2016, 2029, 2031, 2024, 2032, 2023, 2013, 2027, 2022, 2012, 1932, 2001, 1921, 2021, 1999, 1976, -2]
ATCTGGAGCAATATGCATCTTCATTAGAATTGGGCATAATATTTAAACAATGTTGTGATAATTAATCTGGATG
GGAATAGAAAACATGGCTAGTTGGCTTATATCCCACGTAAGTTTTCTTGGTGGTAGTCAAGGTTAATTGAAAC
AACCATATGACCCTTGGAACATTGAACATGAGTGTGGGGGAGCAAAAGAGAAAATACCTACCCAGAGCATCAG
AGAGCATGTGCATTTTCATGACAAATAAGGAATATTTGTGCATCGTGTTGTCACTATTTTATGAAGGAAAGAA
ATCACGGTTATTTAGCTGATTCTGAAAATAACACTCCCATAGTACTGTAGCGTTCAATGAAAAGTAACTGAGG
TGTTGACAAATGTTCAATCATGTCTGGCACGTGAAGACTTGTGTTGTTTACCATGCTTCCTCTTCTTACTTTT
GCGTTCTTCCTTCTCTCTTCTGTATTCTTCCTTCTCTTTGCGTTTCTGCGCTCTCAGCTCCTTCCGTCGCTTT
CTACCCTCCTTATCTTCCCTGTCCATTATTATTTCATCTCCCGCAGAATTGTACTGCTCTGATTGTTGTTGTG
TTTGTGTCCTCCTCTCATCTTTCGCAGGAGCAGCAAATAAATCTTGCCCCGATTGAATATTATACGAACTCGC
CAATTCCTGATCCTTGTAGGCATGACCGGCCGCTCCAGCCTCCCCATCGTCATCATTATCACCGCCATCATCA
TCGTCGCCAGTTTTGAGTAGTTTCTCATCTTCCTTCTCCTGCACATGCTTCGGCAACCGATACTTCTCCACAT
GATCCACCCTCAACGTCCTCCCCAACACGTCACTCCCAGTCAAGTTATCCACCGCCAACACGCAACTCCTTGC
ATCCTCGTACTTGACAAATGCGAATCCCTTACTCTTGCCCGAATCGGCCTCTCGTACCAAGTGTATGTCTTCA
ATTTCTCCGTATTGAGATAAGATACAGAGGACATCGCCTTCGGTTAGATTGTTTGGTAAATTTCCGGCGTACA
CCCATGCGGAATTTTGGTACTTTGTGTGCCAGGAGGCGGAGGTGTGGGTTAGGCCTTTTTCGAGTTCGAGATC
GTTGATTCTTTGGATTTCGGCGATTACGTTCATGATTGAGGAATGATGATTTGTTTATTATTAGGAAGCGCTC
AATATCTCGCGCGTAGAGAATGTGATATATTGTTGTGTATCTTTTATTTGAGTTTAGTCTGCGTTTTGAGGGA
AGGCGCCGTGTGTGTCGTGAGTTGGTGTGACATGCATGAAGGGAGAGTTTGGGAGGAGGATAATATTTCTAAA
TGGATGGATGTCTATTAGGGAAGCAGAGGTGCAATGGAGTATAAAGGATAAAGATTATATTGAGTTCTTAGGT
TTGATCGGTGTTGATGTTGGTGAAGGAAACAGAGCTGGCTGAGTGAGAGTAGAGGTGAGAGGAGCGAG
>TR618|c0_g1_i1 len=2499 path=[2903:0-51 2904:52-98 2957:99-659 2883:660-683 2961:684-1012 2935:1013-1028 @2967@!:1029-1909 2898:1910-1931 2965:1932-2010 2954:2011-2034 2971:2035-2078 2970:2079-2089 2969:2090-2102 2966:2103-2190 2973:2191-2211 2977:2212-2212 2979:2213-2243 2952:2244-2263 2875:2264-2498] [-1, 2903, 2904, 2957, 2883, 2961, 2935, 2967, 2898, 2965, 2954, 2971, 2970, 2969, 2966, 2973, 2977, 2979, 2952, 2875, -2]
GATCTAGCTACTGTACCTTGAACGTCACATCTAACTTTAGGCCCTCATAAAATAAGTATCGAGTGGTATTCAC
GAGAATAACAAAAGCAACGAGTAGCAGCAACTTTCTCAGACCATGTCCGACGCCCCAGATTCCACGAACAAGG
AAAAATCCCGTAAATTTCGGGACACCGGCTCCACCCACTTCGGCGACACCGATTTCGCCGAGGAAACCATAGA
CCAAATCGGCGACGCCACCTGGGGCGAAGTCGCCAGCGCGTGTTGCAACCACGATGCTGAGGGTTGGGGTAAA
ATCTTCATTGGGGCGTGCGGCGCCTTGTTCTTCCTCTACTTTTTCCTCTTCTCTCTCGAATTGCTCGGCAACA
GCGCCAAGGTTTTGGGCGGGTGTTCCGCCGGTGGATTGTTGTCCGAAGATACGAATCCCGTGGCGGGTCTTGT
GATTGGCGAGTTAGCGACGGCGTTGGTGCAGAGTTCTTCGACTACGACGTCGATCGTCGTGACGCTCGTGGGA

GCGGAAGCGGTTGATGTTAAGAGCGGAATCTACCTGATCATGGGTGCTAACATTGGAACCTCTGTCACCAACA
CCATCGTAGCCATGGGCCAGATGGGCAGCGGCGATCAGCTCGAGAGAGCTTTCGCTGGAGCCACCGTCCACGA
TCTGTTCAACCTCCTTTCTGTCGCTGTCCTCTTCCCCATTGAGCTCATCACGGGGTACCTCTACGCCATCACC
AAGGCGATGCTCCCTGATACGGTCGAAAAGGGTGATAAGTGGGCTGGTCCTCTGAAGAAGATTGTCAGCCCAT
TGGCCAGTCGTGTCCTCAAGGCCAATAAGGACGTCATCAAGGACGTTGCTACCAAGAAGGTCGAGTCATGCGA
TTCTTACTACCCTGTCGCTTGCATCGATGGCATTGAGGATTATGCGAACTGTGCCTCCAAGTGCGATAAAGAT
GCTGGAGAGTCATTTGCCAACGGTGACTGCGGACGTGTTGGACTCATCACATGCGACAAAAAAATTGGATGTC
CCGCCTTCTTCCAAAATGGAGCAACCAGGCAAGATGATGCCACCTCTGGAGGTGTATGTCTCTTCCTTTCGTT
GTGTTTGCTCATGGTTTGTTTGATGGGACTGGTCAACGTGTTGCAGCGAGGATTGATGGGTATGTCTACCCGA
ATCATATACAAAGCTACCAAGGTGCCTGGTGTGTTTGCCATCCTTATTGGGGCGGCCATTACTGTTTTGGTAC
AGAGTTCAAGTATTACCACCAGTGTGCTGACTCCCATTGTTGGTCTGGGCGTGATTCAAATTGAGCAGATGCT
TCCGCTCACCCTTGGAGCCAATATCGGTACTACCATTACTGGTCTCCTTGCATCTCTTGTATCAGGCAAGGTT
GAGGCACTCCAAGTAGCTCTTGCTCACCTCTTCTTCAACCTTACTGGAATTCTTATCTGGTACCCAATTCCAT
TCATGCGCATGATTCCCCTAAATGGAGCCAAGGCCCTCGGAAAGTGGACCCAACGCAGTCGCTTAGTCCCTCC
CATTTACATCCTTGTTGCATTCTTCATCATCCCTCTTGGGTTATTGGGATTGTCTGCTTTATTCGCAGAGAAG
ACCACTGGATTCACAGTGCTCGGAGCATTCTTAGTCATTCTCGTGGCAGCCGGAATTGCCCGTTTTGTGTGGT
GGTGGCTGAAGCAAGAAGGAAGGGCTAAGTTCATCACCTTTTTGGACACCCGTTCTGCCATGGCGGAGACGAA
TCGGACATTGCCAGAGGATATGCAATACCTTAAGGCCAAAGTCAGTCAATTGGCAGAACATACGGGTTTGCCT
GAGGACGAGGAAGAAGGTGTGGAGATGGATATTGAAGTGCAGCCAAAGGAGATTAGTGGTGGTACTGCAGAAG
AAGAGGTTGTTGAAGAAGAGGCTGCTGCTGTGACTGAGTCTGCCGATATTGCCAAGTCTGCTGACTGAGACAC
TGAACTTATGACAGGCGCACACGCGGAGTACCAGTAGCAATATTGTGAAGCAAAGCTGATGCAACAGGTTTTC
TGATGCATCGAACGCTGATATTTTGAAGCTTGAATTCTTGGGTCATTATTGGTTGGTCAATGGTGATAGCGAT
TTGAGTACTATGTTGACTGAAAATTAGATCAGTCTGGGCAGTATTCGGCTACAGTTGACTGACGTTTGATGTA
ACCACAAAATTGTACAGTCTGTGGAAATATTATTTGTAACCAGTGAAGTTAGCGTATCAATTGTCAATATGGG
GAAATTTTATATCATTCGCTATTGCTCTTCTATTTCAAAGACATGGTTCCTCATGCCATATCACCCCTATGGT
TTCCATCTATCCATGTATGGTAGAAAACACCAGTATGAATTAACCAACTCAAAATGTTCCTGTCCATTATGAT
GAAGATTGACTGGATCTGTGCCTGTGCACTGCAGTACTCAGTCATAGTCATTAATCCAGGATGAACTAAAAGA
GCCTGAACCACTCGCAG
>TR618|c0_g1_i2 len=2388 path=[2903:0-51 2904:52-98 2957:99-659 2883:660-683 2961:684-1012 2935:1013-1028 @2967@!:1029-1909 2938:1910-1955 2965:1956-2034 2972:2035-2035 2955:2036-2058 2971:2059-2102 2968:2103-2126 2966:2127-2214 2974:2215-2221 2976:2222-2245 2979:2246-2276 2952:2277-2296 2975:2297-2387] [-1, 2903, 2904, 2957, 2883, 2961, 2935, 2967, 2938, 2965, 2972, 2955, 2971, 2968, 2966, 2974, 2976, 2979, 2952, 2975, -2]
GATCTAGCTACTGTACCTTGAACGTCACATCTAACTTTAGGCCCTCATAAAATAAGTATCGAGTGGTATTCAC
GAGAATAACAAAAGCAACGAGTAGCAGCAACTTTCTCAGACCATGTCCGACGCCCCAGATTCCACGAACAAGG
AAAAATCCCGTAAATTTCGGGACACCGGCTCCACCCACTTCGGCGACACCGATTTCGCCGAGGAAACCATAGA
CCAAATCGGCGACGCCACCTGGGGCGAAGTCGCCAGCGCGTGTTGCAACCACGATGCTGAGGGTTGGGGTAAA
ATCTTCATTGGGGCGTGCGGCGCCTTGTTCTTCCTCTACTTTTTCCTCTTCTCTCTCGAATTGCTCGGCAACA
GCGCCAAGGTTTTGGGCGGGTGTTCCGCCGGTGGATTGTTGTCCGAAGATACGAATCCCGTGGCGGGTCTTGT
GATTGGCGAGTTAGCGACGGCGTTGGTGCAGAGTTCTTCGACTACGACGTCGATCGTCGTGACGCTCGTGGGA
GCGGAAGCGGTTGATGTTAAGAGCGGAATCTACCTGATCATGGGTGCTAACATTGGAACCTCTGTCACCAACA
CCATCGTAGCCATGGGCCAGATGGGCAGCGGCGATCAGCTCGAGAGAGCTTTCGCTGGAGCCACCGTCCACGA
TCTGTTCAACCTCCTTTCTGTCGCTGTCCTCTTCCCCATTGAGCTCATCACGGGGTACCTCTACGCCATCACC
AAGGCGATGCTCCCTGATACGGTCGAAAAGGGTGATAAGTGGGCTGGTCCTCTGAAGAAGATTGTCAGCCCAT
TGGCCAGTCGTGTCCTCAAGGCCAATAAGGACGTCATCAAGGACGTTGCTACCAAGAAGGTCGAGTCATGCGA
TTCTTACTACCCTGTCGCTTGCATCGATGGCATTGAGGATTATGCGAACTGTGCCTCCAAGTGCGATAAAGAT
GCTGGAGAGTCATTTGCCAACGGTGACTGCGGACGTGTTGGACTCATCACATGCGACAAAAAAATTGGATGTC
CCGCCTTCTTCCAAAATGGAGCAACCAGGCAAGATGATGCCACCTCTGGAGGTGTATGTCTCTTCCTTTCGTT
GTGTTTGCTCATGGTTTGTTTGATGGGACTGGTCAACGTGTTGCAGCGAGGATTGATGGGTATGTCTACCCGA
ATCATATACAAAGCTACCAAGGTGCCTGGTGTGTTTGCCATCCTTATTGGGGCGGCCATTACTGTTTTGGTAC
AGAGTTCAAGTATTACCACCAGTGTGCTGACTCCCATTGTTGGTCTGGGCGTGATTCAAATTGAGCAGATGCT
TCCGCTCACCCTTGGAGCCAATATCGGTACTACCATTACTGGTCTCCTTGCATCTCTTGTATCAGGCAAGGTT
GAGGCACTCCAAGTAGCTCTTGCTCACCTCTTCTTCAACCTTACTGGAATTCTTATCTGGTACCCAATTCCAT
TCATGCGCATGATTCCCCTAAATGGAGCCAAGGCCCTCGGAAAGTGGACCCAACGCAGTCGCTTAGTCCCTCC
CATTTACATCCTTGTTGCATTCTTCATCATCCCTCTTGGGTTATTGGGATTGTCTGCTTTATTCGCAGAGAAG
ACCACTGGATTCACAGTGCTCGGAGCATTCTTAGTCATTCTCGTGGCAGCCGGAATTGCCCGTTTTGTGTGGT
GGTGGCTGAAGCAAGAAGGAAGGGCTAAGTTCATCACCTTTTTGGACACCCGTTCTGCCATGGCGGAGACGAA
TCGGACATTGCCAGAGGATATGCAATACCTTAAGGCCAAAGTCAGTCAATTGGCAGAACATACGGGTTTGCCT

GAGGACGAGGAAGAAGGTGTGGAGATGGATATTGAAGTGCAGCCAAAGGAGATTAGTGGTGGTACTGCAGAAG
AAGAGGTTGTTGTTGTGGAAGAAGCTGCTGCTGTGGAAGAAGAGGCTGCTGCTGTGACTGAGTCTGCCGATAT
TGCCAAGTCTGCTGACTGAGACACTGAACTTATGACAGGCGCACACGCGGAGTACCAGTAGCAAGATTGTGAA
GCAAAGCTGATGCAACAGGTTTTCTGATGCATCGAACGCTGATATTTTGAAGCTTGAATACTTGGGTCATTAT
TGGTTGGTCAATGGTGATAGCGATTTGAGTACTATGTTGACTGAAAATTAGATCAGTCTGGGCAGTATTCGGC
TACAGTTGACTGACGTTTGATGTAAGCTCAACTACCACAAAATTGTACAGTCTGTGGAAATATTATTTGTAAC
CAGTGAAGTTAGCGTATCAATTGTCAATATGGGGCAATCTTTTATCATTAAACATACTTTATTGCTCCTTTGT
TTTCGTGTCATGGTTCCTCATGAACGATGCCGTTGATGAGAACCAATTACGT
>TR618|c0_g1_i3 len=2477 path=[2900:0-51 2901:52-98 2957:99-659 2883:660-683 2961:684-1012 2935:1013-1028 @2967@!:1029-1909 2938:1910-1955 2965:1956-2034 2972:2035-2035 2908:2036-2067 2969:2068-2080 2966:2081-2168 2973:2169-2189 2977:2190-2190 2979:2191-2221 2952:2222-2241 2875:2242-2476] [-1, 2900, 2901, 2957, 2883, 2961, 2935, 2967, 2938, 2965, 2972, 2908, 2969, 2966, 2973, 2977, 2979, 2952, 2875, -2]
GATCTAGCTACTGTACCTTGAACGTCACATCTAACTTTAGGCCCTCATAAAAAAAGTATCGAGTGGTATTCAC
GAAAATAACAAAAGCAACGAGTAGCAGCAACTTTCTCAGACCATGTCCGACGCCCCAGATTCCACGAACAAGG
AAAAATCCCGTAAATTTCGGGACACCGGCTCCACCCACTTCGGCGACACCGATTTCGCCGAGGAAACCATAGA
CCAAATCGGCGACGCCACCTGGGGCGAAGTCGCCAGCGCGTGTTGCAACCACGATGCTGAGGGTTGGGGTAAA
ATCTTCATTGGGGCGTGCGGCGCCTTGTTCTTCCTCTACTTTTTCCTCTTCTCTCTCGAATTGCTCGGCAACA
GCGCCAAGGTTTTGGGCGGGTGTTCCGCCGGTGGATTGTTGTCCGAAGATACGAATCCCGTGGCGGGTCTTGT
GATTGGCGAGTTAGCGACGGCGTTGGTGCAGAGTTCTTCGACTACGACGTCGATCGTCGTGACGCTCGTGGGA
GCGGAAGCGGTTGATGTTAAGAGCGGAATCTACCTGATCATGGGTGCTAACATTGGAACCTCTGTCACCAACA
CCATCGTAGCCATGGGCCAGATGGGCAGCGGCGATCAGCTCGAGAGAGCTTTCGCTGGAGCCACCGTCCACGA
TCTGTTCAACCTCCTTTCTGTCGCTGTCCTCTTCCCCATTGAGCTCATCACGGGGTACCTCTACGCCATCACC
AAGGCGATGCTCCCTGATACGGTCGAAAAGGGTGATAAGTGGGCTGGTCCTCTGAAGAAGATTGTCAGCCCAT
TGGCCAGTCGTGTCCTCAAGGCCAATAAGGACGTCATCAAGGACGTTGCTACCAAGAAGGTCGAGTCATGCGA
TTCTTACTACCCTGTCGCTTGCATCGATGGCATTGAGGATTATGCGAACTGTGCCTCCAAGTGCGATAAAGAT
GCTGGAGAGTCATTTGCCAACGGTGACTGCGGACGTGTTGGACTCATCACATGCGACAAAAAAATTGGATGTC
CCGCCTTCTTCCAAAATGGAGCAACCAGGCAAGATGATGCCACCTCTGGAGGTGTATGTCTCTTCCTTTCGTT
GTGTTTGCTCATGGTTTGTTTGATGGGACTGGTCAACGTGTTGCAGCGAGGATTGATGGGTATGTCTACCCGA
ATCATATACAAAGCTACCAAGGTGCCTGGTGTGTTTGCCATCCTTATTGGGGCGGCCATTACTGTTTTGGTAC
AGAGTTCAAGTATTACCACCAGTGTGCTGACTCCCATTGTTGGTCTGGGCGTGATTCAAATTGAGCAGATGCT
TCCGCTCACCCTTGGAGCCAATATCGGTACTACCATTACTGGTCTCCTTGCATCTCTTGTATCAGGCAAGGTT
GAGGCACTCCAAGTAGCTCTTGCTCACCTCTTCTTCAACCTTACTGGAATTCTTATCTGGTACCCAATTCCAT
TCATGCGCATGATTCCCCTAAATGGAGCCAAGGCCCTCGGAAAGTGGACCCAACGCAGTCGCTTAGTCCCTCC
CATTTACATCCTTGTTGCATTCTTCATCATCCCTCTTGGGTTATTGGGATTGTCTGCTTTATTCGCAGAGAAG
ACCACTGGATTCACAGTGCTCGGAGCATTCTTAGTCATTCTCGTGGCAGCCGGAATTGCCCGTTTTGTGTGGT
GGTGGCTGAAGCAAGAAGGAAGGGCTAAGTTCATCACCTTTTTGGACACCCGTTCTGCCATGGCGGAGACGAA
TCGGACATTGCCAGAGGATATGCAATACCTTAAGGCCAAAGTCAGTCAATTGGCAGAACATACGGGTTTGCCT
GAGGACGAGGAAGAAGGTGTGGAGATGGATATTGAAGTGCAGCCAAAGGAGATTAGTGGTGGTACTGCAGAAG
AAGAGGTTGTTGTTGTGGAAGAAGCTGCTGCTGTGGAAGAAGAGGCTGCTGCTGTGACTGAGTCTGCCGATAT
TGCCAAGTCTGCTGACTGAGACACTGAACTTATGACAGGCGCACACGCGGAGTACCAGTAGCAAGGTTGTTAT
CTGAAGCTTGAATTCTTGGGTCATTATTGGTTGGTCAATGGTGATAGCGATTTGAGTACTATGTTGACTGAAA
ATTAGATCAGTCTGGGCAGTATTCGGCTACAGTTGACTGACGTTTGATGTAACCACAAAATTGTACAGTCTGT
GGAAATATTATTTGTAACCAGTGAAGTTAGCGTATCAATTGTCAATATGGGGAAATTTTATATCATTCGCTAT
TGCTCTTCTATTTCAAAGACATGGTTCCTCATGCCATATCACCCCTATGGTTTCCATCTATCCATGTATGGTA
GAAAACACCAGTATGAATTAACCAACTCAAAATGTTCCTGTCCATTATGATGAAGATTGACTGGATCTGTGCC
TGTGCACTGCAGTACTCAGTCATAGTCATTAATCCAGGATGAACTAAAAGAGCCTGAACCACTCGCAG
>TR619|c0_g1_i1 len=358 path=[2602:0-39 2603:40-73 2604:74-80 2605:81-357] [-1, 2602, 2603, 2604, 2605, -2]
AACTCAGGCCCAAGTGCTTTTTTGAGTGTCGTGTACATTCTGGTTGTGAGGAAGGTGAGCACGCCCATCGCCA
GGAACGTCTGTTGTGATTGTATTGTGTTGTTTTCGTTGTTGAATGATTGTGGGAACATGGGAGGAAATTGCGT
CATTGATGGCACGGAGTTAGAGGAAAAGAAGCGGAAAATGACTAATCAAGGCAAAGTCAAAAATTGGCGGACA
GGGAAAAATCAAATTATCATACGTAAACTTGAACCCCAACCGCGGAACAAGGAAAAGATCCTCTGTGATGCAC
AAAAAGCATACAAACCAACGGCATACGCGACGACAGACAGATTCGACGGCATGTAATAACATACGC
>TR619|c0_g2_i1 len=2102 path=[2591:0-221 2592:222-245 2668:246-728 2617:729-752 2664:753-764 2656:765-828 2607:829-852 2663:853-882 2671:883-906 2670:907-1269 2662:1270-1286 2654:1287-1320 2666:1321-1327 2659:1328-1344 2652:1345-1410 2638:1411-1434 2658:1435-1536 2649:1537-1560 2651:1561-1596 2641:1597-1620 2642:1621-2101] [-1, 2591, 2592, 2668,

2617, 2664, 2656, 2607, 2663, 2671, 2670, 2662, 2654, 2666, 2659, 2652, 2638, 2658, 2649, 2651, 2641, 2642, -2]
CCAAACTACCATTGCTACTCAATTTTTCATATGTCTCAACATCGCCTTTGAGATAGGGCTAGGCTCTATTGTA
CTGTGGGAACAATATAGGAACTCAAAATTATGAACTTGGGTTTGTGCTCCTGTGCTTAATGCTGCTTTCTTGC
CACTGAATAGAATTGTGACCACCCTTGTCGTCGTGTTGCTTGTATCTGCCGTGAACATATGGAATGGCAATTC
CTCCGTAAGTCAAAACAAAACTGATCAGTGGATGCCGAGGATGGTCCAACCGGGATGGCCGTCAGCTGGGACA
AAAGTGTGATTATTCCTGCAAAAAAAGGGCGAAGAATTATCGTTCGATTTCTGCCCTCTGGTTCTCTCACTCC
AATGGAAGGATTAAAATTCCACCTCAATTATGGCTGACTACCACGTCCACCGTATTTCCCAATATACCATTAA
ATCGTGTACGGCTTGGTATATATTTTGGTAAGGTGATGCTCTACCCAACCAAATGCAAAAAAAGTTGGCTTGC
TATGAATCTCGGTATTGCATGTCGTATTTGAGGGGAACACCCATAAGGTTAATGCAGTGGAATTCCTCGCTCT
TGAGTGAGGCTAAGGATCTATTCCAAAGATTCAATCCTACAATAAATATGCGCACTACCAGGGCTACTAAAAA
AATTGGGTTTATTGTCACCGCATTCCATTCGGACTGTATTAGTAACACAAAAAGTTCCCTACCTCGAATCACT
ACCACTCTCCTGTTTTTTCCTCTTCAACATCTCCTTCATCCTTACGTCGTGCGCCCTAATCTTCTCCCCCACC
TTCCTCATTTTCTCATCCACAATGCTCTCCAATATCATTTCCATAATGGGGAATGCCATGACCACTAGAATTG
CAATGATACCCTCGAACCCAAGTATTGTCGCGTATCTCCAGAATCTACCCCGGAGCCACAGCGATAACACGAA
GGAAATTGCGGAGAACACGGCGAGCAGGGCGGTTGGTTGGTTTTCGAACGAGTTGGAGATGAAGGATGTCATG
ACGAGAGTTTCTCCGAAGAGGAAGGTCCAGGTCCATTCGACGTATTGTATGACGCTCTCCGTGGCGGCGTCGG
TGGTGCTATCTCCGGCCACTGCACTGTCCACTCCCACATCGTCTGTGGTGGAGGCCTCGACGATGAATCGTCG
GCCACGGTACATGGCGAATATTCCGAGCGCAATGATCATTGGAGATATCACATCCTTCATTGTGGCCAGGTTC
AATTTTAACTCAGGCCCAAGTGCTTTTTTGAGTGTCGTGTACATTCTGGTTGTGAGGAAGGTGAGCACGCCCA
TCGCCAGGAACGTCAAAATAGTATACCTCGATATGACCACCGAAACCAACCCCATCATCCCCGTGCGCACCAC
CACCTGCAACACCGCCGCCAAAGTAGCACTCGCCAAATGACCCATCACCGTCTCCGTCTTGCTACTCCCTCCA
TCCTTCCCTGCATCCTCCTGATGAGCCTCCCCACTATCATTCCCTTCCCCATCCTTCTCCGACTTGGCAGCTG
TTGCAGTTGTTTTCGCGTTGTTGTTATTACCTTCCTCGTGATGATTATCCTCCTCGGCATCGTCCTCCAAATC
CAACCCTACCAAATCGTACTCCCGTCGAAGCTTCGCGCTGCTCAGCACGACCTTGGCGCGGTTGAGCATCCGG
AACGTTTCGGCATCGCCCGTTTTGCGATCGGGATGGTGTTTGAGCGAAAGACGACGATAGTTCTTTTTGACCT
GATCGAGGTCTATCCCCCCTCCTTCGGCAGTGGCACCGCTCCCGCCTCCTCCGGCGTCCATGGCGGCTTGGTA
TTTCAATCCCATGGCCTCCGGCGCGGGTTTGGTGATGCCGAGGTAGTTGTAGTATCCATCCGGACTTAGTAGG
ATTTGTATTGCTTCCTCGGTGGGGAGGGCGGTGGGCGTAGTAGTGGTCGTGGAGGCACTGCTGCCGGTGGAGG
AGAAGCTGGCGGATGAGGATGACATGGGTTCGGCACGCGAGGAGGAGGTCGAGTTTTCTGGCATGTTCTATTA
TACGCTGCCGATAGGTATCGTACGATATGCCACTATGTATGTGTTGTAGTACTTGTGT
>TR620|c0_g1_i1 len=1043 path=[1205:0-199 1265:200-216 1275:217-240 1280:241-257 1279:258-268 1277:269-292 1272:293-832 1259:833-865 1231:866-1042] [-1, 1205, 1265, 1275, 1280, 1279, 1277, 1272, 1259, 1231, -2]
TGGCGGACGAAGCAGTGGAGGGGCGTGAGCCACTCCTCGTCTTCGGGCATGGCGAGGGGAAAGTAGTCCGCAG
TTCTATTGTTGGAAGCGGAGGTGGTAGAGGTGAAGGAGGACGAGAGCGGCTTGGGATCGAAGGCTGGCAGGCC
ATTGATGGCTGTTTCGAATTGATCTTCATGGTTACTTCGCAAGTTGTCGAGAGAGCTGCTAGTGGCTGGGGGG
GATGTTTGGGCGGATACTTGGGTAGATGCTTGATGGCAAACTTGATGTTTATGGTACGTTGTGTGCTGCCCAT
CTCTCGTAGCGCTGGCATTGTTCTGGTTCTGCTGCTGTTGTAGTAAGCTATTAAGATATTGACTCTGTGGCGA
CGACAAGTGCGCTTGAGATGCTTGGAGATCATTCAACTGCCTTTGGAAGGAATTGCCGTTGCTTGACATGTAT
TGCCCGATACCGCCTCCCAATGCATTGGACATACCGTACGACGAGTCCACCTGCTGCACCTGTTGCTGTTGCT
GGGCTTGCTGCTGGGCTTGTTGTTGCTGGATATTGAATTGCTGTTCAAGTTGATCCTGCTGCATCTGAAGCTG
GCGCTGCATTTCGCTCTGCTGCTGCTTCTGGAGGAACTGCTGGACCTGAAGATGCATCGGGCAGGAAATCTCG
TGCTCGAGTAGTCGCTCCGATGTGGTGTAGAACTTTTGTTGGCAAAAGCGGCACGACTGACAGCAGGTCTGCG
ACGCTTGATTGGGGCTACTCGACATGGCACTGAGCATGACGTCTATAGCGGAGGCGCCACCGGCGCCGCCGCC
GGGTTGCTGCTGCTGAGAATTGGAAAACCGATGATCATTATGCGATTGACCAATGACATTGATGCCGACGCTG
CCTTGGGGACTGGAGGACGGAGCGGTCGAAATATTCTGACAGTACGAAGAACCATCCATGGCGGCGGCGGCAG
ATGTCGAAAAATTGAAAGGGTGTGCGTTGTTGTTGTTGTTGTTATTGTTGCTGTTGCTTGAGTTATTCATGAT
AACGGAAGAGGGTGCGAACTG
>TR620|c1_g1_i1 len=562 path=[1284:0-151 1285:152-162 1286:163-163 1301:164-206 1320:207-208 1316:209-232 1312:233-387 1236:388-427 1297:428-498 1279:499-522 1280:523-525 1281:526-526 1282:527-550 1283:551-561] [-1, 1284, 1285, 1286, 1301, 1320, 1316, 1312, 1236, 1297, 1279, 1280, 1281, 1282, 1283]
CGGCGCCATTGCTGCTGTTGTTGTTGCTATTGCTGTTGCTGCTTCTTCCCGGATTGTTTTCTCCGTCGGATGA
TCGACGACGATGCGCACTGCCGTTCGAACGATTGTTGTTGTTATTGTTATTGTTGGATTGTTGTTGGGGTTGA
TGCTTGCGAGGGGACGACGACGAAGGTTGGTGGTGAGTGGTGCTGTGGGTACTGTGGTTGCTGTGTTGGGAGG
CAGAATGACCACCGCCGCCGCCACCGCTGTTTCGGCTGTTGGTATTCGAGTGTGGCGGTGGTCTGCTACCACT
GCCAAAGTTTGCATTGGCATTATTAACACGCACGTCGGGAGATACGGGTATCTCGATACTCAACCTCGACGAC

TCGTTCGAATTCGATTGAGGTACGTCGTGGTAATGCTTGAGAGGAGGAGGAGGAGGAGGTGCTTGATGATGTT
GATGATCGTTGTTCGTACGAGAGTACATGCTGTCATACTAATTCGTCGAAGCAGTCGAAGGTGGGGCCTGTCG
CTGTCGTTGCTGGCTCAAGGAAGTGCGAATGGTCGAGGAGCCGCTGTACTC
>TR620|c1_g1_i2 len=497 path=[1226:0-70 1227:71-83 1228:84-94 1307:95-95
1300:96-138 1320:139-140 1316:141-164 1312:165-319 1239:320-362 1297:363-
433 1279:434-457 1280:458-460 1281:461-461 1282:462-485 1283:486-496] [-
1, 1226, 1227, 1228, 1307, 1300, 1320, 1316, 1312, 1239, 1297, 1279,
1280, 1281, 1282, 1283]
TTGGAGGGCTCTTGGGACGACGACGATTCGTTGTTGTTGTTATTGTTATTGTTATTGTTGCATTGTTGTTGGG
GTTGATGCTTGCGAGGGGACGACAACGAAGGTTGGTGGTGAGCGGTGCTGTGGGTACTGTGGTTGCTGTGTTG
GGAGGCAGAATGACCACCGCCGCCGCCACCGCTGTTTCGGCTGTTGGTATTCGAGTGTGGCGGTGGTCTGCTA
CCACTGCCAAAGTTTGCATTGGCATTATTAACACGCACGTCGGGAGATACGGGTATCTCGATACTCAACCTCG
ACGACTCGTTCGAATTCGATTGAGGTACATCGTGGTAATGCTTGCGAGGAGGAGGAGGAGGAGGAGGTGCTTG
ATGATGTTGATGATCGTTGTTCGTACGAGAGTACATGCTGTCATACTAATTCGTCGAAGCAGTCGAAGGTGGG
GCCTGTCGCTGTCGTTGCTGGCTCAAGGAAGTGCGAATGGTCGAGGAGCCGCTGTACTC
>TR620|c1_g2_i1 len=378 path=[1245:0-29 1246:30-30 1247:31-54 1248:55-65
1249:66-89 1299:90-116 1306:117-143 1313:144-171 1308:172-195 1302:196-
206 1273:207-230 1292:231-286 1252:287-310 1253:311-377] [-1, 1245, 1246,
1247, 1248, 1249, 1299, 1306, 1313, 1308, 1302, 1273, 1292, 1252, 1253, -
2]
CCGATCTGCCTGTCGCTGTCGTTGCTGGCTCAAGGAAGTGCGAATGGTCGAGGAGCCGCTGTACTCTTCCCTC
CGCTTCGAGGAACCACCGCTGTACTCGTCCCGCCGCTCTTGGGGTGGTGGCGGTGGCGGCGGCGTAGACTTGT
CATAGTATCTATCGGTGGAAGTACGTTCCTCCTTCGAGTAGACCACCTGACTGGGGATGTGCTCGTTTTCGTT
GTCGTCGTACCCACGAGACGCCAGCGATTCGTACCCCCTATCTTTCGTTCCGCTCGAAGATCGACCACGATGG
TGGTTGTCGTCGTACGATGAATCCTGGTGACGATGATGATGATGATGATGGCGATGTTTCGAAGAGTGCGGAT
GATATCCATACCG
>TR621|c0_g1_i1 len=1632 path=[2130:0-68 2202:69-99 2210:100-116
2188:117-140 2201:141-396 2120:397-420 2121:421-432 2122:433-456
2200:457-501 2141:502-525 2209:526-537 2199:538-561 2219:562-579
2208:580-603 2215:604-651 2146:652-696 2207:697-771 2191:772-795
2197:796-847 2128:848-871 2214:872-916 2170:917-940 2206:941-1189
2220:1190-1220 2213:1221-1282 2205:1283-1371 2167:1372-1409 2216:1410-
1481 2203:1482-1521 2193:1522-1546 2184:1547-1570 2185:1571-1631] [-1,
2130, 2202, 2210, 2188, 2201, 2120, 2121, 2122, 2200, 2141, 2209, 2199,
2219, 2208, 2215, 2146, 2207, 2191, 2197, 2128, 2214, 2170, 2206, 2220,
2213, 2205, 2167, 2216, 2203, 2193, 2184, 2185, -2]
CGCCGCTGGTATCGTATCGTATCGCAGCGAGCAGCGACGCCGCAAATTAAACATTGCCTGCTGCGCCTACCAT
TCCTATCATTATTTTTTACCCAGCCTCGGATCTTTGGACCTCACCTATTTGATCTGATAAAACAACCATGCCA
TAATAGATATTGCGATATTTCCTAAGACGGCACACTGCAACCTGCAACATGTCTCCGATGAATGTCATAGCGA
CCATCCTTCTCGCAACCGCCATCCCTGGCAGTTTTAATGATGTGCTATTCTCAGATGCGTTCCACACACCCCA
TGGTCGCCATCGCACCGCAGCCGCCGCGAACGCCAGAGTCCGTCCTGTTCTGTCAAGCACAATCCTCAGACCC
GAGGTAGAAATCGACGAGACTGCAACCGATTGCACCGGTGACTTTGATACCTCCTTGTCGATGATCGATGACA
ATGAGACCTCCTCGACATCGACAAACGATGAAAAGGAGAATTCCTCAACAGCGAGCTTGAAATTCGCCGGAGA
AGTAATTGTCACTTCCGAACCAGCACCCATGGCAAATAAAGATAAATCTCTGCATGACTTTTTCGCACTTCCT
CAATCGGCCATATTACTGATGCGCGGATCCAAAAATAATCAAGTGAACGAACTTGAAAGCATCGATGCGAAGC
TTTTGAAACAGTATCGCCAAAGCTGTGATAGTGTTAATGCATTGCCACCTACATCTGATGATCGATTCTTTGA
AGTCACTACCTCTGGCGTGAAGTTCCTCCCCGGTCTTCAAGTAATGAATGTTGCAACCATTGGAGTGAAAGTA
ACTAATCAAGAACTCCCTGGATACGAGCTTGTGTTCATCCGAAATCTGACCCGAGCGAAGGGAAACCGTCTCT
TCGTTTGGTTTTTCGATAAGGTGACAGGAAAAGACAAGGAACATAGCAACAAAAAAGACGAGCCTTCCAATGG
CGGGCAGACTACGTTATCTCTCAACAAGATCAGGGTAGTCCCAGCGGAAAATAGCAGCAGCATGATATCCTTT
GAGTCCAATACGTCTTTGAGCATACAAATAGAATTCCCTGCAATTCTTATGAAGGCGATCCCGGGTGCTTCAA
AAGAAAAGTCGGAAAAGACTGGTGGCGATACCCTTCGTAAGGTATTGGAGGATGATTGTCCGGGGTCGCTGGA
AGCGTTTCGACGAGAGTATGTGCGGTGGCTAGAGAGTTAATTGGGAGTTTATTCAAGGCGGTAGATGTTCAAA
AAACCGAAAGTATTGATTTAGGGGAACCGTCCAATCATCATTCTGAAAGATGGTGCGATATGTGACTCAACTT
ATTGAGAATACACGGCGCCTTCATTCGAAGAAATTACCTAGAGCTATCCTATACTTTTATCGAGTTTGGGTTA
TATACCTATGGGGTGAAGTGCACTATCTCTTGACAGAACCTCCGTGCGCCCTAGCAATTTTCATCTTAGCAAC
GTTTGCCATCGCATCGTAAGTAGGTTCGCAACTTGACTGCAACGCAAATGGGGCATACGAACCCTATCAATCT
TAACTTGTGGCTCTTCGATATTCTGACTAGTGCCCTAACAAACAGGAGTAGATAATTGGGAAATACTTAATAT
ATATTCAATTCAAAAAAAAAGATCGG

>TR622|c0_g1_i1 len=278 path=[6705:0-54 6706:55-85 7064:86-139 6525:140-142 6526:143-165 7060:166-246 6621:247-249 6622:250-277] [-1, 6705, 6706, 7064, 6525, 6526, 7060, 6621, 6622, -2]
ATCATTCCCAGTGATAATTTCGGCAGGATGCGTTTGGGCAGGTCTTGATTCTTCGGCTGGAACTTGACAAGCA
ACATTCCCAAGGACAGCTTTTGTAGGAAGCGTTTTTGCAAGTCTCGATTCTTCGGCTAAAACTCGACCGTCAT
CATTCCCAGTGATAATTTCGATAGGAAGGGTTTGGCAGGTCTCGATTCTTTGGCCAAAACTCGACTAGCAACA
TTCCCAGGGATACCTTTCGTATCGAGCGTTTCGGTAGGTATCGATTCTTCAGATCGGAA
>TR622|c0_g2_i1 len=350 path=[6942:0-81 7127:82-93 7066:94-105 7179:106-106 7124:107-130 7237:131-132 7213:133-156 7175:157-190 7057:191-214 7212:215-225 7173:226-227 7055:228-251 7234:252-256 7209:257-259 7168:260-277 7111:278-279 7045:280-314 6488:315-349] [-1, 6942, 7127, 7066, 7179, 7124, 7237, 7213, 7175, 7057, 7212, 7173, 7055, 7234, 7209, 7168, 7111, 7045, 6488, -2]
ATTTCGCGGCGAAGAATCATTGGCGGCGAAGAATTATTGTTGGGCTTTGACGACGACGACGACGACGATATTT
TGGAAACTTGATCTGTCGAGTCAGCTGTGTCGTCGTATTCACCATTACCTAATGTTCGAAGAAAACGTTTCAC
ATTTGATATTAAGTCGCCACCGTCATCTTCGATCGAACTCTCTTCCGGAATTGTTGCATGAATCGGCGTGGGT
TTGTAGTGACTTTCAGACGTGGATGTGTTGGGCGGGGAGTCACGAAGCGTTTTGATAGGACATGATCCTTTAA
CCGAAACTCGATCGACACCACTGGTACGAATAGCCTTTGCTCTGTCAGTATCCGATTT
>TR622|c0_g3_i1 len=2645 path=[6460:0-60 7217:61-88 7239:89-142 7125:143-159 7299:160-193 7177:194-221 @7121@!:222-308 6646:309-317 7172:318-332 7052:333-521 7114:522-1159 6522:1160-1183 7207:1184-1451 7164:1452-1500 7107:1501-1524 7042:1525-1562 6584:1563-1586 7104:1587-1835 6383:1836-1859 7039:1860-1913 7159:1914-1991 7102:1992-2037 7036:2038-2069 @7202@!:2070-2375 6560:2376-2393 @7099@!:2394-2517 6638:2518-2546 6639:2547-2547 7032:2548-2599 6783:2600-2644] [-1, 6460, 7217, 7239, 7125, 7299, 7177, 7121, 6646, 7172, 7052, 7114, 6522, 7207, 7164, 7107, 7042, 6584, 7104, 6383, 7039, 7159, 7102, 7036, 7202, 6560, 7099, 6638, 6639, 7032, 6783, -2]
TCAATTATGATCTATCGATGGTAAATACACTAAAAATGAGATGTGCCTTAATTCTTCTGAAAAAACGAACCTG
GAAAAATAATTACTCAATACAGACATAAGCTGCTACGCACTTTCTCAAATTCAACATCCAAAACAAAAAATTA
TTAAGTACCATTTCCAAAATCAGAGCCATTGATCGCCAAATGAACAGTACAGCCATCACTCAAACTACACCTC
ACATAACACCGCAATCGCCCCTTACTAGTCCACCTTGTCCAACGGGACGCGTCCAACCATTAAATACATGGGC
GTAAAGATGCCCAATTCACCTCCCCGTGCCAAGCCCATGCCGCCGCTCTGAAGCAGAATCTGCGTCCTACTCG
TTCCGGCAGGCGCCAGGCGAAGGAATTCCATTACCTTCAAGGAGTACTTCGTGAGCCTGAGACCAAACCAGTT
GAATTGGAATCGCTGCGTGAACGGATTCCATGACGGCATCAACGGAAGCATCCACGGCTTACCTCCCCGATAG
TGCTGTTTCGCCTCCTTGCTCACCGAATATTCTCCAGTGGCGGGCACCTGCCACCCTCCGTACTCATCATTCA
CCAAATCCCTCTCCTCCAAAATCTCGAACCCGGCCTTCTTCAGCGCCGTGAGACAGTGATGCGTGGACGCAAT
GTCCGGCAACCCATCTCCCTCCTCGATCTGCTTCTTGATCGATACGTGCTCCGGATTATTGGCATCGTACTTG
TCCGTGAGACACCACTCGTAACAGGCGAAGATGGCGCCCGGCTTCAACACGCGGTGAATTTCCGAATAGCAAC
CGACACGGTCGGGAGCGTGGCACGTGGCCTCGATCGCGTAGGCGGCATCGAAGCTCGACGGCTCGAAGGGCTG
CTTCATGAAGTCTCCCTGCACGGACCGGCAGCGCACGTCCGGCAGTCCGTCACCCTTGGCATTCTTAAAGTGG
GCATCGGCGCGACAGAGCTCGTTTCCTCTGTCCACCTGGTACTGATTCAACGTGAGTCCCGTGACGTCGACCC
CGGTGAACTTGCAAATGTTGCGCATGGGCCCGCCGATGCCGCATCCGACGTCCAGCACTTTGACGTTGGCCAT
GGAGGGCGTGCTCCCCTCCCTGGCCTCGTCGCTGTCCACGTAATCTCCGTTCATCTGCCTCCGCACGGCGTCG
CCCACCCTCCCCAATGCAGAGGAAAGGCCCAGTTTGCTCGCGAGGTAGAACTCATGCCGCCGAGTGGCCTCGT
CGAAGGATTCGTGAGGATGGCGATTGGAGAAGTGGAAGGAGGCGCCCCAGCCCCATTCGTAGAAGAGGGTGGC
GAGGTCGTAGTAGGAGTTGACCAGGTTGGTGTAGTTGGAGTTGCGTTCTTCGACCGAGGTTTCGTTCGCGTGG
AGGCGTTCGTACTCGCCCACGGAGGATTTGACATCGGATGCCGAGGTTAGATTTCCCTTCTCCTGTGTGTTAC
GCAGTGTGGATTGTGTGTGGTGTTTGTTCGTGTGAGAAAATAAATGCCATGATAAATCGGAATACATGGTGGG
GTCGCGTTCGATTGTAGATCAATATATAGCATGTTGACATCTTCCTCATTCACCAGAATACAACACGAGGGAG
TCATCATGATGTCATCTAAACTGATCTACTCCATTCATCCTCGGATGGAGAGTTCGAAATCCACGAAGACTCA
ATCTGACATGAGGAATGACAAATTGAAATAAGTTGCCTAACCCTATAACCCTTGGGAAGCGCCCAGCACTTAT
CCAAGATGGATTATCTGCCCTGTAATTGCCACCAGTATCATTCGCAACGACTATCGTGCGGCCAAGCACAGTA
ATGGTCACATTTTCATGTGCCCAAAACTGGAACAGGTCATAATGATGTCATCTAAATTCACCAACTCCATCCA
TGCTAGGATGGAGTTACAAATCCAGGAAGACTCGATCTGACACGAGGATGAAAAAGACGATTTTGACCGTCTC
CGTATGCCCCTGAGAAGCGCCTTCGAATCGCAGTCCTGCTGCCAGATAAAGTAAGGGACAAAAACCACCGTGC
TAACAAACTGAAACAATACACTCTCTATGTCCCTGCCTAGCTGAAGAATCGTTGTCTACTCCACATGAACGAA
AGAGAAGGCATGTACAGAAGCATTTCATCATCGTGCCATCTAAATTCATCAATCCATCCAAAGAATTTGACCT
TAGAAGGATAAGTTCAAATGAAGTTCGGATATCGCGAATGAATCTATCCAACACAGTTGAGCACCATTCCAAT
TTGAACATGATCCTCTTCCGTGCATGTCACCACTTCATCACCGACGTTCACTCGATGTGCGTCCATTCGCACT

GAAACCATCATCCATTCCCTCGGCCCAGCATCTAAAAAAAAACAGTGATCATACATATCGCGTGCCCAAAAAC
TGACGCAGGCCACTTTCTTTGACGCATCGCCACCCGACCACAAAACAGTCTGGATCAATGCTCTCTCAAATCC
TTCCGCGCCGATACTGTGGCACCAACTTCGGTGTGTAGATATTTGAGACGCACAATGCCGAATTCAAGGTTAT
TTCACTTGAAAACCCCATATCTATCTCGACCTGAAAGTACATCGTGTTGCGCCCAGAAGCCACAGAACCATAT
GATAAACACTGATCACT
>TR622|c0_g3_i2 len=1331 path=[6670:0-81 6671:82-93 7065:94-117 7214:118-130 7176:131-132 7252:133-156 7277:157-190 7265:191-195 7251:196-201 7300:202-204 7170:205-228 7233:229-232 7208:233-256 7166:257-259 7108:260-277 7301:278-279 6472:280-283 7231:284-289 7103:290-338 7203:339-357 7100:358-360 7035:361-404 7230:405-427 7156:428-451 7201:452-519 7303:520-529 6969:530-558 7154:559-570 7096:571-765 7304:766-789 7318:790-796 7331:797-803 7200:804-820 7152:821-827 7094:828-846 7305:847-860 7151:861-918 7093:919-932 6974:933-934 7026:935-974 7198:975-1001 7092:1002-1016 7025:1017-1042 6496:1043-1050 7197:1051-1055 7091:1056-1128 6910:1129-1145 6911:1146-1160 6912:1161-1162 7090:1163-1170 7024:1171-1194 6393:1195-1199 7089:1200-1262 6563:1263-1289 7023:1290-1304 7088:1305-1306 7148:1307-1314 7022:1315-1330] [-1, 6670, 6671, 7065, 7214, 7176, 7252, 7277, 7265, 7251, 7300, 7170, 7233, 7208, 7166, 7108, 7301, 6472, 7231, 7103, 7203, 7100, 7035, 7230, 7156, 7201, 7303, 6969, 7154, 7096, 7304, 7318, 7331, 7200, 7152, 7094, 7305, 7151, 7093, 6974, 7026, 7198, 7092, 7025, 6496, 7197, 7091, 6910, 6911, 6912, 7090, 7024, 6393, 7089, 6563, 7023, 7088, 7148, 7022]
ATTTCGCGGCGAAGAATCATTGGCGGCGAAGAATTATTGTTGGGCTTTGACGACGACGACGACGACGATATTT
TGGAAACTTGATCTGTCGAGTGAGCTGTGTCGTCATATTCACCATTACCTAATGTTCGAAGAAAACGTTTCAC
ATTTGATATTAAGTCGCCACCGTCATCTTCGATCGAACTCTCTTCTGGAATTGTTGCATAAATCGGCGTGGGT
TTGTAGTGAGTTTCTGACGTGGATGTGTTGGGCGGGGAGTCACGAAGCGTTTTGATAGGACCTGATCCTTTCG
CTGAAACTCGATCGACACTTCTGATACGAATAGCTTTTGCTCTGTCAGTATCCGATTTTGGTGTATCCGAAGA
GTTCCTATCAGCAACAATCAGGGCTCCAGATCGGGAAGGATCTGCAACCAAATTATTTTCAATCGTTTTGCCT
CTCAAGTTGCTTCTTTTCTCGCATTCATTTGGATCTTTTTCCAAACCACGAAGGCCTTTGGCGGGATCATTTT
CGTGTCCCGAAACTACCTGATGACCAGCAACAGCATCTGATATCGCTCCAGTGTCCCTGATCTTCCTTGTAGT
ATTATTGTTAGAATTCTTCAACGGCATAATTGATGAATCAAACAATGAAACGTTGTGGCCACTTTGTAAATCG
CCAATGCTATTCGAGTTGCGTCTAGGTGGCGGCGGCGGCGGCGGAGAAGGATGTGACGATTTGATCTTTGCCG
GGGACACAGGGAGGCTGCTATTACTGATATCGTTGGTCGACGATGAGAAGCCATTGAAGGATCGTCCCGTTTT
ATCAAGACCTGAATCTTTGGCCAAAACTCGACCAGCATCATCCCTAGGGATAGCTTTCTCATGAAGCGTTGTG
GCAGGAAGTGATTCTTCGGTCAAAACTCGACCAGCATCATCCCTAGGGATAGCTTTCGTGGGAAGCGTTATGG
CAAGGCCTGATTCGTTGGCCAGTACTCGATCAGCGACATTAGTAGGAATAGCTTTCGTAGGAAGCGTTATGGC
AGGGCCAGATTCGTTGACCAGTACTCGATCCGCAGCATCCGTAGAGAAAGTGTTCGTCGGAAGTGTTTTGGCA
GGGCCCGATTCGTTGGCCAGTACTCGACCAGCGACATTAGTAGGAATAGCTTTCGTAGGAAGCGTTTTCGCAG
GGCTAGATTCGTTGACCAGTACTCGATCCGCAACATCCGTAGAGAAAGTTTTCGTGGGAAGTGTTTTGGCAGG
GCCTGATTCGTTGGCCAGTACTCGATCAGCGACATTAGTAGGAATAGCTTTCGTAGGAAGCGTTTTCGCAGGG
CCAGATTCGTTGACCAG
>TR622|c0_g3_i3 len=1561 path=[6460:0-60 7217:61-88 7239:89-142 7125:143-159 7299:160-193 7177:194-221 @7121@!:222-308 6646:309-317 7172:318-332 7052:333-521 7114:522-1159 6522:1160-1183 7207:1184-1451 6644:1452-1560] [-1, 6460, 7217, 7239, 7125, 7299, 7177, 7121, 6646, 7172, 7052, 7114, 6522, 7207, 6644, -2]
TCAATTATGATCTATCGATGGTAAATACACTAAAAATGAGATGTGCCTTAATTCTTCTGAAAAAACGAACCTG
GAAAAATAATTACTCAATACAGACATAAGCTGCTACGCACTTTCTCAAATTCAACATCCAAAACAAAAAATTA
TTAAGTACCATTTCCAAAATCAGAGCCATTGATCGCCAAATGAACAGTACAGCCATCACTCAAACTACACCTC
ACATAACACCGCAATCGCCCCTTACTAGTCCACCTTGTCCAACGGGACGCGTCCAACCATTAAATACATGGGC
GTAAAGATGCCCAATTCACCTCCCCGTGCCAAGCCCATGCCGCCGCTCTGAAGCAGAATCTGCGTCCTACTCG
TTCCGGCAGGCGCCAGGCGAAGGAATTCCATTACCTTCAAGGAGTACTTCGTGAGCCTGAGACCAAACCAGTT
GAATTGGAATCGCTGCGTGAACGGATTCCATGACGGCATCAACGGAAGCATCCACGGCTTACCTCCCCGATAG
TGCTGTTTCGCCTCCTTGCTCACCGAATATTCTCCAGTGGCGGGCACCTGCCACCCTCCGTACTCATCATTCA
CCAAATCCCTCTCCTCCAAAATCTCGAACCCGGCCTTCTTCAGCGCCGTGAGACAGTGATGCGTGGACGCAAT
GTCCGGCAACCCATCTCCCTCCTCGATCTGCTTCTTGATCGATACGTGCTCCGGATTATTGGCATCGTACTTG
TCCGTGAGACACCACTCGTAACAGGCGAAGATGGCGCCCGGCTTCAACACGCGGTGAATTTCCGAATAGCAAC
CGACACGGTCGGGAGCGTGGCACGTGGCCTCGATCGCGTAGGCGGCATCGAAGCTCGACGGCTCGAAGGGCTG
CTTCATGAAGTCTCCCTGCACGGACCGGCAGCGCACGTCCGGCAGTCCGTCACCCTTGGCATTCTTAAAGTGG
GCATCGGCGCGACAGAGCTCGTTTCCTCTGTCCACCTGGTACTGATTCAACGTGAGTCCCGTGACGTCGACCC

CGGTGAACTTGCAAATGTTGCGCATGGGCCCGCCGATGCCGCATCCGACGTCCAGCACTTTGACGTTGGCCAT
GGAGGGCGTGCTCCCCTCCCTGGCCTCGTCGCTGTCCACGTAATCTCCGTTCATCTGCCTCCGCACGGCGTCG
CCCACCCTCCCCAATGCAGAGGAAAGGCCCAGTTTGCTCGCGAGGTAGAACTCATGCCGCCGAGTGGCCTCGT
CGAAGGATTCGTGAGGATGGCGATTGGAGAAGTGGAAGGAGGCGCCCCAGCCCCATTCGTAGAAGAGGGTGGC
GAGGTCGTAGTAGGAGTTGACCAGGTTGGTGTAGTTGGAGTTGCGTTCTTCGACCGAGGTTTCGTTCGCGTGG
AGGCGTTCGTACTCGCCCACGGAGGATTTGACATCGGATGCCGAGGTTAGATTTCCCTTCTCCTGGCGTTTCA
ACTGCTTCAAAAAACGGCAGCTCTTCCCCCATGGATCGGCAGGCGTTACGACCTGTACGATGATCGTGGCGGC
GAGCGTCGCGGCCGTGGATAATACGAGG
>TR622|c0_g3_i4 len=883 path=[6798:0-81 6799:82-93 7181:94-105 7253:106-106 7238:107-117 7214:118-130 7176:131-132 7252:133-156 7277:157-190 7265:191-195 7251:196-201 7171:202-225 7288:226-227 7276:228-228 7264:229-232 7250:233-251 7232:252-256 7302:257-259 7041:260-277 7106:278-338 7203:339-357 7037:358-404 7230:405-427 7156:428-451 7201:452-519 7303:520-529 6969:530-558 7154:559-570 7096:571-765 7304:766-789 7318:790-796 7331:797-803 7200:804-820 7152:821-827 7094:828-846 7305:847-860 6379:861-862 6380:863-874 7090:875-882] [-1, 6798, 6799, 7181, 7253, 7238, 7214, 7176, 7252, 7277, 7265, 7251, 7171, 7288, 7276, 7264, 7250, 7232, 7302, 7041, 7106, 7203, 7037, 7230, 7156, 7201, 7303, 6969, 7154, 7096, 7304, 7318, 7331, 7200, 7152, 7094, 7305, 6379, 6380, 7090]
ATTTCGCGGCGAAGAATCATTGGCGGCGAAGAATTATTGTTGGGCTTTGACGACGACGACGACGACGATATTT
TGGAAACTTGATCTGTCGAGTCAGCTGTGTCGTCATATTCACCATTACCTAATGTTCGAAGAAAACGTTTCAC
ATTTGATATTAAGTCGCCACCGTCATCTTCGATCGAACTCTCTTCTGGAATTGTTGTATGAATCGGCGTGGGT
TTGTAGTGAGTTTCAGACGTGGATGTGTTGGGCGGGGAGTCACGAAGCGTTTTGATAGGGCTTGATTCTTTGG
CCGAAACTCGATGGACACCACTGGTACGAATAGCTTTTGCTCTGTCAGTATCCGATTTTGGTGTATATGAAGA
GTTCCTATCAGCAACAGTCAGGGCTCCAGATCGGGAAGGATCTGCAACCAAATTATTTTCAATCGTTTTGCCT
CTCAAGTTGCTTCTTTTCTCGCATTCATTTGGATCTTTTTCCAAACCACGAAGGCCTTTGGCGGGATCATTTT
CGTGTCCCGAAACTACCTGATGACCAGCAACAGCATCTGATATCGCTCCAGTGTCCCTGATCTTCCTTGTAGT
ATTATTGTTAGAATTCTTCAACGGCATAATTGATGAATCAAACAATGAAACGTTGTGGCCACTTTGTAAATCG
CCAATGCTATTCGAGTTGCGTCTAGGTGGCGGCGGCGGCGGCGGAGAAGGATGTGACGATTTGATCTTTGCCG
GGGACACAGGGAGGCTGCTATTACTGATATCGTTGGTCGACGATGAGAAGCCATTGAAGGATCGTCCCGTTTT
ATCAAGACCTGAATCTTTGGCCAAAACTCGACCAGCATCATCCCTAGGGATAGCTTTCGTAGGAAGCGTTTTC
GCAGGGC
>TR622|c0_g4_i1 len=586 path=[6608:0-95 7308:96-140 7085:141-165 7325:166-166 7338:167-167 7220:168-190 7314:191-196 7327:197-203 7010:204-228 7136:229-246 7077:247-280 7008:281-303 6709:304-305 7075:306-363 6579:364-382 7007:383-419 7134:420-423 7074:424-467 7184:468-469 7133:470-494 7073:495-497 7005:498-521 6859:522-525 6860:526-527 7317:528-585] [-1, 6608, 7308, 7085, 7325, 7338, 7220, 7314, 7327, 7010, 7136, 7077, 7008, 6709, 7075, 6579, 7007, 7134, 7074, 7184, 7133, 7073, 7005, 6859, 6860, 7317]
CGTTGACCAGTACTCGATCCGCAGCATCCGTAGAGAAAGTGTTCGTCGGAAGTGTTTTGGCAGGGCCCGATTC
GTTGGCCAGTACTCGACCAGCGAAATCGTTTCTAGGAATAGCTTTCGTATCAGGCGTTTTGGAAGGACGTGGT
TCTTCGGCTAAAACTCGACCGTCATCATTCCCAGTGATAATTTCGGTAGGAAGCGTTTTTGCAGGTCTCGATT
CTTCGGCTGGAACTTGACCCGCAACATTTCTATGGATGGCTTTCGTATCGAGCGTTTTGGCAGGACCCGATTC
TTCGGCCAAAACTCGATCAGCGCCATTCCTACTTCTAGTGATGATTTCTGTAGGAAGCGTTTCGGCAGGTATC
GATTCTTCGGCAGGAACTTGACCCGCAACATTCGTAGCGATGGCTTTGACGTGATGCATACTACGAGGATCCG
ACTCTTTGGCCGGTATTCGACCGGCAACATCCACAGGGATGGCATTCGCGTCAAGCATTCCATTGGGACCCGA
TTCTTCGGCCAAAACTCGATCAGCGCCATTCCTACTTCTAGTGATGATTTCTGTAGGAAGCGTTTCGGCAGGT
AT
>TR622|c0_g4_i2 len=730 path=[7091:0-95 7308:96-140 7086:141-165 7325:166-166 7338:167-167 7140:168-190 7082:191-196 7015:197-223 7187:224-254 7139:255-308 7081:309-311 7220:312-334 7314:335-340 7327:341-347 7185:348-355 7137:356-372 7078:373-390 7009:391-416 7135:417-419 7076:420-424 7008:425-447 6709:448-449 7075:450-507 6579:508-526 7007:527-563 7134:564-567 7074:568-611 7184:612-613 7133:614-638 7073:639-641 7005:642-665 6859:666-669 6860:670-671 7317:672-729] [7091, 7308, 7086, 7325, 7338, 7140, 7082, 7015, 7187, 7139, 7081, 7220, 7314, 7327, 7185, 7137, 7078, 7009, 7135, 7076, 7008,

6709, 7075, 6579, 7007, 7134, 7074, 7184, 7133, 7073, 7005, 6859, 6860, 7317]
CGTTGACCAGTACTCGATCCGCAGCATCCGTAGAGAAAGTGTTCGTCGGAAGTGTTTTGGCAGGGCCCGATTC
GTTGGCCAGTACTCGACCAGCGAAATCGTTTCTAGGAATAGCTTTCGTATCAGGCGTTTTGGAAGGACCCGGT
TCTTCGGCTAAAACTCGACCGTCATCATTCCCAGTGATAATTTCGGTAGGATGCTTTTGGGCAGGTCTTGATT
CTTCGGCTGGAACTTGACAAGCAACATTCCCAAGGATAGCTTTTGTAGGAAGCGTTTTGGCAGGACACGATTC
TTCGGCTAAAACTCGACCGTCATCATTCCCAGTGATAATTTCGGTAGGAAGCGTTTCGGCAGGTCTCGATTCT
TCGGCTGGAACTTGACCCGCAACATTCCAAGGGATACCTTTCGTATCGAGCGTTTTGGCAGGACCCGATTCTT
CGGCCAAAACTCGATCAGCGCCATTCCTACTTCTAGTGATGATTTCTGTAGGAAGCGTTTCGGCAGGTATCGA
TTCTTCGGCAGGAACTTGACCCGCAACATTCGTAGCGATGGCTTTGACGTGATGCATACTACGAGGATCCGAC
TCTTTGGCCGGTATTCGACCGGCAACATCCACAGGGATGGCATTCGCGTCAAGCATTCCATTGGGACCCGATT
CTTCGGCCAAAACTCGATCAGCGCCATTCCTACTTCTAGTGATGATTTCTGTAGGAAGCGTTTCGGCAGGTAT
>TR622|c0_g3_i5 len=350 path=[6798:0-81 6799:82-93 7181:94-105 7253:106-106 7238:107-117 7214:118-130 7176:131-132 7252:133-156 7277:157-190 7265:191-195 7251:196-201 7300:202-204 7170:205-228 7233:229-232 7208:233-256 7166:257-259 7108:260-277 7301:278-279 6820:280-314 6821:315-349] [-1, 6798, 6799, 7181, 7253, 7238, 7214, 7176, 7252, 7277, 7265, 7251, 7300, 7170, 7233, 7208, 7166, 7108, 7301, 6820, 6821, -2]
ATTTCGCGGCGAAGAATCATTGGCGGCGAAGAATTATTGTTGGGCTTTGACGACGACGACGACGACGATATTT
TGGAAACTTGATCTGTCGAGTCAGCTGTGTCGTCATATTCACCATTACCTAATGTTCGAAGAAAACGTTTCAC
ATTTGATATTAAGTCGCCACCGTCATCTTCGATCGAACTCTCTTCTGGAATTGTTGCATAAATCGGCGTGGGT
TTGTAGTGAGTTTCTGACGTGGATGTGTTGGGCGGGGAGTCACGAAGCGTTTTGATAGGACTTGATCCTCTCG
CCGAAACTCGATCGACACCACTGGTACGAATAGCCTTTGCTCTGTCAGTATCCGATTT
>TR622|c0_g3_i6 len=1460 path=[6798:0-81 6799:82-93 7181:94-105 7253:106-106 7238:107-117 7214:118-130 7176:131-132 7252:133-156 7277:157-190 7265:191-195 7118:196-216 7172:217-231 7052:232-420 7114:421-1058 6522:1059-1082 7207:1083-1350 6644:1351-1459] [-1, 6798, 6799, 7181, 7253, 7238, 7214, 7176, 7252, 7277, 7265, 7118, 7172, 7052, 7114, 6522, 7207, 6644, -2]
ATTTCGCGGCGAAGAATCATTGGCGGCGAAGAATTATTGTTGGGCTTTGACGACGACGACGACGACGATATTT
TGGAAACTTGATCTGTCGAGTCAGCTGTGTCGTCATATTCACCATTACCTAATGTTCGAAGAAAACGTTTCAC
ATTTGATATTAAGTCGCCACCGTCATCTTCGATCGAACTCTCTTCTGGAAGATGCCCAATTCACCTCCCCGTG
CCAAGCCCATGCCGCCGCTCTGAAGCAGAATCTGCGTCCTACTCGTTCCGGCAGGCGCCAGGCGAAGGAATTC
CATTACCTTCAAGGAGTACTTCGTGAGCCTGAGACCAAACCAGTTGAATTGGAATCGCTGCGTGAACGGATTC
CATGACGGCATCAACGGAAGCATCCACGGCTTACCTCCCCGATAGTGCTGTTTCGCCTCCTTGCTCACCGAAT
ATTCTCCAGTGGCGGGCACCTGCCACCCTCCGTACTCATCATTCACCAAATCCCTCTCCTCCAAAATCTCGAA
CCCGGCCTTCTTCAGCGCCGTGAGACAGTGATGCGTGGACGCAATGTCCGGCAACCCATCTCCCTCCTCGATC
TGCTTCTTGATCGATACGTGCTCCGGATTATTGGCATCGTACTTGTCCGTGAGACACCACTCGTAACAGGCGA
AGATGGCGCCCGGCTTCAACACGCGGTGAATTTCCGAATAGCAACCGACACGGTCGGGAGCGTGGCACGTGGC
CTCGATCGCGTAGGCGGCATCGAAGCTCGACGGCTCGAAGGGCTGCTTCATGAAGTCTCCCTGCACGGACCGG
CAGCGCACGTCCGGCAGTCCGTCACCCTTGGCATTCTTAAAGTGGGCATCGGCGCGACAGAGCTCGTTTCCTC
TGTCCACCTGGTACTGATTCAACGTGAGTCCCGTGACGTCGACCCCGGTGAACTTGCAAATGTTGCGCATGGG
CCCGCCGATGCCGCATCCGACGTCCAGCACTTTGACGTTGGCCATGGAGGGCGTGCTCCCCTCCCTGGCCTCG
TCGCTGTCCACGTAATCTCCGTTCATCTGCCTCCGCACGGCGTCGCCCACCCTCCCCAATGCAGAGGAAAGGC
CCAGTTTGCTCGCGAGGTAGAACTCATGCCGCCGAGTGGCCTCGTCGAAGGATTCGTGAGGATGGCGATTGGA
GAAGTGGAAGGAGGCGCCCCAGCCCCATTCGTAGAAGAGGGTGGCGAGGTCGTAGTAGGAGTTGACCAGGTTG
GTGTAGTTGGAGTTGCGTTCTTCGACCGAGGTTTCGTTCGCGTGGAGGCGTTCGTACTCGCCCACGGAGGATT
TGACATCGGATGCCGAGGTTAGATTTCCCTTCTCCTGGCGTTTCAACTGCTTCAAAAAACGGCAGCTCTTCCC
CCATGGATCGGCAGGCGTTACGACCTGTACGATGATCGTGGCGGCGAGCGTCGCGGCCGTGGATAATACGAGG
>TR622|c0_g3_i7 len=2542 path=[6798:0-81 6799:82-93 7181:94-105 7253:106-106 7238:107-117 7214:118-130 7176:131-132 7252:133-156 7277:157-190 7265:191-195 7118:196-216 7172:217-231 7052:232-420 7114:421-1058 6522:1059-1082 7207:1083-1350 7164:1351-1399 7107:1400-1423 7042:1424-1461 6584:1462-1485 7104:1486-1734 6383:1735-1758 7039:1759-1812 7159:1813-1890 7102:1891-1936 7036:1937-1968 @7202@!:1969-2274 6437:2275-2290 @7099@!:2291-2414 6638:2415-2443 6639:2444-2444 7032:2445-2496 6783:2497-2541] [-1, 6798, 6799, 7181, 7253, 7238, 7214, 7176, 7252, 7277, 7265, 7118, 7172, 7052, 7114, 6522, 7207, 7164, 7107, 7042, 6584, 7104, 6383, 7039, 7159, 7102, 7036, 7202, 6437, 7099, 6638, 6639, 7032, 6783, -2]

ATTTCGCGGCGAAGAATCATTGGCGGCGAAGAATTATTGTTGGGCTTTGACGACGACGACGACGACGATATTT
TGGAAACTTGATCTGTCGAGTCAGCTGTGTCGTCATATTCACCATTACCTAATGTTCGAAGAAAACGTTTCAC
ATTTGATATTAAGTCGCCACCGTCATCTTCGATCGAACTCTCTTCTGGAAGATGCCCAATTCACCTCCCCGTG
CCAAGCCCATGCCGCCGCTCTGAAGCAGAATCTGCGTCCTACTCGTTCCGGCAGGCGCCAGGCGAAGGAATTC
CATTACCTTCAAGGAGTACTTCGTGAGCCTGAGACCAAACCAGTTGAATTGGAATCGCTGCGTGAACGGATTC
CATGACGGCATCAACGGAAGCATCCACGGCTTACCTCCCCGATAGTGCTGTTTCGCCTCCTTGCTCACCGAAT
ATTCTCCAGTGGCGGGCACCTGCCACCCTCCGTACTCATCATTCACCAAATCCCTCTCCTCCAAAATCTCGAA
CCCGGCCTTCTTCAGCGCCGTGAGACAGTGATGCGTGGACGCAATGTCCGGCAACCCATCTCCCTCCTCGATC
TGCTTCTTGATCGATACGTGCTCCGGATTATTGGCATCGTACTTGTCCGTGAGACACCACTCGTAACAGGCGA
AGATGGCGCCCGGCTTCAACACGCGGTGAATTTCCGAATAGCAACCGACACGGTCGGGAGCGTGGCACGTGGC
CTCGATCGCGTAGGCGGCATCGAAGCTCGACGGCTCGAAGGGCTGCTTCATGAAGTCTCCCTGCACGGACCGG
CAGCGCACGTCCGGCAGTCCGTCACCCTTGGCATTCTTAAAGTGGGCATCGGCGCGACAGAGCTCGTTTCCTC
TGTCCACCTGGTACTGATTCAACGTGAGTCCCGTGACGTCGACCCCGGTGAACTTGCAAATGTTGCGCATGGG
CCCGCCGATGCCGCATCCGACGTCCAGCACTTTGACGTTGGCCATGGAGGGCGTGCTCCCCTCCCTGGCCTCG
TCGCTGTCCACGTAATCTCCGTTCATCTGCCTCCGCACGGCGTCGCCCACCCTCCCCAATGCAGAGGAAAGGC
CCAGTTTGCTCGCGAGGTAGAACTCATGCCGCCGAGTGGCCTCGTCGAAGGATTCGTGAGGATGGCGATTGGA
GAAGTGGAAGGAGGCGCCCCAGCCCCATTCGTAGAAGAGGGTGGCGAGGTCGTAGTAGGAGTTGACCAGGTTG
GTGTAGTTGGAGTTGCGTTCTTCGACCGAGGTTTCGTTCGCGTGGAGGCGTTCGTACTCGCCCACGGAGGATT
TGACATCGGATGCCGAGGTTAGATTTCCCTTCTCCTGTGTGTTACGCAGTGTGGATTGTGTGTGGTGTTTGTT
CGTGTGAGAAAATAAATGCCATGATAAATCGGAATACATGGTGGGGTCGCGTTCGATTGTAGATCAATATATA
GCATGTTGACATCTTCCTCATTCACCAGAATACAACACGAGGGAGTCATCATGATGTCATCTAAACTGATCTA
CTCCATTCATCCTCGGATGGAGAGTTCGAAATCCACGAAGACTCAATCTGACATGAGGAATGACAAATTGAAA
TAAGTTGCCTAACCCTATAACCCTTGGGAAGCGCCCAGCACTTATCCAAGATGGATTATCTGCCCTGTAATTG
CCACCAGTATCATTCGCAACGACTATCGTGCGGCCAAGCACAGTAATGGTCACATTTTCATGTGCCCAAAACT
GGAACAGGTCATAATGATGTCATCTAAATTCACCAACTCCATCCATGCTAGGATGGAGTTACAAATCCAGGAA
GACTCGATCTGACACGAGGATGAAAAAGACGATTTTGACCGTCTCCGTATGCCCCTGAGAAGCGCCTTCGAAT
CGCAGTCCTGCTGCCAGATAAAGTAAGGGACAAAAACCACCGTGCTAACAAACTGAAACAATACACTCTCTAT
GTCCCTGCCTAGCTGAAGAATCGTTGTCTACTCCACATGAACGAAAGAGAAGGCATGTACAGAAGCATTTCAT
CATCGTGCCATCTAAATTCATCAATCCATCCAAAGAATTTGACCTTAGAAGGATAAGTTCAAATGAAGTTCGG
ATATCGCGAATGAATCTATCCAACACAGTTGAGCACCATTCCAATTTGAACATGATCCTCTTCCGTGCATGTC
ACCACTTCATCACCGACGTTCACTCGATGTGCGTCCATTCGCACTGAAACCATCATCCATTCCCTCGGCCCAG
CATCTAAAAAAACAGTGATCATACATATCGCGTGCCCAAAAACTGACGCAGGCCACTTTCTTTGACGCATCGC
CACCCGACCACAAAACAGTCTGGATCAATGCTCTCTCAAATCCTTCCGCGCCGATACTGTGGCACCAACTTCG
GTGTGTAGATATTTGAGACGCACAATGCCGAATTCAAGGTTATTTCACTTGAAAACCCCATATCTATCTCGAC
CTGAAAGTACATCGTGTTGCGCCCAGAAGCCACAGAACCATATGATAAACACTGATCACT
>TR622|c0_g3_i8 len=1090 path=[6798:0-81 6799:82-93 7181:94-105 7253:106-106 7238:107-117 7214:118-130 7176:131-132 7252:133-156 7277:157-190 7265:191-195 7251:196-201 7300:202-204 7169:205-214 7113:215-225 7288:226-227 7276:228-228 7264:229-232 7250:233-251 7232:252-256 7302:257-259 7040:260-283 7231:284-289 7101:290-321 7035:322-365 7230:366-388 7156:389-412 7201:413-480 6452:481-519 7154:520-531 7096:532-726 7304:727-750 7318:751-757 7331:758-764 7200:765-781 7152:782-788 7094:789-807 7305:808-821 7151:822-879 7093:880-893 6974:894-895 7026:896-935 7198:936-962 7092:963-977 7025:978-1003 6496:1004-1011 7197:1012-1016 7091:1017-1089] [-1, 6798, 6799, 7181, 7253, 7238, 7214, 7176, 7252, 7277, 7265, 7251, 7300, 7169, 7113, 7288, 7276, 7264, 7250, 7232, 7302, 7040, 7231, 7101, 7035, 7230, 7156, 7201, 6452, 7154, 7096, 7304, 7318, 7331, 7200, 7152, 7094, 7305, 7151, 7093, 6974, 7026, 7198, 7092, 7025, 6496, 7197, 7091]
ATTTCGCGGCGAAGAATCATTGGCGGCGAAGAATTATTGTTGGGCTTTGACGACGACGACGACGACGATATTT
TGGAAACTTGATCTGTCGAGTCAGCTGTGTCGTCATATTCACCATTACCTAATGTTCGAAGAAAACGTTTCAC
ATTTGATATTAAGTCGCCACCGTCATCTTCGATCGAACTCTCTTCTGGAATTGTTGCATGAATCGGCGTGGGT
TTGTAGTGAGTTTCAGACGTGGATGTGTTGGGCGGGGAGTCGCGAAGCGTTTTGATAGGACCTGATCCTTTTG
CTCTATTAGTATCCGATTTTGGTGTATCCGAAGAGTTCCTATCAGCAACAATCAGGGCTCCAGATCGGGAAGG
ATCTGCAACCAAATTATTTTCAATCGTTTTGCCTCTCAAGTTGCTTCTTTTCTCGCATTCATTTGGATCTTTT
TCCAAACCACGAAGGCCTTTGGCGGGATCATTTTCGTGTCCCGTAACTACCTGATGACAAGCAACAGCATCTG
ATATCGCTCCAGTGTCCCTGATCTTCCTTGTAGTATTATTGTTAGAATTCTTCAACGGCATAATTGATGAATC
AAACAATGAAACGTTGTGGCCACTTTGTAAATCGCCAATGCTATTCGAGTTGCGTCTAGGTGGCGGCGGCGGC
GGCGGAGAAGGATGTGACGATTTGATCTTTGCCGGGGACACAGGGAGGCTGCTATTACTGATATCGTTGGTCG
ACGATGAGAAGCCATTGAAGGATCGTCCCGTTTTATCAAGACCTGAATCTTTGGCCAAAACTCGACCAGCATC

ATCCCTAGGGATAGCTTTCTCATGAAGCGTTGTGGCAGGAAGTGATTCTTCGGTCAAAACTCGACCAGCATCA
TCCCTAGGGATAGCTTTCGTGGGAAGCGTTATGGCAAGGCCTGATTCGTTGGCCAGTACTCGATCAGCGACAT
TAGTAGGAATAGCTTTCGTAGGAAGCGTTATGGCAGGGCCAGATTCGTTGACCAGTACTCGATCCGCAGCATC
CGTAGAGAAAGTGTTCGTCGGAAGTGTTTTGGCAGGGCCCGATTCGTTGGCCAGTACTCGACCAGCGA
>TR623|c0_g1_i1 len=1176 path=[5434:0-166 6269:167-251 6499:252-279
6473:280-280 6441:281-311 6393:312-312 6333:313-358 6515:359-389
6538:390-413 6559:414-415 6329:416-432 6436:433-435 6387:436-441
6325:442-450 6540:451-458 6524:459-463 6513:464-478 6495:479-483
6530:484-502 6523:503-511 6512:512-535 6541:536-536 6429:537-554
6315:555-587 6522:588-600 6511:601-604 6542:605-605 6373:606-629
6491:630-640 6543:641-652 6490:653-676 6461:677-706 6544:707-711
6369:712-735 6487:736-752 6528:753-776 6520:777-785 6485:786-798
6417:799-827 6547:828-840 6561:841-841 6073:842-851 6222:852-1001
6168:1002-1011 6169:1012-1020 6356:1021-1066 6288:1067-1096 6209:1097-
1156 5617:1157-1157 5618:1158-1175] [-1, 5434, 6269, 6499, 6473, 6441,
6393, 6333, 6515, 6538, 6559, 6329, 6436, 6387, 6325, 6540, 6524, 6513,
6495, 6530, 6523, 6512, 6541, 6429, 6315, 6522, 6511, 6542, 6373, 6491,
6543, 6490, 6461, 6544, 6369, 6487, 6528, 6520, 6485, 6417, 6547, 6561,
6073, 6222, 6168, 6169, 6356, 6288, 6209, 5617, 5618, -2]
TATAAGTTGTAAAGCTTAAAAAACAAAACGGGTAAAAATCCCTCAACTTCAGTAGAAACACATCGCAGTATCG
TGTATCGTACTTACGAGTAGCGAATACCACACCAATCAAGTTGACGAAGGAAAATGGCGAGCATGAGAACGAG
CACCAAAGAAGCTGGAGGGGACGGCGGGGAAGCAATTATCATAGCCGCAGGAGGGGAGTCCGCGACGACGGAA
ACAAAGTCATTCGAGGATGCCGAGAAGAGGGGCAAGGGCAAACGCAGCAGGAAAGGCAGACGGCCCAAACATC
GCACAGCCAGCATGGACATGGACTCTTGCGACGACGACATCAGAATCCTCTCTGGCGAAGGAAAGATCGAGTC
TGCAAACGATACTTGTGAAATACATTACGACGATTCGAACATCTGCTTTAATGATTTATTCGCCGACCTCCAA
CACTTCAAGGAGGATAATAATAGTCTAACCATATCAATATCGCATCCTACCTTCATCTCAATCATGGATAACT
TCTCGGCAGTCGGTATGGAAGAGCTACTTAAAAAACGATGGGAAGATCAGTTCGATTCTCTCAAAGACTTTAC
GAAGAAGCATGGCGATTGCAACATGCCTCCGGAACATCCCACCCTCGGTGAATGGGTGAGAATCCAACGTGGA
CATTATGAACTATACAAAAAGCGATTGCCAACTCCATTGACGAAGAAACGGTTCATGAAACTAGAAGAAATTT
TAGGATTTGATGAATATAATAAGTCAAAAGAAGAAGACGCTGACAAGAACCTTACGCTGGATGAAAGAATCAA
TCTGAAGACGGCAGCGAACAATGTGAGGTTAATGGGGAGGGCCAACAGGGCATTCTATGCCAGTGCCCCAGCA
AAAAAGAAATCACCATGCAAGGAAATGGTCGCTGACGAAATACAAAAGATTAGGGAAGCAGGTAACTTTCATA
CATCGGCAGTGCACAGATCCCTCCTGCTGAAAGAGGTCTTTGCTTCGATCGAGGAAGATGAAAGTTCGAATTC
TTCTTTTGCCGATAATAATGATGCTGGTAGAGAAAACGAGAAGAAAAAGCGAAAGGAACAGAAAGACAAGGAA
CAGTACAAAATCTTCAGTTCGTCTATGTCTGCAACATCGACGAGTGATGTTTCCGCCAAAGAACACCTCGAAA
AACAGGAT
>TR623|c0_g1_i2 len=1808 path=[5434:0-166 6269:167-251 6499:252-279
6473:280-280 6441:281-311 6393:312-312 6333:313-358 6515:359-389
6538:390-413 6559:414-415 6329:416-432 6436:433-435 6387:436-441
6325:442-450 6540:451-458 6524:459-463 6513:464-478 6495:479-483
6530:484-502 6523:503-511 6512:512-535 6541:536-536 6428:537-575
6314:576-578 6529:579-587 6522:588-600 6511:601-604 6374:605-629
6491:630-640 6543:641-652 6423:653-667 6489:668-676 6461:677-706
6544:707-711 6510:712-721 6488:722-730 6367:731-735 6487:736-752
6528:753-776 6520:777-785 6485:786-798 6455:799-809 6416:810-880
6548:881-882 6579:883-899 6549:900-902 6358:903-924 6215:925-941
6413:942-954 6355:955-1003 6291:1004-1013 6411:1014-1051 6480:1052-1067
6449:1068-1085 6552:1086-1109 6564:1110-1111 6448:1112-1125 6406:1126-
1149 6553:1150-1152 6565:1153-1153 6571:1154-1171 6344:1172-1205
6501:1206-1257 6475:1258-1312 6401:1313-1349 6500:1350-1373 6443:1374-
1397 6474:1398-1424 6556:1425-1427 6192:1428-1451 6398:1452-1512
5881:1513-1536 6338:1537-1596 6557:1597-1597 6567:1598-1606 5514:1607-
1807] [-1, 5434, 6269, 6499, 6473, 6441, 6393, 6333, 6515, 6538, 6559,
6329, 6436, 6387, 6325, 6540, 6524, 6513, 6495, 6530, 6523, 6512, 6541,
6428, 6314, 6529, 6522, 6511, 6374, 6491, 6543, 6423, 6489, 6461, 6544,
6510, 6488, 6367, 6487, 6528, 6520, 6485, 6455, 6416, 6548, 6579, 6549,
6358, 6215, 6413, 6355, 6291, 6411, 6480, 6449, 6552, 6564, 6448, 6406,
6553, 6565, 6571, 6344, 6501, 6475, 6401, 6500, 6443, 6474, 6556, 6192,
6398, 5881, 6338, 6557, 6567, 5514, -2]

TATAAGTTGTAAAGCTTAAAAAACAAAACGGGTAAAAATCCCTCAACTTCAGTAGAAACACATCGCAGTATCG
TGTATCGTACTTACGAGTAGCGAATACCACACCAATCAAGTTGACGAAGGAAAATGGCGAGCATGAGAACGAG
CACCAAAGAAGCTGGAGGGGACGGCGGGGAAGCAATTATCATAGCCGCAGGAGGGGAGTCCGCGACGACGGAA
ACAAAGTCATTCGAGGATGCCGAGAAGAGGGGCAAGGGCAAACGCAGCAGGAAAGGCAGACGGCCCAAACATC
GCACAGCCAGCATGGACATGGACTCTTGCGACGACGACATCAGAATCCTCTCTGGCGAAGGAAAGATCGAGTC
TGCAAACGATACTTGTGAAATACATTACGACGATTCGAACATCTGCTTTAATGATTTATTCGCCGACCTCCAA
CACTTCAAGGAGGATAATAATAGTCTAACCATATCAATATCGCATCCTACCTTCATCTCAATCATGGATAACT
TCTCGGCAGTCGGTATGGAAGAGCTATTAAAGAGACGATGGGAAGATCAGTTCAATTCTCTCAAAGACTTTAC
GAAGAAGCATGGCGATTGCAATGTGCCTCCGGAACATCCCACCCTCGGTGAATGGGTGAGAATCCAACGCGGA
CATTATGAACTATACAAAAAGCGATTGCCAACTCCATTGACGAAGAAACGGTTCACGAAACTAGAAGAAATTT
TAGGATTTGATGAATATAATAAGTCAAAAGAAGAAGACGCTGACAAGAACCTTACGCTGGATGAAAGAACCAG
TCTGAAGACGGCAACGAACAAGGTGAGGTTAATGGGGAGGACCAACAGGGCATTCCATGCCAGTGTCCCAGCA
AAAAAAAGAAACTACCCGACAAGGAAAAGGTTGCTGCCGAAATAAAAAAGAAGAGGGAAGCAGGTAATTTTCA
TAAAACGGCAGCGCACAGATCGCTCCTGCTGGAAGAGGTCTTTGCTTCGATCGAGGAAGATGAAAAATTGAAT
TCTTCTACTGCCGATAATAATGATGCTGGTGGAGAAAACGAGAAGAAGAAGCGACAGGAAGAAACGGAACAAT
GCAAAATCTTCAGTTCGCCAATGCCTGCAATTTCTACGAGTGATGTTTCCGCCAAAGAACACCTCGAAAAACA
GGATTCATTAACAACTGTTAATGTGTACGAGGAGAAACTGGAGAAACTCAGACGAAAGCTATTGGCGGAAACT
AATCGGACCTGCTACGCTCCCACCACTGAAGAGCATGATATGGTCGTTGAAGAAGAAAGTGGATCAGGCTCTG
GAGAAGAAGATGACGAAGAAGGAATCCTCGGTCCGTCCATGCCTGTGTTATCGATGAGTGTTTCGACCAAAGA
AGACAATGAAAGGTCGGATTTGACCGTTGATACATACGGGGAAAAAATGGAGAAGCTAATTAGACGAAAGCTG
TTAGAAGAAGAAAATGGCAGCATGTGTAAACCTACCAGTGAGAGCTCGGCAAAGGATGACATCAAAGATAGCA
AGGGAATCGTTGCTTTAGATGTGAGGGCACACAACAAGAACCCCTACCGACGACGTGCCACGGATAGTTACCT
TGGTACATCAAAACCTCCACAAGCGCCGACTGGCAGTTATATCTCCGAGGGTTGGTTGGATGGTGATGTACAG
GGTGATAGCTTAGAAAAAATCTGCATAGATCGCAACAAAAACGACGATTCGGCTCCAGGATCACACAAAGAGA
AACCCTACCAACGACGCGCCACAGAAGATTACCTCAGTACATCAATGTCCCAGATC
>TR623|c0_g1_i3 len=742 path=[5788:0-166 6395:167-220 6335:221-279
6267:280-280 6394:281-311 6334:312-312 6265:313-364 6515:365-395
6391:396-438 6330:439-441 6437:442-469 6388:470-484 6326:485-508
6523:509-517 6512:518-541 6541:542-542 6428:543-581 6313:582-606
6511:607-610 6542:611-611 6311:612-646 6463:647-658 6424:659-673
6371:674-727 6488:728-736 6460:737-741] [-1, 5788, 6395, 6335, 6267,
6394, 6334, 6265, 6515, 6391, 6330, 6437, 6388, 6326, 6523, 6512, 6541,
6428, 6313, 6511, 6542, 6311, 6463, 6424, 6371, 6488, 6460]
TATAAGTTGTAAAGCTTAAAAAACAAAACGGGTAAAAATCCCTCAACTTCAGTAGAAACACATCGCAGTATCG
TGTATCGTACTTACGAGTAGCGAATACCACACCAATCAAGTTGACGAAGGAAAATGGCGAGCATGAGAACGAG
CACCAAAGAAGCTGGAGGGGATGGCGGGGAAGCAATCATCATGGCTGCAGGAGGGGAGTCCGCGACGATGGAA
GCAAAGTTACTCGACGGTGCCGAGAAGAAGGACAAGGTCAAACGCAGCAGGAAAGGCAGACGCCCGAAAAATC
GCACAGCCAGCATGGACATGGGCTCTTGCGACGACGACATCAGCATCAGCATCCTCTCTGGCGAAGGAAAGAT
CGAGTCTGCAAACGATACTTGTGAAATACATCACGACGCCTCGAACATCTCCTTTAATGATTTATTCGCCGAC
CTCCGAAATTTCAAGGAGGATAATAATAGTCTAACCATATCAATATCACATCCTACCTTCATCTCAATCATGG
ATAACTTCTCGGCAGTCGGTATGGAAGAGCTATTAAAGAGACGATGGGAAGATCAGTTCAATTCTCTCAAAAC
CTTTACGAAGAAGCATGGCGATTGCAACGTGCCTCTGGATCATCCCACCCTCGGTGAATGGGTGAGAATCCAA
CGCGGACATTATGAACTGTACAAAATGCGATTGCCAACTCCATTAACAAAGAAACGGTTCACGAAACTAGAAG
AAATTTTGGGAT
>TR623|c0_g1_i4 len=2374 path=[5434:0-166 6269:167-251 6499:252-279
6473:280-280 6441:281-311 6393:312-312 6333:313-358 6515:359-389
6538:390-413 6559:414-415 6439:416-450 6389:451-458 6524:459-463
6513:464-478 6495:479-483 6530:484-502 6523:503-511 6512:512-535
6541:536-536 6428:537-575 6314:576-578 6529:579-587 6522:588-600
6511:601-604 6374:605-629 6491:630-640 6543:641-652 6423:653-667
6489:668-676 6461:677-706 6544:707-711 6510:712-721 6488:722-730
6367:731-735 6487:736-752 6528:753-776 6520:777-785 6485:786-798
6455:799-809 6416:810-880 6484:881-887 6454:888-897 6549:898-900
6358:901-922 6215:923-939 6413:940-952 6355:953-1001 6291:1002-1011
6411:1012-1049 6480:1050-1065 6449:1066-1083 6552:1084-1107 6564:1108-
1109 6448:1110-1123 6405:1124-1145 6516:1146-1147 6553:1148-1150
6565:1151-1151 6571:1152-1169 6403:1170-1201 6343:1202-1203 6278:1204-
1255 6444:1256-1306 6400:1307-1657 5873:1658-1658 6274:1659-1761
6555:1762-1782 5767:1783-1805 5768:1806-1815 6189:1816-1876 5642:1877-
1888 5643:1889-1889 5644:1890-1899 5645:1900-1914 5646:1915-1938

6397:1939-1997 5711:1998-2021 6337:2022-2111 6558:2112-2129 5532:2130-2135 5533:2136-2153 5534:2154-2373] [-1, 5434, 6269, 6499, 6473, 6441, 6393, 6333, 6515, 6538, 6559, 6439, 6389, 6524, 6513, 6495, 6530, 6523, 6512, 6541, 6428, 6314, 6529, 6522, 6511, 6374, 6491, 6543, 6423, 6489, 6461, 6544, 6510, 6488, 6367, 6487, 6528, 6520, 6485, 6455, 6416, 6484, 6454, 6549, 6358, 6215, 6413, 6355, 6291, 6411, 6480, 6449, 6552, 6564, 6448, 6405, 6516, 6553, 6565, 6571, 6403, 6343, 6278, 6444, 6400, 5873, 6274, 6555, 5767, 5768, 6189, 5642, 5643, 5644, 5645, 5646, 6397, 5711, 6337, 6558, 5532, 5533, 5534, -2]
TATAAGTTGTAAAGCTTAAAAAACAAAACGGGTAAAAATCCCTCAACTTCAGTAGAAACACATCGCAGTATCG
TGTATCGTACTTACGAGTAGCGAATACCACACCAATCAAGTTGACGAAGGAAAATGGCGAGCATGAGAACGAG
CACCAAAGAAGCTGGAGGGGACGGCGGGGAAGCAATTATCATAGCCGCAGGAGGGGAGTCCGCGACGACGGAA
ACAAAGTCATTCGAGGATGCCGAGAAGAGGGGCAAGGGCAAACGCAGCAGGAAAGGCAGACGGCCCAAACATC
GCACAGCCAGCATGGACATGGACTCTTGCGACGACGACATCAGAATCCTCTCTGGCGAAGGAAAGATCGAGTC
TGCAAACGATACTTGTGAAATACATTACGACGATTCGAACATCTGCTTTAAAAGTTTATTCGACGACCTCCAA
CACTTCAAGGAGGATAATAATAGTCTAACCATATCAATATCGCATCCTACCTTCATCTCAATCATGGATAACT
TCTCGGCAGTCGGTATGGAAGAGCTATTAAAGAGACGATGGGAAGATCAGTTCAATTCTCTCAAAGACTTTAC
GAAGAAGCATGGCGATTGCAATGTGCCTCCGGAACATCCCACCCTCGGTGAATGGGTGAGAATCCAACGCGGA
CATTATGAACTATACAAAAAGCGATTGCCAACTCCATTGACGAAGAAACGGTTCACGAAACTAGAAGAAATTT
TAGGATTTGATGAATATAATAAGTCAAAAGAAGAAGACGCTGACAAGAACCTTACGCTGGATGAAAGAACCAG
TCTGAAGACGGCAACGAACAAGGTGAGGTTAATGGGGAGGACCAACAGGGCATTCCATGCCAGTGTCCCAGCA
AAAAAGAAACTACCCGACAAGGAAAAGGTTGCTGCCGAAATAAAAAAGAAGAGGGAAGCAGGTAATTTTCATA
AAACGGCAGCGCACAGATCGCTCCTGCTGGAAGAGGTCTTTGCTTCGATCGAGGAAGATGAAAAATTGAATTC
TTCTACTGCCGATAATAATGATGCTGGTGGAGAAAACGAGAAGAAGAAGCGACAGGAAGAAACGGAACAATGC
AAAATCTTCAGTTCGCCAATGCCTGCAATGTCTACGAGTGATGTTTCCGCCAAAGAACACCTCGAAAAACAGG
ATCCATTAATAAGTGTTAATGTGTACGAGGAGAAACTGGAGAAACTCAGACGAAAGCTATTGGCGGAAACTAA
TCGGACCTGCTACGCGCCCACTACTGAAGAGTATGATGCGGTCGTTGAAGAAGAAAGTGGTAGCTCCTTTAAA
AACAAGATCTCGACGAATTCGAAGGTGGCAGCGAACAAGGTGAAGGCAATGAGGAAAGCCGCCAAGGCATTTC
CGTCGCTAAAGAAGAAATCGCCCGACAATGAGGTAGCGGTCGCGTCAAAAATACAAAATAAGAGGGGAGAAGT
CAATATTCACCAACCGACAGTTCACAGTTCGCTTTTACTGAAAGATGTATCTACATCGAGTCCCGGAGCAGAA
AAAGAAGAAGCAACCCTAGGCTCGGCCATGCCTGTGATATCGATGAGTGCTTCGAACAAAAAAGACAATGAAA
GGTCGGATTCGACCCTTGATACATACGAGGAAAAAATGGAGAAACTCGTCAGACGAAAGCTGTCAGAAGAAGA
AAATCGTAGCATCTATGAGCCTGCTAGTAAGAGCTTGGCCGATGATGTCAGGAAAGGCAGCAAGGGGAGCGTT
GATTCGGATGTGGGAGCACACAAGAAAAAAACCTACCGACGACGTGCCACGGATGATTGCCTCACTGCATCGA
CGCCCCCAGAAGCGTCAAATCGGCTGAAGCGCAAAAACAAGCCCTACCGACGACGTGCCACGGATGATTGCCT
CAGTTCATCAATGCCTCGACAAACGCCGGATTTGACCGTTGATACATATGGGGAAAAAATGGAGAAACTCATC
AGGCAAAAGCTGTTAGAAGAAGAAAATCGTAGCATTTATAAGCCTACCAGTAAGAGCTCGGTGAAGGATGTCA
TCAAAGACAGCAAGGGAATCGTTGCTTCAGACGCTGAAGCACACGAAAAGGAATCCTCCCTACGGCGTAGTTC
ATCAATGCCCCCCGAAGCGTCGAGTGGCAGTTCTACCTTCGAGGATCGACTGAATCGCAAATTCCACAGTAAA
AACATCGATTCAAATCCAGGGGCACACGGAAAGAAACCCTACCGACGACGTGCCACAGATGATTACCTCAGTG
CATCAATGTCCGCAAAAGTGTCAAGTAGCAGTTCTACCTTCGAGGATCGGCTGATACGTAAATTACAAGGTGA
TAGTGTGGAAGATATCTACAGAGATCGCAACAAAAAGC
>TR623|c0_g1_i5 len=812 path=[5417:0-142 6499:143-170 6473:171-171 6441:172-202 6393:203-203 6333:204-249 6515:250-280 6538:281-304 6559:305-306 6329:307-323 6436:324-326 6387:327-332 6325:333-341 6540:342-349 6524:350-354 6513:355-369 6495:370-374 6530:375-393 6523:394-402 6512:403-426 6541:427-427 6428:428-466 6314:467-469 6529:470-478 6522:479-491 6511:492-495 6374:496-520 6491:521-531 6543:532-543 6423:544-558 6489:559-567 6461:568-597 6544:598-602 6510:603-612 6488:613-621 6460:622-626 6545:627-652 6509:653-668 6486:669-690 6456:691-700 6416:701-771 6548:772-773 6360:774-811] [-1, 5417, 6499, 6473, 6441, 6393, 6333, 6515, 6538, 6559, 6329, 6436, 6387, 6325, 6540, 6524, 6513, 6495, 6530, 6523, 6512, 6541, 6428, 6314, 6529, 6522, 6511, 6374, 6491, 6543, 6423, 6489, 6461, 6544, 6510, 6488, 6460, 6545, 6509, 6486, 6456, 6416, 6548, 6360]
ATCCACTCCAAATACCTCCATAAGTCAAGGCATAAAACAAGATGAAAATGGCGAGCACGAGAAAGAACACCGA
AGATGGTGGGGACGACGGGGAATCAATCACGACGGAAACAAAGTCAGTCGAGGATGCCGAGAAGAGGGGCAAG
GGCAAACGCAGCAGGAAAGGCAGACGGCCCAAACATCGCACAGCCAGCATGGACATGGACTCTTGCGACGACG
ACATCAGAATCCTCTCTGGCGAAGGAAAGATCGAGTCTGCAAACGATACTTGTGAAATACATTACGACGATTC
GAACATCTGCTTTAATGATTTATTCGCCGACCTCCAACACTTCAAGGAGGATAATAATAGTCTAACCATATCA

ATATCGCATCCTACCTTCATCTCAATCATGGATAACTTCTCGGCAGTCGGTATGGAAGAGCTATTAAAGAGAC
GATGGGAAGATCAGTTCAATTCTCTCAAAGACTTTACGAAGAAGCATGGCGATTGCAATGTGCCTCCGGAACA
TCCCACCCTCGGTGAATGGGTGAGAATCCAACGCGGACATTATGAACTATACAAAAAGCGATTGCCAACTCCA
TTGACGAAGAAACGGTTCACGAAACTAGAAGAAATTTTGGGATTTGATGAATATATTAAGTCAAAAAAAGAAG
ACGCTGACAAAAACCTTACGATGGATGAAAGAACCAGTCTGAAGACGGCAACGAACAAGGTGAGGTTAATGGG
GAGGACCAACAGGGCATTCCATGCCAGTGTCCCAGCAAAAAAAAAATTGAATTCGTCTTCTGCCGATAATAAT
GATGCTGGT
>TR623|c0_g1_i6 len=627 path=[5417:0-142 6499:143-170 6473:171-171
6441:172-202 6393:203-203 6333:204-249 6515:250-280 6538:281-304
6263:305-332 6438:333-341 6328:342-349 6259:350-374 6530:375-393
6523:394-402 6512:403-426 6541:427-427 6429:428-445 5975:446-469
6529:470-478 6522:479-491 6511:492-495 6542:496-496 6311:497-531
6463:532-543 6424:544-558 6371:559-612 6488:613-621 6460:622-626] [-1,
5417, 6499, 6473, 6441, 6393, 6333, 6515, 6538, 6263, 6438, 6328, 6259,
6530, 6523, 6512, 6541, 6429, 5975, 6529, 6522, 6511, 6542, 6311, 6463,
6424, 6371, 6488, 6460]
ATCCACTCCAAATACCTCCATAAGTCAAGGCATAAAACAAGATGAAAATGGCGAGCACGAGAAAGAACACCGA
AGATGGTGGGGACGACGGGGAATCAATCACGACGGAAACAAAGTCAGTCGAGGATGCCGAGAAGAGGGGCAAG
GGCAAACGCAGCAGGAAAGGCAGACGGCCCAAACATCGCACAGCCAGCATGGACATGGACTCTTGCGACGACG
ACATCAGAATCCTCTCTGGCGAAGGAAAGATCGAGTCTGCAAACGATACTTGTGAAATACATTACGACGATTC
GAACATCTGCTTTCAAGGTTTATTCGCCGACCTCCAACACTTCAAGGAGGATAATAATCATCTAACCATATCA
ATATCGCATCCTACCTTCATCTCAATCATGGATAACTTCTCGGCAGTCGGTATGGAAGAGCTACTTAAAAAAC
GATGGGAAAATCAGTTCAATTCTCTCAAAGACTTTACGAAGAAGCATGGCGATTGCAACGTGCCTCTGGATCA
TCCCACCCTCGGTGAATGGGTGAGAATCCAACGCGGACATTATGAACTGTACAAAATGCGATTGCCAACTCCA
TTAACAAAGAAACGGTTCACGAAACTAGAAGAAATTTTGGGAT
>TR623|c0_g1_i7 len=1901 path=[5434:0-166 6269:167-251 6499:252-279
6473:280-280 6441:281-311 6393:312-312 6333:313-358 6515:359-389
6538:390-413 6559:414-415 6329:416-432 6436:433-435 6387:436-441
6325:442-450 6540:451-458 6524:459-463 6513:464-478 6495:479-483
6530:484-502 6523:503-511 6512:512-535 6541:536-536 6428:537-575
6314:576-578 6529:579-587 6522:588-600 6511:601-604 6374:605-629
6491:630-640 6543:641-652 6423:653-667 6489:668-676 6461:677-706
6544:707-711 6510:712-721 6488:722-730 6367:731-735 6487:736-752
6528:753-776 6520:777-785 6485:786-798 6455:799-809 6416:810-880
6548:881-882 6579:883-899 6549:900-902 6217:903-954 6355:955-1003
6291:1004-1013 6411:1014-1051 6480:1052-1067 6449:1068-1085 6552:1086-
1109 6564:1110-1111 6284:1112-1118 6407:1119-1147 6347:1148-1149
6281:1150-1152 6202:1153-1153 6402:1154-1203 6343:1204-1205 6278:1206-
1257 6444:1258-1308 6400:1309-1659 6276:1660-1763 6554:1764-1784
6566:1785-1785 5446:1786-1800 6190:1801-1878 5984:1879-1890 5985:1891-
1891 5986:1892-1900] [-1, 5434, 6269, 6499, 6473, 6441, 6393, 6333, 6515,
6538, 6559, 6329, 6436, 6387, 6325, 6540, 6524, 6513, 6495, 6530, 6523,
6512, 6541, 6428, 6314, 6529, 6522, 6511, 6374, 6491, 6543, 6423, 6489,
6461, 6544, 6510, 6488, 6367, 6487, 6528, 6520, 6485, 6455, 6416, 6548,
6579, 6549, 6217, 6355, 6291, 6411, 6480, 6449, 6552, 6564, 6284, 6407,
6347, 6281, 6202, 6402, 6343, 6278, 6444, 6400, 6276, 6554, 6566, 5446,
6190, 5984, 5985, 5986, -2]
TATAAGTTGTAAAGCTTAAAAAACAAAACGGGTAAAAATCCCTCAACTTCAGTAGAAACACATCGCAGTATCG
TGTATCGTACTTACGAGTAGCGAATACCACACCAATCAAGTTGACGAAGGAAAATGGCGAGCATGAGAACGAG
CACCAAAGAAGCTGGAGGGGACGGCGGGGAAGCAATTATCATAGCCGCAGGAGGGGAGTCCGCGACGACGGAA
ACAAAGTCATTCGAGGATGCCGAGAAGAGGGGCAAGGGCAAACGCAGCAGGAAAGGCAGACGGCCCAAACATC
GCACAGCCAGCATGGACATGGACTCTTGCGACGACGACATCAGAATCCTCTCTGGCGAAGGAAAGATCGAGTC
TGCAAACGATACTTGTGAAATACATTACGACGATTCGAACATCTGCTTTAATGATTTATTCGCCGACCTCCAA
CACTTCAAGGAGGATAATAATAGTCTAACCATATCAATATCGCATCCTACCTTCATCTCAATCATGGATAACT
TCTCGGCAGTCGGTATGGAAGAGCTATTAAAGAGACGATGGGAAGATCAGTTCAATTCTCTCAAAGACTTTAC
GAAGAAGCATGGCGATTGCAATGTGCCTCCGGAACATCCCACCCTCGGTGAATGGGTGAGAATCCAACGCGGA
CATTATGAACTATACAAAAAGCGATTGCCAACTCCATTGACGAAGAAACGGTTCACGAAACTAGAAGAAATTT
TAGGATTTGATGAATATAATAAGTCAAAAGAAGAAGACGCTGACAAGAACCTTACGCTGGATGAAAGAACCAG
TCTGAAGACGGCAACGAACAAGGTGAGGTTAATGGGGAGGACCAACAGGGCATTCCATGCCAGTGTCCCAGCA
AAAAAAAGAAACTACCCGACAAGGAAACGGTCGCTGCCGAAATACAGAAGAAGAGAGAAGCAGGTAATTTTCA

TAAAACGGCAGCGCACAGATCGCTCCTGCTGGAAGAGGTCTTTGCTTCGATCGAGGAAGATGAAAAATTGAAT
TCTTCTACTGCCGATAATAATGATGCTGGTGGAGAAAACGAGAAGAAGAAGCGACAGGAAGAAACGGAACAAT
GCAAAATCTTCAGTTCGTCAATGCCTGCAGTGTCTACGAGTGATGTTTCCGCCAAAGAATACCAAGAGGGACC
GGATTCATCAACCAGTGTTAATGTGTACGAGGAGAAACTGGAGAAACTCAGACGAAAGCTATTGGCGGAAACT
AATCGGACCTGCTACGCGCCCACTACTGAAGAGTATGATGCGGTCGTTGAAGAAGAAAGTGGTAGCTCCTTTA
AAAACAAGATCTCGACGAATTCGAAGGTGGCAGCGAACAAGGTGAAGGCAATGAGGAAAGCCGCCAAGGCATT
TCCGTCGCTAAAGAAGAAATCGCCCGACAATGAGGTAGCGGTCGCGTCAAAAATACAAAATAAGAGGGGAGAA
GTCAATATTCACCAACCGACAGTTCACAGTTCGCTTTTACTGAAAGATGTATCTACATCGAGTCCCGGAGCAG
AAAAAGAAGAAGCAACCCTAGGCTCGGCCATGCCTGTGATATCGATGAGTGCTTCGAACAAAAAAGACAATGA
AAGGTCGGATTCGACCCTTGATACATACGAGGAAAAAATGGAGAAACTCGTCAGGCGAAAGCTGTCAGGAGAA
GAAAATCGTAGAATCTATAAGCCTGCTAGTAAGAGCTCGGCCGATGATGTCAGGAAAGACACCAAGGGGAGCG
TTGATTCGGATGTGGGAGCACACAAGAAAAAAAACTACCGGCGACGTGCCACGGATGATTACCTCAGTGCACC
GACGCCCCCAGAAGCGCTAAATCGGCTGAATCGCAAAAACAAGCCCTACCGACGACGTGCCACGGATAGTTAC
CTT
>TR623|c0_g1_i8 len=1067 path=[5417:0-142 6499:143-170 6473:171-171
6441:172-202 6393:203-203 6333:204-249 6515:250-280 6538:281-304
6559:305-306 6329:307-323 6436:324-326 6387:327-332 6325:333-341
6540:342-349 6524:350-354 6513:355-369 6495:370-374 6530:375-393
6523:394-402 6512:403-426 6541:427-427 6428:428-466 6314:467-469
6529:470-478 6522:479-491 6511:492-495 6374:496-520 6491:521-531
6543:532-543 6423:544-558 6489:559-567 6461:568-597 6544:598-602
6510:603-612 6488:613-621 6367:622-626 6487:627-643 6528:644-667
6520:668-676 6485:677-689 6417:690-718 6547:719-731 6561:732-732
6073:733-742 6222:743-892 6168:893-902 6169:903-911 6356:912-957
6288:958-987 6209:988-1047 5617:1048-1048 5618:1049-1066] [-1, 5417,
6499, 6473, 6441, 6393, 6333, 6515, 6538, 6559, 6329, 6436, 6387, 6325,
6540, 6524, 6513, 6495, 6530, 6523, 6512, 6541, 6428, 6314, 6529, 6522,
6511, 6374, 6491, 6543, 6423, 6489, 6461, 6544, 6510, 6488, 6367, 6487,
6528, 6520, 6485, 6417, 6547, 6561, 6073, 6222, 6168, 6169, 6356, 6288,
6209, 5617, 5618, -2]
ATCCACTCCAAATACCTCCATAAGTCAAGGCATAAAACAAGATGAAAATGGCGAGCACGAGAAAGAACACCGA
AGATGGTGGGGACGACGGGGAATCAATCACGACGGAAACAAAGTCAGTCGAGGATGCCGAGAAGAGGGGCAAG
GGCAAACGCAGCAGGAAAGGCAGACGGCCCAAACATCGCACAGCCAGCATGGACATGGACTCTTGCGACGACG
ACATCAGAATCCTCTCTGGCGAAGGAAAGATCGAGTCTGCAAACGATACTTGTGAAATACATTACGACGATTC
GAACATCTGCTTTAATGATTTATTCGCCGACCTCCAACACTTCAAGGAGGATAATAATAGTCTAACCATATCA
ATATCGCATCCTACCTTCATCTCAATCATGGATAACTTCTCGGCAGTCGGTATGGAAGAGCTATTAAAGAGAC
GATGGGAAGATCAGTTCAATTCTCTCAAAGACTTTACGAAGAAGCATGGCGATTGCAATGTGCCTCCGGAACA
TCCCACCCTCGGTGAATGGGTGAGAATCCAACGCGGACATTATGAACTATACAAAAAGCGATTGCCAACTCCA
TTGACGAAGAAACGGTTCACGAAACTAGAAGAAATTTTAGGATTTGATGAATATAATAAGTCAAAAGAAGAAG
ACGCTGACAAGAACCTTACGCTGGATGAAAGAATCAATCTGAAGACGGCAGCGAACAATGTGAGGTTAATGGG
GAGGGCCAACAGGGCATTCTATGCCAGTGCCCCAGCAAAAAAGAAATCACCATGCAAGGAAATGGTCGCTGAC
GAAATACAAAAGATTAGGGAAGCAGGTAACTTTCATACATCGGCAGTGCACAGATCCCTCCTGCTGAAAGAGG
TCTTTGCTTCGATCGAGGAAGATGAAAGTTCGAATTCTTCTTTTGCCGATAATAATGATGCTGGTAGAGAAAA
CGAGAAGAAAAAGCGAAAGGAACAGAAAGACAAGGAACAGTACAAAATCTTCAGTTCGTCTATGTCTGCAACA
TCGACGAGTGATGTTTCCGCCAAAGAACACCTCGAAAAACAGGAT
>TR623|c0_g1_i9 len=1020 path=[5434:0-166 6269:167-251 6499:252-279
6473:280-280 6441:281-311 6393:312-312 6333:313-358 6515:359-389
6538:390-413 6559:414-415 6329:416-432 6436:433-435 6387:436-441
6325:442-450 6540:451-458 6524:459-463 6513:464-478 6495:479-483
6530:484-502 6523:503-511 6512:512-535 6541:536-536 6428:537-575
6313:576-600 6511:601-604 6542:605-605 6311:606-640 6463:641-652
6424:653-667 6371:668-721 6488:722-730 6460:731-735 6545:736-761
6509:762-777 6486:778-799 6456:800-809 6416:810-880 6548:881-882
6360:883-920 6453:921-936 6414:937-967 6357:968-984 6292:985-1013
6516:1014-1015 6553:1016-1018 6565:1019-1019] [-1, 5434, 6269, 6499,
6473, 6441, 6393, 6333, 6515, 6538, 6559, 6329, 6436, 6387, 6325, 6540,
6524, 6513, 6495, 6530, 6523, 6512, 6541, 6428, 6313, 6511, 6542, 6311,
6463, 6424, 6371, 6488, 6460, 6545, 6509, 6486, 6456, 6416, 6548, 6360,
6453, 6414, 6357, 6292, 6516, 6553, 6565]

TATAAGTTGTAAAGCTTAAAAAACAAAACGGGTAAAAATCCCTCAACTTCAGTAGAAACACATCGCAGTATCG
TGTATCGTACTTACGAGTAGCGAATACCACACCAATCAAGTTGACGAAGGAAAATGGCGAGCATGAGAACGAG
CACCAAAGAAGCTGGAGGGGACGGCGGGGAAGCAATTATCATAGCCGCAGGAGGGGAGTCCGCGACGACGGAA
ACAAAGTCATTCGAGGATGCCGAGAAGAGGGGCAAGGGCAAACGCAGCAGGAAAGGCAGACGGCCCAAACATC
GCACAGCCAGCATGGACATGGACTCTTGCGACGACGACATCAGAATCCTCTCTGGCGAAGGAAAGATCGAGTC
TGCAAACGATACTTGTGAAATACATTACGACGATTCGAACATCTGCTTTAATGATTTATTCGCCGACCTCCAA
CACTTCAAGGAGGATAATAATAGTCTAACCATATCAATATCGCATCCTACCTTCATCTCAATCATGGATAACT
TCTCGGCAGTCGGTATGGAAGAGCTATTAAAGAGACGATGGGAAGATCAGTTCAATTCTCTCAAAACCTTTAC
GAAGAAGCATGGCGATTGCAACGTGCCTCTGGATCATCCCACCCTCGGTGAATGGGTGAGAATCCAACGCGGA
CATTATGAACTGTACAAAATGCGATTGCCAACTCCATTAACAAAGAAACGGTTCACGAAACTAGAAGAAATTT
TGGGATTTGATGAATATATTAAGTCAAAAAAAGAAGACGCTGACAAAAACCTTACGATGGATGAAAGAACCAG
TCTGAAGACGGCAACGAACAAGGTGAGGTTAATGGGGAGGACCAACAGGGCATTCCATGCCAGTGTCCCAGCA
AAAAAAAAATTGAATTCGTCTTCTGCCGATAATAATGATGCTGGTGGAGAAAACGAGAAGATGAAACAGAAAG
AACAGGAACTATACAAAATCTTCAGTTCGTCAATGCCTGCAGTGTCTACGAGTGATGTTTCCGCCAAAGAA
>TR624|c0_g1_i1 len=226 path=[1199:0-77 1200:78-225] [-1, 1199, 1200, -2]
CTATTTTTATTGCATTTCGCTTGCATACCAGTAGATGTTTAGACCATCAAGGACCATGGTATTCTACAATGGT
AGTATCAGGCATATCGAAAAGAGGAAAACGAGGAGAGGAGGACAAAACCGAGATACAGTGTACGAAGTAATTA
AAGGCTGGCCCTGGGATCACCATATTCCAGAGGCATGGAGGATAGGCAGGTCGGTCTAATCACAAGGAAAATG
CACAAAG
>TR624|c0_g2_i1 len=468 path=[1206:0-85 1320:86-124 1347:125-125
1344:126-157 1318:158-180 1336:181-181 1327:182-200 1345:201-228
1342:229-268 1348:269-270 1332:271-297 1323:298-322 1308:323-341
1213:342-342 1214:343-467] [-1, 1206, 1320, 1347, 1344, 1318, 1336, 1327,
1345, 1342, 1348, 1332, 1323, 1308, 1213, 1214, -2]
CGCTCTTCCGATCTTTTTTTTGGACACACTTATATCTATTCTTATTCCCTTTGTGCTCTGCTTGATGAGGGAG
GCAGGCGAGCGGAGAGAGAAATCAATTGCATTCGTCGTCTCCATCAATTCAGTATGGTGCATTATGTTGATAA
TTTCATAATCTACAAAAAGAATCACAGTCTGAATATTGTAATACTCGCGAGGGGTTGTTCGTTCCAATAGGTT
TCGCAGCATGTGTCACCACGTGCATACTATTTCAGTTCCTATTGGCAAAAAGGCACATGGTAGGTGCCGTCAC
TAATTTGTCCGGGGCATCATAATGTTTACTTCAAAAGCGCAACAATTGCAATCATCCCGTAACCCCCAACACC
CCCCCCCCTTTACCATGCCTGCATGTGACCCAACACAACAGCCACGATATCGCTTCATGGCCTCGTTTTCTTT
CCGTGGCAATTATCTCTTTCCGTTGAGAGC
>TR624|c0_g2_i2 len=375 path=[1206:0-85 1320:86-124 1347:125-125
1344:126-157 1318:158-180 1336:181-181 1327:182-200 1345:201-228
1342:229-268 1348:269-270 1311:271-276 1331:277-300 1322:301-341
1349:342-342 1297:343-374] [-1, 1206, 1320, 1347, 1344, 1318, 1336, 1327,
1345, 1342, 1348, 1311, 1331, 1322, 1349, 1297, -2]
CGCTCTTCCGATCTTTTTTTTGGACACACTTATATCTATTCTTATTCCCTTTGTGCTCTGCTTGATGAGGGAG
GCAGGCGAGCGGAGAGAGAAATCAATTGCATTCGTCGTCTCCATCAATTCAGTATGGTGCATTATGTTGATAA
TTTCATAATCTACAAAAAGAATCACAGTCTGAATATTGTAATACTCGCGAGGGGTTGTTCGTTCCAATAGGTT
TCGCAGCATGTGTCACCACGTGCATACTATTTCAGTTCCTATTGGCAAAAAGACACATGGTAGGTGCCGCCAC
TAATTTGCCCGGGGCATCATGATGTTCACTTCAAAAGCGCAACAATTGCAAGCGTCCCGTAACCCCCCACCCC
CCCCCCCCCC
>TR624|c0_g2_i3 len=541 path=[1239:0-77 1321:78-197 1347:198-198
1344:199-230 1318:231-253 1336:254-254 1327:255-273 1345:274-301
1342:302-341 1348:342-343 1332:344-370 1323:371-395 1308:396-414
1213:415-415 1214:416-540] [-1, 1239, 1321, 1347, 1344, 1318, 1336, 1327,
1345, 1342, 1348, 1332, 1323, 1308, 1213, 1214, -2]
CTATTTTTATTGCATTTCGCTTGCATACCAGTAGATGTTTAGACCATCAAGGACCATGGTATTCTACAATGGT
AGTATGAAAATGCACAAAGTTGGACACACTGATATCTATTCTTATTGCCTTTGTGCTCTGCTTGATGAGAGAG
GCAGGCGAACAGAGAGAGAAATCAATTGTATTCGTCGTCTCCATCAATTCAGTATGGTGCATTATGTTGATAA
TTTCATAATCTACAAAAAGAATCACAGTCTGAATATTGTAATACTCGCGAGGGGTTGTTCGTTCCAATAGGTT
TCGCAGCATGTGTCACCACGTGCATACTATTTCAGTTCCTATTGGCAAAAAGGCACATGGTAGGTGCCGTCAC
TAATTTGTCCGGGGCATCATAATGTTTACTTCAAAAGCGCAACAATTGCAATCATCCCGTAACCCCCAACACC
CCCCCCCCTTTACCATGCCTGCATGTGACCCAACACAACAGCCACGATATCGCTTCATGGCCTCGTTTTCTTT
CCGTGGCAATTATCTCTTTCCGTTGAGAGC
>TR624|c0_g2_i4 len=448 path=[1239:0-77 1321:78-197 1347:198-198
1344:199-230 1341:231-254 1335:255-273 1326:274-282 1346:283-292
1334:293-341 1348:342-343 1311:344-349 1331:350-373 1322:374-414
1349:415-415 1297:416-447] [-1, 1239, 1321, 1347, 1344, 1341, 1335, 1326,
1346, 1334, 1348, 1311, 1331, 1322, 1349, 1297, -2]

CTATTTTTATTGCATTTCGCTTGCATACCAGTAGATGTTTAGACCATCAAGGACCATGGTATTCTACAATGGT
AGTATGAAAATGCACAAAGTTGGACACACTGATATCTATTCTTATTGCCTTTGTGCTCTGCTTGATGAGAGAG
GCAGGCGAACAGAGAGAGAAATCAATTGTATTCGTCGTCTCCATCAATTCAGTATGGTGCATTATGTTGATAA
TTTCATAATCTAAAAAAAGAATCACAGTCTGAATATTGTAATACTCGCGAGGGGTCGTTTGTTCCAATAGGTT
TTGCAGCATGTGTCACCACGTGCTGCATATTTCAGTTCCTATTGGCAAAAAGACACATGGTAGGTGCCGCCAC
TAATTTGCCCGGGGCATCATGATGTTCACTTCAAAAGCGCAACAATTGCAAGCGTCCCGTAACCCCCCACCCC
CCCCCCCCCC
>TR624|c0_g2_i5 len=375 path=[1215:0-85 1330:86-124 1319:125-125
1343:126-148 1317:149-209 1346:210-219 1334:220-268 1348:269-270
1311:271-276 1331:277-300 1322:301-341 1349:342-342 1297:343-374] [-1,
1215, 1330, 1319, 1343, 1317, 1346, 1334, 1348, 1311, 1331, 1322, 1349,
1297, -2]
CGCTCTTCCGATCTTTTTTTTGGACACACTTATATCTATTCTTATTCCCTTTGTGCTCTGCTTGATGAGGGAG
GCAGGCGAGCGGAGAGAGAAATCAATTGCATTCGTCGTCTCCATCAATTCAGTGTGGTGCAATATGTCGATAA
TTTAGATATCTTCAAAAGGAATCACAGTCTGAATATTGTAGTACTCGCGAGGGGTCGTTTGTTCCAATAGGTT
TTGCAGCATGTGTCACCACGTGCTGCATATTTCAGTTCCTATTGGCAAAAAGACACATGGTAGGTGCCGCCAC
TAATTTGCCCGGGGCATCATGATGTTCACTTCAAAAGCGCAACAATTGCAAGCGTCCCGTAACCCCCCACCCC
CCCCCCCCCC
>TR624|c0_g2_i6 len=460 path=[1280:0-77 1330:78-116 1319:117-117
1343:118-140 1317:141-201 1346:202-211 1334:212-260 1348:261-262
1311:263-268 1331:269-292 1322:293-333 1349:334-334 1274:335-459] [-1,
1280, 1330, 1319, 1343, 1317, 1346, 1334, 1348, 1311, 1331, 1322, 1349,
1274, -2]
TCTTTTTTTTTCAAGACACACTAATATCTATTTTTATTGCCTTTGTGCTCTGCTAGATGAGGGAGGCAGGCGA
GCGGAGAGAGAAATCAATTGCATTCGTCGTCTCCATCAATTCAGTGTGGTGCAATATGTCGATAATTTAGATA
TCTTCAAAAGGAATCACAGTCTGAATATTGTAGTACTCGCGAGGGGTCGTTTGTTCCAATAGGTTTTGCAGCA
TGTGTCACCACGTGCTGCATATTTCAGTTCCTATTGGCAAAAAGACACATGGTAGGTGCCGCCACTAATTTGC
CCGGGGCATCATGATGTTCACTTCAAAAGCGCAACAATTGCAATCATCCCGTAACCCCCAACACCCCCCCCCC
TTTACCATGCCTGCATGTGACCCAACACAACAGCCACGATATCGCTTCATGGCCTCGTTTTCTTTCCGTGGCA
ATTATCTCTTTCCGTTGAGAGC
>TR624|c0_g2_i7 len=460 path=[1280:0-77 1330:78-116 1319:117-117
1343:118-140 1328:141-172 1316:173-173 1335:174-192 1326:193-201
1346:202-211 1313:212-220 1342:221-260 1348:261-262 1332:263-289
1323:290-314 1308:315-333 1213:334-334 1214:335-459] [-1, 1280, 1330,
1319, 1343, 1328, 1316, 1335, 1326, 1346, 1313, 1342, 1348, 1332, 1323,
1308, 1213, 1214, -2]
TCTTTTTTTTTCAAGACACACTAATATCTATTTTTATTGCCTTTGTGCTCTGCTAGATGAGGGAGGCAGGCGA
GCGGAGAGAGAAATCAATTGCATTCGTCGTCTCCATCAATTCAGTGTGGTGCAATATGTCGATAATTTCATAA
TCTTCAAAAAGAATCACAGTCTGAATATTGTAATACTCGCGAGGGGTCGTTTGTTCCAATAGGTTTCGCAGCA
TGTGTCACCACGTGCATACTATTTCAGTTCCTATTGGCAAAAAGGCACATGGTAGGTGCCGTCACTAATTTGT
CCGGGGCATCATAATGTTTACTTCAAAAGCGCAACAATTGCAATCATCCCGTAACCCCCAACACCCCCCCCCC
TTTACCATGCCTGCATGTGACCCAACACAACAGCCACGATATCGCTTCATGGCCTCGTTTTCTTTCCGTGGCA
ATTATCTCTTTCCGTTGAGAGC
>TR624|c1_g1_i1 len=641 path=[6088:0-147 6719:148-182 6908:183-213
6796:214-233 6716:234-257 6906:258-295 6794:296-322 6714:323-399
6231:400-423 6232:424-640] [-1, 6088, 6719, 6908, 6796, 6716, 6906, 6794,
6714, 6231, 6232, -2]
GCCATTGTCATTGAAATTTAATTCATGGTATGAGTACATACTATTTTTGATTCGTGTTTCTTCTTTCAAGTTT
GTGTACTATCTATTCGATTCGGACGTGACATCGAGGCTCTCGAAATCTCTTTTGCAGCGCGCGCCGTTAAAAA
AAAGCATCAAATGTTGTGTGTAAATAACAAAATATTGTCTGACGCGAATATATTGGCGGTTAACATGTGGTCT
GTATCGTTGCTCTGCCTTTTTTCATTTTAGTCTCTCACACGAAAATAGAATATATCATCATGCCTCATTATAT
ATATGTTGTACACTGATACTATAGATTATTTCCATTTTTTCATTTATATATGCATATACATCCATCCGGCTGG
CAGCAGCATCAGAGAAGCAGGAGGAGGTGCCGATACAATAAAACATACTACTTATACTGATTGAGACTTTCCC
CGTGTGTCAAAATTTAAAATCGAAACCACACAACATATACTTATATACTCCAAAAAGCAAAATACCATGATGA
CTTCTTTGGAAACAACGCCGACAACGTTGGAGTCGTACCACCTCCCTCAACACACTCAACTGTATCGTGATCA
CCAGCAGCACCCACGAACGCTGGAGGCCGACGATAACAACAACAACAACAACAACAA
>TR624|c1_g2_i1 len=2884 path=[6006:0-25 6007:26-28 6008:29-31 6009:32-34
6010:35-37 6907:38-40 6795:41-43 6862:44-46 6940:47-49 6793:50-52
6968:53-63 6939:64-100 6712:101-117 6859:118-129 6790:130-362 6710:363-
545 6042:546-569 6789:570-585 6709:586-609 6858:610-696 6370:697-720

6967:721-802 7010:803-840 6903:841-845 6787:846-916 6107:917-958
6994:959-1034 7012:1035-1051 7028:1052-1056 6936:1057-1067 6853:1068-1091
6963:1092-1100 7000:1101-1115 6993:1116-1141 6981:1142-1179 6934:1180-
1205 6898:1206-1332 7013:1333-1334 6775:1335-1358 6961:1359-1360
6992:1361-1410 6979:1411-1446 6960:1447-1481 6932:1482-1509 6978:1510-
1521 7014:1522-1523 6895:1524-1543 6958:1544-1547 7029:1548-1561
7034:1562-1569 6684:1570-1586 6991:1587-1596 6957:1597-1612 6977:1613-
1628 6891:1629-1636 7016:1637-1637 6976:1638-1652 6956:1653-1661
6926:1662-1709 6832:1710-1724 6754:1725-1845 7017:1846-1869 7030:1870-
1897 6753:1898-1921 7018:1922-1940 7031:1941-1943 6828:1944-1959
6989:1960-2012 6974:2013-2049 6923:2050-2068 6884:2069-2073 6824:2074-
2082 7020:2083-2105 6822:2106-2129 6951:2130-2138 6922:2139-2162
6883:2163-2176 6744:2177-2208 6950:2209-2214 6921:2215-2238 6882:2239-
2254 6949:2255-2304 7005:2305-2305 6920:2306-2348 7021:2349-2349
6814:2350-2390 6918:2391-2414 6877:2415-2420 7022:2421-2422 6916:2423-
2471 7032:2472-2478 6809:2479-2481 6729:2482-2542 6643:2543-2565
6150:2566-2566 6151:2567-2605 6721:2606-2637 6344:2638-2661 6632:2662-
2663 6799:2664-2709 6720:2710-2733 6631:2734-2820 6291:2821-2883] [-1,
6006, 6007, 6008, 6009, 6010, 6907, 6795, 6862, 6940, 6793, 6968, 6939,
6712, 6859, 6790, 6710, 6042, 6789, 6709, 6858, 6370, 6967, 7010, 6903,
6787, 6107, 6994, 7012, 7028, 6936, 6853, 6963, 7000, 6993, 6981, 6934,
6898, 7013, 6775, 6961, 6992, 6979, 6960, 6932, 6978, 7014, 6895, 6958,
7029, 7034, 6684, 6991, 6957, 6977, 6891, 7016, 6976, 6956, 6926, 6832,
6754, 7017, 7030, 6753, 7018, 7031, 6828, 6989, 6974, 6923, 6884, 6824,
7020, 6822, 6951, 6922, 6883, 6744, 6950, 6921, 6882, 6949, 7005, 6920,
7021, 6814, 6918, 6877, 7022, 6916, 7032, 6809, 6729, 6643, 6150, 6151,
6721, 6344, 6632, 6799, 6720, 6631, 6291, -2]
ACAACAACAACAACAACAACAACAACAACAACAACAACAACAACAACAACAACGGCGGATACCCTAGCCAGGA
GTTGGGAGACTGCGAGGAACGCAAAAAATCGGCACATCGCTTCCCCACCATTCCCATGACGAGAAGTCCCCCT
CCGCCCCCCGCCCACTCCTCCGTACAACTGCGTCCGAAACCCCCCACCATCGCCGCCGTCCACCACAGACTCC
ACCGGTACGGCGCCGTCGATGCCGGTGCCGACGACGCTGCCGCAACCGCCACGACGACCGCGGAGGAGGGGGG
AGACCACGACGGCGTCGTTGAATGCCTTGGCCCTGGGACCTGTGCTCTGCCCCTTCCGCCCGGATTGCCTCCT
CCTCCGTTGCCCCCCGGCATGCCCCCTCCCAACTTCGGCGCGCCTCCTCCCAACTTTGCCGTCCCTCCTCCCG
TCCAGAAGGAATTGATCCCGCAGCCATCGGCGCCCAGGGCGGATCTCGCCGTGGCCAGCGCCGTCGTGACGAC
GACGACGACGTCATCGTCCGAGGGGAATGCTACCGCCATCGCTCCCGCCGTCGTGACGGTGTTGTTGGGTTCG
GCGAACAACAACAACAGCAATGCCATCACTGCTTCCAATAATGGTGGTGGTAGTACCGCGGAAGGGAAGAAGA
CTGCGGGGAGCGAGGTGGTATTCGCGGGGGTAGAATTGGACGAGGAACGGGAGGAATTATCGATGGAGGAGAA
GAGGATGCAGGTCGGTAGGTATTGGAATTTGGTGGTCAGGGCTTTGGCGAAACGGTGAAAACAAACGAACGAA
CATGCGATGGAGCGAGAGAGAATGGGGAGGGGAATGGGGGATGAGGATGGGGATCTGTAGGGGATATATTCGA
AGGATATAAGGTGGGTGAGTGGATTAGAGAATTTCGATATCGCCCGGGGCATTGGCGATGTATGAAAATGAGC
AGTTACCGTAGTATGCGAATGGACGTAGGGGGATTGTGTATGTAAAGTCGGAAGAAAAGGTTAGTGATGGAGA
AAGAAGGTTTTTTCGAGATATCGCGCCCCTGTTTTCGTTGGGTATTACGCATCACCAGATTGCGCAGATTATC
AGGAGAGATTATGAGGACATATACATAGGTAGAGTCACACTTTGGCAGTATTTCATCGTATTCCCATCTACAT
ATTGCATTTGGCGTGTGTACCATATCGCATTATTTTCGTTAAAGTAACAAGAGAAGGCGGCAGGTGTTGCATC
ATCTCAATGGGACACACACACCGGGGGGAAGGGAGGTAGATTACCCAGGTATATATGATTGATGGTTACATAT
AAGGTAATGTCATTGTTAATAGAAATTATCTCTACTGGCTCTCACTTCCGGGAGGTGGGGTGGGGGAGCAGAG
GCTACAGTGTACCATACCTACACAACACGCTTTTAGGGACGGTGAGAGGTGCACGAGGATAAAACAGTTTCCT
AGTTCCCTAAAAACGGGCAAGTCGTTTTCTGCGAAGATGAAAATTATCGAACGAATGCGACACAAACACCCCA
AGAGGCAAATAAACTTGTGTCACCCTCAAAACCCCCCTCCCCCTACCCGTTGGGGGGGGGAGGCAACCTCGTA
GGGAAAAAGATATTTTGCGCAAGGAAAACAGGTTCCTCTACCGCTCGGGCAATTTTCGGAAATACCGTCTCGA
ATGGGATTGAAAATTATCGAACGAAAAAAAATGTGACACACGCACCCAAAGAGCCTCGAGGCAGATGGACTAT
ATATGCCGTCCACAACTTGAAATACCCACTTGGTGGCGTTTGGGAGAAAGTTGGCTATGGTACCTACACACAC
ACTTCGCAAATATTGCATCGAGGTATACTAAAACACGAAGCGGCCATCTGCTATCTAGAGGAAGAGAAACCTC
GCCACAGGAGAACAACATCGCAAATTGATGTCGTGGCCGTTTTTTTCTTCGGTCGTGCAGACCACGACGACGG
GGTAAAACGATGCTCGCTTTCCTGCGCTGGGTTGTGCCAAGTGCAGTTTACACGCACTCGTGCTAATTCTTGC
GTGTTCGGATTGAATACAATGCTGGGCAAACCAGTGAAGTAAAATATACGTGGCGACATACGTTGCGCGAAGT
CTACTGGCCGATACGGACCCATCGTGTATCCCACAAGCTCCTTCGCAAACCATGGTGCATGGGAATGTAAGAG
AAATGGGCCCTGCTCTCAACGGAAAGAGATAATTGCCACGGAAAGAAAACGAGGCCATGAAGCGATATCGTGG
CTGTTGTGTTGGGTCACATGCAGGCATGGTAAAGGGGGGGGGGGGTTGGGGGTTACGGACGCTTGCAATTGT
TGCGCTTTTGAAGTGAACATCATGATGCCCCGGGCAAATTAGTGGCGGCACCTACCATGTGTCTTTTTGCCAA

TAGGAACTGAAATATGCAGCACGTGGTGACACATGCTGCAAAACCTATTGGAACAAACGACCCCTCGCGAGTA
CTACAATATTCAGACTGTGATTCCTTTTGAAGATATCTAAATTATCGACATATTGCACCACACTGAATTGATG
GAGACGACGAATGCAATTGATTTCTCTCTCCGCTCGCCTGCCTCCCTCATCAAGCAGAGCACAAAGGGAATAA
GAATAGATATAAGTGTGTCCAACTTTGTGCATTTTCCTTGTGATTAGACCGACCTGCCTATCCTCCATGCCTC
TGGAATATGGTGATCCCAGGGCCAGCCTTTAATTACTTCGTACACTGTATCTCGGTTTTGTCCTCCTCTCCTC
GTTTTCCTCTTTTCGATATGCCTGATACTACCATTGTAGAATACCATGGTCCTTGATGGTCTAAACATCTACT
GGTATGCAAGCGAAATGCAATAAAAATAGATATTAGA
>TR624|c1_g2_i4 len=1016 path=[6399:0-100 6400:101-124 6791:125-242
6710:243-425 6042:426-449 6789:450-465 6709:466-489 6858:490-576
6370:577-600 6967:601-682 7010:683-720 6786:721-746 6706:747-817
6966:818-832 6994:833-908 7012:909-925 7028:926-930 6964:931-974
7000:975-989 6993:990-1015] [-1, 6399, 6400, 6791, 6710, 6042, 6789,
6709, 6858, 6370, 6967, 7010, 6786, 6706, 6966, 6994, 7012, 7028, 6964,
7000, 6993]
GGGCGGCGTTCGCTCTCGTCGCGGCCCACGGCTACGACGATTTCGCTTTGACGGAGGATTGCTTCACGGAGGA
GCCTCCGTTGGTATCGTCGTCGTCAATGTCGGATGGCGGTGCGAGCGGCATGGCGCCCTTGCCTCCTCCTCCG
CCTCCTCCTCCCGCGTTTGGAATGCCGGGCATGCCACCGGGGATGCCCGGAATGCCCGGCATGGGCGGTGCTC
CGCCCCTTCCGCCCGGATTGCCTCCTCCTCCGTTGCCCCCCGGCATGCCCCCTCCCAACTTCGGCGCGCCTCC
TCCCAACTTTGCCGTCCCTCCTCCCGTCCAGAAGGAATTGATCCCGCAGCCATCGGCGCCCAGGGCGGATCTC
GCCGTGGCCAGCGCCGTCGTGACGACGACGACGACGTCATCGTCCGAGGGGAATGCTACCGCCATCGCTCCCG
CCGTCGTGACGGTGTTGTTGGGTTCGGCGAACAACAACAACAGCAATGCCATCACTGCTTCCAATAATGGTGG
TGGTAGTACCGCGGAAGGGAAGAAGACTGCGGGGAGCGAGGTGGTATTCGCGGGGGTAGAATTGGACGAGGAA
CGGGAGGAATTATCGATGGAGGAGAAGAGGATGCAGGTCGGTAGGTATTGGAATTTGGTGGTCAGGGCTTTGG
CGAAACGGTGAAAACAAACGAACGAACATGCGATGGAGCGAGAGAGAATGGGGAGGGGAATGGGAGGTGGGGG
CCTGTGTGGGGTGTGTTCGAAGGATAAAAGGTGGGTGAGTGGATGAGAGAATTTCGATATCGCCAGGGGCATT
GGCGATATATGAAAATGAGCAGTTACCGTAGTATGCGAATGGACGTAGGGGGATTGTGTATGTAAAGTCGGAA
GAAAAGGTTAGTGATGGAGAAAGAAGGTTTTTTCGAGATATCGCGCCCCTGTTTTTGTTGGGTATTACGCATC
ACTAGATTGCGCAGATTATCAGGAGAGATTATGAGGACATATACATAGGTAGAGTCACACTTTGGCA
>TR624|c1_g2_i5 len=1202 path=[6006:0-25 6007:26-28 6008:29-31 6009:32-34
6010:35-37 6907:38-40 6795:41-43 6862:44-46 6940:47-49 6793:50-52
6968:53-63 6939:64-100 6904:101-118 6859:119-130 6790:131-363 6710:364-
546 6042:547-570 6789:571-586 6709:587-610 6858:611-697 6370:698-721
6967:722-803 7010:804-841 6786:842-867 6706:868-938 6966:939-953
6994:954-1029 7011:1030-1047 7028:1048-1052 6936:1053-1063 6853:1064-1087
6963:1088-1096 7000:1097-1111 6852:1112-1137 6981:1138-1175 6934:1176-
1201] [-1, 6006, 6007, 6008, 6009, 6010, 6907, 6795, 6862, 6940, 6793,
6968, 6939, 6904, 6859, 6790, 6710, 6042, 6789, 6709, 6858, 6370, 6967,
7010, 6786, 6706, 6966, 6994, 7011, 7028, 6936, 6853, 6963, 7000, 6852,
6981, 6934]
ACAACAACAACAACAACAACAACAACAACAACAACAACAACAACAACAACAACGGCGGATACCCTAGCCAGGA
GTTGGGAGACTGCGAGGAACGCAAAAAAATCGGCACATCGCTTCCCCACCATTCCCATGACGAGAAGTCCCCC
TCCGCCCCCCGCCCACTCCTCCGTACAACTGCGTCCGAAACCCCCCACCATCGCCGCCGTCCACCACAGACTC
CACCGGTACGGCGCCGTCGATGCCGGTGCCGACGACGCTGCCGCAACCGCCACGACGACCGCGGAGGAGGGGG
GAGACCACGACGGCGTCGTTGAATGCCTTGGCCCTGGGACCTGTGCTCTGCCCCTTCCGCCCGGATTGCCTCC
TCCTCCGTTGCCCCCCGGCATGCCCCCTCCCAACTTCGGCGCGCCTCCTCCCAACTTTGCCGTCCCTCCTCCC
GTCCAGAAGGAATTGATCCCGCAGCCATCGGCGCCCAGGGCGGATCTCGCCGTGGCCAGCGCCGTCGTGACGA
CGACGACGACGTCATCGTCCGAGGGGAATGCTACCGCCATCGCTCCCGCCGTCGTGACGGTGTTGTTGGGTTC
GGCGAACAACAACAACAGCAATGCCATCACTGCTTCCAATAATGGTGGTGGTAGTACCGCGGAAGGGAAGAAG
ACTGCGGGGAGCGAGGTGGTATTCGCGGGGGTAGAATTGGACGAGGAACGGGAGGAATTATCGATGGAGGAGA
AGAGGATGCAGGTCGGTAGGTATTGGAATTTGGTGGTCAGGGCTTTGGCGAAACGGTGAAAACAAACGAACGA
ACATGCGATGGAGCGAGAGAGAATGGGGAGGGGAATGGGAGGTGGGGGCCTGTGTGGGGTGTGTTCGAAGGAT
AAAAGGTGGGTGAGTGGATGAGAGAATTTCGATATCGCCAGGGGCATTGGCGATATATGAAAATGAGCAGTTA
CCGTAGTATGCGAATGGACGTAGGGGGATTGTGTATGTAAAGTCGGAAGAAAAGGTTAGTGATGGAGAAAGAA
GGTTTTTTTCGAGATATCGCGCCCCTGTTTTCGTTGGGTATTACGCATCACCAGATTGCGCAGATTATCAGGA
GAGATTATGAGGACATACATATAGGTAGAGTCACACTTTGGCAGTATTTCATCGTATTCCCATCTACATATTG
CATTTGGCGTGTGTACCATATCGCATTATTTTCG
>TR624|c1_g2_i9 len=1087 path=[6399:0-100 6400:101-124 6791:125-242
6710:243-425 6042:426-449 6789:450-465 6709:466-489 6858:490-576
6370:577-600 6967:601-682 7010:683-720 6903:721-725 6787:726-796
6163:797-823 6966:824-838 6994:839-914 7011:915-932 7028:933-937

6936:938-948 6853:949-972 6963:973-981 7000:982-996 6852:997-1022
6981:1023-1060 6934:1061-1086] [-1, 6399, 6400, 6791, 6710, 6042, 6789,
6709, 6858, 6370, 6967, 7010, 6903, 6787, 6163, 6966, 6994, 7011, 7028,
6936, 6853, 6963, 7000, 6852, 6981, 6934]
GGGCGGCGTTCGCTCTCGTCGCGGCCCACGGCTACGACGATTTCGCTTTGACGGAGGATTGCTTCACGGAGGA
GCCTCCGTTGGTATCGTCGTCGTCAATGTCGGATGGCGGTGCGAGCGGCATGGCGCCCTTGCCTCCTCCTCCG
CCTCCTCCTCCCGCGTTTGGAATGCCGGGCATGCCACCGGGGATGCCCGGAATGCCCGGCATGGGCGGTGCTC
CGCCCCTTCCGCCCGGATTGCCTCCTCCTCCGTTGCCCCCCGGCATGCCCCCTCCCAACTTCGGCGCGCCTCC
TCCCAACTTTGCCGTCCCTCCTCCCGTCCAGAAGGAATTGATCCCGCAGCCATCGGCGCCCAGGGCGGATCTC
GCCGTGGCCAGCGCCGTCGTGACGACGACGACGACGTCATCGTCCGAGGGGAATGCTACCGCCATCGCTCCCG
CCGTCGTGACGGTGTTGTTGGGTTCGGCGAACAACAACAACAGCAATGCCATCACTGCTTCCAATAATGGTGG
TGGTAGTACCGCGGAAGGGAAGAAGACTGCGGGGAGCGAGGTGGTATTCGCGGGGGTAGAATTGGACGAGGAA
CGGGAGGAATTATCGATGGAGGAGAAGAGGATGCAGGTCGGTAGGTATTGGAATTTGGTGGTCAGGGCTTTGG
CGAAACGGTGAAAACAAACGAACGAACATGCGATGGAGCGAGAGAGAATGGGGAGGGGAATGGGGGATGAGGA
TGGGGATCTGTAGGGGATATATTCGAAGGATATAAGGTGGGTGAGTGGATTAGAGAATTTCGATATCTCCCGG
GGCATTGGCGATATATGAAAATGAGCAGTTACCGTAGTATGCGAATGGACGTAGGGGGATTGTGTATGTAAAG
TCGGAAGAAAAGGTTAGTGATGGAGAAAGAAGGTTTTTTTCGAGATATCGCGCCCCTGTTTTCGTTGGGTATT
ACGCATCACCAGATTGCGCAGATTATCAGGAGAGATTATGAGGACATACATATAGGTAGAGTCACACTTTGGC
AGTATTTCATCGTATTCCCATCTACATATTGCATTTGGCGTGTGTACCATATCGCATTATTTTCG
>TR624|c1_g2_i11 len=2751 path=[6006:0-25 6007:26-28 6008:29-31 6009:32-
34 6010:35-37 6907:38-40 6795:41-43 6862:44-46 6940:47-49 6793:50-52
6968:53-63 6939:64-100 6712:101-117 6859:118-129 6790:130-362 6710:363-
545 6042:546-569 6789:570-585 6709:586-609 6858:610-696 6370:697-720
6967:721-802 7010:803-840 6903:841-845 6787:846-916 6163:917-943
6966:944-958 6994:959-1034 7012:1035-1051 7028:1052-1056 6936:1057-1067
6853:1068-1091 6963:1092-1100 7000:1101-1115 6993:1116-1141 6981:1142-
1179 6934:1180-1205 6898:1206-1332 7013:1333-1334 6775:1335-1358
6961:1359-1360 6992:1361-1410 6979:1411-1446 6960:1447-1481 6932:1482-
1509 6978:1510-1521 7014:1522-1523 6895:1524-1543 6958:1544-1547
7029:1548-1561 7034:1562-1569 6684:1570-1586 6991:1587-1596 6957:1597-
1612 6977:1613-1628 6891:1629-1636 7016:1637-1637 6976:1638-1652
6956:1653-1661 6926:1662-1709 6832:1710-1724 6754:1725-1845 7017:1846-
1869 7030:1870-1897 6830:1898-1916 6083:1917-1921 7018:1922-1940
7031:1941-1943 6829:1944-1960 6989:1961-2013 6974:2014-2050 6923:2051-
2069 6884:2070-2074 6824:2075-2083 7020:2084-2106 6822:2107-2130
6951:2131-2139 6922:2140-2163 6883:2164-2177 6743:2178-2209 6950:2210-
2215 6921:2216-2239 6882:2240-2255 6949:2256-2305 7005:2306-2306
6920:2307-2349 7021:2350-2350 6814:2351-2391 6918:2392-2415 6877:2416-
2421 7022:2422-2423 6916:2424-2472 7032:2473-2479 6914:2480-2482
6872:2483-2491 6641:2492-2510 6725:2511-2534 6911:2535-2566 7027:2567-
2567 6636:2568-2662 6517:2663-2664 6518:2665-2687 6519:2688-2750] [-1,
6006, 6007, 6008, 6009, 6010, 6907, 6795, 6862, 6940, 6793, 6968, 6939,
6712, 6859, 6790, 6710, 6042, 6789, 6709, 6858, 6370, 6967, 7010, 6903,
6787, 6163, 6966, 6994, 7012, 7028, 6936, 6853, 6963, 7000, 6993, 6981,
6934, 6898, 7013, 6775, 6961, 6992, 6979, 6960, 6932, 6978, 7014, 6895,
6958, 7029, 7034, 6684, 6991, 6957, 6977, 6891, 7016, 6976, 6956, 6926,
6832, 6754, 7017, 7030, 6830, 6083, 7018, 7031, 6829, 6989, 6974, 6923,
6884, 6824, 7020, 6822, 6951, 6922, 6883, 6743, 6950, 6921, 6882, 6949,
7005, 6920, 7021, 6814, 6918, 6877, 7022, 6916, 7032, 6914, 6872, 6641,
6725, 6911, 7027, 6636, 6517, 6518, 6519, -2]
ACAACAACAACAACAACAACAACAACAACAACAACAACAACAACAACAACAACGGCGGATACCCTAGCCAGGA
GTTGGGAGACTGCGAGGAACGCAAAAAATCGGCACATCGCTTCCCCACCATTCCCATGACGAGAAGTCCCCCT
CCGCCCCCCGCCCACTCCTCCGTACAACTGCGTCCGAAACCCCCCACCATCGCCGCCGTCCACCACAGACTCC
ACCGGTACGGCGCCGTCGATGCCGGTGCCGACGACGCTGCCGCAACCGCCACGACGACCGCGGAGGAGGGGGG
AGACCACGACGGCGTCGTTGAATGCCTTGGCCCTGGGACCTGTGCTCTGCCCCTTCCGCCCGGATTGCCTCCT
CCTCCGTTGCCCCCCGGCATGCCCCCTCCCAACTTCGGCGCGCCTCCTCCCAACTTTGCCGTCCCTCCTCCCG
TCCAGAAGGAATTGATCCCGCAGCCATCGGCGCCCAGGGCGGATCTCGCCGTGGCCAGCGCCGTCGTGACGAC
GACGACGACGTCATCGTCCGAGGGGAATGCTACCGCCATCGCTCCCGCCGTCGTGACGGTGTTGTTGGGTTCG
GCGAACAACAACAACAGCAATGCCATCACTGCTTCCAATAATGGTGGTGGTAGTACCGCGGAAGGGAAGAAGA
CTGCGGGGAGCGAGGTGGTATTCGCGGGGGTAGAATTGGACGAGGAACGGGAGGAATTATCGATGGAGGAGAA

GAGGATGCAGGTCGGTAGGTATTGGAATTTGGTGGTCAGGGCTTTGGCGAAACGGTGAAAACAAACGAACGAA
CATGCGATGGAGCGAGAGAGAATGGGGAGGGGAATGGGGGATGAGGATGGGGATCTGTAGGGGATATATTCGA
AGGATATAAGGTGGGTGAGTGGATTAGAGAATTTCGATATCTCCCGGGGCATTGGCGATATATGAAAATGAGC
AGTTACCGTAGTATGCGAATGGACGTAGGGGGATTGTGTATGTAAAGTCGGAAGAAAAGGTTAGTGATGGAGA
AAGAAGGTTTTTTCGAGATATCGCGCCCCTGTTTTCGTTGGGTATTACGCATCACCAGATTGCGCAGATTATC
AGGAGAGATTATGAGGACATATACATAGGTAGAGTCACACTTTGGCAGTATTTCATCGTATTCCCATCTACAT
ATTGCATTTGGCGTGTGTACCATATCGCATTATTTTCGTTAAAGTAACAAGAGAAGGCGGCAGGTGTTGCATC
ATCTCAATGGGACACACACACCGGGGGGAAGGGAGGTAGATTACCCAGGTATATATGATTGATGGTTACATAT
AAGGTAATGTCATTGTTAATAGAAATTATCTCTACTGGCTCTCACTTCCGGGAGGTGGGGTGGGGGAGCAGAG
GCTACAGTGTACCATACCTACACAACACGCTTTTAGGGACGGTGAGAGGTGCACGAGGATAAAACAGTTTCCT
AGTTCCCTAAAAACGGGCAAGTCGTTTTCTGCGAAGATGAAAATTATCGAACGAATGCGACACAAACACCCCA
AGAGGCAAATAAACTTGTGTCACCCTCAAAACCCCCCTCCCCCTACCCGTTGGGGGGGGGAGGCAACCTCGTA
GGGAAAAAGATATTTTGCGCAAGGAAAACAGGTTCCTCTACCGCTCGGGCAATTTTCGGAAATACCGTCTCGA
ATGGGATTGAAAATTATCGAACGAAAAAAAATGTGACACACGCACCCAAAGAGCCTCGAGGCAGATGGACTAT
ATATGCCGTCCACAACTTGAAATACCCACTTGGTGGCGTTTGGGAGAAAGTTGGCTATGGTACCTACACACAC
ACTTCGCAAATATTGCATCGAGGTATACTAAAACACGAAGCGGCCATCTGCTATCTAGAGGAAGAGAAACCTC
ACCACAGGAGAACAACATCGCAAATTGATGTCGTGGCCGTTTTTTTTCTTCGGTCGTGCAGACCACGACGACG
GGGTAAAACGATGCTCGCTTTCCTGCGCTGGGTTGTGCCAAGTGCAGTTTACACGCACTCGTGCTAATTCTTG
CGTGTTCGGATTGAATACAATGCTGGGCAAACCAGTGAAGTAAAATATACGTGGCGACATACGTTGCGCGAAG
TCTACTGGCCGATACGGACCCATCGTGTATCCCACAAGCTCCTTCGCAAACCATGGTGCATAGGAATGGTAGA
GAAATGGGCCCTGCTCTCAACGGAAAGAGATAATTGCCACGGAAAGAAAACGAGGCCATGAAGCGATATCGTG
GCTGTTGTGTTGGGTCACATGCAGGCATGGTAAAGGGGGGGGGGGGTTGGGGGTTACGGGACGCTTGCAATTG
TTGCGCTTTTGAAGTGAACATCATGATGCCCCGGGCAAATTAGTGGCGGCACCTACCATGTGTCTTTTTGCCA
ATAGGAACTGAAATATGCAGCACGTGGTGACACATGCTGCAAAACCTATTGGAACAAACGACCCCTCGCGAGT
ATTACAATATTCAGACTGTGATTCTTTTTTTAGATTATGAAATTATCAACATAATGCACCATACTGAATTGAT
GGAGACGACGAATACAATTGATTTCTCTCTCTGTTCGCCTGCCTCTCTCATCAAGCAGAGCACAAAGGCAATA
AGAATAGATATCAGTGTGTCCAACTTTGTGCATTTTCATACTACCATTGTAGAATACCATGGTCCTTGATGGT
CTAAACATCTACTGGTATGCAAGCGAAATGCAATAAAAATAGATATTAGA
>TR625|c0_g1_i1 len=385 path=[363:0-384] [-1, 363, -2]
TCCGATCTGCTGAGTCTGCGTCGTCCCCAAATGGATTCGTATACGATTTGACCTTGCGTGAAGGAGATAATGG
ATGTTGCTTCGTCGTCGGAGACTCTGGCTTCAAAGGTGGTGTGATGTCATACTTGGACAGACTTAGCTTCCAT
TTATCCTCCTGTGGTTTCGATTTTTTCGTAGATGCAGACTTAACGTCGAGTGCGATTGGATTACTATGTGATT
CGGCCTTTGATGTTGGAGACCCTGACTTTACAGATCGGATGTCATACTTGGAAACGTCAACCTCGGAAAAAAC
TTGAGCGTACTTGGAAAGACTTTTTCTCCACTTGTCTGGCTGCGATTCTACTGGAGATGCCGACTCAACCAAC
GCGACCAACTCTTCAACCTC
>TR626|c0_g1_i1 len=305 path=[283:0-304] [-1, 283, -2]
GGGACCGCGAATCTTTTTTCCATCGAGGGTGCATTGTCTTTTCAATGTTTCCTCGGACATGGAGAGGGCAGTT
CTAATGGATGATTTTTGGTGGACGCTTGTGAAGGCAGACGAAGATGTGATGCCGGCCAAGGCTATGCAAAGGG
CAGCGTATTTCGAACGTGAGTTCGCCATGGTCGTACGTGAATTTCCGTCTTTTCAAGTCCAACGAGGAATAAG
TGGTGTGATTGATGCGTCTCTAGAGAGAGTGAGGTATGATTTTGCTTCGTAGTGGCCGATTAGTTTCAGGCGG
AAAATCCCATTGT
>TR627|c0_g1_i1 len=421 path=[399:0-420] [-1, 399, -2]
GCGCAGTGCCTGGCTCGTCTTGTCCCACGCGCGTTTGTCCCCGATGTCATAATATAGACCGCCGCGCTTTTCG
TCCGCTTGCATGAAGTTGCCCCCGAACTCGCGGACCGCAGCGACGATGGCCTTGGATATTTTCAGCTTGTCGA
ATTTGGATGATGTCGCATAGAGTTCCTTATTCAAATACACGAGCTTTCGATAGGTGACGTTCCCGCAATGGTT
TTGGGTTCCAGAACCGCGGCCCAGCACTACGTCTTTGCATGTCACGCTGAACATGGCTTCGGTGCCGGGGGGG
AGGCCGGGGGCGGCTTGGTTCTTCACCATGGTTGCTTTGAACCTAAGTATTTGTATCTTTTTGTCGTGGTAGC
GTTGATAGTCTACTGAATTCAGAGAGCGGAACCACTCGGCCGGCCATGGTGTTGGT
>TR628|c0_g1_i1 len=371 path=[349:0-370] [-1, 349, -2]
GGATTTGTCGTCGTCGTCATTGTCGTCGTCATCGGTATCGTTGTCCGCAGTCCAGTTCTCATTGTCTTCCTCG
TTTCTACCCGTTTCTGCGGCACTCTCTCCTTCCATCTCCATCGACTCACCCTCCCCATCTCTCTCGACGCCTT
CGTCTTGGGCGTTGTTTCTGGCAGAACGGCGGAGAAACACTTCGCCCCTCACGTCCACCACCGAGTCAAAGGA
TTTCTTACGGGAGGAGGATGTTGTACTCGTTTCCAACACTGAGGTATTCTCCTCATCGTCCCCACTGCCGTCA
TCTGCTCCCTCCTCGTTTTCGGAATCGTTTCCGGACGTCGGCCTTTCCGCAGCGGCATTCTCACGCCGTTTGG
AAGCAG
>TR630|c0_g1_i1 len=498 path=[452:0-467 453:468-473 454:474-479 455:480-
485 456:486-491 457:492-497] [-1, 452, 453, 454, 455, 456, 457, -2]
TGCATTGTTGTTGTTGTTGTTGTGTTCTCGTTTGATGCGACGATCGTCATCGCGTCCTGCCGATACCGTTCGT
TCACGCGAAGAATTTCTACGATGTTGACGTTGTTGCCCCCCTCTCATTGCGGAGTCGGAGAAGGACGACATCG
CCATGCCCCGTCGCCATTCTCCTCTCTCGTCCGATTGCAATTGGTCATTCCCTGACGACCATCGAGGTGGGTT

GAAATGGTTTCGCTGTTGCTGCTGGGTTTGATGTTGCTGTTGTTGTCGCGAATGGCGATATCCAGTTCCATCA
ACACGCATGTCGTCATGGTGATGATTTCCGTCCCTTCCTCGACTAAATTCCTCGTATTCTCCGCCGCGACTTC
CCCATCGATCTTTCGTATAGTACCCATCTCCGTGATGCTGTTGTTGTTGTCTTTCTCTACTGCGATCTCGAAA
TTCGTGTTGTTGCTGTTGCTGTTGCTGTTGCTGTTGCTGTTGCTGTTGCTGTTGCTGTTG
>TR631|c0_g1_i1 len=291 path=[269:0-290] [-1, 269, -2]
CGGCAGCAGCACCTGCCGCAGCAGCAACCGTTCTCAAGAACAAACCACCATTGCCCAAACTAGACACTGCCGC
TGCTATAGCCACACCAGACGAAAGTATAGAAACGCCACTTTTCACCTACGAACTTCCCTCTGTCGTTGCGAAG
AAGGCAGCAGCAGCATCCGGGAATAACAATTCATCGCGAAGATCCTTGTTTTTTCGTGGGAAAAGTTCTTCCA
AGGAACAAGAATCGGATAATGGCAACAATAGCAGTGATAGCAGCGGTGATAACAAGATGAGTAACAGCAACG
>TR632|c0_g1_i1 len=841 path=[819:0-840] [-1, 819, -2]
TTTTGTTTCTAATTCAAATTCATGTTACATCATCTTTATAGCTATATTTTGATCTCTGTGTCTACCTACCACC
ACAATACCCCATCATCATCTAAAAACTGGTGGCAGTAATCTTGTAGCAAATCTTGGAATTATCCTCGGGCAAA
TTAATCACCAAATTGCCCACGAAGGTCCCCGCCCTCCCCTGCGGATTACACCTAACCTCCAACTCGGTAGGCT
CTCCCCCCCTCCTATCCATCCTCCCGGCACACGGCTTGACGCTAAAGCTCCCGTGACTATCCTCCGTAAACGC
CGCGAAGTAATCCTCGAACCCCATGCAAACCGGCTTCACGCTCAACAGCAACTCGGCACCCTTCGAACTCGCG
GCCACGTCCATGTCGATCTCCCCATCCGGAACGCTCGACATTACGAATTCCATACCGTCGGGAATTTCGGAAT
TCTCCTCGATGCCGCCCATGATCTCGGCGTCAGCTCCCAGGTTTTCCATGCCGGGGAGGGCGGGGCCGCGTTC
TCCTCCTCCACCCATGCCGGAGGATCCTCCCATTCCTTCGGTGAAGTCCACGGCATTTTTGTCGTAGCCTTCG
GGGAAGTAATCTCCCGTGTCGGTGGACCCCATTTTCTCGCTATCGGGCTTCCACGAATTCCCGCCAAACTCTA
CATTACCCTCCTTTCCGAGAGATAAGGCGCTTGCTCTGTTAACACTGGCAGCGGGAGCGAAGGCGGCGGTGCC
GGAGATGGCGGTGGCGGCGAGGATGGCAGTAATCTTCATGGTGTAGGTTTGGGGTGTTGTTGGTGTTCGTGTC
AACGAAGTGAAAAATAAGAGAGGTTACGATGGAGTACA
>TR635|c0_g1_i1 len=1030 path=[1033:0-539 1034:540-563 1035:564-1029] [-
1, 1033, 1034, 1035, -2]
CTTATACATCGTTATCCTTTGAAGGGTTGGATGGGGAATACCTCGCATGCTGCGGGAAGAGCATGTGCCTCAG
CTGCGTCATCCAGGAACATAAAGTCACAAAGGAGAGGATGTATACTAGTCAGCATCAGCATGGGATATCTGGA
CAGCAGAGGAGGAGACTAACTGATCGATGTCCCTTCTGCAGAGCACTGCAATATGTATCGATCGCCGAGGATA
GAAAGAGACTTCAGAAGCGACTGGATGCGAATGATCCCCATGCATATGCAGTGTATGCTAAGAGGGCTGGAGT
ACGCGGGAATCATCGTCGGGCTCTAAAGCTTGAGTTGAAGGCCGCTGAGTTAGGTTGGCTCAAATCATACTCA
GACGTAGCAACATCTCTTCGTATTCTATCTCGCTGTATTGACGATCAAGGAGATAGTAGCAATGGTGTACTAC
TTGAACAACTTCGGATGTCAAGGAAGAATTGGCTGGGAATGGGCGCCAAGAAAGGCAGTATCGAATCTCATCA
TCAACTGGGAGAGGTACATCTTCTAGAGTATACTGACACTTCTACTGCATTGGCGTATAAACATTTTTGTTTT
GCGGCACGACATGGTCACACGGGAAGCTCTAAGATGCTGCTCATGGGGTTCCGCCGAGGTTTTATCAACAAGG
ATGAGTATGCATTGATATTGAGAGCATACAGAGATGCTTATGAATCAATTATGAGTCCGGAGAGAGAATTCGC
TCTTGAGGTATCCCAAGCCTTGATTTGGCGCAACGTCGTCCTAGGAAGAAGCTTGAGTATCTGATAGTCTTCT
GATTCATCGGGAGATTAGTGTTGATGAGGTTGTGAGATCACCATCTCGTTCATGGGGGAAACGGTGCATTGGG
TATTTGTTTGCTAATTTGTCGAGAATGAGACAATTTTGTAAATCTGTCGCTATAAATATATGTGCATTGCTTT
AGCAATGCTGCATTAGTTTGGTCTTGGCGGTTCTCGCATCTCTTGGATTTAGTGAACACCATAACATTTTCAA
GATCGGAA
>TR636|c0_g1_i1 len=922 path=[925:0-694 926:695-718 927:719-921] [-1,
925, 926, 927, -2]
AGCGACATCGAGCTGTAAAGTGAATCAGAAAATGTCTTTTGAAATACTCATTTTCTATGTTTAGCTAGGTACA
CTTATAATGCCATCTCTTAGTAAAGGGCACGGACACCATCGGGATCATATTGTTCACCAGGAGTCTTGATCAC
ATTACCATCGTCATCAATGTATTCGCTCTTCTTCTGCAGAATAAATCGTTCAAAGAATTTTGTCAAGATCCTG
TCTGTGGTAAGGAATTCGACTATGCCTCCGTTGGTCTTAATTCCAAGGATTTTGGGCACAAGATCAGCGGCGA
CCTCCTCCGGTTGTGCGGCCAGAACACCGAAAGGGAATTTGCGAAGCTCAGGAGTAGAATCATCAAGCAAAAG
CTTGGTAAACACCATCCCCGGTGAAATACTGTGAACTTGAATATTACCCTTCGTCTCCTTCTTCTCATACCCC
TGCACACCCTCATCCAATTCCTTCACCAAACTGGCCGTCAATTGCGGCAAACCCCTCTTCGTCGCACCATACG
TCGCATACCCCGGCGTTCCACCACCCTTCACGCCGCTGCCCACGGTGTTAAAAATGTGCCCGGTCATACCCTC
CTGCTCTCCCATGATATCCATGGCAACCTTCGTACAGAAGAAAATCCCCAACAAATTCACTTTCACAACCGTC
TCGACCTGTCCCATGGTCAAATCGGTCAACGCCCTCCTTCCTCCGTTGATACCGGCATTGTTCACCCAATACC
CAATGTTGCCCAGTTTGTCCTTGGCGAATTGTCCCAACTTTTCGCAACTCGCGGAATCGCTAACGTCGCATTT
GGTATGAAAGACGTTTCCGTTGGGATGACGCGCCGTCAAAGCCTTGGCCGCGGCGGCGCAGTCCTTGACATCG
CAGATCACGACGTCGTAGCCACGGTCCATGAATTCTCCGGCGTAGG
>TR637|c0_g1_i1 len=230 path=[228:0-199 229:200-229] [-1, 228, 229, -2]
ACCTACTGTACCTGCTTATAAAAATTCCTTGCTCCGCCACGATTGGATATTGAAATACAAACCATGAATTGTC
AATAATATCTCAATTCGCATTCGCCTTCCATTTCCACACCCTATCCTTATCCCCAACAGATCTGACGATAACA
ACAAAAGCAACTTCAAGGCAAGGATGAAGGTCCACTCTTCGGCGTGGTTGCTCATCGCATCAGTTCCTGCCGT
CGCCGAGATCG

>TR639|c0_g1_i1 len=875 path=[878:0-625 879:626-649 880:650-874] [-1, 878, 879, 880, -2]
CCGATCTTGAAGCTAAGCCGTGAGCCATGGCATACAACATTTCTCTCATGCAACATGCCGCTGACCAACGCCT
CAGAACCAGCGTCAGCTTCCTTCTACAACGCGCTCTTGAACAAGAACACAACCATAAAAAAGCGGTGTCATCA
GCGACGGCGATTTAGATCTTTCGTCCATTCGATTCAGTTTCATTTTCAGACAAGCAACCATTCAATATGACAT
TGACAAACCAACAGGAGCCTATGTTTAGTGGCCAACGCCATCGCTCATATTCTGAGCACATTAGTTCGTCCAC
TCACGATTCAACCGGTATCACAAATGCGAATGACAAGATGACACCCACCACCAAGAAGCATTCACATTCTCAT
CGTCATCATGGCAACCGGCCGCGATCTAAATCATGCGATCGTCATGTCCACAAGAAGAACAGTTCCACCAAGC
ATAATCCCGGCACGCGTTCGCCAGGCTGCCATCATGTTCATGGGCGTCATCATATTCAAGGGCGAGTAAGTCC
CAAGAACTTGCCTTGTCAACAGCTTCATGGAAGATTGTGGATCCGTCCCATGGTCGTGAATAGGAACTCTCAT
CCTTTTAAACCCGTCTCATCAAGCGCTGCGCCACAAGTCGACAACAACAGCACCTTTGCTCCCATCTTGACAA
AGAATGATCCTCCAGTTCGCCAGGTTACTGGTTCCCCAATCTATGACGACAATGAGGAAATGATGTGGGCAAC
ATCGCTAACACCAAGATCGCTGAATTCCTCCTTCCGGTCTCTTCAAAATCGGCATGGACGAACCAGTCCAAAG
AATTTGCGACTCATCTTTGGAGAGAGTGGCAAGCCCATCCAACCGAGGAATGCTTTTTAATTCTATACCAGT
>TR640|c0_g1_i1 len=254 path=[273:0-30 274:31-69 275:70-253] [-1, 273, 274, 275, -2]
TCTCTCTGGCACAATGCAGCCATTGTTTTTTTGCCCCATCATAGGCATGGTTACATTTAATGTCCATCTTAAA
CGCATCATGATATTGATTTTGTCTGATTAGATGTCATATCTCAGTTTCCCGTCTTCAGTTCCTCTCCTTGATC
CAAAGAACTAGAACTGTAATGTTGTCACTTGATCCCCTGTTTAAAGCTTCTTCCACTACATACTCTGCTATGC
CGCGTTGCATCTCGCGCCGGTTCTCCGGAGGCGTG
>TR641|c0_g1_i1 len=1139 path=[1159:0-357 1161:358-978 1155:979-1002 1156:1003-1138] [-1, 1159, 1161, 1155, 1156, -2]
GTATTAATGCCACGCCTTAAATGTGCGAAGTGGATGAATACGAATAGATTCACAAGCCCAACCATAACTCGAA
ATGTTAACCATGTCAGAACAGAAAGCCCATTCCAAGTTAGGTTATTATTCATTGGTTTCCTTCTATTGGTGGT
GCTGCCGCATCATCTGTGTGAAAGGTAGGCTCTGGTATGTATGCTCTATTCCTTTATCCCCTTGTACAGGTAC
CTTGCAATATACTCTAGTAGGTACCATACAAAACAATGTTTTTTAGAAATTCAGCCCCAAGCTATACCTCCAT
GTGTTAATTGCTTCGAAAGAATCAGAATTACTTTCTTCGATCGTGTCACTTCTAACTCGGCACGTCAGGCTTT
TGTTCTCCTCTTATAATGCATCATCCTCGTCCAACATAAGGCATATGGCTCGCCATAACCGTCATATTCAACA
CATTCGCCTCCAACGGCAACGCCGCCATCGCAAAAACGGTCCTCGCCGCATCCTCCACAGAAAACGTGGGCTC
CGGCGCCACCGTCCCGTTCGCCTGTGGCATTCCCGCGCTCATCGAATATGACGAACCTCCCACGGCCTTGGTC
ATGTCGCTGCTTACATTGCCGAAATCGATTTGTCCGCAGGTGACGTTGTACTTCCTGCCGTCGAGGGCGATGC
ATTTGGTTAGGCCGAGGACTGCGTGTTTGGATGCTGTATAGGGAGCGGAGCCAGGACGAGGGCTGAATGCGGA
TATTGAGCCGTTGTTTATGATGCGACCTCCTTGTGGTTGTTGCTTTGACATTAGTTGCATTGCCCCCTTGGCC
ATTCTCCAACAGGCGGTGACGTTTGTGTCCATAACTTTCTGAAAGCCGTCGGTATCAATAATTTCGACTGATG
CAGGCGCAATATTTATCCCGGCATTGTTAAATAAGAGGTCTACCCTTCCGAACTGTGTATCGATGGCTTTCAT
GAGGTTTTCAACATCGCTCTCCTTAGTCACATCGGTGGGCATGGTAAGAATGTCAACCCCAACCTTCGAGCAT
TCTGCACACATTTTTTTCGTTGCGTTCAATGGTTCACATCTTCTACCCACCAGCACCAGGCCCACTCGGAGCG
ATTTCCCGTCGGCACCATTGCCTCTTCCGTCCGCTCCTCCCCAG
>TR642|c0_g1_i1 len=320 path=[334:0-138 335:139-171 336:172-319] [-1, 334, 335, 336, -2]
CCGATCTATTCTCTTCTTTGGTGTTATTTTACAAATAGAGAGCCAATGCAAATAATATGAAATCCATGTAAGA
TAATTCAAAGATCCTCCAAAACCTCATCACGGCAACAGATGCACACATCCCAAAAGAAAGCACAACTATGTCC
TCCTGACAAAAATTACACAGAATTCACAAAACTCCAGCTAACAGACAAGCAAGCTTCTTATCCGGATTCATGT
TCGACGACGAAGACGACTCGCTCTCGTCTTCCGACGACAATCCCACCATCATTATCTTCTCCTCATTCGAAGG
CATAGTTTCGCTACCGGCGAAGAGTAGA
>TR642|c1_g1_i1 len=329 path=[307:0-328] [-1, 307, -2]
TCCGACGACAATCCCACCATCATCATCTTCTCCTCATTCGAAGGCATGGTTTCGCTACCGGCGAAGAGTAGAG
CCTTTTCGGTCCGCGACAATACGCTTTCGTCGCCCTGCGACAACCTCTGATACTTCTCCACCGATGCATCAAT
CGTTCCAATGGTCTCTTTCATCGATTCCAACTTTTCCGTCAAGTTGGAGTCGACATGACGCAACGTCGTCGTT
AGAGTTTGAAGGGCGATGTGCATGTTGTGGTTCGATACTGCACCCCCATTCGTCTCCATACTACCACCATCCT
TCTCCTTCGTTTGTTGTTCATCACCATCACCATCATC
>TR643|c0_g2_i1 len=232 path=[546:0-196 547:197-231] [-1, 546, 547, -2]
CTCTTCCGATCTGCAGTGCGACAGAGTTCCGCACGGCGCTTGAAATCCGTTTTATGTCGCGATTGCGTCGTTC
CTGACGCAGGAAATTGTCTTTAATCGACGCGATGCGGCATGCCTTCAATTCCACGGTTGTCCGGTAGAAGCAG
TGACCGGGCTGACCGATTATCGGAACAACCACTGCAAGATGAGACTGGGAGATTAACAAAAAAAAAAAGTAAG
AAAGAACAGATCG
>TR644|c0_g3_i1 len=847 path=[859:0-176 860:177-177 861:178-192 862:193-193 863:194-846] [-1, 859, 860, 861, 862, 863, -2]
TCCATTTCCACCGTGACCAACATTTTGTACAGTACAAGAGAATTGGTAGATCAATGCGGGAGAAGATTCTATG
AGACGGATTTACTCGTGAAAGAGATGACGAAGCGAGAGCCGGACGAGCCACGTGCCAAGGCGGCAATTGTGAG

GATGAATTACATCCATGCCCAGTAAAAAAAAAATATCGAACGAGGACTATCTACGTTCTCAGCGTGTTCATCA
TGGAGCCGATTCGTTGGGTGGAGCGTTTCGGTCGTTCGCGACCCTCATCCGAAGGAGAGGGTTGCAATACATC
TACGCTGGGAGGATATTGCTAGACAGATGGGAATCAATGATATCCCAAATACGTACGAAGAAACCGAGAAGTA
CCTGGATGATTACGAAGAGAAGTTCATGGTGTATGTTGACACGAATGTCCATATCGCCAATGCGACCATGGAC
CTATTCATCGGTGTCATTCCTGTCAGGCTTCGTTCCATGTGCAAACTGGTAGCGCACGCCCTCTGTCCCCCTC
GTCTACGAAAGTCAATTGGATTTCCGACTCCTCATTGGTCTCTTGCATTTTTCATCGAATATTCTCTCAAGGT
TGCGGGGTTGTTTGTGAGGTATTTCTGTCCGCCTTGCACAGTTCCAAAGTGTCGTACTCCACGTCAACCCATC
GCGGTTTGGTGAAAAACGAATTTATATCCCCTCTGGCACTCATTCGAGAAAACGTATGAGCATGGATATGAGA
TTTCAGAGCTGGGTCCAGAACGCTTTTGCCCTGTAAAATGTTGATTGGAAGGATTTAACACTAAAAATATGAA
TTAATTAAACCTGAATTCTAATCCCAAAACAAATACTATCCAAG
>TR645|c0_g1_i1 len=385 path=[585:0-71 592:72-159 576:160-174 595:175-221 589:222-315 579:316-384] [-1, 585, 592, 576, 595, 589, 579, -2]
TATTTTACGGGATTTTTGGCGCGGCCCAGTCCGTCCTGATGTAGTATTATTTATCGTTGGGGTGTGTATTGTG
AGTGATGGAGAATGGGTGTGTAGTGATGTGTTATTTGGCACACGGAGGGGGAGAGGATAGAGGGGGGCGGGGA
AAGCGTCAATGAAATGTTGACATCCACAGAAAGAGAGATACAAATTGCCAGCGCTGCTCAATGTGACGATGCA
TCGCAATATCCCCCGAATTTCAATATACCACGATATTATAACATAATATCACCATGTAGAACACGAATTCACG
TCACAGTAAGGACCCAAAAATTCGCCCGAATAATGCTCTACGCGATGTGTCACATCACGATGAAACTTTTTAA
TTTCCGCGTGAAAATAATCG
>TR645|c0_g1_i2 len=384 path=[585:0-71 592:72-159 576:160-174 595:175-221 590:222-314 588:315-383] [-1, 585, 592, 576, 595, 590, 588, -2]
TATTTTACGGGATTTTTGGCGCGGCCCAGTCCGTCCTGATGTAGTATTATTTATCGTTGGGGTGTGTATTGTG
AGTGATGGAGAATGGGTGTGTAGTGATGTGTTATTTGGCACACGGAGGGGGAGAGGATAGAGGGGGGCGGGGA
AAGCGTCAATGAAATGTTGACATCCACAGAAAGAGAGATACAAATTGCCAGCGCTGCTCAATGTGACGATGCA
TCGTAATATCCCCGAATTTCAATATACCATGATGCAACATCGTAATATCACCATGTAGAACATGAATTCACGC
CACAGTAAGGACCCAAAAATTCGCCCGAATAATGCTCTACGCGATGTGTCACATCACGATGAAACTTTTTAAT
TTCCGCGTGAAAATAATCG
>TR646|c0_g1_i1 len=348 path=[545:0-263 555:264-287 553:288-334 541:335-347] [-1, 545, 555, 553, 541, -2]
GGTAGACTATGGTATCATATCTAATATTACCTCTTACCTCTTGCACATCAGTGTGCCATCTAGTGCTACTGAC
TGTGCCGTCTAAGTGCCTGCCGCTTGCGGGCGGCGACCTAAATGAGATTTGAAGAGGGTTCAGCAGTGATGGT
GGCCGGGAGCCAGTAGCGGAGTGGTACAGTGGCCTACAGTCTCAAGGTGGTCATTGTGTTCTATGGCGAATAC
TAGTTAGGCAATGCAGGCCTGACACTAACAACAATCTATGGATAAGATGACACCCAGGGGAAAGAAAACAATA
TATGACAGGACCTCAGGAACCATAAGAATTCTGTCAAAATCCCAGATCGGAAGAGC
>TR646|c0_g2_i1 len=505 path=[548:0-263 549:264-269 556:270-287 554:288-334 544:335-504] [-1, 548, 549, 556, 554, 544, -2]
GGTAGACTATGGTATCATATCTAATATTACCTCTTACCTCTTGCACATCAGTGTGCCATCTAGTGCTACTGAC
TGTGCCGTCTAAGTGCCTGCCGCTTGCGGGCGGCGACCTAAATGAGATTTGAAGAGGGTTCAGCAGTGATGGT
GGCCGGGAGCCAGTAGCGGAGTGGTACAGTGGCCTACAGTCTCAAGGTGGTCATTGTGTTCTATGGCGAATAC
TAGTTAGGCAATGCAGGCCTGACACTAACAACAATCTATGGATAAAATGACACCCAGGGGAAAGAAAACAATA
TATGACAGGACCTCAGGAACCATAAGAATTCTGTCAAAATCCCCACAAAGCTGGTGTGCATCATATCAAATGC
ATCAAGTTCTTATTTACACAAATATCATACCTCTAAAATATAATTTATTTCAGCTTATCCATCTAGTACAAGT
AGTACCTAATGCACCCTCCTACACCCTCTCGTTGCTTAGCTCATACTACTACATCAGAAAACAGATC
>TR646|c0_g2_i2 len=271 path=[533:0-35 556:36-53 554:54-100 544:101-270] [-1, 533, 556, 554, 544, -2]
GCTCTTCCGATCTCAACAATCTATGGATAAAATGACACCCAGGGGAAAGAAAACAATATATGACAGGACCTCA
GGAACCATAAGAATTCTGTCAAAATCCCCACAAAGCTGGTGTGCATCATATCAAATGCATCAAGTTCTTATTT
ACACAAATATCATACCTCTAAAATATAATTTATTTCAGCTTATCCATCTAGTACAAGTAGTACCTAATGCACC
CTCCTACACCCTCTCGTTGCTTAGCTCATACTACTACATCAGAAAACAGATC
>TR647|c0_g1_i1 len=537 path=[589:0-341 590:342-366 591:367-390 593:391-464 587:465-488 588:489-536] [-1, 589, 590, 591, 593, 587, 588, -2]
GGTATTGCTGGAGTGATTGATCTGCTCCGTACACAAGGATCATTCGCATTGAGCATTCGATTCGCGTTGTGCC
TGACTTTGCCATCCAGCAAAGTTGAAGCGGCCCCAGCTGTAGATTACCCTACAGTTCGAGAGTATCAAGAGAT
TGCCAAGTCATTTGAAACAGAATTGAACAACCTAACAGATGGTGCCGACATGAATTATGGAAAGTTGTCCTTA
GAGGACATTAGTTCGGACAAAGAAAAACGTAACCGACTTGCATCCATGGTGGTAGAGGCGCTCCGAGAAAAAG
TTGATGTGCCAAATCCCGGGTTGGGCTTTGATAGGTTGTCCAAATTTGTCTCATTCGACTTAGAAGATGCCGA
GTAGACAAACTTCGTGCGAGCAACCGTTGACGCGTTCTTCGACAATGATCTCTTCCATACGGCCCGCCTATTT
TTCCAAGATGCGAAAGGATCCTTTGGGTTGTCAGTTTCATCTTCCCTCGATGCTCATAGACAGGTGTGCTTCG
CTGCACGCGGGCAAACATGCTCCATC
>TR648|c0_g1_i1 len=371 path=[407:0-53 408:54-80 410:81-185 405:186-209 406:210-370] [-1, 407, 408, 410, 405, 406, -2]

TTTATAACATATATATCTCTGATTGAAATTGTAATGTTGCTTGGTCAACGCTCTTGCGTATACCTAGGCATCA
AAACACACCCAAATACGCACTTCTCAACAAGTGTCCATGAGAGGCTTCAAATTATCCATCCCATTTATCGTCG
CCTTATACCACGCCAACTTAAACGCCTGATAAAAATCTTCGTTATTCTCATTCGGACTAGATCCACCCAAATA
CCTCCTCACGGCCCGTGCCGCTTCCCAACGCGGATGAGTATTGTCGAGAACAGCGCATTGATCCATCTGCTCT
CCTTCGAATGTGTTGCAACGGCTCGCTCCATTTTCGTCCGTGATGTCCGTCCGCGTGCGGCACGGGGCGCTCG
TTTGGA
>TR649|c0_g1_i1 len=1011 path=[2643:0-495 @2662@!:496-914 2649:915-1010]
[-1, 2643, 2662, 2649, -2]
CGAAAGAGACTCGATAGATCCCGGAACATATCCCATGCCTAAGCCGCTCGCTCACCGCCATTCAATTTATCAT
TGAGTTGACAAGCAGCAGCTTTCTTCGAAGAATCATTATTTTTGTGAATTGCTCCCGATAGCAAATATTAATA
TAGCTGTACCAATTTAGAGCTACCATGAAAGGCGACATCGAAGCTCCTACTGTTAGCGAAAGATCTCCACTCG
TCGCCGACAACAACGAGTCAACCCCCCGTGAATCGCCGACCGCAAGTTCAGTGGATAGTACTGCTCTCGAGTA
CTCCGTTTCCTCCTCCGCAGAATCTTCTACTATCGATGACATCAAAGACGAATTGGACAAGCCGTGGCCTGCC
ACCTTTGATAGGGGAATACAGATATTGGCAGGACCAGTTTTAAATGAAGCAAAGGTGGCTGATTTGACCAAGA
GTCCTATTGTTAGTGCTCGTTACAGACACAGGAATCAACTCAACCGGGGATACGACACACCAGATCCTCCTCC
TACGTCCCATCTTATTTCACCCGGAACACCCACCTGGCTTAAAAAAGGTCTCGTTCGAATGAAGTCGCTAGAC
TACACGTTCAGCGATCAAAATACAGCAATAAGCACTCCTACCTATCACAGCAAAAAAAAGAACCGACAACAAG
AAAAATTAGAAGCGCATGCATACCGTCAGAAAATACTTGCTGGGAGAGGTGGAGGGAGTTCCTCAGCGGGACA
TTTGGCCATTGATAAATCAGATTTGCCCCAGCTTCCACATGAAAAGTCCCCAGGGTTTCGAAGAGAGAAGCAT
TTGGACAAACAGAGAAAGAAAAAAAAAGAAATGGAAGTGAAGTTAAGTGAGGAGGAAGATCATAAATCAAGTT
TTTCGCAGTGCACCTTCAACATGGCAAATATTTTGATGGTGAGTTTTATGCCTTGTGTACTTTTTGACGAAGC
TATTGACATCCAGTGGTTTGATTATAGCATCTCAAAAAATATATTTAACACAACTATAATCT
>TR649|c0_g1_i2 len=2364 path=[2643:0-495 @2662@!:496-914 2661:915-1542
2646:1543-1565 2647:1566-2363] [-1, 2643, 2662, 2661, 2646, 2647, -2]
CGAAAGAGACTCGATAGATCCCGGAACATATCCCATGCCTAAGCCGCTCGCTCACCGCCATTCAATTTATCAT
TGAGTTGACAAGCAGCAGCTTTCTTCGAAGAATCATTATTTTTGTGAATTGCTCCCGATAGCAAATATTAATA
TAGCTGTACCAATTTAGAGCTACCATGAAAGGCGACATCGAAGCTCCTACTGTTAGCGAAAGATCTCCACTCG
TCGCCGACAACAACGAGTCAACCCCCCGTGAATCGCCGACCGCAAGTTCAGTGGATAGTACTGCTCTCGAGTA
CTCCGTTTCCTCCTCCGCAGAATCTTCTACTATCGATGACATCAAAGACGAATTGGACAAGCCGTGGCCTGCC
ACCTTTGATAGGGGAATACAGATATTGGCAGGACCAGTTTTAAATGAAGCAAAGGTGGCTGATTTGACCAAGA
GTCCTATTGTTAGTGCTCGTTACAGACACAGGAATCAACTCAACCGGGGATACGACACACCAGATCCTCCTCC
TACGTCCCATCTTATTTCACCCGGAACACCCACCTGGCTTAAAAAAGGTCTCGTTCGAATGAAGTCGCTAGAC
TACACGTTCAGCGATCAAAATACAGCAATAAGCACTCCTACCTATCACAGCAAAAAAAAGAACCGACAACAAG
AAAAATTAGAAGCGCATGCATACCGTCAGAAAATACTTGCTGGGAGAGGTGGAGGGAGTTCCTCAGCGGGACA
TTTGGCCATTGATAAATCAGATTTGCCCCAGCTTCCACATGAAAAGTCCCCAGGGTTTCGAAGAGAGAAGCAT
TTGGACAAACAGAGAAAGAAAAAAAAAGAAATGGAAGTGAAGTTAAGTGAGGAGGAAGATCATAAATCAAGTT
TTTCGCAGTGCACCTTCAACATGGCAAATATTTTGATGGGAGTAGGTATGTTGGGCCTTCCCTATGTCTTCAA
ATCTGCTGGATGGATTGGAGGATTCTGTGTCACTGTTGGGTTTTGCTTGGTCACATGGAGAACTTCCTATTAC
CTTGGCAGAGAACTCAATGGCGATCCCAGACCTGTGCATCTGTTTGACAGTGCACTACCAGACAATGCAATAA
CACGAATGCGCAAACCAATCACCAGTTTCCCTGCAATTGCAAGAGAGGCATTTGGAGACAGTGGGTGCATCTG
CCTCTCTGTAGTTCTTTACTTTGAACTATTTTCTTGTCTGAGCATATTCTTCGTATCACTGGGAGACCATTTG
CACGCGTTGTATCCGAGTGTATCACAATCAAAACATATGACAATTGTGGCAGGGATTTTGATTTTGCCATCGG
CACTGCTGCGCACGCCTAAGCTGTTGTCTTACCTTTCTACGGTTGGGACGTTTGCCACCGTGGCAGTGGTTTT
ATCGGTTGTGCTTTCGGCGTTAGTCATGTTCTTTACGGTTGGAAATGCCGGTAGTGAAAGAGAATACAACCTA
TATAGATCCAACGGCTTGCCTCTTGCAGCTGGAATTGTGGCATATTGCTTCTCGGGCCATGCTATAGTTCCTT
CCATTTACCAATCCATGAAAAAGCCACAGGAGTTTGAGCGAATGATAGATTTGACCTATGGAGTAGTTCTTCT
AAGCTGCCTCCTTGTTGCTGGTAGCGGCTATTACATGTTTGGAGATGATGTCGAGGATCAAATCACACTCTCT
CTCGAAGAGGAGTCGGAAGGTGCAGGTATTCTAATGACGGCATTGACATGGTTGATGATCTTAACTGCCATTA
GCAAATTTACACTGACAATGTTTCCTTTGGCATTGGGTTTTGAAGAGATGTTGACAGGTGTCCTCCCGAGTGA
TTTGGCAATGGAACTGTTAGATTCAGTGGTGAAAATTGTACTGATTTTTTCATCTTTGGGGGTGGCAATATTC
TTCCCGTCGTTCAGTTTCTTATGCTCACTGGTTGGTTTGATATGCACAATGATTGTGAGCGTGATATTTCCAG
CATTGGCTCATTTGAAGTTATTTGGGACAACTTTATCCTATCTGGACAAAACGATTGACTGGCTGCTTGTTAT
TGGTGGAAGTGTGATAGCTGTTGTTGGGACAATCGCAACTTTGCAGGAAGATGTGTAGAGAGAGAGAAGTGAT
TAGGTAGGAAGTGTTGAATAATACTGAGTTAATGCTCCGGGAAATGTGGTTTTGTTCTTATTTTTCACATGCG
CTGGCGCTGGAACAGAGCAGGTATTATTCAACCAATTCACCCTGAAGTGCCTCAGACCGCTAGATTGGCAAGA
AAAAGCACACCATGAGCATAATGTCAACCAATCACCTCCTAAAAGGATACACTTCAACATATGAACCAAAAAT
GCAAATAAAATCATCTTACTTACTTATA
>TR650|c0_g1_i1 len=428 path=[538:0-147 539:148-171 556:172-228 552:229-
270 550:271-283 554:284-307 551:308-342 549:343-366 537:367-427] [-1,
538, 539, 556, 552, 550, 554, 551, 549, 537, -2]

CAGTACACCTGCCGAGAGGCTTAGTCGCCAAATCTTCCGACGAAGCCTCCACCAGCGCGTGGACAGATGCATT
TCTCGTCTGTTCGCTCTTCGCTCCCGATGCGAAGACGAGCGCGGTCCCCCTACGAAGGCGAAAGAGGGACAAT
AGCAGTTCTTTGAATACCGGGCTGTATTGTTTGCATGCACCCTTTTTGCGGAGGACTATGATGTCATTCAGTC
CATCTTCCACGATTTGCAGAGGCTCGTGGCTTGAAAAAGTTGGGTTTGGCCGAGATGATGATTGAATGTCAAT
CAATTTCAGTTCCGTGGCAAGACCACTCGTTTTTGGTTTCAGTATCAAATAGGCCAGATAGTGGCCAAGTTGC
GTTGCAGTGCACTTTCCAAGAGGCTTCTCCACCAAATCCTTCGCCGATGCCTCAATCAACGCA
>TR651|c0_g1_i1 len=735 path=[1289:0-650 1290:651-734] [-1, 1289, 1290, -2]
GGTTGCCGTCGTCCTCCTCCTCATCGTCGGCGCCGCCGCCGGGAACGAGGATGGGATCGAGCGGTTCGAAGGA
GAGAATGTACCGCATGGCACTGGCATTGTCGGCTCCCACGCGCCGTGTGTAGTTGTGGAAGGAGTGCGTGCCC
TCGAATAATTTCAGGCCGCGTTTGAGACGTTCCATTTGTTCGGGCGTCGCGCGGTGTTGGAGGAGGATTTCGC
CGGCCTTTTTCAGTACCTCGGGATCGATGGCGTCCTCGTCGGAAGCCTCCGCCGTCGTCTCCGTTTCGCCATC
CTTTTCCTCTCCCATTGAAAACGCCGCCCGCACCTCCTCCCTCGAACAAAGCATGAAACTCGGCACCATGTAC
TGATACCGTACCTTATCCCTGCTCGTCCTCGCGCAGAACCTCCCCGTGACCCTTCCAAAGTCCAACACGCGTA
TGTCCGAGGGTAGGTACGAATTGACCTTGAGCCGCATGGCATCCAATTGTCCGGGTTCGTCGTCTACCAAATC
GTCTTTGTGATGGAAGATCATTTCTCCCTTGAGGGAACATACCTGTGCGGCGGCGTGTACTCCCTTGTCGGTG
CGGGCGGAATTGCTCCAGCCGTATTTGGAGGGGTAGCCAAAGTTCATTTTGGAGATGATGCCCGATCTACGGT
ATTCATGTATTGCATCGTCATAGCATCGTCCAGATAGGTTTGTGTGTTGTCCATACATTGAGTTAGAAATCCA
GACCA
>TR651|c0_g2_i1 len=1225 path=[1287:0-650 1288:651-1224] [-1, 1287, 1288, -2]
GGTTGCCGTCGTCCTCCTCCTCATCGTCGGCGCCGCCGCCGGGAACGAGGATGGGATCGAGCGGTTCGAAGGA
GAGAATGTACCGCATGGCACTGGCATTGTCGGCTCCCACGCGCCGTGTGTAGTTGTGGAAGGAGTGCGTGCCC
TCGAATAATTTCAGGCCGCGTTTGAGACGTTCCATTTGTTCGGGCGTCGCGCGGTGTTGGAGGAGGATTTCGC
CGGCCTTTTTCAGTACCTCGGGATCGATGGCGTCCTCGTCGGAAGCCTCCGCCGTCGTCTCCGTTTCGCCATC
CTTTTCCTCTCCCATTGAAAACGCCGCCCGCACCTCCTCCCTCGAACAAAGCATGAAACTCGGCACCATGTAC
TGATACCGTACCTTATCCCTGCTCGTCCTCGCGCAGAACCTCCCCGTGACCCTTCCAAAGTCCAACACGCGTA
TGTCCGAGGGTAGGTACGAATTGACCTTGAGCCGCATGGCATCCAATTGTCCGGGTTCGTCGTCTACCAAATC
GTCTTTGTGATGGAAGATCATTTCTCCCTTGAGGGAACATACCTGTGCGGCGGCGTGTACTCCCTTGTCGGTG
CGGGCGGAATTGCTCCAGCCGTATTTGGAGGGGTAGCCAAAGTTCATTTTGGAGATGATGCCCGATCGATAGA
GTGCCAACTCGATCTCCGCCTGCAACGTCCGTTTCTCATCATTGATTTGAAATCCAATGTAATTGCTCCCCAG
AAACGATGTGAGGAGGGCGAGTTTCCTTTTGGGTACCTTCTTGACAATTTCATTTTCTTTTACTACATCGACG
CTACCATCAGGATTATTATTCGCGGTGGTTGCTGTTGCTGCATTGTCGCCCTCGCCTTTTTCTTCCTCCCCCC
CGGACGATGATAATGGCGCATCAATATCCACCCCGAACAACTTCTGCATAGTCTCATTCGCAAACGATCCCGA
ATGAGGTAATTTTTCGGCATCGTAATCCTTCTTCCATTCGTTTTGATTCTTTTTCATCTTTTTCTTTTTATTG
TCTTCGTTGTTGTCGTCTTCTTCCCCGTCTTTTGCGAACTTTTGACGACGGCCGTCCTTTCGTTTTTTGTTGA
GTTGTTTCCATCTTGCGGCCTTGCGTTGTTTGCTATTTCCATTTTGGTTGGTTTTGGATTGATCTTTTTGGTC
GTTGTTGTTGTTGTTGTTGTTGGTGTTGTTGGTGTTGGTGTTGATGTCTTGGCCATT
>TR651|c1_g1_i1 len=796 path=[908:0-156 932:157-173 930:174-197 928:198-301 931:302-334 929:335-344 927:345-599 913:600-623 924:624-706 916:707-795] [-1, 908, 932, 930, 928, 931, 929, 927, 913, 924, 916, -2]
GTCGCGATCGCCGTTGGGCGTCAGGTCGTTCCCGCAGATGATGGCGCTGGAGTATTCTTCGGAGTTGAGGTGG
AGGATGGAGTCTGTGGTGAGGTCCGAGGAGGGGATTTTGGTCCATGGGTAGCGATCGTGTTGATTCGGATTGG
ATGTTGTGGTTTGTTGGATGTTGTTATTGTTGTTTGCCAGGTGATTGTTATTGGGAGAGCTGAGCAACATTGA
TTGGGAGGTGAATAAGAGAGCTCCGTCGGGAGATACCAAGACGCTGCAATCGGGTATCACTTCACTATCAGCC
TTCGATCTACGACCTTGTCTTTGTTGTTGTTGTTGTTGGACAATGTCCTTATACTCGTCATTATCATTATTCT
GGTGATTGATATTGTCGTTGCAGTCATCGTCTTCGTCTTTGTGAACATTATTATCCACCGAAGTAGTCGAAGG
TGTTGATTCAACTACGCATTGACGCTGTCGTGACGTTGCCGCCAATGCATCACACGCCTCGATGCACGCGTCG
AACATGGCGAGTGGAGGATGATTCTTATTCCATTGAGTCGTCTGATCATCATGTTGATGATGTTGGTACGAGG
AGGAGTAAGAGCCATTGTTGTTGTTGCGTCGAGAATACGATATCGACGATGATGAATTGTTGCTTGCGGAACG
ACCAGCAAGGAAAGATTTTCCAGGATAGGATCTTCCGGTATTAATGTTGTTCTTGCTGCTGTTGTTATTGGAA
GGAGAAGAGGAGGAAGGCGGAGCGGGAGAGCGGCGACGAAATCGGTCGCGGAAGGAGGGTGAGATC
>TR651|c1_g1_i2 len=489 path=[897:0-37 927:38-292 913:293-316 924:317-399 916:400-488] [-1, 897, 927, 913, 924, 916, -2]
GACGCTCTTCCGATCTGTTGTTGGACAATGTCCTTATACTCGTCATTATCATTATTCTGGTGATTGATATTGT
CGTTGCAGTCATCGTCTTCGTCTTTGTGAACATTATTATCCACCGAAGTAGTCGAAGGTGTTGATTCAACTAC
GCATTGACGCTGTCGTGACGTTGCCGCCAATGCATCACACGCCTCGATGCACGCGTCGAACATGGCGAGTGGA
GGATGATTCTTATTCCATTGAGTCGTCTGATCATCATGTTGATGATGTTGGTACGAGGAGGAGTAAGAGCCAT
TGTTGTTGTTGCGTCGAGAATACGATATCGACGATGATGAATTGTTGCTTGCGGAACGACCAGCAAGGAAAGA

TTTTCCAGGATAGGATCTTCCGGTATTAATGTTGTTCTTGCTGCTGTTGTTATTGGAAGGAGAAGAGGAGGAA
GGCGGAGCGGGAGAGCGGCGACGAAATCGGTCGCGGAAGGAGGGTGAGATC
>TR652|c0_g1_i1 len=321 path=[1396:0-120 @1421@!:121-198 1419:199-250 1415:251-269 1402:270-320] [-1, 1396, 1421, 1419, 1415, 1402, -2]
AGATGCAAAAGCTCACGTTATCTAGATGTTTTCTTCTCTATCAATTATAACCCAAACATAAATCATTACGATC
CCACCAAACAAATTACAATTCATCATCACCAAAGTCCATGTCATCACTGCCACCATCGCCTACTCTCGCGTAA
AAACTCCTCATAATGGGATTGGCTAGGTTCTCTATCTCCCTTTGCTTAGCCAAACACTCATCCTTCTCTGAAT
CAAAATGTCCTCCCATCCAATCCAATGCTTCGTCTGTGATGTCTTGCAAGTTTCTCTTGTCTACAGCTGACAT
ATGATCCTCCACGACATCGTCCTCCAGAC
>TR652|c0_g1_i2 len=345 path=[1391:0-89 @1421@!:90-167 1418:168-219 1423:220-238 1422:239-317 1404:318-344] [-1, 1391, 1421, 1418, 1423, 1422, 1404, -2]
CTTCTCTATCAATTATAACCTAAACATAAATCATTACGCTCTCGACAACACACATTACAATTCATCGTCACCA
AAGTCCATGTCATCACTGCCACCATCGCCTACTCTCGCGTAAAAACTCCTCATAATGGGATTGGCTAGGTTCT
CTATCTCCCTTTGCTTAGCCAAGCATTCATCCTTCTCTGCATCAAAATTTTCTCCCATCCAATCCAATGCTTC
GTCTGTGATGTCTTGCAAGTCTCTCTTGTCTACAGCTGAAAATTTACCCTCCACGTCATCGTCCTCCAGAGTA
TTCTTCAGGTTATACAAGTAAGATTCGAGCCCATTGCGACAATCAATGTTGTC
>TR652|c0_g1_i3 len=290 path=[1391:0-89 @1421@!:90-167 1419:168-219 1415:220-238 1402:239-289] [-1, 1391, 1421, 1419, 1415, 1402, -2]
CTTCTCTATCAATTATAACCTAAACATAAATCATTACGCTCTCGACAACACACATTACAATTCATCGTCACCA
AAGTCCATGTCATCACTGCCACCATCGCCTACTCTCGCGTAAAAACTCCTCATAATGGGATTGGCTAGGTTCT
CTATCTCCCTTTGCTTAGCCAAACACTCATCCTTCTCTGAATCAAAATGTCCTCCCATCCAATCCAATGCTTC
GTCTGTGATGTCTTGCAAGTTTCTCTTGTCTACAGCTGACATATGATCCTCCACGACATCGTCCTCCAGAC
>TR652|c0_g1_i4 len=1159 path=[1396:0-120 @1421@!:121-198 1418:199-250 1423:251-269 1422:270-348 1420:349-934 1389:935-986 1390:987-1158] [-1, 1396, 1421, 1418, 1423, 1422, 1420, 1389, 1390, -2]
AGATGCAAAAGCTCACGTTATCTAGATGTTTTCTTCTCTATCAATTATAACCCAAACATAAATCATTACGATC
CCACCAAACAAATTACAATTCATCATCACCAAAGTCCATGTCATCACTGCCACCATCGCCTACTCTCGCGTAA
AAACTCCTCATAATGGGATTGGCTAGGTTCTCTATCTCCCTTTGCTTAGCCAAGCATTCATCCTTCTCTGCAT
CAAAATTTTCTCCCATCCAATCCAATGCTTCGTCTGTGATGTCTTGCAAGTCTCTCTTGTCTACAGCTGAAAA
TTTACCCTCCACGTCATCGTCCTCCAGAGTATTCTTCAGGTTATACAAGTAAGATTCAAGTCCATTGCGACAA
TCAATGTTGTCCTTCAAAATTTTGTCTTCCTCAGCATATCGGTCAGCTTCTTCGATCATCCTACCAATCTCTT
CTTGCGAGAGCCGACCCGTCTCAGCCTCGGTTGTGATTGATTCGGCCCTACCAGTTCCCTTGTCTTCTGCGGA
CACTTGAAGGATACCATTGGCGTCAACGAAGAATGTAACCTCGATCTGAGGTACACCGCGAGGCGCGGGAGGT
ATGCCTTTGAGGTCAAAACTTCCAAGAAAGTGGTTGTCCTTAGTCATACTTCGCTCGCCTTCAAACACTTGAA
TGTTGACAGATGGTTCCTTGTCAGTCGATGTAGAAAATGTTCGAGACTTTTTTGTTGGAATTGTAGTGCCGCG
ATCGATCAACTTTGTCATGGCACCTCCAACTGTCTCAATTCCTTGAGAAAGCGGAACAACATCGAGTAGTATG
TCAATACCCCAAATTTTTTCACCGGACATTATCCCACCTTGAACAGCGGCGCCATAGGCAACGGCCTCGTCCG
GATTTACTCCCTTGGACGGCTCCTTGCCCTCAAAGTACTCAGAAACCAAGGACTGCACCTTTGGAATGCGCGT
AGAGCCACCAACCAAGACAATATCATCAATCTCCGATTTGCCGACACCTGCATCTTCGAGAACCCTAGAAACA
GGCCCGAGCGTCTTCTTGAACAAATCTTGGTTCAACTCCTCAAAGCGAGCCCGGGTGAGAGTTTCAGAAAAGT
CAAAACCTTTAGCCAAATCTTCGATCTCAAGGCGAACTTGCTGCTGTGACGAGAGGGCCCTCTT
>TR654|c0_g1_i1 len=2006 path=[2884:0-229 2885:230-273 @2949@!:274-878 2935:879-899 2943:900-912 2939:913-950 2933:951-1051 2924:1052-1075 2938:1076-1342 2912:1343-1366 2932:1367-1498 2929:1499-1522 2937:1523-1686 2931:1687-1965 2880:1966-1989 2881:1990-2005] [-1, 2884, 2885, 2949, 2935, 2943, 2939, 2933, 2924, 2938, 2912, 2932, 2929, 2937, 2931, 2880, 2881, -2]
GATACTCAGGGCGGCCTCAATGCCTCAACCTCAAACTAAGTAGATTTATTTTTATTATTTGCTTCAATCGACA
GTACGAAATCATTCCCAACAATGCCATCGCAAAACTCGTCCACGTCCAAGTCCGTCCTCCGAGAATGCCTCTA
CTCGCTAAACCTACCACCCGGCCTCCTCTCCTCCGTCTACCAGAACTACCTTTCCTGCGACTCGCGCATGTTC
CTCATCGACAAGTCCTCCCGCATGAAAATCACAGACAGTCACATCGGACGTCCCAGTACTAACAAACGCAACG
GTTCAACGGGAAACGGTCATGAAATCGAACGAATGGACAATGTCTCGCGTTGGGAGGAATTACATGAGTGTGT
CGCCTTCCACGCCAAGATGGCATCCAAGTGTTGGATCCCCTCCAAATACTGGCTCGTCAACGATCCCGCCGCA
TCTTCCGTGAAGCGAGAGGCATGGCAAAATCACCACGGAAGGAAATTCACTCTGTGCTGGGGAAGTACCAAGG
ATGTTCAGTCCGAATTGAACGTGATGAAACACACGATGAAAAATGCAACGTTGGATCAACGTACGTGTATGTT
GTCGTCCTGTATTCGCCATCTTTCGAAGGGCCTCGTGAGGGAAGGGCCGAGTTTGTCCGCGAGGAATGCTTAT
GTGACGTTGGTGATTTGCACGCAGGGGCAGCCCACCGATAAGGATGGTCGTTCTGGGTCTGACATTTTGCGCG
AGTTTCAGAATGAGATGTCTCGATTTGCTGGTCTACCCGTGAAGGTTATCGTGCGGCTGTGTACTGATAACGA
GAAGGTGCGGGATATGTTCAACACGATGGATGGTCATTTCGATTCCATCGATGTGTTGGATGACTTTTGGGGA

GAGTTATATCTCTGCTTCCCTGAAATGGCACAACAATGATTTGTCTGCTGGTAGGCTTTGGAAGTGTATCTTC
ACAATCCATGGTTGACATACCCAATGGGGCTGCATCGTTTGAGAGAGTCGGGCCTGGCCCCAGGAGTGCTAGA
CGACCTGGACGAACGCCCACTCAGCCTCGATGAAATCCATCAAATGTGCAAAATGCTATTCCTCGGCGAGGAC
GGCCGCACCGACCTGCCCCATCCGCGTAAGAATTGGGACAACTTTTTCCAAGCATTGAAGATACTGGTGGAGA
AGGAGAGGCCACAGTGGAACTCCGTCAAGAAGGGGATGACGCCGTGGATCAACTTGAGCAAGCTGGAGGCCAT
ACACGGCCGGAGAGAATCGTCGCGCTATCACCGCCGGAATTCACACTCGAACGCGGGACAAGCTTCGAGTCGT
CCACGGGAGGACGATCGCTCACGTCACCATCATCGCACTAGTTCGACCCGGAATTCTTTGCGCGATCAGCAGC
GACAGTCTTGGAAGGAATCGCATGGTGCGAGAGTATCACAGCATGCGCAGAGAGACTCGGCTCTCACCCTGCC
GGAAGTGCTCGAACGGTGGTCTCGCCAACCACCCGAGTTCAAAACATTGCATTCGCTGCAGCATATTCTCGTG
ACGGTACCTCAGACCTTTCCGCCAACGAATACCATGGTGGAACCTCACGAGTATTTTGCAAAATGGAAGGAGT
TCGACAAAGAGGCATTTTCTGATGAGAGCGGTGATGAATTGAAGGAACTATTGAAGCGAGCTGTGAGGAAAGC
CAAGTTCTTTTTACACCCGGATAAACTGCCCAAAGATTTAACAGAAAATCAGACACTCCTCTTTAAGACCATT
TGGAATGCTATCCAGGATCAAGAAGCAGCAACCCTTGGTTGAAGTCCCGTTAATGAATTCAATGCTGTTTTTG
GAAACCATGTTTGAACGCCCCCAGTCTTAAGGTCACATAATGCAGAACCTTACATTTTTGTGCATGCTCTACA
CGAGTGAGTTGAGCCTTCCTTGTGATGTTACCTAACTTGGTGTTTTCAGAATTATTATAGTTTTGTAAGTCTG
TTATTAGGAGTATTTAACAACAAAAAAAAAAGATC
>TR654|c0_g1_i2 len=1955 path=[2884:0-229 2885:230-273 @2949@!:274-878
2950:879-879 2877:880-899 2933:900-1000 2924:1001-1024 2938:1025-1291
2912:1292-1315 2932:1316-1447 2929:1448-1471 2937:1472-1635 2931:1636-
1914 2880:1915-1938 2881:1939-1954] [-1, 2884, 2885, 2949, 2950, 2877,
2933, 2924, 2938, 2912, 2932, 2929, 2937, 2931, 2880, 2881, -2]
GATACTCAGGGCGGCCTCAATGCCTCAACCTCAAACTAAGTAGATTTATTTTTATTATTTGCTTCAATCGACA
GTACGAAATCATTCCCAACAATGCCATCGCAAAACTCGTCCACGTCCAAGTCCGTCCTCCGAGAATGCCTCTA
CTCGCTAAACCTACCACCCGGCCTCCTCTCCTCCGTCTACCAGAACTACCTTTCCTGCGACTCGCGCATGTTC
CTCATCGACAAGTCCTCCCGCATGAAAATCACAGACAGTCACATCGGACGTCCCAGTACTAACAAACGCAACG
GTTCAACGGGAAACGGTCATGAAATCGAACGAATGGACAATGTCTCGCGTTGGGAGGAATTACATGAGTGTGT
CGCCTTCCACGCCAAGATGGCATCCAAGTGTTGGATCCCCTCCAAATACTGGCTCGTCAACGATCCCGCCGCA
TCTTCCGTGAAGCGAGAGGCATGGCAAAATCACCACGGAAGGAAATTCACTCTGTGCTGGGGAAGTACCAAGG
ATGTTCAGTCCGAATTGAACGTGATGAAACACACGATGAAAAATGCAACGTTGGATCAACGTACGTGTATGTT
GTCGTCCTGTATTCGCCATCTTTCGAAGGGCCTCGTGAGGGAAGGGCCGAGTTTGTCCGCGAGGAATGCTTAT
GTGACGTTGGTGATTTGCACGCAGGGGCAGCCCACCGATAAGGATGGTCGTTCTGGGTCTGACATTTTGCGCG
AGTTTCAGAATGAGATGTCTCGATTTGCTGGTCTACCCGTGAAGGTTATCGTGCGGCTGTGTACTGATAACGA
GAAGGTGCGGGATATGTTCAACACGATGGATGGTCATTTCGATTCCATCGATGTGTTGGATGACTTTTGGGGA
GAGGCTTTGGAAGTGTATCTTCACAATCCATGGTTGACATACCCAATGGGGCTGCATCGTTTGAGAGAGTCGG
GCCTGGCCCCAGGAGTGCTAGACGACCTGGACGAACGCCCACTCAGCCTCGATGAAATCCATCAAATGTGCAA
AATGCTATTCCTCGGCGAGGACGGCCGCACCGACCTGCCCCATCCGCGTAAGAATTGGGACAACTTTTTCCAA
GCATTGAAGATACTGGTGGAGAAGGAGAGGCCACAGTGGAACTCCGTCAAGAAGGGGATGACGCCGTGGATCA
ACTTGAGCAAGCTGGAGGCCATACACGGCCGGAGAGAATCGTCGCGCTATCACCGCCGGAATTCACACTCGAA
CGCGGGACAAGCTTCGAGTCGTCCACGGGAGGACGATCGCTCACGTCACCATCATCGCACTAGTTCGACCCGG
AATTCTTTGCGCGATCAGCAGCGACAGTCTTGGAAGGAATCGCATGGTGCGAGAGTATCACAGCATGCGCAGA
GAGACTCGGCTCTCACCCTGCCGGAAGTGCTCGAACGGTGGTCTCGCCAACCACCCGAGTTCAAAACATTGCA
TTCGCTGCAGCATATTCTCGTGACGGTACCTCAGACCTTTCCGCCAACGAATACCATGGTGGAACCTCACGAG
TATTTTGCAAAATGGAAGGAGTTCGACAAAGAGGCATTTTCTGATGAGAGCGGTGATGAATTGAAGGAACTAT
TGAAGCGAGCTGTGAGGAAAGCCAAGTTCTTTTTACACCCGGATAAACTGCCCAAAGATTTAACAGAAAATCA
GACACTCCTCTTTAAGACCATTTGGAATGCTATCCAGGATCAAGAAGCAGCAACCCTTGGTTGAAGTCCCGTT
AATGAATTCAATGCTGTTTTTGGAAACCATGTTTGAACGCCCCCAGTCTTAAGGTCACATAATGCAGAACCTT
ACATTTTTGTGCATGCTCTACACGAGTGAGTTGAGCCTTCCTTGTGATGTTACCTAACTTGGTGTTTTCAGAA
TTATTATAGTTTTGTAAGTCTGTTATTAGGAGTATTTAACAACAAAAAAAAAAGATC
>TR654|c0_g1_i3 len=2468 path=[2884:0-229 2885:230-273 @2949@!:274-878
2950:879-879 2951:880-899 2948:900-1233 2946:1234-1342 2942:1343-1374
2939:1375-1412 2933:1413-1513 2924:1514-1537 2938:1538-1804 2912:1805-
1828 2932:1829-1960 2929:1961-1984 2937:1985-2148 2931:2149-2427
2880:2428-2451 2881:2452-2467] [-1, 2884, 2885, 2949, 2950, 2951, 2948,
2946, 2942, 2939, 2933, 2924, 2938, 2912, 2932, 2929, 2937, 2931, 2880,
2881, -2]
GATACTCAGGGCGGCCTCAATGCCTCAACCTCAAACTAAGTAGATTTATTTTTATTATTTGCTTCAATCGACA
GTACGAAATCATTCCCAACAATGCCATCGCAAAACTCGTCCACGTCCAAGTCCGTCCTCCGAGAATGCCTCTA
CTCGCTAAACCTACCACCCGGCCTCCTCTCCTCCGTCTACCAGAACTACCTTTCCTGCGACTCGCGCATGTTC
CTCATCGACAAGTCCTCCCGCATGAAAATCACAGACAGTCACATCGGACGTCCCAGTACTAACAAACGCAACG
GTTCAACGGGAAACGGTCATGAAATCGAACGAATGGACAATGTCTCGCGTTGGGAGGAATTACATGAGTGTGT

CGCCTTCCACGCCAAGATGGCATCCAAGTGTTGGATCCCCTCCAAATACTGGCTCGTCAACGATCCCGCCGCA
TCTTCCGTGAAGCGAGAGGCATGGCAAAATCACCACGGAAGGAAATTCACTCTGTGCTGGGGAAGTACCAAGG
ATGTTCAGTCCGAATTGAACGTGATGAAACACACGATGAAAAATGCAACGTTGGATCAACGTACGTGTATGTT
GTCGTCCTGTATTCGCCATCTTTCGAAGGGCCTCGTGAGGGAAGGGCCGAGTTTGTCCGCGAGGAATGCTTAT
GTGACGTTGGTGATTTGCACGCAGGGGCAGCCCACCGATAAGGATGGTCGTTCTGGGTCTGACATTTTGCGCG
AGTTTCAGAATGAGATGTCTCGATTTGCTGGTCTACCCGTGAAGGTTATCGTGCGGCTGTGTACTGATAACGA
GAAGGTGCGGGATATGTTCAACACGATGGATGGTCATTTCGATTCCATCGATGTGTTGGATGACTTTTGGGGA
GAGGTGAGTTTTTATTCATCTCGCAGAAACATATTTGGGAGAACAAAGAGGCCCATTTTTGACAGATTAATGG
TTAACGATAACCGTGTGGCATCAAGTGCGATAACCTTGCCATAACCCATCTCTTAAAAGTGCAGCATAGAGGT
GTTCCATTCTGTAAGGTTATGACACATGTGTGCTAGTGTTCGGCGAAGTGCTCCGCTTCAGTCGATTTTGCTT
CGCAACAAGTAACACATGATATGTACCCTGTAATCATTTGTATGTTTGAAGAGCGTGGCAAAAATGAGGGGAA
TAGAGCGGTGGCAAATTCAAACTTCGCTCGATTATGACAACTGACATCATGAACGATAGAAAATGTTATGATT
CACGCTCTAGAGTACATTTGGTCTTGCTAGTGCGGCTACATTTGCATGATACCTGAAGCGTCTTGTCTGTTCT
TTGCAATTATTTATTTTGCGCACTCAGTTGTATCTCTTCTTCCCTGAAATGGCACAACAATGATTTGTCTGCT
GGTAGGCTTTGGAAGTGTATCTTCACAATCCATGGTTGACATACCCAATGGGGCTGCATCGTTTGAGAGAGTC
GGGCCTGGCCCCAGGAGTGCTAGACGACCTGGACGAACGCCCACTCAGCCTCGATGAAATCCATCAAATGTGC
AAAATGCTATTCCTCGGCGAGGACGGCCGCACCGACCTGCCCCATCCGCGTAAGAATTGGGACAACTTTTTCC
AAGCATTGAAGATACTGGTGGAGAAGGAGAGGCCACAGTGGAACTCCGTCAAGAAGGGGATGACGCCGTGGAT
CAACTTGAGCAAGCTGGAGGCCATACACGGCCGGAGAGAATCGTCGCGCTATCACCGCCGGAATTCACACTCG
AACGCGGGACAAGCTTCGAGTCGTCCACGGGAGGACGATCGCTCACGTCACCATCATCGCACTAGTTCGACCC
GGAATTCTTTGCGCGATCAGCAGCGACAGTCTTGGAAGGAATCGCATGGTGCGAGAGTATCACAGCATGCGCA
GAGAGACTCGGCTCTCACCCTGCCGGAAGTGCTCGAACGGTGGTCTCGCCAACCACCCGAGTTCAAAACATTG
CATTCGCTGCAGCATATTCTCGTGACGGTACCTCAGACCTTTCCGCCAACGAATACCATGGTGGAACCTCACG
AGTATTTTGCAAAATGGAAGGAGTTCGACAAAGAGGCATTTTCTGATGAGAGCGGTGATGAATTGAAGGAACT
ATTGAAGCGAGCTGTGAGGAAAGCCAAGTTCTTTTTACACCCGGATAAACTGCCCAAAGATTTAACAGAAAAT
CAGACACTCCTCTTTAAGACCATTTGGAATGCTATCCAGGATCAAGAAGCAGCAACCCTTGGTTGAAGTCCCG
TTAATGAATTCAATGCTGTTTTTGGAAACCATGTTTGAACGCCCCCAGTCTTAAGGTCACATAATGCAGAACC
TTACATTTTTGTGCATGCTCTACACGAGTGAGTTGAGCCTTCCTTGTGATGTTACCTAACTTGGTGTTTTCAG
AATTATTATAGTTTTGTAAGTCTGTTATTAGGAGTATTTAACAACAAAAAAAAAAGATC
>TR654|c0_g1_i4 len=441 path=[2917:0-69 2918:70-74 2919:75-93 2944:94-98 2936:99-121 2931:122-400 2880:401-424 2881:425-440] [-1, 2917, 2918, 2919, 2944, 2936, 2931, 2880, 2881, -2]
ATTGAAGGAACTATTGAAGCGAGGTAGGTAGTCATATTGTACATTTGTTTCTCTCCTTCATCTCATTCTCACA
CAAACGCTTTCTTTGACATACTATGTAGCTGTGAGGAAAGCCAAGTTCTTTTTACACCCGGATAAACTGCCCA
AAGATTTAACAGAAAATCAGACACTCCTCTTTAAGACCATTTGGAATGCTATCCAGGATCAAGAAGCAGCAAC
CCTTGGTTGAAGTCCCGTTAATGAATTCAATGCTGTTTTTGGAAACCATGTTTGAACGCCCCCAGTCTTAAGG
TCACATAATGCAGAACCTTACATTTTTGTGCATGCTCTACACGAGTGAGTTGAGCCTTCCTTGTGATGTTACC
TAACTTGGTGTTTTCAGAATTATTATAGTTTTGTAAGTCTGTTATTAGGAGTATTTAACAACAAAAAAAAAAG
ATC
>TR655|c0_g1_i1 len=1530 path=[1719:0-144 1720:145-168 1740:169-451 1744:452-475 1746:476-510 1742:511-611 1745:612-619 1724:620-643 1734:644-919 @1743@!:920-1167 1698:1168-1197 @1737@!:1198-1296 1696:1297-1529] [-1, 1719, 1720, 1740, 1744, 1746, 1742, 1745, 1724, 1734, 1743, 1698, 1737, 1696, -2]
GTAGGAGTACCGTACGATACTTCTAGTGAGACCTGGGCCACGCTTGGGCACCAAATTCGATATCATCAGTTGA
CGCAAACGTATTCATTGTCATTGTGCACCCTAACCATGAGATATGCATCACCCCTTAACCGCCATGTGCGACG
TCGGCAACGCTTCCGCACCGCCATCATCGCGACTGTGGCCATCATAGTCCTTTCCCTCTTCGTTTTCGCCCCC
GCTGCGTCAGCCGCGAGTAGCAACTTCGTCGTTGAGGACAAGCGGTGTATCGAGGTCGTGGATGATTACGATA
TAAGGACTACTGCGCGTGACTCCAACGTATTCGTTATCGTCCACGAAAAGGATGATGTGGTAGCACAGGAGCA
TATCTGCAAGAAGTTGGAGGCCACCCTCAACAAAGTGTATTTGGGTGCCGCGATGAAAGGCAAGGGAGCGGTG
TTTGCCTACCTGGTGCTTGAAGAGCCATCGGAGGGCAAGAATGGCAATAAAAACTTTGTGAAGAATACTCTCG
GCGTGAGGAAATTTCCCTCCTATCTGTTTGTGTCGAAAGGAATGGACAGGTATTCTAAATTTTCGGATCACCT
CACTGAACTCAAGGGTGGAGATTCTCTAGAAATGTCTGATGTCGAAACTTTTATTGAAGATAAAGTTGGTTTC
AGACTCGGGAATGACGTTTTCAGCATTGTCTTCTTTGACAGCGTTGCGTCTCGTTTTGTTTCCTACGGCGATA
CAGCGCCTTGGTCCTCAGATCGCCTAAAACAACGAATCCTGCAACTTCTCGTTCGCTTCTCCACAATTGTCTC
CTTCAAAGAGCCCTTCAGTTCCATTGGAAAATTATACAATCGTGCATTTTCCATGTCATTGGCGAACGGAATG
GACTACAGCGAGAATCAGGTGAAAAAACTACAAAAGAAACTCGACTTAAAGAAGCACGAACTTTCCGATGAGA
AAATTCACGAATTTCAACAAAAGATTAGCATTTTGAAGTCGTTCGAGGAGCCGAAGGAAAATACTGCCGAGGA
TGACAAGCAAATTTTTATCCACGCCGCTCTTCATTTCGGATTGGCAATTGCCACGATTTTGCTGATCATTCTT
CCCGCCGCTGATGAGGAGACTGAGGAAGAGGAAGTTATCAATGCTGAGCCAGTCGTCGCAAAAGCTGTGAGGG

GGGACTATAAGTCCACCAAGAAATCAAGCTAATGAAATGAGAGCCAGAACCGAGAGGCTAGTGATCTTATGTC
CCATTCTGGGTGGTTAGTTTTTAACAGTTATATTACAAACATTACCTCTCTTAAAATGAAAAAGATATCTGTT
GTCACTACAGTGCGTTTATTGGGCTACATTCTCGAACAAATTGTGTTCCAAACTTGATAGATTAGTACCAGAT
CATGAGTGTGTTGTCTCATTTGGTATGCTGTATCTTAAGGTTGGGTAAATGACCTGTTTTGGTGCACATCCTC
TTTCCATGGTAATTTTTGCTCTTCCCCTTTCGGCTCCAGTGTTCAGATTGCCATGGTAACCGAAGAAGAC
>TR656|c0_g1_i1 len=1241 path=[1470:0-142 1471:143-166 1495:167-283 1486:284-307 1497:308-503 1494:504-657 1477:658-681 1490:682-741 1466:742-765 1493:766-804 1463:805-828 1498:829-869 1457:870-895 @1496@!:896-954 1460:955-998 1488:999-1171 1474:1172-1195 1475:1196-1240] [-1, 1470, 1471, 1495, 1486, 1497, 1494, 1477, 1490, 1466, 1493, 1463, 1498, 1457, 1496, 1460, 1488, 1474, 1475, -2]
[truncated: 521,584 more chars]
